# A Computer-Assisted Personal Interview App in Research Electronic Data Capture for Administering Time Trade-off Surveys (REDCap): Development and Pretest

**DOI:** 10.2196/formative.8202

**Published:** 2018-01-23

**Authors:** Mark Oremus, Anis Sharafoddini, Gian Paolo Morgano, Xuejing Jin, Feng Xie

**Affiliations:** ^1^ School of Public Health and Health Systems University of Waterloo Waterloo, ON Canada; ^2^ Department of Health Research Methods, Evidence, and Impact McMaster University Hamilton, ON Canada; ^3^ Program for Health Economics and Outcome Measures Hamilton, ON Canada; ^4^ Centre for Evaluation of Medicines St. Joseph’s Healthcare Hamilton Hamilton, ON Canada

**Keywords:** computer-assisted personal interview, health-related quality-of-life, REDCap, time trade-off

## Abstract

**Background:**

The time trade-off (TTO) task is a method of eliciting health utility scores, which range from 0 (equivalent to death) to 1 (equivalent to perfect health). These scores numerically represent a person’s health-related quality of life. Software apps exist to administer the TTO task; however, most of these apps are poorly documented and unavailable to researchers.

**Objective:**

To fill the void, we developed an online app to administer the TTO task for a research study that is examining general public proxy health-related quality of life estimates for persons with Alzheimer’s disease. This manuscript describes the development and pretest of the app.

**Methods:**

We used Research Electronic Data Capture (REDCap) to build the TTO app. The app’s modular structure and REDCap’s object-oriented environment facilitated development. After the TTO app was built, we recruited a purposive sample of 11 members of the general public to pretest its functionality and ease of use.

**Results:**

Feedback from the pretest group was positive. Minor modifications included clarity enhancements, such as rearranging some paragraph text into bullet points, labeling the app to delineate different question sections, and revising or deleting text. We also added a research question to enable the identification of respondents who know someone with Alzheimer’s disease.

**Conclusions:**

We developed an online app to administer the TTO task. Other researchers may access and customize the app for their own research purposes.

## Introduction

Health-related quality of life (HRQoL) is an individual’s normative perception of how disease and treatment affect their physical, functional, psychological, and social well-being [1]. HRQoL can be measured using health utility scores, which lie on an interval scale ranging from 0 (equivalent to death) to 1 (equivalent to full health).

Numerous methods exist to elicit health utility scores [2,3]. One method is the time trade-off (TTO) method [4], which has three variants: conventional [4], lead-time [5], and composite [6]. Regardless of the variant, the basic premise of TTO remains the same: individuals read a brief description of a health state and choose between two (usually hypothetical) outcomes (ie, Life A or Life B). For example, Life A involves living in a full health state for *x* years, followed by death. Life B involves living in the health state in question for a fixed number of years (*t,* usually 10 years), followed by death. The lead-time and composite TTO approaches involve adding additional years of life in full health (*l*) to both Life A and Life B [3].

After being presented with Life A and Life B, individuals may choose Life A, Life B, or they can say that the two options are equivalent. When individuals choose Life A or B, they are presented with a second iteration where *t* in Life B is held constant, but *x* in Life A is varied to reflect a changing length of time in full health. Once again, individuals are asked to choose between Life A or B, or equivalence. Subsequent iterations involve further variations of *x* in Life A, while *t* in Life B is always held constant. The iterations continue until individuals indicate that Life A and Life B are equivalent, that is, they are indifferent between the two choices. Once the point of indifference is known, researchers use a formula to calculate the health utility score. The formula differs according to the TTO variant [4-6].

Early protocols called for the TTO task to be administered using a paper-and-pencil format in face-to-face interviews [7]. Interviewers used props such as cards, colored envelopes, and custom-made boards with sliding scales to depict health states and the number of years spent in Life A and Life B. The sliding scales permitted interviewers to visually illustrate differences between the various iterations of Life A and Life B.

To standardize the TTO task and ease the burden of using multiple props to administer the activity, researchers developed computer-assisted personal interview (CAPI) software such as U-titer [8] and iMPACT3 [9] to replace the paper-and-pencil format. The EuroQol Group currently uses CAPI software (EuroQol Valuation Technology [EQ-VT]) [10-12] to administer the TTO task in studies designed to value health states for the EQ-5D [13,14] (a 5-item questionnaire created to measure HRQoL). Despite the documented existence of these three software apps, U-titer and iIMPACT3 do not appear to be available any longer. The EQ-VT’s technical specifications have not been published, and the software does not appear to be available on the EuroQol Group’s website [15].

### The Need for a Computer-Assisted Personal Interview Time Trade-Off App

Our interest in TTO emerges from research examining whether the general public can act as a proxy and estimate HRQoL in place of persons with Alzheimer’s disease (AD) [16]. We plan to ask members of the general public to read descriptions of the mild, moderate, and severe health states of AD. Subsequently, they will perform the TTO task for each health state and generate health utility scores for each state. The health utility scores from the general public will be compared to a set of similarly elicited scores from persons with AD. We have already established that the general public can use health state descriptions to discriminate between mild, moderate, and severe AD [16].

To enable the interviewing of participants in multiple locations, we decided the TTO task should be administered using CAPI rather than paper-and-pencil. We wanted interviewers to carry only a laptop between locations, instead of bulky sets of forms, props, and writing materials.

Prior to developing our own CAPI app, we reviewed the literature to assess whether existing software could serve our purpose. We found 13 TTO studies that reported using CAPI software during in-person interviews: 7 studies used the EQ-VT [11,12,17-21] and 6 did not describe the specific software [22-27]. Of these 6 studies, one [23] cited a previous study [28] as the source of the CAPI software. The previous study contained screenshots of the software but no technical details. Another 2 of the 6 studies reported using hard-copy visual aids to conduct the TTO task, with CAPI software reserved for data entry only [24,25]. A total of 22 other studies used Internet-based CAPI software, where participants logged onto websites and completed the TTO task on their own, without an interviewer present [22,29-49]. Five [30-34] of these 22 studies provided screenshots of the software, but none of the 22 studies described the technical details of the software.

The lack of information on existing CAPI software led us to develop our own Web-based app with a point-and-click interface to administer the TTO task. This paper describes the technical details and pretest of the app. At the outset, we specified two prerequisites for the app. First, the mild, moderate, and severe health states of AD would have to be presented to study participants in one of six random orders to help minimize biases due to ordering effects. This is because we will ask members of the general public and persons with AD to perform the TTO task for all three AD health states. Second, the app would start the lead-time TTO iterations at a value of 20 years in perfect health followed by death for Life A (*x*=10; *l*=10). The static Life B comparator would be 10 years in perfect health, followed by 10 years in the AD health state in question (*t*=10), and then death. Multimedia Appendix 1 shows the iterations in our software. Health utility scores would be calculated using the following formula: *(x – l)/t*, with *x* being the amount of time in perfect health (Life A) at the point of indifference between Life A and Life B. The formula permits the calculation of health utility scores less than 0, which represent health states worse than death (eg, persistent vegitative state).

Our TTO app is still in its formative stages. The content of the app is based on established lead-time TTO methods [5] that have been implemented in a large valuation study conducted by one of the co-authors (FX) [50]. We report on the development and initial pretest results for the app, which is Web-based and freely available to all potential users.

## Methods

We used the Research Electronic Data Capture (REDCap) app [51] to build the TTO app. REDCap is a secured Web-based app that allows researchers to create CAPI apps for collecting, storing, and manipulating research data. REDCap is hosted on the server of the University of Waterloo, and we selected it as the preferred platform because of the following features: (1) minimal programming requirements, (2) easy accessibility to the TTO app across academic departments and institutions (at the time of writing, REDCap has 1911 active institutional partners), (3) flexibility in defining user privileges, such as limited/temporary privileges, (4) real-time data format/range validation, (5) data export options for common statistical software packages (eg, SPSS, SAS, R), (6) software and support for no charge, (7) modest hardware and software requirements, and (8) availability as a mobile app.

**Figure 1:**
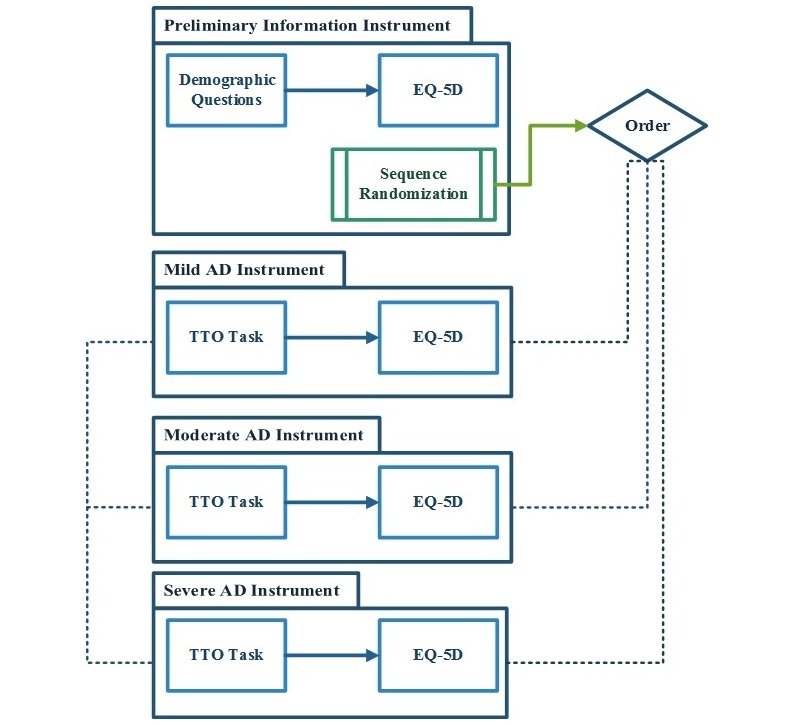
Modular structure of the time trade-off (TTO) app. AD: Alzheimer’s disease; EQ-5D: EuroQol 5-dimension.

**Figure 2:**
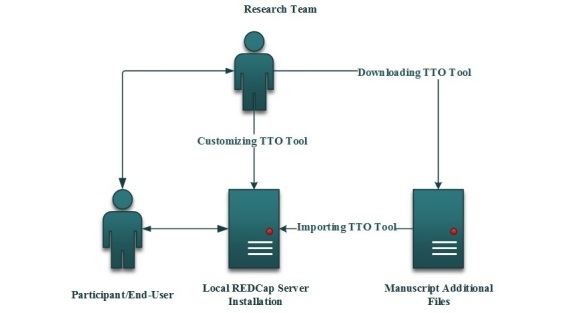
Workflow for accessing the time trade-off (TTO) app. REDCap: Research Electronic Data Capture.

The TTO app was designed in a modular format. Its overall structure is presented in Figure 1. The app’s modular structure and REDCap’s object-oriented environment facilitate the app’s ready customization to meet researchers’ needs (Figure 2). In addition to accessibility through the project home page, the TTO-CAPI eXtensible markup language (XML) file and installation manual are included as Multimedia Appendices 2 and 3 to guarantee the availability of the software in the future. The detailed methodology to construct the TTO app follows.

### Time Trade-Off Software

As shown in Figure 1, there are four data collection instruments in the TTO app:

1. Preliminary information: demographic data (ie, age, gender, education level, income level), knowing anyone with AD, and EQ-5D-5L (5L indicates 5 response options per question); this instrument assigns a unique identification number to each record (study participant) in the background
2. Mild AD: Mild AD TTO data; EQ-5D-5L data (participants answer the EQ-5D-5L as if they had AD according to the health state description just read [all three health states])
3. Moderate AD: Moderate AD TTO data; EQ-5D-5L data
4. Severe AD: Severe AD TTO data; EQ-5D-5L data

In each of the three AD instruments (numbers 2-4 above), questions appear in an order based on the end-user responses to TTO iterations shown in Multimedia Appendix 1. In REDCap, each singular data entry is called a *field*. Various types of project fields were used to house questions and then combined in sequence one after the other to form the instruments. The following project fields were used to design the instruments: text box, multiple-choice radio buttons, multiple-choice drop-down list, yes-no, descriptive text, and calculated field. Hypertext markup language (HTML) tags were used to customize the appearance of the text in each of the instruments.

**Figure 3:**
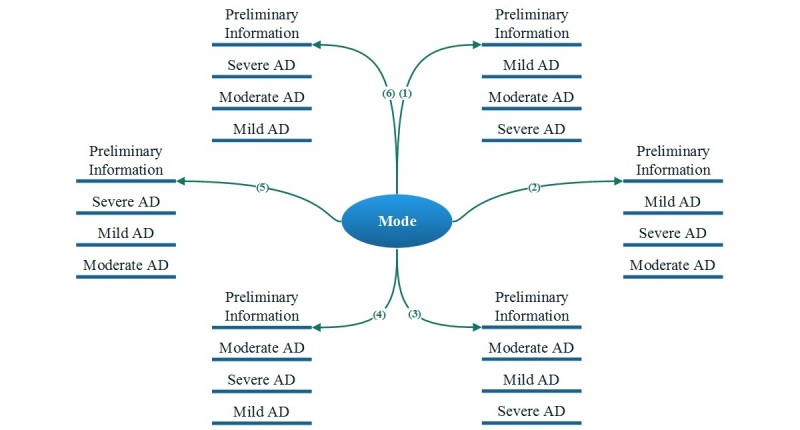
Six possible navigation options between Alzheimer's disease (AD) instruments based on mode.

**Figure 4:**
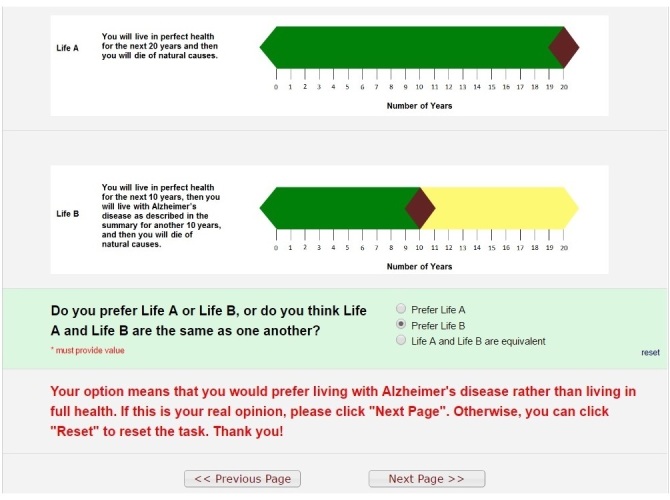
Screenshot of a logical error check for the pop-up text.

We employed two logic options in the TTO app:

1. Survey queue: a set of navigation conditions was defined to ensure the three TTO tasks would appear in a uniformly random sequence (Figure 3). Table 1 shows the conditions fixed for each instrument. We used an “auto start” feature to enable smooth and immediate navigation between modules. Uniform randomization was done by defining a variable named “mod_6” in the Preliminary information instrument. This variable could take a value from 1-6 and represent one of the six possible orders of AD tasks shown in Figure 3. The completion of a task was monitored by a flag named “part *i* _complete”, where *i* is the task number that can take a value from 1-3 for mild, moderate, and severe AD, respectively. The following values were used for the flag status: Complete=2; Incomplete=0.
2. Branching logic: used to (1) control the order of questions in the TTO task, (2) initiate logical error check pop-up texts (eg, in situations when users choose life with AD as being more desirable than a state of full health; see Figure 4), and (3) demonstrate users’ progression toward completing the survey.

**Table 1:** Survey queue conditions for Alzheimer’s disease (AD) instruments.

Instruments	Conditions
Mild AD	WHEN
	Preliminary Information is complete
	AND
	([mod6^a^]<“3”) or ([mod6]=“3” and
	([part2_complete]+[part3_complete^b^]=“2”)) or (([mod6]=“4” or
	[mod6]=“6”) and ([part2_complete^c^]+[part3_complete]=“4”)) or
	([mod6]=“5” and ([part2_complete]+[part3_complete]=“2”))
Moderate AD	WHEN
	Preliminary Information is complete
	AND
	([mod6]=“3”) or ([mod6]=“4”) or ([mod6]=“1” and
	([part1_complete]+[part3_complete]=“2”)) or (([mod6]=“2” or
	[mod6]=“5”) and ([part3_complete]+[part1_complete]=“4”)) or
	([mod6]=“6” and ([part1_complete^d^]+[part3_complete]=“2”))
Severe AD	WHEN
	Preliminary Information is complete
	AND
	([mod6]=“5”) or ([mod6]=“6”) or ([mod6]=“2” and
	([part1_complete]+[part2_complete]=“2”)) or (([mod6]=“1” or
	[mod6]=“3”) and ([part2_complete]+[part1_complete]=“4”)) or
	([mod6]=“4” and ([part1_complete]+[part2_complete]=“2”))

^a^Mod6: randomization variable.

^b^Severe AD task flag.

^c^Moderate AD task flag.

^d^Mild AD task flag.

**Figure 5:**
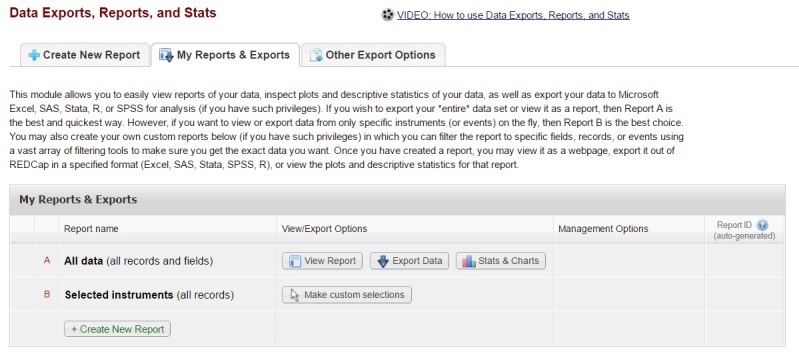
Record export dashboard.

The end-user can get access to the survey through a public uniform resource locator (URL) or quick response (QR) code. Researchers can export the collected data from all instruments at once without being affected by the randomized order of the TTO tasks. Researchers can also export the data from specific instruments or fields separately (Figure 5).

For building the TTO app, REDCap Version 6.12.0 was installed on the isysign webserver, which provides high security for hosted projects. We assigned different levels of data access rights to research team members, who had access to the project only through their individual usernames and passwords.

**Figure 6:**
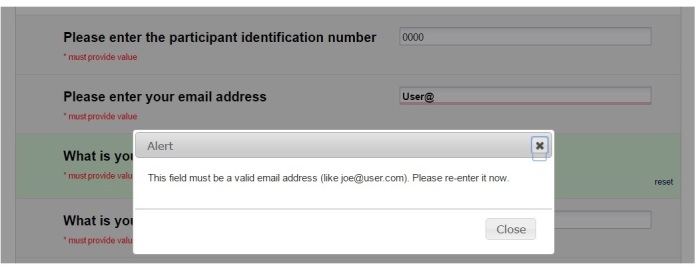
Screenshot of a pop-up alert for incorrect free-text entry by end-user for email address.

One of the issues in the design of the TTO app was the possibility of incorrect data entry in ordinary text fields by the end-user. This problem was avoided using the *validation* option in each field. This option ensures end-users can enter only free-form text in predefined formats. Otherwise, a pop-up alert will inform the end-user of the correct format (Figure 6). This option is not only available for the format of the data but can also be used to minimize the problem of outliers by defining a valid range for numeric free-text fields such as age.

Before publishing the app for pretesting, the research team tested the accuracy and reliability of the app’s iteration and randomization sequences. The team also reviewed the user interface from various perspectives, including consistency of appearance, clarity of cues, and simplicity of user engagement.

### Pretest

Following the development of the software, members of the research team recruited a purposive sample of the general public to provide quality assurance feedback on the survey. These pretest participants were recruited from the researchers’ social networks (eg, friends, family, landlords). None of the pretest participants had an academic background. Each participant met with an interviewer for a one-on-one interview, during which the interviewer explained the purpose of the pretest, that is, to get feedback on the comprehensibility of the survey questions and the TTO task, as well as to assess the appearance and functionality of the online survey. The interviewers gave participants control over a laptop, and the participants completed the survey at their own pace.

The pretests followed the method of cognitive interviewing [52]. As participants conducted the TTO task for the first AD health state, the interviewers asked them a series of scripted questions about whether they understood the description of the health state and the difference between Life A and Life B. After completing the first TTO task, the participants were asked to describe what they had just done using their own words. The interviewers encouraged participants to verbally express any thoughts that came to mind (ie, think aloud [53]) as they performed the TTO task for the remaining two health states. After participants finished the survey, they were asked to comment on the similarities and differences between the three health state descriptions. The interviewers also elicited feedback about the following components of the survey: the visual aids used to depict Life A and Life B, the appearance of the webpages and fonts, and the wording used to articulate the questions. After aggregating and discussing the pretest feedback, the researchers revised the survey.

We considered the pretest participants to be part of the research team. While these individuals had to complete the survey, they were told at the outset that their thoughts about the survey were of interest, not their responses to specific survey questions. The interviewers informed participants that their responses to specific survey questions would not be used in later research, nor would these responses be published anywhere.

### Ethics

Research ethics approval was obtained from the University of Waterloo’s Office of Research Ethics (study #21461) and the Hamilton Integrated Research Ethics Board.

## Results

We pretested the TTO app on 11 members of the general public who had not previously seen the app or responded to a TTO survey. Nine pretest participants were female, the median age of the 11 participants was 46 years (range 21-59 years), and none of the participants had a university degree. A member of the research team (GPM) and a student volunteer who was trained by the research team conducted the pretests. The pretest interviewers summarized their findings, which were discussed by the research team. The findings and discussion led to six modifications of the TTO app:

1. To enhance readability, the scenarios describing the mild, moderate, and severe AD health states were rewritten in bullet-point form, rather than in paragraph form.
2. To assess whether participants’ responses to the TTO task might be influenced by their experiences with AD, a question about knowing someone with AD was inserted into the Preliminary information instrument.
3. To help participants identify different parts of the survey, the scenarios were labeled as Description 1, 2, or 3.
4. To enhance the empathy of the TTO task, a phrase comparing life with AD to a state worse than death was deleted from the app.
5. To enrich the descriptions of Life A and Life B, the text was modified to indicate that death will occur from natural causes after the specified number of years spent in full health or with AD.
6. To enhance comprehensibility, a phrase in the TTO response options was changed from “Life A and Life B are the same” to “Life A and Life B are equivalent”.

The pretest participants reported understanding the substantive differences between scenarios. One participant found the need to value Life A in comparison to Life B as cognitively challenging, though this was not an issue with the online app but with her comprehension of the TTO task itself. The iterations functioned as described in Multimedia Appendix 1.

## Discussion

### Principal Considerations

We will use the TTO app in a research study that examines whether the general public can provide proxy HRQoL estimates in place of persons with AD. Due to cognitive impairment, many persons with AD are unable to estimate their own HRQoL using instruments such as the EQ-5D [54,55]. Family caregivers sometimes act as proxies, but they often integrate their own life experiences (eg, the burdens and stresses of caregiving) into the proxy assessments and underestimate the HRQoL of their loved ones with AD [56-62]. To the best of our knowledge, no one has examined whether the general public can provide a better set of proxy HRQoL estimates than caregivers in AD.

The TTO app is publicly available for other researchers to adapt to their own studies. As described above, existing CAPI software to conduct the TTO task is poorly documented in the literature and not readily available for researchers to access. Our app fills a gap for researchers who require a means of administering the TTO task in their studies.

The pretest interviewers found the app worked well with regard to presenting all of the information necessary to conduct the TTO task, that is, the scenarios describing the three AD health states, the diagrammatic representations of Life A and Life B, and the questions about participants’ preferences for Life A or Life B. Additionally, the app worked quickly on a variety of public (eg, university) and private (eg, personal residence) wireless Internet connections. We did not notice any difference in performance on laptops running Windows, OS X, or Linux operating systems.

The use of laptops provides flexibility in conducting interviews. Interviewers can turn the laptops over to participants and allow the TTO task to be performed in a completely self-administered fashion. Interviewers can also operate the point-and-click interface to record participants’ responses and help them navigate the task. This versatility is especially important when conducting research in populations that may be less familiar with technology (eg, seniors) or that may experience challenges in using technology (eg, persons with AD).

The use of REDCap is timely given other developments in the HRQoL field. Recently, the EuroQoL Group released REDCap versions of the EQ-5D in over 50 languages to facilitate the collection of HRQoL data [63].

The TTO app described in this manuscript will facilitate the conduct of high-quality cost-utility analyses, which are undertaken to assess the costs and benefits of new health technologies. The health utility scores generated from the TTO task are an essential ingredient of these analyses. Cost-utility analyses are increasing in frequency and can influence public sector treatment reimbursement decisions and health program priority setting [64].

### Limitations

The pretest sample was one of convenience and may therefore be unrepresentative of average members of the general public. However, the pretest specifically excluded people with academic backgrounds to enable us to collect feedback on the app from individuals who would reflect a variety of lay opinions. Also, cognitive interviewing methods do not require the recruitment of representative samples nor do they specifically call for the use of formal qualitative analytical approaches [65,66]. In this research, we did not focus on analyzing pretest participants’ specific health utilities because the objective of the work was to develop and test an online TTO app. We have already shown in previous research that members of the general public can differentiate between AD health states [16].

The TTO app was developed to meet the needs of a specific research protocol. Therefore, the app does not contain multiple means of eliciting health utilities (eg, visual analogue scale or standard gamble approach [67]) nor does it include multiple methods of iterating between Life A and Life B (eg, titration, bisection [3]). Some users may find REDCap’s graphic capabilities uncustomizable to their needs.

The TTO app is still preliminary and may undergo further refinement as we move forward with our study of general public proxy HRQoL estimates in AD. However, our work demonstrates that researchers can easily use REDCap to computerize the TTO task and create a useful online CAPI app.

### Conclusions

We developed an online app to administer the TTO task. Other researchers may access and alter the app for their own research purposes. Our app fills an operational gap in eliciting health utility scores because existing TTO apps are poorly documented and inaccessible to researchers. Researchers may contact us for information on how to use the app in their projects.

## Appendix

Multimedia Appendix 1 Time trade-off iteration.

Multimedia Appendix 2 TTO-CAPI XML file.

Multimedia Appendix 3 TTO-CAPI installation on local REDCapTM server (manual).

## Abbreviations

AD: Alzheimer’s disease
CAPI: computer-assisted personal interview
EQ-5D: EuroQol 5-dimension
EQ-5D-5L: EuroQol 5-dimension 5-level
EQ-VT: EuroQol Valuation Technology
HRQoL: health-related quality of life
REDCap: Research Electronic Data Capture
TTO: time trade-off
