# Multimedia Appendix Correction: A Computer-Assisted Personal Interview App in Research Electronic Data Capture for Administering Time Trade-off Surveys (REDCap): Development and Pretest

**DOI:** 10.2196/11436

**Published:** 2018-07-06

**Authors:** Mark Oremus, Anis Sharafoddini, Gian Paolo Morgano, Xuejing Jin, Feng Xie

**Affiliations:** ^1^ School of Public Health and Health Systems University of Waterloo Waterloo, ON Canada; ^2^ Department of Health Research Methods, Evidence, and Impact McMaster University Hamilton, ON Canada; ^3^ Program for Health Economics and Outcome Measures Hamilton, ON Canada; ^4^ Centre for Evaluation of Medicines St. Joseph’s Healthcare Hamilton Hamilton, ON Canada

The authors of the paper “A Computer-Assisted Personal Interview App in Research Electronic Data Capture for Administering Time Trade-off Surveys (REDCap): Development and Pretest” (JMIR Formativ Res 2018;2(1):e3) made an error in Multimedia Appendix 2. The original Multimedia Appendix 2 contained the EQ-5D-5L in XML format which the authors did not have copyright permission to reproduce in the appendix. The EuroQol Group (owners of the EQ-5D) consider the original Multimedia Appendix a copyright infringement of the EQ-5D and asked for removal. The new version of Multimedia Appendix 2 does not contain the EQ-5D-5L. The described app is designed to provide an electronic means of administering the time trade-off task in health economic studies. As such, the presence of EQ-5D in the Multimedia Appendix was illustrative and completely non-essential to the thrust and substance of the app and the article that has been published in the journal.

Editor and publisher remind authors to preferably use open, freely available research instruments and remind authors that JMIR Publications will no longer publish studies which report the development and validation of an instrument or scale without making the instrument itself openly accessible (for example, under a Creative Common license). The Comittee on Publication Ethics (COPE) has also recently discussed the deplorable behavior of some scale developers which appear to comb the literature and ask those who used the scale for research to retract the paper or to pay for a retroactive license, sometimes asking for very large sums of money. COPE emphasized that “holding authors to ransom in this way is not good for the advancement of scientific knowledge or in the public interest,” and while the EuroQol Group has not asked for money in this specific case, the case serves as reminder for researchers to develop and use openly accessible instruments rather than copyrighted ones.

The corrected version of Multimedia Appendix 2 is available below.

The corrected article will appear in the online version of the paper on the JMIR website on July 6, 2017, together with the publication of this correction notice. Because this correction was made after submission to PubMed or PubMed Central and other full-text repositories, the corrected article, with the updated Multimedia Appendix, has also been resubmitted to those repositories.

## Appendix

Multimedia Appendix 2 TTO-CAPI XML file.

