# Supplementary material for: A Computer-Assisted Personal Interview App in Research Electronic Data Capture for Administering Time Trade-off Surveys (REDCap): Development and Pretest
Source: JMIR Form Res. 2018 Jan 23;2(1):e3. doi: 10.2196/formative.8202 (PMC6334703; doi:10.2196/formative.8202)
Supplement: Multimedia Appendix 1 [file formative_v2i1e3_app1.pdf]

Multimedia Appendix 1 Time Trade-off Iterations

Iteration	Years in Life A (Perfect Health)	Participant Response	Next Iteration
	Introduction Page		Life A=20
1	20	Equal	End task? Yes/No
1	20	Prefer A	Life A=10
1	20	Prefer B	Error!
2	10	Equal	End task? Yes/No
2	10	Prefer A	Life A=5
2	10	Prefer B	Life A=15
3	5	Equal	End task? Yes/No
3	5	Prefer A	Life A=4
3	5	Prefer B	Life A=6
3	15	Equal	End task? Yes/No
3	15	Prefer A	Life A=14
3	15	Prefer B	Life A=16
4	4	Equal	End task? Yes/No
4	4	Prefer A	Life A=3
4	4	Prefer B	Life A=4.5
4	6	Equal	End task? Yes/No
4	6	Prefer A	Life A=5.5
4	6	Prefer B	Life A=7
4	14	Equal	End task? Yes/No
4	14	Prefer A	Life A=13
4	14	Prefer B	Life A=14.5
4	16	Equal	End task? Yes/No
4	16	Prefer A	Life A=15.5
4	16	Prefer B	Life A=17
5	3	Equal	End task? Yes/No
5	3	Prefer A	Life A=2
5	3	Prefer B	Life A=3.5
5	4.5	Equal	End task? Yes/No
5	4.5	Prefer A	End task? Yes/No
5	4.5	Prefer B	End task? Yes/No
5	5.5	Equal	End task? Yes/No
5	5.5	Prefer A	End task? Yes/No
5	5.5	Prefer B	End task? Yes/No
5	7	Equal	End task? Yes/No
5	7	Prefer A	Life A=6.5
5	7	Prefer B	Life A=8
5	13	Equal	End task? Yes/No
5	13	Prefer A	Life A=12
5	13	Prefer B	Life A=13.5
5	14.5	Equal	End task? Yes/No
5	14.5	Prefer A	End task? Yes/No
5	14.5	Prefer B	End task? Yes/No

5	15.5	Equal	End task? Yes/No
5	15.5	Prefer A	End task? Yes/No
5	15.5	Prefer B	End task? Yes/No
5	17	Equal	End task? Yes/No
5	17	Prefer A	Life A=16.5
5	17	Prefer B	Life A=18
6	2	Equal	End task? Yes/No
6	2	Prefer A	Life A=1
6	2	Prefer B	Life A=2.5
6	3.5	Equal	End task? Yes/No
6	3.5	Prefer A	End task? Yes/No
6	3.5	Prefer B	End task? Yes/No
6	6.5	Equal	End task? Yes/No
6	6.5	Prefer A	End task? Yes/No
6	6.5	Prefer B	End task? Yes/No
6	8	Equal	End task? Yes/No
6	8	Prefer A	Life A=7.5
6	8	Prefer B	Life A=9
6	12	Equal	End task? Yes/No
6	12	Prefer A	Life A=11
6	12	Prefer B	Life A=12.5
6	13.5	Equal	End task? Yes/No
6	13.5	Prefer A	End task? Yes/No
6	13.5	Prefer B	End task? Yes/No
6	16.5	Equal	End task? Yes/No
6	16.5	Prefer A	End task? Yes/No
6	16.5	Prefer B	End task? Yes/No
6	18	Equal	End task? Yes/No
6	18	Prefer A	Life A=17.5
6	18	Prefer B	Life A=19
7	1	Equal	End task? Yes/No
7	1	Prefer A	Life A=0.5
7	1	Prefer B	Life A=1.5
7	2.5	Equal	End task? Yes/No
7	2.5	Prefer A	End task? Yes/No
7	2.5	Prefer B	End task? Yes/No
7	7.5	Equal	End task? Yes/No
7	7.5	Prefer A	End task? Yes/No
7	7.5	Prefer B	End task? Yes/No
7	9	Equal	End task? Yes/No
7	9	Prefer A	Life A=8.5
7	9	Prefer B	Life A=9.5
7	11	Equal	End task? Yes/No
7	11	Prefer A	Life A=10.5
7	11	Prefer B	Life A=11.5
7	12.5	Equal	End task? Yes/No

7	12.5	Prefer A	End task? Yes/No
7	12.5	Prefer B	End task? Yes/No
7	17.5	Equal	End task? Yes/No
7	17.5	Prefer A	End task? Yes/No
7	17.5	Prefer B	End task? Yes/No
7	19	Equal	End task? Yes/No
7	19	Prefer A	Life A=18.5
7	19	Prefer B	Life A=19.5
8	0.5	Equal	End task? Yes/No
8	0.5	Prefer A	Life A=0
8	0.5	Prefer B	End task? Yes/No
8	1.5	Equal	End task? Yes/No
8	1.5	Prefer A	End task? Yes/No
8	1.5	Prefer B	End task? Yes/No
8	8.5	Equal	End task? Yes/No
8	8.5	Prefer A	End task? Yes/No
8	8.5	Prefer B	End task? Yes/No
8	9.5	Equal	End task? Yes/No
8	9.5	Prefer A	End task? Yes/No
8	9.5	Prefer B	End task? Yes/No
8	10.5	Equal	End task? Yes/No
8	10.5	Prefer A	End task? Yes/No
8	10.5	Prefer B	End task? Yes/No
8	11.5	Equal	End task? Yes/No
8	11.5	Prefer A	End task? Yes/No
8	11.5	Prefer B	End task? Yes/No
8	18.5	Equal	End task? Yes/No
8	18.5	Prefer A	End task? Yes/No
8	18.5	Prefer B	End task? Yes/No
8	19.5	Equal	End task? Yes/No
8	19.5	Prefer A	End task? Yes/No
8	19.5	Prefer B	Life A=20
9	0	Equal	End task? Yes/No
9	0	Prefer A	End task? Yes/No
9	0	Prefer B	End task? Yes/No
9	20	Equal	End task? Yes/No
9	20	Prefer A	End task? Yes/No
9	20	Prefer B	End task? Yes/No
