# Supplementary material for: A Computer-Assisted Personal Interview App in Research Electronic Data Capture for Administering Time Trade-off Surveys (REDCap): Development and Pretest
Source: JMIR Form Res. 2018 Jan 23;2(1):e3. doi: 10.2196/formative.8202 (PMC6334703; doi:10.2196/formative.8202)
Supplement: Multimedia Appendix 3 [file formative_v2i1e3_app3.pdf]

TTO-CAPI Installation on Local REDCap™ Server

(Manual)

Uploading TTO Tool on REDCap™ Server

1. In the local REDCap™ installation, click **Create New Project,** then enter the project title and the purpose of the project.
2. Select **Upload a REDCap project XML file (CDISC ODM format)**, and then click **Choose file**.
3. Upload the **CAPITTO.REDCap.xml** file [Additional file 2].
4. Click the **Create Project** button.

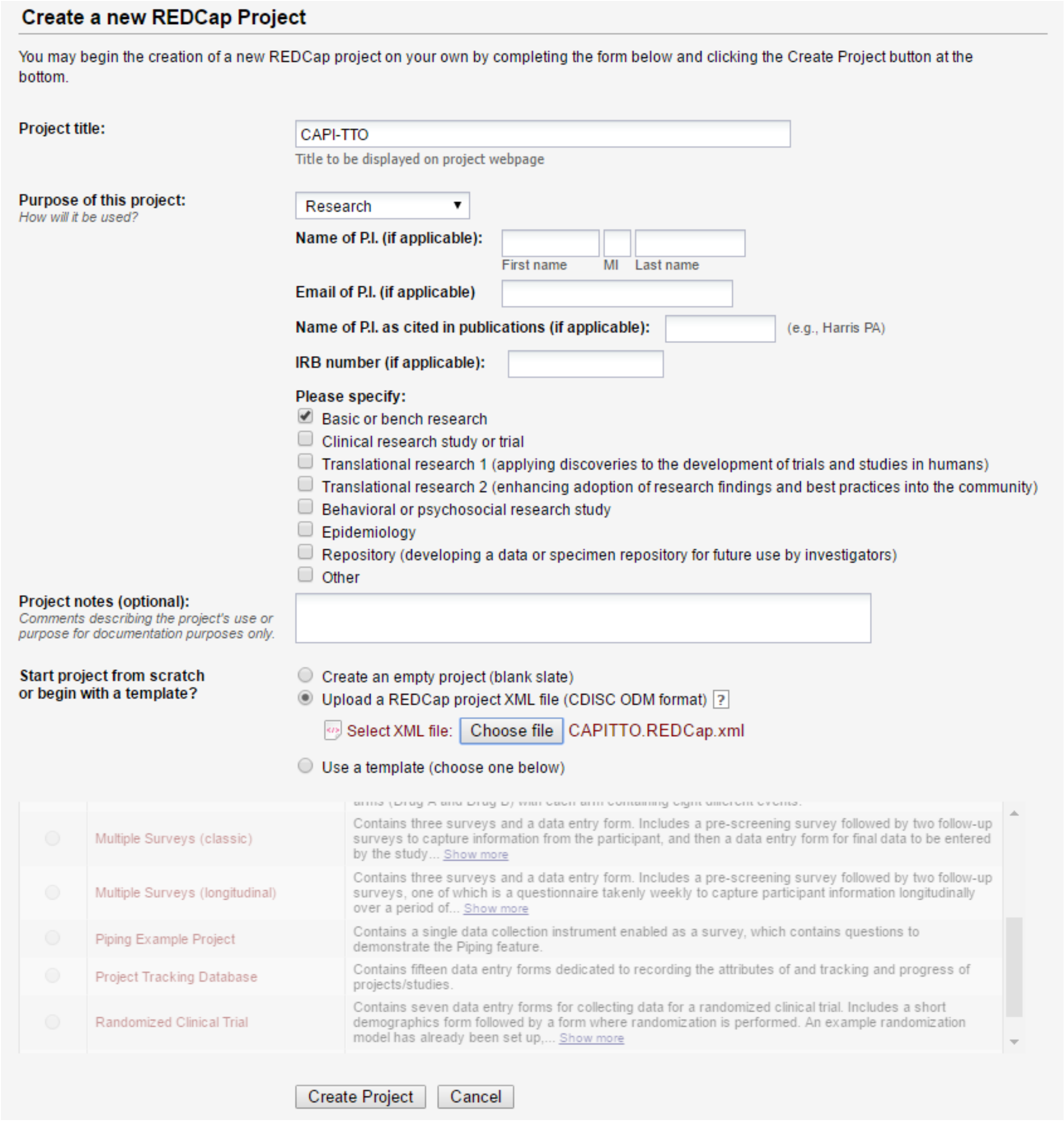

Figure 1 Uploading TTO Tool on REDCapTM Server

Survey Configurations

1. Under **Main project settings**, click the **Enable** button to activate **Use surveys in this project**.
2. Under **Design your data collection instruments**, Click the **Online Designer** button.
3. For all instruments:
    3.1. Under **Enabled as survey**, click the **Enable** button.
    3.2. In the new page, under **Basic Survey Options**, make sure that **Survey Title** and **Survey Instructions** sections are empty.
    3.3. Under **Survey Customizations**, select **Custom numbered** for **Question numbering** and select **One section per page (multiple pages)** for **Question Display Format.**
    3.4. Under **Survey Termination Options**, make sure that **Survey Completion Text** box is empty and then click **Save Changes**.
4. After enabling all surveys, click on the **Survey Queue** button and configure the randomization according to the figure 3S, then enter the conditions in table 1S in the white text box for corresponding instrument.

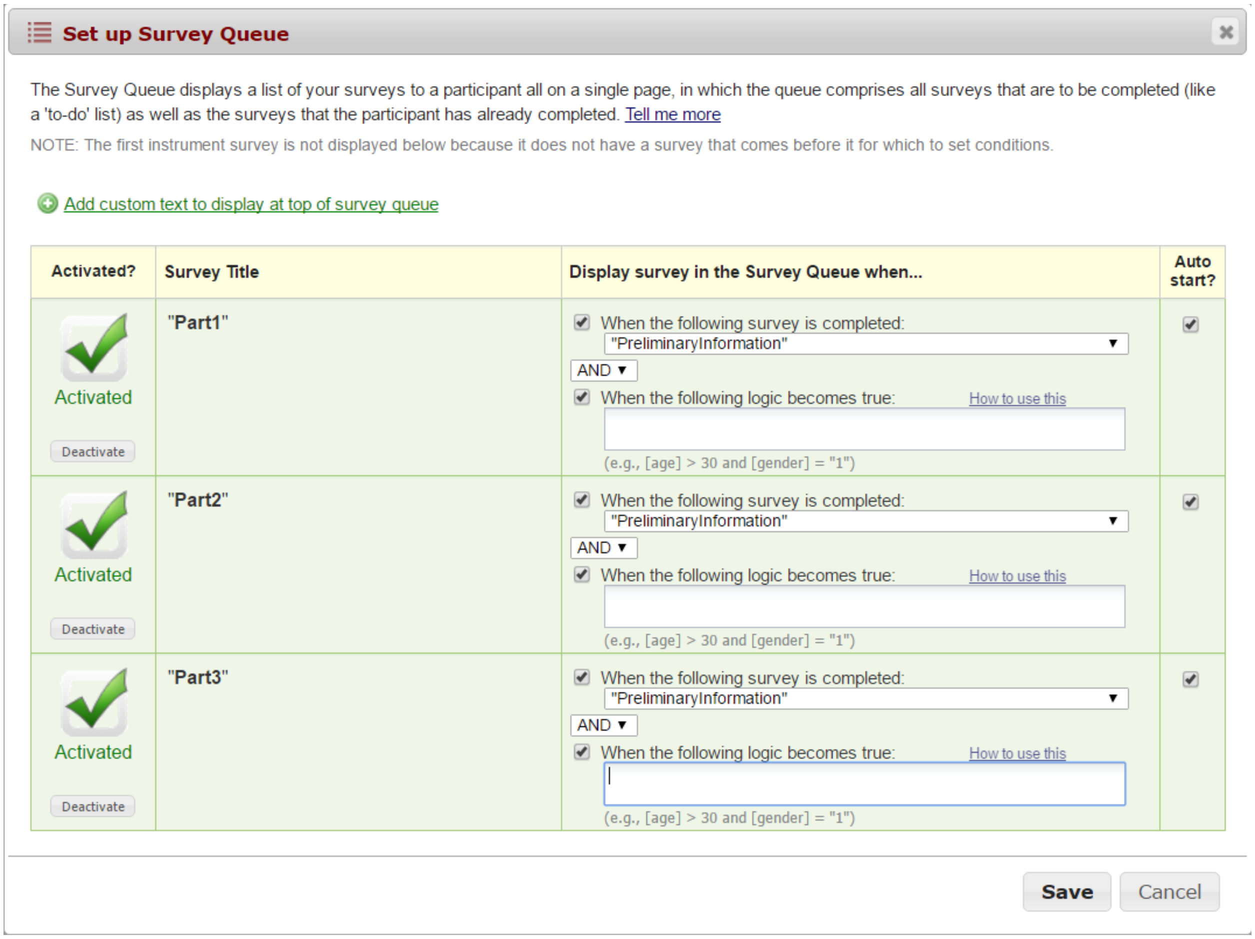

Figure 2 Survey Configurations

Table 1

Conditions in Survey Queue section

Survey	Conditions
**Part 1**	([mod6]<"3") or ([mod6]="3" and ([part2_complete]+[part3_complete]="2")) or (([mod6]="4" or [mod6]="6") and ([part2_complete]+[part3_complete]="4")) or ([mod6]="5" and ([part2_complete]+ [part3_complete]="2"))
**Part 2**	([mod6]="3") or ([mod6]="4") or ([mod6]="1" and ([part1_complete]+[part3_complete]="2")) or (([mod6]="2" or [mod6]="5") and ([part3_complete]+[part1_complete]="4")) or ([mod6]="6" and ([part1_complete]+[part3_complete]="2"))
**Part 3**	([mod6]="5") or ([mod6]="6") or ([mod6]="2" and ([part1_complete]+[part2_complete]="2")) or (([mod6]="1" or [mod6]="3") and ([part2_complete]+[part1_complete]="4")) or ([mod6]="4" and ([part1_complete]+[part2_complete]="2"))
