# Supplementary material for: A Computer-Assisted Personal Interview App in Research Electronic Data Capture for Administering Time Trade-off Surveys (REDCap): Development and Pretest
Source: JMIR Form Res. 2018 Jan 23;2(1):e3. doi: 10.2196/formative.8202 (PMC6334703; doi:10.2196/formative.8202)
Supplement: Multimedia Appendix 2 [file formative_v2i1e3_app2.pdf]

/***/
/*DUE TO COPYRIGHT, THE VERSION OF THE TIME TRADE-OFF APP*/
/*MADE AVAILABLE TO READERS DOES NOT INCLUDE THE EQ-5D-5L*/
/***/


```
<?xml version="1.0" encoding="UTF-8" ?>
<ODM xmlns="http://www.cdisc.org/ns/odm/v1.3"
xmlns:ds="http://www.w3.org/2000/09/xmldsig#"
xmlns:xsi="http://www.w3.org/2001/XMLSchema-instance"
xmlns:redcap="http://projectredcap.org"
xsi:schemaLocation="http://www.cdisc.org/ns/odm/v1.3 schema/odm/ODM1-3-1.xsd"
ODMVersion="1.3.1" FileOID="000-00-0000" FileType="Snapshot" Description="CAPI-TTO
(Paper XML)" AsOfDateTime="2018-06-24T18:53:28" CreationDateTime="2018-06-
24T18:53:28" SourceSystem="REDCap" SourceSystemVersion="6.12.0">
<Study OID="Project.CAPITTOPaperXML">
<GlobalVariables>
	<StudyName>CAPI-TTO (Paper XML)</StudyName>
	<StudyDescription>This file contains the metadata, events, and data for REDCap project
"CAPI-TTO (Paper XML)".</StudyDescription>
	<ProtocolName>CAPI-TTO (Paper XML)</ProtocolName>
	<redcap:RecordAutonumberingEnabled>1</redcap:RecordAutonumberingEnabled>
	<redcap:CustomRecordLabel></redcap:CustomRecordLabel>
	<redcap:SecondaryUniqueField></redcap:SecondaryUniqueField>
	<redcap:SchedulingEnabled>0</redcap:SchedulingEnabled>
	<redcap:Purpose>2</redcap:Purpose>
	<redcap:PurposeOther>1</redcap:PurposeOther>
	<redcap:ProjectNotes></redcap:ProjectNotes>
</GlobalVariables>
<MetaDataVersion OID="Metadata.CAPITTOPaperXML_2018-06-24_1853" Name="CAPI-TTO
(Paper XML)" redcap:RecordIdField="record_id">
	<FormDef OID="Form.preliminaryinformation" Name="PreliminaryInformation"
Repeating="No" redcap:FormName="preliminaryinformation">
		<ItemGroupRef ItemGroupOID="preliminaryinformation.record_id"
Mandatory="No"/>
		<ItemGroupRef ItemGroupOID="preliminaryinformation.mod6"
Mandatory="No"/>
		<ItemGroupRef
ItemGroupOID="preliminaryinformation.preliminaryinformation_complete" Mandatory="No"/>
	</FormDef>
	<FormDef OID="Form.part1" Name="Part1" Repeating="No"
redcap:FormName="part1">
		<ItemGroupRef ItemGroupOID="part1.no" Mandatory="No"/>
		<ItemGroupRef ItemGroupOID="part1.instruction" Mandatory="No"/>
```

<ItemGroupRef ItemGroupOID="part1.part1_complete" Mandatory="No"/>
</FormDef>
<FormDef OID="Form.part2" Name="Part2" Repeating="No" redcap:FormName="part2">
<ItemGroupRef ItemGroupOID="part2.no2" Mandatory="No"/>
<ItemGroupRef ItemGroupOID="part2.instruction_v2" Mandatory="No"/>
<ItemGroupRef ItemGroupOID="part2.part2_complete" Mandatory="No"/>
</FormDef>
<FormDef OID="Form.part3" Name="Part3" Repeating="No" redcap:FormName="part3">
<ItemGroupRef ItemGroupOID="part3.no3" Mandatory="No"/>
<ItemGroupRef ItemGroupOID="part3.instruction_v3" Mandatory="No"/>
<ItemGroupRef ItemGroupOID="part3.part3_complete" Mandatory="No"/>
</FormDef>
<ItemGroupDef OID="preliminaryinformation.record_id" Name="PreliminaryInformation" Repeating="No">
<ItemRef ItemOID="record_id" Mandatory="No" redcap:Variable="record_id"/>
<ItemRef ItemOID="id" Mandatory="Yes" redcap:Variable="id"/>
<ItemRef ItemOID="email" Mandatory="Yes" redcap:Variable="email"/>
<ItemRef ItemOID="gender" Mandatory="Yes" redcap:Variable="gender"/>
<ItemRef ItemOID="age" Mandatory="Yes" redcap:Variable="age"/>
<ItemRef ItemOID="education" Mandatory="Yes" redcap:Variable="education"/>
<ItemRef ItemOID="income" Mandatory="Yes" redcap:Variable="income"/>
<ItemRef ItemOID="someone_with_ad" Mandatory="Yes" redcap:Variable="someone_with_ad"/>
</ItemGroupDef>
<ItemGroupDef OID="preliminaryinformation.mod6" Name=" " Repeating="No">
<ItemRef ItemOID="mod6" Mandatory="No" redcap:Variable="mod6"/>
<ItemRef ItemOID="progress1_4" Mandatory="No" redcap:Variable="progress1_4"/>
</ItemGroupDef>
<ItemGroupDef OID="preliminaryinformation.preliminaryinformation_complete" Name="Form Status" Repeating="No">
<ItemRef ItemOID="preliminaryinformation_complete" Mandatory="No" redcap:Variable="preliminaryinformation_complete"/>
</ItemGroupDef>
<ItemGroupDef OID="part1.no" Name="Part1" Repeating="No">
<ItemRef ItemOID="no" Mandatory="No" redcap:Variable="no"/>
<ItemRef ItemOID="summary" Mandatory="No" redcap:Variable="summary"/>
</ItemGroupDef>
<ItemGroupDef OID="part1.instruction" Name=" " Repeating="No">
<ItemRef ItemOID="instruction" Mandatory="No" redcap:Variable="instruction"/>

<ItemRef ItemOID="summary1" Mandatory="No" redcap:Variable="summary1"/>
<ItemRef ItemOID="a1" Mandatory="No" redcap:Variable="a1"/>
<ItemRef ItemOID="b1" Mandatory="No" redcap:Variable="b1"/>
<ItemRef ItemOID="q1" Mandatory="Yes" redcap:Variable="q1"/>
<ItemRef ItemOID="error" Mandatory="No" redcap:Variable="error"/>
<ItemRef ItemOID="summary2" Mandatory="No" redcap:Variable="summary2"/>
<ItemRef ItemOID="a2" Mandatory="No" redcap:Variable="a2"/>
<ItemRef ItemOID="b2" Mandatory="No" redcap:Variable="b2"/>
<ItemRef ItemOID="q2" Mandatory="Yes" redcap:Variable="q2"/>
<ItemRef ItemOID="summary3" Mandatory="No" redcap:Variable="summary3"/>
<ItemRef ItemOID="a3" Mandatory="No" redcap:Variable="a3"/>
<ItemRef ItemOID="b3" Mandatory="No" redcap:Variable="b3"/>
<ItemRef ItemOID="q3" Mandatory="Yes" redcap:Variable="q3"/>
<ItemRef ItemOID="summary4" Mandatory="No" redcap:Variable="summary4"/>
<ItemRef ItemOID="a4" Mandatory="No" redcap:Variable="a4"/>
<ItemRef ItemOID="b4" Mandatory="No" redcap:Variable="b4"/>
<ItemRef ItemOID="q4" Mandatory="Yes" redcap:Variable="q4"/>
<ItemRef ItemOID="summary5" Mandatory="No" redcap:Variable="summary5"/>
<ItemRef ItemOID="a5" Mandatory="No" redcap:Variable="a5"/>
<ItemRef ItemOID="b5" Mandatory="No" redcap:Variable="b5"/>
<ItemRef ItemOID="q5" Mandatory="Yes" redcap:Variable="q5"/>
<ItemRef ItemOID="summary62_4dc" Mandatory="No" redcap:Variable="summary62_4dc"/>
<ItemRef ItemOID="a6" Mandatory="No" redcap:Variable="a6"/>
<ItemRef ItemOID="b6" Mandatory="No" redcap:Variable="b6"/>
<ItemRef ItemOID="q6" Mandatory="Yes" redcap:Variable="q6"/>
<ItemRef ItemOID="summary6" Mandatory="No" redcap:Variable="summary6"/>
<ItemRef ItemOID="a7" Mandatory="No" redcap:Variable="a7"/>
<ItemRef ItemOID="b7" Mandatory="No" redcap:Variable="b7"/>
<ItemRef ItemOID="q7" Mandatory="Yes" redcap:Variable="q7"/>
<ItemRef ItemOID="summary8" Mandatory="No" redcap:Variable="summary8"/>
<ItemRef ItemOID="a8" Mandatory="No" redcap:Variable="a8"/>
<ItemRef ItemOID="b8" Mandatory="No" redcap:Variable="b8"/>
<ItemRef ItemOID="q8" Mandatory="Yes" redcap:Variable="q8"/>
<ItemRef ItemOID="summary9" Mandatory="No" redcap:Variable="summary9"/>
<ItemRef ItemOID="a9" Mandatory="No" redcap:Variable="a9"/>

<ItemRef ItemOID="b9" Mandatory="No" redcap:Variable="b9"/>
<ItemRef ItemOID="q9" Mandatory="Yes" redcap:Variable="q9"/>
<ItemRef ItemOID="summary10" Mandatory="No" redcap:Variable="summary10"/>
<ItemRef ItemOID="a10" Mandatory="No" redcap:Variable="a10"/>
<ItemRef ItemOID="b10" Mandatory="No" redcap:Variable="b10"/>
<ItemRef ItemOID="q10" Mandatory="Yes" redcap:Variable="q10"/>
<ItemRef ItemOID="summary11" Mandatory="No" redcap:Variable="summary11"/>
<ItemRef ItemOID="a11" Mandatory="No" redcap:Variable="a11"/>
<ItemRef ItemOID="b11" Mandatory="No" redcap:Variable="b11"/>
<ItemRef ItemOID="q11" Mandatory="Yes" redcap:Variable="q11"/>
<ItemRef ItemOID="summary12" Mandatory="No" redcap:Variable="summary12"/>
<ItemRef ItemOID="a12" Mandatory="No" redcap:Variable="a12"/>
<ItemRef ItemOID="b12" Mandatory="No" redcap:Variable="b12"/>
<ItemRef ItemOID="q12" Mandatory="Yes" redcap:Variable="q12"/>
<ItemRef ItemOID="summary13" Mandatory="No" redcap:Variable="summary13"/>
<ItemRef ItemOID="a13" Mandatory="No" redcap:Variable="a13"/>
<ItemRef ItemOID="b13" Mandatory="No" redcap:Variable="b13"/>
<ItemRef ItemOID="q13" Mandatory="Yes" redcap:Variable="q13"/>
<ItemRef ItemOID="summary14" Mandatory="No" redcap:Variable="summary14"/>
<ItemRef ItemOID="a14" Mandatory="No" redcap:Variable="a14"/>
<ItemRef ItemOID="b14" Mandatory="No" redcap:Variable="b14"/>
<ItemRef ItemOID="q14" Mandatory="Yes" redcap:Variable="q14"/>
<ItemRef ItemOID="summary15" Mandatory="No" redcap:Variable="summary15"/>
<ItemRef ItemOID="a15" Mandatory="No" redcap:Variable="a15"/>
<ItemRef ItemOID="b15" Mandatory="No" redcap:Variable="b15"/>
<ItemRef ItemOID="q15" Mandatory="Yes" redcap:Variable="q15"/>
<ItemRef ItemOID="summary16" Mandatory="No" redcap:Variable="summary16"/>
<ItemRef ItemOID="a16" Mandatory="No" redcap:Variable="a16"/>
<ItemRef ItemOID="b16" Mandatory="No" redcap:Variable="b16"/>
<ItemRef ItemOID="q16" Mandatory="Yes" redcap:Variable="q16"/>
<ItemRef ItemOID="summary17" Mandatory="No" redcap:Variable="summary17"/>
<ItemRef ItemOID="a17" Mandatory="No" redcap:Variable="a17"/>
<ItemRef ItemOID="b17" Mandatory="No" redcap:Variable="b17"/>
<ItemRef ItemOID="q17" Mandatory="Yes" redcap:Variable="q17"/>
<ItemRef ItemOID="summary18" Mandatory="No" redcap:Variable="summary18"/>

<ItemRef ItemOID="a18" Mandatory="No" redcap:Variable="a18"/>
<ItemRef ItemOID="b18" Mandatory="No" redcap:Variable="b18"/>
<ItemRef ItemOID="q18" Mandatory="Yes" redcap:Variable="q18"/>
<ItemRef ItemOID="summary19" Mandatory="No" redcap:Variable="summary19"/>
<ItemRef ItemOID="a19" Mandatory="No" redcap:Variable="a19"/>
<ItemRef ItemOID="b19" Mandatory="No" redcap:Variable="b19"/>
<ItemRef ItemOID="q19" Mandatory="Yes" redcap:Variable="q19"/>
<ItemRef ItemOID="summary20" Mandatory="No" redcap:Variable="summary20"/>
<ItemRef ItemOID="a20" Mandatory="No" redcap:Variable="a20"/>
<ItemRef ItemOID="b20" Mandatory="No" redcap:Variable="b20"/>
<ItemRef ItemOID="q20" Mandatory="Yes" redcap:Variable="q20"/>
<ItemRef ItemOID="summary21" Mandatory="No" redcap:Variable="summary21"/>
<ItemRef ItemOID="a21" Mandatory="No" redcap:Variable="a21"/>
<ItemRef ItemOID="b21" Mandatory="No" redcap:Variable="b21"/>
<ItemRef ItemOID="q21" Mandatory="Yes" redcap:Variable="q21"/>
<ItemRef ItemOID="summary22" Mandatory="No" redcap:Variable="summary22"/>
<ItemRef ItemOID="a22" Mandatory="No" redcap:Variable="a22"/>
<ItemRef ItemOID="b22" Mandatory="No" redcap:Variable="b22"/>
<ItemRef ItemOID="q22" Mandatory="Yes" redcap:Variable="q22"/>
<ItemRef ItemOID="summary23" Mandatory="No" redcap:Variable="summary23"/>
<ItemRef ItemOID="a23" Mandatory="No" redcap:Variable="a23"/>
<ItemRef ItemOID="b23" Mandatory="No" redcap:Variable="b23"/>
<ItemRef ItemOID="q23" Mandatory="Yes" redcap:Variable="q23"/>
<ItemRef ItemOID="summary24" Mandatory="No" redcap:Variable="summary24"/>
<ItemRef ItemOID="a24" Mandatory="No" redcap:Variable="a24"/>
<ItemRef ItemOID="b24" Mandatory="No" redcap:Variable="b24"/>
<ItemRef ItemOID="q24" Mandatory="Yes" redcap:Variable="q24"/>
<ItemRef ItemOID="summary25" Mandatory="No" redcap:Variable="summary25"/>
<ItemRef ItemOID="a25" Mandatory="No" redcap:Variable="a25"/>
<ItemRef ItemOID="b25" Mandatory="No" redcap:Variable="b25"/>
<ItemRef ItemOID="q25" Mandatory="Yes" redcap:Variable="q25"/>
<ItemRef ItemOID="summary26" Mandatory="No" redcap:Variable="summary26"/>
<ItemRef ItemOID="a26" Mandatory="No" redcap:Variable="a26"/>
<ItemRef ItemOID="b26" Mandatory="No" redcap:Variable="b26"/>
<ItemRef ItemOID="q26" Mandatory="Yes" redcap:Variable="q26"/>

<ItemRef ItemOID="summary27" Mandatory="No" redcap:Variable="summary27"/>
<ItemRef ItemOID="a27" Mandatory="No" redcap:Variable="a27"/>
<ItemRef ItemOID="b27" Mandatory="No" redcap:Variable="b27"/>
<ItemRef ItemOID="q27" Mandatory="Yes" redcap:Variable="q27"/>
<ItemRef ItemOID="summary28" Mandatory="No" redcap:Variable="summary28"/>
<ItemRef ItemOID="a28" Mandatory="No" redcap:Variable="a28"/>
<ItemRef ItemOID="b28" Mandatory="No" redcap:Variable="b28"/>
<ItemRef ItemOID="q28" Mandatory="Yes" redcap:Variable="q28"/>
<ItemRef ItemOID="summary29" Mandatory="No" redcap:Variable="summary29"/>
<ItemRef ItemOID="a29" Mandatory="No" redcap:Variable="a29"/>
<ItemRef ItemOID="b29" Mandatory="No" redcap:Variable="b29"/>
<ItemRef ItemOID="q29" Mandatory="Yes" redcap:Variable="q29"/>
<ItemRef ItemOID="summary30" Mandatory="No" redcap:Variable="summary30"/>
<ItemRef ItemOID="a30" Mandatory="No" redcap:Variable="a30"/>
<ItemRef ItemOID="b30" Mandatory="No" redcap:Variable="b30"/>
<ItemRef ItemOID="q30" Mandatory="Yes" redcap:Variable="q30"/>
<ItemRef ItemOID="summary31" Mandatory="No" redcap:Variable="summary31"/>
<ItemRef ItemOID="a31" Mandatory="No" redcap:Variable="a31"/>
<ItemRef ItemOID="b31" Mandatory="No" redcap:Variable="b31"/>
<ItemRef ItemOID="q31" Mandatory="Yes" redcap:Variable="q31"/>
<ItemRef ItemOID="summary32" Mandatory="No" redcap:Variable="summary32"/>
<ItemRef ItemOID="a32" Mandatory="No" redcap:Variable="a32"/>
<ItemRef ItemOID="b32" Mandatory="No" redcap:Variable="b32"/>
<ItemRef ItemOID="q32" Mandatory="Yes" redcap:Variable="q32"/>
<ItemRef ItemOID="summary33" Mandatory="No" redcap:Variable="summary33"/>
<ItemRef ItemOID="a33" Mandatory="No" redcap:Variable="a33"/>
<ItemRef ItemOID="b33" Mandatory="No" redcap:Variable="b33"/>
<ItemRef ItemOID="q33" Mandatory="Yes" redcap:Variable="q33"/>
<ItemRef ItemOID="summary34" Mandatory="No" redcap:Variable="summary34"/>
<ItemRef ItemOID="a34" Mandatory="No" redcap:Variable="a34"/>
<ItemRef ItemOID="b34" Mandatory="No" redcap:Variable="b34"/>
<ItemRef ItemOID="q34" Mandatory="Yes" redcap:Variable="q34"/>
<ItemRef ItemOID="summary35" Mandatory="No" redcap:Variable="summary35"/>
<ItemRef ItemOID="a35" Mandatory="No" redcap:Variable="a35"/>
<ItemRef ItemOID="b35" Mandatory="No" redcap:Variable="b35"/>

<ItemRef ItemOID="q35" Mandatory="Yes" redcap:Variable="q35"/>
<ItemRef ItemOID="summary36" Mandatory="No" redcap:Variable="summary36"/>
<ItemRef ItemOID="a36" Mandatory="No" redcap:Variable="a36"/>
<ItemRef ItemOID="b36" Mandatory="No" redcap:Variable="b36"/>
<ItemRef ItemOID="q36" Mandatory="Yes" redcap:Variable="q36"/>
<ItemRef ItemOID="summary37" Mandatory="No" redcap:Variable="summary37"/>
<ItemRef ItemOID="a37" Mandatory="No" redcap:Variable="a37"/>
<ItemRef ItemOID="b37" Mandatory="No" redcap:Variable="b37"/>
<ItemRef ItemOID="q37" Mandatory="Yes" redcap:Variable="q37"/>
<ItemRef ItemOID="summary38" Mandatory="No" redcap:Variable="summary38"/>
<ItemRef ItemOID="a38" Mandatory="No" redcap:Variable="a38"/>
<ItemRef ItemOID="b38" Mandatory="No" redcap:Variable="b38"/>
<ItemRef ItemOID="q38" Mandatory="Yes" redcap:Variable="q38"/>
<ItemRef ItemOID="summary39" Mandatory="No" redcap:Variable="summary39"/>
<ItemRef ItemOID="a39" Mandatory="No" redcap:Variable="a39"/>
<ItemRef ItemOID="b39" Mandatory="No" redcap:Variable="b39"/>
<ItemRef ItemOID="q39" Mandatory="Yes" redcap:Variable="q39"/>
<ItemRef ItemOID="summary40" Mandatory="No" redcap:Variable="summary40"/>
<ItemRef ItemOID="a40" Mandatory="No" redcap:Variable="a40"/>
<ItemRef ItemOID="b40" Mandatory="No" redcap:Variable="b40"/>
<ItemRef ItemOID="q40" Mandatory="Yes" redcap:Variable="q40"/>
<ItemRef ItemOID="summary41" Mandatory="No" redcap:Variable="summary41"/>
<ItemRef ItemOID="a41" Mandatory="No" redcap:Variable="a41"/>
<ItemRef ItemOID="b41" Mandatory="No" redcap:Variable="b41"/>
<ItemRef ItemOID="q41" Mandatory="Yes" redcap:Variable="q41"/>
<ItemRef ItemOID="summary42" Mandatory="No" redcap:Variable="summary42"/>
<ItemRef ItemOID="a42" Mandatory="No" redcap:Variable="a42"/>
<ItemRef ItemOID="b42" Mandatory="No" redcap:Variable="b42"/>
<ItemRef ItemOID="q42" Mandatory="Yes" redcap:Variable="q42"/>
<ItemRef ItemOID="progress2" Mandatory="No" redcap:Variable="progress2"/>
<ItemRef ItemOID="progress22" Mandatory="No" redcap:Variable="progress22"/>
<ItemRef ItemOID="progress222" Mandatory="No" redcap:Variable="progress222"/>
<ItemRef ItemOID="difficulty1" Mandatory="Yes" redcap:Variable="difficulty1"/>
<ItemRef ItemOID="comment1" Mandatory="Yes" redcap:Variable="comment1"/>

</ItemGroupDef>
<ItemGroupDef OID="part1.part1_complete" Name="Form Status" Repeating="No">
<ItemRef ItemOID="part1_complete" Mandatory="No" redcap:Variable="part1_complete"/>
</ItemGroupDef>
<ItemGroupDef OID="part2.no2" Name="Part2" Repeating="No">
<ItemRef ItemOID="no2" Mandatory="No" redcap:Variable="no2"/>
<ItemRef ItemOID="summary_v2" Mandatory="No" redcap:Variable="summary_v2"/>
</ItemGroupDef>
<ItemGroupDef OID="part2.instruction_v2" Name=" " Repeating="No">
<ItemRef ItemOID="instruction_v2" Mandatory="No" redcap:Variable="instruction_v2"/>
<ItemRef ItemOID="summary1_v2" Mandatory="No" redcap:Variable="summary1_v2"/>
<ItemRef ItemOID="a1_v2" Mandatory="No" redcap:Variable="a1_v2"/>
<ItemRef ItemOID="b1_v2" Mandatory="No" redcap:Variable="b1_v2"/>
<ItemRef ItemOID="q1_v2" Mandatory="Yes" redcap:Variable="q1_v2"/>
<ItemRef ItemOID="error_v2" Mandatory="No" redcap:Variable="error_v2"/>
<ItemRef ItemOID="summary2_v2" Mandatory="No" redcap:Variable="summary2_v2"/>
<ItemRef ItemOID="a2_v2" Mandatory="No" redcap:Variable="a2_v2"/>
<ItemRef ItemOID="b2_v2" Mandatory="No" redcap:Variable="b2_v2"/>
<ItemRef ItemOID="q2_v2" Mandatory="Yes" redcap:Variable="q2_v2"/>
<ItemRef ItemOID="summary3_v2" Mandatory="No" redcap:Variable="summary3_v2"/>
<ItemRef ItemOID="a3_v2" Mandatory="No" redcap:Variable="a3_v2"/>
<ItemRef ItemOID="b3_v2" Mandatory="No" redcap:Variable="b3_v2"/>
<ItemRef ItemOID="q3_v2" Mandatory="Yes" redcap:Variable="q3_v2"/>
<ItemRef ItemOID="summary4_v2" Mandatory="No" redcap:Variable="summary4_v2"/>
<ItemRef ItemOID="a4_v2" Mandatory="No" redcap:Variable="a4_v2"/>
<ItemRef ItemOID="b4_v2" Mandatory="No" redcap:Variable="b4_v2"/>
<ItemRef ItemOID="q4_v2" Mandatory="Yes" redcap:Variable="q4_v2"/>
<ItemRef ItemOID="summary5_v2" Mandatory="No" redcap:Variable="summary5_v2"/>
<ItemRef ItemOID="a5_v2" Mandatory="No" redcap:Variable="a5_v2"/>
<ItemRef ItemOID="b5_v2" Mandatory="No" redcap:Variable="b5_v2"/>
<ItemRef ItemOID="q5_v2" Mandatory="Yes" redcap:Variable="q5_v2"/>
<ItemRef ItemOID="summary62_4dc_v2" Mandatory="No" redcap:Variable="summary62_4dc_v2"/>
<ItemRef ItemOID="a6_v2" Mandatory="No" redcap:Variable="a6_v2"/>
<ItemRef ItemOID="b6_v2" Mandatory="No" redcap:Variable="b6_v2"/>
<ItemRef ItemOID="q6_v2" Mandatory="Yes" redcap:Variable="q6_v2"/>

<ItemRef ItemOID="summary6_v2" Mandatory="No" redcap:Variable="summary6_v2"/>
<ItemRef ItemOID="a7_v2" Mandatory="No" redcap:Variable="a7_v2"/>
<ItemRef ItemOID="b7_v2" Mandatory="No" redcap:Variable="b7_v2"/>
<ItemRef ItemOID="q7_v2" Mandatory="Yes" redcap:Variable="q7_v2"/>
<ItemRef ItemOID="summary8_v2" Mandatory="No" redcap:Variable="summary8_v2"/>
<ItemRef ItemOID="a8_v2" Mandatory="No" redcap:Variable="a8_v2"/>
<ItemRef ItemOID="b8_v2" Mandatory="No" redcap:Variable="b8_v2"/>
<ItemRef ItemOID="q8_v2" Mandatory="Yes" redcap:Variable="q8_v2"/>
<ItemRef ItemOID="summary9_v2" Mandatory="No" redcap:Variable="summary9_v2"/>
<ItemRef ItemOID="a9_v2" Mandatory="No" redcap:Variable="a9_v2"/>
<ItemRef ItemOID="b9_v2" Mandatory="No" redcap:Variable="b9_v2"/>
<ItemRef ItemOID="q9_v2" Mandatory="Yes" redcap:Variable="q9_v2"/>
<ItemRef ItemOID="summary10_v2" Mandatory="No" redcap:Variable="summary10_v2"/>
<ItemRef ItemOID="a10_v2" Mandatory="No" redcap:Variable="a10_v2"/>
<ItemRef ItemOID="b10_v2" Mandatory="No" redcap:Variable="b10_v2"/>
<ItemRef ItemOID="q10_v2" Mandatory="Yes" redcap:Variable="q10_v2"/>
<ItemRef ItemOID="summary11_v2" Mandatory="No" redcap:Variable="summary11_v2"/>
<ItemRef ItemOID="a11_v2" Mandatory="No" redcap:Variable="a11_v2"/>
<ItemRef ItemOID="b11_v2" Mandatory="No" redcap:Variable="b11_v2"/>
<ItemRef ItemOID="q11_v2" Mandatory="Yes" redcap:Variable="q11_v2"/>
<ItemRef ItemOID="summary12_v2" Mandatory="No" redcap:Variable="summary12_v2"/>
<ItemRef ItemOID="a12_v2" Mandatory="No" redcap:Variable="a12_v2"/>
<ItemRef ItemOID="b12_v2" Mandatory="No" redcap:Variable="b12_v2"/>
<ItemRef ItemOID="q12_v2" Mandatory="Yes" redcap:Variable="q12_v2"/>
<ItemRef ItemOID="summary13_v2" Mandatory="No" redcap:Variable="summary13_v2"/>
<ItemRef ItemOID="a13_v2" Mandatory="No" redcap:Variable="a13_v2"/>
<ItemRef ItemOID="b13_v2" Mandatory="No" redcap:Variable="b13_v2"/>
<ItemRef ItemOID="q13_v2" Mandatory="Yes" redcap:Variable="q13_v2"/>
<ItemRef ItemOID="summary14_v2" Mandatory="No" redcap:Variable="summary14_v2"/>
<ItemRef ItemOID="a14_v2" Mandatory="No" redcap:Variable="a14_v2"/>
<ItemRef ItemOID="b14_v2" Mandatory="No" redcap:Variable="b14_v2"/>
<ItemRef ItemOID="q14_v2" Mandatory="Yes" redcap:Variable="q14_v2"/>
<ItemRef ItemOID="summary15_v2" Mandatory="No" redcap:Variable="summary15_v2"/>
<ItemRef ItemOID="a15_v2" Mandatory="No" redcap:Variable="a15_v2"/>
<ItemRef ItemOID="b15_v2" Mandatory="No" redcap:Variable="b15_v2"/>

<ItemRef ItemOID="q15_v2" Mandatory="Yes" redcap:Variable="q15_v2"/>
<ItemRef ItemOID="summary16_v2" Mandatory="No" redcap:Variable="summary16_v2"/>
<ItemRef ItemOID="a16_v2" Mandatory="No" redcap:Variable="a16_v2"/>
<ItemRef ItemOID="b16_v2" Mandatory="No" redcap:Variable="b16_v2"/>
<ItemRef ItemOID="q16_v2" Mandatory="Yes" redcap:Variable="q16_v2"/>
<ItemRef ItemOID="summary17_v2" Mandatory="No" redcap:Variable="summary17_v2"/>
<ItemRef ItemOID="a17_v2" Mandatory="No" redcap:Variable="a17_v2"/>
<ItemRef ItemOID="b17_v2" Mandatory="No" redcap:Variable="b17_v2"/>
<ItemRef ItemOID="q17_v2" Mandatory="Yes" redcap:Variable="q17_v2"/>
<ItemRef ItemOID="summary18_v2" Mandatory="No" redcap:Variable="summary18_v2"/>
<ItemRef ItemOID="a18_v2" Mandatory="No" redcap:Variable="a18_v2"/>
<ItemRef ItemOID="b18_v2" Mandatory="No" redcap:Variable="b18_v2"/>
<ItemRef ItemOID="q18_v2" Mandatory="Yes" redcap:Variable="q18_v2"/>
<ItemRef ItemOID="summary19_v2" Mandatory="No" redcap:Variable="summary19_v2"/>
<ItemRef ItemOID="a19_v2" Mandatory="No" redcap:Variable="a19_v2"/>
<ItemRef ItemOID="b19_v2" Mandatory="No" redcap:Variable="b19_v2"/>
<ItemRef ItemOID="q19_v2" Mandatory="Yes" redcap:Variable="q19_v2"/>
<ItemRef ItemOID="summary20_v2" Mandatory="No" redcap:Variable="summary20_v2"/>
<ItemRef ItemOID="a20_v2" Mandatory="No" redcap:Variable="a20_v2"/>
<ItemRef ItemOID="b20_v2" Mandatory="No" redcap:Variable="b20_v2"/>
<ItemRef ItemOID="q20_v2" Mandatory="Yes" redcap:Variable="q20_v2"/>
<ItemRef ItemOID="summary21_v2" Mandatory="No" redcap:Variable="summary21_v2"/>
<ItemRef ItemOID="a21_v2" Mandatory="No" redcap:Variable="a21_v2"/>
<ItemRef ItemOID="b21_v2" Mandatory="No" redcap:Variable="b21_v2"/>
<ItemRef ItemOID="q21_v2" Mandatory="Yes" redcap:Variable="q21_v2"/>
<ItemRef ItemOID="summary22_v2" Mandatory="No" redcap:Variable="summary22_v2"/>
<ItemRef ItemOID="a22_v2" Mandatory="No" redcap:Variable="a22_v2"/>
<ItemRef ItemOID="b22_v2" Mandatory="No" redcap:Variable="b22_v2"/>
<ItemRef ItemOID="q22_v2" Mandatory="Yes" redcap:Variable="q22_v2"/>
<ItemRef ItemOID="summary23_v2" Mandatory="No" redcap:Variable="summary23_v2"/>
<ItemRef ItemOID="a23_v2" Mandatory="No" redcap:Variable="a23_v2"/>
<ItemRef ItemOID="b23_v2" Mandatory="No" redcap:Variable="b23_v2"/>
<ItemRef ItemOID="q23_v2" Mandatory="Yes" redcap:Variable="q23_v2"/>
<ItemRef ItemOID="summary24_v2" Mandatory="No" redcap:Variable="summary24_v2"/>
<ItemRef ItemOID="a24_v2" Mandatory="No" redcap:Variable="a24_v2"/>

<ItemRef ItemOID="b24_v2" Mandatory="No" redcap:Variable="b24_v2"/>
<ItemRef ItemOID="q24_v2" Mandatory="Yes" redcap:Variable="q24_v2"/>
<ItemRef ItemOID="summary25_v2" Mandatory="No" redcap:Variable="summary25_v2"/>
<ItemRef ItemOID="a25_v2" Mandatory="No" redcap:Variable="a25_v2"/>
<ItemRef ItemOID="b25_v2" Mandatory="No" redcap:Variable="b25_v2"/>
<ItemRef ItemOID="q25_v2" Mandatory="Yes" redcap:Variable="q25_v2"/>
<ItemRef ItemOID="summary26_v2" Mandatory="No" redcap:Variable="summary26_v2"/>
<ItemRef ItemOID="a26_v2" Mandatory="No" redcap:Variable="a26_v2"/>
<ItemRef ItemOID="b26_v2" Mandatory="No" redcap:Variable="b26_v2"/>
<ItemRef ItemOID="q26_v2" Mandatory="Yes" redcap:Variable="q26_v2"/>
<ItemRef ItemOID="summary27_v2" Mandatory="No" redcap:Variable="summary27_v2"/>
<ItemRef ItemOID="a27_v2" Mandatory="No" redcap:Variable="a27_v2"/>
<ItemRef ItemOID="b27_v2" Mandatory="No" redcap:Variable="b27_v2"/>
<ItemRef ItemOID="q27_v2" Mandatory="Yes" redcap:Variable="q27_v2"/>
<ItemRef ItemOID="summary28_v2" Mandatory="No" redcap:Variable="summary28_v2"/>
<ItemRef ItemOID="a28_v2" Mandatory="No" redcap:Variable="a28_v2"/>
<ItemRef ItemOID="b28_v2" Mandatory="No" redcap:Variable="b28_v2"/>
<ItemRef ItemOID="q28_v2" Mandatory="Yes" redcap:Variable="q28_v2"/>
<ItemRef ItemOID="summary29_v2" Mandatory="No" redcap:Variable="summary29_v2"/>
<ItemRef ItemOID="a29_v2" Mandatory="No" redcap:Variable="a29_v2"/>
<ItemRef ItemOID="b29_v2" Mandatory="No" redcap:Variable="b29_v2"/>
<ItemRef ItemOID="q29_v2" Mandatory="Yes" redcap:Variable="q29_v2"/>
<ItemRef ItemOID="summary30_v2" Mandatory="No" redcap:Variable="summary30_v2"/>
<ItemRef ItemOID="a30_v2" Mandatory="No" redcap:Variable="a30_v2"/>
<ItemRef ItemOID="b30_v2" Mandatory="No" redcap:Variable="b30_v2"/>
<ItemRef ItemOID="q30_v2" Mandatory="Yes" redcap:Variable="q30_v2"/>
<ItemRef ItemOID="summary31_v2" Mandatory="No" redcap:Variable="summary31_v2"/>
<ItemRef ItemOID="a31_v2" Mandatory="No" redcap:Variable="a31_v2"/>
<ItemRef ItemOID="b31_v2" Mandatory="No" redcap:Variable="b31_v2"/>
<ItemRef ItemOID="q31_v2" Mandatory="Yes" redcap:Variable="q31_v2"/>
<ItemRef ItemOID="summary32_v2" Mandatory="No" redcap:Variable="summary32_v2"/>
<ItemRef ItemOID="a32_v2" Mandatory="No" redcap:Variable="a32_v2"/>
<ItemRef ItemOID="b32_v2" Mandatory="No" redcap:Variable="b32_v2"/>
<ItemRef ItemOID="q32_v2" Mandatory="Yes" redcap:Variable="q32_v2"/>
<ItemRef ItemOID="summary33_v2" Mandatory="No" redcap:Variable="summary33_v2"/>

<ItemRef ItemOID="a33_v2" Mandatory="No" redcap:Variable="a33_v2"/>
<ItemRef ItemOID="b33_v2" Mandatory="No" redcap:Variable="b33_v2"/>
<ItemRef ItemOID="q33_v2" Mandatory="Yes" redcap:Variable="q33_v2"/>
<ItemRef ItemOID="summary34_v2" Mandatory="No"
redcap:Variable="summary34_v2"/>
<ItemRef ItemOID="a34_v2" Mandatory="No" redcap:Variable="a34_v2"/>
<ItemRef ItemOID="b34_v2" Mandatory="No" redcap:Variable="b34_v2"/>
<ItemRef ItemOID="q34_v2" Mandatory="Yes" redcap:Variable="q34_v2"/>
<ItemRef ItemOID="summary35_v2" Mandatory="No"
redcap:Variable="summary35_v2"/>
<ItemRef ItemOID="a35_v2" Mandatory="No" redcap:Variable="a35_v2"/>
<ItemRef ItemOID="b35_v2" Mandatory="No" redcap:Variable="b35_v2"/>
<ItemRef ItemOID="q35_v2" Mandatory="Yes" redcap:Variable="q35_v2"/>
<ItemRef ItemOID="summary36_v2" Mandatory="No"
redcap:Variable="summary36_v2"/>
<ItemRef ItemOID="a36_v2" Mandatory="No" redcap:Variable="a36_v2"/>
<ItemRef ItemOID="b36_v2" Mandatory="No" redcap:Variable="b36_v2"/>
<ItemRef ItemOID="q36_v2" Mandatory="Yes" redcap:Variable="q36_v2"/>
<ItemRef ItemOID="summary37_v2" Mandatory="No"
redcap:Variable="summary37_v2"/>
<ItemRef ItemOID="a37_v2" Mandatory="No" redcap:Variable="a37_v2"/>
<ItemRef ItemOID="b37_v2" Mandatory="No" redcap:Variable="b37_v2"/>
<ItemRef ItemOID="q37_v2" Mandatory="Yes" redcap:Variable="q37_v2"/>
<ItemRef ItemOID="summary38_v2" Mandatory="No"
redcap:Variable="summary38_v2"/>
<ItemRef ItemOID="a38_v2" Mandatory="No" redcap:Variable="a38_v2"/>
<ItemRef ItemOID="b38_v2" Mandatory="No" redcap:Variable="b38_v2"/>
<ItemRef ItemOID="q38_v2" Mandatory="Yes" redcap:Variable="q38_v2"/>
<ItemRef ItemOID="summary39_v2" Mandatory="No"
redcap:Variable="summary39_v2"/>
<ItemRef ItemOID="a39_v2" Mandatory="No" redcap:Variable="a39_v2"/>
<ItemRef ItemOID="b39_v2" Mandatory="No" redcap:Variable="b39_v2"/>
<ItemRef ItemOID="q39_v2" Mandatory="Yes" redcap:Variable="q39_v2"/>
<ItemRef ItemOID="summary40_v2" Mandatory="No"
redcap:Variable="summary40_v2"/>
<ItemRef ItemOID="a40_v2" Mandatory="No" redcap:Variable="a40_v2"/>
<ItemRef ItemOID="b40_v2" Mandatory="No" redcap:Variable="b40_v2"/>
<ItemRef ItemOID="q40_v2" Mandatory="Yes" redcap:Variable="q40_v2"/>
<ItemRef ItemOID="summary41_v2" Mandatory="No"
redcap:Variable="summary41_v2"/>
<ItemRef ItemOID="a41_v2" Mandatory="No" redcap:Variable="a41_v2"/>
<ItemRef ItemOID="b41_v2" Mandatory="No" redcap:Variable="b41_v2"/>
<ItemRef ItemOID="q41_v2" Mandatory="Yes" redcap:Variable="q41_v2"/>

<ItemRef ItemOID="summary42_v2" Mandatory="No" redcap:Variable="summary42_v2"/>
<ItemRef ItemOID="a42_v2" Mandatory="No" redcap:Variable="a42_v2"/>
<ItemRef ItemOID="b42_v2" Mandatory="No" redcap:Variable="b42_v2"/>
<ItemRef ItemOID="q42_v2" Mandatory="Yes" redcap:Variable="q42_v2"/>
<ItemRef ItemOID="progress2_v2" Mandatory="No" redcap:Variable="progress2_v2"/>
<ItemRef ItemOID="progress22_v2" Mandatory="No" redcap:Variable="progress22_v2"/>
<ItemRef ItemOID="progress222_v2" Mandatory="No" redcap:Variable="progress222_v2"/>
<ItemRef ItemOID="difficulty2" Mandatory="Yes" redcap:Variable="difficulty2"/>
<ItemRef ItemOID="comment2" Mandatory="Yes" redcap:Variable="comment2"/>
</ItemGroupDef>
<ItemGroupDef OID="part2.part2_complete" Name="Form Status" Repeating="No">
<ItemRef ItemOID="part2_complete" Mandatory="No" redcap:Variable="part2_complete"/>
</ItemGroupDef>
<ItemGroupDef OID="part3.no3" Name="Part3" Repeating="No">
<ItemRef ItemOID="no3" Mandatory="No" redcap:Variable="no3"/>
<ItemRef ItemOID="summary_v3" Mandatory="No" redcap:Variable="summary_v3"/>
</ItemGroupDef>
<ItemGroupDef OID="part3.instruction_v3" Name=" " Repeating="No">
<ItemRef ItemOID="instruction_v3" Mandatory="No" redcap:Variable="instruction_v3"/>
<ItemRef ItemOID="summary1_v3" Mandatory="No" redcap:Variable="summary1_v3"/>
<ItemRef ItemOID="a1_v3" Mandatory="No" redcap:Variable="a1_v3"/>
<ItemRef ItemOID="b1_v3" Mandatory="No" redcap:Variable="b1_v3"/>
<ItemRef ItemOID="q1_v3" Mandatory="Yes" redcap:Variable="q1_v3"/>
<ItemRef ItemOID="error_v3" Mandatory="No" redcap:Variable="error_v3"/>
<ItemRef ItemOID="summary2_v3" Mandatory="No" redcap:Variable="summary2_v3"/>
<ItemRef ItemOID="a2_v3" Mandatory="No" redcap:Variable="a2_v3"/>
<ItemRef ItemOID="b2_v3" Mandatory="No" redcap:Variable="b2_v3"/>
<ItemRef ItemOID="q2_v3" Mandatory="Yes" redcap:Variable="q2_v3"/>
<ItemRef ItemOID="summary3_v3" Mandatory="No" redcap:Variable="summary3_v3"/>
<ItemRef ItemOID="a3_v3" Mandatory="No" redcap:Variable="a3_v3"/>
<ItemRef ItemOID="b3_v3" Mandatory="No" redcap:Variable="b3_v3"/>
<ItemRef ItemOID="q3_v3" Mandatory="Yes" redcap:Variable="q3_v3"/>

<ItemRef ItemOID="summary4_v3" Mandatory="No" redcap:Variable="summary4_v3"/>
<ItemRef ItemOID="a4_v3" Mandatory="No" redcap:Variable="a4_v3"/>
<ItemRef ItemOID="b4_v3" Mandatory="No" redcap:Variable="b4_v3"/>
<ItemRef ItemOID="q4_v3" Mandatory="Yes" redcap:Variable="q4_v3"/>
<ItemRef ItemOID="summary5_v3" Mandatory="No" redcap:Variable="summary5_v3"/>
<ItemRef ItemOID="a5_v3" Mandatory="No" redcap:Variable="a5_v3"/>
<ItemRef ItemOID="b5_v3" Mandatory="No" redcap:Variable="b5_v3"/>
<ItemRef ItemOID="q5_v3" Mandatory="Yes" redcap:Variable="q5_v3"/>
<ItemRef ItemOID="summary62_4dc_v3" Mandatory="No" redcap:Variable="summary62_4dc_v3"/>
<ItemRef ItemOID="a6_v3" Mandatory="No" redcap:Variable="a6_v3"/>
<ItemRef ItemOID="b6_v3" Mandatory="No" redcap:Variable="b6_v3"/>
<ItemRef ItemOID="q6_v3" Mandatory="Yes" redcap:Variable="q6_v3"/>
<ItemRef ItemOID="summary6_v3" Mandatory="No" redcap:Variable="summary6_v3"/>
<ItemRef ItemOID="a7_v3" Mandatory="No" redcap:Variable="a7_v3"/>
<ItemRef ItemOID="b7_v3" Mandatory="No" redcap:Variable="b7_v3"/>
<ItemRef ItemOID="q7_v3" Mandatory="Yes" redcap:Variable="q7_v3"/>
<ItemRef ItemOID="summary8_v3" Mandatory="No" redcap:Variable="summary8_v3"/>
<ItemRef ItemOID="a8_v3" Mandatory="No" redcap:Variable="a8_v3"/>
<ItemRef ItemOID="b8_v3" Mandatory="No" redcap:Variable="b8_v3"/>
<ItemRef ItemOID="q8_v3" Mandatory="Yes" redcap:Variable="q8_v3"/>
<ItemRef ItemOID="summary9_v3" Mandatory="No" redcap:Variable="summary9_v3"/>
<ItemRef ItemOID="a9_v3" Mandatory="No" redcap:Variable="a9_v3"/>
<ItemRef ItemOID="b9_v3" Mandatory="No" redcap:Variable="b9_v3"/>
<ItemRef ItemOID="q9_v3" Mandatory="Yes" redcap:Variable="q9_v3"/>
<ItemRef ItemOID="summary10_v3" Mandatory="No" redcap:Variable="summary10_v3"/>
<ItemRef ItemOID="a10_v3" Mandatory="No" redcap:Variable="a10_v3"/>
<ItemRef ItemOID="b10_v3" Mandatory="No" redcap:Variable="b10_v3"/>
<ItemRef ItemOID="q10_v3" Mandatory="Yes" redcap:Variable="q10_v3"/>
<ItemRef ItemOID="summary11_v3" Mandatory="No" redcap:Variable="summary11_v3"/>
<ItemRef ItemOID="a11_v3" Mandatory="No" redcap:Variable="a11_v3"/>
<ItemRef ItemOID="b11_v3" Mandatory="No" redcap:Variable="b11_v3"/>
<ItemRef ItemOID="q11_v3" Mandatory="Yes" redcap:Variable="q11_v3"/>
<ItemRef ItemOID="summary12_v3" Mandatory="No" redcap:Variable="summary12_v3"/>
<ItemRef ItemOID="a12_v3" Mandatory="No" redcap:Variable="a12_v3"/>
<ItemRef ItemOID="b12_v3" Mandatory="No" redcap:Variable="b12_v3"/>

<ItemRef ItemOID="q12_v3" Mandatory="Yes" redcap:Variable="q12_v3"/>
<ItemRef ItemOID="summary13_v3" Mandatory="No" redcap:Variable="summary13_v3"/>
<ItemRef ItemOID="a13_v3" Mandatory="No" redcap:Variable="a13_v3"/>
<ItemRef ItemOID="b13_v3" Mandatory="No" redcap:Variable="b13_v3"/>
<ItemRef ItemOID="q13_v3" Mandatory="Yes" redcap:Variable="q13_v3"/>
<ItemRef ItemOID="summary14_v3" Mandatory="No" redcap:Variable="summary14_v3"/>
<ItemRef ItemOID="a14_v3" Mandatory="No" redcap:Variable="a14_v3"/>
<ItemRef ItemOID="b14_v3" Mandatory="No" redcap:Variable="b14_v3"/>
<ItemRef ItemOID="q14_v3" Mandatory="Yes" redcap:Variable="q14_v3"/>
<ItemRef ItemOID="summary15_v3" Mandatory="No" redcap:Variable="summary15_v3"/>
<ItemRef ItemOID="a15_v3" Mandatory="No" redcap:Variable="a15_v3"/>
<ItemRef ItemOID="b15_v3" Mandatory="No" redcap:Variable="b15_v3"/>
<ItemRef ItemOID="q15_v3" Mandatory="Yes" redcap:Variable="q15_v3"/>
<ItemRef ItemOID="summary16_v3" Mandatory="No" redcap:Variable="summary16_v3"/>
<ItemRef ItemOID="a16_v3" Mandatory="No" redcap:Variable="a16_v3"/>
<ItemRef ItemOID="b16_v3" Mandatory="No" redcap:Variable="b16_v3"/>
<ItemRef ItemOID="q16_v3" Mandatory="Yes" redcap:Variable="q16_v3"/>
<ItemRef ItemOID="summary17_v3" Mandatory="No" redcap:Variable="summary17_v3"/>
<ItemRef ItemOID="a17_v3" Mandatory="No" redcap:Variable="a17_v3"/>
<ItemRef ItemOID="b17_v3" Mandatory="No" redcap:Variable="b17_v3"/>
<ItemRef ItemOID="q17_v3" Mandatory="Yes" redcap:Variable="q17_v3"/>
<ItemRef ItemOID="summary18_v3" Mandatory="No" redcap:Variable="summary18_v3"/>
<ItemRef ItemOID="a18_v3" Mandatory="No" redcap:Variable="a18_v3"/>
<ItemRef ItemOID="b18_v3" Mandatory="No" redcap:Variable="b18_v3"/>
<ItemRef ItemOID="q18_v3" Mandatory="Yes" redcap:Variable="q18_v3"/>
<ItemRef ItemOID="summary19_v3" Mandatory="No" redcap:Variable="summary19_v3"/>
<ItemRef ItemOID="a19_v3" Mandatory="No" redcap:Variable="a19_v3"/>
<ItemRef ItemOID="b19_v3" Mandatory="No" redcap:Variable="b19_v3"/>
<ItemRef ItemOID="q19_v3" Mandatory="Yes" redcap:Variable="q19_v3"/>
<ItemRef ItemOID="summary20_v3" Mandatory="No" redcap:Variable="summary20_v3"/>
<ItemRef ItemOID="a20_v3" Mandatory="No" redcap:Variable="a20_v3"/>
<ItemRef ItemOID="b20_v3" Mandatory="No" redcap:Variable="b20_v3"/>
<ItemRef ItemOID="q20_v3" Mandatory="Yes" redcap:Variable="q20_v3"/>
<ItemRef ItemOID="summary21_v3" Mandatory="No" redcap:Variable="summary21_v3"/>
<ItemRef ItemOID="a21_v3" Mandatory="No" redcap:Variable="a21_v3"/>

<ItemRef ItemOID="b21_v3" Mandatory="No" redcap:Variable="b21_v3"/>
<ItemRef ItemOID="q21_v3" Mandatory="Yes" redcap:Variable="q21_v3"/>
<ItemRef ItemOID="summary22_v3" Mandatory="No" redcap:Variable="summary22_v3"/>
<ItemRef ItemOID="a22_v3" Mandatory="No" redcap:Variable="a22_v3"/>
<ItemRef ItemOID="b22_v3" Mandatory="No" redcap:Variable="b22_v3"/>
<ItemRef ItemOID="q22_v3" Mandatory="Yes" redcap:Variable="q22_v3"/>
<ItemRef ItemOID="summary23_v3" Mandatory="No" redcap:Variable="summary23_v3"/>
<ItemRef ItemOID="a23_v3" Mandatory="No" redcap:Variable="a23_v3"/>
<ItemRef ItemOID="b23_v3" Mandatory="No" redcap:Variable="b23_v3"/>
<ItemRef ItemOID="q23_v3" Mandatory="Yes" redcap:Variable="q23_v3"/>
<ItemRef ItemOID="summary24_v3" Mandatory="No" redcap:Variable="summary24_v3"/>
<ItemRef ItemOID="a24_v3" Mandatory="No" redcap:Variable="a24_v3"/>
<ItemRef ItemOID="b24_v3" Mandatory="No" redcap:Variable="b24_v3"/>
<ItemRef ItemOID="q24_v3" Mandatory="Yes" redcap:Variable="q24_v3"/>
<ItemRef ItemOID="summary25_v3" Mandatory="No" redcap:Variable="summary25_v3"/>
<ItemRef ItemOID="a25_v3" Mandatory="No" redcap:Variable="a25_v3"/>
<ItemRef ItemOID="b25_v3" Mandatory="No" redcap:Variable="b25_v3"/>
<ItemRef ItemOID="q25_v3" Mandatory="Yes" redcap:Variable="q25_v3"/>
<ItemRef ItemOID="summary26_v3" Mandatory="No" redcap:Variable="summary26_v3"/>
<ItemRef ItemOID="a26_v3" Mandatory="No" redcap:Variable="a26_v3"/>
<ItemRef ItemOID="b26_v3" Mandatory="No" redcap:Variable="b26_v3"/>
<ItemRef ItemOID="q26_v3" Mandatory="Yes" redcap:Variable="q26_v3"/>
<ItemRef ItemOID="summary27_v3" Mandatory="No" redcap:Variable="summary27_v3"/>
<ItemRef ItemOID="a27_v3" Mandatory="No" redcap:Variable="a27_v3"/>
<ItemRef ItemOID="b27_v3" Mandatory="No" redcap:Variable="b27_v3"/>
<ItemRef ItemOID="q27_v3" Mandatory="Yes" redcap:Variable="q27_v3"/>
<ItemRef ItemOID="summary28_v3" Mandatory="No" redcap:Variable="summary28_v3"/>
<ItemRef ItemOID="a28_v3" Mandatory="No" redcap:Variable="a28_v3"/>
<ItemRef ItemOID="b28_v3" Mandatory="No" redcap:Variable="b28_v3"/>
<ItemRef ItemOID="q28_v3" Mandatory="Yes" redcap:Variable="q28_v3"/>
<ItemRef ItemOID="summary29_v3" Mandatory="No" redcap:Variable="summary29_v3"/>
<ItemRef ItemOID="a29_v3" Mandatory="No" redcap:Variable="a29_v3"/>
<ItemRef ItemOID="b29_v3" Mandatory="No" redcap:Variable="b29_v3"/>
<ItemRef ItemOID="q29_v3" Mandatory="Yes" redcap:Variable="q29_v3"/>
<ItemRef ItemOID="summary30_v3" Mandatory="No" redcap:Variable="summary30_v3"/>

<ItemRef ItemOID="a30_v3" Mandatory="No" redcap:Variable="a30_v3"/>
<ItemRef ItemOID="b30_v3" Mandatory="No" redcap:Variable="b30_v3"/>
<ItemRef ItemOID="q30_v3" Mandatory="Yes" redcap:Variable="q30_v3"/>
<ItemRef ItemOID="summary31_v3" Mandatory="No" redcap:Variable="summary31_v3"/>
<ItemRef ItemOID="a31_v3" Mandatory="No" redcap:Variable="a31_v3"/>
<ItemRef ItemOID="b31_v3" Mandatory="No" redcap:Variable="b31_v3"/>
<ItemRef ItemOID="q31_v3" Mandatory="Yes" redcap:Variable="q31_v3"/>
<ItemRef ItemOID="summary32_v3" Mandatory="No" redcap:Variable="summary32_v3"/>
<ItemRef ItemOID="a32_v3" Mandatory="No" redcap:Variable="a32_v3"/>
<ItemRef ItemOID="b32_v3" Mandatory="No" redcap:Variable="b32_v3"/>
<ItemRef ItemOID="q32_v3" Mandatory="Yes" redcap:Variable="q32_v3"/>
<ItemRef ItemOID="summary33_v3" Mandatory="No" redcap:Variable="summary33_v3"/>
<ItemRef ItemOID="a33_v3" Mandatory="No" redcap:Variable="a33_v3"/>
<ItemRef ItemOID="b33_v3" Mandatory="No" redcap:Variable="b33_v3"/>
<ItemRef ItemOID="q33_v3" Mandatory="Yes" redcap:Variable="q33_v3"/>
<ItemRef ItemOID="summary34_v3" Mandatory="No" redcap:Variable="summary34_v3"/>
<ItemRef ItemOID="a34_v3" Mandatory="No" redcap:Variable="a34_v3"/>
<ItemRef ItemOID="b34_v3" Mandatory="No" redcap:Variable="b34_v3"/>
<ItemRef ItemOID="q34_v3" Mandatory="Yes" redcap:Variable="q34_v3"/>
<ItemRef ItemOID="summary35_v3" Mandatory="No" redcap:Variable="summary35_v3"/>
<ItemRef ItemOID="a35_v3" Mandatory="No" redcap:Variable="a35_v3"/>
<ItemRef ItemOID="b35_v3" Mandatory="No" redcap:Variable="b35_v3"/>
<ItemRef ItemOID="q35_v3" Mandatory="Yes" redcap:Variable="q35_v3"/>
<ItemRef ItemOID="summary36_v3" Mandatory="No" redcap:Variable="summary36_v3"/>
<ItemRef ItemOID="a36_v3" Mandatory="No" redcap:Variable="a36_v3"/>
<ItemRef ItemOID="b36_v3" Mandatory="No" redcap:Variable="b36_v3"/>
<ItemRef ItemOID="q36_v3" Mandatory="Yes" redcap:Variable="q36_v3"/>
<ItemRef ItemOID="summary37_v3" Mandatory="No" redcap:Variable="summary37_v3"/>
<ItemRef ItemOID="a37_v3" Mandatory="No" redcap:Variable="a37_v3"/>
<ItemRef ItemOID="b37_v3" Mandatory="No" redcap:Variable="b37_v3"/>
<ItemRef ItemOID="q37_v3" Mandatory="Yes" redcap:Variable="q37_v3"/>
<ItemRef ItemOID="summary38_v3" Mandatory="No" redcap:Variable="summary38_v3"/>
<ItemRef ItemOID="a38_v3" Mandatory="No" redcap:Variable="a38_v3"/>
<ItemRef ItemOID="b38_v3" Mandatory="No" redcap:Variable="b38_v3"/>
<ItemRef ItemOID="q38_v3" Mandatory="Yes" redcap:Variable="q38_v3"/>

```
<ItemRef ItemOID="summary39_v3" Mandatory="No"
redcap:Variable="summary39_v3"/>
<ItemRef ItemOID="a39_v3" Mandatory="No" redcap:Variable="a39_v3"/>
<ItemRef ItemOID="b39_v3" Mandatory="No" redcap:Variable="b39_v3"/>
<ItemRef ItemOID="q39_v3" Mandatory="Yes" redcap:Variable="q39_v3"/>
<ItemRef ItemOID="summary40_v3" Mandatory="No"
redcap:Variable="summary40_v3"/>
<ItemRef ItemOID="a40_v3" Mandatory="No" redcap:Variable="a40_v3"/>
<ItemRef ItemOID="b40_v3" Mandatory="No" redcap:Variable="b40_v3"/>
<ItemRef ItemOID="q40_v3" Mandatory="Yes" redcap:Variable="q40_v3"/>
<ItemRef ItemOID="summary41_v3" Mandatory="No"
redcap:Variable="summary41_v3"/>
<ItemRef ItemOID="a41_v3" Mandatory="No" redcap:Variable="a41_v3"/>
<ItemRef ItemOID="b41_v3" Mandatory="No" redcap:Variable="b41_v3"/>
<ItemRef ItemOID="q41_v3" Mandatory="Yes" redcap:Variable="q41_v3"/>
<ItemRef ItemOID="summary42_v3" Mandatory="No"
redcap:Variable="summary42_v3"/>
<ItemRef ItemOID="a42_v3" Mandatory="No" redcap:Variable="a42_v3"/>
<ItemRef ItemOID="b42_v3" Mandatory="No" redcap:Variable="b42_v3"/>
<ItemRef ItemOID="q42_v3" Mandatory="Yes" redcap:Variable="q42_v3"/>
<ItemRef ItemOID="progress2_v3" Mandatory="No"
redcap:Variable="progress2_v3"/>
<ItemRef ItemOID="progress22_v3" Mandatory="No"
redcap:Variable="progress22_v3"/>
<ItemRef ItemOID="progress222_v3" Mandatory="No"
redcap:Variable="progress222_v3"/>
<ItemRef ItemOID="difficulty3" Mandatory="Yes" redcap:Variable="difficulty3"/>
<ItemRef ItemOID="comment3" Mandatory="Yes"
redcap:Variable="comment3"/>
</ItemGroupDef>
<ItemGroupDef OID="part3.part3_complete" Name="Form Status" Repeating="No">
<ItemRef ItemOID="part3_complete" Mandatory="No"
redcap:Variable="part3_complete"/>
</ItemGroupDef>
<ItemDef OID="record_id" Name="record_id" DataType="text" Length="999"
redcap:Variable="record_id" redcap:FieldType="text">
<Question><TranslatedText>Record ID</TranslatedText></Question>
</ItemDef>
<ItemDef OID="id" Name="id" DataType="text" Length="999" redcap:Variable="id"
redcap:FieldType="text" redcap:RequiredField="y">
<Question><TranslatedText>Please enter the participant identification
number</TranslatedText><redcap:FormattedTranslatedText>&lt;span style=&quot;line-height:
1.5; font-size: 12pt;&quot;&gt;Please enter the participant identification
number&lt;/span&gt;</redcap:FormattedTranslatedText></Question>
```

```
	</ItemDef>
	<ItemDef OID="email" Name="email" DataType="text" Length="999"
redcap:Variable="email" redcap:FieldType="text" redcap:TextValidationType="email"
redcap:RequiredField="y">
		<Question><TranslatedText>Please enter your email
address</TranslatedText><redcap:FormattedTranslatedText>&lt;span style=&quot;line-height:
1.5; font-size: 12pt;&quot;&gt;Please enter your email
address&lt;/span&gt;</redcap:FormattedTranslatedText></Question>
	</ItemDef>
	<ItemDef OID="gender" Name="gender" DataType="text" Length="1"
redcap:Variable="gender" redcap:FieldType="radio" redcap:RequiredField="y">
		<Question><TranslatedText>What is your gender?
</TranslatedText><redcap:FormattedTranslatedText>&lt;span style=&quot;line-height: 1.5; font-
size: 12pt;&quot;&gt;What is your gender?&lt;/span&gt;
</redcap:FormattedTranslatedText></Question>
		<CodeListRef CodeListOID="gender.choices"/>
	</ItemDef>
	<ItemDef OID="age" Name="age" DataType="integer" Length="999"
redcap:Variable="age" redcap:FieldType="text" redcap:TextValidationType="int"
redcap:RequiredField="y">
		<Question><TranslatedText>What is your age?
</TranslatedText><redcap:FormattedTranslatedText>&lt;span style=&quot;line-height: 1.5; font-
size: 12pt;&quot;&gt;What is your age?
&lt;/span&gt;</redcap:FormattedTranslatedText></Question>
	</ItemDef>
	<ItemDef OID="education" Name="education" DataType="text" Length="1"
redcap:Variable="education" redcap:FieldType="select"
redcap:TextValidationType="autocomplete" redcap:RequiredField="y">
		<Question><TranslatedText>What is the highest level of education that you have
completed? </TranslatedText><redcap:FormattedTranslatedText>&lt;span style=&quot;line-
height: 1.5; font-size: 12pt;&quot;&gt;What is the highest level of education that you have
completed?&lt;/span&gt; </redcap:FormattedTranslatedText></Question>
		<CodeListRef CodeListOID="education.choices"/>
	</ItemDef>
	<ItemDef OID="income" Name="income" DataType="text" Length="1"
redcap:Variable="income" redcap:FieldType="select"
redcap:TextValidationType="autocomplete" redcap:RequiredField="y">
		<Question><TranslatedText>What is your current annual household income from
all sources, before taxes? </TranslatedText><redcap:FormattedTranslatedText>&lt;span
style=&quot;line-height: 1.5; font-size: 12pt;&quot;&gt;What is your current annual household
income from all sources, before taxes?
&lt;/span&gt;</redcap:FormattedTranslatedText></Question>
		<CodeListRef CodeListOID="income.choices"/>
	</ItemDef>
```

<ItemDef OID="someone_with_ad" Name="someone_with_ad" DataType="boolean" Length="1" redcap:Variable="someone_with_ad" redcap:FieldType="yesno" redcap:RequiredField="y">
<Question><TranslatedText>Do you know anyone who has been diagnosed with Alzheimer's disease? This can include a family member, relative, close friend, acquaintance, co-worker, etc. If you knew someone with Alzheimer's disease, but they are now deceased, then you may respond 'yes' to this question. </TranslatedText><redcap:FormattedTranslatedText>Do you know anyone who has been diagnosed with Alzheimer's disease? This can include a family member, relative, close friend, acquaintance, co-worker, etc. If you knew someone with Alzheimer's disease, but they are now deceased, then you may respond 'yes' to this question. </redcap:FormattedTranslatedText></Question>
<CodeListRef CodeListOID="someone_with_ad.choices"/>
</ItemDef>
<ItemDef OID="mod6" Name="mod6" DataType="float" Length="999" redcap:Variable="mod6" redcap:FieldType="calc" redcap:SectionHeader=" " redcap:Calculation="[record_id] - ((rounddown(([record_id]-1) / 6) * 6))">
<Question><TranslatedText></TranslatedText></Question>
</ItemDef>
<ItemDef OID="progress1_4" Name="progress1_4" DataType="text" Length="999" redcap:Variable="progress1_4" redcap:FieldType="descriptive" redcap:SectionHeader=" ">
<Question><TranslatedText>You have successfully finished 25% of the survey. Please click "Submit" in the next page to proceed.</TranslatedText><redcap:FormattedTranslatedText>You have successfully finished 25% of the survey. Please click "Submit" in the next page to proceed.</redcap:FormattedTranslatedText></Question>
</ItemDef>
<ItemDef OID="preliminaryinformation_complete" Name="preliminaryinformation_complete" DataType="text" Length="1" redcap:Variable="preliminaryinformation_complete" redcap:FieldType="select" redcap:SectionHeader="Form Status">
<Question><TranslatedText>Complete?</TranslatedText></Question>
<CodeListRef CodeListOID="preliminaryinformation_complete.choices"/>
</ItemDef>
<ItemDef OID="no" Name="no" DataType="float" Length="999" redcap:Variable="no" redcap:FieldType="calc" redcap:Calculation="([part1_complete]+[part2_complete]+[part3_complete])/2+1">
<Question><TranslatedText></TranslatedText></Question>
</ItemDef>
<ItemDef OID="summary" Name="summary" DataType="text" Length="999" redcap:Variable="summary" redcap:FieldType="descriptive">

<Question><TranslatedText>Please read the summary below, which describes what life is like to have Alzheimer's disease.

Alzheimer's disease - Description #[no]

If you have Alzheimer's disease...

1) You might forget recent events of importance to you. For example, if you are interested in hockey, then you might forget whether your favorite team won their last game, although you may remember watching the game itself.

2) You might forget the names of people you met recently, or the names of local streets.

3) You may no longer be able to handle your personal finances or plan leisure activities such as dinner parties or vacations. Simple decisions such as what to order in restaurants might become difficult to make. Your ability to concentrate may decrease and you might have difficulty driving.

4) You may start having trouble finding words to express your thoughts. You might need to be reminded to shower or brush your teeth, but you could do these activities on your own. You may need to post notes around the home to remind you of simple things like turning off the stove.

5) You may remain aware of the day and your surroundings. You might be able to travel to familiar locations like the supermarket, although probably with the help of a loved one or friend. If you are in a social gathering, then you might have difficulty following the conversation.

6) You may continue to enjoy hobbies such as puzzles or reading.
</TranslatedText><redcap:FormattedTranslatedText>Please read the summary below, which describes what life is like to have Alzheimer's disease.
<p></p>
<p></p>

Alzheimer's disease - Description #[no]

<p></p>

If you have Alzheimer's disease...
<p></p>

1) You might forget recent events of importance to you. For example, if you are interested in hockey, then you might forget whether your favorite team won their last game, although you may remember watching the game itself.
<p></p>
2) You might forget the names of people you met recently, or the names of local streets.
<p></p>
3) You may no longer be able to handle your personal finances or plan leisure activities such as dinner parties or vacations. Simple decisions such as what to order in restaurants might become difficult to make. Your ability to concentrate may decrease and you might have difficulty driving.
<p></p>
4) You may start having trouble finding words to express your thoughts. You might need to be reminded to shower or brush your teeth, but you could do these activities on your own. You may need to post notes around the home to remind you of simple things like turning off the stove.
<p></p>
5) You may remain aware of the day and your surroundings. You might be able to travel to familiar locations like the supermarket, although probably with the help of a loved one or friend. If you are in a social gathering, then you might have difficulty following the conversation.
<p></p>
6) You may continue to enjoy hobbies such as puzzles or reading.
</redcap:FormattedTranslatedText></Question>
</ItemDef>
<ItemDef OID="instruction" Name="instruction" DataType="text" Length="999" redcap:Variable="instruction" redcap:FieldType="descriptive" redcap:SectionHeader=" ">
<Question><TranslatedText>

On the following pages, you will be asked to answer several questions. Please answer these questions as if you have Alzheimer's disease according to the summary you just read. The summary will be available on top of each set of questions. Please click 'Next Page' below to go to the questions.

</TranslatedText><redcap:FormattedTranslatedText>

On the following pages, you will be asked to answer several questions. Please answer these questions as if you have Alzheimer's disease according to the summary you just read. The summary will be available on top of each set of questions. Please click 'Next Page' below to go to the questions.

</redcap:FormattedTranslatedText></Question>
</ItemDef>
<ItemDef OID="summary1" Name="summary1" DataType="text" Length="999" redcap:Variable="summary1" redcap:FieldType="descriptive" redcap:SectionHeader=" ">
<Question><TranslatedText>
Alzheimer's disease - Description #[no]

If you have Alzheimer's disease...

1) You might forget recent events of importance to you. For example, if you are interested in hockey, then you might forget whether your favorite team won their last game, although you may remember watching the game itself.

2) You might forget the names of people you met recently, or the names of local streets.

3) You may no longer be able to handle your personal finances or plan leisure activities such as dinner parties or vacations. Simple decisions such as what to order in restaurants might become difficult to make. Your ability to concentrate may decrease and you might have difficulty driving.

4) You may start having trouble finding words to express your thoughts. You might need to be reminded to shower or brush your teeth, but you could do these activities on your own. You may need to post notes around the home to remind you of simple things like turning off the stove.

5) You may remain aware of the day and your surroundings. You might be able to travel to familiar locations like the supermarket, although probably with the help of a loved one or friend. If you are in a social gathering, then you might have difficulty following the conversation.

6) You may continue to enjoy hobbies such as puzzles or reading.

Now consider two possible lives. In Life A, you will live in perfect health for 20 years and then you will die of natural causes. In Life B, you will live in perfect health for the next 10 years, then you will live with Alzheimer's disease as described in the summary for another 10 years, and then you will die of natural causes.

</TranslatedText><redcap:FormattedTranslatedText>
Alzheimer's disease - Description #[no]

<p></p>

If you have Alzheimer's disease...
<p></p>
1) You might forget recent events of importance to you. For example, if you are interested in hockey, then you might forget whether your favorite team won their last game, although you may remember watching the game itself.
<p></p>
2) You might forget the names of people you met recently, or the names of local streets.
<p></p>
3) You may no longer be able to handle your personal finances or plan leisure activities such as dinner parties or vacations. Simple decisions such as what to order in restaurants might become difficult to make. Your ability to concentrate may decrease and you might have difficulty driving.
<p></p>
4) You may start having trouble finding words to express your thoughts. You might need to be reminded to shower or brush your teeth, but you could do these activities on your own. You may need to post notes around the home to remind you of simple things like turning off the stove.
<p></p>
5) You may remain aware of the day and your surroundings. You might be able to travel to familiar locations like the supermarket, although probably with the help of a loved one or friend. If you are in a social gathering, then you might have difficulty following the conversation.
<p></p>
6) You may continue to enjoy hobbies such as puzzles or reading.
<p></p>
<p></p>
<p></p>
<p></p>
Now consider two possible lives. In Life A, you will live in perfect health for 20 years and then you will die of natural causes. In Life B, you will live in perfect health for the next 10 years, then you will live with Alzheimer's disease as described in the summary for another 10 years, and then you will die of natural causes.
<p></p>
</redcap:FormattedTranslatedText></Question>
</ItemDef>
<ItemDef OID="a1" Name="a1" DataType="text" Length="999" redcap:Variable="a1" redcap:FieldType="descriptive" redcap:InlineImage="1">
<Question><TranslatedText></TranslatedText></Question>
<redcap:Attachment DocName="20.png" MimeType="image/png"><![CDATA[iVBORw0KGgoAAAANSUhEUgAABVEAAAD2CAIAAABQo1B1AAAAAXNSR0IArs4c6QAAAA
RnQU1BAACxjwv8YQUAAAAJcEhZcwAAEnQAABJ0Ad5mH3gAACuuSURBVHhe7d1fyGVnfS/w2PR
PcmoxeIpEe6hTD4WQBBnNzXSQOoWiIghCGhqCLS0twwQqDYE6Ukw5FwmKFDJKIDlQotCek1xER
OiJEgtBKiRQcC5yESGGuYgYSC7GUOQN5qJn77XX3+d96u96Px+

+nDN77Wc9v+dZe8e1ftE9veG/AAAAgCXS8wMAAMAy6fkBAABgmfT8AAAAsEx6fgAAAFgmPT8AA
AAsk54fAAAAlknPDwAAAMuk5wcAAIBl0vMDAADAMun5AQAAYJn0/AAAALBMen4AAABYJj0/AA
AALJOeHwAAAJZJzw8AAADLpOcHAACAZdLzAwAAwDLp+QEAAGCZ9PwAAACwTHp+AAAAWCY9
PwAAACyTnh8AAACWSc8PAAAAy6TnBwAAgGXS8wMAAMAy6fkBAABgmfT8AOzI89eev+F/3SAiIi
LSm/fff/OPvv9c/gzBMej5AdiFa9ev3fKVW4LbuYiIiEicmy/feN+Hz3z+ro+8+dPX8icJxtLzAzC760fXb3
vstuB2LiIiIpLMpz/2gb/6/f+5yiP33P3Lt9/OnycYRc8PwOw+9S+fCu7lIiIiIsl89O73bhr+TZ5+5OH8eYJ
R9PwAzOuB7z0Q3MtFREREknn//TfXG/5N/LD/OPT8AMzoqZeeCu7lIiIiIslsfsYfNPyrPHj+3M/feCN/
tmBLen4A5vLCay/c9PBNwe1cREREJJnyZ/xxvvq5+/LHC7ak5wdgFteuX7v1H28N7uUiIiIiyQQ/44/z
na9/LX/IYBt6fgCmd/TO0dknzgb3chEREZFk/sfF/xZ0+Mn8+MUX80cNBtPzAzC9e5+5N7iXi4iIiCTz7r
/71T+74/eC9j4ZP+wfQc8PwMS+/O9fDu7lIiIiIsnc+NC7PvMHvxP09h159K/
+Mn/gYBg9PwBT+vbL3w7u5SIiIiJtOfeZ3w66+t48940n88cOBtDzAzCZq69f9Rf1i4iIyMB88K9/M+jn
h+TSnbe/evVq/vBBHz0/ANO4fnT9zJUzwb1cREREJJnhP+OPc/nCx3/x1lv5Iwid9PwATODonaML37
wQ3MtFREREktn2Z/xxHrv/Uv4UQic9PwATuPSvl4J7uYiIiEhbRvyMP87z//f/5A8itNPzA3Bcj77waH
AjFxEREWnLuJ/xx7l05+0/e/Un+eMILfT8e/HKlfM3bJy/8kp+rND13t6Ui7r4bP/LNuPOOqbdVDm+Z
y9ulpmZ8pNfXYDOrReFZ/22tXwKzbXtZCXM4vlrzwc3chEREZG2vOfBXxv9M/44X/rkJ3759tv5Qwk
pev49KZugsMOper9DalKDnq37ZZtxZx3TbqocU7nIjcma3nzezq3vq+eP16bnP6GuXb92y1duCe7lIiI
iIsnc+NC7/uSu3w369mPmyctfyJ9LSNHz702yua+av0NuUcMWLtHRpQwcdgrN0+0Ou9776flTa9Pz
n0TXj67f9thtwb1cREREpC1/+Mn3BR37JPnht57Jn06I6Pn3qOz6yyanPHLgXXHQsQ1s5gcOO31muj
DDptXzcxyf+pdPBTdyERERkbb8/p//VtCrTxU/7O+g59+rZo8f/zuASgvkjZYIBiWapp98r3q7OiI8U066P
BNN1v2zTdVa63yuGpDa21hydFK6tXqg+V/fSa2d1n9S1vNokxR+Tqln7N9sYUTs1UaBlh/ULUjsrrta3
mGApjSHle+tFtK1t8Eo4FA9874HgRi4iIiLSlvc8+Gt/cduHgl59wjxyz91+2J+k59+vqk+6eKX4Y9TjJHqk
ldqwerOUazRZCcX7cZNYTpIfyV4H03W/bNN5VlG/PkUxolhSeUZdbdMp4drSF3Ota6JBZ/UtLzHJ+St
X4oPB5alrrDE5IricNfUrWzPJ1lpmKUc0PoW2tQ1aCQfjqZeeCm7kIiIiIm2Z42f8cZ5+5OH8SYUaPf+
+hd1U1N6UnVDxTnlGObQYUju30WSl5AOKU2r9Vn5GMUP2Mpiu+2Wb7rOKFVRzFAPyNUZVEtu
OhWfFG62OVKVD7Zdni+XFk+SiU4fMVo4ojpTTFwcS0yZ0bK11onAxxYDohNYZogMrA1bCoXjhtRd
uevim4F4uIiIi0paZfsYf50fffy5/XqGg59+/WqvTaIE2wvZqLWyYEmNSPVVTPmJzzubFxYvriepHivOD6
bpftuk5q3hdTFK8zncVvFxLHIqEVcqrXa01HJKQOKs6LSs/YHnlJOF64wX0zxafszmUWmD7vlb6L0hQ
eS1xqCEsHS0lOrAy7qNh965dv3brP94a3MhFRERE2jLfz/jjfP6uj7z509fypxYyev5DkOooc8VbzeYqaL
kSgwY0S/lZ2YDsz+evPJudVR0pTw+m637Zpu+s4kD+Ol9BsalikykddcMq5TTVOQOWX5zV+Bga5w1
YXqL0RryA/tmSK2oasK+VxETBiQO2VtMcnb8fLSW1tv6VcACO3jk6+8TZ4EYuIiIi0pb//re/MevP+O
M8cs/d+YMLGT3/Iejo39JvtTRlWzZL+WmrEZvRqz9sDpVHyrOD6bpftuk9qziSHahWt1FsMqWjblhl
3LUqzmqMaJw3YHmJ0hvxAvpna52sMmBfK4mJghMHbK06JZC/Hy0ltbb+lXAA7n3m3uBGLiIiItKW
X//7X/nTsx8MevId5Dtf/1r+7IKe/zB09G/pt4pWKD86tlnanFf81/vZ2dmh4kg1YTBd98s2/WcVh1ZH
8j1V76WvRK+wyrhrlSzeOG/A8lqHxAvon21AvQH7WklMFJw4fDErm0Fh6WgpqbX1r4R9+/K/fzm4
kYuIiIh05I//6NagG99Zfvzii/kTz5z8EHU1V2fTU3ws7ocT5ZRfW1Szl88QQ/498cqc0XVOx+2WbI
WZt1l3+dfe2tgUVC4WnjGsvU5WyetsUk9Y8yE5/bP1tqxLrAdvta6b8gvfPEU4TXK5qibflrW2+B3fj2y
98O7uIiIiIHbnj3vcEffgu8+D5cz9/4438uO8+D5cz9/4438OeZ00/MfgkSzUynbp+Ldsg8qh5dHitaoPKenWapOTJ3a3nt
1v2wz6Kxa/eCdcnxxuBzaVTasUpy0XWOZuCph9f7lJUpvJBbQP1s0ov1ANW1CYlXhiX2LCddWjY+O
5K+TaxuwEvbm6utX/UX9IiIiMjzv+5ubdvwz/jhf/dx9+aPM6abnPwSJZqeubKka6oNrTVaou1mqTiy
nK6slC2ym637ZZthZ1W6jy1Ge0bBV0ZGNZfojWEvN01BNmii9kVxA/2bTI2rTN1fdsrtBF6RnMe2XJ
x8Sz5hY26CVsA/Xj66fuXImuJGLiIiItGVfP+OP44f9K3r+Q5BodkLNpivRAdUHrN4e2izF45JnBge7X7Y
ZeFb31SjPynRcsVxYZVxjWT+r+PNKfELX8lo31rqA/s02R0RDamtNnr4y+IJ0L6ZeaX1a8XozRWrGaG2
DV8JOHb1zdOGbF4IbuYiIiEhH9vgz/iCX7rz91atX88ea00rPD/0S7SicDpf+9VJwFxcRERHpyH5/xh/n8
oWP/+Ktt/Inm1NJzw/99PycTo+

+8GhwFxcRERHpyCH8jD/OY/dfyh9uTiU9P/TT83MKPX/t+eAuLiIiItKRmy/feCA/44/z3DeezB9xTh89
P/TT83PaXLt+7Zav3BLcyEVEREQ68umPfSDotA8np/mH/Xp+ABquH12/7bHbgru4iIiISEc+evd7gzb7
0PKlT37il2+/nT/unCZ6fgAaPvUvnwru4iIiIidef/9NwcN9mHmyctfyB93ThM9PwCVB773QHAXFxER
EenIzZdvvO/DZ4Lu+mDzw289kz/0nBp6fgByT730VHAXFxEREenOIf+MP86lO2//2as/yR99Tgc9/34
VfzdcZpa/IO6VK+cvPpv/eRl/F11zRymNq3rDDYnR9RH+Xr5hBn93lveVOzVeeO2Fmx6+KbiLi4iIiHTk
8H/GH+e0/bBfz79Hq95o0w5tTN8U5QUW1IDFOwoF/X6uvt/gsmd6/iUCK4O+O8v7yp0e165fu/U
fbw3u4iIiIiIdOSk/44/zz//wUP4MdAro+fdo5m6o7G0X04CldtRUdvzFBqMD5ZHNHP1Tkhvw3VneV
+7UOHrn6OwTZ4O7uIiIiEhHTtbP+OP86PvP5U9CS6fn35+5281T2PMnBgSHipfVBWj+OwBa6fmX7
N5n7g3u4iIiIidufGhd33mD34n6KJPVj5/10fe/OIr+cPQoun596NohGrqPWfZO2WCbqnWRFXTBB1
r2/z1Bqw2Ju7HGivobdfq09bPjPro9mmrd8qTygUGqy1EsyfV11a9qs4tCqc32Ty7lDjcd8Ua7680htT
mK/5YrLHzvBZDazUGdn5YqzdbLkWhWncpm7F+Wm1MPEtj0cHbg9fMGF/
+9y8Hd3ERERGR7pz7zG8HLfRJzCP33H0afti/Xc9fPm23P2onhzTagZaWYbDqkf8EP/C3NUjpt1ZqVy
0xILqmbfOnJ1+rTdFovgqdH9sk05bvb44FL9t21Kc8LxtdTlqdmzhUV5zf2H94sG9rLReoGpF4P3szfV
73zkfUyvWveK0+qiZxQrbMQRP1XcCtV8Ng337528EtXERERKQ7H/zr3wya55Obpx95OH8qWq4d9
PzB4/xxn9Hr03W2PgcveTXL5qa4TuWo8sLV+p/OC5CaPz43mj86rTin45OLp62OhHU6pq2PSVVN7
ahbuLvUDEWllkmLU2oLCdYWTRquPZwiXFX9cjWW0TZRdWJkTK3qSFG8PKs4Uh3oqF2Nqm0iLhct
KTot3PegNTPC1dev+ov6RUREZKu8+
+9+9c/u+L2gcz7ReekHP8ifjRZq8p4/Fj2/H0ut91iZZs496WqQ6hsLx5XdTs/uu+avHQuGFS9rkycOBS
abtpwo1ywZzNmrmq04ITVDMapte9E6gxMGbq0uWka50sYZ5bDWiQZor1Vdg3BMdM5KeaxjLan
z+ssVLzsu4IA1s7XrR9fPXDkT3MVFREREOrKAn/HHefD8uZ+/8Ub+hLRE8/f80z6al73A+WZPcBIlrkz
QTebKTW+OJvqfpNSVTxQIhpWzJ7QW7G/JBk9bHxh+uqkdtSoHd2w2U1RsnbQ4Kbj+xfjBW8s0R0
dzNE+o9lAKr0mXwbXCy1IMaRRLHmzqurztn0JzmU3BgjrWzHaO3jm68M0LwV1cREREpDvL+Bl/nK
9+7r78IWmJ5v3f9ice5hPP7GsDO5ninIvPFn/apgU6MImrmW6rgoHpQbHE/KlzW2ZPqU3UNOW0
1chog6kdtahmaYxNzDBg0mJItqBi5nL4kK2VRQLF+8Uc7Vtual/rqFrhNUgup3WNlXCetf5yxYiU9gWl
ajHcpX+9FNzCRURERLqzpJ/xx/nO17+WPyctzp56/nJUXUcvkStOW09S/Ln/rEOVuJrptirYanpQLDH/
gN5p6OwN001bnpYJRqd2lFIUSwws3qreKCbtXFl+2mpMMbyaoH9r1XryQdFGeueoTZEZMnB4rXB
Mcjm9a0x/QP3lBkw8YM1s4dEXHg1u4SIiIiLdWd7P+OP8+MUX86elZZn/7/BLnBMdGvDQv1actzm
teNVz0uHqujL1TYXjBl6t1PwDeqfUWb2mmrYYdf7KldQmB01SDkqOiqco1t65sGLU+YsXN+fXRveuK
r46xZHylMQFLKze2qLYuFrhrKkq5bHEGgup8/rLpc4KDFgzW/B/jV9ERES2zac/9oGgQ15eLl/4eP60t
Cz76PmLI4nn9452Ih406KQDFl+ZlbJDC3dZbTPR/ySl5t+m3ysOlEuqr7Npmmkbp5QvOuZMqMq0XJ
5gjgFTbpTLzTRGVzXzw8HWwp1W48t5EhdwpTyxOpweWBlXK7oK0ZZq87SVXonmWRlQLqoW7m
LQmhnu6J2jc/90LriRi4iIiHTk/fffHHTIy8tz33gyf1paln30/OUDfULH83sxUfXYHx85UVquZvry1PaY6
H/SmjNlVQb1TuWRho6PZpJpy7eLScrlV7MmdtSQLrFRzpIa1LW5XL12OLxna81l1xVDEhcw03Zm+3
pH1SrXX83bfinDNdY1q2ezDSrXcwEHrpktvP6fr/tL+0VERGSrfPTu9wZN8pLy2P2X8uekxTk5PX+6K9
jo6kIOV8fVbG42eD/R/7SpX+ps+ODeqbmCvlLHn7Z6oza+XH41MNpRQ/3dUGN09zRpidXUdV+xes
H1DovX+W6Ll6mp66euNS5oyva1+j+s1Rtda6zUi2cjR5ULqwyehC34P84vIiIi22ap/wv/yxc+/ou33s
ofkhZnnz1/d+8QaDYEoa2mgjGKr6AvG4vx1EtPBTdyERERkY7cfPnG+z58JmiYT3ou3Xn7q1ev5o9HS
7TP3/N3zBIpz2mhD2Ne5TfQV41F+eK/fTG4l4uIiIh0ZHk/7P9/TzyePxgt1MiePylr4aOGPtHhV9Pkx8
r/PXDbvwVITLJRvqETYyb1/7V6+1cUTqzPPv3Z4F4uIiIi0pEl/bD/q5+7L38kWq699PxtE7W2Ux2dfcd
bMIVaz6/hZ4mO3jk6+8TZ4F4uIiIi0pFl/LD/wfPnfv7GG/kj0XLtqedfa07W1bJ39vWdbwLQ69r1a7d
85ZbgXi4iIiLSlmX8sP/HL76YPwwt2nY9PwCL9Py15/01/iIiIjI87/ubm/7itg8FXfQJyne+/rX8MWjp9P
wArD3+H48H93IRERGRjtxx73uCRvqk5DT8jL+k5wcg98D3Hgju5SIiIiId+eM/ujVopw8/n7/rI2/+9LX
86ecU0PPTLfj7GLpfbqX4u/Hyv4jhOFNtq/FX8c/
+F0GsdraDPa3t8hqyWBe+eSG4l4uIiIi05df//lf+9OwHg6b6wPOj7z+XP/ecDnp+unU3+cdpMvfV85

eFNuZt+fNiO+rBd3YNWbLrR9fPXDkT3M5FRERE2nKyftj/9CMP5w89p4aen60EXeVxmsyg59+ZHdb
ddQ+u52caL7/5sr/GX0RERIbnpPyw/5F77v7l22/nTzynhp6frZz8nn+XfbGenxPru698N7iXi4iIiHTk8
H/Yf9p+xl/S8+9FS7ubPFz2cZn+M3r6vuLt6oz4SDHt+kgwXffLbuXgldX4YO3Jqeqn1BfYqXFS46yiZE3
LwuuLC9bd0KxVr9ZWK9j2WrT12phqmvzN9oqJiVoEcwzf1MqQ9a90zrHRGBK/P2AG5vXoC48G93I
RERGRthz+D/t/
+K1n8qecU0bPvx+Jxil1sGr46monDWzA6or3y7fLIuUk+ZHsdTBd98sO6a2s5WWjqcoDdbWNJnVfs
cS7LQvvXe5aZ7G2WsXx2jzR1hPnboZ3Vhz2cSQva+2MnhJj179WW1Xfh9s/Aztx7zP3BrdzERERkbY
c8g/7n7z8hfz55vTR8+9J0fMk2pzyUNn3FEfKRikaUpsmasBC+YDilFp3lZ9RzJC9DKbrftmqHBfWWM
kX0jdzYquRAVcsnjgpvirVkWALW9ZK7CMaFlfP9FZM1WuKTgkuWW+JIesPh0SThCdEZ/TOwI4cvXN
07p/OBbdzERERkbb8/p//VtBsH0K+9MlPnMKf8Zf0/PtSdDFlExP1Uonmqre7Won6qUg+YnPO5sXF
i+uJ6keK84PpuI+2SQ0rj+Vrb44pXiU2VjsUGHLF4gNJxVS1Ub0nxgNSpwz5yMrq7XvNhCeOWOLmU
Nv4jrW1r7982bb8YkBiis2h3hnYodf/83V/jb+IiIgMzx9+8n1By73fXLrz9p+9+pP8yeZU0vPvTdD4FK1
U2X0lequVlrNqg8p+qb2Py8/KBmR/Pn/l2eys6kh5ejBd98s2yb0EB5tTFW+mtNVKVinnLY4OW3JZv
xrVdWJzueWA1CmJVUbDEtVDyYq9e0sUb5fe1ID1l68rjYLNiZs2U/TNwG5dff3qTQ/fFNzORURERJK
58aF3/cldvxs03nvMqf0Zf0nPvz9FX5M1M0UbVDVr6fYsaK8GNGAp+WmrEZvRqz9sDpVHyrOD6bp
ftknuJTjYnKp4M6WtVrJKtMRhS07MFZ9YHgnEA2q1hsyc3slKT8VUvYbWiSt9mzrOlcnfL6ZIKabono
Gde+qlp4LbuYiIiEhb3vPgrx3ID/v/9wN/mz/NnGJ6/n3Ke59V91R0OLWWJt2eFQPzo4MasITNecV/v
Z+dnR0qjlQTBtN1v2yT3EtwsDlV8owe6XOKeYujw5acmCs8sRiyshkVz5yqNWDmtt33VkzVa2iZuNK
/qSHrL9Wmy2xO6l1FTXoG9uGL//bF4HYuIiIi0pZD+GH/5Qsf/8Vbb+WPMqeYnn+v8obm/MWLm6
ap3jOVjVS9ywm7q0T3VDZJbX3fWj5P/DP+zZHafEHF7pdtUsPKY3mt5piBEzeEM2aiiYbNnLiwwYnxi
PjKp2oN+cgSY1b6K/burW1BxZT9JRJD4jErq2O1V826vcvMdM3Annz26c8Gt3MRERGRtuz3h/2X7r
z91atX84eY003Pv19lt5QJ2pnyzaK9Kvuest8qjxTn1ibsbI6qE1On1vq5oNfqftkqWmetfl6sbebijHJ5H
aUGXLGwTotipvYrEa6nWnA1c6pWNS4/WLvuxbBE9ZX+iql6TVH15oEBm4pmiNZfHqiWH2yofY6h
MyQOsANH7xydfeJscDsXERERSWa/P+x/7htP5k8wp56ef8/K7mal6Koq9Xcr9S6n1pSF4unqqhOjLi
pdYDNd98sO7QvNq0VTpc/oKbTFFeucKdFRhiemS61VMzfHbI63X4nyxET1td6KQ/bWUj4vNWBT
W6w/VC0rPUn1fv8MLdeIuV27fu2Wr9wS3NFFRERERktnXD/sfu/9S/uyCnn//yuamrXdpdkdVz1OqD
1i9Xb5MDK2LxyXPDA52v+xWDl5ZjQ+atu7qmYHtXfOkcGXDlpzoKBMn1jvT9dHidW3q+pBysvoKV
2OjmRPVc90Vh+0tvEJBme4Smb71r9WnWYuX1FxF917XmjMU78bnMbfnrz3vr/EXERGRgfnQX74
7aMjnzoPnz/kZf52ef9+KxkfrApwUj//H48HtXERERKQt5z7z20FbPmt+/OKL+SMLGT3/fpX/VaeWHz
hJHvjeA8HtXERERCSZGx9612f+4HeCznymfOfrX8sfVijo+fel+T9cjv+HzwCH7cI3LwR3dBEREZFk3v1
3v/pnd/xe0J9Pnq9+7r78MYUaf9+7r78MYUaPf++1Hp+DT9wAl0/un7mypngji4iIiKSzAf/
+jeDFn3aPHj+3M/feCN/TKFGzw/ASC+/
+bK/xl9EREQGZtYf9r/0gx/kDyg06fkBGO+7r3w3uJ2LiIiIJDPfD/uffCN/TKFGzw/ASC+/
+fr/hp/ERERGZhPf+wDQfc+Iv/8Dw/lDyK00/MDMI2rr1+96eGbgju6iIiISJybL99434fPBD38VvnSJz/
hZ/xD6PkBmMxTLz0V3NFFRERERknn//TcHbfzwXLrz9p+9+pP8+YNOen4ApvTFf/ticEcXERERSWb0D
/t/
+K1n8icP+uj5AZjYZ5/+bHBHFxEREUlmxA/7n7z8hfyZgwH0/ABM7Oido7NPnA3u6CIiIiJxtv1hv5/xb
0vPD8D0rl2/dstXbglu6iIiIiJxhv+w/9Kdt7969Wr+tMEwen4AZvH8tef9Nf4iIiIyJB+
+55agvU/muW88mT9nMJieH4C5PP4fjwd3dBEREZFk/viPbg06/CCP3X8pf8Jg3p+AGb0/AGb0wPceCO7
oIiIiInF+/e9/5U/PfjDo6mStngpu6iIiIiJSw77n1P0PCv8p2vfy1/qmB7en4AZvfymy/7a/xFRERkSIIf9n/1c/flzO
MoucHYBe+

+8p3gzu6iIiISJz6D/sfPH/u52+8kT9MMIqeH4AdefSFR4ObuoiIiEic8of9P37xxfwxgrH0/ADsTnBHFxE
REUnmjnvf42f8k9DzA7AjN9ywo5uOQqMpNJpCoyk0mkKjKTTa8gqdBi4lADviiWQ0hUZTaDSFRlNo
NIVGU4gOLiUAO+KJZDSFRlNoNIVGU2g0hUZTiA4uJQA74olkNIVGU2g0hUZTaDSFRlOIDi4lADviiW
Q0hUZTaDSFRlNoNIVGU4gOLiUAO+KJZDSFRlNoNIVGU2g0hUZTiA4uJQA74olkNIVGU2g0hUZTaD
SFRlOIDi4lADviiWQ0hUZTaDSFRlNoNIVGU4gOLiUAO+KJZDSFRlNoNIVGU2g0hUZTiA4uJQA74olk
NIVGU2g0hUZTaDSFRlOIDi4lADviiWQ0hUZTaDSFRlNoNIVGU4gOLiUAO+KJZDSFRlNoNIVGU2g0h
UZTiA4uJQA74olkNIVGU2g0hUZTaDSFRlOIDi4lADviiWQ0hUZTaDSFRlNoNIVGU4gOLiUAO+KJZDS
FRlNoNIVGU2g0hUZTiA4uJQA74olkNIVGU2g0hUZTaDSFRlOIDi4lADviiWQ0hUZTaDSFRlNoNIVGU
4gOLiUAO+KJZDSFRlNoNIVGU2g0hUZTiA4uJQA74olkNIVGU2g0hUZTaDSFRlOIDi4lADviiWQ0hUZ
TaDSFRlNoNIVGU4gOLiUAO+KJZDSFRlNoNIVGU2g0hUZTiA4uJQA74olkNIVGU2g0hUZTaDSFRlOI
Di4lADviiWQ0hUZTaDSFRlNoNIVGU4gOLiUAO+KJZDSFRlNoNIVGU2g0hUZTiA4uJQA74olkNIVGU
2g0hUZTaDSFRlOIDi4lADviiWQ0hUZTaDSFRlNoNIVGU4gOLiUAO+KJZDSFRlNoNIVGU2g0hUZTiA4
uJQAAACyTnh8AAACWSc8PAAAAy6TnBwAAgGXS8wMAAMAy6fkBAABgmfT8AAAAsEx6fgAAAFg
mPT8AAAAsk54fAAAAlknPDwAAAMuk5weAbT178YbKxWfzowAAh0bPD8CCbJrxebvwV66cryrM
WXDX/2ZhvbGV81deyQ9MqbGZjH9Xsq3GNZznY5pdyz8w+XdvY9pvRsc/oh1vbSs9VeMTm3Zb8+q
8MrP+B8WM2jd1Uj+nlh01tjPVxzSg1gn9z6RTQs8PwLxmfJpvqNXZ5UNbVnaOZ53Vs1S5j+y5auYH
qvVGzl+8uPp/Zqmz3sLOHwnrz6PTfysa3+zKTN+
+bC87+kbUr9t0RVr/CW3+M9Tc6LG0Vux8a1ttU62Plwc2l/S4pdKz1D+uY5fovTLr9yf9D4rUphpbyh
xvXx2byt6a8B+leDu14nUz7SirXx7KXh13d8NqTVBqc+1ywfVpXMXgPQbQ8wMwo+bzVPNpZFJFoe
z/3+UDQXOHc5m9yrpAfvnmKbP+7Ge/THW7+WAasplzffnCueerNfVzfK74POKFh1+NqbbWXrHrr
W0NnaoYl7/cWnb+RrPK6vKVB47/cfVtZ308f/tYdTa6NjXF/LmOTSUOjde6ncDxi7bvKDwyY63ijfzlcT
+19XTl9Nk3uXrZLJW9eaw9nUZ6fgDmEz4DRE8Nk5u/QmA3jx8zb6ucfv2H8Q9tXY73OLi1nX8PVub
9KjQv4Gz7yyauf1BTf3DRyuOtTHwhO67VhJexb6rs/dEXsjh73io16ULl0fUfjl2mWGyi1jz/cREXmmQ
fuY7tNE34/e69dL2LGS6aKtrHhMXKq5m9CL8OUxY6NfT8AMwmvjNP+LSTtuOHgWw/Uz0ztmo8/k
yvds3Wf5ynUHalKjNfs/n20WrmD2llcwlXJTZ/mOdbHv0TWvt2TCKab12xed0mLtkx3YSV+qaKLuwo
fVUm21Bqotqx9R8n+7InasVfiinEhbI66x8qlI5fNXXparK3p9pbslb2VVuX2PyhfSnbiWq1XM5ptla/T
OlCk23slNDzAzCb+Akg+YgypdkL1GUPHlM9vcWyveRm3NJ6F8X065Lzbaiw2dicdbJv3sSP8t1288XL
vnGZuUrF+4j/KT6WqEA8/8TXsmO6CSt1T5V9cBNcxe4q2bvTfFaJQus9FEfWb0/2pWipVTNRqbhQ
8LlkA9qv7zCJ7dR0v7utttmq6zdZqUSt7Eh49Sb6rLId5NXWf25OO+1VPB30/ADMZg+36t09CwRPi7
Oa9GGqab2N6nqtC+1kT/FXY1LBh7O5fnN+LbKCs37v6nvY/Hmeetnc4aWb8KPKJqyvPP4qREOOp2
O6CSt1TRV8HY8hWSU7mJvqskWF1nuoXq/fnuxL0XXpVjbbm6JaXCj68k3wD3LndiaYvy5Ra3O5Ns
c2f56oXnJf2X4KFy82viTHkE1bfjDRp9R9jUnS8wMwmz3cqnf0LLB50tnhM8fm4W36go1HtpopnrC
7ZHXnu37RN2/eetmnM+sliyvMuKPGl2K65/iN6J/Q+B/Z6NM7nrhAqeOtbbVOlV3NqbbTs+Ds7Um
KhYUa34maOWrFJvpGxIWiifvX0qtjiuytqb4La1GtuEL2yR1vRxv9l2Y9Yordhf/IxB9//1oI6fkBmE18Y5
7o2a3dLp4Fsho7fuDIas565TLrMvNXmX87czzKt5vsqbpdXGLWHdVM/ZWI1x1+WFPvrGO+CUulp8
o+twk307vgbMAEBXsKrd+e7FvRu6mp/gmLC2VH6hsJv4wjtG9nom3URLXiEv1Xd6DeidYDjv+lyHb
QnCauPMHHdOro+QGYT3hnnuzxo9X8FTbPJKHJnz9WVWrbSDwHzWKap7bYat7atNmHNOunlFW
ob2TOZ8Tsw5n3Oxd/A3ZSdGXyb0T20TQX3thb9NEdW6JioeOtbSWmyg5N+yH1LjgbMMHl6ym0f
nuyD2mvm2r8gzTJP1Wt25nhn9m4VuMfppXpinZ/TNN8Rtlq4yLrw/W5u5dCkp4fgBk1HkCmeSpIy
+YOnOxngmBHu9nMuugsn9DOd9N41J3uuTc257e6afM8XNrBN2LKvQXfgEy1hfreprqYHRW7F7O
V9qmCz2vjmJvLqjVWuqpSe53VPE6NgVdmPWyyL320qdWB2uSbJSUWMVj3phrvHqdMLtrORnZ4
B9/tyf+Dor1W851jX7rNdMlpGt/rSa/k6aHnB2Be9QcQ92l2pvFAeuzn0TbZ13u22fdg4ud4JtH8VDb
yzyZ46wR9Yu2bOpF76viMVhb3HxSTC/5VxUb1yOBB4pj0/AAAALBMen4AAABYJj0/AAAALJOeHw
AAAJZJzw8AAADLpOcHAACAZdLzAwAAwDLp+QEAAGCZ9PwAAACwTHp+AAAAWCY9PwAAACyT

nh8AAACWSc8PAAAAy6TnBwAAgGXS8wMAAMAy6fkBAABgmfT8AAAAsEx6fgAAAFgmPT8AAAAs
k54fAAAAlknPDwAAAMuk5wcAAIBl0vMDAADAMun5AQAAYJn0/AAAALBMen4AAABYJj0/AAAALJ
OeHwAAAJZJzw8AAADLpOcHAACAZdLzAwAAwDLp+QEAAGCZ9PwAAIfl2Ys35C4+mx/aKN44f+W
V/Mg8ijpB+UNQXZuV6Dq8cuV821ud7wEsl54fAOCw1PraZnt62nv+qm3PpC5Da2dfXdQD/DcZAPP
R8wMAHJbGf5dd71BPe88/aP/J5r76VwE6fuCU0fMDAByWRs9fb3BPec9fNu496yqvX3mdyiM6fu
DU0fMDAByWZs9fa1Sjnj86EDfG+ZD1iNq8+Qm1I/VuuDi8Olb7n9PXihRq70bv19ZWlenpuRvzNSas
rbTQMVc5OhtTvop20LX+teZ6giEdG+w8D2Cn9PwAAIelaiavbP5UtIy1LjN7HR+otZt5S1y1o73KJrr9
nFqfHfa1mdpCEpN0tr7pouHWa2priVSLu3il+GNQvW/9PQtq32D6vM7lAsxHzw8AcFiKpnHVQRaN
6aZhrL2RDYwPdPX8+aBaSxoeKdrS6JzqSHEgLFOOKJdSqzOg3W0vUE4Y1+xSDs5V02R6118MaF9Py
wbbJgpXALAben4AgMPS6BrzF/U/x21nrZ0Mm9liSNWXth8ppolHhGPCjnglPFROUhvTKlzBWriT+EC
3cgFrwSkD1h+Kqqc3WA4btG+A2en5AQAOS7q9XrWaUWecaJXD1jQe0n+keF1vlJvzFiNSgsKNWVr
ES1orShZHw631al/CgPXXNEcX77fMXq6y0twWwE7p+QEADkvYAeevy5/3Vz1kOHJlrp6/ebB4kdJa
uF16bLiV0T1/vIQB608175ni/dbZW04cumqASen5AQAOS9RMhk1k+Uai7Qwb43hI/5HEtMG8qRG
BAUNK6bFFyeJouLVe7UvoX1wxYmUzKKreO0dtikx7MYD56PkBAA5Loplsto/lG10jJ+j56811c8yA9js
u066crj44qjFhz987VXxqdFE6Nrh6qzbx1usGmJCeHwDgsKSaybJvXCvfqI7m7WTZmEZH4ua1/Uj7L
OWQ9spFYxuX6dJeoDp/6965Ywl96w+3U43v2WC0kc5lAMxOzw8AcFh6msn6G7VWNBT0sl0taHi
kfH0+nL3od1fSpasBcZluxfim+tlT9vy9608vZ60Y0jZ725lDVw0wKT0/AMBhaWkmqya18Ua9d121l
WFjHE/Wf6R4XZ8uWs5as29uDmjZRafmfGGTHG6tV+8SutZfnb62Llm7LPW3U7PXT10bumKAyen5
AQAAYJn0/AAAALBMen4AAABYJj0/AAAALJOeHwAAAJZJzw8AAADLpOcHAACAZdLzAwAAwDLp+
QEAAGCZ9PwAAACwTHp+AAAAWCY9PwAAACyTnh8AAACWSc8PAAAAy6TnBwAAgGXS8wMAA
MAy6fkBAABgmfT8AAAAsET/9V//H+XVG5rC/QSQAAAAAElFTkSuQmCC]]></redcap:Attachment>
</ItemDef>
<ItemDef OID="b1" Name="b1" DataType="text" Length="999" redcap:Variable="b1"
redcap:FieldType="descriptive" redcap:InlineImage="1">
<Question><TranslatedText></TranslatedText></Question>
<redcap:Attachment DocName="B.png" MimeType="image/png"><!
[CDATA[iVBORw0KGgoAAAANSUhEUgAABVEAAAEJCAYAAACdcmdaAAAAAXNSR0IArs4c6QAAAA
RnQU1BAACxjwv8YQUAAAAJcEhZcwAAEnQAABJ0Ad5mH3gAAE9sSURBVHhe7d0PjCRnfef/b+/aY
PPHNmBskx+RHat7sZYlGJIg08PxL4egZ+6SJbnbYOVg5Oh3PVyi0zQSkwOxF+mX2wguG4lunS5iWrq
zliQyTEJugrLdIJRgckzHR4IxYTI/Z7rlc3T8wDYGjPljB3u3f/VUPVX1VHd1dVV3VXX39PtltaerpqfqT/T
O/OZ5/k+hb5FAAAAAAAAAAAAAAChjumPAAAAAAAAAAIAQhKgAAAAAAAAAAEIEQFQAAAAAAAAAAiEKIC
AAAAAAAAQARCVAAAAAAAAACIQIgKAAAAAAAAAABEIUQEAAAAAAAAAgAiEqAAAAAAAAAEQgRA
UAAAAAAACACISoAAAAAAAAABCBEBUAAAAAAAAAIhCiAgAAAAAAAEAEQlQAAAAAAAAAiECICg
AAAAAAAARCFEBAAAAAAAAIAIhKgAAAAAAAABEIEQFAAAAAAAAgAiEqAAAAAAAAAAAQgRAVA
AAAAAAAACIQogIAAAAAAAABABEJUAAAAAAAAAIhAiAoAAAAAAAAAAEQhRAQAAAAAAAACACISoA
AAAAAAAARCBEBQAAAAAAAIAIhKgAAAAAAAAAEIEQFQAAAAAAAAAiEKICAAAAAAAAQARCVAA
AAAAAAACIQIgKAAAAAAAAABEIUQEAAAAAAAAgAiEqAAAAAAAAAAEQgRAUAAAAAAACACISoAA
AAAAAAABCBEBUAAAAAAAAAIhCiAgAAAAAAAEAEQlQAAAAAAAAAiECICgAAAAAAAARCFEBAA
AAAAAAIAIhKgAAAAAAAABEIEQFAAAAAAAAgAiEqAAAAAAAAAAAQgRAVAAAAAAAAACIQogIAAA
AAAABABEJUAAAAAAAAAIhAiLp0etJYKUih4DxWGj29PqjXFPPXFFyWo/jMBlSyfnk1Ai3iiMxOm3/hFEN0rT5EK2D
MxLO7LSlg372PzHqPs4Vb2GrGzEO5vtDbdtK5JH08KNuQ9Cjmc+2g0AAAAAwPwhRF06Rdm4UZO1X6mUUS2l3txV9V9OWwfcc8SKDv3xxhvLeTr
XurU1kPCkracr3rq4X0BLBWnqUjjPbie5KzMwwTtSbdU12Q8SI5IiRfCOsUx6leKitJa1Vu7oz8
uytmqfi4dqZ0P9kbrNc75IVW1fXnq3iT4W1mgNdvH0BFPrwuFM8Cd0GVfu9H1x/X3N1y2L9WxkEe8AHBA9PA2ZyiqRRVRtotBpUtRhF7Onl
D7TVk3fijjwsI7ascDAAAAAEOCFHOaFOEZQ2HwHBk05bhfzV0RhVnW4YOQ0jt2
5ms7qo4Kf2qNSRUMjvg4XD2pEOG2nXswPSbZ5twk02OkW1+wGA8z3Gyk9BS3s1aVhIhdp+P43IXL3FHYVDRDt2M0RgqfB11UnqLdxL2ApsWhaVq4xI1oP3ZIj6JNuVt0zNXMTn3CO4fBEbpWhu
cmfaGTq+NgmfYTJFxDmP6/qUJq8C4O9SaMjZrq4z6o8N8AAAAAHAp8VW0sDp0r68nnXzLmOxb4qH8iGyMzEwZ47+06A2urZU/zm3tfIRT1uS+tSj0aFV3DZBpsX58zzD2y2v7t9bDDfqdrOBTS0DHGfD8mMh7bmEq1dOa/PZa8
2kbLr9xC1BZ8xmeYLfjEwW6Mhkfdv0Xfqzx/5hLz0WO6j3xOJ8FTp+bFnfnUNkkaSOWIqODo+DR1JAs
/GQUsdb8AoHFI7HtRPGy4pdDqqDI9DoTApyV7tXfh/uH3JG6qKKDKwiV5TfNRrqIOUwhGpCCzwM0N7/kwGhK6aYrPkCXyv4aBzgCL1smjoY7wqD

AAAAEAuCFGXVkW2W26M6vdGbW/4Q6XL9a3wXoyqlqKuRek+wmpSjqyvaH59ZI1Jo6ZjoC7rqPX
GPu31edcGHd5fZI1J4zwEz5+5nbDPjxN+3INtCdS9tR4xy30ObGewraPOc7xjCm57uPZpYAh6c9VZF
2h4snM3eA7Uw9yc3Z6x+xzPPy71CLkXbAnaHvI9OHq7vqTHE6/dAAAAAAAcfYSoy6yyJXW3OGpz
VTYaDTnnJ6hyIWSM9FAIo3VqpQwmoCrK2hndwM6BdJ1nFmNId2dHLno7bcuubn/5zNpc9FKsnPa
D6gP/AGy9izv6PBo1Pe1wrCSDo63TPb8dqZUKUhrYSXM1aUimtjPY1qasDoZtEx2T2rZR+9S6H+te1
+kREu3HCSwHz4HSXB0dKibnnOtV70AUtW6Kc9TeGFHPNGS7E4vZbgAAAAAAlgQh6lILTjLVrLnBTF
nqF0Imk2pv+KGKV4+yL103ie3UZD3lhKW4dka3b18O3U23d42JhYxw0hjqferEnAz0rpz2yiY0d4M9
Ji/u6BisfEacDLUnjXX3GlSlpc9v3+0xnOr59bfvdUhWIdlAfdzx/O1494Hazrob/E1zTMbr9zZlc9v62PX
vV1Wv1/6cXXIi2X56jXUvsCzXu85r+8MlLiqR+4wr/BzteOl/krZbr3X/0hH2PRjY7rBkxzOu3QAAAAA
ALI+C9QtyXz/HklK9SwM9zlSwEhKq+K8rS71rTubTk8aK24tOBS/OZEYjX6963bk96Ubsy+dvW4Vde
9ZG1BBs1YOwWq1Ks9kcWu+3IaxdcdeNE387w+2yV3rnwG1/6DqbsV0VnO2FBNwB4e0wr3O11fc
nDEt0PSK2E9ivvuaS7JjMbQdfr41qa6JzF35+FOdanQqsS3p+lETnOlHbRwht44jjjDietO4RYBr3Pnyvv
OXCW/QSACAP99/xLnnNW2/SSwCAzB3/NZFjr9ILWBT0REVwkikVtoQGJP5Qeb/npMsYdm/2GE1F
UU6ccp51dJfTrj2WvywnT5+0e9QF11uqp/0AbA74vWmb4nVG7R44gZT1GW8ov7dOdT4sGbUo3S
BMfcIsazBr1jUo6ac28z7QPYSnOKZEvYkT7cec4f+kmIdQ3NyTvhmgTm3wHIWY6rrrurFuwJmaGO0
GMvDwEw/LOz/5Tr0EAMjDDY9fJXd/6AF5/Bs/0msAAJm7dI9I/zt6AYuCEBVBA6FScsO1P6fl1RVt7k
pbenJoj9k/JScqJ6z/D65XGeqc9ZIrrombLbpD+ttuIl09a/TozUtaAZl1DTJrOyHeSKpXqBe0GnVjgQX3x
NNPSOWPKvZHAEA+nvPjY/KWL94oP3ryGWm+/3555p8u6c8AALL1lMilP7A+PussYiEQoiJlGYRfXl3
RfTlsXxS7lKgd9lbEyVeN9XMZvhk9NO3A1+/VOyrwVcO67VqVQ480e0lOK1mv47yOaTHOXbjxbW/
Lhtnr1K2LatY5BRbUnZ+6Ux58/EG9BADIwxv/
+gZ5wQ+vsJ8/9NXvyp/WeR8GgNz0Hxa5/Od6AYuAEBUxleSkN1LbnBFfMSZJitM70Ri+HI+7744c7D
pf686+X7I/4a8fLjUwH4qbZ/0guOFOjFWVQIZacsoTKG6Jgvk22OvYvA/0seV1TIn2Y97LA8Pk1cz3hZ
xnoE/SdmNSNTtwdeukJv6eAubL+z77PvlM7zN6CQCQh1c+eK3c/PXn6yXH5z7+kHzlLx/RSwCAzF3
6gsjlr+kFzDtCVMRUlM2zbuVUc/Z1NbrYnOl8y+vt5wScijnre1s23JlrYvN7cqqJpBS3XmZRF0x117vh
6vxxe81a56Kmj3+wdqsx7F+aq7LhnTIV7Onh297K+dBcNQLH9nm/hqd7bHkdU6L9mLVbm7Lqre/p
me+ta1Qq+NvI2oTnyA9c3XYDi+kT+5+Q+n11vQQAyIOqg/pzD7xELwX94W9/Tb73raf1EgAgc5f/RK
T/fb2AeUaIivgq29LyctSalHTA48w8bynX5YJR4NOfUMnSXNWB0GQ1HAPbsp55Q/a9of6KMUnTHP
Jqu9rKUt8KRKiWomxe8IdlN1d1gOaFzqMm/ZolJ3AMttM8tryOKdl+ipsXpO6/2HmtOZGTdS/7h6Br
7yrua1NNWBO03bzfw9odR+bHA8R339fvk7v+7C69BADIw/FLBXsYv/oYRgWozd+8Xy8BADLXf1Lk
8sf1AuYZISoSqWyH11+stqz17tBiV3FT9gZfW21NVr/R7K0XKBlgDM3OdKKjFJgB2KiyA+qc9bt+wOe
ya1/OW03PstS7fT9Yt1Wl1d8LTpaV1zEl2k9RNvf60h16sXq5MUzeVpHt4EGK7B96PbFTEbvtVlv6Lf
8+sqlzblwHu+5ulByOB4jBnYn/6Wfp7QQAeVr50kvl2iev1Evh/uFL35ZP//6hXgIAZO6y9RvZJcpbzbt
CX81aAgAxtTcK4nSSVCHqQGAKADGo4PT1/+318sAjD+g1AIA8lB56od0LNa6tu8vyiteFD/sHAGTgit
8QKfBL9ryiJyoAAMiVGsJPgAoA+VK9T1Uv1CTUsH7qowJAji79AfVR5xghKgAAyM1HvvgRezIpAEB+
VP3Tt33hZSProI6iAtS7/+NX9RIAIHN2fdR79ALmDSEqAADIxe6Du/LBv/igXgIA5OWOL18/tg7qKPv/
8zH53Mcf0ksAgMxd/n9FLn1BL2CeUBMVAABkTg3fV3VQmUgKAPJ189efL//8Czfppclc+dzjsnX36+
XWV79IrwEAZOtKXR/1Zr2MeUBPVAAAkKknnn6CmfgBYAZe8MMrEk0kNcoz/3RJmu+/X3705DN6
DQAgW9b77aWPWx+fchYxFwhRAQBAZlRwqgLUh594WK8/xdvlOf8OJ1f+R7/xo/k7g8
xKSAA5Kb/HZFL1EedJ4SoAAAgM+/77Pvk3ofv1UsAgLz83AMvkRsev0ovpeMrf/mI3PtJ/igGALm5/
DXr0dELmDVC1KXWlo1CQQrGY6XR05/LSa8hKxttveBob7jtWZG8mzM3Qs5LHL3GinPuVhoy6tT55
9d55H7NjwLuWyCW+n11+djffkwvAQDy8vJvPE9e+eC1eiIdn/zPB/LN//19vQQAyNylXZH+Y3oBs0S
IurR60lhZIaZecpTlzFpRP8+eHfiVasLfVIImPi+9hqzXor5KXfOCrAYvunRqJSlMENguK+5bIB7V+1T1Qg
UA5Ovqp47LG++bvg7qKKo+6u//
+y/bHwEAeVD1Uf+79fFZZxEzQ4i6tLpy4KZA5bp0+33p9/dkM68MdWzgt6QmPS+qZ+S4YK99XtxN

V1vqevelWy87K5qrQo4aA/ctEIuqf6rqoAIA8veWvRvtIDVLqifqH/721/QSACBz/UdFLv2xXsCsEKIuq
96h7OuncuqE5Nf/FGmL1zOyJ41zugtquS5bFedpcfOsVJ2n0twlRQUwPTUTf+WPKvZHAEC+Xvu1F8
vLHr1aL2Vrb/f/2A8AQE4uf8l5YGYIUZeQXbvRDN2aq04tx8GuiKp3o1E7c1T9zGAtyGCd1bDejbH3r
wVreI6qN+kMVfdfl6zW52A9S6+2qH6ENy3GPgPncEP8zZjnydln0vOiqHaWYvWMNHoeB0LzkpzUn
VFl/9A6olGM9obVW21v6GMZPAcJrkvI/RZ2vePcb4PXz35E1ImNY5b37WT3p2vSa2eZ6D1Ar1TMr
49uJI6QOz91pzz4+IN6CQCQFxWevubvXqSX8qF6o1IfFQBydOlPqI86Q4SoCDUUGGl2/cyRYVRHai
WjzqrR43EyanuDNTzVurCgpuQNVXdFt3UUZ5+DwWRzdcJ9Fjflgjtk3joz5/RGeo1z3nkq1y+kUEahK
q1+y+tVOsTseTxK50C6+umwipx2N97ZkYsDJ7W96x2NX1c3yXVRQV7I/eZcj1EBZPj9pu7d0GC5U5
NSIMjOSpb3bcz7M2CCa2eZ7D0Ay07VQP1M7zN6CQCQFzV8Xw3jz5uqi3r3h75KfVQAyI2qj3qP9Z
H6qLNAiLqEKtt96Xfr4kZ7Um3Z9TH72zrxbG/4AZBXL9Won9mpyfrIxEaFec7r+3uboWUCxu4/wN+
et3/pyI6XBPWkse4GPca+Wzo1imzrKP523M2ofdbOu/Fbsn0WNy+If+rOS9v677wbglnn94JOUJOd
F4P9um2ZLK8uyolT+ukYlS23beb5V9ri53BnxMnhkpwj67VGqYGh+21ofyZj2/b9ZrYlbFt+kJ3U/Ny3
/uvD789hya6dZar3ACyrT+x/wp6NHwCQv5UvvTTzOqijPPTV78qf1hmBAAC56T8scvnP9QLyRIiKIW
bPtPoFPwgdDgOHletbE4Z54aotPxwsrp3xAqzOge4z2bsoO14eaey7suW3deeiJIl7zH364ZMh8T6Ls
nnWTbuaslpwe08Gz+8kipt740PW2PblMOpEFdfkTNjxtXf9XrVn1pzjSXSOrPOzp0NEI3gPvd4Dhu43
s8et0evSPk86CNzLYfa0LO/bsfdnmCTXzjLNewCW031fv0/u+rO79BIAIE+vfPBaufnrz9dLs/G5jz8kX/
nLR/QSACBzl74gcpkJ/vJGiIoBI3qm2Yqy5iYxIwK3UyfSDKjKcrKkn47SPdC9+VSoU/LqNhbMYdKRw9
QnMMk+K9tGr0FHOsP403RKoi+fcf2NgNIP3Kpy1j2gqa6LruEZOrw/aOh+M8JCawf20Hd33yNrsaZ
uHu/bBNduyvcALB93Jv6nn31arwEA5OWGx6+Sn3vgJXpptu7+0APy+Dd+pJcAAJlTw/r739ELyAMh
KibUkeHOgTHCo7mX3TFUvMKUSrD+5Oz05HBssVSf36vSHRZuBG7V08FeoUmYExB5PXXHCbtWR
dm8EN470w0rF3t+o8nvz/SvXdh7AJaNCk5VgPrID+h9BAB5e86Pj8lbvnijHL9U0Gtm60dPPiPN99+v
lwAA2XtK5NIf6OfIAyEqJjR/gWm53vWGbAcfk9YLHS/
+Po26n7aO1NZznJyneELGlj4tn5Sxl7S4KW5lAntYuDF8vno6/CyPP0dt2TB7nbo1OM36o0lYbdzT+xj
s/as0z83XpEi53bcTXLtoR+GPJpiWGsL/wCMP6CUAQJ7e+Nc3yAt+eIVemg+qPuqnf/9QLwEAMqfq
o15iYte8EKJiQElOusnV0EzePbnoFnIcO/Q7J6WTXtA2qm5m6ibYZ6+xrodpl6Ved5OsPCfnMa5rc1f8
zphdOfAu6QmJc0m9HrXW/XH+/I4OP6sSyOGSnCOzLqcKFN26qMaQ96TUzPKqx6k9GZQdSHa9W
p7pDpOf0CzuW0usa5fme8AU1xDz7yNf/Ig9mRQAIH+lh1448zqoo3z6v/6D/MOXvq2XAACZu/xZkf
48dRU6ughRMcCcBCnYW9IPAlXYNTChz6yYNTCbq/5Q7faGHhpuPdIev510n72GrLsnrnpWNjeNyY
Nq65JPjmrWsmzKrm5er3HOCzBj90asnBbnDulIs+ke18Bw8Amvix8oDvbcjUlvX80sH+xxaoTFZo9b
oz1p3yaRZnHfKnGu3QTvASU/dZXaee9gZMOb4h9Hze6Du/LBv/igXgIA5OnaJ6+0Z+OfZ83fvF+
+9y1qZQNAbtSw/v739QKyQoiKYeYkSJ2alHSoU/LTE7kw7YxI5vDy5uoUoVGwBmZzVQdQXnhTlVZ
qs9e7kuyzJ411d6h6Wepbav3okCq98zLMnFndbbN3TastiX+aKhIo72oZDmATnCMv2LO4x2xOsJTEi
HvXrLNaPevPNp/YQt+3SpxrZ0n4HuDXW7V41zBubVssGjV8/85P3amXAAB5UvVP3/aFl81NHdRRV
ICqglQAQE76T4pc/rheQFYIURHKHgYdUpOy2rLWu8Otp1KR7cGClfuHRs/BBOwamMZwbZddWzOj
eqgx92n23LN7obonbiCk8of1p3hehhRlc2+4Tqg9hD5hYBeYJMs6ZjsbHhT7uljH3G/5QaqtKq2+0dZ
ACYJo9r07eJA2Z5vTZZMLft9aYl07S6L3AHUsg6+ttiava4u59cTTTzATPwDMkOqBqnqiLgI1pJ/6qAC
Qo8vWb6bUR81Uoa8KBgJAEmo2fT0ZlAph96btmTxL+lhOtaYNWBfEUbp2yJUKTit/VJF7H75XrwE
A5EnVQVWTSS2SK597XLbufr3c+uoX6TUAgGxdKXLFb4gUbtbLSBM9UQEkFCxRcGZtsUO49nnrWC
J6ZB4tR+vaIV/v+
+z7CFABYEbULPx3fPl6vbQ4nvmnS9J8//3yoyef0WsAANmy3m8vqWH9TzmLSBUhKoCY2rJh17o06
pWaJQoWUK+xIqv7demmUqJinh29a4d81e+ry8f+9mN6CQCQJ1X/9C1fvFGe8+PF/NXt8W/8SO7+
0AN6CQCQuf53RC7doxeQJkJUAJNRNS8XfPx7cXMvpRq/C+YIXDvkR/U+Vb1QAQCz8XMPvERueP
wqvbSYvvKXj8jnPv6QXgIAZO7y10QufUEvIC3URAUAAKEefuJhec32a+wJpQAA+bv568+Xf/6Fm/TS
YqM+KgDkjfqoaaMnKgAAGKKCUzWRFAEqAMyGqoO6aBNJRVH1Ue/

+0FftjwCAPKj6qGpY/7POIqZGiAoAAIbc+ak75cHHH9RLAIC8qQB1UeugjvLN//19+cPf/ppeAgBkrv+
oyKU/1guYFiEqAAAIUDVQP9P7jF4CAOTttV97sbzs0av10tGyt/t/7AcAICeXv+Q8MDVCVCTUk8aKm
uVbPTaknWjdOGltZ96MOYZeQ1Y2gmvbG+7rV6TR0yunpfZjb3NEO2zTne9M2m1bkPugvaHbWJC
BSwosjE/sf8KejR8AMBsqPH3N3x3tuqGqN6rqlQoAyMmlPxHpP6YXMClCVGCGeo0VKZRq0tHLWe
pd3DH205RdQj4AA+77+n1y15/dpZcAAHm7+qnj8pa9G/XS0aXqov7+v/8y9VEBIDeqPup/tz5SH3U
ahKhIqCibe33p99VjWyp6bXby3l8WRhxDryHrtTziU6UnF3eC+2qea1hrF8VRuA+A+aZm4n/nJ98pT
z/7tF4DAMibClBVkLoMVE/UT/7nA70EAMicXR91Vy9gEoSowDLoXRQ3Qy2Xy86Tzo5cXJwUdTFUt
qXfrYs+w8DCUMGpClAf+cEjeg0AIG8//ffX2UP5l8m9n3xYvvKX/NsDALm5vGc9mOBvUoSoS8eoLbl
i9kQctd6oc2mvz7s25fD+IutuGnU/VwKfNLcT9vko6Z8z+/PmMP7mqvP5EYU0/WNWj+T1Rv2h/FU
5e+GMDvk6shMnRQ3UUg17jG5PvHbHuTbD59DaumyYrzfqkQa2MbA+/BTHaYN5POpY/P2rh7vd
9nnrupbrsjXQXdYu3WC83n4M3DfArKgh/A888oBeAgDk7YbHr7Ink1pGd3/oAXn8Gz/SSwCAzF26R
6T/Hb2AJAhRl05R1s64PREPpOs8s3TlwE30Aj0U27LbdJ6Vz6xZXz17ldNV/awjB/4B2PywsCxn1nRr7
RCwJIMj5zu1UswQa5bnrCO1UkFW9fYcal2SINUYyl89LZXimniHs3Nx+hCvfEbcU+2L2e6pr43Dfn1w
Z846FVQOrG+uDgSpE7VBHcuqeFvWoakKSlf369Ld2wxcdxW+lsJKN3RqUprnibKwFD7yxY/Yk0kBA
GbjOT8+Jm/54o1y/FJBr1kuP3ryGWm+/37qowJAbp4SufQH1kfqoyZFiLqEimtuT8R9OXQTovauHwi
JEU72Dq1XOU6dmIcI1VI5LW6M2gzMjmSEhV6w15PGutvjsyotu6am9WjpLXRqsh4jjUz7nFW2rTa
Yw76rLadd22HVPv12d+vuV8TsRaoYQ/mrp9X2zVA4xpD+4qbsuefNfnTFa4Z1BPULwcDQN67d6V
wbV7neDX69Frbev2+maYPxeh2aFjf3vOc+P1RXYWtXf41/TppyLsFxAmnafXBXPvgXH9RLAIBZeONf
3yAv+OEVemk5PfTV78qf1h/USwCAzPUfFrn853oBcRGiLiOvJ6IfaPUOndivWnXCo32dFJrDwO38bS
5UZMsNoJq7fi8+s+6n2wPUXFff8ickqmx5QWCs3pgzPGfVlj+Rkh/mWi0Z7IY7gtk792TJfmJsJ0EYq/
Ua636vzepZ2QxPUMe3O61rY6vKWbchRsg+cv3+obPdKdoQeH0UI1Q3Q2s7cNWB6t6okwhkSA3f
v/NTd+olAMAsvPLBa+Xmrz9fLy23z338Idn/n4/pJQBA5i59QeQyf8BKghB1KRXlxCnnmRtode1x6W
U5efqkHXYF11vUMHDn2VzwQ7mmeJ0KuwdeWOgN5ffWWcfkDu+2H8bw7cAQ/VFmdc784HMy
Yb1zLRMP6W/LeT9BlVZoz1klRrtTuzaW8kkJ3d2o9a4p2hC7Z7Zxrq2N2WUO3P3Er8sLpOuJp59gJn
4AmLGXfPe58nMPvEQvQbn7P35Vvvct/m0CgNxcVvVRv68XMA4h6pLy6oraPTl74nSqPCUnKies/w
+uV3ngPEWoFiOYcodmt90x0xG9I6exkOfM6GmphqaXQkPC+LP0tzf8OqBVo6fp8kkSbhdl80L4jP1u
cDtiPjEgE+5M/A8/8bBeAwDIm6p/usx1UEdRAWrzN+/XSwCAzPWfFLn8cb2AcQhRl5U3tHlfDts6aL
N77VXEyQqN9VP3hsyCUdfTDi/9upOjwkuvNubQI2YYuIDnzB/KHyXmkP72hj9RVLUlIzuhTmDqa5O
CTNtg1JUdKNlqa55jln7k532ffZ/c+/C9egkAMAsrX3qpXPvklXoJpn/40rfl079/qJcAAJm7bP02eukze
gFRCFGXVklO2hlkRw52nSHNbh3Rkv0Jf3347OuzV9w864eaDXeSp4E6pCVnqL3i1eKc2KKdM2Mo/
xjjh/S3ZcNLUMtSV1PRTyvVazOhHNugZuhXPU7tScXsQNWYoCtu2QJgSvX76vKxv/2YXgIAzELpoRfa
D4z26f/6D3aYCgDIyeXPivTp2jMOIerS8ntyNptOOObWeCzq4p/uem+Sprnj9gDtSK2mA77BOqRmP
crmqj9sur3h1aUsxB5LvWDnzBzK787+H3iYIV70kP5e45w3jL9cv5BOuYRUr82E8miD3pbKoIM9Trvi
ls8dW7sVSMn/+v/
+l34GAJgF1ftU9ULFeHd/6AH9DACQi0v36CcYhRB1iZkzpqvehd7w88Ds5sYkTXPIq1NqC+shGaxH2
VzVwZg/Ll1GT440LNVzVtS1VJXmauqhoTmUvxxaW8AoiWC9cuSQ/l5D1r0CqtYrAxMwOY/JJkhK99
pMJoc2VLb9IfyBurRGfdmzm7MP3bEU7v7Fu+WOl9+hlwAAeXvjX99AHdSYfv7dP6WfAQByUXijfoJ
RCFGXWWDW8FPiTzbuDltXzPVzyAwvRw2ht+tRGr0uXeW6dJPWu0z1nFVke7BA5v5hSrUxzaH8o0
NdMxQeNaQ/Xl3VCaV5bSaVQxvsIfxhxVBVSNvvp1pfFohy1RVXyf/4lf8ht1x3i14DAMjT373yCf0M
UV7z1pvkbe+5VS8BADJ37FUix9+kFzBKoa/G9QIAgKXxwCMPyOv/2+vtmfoBAPm648vXyysfvFYvYd
D1P/E8+a1PvVGedw0TbwFALgovFrni/daTq51ljERPVAAAlsztN91uD+0HAOTvb27/tjx2PX/ECnPlc4
9L9fdeS4AKALmx3m+Pv8f6SIaByEqAABL6F2n3iUfeMMH9BIAIC+Xjvfl8294VH78nMt6dVz/8t+V
5NZXv0gvAQAyd+ztIoWb9QwvWb9QLGmSpE7TVW9AQpBX9W65F60lhxJ1TZkODL27LhTbbiPCabqGZYeyO
43dBH1jOAAwAwhz788x+W07ed1ksAgLz84PnPyt7rvqWXoLzidS+R1X8bNhkqACATx4oix39eLyCO

OeiJqsJVf5ZqR84zwquZ0VcaKU3oAwDA4rjnl+
+xh/cDAPL10M0/kO6t39dLy+3al14l1d99rV4CAGSucI3IMTWMH0nkGKIWZXOvL2oeq35gxuuuHH
iTiKvZsNXn92QzxwzV1qnJeTqkAgCWjDtj/3VXXafXAADyonqjfu+aZ/TS8lIBqgpSAQA5Of5ukcIL9QLi
mn1P1N6h7OuncuqEZJedlqXedUNc/9Gtl/XnRZq7pKgAgOVzy3W32EGqClQBAPlR9VE/96Zv2h+X1
S/8xivsofwAgJzYdVDz7rl4NOQYog7XRLXrlZZq4nZEtYfVD9UoNb/OeaRVL1Uprp0RP0YFAGA5vfm
WN8tH3/5RvQQAyIvqibqs9VFVePoLv35CLwEAMmfXQX2HXkBSc1ATNUKvISuFktS8lNXRqZVSq2H
aPu+HuNXTfpGB5WVM8uWd46hJwTA7YddqEYTfT/4kcHneY0nPYU7fC+q9b2DCO//8rEiKf0cCAt7
7s+
+V2h01vQQAyIuqjbps9VGfd82VctfvUJMbAPJztcixO/VzTGKmIWpIuy/9bt3vCVptOcPst1WY2ZPGu
htwVqXlDsFvVe01qobpeqIkoSO1khtC+I9Vd0Yra9/2bgFghnqNlWAPfSBnqjeq6pUKAMjXfT/zuD1r/
7JQAer1P/E8vQQAyNzxO0UKL9YLmMT89kTtXZQdnSKU61v+RFSVLXHLmHZ2LqbU+64s9S0S1NFG
TQoGTGKR76eM295ryPpg13tgBlR9VFUnFQCQnx8/57J8/g2PLkV91Le951Z5zVtv0ksAgMwdf5PIsV
fpBUxqfkPU7oHXE8sevu/1HjWG93cOpKufTsfppZpmrVUAABaVmqm//attZuwHgJw9dv3T8je3f1sv
HU23vvpF8ku12/QSACBzhVtEjEv0LvYBpzHdN1FSFz85vlhPo1M7nWItxHgxO2jWqxuPoOpD20GPv6
/VjZG3Jwf1FBNd2Pdzga0fVgsysDVEya98oca+VK/5xJm1f2OsHyncO1O806o7arx19P/kG2p/K9Ux6
DsOEt32wXungORo8P2HiTbTn8/epHqPqpCY5P0DQbdffJvf88j16CQCQl7+/7Xvyjy//oV46WlQd1O
rvvVaufO5xvQYAkK2rRY6/2/p4hbOIqSxEiFqud4fDT/uRwnDa4qac1WVWRfblcGnyBRVsDU7a1ZTV
wqr1/3hUiFMKG3rcqUlpMKBKMklYe2NETUjVYzgYFmXWhihZtW+khNcqwXEma58TyIW9vrlaGBH
2qXNitLNcl/GVM9SxDbR/6us5/f0ej9OrffAcNVdHhZyTcPbh1XO2Dd97qd3vWGrvKL6DGfsBYAb+6v
WPHcn6qL/yH15JHVQAyNPx09RBTdH8hqilk34P0YN0Bu2Ha8tuuinKQug1zvnhkTuhV7/r1Zsdzzhv
5bp0dbDd9TbQlHNeotNLMEmY9dpzesOh2+3IzkX3tVm1IUpW7Rst2bVKcpzJ2tdrrHuBnP+HjZa1F
615bkRQaLRjb1OKem0koz1u06e5ntPf70n47fHb3pHa+ejIPHqivUH+PsLvvbTud0Ds2frfdepdegkAk
AdVH1UFqUfJyumftB8AgJwce53zQGpSC1HtnmjGkFHzEWco65DimpzxspxVfxuqF6C77UQbdnpv
me1yHmYvuTOyFivhWXQ9uejO2qUCFi+kKcrmBSPEidI7lH39VDo74mY3xc09J6yxHnub+mQmmiT
MmLjHCNyKa2eGQ/XM2hAlo/aNlPBaJTnORO0LtuOst74iW/aGVVC3J2GHE2hHTNWz/rmtbPtBrd
/2JNczhfs9gWrL7yFf2Up/+4q5j/B7L8+J+bAM7v7Fu+WOl9+hlwAAefjmjU/JV376u3ppsb3sp14o/
+a3mNAEAHJTuFHk+L/WC0jLHA/nDwYcXkjrjWE1w5B0mMHN0daVAzdTKp+Ukn5qK56QU/ppJDP
kHgioh2ouTjVJmK6nGTZ8Prc2REmpfSMlvFZJjjNR+0a3wwldR5fWOHUi6XdVVU4HNlaSk2473bYn
up4p3O9zpSwnAwcRIteJ+bAMrrriKmbsB4AZuP9V37HD1EWm6p/+
+n/5GeqgAkBurhQ5/mvWR+qgpm2+a6IWN2UvbMitPdQ3hXqoHtWLri8pZ7JH3OhefG5oM1EPZ
MWu5eiGPlE1KzNsQ5R5b19sebQvRuA3VlFOLEzSmcbxAvPpphfcZAepKlAFAOTn8yuPylNXX9JLi0f
1QFU9UQEAOTn+r0QKN+gFpGmqENUc9hv1cMLJoj8M2gxA7aBUrw9NMc2v04+4dRUtdp1B82tD
H2kGskvEuHZ+7Udf89zw5DXjJwlry4bZq9OtjWnWijRl0oYo2bcvLbGOc4btG21wgreeHHp1B4ZlOv
HcEcD5QZpuv+l2e2g/ACA/KkBVQeoiel3l/6IOKgDk6dhrrQd1ULMy3z1RkZGQ4dEus05mDGp2d9V
b0Q+rjZ7D7raTTBLW3vV6dtrhjxuYG8OTB6XehihZtW+khNdqguOM176ldth1itOdfT7Y9JDh+ImO
M737fWHkNjEflpGaZOoDb/iAXgIA5EEN6f+7Vz6hlxaDmoWfOqgAkCM1C7/qhYrMEKIupaKs+bN2
yao3Ztuc0XsMPcGXKlEb7K0YEnhNOEmYH/4YM+KbcmhDlFTbN1LCa5XkOBO1L6Id9rE7NVUTnsK
Rmqsb4jfdL5lQPrPmhNaJrmcK9/uiyeB+B0wf/vkPy+nbTuslAEAeVH3Ux65/Wi/NN1X/tPp7r5XnXX
OlXgMAyJaqg/oe6+PVziIyQYi6pIqbF/zehs1VHawYk86MU9n2h393alJygxmjRqg/UVew9mbkJGGV
09aSNq5dWbUhSmbtGy3ZtUpyrpO1b2w7ynXZSmV8eFnK5aas6vZ4Tbfaftab/j/Z9Zz6fs+LOdGV28
6Jws6U7ncgwj2/fI89vB8AkI9Lx/vy+Tc8Kj9+zmW0e2ufXVL9JLAIDMHVsTKdysF5AVQtSlVZ
TNvcFJu6rS6res/8djD/8OK6Rpb2dgoi679ubg/ixDk4RVZHuoDc72vF01d71eitm0IUqG7Rsp4bVKcJ
zJ2qfa0Zfu0IbVpo3SBlM7JWcHjzfsGiW6ntPf7/mw7q/B67F/aPQSTiCV+x0YzZ2x/7qrrtNrAABZ+8H
zn5W/ev1jemk+veatN8nb3nOrXgIAZO7Yq0SOv0kvIEuFviqCCAAMIF7H75XKn9UkaefXYwhpgBw

FNzx5evllQ9eq5fmx7UvvUr+06ffzDB+AMhL4RqRK9R8BQzjzwM9UQEAwMTefMub5aNv/6heAgD
k4W9u/7Z875pn9NL8qP4udVABIFfH3239jwA1L4SoAABgKu/92fdK7Y6aXgIAZE3VR/3cm75pf5wX
v/Abr5BXvO4legkAkLljbxcppFNUD/EQogIAgKmp3qiqVyoAIB+qJ+re676ll2ZLhae/8Osn9BIAIHPHiiL
H36EXkBdCVAAAkAo10dQt192ilwAAWeve+n37MUuqDqoaxg8AyImqg3rsPXoBeSJEBQAAqVAz9b
d/tc2M/QCQI9UbdZb1Ue/6T6+2g1QAQE6O3SlSeKFeQJ4IUQEAQGpuu/42ueeX79FLAICsqbqon3
/DozOpj/q299wqp/7ZDXoJAJC5428SOXabXkDeCFEBAECq3lF8BzP2A0COvv2if7Jn7M/Tra9+kfxSjV
/kASA3hVtEjv0LvYBZIEQFcteWjUJBCsZjpdHTn1tSvYasbLT1gqO94Z6fFZnX09NrrDhtXGnIqCb6x+E8
lv5aY2mo2frfdepdegkAkLW/v+178o8v/6FeytbzrrlSqr/3Wrnyucf1GgBAtq4WOf5u6+MVziJmghAV
yFVPGiur0tRLjrKcWSvq58vHDiJLNeno5YXRa8h6LarV6loXZDV4saVTK0lhIDAGjqq7f/FuuePld+gIAE
DW/ur1j8kPnv+sXsrOXb9zu1z/E8/TSwCAzB1XdVBfrBcwK4SoQK66cuDmbuW6dPt96ff3ZHNZM9
SxQeScUj1nxwW/7fPiHlq1pa5zX7r1srOiuSrkqFgGV11xFTP2A0COfvycy5nXR33zr9wir3nrTXoJAJC
5YyvW41V6AbNEiArkqXco+/qpnDohy9vV/dHHF6znbk8Y53QW1XJetivO0uHlWqs5Tae6Som153PSC
m+wgVQWqAIDsPXb903L/q76jl9L1sp96ofzKfziplwAAmSvcKHL8tF7ArBGiGvnqP5CKntOLI2peqN
536d163Or/dp135sb+ivNdbZLwuuD/bKS2MbmtlG7zFcYzN4jMM1S+1HWN1Low3jal3a+zDDt+aq
87WDDQ9pc9i2o9o8tpfjROdl+J4ZuZ8YxxD7fGh+W9RjuK0OZwi9/7p0z506/lKsnrNGj+NAWF6Sk7
ozquwfDt9PNqM9ie65eMduS+H7wj1Pcd9HsNxuv+l2e2g/ACAff/fKJ+SbNz6ll9Kh6p/+
+n/5GeqgAkBurhQ5/mvWR+qgzgtC1CWlwpHQMKhTk1JhQ8ZlcHHYtR8HCkLa61TIMrC+uRoeXk2
1DRU2hfYY7EitNCqEU58zapaW61J3uw52duTiYMi0674ynbqmQ8GiZh/zyGBquM1uz8dQE5+X4X
umuToieEt8DFGcfQcvd0hb7WCw5A2hd6V67jxVafVbXq/SIWaP41E6B9LVT4MqcjrpPZfk2FP6vlDn
KY/3ERwdapKpD7zhA3oJAJC1z688Kk9dfUkvTe/f/Nar7J6oAICcHP9XIoUb9ALmASHqUmqLn8O4d
TmNeo3SlHPhSUpi5XrX3na/FYybwtaPGt482TaCw6mHj7EjO4PplEcFZM7r+3ubsrlVF+erBr/GPI9nZ
FyGWtm2ttd1t2Wptpx9bOvUrr3hB4Vhbe7UZH3kdQm2eXRT0jkv/invSO28cd0SHMPY8xHg7zu8r
dZxrbvBoHEu3Iamcu40u53bEitrHVKUE6f00wiVRPdckmNP7/uimOP7CI6OD//8h+X0bQxHAoA8q
ABVBalpWDn9k/YDAJCTY69zHpgrhKjLyOwlZ/R0K27uOQGJ9dhLZaajqpx1t1M5bS25RqwPHd486T
aKsrlnBj6O4toZL7TrHIT3AyzXt4IBWXFNzugv6uxc9NvY3vV65pXPrI0P38YwexjWLxht3rwgfgZ5PrR3
31CbR5r8vFRbfnDoh3xB0xxDFHPfoW3tXZQd3SEycC4qW/5+zWtniH/u1HFY3yOhIe8k9uUwrEFKk
nsu0bGn+H2R2/sIjpp7fvkee3g/ACB7akj/V376u3ppMqr3qeqFCgDIiV0H9V/rBcwTQtRlZAQ0ImqY
bsGrZTiyhuIkyielpJ8GjFofJo1t2HQ9x9BhzEGnTgwGP0VZ8xMtLyzyA0Mj0J1YVK9WY/8jgrfhNscV/7
yMN90xjFaWk+MudvfAa79X7sF+GEPcRwyfn/zcTeuUjN51gntuimNPcv2HzlNe7yM4ctwZ+6+76jq
9BgCQJTXJ1KT1UVX907t+59XUQQWA3Kg6qHdaH6mDOo8IUZdSUTYvhPckdEOYqMl1FkZg8hyjn
mOk8MDO76nnDnc2AsPq6dg9GafXkeGOgjFCRlOK52UyYccwK2keVxw9ORxbLNWR2T2X2vVfkvc
RZOKW625hxn4AyNFfvf4x+fFzLuul+H6pdpvc+uoX6SUAQOaOrYkUbtYLmDeEqMuquCl7esjtQKlR
W/Pcos+u3ZYNs3edW7PRrMGZhHW+zurzZA+NNoYyV0/nF6FOH/qlfF4mkm1w6dXKHXpMWsd0
AsUTMrb06bje1BPcc+OPPf3vi6P9PoIsvfmWN8tH3/5RvQQAyNIPnv+sHaQm8Zq33iRve8tkAkL
ljrxI5/ia9gHlEiLrE1MzaqqeYPcGPHYR0vRqKo4f/GoxhxHPHrB2pgiW3/uMUba64U6Z3duT8+R29n
aqkk6GW5KR37gdnZO/JRbfoZeQQ8BgyOC+
+nI4hTOmkFwKOqumZL+NcNHfF75DZlQPvNJxwzn2EWPdckmPP4PpP/T6Cpfben32v1O6o6SUAQ
Jb+8eU/lL+/7Xt6Kdr1P/E8uet3qF8NALkpvFgP48c8I0RdRu0Ne6itmkU92FPMCHiMXnIlPxkzZmJvy
4Y3Dft884MlY2bySXgTWHWk2dQnKrWh/EXZdLsdqvO87l+XXmPdq22ZZBKkcVI7L578j8Fj1udsrvr
DyPW9bj9yHVtu1oBtyq7eda9xzgsxY/VgjnPPTXjsU1//hO8jZntyvRSYe6o3quqVCgDI3t/c/m157Pqn
9VI4Vf+0+nuvleddc6VeAwDIlqqD+h7r49XOIuYWmz7Q287NSm5QYtRH7F6NnzmbhXSDL52
Lpkz9nttNibamUhF3I6BrlSH8o+4LiU/fZQL005glcl5MSQ9BnPYu9ueiRK2YH3O5qqz34IX9Felldqs+v
EUNy94PTLd9njnodqSeM2Jc88lOPY0r3/C9xEgiqqPquqkAgCydel4Xz7/hkcj66NW/u8idVABIE/H3k
od1AVBiLqk7KG3YUUMVeDS7wcDHlX3cLBmYrWVcx3NpCqy3W/5gZHNOTbvsAPDrOPxhlcr5bpsp

ZzL2dcl5LxWW9Z6d+j1VLI5L6Zkx2C1Z/A+3D80ejUmYNfnNIaSu+y6nznWQ/UUZXNvuFaoPYw+Q
aAb656LfezpXv9E7yNABDVTf/tX28zYDwA5UPVR7/uZx/VS0Cte9xL5hV8/oZcAAJk7Zv2GfPwdegHz
rtBXRewAxKNmNtcT86gwbG/anqHAOEfpntPHcqpFwIpwn+l9Rip/xM0BAHl441/fIKWHXqiXRK596
VXyW3/8z+yPAIAcFK4ROf5+66P/Xoz5Rk9UILaeNNbdmc3LcmaNABVZO1r3XPu8dSwZ9ODG0fGO
4juYsR8AcrL3um/J9655Ri+JVH/3tQSoAJCn4+8mQF0w9EQFxmrLxmANWFXOgK50yMzRu+d6jRU
p7ZyRbiplKXDUFf6fgn4GAMjStU9eKe9s/aS88723MYwfAPJ07O0M419AhKjAWAOBFgEqMsc9h+
WlJiiblx9NaEs42hKOtoSjLeHmqS0nrr1GDr/3pF6aLa5RONoSjraEoy3haAvSQIgKAADmBj/ghqMt4
WhLONoSjraEoy3haEs42hKOtoSjLeHmqS1IhpqoAAAAAAAAABCBEBUAAAAAAAAAIhCiAgAAAAA
AAEAEQlTkSE2WU7DrfxRWGtLTa8fqNWRlo60XHO0NvZ3CijRibwjp6Uljxb0GG9aVTbJuGmH3UNr
7mJbRRv1Y4SYFAAAAAGChEaJirvUaK1Io1aSjl4H5pgJdY1Z9W1nOrBX1cwAAAAAAsIgIUTG/eg1Zrx
GfzqeibO717RkF+/1tqei1szFPbenKgXvLluvStdu0J5tkqAAAAAAALDRCVABIS+9Q9vVTOXVCyE4BA
AAAADgaCFGXmao1OlC7MazG6GD9UXuIvfE1A+VKNbNOpXokq1Vp79Mcxt9cdbYTvjOjjeoxqk7qY
JvCa1VOdrymMbVf2xvetob2H3JN4rTRY359ZEONcxFo46j1xj7t9ea5zKoOadx7KKotg9uYsD5pjOuS
9J4FAAAAAACLgxB1WakgL7TWaEdqpVEhpPpcQUoDQ+ybq4OvVyFiSYIva8pqYbBWZBqcNq0GNh
xyDHYINtgm65W1UnjQaYt7vIMqcrqqn3Z25OLAa9u7bmODtTKHQjgtuo2TKsrambLztHMgXeeZxR
iOHmh7W9xml8+s5dDDMoV7aKJrPizf6wIAAAAAAOYRIepS6knjnJuIuXUb+9Kt61BNOrIzmPx5qtL
Sr2+5QaEKG8/7ve16jXN+0FVt6VqVXfE2H0Nl2/qabl28L3G3sx1W8dJvU/gxWMe77oZg/mv77gF0
arI+MhUdf7xhKltu2wfPpR9GSvmMeBlqe8MPgsOuSWQbJ1NcO6PbuC+H7qbbu0ZI2ZEDN101hq
mfOpF9hDr9PTTNNTckuC7J7lkAAAAAALBICFGXkjERz96m16vQD9VEOl56FlRt+RP3+EGhqScXd9w
+e1VpeQGStc8LYa+fntmm0GPoXRS3SeX6lj/xUGXLC+U6OxdDexSOP94RimvidfQ0t22ElGaPTrN3av
2CcU02L/htrJ0fGKo+Ja+NftDbO3Si0mrVCRv3dbrau7jjBZKnvROYlRTuoSmuuWkm1wUAAAAAAM
wdQlRYdA3P0OH9SZmzk5+Ukn5qK56QU/ppespyMrCTEN0D77jsIdheXUtjqHdgSHsazOHy/rB4P5
SryllvyvYRvVNtxnbMHqOpKMoJfUHcwLlrXzzrnJ4+aYeVwfWW6mk/kMxMCvdQKtd8VtcFAAAAAA
DMG0LUZRWYKCdunckYgeWRMt3x+r1i3Z6eRig3URhpDK9PScUt3trctVrXE6cj6ik5UdFhZWC9anb
2EeriSf+6AAAAAACA+UKIupTasmH2OnVrPZr1HI+ocr3rlDEYevjD9lNT3JSzbglONXTcqCs6WRiZQYh
dOS164L4ctvUQeLv3pzs5lrF+QUP07K/5sv1xAQAAAACA5UOIuozMupwqYHLrohpDoCdXkpNuEjs
4XNoIEXNVcoamK6NqvWbF6+nZ2ZHz50fVFTXP2eBs/mZ90FMydk6nxNfQ3XdHDnadr3VrtZbsT/jr
h4e0ZyWFeyiVa57idQEAAAAAAAuNEHXJ+QGTMWP/VMxakU1Z3XCn3DFnS8+ZMcmTNFfFa1J7w
6+V6a1MmdfTsyPNpj76oaH8Rdl0u6xar6utN6yz5eg11r0anuYESU7AqVivP+8dkGx4U8nH5V+vZtP
5Wnf2/aIumOquNyfCylYK91Aq1zz5dQEAAAAAAEcTIeoy8oI9S3NVh0rGhDtTMmcun2r75iRC7nY
mCjuDs7o3V3WI5gWO5gzwaXOHxftCh/JXtqXl5XU1KdnnrCAlP6mTC95EVCojdOutWrxzHLe2bVBg
W9Yzb2i6eZ9Y68/k0w3VNv09lNI1T3hdAAAAAADA0USIupQqst1vGQGZUpVWv+8HRvaEQpMqyu
Ze1w/BbGr7g/scx2qn1yBt/9DrDZhIcVP2+oNtstj1YDOoh2rwhvQr1v62Ruysst0PrUtbbVnr3ZILLnU
8g6+ttiara2v22gwMTTeGs+c+ZD2Feyila57ougAAAAAAgCOp0FczrADITq8hK3oiL1WDdo+eiwAwk
urtPS8/mtCWcLQlHG0JR1vC0ZZwtCUcbQlHW8LRlnC0BWmgJyqQKbOOZ75D4gEAAAAAAJAOQlQ
gE23ZKAzU8ayeFTqhAgAAAAAALB5CVCAPql5pZpNXAQAAAAAAIEvURAUAAHODelXhaEs42hKOto
SjLeFoSzjaEo62hKMt4WhLuHlqC5KhJyoAAAAAAAAARCBEBQAAAAAAAIAIDOcHAAAAAAAAgAj0R
EV2eg1Z2WjrhVnrSWNFzZavHhsyL60CAAAAAAADA/CNERSZ6jRUplGrS0csAAAAAAADAoiJERfp6D
VmvEZ8CAAAAAADgaCBEBQAAAAAAAIAIhKhLqr3h1gddkUZPD7+3l51HaClTVePUeI3zcL7eZW/XH
MbfXHVepzc4uF+PuW1j58HXt2XDfY318F4Wo12TGjwv6jHpuVGSHr8S1obCSkPCD8+s/eo8VkaciG
TbBQAAAAAAWF6EqEuvI7VSQUoDw+
+bqwMhX3tjRI1T9fXpBJbR1H5WpamXpFyXrYr1MbN2OWHk4HlRmquFYNCZ4blRoWtYG6RTk9Lg
BFl2EFuSwZd3aqWhcDTRdgEAAAAAAJYcISosVWn1+9K3Hq2qXqUCwPNulNaTxjkdX5br0tWv7dbL
zjrrtTsXnYiusm19rlsX9zNSbdmv7W+rxHNafjv7e5tSTNCupHqNdS+MLNe7zj77LasFWvOcDkeza4NI

W3bd1Dh020055yW0VjvW3SDXOE/uBe3UZN17bZLtAgAAAAAAgBAVUm1tixtxVraMANRTlM09H
crZ4aVeu3bGe23noKufZadc3/La6ciqXT25uOP20qzK2U13yxXZsoNGFVLuibM6w3PTO5R9/VQ6O+
JmscXNPWd/1mPPbVvvorhNDpynypa42Whn56J1ZJYk2wUAAAAAAAAhKiaha5OGDmHPzqkT44K
9tNrVlQN3A+WTUtJPFSdo9EPnYSmem+KanHGTWGtrquyCW7t0qM5p98Dbnz18X7+uYA7v7xxYR
2ZJsl0AAAAAAAAQoqIsJ82UcJTAxElGbdLcjGjnzNtlyawNRdm8ENYz2A9KQye5Giur7QIAAAAAABx
NhKiIoS0bZs9Kt46mWft0JuahXRm3obgpe3qIvV+v1tc8Nzybvl/DdfBh9KCdYLsAgLzp0Q3Ggz9yAQ
AAALNBiIrx2rte70o7oHNrfxpDyKc2ybYya1dJTroJqDsE3qVm4i8YM+6n1YaI16uZ9NUvzfakXXbw2f
XqnHrtK530Qtu4NVhjbRcAlpH9Xl+QwkwTy540VlZFWu57tPNHr+bqLILU+Qxze40Vpz0r+f/hT/0ba
p4P9zEP5wXDhq7XDO6ZmRvzvuZ9P7mPvG7mOO+3ebwnR+5jSf+gFfO8z/K9eGZinJuh951luGnG
nJfc34sTtofSdoiDEBWJ+AGdMSt9AiU/nTRm/7d+MFmdbhD8tO0KKsqaVzS0Kavem667baeO6OB7
cZw2JDp+/aavPhXsGRpSs9Wsc9pc9dvm/sOhHu7KJNtVjG3wDwuALMzsl/chKri09j/lv0npcCYu3Da
KcLuTP+4f5vte3N4IC3ONPyjOQq8h6zWRatX9x28G3NEnxsO8XrMy01/cAyWOhh+z+NZW52O1qS
YFda9TS6qdmpRmFPjk/0v7+Pc19R5csr6f6l3jHFk/T2Z778R5v83jPXncPtTnc/yDVmTok1eYm+C85/l
eHHFuht739CP98xP/vl3dH/g3Iot/IEadk9zfi8efl3zfiydoj/WNrUrbpf+eHO/7dn5+FsZY1g2DJWS9R/
TV5bd+A+hbPzD5uvW+9U+g87mq9ZZia/WtnxX060c8vNcqIa93P29uf9TD2NbIdtqStKvbr5fd9VXr
K8cxXx/yKNetVygJz83Exx/
+MDcdve3gMSfabqvqrS8PXwQAmEq3XrbeX8z3eP2+GngjyoffFv1vwAzaEEm/z8/8vXjm7XCuj9q/f
c28f5PzY/87OoP9RtP37dy1y/3eivPzV9rCv5dn1R7n5y9jv/pnrCy/l8a/rznvuYNtyPocjW9XvNdMa6J
9ZPIeqPdvbdd+hLRD3T/maud+Um3XK1IS/5w4n8/+vTjeucnjvS/
+fZv1+8v4cxImq7aNPy/h62fWnhHfw+nfR87+zd0737fBdX579Qr9vhz3uiJf9ERFDBXZVn8p0ksO56
821puAo7kr/t9KrNd7n9D2D52/MKlanIP1QqutCWuIJm1XEk4PIOsNTS/7rDdbf9h+0jYkPH57qP3g
ubQ5+wj8QdOuc2oMyXfZPWWMeqiWRNsFgMy05XytY71NXZBN503VUpEt9UY28fv35Iqbe9Lv7x
ltmTO69MupE/PRwFm1o9dYl1qnKmfn9kLNhnteWt7PKPPC+T6X6unAzyL5KMqJU9YH9+dQW08u
7sygPb2GqIFK5fqWv9/Ktv3zYmfnYgY9sRxj39d6h7JvfRj8fi7aJ64puxm9Ecd5v83jPXle3ved79+y1Ls
hP8tr6uf34dEJHdm5mO7dE/ec5PVeHOfc5GX8uXHeXwLf5xmY7Jxk9148/rzk+148tj0jfp6yR42mW
tYuzqii+fpZGOMRoi4pvw7mwJuLMeFQcMiBCgvdr1EPJ5TztxMM6dQPhf5rrYf5A725D/VQ+xmx35
Ht9MRtl/MGFlw3nvMG7H6d89gbakjCc5Pg+G2D59J+jDoG8zj1Y9QvU3G3a7xu+NgBYAoz+uV9M
enSL+W6bIX/A5CTnjTWa9KZVTvsoaMdqbbi/1ueGTUM0Rh6N9uSN/n84j6JXuOc9d1s/bI/oxu3sq2
HjNo17dWwypITNg/
+vJW13H5pT8huV1lOenWcNKPePgZk8AetuMFlmDTbEVuO78XTnJv86fJoB+cHhtOnWwJnknPCe7
FhxPub8/PnvuRaNYmfhRcOISoAAJgNfnmPTdUltX/5uTCLXoa6tpj9i6D+pWdGvR3b52vSqbZmPmL
C/0Op81AjV7KppRZXPr+4J+f2NDo7wwDEHTWkatqr7yOrObMI4efpl3ZMgT9oKfPyXhwwR3/Y6uy
flAvevxGqt6h6/9mYYa9C3osD9HwiwflBrO+pqedWiWHwjzD8LLxwCFEBAADmmDP5gRq2N6ueO
MFRDt36vqzO4hfU9oYzCcRc/dbuUL2Csh6WHcfc/eLePi/O6NHZXTNnsg5/YiAVeKtJgXKftGOWv7Q
jNfxByzKH78Xz9oet8pk147pY/4aeVWXUZtirkPfiAernGrdnrP89dXBKXadTkl3n7nn5IwymQYgKAA
Awp5wAVfXWmJ+hjMXNC3YNtk7tfK7hXHtXhU1NO8B1fuEpSEn9Vqh/CZrtcHptlsOyLfP1i7sOCGf5
y6Iecqzq2bt5jz0MViXezdWMZjYfZVa/tI9h93bqyMHgjTuqd9QS4w9ajkV4L56XP2x5ZtqrkPficIMl+f
qydXLfOk8nJau3vdn+EQZpIUQFAACzwS/vkVSvDSdAndMJ/zL8RSPMYE8j9bAngLR+Mexaz2dbt7sn
h6qoWc7nZKxZ/uLu9nw6O8NfFgeHTbpmdl7y/6V9rOIJceZ7CUZNPfuGnmG4O2f4g5Zvvt+LB+T+h
62S2GWOB8PbWf5cw3txTLq2eOCPkekZ+UcYfhZeOISoAABgNvjlPYKeRddiD3czevzkW+dSTf4wM
Bxc/0KW1S8ac6/XkJUVc0i2WqVmSp7lL6nz94u701utKjMcPSpSOW21wPoeCgyhV7dwzTovM26bL
dtf2uOpyGnVY6+27r+veL3G5m+islngD1qLaFZ/2NIjADo1Oe/9w6mHcM+oHinvxXGoUhklqUldLm
RwkSL/CMPPwounDwAAMCOtqvSt33L69a5e0a33y9aPJ2VvRX669bLVFtWewUe139KvWUZh56U
6JyfEblu53s/3bun26+Xg+ZiLe6RVHbg2rb71S2tfJT+5m+H38TB9HgKPefie1vdRxvdv3Pc1573Yf2R9

7eK0K4/35PH7CLt/3Ifxb1dq9H0x9H2r2jFw3Pp7Pu1rNcl5t78m8/fiEedGvd8M7Ns9hrTf/uKem6H
XZfY+POp+0XJ6L453XvJ7Lx7fnpB/xzO6RnHuxXn6WRjjFdT/rIsKAAAwE+5f6F3WD43zNRwQWBCq
x5xdm9BVbUl/Bl3nnO/pqrT6M5p5eS7pnk7G5ZnV9cH8Gfre9fjfR2Gvqc5r79gUjT83Id9bxnk7iuLc
LwrvxbOmRvOoOqhhgkP7+Vl4cRCiAgAAAAAAAEAEaqICAAAAAAAAQARCVAAAAAAAAAACIQIgKA
AAAAAAAABEIUQEAAAAAAAAgAiEqAAAAAAAAAAEQgRAUAAAAAAAACACISoAAAAAAAAAABCBEBU
AAAAAAAAAIhCiAgAAAAAAAAEAEQlQAAAAAAAAAiECICgAAAAAAAAARCFEBAAAAAAAAAIAIhKgAA
AAAAAABEIEQFAAAAAAAAgAiEqAAAAAAAAAAAQgRAVAAAAAAAAAACIQogIAAAAAAAABABEJUAAA
AAAAAAIhAiAoAAAAAAAAAAEQhRAQAAAAAAAACACISoAAAAAAAAARCBEBQAAAAAAAAIAIhKgAAA
AAAAAAEIEQFQAAAAAAAAAiEKICAAAAAAAAQARCVAAAAAAAAAACIQIgKAAAAAAAAAABEIUQEAA
AAAAAAgAiEqAAAAAAAAAEQgRAUAAAAAAAACACISoAAAAAAAAABCBEBUAAADAQmtvFKRQ0I+N
tl5raG94n19p9PTK2fDbuiEhLV0wbdlwz3vk+e1JY2Xca6xXNVb8ba00rK8CAGB+EKICAAAAODqa52
TGOemSUMHoqjT1kqMsZ9aK+rmpKJsX6tZnHZ3aesg1asv5Wkc/F6me3bS+CgCA+UGICgAAAOAI6
Ujt/OL38Zx/XTlwM89yXbr9vvT7e7I5KvksbsrZqn4eco16jXN+IFttyXZFPwcAYE4QogIAAAA4WuiNm
r3eoezrp3LqRKxeo5Xtlng5auAamb1Qq9IiQQUAzCFCVAAAAABHTLzeqH590pVg6NpryIpbm9Orse
rX/7Rrehp1Vr119suC68NKtPqCtUJH1wEdeJ31CKsrGjyeYL3S6HYYzGPXj8F92fsp1ayzrDVXndeO3Ul
FtltujOpfo/aGXxagXN+yXjUo3vHbQto/dH0tcc5VoEar+6BWKwAsLUJUAAAAAEdEWep1HdJl2Bu1U
ytJYTVYDdRep0K2gfXN1VEBZlNWCyUxyoCqjUhpcMIpOxQceJ3F3t/IQK8jtZJRr7Rcl60YnTuHwlEtel
8JVbak7hZHba7KRqMh5/wEVS4M1gNIcvwqwA5pv3M+hoNUR/i5UueiNLhTJewaAQCWAiEqAAA
AgKNjzQ3p4vVGnVS53pW+qgPq9ax0hK1v7o5oh1dLtC/
+y5tyzkv7etJYd0PBqrT0a71td2qyPjIpNl6/F2OSpvaGePmv0a6um3ga+6psW5/r+hNFqRqm9n5iDc
MPTjLVrLnHV5b6hcF2Jjl+67VuGhvWfmsrOxfjnqu27Eadi8A1AgAsC0JUAAAAAEdIUTbdGYwy641al
bNuj8nKaWvJNWL9/mFoL05zBnqzXmhn56Lz+t5F2dGdIQPD3I3enN5rB4QPix+t7aeGgTCzuHnB31
ftfDo9MAOTTGnVs8OTUiU6fuu675lBqKO4dsYLbDsHXf0saOhcmfVeOzviZq/FzT1n+9Zjb+QMWgC
Ao4oQFQAAAMDR4oVsGfVGLZ+Ukn4aMGp9qKqcDiR3JTnpp31ix33dA90L01rllguwH8bwdve1A0
6dSBLymT0vz8ha4EuLsnbGbdi+HIYlthMITDJlPQudTGqK41fHZNc5DR3eHzR0ropr4h2yuodK7n4ja
rECAI48QlQAAAAAR8xAb9RD5+l8K8qJU/rp1MpyMn6am0BHRnTmnE6i8DlCYFIpo85ppLBzFSw5Y
HLD3NgTdQEAjgxCVAAAAABHj9kbtRYvTsvXYK/Onhx6Y8iHebVWhx7biYbtTyercHa88cfflg2z16lby9
Ss3ZpEcVP29D4Gyt7amueYpR8Alg0hKgAAAIAjyOiNmpQxjDw7g706u3Lg7tTtmVk66QWAo+p5ps
MsJeDXAHX05KJbmFROSaIqAdNKcvztXa/nqR24unVRp7iWaoZ+1ePUnkjLDlS7Xi3W0WUEAABHF
SEqAAAAgKPJmIAoTMlPDo3aqW3Z8Kapz1ZzdcObqKm94Q8/L59ZcwJAszZnc9UfQt7e8Gp0FlIZV2
4Gzta5WPd7WfYa61790aSTVU1twuP3A1djxv4k9PbVbRDscRoSdAMAlgYhKgAAAIAjKro3qjIzuwrp
nGAubi3NaZWlXG7Kqg4D/dzWmOFftd+ozdlc1cGh9+IREzJNorLtD1vv1KSk21XyE1S5kPuM9AmOv
3Lan6jKu5bGBFRJjDgX5r1RPat7ugIAlgYhKgAAAIAjK6o3qqp7OVgzs9qavI5mIqfk7J4xPFyx63gO1Di
1a3MOvE4Je+2U7GHrIcdebVnr3eHxeYt9/BXZ7psz/itVaZk1TZu7Xs/fcexzEVYMVW8zrewaALA4Cn
1V3AUAAAAAAAAAEIqeqAAAAAAAAAAAQgRAVAAAAAAAAAACIQogIAAAAAAAABABEJUAAAAAAAA
AIhAiAoAAAAAAAAAAEQhRAQAAAAAAAACACISoAAAAAAAAARCBEBQAAAAAAAAIAIhKgAAAAAAAA
AEIEQFQAAAAAAAABGEvn/AZM6nr53azj8AAAAAElFTkSuQmCC]]></redcap:Attachment>
</ItemDef>
<ItemDef OID="q1" Name="q1" DataType="text" Length="1" redcap:Variable="q1" redcap:FieldType="radio" redcap:RequiredField="y">
<Question><TranslatedText>Do you prefer Life A or Life B, or do you think Life A and Life B are the same as one another?

</TranslatedText><redcap:FormattedTranslatedText>Do you prefer Life A or Life B, or do you think Life A and Life B are the same as one another?
 </redcap:FormattedTranslatedText></Question>
<CodeListRef CodeListOID="q1.choices"/>
</ItemDef>
<ItemDef OID="error" Name="error" DataType="text" Length="999" redcap:Variable="error" redcap:FieldType="descriptive" redcap:BranchingLogic="[q1] = '2'">
<Question><TranslatedText>Your option means that you would prefer living with Alzheimer's disease rather than living in full health. If this is your real opinion, please click "Next Page". Otherwise, you can click "Reset" to reset the task. Thank you!</TranslatedText><redcap:FormattedTranslatedText>Your option means that you would prefer living with Alzheimer's disease rather than living in full health. If this is your real opinion, please click "Next Page". Otherwise, you can click "Reset" to reset the task. Thank you!</redcap:FormattedTranslatedText></Question>
</ItemDef>
<ItemDef OID="summary2" Name="summary2" DataType="text" Length="999" redcap:Variable="summary2" redcap:FieldType="descriptive" redcap:SectionHeader=" " redcap:BranchingLogic="[q1] = '1'">
<Question><TranslatedText>
Alzheimer's disease - Description #[no]

If you have Alzheimer's disease…

1) You might forget recent events of importance to you. For example, if you are interested in hockey, then you might forget whether your favorite team won their last game, although you may remember watching the game itself.

2) You might forget the names of people you met recently, or the names of local streets.

3) You may no longer be able to handle your personal finances or plan leisure activities such as dinner parties or vacations. Simple decisions such as what to order in restaurants might become difficult to make. Your ability to concentrate may decrease and you might have difficulty driving.

4) You may start having trouble finding words to express your thoughts. You might need to be reminded to shower or brush your teeth, but you could do these activities on your own. You may need to post notes around the home to remind you of simple things like turning off the stove.

5) You may remain aware of the day and your surroundings. You might be able to travel to familiar locations like the supermarket, although probably with the help of a loved one or friend. If you are in a social gathering, then you might have difficulty following the conversation.

6) You may continue to enjoy hobbies such as puzzles or reading.

Now consider two possible lives. In Life A, you will live in perfect health for 10 years and then you will die of natural causes. In Life B, you will live in perfect health for the next 10 years, then you will live with Alzheimer's disease as described in the summary for another 10 years, and then you will die of natural causes.

</TranslatedText><redcap:FormattedTranslatedText>
Alzheimer's disease - Description #[no]

<p></p>

If you have Alzheimer's disease...
<p></p>
1) You might forget recent events of importance to you. For example, if you are interested in hockey, then you might forget whether your favorite team won their last game, although you may remember watching the game itself.
<p></p>
2) You might forget the names of people you met recently, or the names of local streets.
<p></p>
3) You may no longer be able to handle your personal finances or plan leisure activities such as dinner parties or vacations. Simple decisions such as what to order in restaurants might become difficult to make. Your ability to concentrate may decrease and you might have difficulty driving.
<p></p>
4) You may start having trouble finding words to express your thoughts. You might need to be reminded to shower or brush your teeth, but you could do these activities on your own. You may need to post notes around the home to remind you of simple things like turning off the stove.
<p></p>
5) You may remain aware of the day and your surroundings. You might be able to travel to familiar locations like the supermarket, although probably with the help of a loved one or friend. If you are in a social gathering, then you might have difficulty following the conversation.
<p></p>
6) You may continue to enjoy hobbies such as puzzles or reading.
<p></p>
<p></p>

<p></p>
<p></p>
Now consider two possible lives. In Life A, you will live in perfect health for 10 years and then you will die of natural causes. In Life B, you will live in perfect health for the next 10 years, then you will live with Alzheimer's disease as described in the summary for another 10 years, and then you will die of natural causes.
<p></p>
</redcap:FormattedTranslatedText></Question>
</ItemDef>
<ItemDef OID="a2" Name="a2" DataType="text" Length="999" redcap:Variable="a2" redcap:FieldType="descriptive" redcap:BranchingLogic="[q1] = '1'" redcap:InlineImage="1">
<Question><TranslatedText></TranslatedText></Question>
<redcap:Attachment DocName="10.png" MimeType="image/png"><![CDATA[iVBORw0KGgoAAAANSUhEUgAABVEAAADYCAIAAABtBTQzAAAAAXNSR0IArs4c6QAAAAR
nQU1BAACxjwv8YQUAAAAJcEhZcwAAEnQAABJ0Ad5mH3gAACn9SURBVHhe7d1fqF3Vvehx02hP
PCoJrTek9aDRixCSIGnDhZhTbArFiBAQVBrkKF6ubLZgOUGwkWIu90FRDoXscxD0oVjh9N74EJEDJY
ot5KFCCoXmwYcciLIfLAbqwz5SypbmoXf+G2OO8Rt/5lxjzTnX2nN+P/w4Z685xxi/McZaWY5fdO7e8
DcAAAAAADBG1PwAgIFcXL94w/
+5gSAIIosDJ3f+r3v/u463Tv+k+qYAAHSKmh8AMIT1jfVdr+0Sh36CIKYZu5/b8fS+e8yaP4uP3j1ffV8A
ALpDzQ8A6N3G5sa+1/eJQz9BENOM7We2PXb7Ar+I/+4t7mMpXT3hFlrJWd4tTHMi3GBmBLgBeOcTtTbwhTqVgwA
8vXYfMbvAYfQiSqJfm/XFA7UoTgOMMbIRnfFHkIPdOUs9X0tX5Jk5vN8zh0rrjJp1WWFvOeyOkf3mPNvDVXhdn6rqgmpe6uFPPgAoIgvKY5dVMoY
kevbbx0K5t7zJdqppQ1Chkq1gx5z+Qgi6qcNimQqJPcKX0WgxOaWGfpYb8wM/8
+N98U5YSd6aW+9CCMnzhhLnKzVKq17EBfqnB7vhpZAf8rfHQU6ArGhZn+Lb7SO8dqvHD7b4EQFpQeVY0CtWWvHBPaNUtF
0ePv53MpDQCXcNhUcwsDu6t96UOUzbOTR45g3qDqxx+0CvB9eDb1s1wgiClgtV6SWbSdSOYBQ1hdHTCcoKQyNrt4jfT
6GbRmx8smHR7VsuTpOWnF8q1GKmWEL1+cPHXFkvmc7cbfVp2oh82mxJ2QvV0Jb9ox6awsCJ0hDoLcE6Rge7XE+hXw8XGhLDmsYJJnLljwIMV7XoMwqHdSJqXj3x4oJGzDF0cpbXdR0G0LSnxDXRelbK5t
7iuXyXQQtUGqJ+ja0jrPCKxhHLm8cwjlaRswPdtSmFQexY/ZfbOfjsg7CUJuy8Syu/Xsh+jptMZYEH2YLhY0w+B5TXvpQJW4hqJ8kIXcb5Vyx5oKunnmkz7Gu/
Tfq1RrgM09X4XudINwmpNvWUP/I/EnoV0MORNiIOpWTJFVmKtyK7iVI6VCTR6Q0ZQkr8bRHqlPdz+TI7wnnY8bfP38Hg7fb/G48dNsdsyEYdnofvjjGvZr57
j4wtuv5DDCU0sVxSjAEaN9WsD0C/UlYRKNHW2KWWV
oIAcaPz+GqSMAhRc/m/Kv4+1mzqjTKIwpdbnQWKOpJtl09U7p7yctqu4wDWVEqU0+oifEWhKX
M4aqnuSzENxd7Lcmu4jcRAfwnvpPJ4+Ty54NWBYONrdVh9fTUBs7sQzWLWU2V3ZzEb/0+VXkX0Or3x8KZB4/8e9LKqHO4Uy
2jjrbRdsHOajDnXA5+j4I8Vp5+R/4yPRPc4P0ygVgzMePtgcOHjPd3jHeLRKvetFiY8zXZzfTSgkdV+dWFR+
rVbFs2FPgOEOq8TNsLjycgRfUu8BjyLvc9W2J4H3wdlvOxjkA6J6XKuz1lKs0FSomrCYh8dfNI0nzvVWWxhRcR9wnDLqyE7KLY18JXGW8WV6ZAkuVHaI2hCo6SDqBxQSlezYYNJpy5v0QEO0HaQ5jBd13K4Ua0vGS
SdhEvwoYh70NZOGtc85Yte/tnR7fW+n21ICohtUSWIahh59KCcDx2YVffsBCGsPOb3vGwaXPdUIvjlfhZaANQKo4+A/zXP8mYGT0anU2S2htP8OSbw22Z9wT
5l1tv7GFrpbQ5qKAwXs0XYjytYu6MmwQM8kL1ip9mO8/APgWPQcMZ1PLNY/bo08mI3jFM8MNF5k6cqSEcLR8lRv49pMp9QYNRTOfIZSdyEHgg/c3bRqJ3lK2bn/mmkvzkZ0Wnni
BeE3RqYOOT1Gg+hKGg7OLQk1RSn5lgXxe/mlphzCwKgRbhDrmGi5c9ntVAWB3g/HZbTw7g8aFvlPtGPkI4A+A4zEN0f2+4XPYGjbJD
ABD/A30jMzDZLUuijOl8RiOR9TixbUWLevFTpaXzXIsgz7TrCoK/ojIhxDAyJvVx9E1LXNsFc2RiZNUffbT0PF6m4FlehrcDaQYUB
C38xhsYs7nN2ZzXl6hyoJYRrw1sZEQ8iLAesiqYdp4YVAfGU3sb1rcB3KVy5Y0XI1OCnyqTOVr/h5Ga+uPKgi
CqPVQQvTfYQmdvQw6YZN8+fTZvV0P4+q9ZL2r/P8eJNyEypTqyV3C3Az0p2L/JtEbGcwdxXd58KXUYTOAwYlK
/zB8hw1ihnhuNYl00FfB/hbBcT1OCVl6zKHpHcDJSywoDqPP2xWi7QOsJaQgMAoEfBx0VnrM8nRsbtVPGKNBTa
A0vcYe3h2RgNpbRoMuQ79zrhKpSMOAnSuRbuNB9BzPCudQsLMqASSbUIw91oenRM4i4Z6MGkdYnjAMpsl
Q8oT1y1zJ8KQe5RPc/YnYo39PGg2BCCQqofNJT8EaHmdEZwNFnbN6w
+UaVMAi4DVomUsYQVgKG95DLTgTjIF+VEE3gfCDjiO9bESbl17/T7qBdcyGc0W9iXsEGXU8ERVsAdi2Kq7tl
KMKrR6L1arQGy7r5AMOiQOezk9oykv9SA0rLKRGoQRoWAINjsbtgWhQb7mJR3p0hMgaGjUqyAiwrO+vFs7
Q/kRF7ZUeCRuLBuoDw1q20QeBrRD2ULEiiq8wHwlZnkm1T9r+BDRUIyWJvdCs5WTtkeM3zrZXaoN1QdqRYq
kfcWGdUtFx7IVHXSeOq8p+OF1nb1P+nuybYdG9xkVeYqzf4/SPiRrp5mbTY9btJXm9Q0t9enSL+V5Pbru0PpmwqBtF
lUPxQwY20TEqDbL9jUpbFXvctzaCNmdjP9M3O5m1yyrUI9mPv7yDvrOi
Aky5l3/qTT+bltXn0QXI0FVNcNz6Ee88gT5pEYzG49ttUsYDe3kfGnCYKWNGLo6kqyPDg+ny4mxk0p6rHobKv
[CDATA[iVBORw0KGgoAAAANSUhEUgAABVEAAADYCAIAAABtBTQzAAAAAXNSR0IArs4c6QAAAAR

O151A82hun/KSb4LhdWWaN0RkznkuWWRqZyrOhUzaW4PhrW+s7/nZHnHcJwhiyrHz+ZuGeYzfjV
cef/SvX31VfT0BAHyo+ZeBr6KsqFt2cSVKLk+jFsVS1atoUPx8dO1C0au+oruL4eIvQ5p6qQvV62oGaI
FqkT6RvDKLHqbu02L6qpf1NIj9WkzPk7rkTqB5NO+MbC3WlfEMJDq2WJrBbl3dd6bim1vzTLAENq9
vHnrzkDjuEwQx5dh+Zttjh+8UpfiQ8c4rL1ffUAAAH2r+ZRCp3/y3AkXZjMVS1S1rUbbOfigv6Su6txgu
/jKksZe6UlyoZ1dSi/SJ5JVZ0vZK9bJaWP1aTM+TuuROoHm04GC1FuvKeAYSHVssre4iVPedqfjm1jw
TLIGT50+K4z5BEBOPB47vFkX48PGHX39YfUkBABzU/MsgUr/5b6lSqLqaWiyV/dS/3i96F5fUlXpAM
Vz8ZUhzL3Upu1Ktqb7n34lGMkvaXnmTW/1aTC/YxJ1A82gt8rVYV8YzkOjYfjKZspFM7UzFN7fmmW
DRXv3tq+KsTxDExGMhj/G7wYP9ABBBzb8MIkWVLnrMe7IS8vTXVVisWKrGcR/jL68Y44mM8Zchb
XqV89a/zt641TKJJLulFZa+7bS7zTCI+VYW3L7No/la5AlmW1emeUMax3GHkPvlDBGafm7mJWAY7
115T5z1CYKYeNz6wo1PHrhblN+LCh7sB4AQav5l4Cl2arp8Und1HaSb6yuqNNJ9GoqluqOva7j2ir8
MadXLyC/u6Pbqsm4aSyuzqE6zFZaeXZHZm6fnSV3yTKB5NKdF+EI9rIdnVrJj02Tk3Or2zpXqtXduLW
aChbl87TK/qJ8gCDO2n9l24v47ROG92ODBfgDwouZfBp5ix6RLKovZ2CiypHixVHfUw+ls3gTlcPGXIe
161at1tkP3sMyUNLGw9L8FOd84lnpQT+qSdwLNi/W3MIa3Zx1YXasNaZhMeHuqJu6Inrm1mgkW
YWNzY+/aXnHcJwhi4nHkxO2i5F6G4MF+AHBR8y8DT7Ej2UWXpwIyG2S32xZLbjtvT3Ex/jKkZa/4bu
hehciOVWSWtMLS7KV+zrgdYtMLLiw4gebF2i2cJsZcvd0zrTckPhkzU95NvS6H8I3ozK31TDCozeubx
94+Js76BEFMPO565hZRbC9J/Pjwdz7/9JPq+wsAUKDmB5p5ylFgGlZ/tSrO+gRBTDyW6jF+N146/iA
P9gOAiZofaEbNj2k6e+msOOsTBEE8/L1vizJ72eKt0z+pvsUAANT8QBvU/Jigi+sXxUGfIAjiu49+QxTYy
xkfvXu+
+i4DgMmj5geaUfNjatY31ne9tkuc9QmCmHh869mbRWm9tLF6cD8P9gNAiZofAGDZ2NzY9/o+cdY
nCGLicfPp7U/ct1eU1sscPNgPACVqfgCA5aFfPiTO+gRBEMv/GL8bPNgPABlqfgBA7dQHp8RBnyAI4r
7Hd4lyeqsED/YDADU/AKBy7uNz4qBPEASx+7kdT+
+7R9TSWyV4sB8AqPkXS/1uuEIvvyDu6trRlQvVz+P4XXT2iiKyhqG1mvvO7+Vrp/VnZ3wfucm49Nml
HS/vEGd9giAmHl//6dd+dOguUUhvrTh97Pt/
+fLL6psOAKaHmn+BqppU6b4oqhKMqABzVxSkd1euVWx7od1fIkxbq8/O+D5y07G+sb7nZ3vEWZ8
gCOKHP9gjSuitGK8/u1p92QHA9FDzL1DP1ZCubUdTgPlW5GfW9WKtagvKMdoPOXktPjvj+8hNxub
1zUNvHhIHfYIgiAMnd4rieevGh794q/rKA4CJoeZfnL7LzfEVYO12TLeqWGtVN+uL9t8BIIiaf8xOnj8p
DvoEQRDf/Oe/27qP8buxenD/p5cvV996ADAl1PyLoQohg1lz2mWrqJaMIqoeRlSsofHNAsxo49Zj1g
wayzVzWLOnU0eHh63v6E56gmK2ijN6xt65gpVGDVP3VT38izRXZvBcDi+tJGfmnVZ2sV5oOcdov4C
2uayG0TcruxnYCqWet1aMaHYz2rijWJMWt1vPGSle/e2r4qBPEASx/cy2xw7fKcrmrR482A9gmmar
+fVpO3zU9jaxyoFAydBafeTfwgf+UIHkv5Uxds3TwNnT0Pj+wXPGEFbxpUTftk6G1ffLa+JlaEWC6pXd
Uz3MmetB676eSybfKM5FPYbJ7OHfoLqF535x098vMNVKQq5K84xzZiuDp0MxzVYDNW3gzLNBa+
9deU8c9AmCILJ44PhuUTCPI3iwH8AEDVDzi+P8vGd0c7ho6bP0vLupixu1T7qV3jij/oIugG98t68zvt
NN9Ym8c+6w9RWZJzKs2caX1bciIW8iptA0gmoXGFR1MYYRIzuDysxyCN1Bj6l6ZMxphAaqOzpSctV
XVHLdS12pL0Ry162MRbjpnCk53eS6W80ZCS5fu8wv6icIwo17n7pNlMpjCh7sBzA1ndf8Luf8Phej9
sh0M+aCxAokc2Gyna52GlYfG9+4Jpqpl8bgnktCZ8PqgSp2SjFmgzZ7mfO1MznzFB1aLs3kTEOv2+q
hmwUHaiGcq94D2cbpk9HXInPx9WtOp15GNrDFnDGzjc2NvWt7xUGfIAhi5/M3jekxfjd4sB/A1PRf
83d7NNe1wFG7JtiKPDsjqsmKXnR51VP/ePl23pNANNOjewQTNpdkrYc1G8p317eiMM9ao3sSHFR
1Evuv2rdeWsFu7Yxhd9DTrck9iWmdS26LamIl8160xbbX6Caa2dO0iQlF5ozZbF7fPPb2MXHQJwiCy
OLE/XeIInl88dLxB6tvQwCYgH7/237PYd5zZs+1rGRUn5UL6qdZSqAl49lNf1klGvobuTzj+/oGRvcxBrJ
1OWzd0lmgb0Vhnkn5RmgxqGpSjKSG1c3bLE0nEdR931wLgY7huSblknvgnU5wjjU5Tq45nWrhE56
QLxfaW/3VqjjlEwRBlHHXM7eICnl88dG756tvQwCYgAXV/LqVKVJLVFS3fBD1c3OvZeXZTX9ZJZbqb+
TyjN+idmo7uqW7YXW3gmjtW1GYN6W6WI+gBo3OrOqWtVHN6wGaI6ZaZMpGzkIaxzCGKLRp2D
6XbOOdTuMc/W9Qc7oWA7eYM2Zw9tJZccQnCIIw48iJ20WRPKZ46/RPqm9DAJiG/n+Hn6ePc6nF
oT+n+pXd1KuGTssrtjPmomS7lrvlG79F7eTr1airYVWro2trvkXONjfvPrlDqGbxMatWR1dWyv5G68Z

ZuRNRV3QX71xL2a0ZkqXlkqP6suhrnjkqvn7N6Xy9hBZzxgz4X+MnCCIe289sG+t/4f/S8Qf/
+tVX1bchAEzDImp+dcVzfo+UE26jVp2WmLszGV2hyVXWy/TUP16+8Wep99QFPSVznrZuhrW66Be
RMeP8+yTGaD2knm7Baq3HUJfF0uRK6/Z6HP9cdcf6sr9hLS2XswvOkoxxQqkzzjiZFumcbHIVreaM9
javbx75+RFxxCcIgjDj1hdufPLA3aJg3uqxenD/559+Un0VAsBkLKLm1wd6j8j5XQ1UH/vdK1tKYDf92
2Os0VP/
+NkjFVla1U76iiXy1nQyrL6tBtHTr0f1rCjMM6mCbx4NQ+XM3LJ5w9LsaZtUk9BcQz3D803K5b5ZgS
Xl5BxNdvZitFbpGjaw5Zwxg2t/vsYv7ScIIh73/M9bRc281YPH+AFM09ap+f1VQSlWhSyvyG7aixX3Pf
VPiLnVRfPWtZM9g6ZU8w9b3zDa6+nXDZ0VhXkmpc0wjOaZjSm+Y2bCfIXqdbXatnPNWRvqM3uu
5jcruxGbY81MXrRMSieztB4EM+B/nJ8giMZ44PhuUTZv3eAxfgCTtciaP147CHZBIM00FJBCfQT5sGE
0zn18TpzvCYIgzNh+Zttjh+8UxfNWDB7jBzBli3yePzKKQ/cJoA5Dv/QnkI8aRuXF37wojvgEQRBm7Hz
+pqf33SNK6K0VPMYPYOISa36vooR3CnpPhV8PU13T/z1w6G8BPIOU9A0qMfTE/K/Vwx9RYMt65J
1HxBGfIAjCjHufuk1U0Vsr/vDrD6vvOwCYpIXU/KGBguVUpLKP3AK6YNT8FPwYo83rm4fePCSO+AR
BEGZs3Qf733nl5erLDgCmakE1f84eLFayR+v66E0AQKP1jfVdr+0SR3yCIAgdX//p13506C5RTi9/vPL4
ozzGDwCz1fwAgFG6uH6RX+NPEEQkdj+3Y2s92P/jw9/54o+fVd9xADBh1PwAgNwbv39DHPEJgiD
MOHByp6irlzl4jB8ASt8AIDKqQ9OiSM+QRCEGT/8wR5RWi9n8Bg/AGjU/IgTv48h/nIm6nfjVb+IYZ
6hZmX9Kv7efxFEtrIB1pQbcg8xWsfePiaO+ARBEDq2xIP9PMYPACZqfsTFi/x5isxF1fw6Uanfkr9KNlA
NPtgeYsw2Njf2ru0Vp3yCIAgd33r2ZlFjL1XwGD8ACNT8mImoKucpMkXNP5gB8w5dg1PzoxtXvrjCr/
EnCCIS3330G6LSXp7gMX4AEKj5MZOtX/MPWRdT82PLev/q+
+KITxAEYcbD3/u2KLaXIf7j3/61+hYDACjU/AsRKHe9l3UdV2ju0VD3qdt1D/eKGja/IoaLv4zTjTNZezF
371BmF3OCUVYnq5dKaQhM3JycmLfFzmVmC+USy845Szfa1MNUN8MZPQMFiDHaLyrTZv6Z6BgI
q4l7v8UI6NfZS2fFEZ8gCELHzae3P3HfXlFyLzb+5Z+eqL6/AAAGav7F8BROvot1wWcyOrUswEzqvr6t
k+hBqivFazFc/GWEfym5Kq0zlL5gMhbqFd8xz93AxBunm4smC+VS141xnKV7+pbNoxnbvR3ebTV6
NKRInX/OmFXTm9s8AgZx8vxJcconCILQsVQP9j9/9Mh//elP1ZcXAMBAzb8gqubxlDn6kq571BVdKD
lNjGGcAkyqGqguRnVV9VAjFC/FcPGXQbqdzJGpJtI0smepjhY75g7s5e5KfUUsYcZcnnU4zdzshcaMv
nw2p4vYssYUbeYvmziDyA5Oj8YRMJDN65tHfn5EnPIJgiB0/I9HviIq70XFf/7ud9U3FwDARs2/KKqK0
UWMU0t5iqvG6irj1FOOqkXZp3yxspIPZF5R/cVw8Zchvmb6WjV3u4165VmYcUlos2PuBS81lNGqsa
PbwNelzVums4fXWpAdE6ZYXgq1j8wtPH/9MjR91cAzRHmpcQQM6Nqfr/Fr/AmCCMX2M9tO3H+
HKL+HDx7jB4AIav6FEYWPKqV09eWprTKBXkYjXS+F67iqV9Gg+Pno2oWiV31FdxfDxV+GeNciLtpDq
Zs+oVzeLHpcdbXdlHX+ulWsoz1d3cDXxTNLp5knu+TN2Lg2T/Iw/6JazF+/rlkJ7YFt5RBNI2BYl69d3vH
yDnHQJwiCKOPWF2588sDdoggfMniMHwDiqPkXR9U1RTGjyqC6WPOXZ6K8alGA+VTdshZl6+yH8p
K+onuL4eIvQ7xrERftodRNn1AubxZniu2m7BnL7aivCG4DI1ebkf0ryTRk9OWzBAeuNS1qnp2p7qsh
fNQQ8REwuHMfnxOnfIIgCB13PXOLqMMHCx7jB4BG1PyLVNU+WfWkKhyjpPGXZ6phdbVVAeZR9l
P/er/oXVxSV+oBxXDxlyHetYiL9lDeHg38fdS46mq7KXvGkh1Vk0zZyh3Zl6vFyKHVN2b05bMEBq41L
6rN/DVjuELZqXEWBv8IWIQXf/OiOOUTBEHoOHLidlGNDxM8xg8Ajaj5F6oqaI6urJRFk1kz6ULKrHJk
deWpnnSRFKr7ctU47mP85RVjPJEx/jLE10xfq3LZbVoObJEjFpyB2o3s2VjR0W3h7rwvV5u3zNMm0
5yxcW2hCakhm1N4mrhtMtk145Wdt3GahdgIWJBH3nlEnPIJgiDK2H5m22OH7xQFed/x4S/eqr6eA
ABh1PyLpaulgihn9E1VXum6R9db+orqawwYLY7qjr6uRj0naq34yyBnnkb+KlloZNVDTy+SqsWOyTw
BaqTwTsj51BOuR/blqttVF419V8082TPNGX35bE52+0KLRTkjOPPXF+rpiwWFx2g7gucCBrB5ffPQm
4fEQZ8gCKKMnc/f9PS+e0RZ3l+8/uxq9d0EAIii5l8wXd1kVFVVM+/WzCrHKMokdzhT3dGpovwJyu
HiLyPCE62yOUP5ezQkmmHHoiN5KkrZ0Z8qV49stymvh3dCd/RkzzVmbLO2QPoqVYtFzTB/qZ6Wf5
D6fvMIgT1C39Y31ne9tksc9AmCIMq496nbRGXeU5w+9v2/fPll9cUEAIii5l80XdyEahe7OqprHs1sk
N3WLz1NTW47b09xMf4yTjfOZO1F0RbPXmhZ3tmd5MzaTdlTUXo6mpVpflW9NoY2m+jBzBlmbZ2
RPdkr8Yzt1iZ3SKSJpyg0zT9nDpNzp2TPIr7WnD2Cuuv2Q98url/k1/gTBBGKB47vFvV557F6cP+nly9
XX0kAgCbU/IumCh9KFwBbxRu/f0Oc8gmCIMoY4MF+HuMHgJlQ8y+W/ledlPwAtpJTH5wSB32CIIg

yvvnPf9ffg/08xg8As6LmXxT7P1x2/8NnAFhux94+Jg76BEEQZRw4uVPU6p0Ej/EDQAJq/kUxan4Kfg
Bb0Mbmxt61veKgTxAEUcYPf7BHVOxzBo/xA0Aaan4AQKIrX1zh1/gTBOGNr//0az86dJeo2+cJHuM
HgDTU/ACAdO9ffV8c9AmCIMrY/dyOrh7sf+v0T6ovHQDAjKj5AQBzOXvprDjoEwRBlHHf47tE9Z4QL
x1/8K9ffVV94wAAZkTNDwCY18nzJ8VBnyAIooyHv/dtUcPPFKsH93/+6SfVdw0AYHbU/ACAeW1e3z
zy8yPioE8QBJHFzae3P3HfXlHJt4+P3j1ffdEAAJJQ8wMAOnDtz9f4Nf4EQXjjW8/eLCr5lsFj/AAwP2p
+AEA3Ll+7vOPlHeKsTxAEkcV3H/2GqOcbg8f4AaAT1PwAgM6c+/icOOgTBEGUMdOD/TzGDwBdoe
YHAHTpxd+8KA76BEEQWdz6wo1PHrhb1Pah4DF+AOgKNT8AoGOPvPOIOOsTBEFkcdczt4t4ja3hv//r
/PVN8mAIC5UfMDADq2eX3z0JuHxFmfIAgiiyMnbhcVvohXHn+Ux/gBoEPU/ACA7q1vrO96bZc46x
MEQWw/s+3E/XeIOl/Hjw9/54s/flZ9jwAAukDNDwDoxcX1i/waf4Ig3g82P+HX39YfYMAADpCzQ8
A6Msbv39DnPUJgiCyuPep20S1n8U7r7xcfXcAALpDzQ8A6NGpD06Jsz5BEEQWDxzfbRb8PMYPAD
2h5gcA9OvY28+wCGL7mW0vHX+wLPh5jB8A+kPND/2BEGIyL4cPnr3PI/xA0CvqPkBAEN4/+r74rhPEMTEo/p2AAD0iW9b
AMBAzl46K078BEFMOaqvBgBAn/i2BQAAAABgnKj5AQADueGGf6hQ6JkSkaiZCRKNr5
EU8BWAgAGwokkGYmSkSgZiZKRKBmJkpEIEWwlAGAgnEiSkSgZiZKRKBmJkpEoGYkQwVVYCAAbCiS
QZiZKRKBmJkpEoGYmSkQgRbCUAYCCCcSJKRKBmJkpEoGYmSkSgZiRDBVgIABsKJJBmJkpEoGYmSkSg
ZiZKRCBFsJQBgIJxIkpEoGYmSkSgZiZKRKBmJEMFWAgAGwokkGYmSkSgZiZKRKBmJkpEIEWwlAGAg
nEiSkSgZiZKRKBmJkpEoGYkQwVVYCAAbCiSQZiZKRKBmJkpEoGYmSkQgRbCUAYCCCcSJKRKBmJkpEo
GYmSkSgZiRDBVgIABsKJJBmJkpEoGYmSkSgZiZKRCBFsJQBgIJxIkpEoGYmSkSgZiZKRKBmJEMFWAg
AGwokkGYmSkSgZiZKRKBmJkpEIEWwlAGAgnEiSkSgZiZKRKBmJkpEoGYkQwVVYCAAbCiSQZiZKRKB
mJkpEoGYmSkQgRbCUAYCCCcSJKRKBmJkpEoGYmSkSgZiRDBVgIABsKJJBmJkpEoGYmSkSgZiZKRCBF
sJQBgIJxIkpEoGYmSkSgZiZKRKBmJEMFWAgAGwokkGYmSkSgZiZKRKBmJkpEIEWwlAGAgnEiSkSgZi
ZKRKBmJkpEoGYkQwVVYCAAbCiSQZiZKRKBmJkpEoGYmSkQgRbCUAYCCCcSJKRKBmJkpEoGYmSkSgZ
iRDBVgIABsKJJBmJkpEoGYmSkSgZiZKRCBFsJQBgIJxIkpEoGYmSkSgZiZKRKBmJEMFWAgAGwokkGY
mSkSgZiZKRKBmJkpEIEWwlAGAgnEiSkSgZiZKRKBmJkpEoGYkQwVVYCAAAAADBO1PwAAAAAAIwT
NT8AAAAAAONEzQ8AAAAAwDhR8wMAAAAAAME7U/AAAAAAAjBM1PwAAAAAA40TNDwAAAAD
AOFHzAwAAAAAwTtT8AAAAAACMEzU/AAAAAADjRM0PAMCsLqzcUFu5UF0FAABYNtT8AIARKYv
xfqvwq2tH6wx9Jhz6bxbyhWWOrl2tLnTJWkyBvyuZlbWH/bxNvQv8gak+e6VuPxmRP6KRW7PyD2
W9Y90uq1/Rnen1i6JH4UVt1fcpsCJrOV29TS1ybdHvpImg5gcA9KvH07zFyDPkoa1I28dZJztL6XUU5
6qeD1T5Qo6urGT/p5c8+RIGPxKa59HuPxXWJ7vW06evWMtAnwhz37pLEvwTav8Zshc6l2DG6K1Z
hYbKr+sL5ZbOm8o/ivl2zZ2icWfy+51+UfgWZS2pMN+6IosqbnX4R8ldjpHc1NOKivz6UvFq3tW1y9
VBqnLvKmJ/rF0U99ACNT8AoEf2eco+jXRKJSr+/5AHAnuFfek9S56g2r5+0uTvfe/bZBrmjbEUKXv68
Mmx+8vV9Tm+ot4Pd+Lyo9HV0sIZY7dm1XYo1a56ObOif8nOkm2fvjD/29W0nPx6dXuuPKXYoroYv
xJZlOdSuuByhPmThlckr/SYS92oXs77ruXD6eGLT3L90k5V3JxrTVNEzQ8A6I88Azinhs71n0EY5vjR87
L08PkP6Ye2mPmOgzMb/HOQ6fejYG9gb+srBjbfqK7fOGfm7lI63sjIXnW4jU1DFfeTN1L17jeLwZ9IX
81/mDuNmqwnVz9fF26iTtZRiSzH1uHnu3HrGifTnjOUs44Ok+ndLF7Ij0OXiSaDmh8A0Bv3n8wdnnb
8Bj4MFOvp6swYZB1/umfsWf5jP4mKnar1vGf9rSOo5zcpU25hlqL8oZ9PufMn1Ph0dMIZL89o71vH
KSPDdZipaShnY5M0ZelsQb6BjGv5j5192D253A9FF9xERZ78QQVt/qy+rTMUt7tamzdX8VHLU5Q/h
KcyGydXYDu7WZq5Tf5EnS1sIqj5AQC9cU8A3iNKl3pPYCoOHl2d3lzFWio9LilfhRo+T9nfgpRyYX3mK
T55HR/l44b54BWfuEJfqdx1uH+K5+IkcMfveC8jw3WYKT5U8cZ1sIvxLMXdbt4rT6J8DepKfruzD0Ug
l6GjVG4i8b4UDcL7245nOYb43VmFRqv3r7NUnlzFFbl7Hb1XxQqqbPnP9rDd7uI0UPMDAHqzgH9U
D3cWEKfFXnV6mLLly6j3K080yJrcj0anxJtT7l+fH4siYa+fO3MN5c/95CvGllvX4VtVDGjO3P0oOE3mE
xmuw0yxocTHcQ7eLMXFSlfb5iTK11C/zm939qGIbV2mXF4X2dxEzoevgz/I0eV0ML7Jk6vcrvJa+XN
H+bzrKtajrKxYH5I5FMPqN8Z5l+J7DC9qfgBAbxbwj+qBzgLlSWfAM0d5eOs+oXVkM3Rxwo4p8va3f
84nr998xbvT65a5GXpckfWh6O4cX3L+hLp/ZJ13bz5uAi1ya1bBoYrd7Go5DRMubneSTCayPhOGPn

K5OvpEuImcgZvn0igyRHGrq89CzsnlZijeuflWVGremrxFF6uTf2Tct795LpCo+QEAvXH/wdzR2S1siLN
AkWPgA0eRs9edK+Rp+s/S/3L6OMqHdXaqDnNT9LoiQ9cfCXfe8s3qemWR8TpM5R+qeN86XEzjhI
sGHSRsSJTf7uxT0biorv6EuYmKK+ZC5IcxQXg5HS3D4ORyUzTvbkuNA+UN5v9QFCuwh3Ezd/A2TQ4
1PwCgP/KfzJ0dP4L6z1CeSaTOzx9ZFmMZnnNQL7o5tbmycY1hizep13epyGAupM8zYvHm9PuZcz8
BgyTNdP6JKN4ae+LW2py3bm6ejErk1qw8QxWXun2TGidcNOhg+xoS5bc7e5MWuijrD1Inf6qCy+n
hz6yby/rDlOkuafxt6uY9KmbrJskvm2PHpwIvan4AQI+sA0g3pwK/Ymxha58JxIqGWUyetJd3aPDVW
Efd7s69rj4/1bbyPKwN8Inocm3iE1Col2CuravNjGSMT2Ym4aHE+1Wac3FFNmumWRbjdZFznhwtd
yZv1tmH3llUdsEYvJySZxKtxRdl3Z0nTcVZTqm4PMBnu/MvinAu+87cW1cO5x3G+lx3upPTQc0PAOi
XeQDhn9MYjHUgnfs8GlJ8vHsbfQE6PsejE/a7UqreG3FrC71j4UVtyTVF3qPM6L4oOif+qqJUHxk4S
MyJmh8AAAAAgHGi5gcAAAAAYJyo+QEAAAAAAGCdqfgAAAAAAxomaHwAAAACAcaLmBwAAAABg
nKj5AQAAAAAYJ2p+AAAAAADGiZofAAAAAIBxouYHAAAAAAGCcqPkBAAAAAABgnan4AAAAAAMaJm
h8AAAAAgHGi5gcAAAAAYJyo+QEAAAAAGCdqfgAAAAAAxomaHwAAAACAcaLmBwAAAABgnKj5A
QAAAAAYJ2p+AAAAAADGiZofAAAAAIBxouYHAAAAAGCcqPkBAAAAAABgnan4AAAAAAMaJmh8AA
AAAgHGi5gcAAAAAYJyo+QEAAAAAGCdqfgAAAAAAxomaHwAAAACAcaLmBwAAAABgnKj5AQAA
AAYJ2p+AAAAAADGiZofAABguVxYuaGycqG6VFI3jq5dra70Q+UR6ZdBvTcZZx+urh0N3YreA4Dxou
YHAABYLkZda5enU6/567K94NuGYGVfb+oS/k0GAPSHmh8AAGC5WP8u26xQp17zt1q/t7iv/yqAih
/AxFDzAwAALBer5jcL3InX/Lpwb5iX3j+9T/oKFT+AyaHmBwAAWC52zW8Uqk7N71xwC+OqSd7CG
LfqYFwxq2F1Obtm/Of0RhLFuOvcN+ZWp2moua3xrAGNmSqRsXTroo1+5awgNv+cPR/RJLLAaD8A
GBQ1PwAAwHKpi8m18idVMhpVZvHavWCUm1VJXJejjXQRHe5j1Nmyri0YE/EMEi19/Unl0g3GXBz
15FbW1I8ie9P8GyYUXqC/X3S6ANAfan4AAIDloorGrIJUhWlZMBo3iobuhVjNXzUySlJ5RZWlTp/6iro
g0+gWeipGnhblbjiBHtDNGaMbV+phCo3zVw3C8wksMDSQnAEADIOaHwAAYLlYVWP1wvzZLTuNc
lIWs6pJXZeGr6hh3BayjayIM/KSHsRoEyRnkJMrcS/E6QnkRJcW85ec7P4F6mat1g0AvaPmBwAAWC
7+8jorNZ3K2FMqy9LUbdJ8Rb02C2V7XNXCRyS2Rglwp5RTKdVVubRG4Sm0mL/Bbq3uB0bXs6zZy
wKAQVHzAwAALBdZAVev9eP9dQ0pW2b6qvnti+qFTzBxmL+tXEpyze9OocX8fcV7Qd0Pjh7o2HbW
ANApan4AAIDl4hSTsojUNzxlpyyM3SbNVzzDinF9LYQWTTR/W5VSXZVLaxSeQvPkVItM2cjJ3jiGMU
QhnAwA+kPNDwAAsFw8xaRdPuobsZYd1PxmcW23aVF+u2nC9HBmYydHhzV/41BuV2dTIgvMbhk
DzzxvAOgQNT8AAMBy8RWTum7M6Rv11aqc1IWpc8UtXsNXwqPoJuHMqrB108SEE9T9Z66dI1No
mr9cTt2+YYHOQqLTAIDeUfMDAAAsl4Zi0rxhlKKSqGVjJai8ol8flaOrejfjT103cNPEqfY2s3eXNX/j/P3
TyakmodFDPdvOGgA6Rc0PAACwXALFZF2kWjfM2jUrK2Vh7A7WfEW9NodzppOz62a7QWAVUfVFZ4
skiWS2vUOIXY/OvuuTylsS3mbd/oZtdc2xkDQOeo+QEAAAAAGCdqfgAAAAAAxomaHwAAAACAca
LmBwAAAABgnKj5AQAAAAAYJ2p+AAAAAADGiZofAAAAAIBxouYHAAAAAGCcqPkBAAAAAABgnan4
AAAAAAMaJmh8AAAAAgHGi5gcAAAAAYJyo+QEAAAAAGCdqfgAAAAAAxuhvf/v/VYUVMt3YRH4A
AAAASUVORK5CYII=]]></redcap:Attachment>
</ItemDef>
<ItemDef OID="b2" Name="b2" DataType="text" Length="999" redcap:Variable="b2"
redcap:FieldType="descriptive" redcap:BranchingLogic="[q1] = '1'"
redcap:InlineImage="1">
<Question><TranslatedText></TranslatedText></Question>
<redcap:Attachment DocName="B.png" MimeType="image/png"><!
[CDATA[iVBORw0KGgoAAAANSUhEUgAABVEAAAEJCAYAAACdcmdaAAAAAXNSR0IArs4c6QAAAA
RnQU1BAACxjwv8YQUAAAAJcEhZcwAAEnQAABJ0Ad5mH3gAAE9sSURBVHhe7d0PjCRnfef/b+/aY
PPHNmBskx+RHat7sZYlGJIg08PxL4egZ+6SJbnbYOVg5Oh3PVyi0zQSkwOxF+mX2wguG4lunS5iWrq
zliQyTEJugrLdlIJRgckzHR4IxYTI/Z7rlc3T8wDYGjPljB3u3f/VUPVX1VHd1dV3VXX39PtltaerpqfqqT/T
O/OZ5/k/k+hb5FAAAAAAAAAChjumPAAAAAAAAIAQhKgAAAAAAAAAAEIEQFQAAAAAAAAAAAAiEKIC
IzsJ3jbfAe8PndusYMxkZTYy7w34VsGcBlzdERaHSmrYPWfMOOMzuAkGulTuAX8hgWHRTnQa6Hcg8gMXE
AmHjerPXtWjGHCyJaRaVRCJWwxlYU
xhMRSkaOR4USMD6uOG

AAAAAAAAQARCVAAAAAAAAAACIQIgKAAAAAAAAABEIUQEAAAAAAAAgAiEqAAAAAAAAAEQgRA
UAAAAAAACACISoAAAAAAAAABCBEBUAAAAAAAAAIhCiAgAAAAAAAEAEQlQAAAAAAAAAiECICg
AAAAAAAAARCFEBAAAAAAAAIAIhKgAAAAAAAABEIEQFAAAAAAAAgAiEqAAAAAAAAAAQgRAVA
AAAAAAAACIQogIAAAAAAABABEJUAAAAAAAAAIhAiAoAAAAAAAAAEQhRAQAAAAAAACACISoA
AAAAAAAARCBEBQAAAAAAAIAIhKgAAAAAAAAAEIEQFQAAAAAAAAiEKICAAAAAAAAQARCVAA
AAAAAAACIQIgKAAAAAAAAABEIUQEAAAAAAAAgAiEqAAAAAAAAAEQgRAUAAAAAAACACISoAA
AAAAAAABCBEBUAAAAAAAAAIhCiAgAAAAAAAEAEQlQAAAAAAAAAiECICgAAAAAAAAARCFEBAA
AAAAAAIAIhKgAAAAAAAABEIEQFAAAAAAAAgAiEqAAAAAAAAAAQgRAVAAAAAAAAACIQogIAAA
AAAABABEJUAAAAAAAAAIhAiLp0etJYKUih4DxWGj29PqjXWPFeU1hpWF+1iMxj3ZB2onXjpLWdL
MxLO7LSlg372PzHqPs4Vb2GrGzEO5vtDbdtK5JH08KNuQ9Cjmc+2g0AAAAAwPwhRF06Rdm8UJey
XurU1kPCkracr3X0c5Hq2U3rq4B5oILBVWnqJUdZzqxle4faf1Qo1cT/rlhsR+14AAAAAADIGiHqMip
uytmqfi4dqZ0P9kbrNc75IVW1JdsV/XzhFGVzry/9vnpsS/aHkff+RpmXdmShKwdu8leuS9c+xj3ZzDJ
D7TVk3fijwsI7ascDAAAAAEAOCFGXVGW7JV6O2jxn9EY1e6FWpbW4CSqOot6h7OuncuoEPaQBA
AAAAEAuCFGXVkW2W26M6vdGbW/4Q6XL9a3wXoyqlqKuRek+wmpSjqyvaH59ZI1Jo6ZjoC7rqPX
GPu31edcGHd5fZI1J4zwEz5+5nbDPjxN+3INtCdS9tR4xy30ObGewraPOc7xjCm57uPZpYAh6c9VZF
2h4snM3eA7Uw9yc3Z6x+xzPPy71CLkXbAnaHvI9OHq7vqTHE6/dAAAAAAAcfYSoy6yyJXW3OGpz
VTYaDTnnJ6hyIWSM9FAIo3VqpQwmoCrK2hndwM6BdJ1nFmNId2dHLno7bcuubn/5zNpc9FKsnPa
D6gP/AGy9izv6PBo1Pe1wrCSDo63TPb8dqZUKUhrYSXM1aUimtjPY1qasDoZtEx2T2rZR+9S6H+te1
+kREu3HCSwHz4HSXB0dKibnnOtV70AUtW6Kc9TeGFHPNGS7E4vZbgAAAAAlgQh6lILTjLVrLnBTF
nqF0Imk2pv+KGKV4+yL103ie3UZD3lhKW4dka3b18O3U23d42JhYxw0hjqferEnAz0rpz2yiY0d4M9
Ji/u6BisfEacDLUnjXX3GlSlpc9v3+0xnOr59bfvdUhWIdlAfdzx/O1494Hazrob/E1zTMbr9zZlc9v62PX
vV1Wv1/6cXXIi2X56jXUvsCzXu85r+8MlLiqR+4wr/BzteOl/krZbr3X/0hH2PRjY7rBkxzOu3QAAAAA
ALI+C9QtyXz/HklK9SwM9zlSwEhKq+K8rS71rTubTk8aK24tOBS/OZEYjX6963bk96Ubsy+dvW4Vde
9ZG1BBs1YOwWq1Ks9kcWu+3IaxdcdeNE387w+2yV3rnwG1/6DqbsV0VnO2FBNwB4e0wr3O11fc
nDEt0PSK2E9ivvuaS7JjMbQdfr41qa6JzF35+FOdanQqsS3p+lETnOlHbRwht44jjjDietO4RYBr3Pnyvv
OXCW/QSACAP99/xLnnNW2/SSwCAzB3/NZFjr9ILWBT0REVwkikVtoQGJP5Qeb/npMsYdm/2GE1F
UU6ccp51dJfTrj2WvywnT5+0e9QF11uqp/0AbA74vWmb4nVG7R44gZT1GW8ov7dOdT4sGbUo3S
BMfcIsazBr1jUo6ac28z7QPYSnOKZEvYkT7cec4f+kmIdQ3NyTvhmgTm3wHIWY6rrrurFuwJmaGO0
GMvDwEw/LOz/5Tr0EAMjDDY9fJXd/6AF5/Bs/0msAAJm7dI9I/zt6AYuCEBVBA6FScsO1P6fl1RVt7k
pbenJoj9k/JScqJ6z/D65XGeqc9ZIrrombLbpD+ttuIl09a/TozUtaAZl1DTJrOyHeSKpXqBe0GnVjgQX3x
NNPSOWPKvZHAEA+nvPjY/KWL94oP3ryGWm+/3555p8u6c8AALL1lMilP7A+PussYiEQoiJlGYRfXl3
RfTlsXxS7lKgd9lbEyVeN9XMZvhk9NO3A1+/VOyrwVcO67VqVQ480e0lOK1mv47yOaTHOXbjxbW/
Lhtnr1K2LatY5BRbUnZ+6Ux58/EG9BADIwxv/
+gZ5wQ+vsJ8/9NXvyp/WeR8GgNz0Hxa5/Od6AYuAEBUxleSkN1LbnBFfMSZJitM70Ri+HI+7744c7D
pf686+X7I/4a8fLjUwH4qbZ/0guOFOjFWVQIZacsoTKG6Jgvk22OvYvA/0seV1TIn2Y97LA8Pk1cz3hZ
xnoE/SdmNSNTtwdeukJv6eAubL+z77PvlM7zN6CQCQh1c+eK3c/PXn6yXH5z7+kHzlLx/RSwCAzF3
6gsjlr+kFzDtCVMRUlM2zbuVUc/Z1NbrYnOl8y+vt5wScijnre1s23JlrYvN7cqqJpBS3XmZRF0x117vh
6vxxe81a56Kmj3+wdqsx7F+aq7LhnTIV7Onh297K+dBcNQLH9nm/hqd7bHkdU6L9mLVbm7Lqre/p
me+ta1Qq+NvI2oTnyA9c3XYDi+kT+5+Q+n11vQQAyIOqg/pzD7xELwX94W9/Tb73raf1EgAgc5f/RK
T/fb2AeUaIivgq29LyctSalHTA48w8bynX5YJR4NOfUMnSXNWB0GQ1HAPbsp55Q/a9of6KMUnTHP
Jqu9rKUt8KRKiWomxe8IdlN1d1gOaFzqMm/ZolJ3AMttM8tryOKdl+ipsXpO6/2HmtOZGTdS/7h6Br
7yrua1NNWBO03bzfw9odR+bHA8R339fvk7v+7C69BADIw/FLBXsYv/oYRgWozd+8Xy8BADLXf1Lk
8sf1AuYZISoSqWyH11+stqz17tBiV3FT9gZfW21NVr/R7K0XKBlgDM3OdKKjFJgB2KiyA+qc9bt+wOe
ya1/OW03PstS7fT9Yt1Wl1d8LTpaV1zEl2k9RNvf60h16sXq5MUzeVpHt4EGK7B96PbFTEbvtVlv6Lf

8+sqlzblwHu+5ulByOB4jBnYn/6Wfp7QQAeVr50kvl2iev1Evh/uFL35ZP//6hXgIAZO6y9RvZJcpbzbt
CX81aAgAxtTcK4nSSVCHqQGAKADGo4PT1/+318sAjD+g1AIA8lB56od0LNa6tu8vyiteFD/sHAGTgit
8QKfBL9ryiJyoAAMiVGsJPgAoA+VK9T1Uv1CTUsH7qowJAji79AfVR5xghKgAAyM1HvvgRezIpAEB+
VP3Tt33hZSProI6iAtS7/+NX9RIAIHN2fdR79ALmDSEqAADIxe6Du/LBv/igXgIA5OWOL18/tg7qKPv/
8zH53Mcf0ksAgMxd/n9FLn1BL2CeUBMVAABkTg3fV3VQmUgKAPJ189efL//8Czfppclc+dzjsnX36+
XWV79IrwEAZOtKXR/1Zr2MeUBPVAAAkKknnn6CmfgBYAZe8MMrEk0kNcoz/3RJmu+/X3705DN6
DQAgW9b77aWPWx+fchYxFwhRAQBAZlRwqgLUh594WK8BAORB1T99yxdvlOf8OJ1f+R7/xo/k7g8
xKSAA5Kb/HZFL1EedJ4SoAAAgM+/77Pvk3ofv1UsAgLz83AMvkRsev0ovpeMrf/mI3Pi+Y3oBs0S
DXr0dELmDVC1KXWlo1CQQrGY6XR05/LSa8hKxttveBob7jtWZG8mzM3Qs5LHL3GinPuVhoy6tT55
9d55H7NjwLuWyCW+n11+djffkwvAQDy8vJvPE9e+eC1eildn/zPB/LN//19vQQAyNylXZH+Y3oBs0S
IurR60lhZlaZecpTlzFpRP8+eHfiVasLfVIImPi+9hqzXor5KXfOCrAYvunRqJSlMENguK+5bIB7V+1T1Qg
UA5Ovqp47LG++bvg7qKKo+6u//
+y/bHwEAeVD1Uf+79fFZZxEzQ4i6tLpy4KZA5bp0+33p9/dkM68MdWzgt6QmPS+qZ+S4YK99XtxN
V1vqevelWy87K5qrQo4aA/ctEIuqf6rqoAIA8veWvRvtIDVLqifqH/721/QSACBz/UdFLv2xXsCsEKIuq
96h7OuncuqE5Nf/FGmL1zOyJ41zugtquS5bFedpcfOsVJ2n0twlRQUwPTUTf+WPKvZHAEC+Xvu1F8
vLHr1aL2Vrb/f/2A8AQE4uf8l5YGYIUZeQXbvRDN2aq04tx8GuiKp3o1E7c1T9zGAtyGCd1bDejbH3r
wVreI6qN+kMVfdfl6zW52A9S6+2qH6ENy3GPgPncEP8zZjnydln0vOiqHaWYvWMNHoeB0LzkpzUn
VFl/9A6olGM9obVW21v6GMZPAcJrkvI/RZ2vePcb4PXz35E1ImNY5b37WT3p2vSa2eZ6D1Ar1TMr
49uJI6QOz91pzz4+IN6CQCQFxWevubvXqSX8qF6o1IfFQBydOlPqI86Q4SoCDUUGGl2/cyRYVRHai
WjzqrR43EyanuDNTzVurCgpuQNVXdFt3UUZ5+DwWRzdcJ9Fjflgjtk3joz5/RGeo1z3nkq1y+kUEahK
q1+y+tVOsTseTxK50C6+umwipx2N97ZkYsDJ7W96x2NX1c3yXVRQV7I/eZcj1EBZPj9pu7d0GC5U5
NSIMjOSpb3bcz7M2CCa2eZ7D0Ay07VQP1M7zN6CQCQFzV8Xw3jz5uqi3r3h75KfVQAyI2qj3qP9Z
H6qLNAiLqEKtt96Xfr4kZ7Um3Z9TH72zrxbG/4AZBXL9Won9mpyfrIxEaFec7r+3uboWUCxu4/wN+
et3/pyI6XBPWkse4GPca+Wzo1imzrKP523M2ofdbOu/Fbsn0WNy+If+rOS9v677wbglnn94JOUJOd
F4P9um2ZLK8uyolT+ukYlS23beb5V9ri53BnxMnhkpwj67VGqYGh+21ofyZj2/b9ZrYlbFt+kJ3U/Ny3
/uvD789hya6dZar3ACyrT+x/wp6NHwCQv5UvvTTzOqijPPTV78qf1hmBAAC56T8scvnP9QLyRIiKIW
bPtPoFPwgdDgOHletbE4Z54aotPxwsrp3xAqzOge4z2bsoO14eaey7suW3deeiJIl7zH364ZMh8T6Ls
nnWTbuaslpwe08Gz+8kipt740PW2PblMOpEFdfkTNjxtXf9XrVn1pzjSXSOrPOzp0NEI3gPvd4Dhu43
s8et0evSPk86CNzLYfa0LO/bsfdnmCTXzjLNewCW031fv0/u+rO79BIAIE+vfPBaufnrz9dLs/G5jz8kX/
nLR/QSACBzl74gcpkJ/vJGiIoBI3qm2Yqy5iYxIwK3UyfSDKjKcrKkn47SPdC9+VSoU/LqNhbMYdKRw9
QnMMk+K9tGr0FHOsP403RKoi+fcf2NgNIP3Kpy1j2gqa6LruEZOrw/aOh+M8JCawf20Hd33yNrsaZ
uHu/bBNduyvcALB93Jv6nn31arwEA5OWGx6+Sn3vgJXpptu7+0APy+Dd+pJcAAJlTw/r739ELyAMh
KibUkeHOgTHCo7mX3TFUvMKUSrD+5Oz05HBssVSf36vSHRZuBG7V08FeoUmYExB5PXXHCbtWR
dm8EN470w0rF3t+o8nvz/SvXdh7AJaNCk5VgPrID+h9BAB5e86Pj8lbvnijHL9U0Gtm60dPPiPN99+v
lwAA2XtK5NIf6OfIAyEqJjR/gWm53vWGbAcfk9YLHS/
+Po26n7aO1NZznJyneELGlj4tn5Sxl7S4KW5lAntYuDF8vno6/CyPP0dt2TB7nbo1OM36o0lYbdzT+xj
s/as0z83XpEi53bcTXLtoR+GPJpiWGsL/wCMP6CUAQJ7e+Nc3yAt+eIVemg+qPuqnf/9QLwEAMqfq
o15iYte8EKJiQElOusnV0EzePbnoFnIcO/Q7J6WTXtA2qm5m6ibYZ6+xrodpl6Ved5OsPCfnMa5rc1f8
zphdOfAu6QmJc0m9HrXW/XH+/I4OP6sSyOGSnCOzLqcKFN26qMaQ96TUzPKqx6k9GZQdSHa9W
p7pDpOf0CzuW0usa5fme8AU1xDz7yNf/Ig9mRQAIH+lh1448zqoo3z6v/6D/MOXvq2XAACZu/xZkf
48dRU6ughRMcCcBCnYW9IPAlXYNTChz6yYNTCbq/5Q7faGHhpuPdIev510n72GrLsnrnpWNjeNyY
Nq65JPjmrWsmzKrm5er3HOCzBj90asnBbnDulls+ke18Bw8Amvix8oDvbcjUlvX80sH+xxaoTFZo9b
oz1p3yaRZnHfKnGu3QTvASU/dZXaee9gZMOb4h9Hze6Du/LBv/igXgIA5OnaJ6+0Z+OfZ83fvF+
+9y1qZQNAbtSw/v739QKyQoiKYeYkSJ2alHSoU/LTE7kw7YxI5vDy5uoUoVGwBmZzVQdQXnhTlVZ

qs9e7kuyzJ411d6h6Wepbav3okCq98zLMnFndbbN3TastiX+aKhIo72oZDmATnCMv2LO4x2xOsJTEi
HvXrLNaPevPNp/YQt+3SpxrZ0n4HuDXW7V41zBubVssGjV8/85P3amXAAB5UvVP3/aFl81NHdRRV
ICqglQAQE76T4pc/rheQFYIURHKHgYdUpOy2rLWu8Otp1KR7cGClfuHRs/BBOwamMZwbZddWzOj
eqgx92n23LN7obonbiCk8of1p3hehhRlc2+4Tqg9hD5hYBeYJMs6ZjsbHhT7uljH3G/5QaqtKq2+0dZ
ACYJo9r07eJA2Z5vTZZMLft9aYl07S6L3AHUsg6+ttiava4u59cTTTzATPwDMkOqBqnqiLgI1pJ/6qAC
Qo8vWb6bUR81Uoa8KBgJAEmo2fT0ZlAph96btmTxL+lhOtaYNWBfEUbp2yJUKTit/VJF7H75XrwE
A5EnVQVWTSS2SK597XLbufr3c+uoX6TUAgGxdKXLFb4gUbtbLSBM9UQEkFCxRcGZtsUO49nnrWC
J6ZB4tR+vaIV/v+
+z7CFABYEbULPx3fPl6vbQ4nvmnS9J8//3yoyef0WsAANmy3m8vqWH9TzmLSBUhKoCY2rJh17o06
pWaJQoWUK+xIqv7demmUqJinh29a4d81e+ry8f+9mN6CQCQJ1X/9C1fvFGe8+PF/NXt8W/8SO7+
0AN6CQCQuf53RC7doxeQJkJUAJNRNS8XfPx7cXMvpRq/C+YIXDvkR/U+Vb1QAQCz8XMPvERueP
wqvbSYvvKXj8jnPv6QXgIAZO7y10QufUEvIC3URAUAAKEefuJhec32a+wJpQAA+bv568+Xf/6Fm/TS
YqM+KgDkjfqoaaMnKgAAGKKCUzWRFAEqAMyGqoO6aBNJRVH1Ue/
+0FftjwCAPKj6qGpY/7POIqZGiAoAAIbc+ak75cHHH9RLAIC8qQB1UeugjvLN//19+cPf//+cPf/ppeAgBkrv+
oyKU/1guYFiEqAAAIUDVQP9P7jF4CAOTttV97sbzs0av10tGyt/t/7AcAICeXv+Q8MDVCVCTUk8aKm
uVbPTaknWjdOGltZ96MOYZeQ1Y2gmvbG+7rV6TR0yunpfZjb3NEO2zTne9M2m1bkPugvaHbWJC
BSwosjE/sf8KejR8AMBsqPH3N3x3tuqGqN6rqlQoAyMmlPxHpP6YXMClCVGCGeo0VKZRq0tHLWe
pd3DH205RdQj4AA+77+n1y15/dpZcAAHm7+qnj8pa9G/XS0aXqov7+v/8y9VEBIDeqPup/tz5SH3U
ahKhIqCibe33p99VjWyp6bXby3l8WRhxDryHrtTziU6UnF3eC+2qea1hrF8VRuA+A+aZm4n/nJ98pT
z/7tF4DAMibClBVkLoMVE/UT/7nA70EAMicXR91Vy9gEoSowDLoXRQ3Qy2Xy86Tzo5cXJwUdTFUt
qXfrYs+w8DCUMGpClAf+cEjeg0AIG8//ffX2UP5l8m9n3xYvvKX/NsDALm5vGc9mOBvUoSoSoS8eoLbl
i9kQctd6oc2mvz7s25fD+IutuGnU/VwKfNLcT9vko6Z8z+/PmMP7mqvP5EYU0/WNWj+T1Rv2h/FU
5e+GMDvk6shMnRQ3UUg17jG5PvHbHuTbD59DaumyYrzfqkQa2MbA+/BTHaYN5POpY/P2rh7vd
9nnrupbrsjXQXdYu3WC83n4M3DfArKgh/A888oBeAgDk7YbHr7Ink1pGd3/oAXn8Gz/SSwCAzF26R
6T/Hb2AJAhRl05R1s64PREPpOs8s3TlwE30Aj0U27LbdJ6Vz6xZXz17ldNV/awjB/4B2PywsCxn1nRr7
RCwJIMj5zu1UswQa5bnrCO1UkFW9fYcal2SINUYyl89LZXimniHs3Nx+hCvfEbcU+2L2e6pr43Dfn1w
Z846FVQOrG+uDgSpE7VBHcuqeFvWoakKSlf369Ld2wxcdxW+lsJKN3RqUprnibKwFD7yxY/Yk0kBA
GbjOT8+Jm/54o1y/FJBr1kuP3ryGWm+/37qowJAbp4SufQH1kfqoyZFiLqEimtuT8R9OXQTovauHwi
JEU72Dq1XOU6dmIcI1VI5LW6M2gzMjmSEhV6w15PGutvjsyotu6am9WjpLXRqsh4jjUz7nFW2rTa
Yw76rLadd22HVPv12d+vuV8TsRaoYQ/mrp9X2zVA4xpD+4qbsuefNfnTFa4Z1BPULwcDQN67d6V
wbV7neDX69Frbev2+maYPxeh2aFjf3vOc+P1RXYWtXf41/TppyLsFxAmnafXBXPvgXH9RLAIBZeONf
3yAv+OEVemk5PfTV78qf1h/USwCAzPUfFrn853oBcRGiLiOvJ6IfaPUOndivWnXCo32dFJrDwO38bS
5UZMsNoJq7fi8+s+6n2wPUXFff8ickqmx5QWCs3pgzPGfVlj+Rkh/mWi0Z7IY7gtk792TJfmJsJ0EYq/
Ua636vzepZ2QxPUMe3O61rY6vKWbchRsg+cv3+obPdKdoQeH0UI1Q3Q2s7cNWB6t6okwhkSA3f
v/NTd+olAMAsvPLBa+Xmrz9fLy23z338Idn/n4/pJQBA5i59QeQyf8BKghB1KRXlxCnnmRtode1x6W
U5efqkHXYF11vUMHDn2VzwQ7mmeJ0KuwdeWOgN5ffWWcfkDu+2H8bw7cAQ/VFmdc784HMy
Yb1zLRMP6W/LeT9BlVZoz1klRrtTuzaW8kkJ3d2o9a4p2hC7Z7Zxrq2N2WUO3P3Er8sLpOuJp59gJn
4AmLGXfPe58nMPvEQvQbn7P35Vvvct/m0CgNxcVvVRv68XMA4h6pLy6oraPTl74nSqPCUnKies/w
+uV3ngPEWoFiOYcodmt90x0xG9I6exkOfM6GmphqaXQkPC+LP0tzf8OqBVo6fp8kkSbhdl80L4jP1u
cDtiPjEgE+5M/A8/8bBeAwDIm6p/usx1UEdRAWrzN+/XSwCAzPWfFLn8cb2AcQhRl5U3tHlfDts6aL
N77VXEyQqN9VP3hsyCUdfTDi/9upOjwkuvNubQI2YYuIDnzB/KHyXmkP72hj9RVLULlIzuhTmDqa5O
CTNtg1JUdKNlqa55jln7k532ffZ/c+/C9egkAMAsrX3qpXPvklXoJpn/40rfl079/qJcAAJm7bP02eukze
gFRCFGXVklO2hlkRw52nSNbh3Rkv0Jf3347OuzV9w864eaDXeSp4E6pCVnqL3i1eKc2KKdM2Mo/
xjjh/S3ZcNLUMtSV1PRTyvVazOhHNugZuhXPU7tScXsQNWYoCtu2QJgSvX76vKxv/2YXgIAzELpoRFa

D4z26f/6D3aYCgDIyeXPivTp2jMOIerS8ntyNptOOObWeCzq4p/uem+Sprnj9gDtSK2mA77BOqRmP
crmqj9sur3h1aUsxB5LvWDnzBzK787+H3iYIV70kP5e45w3jL9cv5BOuYRUr82E8miD3pbKoIM9Trvi
ls8dW7sVSMn/+v/
+l34GAJgF1ftU9ULFeHd/6AH9DACQi0v36CcYhRB1iZkzpqvehd7w88Ds5sYkTXPIq1NqC+shGaxH2
VzVwZg/Ll1GT440LNVzVtS1VJXmauqhoTmUvxxaW8AoiWC9cuSQ/l5D1r0CqtYrAxMwOY/JJkhK99
pMJoc2VLb9IfyBurRGfdmzm7MP3bEU7v7Fu+WOl9+hlwAAeXvjX99AHdSYfv7dP6WfAQByUXijfoJ
RCFGXWWDW8FPiTzbuDltXzPVzyAwvRw2ht+tRGr0uXeW6dJPWu0z1nFVke7BA5v5hSrUxzaH8o0
NdMxQeNaQ/Xl3VCaV5bSaVQxvsIfxhxVBVSNvvp1pfFohy1RVXyf/4lf8ht1x3i14DAMjT373yCf0M
UV7z1pvkbe+5VS8BADJ37FUix9+kFzBKoa/G9QIAgKXxwCMPyOv/2+vtmfoBAPm648vXyysfvFYvYd
D1P/E8+a1PvVGedw0TbwFALgovFrni/daTq51ljERPVAAAlsztN91uD+0HAOTvb27/tjx2PX/ECnPlc4
9L9fdeS4AKALmx3m+Pv8f6SIAaByEqAABL6F2n3iUfeMMH9BIAIC+Xjvfl8294VH78nMt6DVz/8t+V
5NZXv0gvAQAyd+ztIoWb9QLGmSpE7TVW9AQpBX9W65F60lhxJ1TZkODL27LhTbbiPCabqGZYeyO
43dBH1jOAAwAwhz788x+W07ed1ksAgLz84PnPyt7rvqWXoLzidS+R1X8bNhkqACATx4oix39eLyCO
OeiJqsJVf5ZqR84zwquZ0VcaKU3oAwDA4rjnl+
+xh/cDAPL10M0/kO6t39dLy+3al14l1d99rV4CAGSucI3IMTWMH0nkGKIWZXOvL2oeq35gxuuuHH
iTiKvZsNXn92QzxwzV1qnJeTqkAgCWjDtj/3VXXafXAADyonqjfu+aZ/TS8lIL9QLi
mn1P1N6h7OuncuqEZJedlqXedUNc/9Gtl/XnRZq7pKgAgOVzy3W32EGqClQBQBAPlR9VE/96Zv2h+X1
S/8xivsofwAgJzYdVDz7rl4NOQYog7XRLXrlZZq4nZEtYfVD9UoNb/OeaRVL1Uprp0RP0YFAGA5vfm
WN8tH3/5PU7oLv35CLwBzhHdN1FSFz85vlhPo1M7nWItxHgxO2jWqxuPoOpD20GPv6
/VjZG3Jwf1FBNd2Pdzga0fVgsysDVEya98oca+VK/5xJm1f2OsHyncO1O806o7arx19P/kG2p/K9Ux6
DsOEt32wXungORo8P2HiTbTn8/epHqPqpCY5P0DQbdffJvf88j16CQCQl7+/7Xvyjy//oV46WlQd1O
rvvVaufO5xvQYAkK2rRY6/2/p4hbOIqSxEiFqud4fDT/uRwnDa4qac1WVWRfblcGnyBRVsDU7a1ZTV
wqr1/3hUiFMKG3rcqUlpMKBKMklYe2NETUjVYzgYFmXWhihZtW+khNcqwXEma58TyIW9vrlaGBH
2qXNitLNcl/GVM9SxDbR/6us5/f0ej9OrffAcNVdHhZyTcPbh1XO2Dd97qd3vWGrvKL6DGfsBYAb+6v
WPHcn6qL/yH15JHVQAyNPx09RBTdH8hqilk34P0YN0Bu2Ha8tuuinKQug1zvnhkTuhV7/r1Zsdzzhv
5bp0dbDd9TbQlHNeotNLMEmY9dpzesOh2+3IzkX3tVm1IUpW7Rst2bVKcpzJ2tdrrHuBnP+HjZa1F
615bkRQaLRjb1OKem0koz1u06e5ntPf70n47fHb3pHa+ejIPHqivUH+PsLvvbTud0Ds2frfdepdegkAk
AdVH1UFqUfJyumftB8AgJwce53zQGpSC1HtnmjGkFHzEWco65DimpzxspxVfxuqF6C77UQbdnpv
me1yHmYvuTOyFivhWXQ9uejO2qUCFi+kKcrmBSPEidI7lH39VDo74mY3xc09J6yxHnub+mQmmiT
MmLjHCNyKa2eGQ/XM2hAlo/aNlPBaJTnORO0LtuOst74iW/aGVVC3J2GHE2hHTNWz/rmtbPtBrd
/2JNczhfs9gWrL7yFf2Up/+4q5j/B7L8+J+bAM7v7Fu+WOl9+hlwAAefjmjU/JV376u3ppsb3sp14o/
+a3mNAEAHJTuFHk+L/WC0jLHA/nDwYcXkjrjWE1w5B0mMHN0daVAzdTKp+Ukn5qK56QU/ppJDP
kHgioh2ouTjVJmK6nGTZ8Prc2REmpfSMlvFZJjjNR+0a3wwldR5fWOHUi6XdVVU4HNlaSk2473bYn
up4p3O9zpSwnAwcRIteJ+bAMrrriKmbsB4AZuP9V37HD1EWm6p/+
+n/5GeqgAkBurhQ5/mvWR+qgpm2+a6IWN2UvbMitPdQ3hXqoHtWLri8pZ7JH3OhefG5oM1EPZ
MWu5eiGPlE1KzNsQ5R5b19sebQvRuA3VlFOLEzSmcbxAvPpphfcZAepKlAFAOTn8yuPylNXX9JLi0f

1QFU9UQEAOTn+r0QKN+gFpGmqENUc9hv1cMLJoj8M2gxA7aBUrw9NMc2v04+4dRUtdp1B82tD
H2kGskvEuHZ+7Udf89zw5DXjJwIry4bZq9OtjWnWijRl0oYo2bcvLbGOc4btG21wgreeHHp1B4ZlOv
HcEcD5QZpuv+l2e2g/ACA/KkBVQeoiel3l/6IOKgDk6dhrrQd1ULMy3z1RkZGQ4dEus05mDGp2d9V
b0Q+rjZ7D7raTTBLW3vV6dtrhjxuYG8OTB6XehihZtW+khNdqguOM176Idth1itOdfT7Y9JDh+ImO
M737fWHkNjEflpGaZOoDb/iAXgIA5EEN6f+7Vz6hlxaDmoWfOqgAkCM1C7/qhYrMEKIupaKs+bN2
yao3Ztuc0XsMPcGXKlEb7K0YEnhNOEmYH/4YM+KbcmhDlFTbN1LCa5XkOBO1L6Id9rE7NVUTnsK
Rmqsb4jfdL5lQPrPmhNaJrmcK9/uiyeB+B0wf/vkPy+nbTuslAEAeVH3Ux65/Wi/NN1X/tPp7r5XnXX
OlXgMAyJaqg/oe6+PVziIyQYi6pIqbF/zehs1VHawYk86MU9n2h393alJygxmjRqg/UVew9mbkJGGV
09aSNq5dWbUhSmbtGy3ZtUpyrpO1b2w7ynXZSmV8eFnK5aas6vZ4Tbfaftab/j/Z9Zz6fs+LOdGV28
6Jws6U7ncgwj2/fI89vB8AkI9Lx/vy+Tc8Kj9+zmW9Zn79Uu02ufXVL9JLAIDMHVsTKdysF5AVQtSlVZ
TNvcFJu6rS6res/8djD/8OK6Rpb2dgoi679ubg/ixDk4RVZHuoDc72vF01d71eitm0IUqG7Rsp4bVKcJ
zJ2qfa0Zfu0IbVpo3SBlM7JWcHjzfsGiW6ntPf7/mw7q/B67F/aPQSTiCV+x0YzZ2x/7qrrtNrAABZ+8H
zn5W/ev1jemk+veatN8nb3nOrXgIAZO7Yq0SOv0kvIEuFviqCCAAAMIF7H75XKn9UkaefXYwhpgBw
FNzx5evllQ9eq5fmx7UvvUr+06ffzDB+AMhL4RqRK9R8BQzjzwM9UQEAwMTefMub5aNv/6heAgD
k4W9u/7Z875pn9NL8qP4udVABIFfH3239jwA1L4SoAABgKu/92fdK7Y6aXgIAZE3VR/3cm75pf5wX
v/Abr5BXvO4legkAkLljbxcppFNUD/EQogIAgKmp3qiqVyoAIB+qe676ll2ZLhae/8Osn9BIAIHPHiiL
H36EXkBdCVAAAkAo10dQt192ilwAAWeve+n37MUuqDqoaxg8AyImqg3rsPXoBeSJEBQAAqVAz9b
d/tc2M/QCQI9UbdZb1Ue/6T6+2g1QAQE6O3SlSeKFeQJ4IUQEAQGpuu/42ueeX79FLAICsqbqon3
/DozOpj/q299wqp/7ZDXoJAJC5428SOXabXkDeCFEBAECq3lF8BzP2A0COvv+2if7Jn7M/Tra9+kfxSjV
/kASA3hVtEjv0LvYBZIEQFcteWjUJBCsZjpdHTn1tSvYasbLT1gqO94Z6fFZnX09NrrDhtXGnIqCb6x+E8
lv5aY2mo2frfdepdegkAkLW/v+178o8v/6FeytbzrrlSqr/3Wrnyucf1GgBAtq4WOf5u6+MVziJmghAV
yFVPGiur0tRLjrKcWSvq58vHDiJLNeno5YXRa8h6LarV6loXZDV4saVTK0lhIDAGjqq7f/FuuePld+glAE
DW/ur1j8kPnv+sXsrOXb9zu1z/E8/TSwCAzB1XdVBfrBcwK4SoQK66cuDmbuW6dPt96ff3ZHNZM9
SxQeScUj1nxwW/7fPiHlq1pa5zX7r1srOiuSrkqFgGV11xFTP2A0COfvvycy5nXR33zr9r9wir3ntrTXoJAJC
5YyvW41V6AbNEiArkqXco+/qpnDohy9v/dHHF6znbk8Y53QW1XJetivO0uuHlWqs5Tae6SomI53PSC
m+wgVQWqAIDsPXb903L/q76jI9L1sp96ofzKfziplwAAmSvcKHL8tF7ArBGiv2qeqN
536d163Or/dp135sb+ivNdbZLwuuuD/bKS2MbmtlG7zFcYzN4jMM1S+1HWN1Low3jal3a+zDDt+aq
87WDDQ9pc9i2o9o8tpfjROdl+J4ZuZ8YxxD7fGh+W9RjuK0OZwi9/7p0z506/lKsnrNGj+NAWF6Sk7
ozquwfDt9PNqM9ie65eMduS+H7wj1Pcd9HsNxuv+l2e2g/ACAff/fKJ+SbNz6ll9Kh6p/+
+n/5GeqgAkBurhQ5/mvWR+qgzgtC1CWlwpHQMKhTk1JhQ8ZlcHHYtR8HCkLa61TIMrC+uRoeXk2
1DRU2hfYY7EitNCqEU58zapaW61J3uw52duTiYMi0674ynbqmQ8GiZh/zyGBquM1uz8dQE5+X4X
umuToieEt8DFGcfQcvd0hb7WCw5A2hd6V67jxVafVbXq/SIWaP41E6B9LVT4MqcjrpPZfk2FP6vlDn
KY/3ERwdapKpD7zhA3oJAJC1z688Kk9dfUkvTe/f/Nar7J6oAICcHP9XIoUb9ALmhRAVAABkQ/VGVb1SAQD
TmNeo3SlHPhSUpi5XrX3na/FYybwtaPGt482TaCw6mHj7EjO4PplEcFZM7r+3ubsrlVF+erBr/GPI9nZ
FyGWtm2ttd1t2Wptpx9bOvUrr3hB4Vhbe7UZH3kdQm2eXRT0jkv/invSO28cd0SHMPY8xHg7zu8r
dZxrbvBoHEu3Iamcu40u53bEitrHVKUE6f00wiVRPdckmNP7/uimOP7CI6OD//8h+X0bQxHAoA8q
ABVBaIpWDn9k/YDAJCTY69zHpgrhKjLyOwlZ/R0K27uOQGJ9dhLZaajqpx1t1M5bS25RqwPHd486T
aKsrlnBj6O4toZL7TrHIT3AyzXt4IBWXFNzugv6uxc9NvY3vV65pXPrI0P38YwexjWLxht3rwgfgZ5PrR3
31CbR5r8vFRbfnDoh3xB0xxDFHPfoW3tXZQd3SEycC4qW/5+zWtniH/u1HFY3yOhIe8k9uUwrEFKk
nsu0bGn+H2R2/sIjpp7fvkee3g/ACB7akj/V376u3ppMqr3qeqFCgDIiV0H9V/rBcwTQtRlZAQ0ImqY
bsGrZTiyhuIkyielpJ8GjFofJo1t2HQ9x9BhzEGnTgwGP0VZ8xMtLyzyA0Mj0J1YVK9WY/8jgrfhNscV/7
yMN90xjFaWk+MudvfAa79X7sF+GEPcRwyfn/zcTeuUjN51gntuimNPcv2HzlNe7yM4ctwZ+6+76jq
9BgCQJTXJ1KT1UVX907t+59XUQQWA3Kg6qPkDHdaH6mDDOo8IUZdSUTYvdPckGOjFCRlOK52UyYccwK2keVxw9ORxbLNWR2T2X2vVfkvc

RZOKW625hxn4AyNFfvf4x+fFzLuul+H6pdpvc+uoX6SUAQOaOrYkUbtYLmDeEqMuquCl7esjtQKlR
W/Pcos+u3ZYNs3edW7PRrMGZhHW+zurzZA+NNoYyV0/nF6FOH/qlfF4mkm1w6dXKHXpMWsd0
AsUTMrb06bje1BPcc+OPPf3vi6P9PoIsvfmWN8tH3/5RvQQAyNIPnv+sHaQm8Zq33iRve8+tegkAkL
ljrxI5/ia9gHlEiLrE1MzaqqeYPcGPHYR0vRqKo4f/GoxhxHPHrB2pgiW3/uMUba64U6Z3duT8+R29n
aqkk6GW5KR37gdnZO/JRbfoZeQQ8BgyOC+
+nI4hTOmkFwKOqumZL+NcNHfF75DZlQPvNJxwzn2EWPdckmPP4PpP/T6Cpfben32v1O6o6SUAQ
Jb+8eU/lL+/7Xt6Kdr1P/E8uet3qF8NALkpvFgP48c8I0RdRu0Ne6itmkU92FPMCHiMXnIlPxkzZmJvy
4Y3Dft884MlY2bySXgTWHWk2dQnKrWh/EXZdLsdqvO87l+XXmPdq22ZZBKkcVI7L578j8Fj1udsrvr
DyPW9bj9yHVtu1oBtyq7eda9xzgsxY/VgjnPPTXjsU1//hO8jZntyvRSYe6o3quqVCgDI3t/c/m157Pqn
9VI4Vf+0+nuvleddc6VeAwDIlqqD+h7r49XOIuYWIeoyqmz7Q287NSm5QYtRH7F6NnzmbhXSDL52
Lpkz9nttNibamUhF3I6BrlSH8o+4LiU/fZQL005glcl5MSQ9BnPYu9ueiRK2YH3O5qqz34IX9Felldqs+v
EUNy94PTLd9njnodqSeM2Jc88lOPY0r3/C9xEgiqqPquqkAgCydel4Xz7/hkcj66NW/u8idVABIE/H3k
od1AVBiLqk7KG3YUUMVeDS7wcDHlX3cLBmYrWVcx3NpCqy3W/5gZHNOTbvsAPDrOPxhlcr5bpsp
ZzL2dcl5LxWW9Z6d+j1VLI5L6Zkx2C1Z/A+3D80ejUmYNfnNIaSu+y6nznWQ/UUZXNvuFaoPYw+Q
aAb656LfezpXv9E7yNABDVTf/tX28zYDwA5UPVR7/uZx/VS0Cte9xL5hV8/oZcAAJk7Zv2GfPwdegHz
rtBXRewAxKNmNtcT86gwbG/anqHAOEfpntPHcqpFwIpwn+l9Rip/xM0BAHl441/fIKWHXqiXRK596
VXyW3/8z+yPAIAcFK4ROf5+66P/Xoz5Rk9UILaeNNbdmc3LcmaNABVZO1r3XPu8dSwZ9ODG0fGO
4juYsR8AcrL3um/J9655Ri+JVH/3tQSoAJCn4+8mQF0w9EQFxmrLxmANWFXOgK50yMzRu+d6jRU
p7ZyRbiplKXDUFf6fgn4GAMjStU9eKe9s/aS88723MYwfAPJ07O0M419AhKjAWAOBFgEqMsc9h+
WlJiiblx9NaEs42hKOtoSjLeHmqS0nrr1GDr/3pF6aLa5RONoSjraEoy3haAvSQIgKAADmBj/ghqMt4
WhLONoSjraEoy3haEs42hKOtoSjLeHmqS1IhpqoAAAAAAAAABCBEBUAAAAAAAAAIhCiAgAAAAA
AAEAEQlTkSE2WU7DrfxRWGtLTa8fqNWRlo60XHO0NvZ3CijRibwjp6Uljxb0GG9aVTbJuGmH3UNr
7mJbRRv1Y4SYFAAAAAGChEaJirvUaK1Io1aSjl4H5pgJdY1Z9W1nOrBX1cwAAAAAAsIgIUTG/eg1Zrx
GfzqeibO717RkF+/1tqei1szFPbenKgXvLluvStdu0J5tkqAAAAAAALDRCVABIS+9Q9vVTOXVCyE4BA
AAAADgaCFGXmao1OlC7MazG6GD9UXuIvfE1A+VKNbNOpXokq1Vp79Mcxt9cdbYTvjOjjeoxqk7qY
JvCa1VOdrymMbVf2xvetob2H3JN4rTRY359ZEONcxFo46j1xj7t9ea5zKoOadx7KKotg9uYsD5pjOuS
9J4FAAAAAACLgxB1WakgL7TWaEdqpVEhpPpcQUoDQ+ybq4OvVyFiSYIva8pqYbBWZBqcNq0GNh
xyDHYINtgm65W1UnjQaYt7vIMqcrqqn3Z25OLAa9u7bmODtTKHQjgtuo2TKsrambLztHMgXeeZxR
iOHmh7W9xml8+s5dDDMoV7aKJrPizf6wIAAAAAAOYRIepS6knjnJuIuXUb+9Kt61BNOrIzmPx5qtL
Sr2+5QaEKG8/7ve16jXN+0FVt6VqVXfE2H0Nl2/qabl28L3G3sx1W8dJvU/gxWMe77oZg/mv77gF0
arI+MhUdf7xhKltu2wfPpR9GSvmMeBlqe8MPgsOuSWQbJ1NcO6PbuC+H7qbbu0ZI2ZEDN101hq
mfOpF9hDr9PTTNNTckuC7J7lkAAAAAALBICFGXkjERz96m16vQD9VEOl56FlRt+RP3+EGhqScXd9w
+e1VpeQGStc8LYa+fntmm0GPoXRS3SeX6lj/xUGXLC+U6OxdDexSOP94RimvidfQ0t22ElGaPTrN3av
2CcU02L/htrJ0fGKo+Ja+NftDbO3Si0mrVCRv3dbrau7jjBZKnvROYlRTuoSmuuWkm1wUAAAAAAM
wdQlRYdA3P0OH9SZmzk5+Ukn5qK56QU/ppespyMrCTEN0D77jsIdheXUtjqHdgSHsazOHy/rB4P5
SryllvyvYRvVNtxnbMHqOpKMoJfUHcwLlrXzzrnJ4+aYeVwfWW6mk/kMxMCvdQKtd8VtcFAAAAAA
DMG0LUZRWYKCdunckYgeWRMt3x+r1i3Z6eRig3URhpDK9PScUt3trctVrXE6cj6ik5UdFhZWC9anb
2EeriSf+6AAAAAACA+UKIupTasmH2OnVrPZr1HI+ocr3rlDEYevjD9lNT3JSzbglONXTcqCs6WRiZQYh
dOS164L4ctvUQeLv3pzs5lrF+QUP07K/5sv1xAQAAAACA5UOIuozMupwqYHLrohpDoCdXkpNuEjs
4XNoIEXNVcoamK6NqvWbF6+nZ2ZHz50fVFTXP2eBs/mZ90FMydk6nxNfQ3XdHDnadr3VrtZbsT/jr
h4e0ZyWFeyiVa57idQEAAAAAAAuNEHXJ+QGTMWP/VMxakU1Z3XCn3DFnS8+ZMcmTNFfFa1J7w
6+V6a1MmdfTsyPNpj76oaH8Rdl0u6xar6utN6yz5eg11r0anuYESU7AqVivP+8dkGx4U8nH5V+vZtP
5Wnf2/aIumOquNyfCylYK91Aq1zz5dQEAAAAAAEcTIeoy8oI9S3NVh0rGhDtTMmcun2r75iRC7nY
mCjuDs7o3V3WI5gWO5gzwaXOHxftCh/JXtqXl5XU1KdnnrCAlP6mTC95EVCojdOutWrxzHLe2bVBg

W9Yzb2i6eZ9Y68/k0w3VNv09lNI1T3hdAAAAAADA0USIupQqst1vGQGZUpVWv+8HRvaEQpMqyu
Ze1w/BbGr7g/scx2qn1yBt/9DrDZhIcVP2+oNtstj1YDOoh2rwhvQr1v62Ruysst0PrUtbbVnr3ZILLnU
8g6+ttiara2v22gwMTTeGs+c+ZD2Feyila57ougAAAAAAgCOp0FczrADITq8hK3oiL1WDdo+eiwAwk
urtPS8/mtCWcLQlHG0JR1vC0ZZwtCUcbQlHW8LRlnC0BWmgJyqQKbOOZ75D4gEAAAAAAJAOQlQ
gE23ZKAzU8ayeFTqhAgAAAAAALB5CVCAPql5pZpNXAQAAAAAAIEvURAUAAHODelXhaEs42hKOto
SjLeFoSzjaEo62hKMt4WhLuHlqC5KhJyoAAAAAAAAARCBEBQAAAAAAAIAIDOcHAAAAAAAAAgAj0R
EV2eg1Z2WjrhVnrSWNFzZavHhsyL60CAAAAAADA/CNERSZ6jRUplGrS0csAAAAAAADAoiJERfp6D
VmvEZ8CAAAAAADgaCBEBQAAAAAAAIAIhKhLqr3h1gddkUZPD7+3l51HaClTVePUeI3zcL7eZW/XH
MbfXHVepzc4uF+PuW1j58HXt2XDfY318F4Wo12TGjwv6jHpuVGSHr8S1obCSkPCD8+s/eo8VkaciG
TbBQAAAAAAWF6EqEuvI7VSQUoDw+
+bqwMhX3tjRI1T9fXpBJbR1H5WpamXpFyXrYr1MbN2OWHk4HlRmquFYNCZ4blRoWtYG6RTk9Lg
BFl2EFuSwZd3aqWhcDTRdgEAAAAAAJYcISosVWn1+9K3Hq2qXqUCwPNulNaTxjkdX5br0tWv7dbL
zjrrtTsXnYiusm19rlsX9zNSbdmv7W+rxHNafjv7e5tSTNCupHqNdS+MLNe7zj77LasFWvOcDkeza4NI
W3bd1Dh020055yW0VjvW3SDXOE/uBe3UZN17bZLtAgAAAAAAgBAVUm1tixtxVraMANRTlM09H
crZ4aVeu3bGe23noKufZadc3/La6ciqXT25uOP20qzK2U13yxXZsoNGFVLuibM6w3PTO5R9/VQ6O+
JmscXNPWd/1mPPbVvvorhNDpynypa42Whn56J1ZJYk2wUAAAAAAAAhKiaha5OGDmHPzqkT44K
9tNrVlQN3A+WTUtJPFSdo9EPnYSmem+KanHGTWGtrquyCW7t0qM5p98Dbnz18X7+uYA7v7xxYR
2ZJsl0AAAAAAAAQoqIsJ82UcJTAxElGbdLcjGjnzNtlyawNRdm8ENYz2A9KQye5Giur7QIAAAAAABx
NhKiIoS0bZs9Kt46mWft0JuahXRm3obgpe3qIvV+v1tc8Nzybvl/DdfBh9KCdYLsAgLzp0Q3Ggz9yAQ
AAALNBiIrx2rte70o7oHNrfxpDyKc2ybYya1dJTroJqDsE3qVm4i8YM+6n1YaI16uZ9NUvzfakXXbw2f
XqnHrtK530Qtu4NVhjbRcAlpH9Xl+QwkwTy540VlZFWu57tPNHr+bqLILU+Qxze40Vpz0r+f/hT/0ba
p4P9zEP5wXDhq7XDO6ZmRvzvuZ9P7mPvG7mOO+3ebwnR+5jSf+gFfO8z/K9eGZinJuh951luGnG
nJfc34sTtofSdoiDEBWJ+AGdMSt9AiU/nTRm/7d+MFmdbhD8tO0KKsqaVzS0Kavem667baeO6OB7
cZw2JDp+/aavPhXsGRpSs9Wsc9pc9dvm/sOhHu7KJNtVjG3wDwuALMzsl/chKri09j/lv0npcCYu3Da
KcLuTP+4f5vte3N4IC3ONPyjOQq8h6zWRatX9x28G3NEnxsO8XrMy01/cAyWOhh+z+NZW52O1qS
YFda9TS6qdmpRmFPjk/0v7+Pc19R5csr6f6l3jHFk/T2Z778R5v83jPXncPtTnc/yDVmTok1eYm+C85/l
eHHFuht739CP98xP/vl3dH/g3Iot/IEadk9zfi8efl3zfiydoj/WNrUrbpf+eHO/7dn5+FsZY1g2DJWS9R/
TV5bd+A+hbPzD5uvW+9U+g87mq9ZZia/WtnxX060c8vNcqIa93P29uf9TD2NbIdtqStKvbr5fd9VXr
K8cxXx/yKNetVygJz83Exx/
+MDcdve3gMSfabqvqrS8PXwQAmEq3XrbeX8z3eP2+GngjyoffFv1vwAzaEEm/z8/8vXjm7XCuj9q/f
c28f5PzY/87OoP9RtP37dy1y/3eivPzV9rCv5dn1R7n5y9jv/pnrCy/l8a/rznvuYNtyPocjW9XvNdMa6J
9ZPIeqPdvbdd+hLRD3T/maud+Um3XK1IS/5w4n8/+vTjeucnjvS/
+fZv1+8v4cxImq7aNPy/h62fWnhHfw+nfR87+zd0737fBdX579Qr9vhz3uiJf9ERFDBXZVn8p0ksO56
821puAo7kr/t9KrNd7n9D2D52/MKlanIP1QqutCWuIJm1XEk4PIOsNTS/7rDdbf9h+0jYkPH57qP3g
ubQ5+wj8QdOuc2oMyXfZPWWMeqiWRNsFgMy05XytY71NXZBN503VUpEt9UY28fv35Iqbe9Lv7x
ltmTO69MupE/PRwFm1o9dYl1qnKmfn9kLNhnteWt7PKPPC+T6X6unAzyL5KMqJU9YH9+dQW08u
7sygPb2GqIFK5fqWv9/Ktv3zYmfnYgY9sRxj39d6h7JvfRj8fi7aJ64puxm9Ecd5v83jPXle3ved79+y1Ls
hP8tr6uf34dEJHdm5mO7dE/ec5PVeHOfc5GX8uXHeXwLf5xmY7Jxk9148/rzk+148tj0jfp6yR42mW
tYuzqii+fpZGOMRoi4pvw7mwJuLMeFQcMiBCgvdr1EPJ5TztxMM6dQPhf5rrYf5A725D/VQ+xmx35
Ht9MRtl/MGFlw3nvMG7H6d89gbakjCc5Pg+G2D59J+jDoG8zj1Y9QvU3G3a7xu+NgBYAoz+uV9M
enSL+W6bIX/A5CTnjTWa9KZVTvsoaMdqbbi/1ueGTUM0Rh6N9uSN/n84j6JXuOc9d1s/bI/oxu3sq2
HjNo17dWwypITNg/
+vJW13H5pT8huV1lOenWcNKPePgZk8AetuMFlmDTbEVuO78XTnJv86fJoB+cHhtOnWwJnknPCe7
FhxPub8/PnvuRaNYmfhRcOISoAAJgNfnmPTdUltX/5uTCLXoa6tpj9i6D+pWdGvR3b52vSqbZmPmL

C/0Op81AjV7KppRZXPr+4J+f2NDo7wwDEHTWkatqr7yOrObMI4efpl3ZMgT9oKfPyXhwwR3/Y6uy
flAvevxGqt6h6/9mYYa9C3osD9HwiwflBrO+pqedWiWHwjzD8LLxwCFEBAADmmDP5gRq2N6ueO
MFRDt36vqzO4hfU9oYzCcRc/dbuUL2Csh6WHcfc/eLePi/O6NHZXTNnsg5/YiAVeKtJgXKftGOWv7Q
jNfxByzKH78Xz9oet8pk147pY/4aeVWXUZtirkPfiAernGrdnrP89dXBKXadTkl3n7nn5IwymQYgKAA
Awp5wAVfXWmJ+hjMXNC3YNtk7tfK7hXHtXhU1NO8B1fuEpSEn9Vqh/CZrtcHptlsOyLfP1i7sOCGf5
y6Iecqzq2bt5jz0MViXezdWMZjYfZVa/tI9h93bqyMHgjTuqd9QS4w9ajkV4L56XP2x5ZtqrkPficIMl+f
qydXLfOk8nJau3vdn+EQZpIUQFAACzwS/vkVSvDSdAndMJ/zL8RSPMYE8j9bAngLR+Mexaz2dbt7sn
h6qoWc7nZKxZ/uLu9nw6O8NfFgeHTbpmdl7y/6V9rOIJceZ7CUZNPfuGnmG4O2f4g5Zvvt+LB+T+h
62S2GWOB8PbWf5cw3txTLq2eOCPkekZ+UcYfhZeOISoAABgNvjlPYKeRddiD3czevzkW+dSTf4wM
Bxc/0KW1S8ac6/XkJUVc0i2WqVmSp7lL6nz94u701utKjMcPSpSOW21wPoeCgyhV7dwzTovM26bL
dtf2uOpyGnVY6+27r+veL3G5m+islngD1qLaFZ/2NIjADo1Oe/9w6mHcM+oHinvxXGoUhklqUldLm
RwkSL/CMPPwounDwAAMCOtqvSt33L69a5e0a33y9aPJ2VvRX669bLVFtWewUe139KvWUZh56U
6JyfEblu53s/3bun26+Xg+ZiLe6RVHbg2rb71S2tfJT+5m+H38TB9HgKPefie1vdRxvdv3Pc1573Yf2R9
7eK0K4/35PH7CLt/3Ifxb1dq9H0x9H2r2jFw3Pp7Pu1rNcl5t78m8/fiEedGvd8M7Ns9hrTf/uKem6H
XZfY+POp+0XJ6L453XvJ7Lx7fnpB/xzO6RnHuxXn6WRjjFdT/rIsKAAAwE+5f6F3WD43zNRwQWBCq
x5xdm9BVbUl/Bl3nnO/pqrT6M5p5eS7pnk7G5ZnV9cH8Gfre9fjfR2Gvqc5r79gUjT83Id9bxnk7iuLc
LwrvxbOmRvOoOqhhgkP7+Vl4cRCiAgAAAAAAAAEAEaqICAAAAAAAAAQARCVAAAAAAAAAACIQIgKA
AAAAAAAABEIUQEAAAAAAAAgAiEqAAAAAAAAAEQgRAUAAAAAAAACACISoAAAAAAAAABCBEBU
AAAAAAAAAIhCiAgAAAAAAAEAEQlQAAAAAAAAAiECICgAAAAAAAAARCFEBAAAAAAAAAIAIhKgAA
AAAAAABEIEQFAAAAAAAAgAiEqAAAAAAAAAAQgRAVAAAAAAAAACIQogIAAAAAAABABEJUAAA
AAAAAAIhAiAoAAAAAAAAAEQhRAQAAAAAAACACISoAAAAAAAAARCBEBQAAAAAAAAIAIhKgAAA
AAAAAAEIEQFQAAAAAAAAiEKICAAAAAAAAQARCVAAAAAAAAAACIQIgKAAAAAAAAABEIUQEAA
AAAAAAgAiEqAAAAAAAAAEQgRAUAAAAAAACACISoAAAAAAAAABCBEBUAAADAQmtvFKRQ0I+N
tl5raG94n19p9PTK2fDbuiEhLV0wbdlwz3vk+e1JY2Xca6xXNVb8ba00rK8CAGB+EKICAAAAODqa52
TGOemSUMHoqjT1kqMsZ9aK+rmpKJsX6tZnHZ3aesg1asv5Wkc/F6me3bS+CgCA+UGICgAAAOAI6
Ujt/OL38Zx/XTlwM89yXbr9vvT7e7I5KvksbsrZqn4eco16jXN+IFttyXZFPwcAYE4QogIAAAA4WuiNm
r3eoezrp3LqRKxeo5Xtlng5auAamb1Qq9IiQQUAzCFCVAAAAAABHTLzeqH590pVg6NpryIpbm9Orse
rX/7Rrehp1Vr119suC68NKtPqCtUJH1wEdeJ31CKsrGjyeYL3S6HYYzGPXj8F92fsp1ayzrDVXndeO3Ul
FtltujOpfo/aGXxagXN+yXjUo3vHbQto/dH0tcc5VoEar+6BWKwAsLUJUAAAAAEdEWep1HdJl2Bu1U
ytJYTVYDdRep0K2gfXN1VEBZlNWCyUxyoCqjUhpcMIpOxQceJ3F3t/IQK8jtZJRr7Rcl60YnTuHwlEtel
8JVbak7hZHba7KRqMh5/wEVS4M1gNIcvwqwA5pv3M+hoNUR/i5UueiNLhTJewaAQCWAiEqAAA
AgKNjzQ3p4vVGnVS53pW+qgPq9ax0hK1v7o5oh1dLtC/
+y5tyzkv7etJYd0PBqrT0a71td2qyPjIpNl6/F2OSpvaGePmv0a6um3ga+6psW5/r+hNFqRqm9n5iDc
MPTjLVrLnHV5b6hcF2Jjl+67VuGhvWfmsrOxfjnqu27Eadi8A1AgAsC0JUAAAAAEdIUTbdGYwy641al
bNuj8nKaWvJNWL9/mFoL05zBnqzXmhn56Lz+t5F2dGdIQPD3I3enN5rB4QPix+t7aeGgTCzuHnB31
ftfDo9MAOTTGnVs8OTUiU6fuu675lBqKO4dsYLbDsHXf0saOhcmfVeOzviZq/FzT1n+9Zjb+QMWgC
Ao4oQFQAAAMDR4oVsGfVGLZ+Ukn4aMGp9qKqcDiR3JTnpp31ix33dA90L01rllguwH8bwdve1A0
6dSBLymT0vz8ha4EuLsnbGbdi+HIYlthMITDJlPQudTGqK41fHZNc5DR3eHzR0ropr4h2yuodK7n4ja
rECAI48QlQAAAAAR8xAb9RD5+l8K8qJU/rp1MpyMn6am0BHRnTmnE6i8DlCYFIpo85ppLBzFSw5Y
HLD3NgTdQEAjgxCVAAAAABHj9kbtRYvTsvXYK/Onhx6Y8iHebVWhx7biYbtTyercHa88cfflg2z16lby9
Ss3ZpEcVP29D4Gyt7amueYpR8AIg0hKgAAAIAjyOiNmpQxjDw7g706u3Lg7tTtmVk66QWAo+p5ps
MsJeDXAHX05KJbmFROSaIqAdNKcvztXa/nqR24unVRp7iWaoZ+1ePUnkjLDlS7Xi3W0WUEAABHF
SEqAAAAgKPJmIAoTMlPDo3aqW3Z8Kapz1ZzdcObqKm94Q8/L59ZcwJAszZnc9UfQt7e8Gp0FlIZV2
4Gzta5WPd7WfYa61790aSTVU1twuP3A1djxv4k9PbVbRDscRoSdAMAlgYhKgAAAIAjKro3qjlzuwrp

nGAubi3NaZWlXG7Kqg4D/dzWmOFftd+ozdlc1cGh9+IREzJNorLtD1vv1KSk21XyE1S5kPuM9AmOv
3Lan6jKu5bGBFRJjDgX5r1RPat7ugIAlgYhKgAAAICjK6o3qqp7OVgzs9qavI5mIqfk7J4xPFyx63gO1Di
1a3MOvE4Je+2U7GHrIcdebVnr3eHxeYt9/BXZ7psz/itVaZk1TZu7Xs/fcexzEVYMVW8zrewaALA4Cn
1V3AUAAAAAAAAAEIqeqAAAAAAAAAAQgRAVAAAAAAAAACIQogIAAAAAAABABEJUAAAAAAAA
AIhAiAoAAAAAAAAAEQhRAQAAAAAAACACISoAAAAAAAARCBEBQAAAAAAAIAIhKgAAAAAAAA
AEIEQFQAAAAAAAABGEvn/AZM6nr53azj8AAAAAElFTkSuQmCC]]></redcap:Attachment>
</ItemDef>
<ItemDef OID="q2" Name="q2" DataType="text" Length="1" redcap:Variable="q2" redcap:FieldType="radio" redcap:RequiredField="y" redcap:BranchingLogic="[q1] = '1'">
<Question><TranslatedText>Do you prefer Life A or Life B, or do you think Life A and Life B are the same as one another?
</TranslatedText><redcap:FormattedTranslatedText>Do you prefer Life A or Life B, or do you think Life A and Life B are the same as one another?
 </redcap:FormattedTranslatedText></Question>
<CodeListRef CodeListOID="q2.choices"/>
</ItemDef>
<ItemDef OID="summary3" Name="summary3" DataType="text" Length="999" redcap:Variable="summary3" redcap:FieldType="descriptive" redcap:SectionHeader=" " redcap:BranchingLogic="[q2] = '2'">
<Question><TranslatedText>
Alzheimer's disease - Description #[no]

If you have Alzheimer's disease...

1) You might forget recent events of importance to you. For example, if you are interested in hockey, then you might forget whether your favorite team won their last game, although you may remember watching the game itself.

2) You might forget the names of people you met recently, or the names of local streets.

3) You may no longer be able to handle your personal finances or plan leisure activities such as dinner parties or vacations. Simple decisions such as what to order in restaurants might become difficult to make. Your ability to concentrate may decrease and you might have difficulty driving.

4) You may start having trouble finding words to express your thoughts. You might need to be reminded to shower or brush your teeth, but you could do these activities on your own. You may need to post notes around the home to remind you of simple things like turning off the stove.

5) You may remain aware of the day and your surroundings. You might be able to travel to familiar locations like the supermarket, although probably with the help of a loved one or friend. If you are in a social gathering, then you might have difficulty following the conversation.

6) You may continue to enjoy hobbies such as puzzles or reading.

Now consider two possible lives. In Life A, you will live in perfect health for 15 years and then you will die of natural causes. In Life B, you will live in perfect health for the next 10 years, then you will live with Alzheimer's disease as described in the summary for another 10 years, and then you will die of natural causes.

</TranslatedText><redcap:FormattedTranslatedText>
Alzheimer's disease - Description #[no]

<p></p>

If you have Alzheimer's disease...
<p></p>
1) You might forget recent events of importance to you. For example, if you are interested in hockey, then you might forget whether your favorite team won their last game, although you may remember watching the game itself.
<p></p>
2) You might forget the names of people you met recently, or the names of local streets.
<p></p>
3) You may no longer be able to handle your personal finances or plan leisure activities such as dinner parties or vacations. Simple decisions such as what to order in restaurants might become difficult to make. Your ability to concentrate may decrease and you might have difficulty driving.
<p></p>
4) You may start having trouble finding words to express your thoughts. You might need to be reminded to shower or brush your teeth, but you could do these activities on your own. You may need to post notes around the home to remind you of simple things like turning off the stove.
<p></p>
5) You may remain aware of the day and your surroundings. You might be able to travel to familiar locations like the supermarket, although probably with the help of a loved one or friend. If you are in a social gathering, then you might have difficulty following the conversation.
<p></p>
6) You may continue to enjoy hobbies such as puzzles or reading.
<p></p>
<p></p>

<p></p>
<p></p>
Now consider two possible lives. In Life A, you will live in perfect health for 15 years and then you will die of natural causes. In Life B, you will live in perfect health for the next 10 years, then you will live with Alzheimer's disease as described in the summary for another 10 years, and then you will die of natural causes.
<p></p>
</redcap:FormattedTranslatedText></Question>
</ItemDef>
<ItemDef OID="a3" Name="a3" DataType="text" Length="999" redcap:Variable="a3" redcap:FieldType="descriptive" redcap:BranchingLogic="[q2] = '2'" redcap:InlineImage="1">
<Question><TranslatedText></TranslatedText></Question>
<redcap:Attachment DocName="150.png" MimeType="image/png"><![CDATA[iVBORw0KGgoAAAANSUhEUgAABVEAAADYCAIAAABtBTQzAAAAAXNSR0IArs4c6QAAAARnQU1BAACxjwv8YQUAAAAJcEhZcwAAEnQAABJ0Ad5mH3gAACpTSURBVHhe7d1fyF3Vue9xY6o7HpWE1h3S9mCiGyEYkbRhQ8wpNoIYEQRBxSBbcXPkJYJlB6GNFHM4F4qyKeTdG0EvihVOz4kXimwoKraKeTdG0EvihVOz4kXimwoKraQiwopFJoLL9xg5b2wNFAv3i2lvNJc9Mx/Y8wxnvFnzjXWmHOtd67vhx/nvGuuOcczxlzrdY3Hut591d8AAAAAAMAU0fMDAEZyYePCVf/7KkIISSU5gMDQD78XgEAxrCxubCxubRa/bnhHnlT1ib0ib0cIYQU2fvsrlv/+Ybih+YzA0A+/F4BAAa3ubV58NWD5vaOEEIqXPtj69+7PD+pw7euvu5a5qPQDDQ50PMDAZ3/+z7n7f9Q5FHjtz816h6+aj45AGRCzw8AGNbpD06SvVR3DX+eNMz9qPjwAzELDwBg/PmRz0xAJfHv/q8RwxzwoJ1f+1//Oz3zQcJ1gLLJrGdJixTYQQ6NlmfjPDKh9ZNkjXRkuZcowdyJKjmRa3OChXXLbI2hFDr4QcMSJxvO3jEHfw5RAsTk89VEKBvuJ9mPjYtcGSzrPlitoKvt7ZSBaaBiUh2nCrIZFWDxh4dAEzzZpFs/FFuwhzWxr3pgjFfI12nchMgP9e1NTEGuXKXvUugKeLEE4ua0Wto/UOnhoKTZiwnzHBGqnLFJSoYHTSgRmqvZBshxNTpD37l/Hx1/fNAsEixFxKA9TIGn1NkoPLZ9u+7qBODj/4cyU0rIWYQUiWOJadXkWRx+eVRlevx0A3frHlYNU0Xb5GjrYqaZgsPvqK+sl1ajFmKXzjK11KU+FmvtAiXkNP6KZvnawfd7HkeV6/aJpSo2hxQ6NEyw7hU5dXfcENX1cA8YFPAgRUCG4sk6OjdtTfRpOQefXmmz08GONTN7SrTp/ahjhpvFd7EFMdVSAK4m+xt1LQqzvMXIyx2ZNEBQBYVKXMrbFuCjZIE5uN6dkJZo9OwarBQMBCJNpRbe3HRNN8lsJ0rVqrgTuNWbunHmOS1DISS1gNZK+hTdbyA47uKqSb6KeEY6dJa09Sq0vPhdXUC5qPe5Fmwkrcv2+xp6vAESGBqqJzC6WT5tlCU3HpmnWD3yfAmcI1m2DL

Gdl6rKvgGUhc2GNpBvvs5nlnKr65dc8ES2Drytbh1w+LvR0hhBCic+2Pr37s8H7RnA8XvtgPdKLnXwa
R/s3/VKApm7FZai4rzqjPLn6oD+kj+moxXPxhSOdV6kh1oJ1dTS3SJ1JXVkm7V+oq6wzruh7T85Suu
RPoHi04WKvHugqegcSFPZbWXiI0zztT8c2teyZYAiffPin2doQQQoiZob/G74Yv9gNx9PzLINK/
+Z9SrVBzNLVZqq9T//N+dXV1SB1pBxTDxR+GdF+lDhVHmjW1z/nvRCdZJe1eeYtb1/WYXvAUdwLd
o/Wo12NdBc9A4sL+kynUJ8nSzlR8c+ueCRbt5V+/LDZ2hBBCiJnbnrxRNOQj5AdHvvPFHz5vPqsAOO
j5l0GkqdJNj/mc7IQ81+suLNYsNeO4X+OvjxjjiYrxhyF9rqrnrf+cvfFUzyKSvCytsfTdTvuyGQYxX8qKe2
33aL4zygKzravQfUM6x3GHkPfLGSI0/dLMS8A43v3kXbGxI4QQQszsfu6acb7G7+alRx/
+61dfNZ9YAGz0/MvA0+y0dPukntV9kD5dH1Gtkb6mo1lqL/RdGu694g9Del1l1BfP6PPVYX1qrKysoi
6arbH03BVZvXt6ntI1zwS6R3POCB9oh/XwzEpe2DUZObf2fOdI89g7tx4zwcJcunyJP9RPCCEkkp1nd
zxy5GbRio+Zt156sfnQAmCj518GnmbHpFsqi3my0WRJ8WapvVAPp6t5C9TDxR+G9LuqXa1zO/QVl
pmKJjaW/peg5BvH0g7qKV3zTqB7sf4zjOHtWQdW1+uGdEwmfHuaU9wRPXPrNRMswubW5oH1A
2JvRwghhJi5+8Re0YSPn9/98sPmowuAgZ5/GXiaHcluujwdkHlC8XTfZsk9z3ulOBh/GNLzqvjd0FdVIn
esIaukNZbmVerngntBbHrBhQUn0L1Y+wznFGOu3ssLvW9IfDJmpfIy9bgewjeiM7feM8Gotq5sHX/z
uNjYEUIIIWYW8jV+N3yxH/Ci5we6edpRYDWc+sUpsbEjhBBCzNzww689cegW0X4vKnyxH3DR8wP
d6Pmxms5dPCc2doQQQoiZnWd3PHjXt0XjvdjwxX5AoOcHutHzYwVd2LggNnaEEEKIyNEHbxIt9zKE
L/YDJnp+oBs9P1bNxubGnlf2iI0dIYQQYmb/09eLZntJ8oMj3/njZ79vPtKAlUfPDwCwbG5tHnz1oNjY
EUIIIWaW6mv8bl44cR9f7Adq9PwAAMv9P79fbOwIIYQQkQe+9y3RZi9b3jjzo+aDDVht9PwAgNbp
D06LXR0hhBAi8t2Hvy4a7OXMR+
+83Xy8ASuMnh8A0Dj/8XmxqyOEEEJEvvnMdaK1XtqcuuN2vtgP0PMvlvrbcJVB/kDcp+vH1t5rfp7G
36KzVxRRnOhfq3XXlX5jrrDe753pveVWxsXPL+56cZfY2BFCCCFmrjuz8/E7D4jWepnDF/sBev4Fanp
SJX9T1BSYUAPmrihI311nrd6Wn56/S6/3zvTecqtjY3Nj30/2iY0dIYQQIrL8X+N3wxf7seLo+Rdo4G5I
N72TacB8K/LTZxbkWtVzNKGz6fHemd5bbmVsXdk6/PphsasjhBBCRO58dI9op7dL+GI/Vhk9/+L072
DTTK8B63fH9FkNuVZ1Dwa67ZNFzz9lJ98+KXZ1hBBCiMjeZ3c9dfBW0Utvl/DFfqwyev7FUI2QwWx
B7bZVdEtGE9UOIxrY0PhmA2ac4/Zj1gw62zVzWPNKp60OD9s+oy/SExSzVZzRC/adq4jZqzN8V7vMl
Rk8h8NLq8mZWacY47ULrWcYvS6gby3rxOiLVTwZuBVKO2+tGtG8zDjHHcWatHi695yR4uVfvyx2dY
QQQojItT++
+rHD+0Ujvb1y5vj3//Lll82HH7BKZuv59W47vNX2nmK1A4GWobd2y7+NN/yhBsn/VMG4a54TnHs
aGt8/eMkYwmq+lOjLlmVY/Xx9TDwMrUhQVxXPqSvsmftmEXsjeUeRB7uWFrhB7Rme56sn/ddFJ5x
Sq9E945J5lsFzQb633MyzQW/vfvKu2NURQgghbu69Z59oobdjXn3mVPP5B6ySEXp+sZ2fd49uDhd
tfZae927q5kbdJ32WvnFG/xO9Ab7x3Wud8Z3L1DWRV84dtj0i60SGNc/xVfWtSChPEVOw521M1R
RenCpqnCFG7lyaHEJfoMc0ZmWuLTRQe6EjpVZ7RBXXV6kj7YFI7fYsYxFuOWdKzmVy3b3mjASXLl/i
D/UTQgjpzKGTu0XzvH3z4c/eaD4FgZWRved3Ofv3uRi9RyHPmAsSa5DMhcnzdLfTsfrY+MYxcZp6aA
zuOSRkG1YP1LBLijE7+O6lO0T33XTmKQbuuTRTz1no04ID9RCu1d5FeY5zTUEfi8zFd113OfUwcgN7
zBkz29zaPLB+QOzqCCGEEJFv/Mvfbd+v8bs5dcftn1261HwWAqth+J4/79Zc9wLH7J5gO/LcGdFNNv
Si66Oe/sfLd+c9BcRpenSPYMHulqz3sOaJ8tX1rSjMs1avzvMC919NovfSKvbZzhj2BXrBra7VmHrXkjd
WnWIV8x60+V4gz2XiNHuaNjGhyJwxm60rW8ffPC52dYQQQojIzrM7Hjlys2ibt3v4Yj9WzbD/bb9n
M+/Zs5d6djLqmrX31E+ztEBLxnM3/W2VONF/kszvu/awOg+xkC2nMO2ZzoL9K0ozDMpr+5R1RnV
SGpYfXafpekagno+ONfAhbEbkFBL3gLvdIJzbMlxSt3l1Bk+4Qn5aqG/U784JXZ1hBBCiJu7T+wVDfM
0whf7sVIW1PPrs0yRXqKhLisHUT93X7WsPHfT31aJpfpPcnnG79E79R3dkm9YfVlFnO1bUdisJWPn
NWMVp6izu140kzqjUJ/kLKRzDGOISp8T+9eS53in0zlH/wvUXa7HwD3mjBmcu3hObOkIIYQQN7c9
eaNolacUvtiP1TH83/DzXOMc6rHpL6nr6svUo46Lllfszpilkuf1vFu+8Xv0Tr6rOuUaVp11bH3dt8jZ5u
a7T74R1HnRQZuTjq2t1QMYJ3fOyp2IOqIv8c21UTw1Q7G0WnJUXxV9zDNHxXdddznfVUKPOWM
G/F/jJ4QQ0pndz10zpa/xu+GL/Vgdi+j51RHP/j3STrgn9bpoibl3pqA7NLnKdpme/sfLN/4s/Z46oKdkz
tOWZ1jrEv0gMmac9z71mIafPq9indw1pizRnq/H8c61vbA97D+xlVbLubHOkoxxQqULzjiFHuWcanI

VveaM/raubB396VGxtyOEEELMPHjXt0WTPL28cOK+5qMRmLRF9Px6Q+8R2b+rgdptv3tkWwncTf
/tMdbo6X/87JGqKr16J33EEnlpsgyrn1aD6Om3o3pWFOaZVMkeQ+kYq2BeJ8/uWJq/ZEmdEphr8
MrwdJNquS9WYEklOUeTXb0arVe5jhvYc86YweU/X+aP9hNCCIlk/9PXiw55evnonbebz0Vg0rZPz+/v
CmqxLmR5Re6mvVjxvKf/CTFvdXV6797JnkFXqfmHbZ8wztfTb090VhTmmZRijuK7/16e2Zjid8ysWN
ZTj5vakbmKyfaY7uy1ul+s4onYHFtm8erMpHKySu9BMAP+j/MTQgiJ5+iDN4kmeUp548yPmk9EYO
oW2fPHewfBbgikmYYCUqi3IG82TMb5j8+L7R0hhBCis/Psjqn+F/4vnLjvr1991XwcAlO3yO/zR0Zx6G
sC6MMwLP0O5K2GSXn+V8+LHR4hhBCic8MPv/bEoVtEw7zdc+qO2//42e+bD0JgBST2/F5VC+809J
4Ovx2mOab/e+DQvwXwDFLTT9CJYSDmf60efosC29ZDbz0kdniEEEKIzq3/fIPombd7+Bo/Vs1Cev7Q
QMF2KtLZR54CcjB6fhp+TNHWla3Drx8WOzxCCCFE5+4Te0XbvH3D1/ixghbU85fswWIte7Svjz4JAO
i0sbmx55U9YodHCCGE1Nl5dscjR24WzfN2DF/jx2qarecHAEzShY0L/Bl/Qgghoex+7pqnDt4qWujtF
b7Gj5VFzw8AKL3229fEDo8QQgjRue3JG0UXvb3yu19+2HzgASuGnh8A0Dj9wWmxwyOEEEJ0tu8X
+9966cXmow5YPfT8iBN/jyH+cCbqb+M1f4hhnqFmZf0p/sH/EESxshHWVBrzHmKyjr95XOzwCCGE
kDrX/vjqxw7vF+308uelRx/ma/xYZfT8iIs3+fM0mYvq+XWh2rAtf1NspB58tHuIKdvc2jywfkBs8gghhJ
A6e5/dtb2+2P+DI9/54g+fNx9ywEqi58dMRFc5T5Mpev7RjFh37B6cnh95fPLFJ/wZf0IIIaEcOrlb9NX
LHL7GD9DzYybbv+cfsy+m58e29f6n74sdHiGEEKJz7z37RGu9nOFr/ECBnn8hAu2u97Du4yrdV3T0f
erp9gr3iBq2PCKGiz+M0ycXivPF3L1DmZeYE4yyLrKuUiUNgYmbkxPztti1zGqhWmLZJWfpxjntMM2
T4YqegQLEGP0XVegz/0J0jJp1ivt8jxEwrHMXz4kdHiGEEFJnW3yxn6/xAzV6/sXwNE6+g23DZzIu6tm
AmdTz+mldRA/SHKkei+HiDyP8Syk1ZZ2h9AGTsVCv+B3zPBuYeOd0S9FioVrquDGOs3TPtfXp0Yr9Xg
7vbTWu6CiROv+SMauuF7d7BIzi5NsnxSaPEEIIqfPNZ64TPfZSha/xAxo9/4KonsfT5uhDuu9RR3Sj5Jx
iDOM0YFJzgrrE6K6aK9QI1UMxXPxhkD5P1ig0E+ka2bNUR4875g7s5d6V9ohYwoy1POtwTnOrVzor
+urZnEvELess0Wf+8hRnEHmBc0XnCBjJ1pWtoz89KjZ5hBBCSJ3vPvx10WkvT/gaP6DR8y+K6mJ0E+
P0Up7mqrO7Kjj9lKM5o76mfrC2Vg5kHlHXi+HiD0N8p+ljzdztc9Qjz8KMQ0KfO+Ye8FJDGWd1Xuie4
Lukz0umq4fXWpEXJkyxPhQ6PzK38Pz1w9D01QmeIepDnSNgRJf/fJk/408IISSUB773LdFsL0P+49//r
fkYA0DPv0Ci8VGtlO6+PL1VIXCVcZLul8J9XHNVdUL187H196qr2iP6cjFc/GGIdy3ioD2UetInVMtbRY
+rjvabsq7fnhW70J6uPsF3iWeWzmme6pK3YufaPMXD/IvqMX/9uGUVtAe21UN0jYBxXbp8adeLu8
QmjxBCCCly3Zmdj995QLTci82//tPjzQcYgAo9/+KovqZqZlQb1DZr/vZMtFc9GjCf5rLijPrs4of6kD6irx
bDxR+GeNciDtpDqSd9QrW8VZwp9puyZyz3Qn1EcE8wavUZ2b+SQkdFXz1LcOBW16LmuTPN82oI
HzVEfASM7vzH58UmjxBCCKmzVF/sf+7Y0f/605+aTy8AFXr+RWp6n6J7Uh2O0dL42zN1YnO0VwP
mUV+n/uf96urqkDrSDiiGiz8M8a5FHLSH8l7RwX+NGlcd7Tdlz1jyQnVKoT7LHdlXq8fIodV3VvTVsw
QGbnUvqs/8NWO4Sn1R5ywM/hGwCM//6nmxySOEEELq/OND3xC996Lyn7/5TfO5BUCh51+opqE
5trZWN01mz6QbKbPLkd2Vp3vSTVKo7ys147hf46+PGOOJivGHIb7T9LGmln1Oz4EtcsSKM1C/kT03
VlzonuHeeV+tPi+Z55xCd8XOtYUmpIbsLuE5xT2nUBwzHtl1O6dZiY2ABXnorYfEJo8QQggpsvPsjgfv
+rZov8cPX+MHvOj5F0t3SxXRzugnVXul+x7db+kj6lpjwGhz1F7ou9To50SvFX8Y5MzTqN8UC42srtD
Ti5TqccdknQA1UvhOyPm0E25H9tVqz2sOGvddneapXuiu6Ktnc6rbB3osyhnBmb8+0E5fLCg8Rt8RP
Acwgq0rW4dfPyz2eYQQQkiRG374tScO3SKa8DHD1/iBEHr+BdPdTUF1VS3z2ZbZ5RhNmeQOZ2ov
dLoof4F6uPjDiPBEm2rOUP4rOgrNcMeiI3k6Snmhv1SpHdk+pz4evhP6Qk/1UmfFPmsLlG9K9VjUD
POX2mn5B2mf7x4hcI8wtI3NjT2v7BH7PEIIIaTI/qevF334aOFr/EAEPf+i6eYm1LvY3VHb82jmCcXT+
qHnVJN7nvdKcTD+ME6fXCjOF01bvHqlZ3tnXyRn1m/Kno7Sc6HZmZZH1WNjaPMUPZg5w+JcZ2RP
9Ua8Yr+1yTskysRLVLrmXzKHKblTsmcRX2vJHkE9616HoV3YuMCf8SeEEOLN0QdvEt34OOFr/EAEPf
+iqcaH1gXAdvHab18TmzxCCCGkyM6zOx45crNoyIfOhz97o/l8AuBDz79Y+n/qpOUHsJ2c/uC02OcR
QgghRXY/d81TB28VbflwefWZU80nE4AAev5Fsf/DZfc/fAaA5Xb8zeNin0cIIYQUue3JG0VnPlDOHP/
+X778svIYAhBAz78oRs9Pww9gG9rc2jywfkDs8wghhJAid5/YK/rz7Dl1x+2fXbrUfCYBCKPnBwAk+uS
LT/gz/oQQQtyM8MV+vsYP9ETPDwBI9/6n74t9HiGEEFLkG//yd8N9sZ+v8QP90fMDAOZy7uI5sc8jh
BBCihw6uVv06lnC1/iBmdDzAwAw6uVv06lnC1/iBmdDzAwDmdfLtk2KfRwghhhBS59559omOfM3yNH5gVPT8AYF5bV7aO/vSo

2OcRQggh1/746scO7xd9+zzha/zArOj5AQAZXP7zZf6MPyGEEDd7n92V64v9b5z5UfOpA6A3en4A
QB6XLl/a9eIusdUjhBBC7nx0j+jeE/LCifv++tVXzUcOgN7o+QEA2Zz/
+LzY5xFCCCFFHvjet0QPP1NO3XH7Hz/7ffNhA2AW9PwAgJye/9XzYp9HCCGEXHdm5+N3HhCdfP98
9M7bzccMgBnR8wMAMnvorYfEVo8QQgj55jPXiU6+Z/gaPzAPen4AQGZbV7YOv35YbPUIIYSQ7z78
ddHPd4av8QNzoucHAOS3sbmx55U9YqtHCCGEzPTFfr7GD8yPnh8AMIgLGxf4M/6EEEJEbvjh1544
dIvo7UPha/zA/Oj5AQBDee23r4mtHiGEELL/6etFb+/N//lfZ5uPEwBzoOcHAAzo9AenxVaPEEIOfrgT
aLDF3np0Yf5Gj+QBT0/AGBYx988LrZ6hBBCVjw7z+548K5viz5f5wdHvvPFHz5vPkUAzIeeHwAwrM2
tzQPrB8RujxBCyIon8sX+3/3yw+YjBMDc6PkBAIP75ItP+DP+hBBCRG578kbR7Rd566UXmw8PADn
Q8wMAxvD+p+
+LrR4hhBBy94m9ZsPP1/iB7Oj5AQAjOXfxnNjqEUIIWfHsPLvjkSM31w3/E4du4Wv8QHb0/ACA8Yit
HiGEELL7uWueOnhr0fPvf/r65tMCQD70/ACAkVx11UgfOhRKRqFkFEpGoWRTKvTRO2+/9dKL3Lpk
FEIEtxIAMBJ2JMkolIxCySiUjELJKJSMQojgVgIARsKOJBmFklEoGYWSUSgZhZJRCBHcSgDASNiRJKNQ
Mgolo1AyCiWjUDIKIYJbCQAYCTuSZBRKRqFkFEpGoWQUSkYhRHArAQAjYUeSjELJKJSMQskolIxCySi
ECG4lAGAk7EiSUSgZhZJRKBmFklEoGYUQwa0EAIyEHUkyCiWjUDIKJaNQMgoIoxAiuJUAgJGwI0lGo
WQUSkahZBRKRqFkFEIEtxIAMBJ2JMkolIxCySiUjELJKJSMQojgVgIARsKOJBmFklEoGYWSUSgZhZJR
CBHcSgDASNiRJKNQMgolo1AyCiWjUDIKIYJbCQAYCTuSZBRKRqFkFEpGoWQUSkYhRHArAQAjYUeS
jELJKJSMQskolIxCySiECG4lAGAk7EiSUSgZhZJRKBmFklEoGYUQwa0EAIyEHUkyCiWjUDIKJaNQMgo
loxAiuJUAgJGwI0lGoWQUSkahZBRKRqFkFEIEtxIAMBJ2JMkolIxCySiUjELJKJSMQojgVgIARsKOJBmF
klEoGYWSUSgZhZJRCBHcSgDASNiRJKNQMgolo1AyCiWjUDIKIYJbCQAYCTuSZBRKRqFkFEpGoWQU
SkYhRHArAQAjYUeSjELJKJSMQskolIxCySiECG4lAGAk7EiSUSgZhZJRKBmFklEoGYUQwa0EAIyEHUk
yCiWjUDIKJaNQMgoloxAiuJUAgJGwI0lGoWQUSkahZBRKRqFkFEIEtxIAMBJ2JMkolIxCySiUjELJKJS
MQojgVgIARsKOJBmFklEoGYWSUSgZhZJRCBHcSgAAAAAApomeHwAAAACAaaLnBwAAAABgmuj5
AQAAAACYJnp+AAAAAACmiZ4fAAAAAIBpoucHAAAAAGCa6PkBAAAAAJgmen4AAAAAAKaJnh8A
AAAAgGmi5wcAAAAAYJro+QEAmNV7a1e11t5rjgIAACwben4AwITUzfiwXfin68faCkMWHPvfLJQL
Kxxb/7Q5kJO1mAr/rmRW1j0c5mUaXOAXpnnv1fK+MyK/opGnZuUfynrF8i5rWNE7M+g/KAYUXtR
2fZ0CK7KWk+tl6lFrm/4zaUXQ8wMAhjXgbt5i1Blz01aVHWKvU+yl9DqqfdXAG6pyIcfW1or/Z5A65
RJG3xKa+9H87wrrnd0a6N1XrWWkd4R53/IVCf6G2r9D9kLnEqwYfWpWoaHK4/pAfUvnLeUfxXy55
i7ReWfK57P+g8K3KGtJlfnWFVlU9VTGXyV3OUZx00ArqurrQ9WjeVfXr1aGUvW9a4j7Y91F8Rx6oO
cHAAzI3k/Zu5GsVKHq/x9zQ2CvcCiDVykLNLdvmDLlaz/4bTKN88JYqpIDvfnk2MPVyr2Pb6jXw524f
GvkWlq4YuypWfUdSp3XPJxZdX3NrlLcPn1g/peraznl8ebpuerUYovKMX4jsijPoXTB5QjzFw2vSB4ZsJ
Z6onk476tWDqeHr97J7UO7VPXkXGtaRfT8AIDhyD2As2vIbvgKwjjbj4GXpYcvf0jftMXMtx2c2ejvg8
KwbwX7Bg62vmpg84XK/cI5M3eXkvlGRu5VxtvYNVT1fPKNVFcPW8XgL6SPlj/MXUZN1lNrmH9cuI
WyrKMRWY4t4/u789Z1TqY/ZyhnHRmL6btZPZBvh5yFVgY9PwBgMO4nc8bdjt/Im4FqPbn2jEHW9i
c/456VPw5TqLpTrYHv2XDrCBr4RSrUt7AoUf8wzLvc+Q013h1ZOOOVFe37lrlkZLiMlbqGcm5skq4q2
RbkG8g4Vv6Y7c3uqeW+KXJwC1V1yi8qaPNX9d06Q/V0rrV5a1VvtbJE/UN4KrNxagVuZ56lmbfJXyj
bwlYEPT8AYDDuDsC7Rclp8AKmauORa/fmqtbSGHBJ5SrU8GXJ4Rak1Asbsk71zsu8lY8b541XveMq
Q5Vy1+H+Fs/FKeCOn/leRobLWCk+VPXCZbiL8SrVs3leK0+hcg3qSPl0tjdFoJYhUym3kHhdqhPC97cf
z3IM8WdnFRqtvX/ZSnlqVUfk3cv0WlUraKqVP9vD5r2Lq4GeHwAwmAV8VI+3FxC7xUFl3UzZymW
096ssNMqa3LdGVuLFqe/fkG+LquCg7ztzDfXPw9Srxpa3LuNLVQ1oztx9KzinzCcyXMZKsaHE23EO3i
rVwUau2+YUKtfQPi6fzvamiN26Qr28HNXcQs6bL8MvcnQ5GcY3eWrVt6s+Vv+cqZ53XdV6lLU1600
yh2pY/cI4r1L8HsOLnh8AMJgFfFSPtBeodzoj7jnqzVv+gtaWzZBjhx1T1R3u/jnvvGHrVa/OoLfMrTDgi
qw3Rb59fM35DXV/ZZ1Xbz5uAS3y1KyCQ1V3M9dyOiZcPZ2lmCxkvScMQ9RyZXpHuIWcgbvn0ikyR
PVUrvdCyanlVqheuflWVOu+NeUZOVYnf2Xcl797LpDo+QEAg3E/mDPt3cLG2AtUNUbecFQ1B71zlb
LM8FWGX84QW/mwbLvqMLfEoCsy5H5LuPOWL1bulUXGy1jKP1T1umVcTOeEqxMyFOwoVD6d7

V3Ruahcv2FuoeqIuRD5ZkwQXk6mZRicWm6J7rvbU+dA5QnzvymqFdjDuJUzvEwrh54fADAc+cmc
bfsRNHyFek8iZd9/FFWMZXj2QYPIs2tzFeMaw1Yv0qCvUlXBXMiQe8TqxRn2Pee+A0YpWsj+jqheGn
vi1tqcl25unopK5KlZeYaqDuV9kTonXJ2Q4fZ1FCqfzvYiLXRR1i9Slt+q4HIG+J11a1m/TIV8ReMvU57X
qJqtW6Q8bI4dnwq86PkBAAOyNiB5dgV+1djC9t4TiBWNs5iy6CCv0Oirsba6+fa9riHf1bZ6P6yN8I7I
uTbxDqi0SzDXlutmRirGJzOT8FDi9arNubiqmjXToorxuKo5T42ed6Y8Ldub3llUccAYvJ6SZxK9xRdlPTt
PmYaznFp1eIT3dvZ/UIRr2c/Mfevq4bzDWO/rrHdyddDzAwCGZW5A+JzGaKwN6dz70ZDq7T3Y6Au
QeR+PLOxXpda8NuKpbfSKhRe1LdcUeY0Kk/sHRXbiX1XU2i0DG4k50fMDAAAAADBN9PwAAAAAA
EwTPT8AAAAAANNEzw8AAAAAwDTR8wMAAAAAME30/AAAAAAATBM9PwAAAAAA00TPDwAA
AADANNHzAwAAAAAwTfT8AAAAAABMEz0/AAAAAADTRM8PAAAAAMA00fMDAAAAADBN9PwA
AAAAAEwTPT8AAAAAANNEzw8AAAAAwDTR8wMAAAAAME30/AAAAAAATBM9PwAAAAAA00TP
DwAAAADANNHzAwAAAAAwTfT8AAAAAABMEz0/AAAAAADTRM8PAAAAAMA00fMDAAAAADB
N9PwAAAAAAEwTPT8AAAAAANNEzw8AAAAAwDTR8wMAAAAAME30/AAAAAAATBM9PwAAAA
AA00TPDwAAAADANNHzAwAALJf31q5qrL3XHKqpJ46tf9ocGYaqI8ovg/beFJz78On6sdBT0ecAYLro
+QEAAJaL0dfa7emq9/xt217x3YZgZ9/e1CX8NxkAMBx6fgAAgOVi/W/ZZoe66j1/r/V7m/v2XwXQ8
QNYMfT8AAAAy8Xq+c0Gd8V7ft24d8xL3z99n/QROn4AK4eeHwAAYLnYPb/RqDo9v3PAbYybU8ozj
HGbC4wjZjesDhfHjP+c3iiiGM86zxtza8t09NzWeNaAxkyVyFj67Ooc/chZQWz+JXs+4pTIAqPXAcCo6
PkBAACWS9tMrtc/qZbR6DKrx+4Bo91sWuK2He2km+jwNUafLfvaijERzyDR1tdfVC7dYMzF0U5ubV
39KKp3zb9jQuEF+q+LThcAhkPPDwAAsFxU01h0kKoxrRtG44nqRPdArOdvTjJaUnlEtaXONe0RdUC
W0WfoqRh1erS74QJ6QLdmjD650Q5T6Zy/OiE8n8ACQwPJGQDAOOj5AQAAlovVNTYPzJ/dttNoJ2
Uzq05p+9LwETWMe4Y8R3bEBXlID2KcEyRnUJIrcQ/E6QmUxCU95i851f0L1Kf1WjcADI6eHwAAYLn
42+ui1XQ6Y0+rLFtT95TuI+qx2Sjb46ozfERha5QAd0olVVIdlUvrFJ5Cj/kb7LPV84HR9Sxb9rIAYFT0/A
AAAMtFdsDNY/31/raHlGcWhur57YPqgU+wcJj/XLmU5J7fnUKP+fua94p6Pjh64MK+swaArOj5AQA
AlovTTMomUj/haTtlY+ye0n3EM6wY13eG0OMUzX+uKqmOyqV1Ck+he3LqjEJ9klO9cwxjiEq4GAA
Mh54fAABguXiaSbt91E/EzszQ85vNtX1Oj/bbLROmhzNPdmpk7Pk7h3IvdW5KZIHFU8bAM88bAD
Ki5wcAAFguvmZS940l/UR7tGkndWPqHHGb1/CR8Cj6lHBl1di6ZWLCBdrrZ+6dI1Pomr9cTnt+xwKd
hUSnAQCDo+cHAABYLh3NpPmE0YpKopeNtaDyiH58TI6u+t2Cv3R7glsmTp1vM6/O2fN3zt8/nZI6J
TR66Mq+swaArOj5AQAAlkugmWybVOsJs3ct2krZGLuDdR9Rj83hnOmU7L7ZPiGwiih7PNkky6V16
pxCbP7t5aWypHFbzKd9o5uXlvrOGACyo+cHAAAAAGCa6PkBAAAAAJgmen4AAAAAAKaJnh8AAAA
AgGmi5wcAAAAAYJro+QEAAAAAmCZ6fgAAAAAApomeHwAAAACAaaLnBwAAAABgmuj5AQAAA
ACYJnp+AAAAAACmiZ4fAAAAAIBpoucHAAAAAGCa6PkBAAAAAJiiv/3t/wPKb2FXPGlLwwAAAABJR
U5ErkJggg==]]></redcap:Attachment>
</ItemDef>
<ItemDef OID="b3" Name="b3" DataType="text" Length="999" redcap:Variable="b3"
redcap:FieldType="descriptive" redcap:BranchingLogic="[q2] = '2'"
redcap:InlineImage="1">
<Question><TranslatedText></TranslatedText></Question>
<redcap:Attachment DocName="B.png" MimeType="image/png"><!
[CDATA[iVBORw0KGgoAAAANSUhEUgAABVEAAAEJCAYAAACdcmdaAAAAAXNSR0IArs4c6QAAAA
RnQU1BAACxjwv8YQUAAAAJcEhZcwAAEnQAABJ0Ad5mH3gAAE9sSURBVHhe7d0PjCRnfef/b+/aY
PPHNmBskx+RHat7sZYlGJIg08PxL4egZ+6SJbnbYOVg5Oh3PVyi0zQSkwOxF+mX2wguG4lunS5iWrq
zliQyTEJugrLdIJRgckzHR4IxYTI/Z7rlc3T8wDYGjPljB3u3f/VUPVX1VHd1dVV3VXX39PtltaerpqfqT/T
O/OZ5/k+hb5FAAAAAAAAAAAAChjumPAAAAAAAAAAIAQhKgAAAAAAAAAAEIEQFQAAAAAAAAAAiEKIC
AAAAAAAAQARCVAAAAAAAAAACIQIgKAAAAAAAAABEIUQEAAAAAAAgAiEqAAAAAAAAAEQgRA
UAAAAAACACISoAAAAAAAABCBEBUAAAAAAAAIhCiAgAAAAAAAEAEQlQAAAAAAAAAiECICg

AAAAAAAAARCFEBAAAAAAAAAIAIhKgAAAAAAAAABEIEQFAAAAAAAAgAiEqAAAAAAAAAAAQgRAVA
AAAAAAAACIQogIAAAAAAABABEJUAAAAAAAAAIhAiAoAAAAAAAAAEQhRAQAAAAAAACACISoA
AAAAAAAARCBEBQAAAAAAAIAIhKgAAAAAAAAAAEIEQFQAAAAAAAAAiEKICAAAAAAAAQARCVAA
AAAAAAACIQIgKAAAAAAAAABEIUQEAAAAAAAAgAiEqAAAAAAAAAAEQgRAUAAAAAAAACACISoAA
AAAAAAABCBEBUAAAAAAAAAIhCiAgAAAAAAAEAEQlQAAAAAAAAAiECICgAAAAAAAAARCFEBAA
AAAAAAIAIhKgAAAAAAAABEIEQFAAAAAAAAgAiEqAAAAAAAAAAAQgRAVAAAAAAAAACIQogIAAA
AAAABABEJUAAAAAAAAAIhAiLp0etJYKUih4DxWGj29PqjXWPFeU1hpWF+1iMxj3ZB2onXjpLWdL
MxLO7LSlg372PzHqPs4Vb2GrGzEO5vtDbdtK5JH08KNuQ9Cjmc+2g0AAAAAwPwhRF06Rdm8UJey
XurU1kPCkracr3X0c5Hq2U3rq4B5oILBVWnqJUdZzqxle4faf1Qo1cT/rlhsR+14AAAAAADIGiHqMip
uytmqfi4dqZ0P9kbrNc75IVW1JdsV/XzhFGVzry/9vnpsS/aHkff+RpmXdmShKwdu8leuS9c+xj3ZzDJ
D7TVk3fijwsI7ascDAAAAAEAOCFGXVGW7JV6O2jxn9EY1e6FWpbW4CSqOot6h7OuncuoEPaQBA
AAAAEAuCFGXVkW2W26M6vdGbW/4Q6XL9a3wXoyqlqKuRek+wmpSjqyvaH59ZI1Jo6ZjoC7rqPX
GPu31edcGHd5fZI1J4zwEz5+5nbDPjxN+3INtCdS9tR4xy30ObGewraPOc7xjCm57uPZpYAh6c9VZF
2h4snM3eA7Uw9yc3Z6x+xzPPy71CLkXbAnaHvI9OHq7vqTHE6/dAAAAAAAcfYSoy6yyJXW3OGpz
VTYaDTnnJ6hyIWSM9FAIo3VqpQwmoCrK2hndwM6BdJ1nFmNId2dHLno7bcuubn/5zNpc9FKsnPa
D6gP/AGy9izv6PBo1Pe1wrCSDo63TPb8dqZUKUhrYSXM1aUimtjPY1qasDoZtEx2T2rZR+9S6H+te1
+kREu3HCSwHz4HSXB0dKibnnOtV70AUtW6Kc9TeGFHPNGS7E4vZbgAAAAAAlgQh6lILTjLVrLnBTF
nqF0Imk2pv+KGKV4+yL103ie3UZD3lhKW4dka3b18O3U23d42JhYxw0hjqferEnAz0rpz2yiY0d4M9
Ji/u6BisfEacDLUnjXX3GlSlpc9v3+0xnOr59bfvdUhWIdlAfdzx/O1494Hazrob/E1zTMbr9zZlc9v62PX
vV1Wv1/6cXXIi2X56jXUvsCzXu85r+8MlLiqR+4wr/BzteOl/krZbr3X/0hH2PRjY7rBkxzOu3QAAAAA
ALI+C9QtyXz/HklK9SwM9zlSwEhKq+K8rS71rTubTk8aK24tOBS/OZEYjX6963bk96Ubsy+dvW4Vde
9ZG1BBs1YOwWq1Ks9kcWu+3IaxdcdeNE387w+2yV3rnwG1/6DqbsV0VnO2FBNwB4e0wr3O11fc
nDEt0PSK2E9ivvuaS7JjMbQdfr41qa6JzF35+FOdanQqsS3p+lETnOlHbRwht44jjjDietO4RYBr3Pnyvv
OXCW/QSACAP99/xLnnNW2/SSwCAzB3/NZFjr9ILWBT0REVwkikVtoQGJP5Qeb/npMsYdm/2GE1F
UU6ccp51dJfTrj2WvywnT5+0e9QF11uqp/0AbA74vWmb4nVG7R44gZT1GW8ov7dOdT4sGbUo3S
BMfcIsazBr1jUo6ac28z7QPYSnOKZEvYkT7cec4f+kmIdQ3NyTvhmgTm3wHIWY6rrrurFuwJmaGO0
GMvDwEw/LOz/5Tr0EAMjDDY9fJXd/6AF5/Bs/0msAAJm7dI9I/zt6AYuCEBVBA6FScsO1P6fl1RVt7k
pbenJoj9k/JScqJ6z/D65XGeqc9ZIrrombLbpD+ttuIl09a/TozUtaAZl1DTJrOyHeSKpXqBe0GnVjgQX3x
NNPSOWPKvZHAEA+nvPjY/KWL94oP3ryGWm+/3555p8u6c8AALL1lMilP7A+PussYiEQoiJlGYRfXl3
RfTlsXxS7lKgd9lbEyVeN9XMZvhk9NO3A1+/VOyrwVcO67VqVQ480e0lOK1mv47yOaTHOXbjxbW/
Lhtnr1K2LatY5BRbUnZ+6Ux58/EG9BADIwxv/
+gZ5wQ+vsJ8/9NXvyp/WeR8GgNz0Hxa5/Od6AYuAEBUxleSkN1LbnBFfMSZJitM70Ri+HI+7744c7D
pf686+X7I/4a8fLjUwH4qbZ/0guOFOjFWVQIZacsoTKG6Jgvk22OvYvA/0seV1TIn2Y97LA8Pk1cz3hZ
xnoE/SdmNSNTtwdeukJv6eAubL+z77PvlM7zN6CQCQh1c+eK3c/PXn6yXH5z7+kHzlLx/RSwCAzF3
6gsjlr+kFzDtCVMRUlM2zbuVUc/Z1NbrYnOl8y+vt5wScijnre1s23JlrYvN7cqqJpBS3XmZRF0x117vh
6vxxe81a56Kmj3+wdqsx7F+aq7LhnTIV7Onh297K+dBcNQLH9nm/hqd7bHkdU6L9mLVbm7Lqre/p
me+ta1Qq+NvI2oTnyA9c3XYDi+kT+5+Q+n11vQQAyIOqg/pzD7xELwX94W9/Tb73raf1EgAgc5f/RK
T/fb2AeUaIivgq29LyctSalHTA48w8bynX5YJR4NOfUMnSXNWB0GQ1HAPbsp55Q/a9of6KMUnTHP
Jqu9rKUt8KRKiWomxe8IdlN1d1gOaFzqMm/ZolJ3AMttM8tryOKdl+ipsXpO6/2HmtOZGTdS/7h6Br
7yrua1NNWBO03bzfw9odR+bHA8R339fvk7v+7C69BADIw/FLBXsYv/oYRgWozd+8Xy8BADLXf1Lk
8sf1AuYZISoSqWyH11+stqz17tBiV3FT9gZfW21NVr/R7K0XKBlgDM3OdKKjFJgB2KiyA+qc9bt+wOe
ya1/OW03PstS7fT9Yt1Wl1d8LTpaV1zEl2k9RNvf60h16sXq5MUzeVpHt4EGK7B96PbFTEbvtVlv6Lf
8+sqlzblwHu+5ulByOB4jBnYn/6Wfp7QQAeVr50kvl2iev1Evh/uFL35ZP//6hXgIAZO6y9RvZJcpbzbt
CX81aAgAxtTcK4nSSVCHqQGAKADGo4PT1/+318sAjD+g1AIA8lB56od0LNa6tu8vyiteFD/sHAGTgit

8QKfBL9ryiJyoAAMiVGsJPgAoA+VK9T1Uv1CTUsH7qowJAji79AfVR5xghKgAAyM1HvvgRezIpAEB+
VP3Tt33hZSProI6iAtS7/+NX9RIAIHN2fdR79ALmDSEqAADIxe6Du/LBv/igXgIA5OWOL18/tg7qKPv/
8zH53Mcf0ksAgMxd/n9FLn1BL2CeUBMVAABkTg3fV3VQmUgKAPJ189efL//8Czfppclc+dzjsnX36+
XWV79IrwEAZOtKXR/1Zr2MeUBPVAAAkKknnn6CmfgBYAZe8MMrEk0kNcoz/3RJmu+/X3705DN6
DQAgW9b77aWPWx+fchYxFwhRAQBAZlRwqgLUh594WK8BAORB1T99yxdvlOf8OJ1f+R7/xo/k7g8
xKSAA5Kb/HZFL1EedJ4SoAAAgM+/77Pvk3ofv1UsAgLz83AMvkRsev0ovpeMrf/mI3PtJ/igGALm5/
DXr0dELmDVC1KXWlo1CQQrGY6XR05/LSa8hKxttveBob7jtWZG8mzM3Qs5LHL3GinPuVhoy6tT55
9d55H7NjwLuWyCW+n11+djffkwvAQDy8vJvPE9e+eC1eildn/zPB/LN//19vQQAyNylXZH+Y3oBs0S
IurR60lhZlaZecpTlzFpRP8+eHfiVasLfVIImPi+9hqzXor5KXfOCrAYvunRqJSlMENguK+5bIB7V+1T1Qg
UA5Ovqp47LG++bvg7qKKo+6u//
+y/bHwEAeVD1Uf+79fFZZxEzQ4i6tLpy4KZA5bp0+33p9/dkM68MdWzgt6QmPS+qZ+S4YK99XtxN
V1vqevelWy87K5qrQo4aA/ctEIuqf6rqoAIA8veWvRvtIDVLqifqH/721/QSACBz/UdFLv2xXsCsEKIuq
96h7OuncuqE5Nf/FGmL1zOyJ41zugtquS5bFedpcfOsVJ2n0twlRQUwPTUTf+WPKvZHAEC+Xvu1F8
vLHr1aL2Vrb/f/2A8AQE4uf8l5YGYIUZeQXbvRDN2aq04tx8GuiKp3o1E7c1T9zGAtyGCd1bDejbH3r
wVreI6qN+kMVfdfl6zW52A9S6+2qH6ENy3GPgPncEP8zZjnydln0vOiqHaWYvWMNHoeB0LzkpzUn
VFl/9A6olGM9obVW21v6GMZPAcJrkvI/RZ2vePcb4PXz35E1ImNY5b37WT3p2vSa2eZ6D1Ar1TMr
49uJI6QOz91pzz4+IN6CQCQFxWevubvXqSX8qF6o1IfFQBydOlPqI86Q4SoCDUUGGl2/cyRYVRHai
WjzqrR43EyanuDNTzVurCgpuQNVXdFt3UUZ5+DwWRzdcJ9Fjflgjtk3joz5/RGeo1z3nkq1y+kUEahK
q1+y+tVOsTseTxK50C6+umwipx2N97ZkYsDJ7W96x2NX1c3yXVRQV7I/eZcj1EBZPj9pu7d0GC5U5
NSIMjOSpb3bcz7M2CCa2eZ7D0Ay07VQP1M7zN6CQCQFzV8Xw3jz5uqi3r3h75KfVQAyI2qj3qP9Z
H6qLNAiLqEKtt96Xfr4kZ7Um3Z9TH72zrxbG/4AZBXL9Won9mpyfrIxEaFec7r+3uboWUCxu4/wN+
et3/pyI6XBPWkse4GPca+Wzo1imzrKP523M2ofdbOu/Fbsn0WNy+If+rOS9v677wbglnn94JOUJOd
F4P9um2ZLK8uyolT+ukYlS23beb5V9ri53BnxMnhkpwj67VGqYGh+21ofyZj2/b9ZrYlbFt+kJ3U/Ny3
/uvD789hya6dZar3ACyrT+x/wp6NHwCQv5UvvTTzOqijPPTV78qf1hmBAAC56T8scvnP9QLyRIiKIW
bPtPoFPwgdDgOHletbE4Z54aotPxwsrp3xAqzOge4z2bsoO14eaey7suW3deeiJIl7zH364ZMh8T6Ls
nnWTbuaslpwe08Gz+8kipt740PW2PblMOpEFdfkTNjxtXf9XrVn1pzjSXSOrPOzp0NEI3gPvd4Dhu43
s8et0evSPk86CNzLYfa0LO/bsfdnmCTXzjLNewCW031fv0/u+rO79BIAIE+vfPBaufnrz9dLs/G5jz8kX/
nLR/QSACBzl74gcpkJ/vJGiIoBI3qm2Yqy5iYxIwK3UyfSDKjKcrKkn47SPdC9+VSoU/LqNhbMYdKRw9
QnMMk+K9tGr0FHOsP403RKoi+fcf2NgNIP3Kpy1j2gqa6LruEZOrw/aOh+M8JCawf20Hd33yNrsaZ
uHu/bBNduyvcALB93Jv6nn31arwEA5OWGx6+Sn3vgJXpptu7+0APy+Dd+pJcAAJlTw/r739ELyAMh
KibUkeHOgTHCo7mX3TFUvMKUSrD+5Oz05HBssVSf36vSHRZuBG7V08FeoUmYExB5PXXHCbtWR
dm8EN470w0rF3t+o8nvz/SvXdh7AJaNCk5VgPrID+h9BAB5e86Pj8lbvnijHL9U0Gtm60dPPiPN99+v
lwAA2XtK5NIf6OfIAyEqJjR/gWm53vWGbAcfk9YLHS/
+Po26n7aO1NZznJyneELGlj4tn5Sxl7S4KW5lAntYuDF8vno6/CyPP0dt2TB7nbo1OM36o0lYbdzT+xj
s/as0z83XpEi53bcTXLtoR+GPJpiWGsL/wCMP6CUAQJ7e+Nc3yAt+eIVemg+qPuqnf/9QLwEAMqfq
o15iYte8EKJiQElOusnV0EzePbnoFnIcO/Q7J6WTXtA2qm5m6ibYZ6+xrodpl6Ved5OsPCfnMa5rc1f8
zphdOfAu6QmJc0m9HrXW/XH+/I4OP6sSyOGSnCOzLqcKFN26qMaQ96TUzPKqx6k9GZQdSHa9W
p7pDpOf0CzuW0usa5fme8AU1xDz7yNf/Ig9mRQAIH+lh1448zqoo3z6v/6D/MOXvq2XAACZu/xZkf
48dRU6ughRMcCcBCnYW9IPAlXYNTChz6yYNTCbq/5Q7faGHhpuPdIev510n72GrLsnrnpWNjeNyY
Nq65JPjmrWsmzKrm5er3HOCzBj90asnBbnDuIIs+ke18Bw8Amvix8oDvbcjUlvX80sH+xxaoTFZo9b
oz1p3yaRZnHfKnGu3QTvASU/dZXaee9gZMOb4h9Hze6Du/LBv/igXgIA5OnaJ6+0Z+OfZ83fvF+
+9y1qZQNAbtSw/v739QKyQoiKYeYkSJ2alHSoU/LTE7kw7YxI5vDy5uoUoVGwBmZzVQdQXnhTlVZ
qs9e7kuyzJ411d6h6Wepbav3okCq98zLMnFndbbN3TastiX+aKhIo72oZDmATnCMv2LO4x2xOsJTEi
HvXrLNaPevPNp/YQt+3SpxrZ0n4HuDXW7V41zBubVssGjV8/85P3amXAAB5UvVP3/aFl81NHdRRV

ICqglQAQE76T4pc/rheQFYIURHKHgYdUpOy2rLWu8Otp1KR7cGClfuHRs/BBOwamMZwbZddWzOj
eqgx92n23LN7obonbiCk8of1p3hehhRlc2+4Tqg9hD5hYBeYJMs6ZjsbHhT7uljH3G/5QaqtKq2+0dZ
ACYJo9r07eJA2Z5vTZZMLft9aYl07S6L3AHUsg6+ttiava4u59cTTTzATPwDMkOqBqnqiLgI1pJ/6qAC
Qo8vWb6bUR81Uoa8KBgJAEmo2fT0ZlAph96btmTxL+lhOtaYNWBfEUbp2yJUKTit/VJF7H75XrwE
A5EnVQVWTSS2SK597XLbufr3c+uoX6TUAgGxdKXLFb4gUbtbLSBM9UQEkFCxRcGZtsUO49nnrWC
J6ZB4tR+vaIV/v+
+z7CFABYEbULPx3fPl6vbQ4nvmnS9J8//3yoyef0WsAANmy3m8vqWH9TzmLSBUhKoCY2rJh17o06
pWaJQoWUK+xIqv7demmUqJinh29a4d81e+ry8f+9mN6CQCQJ1X/9C1fvFGe8+PF/NXt8W/8SO7+
0AN6CQCQuf53RC7doxeQJkJUAJNRNS8XfPx7cXMvpRq/C+YIXDvkR/U+Vb1QAQCz8XMPvERueP
wqvbSYvvKXj8jnPv6QXgIAZO7y10QufUEvIC3URAUAAKEefuJhec32a+wJpQAA+bv568+Xf/6Fm/TS
YqM+KgDkjfqoaaMnKgAAGKKCUzWRFAEqAMyGqoO6aBNJRVH1Ue/
+0FftjwCAPKj6qGpY/7POIqZGiAoAAIbc+ak75cHHH9RLAIC8qQB1UeugjvLN//19+cPf/ppeAgBkrv+
oyKU/1guYFiEqAAAIUDVQP9P7jF4CAOTttV97sbzs0av10tGyt/t/7AcAICeXv+Q8MDVCVCTUk8aKm
uVbPTaknWjdOGltZ96MOYZeQ1Y2gmvbG+7rV6TR0yunpfZjb3NEO2zTne9M2m1bkPugvaHbWJC
BSwosjE/sf8KejR8AMBsqPH3N3x3tuqGqN6rqlQoAyMmlPxHpP6YXMClCVGCGeo0VKZRq0tHLWe
pd3DH205RdQj4AA+77+n1y15/dpZcAAHm7+qnj8pa9G/XS0aXqov7+v/8y9VEBIDeqPup/tz5SH3U
ahKhIqCibe33p99VjWyp6bXby3l8WRhxDryHrtTziU6UnF3eC+2qea1hrF8VRuA+A+aZm4n/nJ98pT
z/7tF4DAMibClBVkLoMVE/UT/7nA70EAMicXR91Vy9gEoSowDLoXRQ3Qy2Xy86Tzo5cXJwUdTFUt
qXfrYs+w8DCUMGpClAf+cEjeg0AIG8//ffX2UP5l8m9n3xYvvKX/NsDALm5vGc9mOBvUoSoS8eoLbl
i9kQctd6oc2mvz7s25fD+IutuGnU/VwKfNLcT9vko6Z8z+/PmMP7mqvP5EYU0/WNWj+T1Rv2h/FU
5e+GMDvk6shMnRQ3UUg17jG5PvHbHuTbD59DaumyYrzfqkQa2MbA+/BTHaYN5POpY/P2rh7vd
9nnrupbrsjXQXdYu3WC83n4M3DfArKgh/A888oBeAgDk7YbHr7Ink1pGd3/oAXn8Gz/SSwCAzF26R
6T/Hb2AJAhRl05R1s64PREPpOs8s3TlwE30Aj0U27LbdJ6Vz6xZXz17ldNV/awjB/4B2PywsCxn1nRr7
RCwJIMj5zu1UswQa5bnrCO1UkFW9fYcal2SINUYyl89LZXimniHs3Nx+hCvfEbcU+2L2e6pr43Dfn1w
Z846FVQOrG+uDgSpE7VBHcuqeFvWoakKSlf369Ld2wxcdxW+lsJKN3RqUprnibKwFD7yxY/Yk0kBA
GbjOT8+Jm/54o1y/FJBr1kuP3ryGWm+/37qowJAbp4SufQH1kfqoyZFiLqEimtuT8R9OXQTovauHwi
JEU72Dq1XOU6dmIcI1VI5LW6M2gzMjmSEhV6w15PGutvjsyotu6am9WjpLXRqsh4jjUz7nFW2rTa
Yw76rLadd22HVPv12d+vuV8TsRaoYQ/mrp9X2zVA4xpD+4qbsuefNfnTFa4Z1BPULwcDQN67d6V
wbV7neDX69Frbev2+maYPxeh2aFjf3vOc+P1RXYWtXf41/TppyLsFxAmnafXBXPvgXH9RLAIBZeONf
3yAv+OEVemk5PfTV78qf1h/USwCAzPUfFrn853oBcRGiLiOvJ6IfaPUOndivWnXCo32dFJrDwO38bS
5UZMsNoJq7fi8+s+6n2wPUXFff8ickqmx5QWCs3pgzPGfVlj+Rkh/mWi0Z7IY7gtk792TJfmJsJ0EYq/
Ua636vzepZ2QxPUMe3O61rY6vKWbchRsg+cv3+obPdKdoQeH0UI1Q3Q2s7cNWB6t6okwhkSA3f
v/NTd+olAMAsvPLBa+Xmrz9fLy23z338Idn/n4/pJQBA5i59QeQyf8BKghB1KRXlxCnnmRtode1x6W
U5efqkHXYF11vUMHDn2VzwQ7mmeJ0KuwdeWOgN5ffWWcfkDu+2H8bw7cAQ/VFmdc784HMy
Yb1zLRMP6W/LeT9BlVZoz1klRrtTuzaW8kkJ3d2o9a4p2hC7Z7Zxrq2N2WUO3P3Er8sLpOuJp59gJn
4AmLGXfPe58nMPvEQvQbn7P35Vvvct/m0CgNxcVvVRv68XMA4h6pLy6oraPTl74nSqPCUnKies/w
+uV3ngPEWoFiOYcodmt90x0xG9I6exkOfM6GmphqaXQkPC+LP0tzf8OqBVo6fp8kkSbhdl80L4jP1u
cDtiPjEgE+5M/A8/8bBeAwDIm6p/usx1UEdRAWrzN+/XSwCAzPWfFLn8cb2AcQhRl5U3tHlfDts6aL
N77VXEyQqN9VP3hsyCUdfTDi/9upOjwkuvNubQI2YYuIDnzB/KHyXmkP72hj9RVLUlIzuhTmDqa5O
CTNtg1JUdKNlqa55jln7k532ffZ/c+/C9egkAMAsrX3qpXPvklXoJpn/40rfl079/qJcAAJm7bP02eukze
gFRCFGXVklO2hlkRw52nSHNbh3Rkv0Jf3347OuzV9w864eaDXeSp4E6pCVnqL3i1eKc2KKdM2Mo/
xjjh/S3ZcNLUMtSV1PRTyvVazOhHNugZuhXPU7tScXsQNWYoCtu2QJgSvX76vKxv/2YXgIAzELpoRfa
D4z26f/6D3aYCgDIyeXPivTp2jMOIerS8ntyNptOOObWeCzq4p/uem+Sprnj9gDtSK2mA77BOqRmP
crmqj9sur3h1aUsxB5LvWDnzBzK787+H3iYIV70kP5e45w3jL9cv5BOuYRUr82E8miD3pbKoIM9Trvi

ls8dW7sVSMn/+v/
+l34GAJgF1ftU9ULFeHd/6AH9DACQi0v36CcYhRB1iZkzpqvehd7w88Ds5sYkTXPIq1NqC+shGaxH2
VzVwZg/Ll1GT440LNVzVtS1VJXmauqhoTmUvxxaW8AoiWC9cuSQ/l5D1r0CqtYrAxMwOY/JJkhK99
pMJoc2VLb9IfyBurRGfdmzm7MP3bEU7v7Fu+WOl9+hlwAAeXvjX99AHdSYfv7dP6WfAQByUXijfoJ
RCFGXWWDW8FPiTzbuDltXzPVzyAwvRw2ht+tRGr0uXeW6dJPWu0z1nFVke7BA5v5hSrUxzaH8o0
NdMxQeNaQ/Xl3VCaV5bSaVQxvsIfxhxVBVSNvvp1pfFohy1RVXyf/4lf8ht1x3i14DAMjT373yCf0M
UV7z1pvkbe+5VS8BADJ37FUix9+kFzBKoa/G9QIAgKXxwCMPyOv/2+vtmfoBAPm648vXyysfvFYvYd
D1P/E8+a1PvVGedw0TbwFALgovFrni/daTq51ljERPVAAAlsztN91uD+0HAOTvb27/tjx2PX/ECnPlc4
9L9fdeS4AKALmx3m+Pv8f6SIAaByEqAABL6F2n3iUfeMMH9BIAlC+Xjvfl8294VH78nMt6DVz/8t+V
5NZXv0gvAQAyd+ztIoWb9QLGmSpE7TVW9AQpBX9W65F60lhxJ1TZkODL27LhTbbiPCabqGZYeyO
43dBH1jOAAwAwhz788x+W07ed1ksAgLz84PnPyt7rvqWXoLzidS+R1X8bNhkqACATx4oix39eLyCO
OeiJqsJVf5ZqR84zwquZ0VcaKU3oAwDA4rjnl+
+xh/cDAPL10M0/kO6t39dLy+3al14l1d99rV4CAGSucI3IMTWMH0nkGKIWZXOvL2oeq35gxuuuHH
iTiKvZsNXn92QzxwzV1qnJeTqkAgCWjDtj/3VXXafXAADyonqjfu+aZ/TS8lIBqgpSAQA5Of5ukcIL9QLi
mn1P1N6h7OuncuqEZJedlqXedUNc/9Gtl/XnRZq7pKgAgOVzy3W32EGqClQBAPlR9VE/96Zv2h+X1
S/8xivsofwAgJzYdVDz7rl4NOQYog7XRLXrlZZq4nZEtYfVD9UoNb/OeaRVL1Uprp0RP0YFAGA5vfm
WN8tH3/5RvQQAyIvqibqs9VFVePoLv35CLwEAMmfXQX2HXkBSc1ATNUKvISuFktS8lNXRqZVSq2H
aPu+HuNXTfpGB5WVM8uWd46hJwTA7YddqEYTfT/4kcHneY0nPYU7fC+q9b2DCO//8rEiKf0cCAt7
7s+
+V2h01vQQAyIuqjbps9VGfd82VctfvUJMbAPJztcixO/VzTGKmIWpluy/9bt3vCVptOcPst1WY2ZPGu
htwVqXlDsFvVe01qobpeqIkoSO1khtC+I9Vd0Yra9/2bgFghnqNlWAPfSBnqjeq6pUKAMjXfT/zuD1r/
7JQAer1P/E8vQQAyNzxO0UKL9YLmMT89kTtXZQdnSKU61v+RFSVLXHLmHZ2LqbU+64s9S0S1NFG
TQoGTGKR76eM295ryPpg13tgBlR9VFUnFQCQnx8/57J8/g2PLkV91Le951Z5zVtv0ksAgMwdf5PIsV
fpBUxqfkPU7oHXE8sevu/1HjWG93cOpKufTsfppZpmrVUAABaVmqm//attZuwHgJw9dv3T8je3f1sv
HU23vvpF8ku12/QSACBzhVtEjv0LvYBpzHdN1FSFz85vlhPo1M7nWItxHgxO2jWqxuPoOpD20GPv6
/VjZG3Jwf1FBNd2Pdzga0fVgsysDVEya98oca+VK/5xJm1f2OsHyncO1O806o7arx19P/kG2p/K9Ux6
DsOEt32wXungORo8P2HiTbTn8/epHqPqpCY5P0DQbdffJvf88j16CQCQl7+/7Xvyjy//oV46WlQd1O
rvvVaufO5xvQYAkK2rRY6/2/p4hbOIqSxEiFqud4fDT/uRwnDa4qac1WVWRfblcGnyBRVsDU7a1ZTV
wqr1/3hUiFMKG3rcqUlpMKBKMklYe2NETUjVYzgYFmXWhihZtW+khNcqwXEma58TyIW9vrlaGBH
2qXNitLNcl/GVM9SxDbR/6us5/f0ej9OrffAcNVdHhZyTcPbh1XO2Dd97qd3vWGrvKL6DGfsBYAb+6v
WPHcn6qL/yH15JHVQAyNPx09RBTdH8hqilk34P0YN0Bu2Ha8tuuinKQug1zvnhkTuhV7/r1Zsdzzhv
5bp0dbDd9TbQlHNeotNLMEmY9dpzesOh2+3IzkX3tVm1IUpW7Rst2bVKcpzJ2tdrrHuBnP+HjZa1F
615bkRQaLRjb1OKem0koz1u06e5ntPf70n47fHb3pHa+ejIPHqivUH+PsLvvbTud0Ds2frfdepdegkAk
AdVH1UFqUfJyumftB8AgJwce53zQGpSC1HtnmjGkFHzEWco65DimpzxspxVfxuqF6C77UQbdnpv
me1yHmYvuTOyFivhWXQ9uejO2qUCFi+kKcrmBSPEidI7lH39VDo74mY3xc09J6yxHnub+mQmmiT
MmLjHCNyKa2eGQ/XM2hAlo/aNlPBaJTnORO0LtuOst74iW/aGVVC3J2GHE2hHTNWz/rmtbPtBrd
/2JNczhfs9gWrL7yFf2Up/+4q5j/B7L8+J+bAM7v7Fu+WOl9+hlwAAefjmjU/JV376u3ppsb3sp14o/
+a3mNAEAHJTuFHk+L/WC0jLHA/nDwYcXkjrjWE1w5B0mMHN0daVAzdTKp+Ukn5qK56QU/ppJDP
kHgioh2ouTjVJmK6nGTZ8Prc2REmpfSMlvFZJjjNR+0a3wwldR5fWOHUi6XdVVU4HNlaSk2473bYn
up4p3O9zpSwnAwcRIteJ+bAMrrriKmbsB4AZuP9V37HD1EWm6p/+
+n/5GeqgAkBurhQ5/mvWR+qgpm2+a6IWN2UvbMitPdQ3hXqoHtWLri8pZ7JH3OhefG5oM1EPZ
MWu5eiGPlE1KzNsQ5R5b19sebQvRuA3VlFOLEzSmcbxAvPpphfcZAepKlAFAOTn8yuPylNXX9JLi0f
1QFU9UQEAOTn+r0QKN+gFpGmqENUc9hv1cMLJoj8M2gxA7aBUrw9NMc2v04+4dRUtdp1B82tD
H2kGskvEuHZ+7Udf89zw5DXjJwlry4bZq9OtjWnWijRl0oYo2bcvLbGOc4btG21wgreeHHp1B4ZlOv

HcEcD5QZpuv+l2e2g/ACA/KkBVQeoiel3l/6IOKgDk6dhrrQd1ULMy3z1RkZGQ4dEus05mDGp2d9V
b0Q+rjZ7D7raTTBLW3vV6dtrhjxuYG8OTB6XehihZtW+khNdqguOM176Idth1itOdfT7Y9JDh+ImO
M737fWHkNjEflpGaZOoDb/iAXgIA5EEN6f+7Vz6hlxaDmoWfOqgAkCM1C7/qhYrMEKIupaKs+bN2
yao3Ztuc0XsMPcGXKlEb7K0YEnhNOEmYH/4YM+KbcmhDlFTbN1LCa5XkOBO1L6Id9rE7NVUTnsK
Rmqsb4jfdL5lQPrPmhNaJrmcK9/uiyeB+B0wf/vkPy+nbTuslAEAeVH3Ux65/Wi/NN1X/tPp7r5XnXX
OlXgMAyJaqg/oe6+PVziIyQYi6pIqbF/zehs1VHawYk86MU9n2h393alJygxmjRqg/UVew9mbkJGGV
09aSNq5dWbUhSmbtGy3ZtUpyrpO1b2w7ynXZSmV8eFnK5aas6vZ4Tbfaftab/j/Z9Zz6fs+LOdGV28
6Jws6U7ncgwj2/fI89vB8AkI9Lx/vy+Tc8Kj9+zmW9Zn79Uu02ufXVL9JLAIDMHVsTKdysF5AVQtSlVZ
TNvcFJu6rS6res/8djD/8OK6Rpb2dgoi679ubg/ixDk4RVZHuoDc72vF01d71eitm0IUqG7Rsp4bVKcJ
zJ2qfa0Zfu0IbVpo3SBlM7JWcHjzfsGiW6ntPf7/mw7q/B67F/aPQSTiCV+x0YzZ2x/7qrrtNrAABZ+8H
zn5W/ev1jemk+veatN8nb3nOrXgIAZO7Yq0SOv0kvIEuFviqCCAAAMIF7H75XKn9UkaefXYwhpgBw
FNzx5evllQ9eq5fmx7UvvUr+06ffzDB+AMhL4RqRK9R8BQzjzwM9UQEAwMTefMub5aNv/6heAgD
k4W9u/7Z875pn9NL8qP4udVABIFfH3239jwA1L4SoAABgKu/92fdK7Y6aXgIAZE3VR/3cm75pf5wX
v/Abr5BXvO4legkAkLljbxcppFNUD/EQogIAgKmp3qiqVyoAIB+qJ+re676ll2ZLhae/8Osn9BIAIHPHiiL
H36EXkBdCVAAAkAo10dQt192ilwAAWeve+n37MUuqDqoaxg8AyImqg3rsPXoBeSJEBQAAqVAz9b
d/tc2M/QCQI9UbdZb1Ue/6T6+2g1QAQE6O3SlSeKFeQJ4IUQEAQGpuu/42ueeX79FLAICsqbqon3
/DozOpj/q299wqp/7ZDXoJAJC5428SOXabXkDeCFEBAECq3lF8BzP2A0COvv2if7Jn7M/Tra9+kfxSjV
/kASA3hVtEjv0LvYBZIEQFcteWjUJBCsZjpdHTn1tSvYasbLT1gqO94Z6fFZnX09NrrDhtXGnIqCb6x+E8
lv5aY2mo2frfdepdegkAkLW/v+178o8v/6FeytbzrrlSqr/3Wrnyucf1GgBAtq4WOf5u6+MVziJmghAV
yFVPGiur0tRLjrKcWSvq58vHDiJLNeno5YXRa8h6LarV6loXZDV4saVTK0lhIDAGjqq7f/FuuePld+glAE
DW/ur1j8kPnv+sXsrOXb9zu1z/E8/TSwCAzB1XdVBfrBcwK4SoQK66cuDmbuW6dPt96ff3ZHNZM9
SxQeScUj1nxwW/7fPiHlq1pa5zX7r1srOiuSrkqFgGV11xFTP2A0COfvycy5nXR33zr9wir3nrTXoJAJC
5YyvW41V6AbNEiArkqXco+/qpnDohy9v/dHHF6znbk8Y53QW1XJetivO0uHlWqs5Tae6SomI53PSC
m+wgVQWqAIDsPXb903L/q76jl9L1sp96ofzKfziplwAAmSvcKHL8tF7ArBGiLjGvnqP5CKntOLI2peqN
536d163Or/dp135sb+ivNdbZLwuuD/bKS2MbmtlG7zFcYzN4jMM1S+1HWN1Low3jal3a+zDDt+aq
87WDDQ9pc9i2o9o8tpfjROdl+J4ZuZ8YxxD7fGh+W9RjuK0OZwi9/7p0z506/lKsnrNGj+NAWF6Sk7
ozquwfDt9PNqM9ie65eMduS+H7wj1Pcd9HsNxuv+l2e2g/ACAff/fKJ+SbNz6ll9Kh6p/+
+n/5GeqgAkBurhQ5/mvWR+qgzgtC1CWlwpHQMKhTk1JhQ8ZlcHHYtR8HCkLa61TIMrC+uRoeXk2
1DRU2hfYY7EitNCqEU58zapaW61J3uw52duTiYMi0674ynbqmQ8GiZh/zyGBquM1uz8dQE5+X4X
umuToieEt8DFGcfQcvd0hb7WCw5A2hd6V67jxVafVbXq/SIWaP41E6B9LVT4MqcjrpPZfk2FP6vlDn
KY/3ERwdapKpD7zhA3oJAJC1z688Kk9dfUkvTe/f/Nar7J6oAICcHP9XIoUb9ALmASHqUmqLn8O4d
TmNeo3SlHPhSUpi5XrX3na/FYybwtaPGt482TaCw6mHj7EjO4PplEcFZM7r+3ubsrlVF+erBr/GPI9nZ
FyGWtm2ttd1t2Wptpx9bOvUrr3hB4Vhbe7UZH3kdQm2eXRT0jkv/invSO28cd0SHMPY8xHg7zu8r
dZxrbvBoHEu3Iamcu40u53bEitrHVKUE6f00wiVRPdckmNP7/uimOP7CI6OD//8h+X0bQxHAoA8q
ABVBalpWDn9k/YDAJCTY69zHpgrhKjLyOwlZ/R0K27uOQGJ9dhLZaajqpx1t1M5bS25RqwPHd486T
aKsrlnBj6O4toZL7TrHIT3AyzXt4IBWXFNzugv6uxc9NvY3vV65pXPrI0P38YwexjWLxht3rwgfgZ5PrR3
31CbR5r8vFRbfnDoh3xB0xxDFHPfoW3tXZQd3SEycC4qW/5+zWtniH/u1HFY3yOhIe8k9uUwrEFKk
nsu0bGn+H2R2/sIjpp7fvkee3g/ACB7akj/V376u3ppMqr3qeqFCgDIiV0H9V/rBcwTQtRlZAQ0ImqY
bsGrZTiyhuIkyielpJ8GjFofJo1t2HQ9x9BhzEGnTgwGP0VZ8xMtLyzyA0Mj0J1YVK9WY/8jgrfhNscV/7
yMN90xjFaWk+MudvfAa79X7sF+GEPcRwyfn/zcTeuUjN51gntuimNPcv2HzlNe7yM4ctwZ+6+76jq
9BgCQJTXJ1KT1UVX907t+59XUQQWA3Kg6qHdaH6mDOo8IUZdSUTYvhPckdEOYqMl1FkZg8hyjn
mOk8MDO76nnDnc2AsPq6dg9GafXkeGOgjFCRlOK52UyYccwK2keVxw9ORxbLNWR2T2X2vVfkvc
RZOKW625hxn4AyNFfvf4x+fFzLuul+H6pdpvc+uoX6SUAQOaOrYkUbtYLmDeEqMuquCl7esjtQKlR
W/Pcos+u3ZYNs3edW7PRrMGZhHW+zurzZA+NNoYyV0/nF6FOH/qlfF4mkm1w6dXKHXpMWsd0

AsUTMrb06bje1BPcc+OPPf3vi6P9PoIsvfmWN8tH3/5RvQQAyNIPnv+sHaQm8Zq33iRve8+tegkAkL
ljrxI5/ia9gHlEiLrE1MzaqqeYPcGPHYR0vRqKo4f/GoxhxHPHrB2pgiW3/uMUba64U6Z3duT8+R29n
aqkk6GW5KR37gdnZO/JRbfoZeQQ8BgyOC+
+nI4hTOmkFwKOqumZL+NcNHfF75DZlQPvNJxwzn2EWPdckmPP4PpP/T6Cpfben32v1O6o6SUAQ
Jb+8eU/lL+/7Xt6Kdr1P/E8uet3qF8NALkpvFgP48c8I0RdRu0Ne6itmkU92FPMCHiMXnIlPxkzZmJvy
4Y3Dft884MlY2bySXgTWHWk2dQnKrWh/EXZdLsdqvO87l+XXmPdq22ZZBKkcVI7L578j8Fj1udsrvr
DyPW9bj9yHVtu1oBtyq7eda9xzgsxY/VgjnPPTXjsU1//hO8jZntyvRSYe6o3quqVCgDI3t/c/m157Pqn
9VI4Vf+0+nuvleddc6VeAwDIlqqD+h7r49XOIuYWIeoyqmz7Q287NSm5QYtRH7F6NnzmbhXSDL52
Lpkz9nttNibamUhF3I6BrlSH8o+4LiU/fZQL005glcl5MSQ9BnPYu9ueiRK2YH3O5qqz34IX9Felldqs+v
EUNy94PTLd9njnodqSeM2Jc88lOPY0r3/C9xEgiqqPquqkAgCydeI4Xz7/hkcj66NW/u8idVABIE/H3k
od1AVBiLqk7KG3YUUMVeDS7wcDHlX3cLBmYrWVcx3NpCqy3W/5gZHNOTbvsAPDrOPxhlcr5bpsp
ZzL2dcl5LxWW9Z6d+j1VLI5L6Zkx2C1Z/A+3D80ejUmYNfnNIaSu+y6nznWQ/UUZXNvuFaoPYw+Q
aAb656LfezpXv9E7yNABDVTf/tX28zYDwA5UPVR7/uZx/VS0Cte9xL5hV8/oZcAAJk7Zv2GfPwdegHz
rtBXRewAxKNmNtcT86gwbG/anqHAOEfpntPHcqpFwIpwn+l9Rip/xM0BAHl441/fIKWHXqiXRK596
VXyW3/8z+yPAIAcFK4ROf5+66P/Xoz5Rk9UILaeNNbdmc3LcmaNABVZO1r3XPu8dSwZ9ODG0fGO
4juYsR8AcrL3um/J9655Ri+JVH/3tQSoAJCn4+8mQF0w9EQFxmrLxmANWFXOgK50yMzRu+d6jRU
p7ZyRbiplKXDUFf6fgn4GAMjStU9eKe9s/aS88723MYwfAPJ07O0M419AhKjAWAOBFgEqMsc9h+
WlJiiblx9NaEs42hKOtoSjLeHmqS0nrr1GDr/3pF6aLa5RONoSjraEoy3haAvSQIgKAADmBj/ghqMt
WhLONoSjraEoy3haEs42hKOtoSjLeHmqS1IhpqoAAAAAAAABCBEBUAAAAAAAAIhCiAgAAAAA
AAEAEQlTkSE2WU7DrfxRWGtLTa8fqNWRlo60XHO0NvZ3CijRibwjp6Uljxb0GG9aVTbJuGmH3UNr
7mJbRRv1Y4SYFAAAAAGChEaJirvUaK1Io1aSjl4H5pgJdY1Z9W1nOrBX1cwAAAAAAsIgIUTG/eg1Zrx
GfzqeibO717RkF+/1tqei1szFPbenKgXvLluvStdu0J5tkqAAAAAAALDRCVABIS+9Q9vVTOXVCyE4BA
AAAADgaCFGXmao1OlC7MazG6GD9UXuIvfE1A+VKNbNOpXokq1Vp79Mcxt9cdbYTvjOjjeoxqk7qY
JvCa1VOdrymMbVf2xvetob2H3JN4rTRY359ZEONcxFo46j1xj7t9ea5zKoOadx7KKotg9uYsD5pjOuS
9J4FAAAAAACLgxB1WakgL7TWaEdqpVEhpPpcQUoDQ+ybq4OvVyFiSYIva8pqYbBWZBqcNq0GNh
xyDHYINtgm65W1UnjQaYt7vIMqcrqqn3Z25OLAa9u7bmODtTKHQjgtuo2TKsrambLztHMgXeeZxR
iOHmh7W9xml8+s5dDDMoV7aKJrPizf6wIAAAAAAOYRIepS6knjnJuIuXUb+9Kt61BNOrIzmPx5qtL
Sr2+5QaEKG8/7ve16jXN+0FVt6VqVXfE2H0Nl2/qabl28L3G3sx1W8dJvU/gxWMe77oZg/mv77gF0
arI+MhUdf7xhKltu2wfPpR9GSvmMeBlqe8MPgsOuSWQbJ1NcO6PbuC+H7qbbu0ZI2ZEDN101hq
mfOpF9hDr9PTTNNTckuC7J7lkAAAAAALBICFGXkjERz96m16vQD9VEOl56FlRt+RP3+EGhqScXd9w
+e1VpeQGStc8LYa+fntmm0GPoXRS3SeX6lj/xUGXLC+U6OxdDexSOP94RimvidfQ0t22ElGaPTrN3av
2CcU02L/htrJ0fGKo+Ja+NftDbO3Si0mrVCRv3dbrau7jjBZKnvROYlRTuoSmuuWkm1wUAAAAAAM
wdQlRYdA3P0OH9SZmzk5+Ukn5qK56QU/ppespyMrCTEN0D77jsIdheXUtjqHdgSHsazOHy/rB4P5
SryllvyvYRvVNtxnbMHqOpKMoJfUHcwLlrXzzrnJ4+aYeVwfWW6mk/kMxMCvdQKtd8VtcFAAAAAA
DMG0LUZRWYKCdunckYgeWRMt3x+r1i3Z6eRig3URhpDK9PScUt3trctVrXE6cj6ik5UdFhZWC9anb
2EeriSf+6AAAAAACA+UKIupTasmH2OnVrPZr1HI+ocr3rlDEYevjD9lNT3JSzbglONXTcqCs6WRiZQYh
dOS164L4ctvUQeLv3pzs5lrF+QUP07K/5sv1xAQAAAACA5UOIuozMupwqYHLrohpDoCdXkpNuEjs
4XNoIEXNVcoamK6NqvWbF6+nZ2ZHz50fVFTXP2eBs/mZ90FMydk6nxNfQ3XdHDnadr3VrtZbsT/jr
h4e0ZyWFeyiVa57idQEAAAAAAAuNEHXJ+QGTMWP/VMxakU1Z3XCn3DFnS8+ZMcmTNFfFa1J7w
6+V6a1MmdfTsyPNpj76oaH8Rdl0u6xar6utN6yz5eg11r0anuYESU7AqVivP+8dkGx4U8nH5V+vZtP
5Wnf2/aIumOquNyfCylYK91Aq1zz5dQEAAAAAAEcTIeoy8oI9S3NVh0rGhDtTMmcun2r75iRC7nY
mCjuDs7o3V3WI5gWO5gzwaXOHxftCh/JXtqXl5XU1KdnnrCAlP6mTC95EVCojdOutWrxzHLe2bVBg
W9Yzb2i6eZ9Y68/k0w3VNv09lNI1T3hdAAAAAADA0USIupQqst1vGQGZUpVWv+8HRvaEQpMqyu
Ze1w/BbGr7g/scx2qn1yBt/9DrDZhIcVP2+oNtstj1YDOoh2rwhvQr1v62Ruysst0PrUtbbVnr3ZILLnU

8g6+ttiara2v22gwMTTeGs+c+ZD2Feyila57ougAAAAAAgCOp0FczrADITq8hK3oiL1WDdo+eiwAwk
urtPS8/mtCWcLQlHG0JR1vC0ZZwtCUcbQlHW8LRlnC0BWmgJyqQKbOOZ75D4gEAAAAAAJAOQIQ
gE23ZKAzU8ayeFTqhAgAAAAAALB5CVCAPql5pZpNXAQAAAAAAIEvURAUAAHODelXhaEs42hKOto
SjLeFoSzjaEo62hKMt4WhLuHlqC5KhJyoAAAAAAAAARCBEBQAAAAAAAIAIDOcHAAAAAAAAAgAj0R
EV2eg1Z2WjrhVnrSWNFzZavHhsyL60CAAAAAAADA/CNERSZ6jRUplGrS0csAAAAAAADAoiJERfp6D
VmvEZ8CAAAAAADgaCBEBQAAAAAAAIAIhKhLqr3h1gddkUZPD7+3l51HaClTVePUeI3zcL7eZW/XH
MbfXHVepzc4uF+PuW1j58HXt2XDfY318F4Wo12TGjwv6jHpuVGSHr8S1obCSkPCD8+s/eo8VkaciG
TbBQAAAAAAWF6EqEuvI7VSQUoDw+
+bqwMhX3tjRI1T9fXpBJbR1H5WpamXpFyXrYr1MbN2OWHk4HlRmquFYNCZ4blRoWtYG6RTk9Lg
BFl2EFuSwZd3aqWhcDTRdgEAAAAAAJYcISosVWn1+9K3Hq2qXqUCwPNulNaTxjkdX5br0tWv7dbL
zjrrtTsXnYiusm19rlsX9zNSbdmv7W+rxHNafjv7e5tSTNCupHqNdS+MLNe7zj77LasFWvOcDkeza4NI
W3bd1Dh020055yW0VjvW3SDXOE/uBe3UZN17bZLtAgAAAAAAgBAVUm1tixtxVraMANRTlM09H
crZ4aVeu3bGe23noKufZadc3/La6ciqXT25uOP20qzK2U13yxXZsoNGFVLuibM6w3PTO5R9/VQ6O+
JmscXNPWd/1mPPbVvvorhNDpynypa42Whn56J1ZJYk2wUAAAAAAAAhKiaha5OGDmHPzqkT44K
9tNrVlQN3A+WTUtJPFSdo9EPnYSmem+KanHGTWGtrquyCW7t0qM5p98Dbnz18X7+uYA7v7xxYR
2ZJsl0AAAAAAAAQoqIsJ82UcJTAxElGbdLcjGjnzNtlyawNRdm8ENYz2A9KQye5Giur7QIAAAAAABx
NhKiIoS0bZs9Kt46mWft0JuahXRm3obgpe3qIvV+v1tc8Nzybvl/DdfBh9KCdYLsAgLzp0Q3Ggz9yAQ
AAALNBiIrx2rte70o7oHNrfxpDyKc2ybYya1dJTroJqDsE3qVm4i8YM+6n1YaI16uZ9NUvzfakXXbw2f
XqnHrtK530Qtu4NVhjbRcAlpH9Xl+QwkwTy540VlZFWu57tPNHr+bqLILU+Qxze40Vpz0r+f/hT/0ba
p4P9zEP5wXDhq7XDO6ZmRvzvuZ9P7mPvG7mOO+3ebwnR+5jSf+gFfO8z/K9eGZinJuh951luGnG
nJfc34sTtofSdoiDEBWJ+AGdMSt9AiU/nTRm/7d+MFmdbhD8tO0KKsqaVzS0Kavem667baeO6OB7
cZw2JDp+/aavPhXsGRpSs9Wsc9pc9dvm/sOhHu7KJNtVjG3wDwuALMzsl/chKri09j/lv0npcCYu3Da
KcLuTP+4f5vte3N4IC3ONPyjOQq8h6zWRatX9x28G3NEnxsO8XrMy01/cAyWOhh+z+NZW52O1qS
YFda9TS6qdmpRmFPjk/0v7+Pc19R5csr6f6l3jHFk/T2Z778R5v83jPXncPtTnc/yDVmTok1eYm+C85/l
eHHFuht739CP98xP/vl3dH/g3Iot/IEadk9zfi8efl3zfiydoj/WNrUrbpf+eHO/7dn5+FsZY1g2DJWS9R/
TV5bd+A+hbPzD5uvW+9U+g87mq9ZZia/WtnxX060c8vNcqIa93P29uf9TD2NbIdtqStKvbr5fd9VXr
K8cxXx/yKNetVygJz83Exx/
+MDcdve3gMSfabqvqrS8PXwQAmEq3XrbeX8z3eP2+GngjyoffFv1vwAzaEEm/z8/8vXjm7XCuj9q/f
c28f5PzY/87OoP9RtP37dy1y/3eivPzV9rCv5dn1R7n5y9jv/pnrCy/l8a/rznvuYNtyPocjW9XvNdMa6J
9ZPIeqPdvbdd+hLRD3T/maud+Um3XK1IS/5w4n8/+vTjeucnjvS/
+fZv1+8v4cxImq7aNPy/h62fWnhHfw+nfR87+zd0737fBdX579Qr9vhz3uiJf9ERFDBXZVn8p0ksO56
821puAo7kr/t9KrNd7n9D2D52/MKlanIP1QqutCWuIJm1XEk4PIOsNTS/7rDdbf9h+0jYkPH57qP3g
ubQ5+wj8QdOuc2oMyXfZPWWMeqiWRNsFgMy05XytY71NXZBN503VUpEt9UY28fv35Iqbe9Lv7x
ltmTO69MupE/PRwFm1o9dYl1qnKmfn9kLNhnteWt7PKPPC+T6X6unAzyL5KMqJU9YH9+dQW08u
7sygPb2GqIFK5fqWv9/Ktv3zYmfnYgY9sRxj39d6h7JvfRj8fi7aJ64puxm9Ecd5v83jPXle3ved79+y1Ls
hP8tr6uf34dEJHdm5mO7dE/ec5PVeHOfc5GX8uXHeXwLf5xmY7Jxk9148/rzk+148tj0jfp6yR42mW
tYuzqii+fpZGOMRoi4pvw7mwJuLMeFQcMiBCgvdr1EPJ5TztxMM6dQPhf5rrYf5A725D/VQ+xmx35
Ht9MRtl/MGFlw3nvMG7H6d89gbakjCc5Pg+G2D59J+jDoG8zj1Y9QvU3G3a7xu+NgBYAoz+uV9M
enSL+W6bIX/A5CTnjTWa9KZVTvsoaMdqbbi/1ueGTUM0Rh6N9uSN/n84j6JXuOc9d1s/bI/oxu3sq2
HjNo17dWwypITNg/
+vJW13H5pT8huV1lOenWcNKPePgZk8AetuMFlmDTbEVuO78XTnJv86fJoB+cHhtOnWwJnknPCe7
FhxPub8/PnvuRaNYmfhRcOISoAAJgNfnmPTdUltX/5uTCLXoa6tpj9i6D+pWdGvR3b52vSqbZmPmL
C/0Op81AjV7KppRZXPr+4J+f2NDo7wwDEHTWkatqr7yOrObMI4efpl3ZMgT9oKfPyXhwwR3/Y6uy
flAvevxGqt6h6/9mYYa9C3osD9HwiwflBrO+pqedWiWHwjzD8LLxwCFEBAADmmDP5gRq2N6ueO

MFRDt36vqzO4hfU9oYzCcRc/dbuUL2Csh6WHcfc/eLePi/O6NHZXTNnsg5/YiAVeKtJgXKftGOWv7Q
jNfxByzKH78Xz9oet8pk147pY/4aeVWXUZtirkPfiAernGrdnrP89dXBKXadTkl3n7nn5IwymQYgKAA
Awp5wAVfXWmJ+hjMXNC3YNtk7tfK7hXHtXhU1NO8B1fuEpSEn9Vqh/CZrtcHptlsOyLfP1i7sOCGf5
y6Iecqzq2bt5jz0MViXezdWMZjYfZVa/tI9h93bqyMHgjTuqd9QS4w9ajkV4L56XP2x5ZtqrkPficIMl+f
qydXLfOk8nJau3vdn+EQZpIUQFAACzwS/vkVSvDSdAndMJ/zL8RSPMYE8j9bAngLR+Mexaz2dbt7sn
h6qoWc7nZKxZ/uLu9nw6O8NfFgeHTbpmdl7y/6V9rOIJceZ7CUZNPfuGnmG4O2f4g5Zvvt+LB+T+h
62S2GWOB8PbWf5cw3txTLq2eOCPkekZ+UcYfhZeOISoAABgNvjlPYKeRddiD3czevzkW+dSTf4wM
Bxc/0KW1S8ac6/XkJUVc0i2WqVmSp7lL6nz94u701utKjMcPSpSOW21wPoeCgyhV7dwzTovM26bL
dtf2uOpyGnVY6+27r+veL3G5m+islngD1qLaFZ/2NIjADo1Oe/9w6mHcM+oHinvxXGoUhklqUldLm
RwkSL/CMPPwounDwAAMCOtqvSt33L69a5e0a33y9aPJ2VvRX669bLVFtWewUe139KvWUZh56U
6JyfEblu53s/3bun26+Xg+ZiLe6RVHbg2rb71S2tfJT+5m+H38TB9HgKPefie1vdRxvdv3Pc1573Yf2R9
7eK0K4/35PH7CLt/3Ifxb1dq9H0x9H2r2jFw3Pp7Pu1rNcl5t78m8/fiEedGvd8M7Ns9hrTf/uKem6H
XZfY+POp+0XJ6L453XvJ7Lx7fnpB/xzO6RnHuxXn6WRjjFdT/rIsKAAAwE+5f6F3WD43zNRwQWBCq
x5xdm9BVbUl/Bl3nnO/pqrT6M5p5eS7pnk7G5ZnV9cH8Gfre9fjfR2Gvqc5r79gUjT83Id9bxnk7iuLc
LwrvxbOmRvOoOqhhgkP7+Vl4cRCiAgAAAAAAAEAEaqICAAAAAAAAQARCVAAAAAAAAAACIQIgKA
AAAAAAAABEIUQEAAAAAAAAgAiEqAAAAAAAAAAEQgRAUAAAAAAAACACISoAAAAAAAAAABCBEBU
AAAAAAAAAIhCiAgAAAAAAAEAEQlQAAAAAAAAAiECICgAAAAAAAAARCFEBAAAAAAAAIAIhKgAA
AAAAAABEIEQFAAAAAAAAgAiEqAAAAAAAAAAAQgRAVAAAAAAAAAACIQogIAAAAAAAABABEJUAAA
AAAAAAIhAiAoAAAAAAAAAAEQhRAQAAAAAAAACACISoAAAAAAAAAARCBEBQAAAAAAAAIAIhKgAAA
AAAAAAEIEQFQAAAAAAAAAiEKICAAAAAAAAAQARCVAAAAAAAAAACIQIgKAAAAAAAAAABEIUQEAA
AAAAAAgAiEqAAAAAAAAAAEQgRAUAAAAAAAACACISoAAAAAAAAAABCBEBUAAADAQmtvFKRQ0I+N
tl5raG94n19p9PTK2fDbuiEhLV0wbdlwz3vk+e1JY2Xca6xXNVb8ba00rK8CAGB+EKICAAAAODqa52
TGOemSUMHoqjT1kqMsZ9aK+rmpKJsX6tZnHZ3aesg1asv5Wkc/F6me3bS+CgCA+UGICgAAAOAI6
Ujt/OL38Zx/XTlwM89yXbr9vvT7e7I5KvksbsrZqn4eco16jXN+IFttyXZFPwcAYE4QogIAAAA4WuiNm
r3eoezrp3LqRKxeo5Xtlng5auAamb1Qq9IiQQUAzCFCVAAAAAABHTLzeqH590pVg6NpryIpbm9Orse
rX/7Rrehp1Vr119suC68NKtPqCtUJH1wEdeJ31CKsrGjyeYL3S6HYYzGPXj8F92fsp1ayzrDVXndeO3Ul
FtltujOpfo/aGXxagXN+yXjUo3vHbQto/dH0tcc5VoEar+6BWKwAsLUJUAAAAAEdEWep1HdJl2Bu1U
ytJYTVYDdRep0K2gfXN1VEBZlNWCyUxyoCqjUhpcMIpOxQceJ3F3t/IQK8jtZJRr7Rcl60YnTuHwlEtel
8JVbak7hZHba7KRqMh5/wEVS4M1gNIcvwqwA5pv3M+hoNUR/i5UueiNLhTJewaAQCWAiEqAAA
AgKNjzQ3p4vVGnVS53pW+qgPq9ax0hK1v7o5oh1dLtC/
+y5tyzkv7etJYd0PBqrT0a71td2qyPjIpNl6/F2OSpvaGePmv0a6um3ga+6psW5/r+hNFqRqm9n5iDc
MPTjLVrLnHV5b6hcF2Jjl+67VuGhvWfmsrOxfjnqu27Eadi8A1AgAsC0JUAAAAAEdIUTbdGYwy641al
bNuj8nKaWvJNWL9/mFoL05zBnqzXmhn56Lz+t5F2dGdIQPD3I3enN5rB4QPix+t7aeGgTCzuHnB31
ftfDo9MAOTTGnVs8OTUiU6fuu675lBqKO4dsYLbDsHXf0saOhcmfVeOzviZq/FzT1n+9Zjb+QMWgC
Ao4oQFQAAAMDR4oVsGfVGLZ+Ukn4aMGp9qKqcDiR3JTnpp31ix33dA90L01rllguwH8bwdve1A0
6dSBLymT0vz8ha4EuLsnbGbdi+HIYlthMITDJlPQudTGqK41fHZNc5DR3eHzR0ropr4h2yuodK7n4ja
rECAI48QlQAAAAAR8xAb9RD5+l8K8qJU/rp1MpyMn6am0BHRnTmnE6i8DlCYFIpo85ppLBzFSw5Y
HLD3NgTdQEAjgxCVAAAAABHj9kbtRYvTsvXYK/Onhx6Y8iHebVWhx7biYbtTyercHa88cfflg2z16lby9
Ss3ZpEcVP29D4Gyt7amueYpR8Alg0hKgAAAIAjyOiNmpQxjDw7g706u3Lg7tTtmVk66QWAo+p5ps
MsJeDXAHX05KJbmFROSaIqAdNKcvztXa/nqR24unVRp7iWaoZ+1ePUnkjLDlS7Xi3W0WUEAABHF
SEqAAAAgKPJmIAoTMlPDo3aqW3Z8Kapz1ZzdcObqKm94Q8/L59ZcwJAszZnc9UfQt7e8Gp0FlIZV2
4Gzta5WPd7WfYa61790aSTVU1twuP3A1djxv4k9PbVbRDscRoSdAMAlgYhKgAAAIAjKro3qjlzuwrp
nGAubi3NaZWlXG7Kqg4D/dzWmOFftd+ozdlc1cGh9+IREzJNorLtD1vv1KSk21XyE1S5kPuM9AmOv
3Lan6jKu5bGBFRJjDgX5r1RPat7ugIAlgYhKgAAAICjK6o3qqp7OVgzs9qavI5mIqfk7J4xPFyx63gO1Di

1a3MOvE4Je+2U7GHrIcdebVnr3eHxeYt9/BXZ7psz/itVaZk1TZu7Xs/fcexzEVYMVW8zrewaALA4Cn
1V3AUAAAAAAAAAEIqeqAAAAAAAAAAQgRAVAAAAAAAAACIQogIAAAAAAABABEJUAAAAAAAA
AIhAiAoAAAAAAAAAEQhRAQAAAAAAACACISoAAAAAAAARCBEBQAAAAAAAIAIhKgAAAAAAAA
AEIEQFQAAAAAAABGEvn/AZM6nr53azj8AAAAAElFTkSuQmCC]]></redcap:Attachment>
</ItemDef>
<ItemDef OID="q3" Name="q3" DataType="text" Length="1" redcap:Variable="q3" redcap:FieldType="radio" redcap:RequiredField="y" redcap:BranchingLogic="[q2] = '2'">
<Question><TranslatedText>Do you prefer Life A or Life B, or do you think Life A and Life B are the same as one another?
</TranslatedText><redcap:FormattedTranslatedText>Do you prefer Life A or Life B, or do you think Life A and Life B are the same as one another?
 </redcap:FormattedTranslatedText></Question>
<CodeListRef CodeListOID="q3.choices"/>
</ItemDef>
<ItemDef OID="summary4" Name="summary4" DataType="text" Length="999" redcap:Variable="summary4" redcap:FieldType="descriptive" redcap:SectionHeader=" " redcap:BranchingLogic="[q3] = '2'">
<Question><TranslatedText>
Alzheimer's disease - Description #[no]

If you have Alzheimer's disease...

1) You might forget recent events of importance to you. For example, if you are interested in hockey, then you might forget whether your favorite team won their last game, although you may remember watching the game itself.

2) You might forget the names of people you met recently, or the names of local streets.

3) You may no longer be able to handle your personal finances or plan leisure activities such as dinner parties or vacations. Simple decisions such as what to order in restaurants might become difficult to make. Your ability to concentrate may decrease and you might have difficulty driving.

4) You may start having trouble finding words to express your thoughts. You might need to be reminded to shower or brush your teeth, but you could do these activities on your own. You may need to post notes around the home to remind you of simple things like turning off the stove.

5) You may remain aware of the day and your surroundings. You might be able to travel to familiar locations like the supermarket, although probably with the help of a loved one or friend. If you are in a social gathering, then you might have difficulty following the conversation.

6) You may continue to enjoy hobbies such as puzzles or reading.

Now consider two possible lives. In Life A, you will live in perfect health for 16 years and then you will die of natural causes. In Life B, you will live in perfect health for the next 10 years, then you will live with Alzheimer's disease as described in the summary for another 10 years, and then you will die of natural causes.

</TranslatedText><redcap:FormattedTranslatedText>
Alzheimer's disease - Description #[no]

<p></p>

If you have Alzheimer's disease...
<p></p>
1) You might forget recent events of importance to you. For example, if you are interested in hockey, then you might forget whether your favorite team won their last game, although you may remember watching the game itself.
<p></p>
2) You might forget the names of people you met recently, or the names of local streets.
<p></p>
3) You may no longer be able to handle your personal finances or plan leisure activities such as dinner parties or vacations. Simple decisions such as what to order in restaurants might become difficult to make. Your ability to concentrate may decrease and you might have difficulty driving.
<p></p>
4) You may start having trouble finding words to express your thoughts. You might need to be reminded to shower or brush your teeth, but you could do these activities on your own. You may need to post notes around the home to remind you of simple things like turning off the stove.
<p></p>
5) You may remain aware of the day and your surroundings. You might be able to travel to familiar locations like the supermarket, although probably with the help of a loved one or friend. If you are in a social gathering, then you might have difficulty following the conversation.
<p></p>
6) You may continue to enjoy hobbies such as puzzles or reading.
<p></p>
<p></p>
<p></p>
<p></p>

Now consider two possible lives. In Life A, you will live in perfect health for 16 years and then you will die of natural causes. In Life B, you will live in perfect health for the next 10 years, then you will live with Alzheimer's disease as described in the summary for another 10 years, and then you will die of natural causes.
<p></p>
</redcap:FormattedTranslatedText></Question>
</ItemDef>
<ItemDef OID="a4" Name="a4" DataType="text" Length="999" redcap:Variable="a4" redcap:FieldType="descriptive" redcap:BranchingLogic="[q3] = '2'" redcap:InlineImage="1">
<Question><TranslatedText></TranslatedText></Question>
<redcap:Attachment DocName="16.png" MimeType="image/png"><![CDATA[iVBORw0KGgoAAAANSUhEUgAABVEAAADYCAIAAABtBTQzAAAAAXNSR0IArs4c6QAAAARnQU1BAACxjwv8YQUAAAAJcEhZcwAAEnQAABJ0Ad5mH3gAACqcSURBVHhe7d1fyF3Vue9xY9Qdj0pC6w5pPWj0IAQjklYOxJxiI4gRQRBUDLIVD0deIlh2EGxEzOFcKMqmkHdvBL0oVjg9J15YZHNKFFvI
RQvpodBceOEGK++FpYF68W4p5ZXmonv+G2O8Yw/c66x5r813+
+HH3u/a645xzPGXCuu8UTX2yv+BgAAAAA5oieHwAwkPMb56/4X1cQQQgghI2bv87v+/8//X/XJBGwD9PwAgCFsbG7seWOP2HgRQgghQ+aal6984+tJ+6844+f/776fALLmjp4fANC7za3NA28eEBsvQg
ghZODcf9+
+/3H7f8nyyrEH/vr119WnFDBr9PwAgN49+NMHxa6LEEIIGTgHj+8uG/4y75z6YfUpBcwaPT8AoF8n
Pzopdl2EEELIwNn7/K5nDtxm9vxZfv2z296vPKmC+6PkBAD06+8lZsesihBBCBs7O0zseu/tm0fBn4Yv9
2A7o+QEAfbnwxYVdr+4SGy9CCCFk4Nx7bK/o9nX4Yj9mj54fANCLjc2NfT/aJrc9XIGDX7bk/ekbRJ
8vwhf7MW/0/ACA7m1d3jr09iXGx6yKEEEIGzu4Xrna/xu/m/P/9P9UHGDA79PwAgO4df/
+42HURQggAyf0NX43J+684/OLF6vPMGBe6kBAB17/Vevi10XYQQMnz+20N/f5fvvq
q+iQDZoSeHwDQpQ8+/UBsuQghhJDhc8uz14muvuvjFvPnei+jADZoSeHwDQmYuXLvKL+gkhhIye61+
86qmDt4qWvk0+/sk71UcaMBf0/ACAbmxube5f3yf3YvHPYBf3/6T6OEThwwR1gxhhJDhc+2pnU/etV/2pnU/etV808Gnhi/2YDXr+UXy2fuSK0pH1z6pj50rOLX/vOtTPiQCAtTeVCa1cv9U/3A/+A5sxM4qa/4XyDxbdOPZVHsdX4Uatk9Oci3Uu3jLQG8h4XYHXfIO4G8J7
u8hQPz1X1ZUX5FtPTg8j5uhNoHs29pjzkm2B4XZnmGyIq5zyHLLK0MxXnQCbtpcHwNjY39v1on9hyEUIIIcPnmpevfOLQLaI57y98sR8rip5/CnwdZU9ZTdXouXynnNSiWaquKk4ofj6yq6j6iLxDxR
NF2lDlSPqxmoRalF+kTqyip6mPqaFtNXV1kvg3Vdi+l5SpfcCTSP5p2RrcW6Mp6BxIUtlmawz66ed6bi

m1vzTDABW5e3Dr19SGy5CCGEkFHS99f43fDFfqwiev4piPRv/qcCTdmCzVJ1WXZGeXb2Q3lIH9FXi+
HiD0Mar1JHigP17EpqkT6RurJK2r1SV1lnWNe1mJ6ndMmdQPNowcFqLdaV8QwkLmyxtPoSoXrem
Ypvbs0zwQQcf/+42G8RQggho+T2p28QDfkA+cHd3/nyD19UH4rAiqDnn4Jl/
+Z/SrVC1dHUZqm8Tv3r/eLq4pA6Ug8ohos/DGm+Sh3KjlRrqp/z34lGskravfIWt65rMb3gKe4Emkdr
Ua/FujKegcSF7SeTKU+SpZ2p+ObWPBOM7fVfvS72W4QQQsgo2f3C1cN8jd/Na48/+tevv64+GoFV
QM8/BZGmSjc95nOyE/Jcr7uwWLNUjeN+jb88YownKsYfhrS5qpy3/nX2xlMti0jysrTG0nc77csWGM
R8KQvutc2j+c7ICyy2rkzzDWkcxx1C3i9niND0cwsvAcP44NMPxH6LEEIIGSU7T+947O6bRSs+ZN577
dXq0xFYBfT8U+Bpdmq6fVLP6j5In66PqNZIX9PQLNUX+i4N917xhyGtrjLqi2f0+eqwPjVWVlZRFy3W
WHruiqzePD1P6ZJnAs2jOWeED9TDenhmJS9smoycW32+c6R67J1bi5lgNBcvXeQX9RNCCJlI7j22Vz
Thw+d3v/i4+owEJo+efwo8zY5Jt1QW82SjyZLizVJ9oR5OV/MWKIeLPwxpd1W9Wud26CssCxVNbC
z9L0HON46lHtRTuuSdQPNi/WcYw9uzDqyu1Q1pmEz49lSnuCN65tZqJhjD5tbm/vX9Yr9FCCGEjJJR
vsbvhi/2Y4XQ80+Bp9mR7KbL0wGZJ2RPt22W3PO8V4qD8YchLa+K3w19VSFyxyqySlpjaV6lfs64F8
SmF1xYcALNi7XPcE4x5uq9PNP6hsQnY1bKL1OPyyF8Izpzaz0TDGrr8tbRd4+K/RYhhBAySq5/8aqnD
t4q2u+xwhf7sSro+YFmnnYU2B5O/PyE2G8RQggho2Tn6R0P33OTaLzHDV/sx0qg5wea0fNjezpz4Yz
YbxFCCCFj5fDDN4qWewrhi/2YPnp+oBk9P7ah8xvnxWaLEEIIGSu3PHudaLYnkh/c/Z0/fv776rMTmC
R6fqAZPT+2m43NjT1v7BH7LUIIIWSUTOpr/G5eOfYAX+zHlNHzAwAsm1ubB948IPZbhBBCyFh56H
vfFm321PLOqR9WH6LA9NDzAwAsD/70QbHZIoQQQsbkKdx/9hmiwp5lf/
+z96nMUmBh6fgBA7eRHJ8VmixBCCBkr33ruWtFaTzYn7ryDL/Zjmuj5AQCVs5+cFZstQgghZKxce2r
nk3ftF631lMMX+zFN9PzjUr8brtDLL4j7bP3I2rnq53n8Ljp7RRHZicG1Wje+3XDbXev3zvzectvGhS8u
7Hp1l9hvEUIIIWNl+l/jd8MX+zFB9PwjqnpSpfumqCowowbMXVGQvrvOWsV9L9CRNmr13pnfW27
72Njc2PejfWKzRQghhIyVux7fI9rpVQlf7MfU0POPqOduSLe2s2nAfCvyM9t6sVb7FtQntvl7hG2txXt
nfm+5bWPr8tahtw+JzRYhhBAyVvY+v+uZA7eJXnpVwhf7MTX0/ONp38GmmV8D1u6O1W18yV6r
ugNqiPLsnl6CWaHnn7Pj7x8Xmy1CCCFkrFzz8pVPHLpFNNKrlVNHv/
+Xr76qPmWBsdHzj0M1Qgaz7bTbVtEtGU1UPYxoWkPjmw2YcY7bj1kzaGzXzGHNK51WOjxs/Yy+S
E9QzFZxRs/Yd65glTFn2kbgfM/hpjsmZxaaVr3Qcn3R6wLa1rJOjL5Y2ZOBW6HU89aKEc3LjHPcUaxJi
6dbzxkpXv/V62KzRQghhIyY+
+/bJ1roVcybz52oPmiBsS3W8+vddnir7T3FagcCLUNr9ZZ/hTf8oQbJ/1TGuGueE5x7GhrfP3jOGMJq
vpToy9bJsPr58ph4GFqRoK7KnlNXmDPXg66dqwc0T3D4RnEONi0tcIPqMzzPF0/6r/OuXEuoVWme
cc48y+C5oLu33MKzQWsffPqB2GkRQgghI+bg8d2ieV7dfPyTd6qPW2BUA/T8Yju/7B7dHC7a+kye9
27q5kbdJ32WvnFG/xO9Ab7x3Wud8Z3L1DWRV84dtj4i60SGNc/xVfWtSMhPEVPwjiDFR8wZw4iR
nWnJynIIfYEeU12RMWcSGqi+0JFSqz6iiuur1JH6QKR2fZaxCLecMyXnMrnuVnNGgouXLvKL+gkhhE
wn3/zHv1vdr/G7OXHnHZ9fvFh96ALj6bzndzn796UYvUemmzFHEmuQzIXJ83S307D62PjGMXGae
mgM7jkkdDasHqhilxRjNojeS33YM3XBmacYuOXSTM5C9CysK+rZhgZqIVyrXrE8x7kmo49F5uK7rrm
cehi5gS3mjIVtbm3uX98vNluEEELIWNl5esdjd98s2uZVD1/sxxT03/N3uzXXvcARuydYRZ47I7rJiI50e
dTT/3j57ryngDhNj+4RLNjckrUe1jxRvrq+FYV51lqPUA/RPKg6Q9x/dX7rpRXss50x7Avq6WrynsS0riX
vgTrFKuY9aPPdS89l4jR7mjYxocicsZity1tH3z0qNluEEELIiLn32F7RMM8jfLEfo+v3v+33bOY9e/Zcy05
GXbN2Tv20SAs0MZ676W+rxIn+k1ye8X3XBkb3MQaydTlsfaazQN+KwjyTqkcwDqrzwoOqq4qLnNP
bLE3XFdTzvrkWAhfGbkBCLX1JdY53OsE51uQ4ueZy6gyf8IR8tdDeiZ+fEDstQgghZMTc/vQNolWeU/
hiP8Y1Us+vzzJFeomKuiwfRP3cfNVUee6mv60SS/Wf5PKM36J3aju6pbth9WUFcbZvRWHekp6D6lB
s0Oqc7Co1h/rs5qWpMzLlSc5CGscwhii0ObF9LXmOdzqNc/S/QM3lWgzcYs5YwJkLZ8ROixBCCBkxu
1+4ek5f43fDF/sxrv5/h5/nGudQi01/Tl1XXqYeNVw0XbE7Yy5KntfybvnGb9E7+a5q1NWw6qwj6+u
+RS42N/99Ukf1EO0GrS47srZWnm2c3DiAOxF1RF/in2she2qBYmm15Ki+KvqYZ46K77rmcr6rhBZzx
gL4X+MnhBAyqTx8z02iSZ5fXjn2QPUxDAxujJ5fHfHs3yPthHtSq4smzL0zGd2hyVXWy/T0P16+8Rfp
99QBPSVznrZuhrUu0Q8iY8YF7pOoGzjLoS8rWBNoWpp4aJyvx/HPQl9YH/afWEur5dxYZ0nGOJE75

YyTaVHOqSZX0WrOaG/r8tbhHx8W+y1CCCFkrNzy7HWiQ55ffv2z96uPYWBwY/T8ekPvEdm/q4Hq
bb97ZKUE7qb/9hhr9PQ/fvZIRZVWvZM+Yom8NJ0Mq59Wg+jp16N6VhTmmVTJHqXUMFbGvEqe3
bA0X8GSOiU019CV4ekm1XJfrMCScs79NNjVi9FalWu4gS3njAVc+vMlfmk/IYSQ6eTwwzeKJnlOeef
UD6sPYGAMq9Pz+7uCUqwLma7I3bQXK5739D8h5q0uTm/dO9kzaCq1/LD1E8b5evr1ic6KwjyT0qx
5uPffyzMbU/yOmfPO66nHVe3YXM1Lc43TXbxW84uVPRGbY80sXpyZVE5WaT0IFsD/OD8hhJDpZ
OfpHXP9L/xfOfbAX7/+uvr0BcYwZs8f7x0EuyGQFhoKSKHegrzZMBtnPzkrtlyEEELIWLn+xaueOniraJ
hXPSfuvOOPn/++
+twFRjLm9/kjozj0NQH0YeiXfgfyVsOsvPTLl8SWixBCCBkrt/3360XPvOrha/yYgsSe36to4Z2G3tPh18
NUx/R/Dxz6WwDPICX9BJ0YemL+1+rhtyiwsh557xGx5SKEEELGyr3H9oq2eXXD1/gxEaP0/KGBgu1
UpLOPPAV0wej5afgxR1uXtw69fUhsuQghhJBRsvP0jsfuvlk0z6sYvsaP6Rip58/Zg8Va9mhfH30SANB
oY3Njzxt7xK6LEEIIGSW7X7j6mQO3iRZ6tcLX+DEpi/X8AIBZOr9xnl/jTwghZCK5/ekbRBe9WvndLz6u
Pl+BCaDnBwDk3vrtW2LLRQghhIyV1f1i/3uvvVp9sgLTQM8PAKic/Oik2HIRQgghoSal6984tAtop2e
fl57/FG+xo+poedHnPh9DPGHC1G/G6/6RQzLDLUo61fx9/6LILKVDbCm3JD3ELN19N2jYtdFCCGEjJ
K9z+9arS/2/+Du73z5hy+qD1RgMuj5ERdv8pdMsfq+XWhUr8tf1VsoB58sHuIOdvc2ty/vl/sugghhJ
BRcvD4btFXTzl8jR/TRM+PhYiucpkmU/T8gxmw7tA9OD0/uvHpl5/ya/wJIYRMJPfft0+01tMMX+PH
ZNHzYyGr3/MP2RfT82NlffjZh2LLRQghhIySlfhiP1/jx5TR848i0O56D+s+rtB8RUPfp56ur3CPqGHzI2
K4+MM4fXImO1/M3TuUeYk5wSjrIusqVdIQmLg5OTFvi13LrBaqJZadc5ZunFMPUz0ZrugZKECM0X
5RmTbzz0THKFmnuM+3GAH9OnPhjNh1EUIIIaPkW89dK3rsSYWv8WPi6PnH4WmcfAfrhs9kXNSy
ATOp5/XTuogepDpSPBbDxR9G+JeSq8o6Q+kDJmOhXvE75nk2MPHG6eaixUK11HFjHGfpnmvL06
MV270c3ttqXNFQInX+OWNWTS9u8wgYxPH3j4tdFyGEEDJKvvvoN0SnPZ3wNX5MHD3/SFTP42lz9
CHd96gjulFyTjGGcRowqTpBXWJ0V9UVaoTioRgu/jBInydrZKqJNI3sWaqjxR1zB/Zy70p9RCxhwVqed
TinudULjRV99WzOJeKWNZZoM395ijOIvMC5onEEDGTr8tbhHx8Wuy5CCCFklDz0vW+LZnsK+dd/
+efqUxOYKnr+saguRjcxTi/laa4au6uM0085qjPKa8oHa2v5QOYRdb0YLv4wxHeaPlbN3T5HPfIszDgk
tLlj7gEvNZRxVuOF7gm+S9q8ZLp6eK0FeWHCFMtDofMjcwvPXz8MTV+d4BmiPNQ4AgZ06c+X+DX
+hBBCppBrT+188q79ouUeN//0D09Wn5fAhNHzj0Y0PqqV0t2Xp7fKBK4yTtL9UriPq64qTih+PrJ+rri
qPqIvF8PFH4Z41yIO2kOpJ31CtbxV9LjqaLsp6/r1WbEL7enqE3yXeGbpnOapLnkrNq7NUzzMv6gW
89ePa1ZBe2BbOUTTCBjWxUsXd726S2y8CCGEkOEzqS/2v3Dk8L//6U/VhyUwYfT841F9TdHMqDao
btb87Zlor1o0YD7VZdkZ5dnZD+UhfURfLYaLPwzxrkUctIdST/qEanmrOFNsN2XPWO6F+ojgnmDUaj
OyfyWZhoq+epbgwLWmRS1zZ6rn1RA+aoj4CBjc2U/Oil0XIYQQMkr+6yPfFL33WPm33/ym+pgEpo
2ef0xV75N1T6rDMVoaf3umTqyOtmrAPMrr1L/eL64uDqkj9YBiuPjDEO9axEF7KO8VDfzXqHHV0XZ
T9owlL1SnZMqz3JF9tVqMHFp9Y0VfPUtg4FrzotrMXzOGK5QXNc7C4B8BY3jply+JXRchhBAyfHae3
vHwPTeJ9nv48DV+rBB6/lFVDc2RtbWyaTJ7Jt1ImV2O7K483ZNukkJ9X64ax/0af3nEGE9UjD8M8Z2
mj1W17HNaDmyRIxacgdqN7Lmx4kL3DPfO+2q1eck852SaKzauLTQhNWRzCc8p7jmZ7JjxyK7bOM
1CbASM5JH3HhEbL0IIIWT4XP/iVU8dvFU04UOGr/FjtdDzj0t3SwXRzugnVXul+x7db+kj6lpjwGhzVF
/ou9To50SvFX8Y5MzTqF8VC42srtDTi5RqccdknQA1UvhOyPnUE65H9tWqz6sOGvddneapnmmu6
Ktnc6rbB1osyhnBmb8+UE9fLCg8RtsRPAcwgK3LW4fePiQ2XoQQQsjwueXZ60QfPlj4Gj9WDj3/yHR
3k1FdVc18tmZ2OUZTJrnDmeoLnS7KX6AcLv4wIjzRqpozlP+KhkIL3LHoSJ6OUl7oL5WrR7bPKY+H74
S+0FM911ixzdoC5atSLRa1wPylelr+Qernm0cI3CP0bWNzY88be8TGixBCCBk+hx+
+UXTjw4Sv8WPl0POPTTc3od7F7o7qnkczT8ie1g89p5rc87xXioPxh3H65Ex2vmja4tULLds7+yI5s3Z
T9nSUngvNzjQ/qh4bQ5un6MHMGWbnOiN7qlfiFdutTd4hUSZeotA0/5w5TM6dkj2L+Fpz9gjqWfc
69O38xnl+jT8hhJDRs/P0jsfuvlk05H3n45+8U30cAquDnn9sqvGhdQGwKt767Vti40UIIYQMn90vXP
3MgdtEW95f3nzuRPVBCKwUev5x6X/VScsPYJWc/Oik2HgRQgghw+f2p28QnXlPOXX0+3/56qvqUx
BYKfT8Y7H/w2X3P3wGgGk7+u5RsfEihBBChs+9x/rzznLjzjs8vXqw+/4BVQ88/FqPnp+EHsII2tzb
3r+8XGy9CCCFk4AzwxX6+xo+VRs8PAEj06Zef8mv8CSGEjJ5v/uPf9ffFfr7Gj1VHzw8ASPfhZx+KjRch

hBAyfA4e3y169U7C1/gxA/T8AIClnLlwRmy8CCGEkOFz/337RMe+ZPgaP+aBnh8AsKzj7x8XGy9CCC
Fk4Fzz8pVPHLpF9O3LhK/xYx7o+QEAy9q6vHX4x4fF3osQQggZOHuf39XVF/vfOfXD6kMOWHH0/A
CADlz68yV+jT8hhJDRc9fje0T3npBXjj3w16+/rj7hgBVHzw8A6MbFSxd3vbpL7L0IIYSQgfPQ974teviF
cuLOO/74+e+rzzZg9dHzAwA6c/aTs2LjRQghhAyca0/tfPKu/aKTb59f/
+z96lMNmAV6fgBAl1765Uti70UIIYQMnG89d63o5FuGr/Fjfuj5AQAde+S9R8TeixBCCBk43330G6K
fbwxf48cs0fMDADq2dXnr0NuHxN6LEEIIGTgLfbGfr/Fjruj5AQDd29jc2PPGHrH3IoQQQobM9S9e9
dTBW0VvHwpf48dc0fMDAHpxfuM8v8afEELIuLnl2etEb+/N//6fp6tPL2B26PkBAH1567dvib0XIYQ
QMnAOP3yj6PBFXnv8Ub7Gjxmj5wcA9OjkRyfF3osQQggZMjtP73j4nptEn6/zg7u/8+Ufvqg+tIA5ou
cHAPTr6LtHxfaLEEIIGTKRL/b/7hcfVx9XwEzR8wMA+rW5tbl/fb/YfhFCCCFD5vanbxDdfpb3Xnu1+q
wC5oueHwDQu0+//JRf408IIWTc3Htsr9nw8zV+bBP0/ACAIXz42Ydi70UIIYQMmZ2ndzx2981lw8/X
+LF90PMDAAZy5sIZsf0ihBBChszuF65+5sBtWc/P1/ixfdDzAwCGI/ZehBBCyMC5/ekb+Bo/thV6fgDA
QK64YqAPHQolo1AyCiWjUDIKJaNQsvkV2g64lQCAgbAjSUahZBRKRqFkFEpGoWQUQgS3EgAwEH
YkySiUjELJKJSMQskolIxCiOBWAgAGwo4kGYWSUSgZhZJRKBmFklEIEdxKAMBA2JEko1AyCiWjUDIK
JaNQMgohglsJABgIO5JkFEpGoWQUSkahZBRKRiFEcCsBAANhR5KMQskolIxCySiUjELJKIQIbiUAYCD
sSJJRKBmFklEoGYWSUSgZhRDBrQQADIQdSTIKJaNQMgolo1AyCiWjECK4lQCAgbAjSUahZBRKRqFk
FEpGoWQUQgS3EgAwEHYkySiUjELJKJSMQskolIxCiOBWAgAGwo4kGYWSUSgZhZJRKBmFklEIEdxK
AMBA2JEko1AyCiWjUDIKJaNQMgohglsJABgIO5JkFEpGoWQUSkahZBRKRiFEcCsBAANhR5KMQsk
olIxCySiUjELJKIQIbiUAYCDsSJJRKBmFklEoGYWSUSgZhRDBrQQADIQdSTIKJaNQMgolo1AyCiWjECK
4lQCAgbAjSUahZBRKRqFkFEpGoWQUQgS3EgAwEHYkySiUjELJKJSMQskolIxCiOBWAgAGwo4kGY
WSUSgZhZJRKBmFklEIEdxKAMBA2JEko1AyCiWjUDIKJaNQMgohglsJABgIO5JkFEpGoWQUSkahZB
RKRiFEcCsBAANhR5KMQskolIxCySiUjELJKIQIbiUAYCDsSJJRKBmFklEoGYWSUSgZhRDBrQQADIQdS
TIKJaNQMgolo1AyCiWjECK4lQCAgbAjSUahZBRKRqFkFEpGoWQUQgS3EgAwEHYkySiUjELJKJSMQ
skolIxCiOBWAgAAAAAwT/T8AAAAAADMEz0/AAAAAADzRM8PAAAAAMA80fMDAAAAADBP9Pw
AAAAAMwTPT8AAAAAAPNEzw8AAAAAwDzR8wMAAAAAME/0/AAAAAAAzBM9PwAAAAAA80
TPDwDAos6tXVFbO1cdBQAAmBp6fgDAjJTNeL9d+GfrR+oKfRYc+m8W8oVljqx/Vh3okrWYAn9Xsijr
HvbzMvUu8Aemeu+Vun1nRP6IRp5alH8o6xXrdln9it6ZXv9B0aPwolb1dQqsyFpOVy9Ti1or+s+kbYK
eHwDQrx538xajzpCbtqJsH3udbC+l11Hsq3reUOULObK2lv2fXurkSxh8S2juR7t/V1jv7FpP775iLQO
9I8z71l2R4J9Q+8+QvdClBCtGn1pUaKj8uD5Q3tJlS/lHMV+upUs03pn8+U7/QeFblLWkwnLriiyqeKr
DP0rucozipp5WVNTXh4pHy66uXa0OSpX3riLuj3UXxXNogZ4fANAjez9l70Y6pQoV/3/IDYG9wr70Xi
UvUN2+fsrkr33vt8k0zAtjKUr29OaTY/dXq+t9fEW9Hu7E5Vujq6WFK8aeWlTbodR51cOFFdeX7CrZ
7dMHln+5mpaTH6+eXqpOKbaoLsavRBblOZQuuBxh+aLhFckjPdZST1QPl33V8uH08MU7uX5oIyqe
XGpN2xE9PwCgP3IP4OwaOtd/BWGY7UfPy9LD5z+kb9piltsOLmzw90Gm37eCfQN7W18xsPlCdf3
COTN3l9LxjYzcqw5vY9NQxfPJN1Jd3W8Vg7+QPpr/sHQZNVlPrX7+ceEW6mQdlchybB2+vxtvXeNk2
nOGctbRYTF9N4sH8u3QZaFtg54fANAb95O5w92O38CbgWI9Xe0Zg6ztT/eMe5b/2E+h4k7Ver5n/
a0jqOcXKVPewqxE+UM/73LnT6jx7uiEM15e0b5vHZeMDNdhpaahnBubpKlKZwvyDWQcy3/s7M3
uqeW+KbrgFirq5F9U0Jav6rt1huLprtbmrVW81fIS5Q/hqSzGqRW4nd0szbxN/kKdLWyboOcHAPTG
3QF4tyhd6r2Aqdh4dLV7cxVrqfS4pHwVavi8ZH8LUsqF9VmneOd1vJWPG+aNV7zjCn2Vctfh/ileilPA
Hb/jexkZrsNK8aGKF66DuxivUjzbzWvlKZSvQR3Jn+7sTRGoZeiolFtIvC7FCeH7245nOYb4s4sKjVbfv8
5KeWoVR+Td6+i1KlZQVct/toft9i5uD/T8AIDejPBRPdxeQOwWe9XpZsqWL6O+X3mhQdbkvjU6JV6
c8v71+bYoCvb6vjPXUP7cT71ibHnrOnypigHNmbtvBeeU5USG67BSbCjxdlyCt0pxsNLVbXMK5Wuo
H+dPd/amiN26TLm8Lqq5hZw3Xwd/kKPL6WB8k6dWebvKY+XPHdXzrqtYj7K2Zr1JlIAMq18Y51WK
32N40fMDAHozwkf1QHuBcqcz4J6j3Lx1X9Dashm62GHHFHX7u3/OO6/fesWr0+stcyv0uCLrTdHdP
r7k/Al1/8g6r95y3AJa5KlFBYcq7mZXy2mYcPF0J8VkIes9YeijlqujdURbyBm4eS6NIkMUT3X1Xsg5td
wKxSu33IpKzbcmP6OL1ck/Mu7L3zwXSPT8AIDeuB/MHe3dwobYCxQ1Bt5wFDV7vXOFvEz/VfpfTh

9b+bDOdtVhboleV2To+i3hzlu+WF2vLDJeh6X8QxWvW4eLaZxwcUIHBRsK5U939q5oXFRXf8LcQs
URcyHyzZggvJyOlmFwarklmu9uS40D5Scs/6YoVmAP41bu4GXaduj5AQD9kZ/MnW0/gvqvUO5Jp
M73H1kVYxmefVAvutm1ubJxjWGLF6nXV6moYC6kzz1i8eL0+55z3wGDFM10/o4oXhp74tbanJdua
Z6KSuSpRXmGKg51+yI1Trg4oYPb11Aof7qzF2nURVl/kDr5UxVcTg9/Zt1a1h+mTHdF4y9TN69RMV
u3SH7YHDs+FXjR8wMAemRtQLrZFfgVYwurvScQKxpmMXnRXl6hwVdjbXW72/e6+nxX28r9sDbAO
6LLtYl3QKFegrm2rm5mpGJ8MgsJDyVer9KSiyuqWTPNqhiPi5rL1Gh5Z/LTOnvTO4vKDhiDl1PyTKK1
+KKsZ5cpU3GWUyoOD/De7vwfFOFa9jNL37pyOO8w1vu60zu5fdDzAwD6ZW5A+JzGYKwN6dL70Z
Di7d3b6CPoeB+PTtivSql6bcRTK/SKhRe1kmuKvEaZ2f2DonPirypK9ZaBjcSS6PkBAAAAAJgnen4AAA
AAAOaJnh8AAAAAgHmi5wcAAAAAYJ7o+QEAAAAAmCd6fgAAAAAA5omeHwAAAACAeaLnBwAA
AABgnuj5AQAAAACYJ3p+AAAAAADmiZ4fAAAAAIB5oucHAAAAAGCe6PkBAAAAAJgnen4AAAAAA
OaJnh8AAAAAgHmi5wcAAAAAYJ7o+QEAAAAAmCd6fgAAAAAA5omeHwAAAACAeaLnBwAAAABg
nuj5AQAAAACYJ3p+AAAAAADmiZ4fAAAAAIB5oucHAAAAAGCe6PkBAAAAAJgnen4AAAAAAOaJn
h8AAAAAgHmi5wcAAAAAYJ7o+QEAAAAAmCd6fgAAAAAA5omeHwAAAACAeaLnBwAAAABgnuj5
AQAAApuXc2hWVtXPVoZJ64sj6Z9WRfqg6ovwU1Pcm49yHz9aPhJ6KPgcA80XPDwAAMC1GX2u3p9
u956/b9oLvNgQ7+/qmTvBvMgCgP/T8AAAA02L9u2yzQ93uPX+r9Xub+/qvAuj4AWwz9PwAAADTY
vX8ZoO7zXt+3bg3zEvfP32f9BE6fgDbDj0/AADAtNg9v9GoOj2/c8BtjKtT8jOMcasLjCNmN6wOZ8e
M/5zeKKIYzzrPG3OryzT03NZ41oDGTJXIWPrs4hz9yFlBbP45ez7ilMgCo9cBwKDo+QEAAKalbibXy5
9Uy2h0mcVj94DRblYtcd2ONtJNdPgao8+WfW3BmIhnkGjr6y8ql24w5uKoJ7e2rn4U1Zvm3zCh8AL
910WnCwD9oecHAACYFtU0Zh2kakzLhtF4ojjRPRDr+auTjJZUHlFtqXNNfUQdkGX0GXoqRp0W7W6
4gB7QrRmjT67UwxQa569OCM8nsMDQQHIGADAMen4AAIBpsbrG6oH5s9t2Gu2kbGbVKXVfGj6i
hnHPkOfIjjgjD+lBjHOC5AxyciXugTg9gZy4pMX8Jae6f4H6tFbrBoDe0fMDAABMi7+9zlpNpzP2tMqy
NXVPaT6iHpuNsj2uOsNHFLZGCXCnlFMl1VG5tEbhKbSYv8E+Wz0fGF3PsmYvCwAGRc8PAAAwLbID
rh7rr/fXPaQ8M9NXz28fVA98goXD/OfKpST3/O4UWszf17wX1PPB0QMXtp01AHSKnh8AAGBanGZ
SNpH6CU/bKRtj95TmI55hxbi+M4QWp2j+c1VJdVQurVF4Cs2TU2dkypOc6o1jGEMUwsUAoD/0/A
AAANPiaSbt9lE/ETuzg57fbK7tc1q0326ZMD2cebJTo8Oev3Eo91LnpkQWmD1lDLzwvAGgQ/T8AAA
A0+JrJnXfmNNP1EerdlI3ps4Rt3kNHwmPok8JV1aNrVsmJlygvn7h3jkyhab5y+XU5zcs0FlIdBoA0Dt6
fgAAgGlpaCbNJ4xWVBK9bKwFlUf04yNydNXvZvyl6xPcMnHqfJt5dZc9f+P8/dPJqVNCo4eubDtrAOg
UPT8AAMC0BJrJukm1njB716ytlI2xO1jzEfXYHM6ZTs7um+0TAquIsseTTbJcWqPGKcTmX1+ey0sat8
V82je6eWmu7YwBoHP0/AAAAAAAAzBM9PwAAAAAA80TPDwAAAADAPNHzAwAAAAAwT/T8AAA
AAADMEz0/AAAAAADzRM8PAAAAAMA80fMDAAAAADBP9PwAAAAAAMwTPT8AAAAAAPNEzw8
AAAAAwDzR8wMAAAAAME/0/AAAAAAAzBM9PwAAAAAc/S3v/0H2zJF2W61WWUAAAAASUV
ORK5CYII=]]></redcap:Attachment>
</ItemDef>
<ItemDef OID="b4" Name="b4" DataType="text" Length="999" redcap:Variable="b4"
redcap:FieldType="descriptive" redcap:BranchingLogic="[q3] = '2'"
redcap:InlineImage="1">
<Question><TranslatedText></TranslatedText></Question>
<redcap:Attachment DocName="B.png" MimeType="image/png"><!
[CDATA[iVBORw0KGgoAAAANSUhEUgAABVEAAAEJCAYAAACdcmdaAAAAAXNSR0IArs4c6QAAAA
RnQU1BAACxjwv8YQUAAAAJcEhZcwAAEnQAABJ0Ad5mH3gAAE9sSURBVHhe7d0PjCRnfef/b+/aY
PPHNmBskx+RHat7sZYlGJIg08PxL4egZ+6SJbnbYOVg5Oh3PVyi0zQSkwOxF+mX2wguG4lunS5iWrq
zliQyTEJugrLdlJRgckzHR4IxYTI/Z7rlc3T8T8wDYGjPljB3u3f/VUPVX1VHd1dVV3VXX39PtltaerpqfqqT/T
O/OZ5/k+hb5FAAAAAAAAAAChjumAAAAAAAAAAIAQhKgAAAAAAAAAAAAAiEKIC
AAAAAAAAAQARCVAAAAAAAAAACIQIgKAAAAAAAAAAAAAAABEIUQEAAAAAAAAAAAAgAiEqAAAAAAAAAAAAEQgRA

UAAAAAAACACISoAAAAAAAAABCBEBUAAAAAAAAAIhCiAgAAAAAAAEAEQlQAAAAAAAAAiECICg
AAAAAAAAARCFEBAAAAAAAAIAIhKgAAAAAAAABEIEQFAAAAAAAAgAiEqAAAAAAAAAAQgRAVA
AAAAAAAACIQogIAAAAAAABABEJUAAAAAAAAAIhAiAoAAAAAAAAAEQhRAQAAAAAAACACISoA
AAAAAAAARCBEBQAAAAAAAIAIhKgAAAAAAAAAEIEQFQAAAAAAAAiEKICAAAAAAAAQARCVAA
AAAAAAACIQIgKAAAAAAAAABEIUQEAAAAAAAAgAiEqAAAAAAAAAEQgRAUAAAAAAACACISoAA
AAAAAAABCBEBUAAAAAAAAAIhCiAgAAAAAAAEAEQlQAAAAAAAAAiECICgAAAAAAAAARCFEBAA
AAAAAAIAIhKgAAAAAAAABEIEQFAAAAAAAAgAiEqAAAAAAAAAAQgRAVAAAAAAAAACIQogIAAA
AAAABABEJUAAAAAAAAAIhAiLp0etJYKUih4DxWGj29PqjXWPFeU1hpWF+1iMxj3ZB2onXjpLWdL
MxLO7LSlg372PzHqPs4Vb2GrGzEO5vtDbdtK5JH08KNuQ9Cjmc+2g0AAAAAwPwhRF06Rdm8UJey
XurU1kPCkracr3X0c5Hq2U3rq4B5oILBVWnqJUdZzqxle4faf1Qo1cT/rlhsR+14AAAAAADIGiHqMip
uytmqfi4dqZ0P9kbrNc75IVW1JdsV/XzhFGVzry/9vnpsS/aHkff+RpmXdmShKwdu8leuS9c+xj3ZzDJ
D7TVk3fijwsI7ascDAAAAAEAOCFGXVGW7JV6O2jxn9EY1e6FWpbW4CSqOot6h7OuncuoEPaQBA
AAAAEAuCFGXVkW2W26M6vdGbW/4Q6XL9a3wXoyqlqKuRek+wmpSjqyvaH59ZI1Jo6ZjoC7rqPX
GPu31edcGHd5fZI1J4zwEz5+5nbDPjxN+3INtCdS9tR4xy30ObGewraPOc7xjCm57uPZpYAh6c9VZF
2h4snM3eA7Uw9yc3Z6x+xzPPy71CLkXbAnaHvI9OHq7vqTHE6/dAAAAAAAcfYSoy6yyJXW3OGpz
VTYaDTnnJ6hyIWSM9FAIo3VqpQwmoCrK2hndwM6BdJ1nFmNId2dHLno7bcuubn/5zNpc9FKsnPa
D6gP/AGy9izv6PBo1Pe1wrCSDo63TPb8dqZUKUhrYSXM1aUimtjPY1qasDoZtEx2T2rZR+9S6H+te1
+kREu3HCSwHz4HSXB0dKibnnOtV70AUtW6Kc9TeGFHPNGS7E4vZbgAAAAAAlgQh6lILTjLVrLnBTF
nqF0Imk2pv+KGKV4+yL103ie3UZD3lhKW4dka3b18O3U23d42JhYxw0hjqferEnAz0rpz2yiY0d4M9
Ji/u6BisfEacDLUnjXX3GlSlpc9v3+0xnOr59bfvdUhWIdlAfdzx/O1494Hazrob/E1zTMbr9zZlc9v62PX
vV1Wv1/6cXXIi2X56jXUvsCzXu85r+8MlLiqR+4wr/BzteOl/krZbr3X/0hH2PRjY7rBkxzOu3QAAAAA
ALI+C9QtyXz/HklK9SwM9zlSwEhKq+K8rS71rTubTk8aK24tOBS/OZEYjX6963bk96Ubsy+dvW4Vde
9ZG1BBs1YOwWq1Ks9kcWu+3IaxdcdeNE387w+2yV3rnwG1/6DqbsV0VnO2FBNwB4e0wr3O11fc
nDEt0PSK2E9ivvuaS7JjMbQdfr41qa6JzF35+FOdanQqsS3p+lETnOlHbRwht44jjjDietO4RYBr3Pnyvv
OXCW/QSACAP99/xLnnNW2/SSwCAzB3/NZFjr9ILWBT0REVwkikVtoQGJP5Qeb/npMsYdm/2GE1F
UU6ccp51dJfTrj2WvywnT5+0e9QF11uqp/0AbA74vWmb4nVG7R44gZT1GW8ov7dOdT4sGbUo3S
BMfcIsazBr1jUo6ac28z7QPYSnOKZEvYkT7cec4f+kmIdQ3NyTvhmgTm3wHIWY6rrrurFuwJmaGO0
GMvDwEw/LOz/5Tr0EAMjDDY9fJXd/6AF5/Bs/0msAAJm7dI9I/zt6AYuCEBVBA6FScsO1P6fl1RVt7k
pbenJoj9k/JScqJ6z/D65XGeqc9ZIrrombLbpD+ttuIl09a/TozUtaAZl1DTJrOyHeSKpXqBe0GnVjgQX3x
NNPSOWPKvZHAEA+nvPjY/KWL94oP3ryGWm+/3555p8u6c8AALL1lMilP7A+PussYiEQoiJlGYRfXl3
RfTlsXxS7lKgd9lbEyVeN9XMZvhk9NO3A1+/VOyrwVcO67VqVQ480e0lOK1mv47yOaTHOXbjxbW/
Lhtnr1K2LatY5BRbUnZ+6Ux58/EG9BADIwxv/
+gZ5wQ+vsJ8/9NXvyp/WeR8GgNz0Hxa5/Od6AYuAEBUxleSkN1LbnBFfMSZJitM70Ri+HI+7744c7D
pf686+X7I/4a8fLjUwH4qbZ/0guOFOjFWVQIZacsoTKG6Jgvk22OvYvA/0seV1TIn2Y97LA8Pk1cz3hZ
xnoE/SdmNSNTtwdeukJv6eAubL+z77PvlM7zN6CQCQh1c+eK3c/PXn6yXH5z7+kHzlLx/RSwCAzF3
6gsjlr+kFzDtCVMRUlM2zbuVUc/Z1NbrYnOl8y+vt5wScijnre1s23JlrYvN7cqqJpBS3XmZRF0x117vh
6vxxe81a56Kmj3+wdqsx7F+aq7LhnTIV7Onh297K+dBcNQLH9nm/hqd7bHkdU6L9mLVbm7Lqre/p
me+ta1Qq+NvI2oTnyA9c3XYDi+kT+5+Q+n11vQQAyIOqg/pzD7xELwX94W9/Tb73raf1EgAgc5f/RK
T/fb2AeUaIivgq29LyctSalHTA48w8bynX5YJR4NOfUMnSXNWB0GQ1HAPbsp55Q/a9of6KMUnTHP
Jqu9rKUt8KRKiWomxe8IdlN1d1gOaFzqMm/ZolJ3AMttM8tryOKdl+ipsXpO6/2HmtOZGTdS/7h6Br
7yrua1NNWBO03bzfw9odR+bHA8R339fvk7v+7C69BADIw/FLBXsYv/oYRgWozd+8Xy8BADLXf1Lk
8sf1AuYZISoSqWyH11+stqz17tBiV3FT9gZfW21NVr/R7K0XKBlgDM3OdKKjFJgB2KiyA+qc9bt+wOe
ya1/OW03PstS7fT9Yt1Wl1d8LTpaV1zEl2k9RNvf60h16sXq5MUzeVpHt4EGK7B96PbFTEbvtVlv6Lf
8+sqlzblwHu+5ulByOB4jBnYn/6Wfp7QQAeVr50kvl2iev1Evh/uFL35ZP//6hXgIAZO6y9RvZJcpbzbt

CX81aAgAxtTcK4nSSVCHqQGAKADGo4PT1/+318sAjD+g1AIA8lB56od0LNa6tu8vyiteFD/sHAGTgit
8QKfBL9ryiJyoAAMiVGsJPgAoA+VK9T1Uv1CTUsH7qowJAji79AfVR5xghKgAAyM1HvvgRezIpAEB+
VP3Tt33hZSProI6iAtS7/+NX9RIAIHN2fdR79ALmDSEqAADIxe6Du/LBv/igXgIA5OWOL18/tg7qKPv/
8zH53Mcf0ksAgMxd/n9FLn1BL2CeUBMVAABkTg3fV3VQmUgKAPJ189efL//8Czfppclc+dzjsnX36+
XWV79IrwEAZOtKXR/1Zr2MeUBPVAAAkKknnn6CmfgBYAZe8MMrEk0kNcoz/3RJmu+/X3705DN6
DQAgW9b77aWPWx+fchYxFwhRAQBAZlRwqgLUh594WK8BAORB1T99yxdvlOf8OJ1f+R7/xo/k7g8
xKSAA5Kb/HZFL1EedJ4SoAAAgM+/77Pvk3ofv1UsAgLz83AMvkRsev0ovpeMrf/mI3PtJ/igGALm5/
DXr0dELmDVC1KXWlo1CQQrGY6XR05/LSa8hKxttveBob7jtWZG8mzM3Qs5LHL3GinPuVhoy6tT55
9d55H7NjwLuWyCW+n11+djffkwvAQDy8vJvPE9e+eC1eildn/zPB/LN//19vQQAyNylXZH+Y3oBs0S
IurR60lhZlaZecpTlzFpRP8+eHfiVasLfVIImPi+9hqzXor5KXfOCrAYvunRqJSlMENguK+5bIB7V+1T1Qg
UA5Ovqp47LG++bvg7qKKo+6u//
+y/bHwEAeVD1Uf+79fFZZxEzQ4i6tLpy4KZA5bp0+33p9/dkM68MdWzgt6QmPS+qZ+S4YK99XtxN
V1vqevelWy87K5qrQo4aA/ctEIuqf6rqoAIA8veWvRvtIDVLqifqH/721/QSACBz/UdFLv2xXsCsEKIuq
96h7OuncuqE5Nf/FGmL1zOyJ41zugtquS5bFedpcfOsVJ2n0twlRQUwPTUTf+WPKvZHAEC+Xvu1F8
vLHr1aL2Vrb/f/2A8AQE4uf8l5YGYIUZeQXbvRDN2aq04tx8GuiKp3o1E7c1T9zGAtyGCd1bDejbH3r
wVreI6qN+kMVfdfl6zW52A9S6+2qH6ENy3GPgPncEP8zZjnydln0vOiqHaWYvWMNHoeB0LzkpzUn
VFl/9A6olGM9obVW21v6GMZPAcJrkvI/RZ2vePcb4PXz35E1ImNY5b37WT3p2vSa2eZ6D1Ar1TMr
49uJI6QOz91pzz4+IN6CQCQFxWevubvXqSX8qF6o1IfFQBydOlPqI86Q4SoCDUUGGl2/cyRYVRHai
WjzqrR43EyanuDNTzVurCgpuQNVXdFt3UUZ5+DwWRzdcJ9Fjflgjtk3joz5/RGeo1z3nkq1y+kUEahK
q1+y+tVOsTseTxK50C6+umwipx2N97ZkYsDJ7W96x2NX1c3yXVRQV7I/eZcj1EBZPj9pu7d0GC5U5
NSIMjOSpb3bcz7M2CCa2eZ7D0Ay07VQP1M7zN6CQCQFzV8Xw3jz5uqi3r3h75KfVQAyI2qj3qP9Z
H6qLNAiLqEKtt96Xfr4kZ7Um3Z9TH72zrxbG/4AZBXL9Won9mpyfrIxEaFec7r+3uboWUCxu4/wN+
et3/pyI6XBPWkse4GPca+Wzo1imzrKP523M2ofdbOu/Fbsn0WNy+If+rOS9v677wbglnn94JOUJOd
F4P9um2ZLK8uyolT+ukYlS23beb5V9ri53BnxMnhkpwj67VGqYGh+21ofyZj2/b9ZrYlbFt+kJ3U/Ny3
/uvD789hya6dZar3ACyrT+x/wp6NHwCQv5UvvTTzOqijPPTV78qf1hmBAAC56T8scvnP9QLyRIiKIW
bPtPoFPwgdDgOHletbE4Z54aotPxwsrp3xAqzOge4z2bsoO14eaey7suW3deeiJIl7zH364ZMh8T6Ls
nnWTbuaslpwe08Gz+8kipt740PW2PblMOpEFdfkTNjxtXf9XrVn1pzjSXSOrPOzp0NEI3gPvd4Dhu43
s8et0evSPk86CNzLYfa0LO/bsfdnmCTXzjLNewCW031fv0/u+rO79BIAIE+vfPBaufnrz9dLs/G5jz8kX/
nLR/QSACBzl74gcpkJ/vJGiIoBI3qm2Yqy5iYxIwK3UyfSDKjKcrKkn47SPdC9+VSoU/LqNhbMYdKRw9
QnMMk+K9tGr0FHOsP403RKoi+fcf2NgNIP3Kpy1j2gqa6LruEZOrw/aOh+M8JCawf20Hd33yNrsaZ
uHu/bBNduyvcALB93Jv6nn31arwEA5OWGx6+Sn3vgJXpptu7+0APy+Dd+pJcAAJlTw/r739ELyAMh
KibUkeHOgTHCo7mX3TFUvMKUSrD+5Oz05HBssVSf36vSHRZuBG7V08FeoUmYExB5PXXHCbtWR
dm8EN470w0rF3t+o8nvz/SvXdh7AJaNCk5VgPrID+h9BAB5e86Pj8lbvnijHL9U0Gtm60dPPiPN99+v
lwAA2XtK5NIf6OfIAyEqJjR/gWm53vWGbAcfk9YLHS/
+Po26n7aO1NZznJyneELGlj4tn5Sxl7S4KW5lAntYuDF8vno6/CyPP0dt2TB7nbo1OM36o0lYbdzT+xj
s/as0z83XpEi53bcTXLtoR+GPJpiWGsL/wCMP6CUAQJ7e+Nc3yAt+eIVemg+qPuqnf/9QLwEAMqfq
o15iYte8EKJiQElOusnV0EzePbnoFnIcO/Q7J6WTXtA2qm5m6ibYZ6+xrodpl6Ved5OsPCfnMa5rc1f8
zphdOfAu6QmJc0m9HrXW/XH+/I4OP6sSyOGSnCOzLqcKFN26qMaQ96TUzPKqx6k9GZQdSHa9W
p7pDpOf0CzuW0usa5fme8AU1xDz7yNf/Ig9mRQAIH+lh1448zqoo3z6v/6D/MOXvq2XAACZu/xZkf
48dRU6ughRMcCcBCnYW9IPAlXYNTChz6yYNTCbq/5Q7faGHhpuPdIev510n72GrLsnrnpWNjeNyY
Nq65JPjmrWsmzKrm5er3HOCzBj90asnBbnDulIs+ke18Bw8Amvix8oDvbcjUlvX80sH+xxaoTFZo9b
oz1p3yaRZnHfKnGu3QTvASU/dZXaee9gZMOb4h9Hze6Du/LBv/igXgIA5OnaJ6+0Z+OfZ83fvF+
+9y1qZQNAbtSw/v739QKyQoiKYeYkSJ2alHSoU/LTE7kw7YxI5vDy5uoUoVGwBmZzVQdQXnhTlVZ
qs9e7kuyzJ411d6h6Wepbav3okCq98zLMnFndbbN3TastiX+aKhIo72oZDmATnCMv2LO4x2xOsJTEi

HvXrLNaPevPNp/YQt+3SpxrZ0n4HuDXW7V41zBubVssGjV8/85P3amXAAB5UvVP3/aFl81NHdRRV
ICqglQAQE76T4pc/rheQFYIURHKHgYdUpOy2rLWu8Otp1KR7cGClfuHRs/BBOwamMZwbZddWzOj
eqgx92n23LN7obonbiCk8of1p3hehhRlc2+4Tqg9hD5hYBeYJMs6ZjsbHhT7uljH3G/5QaqtKq2+0dZ
ACYJo9r07eJA2Z5vTZZMLft9aYl07S6L3AHUsg6+ttiava4u59cTTTzATPwDMkOqBqnqiLgI1pJ/6qAC
Qo8vWb6bUR81Uoa8KBgJAEmo2fT0ZlAph96btmTxL+lhOtaYNWBfEUbp2yJUKTit/VJF7H75XrwE
A5EnVQVWTSS2SK597XLbufr3c+uoX6TUAgGxdKXLFb4gUbtbLSBM9UQEkFCxRcGZtsUO49nnrWC
J6ZB4tR+vaIV/v+
+z7CFABYEbULPx3fPl6vbQ4nvmnS9J8//3yoyef0WsAANmy3m8vqWH9TzmLSBUhKoCY2rJh17o06
pWaJQoWUK+xIqv7demmUqJinh29a4d81e+ry8f+9mN6CQCQJ1X/9C1fvFGe8+PF/NXt8W/8SO7+
0AN6CQCQuf53RC7doxeQJkJUAJNRNS8XfPx7cXMvpRq/C+YIXDvkR/U+Vb1QAQCz8XMPvERueP
wqvbSYvvKXj8jnPv6QXgIAZO7y10QufUEvIC3URAUAAKEefuJhec32a+wJpQAA+bv568+Xf/6Fm/TS
YqM+KgDkjfqoaaMnKgAAGKKCUzWRFAEqAMyGqoO6aBNJRVH1Ue/
+0FftjwCAPKj6qGpY/7POIqZGiAoAAIbc+ak75cHHH9RLAIC8qQB1UeugjvLN//19+cPf/ppeAgBkrv+
oyKU/1guYFiEqAAAIUDVQP9P7jF4CAOTttV97sbzs0av10tGyt/t/7AcAICeXv+Q8MDVCVCTUk8aKm
uVbPTaknWjdOGltZ96MOYZeQ1Y2gmvbG+7rV6TR0yunpfZjb3NEO2zTne9M2m1bkPugvaHbWJC
BSwosjE/sf8KejR8AMBsqPH3N3x3tuqGqN6rqlQoAyMmlPxHpP6YXMClCVGCGeo0VKZRq0tHLWe
pd3DH205RdQj4AA+77+n1y15/dpZcAAHm7+qnj8pa9G/XS0aXqov7+v/8y9VEBIDeqPup/tz5SH3U
ahKhIqCibe33p99VjWyp6bXby3l8WRhxDryHrtTziU6UnF3eC+2qea1hrF8VRuA+A+aZm4n/nJ98pT
z/7tF4DAMibClBVkLoMVE/UT/7nA70EAMicXR91Vy9gEoSowDLoXRQ3Qy2Xy86Tzo5cXJwUdTFUt
qXfrYs+w8DCUMGpClAf+cEjeg0AIG8//ffX2UP5l8m9n3xYvvKX/NsDALm5vGc9mOBvUoSoS8eoLbl
i9kQctd6oc2mvz7s25fD+IutuGnU/VwKfNLcT9vko6Z8z+/PmMP7mqvP5EYU0/WNWj+T1Rv2h/FU
5e+GMDvk6shMnRQ3UUg17jG5PvHbHuTbD59DaumyYrzfqkQa2MbA+/BTHaYN5POpY/P2rh7vd
9nnrupbrsjXQXdYu3WC83n4M3DfArKgh/A888oBeAgDk7YbHr7Ink1pGd3/oAXn8Gz/SSwCAzF26R
6T/Hb2AJAhRl05R1s64PREPpOs8s3TlwE30Aj0U27LbdJ6Vz6xZXz17ldNV/awjB/4B2PywsCxn1nRr7
RCwJIMj5zu1UswQa5bnrCO1UkFW9fYcal2SINUYyl89LZXimniHs3Nx+hCvfEbcU+2L2e6pr43Dfn1w
Z846FVQOrG+uDgSpE7VBHcuqeFvWoakKSlf369Ld2wxcdxW+lsJKN3RqUprnibKwFD7yxY/Yk0kBA
GbjOT8+Jm/54o1y/FJBr1kuP3ryGWm+/37qowJAbp4SufQH1kfqoyZFiLqEimtuT8R9OXQTovauHwi
JEU72Dq1XOU6dmIcI1VI5LW6M2gzMjmSEhV6w15PGutvjsyotu6am9WjpLXRqsh4jjUz7nFW2rTa
Yw76rLadd22HVPv12d+vuV8TsRaoYQ/mrp9X2zVA4xpD+4qbsuefNfnTFa4Z1BPULwcDQN67d6V
wbV7neDX69Frbev2+maYPxeh2aFjf3vOc+P1RXYWtXf41/TppyLsFxAmnafXBXPvgXH9RLAIBZeONf
3yAv+OEVemk5PfTV78qf1h/USwCAzPUfFrn853oBcRGiLiOvJ6IfaPUOndivWnXCo32dFJrDwO38bS
5UZMsNoJq7fi8+s+6n2wPUXFff8ickqmx5QWCs3pgzPGfVlj+Rkh/mWi0Z7IY7gtk792TJfmJsJ0EYq/
Ua636vzepZ2QxPUMe3O61rY6vKWbchRsg+cv3+obPdKdoQeH0UI1Q3Q2s7cNWB6t6okwhkSA3f
v/NTd+olAMAsvPLBa+Xmrz9fLy23z338Idn/n4/pJQBA5i59QeQyf8BKghB1KRXlxCnnmRtode1x6W
U5efqkHXYF11vUMHDn2VzwQ7mmeJ0KuwdeWOgN5ffWWcfkDu+2H8bw7cAQ/VFmdc784HMy
Yb1zLRMP6W/LeT9BlVZoz1klRrtTuzaW8kkJ3d2o9a4p2hC7Z7Zxrq2N2WUO3P3Er8sLpOuJp59gJn
4AmLGXfPe58nMPvEQvQbn7P35Vvvct/m0CgNxcVvVRv68XMA4h6pLy6oraPTl74nSqPCUnKies/w
+uV3ngPEWoFiOYcodmt90x0xG9I6exkOfM6GmphqaXQkPC+LP0tzf8OqBVo6fp8kkSbhdl80L4jP1u
cDtiPjEgE+5M/A8/8bBeAwDIm6p/usx1UEdRAWrzN+/XSwCAzPWfFLn8cb2AcQhRl5U3tHlfDts6aL
N77VXEyQqN9VP3hsyCUdfTDi/9upOjwkuvNubQI2YYuIDnzB/KHyXmkP72hj9RVLUlIzuhTmDqa5O
CTNtg1JUdKNlqa55jln7k532ffZ/c+/C9egkAMAsrX3qpXPvklXoJpn/40rfl079/qJcAAJm7bP02eukze
gFRCFGXVklO2hlkRw52nSHNbh3Rkv0Jf3347OuzV9w864eaDXeSp4E6pCVnqL3i1eKc2KKdM2Mo/
xjjh/S3ZcNLUMtSV1PRTyvVazOhHNugZuhXPU7tScXsQNWYoCtu2QJgSvX76vKxv/2YXgIAzELpoRfa
D4z26f/6D3aYCgDIyeXPivTp2jMOIerS8ntyNptOOObWeCzq4p/uem+Sprnj9gDtSK2mA77BOqRmP

crmqj9sur3h1aUsxB5LvWDnzBzK787+H3iYIV70kP5e45w3jL9cv5BOuYRUr82E8miD3pbKoIM9Trvi
ls8dW7sVSMn/+v/
+l34GAJgF1ftU9ULFeHd/6AH9DACQi0v36CcYhRB1iZkzpqvehd7w88Ds5sYkTXPIq1NqC+shGaxH2
VzVwZg/Ll1GT440LNVzVtS1VJXmauqhoTmUvxxaW8AoiWC9cuSQ/l5D1r0CqtYrAxMwOY/JJkhK99
pMJoc2VLb9IfyBurRGfdmzm7MP3bEU7v7Fu+WOl9+hlwAAeXvjX99AHdSYfv7dP6WfAQByUXijfoJ
RCFGXWWDW8FPiTzbuDltXzPVzyAwvRw2ht+tRGr0uXeW6dJPWu0z1nFVke7BA5v5hSrUxzaH8o0
NdMxQeNaQ/Xl3VCaV5bSaVQxvsIfxhxVBVSNvvp1pfFohy1RVXyf/4lf8ht1x3i14DAMjT373yCf0M
UV7z1pvkbe+5VS8BADJ37FUix9+kFzBKoa/G9QIAgKXxwCMPyOv/2+vtmfoBAPm648vXyysfvFYvYd
D1P/E8+a1PvVGedw0TbwFALgovFrni/daTq51ljERPVAAAlsztN91uD+0HAOTvb27/tjx2PX/ECnPlc4
9L9fdeS4AKALmx3m+Pv8f6SIAaByEqAABL6F2n3iUfeMMH9BIAlC+Xjvfl8294VH78nMt6DVz/8t+V
5NZXv0gvAQAyd+ztIoWb9QLGmSpE7TVW9AQpBX9W65F60lhxJ1TZkODL27LhTbbiPCabqGZYeyO
43dBH1jOAAwAwhz788x+W07ed1ksAgLz84PnPyt7rvqWXoLzidS+R1X8bNhkqACATx4oix39eLyCO
OeiJqsJVf5ZqR84zwquZ0VcaKU3oAwDA4rjnl+
+xh/cDAPL10M0/kO6t39dLy+3al14l1d99rV4CAGSucI3IMTWMH0nkGKIWZXOvL2oeq35gxuuuHH
iTiKvZsNXn92QzxwzV1qnJeTqkAgCWjDtj/3VXXafXAAADyonqjfu+aZ/TS8lIBqgpSAQA5Of5ukcIL9QLi
mn1P1N6h7OuncuqEZJedlqXedUNc/9Gtl/XnRZq7pKgAgOVzy3W32EGqClQBAPlR9VE/96Zv2h+X1
S/8xivsofwAgJzYdVDz7rl4NOQYog7XRLXrlZZq4nZEtYfVD9UoNb/OeaRVL1Uprp0RP0YFAGA5vfm
WN8tH3/5RvQAyIvqibqs9VFVePoLv35CLwEAMmfXAQ2HVXkBSc1ATNUKvISuFktS8lNXRqZVSq2H
aPu+HuNXTfpGB5WVM8uWd46hJwTA7YddqEYTfT/4kcHneY0nPYU7fC+q9b2DCO//8rEiKf0cCAt7
7s+
+V2h01vQQAyIuqjbps9VGfd82VctfvUJMbAPJztcixO/VzTGKmIWpluy/9bt3vCVptOcPst1WY2ZPGu
htwVqXlDsFvVe01qobpeqIkoSO1khtC+I9Vd0Yra9/2bgFghnqNlWAPfSBnqjeq6pUKAMjXfT/zuD1r/
7JQAer1P/E8vQQAyNzxO0UKL9YLmMT89kTtXZQdnSKU61v+RFSVLXHLmHZ2LqbU+64s9S0S1NFG
TQoGTGKR76eM295ryPpg13tgBlR9VFUnFQCQnx8/57J8/g2PLkV91Le951Z5zVtv0ksAgMwdf5PIsV
fpBUxqfkPU7oHXE8sevu/1HjWG93cOpKufTsfppZpmrVUAABaVmqm//attZuwHgJw9dv3T8je3f1sv
HU23vvpF8ku12/QSACBzhVtEjv0LvYBpzHdN1FSFz85vlhPo1M7nWItxHgxO2jWqxuPoOpD20GPv6
/VjZG3Jwf1FBNd2Pdzga0fVgsysDVEya98oca+VK/5xJm1f2OsHyncO1O806o7arx19P/kG2p/K9Ux6
DsOEt32wXungORo8P2HiTbTn8/epHqPqpCY5P0DQbdffJvf88j16CQCQl7+/7Xvyjy//oV46WlQd1O
rvvVaufO5xvQYAkK2rRY6/2/p4hbOIqSxEiFqud4fDT/uRwnDa4qac1WVWRfblcGnyBRVsDU7a1ZTV
wqr1/3hUiFMKG3rcqUlpMKBKMklYe2NETUjVYzgYFmXWhihZtW+khNcqwXEma58TyIW9vrlaGBH
2qXNitLNcl/GVM9SxDbR/6us5/f0ej9OrffAcNVdHhZyTcPbh1XO2Dd97qd3vWGrvKL6DGfsBYAb+6v
WPHcn6qL/yH15JHVQAyNPx09RBTdH8hqilk34P0YN0Bu2Ha8tuuinKQug1zvnhkTuhV7/r1Zsdzzhv
5bp0dbDd9TbQlHNeotNLMEmY9dpzesOh2+3IzkX3tVm1IUpW7Rst2bVKcpzJ2tdrrHuBnP+HjZa1F
615bkRQaLRjb1OKem0koz1u06e5ntPf70n47fHb3pHa+ejIPHqivUH+PsLvvbTud0Ds2frfdepdegkAk
AdVH1UFqUfJyumftB8AgJwce53zQGpSC1HtnmjGkFHzEWco65DimpzxspxVfxuqF6C77UQbdnpv
me1yHmYvuTOyFivhWXQ9uejO2qUCFi+kKcrmBSPEidI7lH39VDo74mY3xc09J6yxHnub+mQmmiT
MmLjHCNyKa2eGQ/XM2hAlo/aNlPBaJTnORO0LtuOst74iW/aGVVC3J2GHE2hHTNWz/rmtbPtBrd
/2JNczhfs9gWrL7yFf2Up/+4q5j/B7L8+J+bAM7v7Fu+WOl9+hlwAAefjmjU/JV376u3ppsb3sp14o/
+a3mNAEAHJTuFHk+L/WC0jLHA/nDwYcXkjrjWE1w5B0mMHN0daVAzdTKp+Ukn5qK56QU/ppJDP
kHgioh2ouTjVJmK6nGTZ8Prc2REmpfSMlvFZJjjNR+0a3wwldR5fWOHUi6XdVVU4HNlaSk2473bYn
up4p3O9zpSwnAwcRIteJ+bAMrrriKmbsB4AZuP9V37HD1EWm6p/+
+n/5GeqgAkBurhQ5/mvWR+qgpm2+a6IWN2UvbMitPdQ3hXqoHtWLri8pZ7JH3OhefG5oM1EPZ
MWu5eiGPlE1KzNsQ5R5b19sebQvRuA3VlFOLEzSmcbxAvPpphfcZAepKlAFAOTn8yuPylNXX9JLi0f
1QFU9UQEAOTn+r0QKN+gFpGmqENUc9hv1cMLJoj8M2gxA7aBUrw9NMc2v04+4dRUtdp1B82tD

H2kGskvEuHZ+7Udf89zw5DXjJwlry4bZq9OtjWnWijRl0oYo2bcvLbGOc4btG21wgreeHHp1B4ZlOv
HcEcD5QZpuv+l2e2g/ACA/KkBVQeoiel3l/6IOKgDk6dhrrQd1ULMy3z1RkZGQ4dEus05mDGp2d9V
b0Q+rjZ7D7raTTBLW3vV6dtrhjxuYG8OTB6XehihZtW+khNdqguOM176Idth1itOdfT7Y9JDh+ImO
M737fWHkNjEflpGaZOoDb/iAXgIA5EEN6f+7Vz6hlxaDmoWfOqgAkCM1C7/qhYrMEKIupaKs+bN2
yao3Ztuc0XsMPcGXKlEb7K0YEnhNOEmYH/4YM+KbcmhDlFTbN1LCa5XkOBO1L6Id9rE7NVUTnsK
Rmqsb4jfdL5lQPrPmhNaJrmcK9/uiyeB+B0wf/vkPy+nbTuslAEAeVH3Ux65/Wi/NN1X/tPp7r5XnXX
OlXgMAyJaqg/oe6+PVziIyQYi6pIqbF/zehs1VHawYk86MU9n2h393alJygxmjRqg/UVew9mbkJGGV
09aSNq5dWbUhSmbtGy3ZtUpyrpO1b2w7ynXZSmV8eFnK5aas6vZ4Tbfaftab/j/Z9Zz6fs+LOdGV28
6Jws6U7ncgwj2/fI89vB8AkI9Lx/vy+Tc8Kj9+zmW9Zn79Uu02ufXVL9JLAIDMHVsTKdysF5AVQtSlVZ
TNvcFJu6rS6res/8djD/8OK6Rpb2dgoi679ubg/ixDk4RVZHuoDc72vF01d71eitm0IUqG7Rsp4bVKcJ
zJ2qfa0Zfu0IbVpo3SBlM7JWcHjzfsGiW6ntPf7/mw7q/B67F/aPQSTiCV+x0YzZ2x/7qrrtNrAABZ+8H
zn5W/ev1jemk+veatN8nb3nOrXgIAZO7Yq0SOv0kvIEuFviqCCAAAMIF7H75XKn9UkaefXYwhpgBw
FNzx5evllQ9eq5fmx7UvvUr+06ffzDB+AMhL4RqRK9R8BQzjzwM9UQEAwMTefMub5aNv/6heAgD
k4W9u/7Z875pn9NL8qP4udVABIFfH3239jwA1L4SoAABgKu/92fdK7Y6aXgIAZE3VR/3cm75pf5wX
v/Abr5BXvO4legkAkLljbxcppFNUD/EQogIAgKmp3qiqVyoAIB+qJ+re676ll2ZLhae/8Osn9BIAIHPHiiL
H36EXkBdCVAAAkAo10dQt192ilwAAWeve+n37MUuqDqoaxg8AyImqg3rsPXoBeSJEBQAAqVAz9b
d/tc2M/QCQI9UbdZb1Ue/6T6+2g1QAQE6O3SlSeKFeQJ4IUQEAQGpuu/42ueeX79FLAICsqbqon3
/DozOpj/q299wqp/7ZDXoaJAJC5428SOXabXkDeCFEBAECq3lF8BzP2A0COvv2if7Jn7M/Tra9+kfxSjV
/kASA3hVtEjv0LvYBZIEQFcteWjUJBCsZjpdHTn1tSvYasblLT1gqO94Z6fFZnX09NrrDhtXGnIqCb6x+E8
lv5aY2mo2frfdepdegkAkLW/v+178o8v/6FeytbzrrlSqr/3Wrnyucf1GgBAtq4WOf5u6+MVziJmghAV
yFVPGiur0tRLjrKcWSvq58vHDiJLNeno5YXRa8h6LarV6loXZDV4saVTK0lhIDAGjqq7f/FuuePld+gIAE
DW/ur1j8kPnv+sXsrOXb9zu1z/E8/TSwCAzB1XdVBfrBcwK4SoQK66cuDmbuW6dPt96ff3ZHNZM9
SxQeScUj1nxwW/7fPiHlq1pa5zX7r1srOiuSrkqFgFgV11xFTP2A0COfvycy5nXR33fP/Pjqnwv
5YyvW41V6AbNEiArkqXco+/qpnDohy9v/dHHF6znbk8Y53QW1XJetivO0uHlWqs5Tae6SomI53PSC
m+wgVQWqAIDsPXb903L/q76jl9L1sp96fzKziplwAAmSvcKHL8tF7ArBGiLjGvnqP5CKntOLI2peqN
536d163Or/dp135sb+ivNdbZLwuuD/bKS2MbmtlG7zFcYzN4jMM1S+1HWN1Low3jal3a+zDDt+aq
87WDDQ9pc9i2o9o8tpfjROdl+J4ZuZ8YxxD7fGh+W9RjuK0OZwi9/7p0z506/lKsnrNGj+NAWF6Sk7
ozquwfDt9PNqM9ie65eMduS+H7wj1Pcd9HsNxuv+l2e2g/ACAff/fKJ+SbNz6ll9Kh6p/+
+n/5GeqgAkBurhQ5/mvWR+qgzgtC1CWlwpHQMKhTk1JhQ8ZlcHHYtR8HCkLa61TIMrC+uRoeXk2
1DRU2hfYY7EitNCqEU58zapaW61J3uw52duTiYMi0674ynbqmQ8GiZh/zyGBquM1uz8dQE5+X4X
umuToieEt8DFGcfQcvd0hb7WCw5A2hd6V67jxVafVbXq/SIWaP41E6B9LVT4MqcjrpPZfk2FP6vlDn
KY/3ERwdapKpD7zhA3oJAJC1z688Kk9dfUkvTe/f/Nar7J6oAICcHP9XIoUb9ALmASHqUmqLn8O4d
TmNeo3SlHPhSUpi5XrX3na/FYybwtaPGt482TaCw6mHj7EjO4PplEcFZM7r+3ubsrlVF+erBr/GPI9nZ
FyGWtm2ttd1t2Wptpx9bOvUrr3hB4Vhbe7UZH3kdQm2eXRT0jkv/invSO28cd0SHMPY8xHg7zu8r
dZxrbvBoHEu3Iamcu40u53bEitrHVKUE6f00wiVRPdckmNP7/uimOP7CI6OD//8h+X0bQxHAoA8q
ABVBalpWDn9k/YDAJCTY69zHpgrhKjLyOwlZ/R0K27uOQGJ9dhLZaajqpx1t1M5bS25RqwPHd486T
aKsrlnBj6O4toZL7TrHIT3AyzXt4IBWXFNzugv6uxc9NvY3vV65pXPrI0P38YwexjWLxht3rwgfgZ5PrR3
31CbR5r8vFRbfnDoh3xB0xxDFHPfoW3tXZQd3SEycC4qW/5+zWtniH/u1HFY3yOhIe8k9uUwrEFKk
nsu0bGn+H2R2/sIjpp7fvkee3g/ACB7akj/V376u3ppMqr3qeqFCgDlIiV0H9V/rBcwTQtRlZAQ0ImqY
bsGrZTiyhuIkyielpJ8GjFofJo1t2HQ9x9BhzEGnTgwGP0VZ8xMtLyzyA0Mj0J1YVK9WY/8jgrfhNscV/7
yMN90xjFaWk+MudvfAa79X7sF+GEPcRwyfn/zcTeuUjN51gntuimNPcv2HzlNe7yM4ctwZ+6+76jq
9BgCQJTXJ1KT1UVX907t+59XUQQWA3Kg6qHdaH6l6mDdOO8IUZdSUTYvhPckdEOYqMl1FkZg8hyjn
mOk8MDO76nnDnc2AsPq6dg9GafXkeGOgjFCRlOK52UyYccwK2keVxw9ORxbLNWR2T2X2vVfkvc
RZOKW625hxn4AyNFfvf4x+fFzLuul+H6pdpvc+uoX6SUAQOaOrYkUbtYLmDeEqMuquCl7Rl

W/Pcos+u3ZYNs3edW7PRrMGZhHW+zurzZA+NNoYyV0/nF6FOH/qlfF4mkm1w6dXKHXpMWsd0
AsUTMrb06bje1BPcc+OPPf3vi6P9PoIsvfmWN8tH3/5RvQQAyNIPnv+sHaQm8Zq33iRve8+tegkAkL
ljrxI5/ia9gHlEiLrE1MzaqqeYPcGPHYR0vRqKo4f/GoxhxHPHrB2pgiW3/uMUba64U6Z3duT8+R29n
aqkk6GW5KR37gdnZO/JRbfoZeQQ8BgyOC+
+nI4hTOmkFwKOqumZL+NcNHfF75DZlQPvNJxwzn2EWPdckmPP4PpP/T6Cpfben32v1O6o6SUAQ
Jb+8eU/lL+/7Xt6Kdr1P/E8uet3qF8NALkpvFgP48c8I0RdRu0Ne6itmkU92FPMCHiMXnIlPxkzZmJvy
4Y3Dft884MlY2bySXgTWHWk2dQnKrWh/EXZdLsdqvO87l+XXmPdq22ZZBKkcVI7L578j8Fj1udsrvr
DyPW9bj9yHVtu1oBtyq7eda9xzgsxY/VgjnPPTXjsU1//hO8jZntyvRSYe6o3quqVCgDI3t/c/m157Pqn
9VI4Vf+0+nuvleddc6VeAwDIlqqD+h7r49XOIuYWIeoyqmz7Q287NSm5QYtRH7F6NnzmbhXSDL52
Lpkz9nttNibamUhF3I6BrlSH8o+4LiU/fZQL005glcl5MSQ9BnPYu9ueiRK2YH3O5qqz34IX9Felldqs+v
EUNy94PTLd9njnodqSeM2Jc88lOPY0r3/C9xEgiqqPquqkAgCydel4Xz7/hkcj66NW/u8idVABIE/H3k
od1AVBiLqk7KG3YUUMVeDS7wcDHlX3cLBmYrWVcx3NpCqy3W/5gZHNOTbvsAPDrOPxhlcr5bpsp
ZzL2dcl5LxWW9Z6d+j1VLI5L6Zkx2C1Z/A+3D80ejUmYNfnNIaSu+y6nznWQ/UUZXNvuFaoPYw+Q
aAb656LfezpXv9E7yNABDVTf/tX28zYDwA5UPVR7/uZx/VS0Cte9xL5hV8/oZcAAJk7Zv2GfPwdegHz
rtBXRewAxKNmNtcT86gwbG/anqHAOEfpntPHcqpFwIpwn+l9Rip/xM0BAHl441/fIKWHXqiXRK596
VXyW3/8z+yPAIAcFK4ROf5+66P/Xoz5Rk9UILaeNNbdmc3LcmaNABVZO1r3XPu8dSwZ9ODG0fGO
4juYsR8AcrL3um/J9655Ri+JVH/3tQSoAJCn4+8mQF0w9EQFxmrLxmANWFXOgK50yMzRu+d6jRU
p7ZyRbiplKXDUFf6fgn4GAMjStU9eKe9s/aS88723MYwfAPJ07O0M419AhKjAWAOBFgEqMsc9h+
WlJiiblx9NaEs42hKOtoSjLeHmqS0nrr1GDr/3pF6aLa5RONoSjraEoy3haAvSQIgKAADmBj/ghqMt4
WhLONoSjraEoy3haEs42hKOtoSjLeHmqS1IhpqoAAAAAAAABCBEBUAAAAAAAAIhCiAgAAAAA
AAEAEQlTkSE2WU7DrfxRWGtLTa8fqNWRlo60XHO0NvZ3CijRibwjp6Uljxb0GG9aVTbJuGmH3UNr
7mJbRRv1Y4SYFAAAAAGChEaJirvUaK1Io1aSjl4H5pgJdY1Z9W1nOrBX1cwAAAAAAsIgIUTG/eg1Zrx
GfzqeibO717RkF+/1tqei1szFPbenKgXvLluvStdu0J5tkqAAAAAAALDRCVABIS+9Q9vVTOXVCyE4BA
AAAADgaCFGXmao1OlC7MazG6GD9UXuIvfE1A+VKNbNOpXokq1Vp79Mcxt9cdbYTvjOjjeoxqk7qY
JvCa1VOdrymMbVf2xvetob2H3JN4rTRY359ZEONcxFo46j1xj7t9ea5zKoOadx7KKotg9uYsD5pjOuS
9J4FAAAAAACLgxB1WakgL7TWaEdqpVEhpPpcQUoDQ+ybq4OvVyFiSYIva8pqYbBWZBqcNq0GNh
xyDHYlNtgm65W1UnjQaYt7vIMqcrqqn3Z25OLAa9u7bmODtTKHQjgtuo2TKsrambLztHMgXeeZxR
iOHmh7W9xml8+s5dDDMoV7aKJrPizf6wIAAAAAAOYRIepS6knjnJuIuXUb+9Kt61BNOrIzmPx5qtL
Sr2+5QaEKG8/7ve16jXN+0FVt6VqVXfE2H0Nl2/qabl28L3G3sx1W8dJvU/gxWMe77oZg/mv77gF0
arI+MhUdf7xhKltu2wfPpR9GSvmMeBlqe8MPgsOuSWQbJ1NcO6PbuC+H7qbbu0ZI2ZEDN101hq
mfOpF9hDr9PTTNNTckuC7J7lkAAAAAALBICFGXkjERz96m16vQD9VEOl56FlRt+RP3+EGhqScXd9w
+e1VpeQGStc8LYa+fntmm0GPoXRS3SeX6lj/xUGXLC+U6OxdDexSOP94RimvidfQ0t22ElGaPTrN3av
2CcU02L/htrJ0fGKo+Ja+NftDbO3Si0mrVCRv3dbrau7jjBZKnvROYlRTuoSmuuWkm1wUAAAAAAM
wdQlRYdA3P0OH9SZmzk5+Ukn5qK56QU/ppespyMrCTEN0D77jsIdheXUtjqHdgSHsazOHy/rB4P5
SryllvyvYRvVNtxnbMHqOpKMoJfUHcwLlrXzzrnJ4+aYeVwfWW6mk/kMxMCvdQKtd8VtcFAAAAAA
DMG0LUZRWYKCdunckYgeWRMt3x+r1i3Z6eRig3URhpDK9PScUt3trctVrXE6cj6ik5UdFhZWC9anb
2EeriSf+6AAAAAACA+UKIupTasmH2OnVrPZr1HI+ocr3rlDEYevjD9lNT3JSzbglONXTcqCs6WRiZQYh
dOS164L4ctvUQeLv3pzs5lrF+QUP07K/5sv1xAQAAAACA5UOIuozMupwqYHLrohpDoCdXkpNuEjs
4XNoIEXNVcoamK6NqvWbF6+nZ2ZHz50fVFTXP2eBs/mZ90FMydk6nxNfQ3XdHDnadr3VrtZbsT/jr
h4e0ZyWFeyiVa57idQEAAAAAAAuNEHXJ+QGTMWP/VMxakU1Z3XCn3DFnS8+ZMcmTNFfFa1J7w
6+V6a1MmdfTsyPNpj76oaH8Rdl0u6xar6utN6yz5eg11r0anuYESU7AqVivP+8dkGx4U8nH5V+vZtP
5Wnf2/aIumOquNyfCylYK91Aq1zz5dQEAAAAAAEcTIeoy8oI9S3NVh0rGhDtTMmcun2r75iRC7nY
mCjuDs7o3V3WI5gWO5gzwaXOHxftCh/JXtqXl5XU1KdnnrCAlP6mTC95EVCojdOutWrxzHLe2bVBg
W9Yzb2i6eZ9Y68/k0w3VNv09lNI1T3hdAAAAAADA0USIupQqst1vGQGZUpVWv+8HRvaEQpMqyu

Ze1w/BbGr7g/scx2qn1yBt/9DrDZhIcVP2+oNtstj1YDOoh2rwhvQr1v62Ruysst0PrUtbbVnr3ZILLnU
8g6+ttiara2v22gwMTTeGs+c+ZD2Feyila57ougAAAAAAgCOp0FczrADITq8hK3oiL1WDdo+eiwAwk
urtPS8/mtCWcLQlHG0JR1vC0ZZwtCUcbQlHW8LRlnC0BWmgJyqQKbOOZ75D4gEAAAAAAJAOQlQ
gE23ZKAzU8ayeFTqhAgAAAAAALB5CVCAPql5pZpNXAQAAAAAAIEvURAUAAHODelXhaEs42hKOto
SjLeFoSzjaEo62hKMt4WhLuHlqC5KhJyoAAAAAAAAARCBEBQAAAAAAAIAIDOcHAAAAAAAAAgAj0R
EV2eg1Z2WjrhVnrSWNFzZavHhsyL60CAAAAAAADA/CNERSZ6jRUplGrS0csAAAAAAADAoiJERfp6D
VmvEZ8CAAAAAADgaCBEBQAAAAAAAIAIhKhLqr3h1gddkUZPD7+3l51HaClTVePUeI3zcL7eZW/XH
MbfXHVepzc4uF+PuW1j58HXt2XDfY318F4Wo12TGjwv6jHpuVGSHr8S1obCSkPCD8+s/eo8VkaciG
TbBQAAAAAAWF6EqEuvI7VSQUoDw+
+bqwMhX3tjRI1T9fXpBJbR1H5WpamXpFyXrYr1MbN2OWHk4HlRmquFYNCZ4blRoWtYG6RTk9Lg
BFl2EFuSwZd3aqWhcDTRdgEAAAAAAJYcISosVWn1+9K3Hq2qXqUCwPNulNaTxjkdX5br0tWv7dbL
zjrrtTsXnYiusm19rlsX9zNSbdmv7W+rxHNafjv7e5tSTNCupHqNdS+MLNe7zj77LasFWvOcDkeza4NI
W3bd1Dh020055yW0VjvW3SDXOE/uBe3UZN17bZLtAgAAAAAAgBAVUm1tixtxVraMANRTlM09H
crZ4aVeu3bGe23noKufZadc3/La6ciqXT25uOP20qzK2U13yxXZsoNGFVLuibM6w3PTO5R9/VQ6O+
JmscXNPWd/1mPPbVvvorhNDpynypa42Whn56J1ZJYk2wUAAAAAAAAhKiaha5OGDmHPzqkT44K
9tNrVlQN3A+WTUtJPFSdo9EPnYSmem+KanHGTWGtrquyCW7t0qM5p98Dbnz18X7+uYA7v7xxYR
2ZJsl0AAAAAAAAQoqIsJ82UcJTAxElGbdLcjGjnzNtlyawNRdm8ENYz2A9KQye5Giur7QIAAAAAABx
NhKiIoS0bZs9Kt46mWft0JuahXRm3obgpe3qIvV+v1tc8Nzybvl/DdfBh9KCdYLsAgLzp0Q3Ggz9yAQ
AAALNBiIrx2rte70o7oHNrfxpDyKc2ybYya1dJTroJqDsE3qVm4i8YM+6n1YaI16uZ9NUvzfakXXbw2f
XqnHrtK530Qtu4NVhjbRcAlpH9Xl+QwkwTy540VlZFWu57tPNHr+bqLILU+Qxze40Vpz0r+f/hT/0ba
p4P9zEP5wXDhq7XDO6ZmRvzvuZ9P7mPvG7mOO+3ebwnR+5jSf+gFfO8z/K9eGZinJuh951luGnG
nJfc34sTtofSdoiDEBWJ+AGdMSt9AiU/nTRm/7d+MFmdbhD8tO0KKsqaVzS0Kavem667baeO6OB7
cZw2JDp+/aavPhXsGRpSs9Wsc9pc9dvm/sOhHu7KJNtVjG3wDwuALMzsl/chKri09j/lv0npcCYu3Da
KcLuTP+4f5vte3N4IC3ONPyjOQq8h6zWRatX9x28G3NEnxsO8XrMy01/cAyWOhh+z+NZW52O1qS
YFda9TS6qdmpRmFPjk/0v7+Pc19R5csr6f6l3jHFk/T2Z778R5v83jPXncPtTnc/yDVmTok1eYm+C85/l
eHHFuht739CP98xP/vl3dH/g3Iot/IEadk9zfi8efl3zfiydoj/WNrUrbpf+eHO/7dn5+FsZY1g2DJWS9R/
TV5bd+A+hbPzD5uvW+9U+g87mq9ZZia/WtnxX060c8vNcqIa93P29uf9TD2NbIdtqStKvbr5fd9VXr
K8cxXx/yKNetVygJz83Exx/
+MDcdve3gMSfabqvqrS8PXwQAmEq3XrbeX8z3eP2+GngjyoffFv1vwAzaEEm/z8/8vXjm7XCuj9q/f
c28f5PzY/87OoP9RtP37dy1y/3eivPzV9rCv5dn1R7n5y9jv/pnrCy/l8a/rznvuYNtyPocjW9XvNdMa6J
9ZPIeqPdvbdd+hLRD3T/maud+Um3XK1IS/5w4n8/+vTjeucnjvS/
+fZv1+8v4cxImq7aNPy/h62fWnhHfw+nfR87+zd0737fBdX579Qr9vhz3uiJf9ERFDBXZVn8p0ksO56
821puAo7kr/t9KrNd7n9D2D52/MKlanIP1QqutCWuIJm1XEk4PIOsNTS/7rDdbf9h+0jYkPH57qP3g
ubQ5+wj8QdOuc2oMyXfZPWWMeqiWRNsFgMy05XytY71NXZBN503VUpEt9UY28fv35Iqbe9Lv7x
ltmTO69MupE/PRwFm1o9dYl1qnKmfn9kLNhnteWt7PKPPC+T6X6unAzyL5KMqJU9YH9+dQW08u
7sygPb2GqIFK5fqWv9/Ktv3zYmfnYgY9sRxj39d6h7JvfRj8fi7aJ64puxm9Ecd5v83jPXle3ved79+y1Ls
hP8tr6uf34dEJHdm5mO7dE/ec5PVeHOfc5GX8uXHeXwLf5xmY7Jxk9148/rzk+148tj0jfp6yR42mW
tYuzqii+fpZGOMRoi4pvw7mwJuLMeFQcMiBCgvdr1EPJ5TztxMM6dQPhf5rrYf5A725D/VQ+xmx35
Ht9MRtl/MGFlw3nvMG7H6d89gbakjCc5Pg+G2D59J+jDoG8zj1Y9QvU3G3a7xu+NgBYAoz+uV9M
enSL+W6bIX/A5CTnjTWa9KZVTvsoaMdqbbi/1ueGTUM0Rh6N9uSN/n84j6JXuOc9d1s/bI/oxu3sq2
HjNo17dWwypITNg/
+vJW13H5pT8huV1lOenWcNKPePgZk8AetuMFlmDTbEVuO78XTnJv86fJoB+cHhtOnWwJnknPCe7
FhxPub8/PnvuRaNYmfhRcOISoAAJgNfnmPTdUltX/5uTCLXoa6tpj9i6D+pWdGvR3b52vSqbZmPmL
C/0Op81AjV7KppRZXPr+4J+f2NDo7wwDEHTWkatqr7yOrObMI4efpl3ZMgT9oKfPyXhwwR3/Y6uy

flAvevxGqt6h6/9mYYa9C3osD9HwiwflBrO+pqedWiWHwjzD8LLxwCFEBAADmmDP5gRq2N6ueO
MFRDt36vqzO4hfU9oYzCcRc/dbuUL2Csh6WHcfc/eLePi/O6NHZXTNnsg5/YiAVeKtJgXKftGOWv7Q
jNfxByzKH78Xz9oet8pk147pY/4aeVWXUZtirkPfiAernGrdnrP89dXBKXadTkl3n7nn5IwymQYgKAA
Awp5wAVfXWmJ+hjMXNC3YNtk7tfK7hXHtXhU1NO8B1fuEpSEn9Vqh/CZrtcHptlsOyLfP1i7sOCGf5
y6Iecqzq2bt5jz0MViXezdWMZjYfZVa/tI9h93bqyMHgjTuqd9QS4w9ajkV4L56XP2x5ZtqrkPficIMl+f
qydXLfOk8nJau3vdn+EQZpIUQFAACzwS/vkVSvDSdAndMJ/zL8RSPMYE8j9bAngLR+Mexaz2dbt7sn
h6qoWc7nZKxZ/uLu9nw6O8NfFgeHTbpmdl7y/6V9rOIJceZ7CUZNPfuGnmG4O2f4g5Zvvt+LB+T+h
62S2GWOB8PbWf5cw3txTLq2eOCPkekZ+UcYfhZeOISoAABgNvjlPYKeRddiD3czevzkW+dSTf4wM
Bxc/0KW1S8ac6/XkJUVc0i2WqVmSp7lL6nz94u701utKjMcPSpSOW21wPoeCgyhV7dwzTovM26bL
dtf2uOpyGnVY6+27r+veL3G5m+islngD1qLaFZ/2NIjADo1Oe/9w6mHcM+oHinvxXGoUhklqUldLm
RwkSL/CMPPwounDwAAMCOtqvSt33L69a5e0a33y9aPJ2VvRX669bLVFtWewUe139KvWUZh56U
6JyfEblu53s/3bun26+Xg+ZiLe6RVHbg2rb71S2tfJT+5m+H38TB9HgKPefie1vdRxvdv3Pc1573Yf2R9
7eK0K4/35PH7CLt/3lfxb1dq9H0x9H2r2jFw3Pp7Pu1rNcl5t78m8/fiEedGvd8M7Ns9hrTf/uKem6H
XZfY+POp+0XJ6L453XvJ7Lx7fnpB/xzO6RnHuxXn6WRjjFdT/rIsKAAAwE+5f6F3WD43zNRwQWBCq
x5xdm9BVbUl/Bl3nnO/pqrT6M5p5eS7pnk7G5ZnV9cH8Gfre9fjfR2Gvqc5r79gUjT83Id9bxnk7iuLc
LwrvxbOmRvOoOqhhgkP7+Vl4cRCiAgAAAAAAAEAEaqICAAAAAAAAQARCVAAAAAAAAACIQIgKA
AAAAAAAABEIUQEAAAAAAAAgAiEqAAAAAAAAAAEQgRAUAAAAAAAACACISoAAAAAAAAAABCBEBU
AAAAAAAAAIhCiAgAAAAAAAEAEQlQAAAAAAAAAiECICgAAAAAAAAARCFEBAAAAAAAAAIAIhKgAA
AAAAAABEIEQFAAAAAAAAgAiEqAAAAAAAAAAAQgRAVAAAAAAAAACIQogIAAAAAAAABABEJUAAA
AAAAAAIhAiAoAAAAAAAAAEQhRAQAAAAAAACACISoAAAAAAAAARCBEBQAAAAAAAIAIhKgAAA
AAAAAAEIEQFQAAAAAAAAiEKICAAAAAAAAQARCVAAAAAAAAACIQIgKAAAAAAAAABEIUQEAA
AAAAAAgAiEqAAAAAAAAAEQgRAUAAAAAAACACISoAAAAAAAAABCBEBUAAADAQmtvFKRQ0I+N
tl5raG94n19p9PTK2fDbuiEhLV0wbdlwz3vk+e1JY2Xca6xXNVb8ba00rK8CAGB+EKICAAAAODqa52
TGOemSUMHoqjT1kqMsZ9aK+rmpKJsX6tZnHZ3aesg1asv5Wkc/F6me3bS+CgCA+UGICgAAAOAI6
Ujt/OL38Zx/XTlwM89yXbr9vvT7e7I5KvksbsrZqn4eco16jXN+IFttyXZFPwcAYE4QogIAAAA4WuiNm
r3eoezrp3LqRKxeo5Xtlng5auAamb1Qq9IiQQUAzCFCVAAAAAABHTLzeqH590pVg6NpryIpbm9Orse
rX/7Rrehp1Vr119suC68NKtPqCtUJH1wEdeJ31CKsrGjyeYL3S6HYYzGPXj8F92fsp1ayzrDVXndeO3Ul
FtltujOpfo/aGXxagXN+yXjUo3vHbQto/dH0tcc5VoEar+6BWKwAsLUJUAAAAAEdEWep1HdJl2Bu1U
ytJYTVYDdRep0K2gfXN1VEBZlNWCyUxyoCqjUhpcMIpOxQceJ3F3t/IQK8jtZJRr7Rcl60YnTuHwlEtel
8JVbak7hZHba7KRqMh5/wEVS4M1gNIcvwqwA5pv3M+hoNUR/i5UueiNLhTJewaAQCWAiEqAAA
AgKNjzQ3p4vVGnVS53pW+qgPq9ax0hK1v7o5oh1dLtC/
+y5tyzkv7etJYd0PBqrT0a71td2qyPjIpNl6/F2OSpvaGePmv0a6um3ga+6psW5/r+hNFqRqm9n5iDc
MPTjLVrLnHV5b6hcF2Jjl+67VuGhvWfmsrOxfjnqu27Eadi8A1AgAsC0JUAAAAAEdIUTbdGYwy641al
bNuj8nKaWvJNWL9/mFoL05zBnqzXmhn56Lz+t5F2dGdIQPD3I3enN5rB4QPix+t7aeGgTCzuHnB31
ftfDo9MAOTTGnVs8OTUiU6fuu675lBqKO4dsYLbDsHXf0saOhcmfVeOzviZq/FzT1n+9Zjb+QMWgC
Ao4oQFQAAAMDR4oVsGfVGLZ+Ukn4aMGp9qKqcDiR3JTnpp31ix33dA90L01rllguwH8bwdve1A0
6dSBLymT0vz8ha4EuLsnbGbdi+HIYlthMITDJlPQudTGqK41fHZNc5DR3eHzR0ropr4h2yuodK7n4ja
rECAI48QlQAAAAAR8xAb9RD5+l8K8qJU/rp1MpyMn6am0BHRnTmnE6i8DlCYFIpo85ppLBzFSw5Y
HLD3NgTdQEAjgxCVAAAAAABHj9kbtRYvTsvXYK/Onhx6Y8iHebVWhx7biYbtTyercHa88cfflg2z16lby9
Ss3ZpEcVP29D4Gyt7amueYpR8Alg0hKgAAAIAjyOiNmpQxjDw7g706u3Lg7tTtmVk66QWAo+p5ps
MsJeDXAHX05KJbmFROSaIqAdNKcvztXa/nqR24unVRp7iWaoZ+1ePUnkjLDlS7Xi3W0WUEAABHF
SEqAAAAgKPJmIAoTMlPDo3aqW3Z8Kapz1ZzdcObqKm94Q8/L59ZcwJAszZnc9UfQt7e8Gp0FlIZV2
4Gzta5WPd7WfYa61790aSTVU1twuP3A1djxv4k9PbVbRDscRoSdAMAlgYhKgAAAIAjKro3qjIzuwrp
nGAubi3NaZWlXG7Kqg4D/dzWmOFftd+ozdlc1cGh9+IREzJNorLtD1vv1KSk21XyE1S5kPuM9AmOv

3Lan6jKu5bGBFRJjDgX5r1RPat7ugIAlgYhKgAAAICjK6o3qqp7OVgzs9qavI5mIqfk7J4xPFyx63gO1Di
1a3MOvE4Je+2U7GHrIcdebVnr3eHxeYt9/BXZ7psz/itVaZk1TZu7Xs/fcexzEVYMVW8zrewaALA4Cn
1V3AUAAAAAAAAAEIqeqAAAAAAAAAAQgRAVAAAAAAAAACIQogIAAAAAAABABEJUAAAAAAAA
AIhAiAoAAAAAAAAAEQhRAQAAAAAAACACISoAAAAAAAAARCBEBQAAAAAAAIAIhKgAAAAAAAA
AEIEQFQAAAAAAAABGEvn/AZM6nr53azj8AAAAAElFTkSuQmCC]]></redcap:Attachment>
</ItemDef>
<ItemDef OID="q4" Name="q4" DataType="text" Length="1" redcap:Variable="q4" redcap:FieldType="radio" redcap:RequiredField="y" redcap:BranchingLogic="[q3] = '2'">
<Question><TranslatedText>Do you prefer Life A or Life B, or do you think Life A and Life B are the same as one another?
</TranslatedText><redcap:FormattedTranslatedText>Do you prefer Life A or Life B, or do you think Life A and Life B are the same as one another?
 </redcap:FormattedTranslatedText></Question>
<CodeListRef CodeListOID="q4.choices"/>
</ItemDef>
<ItemDef OID="summary5" Name="summary5" DataType="text" Length="999" redcap:Variable="summary5" redcap:FieldType="descriptive" redcap:SectionHeader=" " redcap:BranchingLogic="[q4] = '2'">
<Question><TranslatedText>
Alzheimer's disease - Description #[no]

If you have Alzheimer's disease...

1) You might forget recent events of importance to you. For example, if you are interested in hockey, then you might forget whether your favorite team won their last game, although you may remember watching the game itself.

2) You might forget the names of people you met recently, or the names of local streets.

3) You may no longer be able to handle your personal finances or plan leisure activities such as dinner parties or vacations. Simple decisions such as what to order in restaurants might become difficult to make. Your ability to concentrate may decrease and you might have difficulty driving.

4) You may start having trouble finding words to express your thoughts. You might need to be reminded to shower or brush your teeth, but you could do these activities on your own. You may need to post notes around the home to remind you of simple things like turning off the stove.

5) You may remain aware of the day and your surroundings. You might be able to travel to familiar locations like the supermarket, although probably with the help of a loved one or friend. If you are in a social gathering, then you might have difficulty following the conversation.

6) You may continue to enjoy hobbies such as puzzles or reading.

Now consider two possible lives. In Life A, you will live in perfect health for 17 years and then you will die of natural causes. In Life B, you will live in perfect health for the next 10 years, then you will live with Alzheimer's disease as described in the summary for another 10 years, and then you will die of natural causes.

</TranslatedText><redcap:FormattedTranslatedText>
Alzheimer's disease - Description #[no]

<p></p>

If you have Alzheimer's disease...
<p></p>
1) You might forget recent events of importance to you. For example, if you are interested in hockey, then you might forget whether your favorite team won their last game, although you may remember watching the game itself.
<p></p>
2) You might forget the names of people you met recently, or the names of local streets.
<p></p>
3) You may no longer be able to handle your personal finances or plan leisure activities such as dinner parties or vacations. Simple decisions such as what to order in restaurants might become difficult to make. Your ability to concentrate may decrease and you might have difficulty driving.
<p></p>
4) You may start having trouble finding words to express your thoughts. You might need to be reminded to shower or brush your teeth, but you could do these activities on your own. You may need to post notes around the home to remind you of simple things like turning off the stove.
<p></p>
5) You may remain aware of the day and your surroundings. You might be able to travel to familiar locations like the supermarket, although probably with the help of a loved one or friend. If you are in a social gathering, then you might have difficulty following the conversation.
<p></p>
6) You may continue to enjoy hobbies such as puzzles or reading.
<p></p>
<p></p>

<p></p>
<p></p>
Now consider two possible lives. In Life A, you will live in perfect health for 17 years and then you will die of natural causes. In Life B, you will live in perfect health for the next 10 years, then you will live with Alzheimer's disease as described in the summary for another 10 years, and then you will die of natural causes.
<p></p>
</redcap:FormattedTranslatedText></Question>
</ItemDef>
<ItemDef OID="a5" Name="a5" DataType="text" Length="999" redcap:Variable="a5" redcap:FieldType="descriptive" redcap:BranchingLogic="[q4] = '2'" redcap:InlineImage="1">
<Question><TranslatedText></TranslatedText></Question>
<redcap:Attachment DocName="17.png" MimeType="image/png"><![CDATA[iVBORw0KGgoAAAANSUhEUgAABVEAAADYCAIAAABtBTQzAAAAAXNSR0IArs4c6QAAAAR
nQU1BAACxjwv8YQUAAAAJcEhZcwAAEnQAABJ0Ad5mH3gAACpxSURBVHhe7d1fyF1Xve//1qg7P
bpp8EipetiNHoTSlhLtTXyQ3Qhii1AoZBdDqBs3Wx5SUHYIaIrYzblIaClCnpZCe0Dawvac9KJSCp60RC
GIQgJCn4tetBDLc1ExkF48DVKe0Fx45p8xxhzzO/7MucYac671zPV+8EdY64V5/i6XTk3/R
0AAAAAAEwRPT8AYCQXti7c9L9uIoQQQlYk3/3mF//9q/
+zyOlHDn98/bp6HALLjoucHAIxha3tr31P7xGaIEEIImWq+fvhzdcNf55XTp9QTERgXPT8AYHDbO9t3P
nen2AwRQggUBHvjvrPbXb8+q5CIyFnh8AMLgHf/Wg2AwRQgghU80tJ/ccvxXe/aPiLnFg7+OH
Vq+rRCIyFnh8AMKzjbx4XmyFCCCCFkwjE/43fz9KNH1dMRGAs9PwBgQGffPPit2QoQQQsiEI37G7+b1
Z59Rz0hgFPT8AIChXHz/4t5Te8VmiBBCCJlq9jxx80Pf+JLo7SM58+//HdXBkt3Nj58ALB8RmiBBCCJlq9jxx80Pf+JLo7SM58+//ph6ZwM
Do+QEA+R159YjYDBFCCCETzsGHPi+6s6cf+s6f6dfcf+lF9dQEhkTPDwI7Mk/PCl2QoQQQsiEc8cPPyP6+T45
ds9d721uqmcnMBh6fgBATq+985rYCDFCCCCETTv+fbs5eej+z5EmwDDK6tTtbX6buqGMbTU8/hTM2S7BFtm3QzWAB7n7BbZ+Ekv8/m
7RM7IUIIIWTC+eq//qPo1XOFH/Ct8C+Nt7pvmb5lbVNnCeTo6n56nraBhut1+93sxPb9vbvT8u9
H2zvadz90pdkKEEEELIhHPPriU/94M6viF49Y04/cpgf9Mvev4FMl2/aXLMkSXvikXH1rOZ73na6hnoxv
Qblp4f83jwVw+KnRAhhBAy4QzxM343r5w+pR60QA70/AvV7vHd/w6fvnz+Ue/39kwyh6g
etjwihou/DIld5e/39Cm+hZXaZ3vJudmF7LHiU7eui8Um541iP6jVzNq92JbZ1iXegoEVmjfEOsqt1rXZ
MRUWqeY98pJhObWeyZYFsffPC52QoQQQsi0M9DP+N289dv/GaLPPoPOl9Q//R6PVLB
Os1ulpRWk+Wh33ebRDOIOlK9FsPFX4ZEr9L17SH0GXpK5gqbtWgftWmAHFMc0foUQnPrNRMsjbNvnxXbIEIIIWTaGe5n/n
+qhC8yHnn/RZDfltDemE9LvmCvMqfoU69pWk+WjTtCXWP2pYzmwQYH7u7sZyqrZ/Ozq6gDZzXBCY/o/rOhX7a
6VTzLdsmr3IU2R5rSUvj2zDA9dxDFubTPaOYMfcQMrw94hvWILC04kJyMPsG5IDiCc6DQYyZYFhffv
7j31F6xEyKEEEImnP/
+H/8w6M/43Zx5+X+oGyDAWNT/bS78otPR3FT0cCgXfMxsH1AVZpAP8x8k+S6vNTXYiARV+FRfb0YLv4ypOM
q/VoPol+rVYmXJc8hh6xi7nYzV3mKh+eq5rKqfI/pmUHkfN0JdI/mXlMf8k0wvK5C9w0RlUueQy2yt
DMV50Ah7aPB+La2t27/xe1iJ0QIIYRMOJ/

+2Se+d+AO0ZOPkNeffUY9fYE50PMvA19Hqei32s2VaLk8J/VoltRV1QnVn9c2zlVXNUfM5WK4+Mu
Qrqv0AfVazUAvSi/SJ1JXVjHDNNf0mL6+qvUxtK7rMT1P6Zo7ge7RvDNq67GugmcgcWGPpVnaZ6v
3nan45tY9EyyBnRs7B144IHZChBBCyLTz7W/dLrrx0fLupUvqGQykoudfBpH+zf9WoCmbsVlSlxVn1G
cXf6gPmSPmajFc/GVI51X6SHWgmV1NL9InUldWSbtX+qrWGa3rekzPU7rmTqB7tOBgjR7rKngGEhf
2WFpziaDed6bim1v3TLAEjrx6RGyDCCGEkGnn7iO3ij58zJxYO/jh1avqMQwkoedfBpH+zf+WboXU0
dRmqb5O/5/3q6urQ/pIM6AYLv4ypPsqfag4otbUvOe/E51klbR75S3euq7H9IKnuBPoHq1HvR7rKng
GEhf2n0yhPkmWdqbim1v3TLBoT/7hSbENIoQQQqad2360d+Sf8bt5+tGj6kkMJKHnXwaRpso0PfZ
7shPyXG+6sFizpMZxf8ZfH7HGExXjL0P6XFXP2/xz9tZbPYtI8rK0xtJ3O9uXzTCI/VFW3Gu7R/OdURa
YbV2F7hvSOY47hLxfzhCh6ZdmXgLG8do7r4ltECGEEDLtLOpn/G74YT/mQc+/DDzNTsO0T/pd0weZ
080R3RqZazqapeZC36Xh3iv+MqTXVVZ98Y45Xx82p8bKyir6otkaS89dkdW7p+cpXfNMoHs054zwg
WZYD8+s5IVdk5Fza853jqjX3rn1mAkWZvPKJv9QPyGEkFXLAn/GL3Lsnrve29xUT2VgRvT8y8DT7Nh
MS9Vin2w1WVK8WWouNMOZat4C9XDxlyH9rmpW69wOc0XLTEUTG0v/R1DyjdPSDOopXfNOoH
ux/jOs4duzDqyu1w3pmEz49qhT3BE9c+s1EyzC9s72/o39YhtECCGETDuL/Rm/m5OH7v/o2jX1bAZ
mQc+/DDzNjtRuujwdkH1C8XbfZsk9z3ulOBh/GdLzqvjdMFdVIndMkVXSGkv7Kv3ngntBbHrBhQUn
0L3Y9hnOKdZcvZcXet+Q+GTsSuVl+nU9hG9EZ269Z4JR7dzYOfTyIbENIoQQQqadZfgZv5vnHjumHs/
ALOj5gW6edhRYDcd+c0xsgwghhJBp55aTe5bkZ/xuzr/0onpCA73R8wPd6Pmxms5cPCO2QYQQQsj
k891vflF02ssTftiPBPT8QDd6fqygC1sXxB6IEEIImXy+fvhzos1etvz8ge98fP26eIoDPdDzA93o+bFqtra
39j21T2yDCCGEkGnnC4/dIhrs5cyLJ3+qHthAD/T8AICW7Z3tO5+7U2yDCCGEkGnnlpN7jt67X3TXS
5s//vpV9dgGutDzAwBaHvzVg2IbRAghhEw+y/wzfjfH7rnrr+/9WT25gSh6fgBA4/ibx8UeiBBCCJl8lv9
n/G74YT96oucHAChn3z4r9kCEEELI5LNbfsbv5r/
+8wn1CAfC6PkXS//bcJVB/oG4yxtr6+fUn6fxb9G1VxRRnCjXqg559Rt0dfX+7kzvK7cyLr5/ce+pvWIb
RAghhEw7u+tn/G7e+u159SAHAuj5F0g0oPmbIlVgQg2Yu6Igc3fp+fPo9d2Z3ldudWxtb93+i9vFNog
QQgiZdvY8cfND3/iS6KJ3V35839c+
+Mv76nEO+NDzL9DA3ZDpbyfTgPlW5Gf39vG16jtCy9+lx3dnel+5lbFzY+fACwfENogQQgiZfA4+9Hn
RQu/GnH7kMD/sRwQ9/+L072DTrGrPb/f7pdhaaUj7o+efsiOvHhF7IEIIIWTyueOHnxHN8+7NK6dP
qYc64KDnXwzdCFnsPrbdtopuyWqimmFEFxwa327ArHPcfqw1g852zR7WvtLpzcPDNu+Yi8wExWw
1Z/RC+85VwrM3Y/pGqtkrs3gOd90xObPWKdZ4zULrWUWvC+hbq3Vi9MMq3gzcCq2Zt1GNaF9m
neOO0pq0eLv3nJHiyT88KfZAhBBCyOTz2Z988vt3f1l0zrs6b//
+9+rRDrTN1vOb3XZ4q+09pdUOBFqG3pot/y7e8IcaJP9bBeuueU5w7mlofP/gJWuIVvOlRT+2LMOa
9+tj4mVoRYK+qnhPXxGauSgQ4B1FHuxaWuAGNWd43q/e9F8X/
+Yn1FK6Z1yyz7J4Lsj3lZt5NujttXdeE3sgQgghZPKZwM/43ZxYO/jh1avqAQ9YRuj5xXZ+3j26PVy09Vl
63rtpmht9n8xZ5sZZ/U/0BvjGd691xncu09dEPjl32OaIrBMZ1j7HV9W3IqE8RUwhMO8eg1X0edYw
YmRnJFlZDmEuMGPqKwr2dEIDNRc6Umo1R3Rxc5U+0hyI1G7OshbhlnOm5Fwm191rzkiweWWTf
6ifEELICmYaP+N38/SjR9UzHrBk7/ldzv59LlbvUcgz5oLEGiR7YfI80+10rD42vnVMnKZfWoN7DgnZhj
UDKe2SYswOvnvZiL9rc+YpLu25NJuzELPu1hXmtOBAPYRrBT8s95qCORaZi+
+67nL6ZeQG9pgzZra9s71/Y7/YAxFCCCGTz5R+xu/m9WefUU96QBu+58+7NTe9wFq7J9iNPHdGdJ
OKWXR91NP/ePnuvKeAOM2M7hEs2N2S9R7WPlF+ur4VhXnW2oi+KQTuv55E76VV2mc7Y7QvMA
tu9Jmw1ruWvLH6lFYx78E23wfkuUyc1p5mm5hQZM6Yzc6NnUMvHxJ7IEIIIWTymd7P+N28e+mSe
t4DlWH/t/2ezbxnz17q2cnoa9bP6T/N0gItGc/d9LdV4kT/SS7P+L5rA6P7WAO15Ry2OdNZoG9FYZ5J
GbH3XLpudba+1Myhz9LMzAX9fnA+gQtjNyChlryx3ukE59iQ45S6y+kzfMIT8tVCf8d+c0zsgQghhJB
VyHe/
+UXRIU8vJw/dr573QGVBPb85yxbpJRR9WTmI/nP3VcvKczf9bZVYqv8kl2f8Hr1T39Fb8g1rLquIs30r
CouU1AP1XqQaqzhfX9r1odn0GYX6JGchnWNYQ1T6nNi/ljzHO53OOfo/oO5yPQbuMWfM4MzFM
2IDRAghhKxIvvDYLaJDnl7Ov/SieuQDleH/DT/PNc6hHpv+kr6uvky/6rhoecXujL0oeV7Pu+Ubv0fv5L

uqU65h9VlrGxu+Rc42t/B9SlijGmxtfb2+1LqyczR3IvqIuSQ81/KtGYql1ZKj+qqYY545ar7rusv5rhJ6zB
kz4P81fkIIIaucrx/
+nGiSp5TnHjumnveAtoieXx/x7N8j7YR7Uq+Llph7ZwqmQ5OrbJbp6X+8fOPP0u/pA2ZK9jzb8gzbus
S8iIwZF75P4XfCzHQrrQl0LU2utDnfjOOfkbmwOew/sZFWy7mxzpKscSJ3zRmn0KOcU02uotec0d/O
jZ2DvzwoNkCEEELI6mSq/wv/k4fu/
+jaNfW8B7RF9PxmQ+8R2b/rgZptv3tkVwncTf/tsdbo6X/82iNVVXr1TuZIS+SjyTKseVsPYqbfjOpZUZ
hnUop+p2OANru2vLBjae1p2/QpobmGrgzPPKmW+2EFllRy72ejXb0arVe5jhvYc86YwZW/XeEf7SeE
ELKyueXknqP37hcN827PsXvuem9zUz3pAcvu6fn9XUEt1oUsr8jdbC9WvO/pf0LsW12d3rt3as+gq9
T8wzZvWOeb6TcnOisK80yq5p9aJ89sbPE7Zs+7LKtfqykE51qwLy11znr2Wt0fVvFGbI4Nu3h1ZlI5W
aX3IJgB/4/zE0IIWeVM74f9/+
+F59UzHmhbZM8f7x2EdkMgzTQUkEJ/BfmyYTLOvn1WbIAIIYSQ1cmUftj/9KNH1dMdcCzy9/yRUR
zmmgD6MAzLfAP5qmFSHv/d42IDRAghhKxOpvHD/hNrBz+8elU92gFHYs/vVbXwTkPv6fCbYdQx87
8HDv23AJ5BauYNOjEMxP5fq4e/osCu9fArD4sNECGEELIimcYP+9+9dEk91AGfhfT8oYGC7VSks4+8
BeRg9fw0/JiinRs7B144IPZAhBBCyIrkth/t/cGdXxFd9C7K688+o57oQMCCev5Se7BYyx7t66NvAgA6
bW1v7Xtqn9gDEUIIISuSu4/cKhrp3RJ+xo8+Zuv5AQCTdGHrAv+MPyGEkJXNt791u2inlz8/vu9rH/zl
ffUgB8Lo+QEApef/9LzYABFCCCERkk//7BPfO3CHaKqXPG/99rx6hANR9PwAAOX4m8fFHogQQgZhk
eyuH/a/cvqUengDXej5ESf+PYb4ynofxtP/UMM8ww1q9Y/xT/4PwRRrGyENZXGvIeYrEMvHxJ7IEIII
WRFslt+2H/6kcMfX7+untxAF3p+xMWb/HmazEX1/KZQbdiWXxUbqQcf7R5iyrZ3tvdv7Bd7IEIIIWR
Fsvw/7Odn/JgVPT9mIrrKeZpM0fOPZsS6Y/fg9PzI450P3uGf8SeEELKaWf4f9v/x16+qBzbQDz0/ZrL7
e/4x+2J6fuxab1x+Q+yBCCGEkBXJMv+w/8WTP1WPaqA3ev6FCLS73sOmj6t0X9HR9+m3myvcI3rY
8ogYLv4yzpxcKM4Xc/cOZV9iTzCqdVHrKl3SEpi4PTkx75Z2LbtaqJZYdslZunVOM4x6M1zRM1CAGKP
/ogp95l+IjlFrneK+32MEDOvMxTNiD0QIIYSsSL76r/8omu1lyM8f+A4/40cCev7F8DROvoNNw2ezLu
rZgNn0+
+ZtU8QMoo5Ur8Vw8ZcR/qWUVFlnKHPAZi3UK37HPO8GJt453VK0WKiWPm6N4yzdc219erRiv4/
De1utKzpKpM6/ZM2q68PtHgGjOPLqEbEHIoQQQlYk//zAbaLlXmyO3XPXX9/7s3pCA7Og518Q3fN
42hxzyPQ9+ohplJxTrGGcBkxSJ+hLrO5KXaFHqF6K4eIvg8x5skZBTaRrZM9SHT3umDuwl3tXmiNiCT
PW8qzDOc2tXums6KvX5lwibllniT7zl6c4g8gLnCs6R8BIdm7sHPzlQbEHIoQQQlYhe564+V/u+yfReC
8w/Iwfyej5F0V3MaaJcXopT3PV2V0VnH7Koc6or6lfrK+XA9lH9PViuPjLEN9p5piae/sc/cqzMOuQ0O
eOuQe89FDWWZ0Xuif4LunzkZnq4bVW5IUJU6wPhc6PzC08f/MyNH19gmeI+lDnCBjRlb9d4Z/xJ4
QQspq59cSnluSH/f/7+H+oBzMwO3r+hRGNj26lTPfl6a0Kgausk0y/FO7j1FXVCdWf1zbOVVc1R8zlY
rj4yxDvWsTB9lD6TZ9QLW8VM64+2m/Kpn5zVuzC9nTNCb5LPLN0TvNUl7wVO9fmKR7mX1SP+Zv
XjVbB9sBt9RBdI2Bcm1c2957aK7ZBhBBCyCpkGX7Yf/LQ/R9du6aeysDs6PkXR/c1VTOj26CmWfO3
Z6K96tGA+ajLijPqs4s/1IfMEXO1GC7+MsS7FnGwPZR+0ydUy1vFmWK/KXvGci80RwT3BKtWn5H9
Kyl0VPTVawkO3Oha1Dx3Rr2vh/DRQ8RHwOjOvn1W7IEIIYSQFclif9h/7J673tvcVM9jIAk9/yKp3qfo
nnSHY7U0/vZMn6iO9mrAPOrr9P95v7q6OqSPNAOK4eIvQ7xrEQfbQ3mv6OC/Ro+rj/absmcseaE+
pVCf5Y7sq9Vj5NDqOyv66rUEBm50L6rP/A1ruEp9UecsLP4RsAiP/+5xsQcihBBCViGL/WH/
+ZdeVE9iIBU9/0KphmZtfb1umuyeyTRSdpcjuytP92SapFDfV1LjuD/jr49Y44mK8ZchvtPMMVWrfU
7PgVvkiBVnoH4je26suNA9w73zvlp9PjLPOYXuip1rC01ID9ldwnOKe06hOGa9atftnGYlNgIW5OFXH
hbbIEIIIWQVsqgf9j/32DH1DAbmQM+/WKZbqoh2xryp2yvT95h+yxzR11oDRpuj5kLfpVY/J3qt+Ms
gZ55WfVUsNLK+wkwvUqrHHZN1AvRI4Tsh59NMuBnZV6s5Tx207rs+zVO90F3RV6/Nqd4+0GNRzg
jO/M2BZvpiQeEx+o7gOYAR7NzYOfDCAbENIoQQQlYhX/m3z4qGfOicWDvIz/iRBT3/gpnupqC7qob
9bsPucqymTHKHszUXOl2Uv0A9XPxlRHiiqpozlP+KjkIz3LHoSJ6OUl7oL1VqRm6fUx8P3wlzoad6qbN
in7UFyqtSPRY1w/ylZlr+QZr3u0cI3CMMbWt7a99T+8Q2iBBCCFmFHHzo86ItHzTvXrqknr7AfOj5F8
00N6Hepd0dNT2PYZ9QvG1eek61ued5rxQH4y/jzMmF4nzRtMWrV3q2d+2L5Mz6TdnTUXoutDvT

8qh+bQ1tn2lGs2dYnOuM7KmuxCv2W5u8Q6JMvESla/4le5iSO6X2LOJrLbVH0O+612FoF7Yu8M/4
E0IIWcHseeLmh77xJdGZD5TXn31GPXeBudHzL5pufGhdAOwWz//pebENIoQQQlYhn/3JJ79/95dFf
549Tz96VD1xgRzo+RfL/J86afkB7CbH3zwutkGEEELIKuSOH35GtOh5c2Lt4IdXr6rHLZADPf+itP+Hy+
7/8BkAltuhlw+JbRAhhBCyChn0h/1v//736kELZELPvyhWz0/DD2AX2t7Z3r+xX2yDCCGEkMlnuB/2v
3L6lHrKAvnQ8wMAEr3zwTv8M/6EEEJWMEP8sP/0I4c/vn5dPWKBfOj5AQDp3rj8htgGEUIIIauQvD
/s//F9X/vgL+
+rhyuQFT0/AGAuZy6eEdsgQgghZBXy9cOfE617ct767Xn1WAVyo+cHAMzryKtHxDaIEEIIWYV895tf
FN17Qv7rP59QD1RgAPT8AIB57dzYOfjLg2IbRAghhEw+t5zcc/Te/aKHnyk/f+A7/Iwfg6LnBwBkcOVv
V/hn/AkhhKxgvvDYLaKN759j99z11/f+rB6lwDDo+QEAeWxe2dx7aq/YCRFCCCGTT/IP+//461fVQxQ
YDD0/ACCbs2+fFdsgQgghZBWS8MP+F0/+VD0+gSHR8wMAcnr8d4+LbRAhhBAy+cz6w35+xo/R0P
MDADJ7+JWHxU6IEEIImXz6/7D/2D13vbe5qZ6awMDo+QEAme3c2DnwwgGxEyKEEEImn3sf2Sfae
2/Ov/SiemQCw6PnBwDkt7W9te+pfWInRAghhEw+3/7W7aLDF3nusWPqYQmMgp4fADCIC1sX+G
f8CSGErFo+/bNPfO/AHaLPNzl56P6Prl1TT0pgFPT8AIChPP+n58VOiBBCCJl8bvvR3h/c+RXR7RfhZ/x
YCHp+AMCAjr95XOyECCGEkMnn7iO3ioa/yOvPPPqOejsCI6PkBAMM69PIhsRMihBCCJh/xw/6nHz2
qnovAuOj5AQDD2t7Z3r+xX+yECCGEkGnH/mH/ibWDH169qp6LwLjo+QEAg3vng3f4Z/wJIYSsWsw
P+9+9dEk9EYHR0fMDAMbwxuU3xE6IEEImXzuPnIrP+PHYtHzAwBGcubiGbETIoQQQiYf9RQEFoSv
IABgPGIbRAghhEw+6hEILAhfQQDASG66aaSHDoWSUSgZhZJRKBmFklEo2fQKrQJuJQBgJOxIklEoG
YWSUSgZhZJRKBmFEMGtBACMhB1JMgolo1AyCiWjUDIKJaMQIriVAICRsCNJRqFkFEpGoWQUSkah
ZBRCBLcSADASdiTJKJSMQskolIxCySiUjEKI4FYCAEbCjiQZhZJRKBmFklEoGYWSUQgR3EoAwEjYkSSj
UDIKJaNQMgolo1AyCiGCWwkAGAk7kmQUSkahZBRKRqFkFEpGlURwKwEAI2FHkoxCySiUjELJKJS
MQskohAhuJQBgJOxIklEoGYWSUSgZhZJRKBmFEMGtBACMhB1JMgolo1AyCiWjUDIKJaMQIriVAIC
RsCNJRqFkFEpGoWQUSkahZBRCBLcSADASdiTJKJSMQskolIxCySiUjEKI4FYCAEbCjiQZhZJRKBmFklE
oGYWSUQgR3EoAwEjYkSSjUDIKJaNQMgolo1AyCiGCWwkAGAk7kmQUSkahZBRKRqFkFEpGlURw
KwEAI2FHkoxCySiUjELJKJSMQskohAhuJQBgJOxIklEoGYWSUSgZhZJRKBmFEMGtBACMhB1JMgolo
1AyCiWjUDIKJaMQIriVAICRsCNJRqFkFEpGoWQUSkahZBRCBLcSADASdiTJKJSMQskolIxCySiUjEKI4
FYCAEbCjiQZhZJRKBmFklEoGYWSUQgR3EoAwEjYkSSjUDIKJaNQMgolo1AyCiGCWwkAGAk7kmQ
USkahZBRKRqFkFEpGlURwKwEAI2FHkoxCySiUjELJKJSMQskohAhuJQBgJOxIklEoGYWSUSgZhZJRK
BmFEMGtBACMhB1JMgolo1AyCiWjUDIKJaMQIriVAAAAAABMEz0/AAAAAADTRM8PAAAAAMA0
0fMDAAAAADBN9PwAAAAAAEwTPT8AAAAAANNEzw8AAAAAwDTR8wMAAAAAME30/AAAAAA
ATBM9PwAAAAAA00TPDwAAAADANNHzAwAwq3PrNzXWz6mjAAAAy4aeHwAwIXUzPmwXfnljra
kwZMGx/5uFcmGFtY3L6kBOrcVU+O9KZtW6h8N8TIML/IVR371a3m9G5K9o5K1Z+YdqfWJ5lzWs6
J0Z9D8oBhRe1G79nAIrai0n18fUo9Yu/c+kFUHPDwAY1oC7+RarzpibtqrsEHudYi9l1lHtqwbeUJULW
VtfL/4/g9QplzD6ltDej+b/VrS+2Y2Bvn3VWkb6Rtj3LV+R4N/Q9t+h9kLnEqwYfWtWoaHK4+ZAfUvn
LeUfxf645i7ReWfK97P+B4VvUa0lVeZbV2RR1VsZ/yq5y7GK2wZaUVXfHKpezbu6frUylKrvnSLuT+su
ivfQAz0/AGBA7f1UezeSlS5U/f/H3BC0VziUwauUBdTtG6ZM+dkPfpts43wwLVXJgb58cuzhauXexyv
683AnLr8auZYWrhh7a1Z9h9LnqZczq66vtasUt88cmP/j6lpOeVy9PVedWmxROcZXIovyHEoXXI4wf
9HwiuSRAWvpN9TLeT+1cjgzfPVNbl62S1VvzrWmVUTPDwAYjtwDOLuG7IavIIyz/Rh4WWb48g/pm
7aY+baDMxv9e1AY9qvQvoGDra8a2P6gcn9wzszdpWS+kZF7lfE2dg1VvZ98I/XVw1ax+AuZo+Uf5i6j
J+upNcx/XLiFsqxDiSynLeP3u/PWdU6mP2coZx0Zi5m7Wb2QX4echVYGPT8AYDDukznjbsdv5M1At
Z5ce8ag1vYnP+ueIX8cplB1pxoD37Ph1hE08IdUqG9hUaL+wzDfcudvqPXtyMIZr6zYvm+ZS0aGy1ip
ayjnxibpqpJtQb6BrGPlH7N92T213C9FDm6hqk75QwVj/qq+W2ep3s61Nm+t6qtWlqj/EJ7KbJxagd
uZZ2n2bfIXyrawFUHPDwAYjLsD8G5Rchq8gK3aeOTavbmqtSgDLqlchR6+LDncgrR6YUPWqb55mbf
yceN88apvXGWoUu463L/Fc3EKuONnvpeR4TJWig9VfXAZ7mK8SvVuns/KU6hcgz5Svp3tSxGoZclU
yi0kPpfqhPD97cezHEv83VmFRmvuX7ZSnlrVEXn3Mn1W1QpUtfLP7WHz3sXVQM8PABjMAh7V4+

0FxG5xUFk3U23lMpr7VRYaZU3uVyMr8eHU92/Ir0VVcNDvnb2G+s/D1KvGlrcu40dVDWjP3P0qOK
fMJzJcxkqxocTXcQ7eKtVBJddtcwqVa2heI29n+1LEbl2hXl6Oam4h58uX4S9ydDkZxrd5atW3qz5W/
zlTPe+6qvVo6+utL8kcqmHNB+N8SvF7DC96fgDAYBbwqB5pL1DvdEbcc9Sbt/wFW1s2S44ddkxVd7
j753zzhq1XfTqD3jK3woAran0p8u3ja87fUPevrPPpzcctYETemlVwqOpu5lpOx4Srt7MUk4Va3wnLEL
Vcmb4RbiFn4O65dIoMUb2V67tQcmq5FapPbr4V1bpvTXlGjtXJvzLux989F0j0/ACAwbgP5kx7t7Ax
9gJVjZE3HFXNQe9cpSwzfJXhlzPEVj4s2646zC0x6Iosub8S7rzlh5V7ZZHxMpbyD1V9bhkX0znh6oQ
MBTsKlW9n+1Z0LirX3zC3UHXEXoj8MiYILyfTMixOLbdE993tqXOg8oT5vxTVCtrDuJUzfEwrh54fAD
Ac+WTOtv0IGr5CvSeRsu8/iirWMjz7oEHk2bW5inGtYasPadBPqapgL2TIPWL14Qz7nXO/AaMULWT
/RlQfTXvirbU5H93cPBW1yFuz8gxVHcr7IXVOuDohw+3rKFS+ne1DWuiiWn+RsvytCi5ngL+zbq3WX
6ZCvqLxjynPZ1TN1i1SHrbHjk8FXvT8AIABtTYgeXYFftXYwu7eE4gVjbOYsuggn9Doq2ltdfPte11Dfqv
b6v2wMcI3IufaxDeg0izBXluumxmpGJ/MTMJDic+rNufiqmqtmRZVrNdVzXlq9Lwz5WnZvvTOoooD
1uD1lDyT6C2+qNa785RRnOXUqsMjfLez/wdFuFb7nblvXT2cd5jW9zrrnVwd9PwAgGHZGxCe0xhN
a0M69340pPp6Dzb6AmTexyOL9qdSU5+NeGsXfWLhRe3KNUU+o8Lk/oMiO/FfVdSaLQMbiTnR8w
MAAAAAME30/AAAAAAATBM9PwAAAAAA00TPDwAAAADANNHzAwAAAAAwTfT8AAAAAAABME
z0/AAAAAADTRM8PAAAAAMA00fMDAAAAADBN9PwAAAAAAEwTPT8AAAAAANNEzw8AAAAAw
DTR8wMAAAAAME30/AAAAAAATBM9PwAAAAAA00TPDwAAAADANNHzAwAAAAAwTfT8AAAA
AABMEz0/AAAAAADTRM8PAAAAAMA00fMDAAAAADBN9PwAAAAAAEwTPT8AAAAAANNEzw8A
AAAAwDTR8wMAAAAAME30/AAAAAAATBM9PwAAAAAA00TPDwAAAADANNHzAwAAAAAwTfT
8AAAAAABMEz0/AAAAAADTRM8PAAAAAMA00fMDAAAsl3PrNynr59Shmn5jbeOyOjIMXUeUXw
bNvSk49+Hyxlroreh7ADBd9PwAAADLxepr2+3pqvf8Tdte8d2GYGff3NQl/G8yAGA49PwAAADLpfV
/y7Y71FXv+Xut39vcN/9VAB0/gBVDzw8AALBcWj2/3eCueM9vGveOeZn7Z+6TOULHD2Dl0PMDAA
Asl3bPbzWqTs/vHHAbY3VKeYY1rrrAOmJ3w/pwccz6n9NbRTTrXed9a25NmY6euzVea0BrplpkLHN
2dY555awgNv9Sez7ilMgCo9cBwKjo+QEAAJZL00xu1H/SLaPVZVav3QNWu6la4qYd7WSa6PA1Vp8t
+9qKNRHPINHW119ULt1izcXRTG59Q/9RVO+af8eEwgv0XxedLgAMh54fAABgueimseggdWNaN4z
WG9WJ7oFYz69OslpSeUS3pc41zRF9QJYxZ5ipWHV6tLvhAmZAt2aMOVlphql0zl+fEJ5PYIGhgeQM
AGAc9PwAAADLpdU1qhf2n92202onZTOrT2n60vARPYx7hjxHdsQFecgMYp0TJGdQkitxD8SZCZTEJ
T3mLznV/Qs0p/VaNwAMjp4fAABgufjb66LVdDpjT6ssW1P3lO4j+rXdKLfH1Wf4iMKtUQLcKZV0SX1
ULq1TeAo95m9pn63fD4xuZtloLwsARkXPDwAAsFxkB6xem5/3Nz2kPLMwVM/fPqhf+AQLh/nPlUtJ
7vndKfSYv695r+j3g6MHLuw7awDIip4fAABguTjNpGwizRuetlM2xu4p3Uc8w4pxfWcIPU4x/Ofqkvq
oXFqn8BS6J6fPKNQnOdU7x7CGqISLAcBw6PkBAACWi6eZbLeP5o3YmRl6fru5bp/To/12y4SZ4eyTn
RoZe/7OodxLnZsSWWDxljXwzPMGgIzo+QEAAJaLr5k0fWPJvNEcVe2kaUydI27zGj4SHsWcEq6sG1
u3TEy4QHP9zL1zZApd85fLac7vWKCzkOg0AGBw9PwAAADLpaOZtN+wWlFJ9LKxFlQeMa/X5Oi63
y34SzcnuGXi9Plt9tU5e/7O+funU9KnhEYPXdl31gCQFT0/AADAcgk0k02T2nrD7l2LtlI2xu5g3Uf0a3s
4Zzqldt/cPiGwiqj2eLJJlkvr1DmF2Pyby0tlSeu22G/7RrcvLfWdMQBkR88PAAAAAMA00fMDAAAAA
DBN9PwAAAAAAEwTPT8AAAAAANNEzw8AAAAAwDTR8wMAAAAAME30/AAAAAAATBM9PwAA
AAAA00TPDwAAAADANNHzAwAAAAAwTfT8AAAAAABMEz0/AAAAAADTRM8PAAAAAMA00fMD
AAAAADBFf//7/wdlKhH78Ney0gAAAABJRU5ErkJggg==]]></redcap:Attachment>
</ItemDef>
<ItemDef OID="b5" Name="b5" DataType="text" Length="999" redcap:Variable="b5"
redcap:FieldType="descriptive" redcap:BranchingLogic="[q4] = '2'"
redcap:InlineImage="1">
<Question><TranslatedText></TranslatedText></Question>
<redcap:Attachment DocName="B.png" MimeType="image/png"><!
[CDATA[iVBORw0KGgoAAAANSUhEUgAABVEAAAEJCAYAAACdcmdaAAAAAXNSR0IArs4c6QAAAA

RnQU1BAACxjwv8YQUAAAAJcEhZcwAAEnQAABJ0Ad5mH3gAAE9sSURBVHhe7d0PjCRnfef/b+/aY
PPHNmBskx+RHat7sZYlGJIg08PxL4egZ+6SJbnbYOVg5Oh3PVyi0zQSkwOxF+mX2wguG4lunS5iWrq
zliQyTEJugrLdIJRgckzHR4IxYTI/Z7rlc3T8wDYGjPljB3u3f/VUPVX1VHd1dVV3VXX39PtltaerpqfqqT/T
O/OZ5/k+hb5FAAAAAAAAAAChjumPAAAAAAAAAIAQhKgAAAAAAAAAEIEQFQAAAAAAAAAiEKIC
AAAAAAAAQARCVAAAAAAAAACIQIgKAAAAAAAAABEIUQEAAAAAAAAgAiEqAAAAAAAAAEQgRA
UAAAAAAACACISoAAAAAAAAABCBEBUAAAAAAAAIhCiAgAAAAAAAEAEQlQAAAAAAAAAiECICg
AAAAAAAAARCFEBAAAAAAAAIAIhKgAAAAAAAABEIEQFAAAAAAAAgAiEqAAAAAAAAAAQgRAVA
AAAAAAAACIQogIAAAAAAABABEJUAAAAAAAAAIhAiAoAAAAAAAAAEQhRAQAAAAAAACACISoA
AAAAAAAARCBEBQAAAAAAAIAIhKgAAAAAAAAAEIEQFQAAAAAAAAAiEKICAAAAAAAAQARCVAA
AAAAAAACIQIgKAAAAAAAAABEIUQEAAAAAAAAgAiEqAAAAAAAAAAEQgRAUAAAAAAACACISoAA
AAAAAAABCBEBUAAAAAAAAIhCiAgAAAAAAAEAEQlQAAAAAAAAAiECICgAAAAAAAAARCFEBAA
AAAAAAIAIhKgAAAAAAAABEIEQFAAAAAAAAgAiEqAAAAAAAAAAQgRAVAAAAAAAAAACIQogIAAA
AAAABABEJUAAAAAAAAAIhAiLp0etJYKUih4DxWGj29PqjXXWFeU1U1hxUMdj9hDpMeKBdL1hpWhL
MxLO7LSlg372PzHqPs4Vb2GrGzEO5vtDbdtK5JH08KNuQ9Cjmc+2g0AAAAAwPwhRF06Rdm8UJey
XurU1kPB4tXv/8HAXTVb7uwCh0XnZmZMqYKWxQUlb58lAdI7cT/A6B8hvrlsW1m6ZDlXBh1nrhFj2rXU1pW
MxLQ7LSlg372PzHqPs4Vb2GrGzEO5vtDbdtK5JH08KNuQ9Cjmc+2g0AAAAAwPwhRF06Rdm8UJey
uytmqfi4dqZ0P9kbrNc75IVW1JdsV/XzFGN1/wqSZxTx6f+fKMNiBvWvV69ZoGH
ZPsnXCP1Z1IPE48D5Xrv5AEk5oXG1bi2gfMgPMf1Ap6VJK4Msx+sF7TdvMw1JgkNvwsD9T4n2Bt1GJxRqhtsqvf5jvRjRTR/Cm
D6gP/AGy9izv6PBo1Pe1wrCSDo63TPb8dqZUKUhrYSXM1aUimtjPY1qasDoZtEx2T2rZR+9S6H+te1
+kREu3HCSwHz4HSXB0dKibnnOtV70AUtW6Kc9TeGFHPNGS7E4vZbgAAAAAlgQh6lIILTjLVrLnBTF
nqF0Imk2pv+KGKV4+yL103ie3UZD3lhKW4dka3b18O3U23d42JhYxw0ohjqferEnAz0rpz2yiY0d4M9
Ji/u6BisfEacDLUnjXX3GlSlpc9v3+0xnOr59bfvdUhWIdlAfdzx/O1494Hazrob/E1zTMbr9zZlc9v62PX
vV1Wv1/6cXXIi2X56jXUvsCzXu85r+8MlLiqR+4wr/BzteOl/krZbr3X/0hH2PRjY7rBkxzOu3QAAAA
ALI+C9QtyXz/HklK9SwM9zlSwEhKq+K8rS71rTubk8aK24tOBS/OBS/OZEYjX6963bk96Ubsy+dvW4Vde
9ZG1BBs1YOwwWq1Ks9kcWu+3IaxdcdeNE387w11fcnDqbsV0VnO2FBNwB4e0wr3O11fc
nDEt0PSK2E9ivvuaS7JjMbdfr41qa6JzF35+FOdanQqsS3p+lETnOlHbHSHWbhr3PnyvB
OXCW/QSACAP99/xLnnNW2/SLnNmqf7ARz/BKicVVnQBZ5xFa3kL8dTr5/ZKhHbX5qf1DKGaPV
BMfcIsazBr1jUo6ac28z7QPYSnOKZEvYkT7cec4f+kmIdQ3NyTvhmgTm3wHIWY6rrrurFuwJmaGO0
GMvDwEw/LOz/5Tr0EAMjDDY9fJXd/6AF5/Bs/zBCmhzmzyJd//ztjrRfbsG+zTl0+z/4+7zP03
pbenJoj9k/JScqJ6z/D65XGeqc9ZIrrombLbpD+ttuIl09a/TozUtaAZl1DTJrOyHeSKpXqBe0GnVjgQX3x
NNPSOWPKvZHAEA+nvPjZYHXXI3RfXl9lbEyVeN9XMZvhk9NO3A1+/VOyrVqVM2zrF4IAAT0lOk2nFfoVt6HWLXk
RfTlsXxS7lKgd9lbEyVeN9XMZvhk9NO3A1+/VOyrwVcO67VqVQ480e0lOK1mv47yOaTHOXbjxbW/
Lhtnr1K2LatY5BRbUnZ+6Ux58/EG9BADIwxv/+gZ5wP+SkN1LbnBFfMSZJitM70Ri+HI+7744c7D
pf686+X7I/4a8fLjUwH4qbZ/0guOFOjFWVQIZacsoTKG6Jgvk22OvYvA/0seV1TIn2Y97LA8Pk1cz3hZ
xnoE/SdmNSNTtwdeukJv6eAubL+z77PvlM7zN6CQCQh1c+kK3+kHHzlLx/RSwCAzF3
6gsjlr+kFzDtCVMRUlM2zbuZYVfX1n0YsMwDdwp2MOk3fsCWi6u7Cza2/uJVm5wZwN76cqqJrYvN7cqqJpBS3XmZRF0x117vh
6vxxe81a56Kmj3+wdqsx7F+aq7LhnTIV7Onh297K+dBcNQLH9nm/hqd7bHkdU6L9mLVbm7Lqre/p
me+ta1Qq+NvI2oTnyA9c3XYDi+kT+5+Q+n11vQQAyIOqg/bOfn/8Tb73raf1EgAgD6+J9t/NvU2R+OzsWSF9k/RHnUXDv4x7cRQeTr1+SkrMEHT+A0vq4f/6VMLFgWJfeaUCZQVqMwSqfsgtdrYN2rlSoyNSsZ17jvV0Rw=/Od7a9

Jqu9rKUt8KRKiWomxe8IdlN1d1gOaFzqMm/ZoIJ3AMttM8tryOKdl+ipsXpO6/2HmtOZGTdS/7h6Br
7yrua1NNWBO03bzfw9odR+bHA8R339fvk7v+7C69BADIw/FLBXsYv/oYRgWozd+8Xy8BADLXf1Lk
8sf1AuYZISoSqWyH11+stqz17tBiV3FT9gZfW21NVr/R7K0XKBlgDM3OdKKjFJgB2KiyA+qc9bt+wOe
ya1/OW03PstS7fT9Yt1Wl1d8LTpaV1zEl2k9RNvf60h16sXq5MUzeVpHt4EGK7B96PbFTEbvtVlv6Lf
8+sqlzblwHu+5ulByOB4jBnYn/6Wfp7QQAeVr50kvl2iev1Evh/uFL35ZP//6hXgIAZO6y9RvZJcpbzbt
CX81aAgAxtTcK4nSSVCHqQGAKADGo4PT1/+318sAjD+g1AIA8lB56od0LNa6tu8vyiteFD/sHAGTgit
8QKfBL9ryiJyoAAMiVGsJPgAoA+VK9T1Uv1CTUsH7qowJAji79AfVR5xghKgAAyM1HvvgRezIpAEB+
VP3Tt33hZSProI6iAtS7/+NX9RIAIHN2fdR79ALmDSEqAADIxe6Du/LBv/igXgIA5OWOL18/tg7qKPv/
8zH53Mcf0ksAgMxd/n9FLn1BL2CeUBMVAABkTg3fV3VQmUgKAPJ189efL//8Czfppclc+dzjsnX36+
XWV79IrwEAZOtKXR/1Zr2MeUBPVAAAkKknnn6CmfgBYAZe8MMrEk0kNcoz/3RJmu+/X3705DN6
DQAgW9b77aWPWx+fchYxFwhRAQBAZlRwqgLUh594WK8BAORB1T99yxdvlOf8OJ1f+R7/xo/k7g8
xKSAA5Kb/HZFL1EedJ4SoAAAgM+/77Pvk3ofv1UsAgLz83AMvkRsev0ovpeMrf/mI3PtJ/igGALm5/
DXr0dELmDVC1KXWlo1CQQrGY6XR05/LSa8hKxttveBob7jtWZG8mzM3Qs5LHL3GinPuVhoy6tT55
9d55H7NjwLuWyCW+n11+djffkwvAQDy8vJvPE9e+eC1eildn/zPB/LN//19vQQAyNylXZH+Y3oBs0S
IurR60lhZlaZecpTlzFpRP8+eHfiVasLfVIImPi+9hqzXor5KXfOCrAYvunRqJSlMENguK+5bIB7V+1T1Qg
UA5Ovqp47LG++bvg7qKKo+6u//
+y/bHwEAeVD1Uf+79fFZZxEzQ4i6tLpy4KZA5bp0+33p9/dkM68MdWzgt6QmPS+qZ+S4YK99XtxN
V1vqevelWy87K5qrQo4aA/ctEIuqf6rqoAIA8veWvRvtIDVLqifqH/721/QSACBz/UdFLv2xXsCsEKIuq
96h7OuncuqE5Nf/FGmL1zOyJ41zugtquS5bFedpcfOsVJ2n0twlRQUwPTUTf+WPKvZHAEC+Xvu1F8
vLHr1aL2Vrb/f/2A8AQE4uf8l5YGYIUZeQXbvRDN2aq04tx8GuiKp3o1E7c1T9zGAtyGCd1bDejbH3r
wVreI6qN+kMVfdfl6zW52A9S6+2qH6ENy3GPgPncEP8zZjnydln0vOiqHaWYvWMNHoeB0LzkpzUn
VFl/9A6olGM9obVW21v6GMZPAcJrkvI/RZ2vePcb4PXz35E1ImNY5b37WT3p2vSa2eZ6D1Ar1TMr
49uJI6QOz91pzz4+IN6CQCQFxWevubvXqSX8qF6o1IfFQBydOlPqI86Q4SoCDUUGGl2/cyRYVRHai
WjzqrR43EyanuDNTzVurCgpuQNVXdFt3UUZ5+DwWRzdcJ9Fjflgjtk3joz5/RGeo1z3nkq1y+kUEahK
q1+y+tVOsTseTxK50C6+umwipx2N97ZkYsDJ7W96x2NX1c3yXVRQV7I/eZcj1EBZPj9pu7d0GC5U5
NSIMjOSpb3bcz7M2CCa2eZ7D0Ay07VQP1M7zN6CQCQFzV8Xw3jz5uqi3r3h75KfVQAyI2qj3qP9Z
H6qLNAiLqEKtt96Xfr4kZ7Um3Z9TH72zrxbG/4AZBXL9Won9mpyfrIxEaFec7r+3uboWUCxu4/wN+
et3/pyI6XBPWkse4GPca+Wzo1imzrKP523M2ofdbOu/Fbsn0WNy+If+rOS9v677wbglnn94JOUJOd
F4P9um2ZLK8uyolT+ukYlS23beb5V9ri53BnxMnhkpwj67VGqYGh+21ofyZj2/b9ZrYlbFt+kJ3U/Ny3
/uvD789hya6dZar3ACyrT+x/wp6NHwCQv5UvvTTzOqijPPTV78qf1hmBAAC56T8scvnP9QLyRIiKIW
bPtPoFPwgdDgOHletbE4Z54aotPxwsrp3xAqzOge4z2bsoO14eaey7suW3deeiJIl7zH364ZMh8T6Ls
nnWTbuaslpwe08Gz+8kipt740PW2PblMOpEFdfkTNjxtXf9XrVn1pzjSXSOrPOzp0NEI3gPvd4Dhu43
s8et0evSPk86CNzLYfa0LO/bsfdnmCTXzjLNewCW031fv0/u+rO79BIAIE+vfPBaufnrz9dLs/G5jz8kX/
nLR/QSACBzl74gcpkJ/vJGiIoBI3qm2Yqy5iYxIwK3UyfSDKjKcrKkn47SPdC9+VSoU/LqNhbMYdKRw9
QnMMk+K9tGr0FHOsP403RKoi+fcf2NgNIP3Kpy1j2gqa6LruEZOrw/aOh+M8JCawf20Hd33yNrsaZ
uHu/bBNduyvcALB93Jv6nn31arwEA5OWGx6+Sn3vgJXpptu7+0APy+Dd+pJcAAJlTw/r739ELyAMh
KibUkeHOgTHCo7mX3TFUvMKUSrD+5Oz05HBssVSf36vSHRZuBG7V08FeoUmYExB5PXXHCbtWR
dm8EN470w0rF3t+o8nvz/SvXdh7AJaNCk5VgPrID+h9BAB5e86Pj8lbvnijHL9U0Gtm60dPPiPN99+v
lwAA2XtK5NIf6OfIAyEqJjR/gWm53vWGbAcfk9YLHS/
+Po26n7aO1NZznJyneELGlj4tn5Sxl7S4KW5lAntYuDF8vno6/CyPP0dt2TB7nbo1OM36o0lYbdzT+xj
s/as0z83XpEi53bcTXLtoR+GPJpiWGsL/wCMP6CUAQJ7e+Nc3yAt+eIVemg+qPuqnf/9QLwEAMqfq
o15iYte8EKJiQElOusnV0EzePbnoFnIcO/Q7J6WTXtA2qm5m6ibYZ6+xrodpl6Ved5OsPCfnMa5rc1f8
zphdOfAu6QmJc0m9HrXW/XH+/I4OP6sSyOGSnCOzLqcKFN26qMaQ96TUzPKqx6k9GZQdSHa9W
p7pDpOf0CzuW0usa5fme8AU1xDz7yNf/Ig9mRQAIH+lh1448zqoo3z6v/6D/MOXvq2XAACZu/xZkf

48dRU6ughRMcCcBCnYW9IPAlXYNTChz6yYNTCbq/5Q7faGHhpuPdIev510n72GrLsnrnpWNjeNyY
Nq65JPjmrWsmzKrm5er3HOCzBj90asnBbnDulIs+ke18Bw8Amvix8oDvbcjUlvX80sH+xxaoTFZo9b
oz1p3yaRZnHfKnGu3QTvASU/dZXaee9gZMOb4h9Hze6Du/LBv/igXgIA5OnaJ6+0Z+OfZ83fvF+
+9y1qZQNAbtSw/v739QKyQoiKYeYkSJ2alHSoU/LTE7kw7YxI5vDy5uoUoVGwBmZzVQdQXnhTlVZ
qs9e7kuyzJ411d6h6Wepbav3okCq98zLMnFndbbN3TastiX+aKhIo72oZDmATnCMv2LO4x2xOsJTEi
HvXrLNaPevPNp/YQt+3SpxrZ0n4HuDXW7V41zBubVssGjV8/85P3amXAAB5UvVP3/aFl81NHdRRV
ICqglQAQE76T4pc/rheQFYIURHKHgYdUpOy2rLWu8Otp1KR7cGClfuHRs/BBOwamMZwbZddWzOj
eqgx92n23LN7obonbiCk8of1p3hehhRlc2+4Tqg9hD5hYBeYJMs6ZjsbHhT7uljH3G/5QaqtKq2+0dZ
ACYJo9r07eJA2Z5vTZZMLft9aYl07S6L3AHUsg6+ttiava4u59cTTTzATPwDMkOqBqnqiLgI1pJ/6qAC
Qo8vWb6bUR81Uoa8KBgJAEmo2fT0ZlAph96btmTxL+lhOtaYNWBfEUbp2yJUKTit/VJF7H75XrwE
A5EnVQVWTSS2SK597XLbufr3c+uoX6TUAgGxdKXLFb4gUbtbLSBM9UQEkFCxRcGZtsUO49nnrWC
J6ZB4tR+vaIV/v+
+z7CFABYEbULPx3fPl6vbQ4nvmnS9J8//3yoyef0WsAANmy3m8vqWH9TzmLSBUhKoCY2rJh17o06
pWaJQoWUK+xIqv7demmUqJinh29a4d81e+ry8f+9mN6CQCQJ1X/9C1fvFGe8+PF/NXt8W/8SO7+
0AN6CQCQuf53RC7doxeQJkJUAJNRNS8XfPx7cXMvpRq/C+YIXDvkR/U+Vb1QAQCz8XMPvERueP
wqvbSYvvKXj8jnPv6QXgIAZO7y10QufUEvIC3URAUAAKEefuJhec32a+wJpQAA+bv568+Xf/6Fm/TS
YqM+KgDkjfqoaaMnKgAAGKKCUzWRFAEqAMyGqoO6aBNJRVH1Ue/
+0FftjwCAPKj6qGpY/7POIqZGiAoAAIbc+ak75cHHH9RLAIC8qQB1UeugjvLN//19+cPf/ppeAgBkrv+
oyKU/1guYFiEqAAAIUDVQP9P7jF4CAOTttV97sbzs0av10tGyt/t/7AcAICeXv+Q8MDVCVCTUk8aKm
uVbPTaknWjdOGltZ96MOYZeQ1Y2gmvbG+7rV6TR0yunpfZjb3NEO2zTne9M2m1bkPugvaHbWJC
BSwosjE/sf8KejR8AMBsqPH3N3x3tuqGqN6rqlQoAyMmlPxHpP6YXMClCVGCGeo0VKZRq0tHLWe
pd3DH205RdQj4AA+77+n1y15/dpZcAAHm7+qnj8pa9G/XS0aXqov7+v/8y9VEBIDeqPup/tz5SH3U
ahKhIqCibe33p99VjWyp6bXby3l8WRhxDryHrtTziU6UnF3eC+2qea1hrF8VRuA+A+aZm4n/nJ98pT
z/7tF4DAMibClBVkLoMVE/UT/7nA70EAMicXR91Vy9gEoSowDLoXRQ3Qy2Xy86Tzo5cXJwUdTFUt
qXfrYs+w8DCUMGpClAf+cEjeg0AIG8//ffX2UP5l8m9n3xYvvKX/NsDALm5vGc9mOBvUoSoS8eoLbl
i9kQctd6oc2mvz7s25fD+IutuGnU/VwKfNLcT9vko6Z8z+/PmMP7mqvP5EYU0/WNWj+T1Rv2h/FU
5e+GMDvk6shMnRQ3UUg17jG5PvHbHuTbD59DaumyYrzfqkQa2MbA+/BTHaYN5POpY/P2rh7vd
9nnrupbrsjXQXdYu3WC83n4M3DfArKgh/A888oBeAgDk7YbHr7Ink1pGd3/oAXn8Gz/SSwCAzF26R
6T/Hb2AJAhRl05R1s64PREPpOs8s3TlwE30Aj0U27LbdJ6Vz6xZXz17ldNV/awjB/4B2PywsCxn1nRr7
RCwJIMj5zu1UswQa5bnrCO1UkFW9fYcal2SINUYyl89LZXimniHs3Nx+hCvfEbcU+2L2e6pr43Dfn1w
Z846FVQOrG+uDgSpE7VBHcuqeFvWoakKSlf369Ld2wxcdxW+lsJKN3RqUprnibKwFD7yxY/Yk0kBA
GbjOT8+Jm/54o1y/FJBr1kuP3ryGWm+/37qowJAbp4SufQH1kfqoyZFiLqEimtuT8R9OXQTovauHwi
JEU72Dq1XOU6dmIcI1VI5LW6M2gzMjmSEhV6w15PGutvjsyotu6am9WjpLXRqsh4jjUz7nFW2rTa
Yw76rLadd22HVPv12d+vuV8TsRaoYQ/mrp9X2zVA4xpD+4qbsuefNfnTFa4Z1BPULwcDQN67d6V
wbV7neDX69Frbev2+maYPxeh2aFjf3vOc+P1RXYWtXf41/TppyLsFxAmnafXBXPvgXH9RLAIBZeONf
3yAv+OEVemk5PfTV78qf1h/USwCAzPUfFrn853oBcRGiLiOvJ6IfaPUOndivWnXCo32dFJrDwO38bS
5UZMsNoJq7fi8+s+6n2wPUXFff8ickqmx5QWCs3pgzPGfVlj+Rkh/mWi0Z7IY7gtk792TJfmJsJ0EYq/
Ua636vzepZ2QxPUMe3O61rY6vKWbchRsg+cv3+obPdKdoQeH0UI1Q3Q2s7cNWB6t6okwhkSA3f
v/NTd+olAMAsvPLBa+Xmrz9fLy23z338Idn/n4/pJQBA5i59QeQyf8BKghB1KRXlxCnnmRtode1x6W
U5efqkHXYF11vUMHDn2VzwQ7mmeJ0KuwdeWOgN5ffWWcfkDu+2H8bw7cAQ/VFmdc784HMy
Yb1zLRMP6W/LeT9BlVZoz1klRrtTuzaW8kkJ3d2o9a4p2hC7Z7Zxrq2N2WUO3P3Er8sLpOuJp59gJn
4AmLGXfPe58nMPvEQvQbn7P35Vvvct/m0CgNxcVvVRv68XMA4h6pLy6oraPTl74nSqPCUnKies/w
+uV3ngPEWoFiOYcodmt90x0xG9I6exkOfM6GmphqaXQkPC+LP0tzf8OqBVo6fp8kkSbhdl80L4jP1u
cDtiPjEgE+5M/A8/8bBeAwDIm6p/usx1UEdRAWrzN+/XSwCAzPWfFLn8cb2AcQhRl5U3tHlfDts6aL

N77VXEyQqN9VP3hsyCUdfTDi/9upOjwkuvNubQI2YYuIDnzB/KHyXmkP72hj9RVLUlIzuhTmDqa5O
CTNtg1JUdKNlqa55jln7k532ffZ/c+/C9egkAMAsrX3qpXPvklXoJpn/40rfl079/qJcAAJm7bP02eukze
gFRCFGXVklO2hlkRw52nSHNbh3Rkv0Jf3347OuzV9w864eaDXeSp4E6pCVnqL3i1eKc2KKdM2Mo/
xjjh/S3ZcNLUMtSV1PRTyvVazOhHNugZuhXPU7tScXsQNWYoCtu2QJgSvX76vKxv/2YXgIAzELpoRfa
D4z26f/6D3aYCgDIyeXPivTp2jMOIerS8ntyNptOOObWeCzq4p/uem+Sprnj9gDtSK2mA77BOqRmP
crmqj9sur3h1aUsxB5LvWDnzBzK787+H3iYIV70kP5e45w3jL9cv5BOuYRUr82E8miD3pbKoIM9Trvi
ls8dW7sVSMn/+v/
+l34GAJgF1ftU9ULFeHd/6AH9DACQi0v36CcYhRB1iZkzpqvehd7w88Ds5sYkTXPIq1NqC+shGaxH2
VzVwZg/Ll1GT440LNVzVtS1VJXmauqhoTmUvxxaW8AoiWC9cuSQ/l5D1r0CqtYrAxMwOY/JJkhK99
pMJoc2VLb9IfyBurRGfdmzm7MP3bEU7v7Fu+WOl9+hlwAAeXvjX99AHdSYfv7dP6WfAQByUXijfoJ
RCFGXWWDW8FPiTzbuDltXzPVzyAwvRw2ht+tRGr0uXeW6dJPWu0z1nFVke7BA5v5hSrUxzaH8o0
NdMxQeNaQ/Xl3VCaV5bSaVQxvsIfxhxVBVSNvvp1pfFohy1RVXyf/4lf8ht1x3i14DAMjT373yCf0M
UV7z1pvkbe+5VS8BADJ37FUix9+kFzBKoa/G9QIAgKXxwCMPyOv/2+vtmfoBAPm648vXyysfvFYvYd
D1P/E8+a1PvVGedw0TbwFALgovFrni/daTq51ljERPVAAAlsztN91uD+0HAOTvb27/tjx2PX/ECnPlc4
9L9fdeS4AKALmx3m+Pv8f6SIAaByEqAABL6F2n3iUfeMMH9BIAIC+Xjvfl8294VH78nMt6DVz/8t+V
5NZXv0gvAQAyd+ztIoWb9QLGmSpE7TVW9AQpBX9W65F60lhxJ1TZkODL27LhTbbiPCabqGZYeyO
43dBH1jOAAwAwhz788x+W07ed1ksAgLz84PnPyt7rvqWXoLzidS+R1X8bNhkqACATx4oix39eLyCO
OeiJqsJVf5ZqR84zwquZZ0VcaKU3oAwDA4rjnl+
+xh/cDAPL10M0/kO6t39dLy+3al14d1d99rV4CAGSucI3IMTWMH0nkGKIXOvL2oeq35gxuuuHH
iTiKvZsNXn92QzxwzV1qnJeTqkAgCWjDtj/3VXXafXAADyonqjfu+aZ/TS8lIBqgpSAQA5Of5ukcIL9QLi
mn1P1N6h7OuncuqEZJedlqXedUNc/9Gtl/XnRZq7pKgAgOVzy3W32EGqClQBAPlR9VE/96Zv2h+X1
S/8xivsofwAgJzYdVDz7rl4NOQYog7XRLXrlZZq4nZEtYfVD9UoNb/OeaRVL1Uprp0RP0YFAGA5vfm
WN8tH3/5RvQQAyIvqibqs9VFVePoLv35CLwEAMmfXQX2HXkBSc1AT1vFAxAHXkBSc1ATNUKvISuFktS8lNXRqZVSq2H
aPu+HuNXTfpGB5WVM8uWd46hJwTA7YddqEYTfT/4kcHneY0nPYU7fC+q9b2DCO//8rEiKf0cCAt7
7s+
+V2h01vQQAyIuqjbps9VGfd82VctfvUJMbAPJztcixO/VzTGKmIWpluy/9bt3vCVptOcPst1WY2ZPGu
htwVqXlDsFvVe01qobpeqIkoSO1khtC+I9Vd0Yra9/2bgFghnqNlWAPfSBnqjeq6pUKAMjXfT/zuD1r/
7JQAer1P/E8vQQAyNzxO0UKL9YLmMT89kTtXZQdnSKU61v+RFSVLXHLmHZ2LqbU+64s9S0S1NFG
TQoGTGKR76eM295ryPpg13tgBlR9VFUnFQCQnx8/57J8/g2PLkV91Le951Z5zVtv0ksAgMwdf5PIsV
fpBUxqfkPU7oHXE8sevu/1HjWG93cOpKufTsfppZpmrVUAABaVmqm//attZuwHgJw9dv3T8je3f1sv
HU23vvpF8ku12/QSACBzhVtEjv0LvYBpzHdN1FSFz85vlhPo1M7nWItxHgxO2jWqxuPoOpD20GPv6
/VjZG3Jwf1FBNd2Pdzga0fVgsysDVEya98oca+VK/5xJm1f2OsHyncO1O806o7arx19P/kG2p/K9Ux6
DsOEt32wXungORo8P2HiTbTn8/epHqPqpCY5P0DQbdffJvf88j16CQCQl7+/7Xvyjy//oV46WlQd1O
rvvVaufO5xvQYAkK2rRY6/2/p4hbOIqSxEiFqud4fDT/uRwnDa4qac1WVWRfblcGnyBRVsDU7a1ZTV
wqr1/3hUiFMKG3rcqUlpMKBKMklYe2NETUjVYzgYFmXWhihZtW+khNcqwXEma58TyIW9vrlaGBH
2qXNitLNcl/GVM9SxDbR/6us5/f0ej9OrffAcNVdHhZyTcPbh1XO2Dd97qd3vWGrvKL6DGfsBYAb+6v
WPHcn6qL/yH15JHVQAyNPx09RBTdH8hqilk34P0YN0Bu2Ha8tuuinKQug1zvnhkTuhV7/r1Zsdzzhv
5bp0dbDd9TbQlHNeotNLMEmY9dpzesOh2+3IzkX3tVm1IUpW7Rst2bVKcpzJ2tdrrHuBnP+HjZa1F
615bkRQaLRjb1OKem0koz1u06e5ntPf70n47fHb3pHa+ejIPHqivUH+PsLvvbTud0Ds2frfdepdegkAk
AdVH1UFqUfJyumftB8AgJwce53zQGpSC1HtnmjGkFHzEWco65DimpzxspxVfxuqF6C77UQbdnpv
me1yHmYvuTOyFivhWXQ9uejO2qUCFi+kKcrmBSPEidI7lH39VDo74mY3xc09J6yxHnub+mQmmiT
MmLjHCNyKa2eGQ/XM2hAlo/aNlPBaJTnORO0LtuOst74iW/aGVVC3J2GHE2hHTNWz/rmtbPtBrd
/2JNczhfs9gWrL7yFf2Up/+4q5j/B7L8+J+bAM7v7Fu+WOl9+hlwAAefjmjU/JV376u3ppsb3sp14o/
+a3mNAEAHJTuFHk+L/WC0jLHA/nDwYcXkjrjWE1w5B0mMHN0daVAzdTKp+Ukn5qK56QU/ppJDP

kHgioh2ouTjVJmK6nGTZ8Prc2REmpfSMlvFZJjjNR+0a3wwldR5fWOHUi6XdVVU4HNlaSk2473bYn
up4p3O9zpSwnAwcRIteJ+bAMrrriKmbsB4AZuP9V37HD1EWm6p/+
+n/5GeqgAkBurhQ5/mvWR+qgpm2+a6IWN2UvbMitPdQ3hXqoHtWLri8pZ7JH3OhefG5oM1EPZ
MWu5eiGPlE1KzNsQ5R5b19sebQvRuA3VlFOLEzSmcbxAvPpphfcZAepKlAFAOTn8yuPylNXX9JLi0f
1QFU9UQEAOTn+r0QKN+gFpGmqENUc9hv1cMLJoj8M2gxA7aBUrw9NMc2v04+4dRUtdp1B82tD
H2kGskvEuHZ+7Udf89zw5DXjJwlry4bZq9OtjWnWijRl0oYo2bcvLbGOc4btG21wgreeHHp1B4ZlOv
HcEcD5QZpuv+l2e2g/ACA/KkBVQeoiel3l/6IOKgDk6dhrrQd1ULMy3z1RkZGQ4dEus05mDGp2d9V
b0Q+rjZ7D7raTTBLW3vV6dtrhjxuYG8OTB6XehihZtW+khNdqguOM176Idth1itOdfT7Y9JDh+ImO
M737fWHkNjEflpGaZOoDb/iAXgIA5EEN6f+7Vz6hlxaDmoWfOqgAkCM1C7/qhYrMEKIupaKs+bN2
yao3Ztuc0XsMPcGXKlEb7K0YEnhNOEmYH/4YM+KbcmhDlFTbN1LCa5XkOBO1L6Id9rE7NVUTnsK
Rmqsb4jfdL5lQPrPmhNaJrmcK9/uiyeB+B0wf/vkPy+nbTuslAEAeVH3Ux65/Wi/NN1X/tPp7r5XnXX
OlXgMAyJaqg/oe6+PVziIyQYi6pIqbF/zehs1VHawYk86MU9n2h393alJygxmjRqg/UVew9mbkJGGV
09aSNq5dWbUhSmbtGy3ZtUpyrpO1b2w7ynXZSmV8eFnK5aas6vZ4Tbfaftab/j/Z9Zz6fs+LOdGV28
6Jws6U7ncgwj2/fI89vB8AkI9Lx/vy+Tc8Kj9+zmW9Zn79Uu02ufXVL9JLAIDMHVsTKdysF5AVQtSlVZ
TNvcFJu6rS6res/8djD/8OK6Rpb2dgoi679ubg/ixDk4RVZHuoDc72vF01d71eitm0IUqG7Rsp4bVKcJ
zJ2qfa0Zfu0IbVpo3SBlM7JWcHjzfsGiW6ntPf7/mw7q/B67F/aPQSTiCV+x0YzZ2x/7qrrtNrAABZ+8H
zn5W/ev1jemk+veatN8nb3nOrXgIAZO7Yq0SOv0kvIEuFviqCCAAAMIF7H75XKn9UkaefXYwhpgBw
FNzx5evllQ9eq5fmx7UvvUr+06ffzDB+AMhL4RqRK9R8BQzjzwM9UQEAwMTefMub5aNv/6heAgD
k4W9u/7Z875pn9NL8qP4udVABIFfH3239jwA1L4SoAABgKu/92fdK7Y6aXgIAZE3VR/3cm75pf5wX
v/Abr5BXvO4legkAkLljbxcppFNUD/EQogIAgKmp3qiqVyoAIB+qJ+re676ll2ZLhae/8Osn9BIAIHPHiiL
H36EXkBdCVAAAAkAo10dQt192ilwAAWeve+n37MUuqDqoaxg8AyImqg3rsPXoBeSJEBQAAqVAz9b
d/tc2M/QCQI9UbdZb1Ue/6T6+2g1QAQE6O3SlSeKFeQJ4IUQEAQGpuu/42ueeX79FLAICsqbqon3
/DozOpj/q299wqp/7ZDXoJAJC5428SOXabXkDeCFEBAECq3lF8BzP2A0COvv2if7Jn7M/Tra9+kfxSjV
/kASA3hVtEjv0LvYBZIEQFcteWjUJBCsZjpdHTn1tSvYasblLT1gqO94Z6fFZnX09NrrDhtXGnIqCb6x+E8
lv5aY2mo2frfdepdegkAkLW/v+178o8v/6FeytbzrrlSqr/3Wrnyucf1GgBAtq4WOf5u6+MVziJmghAV
yFVPGiur0tRLjrKcWSvq58vHDiJLNeno5YXRa8h6LarV6loXZDV4saVTK0lhIDAGjqq7f/FuuePld+glAE
DW/ur1j8kPnv+sXsrOXb9zu1z/E8/TSwCAzB1XdVBfrBcwK4SoQK66cuDmbuW6dI3L3jalm3cDPt96ff3ZHNZM9
SxQeScUj1nxwW/7fPiHlq1pa5zX7r1srOiuSrkqFgGV11xFTP2A0COfvycy5nXR33zr9zr3wir3nrTXoJAJC
5YyvW41V6AbNEiArkqXco+/qpnDohy9v/dHHF6znbk8Y53QW1XJetivO0uHlWqs5Tae6SomI53PSC
m+wgVQWqAIDsPXb903L/q76jl9L1sp96ofzfziplwAAmSvcKHL8tF7ArBGivnqP5CKntOLI2peqN
536d163Or/dp135sb+ivNdbZLwuuD/bKS2MbmtlG7zFcYzN4jMM1S+1HWN1Low3jal3a+zDDt+aq
87WDDQ9pc9i2o9o8tpfjROdl+J4ZuZ8YxxD7fGh+W9RjuK0OZwi9/7p0z506/lKsnrNGj+NAWF6Sk7
ozquwfDt9PNqM9ie65eMduS+H7wj1Pcd9HsNxuv+l2e2g/ACAff/fKJ+SbNz6ll9Kh6p/+
+n/5GeqgAkBurhQ5/mvWR+qgzgtC1CWlwpHQMKhTk1JhQ8ZlcHHYtR8HCkLa61TIMrC+uRoeXk2
1DRU2hfYY7EitNCqEU58zapaW61J3uw52duTiYMi0674ynbqmQ8GiZh/zyGBquM1uz8dQE5+X4X
umuToieEt8DFGcfQcvd0hb7WCw5A2hd6V67jxVafVbXq/SIWaP41E6B9LVT4MqcjrpPZfk2FP6vlDn
KY/3ERwdapKpD7zhA3oJAJC1z688Kk9dfUkvTe/f/Nar7J6oAICcHP9XIoUb9ALmASHqUmqLn8O4d
TmNeo3SlHPhSUpi5XrX3na/FYybwtaPGt482TaCw6mHj7EjO4PplEcFZM7r+3ubsrlVF+erBr/GPI9nZ
FyGWtm2ttd1t2Wptpx9bOvUrr3hB4Vhbe7UZH3kdQm2eXRT0jkv/invSO28cd0SHMPY8xHg7zu8r
dZxrbvBoHEu3Iamcu40u53bEitrHVKUE6f00wiVRPdckmNP7/uimOP7CI6OD//8h+X0bQxHAoA8q
ABVBalpWDn9k/YDAJCTY69zHpgrhKjLyOwlZ/R0K27uOQGJ9dhLZaajqpx1t1M5bS25RqwPHd486T
aKsrlnBj6O4toZL7TrHIT3AyzXt4IBWXFNzugv6uxc9NvY3vV65pXPrI0P38YwexjWLxht3rwgfgZ5PrR3
31CbR5r8vFRbfnDoh3xB0xxDFHPfoW3tXZQd3SEycC4qW/5+zWtniH/u1HFY3yOhIe8k9uUwrEFKk
nsu0bGn+H2R2/sIjpp7fvkee3g/ACB7akj/V376u3ppMqr3qeqFCgDIiV0H9V/rBcwLIeoyKp+vS/V0XW
Ej8SUWZAQ0ImqY

bsGrZTiyhuIkyielpJ8GjFofJo1t2HQ9x9BhzEGnTgwGP0VZ8xMtLyzyA0Mj0J1YVK9WY/8jgrfhNscV/7
yMN90xjFaWk+MudvfAa79X7sF+GEPcRwyfn/zcTeuUjN51gntuimNPcv2HzlNe7yM4ctwZ+6+76jq
9BgCQJTXJ1KT1UVX907t+59XUQQWA3Kg6qHdaH6mDOo8IUZdSUTYvhPckdEOYqMl1FkZg8hyjn
mOk8MDO76nnDnc2AsPq6dg9GafXkeGOgjFCRlOK52UyYccwK2keVxw9ORxbLNWR2T2X2vVfkvc
RZOKW625hxn4AyNFfvf4x+fFzLuul+H6pdpvc+uoX6SUAQOaOrYkUbtYLmDeEqMuquCl7esjtQKlR
W/Pcos+u3ZYNs3edW7PRrMGZhHW+zurzZA+NNoYyV0/nF6FOH/qlfF4mkm1w6dXKHXpMWsd0
AsUTMrb06bje1BPcc+OPPf3vi6P9PoIsvfmWN8tH3/5RvQQAyNIPnv+sHaQm8Zq33iRve8+tegkAkL
ljrxI5/ia9gHlEiLrE1MzaqqeYPcGPHYR0vRqKo4f/GoxhxHPHrB2pgiW3/uMUba64U6Z3duT8+R29n
aqkk6GW5KR37gdnZO/JRbfoZeQQ8BgyOC+
+nI4hTOmkFwKOqumZL+NcNHfF75DZlQPvNJxwzn2EWPdckmPP4PpP/T6Cpfben32v1O6o6SUAQ
Jb+8eU/lL+/7Xt6Kdr1P/E8uet3qF8NALkpvFgP48c8I0RdRu0Ne6itmkU92FPMCHiMXnIlPxkzZmJvy
4Y3Dft884MlY2bySXgTWHWk2dQnKrWh/EXZdLsdqvO87l+XXmPdq22ZZBKkcVI7L578j8Fj1udsrvr
DyPW9bj9yHVtu1oBtyq7eda9xzgsxY/VgjnPPTXjsU1//hO8jZntyvRSYe6o3quqVCgDI3t/c/m157Pqn
9VI4Vf+0+nuvleddc6VeAwDIlqqD+h7r49XOIuYWIeoyqmz7Q287NSm5QYtRH7F6NnzmbhXSDL52
Lpkz9nttNibamUhF3I6BrlSH8o+4LiU/fZQL005glcl5MSQ9BnPYu9ueiRK2YH3O5qqz34IX9Felldqs+v
EUNy94PTLd9njnodqSeM2Jc88lOPY0r3/C9xEgiqqPquqkAgCydel4Xz7/hkcj66NW/u8idVABIE/H3k
od1AVBiLqk7KG3YUUMVeDS7wcDHlX3cLBmYrWVcx3NpCqy3W/5gZHNOTbvsAPDrOPxhlcr5bpsp
ZzL2dcl5LxWW9Z6d+j1VLI5L6Zkx2C1Z/A+3D80ejUmYNfnNIaSu+y6nznWQ/UUZXNvuFaoPYw+Q
aAb656LfezpXv9E7yNABDVTf/tX28zYDwA5UPVR7/uZx/VS0Cte9xL5hV8/oZcAAJk7Zv2GfPwdegHz
rtBXRewAxKNmNtcT86gwbG/anqHAOEfpntPHcqpFwIpwn+l9Rip/xM0BAHl441/fIKWHXqiXRK596
VXyW3/8z+yPAIAcFK4ROf5+66P/Xoz5Rk9UILaeNNbdmc3LcmaNABVZO1r3XPu8dSwZ9ODG0fGO
4juYsR8AcrL3um/J9655Ri+JVH/3tQSoAJCn4+8mQF0w9EQFxmrLxmANWFXOgK50yMzRu+d6jRU
p7ZyRbiplKXDUFf6fgn4GAMjStU9eKe9s/aS88723MYwfAPJ07O0M419AhKjAWAOBFgEqMsc9h+
WlJiiblx9NaEs42hKOtoSjLeHmqS0nrr1GDr/3pF6aLa5RONoSjraEoy3haAvSQIgKAADmBCZhAFm
WhLONoSjraEoy3haEs42hKOtoSjLeHmqS1IhpqoAAAAAAAABCBEBUAAAAAAAAIhCiAgAAAAA
AAEAEQlTkSE2WU7DrfxRWGtLTa8fqNWRlo60XHO0NvZ3CijRibwjp6Uljxb0GG9aVTbJuGmH3UNr
7mJbRRv1Y4SYFAAAAAGChEaJirvUaK1Io1aSjl4H5pgJdY1Z9W1nOrBX1cwAAAAAAsIgIUTG/eg1Zrx
GfzqeibO717RkF+/1tqei1szFPbenKgXvLluvStdu0J5tkqAAAAAAALDRCVABIS+9Q9vVTOXVCyE4BA
AAAADgaCFGXmao1OlC7MazG6GD9UXuIvfE1A+VKNbNOpXokq1Vp79Mcxt9cdbYTvjOjjeoxqk7qY
JvCa1VOdrymMbVf2xvetob2H3JN4rTRY359ZEONcxFo46j1xj7t9ea5zKoOadx7KKotg9uYsD5pjOuS
9J4FAAAAAACLgxB1WakgL7TWaEdqpVEhpPpcQUoDQ+ybq4OvVyFiSYIva8pqYbBWZBqcNq0GNh
xyDHYINtgm65W1UnjQaYt7vIMqcrqqn3Z25OLAa9u7bmODtTKHQjgtuo2TKsrambLztHMgXeeZxR
iOHmh7W9xml8+s5dDDMoV7aKJrPizf6wIAAAAAAOYRIepS6knjnJuIuXUb+9Kt61BNOrIzmPx5qtL
Sr2+5QaEKG8/7ve16jXN+0FVt6VqVXfE2H0Nl2/qabl28L3G3sx1W8dJvU/gxWMe77oZg/mv77gF0
arI+MhUdf7xhKltu2wfPpR9GSvmMeBlqe8MPgsOuSWQbJ1NcO6PbuC+H7qbbu0ZI2ZEDN101hq
mfOpF9hDr9PTTNNTckuC7J7lkAAAAAALBICFGXkjERz96m16vQD9VEOl56FlRt+RP3+EGhqScXd9w
+e1VpeQGStc8LYa+fntmm0GPoXRS3SeX6lj/xUGXLC+U6OxdDexSOP94RimvidfQ0t22ElGaPTrN3av
2CcU02L/htrJ0fGKo+Ja+NftDbO3Si0mrVCRv3dbrau7jjBZKnvROYlRTuoSmuuWkm1wUAAAAAAM
wdQlRYdA3P0OH9SZmzk5+Ukn5qK56QU/ppespyMrCTEN0D77jsIdheXUtjqHdgSHsazOHy/rB4P5
SryllvyvYRvVNtxnbMHqOpKMoJfUHcwLlrXzzrnJ4+aYeVwfWW6mk/kMxMCvdQKtd8VtcFAAAAAA
DMG0LUZRWYKCdunckYgeWRMt3x+r1i3Z6eRig3URhpDK9PScUt3trctVrXE6cj6ik5UdFhZWC9anb
2EeriSf+6AAAAAACA+UKIupTasmH2OnVrPZr1HI+ocr3rlDEYevjD9lNT3JSzbglONXTcqCs6WRiZQYh
dOS164L4ctvUQeLv3pzs5lrF+QUP07K/5sv1xAQAAAACA5UOIuozMupwqYHLrohpDoCdXkpNuEjs
4XNoIEXNVcoamK6NqvWbF6+nZ2ZHz50fVFTXP2eBs/mZ90FMydk6nxNfQ3XdHDnadr3VrtZbsT/jr

h4e0ZyWFeyiVa57idQEAAAAAAAuNEHXJ+QGTMWP/VMxakU1Z3XCn3DFnS8+ZMcmTNFfFa1J7w
6+V6a1MmdfTsyPNpj76oaH8Rdl0u6xar6utN6yz5eg11r0anuYESU7AqVivP+8dkGx4U8nH5V+vZtP
5Wnf2/aIumOquNyfCylYK91Aq1zz5dQEAAAAAAEcTIeoy8oI9S3NVh0rGhDtTMmcun2r75iRC7nY
mCjuDs7o3V3WI5gWO5gzwaXOHxftCh/JXtqXl5XU1KdnnrCAlP6mTC95EVCojdOutWrxzHLe2bVBg
W9Yzb2i6eZ9Y68/k0w3VNv09lNI1T3hdAAAAAADA0USIupQqst1vGQGZUpVWv+8HRvaEQpMqyu
Ze1w/BbGr7g/scx2qn1yBt/9DrDZhIcVP2+oNtstj1YDOoh2rwhvQr1v62Ruysst0PrUtbbVnr3ZILLnU
8g6+ttiara2v22gwMTTeGs+c+ZD2Feyila57ougAAAAAAgCOp0FczrADITq8hK3oiL1WDdo+eiwAwk
urtPS8/mtCWcLQlHG0JR1vC0ZZwtCUcbQlHW8LRlnC0BWmgJyqQKbOOZ75D4gEAAAAAAJAOQlQ
gE23ZKAzU8ayeFTqhAgAAAAAALB5CVCAPql5pZpNXAQAAAAAAIEvURAUAAHODelXhaEs42hKOto
SjLeFoSzjaEo62hKMt4WhLuHlqC5KhJyoAAAAAAAAARCBEBQAAAAAAAIAIDOcHAAAAAAAAAgAj0R
EV2eg1Z2WjrhVnrSWNFzZavHhsyL60CAAAAAADA/CNERSZ6jRUplGrS0csAAAAAAADAoiJERfp6D
VmvEZ8CAAAAAADgaCBEBQAAAAAAAIAIhKhLqr3h1gddkUZPD7+3l51HaClTVePUeI3zcL7eZW/XH
MbfXHVepzc4uF+PuW1j58HXt2XDfY318F4Wo12TGjwv6jHpuVGSHr8S1obCSkPCD8+s/eo8VkaciG
TbBQAAAAAAWF6EqEuvI7VSQUoDw+
+bqwMhX3tjRI1T9fXpBJbR1H5WpamXpFyXrYr1MbN2OWHk4HlRmquFYNCZ4blRoWtYG6RTk9Lg
BFl2EFuSwZd3aqWhcDTRdgEAAAAAAJYcISosVWn1+9K3Hq2qXqUCwPNulNaTxjkdX5br0tWv7dbL
zjrrtTsXnYiusm19rlsX9zNSbdmv7W+rxHNafjv7e5tSTNCupHqNdS+MLNe7zj77LasFWvOcDkeza4NI
W3bd1Dh020055yW0VjvW3SDXOE/uBe3UZN17bZLtAgAAAAAAgBAVUm1tixtxVraMANRTlM09H
crZ4aVeu3bGe23noKufZadc3/La6ciqXT25uOP20qzK2U13yxXZsoNGFVLuibM6w3PTO5R9/VQ6O+
JmscXNPWd/1mPPbVvvorhNDpynypa42Whn56J1ZJYk2wUAAAAAAAAhKiaha5OGDmHPzqkT44K
9tNrVlQN3A+WTUtJPFSdo9EPnYSmem+KanHGTWGtrquyCW7t0qM5p98Dbnz18X7+uYA7v7xxYR
2ZJsl0AAAAAAAAQoqIsJ82UcJTAxElGbdLcjGjnzNtlyawNRdm8ENYz2A9KQye5Giur7QIAAAAAABx
NhKiIoS0bZs9Kt46mWft0JuahXRm3obgpe3qIvV+v1tc8Nzybvl/DdfBh9KCdYLsAgLzp0Q3Ggz9yAQ
AAALNBiIrx2rte70o7oHNrfxpDyKc2ybYya1dJTroJqDsE3qVm4i8YM+6n1YaI16uZ9NUvzfakXXbw2f
XqnHrtK530Qtu4NVhjbRcAlpH9Xl+QwkwTy540VlZFWu57tPNHr+bqLILU+Qxze40Vpz0r+f/hT/0ba
p4P9zEP5wXDhq7XDO6ZmRvzvuZ9P7mPvG7mOO+3ebwnR+5jSf+gFfO8z/K9eGZinJuh951luGnG
nJfc34sTtofSdoiDEBWJ+AGdMSt9AiU/nTRm/7d+MFmdbhD8tO0KKsqaVzS0Kavem667baeO6OB7
cZw2JDp+/aavPhXsGRpSs9Wsc9pc9dvm/sOhHu7KJNtVjG3wDwuALMzsl/chKri09j/lv0npcCYu3Da
KcLuTP+4f5vte3N4IC3ONPyjOQq8h6zWRatX9x28G3NEnxsO8XrMy01/cAyWOhh+z+NZW52O1qS
YFda9TS6qdmpRmFPjk/0v7+Pc19R5csr6f6l3jHFk/T2Z778R5v83jPXncPtTnc/yDVmTok1eYm+C85/l
eHHFuht739CP98xP/vl3dH/g3Iot/IEadk9zfi8efl3zfiydoj/WNrUrbpf+eHO/7dn5+FsZY1g2DJWS9R/
TV5bd+A+hbPzD5uvW+9U+g87mq9ZZia/WtnxX060c8vNcqIa93P29uf9TD2NbIdtqStKvbr5fd9VXr
K8cxXx/yKNetVygJz83Exx/
+MDcdve3gMSfabqvqrS8PXwQAmEq3XrbeX8z3eP2+GngjyoffFv1vwAzaEEm/z8/8vXjm7XCuj9q/f
c28f5PzY/87OoP9RtP37dy1y/3eivPzV9rCv5dn1R7n5y9jv/pnrCy/l8a/rznvuYNtyPocjW9XvNdMa6J
9ZPIeqPdvbdd+hLRD3T/maud+Um3XK1IS/5w4n8/+vTjeucnjvS/
+fZv1+8v4cxImq7aNPy/h62fWnhHfw+nfR87+zd0737fBdX579Qr9vhz3uiJf9ERFDBXZVn8p0ksO56
821puAo7kr/t9KrNd7n9D2D52/MKlanIP1QqutCWuIJm1XEk4PIOsNTS/7rDdbf9h+0jYkPH57qP3g
ubQ5+wj8QdOuc2oMyXfZPWWMeqiWRNsFgMy05XytY71NXZBN503VUpEt9UY28fv35Iqbe9Lv7x
ltmTO69MupE/PRwFm1o9dYl1qnKmfn9kLNhnteWt7PKPPC+T6X6unAzyL5KMqJU9YH9+dQW08u
7sygPb2GqIFK5fqWv9/Ktv3zYmfnYgY9sRxj39d6h7JvfRj8fi7aJ64puxm9Ecd5v83jPXle3ved79+y1Ls
hP8tr6uf34dEJHdm5mO7dE/ec5PVeHOfc5GX8uXHeXwLf5xmY7Jxk9148/rzk+148tj0jfp6yR42mW
tYuzqii+fpZGOMRoi4pvw7mwJuLMeFQcMiBCgvdr1EPJ5TztxMM6dQPhf5rrYf5A725D/VQ+xmx35
Ht9MRtl/MGFlw3nvMG7H6d89gbakjCc5Pg+G2D59J+jDoG8zj1Y9QvU3G3a7xu+NgBYAoz+uV9M

enSL+W6bIX/A5CTnjTWa9KZVTvsoaMdqbbi/1ueGTUM0Rh6N9uSN/n84j6JXuOc9d1s/bI/oxu3sq2
HjNo17dWwypITNg/
+vJW13H5pT8huV1lOenWcNKPePgZk8AetuMFlmDTbEVuO78XTnJv86fJoB+cHhtOnWwJnknPCe7
FhxPub8/PnvuRaNYmfhRcOISoAAJgNfnmPTdUltX/5uTCLXoa6tpj9i6D+pWdGvR3b52vSqbZmPmL
C/0Op81AjV7KppRZXPr+4J+f2NDo7wwDEHTWkatqr7yOrObMI4efpl3ZMgT9oKfPyXhwwR3/Y6uy
flAvevxGqt6h6/9mYYa9C3osD9HwiwflBrO+pqedWiWHwjzD8LLxwCFEBAADmmDP5gRq2N6ueO
MFRDt36vqzO4hfU9oYzCcRc/dbuUL2Csh6WHcfc/eLePi/O6NHZXTNnsg5/YiAVeKtJgXKftGOWv7Q
jNfxByzKH78Xz9oet8pk147pY/4aeVWXUZtirkPfiAernGrdnrP89dXBKXadTkl3n7nn5IwymQYgKAA
Awp5wAVfXWmJ+hjMXNC3YNtk7tfK7hXHtXhU1NO8B1fuEpSEn9Vqh/CZrtcHptlsOyLfP1i7sOCGf5
y6Iecqzq2bt5jz0MViXezdWMZjYfZVa/tI9h93bqyMHgjTuqd9QS4w9ajkV4L56XP2x5ZtqrkPficIMl+f
qydXLfOk8nJau3vdn+EQZpIUQFAACzwS/vkVSvDSdAndMJ/zL8RSPMYE8j9bAngLR+Mexaz2dbt7sn
h6qoWc7nZKxZ/uLu9nw6O8NfFgeHTbpmdl7y/6V9rOIJceZ7CUZNPfuGnmG4O2f4g5Zvvt+LB+T+h
62S2GWOB8PbWf5cw3txTLq2eOCPkekZ+UcYfhZeOISoAABgNvjlPYKeRddiD3czevzkW+dSTf4wM
Bxc/0KW1S8ac6/XkJUVc0i2WqVmSp7lL6nz94u701utKjMcPSpSOW21wPoeCgyhV7dwzTovM26bL
dtf2uOpyGnVY6+27r+veL3G5m+islngD1qLaFZ/2NIjADo1Oe/9w6mHcM+oHinvxXGoUhklqUldLm
RwkSL/CMPPwounDwAAMCOtqvSt33L69a5e0a33y9aPJ2VvRX669bLVFtWewUe139KvWUZh56U
6JyfEblu53s/3bun26+Xg+ZiLe6RVHbg2rb71S2tfJT+5m+H38TB9HgKPefie1vdRxvdv3Pc1573Yf2R9
7eK0K4/35PH7CLt/3Ifxb1dq9H0x9H2r2jFw3Pp7Pu1rNcl5t78m8/fiEedGvd8M7Ns9hrTf/uKem6H
XZfY+POp+0XJ6L453XvJ7Lx7fnpB/xzO6RnHuxXn6WRjjFdT/rIsKAAAwE+5f6F3WD43zNRwQWBCq
x5xdm9BVbUl/Bl3nnO/pqrT6M5p5eS7pnk7G5ZnV9cH8Gfre9fjfR2Gvqc5r79gUjT83Id9bxnk7iuLc
LwrvxbOmRvOoOqhhgkP7+Vl4cRCiAgAAAAAAAEAEaqICAAAAAAAAQARCVAAAAAAAAAACIQIgKA
AAAAAAAABEIUQEAAAAAAAAgAiEqAAAAAAAAAAEQgRAUAAAAAAAACACISoAAAAAAAAAABCBEBU
AAAAAAAAAIhCiAgAAAAAAAAEAEQlQAAAAAAAAAAiECICgAAAAAAAAAARCFEBAAAAAAAAAIAIhKgAA
AAAAAABEIEQFAAAAAAAAgAiEqAAAAAAAAAAAAQgRAVAAAAAAAAAAACIQogIAAAAAAAABABEJUAAA
AAAAAAIhAiAoAAAAAAAAAAEQhRAQAAAAAAAACACISoAAAAAAAAAARCBEBQAAAAAAAAIAIhKgAAA
AAAAAAEIEQFQAAAAAAAAAAiEKICAAAAAAAAAQARCVAAAAAAAAAACIQIgKAAAAAAAAAABEIUQEAA
AAAAAAgAiEqAAAAAAAAAAEQgRAUAAAAAAAACACISoAAAAAAAAAABCBEBUAAADAQmtvFKRQ0I+N
tl5raG94n19p9PTK2fDbuiEhLV0wbdlwz3vk+e1JY2Xca6xXNVb8ba00rK8CAGB+EKICAAAAODqa52
TGOemSUMHoqjT1kqMsZ9aK+rmpKJsX6tZnHZ3aesg1asv5Wkc/F6me3bS+CgCA+UGICgAAAOAI6
Ujt/OL38Zx/XTlwM89yXbr9vvT7e7I5KvksbsrZqn4eco16jXN+IFttyXZFPwcAYE4QogIAAAA4WuiNm
r3eoezrp3LqRKxeo5XtIng5auAamb1Qq9IiQQUAzCFCVAAAAAABHTLzeqH590pVg6NpryIpbm9Orse
rX/7Rrehp1Vr119suC68NKtPqCtUJH1wEdeJ31CKsrGjyeYL3S6HYYzGPXj8F92fsp1ayzrDVXndeO3Ul
FtltujOpfo/aGXxagXN+yXjUo3vHbQto/dH0tcc5VoEar+6BWKwAsLUJUAAAAAEdEWep1HdJl2Bu1U
ytJYTVYDdRep0K2gfXN1VEBZlNWCyUxyoCqjUhpcMIpOxQceJ3F3t/IQK8jtZJRr7Rcl60YnTuHwlEtel
8JVbak7hZHba7KRqMh5/wEVS4M1gNIcvwqwA5pv3M+hoNUR/i5UueiNLhTJewaAQCWAiEqAAA
AgKNjzQ3p4vVGnVS53pW+qgPq9ax0hK1v7o5oh1dLtC/
+y5tyzkv7etJYd0PBqrT0a71td2qyPjIpNl6/F2OSpvaGePmv0a6um3ga+6psW5/r+hNFqRqm9n5iDc
MPTjLVrLnHV5b6hcF2Jjl+67VuGhvWfmsrOxfjnqu27Eadi8A1AgAsC0JUAAAAAEdIUTbdGYwy641al
bNuj8nKaWvJNWL9/mFoL05zBnqzXmhn56Lz+t5F2dGdIQPD3I3enN5rB4QPix+t7aeGgTCzuHnB31
ftfDo9MAOTTGnVs8OTUiU6fuu675lBqKO4dsYLbDsHXf0saOhcmfVeOzviZq/FzT1n+9Zjb+QMWgC
Ao4oQFQAAAMDR4oVsGfVGLZ+Ukn4aMGp9qKqcDiR3JTnpp31ix33dA90L01rllguwH8bwdve1A0
6dSBLymT0vz8ha4EuLsnbGbdi+HIYlthMITDJlPQudTGqK41fHZNc5DR3eHzR0ropr4h2yuodK7n4ja
rECAI48QlQAAAAAR8xAb9RD5+l8K8qJU/rp1MpyMn6am0BHRnTmnE6i8DlCYFIpo85ppLBzFSw5Y
HLD3NgTdQEAjgxCVAAAAABHj9kbtRYvTsvXYK/Onhx6Y8iHebVWhx7biYbtTyercHa88cfflg2z16lby9

Ss3ZpEcVP29D4Gyt7amueYpR8Alg0hKgAAAIAjyOiNmpQxjDw7g706u3Lg7tTtmVk66QWAo+p5ps
MsJeDXAHX05KJbmFROSaIqAdNKcvztXa/nqR24unVRp7iWaoZ+1ePUnkjLDlS7Xi3W0WUEAABHF
SEqAAAAgKPJmIAoTMlPDo3aqW3Z8Kapz1ZzdcObqKm94Q8/L59ZcwJAszZnc9UfQt7e8Gp0FlIZV2
4Gzta5WPd7WfYa61790aSTVU1twuP3A1djxv4k9PbVbRDscRoSdAMAlgYhKgAAAIAjKro3qjlzuwrp
nGAubi3NaZWlXG7Kqg4D/dzWmOFftd+ozdlc1cGh9+IREzJNorLtD1vv1KSk21XyE1S5kPuM9AmOv
3Lan6jKu5bGBFRJjDgX5r1RPat7ugIAlgYhKgAAAICjK6o3qqp7OVgzs9qavI5mIqfk7J4xPFyx63gO1Di
1a3MOvE4Je+2U7GHrIcdebVnr3eHxeYt9/BXZ7psz/itVaZk1TZu7Xs/fcexzEVYMVW8zrewaALA4Cn
1V3AUAAAAAAAAAEIqeqAAAAAAAAAAQgRAVAAAAAAAAAACIQogIAAAAAAABABEJUAAAAAAAA
AIhAiAoAAAAAAAAAEQhRAQAAAAAAACACISoAAAAAAAAARCBEBQAAAAAAAIAIhKgAAAAAAAA
AEIEQFQAAAAAAAABGEvn/AZM6nr53azj8AAAAAElFTkSuQmCC]]></redcap:Attachment>
</ItemDef>
<ItemDef OID="q5" Name="q5" DataType="text" Length="1" redcap:Variable="q5" redcap:FieldType="radio" redcap:RequiredField="y" redcap:BranchingLogic="[q4] = '2'">
<Question><TranslatedText>Do you prefer Life A or Life B, or do you think Life A and Life B are the same as one another?
</TranslatedText><redcap:FormattedTranslatedText>Do you prefer Life A or Life B, or do you think Life A and Life B are the same as one another?
 </redcap:FormattedTranslatedText></Question>
<CodeListRef CodeListOID="q5.choices"/>
</ItemDef>
<ItemDef OID="summary62_4dc" Name="summary62_4dc" DataType="text" Length="999" redcap:Variable="summary62_4dc" redcap:FieldType="descriptive" redcap:SectionHeader=" " redcap:BranchingLogic="[q4] = '1'">
<Question><TranslatedText>
Alzheimer's disease - Description #[no]

If you have Alzheimer's disease…

1) You might forget recent events of importance to you. For example, if you are interested in hockey, then you might forget whether your favorite team won their last game, although you may remember watching the game itself.

2) You might forget the names of people you met recently, or the names of local streets.

3) You may no longer be able to handle your personal finances or plan leisure activities such as dinner parties or vacations. Simple decisions such as what to order in restaurants might become difficult to make. Your ability to concentrate may decrease and you might have difficulty driving.

4) You may start having trouble finding words to express your thoughts. You might need to be reminded to shower or brush your teeth, but you could do these activities on your own. You

may need to post notes around the home to remind you of simple things like turning off the stove.

5) You may remain aware of the day and your surroundings. You might be able to travel to familiar locations like the supermarket, although probably with the help of a loved one or friend. If you are in a social gathering, then you might have difficulty following the conversation.

6) You may continue to enjoy hobbies such as puzzles or reading.

Now consider two possible lives. In Life A, you will live in perfect health for 15.5 years and then you will die of natural causes. In Life B, you will live in perfect health for the next 10 years, then you will live with Alzheimer's disease as described in the summary for another 10 years, and then you will die of natural causes.

</TranslatedText><redcap:FormattedTranslatedText>
Alzheimer's disease - Description #[no]

<p></p>

If you have Alzheimer's disease...
<p></p>
1) You might forget recent events of importance to you. For example, if you are interested in hockey, then you might forget whether your favorite team won their last game, although you may remember watching the game itself.
<p></p>
2) You might forget the names of people you met recently, or the names of local streets.
<p></p>
3) You may no longer be able to handle your personal finances or plan leisure activities such as dinner parties or vacations. Simple decisions such as what to order in restaurants might become difficult to make. Your ability to concentrate may decrease and you might have difficulty driving.
<p></p>
4) You may start having trouble finding words to express your thoughts. You might need to be reminded to shower or brush your teeth, but you could do these activities on your own. You may need to post notes around the home to remind you of simple things like turning off the stove.
<p></p>
5) You may remain aware of the day and your surroundings. You might be able to travel to familiar locations like the supermarket, although probably with the help of a loved one or friend. If you are in a social gathering, then you might have difficulty following the conversation.
<p></p>

6) You may continue to enjoy hobbies such as puzzles or reading.
<p></p>
<p></p>
<p></p>
<p></p>
Now consider two possible lives. In Life A, you will live in perfect health for 15.5 years and then you will die of natural causes. In Life B, you will live in perfect health for the next 10 years, then you will live with Alzheimer's disease as described in the summary for another 10 years, and then you will die of natural causes.
<p></p>
</redcap:FormattedTranslatedText></Question>
</ItemDef>
<ItemDef OID="a6" Name="a6" DataType="text" Length="999" redcap:Variable="a6" redcap:FieldType="descriptive" redcap:BranchingLogic="[q4] = '1'" redcap:InlineImage="1">
<Question><TranslatedText></TranslatedText></Question>
<redcap:Attachment DocName="155.png" MimeType="image/png"><![CDATA[iVBORw0KGgoAAAANSUhEUgAABVEAAADYCAIAAABtBTQzAAAAAXNSR0IArs4c6QAAAARnQU1BAACxjwv8YQUAAAAJcEhZcwAAEnQAABJ0Ad5mH3gAACr5SURBVHhe7d1fyF3Vue9xY9Qdj0pC6w5pPWj0IAQjklYOxPcUG0GMCIKgYpCteDjyEsGyg2AjYg7nQlE2hbx7I+hFscLpOfFCkc0pUWwhFxXSQ6G58MINqbwXlgbqxbuLlFeai+75b4w55jP+zLnGHHOuteb6fvix+665xzhxPGHwuOuteb6fvix+665xhzPGHOtuOYTXe+
+4m8AAAAAAGCK6PkBACM5t3nuiv91BSGEEJIw1WcMAA/
+kAAAxrC5tbnnjT3iRo0QQgjpk73P7/r/v/h/1ScNABd6fgDA4La2tw68eUDcqBFCCF9cs3LVz5x6x6Jbj
d97xxy9+X33eALDQ8wMABvfgzx8UN2qEEEJIz9x/377/cft/yfLK0Qf+
+s031UcOgCZ6fgDAsE58fELLcpRFCCCE9cs/DY7rLhL/POyR9XnzoAmuj5AQADOvPZGXGXRgghhPTM
3ud3PXPgNrPnz/LpB+9Xnz0ADPT8AIChnP/y/y/K5Xd4kbNUIIaRPdp7a8djdN4uGPwtf7Aec6PkBAIP
Y3Nrc95N94kaNEII6Zl7j+4V3b4OX+wHbPT8AID0tz97G+CE2f3xC/sBwR6fg/DY7rLhL/POyR9XnzoAmuj5AQADOvPZGXGXRgghhPTM
MJWHn0/ACAlD78/ENxl0YIlYT0z3976O9Fbx/IySM//MvXX1efTMBqo+cHACRz4dlfFLRohBDSP/95/T+JHj4ifqbvm6efyQ0B4ABAAl
sX94+8u4RcZdGCCGE9MzOUzsevucm0cx3DF/sBzL0/ACABI7/4ri4SyOEOEEEL65/DDN4pOfqbwxX6A
nh8A0Nfp86fFLRohBDSP/95/T+JHj4ifLEfK46efy4ubqxdUVrbuFgdU0LPzY1e1PrZ9oc+cWf1NE6V/
s6ul8sspHzlswsQ3LoqPOi7zfMqNNc2ykowiHOb58QtGiGEENI/157c+eRd+0UDHxe2I9VRs8/J7oJ
kh1O3fstUpMqerbwQ5+4s3oap0pPepGlZE1vNW9w6/Pq+e210fMvqc2tzT1v7BF3aYQQQQkj/PPSD
74rWPTrH77zzm337zm+qjC1gx9Pxz42zu6+ZvkVtU2cI5OjQXjsNW0DDdbrfrPZ+e37U2ev5ltLW9eDe
NA+IWjRBCCOmf7z/6LdG398wLa4f//wLa4f//U9/qj7AgFVCzz9HuuuvXTTY4+suBdsejYOjbzbzHLdp6f
nRx4vM/f1DcohFCCCFH+53nrhUdfrc8vP9e47D0wrtWV/R5xTQQEi1fzz8yS5uX3eGAoWbFd14g8pwm
Nq2vyIuC7S0Cd0lrvfU0NcG8s1RzvJtZvFzLnCSzfRrX/YDDf5vwOB3HW0t4yVYtm/8SPQ4e8PrMjbHGcGYZ
2GLGUxhD9XL4I39o6rwSL4sTHJ8QtGiGEENI/Cb/Gb+dfhnfLfqljHanqSr/YxXCSNL+Q3dF47rSF0O1G1b
pM8wGZt2kWtzX8xcaKJOZ7UtzzTHJ2RNJbLLjNJ9Nvi7BqsFSWn4h3l/7NYKbuf6mVf6HK0RsaPi+A1V4WQ
+kkhnHpX6tnPsgmkWfOdf3oEEkcPx8KWqvcCn4D+ER2wprcvi8PcW9ghDjZgiVQKlsJ3iykPq0yKfp2CsADOMpmMFl4VdpCFb2zRSt5ZlgzpzjGTjy3q6FNzx9CQ0l+eOjP0o1BnKVKVUbf5JdNmzPSpMoo0JexuI9xUNJyrVJ6o4HRFnMWzgLTgKlHe6ZGU7C2cj0mIVvT6ttXmlDWPbyHUFhXrmBYyYLpdnDfGb90vbTh33SD3DepfuGKFGkKJxv17
[sic: continued]

nPKB+vr+UTmEXW+mC780KflLPVYTaIeV7sSD3OOQxZZRV/teq1yiIPjrPq0onyH5elJ5HrtBbTPZp9T
HnIt0L+vTPsFEZVzjkMNsrS1FOtAJu6lwfg2tzb3/WSfuEUjhBBC+mfv87ueOXCbaNGHCF/sx0qh518E
ro6yop5qNlei5XIM6tAsVWcVA4qf1zbOFmfVR/TpYrrwQ5+2s9SB6nG1ArUptUmXQF1ZRU9Tn9Nh
+eqsxsvQOK/D8hylS/YC2mdzrqipw74yjonEiR22ZmiOrp63luJaW/tKsAC2L28fevuQuEUjhBBC+ueal
6984tAtojkfLnyxH6uDnn8RBPo391OepmzGZqk6LRtRjs5+KA/pI/psMV34oU/rWepIcaBeXUlt0iVQ
V1aJu1bqrMaIxnkdlucoXbIX0D6bd7Jah31lHBOJEztsrT5FqJ63luJaW/tKsACOvX9M3KIRQgghSTL01
/jt8MV+rAh6/kUQ6N/cT6lWqDoa2yyV56l/vV+cXRxSR+oJxXThhz7tZ6lD2ZFqT/Vz7ivRSlaJu1bO4o
3zOizPO8ReQPtsHep12FfGMZE4sftiMuUgWdpaimtt7SvBvL3+69fF/RkhhBCSJLc/fYNoyEfIj+7+3ld/
+LL6kAOmi55/EQSaKt30mM/JTshxvu7CQs1SNY/9Nf7yiDGfqBh+6NPlrHLd+tfZG091LCLJ0+IaS9flb
J42wyTmS1mwz22fzTUiLzDbvjLtF6R1HnsKeb2sKXzLz828BYzjw88/FPdnhBBCSJLsfuHqcb7Gb+e1x
x/96zffVB91wETR8y8CR7NT0+2Telb3QXq4PqJaI31OS7NUn+g61d97hR/6dDrLqC+e0ePVYT00VFZ
WUSfN1lg6roqs3r48R+mSYwHts1kj/AfqaR0cq5Inti1Grq0ebx2pHjvX1mElmJsLly7wi/oJIYQMkZ2n
djx2982iFR8z7732avVpB0wUPf8icDQ7Jt1SNZiDjSZLCjdL9Yl6Ol3NWaCcLvzQp9tZ9W6ty6HPaJipa
GRj6X4Jcq55GupJHaVLzgW0b9Y9wpi+uWrP7jpdkJbF+C9PNcSe0bG2TivBPGxtb+3f2C9u0QghhJA
kuffoXtGEj5/f/fKT6jMPmCJ6/kXgaHakZtPl6IDMAdnTXZsle5zzTHEw/NCn41nhq6HPKgSuWEVWi
WsszbPUzxn7hNDyvBvzLqB9s80R1hBjrc7TM50vSHgxZqX8NPW4nMI1o7W2zivBqLYvbx9594i4Py
OEEEKSZC5f47fDF/sxbfT8QDtHOwqshuO/OC7uzwghhJAkuf7Fq546eKtov+cVvtiPCaPnB9rR82M1
nT5/WtyfEUIIIUmy89SOh++5STTe8w1f7MdU0fMD7ej5sYLObZ4T92eEEEJIqhx+
+EbRci9C+GI/JomeH2hHz49Vs7m1ueeNPeL+jBBCCEmSW569TjTbC5If3f29P37x+
+qzEJgKen4AQMPW9taBNw+I+zNCCCEkSRbqa/x2Xjn6AF/sx8TQ8wMAGh78+YPi/owQQghJlYd+8
F3RZi9a3jn54+oTEZgEen4AQO3ExyfEzRkhhBCSKt9/9FuiwV7MfPrB+9XnIrD86PkBAJUzn50RN2eE
EEJIqnznuWtFa72wOX7nHXyxH5NBzz9f6nfDFQb5BXEXN9bWz1Y/T+N30TV3FJANdO+1cdWV8Jw
Rp4Bffbhszn95fteru8T9GSGEEJIk157c+eRd+0Vrvcjhi/2YDHr+Oap6UiV9Y1QVmFDPb+/IS19da6/O
/j08Z8QpoOdfLptbm/t+sk/cnxFCCCGpsvhf47fDF/sxDfT8czRwR6Sb3sn0/K4duemRGblX9dwM1yD
iFGTo+ZfG9uXtQ28fEjdnhBBCSKrc9fge0U4vS/hiPyaAnn9+unewcVzzr0LPr0dV5F7VNZjhskecggw9
/9I49v4xcXNGCCGEpMre53c9c+A20UsvS/hiPyaAnn8+VDNkMPvJZtsqOiajkaqnEd2ob36zCTPG2D
1ZYwWtLZs5rXmm1SP7p62f0SfpBYrVKtbsmeaVK4jVqxGusz1mO8W8GAbHYf/VKMnNNIYY89XXp
lxh8DyPrrUaA60LIp70XAosmNd//bq4OSOEEEJS5ZqXr3zi0C2ikV6unDzyw798/XX1qQksodl6fn1H7
29+nEPqniTT9/6/biv8q1h4jStSUrtxPJUxrppjgHVNffO7J88ZU5iNmxZ82ZJMq58vj4mHvh0J6qzsOX
VGc+WuVbS8kWY9xVlYHnRN2jjDfU3rEY7niyfd54X3GFGr0r7inDkKC+bDzz8UN2eEEEJIwtx/3z7RQ
i9j3nzuePXBCSyhEXp+0dz0vf03p2vp1Rac82rqvkldJz1KXzijtQpeANf89rnW/NZp6pzAK2dPWx+Rd
QLTmmNcVV07EvIhYgnNdRtLNQU2N/spap3GCLGY1qshp9An6DmNVZmXwzdRfaIlpIZ9RBXXZ6kj9
YFAbczXhUsX+EX9hBBChsvBY7tF87y8+eRn71Qfn8CySd7z22QT0o/RSGSWupdwXU3XxZLjdLvVsvv
Q/MYxMUw9NCZ3HBKSTasnqjRLijlbuK6lPUX71exxih4g1uLYuuNQQ8dV6GHeiTrw16ovvBxjnZPRx
3qsBcPZ2t7av7Ff3JwRQgghqfLtf/y75f0av53jd97xxYUL1YcosFSG7/ljzvFTs62tVf+7xM2E48qI1rCiN
10edTRgTq4r7ygghunZHbwF23vCztOaA+Wr69qRn2OvTl3HGVpP8bxkat3mJiV7b83R1hzNE/Q1qs
2wse615GuhhjSKOQ9iIWxf3j7y7hFxc0YIIYSkys5TOx67+2bRNi97+GI/ltSw/21/s4EoOJqGXMemQJ
2zflb9tMTdhONqunskMbBrI+WY33WuZ3YXY6KmlNPWI60Nunbk51iU02yzFtpPUSOK4molenSXq
6FrCOp57/Y8J4Z2F1FLXgLncrxrxNwd/8VxcXNGCCGEJMy9R/eKhnka4Yv9WEZz6vmdPUZ7Y6BOyy
dRPy9vO+G4mu4eSWy1ayPlmN91rhgW1aaIm1afVhCjXTvym7XkDDvuckpVPhuiRre9ziY1IlMOsvb
eOocxRaHLwO615BjnclrXiPk4ff60uDMjhBBCEub2p28QrfKUwhf7sXSG/x1+jnO6tBQu6rzyNPVoaf
uJ0JUxNyXHdW2kHPN3aN5cZ7VKNa0atbax4drkbGtzXSfXDGqcZ9KIU0rVoLX19XICY3DrRuy1qyP6
FHuIlj01Q7G4WnJWVxV9zLFGzBP/3/gJIYQMl90vXD2lr/Hb4Yv9WDrz6PnVEUcDEewN5KBOJy0w+
8pkdLsld1lv09GAObnmn6V5UwesDtCWZtrGKfpBYM4w53XqsAx5XodT3PS4QmNw25yyRD1ezyO

XWdIn1ofdA2txtfQoNcbakjGPrzTmZPvy9uGfHha3aIQQQkiSPHzPTaJJnl5eOfpA9ZkKLIN59Py6x3D
wT6wnqvsH+8hS8VxN9+Ux9tjWw2nNmYoqXZq3+khD4KVJMq1+Wk2il1/P6tiRn2NRueYcijGXfV7r
KR7meXJ0y9Vwl8ypIfYyS74z/cuNqmW/vp4t5eQaMX+X/nyJX9pPCCFkiNzy7HWiQ55ePv3g/eoDF
VgGy9Pz+xuKZW0pAlezuVnxvK/ZczAvdTG8W/OWaa6grVT/aesnjPF6+fVAa0d+jkUp5iwdr2/wFB9
9knup4YtsVszrqcdVbfcyS83Fdlju7LXaX9/sidAaMXf8P+cnhBAyUA4/fKNokqeUd07+uPooBZbEPHv
+mRoBs5mw0VNgEal3Le9PLKYzn50Rd2mEEEJI/
+w8tWOq/4X/K0cf+Os331Sfo8CSmOf3+QOzWPQ5HjRVWDj6Tcu7E4vrpV+9JG7UCCGEkP65/sWr
njp4q2iYlz3H77zjj1/8vvoEBZZHZM/vVLTwVkPv6PDraapj+r8s9v0tgGOSkn6CtgqLQ7+hC753NbAY
HnnvEXGjRgghhPTPbf/9etEzL3v4Gj+W1Fx6ft9E3t4o0NkHngLmxOj5afix8LYvbx96+5C4USOEEEL6
596je0XbvLzha/xYXnPq+XPNyUIte7CvDz4JAGi1ubW554094kaNEEII6Zmdp3Y8dvfNonlexvA1fiy1
2Xp+AMAknds8x6/xJ4QQkjy7X7j6mQO3iRZ6ucLX+LHs6PkBALm3fvuWuFEjhBBC+uf2p28QXfRy5
Xe//KT6pASWEz0/AKBy4uMT4kaNEEII6Z/l/WL/e6+9Wn1GAkuLnn/eLm6sGb/pQP3yN345gY/4/R
DhhzMR177PVLNq/J7/wV/75ltuSGNeQyRz5N0j4kaNEEII6ZlrXr7yiUO3iHZ68fPa44/yNX5MAD3/P
FUtkdEQ0fO3EW1k+OFM5tXz60KlYV/6qtjQe6qMdg2R0tb21v6N/eJejRBCCOmZvc/vWq4v9v/o7u
999Ycvq09HYJnR88+PqyGi55+RuIh9msx5XfsR647dg9PzL6vPv/qcX+NPCCEkeQ4e2y366kUOX+PH
ZNDzzw89fwLL3/OP2ReP3YOPXQ8JfXTxI3GjRgghhPTP/fftE631Yoav8WNK6PnnQ/WXhqItMvtOY4
zdhupuKieeNicxx/kbL0+76zzcqNzhjJa+Tz1dn2EfUdPmR8R04YdhenAmGy/W7pzKPMVcYFDjpMZZ
qqTBs3BzcWLdDc1aZjVfLbHtnLV1Y0w9TfWkv6JjIiyV0+dPixs1QgghpGeW4ov9fI0fE0PPPx9tDZjN1
ZKZjOc7TdJkNHXVkYx90D2zo7RxqK3vU8/rp3URPUl1pHgspgs/DHBvJVeVtabSB0zGRp3CV8zxrGfh
rcvNBYv5aqnjxjzW1h3nlsODFWd5ObCgjr1/TNyrEUIIIT3zneeuFT32QoWv8WN66Pnnx9UQGT1Ud
VSPUo2UtyHTnZY9SX3E13ypWY3Ozz+vtRRriDGNa5sN1QB1ir16NUPxUEwXfuilx8kamWohbTM7t
mrpcMXsiZ3sq1IfEVuYsZZjH9Ywu3qhtaKrHpbK9uXtwz89LO7VCCGEkJ75/qPfEp324oSv8WN66Pn
nJ9SAGcfEMNlnZeSh9kkcrHmtXtDRHFrzOsZ0Ll2eUz5YX88nMo+o88V04Yc+rmH6WLX25hj1yLEx4
5DQ5YrZB5xiXlN7gOuULi+Zru7fa0Ge2LpELIFLf77Er/EnhBCSPA/94Lui2V6E/Ou//HP1+QdMCD3//
MQ1YLr9cqgmiukP6yFVaTWJPsGxtIznLP/6XaqzigHFz2sbZ4uz6iP6dDFd+KGPcy/iYHMq9aSLr5azip
5XHe22ZF2/HhU6sblcPcB1imOV1jBHdclZsdvesPAuXLqw69Vd4l6NEEII6ZNrT+588q79ouWeb/7p
H56sPvmAaaHnn5+4BqzZWzVVE3Xp4hzUmOI0NUc93jFrxrO6GUtXp2UjytHZD+UhfUSfLaYLP/Rx7
kUcbE6lnnTx1XJWsZbYbcmOuewT9RHBHmDU6jKzeyeZloquelhOZz47I+7VCCGEkJ5ZqC/2v7B2+N
//9KfqYw+YFnr+
+YlrwLztV61Tf+hSnZidp8aHl5ZRA6ujsaXL89S/3i/OLg6pI/WEYrrwQx/nXsTB5lTOM1q4z1HzqqPdlu
yYS56ohmTKUfbMrlodZvbtvrWiqx6W1ku/ekncqxFCCCE9818f+bboveeVf/vNb6oPPGBy6PnnJ64B
69BGdeni3Koz19bXy+HmaD2F2fjJeR2ldWMYKl3NY3+NvzxizCcqhh/6uIbpY1Wt5piOEzfIGQvWRN
1mbn9N7RH2lXfV6vKSOcZk2it22xuWxyPvPSLu1QghhJA+2Xlqx8P33CTa7/HD1/gxbfT889OxAZPD
9GN1oEuH1rX50nMVxGD9pJpYz6or+RfXUro+0XWqfyfhh17WOo36VTHfzOoMvbxAqQ5XTNbxUD
P5r4RcT73gemZXrXpcddC47mqYo3qmvaKrHpbZ9uXtQ28fErdrhBBCSJ9c/
+JVTx28VTThY4av8WPy6PnnyGiuMkVb5Giu7L7J6K0MdVvVaRIPc0322OaKFbMTdK+tEC5dn6in09
WcBcrpwg8D/AutqllTuc9oKTTDFQvO1OE1dZfK1TM3x5TH/VdCn+ionmut2G1vWCqbW5t73tgjbtc
IIYSQPrnl2etEHz5a+Bo/VgE9/1yZTVPRTzmaK3ff1GzUmr1Y50lc9JJEf6c1KzvmMwdkT3ctbY9zni
kO
hh+G6cGZbLy4bOHqBd8lEponyZV1W3K311S/eJn8qHpsTG0O0ZOZK8zGWjM7qlfCFbvtDcvm3OY
5fo0/IYSQtDn88I2iGx8nfI0fq4CeH02qTevYzwJYQW/99i1xr0YIIYT0yc5TOx67+2bRkA+dT7372TvXBB
kwaPT9M+l/M0vIDCDnx8Qlxu0YIIYT0ye4Xrn7mwG2iLR8ubz53vPpIA6aOnh8l8z/S5r/FBtDuyLtHx
O0aIYQQ0ie3P32D6MwHyskjP/zL119Xn2fA1NHzo2T0/DT8ADrY2t7av7Ff3K4RQghfXLv0b2iP0+e
43fe8cWFC9WHGbAC6PkBAJE+/+pzfo0/IYSQhBnhi/18jR+rhp4fI4/Y9/N9wX+/kaP1YQPT8AoJfT50+L2zVCCCGkTw4e2y169ST ha/xYTfT8AIC+jr1/TNyuEUIIIX1y/337

RMfeM3yNHyuLnh8A0Nf25e3DPz0sbtcIIYSQ6Fzz8pVPHLpF9O19wtf4sbLo+QEACVz68yV+jT8hhJ
CE2fv8rlRf7H/n5I+rjytg9dDzAwDSuHDpwq5Xd4k7NkIIISQ6dz2+R3TvEXnl6AN//eab6rMKWD30/
ACAZM58dkbcrhFCCCF98tAPvit6+Jly/M47/vjF76tPKWAl0fPP2cWNtSsM62er4xXx9BVrGxerZzJn1
62DerwxUXCOUmOI/XyHGQCg8tKvXhK3a4QQQkh0rj2588m79otOvns+/eD96vMJWFX0/HMkW+
mKbtdVT99UN9xden73HKG/EigYU7bPAABNj7z3iLhjI4QQQqLzneeuFZ18x/A1fiBDzz83utdW/bVur
ssD6nn1tDW+S88vh1iTyBOsM1pnAABp+/L2obcPiTs2QgghJDrff/Rbop9vDV/jB0r0/PNiNdvVIf+/P
29tzzNijH7o69DVAMcU5aHWGQDAZXNrc88be8QdGyGEEBKdmb7Yz9f4AY2ef14cDbufGlzq3vMb
LbvWKNicuEn+tYFG9w+gi3Ob5/g1/oQQQlLl+heveurgraK394Wv8QMaPf+8dOj5He12YYae3ztJ9X
x7z982AwD4vfXbt8QdGyGEEBKdW569TvT2zvzv/3mq+hwCQM8/P609v9GOl2Osfr5Tz6/I7r48qXU
VBvcMABB04uMT4o6NEEIIic7hh28UHb7Ia48/ytf4ARM9/7y4uvO8q1adtN2N65470PNbYzLZMeN
Rs67/7whMoRkAoM2Rd4+IOzZCCCEkLjtP7Xj4nptEn6/zo7u/99Ufvqw+fgAU6PnnRrfOqnduHpDd
e/2s7rWtGfQ56og+YP/NQXXEP0fXGRwHAMC0tb21f2O/uGkjhBBC4hL4Yv/vfvlJ9dkDQKHnnyOjjT
dVrbPuti2q5/dNkFNjfLPoOTyT1M+3z0DPD6DN5199zq/xJ4QQkiq3P32D6PazvPfaq9WnDgADPf+c
iY672Tab7XbeYqvHdbvdOD87rB8aQ6yu3Xyu1FyF3buHZ6DnB9DBRxc/EndshBBCSHTuPbrXbPj5Gj
/gQ88PABjJ6fOnxR0bIYQQEpedp3Y8dvfNZcPP1/iBAHp+AMB4xB0bIYQQEp3dL1z9zIHbsp6fr/EDA
fT8AICRXHHFSB86FIpGoWgUikahaBTKfPrB+xFf4+fSRZteoVXApQQAjIQ7kmgUikahaBSKRqFoFIpGI
QRwKQEAI+GOJBqFolEoGoWiUSgahaJRCAFcSgDASLgjiUahaBSKRqFoFIpGoWgUQgCXEgAwEu5Iol
EoGoWiUSgahaJRKBqFEMClBACMhDuSaBSKRqFoFIpGoWgUikYhBHApAQAj4Y4kGoWiUSgahaJR
KBqFolEIAVxKAMBIuCOJRqFoFIpGoWgUikahaBRCAJcSADAS7kiiUSgahaJRKBqFolEoGoUQwKUEAI
yEO5JoFIpGoWgUikahaBSKRiEEcCkBACPhjiQahaJRKBqFolEoGoWiUQgBXEoAwEi4I4lGoWgUikah
aBSKRqFoFEIAlxIAMBLuSKJRKBqFolEoGoWiUSgahRDApQQAAjIQ7kmgUikahaBSKRqFoFIpGIQRwKQEAI+
GOJBqFolEoGoWiUSgahaJRCAFcSgDASLgjiUahaBSKRqFoFIpGoWgUQgCXEgAwEu5IolEoGoWiUSgahaJR
KBqFEMClBACMhDuSaBSKRqFoFIpGoWgUikYhBHApAQAj4Y4kGoWiUSgahaJRKBqFolEIAVxKAMBIuCOJRqFoFIpGoWgUikahaBRCAJcSADAS7kiiUSgahaJRKBqFolEoGoUQwKUEAI+
GOJBqFolEoGoWiUSgahaJRCAFcSgAAAAApomeHwAAAACAaaLnBwAAAABgmuj5AQAAAACYJnp
+AAAAAACmiZ4fAAAAAIBpoucHAAAAAGCa6PkBAAAAAJgmen4AAAAAAKaJnh8AAAAAgGmi5wc
AAAAAYJro+QEAmNXZ9Stq62erowAAAIuGnh8AMCFlMz5sF35xY62uMGTBsf9mId9YZm3jYnUgpc
ZmCvxdyawa13CYl2lwnj8w1XuvlPadEfgjGnhqVu6pGq9Y2m0NK3hlBv0HxYD8m1rW18mzo8Z2Ur
1MHWot6T+TVgQ9PwBgWAPezTcYdca8aSvKDnGvk91L6X0U91UD31DlG1lbX8/+zyB18i2Mfkto3o
+mf1c03tm1gd59xV5GekeY1y1dEe+f0OafoeZGe/FWDD41K99U+XF9oLykfUu5ZzFfrt4lWq9M/nzS
f1C4NtXYUqHfvgKbKp5K+EfJ3o5R3DTQjor6+lDxqO/uutVKUKq8dhVxfRpXUTyHDuj5AQADat5PNe
9GklKFiv8d84agucOhDF4lL1BdvmHK5K/94JfJNM4L01CUHOjNJ+cerlbq+/iKej3shcu3Rqqt+SuGnp
pV16nUuOrhzIrzS80q2eXTB/q/XG3byY9XT/eqUwptKsX8lcCmHIfiebcj9C/q35E8MmAt9UT1sO+rlk
+npy/eyfXDZqniyV57WkX0/ACA4ch7AOuuIbnhKwjj3H4MvC09ff5D/E1bSL/bwZmN/j7IDPtWaF7A
wfZXTGy+UKlfOGvl9lYSX8jAtUp4GdumKp6PvpDq7GGrGNyF9NH8h95l1GIdtYb5x4VdKMk+KoHtN
CV8f7deutbFdGdNZe0jYTF9NYsH8u2QstDKoOcHAAzG/mROeLfjNvLNQLGfVPeMXo3bn/SMa5b/
OEyh4krVBr5mw+3Da+AXKVNewqxE+cMw73LrT6jx7kjCmi+v2LxuiUsGpktYqW0q68JGaauSbEOui
Yxj+Y/J3uyOWvabIgW7UFEn/6KC1r+q69IZiqdT7c1Zq3ir5SXKH/xLmY1Vy3M502zNvEzuQsk2tiLo+
QEAg7HvAJy3KCkNXsBU3HikunuzFXupDLiIfBdq+rzkcBtSyo0NWad45yW+lQ8b541XvOMKQ5Wy9
2H/Ke7FKmDPn/haBqZLWCk8VfHCJbiK4SrFs2leK0ehfA/qSP50sjeFp5YhUSm7kHhdigH+69uNYzu
G8LOz8s1WX79kpRy1iiPy6iV6rYodVNXyn5vTpr2Kq4GeHwAwmDl8VI93LyDuFgeV9GaqKd9Gfb3y
QqPsyX5rJCVenPL6Dfm2KAoO+r4z91D+PEy9Ym556RK+VMWE5srtt4I1pJ/AdAkrhaYSb8cenFWKg

5VUl80qlO+hfpw/nexNEbp0mXJ7KarZhaw3X4I/yMHtJJjf5KhVXq7yWPlzonrOfRX7UdbXG2+SHop
p9QtjvUrhawwnen4AwGDm8FE90r1Aeacz4j1HefOWvmDjls2Q4g47pKg73PWz3nnD1itenUEvmV
1hwB013hTp7uNL1p9Q+4+s9er1YxfQAk/NyjtVcTVTbadlwcXTSYrJQo33hGGIWrZE7wi7kDVx+1pa
BaYonkr1XshZtewKxSvXb0el9kuTj0ixO/lHxn7529cCiZ4fADAY+4M50b2b3xj3AkWNkW84ipqDXrlC
Xmb4KsNvZ4hbeb9kd9V+dolBd2Rl/Zaw1y1frNQ7C8yXsJR7quJ1S7iZ1gUXAxIUbCmUP53sXdG6q
VR/wuxCxRFzI/LNGMG/nUTbMFi17BLtV7ej1onyAf3fFMUOmtPYlRO8TCuHnh8AMBz5yZzs9sNr+
ArlPYmU/P4jq2Jsw3EfNIg0d222bF5j2uJFGvRVKiqYGxnyHrF4cYZ9z9nvgFGKZpK/I4qXprnwxt6sl64
3R0Ul8NSsHFMVh9K+SK0LLgYkuHwthfKnk71Ic91U4w9Skj9V3u0M8GfWrtX4w5RJVzT8MqV5jYrV
2kXyw+bc4aXAiZ4fADCgxg1ImrsCt2JuYbnvCcSOxtlMXnSQV2j03TRuddPd99qGfFc3lffD2gjviJR7E+
+AQr0Fc2+pLmagYngxM/FPJV6vUs/NFdUaK82qGI+Lmn1qdLwy+bBkb3prU9kBY/JySY5FdBbeVOP
ZPmUq1nZKxeER3tvJ/0Hhr9V8pvelK6dzTtN4Xye9kquDnh8AMCzzBoTPaYymcUPa+37Up3h7Dzb7
HCS+j0cSzVelVL024qklesX8m1rKPQVeo8zk/kGRnPirilJ9y8CNRE/0/AAAAAAATBM9PwAAAAAA00
TPDwAAAADANNHzAwAAAAAwTfT8AAAAAABMEz0/AAAAAADTRM8PAAAAAMA00fMDAAAAAD
BN9PwAAAAAAEwTPT8AAAAAANNEzw8AAAAAwDTR8wMAAAAAME30/AAAAAAATBM9PwAAA
AAA00TPDwAAAADANNHzAwAAAAAwTfT8AAAAAABMEz0/AAAAAADTRM8PAAAAAMA00fMDA
AAAADBN9PwAAAAAAEwTPT8AAAAAANNEzw8AAAAAwDTR8wMAAAAAME30/AAAAAAATBM9
PwAAAAAA00TPDwAAAADANNHzAwAAAAAwTfT8AAAAAABMEz0/AAAAAADTRM8PAAAAAMA0
0fMDAAAAADBN9PwAAACL5ez6FZX1s9WhknpibeNidWQYqo4ovwjqa5OxrsPFjTXfU8HnAGC66P
kBAAAWi9HXNtvTVe/567a94LoM3s6+vqgL+DcZADAcen4AAIDF0vh32WaHuuo9f6f9O5v7+q8C6P
gBrBh6fgAAgMXS6PnNBnfFe37duLesS18/fZ30ETp+ACuHnh8AAGCxNHt+o1G1en7rgN0YV0PyEca
81QnGEbMbVoezY8Z/Tm8UUYxnreeNtdVlWnruxnyNCY2VKoG59OhijH5k7SC0/lxzPWJIYIPB8wBg
VPT8AAAAi6VuJjfKn1TLaHSZxWP7gNFuVi1x3Y620k20/xyjz5Z9bcFYiGOSYOvrLiq3bjDWYqkXt76h
fhTV29bfsiD/Bt3nBZcLAMOh5wcAAFgsqmnMOkjVmJYNo/FEMdA+EOr5q0FGSyqPqLbUOqc+og7I
MnqEXopRp0O76y+gJ7RrhujBlXqaQuv61QD/ejwb9E0kVwAA46DnBwAAWCyNrrF6YP5st51GOy
mbWTWk7kv9R9Q09gg5RnbEGXlIT2KM8ZIryMmd2AfC9AJy4pQO65es6u4N6mGd9g0Ag6PnBw
AAWCzu9jprNa3O2NEqy9bUHtJ+RD02G+XmvGqEiyjcmMXDXlJOlVRH5dZa+ZfQYf2G5mj1vGd2vc
pac1sAMCp6fgAAgMUiO+Dqsf56f91DypGZoXr+5kH1wMVb2M89Vm4luue3l9Bh/a7mvaCe987u
ObHrqgEgKXp+AACAxWI1k7KJ1E842k7ZGNtD2o84phXzukYIHYZo7rGqpDoqt9bKv4T2xakRmXKQ
Vb11DmOKgr8YAAyHnh8AAGCxOJrJZvuonwiNTNDzm811c0yH9tsu46enMwdbNRL2/K1T2adaFy
WwwewpY+KZ1w0ACdHzAwAALBZXM6n7xpx+oj5atZO6MbWO2M2r/4h/Fj3EX1k1tnaZEH+B+vy
Ze+fAEtrWL7dTj2/ZoLWR4DIAYHD0/AAAAIuIpZk0nzBaUUn0sqEWVB7Rj9fk7KrfzbhL1wPsMmFqf
JN5dsqev3X97uXk1BDf7L4zu64aAJKi5wcAAFgsnmaybllbT5i9a9ZWysbYnqz9iHpsTmctJ9fsm5sDP
LsIas4nm2S5tVatSwitvz49l5c0Lov5tGt289Rc1xUDQHL0/AAAAAAATBM9PwAAAAAA00TPDwAAA
ADANNHzAwAAAAAwTfT8AAAAAABMEz0/AAAAAADTRM8PAAAAAMA00fMDAAAAADBN9PwAA
AAAAEwTPT8AAAAAANNEzw8AAAAAwDTR8wMAAAAAME30/AAAAAAATBM9PwAAAAAAU/S3v
/0HGR11c8nx0YgAAAAASUVORK5CYII=]]></redcap:Attachment>
</ItemDef>
<ItemDef OID="b6" Name="b6" DataType="text" Length="999" redcap:Variable="b6" redcap:FieldType="descriptive" redcap:BranchingLogic="[q4] = '1'" redcap:InlineImage="1">
<Question><TranslatedText></TranslatedText></Question>
<redcap:Attachment DocName="B.png" MimeType="image/png"><![CDATA[iVBORw0KGgoAAAANSUhEUgAABVEAAAEJCAYAAACdcmdaAAAAAXNSR0IArs4c6QAAAA
RnQU1BAACxjwv8YQUAAAAJcEhZcwAAEnQAABJ0Ad5mH3gAAE9sSURBVHhe7d0PjCRnfef/b+/aY

PPHNmBskx+RHat7sZYlGJIg08PxL4egZ+6SJbnbYOVg5Oh3PVyi0zQSkwOxF+mX2wguG4lunS5iWrq
zliQyTEJugrLdIJRgckzHR4IxYTI/Z7rlc3T8wDYGjPljB3u3f/VUPVX1VHd1dVV3VXX39PtltaerpqfqqT/T
O/OZ5/k+hb5FAAAAAAAAAAChjumPAAAAAAAAAAIAQhKgAAAAAAAAAEIEQFQAAAAAAAAAiEKIC
AAAAAAAAAQARCVAAAAAAAAAACIQIgKAAAAAAAAAABEIUQEAAAAAAAAAgAiEqAAAAAAAAAAAEQgRA
UAAAAAAAACACISoAAAAAAAAAABCBEBUAAAAAAAAAAIhCiAgAAAAAAAAEAEQlQAAAAAAAAAAiECICg
AAAAAAAAARCFEBAAAAAAAAAAIAIhKgAAAAAAAAABEIEQFAAAAAAAAAgAiEqAAAAAAAAAAAAQgRAVA
AAAAAAAAACIQogIAAAAAAAABABEJUAAAAAAAAAAIhAiAoAAAAAAAAAAEQhRAQAAAAAAAACACISoA
AAAAAAAAARCBEBQAAAAAAAAAIAIhKgAAAAAAAAAAAEIEQFQAAAAAAAAAAiEKICAAAAAAAAAQARCVAA
AAAAAAACIQIgKAAAAAAAAAABEIUQEAAAAAAAAAgAiEqAAAAAAAAAAAEQgRAUAAAAAAAACACISoAA
AAAAAAABCBEBUAAAAAAAAAAIhCiAgAAAAAAAAEAEQlQAAAAAAAAAAiECICgAAAAAAAAARCFEBAA
AAAAAAIAIhKgAAAAAAAAABEIEQFAAAAAAAAAgAiEqAAAAAAAAAAAAQgRAVAAAAAAAAAACIQogIAAA
AAAABABEJUAAAAAAAAAAIhAiLp0etJYKUih4DxWGj29PqjXWPFeU1hpWF+1iMxj3ZB2onXjpLWdL
MxLO7LSlg372PzHqPs4Vb2GrGzEO5vtDbdtK5JH08KNuQ9Cjmc+2g0AAAAAwPwhRF06Rdm8UJey
XurU1kPCkracr3X0c5Hq2U3rq4B5oILBVWnqJUdZzqxle4faf1Qo1cT/rlhsR+14AAAAAADIGiHqMip
uytmqfi4dqZ0P9kbrNc75IVW1JdsV/XzhFGVzry/9vnpsS/aHkff+RpmXdmShKwdu8leuS9c+xj3ZzDJ
D7TVk3fijwsI7ascDAAAAAEAOCFGXVGW7JV6O2jxn9EY1e6FWpbW4CSqOot6h7OuncuoEPaQBA
AAAAEAuCFGXVkW2W26M6vdGbW/4Q6XL9a3wXoyqlqKuRek+wmpSjqyvaH59ZI1Jo6ZjoC7rqPX
GPu31edcGHd5fZI1J4zwEz5+5nbDPjxN+3INtCdS9tR4xy30ObGewraPOc7xjCm57uPZpYAh6c9VZF
2h4snM3eA7Uw9yc3Z6x+xzPPy71CLkXbAnaHvI9OHq7vqTHE6/dAAAAAAAcfYSoy6yyJXW3OGpz
VTYaDTnnJ6hyIWSM9FAIo3VqpQwmoCrK2hndwM6BdJ1nFmNId2dHLno7bcuubn/5zNpc9FKsnPa
D6gP/AGy9izv6PBo1Pe1wrCSDo63TPb8dqZUKUhrYSXM1aUimtjPY1qasDoZtEx2T2rZR+9S6H+te1
+kREu3HCSwHz4HSXB0dKibnnOtV70AUtW6Kc9TeGFHPNGS7E4vZbgAAAAAAlgQh6lILTjLVrLnBTF
nqF0Imk2pv+KGKV4+yL103ie3UZD3lhKW4dka3b18O3U23d42JhYxw0hjqferEnAz0rpz2yiY0d4M9
Ji/u6BisfEacDLUnjXX3GlSlpc9v3+0xnOr59bfvdUhWIdlAfdzx/O1494Hazrob/E1zTMbr9zZlc9v62PX
vV1Wv1/6cXXIi2X56jXUvsCzXu85r+8MlLiqR+4wr/BzteOl/krZbr3X/0hH2PRjY7rBkxzOu3QAAAAA
ALI+C9QtyXz/HklK9SwM9zlSwEhKq+K8rS71rTubTk8aK24tOBS/OZEYjX6963bk96Ubsy+dvW4Vde
9ZG1BBs1YOwWq1Ks9kcWu+3IaxdcdeNE387w+2yV3rnwG1/6DqbsV0VnO2FBNwB4e0wr3O11fc
nDEt0PSK2E9ivvuaS7JjMbQdfr41qa6JzF35+FOdanQqsS3p+lETnOlHbRwht44jjjDietO4RYBr3Pnyvv
OXCW/QSACAP99/xLnnNW2/SSwCAzB3/NZFjr9ILWBT0REVwkikVtoQGJP5Qeb/npMsYdm/2GE1F
UU6ccp51dJfTrj2WvywnT5+0e9QF11uqp/0AbA74vWmb4nVG7R44gZT1GW8ov7dOdT4sGbUo3S
BMfcIsazBr1jUo6ac28z7QPYSnOKZEvYkT7cec4f+kmIdQ3NyTvhmgTm3wHIWY6rrrurFuwJmaGO0
GMvDwEw/LOz/5Tr0EAMjDDY9fJXd/6AF5/Bs/0msAAJm7dI9I/zt6AYuCEBVBA6FScsO1P6fl1RVt7k
pbenJoj9k/JScqJ6z/D65XGeqc9ZIrrombLbpD+ttuIl09a/TozUtaAZl1DTJrOyHeSKpXqBe0GnVjgQX3x
NNPSOWPKvZHAEA+nvPjY/KWL94oP3ryGWm+/3555p8u6c8AALL1lMilP7A+PussYiEQoiJlGYRfXI3
RfTlsXxS7lKgd9lbEyVeN9XMZvhk9NO3A1+/VOyrwVcO67VqVQ480e0lOK1mv47yOaTHOXbjxbW/
Lhtnr1K2LatY5BRbUnZ+6Ux58/EG9BADIwxv/
+gZ5wQ+vsJ8/9NXvyp/WeR8GgNz0Hxa5/Od6AYuAEBUxleSkN1LbnBFfMSZJitM70Ri+HI+7744c7D
pf686+X7I/4a8fLjUwH4qbZ/0guOFOjFWVQIZacsoTKG6Jgvk22OvYvA/0seV1TIn2Y97LA8Pk1cz3hZ
xnoE/SdmNSNTtwdeukJv6eAubL+z77PvlM7zN6CQCQh1c+eK3c/PXn6yXH5z7+kHzlLx/RSwCAzF3
6gsjlr+kFzDtCVMRUlM2zbuVUc/Z1NbrYnOl8y+vt5wScijnre1s23JlrYvN7cqqJpBS3XmZRF0x117vh
6vxxe81a56Kmj3+wdqsx7F+aq7LhnTIV7Onh297K+dBcNQLH9nm/hqd7bHkdU6L9mLVbm7Lqre/p
me+ta1Qq+NvI2oTnyA9c3XYDi+kT+5+Q+n11vQQAyIOqg/pzD7xELwX94W9/Tb73raf1EgAgc5f/RK
T/fb2AeUaIivgq29LytctSalHTA48w8bynX5YJR4NOfUMnSXNWB0GQ1HAPbsp55Q/a9of6KMUnTHP
Jqu9rKUt8KRKiWomxe8IdlN1d1gOaFzqMm/ZolJ3AMttM8tryOKdl+ipsXpO6/2HmtOZGTdS/7h6Br

7yrua1NNWBO03bzfw9odR+bHA8R339fvk7v+7C69BADIw/FLBXsYv/oYRgWozd+8Xy8BADLXf1Lk
8sf1AuYZISoSqWyH11+stqz17tBiV3FT9gZfW21NVr/R7K0XKBlgDM3OdKKjFJgB2KiyA+qc9bt+wOe
ya1/OW03PstS7fT9Yt1Wl1d8LTpaV1zEl2k9RNvf60h16sXq5MUzeVpHt4EGK7B96PbFTEbvtVlv6Lf
8+sqlzblwHu+5ulByOB4jBnYn/6Wfp7QQAeVr50kvl2iev1Evh/uFL35ZP//6hXgIAZO6y9RvZJcpbzbt
CX81aAgAxtTcK4nSSVCHqQGAKADGo4PT1/+318sAjD+g1AIA8lB56od0LNa6tu8vyiteFD/sHAGTgit
8QKfBL9ryiJyoAAMiVGsJPgAoA+VK9T1Uv1CTUsH7qowJAji79AfVR5xghKgAAyM1HvvgRezIpAEB+
VP3Tt33hZSProI6iAtS7/+NX9RIAIHN2fdR79ALmDSEqAADIxe6Du/LBv/igXgIA5OWOL18/tg7qKPv/
8zH53Mcf0ksAgMxd/n9FLn1BL2CeUBMVAABkTg3fV3VQmUgKAPJ189efL//8Czfppclc+dzjsnX36+
XWV79IrwEAZOtKXR/1Zr2MeUBPVAAAkKknnn6CmfgBYAZe8MMrEk0kNcoz/3RJmu+/X3705DN6
DQAgW9b77aWPWx+fchYxFwhRAQBAZlRwqgLUh594WK8BAORB1T99yxdvlOf8OJ1f+R7/xo/k7g8
xKSAA5Kb/HZFL1EedJ4SoAAAgM+/77Pvk3ofv1UsAgLz83AMvkRsev0ovpeMrf/mI3PtJ/igGALm5/
DXr0dELmDVC1KXWlo1CQQrGY6XR05/LSa8hKxttveBob7jtWZG8mzM3Qs5LHL3GinPuVhoy6tT55
9d55H7NjwLuWyCW+n11+djffkwvAQDy8vJvPE9e+eC1eildn/zPB/LN//19vQQAyNylXZH+Y3oBs0S
IurR60lhZlaZecpTlzFpRP8+eHfiVasLfVIImPi+9hqzXor5KXfOCrAYvunRqJSlMENguK+5bIB7V+1T1Qg
UA5Ovqp47LG++bvg7qKKo+6u//
+y/bHwEAeVD1Uf+79fFZZxEzQ4i6tLpy4KZA5bp0+33p9/dkM68MdWzgt6QmPS+qZ+S4YK99XtxN
V1vqevelWy87K5qrQo4aA/ctEIuqf6rqoAIA8veWvRvtIDVLqifqH/721/QSACBz/UdFLv2xXsCsEKIuq
96h7OuncuqE5Nf/FGmL1zOyJ41zugtquS5bFedpcfOsVJ2n0twlRQUwPTUTf+WPKvZHAEC+Xvu1F8
vLHr1aL2Vrb/f/2A8AQE4uf8l5YGYIUZeQXbvRDN2aq04tx8GuiKp3o1E7c1T9zGAtyGCd1bDejbH3r
wVreI6qN+kMVfdfl6zW52A9S6+2qH6ENy3GPgPncEP8zZjnydln0vOiqHaWYvWMNHoeB0LzkpzUn
VFl/9A6olGM9obVW21v6GMZPAcJrkvI/RZ2vePcb4PXz35E1ImNY5b37WT3p2vSa2eZ6D1Ar1TMr
49uJI6QOz91pzz4+IN6CQCQFxWevubvXqSX8qF6o1IfFQBydOlPqI86Q4SoCDUUGGl2/cyRYVRHai
WjzqrR43EyanuDNTzVurCgpuQNVXdFt3UUZ5+DwWRzdcJ9Fjflgjtk3joz5/RGeo1z3nkq1y+kUEahK
q1+y+tVOsTseTxK50C6+umwipx2N97ZkYsDJ7W96x2NX1c3yXVRQV7I/eZcj1EBZPj9pu7d0GC5U5
NSIMjOSpb3bcz7M2CCa2eZ7D0Ay07VQP1M7zN6CQCQFzV8Xw3jz5uqi3r3h75KfVQAyI2qj3qP9Z
H6qLNAiLqEKtt96Xfr4kZ7Um3Z9TH72zrxbG/4AZBXL9Won9mpyfrIxEaFec7r+3uboWUCxu4/wN+
et3/pyI6XBPWkse4GPca+Wzo1imzrKP523M2ofdbOu/Fbsn0WNy+If+rOS9v677wbglnn94JOUJOd
F4P9um2ZLK8uyolT+ukYlS23beb5V9ri53BnxMnhkpwj67VGqYGh+21ofyZj2/b9ZrYlbFt+kJ3U/Ny3
/uvD789hya6dZar3ACyrT+x/wp6NHwCQv5UvvTTzOqijPPTV78qf1hmBAAC56T8scvnP9QLyRIiKIW
bPtPoFPwgdDgOHletbE4Z54aotPxwsrp3xAqzOge4z2bsoO14eaey7suW3deeiJll7zH364ZMh8T6Ls
nnWTbuaslpwe08Gz+8kipt740PW2PblMOpEFdfkTNjxtXf9XrVn1pzjSXSOrPOzp0NEI3gPvd4Dhu43
s8et0evSPk86CNzLYfa0LO/bsfdnmCTXzjLNewCW031fv0/u+rO79BIAIE+vfPBaufnrz9dLs/G5jz8kX/
nLR/QSACBzl74gcpkJ/vJGiIoBI3qm2Yqy5iYxIwK3UyfSDKjKcrKkn47SPdC9+VSoU/LqNhbMYdKRw9
QnMMk+K9tGr0FHOsP403RKoi+fcf2NgNIP3Kpy1j2gqa6LruEZOrw/aOh+M8JCawf20Hd33yNrsaZ
uHu/bBNduyvcALB93Jv6nn31arwEA5OWGx6+Sn3vgJXpptu7+0APy+Dd+pJcAAJlTw/r739ELyAMh
KibUkeHOgTHCo7mX3TFUvMKUSrD+5Oz05HBssVSf36vSHRZuBG7V08FeoUmYExB5PXXHCbtWR
dm8EN470w0rF3t+o8nvz/SvXdh7AJaNCk5VgPrID+h9BAB5e86Pj8lbvnijHL9U0Gtm60dPPiPN99+v
lwAA2XtK5NIf6OfIAyEqJjR/gWm53vWGbAcfk9YLHS/
+Po26n7aO1NZznJyneELGlj4tn5Sxl7S4KW5lAntYuDF8vno6/CyPP0dt2TB7nbo1OM36o0lYbdzT+xj
s/as0z83XpEi53bcTXLtoR+GPJpiWGsL/wCMP6CUAQJ7e+Nc3yAt+eIVemg+qPuqnf/9QLwEAMqfq
o15iYte8EKJiQElOusnV0EzePbnoFnIcO/Q7J6WTXtA2qm5m6ibYZ6+xrodpl6Ved5OsPCfnMa5rc1f8
zphdOfAu6QmJc0m9HrXW/XH+/I4OP6sSyOGSnCOzLqcKFN26qMaQ96TUzPKqx6k9GZQdSHa9W
p7pDpOf0CzuW0usa5fme8AU1xDz7yNf/Ig9mRQAIH+lh1448zqoo3z6v/6D/MOXvq2XAACZu/xZkf
48dRU6ughRMcCcBCnYW9IPAlXYNTChz6yYNTCbq/5Q7faGHhpuPdIev510n72GrLsnrnpWNjeNyY

Nq65JPjmrWsmzKrm5er3HOCzBj90asnBbnDulIs+ke18Bw8Amvix8oDvbcjUlvX80sH+xxaoTFZo9b
oz1p3yaRZnHfKnGu3QTvASU/dZXaee9gZMOb4h9Hze6Du/LBv/igXgIA5OnaJ6+0Z+OfZ83fvF+
+9y1qZQNAbtSw/v739QKyQoiKYeYkSJ2alHSoU/LTE7kw7YxI5vDy5uoUoVGwBmZzVQdQXnhTlVZ
qs9e7kuyzJ411d6h6Wepbav3okCq98zLMnFndbbN3TastiX+aKhIo72oZDmATnCMv2LO4x2xOsJTEi
HvXrLNaPevPNp/YQt+3SpxrZ0n4HuDXW7V41zBubVssGjV8/85P3amXAAB5UvVP3/aFl81NHdRRV
ICqglQAQE76T4pc/rheQFYIURHKHgYdUpOy2rLWu8Otp1KR7cGClfuHRs/BBOwamMZwbZddWzOj
eqgx92n23LN7obonbiCk8of1p3hehhRlc2+4Tqg9hD5hYBeYJMs6ZjsbHhT7uljH3G/5QaqtKq2+0dZ
ACYJo9r07eJA2Z5vTZZMLft9aYl07S6L3AHUsg6+ttiava4u59cTTTzATPwDMkOqBqnqiLgI1pJ/6qAC
Qo8vWb6bUR81Uoa8KBgJAEmo2fT0ZlAph96btmTxL+lhOtaYNWBfEUbp2yJUKTit/VJF7H75XrwE
A5EnVQVWTSS2SK597XLbufr3c+uoX6TUAgGxdKXLFb4gUbtbLSBM9UQEkFCxRcGZtsUO49nnrWC
J6ZB4tR+vaIV/v+
+z7CFABYEbULPx3fPl6vbQ4nvmnS9J8//3yoyef0WsAANmy3m8vqWH9TzmLSBUhKoCY2rJh17o06
pWaJQoWUK+xIqv7demmUqJinh29a4d81e+ry8f+9mN6CQCQJ1X/9C1fvFGe8+PF/NXt8W/8SO7+
0AN6CQCQuf53RC7doxeQJkJUAJNRNS8XfPx7cXMvpRq/C+YIXDvkR/U+Vb1QAQCz8XMPvERueP
wqvbSYvvKXj8jnPv6QXgIAZO7y10QufUEvIC3URAUAAKEefuJhec32a+wJpQAA+bv568+Xf/6Fm/TS
YqM+KgDkjfqoaaMnKgAAGKKCUzWRFAEqAMyGqoO6aBNJRVH1Ue/
+0FftjwCAPKj6qGpY/7POIqZGiAoAAIbc+ak75cHHH9RLAIC8qQB1UeugjvLN//19+cPf/ppeAgBkrv+
oyKU/1guYFiEqAAAIUDVQP9P7jF4CAOTttV97sbzs0av10tGyt/t/7AcAICeXv+Q8MDVCVCTUk8aKm
uVbPTaknWjdOGltZ96MOYZeQ1Y2gmvbG+7rV6TR0yunpfZjb3NEO2zTne9M2m1bkPugvaHbWJC
BSwosjE/sf8KejR8AMBsqPH3N3x3tuqGqN6rqlQoAyMmlPxHpP6YXMClCVGCGeo0VKZRq0tHLWe
pd3DH205RdQj4AA+77+n1y15/dpZcAAHm7+qnj8pa9G/XS0aXqov7+v/8y9VEBIDeqPup/tz5SH3U
ahKhIqCibe33p99VjWyp6bXby3l8WRhxDryHrtTziU6UnF3eC+2qea1hrF8VRuA+A+aZm4n/nJ98pT
z/7tF4DAMibClBVkLoMVE/UT/7nA70EAMicXR91Vy9gEoSowDLoXRQ3Qy2Xy86Tzo5cXJwUdTFUt
qXfrYs+w8DCUMGpClAf+cEjeg0AIG8//ffX2UP5l8m9n3xYvvKX/NsDALm5vGc9mOBvUoSoS8eoLbl
i9kQctd6oc2mvz7s25fD+IutuGnU/VwKfNLcT9vko6Z8z+/PmMP7mqvP5EYU0/WNWj+T1Rv2h/FU
5e+GMDvk6shMnRQ3UUg17jG5PvHbHuTbD59DaumyYrzfqkQa2MbA+/BTHaYN5POpY/P2rh7vd
9nnrupbrsjXQXdYu3WC83n4M3DfArKgh/A888oBeAgDk7YbHr7Ink1pGd3/oAXn8Gz/SSwCAzF26R
6T/Hb2AJAhRl05R1s64PREPpOs8s3TlwE30Aj0U27LbdJ6Vz6xZXz17ldNV/awjB/4B2PywsCxn1nRr7
RCwJIMj5zu1UswQa5bnrCO1UkFW9fYcal2SINUYyl89LZXimniHs3Nx+hCvfEbcU+2L2e6pr43Dfn1w
Z846FVQOrG+uDgSpE7VBHcuqeFvWoakKSlf369Ld2wxcdxW+lsJKN3RqUprnibKwFD7yxY/Yk0kBA
GbjOT8+Jm/54o1y/FJBr1kuP3ryGWm+/37qowJAbp4SufQH1kfqoyZFiLqEimtuT8R9OXQTovauHwi
JEU72Dq1XOU6dmIcI1VI5LW6M2gzMjmSEhV6w15PGutvjsyotu6am9WjpLXRqsh4jjUz7nFW2rTa
Yw76rLadd22HVPv12d+vuV8TsRaoYQ/mrp9X2zVA4xpD+4qbsuefNfnTFa4Z1BPULwcDQN67d6V
wbV7neDX69Frbev2+maYPxeh2aFjf3vOc+P1RXYWtXf41/TppyLsFxAmnafXBXPvgXH9RLAIBZeONf
3yAv+OEVemk5PfTV78qf1h/USwCAzPUfFrn853oBcRGiLiOvJ6IfaPUOndivWnXCo32dFJrDwO38bS
5UZMsNoJq7fi8+s+6n2wPUXFff8ickqmx5QWCs3pgzPGfVlj+Rkh/mWi0Z7IY7gtk792TJfmJsJ0EYq/
Ua636vzepZ2QxPUMe3O61rY6vKWbchRsg+cv3+obPdKdoQeH0UI1Q3Q2s7cNWB6t6okwhkSA3f
v/NTd+olAMAsvPLBa+Xmrz9fLy23z338Idn/n4/pJQBA5i59QeQyf8BKghB1KRXlxCnnmRtode1x6W
U5efqkHXYF11vUMHDn2VzwQ7mmeJ0KuwdeWOgN5ffWWcfkDu+2H8bw7cAQ/VFmdc784HMy
Yb1zLRMP6W/LeT9BlVZoz1klRrtTuzaW8kkJ3d2o9a4p2hC7Z7Zxrq2N2WUO3P3Er8sLpOuJp59gJn
4AmLGXfPe58nMPvEQvQbn7P35Vvvct/m0CgNxcVvVRv68XMA4h6pLy6oraPTl74nSqPCUnKies/w
+uV3ngPEWoFiOYcodmt90x0xG9I6exkOfM6GmphqaXQkPC+LP0tzf8OqBVo6fp8kkSbhdl80L4jP1u
cDtiPjEgE+5M/A8/8bBeAwDIm6p/usx1UEdRAWrzN+/XSwCAzPWfFLn8cb2AcQhRl5U3tHlfDts6aL
N77VXEyQqN9VP3hsyCUdfTDi/9upOjwkuvNubQI2YYuIDnzB/KHyXmkP72hj9RVLUlIzuhTmDqa5O

CTNtg1JUdKNlqa55jln7k532ffZ/c+/C9egkAMAsrX3qpXPvklXoJpn/40rfl079/qJcAAJm7bP02eukze
gFRCFGXVklO2hlkRw52nSHNbh3Rkv0Jf3347OuzV9w864eaDXeSp4E6pCVnqL3i1eKc2KKdM2Mo/
xjjh/S3ZcNLUMtSV1PRTyvVazOhHNugZuhXPU7tScXsQNWYoCtu2QJgSvX76vKxv/2YXgIAzELpoRfa
D4z26f/6D3aYCgDIyeXPivTp2jMOIerS8ntyNptOOObWeCzq4p/uem+Sprnj9gDtSK2mA77BOqRmP
crmqj9sur3h1aUsxB5LvWDnzBzK787+H3iYIV70kP5e45w3jL9cv5BOuYRUr82E8miD3pbKoIM9Trvi
ls8dW7sVSMn/+v/
+l34GAJgF1ftU9ULFeHd/6AH9DACQi0v36CcYhRB1iZkzpqvehd7w88Ds5sYkTXPIq1NqC+shGaxH2
VzVwZg/Ll1GT440LNVzVtS1VJXmauqhoTmUvxxaW8AoiWC9cuSQ/l5D1r0CqtYrAxMwOY/JJkhK99
pMJoc2VLb9IfyBurRGfdmzm7MP3bEU7v7Fu+WOl9+hlwAAeXvjX99AHdSYfv7dP6WfAQByUXijfoJ
RCFGXWWDW8FPiTzbuDltXzPVzyAwvRw2ht+tRGr0uXeW6dJPWu0z1nFVke7BA5v5hSrUxzaH8o0
NdMxQeNaQ/Xl3VCaV5bSaVQxvsIfxhxVBVSNvvp1pfFohy1RVXyf/4lf8ht1x3i14DAMjT373yCf0M
UV7z1pvkbe+5VS8BADJ37FUix9+kFzBKoa/G9QIAgKXxwCMPyOv/2+vtmfoBAPm648vXyysfvFYvYd
D1P/E8+a1PvVGedw0TbwFALgovFrni/daTq51ljERPVAAAlsztN91uD+0HAOTvb27/tjx2PX/ECnPlc4
9L9fdeS4AKALmx3m+Pv8f6SIAaByEqAABL6F2n3iUfeMMH9BIAIC+Xjvfl8294VH78nMt6DVz/8t+V
5NZXv0gvAQAyd+ztIoWb9QLGmSpE7TVW9AQpBX9W65F60lhxJ1TZkODL27LhTbbiPCabqGZYeyO
43dBH1jOAAwAwhz788x+W07ed1ksAgLz84PnPyt7rvqWXoLzidS+R1X8bNhkqACATx4oix39eLyCO
OeiJqsJVf5ZqR84zwquuZ0VcaKU3oAwDA4rjnl+
+xh/cDAPL10M0/kO6t39dLy99d99rV4CAGSucI3IMTWMH0nkGKIWTZXOvL2oeq35gxXUdNc/9Gtl/XnRZq7pKgAgAgOVzy3W32EX/
XnRZq7pKgAgAgOVzy3W32EGqCIQBAPlR9VE/96ZVc/9QLi
mn1P1N6h7OuncuqEZJedlqXedUNc/9Gtl/XnRZq7pKgAgOVzy3W32EGqClQBAPlR9VE/96Zv2h+X1
S/8xivsofwAgJzYdVDz7rl4NOQYog7XRLXrlZZq4nZEtYfVD9UoNb/OeaRVL1Uprp0RP0YFAGA5vfm
WN8tH3/5RvQQAyIvqibqs9VFVePoLv35CLwEAMmfXQX2HXkBSc1ATNUKvISuFktS8lNXRqZVSq2H
aPu+HuNXTfpGB5WVM8uWd46hJwTA7YddqEYTfT/4kcHneY0nPYU7fC+q9b2DCO//8rEiKf0cCAt7
7s+
+V2h01vQQAyIuqjbps9VGfd82VctfvUJMbAPJztcixO/VzTGKmIWpluy/9bt3vCVptOcPst1WY2ZPGu
htwVqXlDsFvVe01qobpeqIkoSO1khtC+I9Vd0Yra9/2bgFghnqNlWAPfSBnqjeq6pUKAMjXfT/zuD1r/
7JQAer1P/E8vQQAyNzxO0UKL9YLmMT89kTtXZQdnSKU61v+RFSVLXHLmHZ2LqbU+64s9S0S1NFG
TQoGTGKR76eM295ryPpg13tgBlR9VFUnFQCQnx8/57J8/g2PLkV91Le951Z5zVtv0ksAgMwdf5PIsV
fpBUxqfkPU7oHXE8sevu/1HjWG93cOpKufTsfppZpmrVUAABaVmqm//attZuwHgJw9dv3T8je3f1sv
HU23vvpF8ku12/QSACBzhVtEjEjv0LvYBpzHdN1FSFz85vlhPo1M7nWItxHgxO2jWqxuPoOpD20GPv6
/VjZG3Jwf1FBNd2Pdzga0fVgsysDVEya98oca+VK/5xJm1f2OsHyncO1O806o7arx19P/kG2p/K9Ux6
DsOEt32wXungORo8P2HiTbTn8/epHqPqpCY5P0DQbdffJvf88j16CQCQl7+/7Xvyjy//oV46WlQd1O
rvvVaufO5xvQYAkK2rRY6/2/p4hbOIqSxEiFqud4fDT/uRwnDa4qac1WVWRfblcGnyBRVsDU7a1ZTV
wqr1/3hUiFMKG3rcqUlpMKBKMklYe2NETUjVYzgYFmXWhihZtW+khNcqwXEma58TyIW9vrlaGBH
2qXNitLNcl/GVM9SxDbR/6us5/f0ej9OrffAcNVdHhZyTcPbh1XO2Dd97qd3vWGrvKL6DGfsBYAb+6v
WPHcn6qL/yH15JHVQAyNPx09RBTdH8hqilk34P0YN0Bu2Ha8tuuinKQug1zvnhkTuhV7/r1Zsdzzhv
5bp0dbDd9TbQlHNeotNLMEmY9dpzesOh2+3IzkX3tVm1IUpW7Rst2bVKcpzJ2tdrrHuBnP+HjZa1F
615bkRQaLRjb1OKem0koz1u06e5ntPf70n47fHb3pHa+ejIPHqivUH+PsLvvbTud0Ds2frfdepdegkAk
AdVH1UFqUfJyumftB8AgJwce53zQGpSC1HtnmjGkFHzEWco65DimpzxspxVfxuqF6C77UQbdnpv
me1yHmYvuTOyFivhWXQ9uejO2qUCFi+kKcrmBSPEidI7lH39VDo74mY3xc09J6yxHnub+mQmmiT
MmLjHCNyKa2eGQ/XM2hAlo/aNlPBaJTnORO0LtuOst74iW/aGVVC3J2GHE2hHTNWz/rmtbPtBrd
/2JNczhfs9gWrL7yFf2Up/+4q5j/B7L8+J+bAM7v7Fu+WOl9+hlwAAefjmjU/JV376u3ppsb3sp14o/
+a3mNAEAHJTuFHk+L/WC0jLHA/nDwYcXkjrjWE1w5B0mMHN0daVAzdTKp+Ukn5qK56QU/ppJDP
kHgioh2ouTjVJmK6nGTZ8Prc2REmpfSMlvFZJjjNR+0a3wwldR5fWOHUi6XdVVU4HNlaSk2473bYn

up4p3O9zpSwnAwcRIteJ+bAMrrriKmbsB4AZuP9V37HD1EWm6p/+
+n/5GeqgAkBurhQ5/mvWR+qgpm2+a6IWN2UvbMitPdQ3hXqoHtWLri8pZ7JH3OhefG5oM1EPZ
MWu5eiGPlE1KzNsQ5R5b19sebQvRuA3VlFOLEzSmcbxAvPpphfcZAepKlAFAOTn8yuPylNXX9JLi0f
1QFU9UQEAOTn+r0QKN+gFpGmqENUc9hv1cMLJoj8M2gxA7aBUrw9NMc2v04+4dRUtdp1B82tD
H2kGskvEuHZ+7Udf89zw5DXjJwIry4bZq9OtjWnWijRl0oYo2bcvLbGOc4btG21wgreeHHp1B4ZlOv
HcEcD5QZpuv+l2e2g/ACA/KkBVQeoiel3l/6IOKgDk6dhrrQd1ULMy3z1RkZGQ4dEus05mDGp2d9V
b0Q+rjZ7D7raTTBLW3vV6dtrhjxuYG8OTB6XehihZtW+khNdqguOM176Idth1itOdfT7Y9JDh+ImO
M737fWHkNjEflpGaZOoDb/iAXgIA5EEN6f+7Vz6hlxaDmoWfOqgAkCM1C7/qhYrMEKIupaKs+bN2
yao3Ztuc0XsMPcGXKlEb7K0YEnhNOEmYH/4YM+KbcmhDlFTbN1LCa5XkOBO1L6Id9rE7NVUTnsK
Rmqsb4jfdL5lQPrPmhNaJrmcK9/uiyeB+B0wf/vkPy+nbTuslAEAeVH3Ux65/Wi/NN1X/tPp7r5XnXX
OlXgMAyJaqg/oe6+PVziIyQYi6pIqbF/zehs1VHawYk86MU9n2h393alJygxmjRqg/UVew9mbkJGGV
09aSNq5dWbUhSmbtGy3ZtUpyrpO1b2w7ynXZSmV8eFnK5aas6vZ4Tbfaftab/j/Z9Zz6fs+LOdGV28
6Jws6U7ncgwj2/fI89vB8AkI9Lx/vy+Tc8Kj9+zmW9Zn79Uu02ufXVL9JLAIDMHVsTKdysF5AVQtSlVZ
TNvcFJu6rS6res/8djD/8OK6Rpb2dgoi679ubg/ixDk4RVZHuoDc72vF01d71eitm0IUqG7Rsp4bVKcJ
zJ2qfa0Zfu0IbVpo3SBlM7JWcHjzfsGiW6ntPf7/mw7q/B67F/aPQSTiCV+x0YzZ2x/7qrrtNrAABZ+8H
zn5W/ev1jemk+veatN8nb3nOrXgIAZO7Yq0SOv0kvIEuFviqCCAAAMIF7H75XKn9UkaefXYwhpgBw
FNzx5evllQ9eq5fmx7UvvUr+06ffzDB+AMhL4RqRK9R8BQzjzwM9UQEAwMTefMub5aNv/6heAgD
k4W9u/7Z875pn9NL8qP4udVABIFfH3239jwA1L4SoAABgKu/92fdK7Y6aXgIAZE3VR/3cm75pf5wX
v/Abr5BXvO4legkAkLljbxcppFNUD/EQogIAgKmp3qiqVyoAIB+qJ+re676ll2ZLhae/8Osn9BIAIHPHiiL
H36EXkBdCVAAAkAo10dQt192ilwAAWeve+n37MUuqDqoaxg8AyImqg3rsPXoBeSJEBQAAqVAz9b
d/tc2M/QCQI9UbdZb1Ue/6T6+2g1QAQE6O3SlSeKFeQJ4IUQEAQGpuu/42ueeX79FLAICsqbqon3
/DozOpj/q299wqp/7ZDXoJAJC5428SOXabXkDeCFEBAECq3lF8BzP2A0COvv2if7Jn7M/Tra9+kfxSjV
/kASA3hVtEjv0LvYBZIEQFcteWjUJBCsZjpdHTn1tSvYasblLT1gqO94Z6fFZnX09NrrDhtXGnIqCb6x+E8
lv5aY2mo2frfdepdegkAkLW/v+178o8v/6FeytbzrrlSqr/3Wrnyucf1GgBAtq4WOf5u6+MVziJmghAV
yFVPGiur0tRLjrKcWSvq58vHDiJLNeno5YXRa8h6LarV6loXZDV4saVTK0lhIDAGjqq7f/FuuePld+glAE
DW/ur1j8kPnv+sXsrOXb9zu1z/E8/TSwCAzB1XdVBfrBcwK4SoQK66cuDmbuW6dPt96ff3ZHNZM9
SxQeScUj1nxwW/7fPiHlq1pa5zX7r1srOiuSrkqFgGV11xFTP2A0COfvycy5nXR33zr9wirl3nrTXoJAJC
5YyvW41V6AbNEiArkqco+/qpnDohy9v/dHHF6znbk8Y53QW1XJetivO0uHlWqs5Tae6SomI53PSC
m+wgVQWqAIDsPXb903L/q76jl9L1sp96ofzKzipplwAAmSvcKHL8tF7ArBGiLjGvnqP0CKntOLI2peqN
536d163Or/dp135sb+ivNdbZLwuuD/bKS2MbmtlG7zFcYzN4jMM1S+1HWN1Low3jal3a+zDDt+aq
87WDDQ9pc9i2o9o8tpfjROdl+J4ZuZ8YxxD7fGh+W9RjuK0OZwi9/7p0z506/lKsnrNGj+NAWF6Sk7
ozquwfDt9PNqM9ie65eMduS+H7wj1Pcd9HsNxuv+l2e2g/ACAff/fKJ+SbNz6ll9Kh6p/+
+n/5GeqgAkBurhQ5/mvWR+qgzgtC1CWlwpHQMKhTk1JhQ8ZlcHHYtR8HCkLa61TIMrC+uRoeXk2
1DRU2hfYY7EitNCqEU58zapaW61J3uw52duTiYMi0674ynbqmQ8GiZh/zyGBquM1uz8dQE5+X4X
umuToieEt8DFGcfQcvd0hb7WCw5A2hd6V67jxVafVbXq/SIWaP41E6B9LVT4MqcjrpPZfk2FP6vlDn
KY/3ERwdapKpD7zhA3oJAJC1z688Kk9dfUkvTe/f/Nar7J6oAICcHP9XIoUb9ALmASHqUmqLn8O4d
TmNeo3SlHPhSUpi5XrX3na/FYybwtaPGt482TaCw6mHj7EjO4PplEcFZM7r+3ubsrlVF+erBr/GPI9nZ
FyGWtm2ttd1t2Wptpx9bOvUrr3hB4Vhbe7UZH3kdQm2eXRT0jkv/invSO28cd0SHMPY8xHg7zu8r
dZxrbvBoHEu3Iamcu40u53bEitrHVKUE6f00wiVRPdckmNP7/uimOP7CI6OD//8h+X0bQxHAoA8q
ABVBalpWDn9k/YDAJCTY69zHpgrhKjLyOwlZ/R0K27uOQGJ9dhLZaajqpx1t1M5bS25RqwPHd486T
aKsrlnBj6O4toZL7TrHIT3AyzXt4IBWXFNzugv6uxc9NvY3vV65pXPrI0P38YwexjWLxht3rwgfgZ5PrR3
31CbR5r8vFRbfnDoh3xB0xxDFHPfoW3tXZQd3SEycC4qW/5+zWtniH/u1HFY3yOhIe8k9uUwrEFKk
nsu0bGn+H2R2/sIjpp7fvkee3g/ACB7akj/V376u3ppMqr3qeqFCgDIiV0H9V/rBcwTQtRlZAQ0ImqY
bsGrZTiyhuIkyielpJ8GjFofJo1t2HQ9x9BhzEGnTgwGP0VZ8xMtLyzyA0Mj0J1YVK9WY/8jgrfhNscV/7

yMN90xjFaWk+MudvfAa79X7sF+GEPcRwyfn/zcTeuUjN51gntuimNPcv2HzlNe7yM4ctwZ+6+76jq
9BgCQJTXJ1KT1UVX907t+59XUQQWA3Kg6qHdaH6mDOo8IUZdSUTYvhPckdEOYqMl1FkZg8hyjn
mOk8MDO76nnDnc2AsPq6dg9GafXkeGOgjFCRlOK52UyYccwK2keVxw9ORxbLNWR2T2X2vVfkvc
RZOKW625hxn4AyNFfvf4x+fFzLuul+H6pdpvc+uoX6SUAQOaOrYkUbtYLmDeEqMuquCl7esjtQKlR
W/Pcos+u3ZYNs3edW7PRrMGZhHW+zurzZA+NNoYyV0/nF6FOH/qlfF4mkm1w6dXKHXpMWsd0
AsUTMrb06bje1BPcc+OPPf3vi6P9PoIsvfmWN8tH3/5RvQQAyNIPnv+sHaQm8Zq33iRve8+tegkAkL
ljrxI5/ia9gHlEiLrE1MzaqqeYPcGPHYR0vRqKo4f/GoxhxHPHrB2pgiW3/uMUba64U6Z3duT8+R29n
aqkk6GW5KR37gdnZO/JRbfoZeQQ8BgyOC+
+nI4hTOmkFwKOqumZL+NcNHfF75DZlQPvNJxwzn2EWPdckmPP4PpP/T6Cpfben32v1O6o6SUAQ
Jb+8eU/lL+/7Xt6Kdr1P/E8uet3qF8NALkpvFgP48c8I0RdRu0Ne6itmkU92FPMCHiMXnIlPxkzZmJvy
4Y3Dft884MlY2bySXgTWHWk2dQnKrWh/EXZdLsdqvO87l+XXmPdq22ZZBKkcVI7L578j8Fj1udsrvr
DyPW9bj9yHVtu1oBtyq7eda9xzgsxY/VgjnPPTXjsU1//hO8jZntyvRSYe6o3quqVCgDI3t/c/m157Pqn
9VI4Vf+0+nuvleddc6VeAwDIlqqD+h7r49XOIuYWIeoyqmz7Q287NSm5QYtRH7F6NnzmbhXSDL52
Lpkz9nttNibamUhF3I6BrlSH8o+4LiU/fZQL005glcl5MSQ9BnPYu9ueiRK2YH3O5qqz34IX9Felldqs+v
EUNy94PTLd9njnodqSeM2Jc88lOPY0r3/C9xEgiqqPquqkAgCydeI4Xz7/hkcj66NW/u8idVABIE/H3k
od1AVBiLqk7KG3YUUMVeDS7wcDHlX3cLBmYrWVcx3NpCqy3W/5gZHNOTbvsAPDrOPxhlcr5bpsp
ZzL2dcl5LxWW9Z6d+j1VLI5L6Zkx2C1Z/A+3D80ejUmYNfnNIaSu+y6nznWQ/UUZXNvuFaoPYw+Q
aAb656LfezpXv9E7yNABDVTf/tX28zYDwA5UPVR7/uZx/VS0Cte9xL5hV8/oZcAAJk7Zv2GfPwdegHz
rtBXRewAxKNmNtcT86gwbG/anqHAOEfpntPHcqpFwIpwn+l9Rip/xM0BAHl441/fIKWHXqiXRK596
VXyW3/8z+yPAIAcFK4ROf5+66P/Xoz5Rk9UILaeNNbdmc3LcmaNABVZO1r3XPu8dSwZ9ODG0fGO
4juYsR8AcrL3um/J9655Ri+JVH/3tQSoAJCn4+8mQF0w9EQFxmrLxmANWFXOgK50yMzRu+d6jRU
p7ZyRbiplKXDUFf6fgn4GAMjStU9eKe9s/aS88723MYwfAPJ07O0M419AhKjAWAOBFgEqMsc9h+
WlJiiblx9NaEs42hKOtoSjLeHmqS0nrr1GDr/3pF6aLa5RONoSjraEoy3haAvSQIgKAADmBj/ghqMt4
WhLONoSjraEoy3haEs42hKOtoSjLeHmqS1IhpqoAAAAAAAAABCBEBUAAAAAAAAAIhCiAgAAAAA
AAEAEQlTkSE2WU7DrfxRWGtLTa8fqNWRlo60XHO0NvZ3CijRibwjp6Uljxb0GG9aVTbJuGmH3UNr
7mJbRRv1Y4SYFAAAAAGChEaJirvUaK1Io1aSjl4H5pgJdY1Z9W1nOrBX1cwAAAAAAsIgIUTG/eg1Zrx
GfzqeibO717RkF+/1tqei1szFPbenKgXvLluvStdu0J5tkqAAAAAAALDRCVABIS+9Q9vVTOXVCyE4BA
AAAADgaCFGXmao1OlC7MazG6GD9UXuIvfE1A+VKNbNOpXokq1Vp79Mcxt9cdbYTvjOjjeoxqk7qY
JvCa1VOdrymMbVf2xvetob2H3JN4rTRY359ZEONcxFo46j1xj7t9ea5zKoOadx7KKotg9uYsD5pjOuS
9J4FAAAAAACLgxB1WakgL7TWaEdqpVEhpPpcQUoDQ+ybq4OvVyFiSYIva8pqYbBWZBqcNq0GNh
xyDHYlNtgm65W1UnjQaYt7vIMqcrqqn3Z25OLAa9u7bmODtTKHQjgtuo2TKsrambLztHMgXeeZxR
iOHmh7W9xml8+s5dDDMoV7aKJrPizf6wIAAAAAAOYRIepS6knjnJuIuXUb+9Kt61BNOrIzmPx5qtL
Sr2+5QaEKG8/7ve16jXN+0FVt6VqVXfE2H0Nl2/qabl28L3G3sx1W8dJvU/gxWMe77oZg/mv77gF0
arI+MhUdf7xhKltu2wfPpR9GSvmMeBlqe8MPgsOuSWQbJ1NcO6PbuC+H7qbbu0ZI2ZEDN101hq
mfOpF9hDr9PTTNNTckuC7J7lkAAAAAALBICFGXkjERz96m16vQD9VEOl56FlRt+RP3+EGhqScXd9w
+e1VpeQGStc8LYa+fntmm0GPoXRS3SeX6lj/xUGXLC+U6OxdDexSOP94RimvidfQ0t22ElGaPTrN3av
2CcU02L/htrJ0fGKo+Ja+NftDbO3Si0mrVCRv3dbrau7jjBZKnvROYlRTuoSmuuWkm1wUAAAAAAM
wdQlRYdA3P0OH9SZmzk5+Ukn5qK56QU/ppespyMrCTEN0D77jsIdheXUtjqHdgSHsazOHy/rB4P5
SryllvyvYRvVNtxnbMHqOpKMoJfUHcwLlrXzzrnJ4+aYeVwfWW6mk/kMxMCvdQKtd8VtcFAAAAAA
DMG0LUZRWYKCdunckYgeWRMt3x+r1i3Z6eRig3URhpDK9PScUt3trctVrXE6cj6ik5UdFhZWC9anb
2EeriSf+6AAAAAACA+UKIupTasmH2OnVrPZr1HI+ocr3rlDEYevjD9lNT3JSzbglONXTcqCs6WRiZQYh
dOS164L4ctvUQeLv3pzs5lrF+QUP07K/5sv1xAQAAAACA5UOIuozMupwqYHLrohpDoCdXkpNuEjs
4XNoIEXNVcoamK6NqvWbF6+nZ2ZHz50fVFTXP2eBs/mZ90FMydk6nxNfQ3XdHDnadr3VrtZbsT/jr
h4e0ZyWFeyiVa57idQEAAAAAAAuNEHXJ+QGTMWP/VMxakU1Z3XCn3DFnS8+ZMcmTNFfFa1J7w

6+V6a1MmdfTsyPNpj76oaH8Rdl0u6xar6utN6yz5eg11r0anuYESU7AqVivP+8dkGx4U8nH5V+vZtP
5Wnf2/aIumOquNyfCylYK91Aq1zz5dQEAAAAAAEcTIeoy8oI9S3NVh0rGhDtTMmcun2r75iRC7nY
mCjuDs7o3V3WI5gWO5gzwaXOHxftCh/JXtqXl5XU1KdnnrCAlP6mTC95EVCojdOutWrxzHLe2bVBg
W9Yzb2i6eZ9Y68/k0w3VNv09lNI1T3hdAAAAAADA0USIupQqst1vGQGZUpVWv+8HRvaEQpMqyu
Ze1w/BbGr7g/scx2qn1yBt/9DrDZhIcVP2+oNtstj1YDOoh2rwhvQr1v62Ruysst0PrUtbbVnr3ZILLnU
8g6+ttiara2v22gwMTTeGs+c+ZD2Feyila57ougAAAAAgCOp0FczrADITq8hK3oiL1WDdo+eiwAwk
urtPS8/mtCWcLQlHG0JR1vC0ZZwtCUcbQlHW8LRlnC0BWmgJyqQKbOOZ75D4gEAAAAAAJAOQlQ
gE23ZKAzU8ayeFTqhAgAAAAAALB5CVCAPql5pZpNXAQAAAAAAIEvURAUAAHODelXhaEs42hKOto
SjLeFoSzjaEo62hKMt4WhLuHlqC5KhJyoAAAAAAAAARCBEBQAAAAAAAIAIDOcHAAAAAAAAAgAj0R
EV2eg1Z2WjrhVnrSWNFzZavHhsyL60CAAAAAAADA/CNERSZ6jRUplGrS0csAAAAAAAADAoiJERfp6D
VmvEZ8CAAAAAADgaCBEBQAAAAAAAIAIhKhLqr3h1gddkUZPD7+3l51HaClTVePUeI3zcL7eZW/XH
MbfXHVepzc4uF+PuW1j58HXt2XDfY318F4Wo12TGjwv6jHpuVGSHr8S1obCSkPCD8+s/eo8VkaciG
TbBQAAAAAAWF6EqEuvI7VSQUoDw+
+bqwMhX3tjRI1T9fXpBJbR1H5WpamXpFyXrYr1MbN2OWHk4HlRmquFYNCZ4blRoWtYG6RTk9Lg
BFl2EFuSwZd3aqWhcDTRdgEAAAAAAJYcISosVWn1+9K3Hq2qXqUCwPNulNaTxjkdX5br0tWv7dbL
zjrrtTsXnYiusm19rlsX9zNSbdmv7W+rxHNafjv7e5tSTNCupHqNdS+MLNe7zj77LasFWvOcDkeza4NI
W3bd1Dh020055yW0VjvW3SDXOE/uBe3UZN17bZLtAgAAAAAAgBAVUm1tixtxVraMANRTlM09H
crZ4aVeu3bGe23noKufZadc3/La6ciqXT25uOP20qzK2U13yxXZsoNGFVLuibM6w3PTO5R9/VQ6O+
JmscXNPWd/1mPPbVvvorhNDpynypa42Whn56J1ZJYk2wUAAAAAAAAhKiaha5OGDmHPzqkT44K
9tNrVlQN3A+WTUtJPFSdo9EPnYSmem+KanHGTWGtrquyCW7t0qM5p98Dbnz18X7+uYA7v7xxYR
2ZJsl0AAAAAAAAQoqIsJ82UcJTAxElGbdLcjGjnzNtlyawNRdm8ENYz2A9KQye5Giur7QIAAAAAABx
NhKiIoS0bZs9Kt46mWft0JuahXRm3obgpe3qIvV+v1tc8Nzybvl/DdfBh9KCdYLsAgLzp0Q3Ggz9yAQ
AAALNBiIrx2rte70o7oHNrfxpDyKc2ybYya1dJTroJqDsE3qVm4i8YM+6n1YaI16uZ9NUvzfakXXbw2f
XqnHrtK530Qtu4NVhjbRcAlpH9Xl+QwkwTy540VlZFWu57tPNHr+bqLILU+Qxze40Vpz0r+f/hT/0ba
p4P9zEP5wXDhq7XDO6ZmRvzvuZ9P7mPvG7mOO+3ebwnR+5jSf+gFfO8z/K9eGZinJuh951luGnG
nJfc34sTtofSdoiDEBWJ+AGdMSt9AiU/nTRm/7d+MFmdbhD8tO0KKsqaVzS0Kavem667baeO6OB7
cZw2JDp+/aavPhXsGRpSs9Wsc9pc9dvm/sOhHu7KJNtVjG3wDwuALMzsl/chKri09j/lv0npcCYu3Da
KcLuTP+4f5vte3N4IC3ONPyjOQq8h6zWRatX9x28G3NEnxsO8XrMy01/cAyWOhh+z+NZW52O1qS
YFda9TS6qdmpRmFPjk/0v7+Pc19R5csr6f6l3jHFk/T2Z778R5v83jPXncPtTnc/yDVmTok1eYm+C85/l
eHHFuht739CP98xP/vl3dH/g3Iot/IEadk9zfi8efl3zfiydoj/WNrUrbpf+eHO/7dn5+FsZY1g2DJWS9R/
TV5bd+A+hbPzD5uvW+9U+g87mq9ZZia/WtnxX060c8vNcqIa93P29uf9TD2NbIdtqStKvbr5fd9VXr
K8cxXx/yKNetVygJz83Exx/
+MDcdve3gMSfabqvqrS8PXwQAmEq3XrbeX8z3eP2+GngjyoffFv1vwAzaEEm/z8/8vXjm7XCuj9q/f
c28f5PzY/87OoP9RtP37dy1y/3eivPzV9rCv5dn1R7n5y9jv/pnrCy/l8a/rznvuYNtyPocjW9XvNdMa6J
9ZPIeqPdvbdd+hLRD3T/maud+Um3XK1IS/5w4n8/+vTjeucnjvS/
+fZv1+8v4cxImq7aNPy/h62fWnhHfw+nfR87+zd0737fBdX579Qr9vhz3uiJf9ERFDBXZVn8p0ksO56
821puAo7kr/t9KrNd7n9D2D52/MKlanIP1QqutCWuIJm1XEk4PIOsNTS/7rDdbf9h+0jYkPH57qP3g
ubQ5+wj8QdOuc2oMyXfZPWWMeqiWRNsFgMy05XytY71NXZBN503VUpEt9UY28fv35Iqbe9Lv7x
ltmTO69MupE/PRwFm1o9dYl1qnKmfn9kLNhnteWt7PKPPC+T6X6unAzyL5KMqJU9YH9+dQW08u
7sygPb2GqIFK5fqWv9/Ktv3zYmfnYgY9sRxj39d6h7JvfRj8fi7aJ64puxm9Ecd5v83jPXle3ved79+y1Ls
hP8tr6uf34dEJHdm5mO7dE/ec5PVeHOfc5GX8uXHeXwLf5xmY7Jxk9148/rzk+148tj0jfp6yR42mW
tYuzqii+fpZGOMRoi4pvw7mwJuLMeFQcMiBCgvdr1EPJ5TztxMM6dQPhf5rrYf5A725D/VQ+xmx35
Ht9MRtl/MGFlw3nvMG7H6d89gbakjCc5Pg+G2D59J+jDoG8zj1Y9QvU3G3a7xu+NgBYAoz+uV9M
enSL+W6bIX/A5CTnjTWa9KZVTvsoaMdqbbi/1ueGTUM0Rh6N9uSN/n84j6JXuOc9d1s/bI/oxu3sq2

HjNo17dWwypITNg/
+vJW13H5pT8huV1lOenWcNKPePgZk8AetuMFlmDTbEVuO78XTnJv86fJoB+cHhtOnWwJnknPCe7
FhxPub8/PnvuRaNYmfhRcOISoAAJgNfnmPTdUltX/5uTCLXoa6tpj9i6D+pWdGvR3b52vSqbZmPmL
C/0Op81AjV7KppRZXPr+4J+f2NDo7wwDEHTWkatqr7yOrObMI4efpl3ZMgT9oKfPyXhwwR3/Y6uy
flAvevxGqt6h6/9mYYa9C3osD9HwiwflBrO+pqedWiWHwjzD8LLxwCFEBAADmmDP5gRq2N6ueO
MFRDt36vqzO4hfU9oYzCcRc/dbuUL2Csh6WHcfc/eLePi/O6NHZXTNnsg5/YiAVeKtJgXKftGOWv7Q
jNfxByzKH78Xz9oet8pk147pY/4aeVWXUZtirkPfiAernGrdnrP89dXBKXadTkl3n7nn5IwymQYgKAA
Awp5wAVfXWmJ+hjMXNC3YNtk7tfK7hXHtXhU1NO8B1fuEpSEn9Vqh/CZrtcHptlsOyLfP1i7sOCGf5
y6Iecqzq2bt5jz0MViXezdWMZjYfZVa/tI9h93bqyMHgjTuqd9QS4w9ajkV4L56XP2x5ZtqrkPficIMl+f
qydXLfOk8nJau3vdn+EQZpIUQFAACzwS/vkVSvDSdAndMJ/zL8RSPMYE8j9bAngLR+Mexaz2dbt7sn
h6qoWc7nZKxZ/uLu9nw6O8NfFgeHTbpmdl7y/6V9rOIJceZ7CUZNPfuGnmG4O2f4g5Zvvt+LB+T+h
62S2GWOB8PbWf5cw3txTLq2eOCPkekZ+UcYfhZeOISoAABgNvjlPYKeRddiD3czevzkW+dSTf4wM
Bxc/0KW1S8ac6/XkJUVc0i2WqVmSp7lL6nz94u701utKjMcPSpSOW21wPoeCgyhV7dwzTovM26bL
dtf2uOpyGnVY6+27r+veL3G5m+islngD1qLaFZ/2NIjADo1Oe/9w6mHcM+oHinvxXGoUhklqUldLm
RwkSL/CMPPwounDwAAMCOtqvSt33L69a5e0a33y9aPJ2VvRX669bLVFtWewUe139KvWUZh56U
6JyfEblu53s/3bun26+Xg+ZiLe6RVHbg2rb71S2tfJT+5m+H38TB9HgKPefie1vdRxvdv3Pc1573Yf2R9
7eK0K4/35PH7CLt/3Ifxb1dq9H0x9H2r2jFw3Pp7Pu1rNcl5t78m8/fiEedGvd8M7Ns9hrTf/uKem6H
XZfY+POp+0XJ6L453XvJ7Lx7fnpB/xzO6RnHuxXn6WRjjFdT/rIsKAAAwE+5f6F3WD43zNRwQWBCq
x5xdm9BVbUl/Bl3nnO/pqrT6M5p5eS7pnk7G5ZnV9cH8Gfre9fjfR2Gvqc5r79gUjT83Id9bxnk7iuLc
LwrvxbOmRvOoOqhhgkP7+Vl4cRCiAgAAAAAAAEAEaqICAAAAAAAAQARCVAAAAAAAAACIQIgKA
AAAAAAAABEIUQEAAAAAAAAgAiEqAAAAAAAAAEQgRAUAAAAAAACACISoAAAAAAAAABCBEBU
AAAAAAAAAIhCiAgAAAAAAAEAEQlQAAAAAAAAAiECICgAAAAAAAARCFEBAAAAAAAAIAIhKgAA
AAAAAABEIEQFAAAAAAAAgAiEqAAAAAAAAAAQgRAVAAAAAAAAACIQogIAAAAAAABABEJUAAA
AAAAAIhAiAoAAAAAAAAAEQhRAQAAAAAAACACISoAAAAAAAAARCBEBQAAAAAAAIAIhKgAAA
AAAAAAEIEQFQAAAAAAAAAiEKICAAAAAAAAQARCVAAAAAAAAACIQIgKAAAAAAAAABEIUQEAA
AAAAAAgAiEqAAAAAAAAAEQgRAUAAAAAAACACISoAAAAAAAAABCBEBUAAADAQmtvFKRQ0I+N
tl5raG94n19p9PTK2fDbuiEhLV0wbdlwz3vk+e1JY2Xca6xXNVb8ba00rK8CAGB+EKICAAAAODqa52
TGOemSUMHoqjT1kqMsZ9aK+rmpKJsX6tZnHZ3aesg1asv5Wkc/F6me3bS+CgCA+UGICgAAAOAI6
Ujt/OL38Zx/XTlwM89yXbr9vvT7e7I5KvksbsrZqn4eco16jXN+IFttyXZFPwcAYE4QogIAAAA4WuiNm
r3eoezrp3LqRKxeo5Xtlng5auAamb1Qq9IiQQUAzCFCVAAAAABHTLzeqH590pVg6NpryIpbm9Orse
rX/7Rrehp1Vr119suC68NKtPqCtUJH1wEdeJ31CKsrGjyeYL3S6HYYzGPXj8F92fsp1ayzrDVXndeO3Ul
FtltujOpfo/aGXxagXN+yXjUo3vHbQto/dH0tcc5VoEar+6BWKwAsLUJUAAAAAEdEWep1HdJl2Bu1U
ytJYTVYDdRep0K2gfXN1VEBZlNWCyUxyoCqjUhpcMIpOxQceJ3F3t/IQK8jtZJRr7Rcl60YnTuHwlEtel
8JVbak7hZHba7KRqMh5/wEVS4M1gNIcvwqwA5pv3M+hoNUR/i5UueiNLhTJewaAQCWAiEqAAA
AgKNjzQ3p4vVGnVS53pW+qgPq9ax0hK1v7o5oh1dLtC/
+y5tyzkv7etJYd0PBqrT0a71td2qyPjIpNl6/F2OSpvaGePmv0a6um3ga+6psW5/r+hNFqRqm9n5iDc
MPTjLVrLnHV5b6hcF2Jjl+67VuGhvWfmsrOxfjnqu27Eadi8A1AgAsC0JUAAAAAEdIUTbdGYwy641al
bNuj8nKaWvJNWL9/mFoL05zBnqzXmhn56Lz+t5F2dGdIQPD3I3enN5rB4QPix+t7aeGgTCzuHnB31
ftfDo9MAOTTGnVs8OTUiU6fuu675lBqKO4dsYLbDsHXf0saOhcmfVeOzviZq/FzT1n+9Zjb+QMWgC
Ao4oQFQAAAMDR4oVsGfVGLZ+Ukn4aMGp9qKqcDiR3JTnpp31ix33dA90L01rllguwH8bwdve1A0
6dSBLymT0vz8ha4EuLsnbGbdi+HIYlthMITDJlPQudTGqK41fHZNc5DR3eHzR0ropr4h2yuodK7n4ja
rECAI48QlQAAAAAR8xAb9RD5+l8K8qJU/rp1MpyMn6am0BHRnTmnE6i8DlCYFIpo85ppLBzFSw5Y
HLD3NgTdQEAjgxCVAAAAABHj9kbtRYvTsvXYK/Onhx6Y8iHebVWhx7biYbtTyercHa88cfflg2z16lby9
Ss3ZpEcVP29D4Gyt7amueYpR8AIg0hKgAAAIAjyOiNmpQxjDw7g706u3Lg7tTtmVk66QWAo+p5ps

MsJeDXAHX05KJbmFROSaIqAdNKcvztXa/nqR24unVRp7iWaoZ+1ePUnkjLDlS7Xi3W0WUEAABHF
SEqAAAAgKPJmIAoTMlPDo3aqW3Z8Kapz1ZzdcObqKm94Q8/L59ZcwJAszZnc9UfQt7e8Gp0FlIZV2
4Gzta5WPd7WfYa61790aSTVU1twuP3A1djxv4k9PbVbRDscRoSdAMAlgYhKgAAAIAjKro3qjlzuwrp
nGAubi3NaZWlXG7Kqg4D/dzWmOFftd+ozdlc1cGh9+IREzJNorLtD1vv1KSk21XyE1S5kPuM9AmOv
3Lan6jKu5bGBFRJjDgX5r1RPat7ugIAlgYhKgAAAICjK6o3qqp7OVgzs9qavI5mIqfk7J4xPFyx63gO1Di
1a3MOvE4Je+2U7GHrIcdebVnr3eHxeYt9/BXZ7psz/itVaZk1TZu7Xs/fcexzEVYMVW8zrewaALA4Cn
1V3AUAAAAAAAAAEIqeqAAAAAAAAAAQgRAVAAAAAAAAACIQogIAAAAAAABABEJUAAAAAAAA
AIhAiAoAAAAAAAAAEQhRAQAAAAAAACACISoAAAAAAAAARCBEBQAAAAAAAIAIhKgAAAAAAAA
AEIEQFQAAAAAAAABGEvn/AZM6nr53azj8AAAAAElFTkSuQmCC]]></redcap:Attachment>
</ItemDef>
<ItemDef OID="q6" Name="q6" DataType="text" Length="1" redcap:Variable="q6" redcap:FieldType="radio" redcap:RequiredField="y" redcap:BranchingLogic="[q4] = '1'">
<Question><TranslatedText>Do you prefer Life A or Life B, or do you think Life A and Life B are the same as one another?
</TranslatedText><redcap:FormattedTranslatedText>Do you prefer Life A or Life B, or do you think Life A and Life B are the same as one another?
 </redcap:FormattedTranslatedText></Question>
<CodeListRef CodeListOID="q6.choices"/>
</ItemDef>
<ItemDef OID="summary6" Name="summary6" DataType="text" Length="999" redcap:Variable="summary6" redcap:FieldType="descriptive" redcap:SectionHeader=" " redcap:BranchingLogic="[q5] = '2'">
<Question><TranslatedText>
Alzheimer's disease - Description #[no]

If you have Alzheimer's disease...

1) You might forget recent events of importance to you. For example, if you are interested in hockey, then you might forget whether your favorite team won their last game, although you may remember watching the game itself.

2) You might forget the names of people you met recently, or the names of local streets.

3) You may no longer be able to handle your personal finances or plan leisure activities such as dinner parties or vacations. Simple decisions such as what to order in restaurants might become difficult to make. Your ability to concentrate may decrease and you might have difficulty driving.

4) You may start having trouble finding words to express your thoughts. You might need to be reminded to shower or brush your teeth, but you could do these activities on your own. You

may need to post notes around the home to remind you of simple things like turning off the stove.

5) You may remain aware of the day and your surroundings. You might be able to travel to familiar locations like the supermarket, although probably with the help of a loved one or friend. If you are in a social gathering, then you might have difficulty following the conversation.

6) You may continue to enjoy hobbies such as puzzles or reading.

Now consider two possible lives. In Life A, you will live in perfect health for 18 years and then you will die of natural causes. In Life B, you will live in perfect health for the next 10 years, then you will live with Alzheimer's disease as described in the summary for another 10 years, and then you will die of natural causes.

</TranslatedText><redcap:FormattedTranslatedText>
Alzheimer's disease - Description #[no]

<p></p>

If you have Alzheimer's disease...
<p></p>
1) You might forget recent events of importance to you. For example, if you are interested in hockey, then you might forget whether your favorite team won their last game, although you may remember watching the game itself.
<p></p>
2) You might forget the names of people you met recently, or the names of local streets.
<p></p>
3) You may no longer be able to handle your personal finances or plan leisure activities such as dinner parties or vacations. Simple decisions such as what to order in restaurants might become difficult to make. Your ability to concentrate may decrease and you might have difficulty driving.
<p></p>
4) You may start having trouble finding words to express your thoughts. You might need to be reminded to shower or brush your teeth, but you could do these activities on your own. You may need to post notes around the home to remind you of simple things like turning off the stove.
<p></p>
5) You may remain aware of the day and your surroundings. You might be able to travel to familiar locations like the supermarket, although probably with the help of a loved one or friend. If you are in a social gathering, then you might have difficulty following the conversation.
<p></p>

6) You may continue to enjoy hobbies such as puzzles or reading.
<p></p>
<p></p>
<p></p>
<p></p>
Now consider two possible lives. In Life A, you will live in perfect health for 18 years and then you will die of natural causes. In Life B, you will live in perfect health for the next 10 years, then you will live with Alzheimer's disease as described in the summary for another 10 years, and then you will die of natural causes.
<p></p>
</redcap:FormattedTranslatedText></Question>
</ItemDef>
<ItemDef OID="a7" Name="a7" DataType="text" Length="999" redcap:Variable="a7" redcap:FieldType="descriptive" redcap:BranchingLogic="[q5] = '2'" redcap:InlineImage="1">
<Question><TranslatedText></TranslatedText></Question>
<redcap:Attachment DocName="18.png" MimeType="image/png"><![CDATA[iVBORw0KGgoAAAANSUhEUgAABVEAAADYCAIAAABtBTQzAAAAAXNSR0IArs4c6QAAAAR
nQU1BAACxjwv8YQUAAAAJcEhZcwAAEnQAABJ0Ad5mH3gAACrHSURBVHhe7d1vqGTVme9xTSe
Z9iaDTW4Qk1zGTi4BUZFOfNM5hLEDIUpAEBxJ05ghw4TDERKmEZKWEIf7QlEk0EcR9EJQYXJv+8Ig
Qq5KG2hCAt0Q8LzwhYJpzgtDGtoXxybIkfSL3P1nrbXXftafvWvV2ruqdn0//Jg5tWvv9ay1q9paT2ud
XPN3AAAAAAAwRfT8AICRnNs9d83/uoYQQggheVN8yL574cK/f/V/FnnpsUfrj12gRs8PABjD7t7uoS
cOiT0KIYQQQubPh5cvP7P7Rxt+Z5MOXxc7zuXC3CHQohhBBCsuTJB06Yhr9I0f9/
ePmy+gzG2qPnBwAM7u5f3S12J4Qe+jSqq/f9zm74a/z5AMn1Gcw1h49PwBgWCffOCl2J4QQgjJkq/f9zm74a/z5AMn1Gcw1h49PwBgWCffOCl2J4QQQgj
Jki88eJ3o9k1effop9UmM9UbPDwAY0Jm3z4jdCSGEEEKy5LpTB07cfli0+nbevXBBfR5jjdHzAwCGcv
798wcfPSg2KIQQQgjJku9+84uiyRfhi/0o0PMDAAaxu7d74y9uFLsTQgghhGSJ92v8bk7/+7+pD2asK
3p+AEB+
+1f3jzx3ROxOCCGEEJIl/2Pzv4nePpKzLzyvPp6xjvj6Auj5AQD5HX/5uNidEIIISRLPvuTT37/1i+Lxj6Srdtuu
bizoz6hsX7o+QEAmT3+
+8fF7oQQQQgghWXLgkWvv+caXRFffmVPH7P+vzoyhX1XdeEbsTQgghhOTK0Xs+L/r5
nnnmwS31UY01Q88PAMhm59lOv6ifEEIIGSg3/fAzop/fAzopOfKef+7/9RH9hhYJ/T8AIA89vb3Dm8fTQg
ghhGTJrF/jd7N12y1/ufgn9bn9bGNtUHPDwDIYP/ybj1rDG+uBnh8BnhNtUHPDwDIYP/q/rEXj4ndCSGEEEKyJO1r/G5+ftu4DhB
8AkMHWb7bE7oQQQQgghuZL8NX43z5/6qfrwxnqg5wcA9Ov0+dNia0IIIYQQQggghgkuS2v2wKd2nl5kGWtmS9Ak3pdKL+nc7vqrzi0kvvttr2f+a0Pt5v2ad5UrWz88SxGa2YaVSwXo2EGa98GLcU7UjYX/nUb7LRnAtJI5bzw8uHKEbCVEc7cH8xsRoS+dmNEjGWyj3rFlPlGjSAjH63ENP5nSU7u62cMbgPaNdXX5/J5i95w0SF8Wa40Syl9dEZ6M7+y3jVF5IpR1UyNHHAHVDJ8evVDuhN1fDuQ8ouD1zGkuo3V10sm0lfH3zZKAg2YIlhbGgvzn8oK6oLrr0QqPb0+eb2nmsTCKWWg9JKGDM0TnNjpSe5obN+5EXjEvFjyCZhWHJiBPaSVGKbPR3mkhW3a92PYqwUnrWzIQlyPFj/vmxBPI5QdMyBwrMC5vH96TsAe9Xy2IzhdFOhdP43lpUWeMqrhP3CQCaHR1gHuAJZh6ExXoz+YWtg5H9krkrGLDy5KJcjW+BV3KQaj2tvH+XmLJPdHRfgcdUoB7jzmvPVc7WEZdXWJYDgwMdGatTMV7ldlwj3eq5hPXYUyBnlAgZT/hFPJbhvmF6m5Tqvh6xb8q6IGCwXtLBfaanLbKRBPPgwKyEdBK6OsxSsn2EgX9DFbCWVnDMd96njNBQTowKbDWkwfB9MrDi7SEfa0pmCNSGrTsgIZXsZjnBifzpSCxLQjTe55T0zLrYMFTT/QW+NaIGhY8ibYWSZLbOhGrgmD0RdpW6Qi9xNZF6RcXx9DGcJpuz82mNh24Q+LmbcgFKelgqfYhyt1n3lzHtdkldaSdr0HVoBmGFHDGw6+LIt3pumVYrCftKemohYhUcjZPuWRfDvs2kUWBd85lPbZzrCLLuBKOC1UF4LVZJ2r/RRR60M9vQTFf7xRBgI1X/3r68cHw7EJ2VYgtgTBdkH81TKwX3QaGsk1xqqxVz3gXNMGbZEz0IKS5rvk8d4cFgsGZq8tvyS+VBwGvlsBKzdJ3GbF9x3H/9Ob2mVfEl03hYxhWeI6M/f0CIUvxRsgFnd9fpbDBTWDOpu64YXIi8ZnN41o8Kfqv6VP3rRZH8pBgl/mX0dT8C/lp2vjYA+yXcvB68lAL0ZBGJ6qL6nZBqTMtQDw4G+bojqpmzvCgw5q00wsttiemAYVn1t5PXn2eKNn4CTR/ox8YwN1fqJXDqOrShh6q/L8ttLHH+4I9P0VPoFRnpEFXyu9Hk9Yi2VnbAkYJHh+XY10Zth8K5dogxRg4ZRB/ps+DvH+jhZlUYQWlkYBSG/1mbeG8zKxHQo4EIZNXsvz9KSDbHuZALJNw4IgJ/3XLWnsl/pzpTrC/BZHsm2Wr7/yuqiJG4BP41+xjqsqvn29ZMTeEQmM1QumfqeJfkMaPthR1SGCdp6ncMF9vnU3xzz+TTObGXj+0PqjqjQ4E5apftoKM/lFDz+vHMHSl8DoMwXi6VVfMvUtp/6OT4udqUIQNc6K53H3X4v98xu0p6OUwtzhmHt3IKRrJswQSgHHnGr+x4GJeEjn0sh1xB7ppuZLg/ZTS3Sap4dS4Bz/yeZLF+pZv6kWSMImqZCzE5VeVkKQPs+MpB+MegejU6Myg34+kMO3qrWYbUuSIu6uwjmyKLxpIT8HC5mOcTAmjX8YbGwhWCu4N7DmoA3ATeHLjRwtb0AwfKiFwlH8rNBKUR6TOXBndFqmrI4dzTB+gh4Svp/8MBItSJL+ceNkCrz+9n2lpjXLiEX6kB5UKY2bqvnqKd4fUfL0dI/kvy/KvYjCPNlxX/7lcyGU4aoHV4w9I3ESX5PGEHgn+N4i9eKHnhOTk9eB4SLjU7zABMkqOkW2b6OrdDbUbHHbS4XF2fn81lBbaNpeBbuvBsp8w6CKG0b9fwYw7/cxQTWk0PJqYbDSoXLaMb+ZXXqzOmp/Xq3bKKnfXDnl6NZwXwdzt2Br9Z9e41mDb/r1Ow16Asfi8nGLHFwUy37TlMWc/cgEbg8EWmH93UA3jEl2tkwOuyJkbX1nVnGuxHWmy6k1eWAwsGp/c00/oz2/ZGdwRZDqZ+hxR5/TQIzdG+1l/pp82zdkvKZcrjJpRFiaUpVTL4rjSPZXmzz3lAbZjkuKsHSfG2EV0UXsnK3HutSOsHw7gdeNU4rpNLBY3JAlsyyh9kqpugsp5ubNhzhXl61Q/d4rwTYmemeKoTLvW2Fy6hngvbOLc9SjBPjRFgrNEEWqetgmXXVHfdeMJ+Qw8HzxaDnmd2MBlMG4IwgmYqKDwYXJ2Gq7/8xSeq30FqpKj2nqKZ8u/UlIsNsMZ6Ln8U05SUzDbt+IhWp1PgVJcIehSS11mNK/Jwxv8PRz/xW08DO/5++yIDrKHoQK9P6fHXTvxuQN6qDh8Nl2rIthvmxe2ClK32oaolg+lFFn1tDkvHHPxa8tjEzlvZY1Nx9MfSdmv4cdOZOlsjIOqRJaR/QtCVSGiSR+sGtSUQ4BpmqV63vkNH4lF7SBXnP4ayCrpSm/4D5CmjiDxDnn8XvIgqMWt01B7VKvR+JcLT9phPXc+RXeQJa5JjrnWyhHr8CUOt2+vQ8M9W02tHBCTMJxsiaCfOhTfFXzjH+XNrfcRO1AIjGHdiPlv8B1jrxYNRPwZB3EjLLKfR1

gTnguAIzoFCj5lgWZx///zBRw+K3QkhhBBCcmXQr/G7efXpp9RnPKaInn/xrFan1QLVZHtVkg2T5xxfT
9WmzqivqR9sbpYD2Uf09WK4+MOQjqv0Yz2IfqxWJR6WPIccsoq5281c5Skenquay6ryPaZnBpHzdS
fQPZp7TX3IN8HwugrdN0RULnkOtcjSzlScA4W0lwbj293bvfEXN4qtCSGEEEJyZYSv8bt598IF9UmPy
aHnXwa+jlLRT7WbK9FyeU7q0Sypq6oTqp83tl+rrmqOmMvFcPGHIV1X6QPqsZqBXpRepE+krqxih
mmu6TF9fVXrZWhd12N6ntI1dwLdo3ln1NZjXQXPQOLCHkuztM9WzztT8c2teyZYAvtX9488d0RsT
QghhBCSK//9P/5hhK/xu3lo4+iHly+rz3tMCz3/Moj0b/6nAk3ZjM2Suqw4oz67+KE+ZI6Yq8Vw8Ych
nVfpI9WBZnY1vUifSF1ZJe1e6ataZ7Su6zE9T+maO4Hu0YKDNXqsq+AZSFzYY2nNJYJ63pmKb27dM
8ESOP7ycbE1IYQQQkiufPpnn/jekZtENz5annzghPq8x7TQ8y+DSP/mf0q3QupoarNUX6f/9X51dXVI
H2kGFMPFH4Z0X6UPFUfUmprn/Heik6ySdq+8xVvX9Zhe8BR3At2j9ajXY10Fz0Diwv6TKdQnydLOV
Hxz654JFu3x3z8utiaEEEIIyZhvf+tG0YePHL7YP0n0/Msg0lSZpsd+TnZCnutNFxZrltQ47tf46yPWeKJi/
GFIn6vqeZtfZ2891bOIJC9Layx9t7N92QyD2C9lxb22ezTfGWWB2dZV6L4hneO4Q8j75QwRmn5p5i
VgHK+884rYlxBCCCEkY249fr3owMfP1m23XNzZUZ/9mAp6/mXgaXYapn3Sz5o+yJxujujWyFzT0Sw
1F/ouDfde8Ychva6y6otnzPn6sDk1VlZW0RfN1lh67oqs3j09T+maZwLdozlnhA80w3p4ZiUv7JqMnF
tzvnNEPfbOrcdMsDA7l3b4Rf2EEELIcLnhRwcX8jV+N6eO3fnRlStqB4BJoOdfBp5mx2Zaqhb7ZKvJku
LNUnOhGc5U8xaoh4s/DOl3VbNa53aYK1pmKprYWPpfgpJvnJZmUE/pmncC3Yv1n2EN3551YHW9
bkjHZMK3R53ijuiZW6+ZYBH29vcObx8WWxNCCCGE5Mpiv8bv5pkHt9QmAJNAz78MPM2O1G66
PB2QfULxdN9myT3Pe6U4GH8Y0vOq+N0wV1Uid0yRVdIaS/sq/XPBvSA2veDCghPoXmz7DOcUa67
eywu9b0h8Mnal8jL9uB7CN6Izt94zwaj2r+4fe/GY2JoQQgghJGMW/jV+N2dfeF5tBbD66PmBbp52
FFgPW7/ZEvsSQgghhGTMMnyN3w1f7J8Sen6gGz0/1tPp86fFvoQQQgghGbM8X+N38/O7vvO3jz9
WewKsMnp+oBs9P9bQud1zYl9CCCGEkIy57tSBpfoav5vnT/1UbQuwyuj5gW70/Fg3u3u7h544JLY
mhBBCCMmY737zi6LHXsL84dcvq80BVhY9PwCgZW9/7+Znbhb7EkIIIYRkzNfv+5zorpczW7fd8peLf
1JbBKwmen4AQMvdv7pb7EsIIYQQkjFfePA60Vovc/hi/6qj5wcANE6+cVLsSwghhBCSMdedOnDi9s
Oir17y/Nd/PqI2ClhB9PwAAOXM22fEvoQQQgghebMSX+N389abZ9V2AauGnn+x9O+GqwzyC+Le
297YfE39PI3fRddeUURxYmCtrdvOL+brp/d7Z3pvubVx/v3zBx89KPYlhBBCCMmYVfkav5sf3/G1D/7
8vto0YKXQ8y+Q6km1/E2RKjChBsxdUZC5u2Kt4q4r/f4WYa31eu9M7y23Pnb3dm/8xY1iX0IIIYSQjF
mtr/G7eez+
+/hi/yqi51+ggbsh09xOpgHzrcjPbuzba9V3QI1hTqQl7dLjvTO9t9za2L+6f+S5I2JfQgghhJCMWcWv8
bt56bFH1e4Bq4Oef3H6d7BppteA9btj5iyltVZ3CHrSnuj5p+z4y8fFvoQQQgghGXPgkWvv+caXRP+8
onn7d79TGwisCHr+xdCNkMXuY9ttq+iWrCaqGUZ0waHx7QbMOsftx1oz6GzX7GHtK8WsYsM2z5iL
zATFbDVn9EL7zlX8ZZwqvtHaK7N4DoeXVpMza51ijdcstJ5R9LqAvrVaJzo3QDwZuBVaM2+jGtG+zDr
HHaU1afF07zkjxeO/f1zsSwghhBCSN0fv+bzonFc3D20c/fDyZbWNwCqYrec3u+3wVtt7SqsdCLQMv
TVb/hXe8IcaJP9TBeuueU5w7mlofP/gJWuIVvOlRV+2LMOa5+tj4mFoRYK+qnhOX9GeuRm2vlo8dH
lHkQfNKDb7Cv8Nas7wPF896b8uONlKQi2le8Yl+yyL54Jqmr0G6rqBM88Gvb3yzitiU0IIIYSQvLnph5
8RbfOq58kHTqidBFbBCD2/2M7Pu0e3h4u2PkvPezdNc6PvkznL3Dir/4neAN/47rXO+M5l+prIK+cO
2xyRdSLD2uf4qvpWJJSniCk48zbDGOHxmrOtYcTIzrRkZTmEucCMqa8o2HMJDdRc6Eip1RzRxc1V+k
hzIFK7OctahFvOmZJzmVx3rzkjwc6lHX5RPyGEEDJoPvuTT37/1i+LnnkCefXpp9R+Aksve8/vcvbvc7F
6j0KeMRck1iDZC5PnmW6nY/Wx8a1j4jT90Brcc0jINqwZSGmXFGN28N3LiixSiA3ozFMM3HNpNmc
hZkqtK8xpwYF6CNdqFi3Pca4pmGORufiu6y6nH0ZuYI85Y2Z7+3uHtw+LfQkhhBBCMmZKX+N38+6
FC2pXgeU2fM+fd2tueoGNdk+wijx3RnSTill0fdTT/3j57ryngDjNjO4RLNjdkvUe1j5Rvrq+FYV51lqQR
/1ntQTuv56EPWPJnWj7bGeM9gVmwY3IPB29a8kbq09pFfMebPO9QJ7LxGntabaJCUXmjNnsX90/
9uIxsS8hhBBCSN5M6Wv8bk4du1NtLLDchv1v+z2bec+evdSzk9HXbL6mf5qlBVoynrvpb6vEif6TXJ7
xfdcGRvexBmrLOWxzprNA34rCPJNqDjYj9BhUn1KN5IzQZ2mmiKCf9861FLgwPNekWvIeeKcTnGN
DjlPqLqfP8AlPyFcL/W39ZktsSgghhBCSN9P7Gr/I2ReeVxsLLLcF9fzmLFukl1D0ZeUg+ufuq5aV52762
yqxVP9JLs/4PXqnvqO35BvWXFYRZ/tWFOYr2fOeONQ5xSl6hGaA7uv1GYX6JGcanWNYQ1T6nNi/lj

zHO53OOfa+veK0HgP3mDNmcPr8abEpIYQQQkjeTPVr/CbPPLilNhZYesP/Dj/PNc6hHpv+kr6uvkw/
6rhoecXujL0oeV7Pu+Ubv0fv5LuqU65h9Vkb29u+Rc42N9998ozQb1A12MbmZn22dXLnAO5E9BFz
iW+uSvHUDMXSaslRfVXMMc8cNd913eV8Vwk95owZ8L/GTwghhAyd737zi6JJnlJOHbvzoytX1MYC
S28RPb8+4tm/R9oJ96ReFy0x984UTIcmV9ks09P/ePnGn6Xf0wfMlOx5tuUZtnWJeRAZM857n3pM
w8+cV2md3DWmLNGcb8bxzrW5sDnsP7GRVsu5sc6SrHFCpQvOOIUe5ZxqchW95oz+9q/uH/3lUb
E1IYQQQkjGfOHB60SfPJls3XbLxZ0dtavAKlhEz2829B6R/bseqNn2u0dWSuBu+m+PtUZP/
+PXHqmq0qt3MkdaIi9NlmHN03oQM/1mVM+KwjyTKs2+uppdW57dMWZ72jZ9SmCuwSvD002q
5b5YgSWV5Bxt7erVaL3Kdb0o/eaMGVz66yV+aT8hhBAyaL5+3+dEtzyN/L/nnlX7CayI1en5/V1BLda
FLK/I3WwvVjzv6X9C7Ftdnd67d2rPoKvU/MM2T1jnm+k3JzorCvNMSpttdYpnNrb4mPa8yxXqx2q1
kbm2Li21bqjP7LW6X6ziidgcG3bx6sykcrJK70EwA/7H+QkhhJChM73/wv/JB06onQRWxyJ7/njvILQ
bAmmmoYAU+i3Imw2TcebtM2JrQgghhJCMue7UgRO3HxZt8+rmoY2jH16+rLYRWB2L/D5/ZBSHu
SaAPgzDMu9A3mqYlId/
+7DYnRBCCCEkY6b0xf53L1xQGwislMSe36tq4Z2G3tPhN8OoY+a/Bw79LYBnkJp5gk4MA7H/a/Xw
WxRYWfe+dK/YnRBCCCEkY6bxxf5Xn35KbR2wahbS84cGCrZTkc4+8hSQg9Xz0/Bjivav7h957ojYnR
BCCCEkY1b9i/18jX+lLajnL7UHi7Xs0b4+
+iQAoNPu3u6hJw6J3QkhhBBCcmWlv9j/4zu+9sGf31ebBqyg2Xp+AMAknds9x6/xJ4QQQobLDT86
+IObvyLa6ZXIW2+eVdsFrCZ6fgBA6dk/Pit2J4QQQgjJmFuPXy/a6eXPS489qjYKWFn0/AAA5eQbJ8X
uhBBCCCEZ8+1v3Sia6mXOY/ff97ePP1a7BKwsen7Eid/HEH84E/278dQvYphnqFm1fhX/4L8IoljZCGs
qjXkPMVnHXjwmdieEEEIIyZVP/
+wT3ztyk2itlzN8jX8y6PkRF2/y52kyF9Xzm0K1YVt+VWykHny0e4gp29vfO7x9WGxQCCGEEJIrq/LF/
j/8+mW1OcCKo+fHTERXOU+TKXr+0YxYd+wenJ4febzzwTv8Gn9CCCFkuCz/F/ufP/VTtS3A6qPnx0x
Wv+cfsy+m58fKev2918XuhBBCCCEZs8xf7P/5Xd/ha/xTQs+/EIF213vY9HGV7is6+j79dHOFe0QPWx
4Rw8UfxpmTC8X5Yu7eoexL7AlGtS5qXaVLWgITtycn5t3SrmVXC9USyy45S7fOaYZRT4YregYKEGP0
X1Shz/wL0TFqrVPc53uMgGGdPn9a7E4IIYQQkitL+8X+rdtu+cvFP6ndACaBnn8xPI2T72DT8Nmsi3o
2YDb9vHnaFDGDqCPVYzFc/GGEfyklVdYZyhywWQv1it8xz7OBiXdOtxQtFqqlj1vjOEv3XFufHq3Y7+
Xw3lbrio4SqfMvWbPqenG7R8Aojr98XGxQCCGEEJIry/nFfr7GPz30/Auiex5Pm2MOmb5HHzGNkn
OKNYzTgEnqBH2J1V2pK/QI1UMxXPxhkDlP1iioiXSN7Fmqo8cdcwf2cu9Kc0QsYcZannU4p7nVK50V
ffXanEvELess0Wf+8hRnEHmBc0XnCBjJ/tX9o788KjYohBBCCMmVr/7rP4qWe7H53yf/Q20CMCH0/
IuiuxjTxDi9lKe56uyuCk4/5VBn1NfUDzY3y4HsI/p6MVz8YYjvNHNMzb19jn7kWZh1SOhzx9wDXno
o66zOC90TfJf0eclM9fBaK/LChCnWh0LnR+YWnr95GJq+PsEzRH2ocwSM6NJfL/Fr/AkhhJDh8s933
SAa70Xl1LE7P7pyRe0AMCH0/AsjGh/dSpnuy9NbFQJXWSeZfincx6mrqhOqnze2X6uuao6Yy8Vw8Y
ch3rWIg+2h9JM+oVreKmZcfbTflE395qzYhe3pmhN8l3hm6ZzmqS55K3auzVM8zL+oHvM3jxutgu2
B2+ohukbAuHYu7Rx89KDYoBBCCCEkSw48cu2/3PFPov0eP1u33XJxZ0d99mNa6PkXR/c1VTOj26C
mWfO3Z6K96tGA+ajLijPqs4sf6kPmiLlaDBd/GOJdizjYHko/6ROq5a3iTLHflD1juReaI4J7glWrz8j+lR
Q6KvrqtQQHbnQtap47o57XQ/joIeIjYHRn3j4jNiiEEEIIyZXrH/rUwr/Yf/aF59WnPiaHnn+RVO9TdE+
6w7FaGn97pk9UR3s1YB71dfpf71dXV4f0kWZAMVz8YYh3LeJgeyjvFR381+hx9dF+U/aMJS/UpxTqs
9yRfbV6jBxafWdFX72WwMCN7kX1mb9hDVepL+qchcU/Ahbh4d8+LDYohBBCCMmVxX6x/5kHt9T
nPaaInn+hVEOzsblZN012z2QaKbvLkd2Vp3syTVKo7yupcdyv8ddHrPFExfjDEN9p5piq1T6n58AtcsS
KM1C/kT03VlzonuHeeV+tPi+Z55xCd8XOtYUmpIfsLuE5xT2nUByzHrXrdk6zEhsBC3LvS/eKDQohhB
BCcmVRX+x/aOMoX+OfNnr+xTLdUkW0M+ZJ3V6Zvsf0W+aIvtYaMNocNRf6LrX6OdFrxR8GOfO06
qtioZH1FWZ6kVI97pisE6BHCt8JOZ9mws3IvIrNeeqgdd/1aZ7qhe6KvnptTvX2gR6LckZw5m8ONN
MXCwqP0XcEzwGMYP/q/pHnjogNCiGEEEKyZFFf7H/3wgX1SY+JoudfMNPdFHRX1bCfbdhdjtWUSe
5wtuZCp4vyF6iHiz+MCE9UVXOG8l/RUWiGOxYdydNRygv9pUrNyO1z6uPhO2Eu9FQvdVbss7ZAeV
Wqx6JmmL/UTMs/SPN89wiBe4Sh7e7tHnrikNijEEIIISRLxv9i/6tPP6U+4zFd9PyLZpqbUO/S7o6ans

ewTyieNg89p9rc87xXioPxh3Hm5EJxvmja4tUrPdu79kVyZv2m7OkoPRfanWl5VD+2hrZPMYPZMyz
OdUb2VFfiFfutTd4hUSZeotI1/5I9TMmdUnsW8bWW2iPoZ93rMLRzu+f4Nf6EEELIQPnKv31WtOX
D5ckHTqhPd0waPf+i6caH1gXAqnj2j8+KDQohhBBCcuXoPZ8XzfkQeWjj6IeXL6uPdkwaPf9imX/VSc
sPYJWcfOOk2KAQQgghJEsOPHLtPd/4kmjRs+ft3/1Ofahj6uj5F6X9Hy67/+EzACy3Yy8eE3sUQgghh
GTJZ3/yye/f+mXRpWfMS489qj7OsQbo+RfF6vlp+AGsoL39vcPbh8UehRBCCCFZctMPPyMa9Vx57
P77/vbxx+rjHGuAnh8AkOidD97h1/gTQgghA2WIL/b/
+I6vffDn99UHOdYDPT8AIN3r770uNiiEEEIIyZIhvtj/1ptn1Uc41gY9PwBgLqfPnxZ7FEIIIYRkSd4v9v/X
fz6iPryxTuj5AQDzOv7ycbFHIYQQQkiW5Ppi/8/v+g5f419P9PwAgHntX90/+sujYo9CCCGEkCz5+n2f
Ew38rNm67Za/XPyT+tjGmqHnBwBkcOmvl/g1/oQQQshA+e43vyja+Jnyh1+/rD6wsX7o+QEAeexc
2jn46EGxRyGEEELI/Lnu1IETtx8WnXzPPH/qp+qjGmuJnh8AkM2Zt8+IPQohhBBCsuQLD14nmvk+4
Wv8oOcHAOT08G8fFnsUQgghhGTJrF/s37rtlos7O+oTGuuKnh8AkNm9L90r9iiEEEIIyZKZvth/9oXn1
Wcz1hg9PwAgs/2r+0eeOyL2KIQQQgiZP/2/2P/Mg1vqgxnrjZ4fAJDf7t7uoScOiW0KIYQQQuZPny/2
nzp250dXrqhPZaw3en4AwCDO7Z7j1/gTQgghQ+T2+w+JJt8OX+OHjZ4fADCUZ//4rNijEEIIISRLvv2t
G0Wrb/Lq00+pT2KAnh8AMKiTb5wUexRCCCGEzJ9P/
+wT3ztyk+j2izz5wAn1GQxU6PkBAMM69uIxsU0hhBBCyPy54UcHf3DzV+yG/6GNox9evqw+gIEKP
T8AYFh7+3uHtw+LbQohhBBC5s+tx6+3e/53L1xQn76ARs8PABjcOx+8w6/xJ4QQQoaI+WI/X+OHFz
0/AGAMr7/3utijEEIIIWT+fPpnnzh17M6i51efuEAbPT8AYCSnz58W2xRCCCGEzJ+LOztbt92iPm6BN
np+AMB4xB6FEEIIIfOn+IQ9+8Lz9UctINDzAwBGcs01I33oUCgZhZJRKBmFklEoGYWSTa/QOuBWAg
BGwo4kGYWSUSgZhZJRKBmFklEIEdxKAMBI2JEko1AyCiWjUDIKJaNQMgohglsJABgJO5JkFEpGoW
QUSkahZBRKRiFEcCsBACNhR5KMQskolIxCySiUjELJKIQIbiUAYCTsSJJRKBmFklEoGYWSUSgZhRDBr
QQAjIQdSTIKJaNQMgolo1AyCiWjECK4lQCAkbAjSUahZBRKRqFkFEpGoWQUQgS3EgAwEnYkySiUj
ELJKJSMQskolIxCiOBWAgBGwo4kGYWSUSgZhZJRKBmFklEIEdxKAMBI2JEko1AyCiWjUDIKJaNQMg
ohglsJABgJO5JkFEpGoWQUSkahZBRKRiFEcCsBACNhR5KMQskolIxCySiUjELJKIQIbiUAYCTsSJJRKBm
FklEoGYWSUSgZhRDBrQQAjIQdSTIKJaNQMgolo1AyCiWjECK4lQCAkbAjSUahZBRKRqFkFEpGoWQ
UQgS3EgAwEnYkySiUjELJKJSMQskolIxCiOBWAgBGwo4kGYWSUSgZhZJRKBmFklEIEdxKAMBI2JEk
o1AyCiWjUDIKJaNQMgohglsJABgJO5JkFEpGoWQUSkahZBRKRiFEcCsBACNhR5KMQskolIxCySiUjE
LJKIQIbiUAYCTsSJJRKBmFklEoGYWSUSgZhRDBrQQAjIQdSTIKJaNQMgolo1AyCiWjECK4lQCAkbAjS
UahZBRKRqFkFEpGoWQUQgS3EgAwEnYkySiUjELJKJSMQskolIxCiOBWAgBGwo4kGYWSUSgZhZJR
KBmFklEIEdxKAMBI2JEko1AyCiWjUDIKJaNQMgohglsJAAAAAMA00fMDAAAAADBN9PwAAAAAAE
wTPT8AAAAAANNEzw8AAAAAwDTR8wMAAAAAME30/AAAAAAATBM9PwAAAAAA00TPDwAAA
ADANNHzAwAAAAAwTfT8AAAAAABMEz0/AACzem3zmsbma+ooAADAsqHnBwBMSN2MD9uFv7
e90VQYsuDYf7NQLqywsf2eOpBTazEV/q5kVq17OMzLNLjAHxj13qvlfWdE/ohGnpqVf6jWK5Z3WcO
K3plB/0ExoPCiVvV1CqyotZxcL1OPWiv6z6Q1Qc8PABjWgLv5FqvOmJu2quwQe51iL2XWUe2rBt5Q
lQvZ2Nws/s8gdcoljL4ltPej+d8VrXd2Y6B3X7WWkd4R9n3LVyT4J7T9Z6i90LkEK0afmlVoqPK4OVDf
0nlL+UexX665S3TemfL5rP+g8C2qtaTKfOuKLKp6KuMfJXc5VnHbQCuq6ptD1aN5V9evVoZS9b1TxP
1p3UXxHHqg5wcADKi9n2rvRrLShar/P+aGoL3CoQxepSygbt8wZcrXfvDbZBvnhWmpSg705pNjD1c
r9z5e0a+HO3H51si1tHDF2FOz6juUPk89nFl1fa1dpbh95sD8L1fXcsrj6um56tRii8oxvhJZlOdQuuBy
hPmLhlckjwxYSz+hHs77qpXDmeGrd3LzsF2qenKuNa0jen4AwHDkHsDZNWQ3fAVhnO3HwMsyw5
c/pG/aYubbDs5s9PdBYdi3QvsGDra+amD7hcr9wjkzd5eS+UZG7lXG29g1VPV88o3UVw9bxeIvZI6W
P8xdRk/WU2uYf1y4hbKsQ4kspy3j+7vz1nVOpj9nKGcdGYuZu1k9kG+HnIXWBj0/AGAw7idzxt2O38
ibgWo9ufaMQa3tT37WPSt/HKZQdacaA9+z4dYRNPCLVKhvYVGi/mGYd7nzJ9R6d2ThjFdWbN+3zC
Ujw2Ws1DWUc2OTdFXJtiDfQNax8sdsb3ZPLfdNkYNbqKpTflHBmL+q79ZZqqdzrc1bq3qrlSXqH8JT
mY1TK3A78yzNvk3+QtkWtibo+QEAg3F3AN4tSk6DF7BVG49cuzdXtRZlwCWVq9DDlyWHW5BWL
2zIOtU7L/NWPm6cN171jqsMVcpdh/uneC5OAXf8zPcyMlzGSvGhqhcuw12MV6mezfNaeQqVa9B

HyqezvSkCtSyZSrmFxOtSnRC+v/14lmOJPzur0GjN/ctWylOrOiLvXqbXqlqBqlb+3B42711cD/T8AIDB
LOCjery9gNgtDirrZqqtXEZzv8pCo6zJfWtkJV6c+v4N+baoCg76vrPXUP88TL1qbHnrMr5U1YD2zN23
gnPKfCLDZawUG0q8HefgrVIdVHLdNqdQuYbmcfl0tjdF7NYV6uXlqOYWct58Gf4gR5eTYXybp1Z9u+
pj9c+Z6nnXVa1H29xsvUnmUA1rXhjnVYrfY3jR8wMABrOAj+qR9gL1TmfEPUe9ectfsLVls+TYYcdUdY
e7f847b9h61asz6C1zKwy4otabIt8+vub8CXX/yDqv3nzcAkbkqVkFh6ruZq7ldEy4ejpLMVmo9Z6wD
FHLlekd4RZyBu6eS6fIENVTud4LJaeWW6F65eZbUa371pRn5Fid/CPjvvzdc4FEzw8AGIz7wZxp7xY2
xl6gqjHyhqOqOeidq5Rlhq8y/HKG2MqHZdtVh7klBl2RJfdbwp23fLFyrywyXsZS/qGq1y3jYjonXJ2Qo
WBHofLpbO+KzkXl+hPmFqqO2AuRb8YE4eVkWobFqeWW6L67PXUOVJ4w/5uiWkF7GLdyhpdp7d
DzAwCGIz+Zs20/goavUO9JpOz7j6KKtQzPPmgQeXZtrmJca9jqRRr0Vaoq2AsZco9YvTjDvufcd8AoR
QvZ3xHVS9OeeGttzks3N09FLfLUrDxDVYfyvkidE65OyHD7OgqVT2d7kRa6qNYfpCx/qoLLGeDPrFur
9YepkK9o/GXK8xpVs3WLlIftseNTgRc9PwBgQK0NSJ5dgV81trDaewKxonEWUxYd5BUafTWtrW6+f
a9ryHd1W70fNkZ4R+Rcm3gHVJol2GvLdTMjFeOTmUl4KPF61eZcXFWtNdOiivW4qjlPjZ53pjwt25v
eWVRxwBq8npJnEr3FF9V6dp4yirOcWnV4hPd29n9QhGu1n5n71tXDeYdpva+z3sn1Qc8PABiWvQ
HhcxqjaW1I596PhlRv78FGX4DM+3hk0X5Vauq1EU+t0CsWXtRKrinyGhUm9w+K7MRfVdSaLQMbi
TnR8wMAAAAAME30/AAAAAAATBM9PwAAAAAA00TPDwAAAADANNHzAwAAAAAwTfT8AAAAA
ABMEz0/AAAAAADTRM8PAAAAAMA00fMDAAAAADBN9PwAAAAAAEwTPT8AAAAAANNEzw8AA
AAAwDTR8wMAAAAAME30/AAAAAAATBM9PwAAAAAA00TPDwAAAADANNHzAwAAAAAwTfT8
AAAAAABMEz0/AAAAAADTRM8PAAAAAMA00fMDAAAAADBN9PwAAAAAAEwTPT8AAAAAANNE
zw8AAAAAwDTR8wMAAAAAME30/AAAAAAATBM9PwAAAAAA00TPDwAAAADANNHzAwAAAAA
wTfT8AAAAAABMEz0/AAAAAADTRM8PAAAAAMA00fMDAAAsl9c2r1E2X1OHavqJje331JFh6Dqi/
DJo7k3BuQ/vbW+Enoo+BwDTRc8PAACwXKy+tt2ernvP37TtFd9tCHb2zU1dwr/JAIDh0PMDAAAsl
9a/y7Y71HXv+Xut39vcN38VQMcPYM3Q8wMAACyXVs9vN7hr3vObxr1jXub+mftkjtDxA1g79PwA
AADLpd3zW42q0/M7B9zGWJ1SnmGNqy6wjtjdsD5cHLP+c3qriGY96zxvza0p09Fzt8ZrDWjNVIuM
Zc6uzjGPnBXE5l9qz0ecEllg9DoAGBU9PwAAwHJpmsnt+ifdMlpdZvXYPWC1m6olbtrRTqaJDl9j9dm
yr61YE/EMEm19/UXl0i3WXBzN5Da39Y+ietf8OyYUXqD/uuh0AWA49PwAAADLRTeNRQepG9O6Y
bSeqE50D8R6fnWS1ZLKI7otda5pjugDsow5w0zFqtOj3Q0XMAO6NWPMyUozTKVz/vqE8HwCCw
wNJGcAAOOg5wcAAFgura5RPbB/dttOq52Uzaw+pelLw0f0MO4Z8hzZERfkITOIdU6QnEFJrsQ9EGc
mUBKX9Ji/5FT3L9Cc1mvdADA4en4AAIDl4m+vi1bT6Yw9rbJsTd1Tuo/ox3aj3B5Xn+EjCrdGCXCnVN
Il9VG5tE7hKfSYv6V9tn4+MLqZZaO9LAAYFT0/AADAcpEdsHpsvt7f9JDyzMJQPX/7oH7gEywc5j9XLi
W553en0GP+vua9op8Pjh64sO+sASAren4AAIDl4jSTsok0T3jaTtkYu6d0H/EMK8b1nSH0OMXwn6t
L6qNyaZ3CU+ienD6jUJ/kVO8cwxqiEi4GAMOh5wcAAFgunmay3T6aJ2JnZuj57ea6fU6P9tstE2aGs0
92amTs+TuHci91bkpkgcVT1sAzzxsAMqLnBwAAWC6+ZtL0jSXzRHNUtZOmMXWOuM1r+Eh4FHNK
uLJubN0yMeECzfUz986RKXTNXy6nOb9jgc5CotMAgMHR8wMAACyXjmbSfsJqRSXRy8ZaUHnEPN
6Qo+t+t+Av3ZzglonT57fZV+fs+Tvn759OSZ8SGj10Zd9ZA0BW9PwAAADLJdBMNk1q6wm7dy3aStk
Yu4N1H9GP7eGc6ZTafXP7hMAqotrjySZZLq1T5xRi828uL5UlrdtiP+0b3b601HfGAJAdPT8AAAAAA
NNEzw8AAAAAwDTR8wMAAAAAME30/AAAAAAATBM9PwAAAAAA00TPDwAAAADANNHzAwAA
AAAwTfT8AAAAAABMEz0/AAAAAADTRM8PAAAAAMA00fMDAAAAADBN9PwAAAAAAEwTPT8A
AAAAANNEzw8AAAAAwBT9/e//H9dfueQWaoYyAAAAAElFTkSuQmCC]]></redcap:Attachment>
</ItemDef>
<ItemDef OID="b7" Name="b7" DataType="text" Length="999" redcap:Variable="b7" redcap:FieldType="descriptive" redcap:BranchingLogic="[q5] = '2'" redcap:InlineImage="1">
<Question><TranslatedText></TranslatedText></Question>

<redcap:Attachment DocName="B.png" MimeType="image/png"><![CDATA[iVBORw0KGgoAAAANSUhEUgAABVEAAAEJCAYAAACdcmdaAAAAAXNSR0IArs4c6QAAAA
RnQU1BAACxjwv8YQUAAAAJcEhZcwAAEnQAABJ0Ad5mH3gAAE9sSURBVHhe7d0PjCRnfef/b+/aY
PPHNmBskx+RHat7sZYlGJIg08PxL4egZ+6SJbnbYOVg5Oh3PVyi0zQSkwOxF+mX2wguG4lunS5iWrq
zliQyTEJugrLdIJRgckzHR4IxYTI/Z7rlc3T8wDYGjPljB3u3f/VUPVX1VHd1dVV3VXX39PtltaerpqfqqT/T
O/OZ5/k+/k+hb5FAAAAAAAAAAChjumPAAAAAAAAIAQhKgAAAAAAAAAAEIEQFQAAAAAAAAAAiEKIC
AAAAAAAAQARCVAAAAAAAACIQIgKAAAAAAAABEIUQEAAAAAAAAgAiEqAAAAAAAAAEQgRA
UAAAAAAACACISoAAAAAAAAABCBEBUAAAAAAAAAIhCiAgAAAAAAAEAEQlQAAAAAAAAAiECICg
AAAAAAAAARCFEBAAAAAAAAIAIhKgAAAAAAAAABEIEQFAAAAAAAAgAiEqAAAAAAAAAAQgRAVA
AAAAAAAACIQogIAAAAAAABABEJUAAAAAAAAAAIhAiAoAAAAAAAAAEQhRAQAAAAAAACACISoA
AAAAAAAARCBEBQAAAAAAAIAIhKgAAAAAAAAAEIEQFQAAAAAAAAiEKICAAAAAAAAAQARCVAA
AAAAAAACIQIgKAAAAAAAABEIUQEAAAAAAAgAiEqAAAAAAAAAEQgRAUAAAAAAAACACISoAA
AAAAAAABCBEBUAAAAAAAAAIhCiAgAAAAAAAEAEQlQAAAAAAAAAiECICgAAAAAAAAARCFEBAA
AAAAAAIAIhKgAAAAAAAABEIEQFAAAAAAAAgAiEqAAAAAAAAAAAQgRAVAAAAAAAAACIQogIAAA
AAAABABEJUAAAAAAAAAAIhAiLp0etJYKUih4DxWGj29PqXWFeU1hpWXW+F+1iMxj3ZB2onXjL
MxLO7LSlg372PzHqPs4Vb2GrGzEO5vtDbdtK5JH08KNuQ9Cjmc+2g0AAAAAwPwhRF06Rdm8UJey
XurU1kPCkracr3X4B5oIrLSZ6qxl4eS1hpWF+1iMxj3ZB2onXjLWdL
MxLO7LSlg372PzHqPs4Vb2GrGzEO5vtDbdtK5JH08KNuQ9Cjmc+2g0AAAAAwPwhRF06Rdm8UJey
XurU1kPCkracr3X0c5Hq2U3rq4B5oILBVWnqJUdZzqxle4faf1Qo1cT/rlhsR+14AAAAAADIGiHqMip
uytmqfi4d4kdzNc75IVW1/dbHHJ6zGjqb0IkmxsGd8M1uk
D7TVk3fijwsI7ascDAAAAAEAOCFGXVGWUOa6ROWKEW6jjYDi4o7oKOcZrS1mUvpN9BzRIYQxoBBLLQDgy0i3EZF7b
2h4snM3eA7Uw9cre7A4EPe8z22cRKXJCEWmPxNoSpTJqxmPBGwHLTaBQD6FA0AUbLoS/1uqbyfZK4hZEE
bF9PnkJF+Xmxt8LaxpVqDgpL
GPu31edcGHd5fZI1J4zwEz5+5nbDPjxN+3INtCdS9tR4xy30ObGewraPOc7xjCm57uPZZpYAh6c9VZF
2h4snM3eA7Uw9yc3Z6x+xzPPy71CLkXbAnaHvI9OHq7vqTHE6/dAAAAAAAcfYSoy6yyJXW3OGpz
VTYaDTnnJ6hyIwM6BdJ1nmdwkd7bcuubn/5zNpc9FKnPaE
D6gP/AGy9izv6PBo1Pe1wrCSDo63TPb8dqZUKUhrYSXM1aUimtjPY1qasDoZtEx2T2T2rZR+9S6H+te1
+kREu3HCSwHz4HSXB0dKibbnnOtV70AUtW6Kc9TeGFHPNGS7E4vZbgAAAAAlgQh6lLLTjBF
nqF0Imk2pv+KGKV4+yL103ie3Gao103ie3e4UhjLVrLnBTF
Ji/u6BisfEacDLUnjXX3GlSlpc9v3+0xnOr59bfvdUhWIdlAfdzx/O1494Hazrob/E1zTMbr9zZlc9v62PX
vV1Wv1/6cXXIi2X56jXUvsCzXu85r+8MlLiqR+4wr/BzteOl/krZbr3X/0hH2PRjY7rBkQzuO3QAAAAA
ALI+C9QtyXz/HklK9SM9zlSEhKwqEq+K8rBzraBs6K9Nfbk96dbsbk3bk96Ubsy+dvW4Vde
9ZG1BBs1YOwWq1Ks9kcWu+3IaxdcdeNE387w+2yV3rnwG1/6DqbsV0VnO2FBNwB4e0wr3O11fc
nDEt0PSK2E9ivvuaS7JjMbQdfr41qa6JzF35+FOdanQqsS3p+lETnOlHbRwht4bCz8ssNkSwEzPCyc
OXCW/QSACAP99/xLnnNW2/SSwCAzB3/NZFjr9/xLnnNW2/SSwCAzB3/NZFjr9lLWBT0REVwkikVtoQGJP5Qeb/npMsYdm/2GE1F
UU6ccp51dJfTrj2WvywnT5+0e9QF11uqp/0AbA74vWmb4nVG7R44gZT1GW8ov7dOdT4sGbUo3S
BMfcIsazBr1jUo6ac28z7QPYSnOKZEvYkT7cec4f+kmIdQ3NyTvhmgTm3wHIWY6rrrurFuwJmaGO0
GMvDwEw/LOz/5Tr0EAMjDDY9fJXd/6AF5/Bs/0msAAs/0msAAJm7dI9I/zt6AYuCEBVBAX1RVt7k
pbenJoj9k/JScqJ6z/D65Xq6c9ZIrrombLbpD+ttull09a/TozUtaAZZl1DTJrOyHeSKpXqBe0GnVjgQX3x
NNPSOWPKvZHAEA+nvPjY/KWL/3WM94oP3+rVGWm+/3555p8u6c8AALL1lMilP7A+YRKiLhpCVASdE
RfTlsXxS7S7lKgd9lbEyVeN9vNVFcO067VZQ480eI0ln1mv47yOaTHOXbjwxOLCN3Ixdz/6pLx2
Lhtnr1K2LatY5BRbUnZ+6Ux58/EG9BADlwwv/RBQ7/
+gZ5wQ+vsJ8/9NXvyp/WeR8GgNz0Hxa5/Od6AYuEBUxleSkN1LbnBFfMSZJitM70RiI+HI+7744c7D
pf686+X7I/4a8fLjUwH4qbZ/0guOFOjFWVQIZacsoTKG6Jgvk22OvYvA/0seV1TIn2Y97LA8Pk1cz3hZ
xnoE/SdmNSNTtwdeukJv6eAubL+z77PvlM7zN6CQCQh1CQ1VT/lucFyz+kHzlLx/RSwC
6gsjlr+kFzleDtCVMRUlM0zh1e93ynbFGDbyjrYnr23JrYvN7cqqqJpDoLVMbH73ZH
6vxxe81a56Kmj3+wdqsx7F+aq7LhnTIV7Onh297K+dBcNQLH9nm/hqd7bHKdU6L9mLVbm7Lqre/p

me+ta1Qq+NvI2oTnyA9c3XYDi+kT+5+Q+n11vQQAyIOqg/pzD7xELwX94W9/Tb73raf1EgAgc5f/RK
T/fb2AeUaIivgq29LyctSalHTA48w8bynX5YJR4NOfUMnSXNWB0GQ1HAPbsp55Q/a9of6KMUnTHP
Jqu9rKUt8KRKiWomxe8IdlN1d1gOaFzqMm/ZolJ3AMttM8tryOKdl+ipsXpO6/2HmtOZGTdS/7h6Br
7yrua1NNWBO03bzfw9odR+bHA8R339fvk7v+7C69BADIw/FLBXsYv/oYRgWozd+8Xy8BADLXf1Lk
8sf1AuYZISoSqWyH11+stqz17tBiV3FT9gZfW21NVr/R7K0XKBlgDM3OdKKjFJgB2KiyA+qc9bt+wOe
ya1/OW03PstS7fT9Yt1Wl1d8LTpaV1zEl2k9RNvf60h16sXq5MUzeVpHt4EGK7B96PbFTEbvtVlv6Lf
8+sqlzblwHu+5ulByOB4jBnYn/6Wfp7QQAeVr50kvl2iev1Evh/uFL35ZP//6hXgIAZO6y9RvZJcpbzbt
CX81aAgAxtTcK4nSSVCHqQGAKADGo4PT1/+318sAjD+g1AIA8lB56od0LNa6tu8vyiteFD/sHAGTgit
8QKfBL9ryiJyoAAMiVGsJPgAoA+VK9T1Uv1CTUsH7qowJAji79AfVR5xghKgAAyM1HvvgRezIpAEB+
VP3Tt33hZSProI6iAtS7/+NX9RIAIHN2fdR79ALmDSEqAADIxe6Du/LBv/igXgIA5OWOL18/tg7qKPv/
8zH53Mcf0ksAgMxd/n9FLn1BL2CeUBMVAABkTg3fV3VQmUgKAPJ189efL//8Czfppclc+dzjsnX36+
XWV79IrwEAZOtKXR/1Zr2MeUBPVAAAAkKknnn6CmfgBYAZe8MMrEk0kNcoz/3RJmu+/X3705DN6
DQAgW9b77aWPWx+fchYxFwhRAQBAZlRwqgLUh594WK8BAORB1T99yxdOf8OJ1f+R7/xo/k7g8
xKSAA5Kb/HZFL1EedJ4SoAAAgM+/77Pvk3ofv1UsAgLz83AMvkRsev0ovpeMrf/mI3PtJ/igGALm5/
DXr0dELmDVC1KXWlo1CQQrGY6XR05/LSa8hKxttveBob7jtWZG8mzM3Qs5LHL3GinPuVhoy6tT55
9d55H7NjwLuWyCW+n11+djffkwvAQDy8vJvPE9e+eC1eildn/zPB/LN//19vQQAyNylXZH+Y3oBs0S
IurR60lhZlaZecpTlzFpRP8+eHfiVasLfVIImPi+9hqzXor5KXfOCrAYvunRqJSlMENguK+5bIB7V+1T1Qg
UA5Ovqp47LG++bvg7qKKo+6u//
+y/bHwEAeVD1Uf+79fFZZxEzQ4i6tLpy4KZA5bp0+33p9/dkM68MdWzgt6QmPS+qZ+S4YK99XtxN
V1vqevelWy87K5qrQo4aA/ctEIuqf6rqoAIA8veWvRvtIDVLqifqH/721/QSACBz/UdFLv2xXsCsEKIuq
96h7OuncuqE5Nf/FGmL1zOyJ41zugtquS5bFedpcfOsVJ2n0twlRQUwPTUTf+WPKvZHAEC+Xvu1F8
vLHr1aL2Vrb/f/2A8AQE4uf8l5YGYIUZeQXbvRDN2aq04tx8GuiKp3o1E7c1T9zGAtyGCd1bDejbH3r
wVreI6qN+kMVfdfl6zW52A9S6+2qH6ENy3GPgPncEP8zZjnydln0vOiqHaWYvWMNHoeB0LzkpzUn
VFl/9A6olGM9obVW21v6GMZPAcJrkvI/RZ2vePcb4PXz35E1ImNY5b37WT3p2vSa2eZ6D1Ar1TMr
49uJI6QOz91pzz4+IN6CQCQFxWevubvXqSX8qF6o1IfFQBydOlPqI86Q4SoCDUUGGl2/cyRYVRHai
WjzqrR43EyanuDNTzVurCgpuQNVXdFt3UUZ5+DwWRzdcJ9Fjflgjtk3joz5/RGeo1z3nkq1y+kUEahK
q1+y+tVOsTseTxK50C6+umwipx2N97ZkYsDJ7W96x2NX1c3yXVRQV7I/eZcj1EBZPj9pu7d0GC5U5
NSIMjOSpb3bcz7M2CCa2eZ7D0Ay07VQP1M7zN6CQCQFzV8Xw3jz5uqi3r3h75KfVQAyI2qj3qP9Z
H6qLNAiLqEKtt96Xfr4kZ7Um3Z9TH72zrxbG/4AZBXL9Won9mpyfrIxEaFec7r+3uboWUCxu4/wN+
et3/pyI6XBPWkse4GPca+Wzo1imzrKP523M2ofdbOu/Fbsn0WNy+If+rOS9v677wbglnn94JOUJOd
F4P9um2ZLK8uyolT+ukYlS23beb5V9ri53BnxMnhkpwj67VGqYGh+21ofyZj2/b9ZrYlbFt+kJ3U/Ny3
/uvD789hya6dZar3ACyrT+x/wp6NHwCQv5UvvTTzOqijPPTV78qf1hmBAAC56T8scvnP9QLyRIiKIW
bPtPoFPwgdDgOHletbE4Z54aotPxwsrp3xAqzOge4z2bsoO14eaey7suW3deeiJIl7zH364ZMh8T6Ls
nnWTbuaslpwe08Gz+8kipt740PW2PblMOpEFdfkTNjxtXf9XrVn1pzjSXSOrPOzp0NEI3gPvd4Dhu43
s8et0evSPk86CNzLYfa0LO/bsfdnmCTXzjLNewCW031fv0/u+rO79BIAIE+vfPBaufnrz9dLs/G5jz8kX/
nLR/QSACBzl74gcpkJ/vJGiIoBI3qm2Yqy5iYxIwK3UyfSDKjKcrKkn47SPdC9+VSoU/LqNhbMYdKRw9
QnMMk+K9tGr0FHOsP403RKoi+fcf2NgNIP3Kpy1j2gqa6LruEZOrw/aOh+M8JCawf20Hd33yNrsaZ
uHu/bBNduyvcALB93Jv6nn31arwEA5OWGx6+Sn3vgJXpptu7+0APy+Dd+pJcAAJlTw/r739ELyAMh
KibUkeHOgTHCo7mX3TFUvMKUSrD+5Oz05HBssVSf36vSHRZuBG7V08FeoUmYExB5PXXHCbtWR
dm8EN470w0rF3t+o8nvz/SvXdh7AJaNCk5VgPrID+h9BAB5e86Pj8lbvnijHL9U0Gtm60dPPiPN99+v
lwAA2XtK5NIf6OfIAyEqJjR/gWm53vWGbAcfk9YLHS/
+Po26n7aO1NZznJyneELGlj4tn5Sxl7S4KW5lAntYuDF8vno6/CyPP0dt2TB7nbo1OM36o0lYbdzT+xj
s/as0z83XpEi53bcTXLtoR+GPJpiWGsL/wCMP6CUAQJ7e+Nc3yAt+eIVemg+qPuqnf/9QLwEAMqfq
o15iYte8EKJiQElOusnV0EzePbnoFnIcO/Q7J6WTXtA2qm5m6ibYZ6+xrodpl6Ved5OsPCfnMa5rc1f8

zphdOfAu6QmJc0m9HrXW/XH+/I4OP6sSyOGSnCOzLqcKFN26qMaQ96TUzPKqx6k9GZQdSHa9W
p7pDpOf0CzuW0usa5fme8AU1xDz7yNf/Ig9mRQAIH+lh1448zqoo3z6v/6D/MOXvq2XAACZu/xZkf
48dRU6ughRMcCcBCnYW9IPAlXYNTChz6yYNTCbq/5Q7faGHhpuPdIev510n72GrLsnrnpWNjeNyY
Nq65JPjmrWsmzKrm5er3HOCzBj90asnBbnDulIs+ke18Bw8Amvix8oDvbcjUlvX80sH+xxaoTFZo9b
oz1p3yaRZnHfKnGu3QTvASU/dZXaee9gZMOb4h9Hze6Du/LBv/igXgIA5OnaJ6+0Z+OfZ83fvF+
+9y1qZQNAbtSw/v739QKyQoiKYeYkSJ2alHSoU/LTE7kw7YxI5vDy5uoUoVGwBmZzVQdQXnhTlVZ
qs9e7kuyzJ411d6h6Wepbav3okCq98zLMnFndbbN3TastiX+aKhIo72oZDmATnCMv2LO4x2xOsJTEi
HvXrLNaPevPNp/YQt+3SpxrZ0n4HuDXW7V41zBubVssGjV8/85P3amXAAB5UvVP3/aFl81NHdRRV
ICqglQAQE76T4pc/rheQFYIURHKHgYdUpOy2rLWu8Otp1KR7cGClfuHRs/BBOwamMZwbZddWzOj
eqgx92n23LN7obonbiCk8of1p3hehhRlc2+4Tqg9hD5hYBeYJMs6ZjsbHhT7uljH3G/5QaqtKq2+0dZ
ACYJo9r07eJA2Z5vTZZMLft9aYl07S6L3AHUsg6+ttiava4u59cTTTzATPwDMkOqBqnqiLgI1pJ/6qAC
Qo8vWb6bUR81Uoa8KBgJAEmo2fT0ZlAph96btmTxL+lhOtaYNWBfEUbp2yJUKTit/VJF7H75XrwE
A5EnVQVWTSS2SK597XLbufr3c+uoX6TUAgGxdKXLFb4gUbtbLSBM9UQEkFCxRcGZtsUO49nnrWC
J6ZB4tR+vaIV/v+
+z7CFABYEbULPx3fPl6vbQ4nvmnS9J8//3yoyef0WsAANmy3m8vqWH9TzmLSBUhKoCY2rJh17o06
pWaJQoWUK+xIqv7demmUqJinh29a4d81e+ry8f+9mN6CQCQJ1X/9C1fvFGe8+PF/NXt8W/8SO7+
0AN6CQCQuf53RC7doxeQJkJUAJNRNS8XfPx7cXMvpRq/C+YIXDvkR/U+Vb1QAQCz8XMPvERueP
wqvbSYvvKXj8jnPv6QXgIAZO7y10QufUEvIC3URAUAAKEefuJhec32a+wJpQAA+bv568+Xf/6Fm/TS
YqM+KgDkjfqoaaMnKgAAGKKCUzWRFAEqAMyGqoO6aBNJRVH1Ue/
+0FftjwCAPKj6qGpY/7POIqZGiAoAAIbc+ak75cHHH9RLAIC8qQB1UeugjvLN//19+cPf/ppeAgBkrv+
oyKU/1guYFiEqAAAIUDVQP9P7jF4CAOTttV97sbzs0av10tGyt/t/7AcAICeXv+Q8MDVCVCTUk8aKm
uVbPTaknWjdOGltZ96MOYZeQ1Y2gmvbG+7rV6TR0yunpfZjb3NEO2zTne9M2m1bkPugvaHbWJC
BSwosjE/sf8KejR8AMBsqPH3N3x3tuqGqN6rqlQoAyMmlPxHpP6YXMClCVGCGeo0VKZRq0tHLWe
pd3DH205RdQj4AA+77+n1y15/dpZcAAHm7+qnj8pa9G/XS0aXqov7+v/8y9VEBIDeqPup/tz5SH3U
ahKhIqCibe33p99VjWyp6bXby3l8WRhxDryHrtTziU6UnF3eC+2qea1hrF8VRuA+A+aZm4n/nJ98pT
z/7tF4DAMibClBVkLoMVE/UT/7nA70EAMicXR91Vy9gEoSowDLoXRQ3Qy2Xy86Tzo5cXJwUdTFUt
qXfrYs+w8DCUMGpClAf+cEjeg0AIG8//ffX2UP5l8m9n3xYvvKX/NsDALm5vGc9mOBvUoSoS8eoLbl
i9kQctd6oc2mvz7s25fD+IutuGnU/VwKfNLcT9vko6Z8z+/PmMP7mqvP5EYU0/WNWj+T1Rv2h/FU
5e+GMDvk6shMnRQ3UUg17jG5PvHbHuTbD59DaumyYrzfqkQa2MbA+/BTHaYN5POpY/P2rh7vd
9nnrupbrsjXQXdYu3WC83n4M3DfArKgh/A888oBeAgDk7YbHr7Ink1pGd3/oAXn8Gz/SSwCAzF26R
6T/Hb2AJAhRl05R1s64PREPpOs8s3TlwE30Aj0U27LbdJ6Vz6xZXz17ldNV/awjB/4B2PywsCxn1nRr7
RCwJIMj5zu1UswQa5bnrCO1UkFW9fYcal2SINUYyl89LZXimniHs3Nx+hCvfEbcU+2L2e6pr43Dfn1w
Z846FVQOrG+uDgSpE7VBHcuqeFvWoakKSlf369Ld2wxcdxW+lsJKN3RqUprnibKwFD7yxY/Yk0kBA
GbjOT8+Jm/54o1y/FJBr1kuP3ryGWm+/37qowJAbp4SufQH1kfqoyZFiLqEimtuT8R9OXQTovauHwi
JEU72Dq1XOU6dmIcI1VI5LW6M2gzMjmSEhV6w15PGutvjsyotu6am9WjpLXRqsh4jjUz7nFW2rTa
Yw76rLadd22HVPv12d+vuV8TsRaoYQ/mrp9X2zVA4xpD+4qbsuefNfnTFa4Z1BPULwcDQN67d6V
wbV7neDX69Frbev2+maYPxeh2aFjf3vOc+P1RXYWtXf41/TppyLsFxAmnafXBXPvgXH9RLAIBZeONf
3yAv+OEVemk5PfTV78qf1h/USwCAzPUfFrn853oBcRGiLiOvJ6IfaPUOndivWnXCo32dFJrDwO38bS
5UZMsNoJq7fi8+s+6n2wPUXFff8ickqmx5QWCs3pgzPGfVlj+Rkh/mWi0Z7IY7gtk792TJfmJsJ0EYq/
Ua636vzepZ2QxPUMe3O61rY6vKWbchRsg+cv3+obPdKdoQeH0UI1Q3Q2s7cNWB6t6okwhkSA3f
v/NTd+olAMAsvPLBa+Xmrz9fLy23z338Idn/n4/pJQBA5i59QeQyf8BKghB1KRXlxCnnmRtode1x6W
U5efqkHXYF11vUMHDn2VzwQ7mmeJ0KuwdeWOgN5ffWWcfkDu+2H8bw7cAQ/VFmdc784HMy
Yb1zLRMP6W/LeT9BlVZoz1klRrtTuzaW8kkJ3d2o9a4p2hC7Z7Zxrq2N2WUO3P3Er8sLpOuJp59gJn
4AmLGXfPe58nMPvEQvQbn7P35Vvvct/m0CgNxcVvVRv68XMA4h6pLy6ora PTl74nSqPCUnKies/w

+uV3ngPEWoFiOYcodmt90x0xG9I6exkOfM6GmphqaXQkPC+LP0tzf8OqBVo6fp8kkSbhdl80L4jP1u
cDtiPjEgE+5M/A8/8bBeAwDIm6p/usx1UEdRAWrzN+/XSwCAzPWfFLn8cb2AcQhRl5U3tHlfDts6aL
N77VXEyQqN9VP3hsyCUdfTDi/9upOjwkuvNubQI2YYuIDnzB/KHyXmkP72hj9RVLUlIzuhTmDqa5O
CTNtg1JUdKNlqa55jln7k532ffZ/c+/C9egkAMAsrX3qpXPvklXoJpn/40rfl079/qJcAAJm7bP02eukze
gFRCFGXVklO2hlkRw52nSHNbh3Rkv0Jf3347OuzV9w864eaDXeSp4E6pCVnqL3i1eKc2KKdM2Mo/
xjjh/S3ZcNLUMtSV1PRTyvVazOhHNugZuhXPU7tScXsQNWYoCtu2QJgSvX76vKxv/2YXgIAzELpoRfa
D4z26f/6D3aYCgDIyeXPivTp2jMOIerS8ntyNptOOObWeCzq4p/uem+Sprnj9gDtSK2mA77BOqRmP
crmqj9sur3h1aUsxB5LvWDnzBzK787+H3iYIV70kP5e45w3jL9cv5BOuYRUr82E8miD3pbKoIM9Trvi
ls8dW7sVSMn/+v/
+l34GAJgF1ftU9ULFeHd/6AH9DACQi0v36CcYhRB1iZkzpqvehd7w88Ds5sYkTXPIq1NqC+shGaxH2
VzVwZg/Ll1GT440LNVzVtS1VJXmauqhoTmUvxxaW8AoiWC9cuSQ/l5D1r0CqtYrAxMwOY/JJkhK99
pMJoc2VLb9IfyBurRGfdmzm7MP3bEU7v7Fu+WOl9+hlwAAeXvjX99AHdSYfv7dP6WfAQByUXijfoJ
RCFGXWWDW8FPiTzbuDltXzPVzyAwvRw2ht+tRGr0uXeW6dJPWu0z1nFVke7BA5v5hSrUxzaH8o0
NdMxQeNaQ/Xl3VCaV5bSaVQxvsIfxhxVBVSNvvp1pfFohy1RVXyf/4lf8ht1x3i14DAMjT373yCf0M
UV7z1pvkbe+5VS8BADJ37FUix9+kFzBKoa/G9QIAgKXxwCMPyOv/2+vtmfoBAPm648vXyysfvFYvYd
D1P/E8+a1PvVGedw0TbwFALgovFrni/daTq51ljERPVAAAlsztN91uD+0HAOTvb27/tjx2PX/ECnPlc4
9L9fdeS4AKALmx3m+Pv8f6SIAaByEqAABL6F2n3iUfeMMH9BIAIC+Xjvfl8294VH78nMt6DVz/8t+V
5NZXv0gvAQAyd+ztIoWb9QLGmSpE7TVW9AQpBX9W65F60lhxJ1TZkODL27LhTbbiPCabqGZYeyO
OeiJqsJVf5ZqR84zwquZ0VcaKU3oAwDA4rjnl+
+xh/cDAPL10M0/kO6t39dLy+3al14l1d99rV4CAGSucI3IMTWMH0nkGKIWZXOvL2oeq35gxuuuHH
iTiKvZsNXn92QzxwzV1qnJeTqkAgCWjDtj/3VXXafXAADyonqjfu+aZ/TS8lIBqgpSAQA5Of5ukcIL9QLi
mn1P1N6h7OuncqEZJedlqXedUNc/9Gtl/XnRZq7pKgAgOVzy3W32EGqClQBAPlR9VE/96Zv2h+X1
S/8xivsofwAgJzYdVDz7rl4NOQYog7XRLXrlZZq4nEZJEtYfVD9UoNb/OeaRVL1Uprp0RP0YFAGA5vfm
WN8tH3/5RvQQAyIvqibqs9VFePoLv35CLwEAMmfXQX2HXkBSc1ATNUKvISuFktS8lNXRqZVSq2H
aPu+HuNXTfpGB5WVM8uWd46hJwTA7YddqEYTfT/4kcHneY0nPYU7fC+q9b2DCO//8rEiKf0cCAt7
7s+
+V2h01vQQAyIuqjbps9VGfd82VctfvUJMbAPJztcixO/VzTGKmIWpluy/9bt3vCVptOcPst1WY2ZPGu
htwVqXlDsFvVe01qobpeqIkoSO1khtC+I9Vd0Yra9/2bgFghnqNlWAPfSBnqjeq6pUKAMjXfT/zuD1r/
7JQAer1P/E8vQQAyNzxO0UKL9YLmMT89kTtXZQdnSKU61v+RFSVLXHLmHZ2LqbU+64s9S0S1NFG
TQoGTGKR76eM295ryPpg13tgBlR9VFUnFQCQnx8/57J8/g2PLkV91Le951Z5zVtv0ksAgMwdf5PIsV
fpBUxqfkPU7oHXE8sevu/1HjWG93cOpKufTsfppZpmrVUAABaVmqm//attZuwHgJw9vdv3T8je3f1sv
HU23vvpF8ku12/QSACBzhVtEjv0LvYBpzHdN1FSFz85vlhPo1M7nWItxHgxO2jWqxuPoOpD20GPv6
/VjZG3Jwf1FBNd2Pdzga0fVgsysDVEya98oca+VK/5xJm1f2OsHyncO1O806o7arx19P/kG2p/K9Ux6
DsOEt32wXungORo8P2HiTbTn8/epHqPqpCY5P0DQbdffJvf88j16CQCQl7+/7Xvyjy//oV46WlQd1O
rvvVaufO5xvQYAkK2rRY6/2/p4hbOIqSxEiFqud4fDT/uRwnDa4qac1WVWRfblcGnyBRVsDU7a1ZTV
wqr1/3hUiFMKG3rcqUlpMKBKMklYe2NETUjVYzgYFmXWhihZtW+khNcqwXEma58TyIW9vrlaGBH
2qXNitLNcl/GVM9SxDbR/6us5/f0ej9OrffAcNVdHhZyTcPbh1XO2Dd97qd3vWGrvKL6DGfsBYAb+6v
WPHcn6qL/yH15JHVQAyNPx09RBTdH8hqilk34P0YN0Bu2Ha8tuuinKQug1zvnhkTuhV7/r1Zsdzzhv
5bp0dbDd9TbQlHNeotNLMEmY9dpzesOh2+3IzkX3tVm1IUpW7Rst2bVKcpzJ2tdrrHuBnP+HjZa1F
615bkRQaLRjb1OKem0koz1u06e5ntPf70n47fHb3pHa+ejIPHqivUH+PsLvvbTud0Ds2frfdepdegkAk
AdVH1UFqUfJyumftB8AgJwce53zQGpSC1HtnmjGkFHzEWco65DimpzxspxVfxuqF6C77UQbdnpv
me1yHmYvuTOyFivhWXQ9uejO2qUCFi+kKcrmBSPEidI7lH39VDo74mY3xc09J6yxHnub+mQmmiT
MmLjHCNyKa2eGQ/XM2hAlo/aNlPBaJTnORO0LtuOst74iW/aGVVC3J2GHE2hHTNWz/rmtbPtBrd

/2JNczhfs9gWrL7yFf2Up/+4q5j/B7L8+J+bAM7v7Fu+WOl9+hlwAAefjmjU/JV376u3ppsb3sp14o/
+a3mNAEAHJTuFHk+L/WC0jLHA/nDwYcXkjrjWE1w5B0mMHN0daVAzdTKp+Ukn5qK56QU/ppJDP
kHgioh2ouTjVJmK6nGTZ8Prc2REmpfSMlvFZJjjNR+0a3wwldR5fWOHUi6XdVVU4HNlaSk2473bYn
up4p3O9zpSwnAwcRIteJ+bAMrrriKmbsB4AZuP9V37HD1EWm6p/+
+n/5GeqgAkBurhQ5/mvWR+qgpm2+a6IWN2UvbMitPdQ3hXqoHtWLri8pZ7JH3OhefG5oM1EPZ
MWu5eiGPlE1KzNsQ5R5b19sebQvRuA3VlFOLEzSmcbxAvPpphfcZAepKlAFAOTn8yuPylNXX9JLi0f
1QFU9UQEAOTn+r0QKN+gFpGmqENUc9hv1cMLJoj8M2gxA7aBUrw9NMc2v04+4dRUtdp1B82tD
H2kGskvEuHZ+7Udf89zw5DXjJwlry4bZq9OtjWnWijRl0oYo2bcvLbGOc4btG21wgreeHHp1B4ZlOv
HcEcD5QZpuv+l2e2g/ACA/KkBVQeoiel3l/6IOKgDk6dhrrQd1ULMy3z1RkZGQ4dEus05mDGp2d9V
b0Q+rjZ7D7raTTBLW3vV6dtrhjxuYG8OTB6XehihZtW+khNdqguOM176Idth1itOdfT7Y9JDh+ImO
M737fWHkNjEflpGaZOoDb/iAXgIA5EEN6f+7Vz6hlxaDmoWfOqgAkCM1C7/qhYrMEKIupaKs+bN2
yao3Ztuc0XsMPcGXKlEb7K0YEnhNOEmYH/4YM+KbcmhDlFTbN1LCa5XkOBO1L6Id9rE7NVUTnsK
Rmqsb4jfdL5lQPrPmhNaJrmcK9/uiyeB+B0wf/vkPy+nbTuslAEAeVH3Ux65/Wi/NN1X/tPp7r5XnXX
OlXgMAyJaqg/oe6+PVziIyQYi6pIqbF/zehs1VHawYk86MU9n2h393alJygxmjRqg/UVew9mbkJGGV
09aSNq5dWbUhSmbtGy3ZtUpyrpO1b2w7ynXZSmV8eFnK5aas6vZ4Tbfaftab/j/Z9Zz6fs+LOdGV28
6Jws6U7ncgwj2/fI89vB8AkI9Lx/vy+Tc8Kj9+zmW9Zn79Uu02ufXVL9JLAIDMHVsTKdysF5AVQtSlVZ
TNvcFJu6rS6res/8djD/8OK6Rpb2dgoi679ubg/ixDk4RVZHuoDc72vF01d71eitm0IUqG7Rsp4bVKcJ
zJ2qfa0Zfu0IbVpo3SBlM7JWcHjzfsGiW6ntPf7/mw7q/B67F/aPQSTiCV+x0YzZ2x/7qrrtNrAABZ+8H
zn5W/ev1jemk+veatN8nb3nOrXgIAZO7Yq0SOv0kvIEuFviqCCAAAMIF7H75XKn9UkaefXYwhpgBw
FNzx5evllQ9eq5fmx7UvvUr+06ffzDB+AMhL4RqRK9R8BQzjzwM9UQEAwMTefMub5aNv/6heAgD
k4W9u/7Z875pn9NL8qP4udVABIFfH3239jwA1L4SoAABgKu/92fdK7Y6aXgIAZE3VR/3cm75pf5wX
v/Abr5BXvO4legkAkLljbxcppFNUD/EQogIAgKmp3qiqVyoAIB+qJ+re676ll2ZLhae/8Osn9BIAIHPHiiL
H36EXkBdCVAAAkAo10dQt192ilwAAWeve+n37MUuqDqoaxg8AyImqg3rsPXoBeSJEBQAAqVAz9b
d/tc2M/QCQI9UbdZb1Ue/6T6+2g1QAQE6O3SlSeKFeQJ4IUQEAQGpuu/42ueeX79FLAICsqbqon3
/DozOpj/q299wqp/7ZDXoJAJC5428SOXabXkDeCFEBAECq3lF8BzP2A0COvv2if7Jn7M/Tra9+kfxSjV
/kASA3hVtEjv0LvYBZIEQFcteWjUJBCsZjpdHTn1tSvYasbLT1gqO94Z6fFZnX09NrrDhtXGnIqCb6x+E8
lv5aY2mo2frfdepdegkAkLW/v+178o8v/6FeytbzrrlSqr/3Wrnyucf1GgBAtq4WOf5u6+MVziJmghAV
yFVPGiur0tRLjrKcWSvq58vHDiJLNeno5YXRa8h6LarV6loXZDV4saVTK0lhIDAGjqq7f/FuuePld+glAE
DW/ur1j8kPnv+sXsrOXb9zu1z/E8/TSwCAzB1XdVBfrBcwK4SoQK66cuDmbuW6dPt96ff3ZHNZM9
SxQeScUj1nxwW/7fPiHlq1pa5zX7r1srOiuSrkqFgGV11xFTP2A0COfvycy5nXR33zr9wir3nrTXoJAJC
5YyvW41V6AbNEiArkqXco+/qpnDohy9/dHHF6znbk8Y53QW1XJetivO0uHlWqs5Tae6SomI53PSC
m+wgVQWqAIDsPXb903L/q76jl9L1sp96ofzKfzziplwAAmSvcKHL8tF7ArBGiLjGvnqP5CKntOLI2peqN
536d163Or/dp135sb+ivNdbZLwuuD/bKS2MbmtlG7zFcYzN4jMM1S+1HWN1Low3jal3a+zDDt+aq
87WDDQ9pc9i2o9o8tpfjROdl+J4ZuZ8YxxD7fGh+W9RjuK0OZwi9/7p0z506/lKsnrNGj+NAWF6Sk7
ozquwfDt9PNqM9ie65eMduS+H7wj1Pcd9HsNxuv+l2e2g/ACAff/fKJ+SbNz6ll9Kh6p/+
+n/5GeqgAkBurhQ5/mvWR+qgzgtC1CWlwpHQMKhTk1JhQ8ZlcHHYtR8HCkLa61TIMrC+uRoeXk2
1DRU2hfYY7EitNCqEU58zapaW61J3uw52duTiYMi0674ynbqmQ8GiZh/zyGBquM1uz8dQE5+X4X
umuToieEt8DFGcfQcvd0hb7WCw5A2hd6V67jxVafVbXq/SIWaP41E6B9LVT4MqcjrpPZfk2FP6vlDn
KY/3ERwdapKpD7zhA3oJAJC1z688Kk9dfUkvTe/f/Nar7J9oAICcHP9XIoUb9ALmASHqcjO4d
TmNeo3SlHPhSUpi5XrX3na/FYybwtaPGt482TaCw6mHj7EjO4PplEcFZM7r+3ubsrlVF+erBr/GPI9nZ
FyGWtm2ttd1t2Wptpx9bOvUrr3hB4Vhbe7UZH3kdQm2eXRT0jkv/invSO28cd0SHMPY8xHg7zu8r
dZxrbvBoHEu3Iamcu40u53bEitrHVKUE6f00wiVRPdckmNP7/uimOP7CI6OD//8h+X0bQxHAoA8q
ABVBalpWDn9k/YDAJCTY69zHpgrhKjLyOwlZ/R0K27uOQGJ9dhLZaajqpx1t1M5bS25RqwPHd486T
aKsrlnBj6O4toZL7TrHIT3AyzXt4IBWXFNzugv6uxc9NvY3vV65pXPrI0P38YwexjWLxht3rwgfgZ5PrR3

31CbR5r8vFRbfnDoh3xB0xxDFHPfoW3tXZQd3SEycC4qW/5+zWtniH/u1HFY3yOhIe8k9uUwrEFKk
nsu0bGn+H2R2/sIjpp7fvkee3g/ACB7akj/V376u3ppMqr3qeqFCgDIiV0H9V/rBcwTQtRlZAQ0ImqY
bsGrZTiyhuIkyielpJ8GjFofJo1t2HQ9x9BhzEGnTgwGP0VZ8xMtLyzyA0Mj0J1YVK9WY/8jgrfhNscV/7
yMN90xjFaWk+MudvfAa79X7sF+GEPcRwyfn/zcTeuUjN51gntuimNPcv2HzlNe7yM4ctwZ+6+76jq
9BgCQJTXJ1KT1UVX907t+59XUQQWA3Kg6qHdaH6mDOo8IUZdSUTYvhPckdEOYqMl1FkZg8hyjn
mOk8MDO76nnDnc2AsPq6dg9GafXkeGOgjFCRlOK52UyYccwK2keVxw9ORxbLNWR2T2X2vVfkvc
RZOKW625hxn4AyNFfvf4x+fFzLuul+H6pdpvc+uoX6SUAQOaOrYkUbtYLmDeEqMuquCl7esjtQKlR
W/Pcos+u3ZYNs3edW7PRrMGZhHW+zurzZA+NNoYyV0/nF6FOH/qlfF4mkm1w6dXKHXpMWsd0
AsUTMrb06bje1BPcc+OPPf3vi6P9PoIsvfmWN8tH3/5RvQQAyNIPnv+sHaQm8Zq33iRve8+tegkAkL
ljrxI5/ia9gHlEiLrE1MzaqqeYPcGPHYR0vRqKo4f/GoxhxHPHrB2pgiW3/uMUba64U6Z3duT8+R29n
aqkk6GW5KR37gdnZO/JRbfoZeQQ8BgyOC+
+nI4hTOmkFwKOqumZL+NcNHfF75DZlQPvNJxwzn2EWPdckmPP4PpP/T6Cpfben32v1O6o6SUAQ
Jb+8eU/lL+/7Xt6Kdr1P/E8uet3qF8NALkpvFgP48c8I0RdRu0Ne6itmkU92FPMCHiMXnIlPxkzZmJvy
4Y3Dft884MlY2bySXgTWHWk2dQnKrWh/EXZdLsdqvO87l+XXmPdq22ZZBKkcVI7L578j8Fj1udsrvr
DyPW9bj9yHVtu1oBtyq7eda9xzgsxY/VgjnPPTXjsU1//hO8jZntyvRSYe6o3quqVCgDI3t/c/m157Pqn
9VI4Vf+0+nuvleddc6VeAwDIlqqD+h7r49XOIuYWIeoyqmz7Q287NSm5QYtRH7F6NnzmbhXSDL52
Lpkz9nttNibamUhF3I6BrlSH8o+4LiU/fZQL005glcl5MSQ9BnPYu9ueiRK2YH3O5qqz34IX9Felldqs+v
EUNy94PTLd9njnodqSeM2Jc88lOPY0r3/C9xEgiqqPquqkAgCydel4Xz7/hkcj66NW/u8idVABIE/H3k
od1AVBiLqk7KG3YUUMVeDS7wcDHlX3cLBmYrWVcx3NpCqy3W/5gZHNOTbvsAPDrOPxhlcr5bpsp
ZzL2dcl5LxWW9Z6d+j1VLI5L6Zkx2C1Z/A+3D80ejUmYNfnNIaSu+y6nznWQ/UUZXNvuFaoPYw+Q
aAb656LfezpXv9E7yNABDVTf/tX28zYDwA5UPVR7/uZx/VS0Cte9xL5hV8/oZcAAJk7Zv2GfPwdegHz
rtBXRewAxKNmNtcT86gwbG/anqHAOEfpntPHcqpFwIpwn+l9Rip/xM0BAHl441/fIKWHXqiXRK596
VXyW3/8z+yPAIAcFK4ROf5+66P/Xoz5Rk9UILaeNNbdmc3LcmaNABVZO1r3XPu8dSwZ9ODG0fGO
4juYsR8AcrL3um/J9655Ri+JVH/3tQSoAJCn4+8mQF0w9EQFxmrLxmANWFXOgK50yMzRu+d6jRU
p7ZyRbiplKXDUFf6fgn4GAMjStU9eKe9s/aS88723MYwfAPJ07O0M419AhKjAWAOBFgEqMsc9h+
WlJiiblx9NaEs42hKOtoSjLeHmqS0nrr1GDr/3pF6aLa5RONoSjraEoy3haAvSQIgKAADmBj/ghqMt4
WhLONoSjraEoy3haEs42hKOtoSjLeHmqS1IhpqoAAAAAAAABCBEBUAAAAAAAAIhCiAgAAAAA
AAEAEQlTkSE2WU7DrfxRWGtLTa8fqNWRlo60XHO0NvZ3CijRibwjp6Uljxb0GG9aVTbJuGmH3UNr
7mJbRRv1Y4SYFAAAAAGChEaJirvUaK1Io1aSjl4H5pgJdY1Z9W1nOrBX1cwAAAAAAsIgIUTG/eg1Zrx
GfzqeibO717RkF+/1tqei1szFPbenKgXvLluvStdu0J5tkqAAAAAAALDRCVABIS+9Q9vVTOXVCyE4BA
AAAADgaCFGXmao1OlC7MazG6GD9UXuIvfE1A+VKNbNOpXokq1Vp79Mcxt9cdbYTvjOjjeoxqk7qY
JvCa1VOdrymMbVf2xvetob2H3JN4rTRY359ZEONcxFo46j1xj7t9ea5zKoOadx7KKotg9uYsD5pjOuS
9J4FAAAAAACLgxB1WakgL7TWaEdqpVEhpPpcQUoDQ+ybq4OvVyFiSYIva8pqYbBWZBqcNq0GNh
xyDHYINtgm65W1UnjQaYt7vIMqcrqqn3Z25OLAa9u7bmODtTKHQjgtuo2TKsrambLztHMgXeeZxR
iOHmh7W9xml8+s5dDDMoV7aKJrPizf6wIAAAAAAOYRIepS6knjnJuIuXUb+9Kt61BNOrIzmPx5qtL
Sr2+5QaEKG8/7ve16jXN+0FVt6VqVXfE2H0Nl2/qabl28L3G3sx1W8dJvU/gxWMe77oZg/mv77gF0
arI+MhUdf7xhKltu2wfPpR9GSvmMeBlqe8MPgsOuSWQbJ1NcO6PbuC+H7qbbu0ZI2ZEDN101hq
mfOpF9hDr9PTTNNTckuC7J7lkAAAAAALBICFGXkjERz96m16vQD9VEOl56FlRt+RP3+EGhqScXd9w
+e1VpeQGStc8LYa+fntmm0GPoXRS3SeX6lj/xUGXLC+U6OxdDexSOP94RimvidfQ0t22ElGaPTrN3av
2CcU02L/htrJ0fGKo+Ja+NftDbO3Si0mrVCRv3dbrau7jjBZKnvROYlRTuoSmuuWkm1wUAAAAAAM
wdQlRYdA3P0OH9SZmzk5+Ukn5qK56QU/ppespyMrCTEN0D77jsIdheXUtjqHdgSHsazOHy/rB4P5
SryllvyvYRvVNtxnbMHqOpKMoJfUHcwLlrXzzrnJ4+aYeVwfWW6mk/kMxMCvdQKtd8VtcFAAAAAA
DMG0LUZRWYKCdunckYgeWRMt3x+r1i3Z6eRig3URhpDK9PScUt3trctVrXE6cj6ik5UdFhZWC9anb
2EeriSf+6AAAAAACA+UKIupTasmH2OnVrPZr1HI+ocr3rlDEYevjD9lNT3JSzbglONXTcqCs6WRiZQYh

dOS164L4ctvUQeLv3pzs5lrF+QUP07K/5sv1xAQAAAACA5UOIuozMupwqYHLrohpDoCdXkpNuEjs
4XNoIEXNVcoamK6NqvWbF6+nZ2ZHz50fVFTXP2eBs/mZ90FMydk6nxNfQ3XdHDnadr3VrtZbsT/jr
h4e0ZyWFeyiVa57idQEAAAAAAAuNEHXJ+QGTMWP/VMxakU1Z3XCn3DFnS8+ZMcmTNFfFa1J7w
6+V6a1MmdfTsyPNpj76oaH8Rdl0u6xar6utN6yz5eg11r0anuYESU7AqVivP+8dkGx4U8nH5V+vZtP
5Wnf2/aIumOquNyfCylYK91Aq1zz5dQEAAAAAAEcTIeoy8oI9S3NVh0rGhDtTMmcun2r75iRC7nY
mCjuDs7o3V3WI5gWO5gzwaXOHxftCh/JXtqXl5XU1KdnnrCAlP6mTC95EVCojdOutWrxzHLe2bVBg
W9Yzb2i6eZ9Y68/k0w3VNv09lNI1T3hdAAAAAADA0USIupQqst1vGQGZUpVWv+8HRvaEQpMqyu
Ze1w/BbGr7g/scx2qn1yBt/9DrDZhIcVP2+oNtstj1YDOoh2rwhvQr1v62Ruysst0PrUtbbVnr3ZILLnU
8g6+ttiara2v22gwMTTeGs+c+ZD2Feyila57ougAAAAAAgCOp0FczrADITq8hK3oiL1WDdo+eiwAwk
urtPS8/mtCWcLQlHG0JR1vC0ZZwtCUcbQlHW8LRlnC0BWmgJyqQKbOOZ75D4gEAAAAAAJAOQlQ
gE23ZKAzU8ayeFTqhAgAAAAAALB5CVCAPql5pZpNXAQAAAAAAIEvURAUAAHODelXhaEs42hKOto
SjLeFoSzjaEo62hKMt4WhLuHlqC5KhJyoAAAAAAAAAARCBEBQAAAAAAAIAIDOcHAAAAAAAAAgAj0R
EV2eg1Z2WjrhVnrSWNFzZavHhsyL60CAAAAAADA/CNERSZ6jRUplGrS0csAAAAAAADAoiJERfp6D
VmvEZ8CAAAAAADgaCBEBQAAAAAAAIAIhKhLqr3h1gddkUZPD7+3l51HaClTVePUeI3zcL7eZW/XH
MbfXHVepzc4uF+PuW1j58HXt2XDfY318F4Wo12TGjwv6jHpuVGSHr8S1obCSkPCD8+s/eo8VkaciG
TbBQAAAAAAWF6EqEuvI7VSQUoDw+
+bqwMhX3tjRI1T9fXpBJbR1H5WpamXpFyXrYr1MbN2OWHk4HlRmquFYNCZ4blRoWtYG6RTk9Lg
BFl2EFuSwZd3aqWhcDTRdgEAAAAAAJYcISosVWn1+9K3Hq2qXqUCwPNulNaTxjkdX5br0tWv7dbL
zjrrtTsXnYiusm19rlsX9zNSbdmv7W+rxHNafjv7e5tSTNCupHqNdS+MLNe7zj77LasFWvOcDkeza4NI
W3bd1Dh020055yW0VjvW3SDXOE/uBe3UZN17bZLtAgAAAAAAgBAVUm1tixtxVraMANRTlM09H
crZ4aVeu3bGe23noKufZadc3/La6ciqXT25uOP20qzK2U13yxXZsoNGFVLuibM6w3PTO5R9/VQ6O+
JmscXNPWd/1mPPbVvvorhNDpynypa42Whn56J1ZJYk2wUAAAAAAAAhKiaha5OGDmHPzqkT44K
9tNrVlQN3A+WTUtJPFSdo9EPnYSmem+KanHGTWGtrquyCW7t0qM5p98Dbnz18X7+uYA7v7xxYR
2ZJsl0AAAAAAAAQoqIsJ82UcJTAxElGbdLcjGjnzNtlyawNRdm8ENYz2A9KQye5Giur7QIAAAAAABx
NhKiIoS0bZs9Kt46mWft0JuahXRm3obgpe3qIvV+v1tc8Nzybvl/DdfBh9KCdYLsAgLzp0Q3Ggz9yAQ
AAALNBiIrx2rte70o7oHNrfxpDyKc2ybYya1dJTroJqDsE3qVm4i8YM+6n1YaI16uZ9NUvzfakXXbw2f
XqnHrtK530Qtu4NVhjbRcAlpH9Xl+QwkwTy540VlZFWu57tPNHr+bqLILU+Qxze40Vpz0r+f/hT/0ba
p4P9zEP5wXDhq7XDO6ZmRvzvuZ9P7mPvG7mOO+3ebwnR+5jSf+gFfO8z/K9eGZinJuh951luGnG
nJfc34sTtofSdoiDEBWJ+AGdMSt9AiU/nTRm/7d+MFmdbhD8tO0KKsqaVzS0Kavem667baeO6OB7
cZw2JDp+/aavPhXsGRpSs9Wsc9pc9dvm/sOhHu7KJNtVjG3wDwuALMzsl/chKri09j/lv0npcCYu3Da
KcLuTP+4f5vte3N4IC3ONPyjOQq8h6zWRatX9x28G3NEnxsO8XrMy01/cAyWOhh+z+NZW52O1qS
YFda9TS6qdmpRmFPjk/0v7+Pc19R5csr6f6l3jHFk/T2Z778R5v83jPXncPtTnc/yDVmTok1eYm+C85/l
eHHFuht739CP98xP/vl3dH/g3Iot/IEadk9zfi8efl3zfiydoj/WNrUrbpf+eHO/7dn5+FsZY1g2DJWS9R/
TV5bd+A+hbPzD5uvW+9U+g87mq9ZZia/WtnxX060c8vNcqIa93P29uf9TD2NbIdtqStKvbr5fd9VXr
K8cxXx/yKNetVygJz83Exx/
+MDcdve3gMSfabqvqrS8PXwQAmEq3XrbeX8z3eP2+GngjyoffFv1vwAzaEEm/z8/8vXjm7XCuj9q/f
c28f5PzY/87OoP9RtP37dy1y/3eivPzV9rCv5dn1R7n5y9jv/pnrCy/l8a/rznvuYNtyPocjW9XvNdMa6J
9ZPIeqPdvbdd+hLRD3T/maud+Um3XK1IS/5w4n8/+vTjeucnjvS/
+fZv1+8v4cxImq7aNPy/h62fWnhHfw+nfR87+zd0737fBdX579Qr9vhz3uiJf9ERFDBXZVn8p0ksO56
821puAo7kr/t9KrNd7n9D2D52/MKlanIP1QqutCWuIJm1XEk4PIOsNTS/7rDdbf9h+0jYkPH57qP3g
ubQ5+wj8QdOuc2oMyXfZPWWMeqiWRNsFgMy05XytY71NXZBN503VUpEt9UY28fv35Iqbe9Lv7x
ltmTO69MupE/PRwFm1o9dYl1qnKmfn9kLNhnteWt7PKPPC+T6X6unAzyL5KMqJU9YH9+dQW08u
7sygPb2GqIFK5fqWv9/Ktv3zYmfnYgY9sRxj39d6h7JvfRj8fi7aJ64puxm9Ecd5v83jPXle3ved79+y1Ls
hP8tr6uf34dEJHdm5mO7dE/ec5PVeHOfc5GX8uXHeXwLf5xmY7Jxk9148/rzk+148tj0jfp6yR42mW

tYuzqii+fpZGOMRoi4pvw7mwJuLMeFQcMiBCgvdr1EPJ5TztxMM6dQPhf5rrYf5A725D/VQ+xmx35
Ht9MRtl/MGFlw3nvMG7H6d89gbakjCc5Pg+G2D59J+jDoG8zj1Y9QvU3G3a7xu+NgBYAoz+uV9M
enSL+W6bIX/A5CTnjTWa9KZVTvsoaMdqbbi/1ueGTUM0Rh6N9uSN/n84j6JXuOc9d1s/bI/oxu3sq2
HjNo17dWwypITNg/
+vJW13H5pT8huV1lOenWcNKPePgZk8AetuMFlmDTbEVuO78XTnJv86fJoB+cHhtOnWwJnknPCe7
FhxPub8/PnvuRaNYmfhRcOISoAAJgNfnmPTdUltX/5uTCLXoa6tpj9i6D+pWdGvR3b52vSqbZmPmL
C/0Op81AjV7KppRZXPr+4J+f2NDo7wwDEHTWkatqr7yOrObMI4efpl3ZMgT9oKfPyXhwwR3/Y6uy
flAvevxGqt6h6/9mYYa9C3osD9HwiwflBrO+pqedWiWHwjzD8LLxwCFEBAADmmDP5gRq2N6ueO
MFRDt36vqzO4hfU9oYzCcRc/dbuUL2Csh6WHcfc/eLePi/O6NHZXTNnsg5/YiAVeKtJgXKftGOWv7Q
jNfxByzKH78Xz9oet8pk147pY/4aeVWXUZtirkPfiAernGrdnrP89dXBKXadTkl3n7nn5IwymQYgKAA
Awp5wAVfXWmJ+hjMXNC3YNtk7tfK7hXHtXhU1NO8B1fuEpSEn9Vqh/CZrtcHptlsOyLfP1i7sOCGf5
y6Iecqzq2bt5jz0MViXezdWMZjYfZVa/tI9h93bqyMHgjTuqd9QS4w9ajkV4L56XP2x5ZtqrkPficIMl+f
qydXLfOk8nJau3vdn+EQZpIUQFAACzwS/vkVSvDSdAndMJ/zL8RSPMYE8j9bAngLR+Mexaz2dbt7sn
h6qoWc7nZKxZ/uLu9nw6O8NfFgeHTbpmdl7y/6V9rOIJceZ7CUZNPfuGnmG4O2f4g5Zvvt+LB+T+h
62S2GWOB8PbWf5cw3txTLq2eOCPkekZ+UcYfhZeOISoAABgNvjlPYKeRddiD3czevzkW+dSTf4wM
Bxc/0KW1S8ac6/XkJUVc0i2WqVmSp7lL6nz94u701utKjMcPSpSOW21wPoeCgyhV7dwzTovM26bL
dtf2uOpyGnVY6+27r+veL3G5m+islngD1qLaFZ/2NIjADo1Oe/9w6mHcM+oHinvxXGoUhklqUldLm
RwkSL/CMPPwounDwAAMCOtqvSt33L69a5e0a33y9aPJ2VvRX669bLVFtWewUe139KvWUZh56U
6JyfEblu53s/3bun26+Xg+ZiLe6RVHbg2rb71S2tfJT+5m+H38TB9HgKPefie1vdRxvdv3Pc1573Yf2R9
7eK0K4/35PH7CLt/3Ifxb1dq9H0x9H2r2jFw3Pp7Pu1rNcl5t78m8/fiEedGvd8M7Ns9hrTf/uKem6H
XZfY+POp+0XJ6L453XvJ7Lx7fnpB/xzO6RnHuxXn6WRjjFdT/rIsKAAAwE+5f6F3WD43zNRwQWBCq
x5xdm9BVbUl/Bl3nnO/pqrT6M5p5eS7pnk7G5ZnV9cH8Gfre9fjfR2Gvqc5r79gUjT83Id9bxnk7iuLc
LwrvxbOmRvOoOqhhgkP7+Vl4cRCiAgAAAAAAAEAEaqICAAAAAAAAQARCVAAAAAAAAACIQIgKA
AAAAAAAABEIUQEAAAAAAAAgAiEqAAAAAAAAAEQgRAUAAAAAAAACACISoAAAAAAAAABCBEBU
AAAAAAAAAIhCiAgAAAAAAAEAEQlQAAAAAAAAAiECICgAAAAAAAAARCFEBAAAAAAAAIAIhKgAA
AAAAAABEIEQFAAAAAAAAgAiEqAAAAAAAAAAQgRAVAAAAAAAAACIQogIAAAAAAAABABEJUAAA
AAAAAAIhAiAoAAAAAAAAAEQhRAQAAAAAAAACACISoAAAAAAAAARCBEBQAAAAAAAAIAIhKgAAA
AAAAAAEIEQFQAAAAAAAAiEKICAAAAAAAAQARCVAAAAAAAAACIQIgKAAAAAAAAAABEIUQEAA
AAAAAAgAiEqAAAAAAAAAEQgRAUAAAAAAAACACISoAAAAAAAAABCBEBUAAADAQmtvFKRQ0I+N
tl5raG94n19p9PTK2fDbuiEhLV0wbdlwz3vk+e1JY2Xca6xXNVb8ba00rK8CAGB+EKICAAAAODqa52
TGOemSUMHoqjT1kqMsZ9aK+rmpKJsX6tZnHZ3aesg1asv5Wkc/F6me3bS+CgCA+UGICgAAAOAI6
Ujt/OL38Zx/XTlwM89yXbr9vvT7e7I5KvksbsrZqn4eco16jXN+IFttyXZFPwcAYE4QogIAAAA4WuiNm
r3eoezrp3LqRKxeo5Xtlng5auAamb1Qq9IiQQUAzCFCVAAAAAABHTLzeqH590pVg6NpryIpbm9Orse
rX/7Rrehp1Vr119suC68NKtPqCtUJH1wEdeJ31CKsrGjyeYL3S6HYYzGPXj8F92fsp1ayzrDVXndeO3Ul
FtltujOpfo/aGXxagXN+yXjUo3vHbQto/dH0tcc5VoEar+6BWKwAsLUJUAAAAAEdEWep1HdJl2Bu1U
ytJYTVYDdRep0K2gfXN1VEBZlNWCyUxyoCqjUhpcMIpOxQceJ3F3t/IQK8jtZJRr7Rcl60YnTuHwlEtel
8JVbak7hZHba7KRqMh5/wEVS4M1gNIcvwqwA5pv3M+hoNUR/i5UueiNLhTJewaAQCWAiEqAAA
AgKNjzQ3p4vVGnVS53pW+qgPq9ax0hK1v7o5oh1dLtC/
+y5tyzkv7etJYd0PBqrT0a71td2qyPjIpNl6/F2OSpvaGePmv0a6um3ga+6psW5/r+hNFqRqm9n5iDc
MPTjLVrLnHV5b6hcF2Jjl+67VuGhvWfmsrOxfjnqu27Eadi8A1AgAsC0JUAAAAAEdIUTbdGYwy641al
bNuj8nKaWvJNWL9/mFoL05zBnqzXmhn56Lz+t5F2dGdIQPD3I3enN5rB4QPix+t7aeGgTCzuHnB31
ftfDo9MAOTTGnVs8OTUiU6fuu675lBqKO4dsYLbDsHXf0saOhcmfVeOzviZq/FzT1n+9Zjb+QMWgC
Ao4oQFQAAAMDR4oVsGfVGLZ+Ukn4aMGp9qKqcDiR3JTnpp31ix33dA90L01rllguwH8bwdve1A0
6dSBLymT0vz8ha4EuLsnbGbdi+HIYlthMITDJlPQudTGqK41fHZNc5DR3eHzR0ropr4h2yuodK7n4ja

rECAI48QlQAAAAAR8xAb9RD5+l8K8qJU/rp1MpyMn6am0BHRnTmnE6i8DlCYFIpo85ppLBzFSw5Y
HLD3NgTdQEAjgxCVAAAAABHj9kbtRYvTsvXYK/Onhx6Y8iHebVWhx7biYbtTyercHa88cfflg2z16lby9
Ss3ZpEcVP29D4Gyt7amueYpR8Alg0hKgAAAIAjyOiNmpQxjDw7g706u3Lg7tTtmVk66QWAo+p5ps
MsJeDXAHX05KJbmFROSaIqAdNKcvztXa/nqR24unVRp7iWaoZ+1ePUnkjLDlS7Xi3W0WUEAABHF
SEqAAAAgKPJmIAoTMlPDo3aqW3Z8Kapz1ZzdcObqKm94Q8/L59ZcwJAszZnc9UfQt7e8Gp0FlIZV2
4Gzta5WPd7WfYa61790aSTVU1twuP3A1djxv4k9PbVbRDscRoSdAMAlgYhKgAAAIAjKro3qjlzuwrp
nGAubi3NaZWlXG7Kqg4D/dzWmOFftd+ozdlc1cGh9+IREzJNorLtD1vv1KSk21XyE1S5kPuM9AmOv
3Lan6jKu5bGBFRJjDgX5r1RPat7ugIAlgYhKgAAAICjK6o3qqp7OVgzs9qavI5mIqfk7J4xPFyx63gO1Di
1a3MOvE4Je+2U7GHrIcdebVnr3eHxeYt9/BXZ7psz/itVaZk1TZu7Xs/fcexzEVYMVW8zrewaALA4Cn
1V3AUAAAAAAAAAEIqeqAAAAAAAAAAQgRAVAAAAAAAAACIQogIAAAAAAABABEJUAAAAAAAA
AIhAiAoAAAAAAAAAEQhRAQAAAAAAACACISoAAAAAAAAARCBEBQAAAAAAAIAIhKgAAAAAAAA
AEIEQFQAAAAAAAABGEvn/AZM6nr53azj8AAAAAElFTkSuQmCC]]></redcap:Attachment>
</ItemDef>
<ItemDef OID="q7" Name="q7" DataType="text" Length="1" redcap:Variable="q7" redcap:FieldType="radio" redcap:RequiredField="y" redcap:BranchingLogic="[q5] = '2'">
<Question><TranslatedText>Do you prefer Life A or Life B, or do you think Life A and Life B are the same as one another?
</TranslatedText><redcap:FormattedTranslatedText>Do you prefer Life A or Life B, or do you think Life A and Life B are the same as one another?
 </redcap:FormattedTranslatedText></Question>
<CodeListRef CodeListOID="q7.choices"/>
</ItemDef>
<ItemDef OID="summary8" Name="summary8" DataType="text" Length="999" redcap:Variable="summary8" redcap:FieldType="descriptive" redcap:SectionHeader=" " redcap:BranchingLogic="[q5] = '1'">
<Question><TranslatedText>
Alzheimer's disease - Description #[no]

If you have Alzheimer's disease…

1) You might forget recent events of importance to you. For example, if you are interested in hockey, then you might forget whether your favorite team won their last game, although you may remember watching the game itself.

2) You might forget the names of people you met recently, or the names of local streets.

3) You may no longer be able to handle your personal finances or plan leisure activities such as dinner parties or vacations. Simple decisions such as what to order in restaurants might become difficult to make. Your ability to concentrate may decrease and you might have difficulty driving.

4) You may start having trouble finding words to express your thoughts. You might need to be reminded to shower or brush your teeth, but you could do these activities on your own. You may need to post notes around the home to remind you of simple things like turning off the stove.

5) You may remain aware of the day and your surroundings. You might be able to travel to familiar locations like the supermarket, although probably with the help of a loved one or friend. If you are in a social gathering, then you might have difficulty following the conversation.

6) You may continue to enjoy hobbies such as puzzles or reading.

Now consider two possible lives. In Life A, you will live in perfect health for 16.5 years and then you will die of natural causes. In Life B, you will live in perfect health for the next 10 years, then you will live with Alzheimer's disease as described in the summary for another 10 years, and then you will die of natural causes.

</TranslatedText><redcap:FormattedTranslatedText>
Alzheimer's disease - Description #[no]

<p></p>

If you have Alzheimer's disease...
<p></p>
1) You might forget recent events of importance to you. For example, if you are interested in hockey, then you might forget whether your favorite team won their last game, although you may remember watching the game itself.
<p></p>
2) You might forget the names of people you met recently, or the names of local streets.
<p></p>
3) You may no longer be able to handle your personal finances or plan leisure activities such as dinner parties or vacations. Simple decisions such as what to order in restaurants might become difficult to make. Your ability to concentrate may decrease and you might have difficulty driving.
<p></p>
4) You may start having trouble finding words to express your thoughts. You might need to be reminded to shower or brush your teeth, but you could do these activities on your own. You may need to post notes around the home to remind you of simple things like turning off the stove.
<p></p>

5) You may remain aware of the day and your surroundings. You might be able to travel to familiar locations like the supermarket, although probably with the help of a loved one or friend. If you are in a social gathering, then you might have difficulty following the conversation.
<p></p>
6) You may continue to enjoy hobbies such as puzzles or reading.
<p></p>
<p></p>
<p></p>
<p></p>
Now consider two possible lives. In Life A, you will live in perfect health for 16.5 years and then you will die of natural causes. In Life B, you will live in perfect health for the next 10 years, then you will live with Alzheimer's disease as described in the summary for another 10 years, and then you will die of natural causes.
<p></p>
</redcap:FormattedTranslatedText></Question>
</ItemDef>
<ItemDef OID="a8" Name="a8" DataType="text" Length="999" redcap:Variable="a8" redcap:FieldType="descriptive" redcap:BranchingLogic="[q5] = '1'" redcap:InlineImage="1">
<Question><TranslatedText></TranslatedText></Question>
<redcap:Attachment DocName="165.png" MimeType="image/png"><![CDATA[iVBORw0KGgoAAAANSUhEUgAABVEAAADYCAIAAABtBTQzAAAAAXNSR0IArs4c6QAAAARnQU1BAACxjwv8YQUAAAAJcEhZcwAAEnQAABJ0Ad5mH3gAAC4wSURBVHhe7d0NjBTnnefxmWAMDjaQGAPeEBk5MwRhEpFs4uAZizNKrHiGuw22JdvcRSJopR7H0e40t4uME5QoOe7siOTouM5pqWIoE0Owso23hXTTtpzIbOQZYzsbo2SMWKaDiETO2MbJmDUxGJO5evd1Xx/cg2/VRXP09VddHuX9XzPN09PT3dBQAAAAAMofMDwBok0MnD63ds1YKAACkysIzs//5jn+8ed1/lDKQEh+QPwEAiNPJqZN3/vROKQAAkCpXvveBtc8v2r31wddO/FYWASlB5gcAxG7q/NTAwaM/0ZAIBUWf
PCwqvPXXHxwoVHH3jA+K8sBdKAzA8AiN2GxzccO3NMCgAApMpNx+bdcGqO/fi1E7/98be1UUosBwX6SkAqAAJA2S/7fB286Nk8KTWHFgP5UL
UoHMDwCI1+ZnNj9dfloKAACkysIzsz975FopWMaeeNz4RwpA4pH5AQAx2jexr3C4IAUAAFJlxqXuNS8I4AIUAAFJlxqXuNS8sNP4rZeXH3/omA/uRFmR+AEBcDp86vOmpTVIAACBt+l+6bt7ZmVJwYWA/UoTMDwCIhT1R//
n3z0sZAIBU6T1xjfGPFAIY2I+0IPMDAKJnRH0j8J9+57SUAEEfBfihvf9XCkBSkfkBA/NHddvHChuDn/p7NnpQwkD5kfABClA8cOPPTzh6Q
AAEDa3HBqzvLJuVKow5nfn9q99UEpAMlD5gcAROObGwbBvXYhMzpnLBBAAAZf5D8sEOhr2ys+effZHu6UAJAlyRAwVKnZ0zEwp3/N88Kk9Dw2d2HJAmIBYDCgf0iC4SMAgeq2RfF0EMUEP2xmAtmeAMAKmeY2C1aFdAS5vnarSCHPFUAhm2Pe37/sOQu1Ie9ZO3ZUpdsBrcATJW3gHBgZpa0dOBgGzPcxIzLyWkFWa6EYFQCBvuedGmM9L3q1crpWGT/Ol3dwMAAMwpGetc2/HNhvB2+BUgXOxyRgYBw2CoY3BddN1+HmR2Vw9I2k1ts2tW+S/ZzWedAzuvmv8wj/a9AoFTjTNaBthHQ8k39iz6wNhpX53zPJ0f8gSD/+6AYy9v4dz/1O+/Py7vW7vVDJ1wgEEWb/uyhKTOSjXhJSJbFIrmKSYaNDn60a7pJxD7HsXkwyoRkpDSh2ZAYJP/cyH+LoWpgoqxLK2iCJCuLyDxxnFf6Hb/+L9AoBABTSIQD6fFzrtTeXCcK+67ML6zKXkTiM8gB5NKH/NOdD2B47RGS/PKHZj2zpJxCM/Uj6MwvyrTW59VF1UsAyohgkwhDNOeyOP2aHLDc64tbbZ2Zvt2xxRu9wH8rXShhYOXFgk2jIsBpbHYJMp6OG0A7yEIzE+QR0xe5OmZODdBOHY0Y3F0rtDJ1S5bu7BXgUWpD/dwBC2dkHwIaCnB2gnTc5r1nD6ajsG6u+N6Fk7ZXI5+UfTmMPe+zfgyOWUL9ySfM+QHoZ91+J0jfIiVdPOmm1CjKA1DEJ7JfsjyEg7mkmzzgpeo1Z5ZBQnTM1l6L6w1Xvcw8JkjbLHZfw5xCl9uwGzbJ/ix2S+wi4rZxXvj7hyUmoNEhyqgm8YVoYzmPvnDekG2ZzT3dnHsxSCUw3SVePmUYnwm8sT4U+jhNwKI+Ey16u8FgSJdTMrLhsxHQZ4TX2OA8G1ESOSEC7XtX4/gv8U6PWvyRV8F4j9KX/vbrk+ibSKnONgN4R5XSu8mk5VMxK6xbLnR1Dv6PZMHWpI7ZkTMTaAdHVJDCvwN6y8L4HwThg7iq87NcAbf8Wn6/eUvyMKMtYmOGf2f07EOBBV7Ui68kl8OskJ8bcLh6Uvtba/8DQsfJNGEPHkhlH8wAA==

+9/+SAtBRZP4O0qlfbvWXhqSDd19hiz/x6wHRFt+I5woDofX6lcdyq6edVwSXqGrNJTWqa4qnzspjuS
tslH6Vybt2Rf4tdzfkrqv6jrleVf1F1TbPVYl6aNNd3IuDZtGptfbOetZwvdRsILTaUK7NCrZWa2N8m1J5
e031bFvVLUFSbH5m89Plp6UAAEAK9b90XVTD+IOMzM/AfiQBmb+jBrbIsP7i4NCI6tbfV9jj7dvtyUi
W8Xxva1GoZ909VsPjRyetstO9fHz/Qam3dMDansgGl1c1sF4uf+gNMpQP7rc2Sm2CmSo9B6KF42C
+1F1XcbCeioxXeV/kbr7ezTMWq9H7fYWCdPYIqF1bYA07QdeR7Ssxq3dNKmAUG9k14xz1bYqvhgZ
U3RIkxr6JfYXDBSkAAJBCNx2bd8OpOVKIBwP7kQRk/s7q0ZP5FfN2ZKqQ+PXdf2uw9/Sk/YrA7H8N
UaF/4rhdhwR8g0rdqsN5m/qbq9BfPODc5fZGfj0EPDdqHYdpu5dE88dB6lFDLDwTK4STV8m74DTf0
OapdcaGh3c5dclic1hH7dr0Guq8UPthDRUZqFxtddKW3jV1+afmxqhpKHynqOsCklsd2xa2JUiKw6c
Ob3pqkxQAAEihhWdmf/bItVKIDQP7kQRk/k5zJvOzBCbrV2HcuRagrxPUGVIrk9AvacoO+LmcuSn2
ZQAVuHPr2zSxgOrzoEO/P/Kroh74oF7RXCLUUybozhb10K9yrtbYzTeyeRXGbvjUrk2t4Zwx9hpGWm
5yCkBn1+R6kLr8U3tj1ESJqmV/DQ0K3RIkgz1R//n3z0sZAIC0ufK9D6x9ftGMS91SjhMD+9FxZP7Oc
ybzc8KOpiO/u4e9jkLqLn0zepatNP+w05TVs79vxfoVRr2uJcYWtSvyO3ulQr9sgdpzPfgg3ysDvXVv8v
Ylwr4VvfLIH0gb2bzaXSdq16bWcG2Rlbz9J1Dd3Lvm1diRt0biq+ebEr4lSAAj6huB//Q7p6UMAEAKrX
lh4dXnrpBC/BjYj84i8ydJw2GnpbArvenNiG3f5l+5bMC6DuAsaWfk94d+udoR6PgQkeaCZSQDHbKY
ac0B/zbXSHxk0aanNh05fUQKAACkUO+Ja+Iexh/0f7721TO/PyUFoL3I/KnWWnqU0D9xvGR13jYrsx
epJe1Op+7QryJ/4KKDGjPu1vTN7UbV7lgR7eZ1dGf9wjemNKRv7Nsr6eH6yJZHnn9k38Q+KQAAkEL
zzs7sf+k6KbTRn86eLW7OX7xwQcpAG5H5E67X7G1v8AwK12O5w287q/7YVdmVjx89YK5s96G3Fs
kS74CCdlCTG0wcHwlEfn0kWunb0Cp3486IenMjo9282rVVWsPsVh/DFPc1N8aZc2JShvTXdfohZQ4
cO/DQzx+SAgAAKTTjUvft/3J9e4bxB504cuSJ739PCkAbkfkTTk/x554kXU/YLnOq6VCmJvXTc/1XJ/fV
i0VzZfv6gT3K317S9shvsDsajOfz/sjvDJ8vDsqv0enfcG/y1+ma4fykX2mH3Nq2NzLazatdW3ANe/J8c
9B9Ew1WU/euqYsCahp/ZMiR00c2PL5BCgAApFP/S9fNOztTCp3w7I92v/KzZ6UAtAuZP/H0FH9qCjX
pRu36UT8dyoxUZq1S75hq54W6G7/6zbzORH53+841DVuP87uGnr3MBSY+jJV6G/QvKMpGRrt5t
WsLrCHnhWuL7Fka1QrNXwmotTH6PfNtSRWRbRvaYer8FBP1AwDSrvfENcY/Uuic3VsfZGA/2ozMn
wKe3zO3mD9f7v5Ftp7hMfcKxtN1Dqh2Qr8eJqA6DUQzX13jdIAMXnMwJ6b37pjZm7ydid9sT//Kg
nWgXW9DtJtXu7bgGtYqzha5fhHC0NKPPFTfGOMMdbdknp5SVj/CEBDdtiFm9kT9J6dOShkAgBS6+
twVq/91gRQ6ioH9aL/u6elpeQggRGnIvrdtJF33tRYg+7568KuP/fIxKQAAkEIzLnUP/uwvFp6ZLeUEuP
0rm+79+jekAMSM+/wAgMoKhwsEfgBA2n32yLWJCvwGBvajncj8AIAKDp08tPmZzVIAACCdbjg156
Zj86SQJLu3Pvjaid9KAYgTmR8A4Hdy6uSdP71TCgAApNPV565Y88JCKSTMn86effSBBxjYjzZgPD8Aw
GPq/NQtP7zl2JljUgYAIJ0Gf/YX179+lRQSqf+uuzc98l0pAPHgPj8AwGPD4xsI/ACAtPv0bz6c8MBvGH
viceMfKQDxIPMDABybn9n8dPlpKQAAkE5G2v/Urz8khWT78be+ycB+xIrMDwAQ+yb2FQ4XpAAA
QDpd9e6MtWOLpJB4Fy9cYGA/YkXm76zSULejf6QsiyNUHukfKsljp71YmmoT7x5VYawYuq+eA19H
dZ71lfo24zKWhdPtsnL41OFNT22SAgAAqWUEfiP2SyENXjvx2x9/65tSAKJG5u8gI5MOFuWxoe+ed
T3yMCJm6O3Nj0spCxrYo/LIxpAVzUrcB76rOFgrlZYOuNcHMsieqP/8+
+elDABAOn3y1fnJH8YfxMB+xIfM30GTR+1M2leYnDaMDUcb+cNDb1rVv0dGrA+7NFAasp+xj/pkoc
9aOp7fUeWmffn4hPWnvFPKrgFrKZB6RtQ3Av/pd05LGQCAdFp4Zvanf/NhKaQNA/sREzJ/56gg2bV
yWcT39y9v1fsCqDv2uW3WJZaedfeYoT83WjXAq6szvFPIqE1PbTpy+ogUAABIpyvf+8Da5xfNuNQt5
bS5eOHCyF//9Z/OnpUyEBEyf2eYA511Li0OmmOe3YPDzdjq8PU7dw2SdkaZ+4aW16jf4ry4Qs92zx
bUHI7t2iTPKwNthlfrPKNfpDfQXLGePTIY1YTnfYOK/H0req0/u3qGx2resVdXZ3Lr67mx7z4YLhUW1z
rInucNnlVc9ekDJYek6utC1NuWZ8Wq72+ltweJ9Mjzj+yb2CcFAABSa80LC68+d4UU0unM70/t3vqg
FICINJb59Tf68K/zFVdxMomhngBSjRMrshkqzIPlja3j+d5KR81YrEal9xW2NNbN3KzSNaTdKLoaMI+
wZwtCNiDIXNH9yuJg3dX2DO+RbvbF7day8sh2ieeFPY2PesiNTo/m5LGLq2/FpD4pa+1a+eB+e6vtS
w2WKufewHq74fH9B1316osNMmtDzYNsnAee5w3et0kYCz2nQeXXVf/L0kBbnhU976+/FuNoeWZ
NQDIdOHbgoZ8/JAUAAFLrpmPzbjg1Rwpp9srPnn32R7ulAEShDff5jXDj/urf6kx1OoAZoeJAakP/wC
5nKLmZT/Wt5tKQHCw1dFzWGs9vrJBM7VdWmAogtH6HvFYPaFcJVQ+aV5Xb2bnyBgTJq1Tg1uPka

1fbo1K/9ZrSDnt1lfjr2CPFfLb6jXszkDonZa1IrHr2uxmBNvRSwcAW2Q9X6PdF/ppHQ13x8J0GvgsJQl
Vinga6Id8L5UpKJc20FXh/dS16Ff1uIbmOnD6y4fENUgAAILWu/eOszx65Vgrp98T3v3fiCGPuEJnIM7
/dVdqgQ1e0M9W5In/VHJNSTmJT97a9Sdij4bv7LrlReYNkQLtR/9FJ8w91gJ3KK0XYMLpa9SKlrmp7
hrfZUVLfIm74Hr95/tXK+zY5HyW8FgdDU7/uG+DN566A7qeOqbNr/shf82iov0fqr4v/bXLxnAZ6Y/0
VVfmL10Bboe+v8xdTr6LPXCTU1PkpJuoHAGTAjEvdqR7GH3TxwoXi5jwD+xGV+O/zRztTnQ5LfZ6El
BX+DuAWHcEmjnt3toUjqoezB6hLNObNb6E6bAcjYP3qrHZgl7qBbGqqV3+dZA4/J7yG9hoJXMbSG
xl6+vlDv3pfVaONHWRztIen17yX5zTQJ4ur7tAOCRXUaCucvrTnOrN6lq2UR0gce6L+k1MnpQwAQG
r1v3TdvLMzpZAVDOxHhCLP/Hq0vXnb1AwQOj/4513Ta5rqjCUq8ue27fGGqsuCLw6G5/aOiWCT1G
h4Q6vDQKpxtrSZYNq7wg7WoVdBvKFfR/665gAUzt+P6qPifce8J3h33U7/1UYvNNkWUmzzM5sPnT
wkBQAAUqv3xDXGP1LIFgb2IyptGM9fiZkxPDcTzVhSO/fryL9+wH8n9XLQ1sSlBne71ddpvqpa1Tqjw
s2Tor4pBBoQ8Z3n8HdEDVMwz8/waf/Dj0ZpSP/9sFeqf3S86pfg7jFhCB8I00JbSKfC4cJjv3xMCgAAp
Na8szP7X7pOClnEwH5EIt7MHzLvWrOzxLkiv/9Oakbo+8fundIjpdvy6/A1b2E3p75q1ZnRVyjUd0o0
TE+qr7dDDz4J4em5IlRH9mrviLQ0vn/HDtdpa6t5NJx5HSZlmL1qsh6lIXNjzb99Jj0lX0hjrbWlVNqlW
ocWnfLi71+URwAApNmaFxZmaRh/0MULF+jhj9Z14j6/HpPf4Cxx+nX2zdUshn49iZ17Ane5QOKZqi
0+ekC4ntXOGuLtC70Nq6da51rQtuFhdUpEnfpV6Ffb4fw+gDq8asOk30mlLZce8NX76qvQXyzaO+V
aue6DrBK0u/tDVVY9xua57upXGmlfScNteQR3Sb+dSJzdX9q9eslqKQAAkFq/vmlKHmXXHTn7uyvQ
vE5kfpVB6pvATNORX43yVikjU3f6nfnh5OjIoWliOjvdj90/k0J1Pc4P5ds/R68CrpqPvTk1q/Vd3Khw+
aPZPfLSR9iuRG2GzK5Xgd6SRg+Ia2oCb+SvfTT0S+Vp9RekpsD5U3sPm27LK7BLTdaDNph9xewn731
y6fylUgYAIJ1+t+Tcq8vflkIW9d91t/GPFIBmdWg8fxN0D3cnzKhQkanQ7x0QYTGHQIT/0lo47yT4/ln/
w5kDwr1bYHb7binxm6pW69wU1tnUFV9V6m92j7yMI+zZDvP4Vt078y1xN1zHSyxO6A920qhxkI0
m3S2a7Uk59PcFRHBja21u8215Gbvk3iOjHv92IDkWX73YiP1G+JcyAADp9PKqt95YkM3fnb3+xo99
+dvfkQLQgu7p6Wl5WIfySL+ds42v8yERIrBK8DWlIfvWo5Fx6k+yupqKGqoKaBN11nJ+Ipn2Tezb8Pg
GKQAAkE5Xn7viztGPXvleeu5l1mHmrFnffOqfjNgvZaAFnfi70cwscfouf2XZutOPbHBGK8T4o4NAC+
5bed/WW7dKAQCAdHpnzvtjN78phaz48re/Q+BHVJrM/DJi16vuAeP1TmDm0JHf7HfspvoSE/qRIPY
JrTqmVJkrAOi0hz//8Prl66UAAEA6nbjhnckb/10K6ccwfkSrI31gehqdJU5H/sDU4/r6AaEfiRQ+DAZIiL
137121eJUUAABIp7Gb33x77kUppBnD+BG5Do17aWyWOCfyB/tIE/qRPOY8eoLAj8Szp/GfP3u+lAE
ASKFLM6af/Q+vGf+VcjrNnDXrgUcfNf4rZSAKjc3hBwDIpEMnDw38ZOD8+9mc+hgAcJnoPXHNmhc
WSiGFvvboDz71hduIAEQkU/NbAgCac9vS23Z+cacUAABIp8kb/z29A/tv/8omAj/iQOYHAJu/8z9+d
V5KQAAkE6H//LMO3Pel0J63Lhq1V1/9/dSACJF5u+08ki/69cK1C8Y9I8wOUFlxvGyD5F91KoXG+I79
q1U1Sj9wxWm2N977ykXp3YeQ0Rj5xd33rb0NikAAJBC71355+dufT1dA/s/OHdubmeBYfyICZm/k8
xIpH7QDZcr4yyQH66wxPtj/pxyqOnJe59cOn+pFAAASKE3Fpx/edVbUkiDTY98d8FHlkgBiBqZv3PKIx
sJXw0zf/KhLRPit62hyaPyqxSFSbO5sRh/zJ9TDnWYP3t+6b+UmMYfAJBqry5/+3dLzkkh2RjGj7iR+YG
OKh+fsB+sXBbj/X2gEcsXLN97914pAACQTr+45Y3kD+xnGD/agMzfGeYAbt3FujhYcciza5B3cIi3Hilt8j
3tHpjuXi98ULVvKLtScbGn5Tpeodev3Lx62nlFcImq1lxSo7pGuPckWFvFhtwv8e97KM+LPK8y96vWa
WBzH9hq2+1ty91aWFv1vGWuddRD/WR4i0i1O3ruYBp/AECqvXfln43YL4VEYhg/2oPMN1pCmq5fz7p7+sw/x49OWm
Wnr7uzMbIhkY50N3bFe2zcx7uieg+7R9RHrOp76tsnk7F+S2+Pl1GbOkp9hS0DbWgRnZRfnb9v5X1
SAAAghV5b9O4rn/yjFJKHYfxoDzJ/Zwzsmp6eLFhht6srN1pp2Li9VK+lE7Aeky0rTI9aeX08v7FC0pJ1
7FXMlXZUvo08sMVuxh36A5G/NCSBT0aeq22r3HS9VOifOG7XoZo16pXLAKr3e4Sd38sj26UV32EO
1dBhV2odsTpOg6Cw91Ttk68t9Z421VaQ2n1z2oEaLSIDdn9p9+olq6UAAEAK/eoTfzCSvxSS5K/
+5m8Zxo/2IPMnVG5UApmKxCoBlw/ut6KnfZ/VVCmv23QlapVw+na7riQY+dWCwh6ZZq5neI80HX
YtoR7StLRsB/xczky09mUAtce59U1E1MpUla7DrHYlRCOHXYvjiIW/p2rSQTUJoP/MiYKz+6Z2tIjOmn
3FbKbxBwCk3XP9r7971SUpJMPHP/c5I/NLAYgZmT+Z+lb0yiM/3fM93ysDqHXv6paSlj/0q7ya2yaBL
nANwKRTnrpL34yeZSvNP+zNt/avb8X6FUa9riWRRn5nqnzXYZatCNHMYY/viNVkDikI9LqPQHhXi7h

aRMctvnqxEfuN8C9lAADSxgj8RuyXQgLMu+663P8sSAGIH5k/w8IvHFTiDf068teZtFu63iDTCRQPlO
Q2/8plA1YCd5ZEGvkTobkjVvU9dabTqzU3QZMCrcfeIhJh1eJVu7+0WwoAAKTQa4ve/fVNU1LoNCP
wG7FfCkD8yPxppcZQuzUzPtvRM7zNHqJuhH41hL7upN3Y9QU/Cf0Tx0tWF3qzMnuRWtJi/dGJ7rB
HvkelIX2b3d5KPXo/Lu1vER1z38r7tt66VQoAAKTQrz7xhzcWnJdC5/zV3/ztxz/3OSkAbUHmT5tes9O
7oaUb6yEkeo/v37EjOIReN+wev64Hxod3+la94quyKx8/esDq2W/1hbcWyRJv9/jWVTqI6jJHZc0c9
maPWHOcyQMmZYB9XUe+kjpfGGGLSIOHP//w+uXrpQAAQNpcmjH93K2vv3fln6XcCQzjR0eQ+dN
GjwcvDsoPpFsjqU3+32pvnAr9xaKV3Tx3+VU3gC5nqno9l72e2k3HXDVFnZ65vjrZq2LRXNlOw/b4e
ntJxJG/wkHUuxKimcNe1xGLnLoq4fw0QXXNvmWORltEeu29e+
+qxaukAABA2rwz5/0O/mI/w/jRKWT+ztGTxhUHq0ZHnx49w7z9OjWSOqcmdG+FhH6bv2P/wC75f
Tg1kZ307HampXeisW/janJeqDu9O9sSdeSvcBB1J/UwTR32Oo5YZPThqrJHlU65pt+yulpEttjT+M+fP
V/KAACkze+WnHt1+dtSaC+G8aNTyPwdpBOhpf553M2fSPOOnDZ7V0eQ+A2uoF3hTrTnN94t5s+1
q59qMxnb5l7BeLrOMd5O9NSd3tUd6Mi7wZuCG+p+Mypp6rDXPmKRMZpy74LZjpTNqRBtlU65pt
+yulpE1iydv5Rp/AEAqfbyqrfenntRCu1y79e/wTB+dEr39PS0PAQM5ZF+636tkWdjSaYA0u+xXz721
YNflQIAAGkz7+zMO0c/OuNSt5Rj9qkv3P61R38gBaDtuM8PN2e8eeTd6QFkxv2fuT+/Oi8FAADS5u
25F8duflMKMVvwkSWbHvmuFIBO4D4/bKUh90Du3GhEYwUAZNbaPWsPnTwkBQAA0mbNCwt7
T1wjhXjMnDVryz/85MZVzICLTuI+PwII/ADq8OS9Ty6dv1QKAACkzdjNb8Y9sP+uv/t7Aj86jvv8AIAm
HTtz7JYf3jJ1fkrKAACkyrV/nPWfnvlITAP7GcaPhOA+PwCgScsXLN97914pAACQNm996MLLq96SQq
QYxo/kIPMDAJp3R88dO7+4UwoAAKTNq8vf/t2Sc1KIyMxZs3I7Cx+cO1fKQEeR+QEALcmvzt+38j4p
AACQNr+45Y135rwvhSgwjB+JQuYHALRq95d2r16yWgoAAKTKe1f+
+blbX780I5ppzvrvuvv2r2ySApAAZH4AQKtmXzGbafwBAOn1xoLzv/rEH6TQgutv/NiXv/0dKQDJQO
YHAERg8dWLjdhvhH8pAwCQKr+
+aeq1Re9KoSkzZ8164NFHjf9KGUgGMj8AIBqrFq/a/aXdUgAAIG2e63/93asuSaFxX/72d66/8WNSA
BKDzN9h5ZH+bpehkiwXvqe7+0fK8oyhNBRYqNd3VVS1DptnIeDzddQAAKb7Vt639datUgAAIFWM
wG/Efik0qP+uu41/pAAkCZm/g8wo3Zsfl5KlOOiK60am9z3dNZ7vbSxwV67Df0nAs4r5vKuR2jUAgM
vDn394/fL1UgAAIFVeW/TuK5/8oxTqxjB+JBmZv2PKIxvtKN1XmJw2jeas5V3F7VbgLo9sL5pF9fRkoc
8sdo3vP1h/6C8dsOoIVCJtuLYiN2qtIFsxnt8oK9SsAQD89t69d9VifqMIAJBKv/rEHxoa2M8wfiQcmb9
Tygf32zfPc9uGe6wHA1vMOG1k7zFrQc/wmBWy7ZJRXnePhP6jk9afdSgfn7AfqAsFqlJVq9qKvsKWA
fNPtRX6FTVrAIAAexr/
+bPnSxkAgFT5xS1vvHfln6VQC8P4kXBk/k6ZPGpH/r4VvdafBitO75Ls7WGN3Pf3sK+Dvk5gdce3ecY
GqK1wntbN2JcWatYAAJUsnb+UafwBACn1zpz3jdgvhapu2/CfGcaPhCPzJ5gzdd6g3b+
+cT3De6QvvmZn97qH47deA4DL1G1Lb9v5xZ1SAAAgVX635Nyry9+WQogbV6269+vfkAKQVGT+x
CoN6Rv79lh6NZK+MaovvpotQPiG46vR+m6qx0F9NQBA0P2fuT+/Oi8FAABS5eVVb72x4LwUAj44d
25uZ4Fh/Eg+Mn+n9K6wE7x7dL7ZhV91nHfPnWcPnVf98KuptI5R61Cpa2CXFdydqQCtdittRVC1Gg
Cgqp1f3Hnb0tukAABAelyaMf3cra+HDezf9Mh3F3xkiRSABCPzd4oeKF8clF7y9kT9Zsd5d7d5IazVNP
4uOrDnd9gvKA35BgFY8wAYy1z35L3zCAS3wnqJyS7XrMGgXsI4fwAVPXnvk0vnL5UCAADp8c6c9w
//5RkpuNz+lU2f+sLtUgCSrXt6eIoeot3KI/2V5uXrK0yaN/aNJB0yjD83Kv3uQyowqXXCatF1hFTiPF+7
BrWGbDgABBw7c+yWH94ydX5KygAApMeaFxb2nrhGCtYw/i3/8BN69SMtuM/fQeZAed8gfacnv9
mV3j2C3ojYqlw8IP0AjArcrzdWCYz5Nzvk+wbiW1VJXDcEt8LcCOf52jUAQC3LFyzfe/deKQAAkCpjN7
/59tyL9mOG8SN1uM8PAGiTwuHC5mc2SwEAgPSYd3bmnaMfnXGp+2uP/oBe/UgXMj8AoH26v90
tjwAASJXeE9f8t48/yI/zIXXI/ACANunubtP/dGioaTTUNBpqGg01jYaaRkNNy15DlwPG8wMAAAAAk
E1kfgAAAAAsonMDwAAAABANpH5AQAAAADIJjI/AAAAAADZROYHAAAAACCbyPwAAAAAGQT
mR8AAAAAgGwi8wMAAAAAkE1kfgAAAAAsonMDwAAAABANpH5AQAAAADIJjI/AAAAAADZROY
HAAAAACCbyPwAAAAAAGQTmR8AAAAAgGwi8wMAAAAAkE1kfgAAAAAsonMDwAAAABANpH
5AQAAAADIJjI/AAAAAADZROYHAAAAACCbyPwAAAAAAGQTmR8AAAAAgGwi8wMAAAAAkE1kfg

AAAAAAsql7enpaHgIAAAAAgAzhPj8AAAAAAANlE5gcAAAAAIJvI/AAAAAAAZBOZHwAAAACAbCLzA
wAAAACQTWR+AAAAAACyicwPAAAAAEA2kfkBAAAAAMgmMj8AAAAAANlE5gcAAAAAIJvI/AAAN
Ko01O0YKslSAACApCHzAwAyxA7j8abw8kj/YNfotG0011UcjKnBdl9ZMHbMbKl/pCwLouTZGQvXSh
rlOYbxvE2xC/kbKueeLdozo8pnQpWnGlW5Ks87Fu1uxavqkYn1gyJG4TuV1vcpZI88uxPV21RHWyn9
TLpMkPkBAPGK8du8h9XOYFFKMeoZHpveNSCFgS2Fvq6uiePRf9cpDfmuLMT8hao8sjHflcsZexObv
sKk7JBJH8P4xPtV3nNmO2I6x419GSzmnDNiPN8b2xkRz/f40L+hxhO9+S51cozmIruKVuUzIcKPi7Cq
jOVRXxysnLuiPc1rHZnIPygq7ZRnlyyt7VeNM2FwwvXR1OIHU3B3rMaDYtojo/2oPyjqayuCpjxvu+/4
eI5ia8fuMmW/TQAAxGHSTMQ66hnfe7u69LeRSKmGrD/jaaIy7x7GJfZWzAbk8MXTjPnex36Y3Nrz
xnhYTcZ08vnrjq8tz99SqxDJUVTvR3DD/adGVLsW3mK1pxpVb1VqPSk2zHq9zduKcfj0gtbfrlq7Yy6X
p1tqx1Ztp6KoX1TZqQqLmhe6Oz6tNxq+R/4lMbaInpBiq+
+aWZ2u3jqTnaK3KevJlvbpcsR9fgBAfEo78uN9hT3DPXbRuilePBDHJXrz5vv0mGqojSaPjnd1rVzWjo
bja8W8dTee29aBwxcXe49G23pGmGd7V259PN0XepatdPcnKR/cH09b5ZHtRePL9RapeWCX8f16fP
/B1u/1h/4NLR+f8J7b1q5G8DFR5TMhwo+L9nzyWOezyl1eA7t83Y5aeruq7060HxRVdipa4Ttl/j1yzv
bW1L07EXxQhO9R9B8UoW0F/t/Xu8I4+45OSqlRZjshHeja90Uiw8j8AIDYxPZtPjFKQ4PuhBQT+TIZV
yv2F/nR+Pvaj+d7pWtm3CM/o/wqXydfWI6cmb7NA2gcudJQd29c71jE3+PrYLbYt6JXSiazycxp7eJg
9Sju03QrNUT9QdHQTsXDeleO7nD1Gm/+k6nO3cnIB4Uh8PfUd70hMtn/ItEOZH4AQGyy/m1eEr+
+/RAxPYDR+t4W23dj8x5K/Il/YJf0MDRMFvoiGGRaTZRf5etj30+LtauEcQjNm+75XnNkbVzvWNu+x
19esnBxsD0fFI52XSIcn1ixx/PJFOtg8ax8UBh61t3T11Xcrt8b62pGVNzXyLL+RaI9yPwAADTDmr6orz
AZ330q676RfBWdGIzpi68ZRtr5Rd5k7FhUPcaraOtX+Vj79VvsebTsOeGM/SkaJ0QcOxTr9/jLVRYuDr
b3g6Kdlwj77lmnjljP8LZcvDeQM/NBYbI+yJ1LM71HVxpHL5JeJu25RnZ5IfMDANAwK/B3tW28eM/
wHnOcbn5H5N/dSgeMRGd+LbQZocG+vRb3bXFTrD3G2/pVPu7uumYL9lhhO3OZV4NGzYngY/gyH
9/3+BDmLTvvqRC8rZdm2bg42MEPivZcIhTx3kDO0geFzX1xZnrLiolI/uLGfI3sMkXmBwDEJqPf5ssj/V
bgb8evzXnEcOQ8X9ms+0LGd63J2EfZWgM023kixPpV3r53F2t3XXdPV1uMexTL9/hQgcEDgdG7KZ
aZi4Md+qBwieUSofW/KPfVhFj/D5W1DwofaxYV50prkypcI8voF4k2I/MDAGKTzW/z1hc39z0vU+S
3u4xvPq57M1abrX+d6pjySL/rCFn3omL97tvWr/LWLdBYu+sakWu92VHB6XLfhj7Ctmi+x1dl7tt4fqP
sW7xj0tsqSxcHOyq+S4RWDyDnGol1gzm2D6Zsf1CM9Pfmu1q9N1/5GlmmLwu2j1yvAwAgBqPGF
xD7fpDBvDekCxGz6vZJ9w/4+vaoPTujb99Fre17Y515qhlPIWJxntVe1l442nBGRLlvvjPA4uyCe9+iOp
hVWqy+MQ0Jr8r3ftla3DmrNc+WGq24ylabrbRR55ExV4vspA/slLHAVbm9SRU2om7Vd8rzbCvNiM
Du2KzFbTi3I/
+gCG/L+0zLh86urmI1nvM60iN5+SDzAwDi5f4Cwv+n0TaeL6Qtfx8NY53esdXeARF/j0ckvO+KTd4b3
1MpesfCdyqV+1TlPTJk7oMicr5LFTbnKwNfJFrUbfwrxw8AAAAAAGQI4/kBAAAAAMgmMj8AAAAA
ANlE5gcAAAAAIJvI/AAAAAAAZBOZHwAAAACAbCLzAwAAAACQTWR+AAAAAACyicwPAAAAAAEA2
kfkBAAAAAMgmMj8AAAAAANlE5gcAAAAAIJvI/AAAAAAAZBOZHwAAAACAbCLzAwAAAACQTWR
+AAAAAACyicwPAAAAAEA2kfkBAAAAAMgmMj8AAAAAANlE5gcAAAAAIJvI/AAAAAAAZBOZHwAA
AACAbCLzAwAAAACQTWR+AAAAAACyicwPAAAAAEA2kfkBAAAAAMgmMj8AAAAAANlE5gcAAAA
AIJvI/AAAAAAAZBOZHwAAAACAbCLzAwAAAACQTWR+AAAAAACyicwPAACQLKWhbjFUkkU29UT
/SFmWxEO142s+CZxjYwgch/JIf9hTVZ8DgOwi8wMAACRVcTvx1MWI7YNFeWzou2ddjzwUPcN7Cn
3Wo/H8Ru+hK+3Ij9uPctuGfS8DgAwj8wMAACTWeH5H8u61d8zkUTu29xUmpw1jFbJ7z/C2nP3Ic+
jKI9vlYkFudNeA/QgALgtkfgAAgATjVr9WPj5hP1i5rMqN+oFdo5L6nUOnb/KT+AFcdsj8AAAASVbtV
n+FAf562LoMxpdVzDVcY+HlBa4lIWP3nVHwlYbBu54NPO/aNqeZGjMEeOrzVGhW0at65xcHzadD6
9KpXw5daUhGBPQVtngTf7XtN3m3x7dKlR2s+joAaCsyPwAAQDL1FQpWdo3iVv94vrfbNRbeLBpcS
4wcHQjRxjKds+3XuFYxc63rWfv5CuHWWKyaCWRuN0+qt4RUWIeBLTKsvzg4NKK69fcV9rgHA9Tcf
mODvNtjrRLcIP8OVn5drcsdABATMj8AAEBSrbOzazSj+mUUvOr5bvAtKR4ItCJrOKuoyw/lkY12rs2N
WivlGoGp8yxqnUoD8IXrXrzd4qSd2lWFA7v0IlVftV76PXoyv2Le3s5A4q+x/WoGAN/2dI3vP1h9B0s

HKu4IgzQAdAiZHwAAILHUlHQRBEY1Xf3AesnvwSUTx32N6Cnune7yduQtH9wvSVrduVe31oOZuOr
dfeEEZZXMdWpv8oKHM5mfxTdZf+3t7xkesyK7uk7Rs+4eCf1HJ60/HZ4d1JMO+CuqdsUDAOJD5gc
AAEgwyaIt3+rvW9Erj5TgEr/ceifL9q5wR141g77ZaV2o/uyBTFx1xj2bjvzuX9/TKTtwKaI+zmR+XYGp
+xrZfnvYQaC/vsOzg3qrXXUznB9AB5H5AQAAksx1q/
+4taAzepatlEcNqn1poZYKKbwxzW2CMxGfa9qDCny1O8MKNDv9M6AfQEeQ+QEAAJJN3+rPV82e
0XPfYNd91j3UoHW3yH8Nr/WrBqHCt780pG/s2yvp2QRqUr35dTcDGwP6AXQEmR8AACDhfGPTq1
K91qPgvsGu6rUDuLenf8t0de7JANSY+3oGBzSq5va7Z+KzB+I3cmBLQ+ZdfXPeQZOe/i+agwUADSHz
AwAAJJ7+8TkvHV3VaH89/X00ioPSId2ZVt8eca9HrbvWsPvBN9WFXV/UcM+bLxPr1zMFYMPq3n6V
09U0/jVZ9RgHy3VX33u5BADai8wPAACQfJVv9TsTxhUHrcAaaeLv6uvr89Wrp793Rq1718j5Z8urk/P
DADLznfSs9/3EXmRqbb/
+LQN5Wnf0ryWwI07V3p8OAID2IPMDAACkQcVb/T3DY+5x5rnRBoad17Zym7t2s5u7K9Cbo9a9jfl
WaJDnF/gt5q/ex/cDdzW239gc93h8c1ukXDxQvSeDuSO+ofzWy5s/NADQgu7p6Wl5CAAAAAAAM
oT7/AAAAAAAZBOZHwAAAACAbCLzAwAAAACQTWR+AAAAAACyicwPAAAAAEA2kfkBAAAAAMg
mMj8AAAAAANlE5gcAAAAAIJvI/AAAAAAAZBOZHwAAAACAbCLzAwAAAACQTWR+AAAAAACyicw
PAAAAAEAWdXX9f2IVRTRan7ojAAAAAElFTkSuQmCC]]></redcap:Attachment>
</ItemDef>
<ItemDef OID="b8" Name="b8" DataType="text" Length="999" redcap:Variable="b8" redcap:FieldType="descriptive" redcap:BranchingLogic="[q5] = '1'" redcap:InlineImage="1">
<Question><TranslatedText></TranslatedText></Question>
<redcap:Attachment DocName="B.png" MimeType="image/png"><!
[CDATA[iVBORw0KGgoAAAANSUhEUgAABVEAAAEJCAYAAACdcmdaAAAAAXNSR0IArs4c6QAAAA
RnQU1BAACxjwv8YQUAAAAJcEhZcwAAEnQAABJ0Ad5mH3gAAE9sSURBVHhe7d0PjCRnfef/b+/aY
PPHNmBskx+RHat7sZYlGJIg08PxL4egZ+6SJbnbYOVg5Oh3PVyi0zQSkwOxF+mX2wguG4lunS5iWrq
zliQyTEJugrLdlJRgckzHR4IxYTI/Z7rlc3T8wDYGjPljB3u3f/VUPVX1VHd1dV3VV3VXX39PtltaerpqfqqT/T
O/OZ5/k+hb5FAAAAAAAAAAChjumPAAAAAAAAAIAQhKgAAAAAAAAAAiEKICAAAAAAAAAAAAAAAAAAAAEQgRA
UAAAAAAAACACISoAAAAAAAABCBEBUAAAAAAAAAIhCiAgAAAAAAAAEAEQlQAAAAAAAAAiECICg
AAAAAAAARCFEBAAAAAAAAIAIhKgAAAAAAABEIEQFAAAAAAAAgAiEqAAAAAAAAAQgRAVA
AAAAAAACIQogIAAAAAABABEJUAAAAAAAAAIhAiAoAAAAAAAAEQhRAQAAAAAAACACISoA
AAAAAAAARCBEBQAAAAAAAIAIhKgAAAAAAAAAEIEQFQAAAAAAAAAiEKICAAAAAAAQARCVAA
AAAAAAACIQIgKAAAAAAAAABEIUQEAAAAAAAAgAiEqAAAAAAAAAAEQgRAUAAAAAAAACACISoAA
AAAAAAABCBEBUAAAAAAAAAIhCiAgAAAAAAAEAEQlQAAAAAAAAAiECICgAAAAAAAARCFEBAA
AAAAAAIAIhKgAAAAAAAABEIEQFAAAAAAAAgAiEqAAAAAAAAAAAQgRAVAAAAAAAAACIQogIAAA
AAAABABEJUAAAAAAAAAIhAiLp0etJYKUih4DxWWj29PeqXPAbEL7AJKpx1WWwdnyUyK8oh2xTxWnrzG3VEfZv
Z5OiMwfaVAvYHC6nYsOKxR2gjVlfMCT6hJLdXl
cZY4tsTm6UqkQ44OEfFxXy11iWX5d4O6fE+vI8aoKBxLFQ6XHmfbkPWF0sn2lY8Zxh97CB9OGXFUFxXEJNJ+
uytmqfi4dqZ0P9kbrNc75IVW1JdsV/XzhFGVzry/9vnpsS/aHkff+RpmXdmShKwdu8leuS9c+xj3ZzDJ
D7TVk3fijwsI7ascDAAAAEAOCFGXVGW7W6W7Oo7Oh0jEGaQBAAAAEAuCFGXVGW26W6Mu6vdGbW/4Q6XL9a3wXoyqqlKuRek+wmpEMjjqyyavaaH59H
GPu31edcGHd5fZI1J4zwPNvbWXgfsgB1UaDtSNb1fqZJZT4o7lmEgrpqrrTDAfMsq2vQddcExmr3iHvjOdq6J
2h4snM3eA7Uw9yc3Z6x+xzPPy71CLkXbAnaHvI9OHq7vqTHE6/dAAAAAcfYSoy6yyJXW3OGpz
VTYaDTnnJ6hyIWSM9FAIo3VqpQwmoCrK2hndwM6Bd5nFmNId2dHLno7bcuubn/5zKsnPa
D6gP/AGy9izv6PBo1Pe1wrCSDo63TPb8dqZUKUhrYSXM1aUimtjPY1qasDoZtEx2T2rZR+9S6H+te1

+kREu3HCSwHz4HSXB0dKibnnOtV70AUtW6Kc9TeGFHPNGS7E4vZbgAAAAAlgQh6lILTjLVrLnBTF
nqF0Imk2pv+KGKV4+yL103ie3UZD3lhKW4dka3b18O3U23d42JhYxw0hjqferEnAz0rpz2yiY0d4M9
Ji/u6BisfEacDLUnjXX3GlSlpc9v3+0xnOr59bfvdUhWIdlAfdzx/O1494Hazrob/E1zTMbr9zZlc9v62PX
vV1Wv1/6cXXIi2X56jXUvsCzXu85r+8MlLiqR+4wr/BzteOl/krZbr3X/0hH2PRjY7rBkxzOu3QAAAAA
ALI+C9QtyXz/HklK9SwM9zlSwEhKq+K8rS71rTubTk8aK24tOBS/OZEYjX6963bk96Ubsy+dvW4Vde
9ZG1BBs1YOwWq1Ks9kcWu+3IaxdcdeNE387w+2yV3rnwG1/6DqbsV0VnO2FBNwB4e0wr3O11fc
nDEt0PSK2E9ivvuaS7JjMbQdfr41qa6JzF35+FOdanQqsS3p+lETnOlHbRwht44jjjDietO4RYBr3Pnyvv
OXCW/QSACAP99/xLnnNW2/SSwCAzB3/NZFjr9ILWBT0REVwkikVtoQGJP5Qeb/npMsYdm/2GE1F
UU6ccp51dJfTrj2WvywnT5+0e9QF11uqp/0AbA74vWmb4nVG7R44gZT1GW8ov7dOdT4sGbUo3S
BMfcIsazBr1jUo6ac28z7QPYSnOKZEvYkT7cec4f+kmIdQ3NyTvhmgTm3wHIWY6rrrurFuwJmaGO0
GMvDwEw/LOz/5Tr0EAMjDDY9fJXd/6AF5/Bs/0msAAJm7dI9I/zt6AYuCEBVBA6FScsO1P6fl1RVt7k
pbenJoj9k/JScqJ6z/D65XGeqc9ZIrrombLbpD+ttuIl09a/TozUtaAZl1DTJrOyHeSKpXqBe0GnVjgQX3x
NNPSOWPKvZHAEA+nvPjY/KWL94oP3ryGWm+/3555p8u6c8AALL1lMilP7A+PussYiEQoiJlGYRfXl3
RfTlsXxS7lKgd9lbEyVeN9XMZvhk9NO3A1+/VOyrwVcO67VqVQ480e0lOK1mv47yOaTHOXbjxbW/
Lhtnr1K2LatY5BRbUnZ+6Ux58/EG9BADIwxv/
+gZ5wQ+vsJ8/9NXvyp/WeR8GgNz0Hxa5/Od6AYuAEBUxleSkN1LbnBFfMSZJitM70Ri+HI+7744c7D
pf686+X7I/4a8fLjUwH4qbZ/0guOFOjFWVQIZacsoTKG6Jgvk22OvYvA/0seV1TIn2Y97LA8Pk1cz3hZ
xnoE/SdmNSNTtwdeukJv6eAubL+z77PvlM7zN6CQCQh1c+eK3c/PXn6yXH5z7+kHzlLx/RSwCAzF3
6gsjlr+kFzDtCVMRUlM2zbuVUc/Z1NbrYnOl8y+vt5wScijnre1s23JlrYvN7cqqJpBS3XmZRF0x117vh
6vxxe81a56Kmj3+wdqsx7F+aq7LhnTIV7Onh297K+dBcNQLH9nm/hqd7bHkdU6L9mLVbm7Lqre/p
me+ta1Qq+NvI2oTnyA9c3XYDi+kT+5+Q+n11vQQAyIOqg/pzD7xELwX94W9/Tb73raf1EgAgc5f/RK
T/fb2AeUaIivgq29LyctSalHTA48w8bynX5YJR4NOfUMnSXNWB0GQ1HAPbsp55Q/a9of6KMUnTHP
Jqu9rKUt8KRKiWomxe8IdlN1d1gOaFzqMm/ZolJ3AMttM8tryOKdl+ipsXpO6/2HmtOZGTdS/7h6Br
7yrua1NNWBO03bzfw9odR+bHA8R339fvk7v+7C69BADIw/FLBXsYv/oYRgWozd+8Xy8BADLXf1Lk
8sf1AuYZISoSqWyH11+stqz17tBiV3FT9gZfW21NVr/R7K0XKBlgDM3OdKKjFJgB2KiyA+qc9bt+wOe
ya1/OW03PstS7fT9Yt1Wl1d8LTpaV1zEl2k9RNvf60h16sXq5MUzeVpHt4EGK7B96PbFTEbvtVlv6Lf
8+sqlzblwHu+5ulByOB4jBnYn/6Wfp7QQAeVr50kvl2iev1Evh/uFL35ZP//6hXgIAZO6y9RvZJcpbzbt
CX81aAgAxtTcK4nSSVCHqQGAKADGo4PT1/+318sAjD+g1AIA8lB56od0LNa6tu8vyiteFD/sHAGTgit
8QKfBL9ryiJyoAAMiVGsJPgAoA+VK9T1Uv1CTUsH7qowJAji79AfVR5xghKgAAyM1HvvgRezIpAEB+
VP3Tt33hZSProI6iAtS7/+NX9RIAIHN2fdR79ALmDSEqAADIxe6Du/LBv/igXgIA5OWOL18/tg7qKPv/
8zH53Mcf0ksAgMxd/n9FLn1BL2CeUBMVAABkTg3fV3VQmUgKAPJ189efL//8Czfppclc+dzjsnX36+
XWV79IrwEAZOtKXR/1Zr2MeUBPVAAAkKknnn6CmfgBYAZe8MMrEk0kNcoz/3RJmu+/X3705DN6
DQAgW9b77aWPWx+fchYxFwhRAQBAZlRwqgLUh594WK8BAORB1T99yxdvlOf8OJ1f+R7/xo/k7g8
xKSAA5Kb/HZFL1EedJ4SoAAAgM+/77Pvk3ofv1UsAgLz83AMvkRsev0ovpeMrf/mI3PtJ/igGALm5/
DXr0dELmDVC1KXWlo1CQQrGY6XR05/LSa8hKxttveBob7jtWZG8mzM3Qs5LHL3GinPuVhoy6tT55
9d55H7NjwLuWyCW+n11+djffkwvAQDy8vJvPE9e+eC1eildn/zPB/LN//19vQQAyNylXZH+Y3oBs0S
IurR60lhZlaZecpTlzFpRP8+eHfiVasLfVIImPi+9hqzXor5KXfOCrAYvunRqJSlMENguK+5bIB7V+1T1Qg
UA5Ovqp47LG++bvg7qKKo+6u//
+y/bHwEAeVD1Uf+79fFZZxEzQ4i6tLpy4KZA5bp0+33p9/dkM68MdWzgt6QmPS+qZ+S4YK99XtxN
V1vqevelWy87K5qrQo4aA/ctEIuqf6rqoAIA8veWvRvtIDVLqifqH/721/QSACBz/UdFLv2xXsCsEKIuq
96h7OuncuqE5Nf/FGmL1zOyJ41zugtquS5bFedpcfOsVJ2n0twlRQUwPTUTf+WPKvZHAEC+Xvu1F8
vLHr1aL2Vrb/f/2A8AQE4uf8l5YGYIUZeQXbvRDN2aq04tx8GuiKp3o1E7c1T9zGAtyGCd1bDejbH3r
wVreI6qN+kMVfdfl6zW52A9S6+2qH6ENy3GPgPncEP8zZjnydln0vOiqHaWYvWMNHoeB0LzkpzUn
VFl/9A6olGM9obVW21v6GMZPAcJrkvI/RZ2vePcb4PXz35E1ImNY5b37WT3p2vSa2eZ6D1Ar1TMr

49uJI6QOz91pzz4+IN6CQCQFxWevubvXqSX8qF6o1IfFQBydOlPqI86Q4SoCDUUGGl2/cyRYVRHai
WjzqrR43EyanuDNTzVurCgpuQNVXdFt3UUZ5+DwWRzdcJ9Fjflgjtk3joz5/RGeo1z3nkq1y+kUEahK
q1+y+tVOsTseTxK50C6+umwipx2N97ZkYsDJ7W96x2NX1c3yXVRQV7I/eZcj1EBZPj9pu7d0GC5U5
NSIMjOSpb3bcz7M2CCa2eZ7D0Ay07VQP1M7zN6CQCQFzV8Xw3jz5uqi3r3h75KfVQAyI2qj3qP9Z
H6qLNAiLqEKtt96Xfr4kZ7Um3Z9TH72zrxbG/4AZBXL9Won9mpyfrIxEaFec7r+3uboWUCxu4/wN+
et3/pyI6XBPWkse4GPca+Wzo1imzrKP523M2ofdbOu/Fbsn0WNy+If+rOS9v677wbglnn94JOUJOd
F4P9um2ZLK8uyolT+ukYlS23beb5V9ri53BnxMnhkpwj67VGqYGh+21ofyZj2/b9ZrYlbFt+kJ3U/Ny3
/uvD789hya6dZar3ACyrT+x/wp6NHwCQv5UvvTTzOqijPPTV78qf1hmBAAC56T8scvnP9QLyRIiKIW
bPtPoFPwgdDgOHletbE4Z54aotPxwsrp3xAqzOge4z2bsoO14eaey7suW3deeiJIl7zH364ZMh8T6Ls
nnWTbuaslpwe08Gz+8kipt740PW2PblMOpEFdfkTNjxtXf9XrVn1pzjSXSOrPOzp0NEI3gPvd4Dhu43
s8et0evSPk86CNzLYfa0LO/bsfdnmCTXzjLNewCW031fv0/u+rO79BIAIE+vfPBaufnrz9dLs/G5jz8kX/
nLR/QSACBzl74gcpkJ/vJGiIoBI3qm2Yqy5iYxIwK3UyfSDKjKcrKkn47SPdC9+VSoU/LqNhbMYdKRw9
QnMMk+K9tGr0FHOsP403RKoi+fcf2NgNIP3Kpy1j2gqa6LruEZOrw/aOh+M8JCawf20Hd33yNrsaZ
uHu/bBNduyvcALB93Jv6nn31arwEA5OWGx6+Sn3vgJXpptu7+0APy+Dd+pJcAAJlTw/r739ELyAMh
KibUkeHOgTHCo7mX3TFUvMKUSrD+5Oz05HBssVSf36vSHRZuBG7V08FeoUmYExB5PXXHCbtWR
dm8EN470w0rF3t+o8nvz/SvXdh7AJaNCk5VgPrID+h9BAB5e86Pj8lbvnijHL9U0Gtm60dPPiPN99+v
lwAA2XtK5NIf6OfIAyEqJjR/gWm53vWGbAcfk9YLHS/
+Po26n7aO1NZznJyneELGlj4tn5Sxl7S4KW5lAntYuDF8vno6/CyPP0dt2TB7nbo1OM36o0lYbdzT+xj
s/as0z83XpEi53bcTXLtoR+GPJpiWGsL/wCMP6CUAQJ7e+Nc3yAt+eIVemg+qPuqnf/9QLwEAMqfq
o15iYte8EKJiQElOusnV0EzePbnoFnIcO/Q7J6WTXtA2qm5m6ibYZ6+xrodpl6Ved5OsPCfnMa5rc1f8
zphdOfAu6QmJc0m9HrXW/XH+/I4OP6sSyOGSnCOzLqcKFN26qMaQ96TUzPKqx6k9GZQdSHa9W
p7pDpOf0CzuW0usa5fme8AU1xDz7yNf/Ig9mRQAIH+lh1448zqoo3z6v/6D/MOXvq2XAACZu/xZkf
48dRU6ughRMcCcBCnYW9IPAlXYNTChz6yYNTCbq/5Q7faGHhpuPdIev510n72GrLsnrnpWNjeNyY
Nq65JPjmrWsmzKrm5er3HOCzBj90asnBbnDulIs+ke18Bw8Amvix8oDvbcjUlvX80sH+xxaoTFZo9b
oz1p3yaRZnHfKnGu3QTvASU/dZXaee9gZMOb4h9Hze6Du/LBv/igXgIA5OnaJ6+0Z+OfZ83fvF+
+9y1qZQNAbtSw/v739QKyQoiKYeYkSJ2alHSoU/LTE7kw7YxI5vDy5uoUoVGwBmZzVQdQXnhTlVZ
qs9e7kuyzJ411d6h6Wepbav3okCq98zLMnFndbbN3TastiX+aKhIo72oZDmATnCMv2LO4x2xOsJTEi
HvXrLNaPevPNp/YQt+3SpxrZ0n4HuDXW7V41zBubVssGjV8/85P3amXAAB5UvVP3/aFl81NHdRRV
ICqglQAQE76T4pc/rheQFYIURHKHgYdUpOy2rLWu8Otp1KR7cGClfuHRs/BBOwamMZwbZddWzOj
eqgx92n23LN7obonbiCk8of1p3hehhRlc2+4Tqg9hD5hYBeYJMs6ZjsbHhT7uljH3G/5QaqtKq2+0dZ
ACYJo9r07eJA2Z5vTZZMLft9aYl07S6L3AHUsg6+ttiava4u59cTTTzATPwDMkOqBqnqiLgI1pJ/6qAC
Qo8vWb6bUR81Uoa8KBgJAEmo2fT0ZlAph96btmTxL+lhOtaYNWBfEUbp2yJUKTit/VJF7H75XrwE
A5EnVQVWTSS2SK597XLbufr3c+uoX6TUAgGxdKXLFb4gUbtbLSBM9UQEkFCxRcGZtsUO49nnrWC
J6ZB4tR+vaIV/v+
+z7CFABYEbULPx3fPl6vbQ4nvmnS9J8//3yoyef0WsAANmy3m8vqWH9TzmLSBUhKoCY2rJh17o06
pWaJQoWUK+xIqv7demmUqJinh29a4d81e+ry8f+9mN6CQCQJ1X/9C1fvFGe8+PF/NXt8W/8SO7+
0AN6CQCQuf53RC7doxeQJkJUAJNRNS8XfPx7cXMvpRq/C+YIXDvkR/U+Vb1QAQCz8XMPvERueP
wqvbSYvvKXj8jnPv6QXgIAZO7y10QufUEvIC3URAUAAKEefuJhec32a+wJpQAA+bv568+Xf/6Fm/TS
YqM+KgDkjfqoaaMnKgAAGKKCUzWRFAEqAMyGqoO6aBNJRVH1Ue/
+0FftjwCAPKj6qGpY/7POIqZGiAoAAIbc+ak75cHHH9RLAIC8qQB1UeugjvLN//19+cPf/ppeAgBkrv+
oyKU/1guYFiEqAAAIUDVQP9P7jF4CAOTttV97sbzs0av10tGyt/t/7AcAICeXv+Q8MDVCVCTUk8aKm
uVbPTaknWjdOGltZ96MOYZeQ1Y2gmvbG+7rV6TR0yunpfZjb3NEO2zTne9M2m1bkPugvaHbWJC
BSwosjE/sf8KejR8AMBsqPH3N3x3tuqGqN6rqlQoAyMmlPxHpP6YXMClVGCGeo0VKZRq0tHLWe
pd3DH205RdQj4AA+77+n1y15/dpZcAAHm7+qnj8pa9G/XS0aXqov7+v/8y9VEBIDeqPup/tz5SH3U

ahKhIqCibe33p99VjWyp6bXby3l8WRhxDryHrtTziU6UnF3eC+2qea1hrF8VRuA+A+aZm4n/nJ98pT
z/7tF4DAMibClBVkLoMVE/UT/7nA70EAMicXR91Vy9gEoSowDLoXRQ3Qy2Xy86Tzo5cXJwUdTFUt
qXfrYs+w8DCUMGpClAf+cEjeg0AIG8//ffX2UP5l8m9n3xYvvKX/NsDALm5vGc9mOBvUoSoS8eoLbl
i9kQctd6oc2mvz7s25fD+IutuGnU/VwKfNLcT9vko6Z8z+/PmMP7mqvP5EYU0/WNWj+T1Rv2h/FU
5e+GMDvk6shMnRQ3UUg17jG5PvHbHuTbD59DaumyYrzfqkQa2MbA+/BTHaYN5POpY/P2rh7vd
9nnrupbrsjXQXdYu3WC83n4M3DfArKgh/A888oBeAgDk7YbHr7Ink1pGd3/oAXn8Gz/SSwCAzF26R
6T/Hb2AJAhRl05R1s64PREPpOs8s3TlwE30Aj0U27LbdJ6Vz6xZXz17ldNV/awjB/4B2PywsCxn1nRr7
RCwJIMj5zu1UswQa5bnrCO1UkFW9fYcal2SINUYyl89LZXimniHs3Nx+hCvfEbcU+2L2e6pr43Dfn1w
Z846FVQOrG+uDgSpE7VBHcuqeFvWoakKSlf369Ld2wxcdxW+lsJKN3RqUprnibKwFD7yxY/Yk0kBA
GbjOT8+Jm/54o1y/FJBr1kuP3ryGWm+/37qowJAbp4SufQH1kfqoyZFiLqEimtuT8R9OXQTovauHwi
JEU72Dq1XOU6dmIcI1VI5LW6M2gzMjmSEhV6w15PGutvjsyotu6am9WjpLXRqsh4jjUz7nFW2rTa
Yw76rLadd22HVPv12d+vuV8TsRaoYQ/mrp9X2zVA4xpD+4qbsuefNfnTFa4Z1BPULwcDQN67d6V
wbV7neDX69Frbev2+maYPxeh2aFjf3vOc+P1RXYWtXf41/TppyLsFxAmnafXBXPvgXH9RLAIBZeONf
3yAv+OEVemk5PfTV78qf1h/USwCAzPUfFrn853oBcRGiLiOvJ6IfaPUOndivWnXCo32dFJrDwO38bS
5UZMsNoJq7fi8+s+6n2wPUXFff8ickqmx5QWCs3pgzPGfVlj+Rkh/mWi0Z7IY7gtk792TJfmJsJ0EYq/
Ua636vzepZ2QxPUMe3O61rY6vKWbchRsg+cv3+obPdKdoQeH0UI1Q3Q2s7cNWB6t6okwhkSA3f
v/NTd+oIAMAsvPLBa+Xmrz9fLy23z338Idn/n4/pJQBA5i59QeQyf8BKghB1KRXlxCnnmRtode1x6W
U5efqkHXYF11vUMHDn2VzwQ7mmeJ0KuwdeWOgN5ffWWcfkDu+2H8bw7cAQ/VFmdc784HMy
Yb1zLRMP6W/LeT9BlVZoz1klRrtTuzaW8kkJ3d2o9a4p2hC7Z7Zxrq2N2WUO3P3Er8sLpOuJp59gJn
4AmLGXfPe58nMPvEQvQbn7P35Vvvct/m0CgNxcVvVRv68XMA4h6pLy6oraPTl74nSqPCUnKies/w
+uV3ngPEWoFiOYcodmt90x0xG9I6exkOfM6GmphqaXQkPC+LP0tzf8OqBVo6fp8kkSbhdl80L4jP1u
cDtiPjEgE+5M/A8/8bBeAwDIm6p/usx1UEdRAWrzN+/XSwCAzPWfFLn8cb2AcQhRl5U3tHlfDts6aL
N77VXEyQqN9VP3hsyCUdfTDi/9upOjwkuvNubQI2YYuIDnzB/KHyXmkP72hj9RVLUlIzuhTmDqa5O
CTNtg1JUdKNlqa55jln7k532ffZ/c+/C9egkAMAsrX3qpXPvklXoJpn/40rfl079/qJcAAJm7bP02eukze
gFRCFGXVklO2hlkRw52nSHNbh3Rkv0Jf3347OuzV9w864eaDXeSp4E6pCVnqL3i1eKc2KKdM2Mo/
xjjh/S3ZcNLUMtSV1PRTyvVazOhHNugZuhXPU7tScXsQNWYoCtu2QJgSvX76vKxv/2YXgIAzELpoRfa
D4z26f/6D3aYCgDIyeXPivTp2jMOIerS8ntyNptOOObWeCzq4p/uem+Sprnj9gDtSK2mA77BOqRmP
crmqj9sur3h1aUsxB5LvWDnzBzK787+H3iYIV70kP5e45w3jL9cv5BOuYRUr82E8miD3pbKoIM9Trvi
ls8dW7sVSMn/+v/
+l34GAJgF1ftU9ULFeHd/6AH9DACQi0v36CcYhRB1iZkzpqvehd7w88Ds5sYkTXPIq1NqC+shGaxH2
VzVwZg/Ll1GT440LNVzVtS1VJXmauqhoTmUvxxaW8AoiWC9cuSQ/l5D1r0CqtYrAxMwOY/JJkhK99
pMJoc2VLb9IfyBurRGfdmzm7MP3bEU7v7Fu+WOl9+hlwAAeXvjX99AHdSYfv7dP6WfAQByUXijfoJ
RCFGXWWDW8FPiTzbuDltXzPVzyAwvRw2ht+tRGr0uXeW6dJPWu0z1nFVke7BA5v5hSrUxzaH8o0
NdMxQeNaQ/Xl3VCaV5bSaVQxvsIfxhxVBVSNvvp1pfFohy1RVXyf/4lf8ht1x3i14DAMjT373yCf0M
UV7z1pvkbe+5VS8BADJ37FUix9+kFzBKoa/G9QIAgKXxwCMPyOv/2+vtmfoBAPm648vXyysfvFYvYd
D1P/E8+a1PvVGedw0TbwFALgovFrni/daTq51ljERPVAAAlsztN91uD+0HAOTvb27/tjx2PX/ECnPlc4
9L9fdeS4AKALmx3m+Pv8f6SIAaByEqAABL6F2n3iUfeMMH9BIAIC+Xjvfl8294VH78nMt6DVz/8t+V
5NZXv0gvAQAyd+ztIoWb9QLGmSpE7TVW9AQpBX9W65F60lhxJ1TZkODL27LhTbbiPCabqGZYeyO
43dBH1jOAAwAwhz788x+W07ed1ksAgLz84PnPyt7rvqWXoLzidS+R1X8bNhkqACATx4oix39eLyCO
OeiJqsJVf5ZqR84zwquZ0VcaKU3oAwDA4rjnl+
+xh/cDAPL10M0/kO6t39dLy+3aI14l1d99rV4CAGSucI3IMTWMH0nkGIWZXOvL2oeq35gxuuuHH
iTiKvZsNXn92QzxwzV1qnJeTqkAgCWjDtj/3VXXafXAyonqjfu+aZ/TS8lIBqgpSAQA5Of5ukcIL9QLi
mn1P1N6h7OuncuqEZJedlqXedUNc/9Gtl/XnRZq7pKgAgOVzy3W32EGqClQBAPlR9VE/Z7/Zz8xivsofwAgJzYdVDz7rl4NOQYog7XRLXrlZZq4nZEtYfVD9UoNb/OeaRVL1Uprp0RP0YFAGA5vfm

WN8tH3/5RvQQAyIvqibqs9VFVePoLv35CLwEAMmfXQX2HXkBSc1ATNUKvISuFktS8lNXRqZVSq2H
aPu+HuNXTfpGB5WVM8uWd46hJwTA7YddqEYTfT/4kcHneY0nPYU7fC+q9b2DCO//8rEiKf0cCAt7
7s+
+V2h01vQQAyIuqjbps9VGfd82VctfvUJMbAPJztcixO/VzTGKmIWpluy/9bt3vCVptOcPst1WY2ZPGu
htwVqXlDsFvVe01qobpeqIkoSO1khtC+I9Vd0Yra9/2bgFghnqNlWAPfSBnqjeq6pUKAMjXfT/zuD1r/
7JQAer1P/E8vQQAyNzxO0UKL9YLmMT89kTtXZQdnSKU61v+RFSVLXHLmHZ2LqbU+64s9S0S1NFG
TQoGTGKR76eM295ryPpg13tgBlR9VFUnFQCQnx8/57J8/g2PLkV91Le951Z5zVtv0ksAgMwdf5PIsV
fpBUxqfkPU7oHXE8sevu/1HjWG93cOpKufTsfppZpmrVUAABaVmqm//attZuwHgJw9dv3T8je3f1sv
HU23vvpF8ku12/QSACBzhVtEjv0LvYBpzHdN1FSFz85vlhPo1M7nWItxHgxO2jWqxuPoOpD20GPv6
/VjZG3Jwf1FBNd2Pdzga0fVgsysDVEya98oca+VK/5xJm1f2OsHyncO1O806o7arx19P/kG2p/K9Ux6
DsOEt32wXungORo8P2HiTbTn8/epHqPqpCY5P0DQbdffJvf88j16CQCQl7+/7Xvyjy//oV46WlQd1O
rvvVaufO5xvQYAkK2rRY6/2/p4hbOIqSxEiFqud4fDT/uRwnDa4qac1WVWRfblcGnyBRVsDU7a1ZTV
wqr1/3hUiFMKG3rcqUlpMKBKMklYe2NETUjVYzgYFmXWhihZtW+khNcqwXEma58TyIW9vrlaGBH
2qXNitLNcl/GVM9SxDbR/6us5/f0ej9OrffAcNVdHhZyTcPbh1XO2Dd97qd3vWGrvKL6DGfsBYAb+6v
WPHcn6qL/yH15JHVQAyNPx09RBTdH8hqilk34P0YN0Bu2Ha8tuuinKQug1zvnhkTuhV7/r1Zsdzzhv
5bp0dbDd9TbQlHNeotNLMEmY9dpzesOh2+3IzkX3tVm1IUpW7Rst2bVKcpzJ2tdrrHuBnP+HjZa1F
615bkRQaLRjb1OKem0koz1u06e5ntPf70n47fHb3pHa+ejIPHqivUH+PsLvvbTud0Ds2frfdepdegkAk
AdVH1UFqUfJyumftB8AgJwce53zQGpSC1HtnmjGkFHzEWco65DimpzxspxVfxuqF6C77UQbdnpv
me1yHmYvuTOyFivhWXQ9uejO2qUCFi+kKcrmBSPEidI7lH39VDo74mY3xc09J6yxHnub+mQmmiT
MmLjHCNyKa2eGQ/XM2hAlo/aNlPBaJTnORO0LtuOst74iW/aGVVC3J2GHE2hHTNWz/rmtbPtBrd
/2JNczhfs9gWrL7yFf2Up/+4q5j/B7L8+J+bAM7v7Fu+WOl9+hlwAAefjmjU/JV376u3ppsb3sp14o/
+a3mNAEAHJTuFHk+L/WC0jLHA/nDwYcXkjrjWE1w5B0mMHN0daVAzdTKp+Ukn5qK56QU/ppJDP
kHgioh2ouTjVJmK6nGTZ8Prc2REmpfSMlvFZJjjNR+0a3wwldR5fWOHUi6XdVVU4HNlaSk2473bYn
up4p3O9zpSwnAwcRIteJ+bAMrrriKmbsB4AZuP9V37HD1EWm6p/+
+n/5GeqgAkBurhQ5/mvWR+qgpm2+a6IWN2UvbMitPdQ3hXqoHtWLri8pZ7JH3OhefG5oM1EPZ
MWu5eiGPlE1KzNsQ5R5b19sebQvRuA3VlFOLEzSmcbxAvPpphfcZAepKlAFAOTn8yuPylNXX9JLi0f
1QFU9UQEAOTn+r0QKN+gFpGmqENUc9hv1cMLJoj8M2gxA7aBUrw9NMc2v04+4dRUtdp1B82tD
H2kGskvEuHZ+7Udf89zw5DXjJwlry4bZq9OtjWnWijRl0oYo2bcvLbGOc4btG21wgreeHHp1B4ZlOv
HcEcD5QZpuv+l2e2g/ACA/KkBVQeoiel3l/6IOKgDk6dhrrQd1ULMy3z1RkZGQ4dEus05mDGp2d9V
b0Q+rjZ7D7raTTBLW3vV6dtrhjxuYG8OTB6XehihZtW+khNdqguOM176Idth1itOdfT7Y9JDh+ImO
M737fWHkNjEflpGaZOoDb/iAXgIA5EEN6f+7Vz6hlxaDmoWfOqgAkCM1C7/qhYrMEKIupaKs+bN2
yao3Ztuc0XsMPcGXKlEb7K0YEnhNOEmYH/4YM+KbcmhDlFTbN1LCa5XkOBO1L6Id9rE7NVUTnsK
Rmqsb4jfdL5lQPrPmhNaJrmcK9/uiyeB+B0wf/vkPy+nbTuslAEAeVH3Ux65/Wi/NN1X/tPp7r5XnXX
OlXgMAyJaqg/oe6+PVziIyQYi6pIqbF/zehs1VHawYk86MU9n2h393alJygxmjRqg/UVew9mbkJGGV
09aSNq5dWbUhSmbtGy3ZtUpyrpO1b2w7ynXZSmV8eFnK5aas6vZ4Tbfaftab/j/Z9Zz6fs+LOdGV28
6Jws6U7ncgwj2/fI89vB8AkI9Lx/vy+Tc8Kj9+zmW9Zn79Uu02ufXVL9JLAIDMHVsTKdysF5AVQtSlVZ
TNvcFJu6rS6res/8djD/8OK6Rpb2dgoi679ubg/ixDk4RVZHuoDc72vF01d71eitm0IUqG7Rsp4bVKcJ
zJ2qfa0Zfu0IbVpo3SBlM7JWcHjzfsGiW6ntPf7/mw7q/B67F/aPQSTiCV+x0YzZ2x/7qrrtNrAABZ+8H
zn5W/ev1jemk+veatN8nb3nOrXgIAZO7Yq0SOv0kvIEuFviqCCAAAMIF7H75XKn9UkaefXYwhpgBw
FNzx5evlIQ9eq5fmx7UvvUr+06ffzDB+AMhL4RqRK9R8BQzjwzM9UQEAwMTefMub5aNv/6heAgD
k4W9u/7Z875pn9NL8qP4udVABIBFfH3239jwA1L4SoAABgKu/92fdK7Y6aXgIAZE3VR/3cm75pf5wX
v/Abr5BXvO4legkAkLLjbxcppFNUD/EQogIAgKmp3qiqVyoAIB+qJ676re676ll2ZLhae/8Osn9BIAIHPHiiL
H36EXkBdCVAAAkAo10dQt192ilwAAWeve+n37MUuqDqoaxg8AyImqg3rsPXoBeSJEBQAAqVAz9b
d/tc2M/QCQI9UbdZb1Ue/6T6+2g1QAQE6O3SlSeKFeQJ4IUQEAQGpuu/42ueeX79FLACIsqbqon3

/DozOpj/q299wqp/7ZDXoJAJC5428SOXabXkDeCFEBAECq3lF8BzP2A0COvv2if7Jn7M/Tra9+kfxSjV
/kASA3hVtEjv0LvYBZIEQFcteWjUJBCsZjpdHTn1tSvYasbLT1gqO94Z6fFZnX09NrrDhtXGnIqCb6x+E8
lv5aY2mo2frfdepdegkAkLW/v+178o8v/6FeytbzrrlSqr/3Wrnyucf1GgBAtq4WOf5u6+MVziJmghAV
yFVPGiur0tRLjrKcWSvq58vHDiJLNeno5YXRa8h6LarV6loXZDV4saVTK0lhIDAGjqq7f/FuuePld+glAE
DW/ur1j8kPnv+sXsrOXb9zu1z/E8/TSwCAzB1XdVBfrBcwK4SoQK66cuDmbuW6dPt96ff3ZHNZM9
SxQeScUj1nxwW/7fPiHlq1pa5zX7r1srOiuSrkqFgGV11xFTP2A0COfvycy5nXR33zr9wir3nrTXoJAJC
5YyvW41V6AbNEiArkqXco+/qpnDohy9v/dHHF6znbk8Y53QW1XJetivO0uHlWqs5Tae6SomI53PSC
m+wgVQWqAIDsPXb903L/q76jl9L1sp96ofzKfziplwAAmSvcKHL8tF7ArBGiLjGvnqP5CKntOLI2peqN
536d163Or/dp135sb+ivNdbZLwuuD/bKS2MbmtlG7zFcYzN4jMM1S+1HWN1Low3jal3a+zDDt+aq
87WDDQ9pc9i2o9o8tpfjROdl+J4ZuZ8YxxD7fGh+W9RjuK0OZwi9/7p0z506/lKsnrNGj+NAWF6Sk7
ozquwfDt9PNqM9ie65eMduS+H7wj1Pcd9HsNxuv+l2e2g/ACAff/fKJ+SbNz6ll9Kh6p/+
+n/5GeqgAkBurhQ5/mvWR+qgzgtC1CWlwpHQMKhTk1JhQ8ZlcHHYtR8HCkLa61TIMrC+uRoeXk2
1DRU2hfYY7EitNCqEU58zapaW61J3uw52duTiYMi0674ynbqmQ8GiZh/zyGBquM1uz8dQE5+X4X
umuToieEt8DFGcfQcvd0hb7WCw5A2hd6V67jxVafVbXq/SIWaP41E6B9LVT4MqcjrpPZfk2FP6vlDn
KY/3ERwdapKpD7zhA3oJAJC1z688Kk9dfUkvTe/f/Nar7J6oAICcHP9XIoUb9ALmASHqUmqLn8O4d
TmNeo3SlHPhSUpi5XrX3na/FYybwtaPGt482TaCw6mHj7EjO4PplEcFZM7r+3ubsrlVF+erBr/GPI9nZ
FyGWtm2ttd1t2Wptpx9bOvUrr3hB4Vhbe7UZH3kdQm2eXRT0jkv/invSO28cd0SHMPY8xHg7zu8r
dZxrbvBoHEu3Iamcu40u53bEitrHVKUE6f00wiVRPdckmNP7/uimOP7CI6OD//8h+X0bQxHAoA8q
ABVBalpWDn9k/YDAJCTY69zHpgrhKjLyOwlZ/R0K27uOQGJ9dhLZaajqpx1t1M5bS25RqwPHd486T
aKsrlnBj6O4toZL7TrHIT3AyzXt4IBWXFNzugv6uxc9NvY3vV65pXPrI0P38YwexjWLxht3rwgfgZ5PrR3
31CbR5r8vFRbfnDoh3xB0xxDFHPfoW3tXZQd3SEycC4qW/5+zWtniH/u1HFY3yOhIe8k9uUwrEFKk
nsu0bGn+H2R2/sIjpp7fvkee3g/ACB7akj/V376u3ppMqr3qeqFCgDIiV0H9V/rBcwTQtRlZAQ0ImqY
bsGrZTiyhuIkyielpJ8GjFofJo1t2HQ9x9BhzEGnTgwGP0VZ8xMtLyzyA0Mj0J1YVK9WY/8jgrfhNscV/7
yMN90xjFaWk+MudvfAa79X7sF+GEPcRwyfn/zcTeuUjN51gntuimNPcv2HzlNe7yM4ctwZ+6+76jq
9BgCQJTXJ1KT1UVX907t+59XUQQWA3Kg6qHdaH6mDOo8IUZdSUTYvhPckdEOYqMl1FkZg8hyjn
mOk8MDO76nnDnc2AsPq6dg9GafXkeGOgjFCRlOK52UyYccwK2keVxw9ORxbLNWR2T2X2vVfkvc
RZOKW625hxn4AyNFfvf4x+fFzLuul+H6pdpvc+uoX6SUAQOaOrYkUbtYLmDeEqMuquCl7esjtQKlR
W/Pcos+u3ZYNs3edW7PRrMGZhHW+zurzZA+NNoYyV0/nF6FOH/qlfF4mkm1w6dXKHXpMWsd0
AsUTMrb06bje1BPcc+OPPf3vi6P9PoIsvfmWN8tH3/5RvQQAyNIPnv+sHaQm8Zq33iRve8+tegkAkL
ljrxI5/ia9gHlEiLrE1MzaqqeYPcGPHYR0vRqKo4f/GoxhxHPHrB2pgiW3/uMUba64U6Z3duT8+R29n
aqkk6GW5KR37gdnZO/JRbfoZeQQ8BgyOC+
+nI4hTOmkFwKOqumZL+NcNHfF75DZlQPvNJxwzn2EWPdckmPP4PpP/T6Cpfben32v1O6o6SUAQ
Jb+8eU/lL+/7Xt6Kdr1P/E8uet3qF8NALkpvFgP48c8I0RdRu0Ne6itmkU92FPMCHiMXnIlPxkzZmJvy
4Y3Dft884MlY2bySXgTWHWk2dQnKrWh/EXZdLsdqvO87l+XXmPdq22ZZBKkcVI7L578j8Fj1udsrvr
DyPW9bj9yHVtu1oBtyq7eda9xzgsxY/VgjnPPTXjsU1//hO8jZntyvRSYe6o3quqVCgDI3t/c/m157Pqn
9VI4Vf+0+nuvleddc6VeAwDIlqqD+h7r49XOIuYWIeoyqmz7Q287NSm5QYtRH7F6NnzmbhXSDL52
Lpkz9nttNibamUhF3I6BrlSH8o+4LiU/fZQL005glcl5MSQ9BnPYu9ueiRK2YH3O5qqz34IX9Felldqs+v
EUNy94PTLd9njnodqSeM2Jc88lOPY0r3/C9xEgiqqPquqkAgCydel4Xz7/hkcj66NW/u8idVABIE/H3k
od1AVBiLqk7KG3YUUMVeDS7wcDHlX3cLBmYrWVcx3NpCqy3W/5gZHNOTbvsAPDrOPxhlcr5bpsp
ZzL2dcl5LxWW9Z6d+j1VLI5L6Zkx2C1Z/A+3D80ejUmYNfnNIaSu+y6nznWQ/UUZXNvuFaoPYw+Q
aAb656LfezpXv9E7yNABDVTf/tX28zYDwA5UPVR7/uZx/VS0Cte9xL5hV8/oZcAAJk7Zv2GfPwdegHz
rtBXRewAxKNmNtcT86gwbG/anqHAOEfpntPHcqpFwIpwn+l9Rip/xM0BAHl441/fIKWHXqiXRK596
VXyW3/8z+yPAIAcFK4ROf5+66P/Xoz5Rk9UILaeNNbdmc3LcmaNABVZO1r3XPu8dSwZ9ODG0fGO
4juYsR8AcrL3um/J9655Ri+JVH/3tQSoAJCn4+8mQF0w9EQFxmrLxmANWFXOgK50yMzRu+d6jRU

p7ZyRbiplKXDUFf6fgn4GAMjStU9eKe9s/aS88723MYwfAPJ07O0M419AhKjAWAOBFgEqMsc9h+
WlJiiblx9NaEs42hKOtoSjLeHmqS0nrr1GDr/3pF6aLa5RONoSjraEoy3haAvSQIgKAADmBj/ghqMt4
WhLONoSjraEoy3haEs42hKOtoSjLeHmqS1IhpqoAAAAAAAAABCBEBUAAAAAAAAAIhCiAgAAAAA
AAEAEQlTkSE2WU7DrfxRWGtLTa8fqNWRlo60XHO0NvZ3CijRibwjp6Uljxb0GG9aVTbJuGmH3UNr
7mJbRRv1Y4SYFAAAAAGChEaJirvUaK1Io1aSjl4H5pgJdY1Z9W1nOrBX1cwAAAAAAsIgIUTG/eg1Zrx
GfzqeibO717RkF+/1tqei1szFPbenKgXvLluvStdu0J5tkqAAAAAAALDRCVABIS+9Q9vVTOXVCyE4BA
AAAADgaCFGXmao1OlC7MazG6GD9UXuIvfE1A+VKNbNOpXokq1Vp79Mcxt9cdbYTvjOjjeoxqk7qY
JvCa1VOdrymMbVf2xvetob2H3JN4rTRY359ZEONcxFo46j1xj7t9ea5zKoOadx7KKotg9uYsD5pjOuS
9J4FAAAAAACLgxB1WakgL7TWaEdqpVEhpPpcQUoDQ+ybq4OvVyFiSYIva8pqYbBWZBqcNq0GNh
xyDHYlNtgm65W1UnjQaYt7vIMqcrqqn3Z25OLAa9u7bmODtTKHQjgtuo2TKsrambLztHMgXeeZxR
iOHmh7W9xml8+s5dDDMoV7aKJrPizf6wIAAAAAAOYRIepS6knjnJuIuXUb+9Kt61BNOrIzmPx5qtL
Sr2+5QaEKG8/7ve16jXN+0FVt6VqVXfE2H0Nl2/qabl28L3G3sx1W8dJvU/gxWMe77oZg/mv77gF0
arI+MhUdf7xhKltu2wfPpR9GSvmMeBlqe8MPgsOuSWQbJ1NcO6PbuC+H7qbbu0ZI2ZEDN101hq
mfOpF9hDr9PTTNNTckuC7J7lkAAAAAALBICFGXkjERz96m16vQD9VEOl56FlRt+RP3+EGhqScXd9w
+e1VpeQGStc8LYa+fntmm0GPoXRS3SeX6lj/xUGXLC+U6OxdDexSOP94RimvidfQ0t22ElGaPTrN3av
2CcU02L/htrJ0fGKo+Ja+NftDbO3Si0mrVCRv3dbrau7jjBZKnvROYlRTuoSmuuWkm1wUAAAAAAM
wdQlRYdA3P0OH9SZmzk5+Ukn5qK56QU/ppespyMrCTEN0D77jsIdheXUtjqHdgSHsazOHy/rB4P5
SryllvyvYRvVNtxnbMHqOpKMoJfUHcwLlrXzzrnJ4+aYeVwfWW6mk/kMxMCvdQKtd8VtcFAAAAAA
DMG0LUZRWYKCdunckYgeWRMt3x+r1i3Z6eRig3URhpDK9PScUt3trctVrXE6cj6ik5UdFhZWC9anb
2EeriSf+6AAAAAACA+UKIupTasmH2OnVrPZr1HI+ocr3rlDEYevjD9lNT3JSzbglONXTcqCs6WRiZQYh
dOS164L4ctvUQeLv3pzs5lrF+QUP07K/5sv1xAQAAAACA5UOIuozMupwqYHLrohpDoCdXkpNuEjs
4XNoIEXNVcoamK6NqvWbF6+nZ2ZHz50fVFTXP2eBs/mZ90FMydk6nxNfQ3XdHDnadr3VrtZbsT/jr
h4e0ZyWFeyiVa57idQEAAAAAAAuNEHXJ+QGTMWP/VMxakU1Z3XCn3DFnS8+ZMcmTNFfFa1J7w
6+V6a1MmdfTsyPNpj76oaH8Rdl0u6xar6utN6yz5eg11r0anuYESU7AqVivP+8dkGx4U8nH5V+vZtP
5Wnf2/aIumOquNyfCylYK91Aq1zz5dQEAAAAAAEcTIeoy8oI9S3NVh0rGhDtTMmcun2r75iRC7nY
mCjuDs7o3V3WI5gWO5gzwaXOHxftCh/JXtqXl5XU1KdnnrCAlP6mTC95EVCojdOutWrxzHLe2bVBg
W9Yzb2i6eZ9Y68/k0w3VNv09lNI1T3hdAAAAAADA0USIupQqst1vGQGZUpVWv+8HRvaEQpMqyu
Ze1w/BbGr7g/scx2qn1yBt/9DrDZhIcVP2+oNtstj1YDOoh2rwhvQr1v62Ruysst0PrUtbbVnr3ZILLnU
8g6+ttiara2v22gwMTTeGs+c+ZD2Feyila57ougAAAAAAgCOp0FczrADITq8hK3oiL1WDdo+eiwAwk
urtPS8/mtCWcLQlHG0JR1vC0ZZwtCUcbQlHW8LRlnC0BWmgJyqQKbOOZ75D4gEAAAAAAJAOQlQ
gE23ZKAzU8ayeFTqhAgAAAAAALB5CVCAPql5pZpNXAQAAAAAAIEvURAUAAHODelXhaEs42hKOto
SjLeFoSzjaEo62hKMt4WhLuHlqC5KhJyoAAAAAAAAAARCBEBQAAAAAAAIAIDOcHAAAAAAAAgAj0R
EV2eg1Z2WjrhVnrSWNFzZavHhsyL60CAAAAAADA/CNERSZ6jRUplGrS0csAAAAAAADAoiJERfp6D
VmvEZ8CAAAAAADgaCBEBQAAAAAAAIAIhKhLqr3h1gddkUZPD7+3l51HaClTVePUeI3zcL7eZW/XH
MbfXHVepzc4uF+PuW1j58HXt2XDfY318F4Wo12TGjwv6jHpuVGSHr8S1obCSkPCD8+s/eo8VkaciG
TbBQAAAAAAWF6EqEuvI7VSQUoDw+
+bqwMhX3tjRI1T9fXpBJbR1H5WpamXpFyXrYr1MbN2OWHk4HlRmquFYNCZ4blRoWtYG6RTk9Lg
BFl2EFuSwZd3aqWhcDTRdgEAAAAAAJYcISosVWn1+9K3Hq2qXqUCwPNulNaTxjkdX5br0tWv7dbL
zjrrtTsXnYiusm19rlsX9zNSbdmv7W+rxHNafjv7e5tSTNCupHqNdS+MLNe7zj77LasFWvOcDkeza4NI
W3bd1Dh020055yW0VjvW3SDXOE/uBe3UZN17bZLtAgAAAAAAgBAVUm1tixtxVraMANRTlM09H
crZ4aVeu3bGe23noKufZadc3/La6ciqXT25uOP20qzK2U13yxXZsoNGFVLuibM6w3PTO5R9/VQ6O+
JmscXNPWd/1mPPbVvvorhNDpynypa42Whn56J1ZJYk2wUAAAAAAAAhKiaha5OGDmHPzqkT44K
9tNrVlQN3A+WTUtJPFSdo9EPnYSmem+KanHGTWGtrquyCW7t0qM5p98Dbnz18X7+uYA7v7xxYR
2ZJsl0AAAAAAAAQoqIsJ82UcJTAxElGbdLcjGjnzNtlyawNRdm8ENYz2A9KQye5Giur7QIAAAAAABx

NhKiIoS0bZs9Kt46mWft0JuahXRm3obgpe3qIvV+v1tc8Nzybvl/DdfBh9KCdYLsAgLzp0Q3Ggz9yAQ
AAALNBiIrx2rte70o7oHNrfxpDyKc2ybYya1dJTroJqDsE3qVm4i8YM+6n1YaI16uZ9NUvzfakXXbw2f
XqnHrtK530Qtu4NVhjbRcAlpH9Xl+QwkwTy540VlZFWu57tPNHr+bqLILU+Qxze40Vpz0r+f/hT/0ba
p4P9zEP5wXDhq7XDO6ZmRvzvuZ9P7mPvG7mOO+3ebwnR+5jSf+gFfO8z/K9eGZinJuh951luGnG
nJfc34sTtofSdoiDEBWJ+AGdMSt9AiU/nTRm/7d+MFmdbhD8tO0KKsqaVzS0Kavem667baeO6OB7
cZw2JDp+/aavPhXsGRpSs9Wsc9pc9dvm/sOhHu7KJNtVjG3wDwuALMzsl/chKri09j/lv0npcCYu3Da
KcLuTP+4f5vte3N4IC3ONPyjOQq8h6zWRatX9x28G3NEnxsO8XrMy01/cAyWOhh+z+NZW52O1qS
YFda9TS6qdmpRmFPjk/0v7+Pc19R5csr6f6l3jHFk/T2Z778R5v83jPXncPtTnc/yDVmTok1eYm+C85/l
eHHFuht739CP98xP/vl3dH/g3Iot/IEadk9zfi8efl3zfiydoj/WNrUrbpf+eHO/7dn5+FsZY1g2DJWS9R/
TV5bd+A+hbPzD5uvW+9U+g87mq9ZZia/WtnxX060c8vNcqIa93P29uf9TD2NbIdtqStKvbr5fd9VXr
K8cxXx/yKNetVygJz83Exx/
+MDcdve3gMSfabqvqrS8PXwQAmEq3XrbeX8z3eP2+GngjyoffFv1vwAzaEEm/z8/8vXjm7XCuj9q/f
c28f5PzY/87OoP9RtP37dy1y/3eivPzV9rCv5dn1R7n5y9jv/pnrCy/l8a/rznvuYNtyPocjW9XvNdMa6J
9ZPIeqPdvbdd+hLRD3T/maud+Um3XK1IS/5w4n8/+vTjeucnjvS/
+fZv1+8v4cxImq7aNPy/h62fWnhHfw+nfR87+zd0737fBdX579Qr9vhz3uiJf9ERFDBXZVn8p0ksO56
821puAo7kr/t9KrNd7n9D2D52/MKlanIP1QqutCWuIJm1XEk4PIOsNTS/7rDdbf9h+0jYkPH57qP3g
ubQ5+wj8QdOuc2oMyXfZPWWMeqiWRNsFgMy05XytY71NXZBN503VUpEt9UY28fv35Iqbe9Lv7x
ltmTO69MupE/PRwFm1o9dYl1qnKmfn9kLNhnteWt7PKPPC+T6X6unAzyL5KMqJU9YH9+dQW08u
7sygPb2GqIFK5fqWv9/Ktv3zYmfnYgY9sRxj39d6h7JvfRj8fi7aJ64puxm9Ecd5v83jPXle3ved79+y1Ls
hP8tr6uf34dEJHdm5mO7dE/ec5PVeHOfc5GX8uXHeXwLf5xmY7Jxk9148/rzk+148tj0jfp6yR42mW
tYuzqii+fpZGOMRoi4pvw7mwJuLMeFQcMiBCgvdr1EPJ5TztxMM6dQPhf5rrYf5A725D/VQ+xmx35
Ht9MRtl/MGFlw3nvMG7H6d89gbakjCc5Pg+G2D59J+jDoG8zj1Y9QvU3G3a7xu+NgBYAoz+uV9M
enSL+W6bIX/A5CTnjTWa9KZVTvsoaMdqbbi/1ueGTUM0Rh6N9uSN/n84j6JXuOc9d1s/bI/oxu3sq2
HjNo17dWwypITNg/
+vJW13H5pT8huV1lOenWcNKPePgZk8AetuMFlmDTbEVuO78XTnJv86fJoB+cHhtOnWwJnknPCe7
FhxPub8/PnvuRaNYmfhRcOISoAAJgNfnmPTdUltX/5uTCLXoa6tpj9i6D+pWdGvR3b52vSqbZmPmL
C/0Op81AjV7KppRZXPr+4J+f2NDo7wwDEHTWkatqr7yOrObMI4efpl3ZMgT9oKfPyXhwwR3/Y6uy
flAvevxGqt6h6/9mYYa9C3osD9HwiwflBrO+pqedWiWHwjzD8LLxwCFEBAADmmDP5gRq2N6ueO
MFRDt36vqzO4hfU9oYzCcRc/dbuUL2Csh6WHcfc/eLePi/O6NHZXTNnsg5/YiAVeKtJgXKftGOWv7Q
jNfxByzKH78Xz9oet8pk147pY/4aeVWXUZtirkPfiAernGrdnrP89dXBKXadTkl3n7nn5IwymQYgKAA
Awp5wAVfXWmJ+hjMXNC3YNtk7tfK7hXHtXhU1NO8B1fuEpSEn9Vqh/CZrtcHptlsOyLfP1i7sOCGf5
y6Iecqzq2bt5jz0MViXezdWMZjYfZVa/tI9h93bqyMHgjTuqd9QS4w9ajkV4L56XP2x5ZtqrkPficIMl+f
qydXLfOk8nJau3vdn+EQZpIUQFAACzwS/vkVSvDSdAndMJ/zL8RSPMYE8j9bAngLR+Mexaz2dbt7sn
h6qoWc7nZKxZ/uLu9nw6O8NfFgeHTbpmdl7y/6V9rOIJceZ7CUZNPfuGnmG4O2f4g5Zvvt+LB+T+h
62S2GWOB8PbWf5cw3txTLq2eOCPkekZ+UcYfhZeOISoAABgNvjlPYKeRddiD3czevzkW+dSTf4wM
Bxc/0KW1S8ac6/XkJUVc0i2WqVmSp7lL6nz94u701utKjMcPSpSOW21wPoeCgyhV7dwzTovM26bL
dtf2uOpyGnVY6+27r+veL3G5m+islngD1qLaFZ/2NIjADo1Oe/9w6mHcM+oHinvxXGoUhklqUldLm
RwkSL/CMPPwounDwAAMCOtqvSt33L69a5e0a33y9aPJ2VvRX669bLVFtWewUe139KvWUZh56U
6JyfEblu53s/3bun26+Xg+ZiLe6RVHbg2rb71S2tfJT+5m+H38TB9HgKPefie1vdRxvdv3Pc1573Yf2R9
7eK0K4/35PH7CLt/3Ifxb1dq9H0x9H2r2jFw3Pp7Pu1rNcl5t78m8/fiEedGvd8M7Ns9hrTf/uKem6H
XZfY+POp+0XJ6L453XvJ7Lx7fnpB/xzO6RnHuxXn6WRjjFdT/rIsKAAAwE+5f6F3WD43zNRwQWBCq
x5xdm9BVbUl/Bl3nnO/pqrT6M5p5eS7pnk7G5ZnV9cH8Gfre9fjfR2Gvqc5r79gUjT83Id9bxnk7iuLc
LwrvxbOmRvOoOqhhgkP7+Vl4cRCiAgAAAAAAAEAEaqICAAAAAAAAQARCVAAAAAAAAACIQIgKA
AAAAAAABEIUQEAAAAAAAgAiEqAAAAAAAAAEQgRAUAAAAAAACACISoAAAAAAAAABCBEBU

AAAAAAAAAIhCiAgAAAAAAAEAEQlQAAAAAAAAAiECICgAAAAAAAAARCFEBAAAAAAAAIAIhKgAA
AAAAAABEIEQFAAAAAAAAgAiEqAAAAAAAAAAQgRAVAAAAAAAAACIQogIAAAAAAABABEJUAAA
AAAAAAIhAiAoAAAAAAAAAEQhRAQAAAAAAACACISoAAAAAAAAARCBEBQAAAAAAAIAIhKgAAA
AAAAAAEIEQFQAAAAAAAAiEKICAAAAAAAAQARCVAAAAAAAAACIQIgKAAAAAAAAABEIUQEAA
AAAAAAgAiEqAAAAAAAAAEQgRAUAAAAAAACACISoAAAAAAAAABCBEBUAAADAQmtvFKRQ0I+N
tl5raG94n19p9PTK2fDbuiEhLV0wbdlwz3vk+e1JY2Xca6xXNVb8ba00rK8CAGB+EKICAAAAODqa52
TGOemSUMHoqjT1kqMsZ9aK+rmpKJsX6tZnHZ3aesg1asv5Wkc/F6me3bS+CgCA+UGICgAAAOAI6
Ujt/OL38Zx/XTlwM89yXbr9vvT7e7I5KvksbsrZqn4eco16jXN+IFttyXZFPwcAYE4QogIAAAA4WuiNm
r3eoezrp3LqRKxeo5Xtlng5auAamb1Qq9IiQQUAzCFCVAAAAAABHTLzeqH590pVg6NpryIpbm9Orse
rX/7Rrehp1Vr119suC68NKtPqCtUJH1wEdeJ31CKsrGjyeYL3S6HYYzGPXj8F92fsp1ayzrDVXndeO3Ul
FtltujOpfo/aGXxagXN+yXjUo3vHbQto/dH0tcc5VoEar+6BWKwAsLUJUAAAAAEdEWep1HdJl2Bu1U
ytJYTVYDdRep0K2gfXN1VEBZlNWCyUxyoCqjUhpcMIpOxQceJ3F3t/IQK8jtZJRr7Rcl60YnTuHwlEtel
8JVbak7hZHba7KRqMh5/wEVS4M1gNIcvwqwA5pv3M+hoNUR/i5UueiNLhTJewaAQCWAiEqAAA
AgKNjzQ3p4vVGnVS53pW+qgPq9ax0hK1v7o5oh1dLtC/
+y5tyzkv7etJYd0PBqrT0a71td2qyPjIpNl6/F2OSpvaGePmv0a6um3ga+6psW5/r+hNFqRqm9n5iDc
MPTjLVrLnHV5b6hcF2Jjl+67VuGhvWfmsrOxfjnqu27Eadi8A1AgAsC0JUAAAAAEdIUTbdGYwy641al
bNuj8nKaWvJNWL9/mFoL05zBnqzXmhn56Lz+t5F2dGdIQPD3I3enN5rB4QPix+t7aeGgTCzuHnB31
ftfDo9MAOTTGnVs8OTUiU6fuu675lBqKO4dsYLbDsHXf0saOhcmfVeOzviZq/FzT1n+9Zjb+QMWgC
Ao4oQFQAAAMDR4oVsGfVGLZ+Ukn4aMGp9qKqcDiR3JTnpp31ix33dA90L01rllguwH8bwdve1A0
6dSBLymT0vz8ha4EuLsnbGbdi+HIYlthMITDJlPQudTGqK41fHZNc5DR3eHzR0ropr4h2yuodK7n4ja
rECAI48QlQAAAAAR8xAb9RD5+l8K8qJU/rp1MpyMn6am0BHRnTmnE6i8DlCYFIpo85ppLBzFSw5Y
HLD3NgTdQEAjgxCVAAAAAABHj9kbtRYvTsvXYK/Onhx6Y8iHebVWhx7biYbtTyercHa88cfflg2z16lby9
Ss3ZpEcVP29D4Gyt7amueYpR8AIg0hKgAAAIAjyOiNmpQxjDw7g706u3Lg7tTtmVk66QWAo+p5ps
MsJeDXAHX05KJbmFROSaIqAdNKcvztXa/nqR24unVRp7iWaoZ+1ePUnkjLDlS7Xi3W0WUEAABHF
SEqAAAAgKPJmIAoTMlPDo3aqW3Z8Kapz1ZzdcObqKm94Q8/L59ZcwJAszZnc9UfQt7e8Gp0FlIZV2
4Gzta5WPd7WfYa61790aSTVU1twuP3A1djxv4k9PbVbRDscRoSdAMAIgYhKgAAAIAjKro3qjlzuwrp
nGAubi3NaZWlXG7Kqg4D/dzWmOFftd+ozdlc1cGh9+IREzJNorLtD1vv1KSk21XyE1S5kPuM9AmOv
3Lan6jKu5bGBFRJjDgX5r1RPat7ugIAIgYhKgAAAICjK6o3qqp7OVgzs9qavI5mIqfk7J4xPFyx63gO1Di
1a3MOvE4Je+2U7GHrIcdebVnr3eHxeYt9/BXZ7psz/itVaZk1TZu7Xs/fcexzEVYMVW8zrewaALA4Cn
1V3AUAAAAAAAAAAEIqeqAAAAAAAAAAAQgRAVAAAAAAAAACIQogIAAAAAAABABEJUAAAAAAAA
AIhAiAoAAAAAAAAAEQhRAQAAAAAAACACISoAAAAAAAAARCBEBQAAAAAAAIAIhKgAAAAAAAA
AEIEQFQAAAAAAAABGEvn/AZM6nr53azj8AAAAAElFTkSuQmCC]]></redcap:Attachment>
</ItemDef>
<ItemDef OID="q8" Name="q8" DataType="text" Length="1" redcap:Variable="q8" redcap:FieldType="radio" redcap:RequiredField="y" redcap:BranchingLogic="[q5] = '1'">
<Question><TranslatedText>Do you prefer Life A or Life B, or do you think Life A and Life B are the same as one another?
</TranslatedText><redcap:FormattedTranslatedText>Do you prefer Life A or Life B, or do you think Life A and Life B are the same as one another?
 </redcap:FormattedTranslatedText></Question>
<CodeListRef CodeListOID="q8.choices"/>
</ItemDef>

<ItemDef OID="summary9" Name="summary9" DataType="text" Length="999" redcap:Variable="summary9" redcap:FieldType="descriptive" redcap:SectionHeader=" " redcap:BranchingLogic="[q7] = '2'">
<Question><TranslatedText>
Alzheimer's disease - Description #[no]

If you have Alzheimer's disease...

1) You might forget recent events of importance to you. For example, if you are interested in hockey, then you might forget whether your favorite team won their last game, although you may remember watching the game itself.

2) You might forget the names of people you met recently, or the names of local streets.

3) You may no longer be able to handle your personal finances or plan leisure activities such as dinner parties or vacations. Simple decisions such as what to order in restaurants might become difficult to make. Your ability to concentrate may decrease and you might have difficulty driving.

4) You may start having trouble finding words to express your thoughts. You might need to be reminded to shower or brush your teeth, but you could do these activities on your own. You may need to post notes around the home to remind you of simple things like turning off the stove.

5) You may remain aware of the day and your surroundings. You might be able to travel to familiar locations like the supermarket, although probably with the help of a loved one or friend. If you are in a social gathering, then you might have difficulty following the conversation.

6) You may continue to enjoy hobbies such as puzzles or reading.

Now consider two possible lives. In Life A, you will live in perfect health for 19 years and then you will die of natural causes. In Life B, you will live in perfect health for the next 10 years, then you will live with Alzheimer's disease as described in the summary for another 10 years, and then you will die of natural causes.

</TranslatedText><redcap:FormattedTranslatedText>
Alzheimer's disease - Description #[no]

<p></p>

If you have Alzheimer's disease...
<p></p>
1) You might forget recent events of importance to you. For example, if you are interested in hockey, then you might forget whether your favorite team won their last game, although you may remember watching the game itself.
<p></p>
2) You might forget the names of people you met recently, or the names of local streets.
<p></p>
3) You may no longer be able to handle your personal finances or plan leisure activities such as dinner parties or vacations. Simple decisions such as what to order in restaurants might become difficult to make. Your ability to concentrate may decrease and you might have difficulty driving.
<p></p>
4) You may start having trouble finding words to express your thoughts. You might need to be reminded to shower or brush your teeth, but you could do these activities on your own. You may need to post notes around the home to remind you of simple things like turning off the stove.
<p></p>
5) You may remain aware of the day and your surroundings. You might be able to travel to familiar locations like the supermarket, although probably with the help of a loved one or friend. If you are in a social gathering, then you might have difficulty following the conversation.
<p></p>
6) You may continue to enjoy hobbies such as puzzles or reading.
<p></p>
<p></p>
<p></p>
<p></p>
Now consider two possible lives. In Life A, you will live in perfect health for 19 years and then you will die of natural causes. In Life B, you will live in perfect health for the next 10 years, then you will live with Alzheimer's disease as described in the summary for another 10 years, and then you will die of natural causes.
<p></p>
</redcap:FormattedTranslatedText></Question>
</ItemDef>
<ItemDef OID="a9" Name="a9" DataType="text" Length="999" redcap:Variable="a9" redcap:FieldType="descriptive" redcap:BranchingLogic="[q7] = '2'" redcap:InlineImage="1">
<Question><TranslatedText></TranslatedText></Question>
<redcap:Attachment DocName="19.png" MimeType="image/png"><![CDATA[iVBORw0KGgoAAAANSUhEUgAABVEAAADrCAIAAADv60EwAAAAAXNSR0IArs4c6QAAAA
RnQU1BAACxjwv8YQUAAAAJcEhZcwAAEnQAABJ0Ad5mH3gAACtnSURBVHhe7d1fyGVnfS/w2PR
PcrQYPEWiPTSjh0JIgox6Mx2kTqGoCIKQhg5BS0tlmEClVBHiinnIkGRQkYJJAdKFNpzkouICD1RYiFIh
QQKzkUuIsQwFxEDycUYirzBXPTsvfZaa6/1PM/68653rb33b33/nF7OM/68653rb33WvP58OWcd68/z+951t6T9/nF7OkN/w
UAAAAskZ4fAAAAlknPDwAAAMuk5wcAAIBl0vMDAADAMun5AQAAYJn0/AAAALBMen4AAABYJj0

/AAAALJOeHwAAAJZJzw8AAADLpOcHAACAZdLzAwAAwDLp+QEAAGCZ9PwAAACwTHp+AAAAW
CY9PwAAACyTnh8AAACWSc8PAAAAy6TnBwAAgGXS8wMAAMAy6fkBAABgmfT8AAAAsEx6fgAAA
FgmPT8AAAAsk54fAAAAlknPDwAAAMuk5wdgR567+twN/
+sGERERmW/ed9/Nt33hnfmvduZAzw/ALly9dvWWr90S7BtERERkRrn50o33fujU5+/8wBs/fzX/Bc/
B0/MDMLlrR9duf/T2YN8gIiIi88qnP/b+v/7D/7nKw/fc/eu33sp/zXPY9PwATO5T//KpYNMgIiIi88pH
7n7PpuHf5KmHH8p/zXPY9PwATOv+H9wfbBpERERkXnnffTdXG/5NfvLDZ/Nf9hwwPT8AE3ryxSeD
TYOIiIjMK5uv8QcN/yoPnD3zy9dfz3/lc6j0/ABM5flXn7/poZuCfYOIiIjMK+XX+ON8/XP35r/1OVR6fg
AmcfXa1Vv/8dZg0yAiIiLzSvA1/jjf++Y38t/9HCQ9PwDjO3r76PTjp4NNg4iIiMwr/+PCfws6/GR+
+sIL+Q6Aw6PnB2B8558+H2waREREZF5519/95ufv/EDQ3ifji/2HTM8PwMi+
+u9fDTYNIiIiMq/c+OA7PvNHvx/09i155K//Kt8HcGD0/ACM6bsvfTfYNIiIiMjscuYzvxd09Z159ltP5Ls
BDomeH4DRXHntir+oX0REZO657QvvDPr5Prl41x2vXLmS7wk4GHp+AMZx7ejaqcungk2DiIiIzCv9v
8Yf59K5j//qzTfznQGHQc8PwAiO3j469+1zwaZBRERE5pXjfo0/zqP3Xcw3BxwGPT8AI7j4rxeDTYOIiIj
MLgO+xh/nuf/7f/L9AQdAzw/AST3y/CPBjkFERERml2Ff449z8a47fvHKz/JdAvum59+Lly+fvWHj7O
WX82OFtnN7U07qwjPdL5sMu+uEdlPl5J65sJlmZsx3fvUAWpdeFJ7009bwLtTntpOZMInnrj4X7BhE
ERkdnn3A781+Gv8cb7yyU/8+q238r0Ce6Xn35OyCQo7nG3vd0hNatCztb9sMuyuE9pNlRMqJ7kx
WtObj9u69H31/PHc9PwzdfXa1Vu+dkuwaRAREZF55cYH3/FnH/2DoG8/YZ649KV8u8Be6fn3Jtncb
5u/Q25RwxYu0dGl9LzsOjRNt9vvee+n50/NTc8/R9eOrt3+6O3BpkFERERmlz/
+5HuDjn2U/Pg7T+ebBvZHz79HZddfNjnlkQPvioOOrWcz3/Oy689ED6bfsHp+TuJT//KpYMcgIiIis8sf/
sXvBr36WPHF/kOg59+reo8f/zuArbJHygQXJJqljn6vOL29Iz5SDLs+EgzX/rJJ213pfq+4JLWwtfrVSeHc
qoWqY7VPvXJX+01t06sMUvyYtB21e7G1Kyq3Jgo0rLD6QCp3xdW6JhNMpXZJeW49iaa59Z4Jh+L+
H9wf7BhERERkdnn3A7/1l7d/MOjVR8zD99zti/37peffr22fdOFy8WPU4yR6pJXKZdVmKVdrshKK83
GTWA6SH8leB8O1v2zSeldRvzpEcUUxpfKOqsqiU8K5pR/mWttAve7qml5ikLOXL8cHg8dTVZtj8orgc
VZUn2zFKEtrGKW8ovYuNM2t10w4GE++
+GSwYxAREZHZZYqv8cd56uGH8g0E+6Dn37ewm4ram7ITKs6Ud5SXFpdU7q01WSn5BcUtlX4rv6M
YIXsZDNf+skn7XcUMtmMUF+RzjKoklh0L74oXuj2yLR1qfjzHmF48SC66tc9o5RXFkXL44kBi2ISWpT
UOFE6muCC6oXGE6MBKj5lwKJ5/9fmbHrop2DSIiIjI7DLR1/jj/OSHz+bbCHZOz79/lVan1gJthO3VW
tgwJa5J9VR1+RWbezYvLlxYD1Q9UtwfDNf+sknHXcXrYpDidb6q4OVa4lAkrFI+7e1cw0sSEndtb8vK9
5heOUg433gC3aPF92wOpSbYvK6V7gcSVF5LHKoJS0dTiQ6sDHtr2L2r167e+o+3BjsGERERmV2m+x
p/nC9+9MNv/PzVfDPBbun5D0Gqo8wVp+rNVdByJS7q0Szld2UXZD+fvfxMdtf2SHl7MFz7yyZddxU
H8tf5DIpFFYtMaakbVimH2d7TY/rFXbW3oXZfj+klSm/EE+geLTmjuh7rWkkMFNzYY2kV9avz89FUU
nPrngkH4Ojto9OPnw52DCIiIjK7/Pe//Z1Jv8Yf5+F77s73E+yWnv8QtPRv6VMNTdkxm6X8ttUVm6tX
P2wOlUfKu4Ph2l826byrOJId2M5uo1hkSkvdsMqwZ1XcVbuidl+P6SVKb8QT6B6tcbCtHutaSQwU3
NhjadtbAvn5aCqpuXXPhANw/unzwY5BREREZpff/vvf+PPTtwU9+Q7yvW9+I99SsEN6/kPQ0r+lTx
WtUH50aLO0ua/4n/ezu7NDxZHtgMFw7S+bdN9VHFodyde0PZd+Ep3CKsOeVbJ47b4e02u8JJ5A9
2g96vVY10pioODG/pNZ2VwUlo6mkppb90zYt6/+
+1eDHYOIiIjMMX/6J7cG3fjO8tMXXsg3FuyKnv8QtDRVZdNTPRd2Qon7yy6srVnKx4m/xr85Uhkvq
Nj+skmfuzbzLv86+8qpnkVC4W3DGsvU46zfdoxBqm9lJr63e7TUFesCx1vXSvcD6RwnHiJ8XtEQTdNf
O/YS2I3vvvTdYLsgIiIic8yd598d9OG7zANnz/zy9dfz7QU7oec/BIlmZ6tsn4qzZR9UXl4eKVqj8p6OZm
l7Y+rW5t6r/WWTXndV6gdnyuuLw+WlbWXDKsVNx2ssE08lrN49vUTpjcQEukeLrmg+sB02ITGr8M
auyYRz214fHclfJ+fWYybszZXXrviL+kVERBaQ9/7NTTv+Gn+cr3/u3nyHwU7o+Q9BotmpKluqmurFlS
Yr1N4sbW8shyurJQtshmt/2aTfXdvVRo+jvKPmWEUHNpbpt2AtNU7NdtBE6Y3kBLoXm76iMnx91g
2r6/VAOibT/HjyS+IRE3PrNRP24drRtVOXTwU7BhEREZld9vU1/ji+2L9Lev5DkGh2QvWmK9EBVS9Y
ne7bLMXXJe8MDra/bNLzrvanUd6VaXliubDKsMayelfx80p8Q9v0GhfWOIHuxdaviC6pzDV5+0rvB9I
+mWql9W3F680QqRGjufWeCTt19PbRuW+fC3YMIiIiMsfs8Wv8QS7edccrV67kuw0mpueHbol2FK

4PF//1YrBdEBERkTlmv1/jj3Pp3Md/9eab+YaDKen5oZuen+vTI88/EmwXREREZI45hK/xx3n0vov5n
oMp6fmhm56f69BzV58LtgsiIiIyx9x86cYD+Rp/nGe/9US+82Ayen7opufnenP12tVbvnZLsGMQERG
ROebTH3t/0GkfTnyxfwf0/ADUXDu6dvujtwfbBREREZljPnL3e4I2+9DylU9+4tdvvZXvQpiAnh+Amk/
9y6eC7YKIiIjMMe+77+agwT7MPHHpS/kuhAno+QHYuv8H9wfbBREREZljbr50470fOhV01webH3/
n6Xwvwtj0/ADknnzxyWC7ICIiIjPNIX+NP87Fu+74xSs/y3ckjErPv1/F3w2XmeQviHv58tkLz+Q/L+Pv
oquvqMXqwoa15mc2+g1G78/O8j5y143nX33+poduCrYLIiIiMscc/tf44/hi/0T0/HtU6zynaIryAgtqw
OIVNSqfbrjW2r9n2dCP9tDrs7O8j9z14+q1q7f+463BdkFEREtmmLl8jT/OP//Dg/nWhPHo+fdo4m6
obHoX04ClVpRWXrlSX2v9CTT+iwEiPT47y/vIXTeO3j46/fjpYLsgIiIic8y8vsYf5yc/fDbfoDASPf/
+9O9gh1leA9bviZVX5WprLR5AOcLU78Jy6PmX7PzT54PtgoiIiMwxNz74js/80e8HXfS88sWPfviNn7
+a71EYg55/P4pGqKLac9bb1qBbqjRR22GCjrVp/GoDVrkm7sdqM+hs16rDVu+M+ujmYbdnypvKCQ
azLUSjr9SfXKZapjy9vbc691jD2cTh5qVthDOrXVIZb7vQzRxb72vQt1btwtY3a3Wy4VEUtvMuZSNW
b6tcE49Sm3RwuvecGeKr//7VYLsgIiIiM82Zz/xe0ELPMQ/fc7cv9o/oeD1/udtu3monL6m1Aw0tQ2/
bLf+MN/xNDVL61ErlqSUuiJ5p0/jpwdcqQ9Sar0Lr2zbKsOX5zbHgZdOKAsVdq3PFHdWZl4Nu7y0HT
g23PV0dJTrYtbTU7Fe2VyTOZyfT9zVMNTegVq57xmvVqyoSN2TT7DVQ1wM89mzo7bsvfTfYK4iIiM
hMc9sX3hk0z/PNUw8/lG9WOLEd9PzBdv6ke/TqcK2tz8FLPs2yuSmeU3lV+eAq/U/rA0iNH98bjR/d
VtzT8s7Fw26PhHVahq1ek6qaWlFgfUkwhdq8o4PlmE2DFhdUhgkGiaYVFgmHKG8oxyzuWKlOo2m
g7Y2RIbW2R4ri5V3Fke2BltrbqyqLiMtFU4puC9fda84McOW1K/6ifhERkWXkXX/3m5+/8wNB5zzrv
PijH+VbFk5m9J4/Fu3fT6TSe6yMM+aetDVI1YWF15XdTsfq28avHAsuK15WBk8cCow2bDlQrl4yGL
ND6llGU60UbFpeNM9g4J5Lq4oWUk6jdkd5WeNAPTTX2j7F8JronpXyWMtcUvd1lytetjzAHnPm2K
4dXTt1+VSwXRAREZE5ZgFf44/zwNkzv3z99XzjwglM3/OPuzUve4Gz9Z5gjhJPJugmc+WiN0cT/U9S6
sknCgSXlaMnNBbsbsl6D1u9MHx3UytqlljrWjlIqHHQ4o7g+RfX915apn51NEb9hsRcw2fSpnet8ME
Wl9SKJQ/Wpd6gxG3BZfVp1gUTapkzx3P09tG5b58LtgsiIiIy0yzja/xxvv65e/O9Cycw7X/bn9jMJ/bsa
z07meKeC88UPx2nBTowiaeZbquCC9MXxRLjp+5tGD2lMlDdmMNur4wWmFpRs8SkCtUaz3QPWt
TNRiruLS/vs7Ry5oHifONcG25sewADaoUPNjmdxjluheOsdZcrrkhpnlCqFv1d/NeLwV5BREREZpolfY
0/zve+
+Y18+8JQe+r5y6uqWnqJXHHbepDi5+67DlXiaabbqmCp6YtiifF79E59R68Zb9jytkxwdWpFzXqWLA
ZtvSwfa3VNcXnXm1ZVXLGyuShaSOcYlSEyfS7sXyu8Jjmdzjmm36Ducj0G7jFnjuGR5x8J9goIiIy0yzv
a/xxfvrCC/kmhkGm/zv8EvdEh3ps+teK+za3Fa86bjpcbU+muqjwup5PKzV+j94pdVensYYtrjp7+XJqk
cebW/I5RUNsS7Y+zXywsxcubC6vzKBzVvFEiiPlLcm5bqxOHaPYsFrhqKkq5bGWJ5W6r7tc6q5AjzlzD
P6v8YuIiCwmn/7Y+4MOeXm5dO7j+SaGQfbR8xdHEvv3lnYivqjXTQcsfjIrZYcWrnK7zET/k5Qa/zj9Xn
GgnFJ1nnXjDFu7pXzRMma79HMKyqYvSijvy9Qm0LW04GXl+nKc9DTKG7eH0xduDasVPdhoSZVxW
h5VNM5Kj3JRtXAVveZMf0dvH535pzPBjkFERETmmPfdd3PQIS8vz37riXwTwyD76PnLDX1Cy/69GG
i77Y+PzErD00w/nsoaE/1PWn2krEqv3qk8UtPy1owybHm6GKSc/nbUxIqaJSa1lppGcElStXZYuWNp
9WlXFZc0zLXxzualD6oVv1kNS1pre1j16tlovcp1PMCec+YYXvvP1/yl/SIiIsvIR+5+T9AkLymP3ncx374
w1Hx6/nRXsNHWhRyulqdZX2xwPtH/NKk+6uzy3r1TfQZdpU4+7PZE5fpy+tsLoxU1S0yqcIxRthKzq
Wp/YtWK6xUWr/PVtsy1duta7YGmHL9W95u1OtE2x61q8ezKQeXCKr0H4Rj8H+cXERFZTJb6X/hfO
vfxX735Zr53Yah99vztvUOg3hCEjjUUDFF8BH3YWIwnX3wy2DGIiIjIHHPzpRvv/dCpoGGeey7edccr
V67kuxZOYJ/f528ZJVLe00AfxrTKT6CPGovy5X/7crBpEBERkTlmeV/s/3+PP5bvVziZgT1/UtbCRw19o
sPfDpMfK/974KZ/C5AYZKM8oRNjItX/Wr35Iwqz9dmnPhtsGkRERGSOWdIX+7/+uXvznQontpeev2
mgxnaqpbNvOQVjqPT8Gn6W6Ojto9OPnw42DSIiIjLHLOOL/Q+cPfPL11/Pdyqc2J56/rX6YG0te2tf3
3oSgE5Xr1295Wu3BJsGERERmV2W8cX+n77wQr5HYQzH6/kBWKTnrj7nr/EXERFZQN77Nzf95e0f
DLroGeV73/xGvjthJHp+ANYe+4/Hgk2DiIiIzDF3nn930EjPJb7GPwU9PwC5+39wf7BpEBERkTnmT//
k1qCdPvx88aMffuPnr+abEsaj56dd8PcxtL88luLvxsv/IoaTDHVctb+Kf/K/CGK1sh2saW2Xz5DFOvftc
8GmQUREREGaX3/773/jz07cFTfWB5yc/fDbfjjAqPT/t2pv8kzSZ+

+r5y0Ib07b8ebEd9eA7e4Ys2bWja6cunwr2DSIiIjK7zOuL/U89/FC+F2Fsen6OJegqT9JkBj3/zuyw7q
57cD0/43jpjZf8Nf4iIiILyFy+2P/wPXf/
+q238o0IY9Pzcyzz7/l32Rfr+Zmt77/8/WDTICIiInPM4X+x39f4p6bn34uGdjd5uOzjMt13dPR9xentH
fGRYtj1kWC49pftyotXVtcHc08OVb2lOsFWtZtqdxUlKxomXp1cMO+aeq1qtaZawbLXoqVXrtkOk59
srpgYqEEwRv9FrfSZ/0rrGBu1S+LzPUZgWo88/0iwaRAREZHZ5fC/2P/j7zydbz6Yhp5/PxKNU+rgtuGr
qtzUswGrKs6Xp8si5SD5kex1MFz7yxbppazlZaOhygNVlYUmtT+xxNmGiXdOd621WFOt4nhlnGjpiX
s3l7dW7Pd2JB9r5Y6OEkPnv1aZVdeb2z0CO3H+6fPBvkFERERml0P+Yv8Tl76UbzuYjJ5/T4qeJ9HmlI
fKvqc4UjZK0SWVYaIGLJRfUNxS6a7yO4oRspfBcO0vG5XXhTVW8ol0jZxYaqTHE4sHToqfyvZIsIRj1kq
sI7osrp7prJiqVxfdEjyyzhJ95h9eEg0S3hDd0TkCO3L09tGZfzoT7BtERERkdvnDv/jdoNk+hHzlk5/wNf
4d0PPvS9HFlE1M1EslmqvO7mol6qci+RWbezYvLlxYD1Q9UtwfDNf+sknqsvJYPvf6NcWrxMIqhwJ9
nlh8IKkYqnJV543xBalb+rxlZfXmtWbCGwdMcXOo6fqWuTXPv3zZNP3igsQQm0OdI7BDr/3na/4afx
ERkQXkjz/53qDl3m8u3nXHL175Wb7hYEp6/r0JGp+ilSq7r0RvtdJwV+Wisl9q7uPyu7ILsp/PXn4mu
2t7pLw9GK79ZZPkWoKD9aGKkylNtZJVynGLo/2mXNbfXtV2Y3265QWpWxKzjC5LVA8lK3auLVG8
WXpRPeZfvt6qFawPXLcZomsEduvKa1dueuimYN8gIiIi88qND77jzz76B0Hjvcf4Gv/O6Pn3p+hrsma
maIO2zVq6PQvaqx4NWEp+2+qKzdWrHzaHyiPl3cFw7S+bJNcSHKwPVZxMaaqVrBJNsd+UE2PFN5
ZHAvEFlVp9Rk6vZKWjYqpeTePAW12LOsmTyc8XQ6QUQ7SPwM49+eKTwb5BREREZpd3P/BbB/LF/
v99/9/mmwymp+ffp7z3WXVPRYdTaWnS7VlxYX60VwOWsLmv+J/3s7uzQ8WR7YDBcO0vmyTXEh
ysD5W8o0P6nmLc4mi/KSfGCm8sLlnZXBWPnKrVY+Sm1XdWTNWraRh4q3tRfeZfqgyX2dzUOYuK9
Ajsw5f/7cvBvkFERERml0P4Yv+lcx//1Ztv5jsMpqfn36u8oTl74cKmaar2TGUjVe1ywu4q0T2VTVJT37
eWjxN/jX9zpDJeULH9ZZPUZeWxvFb9mp4D14QjZqKB+o2ceLDBjfEV8ZNP1erzliWuWemu2Lm2pg
kVQ3aXSFwSX7OyOlZ5Va/bOc1M2wjsyWef+mywbxAREZHZZb9f7L941x2vXLmS7y3YCT3/fpXdUiZ
oZ8qTRXtV9j1lv1UeKe6tDNjaHG1vTN1a6eeCXqv9ZaNonpX6ebGmkYs7yum1lOrxxMI6DYqRmp9
EOJ/thLcjp2ptr8sPVp57cVmi+kp3xVS9uqh6/UCPRUUjRPMvD2ynHyyoeYy+IyQOsANHbx+dfvx0s
G8QERGReWW/X+x/9ltP5BsLdkXPv2dld7NSdFVb1bNb1S6n0pSF4uGqtjdGXVS6wGa49pctmieaV
4uGSt/RUegYT6x1pERHGd6YLrW2Hbl+zeZ485Mob0xUX+us2GdtDeXzUj0WdYz5h7bTSg+yPd89Q
sMzYmpXr1295Wu3BFsHERERmVf29cX+R+
+7mG8p2CE9/76VzU1T71LvjrY9T6l6wep0+TJxaVV8XfLO4GD7y3blxSur64Omrb16pmd7V78pnFm
/KSc6ysSN1c50fbR4XRm6ekk5WHWGq2ujkRPVc+0V+60tfEJBmfYSma75r1WHWYunVJ9F+1rX6iM
UZ+P7mNpzV5/z1/iLiIjMPR/8q3cFDfnUeeDsGV/j3ws9/74VjY/WBZiLx/7jsWDfICIiIrPLmc/8XtCWT
5qfvvBCvpNgt/T8+1X+T51afmBO7v/B/cG+QUREROaVGx98x2f+6PeDznyifO+b38j3EOycnn9f6v/h
cvwfPgMctnPfPhdsHURERGReedff/ebn7/xA0J+Pnq9/7t5898A+6Pn3pdLza/iBGbp2dO3U5VPB1k
FERETmldu+8M6gRR83D5w988vXX893D+yDnh+AgV564yV/jb+IiMjcM+kX+1/80Y/yfQN7ouchYLj
vv/z9YN8gIiIi88p0X+x/6uGH8h0D+6PnB+EHnn+kWDrICIiIvPKFF/sf/ieu3/91lv5doH90fMDcFLnn
z4fbB1ERERkXhn3i/1f/OiH3/j5q/bB1EERERkXhn3i/1f/OiH3/j5q/lGgb3S8wNwUkvdH90fMDcFLnn
L7p+QEYwWv/

+Zq/xl9ERGTu+fTH3h907wPyz//wYL4/4ADo+QEYx5XXrtz00E3B1kFERERmlJsv3Xjvh04FPfyx8pVPf
sLX+A+Knh+A0Tz54pPB1kFERETmlffdd3PQxvfPxbvu+MUrP8u3BRwGPT8AY/ryv3052DqIiIjIvDL4i/
0//s7T+YaAg6HnB2Bkn33qs8HWQUREROaVAV/sf+LSl/KtAIdEzw/AyI7ePjr9+Olg6yAiIiIzynG/2O9r
/AdLzw/A+K5eu3rL124Jdg8iIiIyo/T/Yv/Fu+545cqVfBPAgdHzXAzCJ564+56/xFxERmXU+dM8tQXufzL
PfeiL/9c/h0fMDMJXH/uOxYOsgIiIi88qf/smtQYcf5NH7Lua/+DlIen4AJnT/D+4Ptg4iiIiIyo/z23//Gn5+
+Lejzy1w69/Ffvflm/lufg6TnB2Ba5759Ltg9iIiIyIzy3r+56S9v2/2DQ7a/ia/yzoOcHYFrXjq6dunwq2D2Ii
IjIjHLn+XcHDf8q3/vmN/Jf9hwwPT8Ak3vpjZf8NF4iIiKzTvDF/q9/7t781zyHTc8Pwp58/+XvB1sHERE
RmVGGqX+x/4OyZX77+ev47nsOm5wdgRx55/pFg6yAiIiIzSvnF/p+

+8EL+252Dp+cHYHeCrYOIiIjMK3eef/dH7n5P/nudOdDzA7AjN9ywo186Cg2m0GAKDabQYAoNptB

gyyt0PfAoAdgRO5LBFBpMocEUGkyhwRQaTCFaeJQA7IgdyWAKDabQYAoNptBgCg2mEC08SgB2xI
5kMIUGU2gwhQZTaDCFBlOIFh4lADtiRzKYQoMpNJhCgyk0mEKDKUQLjxKAHbEjGUyhwRQaTKHBF
BpMocEUooVHCcCO2JEMptBgCg2m0GAKDabQYArRwqMEYEfsSAZTaDCFBlNoMIUGU2gwhWjhU
QKwI3Ykgyk0mEKDKTSYQoMpNJhCtPAoAdgRO5LBFBpMocEUGkyhwRQaTCFaeJQA7IgdyWAKDa
bQYAoNptBgCg2mEC08SgB2xI5kMIUGU2gwhQZTaDCFBlOIFh4lADtiRzKYQoMpNJhCgyk0mEKDK
UQLjxKAHbEjGUyhwRQaTKHBFBpMocEUooVHCcCO2JEMptBgCg2m0GAKDabQYArRwqMEYEfsSA
ZTaDCFBlNoMIUGU2gwhWjhUQKwI3Ykgyk0mEKDKTSYQoMpNJhCtPAoAdgRO5LBFBpMocEUGky
hwRQaTCFaeJQA7IgdyWAKDabQYAoNptBgCg2mEC08SgB2xI5kMIUGU2gwhQZTaDCFBlOIFh4lAD
tiRzKYQoMpNJhCgyk0mEKDKUQLjxKAHbEjGUyhwRQaTKHBFBpMocEUooVHCcCO2JEMptBgCg2
m0GAKDabQYArRwqMEYEfsSAZTaDCFBlNoMIUGU2gwhWjhUQKwI3Ykgyk0mEKDKTSYQoMpNJh
CtPAoAdgRO5LBFBpMocEUGkyhwRQaTCFaeJQA7IgdyWAKDabQYAoNptBgCg2mEC08SgAAAFgm
PT8AAAAsk54fAAAAlknPDwAAAMuk5wcAAIBl0vMDAADAMun5AQAAYJn0/AAAALBMen4AAABY
Jj0/AAAALJOeHwAAAJZJzw8Ax/XMhRu2LjyTHwUAODR6fgAWZNOMT9uFv3z57LbClAV3/W8W1g
tbOXv55fzAmGqLyfh3JcdVe4bTvE2Ta/gDk3/2Nsb9ZLT8EW05dVzpoWrv2LjLmlbrk5n0HxQTal7UX
N+nhhXVljPW29Sj1kz/mXSd0PMDMK0Jd/M1lTq73LRlZafY66z2UuU6sn3VxBuq9ULOXriw+n8mq
bNews63hNX96Pifitone2uiT1+2lh19IqrPbbwijX9C63+G6gs9kcaKraeOq2mo9fHywOaRnrRUepTq
23XiEp1PZn1+1H9QpBZVW1LmZOtqWVR2asQ/SvFyKsWrJlpRVr88lL066er61Rqh1ObZ5YLnU3uK
wTl60PMDMKH6fqq+GxlVUSj7/3e5IaivcCqTV1kXyB/fNGXW7/3kj6lqN29MTVZyog9fOPZ0tcbex+
eK9yOeePjRGGtpzRXbTh1X36GK6/KXx5bdv1Gvsnp85YGTv11dy1kfz0+fqM5G26LGGD/XsqjEoeEa
lxM4edHmFYVHJqxVnMhfnvRdWw9XDp99krcv66Wykyda0/VIzw/AdMI9QLRrGN30FQK72X5MvK
xy+PUPwzdtbU62HTy2nX8OVqb9KNQf4GTrywauvlFjv3HRzOOljPwgW57ViI+xa6js/OAHWdw9bZ
WKdKHy6PqHE5cpJpuoNc0/LuJCo6wj17KcuhE/352PrnMy/UVDResYsVj5NLMX4cdhzELXDT0/AJO
JfzOPuNtJ2/FmIFvPWHvGRrXtz/gqz2z94zSFsie1NfEzm24djSZ+k1Y2j3BVYvPDNJ/y6E9o5dMximi8
dcX6cxu5ZMtwI1bqGip6sIN0VRltQamBKsfWP472YU/Uij8UY4gLZXXWX1Qonbxq6tFVZKfHWluyV
vZRW5fY/NA8leOJajU8znGWVn1M6UKjLew6oecHYDLxDiC5RRnT5AWqso3HWLu3WLaW3IRLWq
+iGH5dcroFFTYLm7JO9skbeSvfbjcfvOwTl5mqVLyO+E/xiUQF4vFHfpYtw41YqX2o7I0b4Sm2V8nOj
vNeJQqt11AcWZ8e7UPRUKtipFJxoeB9yS5ofr79JJZT0X72uJpG2z6/0UolamVHwqc30nuVrSCvtv65
Puy4T/H6oOcHYDJ7+FW9u71AsFuc1Kibqbr1MrbPa11oJ2uKPxqjCt6czfOb8mORFZz0c1ddw+bna
eplY4ePbsS3KhuwOvP4oxBdcjItw41YqW2o4ON4Askq2cHcWI8tKrRew/b1+vRoH4q2R7eyWd4Y1
eJC0YdvhD/IrcsZYfyqRK3N49oc2/w8Ur3kurL1FC5cqH1ITiAbtnxjonep/RmTpOcHYDJ7+FW9o73AZ
qezwz3HZvM2fsHalq1ijB12m6zudM8v+uRNWy97dyZ9ZHGFCVdU+1CMt4/fiP6Exn9ko3fvZOICpZ
ZTx9U4VPY0x1pOx4Sz06MUCwvVPhMVU9SKjfSJiAtFA3fPpVPLENmpsT4La1GtuEL2zp1sRRvdj2Z9
xRirC//IxG9/91wI6fkBmEz8i3mkvVuzXewFsho73nBkNSd9cpl1memrTL+cKbbyzUbbVTeLS0y6ooq
xPxLxvMM3a+yVtYw3Yqn0UNn7NuJiOiecXTBCwY5C69OjfSo6FzXWn7C4UHakupDwwzhA83JGW
kZFVCsu0f10e+ocaH3ByT8U2Qrqw8SVR3ibrjt6fgCmE/5mHm370Wj6Cps9SWj0/ceqSmUZiX3QJ
MbZtcVW41aGzd6kSd+lrEJ1IVPuEbM3Z9rPXPwJ2EnRldE/EdlbU594bW3RW3diiYqFllPHlRgqOzTu
m9Q54eyCER5fR6H16dHepL0uqvYHaZQ/VY3LmeDPbFyr9odpZbyi7W/TOO9RNtu4yPpwdez2qZC
k5wdgQrUNyDi7grRs7MC89wTBinazmHXRSd6hna+mttUdb98bm/JTXbfZD5d28IkYc23BJyCzXUJ1
bWM9zJaK7ZM5luahgvdr44SLy6rVZrqqUnmd1TxJjZ5PZn3ZaB/6aFGrA5XBN1NKTKK39kXVzp6kT
C5azkZ2eAef7dH/QdFcq37mxI9uM1xymNrnetQnef3Q8wMwreoGxO9pdqa2IT3xfrRJ9vGebPQ9G
Hkfzyjq78pG/t4Ep2b0jjUvapZranmPVhb3D4rRBf+qYmO7ZbCROCE9PwAAACyTnh8AAACWSc8PA
AAAy6TnBwAAgGXS8wMAAAMAy6fkBAABgmfT8AAAAsEx6fgAAAFgmPT8AAAAsk54fAAAAlknPDw
AAAMuk5wcAAIBl0vMDAADAMun5AQAAYJn0/AAAALBMen4AAABYJj0/AAAALJOeHwAAAJZJzw8
AAADLpOcHAACAZdLzAwAAwDLp+QEAAGCZ9PwAAACwTHp+AAAAWCY9PwAAACyTnh8AAACW

Sc8PAAAAy6TnBwAAgGXS8wMAAMAy6fkBAABgmfT8AAAAsEx6fgAAAFgmPT8AwGF55sINuQvP5
Ic2ihNnL7+cH5lGUScofwi2z2Yleg4vXz7bdKr1HMBy6fkBAA5Lpa+tt6fXe8+/bdszqcfQ2NlvH+oB/ps
MgOno+QEADkvtf8uudqjXe8/fa/3J5n77rwJ0/MB1Rs8PAHBYaj1/tcG9znv+snHvmFf5/MrnVB7R8
QPXHT0/AMBhqff8lUY16vmjA3FjnF+yvqIybn5D5Ui1Gy4Or45V/nP6SpFC5Wx0vjK3bZmOnrs2Xm
3AykwLLWOVV2fXlK+iFbTNf60+n+CSlgW23gewU3p+AIDDsm0mL29+KlrGSpeZvY4PVNrNvCXetq
Odyia6+Z5Knx32tZnKRBKDtLa+6aLh0isqc4lsJ3fhcvFjUL1r/h0Tal5g+r7W6QJMR88PAHBYiqZx1UE
WjemmYaycyC6MD7T1/PlFlZY0PFK0pdE92yPFgbBMeUU5lUqdHu1uc4FywLhmm/Li3HaYTOf8iw
ua59OwwKaBwhkA7IaeHwDgsNS6xvxF9ee47ay0k2EzW1yy7UubjxTDxFeE14Qd8Up4qBykck2jcA
Zr4UriA+3KCawFt/SYfyiqnl5geVmvdQNMTs8PAHBY0u31qtWMOuNEqxy2pvEl3UeK19VGuT5ucU
VKULg2SoN4SmtFyeJouLROzVPoMf+K+tXF+YbRy1lu1ZcFsFN6fgCAwxJ2wPnr8uv92x4yvHJlqp6/fr
B4kdJYuFn62nApg3v+eAo95p9q3jPF+cbRG27sO2uAUen5AQAOS9RMhk1keSLRdoaNcXxJ95HEs
MG4qSsCPS4ppa8tShZHw6V1ap5C9+SKK1Y2F0XVO8eoDJFpLgYwHT0/AMBhSTST9faxPNF25Qg9
f7W5rl/To/2OyzQrh6teHNUYsefvHCq+NXooLQtcnaoMfOx5A4xIzw8AcFhSzWTZN66VJ7ZH83aybE
yjI3Hz2nykeZTykubKRWMbl2nTXGB7/7F755YpdM0/XM72+o4FRgtpnQbA5PT8AACHpaOZrJ6otK
KhoJdta0HDI+Xrs+HoRb+7ki69vSAu0664vq5695g9f+f809NZKy5pGr3pzr6zBhiVnh8A4LA0NJPbJrV
2otq7rtrKsDGOB+s+UryuDhdNZ63eN9cvaFhFq/p4YZMcLq1T5xTa5r+9fW1dsvJYqqdTo1dvXes7Y4
DR6fkBAABgmfT8AAAAsEx6fgAAAFgmPT8AAAAsk54fAAAAlknPDwAAAMuk5wcAAIAl+q//
+v8w1YanVjLS7AAAAABJRU5ErkJggg==]]></redcap:Attachment>
</ItemDef>
<ItemDef OID="b9" Name="b9" DataType="text" Length="999" redcap:Variable="b9" redcap:FieldType="descriptive" redcap:BranchingLogic="[q7] = '2'" redcap:InlineImage="1">
<Question><TranslatedText></TranslatedText></Question>
<redcap:Attachment DocName="B.png" MimeType="image/png"><!
[CDATA[iVBORw0KGgoAAAANSUhEUgAABVEAAAEJCAYAAACdcmdaAAAAAXNSR0IArs4c6QAAAA
RnQU1BAACxjwv8YQUAAAAJcEhZcwAAEnQAABJ0Ad5mH3gAAE9sSURBVHhe7d0PjCRnfef/b+/aY
PPHNmBskx+RHat7sZYlGJIg08PxL4egZ+6SJbnbYOVg5Oh3PVyi0zQSkwOxF+mX2wguG4lunS5iWrq
zliQyTEJugrLdIJRgckzHR4IxYTI/Z7rlc3T8wDYGjPljB3u3f/VUPVX1VHd1dVV3VXX39PtltaerpqfqT/T
O/OZ5/k+hb5FAAAAAAAAAAChjumPAAAAAAAAAIAQhKgAAAAAAAAAAEIEQFQAAAAAAAAAAiEKIC
AAAAAAAAQARCVAAAAAAAAACIQIgKAAAAAAAAABEIUQEAAAAAAAgAiEqAAAAAAAAAEQgRA
UAAAAAAACACISoAAAAAAAAABCBEBUAAAAAAAAAIhCiAgAAAAAAAEAEQlQAAAAAAAAAAiECICg
AAAAAAAAARCFEBAAAAAAAAIAIhKgAAAAAAAABEIEQFAAAAAAAAAgAiEqAAAAAAAAAAAQgRAVA
AAAAAAAACIQogIAAAAAAABABEJUAAAAAAAAAIhAiAoAAAAAAAAAAEQhRAQAAAAAAACACISoA
AAAAAAAARCBEBQAAAAAAAIAIhKgAAAAAAAAAAEIEQFQAAAAAAAAAiEKICAAAAAAAAQARCVAA
AAAAAAACIQIgKAAAAAAAAABEIUQEAAAAAAAAAgAiEqAAAAAAAAAEQgRAUAAAAAAAACACISoAA
AAAAAAABCBEBUAAAAAAAAAIhCiAgAAAAAAAEAEQlQAAAAAAAAAAiECICgAAAAAAAAARCFEBAA
AAAAAAIAIhKgAAAAAAAABEIEQFAAAAAAAAAgAiEqAAAAAAAAAAAQgRAVAAAAAAAAACIQogIAAA
AAAABABEJUAAAAAAAAAIhAiLp0etJYKUih4DxWGj29hbLpe1LZsIrqibwpX2YGxgU41NxXHd4u
MxLO7LSlg372PzHqPs4Vb2GrGzEO5vtDbdtK5JH08Knjmc+2g0AAAAAwPwhRF06Rdm+8rtvWepuVm
XurU1kFsAAEGJGqFQqDRrPGtavNaNIBROPERwnMCeuw6RHpsq0tAAAAAAEAFJ9mtW16HuF/6bk9B0bSM
wdPw1Le6X9nyc4QSA8Xoa57EXbaGrGBp5xhp1iZo6UpRa/cJDxbxsCnYJo3e7oBbHxGVOxYpXJQhLtx
1sgmfhTlqPrWHlZkknDTPmDyuDd+R1h9EJ6LhVuTjaRMPhy9S94SDDaHu8ez+5+nbDPy+QP2/aZJZ9I2Ot
zvZOOyqGxbpdBXzsfCTu0FvLXjx6Pe4rKLGhs39DHz11tbX7xAnJHWDP1m5ORKwxmXJ2ClWGvfm0ImQ
AAAAAEAucFXVkWbMhOmjGnBRqpmL75wDZ8xC+i2tN38F+M0dGPWOuTW2

2h4snM3eA7Uw9yc3Z6x+xzPPy71CLkXbAnaHvI9OHq7vqTHE6/dAAAAAAAcfYSoy6yyJXW3OGpz
VTYaDTnnJ6hyIWSM9FAIo3VqpQwmoCrK2hndwM6BdJ1nFmNId2dHLno7bcuubn/5zNpc9FKsnPa
D6gP/AGy9izv6PBo1Pe1wrCSDo63TPb8dqZUKUhrYSXM1aUimtjPY1qasDoZtEx2T2rZR+9S6H+te1
+kREu3HCSwHz4HSXB0dKibnnOtV70AUtW6Kc9TeGFHPNGS7E4vZbgAAAAAAlgQh6lILTjLVrLnBTF
nqF0Imk2pv+KGKV4+yL103ie3UZD3lhKW4dka3b18O3U23d42JhYxw0hjqferEnAz0rpz2yiY0d4M9
Ji/u6BisfEacDLUnjXX3GlSlpc9v3+0xnOr59bfvdUhWIdlAfdzx/O1494Hazrob/E1zTMbr9zZlc9v62PX
vV1Wv1/6cXXIi2X56jXUvsCzXu85r+8MlLiqR+4wr/BzteOl/krZbr3X/0hH2PRjY7rBkxzOu3QAAAAA
ALI+C9QtyXz/HklK9SwM9zlSwEhKq+K8rS71rTubTk8aK24tOBS/OZEYjX6963bk96Ubsy+dvW4Vde
9ZG1BBs1YOwWq1Ks9kcWu+3IaxdcdeNE387w+2yV3rnwG1/6DqbsV0VnO2FBNwB4e0wr3O11fc
nDEt0PSK2E9ivvuaS7JjMbQdfr41qa6JzF35+FOdanQqsS3p+lETnOlHbRwht44jjjDietO4RYBr3Pnyvv
OXCW/QSACAP99/xLnnNW2/SSwCAzB3/NZFjr9ILWBT0REVwkikVtoQGJP5Qeb/npMsYdm/2GE1F
UU6ccp51dJfTrj2WvywnT5+0e9QF11uqp/0AbA74vWmb4nVG7R44gZT1GW8ov7dOdT4sGbUo3S
BMfcIsazBr1jUo6ac28z7QPYSnOKZEvYkT7cec4f+kmIdQ3NyTvhmgTm3wHIWY6rrrurFuwJmaGO0
GMvDwEw/LOz/5Tr0EAMjDDY9fJXd/6AF5/Bs/0msAAJm7dI9I/zt6AYuCEBVBA6FScsO1P6fl1RVt7k
pbenJoj9k/JScqJ6z/D65XGeqc9ZIrrombLbpD+ttuIl09a/TozUtaAZl1DTJrOyHeSKpXqBe0GnVjgQX3x
NNPSOWPKvZHAEA+nvPjY/KWL94oP3ryGWm+/3555p8u6c8AALL1lMilP7A+PussYiEQoiJlGYRfXl3
RfTlsXxS7lKgd9lbEyVeN9XMZvhk9NO3A1+/VOyrwVcO67VqVQ480e0lOK1mv47yOaTHOXbjxbW/
Lhtnr1K2LatY5BRbUnZ+6Ux58/EG9BADIwxv/
+gZ5wQ+vsJ8/9NXvyp/WeR8GgNz0Hxa5/Od6AYuAEBUxleSkN1LbnBFfMSZJitM70Ri+HI+7744c7D
pf686+X7I/4a8fLjUwH4qbZ/0guOFOjFWVQIZacsoTKG6Jgvk22OvYvA/0seV1TIn2Y97LA8Pk1cz3hZ
xnoE/SdmNSNTtwdeukJv6eAubL+z77PvlM7zN6CQCQh1c+eK3c/PXn6yXH5z7+kHzlLx/RSwCAzF3
6gsjlr+kFzDtCVMRUlM2zbuVUc/Z1NbrYnOl8y+vt5wScijnre1s23JlrYvN7cqqJpBS3XmZRF0x117vh
6vxxe81a56Kmj3+wdqsx7F+aq7LhnTIV7Onh297K+dBcNQLH9nm/hqd7bHkdU6L9mLVbm7Lqre/p
me+ta1Qq+NvI2oTnyA9c3XYDi+kT+5+Q+n11vQQAyIOqg/pzD7xELwX94W9/Tb73raf1EgAgc5f/RK
T/fb2AeUaIivgq29LyctSalHTA48w8bynX5YJR4NOfUMnSXNWB0GQ1HAPbsp55Q/a9of6KMUnTHP
Jqu9rKUt8KRKiWomxe8IdlN1d1gOaFzqMm/ZolJ3AMttM8tryOKdl+ipsXpO6/2HmtOZGTdS/7h6Br
7yrua1NNWBO03bzfw9odR+bHA8R339fvk7v+7C69BADIw/FLBXsYv/oYRgWozd+8Xy8BADLXf1Lk
8sf1AuYZISoSqWyH11+stqz17tBiV3FT9gZfW21NVr/R7K0XKBlgDM3OdKKjFJgB2KiyA+qc9bt+wOe
ya1/OW03PstS7fT9Yt1Wl1d8LTpaV1zEl2k9RNvf60h16sXq5MUzeVpHt4EGK7B96PbFTEbvtVlv6Lf
8+sqlzblwHu+5ulByOB4jBnYn/6Wfp7QQAeVr50kvl2iev1Evh/uFL35ZP//6hXgIAZO6y9RvZJcpbzbt
CX81aAgAxtTcK4nSSVCHqQGAKADGo4PT1/+318sAjD+g1AIA8lB56od0LNa6tu8vyiteFD/sHAGTgit
8QKfBL9ryiJyoAAMiVGsJPgAoA+VK9T1Uv1CTUsH7qowJAji79AfVR5xghKgAAyM1HvvgRezIpAEB+
VP3Tt33hZSProI6iAtS7/+NX9RIAIHN2fdR79ALmDSEqAADIxe6Du/LBv/igXgIA5OWOL18/tg7qKPv/
8zH53Mcf0ksAgMxd/n9FLn1BL2CeUBMVAABkTg3fV3VQmUgKAPJ189efL//8Czfppclc+dzjsnX36+
XWV79IrwEAZOtKXR/1Zr2MeUBPVAAAkKknnn6CmfgBYAZe8MMrEk0kNcoz/3RJmu+/X3705DN6
DQAgW9b77aWPWx+fchYxFwhRAQBAZlRwqgLUh594WK8BAORB1T99yxdvlOf8J1f+R7/xo/k7g8
xKSAA5Kb/HZFL1EedJ4SoAAAgM+/77Pvk3ofvJ/igGALm5/DXr0dELmDVC1KXWlo1CQQrGY6XR05/LSa8hKxttveBob7jtWZG8mzM3Qs5LHL3GinPuVhoy6tT55
9d55H7NjwLuWyCW+n11+djffkwvAQDy8vJvPE9e+eC1eildn/zPB/LN//19vQQAyNylXZH+Y3oBs0S
IurR60lhZlaZecpTlzFpRP8+eHfiVasLfVIImPi+9hqzXor5KXfOCrAYvunRqJSlMENguK+5bIB7V+1T1Qg
UA5Ovqp47LG++bvg7qKKo+6u//
+y/bHwEAeVD1Uf+79fFZZxEzQ4i6tLpy4KZA5bp0+33p9/dkM68MdWzgt6QmPS+qZ+S4YK99XtxN
V1vqevelWy87K5qrQo4aA/ctEIuqf6rqoAIA8veWvRvtIDVLqifqH/721/QSACBz/UdFLv2xXsCsEKIuq
96h7OuncuqE5Nf/FGmL1zOyJ41zugtquS5bFedpcfOsVJ2n0twlRQUwPTUTf+WPKvZHAEC+Xvu1F8

vLHr1aL2Vrb/f/2A8AQE4uf8l5YGYIUZeQXbvRDN2aq04tx8GuiKp3o1E7c1T9zGAtyGCd1bDejbH3r
wVreI6qN+kMVfdfl6zW52A9S6+2qH6ENy3GPgPncEP8zZjnydln0vOiqHaWYvWMNHoeB0LzkpzUn
VFl/9A6olGM9obVW21v6GMZPAcJrkvI/RZ2vePcb4PXz35E1ImNY5b37WT3p2vSa2eZ6D1Ar1TMr
49uJI6QOz91pzz4+IN6CQCQFxWevubvXqSX8qF6o1IfFQBydOlPqI86Q4SoCDUUGGl2/cyRYVRHai
WjzqrR43EyanuDNTzVurCgpuQNVXdFt3UUZ5+DwWRzdcJ9Fjflgjtk3joz5/RGeo1z3nkq1y+kUEahK
q1+y+tVOsTseTxK50C6+umwipx2N97ZkYsDJ7W96x2NX1c3yXVRQV7I/eZcj1EBZPj9pu7d0GC5U5
NSIMjOSpb3bcz7M2CCa2eZ7D0Ay07VQP1M7zN6CQCQFzV8Xw3jz5uqi3r3h75KfVQAyI2qj3qP9Z
H6qLNAiLqEKtt96Xfr4kZ7Um3Z9TH72zrxbG/4AZBXL9Won9mpyfrIxEaFec7r+3uboWUCxu4/wN+
et3/pyI6XBPWkse4GPca+Wzo1imzrKP523M2ofdbOu/Fbsn0WNy+If+rOS9v677wbglnn94JOUJOd
F4P9um2ZLK8uyolT+ukYlS23beb5V9ri53BnxMnhkpwj67VGqYGh+21ofyZj2/b9ZrYlbFt+kJ3U/Ny3
/uvD789hya6dZar3ACyrT+x/wp6NHwCQv5UvvTTzOqijPPTV78qf1hmBAAC56T8scvnP9QLyRIiKIW
bPtPoFPwgdDgOHletbE4Z54aotPxwsrp3xAqzOge4z2bsoO14eaey7suW3deeiJll7zH364ZMh8T6Ls
nnWTbuaslpwe08Gz+8kipt740PW2PblMOpEFdfkTNjxtXf9XrVn1pzjSXSOrPOzp0NEI3gPvd4Dhu43
s8et0evSPk86CNzLYfa0LO/bsfdnmCTXzjLNewCW031fv0/u+rO79BIAIE+vfPBaufnrz9dLs/G5jz8kX/
nLR/QSACBzl74gcpkJ/vJGiIoBI3qm2Yqy5iYxIwK3UyfSDKjKcrKkn47SPdC9+VSoU/LqNhbMYdKRw9
QnMMk+K9tGr0FHOsP403RKoi+fcf2NgNIP3Kpy1j2gqa6LruEZOrw/aOh+M8JCawf20Hd33yNrsaZ
uHu/bBNduyvcALB93Jv6nn31arwEA5OWGx6+Sn3vgJXpptu7+0APy+Dd+pJcAAJlTw/r739ELyAMh
KibUkeHOgTHCo7mX3TFUvMKUSrD+5Oz05HBssVSf36vSHRZuBG7V08FeoUmYExB5PXXHCbtWR
dm8EN470w0rF3t+o8nvz/SvXdh7AJaNCk5VgPrID+h9BAB5e86Pj8lbvnijHL9U0Gtm60dPPiPN99+v
lwAA2XtK5NIf6OfIAyEqJjR/gWm53vWGbAcfk9YLHS/
+Po26n7aO1NZznJyneELGlj4tn5Sxl7S4KW5lAntYuDF8vno6/CyPP0dt2TB7nbo1OM36o0lYbdzT+xj
s/as0z83XpEi53bcTXLtoR+GPJpiWGsL/wCMP6CUAQJ7e+Nc3yAt+eIVemg+qPuqnf/9QLwEAMqfq
o15iYte8EKJiQElOusnV0EzePbnoFnIcO/Q7J6WTXtA2qm5m6ibYZ6+xrodpl6Ved5OsPCfnMa5rc1f8
zphdOfAu6QmJc0m9HrXW/XH+/I4OP6sSyOGSnCOzLqcKFN26qMaQ96TUzPKqx6k9GZQdSHa9W
p7pDpOf0CzuW0usa5fme8AU1xDz7yNf/Ig9mRQAIH+lh1448zqoo3z6v/6D/MOXvq2XAACZu/xZkf
48dRU6ughRMcCcBCnYW9IPAlXYNTChz6yYNTCbq/5Q7faGHhpuPdIev510n72GrLsnrnpWNjeNyY
Nq65JPjmrWsmzKrm5er3HOCzBj90asnBbnDulls+ke18Bw8Amvix8oDvbcjUlvX80sH+xxaoTFZo9b
oz1p3yaRZnHfKnGu3QTvASU/dZXaee9gZMOb4h9Hze6Du/LBv/igXgIA5OnaJ6+0Z+OfZ83fvF+
+9y1qZQNAbtSw/v739QKyQoiKYeYkSJ2alHSoU/LTE7kw7YxI5vDy5uoUoVGwBmZzVQdQXnhTlVZ
qs9e7kuyzJ411d6h6Wepbav3okCq98zLMnFndbbN3TastiX+aKhIo72oZDmATnCMv2LO4x2xOsJTEi
HvXrLNaPevPNp/YQt+3SpxrZ0n4HuDXW7V41zBubVssGjV8/85P3amXAAB5UvVP3/aFl81NHdRRV
ICqglQAQE76T4pc/rheQFYIURHKHgYdUpOy2rLWu8Otp1KR7cGClfuHRs/BBOwamMZwbZddWzOj
eqgx92n23LN7obonbiCk8of1p3hehhRlc2+4Tqg9hD5hYBeYJMs6ZjsbHhT7uljH3G/5QaqtKq2+0dZ
ACYJo9r07eJA2Z5vTZZMLft9aYl07S6L3AHUsg6+ttiava4u59cTTTzATPwDMkOqBqnqiLgI1pJ/6qAC
Qo8vWb6bUR81Uoa8KBgJAEmo2fT0ZlAph96btmTxL+lhOtaYNWBfEUbp2yJUKTit/VJF7H75XrwE
A5EnVQVWTSS2SK597XLbufr3c+uoX6TUAgGxdKXLFb4gUbtbLSBM9UQEkFCxRcGZtsUO49nnrWC
J6ZB4tR+vaIV/v+
+z7CFABYEbULPx3fPl6vbQ4nvmnS9J8//3yoyef0WsAANmy3m8vqWH9TzmLSBUhKoCY2rJh17o06
pWaJQoWUK+xIqv7demmUqJinh29a4d81e+ry8f+9mN6CQCQJ1X/9C1fvFGe8+PF/NXt8W/8SO7+
0AN6CQCQuf53RC7doxeQJkJUAJNRNS8XfPx7cXMvpRq/C+YIXDvkR/U+Vb1QAQCz8XMPvERueP
wqvbSYvvKXj8jnPv6QXgIAZO7y10QufUEvIC3URAUAAKEefuJhec32a+wJpQAA+bv568+Xf/6Fm/TS
YqM+KgDkjfqoaaMnKgAAGKKCUzWRFAEqAMyGqoO6aBNJRVH1Ue/
+0FftjwCAPKj6qGpY/7POIqZGiAoAAIbc+ak75cHHH9RLAIC8qQB1UeugjvLN//19+cPf/ppeAgBkrv+
oyKU/1guYFiEqAAAAIUDVQP9P7jF4CAOTtV97sbzs0av10tGyt/t/7AcAICeXv+Q8MDVCVCTUk8aKm

uVbPTaknWjdOGltZ96MOYZeQ1Y2gmvbG+7rV6TR0yunpfZjb3NEO2zTne9M2m1bkPugvaHbWJC
BSwosjE/sf8KejR8AMBsqPH3N3x3tuqGqN6rqlQoAyMmlPxHpP6YXMClCVGCGeo0VKZRq0tHLWe
pd3DH205RdQj4AA+77+n1y15/dpZcAAHm7+qnj8pa9G/XS0aXqov7+v/8y9VEBIDeqPup/tz5SH3U
ahKhIqCibe33p99VjWyp6bXby3l8WRhxDryHrtTziU6UnF3eC+2qea1hrF8VRuA+A+aZm4n/nJ98pT
z/7tF4DAMibClBVkLoMVE/UT/7nA70EAMicXR91Vy9gEoSowDLoXRQ3Qy2Xy86Tzo5cXJwUdTFUt
qXfrYs+w8DCUMGpClAf+cEjeg0AIG8//ffX2UP5l8m9n3xYvvKX/NsDALm5vGc9mOBvUoSoS8eoLbl
i9kQctd6oc2mvz7s25fD+IutuGnU/VwKfNLcT9vko6Z8z+/PmMP7mqvP5EYU0/WNWj+T1Rv2h/FU
5e+GMDvk6shMnRQ3UUg17jG5PvHbHuTbD59DaumyYrzfqkQa2MbA+/BTHaYN5POpY/P2rh7vd
9nnrupbrsjXQXdYu3WC83n4M3DfArKgh/A888oBeAgDk7YbHr7Ink1pGd3/oAXn8Gz/SSwCAzF26R
6T/Hb2AJAhRl05R1s64PREPpOs8s3TlwE30Aj0U27LbdJ6Vz6xZXz17ldNV/awjB/4B2PywsCxn1nRr7
RCwJIMj5zu1UswQa5bnrCO1UkFW9fYcal2SINUYyl89LZXimniHs3Nx+hCvfEbcU+2L2e6pr43Dfn1w
Z846FVQOrG+uDgSpE7VBHcuqeFvWoakKSlf369Ld2wxcdxW+lsJKN3RqUprnibKwFD7yxY/Yk0kBA
GbjOT8+Jm/54o1y/FJBr1kuP3ryGWm+/37qowJAbp4SufQH1kfqoyZFiLqEimtuT8R9OXQTovauHwi
JEU72Dq1XOU6dmIcI1VI5LW6M2gzMjmSEhV6w15PGutvjsyotu6am9WjpLXRqsh4jjUz7nFW2rTa
Yw76rLadd22HVPv12d+vuV8TsRaoYQ/mrp9X2zVA4xpD+4qbsuefNfnTFa4Z1BPULwcDQN67d6V
wbV7neDX69Frbev2+maYPxeh2aFjf3vOc+P1RXYWtXf41/TppyLsFxAmnafXBXPvgXH9RLAIBZeONf
3yAv+OEVemk5PfTV78qf1h/USwCAzPUfFrn853oBcRGiLiOvJ6IfaPUOndivWnXCo32dFJrDwO38bS
5UZMsNoJq7fi8+s+6n2wPUXFff8ickqmx5QWCs3pgzPGfVlj+Rkh/mWi0Z7IY7gtk792TJfmJsJ0EYq/
Ua636vzepZ2QxPUMe3O61rY6vKWbchRsg+cv3+obPdKdoQeH0UI1Q3Q2s7cNWB6t6okwhkSA3f
v/NTd+olAMAsvPLBa+Xmrz9fLy23z338Idn/n4/pJQBA5i59QeQyf8BKghB1KRXlxCnnmRtode1x6W
U5efqkHXYF11vUMHDn2VzwQ7mmeJ0KuwdeWOgN5ffWWcfkDu+2H8bw7cAQ/VFmdc784HMy
Yb1zLRMP6W/LeT9BlVZoz1klRrtTuzaW8kkJ3d2o9a4p2hC7Z7Zxrq2N2WUO3P3Er8sLpOuJp59gJn
4AmLGXfPe58nMPvEQvQbn7P35Vvvct/m0CgNxcVvVRv68XMA4h6pLy6oraPTl74nSqPCUnKies/w
+uV3ngPEWoFiOYcodmt90x0xG9I6exkOfM6GmphqaXQkPC+LP0tzf8OqBVo6fp8kkSbhdl80L4jP1u
cDtiPjEgE+5M/A8/8bBeAwDIm6p/usx1UEdRAWrzN+/XSwCAzPWfFLn8cb2AcQhRl5U3tHlfDts6aL
N77VXEyQqN9VP3hsyCUdfTDi/9upOjwkuvNubQI2YYuIDnzB/KHyXmkP72hj9RVLUlIzuhTmDqa5O
CTNtg1JUdKNlqa55jln7k532ffZ/c+/C9egkAMAsrX3qpXPvklXoJpn/40rfl079/qJcAAJm7bP02euk
gFRCFGXVklO2hlkRw52nSHNbh3Rkv0Jf3347OuzV9w864eaDXeSp4E6pCVnqL3i1eKc2KKdM2Mo/
xjjh/S3ZcNLUMtSV1PRTyvVazOhHNugZuhXPU7tScXsQNWYoCtu2QJgSvX76vKxv/2YXgIAzELpoRfa
D4z26f/6D3aYCgDIyeXPivTp2jMOIerS8ntyNptOOObWeCzq4p/uem+Sprnj9gDtSK2mA77BOqRmP
crmqj9sur3h1aUsxB5LvWDnzBzK787+H3iYIV70kP5e45w3jL9cv5BOuYRUr82E8miD3pbKoIM9Trvi
ls8dW7sVSMn/+v/
+l34GAJgF1ftU9ULFeHd/6AH9DACQi0v36CcYhRB1iZkzpqvehd7w88Ds5sYkTXPIq1NqC+shGaxH2
VzVwZg/Ll1GT440LNVzVtS1VJXmauqhoTmUvxxaW8AoiWC9cuSQ/l5D1r0CqtYrAxMwOY/JJkhK99
pMJoc2VLb9IfyBurRGfdmzm7MP3bEU7v7Fu+WOl9+hlwAAeXvjX99AHdSYfv7dP6WfAQByUXijfoJ
RCFGXWWDW8FPiTzbuDltXzPVzyAwvRw2ht+tRGr0uXeW6dJPWu0z1nFVke7BA5v5hSrUxzaH8o0
NdMxQeNaQ/Xl3VCaV5bSaVQxvsIfxhxVBVSNvvp1pfFohy1RVXyf/4lf8ht1x3i14DAMjT373yCf0M
UV7z1pvkbe+5VS8BADJ37FUix9+kFzBKoa/G9QIAgKXxwCMPyOv0ov/2+vtmfoBAPm648yXVyysfvFYvYd
D1P/E8+a1PvVGedw0TbwAL6govFrni/daTq51ljERPVAAAlsztN91uHAOTvb27/0K9/
9L9fdeS4AKALmx3m+Pv8f6SIAaByEqAABL6F2n3iUfeMMH9BIAIC+Xjvfl8294VH78nMt6DVz/8t+V
5NZXv0gvAQAyd+ztIoWb9QLGmSpE7TVW9AQpBX9W65F60lhxJ1TZkODL27LhTbbiPCabqGZYeyO
43dBH1jOAAwAwhz788x+W07ed1ksAgLz84PnPyt7rvqWoLzidS+R1X8bNhkqACATx4oix39eLyCO
OeiJqsJVf5ZqR84zwquuZ0VcaKU3oAwDA4rjnl+
+xh/cDAPL10M0/kO6O3MGGdIWnkGIWZXOvL2oeq35gxuuuHH

iTiKvZsNXn92QzxwzV1qnJeTqkAgCWjDtj/3VXXafXAADyonqjfu+aZ/TS8lIBqgpSAQA5Of5ukcIL9QLi
mn1P1N6h7OuncuqEZJedlqXedUNc/9Gtl/XnRZq7pKgAgOVzy3W32EGqClQBAPlR9VE/96Zv2h+X1
S/8xivsofwAgJzYdVDz7rl4NOQYog7XRLXrlZZq4nZEtYfVD9UoNb/OeaRVL1Uprp0RP0YFAGA5vfm
WN8tH3/5RvQQAyIvqibqs9VFVePoLv35CLwEAMmfXQX2HXkBSc1ATNUKvISuFktS8lNXRqZVSq2H
aPu+HuNXTfpGB5WVM8uWd46hJwTA7YddqEYTfT/4kcHneY0nPYU7fC+q9b2DCO//8rEiKf0cCAt7
7s+
+V2h01vQQAyIuqjbps9VGfd82VctfvUJMbAPJztcixO/VzTGKmIWpluy/9bt3vCVptOcPst1WY2ZPGu
htwVqXlDsFvVe01qobpeqIkoSO1khtC+I9Vd0Yra9/2bgFghnqNlWAPfSBnqjeq6pUKAMjXfT/zuD1r/
7JQAer1P/E8vQQAyNzxO0UKL9YLmMT89kTtXZQdnSKU61v+RFSVLXHLmHZ2LqbU+64s9S0S1NFG
TQoGTGKR76eM295ryPpg13tgBlR9VFUnFQCQnx8/57J8/g2PLkV91Le951Z5zVtv0ksAgMwdf5PIsV
fpBUxqfkPU7oHXE8sevu/1HjWG93cOpKufTsfppZpmrVUAABaVmqm//attZuwHgJw9dv3T8je3f1sv
HU23vvpF8ku12/QSACBzhVtEjv0LvYBpzHdN1FSFz85vlhPo1M7nWItxHgxO2jWqxuPoOpD20GPv6
/VjZG3Jwf1FBNd2Pdzga0fVgsysDVEya98oca+VK/5xJm1f2OsHyncO1O806o7arx19P/kG2p/K9Ux6
DsOEt32wXungORo8P2HiTbTn8/epHqPqpCY5P0DQbdffJvf88j16CQCQl7+/7Xvyjy//oV46WlQd1O
rvvVaufO5xvQYAkK2rRY6/2/p4hbOIqSxEiFqud4fDT/uRwnDa4qac1WVWRfblcGnyBRVsDU7a1ZTV
wqr1/3hUiFMKG3rcqUlpMKBKMklYe2NETUjVYzgYFmXWhihZtW+khNcqwXEma58TyIW9vrlaGBH
2qXNitLNcl/GVM9SxDbR/6us5/f0ej9OrffAcNVdHhZyTcPbh1XO2Dd97qd3vWGrvKL6DGfsBYAb+6v
WPHcn6qL/yH15JHVQAyNPx09RBTdH8hqilk34P0YN0Bu2Ha8tuuinKQug1zvnhkTuhV7/r1Zsdzzhv
5bp0dbDd9TbQlHNeotNLMEmY9dpzesOh2+3IzkX3tVm1IUpW7Rst2bVKcpzJ2tdrrHuBnP+HjZa1F
615bkRQaLRjb1OKem0koz1u06e5ntPf70n47fHb3pHa+ejIPHqivUH+PsLvvbTud0Ds2frfdepdegkAk
AdVH1UFqUfJyumftB8AgJwce53zQGpSC1HtnmjGkFHzEWco65DimpzxspxVfxuqF6C77UQbdnpv
me1yHmYvuTOyFivhWXQ9uejO2qUCFi+kKcrmBSPEidI7lH39VDo74mY3xc09J6yxHnub+mQmmiT
MmLjHCNyKa2eGQ/XM2hAlo/aNlPBaJTnORO0LtuOst74iW/aGVVC3J2GHE2hHTNWz/rmtbPtBrd
/2JNczhfs9gWrL7yFf2Up/+4q5j/B7L8+J+bAM7v7Fu+WOl9+hlwAAefjmjU/JV376u3ppsb3sp14o/
+a3mNAEAHJTuFHk+L/WC0jLHA/nDwYcXkjrjWE1w5B0mMHN0daVAzdTKp+Ukn5qK56QU/ppJDP
kHgioh2ouTjVJmK6nGTZ8Prc2REmpfSMlvFZJjjNR+0a3wwldR5fWOHUi6XdVVU4HNlaSk2473bYn
up4p3O9zpSwnAwcRIteJ+bAMrrriKmbsB4AZuP9V37HD1EWm6p/+
+n/5GeqgAkBurhQ5/mvWR+qgpm2+a6IWN2UvbMitPdQ3hXqoHtWLri8pZ7JH3OhefG5oM1EPZ
MWu5eiGPlE1KzNsQ5R5b19sebQvRuA3VlFOLEzSmcbxAvPpphfcZAepKlAFAOTn8yuPylNXX9JLi0f
1QFU9UQEAOTn+r0QKN+gFpGmqENUc9hv1cMLJoj8M2gxA7aBUrw9NMc2v04+4dRUtdp1B82tD
H2kGskvEuHZ+7Udf89zw5DXjJwIry4bZq9OtjWnWijRl0oYo2bcvLbGOc4btG21wgreeHHp1B4ZlOv
HcEcD5QZpuv+l2e2g/ACA/KkBVQeoieI3l/6IOKgDk6dhrrQd1ULMy3z1RkZGQ4dEus05mDGp2d9V
b0Q+rjZ7D7raTTBLW3vV6dtrhjxuYG8OTB6XehihZtW+khNdqguOM176Idth1itOdfT7Y9JDh+ImO
M737fWHkNjEflpGaZOoDb/iAXgIA5EEN6f+7Vz6hlxaDmoWfOqgAkCM1C7/qhYrMEKIupaKs+bN2
yao3Ztuc0XsMPcGXKlEb7K0YEnhNOEmYH/4YM+KbcmhDlFTbN1LCa5XkOBO1L6Id9rE7NVUTnsK
Rmqsb4jfdL5lQPrPmhNaJrmcK9/uiyeB+B0wf/vkPy+nbTuslAEAeVH3Ux65/Wi/NN1X/tPp7r5XnXX
OlXgMAyJaqg/oe6+PVziIyQYi6pIqbF/zehs1VHawYk86MU9n2h393alJygxmjRqg/UVew9mbkJGGV
09aSNq5dWbUhSmbtGy3ZtUpyrpO1b2w7ynXZSmV8eFnK5aas6vZ4Tbfaftab/j/Z9Zz6fs+LOdGV28
6Jws6U7ncgwj2/fI89vB8AkI9Lx/vy+Tc8Kj9+zmW9Zn79Uu02ufXVL9JLAIDMHVsTKdysF5AVQtSlVZ
TNvcFJu6rS6res/8djD/8OK6Rpb2dgoi679ubg/ixDk4RVZHuoDc72vF01d71eitm0IUqG7Rsp4bVKcJ
zJ2qfa0Zfu0IbVpo3SBlM7JWcHjzfsGiW6ntPf7/mw7q/B67F/aPQSTiCV+x0YzZ2x/7qrrtNrAABZ+8H
zn5W/ev1jemk+veatN8nb3nOrXgIAZO7Yq0SOv0kvIEuFviqCCAAAMIF7H75XKn9UkaefXYwhpgBw
FNzx5evllQ9eq5fmx7UvvUr+06ffzDB+AMhL4RqRK9R8BQzjzwM9UQEAwMTefMub5aNv/6heAgD
k4W9u/7Z875pn9NL8qP4udVABIFfH3239jwA1L4SoAAAZE3VR/3cm75pf5wX

v/Abr5BXvO4legkAkLljbxcppFNUD/EQogIAgKmp3qiqVyoAIB+qJ+re676ll2ZLhae/8Osn9BIAIHPHiiL
H36EXkBdCVAAAkAo10dQt192ilwAAWeve+n37MUuqDqoaxg8AyImqg3rsPXoBeSJEBQAAqVAz9b
d/tc2M/QCQI9UbdZb1Ue/6T6+2g1QAQE6O3SlSeKFeQJ4IUQEAQGpuu/42ueeX79FLAICsqbqon3
/DozOpj/q299wqp/7ZDXoJAJC5428SOXabXkDeCFEBAECq3lF8BzP2A0COvv2if7Jn7M/Tra9+kfxSjV
/kASA3hVtEjv0LvYBZIEQFcteWjUJBCsZjpdHTn1tSvYasbLT1gqO94Z6fFZnX09NrrDhtXGnIqCb6x+E8
lv5aY2mo2frfdepdegkAkLW/v+178o8v/6FeytbzrrlSqr/3Wrnyucf1GgBAtq4WOf5u6+MVziJmghAV
yFVPGiur0tRLjrKcWSvq58vHDiJLNeno5YXRa8h6LarV6loXZDV4saVTK0lhIDAGjqq7f/FuuePld+glAE
DW/ur1j8kPnv+sXsrOXb9zu1z/E8/TSwCAzB1XdVBfrBcwK4SoQK66cuDmbuW6dPt96ff3ZHNZM9
SxQeScUj1nxwW/7fPiHlq1pa5zX7r1srOiuSrkqFgGV11xFTP2A0COfvycy5nXR33zr9wir3nrTXoJAJC
5YyvW41V6AbNEiArkqXco+/qpnDohy9v/dHHF6znbk8Y53QW1XJetivO0uHlWqs5Tae6SomI53PSC
m+wgVQWqAIDsPXb903L/q76jl9L1sp96ofzKfziplwAAmSvcKHL8tF7ArBGiLjGvnqP5CKntOLI2peqN
536d163Or/dp135sb+ivNdbZLwuuD/bKS2MbmtlG7zFcYzN4jMM1S+1HWN1Low3jal3a+zDDt+aq
87WDDQ9pc9i2o9o8tpfjROdl+J4ZuZ8YxxD7fGh+W9RjuK0OZwi9/7p0z506/lKsnrNGj+NAWF6Sk7
ozquwfDt9PNqM9ie65eMduS+H7wj1Pcd9HsNxuv+l2e2g/ACAff/fKJ+SbNz6ll9Kh6p/+
+n/5GeqgAkBurhQ5/mvWR+qgzgtC1CWlwpHQMKhTk1JhQ8ZlcHHYtR8HCkLa61TIMrC+uRoeXk2
1DRU2hfYY7EitNCqEU58zapaW61J3uw52duTiYMi0674ynbqmQ8GiZh/zyGBquM1uz8dQE5+X4X
umuToieEt8DFGcfQcvd0hb7WCw5A2hd6V67jxVafVbXq/SIWaP41E6B9LVT4MqcjrpPZfk2FP6vlDn
KY/3ERwdapKpD7zhA3oJAJC1z688Kk9dfUkvTe/f/Nar7J6oAICcHP9XIoUb9ALmASHqUmqLn8O4d
TmNeo3SlHPhSUpi5XrX3na/FYybwtaPGt482TaCw6mHj7EjO4PplEcFZM7r+3ubsrlVF+erBr/GPI9nZ
FyGWtm2ttd1t2Wptpx9bOvUrr3hB4Vhbe7UZH3kdQm2eXRT0jkv/invSO28cd0SHMPY8xHg7zu8r
dZxrbvBoHEu3Iamcu40u53bEitrHVKUE6f00wiVRPdckmNP7/uimOP7CI6OD//8h+X0bQxHAoA8q
ABVBalpWDn9k/YDAJCTY69zHpgrhKjLyOwlZ/R0K27uOQGJ9dhLZaajqpx1t1M5bS25RqwPHd486T
aKsrlnBj6O4toZL7TrHIT3AyzXt4IBWXFNzugv6uxc9NvY3vV65pXPrI0P38YwexjWLxht3rwgfgZ5PrR3
31CbR5r8vFRbfnDoh3xB0xxDFHPfoW3tXZQd3SEycC4qW/5+zWtniH/u1HFY3yOhIe8k9uUwrEFKk
nsu0bGn+H2R2/sIjpp7fvkee3g/ACB7akj/V376u3ppMqr3qeqFCgDIiV0H9V/rBcwTQtRlZAQ0ImqY
bsGrZTiyhuIkyielpJ8GjFofJo1t2HQ9x9BhzEGnTgwGP0VZ8xMtLyzyA0Mj0J1YVK9WY/8jgrfhNscV/7
yMN90xjFaWk+MudvfAa79X7sF+GEPcRwyfn/zcTeuUjN51gntuimNPcv2HzlNe7yM4ctwZ+6+76jq
9BgCQJTXJ1KT1UVX907t+59XUQQWA3Kg6qHdaH6mDOo8IUZdSUTYvhPckdEOYqMl1FkZg8hyjn
mOk8MDO76nnDnc2AsPq6dg9GafXkeGOgjFCRlOK52UyYccwK2keVxw9ORxbLNWR2T2X2vVfkvc
RZOKW625hxn4AyNFfvf4x+fFzLuul+H6pdpvc+uoX6SUAQOaOrYkUbtYLmDeEqMuquCl7esjtQKlR
W/Pcos+u3ZYNs3edW7PRrMGZhHW+zurzZA+NNoYyV0/nF6FOH/qlfF4mkm1w6dXKHXpMWsd0
AsUTMrb06bje1BPcc+OPPf3vi6P9PoIsvfmWN8tH3/5RvQQAyNIPnv+sHaQm8Zq33iRve8+tegkAkL
ljrxI5/ia9gHlEiLrE1MzaqqeYPcGPHYR0vRqKo4f/GoxhxHPHrB2pgiW3/uMUba64U6Z3duT8+R29n
aqkk6GW5KR37gdnZO/JRbfoZeQQ8BgyOC+
+nI4hTOmkFwKOqumZL+NcNHfF75DZlQPvNJxwzn2EWPdckmPP4PpP/T6Cpfben32v1O6o6SUAQ
Jb+8eU/lL+/7Xt6Kdr1P/E8uet3qF8NALkpvFgP48c8l0RdRu0Ne6itmkU92FPMCHiMXnIlPxkzZmJvy
4Y3Dft884MlY2bySXgTWHWk2dQnKrWh/EXZdLsdqvO87l+XXmPdq22ZZBKkcVI7L578j8Fj1udsrvr
DyPW9bj9yHVtu1oBtyq7eda9xzgsxY/VgjnPPTXjsU1//hO8jZntyvRSYe6o3quqVCgDI3t/c/m157Pqn
9VI4Vf+0+nuvleddc6VeAwDIlqqD+h7r49XOIuYWIeoyqmz7Q287NSm5QYtRH7F6NnzmbhXSDL52
Lpkz9nttNibamUhF3I6BrlSH8o+4LiU/fZQL005glcl5MSQ9BnPYu9ueiRK2YH3O5qqz34IX9Felldqs+v
EUNy94PTLd9njnodqSeM2Jc88lOPY0r3/C9xEgiqqPquqkAgCydel4Xz7/hkcj66NW/u8idVABIE/H3k
od1AVBiLqk7KG3YUUMVeDS7wcDHlX3cLBmYrWVcx3NpCqy3W/5gZHNOTbvsAPDrOPxhlcr5bpsp
ZzL2dcl5LxWW9Z6d+j1VLI5L6Zkx2C1Z/A+3D80ejUmYNfnNIaSu+y6nznWQ/UUZXNvuFaoPYw+Q
aAb656LfezpXv9E7yNABDVTf/tX28zYDwA5UPVR7/uZx/VS0Cte9xL5hV8/oZcAAJk7Zv2GfPwdegHz

rtBXRewAxKNmNtcT86gwbG/anqHAOEfpntPHcqpFwIpwn+l9Rip/xM0BAHl441/fIKWHXqiXRK596
VXyW3/8z+yPAIAcFK4ROf5+66P/Xoz5Rk9UILaeNNbdmc3LcmaNABVZO1r3XPu8dSwZ9ODG0fGO
4juYsR8AcrL3um/J9655Ri+JVH/3tQSoAJCn4+8mQF0w9EQFxmrLxmANWFXOgK50yMzRu+d6jRU
p7ZyRbiplKXDUFf6fgn4GAMjStU9eKe9s/aS88723MYwfAPJ07O0M419AhKjAWAOBFgEqMsc9h+
WlJiiblx9NaEs42hKOtoSjLeHmqS0nrr1GDr/3pF6aLa5RONoSjraEoy3haAvSQIgKAADmBj/ghqMt4
WhLONoSjraEoy3haEs42hKOtoSjLeHmqS1IhpqoAAAAAAAAABCBEBUAAAAAAAAAIhCiAgAAAAA
AAEAEQlTkSE2WU7DrfxRWGtLTa8fqNWRlo60XHO0NvZ3CijRibwjp6Uljxb0GG9aVTbJuGmH3UNr
7mJbRRv1Y4SYFAAAAAGChEaJirvUaK1Io1aSjl4H5pgJdY1Z9W1nOrBX1cwAAAAAAsIgIUTG/eg1Zrx
GfzqeibO717RkF+/1tqei1szFPbenKgXvLluvStdu0J5tkqAAAAAAALDRCVABIS+9Q9vVTOXVCyE4BA
AAAADgaCFGXmao1OlC7MazG6GD9UXuIvfE1A+VKNbNOpXokq1Vp79Mcxt9cdbYTvjOjjeoxqk7qY
JvCa1VOdrymMbVf2xvetob2H3JN4rTRY359ZEONcxFo46j1xj7t9ea5zKoOadx7KKotg9uYsD5pjOuS
9J4FAAAAAACLgxB1WakgL7TWaEdqpVEhpPpcQUoDQ+ybq4OvVyFiSYIva8pqYbBWZBqcNq0GNh
xyDHYlNtgm65W1UnjQaYt7vIMqcrqqn3Z25OLAa9u7bmODtTKHQjgtuo2TKsrambLztHMgXeeZxR
iOHmh7W9xml8+s5dDDMoV7aKJrPizf6wIAAAAAAOYRIepS6knjnJuIuXUb+9Kt61BNOrIzmPx5qtL
Sr2+5QaEKG8/7ve16jXN+0FVt6VqVXfE2H0Nl2/qabl28L3G3sx1W8dJvU/gxWMe77oZg/mv77gF0
arI+MhUdf7xhKltu2wfPpR9GSvmMeBlqe8MPgsOuSWQbJ1NcO6PbuC+H7qbbu0ZI2ZEDN101hq
mfOpF9hDr9PTTNNTckuC7J7lkAAAAAALBICFGXkjERz96m16vQD9VEOl56FlRt+RP3+EGhqScXd9w
+e1VpeQGStc8LYa+fntmm0GPoXRS3SeX6lj/xUGXLC+U6OxdDexSOP94RimvidfQ0t22ElGaPTrN3av
2CcU02L/htrJ0fGKo+Ja+NftDbO3Si0mrVCRv3dbrau7jjBZKnvROYlRTuoSmuuWkm1wUAAAAAAM
wdQlRYdA3P0OH9SZmzk5+Ukn5qK56QU/ppespyMrCTEN0D77jsIdheXUtjqHdgSHsazOHy/rB4P5
SryllvyvYRvVNtxnbMHqOpKMoJfUHcwLlrXzzrnJ4+aYeVwfWW6mk/kMxMCvdQKtd8VtcFAAAAAAA
DMG0LUZRWYKCdunckYgeWRMt3x+r1i3Z6eRig3URhpDK9PScUt3trctVrXE6cj6ik5UdFhZWC9anb
2EeriSf+6AAAAAACA+UKIupTasmH2OnVrPZr1HI+ocr3rlDEYevjD9lNT3JSzbglONXTcqCs6WRiZQYh
dOS164L4ctvUQeLv3pzs5lrF+QUP07K/5sv1xAQAAAACA5UOIuozMupwqYHLrohpDoCdXkpNuEjs
4XNoIEXNVcoamK6NqvWbF6+nZ2ZHz50fVFTXP2eBs/mZ90FMydk6nxNfQ3XdHDnadr3VrtZbsT/jr
h4e0ZyWFeyiVa57idQEAAAAAAAAuNEHXJ+QGTMWP/VMxakU1Z3XCn3DFnS8+ZMcmTNFfFa1J7w
6+V6a1MmdfTsyPNpj76oaH8Rdl0u6xar6utN6yz5eg11r0anuYESU7AqVivP+8dkGx4U8nH5V+vZtP
5Wnf2/aIumOquNyfCylYK91Aq1zz5dQEAAAAAAAEcTIeoy8oI9S3NVh0rGhDtTMmcun2r75iRC7nY
mCjuDs7o3V3WI5gWO5gzwaXOHxftCh/JXtqXl5XU1KdnnrCAlP6mTC95EVCojdOutWrxzHLe2bVBg
W9Yzb2i6eZ9Y68/k0w3VNv09lNI1T3hdAAAAAADA0USIupQqst1vGQGZUpVWv+8HRvaEQpMqyu
Ze1w/BbGr7g/scx2qn1yBt/9DrDZhIcVP2+oNtstj1YDOoh2rwhvQr1v62Ruysst0PrUtbbVnr3ZILLnU
8g6+ttiara2v22gwMTTeGs+c+ZD2Feyila57ougAAAAAAgCOp0FczrADITq8hK3oiL1WDdo+eiwAwk
urtPS8/mtCWcLQlHG0JR1vC0ZZwtCUcbQlHW8LRlnC0BWmgJyqQKbOOZ75D4gEAAAAAAAJAOQlQ
gE23ZKAzU8ayeFTqhAgAAAAAAALB5CVCAPql5pZpNXAQAAAAAAAIEvURAUAAHODelXhaEs42hKOto
SjLeFoSzjaEo62hKMt4WhLuHlqC5KhJyoAAAAAAAAAARCBEBQAAAAAAAAIAIDOcHAAAAAAAAAAgAj0R
EV2eg1Z2WjrhVnrSWNFzZavHhsyL60CAAAAAAADA/CNERSZ6jRUplGrS0csAAAAAAAAADAoiJERfp6D
VmvEZ8CAAAAAAADgaCBEBQAAAAAAAAAAIAIhKhLqr3h1gddkUZPD7+3l51HaClTVePUeI3zcL7eZW/XH
MbfXHVepzc4uF+PuW1j58HXt2XDfY318F4Wo12TGjwv6jHpuVGSHr8S1obCSkPCD8+s/eo8VkaciG
TbBQAAAAAAWF6EqEuvI7VSQUoDw+
+bqwMhX3tjRI1T9fXpBJbR1H5WpamXpFyXrYr1MbN2OWHk4HlRmquFYNCZ4blRoWtYG6RTk9Lg
BFl2EFuSwZd3aqWhcDTRdgEAAAAAAJYcISosVWn1+9K3Hq2qXqUCwPNulNaTxjkdX5br0tWv7dbL
zjrrtTsXnYiusm19rlsX9zNSbdmv7W+rxHNafjv7e5tSTNCupHqNdS+MLNe7zj77LasFWvOcDkeza4NI
W3bd1Dh020055yW0VjvW3SDXOE/uBe3UZN17bZLtAgAAAAAAgBAVUm1tixtxVraMANRTlM09H
crZ4aVeu3bGe23noKufZadc3/La6ciqXT25uOP20qzK2U13yxXZsoNGFVLuibM6w3PTO5R9/VQ6O+

JmscXNPWd/1mPPbVvvorhNDpynypa42Whn56J1ZJYk2wUAAAAAAAAhKiaha5OGDmHPzqkT44K
9tNrVlQN3A+WTUtJPFSdo9EPnYSmem+KanHGTWGtrquyCW7t0qM5p98Dbnz18X7+uYA7v7xxYR
2ZJsl0AAAAAAAAQoqIsJ82UcJTAxElGbdLcjGjnzNtlyawNRdm8ENYz2A9KQye5Giur7QIAAAAAABx
NhKiIoS0bZs9Kt46mWft0JuahXRm3obgpe3qIvV+v1tc8Nzybvl/DdfBh9KCdYLsAgLzp0Q3Ggz9yAQ
AAALNBiIrx2rte70o7oHNrfxpDyKc2ybYya1dJTroJqDsE3qVm4i8YM+6n1YaI16uZ9NUvzfakXXbw2f
XqnHrtK530Qtu4NVhjbRcAlpH9Xl+QwkwTy540VlZFWu57tPNHr+bqLILU+Qxze40Vpz0r+f/hT/0ba
p4P9zEP5wXDhq7XDO6ZmRvzvuZ9P7mPvG7mOO+3ebwnR+5jSf+gFfO8z/K9eGZinJuh951luGnG
nJfc34sTtofSdoiDEBWJ+AGdMSt9AiU/nTRm/7d+MFmdbhD8tO0KKsqaVzS0Kavem667baeO6OB7
cZw2JDp+/aavPhXsGRpSs9Wsc9pc9dvm/sOhHu7KJNtVjG3wDwuALMzsl/chKri09j/lv0npcCYu3Da
KcLuTP+4f5vte3N4IC3ONPyjOQq8h6zWRatX9x28G3NEnxsO8XrMy01/cAyWOhh+z+NZW52O1qS
YFda9TS6qdmpRmFPjk/0v7+Pc19R5csr6f6l3jHFk/T2Z778R5v83jPXncPtTnc/yDVmTok1eYm+C85/l
eHHFuht739CP98xP/vl3dH/g3Iot/IEadk9zfi8efl3zfiydoj/WNrUrbpf+eHO/7dn5+FsZY1g2DJWS9R/
TV5bd+A+hbPzD5uvW+9U+g87mq9ZZia/WtnxX060c8vNcqIa93P29uf9TD2NbIdtqStKvbr5fd9VXr
K8cxXx/yKNetVygJz83Exx/
+MDcdve3gMSfabqvqrS8PXwQAmEq3XrbeX8z3eP2+GngjyoffFv1vwAzaEEm/z8/8vXjm7XCuj9q/f
c28f5PzY/87OoP9RtP37dy1y/3eivPzV9rCv5dn1R7n5y9jv/pnrCy/l8a/rznvuYNtyPocjW9XvNdMa6J
9ZPIeqPdvbdd+hLRD3T/maud+Um3XK1IS/5w4n8/+vTjeucnjvS/
+fZv1+8v4cxImq7aNPy/h62fWnhHfw+nfR87+zd0737fBdX579Qr9vhz3uiJf9ERFDBXZVn8p0ksO56
821puAo7kr/t9KrNd7n9D2D52/MKlanIP1QqutCWuIJm1XEk4PIOsNTS/7rDdbf9h+0jYkPH57qP3g
ubQ5+wj8QdOuc2oMyXfZPWWMeqiWRNsFgMy05XytY71NXZBN503VUpEt9UY28fv35Iqbe9Lv7x
ltmTO69MupE/PRwFm1o9dYl1qnKmfn9kLNhnteWt7PKPPC+T6X6unAzyL5KMqJU9YH9+dQW08u
7sygPb2GqIFK5fqWv9/Ktv3zYmfnYgY9sRxj39d6h7JvfRj8fi7aJ64puxm9Ecd5v83jPXle3ved79+y1Ls
hP8tr6uf34dEJHdm5mO7dE/ec5PVeHOfc5GX8uXHeXwLf5xmY7Jxk9148/rzk+148tj0jfp6yR42mW
tYuzqii+fpZGOMRoi4pvw7mwJuLMeFQcMiBCgvdr1EPJ5TztxMM6dQPhf5rrYf5A725D/VQ+xmx35
Ht9MRtl/MGFlw3nvMG7H6d89gbakjCc5Pg+G2D59J+jDoG8zj1Y9QvU3G3a7xu+NgBYAoz+uV9M
enSL+W6bIX/A5CTnjTWa9KZVTvsoaMdqbbi/1ueGTUM0Rh6N9uSN/n84j6JXuOc9d1s/bI/oxu3sq2
HjNo17dWwyplTNg/
+vJW13H5pT8huV1lOenWcNKPePgZk8AetuMFlmDTbEVuO78XTnJv86fJoB+cHhtOnWwJnknPCe7
FhxPub8/PnvuRaNYmfhRcOISoAAJgNfnmPTdUltX/5uTCLXoa6tpj9i6D+pWdGvR3b52vSqbZmPmL
C/0Op81AjV7KppRZXPr+4J+f2NDo7wwDEHTWkatqr7yOrObMI4efpl3ZMgT9oKfPyXhwwR3/Y6uy
flAvevxGqt6h6/9mYYa9C3osD9HwiwflBrO+pqedWiWHwjzD8LLxwCFEBAADmmDP5gRq2N6ueO
MFRDt36vqzO4hfU9oYzCcRc/dbuUL2Csh6WHcfc/eLePi/O6NHZXTNnsg5/YiAVeKtJgXKftGOWv7Q
jNfxByzKH78Xz9oet8pk147pY/4aeVWXUZtirkPfiAernGrdnrP89dXBKXadTkl3n7nn5IwymQYgKAA
Awp5wAVfXWmJ+hjMXNC3YNtk7tfK7hXHtXhU1NO8B1fuEpSEn9Vqh/CZrtcHptlsOyLfP1i7sOCGf5
y6Iecqzq2bt5jz0MViXezdWMZjYfZVa/tI9h93bqyMHgjTuqd9QS4w9ajkV4L56XP2x5ZtqrkPficIMl+f
qydXLfOk8nJau3vdn+EQZpIUQFAACzwS/vkVSvDSdAndMJ/zL8RSPMYE8j9bAngLR+Mexaz2dbt7sn
h6qoWc7nZKxZ/uLu9nw6O8NfFgeHTbpmdl7y/6V9rOIJceZ7CUZNPfuGnmG4O2f4g5Zvvt+LB+T+h
62S2GWOB8PbWf5cw3txTLq2eOCPkekZ+UcYfhZeOISoAABgNvjlPYKeRddiD3czevzkW+dSTf4wM
Bxc/0KW1S8ac6/XkJUVc0i2WqVmSp7lL6nz94u701utKjMcPSpSOW21wPoeCgyhV7dwzTovM26bL
dtf2uOpyGnVY6+27r+veL3G5m+islngD1qLaFZ/2NIjADo1Oe/9w6mHcM+oHinvxXGoUhklqUldLm
RwkSL/CMPPwounDwAAMCOtqvSt33L69a5e0a33y9aPJ2VvRX669bLVFtWewUe139KvWUZh56U
6JyfEblu53s/3bun26+Xg+ZiLe6RVHbg2rb71S2tfJT+5m+H38TB9HgKPefie1vdRxvdv3Pc1573Yf2R9
7eK0K4/35PH7CLt/3Ifxb1dq9H0x9H2r2jFw3Pp7Pu1rNcl5t78m8/fiEedGvd8M7Ns9hrTf/uKem6H
XZfY+POp+0XJ6L453XvJ7Lx7fnpB/xzO6RnHuxXn6WRjjFdT/rIsKAAAwE+5f6F3WD43zNRwQWBCq

x5xdm9BVbUl/Bl3nnO/pqrT6M5p5eS7pnk7G5ZnV9cH8Gfre9fjfR2Gvqc5r79gUjT83Id9bxnk7iuLc
LwrvxbOmRvOoOqhhgkP7+Vl4cRCiAgAAAAAAAEAEaqICAAAAAAAAQARCVAAAAAAAAACIQIgKA
AAAAAAAABEIUQEAAAAAAAAgAiEqAAAAAAAAAEQgRAUAAAAAAACACISoAAAAAAAAABCBEBU
AAAAAAAAAIhCiAgAAAAAAAEAEQlQAAAAAAAAAiECICgAAAAAAAAARCFEBAAAAAAAAIAIhKgAA
AAAAAABEIEQFAAAAAAAAgAiEqAAAAAAAAAAAQgRAVAAAAAAAAACIQogIAAAAAAAABABEJUAAA
AAAAAAIhAiAoAAAAAAAAAEQhRAQAAAAAAACACISoAAAAAAAAARCBEBQAAAAAAAIAIhKgAAA
AAAAAAEIEQFQAAAAAAAAAiEKICAAAAAAAAQARCVAAAAAAAAACIQIgKAAAAAAAAABEIUQEAA
AAAAAAgAiEqAAAAAAAAAEQgRAUAAAAAAACACISoAAAAAAAAABCBEBUAAADAQmtvFKRQ0I+N
tl5raG94n19p9PTK2fDbuiEhLV0wbdlwz3vk+e1JY2Xca6xXNVb8ba00rK8CAGB+EKICAAAAODqa52
TGOemSUMHoqjT1kqMsZ9aK+rmpKJsX6tZnHZ3aesg1asv5Wkc/F6me3bS+CgCA+UGICgAAAOAI6
Ujt/OL38Zx/XTlwM89yXbr9vvT7e7I5KvksbsrZqn4eco16jXN+IFttyXZFPwcAYE4QogIAAAA4WuiNm
r3eoezrp3LqRKxeo5Xtlng5auAamb1Qq9IiQQUAzCFCVAAAAAABHTLzeqH590pVg6NpryIpbm9Orse
rX/7Rrehp1Vr119suC68NKtPqCtUJH1wEdeJ31CKsrGjyeYL3S6HYYzGPXj8F92fsp1ayzrDVXndeO3Ul
FtltujOpfo/aGXxagXN+yXjUo3vHbQto/dH0tcc5VoEar+6BWKwAsLUJUAAAAAEdEWep1HdJl2Bu1U
ytJYTVYDdRep0K2gfXN1VEBZlNWCyUxyoCqjUhpcMIpOxQceJ3F3t/IQK8jtZJRr7RcI60YnTuHwlEtel
8JVbak7hZHba7KRqMh5/wEVS4M1gNIcvwqwA5pv3M+hoNUR/i5UueiNLhTJewaAQCWAiEqAAA
AgKNjzQ3p4vVGnVS53pW+qgPq9ax0hK1v7o5oh1dLtC/
+y5tyzkv7etJYd0PBqrT0a71td2qyPjIpNl6/F2OSpvaGePmv0a6um3ga+6psW5/r+hNFqRqm9n5iDc
MPTjLVrLnHV5b6hcF2Jjl+67VuGhvWfmsrOxfjnqu27Eadi8A1AgAsC0JUAAAAAEdIUTbdGYwy641al
bNuj8nKaWvJNWL9/mFoL05zBnqzXmhn56Lz+t5F2dGdIQPD3I3enN5rB4QPix+t7aeGgTCzuHnB31
ftfDo9MAOTTGnVs8OTUiU6fuu675lBqKO4dsYLbDsHXf0saOhcmfVeOzviZq/FzT1n+9Zjb+QMWgC
Ao4oQFQAAAMDR4oVsGfVGLZ+Ukn4aMGp9qKqcDiR3JTnpp31ix33dA90L01rllguwH8bwdve1A0
6dSBLymT0vz8ha4EuLsnbGbdi+HIYlthMITDJlPQudTGqK41fHZNc5DR3eHzR0ropr4h2yuodK7n4ja
rECAI48QlQAAAAAR8xAb9RD5+l8K8qJU/rp1MpyMn6am0BHRnTmnE6i8DlCYFIpo85ppLBzFSw5Y
HLD3NgTdQEAjgxCVAAAAAABHj9kbtRYvTsvXYK/Onhx6Y8iHebVWhx7biYbtTyercHa88cfflg2z16lby9
Ss3ZpEcVP29D4Gyt7amueYpR8Alg0hKgAAAIAjyOiNmpQxjDw7g706u3Lg7tTtmVk66QWAo+p5ps
MsJeDXAHX05KJbmFROSaIqAdNKcvztXa/nqR24unVRp7iWaoZ+1ePUnkjLDlS7Xi3W0WUEAABHF
SEqAAAAgKPJmIAoTMlPDo3aqW3Z8Kapz1ZzdcObqKm94Q8/L59ZcwJAszZnc9UfQt7e8Gp0FlIZV2
4Gzta5WPd7WfYa61790aSTVU1twuP3A1djxv4k9PbVbRDscRoSdAMAlgYhKgAAAIAjKro3qjlzuwrp
nGAubi3NaZWlXG7Kqg4D/dzWmOFftd+ozdlc1cGh9+IREzJNorLtD1vv1KSk21XyE1S5kPuM9AmOv
3Lan6jKu5bGBFRJjDgX5r1RPat7ugIAlgYhKgAAAICjK6o3qqp7OVgzs9qavI5mIqfk7J4xPFyx63gO1Di
1a3MOvE4Je+2U7GHrIcdebVnr3eHxeYt9/BXZ7psz/itVaZk1TZu7Xs/fcexzEVYMVW8zrewaALA4Cn
1V3AUAAAAAAAAAAEIqeqAAAAAAAAAAAQgRAVAAAAAAAAACIQogIAAAAAAAABABEJUAAAAAAAA
AIhAiAoAAAAAAAAAEQhRAQAAAAAAACACISoAAAAAAAAARCBEBQAAAAAAAIAIhKgAAAAAAAA
AEIEQFQAAAAAAAABGEvn/AZM6nr53azj8AAAAAElFTkSuQmCC]]></redcap:Attachment>
</ItemDef>
<ItemDef OID="q9" Name="q9" DataType="text" Length="1" redcap:Variable="q9" redcap:FieldType="radio" redcap:RequiredField="y" redcap:BranchingLogic="[q7] = '2'">
<Question><TranslatedText>Do you prefer Life A or Life B, or do you think Life A and Life B are the same as one another?
</TranslatedText><redcap:FormattedTranslatedText>Do you prefer Life A or Life B, or do you think Life A and Life B are the same as one another?

 </redcap:FormattedTranslatedText></Question>
<CodeListRef CodeListOID="q9.choices"/>
</ItemDef>
<ItemDef OID="summary10" Name="summary10" DataType="text" Length="999" redcap:Variable="summary10" redcap:FieldType="descriptive" redcap:SectionHeader=" " redcap:BranchingLogic="[q7] = '1'">
<Question><TranslatedText>
Alzheimer's disease - Description #[no]

If you have Alzheimer's disease...

1) You might forget recent events of importance to you. For example, if you are interested in hockey, then you might forget whether your favorite team won their last game, although you may remember watching the game itself.

2) You might forget the names of people you met recently, or the names of local streets.

3) You may no longer be able to handle your personal finances or plan leisure activities such as dinner parties or vacations. Simple decisions such as what to order in restaurants might become difficult to make. Your ability to concentrate may decrease and you might have difficulty driving.

4) You may start having trouble finding words to express your thoughts. You might need to be reminded to shower or brush your teeth, but you could do these activities on your own. You may need to post notes around the home to remind you of simple things like turning off the stove.

5) You may remain aware of the day and your surroundings. You might be able to travel to familiar locations like the supermarket, although probably with the help of a loved one or friend. If you are in a social gathering, then you might have difficulty following the conversation.

6) You may continue to enjoy hobbies such as puzzles or reading.

Now consider two possible lives. In Life A, you will live in perfect health for 17.5 years and then you will die of natural causes. In Life B, you will live in perfect health for the next 10 years, then you will live with Alzheimer's disease as described in the summary for another 10 years, and then you will die of natural causes.

</TranslatedText><redcap:FormattedTranslatedText>

Alzheimer's disease - Description #[no]

<p></p>

If you have Alzheimer's disease...
<p></p>
1) You might forget recent events of importance to you. For example, if you are interested in hockey, then you might forget whether your favorite team won their last game, although you may remember watching the game itself.
<p></p>
2) You might forget the names of people you met recently, or the names of local streets.
<p></p>
3) You may no longer be able to handle your personal finances or plan leisure activities such as dinner parties or vacations. Simple decisions such as what to order in restaurants might become difficult to make. Your ability to concentrate may decrease and you might have difficulty driving.
<p></p>
4) You may start having trouble finding words to express your thoughts. You might need to be reminded to shower or brush your teeth, but you could do these activities on your own. You may need to post notes around the home to remind you of simple things like turning off the stove.
<p></p>
5) You may remain aware of the day and your surroundings. You might be able to travel to familiar locations like the supermarket, although probably with the help of a loved one or friend. If you are in a social gathering, then you might have difficulty following the conversation.
<p></p>
6) You may continue to enjoy hobbies such as puzzles or reading.
<p></p>
<p></p>
<p></p>
<p></p>
Now consider two possible lives. In Life A, you will live in perfect health for 17.5 years and then you will die of natural causes. In Life B, you will live in perfect health for the next 10 years, then you will live with Alzheimer's disease as described in the summary for another 10 years, and then you will die of natural causes.
<p></p>
</redcap:FormattedTranslatedText></Question>
</ItemDef>
<ItemDef OID="a10" Name="a10" DataType="text" Length="999" redcap:Variable="a10" redcap:FieldType="descriptive" redcap:BranchingLogic="[q7] = '1'" redcap:InlineImage="1">
<Question><TranslatedText></TranslatedText></Question>
<redcap:Attachment DocName="175.png" MimeType="image/png"><![CDATA[iVBORw0KGgoAAAANSUhEUgAABVEAAADYCAIAAABtBTQzAAAAAXNSR0IArs4c6QAAAARnQU1BAACxjwv8YQUAAAAJcEhZcwAAEnQAABJ0Ad5mH3gAACtqSURBVHhe7d1/qF3Vnffx2Dh94

oydhrbjo22Zpn0ohBgkVuYhvVPGFIqR8vggODLBsaXDDJcrVCYIGikK80eCIoVcRdAHivpHnyf+ERGh
E0ULYaiQQMH7h38oxHD/UAzoH9dQ5AbzR5/9Y6211/6uH3ufdfbe55x93i8+zNyzz9rru9Y+J5791e
aeHX8GAAAAAABjRM8PABjI2c2zO/5jByGEEEJC+f7Pv/Kv3/8fJgfv+ob6EAVS0fMDAIawubW5+8nd
4s6GEEIIISbXP3ztz27+rt3zZ3n3v/5LfZQCSej5AQC929re2vvsXnFnQwghhBA7d/3wW6Lhz/LQysHP
PvlEfaACk6PnBwD07s7f3iluawghhBBi5wf3fE10+yZP3X+f+kAFJkfPDwDo19E3jorbGkIIIYTYuemB6
0SfL/LaM0+rj1VgQvT8AIAenXr3lLitIYQQQoidL//qS/904DuiyXfz/vnz6sMVVmAQ9PwCgL+c+PLfr+C5
xZ0MIIYQQOz/
+yX8X7b03xw7d/vnly+ojFmiNnh8A0IvNrc0bf32juK0hhBBBCiB3x5XzxPPvAmvqUBVqj5wcAdG/76v
aB5w+I2xpCCCGE2Ln+4Wt/sfd7orGP580XX1CftUA79PwAgO4dOX1E3NYQQggxM7Ox6/xfjlfPGv
7913c2FAft0AL9PwAgI498YcnxG0NIYQQQkT+7u6vi36+ZR47fMcXV66oD12gCT0/AKBLr773qrinIY
QQQohI45fzxfPCsUfU5y7QhJ4fANCZjUsb/KJ+QgghJJ4v/
+pL992yR7Txk+btV06rT18gip4fANCNre2tPet7xG0NIYQQQkR+8uMbRQOfkLX9+z6+
+IH6DAbC6PkBAB3Yvrp96KVD4p6GEEIIISITfTlfPPzFfrRBzw8A6MDa79bEPQ0hhBBCRL760F9M+u
V88bx84rj6JAYC6PkBANM6ee6kuKchhBBCiEjal/M15p233lSfx4APPf9MXFhf2VFaWb+gjmmx52bG
LGr1TPPDkLSzpjRMlemdWS2XWejylc8uQHTrunCv77bAq1Bf2yArQS/Obp4V9zSEEEIIcXPwrm+Idr
2TPHjbrZ9+9KH6VAYc9PwzYpog2eFUvd88NamiZ4s/DEk7a0rDVJmSWWSps6ZXzRvd+qx6fndt9Pw
LanNrc/eTu8U9DSGEEEJEpvxyvnhO3HsPf7EfIfT8M+Nt7qvmb55bVNnCeTo6n5bDllA/3W676z2bn
t+3Nnr+RbS1vbX32b3inoYQQgghItcd2zn9l/PF89ozT6uPZ6COnn+GTNdvmhxzZM67YtGxtWzmW
w5bPj1dmHbT0vNjGnf+9k5xT0MIIYQQN518OV9j3j9/Xn1CAxZ6/pmq9/juvwOomB6pIAZ4mqWG
fk8/XZ3hHtHT5kfEdPGHIbGz/P2eHuLbWK4+2kuuzS5kzxVfunVW/KTY8qxJ9I9e1azNm62NsE71FA
js0L4g1llutabFiKXUhpjn8kWE1tZ6JZgXR984Km5oCCGEEOJm7z//tWjOe8pDKwc/+
+QT9TkNaPT8s1X1Savr+kenx/H0SBlrmN0sKbUmy0M/7zaJZhJ1pHgspos/DImepevbU+gReknmDJ
u1aR+5Nv/FzMUmanVW0/I8k6ysr7sHxeWx1dboHSEup8W+spZOthaYxYyovQqhtbVaCebGqXdPi
RsaQgghhLjp/Mv54nnq/vvURzWg0fPPmuymnPbGdEL6GXOGGaqHWOfWmiwfNUCfYvVb6gw9Q
/FQTBd/GBI/S6+gmkMPUGt0qni27ZJnuRutjlSlpfDlmWB57iSKc2qb2cwIfcRMrw94pvWIbC04kVy
MHuCcEJzBOZBpsRLMi3Mfntt1fJe4pyGEEEKIyM7Hr/nH2/5WtOV95z+ff059YAMFev7Zs1qdWgtU
ku1VTjZMnjG+nqpOjSjPKR+sruYT2Uf0+WK6+MOQhrP0Yz2Jfqx2JR7mPIccsoq52tVa5RAPz1nVaU
X5Fsszk8j1ugtons09pzzkW2B4X5nmCyIq5zyHamRpZynOgUzaS4PhbW5t3vjrG8U9DSGEEELc9PTlf
PGs7d93cWNDfWwD9PzzwddRKvqpenMlWi7PoBbNkjqrGFD8vLJ+pjirOmJOF9PFH4Y0naUPqMd
qBXpTepM+kbqyipmmOqfF8vVZtZehdl6L5XlKl9wFNM/mXVFdi31lPBOJE1tszVIfrZ53luJbW/NKM
Ae2r24feP6AuKEhhBBCiJtvr/6l6MYHy7FDt39+
+bL68MbSo+efB5H+zf9UoCmbsFlSp2UjytHZD+Uhc8ScLaaLPwxpPEsfKQ5UqyvpTfpE6soqaddKn1
UbUTuvxfI8pUvuAppnC05WabGvjGcicWKLrVWnCOp5Zym+tTWvBHPgyOkj4oaGEEIIIW4G+HK+eJ
59YE19eGPp0fPPg0j/5n9Kt0LqaGqzVJ6n//N+cXZxSB+pJhTTxR+GNJ+lD2VH1J6q5/xXopGsknatvM
Vr57VYXnCIu4Dm2VrUa7GvjGcicWL7xWTKQbK0sxTf2ppXgll74g9PiBsaQgghhHhz+PabRBM+fN58
8QX1EY7lRs8/DyJNlWl67OdkJ+Q533RhsWZJzeP+Nf7yiDWfqBh/GNLmrHLd5tfZW0+1LCLJ09IaS9/
lrJ82wST2S1lwz22ezTciLzDZvjLNF6RxHncKeb2cKULLz028BQzj1fdeFXczhBBBCCPHm5iNfFe33TLK2f
9/HFz9QH+RYYvT888DT7FRM+6SfNX2QGW6O6NbInNPQLFUn+k4N917xhyGtzrLqi2fMeH3YDI2V
lVX0SZM1lp6rIqs3L89TuuRZQPNszojwgWpaD8+q5IlNi5Frq8Y7R9Rj79parAQzs3Fpg1/UTwghhLTJ
wF/OF89jh+/44soV9XGOZUXPPw88zY7NtFQ19mCryZLizVJ1opnOVPMWKKeLPwxpd1a1W+dymD
NqJiqa2Fj6X4Kcb56aalJP6ZJ3Ac2b9Y+wpq+vOrC7VhekYTHHy6OGuDN61tZqJZiFre2tPet7xA0NIY
QQQtzM5Mv54nnh2CPqEx3Lip5/HniaHanedHk6IHtA9nTbZskd5z1THIw/DGl5VvxqmLMKkSumyC
ppjaV9lv45454QW15wY8EFNG+2PsIZYq3Ve3qm9QWJL8aulJ+mH5dT+GZ01tZ6JRjU9tXtQy8dEjc0
hBBBCPHm73/6N6Lllnoe8/cpp9bmOpUTPDzTztKPAclj73Zq4myGEEEKIN9/5t78SzfacZG3/vk8/+lB

9tGP50PMDzej5sZxOnjsp7mYIIYQQ4s3Mv5wvnhP33sNf7F9a9PxAM3p+LKGzm2fF3QwhhBBCQv
npj74p2ux5y8snjqvPeCwZen6gGT0/ls3m1ubuJ3eLuxlCCCGEeDMnX87XmHfeelN90mOZ0PMDAG
q2trf2PrtX3M0QQgghxJsbfrlrfr6cL54Hb7v1s08+UZ/3WBr0/ACAmjt/e6e4myGEEEKIN3P45XzxPH
X/ferzHkuDnh8AUDn6xlFxN0MIIYSQUP7h8A2iqZ7/vPbM0+pTH8uBnh8AoJx695S4lSGEEEJIKHP7
5XyNef/8efXZjyVAzz9b+nfDFXr5BXEX1ldWz6ifx/G76Oo7isgGyr2qQ17RSWsvlNZuGUuMX324YM
59eG7X8V3iboYQQggh3lx3bOfPbv6u6KUXJQ+tHOQv9i8Pev4ZEg1o942RKjCint/dUZC5ul30/N6W
n56/CT3/Itnc2rzx1zeKuxlCCCGEhDL/X84Xz8l//Rd1E4Cxo+efoZ47ItPfjqbn9+3Iz+7t43s13XxsTj0dv
etk6PkXxvbV7QPPHxC3MoQQQggJ5ZZ7d4sWehHz5osvqFsBjBo9/+y072DTLGvPb/f7udheW14PP
aynV2q06PkXxpHTR8StDCGEEEJCWaAv54tnbf+
+ixsb6m4A40XPPxu6GbLY/WS9bRUdk9VIVdOIbjQ0v92EWWPcnqy2gsaWzZ7WPtPpkcPTVs+Yk8
wCxWo1Z/ZM/coVwqs3c/pmsuhJG4Yp9sWweA6Hr0ZJbqY2xJqvujblCqPnBbStVRvoXBDxZOBSYM
488YcnxK0MIYQQQkLZ+fg1/3TgO6J5XtwcO3T755cvq3sCjNRkPb+5ow83P94hVU+Smfb+v2orwqu
Ye7UrUtK78TyVsa6aZ4BzTUPz+yfPWVPYjZsRfdk6mdY8Xx4TD0M7EvRZ2XP6jNDKRYEw38Jj7z1vY
XnQN2ntDP81rUZ4ni+e9J8XXXBKLaV5xTl7FObMq+
+9Km5lCCGEEBLJIn45Xzz/5+i/q9sCjNQAPb9obqa9/beni/Yxc897NU3fpK+TGWUunNVaRS+Ab373
XGd+5zR9TuSVc6etjsg6kWntMb6qvh0J+RCxhMC6W0ymWLuzha+HntoaIRbjFJeLlVOYE8yc1qrsHY
Qmqk50pNSqjuji5ix9pDoQqY3Z2ri0wS/qJ4QQQtrne/9yvWiYx5G3Xzmtbg4wRp33/C7ZhEzHaiQy
C91L+K6m72LJcabdath9bH7rmBimH1qTew4JnU1rJlLqJcWcDXzXshJ/1uZUNYsMnutsTVRreTVsLVd
hhgUnaiFcq7rwcoxzTsYcm2It6M/W9tae9T3iVoYQQgghoVz/8LWL++V88azt3/fxxQ/ULQJGp/
+eP+WcMD3byor6/wvcTHiujL8RNZsuj3oaMC/flfcUEMPM7B7Bgs09Yetp7YHy1fXtKMyz10r0yUa
NZwdeMr1ue5OSu7f6aGeO+gnmGlUm2WPrWvK10ENqxbwHMRe2r24feumQuJUhhBBCSCSL/uV
88Tx2+I4vrlxRNwoYl37/t/31BqLgaRpyLZsCfc7qGf3TAncTnqvp75HEwLaNlGd+37mB2X2sieq6nLYa
6WzQt6Mwz6KM2HMtNC9EjygK6GpmdJurYWoI+vngFgInxq5ZQi15CbzLCa4RM7f2uzVxH0MIIYS
QSH5wz9dEkzy+vHDsEXWjgHGZUc/v7TGaGwN9Wj6J/nlx2wnP1fT3SGKrbRspz/y+c8WwpDatu2n
NaQUx2rejsEhJPdFEm7S0OV+Vz4bo0U2vs02PyJSDnL03zmFNUWgzsH0tOca7nMY1YjZOnjsp7m
MIIYQQEs9Pfnyj6JDHlxP33qPuFTAu/f8OP885bVoKH31eeZp+tLD9ROzK2JuS49o2Up75WzRvvrMa
dTWtHrWyvu7b5GRrC1+niebxDdZTR89Xg1ZWV8sJrMGNC3DXro+YU8Lby5+aoFhaLTmrr4o55lkjZ
olv4yeEEEImzZd/9aUxfUWfmwdvu/XTjz5U9woYl1n0/PqIp4GI9gZyUKuT5ph7ZTKm3ZK7rLbpacC8
fPNP0rzpA04H6Opm2top5kFkzrjwdQo/k5PPtli5nxlXqA1umlOWqMabeeQyS+bE6rB/YCWtlvNaO
Fuy5gmVxoxsX90+
+JuD4laGEEIIIfHc9MB1ok8eU9556011o4DRmUXPb3oMj/DEZqKqf3CPLJTA1fRfHmuPTT2cUZ+pq
NKmeauO1ERemk6mNU/rSczyq1k9OwrzLErRz/gncM+rl9Uaymfs8+TohqvhL5nTQ9xllkJnhpebVM
t9fQNbyrkvAWbt0p8u8Uv7CSGEkEkz1r/V//KJ4+oWAWO0OD1/uKFY1JYicjXrmxXPh5o9D/tSF8Pb
NW+Z+gqaSk0/bfWENd4svxro7CjMs6iSf2mG/zy7cOhMh2cDtvhFtivm9fRjVdu/zFJ9sS2WO3mt5t
c3eyK2RswcX85PCCGETJqdj19z1w+/JRrmRc9//O//xW/sH7dZ9vwTNQJ2M+Gip8A80u9a3p+YT6f
ePSVuZQghhBASz/UPX/uLvd8TbfPihm/mXwaz/Pv8kVkc5pwAmirMHfOm5d2J+fXo7x8VtzKEEEEIIie
f7P/
+K6JwXN2+/clrdE2C8Ent+r6KFdxp6T4dfTaOOmf9lcejfAngmKZknaKswP8wbuhB6VwPz4e6X7xa3
MoQQQQgiJ5x8O3yCa50UMX8i/JGbS84cmCvZGkc4+8hQwI1bPT8OPubd9dfvA8wfErQwhhBBCIhn
BV/c9dvgO/hr/kphRz5+rTxZr2aN9ffRJAECjza3N3U/uFnczhBBBCCInkhl/uEl30AmVt/76LGxvqPgBjN
1nPDwAYpbObZ/k1/oQQQshEWdyv7nvzxRfUHQCWAD0/ACD33B+fE7cyhBBBCCIlnEb+679kH1tRn
P5YDPT8AQDn6xlFxK0MIIYSQSK5/+Nqf3fxd0VTPc44duv3zy5fVBz+WAz3/rF1YX7F+04H+5W/8coI
Q8fsh4g8nIq79NFNNqvZ7/nt/7etvuT4NeQ3RmUMvHRJ3M4QQQQgiJZLG+uo+/xr+E6PlnSbVEVkN
Ez99EtJHxhxOZVc9vCpX6felVsb73pAx2DdGlre2tPet7xN0MIYQQQiJZlK/ue+2Zp9XnPZYJPf/s+Boie

v4JiYs4TZM5q2s/YN2he3B6/kX13qfv8Wv8CSGEkPbZ+fg18//VfU/df5/6pMeSoeefHXr+Dix+zz9kX
zx0Dz50PXTo9Quvi7sZQgghhERywy93/WLv90SbPT95aOXgZ598oj7msWTo+WdD95eWoi2y+05rj
NuGmm4qJ562J7HHhRuvQLvrPVyr3OKMhr5PP12d4R7R0+ZHxHTxh3FmcCYbL9bunco+xV5gVO2
k2lm6pCWwcHtxYt019Vp2tVAtse2cs3VrTDWNejJc0TMRFsrJcyfF3QwhhBBCIpnnr+57//x59QGP5
UPPPxtNDZjL15LZrOdbTVJnNXXqSMY96J/ZU9o61NT36efN06aImUQdKR6L6eIPI/xbyamyzlTmgM
3aqFf8inmeDSy8cbm5aLFQLX3cmsfZuufccni04iQvB+bUkdNHxN0MIYQQQiL56Y+
+KZrtecjLJ46rj3YsJXr+2fE1RFYPpY6aUbqRCjZkptNyJ6mOhJovPavV+YXndZbiDLGm8W2zRg3Qp7ir
1zMUD8V08YdBZpyskVELaZrZs1VHiyvmTuzlXpXqiNjChLU8+3CGudULjRV99bBQtq9uH/zNQXE3Q
wghhJBQ5vCr+07ce88XV66oj3YsJXr+2Yk1YNYxMUz2WRl5qHkSD2depxf0NIfOvJ4xrUuX55QPVlfzi
ewj+nwxXfxhiG+YOabWXh+jH3k2Zh0S2lwx94BXymvqDvCd0uYlM9XDey3IExuXiAVw6U+X+DX+h
BBCSPt859/+SnTdM8yDt9366Ucfqg91LCt6/tlJa8BM+
+WhJkrpD6shqrSexJzgWVomcFZ4/T7qrGJA8fPK+pnirOqIOV1MF38Y4t2LOFifSj/pE6rlrWLm1UfbL
dnUr0bFTqwv1wzwneJZpTPMU13yVmy3N8y9jUsbu47vEjc0hBBCCAllfr6675233lQf51hi9Pyzk9aA
1XurOjVRmy7OQ48pTtNzVOM9s2YCq5uwtDotG1GOzn4oD5kj5mwxXfxhiHcv4mB9Kv2kT6iWt4q
zxHZL9szlnmiOCO4Aq1abmf07yTRU9NXDYjr17ilxN0MIIYSQUHY+fs0/3va3ov0ePi8ce0R9kGO50fP
PTloDFmy/Kq36Qx91YnaeHh9fWkYPVEdTS5fn6f+8X5xdHNJHqgnFdPGHId69iIP1qbxnNPCfo+fVR
9st2TOXPFEPyZSj3Jl9tVrMHNp9Y0VfPSysR3//qLihIYQQQkgoM//qvscO38Ff40eJnn920hqwFm1U
my7OT525srpaDrdHmynsxk/O6yltGsNYaTWP+9f4yyPWfKJi/GGIb5g5pmrVx7ScuEbOWHAmajdz8
2vqjnCvvK9Wm5fMMybTXLHd3rA47n75bnFDQwghhJBQbrl3t+jDB8va/n0fX/xAfX5j6dHzz07LBk
wOM4/1gTYdWtvmy8xVEIPNk3piM6upFF5cQ+nqRN+p4Z3EHwY567Tqq2KhmfUZZnmRUi2umK
wToGcKXwm5nmrB1cy+WtU4ddC67nqYp3qmuaKvHhbZ9tXtA88fEDc0hBBCCAllVl/d9/Yrp9WHN
0DPP1NWc5Up2iJPc+X2TVZvZanaqlaTBNhrcsfWV6zZnaB/bYV46epEM52p5i1QThd/GBFeqKrmT
OU/o6HQBFcsOlOL19RfKlfNXB9THg9fCXOip3qusWK7vWGhbG5t7n5yt7ihIYQQQog31x3bOfxX9/
HX+CHQ88+U3TQV/ZSnufL3TfVGrd6LtZ7ExyxJ9HdGvbJnPntA9nTb0u4475niYPxhnBmcycaLyxavX
ghdIqF+klxZuyW3e03Ni5fJj+rH1tT2EDOZvcJsrDOzp7oSr9hub1g0ZzfP8mv8CSGEkJYZ+Kv7jh26/fP
Ll9VnNlCg50edbtNa9rMAltBzf3xO3NAQQgghJJS//
+nfiM68p6zt33dxY0N9WgMaPT9s5j/M0vIDiDn6xlFxQ0MIIYQQbwb76r43X3xBfU4DFnp+lOz/kTb
/W2wAzQ69dEjc0xBCCCHEm6//+3/r+6v7nn1gTX1CA3X0/ChZPT8NP4AWtra39qzvEfc0hBBCCPH
m5iNfFV16h3lo5eBnn3yiPqGBOnp+AECi9z59j1/jTwghhLRMf1/d9/758+qzGXDQ8wMA0r1+4XVx
Q0MIIYQQbwv647tvO+W/au+dt95Un8Fnn6CeyZxZdQ6a8dZE0TlKtSHu8y1mAADl0d8/Ku5pCCGEE
Om4+m+uu+xw3fw1/gxKXr+WXGabXUo/N/PG9vzjBhjHoY6dD3AM0V5qHEGAPDZ3Nrc/eRucVtDC
CGEEJGdj19z//9SXvV/et7d93cWNDfDfaYKQ6k4/Ku5pCCCGEEEJIJdn4Y6cA5OQ+dRGCGEEEKIy3q/u+8/
nn1MfqEASev4Zstp4m2qdTbft0D1/aIKcHhOaxcwRmKR
6vnkGen4ATd779D1+jT8hhBDSmL+7+

+t2w//TH31TfZQCqej5Z0x03PW22W638xZbP67a7dr52WHz0BridO32c6X6KtzePT4DPT+AFl6/8Lq
4rSGEEEKIyM7Hr7nrh98qG/77btlz3bGd6nMUSEXPDwAYyMlzJ8WdDSGEEEJEzFf33fTAddlD9SEKp
OI9BAAYjn1PQwghhBBvvv/zr/zgnq+VP6tPUCAV7yEAwEB27BjoQ4dCySiUjELJKJSMQskolGx8hZYB
lxIAMBDuSJJRKBmFklEoGYWSUSgZhRDBpQQADIQ7kmQUSkahZBRKRqFkFEpGIURwKQEAA+GOJ
BmFklEoGYWSUSgZhZJRCBFcSgDAQLgjSUahZBRKRqFkFEpGoWQUQgSXEgAwEO5IklEoGYWSUSg
ZhZJRKBmFEMGlBAAMhDuSZBRKRqFkFEpGoWQUSkYhRHApAQAD4Y4kGYWSUSgZhZJRKBmFklE
lEVxKAMBAuCNJRqFkFEpGoWQUSkahZBRCBJcSADAQ7kiSUSgZhZJRKBmFklEoGYUQwaUEAAyEO
5JkFEpGoWQUSkahZBRKRiFEcCkBAAPhjiQZhZJRKBmFklEoGYWSUQgRXEoAwEC4I0lGoWQUSkah
ZBRKRqFkFElElxIAMBDuSJJRKBmFklEoGYWSUSgZhRDBpQQADIQ7kmQUSkahZBRKRqFkFEpGIUR
wKQEAA+GOJBmFklEoGYWSUSgZhZJRCBFcSgDAQLgjSUahZBRKRqFkFEpGoWQUQgSXEgAwEO5Ik
lEoGYWSUSgZhZJRKBmFEMGlBAAMhDuSZBRKRqFkFEpGoWQUSkYhRHApAQAD4Y4kGYWSUSgZ
hZJRKBmFklElEVxKAMBAuCNJRqFkFEpGoWQUSkahZBRCBJcSADAQ7kiSUSgZhZJRKBmFklEoGYU
QwaUEAAyEO5JkFEpGoWQUSkahZBRKRiFEcCkBAAPhjiQZhZJRKBmFklEoGYWSUQgRXEoAwEC4I0
lGoWQUSkahZBRKRqFkFElElxIAMBDuSJJRKBmFklEoGYWSUSgZhRDBpQQADIQ7kmQUSkahZBRK
RqFkFEpGIURwKQEAAAAAGCd6fgAAAAAAxomeHwAAAACAcaLnBwAAAABgnOj5AQAAAAAYJ3p+
AAAAAADGiZ4fAAAAAIBxoucHAAAAAGCc6PkBAAAAABgnen4AAAAAAMaJnh8AAAAAgHGi5wcA
YFJnVndUVs+oowAAAPOGnh8AMCJlM95vF35hfaWq0GfBof/NQr6xzMr6BXWgS7XNFPh3JZOqXc
N+XqbeBf7AqPdeqdt3RuSPaOSpSfmnqr1i3W6rX9Er0+s/KHoU3tSivk6BHdW209XL1KLWgv4zaUn
Q8wMA+tXj3XyNVWfIm7aibB/3Otm9lNlHcV/V8w1VvpGV1dXs//RSJ9/C4LeE9v1o9+
+K2ju70tO7r9jLQO8I+7p1VyT4J7T+Z6i+0akEK0afmlRoqvy4OVBe0mlL+WexX66pSzRemfz5Tv9B4
dtUbUuF6fYV2VTxVId/lNztWMVtPe2oqG8OFY+m3V27Wh2UKq+dIq5P7SqK59ACPT8AoEf1+6n6
3UindKHi/w95Q1DfYV96r5lXUJevnzL5a9/7ZbIN88LUFCV7evPJufur1fV9vKJfD3fh8q3R1dbCFWNP
TartVHqcejix4vxSvUp2+cyB6V+upu3kx9XTU9UpxTbVxfxKZFOeQ+mC2xGmLxrekTzSYy39hHo47a
uWT2emL97J1cN6qeLJqfa0jOj5AQD9kfcAzl1D5/qvIAxz+9Hztsz0+Q/pN20x090OTmzw90Gm37dC
/QL2tr9iYvuF6vqFc1bubqXjCxm5Vh1exqapiueTL6Q+u98qFn8hczT/YeoyerGeWv3848It1Mk+lMh
26jp8fzdeusbFtOdM5eyjw2LmahYP5Nuhy0JLg54fANAb95O5w7sdv4FvBor9dHXPGFS7/emedc3y
H/spVFypSs/XrL99BPX8ImXKS5iVKH/o513u/Am13h2dcObLK9avW8clI9N1WKlpKufCJmmq0tmGf
BNZx/IfO3uze2q5b4ouuIWKOvlfVDCmr+q7dJbi6a725q1VvNXyEuUP4aVMxqkVuJzdbM2+TP5Cn
W1sSdDzAwB6494BeG9RutR7AVtx49HV3Zur2IvS45byXejp85L9bUgrN9ZnneKd1/GtfNwwb7ziHV
foq5S7D/dP8VScAu78HV/LyHQdVopPVbxwHVzFeJXi2W5eK0+hfA/6SP50Z2+KQC1LR6XcQuJ1KQa
Er287nu1Y4s9OKjRbdf06K+WpVRyRV6+j16rYgaqW/1yftturuBzo+QEAvZnBR/Vw9wLibrFXnd5M1
eXbqK5XXmiQPblvjU6JF6e8fn2+LYqCvb7v7D2UP/dTr5hbXroOX6piQnvl7lvBGTKdyHQdVopNJd6O
U/BWKQ4qXV02p1C+h+px/nRnb4rYpcuU2+uimlvIefN18Ac5up0O5rd5apWXqzxW/txRPe+
+iv1oq6u1N8kUimnNC+O8SvFrDC96fgBAb2bwUT3QvUB5pzPgPUd589Z9wdotm6WLO+yYom5/1
8955/Vbr3h1er1kboUed1R7U3R3H19y/oS6f2SdV286bgEj8tSkglMVV7Or7TQsuHi6k2KyUO09Ye
mjlqujd4RbyJm4eS2NIlMUT3X1Xsg5tdwKxSs33Y5KzZcmH9HF7uQfGfflb14LJHp+AEBv3A/mju7d
woa4FyhqDHzDUdTs9coV8jL9V+l/O33cyod1dlcd5pbodUeWrt8S7rrli9X1ziLzdVjKP1XxunW4mcYF
FwM6KNhQKH+6s3dF46a6+hPmFiqO2BuRb8YE4e10tA2LU8st0Xx1W2qcKB8w/Zui2EF9GrdyBy/T
0qHnBwD0R34yd3b7EdR/hfKeROr8/iOrYm3Dcx/Ui27u2lzZvNa0xYvU66tUVLA30uc9YvHi9Puec98
BgxTNdP6OKF6a+sJre3Neuql5KmqRpyblmao41O2L1LjgYkAHl6+hUP50Zy/STDdV+4PUyZ+q4HZ6
+DPr1qr9Ycp0VzT+MnXzGhWrdYvkh+2540uBFz0/AKBHtRuQbu4K/Iq5hcW+JxA7GmYzedFeXqHB
d1O71e3uvtfV57u6rrwfNgZ4R3S5N/EOKFRbsPfW1cWMVIwvZiLhqcTrVZpyc0W12kqzKtbjouY0N
VpemXxYZ296Z1PZAWvyckmeRbQW31Tt2WnKKM52SsXhAd7bnf+DIlyr/szUl66czjtN7X3d6ZVcHv
T8AIB+2TcgfE5jMLUb0qnvR0OKt3dvs89Ax/fx6ET9VSmp10Y8tUCvWHhTC7mnyGuUGd0/KDon/l

VFqbpl4EZiSvT8AAAAAACMEz0/AAAAAADjRM8PAAAAAMA40fMDAAAAADBO9PwAAAAAAIwTP
T8AAAAAAONEzw8AAAAAwDjR8wMAAAAAME70/AAAAAAAjBM9PwAAAAAA40TPDwAAAADAO
NHzAwAAAAAwTvT8AAAAAACMEz0/AAAAAADjRM8PAAAAAMA40fMDAAAAADBO9PwAAAAAAI
wTPT8AAAAAAONEzw8AAAAAwDjR8wMAAAAAME70/AAAAAAAjBM9PwAAAAAA40TPDwAAAA
DAONHzAwAAAAAwTvT8AAAAAACMEz0/AAAAAADjRM8PAAAAAMA40fMDAAAAADBO9PwAAA
AAAIwTPT8AAAAAAONEzw8AAAAAwDjR8wMAAAAAME70/AAAAPPlzOoOZfWMOlTST6ysX1BH+
qHriPLzoLo2Gec6XFhfCT0VfQ4AxoueHwAAYL5YfW29PV32nr9q2wu+yxDs7KuLOof/JgMA+kPPDw
AAMF9q/y3b7lCXvedvtX9vc1/9qwA6fgBLhp4fAABgvtR6frvBXfKe3zTuDesy189cJ3OEjh/A0qHnBw
AAmC/1nt9qVJ2e3zngNsZqSD7CmledYB2xu2F9ODtm/c/prSKa9azzvLW2qkxDz12brzahtVItMpcZX
Ywxj5wdxNafq69HDIlsMHoeAAyKnh8AAGC+VM3kevmTbhmtLrN47B6w2k3VElftaCPTRIfPsfps2dc
WrIV4Jom2vv6icusWay2OanGr6/pHUb1p/Q0LCm/Qf150uQDQH3p+AACA+aKbxqyD1I1p2TBaTx
QD3QOxnl8NslpSeUS3pc451RF9QJYxI8xSrDot2t1wATOhWzPGDFaqaQqN69cDwusJbDA0kVwBA
AyDnh8AAGC+1LpG9cD+2W07rXZSNrN6SNWXho/oadwRcozsiDPykJnEGhMkV5CTO3EPxJkF5MQ
pLdYvOdX9GzTDWu0bAHpHzw8AADBf/O111mo6nbGnVZatqTuk+Yh+bDfK9Xn1CB9RuDZLgLukn
C6pj8qtNQovocX6LfXR+vnA7GaVlfq2AGBQ9PwAAADzRXbA6rH56/1VDylHZvrq+esH9QOfYOEw/
1i5leSe311Ci/X7mveCfj44e+DEtqsGgE7R8wMAAMwXp5mUTaR5wtN2ysbYHdJ8xDOtmNc3Qmgx
xPCP1SX1Ubm1RuElNC9Oj8iUg5zqjXNYUxTCxQCgP/T8AAAA88XTTNbbR/NEbGQHPb/dXNfHtGi/
3TJhZjp7sFOjw56/cSr3VOeiRDaYPWVNPPG6AaBD9PwAAADzxddMmr4xZ56ojqp20jSmzhG3eQ0f
Cc9ihoQr68bWLRMTLlCdP3HvHFlC0/rldqrxDRt0NhJdBgD0jp4fAABgvjQ0k/YTVisqiV421oLKI+bxip
xd97sZf+lqgFsmTo+vs8/usudvXL9/OTk9JDR76My2qwaATtHzAwAAzJdAM1k1qbUn7N41aytlY+xO
1nxEP7anc5aTq/fN9QGBXUTV55NNstxao8YlxNZfnZ7LS1qXxX7aN7t9aq7tigGgc/T8AAAAAACMEz
0/AAAAAADjRM8PAAAAAMA40fMDAAAAADBO9PwAAAAAAIwTPT8AAAAAAONEzw8AAAAAwDj
R8wMAAAAAME70/AAAAAAAjBM9PwAAAAAA40TPDwAAAADAONHzAwAAAAAwTvT8AAAAAA
CMEz0/AAAAAABj9Oc//3+nSw9g3ifoEQAAAABJRU5ErkJggg==]]></redcap:Attachment>
</ItemDef>
<ItemDef OID="b10" Name="b10" DataType="text" Length="999" redcap:Variable="b10" redcap:FieldType="descriptive" redcap:BranchingLogic="[q7] = '1'" redcap:InlineImage="1">
<Question><TranslatedText></TranslatedText></Question>
<redcap:Attachment DocName="B.png" MimeType="image/png"><![CDATA[iVBORw0KGgoAAAANSUhEUgAABVEAAAEJCAYAAACdcmdaAAAAAXNSR0IArs4c6QAAAA
RnQU1BAACxjwv8YQUAAAAJcEhZcwAAEnQAABJ0Ad5mH3gAAE9sSURBVHhe7d0PjCRnfef/b+/aY
PPHNmBskx+RHat7sZYlGJIg08PxL4egZ+6SJbnbYOVg5Oh3PVyi0zQSkwOxF+mX2wguG4lunS5iWrq
zliQyTEJugrLdIJRgckzHR4IxYTI/Z7rlc3T8wDYGjPljB3u3f/VUPVX1VHd1dVV3VX3Ptltaerpqfqq
O/OZ5/k+hb5FAAAAAAAAAAAAChjumPAAAAAAAAIAQhKgAAAAAAAAAAAEIEQFQAAAAAAAAAiEKIC
AAAAAAAAQARCVAAAAAAAAACIQIgKAAAAAAAAAABEIUQEAAAAAAAAgAiEqAAAAAAAAAAEQgRA
UAAAAAAACACISoAAAAAAAABCBEBUAAAAAAAAIhCiAgAAAAAAAEAEQlQAAAAAAAAAiECICg
AAAAAAAAARCFEBAAAAAAAAIAIhKgAAAAAAAABEIEQFAAAAAAAAAgAiEqAAAAAAAAAAAQgRAVA
AAAAAAAACIQogIAAAAAAABABEJUAAAAAAAAAIhAiAoAAAAAAAAAEQhRAQAAAAAAACACISoA
AAAAAAARCBEBQAAAAAAAIAIhKgAAAAAAAAAEIEQFQAAAAAAAAAiEKICAAAAAAAAQARCVAA
AAAAAAACIQIgKAAAAAAAAABEIUQEAAAAAAAAgAiEqAAAAAAAAAEQgRAUAAAAAAACACISoAA
AAAAAABCBEBUAAAAAAAAIhCiAgAAAAAAAEAEQlQAAAAAAAAAiECICgAAAAAAAARCFEBAA
AAAAAAIAIhKgAAAAAAAABEIEQFAAAAAAAAgAiEqAAAAAAAAAAAAQgRAVAAAAAAAAACIQogIAAA
AAAABABEJUAAAAAAAAAIhAiLp0etJYKUih4DxWGj29PqjXWPFeU1FeU1hpWF+iMxj3ZB2onXjL

MxLO7LSlg372PzHqPs4Vb2GrGzEO5vtDbdtK5JH08KNuQ9Cjmc+2g0AAAAAwPwhRF06Rdm8UJey
XurU1kPCkracr3X0c5Hq2U3rq4B5oILBVWnqJUdZzqxle4faf1Qo1cT/rlhsR+14AAAAAADIGiHqMip
uytmqfi4dqZ0P9kbrNc75IVW1JdsV/XzhFGVzry/9vnpsS/aHkff+RpmXdmShKwdu8leuS9c+xj3ZzDJ
D7TVk3fijwsI7ascDAAAAAEAOCFGXVGW7JV6O2jxn9EY1e6FWpbW4CSqOot6h7OuncuoEPaQBA
AAAAEAuCFGXVkW2W26M6vdGbW/4Q6XL9a3wXoyqlqKuRek+wmpSjqyvaH59ZI1Jo6ZjoC7rqPX
GPu31edcGHd5fZI1J4zwEz5+5nbDPjxN+3INtCdS9tR4xy30ObGewraPOc7xjCm57uPZpYAh6c9VZF
2h4snM3eA7Uw9yc3Z6x+xzPPy71CLkXbAnaHvI9OHq7vqTHE6/dAAAAAAAcfYSoy6yyJXW3OGpz
VTYaDTnnJ6hyIWSM9FAIo3VqpQwmoCrK2hndwM6BdJ1nFmNId2dHLno7bcuubn/5zNpc9FKsnPa
D6gP/AGy9izv6PBo1Pe1wrCSDo63TPb8dqZUKUhrYSXM1aUimtjPY1qasDoZtEx2T2rZR+9S6H+te1
+kREu3HCSwHz4HSXB0dKibnnOtV70AUtW6Kc9TeGFHPNGS7E4vZbgAAAAAAlgQh6lILTjLVrLnBTF
nqF0Imk2pv+KGKV4+yL103ie3UZD3lhKW4dka3b18O3U23d42JhYxw0hjqferEnAz0rpz2yiY0d4M9
Ji/u6BisfEacDLUnjXX3GlSlpc9v3+0xnOr59bfvdUhWIdlAfdzx/O1494Hazrob/E1zTMbr9zZlc9v62PX
vV1Wv1/6cXXIi2X56jXUvsCzXu85r+8MlLiqR+4wr/BzteOl/krZbr3X/0hH2PRjY7rBkxzOu3QAAAAA
ALI+C9QtyXz/HklK9SwM9zlSwEhKq+K8rS71rTubTk8aK24tOBS/OZEYjX6963bk96Ubsy+dvW4Vde
9ZG1BBs1YOwWq1Ks9kcWu+3IaxdcdeNE387w+2yV3rnwG1/6DqbsV0VnO2FBNwB4e0wr3O11fc
nDEt0PSK2E9ivvuaS7JjMbQdfr41qa6JzF35+FOdanQqsS3p+lETnOlHbRwht44jjjDietO4RYBr3Pnyvv
OXCW/QSACAP99/xLnnNW2/SSwCAzB3/NZFjr9ILWBT0REVwkikVtoQGJP5Qeb/npMsYdm/2GE1F
UU6ccp51dJfTrj2WvywnT5+0e9QF11uqp/0AbA74vWmb4nVG7R44gZT1GW8ov7dOdT4sGbUo3S
BMfcIsazBr1jUo6ac28z7QPYSnOKZEvYkT7cec4f+kmIdQ3NyTvhmgTm3wHIWY6rrrurFuwJmaGO0
GMvDwEw/LOz/5Tr0EAMjDDY9fJXd/6AF5/Bs/0msAAJm7dI9I/zt6AYuCEBVBA6FScsO1P6fl1RVt7k
pbenJoj9k/JScqJ6z/D65XGeqc9ZIrrombLbpD+ttuIl09a/TozUtaAZl1DTJrOyHeSKpXqBe0GnVjgQX3x
NNPSOWPKvZHAEA+nvPjY/KWL94oP3ryGWm+/3555p8u6c8AALL1lMilP7A+PussYiEQoiJlGYRfXl3
RfTlsXxS7lKgd9lbEyVeN9XMZvhk9NO3A1+/VOyrwVcO67VqVQ480e0lOK1mv47yOaTHOXbjxbW/
Lhtnr1K2LatY5BRbUnZ+6Ux58/EG9BADIwxv/
+gZ5wQ+vsJ8/9NXvyp/WeR8GgNz0Hxa5/Od6AYuAEBUxleSkN1LbnBFfMSZJitM70Ri+HI+7744c7D
pf686+X7I/4a8fLjUwH4qbZ/0guOFOjFWVQIZacsoTKG6Jgvk22OvYvA/0seV1TIn2Y97LA8Pk1cz3hZ
xnoE/SdmNSNTtwdeukJv6eAubL+z77PvlM7zN6CQCQh1c+eK3c/PXn6yXH5z7+kHzlLx/RSwCAzF3
6gsjlr+kFzDtCVMRUlM2zbuVUc/Z1NbrYnOl8y+vt5wScijnre1s23JlrYvN7cqqJpBS3XmZRF0x117vh
6vxxe81a56Kmj3+wdqsx7F+aq7LhnTIV7Onh297K+dBcNQLH9nm/hqd7bHkdU6L9mLVbm7Lqre/p
me+ta1Qq+NvI2oTnyA9c3XYDi+kT+5+Q+n11vQQAyIOqg/pzD7xELwX94W9/Tb73raf1EgAgc5f/RK
T/fb2AeUaIivgq29LyctSalHTA48w8bynX5YJR4NOfUMnSXNWB0GQ1HAPbsp55Q/a9of6KMUnTHP
Jqu9rKUt8KRKiWomxe8IdlN1d1gOaFzqMm/ZolJ3AMttM8tryOKdl+ipsXpO6/2HmtOZGTdS/7h6Br
7yrua1NNWBO03bzfw9odR+bHA8R339fvk7v+7C69BADIw/FLBXsYv/oYRgWozd+8Xy8BADLXf1Lk
8sf1AuYZISoSqWyH11+stqz17tBiV3FT9gZfW21NVr/R7K0XKBlgDM3OdKKjFJgB2KiyA+qc9bt+wOe
ya1/OW03PstS7fT9Yt1Wl1d8LTpaV1zEl2k9RNvf60h16sXq5MUzeVpHt4EGK7B96PbFTEbvtVlv6Lf
8+sqlzblwHu+5ulByOB4jBnYn/6Wfp7QQAeVr50kvl2iev1Evh/uFL35ZP//6hXgIAZO6y9RvZJcpbzbt
CX81aAgAxtTcK4nSSVCHqQGAKADGo4PT1/+318sAjD+g1AIA8lB56od0LNa6tu8vyiteFD/sHAGTgit
8QKfBL9ryiJyoAAMiVGsJPgAoA+VK9T1Uv1CTUsH7qowJAji79AfVR5xghKgAAyM1HvvgRezIpAEB+
VP3Tt33hZSProI6iAtS7/+NX9RIAIHN2fdR79ALmDSEqAADIxe6Du/LBv/igXgIA5OWOL18/tg7qKPv/
8zH53Mcf0ksAgMxd/n9FLn1BL2CeUBMVAABkTg3fV3VQmUgKAPJ189efL//8Czfppclc+dzjsnX36+
XWV79IrwEAZOtKXR/1Zr2MeUBPVAAAkKknnn6CmfgBYAZe8MMrEk0kNcoz/3RJmu+/X3705DN6
DQAgW9b77aWPWx+fchYxFwhRAQBAZlRwqgLUh594WK8BAORB1T99yxdvlOf8OJ1f+R7/xo/k7g8
xKSAA5Kb/HZFL1EedJ4SoAAAgM+/77Pvk3ofv1UsAgLz83AMvkRsev0ovpeMrf/mI3PtJ/igGALm5/
DXr0dELmDVC1KXWlo1CQQrGY6XR05/LSa8hKxttveBob7jtWZG8mzM3Qs5LHL3GinPuVhoy6tT55

9d55H7NjwLuWyCW+n11+djffkwvAQDy8vJvPE9e+eC1eildn/zPB/LN//19vQQAyNylXZH+Y3oBs0S
IurR60lhZlaZecpTlzFpRP8+eHfiVasLfVIImPi+9hqzXor5KXfOCrAYvunRqJSlMENguK+5bIB7V+1T1Qg
UA5Ovqp47LG++bvg7qKKo+6u//
+y/bHwEAeVD1Uf+79fFZZxEzQ4i6tLpy4KZA5bp0+33p9/dkM68MdWzgt6QmPS+qZ+S4YK99XtxN
V1vqevelWy87K5qrQo4aA/ctEIuqf6rqoAIA8veWvRvtIDVLqifqH/721/QSACBz/UdFLv2xXsCsEKIuq
96h7OuncuqE5Nf/FGmL1zOyJ41zugtquS5bFedpcfOsVJ2n0twlRQUwPTUTf+WPKvZHAEC+Xvu1F8
vLHr1aL2Vrb/f/2A8AQE4uf8l5YGYIUZeQXbvRDN2aq04tx8GuiKp3o1E7c1T9zGAtyGCd1bDejbH3r
wVreI6qN+kMVfdfl6zW52A9S6+2qH6ENy3GPgPncEP8zZjnydln0vOiqHaWYvWMNHoeB0LzkpzUn
VFl/9A6olGM9obVW21v6GMZPAcJrkvI/RZ2vePcb4PXz35E1ImNY5b37WT3p2vSa2eZ6D1Ar1TMr
49uJI6QOz91pzz4+IN6CQCQFxWevubvXqSX8qF6o1IfFQBydOlPqI86Q4SoCDUUGGl2/cyRYVRHai
WjzqrR43EyanuDNTzVurCgpuQNVXdFt3UUZ5+DwWRzdcJ9Fjflgjtk3joz5/RGeo1z3nkq1y+kUEahK
q1+y+tVOsTseTxK50C6+umwipx2N97ZkYsDJ7W96x2NX1c3yXVRQV7I/eZcj1EBZPj9pu7d0GC5U5
NSlMjOSpb3bcz7M2CCa2eZ7D0Ay07VQP1M7zN6CQCQFzV8Xw3jz5uqi3r3h75KfVQAyI2qj3qP9Z
H6qLNAiLqEKtt96Xfr4kZ7Um3Z9TH72zrxbG/4AZBXL9Won9mpyfrIxEaFec7r+3uboWUCxu4/wN+
et3/pyI6XBPWkse4GPca+Wzo1imzrKP523M2ofdbOu/Fbsn0WNy+If+rOS9v677wbglnn94JOUJOd
F4P9um2ZLK8uyolT+ukYlS23beb5V9ri53BnxMnhkpwj67VGqYGh+21ofyZj2/b9ZrYlbFt+kJ3U/Ny3
/uvD789hya6dZar3ACyrT+x/wp6NHwCQv5UvvTTzOqijPPTV78qf1hmBAAC56T8scvnP9QLyRIiKIW
bPtPoFPwgdDgOHletbE4Z54aotPxwsrp3xAqzOge4z2bsoO14eaey7suW3deeiJIl7zH364ZMh8T6Ls
nnWTbuaslpwe08Gz+8kipt740PW2PblMOpEFdfkTNjxtXf9XrVn1pzjSXSOrPOzp0NEI3gPvd4Dhu43
s8et0evSPk86CNzLYfa0LO/bsfdnmCTXzjLNewCW031fv0/u+rO79BIAIE+vfPBaufnrz9dLs/G5jz8kX/
nLR/QSACBzl74gcpkJ/vJGiIoBI3qm2Yqy5iYxIwK3UyfSDKjKcrKkn47SPdC9+VSoU/LqNhbMYdKRw9
QnMMk+K9tGr0FHOsP403RKoi+fcf2NgNIP3Kpy1j2gqa6LruEZOrw/aOh+M8JCawf20Hd33yNrsaZ
uHu/bBNduyvcALB93Jv6nn31arwEA5OWGx6+Sn3vgJXpptu7+0APy+Dd+pJcAAJlTw/r739ELyAMh
KibUkeHOgTHCo7mX3TFUvMKUSrD+5Oz05HBssVSf36vSHRZuBG7V08FeoUmYExB5PXXHCbtWR
dm8EN470w0rF3t+o8nvz/SvXdh7AJaNCk5VgPrID+h9BAB5e86Pj8lbvnijHL9U0Gtm60dPPiPN99+v
lwAA2XtK5NIf6OfIAyEqJjR/gWm53vWGbAcfk9YLHS/
+Po26n7aO1NZznJyneELGlj4tn5Sxl7S4KW5lAntYuDF8vno6/CyPP0dt2TB7nbo1OM36o0lYbdzT+xj
s/as0z83XpEi53bcTXLtoR+GPJpiWGsL/wCMP6CUAQJ7e+Nc3yAt+eIVemg+qPuqnf/9QLwEAMqfq
o15iYte8EKJiQElOusnV0EzePbnoFnIcO/Q7J6WTXtA2qm5m6ibYZ6+xrodpl6Ved5OsPCfnMa5rc1f8
zphdOfAu6QmJc0m9HrXW/XH+/I4OP6sSyOGSnCOzLqcKFN26qMaQ96TUzPKqx6k9GZQdSHa9W
p7pDpOf0CzuW0usa5fme8AU1xDz7yNf/Ig9mRQAIH+lh1448zqoo3z6v/6D/MOXvq2XAACZu/xZkf
48dRU6ughRMcCcBCnYW9IPAlXYNTChz6yYNTCbq/5Q7faGHhpuPdIev510n72GrLsnrnpWNjeNyY
Nq65JPjmrWsmzKrm5er3HOCzBj90asnBbnDulIs+ke18Bw8Amvix8oDvbcjUlvX80sH+xxaoTFZo9b
oz1p3yaRZnHfKnGu3QTvASU/dZXaee9gZMOb4h9Hze6Du/LBv/igXgIA5OnaJ6+0Z+OfZ83fvF+
+9y1qZQNAbtSw/v739QKyQoiKYeYkSJ2alHSoU/LTE7kw7YxI5vDy5uoUoVGwBmZzVQdQXnhTlVZ
qs9e7kuyzJ411d6h6Wepbav3okCq98zLMnFndbbN3TastiX+aKhIo72oZDmATnCMv2LO4x2xOsJTEi
HvXrLNaPevPNp/YQt+3SpxrZ0n4HuDXW7V41zBubVssGjV8/85P3amXAAB5UvVP3/aFl81NHdRRV
lCqglQAQE76T4pc/rheQFYIURHKHgYdUpOy2rLWu8Otp1KR7cGClfuHRs/BBOwamMZwbZddWzOj
eqgx92n23LN7obonbiCk8of1p3hehhRlc2+4Tqg9hD5hYBeYJMs6ZjsbHhT7uljH3G/5QaqtKq2+0dZ
ACYJo9r07eJA2Z5vTZZMLft9aYl07S6L3AHUsg6+ttiava4u59cTTTzATPwDMkOqBqnqiLgI1pJ/6qAC
Qo8vWb6bUR81Uoa8KBgJAEmo2fT0ZlAph96btmTxL+lhOtaYNWBfEUbp2yJUKTit/VJF7H75XrwE
A5EnVQVWTSS2SK597XLbufr3c+uoX6TUAgGxdKXLFb4gUbtbLSBM9UQEkFCxRcGZtsUO49nnrWC
J6ZB4tR+vaIV/v+
+z7CFABYEbULPx3fPl6vbQ4nvmnS9J8//3yoyef0WsAANmy3m8vqWH9TzmLSBUhKoCY2rJh17o06

pWaJQoWUK+xIqv7demmUqJinh29a4d81e+ry8f+9mN6CQCQJ1X/9C1fvFGe8+PF/NXt8W/8SO7+
0AN6CQCQuf53RC7doxeQJkJUAJNRNS8XfPx7cXMvpRq/C+YIXDvkR/U+Vb1QAQCz8XMPvERueP
wqvbSYvvKXj8jnPv6QXgIAZO7y10QufUEvIC3URAUAAKEefuJhec32a+wJpQAA+bv568+Xf/6Fm/TS
YqM+KgDkjfqoaaMnKgAAGKKCUzWRFAEqAMyGqoO6aBNJRVH1Ue/
+0FftjwCAPKj6qGpY/7POIqZGiAoAAIbc+ak75cHHH9RLAIC8qQB1UeugjvLN//19+cPf/ppeAgBkrv+
oyKU/1guYFiEqAAAIUDVQP9P7jF4CAOTttV97sbzs0av10tGyt/t/7AcAICeXv+Q8MDVCVCTUk8aKm
uVbPTaknWjdOGltZ96MOYZeQ1Y2gmvbG+7rV6TR0yunpfZjb3NEO2zTne9M2m1bkPugvaHbWJC
BSwosjE/sf8KejR8AMBsqPH3N3x3tuqGqN6rqlQoAyMmlPxHpP6YXMClCVGCGeo0VKZRq0tHLWe
pd3DH205RdQj4AA+77+n1y15/dpZcAAHm7+qnj8pa9G/XS0aXqov7+v/8y9VEBIDeqPup/tz5SH3U
ahKhIqCibe33p99VjWyp6bXby3l8WRhxDryHrtTziU6UnF3eC+2qea1hrF8VRuA+A+aZm4n/nJ98pT
z/7tF4DAMibClBVkLoMVE/UT/7nA70EAMicXR91Vy9gEoSowDLoXRQ3Qy2Xy86Tzo5cXJwUdTFUt
qXfrYs+w8DCUMGpClAf+cEjeg0AIG8//ffX2UP5l8m9n3xYvvKX/NsDALm5vGc9mOBvUoSoS8eoLbl
i9kQctd6oc2mvz7s25fD+IutuGnU/VwKfNLcT9vko6Z8z+/PmMP7mqvP5EYU0/WNWj+T1Rv2h/FU
5e+GMDvk6shMnRQ3UUg17jG5PvHbHuTbD59DaumyYrzfqkQa2MbA+/BTHaYN5POpY/P2rh7vd
9nnrupbrsjXQXdYu3WC83n4M3DfArKgh/A888oBeAgDk7YbHr7Ink1pGd3/oAXn8Gz/SSwCAzF26R
6T/Hb2AJAhRl05R1s64PREPpOs8s3TlwE30Aj0U27LbdJ6Vz6xZXz17ldNV/awjB/4B2PywsCxn1nRr7
RCwJIMj5zu1UswQa5bnrCO1UkFW9fYcal2SINUYyl89LZXimniHs3Nx+hCvfEbcU+2L2e6pr43Dfn1w
Z846FVQOrG+uDgSpE7VBHcuqeFvWoakKSlf369Ld2wxcdxW+lsJKN3RqUprnibKwFD7yxY/Yk0kBA
GbjOT8+Jm/54o1y/FJBr1kuP3ryGWm+/37qowJAbp4SufQH1kfqoyZFiLqEimtuT8R9OXQTovauHwi
JEU72Dq1XOU6dmIcI1VI5LW6M2gzMjmSEhV6w15PGutvjsyotu6am9WjpLXRqsh4jjUz7nFW2rTa
Yw76rLadd22HVPv12d+vuV8TsRaoYQ/mrp9X2zVA4xpD+4qbsuefNfnTFa4Z1BPULwcDQN67d6V
wbV7neDX69Frbev2+maYPxeh2aFjf3vOc+P1RXYWtXf41/TppyLsFxAmnafXBXPvgXH9RLAIBZeONf
3yAv+OEVemk5PfTV78qf1h/USwCAzPUfFrn853oBcRGiLiOvJ6IfaPUOndivWnXCo32dFJrDwO38bS
5UZMsNoJq7fi8+s+6n2wPUXFff8ickqmx5QWCs3pgzPGfVlj+Rkh/mWi0Z7IY7gtk792TJfmJsJ0EYq/
Ua636vzepZ2QxPUMe3O61rY6vKWbchRsg+cv3+obPdKdoQeH0UI1Q3Q2s7cNWB6t6okwhkSA3f
v/NTd+olAMAsvPLBa+Xmrz9fLy23z338Idn/n4/pJQBA5i59QeQyf8BKghB1KRXlxCnnmRtode1x6W
U5efqkHXYF11vUMHDn2VzwQ7mmeJ0KuwdeWOgN5ffWWcfkDu+2H8bw7cAQ/VFmdc784HMy
Yb1zLRMP6W/LeT9BlVZoz1klRrtTuzaW8kkJ3d2o9a4p2hC7Z7Zxrq2N2WUO3P3Er8sLpOuJp59gJn
4AmLGXfPe58nMPvEQvQbn7P35Vvvct/m0CgNxcVvVRv68XMA4h6pLy6oraPTl74nSqPCUnKies/w
+uV3ngPEWoFiOYcodmt90x0xG9I6exkOfM6GmphqaXQkPC+LP0tzf8OqBVo6fp8kkSbhdl80L4jP1u
cDtiPjEgE+5M/A8/8bBeAwDIm6p/usx1UEdRAWrzN+/XSwCAzPWfFLn8cb2AcQhRl5U3tHlfDts6aL
N77VXEyQqN9VP3hsyCUdfTDi/9upOjwkuvNubQI2YYuIDnzB/KHyXmkP72hj9RVLUlIzuhTmDqa5O
CTNtg1JUdKNlqa55jln7k532ffZ/c+/C9egkAMAsrX3qpXPvklXoJpn/40rfl079/qJcAAJm7bP02eukze
gFRCFGXVklO2hlkRw52nSHNbh3Rkv0Jf3347OuzV9w864eaDXeSp4E6pCVnqL3i1eKc2KKdM2Mo/
xjjh/S3ZcNLUMtSV1PRTyvVazOhHNugZuhXPU7tScXsQNWYoCtu2QJgSvX76vKxv/2YXgIAzELpoRfa
D4z26f/6D3aYCgDIyeXPivTp2jMOIerS8ntyNptOOObWeCzq4p/uem+Sprnj9gDtSK2mA77BOqRmP
crmqj9sur3h1aUsxB5LvWDnzBzK787+H3iYIV70kP5e45w3jL9cv5BOuYRUr82E8miD3pbKoIM9Trvi
ls8dW7sVSMn/+v/
+l34GAJgF1ftU9ULFeHd/6AH9DACQi0v36CcYhRB1iZkzpqvehd/Zw88Ds5sYkTXPIq1NqC+shGaxH2
VzVwZg/LI1GT440LNVzVtS1VJXmauqhoTmUvxxaW8AoiWC9cuSQ/l5D1r0CqtYrAxMwOY/JJkhK99
pMJoc2VLb9IfyBurRGfdmzm7MP3bEU7v7Fu+WOl9+hlwAAeXvjX99AHdSYfv7dP6WfAQByUXijfoJ
RCFGXWWDW8FPiTzbuDltXzPVzyAwvRw2ht+tRGr0uXeW6dJPWu0z1nFVke7BA5v5hSrUxzaH8o0
NdMxQeNaQ/Xl3VCaV5bSaVQxvsIfxhxVBVSNvvp1pfFohy1RVXyf/4lf8ht1x3i14DAMjT373yCf0M
UV7z1pvkbe+5VS8BADJ37FUix9+kFzBKoa/G9QIAgKXxwCMPyOv/2+vtmfoBAPm648vvYFYd

D1P/E8+a1PvVGedw0TbwFALgovFrni/daTq51ljERPVAAAlsztN91uD+0HAOTvb27/tjx2PX/ECnPlc4
9L9fdeS4AKALmx3m+Pv8f6SIAaByEqAABL6F2n3iUfeMMH9BIAIC+Xjvfl8294VH78nMt6DVz/8t+V
5NZXv0gvAQAyd+ztIoWb9QLGmSpE7TVW9AQpBX9W65F60lhxJ1TZkODL27LhTbbiPCabqGZYeyO
43dBH1jOAAwAwhz788x+W07ed1ksAgLz84PnPyt7rvqWXoLzidS+R1X8bNhkqACATx4oix39eLyCO
OeiJqsJVf5ZqR84zwquZ0VcaKU3oAwDA4rjnl+
+xh/cDAPL10M0/kO6t39dLy+3al14l1d99rV4CAGSucI3IMTWMH0nkGKIWZXOvL2oeq35gxuuuHH
iTiKvZsNXn92QzxwzV1qnJeTqkAgCWjDtj/3VXXafXAADyonqjfu+aZ/TS8lIBqgpSAQA5Of5ukcIL9QLi
mn1P1N6h7OuncuqEZJedlqXedUNc/9Gtl/XnRZq7pKgAgOVzy3W32EGqClQBAPlR9VE/96Zv2h+X1
S/8xivsofwAgJzYdVDz7rl4NOQYog7XRLXrlZZq4nZEtYfVD9UoNb/OeaRVL1Uprp0RP0YFAGA5vfm
WN8tH3/5RvQQAyIvqibqs9VFVePoLv35CLwEAMmfXQX2HXkBSc1ATNUKvISuFktS8lNXRqZVSq2H
aPu+HuNXTfpGB5WVM8uWd46hJwTA7YddqEYTfT/4kcHneY0nPYU7fC+q9b2DCO//8rEiKf0cCAt7
7s+
+V2h01vQQAyIuqjbps9VGfd82VctfvUJMbAPJztcixO/VzTGKmIWpluy/9bt3vCVptOcPst1WY2ZPGu
htwVqXlDsFvVe01qobpeqIkoSO1khtC+I9Vd0Yra9/2bgFghnqNlWAPfSBnqjeq6pUKAMjXfT/zuD1r/
7JQAer1P/E8vQQAyNzxO0UKL9YLmMT89kTtXZQdnSKU61v+RFSVLXHLmHZ2LqbU+64s9S0S1NFG
TQoGTGKR76eM295ryPpg13tgBlR9VFUnFQCQnx8/57J8/g2PLkV91Le951Z5zVtv0ksAgMwdf5PIsV
fpBUxqfkPU7oHXE8sevu/1HjWG93cOpKufTsfppZpmrVUAABaVmqm//attZuwHgJw9dv3T8je3f1sv
HU23vvpF8ku12/QSACBzhVtEjv0LvYBpzHdN1FSFz85vlhPo1M7nWItxHgxO2jWqxuPoOpD20GPv6
/VjZG3Jwf1FBNd2Pdzga0fVgsysDVEya98oca+VK/5xJm1f2OsHyncO1O806o7arx19P/kG2p/K9Ux6
DsOEt32wXungORo8P2HiTbTn8/epHqPqpCY5P0DQbdffJvf88j16CQCQl7+/7Xvyjy//oV46WlQd1O
rvvVaufO5xvQYAkK2rRY6/2/p4hbOIqSxEiFqud4fDT/uRwnDa4qac1WVWRfblcGnyBRVsDU7a1ZTV
wqr1/3hUiFMKG3rcqUlpMKBKMklYe2NETUjVYzgYFmXWhihZtW+khNcqwXEma58TyIW9vrlaGBH
2qXNitLNcl/GVM9SxDbR/6us5/f0ej9OrffAcNVdHhZyTcPbh1XO2Dd97qd3vWGrvKL6DGfsBYAb+6v
WPHcn6qL/yH15JHVQAyNPx09RBTdH8hqilk34P0YN0Bu2Ha8tuuinKQug1zvnhkTuhV7/r1Zsdzzhv
5bp0dbDd9TbQlHNeotNLMEmY9dpzesOh2+3IzkX3tVm1IUpW7Rst2bVKcpzJ2tdrrHuBnP+HjZa1F
615bkRQaLRjb1OKem0koz1u06e5ntPf70n47fHb3pHa+ejIPHqivUH+PsLvvbTud0Ds2frfdepdegkAk
AdVH1UFqUfJyumftB8AgJwce53zQGpSC1HtnmjGkFHzEWco65DimpzxspxVfxuqF6C77UQbdnpv
me1yHmYvuTOyFivhWXQ9uejO2qUCFi+kKcrmBSPEidI7lH39VDo74mY3xc09J6yxHnub+mQmmiT
MmLjHCNyKa2eGQ/XM2hAlo/aNlPBaJTnORO0LtuOst74iW/aGVVC3J2GHE2hHTNWz/rmtbPtBrd
/2JNczhfs9gWrL7yFf2Up/+4q5j/B7L8+J+bAM7v7Fu+WOl9+hlwAAefjmjU/JV376u3ppsb3sp14o/
+a3mNAEAHJTuFHk+L/WC0jLHA/nDwYcXkjrjWE1w5B0mMHN0daVAzdTKp+Ukn5qK56QU/ppJDP
kHgioh2ouTjVJmK6nGTZ8Prc2REmpfSMlvFZJjjNR+0a3wwldR5fWOHUi6XdVVU4HNlaSk2473bYn
up4p3O9zpSwnAwcRIteJ+bAMrrriKmbsB4AZuP9V37HD1EWm6p/+
+n/5GeqgAkBurhQ5/mvWR+qgpm2+a6IWN2UvbMitPdQ3hXqoHtWLri8pZ7JH3OhefG5oM1EPZ
MWu5eiGPlE1KzNsQ5R5b19sebQvRuA3VlFOLEzSmcbxAvPpphfcZAepKlAFAOTn8yuPylNXX9JLi0f
1QFU9UQEAOTn+r0QKN+gFpGmqENUc9hv1cMLJoj8M2gxA7aBUrw9NMc2v04+4dRUtdp1B82tD
H2kGskvEuHZ+7Udf89zw5DXjJwlry4bZq9OtjWnWijRl0oYo2bcvLbGOc4btG21wgreeHHp1B4ZlOv
HcEcD5QZpuv+l2e2g/ACA/KkBVQeoiel3l/6IOKgDk6dhrrQd1ULMy3z1RkZGQ4dEus05mDGp2d9V
b0Q+rjZ7D7raTTBLW3vV6dtrhjxuYG8OTB6XehihZtW+khNdqguOM176Idth1itOdfT7Y9JDh+ImO
M737fWHkNjEflpGaZOoDb/iAXgIA5EEN6f+7Vz6hlxaDmoWfOqgAkCM1C7/qhYrMEKIupaKs+bN2
yao3Ztuc0XsMPcGXKlEb7K0YEnhNOEmYH/4YM+KbcmhDlFTbN1LCa5XkOBO1L6Id9rE7NVUTnsK
Rmqsb4jfdL5lQPrPmhNaJrmcK9/uiyeB+B0wf/vkPy+nbTuslAEAeVH3Ux65/Wi/NN1X/tPp7r5XnXX
OlXgMAyJaqg/oe6+PVziIyQYi6pIqbF/zehs1VHawYk86MU9n2h393alJygxmjRqg/UVew9mbkJGGV
09aSNq5dWbUhSmbtGy3ZtUpyrpO1b2w7ynXZSmV8eFnK5aas6vZ4Tbfaftab/j/Z9Zz6fs+LOdGV28

6Jws6U7ncgwj2/fI89vB8AkI9Lx/vy+Tc8Kj9+zmW9Zn79Uu02ufXVL9JLAIDMHVsTKdysF5AVQtSlVZ
TNvcFJu6rS6res/8djD/8OK6Rpb2dgoi679ubg/ixDk4RVZHuoDc72vF01d71eitm0IUqG7Rsp4bVKcJ
zJ2qfa0Zfu0IbVpo3SBlM7JWcHjzfsGiW6ntPf7/mw7q/B67F/aPQSTiCV+x0YzZ2x/7qrrtNrAABZ+8H
zn5W/ev1jemk+veatN8nb3nOrXgIAZO7Yq0SOv0kvIEuFviqCCAAAMIF7H75XKn9UkaefXYwhpgBw
FNzx5evllQ9eq5fmx7UvvUr+06ffzDB+AMhL4RqRK9R8BQzjzwM9UQEAwMTefMub5aNv/6heAgD
k4W9u/7Z875pn9NL8qP4udVABIFfH3239jwA1L4SoAABgKu/92fdK7Y6aXgIAZE3VR/3cm75pf5wX
v/Abr5BXvO4legkAkLljbxcppFNUD/EQogIAgKmp3qiqVyoAIB+qJ+re676ll2ZLhae/8Osn9BIAIHPHiiL
H36EXkBdCVAAAkAo10dQt192ilwAAWeve+n37MUuqDqoaxg8AyImqg3rsPXoBeSJEBQAAqVAz9b
d/tc2M/QCQI9UbdZb1Ue/6T6+2g1QAQE6O3SlSeKFeQJ4IUQEAQGpuu/42ueeX79FLAICsqbqon3
/DozOpj/q299wqp/7ZDXoJAJC5428SOXabXkDeCFEBAECq3lF8BzP2A0COvv2if7Jn7M/Tra9+kfxSjV
/kASA3hVtEjv0LvYBZIEQFcteWjUJBCsZjpdHTn1tSvYasbLT1gqO94Z6fFZnX09NrrDhtXGnIqCb6x+E8
lv5aY2mo2frfdepdegkAkLW/v+178o8v/6FeytbzrrlSqr/3Wrnyucf1GgBAtq4WOf5u6+MVziJmghAV
yFVPGiur0tRLjrKcWSvq58vHDiJLNeno5YXRa8h6LarV6loXZDV4saVTK0lhIDAGjqq7f/FuuePld+glAE
DW/ur1j8kPnv+sXsrOXb9zu1z/E8/TSwCAzB1XdVBfrBcwK4SoQK66cuDmbuW6dPt96ff3ZHNZM9
SxQeScUj1nxwW/7fPiHlq1pa5zX7r1srOiuSrkqFgGV11xFTP2A0COfvycy5nXR33zr9wir3nrTXoJAJC
5YyvW41V6AbNEiArkqXco+/qpnDohy9v/dHHF6znbk8Y53QW1XJetivO0uHlWqs5Tae6SomI53PSC
m+wgVQWqAIDsPXb903L/q76jl9L1sp96ofzKfziplwAAmSvcKHL8tF7ArBGiLjGvnqP5CKntOLI2peqN
536d163Or/dp135sb+ivNdbZLwuuD/bKS2MbmtlG7zFcYzN4jMM1S+1HWN1Low3jal3a+zDDt+aq
87WDDQ9pc9i2o9o8tpfjROdl+J4ZuZ8YxxD7fGh+W9RjuK0OZwi9/7p0z506/lKsnrNGj+NAWF6Sk7
ozquwfDt9PNqM9ie65eMduS+H7wj1Pcd9HsNxuv+l2e2g/ACAff/fKJ+SbNz6ll9Kh6p/+
+n/5GeqgAkBurhQ5/mvWR+qgzgtC1CWlwpHQMKhTk1JhQ8ZlcHHYtR8HCkLa61TIMrC+uRoeXk2
1DRU2hfYY7EitNCqEU58zapaW61J3uw52duTiYMi0674ynbqmQ8GiZh/zyGBquM1uz8dQE5+X4X
umuToieEt8DFGcfQcvd0hb7WCw5A2hd6V67jxVafVbXq/SIWaP41E6B9LVT4MqcjrpPZfk2FP6vlDn
KY/3ERwdapKpD7zhA3oJAJC1z688Kk9dfUkvTe/f/Nar7J6oAICcHP9XIoUb9ALmASHqUmqLn8O4d
TmNeo3SlHPhSUpi5XrX3na/FYybwtaPGt482TaCw6mHj7EjO4PplEcFZM7r+3ubsrlVF+erBr/GPI9nZ
FyGWtm2ttd1t2Wptpx9bOvUrr3hB4Vhbe7UZH3kdQm2eXRT0jkv/invSO28cd0SHMPY8xHg7zu8r
dZxrbvBoHEu3Iamcu40u53bEitrHVKUE6f00wiVRPdckmNP7/uimOP7CI6OD//8h+X0bQxHAoA8q
ABVBalpWDn9k/YDAJCTY69zHpgrhKjLyOwlZ/R0K27uOQGJ9dhLZaajqpx1t1M5bS25RqwPHd486T
aKsrlnBj6O4toZL7TrHIT3AyzXt4IBWXFNzugv6uxc9NvY3vV65pXPrI0P38YwexjWLxht3rwgfgZ5PrR3
31CbR5r8vFRbfnDoh3xB0xxDFHPfoW3tXZQd3SEycC4qW/5+zWtniH/u1HFY3yOhIe8k9uUwrEFKk
nsu0bGn+H2R2/sIjpp7fvkee3g/ACB7akj/V376u3ppMqr3qeqFCgDIiV0H9V/rBcwTQtRlZAQ0ImqY
bsGrZTiyhuIkyieIpJ8GjFofJo1t2HQ9x9BhzEGnTgwGP0VZ8xMtLyzyA0Mj0J1YVK9WY/8jgrfhNscV/7
yMN90xjFaWk+MudvfAa79X7sF+GEPcRwyfn/zcTeuUjN51gntuimNPcv2HzlNe7yM4ctwZ+6+76jq
9BgCQJTXJ1KT1UVX907t+59XUQQWA3Kg6qHdaH6mDOo8IUZdSUTYvhPckdEOYqMl1FkZg8hyjn
mOk8MDO76nnDnc2AsPq6dg9GafXkeGOgjFCRlOK52UyYccwK2keVxw9ORxbLNWR2T2X2vVfkvc
RZOKW625hxn4AyNFfvf4x+fFzLuul+H6pdpvc+uoX6SUAQOaOrYkUbtYLmDeEqMuquCl7esjtQKlR
W/Pcos+u3ZYNs3edW7PRrMGZhHW+zurzZA+NNoYyV0/nF6FOH/qlfF4mkm1w6dXKHXpMWsd0
AsUTMrb06bje1BPcc+OPPf3vi6P9PoIsvfmWN8tH3/5RvQQAyNIPnv+sHaQm8Zq33iRve8+tegkAkL
ljrxI5/ia9gHlEiLrE1MzaqqeYPcGPHYR0vRqKo4f/GoxhxHPHrB2pgiW3/uMUba64U6Z3duT8+R29n
aqkk6GW5KR37gdnZO/JRbfoZeQQ8BgyOC+
+nI4hTOmkFwKOqumZL+NcNHfF75DZlQPvNJxwzn2EWPdckmPP4PpP/T6Cpfben32v1O6o6SUAQ
Jb+8eU/lL+/7Xt6Kdr1P/E8uet3qF8NALkpvFgP48c8I0RdRu0Ne6itmkU92FPMCHiMXnIlPxkzZmJvy
4Y3Dft884MlY2bySXgTWHWk2dQnKrWh/EXZdLsdqvO87l+XXmPdq22ZZBKkcVI7L578j8Fj1udsrvr
DyPW9bj9yHVtu1oBtyq7eda9xzgsxY/VgjnPPTXjsU1//hO8jZntyvRSYe6o3quqVCgDI3t/c/m157Pqn

9VI4Vf+0+nuvleddc6VeAwDIlqqD+h7r49XOIuYWIeoyqmz7Q287NSm5QYtRH7F6NnzmbhXSDL52
Lpkz9nttNibamUhF3I6BrlSH8o+4LiU/fZQL005glcl5MSQ9BnPYu9ueiRK2YH3O5qqz34IX9FeIldqs+v
EUNy94PTLd9njnodqSeM2Jc88lOPY0r3/C9xEgiqqPquqkAgCydel4Xz7/hkcj66NW/u8idVABIE/H3k
od1AVBiLqk7KG3YUUMVeDS7wcDHlX3cLBmYrWVcx3NpCqy3W/5gZHNOTbvsAPDrOPxhlcr5bpsp
ZzL2dcl5LxWW9Z6d+j1VLI5L6Zkx2C1Z/A+3D80ejUmYNfnNIaSu+y6nznWQ/UUZXNvuFaoPYw+Q
aAb656LfezpXv9E7yNABDVTf/tX28zYDwA5UPVR7/uZx/VS0Cte9xL5hV8/oZcAAJk7Zv2GfPwdegHz
rtBXRewAxKNmNtcT86gwbG/anqHAOEfpntPHcqpFwIpwn+l9Rip/xM0BAHl441/fIKWHXqiXRK596
VXyW3/8z+yPAIAcFK4ROf5+66P/Xoz5Rk9UILaeNNbdmc3LcmaNABVZO1r3XPu8dSwZ9ODG0fGO
4juYsR8AcrL3um/J9655Ri+JVH/3tQSoAJCn4+8mQF0w9EQFxmrLxmANWFXOgK50yMzRu+d6jRU
p7ZyRbiplKXDUFf6fgn4GAMjStU9eKe9s/aS88723MYwfAPJ07O0M419AhKjAWAOBFgEqMsc9h+
WlJiiblx9NaEs42hKOtoSjLeHmqS0nrr1GDr/3pF6aLa5RONoSjraEoy3haAvSQIgKAADmBj/ghqMt4
WhLONoSjraEoy3haEs42hKOtoSjLeHmqS1IhpqoAAAAAAAAABCBEBUAAAAAAAAAIhCiAgAAAAA
AAEAEQlTkSE2WU7DrfxRWGtLTa8fqNWRlo60XHO0NvZ3CijRibwjp6Uljxb0GG9aVTbJuGmH3UNr
7mJbRRv1Y4SYFAAAAAGChEaJirvUaK1Io1aSjl4H5pgJdY1Z9W1nOrBX1cwAAAAAAsIgIUTG/eg1Zrx
GfzqeibO717RkF+/1tqei1szFPbenKgXvLluvStdu0J5tkqAAAAAAALDRCVABIS+9Q9vVTOXVCyE4BA
AAAADgaCFGXmao1OlC7MazG6GD9UXuIvfE1A+VKNbNOpXokq1Vp79Mcxt9cdbYTvjOjjeoxqk7qY
JvCa1VOdrymMbVf2xvetob2H3JN4rTRY359ZEONcxFo46j1xj7t9ea5zKoOadx7KKotg9uYsD5pjOuS
9J4FAAAAAACLgxB1WakgL7TWaEdqpVEhpPpcQUoDQ+ybq4OvVyFiSYIva8pqYbBWZBqcNq0GNh
xyDHYlNtgm65W1UnjQaYt7vIMqcrqqn3Z25OLAa9u7bmODtTKHQjgtuo2TKsrambLztHMgXeeZxR
iOHmh7W9xml8+s5dDDMoV7aKJrPizf6wIAAAAAAOYRIepS6knjnJuIuXUb+9Kt61BNOrIzmPx5qtL
Sr2+5QaEKG8/7ve16jXN+0FVt6VqVXfE2H0Nl2/qabl28L3G3sx1W8dJvU/gxWMe77oZg/mv77gF0
arI+MhUdf7xhKltu2wfPpR9GSvmMeBlqe8MPgsOuSWQbJ1NcO6PbuC+H7qbbu0ZI2ZEDN101hq
mfOpF9hDr9PTTNNTckuC7J7lkAAAAAALBICFGXkjERz96m16vQD9VEOl56FlRt+RP3+EGhqScXd9w
+e1VpeQGStc8LYa+fntmm0GPoXRS3SeX6lj/xUGXLC+U6OxdDexSOP94RimvidfQ0t22ElGaPTrN3av
2CcU02L/htrJ0fGKo+Ja+NftDbO3Si0mrVCRv3dbrau7jjBZKnvROYlRTuoSmuuWkm1wUAAAAAAM
wdQlRYdA3P0OH9SZmzk5+Ukn5qK56QU/ppespyMrCTEN0D77jsIdheXUtjqHdgSHsazOHy/rB4P5
SryllvyvYRvVNtxnbMHqOpKMoJfUHcwLlrXzzrnJ4+aYeVwfWW6mk/kMxMCvdQKtd8VtcFAAAAAA
DMG0LUZRWYKCdunckYgeWRMt3x+r1i3Z6eRig3URhpDK9PScUt3trctVrXE6cj6ik5UdFhZWC9anb
2EeriSf+6AAAAAACA+UKIupTasmH2OnVrPZr1HI+ocr3rlDEYevjD9lNT3JSzbglONXTcqCs6WRiZQYh
dOS164L4ctvUQeLv3pzs5lrF+QUP07K/5sv1xAQAAAACA5UOIuozMupwqYHLrohpDoCdXkpNuEjs
4XNoIEXNVcoamK6NqvWbF6+nZ2ZHz50fVFTXP2eBs/mZ90FMydk6nxNfQ3XdHDnadr3VrtZbsT/jr
h4e0ZyWFeyiVa57idQEAAAAAAAuNEHXJ+QGTMWP/VMxakU1Z3XCn3DFnS8+ZMcmTNFfFa1J7w
6+V6a1MmdfTsyPNpj76oaH8Rdl0u6xar6utN6yz5eg11r0anuYESU7AqVivP+8dkGx4U8nH5V+vZtP
5Wnf2/aIumOquNyfCylYK91Aq1zz5dQEAAAAAAEcTIeoy8oI9S3NVh0rGhDtTMmcun2r75iRC7nY
mCjuDs7o3V3WI5gWO5gzwaXOHxftCh/JXtqXl5XU1KdnnrCAlP6mTC95EVCojdOutWrxzHLe2bVBg
W9Yzb2i6eZ9Y68/k0w3VNv09lNI1T3hdAAAAAADA0USIupQqst1vGQGZUpVWv+8HRvaEQpMqyu
Ze1w/BbGr7g/scx2qn1yBt/9DrDZhIcVP2+oNtstj1YDOoh2rwhvQr1v62Ruysst0PrUtbbVnr3ZILLnU
8g6+ttiara2v22gwMTTeGs+c+ZD2Feyila57ougAAAAAAgCOp0FczrADITq8hK3oiL1WDdo+eiwAwk
urtPS8/mtCWcLQlHG0JR1vC0ZZwtCUcbQlHW8LRlnC0BWmgJyqQKbOOZ75D4gEAAAAAAJAOQlQ
gE23ZKAzU8ayeFTqhAgAAAAAALB5CVCAPql5pZpNXAQAAAAAAIEvURAUAHODelXhaEs42hKOto
SjLeFoSzjaEo62hKMt4WhLuHlqC5KhJyoAAAAAAAAARCBEBQAAAAAAAAAIAIDOcHAAAAAAAAgAj0R
EV2eg1Z2WjrhVnrSWNFzZavHhsyL60CAAAAAADA/CNERSZ6jRUplGrS0csAAAAAAADAoiJERfp6D
VmvEZ8CAAAAAADgaCBEBQAAAAAAAIAIhKhLqr3h1gddkUZPD7+3l51HaClTVePUeI3zcL7eZW/XH
MbfXHVepzc4uF+PuW1j58HXt2XDfY318F4Wo12TGjwv6jHpuVGSHr8S1obCSkPCD8+s/eo8VkaciG

TbBQAAAAAAWF6EqEuvI7VSQUoDw+
+bqwMhX3tjRI1T9fXpBJbR1H5WpamXpFyXrYr1MbN2OWHk4HlRmquFYNCZ4blRoWtYG6RTk9Lg
BFl2EFuSwZd3aqWhcDTRdgEAAAAAAJYcISosVWn1+9K3Hq2qXqUCwPNulNaTxjkdX5br0tWv7dbL
zjrrtTsXnYiusm19rlsX9zNSbdmv7W+rxHNafjv7e5tSTNCupHqNdS+MLNe7zj77LasFWvOcDkeza4NI
W3bd1Dh020055yW0VjvW3SDXOE/uBe3UZN17bZLtAgAAAAAAgBAVUm1tixtxVraMANRTlM09H
crZ4aVeu3bGe23noKufZadc3/La6ciqXT25uOP20qzK2U13yxXZsoNGFVLuibM6w3PTO5R9/VQ6O+
JmscXNPWd/1mPPbVvvorhNDpynypa42Whn56J1ZJYk2wUAAAAAAAAhKiaha5OGDmHPzqkT44K
9tNrVlQN3A+WTUtJPFSdo9EPnYSmem+KanHGTWGtrquyCW7t0qM5p98Dbnz18X7+uYA7v7xxYR
2ZJsl0AAAAAAAAQoqIsJ82UcJTAxElGbdLcjGjnzNtlyawNRdm8ENYz2A9KQye5Giur7QIAAAAAABx
NhKiIoS0bZs9Kt46mWft0JuahXRm3obgpe3qIvV+v1tc8Nzybvl/DdfBh9KCdYLsAgLzp0Q3Ggz9yAQ
AAALNBiIrx2rte70o7oHNrfxpDyKc2ybYya1dJTroJqDsE3qVm4i8YM+6n1YaI16uZ9NUvzfakXXbw2f
XqnHrtK530Qtu4NVhjbRcAlpH9Xl+QwkwTy540VlZFWu57tPNHr+bqLILU+Qxze40Vpz0r+f/hT/0ba
p4P9zEP5wXDhq7XDO6ZmRvzvuZ9P7mPvG7mOO+3ebwnR+5jSf+gFfO8z/K9eGZinJuh951luGnG
nJfc34sTtofSdoiDEBWJ+AGdMSt9AiU/nTRm/7d+MFmdbhD8tO0KKsqaVzS0Kavem667baeO6OB7
cZw2JDp+/aavPhXsGRpSs9Wsc9pc9dvm/sOhHu7KJNtVjG3wDwuALMzsl/chKri09j/lv0npcCYu3Da
KcLuTP+4f5vte3N4IC3ONPyjOQq8h6zWRatX9x28G3NEnxsO8XrMy01/cAyWOhh+z+NZW52O1qS
YFda9TS6qdmpRmFPjk/0v7+Pc19R5csr6f6l3jHFk/T2Z778R5v83jPXncPtTnc/yDVmTok1eYm+C85/l
eHHFuht739CP98xP/vl3dH/g3Iot/IEadk9zfi8efl3zfiydoj/WNrUrbpf+eHO/7dn5+FsZY1g2DJWS9R/
TV5bd+A+hbPzD5uvW+9U+g87mq9ZZia/WtnxX060c8vNcqIa93P29uf9TD2NbIdtqStKvbr5fd9VXr
K8cxXx/yKNetVygJz83Exx/
+MDcdve3gMSfabqvqrS8PXwQAmEq3XrbeX8z3eP2+GngjyoffFv1vwAzaEEm/z8/8vXjm7XCuj9q/f
c28f5PzY/87OoP9RtP37dy1y/3eivPzV9rCv5dn1R7n5y9jv/pnrCy/l8a/rznvuYNtyPocjW9XvNdMa6J
9ZPIeqPdvbdd+hLRD3T/maud+Um3XK1IS/5w4n8/+vTjeucnjvS/
+fZv1+8v4cxImq7aNPy/h62fWnhHfw+nfR87+zd0737fBdX579Qr9vhz3uiJf9ERFDBXZVn8p0ksO56
821puAo7kr/t9KrNd7n9D2D52/MKlanIP1QqutCWuIJm1XEk4PIOsNTS/7rDdbf9h+0jYkPH57qP3g
ubQ5+wj8QdOuc2oMyXfZPWWMeqiWRNsFgMy05XytY71NXZBN503VUpEt9UY28fv35Iqbe9Lv7x
ltmTO69MupE/PRwFm1o9dYl1qnKmfn9kLNhnteWt7PKPPC+T6X6unAzyL5KMqJU9YH9+dQW08u
7sygPb2GqIFK5fqWv9/Ktv3zYmfnYgY9sRxj39d6h7JvfRj8fi7aJ64puxm9Ecd5v83jPXle3ved79+y1Ls
hP8tr6uf34dEJHdm5mO7dE/ec5PVeHOfc5GX8uXHeXwLf5xmY7Jxk9148/rzk+148tj0jfp6yR42mW
tYuzqii+fpZGOMRoi4pvw7mwJuLMeFQcMiBCgvdr1EPJ5TztxMM6dQPhf5rrYf5A725D/VQ+xmx35
Ht9MRtl/MGFlw3nvMG7H6d89gbakjCc5Pg+G2D59J+jDoG8zj1Y9QvU3G3a7xu+NgBYAoz+uV9M
enSL+W6bIX/A5CTnjTWa9KZVTvsoaMdqbbi/1ueGTUM0Rh6N9uSN/n84j6JXuOc9d1s/bI/oxu3sq2
HjNo17dWwypITNg/
+vJW13H5pT8huV1lOenWcNKPePgZk8AetuMFlmDTbEVuO78XTnJv86fJoB+cHhtOnWwJnknPCe7
FhxPub8/PnvuRaNYmfhRcOISoAAJgNfnmPTdUltX/5uTCLXoa6tpj9i6D+pWdGvR3b52vSqbZmPmL
C/0Op81AjV7KppRZXPr+4J+f2NDo7wwDEHTWkatqr7yOrObMI4efpl3ZMgT9oKfPyXhwwR3/Y6uy
flAvevxGqt6h6/9mYYa9C3osD9HwiwflBrO+pqedWiWHwjzD8LLxwCFEBAADmmDP5gRq2N6ueO
MFRDt36vqzO4hfU9oYzCcRc/dbuUL2Csh6WHcfc/eLePi/O6NHZXTNnsg5/YiAVeKtJgXKftGOWv7Q
jNfxByzKH78Xz9oet8pk147pY/4aeVWXUZtirkPfiAernGrdnrP89dXBKXadTkl3n7nn5IwymQYgKAA
Awp5wAVfXWmJ+hjMXNC3YNtk7tfK7hXHtXhU1NO8B1fuEpSEn9Vqh/CZrtcHptlsOyLfP1i7sOCGf5
y6Iecqzq2bt5jz0MViXezdWMZjYfZVa/tI9h93bqyMHgjTuqd9QS4w9ajkV4L56XP2x5ZtqrkPficIMl+f
qydXLfOk8nJau3vdn+EQZpIUQFAACzwS/vkVSvDSdAndMJ/zL8RSPMYE8j9bAngLR+Mexaz2dbt7sn
h6qoWc7nZKxZ/uLu9nw6O8NfFgeHTbpmdl7y/6V9rOIJceZ7CUZNPfuGnmG4O2f4g5Zvvt+LB+T+h
62S2GWOB8PbWf5cw3txTLq2eOCPkekZ+UcYfhZeOISoAABgNvjlPYKeRddiD3czevzkW+dSTf4wM

Bxc/0KW1S8ac6/XkJUVc0i2WqVmSp7lL6nz94u701utKjMcPSpSOW21wPoeCgyhV7dwzTovM26bL
dtf2uOpyGnVY6+27r+veL3G5m+islngD1qLaFZ/2NIjADo1Oe/9w6mHcM+oHinvxXGoUhklqUldLm
RwkSL/CMPPwounDwAAMCOtqvSt33L69a5e0a33y9aPJ2VvRX669bLVFtWewUe139KvWUZh56U
6JyfEblu53s/3bun26+Xg+ZiLe6RVHbg2rb71S2tfJT+5m+H38TB9HgKPefie1vdRxvdv3Pc1573Yf2R9
7eK0K4/35PH7CLt/3Ifxb1dq9H0x9H2r2jFw3Pp7Pu1rNcl5t78m8/fiEedGvd8M7Ns9hrTf/uKem6H
XZfY+POp+0XJ6L453XvJ7Lx7fnpB/xzO6RnHuxXn6WRjjFdT/rIsKAAAwE+5f6F3WD43zNRwQWBCq
x5xdm9BVbUl/Bl3nnO/pqrT6M5p5eS7pnk7G5ZnV9cH8Gfre9fjfR2Gvqc5r79gUjT83Id9bxnk7iuLc
LwrvxbOmRvOoOqhhgkP7+Vl4cRCiAgAAAAAAAEAEaqICAAAAAAAAQARCVAAAAAAAAACIQIgKA
AAAAAAAABEIUQEAAAAAAAAgAiEqAAAAAAAAAEQgRAUAAAAAAACACISoAAAAAAAAABCBEBU
AAAAAAAAAIhCiAgAAAAAAAEAEQlQAAAAAAAAAiECICgAAAAAAAAARCFEBAAAAAAAAIAIhKgAA
AAAAAABEIEQFAAAAAAAAgAiEqAAAAAAAAAAQgRAVAAAAAAAAACIQogIAAAAAAABABEJUAAA
AAAAAAIhAiAoAAAAAAAAAEQhRAQAAAAAAACACISoAAAAAAAAARCBEBQAAAAAAAIAIhKgAAA
AAAAAAEIEQFQAAAAAAAAiEKICAAAAAAAAQARCVAAAAAAAAACIQIgKAAAAAAAAABEIUQEAA
AAAAAAgAiEqAAAAAAAAAEQgRAUAAAAAAACACISoAAAAAAAAABCBEBUAAADAQmtvFKRQ0I+N
tl5raG94n19p9PTK2fDbuiEhLV0wbdlwz3vk+e1JY2Xca6xXNVb8ba00rK8CAGB+EKICAAAAODqa52
TGOemSUMHoqjT1kqMsZ9aK+rmpKJsX6tZnHZ3aesg1asv5Wkc/F6me3bS+CgCA+UGICgAAAOAI6
Ujt/OL38Zx/XTlwM89yXbr9vvT7e7I5KvksbsrZqn4eco16jXN+IFttyXZFPwcAYE4QogIAAAA4WuiNm
r3eoezrp3LqRKxeo5Xtlng5auAamb1Qq9IiQQUAzCFCVAAAAAABHTLzeqH590pVg6NpryIpbm9Orse
rX/7Rrehp1Vr119suC68NKtPqCtUJH1wEdeJ31CKsrGjyeYL3S6HYYzGPXj8F92fsp1ayzrDVXndeO3Ul
FtltujOpfo/aGXxagXN+yXjUo3vHbQto/dH0tcc5VoEar+6BWKwAsLUJUAAAAAEdEWep1HdJl2Bu1U
ytJYTVYDdRep0K2gfXN1VEBZlNWCyUxyoCqjUhpcMIpOxQceJ3F3t/IQK8jtZJRr7Rcl60YnTuHwlEtel
8JVbak7hZHba7KRqMh5/wEVS4M1gNIcvwqwA5pv3M+hoNUR/i5UueiNLhTJewaAQCWAiEqAAA
AgKNjzQ3p4vVGnVS53pW+qgPq9ax0hK1v7o5oh1dLtC/
+y5tyzkv7etJYd0PBqrT0a71td2qyPjIpNl6/F2OSpvaGePmv0a6um3ga+6psW5/r+hNFqRqm9n5iDc
MPTjLVrLnHV5b6hcF2Jjl+67VuGhvWfmsrOxfjnqu27Eadi8A1AgAsC0JUAAAAAEdIUTbdGYwy641al
bNuj8nKaWvJNWL9/mFoL05zBnqzXmhn56Lz+t5F2dGdIQPD3I3enN5rB4QPix+t7aeGgTCzuHnB31
ftfDo9MAOTTGnVs8OTUiU6fuu675lBqKO4dsYLbDsHXf0saOhcmfVeOzviZq/FzT1n+9Zjb+QMWgC
Ao4oQFQAAAMDR4oVsGfVGLZ+Ukn4aMGp9qKqcDiR3JTnpp31ix33dA90L01rllguwH8bwdve1A0
6dSBLymT0vz8ha4EuLsnbGbdi+HIYlthMITDJlPQudTGqK41fHZNc5DR3eHzR0ropr4h2yuodK7n4ja
rECAI48QlQAAAAAR8xAb9RD5+l8K8qJU/rp1MpyMn6am0BHRnTmnE6i8DlCYFIpo85ppLBzFSw5Y
HLD3NgTdQEAjgxCVAAAAAABHj9kbtRYvTsvXYK/Onhx6Y8iHebVWhx7biYbtTyercHa88cfflg2z16lby9
Ss3ZpEcVP29D4Gyt7amueYpR8Alg0hKgAAAIAjyOiNmpQxjDw7g706u3Lg7tTtmVk66QWAo+p5ps
MsJeDXAHX05KJbmFROSaIqAdNKcvztXa/nqR24unVRp7iWaoZ+1ePUnkjLDlS7Xi3W0WUEAABHF
SEqAAAAgKPJmIAoTMlPDo3aqW3Z8Kapz1ZzdcObqKm94Q8/L59ZcwJAszZnc9UfQt7e8Gp0FlIZV2
4Gzta5WPd7WfYa61790aSTVU1twuP3A1djxv4k9PbVbRDscRoSdAMAlgYhKgAAAIAjKro3qjlzuwrp
nGAubi3NaZWlXG7Kqg4D/dzWmOFftd+ozdlc1cGh9+IREzJNorLtD1vv1KSk21XyE1S5kPuM9AmOv
3Lan6jKu5bGBFRJjDgX5r1RPat7ugIAlgYhKgAAAICjK6o3qqp7OVgzs9qavI5mIqfk7J4xPFyx63gO1Di
1a3MOvE4Je+2U7GHrIcdebVnr3eHxeYt9/BXZ7psz/itVaZk1TZu7Xs/fcexzEVYMVW8zrewaALA4Cn
1V3AUAAAAAAAAAEIqeqAAAAAAAAAAQgRAVAAAAAAAAACIQogIAAAAAAABABEJUAAAAAAAA
AIhAiAoAAAAAAAAAEQhRAQAAAAAAACACISoAAAAAAAAARCBEBQAAAAAAAIAIhKgAAAAAAAA
AEIEQFQAAAAAAABGEvn/AZM6nr53azj8AAAAAElFTkSuQmCC]]></redcap:Attachment>
</ItemDef>

<ItemDef OID="q10" Name="q10" DataType="text" Length="1" redcap:Variable="q10" redcap:FieldType="radio" redcap:RequiredField="y" redcap:BranchingLogic="[q7] = '1'">
<Question><TranslatedText>Do you prefer Life A or Life B, or do you think Life A and Life B are the same as one another?
</TranslatedText><redcap:FormattedTranslatedText>Do you prefer Life A or Life B, or do you think Life A and Life B are the same as one another?
 </redcap:FormattedTranslatedText></Question>
<CodeListRef CodeListOID="q10.choices"/>
</ItemDef>
<ItemDef OID="summary11" Name="summary11" DataType="text" Length="999" redcap:Variable="summary11" redcap:FieldType="descriptive" redcap:SectionHeader=" " redcap:BranchingLogic="[q9] = '2'">
<Question><TranslatedText>
Alzheimer's disease - Description #[no]

If you have Alzheimer's disease...

1) You might forget recent events of importance to you. For example, if you are interested in hockey, then you might forget whether your favorite team won their last game, although you may remember watching the game itself.

2) You might forget the names of people you met recently, or the names of local streets.

3) You may no longer be able to handle your personal finances or plan leisure activities such as dinner parties or vacations. Simple decisions such as what to order in restaurants might become difficult to make. Your ability to concentrate may decrease and you might have difficulty driving.

4) You may start having trouble finding words to express your thoughts. You might need to be reminded to shower or brush your teeth, but you could do these activities on your own. You may need to post notes around the home to remind you of simple things like turning off the stove.

5) You may remain aware of the day and your surroundings. You might be able to travel to familiar locations like the supermarket, although probably with the help of a loved one or friend. If you are in a social gathering, then you might have difficulty following the conversation.

6) You may continue to enjoy hobbies such as puzzles or reading.

Now consider two possible lives. In Life A, you will live in perfect health for 19.5 years and then you will die of natural causes. In Life B, you will live in perfect health for the next 10 years, then you will live with Alzheimer's disease as described in the summary for another 10 years, and then you will die of natural causes.

</TranslatedText><redcap:FormattedTranslatedText>
Alzheimer's disease - Description #[no]

<p></p>

If you have Alzheimer's disease...
<p></p>
1) You might forget recent events of importance to you. For example, if you are interested in hockey, then you might forget whether your favorite team won their last game, although you may remember watching the game itself.
<p></p>
2) You might forget the names of people you met recently, or the names of local streets.
<p></p>
3) You may no longer be able to handle your personal finances or plan leisure activities such as dinner parties or vacations. Simple decisions such as what to order in restaurants might become difficult to make. Your ability to concentrate may decrease and you might have difficulty driving.
<p></p>
4) You may start having trouble finding words to express your thoughts. You might need to be reminded to shower or brush your teeth, but you could do these activities on your own. You may need to post notes around the home to remind you of simple things like turning off the stove.
<p></p>
5) You may remain aware of the day and your surroundings. You might be able to travel to familiar locations like the supermarket, although probably with the help of a loved one or friend. If you are in a social gathering, then you might have difficulty following the conversation.
<p></p>
6) You may continue to enjoy hobbies such as puzzles or reading.
<p></p>
<p></p>
<p></p>
<p></p>
Now consider two possible lives. In Life A, you will live in perfect health for 19.5 years and then you will die of natural causes. In Life B, you will live in perfect health for the next 10 years, then you will live with Alzheimer's disease as described in the summary for another 10 years, and then you will die of natural causes.
<p></p>
</redcap:FormattedTranslatedText></Question>

</ItemDef>
<ItemDef OID="a11" Name="a11" DataType="text" Length="999" redcap:Variable="a11" redcap:FieldType="descriptive" redcap:BranchingLogic="[q9] = '2'" redcap:InlineImage="1">
<Question><TranslatedText></TranslatedText></Question>
<redcap:Attachment DocName="195.png" MimeType="image/png"><![CDATA[iVBORw0KGgoAAAANSUhEUgAABVEAAADYCAIAAABtBTQzAAAAAXNSR0IArs4c6QAAAARnQU1BAACxjwv8YQUAAAAJcEhZcwAAEnQAABJ0Ad5mH3gAACs+SURBVHhe7d1fyF3Vue/xxKg7HpWE1h3SetDoQQgqklYOxPcUG0GMCIKg0iBb8XDkJYJlB8FGxBzOhaJsCnn3RtCLYoXTc+JFRDanRLG
FXLSQHgrNhRduSOW9sDRQL94tpbzSXHTPf2PMMZ7xZ8411pxzrTXn98OP3XfNf98OP3XfNNeZ4xphrxTWf6H
r3jr8BAAAAAIAxoucHAAzk/Ob5Hf9rByGEEEKmls8vXxvz/P/P/9/1Q0BhkXPDwAYwubW5t4394o7AEIII
YSMPte+ctXJI98/fvedf/z899VtAQZEzw8A6N3W9tbBtw6KOwBCCCGETCEEPPrD/f9zxX7K8evShv37evShv37
9dXVzgKHQ8wMAevfwzx4WH/
+EEEIImULuOranbPjLvHvyR9XNAYZCzw8A6NeJj0+Ij39CCCGETCH7Xtj97MHbzZ4/y68/OFvdAQ9
PwAgB6d+fSM+PgnhBBCyBSy69TOJ+69RTT8Wfhi/8Do+QEAfbnwxYXdr+0WdwCEEEIImULuP7pPzP
dPs6fLF/SPT8AIBebG5t7v/xfvHxTwghhJAp5I5nbhR9vghf7f7Fb8MPT8AoHvbV7YPvXNlfPwTQgghZAr
Z8+I17tf43Zz/v/
+num9An+j5AQDdO3b2mPj4J4QQQsgUEvoav5vjd9/5+cWL1a0DekPPDwDo2Bu/ekN8/BNCCCFkI
vlvj/y96O0jOXnk+3/56qvBgL9oOcHAHTpw88+FJ/9hBBBCJlIbn3uetHn
y9f5Bf1E0IIIdPMDS9d/fRdt4mWvk0+
+em71Z0EekDPDwDoxtb21oGNA+LjnxBCCCFTyK5TOx+972bR2j/vX7LcMX+zvFT0/AKAD21e2j7j7x3RHz8
E0IIIWQiOfzoaTnyl8sb8/9PwAgA4c//lx8dlPCCGEkHnkoP6UjwW08UfhkTPUvXNKd
+em71Z0EekDPDwDoxtb21oGNA+LjnxBCCCFTyK5TOx+972bRzLcMX+zvFT0/AKAD21e2j7x3RHz8
E0IIIWQiOfzoaTaKTnyl8sb8/9PwAgA4c//lx8dlPCCGEkInkP6//J9HDJ4Qv9veEnh8AMK/TF06Lz35CC
CGETCTXndz1xnV11D0HRAOfFr7Y3wd6/oW4e6wGtLG2o2o6e6ak6psWVzwMoCV3g2M+ee/e6axOr6rsF
ctXPrsA0a2rwr2+wKvgr22QVaCXpzfPC8++wkKvgr22QVaCXpzfPC8++wkhhBAynTzyvW+L1j7+gLB9Vr
+899+85vqDgMdoedfEN0EyQ6n7v2WqUkVPVv8YUjaWXMapsqc9CJLnTW91bzRrS+q53fXRs+/oja
3Nve+uVd89hNCCCFkCtnk/lPzZaxcCqw6JZK9V+6qoxgeoxnzUzDhHdYT1Bu1nPe6K9BIUX0+tEbeE+VrdW
d78X0/L610fOvoq3trYNVNvHRSf/YQQQgiZSL71aFN6oTRj//MzGjy+8DDMI1qrrT9Ohow4H9l7xNb1tJ1EA/
MtxW2PT1dmHbT0vNjHg//7GHx2U8IIYSQKeS6ibGXSLVu+HWX9o2FTszVFV9G5eVFR9Dj2gx1k4iDAJoYrkDu6kMfOX7rOIhjxeLwJdTTMWWF0V1WDV0Df0cwmnaZcI8ZWN4I/PQy3Fm
Mtxw2PT1dmHbT0vNjHg//7GHx2U8IIYSQiaTDr/G7+dd/
+efqbgNzo+dfKLvHd/8OoKZ7pIY4GmWGvo99ZsfEdL3e2qIb2M5e7SXXJtZyJ
rvnTjrPhJseUZk6gfvepZmzdrjTBO9RQI7NC8IMZZbrWmxYiIWEP0c/kiQmtrvRIsixMfnxCf/YQQQgiZ
Th58YL9o9o1cmYtI9cm/nI4fbfrn5YJzsYRXyXqtDXq5JvF5IUbeQ2AZKRDS5/iuXZlHSdNrSCgRF6oZS8e
QItSR9hsnYtI9cm/nI4fbfrn5YJzsYRXyXqtDXq5JvF5Im9cm/9i5mITtTqraXmeSdY2NtyD4vKYrDV6R4jLaTCvrKGTrQVm0SOsVyG0tlYrwdI48+k
Z8cFPCCGEkOnkrmN7RIveefhif1fo+RdNdlNOe6M7IfWMPcMBOHzM7IfWMPkMPVUOMc60my6caoE4x+q3qDDVD8
VBMF38YEj9LraCeQw2o1uhU8WzbJc9yN1ofqUtL4cszw/LcSSrOqW1m0yPUET29OuCZ1iOyteBEcj
FqgHNCcAbnQKbFSrAsLnxxYfdru8VnPyGEEEImkn0v7H724O2KiRe8jfLG/E/T8i2e0OlYLVJLtVU42TJ4
xvp7KVo0ozykfrK/nE5IH1PliuvjDkIaz1GM1iXpc7UJo8zHkOOWVfbXrtcohHp6z6tOK8i2WpyeR63
UX0Dybe055yLfA8L4yzRdEVM55DllkaWcpzoFM2kuD4W1ube7/8X7X7x2U8IIYSQieTaV6X3876waFbRXP
eX/hi//zo+ZeBr6OsqKfs5kq0XJ5BLZqI6qxiQPHz2sa54qz6iD5D5dTBd/GNJD1LEyk2UwtFXRWyq95DxaR
Gnqc1osX51lvQzWeS2W5ydchfQPJt3RbYW+8p4JhInttiawR5dyD4vWdMVsiqUtiUw0ZhyXa0sN6ae+Z
YSQ6aTvr/G7+dd/+efqbgNzo+dfKLvHd/8OoKZ7pIY4GmWGvo99ZsfEdL3e2qIb2M5e7SXXJtZyJw
dKulTrLGmGd12J5ntIldwHNswUnq7XYV8YzkTixxdbqU4TqeWcpvrU1rwRL4NjZY+KDnxBCCCHTyR
3P3Cga8g8gHyw3u/8+UfvqjuRTA7ev5lEOnf/E+pVqg6mtosleepf71fnF0cUkfqcCV08YchcfgqQ9mRa
k/1c/4r0UhWSbtW3uLWeS2WFxziLqB5thb1Wuwr45lLnNh+MZlykCztLMW3tuaVYNHe+NUb4Q9k+rv1Y+2j/e
E1IIdPJnhevEQb/G4fn+KH+/Lxv379v379v389v8/oxyE6+DrmD8X/e+EaGbjK7x5Zz+Xx4b9XQZ5b1=
EEIIdPJnhevGeZr/G5ef/Lxv379dXVHghnR8y+DSFOlmx7zOdkJec7XXVisWarmcb/GXx4x5hMV4w9

D2pxVrlv/OnvjqZZFJHlaWmPpu5z2aTNMYr6UBffc5tl8I/ICs+0r03xBGudxp5DXy5kitPzczFvAMD78
7EPxwU8IIYSQ6WTXqZ1P3HuLaMWHzPuvv1bdlGBG9PzLwNPs1HT7pJ7VfZAero+o1kif09As1Sf6T
g33XvGHIa3OMuqLZ/R4dVgPjZWVVdRJszWWnqsiqzcvz1O65FlA82zOiPCBeloPz6rkiU2LkWurxztH
qsfetbVYCRbm4uWL/KJ+QgghZMq5/+g+0YQPn9/94pPq1gSzoOdfBp5mx6RbKos52GiypHizVJ+op
9PVvAXK6eIPQ9qdVe/WuRz6DMtMRRMbS/9LkPPNY6kn9ZQueRfQvFn/CGN6e9WB3bW6IA2LCV
+eaog7o2dtrVaCRdja3jqwcUB88BNCCCFkOlnI1/jd8MX+NPT8y8DT7Eh20+XpgMwB2dNtmyV3nP
dMcTD+MKTlWfGroc8qRK5YRVZJayzNs9TPGfeE2PKCGwsuoHmz9ghniLFW7+mZ1hckvhizUn6ael
xO4ZvRWVvrlWBQ21e2j7x3RHzwE0IIIWQ6ueGlq5+
+6zbRfi8qfLE/AT0/0MzTjgLTcPznx8UHPyGEEEKmk12ndj56382i8V5s+GL/rOj5gWb0/Jim0xdOiw9+
QgghhEwqhx+9SbTcyxC+2D8Ten6gGT0/Juj85nnxqU8IIYSQSeXW564XzfaS5If3fuePn/+
+umVBE3p+oBk9P6Zmc2tz75t7xQc/IYQQQqaTpfoav5tXjz7EF/tboucHAFi2trcOvnVQfPATQgghZFJ
55HvfFm32suXdkz+q7l0QRc8PALA8/LOHxac+IYQQQiaV7z7+DdFgL2d+/cHZ6vYFYfT8AIDaiY9PiE9
9QgghhEwq33r+OtFaL22O330nX+xvRM8PAKic+fSM+NQnhBBCyKRy3cldT91zQLTWyxy+2N+Inn+
x1O+GK/TyC+Iubaytn6t+HsfvorN3FJENDOy1eqbUajLrhVLaLWPC+NWHK+bCFxd2v7ZbfPATQgghZ
FJZ/q/xu+GL/XH0/AtkdZ59NEZVgRH1/O6OgvTVlXv1tO+Nl8Pb8tPzN6HnXyWbW5v7f7xffOoTQgg
hZFK558m9op1elfDF/gh6/gXquSPSTe9oen7fjvz0yIy9V/sKBP9iwKaGreZlWxx6/pWxfWX70DuHxKc
+IYQQQiaVfS/sfvbg7aKXXpXwxf4Iev7Fad/BpvHNP4WeX4+qWHtVF0DP0GpO5yy0Qs+/Mo6dPSY
+9QkhhBAyqVz7ylU/OHSraKRXKyePfP8vX31V3dzAQM+/GKoZMpj9pN22io7JaKTqaUQ3GprfbMK
MMW5PZq2gsWUzpzXPdHrk8LT1M/okvUCxWsWZPWNfuYJZRj9dn2uuPUCd5SvoCkzoORy+GiW5
GWuIMV99bcoVRs8LaFvLGuhcEPFk4FJgybzxqzfEpz4hhBBCppYHH9gvWuhVzFvPH6/ub2CYrefXd/
Th5sc7pO5JMvPe/9dtRXgVS8+6IiW1G89TGeOqeQY41zQ0v3/ynDGF2bhp0Zetk2n18+Ux8TC0I0G
dlT2nzjBXrietz9UT+6Yr+BYeHJzxFXYO+ia1zvBsOFOP8DxfPOk/L7rglFqV5hXnzFFYMh9+9qH4yCeE
EELI1HLXsT2ieV7dfPLTd6u7HCgD9PyiuZn39t+cLtrHLD3v1dR9k7pOepS+cEZrFb0Avvndc535ndPU
OZFXzp22PiLrRKY1x/iq+nYk5EPEEqx1Owf1nOFJ671YwtdDzWmMEHWdnch1ySn0CXpOY1XmykM
T1Sc6UmrVR1RxfZY6Uh+I1MZiXbx8kV/UTwghhEw83/zHv1vdr/G7OX73nZ9fvFjd66DQec/vkk3IfIx
GIrPSvYTvavoulhyn262G3cfmN46JYeqhMbnnkNDZtHqiil1SzNnAdy2dpRoFQ9tzqupzQme4WxNra
Xk1TC1XoYcFJ2ohXKu+8HKMc05GH5tjLejP1vbWgY0D4lOfEEIIIZPKrlM7n7j3FtE2r3r4Yr/Qf8+fck6Y
mm1trfrfFW4mPFdGtIYVvenyqKcB8/JdeU8BMUzP7hEs2NwTtp7WHChfXd+Owjx7zelJpFaTlgJT11
QR8ZKpEq2vRsEe7cxhn+DZXnCVHq1ryddCDbGKeQ9iKWxf2T7y3hHxqU8IIYSQqeX+o/tEwzyO8M
V+U7//bb/dQBQ8TUOuZVOgzlk/p35a4W7CczX9PZIY2LaR8szvOzcwu48xka3LaeuRzgZ9OwrzLEox
a5ybadJS80LUiKK4KqdHt7kauoagng9uL3BibHsJteQl8C4nuEYs3PGfHxcf+YQQQgiZWu545kbRKo8
pfLFfW1DP7+0xmhsDdVo+ifp5ddsJz9X090hiq20bKc/8vnPFsKQ2rbtp9WkFMdq3o7CWJdWks2y4
zTlV+WyIGt30OpvUiEw5yNl74xzGFIU2A9vXkmO8y2lcIxbj9IXT4iOfEEIIIVPLnhevGdPX+N3wxX6t/
9/h5zmnTUvho84rT1OPVrafiF0Zc1NyXNtGyjN/i+bNd1ajrqZVo9Y2NnybnG1t3uvkTFGXDFxNX1E1
dXQd1aC19fVyAmNw40bctasj+hTv9krZUzMUS6slZ/VV0ceCFxeLwf83fkIIIYQ8et/NokkeX149+lB19
zNti+j51RFPAxHtDeSgVictMffKZHS7JXdZb9PTgHn55p+leVMHnA7Q1c201in6QWTOOP91EmU9g+
ShFiv30+MK1uCmOWWJeryeRy6zpE+sD/sH1tJqOa+FsyVjnlBpLMj2le3DPzksPvgJIYQQMqnc+tz1o
kMeX379wdnq7mfaFtHz6x7DIzyxnqjuH9wjKyVwNf2Xx9hjUw+n2TMVVdo0b/URS+Sl6WRa/bSaR
C+/ntWzozDPonK+ZVhD3PPsskpD+Yx5nhzdcDX8JXNqSGB7wTPDy02q5b6+gS3l5BqxeJf/fJlf2k8IIY
RMPIcfvUk0yWPKuyd/VN33TN7q9PzhhmJVW4rI1bQ3K54PNXse5qUuhrdr3jL2CppKzT9t/YQxXi+
/HujsKMyzKCU6i/888xTfS+alT/IvNX6RzYp5PfW4qu1fZslebIvlzl6r+fXNnoitEQvH/3N+QgghZOLZd
WrnWP8L/1ePPvTXr7+ubnomb5E9/0yNgNlMuOgpsIzUu5b3J5bTmU/PiM9+QgghhEwqN7x09dN
33SYa5lXP8bvv/OPnv69ud7DY7/NHZnHocwJoqrB09JuWdyeW18u/fFl89hNCCCFkUrn9v98geuZV
D1/jFxJ7fq+ihXcaek+HX09THdP/ZXHobwE8k5T0E7RVWB76DV0IvauB5fDY+4+Jz35CCCGETCr3H9

0n2ubVDV/jdy2k5w9NFOyNIp195ClgQYyen4YfS2/7yvahdw6Jz35CCCGETCe7Tu184t5bRPO8iuFr/
F4L6vlz9mSxlj3a10efBAA02tza3PvmXvHxTwghhJDpZM+L1zx78HbRQq9W+Bp/yGw9PwBglM5vn
ufX+BNCCCFTzh3P3Ci66NXK737xSXVbAxs9PwAg9/Zv3xaf/YQQQgiZVFb3i/3vv/5adUMDBz0/AKB
y4uMT4rOfEEIIIdPJta9c9YNDt4p2evnz+pOP8zX+CHr+Rbu0sWb8pgP1y9/45QQh4vdDxB/ORFz7e
aaalfV7/nt/7e23XJ+GvIbozJH3joiPf0IIIYRMJ/te2L1aX+z/4b3f+fIPX1T3MfCh51+kqiUyGiJ6/iaijYw/
nMmien5dqNTvS18V63tPlcGuIbq0tb11YOOA+PgnhBBCyHRy17E9oq9e5vA1/kb0/Ivja4jo+WckLuI
8Teairv2AdYfuwen5V9VnX37Gr/EnhBBCppwHH9gvWuvlDF/jb4Oef3Ho+Tuw+j3/kH3x0D340PXQ
oY8ufSQ+
+wkhhBAynazEF/v5Gn9L9PyLofpLQ9EWmX2nMcZtQ3U3lRNPm5OY48KNV6Dd9R62Krc4o6HvU0
/XZ7hH1LT5ETFd/GGcHpzJxou1e6cyTzEXGGWdZJ2lShoCCzcXJ9ZtsWuZ1UK1xLZzztaNMfU01ZPhi
p6JsFJOXzgtPv4JIYQQMp186/nrRI+9VOFr/O3R8y9GUwPm8rVkJuP5VpPYjKauOpJxD/pn9pQ2DjX
1fep5/bQuoiepjhSPxXTxhxH+reSqss5U+oDJ2KhX/Ip5ng0svHG5uWixUC113JjH2brn3HJ4tOIsLwe
W1LGzx8THPyGEEEKmk+8+/g3RaS9P+Bp/e/T8i+NriIweqjqqR6lGKtiQ6U7LnaQ+Emq+1KxG5xee1
1mKM8SYxrdNSzVAneKuXs1QPBTTxR8G6XGyRqZaSNPMnq06Wlwxd2Iv96rUR8QWZqzl2YczzK1e
aKzoq4eVsn1l+/BPDouPf0IIIYRMJ49879ui2V6G/Ou//HN1s4IW6PkXJ9aAGcfEMNlnZeSh5kk8nHm
dXtDTHDrzesa0Ll2eUz5YX88nMo+o88V08YchvmH6WLV2e4x65NmYcUhoc8XcA14pr6k7wHdKm5
dMVw/vtSBPbFwiVsDlP1/m1/gTQgghk811J3c9dc8B0XIvNv/0D09Vtyloh55/cdIaMN1+eVQTpfSH
9ZCqtJpEn+BZWiZwVnj9PtVZxYDi57WNc8VZ9RF9upgu/jDEuxdx0J5KPekTquWtoudVR9stWdevR8
VOtJerB/hO8azSGeapLnkrttsblt7Fyxd3v7Zb3AEQQgghZCJZqi/2v7h2+N//9KfqHgXt0PMvTloDZvd
WtmqiNl2chxpTnKbmqMd7Zs0EVjdj6eq0bEQ5OvuhPKSP6LPFdPGHId69iIP2VOpJn1AtbxVnie2W
7JnLPVEfEdwBRq02M/t3kmmo6KuH1XTm0zPi458QQggh08l/feybovdeVP7tN7+p7k7QGj3/4qQ1
YMH2q9aqP/SpTszOU+PjS8uogdXR1NLleepf7xdnF4fUkXpCMV38YYh3L+KgPZX3jAb+c9S86mi7JX
vmkieqIZlylDuzr1aLmUO7b6zoq4eV9fIvXxYf/4QQQgiZSHad2vnofTeL9nv48DX+NPT8i5PWgLVoo9
p0cX7VmWvr6+Vwc7Sewmz85Lye0roxjJWu5nG/xl8eMeYTFeMPQ3zD9LGqlj2m5cQWOWPBmaj
dzM2vqTvCvfK+Wm1eMs+YTHPFdnvD6njs/cfEHQAhhBBCJpIbXrr66btuE034kOFr/Mno+RenZQM
mh+nH6kCbDq1t86XnKojB+kk1sZ5VVwovrqF0faLv1PBO4g+DnHUa9atioZnVGXp5kVItrpisE6BmCl
8JuZ56wfXMvlr1uOqgcd3VME/1THNFXz2ssu0r24feOSTuAAghhBAykdz63PWiDx8sfI1/HvT8C2Q0
V5miLfI0V27fZPRWhrqtajVJgLkmd6y9YsXsBP1rK8RL1yfq6XQ1b4FyuvjDiPBCq2rOVP4zGgrNcMWi
M7V4Tf2lcvXM9pjyePhK6BM91XONFdvtDStlc2tz75t7xR0AIYQQQiaSw4/eJLrxYcLX+OdBz79QZtN
U9FOe5srfN9mNmt2LtZ7ERy9J9HeaXdkznzkge7ptaXec90xxMP4wTg/OZOPFZYtXL4QukWCfJFfW
bsntXlP94mXyo+qxMbU5RE9mrjAb68zsqV6JV2y3N6ya85vn+TX+hBBCyDSz69TOJ+69RTTkfeeTn7
5b3YUgCT0/bKpNa9nPApigt3/7trgDIIQQQshEsufFa549eLtoy/vLW88fr+4/kIqeHyb9L2Zp+QHEnPj
4hLgDIIQQQshEcsczN4rOvKecPPL9v3z1VXXzgVT0/CiZ/5E2/y02gGZH3jsi7gAIIYQQMpHcf3Sf6M8
7z/G77/z84sXqtgNzoOdHyej5afgBtLC1vXVg44C4AyCEEELIFDLAF/v5Gn9X6PkBAIk+
+/Izfo0/IYQQMs188x//rr8v9vM1/g7R8wMA0n106SNxB0AIIYSQieSuY3tEr95J+Bp/t+j5AQBzOX3h
tLgDIIQQQshE8uAD+0XHPmf4Gn/n6PkBAPM6dvaYuAMghBBCyBRy7StX/eDQraJvnyd8jb9z9PwA
gHltX9k+/JPD4iaAEEIIIVPIvhd2d/XF/ndP/qi6t0B36PkBAB24/OfL/Bp/QgghZJq558m9ontPyKtHH/r
r119XNxboDj0/AKAbFy9f3P3abnETQAghhJAp5JHvfVv08DPl+N13/vHz31e3FOgUPT8AoDNnPj0j7
gAIIYQQMoVcd3LXU/ccEJ18+/z6g7PVzQS6Rs+/YJc21nYY1s9Vxyvi6R1rG5eqZZLn1p2DerwxUXSOk
jXEfb7FDABQefmXL4ubAEIIIYRMId96/jrRybcMX+PvFT3/AslWuqLbddXT2+qGu03P758j9lcCBWPK
5hkAwPbY+4+JmwBCCCGETCHfffwbop9vDF/j7xs9/8LoXlv117q5Lg+o59XTzvg2Pb8c4kwiT3DOaJ
wBAKTtK9uH3jkkbgIIIYQQMoXM9MV+vsY/AHr+RXGa7epQ+N+fN7bnGTFGPwx16GqAZ4ryUOM
MAOCzubW598294iaAEEIIIaPPDS9d/fRdt4nePhS+xj8Aev5F8TTsYWpwqX3Pb7TsmlXQntgm/9pA
o/sH0Mb5zfP8Gn9CCCFkgrn1uetFb+/N//6fp6qbBvSJnn9RWvT8na7MEPH5yker6552+aAQDC3

v7t2+ImgBBCCCFTyOFHbxIdvsjrTz7O1/iHQc+/KI09v9GOl2Ocfr5Vz6/I7r48qXEVBv8MABB14uMT4
iaAEEIIIaPPrlM7H73vZtHn6/zw3u98+YcvqnsF9Iyef1F83XneVatO2u3Gdc8d6fmdMZnsmPHIrhv+O
wJTbAYAaHLkvSPiPoAQQggho0/ki/2/+8Un1V0C+kfPvzC6dVa9s31Adu/1s7rXdmbQ56gj+oD7Nwf
VkfAcbWfwHAAA09b21oGNA+I+gBBCCCGjzx3P3Ci6/Szvv/5adYuAQdDzL5DRxpuq1ll32w7V84cm
yKkxoVn0HIFJ6uebZ6DnB9Dksy8/49f4E0IIIRPM/Uf3mQ0/X+MfHj3/gomO226bzXY7b7HV47rdts7
PDuuHxhCnazefK9mrcHv3+Az0/ABa+OjSR+ImgBBCCCGjz65TO5+495ay4edr/AtBzw8AGMjpC6fFf
QAhhBBCRp89L15z/O47s56fr/EvBD0/AGA44iaAEEIIIVPIrz84y9f4F4WeHwAwkB07BvrQoVAyCiWj
UDIKJaNQMgolG1+hKeBSAgAGwh1JMgolo1AyCiWjUDIKJaMQIriUAICBcEeSjELJKJSMQskolIxCySiE
CC4lAGAg3JEko1AyCiWjUDIKJaNQMgohgksJABgIdyTJKJSMQskolIxCySiUjEKI4FICAAbCHUkyCiWj
UDIKJaNQMgoIoxAiuJQAgIFwR5KMQskolIxCySiUjELJKIQILiUAYCDckSSjUDIKJaNQMgolo1AyCiGCS
wkAGAh3JMkolIxCySiUjELJKJSMQojgUgIABsIdSTIKJaNQMgolo1AyCiWjECK4lACAgXBHkoxCySiUjE
LJKJSMQskohAguJQBgINyRJKNQMgolo1AyCiWjUDIKIYJLCQAYCHckySiUjELJKJSMQskolIxCiOBSAg
AGwh1JMgolo1AyCiWjUDIKJaMQIriUAICBcEeSjELJKJSMQskolIxCySiECC4lAGAg3JEko1AyCiWjUDI
KJaNQMgohgksJABgIdyTJKJSMQskolIxCySiUjEKI4FICAAbCHUkyCiWjUDIKJaNQMgoIoxAiuJQAgIF
wR5KMQskolIxCySiUjELJKIQILiUAYCDckSSjUDIKJaNQMgolo1AyCiGCSwkAGAh3JMkolIxCySiUjELJK
JSMQojgUgIABsIdSTIKJaNQMgolo1AyCiWjECK4lACAgXBHkoxCySiUjELJKJSMQskohAguJQBgINyRJ
KNQMgolo1AyCiWjUDIKIYJLCQAYCHckySiUjELJKJSMQskolIxCiOBSAgAGwh1JMgolo1AyCiWjUDIKJ
aMQIriUAICBcEeSjELJKJSMQskolIxCySiECC4lAAAAAADjRM8PAAAAAMA40fMDAAAAADBO9PwA
AAAAAIwTPT8AAAAAAONEzw8AAAAAwDjR8wMAAAAAME70/AAAAAAAjBM9PwAAAAAA40TPD
wAAAADAONHzAwAAAAAwTvT8AADM6tz6jtr6ueooAADAsqHnBwCMSNmM99uFX9pYqyv0WXD
ov1nIN5ZZ27hUHeiStZkCf1cyK+sa9vMy9S7wB6Z675W6fWdE/ohGnpqVfyrrFet2W/2KXple/0HRo
/CmVvV1CuzI2k5XL1OLWiv6z6SJoOcHAPSrx7t5i1FnyJu2omwf9zrZvZTeR3Ff1fMNVb6RtfX17P/0U
iffwuC3hOb9aPfvCuudXevp3VfsZaB3hHnduisS/BNq/xmyNzqXYMXoU7MKTZUf1wfKSzpvKf8s5ss
1d4nGK5M/3+k/KHybsrZUmG9fkU0VT3X4R8ndjlHc1NOOivr6UPFo3t21q9VBqfLaVcT1sa6ieA4t0
PMDAHpk30/ZdyOdUoWK/x3yhsDeYV96r5IXqC5fP2Xy1773y2Qa5oWxFCV7evPJufur1fV9fEW9H
u7C5Vujq62FK8aemlXbqdS46uHMivNLdpXs8ukD879cTdvJj1dPz1WnFNtUF/NXIpvyHEoX3I4wf9H
wjuSRHmupJ6qH875q+XR6+uKdXD+0SxVPzrWnKaLnBwD0R94DOHcNneu/gjDM7UfP29LT5z+k37
TFzHc7OLPB3weZft8K9gXsbX/FxOYL1fUL56zc3UrHFzJyrTq8jE1TFc8nX0h1dr9VDP5C+mj+w9xl1GI
9tfr5x4VbqJN9VCLbsXX4/m68dI2Lac+ZytlHh8X01SweyLdDl4Umg54fANAb95O5w7sdv4FvBor9d
HXPGGTd/nTPuGb5j/0UKq5Uredr1t8+gnp+kTLlJcxKlD/08y53/oQa745OOPPlFe3r1nHJyHQdVmq
ayrmwSZqqdLYh30TGsfzHzt7snlrum6ILbqGiTv5FBW3+qr5LZyie7mpv3lrFWy0vUf4QXspsnFqBy9n
N1szL5C/U2cYmgp4fANAb9w7Ae4vSpd4LmIobj67u3lzFXio9binfhZo+L9nfhpRyY33WKd55Hd/Kx
w3zxivecYW+Srn7cP8Uz8Up4M7f8bWMTNdhpfhUxQvXwVWMVyme7ea18hTK96CO5E939qYI1
DJ0VMotJF6XYkD4+rbj2Y4h/uysQrPV16+zUp5axRF59Tp6rYodVNXyn+1pu72K00DPDwDozQI+qo
e7FxB3i73q9GbKlm+jvl55oUH25L41OiVenPL69fm2KAr2+r4z91D+3E+9Ym556Tp8qYoJzZW7bwVn
yHwi03VYKTaVeDvOwVulOFjp6rI5hfI91I/zpzt7U8QuXabcXhfV3ELOm6+DP8jR7XQwv8lTq7xc5bH
y547qefdV7EdZX7feJHMoptUvjPMqxa8xvOj5AQC9WcBH9UD3AuWdzoD3HOXNW/cFrVs2Qxd32
DFF3f6un/PO67de8er0esncCj3uyHpTdHcfX3L+hLp/ZJ1Xbz5uAS3y1KyCUxVXs6vtNCy4eLqTYrKQ9
Z4w9FHL1dE7wi3kTNy8lkaRKYqnunov5JxaboXilZtvR6XmS5OP6GJ38o+M+/I3rwUSPT8AoDfuB3N
H925hQ9wLFDUGvuEoavZ65Qp5mf6r9L+dPm7lwzq7qw5zS/S6I0PXbwl33fLF6npnkfk6LOWfqnjd
OtxM44KLAR0UbCiUP93Zu6JxU139CXMLFUfMjcg3Y4LwdjrahsGp5ZZovrotNU6UD5j/TVHswJ7Gr
dzByzQ59PwAgP7IT+bObj+C+q9Q3pNInd9/ZFWMbXjug3rRzV2bK5vXmLZ4kXp9lYoK5kb6vEcsXpx
+33PuO2CQopnO3xHFS2Mv3Nqb89LNzVNRiTw1K89UxaFuX6TGBRcDOrh8DYXypzt7kRa6KesPUi
d/qoLb6eHPrFvL+sOU6a5o/GXq5jUqVusWyQ+bc8eXAi96fgBAj6wbkG7uCvyKuYXVvicQOxpmM3

nRXl6hwXdj3ep2d9/r6vNdbSvvh7UB3hFd7k28Awr1Fsy9dXUxIxXji5lJeCrxepXm3FxRzVppVsV4XN
Scp0bLK5MP6+xN72wqO2BMXi7Js4jW4puynp2nTMXZTqk4PMB7u/N/UIRr2c/MfenK6bzTWO/rT
q/kdNDzAwD6Zd6A8DmNwVg3pHPfj4YUb+/eZl+Aju/j0Qn7VSlVr414aoVesfCmVnJPkdcoM7p/UH
RO/FVFqb5l4EZiTvT8AAAAAACMEz0/AAAAAADjRM8PAAAAAMA40fMDAAAAADBO9PwAAAAAAI
wTPT8AAAAAAONEzw8AAAAAwDjR8wMAAAAAME70/AAAAAAAjBM9PwAAAAAA40TPDwAAAA
DAONHzAwAAAAAwTvT8AAAAAACMEz0/AAAAAADjRM8PAAAAAMA40fMDAAAAADBO9PwAAA
AAAIwTPT8AAAAAAONEzw8AAAAAwDjR8wMAAAAAME70/AAAAAAAjBM9PwAAAAAA40TPDw
AAAADAONHzAwAAAAAwTvT8AAAAAACMEz0/AAAAAADjRM8PAAAAAMA40fMDAAAAADBO9P
wAAAAAAIwTPT8AAAAAAONEzw8AAAAAwDjR8wMAAAAAME70/AAAAMvl3PqOyvq56lBJPbG2c
ak60g9VR5RfBvW1yTjX4dLGWuip6HMAMF70/AAAAMvF6Gvt9nTqPX/dthd8lyHY2dcXdQn/JgMA
+kPPDwAAsFysf5dtdqhT7/lb7d/b3Nd/FUDHD2Bi6PkBAACWi9Xzmw3uxHt+3bg3rEtfP32d9BE6fg
CTQ88PAACwXOye32hUnZ7fOeA2xtWQfIQxb3WCccTshtXh7Jjxn9MbRRTjWed5Y211mYae25rPm
tBYqRKZS48uxuhHzg5i68/Z6xFDIhuMngcAg6LnBwAAWC51M7lR/qRaRqPLLB67B4x2s2qJ63a0kW
6iw+cYfbbsawvGQjyTRFtff1G5dYOxFke9uPUN9aOo3rT+hgWFN+g/L7pcAOgPPT8AAMByUU1j1k
GqxrRsGI0nioHugVjPXw0yWlJ5RLWlzjn1EXVAltEj9FKMOi3a3XABPaFbM0YPrtTTFBrXrwaE1xPYYG
giuQIAGAY9PwAAwHKxusbqgfmz23Ya7aRsZtWQui8NH1HTuCPkGNkRZ+QhPYkxJkiuICd34h6I0wv
IiVNarF9yqvs3qIe12jcA9I6eHwAAYLn42+us1XQ6Y0+rLFtTd0jzEfXYbJTtedUIH1HYmiXAXVJOlVRH5
dYahZfQYv0Ge7R6PjC7XmXN3hYADIqeHwAAYLnIDrh6rL/eX/eQcmSmr57fPqge+AQLh/nHyq0k9/
zuElqs39e8F9TzwdkDJ7ZdNQB0ip4fAABguTjNpGwi9ROetlM2xu6Q5iOeacW8vhFCiyGaf6wqqY7K
rTUKL6F5cWpEphzkVG+cw5iiEC4GAP2h5wcAAFgunmbSbh/1E7GRHfT8ZnNtj2nRfrtlwvR05mCnR
oc9f+NU7qnORYlsMHvKmHjmdQNAh+j5AQAAlouvmdR9Y04/UR+t2kndmDpH3OY1fCQ8ix4Srqw
aW7dMTLhAff7MvXNkCU3rl9upxzds0NlIdBkA0Dt6fgAAgOXS0EyaTxitqCR62VgLKo/ox2tydtXvZvyl
6wFumTg13mae3WXP37h+/3Jyakho9tCZbVcNAJ2i5wcAAFgugWayblKtJ8zeNWsrZWPsTtZ8RD02
p3OWk7P7ZntAYBdR9nyySZZba9S4hNj669NzeUnjsphP+2Y3T821XTEAdI6eHwAAAACAcaLnBwAA
AABgnOj5AQAAAAAYJ3p+AAAAAAADGiZ4fAAAAAIBxoucHAAAAAGCc6PkBAAAAAABgnen4AAAAAA
MaJnh8AAAAAgHGi5wcAAAAAYJzo+QEAAAAAGCd6fgAAAAAAxomeHwAAAACAcaLnBwAAAABgj
P72t/8AmVYovm/3qE8AAAAASUVORK5CYII=]]></redcap:Attachment>
</ItemDef>
<ItemDef OID="b11" Name="b11" DataType="text" Length="999" redcap:Variable="b11" redcap:FieldType="descriptive" redcap:BranchingLogic="[q9] = '2'" redcap:InlineImage="1">
<Question><TranslatedText></TranslatedText></Question>
<redcap:Attachment DocName="B.png" MimeType="image/png"><!
[CDATA[iVBORw0KGgoAAAANSUhEUgAABVEAAAEJCAYAAACdcmdaAAAAAXNSR0IArs4c6QAAAA
RnQU1BAACxjwv8YQUAAAAJcEhZcwAAEnQAABJ0Ad5mH3gAAE9sSURBVHhe7d0PjCRnfef/b+/aY
PPHNmBskx+RHat7sZYlGJIg08PxL4egZ+6SJbnbYOVg5Oh3PVyi0zQSkwOxF+mX2wguG4lunS5iWrq
zliQyTEJugrLdIJRgckzHR4IxYTI/Z7rlc3T8wDYGjPljB3u3f/VUPVX1VHd1dVV3VXX39PtltaerpqfqT/T
O/OZ5/k+hb5FAAAAAAAAAAAChjumPAAAAAAAAIAQhKgAAAAAAAAAAEIEQFQAAAAAAAAAAiEKIC
AAAAAAAAQARCVAAAAAAAACIQIgKAAAAAAAABEIUQEAAAAAAAgAiEqAAAAAAAAEQgRA
UAAAAAAACACISoAAAAAAAABCBEBUAAAAAAAAIhCiAgAAAAAAAEAEQlQAAAAAAAAAiECICg
AAAAAAAARCFEBAAAAAAAAIAIhKgAAAAAAABEIEQFAAAAAAAAgAiEqAAAAAAAAAAQgRAVA
AAAAAAAACIQogIAAAAAAABABEJUAAAAAAAAAIhAiAoAAAAAAAAAEQhRAQAAAAAAACACISoA
AAAAAAAARCBEBQAAAAAAAIAIhKgAAAAAAAAAEIEQFQAAAAAAAAAiEKICAAAAAAAAQARCVAA
AAAAAAACIQIgKAAAAAAAAABEIUQEAAAAAAAgAiEqAAAAAAAAAEQgRAUAAAAAAACACISoAA

AAAAAAABCBEBUAAAAAAAAAAIhCiAgAAAAAAAEAEQlQAAAAAAAAAAiECICgAAAAAAAAARCFEBAA
AAAAAAIAIhKgAAAAAAAABEIEQFAAAAAAAAgAiEqAAAAAAAAAAQgRAVAAAAAAAAACIQogIAAA
AAAABABEJUAAAAAAAAAIhAiLp0etJYKUih4DxWGj29PqjXWPFeU1hpWF+1iMxj3ZB2onXjpLWdL
MxLO7LSlg372PzHqPs4Vb2GrGzEO5vtDbdtK5JH08KNuQ9Cjmc+2g0AAAAAwPwhRF06Rdm8UJey
XurU1kPCkracr3X0c5Hq2U3rq4B5oILBVWnqJUdZzqxle4faf1Qo1cT/rlhsR+14AAAAAADIGiHqMip
uytmqfi4dqZ0P9kbrNc75IVW1JdsV/XzhFGVzry/9vnpsS/aHkff+RpmXdmShKwdu8leuS9c+xj3ZzDJ
D7TVk3fijwsI7ascDAAAAAEAOCFGXVGW7JV6O2jxn9EY1e6FWpbW4CSqOot6h7OuncuoEPaQBA
AAAAEAuCFGXVkW2W26M6vdGbW/4Q6XL9a3wXoyqlqKuRek+wmpSjqyvaH59ZI1Jo6ZjoC7rqPX
GPu31edcGHd5fZI1J4zwEz5+5nbDPjxN+3INtCdS9tR4xy30ObGewraPOc7xjCm57uPZpYAh6c9VZF
2h4snM3eA7Uw9yc3Z6x+xzPPy71CLkXbAnaHvI9OHq7vqTHE6/dAAAAAAAcfYSoy6yyJXW3OGpz
VTYaDTnnJ6hyIWSM9FAIo3VqpQwmoCrK2hndwM6BdJ1nFmNId2dHLno7bcuubn/5zNpc9FKsnPa
D6gP/AGy9izv6PBo1Pe1wrCSDo63TPb8dqZUKUhrYSXM1aUimtjPY1qasDoZtEx2T2rZR+9S6H+te1
+kREu3HCSwHz4HSXB0dKibnnOtV70AUtW6Kc9TeGFHPNGS7E4vZbgAAAAAAlgQh6lILTjLVrLnBTF
nqF0Imk2pv+KGKV4+yL103ie3UZD3lhKW4dka3b18O3U23d42JhYxw0hjqferEnAz0rpz2yiY0d4M9
Ji/u6BisfEacDLUnjXX3GlSlpc9v3+0xnOr59bfvdUhWIdlAfdzx/O1494Hazrob/E1zTMbr9zZlc9v62PX
vV1Wv1/6cXXIi2X56jXUvsCzXu85r+8MlLiqR+4wr/BzteOl/krZbr3X/0hH2PRjY7rBkxzOu3QAAAAA
ALI+C9QtyXz/HklK9SwM9zlSwEhKq+K8rS71rTubTk8aK24tOBS/OZEYjX6963bk96Ubsy+dvW4Vde
9ZG1BBs1YOwWq1Ks9kcWu+3IaxdcdeNE387w+2yV3rnwG1/6DqbsV0VnO2FBNwB4e0wr3O11fc
nDEt0PSK2E9ivvuaS7JjMbQdfr41qa6JzF35+FOdanQqsS3p+lETnOlHbRwht44jjjDietO4RYBr3Pnyvv
OXCW/QSACAP99/xLnnNW2/SSwCAzB3/NZFjr9ILWBT0REVwkikVtoQGJP5Qeb/npMsYdm/2GE1F
UU6ccp51dJfTrj2WvywnT5+0e9QF11uqp/0AbA74vWmb4nVG7R44gZT1GW8ov7dOdT4sGbUo3S
BMfcIsazBr1jUo6ac28z7QPYSnOKZEvYkT7cec4f+kmIdQ3NyTvhmgTm3wHIWY6rrrurFuwJmaGO0
GMvDwEw/LOz/5Tr0EAMjDDY9fJXd/6AF5/Bs/0msAAJm7dI9I/zt6AYuCEBVBA6FScsO1P6fl1RVt7k
pbenJoj9k/JScqJ6z/D65XGeqc9ZIrrombLbpD+ttuIl09a/TozUtaAZl1DTJrOyHeSKpXqBe0GnVjgQX3x
NNPSOWPKvZHAEA+nvPjY/KWL94oP3ryGWm+/3555p8u6c8AALL1lMilP7A+PussYiEQoiJlGYRfXl3
RfTlsXxS7lKgd9lbEyVeN9XMZvhk9NO3A1+/VOyrwVcO67VqVQ480e0lOK1mv47yOaTHOXbjxbW/
Lhtnr1K2LatY5BRbUnZ+6Ux58/EG9BADIwxv/
+gZ5wQ+vsJ8/9NXvyp/WeR8GgNz0Hxa5/Od6AYuAEBUxleSkN1LbnBFfMSZJitM70Ri+HI+7744c7D
pf686+X7I/4a8fLjUwH4qbZ/0guOFOjFWVQIZacsoTKG6Jgvk22OvYvA/0seV1TIn2Y97LA8Pk1cz3hZ
xnoE/SdmNSNTtwdeukJv6eAubL+z77PvlM7zN6CQCQh1c+eK3c/PXn6yXH5z7+kHzlLx/RSwCAzF3
6gsjlr+kFzDtCVMRUlM2zbuVUc/Z1NbrYnOl8y+vt5wScijnre1s23JlrYvN7cqqJpBS3XmZRF0x117vh
6vxxe81a56Kmj3+wdqsx7F+aq7LhnTIV7Onh297K+dBcNQLH9nm/hqd7bHkdU6L9mLVbm7Lqre/p
me+ta1Qq+NvI2oTnyA9c3XYDi+kT+5+Q+n11vQQAyIOqg/pzD7xELwX94W9/Tb73raf1EgAgc5f/RK
T/fb2AeUaIivgq29LyctSalHTA48w8bynX5YJR4NOfUMnSXNWB0GQ1HAPbsp55Q/a9of6KMUnTHP
Jqu9rKUt8KRKiWomxe8IdlN1d1gOaFzqMm/ZolJ3AMttM8tryOKdl+ipsXpO6/2HmtOZGTdS/7h6Br
7yrua1NNWBO03bzfw9odR+bHA8R339fvk7v+7C69BADIw/FLBXsYv/oYRgWozd+8Xy8BADLXf1Lk
8sf1AuYZISoSqWyH11+stqz17tBiV3FT9gZfW21NVr/R7K0XKBlgDM3OdKKjFJgB2KiyA+qc9bt+wOe
ya1/OW03PstS7fT9Yt1Wl1d8LTpaV1zEl2k9RNvf60h16sXq5MUzeVpHt4EGK7B96PbFTEbvtVlv6Lf
8+sqIzblwHu+5ulByOB4jBnYn/6Wfp7QQAeVr50kvl2iev1Evh/uFL35ZP//6hXgIAZO6y9RvZJcpbzbt
CX81aAgAxtTcK4nSSVCHqQGAKADGo4PT1/+318sAjD+g1AIA8lB56od0LNa6tu8vyiteFD/sHAGTgit
8QKfBL9ryiJyoAAMiVGsJPgAoA+VK9T1Uv1CTUsH7qowJAji79AfVR5xghKgAAyM1HvvgRezIpAEB+
VP3Tt33hZSProI6iAtS7/+NX9RIAIHN2fdR79ALmDSEqAADIxe6Du/LBv/igXgIA5OWOL18/tg7qKPv/
8zH53Mcf0ksAgMxd/n9FLn1BL2CeUBMVAABkTg3fV3VQmUgKAPJ189efL//8Czfppclc+dzjsnX36+
XWV79IrwEAZOtKXR/1Zr2MeUBPVAAAkKknnn6CmfgBYAZe8MMrEk0kNcoz/3RJmu+/X3705DN6

DQAgW9b77aWPWx+fchYxFwhRAQBAZlRwqgLUh594WK8BAORB1T99yxdvlOf8OJ1f+R7/xo/k7g8
xKSAA5Kb/HZFL1EedJ4SoAAAgM+/77Pvk3ofv1UsAgLz83AMvkRsev0ovpeMrf/mI3PtJ/igGALm5/
DXr0dELmDVC1KXWlo1CQQrGY6XR05/LSa8hKxttveBob7jtWZG8mzM3Qs5LHL3GinPuVhoy6tT55
9d55H7NjwLuWyCW+n11+djffkwvAQDy8vJvPE9e+eC1eildn/zPB/LN//19vQQAyNylXZH+Y3oBs0S
IurR60lhZlaZecpTlzFpRP8+eHfiVasLfVIImPi+9hqzXor5KXfOCrAYvunRqJSlMENguK+5bIB7V+1T1Qg
UA5Ovqp47LG++bvg7qKKo+6u//
+y/bHwEAeVD1Uf+79fFZZxEzQ4i6tLpy4KZA5bp0+33p9/dkM68MdWzgt6QmPS+qZ+S4YK99XtxN
V1vqevelWy87K5qrQo4aA/ctEIuqf6rqoAIA8veWvRvtIDVLqifqH/721/QSACBz/UdFLv2xXsCsEKIuq
96h7OuncuqE5Nf/FGmL1zOyJ41zugtquS5bFedpcfOsVJ2n0twlRQUwPTUTf+WPKvZHAEC+Xvu1F8
vLHr1aL2Vrb/f/2A8AQE4uf8l5YGYIUZeQXbvRDN2aq04tx8GuiKp3o1E7c1T9zGAtyGCd1bDejbH3r
wVreI6qN+kMVfdfl6zW52A9S6+2qH6ENy3GPgPncEP8zZjnydln0vOiqHaWYvWMNHoeB0LzkpzUn
VFl/9A6olGM9obVW21v6GMZPAcJrkvI/RZ2vePcb4PXz35E1ImNY5b37WT3p2vSa2eZ6D1Ar1TMr
49uJI6QOz91pzz4+IN6CQCQFxWevubvXqSX8qF6o1IfFQBydOlPqI86Q4SoCDUUGGl2/cyRYVRHai
WjzqrR43EyanuDNTzVurCgpuQNVXdFt3UUZ5+DwWRzdcJ9Fjflgjtk3joz5/RGeo1z3nkq1y+kUEahK
q1+y+tVOsTseTxK50C6+umwipx2N97ZkYsDJ7W96x2NX1c3yXVRQV7I/eZcj1EBZPj9pu7d0GC5U5
NSIMjOSpb3bcz7M2CCa2eZ7D0Ay07VQP1M7zN6CQCQFzV8Xw3jz5uqi3r3h75KfVQAyI2qj3qP9Z
H6qLNAiLqEKtt96Xfr4kZ7Um3Z9TH72zrxbG/4AZBXL9Won9mpyfrIxEaFec7r+3uboWUCxu4/wN+
et3/pyI6XBPWkse4GPca+Wzo1imzrKP523M2ofdbOu/Fbsn0WNy+If+rOS9v677wbglnn94JOUJOd
F4P9um2ZLK8uyolT+ukYlS23beb5V9ri53BnxMnhkpwj67VGqYGh+21ofyZj2/b9ZrYlbFt+kJ3U/Ny3
/uvD789hya6dZar3ACyrT+x/wp6NHwCQv5UvvTTzOqijPPTV78qf1hmBAAC56T8scvnP9QLyRIiKIW
bPtPoFPwgdDgOHletbE4Z54aotPxwsrp3xAqzOge4z2bsoO14eaey7suW3deeiJIl7zH364ZMh8T6Ls
nnWTbuaslpwe08Gz+8kipt740PW2PblMOpEFdfkTNjxtXf9XrVn1pzjSXSOrPOzp0NEI3gPvd4Dhu43
s8et0evSPk86CNzLYfa0LO/bsfdnmCTXzjLNewCW031fv0/u+rO79BIAIE+vfPBaufnrz9dLs/G5jz8kX/
nLR/QSACBzl74gcpkJ/vJGiIoBI3qm2Yqy5iYxIwK3UyfSDKjKcrKkn47SPdC9+VSoU/LqNhbMYdKRw9
QnMMk+K9tGr0FHOsP403RKoi+fcf2NgNIP3Kpy1j2gqa6LruEZOrw/aOh+M8JCawf20Hd33yNrsaZ
uHu/bBNduyvcALB93Jv6nn31arwEA5OWGx6+Sn3vgJXpptu7+0APy+Dd+pJcAAJlTw/r739ELyAMh
KibUkeHOgTHCo7mX3TFUvMKUSrD+5Oz05HBssVSf36vSHRZuBG7V08FeoUmYExB5PXXHCbtWR
dm8EN470w0rF3t+o8nvz/SvXdh7AJaNCk5VgPrID+h9BAB5e86Pj8lbvnijHL9U0Gtm60dPPiPN99+v
lwAA2XtK5NIf6OfIAyEqJjR/gWm53vWGbAcfk9YLHS/
+Po26n7aO1NZznJyneELGlj4tn5Sxl7S4KW5lAntYuDF8vno6/CyPP0dt2TB7nbo1OM36o0lYbdzT+xj
s/as0z83XpEi53bcTXLtoR+GPJpiWGsL/wCMP6CUAQJ7e+Nc3yAt+eIVemg+qPuqnf/9QLwEAMqfq
o15iYte8EKJiQElOusnV0EzePbnoFnIcO/Q7J6WTXtA2qm5m6ibYZ6+xrodpl6Ved5OsPCfnMa5rc1f8
zphdOfAu6QmJc0m9HrXW/XH+/I4OP6sSyOGSnCOzLqcKFN26qMaQ96TUzPKqx6k9GZQdSHa9W
p7pDpOf0CzuW0usa5fme8AU1xDz7yNf/Ig9mRQAIH+lh1448zqoo3z6v/6D/MOXvq2XAACZu/xZkf
48dRU6ughRMcCcBCnYW9IPAlXYNTChz6yYNTCbq/5Q7faGHhpuPdIev510n72GrLsnrnpWNjeNyY
Nq65JPjmrWsmzKrm5er3HOCzBj90asnBbnDulIs+ke18Bw8Amvix8oDvbcjUlvX80sH+xxaoTFZo9b
oz1p3yaRZnHfKnGu3QTvASU/dZXaee9gZMOb4h9Hze6Du/LBv/igXgIA5OnaJ6+0Z+OfZ83fvF+
+9y1qZQNAbtSw/v739QKyQoiKYeYkSJ2alHSoU/LTE7kw7YxI5vDy5uoUoVGwBmZzVQdQXnhTlVZ
qs9e7kuyzJ411d6h6Wepbav3okCq98zLMnFndbbN3TastiX+aKhIo72oZDmATnCMv2LO4x2xOsJTEi
HvXrLNaPevPNp/YQt+3SpxrZ0n4HuDXW7V41zBubVssGjV8/85P3amXAAB5UvVP3/aFl81NHdRRV
ICqglQAQE76T4pc/rheQFYIURHKHgYdUpOy2rLWu8Otp1KR7cGClfuHRs/BBOwamMZwbZddWzOj
eqgx92n23LN7obonbiCk8of1p3hehhRlc2+4Tqg9hD5hYBeYJMs6ZjsbHhT7uljH3G/5QaqtKq2+0dZ
ACYJo9r07eJA2Z5vTZZMLft9aYl07S6L3AHUsg6+ttiava4u59cTTTzATPwDMkOqBqnqiLgI1pJ/6qAC
Qo8vWb6bUR81Uoa8KBgJAEmo2fT0ZlAph96btmTxL+lhOtaYNWBfEUbp2yJUKTit/VJF7H75XrwE

A5EnVQVWTSS2SK597XLbufr3c+uoX6TUAgGxdKXLFb4gUbtbLSBM9UQEkFCxRcGZtsUO49nnrWC
J6ZB4tR+vaIV/v+
+z7CFABYEbULPx3fPl6vbQ4nvmnS9J8//3yoyef0WsAANmy3m8vqWH9TzmLSBUhKoCY2rJh17o06
pWaJQoWUK+xIqv7demmUqJinh29a4d81e+ry8f+9mN6CQCQJ1X/9C1fvFGe8+PF/NXt8W/8SO7+
0AN6CQCQuf53RC7doxeQJkJUAJNRNS8XfPx7cXMvpRq/C+YIXDvkR/U+Vb1QAQCz8XMPvERueP
wqvbSYvvKXj8jnPv6QXgIAZO7y10QufUEvIC3URAUAAKEefuJhec32a+wJpQAA+bv568+Xf/6Fm/TS
YqM+KgDkjfqoaaMnKgAAGKKCUzWRFAEqAMyGqoO6aBNJRVH1Ue/
+0FftjwCAPKj6qGpY/7POIqZGiAoAAIbc+ak75cHHH9RLAIC8qQB1UeugjvLN//19+cPf/ppeAgBkrv+
oyKU/1guYFiEqAAAIUDVQP9P7jF4CAOTttV97sbzs0av10tGyt/t/7AcAICeXv+Q8MDVCVCTUk8aKm
uVbPTaknWjdOGltZ96MOYZeQ1Y2gmvbG+7rV6TR0yunpfZjb3NEO2zTne9M2m1bkPugvaHbWJC
BSwosjE/sf8KejR8AMBsqPH3N3x3tuqGqN6rqlQoAyMmlPxHpP6YXMClCVGCGeo0VKZRq0tHLWe
pd3DH205RdQj4AA+77+n1y15/dpZcAAHm7+qnj8pa9G/XS0aXqov7+v/8y9VEBIDeqPup/tz5SH3U
ahKhIqCibe33p99VjWyp6bXby3l8WRhxDryHrtTziU6UnF3eC+2qea1hrF8VRuA+A+aZm4n/nJ98pT
z/7tF4DAMibClBVkLoMVE/UT/7nA70EAMicXR91Vy9gEoSowDLoXRQ3Qy2Xy86Tzo5cXJwUdTFUt
qXfrYs+w8DCUMGpClAf+cEjeg0AIG8//ffX2UP5l8m9n3xYvvKX/NsDALm5vGc9mOBvUoSoS8eoLbl
i9kQctd6oc2mvz7s25fD+IutuGnU/VwKf6NLcT9vko6Z8z+/PmMP7mqvP5EYU0/WNWj+T1Rv2h/FU
5e+GMDvk6shMnRQ3UUg17jG5PvHbHuTbD59DaumyYrzfqkQa2MbA+/BTHaYN5POpY/P2rh7vd
9nnrupbrsjXQXdYu3WC83n4M3DfArKgh/A888oBeAgDk7YbHr7Ink1pGd3/oAXn8Gz/SSwCAzF26R
6T/Hb2AJAhRl05R1s64PREPpOs8s3TlwE30Aj0U27LbdJ6Vz6xZXz17ldNV/awjB/4B2PywsCxn1nRr7
RCwJIMj5zu1UswQa5bnrCO1UkFW9fYcal2SINUYyl89LZXimniHs3Nx+hCvfEbcU+2L2e6pr43Dfn1w
Z846FVQOrG+uDgSpE7VBHcuqeFvWoakKSlf369Ld2wxcdxW+lsJKN3RqUprnibKwFD7yxY/Yk0kBA
GbjOT8+Jm/54o1y/FJBr1kuP3ryGWm+/37qowJAbp4SufQH1kfqoyZFiLqEimtuT8R9OXQTovauHwi
JEU72Dq1XOU6dmIcI1VI5LW6M2gzMjmSEhV6w15PGutvjsyotu6am9WjpLXRqsh4jjUz7nFW2rTa
Yw76rLadd22HVPv12d+vuV8TsRaoYQ/mrp9X2zVA4xpD+4qbsuefNfnTFa4Z1BPULwcDQN67d6V
wbV7neDX69Frbev2+maYPxeh2aFjf3vOc+P1RXYWtXf41/TppyLsFxAmnafXBXPvgXH9RLAIBZeONf
3yAv+OEVemk5PfTV78qf1h/USwCAzPUfFrn853oBcRGiLiOvJ6IfaPUOndivWnXCo32dFJrDwO38bS
5UZMsNoJq7fi8+s+6n2wPUXFff8ickqmx5QWCs3pgzPGfVlj+Rkh/mWi0Z7IY7gtk792TJfmJsJ0EYq/
Ua636vzepZ2QxPUMe3O61rY6vKWbchRsg+cv3+obPdKdoQeH0UI1Q3Q2s7cNWB6t6okwhkSA3f
v/NTd+olAMAsvPLBa+Xmrz9fLy23z338Idn/n4/pJQBA5i59QeQyf8BKghB1KRXlxCnnmRtode1x6W
U5efqkHXYF11vUMHDn2VzwQ7mmeJ0KuwdeWOgN5ffWWcfkDu+2H8bw7cAQ/VFmdc784HMy
Yb1zLRMP6W/LeT9BlVZoz1klRrtTuzaW8kkJ3d2o9a4p2hC7Z7Zxrq2N2WUO3P3Er8sLpOuJp59gJn
4AmLGXfPe58nMPvEQvQbn7P35Vvvct/m0CgNxcVvVRv68XMA4h6pLy6oraPTl74nSqPCUnKies/w
+uV3ngPEWoFiOYcodmt90x0xG9I6exkOfM6GmphqaXQkPC+LP0tzf8OqBVo6fp8kkSbhdl80L4jP1u
cDtiPjEgE+5M/A8/8bBeAwDIm6p/usx1UEdRAWrzN+/XSwCAzPWfFLn8cb2AcQhRl5U3tHlfDts6aL
N77VXEyQqN9VP3hsyCUdfTDi/9upOjwkuvNubQI2YYuIDnzB/KHyXmkP72hj9RVLUlIzuhTmDqa5O
CTNtg1JUdKNlqa55jln7k532ffZ/c+/C9egkAMAsrX3qpXPvklXOJpn/40rfl079/qJcAAJm7bP02eukze
gFRCFGXVklO2hlkRw52nSHNbh3Rkv0Jf3347OuzV9w864eaDXeSp4E6pCVnqL3i1eKc2KKdM2Mo/
xjjh/S3ZcNLUMtSV1PRTyvVazOhHNugZuhXPU7tScXsQNWYoCtu2QJgSvX76vKxv/2YXgIAzELpoRfa
D4z26f/6D3aYCgDIyeXPivTp2jMOIerSx8ntyNpNptOObWeCzq4p/uem+BnHyUoNXXUr82E8miD3pbKoIM9Trvi
ls8dW7sVSMn/+v/
+l34GAJgF1ftU9ULFeHd/iOBv3tB3QLBBoBdDcxvRd/ofsEdHJx1NRR14sZEBBVZbHTDXX4zxTuQvv57V
VzVwZg/Ll1GT440LNVzVtS1VJXmauqhoTmUvxxaW8AoiWC9cuSQ/l5D1r0CqtYrAxMwOY/JJkhK99
pMJoc2VLb9IfyBurRGfdmzm7MP3bEU7v7Fu+WOl9+hlwAAeXvjX99AHdSYfv7dP6WfAQByUXijfoJ

RCFGXWWDW8FPiTzbuDltXzPVzyAwvRw2ht+tRGr0uXeW6dJPWu0z1nFVke7BA5v5hSrUxzaH8o0
NdMxQeNaQ/Xl3VCaV5bSaVQxvsIfxhxVBVSNvvp1pfFohy1RVXyf/4lf8ht1x3i14DAMjT373yCf0M
UV7z1pvkbe+5VS8BADJ37FUix9+kFzBKoa/G9QIAgKXxwCMPyOv/2+vtmfoBAPm648vXyysfvFYvYd
D1P/E8+a1PvVGedw0TbwFALgovFrni/daTq51ljERPVAAAlsztN91uD+0HAOTvb27/tjx2PX/ECnPlc4
9L9fdeS4AKALmx3m+Pv8f6SIAaByEqAABL6F2n3iUfeMMH9BIAIC+Xjvfl8294VH78nMt6DVz/8t+V
5NZXv0gvAQAyd+ztIoWb9QLGmSpE7TVW9AQpBX9W65F60lhxJ1TZkODL27LhTbbiPCabqGZYeyO
43dBH1jOAAwAwhz788x+W07ed1ksAgLz84PnPyt7rvqWXoLzidS+R1X8bNhkqACATx4oix39eLyCO
OeiJqsJVf5ZqR84zwquZ0VcaKU3oAwDA4rjnl+
+xh/cDAPL10M0/kO6t39dLy+3al14l1d99rV4CAGSucI3IMTWMH0nkGKIWZXOvL2oeq35gxuuuHH
iTiKvZsNXn92QzxwzV1qnJeTqkAgCWjDtj/3VXXafXAADyonqjfu+aZ/TS8lIBqgpSAQA5Of5ukcIL9QLi
mn1P1N6h7OuncuqEZJedlqXedUNc/9Gtl/XnRZq7pKgAgOVzy3W32EGqClQBAPlR9VE/96Zv2h+X1
S/8xivsofwAgJzYdVDz7rl4NOQYog7XRLXrlZZq4nZEtYfVD9UoNb/OeaRVL1Uprp0RP0YFAGA5vfm
WN8tH3/5RvQQAyIvqibqs9VFVePoLv35CLwEAMmfXQX2HXkBSc1ATNUKvISuFktS8lNXRqZVSq2H
aPu+HuNXTfpGB5WVM8uWd46hJwTA7YddqEYTfT/4kcHneY0nPYU7fC+q9b2DCO//8rEiKf0cCAt7
7s+
+V2h01vQQAyIuqjbps9VGfd82VctfvUJMbAPJztcixO/VzTGKmIWpluy/9bt3vCVptOcPst1WY2ZPGu
htwVqXlDsFvVe01qobpeqIkoSO1khtC+I9Vd0Yra9/2bgFghnqNlWAPfSBnqjeq6pUKAMjXfT/zuD1r/
7JQAer1P/E8vQQAyNzxO0UKL9YLmMT89kTtXZQdnSKU61v+RFSVLXHLmHZ2LqbU+64s9S0S1NFG
TQoGTGKR76eM295ryPpg13tgBlR9VFUnFQCQnx8/57J8/g2PLkV91Le951Z5zVtv0ksAgMwdf5PIsV
fpBUxqfkPU7oHXE8sevu/1HjWG93cOpKufTsfppZpmrVUAABaVmqm//attZuwHgJw9dv3T8je3f1sv
HU23vvpF8ku12/QSACBzhVtEjv0LvYBpzHdN1FSFz85vlhPo1M7nWItxHgxO2jWqxuPoOpD20GPv6
/VjZG3Jwf1FBNd2Pdzga0fVgsysDVEya98oca+VK/5xJm1f2OsHyncO1O806o7arx19P/kG2p/K9Ux6
DsOEt32wXungORo8P2HiTbTn8/epHqPqpCY5P0DQbdffJvf88j16CQCQl7+/7Xvyjy//oV46WlQd1O
rvvVaufO5xvQYAkK2rRY6/2/p4hbOIqSxEiFqud4fDT/uRwnDa4qac1WVWRfblcGnyBRVsDU7a1ZTV
wqr1/3hUiFMKG3rcqUlpMKBKMklYe2NETUjVYzgYFmXWhihZtW+khNcqwXEma58TyIW9vrlaGBH
2qXNitLNcl/GVM9SxDbR/6us5/f0ej9OrffAcNVdHhZyTcPbh1XO2Dd97qd3vWGrvKL6DGfsBYAb+6v
WPHcn6qL/yH15JHVQAyNPx09RBTdH8hqilk34P0YN0Bu2Ha8tuuinKQug1zvnhkTuhV7/r1Zsdzzhv
5bp0dbDd9TbQlHNeotNLMEmY9dpzesOh2+3IzkX3tVm1IUpW7Rst2bVKcpzJ2tdrrHuBnP+HjZa1F
615bkRQaLRjb1OKem0koz1u06e5ntPf70n47fHb3pHa+ejIPHqivUH+PsLvvbTud0Ds2frfdepdegkAk
AdVH1UFqUfJyumftB8AgJwce53zQGpSC1HtnmjGkFHzEWco65DimpzxspxVfxuqF6C77UQbdnpv
me1yHmYvuTOyFivhWXQ9uejO2qUCFi+kKcrmBSPEidI7lH39VDo74mY3xc09J6yxHnub+mQmmiT
MmLjHCNyKa2eGQ/XM2hAlo/aNlPBaJTnORO0LtuOst74iW/aGVVC3J2GHE2hHTNWz/rmtbPtBrd
/2JNczhfs9gWrL7yFf2Up/+4q5j/B7L8+J+bAM7v7Fu+WOl9+hlwAAefjmjU/JV376u3ppsb3sp14o/
+a3mNAEAHJTuFHk+L/WC0jLHA/nDwYcXkjrjWE1w5B0mMHN0daVAzdTKp+Ukn5qK56QU/ppJDP
kHgioh2ouTjVJmK6nGTZ8Prc2REmpfSMlvFZJjjNR+0a3wwldR5fWOHUi6XdVVU4HNlaSk2473bYn
up4p3O9zpSwnAwcRIteJ+bAMrrriKmbsB4AZuP9V37HD1EWm6p/+
+n/5GeqgAkBurhQ5/mvWR+qgpm2+a6IWN2UvbMitPdQ3hXqoHtWLri8pZ7JH3OhefG5oM1EPZ
MWu5eiGPlE1KzNsQ5R5b19sebQvRuA3VlFOLEzSmcbxAvPpphfcZAepKlAFAOTn8yuPylNXX9JLi0f
1QFU9UQEAOTn+r0QKN+gFpGmqENUc9hv1cMLJoj8M2gxA7aBUrw9NMc2v04+4dRUtdp1B82tD
H2kGskvEuHZ+7Udf89zw5DXjJwlry4bZq9OtjWnWijRl0oYo2bcvLbGOc4btG21wgreeHHp1B4ZlOv
HcEcD5QZpuv+l2e2g/ACA/KkBVQeoiel3l/6IOKgDk6dhrrQd1ULMy3z1RkZGQ4dEus05mDGp2d9V
b0Q+rjZ7D7raTTBLW3vV6dtrhjxuYG8OTB6XehihZtW+khNdqguOM176Idth1itOdfT7Y9JDh+ImO
M737fWHkNjEflpGaZOoDb/iAXgIA5EEN6f+7Vz6hlxaDmoWfOqgAkCM1C7/qhYrMEKIupaKs+bN2
yao3Ztuc0XsMPcGXKlEb7K0YEnhNOEmYH/4YM+KbcmhDlFTbN1LCa5XkOBO1L6Id9rE7NVUTnsK

Rmqsb4jfdL5lQPrPmhNaJrmcK9/uiyeB+B0wf/vkPy+nbTuslAEAeVH3Ux65/Wi/NN1X/tPp7r5XnXX
OlXgMAyJaqg/oe6+PVziIyQYi6pIqbF/zehs1VHawYk86MU9n2h393alJygxmjRqg/UVew9mbkJGGV
09aSNq5dWbUhSmbtGy3ZtUpyrpO1b2w7ynXZSmV8eFnK5aas6vZ4Tbfaftab/j/Z9Zz6fs+LOdGV28
6Jws6U7ncgwj2/fI89vB8AkI9Lx/vy+Tc8Kj9+zmW9Zn79Uu02ufXVL9JLAIDMHVsTKdysF5AVQtSlVZ
TNvcFJu6rS6res/8djD/8OK6Rpb2dgoi679ubg/ixDk4RVZHuoDc72vF01d71eitm0IUqG7Rsp4bVKcJ
zJ2qfa0Zfu0IbVpo3SBlM7JWcHjzfsGiW6ntPf7/mw7q/B67F/aPQSTiCV+x0YzZ2x/7qrrtNrAABZ+8H
zn5W/ev1jemk+veatN8nb3nOrXgIAZO7Yq0SOv0kvIEuFviqCCAAAMIF7H75XKn9UkaefXYwhpgBw
FNzx5evllQ9eq5fmx7UvvUr+06ffzDB+AMhL4RqRK9R8BQzjzwM9UQEAwMTefMub5aNv/6heAgD
k4W9u/7Z875pn9NL8qP4udVABIFfH3239jwA1L4SoAABgKu/92fdK7Y6aXgIAZE3VR/3cm75pf5wX
v/Abr5BXvO4legkAkLljbxcppFNUD/EQogIAgKmp3qiqVyoAIB+qJ+re676ll2ZLhae/8Osn9BIAIHPHiiL
H36EXkBdCVAAAAkAo10dQt192ilwAAWeve+n37MUuqDqoaxg8AyImqg3rsPXoBeSJEBQAAqVAz9b
d/tc2M/QCQI9UbdZb1Ue/6T6+2g1QAQE6O3SlSeKFeQJ4lUQEAQGpuu/42ueeX79FLAICsqbqon3
/DozOpj/q299wqp/7ZDXoJAJC5428SOXabXkdeCFEBAECq3lF8BzP2A0COWjUJBUCsZjpdHTn1tSvYasbLT1gqO9NrrDhtXGnIqCb6x+E8
lv5aY2mo2frfdepdegkAkLW/v+178o8v8/6Feytbzrrl5qr/3Wrnyucf1GgBAtq4WOf5u6+MVziJmghAV
yFVPGiur0tRLjrKcWSvq58vHDiJLNeno5YXRa8h6LarV6loXZDV4saVTK0lhIDAGjqq7f/FuuePld+glAE
DW/ur1j8kPnv+sXsOXb9zu1z/E8/TSwCAzB1XdVBfrBcwK66uG6bzN8wbs/F
DW/ur1j8kPnv+sXsrOXb9zu1z/E8/TSwCAzB1XdVBfrBcwK4SoQK66cuDmbuW6dPt96ff3ZHNZM9
SxQeScUj1nxwW/7fPiHlq1pa5zX7r1srOiuSrkqFgGV11xFTP2A0COfvycy5nXR33zr9wir3nrTXoJAJC
5YyvW41V6AbNEiArkqXco+/qpnDohy9v/dHHF6znbk8Y53QW1XJetivO0uHlWqs5Tae6SomI53PSC
m+wgVQWqAIDsPXb903L/q76jl9L1sp96ofzKwAAmSvcKHL8tF7ArBGivnqP5CKntOLI2peqN
536d163Or/dp135sb+ivNdbZLwuuD/bKS2MbmtlG7zFcYzN4jMM1S+1HWN1Low3jal3a+zDDt+aq
87WDDQ9pc9i2o9o8tpfjROdl+J4ZuZ8YxxD7fGh+W9RjuK0OZwi9/7p0z506/lKsnrNGj+NAWF6Sk7
ozquwfDt9PNqM9ie65eMduS+H7wj1Pcd9HsNxuv+l2e2g/ACAff/fKJ+SbNz6ll9Kh6p/+
+n/5GeqgAkBurhQ5/mvWR+qgzgtC1CWlwpHQMKhTk1JhQ8ZlcHHYtR8HCkLa61TIMrC+uRoeXk2
1DRU2hfYY7EitNCqEU58zapaW61J3uw52duTiYMi0674ynbqmQ8GiZh/zyGBquM1uz8dQE5+X4X
umuToieEt8DFGcfQcvd0hb7WCw5A2hd6V67jxVafVbXq/SIWaP41E6B9LVT4MqcjrpPZfk2FP6vlDn
KY/3ERwdapKpD7zhA3oJAJC1z688Kk9dfUkvTe/f/Nar7J6oAICcHP9XIoUb9ALmASHqUmqLn8O4d
TmNeo3SlHPhSUpi5XrX3na/FYybwtaPGt482TaCw6mHj7EjO4PplEcFZM7r+3ubsrlVF+erBr/GPI9nZ
FyGWtm2ttd1t2Wptpx9bOvUrr3hB4Vhbe7UZH3kdQm2eXRT0jkv/invSO28cd0SHMPY8xHg7zu8r
dZxrbvBoHEu3Iamcu40u53bEitrHVKUE6f00wiVRPdckmNP7/uimOP7CI6OD//8h+X0bQxHAoA8q
ABVBalpWDn9k/YDAJCTY69zHpgrhKjLyOwlZ/R0K27uOQGJ9dhLZaajqpx1t1M5bS25RqwPHd486T
aKsrlnBj6O4toZL7TrHIT3AyzXt4IBWXFNzugv6uxc9NvY3vV65pXPrI0P38YwexjWLxht3rwgfgZ5PrR3
31CbR5r8vFRbfnDoh3xB0xxDFHPfoW3tXZQd3SEycC4qW/5+zWtniH/u1HFY3yOhIe8k9uUwrEFKk
nsu0bGn+H2R2/sIjpp7fvkee3g/ACB7akj/V376u3ppMqr3qeqFCgDIiV0H9V/rBcwTQtQRlZAQ0ImqY
bsGrZTiyhuIkyielpJ8GjFofJo1t2HQ9x9BhzEGnTgwGP0VZ8xMtLyzyA0Mj0J1YVK9WY/8jgrfhNscV/7
yMN90xjFaWk+MudvfAa79X7sF+GEPcRwyfn/zcTeuUjN51gntuimNPcv2HzlNe7yM4ctwZ+6+76jq
9BgCQJTXJ1KT1UVX907t+59XUQQWA3Kg6qHdaH6mDOo8IUZdSUTYvhPckdEOYqMl1FkZg8hyjn
mOk8MDO76nnDnc2AsPq6dg9GafXkeGOgjFCRlOK52UyYccwK2keVxw9ORxbLNWR2T2X2vVfkvc
RZOKW625hxn4AyNFfvf4x+fFzLuul+H6pdpvc+uoX6SUAQOaOrYkUbtYLmDeEqMuquCl7esjtQKlR
W/Pcos+u3ZYNs3edW7PRrMGZhHW+zurzZA+NNoYyV0/nF6FOH/qlfF4mkm1w6dXKHXpMWsd0
AsUTMrb06bje1BPcc+OPPf3vi6P9PoIsvfmWN8tH3/5RvQQAyNIPnv+sHaQm8zTaqqe4f/GoxhxHPHrB2pgiW3/uMUba64U6Z3duT8+R29n
aqkk6GW5KR37gdnZO/JRbfoZeQQ8BgyOC+
+nI4hTOmkFwKOqumZL+NcNHfF75DZlQPvNJxwzn2EWPdckmPP4PpP/T6Cpfben32v1O6o6SUAQ

Jb+8eU/lL+/7Xt6Kdr1P/E8uet3qF8NALkpvFgP48c8I0RdRu0Ne6itmkU92FPMCHiMXnIlPxkzZmJvy
4Y3Dft884MlY2bySXgTWHWk2dQnKrWh/EXZdLsdqvO87l+XXmPdq22ZZBKkcVI7L578j8Fj1udsrvr
DyPW9bj9yHVtu1oBtyq7eda9xzgsxY/VgjnPPTXjsU1//hO8jZntyvRSYe6o3quqVCgDI3t/c/m157Pqn
9VI4Vf+0+nuvleddc6VeAwDIlqqD+h7r49XOIuYWIeoyqmz7Q287NSm5QYtRH7F6NnzmbhXSDL52
Lpkz9nttNibamUhF3I6BrlSH8o+4LiU/fZQL005glcl5MSQ9BnPYu9ueiRK2YH3O5qqz34IX9Felldqs+v
EUNy94PTLd9njnodqSeM2Jc88lOPY0r3/C9xEgiqqPquqkAgCydel4Xz7/hkcj66NW/u8idVABIE/H3k
od1AVBiLqk7KG3YUUMVeDS7wcDHlX3cLBmYrWVcx3NpCqy3W/5gZHNOTbvsAPDrOPxhlcr5bpsp
ZzL2dcl5LxWW9Z6d+j1VLI5L6Zkx2C1Z/A+3D80ejUmYNfnNIaSu+y6nznWQ/UUZXNvuFaoPYw+Q
aAb656LfezpXv9E7yNABDVTf/tX28zYDwA5UPVR7/uZx/VS0Cte9xL5hV8/oZcAAJk7Zv2GfPwdegHz
rtBXRewAxKNmNtcT86gwbG/anqHAOEfpntPHcqpFwIpwn+l9Rip/xM0BAHl441/fIKWHXqiXRK596
VXyW3/8z+yPAIAcFK4ROf5+66P/Xoz5Rk9UILaeNNbdmc3LcmaNABVZO1r3XPu8dSwZ9ODG0fGO
4juYsR8AcrL3um/J9655Ri+JVH/3tQSoAJCn4+8mQF0w9EQFxmrLxmANWFXOgK50yMzRu+d6jRU
p7ZyRbiplKXDUFf6fgn4GAMjStU9eKe9s/aS88723MYwfAPJ07O0M419AhKjAWAOBFgEqMsc9h+
WlJiiblx9NaEs42hKOtoSjLeHmqS0nrr1GDr/3pF6aLa5RONoSjraEoy3haAvSQIgKAADmBj/ghqMt4
WhLONoSjraEoy3haEs42hKOtoSjLeHmqS1IhpqoAAAAAAAAABCBEBUAAAAAAAAAIhCiAgAAAAA
AAEAEQlTkSE2WU7DrfxRWGtLTa8fqNWRlo60XHO0NvZ3CijRibwjp6Uljxb0GG9aVTbJuGmH3UNr
7mJbRRv1Y4SYFAAAAAGChEaJirvUaK1Io1aSjl4H5pgJdY1Z9W1nOrBX1cwAAAAAAsIgIUTG/eg1Zrx
GfzqeibO717RkF+/1tqei1szFPbenKgXvLluvStdu0J5tkqAAAAAAALDRCVABIS+9Q9vVTOXVCyE4BA
AAAADgaCFGXmao1OlC7MazG6GD9UXuIvfE1A+VKNbNOpXokq1Vp79Mcxt9cdbYTvjOjjeoxqk7qY
JvCa1VOdrymMbVf2xvetob2H3JN4rTRY359ZEONcxFo46j1xj7t9ea5zKoOadx7KKotg9uYsD5pjOuS
9J4FAAAAAACLgxB1WakgL7TWaEdqpVEhpPpcQUoDQ+ybq4OvVyFiSYIva8pqYbBWZBqcNq0GNh
xyDHYlNtgm65W1UnjQaYt7vIMqcrqqn3Z25OLAa9u7bmODtTKHQjgtuo2TKsrambLztHMgXeeZxR
iOHmh7W9xml8+s5dDDMoV7aKJrPizf6wIAAAAAAOYRIepS6knjnJuIuXUb+9Kt61BNOrIzmPx5qtL
Sr2+5QaEKG8/7ve16jXN+0FVt6VqVXfE2H0Nl2/qabl28L3G3sx1W8dJvU/gxWMe77oZg/mv77gF0
arI+MhUdf7xhKltu2wfPpR9GSvmMeBlqe8MPgsOuSWQbJ1NcO6PbuC+H7qbbu0ZI2ZEDN101hq
mfOpF9hDr9PTTNNTckuC7J7lkAAAAAALBICFGXkjERz96m16vQD9VEOl56FlRt+RP3+EGhqScXd9w
+e1VpeQGStc8LYa+fntmm0GPoXRS3SeX6lj/xUGXLC+U6OxdDexSOP94RimvidfQ0t22ElGaPTrN3av
2CcU02L/htrJ0fGKo+Ja+NftDbO3Si0mrVCRv3dbrau7jjBZKnvROYlRTuoSmuuWkm1wUAAAAAAM
wdQlRYdA3P0OH9SZmzk5+Ukn5qK56QU/ppespyMrCTEN0D77jsIdheXUtjqHdgSHsazOHy/rB4P5
SryllvyvYRvVNtxnbMHqOpKMoJfUHcwLlrXzzrnJ4+aYeVwfWW6mk/kMxMCvdQKtd8VtcFAAAAAA
DMG0LUZRWYKCdunckYgeWRMt3x+r1i3Z6eRig3URhpDK9PScUt3trctVrXE6cj6ik5UdFhZWC9anb
2EeriSf+6AAAAAACA+UKIupTasmH2OnVrPZr1HI+ocr3rlDEYevjD9lNT3JSzbglONXTcqCs6WRiZQYh
dOS164L4ctvUQeLv3pzs5lrF+QUP07K/5sv1xAQAAAACA5UOIuozMupwqYHLrohpDoCdXkpNuEjs
4XNoIEXNVcoamK6NqvWbF6+nZ2ZHz50fVFTXP2eBs/mZ90FMydk6nxNfQ3XdHDnadr3VrtZbsT/jr
h4e0ZyWFeyiVa57idQEAAAAAAAuNEHXJ+QGTMWP/VMxakU1Z3XCn3DFnS8+ZMcmTNFfFa1J7w
6+V6a1MmdfTsyPNpj76oaH8Rdl0u6xar6utN6yz5eg11r0anuYESU7AqVivP+8dkGx4U8nH5V+vZtP
5Wnf2/aIumOquNyfCylYK91Aq1zz5dQEAAAAAAEcTIeoy8oI9S3NVh0rGhDtTMmcun2r75iRC7nY
mCjuDs7o3V3WI5gWO5gzwaXOHxftCh/JXtqXl5XU1KdnnrCAlP6mTC95EVCojdOutWrxzHLe2bVBg
W9Yzb2i6eZ9Y68/k0w3VNv09lNI1T3hdAAAAAADA0USIupQqst1vGQGZUpVWv+8HRvaEQpMqyu
Ze1w/BbGr7g/scx2qn1yBt/9DrDZhIcVP2+oNtstj1YDOoh2rwhvQr1v62Ruysst0PrUtbbVnr3ZILLnU
8g6+ttiara2v22gwMTTeGs+c+ZD2Feyila57ougAAAAAAgCOp0FczrADITq8hK3oiL1WDdo+eiwAwk
urtPS8/mtCWcLQlHG0JR1vC0ZZwtCUcbQlHW8LRlnC0BWmgJyqQKbOOZ75D4gEAAAAAAJAOQlQ
gE23ZKAzU8ayeFTqhAgAAAAAALB5CVCAPql5pZpNXAQAAAAAAIEvURAUAAOcHlXhaEs42hKOtoSjLeFoSzjaEo62hKMt4WhLuHlqC5KhJyoAAAAAAAAAREBCVAAAAAAAAAAIAIIDOcHAAAAAAAAAAgAj0R

EV2eg1Z2WjrhVnrSWNFzZavHhsyL60CAAAAAADA/CNERSZ6jRUplGrS0csAAAAAAADAoiJERfp6D
VmvEZ8CAAAAAADgaCBEBQAAAAAAIAIhKhLqr3h1gddkUZPD7+3l51HaClTVePUeI3zcL7eZW/XH
MbfXHVepzc4uF+PuW1j58HXt2XDfY318F4Wo12TGjwv6jHpuVGSHr8S1obCSkPCD8+s/eo8VkaciG
TbBQAAAAAAWF6EqEuvI7VSQUoDw+
+bqwMhX3tjRI1T9fXpBJbR1H5WpamXpFyXrYr1MbN2OWHk4HlRmquFYNCZ4blRoWtYG6RTk9Lg
BFl2EFuSwZd3aqWhcDTRdgEAAAAAAJYcISosVWn1+9K3Hq2qXqUCwPNulNaTxjkdX5br0tWv7dbL
zjrrtTsXnYiusm19rlsX9zNSbdmv7W+rxHNafjv7e5tSTNCupHqNdS+MLNe7zj77LasFWvOcDkeza4NI
W3bd1Dh020055yW0VjvW3SDXOE/uBe3UZN17bZLtAgAAAAAAgBAVUm1tixtxVraMANRTlM09H
crZ4aVeu3bGe23noKufZadc3/La6ciqXT25uOP20qzK2U13yxXZsoNGFVLuibM6w3PTO5R9/VQ6O+
JmscXNPWd/1mPPbVvvorhNDpynypa42Whn56J1ZJYk2wUAAAAAAAAhKiaha5OGDmHPzqkT44K
9tNrVlQN3A+WTUtJPFSdo9EPnYSmem+KanHGTWGtrquyCW7t0qM5p98Dbnz18X7+uYA7v7xxYR
2ZJsl0AAAAAAAAQoqIsJ82UcJTAxElGbdLcjGjnzNtlyawNRdm8ENYz2A9KQye5Giur7QIAAAAAABx
NhKiIoS0bZs9Kt46mWft0JuahXRm3obgpe3qIvV+v1tc8Nzybvl/DdfBh9KCdYLsAgLzp0Q3Ggz9yAQ
AAALNBiIrx2rte70o7oHNrfxpDyKc2ybYya1dJTroJqDsE3qVm4i8YM+6n1YaI16uZ9NUvzfakXXbw2f
XqnHrtK530Qtu4NVhjbRcAlpH9Xl+QwkwTy540VlZFWu57tPNHr+bqLILU+Qxze40Vpz0r+f/hT/0ba
p4P9zEP5wXDhq7XDO6ZmRvzvuZ9P7mPvG7mOO+3ebwnR+5jSf+gFfO8z/K9eGZinJuh951luGnG
nJfc34sTtofSdoiDEBWJ+AGdMSt9AiU/nTRm/7d+MFmdbhD8tO0KKsqaVzS0Kavem667baeO6OB7
cZw2JDp+/aavPhXsGRpSs9Wsc9pc9dvm/sOhHu7KJNtVjG3wDwuALMzsl/chKri09j/lv0npcCYu3Da
KcLuTP+4f5vte3N4lC3ONPyjOQq8h6zWRatX9x28G3NEnxsO8XrMy01/cAyWOhh+z+NZW52O1qS
YFda9TS6qdmpRmFPjk/0v7+Pc19R5csr6f6l3jHFk/T2Z778R5v83jPXncPtTnc/yDVmTok1eYm+C85/l
eHHFuht739CP98xP/vl3dH/g3Iot/IEadk9zfi8efl3zfiydoj/WNrUrbpf+eHO/7dn5+FsZY1g2DJWS9R/
TV5bd+A+hbPzD5uvW+9U+g87mq9ZZia/WtnxX060c8vNcqIa93P29uf9TD2NbIdtqStKvbr5fd9VXr
K8cxXx/yKNetVygJz83Exx/
+MDcdve3gMSfabqvqrS8PXwQAmEq3XrbeX8z3eP2+GngjyoffFv1vwAzaEEm/z8/8vXjm7XCuj9q/f
c28f5PzY/87OoP9RtP37dy1y/3eivPzV9rCv5dn1R7n5y9jv/pnrCy/l8a/rznvuYNtyPocjW9XvNdMa6J
9ZPIeqPdvbdd+hLRD3T/maud+Um3XK1lS/5w4n8/+vTjeucnjvS/
+fZv1+8v4cxImq7aNPy/h62fWnhHfw+nfR87+zd0737fBdX579Qr9vhz3uiJf9ERFDBXZVn8p0ksO56
821puAo7kr/t9KrNd7n9D2D52/MKlanIP1QqutCWuIJm1XEk4PIOsNTS/7rDdbf9h+0jYkPH57qP3g
ubQ5+wj8QdOuc2oMyXfZPWWMeqiWRNsFgMy05XytY71NXZBN503VUpEt9UY28fv35Iqbe9Lv7x
ltmTO69MupE/PRwFm1o9dYl1qnKmfn9kLNhnteWt7PKPPC+T6X6unAzyL5KMqJU9YH9+dQW08u
7sygPb2GqIFK5fqWv9/Ktv3zYmfnYgY9sRxj39d6h7JvfRj8fi7aJ64puxm9Ecd5v83jPXle3ved79+y1Ls
hP8tr6uf34dEJHdm5mO7dE/ec5PVeHOfc5GX8uXHeXwLf5xmY7Jxk9148/rzk+148tj0jfp6yR42mW
tYuzqii+fpZGOMRoi4pvw7mwJuLMeFQcMiBCgvdr1EPJ5TztxMM6dQPhf5rrYf5A725D/VQ+xmx35
Ht9MRtl/MGFlw3nvMG7H6d89gbakjCc5Pg+G2D59J+jDoG8zj1Y9QvU3G3a7xu+NgBYAoz+uV9M
enSL+W6bIX/A5CTnjTWa9KZVTvsoaMdqbbi/1ueGTUM0Rh6N9uSN/n84j6JXuOc9d1s/bI/oxu3sq2
HjNo17dWwypITNg/
+vJW13H5pT8huV1lOenWcNKPePgZk8AetuMFlmDTbEVuO78XTnJv86fJoB+cHhtOnWwJnknPCe7
FhxPub8/PnvuRaNYmfhRcOISoAAJgNfnmPTdUltX/5uTCLXoa6tpj9i6D+pWdGvR3b52vSqbZmPmL
C/0Op81AjV7KppRZXPr+4J+f2NDo7wwDEHTWkatqr7yOrObMI4efpl3ZMgT9oKfPyXhwwR3/Y6uy
flAvevxGqt6h6/9mYYa9C3osD9HwiwflBrO+pqedWiWHwjzD8LLxwCFEBAADmmDP5gRq2N6ueO
MFRDt36vqzO4hfU9oYzCcRc/dbuUL2Csh6WHcfc/eLePi/O6NHZXTNnsg5/YiAVeKtJgXKftGOWv7Q
jNfxByzKH78Xz9oet8pk147pY/4aeVWXUZtirkPfiAernGrdnrP89dXBKXadTkl3n7nn5IwymQYgKAA
Awp5wAVfXWmJ+hjMXNC3YNtk7tfK7hXHtXhU1NO8B1fuEpSEn9Vqh/CZrtcHptlsOyLfP1i7sOCGf5
y6Iecqzq2bt5jz0MViXezdWMZjYfZVa/tI9h93bqyMHgjTuqd9QS4w9ajkV4L56XP2x5ZtqrkPficIMl+f

qydXLfOk8nJau3vdn+EQZpIUQFAACzwS/vkVSvDSdAndMJ/zL8RSPMYE8j9bAngLR+Mexaz2dbt7sn
h6qoWc7nZKxZ/uLu9nw6O8NfFgeHTbpmdl7y/6V9rOIJceZ7CUZNPfuGnmG4O2f4g5Zvvt+LB+T+h
62S2GWOB8PbWf5cw3txTLq2eOCPkekZ+UcYfhZeOISoAABgNvjlPYKeRddiD3czevzkW+dSTf4wM
Bxc/0KW1S8ac6/XkJUVc0i2WqVmSp7lL6nz94u701utKjMcPSpSOW21wPoeCgyhV7dwzTovM26bL
dtf2uOpyGnVY6+27r+veL3G5m+islngD1qLaFZ/2NIjADo1Oe/9w6mHcM+oHinvxXGoUhklqUldLm
RwkSL/CMPPwounDwAAMCOtqvSt33L69a5e0a33y9aPJ2VvRX669bLVFtWewUe139KvWUZh56U
6JyfEblu53s/3bun26+Xg+ZiLe6RVHbg2rb71S2tfJT+5m+H38TB9HgKPefie1vdRxvdv3Pc1573Yf2R9
7eK0K4/35PH7CLt/3Ifxb1dq9H0x9H2r2jFw3Pp7Pu1rNcl5t78m8/fiEedGvd8M7Ns9hrTf/uKem6H
XZfY+POp+0XJ6L453XvJ7Lx7fnpB/xzO6RnHuxXn6WRjjFdT/rIsKAAAwE+5f6F3WD43zNRwQWBCq
x5xdm9BVbUl/Bl3nnO/pqrT6M5p5eS7pnk7G5ZnV9cH8Gfre9fjfR2Gvqc5r79gUjT83Id9bxnk7iuLc
LwrvxbOmRvOoOqhhgkP7+Vl4cRCiAgAAAAAAAEAEaqICAAAAAAAAQARCVAAAAAAAAACIQIgKA
AAAAAAAABEIUQEAAAAAAAAgAiEqAAAAAAAAAEQgRAUAAAAAAAACACISoAAAAAAAAABCBEBU
AAAAAAAAAIhCiAgAAAAAAAEAEQlQAAAAAAAAAiECICgAAAAAAAAARCFEBAAAAAAAAIAIhKgAA
AAAAAABEIEQFAAAAAAAAgAiEqAAAAAAAAAAAQgRAVAAAAAAAAAACIQogIAAAAAAAABABEJUAAA
AAAAAAIhAiAoAAAAAAAAAAEQhRAQAAAAAAACACISoAAAAAAAAARCBEBQAAAAAAAIAIhKgAAA
AAAAAAEIEQFQAAAAAAAAAiEKICAAAAAAAAQARCVAAAAAAAAACIQIgKAAAAAAAAABEIUQEAA
AAAAAAgAiEqAAAAAAAAAAEQgRAUAAAAAAACACISoAAAAAAAAABCBEBUAAADAQmtvFKRQ0I+N
tl5raG94n19p9PTK2fDbuiEhLV0wbdlwz3vk+e1JY2Xca6xXNVb8ba00rK8CAGB+EKICAAAAODqa52
TGOemSUMHoqjT1kqMsZ9aK+rmpKJsX6tZnHZ3aesg1asv5Wkc/F6me3bS+CgCA+UGICgAAAOAI6
Ujt/OL38Zx/XTlwM89yXbr9vvT7e7I5KvksbsrZqn4eco16jXN+IFttyXZFPwcAYE4QogIAAAA4WuiNm
r3eoezrp3LqRKxeo5Xtlng5auAamb1Qq9IiQQUAzCFCVAAAAAABHTLzeqH590pVg6NpryIpbm9Orse
rX/7Rrehp1Vr119suC68NKtPqCtUJH1wEdeJ31CKsrGjyeYL3S6HYYzGPXj8F92fsp1ayzrDVXndeO3Ul
FtltujOpfo/aGXxagXN+yXjUo3vHbQto/dH0tcc5VoEar+6BWKwAsLUJUAAAAAEdEWep1HdJl2Bu1U
ytJYTVYDdRep0K2gfXN1VEBZlNWCyUxyoCqjUhpcMIpOxQceJ3F3t/IQK8jtZJRr7Rcl60YnTuHwlEtel
8JVbak7hZHba7KRqMh5/wEVS4M1gNIcvwqwA5pv3M+hoNUR/i5UueiNLhTJewaAQCWAiEqAAA
AgKNjzQ3p4vVGnVS53pW+qgPq9ax0hK1v7o5oh1dLtC/
+y5tyzkv7etJYd0PBqrT0a71td2qyPjIpNl6/F2OSpvaGePmv0a6um3ga+6psW5/r+hNFqRqm9n5iDc
MPTjLVrLnHV5b6hcF2Jjl+67VuGhvWfmsrOxfjnqu27Eadi8A1AgAsC0JUAAAAAEdIUTbdGYwy641al
bNuj8nKaWvJNWL9/mFoL05zBnqzXmhn56Lz+t5F2dGdIQPD3I3enN5rB4QPix+t7aeGgTCzuHnB31
ftfDo9MAOTTGnVs8OTUiU6fuu675lBqKO4dsYLbDsHXf0saOhcmfVeOzviZq/FzT1n+9Zjb+QMWgC
Ao4oQFQAAAMDR4oVsGfVGLZ+Ukn4aMGp9qKqcDiR3JTnpp31ix33dA90L01rllguwH8bwdve1A0
6dSBLymT0vz8ha4EuLsnbGbdi+HIYlthMITDJlPQudTGqK41fHZNc5DR3eHzR0ropr4h2yuodK7n4ja
rECAI48QlQAAAAAR8xAb9RD5+l8K8qJU/rp1MpyMn6am0BHRnTmnE6i8DlCYFIpo85ppLBzFSw5Y
HLD3NgTdQEAjgxCVAAAAABHj9kbtRYvTsvXYK/Onhx6Y8iHebVWhx7biYbtTyercHa88cfflg2z16lby9
Ss3ZpEcVP29D4Gyt7amueYpR8Alg0hKgAAAIAjyOiNmpQxjDw7g706u3Lg7tTtmVk66QWAo+p5ps
MsJeDXAHX05KJbmFROSaIqAdNKcvztXa/nqR24unVRp7iWaoZ+1ePUnkjLDlS7Xi3W0WUEAABHF
SEqAAAAgKPJmIAoTMlPDo3aqW3Z8Kapz1ZzdcObqKm94Q8/L59ZcwJAszZnc9UfQt7e8Gp0FlIZV2
4Gzta5WPd7WfYa61790aSTVU1twuP3A1djxv4k9PbVbRDscRoSdAMAlgYhKgAAAIAjKro3qjlzuwrp
nGAubi3NaZWlXG7Kqg4D/dzWmOFftd+ozdlc1cGh9+IREzJNorLtD1vv1KSk21XyE1S5kPuM9AmOv
3Lan6jKu5bGBFRJjDgX5r1RPat7ugIAlgYhKgAAAICjK6o3qqp7OVgzs9qavI5mIqfk7J4xPFyx63gO1Di
1a3MOvE4Je+2U7GHrIcdebVnr3eHxeYt9/BXZ7psz/itVaZk1TZu7Xs/fcexzEVYMVW8zrewaALA4Cn
1V3AUAAAAAAAAAEIqeqAAAAAAAAAAQgRAVAAAAAAAAACIQogIAAAAAAAABABEJUAAAAAAAA
AIhAiAoAAAAAAAAAAEQhRAQAAAAAAACACISoAAAAAAAAARCBEBQAAAAAAAIAIhKgAAAAAAAA
AEIEQFQAAAAAAAABGEvn/AZM6nr53azj8AAAAAElFTkSuQmCC]]></redcap:Attachment>

</ItemDef>
<ItemDef OID="q11" Name="q11" DataType="text" Length="1" redcap:Variable="q11" redcap:FieldType="radio" redcap:RequiredField="y" redcap:BranchingLogic="[q9] = '2'">
<Question><TranslatedText>Do you prefer Life A or Life B, or do you think Life A and Life B are the same as one another?
</TranslatedText><redcap:FormattedTranslatedText>Do you prefer Life A or Life B, or do you think Life A and Life B are the same as one another?
 </redcap:FormattedTranslatedText></Question>
<CodeListRef CodeListOID="q11.choices"/>
</ItemDef>
<ItemDef OID="summary12" Name="summary12" DataType="text" Length="999" redcap:Variable="summary12" redcap:FieldType="descriptive" redcap:SectionHeader=" " redcap:BranchingLogic="[q9] = '1'">
<Question><TranslatedText>
Alzheimer's disease - Description #[no]

If you have Alzheimer's disease...

1) You might forget recent events of importance to you. For example, if you are interested in hockey, then you might forget whether your favorite team won their last game, although you may remember watching the game itself.

2) You might forget the names of people you met recently, or the names of local streets.

3) You may no longer be able to handle your personal finances or plan leisure activities such as dinner parties or vacations. Simple decisions such as what to order in restaurants might become difficult to make. Your ability to concentrate may decrease and you might have difficulty driving.

4) You may start having trouble finding words to express your thoughts. You might need to be reminded to shower or brush your teeth, but you could do these activities on your own. You may need to post notes around the home to remind you of simple things like turning off the stove.

5) You may remain aware of the day and your surroundings. You might be able to travel to familiar locations like the supermarket, although probably with the help of a loved one or friend. If you are in a social gathering, then you might have difficulty following the conversation.

6) You may continue to enjoy hobbies such as puzzles or reading.

Now consider two possible lives. In Life A, you will live in perfect health for 18.5 years and then you will die of natural causes. In Life B, you will live in perfect health for the next 10 years, then you will live with Alzheimer's disease as described in the summary for another 10 years, and then you will die of natural causes.

</TranslatedText><redcap:FormattedTranslatedText>
Alzheimer's disease - Description #[no]

<p></p>

If you have Alzheimer's disease...
<p></p>
1) You might forget recent events of importance to you. For example, if you are interested in hockey, then you might forget whether your favorite team won their last game, although you may remember watching the game itself.
<p></p>
2) You might forget the names of people you met recently, or the names of local streets.
<p></p>
3) You may no longer be able to handle your personal finances or plan leisure activities such as dinner parties or vacations. Simple decisions such as what to order in restaurants might become difficult to make. Your ability to concentrate may decrease and you might have difficulty driving.
<p></p>
4) You may start having trouble finding words to express your thoughts. You might need to be reminded to shower or brush your teeth, but you could do these activities on your own. You may need to post notes around the home to remind you of simple things like turning off the stove.
<p></p>
5) You may remain aware of the day and your surroundings. You might be able to travel to familiar locations like the supermarket, although probably with the help of a loved one or friend. If you are in a social gathering, then you might have difficulty following the conversation.
<p></p>
6) You may continue to enjoy hobbies such as puzzles or reading.
<p></p>
<p></p>
<p></p>
<p></p>
Now consider two possible lives. In Life A, you will live in perfect health for 18.5 years and then you will die of natural causes. In Life B, you will live in perfect health for the next 10 years, then you will live with Alzheimer's disease as described in the summary for another 10 years, and then you will die of natural causes.
<p></p>

</redcap:FormattedTranslatedText></Question>
</ItemDef>
<ItemDef OID="a12" Name="a12" DataType="text" Length="999" redcap:Variable="a12" redcap:FieldType="descriptive" redcap:BranchingLogic="[q9] = '1'" redcap:InlineImage="1">
<Question><TranslatedText></TranslatedText></Question>
<redcap:Attachment DocName="185.png" MimeType="image/png"><![CDATA[iVBORw0KGgoAAAANSUhEUgAABVEAAADYCAIAAABtBTQzAAAAAXNSR0IArs4c6QAAAARnQU1BAACxjwv8YQUAAAAJcEhZcwAAEnQAABJ0Ad5mH3gAACxQSURBVHhe7d1vqF1Xue/xxlhPetuSoL0h6qWJPRRCGko0XIj7So0gTREjhbYYipYeTtnsgmIIaIo04IuWFo+QrRTaA1L7wnvTFy1FjjctVcgLhfQZoXnhnWvOMcYc8xl/5txjzX9r5vvvhxzlZc405njHmWukTzXxhn8Ax/AAAAAAAIApoucHAPPTkwvvqFG350AyGEEEELGkN3f2fHo/jt2nrpR/ZzGRNHzAwD6sL6xvuvZXeK0QQgh/b+653//ODh2z+6X9f6elX9tMYU0fMDADq3sbmx/7n94rRBCCGEkKHFy1a/yJu/eFH9zMbk0PMDADLp2o18FJ/2DCwA0fzkWe5ufoPjsxq2vWuC7tqa+lmJOYb5BBCCGGDFS2F5T7r6/p29SrfcrYjUbL2V2B1+/D5rVe/tUhxJ4SCFsQjiFetmUbV0E7VZe80mSjQlNiq1QmezwypxzEmVIjz9Cy5XxJFYzt/92vfJUTqFc5tvsmkk7x7nKCa/rvp7NyP1grZ/1cbn6bmjKUOmKdJVwmSnK41XlMmqOYwXV93YpsmeT/9XBJAyAObs9aXkxLH6rGYy+1ov8QrW7/TyYHLTx2DJWYHIrqUQSoyXVp6FfDyO9cZGv30IKMHI7CMkufLynAMOB3uZRtOl9ZDRKG40tC9XZNkRQzgsqjpI9zm8OUCgB2F8KMuwCKKsXwA2s8xkWqHS3Gbv9SKa3RnJ7o/2Qi6NTYQGRuA34RbBN1ULoZ81YFk0pq+xuEAl2amxH2GnV66kXmezRt9+bXBoRo2Kg7gXgTo5lEnqMDpdUN1/k9pNq4cOhdKFtRfSv7A44s8DADqsGQiwuETGBQAPNkKp5BBKJ3/OOQ3mOZJEJEEHB0p4zMDrJ3X1/QFTJ0HqHMlaQUCNqpfHc9AO48BBu5sWsk0Nq4h8mM2NBkRpD+hwc2LLeKxL7UhvHuhJmrgPLTkjt6tldaYOoz+/4T6I1PCRqmrC0M8YJRcW8I6JTLc91pLKsjvygJh9u1k+jVgVoDJyWBFOEpJFhmB9QnTkvDaLXZYiCOW99W3SmJ+jb9rT0JYkwewoVwZeSSk0atA7fC9gnP43xwn7c4cMAEMcxOtzQSPd6dKlBeS48wwpfU8FdeBijwBKpZAODAGwrXjfU6IIybfHvJp0MLpbrvTC6wnA8Sns8sNbSO+d2vQxBBPoy/Fm1iH8f4A09iUT1A/p5+gTiRTDwxXYT4IJFIhNwA9aAr3yLkOuKEt7NqwM9d6yQKg5i4OPJeVyR4tf3XW7mvpCkwPCszIKc1eBtmv/qpS13V0R1h5oqpTrkYDf8iuyQqi+LAWnIV2VD6Q9uIWMHfGYPcQXvt8ehDtsEUUhdiEl8Cdn2S4y/4kpd4CzRnAeeMedT0qoL0VnNH4rVfUqDHQ7ELVZKzyYHg0fd5n8VCCyjBgJHf/+5xw5iowqjR6aDC3IWGGOmvrUFn0h9TD4rpiTiPN6WXVhfMnfBvnBhW2nGoGdlYRLrYlVpXPs+m1OnGdVnE8UX/Xf+Dfq8rz6fAfX5O5VxXn7Qgn6WwUEPBO5UQXLERK2Gv9Q6zi3KyAWW9pyhqXOPpqM0HQDtnw/AMN7r6QOzLtHt8TZIf7sKnpZVSpKTAwDbaAUzlELtL+3GEr/XLsAd2YcEqDMiYsRjKvTrfVZbWh+ORXgy2bVS4VqNQhCQkUEmEcqoaX26DMLRhiK6jwZgzGEC8NxF8u4fdzRwJlnqsBxUeuWWo/tIbMXCYvkkV0nTnRSItmv1egPHfh

DBW9PxjEGmqTNNjvyc7Ic/9pguLNUtqHveP8RdXrPlExfjLkCZ3Fes2f5299VbDIpK8La2x9D3O6m1b
mMT+KHPuvfWz+UbMCmxtX5n6B1I7jzuFfF7OFKHlz2x5C+jHa+
+8Jg4ZhBBCCBk2289se/Dw7aIP7zk//tbD6qyAsaLnHwNPs1My7ZN+1/RBZri5olsjc09Ns1Te6Ls13H
vFX4Y0usuqL94x4/VlMzRWVlbRN22tsfQ8FVm9fnme0gXPAupnc0aEL5TTenhWJW+sW4xcWzneu
aJee9fWYCUYzNrlNf6ifkIIIWRsuefYbtGBD5Jf/eyn6sSAUaLnHwNPs2MzLVWFPdhqsqR4s1TeaKYz1
bwFiuniL0Oa3VXu1nkc5o6KLRVNbCz9H8GMb56KcIJP6YJ3AfWb9Y+wpq+uOrC7Rg+kZjHhx6OGu
DN61tZoJRjCxubGvtV94pBBCCGEkGEz1B/j9+aPv3lTnRswPvT8Y+BpdqRq0+XpgOwB2dtNmyV3nP
dOcTH+MqThXfGnYe7KRZ6YIqukNZb2XfrXGfeG2PKCGwsuoH6z1RHOEGut3tszjR9IfDF2pdlt+nUx
hW9GZ22NV4JebV7bPPrSUXHIIIQQQsiwueX7H//2XZ8TjfeA+e7hz195/z11esDI0PMD9TztKHB9W
Pn1ijhkEEIIIWTYbD+z7fgXPyu67sHz9EMPfHT1qjpAYEzo+YF69Py4Pp29eFYcMgghhBAyeI4cv0302
yPJi6d/oM4QGBN6fqAePT+uQxfWL4gTBiGEEEIGz97Hbhad9qjy+1dfUScJjAY9P1CPnh/Xm/WN9V
3P7hKHDEIIIYQMm7H9MX43KwcP/L//
+i91nsA40PMDACo2Njf2P7dfHDIIIYQQMni+9qXPiB57hDl99Mt///BDdarACNDzAwAq7vvlfeKEQQ
ghhJDB84UHPim669HmucdX1KkCI0DPDwAonXzjpDhhEEIIIWTwfPrxm0RfPfL83xeeV2cLDI2eHwC
gnHv7nDhhEEIIIWTw3HR6+8N37xNN9fjzp7feUicMDIqef1j674bLdfIXxF1aXVo+r349jb+LrrqjiGxgY
K+Vx97oYVTvUJot4zrGX324YC6+d3HHUzvEIYMQQgghg2ch/hi/m1NLR/76wQfqnIHh0PMPSPWk
WvuNkSowoZ7f3VGQebpir+KpKzUzelt+ev469PyLZH1jfc9P9ogTBiGEEEIGz90P7RK99ALl6Yce+Ojq
VXXawEDo+QfUcUdkmtvJ9Py+HfnZjX11r6Z7L+YI/ZuBKj2K3nVr6PkXxua1zUMvHBInDEIIIYQMnt3f
2fHo/jtEI71Yefnpp9SBAwOh5x9O8w42jW/
+66HnN6OUyl7dKZo8Ej2mo09qsuj5F8aJV06IEwYhhBBCBs8nfvixbx7aK1roRcwff/OmOnNgCPT8w
9DNkMXuJ6ttq+iYrEaqnEZ0o6H57SbMGuP2ZJUV1LZs9rT2nU6PHJ62fMfcZBYoVqs5s2eqTy7nL+N
U8c2m6JsiQyz2w7B4LoefRkFupjLEmq98NsUKo/cFNK1VGeg8EPFm4FFgZJ753TPihEEIIYSQMeSrX
9kjmucFzcrBA39598/q5IHeba3nNyf6cPPjHVL2JJl5z/9lWxFexehVnkhB78bzVsZ6ap4BzjMNze+ffM
aawm7cjOjH1sq05v3imngZ2pGg78re03dUV26mLe4WL73MGEtkuL+wvOibtHKH/5mWIzzv52/67
4suOKWWUr/iGXsURua1d14TxwtCCCGEjCF3ndgpOueFzpPH7uUP9g+lh55fNDfzHv/t6aJ9zOh5n6
bpm/RzMqPMg7Naq+gD8M3v3uvM79ym74l8cu605RVZJzKtPcZX1bcjYTZELMFZt5nGCM83Y+3O
Fn4euoA1QizG2YlcrJzC3GDmtFZlLz80UXmjI6VWeUUXN3fpK+WFSG0Ma+3yGn9RPyGEEDLCfOp7
/7Tof4zfzb+f/J46gqBfrff8LtmEzMdqJDIL3Uv4nqbvYclxpt2q2X1sfuuaGKZfWpN7LgmtTWsmUqolx
Zw1fM8yJ4tkYhM6Vc39wQfibE2speHTsDVchRkWnKiBcK3yOckxzj0Zc22OtaA7G5sb+1b3iRMGIYQ
QQgbP9jPbHjx8u2iYp5E3f/GiOoigR933/Cn3hOnZlpbU/1/gZsLzZERrqJhNF1c9DZiX78l7CohhZnaP
YMH6nrDxtPZA+en6dhTm2WtGXvWPqlF7U+Aj0+vWr33cvVVHO3NUbzDPqLSVrTWuJT8LPaRSzH
sRo7B5bfPoS0fFCYMQQgghY8g9x3aLVnkyWTl44N21NXUcQV+6/e/2VxuInKdpmGnYFOh7ls/rXy1
wN+F5mv4eSQxs2kh55vfdG5jdx5qoqs1py5HOBn07CvMsqrxYzrC1SQv19+gReXGnaJOnYWoI+n3
v9mYCN8a2l1BLPgLvcoJrxOBWfr0ijheEEEIIGUPufORW0SdPLE8eu1cdR9CXgXp+b49R3xjo22aT6F
8vbjvheZr+HklstWkj5Znfd68YltSmtTetuS0nRvt2FOYr2fCZ1NHTxG5R02ZD9OiyZn1JPSJTDHJWXju
HNUWuycDmteQY73Jq14hhnL14VhwvCCGEEDKG7Dx14/T+GL/I7199RZ1l0Jfu/w4/zz1NWgoffV9
xm361sP1E7MnYm5LjmjZSnvkbNG+
+u2q1Na0etbS66tvk1tbme06eGeom9b2vp46uQw1aWl4uJrAG127EXbu+Ym7xbU/J3tpCsbRaclZf
FXPNs0YMif81fkIIIWScOf7Fz4oOeWJ58fQP1HEEPRqi59dXPA1EtDeQgxrdNGLuk8mYdkvustympw
Hz8s2/leZNX3A6QFc701ZuMS8ic8Z5n1ODZcj7GtziZ8blKoPr5pQlyvFmHrnMgrmxvOwfWEqrZUbp
Mc6WrHlCpTGQzWubR35+RBwyCCGEEDJ49j52s2iSp5QffePr/M/1DWKInt/0GB7hic1EZf/gXlkogaf
pfzzWHut6OKM6U16lSfNWXqmIfDStTGve1pOY5ZezenYU5lnUTO3u3PuqZbWa8hn7Pjm6Zhn+kj
N6SGB7wTvDy02q5X6+gS3NyDVieJf/dpm/tJ8QQggZYY4cv020ytPIdw9//sr776mDCPq1OD1/uKF
Y1JYi8jSrmxXvh5o9D/tR58ObNW+Z6grqSs0/bfmGNd4svxzo7CjMsygtujv/fXZh30fm5dmALf6Q7Y

qzevq1qu1fZqG62AbL3Xqt+s83eyO2RgyO/3F+QgghZITZfmbbJP8b/n/8zZvqCILeDdnzb6kRsJsJFz0
Fxkh/a/l+YpzOvX1OnDMIIYQQMnhu+f7Hv33X50TPvNB59Sf/pg4fGMKQf54/MovD3BNAU4XRMV
9avp0Yryd+
+4Q4ZxBCCCFk8NzxL7eItnlx8+NvPayOHRhIYs/vlbfwTkPv6fDLadQ1898sDv1bAM8kBfMGbRXGw3
yhc6FvNTAO9798vzhnEEIIIWTw3HNst2ieFzGnlo789YMP1JkDAxmk5w9NFOyNIp195C1gIFbPT8O
P0du8tnnohUPinEEIIYSQYbP9zLYHD98uWujFysrBA+
+urakDB4YzUM8/U50s1rJH+/romwCAWusb67ue3SWOGoQQQggZNjtP3fjo/jtEI71AefMXL6qjBga
1tZ4fADBJF9Yv8Nf4E0IIIIWPLnY/cKhrpRclzj6+oQwaGRs8PAJh5/g/Pi3MGIYQQQgbPIv7B/ieP3fvR1
avqhIGh0fMDAJSTb5wU5wxCCCGEDJtP/PBj3zy0VzTVY87KwQN/effP6myBEaDnH9ql1SXrbzrQf/k
bfzlBiPj7IeIvt0Q8+3mm2qrK3/Pf+Wdf/cp1qc9niNYcfemoOGoQQQgghZNjs/s6OBfqD/f/56/9Qpwq
MAz3/kFRLZDVE9Px1RBsZf7klQ/X8plCh249eFet6T0pvzxBt2tjc2Le6Txw1CCGEEDJs7jqxU7TW48z
LTz+ljhQYDXr+4fgaInr+LRIPcZ4mc6hn32Pdvntwev5F9c6Vd/hr/AkhhJCx5atf2SMa7LHl6Yce4I/xjxA
9/3Do+Vuw+D1/n31x3z143/XQotcvvS7OGYQQQggZNiP/g/2nlo5cef89dZLAmNDzD0P3l5a8LbL7T
muM24aabmpGvG1PYo8LN16Bdtd7uVK5wR01fZ9+u7zDvaKnnV0R08VfxpnBmWy8WLt3KvsWe4
FRlZsqd+mSlsDC7cWJdVdUa9nVQrXEtmecrVtjymnUm+GKnomwUM5ePCuOGoQQQggZNp9+/Cb
RaY8nf3rrLXWGwMjQ8w+jrgFz+Voym/V+o0mqrKZOXcm4F/0ze0pbl+r6Pv2+edsUMZOoK/lrMV3
8ZYR/KzOqrDOVuWCzNuoVf2KedwMLr13uTLRYqJa+bs3jbN1zbzE8WnErHwdG6sQrJ8RRgxBCCCH
D5gsPfFI022PIr372U3V6wPjQ8w/H1xBZPZS6akbpRirYkJlOy52kvBJqvvSsVucXntdZijPEmsa3zQo1
QN/irl7PkL8U08VfBplxskZGLaRuZs9WHQ2emDuxl/tUyitiC1us5dmHM8ytnqut6KuHhbJ5bfPIz4+I
owYhhBBChs3XvvQZ0XIPm+ceX1FHB4wSPf9wYg2YdU0Mk31WRl6qn8TDmdfpBT3NoTOvZ0zj0sU
9xYvl5dlE9hV9v5gu/jLEN8xcU2uvjtGvPBuzLglNnph7wSvlM3UH+G5p8pGZ6uG95uSNtUvEArj8t8v
8Nf6EEELIqHLT6e0P371PNN5D5fTRL//9ww/VuQGjRM8/nLQGzLRfHmqilP6wHKJK60nMDZ6lZQJ
3hdfvo+7KB+S/Xlo9n99VXjG3i+niL0O8exEXq1PpN31CtbxVzLz6arMlm/rlqNiN1eWaAb5bPKt0hn
mqS96KzfaG0Vu7vLbjqR3itEEIIYSQATOSP9i/cvDAu2tr6sSAsaLnH05aA1btrarURE26OA89Jr9Nz1G
O98yaCaxui6XVbdmIYnT2i+KSuWLuFtPFX4Z49yIuVqfSb/qEanmrOEtstmTPXO6N5orgDrBqNZnZv
5NMTUVfPSymc2+fE0cNQgghhAyb/3n/p0QH3n9+/
+or6qyAEaPnH05aAxZsv0qN+kMfdWN2nx4fX1pGD1RXU0sX9+n/eD+/O7+kr5QTiuniL0O8exEXq1
N576jhv0fPq682W7JnLnmjHpIpRrkz+2o1mDm0+9qKvnpYWE/89glx1CCEEELIgNl+ZtvxL35WNOF
95sXTP1CnBIwbPf9w0hqwBm1Uky7OT925tLxcDLdHmynsxk/O6yltGsNYaTWP+8f4iyvWfKJi/GWI
b5i5pmpVxzScuELOmHMmajZz/WfqjnCfvK9Wk4/MMyZTX7HZ3rA47n/5fnHaIIQQQsiAueX7H//2
XZ8TrXg/
+dE3vv7R1avqiIBxo+cfTsMGTA4zr/WFJh1a0+bLzJUTg82bemIzq6kUXlxN6fJG363hncRfBjnrtOqrYq
GZ9R1meZFSDZ6YrBOgZwo/CbmecsHlzL5a5Th10Xruepineqa+oq8eFtnmtc1DLxwSpw1CCCGEDJi
9j90suvEe8t3Dn7/y/nvqfIDRo+cfkNVcZfK2yNNcuX2T1VtZyraq0SQB9prcsdUVa3Yn6F9bLl66vNFM
Z6p5CxTTxV9GhBeqqjlT+e+oKbSFJxadqcFn6i81U85cHVNcDz8Jc6On+kxtxWZ7w0JZ31jf9ewucdo
ghBBCyIA5cvw20ZN3nT/
+5k11MsAiocflN005f2Up7ny903VRq3aizWexMcsSfR3RrWyZz57QPZ209LuOO+d4mL8ZZwZnM
nGi8cWr54LPSKhepNcWbMlN/tMzYeXmV3Vr62p7SFmMnuF2VhnZk91JV6x2d6waC6sX+Cv8SeEE
ELGk+1ntj14+HbRlneXl59+Sp0JsCDo+VGl27SG/SyA69Dzf3henDYIIYQQMmB2nrrx0f13iOa8i/z4W
w+r0wAWBz0/bOY/mKXlBxBz8o2T4rRBCCGEkAFz5yO3iv689ZxaOvLXDz5QRwEsDnp+FOz/kjb/X
WwA9Y6+dFScNgghhBAyYO45tlt06S1m5eCBP731ljoEYKHQ86Ng9fw0/AAa2Njc2Le6T5w2CCGEE
DJUOv2D/W/
+4kV1AsCioecHACR658o7/DX+hBBCyHjyqe/9Uxd/sP+5x1fUz34sIHp+AEC61y+9Lk4bhBBBCCBkwd
53YKTr2OfPksXv//uGH6gc/FhA9PwBgLmcvnhWnDUIIIYQMmK9+ZY/o25OzcvDAX979s/qRj8VEzw

8AmNeJV06I0wYhhBBChsonfvixbx7aK7r3tPz+1VfUD3ssLHp+AMC8Nq9tHvn5EXHgIIQQQshQ2f2
dHfP/wf6Xn35K/aTHIqPnBwC04PLfLvPX+BNCCCHjyd0P7RI9/Jby9EMPfHT1qvoxj0VGzw8AaMfa5
bUdT+0QBw5CCCGEDJWvfekzopNvmO8e/vyV999TP+Cx4Oj5AQCtOff2OXHaIIQQQshQuen09ofv
3if6+Sb501tvqR/tWHz0/AO7tLp0g2X5vLquiLdvWFq9pN7JnF92Lprx1kTROQqVIe77DWYAAOWJ3z
4hDhyEEEIIGSqffvwm0c/X5lc/
+6n6oY5JoOcfkGylFdOu656+qmy4m/T8/jli/0ogZ01ZPwMAVN3/8v3iwEEIIYSQofKFBz4puvpIzv7r
v6gf55gKev7BmF5b99emuS4u6Pf12874Jj2/HOJMIm9w7qidAQCkzWubh144JA4chBBCCBkqDf9g
/
+mjX/77hx+qH+eYCnr+oTjNtroU/s/Pa9vzjBhjXoY6dD3AM0VxqXYGAPBZ31jf9ewuceAghBBCyCC5
5fsf//ZdnxMdvsjKwQPvrq2pH+SYEHr+oXga9jA9uNC857dadqNSsDpxlfzXBgbdP4AmLqxf4K/xJ4Q
QQkaSvY/dLJp8kQv/53+rH+GYFnr+oTTo+T3tdm4LPX9wEvV+fc9fNwMAhD3/h+fFgYMQQgghQ+X
I8dtEn2/y4ukfqB/emBx6/qHU9vxWO16Mcfr5Rj2/Jrv74qbaVVj8MwBA1Mk3TooDByGEEEIGyfYz2
45/8bOi28/y5LF7P7p6Vf3kxuTQ8w/F153PumrdSbvduOm5Iz2/MyaTXbNeVeuG/x2BLTYDANQ5+t
JRceYghBBCyCBx/2B/9vLK++
+pn9mYInr+wZjWWffO1Quyey/fNb22M4O5R18xF9x/c6CuhOdoOoPnAgDYNjY39q3uE2cOQggh
AySOx+51e759z52s/qBjYmi5x+Q1cbbVOtsum2H7vlDE8zoMaFZzByBScr362eg5wdQ550r7/DX+B
NCCCEjyT3HdhcN/5Hjt2Uv1U9rTBQf8MBEx11tm+12e9Zi69dlu125P7tsXlpDnK7dfq9QXYXbu8dn
oOcH0MDrl163TxuEEEIIGSrbz2x78PDtWc9fvFQ/qjFRfMAAgJ6cvXjWnDYIIYQQMmB2nrrx0f13FL9
WP6cxUXzAAID+mKMGIYQQQobNnY/cWvxC/ZDGRPEBAwB6csMNPf3QoVAyCiWjUDIKJaNQMgo
lm16h6wGPEgDQE04kySiUjELJKJSMQskoIIxCiOBRAgB6wokkGYWSUSgZhZJRKBmFklEIETxKAEBPO
JEko1AyCiWjUDIKJaNQMgohgkcJAOgJJ5JkFEpGoWQUSkahZBRKRiFE8CgBAD3hRJKMQskoIIxCySi
UjELJKIQIHiUAoCecSJJRKBmFklEoGYWSUSgZhRDBowQA9IQTSTIKJaNQMgolo1AyCiWjECJ4lACAn
nAiSUahZBRKRqFkFEpGoWQUQgSPEgDQE04kySiUjELJKJSMQskoIIxCiOBRAgB6wokkGYWSUSgZh
ZJRKBmFklEIETxKAEBPOJEko1AyCiWjUDIKJaNQMgohgkcJAOgJJ5JkFEpGoWQUSkahZBRKRiFE8Cg
BAD3hRJKMQskoIIxCySiUjELJKIQIHiUAoCecSJJRKBmFklEoGYWSUSgZhRDBowQA9IQTSTIKJaNQM
golo1AyCiWjECJ4lACAnnAiSUahZBRKRqFkFEpGoWQUQgSPEgDQE04kySiUjELJKJSMQskoIIxCiOBR
AgB6wokkGYWSUSgZhZJRKBmFklEIETxKAEBPOJEko1AyCiWjUDIKJaNQMgohgkcJAOgJJ5JkFEpGo
WQUSkahZBRKRiFE8CgBAD3hRJKMQskoIIxCySiUjELJKIQIHiUAoCecSJJRKBmFklEoGYWSUSgZhRD
BowQA9IQTSTIKJaNQMgolo1AyCiWjECJ4lACAnnAiSUahZBRKRqFkFEpGoWQUQgSPEgDQE04kySi
UjELJKJSMQskoIIxCiOBRAgB6wokkGYWSUSgZhZJRKBmFklEIETxKAAAAAACmiZ4fAAAAAIBpoucH
AAAAAGCa6PkBAAAAAJgmen4AAAAAAKaJnh8AAAAAgGmi5wcAAAAAYJro+QEAAAAAmCZ6fgAA
AAAApomeHwAAAACAaaLnBwAAAABgmuj5AQDYqvPLN5SWz6urAAAAY0PPDwCYkKIZ77YLv7S6
VFbosmDf/2ZhtrHM0uoldaFNlc3k+HclW1V5ht18TJ0L/IZR371Cu9+MyG/RyFtb5Z+q8om1u61uRZ
9Mp/
+g6FB4U4v6OQV2VNlOWx9Tg1oL+s+k6wQ9PwCgWx2e5iusOn0e2vKyXZx1srOU2Ud+rur4QDXby
NLycvZ/Oqkz20LvR0L7PNr+t6LyzS519O3L99LTN8J+bu0VCf4Orf4eqm50LsGK0be2KjTV7Lq5UDzS
eUv5Z7E/rrlL1D6Z2fut/oPCt6nKlnLz7SuyqfytFn8ruduxits62lFe31zKX827u2a1WihVPDtFPJ/KUxTv
oQF6fgBAh6rnqepppFW6UP7/+zwQVHfYlc6rzAqox9dNmdln3/ljsvXzwVTkJTv68sm5u6vV9jle0Z+
Hu3D51Whra+GKsbe2quIUepx6uWX5/YVqlezxmQvzf1x125ldV2/PVacQ21Qb8yuRTXkupQtuR5i/
aHhH8kqHtfQb6uW8n9psOjN9/k0uX1ZL5W/OtafrET0/AKA78gzgnBpa130FoZ/jR8fbMtPPfpF+aIu
Z7zi4Zb1/DzLdfhWqD7Cz/eUT2x9U2x+cs3J3Ky0/yMizavEx1k2Vv5/8IPXd3Vax+AuZq7NfzF1GL9ZT
q5t/XLiFWtmHEtlOVYvf79pHV7uY5pypnH20WMw8zfyF/Dq0Wei6Qc8PAOiM+5O5xdOOX8+HgX
w/bZ0ZgyrHn/ZZz2z2y24K5U+q1PEz624fQR1/SJniEWYlil908y13foda345WOPPNKlafW8slI9O1W
KluKufBJqmr0tqGfBNZ12a/bO3L7qnlfina4BbK68z+oIIxf1Xfo7Pkb7e1N2+t/Ks2K1H8IryUrXFqBR5

nO1uzH5O/UGsbu07Q8wMAOuOeALxHlDZ1XsCWHzzaOr258r0oHW5ptgs9/axkdxvSio11WSf/5r
V8lI/r54uXf+NyXZVy9+H+Lp6LU8Cdv+VnGZmuxUrxqfIProWnGK+Sv9vOZ+UpNNuDvjJ7u7UvRaC
WpaVSbiHxueQDws+3Gc92LPF3tyo0W/n8WivlqZVfkU+vpc8q34GqNvt1ddp2n+L1gZ4fANCZAX5
U93cWEKfFTrV6mKqabaN8XrNCvezJ/Wq0Snw4xfPr8muRF+z0e2fvofh1N/XyueWja/Gjyie0V+5+F
Zwh84lM12Kl2FTi6zgHb5X8otLWY3MKzfZQvp693dqXIvboMsX22qjmFnK+fC38Ro5up4X5bZ5axe
MqrhW/bqmed1/5frTl5cqXZA75tOaDcT6l+DOGFz0/AKAzA/yo7uksUJx0ejxzFIe39gtWjmyWNk7Y
MXnd7p6f883rtl7+6XT6yNwKHe6o8qVo7xxfcH6Hur9lnU9vPm4BI/LWVgWnyp9mW9upWXD+div
FZKHKd8LSRS1XS98It5Azcf1aakWmyN9q67sw49RyK+Sf3Hw7KtQ/mtmINnYnf8u4H3/9WiDR8w
MAOuP+YG7p7BbWx1kgr9HzgSOv2emTy83KdF+l+
+10cZQPa+1UHeaW6HRHlra/Eu665YfV9s4i87VYyj9V/rm1uJnaBecDWihYU2j2dmvfitpNtfU7zC2U
X7E3Ir+MCcLbaWkbFqeWW6L+6TZUO9FswPxfinwH1Wncyi18TNcden4AQHfkT+bWjh9B3VcoziR
S6+ePrIq1Dc85qBPtnNpc2bzWtPmH1OmnlFewN9LlGTH/cLr9zrnfgF6KZlr/RuQfTXXhlb05H93cPB
W1yFtb5Zkqv9Tuh1S74HxAC4+vptDs7dY+pEE3VfmN1MrvquB2Ovg969aq/GbKtFc0/jG18xnlq3W
LzC7bc8eXAi96fgBAhyoHkHZOBX753MJinwnEjvrZzKxoJ59Q77upHHXbO/e6uvxWVxXnYaOHb0Sb
exPfgFy5BXtvbT3MSMX4YrYkPJX4vApzbi6vVllpVsV6ndecp0bDJzMb1tqX3tlUdsGavFiSZxGNxTdVe
XeeMoqznUJ+uYfvduv/oAjXqr4z96MrpvNOU/let/okrx/0/ACAbtkHEH5OozeVA+nc59GQ/Ovd2ew
DaPkcj1ZUP5WC+mzEWwv0iYU3tZB7inxGmcn9g6J14l9VFMojAweJOdHzAwAAAAAwTfT8AAAAA
ABMEz0/AAAAAADTRM8PAAAAAMA00fMDAAAAADBN9PwAAAAAAEwTPT8AAAAAANNEzw8AA
AAAwDTR8wMAAAAAME30/AAAAAAATBM9PwAAAAAA00TPDwAAAADANNHzAwAAAAAwTfT8
AAAAAABMEz0/AAAAAADTRM8PAAAAAMA00fMDAAAAADBN9PwAAAAAAEwTPT8AAAAAANNE
zw8AAAAAwDTR8wMAAAAAME30/AAAAAAATBM9PwAAAAAA00TPDwAAAADANNHzAwAAAAA
wTfT8AAAAAABMEz0/AAAAAADTRM8PAAAAAMA00fMDAAAAADBN9PwAAAAAAEwTPT8AAAA
AANNEzw8AAAAAwDTR8wMAAIzL+eUblOXz6lJBv7G0ekld6YauI8qPQflsMs5zuLS6FHor+h4ATBc9
PwAAwLhYfW21Pb3ee/6ybc/5HkOwsy8f6gj/TQYAdIeeHwAAYFwq/1m23aFe7z1/o/17m/vyXwXQ
8QO4ztDzAwAAjEuI57cb3Ou85zeNe826zPMzz8lcoeMHcN2h5wcAABiXas9vNapOz+9ccBtjNWQ2
wppX3WBdsbthfTm7Zv3X6a0imvWu8761trJMTc9dma8yobVSLTKXGZ2PMa+cHcTWP1NdjxgS2W
D0PgDoFT0/AADAuJTN5GrxK90yWl1m/tq9YLWbqiUu29FapokO32P12bKvzVkL8UwSbX39ReXWL
dZaHOXillf1L0X1uvXXLCi8Qf990eUCQHfo+QEAAMZFN41ZB6kb06JhtN7IB7oXYj2/GmS1pPKKbku
de8or+oIsY0aYpVh1GrS74QJmQrdmjBmslNPkatevB4TXE9hgaCK5AgDoBz0/AADAuFS6RvXC/rXbd
lrtpGxm9ZCyLw1f0dO4I+QY2RFn5CUziTUmSK5gRu7EvRBnFjAjbmmwfsmp7t+gGdZo3wDQOXp+
AACAcfG311mr6XTGnlZZtqbukPor+rXdKFFfn1SN8ROHKLAHukmZ0SX1Vbq1WeAkN1m+pjtbvB2Y3
qyxVtwUAvaLnBwAAGBfZAavX5o/3lz2kHJnpquevXtQvfIKFw/xj5VaSe353CQ3W72vec/r94OyBG5
uuGgBaRc8PAAAwLk4zKZtI84an7ZSNsTuk/opnWjGvb4TQYIjH6tL6qtya7XCS6hfnB6RKQY51Wvn
sKbIhYsBQHfo+QEAAMbF00xW20fzRmxkCz2/3VxXxzRov90yYWY6e7BTo8Wev3Yq91bnoUQ2mL1
lTbzldQNAi+j5AQAAxsXXTJq+cca8UV5V7aRpTJ0rbvMavhKexQwJV9aNrVsmJlygvH/LvXNkCXXrl9s
px9ds0NlIdBkA0Dl6fgAAgHGpaSbtN6xWVBK9bKwFlVfM6yU5u+53M/7S5QC3TJweX2Xf3WbPX7t
+/3Jm9JDQ7KE7m64aAFpFzw8AADAugWaybFIrb9i9a9ZWysbYnaz+in5tT+csZ6baN1cHBHYRVZ1P
Nslya7VqlxBbf3n7zKyk9Vjst32z27fONF0xALSOnh8AAAAAgGmi5wcAAAAAYJro+QEAAAAAmCZ6fg
AAAAAApomeHwAAAACAaaLnBwAAAABgmuj5AQAAAACYJnp+AAAAAACmiZ4fAAAAAIBpoucHA
AAAAGCa6PkBAAAAAJgmen4AAAAAAKaJnh8AAAAAgGmi5wcAAAAAYIr+8Y//D36DcJhawniMAA
AAAElFTkSuQmCC]]></redcap:Attachment>
</ItemDef>

<ItemDef OID="b12" Name="b12" DataType="text" Length="999" redcap:Variable="b12" redcap:FieldType="descriptive" redcap:BranchingLogic="[q9] = '1'" redcap:InlineImage="1">
<Question><TranslatedText></TranslatedText></Question>
<redcap:Attachment DocName="B.png" MimeType="image/png"><![CDATA[iVBORw0KGgoAAAANSUhEUgAABVEAAAEJCAYAAACdcmdaAAAAAXNSR0IArs4c6QAAAA
RnQU1BAACxjwv8YQUAAAAJcEhZcwAAEnQAABJ0Ad5mH3gAAE9sSURBVHhe7d0PjCRnfef/b+/aY
PPHNmBskx+RHat7sZYlGJIg08PxL4egZ+6SJbnbYOVg5Oh3PVyi0zQSkwOxF+mX2wguG4lunS5iWrq
zliQyTEJugrLdIJRgckzHR4IxYTI/Z7rlc3T8wDYGjPljB3u3f/VUPVX1VHd1dV3VV3VXX39PtltaerpqfqqT/T
O/OZ5/k+hb5FAAAAAAAAAAAChjumPAAAAAAAAAAIAQhKgAAAAAAAAAAAEIEQFQAAAAAAAAAAiEKIC
AAAAAAAAAQARCVAAAAAAAAAACIQIgKAAAAAAAAAABEIUQEAAAAAAAAAgAiEqAAAAAAAAAAEQgRA
UAAAAAAAACACISoAAAAAAAAAABCBEBUAAAAAAAAAAIhCiAgAAAAAAAAEAEQlQAAAAAAAAAAiECICg
AAAAAAAAARCFEBAAAAAAAAAAIAIhKgAAAAAAAAABEIEQFAAAAAAAAAgAiEqAAAAAAAAAAAAQgRAVA
AAAAAAAACIQogIAAAAAAAABABEJUAAAAAAAAAAIhAiAoAAAAAAAAAAEQhRAQAAAAAAAACACISoA
AAAAAAAARCBEBQAAAAAAAAIAIhKgAAAAAAAAAAEIEQFQAAAAAAAAAiEKICAAAAAAAAAQARCVAA
AAAAAAACIQIgKAAAAAAAAAABEIUQEAAAAAAAAgAiEqAAAAAAAAAAAEQgRAUAAAAAAAAACACISoAA
AAAAAAABCBEBUAAAAAAAAAAIhCiAgAAAAAAAAEAEQlQAAAAAAAAAiECICgAAAAAAAAAARCFEBAA
AAAAAAIAIhKgAAAAAAAABEIEQFAAAAAAAAgAiEqAAAAAAAAAAAAQgRAVAAAAAAAAAACIQogIAAA
AAAABABEJUAAAAAAAAAIhAiLp0etJYKUih4DxWGj29PjdPPFeU1hpWF+1iMxj3ZB2onXjqXjpHWdL
MxLO7LSlg372PzHqPs4Vb2GrGzEO5vtDbdtK5JH08KNuQ9CjmjNj+2g0AAAAAwPwhRF06Rdm8UJey
XurU1kPKe9Pc44q9+r+xzoDRsr0V1dSKdxX/XzaF/vwA7OMAJK++2rh6csLrj7F51V/XzDWaW+YFr3F3
Nt+iP0ibCz0/ewV/RPkJn/TDq2fXsHF/I4Ylp74BZJMAX/v2V/+pzGlepGN9vUI9bJeUuBM/sUUVYqJQ
gP5PdtoTbJPbMfWFi1tpJxUuT6wRrUfoQt3zbuKyyQcWQPrsDDzAUZLNNfvQwXJ0h8tK/OdDqnKJyrQu
ULTR/ocaBJK7+bbdRX0VIhyn4JXGw1ZGbhH2CI5ui3bKasF/2MDFTTJxVZ3xMTqMvU46xZ8wfVsgp/mYj
7ob3o6QysjMkWq7gbQN97PZWN/8sFdTzNP1xLZqYeJJBvNGvXs9vvlH+oQIJTJ5Wq5ufwGfSePRH1C
B9q57MpqEbG2dfkr9iuH+pYjXGQ68uSeGfEnd8+hrYRSmOc7y/YPH2ZiQKGhHnCEk2jZbOOLUytNoTJJJ
kWPdJc3UMAnjWSyiFEM3vz7KqxsNC2kdmmcqs8Dx6D3PMbsZ6jjbN1rsCOcYWUkVfvMaPgPC+/PQcbS
GpMASnWYC6ouKbRg8mVG2fENEpQC2ODtGkKPTFmtATOchHbw1ejeNoOMP+DDSZ/kbL/iEAkWx38rN3
YrD+o3LIFWyC0C9P2T09CFRtNp6nSUyNd5NCcPWCP6ur58c7GBG3gV1fY+EpgnAA+HZwTL0MhNTsnT
vz8aZ3lVA6jEcTd/xC4ITXS3TAm7NKLGZR/qOFRKbOtQcCzeLrtgDDVqhYiwMmPk1kKH4xQkUJ2ACsTg
QERHswX2a1/WWB0iAtOJDN0XuALvRWqqY6tw/wxYjTanU87Sy8VWaDaHbdvuv/nAEKrSnO6+SVlVYXDuZ
mt6kmRvdf3cpW1iRrv/BTTtt9K1FBUkDmp00Se3G7l8ZqlnvelzUN7Ef7r3R2DP5r7A6EyHpeigV09fDiv
/jxFwEk9KHWy7Arre6ZiNeejWUP0/fuaE9x2ex7ULfo8OzrTZvg4EwrdOwGp8dAywnQzaBacKfRFDYk
zmRBJlj9GaIw3X2lnSYPcpUwJaLeuDO5gDVuoXQnfVXOGmN9X0nDH/VzrdJ4qEmA67Z5xsHSK79ZvMiT
KBnRRLPXhRgxQCcZTLA1J6eGsGtgnwRIdLOnmLSCrUvCwh7yEgtSP06hAA3ZrgiT9e6XAFBgGjNjd/OFT/
AxHnSeppU60J2ukhh0Kvr/ZIovhXYchlv/UGyu/OnpnZ3aJwWqrp6HHRoUoejTAfhFYc6dN+vZI6NNgi
6WTaQqkBvgtHwVoeXlcJtv5gxuGoMuy9tb33IHTgYzn6jvz/UiIiEyVgQw8sPMtQVUxvBA0bT/iNHZbv
ihoHlI/qBfXgrPFtSo4+CeB8ulBvtqOkqq7KXSbrT8jvf//SbPJOm1/V29lJz13Bb+QL3/u8xQBmU+Pu
DKnxy8P99/RZbaVNXAc0elqWvoN1g0wmhjiRUEbc4WqMbi2W4Z5j9XxcKtDwjk68fNGaPhfqI38EzFo
mVgkr7mqAifTqKiNp7bJOXxc54T3cIeegsMlKKS/j3WDAXncw5XHJ2RiM4QVAYjeX40DKhu3zEv1aiK2
n+ll6g7IJwK1Fe8V76Zw1z4MwH/Or1W/iyY2c3WNx1/5C6AeE4sL/iTP64CmdvGS2yHJtpWSQrr6ybfX9
4u3PMBEC6GJiaRBXl6TkHm3NHXSekIRf/3Tqh2VzIgmwBXUcR/OFcDsHP4PLm2mJNmfiN91H4RADRdk
Pc8oaJhgMpeEoRrPXnGQRVuy2tyYyCf5ryDY8Lj1QJzpZdGP0j/R8cxGVw4LvLmjJ0X6I1AtTcbePAaIt
yyYymftWfnrWewyFzUkUh4R5VI47UZqHwj2SI/bZQE8G92+KZaz3mtdHGJ5UD7ufLmgOrmDFX1a7ye+
wd3IKUdcVgPi51vOVxpTHjKQgoJxVvpCwk2Ybm32EPAhUIPPcoK1nOZ7jxgPgLwChVkB+aXR41Sdm
GYYlTXp7QFCAp1o3kOZANjw+gmk8wOOiFgmtUzVPGX5O5ZVBEaJXybILdUbS1k82lx4ZrrdTD30C5
9WkK9NwM2ZY5aBIBlE5ZL4CZFfJ3bR13Wxeo1dqR6/dLQvt2EVCN7UfeqzqyPxdkOTQxG9CDQVBL2
0VcSQpPXd2KHUkEGD4UuMp5hBQ4HDnx50oj/mlRU/cWdSi6hOZg/o/IzN/1pFmmYDu7jnnWBgdP0jfbaL
0FSKEk0YkF0o/a6QgJ3NMCqcQUXlFf11MKXjo70zPOvDjA3FcbF7FU8NbQVlIgwX7Zvnxmdxgn+CkoI
HxqnJpvhlgiEwpSnfNS6rbUkePVupLDJr/ssV6hm0R08ELrG0XcQxapB0c/dOn+Nw/hdYDkStDrQIaLO
eQmJSdA8JPFXzBXSTm64Id8hi+s5WfsrCt7SM8rbU4hbsDJU6+5/5f7ysMk5xfHEqPtv5j4Zo+QrDL2G8
w1Ne1L5+b/G2VdEMs2jx3ohiC4PYo/bXkRy5n2CCVW1GEe1/8l/0R6Jb48Wb9Ljg+fj0glsOazKW/a4Rs
j/9BTgH0+VMhAJEBITVbf0HS2Zb/OfAVr/pzp+rFMpY6h2i9H7SwpHVmL+1KGXS+KAY7K36hc7+99U
u/Y+oeYQtx+ssbt0KE5Xh1xNOO2sOaNw69OE/GSQhYj6ZbjTKT/dYq6zncq/F7hTrtoHWe/Qt8d1uDF
YbMyLGoxeozC+K2DK/QaMdsonc2+bzMIVG5nr8l/WGIG0pm8Y/FGDk8Hrn4dQA0g8/wKw3AtJ/Hcqnd
P8VxBZvA8wHBPqVgwsDtOYnnXWIN/Q3kEeQy/fBWqGMRE2ngpxnf9Ptb3wUeMgMwAgCd28oJQW2Uy
ug21f9mN3EmvOhjnNlyeFyhAyeUxSbXkOm5+Kw7CNgyL1svN3KxvuuPdUZeYsGA1C3XOzYJ/DgQrw+k
4F6GwSEw3RXXkm1ANxDTwfl4tYvnF6TM3bT+ggiHiXRQMwj/s4BSl8Xju8uWpQHbTWG2eb8VLx8kgjTcu
Gy8v8fz2pNOtM1S+VG7fqXGNjzh7Ds6nZY12kf3mxO1t8EbZwhs8tbLSg3Vfjvh2RRkjWWVpTBBIsCu
NHegMh23NRnHgscvThPt3HOgW/dbfvIm7fxg8Z3TQtwHjIpaIMRtnVbNhj8nNbU75YH34zoUPEAgIZ2zi
ZUaS6rv5TW4VmCMM8nxHSpm8PlhSKOAB28VFe7xi9UNVp52ZbbjpgdxL9tQm3NI4x2nTfNDfGP2LxRW
GBJz/o0pudp1TRGBfHbB8qfxDkmBuP+Y+QrJB6/WTuvYnNgKtE+1ikMiSJNktmrGPHCbmetlH2YWOB+g
R8LqIXhc1vuUWJ2V3+4v57ZD0a72OHsq9DfmQFXSLn6mfHtFIVCyKKxVW9IqSY6p73nwmtZ/UKzRoLkCu
Cv5N+XNvNK21OqfdV9nwkVnEpEKy+ZnJ0iR3MJBkjpmSr1LmVY2uHNdQzaT7yUHjhzYP3LoZM2sHeXFD
nI/6v5VMYUc1ruWZrP8zbZEDP/eAcXuhOPZi4TlXuZA3znt7YzVNGsOFKmEm0PRfpOk2K8m3NGbiAffd
X92z41kXlKAh5PnHfd/9RkJQ7DWEEJmM9PAdTKSc9qsctlQxnkz8oq4DZFQhxgU0IiNnwvNlehyX8P
0cCdmvkbs/HgnjAuIKQyQfCrMatzUnB1TBftNmAWmwIlRtfIeKqfTdmWn+FgDBrPOZ9sigH59tC45Bo
R6iQVXrqmxLT5flNkOvj1p75cVD/RIQh0d0gP39w8YWQCbOLtyh6p/uvHIfsz9NHtOncHG6mcZc3WvNj
hbsuXHDPdSyPSA8ZVsMCKNvPbvD1h02NxnXUe/IkTrSZBMR6Yx/Zqt8YX1R8CWZjz3/v8AsdmC7mUv
9DVQ+DIDTgl7HWWn3W3QG0H2vVQhBaR4JDWz8h9hYXtUR0iZRyW48eCP+8jGLpxs4R/eRZAPRmPRtkjX
+6w+b9BcXwObmFXVA6GDCjRCzS3BStiEv5xuxzwsr7CAgDvoUTMDNVaJzJIqaA7Q8y+iGvCRnmhg75G
bWWzvu/z2KStR75e5vRE0rz9z+VFT9XWPqF7/sGUOz9uv+5PCwv11uh9vaF6zwFAC6Kwl4k8y2hEUuEX/
yyWrjG3kJpj/WqhGvG/z+cqbrXymN5c6LYppOsoQ17eEUFu/MeX/1wMN9OUQzBuXt/p2vSWfcgDZx2/
Cff/Lgh/ZbkNDJpZLCTC8S4uXOqKxPeQsOeqFh8a8cQOrMa/vM/85iC4l/nfH3Hyhpz0TTQyHvM5j2y
ewE6TZSgLhhtZPsTmMnlqyCkl3rmwjC9AmjGP7nszqj+gtzMNhBIg7cwY9R6YbOzu1jd/6GqxzPyP3PvQ
2dzwhnh3y7V54GMD/eOi3F7uhsPIY3fcdRWfqPfP7xvpzsSDmO98e9M6U8kdz9sW+hFn2d/xPtgsAVIM
tt3bBCSDSKAzbnN3jwbHXvBC6EJ3pzm5MPfLnnm13/pg3ALmhtZ/d4DM8x1EaQtsu/ljmlxD+j8A7/W6L+
2OAcG3v53eSh8L3f85Nj8I1fUP1CfdT+jDw5W7rQHXMmeenj4ja86CjJdWvMDVb1Xf46L6/tv2iMZ6
dvzyJ60Pi05Q9H6GxfyzEkh8mLeNj0fC7l/kvWxa9idfSPSADC8eu+Oq4e+q7s+mfrTtOdO/Q/XrFj
dSk2/hD/3rhWvZUMLZPyiCjOTJfGm8RZLH8dXLe2+tWSYsXXr3Mjr3/jPz+5V5e7t5v+94RfTaGm5WP
xxf6+bh/BoyzHVl5zsH7TrlJWsOjWLVPFzZwvq6zLR3c5ru/5qc+RDKgfMa5/BYFWsjTNNz+gWmNzfK
1/DG18fW8hbeH3/sGt4Ln1qo/v23XBbezhjDN8qNJQb2/99yz+BnJeJr2Y6B5exyKrB3kJXyxjlfTSFb
CM8rtsnWvm1vrXiCcwQSgvrl7SCE7ByyznGzxybMNd8ymxNlPrO9/gyDufbHL2eVQbexB59bOZn8e4
48u9oE5pg9qP7DJb7MjoMtB3tI4FI3xwK5ewUuOzTA/MFfcb6lZcvoCWtkKpdBMlawWpdGXfGbRDCXj
R1bsaOt+sC6tu95ZA99Ab/eKo+e1utzL7e4j5C6uzDfXzK9t+ZQlgmBm/AU+1uTR4NpMbqoFH+Lu7r0d
QBV+qI43LaxrBWT7PCDkXbs/d48YfmR/HGu4b3HaLr1adndKfhFRbeHmCoYVY9k09pNKbrQSJ/aeRZQsv
7tpSEThkdBRVIcBbbjTSsrlnlEltmk9vbzGVCDm0Jk3LwmsXLjm5sE+1lUIg0HuRX+7RfJe9ydE09YS
EO6IW5CiiydYjp7KB6isTDGY7ciXyr9NtTTYQd/BAwAUBCjeKB0ihn/pfa5d/hdP2q/dZgxP6XhWmKv
C8Aw+uDjxeYfQxHNG1afNTr9L3U+Y/3FgWQBDc6UR9JdvtIYWYK2WnsCo5G8evNKzJWXfIdCmQFlzn6
u1ncyEF51MtOGbSuoNR0Fn/yp3ORt3sVHJwXQbYDIbftq2PY9DYPTPWQ0F/PZVSvfTTSlIn0aefvU7
pnM3SXVAvc89Gk4+6m/nvdQrpuaDXnmxTSf4Xll4xh4YUR2kYN+u2wtNwzgF8WAL95nS4o2Q7LfL4OBd
4gLR4fnq0BxQRb7sU5yjQsOnjxqFYpP2z6MRU5ytjnr7O42yHNxa6PWhw6BR++8wdbP/5BUf4xnkFh8X+
0zi/GdE+9oHvKQ/+/4TxXTFEy6tUtM4w0xapZQCv+sXT/fhHrh/v/A/uJ5hb+rujJd0+bX+OcmFZEe9FB1n
2itPpbYSjkdkOZ7ffqGG7vkJt+8cRS2vd4M4vIh0J5f/hHTJ7XLZ9+eBd9Hx0Hs1pjPW8y/7R3Kyc0F/gbu
A0ekf3gLG9V9n7fqd+r7IZ6wt70dCfMTp6q8XmPRGyJaWe0q0F/k2tM//5r34HmmIE8R5SGKHt3+eHgfr
7BeZ0n/YZj7ca7wNZOz16H07LkdSjMbJXwZ3yU7PiYjK7Dhh1h2s6n6p/1NSRWO0+bvS12R8JxDoZsr
zuT10Qbk8qaWw5v8f5F0d8fwRzF/uNeCufd2xZC1oBB76OvPK8Dy/yp9FdaA6yJNj9PNQPbm0n2tMdGp
jo1frx0HHIhntbkNBBxDpMjK+XDzpLmR+ymnL+1HL8S9CldY5tmPknXW9+cXYtVPB7rXK1nlJn+F2DE
I+n0G4avxM9M/MJXvrBbrqSOm0zC3sc9+cKLjqn8nKapNQiqr6e4YDM1y+Lhy4LDbAzMOYvctNyu6
mwuJD9y9LIaI3GIeW7RrMSQswh1B3FLsKhpLRtsd85BmGY9nUvwr0Ziurgb2bBoXmtiFZ6dpdVIN3Xs
t6irTc5Y9K0hhcbb8E4z1AtSUNpUYq7RiAVfHzBj8rc1r6AvW2ZPs3Lf9XTYlUJs16Dj2lbxJ9yHa1Gi/
3UcYAaH1rSYSpyxXVPjNqw5XSDO7ZC8RN8+Qe3PcHY5Epp1aZvQfXn9dTyYDHCUq0AC6ymA5SpDc6
cyCuXZ9ffV1tW8DJG7jn6/BlM+3w7ke8/U1WHajK6D5LNDnjvf6P/FBOR9Dd/Xr/Q3UbfP1VnRw5MeU
sGT9Ue0H2SNcL1g5UgGQVDkkFU2VSeeMF/rGGRHKhy/RqIENKn81dXRWqKiz/Q9/8tL5ibaUTXnLCC
zJvOoBEUVqaIyF3n8iHcTZT/5I1+0OO6+9tPkjpD2TbHFDVtvqbeEvrxm9D5JT8R7Uh1NfP2xkOqD1+
+VUEfw2jr6kR5brqPqaqt3l1F/5AlrsHpeFk8e+mGTvK5TBq1ETeS/RjPoUUUh+/fVC7HEv6yF8l5/jTL
sj2UvvAPgaRxYMTEDzVUUTR/eywqV/WUsA8fvgm6kbd2DE4wAkwXGIPbeBiVdjr3M1BT8P9JxhcDGFrVHc
Mh6Ke36+bNUwAbMH/ScnD2vejnvWkwqr+C8zHN0UTZTTtyA9RCt/0gtZ8Jf4Ao7AEftpt1pQZ1Pp31vLt6
gjR8qxbbNZXljf6fV4L8a7IJJPNP3XcAFctbAupdCcG0Vzrz3YU63b+vff0JkutdxJPTywR3A+Wsb/NeDc
bjtiiLuPmp2IqPYeJU89JKEdQmbsaR8fZ3X2XRmbPV6ctmFnvHPvRLJPm7Tw2G3hRnLO7u+8fTrtuf+yy
u6k92hJqwa5xNDDYgPsTNFjW/cbaFyrNqPHHTTaeFd0htfxd0wUmQK73NGa9bBLPTr9H7cBrzunl86Yg
VgrRhM8T3P0Zj0SE6gm+lE76eVjVmkOJB+cqQOMDv0NQOH5VX/kyLe+cZkfUePNv8pNdfzuFaf3H6y
+4TMzr6QJ+NsZZ6r2Mh3FhD9eFyY3m8B6v8WQqwm/xvnOvndj6bMdhiOjrP/nIDr5GNQdCzV5zfPK0s
rRx4ryDzdutOSG99Y3uHHX+v4I1ypwzXCl2vDzRLm+SGqhBaDGpbSJs6wf1K45LeS0nsZYvYIJk4VX0tX
A3ub9CxD8qI1mhtbNJMDJXW4qWaWmljzOgaLTM1GgmS1Vm8uTRzXeHpw6xuljmD+X+U2mhTOeyMgeP
aWeWMaAhVJhY1VhnGAFPgY7BPSsgNzP/z9vA4eJE6UR4uwuGS6F7+DYiAozA/R1Wz7+t5HV/XaeY7nG
OlVUJ6TKEQ2sZhvXKS+H0J6Xaw/NAwwhQ/a+Ts8MBNkVTWjAMuHC4g2xbWhe0lhCY+ouXSrrnqqLM
DQjPlXjVkgalNGY/XJrENvRO4wNl/8rYf1Xg2TrhjpJuFi6+MjGX4e5oVqGYVRbfDq6y6/pbpjg2TSm8
Mt0mn9O1XUawzmNBmcTBf0Ovz8yrdtmAtkLxKrKKvuHq4L34UiVF0Cy7bS2C2qn9Ef7z8BuhXRAbfZ
DqJaIyiHM3vyNZrIbpPOwv/EWcuZbtHrZ9Td6V1UB4wBNHCBuytehAlFqa7p3Bj2vZNefv2ruD31ZIw
dDjY0jx8qEJe8RtK16glb/NRc52KU3/9mEtHNjO8vLn5kC7UCZV1AkaYbmgsbxPE4FP+/XgS/Sn7zKG
v3/rj6Jtkw1tmOIrGddNAQRzlRxePSyEYvX7ElhtBUe7r8XZncQUWO6/cS6+v/wP4t4/qOwjYIc6H/4
e9gdqK3PycVbS1tM/ZD9Qe/Dhm4MzAxhebv+yDl7e7zcJ8+P3b+MFuqu5OCGuiLfD06T4pe9/4n/TMcu
Ho78DprXWyTbwOf8c44XgQUZuJ9fUxGy9zSSu7w87ne1SYnh+OQ2KxSB+BrSugjnzLTyc5owlNFUoPl
WCVTcL0TdqUPlPZCfSDyaP6+mJDcqmpDJpu5PJHxyuntaTzo6g40zVXXLEdgcTbgfsL5m9Nu1kYhvl6ZP
VeYoapgu0rOCbbGmlfTvgXwb/lrWZbtZ0ZrQ67JkUhiM1h8fnnv7bWwwKOzX9qG2AmVfdDZTMfSw5n8a
Jj2PfPMLr7z+fFRD/6xiL5AytFsk6V7rIq/Yzfx5Et0u5PHCMG4ssCy8AYZ+1ppCbMPPtJdvAtfIyVdS
qP1HvveZcgxvKEiK9xfjjuv91vDpPHaR7GCmDq79Qf8Ze9mdYvLZNW1xoTjpK0/sg29XFnO4VhhGGKyJI
n9QEtenMiqMsWpuaxxs0aXU3hrjmtmvjb/Hdlek+ebcxLzLNgOhN+N/vjMAsLDcN2UT6UMWOaCV9xD7
4lg20wMhKHX1CHUzDt9BSmU37qvIjlc3cm9D/ZSW9M8N86+yjK2XWhzlqzUXnbvvpM7qU0eW/YyH5
w3WZdnRyzcbwXxB5TQVxS/JwCF6bXGYbRa1Mmd3kq4aNtxhzqOLY1yeqxEL2BuJr8Xy8Id7+4Ygn9
xYzQ1EsfF/KZK73M1KyqCtdyz/4AAAAAKEmOgnkSXJ3VcIJxBvMLXKAUnbx99dh5LLOD1MZwb8NBoZ6
T41tZ3b4CfOAq1fvXWBPzhWW2+nx6KvO7Q+/+j+TPCf9i8+PqDT/ppf9//tZunTP7TxWkZbfhvqxrDQ
vRAoTlWrrPafMlVv5VUKfpLqmbPXxYeyWphtEg8oPUSKKDwjB6ZLOUwLJm9Wd/Fw2e7VtlWMvk6FmzC
fjIoh5otBaHDJPjWDGSplAyzzfYpnX//nQyfKyM+CeHKOuDkeF+B/nZFs/gkxQf2ihPDgLUxZBNTAWW8
EIrJ9FBgt8++1M6PB9Y4VGgz0eyRdZNO9NpvpKsPeLvf/Dm3l1fXv/SL/9GbKf5VlSBn9OqNnCsM4rfp
+Un9u9T/M5fVHnz6pT+Xn5GvD8n90sK3Qj4/7cW/HAXFXbT57AfsRKuJFS9gsXY+SonLvWK5nNwcK
iWFRfkQ+6U4QgiYhGfwJ5/hv9Jzmf5b0KgFkMJ7+yqoBW/4cVPXr/ORvr0nE/V3rWWM4jlnQjRZr4b
rHnDBPKUfkWDqOGlVMiIbhPE8TmyF9/LL/Fi4e37u9k+kTx9TwTyXnMEv+IKNoVjd5Vhl6lovX/n0wfpc
x1JGQ3+P3Lgp1vaUxnjDD1d1vKDKAd5MXYfEVNkwqt7+S4RIa3vbhHx7v2jKqPzKnIX5LORoC2r0x/lvf
x9XQsf+pKCbc9bIhHjZZWGiN/FcC4yxp3veeHTIxSrS8dsgwaTxZHd93LKDMBFhy/lpn3JYNH0gxJl3MUh
IBiaEJ0uSkLVGzFJbHHTdtqtD97Lxa/GefPmnZMNp0n5vxEnzeobXrvz/p5bvF1qyeb9uNvUr/8/Ho++
nsA/cKfZ5ixaLTu8g/9E3l1EnT3LrLTxjKrtrQvNbXXzjdy6dbjcNlYoIO5l5nW9R9dGlq4e9RfeH5e
wsg8SZQeZefAWWb9y7bvb6+3Pp8fzGcObt9H9s9UgF+1U3yyHmdC0f4v2gNu1QyVh6xoafN+4ZmO6Fq
a+EUZqh4BLGOvRTjl9nrn6v2lJ0xjFPmHv6VjFx49y5a+4CRJ2Xb/XXU9iu65dcv3KzEwFxHZk9zS2
3gPzV0/mOyMiyQ0yXHfqnjMG7U+9KpLlt6bxpPkkjrPHWFM5htLFYvAu5vTOPGuPqmnSbWXfpkKWS
8WL6W7sVqddeDWR+WfcTJB8xJjrWhbLyfvHGT6/ndufLC7BUYpbYwOS/sOfbMBIkzRS/rWqD8a8dtrf
y9pfhNoYmwc0/XHfxwl+4cJxk4Oa9z0+qnZvmbZnmr3lclyrmrQ5NlSLq+Y9eZlnkT1nY5bmV7UnPUm
oVLVK2ruDF7CT9mDX0b3C6hvf6Y6tm8bT8kNjmMvs0YPbPUNyjk6xIvUmDeRyxT2k/47t9jtvVVBVdun
1aMGfOOYSHvAryJ9QhQq8qV1+w8/9Bt/OjAOqOZSfM4ABbA67gw7EfUlYnBqvXzSjiBXhoIPN1Yv+j8fcb
8pYx4rlpdbnDOEsh69mzxfqRZ54jAqxp7yxzyBJMm4NRBXenmfZyaKf5zxxy7aDbmNt8f8HjvE9Gz7
dufA1XM+SbM6k8/yfO87qI9hE9d2nDj/zzRnsucj2B9SjHyfLq4MwOjuV+DWcn4W2iA9g5rjh+ojOT8y
Cvqo96Jp+Ghk0SPAX9f4CZdmJjG8Lq1A3oxwZzuzIKn17W3H9vEcmZFz0bLmn3tZPrQ58LgmmQVxa3
4fw2pnnwXJVv2HJBjHZ/7eZfy3mj7bMm2l8GnhJ9NbC1VpEq9Tb9K2mfVdhbqqZdWEGX4u9o1POy0+tu
iYbGv0j6rudFAPZfUyaL7NlQVUIeqxzNPlK10Ej0+ZDb5yQGqxTuc5tmp0sqsvSbTu1+vzhu2fTnDW
Tt+TUuoXDxjJ6/Kzgw5dTZNYtmxtj0wz8cbl/fPt51b5ksixaf+4eS9GXGfRn8QIlO9HBuXctVZNdvO9+
8oP9TOAAAAAPOAygpFuEoF2/Rn9Z0Ldmvbbfz1ZysbuL7aHQ9q+w6UuNW0n2p4uAhH0l6l7GqFiRkwmQFd
v4fFvPzO3Z6Oh9V3/79HbmfmQhsA3ljrkW0rQlO1W0l3U9CXfzKBDXWfG0G5a0i3Swq3Lk9Cgb+bGvdL
8qLvvSpzpn1I6+xr3u1u7fTZKjxPfFX5w5cjRs0WCdwXl2zrKEB3X9y2eHjp2xX5V6qx+Z3+PqvVLlb
+Nwq3Kgc2M1p6Vx5RtLAs+/u1ri76CqYTPJSwnSh3p1UyXv78hWk+NKM3/2Ds3pbXhEU1XZm96mmh
b28+B89pmYyc2SSObm7Iv0jmaajdvjWzzaGZkZ94Is1vVq2rrDUS+u+u1+/BbLpyuT3rszzZf4yxPVn6z
uGmgz7Ez3fX8XPWdqt9H5lznHAb1b7Gvrvf0b5PuxndzSuaDadC3fUbAu2lMtkCWPqOuRvjPx2c22WlT
2y+ysOKdncXVxWSS/7Y/vCB0OtFSeU1PbS0vuFN9ybNp1dXPPj7m0I7YH8V28dLb65gUAV2o+v+F7dcz
ksHsjY+S7e5ZY21/ytPsCBMPtlqsc4N9ubQ2iXpwYD0d8XstdtJ+7Jl0B9jdz7+R1yGXXWd8NfWpk1iZrr
8MfLX2iB23cfUuAd93xP0KL5sgFXqbqzKyWTJIxbRe55JbMT8fqt/3Tjbw3BQ/Ex3e5ouTt2vLN8jxvlK
PBs/fOh6s/KyTD4zvx2jFFuWA+vQeOifmrxEU+5vvPfjurLl2KehOnHCwrd1Opq9dttYiL0NEXGG3n2
5Yy/8KsBU/1/TZtkN/9MXWmTesqhmN/vrJJvMmaPuL49f9sbN/+Ywa9hxmu5WNL+2C5hP0TuenS/1BxtUw/
i/OX0Pu43NTYgHfUHcLxuSzfm3h4Gsufa0fnNU/vr5G24QdAVnbGj//3d+9M7NXxNJTZ0q4Ua/c3T/nPXjI
u6SstbbN/5ZIP1Y3VuRNyb+1d1Hq5lpqsRf7rutDTy5ryTZr7LxY1qgf+P3rXbH6lPlb4uUKrhHpKYPfzP
kzpYy33N7PSW7VXqZ8gbN+Xer4oymLbntNJ8VaD9Q/O1f6n3WVtJw3P7dw+IaXuyfSvoLpzMfaOdtlpPF
Nkx/3nv3L1C+NnxUWl0FzBAHP9J/ub3ZLh/0s/VdwdR15m5nNy+H5PMU/bXQ5fnGl6H7p2vqTf38qOX/
T2RPWq8+3ugMjycw8bgKh+tGQ54uK/vvqsY99zazeyqhBy3MR6vuqfC+3ZC46hzJvYsD2UbwSAZ5Ie
h7X78Qe/5cgD1Ej7z0BBgWO+ZmUqPbiQeeN2DODIyn/u4iC+0WAKxR+GnuD4NWKuIeYqrf3ZzlNmD
3MxfTiJ/bn0ZxcSSIvXu28zkbyr5zYGIXi2n1v9hJy1PNqwNEoTqPvbM0y41VLsHvUSkvnOVaL3vjPZd
/HmcaZ6W/nt68DcTc1tX+Ye+Lq9P3z0W2/y8yq99v3Dqtk+PpmxZ+NjMdXnfUiX8jf/v0N+mGQqrrdnnIl
sGgPO0TYIgn+JWzz8GGctY1YNjqNf0Y/bWGdyu/Hz3GP4WThgkz/nF/Vr0x/Dm1nv62qpqH47eqfD6ld
wXnWgXyhyRfv18bH0+lqFKe6x7eHLr6dHG7DXvAJUd0gKUhPlMmv7Fzh9QbgsEM5h5NeV8ZMd8Pou/
NDLdqnDMXP/cgLyG6kpLuQMdWfl2YSsyqyXWy+pU5+mxfWkR5ZvfO9wvL3q9Ffj+3j0Lp9G+vNq9yN8/qS
zlZ1M9v8VVf/vpL/vb/FVqc9cf+5u/PE4a6ibtfj0bqnN1zcLb+S/3SwN/wsKFdljJ3zHzX25qaoVhdB/n
8Z/PwHIuvMg3vu2MFBcHB2fuVwNb7p3+XqNRTfatzv2fvSJcJ9ntlk2zTcMnu+pp9Znz11Vuf9WM6YYwn7J
EMDbBjbZ2PxzcO/+mf55DjlDbeqhN3tbvB0+5sy+5e2tK6SX8X0fmjZw9+mOY/+s9k0fnfDM4ngXMMU
6/ynfSs4P4aXk/8fdvWZPOC03Nqd47xG/kJ/Wp3T93Pc5nJUbuAvrLJx8bb7lh8lf8XD7eknK/Cnf+ecv2G9
/fy1sAcAzDkZ2q6ur+V+2b7+FjpzuZmfu/Pv2ZjNm7G8feX2+sq6dazD85lhzzTFb+7cx3kTyCusv4ujR9
33lX/nQQ6yLNvEXmvNsnmgPtnl1gfwxe3+PXQc+P/ZtD9M+7b2bHtuhHEWHiz4CXN9dj1btv5aucN9zz
2L3JfL+b+p5j3aVVaj/y/yrtuJj+/p+/ws7b+HzB1DF/OwTmjlrctb7VMkE8fuWs3r3OwVvOv/fp9S+t5PnV
y/zpWXfVOsvQA7uC9z6lL+O/U6n7dr+tLAeQ7c33eWVVJxr9vTL+k1n0f5Ouj/ZWPBzg+zd4mrn+Dq0f
ttfvO3dwjK4fq1Fsd23mfV83rvnn/4VX+8z7w8VjDJjB3Nrc6f/s76dvW97TW18xXrv39uf/X9ZbdWTnP
r/kE7rvaT31/1qPZ6tldzMKuGzYuHDmXXr3M62bZ8/f6ZqF7UB9dVy/8ARLX2fMYfH9+mBuUXWQ4XbvWX
+eWfNdmvW7/8D8T4+/ff/5dN5t6r/PkdRRt/Wbl7VGb1TwAAcsyW6bsvxz5svuXh5Q9q/rb1s4xjs3a6P
1Nj/8cf/z/4PSLC7H9+ufvd3V1A8M/9fY3bt/d3H6P95dfvP7/0j/+Q6Xwjm/nR++s2Tg2uOVzWC/m9
9+/HbP+f9xutvnnwwAf+ZqyOPWMQw7X7r2P1q/99L+PAHPsW/+Z6OFYHr35P/76ZsbyP/dVdt+sLv/E5
YDfv8QqT9i3f8T/9j77eYf/jX7o3Mdf/xv6/8RwvY/f+pHl/P3r+u9n+IIYA8eKXxuL4OzbVvz+cPdu/e
H/wt8f8/88Du93Lf/r8Kg8+Y9//D/uv/vnO3p7bf3d3z7+O5v2c3z+b/47P/t6/kv/sGf/eX/8+d27f
+5XfzX/I/mP/3x+E/1/P7+3i9Fs4/BP/+I/7v/v7b/yH/6+f+n+4cX/qJP/df+J9/3u3v+bTl/XAP+//
7uPxrR3/vvv/yz/8h4s7Hl9+7b//3//uLfHX/8n+v/7W2/3sf/3/+83O/8v/13/r/6f9f/27/6//9//4
3//f//f/9/vP3/rvT/9O94z//fPpgP/+/P+d/8/9//0PvvO/+f/8f/zvf/zv/7/P/+/8p//+zvP/9H/8d//
9/+f/5z/6X/93/+///8///3/3///3/////////////////f/9//7/5/bnrCQ3AAAAAElFTkSuQmCC

pf686+X7I/4a8fLjUwH4qbZ/0guOFOjFWVQIZacsoTKG6Jgvk22OvYvA/0seV1TIn2Y97LA8Pk1cz3hZ
xnoE/SdmNSNTtwdeukJv6eAubL+z77PvlM7zN6CQCQh1c+eK3c/PXn6yXH5z7+kHzlLx/RSwCAzF3
6gsjlr+kFzDtCVMRUlM2zbuVUc/Z1NbrYnOl8y+vt5wScijnre1s23JlrYvN7cqqJpBS3XmZRF0x117vh
6vxxe81a56Kmj3+wdqsx7F+aq7LhnTIV7Onh297K+dBcNQLH9nm/hqd7bHkdU6L9mLVbm7Lqre/p
me+ta1Qq+NvI2oTnyA9c3XYDi+kT+5+Q+n11vQQAyIOqg/pzD7xELwX94W9/Tb73raf1EgAgc5f/RK
T/fb2AeUaIivgq29LyctSalHTA48w8bynX5YJR4NOfUMnSXNWB0GQ1HAPbsp55Q/a9of6KMUnTHP
Jqu9rKUt8KRKiWomxe8IdlN1d1gOaFzqMm/ZolJ3AMttM8tryOKdl+ipsXpO6/2HmtOZGTdS/7h6Br
7yrua1NNWBO03bzfw9odR+bHA8R339fvk7v+7C69BADIw/FLBXsYv/oYRgWozd+8Xy8BADLXf1Lk
8sf1AuYZISoSqWyH11+stqz17tBiV3FT9gZfW21NVr/R7K0XKBlgDM3OdKKjFJgB2KiyA+qc9bt+wOe
ya1/OW03PstS7fT9Yt1Wl1d8LTpaV1zEl2k9RNvf60h16sXq5MUzeVpHt4EGK7B96PbFTEbvtVlv6Lf
8+sqlzblwHu+5ulByOB4jBnYn/6Wfp7QQAeVr50kvl2iev1Evh/uFL35ZP//6hXgIAZO6y9RvZJcpbzbt
CX81aAgAxtTcK4nSSVCHqQGAKADGo4PT1/+318sAjD+g1AIA8lB56od0LNa6tu8vyiteFD/sHAGTgit
8QKfBL9ryiJyoAAMiVGsJPgAoA+VK9T1Uv1CTUsH7qowJAji79AfVR5xghKgAAyM1HvvgRezIpAEB+
VP3Tt33hZSProI6iAtS7/+NX9RIAIHN2fdR79ALmDSEqAADIxe6Du/LBv/igXgIA5OWOL18/tg7qKPv/
8zH53Mcf0ksAgMxd/n9FLn1BL2CeUBMVAABkTg3fV3VQmUgKAPJ189efL//8Czfppclc+dzjsnX36+
XWV79IrwEAZOtKXR/1Zr2MeUBPVAAAAkKknnn6CmfgBYAZe8MMrEk0kNcoz/3RJmu+/X3705DN6
DQAgW9b77aWPWx+fchYxFwhRAQBAZlRwqgLUh594WK8BAORB1T99yxdvlOf8OJ1f+R7/xo/k7g8
xKSAA5Kb/HZFL1EedJ4SoAAAgM+/77Pvk3ofv1UsAgLz83AMvkRsev0ovpeMrf/mI3PtJ/igGALm5/
DXr0dELmDVC1KXWlo1CQQrGY6XR05/LSa8hZecpTlzFpRP8+eHfiVasLfVIImPi+9hqzXor5KXfOCrAYvunRqJSlMENguK+5bIB7V+1T1Qg
UA5Ovqp47LG++bvg7qKKo+6u//
+y/bHwEAeVD1Uf+79fFZZxEzQ4i6tLpy4KZA5bp0+33p9/dkM68MdWzgt6QmPS+qZ+S4YK99XtxN
V1vqevelWy87K5qrQo4aA/ctEIuqf6rqoAIA8veWvRvtIDVLqifqH/721/QSACBz/UdFLv2xXsCsEKIuq
96h7OuncuqE5Nf/FGmL1zOyJ41zugtquS5bFedpcfOsVJ2n0twlRQUwPTUTf+WPKvZHAEC+Xvu1F8
vLHr1aL2Vrb/f/2A8AQE4uf8l5YGYIUZeQXbvRDN2aq04tx8GuiKp3o1E7c1T9zGAtyGCd1bDejbH3r
wVreI6qN+kMVfdfl6zW52A9S6+2qH6ENy3GPgPncEP8zZjnydln0vOiqHaWYvWMNHoeB0LzkpzUn
VFl/9A6olGM9obVW21v6GMZPAcJrkvI/RZ2vePcb4PXz35E1ImNY5b37WT3p2vSa2eZ6D1Ar1TMr
49uJI6QOz91pzz4+IN6CQCQFxWevubvXqSX8qF6o1IfFQBydOlPqI86Q4SoCDUUGGl2/cyRYVRHai
WjzqrR43EyanuDNTzVurCgpuQNVXdFt3UUZ5+DwWRzdcJ9Fjflgjtk3joz5/RGeo1z3nkq1y+kUEahK
q1+y+tVOsTseTxK50C6+umwipx2N97ZkYsDJ7W96x2NX1c3yXVRQV7I/eZcj1EBZPj9pu7d0GC5U5
NSIMjOSpb3bcz7M2CCa2eZ7D0Ay07VQP1M7zN6CQCQFzV8Xw3jz5uqi3r3h75KfVQAyI2qj3qP9Z
H6qLNAiLqEKtt96Xfr4kZ7Um3Z9TH72zrxbG/4AZBXL9Won9mpyfrIxEaFec7r+3uboWUCxu4/wN+
et3/pyI6XBPWkse4GPca+Wzo1imzrKP523M2ofdbOu/Fbsn0WNy+If+rOS9v677wbglnn94JOUJOd
F4P9um2ZLK8uyolT+ukYlS23beb5V9ri53BnxMnhkpwj67VGqYGh+21ofyZj2/b9ZrYlbFt+kJ3U/Ny3
/uvD789hya6dZar3ACyrT+x/wp6NHwCQv5UvvTTzOqijPPTV78qf1hmBAAC56T8scvnP9QLyRIiKIW
bPtPoFPwgdDgOHletbE4Z54aotPxwsrp3xAqzOge4z2bsoO14eaey7suW3deeiJIl7zH364ZMh8T6Ls
nnWTbuaslpwe08Gz+8kipt740PW2PblMOpEFdfkTNjxtXf9XrVn1pzjSXSOrPOzp0NEI3gPvd4Dhu43
s8et0evSPk86CNzLYfa0LO/bsfdnmCTXzjLNewCW031fv0/u+rO79BIAIE+vfPBaufnrz9dLs/G5jz8kX/
nLR/QSACBzl74gcpkJ/vJGiIoBI3qm2Yqy5iYxIwK3UyfSDKjKcrKkn47SPdC9+VSoU/LqNhbMYdKRw9
QnMMk+K9tGr0FHOsP403RKoi+fcf2NgNIP3Kpy1j2gqa6LruEZOrw/aOh+M8JCawf20Hd33yNrsaZ
uHu/bBNduyvcALB93Jv6nn31arwEA5OWGx6+Sn3vgJXpptu7+0APy+Dd+pJcAAJlTw/r739ELyAMh
KibUkeHOgTHCo7mX3TFUvMKUSrD+5Oz05HBssVSf36vSHRZuBG7V08FeoUmYExB5PXXHCbtWR
dm8EN470w0rF3t+o8nvz/SvXdh7AJaNCk5VgPrID+h9BAB5e86Pj8lbvnijHL9U0Gtm60dPPiPN99+v

lwAA2XtK5NIf6OfIAyEqJjR/gWm53vWGbAcfk9YLHS/
+Po26n7aO1NZznJyneELGlj4tn5Sxl7S4KW5lAntYuDF8vno6/CyPP0dt2TB7nbo1OM36o0lYbdzT+xj
s/as0z83XpEi53bcTXLtoR+GPJpiWGsL/wCMP6CUAQJ7e+Nc3yAt+eIVemg+qPuqnf/9QLwEAMqfq
o15iYte8EKJiQElOusnV0EzePbnoFnIcO/Q7J6WTXtA2qm5m6ibYZ6+xrodpl6Ved5OsPCfnMa5rc1f8
zphdOfAu6QmJc0m9HrXW/XH+/I4OP6sSyOGSnCOzLqcKFN26qMaQ96TUzPKqx6k9GZQdSHa9W
p7pDpOf0CzuW0usa5fme8AU1xDz7yNf/Ig9mRQAIH+lh1448zqoo3z6v/6D/MOXvq2XAACZu/xZkf
48dRU6ughRMcCcBCnYW9IPAlXYNTChz6yYNTCbq/5Q7faGHhpuPdIev510n72GrLsnrnpWNjeNyY
Nq65JPjmrWsmzKrm5er3HOCzBj90asnBbnDulIs+ke18Bw8Amvix8oDvbcjUlvX80sH+xxaoTFZo9b
oz1p3yaRZnHfKnGu3QTvASU/dZXaee9gZMOb4h9Hze6Du/LBv/igXgIA5OnaJ6+0Z+OfZ83fvF+
+9y1qZQNAbtSw/v739QKyQoiKYeYkSJ2alHSoU/LTE7kw7YxI5vDy5uoUoVGwBmZzVQdQXnhTlVZ
qs9e7kuyzJ411d6h6Wepbav3okCq98zLMnFndbbN3TastiX+aKhIo72oZDmATnCMv2LO4x2xOsJTEi
HvXrLNaPevPNp/YQt+3SpxrZ0n4HuDXW7V41zBubVssGjV8/85P3amXAAB5UvVP3/aFl81NHdRRV
lCqglQAQE76T4pc/rheQFYIURHKHgYdUpOy2rLWu8Otp1KR7cGClfuHRs/BBOwamMZwbZddWzOj
eqgx92n23LN7obonbiCk8of1p3hehhRlc2+4Tqg9hD5hYBeYJMs6ZjsbHhT7uljH3G/5QaqtKq2+0dZ
ACYJo9r07eJA2Z5vTZZMLft9aYl07S6L3AHUsg6+ttiava4u59cTTTzATPwDMkOqBqnqiLgI1pJ/6qAC
Qo8vWb6bUR81Uoa8KBgJAEmo2fT0ZlAph96btmTxL+lhOtaYNWBfEUbp2yJUKTit/VJF7H75XrwE
A5EnVQVWTSS2SK597XLbufr3c+uoX6TUAgGxdKXLFb4gUbtbLSBM9UQEkFCxRcGZtsUO49nnrWC
J6ZB4tR+vaIV/v+
+z7CFABYEbULPx3fPl6vbQ4nvmnS9J8//3yoyef0WsAANmy3m8vqWH9TzmLSBUhKoCY2rJh17o06
pWaJQoWUK+xIqv7demmUqJinh29a4d81e+ry8f+9mN6CQCQJ1X/9C1fvFGe8+PF/NXt8W/8SO7+
0AN6CQCQuf53RC7doxeQJkJUAJNRNS8XfPx7cXMvpRq/C+YIXDvkR/U+Vb1QAQCz8XMPvERueP
wqvbSYvvKXj8jnPv6QXgIAZO7y10QufUEvIC3URAUAAKEefuJhec32a+wJpQAA+bv568+Xf/6Fm/TS
YqM+KgDkjfqoaaMnKgAAGKKCUzWRFAEqAMyGqoO6aBNJRVH1Ue/
+0FftjwCAPKj6qGpY/7POIqZGiAoAAIbc+ak75cHHH9RLAIC8qQB1UeugjvLN//19+cPf/ppeAgBkrv+
oyKU/1guYFiEqAAAIUDVQP9P7jF4CAOTttV97sbzs0av10tGyt/t/7AcAICeXv+Q8MDVCVCTUk8aKm
uVbPTaknWjdOGltZ96MOYZeQ1Y2gmvbG+7rV6TR0yunpfZjb3NEO2zTne9M2m1bkPugvaHbWJC
BSwosjE/sf8KejR8AMBsqPH3N3x3tuqGqN6rqlQoAyMmlPxHpP6YXMClCVGCGeo0VKZRq0tHLWe
pd3DH205RdQj4AA+77+n1y15/dpZcAAHm7+qnj8pa9G/XS0aXqov7+v/8y9VEBIDeqPup/tz5SH3U
ahKhIqCibe33p99VjWyp6bXby3l8WRhxDryHrtTziU6UnF3eC+2qea1hrF8VRuA+A+aZm4n/nJ98pT
z/7tF4DAMibClBVkLoMVE/UT/7nA70EAMicXR91Vy9gEoSowDLoXRQ3Qy2Xy86Tzo5cXJwUdTFUt
qXfrYs+w8DCUMGpClAf+cEjeg0AIG8//ffX2UP5l8m9n3xYvvKX/NsDALm5vGc9mOBvUoSoS8eoLbl
i9kQctd6oc2mvz7s25fD+IutuGnU/VwKfNLcT9vko6Z8z+/PmMP7mqvP5EYU0/WNWj+T1Rv2h/FU
5e+GMDvk6shMnRQ3UUg17jG5PvHbHuTbD59DaumyYrzfqkQa2MbA+/BTHaYN5POpY/P2rh7vd
9nnrupbrsjXQXdYu3WC83n4M3DfArKgh/A888oBeAgDk7YbHr7Ink1pGd3/oAXn8Gz/SSwCAzF26R
6T/Hb2AJAhRl05R1s64PREPpOs8s3TlwE30Aj0U27LbdJ6Vz6xZXz17ldNV/awjB/4B2PywsCxn1nRr7
RCwJIMj5zu1UswQa5bnrCO1UkFW9fYcal2SINUYyl89LZXimniHs3Nx+hCvfEbcU+2L2e6pr43Dfn1w
Z846FVQOrG+uDgSpE7VBHcuqeFvWoakKSlf369Ld2wxcdxW+lsJKN3RqUprnibKwFD7yxY/Yk0kBA
GbjOT8+Jm/54o1y/FJBr1kuP3ryGWm+/37qowJAbp4SufQH1kfqoyZFiLqEimtuT8R9OXQTovauHwi
JEU72Dq1XOU6dmIcI1VI5LW6M2gzMjmSEhV6w15PGutvjsyotu6am9WjpLXRqsh4jjUz7nFW2rTa
Yw76rLadd22HVPv12d+vuV8TsRaoYQ/mrp9X2zVA4xpD+4qbsuefNfnTFa4Z1BPULwcDQN67d6V
wbV7neDX69Frbev2+maYPxeh2aFjf3vOc+P1RXYWtXf41/TppyLsFxAmnafXBXPvgXH9RLAIBZeONf
3yAv+OEVemk5PfTV78qf1h/USwCAzPUfFrn853oBcRGiLiOvJ6IfaPUOndivWnXCo32dFJrDwO38bS
5UZMsNoJq7fi8+s+6n2wPUXFff8ickqmx5QWCs3pgzPGfVlj+Rkh/mWi0Z7IY7gtk792TJfmJsJ0EYq/
Ua636vzepZ2QxPUMe3O61rY6vKWbchRsg+cv3+obPdKdoQeH0UI1Q3Q2s7cNWB6t6okwhkSA3f

v/NTd+oIAMAsvPLBa+Xmrz9fLy23z338Idn/n4/pJQBA5i59QeQyf8BKghB1KRXlxCnnmRtode1x6W
U5efqkHXYF11vUMHDn2VzwQ7mmeJ0KuwdeWOgN5ffWWcfkDu+2H8bw7cAQ/VFmdc784HMy
Yb1zLRMP6W/LeT9BlVZoz1klRrtTuzaW8kkJ3d2o9a4p2hC7Z7Zxrq2N2WUO3P3Er8sLpOuJp59gJn
4AmLGXfPe58nMPvEQvQbn7P35Vvvct/m0CgNxcVvVRv68XMA4h6pLy6oraPTl74nSqPCUnKies/w
+uV3ngPEWoFiOYcodmt90x0xG9I6exkOfM6GmphqaXQkPC+LP0tzf8OqBVo6fp8kkSbhdl80L4jP1u
cDtiPjEgE+5M/A8/8bBeAwDIm6p/usx1UEdRAWrzN+/XSwCAzPWfFLn8cb2AcQhRl5U3tHlfDts6aL
N77VXEyQqN9VP3hsyCUdfTDi/9upOjwkuvNubQI2YYuIDnzB/KHyXmkP72hj9fRVLUlIzuhTmDqa5O
CTNtg1JUdKNlqa55jln7k532ffZ/c+/C9egkAMAsrX3qpXPvklXoJpn/40rfl079/qJcAAJm7bP02eukze
gFRCFGXVklO2hlkRw52nSHNbh3Rkv0Jf3347OuzV9w864eaDXeSp4E6pCVnqL3i1eKc2KKdM2Mo/
xjjh/S3ZcNLUMtSV1PRTyvVazOhHNugZuhXPU7tScXsQNWYoCtu2QJgSvX76vKxv/2YXgIAzELpoRfa
D4z26f/6D3aYCgDIyeXPivTp2jMOIerS8ntyNptOOObWeCzq4p/uem+Sprnj9gDtSK2mA77BOqRmP
crmqj9sur3h1aUsxB5LvWDnzBzK787+H3iYIV70kP5e45w3jL9cv5BOuYRUr82E8miD3pbKoIM9Trvi
ls8dW7sVSMn/+v/
+l34GAJgF1ftU9ULFeHd/6AH9DACQi0v36CcYhRB1iZkzpqvehd7w88Ds5sYkTXPIq1NqC+shGaxH2
VzVwZg/Ll1GT440LNVzVtS1VJXmauqhoTmUvxxaW8AoiWC9cuSQ/l5D1r0CqtYrAxMwOY/JJkhK99
pMJoc2VLb9IfyBurRGfdmzm7MP3bEU7v7Fu+WOl9+hlwAAeXvjX99AHdSYfv7dP6WfAQByUXijfoJ
RCFGXWWDW8FPiTzbuDltXzPVzyAwvRw2ht+tRGr0uXeW6dJPWu0z1nFVke7BA5v5hSrUxzaH8o0
NdMxQeNaQ/Xl3VCaV5bSaVQxvsIfxhxVBVSNvvp1pfFohy1RVXyf/4lf8ht1x3i14DAMjT373yCf0M
UV7z1pvkbe+5VS8BADJ37FUix9+kFzBKoa/G9QIAgKXxwCMPyOv/2+vtmfoBAPm648vXyysfvFYvYd
D1P/E8+a1PvVGedw0TbwFALgovFrni/daTq51ljERPVAAAlsztN91uD+0HAOTvb27/tjx2PX/ECnPlc4
9L9fdeS4AKALmx3m+Pv8f6SIAaByEqAABL6F2n3iUfeMMH9BIAIC+Xjvfl8294VH78nMt6DVz/8t+V
5NZXv0gvAQAyd+ztIoWb9QLGmSpE7TVW9AQpBX9W65F60lhxJ1TZkODL27LhTbbiPCabqGZYeyO
43dBH1jOAAwAwhz788x+W07ed1ksAgLz84PnPyt7rvqWXoLzidS+R1X8bNhkqACATx4oix39eLyCO
OeiJqsJVf5ZqR84zwquZ0VcaKU3oAwDA4rjnl+
+xh/cDAPL10M0/kO6t39dLy+3al4l14l1d99rV4CAGSucI3IMTWMH0nkGKIWZXOvL2oeq35gxuuuHH
iTiKvZsNXn92QzxwzV1qnJeTqkAgCWjDtj/3VXXafXAyonqjfu+aZ/TS8lIBqgpSAQA5Of5ukcIL9QLi
mn1P1N6h7OuncuqEZJedlqXedUNc/9Gtl/XnRZq7pKgAgOVzy3W32EGqClQBAPlR9E/96Zv2h+X1
S/8xivsofwAgJzYdVDz7rl4NOQYog7XRLxrlZZq4nZEtYfVD9UoNb/OeaRVL1Uprp0RP0YFAGA5vfm
WN8tH/5RvQQAyIvqibvqeqCKbwCGJDIYQ6qAxwHneY0nPYU7fC+q9b2DCO//8rEiKf0cCAt7
7s+
+V2h01vQQAyIuqjbps9VGfd82VctfvUJMbAPJztcixO/VzTGKmIWpluy/9bt3vCVptOcPst1WY2ZPGu
htwVqXlDsFvVe01qobpeqIkoSO1khtC+I9Vd0Yra9/2bgFghnqNlWAPfSBnqjeq6pUKAMjXfT/zuD1r/
7JQAer1P/E8vQQAyNzxO0UKL9YLmMT89kTtXZQdnSKU61v+RFSVLXHLmHZ2LqbU+64s9S0S1NFG
TQoGTGKR76eM295ryPpg13tgBlR9VFUnFQCQnx8/57J8/g2PLkV91Le951Z5zVtv0ksAgMwdf5PIsV
fpBUxqfkPU7oHXE8sevu/1HjWG93cOpKufTsfppZpmrVUAABaVmqm//attZuwHgJw9dv3T8je3f1sv
HU23vvpF8ku12/QSACBzhVtEjv0LvYBpzHdN1FSFz85vlhPo1M7nWItxHgxO2jWqxuPoOpD20GPv6
/VjZG3Jwf1FBNd2Pdzga0fVgsysDVEya98oca+VK/5xJm1f2OsHyncO1O806o7arx19P/kG2p/K9Ux6
DsOEt32wXungORo8P2HiTbTn8/epHqPqpCY5P0DQbdffJvf88j16CQCQl7+/7Xvyjy//oV46WlQd1O
rvvVaufO5xvQYAkK2rRY6/2/p4hbOIqSxEiFqud4fDT/uRwnDa4qac1WVWRfblcGnyBRVsDU7a1ZTV
wqr1/3hUiFMKG3rcqUlpMKBKMklYe2NETUjVYzgYFmXWhihZtW+khNcqwXEma58TyIW9vrlaGBH
2qXNitLNcl/GVM9SxDbR/6us5/f0ej9OrffAcNVdHhZyTcPbh1XO2Dd97qd3vWGrvKL6DGfsBYAb+6v
WPHcn6qL/yH15JHVQAyNPx09RBTdH8hqilk34P0YN0Bu2Ha8tuuinKQug1zvnhkTuhV7/r1Zsdzzhv
5bp0dbDd9TbQlHNeotNLMEmY9dpzesOh2+3IzkX3tVm1IUpW7Rst2bVKcpzJ2tdrrHuBnP+HjZa1F

615bkRQaLRjb1OKem0koz1u06e5ntPf70n47fHb3pHa+ejIPHqivUH+PsLvvbTud0Ds2frfdepdegkAk
AdVH1UFqUfJyumftB8AgJwce53zQGpSC1HtnmjGkFHzEWco65DimpzxspxVfxuqF6C77UQbdnpv
me1yHmYvuTOyFivhWXQ9uejO2qUCFi+kKcrmBSPEidI7lH39VDo74mY3xc09J6yxHnub+mQmmiT
MmLjHCNyKa2eGQ/XM2hAlo/aNlPBaJTnORO0LtuOst74iW/aGVVC3J2GHE2hHTNWz/rmtbPtBrd
/2JNczhfs9gWrL7yFf2Up/+4q5j/B7L8+J+bAM7v7Fu+WOl9+hlwAAefjmjU/JV376u3ppsb3sp14o/
+a3mNAEAHJTuFHk+L/WC0jLHA/nDwYcXkjrjWE1w5B0mMHN0daVAzdTKp+Ukn5qK56QU/ppJDP
kHgioh2ouTjVJmK6nGTZ8Prc2REmpfSMlvFZJjjNR+0a3wwldR5fWOHUi6XdVVU4HNlaSk2473bYn
up4p3O9zpSwnAwcRIteJ+bAMrrriKmbsB4AZuP9V37HD1EWm6p/+
+n/5GeqgAkBurhQ5/mvWR+qgpm2+a6IWN2UvbMitPdQ3hXqoHtWLri8pZ7JH3OhefG5oM1EPZ
MWu5eiGPlE1KzNsQ5R5b19sebQvRuA3VlFOLEzSmcbxAvPpphfcZAepKlAFAOTn8yuPylNXX9JLi0f
1QFU9UQEAOTn+r0QKN+gFpGmqENUc9hv1cMLJoj8M2gxA7aBUrw9NMc2v04+4dRUtdp1B82tD
H2kGskvEuHZ+7Udf89zw5DXjJwlry4bZq9OtjWnWijRl0oYo2bcvLbGOc4btG21wgreeHHp1B4ZlOv
HcEcD5QZpuv+l2e2g/ACA/KkBVQeoiel3l/6IOKgDk6dhrrQd1ULMy3z1RkZGQ4dEus05mDGp2d9V
b0Q+rjZ7D7raTTBLW3vV6dtrhjxuYG8OTB6XehihZtW+khNdqguOM176Idth1itOdfT7Y9JDh+ImO
M737fWHkNjEflpGaZOoDb/iAXgIA5EEN6f+7Vz6hlxaDmoWfOqgAkCM1C7/qhYrMEKIupaKs+bN2
yao3Ztuc0XsMPcGXKlEb7K0YEnhNOEmYH/4YM+KbcmhDlFTbN1LCa5XkOBO1L6Id9rE7NVUTnsK
Rmqsb4jfdL5lQPrPmhNaJrmcK9/uiyeB+B0wf/vkPy+nbTuslAEAeVH3Ux65/Wi/NN1X/tPp7r5XnXX
OlXgMAyJaqg/oe6+PVziIyQYi6pIqbF/zehs1VHawYk86MU9n2h393alJygxmjRqg/UVew9mbkJGGV
09aSNq5dWbUhSmbtGy3ZtUpyrpO1b2w7ynXZSmV8eFnK5aas6vZ4Tbfaftab/j/Z9Zz6fs+LOdGV28
6Jws6U7ncgwj2/fI89vB8AkI9Lx/vy+Tc8Kj9+zmW9Zn79Uu02ufXVL9JLAIDMHVsTKdysF5AVQtSlVZ
TNvcFJu6rS6res/8djD/8OK6Rpb2dgoi679ubg/ixDk4RVZHuoDc72vF01d71eitm0IUqG7Rsp4bVKcJ
zJ2qfa0Zfu0IbVpo3SBlM7JWcHjzfsGiW6ntPf7/mw7q/B67F/aPQSTiCV+x0YzZ2x/7qrrtNrAABZ+8H
zn5W/ev1jemk+veatN8nb3nOrXgIAZO7Yq0SOv0kvIEuFviqCCAAAMIF7H75XKn9UkaefXYwhpgBw
FNzx5evllQ9eq5fmx7UvvUr+06ffzDB+AMhL4RqRK9R8BQzjzwM9UQEAwMTefMub5aNv/6heAgD
k4W9u/7Z875pn9NL8qP4udVABIFfH3239jwA1L4SoAABgKu/92fdK7Y6aXgIAZE3VR/3cm75pf5wX
v/Abr5BXvO4legkAkLljbxcppFNUD/EQogIAgKmp3qiqVyoAIB+qJ+re676ll2ZLhae/8Osn9BIAIHPHiiL
H36EXkBdCVAAAkAo10dQt192ilwAAWeve+n37MUuqDqoaxg8Aylmqg3rsPXoBeSJEBQAAqVAz9b
d/tc2M/QCQI9UbdZb1Ue/6T6+2g1QAQE6O3SlSeKFeQJ4IUQEAQGpuu/42ueeX79FLAICsqbqon3
/DozOpj/q299wqp/7ZDXoJAJC5428SOXabXkDeCFEBAECq3lF8BzP2A0A0COOvv2if7Jn7M/Tra9+kfxSjV
/kASA3hVtEjv0LvYBZIEQFcteWjUJBCsZjpdHTn1tSvYasblLT1gqO94Z6fFZnX09NrrDhtXGnIqCb6x+E8
lv5aY2mo2frfdepdegkAkLW/v+178o8v/6FeytbzrrlSqr/3Wrnyucf1GgBAtq4WOf5u6+MVziJmghAV
yFVPGiur0tRLjrKcWSvq58vHDiJLNeno5YXRa8h6LarV6loXZDV4saVTK0lhIDAGjqq7f/FuuePld+glAE
DW/ur1j8kPnv+sXsrOXb9zu1z/E8/TSwCAzB1XdVBfrBcwK4SoAABgV11xFTP2A0A0COOfvycy5nXR33zr9rzTXoJAJC
5YyvW41V6AbNEiArkqco+/qpnDohy9v/dHHF6znbk8k8Q1W61XJetiQqs5Tae6SomI53PSC
m+wgVQWqAIDsPXb903L/q76jl1RNBb9fL/P7zLMQkJqgpIKrEPsR+N
536d163Or/dp135sb+ivNdbZLwuuD/bKS2MbmtlG7zFcYzN4jMM1S+1HWN1Low3jal3a+zDDt+aq
87WDDQ9pc9i2o9o8tpfjROdl+J4ZuZ8YxxD7fGh+W9RjuK0OZwi9/7p0z506/lKsnrNGj+NAWF6Sk7
ozquwfDt9PNqM9ie65eMduS+H7wj1Pcd9HsNxuv+l2e2g/ACAff/fKJ+SbNz6ll9Kh6p/+
+n/5GeqgAkBurhQ5/mvWR+qgzgtC1CWlwpHQMKhTk1JhQ8ZlcHHYtR8HCkLa61TIMrC+uRoeXk2
1DRU2hfYY7EitNCqEU58zapaW61J3uw52duTiYMi0674ynbqmQ8GiZh/zyGBquM1uz8dQE5+X4X
umuToieEt8DFGcfQcvd0hb7WCw5A2hd6V67jxVafVbXq/SIWaP41E6B9LVT4MqcjrpPZfk2FP6vlDn
KY/3ERwdapKpD7zhA3oJAJC1z688Kk9dfUkvTe/f/Nar7J6oAICcHP9XIoUb9ALmASHqWqUmqLn8O4d
TmNeo3SlHPhSUpi5XrX3na/FYybwtaPGt482TaCw6mHj7EjO4PplEcFZM7r+3ubsrlVF+erBr/GPI9nZ

FyGWtm2ttd1t2Wptpx9bOvUrr3hB4Vhbe7UZH3kdQm2eXRT0jkv/invSO28cd0SHMPY8xHg7zu8r
dZxrbvBoHEu3Iamcu40u53bEitrHVKUE6f00wiVRPdckmNP7/uimOP7CI6OD//8h+X0bQxHAoA8q
ABVBalpWDn9k/YDAJCTY69zHpgrhKjLyOwlZ/R0K27uOQGJ9dhLZaajqpx1t1M5bS25RqwPHd486T
aKsrlnBj6O4toZL7TrHIT3AyzXt4IBWXFNzugv6uxc9NvY3vV65pXPrI0P38YwexjWLxht3rwgfgZ5PrR3
31CbR5r8vFRbfnDoh3xB0xxDFHPfoW3tXZQd3SEycC4qW/5+zWtniH/u1HFY3yOhIe8k9uUwrEFKk
nsu0bGn+H2R2/sIjpp7fvkee3g/ACB7akj/V376u3ppMqr3qeqFCgDIiV0H9V/rBcwTQtRlZAQ0ImqY
bsGrZTiyhuIkyielpJ8GjFofJo1t2HQ9x9BhzEGnTgwGP0VZ8xMtLyzyA0Mj0J1YVK9WY/8jgrfhNscV/7
yMN90xjFaWk+MudvfAa79X7sF+GEPcRwyfn/zcTeuUjN51gntuimNPcv2HzlNe7yM4ctwZ+6+76jq
9BgCQJTXJ1KT1UVX907t+59XUQQWA3Kg6qHdaH6mDOo8IUZdSUTYvhPckdEOYqMl1FkZg8hyjn
mOk8MDO76nnDnc2AsPq6dg9GafXkeGOgjFCRlOK52UyYccwK2keVxw9ORxbLNWR2T2X2vVfkvc
RZOKW625hxn4AyNFfvf4x+fFzLuul+H6pdpvc+uoX6SUAQOaOrYkUbtYLmDeEqMuquCl7esjtQKlR
W/Pcos+u3ZYNs3edW7PRrMGZhHW+zurzZA+NNoYyV0/nF6FOH/qlfF4mkm1w6dXKHXpMWsd0
AsUTMrb06bje1BPcc+OPPf3vi6P9PoIsvfmWN8tH3/5RvQQAyNIPnv+sHaQm8Zq33iRve8+tegkAkL
ljrxI5/ia9gHlEiLrE1MzaqqeYPcGPHYR0vRqKo4f/GoxhxHPHrB2pgiW3/uMUba64U6Z3duT8+R29n
aqkk6GW5KR37gdnZO/JRbfoZeQQ8BgyOC+
+nI4hTOmkFwKOqumZL+NcNHfF75DZlQPvNJxwzn2EWPdckmPP4PpP/T6Cpfben32v1O6o6SUAQ
Jb+8eU/lL+/7Xt6Kdr1P/E8uet3qF8NALkpvFgP48c8I0RdRu0Ne6itmkU92FPMCHiMXnIlPxkzZmJvy
4Y3Dft884MlY2bySXgTWHWk2dQnKrWh/EXZdLsdqvO87l+XXmPdq22ZZBKkcVI7L578j8Fj1udsrvr
DyPW9bj9yHVtu1oBtyq7eda9xzgsxY/VgjnPPTXjsU1//hO8jZntyvRSYe6o3quqVCgDI3t/c/m157Pqn
9VI4Vf+0+nuvleddc6VeAwDIlqqD+h7r49XOIuYWIeoyqmz7Q287NSm5QYtRH7F6NnzmbhXSDL52
Lpkz9nttNibamUhF3I6BrlSH8o+4LiU/fZQL005glcl5MSQ9BnPYu9ueiRK2YH3O5qqz34IX9Felldqs+v
EUNy94PTLd9njnodqSeM2Jc88lOPY0r3/C9xEgiqqPquqkAgCydel4Xz7/hkcj66NW/u8idVABIE/H3k
od1AVBiLqk7KG3YUUMVeDS7wcDHlX3cLBmYrWVcx3NpCqy3W/5gZHNOTbvsAPDrOPxhlcr5bpsp
ZzL2dcl5LxWW9Z6d+j1VLI5L6Zkx2C1Z/A+3D80ejUmYNfnNIaSu+y6nznWQ/UUZXNvuFaoPYw+Q
aAb656LfezpXv9E7yNABDVTf/tX28zYDwA5UPVR7/uZx/VS0Cte9xL5hV8/oZcAAJk7Zv2GfPwdegHz
rtBXRewAxKNmNtcT86gwbG/anqHAOEfpntPHcqpFwIpwn+l9Rip/xM0BAHl441/fIKWHXqiXRK596
VXyW3/8z+yPAIAcFK4ROf5+66P/Xoz5Rk9UILaeNNbdmc3LcmaNABVZO1r3XPu8dSwZ9ODG0fGO
4juYsR8AcrL3um/J9655Ri+JVH/3tQSoAJCn4+8mQF0w9EQFxmrLxmANWFXOgK50yMzRu+d6jRU
p7ZyRbiplKXDUFf6fgn4GAMjStU9eKe9s/aS88723MYwfAPJ07O0M419AhKjAWAOBFgEqMsc9h+
WlJiiblx9NaEs42hKOtoSjLeHmqS0nrr1GDr/3pF6aLa5RONoSjraEoy3haAvSQIgKAADmbJ/ghqMt4
WhLONoSjraEoy3haEs42hKOtoSjLeHmqS1IhpqoAAAAAAAABCBEBUAAAAAAAAIhCiAgAAAAA
AAEAEQlTkSE2WU7DrfxRWGtLTa8fqNWRlo60XHO0NvZ3CijRibwjp6Uljxb0GG9aVTbJuGmH3UNr
7mJbRRv1Y4SYFAAAAAGChEaJirvUaK1Io1aSjl4H5pgJdY1Z9W1nOrBX1cwAAAAAAsIgIUTG/eg1Zrx
GfzqeibO717RkF+/1tqei1szFPbenKgXvLluvStdu0J5tkqAAAAAAALDRCVABIS+9Q9vVTOXVCyE4BA
AAAADgaCFGXmao1OlC7MazG6GD9UXuIvfE1A+VKNbNOpXokq1Vp79Mcxt9cdbYTvjOjjeoxqk7qY
JvCa1VOdrymMbVf2xvetob2H3JN4rTRY359ZEONcxFo46j1xj7t9ea5zKoOadx7KKotg9uYsD5pjOuS
9J4FAAAAAACLgxB1WakgL7TWaEdqpVEhpPpcQUoDQ+ybq4OvVyFiSYIva8pqYbBWZBqcNq0GNh
xyDHYINtgm65W1UnjQaYt7vIMqcrqqn3Z25OLAa9u7bmODtTKHQjgtuo2TKsrambLztHMgXeeZxR
iOHmh7W9xml8+s5dDDMoV7aKJrPizf6wIAAAAAAOYRIepS6knjnJuIuXUb+9Kt61BNOrIzmPx5qtL
Sr2+5QaEKG8/7ve16jXN+0FVt6VqVXfE2H0Nl2/qabl28L3G3sx1W8dJvU/gxWMe77oZg/mv77gF0
arI+MhUdf7xhKltu2wfPpR9GSvmMeBlqe8MPgsOuSWQbJ1NcO6PbuC+H7qbbu0ZI2ZEDN101hq
mfOpF9hDr9PTTNNTckuC7J7lkAAAAAALBICFGXkjERz96m16vQD9VEOl56FlRt+RP3+EGhqScXd9w
+e1VpeQGStc8LYa+fntmm0GPoXRS3SeX6lj/xUGXLC+U6OxdDexSOP94RimvidfQ0t22ElGaPTrN3av
2CcU02L/htrJ0fGKo+Ja+NftDbO3Si0mrVCRv3dbrau7jjBZKnvROYlRTuoSmuuWkm1wUAAAAAAM

wdQlRYdA3P0OH9SZmzk5+Ukn5qK56QU/ppespyMrCTEN0D77jsIdheXUtjqHdgSHsazOHy/rB4P5
SryllvyvYRvVNtxnbMHqOpKMoJfUHcwLlrXzzrnJ4+aYeVwfWW6mk/kMxMCvdQKtd8VtcFAAAAAA
DMG0LUZRWYKCdunckYgeWRMt3x+r1i3Z6eRig3URhpDK9PScUt3trctVrXE6cj6ik5UdFhZWC9anb
2EeriSf+6AAAAAACA+UKIupTasmH2OnVrPZr1HI+ocr3rlDEYevjD9lNT3JSzbglONXTcqCs6WRiZQYh
dOS164L4ctvUQeLv3pzs5lrF+QUP07K/5sv1xAQAAAACA5UOIuozMupwqYHLrohpDoCdXkpNuEjs
4XNoIEXNVcoamK6NqvWbF6+nZ2ZHz50fVFTXP2eBs/mZ90FMydk6nxNfQ3XdHDnadr3VrtZbsT/jr
h4e0ZyWFeyiVa57idQEAAAAAAAuNEHXJ+QGTMWP/VMxakU1Z3XCn3DFnS8+ZMcmTNFfFa1J7w
6+V6a1MmdfTsyPNpj76oaH8Rdl0u6xar6utN6yz5eg11r0anuYESU7AqVivP+8dkGx4U8nH5V+vZtP
5Wnf2/aIumOquNyfCylYK91Aq1zz5dQEAAAAAAEcTIeoy8oI9S3NVh0rGhDtTMmcun2r75iRC7nY
mCjuDs7o3V3WI5gWO5gzwaXOHxftCh/JXtqXl5XU1KdnnrCAlP6mTC95EVCojdOutWrxzHLe2bVBg
W9Yzb2i6eZ9Y68/k0w3VNv09lNI1T3hdAAAAAADA0USIupQqst1vGQGZUpVWv+8HRvaEQpMqyu
Ze1w/BbGr7g/scx2qn1yBt/9DrDZhIcVP2+oNtstj1YDOoh2rwhvQr1v62Ruysst0PrUtbbVnr3ZILLnU
8g6+ttiara2v22gwMTTeGs+c+ZD2Feyila57ougAAAAAAgCOp0FczrADITq8hK3oiL1WDdo+eiwAwk
urtPS8/mtCWcLQlHG0JR1vC0ZZwtCUcbQlHW8LRlnC0BWmgJyqQKbOOZ75D4gEAAAAAAJAOQlQ
gE23ZKAzU8ayeFTqhAgAAAAAALB5CVCAPql5pZpNXAQAAAAAAIEvURAUAAHODelXhaEs42hKOto
SjLeFoSzjaEo62hKMt4WhLuHlqC5KhJyoAAAAAAAAARCBEBQAAAAAAAIAIDOcHAAAAAAAAAgAj0R
EV2eg1Z2WjrhVnrSWNFzZavHhsyL60CAAAAAADA/CNERSZ6jRUplGrS0csAAAAAAADAoiJERfp6D
VmvEZ8CAAAAAADgaCBEBQAAAAAAAIAIhKhLqr3h1gddkUZPD7+3l51HaClTVePUeI3zcL7eZW/XH
MbfXHVepzc4uF+PuW1j58HXt2XDfY318F4Wo12TGjwv6jHpuVGSHr8S1obCSkPCD8+s/eo8VkaciG
TbBQAAAAAAWF6EqEuvI7VSQUoDw+
+bqwMhX3tjRI1T9fXpBJbR1H5WpamXpFyXrYr1MbN2OWHk4HlRmquFYNCZ4blRoWtYG6RTk9Lg
BFl2EFuSwZd3aqWhcDTRdgEAAAAAAJYcISosVWn1+9K3Hq2qXqUCwPNulNaTxjkdX5br0tWv7dbL
zjrrtTsXnYiusm19rlsX9zNSbdmv7W+rxHNafjv7e5tSTNCupHqNdS+MLNe7zj77LasFWvOcDkeza4NI
W3bd1Dh020055yW0VjvW3SDXOE/uBe3UZN17bZLtAgAAAAAAgBAVUm1tixtxVraMANRTlM09H
crZ4aVeu3bGe23noKufZadc3/La6ciqXT25uOP20qzK2U13yxXZsoNGFVLuibM6w3PTO5R9/VQ6O+
JmscXNPWd/1mPPbVvvorhNDpynypa42Whn56J1ZJYk2wUAAAAAAAAhKiaha5OGDmHPzqkT44K
9tNrVlQN3A+WTUtJPFSdo9EPnYSmem+KanHGTWGtrquyCW7t0qM5p98Dbnz18X7+uYA7v7xxYR
2ZJsl0AAAAAAAAQoqIsJ82UcJTAxElGbdLcjGjnzNtlyawNRdm8ENYz2A9KQye5Giur7QIAAAAAABx
NhKiIoS0bZs9Kt46mWft0JuahXRm3obgpe3qIvV+v1tc8Nzybvl/DdfBh9KCdYLsAgLzp0Q3Ggz9yAQ
AAALNBiIrx2rte70o7oHNrfxpDyKc2ybYya1dJTroJqDsE3qVm4i8YM+6n1YaI16uZ9NUvzfakXXbw2f
XqnHrtK530Qtu4NVhjbRcAlpH9Xl+QwkwTy540VlZFWu57tPNHr+bqLILU+Qxze40Vpz0r+f/hT/0ba
p4P9zEP5wXDhq7XDO6ZmRvzvuZ9P7mPvG7mOO+3ebwnR+5jSf+gFfO8z/K9eGZinJuh951luGnG
nJfc34sTtofSdoiDEBWJ+AGdMSt9AiU/nTRm/7d+MFmdbhD8tO0KKsqaVzS0Kavem667baeO6OB7
cZw2JDp+/aavPhXsGRpSs9Wsc9pc9dvm/sOhHu7KJNtVjG3wDwuALMzsl/chKri09j/lv0npcCYu3Da
KcLuTP+4f5vte3N4IC3ONPyjOQq8h6zWRatX9x28G3NEnxsO8XrMy01/cAyWOhh+z+NZW52O1qS
YFda9TS6qdmpRmFPjk/0v7+Pc19R5csr6f6l3jHFk/T2Z778R5v83jPXncPtTnc/yDVmTok1eYm+C85/l
eHHFuht739CP98xP/vl3dH/g3Iot/IEadk9zfi8efl3zfiydoj/WNrUrbpf+eHO/7dn5+FsZY1g2DJWS9R/
TV5bd+A+hbPzD5uvW+9U+g87mq9ZZia/WtnxX060c8vNcqIa93P29uf9TD2NbIdtqStKvbr5fd9VXr
K8cxXx/yKNetVygJz83Exx/
+MDcdve3gMSfabqvqrS8PXwQAmEq3XrbeX8z3eP2+GngjyoffFv1vwAzaEEm/z8/8vXjm7XCuj9q/f
c28f5PzY/87OoP9RtP37dy1y/3eivPzV9rCv5dn1R7n5y9jv/pnrCy/l8a/rznvuYNtyPocjW9XvNdMa6J
9ZPIeqPdvbdd+hLRD3T/maud+Um3XK1IS/5w4n8/+vTjeucnjvS/
+fZv1+8v4cxImq7aNPy/h62fWnhHfw+nfR87+zd0737fBdX579Qr9vhz3uiJf9ERFDBXZVn8p0ksO56
821puAo7kr/t9KrNd7n9D2D52/MKlanIP1QqutCWuIJm1XEk4PIOsNTS/7rDdbf9h+0jYkPH57qP3g

ubQ5+wj8QdOuc2oMyXfZPWWMeqiWRNsFgMy05XytY71NXZBN503VUpEt9UY28fv35Iqbe9Lv7x
ltmTO69MupE/PRwFm1o9dYl1qnKmfn9kLNhnteWt7PKPPC+T6X6unAzyL5KMqJU9YH9+dQW08u
7sygPb2GqIFK5fqWv9/Ktv3zYmfnYgY9sRxj39d6h7JvfRj8fi7aJ64puxm9Ecd5v83jPXle3ved79+y1Ls
hP8tr6uf34dEJHdm5mO7dE/ec5PVeHOfc5GX8uXHeXwLf5xmY7Jxk9148/rzk+148tj0jfp6yR42mW
tYuzqii+fpZGOMRoi4pvw7mwJuLMeFQcMiBCgvdr1EPJ5TztxMM6dQPhf5rrYf5A725D/VQ+xmx35
Ht9MRtl/MGFlw3nvMG7H6d89gbakjCc5Pg+G2D59J+jDoG8zj1Y9QvU3G3a7xu+NgBYAoz+uV9M
enSL+W6bIX/A5CTnjTWa9KZVTvsoaMdqbbi/1ueGTUM0Rh6N9uSN/n84j6JXuOc9d1s/bI/oxu3sq2
HjNo17dWwypITNg/
+vJW13H5pT8huV1lOenWcNKPePgZk8AetuMFlmDTbEVuO78XTnJv86fJoB+cHhtOnWwJnknPCe7
FhxPub8/PnvuRaNYmfhRcOISoAAJgNfnmPTdUltX/5uTCLXoa6tpj9i6D+pWdGvR3b52vSqbZmPmL
C/0Op81AjV7KppRZXPr+4J+f2NDo7wwDEHTWkatqr7yOrObMI4efpl3ZMgT9oKfPyXhwwR3/Y6uy
flAvevxGqt6h6/9mYYa9C3osD9HwiwflBrO+pqedWiWHwjzD8LLxwCFEBAADmmDP5gRq2N6ueO
MFRDt36vqzO4hfU9oYzCcRc/dbuUL2Csh6WHcfc/eLePi/O6NHZXTNnsg5/YiAVeKtJgXKftGOWv7Q
jNfxByzKH78Xz9oet8pk147pY/4aeVWXUZtirkPfiAernGrdnrP89dXBKXadTkl3n7nn5IwymQYgKAA
Awp5wAVfXWmJ+hjMXNC3YNtk7tfK7hXHtXhU1NO8B1fuEpSEn9Vqh/CZrtcHptlsOyLfP1i7sOCGf5
y6Iecqzq2bt5jz0MViXezdWMZjYfZVa/tI9h93bqyMHgjTuqd9QS4w9ajkV4L56XP2x5ZtqrkPficIMl+f
qydXLfOk8nJau3vdn+EQZpIUQFAACzwS/vkVSvDSdAndMJ/zL8RSPMYE8j9bAngLR+Mexaz2dbt7sn
h6qoWc7nZKxZ/uLu9nw6O8NfFgeHTbpmdl7y/6V9rOIJceZ7CUZNPfuGnmG4O2f4g5Zvvt+LB+T+h
62S2GWOB8PbWf5cw3txTLq2eOCPkekZ+UcYfhZeOISoAABgNvjlPYKeRddiD3czevzkW+dSTf4wM
Bxc/0KW1S8ac6/XkJUVc0i2WqVmSp7lL6nz94u701utKjMcPSpSOW21wPoeCgyhV7dwzTovM26bL
dtf2uOpyGnVY6+27r+veL3G5m+islngD1qLaFZ/2NIjADo1Oe/9w6mHcM+oHinvxXGoUhklqUldLm
RwkSL/CMPPwounDwAAMCOtqvSt33L69a5e0a33y9aPJ2VvRX669bLVFtWewUe139KvWUZh56U
6JyfEblu53s/3bun26+Xg+ZiLe6RVHbg2rb71S2tfJT+5m+H38TB9HgKPefie1vdRxvdv3Pc1573Yf2R9
7eK0K4/35PH7CLt/3Ifxb1dq9H0x9H2r2jFw3Pp7Pu1rNcl5t78m8/fiEedGvd8M7Ns9hrTf/uKem6H
XZfY+POp+0XJ6L453XvJ7Lx7fnpB/xzO6RnHuxXn6WRjjFdT/rIsKAAAwE+5f6F3WD43zNRwQWBCq
x5xdm9BVbUl/Bl3nnO/pqrT6M5p5eS7pnk7G5ZnV9cH8Gfre9fjfR2Gvqc5r79gUjT83Id9bxnk7iuLc
LwrvxbOmRvOoOqhhgkP7+Vl4cRCiAgAAAAAAAEAEaqICAAAAAAAAQARCVAAAAAAAAAACIQIgKA
AAAAAAAABEIUQEAAAAAAAAgAiEqAAAAAAAAAEQgRAUAAAAAAAACACISoAAAAAAAAAABCBEBU
AAAAAAAAAIhCiAgAAAAAAAAEAEQlQAAAAAAAAAiECICgAAAAAAAAARCFEBAAAAAAAAIAIhKgAA
AAAAAABEIEQFAAAAAAAAgAiEqAAAAAAAAAAQgRAVAAAAAAAAACIQogIAAAAAAABABEJUAAA
AAAAAIhAiAoAAAAAAAAAAEQhRAQAAAAAAACACISoAAAAAAAARCBEBQAAAAAAAIAIhKgAAA
AAAAAAEIEQFQAAAAAAAAiEKICAAAAAAAAQARCVAAAAAAAAACIQIgKAAAAAAAAABEIUQEAA
AAAAAAgAiEqAAAAAAAAAEQgRAUAAAAAAACACISoAAAAAAAAABCBEBUAAADAQmtvFKRQ0I+N
tl5raG94n19p9PTK2fDbuiEhLV0wbdlwz3vk+e1JY2Xca6xXNVb8ba00rK8CAGB+EKICAAAAODqa52
TGOemSUMHoqjT1kqMsZ9aK+rmpKJsX6tZnHZ3aesg1asv5Wkc/F6me3bS+CgCA+UGICgAAAOAI6
Ujt/OL38Zx/XTlwM89yXbr9vvT7e7I5KvksbsrZqn4eco16jXN+IFttyXZFPwcAYE4QogIAAAA4WuiNm
r3eoezrp3LqRKxeo5Xtlng5auAamb1Qq9IiQQUAzCFCVAAAAAABHTLzeqH590pVg6NpryIpbm9Orse
rX/7Rrehp1Vr119suC68NKtPqCtUJH1wEdeJ31CKsrGjyeYL3S6HYYzGPXj8F92fsp1ayzrDVXndeO3Ul
FtltujOpfo/aGXxagXN+yXjUo3vHbQto/dH0tcc5VoEar+6BWKwAsLUJUAAAAAEdEWep1HdJl2Bu1U
ytJYTVYDdRep0K2gfXN1VEBZlNWCyUxyoCqjUhpcMIpOxQceJ3F3t/IQK8jtZJRr7RcI60YnTuHwlEtel
8JVbak7hZHba7KRqMh5/wEVS4M1gNIcvwqwA5pv3M+hoNUR/i5UueiNLhTJewaAQCWAiEqAAA
AgKNjzQ3p4vVGnVS53pW+qgPq9ax0hK1v7o5oh1dLtC/
+y5tyzkv7etJYd0PBqrT0a71td2qyPjIpNl6/F2OSpvaGePmv0a6um3ga+6psW5/r+hNFqRqm9n5iDc
MPTjLVrLnHV5b6hcF2Jjl+67VuGhvWfmsrOxfjnqu27Eadi8A1AgAsC0JUAAAAAEdIUTbdGYwy641al

bNuj8nKaWvJNWL9/mFoL05zBnqzXmhn56Lz+t5F2dGdIQPD3I3enN5rB4QPix+t7aeGgTCzuHnB31
ftfDo9MAOTTGnVs8OTUiU6fuu675lBqKO4dsYLbDsHXf0saOhcmfVeOzviZq/FzT1n+9Zjb+QMWgC
Ao4oQFQAAAMDR4oVsGfVGLZ+Ukn4aMGp9qKqcDiR3JTnpp31ix33dA90L01rllguwH8bwdve1A0
6dSBLymT0vz8ha4EuLsnbGbdi+HIYlthMITDJlPQudTGqK41fHZNc5DR3eHzR0ropr4h2yuodK7n4ja
rECAI48QlQAAAAAR8xAb9RD5+l8K8qJU/rp1MpyMn6am0BHRnTmnE6i8DlCYFIpo85ppLBzFSw5Y
HLD3NgTdQEAjgxCVAAAAABHj9kbtRYvTsvXYK/Onhx6Y8iHebVWhx7biYbtTyercHa88cfflg2z16lby9
Ss3ZpEcVP29D4Gyt7amueYpR8AIg0hKgAAAIAjyOiNmpQxjDw7g706u3Lg7tTtmVk66QWAo+p5ps
MsJeDXAHX05KJbmFROSaIqAdNKcvztXa/nqR24unVRp7iWaoZ+1ePUnkjLDlS7Xi3W0WUEAABHF
SEqAAAAgKPJmIAoTMlPDo3aqW3Z8Kapz1ZzdcObqKm94Q8/L59ZcwJAszZnc9UfQt7e8Gp0FlIZV2
4Gzta5WPd7WfYa61790aSTVU1twuP3A1djxv4k9PbVbRDscRoSdAMAlgYhKgAAAIAjKro3qjlzuwrp
nGAubi3NaZWlXG7Kqg4D/dzWmOFftd+ozdlc1cGh9+IREzJNorLtD1vv1KSk21XyE1S5kPuM9AmOv
3Lan6jKu5bGBFRJjDgX5r1RPat7ugIAlgYhKgAAAICjK6o3qqp7OVgzs9qavI5mIqfk7J4xPFyx63gO1Di
1a3MOvE4Je+2U7GHrIcdebVnr3eHxeYt9/BXZ7psz/itVaZk1TZu7Xs/fcexzEVYMVW8zrewaALA4Cn
1V3AUAAAAAAAAAEIqeqAAAAAAAAAAQgRAVAAAAAAAAACIQogIAAAAAAABABEJUAAAAAAAA
AIhAiAoAAAAAAAAAAEQhRAQAAAAAAACACISoAAAAAAAAARCBEBQAAAAAAAIAIhKgAAAAAAAA
AEIEQFQAAAAAAAABGEvn/AZM6nr53azj8AAAAAElFTkSuQmCC]]></redcap:Attachment>
</ItemDef>
<ItemDef OID="q12" Name="q12" DataType="text" Length="1" redcap:Variable="q12" redcap:FieldType="radio" redcap:RequiredField="y" redcap:BranchingLogic="[q9] = '1'">
<Question><TranslatedText>Do you prefer Life A or Life B, or do you think Life A and Life B are the same as one another?
</TranslatedText><redcap:FormattedTranslatedText>Do you prefer Life A or Life B, or do you think Life A and Life B are the same as one another?
 </redcap:FormattedTranslatedText></Question>
<CodeListRef CodeListOID="q12.choices"/>
</ItemDef>
<ItemDef OID="summary13" Name="summary13" DataType="text" Length="999" redcap:Variable="summary13" redcap:FieldType="descriptive" redcap:SectionHeader=" " redcap:BranchingLogic="[q11] = '2'">
<Question><TranslatedText>
Alzheimer's disease - Description #[no]

If you have Alzheimer's disease...

1) You might forget recent events of importance to you. For example, if you are interested in hockey, then you might forget whether your favorite team won their last game, although you may remember watching the game itself.

2) You might forget the names of people you met recently, or the names of local streets.

3) You may no longer be able to handle your personal finances or plan leisure activities such as dinner parties or vacations. Simple decisions such as what to order in restaurants might become difficult to make. Your ability to concentrate may decrease and you might have difficulty driving.

4) You may start having trouble finding words to express your thoughts. You might need to be reminded to shower or brush your teeth, but you could do these activities on your own. You may need to post notes around the home to remind you of simple things like turning off the stove.

5) You may remain aware of the day and your surroundings. You might be able to travel to familiar locations like the supermarket, although probably with the help of a loved one or friend. If you are in a social gathering, then you might have difficulty following the conversation.

6) You may continue to enjoy hobbies such as puzzles or reading.

Now consider two possible lives. In Life A, you will live in perfect health for 20 years and then you will die of natural causes. In Life B, you will live in perfect health for the next 10 years, then you will live with Alzheimer's disease as described in the summary for another 10 years, and then you will die of natural causes.

</TranslatedText><redcap:FormattedTranslatedText>
Alzheimer's disease - Description #[no]

<p></p>

If you have Alzheimer's disease...
<p></p>
1) You might forget recent events of importance to you. For example, if you are interested in hockey, then you might forget whether your favorite team won their last game, although you may remember watching the game itself.
<p></p>
2) You might forget the names of people you met recently, or the names of local streets.
<p></p>
3) You may no longer be able to handle your personal finances or plan leisure activities such as dinner parties or vacations. Simple decisions such as what to order in restaurants might become difficult to make. Your ability to concentrate may decrease and you might have difficulty driving.
<p></p>
4) You may start having trouble finding words to express your thoughts. You might need to be reminded to shower or brush your teeth, but you could do these activities on your own. You

may need to post notes around the home to remind you of simple things like turning off the stove.
<p></p>
5) You may remain aware of the day and your surroundings. You might be able to travel to familiar locations like the supermarket, although probably with the help of a loved one or friend. If you are in a social gathering, then you might have difficulty following the conversation.
<p></p>
6) You may continue to enjoy hobbies such as puzzles or reading.
<p></p>
<p></p>
<p></p>
<p></p>
Now consider two possible lives. In Life A, you will live in perfect health for 20 years and then you will die of natural causes. In Life B, you will live in perfect health for the next 10 years, then you will live with Alzheimer's disease as described in the summary for another 10 years, and then you will die of natural causes.
<p></p>
</redcap:FormattedTranslatedText></Question>
</ItemDef>
<ItemDef OID="a13" Name="a13" DataType="text" Length="999" redcap:Variable="a13" redcap:FieldType="descriptive" redcap:BranchingLogic="[q11] = '2'" redcap:InlineImage="1">
<Question><TranslatedText></TranslatedText></Question>
<redcap:Attachment DocName="20.png" MimeType="image/png"><![CDATA[iVBORw0KGgoAAAANSUhEUgAABVEAAAD2CAIAAABQo1B1AAAAAXNSR0IArs4c6QAAAA
RnQU1BAACxjwv8YQUAAAAJcEhZcwAAEnQAABJ0Ad5mH3gAACuuSURBVHhe7d1fyGVnfS/w2PR
PcmoxeIpEe6hTD4WQBBnNzXSQOoWiIghCGhqCLS0twQqDYE6Ukw5FwmKFDJKIDlQotCek1xER
OiJEgtBKiRQcC5yESGGuYgYSC7GUOQN5qJn77XX3+d51p+93rX23u96Px+
+nDN77Wc9v+dZe8e1ftE9veG/AAAAgCXS8wMAAMAy6fkBAABgmfT8AAAAsEx6fgAAAFgmPT8AA
AAsk54fAAAAlknPDwAAAMuk5wcAAIBl0vMDAADAMun5AQAAYJn0/AAAALBMen4AAABYJj0/AA
AALJOeHwAAAJZJzw8AAADLpOcHAACAZdLzAwAAwDLp+QEAAGCZ9PwAAACwTHp+AAAAWCY9
PwAAACyTnh8AAACWSc8PAAAAy6TnBwAAgGXS8wMAAMAy6fkBAABgmfT8AOzI89eev+F/3SAiIi
LSm/fff/OPvv7/gzBMej5AdiFa9ev3fKVW4LbuZYiIiEicmy/feN/23D80b60fuJdWv76jB2LiLiIi
vstuB2LiIiIpLMpz/2gb/6/f+5yiP33P3Lt9/OnycYRc8PwOw73bh+TMX8cBXxt8cRc7PZfyEJmX9
R9PwAzOuB7z0Q3MtFREREktn/Tf/L/4XTXa7TRcgBcwu+sbSn3/jrifs7mQ4fl8vqMgT/ss/yfPZXP
ILfGH6wC9PjAFvA9M9r7PA1tpoArmOq1hm4wQUpdeuEJyrMlktLnUaaXj/rqm3v0e0f7vNgKpPjk3/fehtfj
m6Kz8N37Mx7lW+VSz4hFdzfAyyU1Bf/D+v0v3M5q8CaNb1pRu/6c8ifU+7y/b5nAs+hZgsD0g8DIIM
3Xe/jDnTWHX1qDHXC/cXr6Gj0GFcmq2cFF/IvnnLF+AKgu0EVo/lOLzAWmFcOfEKjO0wIFNuFeP
wrK5P9ufpdJLMqk34nxB/eP8rBKyy/pdPMF2lTuvXc8WwXekLTYqBz+vXhYPfuvtTaocLnJKXgIj0ahdv+
AzXm2I/Bq3N4w7/pgBWSsdX8tH5QAE8sNOh6ZjzAkcKbIYxlCCvHHaFBIIoHxjcJsI+T6LfJ1m/Rf0NJOh3
wDOv0jnA13cjBt5FT8oQeKpzuoabwLUw1dVLDkHnbNf0eTmZjNZmIN8qbcNfeX8F3XwzaWdmi+S
7+fMxFAIUCDrlelK0cjGlhaxWK7aowpzcuLmwthi7wNN5yJPvH
m4d9g9MJHjlYBOeIpRVTFgDuDIuWrJiCIxWRUCrQBuEdfN1UAuOXWlxVrRNEg0+ou1u1Lc
Hyvw7p5tHVZCGEEYvCjD0Wr0vHpyVoKNlJtFDw9+a5r2Q8FKE9A6pI1NLmTKIvJyjP/2SwUbL6/8V3VNPXf0
XK/2sOakZjRvf4dAwZe/EPQqPaQG4RpefDFzswqnX4Rgz5oZMOvNSKhMCNqwajfOZDdNdBSJbhrw5kYBLm+
rFd/QVSvp+LOvfbS9TYM1A7NP6cy9Ze/M6YrAYAKj6vq+Wn38f9t28AAOThzNrSNH8O53QnD87b+mn8
TbRgcBu0BsOSk95uPKj8+C7mfUHn3AG0vlrHq6jDrCLdlr9fqG9k0qaPcE+oa5SNFSv/C2LD5sD0Nq5P
qTDvWdgzy2aeXzpt5vOfVwJ+q2TlNDUfMoA51VPJP7VAN2/tYcOLvGZ3fmgmZ3Qdld8/9kjPbXqEkr
hTbmT8sfbRBE3cN5VTHT+ez2y4yvrf+lV0XnRqw2AnNJM1btm3fAHsVpuUIXNi8fj/BcFs9uQB7Xj9+hH
2RCVKWGfQUBXxgPeWZUNMxUcYeudpiZ6KT5FecRDdg+O6WtNwyiHjN+G8OdsLIHf+KqaX6WhH3/Zm
wYKEm4g2SwG1sqHkH1ar0vCUaMWAMCJcP7PPxgEDQMLuEhxtjszdPGq/MXxmaBFKyLQMFyHOq8vt
nIbpVyHXMn1jjk+8Rfzm54Yi5HUoFdGRaHE/mjcNwKD3xzuapKBFn6IdVq53z0S0CJqnnKq30TiNJfw
ZWAL1Bi0SBwnhSwMFw1dXEGhHrmKd/AL3/rPqyGBeSkT3I3n6dHVlHQm2v7fV/xtRD2L+lRDAiT3
B5RUr80fMvZ2Qa79tvKLOOWPqZB8MMOBsr4q9MZPG8TKeu6Lco/VG0gexF6qYc5AdCNGxPOQtUqg
oIEyoBxxwj79U+iA9JXh/QcupkhEDrnH9sOIg8jPx+ZGqngaHr6XmqyJxfQJRDYrH1uNOHsl7YQG/6TOjb
dTK3YUCSjQ0M3kFmZLtgM6+dZoNwFjg/s/OgjBqPnj3hxyYp2cWW3zqWoZ2rrH08lqamHFpRs8+uP
ZgwCKjE4VJYJd5Yby2v1WjjNqoNM5nffwHmM3ps+zWG4+HCMYKzBeLg4Oh2gu8dw1qSnGAf/+kYQmXp
hsgemgb1+y+LzCx1UZQbFBrhmUOwl0UGrT2+9/k8vVp6ngfSo9F3X3HqLgFRIFBl7Tvx7avZpn3ROEd
h7/4U9RvbQELGW2a0QHnn7a/ElALM1/v9p9etn/BHWZ1ia3Afy9o/L8ua38cF+lNnbwLBNhRoWVNBqv
94GUvKKBlLtrkYbrW3/PSt8dHKzTKQLPrb8V4Nmr2HWpCvHRcl7OF2fN5v9lt2SGA/cbPZLq/YXC/Th
t3D7Kyzn8gQydaRd6NBMGpP4W6qTbdDeerU9tzYWhEX4fk4Drfvrxs5NqzOxRC5gv1YF4H/GNsV
JrrwZ6oROZLuqXu/DNW3lyOFV+fzONvyl8NbuTR89AAdixCNq3QD/J4fFbjlvG9UZ3NXtFwZBI5Tt0
0yG2VJdC4lbtZWhJp1CC13bgfk9XBbUYVSLlHuRjtRkZ7RTDy9WHLLKphGQ3dHbrrrcEhVGsazSYWvz
uLb7tH5tbDUM+rJO/KZaLe9r3Zyn5dh+v+NP8DAP9+y5r6nKFA+tGtKSwNW1PeLPVdYeNM6Kzaf1uZ
G2vhkLQcOtt4hzDGb5jwX/oajcrbkNxx4Elo/K2AUx35OFGv5NymFFj42FqJbpZfvWvPqCWkAkvlf3pRw5
XXZFEtzPJKWaghtHj+fX/M6OeP/Ji46ltKTe5g9d/t4EOiwqsmw8MNqsgmhqSnFHwuiejFHGlHG
4dH7JEfH/b/u5h7+ca/+Mo6/a0e9ahmPBb/uL/bq/n9TdWqatpGPmoMoRgTp0fDLjPL1XbxzRGB
gLegRu12g6lc3vJJrrk5ebbG/jfcT93Hu1ltCtTvlbe1ZrXq5ZnSJm2cgWnp/ZOb8+RW8mbZBOuFQ
LHe+5cT1PX8d7HXNHpXXfdDtP6PdUh3AFc7ujX8xfkjYh6mfmf5IhHrNi5wtuGQ5rtbH6//SAUr7hx
6W+/f3K+M7Da9fDUb3MM9vWvrD4N7fL6cbwFbD8pTF1y4aWLMVc8OP6sD6QjRbjUQG7n6P0Ib
8OI39l05j3Jl98dPzN6hQNIoP52kULB6fVS6hzwZ4GRuf+dx1p81yxU6UB7yG0LOlKJt0ovvGLzMHNmv
mM3+9Hz9CUa/SfsKm/0zvX8zSXibh8tRfi3s7WCL7yJtLn0CpwVfr0ztfBZcuTvvsM1x/KFMfR4fe0N
Jk7FfpZ8BubFgXsUanzHvYYkH1ryXrg3J2fXbkA8X4Q4l8wmrOspw+bf08YaXsq3yEmyAz8zh/XX
/vLeO2zm/BZq/C+cMf/eWPmPZKgx9cXz8FZ+Cb1bDpcCJp+t+ul+hP/2e+PJWi/eJp5ue9uen/9nd
rRMHw1fTfB9Dh8N83pGmmYfAuvtK9Wi2nTkeRjn+o5QZB99DBv0FwCTP6EF3ebJqHFIFfEIxnqLZ
VKF8HDeuyfC69fEdbC7A6tvtLHEdM0ycOoeQ9jzC39VIfANWvhJr6lj61WFMP6f5XBvU30aUsoZ
LpVS3C+3+nvq52TOTmmjtPQ5ELxvrP/TPYUXlrtPQ4A3jrqXepU19nt+vZ+Dpfb9xJ41MfSXurr9g3ePs
L/IX9tTpXbZ+x/BBs3ldOdjppJrmEIkpHsCv7b1EJ/Az7CdO7fYB/dHG6O64TYbmZ8GJD+fvT/+9vz
SSbdq3DytvbDK74eB67eGCk+VYE/a9T9XdR5rvLfdq57p/v8iuvdUAKtwwMzeGUn6M/nbUW1mubt
rkOc+39bcfwnVbfj5y5b0pmbtx3tAoKwjJo7F1XlZ+bM1Ap+z7jyNvGn4/VvdxuIILK2F3PjfMU
MUmeZyS/lZn9y79hfv9Pz/MfPAd8NQmQ6xHBHHqg5Qv8lt+Y8wYGD5FM2VzfLL9Xl2JRrwyNr2P5/yc
Ra+8t/ktaVD25j1DlKOjhSBu/i7o6O6Bu2+kvFrZfyr4b8azQ1W/LTjszddWi7T7hq9UjuXr6wo
vV3jC+4e8Y7mBWl+3Wex2oUMZrAu3Dyb/v87/YMHnUXKHzy0F2rqeZX11xQ/nxbqNJ+JY81CPy
pVFl6kgXPV2/Yj3nr7fu1LQv8X5NAv8FHd4+PjOPpqDAJGMQVj4+tRdhDCiUJf91eO/9n7k+6VOm
9yHD+h/dDm9YnyF+G2dw4t7vGk1YfwIOx9w6F5M/xOv/LuunegDgL8vrL9NNnePo+0wf7KKOf
Ab09fgNrjXjKvvO1gDYQ0gqxn9eKr8XNuSjGx9V4Df4D2f2/DfHvwzr8+nGl8CAX4uVOqdpqP1cjOPT
1bBOTtL3T+x88dH8bYMUd/XHvc/yx6+2Hg7fBQoxPjnh77/7WcLU2/9fpp4e80u4Cy9fKxzfp2l9dP9/m/nsbfW
F7ZMGVu8f0xbh6hYJ72MtXfPvdQGfjZtnA0XVVdEqxVBMZwMqtyO5cjQH8yyH6yzP6z+T5TQ8IXVS2Ccz
wy66u18eqpo4hv1t7b1cgr/JxhSwHJEURdh0qHJz7d7vhJ7VB/GbYPV2aj2t7Mw0ZGrfCL8nwMr5TU4YH
LzR7WdZgVWmU5rn+vNnqfH3G6jO4j45JmvyIbvzj7oOwiYF5ntvjNPgOn39GlCnIQP7x4fXBmxAcqC2
cXZ/c2Jq2RKgxANz+u2N8P0iS8gOGvcPf8w0H0PaZDK2i59IjQr6nBubi4MzDj+6Ouj+sPeMbnvsFFmfP
2s9z7AD16b6yfQ+29rB8uKFrmtsIEDbfQdpTDMsJtrfWf3aNiSd3D91DIENdIDpvOLNXvegC+xn
vgnnvXxdZUUNRVZepHDFYnI1Zvc7/ZunqFeBg0Tx36wHCK7fH+O+oPHbPhonmnj9y6JGhHPpmd7mO
iXUvvo82HwDXFhbW8wB9zjxnduX3c+p9jRjnqP9kT7vgPfv7hU6fsWX21fPuH2Z57fs/IHm/ZWc/ZM
kbHXmSNxjfH8d4zTmX3mj7fAZ3X9UewOTnkKZKNCHzT7+k5x9hF1uTYFD+/jvJ9y4Dy0qW6Nnsa0KS9X
4CXo0nVjC+Y8vMnXv2+f/RGyw0qPwrU5AwoszEqbGwaXRvP2cCC68uHb3iv+f+FTHNwAxcyuT4JN
GTGZxpi7zVwfe2ZfHPDPR+fnqmjfqQ6WOCAcTq/YOgSx4eS7Nw+SlUe+c5+XZYx3WqDcTTW1w6l
pd0vFaUW1IDFONoOaafiVb6+s2X3/iuk3yS+WXSIWe3u9nZnYG2XKoWk+IaQ+zVP8AzTEwFbfFO
rF6wUJeF7P+UybD7mQCLPzl13RzsOzRPhqxzhwcK66EOM/N5b+ZlMy1ebD0bgSNjcnLUj+Ku8Y
C/aFsh2dvH/qgCKsTBN6J9/7L9+/eVw9AOsWKm5hsrDhjmgx2M7O8a4Axx0KbVVW9tdnfFz75cd
CRrusz1j4+tktIqbyEDn8ZMFO1+bA6PEJk/6N4uPcWrtn78ZM7XEJgSlB+vLp+r2fHD2/NFWpOj
uTekLtr5vlh7U48HyBKMqRR9HOEVV3J0M4dz/8JD3T4wT/lg1nxUL5/h7+X/f7UZD1/vr87f/Z/z1z
nF14X6m+5ENRkYRXDgJZYbfxbDVnpTBcJvhqT9r0ha+xQjdfaGNoW/X1zM8w/ddBmVc+tdM5U
yr+wlpEL+g4ieCuW8XMtpLfT3+/cbTaHdVcVeCB13m/0KSjtCxLCYg/XG3cNqdG+5OR5gY7uLnhCfhYE
f9jLd/Zm3LK8ufbn15K7iPyvsMOLfhiE+Ewz7V/zkDvi/aKo8ZrmYJaCtT/9a+hbfM5Lav/K03fN4mvU
Hk42czVz92jcfpmzpBPbeWXrd2tWsMZ9l/5J68P0Sa0V/lD0tVN7wCDtgmR78vdNcb2aeRfgs/sIL+
OTY2+Tq/kbr+82bfWTYr5DC7T65PQG8fC/8qenXTqxzd3uMlI+LiilI0CAeeXEX0VkvsOcl5eQ39ZG
SpuJMNBybjiA1Hse9HwL9FJrscqGV2TjvjMMgF+DWtivrv4b+eeQcWI/dL5v5+PNM8fNNl2GxWW
Mb0zfMGJqGPaPNxKBltqJ1tqs27/YnNbXRPGhNjrHkvAEaJbp9yuE9sTdAb/Q9fFDMZemRIWMRq
wnQ0AQpjHqOx8QuvVtyU3YKcFDQPEE9ArlZPLC4+h4Dz4DsnNdXA3jhX9pOv1Af5DBHHmBr9W7f
sM8e7/4i6XLd2wwi99ScM9xl7X/65fDNa9y/oVA+cYjtBl7/2yhWzqhZ36TJhLyeHAd4LbHsiYHXI
Y3Dq30xg9GNbuv9c48n7v79aPf6k6BQsWpVbg0gRH9YuwNZcWabVfCXMIrm5hfvQeO/+GJb8
v/wz6v2Ft24Zd2VSZJ1YdGmlSBL8KY7d+C6IaGl8ExPLy0/n/3wDh+s5Sy6ArXE/3iPPJJ9GW7Fn2fMn
W5BX8sTuq5xg4vJ8K7K34PdnUn4MCqx4oD6ofoV9P5Wuo6f0jbTh9Uu+f2bLBd8f1nFEBXnVeyZ
OKMj9ZMD6ROZ08hcvB9FdejmlPMZBpY2+zwUTbrxmOWazz12mWN8/8dvz6feBBkrDCHNnZz9jR
dSvpcvXfVezpmJkyN6tvmHHXb/e3OPfQuGesbE+GDF7KlZ8PT7GoE3O0fEp1uxzmzrUN+7g8h6
vi91ecFBC1euZBJ3ZOHXz/ZvxZ8e6+/x3+S6MzV2/6d9uSn9oP9OA/2eybKRzj5fjoLerU65G8ta93
V4Lx/bo0H+Y2B0uLhVWBPMB8+v74TQ1M9efxAP5SGB1kn2nDl9Mf+E1YHA/+Lvp5YLBQVAhz
UfinUxjJS/v/efr8qmSmBuWX0t8PHL3NOLhbQwtmsjaeP+nLvHxQa6qoZXWN71pAAJsPOQdH4wA
N8nl3dC0hJ5gxLqM8lM5h3rRvAtOUNKEJMqPlu4vRFW/TROFAR/t/Hw2ryPAjrvRf5PcHxZHgpUOY
f2L/82YtmgSuYW3oXj8MTb9w+/f13l+e4xs89i/G5vF0TxNrYxFhFLNfqeZJ22Wf0TyFwhL6c8c0+Qm
+3/vt8U+PxTYAcjtntI58o+JuDtZHytLZtXXuVLGYuxCd/zjfcNeLfrUhD7cv7xW+LAuE1/q4Ou9X
sIl+FqVLAcTVLx5yf8GVA6FwxB1Z7lXZlG+hbf6dfvd5v/9+dVFo4EPtc3wQtr/L7jnl8v7PDE5+Xd+
YRvuXpOKPdP/vbB23TQpUj+n82+b2sRsIpl/m9xhwZ8/JW/ZYxPPTXrgMs/N/0n+iwO/bz3mBeuKkXX
55+9+zPNzNlyb7Xbat1Kx+XwU+qBxJw5+5L5nd58+84j7+5+3EqaTadQrN5WxVrOOMrD84tYaAgst
c1r+f57WnR7VjqjeMPKy7dj9nJ+c+AO4evaDj7L8b6+bjhOp++fedPLvf/9+i0x8fe7pOjYiMHc+97t7
9dHvy75tEBfd0ijlGUdo8Zn8PyfEzcfPpbfvPJtP5ZWKvrzy+FePO7pxz93nPxaMsvYcPfnrbM8
1d1Nl62+HfSbHxQ2xdUtxPWa3n1//KL8/2Sm1Owvy3O5K37b/32aY9d0c9ZqMp5ZD/7c6l8h7+ogpp
er/qp3MfK/nMdNtMy1jp1XXX9a4fyVX2jPQ1cvo7f+6vBb2QSBJB2HTHgXXd7xPQn6mrtq22ndj/5
71drexdMKp1ojfjtuWUcVkhLSvKLjZ//vx55Iiv5znH5pg67WxYLkZRzZLz9sfDvn28S8k9vv8p+bb
Sbi7duJLEv/WrsbePMev+PZs8+dPs8nH3MmxkR8LLz2lh4vGVhufpZD9jmmcOEb7/kt/8Xe3T/
PC0rVq1flbpvF1erMNbv0vfC/tblxP/bdUZvUnXDdmRYZdb1KRM/KbwHtcQXz/+/tzzzX9u1UmrQXJU
81vL8HDjDN2sxo27NN9Nr/n/L0WvrI/PYlfX/v/ffHNNdMHPpsFvxUrPbyM1P+7dxf4sdPfOxSRX
Xpn/+63FJU1lhcH6dz91eMP/rl/1Lvn8vP/X/2tnmsf+Mcmh9c8dDnIcK3F4UEz2pTJPQ3KYNJm5f8
8V8e6vwRyd3u7+6rNlu6rxZ94MfvSZ/8NkuI4vIzzs+76dN9uamXzvVovduGLs6O0/0RJ3bF7y7VvX8
vhTv8qf6I1VNo7zUYn1jltPvl3qvpf3qd/MFF59CsXkp/9/3X7tkGF1r1L+FhNz4+/JWuq9Yv+Mbd/t
f+Ln48n/WpfMj/9VK/zdX/s5sa4fOt+XlWkR28tlAD1fxT+2X+V2RGKwtrZetdP9/9vvdOpNKDLD3c
WlxpG3wx9R/XXdbt/ln/L9g+2m5Ot7IcNy5Wuy3z+tD1pPKrytUpMXL6fS6/r3U2Xlf2utNdFfLPL+
eGbi7j66YiJ+e35XT3W9V3Tmea+cvGvvPn6hfn+bqt/pOH2HK2F59/6ecP1F6l/9YtN9DA/7/+vv/Lt
r3ad1zt34tbf/vt3v+8R7T1/mjfPbH+9c+oS/89F2/l/GwZ7nqm2qIZjdUjjJ/+tbsteaHRJfGeIhU
C5tbzf/9c///3Xd8OrXrd1f1/fOfn96YUbvPP6Pyf/S/4kb35f2fp7zf/Fz85/z7nPnrV/uC+vuxQ/d7
P55HYpEsKo7zmfb/3X3aPWtW3+BXj/v63z3XPv9uVPn35mffNl/uE34r/ePq92iTqr/9KoUcbeeutaPTTj4
+zn/9A+k5e7a/eHAvd6b35rWf9Xbcf+Nys+W6ctF97YnrT3vI8fPqr/fDu/3rY9vX3+W+y2f9/MvA1
0ZDwp+vcr7oU/GvfVi+VS6rc3/99X9Jff5nuDmHPe1j8Un/Jd79/u/4+7Np77n3+g/Nv7/9GdLlT9/pq/W
RsE+9j/b3+9s5/XPv96b6q0m7MD1Ft+rzM7o5yrXtM6v9oSZp7pz8L33+77Aw5IkLZ3/NP9n3f8j/r2ivN
vHP+VcAAAAAA5JbmXf3v2Le/XZ/zyO5Xf5fXf77rmfu+fdn+NPfeYlz/+Bvn/N3tf3XLf5q/PX/1p/
8+d/w97/8fP/uV3/9TDf/zN/+ePfTsHVtd9/+/Pd2/5f9DbZ8/9c30p/+9uvPO/zC/evf3v99vu6Fv6v
/r3d3T9ze7f/9cve+s/Av+Td39rvnH/0e+zX/13uv73Hf0v/Vu/7/O/Fz+v/3+6p/f/1uOvrzv/7Pm/6
/Pnm9eo/A+/Z9+/zdf/fU/Dp/f/j3e5//3/iVt+t/fXP3v/v++d/3f+g

iIsnc+NC7/uSu3w369mPmyctfyJ9LSNHz702yua+av0NuUcMWLtHRpQwcdgrN0+0Ou9776flTa9Pz
n0TXj67f9thtwb1cREREpC1/+Mn3BR37JPnht57Jn06I6Pn3qOz6yyanPHLgXXHQsQ1s5gcOO31muj
DDptXzcxyf+pdPBTdyERERkbb8/p//VtCrTxU/7O+g59+rZo8f/zuAStkjZYIBiWapp98r3q7OiI8U066P
BNN1v2zTdVa63yuGpDa21hydFK6tXqg+V/fSa2d1n9S1vNokxR+Tqln7N9sYUTs1UaBlh/ULUjsrrta3
mGApjSHle+tFtK1t8Eo4FA9874HgRi4iIiLSlvc8+Gt/cduHgl59wjxyz91+2J+k59+vqk+6eKX4Y9TjJHqk
ldqwerOUazRZCcX7cZNYTpIfyV4H03W/bNN5VlG/PkUxolhSeUZdbdMp4drSF3Ota6JBZ/UtLzHJ+St
X4oPB5alrrDE5IricNfUrWzPJ1lpmKUc0PoW2tQ1aCQfjqZeeCm7kIiIiIm2Z42f8cZ5+5OH8SYUaPf+
+hd1U1N6UnVDxTnlGObQYUju30WSl5AOKU2r9Vn5GMUP2Mpiu+2Wb7rOKFVRzFAPyNUZVEtu
OhWfFG62OVKVD7Zdni+XFk+SiU4fMVo4ojpTTFwcS0yZ0bK11onAxxYDohNYZogMrA1bCoXjhtRd
uevim4F4uIiIi0paZfsYf50fffy5/XqGg59+/WqvTaIE2wvZqLWyYEmNSPVVTPmJzzubFxYvriepHivOD6
bpftuk5q3hdTFK8zncVvFxLHIqEVcqrXa01HJKQOKs6LSs/YHnlJOF64wX0zxafszmUWmD7vlb6L0hQ
eS1xqCEsHS0lOrAy7qNh965dv3brP94a3MhFRERE2jLfz/jjfP6uj7z509fypxYyev5DkOooc8VbzeYqaL
kSgwY0S/lZ2YDsz+evPJudVR0pTw+m637Zpu+s4kD+Ol9BsalikykddcMq5TTVOQOWX5zV+Bga5w1
YXqL0RryA/tmSK2oasK+VxETBiQO2VtMcnb8fLSW1tv6VcACO3jk6+8TZ4EYuIiIi0pb//re/MevP+O
M8cs/d+YMLGT3/Iejo39JvtTRlWzZL+WmrEZvRqz9sDpVHyrOD6bpftuk9qziSHahWt1FsMqWjblhl
3LUqzmqMaJw3YHmJ0hvxAvpna52sMmBfK4mJghMHbK06JZC/Hy0ltbb+lXAA7n3m3uBGLiIiItKW
X//7X/nTsx8MevId5Dtf/1r+7IKe/zB09G/pt4pWKD86tlnanFf81/vZ2dmh4kg1YTBd98s2/WcVh1ZH
8j1V76WvRK+wyrhrlSzeOG/A8lqHxAvon21AvQH7WklMFJw4fDErm0Fh6WgpqbX1r4R9+/K/fzm4
kYuIiIh05I//6NagG99Zfvzii/kTzKmn5z8EHU1V2fTU3ws7ocT5ZRfW1Szl88Q/498cqc0XVOx+2WbI
WZt1l3+dfe2tgUVC4WnjGsvU5WyetsUk9Y8yE5/bP1tqxLrAdvta6b8gvfPEU4TXK5qibflrW2+B3fj2y
98O7uIiIiIiHbnj3vcEffgu8+D5cz9/4438OeZ00/MfgkSzUynbp+Ldsg8qh5dHitaoPKenWapOTJ3a3nt
1v2wz6Kxa/eCdcnxxuBzaVTasUpy0XWOZuCph9f7lJUpvJBbQP1s0ov1ANW1CYlXhiX2LCddWjY+O
5K+TaxuwEvbm6utX/UX9IiIiMjzv+5ubdvwz/jhf/dx9+aPM6abnPwSJZqeubKka6oNrTVaou1mqTiy
nK6slC2ym637ZZthZ1W6jy1Ge0bBV0ZGNZfojWEvN01BNmii9kVxA/2bTI2rTN1fdsrtBF6RnMe2XJ
x8Sz5hY26CVsA/Xj66fuXImuJGLiIiItGVfP+OP44f9K3r+Q5BodkLNpivRAdUHrN4e2izF45JnBge7X7Y
ZeFb31SjPynRcsVxYZVxjWT+r+PNKfELX8lo31rqA/s02R0RDamtNnr4y+IJ0L6ZeaX1a8XozRWrGaG2
DV8JOHb1zdOGbF4IbuYiIiEhH9vgz/iCX7rz91atX88ea00rPD/0S7SicDpf+9VJwFxcRERHpyH5/xh/n8
oWP/+Ktt/Inm1NJzw/99PycTo+
+8GhwFxcRERHpyCH8jD/OY/dfyh9uTiU9P/TT83MKPX/t+eAuLiIiItKRmy/feCA/44/z3DeezB9xTh89
P/TT83PaXLt+7Zav3BLcyEVEREQ68umPfSDotA8np/mH/Xp+ABquH12/7bHbgru4iIiISEc+evd7gzb7
0PKlT37il2+/nT/unCZ6fgAaPvUvnwru4iIiIiIdef/9NwcN9mHmyctfyB93ThM9PwCVB773QHAXFxEREenOIf+MP86lO2//2as/yR99Tgc9/34
VfzdcZpa/IO6VK+cvPpv/eRl/F11zRymNq3rDDYnR9RH+Xr5hBn93lveVOzVeeO2Fmx6+KbiLi4iIiHTk
8H/GH+e0/bBfz79Hq95o0w5tTN8U5QUW1IDFOwoF/X6uvt/gsmd6/iUCK4O+O8v7yp0e165fu/U
fbw3u4iIiIiIdOSk/44/zz//wUP4MdAro+fdo5m6o7G0X04CldtRUdvzFBqMD5ZHNHP1Tkhvw3VneV
+7UOHrn6OwTZ4O7uIiIiEhHTtbP+OP86PvP5U9CS6fn35+5281T2PMnBgSHipfVBWj+OwBa6fmX7
N5n7g3u4iIiIiIdufGhd33mD34n6KJPVj5/10fe/Olr+cPQoun596NohGrqPWfZO2WCbqnWRFXTBB1
r2/z1Bqw2Ju7HGivobdfq09bPjPro9mmrd8qTygUGqy1EsyfV11a9qs4tCqc32Ty7lDjcd8Ua7680htT
mK/5YrLHzvBZDazUGdn5YqzdbLkWhWncpm7F+Wm1MPEtj0cHbg9fMGF/
+9y8Hd3ERERGR7pz7zG8HLfRJzCP33H0afti/Xc9fPm23P2onhzTagZaWYbDqkf8EP/C3NUjpt1ZqVy
0xILqmbfOnJ1+rTdFovgqdH9sk05bvb44FL9t21Kc8LxtdTlqdmzhUV5zf2H94sG9rLReoGpF4P3szfV
73zkfUyvWveK0+qiZxQrbMQRP1XcCtV8Ng337528EtXERERKQ7H/zr3wya55Obpx95OH8qWq4d9
PzB4/xxn9Hr03W2PgcveTXL5qa4TuWo8sLV+p/OC5CaPz43mj86rTin45OLp62OhHU6pq2PSVVN7
ahbuLvUDEWllkmLU2oLCdYWTRquPZwiXFX9cjWW0TZRdWJkTK3qSFG8PKs4Uh3oqF2Nqm0iLhct

KTot3PegNTPC1dev+ov6RUREZKu8+
+9+9c/u+L2gcz7ReekHP8ifjRZq8p4/Fj2/H0ut91iZZs496WqQ6hsLx5XdTs/uu+avHQuGFS9rkycOBS
abtpwo1ywZzNmrmq04ITVDMapte9E6gxMGbq0uWka50sYZ5bDWiQZor1Vdg3BMdM5KeaxjLan
z+ssVLzsu4IA1s7XrR9fPXDkT3MVFREREOrKAn/HHefD8uZ+/8Ub+hLRE8/f80z6al73A+WZPcBIlrkz
QTebKTW+OJvqfpNSVTxQIhpWzJ7QW7G/JBk9bHxh+uqkdtSoHd2w2U1RsnbQ4Kbj+xfjBW8s0R0
dzNE+o9lAKr0mXwbXCy1IMaRRLHmzqurztn0JzmU3BgjrWzHaO3jm68M0LwV1cREREpDvL+Bl/nK
9+7r78IWmJ5v3f9ice5hPP7GsDO5ninIvPFn/apgU6MImrmW6rgoHpQbHE/KlzW2ZPqU3UNOW0
1chog6kdtahmaYxNzDBg0mJItqBi5nL4kK2VRQLF+8Uc7Vtual/rqFrhNUgup3WNlXCetf5yxYiU9gWl
ajHcpX+9FNzCRURERLqzpJ/xx/nO17+WPyctzp56/nJUXUcvkStOW09S/Ln/rEOVuJrptirYanpQLDH/
gN5p6OwN001bnpYJRqd2lFIUSwws3qreKCbtXFl+2mpMMbyaoH9r1XryQdFGeueoTZEZMnB4rXB
Mcjm9a0x/QP3lBkw8YM1s4dEXHg1u4SIiIiLdWd7P+OP8+MUX86elZZn/7/BLnBMdGvDQv1actzm
teNVz0uHqujL1TYXjBl6t1PwDeqfUWb2mmrYYdf7KldQmB01SDkqOiqco1t65sGLU+YsXN+fXRveuK
r46xZHylMQFLKze2qLYuFrhrKkq5bHEGgup8/rLpc4KDFgzW/B/jV9ERES2zac/9oGgQ15eLl/4eP60t
Cz76PmLI4nn9452Ih406KQDFl+ZlbJDC3dZbTPR/ySl5t+m3ysOlEuqr7Npmmkbp5QvOuZMqMq0XJ
5gjgFTbpTLzTRGVzXzw8HWwp1W48t5EhdwpTyxOpweWBlXK7oK0ZZq87SVXonmWRlQLqoW7m
LQmhnu6J2jc/90LriRi4iIiHTk/fffHHTIy8tz33gyf1paln30/OUDfULH83sxUfXYHx85UVquZvry1PaY6
H/SmjNlVQb1TuWRho6PZpJpy7eLScrlV7MmdtSQLrFRzpIa1LW5XL12OLxna81l1xVDEhcw03Zm+3
pH1SrXX83bfinDNdY1q2ezDSrXcwEHrpktvP6fr/tL+0VERGSrfPTu9wZN8pLy2P2X8uekxTk5PX+6K9
jo6kIOV8fVbG42eD/R/7SpX+ps+ODeqbmCvlLHn7Z6oza+XH41MNpRQ/3dUGN09zRpidXUdV+xes
H1DovX+W6Ll6mp66euNS5oyva1+j+s1Rtda6zUi2cjR5ULqwyehC34P84vIiIi22ap/wv/yxc+/ou33s
ofkhZnnz1/d+8QaDYEoa2mgjGKr6AvG4vx1EtPBTdyERERkY7cfPnG+z58JmiYT3ou3Xn7q1ev5o9HS
7TP3/N3zBIpz2mhD2Ne5TfQV41F+eK/fTG4l4uIiIh0ZHk/7P9/TzyePxgt1MiePylr4aOGPtHhV9Pkx8
r/PXDbvwVITLJRvqETYyb1/7V6+1cUTqzPPv3Z4F4uIiIi0pEl/bD/q5+7L38kWq699PxtE7W2Ux2dfcd
bMIVaz6/hZ4mO3jk6+8TZ4F4uIiIi0pFl/LD/wfPnfv7GG/kj0XLtqedfa07W1bJ39vWdbwLQ69r1a7d
85ZbgXi4iIiLSlmX8sP/HL76YPwwt2nY9PwCL9Py15/01/iIiIjI87/ubm/7itg8FXfQJyne+/rX8MWjp9P
wArD3+H48H93IRERGRjtxx73uCRvqk5DT8jL+k5wcg98D3Hgju5SIiIiId+eM/ujVopw8/n7/rI2/+9LX
86ecU0PPTLfj7GLpfbqX4u/Hyv4jhOFNtq/FX8c/
+F0GsdraDPa3t8hqyWBe+eSG4l4uIiIi05df//lf+9OwHg6b6wPOj7z+XP/ecDnp+unU3+cdpMvfV85
eFNuZt+fNiO+rBd3YNWbLrR9fPXDkT3M5FRERE2nKyftj/9CMP5w89p4aen60EXeVxmsyg59+ZHdb
ddQ+u52caL7/5sr/GX0RERIbnpPyw/5F77v7l22/nTzynhp6frZz8nn+XfbGenxPru698N7iXi4iIiHTk8
H/Yf9p+xl/S8+9FS7ubPFz2cZn+M3r6vuLt6oz4SDHt+kgwXffLbuXgldX4YO3Jqeqn1BfYqXFS46yiZE3
LwuuLC9bd0KxVr9ZWK9j2WrT12phqmvzN9oqJiVoEcwzf1MqQ9a90zrHRGBK/P2AG5vXoC48G93I
RERGRthz+D/t/
+K1n8qecU0bPvx+Jxil1sGr46monDWzA6or3y7fLIuUk+ZHsdTBd98sO6a2s5WWjqcoDdbWNJnVfs
cS7LQvvXe5aZ7G2WsXx2jzR1hPnboZ3Vhz2cSQva+2MnhJj179WW1Xfh9s/Aztx7zP3BrdzERERkbY
c8g/7n7z8hfz55vTR8+9J0fMk2pzyUNn3FEfKRikaUpsmasBC+YDilFp3lZ9RzJC9DKbrftmqHBfWWM
kX0jdzYquRAVcsnjgpvirVkWALW9ZK7CMaFlfP9FZM1WuKTgkuWW+JIesPh0SThCdEZ/TOwI4cvXN
07p/OBbdzERERkbb8/p//VtBsH0K+9MlPnMKf8Zf0/PtSdDFlExP1Uonmqre7Won6qUg+YnPO5sXF
i+uJ6keK84PpuI+2SQ0rj+Vrb44pXiU2VjsUGHLF4gNJxVS1Ub0nxgNSpwz5yMrq7XvNhCeOWOLmU
Nv4jrW1r7982bb8YkBiis2h3hnYodf/83V/jb+IiIgMzx9+8n1By73fXLrz9p+9+pP8yeZU0vPvTdD4FK1
U2X0lequVlrNqg8p+qb2Py8/KBmR/Pn/l2eys6kh5ejBd98s2yb0EB5tTFW+mtNVKVinnLY4OW3JZv
xrVdWJzueWA1CmJVUbDEtVDyYq9e0sUb5fe1ID1l68rjYLNiZs2U/TNwG5dff3qTQ/fFNzORURERJK
58aF3/cldvxs03nvMqf0Zf0nPvz9FX5M1M0UbVDVr6fYsaK8GNGAp+WmrEZvRqz9sDpVHyrOD6bp
ftknuJTjYnKp4M6WtVrJKtMRhS07MFZ9YHgnEA2q1hsyc3slKT8VUvYbWiSt9mzrOlcnfL6ZIKabono

Gde+qlp4LbuYiIiEhb3vPgrx3ID/v/9wN/mz/NnGJ6/n3Ke59V91R0OLWWJt2eFQPzo4MasITNecV/v
Z+dnR0qjlQTBtN1v2yT3EtwsDlV8owe6XOKeYujw5acmCs8sRiyshkVz5yqNWDmtt33VkzVa2iZuNK
/qSHrL9Wmy2xO6l1FTXoG9uGL//bF4HYuIiIi0pZD+GH/5Qsf/8Vbb+WPMqeYnn+v8obm/MWLm6
ap3jOVjVS9ywm7q0T3VDZJbX3fWj5P/DP+zZHafEHF7pdtUsPKY3mt5piBEzeEM2aiiYbNnLiwwYnxi
PjKp2oN+cgSY1b6K/burW1BxZT9JRJD4jErq2O1V826vcvMdM3Annz26c8Gt3MRERGRtuz3h/2X7r
z91atX84eY003Pv19lt5QJ2pnyzaK9Kvuest8qjxTn1ibsbI6qE1On1vq5oNfqftkqWmetfl6sbebijHJ5H
aUGXLGwTotipvYrEa6nWnA1c6pWNS4/WLvuxbBE9ZX+iql6TVH15oEBm4pmiNZfHqiWH2yofY6h
MyQOsANH7xydfeJscDsXERERSWa/P+x/7htP5k8wp56ef8/K7mal6Koq9Xcr9S6n1pSF4unqqhOjLi
pdYDNd98sO7QvNq0VTpc/oKbTFFeucKdFRhiemS61VMzfHbI63X4nyxET1td6KQ/bWUj4vNWBT
W6w/VC0rPUn1fv8MLdeIuV27fu2Wr9wS3NFFEREREktnXD/sfu/9S/uyCnn//yuamrXdpdkdVz1OqD
1i9Xb5MDK2LxyXPDA52v+xWDl5ZjQ+atu7qmYHtXfOkcGXDlpzoKBMn1jvT9dHidW3q+pBysvoKV
2OjmRPVc90Vh+0tvEJBme4Smb71r9WnWYuX1FxF917XmjMU78bnMbfnrz3vr/EXERGRgfnQX74
7aMjnzoPnz/kZf52ef9+KxkfrApwUj//H48HtXERERKQt5z7z20FbPmt+/OKL+SMLGT3/fpX/VaeWHz
hJHvjeA8HtXERERCSZGx9612f+4HeCznymfOfrX8sfVijo+feI+T9cjv+HzwCH7cI3LwR3dBEREZFk3v1
3v/pnd/xe0J9Pnq9+7r78MYUaPf++1Hp+DT9wAl0/un7mypngji4iIiKSzAf/
+jeDFn3aPHj+3M/feCN/TKFGzw/ASC+/
+bK/xl9EREQGZtYf9r/0gx/kDyg06fkBGO+7r3w3uJ2LiIiIJDPfD/uffuTh/NGEiJ4fgGN59IVHgzu6iIiISD
Jz/LD/kXvu/uXbb+fPJUT0/AAc173P3Bvc0UVERESSmfaH/Z+/6yNv/vS1/ImEFD0/AMd19M7RuX86
F9zRRURERJL56N3vDVr30fnR95/LH0dooecHYAKv/
+fr/hp/ERERGZhPf+wDQfc+Iv/8Dw/lDyK00/MDMI2rr1+96eGbgju6iIiISJybL99434fPBD38VvnSJz/
hZ/xD6PkBmMxTLz0V3NFFERERknn//TcHbfzwXLrz9p+9+pP8+YNOen4ApvTFf/ticEcXERERSWb0D
/t/
+K1n8icP+uj5AZjYZ5/+bHBHFxEREUlmxA/7n7z8hfyZgwH0/ABM7Oido7NPnA3u6CIiIiJxtv1hv5/xb
0vPD8D0rl2/dstXbglu6iIiIiJxhv+w/9Kdt7969Wr+tMEwen4AZvH8fef9Nf4iIiIyJB+
+55agvU/muW88mT9nMJieH4C5PP4fjwd3dBEREZFk/viPbg06/CCP3X8pf8JgG3p+AGb0wPceCO7
oIiIiInF+/e9/5U/PfjDo88tcvvDxX7z1v1Vv54wTb0/ADM68I3LwQ3dRERERZE47/ubm/7itg8f3f4AZvfymy/7a/xFRERkSIIf9n/1c/flzxO
MoucHYBe+
+8p3gzu6iIiISJz6D/sfPH/u52+8kT9MMIqeH4AdefeBXfSFR4ObuoiIiEic8of9f9P37fxIqeH4AdefSFR4ObuoiIiEic8of9P37xxfwxgrH0/ADsTnBHFxE
REUnmjnvf42f8k9DzA7AjN9ywo5uOQqMpNJpCoyk0mkKjKTTa8gqdBi4lADviiWQ0hUZTaDSFRlNo
NIVGU4gOLiUAO+KJZDSFRlNoNIVGU2g0hUZTiA4uJQA74olkNIVGU2g0hUZTaDSFRlOIDi4lADviiW
Q0hUZTaDSFRlNoNIVGU4gOLiUAO+KJZDSFRlNoNIVGU2g0hUZTiA4uJQA74olkNIVGU2g0hUZTaD
SFRlOIDi4lADviiWQ0hUZTaDSFRlNoNIVGU4gOLiUAO+KJZDSFRlNoNIVGU2g0hUZTiA4uJQA74olk
NIVGU2g0hUZTaDSFRlOIDi4lADviiWQ0hUZTaDSFRlNoNIVGU4gOLiUAO+KJZDSFRlNoNIVGU2g0h
UZTiA4uJQA74olkNIVGU2g0hUZTaDSFRlOIDi4lADviiWQ0hUZTaDSFRlNoNIVGU4gOLiUAO+KJZDS
FRlNoNIVGU2g0hUZTiA4uJQA74olkNIVGU2g0hUZTaDSFRlOIDi4lADviiWQ0hUZTaDSFRlNoNIVGU
4gOLiUAO+KJZDSFRlNoNIVGU2g0hUZTiA4uJQA74olkNIVGU2g0hUZTaDSFRlOIDi4lADviiWQ0hUZ
TaDSFRlNoNIVGU4gOLiUAO+KJZDSFRlNoNIVGU2g0hUZTiA4uJQA74olkNIVGU2g0hUZTaDSFRlOI
Di4lADviiWQ0hUZTaDSFRlNoNIVGU4gOLiUAO+KJZDSFRlNoNIVGU2g0hUZTiA4uJQA74olkNIVGU
2g0hUZTaDSFRlOIDi4lADviiWQ0hUZTaDSFRlNoNIVGU4gOLiUAO+KJZDSFRlNoNIVGU2g0hUZTiA4
uJQAAACyTnh8AAACWSc8PAAAAy6TnBwAAgGXS8wMAAMAy6fkBAABgmfT8AAAAsEx6fgAAAFg
mPT8AAAAsk54fAAAAlknPDwAAAMuk5weAbT178YbKxWfzowAAh0bPD8CCbJrxebvwV66cryrM
WXDX/2ZhvbGV81deyQ9MqbGZjH9Xsq3GNZznY5pdyz8w+XdvY9pvRsc/oh1vbSs9VeMTm3Zb8+q
8MrP+B8WM2jd1Uj+nlh01tjPVxzSg1gn9z6RTQs8PwLxmfJpvqNXZ5UNbVnaOZ53Vs1S5j+y5auYH

qvVGzl+8uPp/Zqmz3sLOHwnrz6PTfysa3+zKTN+
+bC87+kbUr9t0RVr/CW3+M9Tc6LG0Vux8a1ttU62Plwc2l/S4pdKz1D+uY5fovTLr9yf9D4rUphpbyh
xvXx2byt6a8B+leDu14nUz7SirXx7KXh13d8NqTVBqc+1ywfVpXMXgPQbQ8wMwo+bzVPNpZFJFoe
z/3+UDQXOHc5m9yrpAfvnmKbP+7Ge/THW7+WAaspIzffnCueerNfVzfK74POKFh1+NqbbWXrHrr
W0NnaoYl7/cWnb+RrPK6vKVB47/cfVtZ308f/tYdTa6NjXF/LmOTSUOjde6ncDxi7bvKDwyY63ijfzlcT
+19XTl9Nk3uXrZLJW9eaw9nUZ6fgDmEz4DRE8Nk5u/QmA3jx8zb6ucfv2H8Q9tXY73OLi1nX8PVub
9KjQv4Gz7yyauf1BTf3DRyuOtTHwhO67VhJexb6rs/dEXsjh73io16ULl0fUfjl2mWGyi1jz/cRExmmQ
fuY7tNE34/e69dL2LGS6aKtrHhMXKq5m9CL8OUxY6NfT8AMwmvjNP+LSTtuOHgWw/Uz0ztmo8/k
yvds3Wf5ynUHalKjNfs/n20WrmD2llcwlXJTZ/mOdbHv0TWvt2TCKab12xed0mLtkx3YSV+qaKLuwo
fVUm21Bqotqx9R8n+7InasVfiinEhbI66x8qlI5fNXXparK3p9pbslb2VVuX2PyhfSnbiWq1XM5ptla/T
OlCk23slNDzAzCb+Akg+YgypdkL1GUPHlM9vcWyveRm3NJ6F8X065Lzbaiw2dicdbJv3sSP8t1288XL
vnGZuUrF+4j/KT6WqEA8/8TXsmO6CSt1T5V9cBNcxe4q2bvTfFaJQus9FEfWb0/2pWipVTNRqbhQ
8LlkA9qv7zCJ7dR0v7utttmq6zdZqUSt7Eh49Sb6rLId5NXWf25OO+1VPB30/ADMZg+36t09CwRPi7
Oa9GGqab2N6nqtC+1kT/FXY1LBh7O5fnN+LbKCs37v6nvY/Hmeetnc4aWb8KPKJqyvPP4qREOOp2
O6CSt1TRV8HY8hWSU7mJvqskWF1nuoXq/fnuxL0XXpVjbbm6JaXCj68k3wD3LndiaYvy5Ra3O5Ns
c2f56oXnJf2X4KFy82viTHkE1bfjDRp9R9jUnS8wMwmz3cqnf0LLB50tnhM8fm4W36go1HtpopnrC
7ZHXnu37RN2/eetmnM+sliyvMuKPGl2K65/iN6J/Q+B/Z6NM7nrhAqeOtbbVOlV3NqbbTs+Ds7Um
KhYUa34maOWrFJvpGxIWiifvX0qtjiuytqb4La1GtuEL2yR1vRxv9l2Y9Yordhf/IxB9//1oI6fkBmE18Y5
7o2a3dLp4Fsho7fuDIas565TLrMvNXmX87czzKt5vsqbpdXGLWHdVM/ZWI1x1+WFPvrGO+CUulp8
o+twk307vgbMAEBXsKrd+e7FvRu6mp/gmLC2VH6hsJv4wjtG9nom3URLXiEv1Xd6DeidYDjv+lyHb
QnCauPMHHdOro+QGYT3hnnuzxo9X8FTbPJKHJnz9WVWrbSDwHzWKap7bYat7atNmHNOunlFW
ob2TOZ8Tsw5n3Oxd/A3ZSdGXyb0T20TQX3thb9NEdW6JioeOtbSWmyg5N+yH1LjgbMMHl6ym0f
nuyD2mvm2r8gzTJP1Wt25nhn9m4VuMfppXpinZ/TNN8Rtlq4yLrw/W5u5dCkp4fgBk1HkCmeSpIy
+YOnOxngmBHu9nMuugsn9DOd9N41J3uuTc257e6afM8XNrBN2LKvQXfgEy1hfreprqYHRW7F7O
V9qmCz2vjmJvLqjVWuqpSe53VPE6NgVdmPWyyL320qdWB2uSbJSUWMVj3phrvHqdMLtrORnZ4
B9/tyf+Dor1W851jX7rNdMlpGt/rSa/k6aHnB2Be9QcQ92l2pvFAeuzn0TbZ13u22fdg4ud4JtH8VDb
yzyZ46wR9Yu2bOpF76viMVhb3HxSTC/5VxUb1yOBB4pj0/AAAALBMen4AAABYJj0/AAAALJOeHw
AAAJZJzw8AAADLpOcHAACAZdLzAwAAwDLp+QEAAGCZ9PwAAACwTHp+AAAAWCY9PwAAACyT
nh8AAACWSc8PAAAAy6TnBwAAgGXS8wMAAMAy6fkBAABgmfT8AAAAsEx6fgAAAFgmPT8AAAAs
k54fAAAAlknPDwAAAMuk5wcAAIBl0vMDAADAMun5AQAAYJn0/AAAALBMen4AAABYJj0/AAAALJ
OeHwAAAJZJzw8AAADLpOcHAACAZdLzAwAAwDLp+QEAAGCZ9PwAAIfl2Ys35C4+mx/aKN44f+W
V/Mg8ijpB+UNQXZuV6Dq8cuV821ud7wEsl54fAOCw1PraZnt62nv+qm3PpC5Da2dfXdQD/DcZAPP
R8wMAHJbGf5dd71BPe88/aP/J5r76VwE6fuCU0fMDAByWRs9fb3BPec9fNu496yqvX3mdyiM6fu
DU0fMDAByWZs9fa1Sjnj86EDfG+ZD1iNq8+Qm1I/VuuDi8Olb7n9PXihRq70bv19ZWlenpuRvzNSas
rbTQMVc5OhtTvop20LX+teZ6giEdG+w8D2Cn9PwAAIelaiavbP5UtIy1LjN7HR+otZt5S1y1o73KJrr9
nFqfHfa1mdpCEpN0tr7pouHWa2priVSLu3il+GNQvW/9PQtq32D6vM7lAsxHzw8AcFiKpnHVQRaN
6aZhrL2RDYwPdPX8+aBaSxoeKdrS6JzqSHEgLFOOKJdSqzOg3W0vUE4Y1+xSDs5V02R6118MaF9Py
wbbJgpXALAben4AgMPS6BrzF/U/x21nrZ0Mm9liSNWXth8ppolHhGPCjnglPFROUhvTKlzBWriT+EC
3cgFrwSkD1h+Kqqc3WA4btG+A2en5AQAOS7q9XrWaUWecaJXD1jQe0n+keF1vlJvzFiNSgsKNWVr
ES1orShZHw631al/CgPXXNEcX77fMXq6y0twWwE7p+QEADkvYAeevy5/3Vz1kOHJlrp6/ebB4kdJa
uF16bLiV0T1/vIQB608175ni/dbZW04cumqASen5AQAOS9RMhk1k+Uai7Qwb43hI/5HEtMG8qRG
BAUNK6bFFyeJouLVe7UvoX1wxYmUzKKreO0dtikx7MYD56PkBAA5Loplsto/lG10jJ+j56811c8yA9js
u066crj44qjFhz987VXxqdFE6Nrh6qzbx1usGmJCeHwDgsKSaybJvXCvfqI7m7WTZmEZH4ua1/Uj7L
OWQ9spFYxuX6dJeoDp/6965Ywl96w+3U43v2WC0kc5lAMxOzw8AcFh6msn6G7VWNBT0sl0taHi

kfH0+nL3od1fSpasBcZluxfim+tlT9vy9608vZ60Y0jZ725lDVw0wKT0/AMBhaWkmqya18Ua9d121l
WFjHE/Wf6R4XZ8uWs5as29uDmjZRafmfGGTHG6tV+8SutZfnb62Llm7LPW3U7PXT10bumKAyen5
AQAAYJn0/AAAALBMen4AAABYJj0/AAAALJOeHwAAAJZJzw8AAADLpOcHAACAZdLzAwAAwDLp+
QEAAGCZ9PwAAACwTHp+AAAAWCY9PwAAACyTnh8AAACWSc8PAAAAy6TnBwAAgGXS8wMAA
MAy6fkBAABgmfT8AAAAsET/9V//H+XVG5rC/QSQAAAAAElFTkSuQmCC]]></redcap:Attachment>
</ItemDef>
<ItemDef OID="b13" Name="b13" DataType="text" Length="999" redcap:Variable="b13"
redcap:FieldType="descriptive" redcap:BranchingLogic="[q11] = '2'"
redcap:InlineImage="1">
<Question><TranslatedText></TranslatedText></Question>
<redcap:Attachment DocName="B.png" MimeType="image/png"><!
[CDATA[iVBORw0KGgoAAAANSUhEUgAABVEAAAEJCAYAAACdcmdaAAAAAXNSR0IArs4c6QAAAA
RnQU1BAACxjwv8YQUAAAAJcEhZcwAAEnQAABJ0Ad5mH3gAAE9sSURBVHhe7d0PjCRnfef/b+/aY
PPHNmBskx+RHat7sZYlGJIg08PxL4egZ+6SJbnbYOVg5Oh3PVyi0zQSkwOxF+mX2wguG4lunS5iWrq
zIiQyTEJugrLdIJRgckzHR4IxYTI/Z7rlc3T8wDYGjPljB3u3f/VUPVX1VHd1dVV3VXX39PtltaerpfqqT/T
O/OZ5/k+hb5FAAAAAAAAAAChjumPAAAAAAAAAIAQhKgAAAAAAAAAEIEQFAAAAAAAAAAiEKIC
AAAAAAAAAQARCVAAAAAAAAAACIQIgKAAAAAAAABEIUQEAAAAAAAAgAiEqAAAAAAAAAEQgRA
UAAAAAAACACISoAAAAAAAAABCBEBUAAAAAAAAIhCiAgAAAAAAAEAEQlQAAAAAAAAAiECICg
AAAAAAAARCFEBAAAAAAAAIAIhKgAAAAAAAABEIEQFAAAAAAAAgAiEqAAAAAAAAAAAQgRAVA
AAAAAAAACIQogIAAAAAAABABEJUAAAAAAAAAIhAiAoAAAAAAAAAEQhRAQAAAAAAACACISoA
AAAAAAAARCBEBQAAAAAAAIAIhKgAAAAAAAAAEIEQFQAAAAAAAAiEKICAAAAAAAAQARCVAA
AAAAAAACIQIgKAAAAAAAABEIUQEAAAAAAAAgAiEqAAAAAAAAAEQgRAUAAAAAAACACISoAA
AAAAAAABCBEBUAAAAAAAAIhCiAgAAAAAAAEAEQlQAAAAAAAAAiECICgAAAAAAAARCFEBAA
AAAAAAIAIhKgAAAAAAAABEIEQFAAAAAAAAgAiEqAAAAAAAAAAQgRAVAAAAAAAAAACIQogIAAA
AAAABABEJUAAAAAAAAAIhAiLp0etJYKUih4DxWWGj29pXWPqjXXWPFeU1hpWF1+1iMxj3ZB2onXjdL
MxLO7LSlg372PzHqPs4Vb2GrGzEO5vtDbdtK5JH08KNuQ9Cjmc+2g0AAAAAAwPwhRF06Rdm8jEUn
XurU1cTiR7aSfT7FXfXH+DAiZyh6UmnuWe/PJV4Jk6rVoDZ3Fo+4bCTNSqSkGXvUjpVQ3q7ZPpgNoJo
uytmqfi4daf1qkTl/XxxSlGgUtDgS5PE5y0v1vdu+aFGDPqzGtUEYbF7+sMIWMz11fcW
D7TVk3fijwsI7ascDAAAAAAEAOCFGXVGW7JV6JV6O2jvHbGbL1aN6V1OuuacZXBTU+SPW6uYDaA0Gb
AAAAEAuCFGXVkW2W26M6vdGbW/GGbD+M6vdGbW/4Q6XL9a3wXoyqlqRek+wmpSjvaqHguRek+wmpSjqyvaH59ZI1Jo6ZjoC7rqPX
GPu31edcGHd5fZI1J4zwEz5+5nbDPjxN+3IClCtS9tR4xy30ObGewraPOc7xjCm57uPZpYAh6c9VZF
2h4snM3eA7Uw9yc3Z6x+xzPPy71CLkXbAnaHvI9O2jxaTOjUYZfrzwRNWAz/kBZFBBjs/t7RybU
Ji/u6BisfEacDLUnjXX3GlSlpc9v3+0xnOr59bfvdUhWIdlAfdzx/O1494Hazrob/E1zTMbr9kPbPX
vV1Wv1/6cXXIi2X56jXUvsCzXu85r+8MlLiqR+4wr/BzteOl/krZbr3X/0hH2PRjY7rBkxzOu3QAAAAA
ALI+C9QtyXz/HklK9SwM9zlZjT2Wd45l+e1i3jXw0Vqd6P8w2e0+BqnbxOW2+rnkGiT3wOBhNNXWfE+E+2ltwcZ
9ZG1BBs1YOwWq1Ks9kcWu+3Iaxdc dcdeNE387w+2yV3rnwG1/6DqbsV0VnO2FBNwB4e0wr3O11fc
nDEt0PSK2E9ivvuaS7JjMbQdfr41qa6JzF35+FOdanQqsS3p+lETnOlHbRwht44jjjjDietO4RYBr3Pnyvv
OXCW/QSACAP99/xLnnNW2/SSwCAzB3/NZFjr9ILWBT0REVwkikVtoQGJP5Qeb/npMsYdm/2GE1F
UU6ccp51dJfTrj2WvywnT5+0e9QF11uqp/0AbA74vWmb4nVG7R44gZT1GW8ov7dOdT4sGbUo3S
BMfclsazBr1jUo6ac28z7QPYSnOKZEvYkT7cec4f+kmIdQ3NyTvhmgTm3wHIWY6rrurFuwJmaGO0

GMvDwEw/LOz/5Tr0EAMjDDY9fJXd/6AF5/Bs/0msAAJm7dI9I/zt6AYuCEBVBA6FScsO1P6fl1RVt7k
pbenJoj9k/JScqJ6z/D65XGeqc9ZIrrombLbpD+ttuIl09a/TozUtaAZl1DTJrOyHeSKpXqBe0GnVjgQX3x
NNPSOWPKvZHAEA+nvPjY/KWL94oP3ryGWm+/3555p8u6c8AALL1lMilP7A+PussYiEQoiJlGYRfXl3
RfTlsXxS7lKgd9lbEyVeN9XMZvhk9NO3A1+/VOyrwVcO67VqVQ480e0lOK1mv47yOaTHOXbjxbW/
Lhtnr1K2LatY5BRbUnZ+6Ux58/EG9BADIwxv/
+gZ5wQ+vsJ8/9NXvyp/WeR8GgNz0Hxa5/Od6AYuAEBUxleSkN1LbnBFfMSZJitM70Ri+HI+7744c7D
pf686+X7I/4a8fLjUwH4qbZ/0guOFOjFWVQIZacsoTKG6Jgvk22OvYvA/0seV1TIn2Y97LA8Pk1cz3hZ
xnoE/SdmNSNTtwdeukJv6eAubL+z77PvlM7zN6CQCQh1c+eK3c/PXn6yXH5z7+kHzlLx/RSwCAzF3
6gsjlr+kFzDtCVMRUlM2zbuVUc/Z1NbrYnOl8y+vt5wScijnre1s23JlrYvN7cqqJpBS3XmZRF0x117vh
6vxxe81a56Kmj3+wdqsx7F+aq7LhnTIV7Onh297K+dBcNQLH9nm/hqd7bHkdU6L9mLVbm7Lqre/p
me+ta1Qq+NvI2oTnyA9c3XYDi+kT+5+Q+n11vQQAyIOqg/pzD7xELwX94W9/Tb73raf1EgAgc5f/RK
T/fb2AeUaIivgq29LyctSalHTA48w8bynX5YJR4NOfUMnSXNWB0GQ1HAPbsp55Q/a9of6KMUnTHP
Jqu9rKUt8KRKiWomxe8IdlN1d1gOaFzqMm/ZolJ3AMttM8tryOKdl+ipsXpO6/2HmtOZGTdS/7h6Br
7yrua1NNWBO03bzfw9odR+bHA8R339fvk7v+7C69BADIw/FLBXsYv/oYRgWozd+8Xy8BADLXf1Lk
8sf1AuYZISoSqWyH11+stqz17tBiV3FT9gZfW21NVr/R7K0XKBlgDM3OdKKjFJgB2KiyA+qc9bt+wOe
ya1/OW03PstS7fT9Yt1Wl1d8LTpaV1zEl2k9RNvf60h16sXq5MUzeVpHt4EGK7B96PbFTEbvtVlv6Lf
8+sqlzblwHu+5ulByOB4jBnYn/6Wfp7QQAeVr50kvl2iev1Evh/uFL35ZP//6hXgIAZO6y9RvZJcpbzbt
CX81aAgAxtTcK4nSSVCHqQGAKADGo4PT1/+318sAjD+g1AIA8lB56od0LNa6tu8vyiteFD/sHAGTgit
8QKfBL9ryiJyoAAMiVGsJPgAoA+VK9T1Uv1CTUsH7qowJAji79AfVR5xghKgAAyM1HvvgRezIpAEB+
VP3Tt33hZSProI6iAtS7/+NX9RIAIHN2fdR79ALmDSEqAADIxe6Du/LBv/igXgIA5OWOL18/tg7qKPv/
8zH53Mcf0ksAgMxd/n9FLn1BL2CeUBMVAABkTg3fV3VQmUgKAPJ189efL//8Czfppclc+dzjsnX36+
XWV79IrwEAZOtKXR/1Zr2MeUBPVAAAkKknnn6CmfgBYAZe8MMrEk0kNcoz/3RJmu+/X3705DN6
DQAgW9b77aWPWx+fchYxFwhRAQBAZlRwqgLUh594WK8BAORB1T99yxdvlOf8BJOf8OJ1f+R7/xo/k7g8
xKSAA5Kb/HZFL1EedJ4SoAAAgM+/77Pvk3ofv1UsAgLz83AMvkRsev0ovpeMrf/mI3PtJ/igGALm5/
DXr0dELmDVC1KXWlo1CQQrGY6XR05/LSa8hKxttveBob7jtWZG8mzM3Qs5LHL3GinPuVhoy6tT55
9d55H7NjwLuWyCW+n11+djffkwvAQDy8vJvPE9e+eC1eildn/zPB/LN//19vQQAyNylXZH+Y3oBs0S
IurR60lhZlaZecpTlzFpRP8+eHfiVasLfVIImPi+9hqzXor5KXfOCrAYvunRqJSlMENguK+5bIB7V+1T1Qg
UA5Ovqp47LG++bvg7qKKo+6u//
+y/bHwEAeVD1Uf+79fFZxEzQ4i6tLpy4KZA5bp0+33p9/dkM68MdWzgt6QmPS+qZ+S4YK99XtxN
V1vqevelWy87K5qrQo4aA/ctEIuqf6rqoAIA8veWvRvtIDVLqifqH/721/QSACBz/UdFLv2xXsCsEKIuq
96h7OuncuqE5Nf/FGmL1zOyJ41zugtquS5bFedpcfOsVJ2n0twlRQUwPTUTf+WPKvZHAEC+Xvu1F8
vLHr1aL2Vrb/f/2A8AQE4uf8l5YGYIUZeQXbvRDN2aq04tx8GuiKp3o1E7c1T9zGAtyGCd1bDejbH3r
wVreI6qN+kMVfdfl6zW52A9S6+2qH6ENy3GPgPncEP8zZjnydln0vOiqHaWYvWMNHoeB0LzkpzUn
VFl/9A6olGM9obVW21v6GMZPAcJrkvI/RZ2vePcb4PXz35E1ImNY5b37WT3p2vSa2eZ6D1Ar1TMr
49uJI6QOz91pzz4+IN6CQCQFxWevubvXqSX8qF6o1IfFQBydOlPqI86Q4SoCDUUGGl2/cyRYVRHai
WjzqrR43EyanuDNTzVurCgpuQNVXdFt3UUZ5+DwWRzdcJ9Fjflgjtk3joz5/RGeo1z3nkq1y+kUEahK
q1+y+tVOsTseTxK50C6+umwipx2N97ZkYsDJ7W96x2NX1c3yXVRQV7I/eZcj1EBZPj9pu7d0GC5U5
NSIMjOSpb3bcz7M2CCa2eZ7D0Ay07VQP1M7zN6CQCQFzV8Xw3jz5uqi3r3h75KfVQAyI2qj3qP9Z
H6qLNAiLqEKtt96Xfr4kZ7Um3Z9TH72zrxbG/4AZBXL9Won9mpyfrIxEaFec7r+3uboWUCxu4/wN+
et3/pyI6XBPWkse4GPca+Wzo1imzrKP523M2ofdbOu/Fbsn0WNy+If+rOS9v677wbglnn94JOUJOd
F4P9um2ZLK8uyolT+ukYlS23beb5V9ri53BnxMnhkpwj67VGqYGh+21ofyZj2/b9ZrYlbFt+kJ3U/Ny3
/uvD789hya6dZar3ACyrT+x/wp6NHwCQv5UvvTTzOqijPPTV78qf1hmBAAC56T8scvnP9QLyRIiKlW
bPtPoFPwgdDgOHletbE4Z54aotPxwsrp3xAqzOge4z2bsoO14eaey7suW3deeiJll7zH364ZMh8T6Ls
nnWTbuaslpwe08Gz+8kipt740PW2PblMOpEFdfkTNjxtXf9XrVn1pzjSXSOrPOzp0NEI3gPvd4Dhu43

s8et0evSPk86CNzLYfa0LO/bsfdnmCTXzjLNewCW031fv0/u+rO79BIAIE+vfPBaufnrz9dLs/G5jz8kX/
nLR/QSACBzl74gcpkJ/vJGiIoBI3qm2Yqy5iYxIwK3UyfSDKjKcrKkn47SPdC9+VSoU/LqNhbMYdKRw9
QnMMk+K9tGr0FHOsP403RKoi+fcf2NgNIP3Kpy1j2gqa6LruEZOrw/aOh+M8JCawf20Hd33yNrsaZ
uHu/bBNduyvcALB93Jv6nn31arwEA5OWGx6+Sn3vgJXpptu7+0APy+Dd+pJcAAJlTw/r739ELyAMh
KibUkeHOgTHCo7mX3TFUvMKUSrD+5Oz05HBssVSf36vSHRZuBG7V08FeoUmYExB5PXXHCbtWR
dm8EN470w0rF3t+o8nvz/SvXdh7AJaNCk5VgPrID+h9BAB5e86Pj8lbvnijHL9U0Gtm60dPPiPN99+v
lwAA2XtK5NIf6OfIAyEqJjR/gWm53vWGbAcfk9YLHS/
+Po26n7aO1NZznJyneELGlj4tn5Sxl7S4KW5lAntYuDF8vno6/CyPP0dt2TB7nbo1OM36o0lYbdzT+xj
s/as0z83XpEi53bcTXLtoR+GPJpiWGsL/wCMP6CUAQJ7e+Nc3yAt+eIVemg+qPuqnf/9QLwEAMqfq
o15iYte8EKJiQElOusnV0EzePbnoFnIcO/Q7J6WTXtA2qm5m6ibYZ6+xrodpl6Ved5OsPCfnMa5rc1f8
zphdOfAu6QmJc0m9HrXW/XH+/I4OP6sSyOGSnCOzLqcKFN26qMaQ96TUzPKqx6k9GZQdSHa9W
p7pDpOf0CzuW0usa5fme8AU1xDz7yNf/Ig9mRQAIH+lh1448zqoo3z6v/6D/MOXvq2XAACZu/xZkf
48dRU6ughRMcCcBCnYW9IPAlXYNTChz6yYNTCbq/5Q7faGHhpuPdIev510n72GrLsnrnpWNjeNyY
Nq65JPjmrWsmzKrm5er3HOCzBj90asnBbnDulIs+ke18Bw8Amvix8oDvbcjUlvX80sH+xxaoTFZo9b
oz1p3yaRZnHfKnGu3QTvASU/dZXaee9gZMOb4h9Hze6Du/LBv/igXgIA5OnaJ6+0Z+OfZ83fvF+
+9y1qZQNAbtSw/v739QKyQoiKYeYkSJ2alHSoU/LTE7kw7YxI5vDy5uoUoVGwBmZzVQdQXnhTlVZ
qs9e7kuyzJ411d6h6Wepbav3okCq98zLMnFndbbN3TastiX+aKhIo72oZDmATnCMv2LO4x2xOsJTEi
HvXrLNaPevPNp/YQt+3SpxrZ0n4HuDXW7V41zBubVssGjV8/85P3amXAAB5UvVP3/aFl81NHdRRV
lCqglQAQE76T4pc/rheQFYIURHKHgYdUpOy2rLWu8Otp1KR7cGClfuHRs/BBOwamMZwbZddWzOj
eqgx92n23LN7obonbiCk8of1p3hehhRlc2+4Tqg9hD5hYBeYJMs6ZjsbHhT7uljH3G/5QaqtKq2+0dZ
ACYJo9r07eJA2Z5vTZZMLft9aYl07S6L3AHUsg6+ttiava4u59cTTTzATPwDMkOqBqnqiLgI1pJ/6qAC
Qo8vWb6bUR81Uoa8KBgJAEmo2fT0ZlAph96btmTxL+lhOtaYNWBfEUbp2yJUKTit/VJF7H75XrwE
A5EnVQVWTSS2SK597XLbufr3c+uoX6TUAgGxdKXLFb4gUbtbLSBM9UQEkFCxRcGZtsUO49nnrWC
J6ZB4tR+vaIV/v+
+z7CFABYEbULPx3fPl6vbQ4nvmnS9J8//3yoyef0WsAANmy3m8vqWH9TzmLSBUhKoCY2rJh17o06
pWaJQoWUK+xIqv7demmUqJinh29a4d81e+ry8f+9mN6CQCQJ1X/9C1fvFGe8+PF/NXt8W/8SO7+
0AN6CQCQuf53RC7doxeQJkJUAJNRNS8XfPx7cXMvpRq/C+YIXDvkR/U+Vb1QAQCz8XMPvERueP
wqvbSYvvKXj8jnPv6QXgIAZO7y10QufUEvIC3URAUAAKEefuJhec32a+wJpQAA+bv568+Xf/6Fm/TS
YqM+KgDkjfqoaaMnKgAAGKKCUzWRFAEqAMyGqoO6aBNJRVH1Ue/
+0FftjwCAPKj6qGpY/7POIqZGiAoAAIbc+ak75cHHH9RLAIC8qQB1UeugjvLN//19+cPf/ppeAgBkrv+
oyKU/1guYFiEqAAAIUDVQP9P7jF4CAOTtV97sbzs0av10y1tGt0qMWKbGFKKMsq9wFzdHL6SBqa4h4UpBWTWWBK/v3VE0RErJq+LCc3sW6FbHaY1pWZEX6nxbQdtgGqC2Q
....

JEU72Dq1XOU6dmIcI1VI5LW6M2gzMjmSEhV6w15PGutvjsyotu6am9WjpLXRqsh4jjUz7nFW2rTa
Yw76rLadd22HVPv12d+vuV8TsRaoYQ/mrp9X2zVA4xpD+4qbsuefNfnTFa4Z1BPULwcDQN67d6V
wbV7neDX69Frbev2+maYPxeh2aFjf3vOc+P1RXYWtXf41/TppyLsFxAmnafXBXPvgXH9RLAIBZeONf
3yAv+OEVemk5PfTV78qf1h/USwCAzPUfFrn853oBcRGiLiOvJ6IfaPUOndivWnXCo32dFJrDwO38bS
5UZMsNoJq7fi8+s+6n2wPUXFff8ickqmx5QWCs3pgzPGfVlj+Rkh/mWi0Z7IY7gtk792TJfmJsJ0EYq/
Ua636vzepZ2QxPUMe3O61rY6vKWbchRsg+cv3+obPdKdoQeH0UI1Q3Q2s7cNWB6t6okwhkSA3f
v/NTd+olAMAsvPLBa+Xmrz9fLy23z338Idn/n4/pJQBA5i59QeQyf8BKghB1KRXlxCnnmRtode1x6W
U5efqkHXYF11vUMHDn2VzwQ7mmeJ0KuwdeWOgN5ffWWcfkDu+2H8bw7cAQ/VFmdc784HMy
Yb1zLRMP6W/LeT9BlVZoz1klRrtTuzaW8kkJ3d2o9a4p2hC7Z7Zxrq2N2WUO3P3Er8sLpOuJp59gJn
4AmLGXfPe58nMPvEQvQbn7P35Vvvct/m0CgNxcVvVRv68XMA4h6pLy6oraPTl74nSqPCUnKies/w
+uV3ngPEWoFiOYcodmt90x0xG9I6exkOfM6GmphqaXQkPC+LP0tzf8OqBVo6fp8kkSbhdl80L4jP1u
cDtiPjEgE+5M/A8/8bBeAwDIm6p/usx1UEdRAWrzN+/XSwCAzPWfFLn8cb2AcQhRl5U3tHlfDts6aL
N77VXEyQqN9VP3hsyCUdfTDi/9upOjwkuvNubQI2YYuIDnzB/KHyXmkP72hj9RVLUlIzuhTmDqa5O
CTNtg1JUdKNlqa55jln7k532ffZ/c+/C9egkAMAsrX3qpXPvklXoJpn/40rfl079/qJcAAJm7bP02eukze
gFRCFGXVklO2hlkRw52nSHNbh3Rkv0Jf3347OuzV9w864eaDXeSp4E6pCVnqL3i1eKc2KKdM2Mo/
xjjh/S3ZcNLUMtSV1PRTyvVazOhHNugZuhXPU7tScXsQNWYoCtu2QJgSvX76vKxv/2YXgIAzELpoRfa
D4z26f/6D3aYCgDIyeXPivTp2jMOIerS8ntyNptOOObWeCzq4p/uem+Sprnj9gDtSK2mA77BOqRmP
crmqj9sur3h1aUsxB5LvWDnzBzK787+H3iYIV70kP5e45w3jL9cv5BOuYRUr82E8miD3pbKoIM9Trvi
ls8dW7sVSMn/+v/
+l34GAJgF1ftU9ULFeHd/6AH9DACQi0v36CcYhRB1iZkzpqvehd7w88Ds5sYkTXPIq1NqC+shGaxH2
VzVwZg/Ll1GT440LNVzVtS1VJXmauqhoTmUvxxaW8AoiWC9cuSQ/l5D1r0CqtYrAxMwOY/JJkhK99
pMJoc2VLb9IfyBurRGfdmzm7MP3bEU7v7Fu+WOl9+hlwAAeXvjX99AHdSYfv7dP6WfAQByUXijfoJ
RCFGXWWDW8FPiTzbuDltXzPVzyAwvRw2ht+tRGr0uXeW6dJPWu0z1nFVke7BA5v5hSrUxzaH8o0
NdMxQeNaQ/Xl3VCaV5bSaVQxvsIfxhxVBVSNvvp1pfFohy1RVXyf/4lf8ht1x3i14DAMjT373yCf0M
UV7z1pvkbe+5VS8BADJ37FUix9+kFzBKoa/G9QIAgKXxwCMPyOv/2+vtmfoBAPm648vXyysfvFYvYd
D1P/E8+a1PvVGedw0TbwFALgovFrni/daTq51ljERPVAAAlsztN91uD+0HAOTvb27/tjx2PX/ECnPlc4
9L9fdeS4AKALmx3m+Pv8f6SIAaByEqAABL6F2n3iUfeMMH9BIAIC+Xjvfl8294VH78nMt6DVz/8t+V
5NZXv0gvAQAydztIoWb9QLGmSpE7TVW9AQpBX9W65F60lhxJ1TZkODL27LhTbbiPCabqGZYeyO
43dBH1jOAAwAwhz788x+W07ed1ksAgLz84PnPyt7rvqWXoLzidS+R1X8bNhkqACATx4oix39eLyCO
OeiJqsJVf5ZqR84zwquZ0VcaKU3oAwDA4rjnl+
+xh/cDAPL10M0/kO6t39dLy+3al14l1d99rV4CAGSucI3IMTWMH0nkGKIWZXOvL2oeq35gxuuuHH
iTiKvZsNXn92QzxwzV1qnJeTqkAgCWjDtj/3VXXafXAADyonqjfu+aZ/TS8lIBqgpSAQA5Of5ukcIL9QLi
mn1P1N6h7OuncuqEZJedlqXedUNc/9Gtl/XnRZq7pKgAgOVzy3W32EGqClQBAPlR9VE/96Zv2h+X1
S/8xivsofwAgJzYdVDz7rl4NOQYog7XRLXrlZZq4nZEtYfVD9UoNb/OeaRVL1Uprp0RP0YFAGA5vfm
WN8tH3/5RvQQAyIvqibqs9VFVePoLv35CLwEAMmfXQX2HXkBSc1ATNUKvISuFktS8lNXRqZVSq2H
aPu+HuNXTfpGB5WVM8uWd46hJwTA7YddqEYTfT/4kcHneY0nPYU7fC+q9b2DCO//8rEiKf0cCAt7
7s+
+V2h01vQQAyIuqjbps9VGfd82VctfvUJMbAPJztcixO/VzTGKmIWpluy/9bt3vCVptOcPst1WY2ZPGu
htwVqXlDsFvVe01qobpeqIkoSO1khtC+I9Vd0Yra9/2bgFghnqNlWAPfSBnqjeq6pUKAMjXfT/zuD1r/
7JQAer1P/E8vQQAyNzxO0UKL9YLmMT89kTtXZQdnSKU61v+RFSVLXHLmHZ2LqbU+64s9S0S1NFG
TQoGTGKR76eM295ryPpg13tgBlR9VFUnFQCQnx8/57J8/g2PyrGKy/Ks9+BRl8QUJXXRg8Eg9QE0p+4Q5IFW
R76eM295ryPpg13tgBlR9VFUnFQCQnx8/57J8/g2PLkV91Le951Z5zVtv0ksAgMwdf5PIsV
fpBUxqfkPU7oHXE8sevu/1HjWG93cOpKufTsfppZpmrVUAABaVmqm//attZuwHgJw9dv3T8je3f1sv
HU23vvpF8ku12/QSACBzhVtEjv0LvYBpzHdN1FSFz85vlhPo1M7nWItxHgxO2jWqxuPoOpD20GPv6
/VjZG3Jwf1FBNd2Pdzga0fVgsysDVEya98oca+VK/5xJm1f2OsHyncO1O806o7arx19P/kG2p/K9Ux6

DsOEt32wXungORo8P2HiTbTn8/epHqPqpCY5P0DQbdffJvf88j16CQCQl7+/7Xvyjy//oV46WlQd1O
rvvVaufO5xvQYAkK2rRY6/2/p4hbOIqSxEiFqud4fDT/uRwnDa4qac1WVWRfblcGnyBRVsDU7a1ZTV
wqr1/3hUiFMKG3rcqUlpMKBKMklYe2NETUjVYzgYFmXWhihZtW+khNcqwXEma58TyIW9vrlaGBH
2qXNitLNcl/GVM9SxDbR/6us5/f0ej9OrffAcNVdHhZyTcPbh1XO2Dd97qd3vWGrvKL6DGfsBYAb+6v
WPHcn6qL/yH15JHVQAyNPx09RBTdH8hqilk34P0YN0Bu2Ha8tuuinKQug1zvnhkTuhV7/r1Zsdzzhv
5bp0dbDd9TbQlHNeotNLMEmY9dpzesOh2+3IzkX3tVm1IUpW7Rst2bVKcpzJ2tdrrHuBnP+HjZa1F
615bkRQaLRjb1OKem0koz1u06e5ntPf70n47fHb3pHa+ejIPHqivUH+PsLvvbTud0Ds2frfdepdegkAk
AdVH1UFqUfJyumftB8AgJwce53zQGpSC1HtnmjGkFHzEWco65DimpzxspxVfxuqF6C77UQbdnpv
me1yHmYvuTOyFivhWXQ9uejO2qUCFi+kKcrmBSPEidI7lH39VDo74mY3xc09J6yxHnub+mQmmiT
MmLjHCNyKa2eGQ/XM2hAlo/aNlPBaJTnORO0LtuOst74iW/aGVVC3J2GHE2hHTNWz/rmtbPtBrd
/2JNczhfs9gWrL7yFf2Up/+4q5j/B7L8+J+bAM7v7Fu+WOl9+hlwAAefjmjU/JV376u3ppsb3sp14o/
+a3mNAEAHJTuFHk+L/WC0jLHA/nDwYcXkjrjWE1w5B0mMHN0daVAzdTKp+Ukn5qK56QU/ppJDP
kHgioh2ouTjVJmK6nGTZ8Prc2REmpfSMlvFZJjjNR+0a3wwldR5fWOHUi6XdVVU4HNlaSk2473bYn
up4p3O9zpSwnAwcRIteJ+bAMrrriKmbsB4AZuP9V37HD1EWm6p/+
+n/5GeqgAkBurhQ5/mvWR+qgpm2+a6IWN2UvbMitPdQ3hXqoHtWLri8pZ7JH3OhefG5oM1EPZ
MWu5eiGPlE1KzNsQ5R5b19sebQvRuA3VlFOLEzSmcbxAvPpphfcZAepKlAFAOTn8yuPylNXX9JLi0f
1QFU9UQEAOTn+r0QKN+gFpGmqENUc9hv1cMLJoj8M2gxA7aBUrw9NMc2v04+4dRUtdp1B82tD
H2kGskvEuHZ+7Udf89zw5DXjJwlry4bZq9OtjWnWijRl0oYo2bcvLbGOc4btG21wgreeHHp1B4ZlOv
HcEcD5QZpuv+l2e2g/ACA/KkBVQeoiel3l/6IOKgDk6dhrrQd1ULMy3z1RkZGQ4dEus05mDGp2d9V
b0Q+rjZ7D7raTTBLW3vV6dtrhjxuYG8OTB6XehihZtW+khNdqguOM176Idth1itOdfT7Y9JDh+ImO
M737fWHkNjEflpGaZOoDb/iAXgIA5EEN6f+7Vz6hlxaDmoWfOqgAkCM1C7/qhYrMEKIupaKs+bN2
yao3Ztuc0XsMPcGXKlEb7K0YEnhNOEmYH/4YM+KbcmhDlFTbN1LCa5XkOBO1L6Id9rE7NVUTnsK
Rmqsb4jfdL5lQPrPmhNaJrmcK9/uiyeB+B0wf/vkPy+nbTuslAEAeVH3Ux65/Wi/NN1X/tPp7r5XnXX
OlXgMAyJaqg/oe6+PVziIyQYi6pIqbF/zehs1VHawYk86MU9n2h393alJygxmjRqg/UVew9mbkJGGV
09aSNq5dWbUhSmbtGy3ZtUpyrpO1b2w7ynXZSmV8eFnK5aas6vZ4Tbfaftab/j/Z9Zz6fs+LOdGV28
6Jws6U7ncgwj2/fI89vB8AkI9Lx/vy+Tc8Kj9+zmW9Zn79Uu02ufXVL9JLAlDMHVsTKdysF5AVQtSlVZ
TNvcFJu6rS6res/8djD/8OK6Rpb2dgoi679ubg/ixDk4RVZHuoDc72vF01d71eitm0IUqG7Rsp4bVKcJ
zJ2qfa0Zfu0IbVpo3SBlM7JWcHjzfsGiW6ntPf7/mw7q/B67F/aPQSTiCV+x0YzZ2x/7qrrtNrAABZ+8H
zn5W/ev1jemk+veatN8nb3nOrXgIAZO7Yq0SOv0kvIEuFviqCCAAAMIF7H75XKn9UkaefXYwhpgBw
FNzx5evllQ9eq5fmx7UvvUr+06ffzDB+AMhL4RqRK9R8BQzjzwM9UQEAwMTefMub5aNv/6heAgD
k4W9u/7Z875pn9NL8qP4udVABIFfH3239jwA1L4SoAABgKu/92fdK7Y6aXgIAZE3VR/3cm75pf5wX
v/Abr5BXvO4lekAkLljbxcppFNUD/EQogIAgKmp3qiqVyoAIB+qJ+re676ll2ZLhae/8Osn9BIAIHPHiiL
H36EXkBdCVAAAkAo10dQt192ilwAAWeve+n37MUuqDqoaxg8AyImqg3rsPXoBeSJEBQAAqVAz9b
d/tc2M/QCQI9UbdZb1Ue/6T6+2g1QAQE6O3SlSeKFeQJ4IUQEAQGpuu/42ueeX79FLAICsqbon3
/DozOpj/q299wqp/7ZDXoJAJC5428SOXabXkDeCFEBAECq3lF8BzP2A0A0Zj2fF5vpd7Jn7M/Tra9+kfxSjV
/kASA3hVtEjv0LvYBZIEQFcteWjUJBCsZjpdHTn1tSvYasbLT1gqO94Z6fFZnX09NrrDhtXGnIqCb6x+E8
lv5aY2mo2frfdepdegkAkLW/v+178o8v/6FeytbzrrlSqr/3Wrnyucf1GgBAtq4WOf5u6+MVziJmghAV
yFVPGiur0tRLjrKcWSvq58vHDiJLNeno5YXRa8h6LarV6loXZDV4saVTK0lhIDAGjqq7f/FuuePld+glAE
DW/ur1j8kPnv+sXsrOXb9zu1z/E8/TSwCAzB1rKq4j+SQWAbctFTP2A0A0COfvvycy5nXR33zr9ziwzu9i9ff3nrTXoJAJC
5YyvW41V6AbNoDXm/qpnDohy9v/dHHF6znbk8Tae6SomI53PSC
m+wgVQWqAIDsPXb903L/q76jl9L1sp96ofzKfzipl8AAGTumK2peqN
536d163Or/dp135sb+ivNdbZLwuuD/bKS2MbmtlG7zFcYzN4jMM1S+1HWN1Low3jal3a+zDDt+aq
87WDDQ9pc9i2o9o8tpfjROdl+J4ZuZ8YxxD7fGh+W9RjuK0OZwi9/7p0z506/lKsnrNGj+NAWF6Sk7

ozquwfDt9PNqM9ie65eMduS+H7wj1Pcd9HsNxuv+l2e2g/ACAff/fKJ+SbNz6ll9Kh6p/+
+n/5GeqgAkBurhQ5/mvWR+qgzgtC1CWlwpHQMKhTk1JhQ8ZlcHHYtR8HCkLa61TIMrC+uRoeXk2
1DRU2hfYY7EitNCqEU58zapaW61J3uw52duTiYMi0674ynbqmQ8GiZh/zyGBquM1uz8dQE5+X4X
umuToieEt8DFGcfQcvd0hb7WCw5A2hd6V67jxVafVbXq/SIWaP41E6B9LVT4MqcjrpPZfk2FP6vlDn
KY/3ERwdapKpD7zhA3oJAJC1z688Kk9dfUkvTe/f/Nar7J6oAICcHP9XIoUb9ALmASHqUmqLn8O4d
TmNeo3SlHPhSUpi5XrX3na/FYybwtaPGt482TaCw6mHj7EjO4PplEcFZM7r+3ubsrlVF+erBr/GPI9nZ
FyGWtm2ttd1t2Wptpx9bOvUrr3hB4Vhbe7UZH3kdQm2eXRT0jkv/invSO28cd0SHMPY8xHg7zu8r
dZxrbvBoHEu3Iamcu40u53bEitrHVKUE6f00wiVRPdckmNP7/uimOP7CI6OD//8h+X0bQxHAoA8q
ABVBalpWDn9k/YDAJCTY69zHpgrhKjLyOwlZ/R0K27uOQGJ9dhLZaajqpx1t1M5bS25RqwPHd486T
aKsrlnBj6O4toZL7TrHIT3AyzXt4IBWXFNzugv6uxc9NvY3vV65pXPrI0P38YwexjWLxht3rwgfgZ5PrR3
31CbR5r8vFRbfnDoh3xB0xxDFHPfoW3tXZQd3SEycC4qW/5+zWtniH/u1HFY3yOhIe8k9uUwrEFKk
nsu0bGn+H2R2/sIjpp7fvkee3g/ACB7akj/V376u3ppMqr3qeqFCgDIiV0H9V/rBcwTQtRlZAQ0ImqY
bsGrZTiyhuIkyielpJ8GjFofJo1t2HQ9x9BhzEGnTgwGP0VZ8xMtLyzyA0Mj0J1YVK9WY/8jgrfhNscV/7
yMN90xjFaWk+MudvfAa79X7sF+GEPcRwyfn/zcTeuUjN51gntuimNPcv2HzlNe7yM4ctwZ+6+76jq
9BgCQJTXJ1KT1UVX907t+59XUQQWA3Kg6qHdaH6mDOo8IUZdSUTYvhPckdEOYqMl1FkZg8hyjn
mOk8MDO76nnDnc2AsPq6dg9GafXkeGOgjFCRlOK52UyYccwK2keVxw9ORxbLNWR2T2X2vVfkvc
RZOKW625hxn4AyNFfvf4x+fFzLuul+H6pdpvc+uoX6SUAQOaOrYkUbtYLmDeEqMuquCl7esjtQKlR
W/Pcos+u3ZYNs3edW7PRrMGZhHW+zurzZA+NNoYyV0/nF6FOH/qlfF4mkm1w6dXKHXpMWsd0
AsUTMrb06bje1BPcc+OPPf3vi6P9PoIsvfmWN8tH3/5RvQQAyNIPnv+sHaQm8Zq33iRve8+tegkAkL
ljrxI5/ia9gHlEiLrE1MzaqqeYPcGPHYR0vRqKo4f/GoxhxHPHrB2pgiW3/uMUba64U6Z3duT8+R29n
aqkk6GW5KR37gdnZO/JRbfoZeQQ8BgyOC+
+nI4hTOmkFwKOqumZL+NcNHfF75DZlQPvNJxwzn2EWPdckmPP4PpP/T6Cpfben32v1O6o6SUAQ
Jb+8eU/lL+/7Xt6Kdr1P/E8uet3qF8NALkpvFgP48c8I0RdRu0Ne6itmkU92FPMCHiMXnIlPxkzZmJvy
4Y3Dft884MlY2bySXgTWHWk2dQnKrWh/EXZdLsdqvO87l+XXmPdq22ZZBKkcVI7L578j8Fj1udsrvr
DyPW9bj9yHVtu1oBtyq7eda9xzgsxY/VgjnPPTXjsU1//hO8jZntyvRSYe6o3quqVCgDI3t/c/m157Pqn
9VI4Vf+0+nuvleddc6VeAwDIlqqD+h7r49XOIuYWIeoyqmz7Q287NSm5QYtRH7F6NnzmbhXSDL52
Lpkz9nttNibamUhF3I6BrlSH8o+4LiU/fZQL005glcl5MSQ9BnPYu9ueiRK2YH3O5qqz34IX9Felldqs+v
EUNy94PTLd9njnodqSeM2Jc88lOPY0r3/C9xEgiqqPquqkAgCydel4Xz7/hkcj66NW/u8idVABIE/H3k
od1AVBiLqk7KG3YUUMVeDS7wcDHlX3cLBmYrWVcx3NpCqy3W/5gZHNOTbvsAPDrOPxhlcr5bpsp
ZzL2dcl5LxWW9Z6d+j1VLI5L6Zkx2C1Z/A+3D80ejUmYNfnNIaSu+y6nznWQ/UUZXNvuFaoPYw+Q
aAb656LfezpXv9E7yNABDVTf/tX28zYDwA5UPVR7/uZx/VS0Cte9xL5hV8/oZcAAJk7Zv2GfPwdegHz
rtBXRewAxKNmNtcT86gwbG/anqHAOEfpntPHcqpFwIpwn+l9Rip/xM0BAHl441/fIKWHXqiXRK596
VXyW3/8z+yPAIAcFK4ROf5+66P/Xoz5Rk9UILaeNNbdmc3LcmaNABVZO1r3XPu8dSwZ9ODG0fGO
4juYsR8AcrL3um/J9655Ri+JVH/3tQSoAJCn4+8mQF0w9EQFxmrLxmANWFXOgK50yMzRu+d6jRU
p7ZyRbiplKXDUFf6fgn4GAMjStU9eKe9s/aS88723MYwfAPJ07O0M419AhKjAWAOBFgEqMsc9h+
WlJiiblx9NaEs42hKOtoSjLeHmqS0nrr1GDr/3pF6aLa5RONoSjraEoy3haAvSQIgKAADmBj/gj
WhLONoSjraEoy3haEs42hKOtoSjLeHmqS1IhpqoAAAAAAAAABCEBUAAAAAAAAAIhCiAgAAAAA
AAEAEQlTkSE2WU7DrfxRWGtLTa8fqNWRlo60XHO0NvZ3CijRibwjp6Uljxb0GG9aVTbJuGmH3UNr
7mJbRRv1Y4SYFAAAAAGChEaJirvUaK1Io1aSjl4H5pgJdY1Z9W1nOrBX1cwAAAAAAsIgIUTG/eg1Zrx
GfzqeibO717RkF+/1tqei1szFPbenKgXvLluvStdu0J5tkqAAAAAAALDRCVABIS+9Q9vVTOXVCyE4BA
AAAADgaCFGXmao1OlC7MazG6GD9UXuIvfE1A+VKNbNOpXokq1Vp79Mcxt9cdbYTvjOjjeoxqk7qY
JvCa1VOdrymMbVf2xvetob2H3JN4rTRY359ZEONcxFo46j1xj7t9ea5zKoOadx7KKotg9uYsD5pjOuS
9J4FAAAAAACLgxB1WakgL7TWaEdqpVEhpPpcQUoDQ+ybq4OvVyFiSYIva8pqYbBWZBqcNq0GNh
xyDHYINtgm65W1UnjQaYt7vIMqcrqqn3Z25OLAa9u7bmODtTKHQjgtuo2TKsrambLztHMgXeeZxR

iOHmh7W9xml8+s5dDDMoV7aKJrPizf6wIAAAAAAOYRIepS6knjnJuIuXUb+9Kt61BNOrIzmPx5qtL
Sr2+5QaEKG8/7ve16jXN+0FVt6VqVXfE2H0Nl2/qabl28L3G3sx1W8dJvU/gxWMe77oZg/mv77gF0
arI+MhUdf7xhKltu2wfPpR9GSvmMeBlqe8MPgsOuSWQbJ1NcO6PbuC+H7qbbu0ZI2ZEDN101hq
mfOpF9hDr9PTTNNTckuC7J7lkAAAAAALBICFGXkjERz96m16vQD9VEOl56FlRt+RP3+EGhqScXd9w
+e1VpeQGStc8LYa+fntmm0GPoXRS3SeX6lj/xUGXLC+U6OxdDexSOP94RimvidfQ0t22ElGaPTrN3av
2CcU02L/htrJ0fGKo+Ja+NftDbO3Si0mrVCRv3dbrau7jjBZKnvROYlRTuoSmuuWkm1wUAAAAAAM
wdQlRYdA3P0OH9SZmzk5+Ukn5qK56QU/ppespyMrCTEN0D77jsIdheXUtjqHdgSHsazOHy/rB4P5
SryllvyvYRvVNtxnbMHqOpKMoJfUHcwLlrXzzrnJ4+aYeVwfWW6mk/kMxMCvdQKtd8VtcFAAAAAA
DMG0LUZRWYKCdunckYgeWRMt3x+r1i3Z6eRig3URhpDK9PScUt3trctVrXE6cj6ik5UdFhZWC9anb
2EeriSf+6AAAAAACA+UKIupTasmH2OnVrPZr1HI+ocr3rlDEYevjD9lNT3JSzbglONXTcqCs6WRiZQYh
dOS164L4ctvUQeLv3pzs5lrF+QUP07K/5sv1xAQAAAACA5UOIuozMupwqYHLrohpDoCdXkpNuEjs
4XNoIEXNVcoamK6NqvWbF6+nZ2ZHz50fVFTXP2eBs/mZ90FMydk6nxNfQ3XdHDnadr3VrtZbsT/jr
h4e0ZyWFeyiVa57idQEAAAAAAAuNEHXJ+QGTMWP/VMxakU1Z3XCn3DFnS8+ZMcmTNFfFa1J7w
6+V6a1MmdfTsyPNpj76oaH8Rdl0u6xar6utN6yz5eg11r0anuYESU7AqVivP+8dkGx4U8nH5V+vZtP
5Wnf2/aIumOquNyfCylYK91Aq1zz5dQEAAAAAAEcTIeoy8oI9S3NVh0rGhDtTMmcun2r75iRC7nY
mCjuDs7o3V3WI5gWO5gzwaXOHxftCh/JXtqXl5XU1KdnnrCAlP6mTC95EVCojdOutWrxzHLe2bVBg
W9Yzb2i6eZ9Y68/k0w3VNv09lNI1T3hdAAAAAADA0USIupQqst1vGQGZUpVWv+8HRvaEQpMqyu
Ze1w/BbGr7g/scx2qn1yBt/9DrDZhIcVP2+oNtstj1YDOoh2rwhvQr1v62Ruysst0PrUtbbVnr3ZILLnU
8g6+ttiara2v22gwMTTeGs+c+ZD2Feyila57ougAAAAAAgCOp0FczrADITq8hK3oiL1WDdo+eiwAwk
urtPS8/mtCWcLQlHG0JR1vC0ZZwtCUcbQlHW8LRlnC0BWmgJyqQKbOOZ75D4gEAAAAAAJAOQlQ
gE23ZKAzU8ayeFTqhAgAAAAAALB5CVCAPql5pZpNXAQAAAAAAIEvURAUAAHODelXhaEs42hKOto
SjLeFoSzjaEo62hKMt4WhLuHlqC5KhJyoAAAAAAAAARCBEBQAAAAAAAIAIDOcHAAAAAAAAAgAj0R
EV2eg1Z2WjrhVnrSWNFzZavHhsyL60CAAAAAADA/CNERSZ6jRUplGrS0csAAAAAAADAoiJERfp6D
VmvEZ8CAAAAAADgaCBEBQAAAAAAAIAIhKhLqr3h1gddkUZPD7+3l51HaClTVePUeI3zcL7eZW/XH
MbfXHVepzc4uF+PuW1j58HXt2XDfY318F4Wo12TGjwv6jHpuVGSHr8S1obCSkPCD8+s/eo8VkaciG
TbBQAAAAAAWF6EqEuvI7VSQUoDw+
+bqwMhX3tjRI1T9fXpBJbR1H5WpamXpFyXrYr1MbN2OWHk4HlRmquFYNCZ4blRoWtYG6RTk9Lg
BFl2EFuSwZd3aqWhcDTRdgEAAAAAAJYcISosVWn1+9K3Hq2qXqUCwPNulNaTxjkdX5br0tWv7dbL
zjrrtTsXnYiusm19rlsX9zNSbdmv7W+rxHNafjv7e5tSTNCupHqNdS+MLNe7zj77LasFWvOcDkeza4NI
W3bd1Dh020055yW0VjvW3SDXOE/uBe3UZN17bZLtAgAAAAAAgBAVUm1tixtxVraMANRTlM09H
crZ4aVeu3bGe23noKufZadc3/La6ciqXT25uOP20qzK2U13yxXZsoNGFVLuibM6w3PTO5R9/VQ6O+
JmscXNPWd/1mPPbVvvorhNDpynypa42Whn56J1ZJYk2wUAAAAAAAAhKiaha5OGDmHPzqkT44K
9tNrVlQN3A+WTUtJPFSdo9EPnYSmem+KanHGTWGtrquyCW7t0qM5p98Dbnz18X7+uYA7v7xxYR
2ZJsl0AAAAAAAAQoqIsJ82UcJTAxElGbdLcjGjnzNtlyawNRdm8ENYz2A9KQye5Giur7QIAAAAAABx
NhKiIoS0bZs9Kt46mWft0JuahXRm3obgpe3qIvV+v1tc8Nzybvl/DdfBh9KCdYLsAgLzp0Q3Ggz9yAQ
AAALNBiIrx2rte70o7oHNrfxpDyKc2ybYya1dJTroJqDsE3qVm4i8YM+6n1YaI16uZ9NUvzfakXXbw2f
XqnHrtK530Qtu4NVhjbRcAlpH9Xl+QwkwTy540VlZFWu57tPNHr+bqLILU+Qxze40Vpz0r+f/hT/0ba
p4P9zEP5wXDhq7XDO6ZmRvzvuZ9P7mPvG7mOO+3ebwnR+5jSf+gFfO8z/K9eGZinJuh951luGnG
nJfc34sTtofSdoiDEBWJ+AGdMSt9AiU/nTRm/7d+MFmdbhD8tO0KKsqaVzS0Kavem667baeO6OB7
cZw2JDp+/aavPhXsGRpSs9Wsc9pc9dvm/sOhHu7KJNtVjG3wDwuALMzsl/chKri09j/lv0npcCYu3Da
KcLuTP+4f5vte3N4IC3ONPyjOQq8h6zWRatX9x28G3NEnxsO8XrMy01/cAyWOhh+z+NZW52O1qS
YFda9TS6qdmpRmFPjk/0v7+Pc19R5csr6f6l3jHFk/T2Z778R5v83jPXncPtTnc/yDVmTok1eYm+C85/l
eHHFuht739CP98xP/vl3dH/g3Iot/IEadk9zfi8efl3zfiydoj/WNrUrbpf+eHO/7dn5+FsZY1g2DJWS9R/
TV5bd+A+hbPzD5uvW+9U+g87mq9ZZia/WtnxX060c8vNcqIa93P29uf9TD2NbIdtqStKvbr5fd9VXr

K8cxXx/yKNetVygJz83Exx/
+MDcdve3gMSfabqvqrS8PXwQAmEq3XrbeX8z3eP2+GngjyoffFv1vwAzaEEm/z8/8vXjm7XCuj9q/f
c28f5PzY/87OoP9RtP37dy1y/3eivPzV9rCv5dn1R7n5y9jv/pnrCy/l8a/rznvuYNtyPocjW9XvNdMa6J
9ZPIeqPdvbdd+hLRD3T/maud+Um3XK1IS/5w4n8/+vTjeucnjvS/
+fZv1+8v4cxImq7aNPy/h62fWnhHfw+nfR87+zd0737fBdX579Qr9vhz3uiJf9ERFDBXZVn8p0ksO56
821puAo7kr/t9KrNd7n9D2D52/MKlanIP1QqutCWuIJm1XEk4PIOsNTS/7rDdbf9h+0jYkPH57qP3g
ubQ5+wj8QdOuc2oMyXfZPWWMeqiWRNsFgMy05XytY71NXZBN503VUpEt9UY28fv35Iqbe9Lv7x
ltmTO69MupE/PRwFm1o9dYl1qnKmfn9kLNhnteWt7PKPPC+T6X6unAzyL5KMqJU9YH9+dQW08u
7sygPb2GqIFK5fqWv9/Ktv3zYmfnYgY9sRxj39d6h7JvfRj8fi7aJ64puxm9Ecd5v83jPXle3ved79+y1Ls
hP8tr6uf34dEJHdm5mO7dE/ec5PVeHOfc5GX8uXHeXwLf5xmY7Jxk9148/rzk+148tj0jfp6yR42mW
tYuzqii+fpZGOMRoi4pvw7mwJuLMeFQcMiBCgvdr1EPJ5TztxMM6dQPhf5rrYf5A725D/VQ+xmx35
Ht9MRtl/MGFlw3nvMG7H6d89gbakjCc5Pg+G2D59J+jDoG8zj1Y9QvU3G3a7xu+NgBYAoz+uV9M
enSL+W6bIX/A5CTnjTWa9KZVTvsoaMdqbbi/1ueGTUM0Rh6N9uSN/n84j6JXuOc9d1s/bI/oxu3sq2
HjNo17dWwypITNg/
+vJW13H5pT8huV1lOenWcNKPePgZk8AetuMFlmDTbEVuO78XTnJv86fJoB+cHhtOnWwJnknPCe7
FhxPub8/PnvuRaNYmfhRcOISoAAJgNfnmPTdUltX/5uTCLXoa6tpj9i6D+pWdGvR3b52vSqbZmPmL
C/0Op81AjV7KppRZXPr+4J+f2NDo7wwDEHTWkatqr7yOrObMI4efpl3ZMgT9oKfPyXhwwR3/Y6uy
flAvevxGqt6h6/9mYYa9C3osD9HwiwflBrO+pqedWiWHwjzD8LLxwCFEBAADmmDP5gRq2N6ueO
MFRDt36vqzO4hfU9oYzCcRc/dbuUL2Csh6WHcfc/eLePi/O6NHZXTNnsg5/YiAVeKtJgXKftGOWv7Q
jNfxByzKH78Xz9oet8pk147pY/4aeVWXUZtirkPfiAernGrdnrP89dXBKXadTkl3n7nn5IwymQYgKAA
Awp5wAVfXWmJ+hjMXNC3YNtk7tfK7hXHtXhU1NO8B1fuEpSEn9Vqh/CZrtcHptlsOyLfP1i7sOCGf5
y6Iecqzq2bt5jz0MViXezdWMZjYfZVa/tI9h93bqyMHgjTuqd9QS4w9ajkV4L56XP2x5ZtqrkPficIMl+f
qydXLfOk8nJau3vdn+EQZpIUQFAACzwS/vkVSvDSdAndMJ/zL8RSPMYE8j9bAngLR+Mexaz2dbt7sn
h6qoWc7nZKxZ/uLu9nw6O8NfFgeHTbpmdl7y/6V9rOIJceZ7CUZNPfuGnmG4O2f4g5Zvvt+LB+T+h
62S2GWOB8PbWf5cw3txTLq2eOCPkekZ+UcYfhZeOISoAABgNvjlPYKeRddiD3czevzkW+dSTf4wM
Bxc/0KW1S8ac6/XkJUVc0i2WqVmSp7lL6nz94u701utKjMcPSpSOW21wPoeCgyhV7dwzTovM26bL
dtf2uOpyGnVY6+27r+veL3G5m+islngD1qLaFZ/2NIjADo1Oe/9w6mHcM+oHinvxXGoUhklqUldLm
RwkSL/CMPPwounDwAAMCOtqvSt33L69a5e0a33y9aPJ2VvRX669bLVFtWewUe139KvWUZh56U
6JyfEblu53s/3bun26+Xg+ZiLe6RVHbg2rb71S2tfJT+5m+H38TB9HgKPefie1vdRxvdv3Pc1573Yf2R9
7eK0K4/35PH7CLt/3lfxb1dq9H0x9H2r2jFw3Pp7Pu1rNcl5t78m8/fiEedGvd8M7Ns9hrTf/uKem6H
XZfY+POp+0XJ6L453XvJ7Lx7fnpB/xzO6RnHuxXn6WRjjFdT/rIsKAAAwE+5f6F3WD43zNRwQWBCq
x5xdm9BVbUl/Bl3nnO/pqrT6M5p5eS7pnk7G5ZnV9cH8Gfre9fjfR2Gvqc5r79gUjT83Id9bxnk7iuLc
LwrvxbOmRvOoOqhhgkP7+Vl4cRCiAgAAAAAAAEAEaqICAAAAAAAAQARCVAAAAAAAAACIQIgKA
AAAAAAAABEIUQEAAAAAAAAgAiEqAAAAAAAAAEQgRAUAAAAAAAACACISoAAAAAAAAABCBEBU
AAAAAAAAIhCiAgAAAAAAAEAEQlQAAAAAAAAAiECICgAAAAAAAAARCFEBAAAAAAAAIAIhKgAA
AAAAAABEIEQFAAAAAAAAgAiEqAAAAAAAAAAQgRAVAAAAAAAAACIQogIAAAAAAABABEJUAAA
AAAAAAIhAiAoAAAAAAAAAEQhRAQAAAAAAACACISoAAAAAAAAARCBEBQAAAAAAAIAIhKgAAA
AAAAAAEIEQFQAAAAAAAAiEKICAAAAAAAAQARCVAAAAAAAAACIQIgKAAAAAAAAABEIUQEAA
AAAAAAgAiEqAAAAAAAAAEQgRAUAAAAAAACACISoAAAAAAAABCBEBUAAADAQmtvFKRQ0I+N
tl5raG94n19p9PTK2fDbuiEhLV0wbdlwz3vk+e1JY2Xca6xXNVb8ba00rK8CAGB+EKICAAAAODqa52
TGOemSUMHoqjT1kqMsZ9aK+rmpKJsX6tZnHZ3aesg1asv5Wkc/F6me3bS+CgCA+UGICgAAAOAI6
Ujt/OL38Zx/XTlwM89yXbr9vvT7e7I5KvksbsrZqn4eco16jXN+IFttyXZFPwcAYE4QogIAAAA4WuiNm
r3eoezrp3LqRKxeo5Xtlng5auAamb1Qq9IiQQUAzCFCVAAAAABHTLzeqH590pVg6NprIpbm9Orse
rX/7Rrehp1Vr119suC68NKtPqCtUJH1wEdeJ31CKsrGjyeYL3S6HYYzGPXj8F92fsp1ayzrDVXndeO3Ul

FtltujOpfo/aGXxagXN+yXjUo3vHbQto/dH0tcc5VoEar+6BWKwAsLUJUAAAAAEdEWep1HdJl2Bu1U
ytJYTVYDdRep0K2gfXN1VEBZlNWCyUxyoCqjUhpcMIpOxQceJ3F3t/IQK8jtZJRr7Rcl60YnTuHwlEtel
8JVbak7hZHba7KRqMh5/wEVS4M1gNIcvwqwA5pv3M+hoNUR/i5UueiNLhTJewaAQCWAiEqAAA
AgKNjzQ3p4vVGnVS53pW+qgPq9ax0hK1v7o5oh1dLtC/
+y5tyzkv7etJYd0PBqrT0a71td2qyPjIpNl6/F2OSpvaGePmv0a6um3ga+6psW5/r+hNFqRqm9n5iDc
MPTjLVrLnHV5b6hcF2Jjl+67VuGhvWfmsrOxfjnqu27Eadi8A1AgAsC0JUAAAAAEdIUTbdGYwy641al
bNuj8nKaWvJNWL9/mFoL05zBnqzXmhn56Lz+t5F2dGdIQPD3I3enN5rB4QPix+t7aeGgTCzuHnB31
ftfDo9MAOTTGnVs8OTUiU6fuu675lBqKO4dsYLbDsHXf0saOhcmfVeOzviZq/FzT1n+9Zjb+QMWgC
Ao4oQFQAAAMDR4oVsGfVGLZ+Ukn4aMGp9qKqcDiR3JTnpp31ix33dA90L01rllguwH8bwdve1A0
6dSBLymT0vz8ha4EuLsnbGbdi+HIYlthMITDJlPQudTGqK41fHZNc5DR3eHzR0ropr4h2yuodK7n4ja
rECAI48QlQAAAAAR8xAb9RD5+l8K8qJU/rp1MpyMn6am0BHRnTmnE6i8DlCYFIpo85ppLBzFSw5Y
HLD3NgTdQEAjgxCVAAAAABHj9kbtRYvTsvXYK/Onhx6Y8iHebVWhx7biYbtTyercHa88cfflg2z16lby9
Ss3ZpEcVP29D4Gyt7amueYpR8Alg0hKgAAAIAjyOiNmpQxjDw7g706u3Lg7tTtmVk66QWAo+p5ps
MsJeDXAHX05KJbmFROSaIqAdNKcvztXa/nqR24unVRp7iWaoZ+1ePUnkjLDlS7Xi3W0WUEAABHF
SEqAAAAgKPJmIAoTMlPDo3aqW3Z8Kapz1ZzdcObqKm94Q8/L59ZcwJAszZnc9UfQt7e8Gp0FlIZV2
4Gzta5WPd7WfYa61790aSTVU1twuP3A1djxv4k9PbVbRDscRoSdAMAlgYhKgAAAIAjKro3qjlzuwrp
nGAubi3NaZWlXG7Kqg4D/dzWmOFftd+ozdlc1cGh9+IREzJNorLtD1vv1KSk21XyE1S5kPuM9AmOv
3Lan6jKu5bGBFRJjDgX5r1RPat7ugIAlgYhKgAAAlCjK6o3qqp7OVgzs9qavI5mIqfk7J4xPFyx63gO1Di
1a3MOvE4Je+2U7GHrIcdebVnr3eHxeYt9/BXZ7psz/itVaZk1TZu7Xs/fcexzEVYMVW8zrewaALA4Cn
1V3AUAAAAAAAAAAEIqeqAAAAAAAAAAAQgRAVAAAAAAAAACIQogIAAAAAAABABEJUAAAAAAAA
AIhAiAoAAAAAAAAAAEQhRAQAAAAAAACACISoAAAAAAAAARCBEBQAAAAAAAIAIhKgAAAAAAAA
AEIEQFQAAAAAAAABGEvn/AZM6nr53azj8AAAAAElFTkSuQmCC]]></redcap:Attachment>
</ItemDef>
<ItemDef OID="q13" Name="q13" DataType="text" Length="1" redcap:Variable="q13" redcap:FieldType="radio" redcap:RequiredField="y" redcap:BranchingLogic="[q11] = '2'">
<Question><TranslatedText>Do you prefer Life A or Life B, or do you think Life A and Life B are the same as one another?
</TranslatedText><redcap:FormattedTranslatedText>Do you prefer Life A or Life B, or do you think Life A and Life B are the same as one another?
 </redcap:FormattedTranslatedText></Question>
<CodeListRef CodeListOID="q13.choices"/>
</ItemDef>
<ItemDef OID="summary14" Name="summary14" DataType="text" Length="999" redcap:Variable="summary14" redcap:FieldType="descriptive" redcap:SectionHeader=" " redcap:BranchingLogic="[q3] = '1'">
<Question><TranslatedText>
Alzheimer's disease - Description #[no]

If you have Alzheimer's disease...

1) You might forget recent events of importance to you. For example, if you are interested in hockey, then you might forget whether your favorite team won their last game, although you may remember watching the game itself.

2) You might forget the names of people you met recently, or the names of local streets.

3) You may no longer be able to handle your personal finances or plan leisure activities such as dinner parties or vacations. Simple decisions such as what to order in restaurants might become difficult to make. Your ability to concentrate may decrease and you might have difficulty driving.

4) You may start having trouble finding words to express your thoughts. You might need to be reminded to shower or brush your teeth, but you could do these activities on your own. You may need to post notes around the home to remind you of simple things like turning off the stove.

5) You may remain aware of the day and your surroundings. You might be able to travel to familiar locations like the supermarket, although probably with the help of a loved one or friend. If you are in a social gathering, then you might have difficulty following the conversation.

6) You may continue to enjoy hobbies such as puzzles or reading.

Now consider two possible lives. In Life A, you will live in perfect health for 14 years and then you will die of natural causes. In Life B, you will live in perfect health for the next 10 years, then you will live with Alzheimer's disease as described in the summary for another 10 years, and then you will die of natural causes.

</TranslatedText><redcap:FormattedTranslatedText>
Alzheimer's disease - Description #[no]

<p></p>

If you have Alzheimer's disease...
<p></p>
1) You might forget recent events of importance to you. For example, if you are interested in hockey, then you might forget whether your favorite team won their last game, although you may remember watching the game itself.
<p></p>
2) You might forget the names of people you met recently, or the names of local streets.
<p></p>

3) You may no longer be able to handle your personal finances or plan leisure activities such as dinner parties or vacations. Simple decisions such as what to order in restaurants might become difficult to make. Your ability to concentrate may decrease and you might have difficulty driving.
<p></p>
4) You may start having trouble finding words to express your thoughts. You might need to be reminded to shower or brush your teeth, but you could do these activities on your own. You may need to post notes around the home to remind you of simple things like turning off the stove.
<p></p>
5) You may remain aware of the day and your surroundings. You might be able to travel to familiar locations like the supermarket, although probably with the help of a loved one or friend. If you are in a social gathering, then you might have difficulty following the conversation.
<p></p>
6) You may continue to enjoy hobbies such as puzzles or reading.
<p></p>
<p></p>
<p></p>
<p></p>
Now consider two possible lives. In Life A, you will live in perfect health for 14 years and then you will die of natural causes. In Life B, you will live in perfect health for the next 10 years, then you will live with Alzheimer's disease as described in the summary for another 10 years, and then you will die of natural causes.
<p></p>
</redcap:FormattedTranslatedText></Question>
</ItemDef>
<ItemDef OID="a14" Name="a14" DataType="text" Length="999" redcap:Variable="a14" redcap:FieldType="descriptive" redcap:BranchingLogic="[q3] = '1'" redcap:InlineImage="1">
<Question><TranslatedText></TranslatedText></Question>
<redcap:Attachment DocName="14.png" MimeType="image/png"><![CDATA[iVBORw0KGgoAAAANSUhEUgAABVEAAADYCAIAAABtBTQzAAAAAXNSR0IArs4c6QAAAARnQU1BAACxjwv8YQUAAAAJcEhZcwAAEnQAABJ0Ad5mH3gAACqPSURBVHhe7d1fqF3VvehxY9QTr0pC6wlpvWj0IgQVSSsXYm6xEcSIIAgqBjmKlyubCJYTBBsRc7kPinIoZJ+DoA/FCrf3xgdFDreo2EIeKqSHQvPggwdS2Q+WBurDPlLKluahd/4bY475G3/mXGONOdecc38//DhnrznHHGL8xxxlrJGj9l72NwA
AAAAAMEfU/ACAgZzdOHvZ/7qMIAiCmFZUf4kDmCb+DAMAhrCxubHn9T3iHEkQBEGMPPY+t+vff
vH/qr/KAUwQNT8AoHebW5sH3jggzpEEQRDEyOOqly5//OBNx+
+47Y9f/L76Cx3A1FDzAwB698DPHxDnSIIgCGL8cd+9+/7Hrf8li5eP3v/Xb76p/k4HMCnU/ACAfp34
IQ4RBIEQRDjj9uP7S4L/jLePvnj6q91AJNCzQ8A6NGZz86IQyRBEAQx/tj73VkfPr+e9Vf7g
Cmg5ofANCXc1+e2/XKLnGOJAiCIEYeO0/tePSuGAQoidvtBnwUP9gNTRM0PAOjFxubGvp/sE+dIgi
9G9otrXwYP9wORQ8wMA0tu6tHXwrYPiEEkQBEGMP2596jpR54vgBEGOOP3c9faT/Gb8fZ//t/qr/uAYweNT8AILHXfv2aOEQSBEEQ4w/fY/x2HL/jti/On6/+0gcwbtT8AIC
UPvj8A3GIJAiCIYR/
+3Bvxe1fSBOHvnhX77+uvqrH8CIUfMDAJI5f/E8E8v6ifIAhiinHTM9eIqr413nj2ePW3P4ARo+YHAKSxubXn32/OEQSBEEQ449rX7jiydtvFiV9l/jkZ29X3wEAxoqaHwCQxr8z9w/HPE/L1l2/KsEB4D5S6mHcbBNHnwbZHvGGrBZC
bW5f32/OEQSBEEQ449rX7jiydtvFiV9l/jkZ29X3wEAxoqaHwCQwNalrSPvHBGHSIIgCGL8sfPUjofuv

kEU8x2DB/uB8aPmBwAkcPwXx8UhkiAIgphEHHroelHJLxQ82A+MHDU/AGBZp8+dFidIgiAIYhLxn9f
+k6jhI4IH+4Exo+ZfiQvrhy8rHV6/UF1TQvdWRk9q7cP2lz5xvZY0TJblfbhWTrOQ8p3PNiC4dJW410+
b511ozm2QmaAXZzfOihMkQRAEMYm4+uTOJ+7cLwr4uODBfmC0qPlXRBdBssKpa78xFamiZgu/9I
nrtaRhsixJT7KUrOitxg0ufVU1vz03av6J2tjc2PP6HnGIJAiCICYRD/7gu6J0j47jd9z277/5TfXdAGBMq
PlXxlnc18XfmEtUWcI5KjqXjs22oX6q3W77vZqa3zU3av4p2tzaPPDGAXGCJAiCICYR33/kW6JuXzKe
P3zoP/70p+obAsBoUPOvkK76dZGjr4y8KhYVW8divmOz7aenjek2LDU/lvHAzx8QJ0iCIAhiEvGdZ68
WFXuS+Kd/eKL6hgAwGtT8K9Ws8e1/BlDTNVJBNHAUSy31nrpd97CvqGHzK2K48EufUC93vaeauBa
Wa7Z2knMzE5ljhadu9Ap3Ck3PGET96FSP2r7YRgujqyOBZ4Xmhhi97GxtkxFTaTTR9/JJ+ObWeSYYix
MfnxAnSIIgCGISkfAxfjv+9V/
+ufqeADAO1PyrVddJa+vqR6vGcdRIGaOZWSxVGkWWg7pvF4l6kOpK8VoMF37pE+yl8ptDqBZqSrq
HyVi0i5ybezNzoYE69WqbnmOQw+vr9kWxPabGHJ0txHYazJ01JFmaZxTdovEu+ObWaSYYjTOfnRE
nSIIgCGIqcd+9+0ShnjZ4sB8YFWr+VZPVlFXe6EpI3dE9dFPVxOjbKLJcqgaqi1FvVT3UCMVLMVz4pU
+4l5pBPYZqUM3RyuJYtk32shdaX6lTS/7tWWB69iAVq2uX0XQLdUUPry44hnUILM07kJyMamB18I
5gXch0mAnG4tyX53a9skucIAmCIIhJxO3HdosSPXnwYD8wKtT8q2eUOo0SqCTLq5wsmBxtXDVVU
9Wi7FO+WFvLBzKvqP5iuPBLn5Ze6rUaRL2uViVe5hyXLDKL3u16rrKJg6NX3a1I32F6ehA5X3sC7aPZ
fcpLrgn615Vp3xCROee41CBTW1OxLmTi3hoMb2NzY99P9okTJEEQBDGJ2PvcrqcP3CJK9D6CB/uB8
aDmHwNXRVlRt5rFlSi5HI06FEtVr6JB8fPh9Q+LXvUV3V0MF37p09ZLXaheVzNQi1KLdAnklVn0MH
WfDtNXvRpvQ6Nfh+k5UpfsCbSP5pxRU4d1ZRwDiY4dlmZotq7uW1Nxza19JhiBrUtbB986KE6QBEE
QxCTiqpcuf/zgTaI47y94sB8YCWr+MQjUb+5bnqJswWKp6pa1KFtnP5SX9BXdWwwXfunT2ktdKS7
UsyupRboE8soscXulejVaNPp1mJ4jdcmeQPto3sFqHdaVcQwkOnZYWt1FqO5bU3HNrX0mGIFj7x0
TJ0iCIAhiKtH3Y/x28GA/MAbU/GMQqN/ct1QpVF2NLZbKfupf7xe9i0vqSj2gGC780qe9l7qUXanWV
N9z70QrmSVur5zJG/06TM/bxJ5A+2gd8nVYV8YxkOjYfTKZspFMbU3FNbf2mWDVXvv1a+L4SBAEQ
Uwlbn3qOlGQDxA/uut7X/3hy+pbBMCKUPOPQaCo0kWPeU9WQo7+ugoLFUvVOPZj/OUVYzyRMf
zSp0uvct7619kbtzomkWS3uMLStZ3NbgsMYr6VBbtv+2iuFnmCxdaVad+Q1nHsIeR+WUP4pp9beA
kYxgeffyCOjwRBEMRUYvfzVw7zGL8drz72yF+/
+ab6LgGwCtT8Y+Aodmq6fFJ3dR2km+srqjTSfVqKpbqjq6u/9gq/9OnUy8gv7uj26rJuGkors6hOixW
Wjl2R2dun50hdckygfTSrhf9CPayDY1ayY9tk5Nzq9taV6rVzbh1mgpU5f/E8v6ifIAhiorHz1I5H77pRl
OJDxruvvlJ9nQBYBWr+MXAUOyZdUjWYjY0iSwoXS3VHPZzO5kxQDhd+6dOtV71aazt0j4aFkkYWlu
63IOcap6Ee1JG65JxA+2LdLYzhm7P2rK7ThrRMxr89VRN7RMfcOs0Eq7C5tbl/fb84QRIEQRBTiXuO7
hVF+PDxu19+Un2pABgcNf8YOIodqVl0OSogs0F2u2uxZLdz9hQXwy99OvYK74buVQjsWEVmiSsszV
7q54zdITQ978K8E2hfbLOF1cSYq7N7pvOGhCdjZsq7qdflEK4Rrbl1ngkGtXVp68g7R8TxkSAIgphKrO
Qxfjt4sB9YIWp+oJ2jHAW2h+O/OC6OjwRBEMRU4toXrnjy9ptF+b2q4MF+YFWo+YF21PzYnk6fOy2
OjwRBEMRUYuepHQ/dfYMovFcbPNgPrAQ1P9COmh/b0NmNs+L4SBAEQUwoDj10vSi5xxA82A8
Mj5ofaEfNj+1mY3Njz+t7xPGRIAiCmErc9Mw1otgeSfzoru/98YvfV182AAZBzQ8AaNjc2jzwxgFxfCQI
giCmEqN6jN+Ol4/ez4P9wJCo+QEADQ/8/AFxfCQIgiAmFA/
+4LuizB5bvH3yx9VXDoD+UfMDAGonPj4hzo4EQRDEhOL7j3xLFNjjjE/ff6/64gHQM2p+AEDlzGdnx
NmRIAiCmFB859mrRWk92jh+x2082A8Mg5p/tdTvhiv08gviLqwfXvuw+nkev4uuuaKArGHbWvndf
N113qv5feS2jXNfntv1yi5xfCQIgiCmElef3PnEnftFaT3m4MF+YBjU/CtU1aRK+qKoSjCjAsxekZfeXf9a
6zeAgrRdp8/O/D5y28fG5sa+n+wTx0eCIAhiQjH+x/jt4MF+YADU/CvUczWkK9rZFGCuFbnV1bx/rV3
aoNbhszO/j9y2sXVp6+BbB8XZkSAIgphQ3PnYHlFOTyV4sB/oGzX/6nSvYOPMrwDrtmNmLZ/zrFVtR
YGCtB01/5wde+
+YODsSBEEQE4q9z+16+sAtopaeSvBgP9A3av7VaBScJbOObZatoloyiqh6GFEF+8Y3CzCjjV2PNWbQ
Wq6Zw5o9rdrcP2x9R3fSExSzVazRM82dKzhnXw53+HDV2rtCc2UGx+W2HZMzazQxxqsXWq4v2M
+ja65Gw+Cbld30bIVSz1srRjS7GW3sURqTFrc7zxkxXvv1a+LsSBAEQUwornrp8scP3iQK6WnFySM//

MvXX1dfSwBSW6zm16dt/1Hb2aRRDnhKhs7qI/
+ED/y+AsI9K2PsmqOBtae+8d2D54whGsWXEnzbkgyr75fXxEvfigTVK7unejhmXt7KbgTaVJwt5MX
WHVMdmuoWjvvFTXe/8Cc/IlelfcY5s5XB0SHdR27h2aCzDz7/QJwdCYIgiGnFfffuEyX0FOONZ49X3
0wAUhug5hfH+WXP6OZwwdJn9Jy7qYsbtU+6ld44o/4JboBrfLuvNb7VTfUJvHP2sPUVmScwrNnGld
W1IiFvIqZgzbsaJm/mbVNTSY0mopc1LTmqHEJ30GOqHhlzbb6B6o6WmFz1FZVc91JX6guB3HUrYx
F2OmtKVje57k5zRoTzF8/zi/oJgiAmHbcf2y2K5+nGJz97u/p+ApBU8prfZp3fl2LUHpk0Y65IqEAyFyb
b6WqnZfWh8Y1ropl6aQzuuCQkG1YPVGmmFGO2cO1lphqkHMLTpsGap+jUcWkmayF63Y0eupl3o
A78uepdlG2sPhl9LTAXV7/2dOplYAM7zBkL29za3L++X5wdCYIgiAnFt//x76b7GL8dx+
+47Yvz56tvKQDp9F/zpz2a61qgehB7iVpo5Rw7I6rJiI50edVR/zi5dt6RQDTTozt4E7aXZJ2HNRvKd9e
1Ij/HWjPlVXXN3Ubw7L+ahDljyZ5os7U1RrODXnAtOFOhcy65sapJI5nzYpPrDXJ0E82a02wSEwrMG
YvZurR15J0j4uxIEARBTCh2ntrx6F03irJ56sGD/UAf+v1v+x2HeceZPdexklF91j5UPy1SAo2MYzfdZZV
o6G5kc4zv6usZ3cUYqCnlsHVLa4GuFfk5JlVdq6+42thU3qKV6qLnUE/YphrpmQvqvnceno6hDYjIJTf
WOR3vHGtynFx7OtXCxT8hVy50d/wXx8XZkSAIgphW3HN0ryiY5xE82A8kt6KaX7cyBWqJiuqWD6J
+bu81Vo7ddJdVYqnuRjbH+B1qp66jN6QbVncriNauFfk5UqpLboG5VR2zFmoObW+ayUhbNrIW0jq
GnHmXht1zyTbO6bTO0f0GtafrMHCHOWMBp8+dFgdHgiAIYlpx61PXiVJ5TsGD/UBa/f8OP0cf61KH
Q39O9Su7qVctncYrtDPmomS7jrvlGr9D7eTq1SrVsKrV4fV11yIXm5tjUuqSW2hDq56H19bKKRgzaJ
2VPRE9D9XFMVclu7VAsrhcclRXFn0tsE2ufu3pXL2EDnPGAvhf4ycIgph07H7+yjk9xm8HD/YDaa2i5l
dXHOf3QDlhN+rUacTsncnoCk2usl6mo/5xco2/SL2nLugpmfNsSjNso4t+ERgzrMs+dWlT0tMtNCbQ
tjS50rq9Hsc9D92xvuxuWIvLZW2stSRjnMBeWeNkOqSzsslVdJozutu6tHXop4fECZIgCIKYSjx09w2iSJ
5fvHz0/upLC8DSVlHz6wO9Q+D8rgaqj/32lUnx7KZ7e4w1Ouoft+ZIRZZOtZO+0hB4a5IMq2+rQfT06
1EdK/JzTMrSpU3FzC0ztyytOW2TauKbh6+nf+lRuew3y7OkXJf9LBWjdUrXsoEd54wFXPzzRX5pP0E
QxETjpmeuERXy/OLT99+rvrEALG06Nb+7KiiFqpDxCuxmc7HivqP+8TG3umjeuXZqzqAt1fLD1jeM9
nr6dUNrRX6OSVm6tFEcszGFd8ycd75C9bpabWgeZtdcY0NdFs/V/mZlN0JzrJnJi5ZR6WSWzoNgAfy
P8xMEQUw3Dj10vSiS5xRvn/xx9V0FIIVV1vzh2kFoFgTSQkMBMdRHkA8bZuPMZ2fEIZIgCIKYROw8
tWOu/4X/y0fv/
+s331RfVABSWOXz/IFRLLqPB3UY+qU/gXzUMCsv/upFcY4kCIIgJhHXvnDFk7ffLArmqcfxO2774xe/r
76iACQSWfM7FSW8VdA7Kvx6mOqa/u+Bff8UwDFISd+gEkNPzP9a3f8RBSbr4XcfFudIgiAIYhJxy3+/
VtTMUw8e4wf6sJKa3zeQt5wKVPaBW0AKRs1PwY852rq0dfCtg+IcSRAEQUwi7jm6V5TN0w0e4wd
6sqKaP9ccLFSyB+v64E0AQKuNzY09r+8R50iCIAhi/LHz1I5H77pRFM9TDB7jB/qzWM0PAJilsxtn+TX
+BEEQU4zdz1/59IFbRAk9reAxfqBX1PwAgNybv31TnCMJgiCIScStT10nquuhpxe9+
+Un1VQSgB9T8AIDKiY9PiHMkQRAEMYmY7oP97776SvUlBKAf1PwIE7+PIfxyIep341W/iGGZoRbV
+FX8vf8iiGxlA6wpN+QeYraOvHNEnCMJgiCI8cdVL13+
+MGbRDk9/nj1sUd4jB/oGzU/wsJF/jJF5qpqfp2o1G/JXyUbqAYfbA8xZ5tbm/vX94ujJEEQBDH+2Pvc
rmk92P+ju7731R+
+rL5+APSGmh8LEVXlMkWmqPkHM2DeoWtwan6k8flXn/Nr/AmCIKYYtx/bLerqMQePP8QPDoObH
QqZf8w9ZF1PzY7I+uvCROEcSBEEQk4j77t0nSutxBo/xA4Oh5l8JT7nrvKzruEJ7j5a6T92ue9hX1LD5F
TFc+GWYbpzJ2ou5O4cyu5gTDGp0avRSKQ2eiZuTE/NuaOYys/lyiWXnrKUbbephqpv+jI6BPMQY3R
eV6TL/THCMUqOJfb/DCOjX6XOnxTmSIAiCGH9M4sF+HuMHhkTNvxqOwsl1sS74TEanjgWYSd3Xt3
USPUh1pXgthgu/DHAvJVeltYbSF0zGQp3CO+a465l463RzwWS+XOq6MY61dEffsnkwY7e3w7mtRo
+WFLHzzxmzantz20fAII69d0wcJQmCIIjxx3eevVrU2KMKHuMHBkbNvyKq5nGUOfqSrnvUFV0oWU
2MYawCTKoaqC5GdVX1UCMUL8Vw4Zdeup3Mkakm0jayY6mWDjtmD+xk70p9RSxhwVyOdVjN7
OyF1oyufE1WF7FlrSm6zF82sQaRHawerSNgIFuXtg799JA4ShIEQRDjj+8/8i1RaY8neIwfGBg1/6qoK
kYXMVYt5SiuWqurjFVPWaoWZZ/yxdpaPpB5RfUXw4Vf+ria6WvV3Jtt1CvHwoxLQpcdsy84qaGMV
q0d7QauLl3eMp3dv9aC7BgxxfKSr31gbv7565e+6asGjiHKS60jYEAX/3yRX+NPEAQxxXjwB98VxfYY

4l//5Z+rLxgAQ6HmXxlR+KhSSldfjtoq4+llNNL1kr+Oq3oVDYqfD69/WPSqr+juYrjwSx/nWsTF5lDqpo
svlzOLHldd7TZlnb9uFerYnK5u4OrimKXVzJFdcmZsXZsjuZ97UR3mr1/XGgmbAzeVQ7SNgGGdv3h+
1yu7xFGSIAiCGHlcfXLnE3fuFyX3auOf/uGJ6qsFwICo+VdH1TVFMaPKoLpYc5dnorzqUIC5VN2yFmX
r7Ifykr6ie4vhwi99nGsRF5tDqZsuvlzOLNYUu03ZMZbdUV8R7AZGri4ju1eSacnoytfgHbjWtqhldqa6
r4ZwUUOER8Dgznx2RhwlCYIgiPHHqB7sf/7wof/405+q7xUAA6LmX6Wq9smqJ1XhGCWNuzxTDau
rnQowh7Kf+tf7Re/ikrpSDyiGC7/0ca5FXGwO5ezRwt1HjauudpuyYyzZUTXJlK3skV25OozsW31rRle
+Bs/AtfZFdZm/ZgxXKDu1zsLgHgGr8OKvXhRHSYIgCGL88V8f/raovVcV//6b31TfKACGRc2/UlVBc3ht
rSyazJpJF1JmlSOrK0f1pIskX92Xq8axH+MvrxjjiYzhlz6uZvpalavZpuPADXLEgjVQt5EdGys62i3snXfl6v
KWOdpk2jO2rs03ITVkewpHE7tNJrtmvGrmbZ1mITQCVuThdx8WR0mCIAhi5LHz1I6H7r5BlN/DB4/
xAytEzb9auIoqiHJG31TlIa57dL2lr6i+xoDB4qju6Opq1HOi1gq/9LLmaeSvkvlGVj309AKpOuyYzOOh
RvLvhJxPPeF6ZFeuuI110dh31cyRPdOe0ZWvycrevNBhUdYI1vz1hXr6YkH+MbqO4LiAAWxd2jr41k
FxmiQIgiBGHte+cMWTt98sivAhg8f4gdWi5l8xXd1kVFVVM+/WzCrHKMokezhT3dGqotwJyuHCLw
P8E62yWUO5e7QkWmDHgiM5KkrZ0Z0qV4/cbFNe9+
+E7ujInmvN2GVtnvRVqg6LWmD+Uj0t9yD1/fYRPHuEvm1sbux5fY84TRIEQRAjj5ueuUbU4YMFj/E
DK0fNv2q6uPHVLs3qqK55NLNBdlu/dDQ12e2cPcXF8Msw3TiTtRdFWzh7oWN51+wkZ9Ztyo6K0t
HRrEzzq+q1MbTZRA9mzjBra43syF4JZ+y2NrlDIk04RaFt/jlzmJw9peYswmvNNUdQd+1+6NvZjbP8
Gn+CIIjJxaGHrhfV+DDBY/zAylHzr5oqfChdAEzFm799UxwlCYIgiJHHzlM7Hr3rRlGQ9x2f/Ozt6psDw
OpQ86+W/ledlPwApuTExyfEaZIgCIIYeex+/sqnD9wiyvL+4o1nj1ffGQBWipp/VZr/4bL9Hz4DwLgde
eeIOE0SBEEQI49bn7pOVOY9xckjP/zL119XXxgAVoqaf1WMmp+CH8AEbW5t7l/fL06TBEEQxMjjnq
N7RX2ePI7fcdsX589X3xYAVo2aHwAQ6fOvPufX+BMEQUwrBniwn8f4gVGh5gcAxPvowkfiNEkQBE
GMPL79j3/X34P9PMYPjA01PwBgKafPnRanSYIgCGLkcfux3aJWTxI8xg+MEDU/AGBZx947Jk6TBEE
QxMjjvnv3iYp9yeAxfmCcqPkBAMvaurR16KeHxGmSIAiCGHNc9dLljx+8SdTtywSP8QPjRM0PAEjg4
p8v8mv8CYIgphV7n9uV6sH+t0/+uPo+ADAy1PwAgDTOXzy/65Vd4kBJEARBjDnufGyPqN4uWj9/
/1m2+qLwMAI0PNDwBI5sxnZ8RpkiAIghh5PPiD74oafqE4fsdtf/zi99XXAIDxoeYHAKT04q9eFKdJgi
AIYsxx9cmdT9T9y5y5X1Ty3ePT99+rvgAAjBI1PwAgCIIgxx3eevVpU8h2Dx/iB8aPmBwAktn
Vp6+BbB8WBkiAIghhzfP+P+Rb4l6vjV4jB+YGp+AEBKAEb6G5sbe17flw6UBEEQxMjhjoQf7eYwfmApq
85unOXX+BMEQUworn3hiidvv1vv1nU9r7gMX5gkqj5gQ9AQB9efO3b4oDJUEQBDHyuOu5faJ6u2j9/
PVm2+qLwMAI0PNDwBI5sxnZ8RpkiAIghh5PPiD74oafqE4fsdtf/zi99XXAIDxoeYHAKT04q9eFKdJgi
AIYsxx9cmdT9T9y5y5X1Ty3ePT99+rvgAAjBI1PwAgCIIgxx3eevVpU8h2Dx/iB8aPmBwAktn
+8GX19zuAKaDmBwD0a3Nrc//6fnGmJAiCIEYbgQf7f/fLT6q/3AFMBDU/AKB3n3/1Ob/GnyAIYlqx97
1PXiWo/i3dffaX6ax3AdFDzAwCG8NGFj8RpkiAIghh5PPiD74oafqE4fsdtf/zi99XXAIDxoeYHAKT04q9
eFKdJgiAIYsxx9cmdT9T9y5y5X1Ty3ePT99+rvgAAjBI1PwAgCIIgxx3eevVpU8h2Dx/iB8aPmBwAktn
Rxs5TOx6968ay4OcxfmC6qPkBAMMRB0qCIAhizLH7+SufPnBLVvPzGD8wXdT8AICBXHbZQF86JIp
GomgkikaiaMMk+vT99w79/fXVi57xHkUjEQLYSgDAQDiRRCNRNBJFI1E0EkUjUTQSIYCtBAAMhBNJ
NBJFI1E0EkUjUTQSRSMRAthKAMBAOJFEI1E0EkUjUTQSRSNRNBIhgK0EAAyEE0k0EkUjUTQSRSNR
NBJFIxEC2EoAwEA4kUQjUTQSRSNRNBJFI1E0EiGArQQADIQTSTQSRSNRNBJFI1E0EkUjEQLYSgDAQ
DiRRCNRNBJFI1E0EkUjUTQSIYCtBAAMhBNJNBJFI1E0EkUjUTQSRSMRAthKAMBAOJFEI1E0EkUjUT
QSRSNRNBIhgK0EAAyEE0k0EkUjUTQSRSNRNBJFIxEC2EoAwEA4kUQjUTQSRSNRNBJFI1E0EiGArQ
QADIQTSTQSRSNRNBJFI1E0EkUjEQLYSgDAQDiRRCNRNBJFI1E0EkUjUTQSIYCtBAAMhBNJNBJFI1E
0EkUjUTQSRSMRAthKAMBAOJFEI1E0EkUjUTQSRSNRNBIhgK0EAAyEE0k0EkUjUTQSRSNRNBJFIxE
C2EoAwEA4kUQjUTQSRSNRNBJFI1E0EiGArQQADIQTSTQSRSNRNBJFI1E0EkUjEQLYSgDAQDiRRCN
RNBJFI1E0EkUjUTQSIYCtBAAMhBNJNBJFI1E0EkUjUTQSRSMRAthKAMBAOJFEI1E0EkUjUTQSRSNR
NBIhgK0EAAyEE0k0EkUjUTQSRSNRNBJFIxEC2EoAwEA4kUQjUTQSRSNRNBJFI1E0EiGArQQADIQT
STQSRSNRNBJFI1E0EkUjEQLYSgDAQDiRRCNRNBJFI1E0EkUjUTQSIYCtBAAMhBNJNBJFI1E0EkUjUT
QSRSMRAthKAAAAAADmiZofAAAAAIB5ouYHAAAAAGCeqPkBAAAAAJgnan4AAAAAAOaJmh8AA

AAAgHmi5gcAAAAAYJ6o+QEAAAAAmCdqfgAAAAAA5omaHwAAAACAeaLmBwAAAABgnqj5AQBY
1Idrl9XWPqyuAgAAjA01PwBgRspivN8q/ML64TpDnwmH/icL+cIyh9cvVBdSaiymwD8rWVRjD/t5m
3rn+QNTffZKaT8ZgT+igVuLcg/VeMfSLqtfwZ3p9S+KHvkXNdX3ybOixnJSvU0dck3076RtgpofANCvH
k/zDUaeIQ9tRdo+zjrZWUqvozhX9XygyhdyeG0t+z+95MmXMPiR0DyPpv9UND7ZtZ4+fcVaBvpEm
PuWLon3T2jzz1BzoUvxZgzeWpRvqPy6vlBu6bKp3KOYb9fSKVp3Jr+f9C8K16IaSyost67AoopbCf8o2
csxkpt6WlGRX18qXi27um65EqQq964i9qexi+IeOqDmBwD0qHmeap5GklKJiv8/5IGgucK+9J4lT1Bt
Xz9p8ve+920yDfPGNBQpe/rwybH7y5X6HF9R74c9cfnRSLU0f8bQrUV1HUq1q14urOhfambJtk9f
WP7taltOfr26vVSeUmhRKcavBBbluBTPuxxh+aT+FckrPeZSN6qXy75r+XB6+OKTXL9spipuLrWm7Yi
aHwDQH3kGsE4NyfWfQRjm+NHzsvTw+Q/xh7aQ5Y6DCxv8c5Dp96PQ3MDe1lcMbL5Rqd84a+b2
UhJvZGCvEm5j21DF/eiNVL37zWJwJ9JX8x+WTqMm68jVz18XdqIk66gEltOU8PPdunWtk+nOGspa
R8JkejeLF/LjkDLRtkHNDwDojf3NnPC04zbwYaBYT6ozo1fj+JOesWf5j/0kKnaq1vOe9bcOr57fpEy5h
VmK8od+PuXWn1Dj05GENV6esblviVMGhkuYqW0oa2OjtGVJtiDXQMa1/MdkH3ZHLvtDkYKdqMiT
P6igLZ/VtXWG4naqtTlzFR+1PEX5g38qi7FyebYzzdLMbXInSrawbYKaHwDQG/sE4DyipNR7AlNx8Eh
1erMVa6n0uKR8FWr4PGV/C1LKhfWZp/jkJT7Khw3zwSs+cYW+UtnrsP8UL8VKYI+feC8DwyXMFB6
qeOMS7GI4S3E3zXvlSJSvQV3Jbyf7UHhyGRKlshOJ96Vo4N/fbhzLMYTvLso3Wr1/yVI5chVX5O4leq
+KFVTZ8p+bw6bdxe2Bmh8A0JsVfFUPdxYQp8VeJT1MNeXLqPcrTzTImuyPRlLizSn3r8+PRZGw18+d
uYby537yFWPLrUv4VhUDmjO3PwpWk+UEhkuYKTSU+DguwZmluFhJtW1WonwN9ev8drIPRWjr
MuXyUmSzE1kfvgR/kIPLSTC+yZGr3K7yWvlzonzOdRXrUdbWGh+SJRTD6jfGepfCewwnan4AQG9
W8FU90FmgPOkMeOYoD2/pEzaObIYUJ+yQIm9/+2d98vrNV7w7vW6ZnaHHFTU+FOnO8SXrT6j9R
9Z695ZjJ9ACtxblHarYzVTLaZlwcTtJMpmo8Zkw9JHLlugTYSeyBm6fS6vAEMWtVJ+FnJXLzlC8c8utq
NS+NXmLFKuTf2Tst799LpCo+QEAvbG/mBOd3fyGOAsUOQY+cBQ5e925Qp6m/yz9L6ePo7xfslO1
n52i1xUZUn8k7HnLNyv1ygLjJUzlHqp43xIupnXCRYMECVsS5beTfSpaF5XqT5idqLhiLkR+GCP4l5No
GQYrl52ifXc7ah0ob7D8h6JYQXMYO3OCt2nboeYHAPRHfjMnO3549Z+hPJNIyc8fWRZjGY5zUC/Sn
Nps2bjGsMWb1Ou7VGQwF9LnGbF4c/r9zNmfgEGSZpJ/Ioq3pjnxxtqst25pjoxK4NaiHEMVl9K+Sa0
TLhok2L6WRPntZG/SShfV+IOU5E+Vdzk9/Jm1czX+MGXSJQ2/TWneo2K2dpL8sjl2eCpwouYHAPSo
cQBJcypwK8YWpn0mECsaZjF50l7eocFX0zjqpjv32vr8VDeV52FtgE9EyrWJT0ChXoK5tlSbGcgYnsxC
/EOJ96u05OKKbI2ZZlmM10XOZXJ03Jm8WbIPvbWo7IIxeDklxyQ6Cy+qcXeZNBVrOaXi8gCf7eR/U
fhzNe8svXXlcM5hGp/rpDu5fVDzAwD6ZR5A+J7GYBoH0qXPoz7Fx7u30Vcg8TkeSTTflVL13ohbE3r
H/Iua5JoC71Fmdn9RJCf+UUWpPjJwkFgSNT8AAAAAAPNEzQ8AAAAAwDxR8wMAAAAAAME/U/AA
AAAAAzBM1PwAAAAAA80TNDwAAAADAPFHzAwAAAAAwT9T8AAAAAAADMEzU/AAAAAADzRM0
PAAAAAMA8UfMDAAAAADBP1PwAAAAAAMwTNT8AAAAAAPNEzQ8AAAAAwDxR8wMAAAAAAM
E/U/AAAAAAAzBM1PwAAAAAA80TNDwAAAADAPFHzAwAAAAAwT9T8AAAAAAADMEzU/AAAAA
ADzRM0PAAAAAMA8UfMDAAAAADBP1PwAAAAAAMwTNT8AAAAAAPNEzQ8AAAAAwDxR8wM
AAAAAME/U/AAAAAAAzBM1PwAAAAAA80TNDwAAAADAPFHzAwAAAAAwT9T8AAAAAAADMEzU
/AADAuHy4dlll7cPqUkndOLx+obrSD5VHpB+Dem8y1j5cWD/suxW8BwDzRc0PAAAwLkZd2yxPt3v
NX5ftBdc2eCv7elNH+E8yAKA/1PwAAADj0vh32WaFut1r/k7rdxb39T8KoOIHsM1Q8wMAAIxLo+Y
3C9xtXvPrwr1lXnr/9D7pK1T8ALYdan4AAIBxadb8RqFq1fzWBbswrprkLYxxqw7GFbMaVpeza8Z/Tm
8kUYy71n1jbnWalpq7MV5jQGOmSmAs3bpoo19ZKwjNP9ecj2gSWGCwHwAMipofAABgXOpicr38
SZWMRpVZvLYvGOVmVRLX5WgrXUT7+xh1tqxrC8ZEHIMES193Url0gzEXSz25tXX1o8jeNv+WCfkX
6O4XnC4A9IeaHwAAYFxU0ZhVkKowLQtG40bR0L4QqvmrRkZJKq+ostTqU19RF2Qa3UJPxcjTodz1J
9AD2jlDdONKPUyhdf6qgX8+ngX6BpIzAIBhUPMDAACMS6NqrF6YP9tlp1FOymJWNanrUv8VNYzd
QraRFXFGXtKDGG285AxyciX2hTA9gZzo0mH+kpXdvUDDrNO6AaB31PwAAADj4i6vs1LTqowdpbIsT
e0m7VfUa7NQbo6rWriIxI1RPOwp5VRKdVUurZV/Ch3mb2i2Vvc9o+tZ1prLAoBBUfMDAACMi6yA
q9f68f66hpQtM33V/M2L6oWLN7Gfu61cSnTNb0+hw/xdxXtB3feO7unYddYAkBQ1PwAAwLhYxaQ

sIvUNR9kpC2O7SfsVx7BiXFcLoUMTzd1WpVRX5dJa+afQPjnVIlM2srK3jmEMUfAnA4D+UPMDAAC
Mi6OYbJaP+kaoZYKa3yyum206lN92Gj89nNnYypGw5m8dyu5qbUpggdktY+CF5w0ACVHzAwAAjI
urmNR1Y07fqK9W5aQuTK0rdvHqv+IfRTfxZ1aFrZ0mxJ+g7r9w7RyYQtv85XLq9i0LtBYSnAYA9I6aH
wAAYFxaiknzhlGKSqKWDZWg8op+fViOrurdjDt13cBOE6baN5m9U9b8rfN3TyenmvhG9/XsOmsASI
qaHwAAYFw8xWRdpDZumLVrVlbKwtgerP2Kem0OZ00n16ybmw08qwhqjieLZLm0Vq1TCM2/7p7L
UxrbYt52jW52zXWdMQAkR80PAAAAAMA8UfMDAAAAADBP1PwAAAAAAMwTNT8AAAAAAPNEz
Q8AAAAAwDxR8wMAAAAAAME/U/AAAAAAAzBM1PwAAAAAA80TNDwAAAADAPFHzAwAAAAAw
T9T8AAAAAADMEzU/AAAAAADzRM0PAAAAAMA8UfMDAAAAADBHf/vb/we0SIBz4Q+0SAAAAA
BJRU5ErkJggg==]]></redcap:Attachment>
</ItemDef>
<ItemDef OID="b14" Name="b14" DataType="text" Length="999" redcap:Variable="b14"
redcap:FieldType="descriptive" redcap:BranchingLogic="[q3] = '1'"
redcap:InlineImage="1">
<Question><TranslatedText></TranslatedText></Question>
<redcap:Attachment DocName="B.png" MimeType="image/png"><!
[CDATA[iVBORw0KGgoAAAANSUhEUgAABVEAAAEJCAYAAACdcmdaAAAAAXNSR0IArs4c6QAAAA
RnQU1BAACxjwv8YQUAAAAJcEhZcwAAEnQAABJ0Ad5mH3gAAE9sSURBVHhe7d0PjCRnfef/b+/aY
PPHNmBskx+RHat7sZYlGJIg08PxL4egZ+6SJbnbYOVg5Oh3PVyi0zQSkwOxF+mX2wguG4lunS5iWrq
zliQyTEJugrLdlJRgckzHR4IxYTI/Z7rlc3T8wDYGjPljB3u3f/VUPVX1VHd1dVV3VX39PtltaerpqfqqT/T
O/OZ5/k+hb5FAAAAAAAAAAChjumPAAAAAAAAAIAQhKgAAAAAAAAAAEIEQFQAAAAAAAAAAiEKIC
AAAAAAAAAQARCVAAAAAAAAACIQIgKAAAAAAAAABEIUQEAAAAAAAAgAiEqAAAAAAAAAEQgRA
UAAAAAAACACISoAAAAAAAAABCBEBUAAAAAAAAIhCiAgAAAAAAAEAEQlQAAAAAAAAAiECICg
AAAAAAAARCFEBAAAAAAAAIAIhKgAAAAAAABEIEQFAAAAAAAAgAiEqAAAAAAAAAAAQgRAVA
AAAAAAAACIQogIAAAAAAABABEJUAAAAAAAAAIhAiAoAAAAAAAAAAEQhRAQAAAAAAACACISoA
AAAAAAAARCBEBQAAAAAAAIAIhKgAAAAAAAAAAEIEQFQAAAAAAAAAiEKICAAAAAAAAQARCVAA
AAAAAAACIQIgKAAAAAAAAABEIUQEAAAAAAAAgAiEqAAAAAAAAAAEQgRAUAAAAAAAACACISoAA
AAAAAAABCBEBUAAAAAAAAAIhCiAgAAAAAAAEAEQlQAAAAAAAAAiECICgAAAAAAAAARCFEBAA
AAAAAAIAIhKgAAAAAAAABEIEQFAAAAAAAAgAiEqAAAAAAAAAAAQgRAVAAAAAAAAAACIQogIAAA
AAAABABEJUAAAAAAAAAIhAiLp0etJYKUih4DxWGj29PpjXWFeU+1iWLpWF+1iMxj3ZB2onXXjpLWdL
MxLO7LSlg372PzHqPs4Vb2GrGzEO5vtDbdtK5JH08KNuQ9Cjmc+2g0AAAAAwPwhRF06Rdm8AUy
XurU1kPcr3c5c5Hq2U3rq4B5oILBVWnqpe4faf1Qo1cT/rlhsR+14AAAAAADIGiHqip
uytmqfi4dqZ0P9kbrNc75IVW/zVuzo/9nzWcsuDRhFEcx1pWRrQm0T4e9kj4Q6SubRqw
D7TVk3fijwsI7ascDAAAAAEAOCFGXVkGW+dzmXNWfl+z9H6b8VDgAV1w6WgdkQ4+6Qjl9f1pj
Ym2ioWM2UpOJrjFHHPktb9PoicAVBqNDajmprPUJGYUb/W8ou+
AAAAAAAOAOCFGXVGW7J6VOcTWPL/Dx9Sv/ZpefdJ5hbBtwhQkZrWdyCrpG2D8YyZ6IsP5gp+WQmLPOa5ObM
+KV1Wv1/6cXXXIi2X56jXUsvCzXu85r+8MILiqR+4wr/BzteOl/krZbr3X/0hH2PRjY7rBkxzOu3QAAAAA
ALI+C9QtyXz/HlHklAxcEKq+K9SM9Kr8M8tDzTXUsI3H/0rnPl0hmxQZEj/4lcAsbzeFBhe
9ZG1BBs1YOwWq1Ks9kcWu+3Iaxdcde/kLVjrF4CdE/HIcdl0rUaYN4Uh/1Ck4SjOsL+VxdRz2+DNqd3MaFZvC
CSDHqsuL3+g4dEDBnYJUBF2fV9qy8T24rq4AaxjnsmkuQFNwaBhkaAfXOjzSs43KFwD
4UOI5FQAD7GE3vKXW/ZDnMaVN+R7TPrrFF/lhDD8DEh8x8Us0cpX8IE0gLA8GsTVD5sm7T3Yx
oi+DEN6wN2ElUPaIBFVhaB3p8v+VAKYHN3O8O0Vhtq8PdjY9lkcfg/YNvg/NOe/uWI
x3qrhxZZaW8XOWjdBiXkVUnEoG3ja9tY+/3e4B+o13rnw/T2+OB6/sPT82TFCrWlV4ZfCwnqnbcYKkmZ09wPfp
Hm4OsyX5DsAhdlk6Hz4dJLfy+AKSxPO2rX3bDKzS0bGDOdLm5hXIWgEI21Va7PkpGIvQE/Lnw2EQD

nDEt0PSK2E9ivvuaS7JjMbQdfr41qa6JzF35+FOdanQqsS3p+lETnOlHbRwht44jjjDietO4RYBr3Pnyvv
OXCW/QSACAP99/xLnnNW2/SSwCAzB3/NZFjr9ILWBT0REVwkikVtoQGJP5Qeb/npMsYdm/2GE1F
UU6ccp51dJfTrj2WvywnT5+0e9QF11uqp/0AbA74vWmb4nVG7R44gZT1GW8ov7dOdT4sGbUo3S
BMfcIsazBr1jUo6ac28z7QPYSnOKZEvYkT7cec4f+kmIdQ3NyTvhmgTm3wHIWY6rrrurFuwJmaGO0
GMvDwEw/LOz/5Tr0EAMjDDY9fJXd/6AF5/Bs/0msAAJm7dI9I/zt6AYuCEBVBA6FScsO1P6fl1RVt7k
pbenJoj9k/JScqJ6z/D65XGeqc9ZIrrombLbpD+ttuIl09a/TozUtaAZl1DTJrOyHeSKpXqBe0GnVjgQX3x
NNPSOWPKvZHAEA+nvPjY/KWL94oP3ryGWm+/3555p8u6c8AALL1lMilP7A+PussYiEQoiJlGYRfXl3
RfTlsXxS7lKgd9lbEyVeN9XMZvhk9NO3A1+/VOyrwVcO67VqVQ480e0lOK1mv47yOaTHOXbjxbW/
Lhtnr1K2LatY5BRbUnZ+6Ux58/EG9BADIwxv/
+gZ5wQ+vsJ8/9NXvyp/WeR8GgNz0Hxa5/Od6AYuAEBUxleSkN1LbnBFfMSZJitM70Ri+HI+7744c7D
pf686+X7I/4a8fLjUwH4qbZ/0guOFOjFWVQIZacsoTKG6Jgvk22OvYvA/0seV1TIn2Y97LA8Pk1cz3hZ
xnoE/SdmNSNTtwdeukJv6eAubL+z77PvlM7zN6CQCQh1c+eK3c/PXn6yXH5z7+kHzlLx/RSwCAzF3
6gsjlr+kFzDtCVMRUlM2zbuVUc/Z1NbrYnOl8y+vt5wScijnre1s23JlrYvN7cqqJpBS3XmZRF0x117vh
6vxxe81a56Kmj3+wdqsx7F+aq7LhnTIV7Onh297K+dBcNQLH9nm/hqd7bHkdU6L9mLVbm7Lqre/p
me+ta1Qq+NvI2oTnyA9c3XYDi+kT+5+Q+n11vQQAyIOqg/pzD7xELwX94W9/Tb73raf1EgAgc5f/RK
T/fb2AeUaIivgq29LyctSalHTA48w8bynX5YJR4NOfUMnSXNWB0GQ1HAPbsp55Q/a9of6KMUnTHP
Jqu9rKUt8KRKiWomxe8IdlN1d1gOaFzqMm/ZolJ3AMttM8tryOKdl+ipsXpO6/2HmtOZGTdS/7h6Br
7yrua1NNWBO03bzfw9odR+bHA8R339fvk7v+7C69BADIw/FLBXsYv/oYRgWozd+8Xy8BADLXf1Lk
8sf1AuYZISoSqWyH11+stqz17tBiV3FT9gZfW21NVr/R7K0XKBlgDM3OdKKjFJgB2KiyA+qc9bt+wOe
ya1/OW03PstS7fT9Yt1Wl1d8LTpaV1zEl2k9RNvf60h16sXq5MUzeVpHt4EGK7B96PbFTEbvtVlv6Lf
8+sqlzblwHu+5ulByOB4jBnYn/6Wfp7QQAeVr50kvl2iev1Evh/uFL35ZP//6hXgIAZO6y9RvZJcpbzbt
CX81aAgAxtTcK4nSSVCHqQGAKADGo4PT1/+318sAjD+g1AIA8lB56od0LNa6tu8vyiteFD/sHAGTgit
8QKfBL9ryiJyoAAMiVGsJPgAoA+VK9T1Uv1CTUsH7qowJAji79AfVR5xghKgAAyM1HvvgRezIpAEB+
VP3Tt33hZSProI6iAtS7/+NX9RIAIHN2fdR79ALmDSEqAADIxe6Du/LBv/igXgIA5OWOL18/tg7qKPv/
8zH53Mcf0ksAgMxd/n9FLn1BL2CeUBMVAABkTg3fV3VQmUgKAPJ189efL//8Czfppclc+dzjsnX36+
XWV79IrwEAZOtKXR/1Zr2MeUBPVAAAkKknnn6CmfgBYAZe8MMrEk0kNcoz/3RJmu+/X3705DN6
DQAgW9b77aWPWx+fchYxFwhRAQBAZlRwqgLUh594WK8BAORB1T99yxdvlOf8J1f+R7/xo/k7g8
xKSAA5Kb/HZFL1EedJ4SoAAAgM+/77Pvk3ofv1UsAgLz83AMvkRsev0ovpeMrf/mI3PtJ/igGALm5/
DXr0dELmDVC1KXWlo1CQQrGY6XR05/LSa8hKxttveBob7jtWZG8mzM3Qs5LHL3GinPuVhoy6tT55
9d55H7NjwLuWyCW+n11+djffkwvAQDy8vJvPE9e+eC1eildn/zPB/LN//19vQQAyNylXZH+Y3oBs0S
IurR60lhZlaZecpTlzFpRP8+eHfiVasLfVIImPi+9hqzXor5KXfOCrAYvunRqJSlMENguK+5bIB7V+1T1Qg
UA5Ovqp47LG++bvg7qKKo+6u//
+y/bHwEAeVD1Uf+79fFZZxEzQ4i6tLpy4KZA5bp0+33p9/dkM68MdWzgt6QmPS+qZ+S4YK99XtxN
V1vqevelWy87K5qrQo4aA/ctEIuqf6rqoAIA8veWvRvtIDVLqifqH/721/QSACBz/UdFLv2xXsCsEKIuq
96h7OuncuqE5Nf/FGmL1zOyJ41zugtquS5bFedpcfOsVJ2n0twlRQUwPTUTf+WPKvZHAEC+Xvu1F8
vLHr1aL2Vrb/f/2A8AQE4uf8l5YGYIUZeQXbvRDN2aq04tx8GuiKp3o1E7c1T9zGAtyGCd1bDejbH3r
wVreI6qN+kMVfdfl6zW52A9S6+2qH6ENy3GPgPncEP8zZjnydln0vOiqHaWYvWMNHoeB0LzkpzUn
VFl/9A6olGM9obVW21v6GMZPAcJrkvI/RZ2vePcb4PXz35E1ImNY5b37WT3p2vSa2eZ6D1Ar1TMr
49uJI6QOz91pzz4+IN6CQCQFxWevubvXqSX8qF6o1IfFQBydOlPqI86Q4SoCDUUGGl2/cyRYVRHai
WjzqrR43EyanuDNTzVurCgpuQNVXdFt3UUZ5+DwWRzdcJ9Fjflgjtk3joz5/RGeo1z3nkq1y+kUEahK
q1+y+tVOsTseTxK50C6+umwipx2N97ZkYsDJ7W96x2NX1c3yXVRQV7I/eZcj1EBZPj9pu7d0GC5U5
NSIMjOSpb3bcz7M2CCa2eZ7D0Ay07VQP1M7zN6CQCQFzV8Xw3jz5uqi3r3h75KfVQAyI2qj3qP9Z
H6qLNAiLqEKtt96Xfr4kZ7Um3Z9TH72zrxbG/4AZBXL9Won9mpyfrIxEaFec7r+3uboWUCxu4/wN+
et3/pyI6XBPWkse4GPca+Wzo1imzrKP523M2ofdbOu/Fbsn0WNy+If+rOS9v677wbglnn94JOUJOd

F4P9um2ZLK8uyolT+ukYlS23beb5V9ri53BnxMnhkpwj67VGqYGh+21ofyZj2/b9ZrYlbFt+kJ3U/Ny3
/uvD789hya6dZar3ACyrT+x/wp6NHwCQv5UvvTTzOqijPPTV78qf1hmBAAC56T8scvnP9QLyRIiKIW
bPtPoFPwgdDgOHletbE4Z54aotPxwsrp3xAqzOge4z2bsoO14eaey7suW3deeiJIl7zH364ZMh8T6Ls
nnWTbuaslpwe08Gz+8kipt740PW2PblMOpEFdfkTNjxtXf9XrVn1pzjSXSOrPOzp0NEI3gPvd4Dhu43
s8et0evSPk86CNzLYfa0LO/bsfdnmCTXzjLNewCW031fv0/u+rO79BIAIE+vfPBaufnrz9dLs/G5jz8kX/
nLR/QSACBzl74gcpkJ/vJGiIoBI3qm2Yqy5iYxIwK3UyfSDKjKcrKkn47SPdC9+VSoU/LqNhbMYdKRw9
QnMMk+K9tGr0FHOsP403RKoi+fcf2NgNIP3Kpy1j2gqa6LruEZOrw/aOh+M8JCawf20Hd33yNrsaZ
uHu/bBNduyvcALB93Jv6nn31arwEA5OWGx6+Sn3vgJXpptu7+0APy+Dd+pJcAAJlTw/r739ELyAMh
KibUkeHOgTHCo7mX3TFUvMKUSrD+5Oz05HBssVSf36vSHRZuBG7V08FeoUmYExB5PXXHCbtWR
dm8EN470w0rF3t+o8nvz/SvXdh7AJaNCk5VgPrID+h9BAB5e86Pj8lbvnijHL9U0Gtm60dPPiPN99+v
lwAA2XtK5NIf6OfIAyEqJjR/gWm53vWGbAcfk9YLHS/
+Po26n7aO1NZznJyneELGlj4tn5Sxl7S4KW5lAntYuDF8vno6/CyPP0dt2TB7nbo1OM36o0lYbdzT+xj
s/as0z83XpEi53bcTXLtoR+GPJpiWGsL/wCMP6CUAQJ7e+Nc3yAt+eIVemg+qPuqnf/9QLwEAMqfq
o15iYte8EKJiQElOusnV0EzePbnoFnIcO/Q7J6WTXtA2qm5m6ibYZ6+xrodpl6Ved5OsPCfnMa5rc1f8
zphdOfAu6QmJc0m9HrXW/XH+/I4OP6sSyOGSnCOzLqcKFN26qMaQ96TUzPKqx6k9GZQdSHa9W
p7pDpOf0CzuW0usa5fme8AU1xDz7yNf/Ig9mRQAIH+lh1448zqoo3z6v/6D/MOXvq2XAACZu/xZkf
48dRU6ughRMcCcBCnYW9IPAlXYNTChz6yYNTCbq/5Q7faGHhpuPdIev510n72GrLsnrnpWNjeNyY
Nq65JPjmrWsmzKrm5er3HOCzBj90asnBbnDulIs+ke18Bw8Amvix8oDvbcjUlvX80sH+xxaoTFZo9b
oz1p3yaRZnHfKnGu3QTvASU/dZXaee9gZMOb4h9Hze6Du/LBv/igXgIA5OnaJ6+0Z+OfZ83fvF+
+9y1qZQNAbtSw/v739QKyQoiKYeYkSJ2alHSoU/LTE7kw7YxI5vDy5uoUoVGwBmZzVQdQXnhTlVZ
qs9e7kuyzJ411d6h6Wepbav3okCq98zLMnFndbbN3TastiX+aKhIo72oZDmATnCMv2LO4x2xOsJTEi
HvXrLNaPevPNp/YQt+3SpxrZ0n4HuDXW7V41zBubVssGjV8/85P3amXAAB5UvVP3/aFl81NHdRRV
ICqglQAQE76T4pc/rheQFYIURHKHgYdUpOy2rLWu8Otp1KR7cGClfuHRs/BBOwamMZwbZddWzOj
eqgx92n23LN7obonbiCk8of1p3hehhRlc2+4Tqg9hD5hYBeYJMs6ZjsbHhT7uljH3G/5QaqtKq2+0dZ
ACYJo9r07eJA2Z5vTZZMLft9aYl07S6L3AHUsg6+ttiava4u59cTTTzATPwDMkOqBqnqiLgI1pJ/6qAC
Qo8vWb6bUR81Uoa8KBgJAEmo2fT0ZlAph96btmTxL+lhOtaYNWBfEUbp2yJUKTit/VJF7H75XrwE
A5EnVQVWTSS2SK597XLbufr3c+uoX6TUAgGxdKXLFb4gUbtbLSBM9UQEkFCxRcGZtsUO49nnrWC
J6ZB4tR+vaIV/v+
+z7CFABYEbULPx3fPl6vbQ4nvmnS9J8//3yoyef0WsAANmy3m8vqWH9TzmLSBUhKoCY2rJh17o06
pWaJQoWUK+xIqv7demmUqJinh29a4d81e+ry8f+9mN6CQCQJ1X/9C1fvFGe8+PF/NXt8W/8SO7+
0AN6CQCQuf53RC7doxeQJkJUAJNRNS8XfPx7cXMvpRq/C+YIXDvkR/U+Vb1QAQCz8XMPvERueP
wqvbSYvvKXj8jnPv6QXgIAZO7y10QufUEvIC3URAUAAKEefuJhec32a+wJpQA+bv568+Xf/6Fm/TS
YqM+KgDkjfqoaaMnKgAAGKKCUzWRFAEqAMyGqoO6aBNJRVH1Ue/
+0FftjwCAPKj6qGpY/7POIqZGiAoAAIbc+ak75cHHH9RLAIC8qQB1UeugjvLN//19+cPf/ppeAgBkrv+
oyKU/1guYFiEqAAAAIUDVQP9P7jF4CAOTttV97sbzs0av10tGyt/t/7AcAICeXv+Q8MDVCVCTUk8aKm
uVbPTaknWjdOGltZ96MOYZeQ1Y2gmvbG+7rV6TR0yunpfZjb3NEO2zTne9M2m1bkPugvaHbWJC
BSwosjE/sf8KejR8AMBsqPH3N3x3tuqGqN6rqlQoAyMmlPxHpP6YXMClCVGCGeo0VKZRq0tHLWe
pd3DH205RdQj4AA+77+n1y15/dpZcAAHm7+qnj8pa9G/XS0aXqov7+v/8y9VEBIDeqPup/tz5SH3U
ahKhIqCibe33p99VjWyp6bXby3l8WRhxDryHrtTziU6UnF3eC+2qea1hrF8VRuA+A+aZm4n/nJ98pT
z/7tF4DAMibClBVkLoMVE/UT/7nA70EAMicXR91Vy9gEoSowDLoXRQ3Qy2Xy86Tzo5cXJwUdTFUt
qXfrYs+w8DCUMGpClAf+cEjeg0AIG8//ffX2UP5l8m9n3xYvKX/NsDALm5vGc9mOBvUoSoS8eoLbl
i9kQctd6oc2mvz7s25fD+IutuGnU/VwKfNLcT9vko6Z8z+/PmMP7mqvP5EYU0/WNWj+T1Rv2h/FU
5e+GMDvk6shMnRQ3UUg17jG5PvHbHuTbD59DaumyYrzfqkQa2MbA+/BTHaYN5POpY/P2rh7vd
9nnrupbrsjXQXdYu3WC83n4M3DfArKgh/A888oBeAgDk7YbHr7Ink1pGd3/oAXn8Gz/SSwCAzF26R

6T/Hb2AJAhRl05R1s64PREPpOs8s3TlwE30Aj0U27LbdJ6Vz6xZXz17ldNV/awjB/4B2PywsCxn1nRr7
RCwJIMj5zu1UswQa5bnrCO1UkFW9fYcal2SINUYyl89LZXimniHs3Nx+hCvfEbcU+2L2e6pr43Dfn1w
Z846FVQOrG+uDgSpE7VBHcuqeFvWoakKSlf369Ld2wxcdxW+lsJKN3RqUprnibKwFD7yxY/Yk0kBA
GbjOT8+Jm/54o1y/FJBr1kuP3ryGWm+/37qowJAbp4SufQH1kfqoyZFiLqEimtuT8R9OXQTovauHwi
JEU72Dq1XOU6dmIcI1VI5LW6M2gzMjmSEhV6w15PGutvjsyotu6am9WjpLXRqsh4jjUz7nFW2rTa
Yw76rLadd22HVPv12d+vuV8TsRaoYQ/mrp9X2zVA4xpD+4qbsuefNfnTFa4Z1BPULwcDQN67d6V
wbV7neDX69Frbev2+maYPxeh2aFjf3vOc+P1RXYWtXf41/TppyLsFxAmnafXBXPvgXH9RLAIBZeONf
3yAv+OEVemk5PfTV78qf1h/USwCAzPUfFrn853oBcRGiLiOvJ6IfaPUOndivWnXCo32dFJrDwO38bS
5UZMsNoJq7fi8+s+6n2wPUXFff8ickqmx5QWCs3pgzPGfVlj+Rkh/mWi0Z7IY7gtk792TJfmJsJ0EYq/
Ua636vzepZ2QxPUMe3O61rY6vKWbchRsg+cv3+obPdKdoQeH0UI1Q3Q2s7cNWB6t6okwhkSA3f
v/NTd+olAMAsvPLBa+Xmrz9fLy23z338Idn/n4/pJQBA5i59QeQyf8BKghB1KRXlxCnnmRtode1x6W
U5efqkHXYF11vUMHDn2VzwQ7mmeJ0KuwdeWOgN5ffWWcfkDu+2H8bw7cAQ/VFmdc784HMy
Yb1zLRMP6W/LeT9BlVZoz1klRrtTuzaW8kkJ3d2o9a4p2hC7Z7Zxrq2N2WUO3P3Er8sLpOuJp59gJn
4AmLGXfPe58nMPvEQvQbn7P35Vvvct/m0CgNxcVvVRv68XMA4h6pLy6oraPTl74nSqPCUnKies/w
+uV3ngPEWoFiOYcodmt90x0xG9I6exkOfM6GmphqaXQkPC+LP0tzf8OqBVo6fp8kkSbhdl80L4jP1u
cDtiPjEgE+5M/A8/8bBeAwDIm6p/usx1UEdRAWrzN+/XSwCAzPWfFLn8cb2AcQhRl5U3tHlfDts6aL
N77VXEyQqN9VP3hsyCUdfTDi/9upOjwkuvNubQI2YYuIDnzB/KHyXmkP72hj9RVLUlIzuhTmDqa5O
CTNtg1JUdKNlqa55jln7k532ffZ/c+/C9egkAMAsrX3qpXPvklXoJpn/40rfl079/qJcAAJm7bP02eukze
gFRCFGXVklO2hlkRw52nSHNbh3Rkv0Jf3347OuzV9w864eaDXeSp4E6pCVnqL3i1eKc2KKdM2Mo/
xjjh/S3ZcNLUMtSV1PRTyvVazOhHNugZuhXPU7tScXsQNWYoCtu2QJgSvX76vKxv/2YXgIAzELpoRfa
D4z26f/6D3aYCgDIyeXPivTp2jMOIerS8ntyNptOOObWeCzq4p/uem+Sprnj9gDtSK2mA77BOqRmP
crmqj9sur3h1aUsxB5LvWDnzBzK787+H3iYIV70kP5e45w3jL9cv5BOuYRUr82E8miD3pbKoIM9Trvi
ls8dW7sVSMn/+v/
+l34GAJgF1ftU9ULFeHd/6AH9DACQi0v36CcYhRB1iZkzpqvehd7w88Ds5sYkTXPIq1NqC+shGaxH2
VzVwZg/LI1GT440LNVzVtS1VJXmauqhoTmUvxxaW8AoiWC9cuSQ/l5D1r0CqtYrAxMwOY/JJkhK99
pMJoc2VLb9IfyBurRGfdmzm7MP3bEU7v7Fu+WOl9+hlwAAeXvjX99AHdSYfv7dP6WfAQByUXijfoJ
RCFGXWWDW8FPiTzbuDltXzPVzyAwvRw2ht+tRGr0uXeW6dJPWu0z1nFVke7BA5v5hSrUxzaH8o0
NdMxQeNaQ/Xl3VCaV5bSaVQxvsIfxhxVBVSNvvp1pfFohy1RVXyf/4lf8ht1x3i14DAMjT373yCf0M
UV7z1pvkbe+5VS8BADJ37FUix9+kFzBKoa/G9QIAgKXxwCMPyOv/2+vtmfoBAPm648vXyysfvFYvYd
D1P/E8+a1PvVGedw0TbwFALgovFrni/daTq51ljERPVAAAlsztN91uD+0HAOTvb27/tjx2PX/ECnPlc4
9L9fdeS4AKALmx3m+Pv8f6SIAaByEqAABL6F2n3iUfeMMH9lhxJ1TZkODL27LhTbbiPCabqGZYeyO
43dBH1jOAAwAwhz788x+W07ed1ksAgLz84PnPyt7rvqWXoLzidS+R1X8bNhkqACATx4oix39eLyCO
OeiJqsJVf5ZqR84zwquZZ0VcaKU3oAwDA4rjnl+
+xh/cDAPL10M0/kO6t39dLy+3al14l1d99rV4CAGSucI3MTWMHH0nkGKIWDCWjDtj/3VXXafXAADyonqjfu+aZ/TS8lIBqgpSAQ5Of5ukcIL9QLi
mn1P1N6h7OuncuqEZJedlqXedUNc/9Gtl/XnRZq7pKgAgOVzy3W32EGqClQBAPlRP9X6/8xivsofwAgJzYdVD7zrl4NOQYog7XRLXrlZZq4nZEtYfVD9UoNb/OeaRVL1Uprp0RP0YFAGA5vfm
WN8tH3/FRe9gsHRoM5E9y3mM+TzHOSuqrnaWsJPoaY68ISIrFWjqX9tvfVuL6tH6ZaBnyndC

TQoGTGKR76eM295ryPpg13tgBlR9VFUnFQCQnx8/57J8/g2PLkV91Le951Z5zVtv0ksAgMwdf5PIsV
fpBUxqfkPU7oHXE8sevu/1HjWG93cOpKufTsfppZpmrVUAABaVmqm//attZuwHgJw9dv3T8je3f1sv
HU23vvpF8ku12/QSACBzhVtEjv0LvYBpzHdN1FSFz85vlhPo1M7nWItxHgxO2jWqxuPoOpD20GPv6
/VjZG3Jwf1FBNd2Pdzga0fVgsysDVEya98oca+VK/5xJm1f2OsHyncO1O806o7arx19P/kG2p/K9Ux6
DsOEt32wXungORo8P2HiTbTn8/epHqPqpCY5P0DQbdffJvf88j16CQCQl7+/7Xvyjy//oV46WlQd1O
rvvVaufO5xvQYAkK2rRY6/2/p4hbOIqSxEiFqud4fDT/uRwnDa4qac1WVWRfblcGnyBRVsDU7a1ZTV
wqr1/3hUiFMKG3rcqUlpMKBKMklYe2NETUjVYzgYFmXWhihZtW+khNcqwXEma58TyIW9vrlaGBH
2qXNitLNcl/GVM9SxDbR/6us5/f0ej9OrffAcNVdHhZyTcPbh1XO2Dd97qd3vWGrvKL6DGfsBYAb+6v
WPHcn6qL/yH15JHVQAyNPx09RBTdH8hqilk34P0YN0Bu2Ha8tuuinKQug1zvnhkTuhV7/r1Zsdzzhv
5bp0dbDd9TbQlHNeotNLMEmY9dpzesOh2+3IzkX3tVm1IUpW7Rst2bVKcpzJ2tdrrHuBnP+HjZa1F
615bkRQaLRjb1OKem0koz1u06e5ntPf70n47fHb3pHa+ejIPHqivUH+PsLvvbTud0Ds2frfdepdegkAk
AdVH1UFqUfJyumftB8AgJwce53zQGpSC1HtnmjGkFHzEWco65DimpzxspxVfxuqF6C77UQbdnpv
me1yHmYvuTOyFivhWXQ9uejO2qUCFi+kKcrmBSPEidI7lH39VDo74mY3xc09J6yxHnub+mQmmiT
MmLjHCNyKa2eGQ/XM2hAlo/aNlPBaJTnORO0LtuOst74iW/aGVVC3J2GHE2hHTNWz/rmtbPtBrd
/2JNczhfs9gWrL7yFf2Up/+4q5j/B7L8+J+bAM7v7Fu+WOl9+hlwAAefjmjU/JV376u3ppsb3sp14o/
+a3mNAEAHJTuFHk+L/WC0jLHA/nDwYcXkjrjWE1w5B0mMHN0daVAzdTKp+Ukn5qK56QU/ppJDP
kHgioh2ouTjVJmK6nGTZ8Prc2REmpfSMlvFZJjjNR+0a3wwldR5fWOHUi6XdVVU4HNlaSk2473bYn
up4p3O9zpSwnAwcRIteJ+bAMrrriKmbsB4AZuP9V37HD1EWm6p/+
+n/5GeqgAkBurhQ5/mvWR+qgpm2+a6IWN2UvbMitPdQ3hXqoHtWLri8pZ7JH3OhefG5oM1EPZ
MWu5eiGPlE1KzNsQ5R5b19sebQvRuA3VlFOLEzSmcbxAvPpphfcZAepKlAFAOTn8yuPylNXX9JLi0f
1QFU9UQEAOTn+r0QKN+gFpGmqENUc9hv1cMLJoj8M2gxA7aBUrw9NMc2v04+4dRUtdp1B82tD
H2kGskvEuHZ+7Udf89zw5DXjJwlry4bZq9OtjWnWijRl0oYo2bcvLbGOc4btG21wgreeHHp1B4ZlOv
HcEcD5QZpuv+l2e2g/ACA/KkBVQeoiel3l/6lOKgDk6dhrrQd1ULMy3z1RkZGQ4dEus05mDGp2d9V
b0Q+rjZ7D7raTTBLW3vV6dtrhjxuYG8OTB6XehihZtW+khNdqguOM176Idth1itOdfT7Y9JDh+ImO
M737fWHkNjEflpGaZOoDb/iAXgIA5EEN6f+7Vz6hlxaDmoWfOqgAkCM1C7/qhYrMEKIupaKs+bN2
yao3Ztuc0XsMPcGXKlEb7K0YEnhNOEmYH/4YM+KbcmhDlFTbN1LCa5XkOBO1L6Id9rE7NVUTnsK
Rmqsb4jfdL5lQPrPmhNaJrmcK9/uiyeB+B0wf/vkPy+nbTuslAEAeVH3Ux65/Wi/NN1X/tPp7r5XnXX
OlXgMAyJaqg/oe6+PVziIyQYi6pIqbF/zehs1VHawYk86MU9n2h393alJygxmjRqg/UVew9mbkJGGV
09aSNq5dWbUhSmbtGy3ZtUpyrpO1b2w7ynXZSmV8eFnK5aas6vZ4Tbfaftab/j/Z9Zz6fs+LOdGV28
6Jws6U7ncgwj2/fI89vB8AkI9Lx/vy+Tc8Kj9+zmW9Zn79Uu02ufXVL9JLAIDMHVsTKdysF5AVQtSlVZ
TNvcFJu6rS6res/8djD/8OK6Rpb2dgoi679ubg/ixDk4RVZHuoDc72vF01d71eitm0IUqG7Rsp4bVKcJ
zJ2qfa0Zfu0IbVpo3SBlM7JWcHjzfsGiW6ntPf7/mw7q/B67F/aPQSTiCV+x0YzZ2x/7qrrtNrAABZ+8H
zn5W/ev1jemk+veatN8nb3nOrXgIAZO7Yq0SOv0kvIEuFviqCCAAAMIF7H75XKn9UkaefXYwhpgBw
FNzx5evllQ9eq5fmx7UvvUr+06ffzDB+AMhL4RqRK9R8BQzjzwM9UQEAwMTefMub5aNv/6heAgD
k4W9u/7Z875pn9NL8qP4udVABIFfH3239jwA1L4SoAABgKu/92fdK2ZLhae/8Osn9BIAIHPHiiL
H36EXkBdCVAAAkAo10dQt192ilwAAWeve+n37MUuqDqoaxg8AyImqg3rsPXoBeSJEBQAqVAz9b
d/tc2M/QCQI9UbdZb1Ue/6T6+2g1QAQE6O3SlSeKFeQJ4IUQEAQGpuu/42ueeX79FLAICsqbqon3
/DozOpj/q299wqp/7ZDXoJAJC5428SOXbnUZFEBAEcq3lF8BzP2A0COvv2if7Jn7M/Tra9+/kfxSjV
/kASA3hVtEjv0LvYBZIEQFcteWjUJBCsZjpdHTn1tSvYasblT1gqO94Z6fFZnX09NrrDhtXGnIqCb6x+E8
lv5aY2mo2frfdepdegkAkLW/v+178o8v/6FeytbzrrlSqr/3Wrnyucf1GgBAtq4WOf5u6+MVziJmghAV
yFVPGiur0tRLjrKcWSvq58vHDiJLNeno5YXRa8h6LarV6loXZDV4saVTK0lhIDAGjqq7f/FuuePld+glAE
DW/ur1j8kPnv+sXsrOXb9zu1z/E8/TSwCAzB1muW6ffThxRZ9SxQeScUj1nxwW/7fPiHlq1pa5zX7r1srOiuSrkqFgGV11xFTP2A0COfvycy5nXR33zr9wir3nrTXoJAJC

5YyvW41V6AbNEiArkqXco+/qpnDohy9v/dHHF6znbk8Y53QW1XJetivO0uHlWqs5Tae6SomI53PSC
m+wgVQWqAIDsPXb903L/q76jl9L1sp96ofzKfziplwAAmSvcKHL8tF7ArBGiLjGvnqP5CKntOLI2peqN
536d163Or/dp135sb+ivNdbZLwuuD/bKS2MbmtlG7zFcYzN4jMM1S+1HWN1Low3jal3a+zDDt+aq
87WDDQ9pc9i2o9o8tpfjROdl+J4ZuZ8YxxD7fGh+W9RjuK0OZwi9/7p0z506/lKsnrNGj+NAWF6Sk7
ozquwfDt9PNqM9ie65eMduS+H7wj1Pcd9HsNxuv+l2e2g/ACAff/fKJ+SbNz6ll9Kh6p/+
+n/5GeqgAkBurhQ5/mvWR+qgzgtC1CWlwpHQMKhTk1JhQ8ZlcHHYtR8HCkLa61TIMrC+uRoeXk2
1DRU2hfYY7EitNCqEU58zapaW61J3uw52duTiYMi0674ynbqmQ8GiZh/zyGBquM1uz8dQE5+X4X
umuToieEt8DFGcfQcvd0hb7WCw5A2hd6V67jxVafVbXq/SIWaP41E6B9LVT4MqcjrpPZfk2FP6vlDn
KY/3ERwdapKpD7zhA3oJAJC1z688Kk9dfUkvTe/f/Nar7J6oAICcHP9XIoUb9ALmASHqUmqLn8O4d
TmNeo3SlHPhSUpi5XrX3na/FYybwtaPGt482TaCw6mHj7EjO4PplEcFZM7r+3ubsrlVF+erBr/GPI9nZ
FyGWtm2ttd1t2Wptpx9bOvUrr3hB4Vhbe7UZH3kdQm2eXRT0jkv/invSO28cd0SHMPY8xHg7zu8r
dZxrbvBoHEu3Iamcu40u53bEitrHVKUE6f00wiVRPdckmNP7/uimOP7CI6OD//8h+X0bQxHAoA8q
ABVBalpWDn9k/YDAJCTY69zHpgrhKjLyOwlZ/R0K27uOQGJ9dhLZaajqpx1t1M5bS25RqwPHd486T
aKsrlnBj6O4toZL7TrHIT3AyzXt4IBWXFNzugv6uxc9NvY3vV65pXPrI0P38YwexjWLxht3rwgfgZ5PrR3
31CbR5r8vFRbfnDoh3xB0xxDFHPfoW3tXZQd3SEycC4qW/5+zWtniH/u1HFY3yOhIe8k9uUwrEFKk
nsu0bGn+H2R2/sIjpp7fvkee3g/ACB7akj/V376u3ppMqr3qeqFCgDIiV0H9V/rBcwTQtRlZAQ0ImqY
bsGrZTiyhuIkyielpJ8GjFofJo1t2HQ9x9BhzEGnTgwGP0VZ8xMtLyzyA0Mj0J1YVK9WY/8jgrfhNscV/7
yMN90xjFaWk+MudvfAa79X7sF+GEPcRwyfn/zcTeuUjN51gntuimNPcv2HzlNe7yM4ctwZ+6+76jq
9BgCQJTXJ1KT1UVX907t+59XUQQWA3Kg6qHdaH6mDOo8IUZdSUTYvhPckdEOYqMl1FkZg8hyjn
mOk8MDO76nnDnc2AsPq6dg9GafXkeGOgjFCRlOK52UyYccwK2keVxw9ORxbLNWR2T2X2vVfkvc
RZOKW625hxn4AyNFfvf4x+fFzLuul+H6pdpvc+uoX6SUAQOaOrYkUbtYLmDeEqMuquCl7esjtQKlR
W/Pcos+u3ZYNs3edW7PRrMGZhHW+zurzZA+NNoYyV0/nF6FOH/qlfF4mkm1w6dXKHXpMWsd0
AsUTMrb06bje1BPcc+OPPf3vi6P9PoIsvfmWN8tH3/5RvQQAyNIPnv+sHaQm8Zq33iRve8+tegkAkL
ljrxI5/ia9gHlEiLrE1MzaqqeYPcGPHYR0vRqKo4f/GoxhxHPHrB2pgiW3/uMUba64U6Z3duT8+R29n
aqkk6GW5KR37gdnZO/JRbfoZeQQ8BgyOC+
+nI4hTOmkFwKOqumZL+NcNHfF75DZlQPvNJxwzn2EWPdckmPP4PpP/T6Cpfben32v1O6o6SUAQ
Jb+8eU/lL+/7Xt6Kdr1P/E8uet3qF8NALkpvFgP48c8I0RdRu0Ne6itmkU92FPMCHiMXnIlPxkzZmJvy
4Y3Dft884MlY2bySXgTWHWk2dQnKrWh/EXZdLsdqvO87l+XXmPdq22ZZBKkcVI7L578j8Fj1udsrvr
DyPW9bj9yHVtu1oBtyq7eda9xzgsxY/VgjnPPTXjsU1//hO8jZntyvRSYe6o3quqVCgDI3t/c/m157Pqn
9VI4Vf+0+nuvleddc6VeAwDIlqqD+h7r49XOIuYWIeoyqmz7Q287NSm5QYtRH7F6NnzmbhXSDL52
Lpkz9nttNibamUhF3I6BrlSH8o+4LiU/fZQL005glcl5MSQ9BnPYu9ueiRK2YH3O5qqz34IX9Felldqs+v
EUNy94PTLd9njnodqSeM2Jc88lOPY0r3/C9xEgiqqPquqkAgCydel4Xz7/hkcj66NW/u8idVABIE/H3k
od1AVBiLqk7KG3YUUMVeDS7wcDHlX3cLBmYrWVcx3NpCqy3W/5gZHNOTbvsAPDrOPxhlcr5bpsp
ZzL2dcl5LxWW9Z6d+j1VLI5L6Zkx2C1Z/A+3D80ejUmYNfnNIaSu+y6nznWQ/UUZXNvuFaoPYw+Q
aAb656LfezpXv9E7yNABDVTf/tX28zYDwA5UPVR7/uZx/VS0Cte9xL5hV8/oZcAAJk7Zv2GfPwdegHz
rtBXRewAxKNmNtcT86gwbG/anqHAOEfpntPHcqpFwIpwn+l9Rip/xM0BAHl441/fIKWHXqiXRK596
VXyW3/8z+yPAIAcFK4ROf5+66P/Xoz5Rk9UILaeNNbdmc3LcmaNABVZO1r3XPu8dSwZ9ODG0fGO
4juYsR8AcrL3um/J9655Ri+JVH/3tQSoAJCn4+8mQF0w9EQFxmrLxmANWFXOgK50yMzRu+d6jRU
p7ZyRbiplKXDUFf6fgn4GAMjStU9eKe9s/aS88723MYwfAPJ07O0M419AhKjAWAOBFgEqMsc9h+
WlJiiblx9NaEs42hKOtoSjLeHmqS0nrr1GDr/3pF6aLa5RONoSjraEoy3haAvSQIgKAADmBj/ghqMt4
WhLONoSjraEoy3haEs42hKOtoSjLeHmqS1IhpqoAAAAAAAAABCBEBUAAAAAAAAIhCiAgAAAAA
AAEAEQlTkSE2WU7DrfxRWGtLTa8fqNWRlo60XHO0NvZ3CijRibwjp6Uljxb0GG9aVTbJuGmH3UNr
7mJbRRv1Y4SYFAAAAAGChEaJirvUaK1Io1aSjl4H5pgJdY1Z9W1nOrBX1cwAAAAAAsIgIUTG/eg1Zrx
GfzqeibO717RkF+/1tqei1szFPbenKgXvLluvStdu0J5tkqAAAAAAALDRCVABIS+9Q9vVTOXVCyE4BA

AAAADgaCFGXmao1OlC7MazG6GD9UXuIvfE1A+VKNbNOpXokq1Vp79Mcxt9cdbYTvjOjjeoxqk7qY
JvCa1VOdrymMbVf2xvetob2H3JN4rTRY359ZEONcxFo46j1xj7t9ea5zKoOadx7KKotg9uYsD5pjOuS
9J4FAAAAAACLgxB1WakgL7TWaEdqpVEhpPpcQUoDQ+ybq4OvVyFiSYIva8pqYbBWZBqcNq0GNh
xyDHYlNtgm65W1UnjQaYt7vIMqcrqqn3Z25OLAa9u7bmODtTKHQjgtuo2TKsrambLztHMgXeeZxR
iOHmh7W9xml8+s5dDDMoV7aKJrPizf6wIAAAAAAOYRIepS6knjnJuIuXUb+9Kt61BNOrIzmPx5qtL
Sr2+5QaEKG8/7ve16jXN+0FVt6VqVXfE2H0Nl2/qabl28L3G3sx1W8dJvU/gxWMe77oZg/mv77gF0
arI+MhUdf7xhKltu2wfPpR9GSvmMeBlqe8MPgsOuSWQbJ1NcO6PbuC+H7qbbu0ZI2ZEDN101hq
mfOpF9hDr9PTTNNTckuC7J7lkAAAAAALBICFGXkjERz96m16vQD9VEOl56FlRt+RP3+EGhqScXd9w
+e1VpeQGStc8LYa+fntmm0GPoXRS3SeX6lj/xUGXLC+U6OxdDexSOP94RimvidfQ0t22ElGaPTrN3av
2CcU02L/htrJ0fGKo+Ja+NftDbO3Si0mrVCRv3dbrau7jjBZKnvROYlRTuoSmuuWkm1wUAAAAAAM
wdQlRYdA3P0OH9SZmzk5+Ukn5qK56QU/ppespyMrCTEN0D77jsIdheXUtjqHdgSHsazOHy/rB4P5
SryllvyvYRvVNtxnbMHqOpKMoJfUHcwLlrXzzrnJ4+aYeVwfWW6mk/kMxMCvdQKtd8VtcFAAAAAA
DMG0LUZRWYKCdunckYgeWRMt3x+r1i3Z6eRig3URhpDK9PScUt3trctVrXE6cj6ik5UdFhZWC9anb
2EeriSf+6AAAAAACA+UKIupTasmH2OnVrPZr1Hl+ocr3rlDEYevjD9lNT3JSzbglONXTcqCs6WRiZQYh
dOS164L4ctvUQeLv3pzs5lrF+QUP07K/5sv1xAQAAAACA5UOIuozMupwqYHLrohpDoCdXkpNuEjs
4XNoIEXNVcoamK6NqvWbF6+nZ2ZHz50fVFTXP2eBs/mZ90FMydk6nxNfQ3XdHDnadr3VrtZbsT/jr
h4e0ZyWFeyiVa57idQEAAAAAAAuNEHXJ+QGTMWP/VMxakU1Z3XCn3DFnS8+ZMcmTNFfFa1J7w
6+V6a1MmdfTsyPNpj76oaH8Rdl0u6xar6utN6yz5eg11r0anuYESU7AqVivP+8dkGx4U8nH5V+vZtP
5Wnf2/aIumOquNyfCylYK91Aq1zz5dQEAAAAAAEcTIeoy8oI9S3NVh0rGhDtTMmcun2r75iRC7nY
mCjuDs7o3V3WI5gWO5gzwaXOHxftCh/JXtqXl5XU1KdnnrCAlP6mTC95EVCojdOutWrxzHLe2bVBg
W9Yzb2i6eZ9Y68/k0w3VNv09lNI1T3hdAAAAAADA0USIupQqst1vGQGZUpVWv+8HRvaEQpMqyu
Ze1w/BbGr7g/scx2qn1yBt/9DrDZhIcVP2+oNtstj1YDOoh2rwhvQr1v62Ruysst0PrUtbbVnr3ZILLnU
8g6+ttiara2v22gwMTTeGs+c+ZD2FeyiIa57ougAAAAAAgCOp0FczrADITq8hK3oiL1WDdo+eiwAwk
urtPS8/mtCWcLQlHG0JR1vC0ZZwtCUcbQlHW8LRlnC0BWmgJyqQKbOOZ75D4gEAAAAAAJAOQlQ
gE23ZKAzU8ayeFTqhAgAAAAAALB5CVCAPql5pZpNXAQAAAAAAIEvURAUAAHODelXhaEs42hKOto
SjLeFoSzjaEo62hKMt4WhLuHlqC5KhJyoAAAAAAAAAARCBEBQAAAAAAAIAIDOcHAAAAAAAAAgAj0R
EV2eg1Z2WjrhVnrSWNFzZavHhsyL60CAAAAAADA/CNERSZ6jRUplGrS0csAAAAAAAADAoiJERfp6D
VmvEZ8CAAAAAADgaCBEBQAAAAAAAIAIhKhLqr3h1gddkUZPD7+3l51HaClTVePUeI3zcL7eZW/XH
MbfXHVepzc4uF+PuW1j58HXt2XDfY318F4Wo12TGjwv6jHpuVGSHr8S1obCSkPCD8+s/eo8VkaciG
TbBQAAAAAAWF6EqEuvI7VSQUoDw+
+bqwMhX3tjRI1T9fXpBJbR1H5WpamXpFyXrYr1MbN2OWHk4HlRmquFYNCZ4blRoWtYG6RTk9Lg
BFl2EFuSwZd3aqWhcDTRdgEAAAAAAJYcISosVWn1+9K3Hq2qXqUCwPNulNaTxjkdX5br0tWv7dbL
zjrrtTsXnYiusm19rlsX9zNSbdmv7W+rxHNafjv7e5tSTNCupHqNdS+MLNe7zj77LasFWvOcDkeza4NI
W3bd1Dh020055yW0VjvW3SDXOE/uBe3UZN17bZLtAgAAAAAAgBAVUm1tixtxVraMANRTlM09H
crZ4aVeu3bGe23noKufZadc3/La6ciqXT25uOP20qzK2U13yxXZsoNGFVLuibM6w3PTO5R9/VQ6O+
JmscXNPWd/1mPPbVvvorhNDpynypa42Whn56J1ZJYk2wUAAAAAAAAAhKiaha5OGDmHPzqkT44K
9tNrVlQN3A+WTUtJPFSdo9EPnYSmem+KanHGTWGtrquyCW7t0qM5p98Dbnz18X7+uYA7v7xxYR
2ZJsl0AAAAAAAAAQoqIsJ82UcJTAxElGbdLcjGjnzNtlyawNRdm8ENYz2A9KQye5Giur7QIAAAAAABx
NhKiIoS0bZs9Kt46mWft0JuahXRm3obgpe3qIvV+v1tc8NzybvI/DdfBh9KCdYLsAgLzp0Q3Ggz9yAQ
AAALNBiIrx2rte70o7oHNrfxpDyKc2ybYya1dJTroJqDsE3qVm4i8YM+6n1YaI16uZ9NUvzfakXXbw2f
XqnHrtK530Qtu4NVhjbRcAlpH9Xl+QwkwTy540VlZFWu57tPNHr+bqLILU+Qxze40Vpz0r+f/hT/0ba
p4P9zEP5wXDhq7XDO6ZmRvzvuZ9P7mPvG7mOO+3ebwnR+5jSf+gFfO8z/K9eGZinJuh951luGnG
nJfc34sTtofSdoiDEBWJ+AGdMSt9AiU/nTRm/7d+MFmdbhD8tO0KKsqaVzS0Kavem667baeO6OB7
cZw2JDp+/aavPhXsGRpSs9Wsc9pc9dvm/sOhHu7KJNtVjG3wDwuALMzsl/chKri09j/lv0npcCYu3Da

KcLuTP+4f5vte3N4IC3ONPyjOQq8h6zWRatX9x28G3NEnxsO8XrMy01/cAyWOhh+z+NZW52O1qS
YFda9TS6qdmpRmFPjk/0v7+Pc19R5csr6f6l3jHFk/T2Z778R5v83jPXncPtTnc/yDVmTok1eYm+C85/l
eHHFuht739CP98xP/vl3dH/g3Iot/IEadk9zfi8efl3zfiydoj/WNrUrbpf+eHO/7dn5+FsZY1g2DJWS9R/
TV5bd+A+hbPzD5uvW+9U+g87mq9ZZia/WtnxX060c8vNcqIa93P29uf9TD2NbIdtqStKvbr5fd9VXr
K8cxXx/yKNetVygJz83Exx/
+MDcdve3gMSfabqvqrS8PXwQAmEq3XrbeX8z3eP2+GngjyoffFv1vwAzaEEm/z8/8vXjm7XCuj9q/f
c28f5PzY/87OoP9RtP37dy1y/3eivPzV9rCv5dn1R7n5y9jv/pnrCy/l8a/rznvuYNtyPocjW9XvNdMa6J
9ZPIeqPdvbdd+hLRD3T/maud+Um3XK1IS/5w4n8/+vTjeucnjvS/
+fZv1+8v4cxImq7aNPy/h62fWnhHfw+nfR87+zd0737fBdX579Qr9vhz3uiJf9ERFDBXZVn8p0ksO56
821puAo7kr/t9KrNd7n9D2D52/MKlanIP1QqutCWuIJm1XEk4PIOsNTS/7rDdbf9h+0jYkPH57qP3g
ubQ5+wj8QdOuc2oMyXfZPWWMeqiWRNsFgMy05XytY71NXZBN503VUpEt9UY28fv35Iqbe9Lv7x
ltmTO69MupE/PRwFm1o9dYl1qnKmfn9kLNhnteWt7PKPPC+T6X6unAzyL5KMqJU9YH9+dQW08u
7sygPb2GqIFK5fqWv9/Ktv3zYmfnYgY9sRxj39d6h7JvfRj8fi7aJ64puxm9Ecd5v83jPXle3ved79+y1Ls
hP8tr6uf34dEJHdm5mO7dE/ec5PVeHOfc5GX8uXHeXwLf5xmY7Jxk9148/rzk+148tj0jfp6yR42mW
tYuzqii+fpZGOMRoi4pvw7mwJuLMeFQcMiBCgvdr1EPJ5TztxMM6dQPhf5rrYf5A725D/VQ+xmx35
Ht9MRtl/MGFlw3nvMG7H6d89gbakjCc5Pg+G2D59J+jDoG8zj1Y9QvU3G3a7xu+NgBYAoz+uV9M
enSL+W6bIX/A5CTnjTWa9KZVTvsoaMdqbbi/1ueGTUM0Rh6N9uSN/n84j6JXuOc9d1s/bI/oxu3sq2
HjNo17dWwypITNg/
+vJW13H5pT8huV1lOenWcNKPePgZk8AetuMFlmDTbEVuO78XTnJv86fJoB+cHhtOnWwJnknPCe7
FhxPub8/PnvuRaNYmfhRcOISoAAJgNfnmPTdUltX/5uTCLXoa6tpj9i6D+pWdGvR3b52vSqbZmPmL
C/0Op81AjV7KppRZXPr+4J+f2NDo7wwDEHTWkatqr7yOrObMI4efpl3ZMgT9oKfPyXhwwR3/Y6uy
flAvevxGqt6h6/9mYYa9C3osD9HwiwflBrO+pqedWiWHwjzD8LLxwCFEBAADmmDP5gRq2N6ueO
MFRDt36vqzO4hfU9oYzCcRc/dbuUL2Csh6WHcfc/eLePi/O6NHZXTNnsg5/YiAVeKtJgXKftGOWv7Q
jNfxByzKH78Xz9oet8pk147pY/4aeVWXUZtirkPfiAernGrdnrP89dXBKXadTkl3n7nn5IwymQYgKAA
Awp5wAVfXWmJ+hjMXNC3YNtk7tfK7hXHtXhU1NO8B1fuEpSEn9Vqh/CZrtcHptlsOyLfP1i7sOCGf5
y6Iecqzq2bt5jz0MViXezdWMZjYfZVa/tI9h93bqyMHgjTuqd9QS4w9ajkV4L56XP2x5ZtqrkPficIMl+f
qydXLfOk8nJau3vdn+EQZpIUQFAACzwS/vkVSvDSdAndMJ/zL8RSPMYE8j9bAngLR+Mexaz2dbt7sn
h6qoWc7nZKxZ/uLu9nw6O8NfFgeHTbpmdl7y/6V9rOIJceZ7CUZNPfuGnmG4O2f4g5Zvvt+LB+T+h
62S2GWOB8PbWf5cw3txTLq2eOCPkekZ+UcYfhZeOISoAABgNvjlPYKeRddiD3czevzkW+dSTf4wM
Bxc/0KW1S8ac6/XkJUVc0i2WqVmSp7lL6nz94u701utKjMcPSpSOW21wPoeCgyhV7dwzTovM26bL
dtf2uOpyGnVY6+27r+veL3G5m+islngD1qLaFZ/2NIjADo1Oe/9w6mHcM+oHinvxXGoUhklqUldLm
RwkSL/CMPPwounDwAAMCOtqvSt33L69a5e0a33y9aPJ2VvRX669bLVFtWewUe139KvWUZh56U
6JyfEblu53s/3bun26+Xg+ZiLe6RVHbg2rb71S2tfJT+5m+H38TB9HgKPefie1vdRxvdv3Pc1573Yf2R9
7eK0K4/35PH7CLt/3Ifxb1dq9H0x9H2r2jFw3Pp7Pu1rNcl5t78m8/fiEedGvd8M7Ns9hrTf/uKem6H
XZfY+POp+0XJ6L453XvJ7Lx7fnpB/xzO6RnHuxXn6WRjjFdT/rIsKAAAwE+5f6F3WD43zNRwQWBCq
x5xdm9BVbUl/Bl3nnO/pqrT6M5p5eS7pnk7G5ZnV9cH8Gfre9fjfR2Gvqc5r79gUjT83Id9bxnk7iuLc
LwrvxbOmRvOoOqhhgkP7+Vl4cRCiAgAAAAAAAEAEaqICAAAAAAAAQARCVAAAAAAAAAACIQIgKA
AAAAAAAABEIUQEAAAAAAAAgAiEqAAAAAAAAAAEQgRAUAAAAAAAACACISoAAAAAAAAAABCBEBU
AAAAAAAAAIhCiAgAAAAAAAAEAEQlQAAAAAAAAAiECICgAAAAAAAAARCFEBAAAAAAAAIAIhKgAA
AAAAAABEIEQFAAAAAAAAgAiEqAAAAAAAAAAQgRAVAAAAAAAAACIQogIAAAAAAAABABEJUAAA
AAAAAAIhAiAoAAAAAAAAAEQhRAQAAAAAAACACISoAAAAAAAAARCBEBQAAAAAAAIAIhKgAAA
AAAAAAEIEQFQAAAAAAAAiEKICAAAAAAAAQARCVAAAAAAAAAACIQIgKAAAAAAAAABEIUQEAA
AAAAAAgAiEqAAAAAAAAAEQgRAUAAAAAAACACISoAAAAAAAAABCBEBUAAADAQmtvFKRQ0I+N
tl5raG94n19p9PTK2fDbuiEhLV0wbdlwz3vk+e1JY2Xca6xXNVb8ba00rK8CAGB+EKICAAAAODqa52

TGOemSUMHoqjT1kqMsZ9aK+rmpKJsX6tZnHZ3aesg1asv5Wkc/F6me3bS+CgCA+UGICgAAAOAI6
Ujt/OL38Zx/XTlwM89yXbr9vvT7e7I5KvksbsrZqn4eco16jXN+IFttyXZFPwcAYE4QogIAAAA4WuiNm
r3eoezrp3LqRKxeo5Xtlng5auAamb1Qq9IiQQUAzCFCVAAAAABHTLzeqH590pVg6NpryIpbm9Orse
rX/7Rrehp1Vr119suC68NKtPqCtUJH1wEdeJ31CKsrGjyeYL3S6HYYzGPXj8F92fsp1ayzrDVXndeO3Ul
FtltujOpfo/aGXxagXN+yXjUo3vHbQto/dH0tcc5VoEar+6BWKwAsLUJUAAAAAEdEWep1HdJl2Bu1U
ytJYTVYDdRep0K2gfXN1VEBZlNWCyUxyoCqjUhpcMIpOxQceJ3F3t/IQK8jtZJRr7Rcl60YnTuHwlEtel
8JVbak7hZHba7KRqMh5/wEVS4M1gNIcvwqwA5pv3M+hoNUR/i5UueiNLhTJewaAQCWAiEqAAA
AgKNjzQ3p4vVGnVS53pW+qgPq9ax0hK1v7o5oh1dLtC/
+y5tyzkv7etJYd0PBqrT0a71td2qyPjIpNl6/F2OSpvaGePmv0a6um3ga+6psW5/r+hNFqRqm9n5iDc
MPTjLVrLnHV5b6hcF2Jjl+67VuGhvWfmsrOxfjnqu27Eadi8A1AgAsC0JUAAAAAEdIUTbdGYwy641al
bNuj8nKaWvJNWL9/mFoL05zBnqzXmhn56Lz+t5F2dGdIQPD3I3enN5rB4QPix+t7aeGgTCzuHnB31
ftfDo9MAOTTGnVs8OTUiU6fuu675lBqKO4dsYLbDsHXf0saOhcmfVeOzviZq/FzT1n+9Zjb+QMWgC
Ao4oQFQAAAMDR4oVsGfVGLZ+Ukn4aMGp9qKqcDiR3JTnpp31ix33dA90L01rllguwH8bwdve1A0
6dSBLymT0vz8ha4EuLsnbGbdi+HIYlthMITDJlPQudTGqK41fHZNc5DR3eHzR0ropr4h2yuodK7n4ja
rECAI48QlQAAAAAR8xAb9RD5+l8K8qJU/rp1MpyMn6am0BHRnTmnE6i8DlCYFIpo85ppLBzFSw5Y
HLD3NgTdQEAjgxCVAAAAAABHj9kbtRYvTsvXYK/Onhx6Y8iHebVWhx7biYbtTyercHa88cfflg2z16lby9
Ss3ZpEcVP29D4Gyt7amueYpR8Alg0hKgAAAIAjyOiNmpQxjDw7g706u3Lg7tTtmVk66QWAo+p5ps
MsJeDXAHX05KJbmFROSaIqAdNKcvztXa/nqR24unVRp7iWaoZ+1ePUnkjLDlS7Xi3W0WUEAABHF
SEqAAAAgKPJmIAoTMlPDo3aqW3Z8Kapz1ZzdcObqKm94Q8/L59ZcwJAszZnc9UfQt7e8Gp0FlIZV2
4Gzta5WPd7WfYa61790aSTVU1twuP3A1djxv4k9PbVbRDscRoSdAMAlgYhKgAAAIAjKro3qjlzuwrp
nGAubi3NaZWlXG7Kqg4D/dzWmOFftd+ozdlc1cGh9+IREzJNorLtD1vv1KSk21XyE1S5kPuM9AmOv
3Lan6jKu5bGBFRJjDgX5r1RPat7ugIAlgYhKgAAAICjK6o3qqp7OVgzs9qavI5mIqfk7J4xPFyx63gO1Di
1a3MOvE4Je+2U7GHrIcdebVnr3eHxeYt9/BXZ7psz/itVaZk1TZu7Xs/fcexzEVYMVW8zrewaALA4Cn
1V3AUAAAAAAAAAEIqeqAAAAAAAAAAQgRAVAAAAAAAAACIQogIAAAAAAABABEJUAAAAAAAA
AIhAiAoAAAAAAAAAEQhRAQAAAAAAACACISoAAAAAAAAARCBEBQAAAAAAAIAIhKgAAAAAAAA
AEIEQFQAAAAAAAABGEvn/AZM6nr53azj8AAAAAElFTkSuQmCC]]></redcap:Attachment>
</ItemDef>
<ItemDef OID="q14" Name="q14" DataType="text" Length="1" redcap:Variable="q14" redcap:FieldType="radio" redcap:RequiredField="y" redcap:BranchingLogic="[q3] = '1'">
<Question><TranslatedText>Do you prefer Life A or Life B, or do you think Life A and Life B are the same as one another?
</TranslatedText><redcap:FormattedTranslatedText>Do you prefer Life A or Life B, or do you think Life A and Life B are the same as one another?
 </redcap:FormattedTranslatedText></Question>
<CodeListRef CodeListOID="q14.choices"/>
</ItemDef>
<ItemDef OID="summary15" Name="summary15" DataType="text" Length="999" redcap:Variable="summary15" redcap:FieldType="descriptive" redcap:SectionHeader=" " redcap:BranchingLogic="[q14] = '2'">
<Question><TranslatedText>
Alzheimer's disease - Description #[no]

If you have Alzheimer's disease...

1) You might forget recent events of importance to you. For example, if you are interested in hockey, then you might forget whether your favorite team won their last game, although you may remember watching the game itself.

2) You might forget the names of people you met recently, or the names of local streets.

3) You may no longer be able to handle your personal finances or plan leisure activities such as dinner parties or vacations. Simple decisions such as what to order in restaurants might become difficult to make. Your ability to concentrate may decrease and you might have difficulty driving.

4) You may start having trouble finding words to express your thoughts. You might need to be reminded to shower or brush your teeth, but you could do these activities on your own. You may need to post notes around the home to remind you of simple things like turning off the stove.

5) You may remain aware of the day and your surroundings. You might be able to travel to familiar locations like the supermarket, although probably with the help of a loved one or friend. If you are in a social gathering, then you might have difficulty following the conversation.

6) You may continue to enjoy hobbies such as puzzles or reading.

Now consider two possible lives. In Life A, you will live in perfect health for 14.5 years and then you will die of natural causes. In Life B, you will live in perfect health for the next 10 years, then you will live with Alzheimer's disease as described in the summary for another 10 years, and then you will die of natural causes.

</TranslatedText><redcap:FormattedTranslatedText>
Alzheimer's disease - Description #[no]

<p></p>

If you have Alzheimer's disease...
<p></p>
1) You might forget recent events of importance to you. For example, if you are interested in hockey, then you might forget whether your favorite team won their last game, although you may remember watching the game itself.

<p></p>
2) You might forget the names of people you met recently, or the names of local streets.
<p></p>
3) You may no longer be able to handle your personal finances or plan leisure activities such as dinner parties or vacations. Simple decisions such as what to order in restaurants might become difficult to make. Your ability to concentrate may decrease and you might have difficulty driving.
<p></p>
4) You may start having trouble finding words to express your thoughts. You might need to be reminded to shower or brush your teeth, but you could do these activities on your own. You may need to post notes around the home to remind you of simple things like turning off the stove.
<p></p>
5) You may remain aware of the day and your surroundings. You might be able to travel to familiar locations like the supermarket, although probably with the help of a loved one or friend. If you are in a social gathering, then you might have difficulty following the conversation.
<p></p>
6) You may continue to enjoy hobbies such as puzzles or reading.
<p></p>
<p></p>
<p></p>
<p></p>
Now consider two possible lives. In Life A, you will live in perfect health for 14.5 years and then you will die of natural causes. In Life B, you will live in perfect health for the next 10 years, then you will live with Alzheimer's disease as described in the summary for another 10 years, and then you will die of natural causes.
<p></p>
</redcap:FormattedTranslatedText></Question>
</ItemDef>
<ItemDef OID="a15" Name="a15" DataType="text" Length="999" redcap:Variable="a15" redcap:FieldType="descriptive" redcap:BranchingLogic="[q14] = '2'" redcap:InlineImage="1">
<Question><TranslatedText></TranslatedText></Question>
<redcap:Attachment DocName="145.png" MimeType="image/png"><![CDATA[iVBORw0KGgoAAAANSUhEUgAABVEAAADYCAYAAADiZ6NkAAAAAXNSR0IArs4c6QAAAARnQU1BAACxjwv8YQUAAAAJcEhZcwAAEnQAABJ0Ad5mH3gAADa1SURBVHhe7d1/jBvnfefx78aOf8Q+2238K42LGC6pGopayEXb2NzAlyA2Gq7uWiUG1OiuwEb/kE5w6VJNFrFbIUF7au1ASU22QOAlUCiLNrUrwOkmgJY7Fjt1WdpNYcezy5hk+M/OQHA5n+GNmdvh+BY
z4jLichzNDWvzs83yfhY5FAAAAAAAAAC+CFEBAEBqPP7c4/L+9ffrFgAAyKrOZ4kiAOwsb9F/AgAAJOq5c8/Jh/7qQ7oFAAACy6B0vXirvev4y3QKAnYMQFQAAJO7c+XNS/HLR/hMAAGTTpa9dIO/fuk5u/
9a18vJ3n9dbAWBnIEQFAACJO/jIQTnz8hndAgAAWaQCVBWkXvT6R+uCI/+fGP9d8AQPoRogIAgEQd/sZh+Xr767oFAACy6Jf+8aftqfyOZ0+dkq984fO6BQDpR4gKAAAAS8/D2w1J9qqpbAAAgi1R4ess//JRueR790nF55rFHHdQsA0o0QFQAAJOKp55+SQ18
9pqpbAAAgi1R4ess//JRueR790nF55rFHdQsA0o0QFQAAJOKp55+SQ189pFsAACCLnDqow/zFZz8jr7z0km4BQHoRogIAgNg5K/Gff+O83gIAALLIqYM6jApQ679T0S0ASC9CVAAAkoAgOgQhRAQBArNQU/lMvnNItACQRfWoKkwFgDQjRAUAILR
ACALOqvgzrMPz/9tHztT/9EtwAgnQhRAQBArNQU/lMvnNItAACQRTf8v7f51kEdRoWoKkwFgDQiR

AUAALG5/8n77cWkAABAdl3+wwsD66AOo6b1Ux8VQFoRogIAgFhsnNmQe//mXt0CAABZdMGbC
/L+J6+Ti16PHjeoAPX4vZ/WLQBIF0JUAAAwc2r6/sFHDuoWAADIql859Xa59uVLdCu67SeekEe/dFy
3ACA9CFEBAMBMnTt/jpX4AQCYA+96/jJ595krdWt8X/nC5+XZU9RPB5AuhKgAAGBmnJX4nzv3nN
4CAACySNVBvf1b1+rWZH7y4x9L/XBFfvTqq3oLACSPEBUAAMzM4W8clsefe1y3AABAFk1SB3WYl7
/7vBy/h/qoANKDEBUAAMxE9amqPPjtB3ULAABk1aR1UId55rFH5fGH/lK3ACBZhKhzpy21xQVZ
OjeFmttvb1Xu7boPmZhsWb91E5kvtayNCJtG2VazzMLaenHrDSkbL827zbsOp6qdk0Wy+GOZqPs9
G1R4uiavxHXgc/rSUe/kRVq9KkahQoAALJtWnVQh/mrP/pD+d6z/6JbAJAcQtS5k5OV9aoUdKtZWfY
JSxpyrNLU90VKR1asnwLSQAWDS1LXra6CHNg32yvU/qVCviLeu2Jny9rrQfqo+qeqDioAAMi2K1996
9TqoA6j6qN+8eMft/8EgCQRos6j3IocKen70pTKsd7RaO3aUS+kKm3KWlHf33FysrLVkU5H3dZk9i8j
7v0Nk5Z+zEJLTjvJX6EqLfs1bsnKLDPUdk2WjV8q7HhZez1IHbUSf/HLRftPAACQXaoO6p1/+46p1kEd
Ro1E/YvPfka3ACAZhKhzqri2KW6OWj9qjEY1R6GWZHPnJqjIovZZ2dZ3Zc8uRkgDKXTwkYNy5uUzug
UAALJq8f9cY49EjcvWVx6xbwCQFELUuVWUtU0nRvVGozbK3lTpQnXVfxSjqqWoa1E6N7+alEPrK5o
/H1hj0qjp2FOXddh2Y5/29rhrgw7uL7DGpHEceo+f+Tx+fz+K/
+vu70tP3VvrFrLcZ9/z9Pd12HEO95p6n3uw9mnPFPT6UndbT8ejHbv+Y6Bu5tPZ/Rm5z9G816VuPt
eCLULffd6Dw5/XE/X1hOs34FE1UL/e/rpuAQCArMo/
+1/sW9zUaFTqowJICiHqPCuuStUpjlpfknKtJke9BFXWfeZID4QwWrOSn8ECVDnZd0B3sHlaWt17Fm
NKd/OEnHR32pAN3f/CgX2pGKVY3O8F1ae9F2Brnzyhj6NR09MOx/LSP9t6use3KZX8guT7dlJfihqS
qefp72tdlvrDtrFek3puo/apdT1W3aHTQ0TaTzew7D8GSn1peKgYXfdYL7kvRFHbJjhGjfKQeqY+zzu2
kP0GDA9vP2yvxg8AALJNjT5Vo1CToOqiHr/n09RHBZAIQtS51rvIVL3iBDMFqa77LCbVKHuhiluPsiMt
J4ltVmR5yglLbt8B3b9tOes8dWPDWFjICCeNqd57dqVkondxv1s2ob7RO2Ly5AkdgxUOSDdDbUtt2
TkHJdnUx7fjjBie6vH1nt8dkKxCsr76uKN5z+NeB+p5lp3gb5LXZDx+a0VW1qw/W971qur12n9nl5yIt
p92bdkNLAvVVvexncESF8XAfYblf4xOuOl/lL5bj3V+0+H3Hux53kHRXs+ofgOep55/Sg599ZBuAQCA
rHLqoKo/k/LsqVPylS98XrcAID6EqPOuZ5EprXTEd6GehjPMsy9kza2suyNam5Vj0502n9sn3cGoXnjT
PtuNSkulbse3dbrqjewsyf7UlHItyqpzcOob3rFpnxQvQ9WjZs1tZikFY8Rw88RJHU5OprTpLThVXDVC
tYjM5zGvA3eE8ASvaWg5CT+R9mME2Na1csS92J1zpcLD6S1W1XOM3F8KWP1x0v9IfTcWDdsy3o
N+zzuhkf0GNGcl/vNvnNdbAABAVsVdB3WYR790XJ557FHdAoB4EKKid5EpFSD5jkrzpsp7IycdxrR7c
8ToVORk157uPSe8adlz+Quye/9uO9jp3W4p7feCqBTwAqi6uINRW6d14GtM5Xe3Wa9JTeN2a1Ea0
7x7yhokzToHeX3XZl4HeoTwBK8p0mjiSPsxV/jfLeZLyK1sSafjhYeT6z9GPiY677purO/0/kmE6DdgUc
GpClBf+MELegsAAMiqpOqgDqOm9b/83ed1CwBmjxAVvfpCpegGa39Oyq0rao/kbEt3IOoe2VXcZf
1//3aVoaYpQrW4o2lVV7spqjuqd8io39maVkBmnYOZ9Z0Qb6ieRaWMurFAAtQU/lMvnNItAACQV
W//94sTq4M6zI9efVXqhyu6BQCzR4iKKZtB+OXWFd2Wsw099dkOe4vSzVeN7akM34wRmnbg643
qHRb4enU6+2/THCU5qWijjuN6TTvj2Pkb3feGlM1Rp05dVLPOKRCT+5+8315MCgAAZNtFr79F7vjb
6xOtgzqMqo/6tT/9E90CgNkiREVIedntztQ2V8RXzBqTIUYnGtOXw3H23ZTTG92fdeqI5u2/8LYPlhpI
h9zKES8IrjkLY/XVbs13yxMoO6PuZP+o495ao/Zri+s1RdqPeS33TZNXK98vxLwCfZS+G4uq2YGrUxc1
8nsKmMzGmQ2592/u1S0AAJBlt3/rWrn8hxfqVvqoEPWfn35atwwBgdghREVJOVtwVqMzV19XsYnOl
c29hnG7AqZirvjek7C7xH5Y3krNe7/6sUy8zpwumOtvdRZpSxxk1ax2Lin79/bVbjWn/Ul+SsnvIVLCn
p2+7G9OhvmQEjo1jXg1P57XF9Zoi7ces3VqXJXe7s/K9dY7yC95zzNqYx8gLXI0V+4EYqOn7Bx85qFs
AACDL3n3mSnnX85fpVnrVf6cir7z0km4BwGwQoiK84ppsujlqRfI64Ml7CaqsGwU+zRW9VTjUDYTG
q+HY81zWPXfKvjvVXzEWaUoht7arrSDV1Z4I1ZKTlXVvWnZ9SQdobug8bNGvJHUDx95+mq8trtcUb
T+5lXV35Xvv2jQWcrKuZe8l6Nq7ivPYqSasEfpuXu9+/Q5j5q8HWXbu/DlW4gcAYE5c+/Il8iun3q5b6
aYCVBWkAsAsEaIikaufaf/3F0qa13Zla7MityFb/Y0ub49VvNEfr9ZQMMKZmz3ShoykwA7BhZQfUMe
u0vIDPYde+TFtNz4JUWx0vWLeVZLOz1btYVlyvKdJ+crKy1ZHWwIPVw41p8rairPW+SJHts+5I7KkI3X
erL51N7zqyqWNunAe77m6QGF4PMslZif+5c8/pLQAAIKtUHdT3P3ldKuugDqOm9FMfFcAsLXTUqi
UAEFKjvCDdQZIqRO0LTAFk1sdOfkwe/PaDugUAALJMLSS1E6bx93vrxRfL6p9/WW7au1dvAYDpYS

QqAAAIVH2qSoAKAMCc2Cl1UP385Mc/lvrhivzo1Vf1FgCYHkJUAAAw1OPPPS6Hv3FYtwAAQJbtpD
qow7z83efl+D2f1i0AmB5CVAAA4EvVP1V1UAEAQPZd+toFO64O6jDPPPaoPPql47oFANNBTVQAA
DBArcR/25/dJmdePqO3AACALFt67GfkHS9eqls7H/VRAUwbI1EBAMCAg48cJEAFAGBO/NI//nSmAl
RF1UdV0/rVnwAwDYSoAACgh6qB+vX213ULAABkmQpPb/mHn9KtbPnes/8if/HZz+gWAEyGEBUA
ALge3n7YXo0fAABkn10Hdes63cqmra88Yt8AYFKEqHOtIeWFBVkwbou1tv67mLRrslhu6EZXo+z0Z1
Hi7k5q+ByXMNq1xe6xW6zJ6ENnnP9Qj9caZX1+fG5j9BnTNOY5BbSnnn9KDn31kG4BAICsUwGqCl
KzTo1GVaNSAWAShKhzqy21xSWp61ZXQQ7sy+n7s2cHfvmKNHUbXWMfl3ZNlivhf6pdO9p3/sNpbI
zzUwDSzlmJ//wb5/UWAACQZVmsgzqMqov6xY9/nPqoACZCiDq3WnLaydsKVWl1OtLpbMlKXBlqx
MBvbox7XNTI1SjB69jHvy1nt/Vd97oxbmtF/ZcAdhIVnKoA9YUfvKC3AACALMtyHdRh1EjUv/qjP9Qt
AIiOEHVetc+Kk4XJnl0S3/hTTNs4I1cbx8YdAWyE71w3QGaoKfynXjilWwAAIMvmoQ7qMI8/9JfyzG
OP6hYAREOIOofsmqNm6FZf8q9nqUY3OvUV9c2vZmpvDdPeOqt+JTJD71/znl/dhtVJVeUJzMdFq+/
aX4fVrS2qb/5dC7HPnmNYFu9pzOPU3WfU46KofuajjihtlGVJzcgvFKTQ3RKeEb6X9kcddTqiXqdRa7
X3OEY4tz7XrN81E+aa7b8G7FvUOqNj9Wdw3+GuP/P6AsK7/8n77cWkAABA9l3w5oLc8cT1c1EHd
Zjj93xaXv7u87oFAOFNFKKaX/QDch7N/MLf/2W/N8RQtygBWGhDAy30Gwj0tGYlHxAkNaWSN+qs
FqqyOtHsbvV8C93Az6W29YVQ9nnNS3+WGNzXYbr77A8m60tj7jO3IutVJ6qsy1H9JGY90kJ1fQplFE
qy2dm0/n8U673WTVClun5E9nQ3htY+ecK9JupL3vs13PupKPudDjZPyMm+E+PVWjVq80Y5tyqE9R
2R63PNuPyvWXX9+4bTzYrkw352jN2fENef/ZnZf1zqsrTQX+cYCLZxZkPu/Zt7dQsAAGTdr5x6u1z78i
W6NZ9+9OqrUj9coT4qgMhSMBJVhavxLHBkBkAqcNiY0xS1uNaRTqvqjUIsbfbWs3RGKipG3cuWE
wY2K7LsnwBZVJin62NurfhO9x65/x7e87n7t87iCTeBs66fZSeoMva9qdO6wL4O4z2P8zR2uHXMu
WCi7TO3si7eoTsmDet/x5z0yzq+6zpBjXZcDPbj1iRMXu2Gt6UjYwW3LXcufz8V4A0LBj3FVef1medQ
aYiXoR6Q7ts/ynG2HntUP4HfNTuwP5Px3PY1a/bF77m8MHy46fTH//ozzqPiXCedlnudAWGo6fsHH
zmoWwAAIOve9fxl8u4zV+rWfHv21Cn5yhc+r1sAEE6MIWpOVra6wUBv4BPXAkdtOXmiNwCqH40
6SnE+mCMCq+teEDoYBg4qVFdDhXlhlTa9ayW374AbMDZPt7p32ifFOa09+y6uen09cTLSeTb36YV
+hsj7tK79I04aZo4W7D2+48itbIVfzKntLCZVks2xFoAyFpXyCzVV0Lc84j2V2ycH/I5RY8MNBQsH9nW
PSaTjbHy+GOG97zXTZ+CaNesFGyNm7WOtX/PWyA+o8fsz8vqzXrH3WWaeS2uf636PBwadO3+Olf
gBAJgjl//wQrn9W9fqFpRHv3Rctp94QrcAYLTkR6LGtcCRGcgU3ARmYEox/EYEOnKyz0nArLN21ufY7
dk1zTNYkN15fXeY1mk9UtE6nWqKtzu93Jjq3Dwt/nHVmMbZZ3HNGFXYNZ1p/OE5i0mNH3QP+U
WI+dpGvqeMa8h4rBfcl+SIc1AmOre6RIjvdPpeA9esEfRaO7Cn1zv7Hr/MSPj+jGb+4mm39LxFcrsil2j
A/HFW4n/u3HN6CwAAyDJVB/X9T14nF73Okij9jt/7aXnlpZd0CwCCxfgp2h6oiRpuIR3z57q3cYIMby
p/SY6sO6PBgqbUIlhTBgfShQg9U292r6HoFgRVZlOyYih3MSmvfMA05Xc7qaPfddHLG43pvP+M4L6
0f8yA19JT8zhsbVC/8z18RKcT5o6uAW2ZWn+A6Tr8jcPy+HOP6xYAAMg66qAOpwLU+u9UdAsAgq
X7V1FRFpUJZEx/VSHNsCnFiCB9YU+h2nKnW/fejFGTUxZ+n0aNTFuIqe9T5I70tBdG8gn29PbJF3QLc
V3kVsSpbmC//0Ks+D/6ODekbP5CxikNYtaYjcLq45beR/8IYmV0KZAp9weYkupTVXnw2w/qFgAAyD
rqoI72z08/LV/70z/RLQAYLtEQNXghnfb0FgwypvJ3Qxr/KcVQ8uINKuw/NmYtxj0y1Zn748rvdq+fYT
Ump26MfbZry/qXAQWpVse4htPAHFnZNxTTW3Aq3HXhjsq1rrFjx7xR4j0ZapTjbNZUVYGrU4fUKAk
QlRopr16m/Tllf/4YCzeNKhExg/54zPdoXz/M8iiAj6e/
+7S+BwAAso46qOGpEFWFqQAQJL0jUae4YJA3ld8bJTc4pRhdOWMRpN7Rkl4Q2HdOkmTWr6wve
dOsG2U92nIw8JtY1H26CzpZ1Kr4K8Y1XFkeuaL9NHhBoHnbFHeQpR4p6S6YZLwWd3RqwOu2SwU
oYafjF/frfTelXneOTd/PjnluvcC1f/RvSPr51WvqHXEaUIs0wMT9GWDWJq7LknsMzF88Af6O/8ZxufW
GW3ULAABkmQpQqYMa3vF7Pq3vAYC/9H6iTm3BIGP0pLlQElP6h+tZKMib/p33EtTJ62qaC+D41sI
Nq7d+ZX1JXydeqifjrUQfJMo+zWCrINVVtX14UD294zILZr8nPdZF6SkRaxmcyh/hOLuhrMU5bj6lQEI
Zcv2b5Q9KR7wV931Nsz8+civr3qjYGTw/suuSCy+Rv/7Nv5Ybr7pRbwEAAFn1zC/8u76HMD6wvKzv
AYC/7P9ayhjR2huImGEsU/r7DZRa0Eqb1nZnavJEirLWX2xy+
+x4YbZdv9KYau2wR1fOqB5qyH2ao3ftUajOgesL6rxp/VM8LrNg9btnBKvDLsUR7Vj3LLRlHTc7X+4X

+txax22gX90yIO7hrG/YC9qFYV///efB1n3O0VnxdPszKCcrW/3HRT2/z7kB+lx/
+fV2kKoCVQAAkF3fu+41eeYXCVLDuOWOO+XOjx7SLQDwt9BR83rH1K4tuqMTVbgWHCy0pbboBJ
fqy74OQFSdRWcBFhXEOE+iptTqEWeqpqA7zTgis49BJtkHgDEY733ef0D8Ht5+WA4+clC3AABAVi09
9jPyjhcv1S30u/qdN8hnvvo1edsVV+gtAOAvvSNRp7JgkLkQUjCm9ANx6i1zcMCtswEgLh/Z8xG557
336BYAAMiqby6+KK9d+qZuwfTWiy+W0gNVAlQAoUwtRHXrFfrcxit1OYUFg8yp/M7K/z03c7VtpvQ
Ds9eQsv3+NcppmGUOAMTqvg/cJ/tv3q9bAAAgi1SAqoJUDPrv/
+sTctPevboFAMFSXBM1yuI9/rxV+UUKzrL8PcxVrlmlH4idWcIDQCIeuush2Xs9Xx4AAMgy6qMO+vn
3vEeWynfrFgCMlu6FpSZaMMicyj98unBu3wGvbABT+oEZUwsuGaPBCVCBxDkr9l91yVV6CwAAyK
K//4V/s8NUiFx5zTVS+uOqbgFAOBMtLAUAALLh8ecel+KXi3L+jfN6CwAAyJpLX7tAPtT4WfvPebb65
1+2R6ICQBTpHokKAABi8b4b3ycP/NoDugUAALJI1Ud97PYX5M0L5ncs1a9/4rcJUAGMhRAVAADY7
v7lu6Vya0W3AABAFn3/6vPyd3v/VbfmiwpPVYgKAOMgRJ137Zoslhu60dUo60W8FhalRpHYBLSltui
cg7J0z07YbZNwVs63bos1XR942vuYlNFHfVtM+iL1eQ8lJ23nCzuRGo2qRqUCAIDs+qebX5Hv3PBD3
ZoPb7viCjl0/+d0CwCiI0SdY+3aoizkK+IsvwWkmwoIl6SuW13DF42LA+8hZJVaaOrGq27ULQAAkEVP
3PZ9+cFlb+hW9qkA9ep33qBbABAdIeq8atdkuUL0k045WdlyVrBfk2TXr09TX1py2rlkC1Vp2X3akp
WkMlTeQ8gwtVJ/4382WLEfAIAMe/2i/5RvvvfFuaiPeudHD8ktd9ypWwAwHkJUADtD+6xs67uyZ5c
kN/4UmA83X32zPHTXQ7oFAACyaB7qo960d698+JOf0i0AGB8h6hyya56aU5DrS90aikPqOno1UtV
tWJ1UsxZj9+ZXq7K/3qo9Hdr4mdGlJf3qdhoaZfe5BvavaIc6PzvsMZahNWHNnw/sqHEsevo4bLuxT
3t7HHUt+8/XsP0E9aX/OcasTxrivES9Zh1jXW8+/em/Fkb1J+o11Pv43rqv7sNC9AuYtg/mPsiK/QAAZ
FyW66OqOqilB6ry1osv1lsAYHyEqAjQlEp+QZZ6ilCqbX7BUF76ZzY3K3n/oNPWfe583w/Vl0aFQkXZ
X9J3myfkZN9jGxtOZ3trZQ6EXlpwH8eVk30HCt27zdPS6t6zGNPRe/reEKfbhQP7YhhhqUK6/vNVl6
WF/nqjAcY654PiOy8hrzcVwvv0p/vzcQSWaj/GeShUZVXVUEi8X5hnarX+j+z5iG4BAIAsymp91N/83
d+jDiqAqSFEnUPFtY50WlXRMZ9IabNb83LNr+JlSTbt2pMdaVWdn2jKCTcBbEtt2Ql3vMd2NnXS2azI
8tCEx3u883A7FDoWPLqwuOr03eyH4oWRUjggbobaKHtBsFtL03g9gX0cT27fAd3HbTnrPHVjwwgp
m3LaSVeNaep7ds0+Qm3Xjnr9cM59pyXu6R1pknNuiHBeol2zw4y63qzXdVR3yK8/xvU2nf4MYxzTr
RXJRegXMCvHf+O43HrDrboFAACyJov1URc/fJd9A4BpIURFoNKmt5iQFwyKNJ0EsH1STujhcYXqqrf
wUHHVDeWaJ076jig0n9sLRkPI7RN3oKf53EZIaY7oNEenVtdVKNWVW1n3+lg5NmQ6+5jcPnoBV/t
sNyotlboJ3rZOV9snT7iB5P5pZHCB2nLSOWEqrHNDv5ysrIc8BxOcc1Pc52X09WYsomWHl3qr33U/
Qz3H1JaOfmG+XXLhJazYDwBAxqn6qFu/
+pJu7WzvuOnn5Ld+/w90CwCmgxAVAQqyO6/vDtM6rQNAFXjljVqNxlTvnint02BOl/emxXuhXEmO
uEu2DxmdajOexxwxOhU52bWne88JuFr2XH7rmO7fbYdfvdstpf194dksmCvc75ae05vbJbrLwaZyzp
M6L1Ho2qS+0+hnZ/Ro5GT6BVx/
+fV2kKoCVQAAkE2tm/7Dvu1kqv7px7/4ReqgApg6QlQkJERAG8AbheeM9DRCubHCSGN6/ZQUne
Kt9Q2rd23pDkTdI7uKOqzs2a66PfsIdeeZ1nkJeb31LN4UoUbs1AzpZ+L9Arr2Xr/XntoPAACyS41Gfe
WKn+jWzqNGoKqRqAAwbYSomJpCtdWdcjxw86ZRT01uRY44JTjV1HGjruh4YeRkoa6v4n7RE/flbEN
PgbdHfzqLYxnbZ7H/GMz+nMd5XBpSNkd3OvVHzdqniUhrvzCv1CJT97z3Ht0CAABZo+qiPvpfv7cj66
P+6r7/Rh1UADNDiIrJ5LtT05W46zK6Iz2bJ+TYsWF1RfOy2+1g/2r+Zn3QPTJyFrUxjT0cZ99NOb3R/V
mnVmve/gtv+
+CU9lkxj0fflHsjiA40lXM+xfMyLWZNXRUOO/VHI5/3AOM8Vxz9AiK67wP3yf6b9+sWAADIGjUSdaf
VR1Wr8FMHFcAsEaJiMsYiT1JfkrKzClCjrKceWzd345S5Iz2bUq/rOGlgKn9OVpwhq9bjKss1d8Gjdm3
ZreFpLubTDTgVc+X2hpTdpeTD8mp71uvdn3XqXeZ0wVRnu7kQ1myZ9UbrsuSeG3PF/RGmcs6jn5c
4eeGwsTJ+BNO7hnpN2i9gmh666yF7ej8AAMimnVQfVdU/LT1QlbddcYXeAgDTR4g6r8xFhOpLE4S
dvau615d0iOaGReYK8NPmTIv3+E7lL67JppvXVSSvg768l9TJursQlcoIvVXP3WMzZh3Knuey7rlT090
AWCnIgXiGodrM1e+912csCjXSlM55xPMyc+Y5CXNcAt5D07yGIvcLiImzYv9Vl1yltwAAgKzZKfVRP/z
JT8lNe/nlLoDZIkSdW0VZcxMsbfusOxowktyKbHVaXjDnsGs3zqAeqsGd0q9Y+1sdsrPimn8NydKmtd

2ZHu1Qr6f/saXN8WpQmqM2e6amG9PZ45yybsvJylb/
+SrJZmfTCHZHmNI5j3ReZs56TwwcA3VcOl7Yay8G5gh4D03zGorcLyA+N151Iyv2AwCQYTuhPuotd9
wpd370kG4BwOwsdNQqMMBOpVYt14vuqHqRW3GOXAQA2B789oPysZMf0y0AAJA1N33ncnn/k
9fpVnpcec018r8b32AaP4BYMBIVO5hZxzPeKfEAAM/dv3y3VG6t6BYAAMiaZ9/1A/mnm1/RrfQo/T
F1UAHEhxAVO1BDyv11IUtHhEGoAJCcB37tAXnfje/TLQAAkDV/t/df5ftXn9et5P36J35bfv4979EtAJg
9QlTsfKrW5MwWrwIAhKXqo6o6qQAAIHtUXdRvvvdFef2i/9RbkqPCUxWiAkCcqIkKAACm5szLZ+S2
P7tNzp0/p7cAAIAsedfzl8kdf3u9bsVP1UH9zMbX7D8BIE6MRAUAAFNz89U3y0N3PaRbAAAga75z
ww8TrY966L7PEaACSAQhKgAAmKoP5j5o10gFAADZlFR91Ds/ekj23H67bgFAvAhRAQDA1KnV+j+y
5yO6BQAAsiSJ+qg37d0rH/7kp3QLAOJHiAoAAGbi+G8cl1tvuFW3AABAlvzgsjfkidu+r1uz9bYrrpDSA
1V568UX6y0AED9CVAAAMBOXXHgJK/YDAJBhqj7qM7/477o1O4fu/5xc/c/c4bdAsAkkGICgAAZub6y
6+3g1QVqAIAgOz5+1/4N/neda/p1vS97+D/kFvuuFO3ACA5hKgAAGCm9l6/157aDwAAsumbiy/Ka
5e+qVvT846bfk5+83d/T7cAIFmEqHOuXVuUhYWF/S1K7JYt/jFhYWpdbWf681ykP+zvz5vh3
49WFhsSZ9T621pbbY+9jF/k5o0Z4XADBLapGpe957j24BAIAsUQGqClKnSdU//fgXv0gdVACpQYg6t
7phZL7S1G1PfWmhN+hslGUhX5HBRzalkh8MUqNQoatfH6RZkfxCWXriVjuIzUv/w5uV/EA4Gul5A
QCxuO8D98n+m/frFgAAyBI1pX+a9VF/6/f/wB6JCgBpQYg6p9q1ZTeMLFRb0ul0rNumlLqbROpHdTj
altrRur3JeqC07Md1pFUtdLdJU06cHDdFbciGfmr/567LUTehtfqx7AS5JdnUj+1s6h43K7LsPjbK8wIA
4vTQXQ/Z0/sBAED2TKs+6uKH77JvAJAmhKhzqS0nTzijNEtyZCWn7xdl1Q4aVUi5Jd3NOVnZ0oHl1o
rV6srtOyBujHq6pe9F1D4r2/quNE+Ik8XmVra6+7NuW07f2ifF6XKhumr1VCuuipONNk+c7I5GjfK8A
IBYOSv2X3XJVXoLAADIkknro6rRp2oUKgCkDSHqXGrJaSdDLeyWvL6rdIPGNS+kHNCQsqot6ju9P6Lc
PjngJLF2aQCvdulAndPWaXd/9vR9p8apOb2/edp6ZZYozwsAiN2NV93Iiv0AAGTUJPVRVf3TQ/d/jjq
oAFKJEBWj9SwqtSTOTPnJ5WRlveqOaDU5QanvIlcjzep5AQDT8r4b3ycP/NoDugUAALJETen/u73/ql
vhffiTn5Kb9lL2B0A6EaJihIaUzVGnTo3Rln9IGVluRbb0FHunvKmpfnRwNX2vhmv/zRhBO8bzAgDidf
cv3y2VWyu6BQAAsuQf3n1OvnPDD3VrtFvuuFPu/Ogh3QKA9CFEnUt52e0koM4UeIdaiX/BWHG/s
eGOPLXDS6cuqjG9PpSAx6uV9NXI0OKaE4a23Dqnbv/yuyPXYA31vACARKnRqGpUKgAAyJ4nbvu+/
OCyN3RruKvfeYM9jR8A0owQdS7lZJ9bNLQuS+7cdmcl/m4d0f4p7154aazY3yfvpbNSOeY8QUPKSz
6PtwPbBVF/1Tsy1Kdmq1nntL7k9U0/h31zNkZ5XsV4DmqmAkD8VH1UVScVAABky+sX/ad8870vyp
sXdPSWQar+aemBqrztiiv0FgBIp4WOGqKHOdSW2qKxKFM/NW3fHnWqFpIaUQe1tCmdNT2RXtV
PHbXolPF4NVrUL191lDY74jx18HOXZNOYzh/peVWIqh+sRtuycj8AxO/My2fktj+7Tc6dP6e3AACArH
j3mSvl1v97tW71+vVP/LZ9A4C0YyTq3MrJylZHWu5ZHWu78dk/PtH0pylpnU3rLiqrA0qg1Wt8Qd9CqqkXa
Xy9VhaZDaqjaU+39ipbqfbhBp2LXOTWm5DvsOq1GPVRLpOcFACTu5qtvlofueki3AABAlvzTza/41kf
9+fe8hwAVwI7BSFQAAJAa1aeqvgbh3ULAABkxUWvv0U+tPmzcvkPL7TbV15zjwCwEx
CiAgCAVFn4/QV9DwAAZMm1L18iS4/9jFzw5oKs/vmX7ZGoALBTEKICAIDUUIv8Rd/9MUfffGXXtb48+qXj8tp//MfE0/g5R/7oiz/6gmkgRAUAAKnBP3D90Rd/9MUfffFHX/zRF3/0xR
998Udf/NEXf2nqC6JhYSkAAAAAAAAACECICgAAAAAAAAAABCFEBAAAAAAAAAIAAhKgAAAAAAAAA
EIEQFAAAAAAAAgACEqAAAAAAAAAAQgBAVAAAAAAAAAAAIQogIAAAAAAAABAAEJUAAAAAAAAAAA
hAiAoAAAAAAAAAAAQhRAQAAAAAAAACAAISoAAAAAAAAAABCBEBQAAAAAAAAIAAhKgAAAAAAAAAA
EIAQFQAAAAAAAAAACEKICAAAAAAAAAQABCVAAAAAAAAAAAIQIgKAAAAAAAAAAAEIUQEAAAAAAAAA
gACEqAAAAAAAAAAAQgRAUAAAAAAAACAAISoAAAAAAAAAABCAEBUAAAAAAAAAAAhCiAgAAAAAAAA
EAAQlQAAAAAAAAAACLDQsej7AAAAAAAAAIA+jEQFAAAAAAAAgACEqAAAAAAAAAAQgBAVAAAAA
AAAAAAIQogIAAAAAAAABAAEJUAAAAAAAAAAAAhAiAoAAAAAAAAAAAQhRAQAAAAAAAACAAISoAAA
AAAAAABCBEBQAAAAAAAAIAAhKgAAAAAAAAAEIAQFQAAIJUaUl5YkAXjVm7ovwIAAAAQK0JUAA
AAU6PcDS0TTSzbUltcEtnsSKfTvW2WROpLSQSp6Qxz27XFbn8Wa9bRilej3Hs8nBshdzoNnK8ErpnE
jfhcc99Pzi2uiznM520cn8mB+5jTX2iFPO5JfhYnJsSxGfjcmYeLZsRxif2zOGJ/Fmtz918GjIEQFQAAJCq
xL+8DVHBp7X+prttJysnKVkfWirppKa5WpWD9uX023n/kN8p+Ye6iJPpdo12T5YpIqaSOSEIKVWnpY

+LczPOVlES/uFvnZdHcd98tibe2Oh5L9ZJ1CTvnaVNKzYrkEwp84v/SPvpzTX0G5633U7VlHKP60oyv
nTCft3F8Jo/ah/r7GH+hlYowN8Jxj/OzOODYDHzu6dv0j0/463Zpu+
+/EbP4D8SwYxL7Z/Ho4xLvZ/EY/bHe2M1KfgafyeHet+n5tzBGIUQFAACJSebLu792bVkqzYLVl5ZUE8
zm0qa45hfmNuXEyaRSVOvLkfrWXl2X1d16EywxfnEfJrciW+a+9a1lv6FKsj/2kLktZ7etP0r7xdt1UVZ
Vf5qnpaW3xCW+L+2e0Z9rDTlWaUrBej+t5PQm5xjVN6y/nY0wn7dxfCaP3kdcv9AKE/rE8wut8Mc
9rs/i0cfGFsMvtsJft9b7fGvFunpmZcQxifmzePRxifezeGR/2jU5ah26QnXV609xzX5PNU+ctHo7Leo8j
f4lTJr+LYzRCFEBAEBCkvnyPkxuZcv6h+uW0ZeUaZ2WpvXHnl3p6GBS/XC+oB5J7YlKRjxf3MfRfZ/3fn
mOS0527bH+2D5rfCluy8kTCfQnti/tvUZ+rrXPiso2+t/POfvA1WVjRh/EYT5v4/hMTsvn/ugQSl0u8fx
CK+wxieuzOMyxicvoY9P9fOl5n8/AeMdkdp/Fo49LvJ/FI/sz5N9T+d3WwZxqqBvmlzDp+rcwRiNEB
QAAyUjoy/vO1JCyGnFSqMrqLL+ZjdQdedRMqh/21NGmlDbXZvoFNRQ1DdGYepdsLbV4vriPo107a
r2brS/7CV24xTU9ZXRBjdhT0yrz3bA5zhG6Smxf2iOy+1WQ3XndduR321/04WMGv9CaJMydZj9Ci/
GzeJJjE7+WnFYXx+ljfdPppztieJxjwmexYcjnW/ffn9sSa9Uk/i284xCiAgCAZPDlPTQ1jdP+8rOexChDP
W3Q/iKov/QkNNqxcawizdLm1KdoRqVGhDlT89RNTZGc9bTsYPF8cY/OGWl0JMEApChramqk9WlT
yav3kdWdJEL4NH1pxwT4hZaSls/iHin6xVZze7esu/
+NUKNF1edPOcFRhXwW98jtkwPWB3L9qFmP1XpPqekCs9b/Sxj+LbzjEKICAACkWLeOopq2l9RInO
50NC8w3JalJL6gNsrdepKp+tbepUYFzXpadhip+
+LeOCbd2aPJnbPuYh1eTToVeKt6dLHXmkvySzumhl9oWVL4WZy2X2wVDuwzzov139Aj1n8gkhxVy
GdxH/XvGmdkrPeeOr1Hnac9MrvB3Wn5JQwmQYgKAACQUt0AVY3WSM9UxtzKul2DrVk5Fms419
hQYVPdDnC7X3gWJK+
+FeovQclOp9eSnJZtSdcXdx0QJvllUU85LlRb7og5expsd2WPGa1sPkxSX9pHsEc7NeV0/4U7bHTUH
OMXWl074bM4Lb/YciU6qpDPYn9qZKz3flK31d3b1nHaLbP62Ev2lzCYFkJUAACQDL68B1KjNroB6v
RXGJ6KGX7R8NM/0kjd7JWGrS+GakXmrURTZr3ycMzHZKQkv7g7I5+OJPhlsX/apCOx4xL/l/aRcruku
95Lb9TUti/oBMPdlOEXWp50fxb3if0XW3mxyxz3h7dJ/ruGz+KQdG3xnl9GTs/QX8Lwb+EdhxAVAA
Akgy/vAfQquhZ7upsx4ifeOpdq8Ye+6eD6C9msvmikXrsmi4vmlGy1Sa2UnOSX1PR9ce+OVitJgrNHR
Yr7rR5Y76GeKfTqEq5YxyXhvtlm+6U9nKLsVyP2Ksve54o7aix9C5UlgV9o7URJ/WJLzwBoVuSY+x9O
PYU7oXqkfBaHoUpl5KUiVVmfwUkK/CUM/xbeeToAAAAJ2SxJx/qW06m29IZWtVOw/nlScDfEp1Ut
WH1R/em/lTqb+jHzyO+4lFJyQOy+FaqdeK+WVqda6D0eqbhGNkt952azY31p7ajkJ3YJvo8H6ePQc0
vDe1pfRzO+fsN+rnU/i73brM9dmH7F8Zk8eh9+149zM/7bNTX6uhh436p+9L1u/Z6f9rka57jbPzPzz
+Ihx0Z93vTt23kN0/74C3tsBh43s8/hYdeLFtNncbjjEt9n8ej+
+Px3fEbnKMy1mKZ/C2O0BfV/1kkFAABIhPMbeof1j8Z0TQcEdgg1Ys6uTegobUongaFz3fd0STY7Ca
28nEp6pJNxepI6P0ifgfeuy3sf+T2mlNbRsVM0+tj4vLeM45ZFYa4Xhc/ipKnZPKoOqp/eqf38W3jnIE
QFAAAAAAAAgADURAUAAAAAAACAAISoAAAAAAAAABCAEBUAAAAAAAAAAhCiAgAAAAAAAEA
AQlQAAAAAAAAACECICgAAAAAAAABCFEBAAAAAAAAIAAhKgAAAAAAAAAEIEQFAAAAAAAAgA
CEqAAAAAAAAAAQgBAVAAAAAAAAAAIQogIAAAAAAABAAEJUAAAAAAAAAAhAiAoAAAAAAAAA
AQhRAQAAAAAAACAAISoAAAAAAAABCBEBQAAAAAAAIAAhKgAAAAAAAAAEIAQFQAAAAAAAA
ACEKICAAAAAAAAQABCVAAAAAAAAAAIQIgKAAAAAAAAAAEIUQEAAAAAAAAgACEqAAAAAAAA
AAQgRAUAAAAAAACAAISoAAAAAAAAABCAEBUAAAAAAAAAAhCiAgAAAAAAAEAAQlQAAAAAAA
AACECICgAAAAAAAABCFEBAAAAAAAAIAAhKgAAAIAdrVFekIUFfSs39FZDo+z+/WKtrTcmw+trW
Xx6usM0pOwc98Dj25ba4qjHWI+qLXrPtVizfgoAgPQgRAUAAACQHfWjknBOOidUMLokdd3qKsiBf
Tl935STlfWq9bddzcqyzzlqyLFKU98XKR1ZsX4KAID0IEQFAAAAkCFNqRzb+WM8068lp53Ms1CVVqc
jnc6WrAxLPnMrcqSk7/uco3btqBfIljZlrajvAwCQEoSoAAAAALKF0aiz1z4r2/qu7NkVatRocW1T3By1
5xyZo1BLskmCCgBIIUJUAAAAABkTbjSqV590sTd0bddk0anN6dZY9ep/2jU9jTqr7jb7Yb3b/Uq0enpr
hQ6vA9r3OOvmV1e09/X01isN7ofBfO361r8vez/5inWUtfpS97Ejd1KUtU0nRvXOUaPslQUoVFetR/
UL9/ptPv0fOL+WMMeqp0arc6NWKwDMLUJUAAAAABlRkGpVh3QzHI3arORlYam3Gqi9TYVsfdvrS

8MCzLosLeTFKAOqnkTy/QtO2aFg3+Ms9v6GBnpNqeSNeqWFqqyGGNw5EI5qwfuKqLgqVac4an1J
yrWaHPUSVFnvrwcQ5fWrANun/93jMRikdvkfK3Us8v07VfzOEQBgLhCiAgAAAMiOfU5IF2406rgK1
ZZ0VB1Qd2Rll9/2+saQfri1RDviPbwuR920ry21ZScULMmmfqz73M2KLA9Nio3Hb4VYpKlRFjf/NfrV
chJPY1/FNevvWt5CUaqGqb2fUNPwexeZqlec11eQ6np/P6O8fuuxThrr13/rWU6cDHusGrIRdCx6zh
EAYF4QogIAAADIkJysOCsYzWw0akmOOCMmi/utlmPI9u2zvqM4zRXozXqhzRMnu49vn5QTejBkzz
R3YzSn+9g+/tPih2t4qWFPmJlbWff2VTk2nRGYPYtMaaUjg4tSRXr91nnfMoPQrty+A25g2zzd0vd6D
Rwrs95r84Q42WtuZav7/NZta+gKWgCArCJEBQAAAJAtbsg2o9Gohd2S13d7DNvuqyT7e5K7vOz20j
6x477WaT0K09rklAuwb8b0duexffbsihLymSMvD8i+nh/Nyb4DTse25axfYjuGnkWmrHu+i0lN8PrVa
7LrnPpO7+81cKxy+8R9yeoayjv7DajFCgDIPEJUAAAAABnTNxr1bPduuuVk1x59d2IF2R0+zY2gKUM
Gc04mUvgcoGdRKaPOaSC/Y9VbcsDkhLmhF+oCAGQGISoAAACA7DFHo1bCxWnx6h/V2Zaz7hzyQ
W6t1YHbWqRp+5OZVTg72ujX35CyOerUqWVq1m6NIrciW3offWVvbfWjrNIPAPOGEBUAAABABh
mjUaMyppHPTv+ozpacdnbqjMzM73YDwGH1PKfDLCXg1QDtastJpzCp7JFIVQImFeX1Nzbckad24O
rURZ3gXKoV+tWIU3shLTtQbbm1WIeXEQAAZBUhKgAAAIBsMhYg8pP3kkOjdmpDyu4y9bNVXyq7
CzU1yt7088KBfd0A0KzNWV/yppA3ym6NzoWpzCs3A2frWCx7oyzbtWW3/mjUxaomNubr9wJXY8
X+KPTzq8ugd8SpT9ANAJgbhKgAAAAAMip4NKq5crsK6brBXNhampMqSKFQlyUdBnq5rbHCv+q/U
ZuzvqSDQ/fBQxZkGkdxzZu23qxIXvcr7yWosh77ivQRXn9xv7dQlXsujQWoohhyLMxro3REj3QFAM
wNQlQAAAAA2RU0GlXVveyvmVnaHL+OZiR75MiWMT1cset49tU4tWtz9j1O8XvshOxp6z6vvbRpb
Xemx8ct9OsvylrHXPFfKcmmWdO0vuGO/B3FPhZ+xVD1c04ruwYA7BwLHVXcBQAAAAAAAADgi5G
oAAAAAAAAABCAEBUAAAAAAAAAAAhCiAgAAAAAAAEAAQlQAAAAAAAAAACECICgAAAAAAAAABC
FEBAAAAAAAAIAAhKgAAAAAAAAAEIEQFAAAAAAAAgACEqAAAAAAAAAAQgBAVAAAAAAAAAAIQ
ogIAAAAAAABAAEJUAAAAAAAAAAhAiAoAAAAAAAAAQ4n8f/Rr2w9C2vPlAAAAAElFTkSuQmCC]]
></redcap:Attachment>
</ItemDef>
<ItemDef OID="b15" Name="b15" DataType="text" Length="999" redcap:Variable="b15" redcap:FieldType="descriptive" redcap:BranchingLogic="[q14] = '2'" redcap:InlineImage="1">
<Question><TranslatedText></TranslatedText></Question>
<redcap:Attachment DocName="B.png" MimeType="image/png"><!
[CDATA[iVBORw0KGgoAAAANSUhEUgAABVEAAAEJCAYAAACdcmdaAAAAAXNSR0IArs4c6QAAAA
RnQU1BAACxjwv8YQUAAAAJcEhZcwAAEnQAABJ0Ad5mH3gAAE9sSURBVHhe7d0PjCRnfef/b+/aY
PPHNmBskx+RHat7sZYlGJIg08PxL4egZ+6SJbnbYOVg5Oh3PVyi0zQSkwOxF+mX2wguG4lunS5iWrq
zliQyTEJugrLdIJRgckzHR4IxYTI/Z7rlc3T8wDYGjPljB3u3f/VUPVX1VHd1dVV3VXX39VHd1dVV3VXX39PtltaerpqfqqT/T
O/OZ5/k+hb5FAAAAAAAAAAChjumPAAAAAAAAAIAQhKgAAAAAAAAAEIEQFAAAAAAAAAAAiEKIC
AAAAAAAAQARCVAAAAAAAAACIQIgKAAAAAAAAABEIUQEAAAAAAAAAgAiEqAAAAAAAAAEQgRA
UAAAAAAACACISoAAAAAAAAABCBEBUAAAAAAAAAIhCiAgAAAAAAAEAEQlQAAAAAAAAAiECICg
AAAAAAAAARCFEBAAAAAAAAAIAIhKgAAAAAAAAABEIEQFAAAAAAAAAgAiEqAAAAAAAAAAAQgRAVA
AAAAAAAACIQogIAAAAAAAABABEJUAAAAAAAAAAIhAiAoAAAAAAAAAEQhRAQAAAAAAAACACISoA
AAAAAAAARCBEBQAAAAAAAAIAIhKgAAAAAAAAAEIEQFQAAAAAAAAAiEKICAAAAAAAAAQARCVAA
AAAAAAACIQIgKAAAAAAAAABEIUQEAAAAAAAAgAiEqAAAAAAAAAAEQgRAUAAAAAAAACACISoAA
AAAAAAABCBEBUAAAAAAAAAAIhCiAgAAAAAAAEAEQlQAAAAAAAAAiECICgAAAAAAAAARCFEBAA
AAAAAAIAIhKgAAAAAAAABEIEQFAAAAAAAAgAiEqAAAAAAAAAAAQgRAVAAAAAAAAAACIQogIAAA
AAAABABEJUAAAAAAAAAIhAiLp0etJYKUih4DxWGj29PpqjXWPFeU1hwaW+1iMxj3ZB2onXQL
MxLO7LSlg372PzHqPs4Vb2GrGzEO5vtDbdtK5JH08KNuQ9Cjmc+2g0AAAAAwPwhRF06Rdm8UJey
XurU1kPCPpGafE4xY6j6ZXf+d0m1zu1cb/rlhsr+14AAAAADIGiHqMip

uytmqfi4dqZ0P9kbrNc75IVW1JdsV/XzhFGVzry/9vnpsS/aHkff+RpmXdmShKwdu8leuS9c+xj3ZzDJ
D7TVk3fijwsI7ascDAAAAAEAOCFGXVGW7JV6O2jxn9EY1e6FWpbW4CSqOot6h7OuncuoEPaQBA
AAAAEAuCFGXVkW2W26M6vdGbW/4Q6XL9a3wXoyqlqKuRek+wmpSjqyvaH59ZI1Jo6ZjoC7rqPX
GPu31edcGHd5fZI1J4zwEz5+5nbDPjxN+3INtCdS9tR4xy30ObGewraPOc7xjCm57uPZpYAh6c9VZF
2h4snM3eA7Uw9yc3Z6x+xzPPy71CLkXbAnaHvI9OHq7vqTHE6/dAAAAAAAcfYSoy6yyJXW3OGpz
VTYaDTnnJ6hyIWSM9FAIo3VqpQwmoCrK2hndwM6BdJ1nFmNId2dHLno7bcuubn/5zNpc9FKsnPa
D6gP/AGy9izv6PBo1Pe1wrCSDo63TPb8dqZUKUhrYSXM1aUimtjPY1qasDoZtEx2T2rZR+9S6H+te1
+kREu3HCSwHz4HSXB0dKibnnOtV70AUtW6Kc9TeGFHPNGS7E4vZbgAAAAAlgQh6lILTjLVrLnBTF
nqF0Imk2pv+KGKV4+yL103ie3UZD3lhKW4dka3b18O3U23d42JhYxw0hjqferEnAz0rpz2yiY0d4M9
Ji/u6BisfEacDLUnjXX3GlSlpc9v3+0xnOr59bfvdUhWIdlAfdzx/O1494Hazrob/E1zTMbr9zZlc9v62PX
vV1Wv1/6cXXIi2X56jXUvsCzXu85r+8MlLiqR+4wr/BzteOl/krZbr3X/0hH2PRjY7rBkxzOu3QAAAAA
ALI+C9QtyXz/HklK9SwM9zlSwEhKq+K8rS71rTubTk8aK24tOBS/OZEYjX6963bk96Ubsy+dvW4Vde
9ZG1BBs1YOwWq1Ks9kcWu+3IaxdcdeNE387w+2yV3rnwG1/6DqbsV0VnO2FBNwB4e0wr3O11fc
nDEt0PSK2E9ivvuaS7JjMbQdfr41qa6JzF35+FOdanQqsS3p+lETnOlHbRwht44jjjDietO4RYBr3Pnyvv
OXCW/QSACAP99/xLnnNW2/SSwCAzB3/NZFjr9ILWBT0REVwkikVtoQGJP5Qeb/npMsYdm/2GE1F
UU6ccp51dJfTrj2WvywnT5+0e9QF11uqp/0AbA74vWmb4nVG7R44gZT1GW8ov7dOdT4sGbUo3S
BMfcIsazBr1jUo6ac28z7QPYSnOKZEvYkT7cec4f+kmIdQ3NyTvhmgTm3wHIWY6rrrurFuwJmaGO0
GMvDwEw/LOz/5Tr0EAMjDDY9fJXd/6AF5/Bs/0msAAJm7dI9I/zt6AYuCEBVBA6FScsO1P6fl1RVt7k
pbenJoj9k/JScqJ6z/D65XGeqc9ZIrrombLbpD+ttuIl09a/TozUtaAZl1DTJrOyHeSKpXqBe0GnVjgQX3x
NNPSOWPKvZHAEA+nvPjY/KWL94oP3ryGWm+/3555p8u6c8AALL1lMilP7A+PussYiEQoiJlGYRfXl3
RfTlsXxS7lKgd9lbEyVeN9XMZvhk9NO3A1+/VOyrwVcO67VqVQ480e0lOK1mv47yOaTHOXbjxbW/
Lhtnr1K2LatY5BRbUnZ+6Ux58/EG9BADIwxv/
+gZ5wQ+vsJ8/9NXvyp/WeR8GgNz0Hxa5/Od6AYuAEBUxleSkN1LbnBFfMSZJitM70Ri+HI+7744c7D
pf686+X7I/4a8fLjUwH4qbZ/0guOFOjFWVQIZacsoTKG6Jgvk22OvYvA/0seV1TIn2Y97LA8Pk1cz3hZ
xnoE/SdmNSNTtwdeukJv6eAubL+z77PvlM7zN6CQCQh1c+eK3c/PXn6yXH5z7+kHzlLx/RSwCAzF3
6gsjlr+kFzDtCVMRUlM2zbuVUc/Z1NbrYnOl8y+vt5wScijnre1s23JlrYvN7cqqJpBS3XmZRF0x117vh
6vxxe81a56Kmj3+wdqsx7F+aq7LhnTIV7Onh297K+dBcNQLH9nm/hqd7bHkdU6L9mLVbm7Lqre/p
me+ta1Qq+NvI2oTnyA9c3XYDi+kT+5+Q+n11vQQAyIOqg/pzD7xELwX94W9/Tb73raf1EgAgc5f/RK
T/fb2AeUaIivgq29LyctSalHTA48w8bynX5YJR4NOfUMnSXNWB0GQ1HAPbsp55Q/a9of6KMUnTHP
Jqu9rKUt8KRKiWomxe8IdlN1d1gOaFzqMm/ZolJ3AMttM8tryOKdl+ipsXpO6/2HmtOZGTdS/7h6Br
7yrua1NNWBO03bzfw9odR+bHA8R339fvk7v+7C69BADIw/FLBXsYv/oYRgWozd+8Xy8BADLXf1Lk
8sf1AuYZISoSqWyH11+stqz17tBiV3FT9gZfW21NVr/R7K0XKBlgDM3OdKKjFJgB2KiyA+qc9bt+wOe
ya1/OW03PstS7fT9Yt1Wl1d8LTpaV1zEl2k9RNvf60h16sXq5MUzeVpHt4EGK7B96PbFTEbvtVlv6Lf
8+sqlzblwHu+5ulByOB4jBnYn/6Wfp7QQAeVr50kvl2iev1Evh/uFL35ZP//6hXgIAZO6y9RvZJcpbzbt
CX81aAgAxtTcK4nSSVCHqQGAKADGo4PT1/+318sAjD+g1AIA8lB56od0LNa6tu8vyiteFD/sHAGTgit
8QKfBL9ryiJyoAAMiVGsJPgAoA+VK9T1Uv1CTUsH7qowJAji79AfVR5xghKgAAyM1HvvgRezIpAEB+
VP3Tt33hZSProI6iAtS7/+NX9RIAIHN2fdR79ALmDSEqAADIxe6Du/LBv/igXgIA5OWOL18/tg7qKPv/
8zH53Mcf0ksAgMxd/n9FLn1BL2CeUBMVAABkTg3fV3VQmUgKAPJ189efL//8Czfppclc+dzjsnX36+
XWV79IrwEAZOtKXR/1Zr2MeUBPVAAAkKknnn6CmfgBYAZe8MMrEk0kNcoz/3RJmu+/X3705DN6
DQAgW9b77aWPWx+fchYxFwhRAQBAZlRwqgLUh594WK8BAORB1T99yxdvlOf8OJ1f+R7/xo/k7g8
xKSAA5Kb/HZFL1EedJ4SoAAAgM+/77Pvk3ofv1UsAgLz83AMvkRsev0ovpeMrf/mI3PtJ/igGALm5/
DXr0dELmDVC1KXWlo1CQQrGY6XR05/LSa8hKxttveBob7jtWZG8mzM3Qs5LHL3GinPuVhoy6tT55
9d55H7NjwLuWyCW+n11+djffkwvAQDy8vJvPE9e+eC1eildn/zPB/LN//19vQQAyNylXZH+Y3oBs0S
IurR60lhZlaZecpTlzFpRP8+eHfiVasLfVIImPi+9hqzXor5KXfOCrAYvunRqJSlMENguK+5bIB7V+1T1Qg

UA5Ovqp47LG++bvg7qKKo+6u//
+y/bHwEAeVD1Uf+79fFZZxEzQ4i6tLpy4KZA5bp0+33p9/dkM68MdWzgt6QmPS+qZ+S4YK99XtxN
V1vqevelWy87K5qrQo4aA/ctEIuqf6rqoAIA8veWvRvtIDVLqifqH/721/QSACBz/UdFLv2xXsCsEKIuq
96h7OuncuqE5Nf/FGmL1zOyJ41zugtquS5bFedpcfOsVJ2n0twlRQUwPTUTf+WPKvZHAEC+Xvu1F8
vLHr1aL2Vrb/f/2A8AQE4uf8l5YGYIUZeQXbvRDN2aq04tx8GuiKp3o1E7c1T9zGAtyGCd1bDejbH3r
wVreI6qN+kMVfdfl6zW52A9S6+2qH6ENy3GPgPncEP8zZjnydln0vOiqHaWYvWMNHoeB0LzkpzUn
VFl/9A6olGM9obVW21v6GMZPAcJrkvI/RZ2vePcb4PXz35E1ImNY5b37WT3p2vSa2eZ6D1Ar1TMr
49uJI6QOz91pzz4+IN6CQCQFxWevubvXqSX8qF6o1IfFQBydOlPqI86Q4SoCDUUGGl2/cyRYVRHai
WjzqrR43EyanuDNTzVurCgpuQNVXdFt3UUZ5+DwWRzdcJ9Fjflgjtk3joz5/RGeo1z3nkq1y+kUEahK
q1+y+tVOsTseTxK50C6+umwipx2N97ZkYsDJ7W96x2NX1c3yXVRQV7I/eZcj1EBZPj9pu7d0GC5U5
NSIMjOSpb3bcz7M2CCa2eZ7D0Ay07VQP1M7zN6CQCQFzV8Xw3jz5uqi3r3h75KfVQAyI2qj3qP9Z
H6qLNAiLqEKtt96Xfr4kZ7Um3Z9TH72zrxbG/4AZBXL9Won9mpyfrIxEaFec7r+3uboWUCxu4/wN+
et3/pyI6XBPWkse4GPca+Wzo1imzrKP523M2ofdbOu/Fbsn0WNy+If+rOS9v677wbglnn94JOUJOd
F4P9um2ZLK8uyolT+ukYlS23beb5V9ri53BnxMnhkpwj67VGqYGh+21ofyZj2/b9ZrYlbFt+kJ3U/Ny3
/uvD789hya6dZar3ACyrT+x/wp6NHwCQv5UvvTTzOqijPPTV78qf1hmBAAC56T8scvnP9QLyRIiKIW
bPtPoFPwgdDgOHletbE4Z54aotPxwsrp3xAqzOge4z2bsoO14eaey7suW3deeiJIl7zH364ZMh8T6Ls
nnWTbuaslpwe08Gz+8kipt740PW2PblMOpEFdfkTNjxtXf9XrVn1pzjSXSOrPOzp0NEI3gPvd4Dhu43
s8et0evSPk86CNzLYfa0LO/bsfdnmCTXzjLNewCW031fv0/u+rO79BIAIE+vfPBaufnrz9dLs/G5jz8kX/
nLR/QSACBzl74gcpkJ/vJGiIoBI3qm2Yqy5iYxIwK3UyfSDKjKcrKkn47SPdC9+VSoU/LqNhbMYdKRw9
QnMMk+K9tGr0FHOsP403RKoi+fcf2NgNIP3Kpy1j2gqa6LruEZOrw/aOh+M8JCawf20Hd33yNrsaZ
uHu/bBNduyvcALB93Jv6nn31arwEA5OWGx6+Sn3vgJXpptu7+0APy+Dd+pJcAAJlTw/r739ELyAMh
KibUkeHOgTHCo7mX3TFUvMKUSrD+5Oz05HBssVSf36vSHRZuBG7V08FeoUmYExB5PXXHCbtWR
dm8EN470w0rF3t+o8nvz/SvXdh7AJaNCk5VgPrID+h9BAB5e86Pj8lbvnijHL9U0Gtm60dPPiPN99+v
lwAA2XtK5NIf6OfIAyEqJjR/gWm53vWGbAcfk9YLHS/
+Po26n7aO1NZznJyneELGlj4tn5Sxl7S4KW5lAntYuDF8vno6/CyPP0dt2TB7nbo1OM36o0lYbdzT+xj
s/as0z83XpEi53bcTXLtoR+GPJpiWGsL/wCMP6CUAQJ7e+Nc3yAt+eIVemg+qPuqnf/9QLwEAMqfq
o15iYte8EKJiQElOusnV0EzePbnoFnIcO/Q7J6WTXtA2qm5m6ibYZ6+xrodpl6Ved5OsPCfnMa5rc1f8
zphdOfAu6QmJc0m9HrXW/XH+/I4OP6sSyOGSnCOzLqcKFN26qMaQ96TUzPKqx6k9GZQdSHa9W
p7pDpOf0CzuW0usa5fme8AU1xDz7yNf/Ig9mRQAIH+lh1448zqoo3z6v/6D/MOXvq2XAACZu/xZkf
48dRU6ughRMcCcBCnYW9IPAlXYNTChz6yYNTCbq/5Q7faGHhpuPdIev510n72GrLsnrnpWNjeNyY
Nq65JPjmrWsmzKrm5er3HOCzBj90asnBbnDulIs+ke18Bw8Amvix8oDvbcjUlvX80sH+xxaoTFZo9b
oz1p3yaRZnHfKnGu3QTvASU/dZXaee9gZMOb4h9Hze6Du/LBv/igXgIA5OnaJ6+0Z+OfZ83fvF+
+9y1qZQNAbtSw/v739QKyQoiKYeYkSJ2alHSoU/LTE7kw7YxI5vDy5uoUoVGwBmZzVQdQXnhTlVZ
qs9e7kuyzJ411d6h6Wepbav3okCq98zLMnFndbbN3TastiX+aKhIo72oZDmATnCMv2LO4x2xOsJTEi
HvXrLNaPevPNp/YQt+3SpxrZ0n4HuDXW7V41zBubVssGjV8/85P3amXAAB5UvVP3/aFl81NHdRRV
lCqglQAQE76T4pc/rheQFYIURHKHgYdUpOy2rLWu8Otp1KR7cGClfuHRs/BBOwamMZwbZddWzOj
eqgx92n23LN7obonbiCk8of1p3hehhRlc2+4Tqg9hD5hYBeYJMs6ZjsbHhT7uljH3G/5QaqtKq2+0dZ
ACYJo9r07eJA2Z5vTZZMLft9aYl07S6L3AHUsg6+ttiava4u59cTTTzATPwDMkOqBqnqiLgI1pJ/6qAC
Qo8vWb6bUR81Uoa8KBgJAEmo2fT0ZlAph96btmTxL+lhOtaYNWBfEUbp2yJUKTit/VJF7H75XrwE
A5EnVQVWTSS2SK597XLbufr3c+uoX6TUAgGxdKXLFb4gUbtbLSBM9UQEkFCxRcGZtsUO49nnrWC
J6ZB4tR+vaIV/v+
+z7CFABYEbULPx3fPl6vbQ4nvmnS9J8//3yoyef0WsAANmy3m8vqWH9TzmLSBUhKoCY2rJh17o06
pWaJQoWUK+xIqv7demmUqJinh29a4d81e+ry8f+9mN6CQCQJ1X/9C1fvFGe8+PF/NXt8W/8SO7+
0AN6CQCQuf53RC7doxeQJkJUAJNRNS8XfPx7cXMvpRq/C+YIXDvkR/U+Vb1QAQCz8XMPvERueP

wqvbSYvvKXj8jnPv6QXgIAZO7y10QufUEvIC3URAUAAKEefuJhec32a+wJpQAA+bv568+Xf/6Fm/TS
YqM+KgDkjfqoaaMnKgAAGKKCUzWRFAEqAMyGqoO6aBNJRVH1Ue/
+0FftjwCAPKj6qGpY/7POIqZGiAoAAIbc+ak75cHHH9RLAIC8qQB1UeugjvLN//19+cPf/ppeAgBkrv+
oyKU/1guYFiEqAAAIUDVQP9P7jF4CAOTttV97sbzs0av10tGyt/t/7AcAICeXv+Q8MDVCVCTUk8aKm
uVbPTaknWjdOGltZ96MOYZeQ1Y2gmvbG+7rV6TR0yunpfZjb3NEO2zTne9M2m1bkPugvaHbWJC
BSwosjE/sf8KejR8AMBsqPH3N3x3tuqGqN6rqlQoAyMmlPxHpP6YXMClCVGCGeo0VKZRq0tHLWe
pd3DH205RdQj4AA+77+n1y15/dpZcAAHm7+qnj8pa9G/XS0aXqov7+v/8y9VEBIDeqPup/tz5SH3U
ahKhIqCibe33p99VjWyp6bXby3l8WRhxDryHrtTziU6UnF3eC+2qea1hrF8VRuA+A+aZm4n/nJ98pT
z/7tF4DAMibClBVkLoMVE/UT/7nA70EAMicXR91Vy9gEoSowDLoXRQ3Qy2Xy86Tzo5cXJwUdTFUt
qXfrYs+w8DCUMGpClAf+cEjeg0AIG8//ffX2UP5l8m9n3xYvvKX/NsDALm5vGc9mOBvUoSoS8eoLbl
i9kQctd6oc2mvz7s25fD+IutuGnU/VwKfNLcT9vko6Z8z+/PmMP7mqvP5EYU0/WNWj+T1Rv2h/FU
5e+GMDvk6shMnRQ3UUg17jG5PvHbHuTbD59DaumyYrzfqkQa2MbA+/BTHaYN5POpY/P2rh7vd
9nnrupbrsjXQXdYu3WC83n4M3DfArKgh/A888oBeAgDk7YbHr7Ink1pGd3/oAXn8Gz/SSwCAzF26R
6T/Hb2AJAhRl05R1s64PREPpOs8s3TlwE30Aj0U27LbdJ6Vz6xZXz17ldNV/awjB/4B2PywsCxn1nRr7
RCwJIMj5zu1UswQa5bnrCO1UkFW9fYcal2SINUYyl89LZXimniHs3Nx+hCvfEbcU+2L2e6pr43Dfn1w
Z846FVQOrG+uDgSpE7VBHcuqeFvWoakKSlf369Ld2wxcdxW+lsJKN3RqUprnibKwFD7yxY/Yk0kBA
GbjOT8+Jm/54o1y/FJBr1kuP3ryGWm+/37qowJAbp4SufQH1kfqoyZFiLqEimtuT8R9OXQTovauHwi
JEU72Dq1XOU6dmIcI1VI5LW6M2gzMjmSEhV6w15PGutvjsyotu6am9WjpLXRqsh4jjUz7nFW2rTa
Yw76rLadd22HVPv12d+vuV8TsRaoYQ/mrp9X2zVA4xpD+4qbsuefNfnTFa4Z1BPULwcDQN67d6V
wbV7neDX69Frbev2+maYPxeh2aFjf3vOc+P1RXYWtXf41/TppyLsFxAmnafXBXPvgXH9RLAIBZeONf
3yAv+OEVemk5PfTV78qf1h/USwCAzPUfFrn853oBcRGiLiOvJ6IfaPUOndivWnXCo32dFJrDwO38bS
5UZMsNoJq7fi8+s+6n2wPUXFff8ickqmx5QWCs3pgzPGfVlj+Rkh/mWi0Z7IY7gtk792TJfmJsJ0EYq/
Ua636vzepZ2QxPUMe3O61rY6vKWbchRsg+cv3+obPdKdoQeH0UI1Q3Q2s7cNWB6t6okwhkSA3f
v/NTd+olAMAsvPLBa+Xmrz9fLy23z338Idn/n4/pJQBA5i59QeQyf8BKghB1KRXlxCnnmRtode1x6W
U5efqkHXYF11vUMHDn2VzwQ7mmeJ0KuwdeWOgN5ffWWcfkDu+2H8bw7cAQ/VFmdc784HMy
Yb1zLRMP6W/LeT9BlVZoz1klRrtTuzaW8kkJ3d2o9a4p2hC7Z7Zxrq2N2WUO3P3Er8sLpOuJp59gJn
4AmLGXfPe58nMPvEQvQbn7P35Vvvct/m0CgNxcVvVRv68XMA4h6pLy6oraPTl74nSqPCUnKies/w
+uV3ngPEWoFiOYcodmt90x0xG9I6exkOfM6GmphqaXQkPC+LP0tzf8OqBVo6fp8kkSbhdl80L4jP1u
cDtiPjEgE+5M/A8/8bBeAwDIm6p/usx1UEdRAWrzN+/XSwCAzPWfFLn8cb2AcQhRl5U3tHlfDts6aL
N77VXEyQqN9VP3hsyCUdfTDi/9upOjwkuvNubQI2YYuIDnzB/KHyXmkP72hj9RVLUlIzuhTmDqa5O
CTNtg1JUdKNIqa55jIn7k532ffZ/c+/C9egkAMAsrX3qpXPvklXoJpn/40rfl079/qJcAAJm7bP02eukze
gFRCFGXVkIO2hlkRw52nSHNbh3Rkv0Jf3347OuzV9w864eaDXeSp4E6pCVnqL3i1eKc2KKdM2Mo/
xjjh/S3ZcNLUMtSV1PRTyvVazOhHNugZuhXPU7tScXsQNWYoCtu2QJgSvX76vKxv/2YXgIAzELpoRfa
D4z26f/6D3aYCgDIyeXPivTp2jMOIerS8ntyNptNptOOObWeCzq4p/uem+Sprnj9gDtSK2mA77BOqRmP
crmqj9sur3h1aUsxB5LvWDnzBzK787+H3iYIV70kP5e45w3jL9cv5BOuYRUr82E8miD3pbKoIM9Trvi
ls8dW7sVSMn/+v/
+l34GAJgF1ftU9ULFeHd/6AH9DACQi0v36CcYhRB1iZkzpqvehTmMvehWGCjXDmqfAwWEdHhhl1QFmzwTRMVXfPIq1NqC+shGaxH2
VzVwZg/Ll1GT440LNVzVtS1VJXmauqhoTmUvxxaW8AoiWC9cuSQ/l5D1r0CqtYrAxMwOY/JJkhK99
pMJoc2VLb9IfyBurRGfdmzm7MP3bEU7v7Fu+WOl9+hlwAAeXvjX99AHdSYfv7dP6WfAQByUXijfoJ
RCFGXWWDW8FPiTzbuDltXzPVzyAwvRw2ht+tRGr0uXeW6dJPWu0z1nFVke7BA5v5hSrUxzaH8o0
NdMxQeNaQ/Xl3VCaV5bSaVQxvsIfxhxVBVSNvvp1pfFohy1RVXyf/4lf8ht1x3i14DAMjT373yCf0M
UV7z1pvkbe+5VS8BADJ37FUix9+kFzBKoa/G9QIAgKXxwCMPyOv/2+vtmfoBAPm648vxXyysfvFYvYd
D1P/E8+a1PvVGedw0TbwFALgovFrni/daTq51ljERPVAAAlsztN91uD+0HAOTvb27/tjx2PX/ECnPlc4
9L9fdeS4AKALmx3m+Pv8f6SIAaByEqAABL6F2n3iUfeMMH9BIAIC+Xjvfl8294VH78nMt6DVz/8t+V

5NZXv0gvAQAyd+ztIoWb9QLGmSpE7TVW9AQpBX9W65F60lhxJ1TZkODL27LhTbbiPCabqGZYeyO
43dBH1jOAAwAwhz788x+W07ed1ksAgLz84PnPyt7rvqWXoLzidS+R1X8bNhkqACATx4oix39eLyCO
OeiJqsJVf5ZqR84zwquZ0VcaKU3oAwDA4rjnl+
+xh/cDAPL10M0/kO6t39dLy+3al14l1d99rV4CAGSucI3IMTWMH0nkGKIWZXOvL2oeq35gxuuuHH
iTiKvZsNXn92QzxwzV1qnJeTqkAgCWjDtj/3VXXafXAADyonqjfu+aZ/TS8lIBqgpSAQA5Of5ukcIL9QLi
mn1P1N6h7OuncuqEZJedlqXedUNc/9Gtl/XnRZq7pKgAgOVzy3W32EGqClQBAPlR9VE/96Zv2h+X1
S/8xivsofwAgJzYdVDz7rl4NOQYog7XRLXrlZZq4nZEtYfVD9UoNb/OeaRVL1Uprp0RP0YFAGA5vfm
WN8tH3/5RvQQAyIvqibqs9VFVePoLv35CLwEAMmfXQX2HXkBSc1ATNUKvISuFktS8lNXRqZVSq2H
aPu+HuNXTfpGB5WVM8uWd46hJwTA7YddqEYTfT/4kcHneY0nPYU7fC+q9b2DCO//8rEiKf0cCAt7
7s+
+V2h01vQQAyIuqjbps9VGfd82VctfvUJMbAPJztcixO/VzTGKmIWpluy/9bt3vCVptOcPst1WY2ZPGu
htwVqXlDsFvVe01qobpeqIkoSO1khtC+I9Vd0Yra9/2bgFghnqNlWAPfSBnqjeq6pUKAMjXfT/zuD1r/
7JQAer1P/E8vQQAyNzxO0UKL9YLmMT89kTtXZQdnSKU61v+RFSVLXHLmHZ2LqbU+64s9S0S1NFG
TQoGTGKR76eM295ryPpg13tgBlR9VFUnFQCQnx8/57J8/g2PLkV91Le951Z5zVtv0ksAgMwdf5PIsV
fpBUxqfkPU7oHXE8sevu/1HjWG93cOpKufTsfppZpmrVUAABaVmqm//attZuwHgJw9dv3T8je3f1sv
HU23vvpF8ku12/QSACBzhVtEjv0LvYBpzHdN1FSFz85vlhPo1M7nWItxHgxO2jWqxuPoOpD20GPv6
/VjZG3Jwf1FBNd2Pdzga0fVgsysDVEya98oca+VK/5xJm1f2OsHyncO1O806o7arx19P/kG2p/K9Ux6
DsOEt32wXungORo8P2HiTbTn8/epHqPqpCY5P0DQbdffJvf88j16CQCQl7+/7Xvyjy//oV46WlQd1O
rvvVaufO5xvQYAkK2rRY6/2/p4hbOIqSxEiFqud4fDT/uRwnDa4qac1WVWRfblcGnyBRVsDU7a1ZTV
wqr1/3hUiFMKG3rcqUlpMKBKMklYe2NETUjVYzgYFmXWhihZtW+khNcqwXEma58TyIW9vrlaGBH
2qXNitLNcl/GVM9SxDbR/6us5/f0ej9OrffAcNVdHhZyTcPbh1XO2Dd97qd3vWGrvKL6DGfsBYAb+6v
WPHcn6qL/yH15JHVQAyNPx09RBTdH8hqilk34P0YN0Bu2Ha8tuuinKQug1zvnhkTuhV7/r1Zsdzzhv
5bp0dbDd9TbQlHNeotNLMEmY9dpzesOh2+3IzkX3tVm1IUpW7Rst2bVKcpzJ2tdrrHuBnP+HjZa1F
615bkRQaLRjb1OKem0koz1u06e5ntPf70n47fHb3pHa+ejIPHqivUH+PsLvvbTud0Ds2frfdepdegkAk
AdVH1UFqUfJyumftB8AgJwce53zQGpSC1HtnmjGkFHzEWco65DimpzxspxVfxuqF6C77UQbdnpv
me1yHmYvuTOyFivhWXQ9uejO2qUCFi+kKcrmBSPEidI7lH39VDo74mY3xc09J6yxHnub+mQmmiT
MmLjHCNyKa2eGQ/XM2hAlo/aNlPBaJTnORO0LtuOst74iW/aGVVC3J2GHE2hHTNWz/rmtbPtBrd
/2JNczhfs9gWrL7yFf2Up/+4q5j/B7L8+J+bAM7v7Fu+WOl9+hlwAAefjmjU/JV376u3ppsb3sp14o/
+a3mNAEAHJTuFHk+L/WC0jLHA/nDwYcXkjrjWE1w5B0mMHN0daVAzdTKp+Ukn5qK56QU/ppJDP
kHgioh2ouTjVJmK6nGTZ8Prc2REmpfSMlvFZJjjNR+0a3wwldR5fWOHUi6XdVVU4HNlaSk2473bYn
up4p3O9zpSwnAwcRIteJ+bAMrrriKmbsB4AZuP9V37HD1EWm6p/+
+n/5GeqgAkBurhQ5/mvWR+qgpm2+a6IWN2UvbMitPdQ3hXqoHtWLri8pZ7JH3OhefG5oM1EPZ
MWu5eiGPlE1KzNsQ5R5b19sebQvRuA3VlFOLEzSmcbxAvPpphfcZAepKlAFAOTn8yuPylNXX9JLi0f
1QFU9UQEAOTn+r0QKN+gFpGmqENUc9hv1cMLJoj8M2gxA7aBUrw9NMc2v04+4dRUtdp1B82tD
H2kGskvEuHZ+7Udf89zw5DXjJwlry4bZq9OtjWnWijRl0oYo2bcvLbGOc4btG21wgreeHHp1B4ZlOv
HcEcD5QZpuv+l2e2g/ACA/KkBVQeoiel3l/6IOKgDk6dhrrQd1ULMy3z1RkZGQ4dEus05mDGp2d9V
b0Q+rjZ7D7raTTBLW3vV6dtrhjxuYG8OTB6XehihZtW+khNdqguOM176ldth1itOdfT7Y9JDh+ImO
M737fWHkNjEflpGaZOoDb/iAXgIA5EEN6f+7Vz6hlxaDmoWfOqgAkCM1C7/qhYrMEKIupaKs+bN2
yao3Ztuc0XsMPcGXKlEb7K0YEnhNOEmYH/4YM+KbcmhDlFTbN1LCa5XkOBO1L6Id9rE7NVUTnsK
Rmqsb4jfdL5lQPrPmhNaJrmcK9/uiyeB+B0wf/vkPy+nbTuslAEAeVH3Ux65/Wi/NN1X/tPp7r5XnXX
OlXgMAyJaqg/oe6+PVziIyQYi6pIqbF/zehs1VHawYk86MU9n2h393alJygxmjRqg/UVew9mbkJGGV
09aSNq5dWbUhSmbtGy3ZtUpyrpO1b2w7ynXZSmV8eFnK5aas6vZ4Tbfaftab/j/Z9Zz6fs+LOdGV28
6Jws6U7ncgwj2/fI89vB8AkI9Lx/vy+Tc8Kj9+zmW9Zn79Uu02ufXVL9JLAIDMHVsTKdysF5AVQtSlVZ
TNvcFJu6rS6res/8djD/8OK6Rpb2dgoi679ubg/ixDk4RVZHuoDc72vF01d71eitm0IUqG7Rsp4bVKcJ

zJ2qfa0Zfu0IbVpo3SBlM7JWcHjzfsGiW6ntPf7/mw7q/B67F/aPQSTiCV+x0YzZ2x/7qrrtNrAABZ+8H
zn5W/ev1jemk+veatN8nb3nOrXgIAZO7Yq0SOv0kvIEuFviqCCAAAMIF7H75XKn9UkaefXYwhpgBw
FNzx5evllQ9eq5fmx7UvvUr+06ffzDB+AMhL4RqRK9R8BQzjzwM9UQEAwMTefMub5aNv/6heAgD
k4W9u/7Z875pn9NL8qP4udVABIFfH3239jwA1L4SoAABgKu/92fdK7Y6aXgIAZE3VR/3cm75pf5wX
v/Abr5BXvO4legkAkLljbxcppFNUD/EQogIAgKmp3qiqVyoAIB+qJ+re676ll2ZLhae/8Osn9BIAIHPHiiL
H36EXkBdCVAAAkAo10dQt192ilwAAWeve+n37MUuqDqoaxg8AyImqg3rsPXoBeSJEBQAAqVAz9b
d/tc2M/QCQI9UbdZb1Ue/6T6+2g1QAQE6O3SlSeKFeQJ4IUQEAQGpuu/42ueeX79FLAICsqbqon3
/DozOpj/q299wqp/7ZDXoJAJC5428SOXabXkDeCFEBAECq3lF8BzP2A0COvv2if7Jn7M/Tra9+kfxSjV
/kASA3hVtEjv0LvYBZIEQFcteWjUJBCsZjpdHTn1tSvYasbLT1gqO94Z6fFZnX09NrrDhtXGnIqCb6x+E8
lv5aY2mo2frfdepdegkAkLW/v+178o8v/6FeytbzrrlSqr/3Wrnyucf1GgBAtq4WOf5u6+MVziJmghAV
yFVPGiur0tRLjrKcWSvq58vHDiJLNeno5YXRa8h6LarV6loXZDV4saVTK0lhIDAGjqq7f/FuuePld+glAE
DW/ur1j8kPnv+sXsrOXb9zu1z/E8/TSwCAzB1XdVBfrBcwK4SoQK66cuDmbuW6dPt96ff3ZHNZM9
SxQeScUj1nxwW/7fPiHlq1pa5zX7r1srOiuSrkqFgGV11xFTP2A0COfvycy5nXR33zr9wir3nrTXoJAJC
5YyvW41V6AbNEiArkqXco+/qpnDohy9v/dHHF6znbk8Y53QW1XJetivO0uHlWqs5Tae6SomI53PSC
m+wgVQWqAIDsPXb903L/q76jl9L1sp96ofzKfziplwAAmSvcKHL8tF7ArBGiLjGvnqP5CKntOLI2peqN
536d163Or/dp135sb+ivNdbZLwuuD/bKS2MbmtlG7zFcYzN4jMM1S+1HWN1Low3jal3a+zDDt+aq
87WDDQ9pc9i2o9o8tpfjROdl+J4ZuZ8YxxD7fGh+W9RjuK0OZwi9/7p0z506/lKsnrNGj+NAWF6Sk7
ozquwfDt9PNqM9ie65eMduS+H7wj1Pcd9HsNxuv+l2e2g/ACAff/fKJ+SbNz6ll9Kh6p/+
+n/5GeqgAkBurhQ5/mvWR+qgzgtC1CWlwpHQMKhTk1JhQ8ZlcHHYtR8HCkLa61TIMrC+uRoeXk2
1DRU2hfYY7EitNCqEU58zapaW61J3uw52duTiYMi0674ynbqmQ8GiZh/zyGBquM1uz8dQE5+X4X
umuToieEt8DFGcfQcvd0hb7WCw5A2hd6V67jxVafVbXq/SIWaP41E6B9LVT4MqcjrpPZfk2FP6vlDn
KY/3ERwdapKpD7zhA3oJAJC1z688Kk9dfUkvTe/f/Nar7J6oAICcHP9XIoUb9ALmASHqUmqLn8O4d
TmNeo3SlHPhSUpi5XrX3na/FYybwtaPGt482TaCw6mHj7EjO4PplEcFZM7r+3ubsrlVF+erBr/GPI9nZ
FyGWtm2ttd1t2Wptpx9bOvUrr3hB4Vhbe7UZH3kdQm2eXRT0jkv/invSO28cd0SHMPY8xHg7zu8r
dZxrbvBoHEu3Iamcu40u53bEitrHVKUE6f00wiVRPdckmNP7/uimOP7CI6OD//8h+X0bQxHAoA8q
ABVBalpWDn9k/YDAJCTY69zHpgrhKjLyOwlZ/R0K27uOQGJ9dhLZaajqpx1t1M5bS25RqwPHd486T
aKsrlnBj6O4toZL7TrHIT3AyzXt4IBWXFNzugv6uxc9NvY3vV65pXPrI0P38YwexjWLxht3rwgfgZ5PrR3
31CbR5r8vFRbfnDoh3xB0xxDFHPfoW3tXZQd3SEycC4qW/5+zWtniH/u1HFY3yOhIe8k9uUwrEFKk
nsu0bGn+H2R2/sIjpp7fvkee3g/ACB7akj/V376u3ppMqr3qeqFCgDIiV0H9V/rBcwTQtRlZAQ0ImqY
bsGrZTiyhuIkyielpJ8GjFofJo1t2HQ9x9BhzEGnTgwGP0VZ8xMtLyzyA0Mj0J1YVK9WY/8jgrfhNscV/7
yMN90xjFaWk+MudvfAa79X7sF+GEPcRwyfn/zcTeuUjN51gntuimNPcv2HzlNe7yM4ctwZ+6+76jq
9BgCQJTXJ1KT1UVX907t+59XUQQWA3Kg6qHdaH6mDOo8IUZdSUTYvhPckdEOYqMl1FkZg8hyjn
mOk8MDO76nnDnc2AsPq6dg9GafXkeGOgjFCRlOK52UyYccwK2keVxw9ORxbLNWR2T2X2vVfkvc
RZOKW625hxn4AyNFfvf4x+fFzLuul+H6pdpvc+uoX6SUAQOaOrYkUbtYLmDeEqMuquCl7esjtQKlR
W/Pcos+u3ZYNs3edW7PRrMGZhHW+zurzZA+NNoYyV0/nF6FOH/qlfF4mkm1w6dXKHXpMWsd0
AsUTMrb06bje1BPcc+OPPf3vi6P9PoIsvfmWN8tH3/5RvQQAyNIPnv+sHaQm8Zq33iRve8+tegkAkL
ljrxI5/ia9gHlEiLrE1MzaqqeYPcGPHYR0vRqKo4f/GoxhxHPHrB2pgiW3/uMUba64U6Z3duT8+R29n
aqkk6GW5KR37gdnZO/JRbfoZeQQ8BgyOC+
+nI4hTOmkFwKOqumZL+NcNHfF75DZlQPvNJxwzn2EWPdckmPP4PpP/T6Cpfben32v1O6o6SUAQ
Jb+8eU/lL+/7Xt6Kdr1P/E8uet3qF8NALkpvFgP48c8I0RdRu0Ne6itmkU92FPMCHiMXnIlPxkzZmJvy
4Y3Dft884MlY2bySXgTWHWk2dQnKrWh/EXZdLsdqvO87l+XXmPdq22ZZBKkcVI7L578j8Fj1udsrvr
DyPW9bj9yHVtu1oBtyq7eda9xzgsxY/VgjnPPTXjsU1//hO8jZntyvRSYe6o3quqVCgDI3t/c/m157Pqn
9VI4Vf+0+nuvleddc6VeAwDIlqqD+h7r49XOIuYWIeoyqmz7Q287NSm5QYtRH7F6NnzmbhXSDL52
Lpkz9nttNibamUhF3I6BrlSH8o+4LiU/fZQL005glcl5MSQ9BnPYu9ueiRK2YH3O5qqz34IX9Felldqs+v

EUNy94PTLd9njnodqSeM2Jc88lOPY0r3/C9xEgiqqPquqkAgCydeI4Xz7/hkcj66NW/u8idVABIE/H3k
od1AVBiLqk7KG3YUUMVeDS7wcDHlX3cLBmYrWVcx3NpCqy3W/5gZHNOTbvsAPDrOPxhlcr5bpsp
ZzL2dcl5LxWW9Z6d+j1VLI5L6Zkx2C1Z/A+3D80ejUmYNfnNIaSu+y6nznWQ/UUZXNvuFaoPYw+Q
aAb656LfezpXv9E7yNABDVTf/tX28zYDwA5UPVR7/uZx/VS0Cte9xL5hV8/oZcAAJk7Zv2GfPwdegHz
rtBXRewAxKNmNtcT86gwbG/anqHAOEfpntPHcqpFwIpwn+l9Rip/xM0BAHl441/fIKWHXqiXRK596
VXyW3/8z+yPAIAcFK4ROf5+66P/Xoz5Rk9UILaeNNbdmc3LcmaNABVZO1r3XPu8dSwZ9ODG0fGO
4juYsR8AcrL3um/J9655Ri+JVH/3tQSoAJCn4+8mQF0w9EQFxmrLxmANWFXOgK50yMzRu+d6jRU
p7ZyRbiplKXDUFf6fgn4GAMjStU9eKe9s/aS88723MYwfAPJ07O0M419AhKjAWAOBFgEqMsc9h+
WlJiiblx9NaEs42hKOtoSjLeHmqS0nrr1GDr/3pF6aLa5RONoSjraEoy3haAvSQIgKAADmBj/ghqMt4
WhLONoSjraEoy3haEs42hKOtoSjLeHmqS1IhpqoAAAAAAAAABCBEBUAAAAAAAAAIhCiAgAAAAA
AAEAEQlTkSE2WU7DrfxRWGtLTa8fqNWRlo60XHO0NvZ3CijRibwjp6Uljxb0GG9aVTbJuGmH3UNr
7mJbRRv1Y4SYFAAAAAGChEaJirvUaK1Io1aSjl4H5pgJdY1Z9W1nOrBX1cwAAAAAAsIgIUTG/eg1Zrx
GfzqeibO717RkF+/1tqei1szFPbenKgXvLluvStdu0J5tkqAAAAAAALDRCVABIS+9Q9vVTOXVCyE4BA
AAAADgaCFGXmao1OlC7MazG6GD9UXuIvfE1A+VKNbNOpXokq1Vp79Mcxt9cdbYTvjOjjeoxqk7qY
JvCa1VOdrymMbVf2xvetob2H3JN4rTRY359ZEONcxFo46j1xj7t9ea5zKoOadx7KKotg9uYsD5pjOuS
9J4FAAAAAACLgxB1WakgL7TWaEdqpVEhpPpcQUoDQ+ybq4OvVyFiSYIva8pqYbBWZBqcNq0GNh
xyDHYINtgm65W1UnjQaYt7vIMqcrqqn3Z25OLAa9u7bmODtTKHQjgtuo2TKsrambLztHMgXeeZxR
iOHmh7W9xml8+s5dDDMoV7aKJrPizf6wIAAAAAAOYRIepS6knjnJuIuXUb+9Kt61BNOrIzmPx5qtL
Sr2+5QaEKG8/7ve16jXN+0FVt6VqVXfE2H0Nl2/qabl28L3G3sx1W8dJvU/gxWMe77oZg/mv77gF0
arI+MhUdf7xhKltu2wfPpR9GSvmMeBlqe8MPgsOuSWQbJ1NcO6PbuC+H7qbbu0ZI2ZEDN101hq
mfOpF9hDr9PTTNNTckuC7J7lkAAAAAALBICFGXkjERz96m16vQD9VEOl56FlRt+RP3+EGhqScXd9w
+e1VpeQGStc8LYa+fntmm0GPoXRS3SeX6lj/xUGXLC+U6OxdDexSOP94RimvidfQ0t22ElGaPTrN3av
2CcU02L/htrJ0fGKo+Ja+NftDbO3Si0mrVCRv3dbrau7jjBZKnvROYlRTuoSmuuWkm1wUAAAAAAM
wdQlRYdA3P0OH9SZmzk5+Ukn5qK56QU/ppespyMrCTEN0D77jsIdheXUtjqHdgSHsazOHy/rB4P5
SryllvyvYRvVNtxnbMHqOpKMoJfUHcwLlrXzzrnJ4+aYeVwfWW6mk/kMxMCvdQKtd8VtcFAAAAAA
DMG0LUZRWYKCdunckYgeWRMt3x+r1i3Z6eRig3URhpDK9PScUt3trctVrXE6cj6ik5UdFhZWC9anb
2EeriSf+6AAAAAACA+UKIupTasmH2OnVrPZr1HI+ocr3rlDEYevjD9lNT3JSzbglONXTcqCs6WRiZQYh
dOS164L4ctvUQeLv3pzs5lrF+QUP07K/5sv1xAQAAAACA5UOIuozMupwqYHLrohpDoCdXkpNuEjs
4XNoIEXNVcoamK6NqvWbF6+nZ2ZHz50fVFTXP2eBs/mZ90FMydk6nxNfQ3XdHDnadr3VrtZbsT/jr
h4e0ZyWFeyiVa57idQEAAAAAAAuNEHXJ+QGTMWP/VMxakU1Z3XCn3DFnS8+ZMcmTNFfFa1J7w
6+V6a1MmdfTsyPNpj76oaH8Rdl0u6xar6utN6yz5eg11r0anuYESU7AqVivP+8dkGx4U8nH5V+vZtP
5Wnf2/aIumOquNyfCylYK91Aq1zz5dQEAAAAAAEcTIeoy8oI9S3NVh0rGhDtTMmcun2r75iRC7nY
mCjuDs7o3V3WI5gWO5gzwaXOHxftCh/JXtqXl5XU1KdnnrCAlP6mTC95EVCojdOutWrxzHLe2bVBg
W9Yzb2i6eZ9Y68/k0w3VNv09lNI1T3hdAAAAAADA0USIupQqst1vGQGZUpVWv+8HRvaEQpMqyu
Ze1w/BbGr7g/scx2qn1yBt/9DrDZhIcVP2+oNtstj1YDOoh2rwhvQr1v62Ruysst0PrUtbbVnr3ZILLnU
8g6+ttiara2v22gwMTTeGs+c+ZD2Feyila57ougAAAAAAgCOp0FczrADITq8hK3oiL1WDdo+eiwAwk
urtPS8/mtCWcLQlHG0JR1vC0ZZwtCUcbQlHW8LRlnC0BWmgJyqQKbOOZ75D4gEAAAAAAJAOQlQ
gE23ZKAzU8ayeFTqhAgAAAAAALB5CVCAPql5pZpNXAQAAAAAAIEvURAUAAHODelXhaEs42hKOto
SjLeFoSzjaEo62hKMt4WhLuHlqC5KhJyoAAAAAAAAARCBEBQAAAAAAAAAIAIDOcHAAAAAAAAAgAj0R
EV2eg1Z2WjrhVnrSWNFzZavHhsyL60CAAAAAAADA/CNERSZ6jRUplGrS0csAAAAAAADAoiJERfp6D
VmvEZ8CAAAAAADgaCBEBQAAAAAAAAIAIhKhLqr3h1gddkUZPD7+3l51HaClTVePUeI3zcL7eZW/XH
MbfXHVepzc4uF+PuW1j58HXt2XDfY318F4Wo12TGjwv6jHpuVGSHr8S1obCSkPCD8+s/eo8VkaciG
TbBQAAAAAAWF6EqEuvI7VSQUoDw+
+bqwMhX3tjRI1T9fXpBJbR1H5WpamXpFyXrYr1MbN2OWHk4HlRmquFYNCZ4blRoWtYG6RTk9Lg

BFl2EFuSwZd3aqWhcDTRdgEAAAAAAJYcISosVWn1+9K3Hq2qXqUCwPNulNaTxjkdX5br0tWv7dbL
zjrrtTsXnYiusm19rlsX9zNSbdmv7W+rxHNafjv7e5tSTNCupHqNdS+MLNe7zj77LasFWvOcDkeza4NI
W3bd1Dh020055yW0VjvW3SDXOE/uBe3UZN17bZLtAgAAAAAAgBAVUm1tixtxVraMANRTlM09H
crZ4aVeu3bGe23noKufZadc3/La6ciqXT25uOP20qzK2U13yxXZsoNGFVLuibM6w3PTO5R9/VQ6O+
JmscXNPWd/1mPPbVvvorhNDpynypa42Whn56J1ZJYk2wUAAAAAAAAhKiaha5OGDmHPzqkT44K
9tNrVlQN3A+WTUtJPFSdo9EPnYSmem+KanHGTWGtrquyCW7t0qM5p98Dbnz18X7+uYA7v7xxYR
2ZJsl0AAAAAAAAQoqIsJ82UcJTAxElGbdLcjGjnzNtlyawNRdm8ENYz2A9KQye5Giur7QIAAAAAABx
NhKiIoS0bZs9Kt46mWft0JuahXRm3obgpe3qIvV+v1tc8Nzybvl/DdfBh9KCdYLsAgLzp0Q3Ggz9yAQ
AAALNBiIrx2rte70o7oHNrfxpDyKc2ybYya1dJTroJqDsE3qVm4i8YM+6n1YaI16uZ9NUvzfakXXbw2f
XqnHrtK530Qtu4NVhjbRcAlpH9Xl+QwkwTy540VlZFWu57tPNHr+bqLILU+Qxze40Vpz0r+f/hT/0ba
p4P9zEP5wXDhq7XDO6ZmRvzvuZ9P7mPvG7mOO+3ebwnR+5jSf+gFfO8z/K9eGZinJuh951luGnG
nJfc34sTtofSdoiDEBWJ+AGdMSt9AiU/nTRm/7d+MFmdbhD8tO0KKsqaVzS0Kavem667baeO6OB7
cZw2JDp+/aavPhXsGRpSs9Wsc9pc9dvm/sOhHu7KJNtVjG3wDwuALMzsl/chKri09j/lv0npcCYu3Da
KcLuTP+4f5vte3N4IC3ONPyjOQq8h6zWRatX9x28G3NEnxsO8XrMy01/cAyWOhh+z+NZW52O1qS
YFda9TS6qdmpRmFPjk/0v7+Pc19R5csr6f6l3jHFk/T2Z778R5v83jPXncPtTnc/yDVmTok1eYm+C85/l
eHHFuht739CP98xP/vl3dH/g3Iot/IEadk9zfi8efl3zfiydoj/WNrUrbpf+eHO/7dn5+FsZY1g2DJWS9R/
TV5bd+A+hbPzD5uvW+9U+g87mq9ZZia/WtnxX060c8vNcqIa93P29uf9TD2NbIdtqStKvbr5fd9VXr
K8cxXx/yKNetVygJz83Exx/
+MDcdve3gMSfabqvqrS8PXwQAmEq3XrbeX8z3eP2+GngjyoffFv1vwAzaEEm/z8/8vXjm7XCuj9q/f
c28f5PzY/87OoP9RtP37dy1y/3eivPzV9rCv5dn1R7n5y9jv/pnrCy/l8a/rznvuYNtyPocjW9XvNdMa6J
9ZPIeqPdvbdd+hLRD3T/maud+Um3XK1IS/5w4n8/+vTjeucnjvS/
+fZv1+8v4cxImq7aNPy/h62fWnhHfw+nfR87+zd0737fBdX579Qr9vhz3uiJf9ERFDBXZVn8p0ksO56
821puAo7kr/t9KrNd7n9D2D52/MKlanIP1QqutCWuIJm1XEk4PIOsNTS/7rDdbf9h+0jYkPH57qP3g
ubQ5+wj8QdOuc2oMyXfZPWWMeqiWRNsFgMy05XytY71NXZBN503VUpEt9UY28fv35Iqbe9Lv7x
ltmTO69MupE/PRwFm1o9dYl1qnKmfn9kLNhnteWt7PKPPC+T6X6unAzyL5KMqJU9YH9+dQW08u
7sygPb2GqIFK5fqWv9/Ktv3zYmfnYgY9sRxj39d6h7JvfRj8fi7aJ64puxm9Ecd5v83jPXle3ved79+y1Ls
hP8tr6uf34dEJHdm5mO7dE/ec5PVeHOfc5GX8uXHeXwLf5xmY7Jxk9148/rzk+148tj0jfp6yR42mW
tYuzqii+fpZGOMRoi4pvw7mwJuLMeFQcMiBCgvdr1EPJ5TztxMM6dQPhf5rrYf5A725D/VQ+xmx35
Ht9MRtl/MGFlw3nvMG7H6d89gbakjCc5Pg+G2D59J+jDoG8zj1Y9QvU3G3a7xu+NgBYAoz+uV9M
enSL+W6bIX/A5CTnjTWa9KZVTvsoaMdqbbi/1ueGTUM0Rh6N9uSN/n84j6JXuOc9d1s/bI/oxu3sq2
HjNo17dWwypITNg/
+vJW13H5pT8huV1lOenWcNKPePgZk8AetuMFlmDTbEVuO78XTnJv86fJoB+cHhtOnWwJnknPCe7
FhxPub8/PnvuRaNYmfhRcOISoAAJgNfnmPTdUltX/5uTCLXoa6tpj9i6D+pWdGvR3b52vSqbZmPmL
C/0Op81AjV7KppRZXPr+4J+f2NDo7wwDEHTWkatqr7yOrObMI4efpl3ZMgT9oKfPyXhwwR3/Y6uy
flAvevxGqt6h6/9mYYa9C3osD9HwiwflBrO+pqedWiWHwjzD8LLxwCFEBAADmmDP5gRq2N6ueO
MFRDt36vqzO4hfU9oYzCcRc/dbuUL2Csh6WHcfc/eLePi/O6NHZXTNnsg5/YiAVeKtJgXKftGOWv7Q
jNfxByzKH78Xz9oet8pk147pY/4aeVWXUZtirkPfiAernGrdnrP89dXBKXadTkl3n7nn5IwymQYgKAA
Awp5wAVfXWmJ+hjMXNC3YNtk7tfK7hXHtXhU1NO8B1fuEpSEn9Vqh/CZrtcHptlsOyLfP1i7sOCGf5
y6Iecqzq2bt5jz0MViXezdWMZjYfZVa/tI9h93bqyMHgjTuqd9QS4w9ajkV4L56XP2x5ZtqrkPficIMl+f
qydXLfOk8nJau3vdn+EQZpIUQFAACzwS/vkVSvDSdAndMJ/zL8RSPMYE8j9bAngLR+Mexaz2dbt7sn
h6qoWc7nZKxZ/uLu9nw6O8NfFgeHTbpmdl7y/6V9rOIJceZ7CUZNPfuGnmG4O2f4g5Zvvt+LB+T+h
62S2GWOB8PbWf5cw3txTLq2eOCPkekZ+UcYfhZeOISoAABgNvjlPYKeRddiD3czevzkW+dSTf4wM
Bxc/0KW1S8ac6/XkJUVc0i2WqVmSp7lL6nz94u701utKjMcPSpSOW21wPoeCgyhV7dwzTovM26bL
dtf2uOpyGnVY6+27r+veL3G5m+islngD1qLaFZ/2NIjADo1Oe/9w6mHcM+oHinvxXGoUhklqUldLm

RwkSL/CMPPwounDwAAMCOtqvSt33L69a5e0a33y9aPJ2VvRX669bLVFtWewUe139KvWUZh56U
6JyfEblu53s/3bun26+Xg+ZiLe6RVHbg2rb71S2tfJT+5m+H38TB9HgKPefie1vdRxvdv3Pc1573Yf2R9
7eK0K4/35PH7CLt/3Ifxb1dq9H0x9H2r2jFw3Pp7Pu1rNcl5t78m8/fiEedGvd8M7Ns9hrTf/uKem6H
XZfY+POp+0XJ6L453XvJ7Lx7fnpB/xzO6RnHuxXn6WRjjFdT/rIsKAAAwE+5f6F3WD43zNRwQWBCq
x5xdm9BVbUl/Bl3nnO/pqrT6M5p5eS7pnk7G5ZnV9cH8Gfre9fjfR2Gvqc5r79gUjT83Id9bxnk7iuLc
LwrvxbOmRvOoOqhhgkP7+Vl4cRCiAgAAAAAAAEAEaqICAAAAAAAAQARCVAAAAAAAAAACIQIgKA
AAAAAAAABEIUQEAAAAAAAAgAiEqAAAAAAAAAEQgRAUAAAAAAAACACISoAAAAAAAAABCBEBU
AAAAAAAAAIhCiAgAAAAAAAEAEQlQAAAAAAAAAiECICgAAAAAAAAARCFEBAAAAAAAAIAIhKgAA
AAAAAABEIEQFAAAAAAAAgAiEqAAAAAAAAAAQgRAVAAAAAAAAAACIQogIAAAAAAABABEJUAAA
AAAAAAIhAiAoAAAAAAAAAEQhRAQAAAAAAACACISoAAAAAAAAARCBEBQAAAAAAAIAIhKgAAA
AAAAAAEIEQFQAAAAAAAAAiEKICAAAAAAAAQARCVAAAAAAAAAACIQIgKAAAAAAAAABEIUQEAA
AAAAAAgAiEqAAAAAAAAAEQgRAUAAAAAAACACISoAAAAAAAAABCBEBUAAADAQmtvFKRQ0I+N
tl5raG94n19p9PTK2fDbuiEhLV0wbdlwz3vk+e1JY2Xca6xXNVb8ba00rK8CAGB+EKICAAAAODqa52
TGOemSUMHoqjT1kqMsZ9aK+rmpKJsX6tZnHZ3aesg1asv5Wkc/F6me3bS+CgCA+UGICgAAAOAI6
Ujt/OL38Zx/XTlwM89yXbr9vvT7e7I5KvksbsrZqn4eco16jXN+IFttyXZFPwcAYE4QogIAAAA4WuiNm
r3eoezrp3LqRKxeo5Xtlng5auAamb1Qq9IiQQUAzCFCVAAAAAABHTLzeqH590pVg6NpryIpbm9Orse
rX/7Rrehp1Vr119suC68NKtPqCtUJH1wEdeJ31CKsrGjyeYL3S6HYYzGPXj8F92fsp1ayzrDVXndeO3Ul
FtltujOpfo/aGXxagXN+yXjUo3vHbQto/dH0tcc5VoEar+6BWKwAsLUJUAAAAAEdEWep1HdJl2Bu1U
ytJYTVYDdRep0K2gfXN1VEBZlNWCyUxyoCqjUhpcMIpOxQceJ3F3t/IQK8jtZJRr7Rcl60YnTuHwlEtel
8JVbak7hZHba7KRqMh5/wEVS4M1gNIcvwqwA5pv3M+hoNUR/i5UueiNLhTJewaAQCWAiEqAAA
AgKNjzQ3p4vVGnVS53pW+qgPq9ax0hK1v7o5oh1dLtC/
+y5tyzkv7etJYd0PBqrT0a71td2qyPjIpNl6/F2OSpvaGePmv0a6um3ga+6psW5/r+hNFqRqm9n5iDc
MPTjLVrLnHV5b6hcF2Jjl+67VuGhvWfmsrOxfjnqu27Eadi8A1AgAsC0JUAAAAAEdIUTbdGYwy641al
bNuj8nKaWvJNWL9/mFoL05zBnqzXmhn56Lz+t5F2dGdIQPD3I3enN5rB4QPix+t7aeGgTCzuHnB31
ftfDo9MAOTTGnVs8OTUiU6fuu675lBqKO4dsYLbDsHXf0saOhcmfVeOzviZq/FzT1n+9Zjb+QMWgC
Ao4oQFQAAAMDR4oVsGfVGLZ+Ukn4aMGp9qKqcDiR3JTnpp31ix33dA90L01rllguwH8bwdve1A0
6dSBLymT0vz8ha4EuLsnbGbdi+HIYlthMITDJlPQudTGqK41fHZNc5DR3eHzR0ropr4h2yuodK7n4ja
rECAI48QlQAAAAAR8xAb9RD5+l8K8qJU/rp1MpyMn6am0BHRnTmnE6i8DlCYFIpo85ppLBzFSw5Y
HLD3NgTdQEAjgxCVAAAAABHj9kbtRYvTsvXYK/Onhx6Y8iHebVWhx7biYbtTyercHa88cfflg2z16lby9
Ss3ZpEcVP29D4Gyt7amueYpR8AIg0hKgAAAIAjyOiNmpQxjDw7g706u3Lg7tTtmVk66QWAo+p5ps
MsJeDXAHX05KJbmFROSaIqAdNKcvztXa/nqR24unVRp7iWaoZ+1ePUnkjLDlS7Xi3W0WUEAABHF
SEqAAAAgKPJmIAoTMlPDo3aqW3Z8Kapz1ZzdcObqKm94Q8/L59ZcwJAszZnc9UfQt7e8Gp0FlIZV2
4Gzta5WPd7WfYa61790aSTVU1twuP3A1djxv4k9PbVbRDscRoSdAMAlgYhKgAAAIAjKro3qjlzuwrp
nGAubi3NaZWlXG7Kqg4D/dzWmOFftd+ozdlc1cGh9+IREzJNorLtD1vv1KSk21XyE1S5kPuM9AmOv
3Lan6jKu5bGBFRJjDgX5r1RPat7ugIAlgYhKgAAAlCjK6o3qqp7OVgzs9qavI5mIqfk7J4xPFyx63gO1Di
1a3MOvE4Je+2U7GHrIcdebVnr3eHxeYt9/BXZ7psz/itVaZk1TZu7Xs/fcexzEVYMVW8zrewaALA4Cn
1V3AUAAAAAAAAAEIqeqAAAAAAAAAAQgRAVAAAAAAAAAACIQogIAAAAAAABABEJUAAAAAAAA
AIhAiAoAAAAAAAAAEQhRAQAAAAAAACACISoAAAAAAAAARCBEBQAAAAAAAIAIhKgAAAAAAAA
AEIEQFQAAAAAAABGEvn/AZM6nr53azj8AAAAAElFTkSuQmCC]]></redcap:Attachment>
</ItemDef>
<ItemDef OID="q15" Name="q15" DataType="text" Length="1" redcap:Variable="q15" redcap:FieldType="radio" redcap:RequiredField="y" redcap:BranchingLogic="[q14] = '2'">

<Question><TranslatedText>Do you prefer Life A or Life B, or do you think Life A and Life B are the same as one another?
</TranslatedText><redcap:FormattedTranslatedText>Do you prefer Life A or Life B, or do you think Life A and Life B are the same as one another?
 </redcap:FormattedTranslatedText></Question>
<CodeListRef CodeListOID="q15.choices"/>
</ItemDef>
<ItemDef OID="summary16" Name="summary16" DataType="text" Length="999" redcap:Variable="summary16" redcap:FieldType="descriptive" redcap:SectionHeader=" " redcap:BranchingLogic="[q14] = '1'">
<Question><TranslatedText>
Alzheimer's disease - Description #[no]

If you have Alzheimer's disease...

1) You might forget recent events of importance to you. For example, if you are interested in hockey, then you might forget whether your favorite team won their last game, although you may remember watching the game itself.

2) You might forget the names of people you met recently, or the names of local streets.

3) You may no longer be able to handle your personal finances or plan leisure activities such as dinner parties or vacations. Simple decisions such as what to order in restaurants might become difficult to make. Your ability to concentrate may decrease and you might have difficulty driving.

4) You may start having trouble finding words to express your thoughts. You might need to be reminded to shower or brush your teeth, but you could do these activities on your own. You may need to post notes around the home to remind you of simple things like turning off the stove.

5) You may remain aware of the day and your surroundings. You might be able to travel to familiar locations like the supermarket, although probably with the help of a loved one or friend. If you are in a social gathering, then you might have difficulty following the conversation.

6) You may continue to enjoy hobbies such as puzzles or reading.

Now consider two possible lives. In Life A, you will live in perfect health for 13 years and then you will die of natural causes. In Life B, you will live in perfect health for the next 10 years, then

you will live with Alzheimer's disease as described in the summary for another 10 years, and then you will die of natural causes.

</TranslatedText><redcap:FormattedTranslatedText>
Alzheimer's disease - Description #[no]

<p></p>

If you have Alzheimer's disease...
<p></p>
1) You might forget recent events of importance to you. For example, if you are interested in hockey, then you might forget whether your favorite team won their last game, although you may remember watching the game itself.
<p></p>
2) You might forget the names of people you met recently, or the names of local streets.
<p></p>
3) You may no longer be able to handle your personal finances or plan leisure activities such as dinner parties or vacations. Simple decisions such as what to order in restaurants might become difficult to make. Your ability to concentrate may decrease and you might have difficulty driving.
<p></p>
4) You may start having trouble finding words to express your thoughts. You might need to be reminded to shower or brush your teeth, but you could do these activities on your own. You may need to post notes around the home to remind you of simple things like turning off the stove.
<p></p>
5) You may remain aware of the day and your surroundings. You might be able to travel to familiar locations like the supermarket, although probably with the help of a loved one or friend. If you are in a social gathering, then you might have difficulty following the conversation.
<p></p>
6) You may continue to enjoy hobbies such as puzzles or reading.
<p></p>
<p></p>
<p></p>
<p></p>
Now consider two possible lives. In Life A, you will live in perfect health for 13 years and then you will die of natural causes. In Life B, you will live in perfect health for the next 10 years, then you will live with Alzheimer's disease as described in the summary for another 10 years, and then you will die of natural causes.
<p></p>
</redcap:FormattedTranslatedText></Question>
</ItemDef>

<ItemDef OID="a16" Name="a16" DataType="text" Length="999" redcap:Variable="a16" redcap:FieldType="descriptive" redcap:BranchingLogic="[q14] = '1'" redcap:InlineImage="1">
<Question><TranslatedText></TranslatedText></Question>
<redcap:Attachment DocName="13.png" MimeType="image/png"><![CDATA[iVBORw0KGgoAAAANSUhEUgAABVEAAADYCAIAAABtBTQzAAAAAXNSR0IArs4c6QAAAAR
nQU1BAACxjwv8YQUAAAAJcEhZcwAAEnQAABJ0Ad5mH3gAACsgSURBVHhe7d1tqF3Vve9x09See
FUS2p6QthdNPcgJKpJWDqT7Fk2hVCkIgkqDWOm5RzYRWhoCbaQ0h/vCoEghWxH0QrHC6b3xhU
WEXhVbCKVCAoXmhS8UrOwXlgbii60U2WJe9M6HMcYc8z8e5lxjPqy15vp+
+HFv1nwY/zHmWll7/NvunCv+AQAAAAAApoieHwAwkrObZ6/4X1cQQgjpnusfvlp9twJAFD0/AGA
Mm1ube57YI/ashBBC0vLQTTd88Nf31TcsAITR8wMABre1vXXgmQNiw0oIISQtn/vZZ/7jxn85df+9n
37yifqeBYAAen4AwODu+vVdYsNKCCEkOd/
+1r6s58/y4qnH1PcsAATQ8wMAhnXs9WNit0oIISQ5Nx/ZXTb8Zf78uzfUty0A+NDzAwAGdOatM2K
3SgghJDl7f7jrBwdusHv+42uPrx0SX3nAoCDnh8AMJRz75/b9dgusWElhBCSlp0nd9x323V2w1/my
QcfUF+7AOCg5wcADGJza3PfL/aJ7FihJDk3H7nXtHtm7zy9FPqyxcA6uj5AQD92768ffC5C5g2K3Sggh
JDk3PnSt6PNF3jl/Xn0FA4CFnh8A0L8jLx0Ru1VCCCHJ2X38SvFr/G74xX4vT8AICePf7Hx8VulRBCS
HJCv8bv5vR//Lv6IgYAjZ4fANCnl99+WexWCSGEdMn/
+O4/i94+kjd+9bz6OGFCDo+pLGQDo+QEAfdna3tq/sV/sV09Y05cfiOjz/6SH01A1h59PwAgB5sX94+/MJhsVslhBCSnJ0nd9z9z9ja+If
r5lnnnkqPp2BrDy6PkBAD04+tujYrdKCCGkOkSw7d/UXRyc+Us//3/JNP1Jc1gFVFzz
8npgmSHU7V+y1Skyp6tvjLkLS7OhqnSkdmkqXeml41bnTp8+r53bnR8y+pza3NPU/sEbtVQgghXfLd
b35ZdO/Jef7ET9X3NYBVRc8/N97mvmr+FrlFlS2cp6PzaXnZChqm2233vOfT8/vmRs+/jLa2tw48cw
sVQkhhHTJ1+/9OjbO/bN37ykvbUBCSJd86ZGGrRMfxPUbkwzrVzMsfdc8+PQdmkqXeml41bnTp8+r
53bnR8y+pza3NPU/sEbtVQgghXfLd
b35ZdO/Jef7ET9X3NYBVRc8/N97mvmr+FrlFlS2cp6PzaXnZChqm2233vOfT8/vmRs+/jLa2tw48c0B
sVQkhhHTJ1+/9vOjbO+bN37ykvrUBrCR6/jkyXb9pcsyRBe+KRcfWsplvednqGejBtBuWnh9d3PXru8
RWlRBCSJd86ZGGrRMfePfxiP7Di6Pnnqt7ju/8ZQMX0SAVxgadZauj39OnqDveIHjY/IoaLvwyJ3eXv9/
QlvoXl6ld7ybnZheyx4lO37orfFJueNYj1avNia1dYt3oKBFZoPxDrLrda02TEVGqXmHP5JEJzaz0T
LIpjrx8TW1VCCCFd0tev8bs5df+df+9/GI/sLLo+eer6pPWN/QfnR7H0ylMvsZkmpNVke+rzbBJJpB1JHit
Rgu/jIkepeubw+hr9BTMnfYrEX7yLn5H2YuNlCru5qm5xlkbWPDPSgej602R+8V4nFa7Cdr6WVpg
HMFbV3lTS3VjPBwjjz1hmxVSWEENIx3/7WPtGr18bXeRecmGWYx9tzvsEBrBh6/nmaoUIHKOdDq79TyZjfWD
WvbUmy0uGLu5K9ap79Wd1VHzO1Kh11GapvE//Ads1OXuq95cV16ZBzUXbyW6xhaIH2oeqbL8Odi7nJFR
6r+tYf1/S6wQRkGjHcvAAtbyhAPN+V/EmS5jgQUX9Y+fgBP89DDX9/EHtqAMPKsDswSUx7NzeOcuFgY1QpgvWk3cB
YTQHpXbrb2zU3tUlvr3GrDAGmWgBHeXV9QCJwPkFaKGGpxF6EYgN9Gb0Y2qetNuY2Y8IEZKzxO2ii3nv
lYJEJEspqrTtLtSqVBa0r/8VbYKYVJHQDEbs3ANNzRJzTVvfZhXmfhY7XsPNS5WWX4n1mvtapKZW/5ZrCjPy
6ZM/HpabMrYKGwgfr3GTZvJ1yQl6spl4C0I9uggrXY1TKnb/eQoglzT/GacEVsFLomzUeLjSUjvpnDMiM6gBbw
Il9btb+7N0aEQFeXMbDR4aGVd0Eh2OvAMY3Knc41KTfSGMkEZu6P89T39qF3nPcYiE3nlLh3rGDx1EBa
ofg9N2P7yKZnSbDKwfS/DxXd6BkzjEcmGe7LNDGWnDZIeu7Vn6RTZr2bbSH/YgpHyvLHM9dEWXfRP
PrZnnNWDnbzKwQkZAyThhRMKtrbL1gRUPVxf3M2znJjp+fu7i58+c5LeiNr8tr9BbJn9IqbFubtD2MQE9b
SSW+pvqvLOSD9i+cFnTiC8yb6u9bypH4Jf7KnLB7SfeP/sdKLQlLNG+JlWPQOzbjiA+C

6s3T85QueSbQPJpzRfhANayHZ1byxqbJyLlV1ztH1Gvv3FrMBHNz4eIF/qF+QgjpNztP7rjvtutENz5an
nzwAfUVD2Dq6PkXgafZsZmWqsa+2GqypHizVN1ohjPVvAXK4eIvQ9rdVa3WeRzmjpqZiiY2lv63IOc
bp6Ya1FO65J1A82L9V1jD12cdWF2rB9IwmfDjUZe4I3rm1mommIet7a39G/vFVpUQQkjH3H7nXtG
Hjxx+sR9YEfT8i8DT7Ej1psvTAdkXZKfbNkvudd47xcH4y5CWd8WfhrmrEHIiiqyS1ljad+k/Z9wbYtML
Liw4gebF1q9wLrHm6r090/qBxCdjV8pv06/LIXwjOnNrPROMavvy9uEXDot9KiGEkI6Zy6/xixy95ab3
LlxQX/cApoueH2jmaUeB1XD0t0fFPpUQQkjHXPOTz37/5q+KDnwuOXH4jo8/+kh94wOYKHp+oBk9
P1bT6XOnxT6VEEJIx+w8uePub3xF9N5zzDOPHFVf+gAmip4faEbPjxV0dvOs2KcSQgjpnkN3f1F03X
PPG796Xn31A5gien6gGT0/Vs3m1uaeJ/aIfSohhJCOuf7hq0W/vQjhF/uBaaPnBwDUbG1vHXjmgNi
nEkII6ZjF+TV+Nz+/8zuffvKJ+jEAYFro+QEANXf9+i6xTyWEENI93/3ml0WnvvVB5/sRP1Y8BANNCzw8
AqBx7/ZjYpBJCCOmer9/7edFjL2De/M1L6ocBgAmh5wcAKGfeOiM2qYQQQQrrnS49cJbrrxczRW276
23t/UT8SAEwFPf986X8brjDIPxD37sba+qvqz9P4t+jqK4rILgysVZ0p8e/ytdP6szO9j9zKOPf+uV2P7R
L7VEIIIR1z1YmdD9y6X3TXCxt+sR+YHnr+Oap1nkM0RarAhBowd0VB5umKtYqnXmr3HyKstlafnel9
5FbH5tbmvl/sE/tUQggh3bPgv8bv5r/
+86T62QBgEuj552jgbsg0t5NpwHwr8rMb+/paxROgI22txaOa3kduZWxf3j743EGxSSWEENI9t96/R
3TUS5E//
+4N9RMCwPKj55+f9h1smlXt+e1+P1dbqz5pRqAlbYuef8qOvHREbFIJlYR0z94f7vrBgRtEO70U+dFt
X/vgr+
+rHxIAlhw9/3zoRshi97H1tlV0S1YTVQ0juuDQ+HYDZl3j9mO1GTS2a/aw9p1iVrFhqzPmJjNBMVvN
GT1Tf3KF+Oz1uL7BMvbKLJ7DTU9Mzqx2iTVetdBySt H7AtrWql0YfbOyk4FHoVXzNooR7dusa9xRap
MWp1vPGSke/
+PjYpNKCCGkez73s8987+D1opdeopy6/15+sR+Yhtl6frPbDm+1vZfU2oFAy9BateVf4g1/qEHyn8pY
T81zgfNMQ+P7B89ZQ9SaLy36tvUyrDlfHhMvQysS9F3ZOX1HbOamSPCz5B1FHmxaWuABVVd4zhc
n/feFZ5tLqKU0zzhnX2Xx3NDfR27m2aC1l99+WWxSCSGE9JJvf2uf6KKXLi+eekz9tACwzEbo+cV2vu
se3R4u2vosPO/TNM2Nfk7mKvPgrP4n+gB847v3OuM7t+l7Iu+cO2x1RNaJDGtf46vqW5GQXyKmEJ
p3NcXIyqqi1kVi5MalySHMDWbMai61tYUGqm50pNSqjuji5i59pDoQqV1dZS3CLedMyblNrrvVnJH
gwsUL/EP9hBAyRG4+slv0z0uat/7wB/UzA8DS6r3ndzn7906s3iPTz5hzEmuQ7IXJ60y307D62PjWM
XGZfmkN7jkk9DasGUiplxRjNvA9S4sZLBcb0JmnGLjl0mzOQsy6a3dUUwwN1EK4VrVoeY1zT8Yci8zF
d19zOf0y8gBbzBkz29re2r+xX2xSCSGEdM8XfvxPS/pr/G6Orx368NIl9ZMDwHIavufvd2tueoG1ek+
wjDxPRnSTill0edTT/3j5nryngLjMjO4RLNjckrUe1r5Qvru+FYV51urR4qrA89eTaL20Qv1qZ4z6DWbB
lfhq6lrXkg9WX1Ir5j1Y53uDPLeJy+rTrBMTiswZs9m+vH34hcNik0oIIaR7dp7ccd9t14nOeanz5IMPq
B8eAJbTsP/bfs9m3rNnz7XsZPQ966/qP83SAi0Yz9P0t1XiQv9FLs/4vnsDo/tYA9X1OWx1pbNA34rCP
JPyaTGovqQYSQ9rLm+zNFNE0OeDcw3cGHsACbXkM/BOJzjHihwn11xOX+ETnpCvFto7+tujYpNKC
CGkl9x+517RM08grzz9lPr5AWAJzannN1fZIr2Eom/LB9F/br5rUXmepr+tEkv1X+TyjN+id2o7ek1/w
5rbCuJq34rC2pZsc526JrtEz6HpTbPpKzLlRc5CGsewhii0ubB9LXmNdzqNc/S/Qc3lWgzcYs6YweIzp8
UOlRBCSC+58aFrRbc8mbxz/rz6KQJg2Qz/b/h57nEOtdj05/R95W36VcNNiyv2ZOxFyetaPi3f+C16J9
9djfoaVl+1trHhW+Rsc/M9J88I7QZVg62tr5dXWxc3DuBORB8xt/jmqmSnZiiWVkuO6qtijnnmqPnu
ay7nu0toMWfMgP9r/IQQMkR2H79yMr/G7+bE4TvUTxEAy2YePb8+4tm/R9oJ96JWNy0w98lkTIc
mV1kt09P/ePnGn6Xf0wfMlOx51vUzbO0W8yIyZpz3OclhnWmFmOkWahc7Y4ilyZVW15txvHOtbq
wO+y+spNUyV+lrnCVZ44RKZ5xxMi3KOdXkKlrNGe1tX94+9MtDYqtKCCGkY+7+xldEnzylvPGr59VP
EQDLZh49v9nQe0T273qgatvvHlkqgafpfzzWGj39j199pKJKq97JHKmJvDW9DGtO60HM9KtRPSsK8
0wqVx9DEdf42PfJyg1L85Ys6EsCcw3eGV56Ui33zQosKRd7VvXqxWityjU8wJZzxgwu/v0i/2g/IYT0m
+sfvlr0yZPJM48cVT8/ACyh5en5/V1BKdaFLK7I06wvVpz39D8h9qMuLm/dO9Vn0FSq+7DVCet6M/
3qQmdFYZ5JafYovufv5ZmNLf7E7Ip5Pf1a1Y7MVUy2xXRnr9X8ZmUnYnOs2MWLK5PKySqtB8EM+
D/OTwghvefQ3V8U3fIEcuLwHR9/9JH64QFgCc2z54/3DkK9IZBmGgpIoT+CfNgwGWfeOiN2q4QQQ

rpk58kdE/tf+B+95ab3LlxQPzYALKd5/j5/ZBSHuSeAPgzDMp9APmqYlEd//6jYsBJCCOmSa37y2e/f/F
XROS9v/t9zz6ofGACWVmLP71W08E5D7+nwq2HUMfO/Bw79pwCeQUrmBJ0YBmL/r9XDH1Fgad
3z4j1iw0oIIaRLbvj3a0TnvKR58sEH1I8KAMtsLj1/aKBgOxXp7COngD5YPT8NP6Zo+/L2wecOig0rIYS
QLrn9zr2if166HF879OGlS+pHBYBlNqeeP1cfLNayR/v66EkAQKPNrc09T+wRG1ZCCCHJ2Xlyx323XS
e66OXKO+fPqx8SAJbcbD0/AGCSzm6e5Z/xJ4SQHrP7+JU/OHCDaKSXJa88/ZT68QBg+dHzAwByz/7
pWbFhJYQQ0iU3PnSt6KWXIvwaPzAx9PwAAOXY68fEhpUQQkiXLN0v9v/otq998Nf31U8FAJNAz48
48e8xxF/ORP/beOofYugy1Kxq/xT/4P8QRLayEdaUG/MZYrIOv3BYbFgJIYQk53M/
+8z3Dl4v+upFzp9/94b6eQBgKuj5ERdv8rs0mfPq+U2h0rAtvyo2Ug8+2jPElG1tb+3f2C/2rIQQQpKz
94e7luUX+1889Zj6YQBgQuj5MRPRVXZpMkXPP5oR647dg9Pzox9vf/A2/4w/IYT0mJuP7Bbd9QLm
1P33fvrJJ+onAYAJoefHTJa/5x+zL6bnx9J67d3XxIaVEEJIl3z7W/tEj71Q4df4gQmj55+LQLvrPWz6uEL
zHQ19nz5d3eEe0cPmR8Rw8Zdx5uJMdr2Yu3co+xZ7glG1m2p36ZKWwMTtyYl519Rr2dVCtcSyc87
SrWuqYdTJcEXPQAFijPaLyrSZfyY6Rql2iXu+xQgY1ulzp8WGlRBCSHIW/Bf73/zNS+rbH8Dk0PPPh6d
x8h2sGj6bdVPLBsymz5vTpogZRB0pXovh4i8j/EvJqbLOUOaAzVqoV/yJec4GJt443Vy0WKiWPm6N4
yzdc295ebRiu7fD+1itOxpKpM4/Z82q6c1tHgGjOPLSEbFnJYQQkpwvPXKV6LQXJM+f+Kn63gcwRfT
8c6J7Hk+bYw6ZvkcfMY2Sc4k1jNOASeoCfYvVXak79AjFSzFc/GWQuU7WyKiJNI3sWaqjxRNzB/Zyn
0p1RCxhxlqedTiXudULjRV99eqcW8QjayzRZv7yEmcQeYNzR+MIGMn25e1Dvzwk9qyEEEKS8/V7Py
/67bnn53d+h1/jB6aNnn9edBdjmhinl/I0V43dVcbppxzqivKe8sX6ej6QfUTfL4aLvwzxXWaOqbnXr9
GvPAuzDgltnph7wEsPZV3VeKN7ge+WNm+ZqR5ea0HemDDF8lDo+sjcwvM3L0PT1xd4higPNY6AE
V38+0X+GX9CCOkx3/3ml0XXPcccveWmv733F/WND2Ci6PnnRjQ+upUy3Zent8oE7rIuMv1SuI9Tdx
UXFH9e23i1uKs6Ym4Xw8VfhnjXIg7Wh9InfUK1vFXMuPpouymb+tVVsRvr0zUX+G7xzNK5zFNd8lZ
sXJuneJh/US3mb15XagXrA9eVQzSNgHFduHhh12O7xJ6VEEJIWq46sfOBW/eL3nte4df4gVVAzz8/u
q8pmhndBlXNmr89E+1ViwbMR92WXVFenf2hPGSOmLvFcPGXId61iIP1ofRJn1AtbxVniu2m7BnLv
dEcEdwLrFptRvavJNNQ0VevJjhwpWlRXZ6MOq+H8NFDxEfA6M68dUbsWQkhhCRnQX6x/38f+7H
6lgcwafT886R6n6x70h2O1dL42zN9oTraqgHzKO/T//V+cXdxSB+pBhTDxV+GeNciDtaH8t7RwH+PH
lcfbTdlz1jyRn1JprzKHdlXq8XIodU3VvTVqwkMXGleVJv5G9ZwhfKmxllY/CNgHh79/aNiz0oIISQ5/3b
PF0QHPnJOHL7j448+Ul/xACaNnn+uVEOztr5eNk12z2QaKbvLkd2Vp3syTVKo78upcdxf4y+PWOOJi
vGXIb7LzDFVq35Ny4Fr5IgFZ6B2I3serLjRvcJ98r5abd4yzzWZ5oqNawtNSA/ZXMJziXtNJjtmvarXbZx
mITYC5uSeF+8Re1ZCCCFp2Xlyx93f+Irow0fL0Vtueu/CBfXlDmDq6Pnny3RLBdHOmJO6vTJ9j+m3zB
F9rzVgtDmqbvTdavVzoteKvwxy5mnVV8VCI+s7zPQipVo8MVknQI8UfhJyPtWEq5F9tarr1EHruevLP
NUzzRV99eqc6vUDLRbljODM3xyopi8WFB6j7QieAxjB9uXtg88dFNtWQgghabnmJ5/9/s1fFd34OH
njV8+rb3YAK4Cef85Md5PRXVXFPluxuxyrKZPc4WzVjU4X5S9QDhd/GRGeqKrmDOW/o6HQDE8sO
pKno5Q3+kvlqpHr15THw0/C3Oipnmus2GZtgfKqVItFzTB/qZqWf5DqfPMIgWeEoW1ube55Yo/Yth
JCCEnL9Q9fLbrxEfLMI0fVdzqA1UDPP2+muQn1LvXuqOp5DPuC7LR56bnU5l7nvVMcjL+MMxdnsu
tF0xavXmjZ3tVvkjNrN2VPR+m50e5M86P6tTW0fYkZzJ5hdq0zsqe6Eq/Ybm3yCYky8RKFpvnn7GFy
7pTqs4ivNVcfQZ9178PQzm6e5Z/xJ4SQvnLo7i+KnnzQHF87xK/xA6uGnn/edOND6wJgWTz7p2fFn
pUQQkhadp7ccd9t14nOfLi8c/68+ioHsDLo+efL/FedtPwAlsmx14+JbSshhJC07D5+5Q8O3CCa8yHy
ytNPqS9xAKuEnn9e6v/DZfd/
+AwAi+3wC4fFtpUQQkhabnzoWtGf954nH3xAfX0DWDH0/PNi9fw0/ACW0Nb21v6N/WLbSgghJC
2337lXdOk95vjaoQ8vXVJf3wBWDD0/ACDR2x+8zT/jTwghvWTQX+x/6w9/UF/cAFYPPT8AIN1r774
mtq2EEELS8oUf/9MQv9j/4qnH1Fc2gJVEzw8A6OT0udNi20oIISQtNx/ZLTr2jjl1/72ffvKJ+r4GsJLo+
QEAXR156YjYthJCCEnLt7+1T/TtyfnRbV/74K/vq29qAKuKnh8A0NX25e1Dvzwktq2EEEIS8rmffeZ7B
68X3Xta/vy7N9TXNIAVRs8PAOjBxb9f5J/xJ4SQXrL3h7u6/2L/f/3nSfUFDWC10fMDAPpx4eKFXY/tEj
tXQgghCbn1/j2ih58pP7/zO/waPAoDn3jojtq2EEELS8t1vfll08i1z9Jab/vbeX9T3MoMoCVR88
PAOjTo79/VGxbCSGEJOSqEzsfuHW/6Ofb5M3fvKS+kQkgp/nKS+kQGAnh8A0Lt7XrxH7FwJIYQk5EuPXCX6+cY8f

+Kn6rsYAAr0/ACAnm1f3j743EGxcyWEEJKQr9/7edHVR8Kv8QNw0fMDAPq3ubW554k9YudKCCEkI
S1/sf/oLTe9d+GC+hYGAI2eHwAwiLObZ/ln/AkhpHuu+clnv3/zV0WH7+aNXz2vvn8BwELPDwAYyrN
/eIbsXAkhhCTk+oevFh2+yDOPHFXfvABQR88PABjQsdePiZ0rIYSQhBy6+4uizzc5cfiOjz/6SH3tAkAd
PT8AYFiHXzgsdq6EEEJmzc6TO+7+xldEt5+FX+MHEEfPDwAY1tb21v6N/WLzSgghZNZ4f7H/laefUt+
2AOBDzw8AGNzbH7zNP+NPCCHdc+ND19oN/5MPPqC+ZwEggJ4fADCG1959TexcCSGEJOT2O/eW
Df/xtUMfXrqkvmQBIICeHwAwktPnToudKyGEkFmz8+SO+267Luv53zl/Xn29AkAYPT8AYDxi50oISQ
hu49f+T//9V/UFysARNHzAwBGcsUVI/3QoVAyCiWjUDIKpXnzNy/x6JJRKNn0Cq0CHiUAYCTsSJJRKB
mFklEoGYWSUSgZhRDBowQAjIQdSTIKJaNQMgolo1AyCiWjECJ4lACAkbAjSUahZBRKRqFkFEpGoW
QUQgSPEgAwEnYkySiUjELJKJSMQskolIxCiOBRAgBGwo4kGYWSUSgZhZJRKBmFklEIETxKAMBI2JEk
o1AyCiWjUDIKJaNQMgohgkcJABgJO5JkFEpGoWQUSkahZBRKRiFE8CgBACNhR5KMQskolIxCySiUj
ELJKIQIHiUAYCTsSJJRKBmFklEoGYWSUSgZhRDBowQAjIQdSTIKJaNQMgolo1AyCiWjECJ4lACAkbAj
SUahZBRKRqFkFEpGoWQUQgSPEgAwEnYkySiUjELJKJSMQskolIxCiOBRAgBGwo4kGYWSUSgZhZJR
KBmFklEIETxKAMBI2JEko1AyCiWjUDIKJaNQMgohgkcJABgJO5JkFEpGoWQUSkahZBRKRiFE8CgBA
CNhR5KMQskolIxCySiUjELJKIQIHiUAYCTsSJJRKBmFklEoGYWSUSgZhRDBowQAjIQdSTIKJaNQMgol
o1AyCiWjECJ4lACAkbAjSUahZBRKRqFkFEpGoWQUQgSPEgAwEnYkySiUjELJKJSMQskolIxCiOBRAg
BGwo4kGYWSUSgZhZJRKBmFklEIETxKAMBI2JEko1AyCiWjUDIKJaNQMgohgkcJABgJO5JkFEpGoW
QUSkahZBRKRiFE8CgBACNhR5KMQskolIxCySiUjELJKIQIHiUAYCTsSJJRKBmFklEoGYWSUSgZhRDBo
wQAjIQdSTIKJaNQMgolo1AyCiWjECJ4lAAAAAAATBM9PwAAAAAA00TPDwAAAADANNHzAwAAA
AAwTfT8AAAAAABMEz0/AAAAAADTRM8PAAAAAMA00fMDAAAAADBN9PwAAAAAAEwTPT8AA
AAAANNEzw8AAAAAwDTR8wMAMKtX16+orL+qjgIAACwaen4AwISUzfiwXfi7G2tVhSELjv2fLOQLy
6xtvKsO9Km2mAL/Wcmsas9wmLdpcIG/MOqzV+r3kxH5Kxo5NSv/ULV3rN9lDSv6ZAb9ohhQeFHL
+j4FVlRbTl9vU4taS/qdtCLo+QEAwxpwN19j1Rlz01aUHWKvk+2lzDqKfdXAG6p8IWvr69n/M0idfA
mjbwnt/Wj/n4raJ7sy0KevWMtInwj7ufVXJPg3tP53qL7QToIVo6dmFRoqP24OlI+0ayn/KPbb1blE45
PJz/f6ReFbVG1JhW7riiyqONXjXyV3OVZx20ArKuqbQ8WrrqtrV6uHUuWzU8TzqT1FcQ4t0PMDAAZ
U30/VdyO90oWK/3/MDUF9hUMZvEpeQD2+Ycrk7/3gj8k2zhtTU5Qc6MMnxx6uVt/7eEW/H+7E5
Uejr6WFK8ZOzartUPo69XJmxf2lepXs8ZkD3d+upuXkx9XpTnVKsUX1Mb4SWZTnULrgcoTuRcMrkk
cGrKVPqJdd37V8ODN88UmuXtZLFSc7rWkV0fMDAIYj9wDOrqF3w1cQxtl+DLwsM3z+h/RNW0y37
eDMRv8cZIb9KNQf4GDrKwa236i+3zhn5u5Sen6QkWfV42NsGqo4n/wg9d3DVrH4C5mj+R86l9GT
9dQa5uvCLdTLOpTIcup6/Hw3PrrGybTnDOWso8di5mkWL+THoc9CK4OeHwAwGPcnc4+7Hb+RN
wPFevraMwbVtj/9s55Z/sdhChVPqjLwMxtuHUEDv0mZ8hFmJco/DPMpd/6GWp+OXjjj5RXrz63nk
pHheqzUNJTzYJM0VeltQb6BrGP5H3v7sHtquR+KPriFijr5LyoY3av6Hp2lON3X2ry1io9aXqL8Q3gqs3
FqBR5nP0uzH5O/UG8LWxH0/ACAwbg7AO8WpU+DF7AVG4+
+dm+uYi3KgEvKV6GHz0sOtyCtXNiQdYpPXs9b+bhxPnjFJ64wVCl3He7f4k6cAu74PT/LyHA9VooPVb
xxPTzFeJXibD/vladQvgZ9JD/d24ciUMvSUym3kHhfigvCz7cdz3Is8bOzCo1WPb/eSnlqFUfk0+vpvSp
WoKrlf64P2+9TXA30/ACAwczhR/V4ewGxWxxUr5upunwZ1fPKC42yJvej0Svx5pTPb8iPRVFw0M+d
vYbyz8PUK8aWj67Ht6oY0J65+1FwLukmMlyPlWJDiY9jB94qxUGlr8fmFMrXUL3OT/f2oYg9uky5vD
6quYWcD18Pf5Gjy+lhfJunVvm4ymPln3uq511XsR5tfb32IemgGNa8Mc67FH/G8KLnBwAMZg4/qkf
aC5Q7nRH3HOXmrf+CtS2bpY8ddkxRd7jn53zyhq1XvDuDPjK3woArqn0o+tvHl5y/oe5fWefd68YtY
EROzSo4VPE0+1pOw4SL070Uk4VqnwnLELVcPX0i3ELOwM1zaRQZojjV12ch59RyKxTvXLcVlZofTX5
FH6uTf2Xct795LpDo+QEAg3F/MPe0dwsbYy9Q1Bh5w1HUHPTJFfIyw1cZfjlDbOXDettVh7klBI2Rpe
+PhDtv+Wb1vbLIeD2W8g9VvG89LqZxwsUFPRRsKJSf7u1T0biovv6GuYWKI/ZC5IcxQXg5PS3D4tRy
SzQ/3ZYaB8ov6P6hKFZQH8at3MPbtHLo+QEAw5E/mXvbfgQNX6Hck0i97z+yKtYyPPugQfSza3Nl41
rDFm/SoO9SUcFeyJB7xOLNGfYz534CRima6f0TUbw19YnX1ua8dZ15KmqRU7PyDFUc6vdNapxwc
UEPj6+hUH66tzdprouq/UXq5W9VcDkD/J11a9X+MmX6Kxp/m/p5j4rZukXyw/bY8anAi54fADCg2g

akn12BXzG2sNx7ArGicRaTFx3kHRp9NbWtbn/7XteQn+q6cj9sjPCJ6HNt4hNQqJZgr62vhxmpGJ/M
TMJDifer1HFxRbXaTLMq1uuiZpcaLZ9MfllvH3pnUdkBa/BySp5JtBZfVO1slzKKs5xScXiEz3bvXxThW
vUznR9dOZx3mNrnutcnuTro+QEAw7I3IPycxmhqG9LO+9GQ4uM92Ohz0PM+Hr2ovysl9d6IU0v0j
oUXtZRrirxHmcl9UfRO/EcVpWrLwEaiI3p+AAAAAACmiZ4fAAAAAIBpoucHAAAAAGCa6PkBAAAAA
Jgmen4AAAAAAKaJnh8AAAAAgGmi5wcAAAAAYJro+QEAAAAAmCZ6fgAAAAAApomeHwAAAACA
aaLnBwAAAABgmuj5AQAAAACYJnp+AAAAAACmiZ4fAAAAAIBpoucHAAAAAGCa6PkBAAAAAJgm
en4AAAAAAKaJnh8AAAAAgGmi5wcAAAAAYJro+QEAAAAAmCZ6fgAAAAAApomeHwAAAACAaaL
nBwAAAABgmuj5AQAAAACYJnp+AAAAAACmiZ4fAAAAAIBpoucHAAAAAGCa6PkBAAAAAJgmen4
AAAAAAKaJnh8AAAAAgGmi5wcAAAAAYJro+QEAAAAAmCZ6fgAAgMXy6voVyvqr6lBJn1jbeFcdGY
auI8ovgurZZJzn8O7GWuhU9BwATBc9PwAAwGKx+tp6e7rqPX/Vthd8jyHY2VcPdQH/kwwAGA49P
wAAwGKp/XfZdoe66j1/q/V7m/vqPwqg4wewYuj5AQAAFkut57cb3BXv+U3j3jAv8/zMczJH6PgBrB
x6fgAAgMVS7/mtRtXp+Z0DbmOsLsmvsMZVN1hH7G5YH86OWf9zequIZp11zltzq8o09Ny18WoD
WjPVImOZq4trzCtnBbH55+rzEZdEFhi9DwBGRc8PAACwWKpmcqP8k24ZrS6zeO0esNpN1RJX7Wgj
00SH77H6bNnXFqyJeAaJtr7+onLpFmsujmpy6xv6j6J60/wbJhReoP+
+6HQBYDj0/AAAAItFN41ZB6kb07JhtE4UF7oHYj2/ushqSeUR3ZY691RH9AFZxlxhpmLVadHuhguYA
d2aMeZipRqm0Dh/fUF4PoEFhgaSMwCAcdDzAwAALJZa16he2H92206rnZTNrL6k6kvDR/Qw7hXy
GtkRZ+QhM4h1TZCcQU6uxD0QZyaQE7e0mL/kVPcv0FzWat0AMDh6fgAAgMXib6+zVtPpjD2tsmx
N3Uuaj+jXdqNcH1df4SMK10YJcKeU0yX1Ubm0RuEptJi/pX61Ph8Y3cyyUl8WAIyKnh8AAGCxyA5Y
vTa/3l/1kPLKzFA9f/2gfuETLBzmv1YuJbnnd6fQYv6+5r2gzwdHD9zYdtYA0Ct6fgAAgMXiNJOyiTQnP
G2nbIzdS5qPeIYV4/quEFpcYviv1SX1Ubm0RuEpNE9OX5EpL3KqN45hDVEIFwOA4dDzAwAALBZP
M1lvH82J2JU99Px2c12/pkX77ZYJM8PZFzs1euz5G4dyb3UeSmSB2Slr4JnnDQA9oucHAABYLL5m0
vSNOXOiOqraSdOYOkfc5jV8JDyKuSRcWTe2bpmYcIHq/pl758gUmuYvI1Nd37BAZyHRaQDA4Oj5A
QAAFktDM2mfsFpRSfSysRZUHjGv1+Tout/N+EtXF7hl4vT1dfbdffb8jfP3TyenLwmNHrqz7awBoFf0/
AAAAIsl0ExWTWrthN27Zm2lbIzdwZqP6Nf2cM50cvW+uX5BYBVR9fFkkyyX1qhxCrH5V7fn8pLWY
7FP+0a3b821nTEA9I6eHwAAAACAaaLnBwAAAABgmuj5AQAAAACYJnp+AAAAAACmiZ4fAAAAAI
BpoucHAAAAAGCa6PkBAAAAAJgmen4AAAAAAKaJnh8AAAAAgGmi5wcAAAAAYJro+QEAAAAAm
CZ6fgAAAAAApomeHwAAAACAaaLnBwAAAABgiv7xj/8P9RMAaNmaS9cAAAAASUVORK5CYII=]]>
</redcap:Attachment>
</ItemDef>
<ItemDef OID="b16" Name="b16" DataType="text" Length="999" redcap:Variable="b16"
redcap:FieldType="descriptive" redcap:BranchingLogic="[q14] = '1'"
redcap:InlineImage="1">
<Question><TranslatedText></TranslatedText></Question>
<redcap:Attachment DocName="B.png" MimeType="image/png"><!
[CDATA[iVBORw0KGgoAAAANSUhEUgAABVEAAAEJCAYAAACdcmdaAAAAAXNSR0IArs4c6QAAAA
RnQU1BAACxjwv8YQUAAAAJcEhZcwAAEnQAABJ0Ad5mH3gAAE9sSURBVHhe7d0PjCRnfef/b+/aY
PPHNmBskx+RHat7sZYlGJIg08PxL4egZ+6SJbnbYOVg5Oh3PVyi0zQSkwOxF+mX2wguG4lunS5iWrq
zliQyTEJugrLdIJRgckzHR4IxYTI/Z7rlc3T8wDYGjPljB3u3f/VUPVX1VHd1dVV3VX39PtltaerpqfqqT/T
O/OZ5/k+hb5FAAAAAAAAAAChjumPAAAAAAAAAIQhKgAAAAAAAAAAEIEQFQAAAAAAAAAAiEKIC
AAAAAAAARCFEBAAAAAAAAAAIQIhKgAAAAAAAABEIUQEAAAAAAAAAAiEKICAAAAAAAARCFEBAAAAAAA
AAAAAAAARCFEBAAAAAAAAAIAIhKgAAAAAAAABEIEQFAAAAAAAAAgAiEqAAAAAAAAAAAQgRAVA
AAAAAAAACIQogIAAAAAABABEJUAAAAAAAAAIhAiAoAAAAAAAAAAEQhRAQAAAAAAAACACISoA
AAAAAAAARCBEBQAAAAAAAIAIhKgAAAAAAAAAAEIEQFQAAAAAAAAAiEKICAAAAAAAAQARCVAA

AAAAAAACIQIgKAAAAAAAAABEIUQEAAAAAAAAgAiEqAAAAAAAAAEQgRAUAAAAAAAACACISoAA
AAAAAAABCBEBUAAAAAAAAAIhCiAgAAAAAAAEAEQlQAAAAAAAAAiECICgAAAAAAAAARCFEBAA
AAAAAAIAIhKgAAAAAAAABEIEQFAAAAAAAAgAiEqAAAAAAAAAAQgRAVAAAAAAAAACIQogIAAA
AAAABABEJUAAAAAAAAAIhAiLp0etJYKUih4DxWGj29PqjXWPFeU1hpWF+1iMxj3ZB2onXjpLWdL
MxLO7LSlg372PzHqPs4Vb2GrGzEO5vtDbdtK5JH08KNuQ9Cjmc+2g0AAAAAwPwhRF06Rdm8UJey
XurU1kPCkracr3X0c5Hq2U3rq4B5oILBVWnqJUdZzqxle4faf1Qo1cT/rlhsR+14AAAAAADIGiHqMip
uytmqfi4dqZ0P9kbrNc75IVW1JdsV/XzhFGVzry/9vnpsS/aHkff+RpmXdmShKwdu8leuS9c+xj3ZzDJ
D7TVk3fijwsI7ascDAAAAAEAOCFGXVGW7JV6O2jxn9EY1e6FWpbW4CSqOot6h7OuncuoEPaQBA
AAAAEAuCFGXVkW2W26M6vdGbW/4Q6XL9a3wXoyqlqKuRek+wmpSjqyvaH59ZI1Jo6ZjoC7rqPX
GPu31edcGHd5fZI1J4zwEz5+5nbDPjxN+3INtCdS9tR4xy30ObGewraPOc7xjCm57uPZpYAh6c9VZF
2h4snM3eA7Uw9yc3Z6x+xzPPy71CLkXbAnaHvI9OHq7vqTHE6/dAAAAAAAcfYSoy6yyJXW3OGpz
VTYaDTnnJ6hyIWSM9FAIo3VqpQwmoCrK2hndwM6BdJ1nFmNId2dHLno7bcuubn/5zNpc9FKsnPa
D6gP/AGy9izv6PBo1Pe1wrCSDo63TPb8dqZUKUhrYSXM1aUimtjPY1qasDoZtEx2T2rZR+9S6H+te1
+kREu3HCSwHz4HSXB0dKibnnOtV70AUtW6Kc9TeGFHPNGS7E4vZbgAAAAAAlgQh6lILTjLVrLnBTF
nqF0Imk2pv+KGKV4+yL103ie3UZD3lhKW4dka3b18O3U23d42JhYxw0hjqferEnAz0rpz2yiY0d4M9
Ji/u6BisfEacDLUnjXX3GlSlpc9v3+0xnOr59bfvdUhWIdlAfdzx/O1494Hazrob/E1zTMbr9zZlc9v62PX
vV1Wv1/6cXXIi2X56jXUvsCzXu85r+8MlLiqR+4wr/BzteOl/krZbr3X/0hH2PRjY7rBkxzOu3QAAAAA
ALI+C9QtyXz/HklK9SwM9zlSwEhKq+K8rS71rTubTk8aK24tOBS/OZEYjX6963bk96Ubsy+dvW4Vde
9ZG1BBs1YOwWq1Ks9kcWu+3IaxdcdeNE387w+2yV3rnwG1/6DqbsV0VnO2FBNwB4e0wr3O11fc
nDEt0PSK2E9ivvuaS7JjMbQdfr41qa6JzF35+FOdanQqsS3p+lETnOlHbRwht44jjjDietO4RYBr3Pnyvv
OXCW/QSACAP99/xLnnNW2/SSwCAzB3/NZFjr9ILWBT0REVwkikVtoQGJP5Qeb/npMsYdm/2GE1F
UU6ccp51dJfTrj2WvywnT5+0e9QF11uqp/0AbA74vWmb4nVG7R44gZT1GW8ov7dOdT4sGbUo3S
BMfcIsazBr1jUo6ac28z7QPYSnOKZEvYkT7cec4f+kmIdQ3NyTvhmgTm3wHIWY6rrrurFuwJmaGO0
GMvDwEw/LOz/5Tr0EAMjDDY9fJXd/6AF5/Bs/0msAAJm7dI9I/zt6AYuCEBVBA6FScsO1P6fl1RVt7k
pbenJoj9k/JScqJ6z/D65XGeqc9ZIrrombLbpD+ttuIl09a/TozUtaAZl1DTJrOyHeSKpXqBe0GnVjgQX3x
NNPSOWPKvZHAEA+nvPjY/KWL94oP3ryGWm+/3555p8u6c8AALL1lMilP7A+PussYiEQoiJlGYRfXl3
RfTlsXxS7lKgd9lbEyVeN9XMZvhk9NO3A1+/VOyrwVcO67VqVQ480e0lOK1mv47yOaTHOXbjxbW/
Lhtnr1K2LatY5BRbUnZ+6Ux58/EG9BADIwxv/
+gZ5wQ+vsJ8/9NXvyp/WeR8GgNz0Hxa5/Od6AYuAEBUxleSkN1LbnBFfMSZJitM70Ri+HI+7744c7D
pf686+X7I/4a8fLjUwH4qbZ/0guOFOjFWVQIZacsoTKG6Jgvk22OvYvA/0seV1TIn2Y97LA8Pk1cz3hZ
xnoE/SdmNSNTtwdeukJv6eAubL+z77PvlM7zN6CQCQh1c+eK3c/PXn6yXH5z7+kHzlLx/RSwCAzF3
6gsjlr+kFzDtCVMRUlM2zbuVUc/Z1NbrYnOl8y+vt5wScijnre1s23JlrYvN7cqqJpBS3XmZRF0x117vh
6vxxe81a56Kmj3+wdqsx7F+aq7LhnTIV7Onh297K+dBcNQLH9nm/hqd7bHkdU6L9mLVbm7Lqre/p
me+ta1Qq+NvI2oTnyA9c3XYDi+kT+5+Q+n11vQQAyIOqg/pzD7xELwX94W9/Tb73raf1EgAgc5f/RK
T/fb2AeUaIivgq29LyctSalHTA48w8bynX5YJR4NOfUMnSXNWB0GQ1HAPbsp55Q/a9of6KMUnTHP
Jqu9rKUt8KRKiWomxe8IdlN1d1gOaFzqMm/ZolJ3AMttM8tryOKdl+ipsXpO6/2HmtOZGTdS/7h6Br
7yrua1NNWBO03bzfw9odR+bHA8R339fvk7v+7C69BADIw/FLBXsYv/oYRgWozd+8Xy8BADLXf1Lk
8sf1AuYZISoSqWyH11+stqz17tBiV3FT9gZfW21NVr/R7K0XKBlgDM3OdKKjFJgB2KiyA+qc9bt+wOe
ya1/OW03PstS7fT9Yt1Wl1d8LTpaV1zEl2k9RNvf60h16sXq5MUzeVpHt4EGK7B96PbFTEbvtVlv6Lf
8+sqlzblwHu+5ulByOB4jBnYn/6Wfp7QQAeVr50kvI2iev1Evh/uFL35ZP//6hXgIAZO6y9RvZJcpbzbt
CX81aAgAxtTcK4nSSVCHqQGAKADGo4PT1/+318sAjD+g1AIA8lB56od0LNa6tu8vyiteFD/sHAGTgit
8QKfBL9ryiJyoAAMiVGsJPgAoA+VK9T1Uv1CTUsH7qowJAji79AfVR5xghKgAAyM1HvvgRezIpAEB+
VP3Tt33hZSProI6iAtS7/+NX9RIAIHN2fdR79ALmDSEqAADIxe6Du/LBv/igXgIA5OWOL18/tg7qKPv/
8zH53Mcf0ksAgMxd/n9FLn1BL2CeUBMVAABkTg3fV3VQmUgKAPJ189efL//8Czfppclc+dzjsnX36+

XWV79IrwEAZOtKXR/1Zr2MeUBPVAAAkKknnn6CmfgBYAZe8MMrEk0kNcoz/3RJmu+/X3705DN6
DQAgW9b77aWPWx+fchYxFwhRAQBAZlRwqgLUh594WK8BAORB1T99yxdvlOf8OJ1f+R7/xo/k7g8
xKSAA5Kb/HZFL1EedJ4SoAAAgM+/77Pvk3ofv1UsAgLz83AMvkRsev0ovpeMrf/mI3PtJ/igGALm5/
DXr0dELmDVC1KXWlo1CQQrGY6XR05/LSa8hKxttveBob7jtWZG8mzM3Qs5LHL3GinPuVhoy6tT55
9d55H7NjwLuWyCW+n11+djffkwvAQDy8vJvPE9e+eC1eildn/zPB/LN//19vQQAyNylXZH+Y3oBs0S
IurR60lhZlaZecpTlzFpRP8+eHfiVasLfVIImPi+9hqzXor5KXfOCrAYvunRqJSlMENguK+5bIB7V+1T1Qg
UA5Ovqp47LG++bvg7qKKo+6u//
+y/bHwEAeVD1Uf+79fFZZxEzQ4i6tLpy4KZA5bp0+33p9/dkM68MdWzgt6QmPS+qZ+S4YK99XtxN
V1vqevelWy87K5qrQo4aA/ctEIuqf6rqoAIA8veWvRvtIDVLqifqH/721/QSACBz/UdFLv2xXsCsEKIuq
96h7OuncuqE5Nf/FGmL1zOyJ41zugtquS5bFedpcfOsVJ2n0twlRQUwPTUTf+WPKvZHAEC+Xvu1F8
vLHr1aL2Vrb/f/2A8AQE4uf8l5YGYIUZeQXbvRDN2aq04tx8GuiKp3o1E7c1T9zGAtyGCd1bDejbH3r
wVreI6qN+kMVfdfl6zW52A9S6+2qH6ENy3GPgPncEP8zZjnydln0vOiqHaWYvWMNHoeB0LzkpzUn
VFl/9A6olGM9obVW21v6GMZPAcJrkvI/RZ2vePcb4PXz35E1ImNY5b37WT3p2vSa2eZ6D1Ar1TMr
49uJI6QOz91pzz4+IN6CQCQFxWevubvXqSX8qF6o1IfFQBydOlPqI86Q4SoCDUUGGl2/cyRYVRHai
WjzqrR43EyanuDNTzVurCgpuQNVXdFt3UUZ5+DwWRzdcJ9Fjflgjtk3joz5/RGeo1z3nkq1y+kUEahK
q1+y+tVOsTseTxK50C6+umwipx2N97ZkYsDJ7W96x2NX1c3yXVRQV7I/eZcj1EBZPj9pu7d0GC5U5
NSIMjOSpb3bcz7M2CCa2eZ7D0Ay07VQP1M7zN6CQCQFzV8Xw3jz5uqi3r3h75KfVQAyI2qj3qP9Z
H6qLNAiLqEKtt96Xfr4kZ7Um3Z9TH72zrxbG/4AZBXL9Won9mpyfrIxEaFec7r+3uboWUCxu4/wN+
et3/pyI6XBPWkse4GPca+Wzo1imzrKP523M2ofdbOu/Fbsn0WNy+If+rOS9v677wbglnn94JOUJOd
F4P9um2ZLK8uyolT+ukYlS23beb5V9ri53BnxMnhkpwj67VGqYGh+21ofyZj2/b9ZrYlbFt+kJ3U/Ny3
/uvD789hya6dZar3ACyrT+x/wp6NHwCQv5UvvTTzOqijPPTV78qf1hmBAAC56T8scvnP9QLyRIiKIW
bPtPoFPwgdDgOHletbE4Z54aotPxwsrp3xAqzOge4z2bsoO14eaey7suW3deeiJIl7zH364ZMh8T6Ls
nnWTbuaslpwe08Gz+8kipt740PW2PblMOpEFdfkTNjxtXf9/XrVn1pzjSXSOrPOzp0NEI3gPvd4Dhu43
s8et0evSPk86CNzLYfa0LO/bsfdnmCTXzjLNewCW031fv0/u+rO79BIAIE+vfPBaufnrz9dLs/G5jz8kX/
nLR/QSACBzl74gcpkJ/vJGiIoBI3qm2Yqy5iYxIwK3UyfSDKjKcrKkn47SPdC9+VSoU/LqNhbMYdKRw9
QnMMk+K9tGr0FHOsP403RKoi+fcf2NgNIP3Kpy1j2gqa6LruEZOrw/aOh+M8JCawf20Hd33yNrsaZ
uHu/bBNduyvcALB93Jv6nn31arwEA5OWGx6+Sn3vgJXpptu7+0APy+Dd+pJcAAJlTw/r739ELyAMh
KibUkeHOgTHCo7mX3TFUvMKUSrD+5Oz05HBssVSf36vSHRZuBG7V08FeoUmYExB5PXXHCbtWR
dm8EN470w0rF3t+o8nvz/SvXdh7AJaNCk5VgPrID+h9BAB5e86Pj8lbvnijHL9U0Gtm60dPPiPN99+v
lwAA2XtK5NIf6OfIAyEqJjR/gWm53vWGbAcfk9YLHS/
+Po26n7aO1NZznJyneELGlj4tn5Sxl7S4KW5lAntYuDF8vno6/CyPP0dt2TB7nbo1OM36o0lYbdzT+xj
s/as0z83XpEi53bcTXLtoR+GPJpiWGsL/wCMP6CUAQJ7e+Nc3yAt+eIVemg+qPuqnf/9QLwEAMqfq
o15iYte8EKJiQElOusnV0EzePbnoFnIcO/Q7J6WTXtA2qm5m6ibYZ6+xrodpl6Ved5OsPCfnMa5rc1f8
zphdOfAu6QmJc0m9HrXW/XH+/I4OP6sSyOGSnCOzLqcKFN26qMaQ96TUzPKqx6k9GZQdSHa9W
p7pDpOf0CzuW0usa5fme8AU1xDz7yNf/Ig9mRQAIH+lh1448zqoo3z6v/6D/MOXvq2XAACZu/xZkf
48dRU6ughRMcCcBCnYW9IPAlXYNTChz6yYNTCbq/5Q7faGHhpuPdIev510n72GrLsnrnpWNjeNyY
Nq65JPjmrWsmzKrm5er3HOCzBj90asnBbnDulIs+ke18Bw8Amvix8oDvbcjUlvX80sH+xxaoTFZo9b
oz1p3yaRZnHfKnGu3QTvASU/dZXaee9gZMOb4h9Hze6Du/LBv/igXgIA5OnaJ6+0Z+OfZ83fvF+
+9y1qZQNAbtSw/v739QKyQoiKYeYkSJ2alHSoU/LTE7kw7YxI5vDy5uoUoVGwBmZzVQdQXnhTlVZ
qs9e7kuyzJ411d6h6Wepbav3okCq98zLMnFndbbN3TastiX+aKhIo72oZDmATnCMv2LO4x2xOsJTEi
HvXrLNaPevPNp/YQt+3SpxrZ0n4HuDXW7V41zBubVssGjV8/85P3amXAAB5UvVP3/aFl81NHdRRV
ICqglQAQE76T4pc/rheQFYIURHKHgYdUpOy2rLWu8Otp1KR7cGClfuHRs/BBOwamMZwbZddWzOj
eqgx92n23LN7obonbiCk8of1p3hehhRlc2+4Tqg9hD5hYBeYJMs6ZjsbHhT7uljH3G/5QaqtKq2+0dZ
ACYJo9r07eJA2Z5vTZZMLft9aYl07S6L3AHUsg6+ttiava4u59cTTTzATPwDMkOqBqnqiLgI1pJ/6qAC

Qo8vWb6bUR81Uoa8KBgJAEmo2fT0ZlAph96btmTxL+lhOtaYNWBfEUbp2yJUKTit/VJF7H75XrwE
A5EnVQVWTSS2SK597XLbufr3c+uoX6TUAgGxdKXLFb4gUbtbLSBM9UQEkFCxRcGZtsUO49nnrWC
J6ZB4tR+vaIV/v+
+z7CFABYEbULPx3fPl6vbQ4nvmnS9J8//3yoyef0WsAANmy3m8vqWH9TzmLSBUhKoCY2rJh17o06
pWaJQoWUK+xIqv7demmUqJinh29a4d81e+ry8f+9mN6CQCQJ1X/9C1fvFGe8+PF/NXt8W/8SO7+
0AN6CQCQuf53RC7doxeQJkJUAJNRNS8XfPx7cXMvpRq/C+YIXDvkR/U+Vb1QAQCz8XMPvERueP
wqvbSYvvKXj8jnPv6QXgIAZO7y10QufUEvIC3URAUAAKEefuJhec32a+wJpQAA+bv568+Xf/6Fm/TS
YqM+KgDkjfqoaaMnKgAAGKKCUzWRFAEqAMyGqoO6aBNJRVH1Ue/
+0FftjwCAPKj6qGpY/7POIqZGiAoAAIbc+ak75cHHH9RLAIC8qQB1UeugjvLN//19+cPf/ppeAgBkrv+
oyKU/1guYFiEqAAAIUDVQP9P7jF4CAOTtV97sbzs0av10tGyt/t/7AcAICeXv+Q8MDVCVCTUk8aKm
uVbPTaknWjdOGltZ96MOYZeQ1Y2gmvbG+7rV6TR0yunpfZjb3NEO2zTne9M2m1bkPugvaHbWJC
BSwosjE/sf8KejR8AMBsqPH3N3x3tuqGqN6rqlQoAyMmlPxHpP6YXMClCVGCGeo0VKZRq0tHLWe
pd3DH205RdQj4AA+77+n1y15/dpZcAAHm7+qnj8pa9G/XS0aXqov7+v/8y9VEBIDeqPup/tz5SH3U
ahKhIqCibe33p99VjWyp6bXby3l8WRhxDryHrtTziU6UnF3eC+2qea1hrF8VRuA+A+aZm4n/nJ98pT
z/7tF4DAMibClBVkLoMVE/UT/7nA70EAMicXR91Vy9gEoSowDLoXRQ3Qy2Xy86Tzo5cXJwUdTFUt
qXfrYs+w8DCUMGpClAf+cEjeg0AIG8//ffX2UP5l8m9n3xYvvKX/NsDALm5vGc9mOBvUoSoS8eoLbl
i9kQctd6oc2mvz7s25fD+IutuGnU/VwKfNLcT9vko6Z8z+/PmMP7mqvP5EYU0/WNWj+T1Rv2h/FU
5e+GMDvk6shMnRQ3UUg17jG5PvHbHuTbD59DaumyYrzfqkQa2MbA+/BTHaYN5POpY/P2rh7vd
9nnrupbrsjXQXdYu3WC83n4M3DfArKgh/A888oBeAgDk7YbHr7Ink1pGd3/oAXn8Gz/SSwCAzF26R
6T/Hb2AJAhRl05R1s64PREPpOs8s3TlwE30Aj0U27LbdJ6Vz6xZXz17ldNV/awjB/4B2PywsCxn1nRr7
RCwJIMj5zu1UswQa5bnrCO1UkFW9fYcal2SINUYyl89LZXimniHs3Nx+hCvfEbcU+2L2e6pr43Dfn1w
Z846FVQOrG+uDgSpE7VBHcuqeFvWoakKSlf369Ld2wxcdxW+lsJKN3RqUprnibKwFD7yxY/Yk0kBA
GbjOT8+Jm/54o1y/FJBr1kuP3ryGWm+/37qowJAbp4SufQH1kfqoyZFiLqEimtuT8R9OXQTovauHwi
JEU72Dq1XOU6dmIcI1VI5LW6M2gzMjmSEhV6w15PGutvjsyotu6am9WjpLXRqsh4jjUz7nFW2rTa
Yw76rLadd22HVPv12d+vuV8TsRaoYQ/mrp9X2zVA4xpD+4qbsuefNfnTFa4Z1BPULwcDQN67d6V
wbV7neDX69Frbev2+maYPxeh2aFjf3vOc+P1RXYWtXf41/TppyLsFxAmnafXBXPvgXH9RLAIBZeONf
3yAv+OEVemk5PfTV78qf1h/USwCAzPUfFrn853oBcRGiLiOvJ6IfaPUOndivWnXCo32dFJrDwO38bS
5UZMsNoJq7fi8+s+6n2wPUXFff8ickqmx5QWCs3pgzPGfVlj+Rkh/mWi0Z7lY7gtk792TJfmJsJ0EYq/
Ua636vzepZ2QxPUMe3O61rY6vKWbchRsg+cv3+obPdKdoQeH0UI1Q3Q2s7cNWB6t6okwhkSA3f
v/NTd+olAMAsvPLBa+Xmrz9fLy23z338Idn/n4/pJQBA5i59QeQyf8BKghB1KRXlxCnnmRtode1x6W
U5efqkHXYF11vUMHDn2VzwQ7mmeJ0KuwdeWOgN5ffWWcfkDu+2H8bw7cAQ/VFmdc784HMy
Yb1zLRMP6W/LeT9BlVZoz1klRrtTuzaW8kkJ3d2o9a4p2hC7Z7Zxrq2N2WUO3P3Er8sLpOuJp59gJn
4AmLGXfPe58nMPvEQvQbn7P35Vvvct/m0CgNxcVvVRv68XMA4h6pLy6oraPTl74nSqPCUnKies/w
+uV3ngPEWoFiOYcodmt90x0xG9I6exkOfM6GmphqaXQkPC+LP0tzf8OqBVo6fp8kkSbhdl80L4jP1u
cDtiPjEgE+5M/A8/8bBeAwDIm6p/usx1UEdRAWrzN+/XSwCAzPWfFLn8cb2AcQhRl5U3tHlfDts6aL
N77VXEyQqN9VP3hsyCUdfTDi/9upOjwkuvNubQI2YYuIDnzB/KHyXmkP72hj9RVLUlIzuhTmDqa5O
CTNtg1JUdKNlqa55jln7k532ffZ/c+/C9egkAMAsrX3qpXPvklXoJpn/40rfl079/qJcAAm7bP02eukze
gFRCFGXVklO2hlkRw52nSHNbh3Rkv0Jf3347OuzV9w864eaDXeSp4E6pCVnqL3i1eKc2KKdM2Mo/
xjjh/S3ZcNLUMtSV1PRTyvVazOhHNugZuhXPU7tScXsQNWYoCtu2QJgSvX76vKxv/2YXgIAzELpoRfa
D4z26f/6D3aYCgDIyeXPivTp2jMOIerS8ntyNptOObWeCzq4p/uem+Sprnj9gDtSK2mA77BOqRmP
crmqj9sur3h1aUsxB5LvWDnzBzK787+H3iYIV70kP5e45w3jL9cv5BOuYRUr82E8miD3pbKoIM9Trvi
ls8dW7sVSMn/+v/
+l34GAJgF1ftU9ULFeHd/6AH9DACQi0v36CqCtYrAxMwCqtYrAxMwOY/JJkhK99
VzVwZg/Ll1GT440LNVzVtS1VJXmauqhoTmUvxxaW8AoiWC9cuSQ/l5D1r0CqtYrAxMwOY/JJkhK99

pMJoc2VLb9IfyBurRGfdmzm7MP3bEU7v7Fu+WOl9+hlwAAeXvjX99AHdSYfv7dP6WfAQByUXijfoJ
RCFGXWWDW8FPiTzbuDltXzPVzyAwvRw2ht+tRGr0uXeW6dJPWu0z1nFVke7BA5v5hSrUxzaH8o0
NdMxQeNaQ/Xl3VCaV5bSaVQxvsIfxhxVBVSNvvp1pfFohy1RVXyf/4lf8ht1x3i14DAMjT373yCf0M
UV7z1pvkbe+5VS8BADJ37FUix9+kFzBKoa/G9QIAgKXxwCMPyOv/2+vtmfoBAPm648vXyysfvFYvYd
D1P/E8+a1PvVGedw0TbwFALgovFrni/daTq51ljERPVAAAlsztN91uD+0HAOTvb27/tjx2PX/ECnPlc4
9L9fdeS4AKALmx3m+Pv8f6SIAaByEqAABL6F2n3iUfeMMH9BIAIC+Xjvfl8294VH78nMt6DVz/8t+V
5NZXv0gvAQAyd+ztIoWb9QLGmSpE7TVW9AQpBX9W65F60lhxJ1TZkODL27LhTbbiPCabqGZYeyO
43dBH1jOAAwAwhz788x+W07ed1ksAgLz84PnPyt7rvqWXoLzidS+R1X8bNhkqACATx4oix39eLyCO
OeiJqsJVf5ZqR84zwquZ0VcaKU3oAwDA4rjnl+
+xh/cDAPL10M0/kO6t39dLy+3al14l1d99rV4CAGSucI3IMTWMH0nkGKIWZXOvL2oeq35gxuuuHH
iTiKvZsNXn92QzxwzV1qnJeTqkAgCWjDtj/3VXXafXAADyonqjfu+aZ/TS8lIBqgpSAQA5Of5ukcIL9QLi
mn1P1N6h7OuncuqEZJedlqXedUNc/9Gtl/XnRZq7pKgAgOVzy3W32EGqClQBAPlR9VE/96Zv2h+X1
S/8xivsofwAgJzYdVDz7rl4NOQYog7XRLXrlZZq4nZEtYfVD9UoNb/OeaRVL1Uprp0RP0YFAGA5vfm
WN8tH3/5Q2dzga0fVgsysDVEa98oca+VK/5xJm1f2OsHyncO1O806o7arx19P/kG2p/K9Ux6
DsOEt32wXungORo8P2HiTbTn8/epHqPqpCY5P0DQbdffJvf88j16CQCQl7+/7Xvyjy//oV46WlQd1O
rvvVaufO5xvQYAkK2rRY6/2/p4hbOIqSxEiFqud4fDT/uRwnDa4qac1WVWRfblcGnyBRVsDU7a1ZTV
wqr1/3hUiFMKG3rcqUlpMKBKMklYe2NETUjVYzgYFmXWhihZtW+khNcqwXEma58TyIW9vrlaGBH
2qXNitLNcl/GVM9SxDbR/6us5/f0ej9OrffAcNVdHhZyTcPbh1XO2Dd97qd3vWGrvKL6DGfsBYAb+6v
WPHcn6qL/yH15JHVQAyNPx09RBTdH8hqilk34P0YN0Bu2Ha8tuuinKQug1zvnhkTuhV7/r1Zsdzzhv
5bp0dbDd9TbQlHNeotNLMEmY9dpzesOh2+3IzkX3tVm1IUpW7Rst2bVKcpzJ2tdrrHuBnP+HjZa1F
615bkRQaLRjb1OKem0koz1u06e5ntPf70n47fHb3pHa+ejIPHqivUH+PsLvvbTud0Ds2frfdepdegkAk
AdVH1UFqUfJyumftB8AgJwce53zQGpSC1HtnmjGkFHzEWco65DimpzxspxVfxuqF6C77UQbdnpv
me1yHmYvuTOyFivhWXQ9uejO2qUCFi+kKcrmBSPEidI7lH39VDo74mY3xc09J6yxHnub+mQmmiT
MmLjHCNyKa2eGQ/XM2hAlo/aNlPBaJTnORO0LtuOst74iW/aGVVC3J2GHE2hHTNWz/rmtbPtBrd
/2JNczhfs9gWrL7yFf2Up/+4q5j/B7L8+J+bAM7v7Fu+WOl9+hlwAAefjmjU/JV376u3ppsb3sp14o/
+a3mNAEAHJTuFHk+L/WC0jLHA/nDwYcXkjrjWE1w5B0mMHN0daVAzdTKp+Ukn5qK56QU/ppJDP
kHgioh2ouTjVJmK6nGTZ8Prc2REmpfSMlvFZJjjNR+0a3wwldR5fWOHUi6XdVVU4HNlaSk2473bYn
up4p3O9zpSwnAwcRIteJ+bAMrrriKmbsB4AZuP9V37HD1EWm6p/+
+n/5GeqgAkBurhQ5/mvWR+qgpm2+a6IWN2UvbMitPdQ3hXqoHtWLri8pZ7JH3OhefG5oM1EPZ
MWu5eiGPlE1KzNsQ5R5b19sebQvRuA3VlFOLEzSmcbxAvPpphfcZAepKlAFAOTn8yuPylNXX9JLi0f
1QFU9UQEAOTn+r0QKN+gFpGmqENUc9hv1cMLJoj8M2gxA7aBUrw9NMc2v04+4dRUtdp1B82tD
H2kGskvEuHZ+7Udf89zw5DXjJwlry4bZq9OtjWnWijRl0oYo2bcvLbGOc4btG21wgreeHHp1B4ZlOv
HcEcD5QZpuv+l2e2g/ACA/KkBVQeoiel3l/6IOKgDk6dhrrQd1ULMy3z1RkZGQ4dEus05mDGp2d9V
b0Q+rjZ7D7raTTBLW3vV6dtrhjxuYG8OTB6XehihZtW+khNdqguOM176Idth1itOdfT7Y9JDh+ImO
M737fWHkNjEflpGaZOoDb/iAXgIA5EEN6f+7Vz6hlxaDmoWfOqgAkCM1C7/qhYrMEKIupaKs+bN2

yao3Ztuc0XsMPcGXKlEb7K0YEnhNOEmYH/4YM+KbcmhDlFTbN1LCa5XkOBO1L6Id9rE7NVUTnsK
Rmqsb4jfdL5lQPrPmhNaJrmcK9/uiyeB+B0wf/vkPy+nbTuslAEAeVH3Ux65/Wi/NN1X/tPp7r5XnXX
OlXgMAyJaqg/oe6+PVziIyQYi6pIqbF/zehs1VHawYk86MU9n2h393alJygxmjRqg/UVew9mbkJGGV
09aSNq5dWbUhSmbtGy3ZtUpyrpO1b2w7ynXZSmV8eFnK5aas6vZ4Tbfaftab/j/Z9Zz6fs+LOdGV28
6Jws6U7ncgwj2/fI89vB8AkI9Lx/vy+Tc8Kj9+zmW9Zn79Uu02ufXVL9JLAIDMHVsTKdysF5AVQtSlVZ
TNvcFJu6rS6res/8djD/8OK6Rpb2dgoi679ubg/ixDk4RVZHuoDc72vF01d71eitm0IUqG7Rsp4bVKcJ
zJ2qfa0Zfu0IbVpo3SBlM7JWcHjzfsGiW6ntPf7/mw7q/B67F/aPQSTiCV+x0YzZ2x/7qrrtNrAABZ+8H
zn5W/ev1jemk+veatN8nb3nOrXgIAZO7Yq0SOv0kvIEuFviqCCAAAMIF7H75XKn9UkaefXYwhpgBw
FNzx5evllQ9eq5fmx7UvvUr+06ffzDB+AMhL4RqRK9R8BQzjzwM9UQEAwMTefMub5aNv/6heAgD
k4W9u/7Z875pn9NL8qP4udVABIFfH3239jwA1L4SoAABgKu/92fdK7Y6aXgIAZE3VR/3cm75pf5wX
v/Abr5BXvO4legkAkLljbxcppFNUD/EQogIAgKmp3qiqVyoAIB+qJ+re676ll2ZLhae/8Osn9BIAIHPHiiL
H36EXkBdCVAAAkAo10dQt192ilwAAWeve+n37MUuqDqoaxg8AyImqg3rsPXoBeSJEBQAAqVAz9b
d/tc2M/QCQI9UbdZb1Ue/6T6+2g1QAQE6O3SlSeKFeQJ4IUQEAQGpuu/42ueeX79FLAICsqbqon3
/DozOpj/q299wqp/7ZDXoAJC5428SOXabXkDeCFEBAECq3lF8BzP2A0COvv2if7Jn7M/Tra9+kfxSjV
/kASA3hVtEjv0LvYBZIEQFcteWjUJBCsZjpdHTn1tSvYasbLT1gqO94Z6fFZnX09NrrDhtXGnIqCb6x+E8
lv5aY2mo2frfdepdegkAkLW/v+178o8v/6FeytbzrrlSqr/3Wrnyucf1GgBAtq4WOf5u6+MVziJmghAV
yFVPGiur0tRLjrKcWSvq58vHDiJLNeno5YXRa8h6LarV6loXZDV4saVTK0lhIDAGjqq7f/FuuePld+glAE
DW/ur1j8kPnv+sXsrOXb9zu1z/E8/TSwCAzB1XdVBfrBcwK4SoQK66cuDmbuW6dPt96ff3ZHNZM9
SxQeScUj1nxwW/7fPiHlq1pa5zX7r1srOiuSrkqFgGV11xFTP2A0COfvyycy5nXR33zr9ivrTXoJhuHIDAGjqq7f/FuuePld+glAE
DW/ur1j8kPnv+sXsrOXb9zu1z/E8/TSwCAzB1XdVBfrBcwK4SoQK66cuDmbuW6dPt96ff3ZHNZM9
SxQeScUj1nxwW/7fPiHlq1pa5zX7r1srOiuSrkqFgGV11xFTP2A0COfvycy5nXR33zr9wir3nrTXoJAJC
5YyvW41V6AbNEiArkqXco+/qpnDohy9v/dHHF6znbk8Y53QW1XJetivO0uHlWqs5Tae6SomI53PSC
m+wgVQWqAIDsPXb903L/q76jl9L1sp96ofzKfziplwAAmSvcKHL8tF7ArBGiLjGvnqP5CKntOLI2peqN
536d163Or/dp135sb+ivNdbZLwuuD/bKS2MbmtlG7zFcYzN4jMM1S+1HWN1Low3jal3a+zDDt+aq
87WDDQ9pc9i2o9o8tpfjROdl+J4ZuZ8YxxD7fGh+W9RjuK0OZwi9/7p0z506/lKsnrNGj+NAWF6Sk7
ozquwfDt9PNqM9ie65eMduS+H7wj1Pcd9HsNxuv+l2e2g/ACAff/fKJ+SbNz6ll9Kh6p/+
+n/5GeqgAkBurhQ5/mvWR+qgzgtC1CWlwpHQMKhTk1JhQ8ZlcHHYtR8HCkLa61TIMrC+uRoeXk2
1DRU2hfYY7EitNCqEU58zapaW61J3uw52duTiYMi0674ynbqmQ8GiZh/zyGBquM1uz8dQE5+X4X
umuToieEt8DFGcfQcvd0hb7WCw5A2hd6V67jxVafVbXq/SIWaP41E6B9LVT4MqcjrpPZfk2FP6vlDn
KY/3ERwdapKpD7zhA3oJAJC1z688Kk9dfUkvTe/f/Nar7J6oAICcHP9XIoUb9ALmASHqUmqLn8O4d
TmNeo3SlHPhSUpi5XrX3na/FYybwtaPGt482TaCw6mHj7EjO4PplEcFZM7r+3ubsrlVF+erBr/GPI9nZ
FyGWtm2ttd1t2Wptpx9bOvUrr3hB4Vhbe7UZH3kdQm2eXRT0jkv/invSO28cd0SHMPY8xHg7zu8r
dZxrbvBoHEu3Iamcu40u53bEitrHVKUE6f00wiVRPdckmNP7/uimOP7CI6OD//8h+X0bQxHAoA8q
ABVBalpWDn9k/YDAJCTY69zHpgrhKjLyOwlZ/R0K27uOQGJ9dhLZaajqpx1t1M5bS25RqwPHd486T
aKsrlnBj6O4toZL7TrHIT3AyzXt4IBWXFNzugv6uxc9NvY3vV65pXPrI0P38YwexjWLxht3rwgfgZ5PrR3
31CbR5r8vFRbfnDoh3xB0xxDFHPfoW3tXZQd3SEycC4qW/5+zWtniH/u1HFY3yOhIe8k9uUwrEFKk
nsu0bGn+H2R2/sIjpp7fvkee3g/ACB7akj/V376u3ppMqr3qeqFCgDIiV0H9V/rBcwTQtSlZAQ0ImqY
bsGrZTiyhuIkyielpJ8GjFofJo1t2HQ9x9BhzEGnTgwGP0VZ8xMtLyzyA0Mj0J1YVK9WY/8jgrfhNscV/7
yMN90xjFaWk+MudvfAa79X7sF+GEPcRwyfn/zcTeuUjN51gntuimNPcv2HzlNe7yM4ctwZ+6+76jq
9BgCQJTXJ1KT1UVX907t+59XUQQWA3Kg6qHdaH6mDOo8IUZdSUTYvhPckdEOYqMl1FkZg8hyjn
mOk8MDO76nnDnc2AsPq6dg9GafXkeGOgjFCRlOK52UyYccwK2keVxw9ORxbLNWR2T2X2vVfkvc
RZOKW625hxn4AyNFfvf4x+fFzLuul+H6pdpvc+uoX6SUAQOaOrYkUbtYLmDeEqMuquCl7esjtQKlR
W/Pcos+u3ZYNs3edW7PRrMGZhHW+zurzZA+NNoYyV0/nF6FOH/qlfF4mkm1w6dXKHXpMWsd0
AsUTMrb06bje1BPcc+OPPf3vi6P9PoIsvfmWN8tH3/5RvQQAyNIPnv+sHaQm8zerjPkAkL
ljrxI5/ia9gHlEiLrE1MzaqqeYPcGPHYR0vRqKo4f/GoxhxHPHrB2pgiW3/uMUba64U6Z3duT8+R29n
aqkk6GW5KR37gdnZO/JRbfoZeQQ8BgyOC+

+nI4hTOmkFwKOqumZL+NcNHfF75DZlQPvNJxwzn2EWPdckmPP4PpP/T6Cpfben32v1O6o6SUAQ
Jb+8eU/lL+/7Xt6Kdr1P/E8uet3qF8NALkpvFgP48c8I0RdRu0Ne6itmkU92FPMCHiMXnIlPxkzZmJvy
4Y3Dft884MlY2bySXgTWHWk2dQnKrWh/EXZdLsdqvO87l+XXmPdq22ZZBKkcVI7L578j8Fj1udsrvr
DyPW9bj9yHVtu1oBtyq7eda9xzgsxY/VgjnPPTXjsU1//hO8jZntyvRSYe6o3quqVCgDI3t/c/m157Pqn
9VI4Vf+0+nuvleddc6VeAwDIlqqD+h7r49XOIuYWIeoyqmz7Q287NSm5QYtRH7F6NnzmbhXSDL52
Lpkz9nttNibamUhF3I6BrlSH8o+4LiU/fZQL005glcl5MSQ9BnPYu9ueiRK2YH3O5qqz34IX9Felldqs+v
EUNy94PTLd9njnodqSeM2Jc88lOPY0r3/C9xEgiqqPquqkAgCydel4Xz7/hkcj66NW/u8idVABIE/H3k
od1AVBiLqk7KG3YUUMVeDS7wcDHlX3cLBmYrWVcx3NpCqy3W/5gZHNOTbvsAPDrOPxhlcr5bpsp
ZzL2dcl5LxWW9Z6d+j1VLI5L6Zkx2C1Z/A+3D80ejUmYNfnNIaSu+y6nznWQ/UUZXNvuFaoPYw+Q
aAb656LfezpXv9E7yNABDVTf/tX28zYDwA5UPVR7/uZx/VS0Cte9xL5hV8/oZcAAJk7Zv2GfPwdegHz
rtBXRewAxKNmNtcT86gwbG/anqHAOEfpntPHcqpFwIpwn+l9Rip/xM0BAHl441/fIKWHXqiXRK596
VXyW3/8z+yPAIAcFK4ROf5+66P/Xoz5Rk9UILaeNNbdmc3LcmaNABVZO1r3XPu8dSwZ9ODG0fGO
4juYsR8AcrL3um/J9655Ri+JVH/3tQSoAJCn4+8mQF0w9EQFxmrLxmANWFXOgK50yMzRu+d6jRU
p7ZyRbiplKXDUFf6fgn4GAMjStU9eKe9s/aS88723MYwfAPJ07O0M419AhKjAWAOBFgEqMsc9h+
WlJiiblx9NaEs42hKOtoSjLeHmqS0nrr1GDr/3pF6aLa5RONoSjraEoy3haAvSQIgKAADmBj/ghqMt4
WhLONoSjraEoy3haEs42hKOtoSjLeHmqS1IhpqoAAAAAAAABCBEBUAAAAAAAAAIhCiAgAAAAA
AAEAEQlTkSE2WU7DrfxRWGtLTa8fqNWRlo60XHO0NvZ3CijRibwjp6Uljxb0GG9aVTbJuGmH3UNr
7mJbRRv1Y4SYFAAAAAGChEaJirvUaK1Io1aSjl4H5pgJdY1Z9W1nOrBX1cwAAAAAAsIgIUTG/eg1Zrx
GfzqeibO717RkF+/1tqei1szFPbenKgXvLluvStdu0J5tkqAAAAAAALDRCVABIS+9Q9vVTOXVCyE4BA
AAAADgaCFGXmao1OlC7MazG6GD9UXuIvfE1A+VKNbNOpXokq1Vp79Mcxt9cdbYTvjOjjeoxqk7qY
JvCa1VOdrymMbVf2xvetob2H3JN4rTRY359ZEONcxFo46j1xj7t9ea5zKoOadx7KKotg9uYsD5pjOuS
9J4FAAAAAACLgxB1WakgL7TWaEdqpVEhpPpcQUoDQ+ybq4OvVyFiSYIva8pqYbBWZBqcNq0GNh
xyDHYINtgm65W1UnjQaYt7vIMqcrqqn3Z25OLAa9u7bmODtTKHQjgtuo2TKsrambLztHMgXeeZxR
iOHmh7W9xml8+s5dDDMoV7aKJrPizf6wIAAAAAAOYRIepS6knjnJuIuXUb+9Kt61BNOrIzmPx5qtL
Sr2+5QaEKG8/7ve16jXN+0FVt6VqVXfE2H0Nl2/qabl28L3G3sx1W8dJvU/gxWMe77oZg/mv77gF0
arI+MhUdf7xhKltu2wfPpR9GSvmMeBlqe8MPgsOuSWQbJ1NcO6PbuC+H7qbbu0ZI2ZEDN101hq
mfOpF9hDr9PTTNNTckuC7J7lkAAAAAALBICFGXkjERz96m16vQD9VEOl56FlRt+RP3+EGhqScXd9w
+e1VpeQGStc8LYa+fntmm0GPoXRS3SeX6lj/xUGXLC+U6OxdDexSOP94RimvidfQ0t22ElGaPTrN3av
2CcU02L/htrJ0fGKo+Ja+NftDbO3Si0mrVCRv3dbrau7jjBZKnvROYlRTuoSmuuWkm1wUAAAAAAM
wdQlRYdA3P0OH9SZmzk5+Ukn5qK56QU/ppespyMrCTEN0D77jsIdheXUtjqHdgSHsazOHy/rB4P5
SryllvyvYRvVNtxnbMHqOpKMoJfUHcwLlrXzzrnJ4+aYeVwfWW6mk/kMxMCvdQKtd8VtcFAAAAAA
DMG0LUZRWYKCdunckYgeWRMt3x+r1i3Z6eRig3URhpDK9PScUt3trctVrXE6cj6ik5UdFhZWC9anb
2EeriSf+6AAAAAACA+UKIupTasmH2OnVrPZr1HI+ocr3rlDEYevjD9lNT3JSzbglONXTcqCs6WRiZQYh
dOS164L4ctvUQeLv3pzs5lrF+QUP07K/5sv1xAQAAAACA5UOIuozMupwqYHLrohpDoCdXkpNuEjs
4XNoIEXNVcoamK6NqvWbF6+nZ2ZHz50fVFTXP2eBs/mZ90FMydk6nxNfQ3XdHDnadr3VrtZbsT/jr
h4e0ZyWFeyiVa57idQEAAAAAAAuNEHXJ+QGTMWP/VMxakU1Z3XCn3DFnS8+ZMcmTNFfFa1J7w
6+V6a1MmdfTsyPNpj76oaH8Rdl0u6xar6utN6yz5eg11r0anuYESU7AqVivP+8dkGx4U8nH5V+vZtP
5Wnf2/aIumOquNyfCylYK91Aq1zz5dQEAAAAAAEcTIeoy8oI9S3NVh0rGhDtTMmcun2r75iRC7nY
mCjuDs7o3V3WI5gWO5gzwaXOHxftCh/JXtqXl5XU1KdnnrCAlP6mTC95EVCojdOutWrxzHLe2bVBg
W9Yzb2i6eZ9Y68/k0w3VNv09lNI1T3hdAAAAAADA0USIupQqst1vGQGZUpVWv+8HRvaEQpMqyu
Ze1w/BbGr7g/scx2qn1yBt/9DrDZhIcVP2+oNtstj1YDOoh2rwhvQr1v62Ruysst0PrUtbbVnr3ZILLnU
8g6+ttiara2v22gwMTTeGs+c+ZD2Feyila57ougAAAAAAgCOp0FczrADITq8hK3oiL1WDdo+eiwAwk
urtPS8/mtCWcLQlHG0JR1vC0ZZwtCUcbQlHW8LRlnC0BWmgJyqQKbOOZ75D4gEAAAAAAJAOQlQ
gE23ZKAzU8ayeFTqhAgAAAAAALB5CVCAPql5pZpNXAQAAAAAAIEvURAUAAHODelXhaEs42hKOto

SjLeFoSzjaEo62hKMt4WhLuHlqC5KhJyoAAAAAAAAARCBEBQAAAAAAAIAIDOcHAAAAAAAAgAj0R
EV2eg1Z2WjrhVnrSWNFzZavHhsyL60CAAAAAADA/CNERSZ6jRUplGrS0csAAAAAAADAoiJERfp6D
VmvEZ8CAAAAAADgaCBEBQAAAAAAAIAIhKhLqr3h1gddkUZPD7+3l51HaClTVePUeI3zcL7eZW/XH
MbfXHVepzc4uF+PuW1j58HXt2XDfY318F4Wo12TGjwv6jHpuVGSHr8S1obCSkPCD8+s/eo8VkaciG
TbBQAAAAAAWF6EqEuvI7VSQUoDw+
+bqwMhX3tjRI1T9fXpBJbR1H5WpamXpFyXrYr1MbN2OWHk4HlRmquFYNCZ4blRoWtYG6RTk9Lg
BFl2EFuSwZd3aqWhcDTRdgEAAAAAAJYcISosVWn1+9K3Hq2qXqUCwPNulNaTxjkdX5br0tWv7dbL
zjrrtTsXnYiusm19rlsX9zNSbdmv7W+rxHNafjv7e5tSTNCupHqNdS+MLNe7zj77LasFWvOcDkeza4NI
W3bd1Dh020055yW0VjvW3SDXOE/uBe3UZN17bZLtAgAAAAAAgBAVUm1tixtxVraMANRTlM09H
crZ4aVeu3bGe23noKufZadc3/La6ciqXT25uOP20qzK2U13yxXZsoNGFVLuibM6w3PTO5R9/VQ6O+
JmscXNPWd/1mPPbVvvorhNDpynypa42Whn56J1ZJYk2wUAAAAAAAAhKiaha5OGDmHPzqkT44K
9tNrVlQN3A+WTUtJPFSdo9EPnYSmem+KanHGTWGtrquyCW7t0qM5p98Dbnz18X7+uYA7v7xxYR
2ZJsl0AAAAAAAAQoqIsJ82UcJTAxElGbdLcjGjnzNtlyawNRdm8ENYz2A9KQye5Giur7QIAAAAAABx
NhKiIoS0bZs9Kt46mWft0JuahXRm3obgpe3qIvV+v1tc8Nzybvl/DdfBh9KCdYLsAgLzp0Q3Ggz9yAQ
AAALNBiIrx2rte70o7oHNrfxpDyKc2ybYya1dJTroJqDsE3qVm4i8YM+6n1YaI16uZ9NUvzfakXXbw2f
XqnHrtK530Qtu4NVhjbRcAlpH9Xl+QwkwTy540VlZFWu57tPNHr+bqLILU+Qxze40Vpz0r+f/hT/0ba
p4P9zEP5wXDhq7XDO6ZmRvzvuZ9P7mPvG7mOO+3ebwnR+5jSf+gFfO8z/K9eGZinJuh951luGnG
nJfc34sTtofSdoiDEBWJ+AGdMSt9AiU/nTRm/7d+MFmdbhD8tO0KKsqaVzS0Kavem667baeO6OB7
cZw2JDp+/aavPhXsGRpSs9Wsc9pc9dvm/sOhHu7KJNtVjG3wDwuALMzsl/chKri09j/lv0npcCYu3Da
KcLuTP+4f5vte3N4IC3ONPyjOQq8h6zWRatX9x28G3NEnxsO8XrMy01/cAyWOhh+z+NZW52O1qS
YFda9TS6qdmpRmFPjk/0v7+Pc19R5csr6f6l3jHFk/T2Z778R5v83jPXncPtTnc/yDVmTok1eYm+C85/l
eHHFuht739CP98xP/vl3dH/g3Iot/IEadk9zfi8efl3zfiydoj/WNrUrbpf+eHO/7dn5+FsZY1g2DJWS9R/
TV5bd+A+hbPzD5uvW+9U+g87mq9ZZia/WtnxX060c8vNcqIa93P29uf9TD2NbIdtqStKvbr5fd9VXr
K8cxXx/yKNetVygJz83Exx/
+MDcdve3gMSfabqvqrS8PXwQAmEq3XrbeX8z3eP2+GngjyoffFv1vwAzaEEm/z8/8vXjm7XCuj9q/f
c28f5PzY/87OoP9RtP37dy1y/3eivPzV9rCv5dn1R7n5y9jv/pnrCy/l8a/rznvuYNtyPocjW9XvNdMa6J
9ZPIeqPdvbdd+hLRD3T/maud+Um3XK1IS/5w4n8/+vTjeucnjvS/
+fZv1+8v4cxImq7aNPy/h62fWnhHfw+nfR87+zd0737fBdX579Qr9vhz3uiJf9ERFDBXZVn8p0ksO56
821puAo7kr/t9KrNd7n9D2D52/MKlanIP1QqutCWuIJm1XEk4PIOsNTS/7rDdbf9h+0jYkPH57qP3g
ubQ5+wj8QdOuc2oMyXfZPWWMeqiWRNsFgMy05XytY71NXZBN503VUpEt9UY28fv35Iqbe9Lv7x
ltmTO69MupE/PRwFm1o9dYl1qnKmfn9kLNhnteWt7PKPPC+T6X6unAzyL5KMqJU9YH9+dQW08u
7sygPb2GqIFK5fqWv9/Ktv3zYmfnYgY9sRxj39d6h7JvfRj8fi7aJ64puxm9Ecd5v83jPXle3ved79+y1Ls
hP8tr6uf34dEJHdm5mO7dE/ec5PVeHOfc5GX8uXHeXwLf5xmY7Jxk9148/rzk+148tj0jfp6yR42mW
tYuzqii+fpZGOMRoi4pvw7mwJuLMeFQcMiBCgvdr1EPJ5TztxMM6dQPhf5rrYf5A725D/VQ+xmx35
Ht9MRtl/MGFlw3nvMG7H6d89gbakjCc5Pg+G2D59J+jDoG8zj1Y9QvU3G3a7xu+NgBYAoz+uV9M
enSL+W6bIX/A5CTnjTWa9KZVTvsoaMdqbbi/1ueGTUM0Rh6N9uSN/n84j6JXuOc9d1s/bI/oxu3sq2
HjNo17dWwypITNg/
+vJW13H5pT8huV1lOenWcNKPePgZk8AetuMFlmDTbEVuO78XTnJv86fJoB+cHhtOnWwJnknPCe7
FhxPub8/PnvuRaNYmfhRcOISoAAJgNfnmPTdUltX/5uTCLXoa6tpj9i6D+pWdGvR3b52vSqbZmPmL
C/0Op81AjV7KppRZXPr+4J+f2NDo7wwDEHTWkatqr7yOrObMI4efpl3ZMgT9oKfPyXhwwR3/Y6uy
flAvevxGqt6h6/9mYYa9C3osD9HwiwflBrO+pqedWiWHwjzD8LLxwCFEBAADmmDP5gRq2N6ueO
MFRDt36vqzO4hfU9oYzCcRc/dbuUL2Csh6WHcfc/eLePi/O6NHZXTNnsg5/YiAVeKtJgXKftGOWv7Q
jNfxByzKH78Xz9oet8pk147pY/4aeVWXUZtirkPfiAernGrdnrP89dXBKXadTkl3n7nn5IwymQYgKAA
Awp5wAVfXWmJ+hjMXNC3YNtk7tfK7hXHtXhU1NO8B1fuEpSEn9Vqh/CZrtcHptlsOyLfP1i7sOCGf5

y6Iecqzq2bt5jz0MViXezdWMZjYfZVa/tI9h93bqyMHgjTuqd9QS4w9ajkV4L56XP2x5ZtqrkPficIMl+f
qydXLfOk8nJau3vdn+EQZpIUQFAACzwS/vkVSvDSdAndMJ/zL8RSPMYE8j9bAngLR+Mexaz2dbt7sn
h6qoWc7nZKxZ/uLu9nw6O8NfFgeHTbpmdl7y/6V9rOIJceZ7CUZNPfuGnmG4O2f4g5Zvvt+LB+T+h
62S2GWOB8PbWf5cw3txTLq2eOCPkekZ+UcYfhZeOISoAABgNvjlPYKeRddiD3czevzkW+dSTf4wM
Bxc/0KW1S8ac6/XkJUVc0i2WqVmSp7lL6nz94u701utKjMcPSpSOW21wPoeCgyhV7dwzTovM26bL
dtf2uOpyGnVY6+27r+veL3G5m+islngD1qLaFZ/2NIjADo1Oe/9w6mHcM+oHinvxXGoUhklqUldLm
RwkSL/CMPPwounDwAAMCOtqvSt33L69a5e0a33y9aPJ2VvRX669bLVFtWewUe139KvWUZh56U
6JyfEblu53s/3bun26+Xg+ZiLe6RVHbg2rb71S2tfJT+5m+H38TB9HgKPefie1vdRxvdv3Pc1573Yf2R9
7eK0K4/35PH7CLt/3Ifxb1dq9H0x9H2r2jFw3Pp7Pu1rNcl5t78m8/fiEedGvd8M7Ns9hrTf/uKem6H
XZfY+POp+0XJ6L453XvJ7Lx7fnpB/xzO6RnHuxXn6WRjjFdT/rIsKAAAwE+5f6F3WD43zNRwQWBCq
x5xdm9BVbUl/Bl3nnO/pqrT6M5p5eS7pnk7G5ZnV9cH8Gfre9fjfR2Gvqc5r79gUjT83Id9bxnk7iuLc
LwrvxbOmRvOoOqhhgkP7+Vl4cRCiAgAAAAAAAEAEaqICAAAAAAAAQARCVAAAAAAAAACIQIgKA
AAAAAAAABEIUQEAAAAAAAAgAiEqAAAAAAAAAEQgRAUAAAAAAAACACISoAAAAAAAAABCBEBU
AAAAAAAAAIhCiAgAAAAAAAEAEQlQAAAAAAAAAiECICgAAAAAAAARCFEBAAAAAAAAIAIhKgAA
AAAAAABEIEQFAAAAAAAAgAiEqAAAAAAAAAAQgRAVAAAAAAAAACIQogIAAAAAAAABABEJUAAA
AAAAAAIhAiAoAAAAAAAAAEQhRAQAAAAAAACACISoAAAAAAAAARCBEBQAAAAAAAIAIhKgAAA
AAAAAAEIEQFQAAAAAAAAAiEKICAAAAAAAAQARCVAAAAAAAAACIQIgKAAAAAAAAABEIUQEAA
AAAAAAgAiEqAAAAAAAAAEQgRAUAAAAAAAACACISoAAAAAAAAABCBEBUAAADAQmtvFKRQ0I+N
tl5raG94n19p9PTK2fDbuiEhLV0wbdlwz3vk+e1JY2Xca6xXNVb8ba00rK8CAGB+EKICAAAAODqa52
TGOemSUMHoqjT1kqMsZ9aK+rmpKJsX6tZnHZ3aesg1asv5Wkc/F6me3bS+CgCA+UGICgAAAOAI6
Ujt/OL38Zx/XTlwM89yXbr9vvT7e7I5KvksbsrZqn4eco16jXN+IFttyXZFPwcAYE4QogIAAAA4WuiNm
r3eoezrp3LqRKxeo5XtIng5auAamb1Qq9IiQQUAzCFCVAAAAAABHTLzeqH590pVg6NpryIpbm9Orse
rX/7Rrehp1Vr119suC68NKtPqCtUJH1wEdeJ31CKsrGjyeYL3S6HYYzGPXj8F92fsp1ayzrDVXndeO3Ul
FtltujOpfo/aGXxagXN+yXjUo3vHbQto/dH0tcc5VoEar+6BWKwAsLUJUAAAAAEdEWep1HdJl2Bu1U
ytJYTVYDdRep0K2gfXN1VEBZlNWCyUxyoCqjUhpcMIpOxQceJ3F3t/IQK8jtZJRr7Rcl60YnTuHwlEtel
8JVbak7hZHba7KRqMh5/wEVS4M1gNIcvwqwA5pv3M+hoNUR/i5UueiNLhTJewaAQCWAiEqAAA
AgKNjzQ3p4vVGnVS53pW+qgPq9ax0hK1v7o5oh1dLtC/
+y5tyzkv7etJYd0PBqrT0a71td2qyPjIpNl6/F2OSpvaGePmv0a6um3ga+6psW5/r+hNFqRqm9n5iDc
MPTjLVrLnHV5b6hcF2Jjl+67VuGhvWfmsrOxfjnqu27Eadi8A1AgAsC0JUAAAAAEdIUTbdGYwy641al
bNuj8nKaWvJNWL9/mFoL05zBnqzXmhn56Lz+t5F2dGdIQPD3I3enN5rB4QPix+t7aeGgTCzuHnB31
ftfDo9MAOTTGnVs8OTUiU6fuu675lBqKO4dsYLbDsHXf0saOhcmfVeOzviZq/FzT1n+9Zjb+QMWgC
Ao4oQFQAAAMDR4oVsGfVGLZ+Ukn4aMGp9qKqcDiR3JTnpp31ix33dA90L01rllguwH8bwdve1A0
6dSBLymT0vz8ha4EuLsnbGbdi+HIYlthMITDJlPQudTGqK41fHZNc5DR3eHzR0ropr4h2yuodK7n4ja
rECAI48QlQAAAAAR8xAb9RD5+l8K8qJU/rp1MpyMn6am0BHRnTmnE6i8DlCYFIpo85ppLBzFSw5Y
HLD3NgTdQEAjgxCVAAAAABHj9kbtRYvTsvXYK/Onhx6Y8iHebVWhx7biYbtTyercHa88cfflg2z16lby9
Ss3ZpEcVP29D4Gyt7amueYpR8Alg0hKgAAAIAjyOiNmpQxjDw7g706u3Lg7tTtmVk66QWAo+p5ps
MsJeDXAHX05KJbmFROSaIqAdNKcvztXa/nqR24unVRp7iWaoZ+1ePUnkjLDlS7Xi3W0WUEAABHF
SEqAAAAgKPJmIAoTMlPDo3aqW3Z8Kapz1ZzdcObqKm94Q8/L59ZcwJAszZnc9UfQt7e8Gp0FlIZV2
4Gzta5WPd7WfYa61790aSTVU1twuP3A1djxv4k9PbVbRDscRoSdAMAlgYhKgAAAIAjKro3qjlzuwrp
nGAubi3NaZWlXG7Kqg4D/dzWmOFftd+ozdlc1cGh9+IREzJNorLtD1vv1KSk21XyE1S5kPuM9AmOv
3Lan6jKu5bGBFRJjDgX5r1RPat7ugIAlgYhKgAAAICjK6o3qqp7OVgzs9qavI5mIqfk7J4xPFyx63gO1Di
1a3MOvE4Je+2U7GHrIcdebVnr3eHxeYt9/BXZ7psz/itVaZk1TZu7Xs/fcexzEVYMVW8zrewaALA4Cn
1V3AUAAAAAAAAAEIqeqAAAAAAAAAAQgRAVAAAAAAAAACIQogIAAAAAAAABABEJUAAAAAAAA

AIhAiAoAAAAAAAAAEQhRAQAAAAAAACACISoAAAAAAAAARCBEBQAAAAAAAIAIhKgAAAAAAAAEIEQFQAAAAAAAABGEvn/AZM6nr53azj8AAAAAElFTkSuQmCC]]></redcap:Attachment>
</ItemDef>
<ItemDef OID="q16" Name="q16" DataType="text" Length="1" redcap:Variable="q16" redcap:FieldType="radio" redcap:RequiredField="y" redcap:BranchingLogic="[q14] = '1'">
<Question><TranslatedText>Do you prefer Life A or Life B, or do you think Life A and Life B are the same as one another?
</TranslatedText><redcap:FormattedTranslatedText>Do you prefer Life A or Life B, or do you think Life A and Life B are the same as one another?
 </redcap:FormattedTranslatedText></Question>
<CodeListRef CodeListOID="q16.choices"/>
</ItemDef>
<ItemDef OID="summary17" Name="summary17" DataType="text" Length="999" redcap:Variable="summary17" redcap:FieldType="descriptive" redcap:SectionHeader=" " redcap:BranchingLogic="[q16] = '2'">
<Question><TranslatedText>
Alzheimer's disease - Description #[no]

If you have Alzheimer's disease...

1) You might forget recent events of importance to you. For example, if you are interested in hockey, then you might forget whether your favorite team won their last game, although you may remember watching the game itself.

2) You might forget the names of people you met recently, or the names of local streets.

3) You may no longer be able to handle your personal finances or plan leisure activities such as dinner parties or vacations. Simple decisions such as what to order in restaurants might become difficult to make. Your ability to concentrate may decrease and you might have difficulty driving.

4) You may start having trouble finding words to express your thoughts. You might need to be reminded to shower or brush your teeth, but you could do these activities on your own. You may need to post notes around the home to remind you of simple things like turning off the stove.

5) You may remain aware of the day and your surroundings. You might be able to travel to familiar locations like the supermarket, although probably with the help of a loved one or friend. If you are in a social gathering, then you might have difficulty following the conversation.

6) You may continue to enjoy hobbies such as puzzles or reading.

Now consider two possible lives. In Life A, you will live in perfect health for 13.5 years and then you will die of natural causes. In Life B, you will live in perfect health for the next 10 years, then you will live with Alzheimer's disease as described in the summary for another 10 years, and then you will die of natural causes.

</TranslatedText><redcap:FormattedTranslatedText>
Alzheimer's disease - Description #[no]

<p></p>

If you have Alzheimer's disease...
<p></p>
1) You might forget recent events of importance to you. For example, if you are interested in hockey, then you might forget whether your favorite team won their last game, although you may remember watching the game itself.
<p></p>
2) You might forget the names of people you met recently, or the names of local streets.
<p></p>
3) You may no longer be able to handle your personal finances or plan leisure activities such as dinner parties or vacations. Simple decisions such as what to order in restaurants might become difficult to make. Your ability to concentrate may decrease and you might have difficulty driving.
<p></p>
4) You may start having trouble finding words to express your thoughts. You might need to be reminded to shower or brush your teeth, but you could do these activities on your own. You may need to post notes around the home to remind you of simple things like turning off the stove.
<p></p>
5) You may remain aware of the day and your surroundings. You might be able to travel to familiar locations like the supermarket, although probably with the help of a loved one or friend. If you are in a social gathering, then you might have difficulty following the conversation.
<p></p>
6) You may continue to enjoy hobbies such as puzzles or reading.
<p></p>
<p></p>
<p></p>
<p></p>
Now consider two possible lives. In Life A, you will live in perfect health for 13.5 years and then you will die of natural causes. In Life B, you will live in perfect health for the next 10 years, then

you will live with Alzheimer's disease as described in the summary for another 10 years, and then you will die of natural causes.
<p></p>
</redcap:FormattedTranslatedText></Question>
</ItemDef>
<ItemDef OID="a17" Name="a17" DataType="text" Length="999" redcap:Variable="a17" redcap:FieldType="descriptive" redcap:BranchingLogic="[q16] = '2'" redcap:InlineImage="1">
<Question><TranslatedText></TranslatedText></Question>
<redcap:Attachment DocName="135.png" MimeType="image/png"><![CDATA[iVBORw0KGgoAAAANSUhEUgAABVEAAADYCAIAAABtBTQzAAAAAXNSR0IArs4c6QAAAAR
nQU1BAACxjwv8YQUAAAAJcEhZcwAAEnQAABJ0Ad5mH3gAACsNSURBVHhe7d1fqF7Vue9x02h3
PCqG1hNsPWh0IycYkbRyIK5TbArFiCAIKg2yFfc5sohg2UGwkWIO50JRNoWsvRH0oljh9Jx4EZENJYo
t5KJCCoXmwgs3pLIuLA3Ui7WllCXNRc/8N8Yc4xl/5nzHO/
+9c30//NjNO+eY4xljvq9rzcf2zb7qbwAAAAAAYI7o+QEAAzm/ef6q/30VIYSQsXLHP16f/d/qhzKAnY
F/5gEAQ9jc2tz72l7z0ZMQQsjAefrAHVnbX/1cBrAz0PMDAHq3tb114PUD4tGTEELIwPmfd/79//ivf/
/XL7+sfjoD2AHo+QEAvXvw5w+K505CCCHDJ+v5s7x18kfVT2cAOwA9PwCgXc+OCEeOgkhhAyfg8
duLHv+LB+9e7b6GQ1g7uj5AQA9OvPxGfHQSghZPjse27P0wfu0D3/8bvv+uOnv69+UgOYNXp+A
EBfLnx2Yc/Le8RzJyGEkIGz+9Sux+69VTf8ZV46+gBf7Ad2Anp+AEAvNrc2b/7JzeK5/+/+g0f
CX4Yv9wE5Azw8A6N72le1Dbx4SD52EEEKGz51P3SBafTPn/9//rX5wA5gpen4AQPeOnT3mBw
B06b1P3hMPnYQQQkbJf3/rvX/ACAzly8fJG/qJ8QQqaQ2565TjT28Xz4s7e
qH+UA5oWeHwDQja3trf0b+8VDJyGEkOFz/QtXP3nwdtHVx8MX+4Rg+8r2kbePiQddQg
ghw2f3qV33P3HVF3eLaOnbhC/2DA7NEzw8A6MDxXxwXD52EEEJGyeGHbxLNfPvwxX5gfuj5AQA9OnH3ht
HjiJIQQMkr+y/p/Em38ouGL/LG2lWltY1L1TEldm40elHr55pfhqRdtaRhqizv3Hq5zEKX
73x2A6JbV4V7/bQF3gV7bYOsBL04v3lePHESQggZJdee3P3EPftFD79o+F/qjt9917//5jfVj3k
ZIdTt37TalJFT1b/GVI2lVLGqbKkvQiS501vdW80a2P1fO7a6PnX1GbW5t7X9srHjoz5zEK
wKfl+bXD//GnP1U/6AGsOHr+GsOHr+0Xib+7r5m3KLKls4T0fn03LYDtRPt9vufo/T8vWRs+/ira2tw68fkA8c
RJCCBkl3370a6J3Xzh8sZ8QMkpB2hJv3xH3U4xxF3JxaPnU5Z0tJVW8+3Ij5TCFr67Ck+QFq9ZM
z/RO4LEVZA/UvxjdzmZQxUVLuSJr1a8TdJZtlDyZWxgwPaPJWX/wR+U+XSG0eO3q1+P0Dvcq8/xg
Zfcf9Lnsx3yNzCdO2avfn5hUwjUwcbexeqnS0UdM0yYHQRaJKalkOM7hEw8MBptAFz+FWcmcwaqngZeSWR
tTt37TalJFT1b/GVI2lVLGqbKkvQiS501vdW80a2P1fO7a6PnX1GbW5t7X9srHjoJIYSMkoe+803R
wKfl+bXD//GnP1U/6AGsOHr+0Xib+7r5m3KLKls4T0fn03LYDtRPt9vufo/T8vWRs+/ira2tw68fkA8c
RJCCBkl3370a6J1Xyb//A9PVD/rAaw4ev4R6a5fNzn6yMS7YtGxtWzmWw7beXq6Me2mpefHMh7
8+YPiiZMQQsgo+caz14oWfdnwmj5FY9jZO...

YSF7ZfTKYcJEs7S/GtrXklGNurv35VPG4SQggZK3c+dYNoy/vOK48/+tcvv6x+JQBYHfT8UxBpqnTTY5
6TnZDnet2FxZqlah73a/zlEWM+UTH+MqTNVeW69V9nb5xqWUSSl6U1lr7baV+2wCTmW1lwr22e
zTciL7DYvjLNN6RxHncKeb+cKULLzy28BQzjvU/eE4+bhBBCxsqNz18zwNf43bzzysvVbwUAq4Oefwo
8zU5Nt0/qrO6D9HB9RLVG+pqGZqm+0HdpuPeKvwxpdZVRX5zR49VhPTRWVlZRFy3WWHruiqzev
DxP6ZJnAc2zOSPCB+ppPTyrkhc2LUaurR7vHKlee9fWYiUYzcXLF/mL+gkhZCLZfWrXY/feKrrxwfK7X3
5Y/W4AsCLo+afA0+yYdEtlMQcbTZYUb5bqC/V0upq3QDld/GVIu6vq3Tq3Q19hWahoYmPpfwtyvn
ks9aSe0iXvApo36x9hTG+vOrC7VjekYTHh21MNcWf0rK3VSjCGre2t/Rv7xRMnIYSQsXL/0X2iDx8yfL
EfWDn0/FPgaXYku+nydEDmgOx022bJHee9UhyMvwxpeVX8buirCpE7VpFV0hpL8yr154x7QWx5w
Y0FF9C8WXuEM8RYq/fyTOsbEl+MWSm/TL0up/DN6Kyt9UowqO0r20fePiIeNwkhhIyV4b/G74Yv9
gOrhZ4faOZpR4Gd4fgvjovHTUIIIWPl+heufvLg7aIDHyV8sR9YIfT8QDN6fuxMpy+cFo+bhBBCxsruU
7sevu8W0XuPGL7YD6wKen6gGT0/dqDzm+fF4yYhhJARc/jhm0TXPW5+eO+3/vjp76vfGQAmjJ4fa
EbPj51mc2tz72t7xeMmIYSQsXLbM9eJlnsKeenoA3yxH5g+en4AgGVre+vA6wfE4yYhhJCxMp2v8bt
56+SPql8eAKaKnh8AYHnw5w+Kx01CCCEj5qHvfFN02pPKR+
+erX5/AJgken4AQO3EByfEsyYhhJAR8+1HvyZ67Knl+N138cV+YMro+QEAlTMfnxHPmoQQQkbMN
569VjTY0wxf7AemjJ5/XOrvhiv08hfEXdpYWz9X/XkefxedvaOIbGBgr9WZUqt7Yb1RSrtl7GD81Ycr5s
JnF/a8vEc8bhJCCBkr157c/cQ9+0V3PdnwxX5gsuj5R2R1nn00RlWBGfX87o6C9N0VexV3vdQ0obflp
+dvQs+/Sja3Nm/
+yc3icZMQQsiImfjX+N3wxX5gmuj5R9RzR6Sb29n0/L4d+ZmNvb1XcQfa3RA13WretvHQ86+M7Sv
bh948JJ41CSGEjJh7Ht8rOurphy/2A9NEzz+e9h1smp3a85v9fs7aqzqpZ2h1R9Sgnt6p2aLnXxnHzh4
Tz5qEEEJGzL7n9jx94A7RUa9ETh757l++
+KL67QJgGuj5x6GaIYPZT9ptq+iYjEaqnkZ0o6H5zSbMGOP2ZNYKGls2c1rzSqdHDk9bn9EX6QWK1S
rO7Bn7zhXiq1fz+iZT1KSxMTXzZhg8h8N3oyQ3Yw0x5qvvTbnC6HUBbWtZA50bIk4GbgUm5tVfvyq
eNQkhhIyYr/74Kz84dJvopVcorz97vPoFA2AaFuv59RN9uPnxDql7ksyyz/91WxFexeRZd6SkduM5lT
HummeAc09D8/snzxlTmI2bFn3bOplWny+PiZehHQnqquycuiK2cl0k9lnyLTw23ltYHvRNal3hv6f1C
M/54qT/uuiCU2pVmlecM0dhYt775D3xrEkIIWTcfP97N4sueuXy4c/eqn7NAJiAAXp+0dws+/hvThft
YybPezd136Tukx6lb5zRWkVvgG9+91pnfucydU3knXOnrY/IOpFpzTG+qr4dCfkQsYTQuuslRnaWqw
dawlepdRojxGIa74acQl+g5zRWZd6O0ET1hY6UWvURVVxfpY7UByK1Ma6Lly/yF/UTQsikcvDYjaJ/X
sUcv/uuTy9erH7ZABhb5z2/SzYhyzEaicxK9xK+u+m7WXKcbrcadh+b3zgmhqmXxuSeQ0Jn0+qJKnZJ
MWcD37006MlysQmdqs1vgLM1sRbP1j2HLC1XoYcFJ2ohXKu+T3KMc01GH1tiLejP1vbW/o394lmT
EELIiPn6P/3din6N3w1f7Aemo/
+eP+WaMDXb2lr1nyvcTHjujL9N1Zsuj3oaMC/fnfcUEMP07B7Bgs09YetpzYHy3fXtKMyzV492o4TGi
wJvmVq3uUnJ3Zs92pnDvkDfo9oiW2tdS74XaohVzHsQk7B9ZfvI20fEsyYhhJARs/vUrsfuvVV0zisdvt
gPTES//9t+u4EoeJqGXMumQF2zfk79aYW7Cc/d9PdIYmDbRsozv+/awOw+xkS2LqetRzob9O0ozL
Mon8UmLTVfo0YUxdVK9Og2d0PXENT54PYCF8a2l1BL3gLvcoJrxOiO/
+K4eNYkhBAybu4/uk/0zDMIX+wHpmCknt/bYzQ3BuqyfBL159VtJzx3098jia22baQ88/uuFcOS2rTu
ptWXFcRo347C2pZM2LFaSOySatpsiBrd9D6b1IhMOcjZe+McxhSFNgPb15JjvMtpXCPGcfrCafGgSQ
ghZNzc+dQNolueR/hiPzAF/f8dfp5r2rQUPuq68jL1amX7ididMTclx7VtpDzzt2jefFc16mpaNWptY8
O3ycXW5rtPnhmaJvWdV1NH11ENWltfLycwBjduxF27OqIv8W2vkp1aoFhaLTmrr4o+5lkjxsT/N35C
CJlUbnz+mtl8jd/NS0cfqH79ABjJGD2/OuJpIKK9gRzU6qIJc+9MRrdbcpf1Nj0NmJdv/kWaN3XA6QB
d3UxrXaJfROaM894nOa2zLPc6Z0iLG1LQ4wrW4KY5ZYl6vJ5HLrOkL6wP+wfW0mrpUWqMsyVjnlB
pjGT7yvbhnx4WT5yEEELGysP33SL65Dnlo3fPVr9+AIxkjJ5f9xge4Yn1RHX/4B5ZKYG76b89xh6bejjN
nqmo0qZ5q49YIm9NJ9Pq02oSvfx6Vs+OwjyLytlzVMwx7nXeS5rKZ8zr5OiGu+EvmVND3GWWQle
Gl5tUy31/A1vKyTVifJf/fJm/tJ8QQiaS2565TvTJs8lbJ39U/eIBMJ7V6fnDDcWqthSRu2lvVpwPNXse5
q0uhrdr3jL2CppKLT9tfcIYr5dfD3R2FOZZlGLO0vL+Ri8J0Rf5lxq/yWbFvJ56XdX2L7NkL7bFchev1fz+Z

idia8To+H/OTwgh08nhh28S3fIM8tLRB/765ZfVbx0A4xmz51+oETCbCRc9BaZIfWr5fGKaznx8Rjx0E
kIIGSW7T+2a2f/C//jdd/3x099Xv28AjGrM7/NHZnHoawJoqjA5+kPLpxPT9eKvXhTPnYQQQkbJ9S9c
/eTB20XnvLrha/zAdCT2/F5FC+809J4Ov56mOqb/l8WhfwvgmaSkT9BWYTr0B7oQ+lQD0/DIO4+I5
05CCCGj5I5/vF50zisavsYPTMooPX9oomBvFOnsI6eAkRg9Pw0/Jm/7yvahNw+J505CCCGj5P6j+0T/
vHLha/zA1IzU8+fsyWIte7Svj54EADTa3Nrc+9pe8dxJCCFk+Ow+teuxe28VXfQKha/xAxO0WM8PAJil
85vn+Wv8CSFkCrnx+WuePnCH6KVXJb/75YfV7xUAk0HPDwDIvfHbN8RzJyGEkFFy51M3iF56JfLOK
y9Xv1EATAk9PwCgcuKDE+K5kxBCyChZuS/2v/L4o3yNH5gmev6xXdpYM/6mA/WXv/GXE4SIvx8i/n
Ih4t4vM9WirL/nv/f33v7I9WnIe4jOHHn7iHjuJIQQMny+
+uOv/ODQbaKvnmx+eO+3Pv/DZ9UvEgATQ88/pqolMhoiev4moo2Mv1zIWD2/LlTq962vivW9p8p
g9xBd2tre2r+xXzx6EkIIGT77ntuzKl/s52v8wJTR84/H1xDR8y9I3MRlmsyx7v2AdYfuwen5V9Unn3/C
X+NPCCFTyMFjN4rueoLha/zAxNHzj4eevwOr3/MP2RcP3YMPXQ8dev/S+
+K5kxBCyCj5/vduFj32pMLX+IHpo+cfh+ovDUVbZPadxhi3DdXdVE6cNicxx4Ubr0C76z1sVW5xRUPf
p07XV7hH1LT5ETFd/GWcHpzJxou1e6cyLzEXGGVdZF2lShoCCzcXJ9ZtsWuZ1UK1xLZzztaNMfU01cl
wRc9EWCmnL5wWz52EEEKGz5S/2M/X+IGVQM8/jqYGzOVryUzG+VaT2IymrjqScQ/6Z/aUNg419X
3qvD6ti+hJqiPFazFd/GWEfyu5qqwzlT5gMjbqFb9jnrOBhTcuNxctFqqljhvzOFv3XFsOj1Zc5O3ARB0
7e0w8ehJCCBk+33j2WtFsTyR8jR9YCfT84/E1REYPVR3Vo1QjFWzIdKflTlIfCTVfalaj8wvP6yzFGWJM
49umpRqgLnFXr2YoXorp4i+D9DhZI1MtpGlmz1YdLe6YO7GXe1fqI2ILC9by7MMZ5lYvNFb01cNK2b
6yffinh8WjJyGEkOHz7Ue/Jvrt0fNv//ov1W8LANNGzz+eWANmHBPDZJ+VkYeaJ/Fw5nV6QU9z6M
zrGdO6dHlN+WJ9PZ/IPKKuF9PFX4b4hulj1drtMeqVZ2PGIaHNHXMPeKW8p+4A3yVt3jJdPbzXgry
wcYlYAZf/fJm/xp8QQqaQh77zTdF1j5h//ocnqt8TACaPnn88aQ2Ybr88qolS+sN6SFVaTaIv8CwtE7gq
vH6f6qpiQPHntY1zxVX1EX25mC7+MsS7F3HQnkqd9AnV8lbR86qj7Zas69ejYhfay9UDfJd4VukM81
SXvBXb7Q2Td/HyxT0v7xGPnoQQQgbOtSd3P3HPftF7j5Ln1w7/x5/+VP2SADB59PzjSWvA7N7KVk3
UpovzUGOKy9Qc9XjPrJnA6hYsXV2WjShHZ38oD+kj+moxXfxliHcv4qA9lTrpE6rlreIssd2SPXO5F+ojg
jvAqNVmZv9OMg0VffWwms58fEY8ehJCCBk+E/li/7//5jfVrwcAq4CefzxpDViw/aq16g99qguz69T4
+NIyamB1NLV0eZ36r/eLq4tD6kg9oZgu/jLEuxdx0J7Ke0UD/zVqXnW03ZI9c8kL1ZBMOcqd2Verxcy
h3TdW9NXDynrxVy+KR09CCCHD57898nXRgQ8cvsYPrBx6/vGkNWAt2qg2XZxfdeXa+no53BytpzA
bPzmvp7RuDGOlq3ncr/GXR4z5RMX4yxDfMH2sqmWPaTmxRc5YcCZqN3Pze+qOcO+8r1abt8wzJt
Ncsd3esDoeeecR8ehJCCFk4Ow+tevh+24Rffhg4Wv8wCqi5x9PywZMDtOv1YE2HVrb5kvPVRCD9U
k1sZ5VVwovrqF0faHv0vBO4i+DnHUa9atioZnVFXp5kVIt7pisE6BmCt8JuZ56wfXMvlr1uOqgcd/VM
E/1THNFXz2ssu0r24fePCSePgkhhAyc61+4+smDt4tuflDwNX5gRdHzj8horjJFW+Rprty+yeitDHVb1
WqSAHNN7lh7xYrZCfrXVoiXri/U0+lq3gLldPGXEeGFVtWcqfxXNBRa4I5FZ2rxnvpL5eqZ7THl8fCd0B
d6qucaK7bbG1bK5tbm3tf2iqdPQgghA+e2Z64TDfkA4Wv8wIqi5x+V2TQV/ZSnufL3TXajZvdirSfx0U
sS/Z1mV/bMZw7ITrct7Y7zXikOxl/G6cGZbLy4bfHqhdAtEuyL5MraLbnde6rfvEx+VL02pjaH6MnMF
WZjnZk91Svxiu32hlVzfvM8f40/IYSMnsMP3yR68l7z4c/eqn4NAFg19PywqTatZT8LYAd647dviEdPQ
gghA2f3qV2P3Xur6Mx7yuvPHq9+AQBYQfT8MOn/YpaWH0DMiQ9OiKdPQgghA+fG5695+sAdoj/v
PCePfPcvX3xR/fQHsILo+VEy/0fa/G+xATQ78vYR8fRJCCFk4Nz51A2iRe82x++
+69OLF6uf+wBWEz0/SkbPT8MPoIWt7a39G/vF0ychhJCBc//RfaJR7zB8jR+YAXp+AECiTz7/hL/GnxB
Cxk1/X+zna/zAPNDzAwDSvX/pffH0SQghZOB8/Z/
+rvMv9vM1fmA26PkBAEs5feG0ePokhBAycA4eu1E07cuEr/EDc0LPDwBY1rGzx8TTJyGEkIHz/e/dL
Fr35PA1fmBO6PkBAMvavrJ9+KeHxdMnIYSQIfPVH3/lB4duE917Qt46+aPqhzuAWaDnBwB04PKfL/
PX+BNCyLjZ99yeJb/Y/9LRB/765ZfVT3YAs0DPDwDoxsXLF/e8vEc8gBJCCBky9zy+V7Tx7XP87rv+
+Onvq5/pAOaCnh8A0JkzH58RT5+EEEIGzkPf+aZo5lvmo3fPVj/NAcw1cdr4jTV61t
XKrOZM6tOwf1eGOi6Bwla4h7vsUMAFB58VcviqdPQgghQ+bak7ufuGe/6Ocbw9f4gbmi5x+RbKUr
ul1XPb2tbrjb9Pz+OWL/SqBgTNk8AwDYHnnnEfEASgghZMh849lrRUsfD1/jB2aMnn80utdW/bVurs

sD6rw67Yxv0/PLIc4k8gLnisYZAEDavrJ96M1D4gGUEELIkPn2o18TjX0ofI0fmDd6/rE4zXZ1KPzfnze2
5xkxRr8MdehqgGeK8lDjDADgs7m1ufe1veIBlBBCyJBp+cV+vsYPzBs9/1g8DXuYGlxq3/MbLbtmFbQ
ntsl/baDR/QNo4/zmef4af0IIGTHXv3D1kwdvFx2+yP/5X6eqn9oAZoqefywten5Pu11YoOcPTlKdb+7
5m2YAgLA3fvuGeAAlhBAyZG575jrR5Jt55fFH+Ro/MHv0/GNp7PmNdrwc4/TzrXp+RXb35UWNqzD
4ZwCAqBMfnBAPoIQQQobM4YdvEq1+mR/e+63P//BZ9cMawHzR84/F153nXbXqpN1uXPfckZ7fG
ZPJjhmv7Lrhf0dgis0AAE2OvH1EPIASQggZLLtP7Xr4vltEw5/ld7/8sPoxDWDW6PlHo1tn1TvbB2T3X
p/VvbYzg75GHdEH3H9zUB0Jz9F2Bs8BADBtbW/t39gvnkEJIYQMFveL/e+88nL1MxrA3NHzj8ho40
1V66y7bYfq+UMT5NSY0Cx6jsAk9fnmGej5ATT55PNP+Gv8CSFkxNz51A264edr/MCOQs8/MtFx22
2z2W7nLbZ6Xbfb1vXZYf3SGOJ07ea5kr0Kt3ePz0DPD6CF9y+9Lx5ACSGEDJn7j+4re36+xg/sKPT8AI
CBnL5wWjyAEkIIGSy7T+0qe/7qhzKAnYGeHwAwHPEASgghZMh89+i+pw/cUf1EBrAz0PMDAAZy1
VUD/dKhUDIKJaNQMgolSyv00btnqz+1xq1LRqFkgxXaCbiVAICB8ESSjELJKJSMQskolIxCySiECG4lAG
AgPJEko1AyCiWjUDIKJaNQMgohglsJABgITyTJKJSMQskolIxCySiUjEKI4FYCAAbCE0kyCiWjUDIKJaN
QMgoloxAiuJUAgIHwRJKMQskolIxCySiUjELJKIQIbiUAYCA8kSSjUDIKJaNQMgolo1AyCiGCWwkAGA
hPJMkolIxCySiUjELJKJSMQojgVgIABsITSTIKJaNQMgolo1AyCiWjECK4lQCAgfBEkoxCySiUjELJKJSM
QskohAhuJQBgIDyRJKNQMgolo1AyCiWjUDIKIYJbCQAYCE8kySiUjELJKJSMQskolIxCiOBWAgAGwh
NJMgolo1AyCiWjUDIKJaMQIriVAICB8ESSjELJKJSMQskolIxCySiECG4lAGAgPJEko1AyCiWjUDIKJaN
QMgohglsJAAAAAMA80fM
DAAAAADBP9PwAAAAAAMwTPT8AAAAAAPNEzw8AAAAAwDzR8wMAAAAAAME/0/AAAAAAAAzB
M9PwAAAAAA80TPDwAAAADAPNHzAwAAAAAwT/T8AAAAAADMEz0/AACLOrd+VW39XHUUAA
Bgauj5AQAzUjbj/XbhlzbW6gp9Fhz63yzkG8usbVyqDnTJ2kyBf1eyKOse9vM29S7wD0z12St1+8mI/
CMaObUo/1TWO9bttvoVvTO9/qDoUXhTq/o+BXZkbaert6lFrRX9mbRD0PMDAPrV49O8xagz5ENb
UbaPZ53sWUrvo3iu6vmBKt/I2vp69n96qZNvYfBHQvN5tPtPhfXJrvX06Sv2MtAnwrxv3RUJ/hNq/z
Nkb3QpwYrRU4sKTZUf1wfKW7psKf8s5tu1dInGO5Of7/QHhW9T1pYKy+0rsqniVIf/KLnbMYqbetp
RUV8fKl4tu7t2tTooVd67irg/1l0U59ACPT8AoEf285T9NNIpVaj4zyEfCOwd9qX3KnmB6vb1UyZ/73u
/TaZh3hhLUbKnD5+cu79aXT/HV9T74S5cfjS62lq4YuzUotpOpcZVLxdWXF+yq2S3Tx9Y/u1q2k5+vD
q9VJ1SbFNdzF+JbMpzKF1wO8LyRcM7kkd6rKVOVC+Xfdfy6fT0xSe5fmmXKk4utaediJ4fANAf+Qzg
PDV0rv8KwjCPHz1vS0+f/yH9oS1mucfBhQ3+Ocj0+1Gwb2Bv+ysmNt+ort84Z+XuVjq+kZF71eFtbJq
qOJ98I9XV/VYx+Avpo/kfli6jFuup1c+PC7dQJ/uoRLZj6/Dz3XjrGhfTnjOVs48Oi+m7WbyQH4cuC+0Y
9PwAgN64v5k7fNrxG/hhoNhPV8+MQdbjT/eMe5b/sZ9CxZ2q9XzP+ttHUM9vUqa8hVmJ8g/9fMq
df0KNT0cnnPnyivZ967hkZLoOKzVN5dzYJE1VOtuQbyLjWP7Hzj7snlruh6ILbqGiTv5FBW35qr5bZyh
Od7U3b63io5aXKP8QXspinFqB29nN1szb5C/U2cZ2CHp+AEBv3CcA7yNKl3ovYCoePLp6enMVe6n
0uKV8F2r6vGR/G1LKjfVZp/jkdfwoHzfMB6/4xBX6KuXuw/2neClOAXf+ju9lZLoOK8WnKt64Du5ivE
pxtpv3ylMo34M6kp/u7EMRqGXoqJRbSLwvxYDw/W3Hsx1D/OyiQrPV96+zUp5axRF59zp6r4odV
NXyP9vTdnsXdwZ6fgBAb0b4VT3cs4B4WuxVpw9Ttnwb9f3KCw2yJ/ej0Snx5pT3r8+PRVGw18+duY
fyz/3UK+aWt67Dt6qY0Fy5+1FwhiwnMl2HlWJTiY/jErxVioOVrm6bUyjfQ/06P93ZhyJ26zLl9rqo5hZ
yPnwd/IMc3U4H85s8tcrbVR4r/9xRPe+
+iv0o6+vWh2QJxbT6jXHepfg9hhc9PwCgNyP8qh7oWaB80hnwmaN8eOu+oPXIZujiCTumqNvf/X
M+ef3WK96dXm+ZW6HHHVkfiu6e40vOP6HuP7LOu7cct4AWObWo4FTF3exqOw0LLk53UkwWsj
4Thj5quTr6RLiFnImb19IoMkVxqqvPQs6p5VYo3rnldlRqvjX5iC52J/+Rcd/
+5rVAoucHAPTG/cXc0bNb2BDPAkWNgR84ipq93rlCXqb/Kv1vp49H+bDOnqrD3BK97sjQ9UfCXbd

8s7reWWS+Dkv5pyretw4307jgYkAHBRsK5ac7+1Q0bqqrf8LcQsURcyPyw5ggvJ2OtmFwarklmu9u
S40T5QOW/1AUO7CncSt38DbtOPT8AID+yN/MnT1+BPVfoXwmkTp//siqGNvwPAf1opunNlc2rzFt
8Sb1+i4VFcyN9PmMWLw5/X7m3E/AIEUznX8iirfGXri1N+etW5qnohI5tSjPVMWhbt+kxgUXAzq4f
Q2F8tOdvUmjbsr6B6mTf6qC2+nhn1m3lvUPU6a7ovG3qZv3qFitWyQ/bM4dXwq86PkBAD2yHkC
6eSrwK+YWVvuZQOxomM3kRXt5hwbfjfWo291zr6vPT7WtfB7WBvhEdLk38Qko1Fsw99bVzYxUjC
9mIeGpxPtVWnJzRTVrpVkV43VRc5kaLe9MPqyzD72zqeyAMXm5JM8iWotvyjq7TJmKs51ScXiAz3b
nPyjCtewzS9+6cjrvNNbnutM7uXPQ8wMA+mU+gPB7GoOxHkiXfh4NKT7evc0+go6f49EJ+10pVe+
NOLVC71h4Uyu5p8h7lJndD4rOiX9VUaofGXiQWBI9PwAAAAAA80TPDwAAAADAPNHzAwAAAAA
wT/T8AAAAAADMEz0/AAAAAADzRM8PAAAAAMA80fMDAAAAADBP9PwAAAAAAMwTPT8AAAA
AAPNEzw8AAAAAwDzR8wMAAAAAME/0/AAAAAAAzBM9PwAAAAAA80TPDwAAAADAPNHzAw
AAAAAwT/T8AAAAAADMEz0/AAAAAADzRM8PAAAAAMA80fMDAAAAADBP9PwAAAAAAMwTPT
8AAAAAAPNEzw8AAAAAwDzR8wMAAAAAME/0/AAAAAAAzBM9PwAAAAAA80TPDwAAAADAP
NHzAwAAAAAwT/T8AAAAAADMEz0/AAAAAADzRM8PAAAAAMA80fMDAAAAADBP9PwAAAAAA
MwTPT8AAMC0nFu/qrJ+rjpUUifWNi5VR/qh6ojyU1Dfm4xzHy5trIVORc8BwHzR8wMAAEyL0dfa7
elO7/nrtr3guw3Bzr6+qRP8NxkA0B96fgAAgGmx/rtss0Pd6T1/q/17m/v6XwXQ8QPYYej5AQAApsX
q+c0Gd4f3/Lpxb1iXvn/6PukjdPwAdhx6fgAAgGmxe36jUXV6fueA2xhXQ/IRxrzVBcYRsxtWh7Njxv+
c3iiiGGed88ba6jINPbc1nzWhsVIlMpceXYzRr5wdxNafs9cjhkQ2GL0OAAZFzw8AADAtdTO5Uf5JtYx
Gl1m8dg8Y7WbVEtftaCPdRIevMfps2dcWjIV4Jom2vv6icusGYy2OenHrG+qPonrT+hsWFN6g/7roc
gGgP/T8AAAA06KaxqyDVI1p2TAaJ4qB7oFYz18NMlpSeUS1pc419RF1QJbRI/RSjDot2t1wAT2hWz
NGD67U0xQa168GhNcT2GBoIrkCABgGPT8AAMC0WF1j9cL8s9t2Gu2kbGbVkLovDR9R07gj5BjZE
WfkIT2JMSZIriAnd+IeiNMLyIlLWqxfcqr7N6iHtdo3APSOnh8AAGBa/O111mo6nbGnVZatqTuk+Yh6
bTbK9rxqhI8obM0S4C4pp0qqo3JrjcJLaLF+gz1anQ/MrldZs7cFAIOi5wcAAJgW2QFXr/XX+
+seUo7M9NXz2wfVC59g4TD/WLmV5J7fXUKL9fua94I6H5w9cGHbVQNAp+j5AQAApsVpJmUTqU
942k7ZGLtDmo94phXz+kYILYZo/rGqpDoqt9YovITmxakRmXKQU71xDmOKQrgYAPSHnh8AAGBaP
M2k3T7qE7GRHfT8ZnNtj2nRfrtlwvR05mCnRoc9f+NU7qXOTYlsMDtlTLzwugGgQ/T8AAAA0+JrJnX
fmNMn6qNVO6kbU+eI27yGj4Rn0UPClVVj65aJCReor1+4d44soWn9cjv1+IYNOhuJLgMAekfPDwA
AMC0NzaR5wmhFJdHLxlpQeUS/XpOzq3434y9dD3DLxKnxNvPqLnv+xvX7l5NTQ0Kzh65su2oA6BQ
9PwAAwLQEmsm6SbVOmL1r1lbKxtidrPmIem1O5ywnZ/fN9oDALqLs+WSTLLfWqHEJsfXXl+fyksZt
MU/7ZjcvzbVdMQB0jp4fAAAAAIB5oucHAAAAAGCe6PkBAAAAAJgnen4AAAAAAOaJnh8AAAAAg
Hmi5wcAAAAAYJ7o+QEAAAAAmCd6fgAAAAAA5omeHwAAAACAeaLnBwAAAABgnuj5AQAAAACY
J3p+AAAAAADmiZ4fAAAAAIB5oucHAAAAAGCO/va3/w8Vt3ZylidQMgAAAABJRU5ErkJggg==]]></
redcap:Attachment>
</ItemDef>
<ItemDef OID="b17" Name="b17" DataType="text" Length="999" redcap:Variable="b17"
redcap:FieldType="descriptive" redcap:BranchingLogic="[q16] = '2'"
redcap:InlineImage="1">
<Question><TranslatedText></TranslatedText></Question>
<redcap:Attachment DocName="B.png" MimeType="image/png"><!
[CDATA[iVBORw0KGgoAAAANSUhEUgAABVEAAAEJCAYAAACdcmdaAAAAAXNSR0IArs4c6QAAAA
RnQU1BAACxjwv8YQUAAAAJcEhZcwAAEnQAABJ0Ad5mH3gAAE9sSURBVHhe7d0PjCRnfef/b+/aY
PPHNmBskx+RHat7sZYlGJIg08PxL4egZ+6SJbnbYOVg5Oh3PVyi0zQSkwOxF+mX2wguG4lunS5iWrq
zliQyTEJugrLdIJRgckzHR4IxYTI/Z7rlc3T8wDYGjPljB3u3f/VUPVX1VHd1dVV3VXX39PtltaerpqfqqT/T
O/OZ5/k+hb5FAAAAAAAAAAChjumPAAAAAAAAIAQhKgAAAAAAAAAAEIEQFAAAAAAAAAAAiEKIC
AAAAAAAAQARCVAAAAAAAAACIQIgKAAAAAAAAABEIUQEAAAAAAAAAgAiEqAAAAAAAAAEQgRA

UAAAAAAACACISoAAAAAAAAABCBEBUAAAAAAAAAIhCiAgAAAAAAAEAEQlQAAAAAAAAAiECICg
AAAAAAAAARCFEBAAAAAAAAIAIhKgAAAAAAAABEIEQFAAAAAAAAgAiEqAAAAAAAAAAQgRAVA
AAAAAAAACIQogIAAAAAAABABEJUAAAAAAAAAIhAiAoAAAAAAAAAEQhRAQAAAAAAACACISoA
AAAAAAAARCBEBQAAAAAAAIAIhKgAAAAAAAAAEIEQFQAAAAAAAAiEKICAAAAAAAAQARCVAA
AAAAAAACIQIgKAAAAAAAAABEIUQEAAAAAAAgAiEqAAAAAAAAAEQgRAUAAAAAAACACISoAA
AAAAAAABCBEBUAAAAAAAAAIhCiAgAAAAAAAEAEQlQAAAAAAAAAiECICgAAAAAAAARCFEBAA
AAAAAAIAIhKgAAAAAAAABEIEQFAAAAAAAAgAiEqAAAAAAAAAAQgRAVAAAAAAAAACIQogIAAA
AAAABABEJUAAAAAAAAAIhAiLp0etJYKUih4DxWGj29PqjXWPFeU1hpWF+1iMxj3ZB2onXjpLWdL
MxLO7LSlg372PzHqPs4Vb2GrGzEO5vtDbdtK5JH08KNuQ9Cjmc+2g0AAAAAwPwhRF06Rdm8UJey
XurU1kPCkracr3X0c5Hq2U3rq4B5oILBVWnqJUdZzqxle4faf1Qo1cT/rlhsR+14AAAAAADIGiHqMip
uytmqfi4dqZ0P9kbrNc75IVW1JdsV/XzhFGVzry/9vnpsS/aHkff+RpmXdmShKwdu8leuS9c+xj3ZzDJ
D7TVk3fijwsI7ascDAAAAAEAOCFGXVGW7JV6O2jxn9EY1e6FWpbW4CSqOot6h7OuncuoEPaQBA
AAAAEAuCFGXVkW2W26M6vdGbW/4Q6XL9a3wXoyqlqKuRek+wmpSjqyvaH59ZI1Jo6ZjoC7rqPX
GPu31edcGHd5fZI1J4zwEz5+5nbDPjxN+3INtCdS9tR4xy30ObGewraPOc7xjCm57uPZpYAh6c9VZF
2h4snM3eA7Uw9yc3Z6x+xzPPy71CLkXbAnaHvI9OHq7vqTHE6/dAAAAAAAcfYSoy6yyJXW3OGpz
VTYaDTnnJ6hyIWSM9FAIo3VqpQwmoCrK2hndwM6BdJ1nFmNId2dHLno7bcuubn/5zNpc9FKsnPa
D6gP/AGy9izv6PBo1Pe1wrCSDo63TPb8dqZUKUhrYSXM1aUimtjPY1qasDoZtEx2T2rZR+9S6H+te1
+kREu3HCSwHz4HSXB0dKibnnOtV70AUtW6Kc9TeGFHPNGS7E4vZbgAAAAAAlgQh6lILTjLVrLnBTF
nqF0Imk2pv+KGKV4+yL103ie3UZD3lhKW4dka3b18O3U23d42JhYxw0hjqferEnAz0rpz2yiY0d4M9
Ji/u6BisfEacDLUnjXX3GlSlpc9v3+0xnOr59bfvdUhWIdlAfdzx/O1494Hazrob/E1zTMbr9zZlc9v62PX
vV1Wv1/6cXXIi2X56jXUvsCzXu85r+8MlLiqR+4wr/BzteOl/krZbr3X/0hH2PRjY7rBkxzOu3QAAAAA
ALI+C9QtyXz/HklK9SwM9zlSwEhKq+K8rS71rTubTk8aK24tOBS/OZEYjX6963bk96Ubsy+dvW4Vde
9ZG1BBs1YOwWq1Ks9kcWu+3IaxdcdeNE387w+2yV3rnwG1/6DqbsV0VnO2FBNwB4e0wr3O11fc
nDEt0PSK2E9ivvuaS7JjMbQdfr41qa6JzF35+FOdanQqsS3p+lETnOlHbRwht44jjjDietO4RYBr3Pnyvv
OXCW/QSACAP99/xLnnNW2/SSwCAzB3/NZFjr9ILWBT0REVwkikVtoQGJP5Qeb/npMsYdm/2GE1F
UU6ccp51dJfTrj2WvywnT5+0e9QF11uqp/0AbA74vWmb4nVG7R44gZT1GW8ov7dOdT4sGbUo3S
BMfcIsazBr1jUo6ac28z7QPYSnOKZEvYkT7cec4f+kmIdQ3NyTvhmgTm3wHIWY6rrrurFuwJmaGO0
GMvDwEw/LOz/5Tr0EAMjDDY9fJXd/6AF5/Bs/0msAAJm7dI9I/zt6AYuCEBVBA6FScsO1P6fl1RVt7k
pbenJoj9k/JScqJ6z/D65XGeqc9ZIrrombLbpD+ttuIl09a/TozUtaAZl1DTJrOyHeSKpXqBe0GnVjgQX3x
NNPSOWPKvZHAEA+nvPjY/KWL94oP3ryGWm+/3555p8u6c8AALL1lMilP7A+PussYiEQoiJlGYRfXl3
RfTlsXxS7lKgd9lbEyVeN9XMZvhk9NO3A1+/VOyrwVcO67VqVQ480e0lOK1mv47yOaTHOXbjxbW/
Lhtnr1K2LatY5BRbUnZ+6Ux58/EG9BADIwxv/
+gZ5wQ+vsJ8/9NXvyp/WeR8GgNz0Hxa5/Od6AYuAEBUxleSkN1LbnBFfMSZJitM70Ri+HI+7744c7D
pf686+X7I/4a8fLjUwH4qbZ/0guOFOjFWVQIZacsoTKG6Jgvk22OvYvA/0seV1TIn2Y97LA8Pk1cz3hZ
xnoE/SdmNSNTtwdeukJv6eAubL+z77PvlM7zN6CQCQh1c+eK3c/PXn6yXH5z7+kHzlLx/RSwCAzF3
6gsjlr+kFzDtCVMRUlM2zbuVUc/Z1NbrYnOl8y+vt5wScijnre1s23JlrYvN7cqqJpBS3XmZRF0x117vh
6vxxe81a56Kmj3+wdqsx7F+aq7LhnTIV7Onh297K+dBcNQLH9nm/hqd7bHkdU6L9mLVbm7Lqre/p
me+ta1Qq+NvI2oTnyA9c3XYDi+kT+5+Q+n11vQQAyIOqg/pzD7xELwX94W9/Tb73raf1EgAgc5f/RK
T/fb2AeUaIivgq29LyctSalHTA48w8bynX5YJR4NOfUMnSXNWB0GQ1HAPbsp55Q/a9of6KMUnTHP
Jqu9rKUt8KRKiWomxe8IdlN1d1gOaFzqMm/ZolJ3AMttM8tryOKdl+ipsXpO6/2HmtOZGTdS/7h6Br
7yrua1NNWBO03bzfw9odR+bHA8R339fvk7v+7C69BADIw/FLBXsYv/oYRgWozd+8Xy8BADLXf1Lk
8sf1AuYZISoSqWyH11+stqz17tBiV3FT9gZfW21NVr/R7K0XKBlgDM3OdKKjFJgB2KiyA+qc9bt+wOe
ya1/OW03PstS7fT9Yt1Wl1d8LTpaV1zEl2k9RNvf60h16sXq5MUzeVpHt4EGK7B96PbFTEbvtVlv6Lf
8+sqlzblwHu+5ulByOB4jBnYn/6Wfp7QQAeVr50kvl2iev1Evh/uFL35ZP//6hXgIAZO6y9RvZJcpbzbt

CX81aAgAxtTcK4nSSVCHqQGAKADGo4PT1/+318sAjD+g1AIA8lB56od0LNa6tu8vyiteFD/sHAGTgit
8QKfBL9ryiJyoAAMiVGsJPgAoA+VK9T1Uv1CTUsH7qowJAji79AfVR5xghKgAAyM1HvvgRezIpAEB+
VP3Tt33hZSProI6iAtS7/+NX9RIAIHN2fdR79ALmDSEqAADIxe6Du/LBv/igXgIA5OWOL18/tg7qKPv/
8zH53Mcf0ksAgMxd/n9FLn1BL2CeUBMVAABkTg3fV3VQmUgKAPJ189efL//8Czfppclc+dzjsnX36+
XWV79IrwEAZOtKXR/1Zr2MeUBPVAAAkKknnn6CmfgBYAZe8MMrEk0kNcoz/3RJmu+/X3705DN6
DQAgW9b77aWPWx+fchYxFwhRAQBAZlRwqgLUh594WK8BAORB1T99yxdvlOf8OJ1f+R7/xo/k7g8
xKSAA5Kb/HZFL1EedJ4SoAAAgM+/77Pvk3ofv1UsAgLz83AMvkRsev0ovpeMrf/mI3PtJ/igGALm5/
DXr0dELmDVC1KXWlo1CQQrGY6XR05/LSa8hKxttveBob7jtWZG8mzM3Qs5LHL3GinPuVhoy6tT55
9d55H7NjwLuWyCW+n11+djffkwvAQDy8vJvPE9e+eC1eildn/zPB/LN//19vQQAyNylXZH+Y3oBs0S
IurR60lhZlaZecpTlzFpRP8+eHfiVasLfVIImPi+9hqzXor5KXfOCrAYvunRqJSlMENguK+5bIB7V+1T1Qg
UA5Ovqp47LG++bvg7qKKo+6u//
+y/bHwEAeVD1Uf+79fFZZxEzQ4i6tLpy4KZA5bp0+33p9/dkM68MdWzgt6QmPS+qZ+S4YK99XtxN
V1vqevelWy87K5qrQo4aA/ctEIuqf6rqoAIA8veWvRvtIDVLqifqH/721/QSACBz/UdFLv2xXsCsEKIuq
96h7OuncuqE5Nf/FGmL1zOyJ41zugtquS5bFedpcfOsVJ2n0twlRQUwPTUTf+WPKvZHAEC+Xvu1F8
vLHr1aL2Vrb/f/2A8AQE4uf8l5YGYIUZeQXbvRDN2aq04tx8GuiKp3o1E7c1T9zGAtyGCd1bDejbH3r
wVreI6qN+kMVfdfl6zW52A9S6+2qH6ENy3GPgPncEP8zZjnydln0vOiqHaWYvWMNHoeB0LzkpzUn
VFl/9A6olGM9obVW21v6GMZPAcJrkvI/RZ2vePcb4PXz35E1ImNY5b37WT3p2vSa2eZ6D1Ar1TMr
49uJI6QOz91pzz4+IN6CQCQFxWevubvXqSX8qF6o1IfFQBydOlPqI86Q4SoCDUUGGl2/cyRYVRHai
WjzqrR43EyanuDNTzVurCgpuQNVXdFt3UUZ5+DwWRzdcJ9Fjflgjtk3joz5/RGeo1z3nkq1y+kUEahK
q1+y+tVOsTseTxK50C6+umwipx2N97ZkYsDJ7W96x2NX1c3yXVRQV7I/eZcj1EBZPj9pu7d0GC5U5
NSIMjOSpb3bcz7M2CCa2eZ7D0Ay07VQP1M7zN6CQCQFzV8Xw3jz5uqi3r3h75KfVQAyI2qj3qP9Z
H6qLNAiLqEKtt96Xfr4kZ7Um3Z9TH72zrxbG/4AZBXL9Won9mpyfrIxEaFec7r+3uboWUCxu4/wN+
et3/pyI6XBPWkse4GPca+Wzo1imzrKP523M2ofdbOu/Fbsn0WNy+If+rOS9v677wbglnn94JOUJOd
F4P9um2ZLK8uyolT+ukYlS23beb5V9ri53BnxMnhkpwj67VGqYGh+21ofyZj2/b9ZrYlbFt+kJ3U/Ny3
/uvD789hya6dZar3ACyrT+x/wp6NHwCQv5UvvTTzOqijPPTV78qf1hmBAAC56T8scvnP9QLyRIiKIW
bPtPoFPwgdDgOHletbE4Z54aotPxwsrp3xAqzOge4z2bsoO14eaey7suW3deeiJIl7zH364ZMh8T6Ls
nnWTbuaslpwe08Gz+8kipt740PW2PblMOpEFdfkTNjxtXf9XrVn1pzjSXSOrPOzp0NEI3gPvd4Dhu43
s8et0evSPk86CNzLYfa0LO/bsfdnmCTXzjLNewCW031fv0/u+rO79BIAIE+vfPBaufnrz9dLs/G5jz8kX/
nLR/QSACBzl74gcpkJ/vJGiIoBI3qm2Yqy5iYxIwK3UyfSDKjKcrKkn47SPdC9+VSoU/LqNhbMYdKRw9
QnMMk+K9tGr0FHOsP403RKoi+fcf2NgNIP3Kpy1j2gqa6LruEZOrw/aOh+M8JCawf20Hd33yNrsaZ
uHu/bBNduyvcALB93Jv6nn31arwEA5OWGx6+Sn3vgJXpptu7+0APy+Dd+pJcAAJlTw/r739ELyAMh
KibUkeHOgTHCo7mX3TFUvMKUSrD+5Oz05HBssVSf36vSHRZuBG7V08FeoUmYExB5PXXHCbtWR
dm8EN470w0rF3t+o8nvz/SvXdh7AJaNCk5VgPrID+h9BAB5e86Pj8lbvnijHL9U0Gtm60dPPiPN99+v
lwAA2XtK5NIf6OfIAyEqJjR/gWm53vWGbAcfk9YLHS/
+Po26n7aO1NZznJyneELGlj4tn5Sxl7S4KW5lAntYuDF8vno6/CyPP0dt2TB7nbo1OM36o0lYbdzT+xj
s/as0z83XpEi53bcTXLtoR+GPJpiWGsL/wCMP6CUAQJ7e+Nc3yAt+eIVemg+qPuqnf/9QLwEAMqfq
o15iYte8EKJiQElOusnV0EzePbnoFnIcO/Q7J6WTXtA2qm5m6ibYZ6+xrodpl6Ved5OsPCfnMa5rc1f8
zphdOfAu6QmJc0m9HrXW/XH+/I4OP6sSyOGSnCOzLqcKFN26qMaQ96TUzPKqx6k9GZQdSHa9W
p7pDpOf0CzuW0usa5fme8AU1xDz7yNf/Ig9mRQAIH+lh1448zqoo3z6v/6D/MOXvq2XAACZu/xZkf
48dRU6ughRMcCcBCnYW9IPAlXYNTChz6yYNTCbq/5Q7faGHhpuPdIev510n72GrLsnrnpWNjeNyY
Nq65JPjmrWsmzKrm5er3HOCzBj90asnBbnDulIs+ke18Bw8Amvix8oDvbcjUlvX80sH+xxaoTFZo9b
oz1p3yaRZnHfKnGu3QTvASU/dZXaee9gZMOb4h9Hze6Du/LBv/igXgIA5OnaJ6+0Z+OfZ83fvF+
+9y1qZQNAbtSw/v739QKyQoiKYeYkSJ2alHSoU/LTE7kw7YxI5vDy5uoUoVGwBmZzVQdQXnhTlVZ
qs9e7kuyzJ411d6h6Wepbav3okCq98zLMnFndbbN3TastiX+aKhIo72oZDmATnCMv2LO4x2xOsJTEi

HvXrLNaPevPNp/YQt+3SpxrZ0n4HuDXW7V41zBubVssGjV8/85P3amXAAB5UvVP3/aFl81NHdRRV
ICqglQAQE76T4pc/rheQFYIURHKHgYdUpOy2rLWu8Otp1KR7cGClfuHRs/BBOwamMZwbZddWzOj
eqgx92n23LN7obonbiCk8of1p3hehhRlc2+4Tqg9hD5hYBeYJMs6ZjsbHhT7uljH3G/5QaqtKq2+0dZ
ACYJo9r07eJA2Z5vTZZMLft9aYl07S6L3AHUsg6+ttiava4u59cTTTzATPwDMkOqBqnqiLgI1pJ/6qAC
Qo8vWb6bUR81Uoa8KBgJAEmo2fT0ZlAph96btmTxL+lhOtaYNWBfEUbp2yJUKTit/VJF7H75XrwE
A5EnVQVWTSS2SK597XLbufr3c+uoX6TUAgGxdKXLFb4gUbtbLSBM9UQEkFCxRcGZtsUO49nnrWC
J6ZB4tR+vaIV/v+
+z7CFABYEbULPx3fPl6vbQ4nvmnS9J8//3yoyef0WsAANmy3m8vqWH9TzmLSBUhKoCY2rJh17o06
pWaJQoWUK+xIqv7demmUqJinh29a4d81e+ry8f+9mN6CQCQJ1X/9C1fvFGe8+PF/NXt8W/8SO7+
0AN6CQCQuf53RC7doxeQJkJUAJNRNS8XfPx7cXMvpRq/C+YIXDvkR/U+Vb1QAQCz8XMPvERueP
wqvbSYvvKXj8jnPv6QXgIAZO7y10QufUEvIC3URAUAAKEefuJhec32a+wJpQAA+bv568+Xf/6Fm/TS
YqM+KgDkjfqoaaMnKgAAGKKCUzWRFAEqAMyGqoO6aBNJRVH1Ue/
+0FftjwCAPKj6qGpY/7POIqZGiAoAAIbc+ak75cHHH9RLAIC8qQB1UeugjvLN//19+cPf/ppeAgBkrv+
oyKU/1guYFiEqAAAIUDVQP9P7jF4CAOTttV97sbzs0av10tGyt/t/7AcAICeXv+Q8MDVCVCTUk8aKm
uVbPTaknWjdOGltZ96MOYZeQ1Y2gmvbG+7rV6TR0yunpfZjb3NEO2zTne9M2m1bkPugvaHbWJC
BSwosjE/sf8KejR8AMBsqPH3N3x3tuqGqN6rqlQoAyMmlPxHpP6YXMClVGCGeo0VKZRq0tHLWe
pd3DH205RdQj4AA+77+n1y15/dpZcAAHm7+qnj8pa9G/XS0aXqov7+v/8y9VEBIDeqPup/tz5SH3U
ahKhIqCibe33p99VjWyp6bXby3l8WRhxDryHrtTziU6UnF3eC+2qea1hrF8VRuA+A+aZm4n/nJ98pT
z/7tF4DAMibClBVkLoMVE/UT/7nA70EAMicXR91Vy9gEoSowDLoXRQ3Qy2Xy86Tzo5cXJwUdTFUt
qXfrYs+w8DCUMGpClAf+cEjeg0AIG8//ffX2UP5l8m9n3xYvvKX/NsDALm5vGc9mOBvUoSoS8eoLbl
i9kQctd6oc2mvz7s25fD+IutuGnU/VwKfNLcT9vko6Z8z+/PmMP7mqvP5EYU0/WNWj+T1Rv2h/FU
5e+GMDvk6shMnRQ3UUg17jG5PvHbHuTbD59DaumyYrzfqkQa2MbA+/BTHaYN5POpY/P2rh7vd
9nnrupbrsjXQXdYu3WC83n4M3DfArKgh/A888oBeAgDk7YbHr7Ink1pGd3/oAXn8Gz/SSwCAzF26R
6T/Hb2AJAhRl05R1s64PREPpOs8s3TlwE30Aj0U27LbdJ6Vz6xZXz17ldNV/awjB/4B2PywsCxn1nRr7
RCwJIMj5zu1UswQa5bnrCO1UkFW9fYcal2SINUYyl89LZXimniHs3Nx+hCvfEbcU+2L2e6pr43Dfn1w
Z846FVQOrG+uDgSpE7VBHcuqeFvWoakKSlf369Ld2wxcdxW+lsJKN3RqUprnibKwFD7yxY/Yk0kBA
GbjOT8+Jm/54o1y/FJBr1kuP3ryGWm+/37qowJAbp4SufQH1kfqoyZFiLqEimtuT8R9OXQTovauHwi
JEU72Dq1XOU6dmIcI1VI5LW6M2gzMjmSEhV6w15PGutvjsyotu6am9WjpLXRqsh4jjUz7nFW2rTa
Yw76rLadd22HVPv12d+vuV8TsRaoYQ/mrp9X2zVA4xpD+4qbsuefNfnTFa4Z1BPULwcDQN67d6V
wbV7neDX69Frbev2+maYPxeh2aFjf3vOc+P1RXYWtXf41/TppyLsFxAmnafXBXPvgXH9RLAIBZeONf
3yAv+OEVemk5PfTV78qf1h/USwCAzPUfFrn853oBcRGiLiOvJ6IfaPUOndivWnXCo32dFJrDwO38bS
5UZMsNoJq7fi8+s+6n2wPUXFff8ickqmx5QWCs3pgzPGfVlj+Rkh/mWi0Z7IY7gtk792TJfmJsJ0EYq/
Ua636vzepZ2QxPUMe3O61rY6vKWbchRsg+cv3+obPdKdoQeH0UI1Q3Q2s7cNWB6t6okwhkSA3f
v/NTd+oIAMAsvPLBa+Xmrz9fLy23z338Idn/n4/pJQBA5i59QeQyf8BKghB1KRXlxCnnmRtode1x6W
U5efqkHXYF11vUMHDn2VzwQ7mmeJ0KuwdeWOgN5ffWWcfkDu+2H8bw7cAQ/VFmdc784HMy
Yb1zLRMP6W/LeT9BlVZoz1klRrtTuzaW8kkJ3d2o9a4p2hC7Z7Zxrq2N2WUO3P3Er8sLpOuJp59gJn
4AmLGXfPe58nMPvEQvQbn7P35Vvvct/m0CgNxcVvVRv68XMA4h6pLy6oraPTl74nSqPCUnKies/w
+uV3ngPEWoFiOYcodmt90x0xG9I6exkOfM6GmphqaXQkPC+LP0tzf8OqBVo6fp8kkSbhdl80L4jP1u
cDtiPjEgE+5M/A8/8bBeAwDIm6p/usx1UEdRAWrzN+/XSwCAzPWfFLn8cb2AcQhRl5U3tHlfDts6aL
N77VXEyQqN9VP3hsyCUdfTDi/9upOjwkuvNubQI2YYuIDnzB/KHyXmkP72hj9RVLUlIzuhTmDqa5O
CTNtg1JUdKNlqa55jln7k532ffZ/c+/C9egkAMAsrX3qpXPvklXoJpn/40rfl079/qJcAAJm7bP02eukze
gFRCFGXVklO2hlkRw52nSHNbh3Rkv0Jf3347OuzV9w864eaDXeSp4E6pCVnqL3i1eKc2KKdM2Mo/
xjjh/S3ZcNLUMtSV1PRTyvVazOhHNugZuhXPU7tScXsQNWYoCtu2QJgSvX76vKxv/2YXgIAzELpoRfa
D4z26f/6D3aYCgDIyeXPivTp2jMOIerS8ntyNptOObWeCzq4p/uem+Sprnj9gDtSK2mA77BOqRmP

crmqj9sur3h1aUsxB5LvWDnzBzK787+H3iYIV70kP5e45w3jL9cv5BOuYRUr82E8miD3pbKoIM9Trvi
ls8dW7sVSMn/+v/
+l34GAJgF1ftU9ULFeHd/6AH9DACQi0v36CcYhRB1iZkzpqvehd7w88Ds5sYkTXPIq1NqC+shGaxH2
VzVwZg/Ll1GT440LNVzVtS1VJXmauqhoTmUvxxaW8AoiWC9cuSQ/l5D1r0CqtYrAxMwOY/JJkhK99
pMJoc2VLb9IfyBurRGfdmzm7MP3bEU7v7Fu+WOl9+hlwAAeXvjX99AHdSYfv7dP6WfAQByUXijfoJ
RCFGXWWDW8FPiTzbuDltXzPVzyAwvRw2ht+tRGr0uXeW6dJPWu0z1nFVke7BA5v5hSrUxzaH8o0
NdMxQeNaQ/Xl3VCaV5bSaVQxvsIfxhxVBVSNvvp1pfFohy1RVXyf/4lf8ht1x3i14DAMjT373yCf0M
UV7z1pvkbe+5VS8BADJ37FUix9+kFzBKoa/G9QIAgKXxwCMPyOv/2+vtmfoBAPm648vXyysfvFYvYd
D1P/E8+a1PvVGedw0TbwFALgovFrni/daTq51ljERPVAAAlsztN91uD+0HAOTvb27/tjx2PX/ECnPlc4
9L9fdeS4AKALmx3m+Pv8f6SIAaByEqAABL6F2n3iUfeMMH9BIAIC+Xjvfl8294VH78nMt6DVz/8t+V
5NZXv0gvAQAyd+ztIoWb9QLGmSpE7TVW9AQpBX9W65F60lhxJ1TZkODL27LhTbbiPCabqGZYeyO
43dBH1jOAAwAwhz788x+W07ed1ksAgLz84PnPyt7rvqWXoLzidS+R1X8bNhkqACATx4oix39eLyCO
OeiJqsJVf5ZqR84zwquZ0VcaKU3oAwDA4rjnl+
+xh/cDAPL10M0/kO6t39dLy+3al14l1d99rV4CAGSucI3IMTWMH0nkGKIWZXOvL2oeq35gxuuuHH
iTiKvZsNXn92QzxwzV1qnJeTqkAgCWjDtj/3VXXafXAADyonqjfu+aZ/TS8lIBqgpSAQA5Of5ukcIL9QLi
mn1P1N6h7OuncuqEZJedlqXedUNc/9Gtl/XnRZq7pKgAgOVzy3W32EGqClQBAPlR9VE/96Zv2h+X1
S/8xivsofwAgJzYdVDz7rl4NOQYog7XRLXrlZZq4nZEtYfVD9UoNb/OeaRVL1Uprp0RP0YFAGA5vfm
WN8tH3/5RvQQAyIvqibqs9VFVePoLv35CLwEAMmfXQX2HXkBSc1ATNUKvISuFktS8lNXRqZVSq2H
aPu+HuNXTfpGB5WVM8uWd46hJwTA7YddqEYTfT/4kcHneY0nPYU7fC+q9b2DCO//8rEiKf0cCAt7
7s+
+V2h01vQQAyIuqjbps9VGfd82VctfvUJMbAPJztcixO/VzTGKmIWpluy/9bt3vCVptOcPst1WY2ZPGu
htwVqXlDsFvVe01qobpeqIkoSO1khtC+I9Vd0Yra9/2bgFghnqNlWAPfSBnqjeq6pUKAMjXfT/zuD1r/
7JQAer1P/E8vQQAyNzxO0UKL9YLmMT89kTtXZQdnSKU61v+RFSVLXHLmHZ2LqbU+64s9S0S1NFG
TQoGTGKR76eM295ryPpg13tgBlR9VFUnFQCQnx8/57J8/g2PLkV91Le951Z5zVtv0ksAgMwdf5PIsV
fpBUxqfkPU7oHXE8sevu/1HjWG93cOpKufTsfppZpmrVUAABaVmqm//attZuwHgJw9dv3T8je3f1sv
HU23vvpF8ku12/QSACBzhVtEjv0LvYBpzHdN1FSFz85vlhPo1M7nWItxHgxO2jWqxuPoOpD20GPv6
/VjZG3Jwf1FBNd2Pdzga0fVgsysDVEya98oca+VK/5xJm1f2OsHyncO1O806o7arx19P/kG2p/K9Ux6
DsOEt32wXungORo8P2HiTbTn8/epHqPqpCY5P0DQbdffJvf88j16CQCQl7+/7Xvyjy//oV46WlQd1O
rvvVaufO5xvQYAkK2rRY6/2/p4hbOIqSxEiFqud4fDT/uRwnDa4qac1WVWRfblcGnyBRVsDU7a1ZTV
wqr1/3hUiFMKG3rcqUlpMKBKMklYe2NETUjVYzgYFmXWhihZtW+khNcqwXEma58TyIW9vrlaGBH
2qXNitLNcl/GVM9SxDbR/6us5/f0ej9OrffAcNVdHhZyTcPbh1XO2Dd97qd3vWGrvKL6DGfsBYAb+6v
WPHcn6qL/yH15JHVQAyNPx09RBTdH8hqilk34P0YN0Bu2Ha8tuuinKQug1zvnhkTuhV7/r1Zsdzzhv
5bp0dbDd9TbQlHNeotNLMEmY9dpzesOh2+3IzkX3tVm1IUpW7Rst2bVKcpzJ2tdrrHuBnP+HjZa1F
615bkRQaLRjb1OKem0koz1u06e5ntPf70n47fHb3pHa+ejIPHqivUH+PsLvvbTud0Ds2frfdepdegkAk
AdVH1UFqUfJyumftB8AgJwce53zQGpSC1HtnmjGkFHzEWco65DimpzxspxVfxuqF6C77UQbdnpv
me1yHmYvuTOyFivhWXQ9uejO2qUCFi+kKcrmBSPEidI7lH39VDo74mY3xc09J6yxHnub+mQmmiT
MmLjHCNyKa2eGQ/XM2hAlo/aNlPBaJTnORO0LtuOst74iW/aGVVC3J2GHE2hHTNWz/rmtbPtBrd
/2JNczhfs9gWrL7yFf2Up/+4q5j/B7L8+J+bAM7v7Fu+WOl9+hlwAAefjmjU/JV376u3ppsb3sp14o/
+a3mNAEAHJTuFHk+L/WC0jLHA/nDwYcXkjrjWE1w5B0mMHN0daVAzdTKp+Ukn5qK56QU/ppJDP
kHgioh2ouTjVJmK6nGTZ8Prc2REmpfSMlvFZJjjNR+0a3wwldR5fWOHUi6XdVVU4HNlaSk2473bYn
up4p3O9zpSwnAwcRIteJ+bAMrrriKmbsB4AZuP9V37HD1EWm6p/+
+n/5GeqgAkBurhQ5/mvWR+qgpm2+a6IWN2UvbMitPdQ3hXqoHtWLri8pZ7JH3OhefG5oM1EPZ
MWu5eiGPlE1KzNsQ5R5b19sebQvRuA3VlFOLEzSmcbxAvPpphfcZAepKlAFAOTn8yuPylNXX9JLi0f
1QFU9UQEAOTn+r0QKN+gFpGmqENUc9hv1cMLJoj8M2gxA7aBUrw9NMc2v04+4dRUtdp1B82tD

H2kGskvEuHZ+7Udf89zw5DXjJwlry4bZq9OtjWnWijRl0oYo2bcvLbGOc4btG21wgreeHHp1B4ZlOv
HcEcD5QZpuv+l2e2g/ACA/KkBVQeoiel3l/6IOKgDk6dhrrQd1ULMy3z1RkZGQ4dEus05mDGp2d9V
b0Q+rjZ7D7raTTBLW3vV6dtrhjxuYG8OTB6XehihZtW+khNdqguOM176Idth1itOdfT7Y9JDh+ImO
M737fWHkNjEflpGaZOoDb/iAXgIA5EEN6f+7Vz6hlxaDmoWfOqgAkCM1C7/qhYrMEKIupaKs+bN2
yao3Ztuc0XsMPcGXKlEb7K0YEnhNOEmYH/4YM+KbcmhDlFTbN1LCa5XkOBO1L6Id9rE7NVUTnsK
Rmqsb4jfdL5lQPrPmhNaJrmcK9/uiyeB+B0wf/vkPy+nbTuslAEAeVH3Ux65/Wi/NN1X/tPp7r5XnXX
OlXgMAyJaqg/oe6+PVziIyQYi6pIqbF/zehs1VHawYk86MU9n2h393alJygxmjRqg/UVew9mbkJGGV
09aSNq5dWbUhSmbtGy3ZtUpyrpO1b2w7ynXZSmV8eFnK5aas6vZ4Tbfaftab/j/Z9Zz6fs+LOdGV28
6Jws6U7ncgwj2/fI89vB8AkI9Lx/vy+Tc8Kj9+zmW9Zn79Uu02ufXVL9JLAIDMHVsTKdysF5AVQtSlVZ
TNvcFJu6rS6res/8djD/8OK6Rpb2dgoi679ubg/ixDk4RVZHuoDc72vF01d71eitm0IUqG7Rsp4bVKcJ
zJ2qfa0Zfu0IbVpo3SBlM7JWcHjzfsGiW6ntPf7/mw7q/B67F/aPQSTiCV+x0YzZ2x/7qrrtNrAABZ+8H
zn5W/ev1jemk+veatN8nb3nOrXgIAZO7Yq0SOv0kvIEuFviqCCAAAMIF7H75XKn9UkaefXYwhpgBw
FNzx5evllQ9eq5fmx7UvvUr+06ffzDB+AMhL4RqRK9R8BQzjzwM9UQEAwMTefMub5aNv/6heAgD
k4W9u/7Z875pn9NL8qP4udVABIFfH3239jwA1L4SoAABgKu/92fdK7Y6aXgIAZE3VR/3cm75pf5wX
v/Abr5BXvO4legkAkLljbxcppFNUD/EQogIAgKmp3qiqVyoAIB+qJ+re676ll2ZLhae/8Osn9BIAIHPHiiL
H36EXkBdCVAAAkAo10dQt192ilwAAWeve+n37MUuqDqoaxg8AyImqg3rsPXoBeSJEBQAAqVAz9b
d/tc2M/QCQI9UbdZb1Ue/6T6+2g1QAQE6O3SlSeKFeQJ4IUQEAQGpuu/42ueeX79FLAICsqbqon3
/DozOpj/q299wqp/7ZDXoJAJC5428SOXabXkDeCFEBAECq3lF8BzP2A0COvv2if7Jn7M/Tra9+kfxSjV
/kASA3hVtEjv0LvYBZIEQFcteWjUJBCsZjpdHTn1tSvYasbLT1gqO94Z6fFZnX09NrrDhtXGnIqCb6x+E8
lv5aY2mo2frfdepdegkAkLW/v+178o8v/6FeytbzrrlSqr/3Wrnyucf1GgBAtq4WOf5u6+MVziJmghAV
yFVPGiur0tRLjrKcWSvq58vHDiJLNeno5YXRa8h6LarV6loXZDV4saVTK0lhIDAGjqq7f/FuuePld+glAE
DW/ur1j8kPnv+sXsrOXb9zu1z/E8/TSwCAzB1XdVBfrBcwK4SoQK66cuDmbuW6dPt96ff3ZHNZM9
SxQeScUj1nxwW/7fPiHlq1pa5zX7r1srOiuSrkqFgGV11xFTP2A0COfvycy5nXR33zr9wir3nrTXoJAJC
5YyvW41V6AbNEiArkqXco+/qpnDohy9V6WJetivO0uHlWqs5Tae6SomI53PSC
m+wgVQWqAIDsPXb903L/q76jl9L1sp96ofzKfziplwAAmSvcKHL8tF7ArBGiLjGvnqP5CKntOLI2peqN
536d163Or/dp135sb+ivNdbZLwuuD/bKS2MbmtlG7zFcYzN4jMM1S+1HWN1Low3jal3a+zDDt+aq
87WDDQ9pc9i2o9o8tpfjROdl+J4ZuZ8YxxD7fGh+W9RjuK0OZwi9/7p0z506/lKsnrNGj+NAWF6Sk7
ozquwfDt9PNqM9ie65eMduS+H7wj1Pcd9HsNxuv+l2e2g/ACAff/fKJ+SbNz6ll9Kh6p/+
+n/5GeqgAkBurhQ5/mvWR+qgzgtC1CWlwpHQMKhTk1JhQ8ZlcHHYtR8HCkLa61TIMrC+uRoeXk2
1DRU2hfYY7EitNCqEU58zapaW61J3uw52duTiYMi0674ynbqmQ8GiZh/zyGBquM1uz8dQE5+X4X
umuToieEt8DFGcfQcvd0hb7WCw5A2hd6V67jxVafVbXq/SIWaP41E6B9LVT4MqcjrpPZfk2FP6vlDn
KY/3ERwdapKpD7zhA3oJAJC1z688Kk9dfUkvTe/f/Nar7J6oAICcHP9XIoUb9ALmASHqEsu0GNkvt7n8J3Gsha7wpH5ncaWDI0uVW5VRN4xzmo0I53PSC
FyGWtm2ttd1t2Wptpx9bOvUrr3hB4Vhbe7UZH3kdQm2eXRT0jkv/invSO28cd0SHMPY8xHg7zu8r
dZxrbvBoHEu3Iamcu40u53bEitrHVKUE6f00wiVRPdckmNP7/uimOP7CI6OD//8h+X0bQxHAoA8q
ABVBaIpWDn9k/YDAJCTY69zHpgrhKjLyOwlZ/R0K27uOQGJ9dhLZaajqpx1t1M5bS25RqwPHd486T
aKsrlnBj6O4toZL7TrHIT3AyzXt4IBWXFNzugv6uxc9NvY3vV65pXPrI0P38YwexjWLxht3rwgfgZ5PrR3
31CbR5r8vFRbfnDoh3xB0xxDFHPfoW3tXZQd3SEycC4qW/5+zWtniH/u1HFY3yOhIe8k9uUwrEFKk
nsu0bGn+H2R2/sIjpp7fvkee3g/ACB7akj/V376u3ppMqr3qeqFCgDIiV0H9V/rBcwTQtRlZAQ0ImqY
bsGrZTiyhuIkyielpJ8GjFofJo1t2HQ9x9BhzEGnTgwGP0VZ8xMtLyzyA0Mj0J1YVK9WY/8jgrfhNscV/7
yMN90xjFaWk+MudvfAa79X7sF+GEPcRwyfn/zcTeuUjN51gntuimNPcv2HzlNe7yM4ctwZ+6+76jq
9BgCQJTXJ1KT1UVX907t+59XUQQWA3Kg6qHdaH6mDOo8IUDEYqMl1FkZg8hyjn
mOk8MDO76nnDnc2AsPq6dg9GafXkeGOgjFCRlOK52UyYccwK2keVxw9ORxbLNWR2T2X2vVfkvc
RZOKW625hxn4AyNFfvf4x+fFzLuul+H6pdpvc+uoX6SUAQOaOrYkUbtYLmDeEqUeClI7esjtQKlR

W/Pcos+u3ZYNs3edW7PRrMGZhHW+zurzZA+NNoYyV0/nF6FOH/qlfF4mkm1w6dXKHXpMWsd0
AsUTMrb06bje1BPcc+OPPf3vi6P9PoIsvfmWN8tH3/5RvQQAyNIPnv+sHaQm8Zq33iRve8+tegkAkL
ljrxI5/ia9gHlEiLrE1MzaqqeYPcGPHYR0vRqKo4f/GoxhxHPHrB2pgiW3/uMUba64U6Z3duT8+R29n
aqkk6GW5KR37gdnZO/JRbfoZeQQ8BgyOC+
+nI4hTOmkFwKOqumZL+NcNHfF75DZlQPvNJxwzn2EWPdckmPP4PpP/T6Cpfben32v1O6o6SUAQ
Jb+8eU/lL+/7Xt6Kdr1P/E8uet3qF8NALkpvFgP48c8I0RdRu0Ne6itmkU92FPMCHiMXnIlPxkzZmJvy
4Y3Dft884MlY2bySXgTWHWk2dQnKrWh/EXZdLsdqvO87l+XXmPdq22ZZBKkcVI7L578j8Fj1udsrvr
DyPW9bj9yHVtu1oBtyq7eda9xzgsxY/VgjnPPTXjsU1//hO8jZntyvRSYe6o3quqVCgDI3t/c/m157Pqn
9VI4Vf+0+nuvleddc6VeAwDIlqqD+h7r49XOIuYWIeoyqmz7Q287NSm5QYtRH7F6NnzmbhXSDL52
Lpkz9nttNibamUhF3I6BrlSH8o+4LiU/fZQL005glcl5MSQ9BnPYu9ueiRK2YH3O5qqz34IX9Felldqs+v
EUNy94PTLd9njnodqSeM2Jc88lOPY0r3/C9xEgiqqPquqkAgCydel4Xz7/hkcj66NW/u8idVABIE/H3k
od1AVBiLqk7KG3YUUMVeDS7wcDHlX3cLBmYrWVcx3NpCqy3W/5gZHNOTbvsAPDrOPxhlcr5bpsp
ZzL2dcl5LxWW9Z6d+j1VLI5L6Zkx2C1Z/A+3D80ejUmYNfnNIaSu+y6nznWQ/UUZXNvuFaoPYw+Q
aAb656LfezpXv9E7yNABDVTf/tX28zYDwA5UPVR7/uZx/VS0Cte9xL5hV8/oZcAAJk7Zv2GfPwdegHz
rtBXRewAxKNmNtcT86gwbG/anqHAOEfpntPHcqpFwIpwn+l9Rip/xM0BAHl441/fIKWHXqiXRK596
VXyW3/8z+yPAIAcFK4ROf5+66P/Xoz5Rk9UILaeNNbdmc3LcmaNABVZO1r3XPu8dSwZ9ODG0fGO
4juYsR8AcrL3um/J9655Ri+JVH/3tQSoAJCn4+8mQF0w9EQFxmrLxmANWFXOgK50yMzRu+d6jRU
p7ZyRbiplKXDUFf6fgn4GAMjStU9eKe9s/aS88723MYwfAPJ07O0M419AhKjAWAOBFgEqMsc9h+
WlJiiblx9NaEs42hKOtoSjLeHmqS0nrr1GDr/3pF6aLa5RONoSjraEoy3haAvSQIgKAADmBj/ghqMt4
WhLONoSjraEoy3haEs42hKOtoSjLeHmqS1IhpqoAAAAAAAAAABCBEBUAAAAAAAAAIhCiAgAAAAA
AAEAEQlTkSE2WU7DrfxRWGtLTa8fqNWRlo60XHO0NvZ3CijRibwjp6Uljxb0GG9aVTbJuGmH3UNr
7mJbRRv1Y4SYFAAAAAGChEaJirvUaK1Io1aSjl4H5pgJdY1Z9W1nOrBX1cwAAAAAAsIgIUTG/eg1Zrx
GfzqeibO717RkF+/1tqei1szFPbenKgXvLluvStdu0J5tkqAAAAAAALDRCVABIS+9Q9vVTOXVCyE4BA
AAAADgaCFGXmao1OlC7MazG6GD9UXuIvfE1A+VKNbNOpXokq1Vp79Mcxt9cdbYTvjOjjeoxqk7qY
JvCa1VOdrymMbVf2xvetob2H3JN4rTRY359ZEONcxFo46j1xj7t9ea5zKoOadx7KKotg9uYsD5pjOuS
9J4FAAAAAACLgxB1WakgL7TWaEdqpVEhpPpcQUoDQ+ybq4OvVyFiSYIva8pqYbBWZBqcNq0GNh
xyDHYlNtgm65W1UnjQaYt7vIMqcrqqn3Z25OLAa9u7bmODtTKHQjgtuo2TKsrambLztHMgXeeZxR
iOHmh7W9xml8+s5dDDMoV7aKJrPizf6wIAAAAAAOYRIepS6knjnJuIuXUb+9Kt61BNOrIzmPx5qtL
Sr2+5QaEKG8/7ve16jXN+0FVt6VqVXfE2H0Nl2/qabl28L3G3sx1W8dJvU/gxWMe77oZg/mv77gF0
arI+MhUdf7xhKltu2wfPpR9GSvmMeBlqe8MPgsOuSWQbJ1NcO6PbuC+H7qbbu0ZI2ZEDN101hq
mfOpF9hDr9PTTNNTckuC7J7lkAAAAAALBICFGXkjERz96m16vQD9VEOl56FlRt+RP3+EGhqScXd9w
+e1VpeQGStc8LYa+fntmm0GPoXRS3SeX6lj/xUGXLC+U6OxdDexSOP94RimvidfQ0t22ElGaPTrN3av
2CcU02L/htrJ0fGKo+Ja+NftDbO3Si0mrVCRv3dbrau7jjBZKnvROYlRTuoSmuuWkm1wUAAAAAAM
wdQlRYdA3P0OH9SZmzk5+Ukn5qK56QU/ppespyMrCTEN0D77jsIdheXUtjqHdgSHsazOHy/rB4P5
SryllvyvYRvVNtxnbMHqOpKMoJfUHcwLlrXzzrnJ4+aYeVwfWW6mk/kMxMCvdQKtd8VtcFAAAAAA
DMG0LUZRWYKCdunckYgeWRMt3x+r1i3Z6eRig3URhpDK9PScUt3trctVrXE6cj6ik5UdFhZWC9anb
2EeriSf+6AAAAAACA+UKIupTasmH2OnVrPZr1HI+ocr3rlDEYevjD9lNT3JSzbglONXTcqCs6WRiZQYh
dOS164L4ctvUQeLv3pzs5lrF+QUP07K/5sv1xAQAAAACA5UOIuozMupwqYHLrohpDoCdXkpNuEjs
4XNoIEXNVcoamK6NqvWbF6+nZ2ZHz50fVFTXP2eBs/mZ90FMydk6nxNfQ3XdHDnadr3VrtZbsT/jr
h4e0ZyWFeyiVa57idQEAAAAAAAuNEHXJ+QGTMWP/VMxakU1Z3XCn3DFnS8+ZMcmTNFfFa1J7w
6+V6a1MmdfTsyPNpj76oaH8Rdl0u6xar6utN6yz5eg11r0anuYESU7AqVivP+8dkGx4U8nH5V+vZtP
5Wnf2/aIumOquNyfCylYK91Aq1zz5dQEAAAAAAEcTIeoy8oI9S3NVh0rGhDtTMmcun2r75iRC7nY
mCjuDs7o3V3WI5gWO5gzwaXOHxftCh/JXtqXl5XU1KdnnrCAlP6mTC95EVCojdOutWrxzHLe2bVBg
W9Yzb2i6eZ9Y68/k0w3VNv09lNI1T3hdAAAAAADA0USIupQqst1vGQGZUpVWv+8HRvaEQpMqyu

Ze1w/BbGr7g/scx2qn1yBt/9DrDZhIcVP2+oNtstj1YDOoh2rwhvQr1v62Ruysst0PrUtbbVnr3ZILLnU
8g6+ttiara2v22gwMTTeGs+c+ZD2Feyila57ougAAAAAAgCOp0FczrADITq8hK3oiL1WDdo+eiwAwk
urtPS8/mtCWcLQlHG0JR1vC0ZZwtCUcbQlHW8LRlnC0BWmgJyqQKbOOZ75D4gEAAAAAAAJAOQlQ
gE23ZKAzU8ayeFTqhAgAAAAAALB5CVCAPql5pZpNXAQAAAAAAIEvURAUAAHODelXhaEs42hKOto
SjLeFoSzjaEo62hKMt4WhLuHlqC5KhJyoAAAAAAAAARCBEBQAAAAAAAIAIDOcHAAAAAAAAAgAj0R
EV2eg1Z2WjrhVnrSWNFzZavHhsyL60CAAAAAAADA/CNERSZ6jRUplGrS0csAAAAAAAADAoiJERfp6D
VmvEZ8CAAAAAADgaCBEBQAAAAAAAIAIhKhLqr3h1gddkUZPD7+3l51HaClTVePUeI3zcL7eZW/XH
MbfXHVepzc4uF+PuW1j58HXt2XDfY318F4Wo12TGjwv6jHpuVGSHr8S1obCSkPCD8+s/eo8VkaciG
TbBQAAAAAAWF6EqEuvI7VSQUoDw+
+bqwMhX3tjRI1T9fXpBJbR1H5WpamXpFyXrYr1MbN2OWHk4HlRmquFYNCZ4blRoWtYG6RTk9Lg
BFl2EFuSwZd3aqWhcDTRdgEAAAAAAJYcISosVWn1+9K3Hq2qXqUCwPNulNaTxjkdX5br0tWv7dbL
zjrrtTsXnYiusm19rlsX9zNSbdmv7W+rxHNafjv7e5tSTNCupHqNdS+MLNe7zj77LasFWvOcDkeza4NI
W3bd1Dh020055yW0VjvW3SDXOE/uBe3UZN17bZLtAgAAAAAAgBAVUm1tixtxVraMANRTlM09H
crZ4aVeu3bGe23noKufZadc3/La6ciqXT25uOP20qzK2U13yxXZsoNGFVLuibM6w3PTO5R9/VQ6O+
JmscXNPWd/1mPPbVvvorhNDpynypa42Whn56J1ZJYk2wUAAAAAAAAhKiaha5OGDmHPzqkT44K
9tNrVlQN3A+WTUtJPFSdo9EPnYSmem+KanHGTWGtrquyCW7t0qM5p98Dbnz18X7+uYA7v7xxYR
2ZJsl0AAAAAAAAQoqIsJ82UcJTAxElGbdLcjGjnzNtlyawNRdm8ENYz2A9KQye5Giur7QIAAAAAABx
NhKiIoS0bZs9Kt46mWft0JuahXRm3obgpe3qIvV+v1tc8Nzybvl/DdfBh9KCdYLsAgLzp0Q3Ggz9yAQ
AAALNBiIrx2rte70o7oHNrfxpDyKc2ybYya1dJTroJqDsE3qVm4i8YM+6n1YaI16uZ9NUvzfakXXbw2f
XqnHrtK530Qtu4NVhjbRcAlpH9Xl+QwkwTy540VlZFWu57tPNHr+bqLILU+Qxze40Vpz0r+f/hT/0ba
p4P9zEP5wXDhq7XDO6ZmRvzvuZ9P7mPvG7mOO+3ebwnR+5jSf+gFfO8z/K9eGZinJuh951luGnG
nJfc34sTtofSdoiDEBWJ+AGdMSt9AiU/nTRm/7d+MFmdbhD8tO0KKsqaVzS0Kavem667baeO6OB7
cZw2JDp+/aavPhXsGRpSs9Wsc9pc9dvm/sOhHu7KJNtVjG3wDwuALMzsl/chKri09j/lv0npcCYu3Da
KcLuTP+4f5vte3N4IC3ONPyjOQq8h6zWRatX9x28G3NEnxsO8XrMy01/cAyWOhh+z+NZW52O1qS
YFda9TS6qdmpRmFPjk/0v7+Pc19R5csr6f6l3jHFk/T2Z778R5v83jPXncPtTnc/yDVmTok1eYm+C85/l
eHHFuht739CP98xP/vl3dH/g3Iot/IEadk9zfi8efl3zfiydoj/WNrUrbpf+eHO/7dn5+FsZY1g2DJWS9R/
TV5bd+A+hbPzD5uvW+9U+g87mq9ZZia/WtnxX060c8vNcqIa93P29uf9TD2NbIdtqStKvbr5fd9VXr
K8cxXx/yKNetVygJz83Exx/
+MDcdve3gMSfabqvqrS8PXwQAmEq3XrbeX8z3eP2+GngjyoffFv1vwAzaEEm/z8/8vXjm7XCuj9q/f
c28f5PzY/87OoP9RtP37dy1y/3eivPzV9rCv5dn1R7n5y9jv/pnrCy/l8a/rznvuYNtyPocjW9XvNdMa6J
9ZPIeqPdvbdd+hLRD3T/maud+Um3XK1IS/5w4n8/+vTjeucnjvS/
+fZv1+8v4cxImq7aNPy/h62fWnhHfw+nfR87+zd0737fBdX579Qr9vhz3uiJf9ERFDBXZVn8p0ksO56
821puAo7kr/t9KrNd7n9D2D52/MKlanIP1QqutCWuIJm1XEk4PIOsNTS/7rDdbf9h+0jYkPH57qP3g
ubQ5+wj8QdOuc2oMyXfZPWWMeqiWRNsFgMy05XytY71NXZBN503VUpEt9UY28fv35Iqbe9Lv7x
ltmTO69MupE/PRwFm1o9dYl1qnKmfn9kLNhnteWt7PKPPC+T6X6unAzyL5KMqJU9YH9+dQW08u
7sygPb2GqIFK5fqWv9/Ktv3zYmfnYgY9sRxj39d6h7JvfRj8fi7aJ64puxm9Ecd5v83jPXle3ved79+y1Ls
hP8tr6uf34dEJHdm5mO7dE/ec5PVeHOfc5GX8uXHeXwLf5xmY7Jxk9148/rzk+148tj0jfp6yR42mW
tYuzqii+fpZGOMRoi4pvw7mwJuLMeFQcMiBCgvdr1EPJ5TztxMM6dQPhf5rrYf5A725D/VQ+xmx35
Ht9MRtl/MGFlw3nvMG7H6d89gbakjCc5Pg+G2D59J+jDoG8zj1Y9QvU3G3a7xu+NgBYAoz+uV9M
enSL+W6bIX/A5CTnjTWa9KZVTvsoaMdqbbi/1ueGTUM0Rh6N9uSN/n84j6JXuOc9d1s/bI/oxu3sq2
HjNo17dWwypITNg/
+vJW13H5pT8huV1lOenWcNKPePgZk8AetuMFlmDTbEVuO78XTnJv86fJoB+cHhtOnWwJnknPCe7
FhxPub8/PnvuRaNYmfhRcOISoAAJgNfnmPTdUltX/5uTCLXoa6tpj9i6D+pWdGvR3b52vSqbZmPmL
C/0Op81AjV7KppRZXPr+4J+f2NDo7wwDEHTWkatqr7yOrObMI4efpl3ZMgT9oKfPyXhwwR3/Y6uy

flAvevxGqt6h6/9mYYa9C3osD9HwiwflBrO+pqedWiWHwjzD8LLxwCFEBAADmmDP5gRq2N6ueO
MFRDt36vqzO4hfU9oYzCcRc/dbuUL2Csh6WHcfc/eLePi/O6NHZXTNnsg5/YiAVeKtJgXKftGOWv7Q
jNfxByzKH78Xz9oet8pk147pY/4aeVWXUZtirkPfiAernGrdnrP89dXBKXadTkl3n7nn5IwymQYgKAA
Awp5wAVfXWmJ+hjMXNC3YNtk7tfK7hXHtXhU1NO8B1fuEpSEn9Vqh/CZrtcHptlsOyLfP1i7sOCGf5
y6Iecqzq2bt5jz0MViXezdWMZjYfZVa/tI9h93bqyMHgjTuqd9QS4w9ajkV4L56XP2x5ZtqrkPficIMl+f
qydXLfOk8nJau3vdn+EQZpIUQFAACzwS/vkVSvDSdAndMJ/zL8RSPMYE8j9bAngLR+Mexaz2dbt7sn
h6qoWc7nZKxZ/uLu9nw6O8NfFgeHTbpmdl7y/6V9rOIJceZ7CUZNPfuGnmG4O2f4g5Zvvt+LB+T+h
62S2GWOB8PbWf5cw3txTLq2eOCPkekZ+UcYfhZeOISoAABgNvjlPYKeRddiD3czevzkW+dSTf4wM
Bxc/0KW1S8ac6/XkJUVc0i2WqVmSp7lL6nz94u701utKjMcPSpSOW21wPoeCgyhV7dwzTovM26bL
dtf2uOpyGnVY6+27r+veL3G5m+islngD1qLaFZ/2NIjADo1Oe/9w6mHcM+oHinvxXGoUhklqUldLm
RwkSL/CMPPwounDwAAMCOtqvSt33L69a5e0a33y9aPJ2VvRX669bLVFtWewUe139KvWUZh56U
6JyfEblu53s/3bun26+Xg+ZiLe6RVHbg2rb71S2tfJT+5m+H38TB9HgKPefie1vdRxvdv3Pc1573Yf2R9
7eK0K4/35PH7CLt/3Ifxb1dq9H0x9H2r2jFw3Pp7Pu1rNcl5t78m8/fiEedGvd8M7Ns9hrTf/uKem6H
XZfY+POp+0XJ6L453XvJ7Lx7fnpB/xzO6RnHuxXn6WRjjFdT/rIsKAAAwE+5f6F3WD43zNRwQWBCq
x5xdm9BVbUl/Bl3nnO/pqrT6M5p5eS7pnk7G5ZnV9cH8Gfre9fjfR2Gvqc5r79gUjT83Id9bxnk7iuLc
LwrvxbOmRvOoOqhhgkP7+Vl4cRCiAgAAAAAAAEAEaqICAAAAAAAAQARCVAAAAAAAAACIQIgKA
AAAAAAAABEIUQEAAAAAAAAgAiEqAAAAAAAAAAEQgRAUAAAAAAAACACISoAAAAAAAAABCBEBU
AAAAAAAAAIhCiAgAAAAAAAEAEQlQAAAAAAAAAiECICgAAAAAAAAARCFEBAAAAAAAAIAIhKgAA
AAAAAABEIEQFAAAAAAAAgAiEqAAAAAAAAAAQgRAVAAAAAAAAACIQogIAAAAAAAABABEJUAAA
AAAAAAIhAiAoAAAAAAAAAEQhRAQAAAAAAACACISoAAAAAAAAARCBEBQAAAAAAAIAIhKgAAA
AAAAAAEIEQFQAAAAAAAAiEKICAAAAAAAAQARCVAAAAAAAAACIQIgKAAAAAAAAABEIUQEAA
AAAAAAgAiEqAAAAAAAAAEQgRAUAAAAAAACACISoAAAAAAAAABCBEBUAAADAQmtvFKRQ0I+N
tl5raG94n19p9PTK2fDbuiEhLV0wbdlwz3vk+e1JY2Xca6xXNVb8ba00rK8CAGB+EKICAAAAODqa52
TGOemSUMHoqjT1kqMsZ9aK+rmpKJsX6tZnHZ3aesg1asv5Wkc/F6me3bS+CgCA+UGICgAAAOAI6
Ujt/OL38Zx/XTlwM89yXbr9vvT7e7I5KvksbsrZqn4eco16jXN+IFttyXZFPwcAYE4QogIAAAA4WuiNm
r3eoezrp3LqRKxeo5Xtlng5auAamb1Qq9IiQQUAzCFCVAAAAAABHTLzeqH590pVg6NpryIpbm9Orse
rX/7Rrehp1Vr119suC68NKtPqCtUJH1wEdeJ31CKsrGjyeYL3S6HYYzGPXj8F92fsp1ayzrDVXndeO3Ul
FtltujOpfo/aGXxagXN+yXjUo3vHbQto/dH0tcc5VoEar+6BWKwAsLUJUAAAAAEdEWep1HdJl2Bu1U
ytJYTVYDdRep0K2gfXN1VEBZlNWCyUxyoCqjUhpcMIpOxQceJ3F3t/IQK8jtZJRr7Rcl60YnTuHwlEtel
8JVbak7hZHba7KRqMh5/wEVS4M1gNIcvwqwA5pv3M+hoNUR/i5UueiNLhTJewaAQCWAiEqAAA
AgKNjzQ3p4vVGnVS53pW+qgPq9ax0hK1v7o5oh1dLtC/
+y5tyzkv7etJYd0PBqrT0a71td2qyPjIpNl6/F2OSpvaGePmv0a6um3ga+6psW5/r+hNFqRqm9n5iDc
MPTjLVrLnHV5b6hcF2Jjl+67VuGhvWfmsrOxfjnqu27Eadi8A1AgAsC0JUAAAAAEdIUTbdGYwy641al
bNuj8nKaWvJNWL9/mFoL05zBnqzXmhn56Lz+t5F2dGdIQPD3I3enN5rB4QPix+t7aeGgTCzuHnB31
ftfDo9MAOTTGnVs8OTUiU6fuu675lBqKO4dsYLbDsHXf0saOhcmfVeOzviZq/FzT1n+9Zjb+QMWgC
Ao4oQFQAAAMDR4oVsGfVGLZ+Ukn4aMGp9qKqcDiR3JTnpp31ix33dA90L01rllguwH8bwdve1A0
6dSBLymT0vz8ha4EuLsnbGbdi+HIYlthMITDJlPQudTGqK41fHZNc5DR3eHzR0ropr4h2yuodK7n4ja
rECAI48QlQAAAAAR8xAb9RD5+l8K8qJU/rp1MpyMn6am0BHRnTmnE6i8DlCYFIpo85ppLBzFSw5Y
HLD3NgTdQEAjgxCVAAAAABHj9kbtRYvTsvXYK/Onhx6Y8iHebVWhx7biYbtTyercHa88cfflg2z16lby9
Ss3ZpEcVP29D4Gyt7amueYpR8Alg0hKgAAAIAjyOiNmpQxjDw7g706u3Lg7tTtmVk66QWAo+p5ps
MsJeDXAHX05KJbmFROSaIqAdNKcvztXa/nqR24unVRp7iWaoZ+1ePUnkjLDlS7Xi3W0WUEAABHF
SEqAAAAgKPJmIAoTMlPDo3aqW3Z8Kapz1ZzdcObqKm94Q8/L59ZcwJAszZnc9UfQt7e8Gp0FlIZV2
4Gzta5WPd7WfYa61790aSTVU1twuP3A1djxv4k9PbVbRDscRoSdAMAlgYhKgAAAIAjKro3qjIzuwrp
nGAubi3NaZWlXG7Kqg4D/dzWmOFftd+ozdlc1cGh9+IREzJNorLtD1vv1KSk21XyE1S5kPuM9AmOv

3Lan6jKu5bGBFRJjDgX5r1RPat7ugIAlgYhKgAAAICjK6o3qqp7OVgzs9qavI5mIqfk7J4xPFyx63gO1Di
1a3MOvE4Je+2U7GHrIcdebVnr3eHxeYt9/BXZ7psz/itVaZk1TZu7Xs/fcexzEVYMVW8zrewaALA4Cn
1V3AUAAAAAAAAAAEIqeqAAAAAAAAAAQgRAVAAAAAAAAAACIQogIAAAAAAABABEJUAAAAAAAA
AIhAiAoAAAAAAAAAEQhRAQAAAAAAACACISoAAAAAAAAARCBEBQAAAAAAAIAIhKgAAAAAAAA
AEIEQFQAAAAAAAABGEvn/AZM6nr53azj8AAAAAElFTkSuQmCC]]></redcap:Attachment>
</ItemDef>
<ItemDef OID="q17" Name="q17" DataType="text" Length="1" redcap:Variable="q17" redcap:FieldType="radio" redcap:RequiredField="y" redcap:BranchingLogic="[q16] = '2'">
<Question><TranslatedText>Do you prefer Life A or Life B, or do you think Life A and Life B are the same as one another?
</TranslatedText><redcap:FormattedTranslatedText>Do you prefer Life A or Life B, or do you think Life A and Life B are the same as one another?
 </redcap:FormattedTranslatedText></Question>
<CodeListRef CodeListOID="q17.choices"/>
</ItemDef>
<ItemDef OID="summary18" Name="summary18" DataType="text" Length="999" redcap:Variable="summary18" redcap:FieldType="descriptive" redcap:BranchingLogic="[q16] = '1'">
<Question><TranslatedText>
Alzheimer's disease - Description #[no]

If you have Alzheimer's disease...

1) You might forget recent events of importance to you. For example, if you are interested in hockey, then you might forget whether your favorite team won their last game, although you may remember watching the game itself.

2) You might forget the names of people you met recently, or the names of local streets.

3) You may no longer be able to handle your personal finances or plan leisure activities such as dinner parties or vacations. Simple decisions such as what to order in restaurants might become difficult to make. Your ability to concentrate may decrease and you might have difficulty driving.

4) You may start having trouble finding words to express your thoughts. You might need to be reminded to shower or brush your teeth, but you could do these activities on your own. You may need to post notes around the home to remind you of simple things like turning off the stove.

5) You may remain aware of the day and your surroundings. You might be able to travel to familiar locations like the supermarket, although probably with the help of a loved one or friend. If you are in a social gathering, then you might have difficulty following the conversation.

6) You may continue to enjoy hobbies such as puzzles or reading.

Now consider two possible lives. In Life A, you will live in perfect health for 12 years and then you will die of natural causes. In Life B, you will live in perfect health for the next 10 years, then you will live with Alzheimer's disease as described in the summary for another 10 years, and then you will die of natural causes.

</TranslatedText><redcap:FormattedTranslatedText>
Alzheimer's disease - Description #[no]

<p></p>

If you have Alzheimer's disease...
<p></p>
1) You might forget recent events of importance to you. For example, if you are interested in hockey, then you might forget whether your favorite team won their last game, although you may remember watching the game itself.
<p></p>
2) You might forget the names of people you met recently, or the names of local streets.
<p></p>
3) You may no longer be able to handle your personal finances or plan leisure activities such as dinner parties or vacations. Simple decisions such as what to order in restaurants might become difficult to make. Your ability to concentrate may decrease and you might have difficulty driving.
<p></p>
4) You may start having trouble finding words to express your thoughts. You might need to be reminded to shower or brush your teeth, but you could do these activities on your own. You may need to post notes around the home to remind you of simple things like turning off the stove.
<p></p>
5) You may remain aware of the day and your surroundings. You might be able to travel to familiar locations like the supermarket, although probably with the help of a loved one or friend. If you are in a social gathering, then you might have difficulty following the conversation.
<p></p>
6) You may continue to enjoy hobbies such as puzzles or reading.
<p></p>
<p></p>

<p></p>
<p></p>
Now consider two possible lives. In Life A, you will live in perfect health for 12 years and then you will die of natural causes. In Life B, you will live in perfect health for the next 10 years, then you will live with Alzheimer's disease as described in the summary for another 10 years, and then you will die of natural causes.
<p></p>
</redcap:FormattedTranslatedText></Question>
</ItemDef>
<ItemDef OID="a18" Name="a18" DataType="text" Length="999" redcap:Variable="a18" redcap:FieldType="descriptive" redcap:BranchingLogic="[q16] = '1'" redcap:InlineImage="1">
<Question><TranslatedText></TranslatedText></Question>
<redcap:Attachment DocName="12.png" MimeType="image/png"><!
[CDATA[iVBORw0KGgoAAAANSUhEUgAABVEAAADYCAIAAABtBTQzAAAAAXNSR0IArs4c6QAAAAR
nQU1BAACxjwv8YQUAAAAJcEhZcwAAEnQAABJ0Ad5mH3gAACpASURBVHhe7d1fqF3VvejxxKgnX
pWE1hPSejHRixCMSNpwIeYWG0GMCAFBxSBH8XJlE8FygmAjYi73QVEOhexzEPShWOH23vhgkcM
tKraQhxbSS6F58MEDqewHSwP1YR8pZUvz0DP/jTHH+I0/c66x5pxr7bG/H37cu9Z1/fqF3VvejxxKgnX
69xn298AAAAAAECOqPkBABO5sHZh2//aRhAEkXHseX7n///2u+9QBgCVDzAwCmsLa+tvuN3eJ
wTBAEkVNc//I1Txzad+ruu/74+e+b7z4AWDRqfgDA6NY31g+8eUAcjgmCIDKLB+7f+z/u/C9FvHL8w
b9+/XXzDQgAC0XNDwAY3UM/fUicjgmCIDKLgyd31QV/He+c+WQVicjAmCIDKLgyd31QV/He+c+WHzDQgAC0XNDwAY1+mPT4uTM
UEQRGax5/mdzxy4w6z5i/j1z95vgcBYHGo+QEAIzr/6X1gsgsdpzd/tjh20TBXwQP9gNYBtT8AI
CxXPzi4s5Xd4rDMUEQRGZx3/E9otrXwYP9ABaOmh8AMIq19bW9P9orTsYEQRDT82A
9gsaj5AQDD27i6cejtQ+JkTBAEkVnseuE69zF+Ny783//TfDkCwOSo+QEAwzv5/klxMiYIgsgsQo/xu3
Hq7rs+v3Sp+X4EgGlR8wMABvb6r14v/Peito/EmWPf/8tXXzXzfkgAwIWp+AMCQPvjsg3
3EsJgiCyC/2PXujqOo7483nTjVflAAwIWp+AMBgLl25xB/qJwgi+7jpxWufaf/ce+/topjvGTzYD2B61PwAgAFsXN049fap4
VDzAwCGsb6xvn91vzgZEwRB5BZ4ZXlNrjZDoUa8BwGS+pcn0/5++ZL8l6r4PhzEnRUN/qwZfY3P9yhk/6E3+XuHw/ABCnO/epVs4gbQEcp0hDfedRSGUOVXfdPJ/ciVFc+UDSWp3BuxgF1ajxDz55RHWUKdLzgZ62X7KKUkDGSm4ZMvBvLuNfx1Gwuth4G2w+8swxkJHB6x5v8GNAZndrMUtyJGkvU4RlCrGzwGN9Fn8cBK3hM+Y9V7XaAmv5n74WqbLEAHtQgpMTGmPAXVqBhbhgXjxf6mgq9/VDUtaSIgQgIWWYEjB+l1pM2I6RHwPNZFZu3Rv23CRZhXe5G+f66PbECEmX3OA3jIKmUhtf+PbQUrQDOnSgKfK4Rnpo
BAuSxEdGvNqjg2/OYRsrMAn96S71J7yhkNDRWUOc/VEcS7f2vuvsx9v+ndtvpcsmfqMn8ttk8Ru4KF
u8ee91TIWe/F/C8TWngO7N0tQRmWMA1vW5kGYzkIx1jTXIzV6knvtw8lFPcSkfI8V8T0/ny8MnRNe1O3CQ0
UWTSIG5kqZEEAJJkIoVG8mqxwD3/i0TeJ+dp+zzrf3n+Jj2YyX1x9ZKx9uVnOl/P9PFBrl1g23T+Ynz3/s
3r8BAAU6RT5a2z3F8VzFgxvqsJ3dXsf/LPyLGIXQXtD+YJT7HxiNT/O7+GK4hpaUw5v9AkQrjaD2p
bf3/UTcfoS33rNjf+fxkRyjbGGeRCG+n/bYLaNdx/vKLSzeZTHwpJwlv//5TMUxoQYLaJkgvwTLt
vCHaOSw9AKfXrGrtF4ECJKDWLUvOS5kVKT8+Ytwa/Xxfn6/R0pNSLLVj+T4TczKj7Ib/TzqxkGb4Wmt/
p6FHRRL9WWV8PHcNxYf+Uj3sVu/jtjeDSs3uHk9EAIJCS8Qm4RdHuyMnrlkOw1fbTq1+XaTJ7rq9ahTnaM
1mbWn8xomwZMnEUhXxR4HaHDFgdG6bD+Fq92G6KqbpSMz6pz9cuwAHvMKOwzVIbQ+T3NzCt2hDAtBL
ZtKaNRZrYSQ6ZNpXRvQcsNu63ik8aMBWA+0bXL+iIiHEpQybm/7Yy0Ex5o8g1pZeNgOLtGT8ZN+bOe
XyH+gkqo9JIFi3ZQJFNMrsUKwo+sjw1J7uuYKbmeF37SOKVYY/C5fBPEvlq1ewSp9c3l60/e2q4VdDxo
ONn7VEKEYt1pZK0a/E5XPiHe1Y6Ef1G2I6AT9cOGvMpyZwrQ9cNBR5k6nsAEUXvK5T1+cnhAP
3ChZPVeBt8t0Vr5pAVDDmXHbz4jfOhmtjv0nMMV4XFXIuiZntbq7yW/UeQBEEf12fvFkY+iYtivbRP36ROR
tfxY7GXXy+YkNdTTXrFG5/AWWymIBSD4/mCszy+Sc0Z8ZO/oLPAhJVB4/SApdk58ZZU2tb9MvIHsFH
6NaaL4XK9yX/QFVJL5s1m4m5aDvHNd2PL/WXSJrXKKrN7iC9cbfbCg5hqfKyNhoULOOBP/Q1CvkL4I0Y
BzRT0RvDO1w7UNLjjtHT4UpqH1ihsBKNx1VV8Cl1fyJnymEMBQ1ae0qU/uT5ZIrxL6ppf75hBzL38Gt6
6xmBqX5eM61JndJLbt6LvH4XeqkaTO+UT+LL2wmq8UvzKiy/CuZK4tPhPObgJ55CTHQkfqVBvZfYXSQ
T5oiyqtcHMddMSBI5/Ar8GYz3Ko2vE9Vjk4AFnO6p0VWxqnAJKfXRS0xk+cf8/tyqyt4f6xl9HWxd9HeJCCjtb
jUyuLvXjvFQzcS+wZ6KHCt0DLVh0lsDEHuytEdlmr8jdTTeQMvpyZ0VC6HuzQOXdO/qJQGszLoQaAcy
Jbd3eQTW/xhGmQewA3tnmsNZ6YoMr/w0j6Mj93L82Q/oR4mBKM2xWhH5d1NVnPhc5HMZzRgZGR4Z4U/Z1
8iCcMNpjdl5YX01SB2oFoGMC5EqpXl3gBJnYNLqUQwnGwcfv4wE7V9WkjEq8JUqrRbJxrzDkYfLYS
w0Aw23y7zyLk2uQ4WUdH/vbvPmN6jmu1iOMqtmS/D3q4lRFv2+C71Nq3b6sQfMtbg0vB3t+OcC1n
ll1LB5GjDGBDSchxUPdsKh5Jg0HcngkB9vf5QFGDO1t9AUL5yO+1wHbCpM+NWNzh+EuTG+bn8G
DqhZlvOYfxCvNSPX/wUKsXKIxN5K9aoyvjnY+J7sw93d0GaAWc9SiYoNCdvhrvOYVDr60XY8gPDsG/S
KHlmtEVSq4UN6hYR5gWYHJP55xSFYCLxAKrhYQk7ZxjlvzUxvjhdHTtx0s7l6m2Y5bnwCmFIwRDx7d2AW
NxlwCE2iijugp5eLB/Cy/CcwArgNc2ISQMmCr3onNFyOiBgdpfdC3pjaemCEyMKE8bqIrwDbyU6gA
M5shAzL+mZb3hX+H5xyCS8DMnsUrYeESK3Vh9MEwlY6EQd4bqGMGEd6Yhs5l/PRp+k0QiljHoj7Ju
Tyg5/xLt/PjD6jw7/oL0gB7NU2GaNQOhdl3A/mi57ZGrPnbVwT2wJUMgCDx5ipoFXU0K0L7VHhs
W2FsAS7HEwk+P9ov0J8XVqRyjYW3PHmLmbFvxxKW3tNMqSWwbE9h3A6BvzY6ar4+Dw4vMTwwMw6Mi
+PXhIx2sgGF58HbzkHgQLGTH5TQ0G0afb+e7Pr1pbyULh6YJ+3zOfVxi+7WlTnxL9imjWJPlz9g3Zim8fqd6
Rpp+8dxU6o3VkS4GoKuEG/jd5zSmqYFbGc49JGwctW33ErZyOgL3ZkRZUs8yU6+ESDvKNCw7lnMSt1sJ
3wK8q2Ttk9vPxqwu+bnw2FqW+E7P0GXt4rdEpqx12zzAz2fUw8p+t6D6BE/K3MtOCjsvZ9iHbvu1AYPtb
fWoxGmxcgGyybG5Pz87O5EGq5Dtx7xgsT0AS7WIJKOuIBW5/aBZWdVKZfkhnB7mqR/7QzF8bzwaAMYr
NQLDHGvzF/e4sb/i2OhPQzGGzvB2CzVZGqLXLc6VcHsTmYGxJpcVXH1b0rwdq7fm1pTzDOTlVH6a
KNFW5PW1wtP1/wkK5T9WP9bOrtLqr3/D6Mk3itLkyTCQffQ20Mpi0VItSSXOxI1bjstwNmgqkvCuJdOEF
mLVImNpx3hd7NEjYrlKUN4zCXN4jo/6O2eLnPPV4bp/kZMw3ugqY9xpNXh8A5r5iVHGzHrvWPt4Kw8
l4jX5xlJHXfj/xmzkMc5wxh/ySnVJg3ZL3oZkn9Mki8grA3WjUxJ5ph8VHXHQMaG2ukmFCCbwDMKlm
RRQuOHm5sb9CKF/EvT/gfGvD14PzwwdufnV0lDanzj6cxNDJ6Q9VzVtGsh0KL9A0Uk1DFoprH3HmGp
rXF4DJ7ohK7JWEpHS4CgfjH91gRcLv6eRn6CIOw08I1UnFq5f0/PLDMQ+PeJCytO4e4kprRvO8vAGV3
HwzSfzw2g0Sn9Tfu0sd5VQU6xjdU8AD93PTOIAn7L1nGIkBWUwGH7V9Bze36PLsB8yyRE0MgcSnNw
cLa1hsRaP/zDyD7u3ncn9BeO0G6tNkEnUHjVugXEg9I/V8Q9oQJ1uFmJm2ivk35u/ElGOcdLsB2/XGh
wobW/MNbj/AM4IIkDE2aGn8QQbm1/LPEUyo8GP1uaAjpwWpBqb5S9T5yJyPjwv9/U7+uz98aHPl8
KIUkNNOVKg+8cOWY6rxnqhMu0nKpHv52RMqLMkT30uW4Fov3Dm1fUaTb0IG8GGMbVWIlzp2ecvqAm
mAHM0ABaCYvzbUQxY/yuHsZJcQ6ez4+tCP6H5SawuNp6+l1jw1/RJ3jVs9UhPaaK4PWOsm3wvOeHS
U1lyP5UDTDGm2FAnbnl6EeE1xyqTCxFPIu0RVs+aNKOMBtjvbEERGWhw4TRy3cEr9UOmVX1HPL/+9tXKw
GxUI/D3H4Fa6Gud+IQs6O/uuF9ZcKsuH2Qtxan1oUxZD9hRWhYd+PnvlD2+JOF4IKshkJwHW0vbYeAs
/N7AInz2kyXQ14SVBO2AHqfjqjNnH+qBqu6x1KKXFv7mtTpbUUt1Sv2EJHI+hd+U9pqKTRmuMVunr8mZ
JtL8ht7AwpLjTeCCQQF14rIGtZQEuXNzkWV1bM93p7Ajdd4E0b4GunmM8o5LN2J1oFn7J7Fxv2NXlBwBg
1UqrW/K4sMJDvsIkRdcu9qFTERZ6z9FqxwyZeJ4yuomIHSH8ld8YQZcWfBwLyJ+vRfYtWHzjzRFbAZEo
3C0oiDrcq4yUGm4WNAJbAqjrQM1jPeH6WCDjxpYgXi1+7+e5WmUUrQ75uWWYrjv5xtmLb/Ff6FGSZ5jK
5uycZY0uGTsGL5W66JJOlBZUflxUO7VDcdg2OwywVzuYU6oUmBNVEOpNVQ8IlfUxTV/QbdQ6uI2ok73VXK/
hDhWQWKuyOIeZoGnw/cfI06Dv8ANdhCUvBQfJxU/ykkXFKdeKr2BwFMBsCyIwNMHBe2xNvrySWLrJJ/B3
M2vZ6d/Tt+Q7IP4cgDcljdnajFpfVi8iZYMWo7eVP4HFbKJPqRGyzJlaKmtijpbkbHedzOHl7rkLLlBbOkKTJdf
TRs3gaxiqMBfyY1RAe+fQ5PwRSZuZlS3/gt5nVh5yqQBbZ6k+UQsfGaDx1sSqrWtFKuqF0NBFbvC+SdU
RKjYN0rhgMGoEQeUkxk6oS+z8AwyewRUqUcHgRx2IG3j6mBPr89Xr7P72OzLMVE+I0H9+RePXQWeqbuB
rXn4T8Ed0+r3/Vz9uHmQeMVBn4r0onR2xgCIzFMgEkdMDJbnnf0AcryrFuTyYeM9NrsITbC4D0z
hV3cDdTn3zCkfUr+uOufzOY6vMuGrqIwbr0V+zTjrGe1u9Dtbn+vRC/ib+jdbW3TVWqW2OqDYsFfwhY+d
WKY74d82dRdW0/mQaB/bb+KrWM5zkl1ZnXnzk1zqZ4gj4FwxeK+H1a8xdTa0xdGaBYcvW7pgHfPbG
KoQE/5n5wZoN9IbAb5g9EmjR0wTnK9PqTo1mLXh/t/I1bQ+tBUIg6DkYhgpxNgadmyyXmBHfjdTGfW
Y7gcS1JxhAbh0ALP7l7Ghe4ffzm54pnLEQs2lbVO9EW7B1WdgW0+TWyC2u+OeC2Op2xNdSg+qLKvh
OPQ5/qDpgT8a4Q0eOr3d55L+/9DJhD8Wb7QmVd3xJ7wdrZkNI+f6pGJ0d2rHvZwkKzwgd8/dAzjiMLth5xa
9OOZzkcRCN0SrnMNhQm1HB/dbX3rTuySLD1+aIV3F3eDBr1C/7/fX5R5bGZGZw2HZeN8U/4nUNtJ7QS
Gx2LUdaH9g3YSWcwZ+fR2S16jMR/aG5DBXsC2V62JQO5iqXC6V4BsYMkpbbD9lR9RgXrUjxL6OzWfMh
RxMiDDc7mlWiQxtMfYGeHl0sfRiAg0tvAH0jwWhDIvEvmlu8YwZk6YMK5+AvUlJkd3jl0b4IKCgbJDE
3lZaRGP9CHvu7Dz/RZ45TdjBNvYWXYbH/lbLLtdm5K8Cd/HO5FS4rZHHKM+9yJPiL/m8gsmDqCtiWJbz
ZHUltGW3yQIU8HHDv9b9X+fGwDf3J9D8k6x2zPwJNoESO1mLPbaQSyZR+H+Hf3LdO2HBz/GwK3FvFOM
Bb3lb1xV6g5pjyffbwDvkeZxkKQT8e/Q/rB/5WSnUFI/6N4/U39Ps8U76sWv69dp0eBm9wlW6HmcrqP8
3hmGcb37S9tx+sDWyLR5yL+f32rPcC2EzKPSWxvPnRhTnndxPq8iRUcsK43G3X1zvz4+6t8DF+ZYj0B
YjG/bvYR9hsNmFkhuUbdnlCoM6SYfjqCB0ivDCPtzJw2b1NBHZVvv7xSZkpWnDPh3bc3fWt1P2GH1Zn
zU+Je2qVn3wlgXnuJmrfm9f+/sE8Nj83ApJT4nWOeXmqDdBrP4jTOF/+k7KuXXavdsX9uDVY9nX9VXP
ynGxH/PZ/79bbeXBTzrbHGhqX51vV3T2N8XvW/78XtyG5P57bN9D3+ItODAcEMcb1KjvrfLPhzTOQh
YBjB1lVCOj76eDgE1+o0zOuUPuUO4FWXaq2G6m5gvW+W5/qD3+xhHQN3DTHPnBR6/XoW55HRqMpQ
Pjb+4Oi0VuGDXaRlAyS5cL/jkhbT7FZiXPzfOM+E3pzwbtcf7dqBZhhb99m4EMf5ysTUJbn7Ta62e7e
Vf5vUZ0q/3a2JOLVbrA3Upf99/sMXcl7IYrzPv1hfB7vxmEyw9qk+Lz0ZPvRjp/6rvaM16uWBN+QdA4Z
aJz6mOSBrAzbfv8Wfk9DrmcmLyLjetbJL9bxyx83T9fUf+aSIW2RSsf0tgbiHXSJB/nTVj3RTfQtH+bv
Jr37R11TfVW+k/ltsWFRGtZy9bOSJbh/qL67kOmgfr/3pX9+Jcfp76O/tT5V7mb8+DP7oVr/9ftrZ3YpPxb
7MBkzUIRvmR+MBCh93GcxSLbbZ6dbvUe1wtCybwJ5Rd6nSo6q5jEAXz3zVt2RBWc91jtj1I+dKrrD
ew5VatPCbFNTkUg4Or8NPdRzLwz9L1WdeYYjJW9W+CnR7uktIbE4CXzg17rwIHb/zk8xmIqWWbQNc/
57X1U5PVLq45KRpyqdMdt0qLdQy2GqfXX4WSdV/ArqYHqxZl2DLIj7Hj7j3M2bMnK31EKddL5NavYD
nU8Ibt1fRH1+sJVK2m1Gx+qPewUzi2O5ohRxRhq1sD32WgGaH7Jqr3GjyqTXE/eYT62MDO8+D1Jrsh
gIWG7Y/ln6TxaTyS8LZdTuGlgq/3p/Tc56XTLY+cFmxXAWQ+Oz6lHf9ZwKHP9ytBytQr5U4XS/4
bJXLqEo/UVu/vPZSw3NBGvrqjC6M5gMhKQ+a0VaurXtX3v0SlkHiO5lwiN9mAp7i6Kwu1XGbMx9hx
HTLb2nPGGazgmqgsgShTuqmo61VtGq/JjF8nh2+8hHcMb6NkIW3vwyV1IewGOEn0VYnkzlwNmW9iEdK
Rwou8GTLJUgzlAaNTaKMG7I29vWXn1YdZyEWTQf7Oe+f7zVt09m2sj4bfMoLd5EXfdp3/CdX/yMpHHWcu
NgnlWz38e5TZa2+PnHdtVj0x5xnJPjSZhAodXBqZKkuaKzIfnUw26pYsvnWeOeHp5yF0LaMWzeaDN3J9Q
K71gGNyUn5/wYB7SyXeF3LuQyBobtNZfj1aRBYXsT/v7XLZeDMCpWFKJ01Yl0WXvN9YHNlnM5OvsM
o1l+uRzXpp8O+Z4jZnmaKkYPIAMymTu7H24wg8pDuc+fkykOzFMwoOqIOmW0WVVbCk3mmaXfbPQ
qZ2v3wZTs8sqPjaU4xHkL+sY1o14YTrv/ajoYZnI0R1qfxrYp8F1HMpX8mlTjrn+fDMOPXmr1bMDzOLd
KhCtvBo3i9jHvyUrbe5pX59ncoyZGm4pmRH9Y/73/nupV3nU78PfrgbDfDMNaIt1FtWHy0Mmcw0jGEH
BHOazaDR3j0ne3XnaoVDvo0xL3pVw7kGtLV8lJPNiHPUdV+w+TMU1u/uprm5PmuJC6o4pnqF8E+4b8qIJU
ACZvyfCd7N9XPJBN9PtqT5rb8Q3HOZXbwYrnwRBRkdzNnhhtKCNZZPJ+RMx7/PBRGm88L5Kf+NFfqD3
N/u5Q8i3v7OH/+eizNOiJJYHxNaTL3vRXkP2/FHXdyZl1UfRt8AMI3mJVmgX7Wxr+YS/3YbiGdWuq
cwlNPEodhK+lcMnLV4Cs7SOdjDqoOJhnxjfswH7RTyBZ5HYSGp0FvsiCo7M4iOj9j4l6KoR3+Q9N4Y
FXXKHc7gT1XL8Tu3cMf+fMX9Wpn79S15+h4nzfXXT+vBXdgW8zEvBTzsZ4YLeitojgEy+FvI4uXf/
o3bPf+2Uos+17KNj/1UlHdPQRWX6gl8oF4jB1wNU4wOtlzOSBc5iRfS6pYyA4ND4nJqCdP3dgfQfH
35VTl0flzBqrXaZGDYq5WeYQZswiVQ3/QF0uwiOr2s0Cj1jaJQ5CxkBSh4UeGYsWwfoCgPBAX/Vz8kL1TqV
Gxk5KbsSXEN1yxlfPmGQWHzVxbLGO1G4y3CWh+zfiCqPb4WZ7+kCUlbwrcN8fhlwLwVB2EowLmW/Y
+7FBPm0stIb3+YLMEj9gVf+2GCMeRgrw2MpMzRdTjWzAsKu8R/NmFnOM2wlMnQxEGymKv+KVsOBK
hDgjSzs28Smo9nlKAcaS9+9tXpcoEEAh/+7MF/Phu53FH5+XbADffW3dmzIPQuvjb6/rCOrWGXlqxCg
2T+8hOd7PUhJZx6Acx7bAv+PW2VnlOvvLJdRq+5yfcM5Wz0QAM53rs+ibQfI3rMlfWX7w05fuPtH8cUvJ
EMU3atO+z3B9p5L0D4fz6IqHU3Ni6uLA6mQdoIxTeKYdvuTSqbDCnUdnUxoP7D1D4j2ZxOv8M7m3ta
A07fy/tf/9V18S+AxlpYJK/ueeN3zvf6Uvm08Bd7POJVbL8tnubVLKpDv/rjT5CpyX4O12bnjt8+0uuN
W6g15+v8y/HI1VqCKl/7LtBNLQDjUKWuM2Fmn0c3nbhaOLcTIxzHXvhWchvmuyWxPuB/0afRfhaMGm
X0CEcXkZpfXBc7PnxWrxBbAzfr6vdbPtL6Wv8xk4v9eT8lYmHZmUj/oOYVYWU+6ZROUJ23afoJ/oA7a
3pXW98rjPyVWdfs2yBrT8mRxsRFbPNTcVX7WdgFSvv8bt+aJ/eXNHGBiLqvf8T/A9LiGUbNhCtfHR6Q
1KHpIqAAVqj4mCfMyeZ6UXl8Yxfl2uzx0u0G3yIpt9uZsMJqFWoyGHKLYQf2vbVpUeMXe8OXJHIUvaz2X
EMO6ofd4ow2BBZdMmuQaYwMhhiXb9VjUhGOpBYlJL/lf3r7fyZy2Ltc+85p8UzrN7K0kR7u2GJUa46M
/+qkCVZvSIJ9ieqYuMjZ7KyP+f1P2gsUTcDvPn54FPCC22Etly7X+n/QjD8lxJ9TMepWCsfK7t6ds6N9i
6CwWw35yEpuitrmtm7rHxr5ZhtK+l5JbEIj6TXMQhcZvS3euNF4HVZtm6OwWwaJGxTMJrv5E2ZgOI
/zLyBhdz4XvOdY98uIZx6QTnsp7Kqk67LJ7n+/MAOxebd1LvftcUyXhAYcvd1Mbb4AQkr2zIzxp7iV
BcfjtoGxf5Mvm1RXaMFqfQWs4wsCThaNcuovhuE9GnA87ncZNdHQa/yoyv3iC/zTYtWaqUgaxI5Dnb
VIDlvFd5qVT99pmXUqvw7JFO01f2hYcE7E3P6gucNuNDLjEkrSdwx7BD+6ERWlebuRmWnZ9VIu+SNcN
QfVa2X8ydPQHM6P8wKPjf6iNzP3RH2P0/yzl5bbjZ2OUoe39RtT6e0nN4E8e6b8j1Vr4k4EbBQcRsA
8GJ89s3PM05aiz5IsTJv7ubg8a1d+3zqrmcdvuHFIgGaOG+Db37ePhZhyzmhfaNqW1yf+LnWsVHc1ZC
uMLa+2vR2DeZ7mNxGCVO6FEZAv7MqsvqL6zbI6Et06P5yWLCK2tg3uI+sTlPHtZhZw3B0V/lSxWtM
19axV9Wf2dw2R5tP9d6lP+vgbFdBncthtOaTuxyVDZh8rlt0ADY5fdr9/t1PyNb6v8R4LLE5nmY+
ZHI9f5Q92e3iMTsVvY5IEjZbuU7b2qtxhWTHD3FQgFqEXSL3ySBNxgMfAsHBRABmB4E6vqh8ecLbH
TRzqRwiGfl8oFJmkmAljD9VP/F/6m1VmHSYGsIfNN3H1NHEDC2N5EoTI/AMF/3nlS1PpXcYtUpDdyDe
MZ2YWLM3b0Sf0q5lvnPSC9txfEi5KqCsGA58cKCjwWbJtXjAuiAXJaIH33UrfEVfhWG3lcp/k5wyd1d
VecjiKSIbZMfGi7hM0cx7eYJu1RQrsBuJmQx3kLTIxFLG3L2t8H1rs37Vf0fF56W0yhzyvK0dLiemPdJ
sTbc9gPC0cUMG19P5gd0o45CxU8XafXdugcaiCZVA+sdwgn6AWNbCbt5u1r3vPt16uRBcjfcwLLl8Rfm
GL8eWU/AocDHq3zP2d7LIYRvS2YfxQA9YpyOaaUSR6aiG6BqRT/wDHDuWbRhPc0IiQE7sLr8Dl8Y
KDAVmIWfFXb/W4vF7vMBaXUpd08KY8Z+T7eVMS81i1dgq3e+xcO81ajk/20+NXbmkcj0MKT1eQ0c/k
z0PYK1SdbrtlKb3K2/yj8jyXH/GuPmX3I/lRlbP6gGdFa2uQVHzIfe4hVbmGXw1oNxKNFK/wzfl+yUP31u
B3W53RvDYN4HNMamxngIyf5eUsfydUwsAKsN+zgSNZ8pfAhTU/pHPF8RMNqw58eVhVf4xA2lK9ZwPX
9TMtrbnwk9FY/L1DKsfhRjVvnZUnumq2Z+wq4bN4pVkZBZk4XHX5KAE7sCI2+pTpAvzmjZbjmmPMxmTZ
RtpVfU4rkSCvJWn9jiUaHR0Wm5FZNYBuTy/E6IJ1UsBGgj2JbYBeHtPDXrftmgctk7jTw4MZAMc5uKIq
gGZyQrHuGw1jVtXmdV57aAg0iHrcIGM9VEJa60eFyc3x4Ml9kutZ7HzvCwYdyKjEnXKYX3zHVvpTSi
mPb7ScAgRAWJuyV/Lpb69c7zvs1GaY3U2HyXXsqZl2BAZ3/EJYOmhVKqMQQzAY3v44Pu/FhLKtfbC
Wq48Aw6GPpuTLsqovzl7ZX7CE1KBw9nmPBh3kvJEeYsVJFP7DWodXb04SLA3G93L6Lbi7EvDcX8r2Qn
GPWbkuyOiQMhZeHp4iwl/VP0GgH8z0+1DJ5p/k7dBdLqB/y7xmeMjTVHyfNEgHgq4LHYWEAL+HBqT
BvAafG9xcxqYy/TBlqeCvNufCn3wu6AK7nwXDy6W7xdQgt7vuzbPLxpP4pj8L8xf9aTR8MWtuAAqX3W
6fyrHhbA16/bHmoZ9K41BzG6cfjEHd1jtcZfh4B3XzO9Im5mzf19kpnz78O0Nx3qnzxb5rr+h7A0hqg
y9BRcYv33zMZy9CcgmXAKH6pw8NWhaoY7/eMbbnwcymXj6tMbF4Rb3M7O8JtvDQ0RuQRJHBOGMNc
3hyOSy3Etp8G6zHnSf7LbaQUu4LkAuI7eEoqOzffgJ4xdnmgdVpr7T+X6jD0RQr8ZlIH2KD2Ij5U3CLl+3
h7nHJmZqQx6vvT6lG4gn3b9cRoCwIDmZiUqK2oRcOvbxNwv+YlF78FyjoEp83mmNMRyrohBh1tN4Dk
SbqDqQ7WkGAdl15zYGp66CbOwOu3vyXq95wh2kHS1fq/a/tSDTEjYNnjtnnPzCfvOtTgP7pMVvU4Qqi
2kVhnNTePHf/EApHWDjG7yJt8F5Bgno72MuPOxpl43awqYZdmfzGgnqDcfKVl9Tz3D1ozKTtG0n/HmBG
cf0lH1a90zcOTPdFR7iRYxWmq+EtWnHGgCfqMz6MTBaCGNcaOA2ZCYr8A+kOUfajabGzDtoMhDH3jr
VkvR5FJ5kSo2PzkFxAgtEuO+lJSwv45YNLtZOhIE2XmK7H6/9iypEJfRpbwt3FkpvzcWCnVBBR99Eyx
ApUwIhiPc8lgZXQMMDb5i9ciZ+ahaHPtgChzG4ZNZuYaCmsNIPf79D2gupa8UUbH9zotLIXWMudPnHqgi
EDmXp9btvW1EqYe43iPeRoTmPtE5JO6nO23ld3S8Pu9wZlK5dk7Q9ycqJPr04uGpyF2n9pDk3ppl67f
p6WbbqbiJfFkT+xuG4HQMkfcTtyetYR9f/OCKqtj9AXfNPXh6/ntLhTPHl0Ov7o1r5dU9WrAHqfyhgxdK
ncmBT1kqQqQMHtSOSXoxxlGx5dwLBnNOqhp/O9aiMjWVLcP6+fmeCo+c1MtgIMiZi3nHS1+ikFn+mZ
GaYuUOatpBOGnbQzuvUXsefqlsY84uP8m97iPqG/ksJ28mPDAA1uDaM7tpEXOhH1wrhZcwtqJy2tbKhGF
aK0aUU+7KpiR9Pj0FBH+ZdezZ9AovR6mTeYzVyKBaRdRUivnTy8Kp2iAHl7Tg+nyyzG2vj3cNk/SU1r+
Njw4aJj+tHjZJLfv4+VEYpp4U9A5PuU6SVNyf/9lf/ygAUGkUQnk8LN0tIFe3WRewk6Rm5BAlqNeFVm
U2hw9g+rtA/yr3Q3Pg6CNrH6KDFUDjSvTf6KhYMHaRZ3ceQgkgC1D0MFn9kt1I5FxbqYa59l0CqvE6aA
bH/5P5j6gyU9VrpRqbfQXaCU8JP1cyahDCmK8IzdSeIRZAE8EjHZUdUIW1oHY/Sv8CXybeUNNBUn6Po
dYy6kKlApd1v2tO6ZKHGknjN2A2wO1bkwIFnQdGwSmq0WdrYqVZ3sNanR+FynFSK2eP2GLLJWdu5ceOt
gVXpkmMIObvUpPlcxgbfOM+LWnVhzsFb1wvuOkUwHG9AoF4iv/gI+wHCzbLDFQFgPw3zLn2JmbS7dM+y
mGqaNgIBQyjfQqY76/7efr6Vy3lkwddDLEoETxw6qEFGvAdK+HJl4mqyJnWaPi9bVu+vr2DHo0C0kLdb
cNFTWPADOH1Y8HTm/zyMrfUPXdJzG/ktoV1mfVohMqGIHCEhRT6SfFIVXzo6Ic+4RoqMbf7nkAVwv/tLK6
iQtSbkBXmJXm1Q/R2w+tv8gJ0pgS8UgO/PVSvhx6Nbsk10BevEgNS/IrCt1C+EVQ2IqTdnn7o/u4aD4jQ
vUfe7iL+Jnn0cpxUejzk/sIlmu8OqJl6ZbS6tXqd6BKu+cR8JP2aXLFJV7Ac8rlcxS5a2KJlZNtjPSJM
3kX9J5EnHq6L95bBjuCc2d4M3hFMYqY2RPm9DcjzdPi+OF6ptKPNFw9fBZGdwSy4dMTg+ICs1L81dy2T
sFvETPKx9g83l5Zt9Fwn9rY0QR3ZoeBZaKYSQl8IGfvPBP2nFt8NmjnbxhoHvO4sWrqJvh9AL8MNIUS
7wdUr8zNkwYB6fNwFxhCQ8u2OBuQVLJ0bAJjvVlzWQCNvDTrQ9KxVqvu01uMe4m+oz9tn7yC0a5IOQ
VX8zHEN0m2xFsBd2MVhE7kQtXHyZAeGaTKx2HmM0CEAFvVXH6M6jCjsm9uhYkX3qxW1ODmQWuT7x
TZqOWCaTQ7UQ/Uh4+rLlnPAy7z6BO/gkAXqyDpnsJGFX7cMxoJzaiAuKM9aOSm6Tb3AUwQ0RS25RSt
Gx1a28Ri8J6Yl0dT4kQoU7T9vM9hW3qOSNKQvkW9c2Vyvb33dl1u3LHp6H9Mm5bGVa8nqS8WmRzZJu
02iEgWhf3A1ZpqHAmdQA3dW8aaN81OUfrwGtRuLFtJRHVxlyxX71C49M6YNvDtW93bTNVaR4xv0uyYA
7AGiXCyBYO1UJpS9hB5z4RO2rKPPS5qWmhH2u5Fhcw5l6nk4DYUBXh8TYdA2eJzw2pR6LtYMMLD1j7
ktjMQRtbx/zpHMbJQtMM2AQcvRB1R8z3nQKrErQ0w/qM7DN4A/tPbc9k5fXBpKn4YXRm+OQDU8etqYla9
hzL7dfsqH/e6uEsB2GhgR77bBh8N+j0KUwXmMR5RBHQ7FMzA5d7ZAQNZU8rA7MaPi3P8jp7KIyzrqCmbv
OzFx7nzzIiG6mfmxgM9AJPhIZgX89EC2tv4gWHdSmRCVXLVdR1p1nj/oJVBtR/2VgfMtMd0ZtBRM4Gp
XwSMDJIIyv/pedvoHFpJrWd1RQFn1SFOl1jbC/TuXHvbbXBNMONXr1SsE3uc1C/jB3ynZMwGtQHgHK4x
dRUwvoiQR7etLBNH6QvnqKtO0TZajPj4SVugN6TlXBSWDRyQ/6xcfQ+ACqrd8r6Gmm+YdYB38I0YbGFw
dRZJGFBC+57MXQFTSGVYIuoUK/7lY1xvBDVR/7jLlmpBGCVLg5rkN8i5Dcc7ZZkz7gmk1NSFS3hqTM/i
vqb2sY2x+uThHNPtfqhxWuH4tV0zx3X+IDwDWQ3hDkTrmyy6cTPgtfN0ZYhpGUXvGkaK8yNKHR2sqr2D
XmTyoJFs8LGUJgXHtu3f+ZRI0jwbBm9KhA9GMn5GiYAPK7bwODb3SJPO8iXMG7h8sLb99pGafLpBBSw
xVvOzaPvRFjTkGm4C1VrRbEE8uz0Lih/o54nGWB1Ruqg7VL7sQ1I71kxiNWlIIgYD2vwXaw5RlPP9WXV
a8qA+VBN1wU6/t9I5Zr3rvSDAe0hvZ/fwtbiFWm0mcx6/Ab+NqN9K9JN2XjWkpI12LpLRXySofxl/Tfu
fLyE7bnVKJlRKuHv2ogMyDvDDwKoRZKVjvWxBJx1EUXu7nAr7fgF/fvHIFe6D1QM6fozLrNYwjG3KbMpw
6P0V6uT1qtu+0BIXS4BYu0DwjhXP45gbhybILrL2xwp6LAcFa9jFWlyrxpGUmzofbf1Fff4HOR2wKKn5ZU
N4HF7WyobeVRMqK/S4N3w2WIyvpsWUoOwfrHDzmeMdc7mdgNeezn6ujy3LoI0W93gqqqkNNkOuXkVLe
jC7xdRVrSx6X8vhuFa0uFUZXk05h3WZCEkBvUk2rmmqxtVAJbHVEtNO1P+yOKRHQ1lZDrrG4bsWgJ4a
CsKWb1SBuRG0LpBjNT9IFl4KVn6N2hyPvdSdqXx7oXx1uPpSoekjJNsCNjLSPTPudR9BiF2lWqUXSqqiBs
xRNtwGfzv3w4RbKyEpYEEvRXEqKVh+IiZGKtvNqwD3Dz5iOTSCXWHtd6CEHvjRkESGeISfyWqYiAfD/b
2enP5h4UpYS+dZ/Wqfnv37u8RoL0ZAGqH8zNYEDaW7ZNNb9grbdyN5yf16xeSM0lgcCWoNW4yvaJNUT
0uubSkmlwVI8Q6wxuNXgAFqcE7PZUK7XXpSFS9MqLpU9wqr3bc9C8UV3xsOL4i5N8NjFQjvCVQWpbB8Bs
D5xMFm9G/Bh8XG4gfPJd1Hy0EYR8IowX1eSfJ+dY1lmb1gMYGNJyRbAb7Ptu0DJ6oLdEakpoMmoLSvy
ukm6xRHAlBeBhX56gvBmI2h9j28ehFPuLmI+jPy6B8Xc/Hkk+I2LtpUl3BKZUFWhJ7q9yvnLaB5u/h6jc
3yfmGwRp9mcGqX5M5AGmChZ3X4QAAAABJRU5ErkJggg==]]></redcap:Attachment>

ngmWwMbVjUNvHxLHYoIgiCxj7Mf43eDBfgBjoOZfBpH6zX8rUJTNWCw13YoWdevih/qSvqJ7i+HiL
0M6e6kr1YV2djW1SJ9IXpklba9UL6uF1a/H9Dypa+4EukcLDtbqsa6CZyDRscfS2i5Cc9+Zim9u3TPBE
jj5/klxJiYIgsgy7nz6ZlGQTxA/OPydL//wRfOFCwADoeZfBpH6zX9LlULN1dRiqe6n/uf9qnd1SV1pBxT
DxV+GdPdSl4orzZrae/6d6CSzpO2VN7nVr8f0gk3cCXSP1iNfj3UVPAOJjv0nU6gbydTOVHxz654JFu3
1X70uzsQEQRBZxq4XrpvmMX43Xnv80b9+/XXztQsAQ6DmXwaRokoXPeY9WQl5+usqLFYsNeO4j/
HXV4zxRMb4y5A+vep56z9nb9zqmUSS3dIKS9922t1mGMR8Kytu3+7RfC3KBLOtq9C9IZ3juEPI/XK
GCE2/NPMSMI0PPvtAnIkJgiCyjB1ntz92+DZRik8Z7732avPNCwBDoOZfBp5ip6XLJ3VX10G6ub6iSiP
dp6NYajv6uoZrr/jLkF69jPzijm6vLuumsbQyi+o0W2Hp2RWZvXt6ntQ1zwS6R3NahC+0w3p4ZiU7d
k1Gzq1t71xpXnvn1mMmWJhLVy7xh/oJgtgicd/xPaIInz5+94tPmu9fAJgbNf8y8BQ7Jl1SWczGRpElx
YultqMeTmfzJqiHi78M6derXa2zHbqHZaakiYWl/y0o+caxtIN6Ute8E+herL+FMbw968Dqem1Ix2TC
29M0cUf0zK3XTLAI6xvr+1f3izMxQRBElrGQx/jd4MF+AAOi5l8GnmJHsosuTwVkNihu9y2W3Hben
uJi/GVIz17x3dC9KpEda8gsaYWl2Uv9XHA7xKYXXFhwAt2LtVs4TYy5ersXem9IfDJmprKbel0P4RvR
mVvvmWBSG1c3jr17TJyJCYIgsoybXrz2qYO3i/J7UcGD/QCGQs0PdPOUo8DWcOrnp8SZmCAIIsvYc
Xb7iXtvFYX3YoMH+wEMgpof6EbNj63p3MVz4kxMEASRaxw5cYsouZcheLAfwPyo+YFu1PzYgi6sXR
AHYoIgiFxj37M3imJ7SeIHh7/zx89/33wvA0ASan6gGzU/tpq19bXdb+wWZ2KCIIgsY6ke43fjleMP8
mA/gHlQ8wMALOsb6wfePCDOxARBELnGw9/7tiizly3eOfPD5gsaAGZHzQ8AsDz004fEgZggCCLX+O
6j3xAF9nLGr3/2fvMdDQAzouYHALROf3xaHIgJgiCyDWW/zF+N3iwH0ACav4FampSZfiiqEmQUQHmrihI765Yq6j
3G1SknXp9dvL7yG0da+tre3+0VxyICYIgco17Ht8tyunNEjzYD2BW1PwLNHI1pIvebAow34r8dMuCt
VZd8aurzgUE9Pjs5PeR2zI2rm4cevuQOBATBEHkGnue3/nMgTtELb1Zggf7AcyKmn9x+lewabZqzW/
W+yVzrZ4Rxn4bskHNn7OT758UB2KCIIhc4/qXr3ni0D5RSG+uOHPs+3/56qvmGxwAulDzL4YqhAx
myWmXraJaMoqodhhRsIbGNwswo41bj1kz6CzXzGHNnk4ZHR62vaM76QmK2SrO6AV75ypdszcn7
wjc9Fzu2jE5M6uJMV670Hp90X4BfXNZDaNvVnEzsBVKO2+tGtHsZrRxR7EmLW73njNSvP6r18WB
mCAIIuN44P69ooTejPHmc6eaL3EA6DJbza9P2+GjtreJVQ4ESobe2iP/Jj7whwok/62CsWueBs6ehsb
3D14yhrCKLyX6tg0yrL5fXxMvQysSVK/inuoRnbkxsP/j5B1FXuzcMc/sC20Lz/3qpr9fYKqNhFyN7hm
XzFYGT4fhPnIzzwa9ffDZB+I0TBAEkXEcPLlLFM+bNz75yTvNVzkARE1Q84vj/LxndHO4aOmz9Ly7qYs
btU+6ld44o/6JboBvfLevM77TTfWJvHPusO0VmScyrNnGl9W3IqFsIqYQmbcxZLCVamE0ECM705KZ
5RBuUtWjYK4tNFDb0ZGSq72ikute6kp7IZK7bWUswk3nTMnpJtfda85IcOnKJf5QP0EQWye+
+Y9/t3kf43fj1N13fX7pUvOFDgBhg9f8Luf8Phej9igMM+aCxAokc2Gyna52OlYfG9+4Jpqpl8bgnkvC
YMPqgRp2SjFmB99eCm26yIjOPMXAPZdmchaiJ2L10M2CA/UQztWuWbZx+hT0tchcfP2606mXkQ
3sMWfMbH1jff/qfnEgJgiCyDV2nN3+2OHbRNm82YMH+wH0MX7NP+zRXNcCR+2aYDPy7IyoJht6
0fVVT/3j5dt5TwLRTI/uEUzYXZL1HtZsKN9d34rCPGu16NGCLRqqodh/NYneS6vYrZ0x7A7tFLWOuV
p655Ibq5pYybwXbb43yNNNNLOnaRMTiswZs9m4unHs3WPiQEwQBJFx3Hd8jyiY8wge7AfQadz/tt
9zmPec2Us9KxnVZ+VD9dMsJdCS8eymv6wSDf2NXJ7xfX0Do/sYA9mGHLZt6SzQt6Iwz6QMbZruw
VTeaiTVUXfrszQ9c0HdD8410DE254RccmO90wnOsSXHKXWnUy18whPy5UJ/p35+SpyGCYIgMo47
n75ZlMo5BQ/2A4hbUM2vW5kitURDdSsHUT9391pWnt30l1Viqf5GLs/4PWqnvqNbhhtWd6uI1r4
VhUVSqls9R9IdiqHUHNqO3Utr0zWNnIV0jmEMUenTsH8u2cY7nc45+t+g7nQ9Bu4xZ8zg3MVz4jR
MEASRcex64bqcHuN3gwf7AcSN/zf8PH2cSz0O/SXVr+6mXnV0Wl6xnTEXJdv13C3f+D1qJ1+vTkM
Nq1odXV31LXK2uYX2SY8yyxKbwY6urNSdja6ds3Inoq7oLqG5FopbMyRLyyVH9WXR1zxzVHz9utP5
egk95owZ8H+NnyCILRUn7r1VFMn5xSvHH2y+4gHAsYiaX13xnN8j5YTbqFenJebuTEFXaHKV7TI99Y
+Xb/xZ6j11QU/JnKdtmGGtLvpFZMw4/z618+jaP5uebsWaQNfS5Erb9noc/1x1x/ayv2ErLZezsc6SjH
Ei++aMU+iRzskmV9Frzuhv4+rGkR8fEWdigiCIXGPfszeKCjm/+PXP3m+
+4gHAsYiaXx/oPSLndzVQe+x3r2wqgd30b4+xRk/942ePVGXpVTvpK5bIWzPIsPq2GkRPvx3Vs6Iwz6

QCc6iZ7TzM3DJzx9LsaZtUE99cS6Ge4aUn5XLfrMhOxfbJzl6N1itdxwb2nDNmcOXPV/ij/QRBbJ04cu
IWUSTnFO+c+WHz5Q4APpun5vdXBbVYFbK8IrtpL1bc99Q/IeZWV8171072DLpSzT9se8Nor6ffNnR
WFOaZlNVd6hjONxtTfMfMxOUK1etmtb65KnLO1ob6zJ6r+80qbsTm2DKTVy2T0sksvQfBDPg/zk8
QxNaJHWe35/pf+L9y/MG/fv11880OAD6LrPnjtYNgFwTSTEMBKdRHkA8bsnH+0/PiWEwQBJFr3PT
itU8dvF0UzJs9Tt191x8//33znQ4AAYt8nj8yikP3CaAOw7j0J5CPGrLy0i9fEsdigiCIXOOO/36TqJk3e/A
YP4A+Emt+r6qEdwp6T4XfDtNc0/89cOjfAngGqekbVGIYiflfq4c/osCm9ch7j4hjMUEQRK5x3/E9om
zevMFj/AB6WkjNHxooWE5FKvvILWAIRs1PwY8cbVzdOPT2IXEsJgiCyDJ2nN3+2OHbRPG8GYPH+A
H0t6Cav2QPFivZo3V99CYAoNPa+truN3aLkzFBEESWseuF6545cIcooTdX8Bg/gJnMVvMDALJ0Ye0C
f8afIIgtEnc+fbOoojdX/O4XnzTf3QDQAzU/AKD01m/fEsdigiCIXGPzPtj/3muvNt/aANAPNT8AoHH6
49PiWEwQBJFlXP/yNU8c2ifK6eWP1x5/lMf4AcyKmh9x4u8xxF/ORP1tvOYPMcwz1KysP8U/
+h+CKFY2wZpKU+4hsnXs3WPiZEwQBJFl7Hl+5+Z6sP8Hh7/z5R+
+aL6sAaA3an7ExYv8eYrMRdX8OlFt3JK/STZRDT7ZHiJn6xvr+1f3i5MxQRBElnHw5C5RVy9z8Bg/gD
TU/JiJqCrnKTJFzT+ZCfNOXYNT82MYn335GX/GnyCILRIP3L9XlNbLGTzGDyAZNT9msvlr/inrYmp+bF
ofXf5IHIsJgiCyjE3xYD+P8QOYBzX/QgTKXe9lXcdVunt01H3qdtvDvaKGLa+I4eIv43TjQtFezN07lNnF
nGCU1cnqpVIaAhM3JyfmbbFzmdlCucSyS87SjTbtMM3NcEbPQAFijP6LKvSZfyE6Rs1q4t7vMQLGd
e7iOXEyJgiCyDK+9dwNosZequAxfgBzouZfDE/h5LvYFnwmo1PPAsyk7uvbOokepLlSvRbDxV9G+JdS
atI6Q+kLJmOhXvEd89wNTLxzuqVoslAudd0Yx1m6p2/dPJqx39vh3VajR0eK1PmXjFl1vbndI2ASJ98/
KU7GBEEQWcZ3H/2GqLSXJ3iMH8CcqPkXRNU8njJHX9J1j7qiCyWniTGMU4BJTQPVxaiumh5qhOql
GC7+Mki3kzkKzUS6RvYs1dFjx9yBvdxdaa+IJcyYy7MOp5mbvdKZ0ZfP5nQRW9aZos/8ZRNnENnB6
dE5AiaycXXjyI+PiJMxQRBElvHw974tiu1liH/9l39uvpEBIBU1/6KoKkYXMU4t5SmuOqurglNPOZoWd
Z/6xcpKOZB5RfUXw8Vfhvia6WvN3O026pVnYcYloc+OuRe81FBGq86ObgNflz5vmc4eXmtFdkyYYn
0p1D4yt/D89cvQ9FUDzxD1pc4RMKErf77Cn/EnCGIrxA1ndjx5z35Rci82/ukfnmy+iwFgDtT8CyMK
H1VK6erLU1sVAr2MRrpeCtdxTa+qQfXz0dUPq17tFd1dDBd/GeJdi7hoD6Vu+oRyebPocdXVflPW+d
tWsY72dHUDXxfPLJ1mnuySN2Pn2jzJw/yL6jF//bplJbQHttVDdI2AaV26cmnnqzvF4ZggCCK/WKoH
+184euTf//Sn5osYAOZAzb84qq6pihlVBrXFmr88E+VVjwLMp+lWtKhbFz/Ul/QV3VsMF38Z4l2LuGg
PpW76hHJ5szhT7Ddlz1huR31FcBsYufqM7F9JoSOjL58lOHCra1Hz7ExzXw3ho4aIj4DJnf/0vDgZEwR
BZBn/9ZFvitp7UfFvv/lN8xUMAPOh5l+kpvYpqidV4Rgljb88Uw2bq70KMI+6n/qf96ve1SV1pR1QDB
d/GeJdi7hoD+Xt0cHfR42rrvabsmcs2VE1KdSt3JF9uXqMHFp9Z0ZfPktg4Fb3ovrMXzOGq9SdOmdh
8I+ARXjply+JkzFBEER+sePs9hP33irK7+mDx/gBDIiaf6GaguboykpdNJk1ky6kzCpHVlee6kkXSaG6r9
SM4z7GX18xxhMZ4y9DfM30tSaX3abnwBY5YsUZqN/Ino0VHd0W7s77cvV5yzxtCt0ZO9cWmpAas
juFp4nbplBcM17ZeTunWYmNgAV55L1HxOGYIAgiv7jpxWufOni7KMKnDB7jBzAsav7F0tVSRZQz+q
Yqr3Tdo+stfUX1NQaMFkdtR19Xo54TtVb8ZZAzTyN/kyw0suqhpxdJ1WPHZJ4ANVJ4J+R82gm3I/tyt
e2ai8a+q2ae7IXujL58Nie7faHHopwRnPnrC+30xYLCY/QdwXMBE9i4unHo7UPicEwQBJFf7Hv2RlG
HTxY8xg9gcNT8C6arm4Kqqlrm3ZZZ5RhFmeQOZ2o7OlWUP0E9XPxlRHiiTTZnKH+PjkQz7Fh0JE9F
KTv6U5Xake029fXwTuiOnuylzox91hZI36TqsagZ5i+10/IP0t7vHiGwRxjb2vra7jd2i8MxQRBEfnHkxC
2iGp8meIwfwOCo+RdNFzeh2sWujtqaRzMbFLf1S09Tk9vO21NcjL+M040LRXtRtMWzV3qWd3YnO
bN+U/ZUlJ6OZmVaXlWvjaHNJnowc4ZFW2dkT/ZGPGO/tckdEmniKSpd8y+Zw5TcKdmziK+1ZI+g7rr
9MLYLaxf4M/4EQWQfO85uf+zwbaIgHzs+
+ck7zVctAAyHmn/RVOFD6QJgs3jrt2+JwzFBEER+seuF6545cIcoy8eLN5871XzJAsCgqPkXS/9PnZT8
ADaT0x+fFodjgiCI/OLOp28WlflIcebY9//y1VfNNywADIqaf1Hs/3DZ/Q+fAWC5HXv3mDgcEwRB5Bf
3Hd8j6vPB49Tdd31+6VLz3QoAQ6PmXxSj5qfgB7AJrW+s71/dLw7HBEEQmcUED/bzGD+AUVHzAw
ASffblZ/wZf4Igso9v/uPfjfdgP4/xAxgbNT8AIN1Hlz8Sh2OCIIj84uDJXaJWHyR4jB/ABKj5AQBzOXfxn
DgcEwRB5BcP3L9XVOxzBo/xA5gGNT8AYF4n3z8pDscEQRCZxfUvX/PEoX2ibp8neIwfwDSo+QEA89
q4unHkx0fE+ZggCCKz2PP8zqEe7H/nzA+bL1AAGBk1PwBgAFf+fIU/408QRPZxz+O7RfWeEK8cf/Cv

X3/dfHsCwMio+QEAw7h05dLOV3eK8zFBEERm8fD3vi1q+Jni1N13/fHz3zffmwAwPmp+AMBgzn9
6XhyOCYIgMosbzux48p79opLvH7/+2fvNNyYATIKaHwAwpJd+
+ZI4HxMEQWQW33ruBlHJ9wwe4wcwPWp+AMDAHnnvEXE+JgiCyCy++
+g3RD3fGTzGD2AhqPkBAAPbuLpx6O1D4nxMEASRWcz0YD+P8QNYFGp+AMDw1tbXdr+xW5yPC
YIgcoqbXrz2qYO3i9o+FDzGD2BRqPkBAKO4sHaBP+NPEETese/ZG0Vt743//T/PNt+MADA5an4AwF
je+u1b4nxMEASRWRw5cYuo8EW89vijPMYPYIGo+QEAIzr98WlxPiYIgsgpdpzdfuLeW0Wdr+MHh7
/z5R+
+aL4QAWARqPkBAOM69u4xcUQmCILIKSIP9v/uF580X4UAsCDU/ACAca1vrO9f3S+OyARBEDnFnU
/fLKr9It577dXmexAAFoeaHwAwus+
+/Iw/408QRN5x3/E9ZsHPY/wAlgQ1PwBgCh9d/kicjwmCIHKKHWe3P3b4zF+AMuDmh8AMJF
zF8+JIzJBEEROseuF6545cEdR8/MYP4DlQc0PAJiOOB8TBEFkFnc+fTOP8QNYKtT8AICJbNs20T90SJS
MRMlIlIxEyUiUjETJ8ku0FbCVAICJcCJJRqJkJEpGomQkSkaiZCRCBFsJAJgIJ5JkJEpGomQkSkaiZCRKRiJ
EsJUAgIlwIklGomQkSkaiZCRKRqJkJEIEWwkAmAgnkmQkSkaiZCRKRqJkJEpGIkSwlQCAiXAiSUaiZCR
KRqJkJEpGomQkQgRbCQCYCCeSZCRKRqJkJEpGomQkSkYiRLCVAICJcCJJRqJkJEpGomQkSkaiZCRCB
FsJAJgIJ5JkJEpGomQkSkaiZCRKRiJEsJUAgIlwIklGomQkSkaiZCRKRqJkJEIEWwkAmAgnkmQkSkaiZC
RKRqJkJEpGIkSwlQCAiXAiSUaiZCRKRqJkJEpGomQkQgRbCQCYCCeSZCRKRqJkJEpGomQkSkYiRLCV
AICJcCJJRqJkJEpGomQkSkaiZCRCBFsJAJgIJ5JkJEpGomQkSkaiZCRKRiJEsJUAgIlwIklGomQkSkaiZCR
KRqJkJEIEWwkAmAgnkmQkSkaiZCRKRqJkJEpGIkSwlQCAiXAiSUaiZCRKRqJkJEpGomQkQgRbCQCY
CCeSZCRKRqJkJEpGomQkSkYiRLCVAICJcCJJRqJkJEpGomQkSkaiZCRCBFsJAJgIJ5JkJEpGomQkSkaiZ
CRKRiJEsJUAgIlwIklGomQkSkaiZCRKRqJkJEIEWwkAmAgnkmQkSkaiZCRKRqJkJEpGIkSwlQCAiXAiS
UaiZCRKRqJkJEpGomQkQgRbCQCYCCeSZCRKRqJkJEpGomQkSkYiRLCVAICJcCJJRqJkJEpGomQkSka
iZCRCBFsJAJgIJ5JkJEpGomQkSkaiZCRKRiJEsJUAAAAAAOSJmh8AAAAAgDxR8wMAAAAAkCdqfgAA
AAAA8kTNDwAAAABAnqj5AQAAAADIEzU/AAAAAAB5ouYHAAAAAACBP1PwAAAAAAOSJmh8AAA
AAgDxR8wMAAAAAkCdqfgAAZvXhyrbWyofNVQAAgGVDzQ8AyEhdjI9bhV9ePdpmGDPh1P9moV
xY4ejq5ebCkKzFVPh3JbOy9nCct2l0gV+Y5rNXG/aTEfkVjdyalX8o6x0bdlnjiu7MqF8UIwovarO+T4E
VWcsZ6m3qkWuTfidtEdT8AIBxjXiatxh5pjy0VWnHOOsUZym9jupcNfKBqlzI0ZWV4v8ZJU+5hMmP
hOZ5dPhPhfXJbo306avWMtEnwty34ZIEf0Pt3yF7oXMJZozemlVoqPK6vlBv6byp/KOYb9fcKTp3prw
/6BeFb1HWkirzrSuyqOrWgL9K7nKM5KaRVlTl15eqV/OurI+uAVLVe9cQ+2PtoriHHqj5AQAjss9T9m
lkUCpR9f9PeSCwVziW0bOUCZrtGydN+d6Pvk2mad4YS5VypA+fHHu8XEOf4xvq/XAnLj8aQy0tnDF
2a1Z9h1Ltmpczq/rX7CzF9ukL879dXcsprze358pTiy1qiPEbkUV5LqULkeYP2l4RfLKiLnUjeblvO9aO
Zwevvokty/tVNXNuda0FVHzAwDGI88AzqlhcONnEKY5foy8LD18+UP6oS1mvuPgzCb/HBTG/SjYGzj
a+qqBzTdq6DfOmbm7lIE3MrJXA25j11DV/eSNVL3HzWLwJ9JXyx/mTqMm68k1zteFm2iQdTQiy7E
N+Pnu3LrOyfTnDOWsY8BkejerF/LjMGSiLYOaHwAwGvefzAOedvwmPgxU6xnqzBhkHX+GZ+xZ+e
M4iaqdao28Z+OtI2jkN6lQb2GRov5hnE+58xtqfDoG4YxXZrT3beCUkeEGzNQ1lLOxSbqyDLYg30DG
tfLHwT7snlzuh2IIbqIqT/mggjZ/Vt/WGarbQ63Nm6v6qJUp6h/CU5mNkyuwncMszdwmf6LBFrZFU
PMDAEbjngC8R5QhjZ7AVB08hjq9uaq1NEZcUrkKNXyZcrwFKfXCxsxTffIGPsrHTfPBqz5xlbFSuetwf4
vn4iRwxx94LyPDDZgpPlT1xg2wi/Es1d1h3itPonIN6kp5e7APRSCXYaBUbiLxvlQNwvvbj2c5hvjdWY
VGa/dvsFSeXNUVuXsDvVfVCpps5c/2sMPu4tZAzQ8AGM0C/lE93VlAnBZHNehhylYuo92vMtEka3I/
GoMSb069f2N+LKqEo37uzDXUP4+Trxpbbt2Ab1U1oDlz96PgNJlPZLgBM8WGEh/HOXizVBcbQ22b
k6hcQ/u6vD3YhyK2dYV6eUNkcxM5H74BfpGjyxlgfJMnV71d9bX654HyeddVrUdZWbE+JHOohtVv
jPMuxfcYXtT8AIDRLOAf1ROdBeqTzoRnjvrwNnxC68hmGOKEHVPlHW//nE/euPmqd2fULXMzjLgi6
0Mx3Dm+5vyGur+yzrs3HzeBFrk1q+BQ1W4OtZyOCVe3B0kmE1mfCcMYuVwDfSLcRM7A3XPpFB
miujXUZ6Hk5HIzVO/cfCuqdW9N2WKI1clfGfft754LJGp+AMBo3H8wD3R2C5viLFDlmPjAUeUcdec
qZZrxs4y/nDGO8mGDnarD3BSjrsgw9EfCnbd8s4ZeWWS8AVP5h6retwEX0znhqsEACTsSlbcH+1R0

Lmqo3zA3UXXFXIj8MCYIL2egZRicXG6K7t3tqXOgssH8H4pqBfYwbuYB3qYth5ofADAe+U/mwY4fQ
eNnqM8k0uDnjyKLsQzPOWgUw5zaXMW4xrDVmzTqu1RlMBcy5hmxenPG/cy5n4BJkhYG/0RUb4
09cWttzls3N09GJXJrVp6hqkvDvkmdE64aDLB9HYnK24O9SQtdlPWLNMhvVXA5I/zOurmsX6bCcEn
jb9Mw71E1WzdJedkcOz4VeFHzAwBGZB1AhjkV+FVjC5v7TCBWNM1iyqSjvEOTr8Y66g537nWN+a
m21edhbYJPxJBrE5+ASrsEc21DbWYkY3wyMwkPJd6v2pyLq7JZMy2yGK+rnPPk6LkzZbPBPvTOooo
LxuD1lDyT6C2+KOvuPGkaznJq1eUJPtuDf1GEc9l35t66ejjvMNbnetCd3Dqo+QEA4zIPIPxzGpOxDq
Rzn0dDqo/3aKMvwMDneAzCfldqzXsjbm2idyy8qE25psh7VMjui2Jw4l9V1NojAweJOVHzAwAAAA
CQJ2p+AAAAAADyRM0PAAAAAECeqPkBAAAAAMgTNT8AAAAAAHmi5gcAAAAAIE/U/AAAAAAAA5
ImaHwAAAACAPFHzAwAAAACQJ2p+AAAAAADyRM0PAAAAAECeqPkBAAAAAMgTNT8AAAAAAH
mi5gcAAAAAIE/U/AAAAAAAA5ImaHwAAAACAPFHzAwAAAACQJ2p+AAAAAADyRM0PAAAAAECeq
PkBAAAAAMgTNT8AAAAAAHmi5gcAAAAAIE/U/AAAAAAAA5ImaHwAAAACAPFHzAwAAAACQJ2p+
AAAAAADyRM0PAAAAAECeqPkBAAAAAMgTNT8AAAAAAHmi5gcAAAAAIE/U/AAAAAAAA5ImaHw
AAAACAPFHzAwAALJcPV7Y1Vj5sLtXUjaOrl5sr41B5RPpl0O5NwdmHy6tHQ7ei9wAgX9T8AAAAy8
Woa+3ydKvX/G3ZXvFtQ7Cybzd1Cf9NBgCMh5ofAABguVj/W7ZZoW71mr/X+r3FffuvAqj4AWwx1P
wAAADLxar5zQJ3i9f8unDvmJfeP71P+goVP4Ath5ofAABgudg1v1GoOjW/c8EtjJsmZQtj3KaDccWsh
tXl4prxn9MbSRTjrnPfmFubpqPmtsazBjRmqkTG0q2rNvqVs4LY/Ev2fESTyAKj/QBgUtT8AAAAy6UtJl
frn1TJaFSZ1Wv3glFuNiVxW4520kV0uI9RZ8u6tmJMxDNItPT1J5VLNxhzcbSTW1lVP4rsXfPvmFB4g
f5+0ekCwHio+QEAAJaLKhqLClIVpnXBaNyoGroXYjV/08goSeUVVZY6fdor6oJMo1voqRh5epS74QR
6QDdnjG7caIepdM5fNQjPJ7DA0EByBgAwDWp+AACA5WJVjc0L82e37DTKSVnMqiZtXRq+ooZxW8
g2siIuyEt6EKNNkJxBSa7EvRCnJ1ASXXrMX3Ky+xeom/VaNwCMjpofAABgufjL66LUdCpjT6ksS1O3Sf
cV9doslO1xVQsfkdgaJcCdUkmlVFfl0jqFp9Bj/ga7tbofGF3PsmUvCwAmRc0PAACwXGQF3LzWj/e3
NaRsWRir5rcvqhc+wcRh/rZyKck1vzuFHvP3Fe8VdT84eqBj31kDwKCo+QEAAJaLU0zKIlLf8JSdsjB2
m3Rf8QwrxvW1EHo00fxtVUp1VS6tU3gK3ZNTLQp1Iyd75xjGEJVwMgAYDzU/AADAcvEUk3b5qG/
EWg5Q85vFtd2mR/ntpgnTw5mNnRwD1vydQ7ldnU2JLLC4ZQw887wBYEDU/AAAAMvFV0zqurGk
b7RXm3JSF6bOFbd4DV8Jj6KbhDOrwtZNExNO0PafuXaOTKFr/nI5bfuOBToLiU4DAEZHzQ8AALBcO
opJ84ZRikqilo2VoPKKfn1Ujq7q3YI/ddvATROn2tvM3kPW/J3z90+npJqERg/17DtrABgUNT8AAMBy
CRSTbZFq3TBr16KslIWxO1j3FfXaHM6ZTsmum+0GgVVE2ePJIlkurVPnFGLzb7uXypTGtpi3faObXUt
9ZwwAg6PmBwAAAAAgT9T8AAAAAADkiZofAAAAAIA8UfMDAAAAAJAnan4AAAAAAPJEzQ8AAAAA
AQJ6o+QEAAAAAyBM1PwAAAAAAeaLmBwAAAAAgT9T8AAAAAADkiZofAAAAAIA8UfMDAAAAAJ
Anan4AAAAAAPJEzQ8AAAAAQI7+9rf/AFPHm7jIjUyYAAAAAElFTkSuQmCC]]></redcap:Attachmen
t>
</ItemDef>
<ItemDef OID="b18" Name="b18" DataType="text" Length="999" redcap:Variable="b18"
redcap:FieldType="descriptive" redcap:BranchingLogic="[q16] = '1'"
redcap:InlineImage="1">
<Question><TranslatedText></TranslatedText></Question>
<redcap:Attachment DocName="B.png" MimeType="image/png"><!
[CDATA[iVBORw0KGgoAAAANSUhEUgAABVEAAAEJCAYAAACdcmdaAAAAAXNSR0IArs4c6QAAAA
RnQU1BAACxjwv8YQUAAAAJcEhZcwAAEnQAABJ0Ad5mH3gAAE9sSURBVHhe7d0PjCRnfef/b+/aY
PPHNmBskx+RHat7sZYlGJIg08PxL4egZ+6SJbnbYOVg5Oh3PVyi0zQSkwOxF+mX2wguG4lunS5iWrq
zliQyTEJugrLdlJRgckzHR4IxYTI/Z7rlc3T8wDYGjPljB3u3f/VUPVX1VHd1dVV3VXX39PtItaerpfqfqT/T
O/OZ5/k+hb5FAAAAAAAAAAAChjumPAAAAAAAAAAAIAQhKgAAAAAAAAAAAEiEQFQAQAAAAAAAAAAAAgKAAAAAAAAAAAAAAiECgEAAAAAAAAAAAEEQlQAQAAAAAAAAAAAAgKAAAAAAAAAAAAAAIhCiAgAAAAAAAAAAAAAAAAABCEBUAAAAAAAAAAAAAIhCiAgAAAAAAAAAAAgAAAAAEAEQlQAAAAAAAAAAAAAiECICg

AAAAAAAAARCFEBAAAAAAAAAIAIhKgAAAAAAAABEIEQFAAAAAAAAgAiEqAAAAAAAAAAQgRAVA
AAAAAAAACIQogIAAAAAAABABEJUAAAAAAAAAIhAiAoAAAAAAAAAEQhRAQAAAAAAACACISoA
AAAAAAAARCBEBQAAAAAAAIAIhKgAAAAAAAAAEIEQFQAAAAAAAAiEKICAAAAAAAAQARCVAA
AAAAAAACIQIgKAAAAAAAAABEIUQEAAAAAAAAgAiEqAAAAAAAAAEQgRAUAAAAAAACACISoAA
AAAAAAABCBEBUAAAAAAAAAIhCiAgAAAAAAAEAEQlQAAAAAAAAAiECICgAAAAAAAARCFEBAA
AAAAAAIAIhKgAAAAAAAABEIEQFAAAAAAAAgAiEqAAAAAAAAAAQgRAVAAAAAAAAACIQogIAAA
AAAABABEJUAAAAAAAAAIhAiLp0etJYKUih4DxWGj29PqjXWPFeU1hpWF+1iMxj3ZB2onXjpLWdL
MxLO7LSlg372PzHqPs4Vb2GrGzEO5vtDbdtK5JH08KNuQ9Cjmc+2g0AAAAAwPwhRF06Rdm8UJey
XurU1kPCkracr3X0c5Hq2U3rq4B5oILBVWnqJUdZzqxle4faf1Qo1cT/rlhsR+14AAAAAADIGiHqMip
uytmqfi4dqZ0P9kbrNc75IVW1JdsV/XzhFGVzry/9vnpsS/aHkff+RpmXdmShKwdu8leuS9c+xj3ZzDJ
D7TVk3fijwsI7ascDAAAAAEAOCFGXVGW7JV6O2jxn9EY1e6FWpbW4CSqOot6h7OuncuoEPaQBA
AAAAEAuCFGXVkW2W26M6vdGbW/4Q6XL9a3wXoyqlqKuRek+wmpSjqyvaH59ZI1Jo6ZjoC7rqPX
GPu31edcGHd5fZI1J4zwEz5+5nbDPjxN+3INtCdS9tR4xy30ObGewraPOc7xjCm57uPZpYAh6c9VZF
2h4snM3eA7Uw9yc3Z6x+xzPPy71CLkXbAnaHvI9OHq7vqTHE6/dAAAAAAAcfYSoy6yyJXW3OGpz
VTYaDTnnJ6hyIWSM9FAIo3VqpQwmoCrK2hndwM6BdJ1nFmNId2dHLno7bcuubn/5zNpc9FKsnPa
D6gP/AGy9izv6PBo1Pe1wrCSDo63TPb8dqZUKUhrYSXM1aUimtjPY1qasDoZtEx2T2rZR+9S6H+te1
+kREu3HCSwHz4HSXB0dKibnnOtV70AUtW6Kc9TeGFHPNGS7E4vZbgAAAAAAlgQh6lILTjLVrLnBTF
nqF0Imk2pv+KGKV4+yL103ie3UZD3lhKW4dka3b18O3U23d42JhYxw0hjqferEnAz0rpz2yiY0d4M9
Ji/u6BisfEacDLUnjXX3GlSlpc9v3+0xnOr59bfvdUhWIdlAfdzx/O1494Hazrob/E1zTMbr9zZlc9v62PX
vV1Wv1/6cXXIi2X56jXUvsCzXu85r+8MlLiqR+4wr/BzteOl/krZbr3X/0hH2PRjY7rBkxzOu3QAAAAA
ALI+C9QtyXz/HklK9SwM9zlSwEhKq+K8rS71rTubTk8aK24tOBS/OZEYjX6963bk96Ubsy+dvW4Vde
9ZG1BBs1YOwWq1Ks9kcWu+3IaxdcdeNE387w+2yV3rnwG1/6DqbsV0VnO2FBNwB4e0wr3O11fc
nDEt0PSK2E9ivvuaS7JjMbQdfr41qa6JzF35+FOdanQqsS3p+lETnOlHbRwht44jjjDietO4RYBr3Pnyvv
OXCW/QSACAP99/xLnnNW2/SSwCAzB3/NZFjr9ILWBT0REVwkikVtoQGJP5Qeb/npMsYdm/2GE1F
UU6ccp51dJfTrj2WvywnT5+0e9QF11uqp/0AbA74vWmb4nVG7R44gZT1GW8ov7dOdT4sGbUo3S
BMfcIsazBr1jUo6ac28z7QPYSnOKZEvYkT7cec4f+kmIdQ3NyTvhmgTm3wHIWY6rrrurFuwJmaGO0
GMvDwEw/LOz/5Tr0EAMjDDY9fJXd/6AF5/Bs/0msAAJm7dI9I/zt6AYuCEBVBA6FScsO1P6fl1RVt7k
pbenJoj9k/JScqJ6z/D65XGeqc9ZIrrombLbpD+ttuIl09a/TozUtaAZl1DTJrOyHeSKpXqBe0GnVjgQX3x
NNPSOWPKvZHAEA+nvPjY/KWL94oP3ryGWm+/3555p8u6c8AALL1lMilP7A+PussYiEQoiJlGYRfXl3
RfTlsXxS7lKgd9lbEyVeN9XMZvhk9NO3A1+/VOyrwVcO67VqVQ480e0lOK1mv47yOaTHOXbjxbW/
Lhtnr1K2LatY5BRbUnZ+6Ux58/EG9BADIwxv/
+gZ5wQ+vsJ8/9NXvyp/WeR8GgNz0Hxa5/Od6AYuAEBUxleSkN1LbnBFfMSZJitM70Ri+HI+7744c7D
pf686+X7I/4a8fLjUwH4qbZ/0guOFOjFWVQIZacsoTKG6Jgvk22OvYvA/0seV1TIn2Y97LA8Pk1cz3hZ
xnoE/SdmNSNTtwdeukJv6eAubL+z77PvlM7zN6CQCQh1c+eK3c/PXn6yXH5z7+kHzlLx/RSwCAzF3
6gsjlr+kFzDtCVMRUlM2zbuVUc/Z1NbrYnOl8y+vt5wScijnre1s23JlrYvN7cqqJpBS3XmZRF0x117vh
6vxxe81a56Kmj3+wdqsx7F+aq7LhnTIV7Onh297K+dBcNQLH9nm/hqd7bHkdU6L9mLVbm7Lqre/p
me+ta1Qq+NvI2oTnyA9c3XYDi+kT+5+Q+n11vQQAyIOqg/pzD7xELwX94W9/Tb73raf1EgAgc5f/RK
T/fb2AeUaIivgq29LyctSalHTA48w8bynX5YJR4NOfUMnSXNWB0GQ1HAPbsp55Q/a9of6KMUnTHP
Jqu9rKUt8KRKiWomxe8IdlN1d1gOaFzqMm/ZolJ3AMttM8tryOKdl+ipsXpO6/2HmtOZGTdS/7h6Br
7yrua1NNWBO03bzfw9odR+bHA8R339fvk7v+7C69BADIw/FLBXsYv/oYRgWozd+8Xy8BADLXf1Lk
8sf1AuYZISoSqWyH11+stqz17tBiV3FT9gZfW21NVr/R7K0XKBlgDM3OdKKjFJgB2KiyA+qc9bt+wOe
ya1/OW03PstS7fT9Yt1Wl1d8LTpaV1zEl2k9RNvf60h16sXq5MUzeVpHt4EGK7B96PbFTEbvtVlv6Lf
8+sqlzblwHu+5ulByOB4jBnYn/6Wfp7QQAeVr50kvl2iev1Evh/uFL35ZP//6hXgIAZO6y9RvZJcpbzbt
CX81aAgAxtTcK4nSSVCHqQGAKADGo4PT1/+318sAjD+g1AIA8lB56od0LNa6tu8vyiteFD/sHAGTgit

8QKfBL9ryiJyoAAMiVGsJPgAoA+VK9T1Uv1CTUsH7qowJAji79AfVR5xghKgAAyM1HvvgRezIpAEB+
VP3Tt33hZSProI6iAtS7/+NX9RIAIHN2fdR79ALmDSEqAADIxe6Du/LBv/igXgIA5OWOL18/tg7qKPv/
8zH53Mcf0ksAgMxd/n9FLn1BL2CeUBMVAABkTg3fV3VQmUgKAPJ189efL//8Czfppclc+dzjsnX36+
XWV79IrwEAZOtKXR/1Zr2MeUBPVAAAkKknnn6CmfgBYAZe8MMrEk0kNcoz/3RJmu+/X3705DN6
DQAgW9b77aWPWx+fchYxFwhRAQBAZlRwqgLUh594WK8BAORB1T99yxdvlOf8OJ1f+R7/xo/k7g8
xKSAA5Kb/HZFL1EedJ4SoAAAgM+/77Pvk3ofv1UsAgLz83AMvkRsev0ovpeMrf/mI3PtJ/igGALm5/
DXr0dELmDVC1KXWlo1CQQrGY6XR05/LSa8hKxttveBob7jtWZG8mzM3Qs5LHL3GinPuVhoy6tT55
9d55H7NjwLuWyCW+n11+djffkwvAQDy8vJvPE9e+eC1eildn/zPB/LN//19vQQAyNylXZH+Y3oBs0S
IurR60lhZlaZecpTlzFpRP8+eHfiVasLfVIImPi+9hqzXor5KXfOCrAYvunRqJSlMENguK+5bIB7V+1T1Qg
UA5Ovqp47LG++bvg7qKKo+6u//
+y/bHwEAeVD1Uf+79fFZZxEzQ4i6tLpy4KZA5bp0+33p9/dkM68MdWzgt6QmPS+qZ+S4YK99XtxN
V1vqevelWy87K5qrQo4aA/ctEIuqf6rqoAIA8veWvRvtIDVLqifqH/721/QSACBz/UdFLv2xXsCsEKIuq
96h7OuncuqE5Nf/FGmL1zOyJ41zugtquS5bFedpcfOsVJ2n0twlRQUwPTUTf+WPKvZHAEC+Xvu1F8
vLHr1aL2Vrb/f/2A8AQE4uf8l5YGYIUZeQXbvRDN2aq04tx8GuiKp3o1E7c1T9zGAtyGCd1bDejbH3r
wVreI6qN+kMVfdfl6zW52A9S6+2qH6ENy3GPgPncEP8zZjnydln0vOiqHaWYvWMNHoeB0LzkpzUn
VFl/9A6olGM9obVW21v6GMZPAcJrkvI/RZ2vePcb4PXz35E1ImNY5b37WT3p2vSa2eZ6D1Ar1TMr
49uJI6QOz91pzz4+IN6CQCQFxWevubvXqSX8qF6o1IfFQBydOlPqI86Q4SoCDUUGGl2/cyRYVRHai
WjzqrR43EyanuDNTzVurCgpuQNVXdFt3UUZ5+DwWRzdcJ9Fjflgjtk3joz5/RGeo1z3nkq1y+kUEahK
q1+y+tVOsTseTxK50C6+umwipx2N97ZkYsDJ7W96x2NX1c3yXVRQV7I/eZcj1EBZPj9pu7d0GC5U5
NSIMjOSpb3bcz7M2CCa2eZ7D0Ay07VQP1M7zN6CQCQFzV8Xw3jz5uqi3r3h75KfVQAyI2qj3qP9Z
H6qLNAiLqEKtt96Xfr4kZ7Um3Z9TH72zrxbG/4AZBXL9Won9mpyfrIxEaFec7r+3uboWUCxu4/wN+
et3/pyI6XBPWkse4GPca+Wzo1imzrKP523M2ofdbOu/Fbsn0WNy+If+rOS9v677wbglnn94JOUJOd
F4P9um2ZLK8uyolT+ukYlS23beb5V9ri53BnxMnhkpwj67VGqYGh+21ofyZj2/b9ZrYlbFt+kJ3U/Ny3
/uvD789hya6dZar3ACyrT+x/wp6NHwCQv5UvvTTzOqijPPTV78qf1hmBAAC56T8scvnP9QLyRIiKIW
bPtPoFPwgdDgOHletbE4Z54aotPxwsrp3xAqzOge4z2bsoO14eaey7suW3deeiJIl7zH364ZMh8T6Ls
nnWTbuaslpwe08Gz+8kipt740PW2PblMOpEFdfkTNjxtXf9XrVn1pzjSXSOrPOzp0NEI3gPvd4Dhu43
s8et0evSPk86CNzLYfa0LO/bsfdnmCTXzjLNewCW031fv0/u+rO79BIAIE+vfPBaufnrz9dLs/G5jz8kX/
nLR/QSACBzl74gcpkJ/vJGiIoBI3qm2Yqy5iYxIwK3UyfSDKjKcrKkn47SPdC9+VSoU/LqNhbMYdKRw9
QnMMk+K9tGr0FHOsP403RKoi+fcf2NgNIP3Kpy1j2gqa6LruEZOrw/aOh+M8JCawf20Hd33yNrsaZ
uHu/bBNduyvcALB93Jv6nn31arwEA5OWGx6+Sn3vgJXpptu7+0APy+Dd+pJcAAJlTw/r739ELyAMh
KibUkeHOgTHCo7mX3TFUvMKUSrD+5Oz05HBssVSf36vSHRZuBG7V08FeoUmYExB5PXXHCbtWR
dm8EN470w0rF3t+o8nvz/SvXdh7AJaNCk5VgPrID+h9BAB5e86Pj8lbvnijHL9U0Gtm60dPPiPN99+v
lwAA2XtK5NIf6OfIAyEqJjR/gWm53vWGbAcfk9YLHS/
+Po26n7aO1NZznJyneELGlj4tn5Sxl7S4KW5lAntYuDF8vno6/CyPP0dt2TB7nbo1OM36o0lYbdzT+xj
s/as0z83XpEi53bcTXLtoR+GPJpiWGsL/wCMP6CUAQJ7e+Nc3yAt+eIVemg+qPuqnf/9QLwEAMqfq
o15iYte8EKJiQElOusnV0EzePbnoFnIcO/Q7J6WTXtA2qm5m6ibYZ6+xrodpl6Ved5OsPCfnMa5rc1f8
zphdOfAu6QmJc0m9HrXW/XH+/I4OP6sSyOGSnCOzLqcKFN26qMaQ96TUzPKqx6k9GZQdSHa9W
p7pDpOf0CzuW0usa5fme8AU1xDz7yNf/Ig9mRQAIH+lh1448zqoo3z6v/6D/MOXvq2XAACZu/xZkf
48dRU6ughRMcCcBCnYW9IPAlXYNTChz6yYNTCbq/5Q7faGHhpuPdIev510n72GrLsnrnpWNjeNyY
Nq65JPjmrWsmzKrm5er3HOCzBj90asnBbnDulIs+ke18Bw8Amvix8oDvbcjUlvX80sH+xxaoTFZo9b
oz1p3yaRZnHfKnGu3QTvASU/dZXaee9gZMOb4h9Hze6Du/LBv/igXgIA5OnaJ6+0Z+OfZ83fvF+
+9y1qZQNAbtSw/v739QKyQoiKYeYkSJ2alHSoU/LTE7kw7YxI5vDy5uoUoVGwBmZzVQdQXnhTlVZ
qs9e7kuyzJ411d6h6Wepbav3okCq98zLMnFndbbN3TastiX+aKhIo72oZDmATnCMv2LO4x2xOsJTEi
HvXrLNaPevPNp/YQt+3SpxrZ0n4HuDXW7V41zBubVssGjV8/85P3amXAAB5UvVP3/aFl81NHdRRV

ICqglQAQE76T4pc/rheQFYIURHKHgYdUpOy2rLWu8Otp1KR7cGClfuHRs/BBOwamMZwbZddWzOj
eqgx92n23LN7obonbiCk8of1p3hehhRlc2+4Tqg9hD5hYBeYJMs6ZjsbHhT7uljH3G/5QaqtKq2+0dZ
ACYJo9r07eJA2Z5vTZZMLft9aYl07S6L3AHUsg6+ttiava4u59cTTTzATPwDMkOqBqnqiLgI1pJ/6qAC
Qo8vWb6bUR81Uoa8KBgJAEmo2fT0ZlAph96btmTxL+lhOtaYNWBfEUbp2yJUKTit/VJF7H75XrwE
A5EnVQVWTSS2SK597XLbufr3c+uoX6TUAgGxdKXLFb4gUbtbLSBM9UQEkFCxRcGZtsUO49nnrWC
J6ZB4tR+vaIV/v+
+z7CFABYEbULPx3fPl6vbQ4nvmnS9J8//3yoyef0WsAANmy3m8vqWH9TzmLSBUhKoCY2rJh17o06
pWaJQoWUK+xIqv7demmUqJinh29a4d81e+ry8f+9mN6CQCQJ1X/9C1fvFGe8+PF/NXt8W/8SO7+
0AN6CQCQuf53RC7doxeQJkJUAJNRNS8XfPx7cXMvpRq/C+YIXDvkR/U+Vb1QAQCz8XMPvERueP
wqvbSYvvKXj8jnPv6QXgIAZO7y10QufUEvIC3URAUAAKEefuJhec32a+wJpQAA+bv568+Xf/6Fm/TS
YqM+KgDkjfqoaaMnKgAAGKKCUzWRFAEqAMyGqoO6aBNJRVH1Ue/
+0FftjwCAPKj6qGpY/7POIqZGiAoAAIbc+ak75cHHH9RLAIC8qQB1UeugjvLN//19+cPf/ppeAgBkrv+
oyKU/1guYFiEqAAAIUDVQP9P7jF4CAOTttV97sbzs0av10tGyt/t/7AcAICeXv+Q8MDVCVCTUk8aKm
uVbPTaknWjdOGltZ96MOYZeQ1Y2gmvbG+7rV6TR0yunpfZjb3NEO2zTne9M2m1bkPugvaHbWJC
BSwosjE/sf8KejR8AMBsqPH3N3x3tuqGqN6rqlQoAyMmlPxHpP6YXMClCVGCGeo0VKZRq0tHLWe
pd3DH205RdQj4AA+77+n1y15/dpZcAAHm7+qnj8pa9G/XS0aXqov7+v/8y9VEBIDeqPup/tz5SH3U
ahKhIqCibe33p99VjWyp6bXby3l8WRhxDryHrtTziU6UnF3eC+2qea1hrF8VRuA+A+aZm4n/nJ98pT
z/7tF4DAMibClBVkLoMVE/UT/7nA70EAMicXR91Vy9gEoSowDLoXRQ3Qy2Xy86Tzo5cXJwUdTFUt
qXfrYs+w8DCUMGpClAf+cEjeg0AIG8//ffX2UP5l8m9n3xYvvKX/NsDALm5vGc9mOBvUoSoS8eoLbl
i9kQctd6oc2mvz7s25fD+IutuGnU/VwKfNLcT9vko6Z8z+/PmMP7mqvP5EYU0/WNWj+T1Rv2h/FU
5e+GMDvk6shMnRQ3UUg17jG5PvHbHuTbD59DaumyYrzfqkQa2MbA+/BTHaYN5POpY/P2rh7vd
9nnrupbrsjXQXdYu3WC83n4M3DfArKgh/A888oBeAgDk7YbHr7Ink1pGd3/oAXn8Gz/SSwCAzF26R
6T/Hb2AJAhRl05R1s64PREPpOs8s3TlwE30Aj0U27LbdJ6Vz6xZXz17ldNV/awjB/4B2PywsCxn1nRr7
RCwJIMj5zu1UswQa5bnrCO1UkFW9fYcal2SINUYyl89LZXimniHs3Nx+hCvfEbcU+2L2e6pr43Dfn1w
Z846FVQOrG+uDgSpE7VBHcuqeFvWoakKSlf369Ld2wxcdxW+lsJKN3RqUprnibKwFD7yxY/Yk0kBA
GbjOT8+Jm/54o1y/FJBr1kuP3ryGWm+/37qowJAbp4SufQH1kfqoyZFiLqEimtuT8R9OXQTovauHwi
JEU72Dq1XOU6dmIcI1VI5LW6M2gzMjmSEhV6w15PGutvjsyotu6am9WjpLXRqsh4jjUz7nFW2rTa
Yw76rLadd22HVPv12d+vuV8TsRaoYQ/mrp9X2zVA4xpD+4qbsuefNfnTFa4Z1BPULwcDQN67d6V
wbV7neDX69Frbev2+maYPxeh2aFjf3vOc+P1RXYWtXf41/TppyLsFxAmnafXBXPvgXH9RLAIBZeONf
3yAv+OEVemk5PfTV78qf1h/USwCAzPUfFrn853oBcRGiLiOvJ6lfaPUOndivWnXCo32dFJrDwO38bS
5UZMsNoJq7fi8+s+6n2wPUXFff8ickqmx5QWCs3pgzPGfVlj+Rkh/mWi0Z7IY7gtk792TJfmJsJ0EYq/
Ua636vzepZ2QxPUMe3O61rY6vKWbchRsg+cv3+obPdKdoQeH0UI1Q3Q2s7cNWB6t6okwhkSA3f
v/NTd+olAMAsvPLBa+Xmrz9fLy23z338Idn/n4/pJQBA5i59QeQyf8BKghB1KRXlxCnnmRtode1x6W
U5efqkHXYF11vUMHDn2VzwQ7mmeJ0KuwdeWOgN5ffWWcfkDu+2H8bw7cAQ/VFmdc784HMy
Yb1zLRMP6W/LeT9BlVZoz1klRrtTuzaW8kkJ3d2o9a4p2hC7Z7Zxrq2N2WUO3P3Er8sLpOuJp59gJn
4AmLGXfPe58nMPvEQvQbn7P35Vvvct/m0CgNxcVvVRv68XMA4h6pLy6oraPTl74nSqPCUnKies/w
+uV3ngPEWoFiOYcodmt90x0xG9I6exkOfM6GmphqaXQkPC+LP0tzf8OqBVo6fp8kkSbhdl80L4jP1u
cDtiPjEgE+5M/A8/8bBeAwDIm6p/usx1UEdRAWrzN+/XSwCAzPWfFLn8cb2AcQhRl5U3tHlfDts6aL
N77VXEyQqN9VP3hsyCUdfTDi/9upOjwkuvNubQI2YYuIDnzB/KHyXmkP72hj9RVLUlIzuhTmDqa5O
CTNtg1JUdKNlqa55jln7k532ffZ/c+/C9egkAMAsrX3qpXPvklXoJpn/40rfl079/qJcAAJm7bP02eukze
gFRCFGXVklO2hlkRw52nSHNbh3Rkv0Jf3347OuzV9w864eaDXeSp4E6pCVnqL3i1eKc2KKdM2Mo/
xjjh/S3ZcNLUMtSV1PRTyvVazOhHNugZuhXPU7tScXsQNWYoCtu2QJgSvX76vKxv/2YXgIAzELpoRfa
D4z26f/6D3aYCgDIyeXPivTp2jMOIerS8ntyNptOOObWeCzq4p/uem+Sprnj9gDtSK2mA77BOqRmP
crmqj9sur3h1aUsxB5LvWDnzBzK787+H3iYIV70kP5e45w3jL9cv5BOuYRUr82E8miD3pbKoIM9Trvi

ls8dW7sVSMn/+v/
+l34GAJgF1ftU9ULFeHd/6AH9DACQi0v36CcYhRB1iZkzpqvehd7w88Ds5sYkTXPIq1NqC+shGaxH2
VzVwZg/Ll1GT440LNVzVtS1VJXmauqhoTmUvxxaW8AoiWC9cuSQ/l5D1r0CqtYrAxMwOY/JJkhK99
pMJoc2VLb9IfyBurRGfdmzm7MP3bEU7v7Fu+WOl9+hlwAAeXvjX99AHdSYfv7dP6WfAQByUXijfoJ
RCFGXWWDW8FPiTzbuDltXzPVzyAwvRw2ht+tRGr0uXeW6dJPWu0z1nFVke7BA5v5hSrUxzaH8o0
NdMxQeNaQ/Xl3VCaV5bSaVQxvsIfxhxVBVSNvvp1pfFohy1RVXyf/4lf8ht1x3i14DAMjT373yCf0M
UV7z1pvkbe+5VS8BADJ37FUix9+kFzBKoa/G9QIAgKXxwCMPyOv/2+vtmfoBAPm648vXyysfvFYvYd
D1P/E8+a1PvVGedw0TbwFALgovFrni/daTq51ljERPVAAAlsztN91uD+0HAOTvb27/tjx2PX/ECnPlc4
9L9fdeS4AKALmx3m+Pv8f6SIAaByEqAABL6F2n3iUfeMMH9BIAlC+Xjvfl8294VH78nMt6DVz/8t+V
5NZXv0gvAQAyd+ztIoWb9QLGmSpE7TVW9AQpBX9W65F60lhxJ1TZkODL27LhTbbiPCabqGZYeyO
43dBH1jOAAwAwhz788x+W07ed1ksAgLz84PnPyt7rvqWXoLzidS+R1X8bNhkqACATx4oix39eLyCO
OeiJqsJVf5ZqR84zwquZ0VcaKU3oAwDA4rjnl+
+xh/cDAPL10M0/kO6t39dLy+3al14l1d99rV4CAGSucI3IMTWMH0nkGKIWZXOvL2oeq35gxuuuHH
iTiKvZsNXn92QzxwzV1qnJeTqkAgCWjDtj/3VXXafXAADyonqjfu+aZ/TS8lIBqgpSAQA5Of5ukcIL9QLi
mn1P1N6h7OuncuqEZJedlqXedUNc/9Gtl/XnRZq7pKgAgOVzy3W32EGqClQBAPlR9VE/96Zv2h+X1
S/8xivsofwAgJzYdVDz7rl4NOQYog7XRLXrlZZq4nZEtYfVD9UoNb/OeaRVL1Uprp0RP0YFAGA5vfm
WN8tH3/5RvQQAyIvqibqs9VFVePoLv35CLwEAMmfXQX2HXkBSc1ATNUKvISuFktS8lNXRqZVSq2H
aPu+HuNXTfpGB5WVM8uWd46hJwTA7YddqEYTfT/4kcHneY0nPYU7fC+q9b2DCO//8rEiKf0cCAt7
7s+
+V2h01vQQAyIuqjbps9VGfd82VctfvUJMbAPJztcixO/VzTGKmIWpluy/9bt3vCVptOcPst1WY2ZPGu
htwVqXlDsFvVe01qobpeqIkoSO1khtC+I9Vd0Yra9/2bgFghnqNlWAPfSBnqjeq6pUKAMjXfT/zuD1r/
7JQAer1P/E8vQQAyNzxO0UKL9YLmMT89kTtXZQdnSKU61v+RFSVLXHLmHZ2LqbU+64s9S0S1NFG
TQoGTGKR76eM295ryPpg13tgBlR9VFUnFQCQnx8/57J8/g2PLkV91Le951Z5zVtv0ksAgMwdf5PIsV
fpBUxqfkPU7oHXE8sevu/1HjWG93cOpKufTsfppZpmrVUAABaVmqm//attZuwHgJw9dv3T8je3f1sv
HU23vvpF8ku12/QSACBzhVtEjv0LvYBpzHdN1FSFz85vlhPo1M7nWItxHgxO2jWqxuPoOpD20GPv6
/VjZG3Jwf1FBNd2Pdzga0fVgsysDVEya98oca+VK/5xJm1f2OsHyncO1O806o7arx19P/kG2p/K9Ux6
DsOEt32wXungORo8P2HiTbTn8/epHqPqpCY5P0DQbdffJvf88j16CQCQl7+/7Xvyjy//oV46WlQd1O
rvvVaufO5xvQYAkK2rRY6/2/p4hbOIqSxEiFqud4fDT/uRwnDa4qac1WVWRfblcGnyBRVsDU7a1ZTV
wqr1/3hUiFMKG3rcqUlpMKBKMklYe2NETUjVYzgYFmXWhihZtW+khNcqwXEma58TyIW9vrlaGBH
2qXNitLNcl/GVM9SxDbR/6us5/f0ej9OrffAcNVdHhZyTcPbh1XO2Dd97qd3vWGrvKL6DGfsBYAb+6v
WPHcn6qL/yH15JHVQAyNPx09RBTdH8hqilk34P0YN0Bu2Ha8tuuinKQug1zvnhkTuhV7/r1Zsdzzhv
5bp0dbDd9TbQlHNeotNLMEmY9dpzesOh2+3IzkX3tVm1IUpW7Rst2bVKcpzJ2tdrrHuBnP+HjZa1F
615bkRQaLRjb1OKem0koz1u06e5ntPf70n47fHb3pHa+ejIPHqivUH+PsLvvbTud0Ds2frfdepdegkAk
AdVH1UFqUfJyumftB8AgJwce53zQGpSC1HtnmjGkFHzEWco65DimpzxspxVfxuqF6C77UQbdnpv
me1yHmYvuTOyFivhWXQ9uejO2qUCFi+kKcrmBSPEidI7lH39VDo74mY3xc09J6yxHnub+mQmmiT
MmLjHCNyKa2eGQ/XM2hAlo/aNlPBaJTnORO0LtuOst74iW/aGVVC3J2GHE2hHTNWz/rmtbPtBrd
/2JNczhfs9gWrL7yFf2Up/+4q5j/B7L8+J+bAM7v7Fu+WOl9+hlwAAefjmjU/JV376u3ppsb3sp14o/
+a3mNAEAHJTuFHk+L/WC0jLHA/nDwYcXkjrjWE1w5B0mMHN0daVAzdTKp+Ukn5qK56QU/ppJDP
kHgioh2ouTjVJmK6nGTZ8Prc2REmpfSMlvFZJjjNR+0a3wwldR5fWOHUi6XdVVU4HNlaSk2473bYn
up4p3O9zpSwnAwcRIteJ+bAMrrriKmbsB4AZuP9V37HD1EWm6p/+
+n/5GeqgAkBurhQ5/mvWR+qgpm2+a6IWN2UvbMitPdQ3hXqoHtWLri8pZ7JH3OhefG5oM1EPZ
MWu5eiGPlE1KzNsQ5R5b19sebQvRuA3VlFOLEzSmcbxAvPpphfcZAepKlAFAOTn8yuPylNXX9JLi0f
1QFU9UQEAOTn+r0QKN+gFpGmqENUc9hv1cMLJoj8M2gxA7aBUrw9NMc2v04+4dRUtdp1B82tD
H2kGskvEuHZ+7Udf89zw5DXjJwlry4bZq9OtjWnWijRl0oYo2bcvLbGOc4btG21wgreeHHp1B4ZlOv

HcEcD5QZpuv+l2e2g/ACA/KkBVQeoiel3l/6IOKgDk6dhrrQd1ULMy3z1RkZGQ4dEus05mDGp2d9V
b0Q+rjZ7D7raTTBLW3vV6dtrhjxuYG8OTB6XehihZtW+khNdqguOM176Idth1itOdfT7Y9JDh+ImO
M737fWHkNjEflpGaZOoDb/iAXgIA5EEN6f+7Vz6hlxaDmoWfOqgAkCM1C7/qhYrMEKIupaKs+bN2
yao3Ztuc0XsMPcGXKlEb7K0YEnhNOEmYH/4YM+KbcmhDlFTbN1LCa5XkOBO1L6Id9rE7NVUTnsK
Rmqsb4jfdL5lQPrPmhNaJrmcK9/uiyeB+B0wf/vkPy+nbTuslAEAeVH3Ux65/Wi/NN1X/tPp7r5XnXX
OlXgMAyJaqg/oe6+PVziIyQYi6pIqbF/zehs1VHawYk86MU9n2h393alJygxmjRqg/UVew9mbkJGGV
09aSNq5dWbUhSmbtGy3ZtUpyrpO1b2w7ynXZSmV8eFnK5aas6vZ4Tbfaftab/j/Z9Zz6fs+LOdGV28
6Jws6U7ncgwj2/fI89vB8AkI9Lx/vy+Tc8Kj9+zmW9Zn79Uu02ufXVL9JLAIDMHVsTKdysF5AVQtSlVZ
TNvcFJu6rS6res/8djD/8OK6Rpb2dgoi679ubg/ixDk4RVZHuoDc72vF01d71eitm0IUqG7Rsp4bVKcJ
zJ2qfa0Zfu0IbVpo3SBlM7JWcHjzfsGiW6ntPf7/mw7q/B67F/aPQSTiCV+x0YzZ2x/7qrrtNrAABZ+8H
zn5W/ev1jemk+veatN8nb3nOrXgIAZO7Yq0SOv0kvIEuFviqCCAAAMIF7H75XKn9UkaefXYwhpgBw
FNzx5evllQ9eq5fmx7UvvUr+06ffzDB+AMhL4RqRK9R8BQzjzwM9UQEAwMTefMub5aNv/6heAgD
k4W9u/7Z875pn9NL8qP4udVABIFfH3239jwA1L4SoAABgKu/92fdK7Y6aXgIAZE3VR/3cm75pf5wX
v/Abr5BXvO4legkAkLljbxcppFNUD/EQogIAgKmp3qiqVyoAIB+qJ+re676ll2ZLhae/8Osn9BIAIHPHiiL
H36EXkBdCVAAAkAo10dQt192ilwAAWeve+n37MUuqDqoaxg8AyImqg3rsPXoBeSJEBQAAqVAz9b
d/tc2M/QCQI9UbdZb1Ue/6T6+2g1QAQE6O3SlSeKFeQJ4IUQEAQGpuu/42ueeX79FLAICsqbqon3
/DozOpj/q299wqp/7ZDXoJAJC5428SOXabXkDeCFEBAECq3lF8BzP2A0COvv2if7Jn7M/Tra9+kfxSjV
/kASA3hVtEjv0LvYBZIEQFcteWjUJBCsZjpdHTn1tSvYasbLT1gqO94Z6fFZnX09NrrDhtXGnIqCb6x+E8
lv5aY2mo2frfdepdegkAkLW/v+178o8v/6FeytbzrrlSqr/3Wrnyucf1GgBAtq4WOf5u6+MVziJmghAV
yFVPGiur0tRLjrKcWSvq58vHDiJLNeno5YXRa8h6LarV6loXZDV4saVTK0lhIDAGjqq7f/FuuePld+gIAE
DW/ur1j8kPnv+sXsrOXb9zu1z/E8/TSwCAzB1XdVBfrBcwK4SoQK66cuDmbuW6dPt96ff3ZHNZM9
SxQeScUj1nxwW/7fPiHlq1pa5zX7r1srOiuSrkqFgGV11xFTP2A0COfvycy5nXR33zr9wir3nrTXoJAJC
5YyvW41V6AbNEiArkqXco+/qpnDohy9v/dHHF6znbk8Y53QW1XJetivO0uHlWqs5Tae6SomI53PSC
m+wgVQWqAIDsPXb903L/q76jl9L1sp96ofzKfziplwAAmSvcKHL8tF7ArBGiLjGvnqP5CKntOLI2peqN
536d163Or/dp135sb+ivNdbZLwuuD/bKS2MbmtlG7zFcYzN4jMM1S+1HWN1Low3jal3a+zDDt+aq
87WDDQ9pc9i2o9o8tpfjROdl+J4ZuZ8YxxD7fGh+W9RjuK0OZwi9/7p0z506/lKsnrNGj+NAWF6Sk7
ozquwfDt9PNqM9ie65eMduS+H7wj1Pcd9HsNxuv+l2e2g/ACAff/fKJ+SbNz6ll9Kh6p/+
+n/5GeqgAkBurhQ5/mvWR+qgzgtC1CWlwpHQMKhTk1JhQ8ZlcHHYtR8HCkLa61TIMrC+uRoeXk2
1DRU2hfYY7EitNCqEU58zapaW61J3uw52duTiYMi0674ynbqmQ8GiZh/zyGBquM1uz8dQE5+X4X
umuToieEt8DFGcfQcvd0hb7WCw5A2hd6V67jxVafVbXq/SIWaP41E6B9LVT4MqcjrpPZfk2FP6vlDn
KY/3ERwdapKpD7zhA3oJAJC1z688Kk9dfUkvTe/f/Nar7J6oAICcHP9XIoUb9ALmASHqUmqLn8O4d
TmNeo3SlHPhSUpi5XrX3na/FYybwtaPGt482TaCw6mHj7EjO4PplEcFZM7r+3ubsrlVF+erBr/GPI9nZ
FyGWtm2ttd1t2Wptpx9bOvUrr3hB4Vhbe7UZH3kdQm2eXRT0jkv/invSO28cd0SHMPY8xHg7zu8r
dZxrbvBoHEu3Iamcu40u53bEitrHVKUE6f00wiVRPdckmNP7/uimOP7CI6OD//8h+X0bQxHAoA8q
ABVBalpWDn9k/YDAJCTY69zHpgrhKjLyOwlZ/R0K27uOQGJ9dhLZaajqpx1t1M5bS25RqwPHd486T
aKsrlnBj6O4toZL7TrHIT3AyzXt4IBWXFNzugv6uxc9NvY3vV65pXPrI0P38YwexjWLxht3rwgfgZ5PrR3
31CbR5r8vFRbfnDoh3xB0xxDFHPfoW3tXZQd3SEycC4qW/5+zWtniH/u1HFY3yOhIe8k9uUwrEFKk
nsu0bGn+H2R2/sIjpp7fvkee3g/ACB7akj/V376u3ppMqr3qeqFCgDIiV0H9V/rBcwTQtRlZAQ0ImqY
bsGrZTiyhuIkyielpJ8GjFofJo1t2HQ9x9BhzEGnTgwGP0VZ8xMtLyzyA0Mj0J1YVK9WY/8jgrfhNscV/7
yMN90xjFaWk+MudvfAa79X7sF+GEPcRwyfn/zcTeuUjN51gntuimNPcv2HzlNe7yM4ctwZ+6+76jq
9BgCQJTXJ1KT1UVX907t+59XUQQWA3Kg6qHdaH6mDOo8IUZdSUTYvhPckdEOYqMl1FkZg8hyjn
mOk8MDO76nnDnc2AsPq6dg9GafXkeGOgjFCRlOK52UyYccwK2keVxw9ORxbLNWR2T2X2vVfkvc
RZOKW625hxn4AyNFfvf4x+fFzLuul+H6pdpvc+uoX6SUAQOaOrYkUbtYLmDeEqMuquCl7esjtQKlR
W/Pcos+u3ZYNs3edW7PRrMGZhHW+zurzZA+NNoYyV0/nF6FOH/qlfF4mkm1w6dXKHXpMWsd0

AsUTMrb06bje1BPcc+OPPf3vi6P9PoIsvfmWN8tH3/5RvQQAyNIPnv+sHaQm8Zq33iRve8+tegkAkL
ljrxI5/ia9gHlEiLrE1MzaqqeYPcGPHYR0vRqKo4f/GoxhxHPHrB2pgiW3/uMUba64U6Z3duT8+R29n
aqkk6GW5KR37gdnZO/JRbfoZeQQ8BgyOC+
+nI4hTOmkFwKOqumZL+NcNHfF75DZlQPvNJxwzn2EWPdckmPP4PpP/T6Cpfben32v1O6o6SUAQ
Jb+8eU/lL+/7Xt6Kdr1P/E8uet3qF8NALkpvFgP48c8I0RdRu0Ne6itmkU92FPMCHiMXnIlPxkzZmJvy
4Y3Dft884MlY2bySXgTWHWk2dQnKrWh/EXZdLsdqvO87l+XXmPdq22ZZBKkcVI7L578j8Fj1udsrvr
DyPW9bj9yHVtu1oBtyq7eda9xzgsxY/VgjnPPTXjsU1//hO8jZntyvRSYe6o3ququVCgDI3t/c/m157Pqn
9VI4Vf+0+nuvleddc6VeAwDIlqqD+h7r49XOIuYWIeoyqmz7Q287NSm5QYtRH7F6NnzmbhXSDL52
Lpkz9nttNibamUhF3I6BrlSH8o+4LiU/fZQL005glcl5MSQ9BnPYu9ueiRK2YH3O5qqz34IX9Felldqs+v
EUNy94PTLd9njnodqSeM2Jc88lOPY0r3/C9xEgiqqPquqkAgCydeI4Xz7/hkcj66NW/u8idVABIE/H3k
od1AVBiLqk7KG3YUUMVeDS7wcDHlX3cLBmYrWVcx3NpCqy3W/5gZHNOTbvsAPDrOPxhlcr5bpsp
ZzL2dcl5LxWW9Z6d+j1VLI5L6Zkx2C1Z/A+3D80ejUmYNfnNIaSu+y6nznWQ/UUZXNvuFaoPYw+Q
aAb656LfezpXv9E7yNABDVTf/tX28zYDwA5UPVR7/uZx/VS0Cte9xL5hV8/oZcAAJk7Zv2GfPwdegHz
rtBXRewAxKNmNtcT86gwbG/anqHAOEfpntPHcqpFwIpwn+l9Rip/xM0BAHl441/fIKWHXqiXRK596
VXyW3/8z+yPAIAcFK4ROf5+66P/Xoz5Rk9UILaeNNbdmc3LcmaNABVZO1r3XPu8dSwZ9ODG0fGO
4juYsR8AcrL3um/J9655Ri+JVH/3tQSoAJCn4+8mQF0w9EQFxmrLxmANWFXOgK50yMzRu+d6jRU
p7ZyRbiplKXDUFf6fgn4GAMjStU9eKe9s/aS88723MYwfAPJ07O0M419AhKjAWAOBFgEqMsc9h+
WlJiiblx9NaEs42hKOtoSjLeHmqS0nrr1GDr/3pF6aLa5RONoSjraEoy3haAvSQIgKAADmBj/ghqMt4
WhLONoSjraEoy3haEs42hKOtoSjLeHmqS1IhpqoAAAAAAAAABCBEBUAAAAAAAAAIhCiAgAAAAA
AAEAEQlTkSE2WU7DrfxRWGtLTa8fqNWRlo60XHO0NvZ3CijRibwjp6Uljxb0GG9aVTbJuGmH3UNr
7mJbRRv1Y4SYFAAAAAGChEaJirvUaK1Io1aSjl4H5pgJdY1Z9W1nOrBX1cwAAAAAAsIgIUTG/eg1Zrx
GfzqeibO717RkF+/1tqei1szFPbenKgXvLluvStdu0J5tkqAAAAAAALDRCVABIS+9Q9vVTOXVCyE4BA
AAAADgaCFGXmao1OlC7MazG6GD9UXuIvfE1A+VKNbNOpXokq1Vp79Mcxt9cdbYTvjOjjeoxqk7qY
JvCa1VOdrymMbVf2xvetob2H3JN4rTRY359ZEONcxFo46j1xj7t9ea5zKoOadx7KKotg9uYsD5pjOuS
9J4FAAAAAACLgxB1WakgL7TWaEdqpVEhpPpcQUoDQ+ybq4OvVyFiSYIva8pqYbBWZBqcNq0GNh
xyDHYINtgm65W1UnjQaYt7vIMqcrqqn3Z25OLAa9u7bmODtTKHQjgtuo2TKsrambLztHMgXeeZxR
iOHmh7W9xml8+s5dDDMoV7aKJrPizf6wIAAAAAAOYRIepS6knjnJuIuXUb+9Kt61BNOrIzmPx5qtL
Sr2+5QaEKG8/7ve16jXN+0FVt6VqVXfE2H0Nl2/qabl28L3G3sx1W8dJvU/gxWMe77oZg/mv77gF0
arI+MhUdf7xhKltu2wfPpR9GSvmMeBlqe8MPgsOuSWQbJ1NcO6PbuC+H7qbbu0ZI2ZEDN101hq
mfOpF9hDr9PTTNNTckuC7J7lkAAAAAALBICFGXkjERz96m16vQD9VEOl56FlRt+RP3+EGhqScXd9w
+e1VpeQGStc8LYa+fntmm0GPoXRS3SeX6lj/xUGXLC+U6OxdDexSOP94RimvidfQ0t22ElGaPTrN3av
2CcU02L/htrJ0fGKo+Ja+NftDbO3Si0mrVCRv3dbrau7jjBZKnvROYlRTuoSmuuWkm1wUAAAAAAM
wdQlRYdA3P0OH9SZmzk5+Ukn5qK56QU/ppespyMrCTEN0D77jsIdheXUtjqHdgSHsazOHy/rB4P5
SryllvyvYRvVNtxnbMHqOpKMoJfUHcwLlrXzzrnJ4+aYeVwfWW6mk/kMxMCvdQKtd8VtcFAAAAAA
DMG0LUZRWYKCdunckYgeWRMt3x+r1i3Z6eRig3URhpDK9PScUt3trctVrXE6cj6ik5UdFhZWC9anb
2EeriSf+6AAAAAACA+UKIupTasmH2OnVrPZr1HI+ocr3rlDEYevjD9lNT3JSzbglONXTcqCs6WRiZQYh
dOS164L4ctvUQeLv3pzs5lrF+QUP07K/5sv1xAQAAAACA5UOIuozMupwqYHLrohpDoCdXkpNuEjs
4XNoIEXNVcoamK6NqvWbF6+nZ2ZHz50fVFTXP2eBs/mZ90FMydk6nxNfQ3XdHDnadr3VrtZbsT/jr
h4e0ZyWFeyiVa57idQEAAAAAAAuNEHXJ+QGTMWP/VMxakU1Z3XCn3DFnS8+ZMcmTNFfFa1J7w
6+V6a1MmdfTsyPNpj76oaH8Rdl0u6xar6utN6yz5eg11r0anuYESU7AqVivP+8dkGx4U8nH5V+vZtP
5Wnf2/aIumOquNyfCylYK91Aq1zz5dQEAAAAAAEcTIeoy8oI9S3NVh0rGhDtTMmcun2r75iRC7nY
mCjuDs7o3V3WI5gWO5gzwaXOHxftCh/JXtqXl5XU1KdnnrCAlP6mTC95EVCojdOutWrxzHLe2bVBg
W9Yzb2i6eZ9Y68/k0w3VNv09lNI1T3hdAAAAAADA0USIupQqst1vGQGZUpVWv+8HRvaEQpMqyu
Ze1w/BbGr7g/scx2qn1yBt/9DrDZhIcVP2+oNtstj1YDOoh2rwhvQr1v62Ruysst0PrUtbbVnr3ZILLnU

8g6+ttiara2v22gwMTTeGs+c+ZD2Feyila57ougAAAAAAgCOp0FczrADITq8hK3oiL1WDdo+eiwAwk
urtPS8/mtCWcLQlHG0JR1vC0ZZwtCUcbQlHW8LRlnC0BWmgJyqQKbOOZ75D4gEAAAAAAJAOQlQ
gE23ZKAzU8ayeFTqhAgAAAAAALB5CVCAPql5pZpNXAQAAAAAAIEvURAUAAHODelXhaEs42hKOto
SjLeFoSzjaEo62hKMt4WhLuHlqC5KhJyoAAAAAAAAARCBEBQAAAAAAAIAIDOcHAAAAAAAAAgAj0R
EV2eg1Z2WjrhVnrSWNFzZavHhsyL60CAAAAAADA/CNERSZ6jRUplGrS0csAAAAAAADAoiJERfp6D
VmvEZ8CAAAAAADgaCBEBQAAAAAAAIAIhKhLqr3h1gddkUZPD7+3l51HaClTVePUeI3zcL7eZW/XH
MbfXHVepzc4uF+PuW1j58HXt2XDfY318F4Wo12TGjwv6jHpuVGSHr8S1obCSkPCD8+s/eo8VkaciG
TbBQAAAAAAWF6EqEuvI7VSQUoDw+
+bqwMhX3tjRI1T9fXpBJbR1H5WpamXpFyXrYr1MbN2OWHk4HlRmquFYNCZ4blRoWtYG6RTk9Lg
BFl2EFuSwZd3aqWhcDTRdgEAAAAAAJYcISosVWn1+9K3Hq2qXqUCwPNulNaTxjkdX5br0tWv7dbL
zjrrtTsXnYiusm19rlsX9zNSbdmv7W+rxHNafjv7e5tSTNCupHqNdS+MLNe7zj77LasFWvOcDkeza4NI
W3bd1Dh020055yW0VjvW3SDXOE/uBe3UZN17bZLtAgAAAAAAgBAVUm1tixtxVraMANRTlM09H
crZ4aVeu3bGe23noKufZadc3/La6ciqXT25uOP20qzK2U13yxXZsoNGFVLuibM6w3PTO5R9/VQ6O+
JmscXNPWd/1mPPbVvvorhNDpynypa42Whn56J1ZJYk2wUAAAAAAAAhKiaha5OGDmHPzqkT44K
9tNrVlQN3A+WTUtJPFSdo9EPnYSmem+KanHGTWGtrquyCW7t0qM5p98Dbnz18X7+uYA7v7xxYR
2ZJsl0AAAAAAAAQoqIsJ82UcJTAxElGbdLcjGjnzNtlyawNRdm8ENYz2A9KQye5Giur7QIAAAAAABx
NhKiIoS0bZs9Kt46mWft0JuahXRm3obgpe3qIvV+v1tc8Nzybvl/DdfBh9KCdYLsAgLzp0Q3Ggz9yAQ
AAALNBiIrx2rte70o7oHNrfxpDyKc2ybYya1dJTroJqDsE3qVm4i8YM+6n1YaI16uZ9NUvzfakXXbw2f
XqnHrtK530Qtu4NVhjbRcAlpH9Xl+QwkwTy540VlZFWu57tPNHr+bqLILU+Qxze40Vpz0r+f/hT/0ba
p4P9zEP5wXDhq7XDO6ZmRvzvuZ9P7mPvG7mOO+3ebwnR+5jSf+gFfO8z/K9eGZinJuh951luGnG
nJfc34sTtofSdoiDEBWJ+AGdMSt9AiU/nTRm/7d+MFmdbhD8tO0KKsqaVzS0Kavem667baeO6OB7
cZw2JDp+/aavPhXsGRpSs9Wsc9pc9dvm/sOhHu7KJNtVjG3wDwuALMzsl/chKri09j/lv0npcCYu3Da
KcLuTP+4f5vte3N4IC3ONPyjOQq8h6zWRatX9x28G3NEnxsO8XrMy01/cAyWOhh+z+NZW52O1qS
YFda9TS6qdmpRmFPjk/0v7+Pc19R5csr6f6l3jHFk/T2Z778R5v83jPXncPtTnc/yDVmTok1eYm+C85/l
eHHFuht739CP98xP/vl3dH/g3Iot/IEadk9zfi8efl3zfiydoj/WNrUrbpf+eHO/7dn5+FsZY1g2DJWS9R/
TV5bd+A+hbPzD5uvW+9U+g87mq9ZZia/WtnxX060c8vNcqIa93P29uf9TD2NbIdtqStKvbr5fd9VXr
K8cxXx/yKNetVygJz83Exx/
+MDcdve3gMSfabqvqrS8PXwQAmEq3XrbeX8z3eP2+GngjyoffFv1vwAzaEEm/z8/8vXjm7XCuj9q/f
c28f5PzY/87OoP9RtP37dy1y/3eivPzV9rCv5dn1R7n5y9jv/pnrCy/l8a/rznvuYNtyPocjW9XvNdMa6J
9ZPIeqPdvbdd+hLRD3T/maud+Um3XK1IS/5w4n8/+vTjeucnjvS/
+fZv1+8v4cxImq7aNPy/h62fWnhHfw+nfR87+zd0737fBdX579Qr9vhz3uiJf9ERFDBXZVn8p0ksO56
821puAo7kr/t9KrNd7n9D2D52/MKlanIP1QqutCWuIJm1XEk4PIOsNTS/7rDdbf9h+0jYkPH57qP3g
ubQ5+wj8QdOuc2oMyXfZPWWMeqiWRNsFgMy05XytY71NXZBN503VUpEt9UY28fv35Iqbe9Lv7x
ltmTO69MupE/PRwFm1o9dYl1qnKmfn9kLNhnteWt7PKPPC+T6X6unAzyL5KMqJU9YH9+dQW08u
7sygPb2GqIFK5fqWv9/Ktv3zYmfnYgY9sRxj39d6h7JvfRj8fi7aJ64puxm9Ecd5v83jPXle3ved79+y1Ls
hP8tr6uf34dEJHdm5mO7dE/ec5PVeHOfc5GX8uXHeXwLf5xmY7Jxk9148/rzk+148tj0jfp6yR42mW
tYuzqii+fpZGOMRoi4pvw7mwJuLMeFQcMiBCgvdr1EPJ5TztxMM6dQPhf5rrYf5A725D/VQ+xmx35
Ht9MRtl/MGFlw3nvMG7H6d89gbakjCc5Pg+G2D59J+jDoG8zj1Y9QvU3G3a7xu+NgBYAoz+uV9M
enSL+W6bIX/A5CTnjTWa9KZVTvsoaMdqbbi/1ueGTUM0Rh6N9uSN/n84j6JXuOc9d1s/bI/oxu3sq2
HjNo17dWwypITNg/
+vJW13H5pT8huV1lOenWcNKPePgZk8AetuMFlmDTbEVuO78XTnJv86fJoB+cHhtOnWwJnknPCe7
FhxPub8/PnvuRaNYmfhRcOISoAAJgNfnmPTdUltX/5uTCLXoa6tpj9i6D+pWdGvR3b52vSqbZmPmL
C/0Op81AjV7KppRZXPr+4J+f2NDo7wwDEHTWkatqr7yOrObMI4efpl3ZMgT9oKfPyXhwwR3/Y6uy
flAvevxGqt6h6/9mYYa9C3osD9HwiwflBrO+pqedWiWHwjzD8LLxwCFEBAADmmDP5gRq2N6ueO

MFRDt36vqzO4hfU9oYzCcRc/dbuUL2Csh6WHcfc/eLePi/O6NHZXTNnsg5/YiAVeKtJgXKftGOWv7Q
jNfxByzKH78Xz9oet8pk147pY/4aeVWXUZtirkPfiAernGrdnrP89dXBKXadTkl3n7nn5IwymQYgKAA
Awp5wAVfXWmJ+hjMXNC3YNtk7tfK7hXHtXhU1NO8B1fuEpSEn9Vqh/CZrtcHptlsOyLfP1i7sOCGf5
y6Iecqzq2bt5jz0MViXezdWMZjYfZVa/tI9h93bqyMHgjTuqd9QS4w9ajkV4L56XP2x5ZtqrkPficIMl+f
qydXLfOk8nJau3vdn+EQZpIUQFAACzwS/vkVSvDSdAndMJ/zL8RSPMYE8j9bAngLR+Mexaz2dbt7sn
h6qoWc7nZKxZ/uLu9nw6O8NfFgeHTbpmdl7y/6V9rOIJceZ7CUZNPfuGnmG4O2f4g5Zvvt+LB+T+h
62S2GWOB8PbWf5cw3txTLq2eOCPkekZ+UcYfhZeOISoAABgNvjlPYKeRddiD3czevzkW+dSTf4wM
Bxc/0KW1S8ac6/XkJUVc0i2WqVmSp7lL6nz94u701utKjMcPSpSOW21wPoeCgyhV7dwzTovM26bL
dtf2uOpyGnVY6+27r+veL3G5m+islngD1qLaFZ/2NIjADo1Oe/9w6mHcM+oHinvxXGoUhklqUldLm
RwkSL/CMPPwounDwAAMCOtqvSt33L69a5e0a33y9aPJ2VvRX669bLVFtWewUe139KvWUZh56U
6JyfEblu53s/3bun26+Xg+ZiLe6RVHbg2rb71S2tfJT+5m+H38TB9HgKPefie1vdRxvdv3Pc1573Yf2R9
7eK0K4/35PH7CLt/3Ifxb1dq9H0x9H2r2jFw3Pp7Pu1rNcl5t78m8/fiEedGvd8M7Ns9hrTf/uKem6H
XZfY+POp+0XJ6L453XvJ7Lx7fnpB/xzO6RnHuxXn6WRjjFdT/rIsKAAAwE+5f6F3WD43zNRwQWBCq
x5xdm9BVbUl/Bl3nnO/pqrT6M5p5eS7pnk7G5ZnV9cH8Gfre9fjfR2Gvqc5r79gUjT83Id9bxnk7iuLc
LwrvxbOmRvOoOqhhgkP7+Vl4cRCiAgAAAAAAAAEAEaqICAAAAAAAAAQARCVAAAAAAAAAACIQIgKA
AAAAAAAABEIUQEAAAAAAAAAAgAiEqAAAAAAAAAAAEQgRAUAAAAAAAAACACISoAAAAAAAAAABCBEBU
AAAAAAAAAAIhCiAgAAAAAAAEAEQlQAAAAAAAAAAiECICgAAAAAAAAAARCFEBAAAAAAAAAIAIhKgAA
AAAAAABEIEQFAAAAAAAAAgAiEqAAAAAAAAAAAQgRAVAAAAAAAAAAACIQogIAAAAAAAABABEJUAAA
AAAAAAIhAiAoAAAAAAAAAEQhRAQAAAAAAAACACISoAAAAAAAAAARCBEBQAAAAAAAAIAIhKgAAA
AAAAAAEIEQFQAAAAAAAAAAiEKICAAAAAAAAQARCVAAAAAAAAACIQIgKAAAAAAAAAABEIUQEAA
AAAAAAgAiEqAAAAAAAAAAEQgRAUAAAAAAAACACISoAAAAAAAAAABCBEBUAAADAQmtvFKRQ0I+N
tl5raG94n19p9PTK2fDbuiEhLV0wbdlwz3vk+e1JY2Xca6xXNVb8ba00rK8CAGB+EKICAAAAODqa52
TGOemSUMHoqjT1kqMsZ9aK+rmpKJsX6tZnHZ3aesg1asv5Wkc/F6me3bS+CgCA+UGICgAAAOAI6
Ujt/OL38Zx/XTlwM89yXbr9vvT7e7I5KvksbsrZqn4eco16jXN+IFttyXZFPwcAYE4QogIAAAA4WuiNm
r3eoezrp3LqRKxeo5Xtlng5auAamb1Qq9IiQQUAzCFCVAAAAABHTLzeqH590pVg6NpryIpbm9Orse
rX/7Rrehp1Vr119suC68NKtPqCtUJH1wEdeJ31CKsrGjyeYL3S6HYYzGPXj8F92fsp1ayzrDVXndeO3Ul
FtltujOpfo/aGXxagXN+yXjUo3vHbQto/dH0tcc5VoEar+6BWKwAsLUJUAAAAAEdEWep1HdJl2Bu1U
ytJYTVYDdRep0K2gfXN1VEBZlNWCyUxyoCqjUhpcMIpOxQceJ3F3t/IQK8jtZJRr7Rcl60YnTuHwlEtel
8JVbak7hZHba7KRqMh5/wEVS4M1gNIcvwqwA5pv3M+hoNUR/i5UueiNLhTJewaAQCWAiEqAAA
AgKNjzQ3p4vVGnVS53pW+qgPq9ax0hK1v7o5oh1dLtC/
+y5tyzkv7etJYd0PBqrT0a71td2qyPjIpNl6/F2OSpvaGePmv0a6um3ga+6psW5/r+hNFqRqm9n5iDc
MPTjLVrLnHV5b6hcF2Jjl+67VuGhvWfmsrOxfjnqu27Eadi8A1AgAsC0JUAAAAAEdIUTbdGYwy641al
bNuj8nKaWvJNWL9/mFoL05zBnqzXmhn56Lz+t5F2dGdIQPD3I3enN5rB4QPix+t7aeGgTCzuHnB31
ftfDo9MAOTTGnVs8OTUiU6fuu675lBqKO4dsYLbDsHXf0saOhcmfVeOzviZq/FzT1n+9Zjb+QMWgC
Ao4oQFQAAAMDR4oVsGfVGLZ+Ukn4aMGp9qKqcDiR3JTnpp31ix33dA90L01rllguwH8bwdve1A0
6dSBLymT0vz8ha4EuLsnbGbdi+HIYlthMITDJlPQudTGqK41fHZNc5DR3eHzR0ropr4h2yuodK7n4ja
rECAI48QlQAAAAAR8xAb9RD5+l8K8qJU/rp1MpyMn6am0BHRnTmnE6i8DlCYFIpo85ppLBzFSw5Y
HLD3NgTdQEAjgxCVAAAAABHj9kbtRYvTsvXYK/Onhx6Y8iHebVWhx7biYbtTyercHa88cfflg2z16lby9
Ss3ZpEcVP29D4Gyt7amueYpR8AIg0hKgAAAIAjyOiNmpQxjDw7g706u3Lg7tTtmVk66QWAo+p5ps
MsJeDXAHX05KJbmFROSaIqAdNKcvztXa/nqR24unVRp7iWaoZ+1ePUnkjLDlS7Xi3W0WUEAABHF
SEqAAAAgKPJmIAoTMlPDo3aqW3Z8Kapz1ZzdcObqKm94Q8/L59ZcwJAszZnc9UfQt7e8Gp0FlIZV2
4Gzta5WPd7WfYa61790aSTVU1twuP3A1djxv4k9PbVbRDscRoSdAMAlgYhKgAAAIAjKro3qjlzuwrp
nGAubi3NaZWlXG7Kqg4D/dzWmOFftd+ozdlc1cGh9+IREzJNorLtD1vv1KSk21XyE1S5kPuM9AmOv
3Lan6jKu5bGBFRJjDgX5r1RPat7ugIAlgYhKgAAAICjK6o3qqp7OVgzs9qavI5mIqfk7J4xPFyx63gO1Di

1a3MOvE4Je+2U7GHrIcdebVnr3eHxeYt9/BXZ7psz/itVaZk1TZu7Xs/fcexzEVYMVW8zrewaALA4Cn
1V3AUAAAAAAAAAEIqeqAAAAAAAAAAQgRAVAAAAAAAAACIQogIAAAAAAABABEJUAAAAAAAA
AIhAiAoAAAAAAAAAEQhRAQAAAAAAACACISoAAAAAAAAARCBEBQAAAAAAAIAIhKgAAAAAAAA
AEIEQFQAAAAAAAABGEvn/AZM6nr53azj8AAAAAElFTkSuQmCC]]></redcap:Attachment>
</ItemDef>
<ItemDef OID="q18" Name="q18" DataType="text" Length="1" redcap:Variable="q18" redcap:FieldType="radio" redcap:RequiredField="y" redcap:BranchingLogic="[q16] = '1'">
<Question><TranslatedText>Do you prefer Life A or Life B, or do you think Life A and Life B are the same as one another?
</TranslatedText><redcap:FormattedTranslatedText>Do you prefer Life A or Life B, or do you think Life A and Life B are the same as one another?
 </redcap:FormattedTranslatedText></Question>
<CodeListRef CodeListOID="q18.choices"/>
</ItemDef>
<ItemDef OID="summary19" Name="summary19" DataType="text" Length="999" redcap:Variable="summary19" redcap:FieldType="descriptive" redcap:SectionHeader=" " redcap:BranchingLogic="[q18] = '2'">
<Question><TranslatedText>
Alzheimer's disease - Description #[no]

If you have Alzheimer's disease…

1) You might forget recent events of importance to you. For example, if you are interested in hockey, then you might forget whether your favorite team won their last game, although you may remember watching the game itself.

2) You might forget the names of people you met recently, or the names of local streets.

3) You may no longer be able to handle your personal finances or plan leisure activities such as dinner parties or vacations. Simple decisions such as what to order in restaurants might become difficult to make. Your ability to concentrate may decrease and you might have difficulty driving.

4) You may start having trouble finding words to express your thoughts. You might need to be reminded to shower or brush your teeth, but you could do these activities on your own. You may need to post notes around the home to remind you of simple things like turning off the stove.

5) You may remain aware of the day and your surroundings. You might be able to travel to familiar locations like the supermarket, although probably with the help of a loved one or friend. If you are in a social gathering, then you might have difficulty following the conversation.

6) You may continue to enjoy hobbies such as puzzles or reading.

Now consider two possible lives. In Life A, you will live in perfect health for 12.5 years and then you will die of natural causes. In Life B, you will live in perfect health for the next 10 years, then you will live with Alzheimer's disease as described in the summary for another 10 years, and then you will die of natural causes.

</TranslatedText><redcap:FormattedTranslatedText>
Alzheimer's disease - Description #[no]

<p></p>

If you have Alzheimer's disease...
<p></p>
1) You might forget recent events of importance to you. For example, if you are interested in hockey, then you might forget whether your favorite team won their last game, although you may remember watching the game itself.
<p></p>
2) You might forget the names of people you met recently, or the names of local streets.
<p></p>
3) You may no longer be able to handle your personal finances or plan leisure activities such as dinner parties or vacations. Simple decisions such as what to order in restaurants might become difficult to make. Your ability to concentrate may decrease and you might have difficulty driving.
<p></p>
4) You may start having trouble finding words to express your thoughts. You might need to be reminded to shower or brush your teeth, but you could do these activities on your own. You may need to post notes around the home to remind you of simple things like turning off the stove.
<p></p>
5) You may remain aware of the day and your surroundings. You might be able to travel to familiar locations like the supermarket, although probably with the help of a loved one or friend. If you are in a social gathering, then you might have difficulty following the conversation.
<p></p>
6) You may continue to enjoy hobbies such as puzzles or reading.
<p></p>
<p></p>
<p></p>
<p></p>

Now consider two possible lives. In Life A, you will live in perfect health for 12.5 years and then you will die of natural causes. In Life B, you will live in perfect health for the next 10 years, then you will live with Alzheimer's disease as described in the summary for another 10 years, and then you will die of natural causes.
<p></p>
</redcap:FormattedTranslatedText></Question>
</ItemDef>
<ItemDef OID="a19" Name="a19" DataType="text" Length="999" redcap:Variable="a19" redcap:FieldType="descriptive" redcap:BranchingLogic="[q18] = '2'" redcap:InlineImage="1">
<Question><TranslatedText></TranslatedText></Question>
<redcap:Attachment DocName="125.png" MimeType="image/png"><![CDATA[iVBORw0KGgoAAAANSUhEUgAABVEAAADYCAIAAABtBTQzAAAAAXNSR0IArs4c6QAAAARnQU1BAACxjwv8YQUAAAAJcEhZcwAAEnQAABJ0Ad5mH3gAACraSURBVHhe7d1v6F3Vne/xxKgTRyWhdUJaLyZ6EUISJG24EH9TbAQxIgYZxcuVHxEsEwQbEXOZB4oyFPKbQdAHxQrTe+ODiAy3RLGFPKiQXgrNAx9kIJXfA0sD9cFvpJSfNA86+99e63v+rP3WWfvc7Z5/3iw/R39ll7fdfa5+Rkf9Xzmy1/BQAAAAAAAY0TTPDwAYyMX1i1v+aQshhBBvPr98+f///P9Vn5gA0BF6gDAENY31ne+uVPc4BJCCClz8ys3nD76/ZMH9d//+ve/e/57l1WMP/+qt4h/+rR4BpOwc6hHsJAgAdq8i56CzMP6yNPbPqhJTx2ae0gWbDm9rF0fb4Y7ckY/79HVjDSqEhB6A9Cv/iCW9H1Bxh7D09rY/9Q+GcLRcYkVc/WuUZ3Ae1QWRrCdTRbc3UiF81P3S9NiHVqPXY5Bo6/KcUf5yPiy7EH1BfhM7m6fYJ3d2iRXSh/Xvqn9Oezdb1A8fR2FeIE7iHlZ0bAXLDy12ZK0M3G6CsPsYJ7YVbR40Wo2/8VQG5ZrL6MRdUazb7EXB3I1GxUwVRNzbcZowBgeNFQu7pBAC5w16wG71OJJpZ1G5bbSaKkIF5lLHO7lHWLlydNBT1Dud2mJ5lgnUUK1R8gMLH1g0QoLS1gK4ZOoqUk3gL3xJS1v66cAUBkbzQHHjdi3wDXRkGGSOa+xEyB3ZwMmSQRLgCsfPy9N9W8MOkgOJoXkqOSLKWKM1M1bJj1+nOnjXeJpYTnCj+RR7MrddQ0Ba9Ws4Nw2a1gPDzYnHbWpr3vUdDCkpNZQTjKMglJPSvGcFnPbZcEJUZlUQNDiybSLUmEcK2pV+mTwF+9akLD0jnKuGc/FVhHm1cYgYGkFWNpkUvDOB5pF9c9HR64vqOZI6VTWsKWXNbsIZjcpUbyoMy7NyWE4U4WeOQiPhUEkvOBMsyNqnudu8YL6gRPGU75uWkDd4GdxjgnPo/LdhNlVQT1bdqsH4ri5Qvzc7jpdwimjdB+dsxF7PYOTO0mAt+0Wi4gUPt/9ap+IxohhAmr6h3LZIoVdBHqnuSWNS2Z62BTHgLjyFVnJGbzuXwnbLWmPWJDCfwSTaLBQpt3Fp/TlzzfAmd8L1aFXjkrLCwPm1Yx5Ma17jBRPLcETQVTR0CBqYbSdo5E+RZRudfM2DQeyM0N2wbxeG28QW5frP4ogJ+NlqOoA1MAs+Ya3dYA0R7XNg/5e4NTMHiSxZzRlLE75sMAgdHz3c0DWvbwjJpPeXDSwcP19jTsVwr/eRMYD1oE6SDTYLdnWkBmIsz3/hX9jeYzKO/K9CoYJb6hPy/hr2R/53TJRfmdjqbYeAk3MXTbB7V1QQ4OSrNzvJlAlCfMIu3+cxIAx/PmLe6Ajbzrf0uzYw6MyGbhTOxpgNWq2rvptxLVmxkKmNT5Rz6oxqEtwnsIaz0P+v0kVl3dtrhjeE7uWsmpWR4IUr0TwZnrP28MUyYeKVQ8AM6A0DAkbE4QDn5RzmabswmQxodu0zpKMOJJ20Ze21K0W7lK5EZvy73ozBPY7CRenmyxKK1m36h2W3kW6TQ0PZF8RVAQhWn8v2Y4xr5JbcTBf3GHh5KecAHsaxB1C6rX6PIjTxRdaZDfD6uWvz4a4JNekNLuTo9fgEzNVXXOe5B3M4DVTAGcHEHxvwXTyBs9e8zt3tkTE+jZDo3XsDUEUu2CbbSxXAyvG9ILc3Al51nHu+6urnUzVbXMSGwnzrRWAiqPLlOTMfklJDWU3uzWHOt8ztiCLAcs/Bs5e5+12e31drrSG7I+v/CHlNpJbqCrqG+ff1Gw3tlFI3SQm7IzRCB2Lv/LEg7+30Brh4FSC3u+R6tVpV0tqy9VoIHzfi8yMNcAt2lqbN0MT0ow6OIPtKhcBsH1XAcwf4pHE8Yfm1mrBBv5V/dwTTxi8GygntaUfD/WpsAx6SV7Sdqjq5TaeCyNlf6grrl26g5DKRRm7ILr8eupRrV/AqlyuoN3Dp1VjjhYNoIPWIUJgkmOP5Sad3PEMmW4eqgvMVHFvTFQ80yqmKvyGkSHzywUTtd5wiMgpG0PVnVo5HXL5A2mrLbzZNi8kc5+XmGIR6u4znRDB4SkDcIhwMGFbLbVG1Tt5LJDbYeTCNUvYz0LPiLWnqyXRb2Ap25qMF0Wwij36OjFXeFOV2uIgIpHd8etJ3XXGx0MnF86MQQrf2hWx5o/ytyOL4gsJbMPazl8YOYkLT/jmn4Nt7NQYwAtyAA14vyR/ZqsKu4dz2NKp7RFIUvqwRaJFAuyN6X4+zzNiW8uHgc9/A1sWXUrLa2HhdAvKkxtRT7U5LN5lFkwE7rIbf4FJ+y4CgHJbJXqVWQJMILCNFA7f4Kp3DcX3yjWkQNt0Pq72v7ZTLGSXQ9K4Ykpbu5czM9ojH8ix83FRXH12Lt3xmmfeLm3oAuvX+mPUCaaCgv/1rfOdbCg6JiM6XmTWtDb/HLinAMgiAR2nyRtFWBm3B+/0RBGS1HvKqUj5jvI6CVH4u3szTV/qmT/AwZ+0vKgxFSQ26E3yurh+qfvvf8qJ/YPZvzsWGDk5+Rl07J1X2OH1u2RHvGgRy7FDXO3YdXczpsSFO4GzcK4jcM6+FCnefHjgSqvOk0+MhRaWbbGZz4XjiLM8DzMQs5EHBRHNKwyo8KG3GiuAuJqv7RPXDLJiO8S4f/hvmYQWT3tAGmSn+bvrRFnvRbabbF+16F6pHdsPcMOAHG3MDH5WTbrcCtfrYxoh4LZeIStyGP2x8xi08cHSBAdQLGvk1DmtuHJprIVsHQDjqUG6oPLUrJcujIYnuEwGsuzYnuW8y9gb9HELyaSEU05nEvVAjhdVO7VjtM7jN1RnGB9QBk3vbt/u0ezaWbp+0hZi7UoHdRu75iz7dL7KaRQmZXVm8LKhvrxwFydT85ZQmR1Oz7oLGLtv46rp7pRhAfIQPOuX9zGDlN21xC9UiyGBmrhQ7XDFsutNhxOQoW54QMhdXVgH0wTazUs75hX19eg5QZtWwY7zJUAqgg91g0dRiyWHB4P0IT8IDBlxjQtQxU8EWLBdm/qP8/a5Aanv4iAXBCc4C6dSm9U1Q/ysWwkvHlq2S6/kWF8W5d9p8eW1anmDMiPaYp1N52+rbDdyu5imE85BVEtYOSUJhYz6+BYEp2JcYkLnJ8FfVwNvmv/VhkJ8GRQ85UsApvABv0MaYA2KgQeXeyIw9LsOOCsiBqyKMbH2pkR4UHqE5NlU6IZzJQbEuhr7B/6cClekVJKKC/bXVs2c0EKY2N/YVnn/UyTsrZChfmZcOuEnL3KdM/a5bpJfBNLk9JexcnE7E5yi01IvYedoFKVRDwVUW/P3Z44XmxHDR2sFaAv3sMpDLq+MVQ/4MZJCcc4m1XWGDnlopd5O56ysDWBhAXxx/SQDFsy9V5VqLOS3QH4pFN4bRWQpfDg5pVUiDq+/32MXI5/qxODEakrJawcwafDsCJyDjlf3hOpjasRSEVQgfMDg7jcbc+FzH6WmP6hHC+5TOLw6hbJ09nw5WDlHWRBCaDvNEdLJLjBvThHP8WGFb/zYvrKIQS3ZprT3ddIKQNRJc6YHN3UYzXH5ChsZwZzKXd0HnjpbT2V7DqkXrZYlyf7pWbePz7+tedwl0Fr9Q0mFJf/u76sGkMknVK4oqyPiCs/wd2UxMqMMB2aenjzHhjTY/hh7SzXy8Iw+xhDMLXg/D8g4wJc3vGKcTaWC9h1bDnWVYpf9E+rvrtVnj6DGLPQyYuOrSbs20bLfZwwkqi+ZXX+0RMOtuj6CxspN3ksqh45IV7Kzdi0ZrvHmN+C/NYaB6AqimWhB8AWhMR2RyD4qVMfqM/T2BGEU+rvmt/ok/BDsf60A2MgxaC1X7Y4Fh/5+V3t9Pz3W/Fq7hoNqfIQLr7YtP9SfHbaWc3CpNPX0Ka1xl9vZwcQ+1owH7JI9ROz1MR+cebTz9V5vDbf/yXz7JVT3yKCNNi2LQwnzzV0xc2/fVm0vRMKKVEd/hvmd7ONa2Ec5ggmSvdy7xCUmj4BbGwrJ0ny+IRqxPUhzEqk2kvvvdS/cSFTUKkbszs71d1zp2Z6xiqunjZi5ZiNyV/H0cgx6hbH3YRbFpQ5KL5zoykK6oeCFlq9rOEYLSJ/QBhwX6sA3xJ0Y3lKYrZv0LcwAyEBD+1NdYPd/0oBdBSkzvDqGtfs4h+tbdj+c9bcUkTDQ8Gp9FgW+gMk9UzcMdw0iF4SNXX3c32cPfzfsdNRVuGsCkrLkxQXqZ05vcXAPXvp9f05i0EEzlrPa2zD79zbUDPrPYiyuUcnPAhp6VSi8BvhvK7ZSZkIxE5F5GdgQ4XpA8Rqlt7Ii59/ltDMnljRhgSXpZNVZLgwmxwsSlr2tsg2PnwkHLy1HzZxtq65XK+M6Zs4j7XLH+NmTWtI6KXUS8Xvd/6mAO6f8mgHzE+oowa5G0lIM5XXhVHqjCxzRqrNnWFBP/XN5ng2vYrQHSyY+wfz5H7CxtjGCkQO7/aiH6LbMwXGeixpy2WjkzdUvp9X2Q5K3NPq+TyvmF3ZYxwojL0Y0zOVZOQ6fpntsDy01IxnOyoIhlbt7ZQt5wvSa7lBKtoMxLNlqK23nl7X+vMm5Y/tNZbDm7ey4PbbWqaHd3p4iVqBh4qFvStrbX4gJYY2A6G2pJbrNsdZ+CNK3Xd/bxDkcHsHeddy3AjZa8K1/Yn2lbmKuZi3lOT7TCp5i9YyqcNuaAK1hcA+7e5PiFLeK2l3sdPYVd14+yE9Sxti1Tf/x18qwIPwcc7iyx0UtL+3R0Yqsa+WUhDVHQ5F4MCfelVGdrX0p4UiGSMRLywZqYSk5hQ5C+QeRG+nzm/uyMkLTDuUTBtyrlJo/mnOSnxwmLQkJMdfOdrkdqVNxQ8Q4G/0QhkrsAPC1Ny/zGhoi7hWlTLjoqSLEjn9O6xm+1O0mHG+Jxc/XjxqzTexlIbPg8CyMLt1o3Ie2R4d96m2LUHE/GNaKkU0G5r/f9GDpiaN1VeTIpqjPsqFSBGS4W/nNaNu6bOSRcwV+lULp92VYOVkR7bxNwGz2Jl2ffjb22E6QVNH2yukHaeQ8mwVV9ruZKIxuKQOL89ky5MxVyLK8uENbnkxRQzODE3OAmLw5c+nQBVmWYJR1wEB+0xZQaqq7v5iRhVRpTTbnwVSbpWdgNUrGoPWpY8pbZA5aUt5ujFwrEnk5itLhjJFjXG/RGUqw7lY3mOSyhcTcYz3kM3qdLdmJO1ZowlW5RcimHx7rqtqPLuuoKMmzq4KPtVl+Jv0szNXfJuqvz/HEKeS0kKHE+Skh6gf+cvcNp7F/YdSaKgHdyebC5kp5aHsUlL+N8vbyc/Sat5Jft2RX3x8sLgpNNpUL+KaH3NzJ7lW1JWXIbJ6hgjUo5ggBHY/pqcOEBwvoy0fvPaadUjtd5CO5lDNh6+ZhG1Yw4n6AfOR7wjiSNGY6AnwIMlk+W3dmgHbBKYyaxjOFfl21zbNwcSEP0uBocjGsqlp3D5Fe5Dkn2mJ6oQOozjfsE7ycy5Do8jwNPQFK3lG9L21rXJpjTTBTD8rvxXjnzOCKA1P1WF3lqMd9V72Zvw1W6HOrzhKxEHvj+cHgXAX5VzNHCMgoPTGNzglfZLmxlVlCzeJZCkIR1Gm+1QRwOL0cHfg/nFqn70W9i2eXiLvFypnGBTv/RH1R/6B8rbw2KkSo2l8vIvYJN6aQeOA2wJFmjLIMVT4dp2rqDgvvq8iEbx0Bmc0+2qvwMgsMd1uGaa0DmDbJa4rp96u7grtx2OfM8T2aUCQN/d8b7CNlzOMT9cI7xXvIvXP8ZhgDmNgB0DKTYFG4vgXtfyNLkVu3VxWWUWfAs/QNj4T6IJOVRmtqEdFsdbNf7jcnl2NbKjkR1QiyiqIuxGsMiEdkdt3dOl5M9KGtNE7g9F0fSzzvFC/ecUBDgewCH6JB20xlRfFDBa7qL6HZSdyMjCqchaebv+ESfvnbHh9+Pn5jq+MBrZxjeE2x5wmfXmgE9LjonOU1FFbUmHgVTBIhelw7aUxkrTAJ5G7Tkr/A3L0Tyw2bDTanTnfsa+aq+5Bin6MKNhxoPwX1dLoQvzzfr5/vxD9RAOmcXMZaN3G+XVZTQ==

9c0jc2hJCCBHp+2v8bvhiP4AQev55EOnf/E8FmrIJm6XqtGxEOTr7oTykj+izxXTxhyGNZ6kjxYF6dSW1
SZ9IXVkl7Vqps6wR1nktlucpXXlX0DxbcLJai31lPBOJE1tsrT5FqJ53luJbW/NKMAdOnD8h7msJIYSI3P
vM7aIhHyA/PPydL3//RfVhDQAGev55EOnf/E+pVqg6mtosleepf71fnF0cUkfqCcV08YchzWepQ9mR
ak/1c/4r0UhWSbtW3uLWeS2WFxziLqB5thb1Wuwr45lInNh+MZlykCztLMW3tuaVYNbe+NUb4r6
WEEKIyI4Xbxrma/xuXn/y8b98/XX1kQ0ACj3/PIg0VbrpMZ+TnZDnfN2FxZqlah73a/zlEWM+UTH+M
KTNWeW69a+zN55qWUSSp6U1lr7LaZ82wSTmS1lwz22ezTciLzDZvjLNF6RxHncKeb2cKULLz028BQ
zjwysfivtaQgghItvObH3i8F2iFR8y77/+WvWpDQAKPf888DQ7Nd0+qWd1H6SH6yOqNdLnNDRL9Y
m+U8O9V/xhSKuzjPriGT1eHdZDY2VlFXXSZI2l56rI6s3L85QueRbQPJszInygntbDsyp5YtNi5Nrq8c6R
6rF3bS1Wgpm5fO0yv6ifEEIa88CxXaIJHz6//cUn1Wc3ABTo+eeBp9kx6ZbKYg42miwp3izVJ+rpdDVv
gXK6+MOQdmfVu3Uuhz7DMlHRxMbS/xLkfPNY6kk9pUveBTRv1j/CmN5edWB3rS5Iw2LCl6ca4s7
oWVurlWAWNjY39q7tFfe1hBBCRGbyNX43fLEfgEDPPw88zY5kN12eDsgckD3dtllyx3nPFAfjD0Nan
hW/GvqsQuSKVWSVtMbSPEv9nHFPiC0vuLHgApo3a49whhhr9Z6eaX1B4osxK+WnqcflFL4ZnbW1
XgkGtXl98+h7R8V9LSGEEJHbXrrx6QN3i/Z7VuGL/QBM9PxAM087CiyHkz8/Ke5rCSGEiGw7s/X4/X
eKxnu24Yv9ADR6fqAZPT+W09lLZ8V9LSGEEDdHjt8hWu55CF/sB1Ci5wea0fNjCV1cvyhuagkhhLjZ8
9ytotmek/zw8Hf+8Pnvqs90AEuMnh9oRs+PZbO+sb7zzZ3ivpYQQojIXH2N382rxx7mi/0A6PkBAJa
NzY19b+0T97WEEELcPPq9b4s2e97y7ukfVR/uAJYVPT8AwPLIzx4RN7WEEELcfPfxb4gGez7z6Qfnq
893AEuJnh8AUDv18SlxU0sIIcTNt56/RbTWc5uTB/fzxX5gmdHzAwAq5z47J25qCSGEuLnl9Lan7tsr
Wut5Dl/sB5YZPf9sqd8NV+jlF8RdXVtZvVD9PI7fRWfvKCIbGNirddm3bGkxnTij1G4ZS4xffbhgLn1xa
ftr28V9LSGEEDfz/zV+N3yxH1ha9PwzVPWkSveNUVVgRD2/u6MgfXXFXr3de9MF8Z9Ez9+Ann+Rr
G+s7/7xbnFTSwghxM19T+4U7fSihC/2A8uJnn+Geu6IdNM7mp7ftyM/PTJj7VU37+qoc8BHTbeYl2
126PkXxub1zUPvHBI3tYQQQtzsemH7s/vuEb30ooQv9gPLiZ5/dtp3sGmWtec3+/2cuVfPDG0mVZe
tp1dqtOj5F8aJ8yfETS0hhBA3N79yww8O7RGN9GLl9NHv//mrr6pPfwDLgZ5/NvS/Xa6Z/aTdtoqOy
Wik6mlENxqa32zCjDFuT2atoLFlM6c1z3R65PC09TP6JL1AsVrFmT1jX7lC0+rNxfupSX0FXYH5PIfDV
6MkN2MNMearr025wuh5AW1rWQOjr2/2ZOBSYM688as3xE0tIYQQbx56cLdooRcxbz1/svoLAMB
ymKzn13f04ebHO6TuSTLT3v/XbUV4FXPPuiIltRvPUxnjqnkGONc0NL9/8pwxhdm4adGXrZNp9fPlM
fEwtCNBnZU9p86IrtyYOPh28i08ODjjLSwPNl0N34Yz9QjP88WT/vOiC06pVWlecc4chTnz4ZUPxR0tI
YQQbw6c2CGa58XNJz99t/prAMASGKDnF83NtLf/5nTRPmbuea+m7pvUddKj9lUzWqvoBfDN757r
zO+cps6JvHLutPURWScyrTnGV9W3IyEfIpYQWbcxZbvdmcJnqEmNEWIxzk7kYuUU7jqNVZmXIzRRf
aIjpVZ9RBXXZ6kj9YFIbczW5WuX+UX9hBDSJt/8x79Z3K/xuzl5cP/nly9XfxkAGLvOe36XbEKmYzQSm
YXuJXxX03ex5DjdbjXsPja/cUwMUw+NyT2HhM6m1RNV7JJizga+aynU5SIzOlX1WcGZna2JtbS8Gqa
Wq9DDghO1EK5VXyY5xjkno49NsRb0Z2NzY+/aXnFTSwghxM22M1ufOHyXaJsXPXyxH1ge/ff8KeeE
qdlWVqr/XeBmwnNlRGtY0Zsuj3oaMC/flfcUEMP07B7Bgs09YetpzYHy1fXtKMyzV4ueLTgiqGlqPXc
1QI1X6259NQr2aGcO+4R6V9ok22tdS74WaohVzHsQc2Hz+ubR946Km1pCCCHePHBsl2iYxxG+2A8
siX7/2367gSh4moZcy6ZAnbN6Qf20wN2E52r6eyQxsG0j5Znfd25gdh9jIluX09YjnQ36dhTmWZShLt
NqMlvzQtSIoriqpUe3uRq6hqCeD24vcGJsmwm15CXwLie4RszcyZ+fFHe0hBBCvLn3mdtFqzym8M
V+YBnMqOf39hjNjYE6LZ9E/by47YTnavp7JLHVto2UZ37fuWJYUpvW3bT6tIIY7dtRWKSkeqrlTA61k
NhuqhrZEDW6rtV8NeoVVoOcvTfOYUxRaDOwfS05xrucxjViNs5eOivuaAkhhHiz48WbxvQ1fjd8sR9
YBv3/Dj/POW1aCh91XnmaerSw/UTsypibkuPaNlKe+Vs0b76zGnU1rRq1srbm2+RkawtdJz1Lu2l8R
dXU0QmqQSurq+UExuDGjbhrV0f0KaHtZbKnJiiWVkvO6quij3nWiFni/xs/IYS0zPH77xRN8vjy6rGHq
78eAIzULHp+dcTTQER7Azmo1UlzzL0yGd1uyV3W2/Q0YF6+
+Sdp3tQBpwN0dTOtdYp+EJkzzn+d6nWErp88r8XK/fS4gjW4aU5Zoh6v55HLLOkT68P+gbW0Wnq
UGuNsyZgnVBozsnl988hPjoj7WkIIIW72PHer6JDHl08/OF/99QBgpGbR8+sewyM8sZ6o7h/cIwslcD
X9l8fYY1MPp9kzFVXaNG/1EUvkpelkWv20mkQvv57Vs6Mwz6ICayjJuqGySkP5jHmeHN1wNfwlc2
qIb3u50Jnh5SbVcl/fyMWVa8TsXfvTNX5pPyGEtMmR43eIJnlMeff0j6q/GACM1+L0/OGGYlFbisjVtD

crng81ex7mpS6Gt2veMvYKmkpNP239hDFeL78e6OwozLMo63RJjfOdJ890XzIvfZJ/qfGLbFbM66n
HVW3/Mkv2Ylssd/Jaza9v9kRsjZg5/p/zE0JIm2w7s3Ws/4X/q8ce/svXX1d/KwAYr1n2/BM1AmYz4a
KnwDxS71ren5hP5z47J25tCSGEuLntpRufPnC3aJgXPScP7v/D57+r/j4AMGqz/D5/ZBaHPieApgpzR
79peXdifr38y5fFrS0hhBA39/zP20TPvOjha/zA8kjs+b2KFt5p6D0dfj1NdUz/l8WhfwrgmaSkn6Ctwv
zQb+hC6F0NzIfH3n9M3NoSQghx88CxXaJtXtzwNX5gqcyk5w9NFOyNIp195ClgRoyen4Yfc2/z+uah
dw6JW1tCCCEi285sfeLwXaJ5XsTwNX5g2cyo58/Zk8Va9mhfH30SANBofWN955s7xd0tIYQQkR0v3
vTsvntEC71Y4Wv8wBKarOcHAIzSxfWL/Bp/QghpzL3P3C666MXKb3/xSfW5D2Bp0PMDAHJv/
+ZtcWtLCCHEzeJ+sf/911+rPvEBLBN6fgBA5dTHp8StLSGEEJGbX7nhB4f2iHZ6/vP6k4/zNX5gOdHzz
9rVtRXjNx2oX/7GLycIEb8fIv5wIuLaTzPVpKzf89/7a2+/5fo05DVEZ46+d1Tc3RJCCBHZ9cL2xfpi/w8P
f+fL339RfdADWDL0/LNUtURGQ0TP30S0kfGHE5lVz68Llfp96atife+pMtg1RJc2Njf2ru0Vd7eEEEJED
pzYIfrqeQ5f4weWGT3/7PgaInr+CYmLOE2TOatrP2DdoXtwev5FdeXLK/waf0IIacxDD+4WrfV8hq/x
A0uOnn926Pk7sPg9/5B98dA9+ND10KGPrn4kbm0JIYSILMQX+/kaPwB6/tlQ/aWhaIvMvtMY47ah
upvKiafNScxx4cYr0O56D1uVW5zR0Pepp+sz3CNq2vyImC7+ME4PzmTjxdq9U5mnmAuMsk6yzlIlD
YGFm4sT67bYtcxqoVpi2zIn68aYeprqyXBFz0RYKGcvnRV3t4QQQkS+9fwtoseeq/A1fgAZev7ZaGrAX
L6WzGQ832oSm9HUVUcy7kH/zJ7SxqGmvk89r5/WRfQk1ZHisZgu/jDCv5VcVdaZSh8wGRv1il8xz7
OBhTcuNxctFqqljhvzOFv3nFsOj1ac5OXAnDpx/oS4uyWEECLy3ce/ITrt+Qlf4weQoeefHV9DZPRQ1V
E9SjVSwYZMd1ruJPWRUPOlZjU6v/C8zlKcIcY0vm1aqgHqFHf1aobioZgu/jBIj5M1MtVCmmb2bNX
R4oq5E3u5V6U+IrYwYS3PPpxhbvVCY0VfPSyUzeubR35yRNzdEkIIEXn0e98WzfY85N//9V+qT3MA
y42ef3ZiDZhxTAyTfVZGHmqexMOZ1+kFPc2hM69nTOvS5Tnlg9XVfCLziDpfTBd/GOIbpo9Va7fHqE
eejRmHhDZXzD3glfKaugN8p7R5yXT18F4L8sTGJWIBXPvTNX6NPyGExHPL6W1P3bdXtNyzzT//w1P
V5ziApUfPPztpDZhuvzyqiVL6w3pIVVpNok/wLC0TOCu8fp/qrGJA8fPK2oXirPqIPl1MF38Y4t2LOGh
PpZ70CdXyVtHzqqPtlqzr16NiJ9rL1QN8p3hW6QzzVJe8FdvtDXPv8rXL21/bLm5wCSGEmJmrL/a/u
HLkP//4x+pDHMDSo+efnbQGzO6tbNVEbbo4DzWmOE3NUY/3zJoJrG7C0tVp2YhydPZDeUgf0WeL
6eIPQ7x7EQftqdSTPqFa3irOEtst2TOXe6I+IrgDjFptZvbvJNNQ0VcPi+ncZ+fE3S0hhBCR//HYN0XvPav
8x69/XX18AwA9/yylNWDB9qvWqj/0qU7MzlPj40vLqIHV0dTS5XnqX+8XZxeH1JF6QjFd/GGIdy/io
D2V94wG/nPUvOpouyV75pInqiGZcpQ7s69Wi5lDu2+s6KuHhfXyL18Wd7eEEELMbDuz9fj9d4r2e/
jwNX4AAj3/7KQ1YC3aqDZdnF915srqajncHK2nMBs/Oa+ntG4MY6Wredyv8ZdHjPlExfjDEN8wfayq
ZY9pObFFzlhwJmo3c/Nr6o5wr7yvVpuXzDMm01yx3d6wOB57/zFxg0sIIcTMbS/d+PSBu0UTPmT4
Gj8AFz3/7LRswOQw/VgdaNOhtW2+9FwFMVg/qSbWs+pK4cU1lK5P9J0a3kn8YZCzTqN+VSw0szp
DLy9SqsUVk3UC1EzhKyHXUy+4ntlXqx5XHTSuuxrmqZ5pruirh0W2eX3z0DuHxA0uIYQQM3ueu1X
04YOFr/ED8KLnnyGjucoUbZGnuXL7JqO3MtRtVatJAsw1uWPtFStmJ+hfWyFeuj5RT6ereQuU08UfR
oQXWlVzpvKf0VBogisWnanFa+ovIatntseUx8NXQp/oqZ5rrNhub1go6xvrO9/cKW5wCSGEmDly/A
7RjQ8TvsYPwIuef6bMpqnopzzNlb9vshs1uxdrPYmPXpLo7zS7smc+c0D2dNvS7jjvmeJg/GGcHpzJx
ovLFq9eCF0iwT5Jrqzdktu9pvrFy+RH1WNjanOInsxcYTbWmdlTvRKv2G5vWDQX1y/ya/wJISSSbWe
2PnH4LtGQ951Pfvpu9TENADZ6fthUm9aynwWwhN7+zdviBpcQQoiZHS/e9Oy+e0Rb3l/eev5k9QE
NAA56fpj0v5iI5QcQc+rjU+IGlxBCiJl7n7lddOY95fTR7//5q6+qT2cAcNDzo2T+R9r8t9gAmh1976i4w
SWEEGLmgWO7RH/eeU4e3P/55cvV5zIA+NDzo2T0/DT8AFrY2NzYu7ZX3OASQgjRGeCL/XyNH0Aj
en4AQKIrX17h1/gTQkgk3/zHv+nvi/18jR9AG/T8AIB0H139SNzgEkIIMXPgxA7Rq3cSvsYPoCV6fgDA
VM5eOitucAkhhJh56MHdomOfMnyNH0B79PwAgGmdOH9C3OASQgjRufmVG35waI/o26cJX+M
H0B49PwBgWpvXN4/85Ii4xyWEEKKz64XtXX2x/93TP6o+fAGgBXp+AEAHrv3pGr/GnxBCIrnvyZ2ie
0/Iq8ce/svXX1efvADQAj0/AKAbl69d3v7adnGPSwghROfR731b9PAT5eTB/X/4/HfVZy4AtEPPDwDo
zLnPzokbXEIIITq3nN721H17RSffPp9+cL76tAWA1uj5Z+zq2soWw+qF6nhFPL1lZe1q9UzmwqpzUI8
3JorOUbKGuM+3mAEAKi//8mVxj0sIIUTnW8/fIjr5luFr/ADS0PPPkGylK7pdVz29rW642/T8/jli/0igY
EzZPAMA2B57/zFxj0sIIUTnu49/Q/TzjeFr/ACS0fPPjO61VX+tm+vygHpePe2Mb9PzyyHOJPIE54zGG

QBA2ry+eeidQ+IelxBCiM5EX+zna/wApkHPPytOs10dCv/788b2PCPG6IehDl0N8ExRHmqcAQB81jf
Wd765U9zjEkIIKXPbSzc+feBu0duHwtf4AUyDnn9WPA17mBpcat/zGy27ZhW0J7bJf2yg0f0DaOPi+
kV+jT8hhISy57lbRW/vzb/97zPVpyoAJKHnn5UWPb+n3S5M0PMHJ6meb+75m2YAgLC3f/O2uMcl
hBCic+T4HaLDF3n9ycf5Gj+AKdHzz0pjz2+04+UYp59v1fMrsrsvT2pchcE/AwBEnfr4lLjHJYQQUmbb
ma3H779T9Pk6Pzz8nS9//0X1YQoAqej5Z8XXneddteqk3W5c99yRnt8Zk8mOGY/suuF/RmCKzQAA
TY6+d1Tc5hJCCCkT+WL/b3/xSfUxCgBToOefGd06q97ZPiC79/pZ3Ws7M+hz1BF9wP0nB9WR8BxtZ
/AcAADTxubG3rW94jaXEEJImXufuV10+1nef/216jMUAKZDzz9DRhtvqlpn3W07VM8fmiCnxoRm0
XMEJqmfb56Bnh9AkytfXuHX+BNCSCgPHNtlNvx8jR9Ah+j5Z0x03HbbbLbbeYutHtfttnV+dlg/NIY4X
bv5XMlehdu7x2eg5wfQwkdXPxL3uIQQQspsO7P1icN3lQ0/X+MH0C16fgDAQM5eOitucwkkhhJTZ8
eJNJw/uz3p+vsYPoFv0/ACA4Yh7XEIIITqffnCer/ED6Bw9PwBgIFu2DPSXDoWSUSgZhZJRKBmFklEo
2fgKLQMuJQBgINyRJKNQMgolo1AyCiWjUDIKIYJLCQAYCHckySiUjELJKJSMQskolIxCiOBSAgAGwh1J
Mgolo1AyCiWjUDIKJaMQIriUAICBcEeSjELJKJSMQskolIxCySiECC4lAGAg3JEko1AyCiWjUDIKJaNQ
MgohgksJABgIdyTJKJSMQskolIxCySiUjEKI4FICAAbCHUkyCiWjUDIKJaNQMgoloxAiuJQAgIFwR5KM
QskolIxCySiUjELJKIQILiUAYCDckSSjUDIKJaNQMgolo1AyCiGCSwkAGAh3JMkolIxCySiUjELJKJSMQoj
gUgIABsIdSTIKJaNQMgolo1AyCiWjECK4lACAgXBHkoxCySiUjELJKJSMQskohAguJQBgINyRJKNQMg
olo1AyCiWjUDIKIYJLCQAYCHckySiUjELJKJSMQskolIxCiOBSAgAGwh1JMgolo1AyCiWjUDIKJaMQIri
UAICBcEeSjELJKJSMQskolIxCySiECC4lAGAg3JEko1AyCiWjUDIKJaNQMgohgksJABgIdyTJKJSMQskol
IxCySiUjEKI4FICAAbCHUkyCiWjUDIKJaNQMgoloxAiuJQAgIFwR5KMQskolIxCySiUjELJKIQILiUAYCD
ckSSjUDIKJaNQMgolo1AyCiGCSwkAGAh3JMkolIxCySiUjELJKJSMQojgUgIABsIdSTIKJaNQMgolo1A
yCiWjECK4lACAgXBHkoxCySiUjELJKJSMQskohAguJQBgINyRJKNQMgolo1AyCiWjUDIKIYJLCQAYCH
ckySiUjELJKJSMQskolIxCiOBSAgAAAAAwTvT8AAAAAACMEz0/AAAAAADjRM8PAAAAAMA40fMD
AAAAADBO9PwAAAAAIwTPT8AAAAAAONEzw8AAAAAwDjR8wMAAAAAME70/AAAAAAAjBM9
PwAAAAAA40TPDwDApC6sbqmtXqiOAgAAzBt6fgDAiJTNeL9d+NW1lbpCnwWH/icL+cYyK2tXqwN
dsjZT4J+VTMq6hv28TL0L/IGp3nulbt8ZkT+ikacm5Z/KesW63Va/olem1w+KHoU3taivU2BH1na6ep
la1FrQz6QlQc8PAOhXj3fzFqPOkDdtRdk+7nWyeym9j+K+qucbqnwjK6ur2f/ppU6+hcFvCc370e7fF
dY7u9bTu6/Yy0DvCPO6dVck+CfU/jNkb3QqwYrRpyYVmio/rg+Ul3TaUv5ZzJdr6hKNVyZ/vtMPCt+
mrC0VpttXZFPFUx3+UXK3YxQ39bSjor4+VDyadnftanVQqrx2FXF9rKsonkML9PwAgB7Z91P23UinV
KHif4e8IbB32Jfeq+QFqsvXT5n8te/9MpmGeWEsRcme3nxy7v5qdX0fX1Gvh7tw+dboamvhirGnJtV
2KjWuejix4vySXSW7fPrA9C9X03by49XTU9UpxTbVxfyVyKY8h9IFtyNMXzS8I3mkx1rqierhtK9aPp2
evngn1w/tUsWTU+1pGdHzAwD6I+8BnLuGzvVfQRjm9qPnbenp8x/Sb9piprsdnNjg74NMv28F+wL
2tr9iYvOF6vqFc1bubqXjCxm5Vh1exqapiueTL6Q6u98qBn8hfTT/YeoyarGeWv18XLiFOtlHJbIdW4f
v78ZL17iY9pypnH10WExfzeKBfDt0WWhp0PMDAHrj/s3c4d2O38A3A8V+urpnDLJuf7pnXLP8x34K
FVeq1vM1628fQT2/SJnyEmYlyh/6eZc7f0KNd0cnnPnyivZ167hkZLoOKzVN5VzYJE1VOtuQbyLjWP5
jZ292Ty33TdEFt1BRJ/
+igjZ9Vd+lMxRPd7U3b63irZaXKH8IL2UyTq3A5exma+Zl8hfqbGNLgp4fANAb9w7Ae4vSpd4LmIobj
67u3lzFXio9binfhZo+L9nfhpRyY33WKd55Hd/Kxw3zxivecYW+Srn7cP8UT8Up4M7f8bWMTNdhp
fhUxQvXwVWMVyme7ea18hTK96CO5E939qYI1DJ0VMotJF6XYkD4+rbj2Y4h/uykQrPV16+zUp5ax
RF59Tp6rYodVNXyn+1pu72Ky4GeHwDQmxn8VT3cvYC4W+xVpzdTtnwb9fXKCw2yJ/et0Snx4pTXr
8+3RVGw1/eduYfy537qFXPLS9fhS1VMaK7cfSs4Q6YTma7DSrGpxNtxCt4qxcFKV5fNKZTvoX6cP93
ZmyJ26TLl9rqo5hZy3nwd/EGObqeD+U2eWuXlKo+VP3dUz7uvYj/K6qr1JplCMa1+YZxXKX6N4UXP
DwDozQz+qh7oXqC80xnwnqO8eeu+oHXLZujiDjumqNvf9XPeef3WK16dXi+ZW6HHHVlviu7u40v
On1D3j6zz6k3HLaBFnppUcKriana1nYYFF093UkwWst4Thj5quTp6R7iFnImb19IoMkXxVFfvhZxTy6
1QvHLT7ajUfGnyEV3sTv6RcV/
+5rVAoucHAPTG/Yu5o3u3sCHuBYoaA99wFDV7vXKFvEz/VfrfTh+38mGd3VWHuSV63ZGh67eEu2

75YnW9s8h8HZbyT1W8bh1upnHBxYAOCjYUyp/u7F3RuKmu/oS5hYoj5kbkmzFBeDsdbcPg1HJLNF
/dlhonygdM/6YodmBP41bu4GVaOvT8AID+yL+ZO7v9COq/QnlPInV+/5FVMbbhuQ/qRTd3ba5sXm
Pa4kXq9VUqKpgb6fMesXhx+n3Pue+AQYpmOn9HFC+NvXBrb85LNzVPRSXy1KQ8UxWHun2RGhd
cDOjg8jUUyp/u7EWa6aasP0id/KkKbqeHP7NuLesPU6a7ovGXqZvXqFitWyQ/bM4dXwq86PkBAD2
ybkC6uSvwK+YWFvueQOxomM3kRXt5hQbfjXWr2919r6vPd7WtvB/WBnhHdLk38Q4o1Fsw99bVx
YxUjC9mIuGpxOtVmnJzRTVrpVkV43FRc5oaLa9MPqyzN72zqeyAMXm5JM8iWotvynp2mjIVZzul4v
AA7+3OPyjCtexnpr505XTeaaz3dadXcnnQ8wMA+mXegPD3NAZj3ZBOfT8aUry9e5t9Bjq+j0cn7Fel
VL024qkFesXCm1rIPUVeo8zoPig6J/5RRam+ZeBGYkr0/AAAAAAAjBM9PwAAAAAA40TPDwAAAA
DAONHzAwAAAAAwTvT8AAAAAACMEz0/AAAAAADjRM8PAAAAAMA40fMDAAAAAADBO9PwAAA
AAAIwTPT8AAAAAAONEzw8AAAAAwDjR8wMAAAAAME70/AAAAAAAjBM9PwAAAAAA40TPDw
AAAADAONHzAwAAAAAwTvT8AAAAAACMEz0/AAAAAADjRM8PAAAAAMA40fMDAAAAAADBO9P
wAAAAAAIwTPT8AAAAAAONEzw8AAAAAwDjR8wMAAAAAME70/AAAAAAAjBM9PwAAAAAA40
TPDwAAAADAONHzAwAAAAAwTvT8AAAAAACMEz0/AAAAAADjRM8PAAAAAMA40fMDAAAAAD
BO9PwAAADz5cLqlsrqhepQST2xsna1OtIPVUeUnwf1tck41+Hq2kroqehzADBe9PwAAADzxehr7fZ
02Xv+um0v+C5DsLOvL+oc/pMMAOgPPT8AAMB8sf5dttmhLnvP32r/3ua+/kcBdPwAlgw9PwAAw
Hyxen6zwV3ynl837g3r0tdPXyd9hI4fwNKh5wcAAJgvds9vNKpOz+8ccBvjakg+wpi3OsE4YnbD6nB2
zPjP6Y0iivGs87yxtrpMQ89tzWdNaKxUicylRxdj9CNnB7H15+z1iCGRDUbPA4BB0fMDAADMl7qZXC
t/Ui2j0WUWj90DRrtZtcR1O9pIN9Hhc4w+W/a1BWMhnkmira+/qNy6wViLo17c6pr6UVRvWn/Dg
sIb9J8XXS4A9IeeHwAAYL6opjHrIFVjWjaMxhPFQPdArOevBhktqTyi2lLnnPqIOiDL6BF6KUadFu1uuI
Ce0K0ZowdX6mkKjetXA8LrCWwwNJFcAQAMg54fAABgvlhdY/XA/NltO412UjazakjdI4aPqGncEXK
M7Igz8pCexBgTJFeQkztxD8TpBeTEKS3WLznV/RvUw1rtGwB6R88PAAAwX/ztddZqOp2xp1WWrak
7pPmIemw2yva8aoSPKGzNEuAuKadKqqNya43CS2ixfoM9Wj0fmF2vsmZvCwAGRc8PAAAwX2QH
XD3WX+
+ve0g5MtNXz28fVA98goXD/GPlVpJ7fncJLdbva94L6vng7IET264aADpFzw8AADBfnGZSNpH6CU/b
KRtjd0jzEc+0Yl7fCKHFEM0/VpVUR+XWGoWX0Lw4NSJTDnKqN85hTFEIFwOA/tDzAwAAzBdPM2
m3j/qJ2MgOen6zubbHtGi/3TJhejpzsFOjw56/cSr3VOeiRDaYPWVMPPG6AaBD9PwAAADzxddM6r
4xp5+oj1btpG5MnSNu8xo+Ep5FDwlXVo2tWyYmXKA+f+LeObKEpvXL7dTjGzbobCS6DADoHT0/AA
DAfGloJs0njFZUEr1srAWVR/TjFTm76ncz/tL1ALdMnBpvM8/usudvXL9/OTk1JDR76My2qwaATtHz
AwAAzJdAM1k3qdYTZu+atZWyMXYnaz6iHpvTOcvJ2X2zPSCwiyh7Ptkky601alxCbP316bm8pHFZz
Kd9s5un5tquGAA6R88PAAAAAMA40fMDAAAAAADBO9PwAAAAAAIwTPT8AAAAAAONEzw8AAAA
AwDjR8wMAAAAAME70/AAAAAAAjBM9PwAAAAAA40TPDwAAAADAONHzAwAAAAAwTvT8AAA
AAACMEz0/AAAAAADjRM8PAAAAAMA40fMDAAAAAADBGf/3rfwGin6XWhQU6/gAAAABJRU5ErkJ
ggg==]]></redcap:Attachment>
</ItemDef>
<ItemDef OID="b19" Name="b19" DataType="text" Length="999" redcap:Variable="b19"
redcap:FieldType="descriptive" redcap:BranchingLogic="[q18] = '2'"
redcap:InlineImage="1">
<Question><TranslatedText></TranslatedText></Question>
<redcap:Attachment DocName="B.png" MimeType="image/png"><!
[CDATA[iVBORw0KGgoAAAANSUhEUgAABVEAAAEJCAYAAACdcmdaAAAAAXNSR0IArs4c6QAAAA
RnQU1BAACxjwv8YQUAAAAJcEhZcwAAEnQAABJ0Ad5mH3gAAE9sSURBVHhe7d0PjCRnfef/b+/aY
PPHNmBskx+RHat7sZYlGJIg08PxL4egZ+6SJbnbYOVg5Oh3PVyi0zQSkwOxF+mX2wguG4lunS5iWrq
zliQyTEJugrLdlJRgckzHR4IxYTI/Z7rlc3T8wDYGjPljB3u3f/VUPVX1VHd1dVV3VXX39PtltaerpfqT/T
O/OZ5/k+hb5FAAAAAAAAAAAChjumPAAAAAAAAAAIAQhKgAAAAAAAAAAEIEQFQAAAAAAAAAAiEKIC

AAAAAAAAQARCVAAAAAAAAAACIQIgKAAAAAAAAABEIUQEAAAAAAAAgAiEqAAAAAAAAAEQgRA
UAAAAAAACACISoAAAAAAAAABCBEBUAAAAAAAAAIhCiAgAAAAAAAEAEQlQAAAAAAAAAiECICg
AAAAAAAAARCFEBAAAAAAAAIAIhKgAAAAAAAABEIEQFAAAAAAAAgAiEqAAAAAAAAAAQgRAVA
AAAAAAAACIQogIAAAAAAABABEJUAAAAAAAAAIhAiAoAAAAAAAAAEQhRAQAAAAAAACACISoA
AAAAAAAARCBEBQAAAAAAAIAIhKgAAAAAAAAAEIEQFQAAAAAAAAiEKICAAAAAAAAAQARCVAA
AAAAAAACIQIgKAAAAAAAAABEIUQEAAAAAAAAgAiEqAAAAAAAAAEQgRAUAAAAAAACACISoAA
AAAAAAABCBEBUAAAAAAAAAIhCiAgAAAAAAAEAEQlQAAAAAAAAAiECICgAAAAAAAAARCFEBAA
AAAAAAIAIhKgAAAAAAAABEIEQFAAAAAAAAgAiEqAAAAAAAAAAQgRAVAAAAAAAAAACIQogIAAA
AAAABABEJUAAAAAAAAAIhAiLp0etJYKUih4DxWGj29PqjXWPFeU1hpWF+1iMxj3ZB2onXjpLWdL
MxLO7LSlg372PzHqPs4Vb2GrGzEO5vtDbdtK5JH08KNuQ9Cjmc+2g0AAAAAwPwhRF06Rdm8UJey
XurU1kPCkracr3X0c5Hq2U3rq4B5oILBVWnqJUdZzqxle4faf1Qo1cT/rlhsR+14AAAAAADIGiHqMip
uytmqfi4dqZ0P9kbrNc75IVW1JdsV/XzhFGVzry/9vnpsS/aHkff+RpmXdmShKwdu8leuS9c+xj3ZzDJ
D7TVk3fijwsI7ascDAAAAAEAOCFGXVGW7JV6O2jxn9EY1e6FWpbW4CSqOot6h7OuncuoEPaQBA
AAAAEAuCFGXVkW2W26M6vdGbW/4Q6XL9a3wXoyqlqKuRek+wmpSjqyvaH59ZI1Jo6ZjoC7rqPX
GPu31edcGHd5fZI1J4zwEz5+5nbDPjxN+3INtCdS9tR4xy30ObGewraPOc7xjCm57uPZpYAh6c9VZF
2h4snM3eA7Uw9yc3Z6x+xzPPy71CLkXbAnaHvI9OHq7vqTHE6/dAAAAAAAcfYSoy6yyJXW3OGpz
VTYaDTnnJ6hyIWSM9FAIo3VqpQwmoCrK2hndwM6BdJ1nFmNId2dHLno7bcuubn/5zNpc9FKsnPa
D6gP/AGy9izv6PBo1Pe1wrCSDo63TPb8dqZUKUhrYSXM1aUimtjPY1qasDoZtEx2T2rZR+9S6H+te1
+kREu3HCSwHz4HSXB0dKibnnOtV70AUtW6Kc9TeGFHPNGS7E4vZbgAAAAAlgQh6lILTjLVrLnBTF
nqF0Imk2pv+KGKV4+yL103ie3UZD3lhKW4dka3b18O3U23d42JhYxw0hjqferEnAz0rpz2yiY0d4M9
Ji/u6BisfEacDLUnjXX3GlSlpc9v3+0xnOr59bfvdUhWIdlAfdzx/O1494Hazrob/E1zTMbr9zZlc9v62PX
vV1Wv1/6cXXIi2X56jXUvsCzXu85r+8MlLiqR+4wr/BzteOl/krZbr3X/0hH2PRjY7rBkxzOu3QAAAAA
ALI+C9QtyXz/HklK9SwM9zlSwEhKq+K8rS71rTubTk8aK24tOBS/OZEYjX6963bk96Ubsy+dvW4Vde
9ZG1BBs1YOwWq1Ks9kcWu+3IaxdcdeNE387w+2yV3rnwG1/6DqbsV0VnO2FBNwB4e0wr3O11fc
nDEt0PSK2E9ivvuaS7JjMbQdfr41qa6JzF35+FOdanQqsS3p+lETnOlHbRwht44jjjDietO4RYBr3Pnyvv
OXCW/QSACAP99/xLnnNW2/SSwCAzB3/NZFjr9ILWBT0REVwkikVtoQGJP5Qeb/npMsYdm/2GE1F
UU6ccp51dJfTrj2WvywnT5+0e9QF11uqp/0AbA74vWmb4nVG7R44gZT1GW8ov7dOdT4sGbUo3S
BMfcIsazBr1jUo6ac28z7QPYSnOKZEvYkT7cec4f+kmIdQ3NyTvhmgTm3wHIWY6rrrurFuwJmaGO0
GMvDwEw/LOz/5Tr0EAMjDDY9fJXd/6AF5/Bs/0msAAJm7dI9I/zt6AYuCEBVBA6FScsO1P6fl1RVt7k
pbenJoj9k/JScqJ6z/D65XGeqc9ZIrrombLbpD+ttuIl09a/TozUtaAZl1DTJrOyHeSKpXqBe0GnVjgQX3x
NNPSOWPKvZHAEA+nvPjY/KWL94oP3ryGWm+/3555p8u6c8AALL1lMilP7A+PussYiEQoiJlGYRfXl3
RfTlsXxS7lKgd9lbEyVeN9XMZvhk9NO3A1+/VOyrwVcO67VqVQ480e0lOK1mv47yOaTHOXbjxbW/
Lhtnr1K2LatY5BRbUnZ+6Ux58/EG9BADIwxv/
+gZ5wQ+vsJ8/9NXvyp/WeR8GgNz0Hxa5/Od6AYuAEBUxleSkN1LbnBFfMSZJitM70Ri+HI+7744c7D
pf686+X7I/4a8fLjUwH4qbZ/0guOFOjFWVQIZacsoTKG6Jgvk22OvYvA/0seV1TIn2Y97LA8Pk1cz3hZ
xnoE/SdmNSNTtwdeukJv6eAubL+z77PvlM7zN6CQCQh1c+eK3c/PXn6yXH5z7+kHzlLx/RSwCAzF3
6gsjlr+kFzDtCVMRUlM2zbuVUc/Z1NbrYnOl8y+vt5wScijnre1s23JlrYvN7cqqJpBS3XmZRF0x117vh
6vxxe81a56Kmj3+wdqsx7F+aq7LhnTIV7Onh297K+dBcNQLH9nm/hqd7bHkdU6L9mLVbm7Lqre/p
me+ta1Qq+NvI2oTnyA9c3XYDi+kT+5+Q+n11vQQAyIOqg/pzD7xELwX94W9/Tb73raf1EgAgc5f/RK
T/fb2AeUaIivgq29LyctSalHTA48w8bynX5YJR4NOfUMnSXNWB0GQ1HAPbsp55Q/a9of6KMUnTHP
Jqu9rKUt8KRKiWomxe8IdlN1d1gOaFzqMm/ZolJ3AMttM8tryOKdl+ipsXpO6/2HmtOZGTdS/7h6Br
7yrua1NNWBO03bzfw9odR+bHA8R339fvk7v+7C69BADIw/FLBXsYv/oYRgWozd+8Xy8BADLXf1Lk
8sf1AuYZISoSqWyH11+stqz17tBiV3FT9gZfW21NVr/R7K0XKBlgDM3OdKKjFJgB2KiyA+qc9bt+wOe
ya1/OW03PstS7fT9Yt1Wl1d8LTpaV1zEl2k9RNvf60h16sXq5MUzeVpHt4EGK7B96PbFTEbvtVlv6Lf

8+sqlzblwHu+5ulByOB4jBnYn/6Wfp7QQAeVr50kvl2iev1Evh/uFL35ZP//6hXgIAZO6y9RvZJcpbzbt
CX81aAgAxtTcK4nSSVCHqQGAKADGo4PT1/+318sAjD+g1AIA8lB56od0LNa6tu8vyiteFD/sHAGTgit
8QKfBL9ryiJyoAAMiVGsJPgAoA+VK9T1Uv1CTUsH7qowJAji79AfVR5xghKgAAyM1HvvgRezIpAEB+
VP3Tt33hZSProI6iAtS7/+NX9RIAIHN2fdR79ALmDSEqAADIxe6Du/LBv/igXgIA5OWOL18/tg7qKPv/
8zH53Mcf0ksAgMxd/n9FLn1BL2CeUBMVAABkTg3fV3VQmUgKAPJ189efL//8Czfppclc+dzjsnX36+
XWV79IrwEAZOtKXR/1Zr2MeUBPVAAAkKknnn6CmfgBYAZe8MMrEk0kNcoz/3RJmu+/X3705DN6
DQAgW9b77aWPWx+fchYxFwhRAQBAZlRwqgLUh594WK8BAORB1T99yxdvlOf8OJ1f+R7/xo/k7g8
xKSAA5Kb/HZFL1EedJ4SoAAAgM+/77Pvk3ofv1UsAgLz83AMvkRsev0ovpeMrf/mI3PtJ/igGALm5/
DXr0dELmDVC1KXWlo1CQQrGY6XR05/LSa8hKxttveBob7jtWZG8mzM3Qs5LHL3GinPuVhoy6tT55
9d55H7NjwLuWyCW+n11+djffkwvAQDy8vJvPE9e+eC1eildn/zPB/LN//19vQQAyNylXZH+Y3oBs0S
IurR60lhZlaZecpTlzFpRP8+eHfiVasLfVIImPi+9hqzXor5KXfOCrAYvunRqJSlMENguK+5bIB7V+1T1Qg
UA5Ovqp47LG++bvg7qKKo+6u//
+y/bHwEAeVD1Uf+79fFZZxEzQ4i6tLpy4KZA5bp0+33p9/dkM68MdWzgt6QmPS+qZ+S4YK99XtxN
V1vqevelWy87K5qrQo4aA/ctEIuqf6rqoAIA8veWvRvtIDVLqifqH/721/QSACBz/UdFLv2xXsCsEKIuq
96h7OuncuqE5Nf/FGmL1zOyJ41zugtquS5bFedpcfOsVJ2n0twlRQUwPTUTf+WPKvZHAEC+Xvu1F8
vLHr1aL2Vrb/f/2A8AQE4uf8l5YGYIUZeQXbvRDN2aq04tx8GuiKp3o1E7c1T9zGAtyGCd1bDejbH3r
wVreI6qN+kMVfdfl6zW52A9S6+2qH6ENy3GPgPncEP8zZjnydln0vOiqHaWYvWMNHoeB0LzkpzUn
VFl/9A6olGM9obVW21v6GMZPAcJrkvI/RZ2vePcb4PXz35E1ImNY5b37WT3p2vSa2eZ6D1Ar1TMr
49uJI6QOz91pzz4+IN6CQCQFxWevubvXqSX8qF6o1IfFQBydOlPqI86Q4SoCDUUGGl2/cyRYVRHai
WjzqrR43EyanuDNTzVurCgpuQNVXdFt3UUZ5+DwWRzdcJ9Fjflgjtk3joz5/RGeo1z3nkq1y+kUEahK
q1+y+tVOsTseTxK50C6+umwipx2N97ZkYsDJ7W96x2NX1c3yXVRQV7I/eZcj1EBZPj9pu7d0GC5U5
NSIMjOSpb3bcz7M2CCa2eZ7D0Ay07VQP1M7zN6CQCQFzV8Xw3jz5uqi3r3h75KfVQAyI2qj3qP9Z
H6qLNAiLqEKtt96Xfr4kZ7Um3Z9TH72zrxbG/4AZBXL9Won9mpyfrIxEaFec7r+3uboWUCxu4/wN+
et3/pyI6XBPWkse4GPca+Wzo1imzrKP523M2ofdbOu/Fbsn0WNy+If+rOS9v677wbglnn94JOUJOd
F4P9um2ZLK8uyolT+ukYlS23beb5V9ri53BnxMnhkpwj67VGqYGh+21ofyZj2/b9ZrYlbFt+kJ3U/Ny3
/uvD789hya6dZar3ACyrT+x/wp6NHwCQv5UvvTTzOqijPPTV78qf1hmBAAC56T8scvnP9QLyRIiKIW
bPtPoFPwgdDgOHletbE4Z54aotPxwsrp3xAqzOge4z2bsoO14eaey7suW3deeiJIl7zH364ZMh8T6Ls
nnWTbuaslpwe08Gz+8kipt740PW2PblMOpEFdfkTNjxtXf9XrVn1pzjSXSOrPOzp0NEI3gPvd4Dhu43
s8et0evSPk86CNzLYfa0LO/bsfdnmCTXzjLNewCW031fv0/u+rO79BIAIE+vfPBaufnrz9dLs/G5jz8kX/
nLR/QSACBzl74gcpkJ/vJGiIoBI3qm2Yqy5iYxIwK3UyfSDKjKcrKkn47SPdC9+VSoU/LqNhbMYdKRw9
QnMMk+K9tGr0FHOsP403RKoi+fcf2NgNIP3Kpy1j2gqa6LruEZOrw/aOh+M8JCawf20Hd33yNrsaZ
uHu/bBNduyvcALB93Jv6nn31arwEA5OWGx6+Sn3vgJXpptu7+0APy+Dd+pJcAAJlTw/r739ELyAMh
KibUkeHOgTHCo7mX3TFUvMKUSrD+5Oz05HBssVSf36vSHRZuBG7V08FeoUmYExB5PXXHCbtWR
dm8EN470w0rF3t+o8nvz/SvXdh7AJaNCk5VgPrID+h9BAB5e86Pj8lbvnijHL9U0Gtm60dPPiPN99+v
lwAA2XtK5NIf6OfIAyEqJjR/gWm53vWGbAcfk9YLHS/
+Po26n7aO1NZznJyneELGlj4tn5Sxl7S4KW5lAntYuDF8vno6/CyPP0dt2TB7nbo1OM36o0lYbdzT+xj
s/as0z83XpEi53bcTXLtoR+GPJpiWGsL/wCMP6CUAQJ7e+Nc3yAt+eIVemg+qPuqnf/9QLwEAMqfq
o15iYte8EKJiQElOusnV0EzePbnoFnIcO/Q7J6WTXtA2qm5m6ibYZ6+xrodpl6Ved5OsPCfnMa5rc1f8
zphdOfAu6QmJc0m9HrXW/XH+/I4OP6sSyOGSnCOzLqcKFN26qMaQ96TUzPKqx6k9GZQdSHa9W
p7pDpOf0CzuW0usa5fme8AU1xDz7yNf/Ig9mRQAIH+lh1448zqoo3z6v/6D/MOXvq2XAACZu/xZkf
48dRU6ughRMcCcBCnYW9IPAlXYNTChz6yYNTCbq/5Q7faGHhpuPdIev510n72GrLsnrnpWNjeNyY
Nq65JPjmrWsmzKrm5er3HOCzBj90asnBbnDulIs+ke18Bw8Amvix8oDvbcjUlvX80sH+xxaoTFZo9b
oz1p3yaRZnHfKnGu3QTvASU/dZXaee9gZMOb4h9Hze6Du/LBv/igXgIA5OnaJ6+0Z+OfZ83fvF+
+9y1qZQNAbtSw/v739QKyQoiKYeYkSJ2alHSoU/LTE7kw7YxI5vDy5uoUoVGwBmZzVQdQXnhTlVZ

qs9e7kuyzJ411d6h6Wepbav3okCq98zLMnFndbbN3TastiX+aKhIo72oZDmATnCMv2LO4x2xOsJTEi
HvXrLNaPevPNp/YQt+3SpxrZ0n4HuDXW7V41zBubVssGjV8/85P3amXAAB5UvVP3/aFl81NHdRRV
ICqglQAQE76T4pc/rheQFYIURHKHgYdUpOy2rLWu8Otp1KR7cGClfuHRs/BBOwamMZwbZddWzOj
eqgx92n23LN7obonbiCk8of1p3hehhRlc2+4Tqg9hD5hYBeYJMs6ZjsbHhT7uljH3G/5QaqtKq2+0dZ
ACYJo9r07eJA2Z5vTZZMLft9aYl07S6L3AHUsg6+ttiava4u59cTTTzATPwDMkOqBqnqiLgI1pJ/6qAC
Qo8vWb6bUR81Uoa8KBgJAEmo2fT0ZlAph96btmTxL+lhOtaYNWBfEUbp2yJUKTit/VJF7H75XrwE
A5EnVQVWTSS2SK597XLbufr3c+uoX6TUAgGxdKXLFb4gUbtbLSBM9UQEkFCxRcGZtsUO49nnrWC
J6ZB4tR+vaIV/v+
+z7CFABYEbULPx3fPl6vbQ4nvmnS9J8//3yoyef0WsAANmy3m8vqWH9TzmLSBUhKoCY2rJh17o06
pWaJQoWUK+xIqv7demmUqJinh29a4d81e+ry8f+9mN6CQCQJ1X/9C1fvFGe8+PF/NXt8W/8SO7+
0AN6CQCQuf53RC7doxeQJkJUAJNRNS8XfPx7cXMvpRq/C+YIXDvkR/U+Vb1QAQCz8XMPvERueP
wqvbSYvvKXj8jnPv6QXgIAZO7y10QufUEvIC3URAUAAKEefuJhec32a+wJpQAA+bv568+Xf/6Fm/TS
YqM+KgDkjfqoaaMnKgAAGKKCUzWRFAEqAMyGqoO6aBNJRVH1Ue/
+0FftjwCAPKj6qGpY/7POIqZGiAoAAIbc+ak75cHHH9RLAIC8qQB1UeugjvLN//19+cPf/ppeAgBkrv+
oyKU/1guYFiEqAAAIUDVQP9P7jF4CAOTttV97sbzs0av10tGyt/t/7AcAICeXv+Q8MDVCVCTUk8aKm
uVbPTaknWjdOGltZ96MOYZeQ1Y2gmvbG+7rV6TR0yunpfZjb3NEO2zTne9M2m1bkPugvaHbWJC
BSwosjE/sf8KejR8AMBsqPH3N3x3tuqGqN6rqlQoAyMmlPxHpP6YXMClCVGCGeo0VKZRq0tHLWe
pd3DH205RdQj4AA+77+n1y15/dpZcAAHm7+qnj8pa9G/XS0aXqov7+v/8y9VEBIDeqPup/tz5SH3U
ahKhIqCibe33p99VjWyp6bXby3l8WRhxDryHrtTziU6UnF3eC+2qea1hrF8VRuA+A+aZm4n/nJ98pT
z/7tF4DAMibClBVkLoMVE/UT/7nA70EAMicXR91Vy9gEoSowDLoXRQ3Qy2Xy86Tzo5cXJwUdTFUt
qXfrYs+w8DCUMGpClAf+cEjeg0AIG8//ffX2UP5l8m9n3xYvvKX/NsDALm5vGc9mOBvUoSoSoS8eoLbl
i9kQctd6oc2mvz7s25fD+IutuGnU/VwKfNLcT9vko6Z8z+/PmMP7mqvP5EYU0/WNWj+T1Rv2h/FU
5e+GMDvk6shMnRQ3UUg17jG5PvHbHuTbD59DaumyYrzfqkQa2MbA+/BTHaYN5POpY/P2rh7vd
9nnrupbrsjXQXdYu3WC83n4M3DfArKgh/A888oBeAgDk7YbHr7Ink1pGd3/oAXn8Gz/SSwCAzF26R
6T/Hb2AJAhRl05R1s64PREPpOs8s3TlwE30Aj0U27LbdJ6Vz6xZXz17ldNV/awjB/4B2PywsCxn1nRr7
RCwJIMj5zu1UswQa5bnrCO1UkFW9fYcal2SINUYyl89LZXimniHs3Nx+hCvfEbcU+2L2e6pr43Dfn1w
Z846FVQOrG+uDgSpE7VBHcuqeFvWoakKSlf369Ld2wxcdxW+lsJKN3RqUprnibKwFD7yxY/Yk0kBA
GbjOT8+Jm/54o1y/FJBr1kuP3ryGWm+/37qowJAbp4SufQH1kfqoyZFiLqEimtuT8R9OXQTovauHwi
JEU72Dq1XOU6dmIcI1VI5LW6M2gzMjmSEhV6w15PGutvjsyotu6am9WjpLXRqsh4jjUz7nFW2rTa
Yw76rLadd22HVPv12d+vuV8TsRaoYQ/mrp9X2zVA4xpD+4qbsuefNfnTFa4Z1BPULwcDQN67d6V
wbV7neDX69Frbev2+maYPxeh2aFjf3vOc+P1RXYWtXf41/TppyLsFxAmnafXBXPvgXH9RLAIBZeONf
3yAv+OEVemk5PfTV78qf1h/USwCAzPUfFrn853oBcRGiLiOvJ6IfaPUOndivWnXCo32dFJrDwO38bS
5UZMsNoJq7fi8+s+6n2wPUXFff8ickqmx5QWCs3pgzPGfVlj+Rkh/mWi0Z7IY7gtk792TJfmJsJ0EYq/
Ua636vzepZ2QxPUMe3O61rY6vKWbchRsg+cv3+obPdKdoQeH0UI1Q3Q2s7cNWB6t6okwhkSA3f
v/NTd+olAMAsvPLBa+Xmrz9fLy23z338Idn/n4/pJQBA5i59QeQyf8BKghB1KRXlxCnnmRtode1x6W
U5efqkHXYF11vUMHDn2VzwQ7mmeJ0KuwdeWOgN5ffWWcfkDu+2H8bw7cAQ/VFmdc784HMy
Yb1zLRMP6W/LeT9BlVZoz1klRrtTuzaW8kkJ3d2o9a4p2hC7Z7Zxrq2N2WUO3P3Er8sLpOuJp59gJn
4AmLGXfPe58nMPvEQvQbn7P35Vvvct/m0CgNxcVvVRv68XMA4h6pLy6oraPTl74nSqPCUnKies/w
+uV3ngPEWoFiOYcodmt90x0xG9I6exkOfM6GmphqaXQkPC+LP0tzf8OqBVo6fp8kkSbhdl80L4jP1u
cDtiPjEgE+5M/A8/8bBeAwDIm6p/usx1UEdRAWrzN+/XSwCAzPWfFLn8cb2AcQhRl5U3tHlfDts6aL
N77VXEyQqN9VP3hsyCUdfTDi/9upOjwkuvNubQI2YYuIDnzB/KHyXmkP72hj9RVLUlIzuhTmDqa5O
CTNtg1JUdKNlqa55jln7k532ffZ/c+/C9egkAMAsrX3qpXPvklXoJpn/40rfl079/qJcAAJm7bP02eukze
gFRCFGXVklO2hlkRw52nSHNbh3Rkv0Jf3347OuzV9w864eaDXeSp4E6pCVnqL3i1eKc2KKdM2Mo/
xjjh/S3ZcNLUMtSV1PRTyvVazOhHNugZuhXPU7tScXsQNWYoCtu2QJgSvX76vKxv/2YXgIAzELpoRfa

D4z26f/6D3aYCgDIyeXPivTp2jMOIerS8ntyNptOOObWeCzq4p/uem+Sprnj9gDtSK2mA77BOqRmP
crmqj9sur3h1aUsxB5LvWDnzBzK787+H3iYIV70kP5e45w3jL9cv5BOuYRUr82E8miD3pbKoIM9Trvi
ls8dW7sVSMn/+v/
+l34GAJgF1ftU9ULFeHd/6AH9DACQi0v36CcYhRB1iZkzpqvehd7w88Ds5sYkTXPIq1NqC+shGaxH2
VzVwZg/Ll1GT440LNVzVtS1VJXmauqhoTmUvxxaW8AoiWC9cuSQ/l5D1r0CqtYrAxMwOY/JJkhK99
pMJoc2VLb9IfyBurRGfdmzm7MP3bEU7v7Fu+WOl9+hlwAAeXvjX99AHdSYfv7dP6WfAQByUXijfoJ
RCFGXWWDW8FPiTzbuDltXzPVzyAwvRw2ht+tRGr0uXeW6dJPWu0z1nFVke7BA5v5hSrUxzaH8o0
NdMxQeNaQ/Xl3VCaV5bSaVQxvsIfxhxVBVSNvvp1pfFohy1RVXyf/4lf8ht1x3i14DAMjT373yCf0M
UV7z1pvkbe+5VS8BADJ37FUix9+kFzBKoa/G9QIAgKXxwCMPyOv/2+vtmfoBAPm648vXyysfvFYvYd
D1P/E8+a1PvVGedw0TbwFALgovFrni/daTq51ljERPVAAAlsztN91uD+0HAOTvb27/tjx2PX/ECnPlc4
9L9fdeS4AKALmx3m+Pv8f6SIAaByEqAABL6F2n3iUfeMMH9BIAIC+Xjvfl8294VH78nMt6DVz/8t+V
5NZXv0gvAQAyd+ztIoWb9QLGmSpE7TVW9AQpBX9W65F60lhxJ1TZkODL27LhTbbiPCabqGZYeyO
43dBH1jOAAwAwhz788x+W07ed1ksAgLz84PnPyt7rvqWXoLzidS+R1X8bNhkqACATx4oix39eLyCO
OeiJqsJVf5ZqR84zwquZ0VcaKU3oAwDA4rjnl+
+xh/cDAPL10M0/kO6t39dLy+3al14l1d99rV4CAGSucI3IMTWMH0nkGKIWZXOvL2oeq35gxuuuHH
iTiKvZsNXn92QzxwzV1qnJeTqkAgCWjDtj/3VXXafXAADyonqjSfPaqfu+aZ/TS8lIBqgpSAQA5Of5ukcIL9QLi
mn1P1N6h7OuncuqEZJedlqXedUNc/9Gtl/XnRZq7pKgAgOVzy3W32EGqClQBAPlR9VE/96Zv2h+X1
S/8xivsofwAgJzYdVDz7rl4NOQYog7XRLXrlZZq4nZEtYfVD9UoNb/OeaRVL1Uprp0RP0YFAGA5vfm
WN8tH3/5RvQAyIvqibqs9VFePePoLv35CLwEAMmfXQX2HXkBSc1ATNUKvIsUfFktS8lFkFktS8lNXRqZVSq2H
aPu+HuNXTfpGB5WVM8uWd46hJwTA7YddqEYTfT/4kcHneY0nPYU7fC+q9b2DCO//8rEiKf0cCAt7
7s+
+V2h01vQQAyIuqjbps9VGfd82VctfvUJMbAPJztcixO/VzTGKmIWpluy/9bt3vCVptOcPst1WY2ZPGu
htwVqXlDsFvVe01qobpeqIkoSO1khtC+I9Vd0Yra9/2bgFghnqNlWAPfSBnqjeq6pUKAMjXfT/zuD1r/
7JQAer1P/E8vQQAyNzxO0UKL9YLmMT89kTtXZQdnSKU61v+RFSVLXHLmHZ2LqbU+64s9S0S1NFG
TQoGTGKR76eM295ryPpg13tgBlR9VFUnFQCQnx8/57J8/g2PLkV91Le951Z5zVtv0ksAgMwdf5PIsV
fpBUxqfkPU7oHXE8sevu/1HjWG93cOpKufTsfppZpmrVUAABaVmqm//attZuwHgJw9v3T8je3f1sv
HU23vvpF8ku12/QSACBzhVtEjv0LvYBpzHdN1FSFz85vlhPo1M7nWItxHgxO2jWqxuPoOpD20GPv6
/VjZG3Jwf1FBNd2Pdzga0fVgsysDVEya98oca+VK/5xJm1f2OsHyncO1O806o7arx19P/kG2p/K9Ux6
DsOEt32wXungORo8P2HiTbTn8/epHqPqpCY5P0DQbdffJvf88j16CQCQl7+/7Xvyjy//oV46WlQd1O
rvvVaufO5xvQYAkK2rRY6/2/p4hbOIqSxEiFqud4fDT/uRwnDa4qac1WVWRfblcGnyBRVsDU7a1ZTV
wqr1/3hUiFMKG3rcqUlpMKBKMklYe2NETUjVYzgYFmXWhihZtW+khNcqwXEma58TyIW9vrlaGBH
2qXNitLNcl/GVM9SxDbR/6us5/f0ej9OrffAcNVdHhZyTcPbh1XO2Dd97qd3vWGrvKL6DGfsBYAb+6v
WPHcn6qL/yH15JHVQAyNPx09RBTdH8hqilk34P0YN0Bu2Ha8tuuinKQug1zvnhkTuhV7/r1Zsdzzhv
5bp0dbDd9TbQlHNeotNLMEmY9dpzesOh2+3IzkX3tVm1IUpW7Rst2bVKcpzJ2tdrrHuBnP+HjZa1F
615bkRQaLRjb1OKem0koz1u06e5ntPf70n47fHb3pHa+ejIPHqivUH+PsLvvbTud0Ds2frfdepdegkAk
AdVH1UFqUfJyumftB8AgJwce53zQGpSC1HtnmjGkFHzEWco65DimpzxspxVfxuqF6C77UQbdnpv
me1yHmYvuTOyFivhWXQ9uejO2qUCFi+kKcrmBSPEidI7lH39VDo74mY3xc09J6yxHnub+mQmmiT
MmLjHCNyKa2eGQ/XM2hAlo/aNlPBaJTnORO0LtuOst74iW/aGVVC3J2GHE2hHTNWz/rmtbPtBrd
/2JNczhfs9gWrL7yFf2Up/+4q5j/B7L8+J+bAM7v7Fu+WOl9+hlwAAefjmjU/JV376u3ppsb3sp14o/
+a3mNAEAHJTuFHk+L/WC0jLHA/nDwYcXkjrjWE1w5B0mMHN0daVAzdTKp+Ukn5qK56QU/ppJDP
kHgioh2ouTjVJmK6nGTZ8Prc2REmpfSMlvFZJjjNR+0a3wwldR5fWOHUi6XdVVU4HNlaSk2473bYn
up4p3O9zpSwnAwcRIteJ+bAMrrriKmbsB4AZuP9V37HD1EWm6p/+
+n/5GeqgAkBurhQ5/mvWR+qgpm2+a6IWN2UvbMitPdQ3hXqoHtWLri8pZ7JH3OhefG5oM1EPZ
MWu5eiGPlE1KzNsQ5R5b19sebQvRuA3VlFOLEzSmcbxAvPpphfcZAepKlAFAOTn8yuPylNXX9JLi0f

1QFU9UQEAOTn+r0QKN+gFpGmqENUc9hv1cMLJoj8M2gxA7aBUrw9NMc2v04+4dRUtdp1B82tD
H2kGskvEuHZ+7Udf89zw5DXjJwlry4bZq9OtjWnWijRl0oYo2bcvLbGOc4btG21wgreeHHp1B4ZlOv
HcEcD5QZpuv+l2e2g/ACA/KkBVQeoiel3l/6IOKgDk6dhrrQd1ULMy3z1RkZGQ4dEus05mDGp2d9V
b0Q+rjZ7D7raTTBLW3vV6dtrhjxuYG8OTB6XehihZtW+khNdqguOM176Idth1itOdfT7Y9JDh+ImO
M737fWHkNjEflpGaZOoDb/iAXgIA5EEN6f+7Vz6hlxaDmoWfOqgAkCM1C7/qhYrMEKIupaKs+bN2
yao3Ztuc0XsMPcGXKlEb7K0YEnhNOEmYH/4YM+KbcmhDlFTbN1LCa5XkOBO1L6Id9rE7NVUTnsK
Rmqsb4jfdL5lQPrPmhNaJrmcK9/uiyeB+B0wf/vkPy+nbTuslAEAeVH3Ux65/Wi/NN1X/tPp7r5XnXX
OlXgMAyJaqg/oe6+PVziIyQYi6pIqbF/zehs1VHawYk86MU9n2h393alJygxmjRqg/UVew9mbkJGGV
09aSNq5dWbUhSmbtGy3ZtUpyrpO1b2w7ynXZSmV8eFnK5aas6vZ4Tbfaftab/j/Z9Zz6fs+LOdGV28
6Jws6U7ncgwj2/fI89vB8AkI9Lx/vy+Tc8Kj9+zmW9Zn79Uu02ufXVL9JLAIDMHVsTKdysF5AVQtSlVZ
TNvcFJu6rS6res/8djD/8OK6Rpb2dgoi679ubg/ixDk4RVZHuoDc72vF01d71eitm0IUqG7Rsp4bVKcJ
zJ2qfa0Zfu0IbVpo3SBlM7JWcHjzfsGiW6ntPf7/mw7q/B67F/aPQSTiCV+x0YzZ2x/7qrrtNrAABZ+8H
zn5W/ev1jemk+veatN8nb3nOrXgIAZO7Yq0SOv0kvIEuFviqCCAAAMIF7H75XKn9UkaefXYwhpgBw
FNzx5evllQ9eq5fmx7UvvUr+06ffzDB+AMhL4RqRK9R8BQzjzwM9UQEAwMTefMub5aNv/6heAgD
k4W9u/7Z875pn9NL8qP4udVABIFfH3239jwA1L4SoAABgKu/92fdK7Y6aXgIAZE3VR/3cm75pf5wX
v/Abr5BXvO4legkAkLljbxcppFNUD/EQogIAgKmp3qiqVyoAIB+qJ+re676ll2ZLhae/8Osn9BIAIHPHiiL
H36EXkBdCVAAAkAo10dQt192ilwAAWeve+n37MUuqDqoaxg8AyImqg3rsPXoBeSJEBQAAqVAz9b
d/tc2M/QCQI9UbdZb1Ue/6T6+2g1QAQE6O3SlSeKFeQJ4IUQEAQGpuu/42ueeX79FLAICsqbqon3
/DozOpj/q299wqp/7ZDXoJAJC5428SOXabXkDeCFEBAECq3lF8BzP2A0COvv2if7Jn7M/Tra9+kfxSjV
/kASA3hVtEjv0LvYBZIEQFcteWjUJBCsZjpdHTn1tSvYasbLT1gqO94Z6fFZnX09NrrDhtXGnIqCb6x+E8
lv5aY2mo2frfdepdegkAkLW/v+178o8v/6FeytbzrrlSqr/3Wrnyucf1GgBAtq4WOf5u6+MVziJmghAV
yFVPGiur0tRLjrKcWSvq58vHDiJLNeno5YXRa8h6LarV6loXZDV4saVTK0lhIDAGjqq7f/FuuePld+glAE
DW/ur1j8kPnv+sXsrOXb9zu1z/E8/TSwCAzB1XdVBfrBcwK4SoQK66cuDmbuW6dPt96ff3ZHNZM9
SxQeScUj1nxwW/7fPiHlq1pa5zX7r1srOiuSrkqFgGV11xFTP2A0COfvyr3zr9wir3nrTXoJAJC
5YyvW41V6AbNEiArkqXco+/qpnDohy9v/dHHF6znbk8Y53QW1XJetivO0uHlWqs5Tae6SomI53PSC
m+wgVQWqAIDsPXb903L/q76jl9sp96ofzKfziplwAAmSvcKHL8tF7ArBrGiLjGvnqP5CKnntOlI2peqN
536d163Or/dp135sb+ivNdbZLwuuD/bKS2MbmtlG7zFcYzN4jMM1S+1HWN1Low3jal3a+zDDt+aq
87WDDQ9pc9i2o9o8tpfjROdl+J4ZuZ8YxxD7fGh+W9RjuK0OZwi9/7p0z506/lKsnrNGj+NAWF6Sk7
ozquwfDt9PNqM9ie65eMduS+H7wj1Pcd9HsNxuv+l2e2g/ACAff/fKJ+SbNz6ll9Kh6p/+
+n/5GeqgAkBurhQ5/mvWR+qgzgtC1CWlwpHQMKhTk1JhQ8ZlcHHYtR8HCkLa61TIMrC+uRoeXk2
1DRU2hfYY7EitNCqEU58zapaW61J3uw52duTiYMi0674ynbqmQ8GiZh/zyGBquM1uz8dQE5+X4X
umuToieEt8DFGcfQcvd0hb7WCw5A2hd6V67jxVafVbXq/SIWaP41E6B9LVT4MqcjrpPZfk2FP6vlDn
KY/3ERwdapKpD7zhA3oJAJC1z688Kk9dfUkvTe/f/Nar7J6oAICcHP9XIoUb9ALmASHqUmqLn8O4d
TmNeo3SlHPhSUpi5XrX3na/FYybwtaPGt482TaCw6mHj7EjO4PplEcFZM7r+3ubsrlVF+erBr/GPI9nZ
FyGWtm2ttd1t2Wptpx9bOvUrr3hB4Vhbe7UZH3kdQm2eXRT0jkv/invSO28cd0SHMPY8xHg7zu8r
dZxrbvBoHEu3Iamcu40u53bEitrHVKUE6f00wiVRPdckmNP7/uimOP7CI6OD//8h+X0bQxHAoA8q
ABVBalpWDn9k/YDAJCTY69zHpgrhKjLyOwlZ/R0K27uOQGJ9dhLZaajqpx1t1M5bS25RqwPHd486T
aKsrlnBj6O4toZL7TrHIT3AyzXt4IBWXFNzugv6uxc9NvY3vV65pXPrI0P38YwexjWLxht3rwgfgZ5PrR3
31CbR5r8vFRbfnDoh3xB0xxDFHPfoW3tXZQd3SEycC4qW/5+zWtniH/u1HFY3yOhIe8k9uUwrEFKk
nsu0bGn+H2R2/sIjpp7fvkee3g/ACB7akj/V376u3ppMqr3qeqFCgDIiV0H9V/rBcwTQtRlZAQ0ImqY
bsGrZTiyhuIkyielpJ8GjFofJo1t2HQ9x9BhzEGnTgwGP0VZ8xMtLyzyA0Mj0J1YVK9WY/8jgrfhNscV/7
yMN90xjFaWk+MudvfAa79X7sF+GEPcRwyfn/zcTeuUjN51gntuimNPcv2HzlNe7yM4ctwZ+6+76jq
9BgCQJTXJ1KT1UVX907t+59XUQQWA3Kg6qHdaH6mDOo8IUdfSUTYvhPckdEOYqMl1FkZg8hyjn
mOk8MDO76nnDnc2AsPq6dg9GafXkeGOgjFCRlOK52UyYccwK2keVxw9ORxbLNWR2T2X2vVfkvc

RZOKW625hxn4AyNFfvf4x+fFzLuul+H6pdpvc+uoX6SUAQOaOrYkUbtYLmDeEqMuquCl7esjtQKlR
W/Pcos+u3ZYNs3edW7PRrMGZhHW+zurzZA+NNoYyV0/nF6FOH/qlfF4mkm1w6dXKHXpMWsd0
AsUTMrb06bje1BPcc+OPPf3vi6P9PoIsvfmWN8tH3/5RvQQAyNIPnv+sHaQm8Zq33iRve8+tegkAkL
ljrxI5/ia9gHlEiLrE1MzaqqeYPcGPHYR0vRqKo4f/GoxhxHPHrB2pgiW3/uMUba64U6Z3duT8+R29n
aqkk6GW5KR37gdnZO/JRbfoZeQQ8BgyOC+
+nI4hTOmkFwKOqumZL+NcNHfF75DZlQPvNJxwzn2EWPdckmPP4PpP/T6Cpfben32v1O6o6SUAQ
Jb+8eU/lL+/7Xt6Kdr1P/E8uet3qF8NALkpvFgP48c8I0RdRu0Ne6itmkU92FPMCHiMXnIlPxkzZmJvy
4Y3Dft884MlY2bySXgTWHWk2dQnKrWh/EXZdLsdqvO87l+XXmPdq22ZZBKkcVI7L578j8Fj1udsrvr
DyPW9bj9yHVtu1oBtyq7eda9xzgsxY/VgjnPPTXjsU1//hO8jZntyvRSYe6o3quqVCgDI3t/c/m157Pqn
9VI4Vf+0+nuvleddc6VeAwDIlqqD+h7r49XOIuYWIeoyqmz7Q287NSm5QYtRH7F6NnzmbhXSDL52
Lpkz9nttNibamUhF3I6BrlSH8o+4LiU/fZQL005glcl5MSQ9BnPYu9ueiRK2YH3O5qqz34IX9Felldqs+v
EUNy94PTLd9njnodqSeM2Jc88lOPY0r3/C9xEgiqqPquqkAgCydel4Xz7/hkcj66NW/u8idVABIE/H3k
od1AVBiLqk7KG3YUUMVeDS7wcDHlX3cLBmYrWVcx3NpCqy3W/5gZHNOTbvsAPDrOPxhlcr5bpsp
ZzL2dcl5LxWW9Z6d+j1VLI5L6Zkx2C1Z/A+3D80ejUmYNfnNIaSu+y6nznWQ/UUZXNvuFaoPYw+Q
aAb656LfezpXv9E7yNABDVTf/tX28zYDwA5UPVR7/uZx/VS0Cte9xL5hV8/oZcAAJk7Zv2GfPwdegHz
rtBXRewAxKNmNtcT86gwbG/anqHAOEfpntPHcqpFwIpwn+l9Rip/xM0BAHl441/fIKWHXqiXRK596
VXyW3/8z+yPAIAcFK4ROf5+66P/Xoz5Rk9UILaeNNbdmc3LcmaNABVZO1r3XPu8dSwZ9ODG0fGO
4juYsR8AcrL3um/J9655Ri+JVH/3tQSoAJCn4+8mQF0w9EQFxmrLxmANWFXOgK50yMzRu+d6jRU
p7ZyRbiplKXDUFf6fgn4GAMjStU9eKe9s/aS88723MYwfAPJ07O0M419AhKjAWAOBFgEqMsc9h+
WlJiiblx9NaEs42hKOtoSjLeHmqS0nrr1GDr/3pF6aLa5RONoSjraEoy3haAvSQIgKAADmBj/ghqMt4
WhLONoSjraEoy3haEs42hKOtoSjLeHmqS1IhpqoAAAAAAAAABCBEBUAAAAAAAAIhCiAgAAAAA
AAEAEQlTkSE2WU7DrfxRWGtLTa8fqNWRlo60XHO0NvZ3CijRibwjp6Uljxb0GG9aVTbJuGmH3UNr
7mJbRRv1Y4SYFAAAAAGChEaJirvUaK1Io1aSjl4H5pgJdY1Z9W1nOrBX1cwAAAAAAsIgIUTG/eg1Zrx
GfzqeibO717RkF+/1tqei1szFPbenKgXvLluvStdu0J5tkqAAAAAAALDRCVABIS+9Q9vVTOXVCyE4BA
AAAADgaCFGXmao1OlC7MazG6GD9UXuIvfE1A+VKNbNOpXokq1Vp79Mcxt9cdbYTvjOjjeoxqk7qY
JvCa1VOdrymMbVf2xvetob2H3JN4rTRY359ZEONcxFo46j1xj7t9ea5zKoOadx7KKotg9uYsD5pjOuS
9J4FAAAAAACLgxB1WakgL7TWaEdqpVEhpPpcQUoDQ+ybq4OvVyFiSYIva8pqYbBWZBqcNq0GNh
xyDHYlNtgm65W1UnjQaYt7vIMqcrqqn3Z25OLAa9u7bmODtTKHQjgtuo2TKsrambLztHMgXeeZxR
iOHmh7W9xml8+s5dDDMoV7aKJrPizf6wIAAAAAAOYRIepS6knjnJuIuXUb+9Kt61BNOrIzmPx5qtL
Sr2+5QaEKG8/7ve16jXN+0FVt6VqVXfE2H0Nl2/qabl28L3G3sx1W8dJvU/gxWMe77oZg/mv77gF0
arI+MhUdf7xhKltu2wfPpR9GSvmMeBIqe8MPgsOuSWQbJ1NcO6PbuC+H7qbbu0ZI2ZEDN101hq
mfOpF9hDr9PTTNNTckuC7J7lkAAAAAALBICFGXkjERz96m16vQD9VEOl56FlRt+RP3+EGhqScXd9w
+e1VpeQGStc8LYa+fntmm0GPoXRS3SeX6lj/xUGXLC+U6OxdDexSOP94RimvidfQ0t22ElGaPTrN3av
2CcU02L/htrJ0fGKo+Ja+NftDbO3Si0mrVCRv3dbrau7jjBZKnvROYlRTuoSmuuWkm1wUAAAAAAM
wdQlRYdA3P0OH9SZmzk5+Ukn5qK56QU/ppespyMrCTEN0D77jsIdheXUtjqHdgSHsazOHy/rB4P5
SryllvyvYRvVNtxnbMHqOpKMoJfUHcwLlrXzzrnJ4+aYeVwfWW6mk/kMxMCvdQKtd8VtcFAAAAAA
DMG0LUZRWYKCdunckYgeWRMt3x+r1i3Z6eRig3URhpDK9PScUt3trctVrXE6cj6ik5UdFhZWC9anb
2EeriSf+6AAAAAACA+UKIupTasmH2OnVrPZr1HI+ocr3rlDEYevjD9lNT3JSzbglONXTcqCs6WRiZQYh
dOS164L4ctvUQeLv3pzs5lrF+QUP07K/5sv1xAQAAAACA5UOIuozMupwqYHLrohpDoCdXkpNuEjs
4XNoIEXNVcoamK6NqvWbF6+nZ2ZHz50fVFTXP2eBs/mZ90FMydk6nxNfQ3XdHDnadr3VrtZbsT/jr
h4e0ZyWFeyiVa57idQEAAAAAAAuNEHXJ+QGTMWP/VMxakU1Z3XCn3DFnS8+ZMcmTNFfFa1J7w
6+V6a1MmdfTsyPNpj76oaH8Rdl0u6xar6utN6yz5eg11r0anuYESU7AqVivP+8dkGx4U8nH5V+vZtP
5Wnf2/aIumOquNyfCylYK91Aq1zz5dQEAAAAAAEcTIeoy8oI9S3NVh0rGhDtTMmcun2r75iRC7nY
mCjuDs7o3V3WI5gWO5gzwaXOHxftCh/JXtqXl5XU1KdnnrCAlP6mTC95EVCojdOutWrxzHLe2bVBg

W9Yzb2i6eZ9Y68/k0w3VNv09lNI1T3hdAAAAAADA0USIupQqst1vGQGZUpVWv+8HRvaEQpMqyu
Ze1w/BbGr7g/scx2qn1yBt/9DrDZhIcVP2+oNtstj1YDOoh2rwhvQr1v62Ruysst0PrUtbbVnr3ZILLnU
8g6+ttiara2v22gwMTTeGs+c+ZD2Feyila57ougAAAAAAgCOp0FczrADITq8hK3oiL1WDdo+eiwAwk
urtPS8/mtCWcLQlHG0JR1vC0ZZwtCUcbQlHW8LRlnC0BWmgJyqQKbOOZ75D4gEAAAAAAJAOQlQ
gE23ZKAzU8ayeFTqhAgAAAAAALB5CVCAPql5pZpNXAQAAAAAAIEvURAUAAHODelXhaEs42hKOto
SjLeFoSzjaEo62hKMt4WhLuHlqC5KhJyoAAAAAAAAARCBEBQAAAAAAAAIAIDOcHAAAAAAAAAgAj0R
EV2eg1Z2WjrhVnrSWNFzZavHhsyL60CAAAAAADA/CNERSZ6jRUplGrS0csAAAAAAADAoiJERfp6D
VmvEZ8CAAAAAADgaCBEBQAAAAAAAIAIhKhLqr3h1gddkUZPD7+3l51HaClTVePUeI3zcL7eZW/XH
MbfXHVepzc4uF+PuW1j58HXt2XDfY318F4Wo12TGjwv6jHpuVGSHr8S1obCSkPCD8+s/eo8VkaciG
TbBQAAAAAAWF6EqEuvI7VSQUoDw+
+bqwMhX3tjRI1T9fXpBJbR1H5WpamXpFyXrYr1MbN2OWHk4HlRmquFYNCZ4blRoWtYG6RTk9Lg
BFl2EFuSwZd3aqWhcDTRdgEAAAAAAJYcISosVWn1+9K3Hq2qXqUCwPNulNaTxjkdX5br0tWv7dbL
zjrrtTsXnYiusm19rlsX9zNSbdmv7W+rxHNafjv7e5tSTNCupHqNdS+MLNe7zj77LasFWvOcDkeza4NI
W3bd1Dh020055yW0VjvW3SDXOE/uBe3UZN17bZLtAgAAAAAAgBAVUm1tixtxVraMANRTlM09H
crZ4aVeu3bGe23noKufZadc3/La6ciqXT25uOP20qzK2U13yxXZsoNGFVLuibM6w3PTO5R9/VQ6O+
JmscXNPWd/1mPPbVvvorhNDpynypa42Whn56J1ZJYk2wUAAAAAAAAhKiaha5OGDmHPzqkT44K
9tNrVlQN3A+WTUtJPFSdo9EPnYSmem+KanHGTWGtrquyCW7t0qM5p98Dbnz18X7+uYA7v7xxYR
2ZJsl0AAAAAAAAQoqIsJ82UcJTAxElGbdLcjGjnzNtlyawNRdm8ENYz2A9KQye5Giur7QIAAAAAABx
NhKiIoS0bZs9Kt46mWft0JuahXRm3obgpe3qIvV+v1tc8Nzybvl/DdfBh9KCdYLsAgLzp0Q3Ggz9yAQ
AAALNBiIrx2rte70o7oHNrfxpDyKc2ybYya1dJTroJqDsE3qVm4i8YM+6n1YaI16uZ9NUvzfakXXbw2f
XqnHrtK530Qtu4NVhjbRcAlpH9Xl+QwkwTy540VlZFWu57tPNHr+bqLILU+Qxze40Vpz0r+f/hT/0ba
p4P9zEP5wXDhq7XDO6ZmRvzvuZ9P7mPvG7mOO+3ebwnR+5jSf+gFfO8z/K9eGZinJuh951luGnG
nJfc34sTtofSdoiDEBWJ+AGdMSt9AiU/nTRm/7d+MFmdbhD8tO0KKsqaVzS0Kavem667baeO6OB7
cZw2JDp+/aavPhXsGRpSs9Wsc9pc9dvm/sOhHu7KJNtVjG3wDwuALMzsl/chKri09j/lv0npcCYu3Da
KcLuTP+4f5vte3N4IC3ONPyjOQq8h6zWRatX9x28G3NEnxsO8XrMy01/cAyWOhh+z+NZW52O1qS
YFda9TS6qdmpRmFPjk/0v7+Pc19R5csr6f6l3jHFk/T2Z778R5v83jPXncPtTnc/yDVmTok1eYm+C85/l
eHHFuht739CP98xP/vl3dH/g3Iot/IEadk9zfi8efl3zfiydoj/WNrUrbpf+eHO/7dn5+FsZY1g2DJWS9R/
TV5bd+A+hbPzD5uvW+9U+g87mq9ZZia/WtnxX060c8vNcqIa93P29uf9TD2NbIdtqStKvbr5fd9VXr
K8cxXx/yKNetVygJz83Exx/
+MDcdve3gMSfabqvqrS8PXwQAmEq3XrbeX8z3eP2+GngjyoffFv1vwAzaEEm/z8/8vXjm7XCuj9q/f
c28f5PzY/87OoP9RtP37dy1y/3eivPzV9rCv5dn1R7n5y9jv/pnrCy/l8a/rznvuYNtyPocjW9XvNdMa6J
9ZPIeqPdvbdd+hLRD3T/maud+Um3XK1IS/5w4n8/+vTjeucnjvS/
+fZv1+8v4cxImq7aNPy/h62fWnhHfw+nfR87+zd0737fBdX579Qr9vhz3uiJf9ERFDBXZVn8p0ksO56
821puAo7kr/t9KrNd7n9D2D52/MKlanIP1QqutCWuIJm1XEk4PIOsNTS/7rDdbf9h+0jYkPH57qP3g
ubQ5+wj8QdOuc2oMyXfZPWWMeqiWRNsFgMy05XytY71NXZBN503VUpEt9UY28fv35Iqbe9Lv7x
ltmTO69MupE/PRwFm1o9dYl1qnKmfn9kLNhnteWt7PKPPC+T6X6unAzyL5KMqJU9YH9+dQW08u
7sygPb2GqIFK5fqWv9/Ktv3zYmfnYgY9sRxj39d6h7JvfRj8fi7aJ64puxm9Ecd5v83jPXle3ved79+y1Ls
hP8tr6uf34dEJHdm5mO7dE/ec5PVeHOfc5GX8uXHeXwLf5xmY7Jxk9148/rzk+148tj0jfp6yR42mW
tYuzqii+fpZGOMRoi4pvw7mwJuLMeFQcMiBCgvdr1EPJ5TztxMM6dQPhf5rrYf5A725D/VQ+xmx35
Ht9MRtl/MGFlw3nvMG7H6d89gbakjCc5Pg+G2D59J+jDoG8zj1Y9QvU3G3a7xu+NgBYAoz+uV9M
enSL+W6bIX/A5CTnjTWa9KZVTvsoaMdqbbi/1ueGTUM0Rh6N9uSN/n84j6JXuOc9d1s/bI/oxu3sq2
HjNo17dWwypITNg/
+vJW13H5pT8huV1lOenWcNKPePgZk8AetuMFlmDTbEVuO78XTnJv86fJoB+cHhtOnWwJnknPCe7
FhxPub8/PnvuRaNYmfhRcOISoAAJgNfnmPTdUltX/5uTCLXoa6tpj9i6D+pWdGvR3b52vSqbZmPmL

C/0Op81AjV7KppRZXPr+4J+f2NDo7wwDEHTWkatqr7yOrObMI4efpl3ZMgT9oKfPyXhwwR3/Y6uy
flAvevxGqt6h6/9mYYa9C3osD9HwiwflBrO+pqedWiWHwjzD8LLxwCFEBAADmmDP5gRq2N6ueO
MFRDt36vqzO4hfU9oYzCcRc/dbuUL2Csh6WHcfc/eLePi/O6NHZXTNnsg5/YiAVeKtJgXKftGOWv7Q
jNfxByzKH78Xz9oet8pk147pY/4aeVWXUZtirkPfiAernGrdnrP89dXBKXadTkl3n7nn5IwymQYgKAA
Awp5wAVfXWmJ+hjMXNC3YNtk7tfK7hXHtXhU1NO8B1fuEpSEn9Vqh/CZrtcHptlsOyLfP1i7sOCGf5
y6Iecqzq2bt5jz0MViXezdWMZjYfZVa/tI9h93bqyMHgjTuqd9QS4w9ajkV4L56XP2x5ZtqrkPficIMl+f
qydXLfOk8nJau3vdn+EQZpIUQFAACzwS/vkVSvDSdAndMJ/zL8RSPMYE8j9bAngLR+Mexaz2dbt7sn
h6qoWc7nZKxZ/uLu9nw6O8NfFgeHTbpmdl7y/6V9rOIJceZ7CUZNPfuGnmG4O2f4g5Zvvt+LB+T+h
62S2GWOB8PbWf5cw3txTLq2eOCPkekZ+UcYfhZeOISoAABgNvjlPYKeRddiD3czevzkW+dSTf4wM
Bxc/0KW1S8ac6/XkJUVc0i2WqVmSp7lL6nz94u701utKjMcPSpSOW21wPoeCgyhV7dwzTovM26bL
dtf2uOpyGnVY6+27r+veL3G5m+islngD1qLaFZ/2NIjADo1Oe/9w6mHcM+oHinvxXGoUhklqUldLm
RwkSL/CMPPwounDwAAMCOtqvSt33L69a5e0a33y9aPJ2VvRX669bLVFtWewUe139KvWUZh56U
6JyfEblu53s/3bun26+Xg+ZiLe6RVHbg2rb71S2tfJT+5m+H38TB9HgKPefie1vdRxvdv3Pc1573Yf2R9
7eK0K4/35PH7CLt/3Ifxb1dq9H0x9H2r2jFw3Pp7Pu1rNcl5t78m8/fiEedGvd8M7Ns9hrTf/uKem6H
XZfY+POp+0XJ6L453XvJ7Lx7fnpB/xzO6RnHuxXn6WRjjFdT/rIsKAAAwE+5f6F3WD43zNRwQWBCq
x5xdm9BVbUl/Bl3nnO/pqrT6M5p5eS7pnk7G5ZnV9cH8Gfre9fjfR2Gvqc5r79gUjT83Id9bxnk7iuLc
LwrvxbOmRvOoOqhhgkP7+Vl4cRCiAgAAAAAAAAEAEaqICAAAAAAAAAQARCVAAAAAAAAAACIQIgKA
AAAAAAAABEIUQEAAAAAAAAAgAiEqAAAAAAAAAAEQgRAUAAAAAAAACACISoAAAAAAAAAABCBEBU
AAAAAAAAAIhCiAgAAAAAAAAEAEQlQAAAAAAAAAiECICgAAAAAAAAARCFEBAAAAAAAAAIAIhKgAA
AAAAAABEIEQFAAAAAAAAAgAiEqAAAAAAAAAAAQgRAVAAAAAAAAAACIQogIAAAAAAAABABEJUAAA
AAAAAAIhAiAoAAAAAAAAAAEQhRAQAAAAAAAACACISoAAAAAAAAARCBEBQAAAAAAAAIAIhKgAAA
AAAAAAEIEQFQAAAAAAAAAiEKICAAAAAAAAAQARCVAAAAAAAAAACIQIgKAAAAAAAAAABEIUQEAA
AAAAAAgAiEqAAAAAAAAAEQgRAUAAAAAAAACACISoAAAAAAAAABCBEBUAAADAQmtvFKRQ0I+N
tl5raG94n19p9PTK2fDbuiEhLV0wbdlwz3vk+e1JY2Xca6xXNVb8ba00rK8CAGB+EKICAAAAODqa52
TGOemSUMHoqjT1kqMsZ9aK+rmpKJsX6tZnHZ3aesg1asv5Wkc/F6me3bS+CgCA+UGICgAAAOAI6
Ujt/OL38Zx/XTlwM89yXbr9vvT7e7I5KvksbsrZqn4eco16jXN+IFttyXZFPwcAYE4QogIAAAA4WuiNm
r3eoezrp3LqRKxeo5Xtlng5auAamb1Qq9IiQQUAzCFCVAAAAAABHTLzeqH590pVg6NpryIpbm9Orse
rX/7Rrehp1Vr119suC68NKtPqCtUJH1wEdeJ31CKsrGjyeYL3S6HYYzGPXj8F92fsp1ayzrDVXndeO3Ul
FtltujOpfo/aGXxagXN+yXjUo3vHbQto/dH0tcc5VoEar+6BWKwAsLUJUAAAAAEdEWep1HdJl2Bu1U
ytJYTVYDdRep0K2gfXN1VEBZlNWCyUxyoCqjUhpcMIpOxQceJ3F3t/IQK8jtZJRr7Rcl60YnTuHwlEtel
8JVbak7hZHba7KRqMh5/wEVS4M1gNIcvwqwA5pv3M+hoNUR/i5UueiNLhTJewaAQCWAiEqAAA
AgKNjzQ3p4vVGnVS53pW+qgPq9ax0hK1v7o5oh1dLtC/
+y5tyzkv7etJYd0PBqrT0a71td2qyPjIpNl6/F2OSpvaGePmv0a6um3ga+6psW5/r+hNFqRqm9n5iDc
MPTjLVrLnHV5b6hcF2Jjl+67VuGhvWfmsrOxfjnqu27Eadi8A1AgAsC0JUAAAAAEdIUTbdGYwy641al
bNuj8nKaWvJNWL9/mFoL05zBnqzXmhn56Lz+t5F2dGdIQPD3I3enN5rB4QPix+t7aeGgTCzuHnB31
ftfDo9MAOTTGnVs8OTUiU6fuu675lBqKO4dsYLbDsHXf0saOhcmfVeOzviZq/FzT1n+9Zjb+QMWgC
Ao4oQFQAAAMDR4oVsGfVGLZ+Ukn4aMGp9qKqcDiR3JTnpp31ix33dA90L01rllguwH8bwdve1A0
6dSBLymT0vz8ha4EuLsnbGbdi+HIYlthMITDJlPQudTGqK41fHZNc5DR3eHzR0ropr4h2yuodK7n4ja
rECAI48QlQAAAAAR8xAb9RD5+l8K8qJU/rp1MpyMn6am0BHRnTmnE6i8DlCYFIpo85ppLBzFSw5Y
HLD3NgTdQEAjgxCVAAAAABHj9kbtRYvTsvXYK/Onhx6Y8iHebVWhx7biYbtTyercHa88cfflg2z16lby9
Ss3ZpEcVP29D4Gyt7amueYpR8AIg0hKgAAAIAjyOiNmpQxjDw7g706u3Lg7tTtmVk66QWAo+p5ps
MsJeDXAHX05KJbmFROSaIqAdNKcvztXa/nqR24unVRp7iWaoZ+1ePUnkjLDlS7Xi3W0WUEAABHF
SEqAAAAgKPJmIAoTMlPDo3aqW3Z8Kapz1ZzdcObqKm94Q8/L59ZcwJAszZnc9UfQt7e8Gp0FlIZV2
4Gzta5WPd7WfYa61790aSTVU1twuP3A1djxv4k9PbVbRDscRoSdAMAlgYhKgAAAIAjKro3qjlzuwrp

nGAubi3NaZWlXG7Kqg4D/dzWmOFftd+ozdlc1cGh9+IREzJNorLtD1vv1KSk21XyE1S5kPuM9AmOv
3Lan6jKu5bGBFRJjDgX5r1RPat7ugIAlgYhKgAAAICjK6o3qqp7OVgzs9qavI5mIqfk7J4xPFyx63gO1Di
1a3MOvE4Je+2U7GHrIcdebVnr3eHxeYt9/BXZ7psz/itVaZk1TZu7Xs/fcexzEVYMVW8zrewaALA4Cn
1V3AUAAAAAAAAAEIqeqAAAAAAAAAAQgRAVAAAAAAAAACIQogIAAAAAAABABEJUAAAAAAAA
AIhAiAoAAAAAAAAAEQhRAQAAAAAAACACISoAAAAAAAAARCBEBQAAAAAAAIAIhKgAAAAAAAA
AEIEQFQAAAAAAAABGEvn/AZM6nr53azj8AAAAAElFTkSuQmCC]]></redcap:Attachment>
</ItemDef>
<ItemDef OID="q19" Name="q19" DataType="text" Length="1" redcap:Variable="q19" redcap:FieldType="radio" redcap:RequiredField="y" redcap:BranchingLogic="[q18] = '2'">
<Question><TranslatedText>Do you prefer Life A or Life B, or do you think Life A and Life B are the same as one another?
</TranslatedText><redcap:FormattedTranslatedText>Do you prefer Life A or Life B, or do you think Life A and Life B are the same as one another?
 </redcap:FormattedTranslatedText></Question>
<CodeListRef CodeListOID="q19.choices"/>
</ItemDef>
<ItemDef OID="summary20" Name="summary20" DataType="text" Length="999" redcap:Variable="summary20" redcap:FieldType="descriptive" redcap:SectionHeader=" " redcap:BranchingLogic="[q18] = '1'">
<Question><TranslatedText>
Alzheimer's disease - Description #[no]

If you have Alzheimer's disease...

1) You might forget recent events of importance to you. For example, if you are interested in hockey, then you might forget whether your favorite team won their last game, although you may remember watching the game itself.

2) You might forget the names of people you met recently, or the names of local streets.

3) You may no longer be able to handle your personal finances or plan leisure activities such as dinner parties or vacations. Simple decisions such as what to order in restaurants might become difficult to make. Your ability to concentrate may decrease and you might have difficulty driving.

4) You may start having trouble finding words to express your thoughts. You might need to be reminded to shower or brush your teeth, but you could do these activities on your own. You may need to post notes around the home to remind you of simple things like turning off the stove.

5) You may remain aware of the day and your surroundings. You might be able to travel to familiar locations like the supermarket, although probably with the help of a loved one or friend. If you are in a social gathering, then you might have difficulty following the conversation.

6) You may continue to enjoy hobbies such as puzzles or reading.

Now consider two possible lives. In Life A, you will live in perfect health for 11 years and then you will die of natural causes. In Life B, you will live in perfect health for the next 10 years, then you will live with Alzheimer's disease as described in the summary for another 10 years, and then you will die of natural causes.

</TranslatedText><redcap:FormattedTranslatedText>
Alzheimer's disease - Description #[no]

<p></p>

If you have Alzheimer's disease...
<p></p>
1) You might forget recent events of importance to you. For example, if you are interested in hockey, then you might forget whether your favorite team won their last game, although you may remember watching the game itself.
<p></p>
2) You might forget the names of people you met recently, or the names of local streets.
<p></p>
3) You may no longer be able to handle your personal finances or plan leisure activities such as dinner parties or vacations. Simple decisions such as what to order in restaurants might become difficult to make. Your ability to concentrate may decrease and you might have difficulty driving.
<p></p>
4) You may start having trouble finding words to express your thoughts. You might need to be reminded to shower or brush your teeth, but you could do these activities on your own. You may need to post notes around the home to remind you of simple things like turning off the stove.
<p></p>
5) You may remain aware of the day and your surroundings. You might be able to travel to familiar locations like the supermarket, although probably with the help of a loved one or friend. If you are in a social gathering, then you might have difficulty following the conversation.
<p></p>
6) You may continue to enjoy hobbies such as puzzles or reading.
<p></p>
<p></p>

<p></p>
<p></p>
Now consider two possible lives. In Life A, you will live in perfect health for 11 years and then you will die of natural causes. In Life B, you will live in perfect health for the next 10 years, then you will live with Alzheimer's disease as described in the summary for another 10 years, and then you will die of natural causes.
<p></p>
</redcap:FormattedTranslatedText></Question>
</ItemDef>
<ItemDef OID="a20" Name="a20" DataType="text" Length="999" redcap:Variable="a20" redcap:FieldType="descriptive" redcap:BranchingLogic="[q18] = '1'" redcap:InlineImage="1">
<Question><TranslatedText></TranslatedText></Question>
<redcap:Attachment DocName="11.png" MimeType="image/png"><![CDATA[iVBORw0KGgoAAAANSUhEUgAABVEAAADYCAIAAABtBTQzAAAAAXNSR0IArs4c6QAAAARnQU1BAACxjwv8YQUAAAAJcEhZcwAAEnQAABJ0Ad5mH3gAACouSURBVHhe7d1fqGXFveBx21ZvOyrdJJmmEwftOAiNinQiA21PMB0ItgiCoCGN3IjDhEMLhtsISUtID/OgKJdAn3sR9CEYYTLTPhjkQlAxgX5IoAOB9IMPXjByHgxpiA/nSghH0g+Z9a9qVf3qz1q79lq79lq1117lq+1117n+HHzjgnuXXev/vqrto7f5nn6X/PUw8gaSgVedFBFK2Dhxu2Dc+LVZkaeIEAmBK0o8YsuNwj36ow4RNOKVYa2FuAdgYl3WovOA9Dl3EtouhMcncsVRMhjOhzBHinTBdSb/e0VJVjPaEWhl2O+iuWkjRW/Atgmtk/ZrfXY5W5AXUYN8sVrxq6CQ+P5ai+0VgR2pjAAf1NH5/0hJuGsLCR0kCHBrx03f7CvaRmhqBKAF5M02Lat0PJ6SkTLCeL2p26dtQ4IWRbNJPr0OaAK15tF3bMLCvazPrUjaMHGObOK7z+n0mBmDF4aR95gUhk3dkmXrh5O+aoQYF5HxZpa3ZchIGwSjOXXNhzVmFEtycnJlUyjAO6msB/2WXn5iegeKQz6wuiW6qwkR+WTZ9LgP2gWNFpaXvBGCb2b0rZeOXEA7j+1HvcMepsJhmGXz4YpMiNtNu06lILwW5h17W+bdq6T1Fm2j07lBfIzEmV8PQpXQ3mDgavQ8hh5KZvBKxnnIqmsyZLO+mY5O2MMmS0omjHl3qDw1e1aHIzU7eXTgIHp1QtW5+ocpA4wqDaO0RZuA1tXzN+nImxCj6KzzvAwohdaeS/8rLJBZTFlt28T/9Xw0sSC+yRB8RDz7U1/sKY0vMxJwjTk0PTCtEZKwYObZxJVI9xypaCpFodXnOzxDSvGU86QbaY9XnGN/+iD2ZUhZaSJNJbvjp6v0R/9M7UXr+GmDXm9vGsiDH4sdvjO2eM+2f+92Z7xvgC6Pzon2pczjPsVfsFO8iqZlXFxpgQ5otgBb7Ky9+mf9yb2iYLHHNlrmJm+2uGrM0JMXuZfnb2ls0WKw+fG8S05rXaLxzDfO9s2YFE8/GuZpp+I4FLWl2lkG8oH+NMKWy+Lc/QlcQDdpAGpjS6Lw8u0T6U8c4iRdH3qQhwnv8nvtoeppOcEH78+xM8tOxUjWAtLUpgNhF6Gqbpq0nOPCf3Ns9U3DJUVPiffTSJZ7P4O+p8QDwHQVrHXpBuFXrr3Ki+g5mfG9jFRWgGW8g+e+0cC4ZZYNy5C/gnT0JqZhCHtajCDbC3tR3rbSSfh3JBxPUhgLgiVh1I2Mx55qZ4Q5hkM8+F8Qrq8DFo6+aUZxk+XgGTBRN3RqJ4T3GkVhOqx4C2SaImEhxIk6mGv9hqvxh9UJVJNhTBSfepxbDH+7wXRYNvxoUeiBf1NMTH4hCZrNE8k02kKNAo2pmjHgqjBLgtfqh/x1CnxRBGGwOdG8P5ew3i11Ot8z7gzQJrlLbIpyG2+a8yjZwV1Oy4ltzxmlxVKiLU3ApjUC8wzptqzFQt0s8GDB+zfzHg8rVmqpKZvBeE4zDmrP6unDcWyWTt7YKaDCXVYUhK0UbBKPHoK9kg5pO1F5Ey5tySjgZSk7mYwnpuOrm2VAkRykKzZZ6gGp9yTJpUPGgKZBvHBU+0F+uDsvtHuSW5vfiN3Q3aNLw/tuxUeCjrWPpx3wlbr3/XBcJtubPepn5Ry0vrxOmr/K/FI9mQFJLi7LKbZ6Y/tNT/PHNp/MPrWVm0Qzj0+Ee5cvkkcLC2gbYw1dGkhOQAQ4mStKP28qx6/UmD7AApN5rJC7DEdBQnbJSBLMN1rfOycdNtlhCyMrLOVBuMY43FTMTpXU

OpRLDW9qgbVz8c33656tVd0dzFc/GVIVy91oXndzEAtSi3SJ5JXZtHDtH16TF/1st4Gq1+P6XlS19wJd
I/mnZGtx7oKnoFExx5LM9itm/vOVHxz654JJmDn6s7RV4+K8zdBEETOyPMYvxtvvPB881UIAGuFmn
8KIvWb/1agKFuwWGq6FS3q1sUP9SV9RfcWw8VfhnT2UleqC+3samqRPpG8MkvaXqleVgurX4/pe
VLX3Al0jxYcrNVjXQXPQKJjj6W1XYTmvjMV39y6Z4IJOPXmKXH4JgiCyBl3PnmLKMVzxu9/+V7zbQg
A64Oafwoi9Zv/liqFmqupxVLdT/3P+1Xv6pK60g4ohou/DOnupS4VV5o1tff8O9FJZknbK29yq1+P6Q
WbuBPoHq1Hvh7rKngGEh37T6ZQN5Kpnan45tY9E6zai79+URy+CYIgcsb+Z6/P+Ri/GzzYD2AdUfN
PQaSo0kWPeU9WQp7+ugqLFUvNOO5j/PUVYzyRMf4ypE+vet76z9kbt3omkWS3tMLSt512twUG
Md/Kitu3ezRfizLBYusqdG9I5zjuEHK/nCFC0y8tvATk8dYHb4nDN0EQRM7Ye27P4/fdJorw/MGD/Q
DWDjX/FHiKnZYun9RdXQfp5vqKKo10n45iqe3o6xquveIvQ3r1MvKLO7q9uqybxtLKLKrTYoWlZ1dk
9u7peVLXPBPoHs1pEb7QDuvhmZXs2DUZObe2vXOlee2dW4+ZYGUuX7nMH+onCGK18cDJg6L8Xl
XwYD+A9ULNPwWeYsekSyqL2dgosqR4sdR21MPpbN4E9XDxlyH9erWrdbZD97AslDSxsPS/BSXfOJ
Z2UE/qmncC3Yv1tzCGt2cdWF2vDemYTHh7mibuiJ659ZoJVmF7Z/vw5mFx+CYIgsgZq32M3w0e7A
ewRqj5p8BT7Eh20eWpgMwGxe2+xZLbzttTXIy/DOnZK74buIclsmMNmSWtsDR7qZ8LbofY9IILC06
ge7F2C6eJMVdv90LvDYlPxsxUdlOv6yF8Izpz6z0TZLVzdefE6yfE4ZsgCCJn3Pz9675z95dF1b3a+N59
X/nTR39ovigBYNqo+YFunnIU2B1O/
+K0OHwTBEHkjL3n9jxy/62i5J5C/OjkgzzYD2AtUPMD3aj5sTudv3ReHL4JgiAyx7FHviCK7enEa2d/0
HxdAsCEUfMD3aj5sQtd3LooTt4EQRCZ4/bv3iTK7KnFb37+ZvOlCQBTRc0PdKPmx26ztb114KUD4v
BNEASRMyb4GL8bp+
+5iwf7AUwcNT8AwLK9s33k5SPi8E0QBJE5Hv7al0SBPc3gwX4AE0fNDwCwPPSzh8TJmyAIInN89bH
PidJ6ysGD/QCmjJofANA68+4ZcfImCILIHF98+kZRVE8/eLAfwGRR8wMAGhfevyBO3gRBEJnjxrN7n7
j3sKiopx882A9gsqj5V0v9bbjKKH8g7sPN4xtvNz/P42/R2SuKKBrG19rZAIben535feR2jUsfX9r3/D5x
+CYIgsgc6/IYvxtnT3z9r59+2nylAsBkUPOvUFNyKsMXRU2CGRVg7oqC9O6G1trZAJZen535feR2j63t
rUM/PiRO3gRBEJnj3m8dEIX0esXLT59uvlUBYDKo+Vdo5GpI17SzKcB8K/LTLQvetXY2gNTjszO/j9yus
XN15+irR8XJmyAIInMcfGbfU0fuEFX02sV7P32t+W4FgGmg5l+d/hVsmvkVYP12zCznS85aOxvAh5p
/zk69eUqcvAmCIDLHDT+89ttHbxf18zrG6Xvu+ujy5ebrFQAmgJp/NVQhZDDrWLsqFdWSUUS1w4g
qODS+WYAZbdx6zJpBZ7lmDmv2dGrz8LDtHd1JT1DMVnFGL9g7V7Fn39nAZq7M4LkcXlpNJraaGO
O1C63XF+0X0DeX1TD6ZhU3A1uhtPPWqhHNbkYbdxRr0uJ27zkjxYu/flGcvAmCIPLHN79xSBTP6xs
82A9gUhar+fVpO3zU9jaxyoFAydBbe+Rf4wN/qEDy3yoYu+Zp4OxpaHz/4CVjCKv4UqJv2yDD6vv1N
fEytCJB9SruqR72zDsbCN5G8mLX0nyzL7QtPPerm/5+3pVrCbka3TMuma0Mng7DfeQWng16e+uD
t8SxmyAIIn/cfWq/KJvXPXiwH8B0ZKj5xXF+2TO6OVy09Jk8727q4kbtk26lN86of6Ib4Bvf7euM73RTf
SLvnDtse0XmiQxrtvFl9a1IKJuIKdjz7mwgqaRGK9HRmZYcWA6hO+gxVY+CubbQQG1HR0qu9opKrn
upK+2FSO62lbEIN50zJaebXHevOSPB5SuX+UP9BEGsPD7/T/8wg8f43eDBfgATMXjN73LO70sxao/C
MGOuSKxAMhcm2+lqp2P1sfGNa6KZemkM7rkkDDasHqhhpxRjdvDtpaWzQc2Zp+jXc2kmZyF63V
YP3Sw4UA/hXME3y+1T0Ncic/H1606nXkY2sMecsbDtne3Dm4fFyZsgCCJz7D235/H7bhPV8jyCB/s
BTMT4Nf+wR3NdCxy3a4J15NkZUU029KLrq576x8u3854Eopke3SOYsLsk6z2s2VC+u74VhXnWauts
0Ajsv5pE76VV7NbOGHYHveBW12RNvXPJjVVNrGTeizbfG+TpJprZ07SJCUXmjMXsXN058foJcfImCIL
IHw+cPChK5TnFj04+2HztAsDqjPvf9nsO854ze6lnJaP6bLytflqkBJoYz276yyrR0N/I5Rnf1zcwuo8xkG
3IYduWzgJ9KwrzTMrW2UBReauGqpeeQ5+l6ZkL6n5wKoGOsQ1IyCU31jud4BxbcpxSdzrVwic8IV8
u9Hf6F6fFsZsgCCJ/3PnkLaJInln85udvNl+7ALA6K6r5dStTpJZoqG7lIOrn7l5T5dlNf1kllupv5PKM36N
26ju6ZbhhdbeKaO1bUVhnygWW2jQtWqo5dL1pJtWiUDdyFtJ7rkqfhv1zyTbe6XTO0f8GdafrMXCP
OWMB5y+dF8dugiCI/LH/2etn+Ri/jtfO/qD52gWAlRr/b/h5+jiXehz6S6pf3U296ug0XbGdMRcl2/Xc
Ld/4PWonX69OQw2rWh3f3PQtcrG5de5T340sNW2Pb2zUUzBm0DkrN4+6ortEplLcWiBZWi45qi+
LvhbZLl+/7nS+XkKPOWMB/F/jJwhiCvHI/beKInlO8aOTD/7ts8+ar10AWKlV1Pzqiuf8Hikn3Ea9Ok2Y
uzMFXaHJVbbL9NQ/Xr7xF6n31AU9JXOetmGGtbroF5Ex4zr3qbOBSU+3Yk2ga2lypW17PY5/Krpje

9nfsJWWy9lYZ0nGOJHtcsYp9EjnZJOr6DVn9LdzdefYT46JwzdBEETmuP27N4k6eTZx+p67/vTRH5rv
XABYtVXU/PpA7xE5v6uB2mO/e2WtBHbTvz3GGj31j589UpWlV+2kr1gib80gw+rbahA9/XZUz4rCP
JOydTawmLll5o6l2dM2qSahqYR6hpeelMt9swJLKsW2y85ejdYrXccG9pwzFnDlL1f4o/0EQaw8jj3yB
VEtzyN4jB/ApKxPze+vCmqxKmS6IrtpL1bc99Q/IeZWV8171072DLpSLT9se8Nor6ffNnRWFOaZlK2z
gc0zG1N8x8x5lytUr5vVxqZidi1ZG+qzeK7uN6u4EZtjy0xetUxKJ7P0HgQL4P84P0EQK4+95/bM77/
w5zF+AFOzypo/XjsIdkEgLTQUkEJ9BPmwYTYuvH9BnL8JgiAyx83fv+47d39ZlM3rGzzGD2CCVvk8f2
QUh+4TQB2GcelPIB81zMpzv3pOnL8JgiAyxx3/42ZROa9p8Bg/gGlKrPm9qhLeKeg9FX47THNN//fA
oX8L4Bmkpm9QiWEk5n+tHv6IAmvr0TceFedvgiCIzPHAyYOifl7H+P0v32u+WAFgSlZS84cGCpZTkco
+cgsYglHzU/Bjjnau7hx99ag4fxMEQeSMvef2PH7fbaKEXq9444Xnm29VAJiYFdX8JXuwWMkereujN
wEAnba2tw68dEAcwQmCIHLG/mevf+rIHaKQXpd44VuP8Rg/gMIarOYHAMzSxa2L/Bl/giBWG3c+e
YuopdcivnffVz7548fNlykATA81PwCg9MrvXhHnb4IgiMyxjg/28xg/gImj5gcANM68e0acvwmCIHLG
DT+89ttHbxdF9ZSDx/gBTB81P+LE32OIv1yI+tt4zR9iWGaoRVl/in/0PwRRrCzDmko59xCzdeL1E+IIT
hAEkTMOPrNvXR7s5zF+AGuBmh9x8SJ/mSJzVTW/TlQbt+RvkmWqwbPtIeZse2f78OZhcQQnCILIG
Xef2i+q6wkGj/EDWBfU/FiIqCqXKTJFzZ9Nxry5a3Bqfgzjg08+4M/4EwSx2vjmNw6JGntqwWP8ANY
FNT8Wsv41f866mJofa+udD98R52+CIIicMfEH+//tX/
+l+boEgMmj5l+JQLnrvazruEp3j466T91ue7hX1LDlFTFc/GWcblwo2ou5e4cyu5gTjLI6Wb1USkNg4
ubkxLwtdi4zWyiXWHbJWbrRph2muRnO6BkoQIzRf1GFPvMvRMeoWU3c+z1GwLjOXzovjuAEQRA
544tP3ygq7YnEP//jE80XJQCsA2r+1fAUTr6LbcFnMjr1LMBM6r6+rZPoQZor1WsxXPxlhH8ppSatM5
S+YDIW6hXfMc/dwMQ7p1uKJgvlUteNcZyle/rWzaMZ+70d3m01enSkSJ1/yZhV15vbPQKyOPXmK
XEEJwiCyBlffexzot5eeTx7/Nh//PnPzbckAKwDav4VUTWPp8zRl3Tdo67oQslpYgzjFGBS00B1Maqrp
ocaoXophou/DNLtZI5CM5GukT1LdfTYMXdgL3dX2itiCQvm8qzDaeZmr3Rm9OWzOV3ElnWm6DN
/2cQZRHZwenSOgEx2ru4c+8kxcQQnCILIGQ9/7Uui6l5t/Ptvf9t8RQLAmqDmXxVVxegixqmlPMVVZ
3VVcOopR9Oi7lO/2NgoBzKvqP5iuPjLEF8zfa2Zu91GvfIszLgk9Nkx94KXGspo1dnRbeDr0uct09nDa6
3IjglTrC+F2kfmFp6/fhmavmrgGaK+1DkCMrrylyv8GX+CIFYYN57d+8S9h0XhvargMX4A64iaf2VE4a
NKKV19eWqrQqCX0UjXS+E6rulVNah+Pr75dtWrvaK7i+HiL0O8axEX7aHUTZ9QLm8WPa662m/KO
n/bKtbRnq5u4OvimaXTzJNd8mbsXJsneZh/UT3mr1+3rIT2wLZ6iK4RkNflK5f3Pb9PnMIJgiCyxUQe7
OcxfgBripp/dVRdUxUzqgxqizV/eSbKqx4FmE/TrWhRty5+qC/pK7q3GC7+MsS7FnHRHkrd9Anl8mZ
xpthvyp6x3I76iuA2MHL1Gdm/kkJHRl8+S3DgVteiltmZ5r4awkcNER8B2V14/4I4ghMEQeSM//bo5
0UFnjl4jB/A+qLmX6Wm9imqJ1XhGCWNvzxTDZurvQowj7qf+p/3q97VJXWlHVAMF38Z4l2LuGgP5
e3Rwd9Hjauu9puyZyzZUTUp1K3ckX25eowcWn1nRl8+S2DgVvei+sxfM4ar1J06Z2Hwj4BVeO5Xz4
kjOEEQRLbYe27PI/ffKurwnMFj/ADWFzX/SjUFzfGNjbpoMmsmXUiZVY6srjzVky6SQnVfqRnHfYy/v
mKMJzLGX4b4muIrTS67Tc+BLXLEijNQv5E9Gys6ui3cnffl6vOWedoUujN2ri00ITVkdwpPE7dNobhm
vLLzdk6zEhsBK/LoG4+KUzhBEES2uPn7133n7i+LUjxPvPfT15rvQQBYQ9T8q6WrpYooZ/RNVV7pukf
XW/qK6msMGC2O2o6+rkY9J2qt+MsgZ55G/iZZaGTVQ08vkqrHjsk8AWqk8E7I+bQTbkf25WrbNRe
NfVfNPNkL3Rl9+WxOdvtCj0U5Izjz1xfa6YsFhcfoO4LnAjLYubpz9NWj4hROEASRLW7/7k2iGs8QLz9
9uvkSBID1RM2/Yrq6KaiqqmXebZlVjlGUSe5wprajU0X5E9TDxV9GhCfaZHOG8vfoSLTAjkVH8lSUsq
M/Vakd2W5TXw/vhO7oyV7qzNhnbYH0Taoei1pg/lI7Lf8g7f3uEQJ7hLFtbW8deOmAOIUTBEFki2O
PfEHU5KPG2RNf/
+unnzbfgACwnqj5V00XN6Haxa6O2ppHMxsUt/VLT1OT287bU1yMv4zTjQtFe1G0xbNXepZ3dic5s3
5T9lSUno5mZVpeVa+Noc0mejBzhkVbZ2RP9kY8Y7+1yR0SaeIpKl3zL5nDlNwp2bOIr7Vkj6Duuv0w
totbF/kz/gRBrCr2ntvz+H23icp8pDh9z10fXb7cfPcBwNqi5l81VfhQugBYF6/87hVxCicIgsgW+5+9/q
kjd4j6fIzgMX4A80DNv1r6f+qk5AewTs68e0acwgmCILLFnU/eIurzwYPH+AHMBjX/qtj/4bL7Hz4Dw
LSdeP2EOIUTBEFkiwdOHhRV+oDBY/wA5oSaf1WMmp+CH8Aa2t7ZPrx5WJzCCYIg8sR4D/bzGD+A
maHmBwAk+uCTD/gz/gRBrCo+/0//MMaD/TzGD2BmqPkBAOne+fAdcQonCILIFnef2i8q9iXjtbM/a

L7dAGAuqPkBAEs5f+m8OIUTBEFki29+45Co25PjRycf/NtnnzVfbQAwF9T8AIBlnXrzlDiFEwRB5Ikbf
njtt4/eLqr3hDh9z11/+ugPzZcaAMwINT8AYFk7V3eO/eSYOIgTBEHkiYPP7Fv+wf7f/PzN5hsNAOaF
mh8AMIArf7nCn/EnCGJVce+3DogafqHgMX4AM0bNDwAYxuUrl/c9v08cxAmCIPLEw1/7kqjkewaP
8QOYN2p+AMBgLrx/QZzCCYIg8sSNZ/c+ce9hUc93Bo/xA5g9an4AwJCe+9Vz4iBOEASRJ7749I2ipO
8MHuMHMHvU/ACAgT36xqPiIE4QBJEnvvrY50RVH4n/87/ONV9bADBf1PwAgIHtXN05+upRcRAn
CILIEz0f7H/hW4/xGD+A3YCaHwAwvK3trQMvHRAHcYIgixx8/ev+87dXxYVvojv3feVT/74cfOFBQC
zRs0PABjFxa2L/Bl/giBWErd/9yZR5Iv4/S/fa76qAGDuqPkBAGN55XevihM4QRBEnjj2yBdEa/jjReeb
76kAGAXoOYHAIozLtnxEGcIAgiQ+w9t+eR+28V1X4RPMYPYLeh5gcAjOvE6yfEWZwgCCCJDuA/28x
g/gF2Imh8AMK7tne3Dm4fFWZwgCCJD3PnkLWbNz2P8AHYhan4AwOg+
+OQD/ow/QRAriQdOHqwLfh7jB7A7UfMDAHJ458N3xEGcIAgiQ+w9t+fx+24rav7mywgAdhlqfgBAJ
ucvnRdncYIgiAyx/9nrnzpyR/NNBAC7DDU/ACAfcRAnCILIE3c+eUvzNQQAuww1PwAgk2uuyfQPHR
IlI1EyEiUjUTISJSNRsvkl2g3YSgBAJpxIkpEoGYmSkSgZiZKRKBmJEMFWAgAy4USSjETJSJSMRMlIlIxEy
UiECLYSAJAJJ5JkJEpGomQkSkaiZCRKRiJEsJUAgEw4kSQjUTISJSNRMhIlI1EyEiGCrQQAZMKJJBmJkp
EoGYmSkSgZiZKRCBFsJQAgE04kyUiUjETJSJSMRMlIlIxEiGArAQCZcCJJRqJkJEpGomQkSkaiZCRCBFsJ
AMiEE0kyEiUjUTISJSNRMhIlIxEi2EoAQCacSJKRKBmJkpEoGYmSkSgZiRDBVgIAMuFEkoxEyUiUjETJS
JSMRMlIhAi2EgCQCSeSZCRKRqJkJEpGomQkSkYiRLCVAIBMOJEkI1EyEiUjUTISJSNRMhIhgq0EAGTC
iSQZiZKRKBmJkpEoGYmSkQgRbCUAIBNOJMlIlIxEyUiUjETJSJSMRIhgKwEAmXAiSUaiZCRKRqJkJEpG
omQkQgRbCQDIhBNJMhIlI1EyEiUjUTISJSMRIthKAEAmnEiSkSgZiZKRKBmJkpEoGYkQwVYCADLhRJ
KMRMlIlIxEyUiUjETJSIQIthIAkAknkmQkSkaiZCRKRqJkJEpGIkSwlQCATDiRJCNRMhIlI1EyEiUjUTISIY
KtBABkwokkGYmSkSgZiZKRKBmJkpEIEWwlACATTiTJSJSMRMlIlIxEyUiUjESIYCsBAJlwIklGomQkSkai
ZCRKRqJkJEIEWwkAyIQTSTISJSNRMhIlI1EyEiUjESLYSgBAJpxIkpEoGYmSkSgZiZKRKBmJEMFWAgAy
4USSjETJSJSMRMlIlIxEyUiECLYSAAAAAIB5ouYHAAAAAGCeqPkBAAAAAJgnan4AAAAAAOaJmh8A
AAAAgHmi5gcAAAAAYJ6o+QEAAAAAmCdqfgAAAAAA5omaHwAAAACAeaLmBwAAAABgnqj5AQ
AAAACYJ2p+AAAW9fbGNa2Nt5urAAAAU0PNDwCYkboYH7cK/3DzeJthzIS5/81CubDC8c0PmwtDs
hZT4d+VLMraw3HeptEFfmGaz15t2E9G5Fc0cmtR/qGsd2zYZY0rujOjflGMKLyodX2fAiuyljPU29Qj1
5p+J+0S1PwAgHGNeJq3GHlyHtqqtGOcdYqzlF5Hda4a+UBVLuT4xkbx/4ySp1xC9iOheR4d/lNhfbJb
I336qrVk+kSY+zZckuBvqP07ZC90KcGM0VuLCg1VXtcX6i1dNpV/FPPtWjpF586U9wf9ovAtylpSZbl1
RRZV3RrwV8ldjpHcNNKKqvz6UvVq2dX1yzVAqnrvGmJ/rF0U99ADNT8AYET2eco+jQxKJar+/5wHA
nuFYxk9S5mg2b5x0pTv/ejbZMrzxliqlCN9+OTY4+Ua+hzfUO+HO3H50RhqaeGMsVuL6juUate8XFj
Vv2ZnKbZPX1j+7epaTnm9ub1UnlpsUUOM34gsynMpXXA5wvJJwyuSV0bMpW40L5d918rh9PDVJ
7l9aaeqbi61pt2Imh8AMB55BnBODYMbP4OQ5/gx8rL08OUP6Ye2mOWOgwvL/jkojPtRsDdwtPVV
A5tv1NBvnDNzdykDb2Rkrwbcxq6hqvvJG6l6j5vF4E+kr5Y/LJ1GTdaTa5yvCzfRIOtoRJZjG/Dz3bl1nZ
PpzxnKWceAyfRuVi/kx2HIRLsGNT8AYDTuP5kHPO34ZT4MVOsZ6swYZB1/hmfsWfnjOImqnWqNv
GfjrSNo5DepUG9hkaL+YZxPufMbanw6BuGMV2a0923glJHhBszUNZSzsUm6sgy2IN9AxrXyx8E+7J
5c7odiCG6iKk/5oIK2fFbf1hmq20OtzZur+qiVKeofwlNZjJMrsJ3DLM3cJn+iwRa2S1DzAwBG454AvE
eUIY2ewFQdPIY6vbmqtTRGXFK5CjV8mXK8BSn1wsbMU33yBj7Kx+X54FWfuMpYqdx1uL/FS3ESu
OMPvJeR4QbMFB+qeuMG2MV4luruMO+VJ1G5BnWlvD3YhyKQyzBQKjeReF+qBuH97cezHEP87q
JCo7X7N1gqT67qity9gd6ragVNtvJne9hhd3F3oOYHAIxmBf+ozncWEKfFUQ16mLKVy2j3q0yUZU3
uR2NQ4s2p92/Mj0WVcNTPnbmG+udx8lVjy60b8K2qBjRn7n4UnCbLiQw3YKbYUOLjuARvlupiY6ht
cxKVa2hfl7cH+1DEtq5QL2+IbG4i58M3wC9ydDkDjG/y5Kq3q75W/zxQPu+6qvUoGxvWh2QJ1bD6
jXHepfgew4uaHwAwmhX8ozrTWaA+6WQ8c9SHt+ETWkc2wxAn7Jgq73j753zyxs1XvTujbpmbYcQ
VWR+K4c7xNec31P2Vdd695bgJtMitRQWHqnZzqOV0TLi6PUgymcj6TBjGyOUa6BPhJnIG7p5Lp8g
Q1a2hPgslJ5eboXrnlltRrXtryhZDrE7+yrhvf/dcIFHzAwBG4/6DeaCzW1iOs0CVI/OBo8o56s5VyjTjZx
l/OWMc5cMGO1WHuSlGXZFh6I+EO2/5Zg29ssh4A6byD1W9bwMupnPCVYMBEnYkKm8P9qnoX

NRQv2FuouqKuRD5YUwQXs5AyzA4udwU3bvbU+dAZYPlPxTVCuxh3MwDvE27DjU/AGA88p/Mgx
0/gsbPUJ9JpMHPH0UWYxmec9Aohjm1uYpxjWGrN2nUd6nKYC5kzDNi9eaM+5lzPwFZkhYG/0RU
b409cWttzlu3NE9GJXJrUZ6hqkvDvkmdE64aDLB9HYnK24O9SStdlPWLNMhvVXA5I/zOurmsX6bCc
Enjb9Mw71E1WzdJedkcOz4VeFHzAwBGZB1AhjkV+FVjC+t9JhAryrOYMuko71D21VhH3eHOva4xP
9W2+jysZfhEDLk28QmotEsw1zbUZkYyxiezkPBQ4v2qLbm4Kps10yKL8brKuUyOnjtTNhvsQ+8sqrh
gDF5PyTOJ3uKLsu4uk6bhLKdWXc7w2R78iyKcy76z9NbVw3mHsT7Xg+7k7kHNDwAYl3kA4Z/TyM
Y6kC59Hg2pPt6jjb4CA5/jMQj7Xak17424tUbvWHhRa7mmyHtUmN0XxeDEv6qotUcGDhJLouYHA
AAAAGCeqPkBAAAAAJgnan4AAAAAAOaJmh8AAAAAgHmi5gcAAAAAYJ6o+QEAAAAAmCdqfgAAA
AAA5omaHwAAAACAeaLmBwAAAABgnqj5AQAAAACYJ2p+AAAAAADmiZofAAAAAIB5ouYHAAAAA
AGCeqPkBAAAAAJgnan4AAAAAAOaJmh8AAAAAgHmi5gcAAAAAYJ6o+QEAAAAAmCdqfgAAAAAA
5omaHwAAAACAeaLmBwAAAABgnqj5AQAAAACYJ2p+AAAAAADmiZofAAAAAIB5ouYHAAAAAAGC
eqPkBAAAAAJgnan4AAAAAAOaJmh8AAAAAgHmi5gcAAAAAYJ6o+QEAAAAAmCdqfgAAAAAA5om
aHwAAAACAeaLmBwAAmJa3N65pbLzdXKqpG8c3P2yujEPlEemnoN2bgrMPH24eD92K3gOA+aLm
BwAAmBajrrXL091e87dle8W3DcHKvt3UCf6bDAAYDzU/AADAtFj/W7ZZoe72mr/X+r3FffuvAqj4Ae
wy1PwAAADTYtX8ZoG7y2t+Xbh3zEvvn94nfYWKH8CuQ80PAAAwLXbNbxSqTs3vXHAL46ZJ2cIYt+l
gXDGrYXW5uGb85/RGEsW469w35tam6ai5rfGsAY2ZKpGxdOuqjX7lrCA2/5I9H9EkssBoPwDIipofA
ABgWtpicrP+SZWMRpVZvXYvGOVmUxK35WgnXUSH+xh1tqxrK8ZEPINES19/Url0gzEXRzu5jU31o
8jeNf+OCYUX6O8XnS4AjIeaHwAAYFpU0VhUkKowrQtG40bV0L0Qq/mbRkZJKq+ostTp015RF2Qa3
UJPxcjTo9wNJ9ADujljdONGO0ylc/6qQXg+gQWGBpIzAIA8qPkBAACmxaoamxfmz27ZaZSTsphVTd
q6NHxFDeO2kG1kRVyQl/QgRpsgOYOSXIl7IU5PoCS69Ji/5GT3L1A367VuABgdNT8AAMC0+Mvrot
R0KmNPqSxLU7dJ9xX12iyU7XFVCx+R2BolwJ1SSaVUV+XSOoWn0GP+Bru1uh8YXc+yZS8LALKi5gc
AAJgWWQE3r/Xj/W0NKVsWxqr57YvqhU8wcZi/rVxKcs3vTqHH/H3Fe0XdD44e6Nh31gAwKGp+AA
CAaXGKSVlE6hueslMWxm6T7iueYcW4vhZCjyaav61Kqa7KpXUKT6F7cqpFoW7kZO8cwxiiEk4GAO
Oh5gcAAJgWTzFpl4/6RqzlADW/WVzbbXqU326aMD2c2djJMWDN3zmU29XZlMgCi1vGwAvPGwA
GRM0PAAAwLb5iUteNJX2jvdqUk7owda64xWv4SngU3SScWRW2bpqYcIK2/8K1c2QKXfOXy2nbdy
zQWUh0GgAwOmp+AACAaekoJs0bRikqiVo2VoLKK/r1cTm6qncL/tRtAzdNnGpvM3sPWfN3zt8/nZ
JqEho91LPvrAFgUNT8AAAA0xIoJtsi1bph1q5FWSkLY3ew7ivqtTmcM52SXTfbDQKriLLHk0WyXFqn
zinE5t92L5UpjW0xb/tGN7uW+s4YAAZHzQ8AAAAAwDxR8wMAAAAAAME/U/AAAAAAAzBM1PwA
AAAAA80TNDwAAAADAPFHzAwAAAAAwT9T8AAAAAAADMEzU/AAAAAADzRM0PAAAAAMA8Uf
MDAAAAADBP1PwAAAAAAMwTNT8AAAAAAPNEzQ8AAAAAwDxR8wMAAAAAMEd///v/B4p36
MlXpOWqAAAAAElFTkSuQmCC]]></redcap:Attachment>
</ItemDef>
<ItemDef OID="b20" Name="b20" DataType="text" Length="999" redcap:Variable="b20" redcap:FieldType="descriptive" redcap:BranchingLogic="[q18] = '1'" redcap:InlineImage="1">
<Question><TranslatedText></TranslatedText></Question>
<redcap:Attachment DocName="B.png" MimeType="image/png"><![CDATA[iVBORw0KGgoAAAANSUhEUgAABVEAAAEJCAYAAACdcmdaAAAAAXNSR0IArs4c6QAAAA
RnQU1BAACxjwv8YQUAAAAJcEhZcwAAEnQAABJ0Ad5mH3gAAE9sSURBVHhe7d0PjCRnfef/b+/aY
PPHNmBskx+RHat7sZYlGJIg08PxL4egZ+6SJbnbYOVg5Oh3PVyi0zQSkwOxF+mX2wguG4lunS5iWrq
zliQyTEJugrLdIJRgckzHR4IxYTI/Z7rlc3T8wDYGjPljB3u3f/VUPVX1VHd1dVV3VXX39PtltaerpqfqqT/T
O/OZ5/k/k+hb5FAAAAAAAAAAChjumPAAAAAAAAAIAQhKgAAAAAAAAAEIEQFQAAAAAAAAAAiEKlC
AAAAAAAAQARCVAAAAAAAAACIQIgKAAAAAAAAABEIUQEAAAAAAAAAgAiEqAAAAAAAAAEQgRA
UAAAAAAACACISoAAAAAAAABCBEBUAAAAAAAIhCiAgAAAAAAAEAEQlQAAAAAAAAiECICg

AAAAAAAAARCFEBAAAAAAAAAIAIhKgAAAAAAAABEIEQFAAAAAAAAgAiEqAAAAAAAAAAQgRAVA
AAAAAAAACIQogIAAAAAAABABEJUAAAAAAAAAIhAiAoAAAAAAAAAEQhRAQAAAAAAACACISoA
AAAAAAAARCBEBQAAAAAAAIAIhKgAAAAAAAAAEIEQFQAAAAAAAAiEKICAAAAAAAAQARCVAA
AAAAAAACIQIgKAAAAAAAAABEIUQEAAAAAAAAgAiEqAAAAAAAAAEQgRAUAAAAAAAACACISoAA
AAAAAAABCBEBUAAAAAAAAAIhCiAgAAAAAAAEAEQlQAAAAAAAAAiECICgAAAAAAAAARCFEBAA
AAAAAAIAIhKgAAAAAAAABEIEQFAAAAAAAAgAiEqAAAAAAAAAAAQgRAVAAAAAAAAACIQogIAAA
AAAABABEJUAAAAAAAAAIhAiLp0etJYKUih4DxWGj29PqjXWPFeU1hpWF+1iMxj3ZB2onXjpLWdL
MxLO7LSlg372PzHqPs4Vb2GrGzEO5vtDbdtK5JH08KNuQ9Cjmc+2g0AAAAAwPwhRF06Rdm8UJey
XurU1kPCkracr3X0c5Hq2U3rq4B5oILBVWnqJUdZzqxle4faf1Qo1cT/rlhsR+14AAAAAADIGiHqMip
uytmqfi4dqZ0P9kbrNc75IVW1JdsV/XzhFGVzry/9vnpsS/aHkff+RpmXdmShKwdu8leuS9c+xj3ZzDJ
D7TVk3fijwsI7ascDAAAAAEAOCFGXVGW7JV6O2jxn9EY1e6FWpbW4CSqOot6h7OuncuoEPaQBA
AAAAEAuCFGXVkW2W26M6vdGbW/4Q6XL9a3wXoyqlqKuRek+wmpSjqyvaH59ZI1Jo6ZjoC7rqPX
GPu31edcGHd5fZI1J4zwEz5+5nbDPjxN+3INtCdS9tR4xy30ObGewraPOc7xjCm57uPZpYAh6c9VZF
2h4snM3eA7Uw9yc3Z6x+xzPPy71CLkXbAnaHvI9OHq7vqTHE6/dAAAAAAAcfYSoy6yyJXW3OGpz
VTYaDTnnJ6hyIWSM9FAIo3VqpQwmoCrK2hndwM6BdJ1nFmNId2dHLno7bcuubn/5zNpc9FKsnPa
D6gP/AGy9izv6PBo1Pe1wrCSDo63TPb8dqZUKUhrYSXM1aUimtjPY1qasDoZtEx2T2rZR+9S6H+te1
+kREu3HCSwHz4HSXB0dKibnnOtV70AUtW6Kc9TeGFHPNGS7E4vZbgAAAAAAlgQh6lILTjLVrLnBTF
nqF0Imk2pv+KGKV4+yL103ie3UZD3lhKW4dka3b18O3U23d42JhYxw0hjqferEnAz0rpz2yiY0d4M9
Ji/u6BisfEacDLUnjXX3GlSlpc9v3+0xnOr59bfvdUhWIdlAfdzx/O1494Hazrob/E1zTMbr9zZlc9v62PX
vV1Wv1/6cXXIi2X56jXUvsCzXu85r+8MlLiqR+4wr/BzteOl/krZbr3X/0hH2PRjY7rBkxzOu3QAAAAA
ALI+C9QtyXz/HklK9SwM9zlSwEhKq+K8rS71rTubTk8aK24tOBS/OZEYjX6963bk96Ubsy+dvW4Vde
9ZG1BBs1YOwWq1Ks9kcWu+3IaxdcdeNE387w+2yV3rnwG1/6DqbsV0VnO2FBNwB4e0wr3O11fc
nDEt0PSK2E9ivvuaS7JjMbQdfr41qa6JzF35+FOdanQqsS3p+lETnOlHbRwht44jjjDietO4RYBr3Pnyvv
OXCW/QSACAP99/xLnnNW2/SSwCAzB3/NZFjr9ILWBT0REVwkikVtoQGJP5Qeb/npMsYdm/2GE1F
UU6ccp51dJfTrj2WvywnT5+0e9QF11uqp/0AbA74vWmb4nVG7R44gZT1GW8ov7dOdT4sGbUo3S
BMfcIsazBr1jUo6ac28z7QPYSnOKZEvYkT7cec4f+kmIdQ3NyTvhmgTm3wHIWY6rrrurFuwJmaGO0
GMvDwEw/LOz/5Tr0EAMjDDY9fJXd/6AF5/Bs/0msAAJm7dI9I/zt6AYuCEBVBA6FScsO1P6fl1RVt7k
pbenJoj9k/JScqJ6z/D65XGeqc9ZIrrombLbpD+ttuIl09a/TozUtaAZl1DTJrOyHeSKpXqBe0GnVjgQX3x
NNPSOWPKvZHAEA+nvPjY/KWL94oP3ryGWm+/3555p8u6c8AALL1lMilP7A+PussYiEQoiJlGYRfXl3
RfTlsXxS7lKgd9lbEyVeN9XMZvhk9NO3A1+/VOyrwVcO67VqVQ480e0lOK1mv47yOaTHOXbjxbW/
Lhtnr1K2LatY5BRbUnZ+6Ux58/EG9BADIwxv/
+gZ5wQ+vsJ8/9NXvyp/WeR8GgNz0Hxa5/Od6AYuAEBUxleSkN1LbnBFfMSZJitM70Ri+HI+7744c7D
pf686+X7I/4a8fLjUwH4qbZ/0guOFOjFWVQIZacsoTKG6Jgvk22OvYvA/0seV1TIn2Y97LA8Pk1cz3hZ
xnoE/SdmNSNTtwdeukJv6eAubL+z77PvlM7zN6CQCQh1c+eK3c/PXn6yXH5z7+kHzlLx/RSwCAzF3
6gsjlr+kFzDtCVMRUlM2zbuVUc/Z1NbrYnOl8y+vt5wScijnre1s23JlrYvN7cqqJpBS3XmZRF0x117vh
6vxxe81a56Kmj3+wdqsx7F+aq7LhnTIV7Onh297K+dBcNQLH9nm/hqd7bHkdU6L9mLVbm7Lqre/p
me+ta1Qq+NvI2oTnyA9c3XYDi+kT+5+Q+n11vQQAyIOqg/pzD7xELwX94W9/Tb73raf1EgAgc5f/RK
T/fb2AeUaIivgq29LyctSalHTA48w8bynX5YJR4NOfUMnSXNWB0GQ1HAPbsp55Q/a9of6KMUnTHP
Jqu9rKUt8KRKiWomxe8IdlN1d1gOaFzqMm/ZolJ3AMttM8tryOKdl+ipsXpO6/2HmtOZGTdS/7h6Br
7yrua1NNWBO03bzfw9odR+bHA8R339fvk7v+7C69BADIw/FLBXsYv/oYRgWozd+8Xy8BADLXf1Lk
8sf1AuYZISoSqWyH11+stqz17tBiV3FT9gZfW21NVr/R7K0XKBlgDM3OdKKjFJgB2KiyA+qc9bt+wOe
ya1/OW03PstS7fT9Yt1Wl1d8LTpaV1zEl2k9RNvf60h16sXq5MUzeVpHt4EGK7B96PbFTEbvtVlv6Lf
8+sqlzblwHu+5ulByOB4jBnYn/6Wfp7QQAeVr50kvl2iev1Evh/uFL35ZP//6hXgIAZO6y9RvZJcpbzbt
CX81aAgAxtTcK4nSSVCHqQGAKADGo4PT1/+318sAjD+g1AIA8lB56od0LNa6tu8vyiteFD/sHAGTgit

8QKfBL9ryiJyoAAMiVGsJPgAoA+VK9T1Uv1CTUsH7qowJAji79AfVR5xghKgAAyM1HvvgRezIpAEB+
VP3Tt33hZSProI6iAtS7/+NX9RIAIHN2fdR79ALmDSEqAADIxe6Du/LBv/igXgIA5OWOL18/tg7qKPv/
8zH53Mcf0ksAgMxd/n9FLn1BL2CeUBMVAABkTg3fV3VQmUgKAPJ189efL//8Czfppclc+dzjsnX36+
XWV79IrwEAZOtKXR/1Zr2MeUBPVAAAkKknnn6CmfgBYAZe8MMrEk0kNcoz/3RJmu+/X3705DN6
DQAgW9b77aWPWx+fchYxFwhRAQBAZlRwqgLUh594WK8BAORB1T99yxdvlOf8OJ1f+R7/xo/k7g8
xKSAA5Kb/HZFL1EedJ4SoAAAgM+/77Pvk3ofv1UsAgLz83AMvkRsev0ovpeMrf/mI3PtJ/igGALm5/
DXr0dELmDVC1KXWlo1CQQrGY6XR05/LSa8hKxttveBob7jtWZG8mzM3Qs5LHL3GinPuVhoy6tT55
9d55H7NjwLuWyCW+n11+djffkwvAQDy8vJvPE9e+eC1eildn/zPB/LN//19vQQAyNylXZH+Y3oBs0S
IurR60lhZlaZecpTlzFpRP8+eHfiVasLfVIImPi+9hqzXor5KXfOCrAYvunRqJSlMENguK+5bIB7V+1T1Qg
UA5Ovqp47LG++bvg7qKKo+6u//
+y/bHwEAeVD1Uf+79fFZZxEzQ4i6tLpy4KZA5bp0+33p9/dkM68MdWzgt6QmPS+qZ+S4YK99XtxN
V1vqevelWy87K5qrQo4aA/ctEIuqf6rqoAIA8veWvRvtIDVLqifqH/721/QSACBz/UdFLv2xXsCsEKIuq
96h7OuncuqE5Nf/FGmL1zOyJ41zugtquS5bFedpcfOsVJ2n0twlRQUwPTUTf+WPKvZHAEC+Xvu1F8
vLHr1aL2Vrb/f/2A8AQE4uf8l5YGYIUZeQXbvRDN2aq04tx8GuiKp3o1E7c1T9zGAtyGCd1bDejbH3r
wVreI6qN+kMVfdfl6zW52A9S6+2qH6ENy3GPgPncEP8zZjnydln0vOiqHaWYvWMNHoeB0LzkpzUn
VFl/9A6olGM9obVW21v6GMZPAcJrkvI/RZ2vePcb4PXz35E1ImNY5b37WT3p2vSa2eZ6D1Ar1TMr
49uJI6QOz91pzz4+IN6CQCQFxWevubvXqSX8qF6o1IfFQBydOlPqI86Q4SoCDUUGGl2/cyRYVRHai
WjzqrR43EyanuDNTzVurCgpuQNVXdFt3UUZ5+DwWRzdcJ9Fjflgjtk3joz5/RGeo1z3nkq1y+kUEahK
q1+y+tVOsTseTxK50C6+umwipx2N97ZkYsDJ7W96x2NX1c3yXVRQV7I/eZcj1EBZPj9pu7d0GC5U5
NSIMjOSpb3bcz7M2CCa2eZ7D0Ay07VQP1M7zN6CQCQFzV8Xw3jz5uqi3r3h75KfVQAyI2qj3qP9Z
H6qLNAiLqEKtt96Xfr4kZ7Um3Z9TH72zrxbG/4AZBXL9Won9mpyfrIxEaFec7r+3uboWUCxu4/wN+
et3/pyI6XBPWkse4GPca+Wzo1imzrKP523M2ofdbOu/Fbsn0WNy+If+rOS9v677wbglnn94JOUJOd
F4P9um2ZLK8uyolT+ukYlS23beb5V9ri53BnxMnhkpwj67VGqYGh+21ofyZj2/b9ZrYlbFt+kJ3U/Ny3
/uvD789hya6dZar3ACyrT+x/wp6NHwCQv5UvvTTzOqijPPTV78qf1hmBAAC56T8scvnP9QLyRIiKIW
bPtPoFPwgdDgOHletbE4Z54aotPxwsrp3xAqzOge4z2bsoO14eaey7suW3deeiJIl7zH364ZMh8T6Ls
nnWTbuaslpwe08Gz+8kipt740PW2PblMOpEFdfkTNjxtXf9XrVn1pzjSXSOrPOzp0NEI3gPvd4Dhu43
s8et0evSPk86CNzLYfa0LO/bsfdnmCTXzjLNewCW031fv0/u+rO79BIAIE+vfPBaufnrz9dLs/G5jz8kX/
nLR/QSACBzl74gcpkJ/vJGiIoBI3qm2Yqy5iYxIwK3UyfSDKjKcrKkn47SPdC9+VSoU/LqNhbMYdKRw9
QnMMk+K9tGr0FHOsP403RKoi+fcf2NgNIP3Kpy1j2gqa6LruEZOrw/aOh+M8JCawf20Hd33yNrsaZ
uHu/bBNduyvcALB93Jv6nn31arwEA5OWGx6+Sn3vgJXpptu7+0APy+Dd+pJcAAJlTw/r739ELyAMh
KibUkeHOgTHCo7mX3TFUvMKUSrD+5Oz05HBssVSf36vSHRZuBG7V08FeoUmYExB5PXXHCbtWR
dm8EN470w0rF3t+o8nvz/SvXdh7AJaNCk5VgPrID+h9BAB5e86Pj8lbvnijHL9U0Gtm60dPPiPN99+v
lwAA2XtK5NIf6OfIAyEqJjR/gWm53vWGbAcfk9YLHS/
+Po26n7aO1NZznJyneELGlj4tn5Sxl7S4KW5lAntYuDF8vno6/CyPP0dt2TB7nbo1OM36o0lYbdzT+xj
s/as0z83XpEi53bcTXLtoR+GPJpiWGsL/wCMP6CUAQJ7e+Nc3yAt+eIVemg+qPuqnf/9QLwEAMqfq
o15iYte8EKJiQElOusnV0EzePbnoFnIcO/Q7J6WTXtA2qm5m6ibYZ6+xrodpl6Ved5OsPCfnMa5rc1f8
zphdOfAu6QmJc0m9HrXW/XH+/I4OP6sSyOGSnCOzLqcKFN26qMaQ96TUzPKqx6k9GZQdSHa9W
p7pDpOf0CzuW0usa5fme8AU1xDz7yNf/Ig9mRQAIH+lh1448zqoo3z6v/6D/MOXvq2XAACZu/xZkf
48dRU6ughRMcCcBCnYW9IPAlXYNTChz6yYNTCbq/5Q7faGHhpuPdIev510n72GrLsnrnpWNjeNyY
Nq65JPjmrWsmzKrm5er3HOCzBj90asnBbnDulIs+ke18Bw8Amvix8oDvbcjUlvX80sH+xxaoTFZo9b
oz1p3yaRZnHfKnGu3QTvASU/dZXaee9gZMOb4h9Hze6Du/LBv/igXgIA5OnaJ6+0Z+OfZ83fvF+
+9y1qZQNAbtSw/v739QKyQoiKYeYkSJ2alHSoU/LTE7kw7YxI5vDy5uoUoVGwBmZzVQdQXnhTlVZ
qs9e7kuyzJ411d6h6Wepbav3okCq98zLMnFndbbN3TastiX+aKhIo72oZDmATnCMv2LO4x2xOsJTEi
HvXrLNaPevPNp/YQt+3SpxrZ0n4HuDXW7V41zBubVssGjV8/85P3amXAAB5UvVP3/aFl81NHdRRV

ICqglQAQE76T4pc/rheQFYIURHKHgYdUpOy2rLWu8Otp1KR7cGClfuHRs/BBOwamMZwbZddWzOj
eqgx92n23LN7obonbiCk8of1p3hehhRlc2+4Tqg9hD5hYBeYJMs6ZjsbHhT7uljH3G/5QaqtKq2+0dZ
ACYJo9r07eJA2Z5vTZZMLft9aYl07S6L3AHUsg6+ttiava4u59cTTTzATPwDMkOqBqnqiLgI1pJ/6qAC
Qo8vWb6bUR81Uoa8KBgJAEmo2fT0ZlAph96btmTxL+lhOtaYNWBfEUbp2yJUKTit/VJF7H75XrwE
A5EnVQVWTSS2SK597XLbufr3c+uoX6TUAgGxdKXLFb4gUbtbLSBM9UQEkFCxRcGZtsUO49nnrWC
J6ZB4tR+vaIV/v+
+z7CFABYEbULPx3fPl6vbQ4nvmnS9J8//3yoyef0WsAANmy3m8vqWH9TzmLSBUhKoCY2rJh17o06
pWaJQoWUK+xIqv7demmUqJinh29a4d81e+ry8f+9mN6CQCQJ1X/9C1fvFGe8+PF/NXt8W/8SO7+
0AN6CQCQuf53RC7doxeQJkJUAJNRNS8XfPx7cXMvpRq/C+YIXDvkR/U+Vb1QAQCz8XMPvERueP
wqvbSYvvKXj8jnPv6QXgIAZO7y10QufUEvIC3URAUAAKEefuJhec32a+wJpQAA+bv568+Xf/6Fm/TS
YqM+KgDkjfqoaaMnKgAAGKKCUzWRFAEqAMyGqoO6aBNJRVH1Ue/
+0FftjwCAPKj6qGpY/7POIqZGiAoAAIbc+ak75cHHH9RLAIC8qQB1UeugjvLN//19+cPf/ppeAgBkrv+
oyKU/1guYFiEqAAAIUDVQP9P7jF4CAOTttV97sbzs0av10tGyt/t/7AcAICeXv+Q8MDVCVCTUk8aKm
uVbPTaknWjdOGltZ96MOYZeQ1Y2gmvbG+7rV6TR0yunpfZjb3NEO2zTne9M2m1bkPugvaHbWJC
BSwosjE/sf8KejR8AMBsqPH3N3x3tuqGqN6rqlQoAyMmlPxHpP6YXMClCVGCGeo0VKZRq0tHLWe
pd3DH205RdQj4AA+77+n1y15/dpZcAAHm7+qnj8pa9G/XS0aXqov7+v/8y9VEBIDeqPup/tz5SH3U
ahKhIqCibe33p99VjWyp6bXby3l8WRhxDryHrtTziU6UnF3eC+2qea1hrF8VRuA+A+aZm4n/nJ98pT
z/7tF4DAMibClBVkLoMVE/UT/7nA70EAMicXR91Vy9gEoSowDLoXRQ3Qy2Xy86Tzo5cXJwUdTFUt
qXfrYs+w8DCUMGpClAf+cEjeg0AIG8//ffX2UP5l8m9n3xYvvKX/NsDALm5vGc9mOBvUoSoS8eoLbl
i9kQctd6oc2mvz7s25fD+IutuGnU/VwKfNLcT9vko6Z8z+/PmMP7mqvP5EYU0/WNWj+T1Rv2h/FU
5e+GMDvk6shMnRQ3UUg17jG5PvHbHuTbD59DaumyYrzfqkQa2MbA+/BTHaYN5POpY/P2rh7vd
9nnrupbrsjXQXdYu3WC83n4M3DfArKgh/A888oBeAgDk7YbHr7Ink1pGd3/oAXn8Gz/SSwCAzF26R
6T/Hb2AJAhRl05R1s64PREPpOs8s3TlwE30Aj0U27LbdJ6Vz6xZXz17ldNV/awjB/4B2PywsCxn1nRr7
RCwJIMj5zu1UswQa5bnrCO1UkFW9fYcal2SINUYyl89LZXimniHs3Nx+hCvfEbcU+2L2e6pr43Dfn1w
Z846FVQOrG+uDgSpE7VBHcuqeFvWoakKSlf369Ld2wxcdxW+lsJKN3RqUprnibKwFD7yxY/Yk0kBA
GbjOT8+Jm/54o1y/FJBr1kuP3ryGWm+/37qowJAbp4SufQH1kfqoyZFiLqEimtuT8R9OXQTovauHwi
JEU72Dq1XOU6dmIcI1VI5LW6M2gzMjmSEhV6w15PGutvjsyotu6am9WjpLXRqsh4jjUz7nFW2rTa
Yw76rLadd22HVPv12d+vuV8TsRaoYQ/mrp9X2zVA4xpD+4qbsuefNfnTFa4Z1BPULwcDQN67d6V
wbV7neDX69Frbev2+maYPxeh2aFjf3vOc+P1RXYWtXf41/TppyLsFxAmnafXBXPvgXH9RLAIBZeONf
3yAv+OEVemk5PfTV78qf1h/USwCAzPUfFrn853oBcRGiLiOvJ6IfaPUOndivWnXCo32dFJrDwO38bS
5UZMsNoJq7fi8+s+6n2wPUXFff8ickqmx5QWCs3pgzPGfVlj+Rkh/mWi0Z7IY7gtk792TJfmJsJ0EYq/
Ua636vzepZ2QxPUMe3O61rY6vKWbchRsg+cv3+obPdKdoQeH0UI1Q3Q2s7cNWB6t6okwhkSA3f
v/NTd+olAMAsvPLBa+Xmrz9fLy23z338Idn/n4/pJQBA5i59QeQyf8BKghB1KRXlxCnnmRtode1x6W
U5efqkHXYF11vUMHDn2VzwQ7mmeJ0KuwdeWOgN5ffWWcfkDu+2H8bw7cAQ/VFmdc784HMy
Yb1zLRMP6W/LeT9BlVZoz1klRrtTuzaW8kkJ3d2o9a4p2hC7Z7Zxrq2N2WUO3P3Er8sLpOuJp59gJn
4AmLGXfPe58nMPvEQvQbn7P35Vvvct/m0CgNxcVvVRv68XMA4h6pLy6oraPTl74nSqPCUnKies/w
+uV3ngPEWoFiOYcodmt90x0xG9I6exkOfM6GmphqaXQkPC+LP0tzf8OqBVo6fp8kkSbhdl80L4jP1u
cDtiPjEgE+5M/A8/8bBeAwDIm6p/usx1UEdRAWrzN+/XSwCAzPWfFLn8cb2AcQhRl5U3tHlfDts6aL
N77VXEyQqN9VP3hsyCUdfTDi/9upOjwkuvNubQI2YYuIDnzB/KHyXmkP72hj9RVLUlIzuhTmDqa5O
CTNtg1JUdKNIqa55jln7k532ffZ/c+/C9egkAMAsrX3qpXPvklXoJpn/40rfl079/qJcAAJm7bP02eukze
gFRCFGXVklO2hlkRw52nSHNbh3Rkv0Jf3347OuzV9w864eaDXeSp4E6pCVnqL3i1eKc2KKdM2Mo/
xjjh/S3ZcNLUMtSV1PRTyvVazOhHNugZuhXPU7tScXsQNWYoCtu2QJgSvX76vKxv/2YXgIAzELpoRfa
D4z26f/6D3aYCgDIyeXPivTp2jMOIerS8ntyNptOOObWeCzq4p/uem+Sprnj9gDtSK2mA77BOqRmP
crmqj9sur3h1aUsxB5LvWDnzBzK787+H3iYIV70kP5e45w3jL9cv5BOuYRUr82E8miD3pbKoIM9Trvi

ls8dW7sVSMn/+v/
+l34GAJgF1ftU9ULFeHd/6AH9DACQi0v36CcYhRB1iZkzpqvehd7w88Ds5sYkTXPIq1NqC+shGaxH2
VzVwZg/LI1GT440LNVzVtS1VJXmauqhoTmUvxxaW8AoiWC9cuSQ/l5D1r0CqtYrAxMwOY/JJkhK99
pMJoc2VLb9IfyBurRGfdmzm7MP3bEU7v7Fu+WOl9+hlwAAeXvjX99AHdSYfv7dP6WfAQByUXijfoJ
RCFGXWWDW8FPiTzbuDltXzPVzyAwvRw2ht+tRGr0uXeW6dJPWu0z1nFVke7BA5v5hSrUxzaH8o0
NdMxQeNaQ/Xl3VCaV5bSaVQxvsIfxhxVBVSNvvp1pfFohy1RVXyf/4lf8ht1x3i14DAMjT373yCf0M
UV7z1pvkbe+5VS8BADJ37FUix9+kFzBKoa/G9QIAgKXxwCMPyOv/2+vtmfoBAPm648vXyysfvFYvYd
D1P/E8+a1PvVGedw0TbwFALgovFrni/daTq51ljERPVAAAlsztN91uD+0HAOTvb27/tjx2PX/ECnPlc4
9L9fdeS4AKALmx3m+Pv8f6SIAaByEqAABL6F2n3iUfeMMH9BIAlC+Xjvfl8294VH78nMt6DVz/8t+V
5NZXv0gvAQAyd+ztIoWb9QLGmSpE7TVW9AQpBX9W65F60lhxJ1TZkODL27LhTbbiPCabqGZYeyO
43dBH1jOAAwAwhz788x+W07ed1ksAgLz84PnPyt7rvqWXoLzidS+R1X8bNhkqACATx4oix39eLyCO
OeiJqsJVf5ZqR84zwquZ0VcaKU3oAwDA4rjnl+
+xh/cDAPL10M0/kO6t39dLy+3al14l1d99rV4CAGSucI3IMTWMH0nkGKIWZXOvL2oeq35gxuuuHH
iTiKvZsNXn92QzxwzV1qnJeTqkAgCWjDtj/3VXXafXAADyonqjfu+aZ/TS8lIBqgpSAQA5Of5ukcIL9QLi
mn1P1N6h7OuncuqEZJedlqXedUNc/9Gtl/XnRZq7pKgAgOVzy3W32EGqClQBAPlR9VE/96Zv2h+X1
S/8xivsofwAgJzYdVDz7rl4NOQYog7XRLXrlZZq4nZEtYfVD9UoNb/OeaRVL1Uprp0RP0YFAGA5vfm
WN8tH3/5RvQQAyIvqibqs9VFVePoLv35CLwEAMmfXQX2HXkBSc1ATNUKvISuFktS8lNXRqZVSq2H
aPu+HuNXTfpGB5WVM8uWd46hJwTA7YddqEYTfT/4kcHneY0nPYU7fC+q9b2DCO//8rEiKf0cCAt7
7s+
+V2h01vQQAyIuqjbps9VGfd82VctfvUJMbAPJztcixO/VzTGKmIWpluy/9bt3vCVptOcPst1WY2ZPGu
htwVqXlDsFvVe01qobpeqIkoSO1khtC+I9Vd0Yra9/2bgFghnqNlWAPfSBnqjeq6pUKAMjXfT/zuD1r/
7JQAer1P/E8vQQAyNzxO0UKL9YLmMT89kTtXZQdnSKU61v+RFSVLXHLmHZ2LqbU+64s9S0S1NFG
TQoGTGKR76eM295ryPpg13tgBlR9VFUnFQCQnx8/57J8/g2PLkV91Le951Z5zVtv0ksAgMwdf5PIsV
fpBUxqfkPU7oHXE8sevu/1HjWG93cOpKufTsfppZpmrVUAABaVmqm//attZuwHgJw9dv3T8je3f1sv
HU23vvpF8ku12/QSACBzhVtEjv0LvYBpzHdN1FSFz85vlhPo1M7nWItxHgxO2jWqxuPoOpD20GPv6
/VjZG3Jwf1FBNd2Pdzga0fVgsysDVEya98oca+VK/5xJm1f2OsHyncO1O806o7arx19P/kG2p/K9Ux6
DsOEt32wXungORo8P2HiTbTn8/epHqPqpCY5P0DQbdffJvf88j16CQCQl7+/7Xvyjy//oV46WlQd1O
rvvVaufO5xvQYAkK2rRY6/2/p4hbOIqSxEiFqud4fDT/uRwnDa4qac1WVWRfblcGnyBRVsDU7a1ZTV
wqr1/3hUiFMKG3rcqUlpMKBKMklYe2NETUjVYzgYFmXWhihZtW+khNcqwXEma58TyIW9vrlaGBH
2qXNitLNcl/GVM9SxDbR/6us5/f0ej9OrffAcNVdHhZyTcPbh1XO2Dd97qd3vWGrvKL6DGfsBYAb+6v
WPHcn6qL/yH15JHVQAyNPx09RBTdH8hqilk34P0YN0Bu2Ha8tuuinKQug1zvnhkTuhV7/r1Zsdzzhv
5bp0dbDd9TbQlHNeotNLMEmY9dpzesOh2+3IzkX3tVm1IUpW7Rst2bVKcpzJ2tdrrHuBnP+HjZa1F
615bkRQaLRjb1OKem0koz1u06e5ntPf70n47fHb3pHa+ejIPHqivUH+PsLvvbTud0Ds2frfdepdegkAk
AdVH1UFqUfJyumftB8AgJwce53zQGpSC1HtnmjGkFHzEWco65DimpzxspxVfxuqF6C77UQbdnpv
me1yHmYvuTOyFivhWXQ9uejO2qUCFi+kKcrmBSPEidl7lH39VDo74mY3xc09J6yxHnub+mQmmiT
MmLjHCNyKa2eGQ/XM2hAlo/aNlPBaJTnORO0LtuOst74iW/aGVVC3J2GHE2hHTNWz/rmtbPtBrd
/2JNczhfs9gWrL7yFf2Up/+4q5j/B7L8+J+bAM7v7Fu+WOl9+hlwAAefjmjU/JV376u3ppsb3sp14o/
+a3mNAEAHJTuFHk+L/WC0jLHA/nDwYcXkjrjWE1w5B0mMHN0daVAzdTKp+Ukn5qK56QU/ppJDP
kHgioh2ouTjVJmK6nGTZ8Prc2REmpfSMlvFZJjjNR+0a3wwldR5fWOHUi6XdVVU4HNlaSk2473bYn
up4p3O9zpSwnAwcRIteJ+bAMrrriKmbsB4AZuP9V37HD1EWm6p/+
+n/5GeqgAkBurhQ5/mvWR+qgpm2+a6IWN2UvbMitPdQ3hXqoHtWLri8pZ7JH3OhefG5oM1EPZ
MWu5eiGPlE1KzNsQ5R5b19sebQvRuA3VlFOLEzSmcbxAvPpphfcZAepKlAFAOTn8yuPylNXX9JLi0f
1QFU9UQEAOTn+r0QKN+gFpGmqENUc9hv1cMLJoj8M2gxA7aBUrw9NMc2v04+4dRUtdp1B82tD
H2kGskvEuHZ+7Udf89zw5DXjJwlry4bZq9OtjWnWijRl0oYo2bcvLbGOc4btG21wgreeHHp1B4ZlOv

HcEcD5QZpuv+l2e2g/ACA/KkBVQeoiel3l/6IOKgDk6dhrrQd1ULMy3z1RkZGQ4dEus05mDGp2d9V
b0Q+rjZ7D7raTTBLW3vV6dtrhjxuYG8OTB6XehihZtW+khNdqguOM176Idth1itOdfT7Y9JDh+ImO
M737fWHkNjEflpGaZOoDb/iAXgIA5EEN6f+7Vz6hlxaDmoWfOqgAkCM1C7/qhYrMEKIupaKs+bN2
yao3Ztuc0XsMPcGXKlEb7K0YEnhNOEmYH/4YM+KbcmhDlFTbN1LCa5XkOBO1L6Id9rE7NVUTnsK
Rmqsb4jfdL5lQPrPmhNaJrmcK9/uiyeB+B0wf/vkPy+nbTuslAEAeVH3Ux65/Wi/NN1X/tPp7r5XnXX
OlXgMAyJaqg/oe6+PVziIyQYi6pIqbF/zehs1VHawYk86MU9n2h393alJygxmjRqg/UVew9mbkJGGV
09aSNq5dWbUhSmbtGy3ZtUpyrpO1b2w7ynXZSmV8eFnK5aas6vZ4Tbfaftab/j/Z9Zz6fs+LOdGV28
6Jws6U7ncgwj2/fI89vB8AkI9Lx/vy+Tc8Kj9+zmW9Zn79Uu02ufXVL9JLAIDMHVsTKdysF5AVQtSlVZ
TNvcFJu6rS6res/8djD/8OK6Rpb2dgoi679ubg/ixDk4RVZHuoDc72vF01d71eitm0IUqG7Rsp4bVKcJ
zJ2qfa0Zfu0IbVpo3SBlM7JWcHjzfsGiW6ntPf7/mw7q/B67F/aPQSTiCV+x0YzZ2x/7qrrtNrAABZ+8H
zn5W/ev1jemk+veatN8nb3nOrXgIAZO7Yq0SOv0kvIEuFviqCCAAAMIF7H75XKn9UkaefXYwhpgBw
FNzx5evllQ9eq5fmx7UvvUr+06ffzDB+AMhL4RqRK9R8BQzjzwM9UQEAwMTefMub5aNv/6heAgD
k4W9u/7Z875pn9NL8qP4udVABIFfH3239jwA1L4SoAABgKu/92fdK7Y6aXgIAZE3VR/3cm75pf5wX
v/Abr5BXvO4legkAkLljbxcppFNUD/EQogIAgKmp3qiqVyoAIB+qJ+re676ll2ZLhae/8Osn9BIAIHPHiiL
H36EXkBdCVAAAkAo10dQt192ilwAAWeve+n37MUuqDqoaxg8AyImqg3rsPXoBeSJEBQAAqVAz9b
d/tc2M/QCQI9UbdZb1Ue/6T6+2g1QAQE6O3SlSeKFeQJ4IUQEAQGpuu/42ueeX79FLAICsqbqon3
/DozOpj/q299wqp/7ZDXoJAJC5428SOXabXkDeCFEBAECq3lF8BzP2A0COvv2if7Jn7M/Tra9+kfxSjV
/kASA3hVtEjv0LvYBZIEQFcteWjUJBCsZjpdHTn1tSvYasbLT1gqO94Z6fFZnX09NrrDhtXGnIqCb6x+E8
lv5aY2mo2frfdepdegkAkLW/v+178o8v/6FeytbzrrlSqr/3Wrnyucf1GgBAtq4WOf5u6+MVziJmghAV
yFVPGiur0tRLjrKcWSvq58vHDiJLNeno5YXRa8h6LarV6loXZDV4saVTK0lhIDAGjqq7f/FuuePld+glAE
DW/ur1j8kPnv+sXsrOXb9zu1z/E8/TSwCAzB1XdVBfrBcwK4SoQK66cuDmbuW6dPt96ff3ZHNZM9
SxQeScUj1nxwW/7fPiHlq1pa5zX7r1srOiuSrkqFgGV11xFTP2A0COfvycy5nXR33zr9wir3nrTXoJAJC
5YyvW41V6AbNEiArkqXco+/qpnDohy9v/dHHF6znbk8Y53QW1XJetivO0uHlWqs5Tae6SomI53PSC
m+wgVQWqAIDsPXb903L/q76jl9L1sp96ofzKfziplwAAmSvcKHL8tF7ArBGiLjGvnqP5CKntOLI2peqN
536d163Or/dp135sb+ivNdbZLwuuD/bKS2MbmtlG7zFcYzN4jMM1S+1HWN1Low3jaI3a+zDDt+aq
87WDDQ9pc9i2o9o8tpfjROdl+J4ZuZ8YxxD7fGh+W9RjuK0OZwi9/7p0z506/lKsnrNGj+NAWF6Sk7
ozquwfDt9PNqM9ie65eMduS+H7wj1Pcd9HsNxuv+l2e2g/ACAff/fKJ+SbNz6ll9Kh6p/+
+n/5GeqgAkBurhQ5/mvWR+qgzgtC1CWlwpHQMKhTk1JhQ8ZlcHHYtR8HCkLa61TIMrC+uRoeXk2
1DRU2hfYY7EitNCqEU58zapaW61J3uw52duTiYMi0674ynbqmQ8GiZh/zyGBquM1uz8dQE5+X4X
umuToieEt8DFGcfQcvd0hb7WCw5A2hd6V67jxVafVbXq/SIWaP41E6B9LVT4MqcjrpPZfk2FP6vlDn
KY/3ERwdapKpD7zhA3oJAJC1z688Kk9dfUkvTe/f/Nar7J6oAICcHP9XIoUb9ALmASHqUmqLn8O4d
TmNeo3SlHPhSUpi5XrX3na/FYybwtaPGt482TaCw6mHj7EjO4PplEcFZM7r+3ubsrlVF+erBr/GPI9nZ
FyGWtm2ttd1t2Wptpx9bOvUrr3hB4Vhbe7UZH3kdQm2eXRT0jkv/invSO28cd0SHMPY8xHg7zu8r
dZxrbvBoHEu3Iamcu40u53bEitrHVKUE6f00wiVRPdckmNP7/uimOP7CI6OD//8h+X0bQxHAoA8q
ABVBalpWDn9k/YDAJCTY69zHpgrhKjLyOwlZ/R0K27uOQGJ9dhLZaajqpx1t1M5bS25RqwPHd486T
aKsrlnBj6O4toZL7TrHIT3AyzXt4IBWXFNzugv6uxc9NvY3vV65pXPrI0P38YwexjWLxht3rwgfgZ5PrR3
31CbR5r8vFRbfnDoh3xB0xxDFHPfoW3tXZQd3SEycC4qW/5+zWtniH/u1HFY3yOhIe8k9uUwrEFKk
nsu0bGn+H2R2/sIjpp7fvkee3g/ACB7akj/V376u3ppMqr3qeqFCgDIiV0H9V/rBcwTQtRlZAQ0ImqY
bsGrZTiyhuIkyielpJ8GjFofJo1t2HQ9x9BhzEGnTgwGP0VZ8xMtLyzyA0Mj0J1YVK9WY/8jgrfhNscV/7
yMN90xjFaWk+MudvfAa79X7sF+GEPcRwyfn/zcTeuUjN51gntuimNPcv2HzlNe7yM4ctwZ+6+76jq
9BgCQJTXJ1KT1UVX907t+59XUQQWA3Kg6qHdaH6mDOo8IUZdSUTYvhPckdEOYqMl1FkZg8hyjn
mOk8MDO76nnDnc2AsPq6dg9GafXkeGOgjFCRlOK52UyYccwK2keVxw9ORxbLNWR2T2X2vVfkvc
RZOKW625hxn4AyNFfvf4x+fFzLuul+H6pdpvc+uoX6SUAQOaOrYkUbtYLmDeEqMuquCl7esjtQKlR
W/Pcos+u3ZYNs3edW7PRrMGZhHW+zurzZA+NNoYyV0/nF6FOH/qlfF4mkm1w6dXKHXpMWsd0

AsUTMrb06bje1BPcc+OPPf3vi6P9PoIsvfmWN8tH3/5RvQQAyNIPnv+sHaQm8Zq33iRve8+tegkAkL
ljrxI5/ia9gHlEiLrE1MzaqqeYPcGPHYR0vRqKo4f/GoxhxHPHrB2pgiW3/uMUba64U6Z3duT8+R29n
aqkk6GW5KR37gdnZO/JRbfoZeQQ8BgyOC+
+nI4hTOmkFwKOqumZL+NcNHfF75DZlQPvNJxwzn2EWPdckmPP4PpP/T6Cpfben32v1O6o6SUAQ
Jb+8eU/lL+/7Xt6Kdr1P/E8uet3qF8NALkpvFgP48c8I0RdRu0Ne6itmkU92FPMCHiMXnIlPxkzZmJvy
4Y3Dft884MlY2bySXgTWHWk2dQnKrWh/EXZdLsdqvO87l+XXmPdq22ZZBKkcVI7L578j8Fj1udsrvr
DyPW9bj9yHVtu1oBtyq7eda9xzgsxY/VgjnPPTXjsU1//hO8jZntyvRSYe6o3quqVCgDI3t/c/m157Pqn
9VI4Vf+0+nuvleddc6VeAwDIlqqD+h7r49XOIuYWIeoyqmz7Q287NSm5QYtRH7F6NnzmbhXSDL52
Lpkz9nttNibamUhF3I6BrlSH8o+4LiU/fZQL005glcl5MSQ9BnPYu9ueiRK2YH3O5qqz34IX9Felldqs+v
EUNy94PTLd9njnodqSeM2Jc88lOPY0r3/C9xEgiqqPquqkAgCydeI4Xz7/hkcj66NW/u8idVABIE/H3k
od1AVBiLqk7KG3YUUMVeDS7wcDHlX3cLBmYrWVcx3NpCqy3W/5gZHNOTbvsAPDrOPxhlcr5bpsp
ZzL2dcl5LxWW9Z6d+j1VLI5L6Zkx2C1Z/A+3D80ejUmYNfnNIaSu+y6nznWQ/UUZXNvuFaoPYw+Q
aAb656LfezpXv9E7yNABDVTf/tX28zYDwA5UPVR7/uZx/VS0Cte9xL5hV8/oZcAAJk7Zv2GfPwdegHz
rtBXRewAxKNmNtcT86gwbG/anqHAOEfpntPHcqpFwIpwn+l9Rip/xM0BAHl441/fIKWHXqiXRK596
VXyW3/8z+yPAIAcFK4ROf5+66P/Xoz5Rk9UILaeNNbdmc3LcmaNABVZO1r3XPu8dSwZ9ODG0fGO
4juYsR8AcrL3um/J9655Ri+JVH/3tQSoAJCn4+8mQF0w9EQFxmrLxmANWFXOgK50yMzRu+d6jRU
p7ZyRbiplKXDUFf6fgn4GAMjStU9eKe9s/aS88723MYwfAPJ07O0M419AhKjAWAOBFgEqMsc9h+
WlJiiblx9NaEs42hKOtoSjLeHmqS0nrr1GDr/3pF6aLa5RONoSjraEoy3haAvSQIgKAADmBjGy3haEs42hKOtoSjLeHmqS1IhpqoAAAAAAAABCBEBUAAAAAAAAIhCiAgAAAAA
AAEAEQlTkSE2WU7DrfxRWGtLTa8fqNWRlo60XHO0NvZ3CijRibwjp6Uljxb0GG9aVTbJuGmH3UNr
7mJbRRv1Y4SYFAAAAAGChEaJirvUaK1Io1aSjl4H5pgJdY1Z9W1nOrBX1cwAAAAAAsIgIUTG/eg1Zrx
GfzqeibO717RkF+/1tqei1szFPbenKgXvLluvStdu0J5tkqAAAAAAALDRCVABIS+9Q9vVTOXVCyE4BA
AAAADgaCFGXmao1OlC7MazG6GD9UXuIvfE1A+VKNbNOpXokq1Vp79Mcxt9cdbYTvjOjjeoxqk7qY
JvCa1VOdrymMbVf2xvetob2H3JN4rTRY359ZEONcxFo46j1xj7t9ea5zKoOadx7KKotg9uYsD5pjOuS
9J4FAAAAAACLgxB1WakgL7TWaEdqpVEhpPpcQUoDQ+ybq4OvVyFiSYIva8pqYbBWZBqcNq0GNh
xyDHYINtgm65W1UnjQaYt7vIMqcrqqn3Z25OLAa9u7bmODtTKHQjgtuo2TKsrambLztHMgXeeZxR
iOHmh7W9xml8+s5dDDMoV7aKJrPizf6wIAAAAAAOYRIepS6knjnJuIuXUb+9Kt61BNOrIzmPx5qtL
Sr2+5QaEKG8/7ve16jXN+0FVt6VqVXfE2H0Nl2/qabl28L3G3sx1W8dJvU/gxWMe77oZg/mv77gF0
arI+MhUdf7xhKltu2wfPpR9GSvmMeBlqe8MPgsOuSWQbJ1NcO6PbuC+H7qbbu0ZI2ZEDN101hq
mfOpF9hDr9PTTNNTckuC7J7lkAAAAAALBICFGXkjERz96m16vQD9VEOl56FlRt+RP3+EGhqScXd9w
+e1VpeQGStc8LYa+fntmm0GPoXRS3SeX6lj/xUGXLC+U6OxdDexSOP94RimvidfQ0t22ElGaPTrN3av
2CcU02L/htrJ0fGKo+Ja+NftDbO3Si0mrVCRv3dbrau7jjBZKnvROYlRTuoSmuuWkm1wUAAAAAAM
wdQlRYdA3P0OH9SZmzk5+Ukn5qK56QU/ppespyMrCTEN0D77jsIdheXUtjqHdgSHsazOHy/rB4P5
SryllvyvYRvVNtxnbMHqOpKMoJfUHcwLlrXzzrnJ4+aYeVwfWW6mk/kMxMCvdQKtd8VtcFAAAAAA
DMG0LUZRWYKCdunckYgeWRMt3x+r1i3Z6eRig3URhpDK9PScUt3trctVrXE6cj6ik5UdFhZWC9anb
2EeriSf+6AAAAAACA+UKIupTasmH2OnVrPZr1HI+ocr3rlDEYevjD9lNT3JSzbglONXTcqCs6WRiZQYh
dOS164L4ctvUQeLv3pzs5lrF+QUP07K/5sv1xAQAAAACA5UOIuozMupwqYHLrohpDoCdXkpNuEjs
4XNoIEXNVcoamK6NqvWbF6+nZ2ZHz50fVFTXP2eBs/mZ90FMydk6nxNfQ3XdHDnadr3VrtZbsT/jr
h4e0ZyWFeyiVa57idQEAAAAAAAuNEHXJ+QGTMWP/VMxakU1Z3XCn3DFnS8+ZMcmTNFfFa1J7w
6+V6a1MmdfTsyPNpj76oaH8Rdl0u6xar6utN6yz5eg11r0anuYESU7AqVivP+8dkGx4U8nH5V+vZtP
5Wnf2/aIumOquNyfCylYK91Aq1zz5dQEAAAAAAEcTIeoy8oI9S3NVh0rGhDtTMmcun2r75iRC7nY
mCjuDs7o3V3WI5gWO5gzwaXOHxftCh/JXtqXl5XU1KdnnrCAlP6mTC95EVCojdOutWrxzHLe2bVBg
W9Yzb2i6eZ9Y68/k0w3VNv09lNI1T3hdAAAAAADA0USIupQqst1vGQGZUpVWv+8HRvaEQpMqyu
Ze1w/BbGr7g/scx2qn1yBt/9DrDZhIcVP2+oNtstj1YDOoh2rwhvQr1v62Ruysst0PrUtbbVnr3ZILLnU

8g6+ttiara2v22gwMTTeGs+c+ZD2Feyila57ougAAAAAAgCOp0FczrADITq8hK3oiL1WDdo+eiwAwk
urtPS8/mtCWcLQlHG0JR1vC0ZZwtCUcbQlHW8LRlnC0BWmgJyqQKbOOZ75D4gEAAAAAAJAOQlQ
gE23ZKAzU8ayeFTqhAgAAAAAALB5CVCAPql5pZpNXAQAAAAAAIEvURAUAAHODelXhaEs42hKOto
SjLeFoSzjaEo62hKMt4WhLuHlqC5KhJyoAAAAAAAAARCBEBQAAAAAAAIAIDOcHAAAAAAAAAgAj0R
EV2eg1Z2WjrhVnrSWNFzZavHhsyL60CAAAAAADA/CNERSZ6jRUplGrS0csAAAAAAADAoiJERfp6D
VmvEZ8CAAAAAADgaCBEBQAAAAAAAIAIhKhLqr3h1gddkUZPD7+3l51HaClTVePUeI3zcL7eZW/XH
MbfXHVepzc4uF+PuW1j58HXt2XDfY318F4Wo12TGjwv6jHpuVGSHr8S1obCSkPCD8+s/eo8VkaciG
TbBQAAAAAAWF6EqEuvI7VSQUoDw+
+bqwMhX3tjRI1T9fXpBJbR1H5WpamXpFyXrYr1MbN2OWHk4HlRmquFYNCZ4blRoWtYG6RTk9Lg
BFl2EFuSwZd3aqWhcDTRdgEAAAAAAJYcISosVWn1+9K3Hq2qXqUCwPNulNaTxjkdX5br0tWv7dbL
zjrrtTsXnYiusm19rlsX9zNSbdmv7W+rxHNafjv7e5tSTNCupHqNdS+MLNe7zj77LasFWvOcDkeza4NI
W3bd1Dh020055yW0VjvW3SDXOE/uBe3UZN17bZLtAgAAAAAAgBAVUm1tixtxVraMANRTlM09H
crZ4aVeu3bGe23noKufZadc3/La6ciqXT25uOP20qzK2U13yxXZsoNGFVLuibM6w3PTO5R9/VQ6O+
JmscXNPWd/1mPPbVvvorhNDpynypa42Whn56J1ZJYk2wUAAAAAAAAhKiaha5OGDmHPzqkT44K
9tNrVlQN3A+WTUtJPFSdo9EPnYSmem+KanHGTWGtrquyCW7t0qM5p98Dbnz18X7+uYA7v7xxYR
2ZJsl0AAAAAAAAQoqIsJ82UcJTAxElGbdLcjGjnzNtlyawNRdm8ENYz2A9KQye5Giur7QIAAAAAABx
NhKiIoS0bZs9Kt46mWft0JuahXRm3obgpe3qIvV+v1tc8Nzybvl/DdfBh9KCdYLsAgLzp0Q3Ggz9yAQ
AAALNBiIrx2rte70o7oHNrfxpDyKc2ybYya1dJTroJqDsE3qVm4i8YM+6n1YaI16uZ9NUvzfakXXbw2f
XqnHrtK530Qtu4NVhjbRcAlpH9Xl+QwkwTy540VlZFWu57tPNHr+bqLILU+Qxze40Vpz0r+f/hT/0ba
p4P9zEP5wXDhq7XDO6ZmRvzvuZ9P7mPvG7mOO+3ebwnR+5jSf+gFfO8z/K9eGZinJuh951luGnG
nJfc34sTtofSdoiDEBWJ+AGdMSt9AiU/nTRm/7d+MFmdbhD8tO0KKsqaVzS0Kavem667baeO6OB7
cZw2JDp+/aavPhXsGRpSs9Wsc9pc9dvm/sOhHu7KJNtVjG3wDwuALMzsl/chKri09j/lv0npcCYu3Da
KcLuTP+4f5vte3N4IC3ONPyjOQq8h6zWRatX9x28G3NEnxsO8XrMy01/cAyWOhh+z+NZW52O1qS
YFda9TS6qdmpRmFPjk/0v7+Pc19R5csr6f6l3jHFk/T2Z778R5v83jPXncPtTnc/yDVmTok1eYm+C85/l
eHHFuht739CP98xP/vl3dH/g3Iot/IEadk9zfi8efl3zfiydoj/WNrUrbpf+eHO/7dn5+FsZY1g2DJWS9R/
TV5bd+A+hbPzD5uvW+9U+g87mq9ZZia/WtnxX060c8vNcqIa93P29uf9TD2NbIdtqStKvbr5fd9VXr
K8cxXx/yKNetVygJz83Exx/
+MDcdve3gMSfabqvqrS8PXwQAmEq3XrbeX8z3eP2+GngjyoffFv1vwAzaEEm/z8/8vXjm7XCuj9q/f
c28f5PzY/87OoP9RtP37dy1y/3eivPzV9rCv5dn1R7n5y9jv/pnrCy/l8a/rznvuYNtyPocjW9XvNdMa6J
9ZPIeqPdvbdd+hLRD3T/maud+Um3XK1IS/5w4n8/+vTjeucnjvS/
+fZv1+8v4cxImq7aNPy/h62fWnhHfw+nfR87+zd0737fBdX579Qr9vhz3uiJf9ERFDBXZVn8p0ksO56
821puAo7kr/t9KrNd7n9D2D52/MKlanIP1QqutCWuIJm1XEk4PIOsNTS/7rDdbf9h+0jYkPH57qP3g
ubQ5+wj8QdOuc2oMyXfZPWWMeqiWRNsFgMy05XytY71NXZBN503VUpEt9UY28fv35Iqbe9Lv7x
ltmTO69MupE/PRwFm1o9dYl1qnKmfn9kLNhnteWt7PKPPC+T6X6unAzyL5KMqJU9YH9+dQW08u
7sygPb2GqIFK5fqWv9/Ktv3zYmfnYgY9sRxj39d6h7JvfRj8fi7aJ64puxm9Ecd5v83jPXle3ved79+y1Ls
hP8tr6uf34dEJHdm5mO7dE/ec5PVeHOfc5GX8uXHeXwLf5xmY7Jxk9148/rzk+148tj0jfp6yR42mW
tYuzqii+fpZGOMRoi4pvw7mwJuLMeFQcMiBCgvdr1EPJ5TztxMM6dQPhf5rrYf5A725D/VQ+xmx35
Ht9MRtl/MGFlw3nvMG7H6d89gbakjCc5Pg+G2D59J+jDoG8zj1Y9QvU3G3a7xu+NgBYAoz+uV9M
enSL+W6bIX/A5CTnjTWa9KZVTvsoaMdqbbi/1ueGTUM0Rh6N9uSN/n84j6JXuOc9d1s/bI/oxu3sq2
HjNo17dWwypITNg/
+vJW13H5pT8huV1lOenWcNKPePgZk8AetuMFlmDTbEVuO78XTnJv86fJoB+cHhtOnWwJnknPCe7
FhxPub8/PnvuRaNYmfhRcOISoAAJgNfnmPTdUltX/5uTCLXoa6tpj9i6D+pWdGvR3b52vSqbZmPmL
C/0Op81AjV7KppRZXPr+4J+f2NDo7wwDEHTWkatqr7yOrObMI4efpl3ZMgT9oKfPyXhwwR3/Y6uy
flAvevxGqt6h6/9mYYa9C3osD9HwiwflBrO+pqedWiWHwjzD8LLxwCFEBAADmmDP5gRq2N6ueO

MFRDt36vqzO4hfU9oYzCcRc/dbuUL2Csh6WHcfc/eLePi/O6NHZXTNnsg5/YiAVeKtJgXKftGOWv7Q
jNfxByzKH78Xz9oet8pk147pY/4aeVWXUZtirkPfiAernGrdnrP89dXBKXadTkl3n7nn5IwymQYgKAA
Awp5wAVfXWmJ+hjMXNC3YNtk7tfK7hXHtXhU1NO8B1fuEpSEn9Vqh/CZrtcHptlsOyLfP1i7sOCGf5
y6Iecqzq2bt5jz0MViXezdWMZjYfZVa/tI9h93bqyMHgjTuqd9QS4w9ajkV4L56XP2x5ZtqrkPficIMl+f
qydXLfOk8nJau3vdn+EQZpIUQFAACzwS/vkVSvDSdAndMJ/zL8RSPMYE8j9bAngLR+Mexaz2dbt7sn
h6qoWc7nZKxZ/uLu9nw6O8NfFgeHTbpmdl7y/6V9rOIJceZ7CUZNPfuGnmG4O2f4g5Zvvt+LB+T+h
62S2GWOB8PbWf5cw3txTLq2eOCPkekZ+UcYfhZeOISoAABgNvjlPYKeRddiD3czevzkW+dSTf4wM
Bxc/0KW1S8ac6/XkJUVc0i2WqVmSp7lL6nz94u701utKjMcPSpSOW21wPoeCgyhV7dwzTovM26bL
dtf2uOpyGnVY6+27r+veL3G5m+islngD1qLaFZ/2NIjADo1Oe/9w6mHcM+oHinvxXGoUhklqUldLm
RwkSL/CMPPwounDwAAMCOtqvSt33L69a5e0a33y9aPJ2VvRX669bLVFtWewUe139KvWUZh56U
6JyfEblu53s/3bun26+Xg+ZiLe6RVHbg2rb71S2tfJT+5m+H38TB9HgKPefie1vdRxvdv3Pc1573Yf2R9
7eK0K4/35PH7CLt/3Ifxb1dq9H0x9H2r2jFw3Pp7Pu1rNcl5t78m8/fiEedGvd8M7Ns9hrTf/uKem6H
XZfY+POp+0XJ6L453XvJ7Lx7fnpB/xzO6RnHuxXn6WRjjFdT/rIsKAAAwE+5f6F3WD43zNRwQWBCq
x5xdm9BVbUl/Bl3nnO/pqrT6M5p5eS7pnk7G5ZnV9cH8Gfre9fjfR2Gvqc5r79gUjT83Id9bxnk7iuLc
LwrvxbOmRvOoOqhhgkP7+Vl4cRCiAgAAAAAAAEAEaqICAAAAAAAAQARCVAAAAAAAAACIQIgKA
AAAAAAAABEIUQEAAAAAAAAgAiEqAAAAAAAAAEQgRAUAAAAAAAACACISoAAAAAAAAABCBEBU
AAAAAAAAAIhCiAgAAAAAAAEAEQlQAAAAAAAAAiECICgAAAAAAAAARCFEBAAAAAAAAAIAIhKgAA
AAAAAABEIEQFAAAAAAAAgAiEqAAAAAAAAAAQgRAVAAAAAAAAAACIQogIAAAAAAAABABEJUAAA
AAAAAAIhAiAoAAAAAAAAAAEQhRAQAAAAAAACACISoAAAAAAAAARCBEBQAAAAAAAIAIhKgAAA
AAAAAAEIEQFQAAAAAAAAAiEKICAAAAAAAAQARCVAAAAAAAAAACIQIgKAAAAAAAAAABEIUQEAA
AAAAAAgAiEqAAAAAAAAAEQgRAUAAAAAAACACISoAAAAAAAAABCBEBUAAADAQmtvFKRQ0I+N
tl5raG94n19p9PTK2fDbuiEhLV0wbdlwz3vk+e1JY2Xca6xXNVb8ba00rK8CAGB+EKICAAAAODqa52
TGOemSUMHoqjT1kqMsZ9aK+rmpKJsX6tZnHZ3aesg1asv5Wkc/F6me3bS+CgCA+UGICgAAAOAI6
Ujt/OL38Zx/XTlwM89yXbr9vvT7e7I5KvksbsrZqn4eco16jXN+IFttyXZFPwcAYE4QogIAAAA4WuiNm
r3eoezrp3LqRKxeo5Xtlng5auAamb1Qq9IiQQUAzCFCVAAAAAABHTLzeqH590pVg6NpryIpbm9Orse
rX/7Rrehp1Vr119suC68NKtPqCtUJH1wEdeJ31CKsrGjyeYL3S6HYYzGPXj8F92fsp1ayzrDVXndeO3Ul
FtltujOpfo/aGXxagXN+yXjUo3vHbQto/dH0tcc5VoEar+6BWKwAsLUJUAAAAAEdEWep1HdJl2Bu1U
ytJYTVYDdRep0K2gfXN1VEBZlNWCyUxyoCqjUhpcMIpOxQceJ3F3t/IQK8jtZJRr7Rcl60YnTuHwlEtel
8JVbak7hZHba7KRqMh5/wEVS4M1gNIcvwqwA5pv3M+hoNUR/i5UueiNLhTJewaAQCWAiEqAAA
AgKNjzQ3p4vVGnVS53pW+qgPq9ax0hK1v7o5oh1dLtC/
+y5tyzkv7etJYd0PBqrT0a71td2qyPjIpNl6/F2OSpvaGePmv0a6um3ga+6psW5/r+hNFqRqm9n5iDc
MPTjLVrLnHV5b6hcF2Jjl+67VuGhvWfmsrOxfjnqu27Eadi8A1AgAsC0JUAAAAAEdIUTbdGYwy641al
bNuj8nKaWvJNWL9/mFoL05zBnqzXmhn56Lz+t5F2dGdIQPD3I3enN5rB4QPix+t7aeGgTCzuHnB31
ftfDo9MAOTTGnVs8OTUiU6fuu675lBqKO4dsYLbDsHXf0saOhcmfVeOzviZq/FzT1n+9Zjb+QMWgC
Ao4oQFQAAAMDR4oVsGfVGLZ+Ukn4aMGp9qKqcDiR3JTnpp31ix33dA90L01rllguwH8bwdve1A0
6dSBLymT0vz8ha4EuLsnbGbdi+HIYlthMITDJlPQudTGqK41fHZNc5DR3eHzR0ropr4h2yuodK7n4ja
rECAI48QlQAAAAAR8xAb9RD5+l8K8qJU/rp1MpyMn6am0BHRnTmnE6i8DlCYFIpo85ppLBzFSw5Y
HLD3NgTdQEAjgxCVAAAAABHj9kbtRYvTsvXYK/Onhx6Y8iHebVWhx7biYbtTyercHa88cfflg2z16lby9
Ss3ZpEcVP29D4Gyt7amueYpR8AIg0hKgAAAIAjyOiNmpQxjDw7g706u3Lg7tTtmVk66QWAo+p5ps
MsJeDXAHX05KJbmFROSaIqAdNKcvztXa/nqR24unVRp7iWaoZ+1ePUnkjLDlS7Xi3W0WUEAABHF
SEqAAAAgKPJmIAoTMlPDo3aqW3Z8Kapz1ZzdcObqKm94Q8/L59ZcwJAszZnc9UfQt7e8Gp0FlIZV2
4Gzta5WPd7WfYa61790aSTVU1twuP3A1djxv4k9PbVbRDscRoSdAMAlgYhKgAAAIAjKro3qjlzuwrp
nGAubi3NaZWlXG7Kqg4D/dzWmOFftd+ozdlc1cGh9+IREzJNorLtD1vv1KSk21XyE1S5kPuM9AmOv
3Lan6jKu5bGBFRJjDgX5r1RPat7ugIAlgYhKgAAAlCjK6o3qqp7OVgzs9qavI5mIqfk7J4xPFyx63gO1Di

1a3MOvE4Je+2U7GHrIcdebVnr3eHxeYt9/BXZ7psz/itVaZk1TZu7Xs/fcexzEVYMVW8zrewaALA4Cn
1V3AUAAAAAAAAAEIqeqAAAAAAAAAAQgRAVAAAAAAAAACIQogIAAAAAAABABEJUAAAAAAAA
AIhAiAoAAAAAAAAAEQhRAQAAAAAAACACISoAAAAAAAARCBEBQAAAAAAAIAIhKgAAAAAAAA
AEIEQFQAAAAAAAABGEvn/AZM6nr53azj8AAAAAElFTkSuQmCC]]></redcap:Attachment>
</ItemDef>
<ItemDef OID="q20" Name="q20" DataType="text" Length="1" redcap:Variable="q20" redcap:FieldType="radio" redcap:RequiredField="y" redcap:BranchingLogic="[q18] = '1'">
<Question><TranslatedText>Do you prefer Life A or Life B, or do you think Life A and Life B are the same as one another?
</TranslatedText><redcap:FormattedTranslatedText>Do you prefer Life A or Life B, or do you think Life A and Life B are the same as one another?
 </redcap:FormattedTranslatedText></Question>
<CodeListRef CodeListOID="q20.choices"/>
</ItemDef>
<ItemDef OID="summary21" Name="summary21" DataType="text" Length="999" redcap:Variable="summary21" redcap:FieldType="descriptive" redcap:SectionHeader=" " redcap:BranchingLogic="[q20] = '2'">
<Question><TranslatedText>
Alzheimer's disease - Description #[no]

If you have Alzheimer's disease...

1) You might forget recent events of importance to you. For example, if you are interested in hockey, then you might forget whether your favorite team won their last game, although you may remember watching the game itself.

2) You might forget the names of people you met recently, or the names of local streets.

3) You may no longer be able to handle your personal finances or plan leisure activities such as dinner parties or vacations. Simple decisions such as what to order in restaurants might become difficult to make. Your ability to concentrate may decrease and you might have difficulty driving.

4) You may start having trouble finding words to express your thoughts. You might need to be reminded to shower or brush your teeth, but you could do these activities on your own. You may need to post notes around the home to remind you of simple things like turning off the stove.

5) You may remain aware of the day and your surroundings. You might be able to travel to familiar locations like the supermarket, although probably with the help of a loved one or friend. If you are in a social gathering, then you might have difficulty following the conversation.

6) You may continue to enjoy hobbies such as puzzles or reading.

Now consider two possible lives. In Life A, you will live in perfect health for 11.5 years and then you will die of natural causes. In Life B, you will live in perfect health for the next 10 years, then you will live with Alzheimer's disease as described in the summary for another 10 years, and then you will die of natural causes.

</TranslatedText><redcap:FormattedTranslatedText>
Alzheimer's disease - Description #[no]

<p></p>

If you have Alzheimer's disease...
<p></p>
1) You might forget recent events of importance to you. For example, if you are interested in hockey, then you might forget whether your favorite team won their last game, although you may remember watching the game itself.
<p></p>
2) You might forget the names of people you met recently, or the names of local streets.
<p></p>
3) You may no longer be able to handle your personal finances or plan leisure activities such as dinner parties or vacations. Simple decisions such as what to order in restaurants might become difficult to make. Your ability to concentrate may decrease and you might have difficulty driving.
<p></p>
4) You may start having trouble finding words to express your thoughts. You might need to be reminded to shower or brush your teeth, but you could do these activities on your own. You may need to post notes around the home to remind you of simple things like turning off the stove.
<p></p>
5) You may remain aware of the day and your surroundings. You might be able to travel to familiar locations like the supermarket, although probably with the help of a loved one or friend. If you are in a social gathering, then you might have difficulty following the conversation.
<p></p>
6) You may continue to enjoy hobbies such as puzzles or reading.
<p></p>
<p></p>
<p></p>
<p></p>

Now consider two possible lives. In Life A, you will live in perfect health for 11.5 years and then you will die of natural causes. In Life B, you will live in perfect health for the next 10 years, then you will live with Alzheimer's disease as described in the summary for another 10 years, and then you will die of natural causes.
<p></p>
</redcap:FormattedTranslatedText></Question>
</ItemDef>
<ItemDef OID="a21" Name="a21" DataType="text" Length="999" redcap:Variable="a21" redcap:FieldType="descriptive" redcap:BranchingLogic="[q20] = '2'" redcap:InlineImage="1">
<Question><TranslatedText></TranslatedText></Question>
<redcap:Attachment DocName="115.png" MimeType="image/png"><!
[CDATA[iVBORw0KGgoAAAANSUhEUgAABVEAAADYCAIAAABtBTQzAAAAAXNSR0IArs4c6QAAAAR
nQU1BAACxjwv8YQUAAAAJcEhZcwAAEnQAABJ0Ad5mH3gAACvmSURBVHhe7d1vqB7Vvehx02h
PvCoJrTek7UVTD0KIQWLDhXTfYlMoRkotgkqDtOLhlM0WlIZAGykG+iJB6Slktwh6oFhf9N74QpFye
qPEQl60EA8F9wvhWEjDhhtpwLzYlSI7mBe988ystWbNb/2Z2euZmWfm2d8PP87JM7PW+q215sm
4frZ794Z/AAAAAACAeUTNDwDoyfnV8zf89AaCIIgRxc6nty3t2/vXS39RLzIAGBtqfgBAH1bXVne8sE
McpgmCIIYcn/3JZ767/85/vfufnzv8wKfXrqnXGCQCMCjU/AKBza+tre17cwBEEEQA49vfmNXnmNXVvAX8
eJTS+qNBgCjQs0PAOjcg795UJykgBh73HNluCv4izv36FFVSA4DxoOYHAHHTr6Hhi4LHz
6W1P7rlL1PxL+/ZeWllRrzYAGAlqfgBAh868f0acpAcPacpAmCIAYeW09sefTAHaLgL+LYwsG/ffSResEBwBhQ
8wMAunLh8oVtJ7eJwRBEMTA4/7DO0Wpb8fPPve4escBwBwBwBhQ8wMAOrG6trrr17vESQqIGcfcOrrr57vESZogCGLgcfz
Tt4ki3403fv5v/v5v6k0HHAINHzQ8AaN/69fX9L+2X9L+8XJ2mCIIiBx/ZjN7k/xu+N9945p953ADBs1PwAgPYY
eIDgKGi5gcAtOnND94Ux2iCIIjhx50/uEWU+nvjfhuEw/Vu8+ABgqan4AQGtWrqzwi/oJghhd3PqjG79/z
5dFPd8w/vjG6+oNCACDRM0PAGjH2vra7uuXd4iRNEAQx8Nh6YstDX/2SqOvZdWVtR7EACG
h5ofANCC9evrh49JE7SBEEQw4+DD90uyviNxvAxDDQgqXfLYljNEEQxPDjfy
z+N1HAp8WLTy2ptyEADAw1PwBgWqcvnBbHaIIgiOHHzce3Pn7Pn7vblG9J8dvf/kL9U4EgCh5p+Ji8sL
NxQWli+qa1rs3syYSS2erf8YktZrSv1kmd7ZxWKauTaffLYB0aXrxJ1+2wJPoTq3XmaCTpxfPS+O0QRB
EKOIb33ti6JunzL+/O676s0JHAM0fIL6SlAzbDrx/yQ+2oSBU1W/xjSFqvKfWTZUpmkoXWil41bnTps6r5
3blR84/U6trqjhd2iGM0QRDDE8OMrj3xOVTx7GFg1c/vKzejwAwDNT8M+Mt7svvib8glqizhPBWd
8Nmm1A31W6z/Z5Nze+bGzX/GK2trF+15cY84RhMEQQw//vm95v8eD7t+eCv+6uf/F/mZ+ZwXFDnzD+QV4+n+es9cd+
MmarfFDnmysCrYlGxNSzmGzbbfDramGbDUvNjGg/
+5kFxjCYIghh4h/+tPtj68deSCwABQ889UtcZ3/x1AydRIOdHAUyzV1Hv6dtnDvaKHnVwRw8U/h
sR6+es93cS3sIlqay85NzuRPVZ86laveKfY9KxB9B+9ylHrF1tpYXX1JAis0N4Qq5ebrW4yYiqVJubeZBK
huTWeCYbi6NtHxTGaIAhiFPHNb+wSVXr8Z+/
+w/1rgSAWaPmn62yTlpc1n90ahxPjZSxmtnFklIpsjz0fbdINlOoK/lnMVz8Y0i0l85vD6Fb6CmZHjZr0
T5ybv7NnIgN1KhX3fQ8gywsL7sXxfbYKnP0thDbabF31tLK0gKjmBaVpxCaW6OZYDDOvH9GnKEJgi
BGEfcc2S7q8q8y5iad/ev176i3pjAsBMUfPPmqymnPLGVEL6julhmuomVt9KkeWjGuguVr2leugR8o9i
uPjHkHgvPYNyDN1AzdHJ4lm2S/ZyF1peKVNL4e3ZwPTcQRSna5PRTAt9xQyvL3iG9YgsLTiQnIxu4HQ
IjuBcyDSYCYbiwuUL205uv8dogCI4cfOp7c9uecUZ93FM8dfoAf7AcwBNT8s2eVOpUSqCDLqwlZ
MHna+GqqKtWi6FN8WFycDGRf0f3FcPGPITW99Gc9iP6sViU+TnguOWQWs9vlXGUTD0+vsluevH
0zCByvu4E6kdz+xSXfBMMrytTvyEi84TnUoVM7UzFuZBJezTo3+ra6q6f7xLHaIgiOHHzce3Pn7Pn7vblG9J8dvf/kL9U4
NU5p3Gvx/9oXp1AsDsUPMPga+iVPStFkuZFkuqVN8j/vLB8Nu9VXjxHdxDxQF1vfQNVWjWV
QC9KL9InkldmMcOUfRpMX/eqPIZKvwbT86QuuBOoo7p4BhdGp4BhIdGyzNUm0z807QG68o1

PXr6/tf3i+O0QRBEKOIHn6M341zv35FvUABYEao+YcgUr/5bwWKsg0WS6pb1qJonf2huGSumN5iu
PjHkNpe+kp+oZxdQS/SJ5JXZknbK92r0qLSr8H0PKkL7gTqRwsOVmqwroxnINGxwdLKLoK670zFN7f
6mWAAjrx+RJyhCYIgRhF3P3GbqMb7iaV9ey+trKh3KADMAjX/EETqN/8tXQqpq6nFUtFP/8f7ee/8k
r5SDiiGi38Mqe+lL2VX1JrKe/6dqCWzpO2VN3mlX4PpBZu4E6gfrUG+BuvKeAYSHZtPJlM0kqmdqfj
mVj8TzNrzf3henKEJgiBGEduP3dTbj/G7cfzQ1//20UfqTQoAvaPmH4JIUWWKHvuerIQ8/U0VFiuW1
Djuj/EXV6zxRMb4x5AmvYp5m19nb91qmESS3dIKS992VrttYBD7UebcvvWj+VpMEmxsXZn6Dakdx
x1C7pczRGj6ExteAvrx5gdvijM0QRDEKGLriS2PHrhD1OE9x8+
+97h6mQJA76j5h8BT7JRM+aTvmjrINDdXdGlk+tQUS2VHX9dw7RX/GNKol5Vf3DHt9WXTNJZWZt
GdNlZYenZFZq+fnid1wTOB+tGcFuEL5bAenlnJjnWTkXMr2ztX1Gfv3BrMBDOzcmWFX9RPEMRI4/7
DO0UFPpP47S9/oV6pANAvav4h8BQ7NlNSVdiNrSJLihdLZUcznMnmTVAMF/8Y0qxXuVpnO0yPig0l
TSws/Y9gwjdORTmoJ3XBO4H6xfpbWMNXZx1YXaMNqZlMeHtUE3dEz9wazQSzsLa+tnt5tzhDEwR
BjCJm9WP83njvnXPqxQoAPaLmHwJPsSNViy5PBWQ3yG43LZbcdt6e4mL8Y0jDXvHdML1ykR1TZJ
a0wtLupf+ccTvEphdcWHAC9YuttnCaWHP1ds803pD4ZOxMk276czGEb0Rnbo1ngl6tX18/9OohcYY
mCIIYRdz6oxu/f8+XReE9w3jmwH1XP7ysXq8A0BdqfqCepxwFNoel3y2JMzRBEMQoYuuJLQ999Uui
6p55nHrskU+vXVNvWADoBTU/UI+aH5vT6QunxRmaIAhiLHHwodtFvT2QeOX4j9VLFgB6Qc0P1KP
mxyZ0fvW8OEATBEGMJe78wS2i0h5U/PGN19WrFgC6R80P1KPmx2azura644Ud4gxNEAQxihjaj/G
7sbRv7//7r/9SL1wA6Bg1PwCgYm19bc+Le8QZmiAIYizxra99UdTYA4zjh77+yccfq9cuAHSJmh8AUP
Hgbx4UB2iCIIixxFce+ZyorgcbLz61pF67ANAlan4AQOno20fFAZogCGIs8YWnbhZ19cDj/778knr5Ak
BnqPkBAMqZ98+IAzRBEMRY4ubjWx+/d7coqocff373XfUKBoBuUPPPlv7dcLlOfkHcxeWFxbPqz/Px
u+iqK4rIGsbXWtugVHlQWrNpbGL86sORuXD5wraT28QZmiAIYiwxih/jd+PYwsG/ffSRehEDQAeo+
WdIlZxa+4WRSjBHNb+7oiCzu6G11jaweEt+av461Pxjsrq2uuvnu8QBmiAIYixx72M7RC09ojj12COfX
rumXscA0DZq/hnquCIyNe3c1Py+FfmZlhnvWmsbVOjW1K4bQ80/GuvX1/e/vF8coAmCIMYSO5/e9
uSeu0QhPa547dRJ9UYGgLZR889O8wo2jW/8zVDz2+X8hLPW2gYOvW0dPam5Rc0/GkdePyIO0AR
BEGOJz/7kM9/df6cooccY771zTr2UAaBV1PyzoYshi11PVqtSUTFZhVQ5jKhGQ+PbRZjVxq3JKjOoLdn
sYe2eTo0cHra8YzqZCYrZas7omerO5aqzr23gofv4ErrszbB4Lod3oyDnWmlijVfuTTHDaL+AprkqDaPP
N7sZ2AoMzPN/eF4coAmCIEYU3/zGLlE8jzSW9u3966W/qFczALRnYzW/OdGHix9vk7ImyUx7/i/Liv
AsBq+yIwW9Gs+tjLVrngbOnobG9w8+YQ1hF25G9LG1Mqy5X1wTH0MrEnSv7J7uUZ15bQOXb+K+
3Jp3XHnRN2ilh2fBmbKF535+098vOuGUXEr9jCfsVhiYNz94U5yeCYIgRhT3HNkuKudRx3OHH+AH+
wG0roeaXxQ30x7/7eGidczgeXfT1E16n0wrs3FWaRXdAN/4bl9nfKeb7hN5cu6w5RWZJzKs3caX1bc
iYdJETKE679oGrnItFeFuep5WC5HLWYmcixzCdDBjWrOytyM0UNnRkZKrvKKTm176SnkhkhuztXJlh
V/UTxDEeOPzP/ynsf8Yvxv/fvSH6h0NAC1pveZ3ySJkOlYhkRl1LeHbTd9myXam3KpZfWx865popj9a
g3suCa0NawZSqinFmDV8e1lR26DgZDWTDHZ0liZSNdwNW8NZmGbBgRoI5yo3XrZx+mTMtSnmg
u6sra/tXt4tDtAEQRBjia0ntjx64A5RMM9HnPv1K+pNDQBt6L7mT+kTpkdbWFD/f8TFhGdn/FWoW
XRx1VOAefl23pNANDOjewQT1teEjYe1G8qn61tRmGetVbUNgmp7Bh6Znre9SMldW7W1M0a1g9
mj0kbW1ziXfBa6SSWZ9yIGYf36+qFXD4kDNEEQxIji/sM7Rak8N7G0b+
+llRX1vgaAqXX73+2vFhA5T9Ew0bAo0H0Wz+o/jbia8Oymv0YSDZsWUp7xfX0Do/tYA1W1OWzZ0l
mgb0VhnklV1TYIqp+IbpGPrROZ1k12w+QQ9P3g7AMdY3uWkEtugXc6wTli5pZ+tyROzwRBECOKu
5+4TdTJcxbPHX5Ava8BYGozqvm9NUZ9YaC7TQbRfx5vOeHZTX+NJJbatJDyjO/rK5ollWntDWu65UR
r34rCalMmLTWnJxLrqUbPmujWdc/ZpltkikbO2hsvT2vSsHku2cY7ndo5YjZOXzgtTs8EQRAjiu3Hbpq
/H+MX8cc3XlevbACYWve/w8/Tp0lJ4aP7Fd30p9HWE7GdsRcl2zUtpDzjNyjefL1qtTWsbrWwvOxb5
MbmVrtPzTbSl1T3jM5DNVpYXCwGsBrXLsSdmr5iukRmn93aQLK0XHJUXxZzLb7D6B3/a/wEQYw6
Hvrql0SFPGfxyvEfq/c1ALRhFjW/vuIpIKK1gWzUqNOAuTuTMeWWXGW5TE8B5uUbfyPFm77gVIC
udoatdDEfImPG1e5ToIG83GDmfqZdrtK4bkyZomxvxpHTLJiO5WV/w1JaLudZOEuyxgmlxoysX18/+
KuD4gxNEAQxlrjzB7eIInme4qff+Tb/c30A2jWLmt/UGB7hgc1AZf3gXhmVwG76t8daY10NZ1RHyr

M0Kd7KKxWRR9PKsOa2HsRMvxzVs6Iwz6SqAg3cy9W0Wk36jN1Ptq7ZDX/KCd0kMPtgz/B0k3K5z
zewpIngI8DMXPn7FX5pP0EQ442DD90uSuX5iGcO3Hf1w8vqTQ0ALRlPzR8uKMZaUkR2s7pYcT9U7
HnYW503b1a8ZaozqEs1/bDlDau9mX7Z0FlRmGdSVYEG/st2Yt8j8zKd/HOIb7KdcZJPf1a5/dMsVCfb
YLobz1X/fLMbsTli5vgf5ycIYryx9cSWufxv+L/3zjn1jgaA9syy5t9QIWAXEy5qCgyR/tby/cQwnXn/jDh
GEwRBjCVu/dGN37/ny6JmHnW88fN/U29nAGjVLH+ePzKKw/QJoKjC4JgvLd9ODNezv39WHKMJgi
DGEnf9y62ibB5v/Ox7j6v3MgC0LbHm98pLeKeg91T45TDqmvlvFof+LYBnkIK5QVmF4TBf6FzoWw0
Mw8OvPSyO0QRBEGOJ+w/vFMXzGOPYwsG/ffSReikDQNtmUvOHBgrWRpHKPnILmBGr5qfgx+Ct
X1/f//J+cYwmCIIYRWw9seXRA3eIEnpcsbRv76WVFfVGBoAOzKjmn6gOFivZo3V99CYAoNbq2uqOF
3aIkzRBEMQoYvuxm57cc5copEcU5379inoXA0A3NlbzAwDm0vnV8/waf4IgRhp3P3GbKKTHEi8+ta
TewgDQGWp+AMDES396SRyjCYIgxhJj/MH+5w4/8Om1a+oVDACdoeYHAChH3z4qjtEEQRCjiM/
+5DPf3X+nKKqHHEv79v710l/UyxcAukTNP2sXlxes33Sgf/kbv5wgRPx+iPjHDRF7P81QG1X5Pf+dP/v
qV65Lfe4hWnPo1UPiJE0QBDGK2Pn0thH9YP9//u4/1GsXADpGzT9LqiSyCiJq/jqijIx/3JBZ1fwmUaH
bR6+Sdb0mpbc9RJvW1td2L+8WJ2mCIIhRxD1HtovSepjx2qmT6p0LAN2j5p8dX0FEzb9BYhOnKTJn
tfc95u27BqfmH6sPrn7Ar/EnCGKk8c1v7BIF9tDi1GOP8GP8APpEzT871PwtGH/N32dd3HcN3nc+t
Oiti2+JYzRBEMQoYuA/2H9s4eDVDy+rVy0A9IKafzZ0fWnJyyK77rTauGWoqaYmxG17ELtduPAKlLve
y5XMDXrU1H36dtnDvaKHnVwRw8U/xpnGmay9mLt3KLuLPcGoSqdKL53SEpi4PTkx74pqLjtbKJdY
9oSzdKtNOYy6Gc7oGQijcvrCaXGSJgiCGEV84ambRaU9nPjzu+
+qlywA9IWafzbqCjCXrySzWfcbDVJlFXXqSsa96B/Zk9q6VFf36fvmtkliBlFX8s9iuPjHCP9SJlRaZyhzw
WYt1Cu+Y567gYnXTncimiyUS1+3xnGW7ulbNI9m3MjjwEAdef2IOEkTBEGMIr7yyOdEsT2E+O0vf6
FerwDQI2r+2fEVRFYNpa6aVrqQChZkptJyBymvhIovPapV+YXHdabiNLGG8S2zQjXQXdzZ6xHyj2K4+
Mcg007myKiJ1I3sWaqjwY65A3u5u1JeEUvYYC7POpxmbvZcbUZfPozK+vX1g786KE7SBEEQo4hvfe
2LouSebbz41JJ6twJAv6j5ZydWgFnXRDNZZ2XkpfpBPJxxnVrQUxw643raNE5d9Ck+LC5OBrKv6P5iu
PjHEF8zc03NvdpGf/IszLokNNkx94JXyjN1G/i6NHlkJnt4rTnZsXaKGIErf7/Cr/EnCGKMcfPxrY/fu1sU3
rOK44e+/snHH6sXKwD0i5p/dtIKMFN+eaiBUurDsolKrQcxHTxTywR6hefvo3rlDfI/LyyfzXuVV0x3M
Vz8Y4h3LeJidSh90yeUy5vFjKuvNpuyyV+2inWsTtc08HXxzNJp5skueTM2WxsGb+XKyraT28RhmiAI
YvgxkB/sX9q399LKinqlAkDvqPlnJ60Aq9ZWVWqgJlWch26Td9NjlO09o2YCs9tgatUta1G0zv5QXDJX
TG8xXPxjiHct4mJ1KH3TJ5TLm8WZYrMpe8ZyO5orgtvAytVkZP9KMjUZffkwTmfePyNO0gRBEKOI//
nw50UF3n/88Y3X1csUAGaBmn920gqwYPlValQf+qiOWT/dPj61jG6orqamLvrp/3g/751f0lfKAcVw
8Y8h3rWIi9WhvD1q+PvocfXVZlP2jCU76iaZopU7si9Xg5FDq6/N6MuH0Xr298+KkzRBEMTwY+uJL
Q999UuiCO8zXjn+Y/UaBYAZoeafnbQCrEEZ1aSK81M9FxYXi+Z2azOEXfjJcT2pTWEYS63GcX+Mv7hi
jScyxj+G+JqZaypXtU3DgSvkiDlnoGYj1z9Tt4W7875cTR6Zp02mPmOztWE8Hn7tYXGYJgiCGH7c+q
Mbv3/Pl0Up3k/89Dvf/vTaNfUOBYAZoeafnYYFmGxmPusLTSq0psWXGSsnGpubemAzqskUnlxN6rK
jr2t4JfGPQc48rfwqWWhk3cNML5KqwY7JPAF6pPBOyPmUEy5H9uUq26mL1r7rZp7smfqMvnwYs/
Xr6/tf3i8O0wRBEMOPO39wi6jGe4hnDtx39cPL6gUKALNDzT9DVnGVycsiT3Hl1k1WbWUpy6pGg
wTYc3LbVmes2ZWgf265eOqyoxnOZPMmKIaLf4wIT1Rlc4by96hJtIEdi47U4Jn6U02UI1fbFNfDO2E
6erJP1GZstjaMyura6o4XdojDNEEQxPDj4EO3i5q863jvnXPq1QkAM0XNP1N20ZTXU57iyl83VQu1a
i3WeBAfMyVR3xnVzJ7x7AbZ7aap3XbenuJi/GOcaZzJ2otti2fPhbZIqHaSM2s25WbP1Dy8zOSq/mw
NbTcxg9kzzNo6I3uyK/GMzdaGsTm/ep5f408QxOhi64ktjx64Q5Tl3cVrp06qlyYAzBo1P6p0mdawng
WwCb30p5fEYZogCGL4sf3YTU/uuUsU513Ez773uHpdAsAAUPPDZv6DWUp+ADFH3z4qDtMEQRD
Dj7ufuE3U563HsYWDf/voI/WuBIABoOZHwf4vafPfxQZQ79Crh8RhmiAIYvhx/
+GdokpvMZb27f3zu+
+qtyQADAM1PwpWzU/BD6CBtfW13cu7xWGaIAhi4NHpD/af+/Ur6hUJAINBzQ8ASPTB1Q/4Nf4E
QYwuPv/Df+riB/tffGpJvRwBYEio+QEA6d66+JY4TBMEQQw/7jmyXVTsU8Zzhx/45OOP1ZsRAIaEmh
8AMJXTF06LwzRBEMTw45vf2CXq9uRY2rf3r5f+ot6JADAw1PwAgGkdef2IOEwTBEEMPD77k898d/

+donpPiz+
+8bp6GwLA8FDzAwCmtX59/eCvDorzNEEQxMBj59Pbpv/B/tdOnVSvQgAYJGp+AEALrvz9Cr/GnyCI
0cW9j+0QNfyG4tRjj3x67Zp6DwLAIFHzAwDasXJlZdvJbeI8TRAEMfD41te+KCr5hvHMgfuufnhZvQE
BYKio+QEArTnz/hlxmCYIghh43Hx86+P37hb1fJP487vvqncfAAwYNf+MXVxeuMGyeFZdV8TtGxaW
L6o7mbOLzkXT3hooOkah0sS932AEAFCe/f2z4jxNEAQx8PjCUzeLer42fvvLX6i3HgAMGzX/DMlSWj
Hluq7pq8qCu0nN7x8j9q8EctaQ9SMAQNXDrz0sztMEQRADj6888jlR1Ufi9L/
+i3rfAcDgUfPPjKm1dX1tiuvigr6vbzvtm9T8sokziOzg9KgdAQCk9evr+1/eL87TBEEQA4+GP9h//NDX
P/n4Y/W+A4DBo+afFafYVpfC//l5bXmeEW3Mx1CFrht4higu1Y4AAD6ra6s7XtghztMEQRBDjlt/dOP
37/myqPBFLO3be2llRb3pAGAMqPlnxVOwh+nGheY1v1WyG5WE1YGr5L82MKj+ATRxfvU8v8afIIh
xxZ0/uEUU+/SLO/5//rd5xADAS1Pyz0qDm95TbuQ3U/MFB1P36mr9uBAAIe+lPL4nzNEEQxMDj4EO
3izrfxCvHf6zebgAwHtT8s1Jb81vleNHGqecb1fyarO6LTrWzsPhHAICoo28fFedpgiCIIcfWE1se+uqXR
LWfxXOHH/j02jX1agOA8aDmnxUl7VbjuaO1PxOm0x2zfpUzRv+dwS22AgAUOfQq4fEk
ZogCGLI4f5g/zMH7rv64WX1UgOAUaHmnxlTOuvauXpBVu/lXVNrOyOYPvqKueD+mwN1JTxG0xE8
FwDAtra+tnt5tzhSEwRBDDnufuI2u/Z+Z/751z6o0GAGi5p8xUXFXy2a73J6U2PpzWW5X
+meXzUeriVO12/cK1Vm4tXt8BGp+AA28dfEtcZ4mCIIYcmw9seXRA3dkNb96iwHAOFHzAwB6cvrC
aXGkJgiCGHJsP3bTk3vuUq8wBABgnan4AQH/EeZogCGLgcfcTt6n3FwCMEzU/AKAnN9zQ0z90SJSM
RMlIlIlxEyUiUjETJ5i/RZsBWAgB6wokkGYmSkSgZiZKRKBmJkpEIEWwlAKAnnEiSkSgZiZKRKBmJkpEo
GYkQwVYCAHrCiSQZiZKRKBmJkpEoGYmSkQgRbCUAoCecSJKRKBmJkpEoGYmSkSgZiRDBVgIAesKJJ
BmJkpEoGYmSkSgZiZKRCBFsJQCgJ5xIkpEoGYmSkSgZiZKRKBmJEMFWAgB6wokkGYmSkSgZiZKRKB
mJkpEIEWwlAKAnnEiSkSgZiZKRKBmJkpEoGYkQwVYCAHrCiSQZiZKRKBmJkpEoGYmSkQgRbCUAoC
ecSJKRKBmJkpEoGYmSkSgZiRDBVgIAesKJJBmJkpEoGYmSkSgZiZKRCBFsJQCgJ5xIkpEoGYmSkSgZiZ
KRKBmJEMFWAgB6wokkGYmSkSgZiZKRKBmJkpEIEWwlAKAnnEiSkSgZiZKRKBmJkpEoGYkQwVYC
AHrCiSQZiZKRKBmJkpEoGYmSkQgRbCUAoCecSJKRKBmJkpEoGYmSkSgZiRDBVgIAesKJJBmJkpEoG
YmSkSgZiZKRCBFsJQCgJ5xIkpEoGYmSkSgZiZKRKBmJEMFWAgB6wokkGYmSkSgZiZKRKBmJkpEIEW
wlAKAnnEiSkSgZiZKRKBmJkpEoGYkQwVYCAHrCiSQZiZKRKBmJkpEoGYmSkQgRbCUAoCecSJKRKB
mJkpEoGYmSkSgZiRDBVgIAesKJJBmJkpEoGYmSkSgZiZKRCBFsJQCgJ5xIkpEoGYmSkSgZiZKRKBmJE
MFWAgB6wokkGYmSkSgZiZKRKBmJkpEIEWwlAKAnnEiSkSgZiZKRKBmJkpEoGYkQwVYCAAAAADC
fqPkBAAAAAJhP1PwAAAAAAMwnan4AAAAAAOYTNT8AAAAAAPOJmh8AAAAAgPlEzQ8AAAAAw
Hyi5gcAAAAAYD5R8wMAAAAAMJ+o+QEAAAAAmE/U/AAAAAAAzCdqfgAANurs4g2lxbPqKgAAw
NBQ8wMA5khRjHdbhV9cXigzdJmw73+zMFlYZmH5orrQpspicvy7ko2q7GE3j6lzgb8w6rtXaPebEfk
rGrm1Uf6hKk+s3WV1K7oznb4oOhRe1FifU2BFleW09Zga5BrpO2mToOYHAHSrw9N8hZWnz0Nbn
raLs052ljLryM9VHR+oJgtZWFzM/k8neSZL6P1IaJ9H2/9WVL7ZpY6+fflaevpG2PvWXpLg39Dq36Hq
QqcSzBi9tVGhoSbXzYViS6dN5R/FflxTp6jdmcn9Vl8UvkVVlpSbbl2RReW3Wvyr5C7HSm7raEV5fn
Mp/zTt6prlaiFVsXeK2J/KLop7aICaHwDQoep5qnoaaZVOlP//Pg8E1RV2pfMskwRq+7pJM3n2nW+T
rZ8HU5Gn7OjLJ8fuLlfb53hFPw934vKr0dbSwhljtzaq6VC6nfq4YXn/QjVLtn3mwvSPq245k+vq9lR5
CrFFtTG+ElmU51K64HKE6ZOGVySvdJhL31Afp31qk+HM8Pk3ufxYTZXfnGpNmxE1PwCgO/IM4Jwa
Wtd9BqGf40fHyzLDT/6QfmiLme44uGG9fw8y3X4VqhvY2fryge0H1faDc2buLqXljYzsVYvbWDdUfj9
5I3XvbrNY/InM1ckfpk6jJ+vJ1c3rwk3UyjqUyHKqWvx+125d7WSac4Zy1tFiMrOb+Qf5dWgz0aZBzQ
8A6Iz7T+YWTzt+PR8G8vW0dWYMqhx/2mft2eSP3STKd6rU8Z51t46gjh9SptjCLEXxh26+5c7fUOvb
0QpnvEnG6r61nDIyXIuZ6oZyNjZJXZbWFuQbyLo2+WNrX3ZPLvdL0QY3UZ5n8oMKxvRZfVtnyW+3
tTZvrvyrNklR/CE8lY1xcgW2s52I2dvkT9TawjYJan4AQGfcE4D3iNKmzhPY8oNHW6c3V74WpcMlTV
ahh5+k7G5BWrGwLvPk37yWj/Jx/Xzx8m9crqtU7jrcv8VTcRK447e8l5HhWswUHyp/cC3sYjxLfredZ+
VJNFmDvjK53dqXIpDL0lIqN5F4LnmD8P4241mOJX53o0KjlfvXWipPrvyK3L2WnlW+ApVt8ufqsO3u

4uZAzQ8A6MwM/lHd31lAnBY71ephqmqyjHK/Jol6WZP71WiVeDjF/nX5tcgTdvq9s9dQ/LmbfPnYc
utafFT5gPbM3a+C02Q6keFazBQbSnwdp+DNkl9U2to2J9FkDeXnye3WvhSxrcsUy2sjm5vI+fK18Bc
5upwWxrd5chXbVVwr/txSPu+68vVoi4uVL8kU8mHNg3GeUnyP4UXNDwDozAz+Ud3TWaA46fR4
5igOb+0nrBzZLG2csGPyvN3tn/PN6zZf/nQ63TI3Q4crqnwp2jvHF5y/oe5fWefpTcdNYERubVRwqH
w321pOzYTz260kk4kq3wlLF7lcLX0j3ETOwPVzqRUZIr/V1ndhwsnlZsif3HQrKtRvzaRFG6uTf2Xcx18
/F0jU/ACAzrj/YG7p7BbWx1kgz9HzgSPP2enO5SZpus/S/XK6OMqHtXaqDnNTdLoiS9tfCXfe8mG1v
bLIeC2m8g+VP7cWF1M74bxBCwlrEk1ut/atqF1UW3/D3ET5FXsh8suYILyclpZhcXK5Kep3t6HagSY
Npv9S5CuoDuNmbuExbTrU/ACA7sh/Mrd2/AjqPkNxJpFaP39kWaxleM5BnWjn1ObKxrWGzR9Sp0
8pz2AvpMszYv5wuv3Oud+AXpJmWv9G5I+mOvHK2pxHNzVPRi1ya6M8Q+WX2n1ItRPOG7SwfT
WJJrdbe0gzXVTlL1Irf6uCy+ng76ybq/KXKdNe0vhjaucZ5bN1k0wu22PHpwIvan4AQIcqB5B2TgV+
+djCuM8EYkX9LGaStJMn1PtqKkfd9s69ri6/1VXFedjo4RvR5trENyBXLsFeW1ubGckYn8yGhIcSz6sw
5eLybJWZZlmsz3nOaXI03JlJs9a+9M6isgvW4MWUPJNoLL6oyt1p0ijOcgr55R6+262/KMK5qnem3r
piOO8wle91qzu5eVDzAwC6ZR9A+Oc0elM5kE59Hg3Jv96djT4DLZ/j0YrqUymoZyNujeiJhRc1yjVFnl
Fm7l4UrRP/qqJQHhk4SEyJmh8AAAAAgPlEzQ8AAAAAwHyi5gcAAAAAYD5R8wMAAAAAAMJ+o+QE
AAAAAmE/U/AAAAAAAzCdqfgAAAAAA5hM1PwAAAAAA84maHwAAAACA+UTNDwAAAADAfKL
mBwAAAABgPlHzAwAAAAAwn6j5AQAAAACYT9T8AAAAAADMJ2p+AAAAAADmEzU/AAAAAADzi
ZofAAAAAID5RM0PAAAAAMB8ouYHAAAAAGA+UfMDAAAAADCfqPkBAAAAAJhP1PwAAAAAAM
wnan4AAAAAAOYTNT8AAAAAAPOJmh8AAAAAgPlEzQ8AAAAAwHyi5gcAAAAAYD5R8wMAAAAA
MJ+o+QEAAAAAmE/U/AAAAAAAzCdqfgAAAAAA5hM1PwAAAAAA84maHwAAYFjOLt6gLJ5Vlwr6
xsLyRXWlGzqPSD8E5d5knH24uLwQuhW9BwDzi5ofAABgWKy6tlqebvaavyzbc75tCFb25aYO8N9k
AEB3qPkBAACGpfKfZdsV6mav+Rut31vcl/8qgIofwCZDzQ8AADAslZrfLnA3ec1vCveaeZn9M/tkrlDx
A9h0qPkBAACGpVrzW4WqU/M7F9zCWDWZtLDGVR2sK3Y1rC9n16z/Or2VRLPuOvetuZVpamruy
niVAa2ZapGxTOu8jfnkrCA2/4nqfESTyAKj/QCgV9T8AAAAw1IWk8vFn3TJaFWZ+Wf3glVuqpK4LEd
rmSI63Meqs2Vdm7Mm4hkkWvr6k8qlW6y5OMrJLS7rP4rsdfOvmVB4gf5+0ekCQHeo+QEAAIZFF4
1ZBakL06JgtG7kDd0LsZpfNbJKUnlFl6VOn/KKviDTmBZmKlaeBuVuOIEZ0M0ZYxor5TC52vnrBuH5B
BYYGkjOAAD6Qc0PAAAwLJWqUX2w/
+yWnVY5KYtZ3aSsS8NX9DBuC9lGVsQZeckMYrUJkjOYkCtxL8SZCUyILg3mLznZ/Qs0zRqtGwA6R80
PAAAwLP7yOis1ncrYUyrL0tRtUn9Ff7YL5eq4uoWPSFwZJcCd0oROqa/KpdUKT6HB/C3V1vp+YHQz
y1J1WQDQK2p+AACAYZEVsPpsfry/rCFly0xXNX/1ov7gE0wc5m8rl5Jc87tTaDB/X/Ge0/eDowc6Np
01ALSKmh8AAGBYnGJSFpHmhqfslIWx26T+imdYMa6vhdCgieFvq1Pqq3JptcJTqJ+cbpEpGjnZa8ew
hsiFkwFAd6j5AQAAhsVTTFbLR3Mj1rKFmt8urqttGpTfbpowM5zd2MnRYs1fO5Tb1dmUyAKzW9b
AG543ALSImh8AAGBYfMWkqRsnzI3yqionTWHqXHGL1/CV8CimSTizLmzdNDHhBGX/DdfOkSnUzV
8up2xfs0BnIdFpAEDnqPkBAACGpaaYtG9YpagkatlYCSqvmM8LcnRd72b8qcsGbpo43b7K7t1mzV8
7f/90JnST0Oihnk1nDQCtouYHAAAYlkAxWRaplRt27ZqVlbIwdgerv6I/28M505mo1s3VBoFVRFXHk
0WyXFqt2inE5l92n5iktLbFvu0b3e460XTGANA6an4AAAAAAOYTNT8AAAAAAPOJmh8AAAAAgPlE
zQ8AAAAAwHyi5gcAAAAAYD5R8wMAAAAAMJ+o+QEAAAAAmE/U/AAAAAAAzCdqfgAAAAAA5h
M1PwAAAAAA84maHwAAAACA+UTNDwAAAADAfKLmBwAAAABgPlHzAwAAAAAwj/7xj/8PpEFD
RkZ8flsAAAAASUVORK5CYII=]]></redcap:Attachment>
</ItemDef>
<ItemDef OID="b21" Name="b21" DataType="text" Length="999" redcap:Variable="b21" redcap:FieldType="descriptive" redcap:BranchingLogic="[q20] = '2'" redcap:InlineImage="1">
<Question><TranslatedText></TranslatedText></Question>

<redcap:Attachment DocName="B.png" MimeType="image/png"><!
[CDATA[iVBORw0KGgoAAAANSUhEUgAABVEAAAEJCAYAAACdcmdaAAAAAXNSR0IArs4c6QAAAA
RnQU1BAACxjwv8YQUAAAAJcEhZcwAAEnQAABJ0Ad5mH3gAAE9sSURBVHhe7d0PjCRnfef/b+/aY
PPHNmBskx+RHat7sZYlGJIg08PxL4egZ+6SJbnbYOVg5Oh3PVyi0zQSkwOxF+mX2wguG4lunS5iWrq
zliQyTEJugrLdlJRgckzHR4IxYTI/Z7rlc3T8wDYGjPljB3u3f/VUPVX1VHd1dVV3VXX39PtltaerpfqqT/T
O/OZ5/k+hb5FAAAAAAAAAAChjumPAAAAAAAAAAIAQhKgAAAAAAAAAEIEQFQAAAAAAAAAiEKIC
AAAAAAAAAQARCVAAAAAAAAACIQIgKAAAAAAAAABEIUQEAAAAAAAAgAiEqAAAAAAAAAEQgRA
UAAAAAAAACACISoAAAAAAAAABCBEBUAAAAAAAAAIhCiAgAAAAAAAEAEQlQAAAAAAAAAiECICg
AAAAAAAAARCFEBAAAAAAAAAIAIhKgAAAAAAAAABEIEQFAAAAAAAAgAiEqAAAAAAAAAAAQgRAVA
AAAAAAAACIQogIAAAAAAABABEJUAAAAAAAAAIhAiAoAAAAAAAAAEQhRAQAAAAAAACACISoA
AAAAAAAARCBEBQAAAAAAAIAIhKgAAAAAAAAAEIEQFQAAAAAAAAAiEKICAAAAAAAAAQARCVAA
AAAAAAACIQIgKAAAAAAAAABEIUQEAAAAAAAgAiEqAAAAAAAAAAEQgRAUAAAAAAAACACISoAA
AAAAAAABCBEBUAAAAAAAAAIhCiAgAAAAAAAEAEQlQAAAAAAAAAAiECICgAAAAAAAAARCFEBAA
AAAAAAIAIhKgAAAAAAAABEIEQFAAAAAAAAgAiEqAAAAAAAAAAAAQgRAVAAAAAAAACIQogIAAA
AAAABABEJUAAAAAAAAAIhAiLp0etJYKUih4DxWcUGj29PqjXWZWF0hpWnFaLEU
MxLO7LSlg372PzHqPs4Vb2GrGzEO5vtDbdtdK5JH08KNuQ9Cjmc+2g0AAAAAwPwPhRF06Rdm8UJey
XurU1kPCkracr3X0c5Hq4B5oILBVNWnqJUdZzqxe4e4faf1Qo1tcT/rlhsESM1aUimtjPY+iqlWfLSbPVJe
AAAAEAuCFGXVkW2W26M6M6vdGbW/4xqzxaPfy8aiFyL6rSovbYYdk2zxOeX1fMDMt9/n56KoV9Nrd8bubqZ
YJZo32aAg5jNU/VYVjAObWbZQ7Vw+PG+fiZTz5cN74vEqsfigLwIvtSzzaxoxp9k4P5cGDgV5JJ3Po3dAsWzQgebJ1D
yAAAAAAAcfYSoy6yyXW3vU7f++pNaOWLMqauJ8aGEWvrMsCiHJSmjXYA8tyGxWWMZ+WRbEBgvF
6VxFsrpTtBk8Rrk+YmRIYmVyXXHX56KNbfhZZBkqPDAPBdQcZPFB8Q2+xGpG6wyA8OKv7mQOHnQJNd
akmgD0bf9LFi4PSPUqMSthPMVSp1cpCjf39r0u0ks0ycGP1S8abI24+Pko4cEBUAOtOpdWHRw
okOENBTPYBuGqMRDH0aMhoCl8HDmTTRlqz+UgpOn8AM3wKqCN0VRT3ZRh+h8w4GNRoVvrMmEZHk
X+QVaPc0jP3yomMLr6QbxxnZ4KsiZYzMjvmtt4+6yKk4pF+k6iklbNGIu1ldWK2fAgS6ZvDcXy3QOUDzC
C4N3UhxI0S/mnaHbIFbPTRpNC0oeTvY5LBozZqCx8hCMUfZQ0WcC+YH12ICSDEC+YhHa3k72SyNl0Fn4
BW8mmVOOHKgTEVBf/ZLdFFGYD7UkyEX5SlR0bOFMjYUoNw/pyeaxAUhhwnUC5zEU0KHC6DGmAXj
z6vvtBlyk1CnJj/AUROgCG2tOMPOVJi9JeWiQkrhdZ53Oj9JqnVwUH8RwwmdAHtlrvVOB6mpzsmP6xnUDn
OBS6ZI8tpb5PV0DBAAAH5EN/dFmS3rcSL9q6pd9ENKvjOyBMhiq9IRGVdLHDNwbBTqCaEZc0FcK4
l9nDQgG7UW+D5fGACDtM54Cgo4TL1vKAimlqEvjxpPqJjQ0bEqc1QXtLWBrKfbTPlf6RbfYEMFDx+yKxn
Fw8mQzhKqgk7BP18Xu42cK8CF+RTxXNFyyTb35Nz1mbW4LfatGfc4pCbQu3lTgdhEAJhzxVRHJJxtJ
qoCZyJz/amZ41yxkJVZDVhkGGYT/IsVzSp6NgGpnUMHtQfE2PLE9/G0AtpwcY/FmvJAmFxv1RAjjHL6
T/AmfW9nyPzz+nc9jG9DAKFOGt9UJ2t03LZaSMs1GXdsnZ0Y/Bj2M6CHoRb8HmrIOZ5IMsSdF5rhDV
CkPBFy+XYg/+oTtULvPf5lOWSQFxY4ZhhoGvsoALhzFOdmsUBPf5TR0FUU92C9n8rZGR3wNd8tAN
ZE1KZ5yPtegAHRrKtNVDOpj0S2dZv9E4ymfpZpLOnuZFpxvDy2IjP8tL7/J3eo1D3pcEP8aB7MmxeKbhx5Hl
EbAOzDQ/Z8sN+P4IlLoFqiSMinHGDxjJuGyA9lntjIUlNT5zAm53/8xTzuZAFADb+3pgFptipXr8vv6u
MSGyaK2yWE4iuHFaFEvw7xc+a0bkb1CcShjnG0Sufk2V4sn+2G5KSFBY6TA3gIe+8xl8YOR8SDkaX+3gM/Ws
o2MJ0S4wOrhBx99sq4gkh/MeC+xmAt7/eJ52SkPDqblbRSD51jBOsUdCfdu8O4D1shjOG6Rpl08mIUq6
o8z1k4i6hzlaW8n1bbN3z+zYbVj28bDBCACRyOugqRsGn96Y0HFlIaKeFRNAF9JjJtKzS4+TPwT6Bqrs2mBsd+6D
Z0dvajvt0C8htVtw3ZwL4DmMR9SdZJB7AXcRQNjYPKLRDCOmXX9lL4N8PKk3x7DX+/hRbq08/6QD
wVwWptowLAACGgt95ZybsvJNAOzAWm9X7wm2ziLqWSzYjxrlYTR4RxdPaD1Wt2Id2ZhNdyMUYJ0TBg8k41
gpFvqWJeMTx2ubVE2vCLFTZJ7zcBM5qIUz9IGCR0DzOb07IiJqOHpzBSJ2M6OuQ+Gt6Shjsu2L5VN+mcbdGsT
ZG9nU9RS70RdE/mMpaDKtaICtAQ9jCCGRlH66W0+zHpFVUy4pJmHSjSPLTbrTEvLmTqZcXyOn6jqCuXf3aidMxDAmR8HdjQx8kzBvf5lmoe41kPRuj
OkR+wk01PXHTlpe8iHbqdp6Hn/ZRw+uJqJtM+7w27mN39FWkCmrLGRqPvO1kk0gQqeKu22XH7oVDQcT8a2sQ
ZJEyU9Yb9X2L5Rxn0k/S0A6rdO1ILDA36t+xxqXqvYzvzCEqJbNpIAXuOW/1ruoYvswpn/r83zfiHiCSj1Jp
FW/MTRZD8HcTPJCSRxA3lyyZ9SCr5PmPuN6v7DQZYoPCjqpxd3tqJB0M7h9PQb7rxCLL6DpNpsfpXZvt1YU

me+ta1Qq+NvI2oTnyA9c3XYDi+kT+5+Q+n11vQQAyIOqg/pzD7xELwX94W9/Tb73raf1EgAgc5f/RK
T/fb2AeUaIivgq29LyctSalHTA48w8bynX5YJR4NOfUMnSXNWB0GQ1HAPbsp55Q/a9of6KMUnTHP
Jqu9rKUt8KRKiWomxe8IdlN1d1gOaFzqMm/ZolJ3AMttM8tryOKdl+ipsXpO6/2HmtOZGTdS/7h6Br
7yrua1NNWBO03bzfw9odR+bHA8R339fvk7v+7C69BADIw/FLBXsYv/oYRgWozd+8Xy8BADLXf1Lk
8sf1AuYZISoSqWyH11+stqz17tBiV3FT9gZfW21NVr/R7K0XKBlgDM3OdKKjFJgB2KiyA+qc9bt+wOe
ya1/OW03PstS7fT9Yt1Wl1d8LTpaV1zEl2k9RNvf60h16sXq5MUzeVpHt4EGK7B96PbFTEbvtVlv6Lf
8+sqlzblwHu+5ulByOB4jBnYn/6Wfp7QQAeVr50kvl2iev1Evh/uFL35ZP//6hXgIAZO6y9RvZJcpbzbt
CX81aAgAxtTcK4nSSVCHqQGAKADGo4PT1/+318sAjD+g1AIA8lB56od0LNa6tu8vyiteFD/sHAGTgit
8QKfBL9ryiJyoAAMiVGsJPgAoA+VK9T1Uv1CTUsH7qowJAji79AfVR5xghKgAAyM1HvvgRezIpAEB+
VP3Tt33hZSProI6iAtS7/+NX9RIAIHN2fdR79ALmDSEqAADIxe6Du/LBv/igXgIA5OWOL18/tg7qKPv/
8zH53Mcf0ksAgMxd/n9FLn1BL2CeUBMVAABkTg3fV3VQmUgKAPJ189efL//8Czfppclc+dzjsnX36+
XWV79IrwEAZOtKXR/1Zr2MeUBPVAAAAkKknnn6CmfgBYAZe8MMrEk0kNcoz/3RJmu+/X3705DN6
DQAgW9b77aWPWx+fchYxFwhRAQBAZlRwqgLUh594WK8BAORB1T99yxdvlOf8OJ1f+R7/xo/k7g8
xKSAA5Kb/HZFL1EedJ4SoAAAgM+/77Pvk3ofv1Usgf1UsAgLz83AMvkRsev0ovpeMrf/mI3PtJ/igGALm5/
DXr0dELmDVC1KXWlo1CQQrGY6XR05/LSa8hKxttveBob7jtWZG8mzM3Qs5LHL3GinPuVhoy6tT55
9d55H7NjwLuWyCW+n11+djffkwvAQDy8vJvPE9e+eC1eildn/zPB/LN//19vQQAyNylXZH+Y3oBs0S
IurR60lhZlaZecpTlzFpRP8+eHfiVasLfVIImPi+9hqzXor5KXfOCrAYvunRqJSlMENguK+5bIB7V+1T1Qg
UA5Ovqp47LG++bvg7qKKo+6u//
+y/bHwEAeVD1Uf+79fFZZxEzQ4i6tLpy4KZA5bp0+33p9/dkM68MdWzgt6QmPS+qZ+S4YK99XtxN
V1vqevelWy87K5qrQo4aA/ctEIuqf6rqoAIA8veWvRvtIDVLqifqH/721/QSACBz/UdFLv2xXsCsEKIuq
96h7OuncuqE5Nf/FGmL1zOyJ41zugtquS5bFedpcfOsVJ2n0twlRQUwPTUTf+WPKvZHAEC+Xvu1F8
vLHr1aL2Vrb/f/2A8AQE4uf8l5YGYIUZeQXbvRDN2aq04tx8GuiKp3o1E7c1T9zGAtyGCd1bDejbH3r
wVreI6qN+kMVfdfl6zW52A9S6+2qH6ENy3GPgPncEP8zZjnydln0vOiqHaWYvWMNHoeB0LzkpzUn
VFl/9A6olGM9obVW21v6GMZPAcJrkvI/RZ2vePcb4PXz35E1ImNY5b37WT3p2vSa2eZ6D1Ar1TMr
49uJI6QOz91pzz4+IN6CQCQFxWevubvXqSX8qF6o1IfFQBydOlPqI86Q4SoCDUUGGl2/cyRYVRHai
WjzqrR43EyanuDNTzVurCgpuQNVXdFt3UUZ5+DwWRzdcJ9Fjflgjtk3joz5/RGeo1z3nkq1y+kUEahK
q1+y+tVOsTseTxK50C6+umwipx2N97ZkYsDJ7W96x2NX1c3yXVRQV7I/eZcj1EBZPj9pu7d0GC5U5
NSIMjOSpb3bcz7M2CCa2eZ7D0Ay07VQP1M7zN6CQCQFzV8Xw3jz5uqi3r3h75KfVQAyI2qj3qP9Z
H6qLNAiLqEKtt96Xfr4kZ7Um3Z9TH72zrxbG/4AZBXL9Won9mpyfrIxEaFec7r+3uboWUCxu4/wN+
et3/pyI6XBPWkse4GPca+Wzo1imzrKP523M2ofdbOu/Fbsn0WNy+If+rOS9v677wbglnn94JOUJOd
F4P9um2ZLK8uyolT+ukYlS23beb5V9ri53BnxMnhkpwj67VGqYGh+21ofyZj2/b9ZrYlbFt+kJ3U/Ny3
/uvD789hya6dZar3ACyrT+x/wp6NHwCQv5UvvTTzOqijPPTV78qf1hmBAAC56T8scvnP9QLyRIiKIW
bPtPoFPwgdDgOHletbE4Z54aotPxwsrp3xAqzOge4z2bsoO14eaey7suW3deeiJIl7zH364ZMh8T6Ls
nnWTbuaslpwe08Gz+8kipt740PW2PblMOpEFdfkTNjxtXf9XrVn1pzjSXSOrPOzp0NEI3gPvd4Dhu43
s8et0evSPk86CNzLYfa0LO/bsfdnmCTXzjLNewCW031fv0/u+rO79BIAIE+vfPBaufnrz9dLs/G5jz8kX/
nLR/QSACBzl74gcpkJ/vJGiIoBI3qm2Yqy5iYxIwK3UyfSDKjKcrKkn47SPdC9+VSoU/LqNhbMYdKRw9
QnMMk+K9tGr0FHOsP403RKoi+fcf2NgNIP3Kpy1j2gqa6LruEZOrw/aOh+M8JCawf20Hd33yNrsaZ
uHu/bBNduyvcALB93Jv6nn31arwEA5OWGx6+Sn3vgJXpptu7+0APy+Dd+pJcAAJlTw/r739ELyAMh
KibUkeHOgTHCo7mX3TFUvMKUSrD+5Oz05HBssVSf36vSHRZuBG7V08FeoUmYExB5PXXHCbtWR
dm8EN470w0rF3t+o8nvz/SvXdh7AJaNCk5VgPrID+h9BAB5e86Pj8lbvnijHL9U0Gtm60dPPiPN99+v
lwAA2XtK5NIf6OfIAyEqJjR/gWm53vWGbAcfk9YLHS/
+Po26n7aO1NZznJyneELGlj4tn5Sxl7S4KW5lAntYuDF8vno6/CyPP0dt2TB7nbo1OM36o0lYbdzT+xj
s/as0z83XpEi53bcTXLtoR+GPJpiWGsL/wCMP6CUAQJ7e+Nc3yAt+eIVemg+qPuqnf/9QLwEAMqfq
o15iYte8EKJiQElOusnV0EzePbnoFnIcO/Q7J6WTXtA2qm5m6ibYZ6+xrodpl6Ved5OsPCfnMa5rc1f8

zphdOfAu6QmJc0m9HrXW/XH+/I4OP6sSyOGSnCOzLqcKFN26qMaQ96TUzPKqx6k9GZQdSHa9W
p7pDpOf0CzuW0usa5fme8AU1xDz7yNf/Ig9mRQAIH+lh1448zqoo3z6v/6D/MOXvq2XAACZu/xZkf
48dRU6ughRMcCcBCnYW9IPAlXYNTChz6yYNTCbq/5Q7faGHhpuPdIev510n72GrLsnrnpWNjeNyY
Nq65JPjmrWsmzKrm5er3HOCzBj90asnBbnDulIs+ke18Bw8Amvix8oDvbcjUlvX80sH+xxaoTFZo9b
oz1p3yaRZnHfKnGu3QTvASU/dZXaee9gZMOb4h9Hze6Du/LBv/igXgIA5OnaJ6+0Z+OfZ83fvF+
+9y1qZQNAbtSw/v739QKyQoiKYeYkSJ2alHSoU/LTE7kw7YxI5vDy5uoUoVGwBmZzVQdQXnhTlVZ
qs9e7kuyzJ411d6h6Wepbav3okCq98zLMnFndbbN3TastiX+aKhIo72oZDmATnCMv2LO4x2xOsJTEi
HvXrLNaPevPNp/YQt+3SpxrZ0n4HuDXW7V41zBubVssGjV8/85P3amXAAB5UvVP3/aFl81NHdRRV
ICqglQAQE76T4pc/rheQFYIURHKHgYdUpOy2rLWu8Otp1KR7cGClfuHRs/BBOwamMZwbZddWzOj
eqgx92n23LN7obonbiCk8of1p3hehhRlc2+4Tqg9hD5hYBeYJMs6ZjsbHhT7uljH3G/5QaqtKq2+0dZ
ACYJo9r07eJA2Z5vTZZMLft9aYl07S6L3AHUsg6+ttiava4u59cTTTzATPwDMkOqBqnqiLgI1pJ/6qAC
Qo8vWb6bUR81Uoa8KBgJAEmo2fT0ZlAph96btmTxL+lhOtaYNWBfEUbp2yJUKTit/VJF7H75XrwE
A5EnVQVWTSS2SK597XLbufr3c+uoX6TUAgGxdKXLFb4gUbtbLSBM9UQEkFCxRcGZtsUO49nnrWC
J6ZB4tR+vaIV/v+
+z7CFABYEbULPx3fPl6vbQ4nvmnS9J8//3yoyef0WsAANmy3m8vqWH9TzmLSBUhKoCY2rJh17o06
pWaJQoWUK+xIqv7demmUqJinh29a4d81e+ry8f+9mN6CQCQJ1X/9C1fvFGe8+PF/NXt8W/8SO7+
0AN6CQCQuf53RC7doxeQJkJUAJNRNS8XfPx7cXMvpRq/C+YIXDvkR/U+Vb1QAQCz8XMPvERueP
wqvbSYvvKXj8jnPv6QXgIAZO7y10QufUEvIC3URAUAAKEefuJhec32a+wJpQAA+bv568+Xf/6Fm/TS
YqM+KgDkjfqoaaMnKgAAGKKCUzWRFAEqAMyGqoO6aBNJRVH1Ue/
+0FftjwCAPKj6qGpY/7POIqZGiAoAAIbc+ak75cHHH9RLAIC8qQB1UeugjvLN//19+cPf/ppeAgBkrv+
oyKU/1guYFiEqAAAAIUDVQP9P7jF4CAOTttV97sbzs0av10tGyt/t/7AcAICeXv+Q8MDVCVCTUk8aKm
uVbPTaknWjdOGltZ96MOYZeQ1Y2gmvbG+7rV6TR0yunpfZjb3NEO2zTne9M2m1bkPugvaHbWJC
BSwosjE/sf8KejR8AMBsqPH3N3x3tuqGqN6rqlQoAyMmlPxHpP6YXMClCVGCGeo0VKZRq0tHLWe
pd3DH205RdQj4AA+77+n1y15/dpZcAAHm7+qnj8pa9G/XS0aXqov7+v/8y9VEBIDeqPup/tz5SH3U
ahKhIqCibe33p99VjWyp6bXby3l8WRhxDryHrtTziU6UnF3eC+2qea1hrF8VRuA+A+aZm4n/nJ98pT
z/7tF4DAMibClBVkLoMVE/UT/7nA70EAMicXR91Vy9gEoSowDLoXRQ3Qy2Xy86Tzo5cXJwUdTFUt
qXfrYs+w8DCUMGpClAf+cEjeg0AIG8//ffX2UP5l8m9n3xYvvvKX/NsDALm5vGc9mOBvUoSoS8eoLbl
i9kQctd6oc2mvz7s25fD+IutGnU/VwKfNLcT9vko6Z8z+/PmMP7mqvP5EYU0/WNWj+T1Rv2h/FU
5e+GMDvk6shMnRQ3UUg17jG5PvHbHuTbD59DaumyYrzfqkQa2MbA+/BTHaYN5POpY/P2rh7vd
9nnrupbrsjXQXdYu3WC83n4M3DfArKgh/A888oBeAgDk7YbHr7Ink1pGd3/oAXn8Gz/SSwCAzF26R
6T/Hb2AJAhRl05R1s64PREPpOs8s3TlwE30Aj0U27LbdJ6Vz6xZXz17ldNV/awjB/4B2PywsCxn1nRr7
RCwJIMj5zu1UswQa5bnrCO1UkFW9fYcal2SINUYyl89LZXimniHs3Nx+hCvfEbcU+2L2e6pr43Dfn1w
Z846FVQOrG+uDgSpE7VBHcuqeFvWoakKSlf369Ld2wxcdxW+lsJKN3RqUprnibKwFD7yxY/Yk0kBA
GbjOT8+Jm/54o1y/FJBr1kuP3ryGWm+/37qowJAbp4SufQH1kfqoyZFiLqEimtuT8R9OXQTovauHwi
JEU72Dq1XOU6dmIcI1VI5LW6M2gzMjmSEhV6w15PGutvjsyotu6am9WjpLXRqsh4jjUz7nFW2rTa
Yw76rLadd22HVPv12d+vuV8TsRaoYQ/mrp9X2zVA4xpD+4qbsuefNfnTFa4Z1BPULwcDQN67d6V
wbV7neDX69Frbev2+maYPxeh2aFjf3vOc+P1RXYWtXf41/TppyLsFxAmnafXBXPvgXH9RLAIBZeONf
3yAv+OEVemk5PfTV78qf1h/USwCAzPUfFrn853oBcRGiLiOvJ6IfaPUOndivWnXCo32dFJrDwO38bS
5UZMsNoJq7fi8+s+6n2wPUXFff8ickqmx5QWCs3pgzPGfVlj+Rkh/mWi0Z7IY7gtk792TJfmJsJ0EYq/
Ua636vzepZ2QxPUMe3O61rY6vKWbchRsg+cv3+obPdKdoQeH0UI1Q3Q2s7cNWB6t6okwhkSA3f
v/NTd+olAMAsvPLBa+Xmrz9fLy23z338Idn/n4/pJQBA5i59QeQyf8BKghB1KRXlxCnnmRtode1x6W
U5efqkHXYF11vUMHDn2VzwQ7mmeJ0KuwdeWOgN5ffWWcfkDu+2H8bw7cAQ/VFmdc784HMy
Yb1zLRMP6W/LeT9BlVZoz1klRrtTuzaW8kkJ3d2o9a4p2hC7Z7Zxrq2N2WUO3P3Er8sLpOuJp59gJn
4AmLGXfPe58nMPvEQvQbn7P35Vvvct/m0CgNxcVvVRv68XMA4h6pLy6oraPTl74nSqPCUnKies/w

+uV3ngPEWoFiOYcodmt90x0xG9I6exkOfM6GmphqaXQkPC+LP0tzf8OqBVo6fp8kkSbhdl80L4jP1u
cDtiPjEgE+5M/A8/8bBeAwDIm6p/usx1UEdRAWrzN+/XSwCAzPWfFLn8cb2AcQhRl5U3tHlfDts6aL
N77VXEyQqN9VP3hsyCUdfTDi/9upOjwkuvNubQI2YYuIDnzB/KHyXmkP72hj9RVLUlIzuhTmDqa5O
CTNtg1JUdKNlqa55jln7k532ffZ/c+/C9egkAMAsrX3qpXPvklXoJpn/40rfl079/qJcAAJm7bP02eukze
gFRCFGXVklO2hlkRw52nSHNbh3Rkv0Jf3347OuzV9w864eaDXeSp4E6pCVnqL3i1eKc2KKdM2Mo/
xjjh/S3ZcNLUMtSV1PRTyvVazOhHNugZuhXPU7tScXsQNWYoCtu2QJgSvX76vKxv/2YXgIAzELpoRfa
D4z26f/6D3aYCgDIyeXPivTp2jMOIerS8ntyNptOOObWeCzq4p/uem+Sprnj9gDtSK2mA77BOqRmP
crmqj9sur3h1aUsxB5LvWDnzBzK787+H3iYIV70kP5e45w3jL9cv5BOuYRUr82E8miD3pbKoIM9Trvi
ls8dW7sVSMn/+v/
+l34GAJgF1ftU9ULFeHd/6AH9DACQi0v36CcYhRB1iZkzpqvehd7w88Ds5sYkTXPIq1NqC+shGaxH2
VzVwZg/Ll1GT440LNVzVtS1VJXmauqhoTmUvxxaW8AoiWC9cuSQ/l5D1r0CqtYrAxMwOY/JJkhK99
pMJoc2VLb9IfyBurRGfdmzm7MP3bEU7v7Fu+WOl9+hlwAAeXvjX99AHdSYfv7dP6WfAQByUXijfoJ
RCFGXWWDW8FPiTzbuDltXzPVzyAwvRw2ht+tRGr0uXeW6dJPWu0z1nFVke7BA5v5hSrUxzaH8o0
NdMxQeNaQ/Xl3VCaV5bSaVQxvsIfxhxVBVSNvvp1pfFohy1RVXyf/4lf8ht1x3i14DAMjT373yCf0M
UV7z1pvkbe+5VS8BADJ37FUix9+kFzBKoa/G9QIAgKXxwCMPyOv/2+vtmfoBAPm648vXyysfvFYvYd
D1P/E8+a1PvVGedw0TbwFALgovFrni/daTq51ljERPVAAAlsztN91uD+0HAOTvb27/tjx2PX/ECnPlc4
9L9fdeS4AKALmx3m+Pv8f6SIAaByEqAABL6F2n3iUfeMMH9BIAIC+Xjvfl8294VH78nMt6DVz/8t+V
5NZXv0gvAQAyd+ztIoWb9QLGmSpE7TVW9AQpBX9W65F60lhxJ1TZkODL27LhTbbiPCabqGZYeyO
43dBH1jOAAwAwhz788x+W07ed1ksAgLz84PnPyt7rvqWXoLzidS+R1X8bNhkqACATx4oix39eLyCO
OeiJqsJVf5ZqR84zwquZ0VcaKU3oAwDA4rjnl+
+xh/cDAPL10M0/kO6t39dLy+3al14l1d99rV4CAGSucI3IMTWMH0nkGKIqBqpgpSAQA5Of5ukcIL9QLi
mn1P1N6h7OuncuqEZJedlqXedUNc/9Gtl/XnRZq7pKgAgOVzy3W32EGqClQBAPlR9VE/96Zv2h+X1
S/8xivsofwAgJzYdVDz7rl4NOQYog7XRLXrlZZq4nZEtYfVD9UoNb/OeaRVL1Uprp0RP0YFAGA5vfm
WN8tH3/5RvQQQQAyIvqibqs9VFVePoLv35CLwEAMmfXQX2HXkBSc1ATNUKvSuFktS8lSuFktS8lNXRqZVSq2H
aPu+HuNXTfpGB5WVM8uWd46hJwTA7YddqEYTfT/4kcHneY0nPYU7fC+q9b2DCO//8rEiKf0cCAt7
7s+
+V2h01vQQAyIuqjbps9VGfd82VctfvUJMbAPJztcixO/VzTGKmIWpluy/9bt3vCVptOcPst1WY2ZPGu
htwVqXlDsFvVe01qobpeqIkoSO1khtC+I9Vd0Yra9/2bgFghnqNlWAPfSBnqjeq6pUKAMjXfT/zuD1r/
7JQAer1P/E8vQQAyNzxO0UKL9YLmMT89kTtXZQdnSKU61v+RFSVLXHLmHZ2LqbU+64s9S0S1NFG
TQoGTGKR76eM295ryPpg13tgBlR9VFUnFQCQnx8/57J8/g2PLkV91Le951Z5zVtv0ksAgMwdf5PIsV
fpBUxqfkPU7oHXE8sevu/1HjWG93cOpKufTsfppZpmrVUAABaVmqm//attZuwHgJw9dv3T8je3f1sv
HU23vvpF8ku12/QSACBzhVtEjv0LvYBpzHdN1FSFz85vlhPo1M7nWItxHgxO2jWqxuPoOpD20GPv6
/VjZG3Jwf1FBNd2Pdzga0fVgsysDVEya98oca+VK/5xJm1f2OsHyncO1O806o7arx19P/kG2p/K9Ux6
DsOEt32wXungORo8P2HiTbTn8/epHqPqpCY5P0DQbdffJvf88j16CQCQl7+/7Xvyjy//oV46WlQd1O
rvvVaufO5xvQYAkK2rRY6/2/p4hbOIqSxEiFqud4fDT/uRwnDa4qac1WVWRfblcGnyBRVsDU7a1ZTV
wqr1/3hUiFMKG3rcqUlpMKBKMklYe2NETUjVYzgYFmXWhihZtW+khNcqwXEma58TyIW9vrlaGBH
2qXNitLNcl/GVM9SxDbR/6us5/f0ej9OrffAcNVdHhZyTcPbh1XO2Dd97qd3vWGrvKL6DGfsBYAb+6v
WPHcn6qL/yH15JHVQAyNPx09RBTdH8hqilk34P0YN0Bu2Ha8tuuinKQug1zvnhkTuhV7/r1Zsdzzhv
5bp0dbDd9TbQlHNeotNLMEmY9dpzesOh2+3IzkX3tVm1IUpW7Rst2bVKcpzJ2tdrrHuBnP+HjZa1F
615bkRQaLRjb1OKem0koz1u06e5ntPf70n47fHb3pHa+ejIPHqivUH+PsLvvbTud0Ds2frfdepdegkAk
AdVH1UFqUfJyumftB8AgJwce53zQGpSC1HtnmjGkFHzEWco65DimpzxspxVfxuqF6C77UQbdnpv
me1yHmYvuTOyFivhWXQ9uejO2qUCFi+kKcrmBSPEidI7lH39VDo74mY3xc09J6yxHnub+mQmmiT
MmLjHCNyKa2eGQ/XM2hAlo/aNlPBaJTnORO0LtuOst74iW/aGVVC3J2GHE2hHTNWz/rmtbPtBrd

/2JNczhfs9gWrL7yFf2Up/+4q5j/B7L8+J+bAM7v7Fu+WOl9+hlwAAefjmjU/JV376u3ppsb3sp14o/
+a3mNAEAHJTuFHk+L/WC0jLHA/nDwYcXkjrjWE1w5B0mMHN0daVAzdTKp+Ukn5qK56QU/ppJDP
kHgioh2ouTjVJmK6nGTZ8Prc2REmpfSMlvFZJjjNR+0a3wwldR5fWOHUi6XdVVU4HNlaSk2473bYn
up4p3O9zpSwnAwcRIteJ+bAMrrriKmbsB4AZuP9V37HD1EWm6p/+
+n/5GeqgAkBurhQ5/mvWR+qgpm2+a6IWN2UvbMitPdQ3hXqoHtWLri8pZ7JH3OhefG5oM1EPZ
MWu5eiGPlE1KzNsQ5R5b19sebQvRuA3VlFOLEzSmcbxAvPpphfcZAepKlAFAOTn8yuPylNXX9JLi0f
1QFU9UQEAOTn+r0QKN+gFpGmqENUc9hv1cMLJoj8M2gxA7aBUrw9NMc2v04+4dRUtdp1B82tD
H2kGskvEuHZ+7Udf89zw5DXjJwlry4bZq9OtjWnWijRl0oYo2bcvLbGOc4btG21wgreeHHp1B4ZlOv
HcEcD5QZpuv+l2e2g/ACA/KkBVQeoiel3l/6IOKgDk6dhrrQd1ULMy3z1RkZGQ4dEus05mDGp2d9V
b0Q+rjZ7D7raTTBLW3vV6dtrhjxuYG8OTB6XehihZtW+khNdqguOM176Idth1itOdfT7Y9JDh+ImO
M737fWHkNjEflpGaZOoDb/iAXgIA5EEN6f+7Vz6hlxaDmoWfOqgAkCM1C7/qhYrMEKIupaKs+bN2
yao3Ztuc0XsMPcGXKlEb7K0YEnhNOEmYH/4YM+KbcmhDlFTbN1LCa5XkOBO1L6Id9rE7NVUTnsK
Rmqsb4jfdL5lQPrPmhNaJrmcK9/uiyeB+B0wf/vkPy+nbTuslAEAeVH3Ux65/Wi/NN1X/tPp7r5XnXX
OlXgMAyJaqg/oe6+PVziIyQYi6pIqbF/zehs1VHawYk86MU9n2h393alJygxmjRqg/UVew9mbkJGGV
09aSNq5dWbUhSmbtGy3ZtUpyrpO1b2w7ynXZSmV8eFnK5aas6vZ4Tbfaftab/j/Z9Zz6fs+LOdGV28
6Jws6U7ncgwj2/fI89vB8AkI9Lx/vy+Tc8Kj9+zmW9Zn79Uu02ufXVL9JLAIDMHVsTKdysF5AVQtSlVZ
TNvcFJu6rS6res/8djD/8OK6Rpb2dgoi679ubg/ixDk4RVZHuoDc72vF01d71eitm0IUqG7Rsp4bVKcJ
zJ2qfa0Zfu0IbVpo3SBlM7JWcHjzfsGiW6ntPf7/mw7q/B67F/aPQSTiCV+x0YzZ2x/7qrrtNrAABZ+8H
zn5W/ev1jemk+veatN8nb3nOrXgIAZO7Yq0SOv0kvIEuFviqCCAAAMIF7H75XKn9UkaefXYwhpgBw
FNzx5evllQ9eq5fmx7UvvUr+06ffzDB+AMhL4RqRK9R8BQzjzwM9UQEAwMTefMub5aNv/6heAgD
k4W9u/7Z875pn9NL8qP4udVABIFfH3239jwA1L4SoAABgKu/92fdK7Y6aXgIAZE3VR/3cm75pf5wX
v/Abr5BXvO4legkAkLljbxcppFNUD/EQogIAgKmp3qiqVyoAIB+qJ+re676ll2ZLhae/8Osn9BIAIHPHiiL
H36EXkBdCVAAAkAo10dQt192ilwAAWeve+n37MUuqDqoaxg8AyImqg3rsPXoBeSJEBQAAqVAz9b
d/tc2M/QCQI9UbdZb1Ue/6T6+2g1QAQE6O3SlSeKFeQJ4IUQEAQGpuu/42ueeX79FLAICsqbqon3
/DozOpj/q299wqp/7ZDXoJAJC5428SOXabXkDeCFEBAECq3lF8BzP2A0COvv2if7Jn7M/Tra9+kfxSjV
/kASA3hVtEjv0LvYBZIEQFcteWjUJBCsZjpdHTn1tSvYasbLT1gqO94Z6fFZnX09NrrDhtXGnIqCb6x+E8
lv5aY2mo2frfdepdegkAkLW/v+178o8v/6FeytbzrrlSqr/3Wrnyucf1GgBAtq4WOf5u6+MVziJmghAV
yFVPGiur0tRLJrKcWSvq58vHDiJLNeno5YXRa8h6LarV6loXZDV4saVTK0lhIDAGjqq7f/FuuePld+glAE
DW/ur1j8kPnv+sXsrOXb9zu1z/E8/TSwCAzB1XdVBfrBcwK4SoQK66cuDmbuW6dPt96ff3ZHNZM9
SxQeScUj1nxwW/7fPiHlq1pa5sz7r1srOiuSrkqFgGV11xFTP2A0COfvycy5nXR33zr9wirTXoJAJC
5YyvW41V6AbNEiArkqco+/qpnDohy9v/dHHF6znbk8Y53QW1XJetivO0uHlWqs5Tae6SomI53PSC
m+wgVQWqAIDsPXb903L/q76ofzKfziplKwBTP2A0COfvycy5nXR33zr9wir3nrTXoJAJC
536d163Or/dp135sb+ivNdbZLwuuD/bKS2MbmtlG7zFcYzN4jMM1S+1HWN1Low3jal3a+zDDt+aq
87WDDQ9pc9i2o9o8tpfjROdl+J4ZuZ8YxxD7fGh+W9RjuK0OZwi9/7p0z506/lKsnrNGj+NAWF6Sk7
ozquwfDt9PNqM9ie65eMduS+H7wj1Pcd9HsNxuv+l2e2g/ACAff/fKJ+SbNz6ll9Kh6p/+
+n/5GeqgAkBurhQ5/mvWR+qgzgtC1CWlwpHQMKhTk1JhQ8ZlcHHYtR8HCkLa61TIMrC+uRoeXk2
1DRU2hfYY7EitNCqEU58zapaW61J3uw52duTiYMi0674ynbqmQ8GiZh/zyGBquM1uz8dQE5+X4X
umuToieEt8DFGcfQcvd0hb7WCw5A2hd6V67jxVafVbXq/SIWaP41E6B9LVT4MqcjrpPZfk2FP6vlDn
KY/3ERwdapKpD7zhA3oJAJC1z688Kk9dfUkvTe/f/Nar7J6oAICcHP9XIoUb9ALmASHqUmqLn8O4d
TmNeo3SlHPhSUpi5XrX3na/FYybwtaPGt482TaCw6mHj7EjO4PplEcFZM7r+3ubsrlVF+erBr/GPI9nZ
FyGWtm2ttd1t2Wptpx9bOvUrr3hB4Vhbe7UZH3kdQm2eXRT0jkv/invSO28cd0SHMPY8xHg7zu8r
dZxrbvBoHEu3Iamcu40u53bEitrHVKUE6f00wiVRPdckmNP7/uimOP7CI6OD//8h+X0bQxHAoA8q
ABVBalpWDn9k/YDAJCTY69zHpgrhKjLyOwlZ/R0K27uOQGJ9dhLZaajqpx1t1M5bS25RqwPHd486T
aKsrlnBj6O4toZL7TrHIT3AyzXt4IBWXFNzugv6uxc9NvY3vV65pXPrI0P38YwexjWLxht3rwgfgZ5PrR3

31CbR5r8vFRbfnDoh3xB0xxDFHPfoW3tXZQd3SEycC4qW/5+zWtniH/u1HFY3yOhIe8k9uUwrEFKk
nsu0bGn+H2R2/sIjpp7fvkee3g/ACB7akj/V376u3ppMqr3qeqFCgDIiV0H9V/rBcwTQtRlZAQ0ImqY
bsGrZTiyhuIkyielpJ8GjFofJo1t2HQ9x9BhzEGnTgwGP0VZ8xMtLyzyA0Mj0J1YVK9WY/8jgrfhNscV/7
yMN90xjFaWk+MudvfAa79X7sF+GEPcRwyfn/zcTeuUjN51gntuimNPcv2HzlNe7yM4ctwZ+6+76jq
9BgCQJTXJ1KT1UVX907t+59XUQQWA3Kg6qHdaH6mDOo8IUZdSUTYvhPckdEOYqMl1FkZg8hyjn
mOk8MDO76nnDnc2AsPq6dg9GafXkeGOgjFCRlOK52UyYccwK2keVxw9ORxbLNWR2T2X2vVfkvc
RZOKW625hxn4AyNFfvf4x+fFzLuul+H6pdpvc+uoX6SUAQOaOrYkUbtYLmDeEqMuquCl7esjtQKlR
W/Pcos+u3ZYNs3edW7PRrMGZhHW+zurzZA+NNoYyV0/nF6FOH/qlfF4mkm1w6dXKHXpMWsd0
AsUTMrb06bje1BPcc+OPPf3vi6P9PoIsvfmWN8tH3/5RvQQAyNIPnv+sHaQm8Zq33iRve8+tegkAkL
ljrxI5/ia9gHlEiLrE1MzaqqeYPcGPHYR0vRqKo4f/GoxhxHPHrB2pgiW3/uMUba64U6Z3duT8+R29n
aqkk6GW5KR37gdnZO/JRbfoZeQQ8BgyOC+
+nI4hTOmkFwKOqumZL+NcNHfF75DZlQPvNJxwzn2EWPdckmPP4PpP/T6Cpfben32v1O6o6SUAQ
Jb+8eU/lL+/7Xt6Kdr1P/E8uet3qF8NALkpvFgP48c8I0RdRu0Ne6itmkU92FPMCHiMXnIlPxkzZmJvy
4Y3Dft884MlY2bySXgTWHWk2dQnKrWh/EXZdLsdqvO87l+XXmPdq22ZZBKkcVI7L578j8Fj1udsrvr
DyPW9bj9yHVtu1oBtyq7eda9xzgsxY/VgjnPPTXjsU1//hO8jZntyvRSYe6o3quqVCgDI3t/c/m157Pqn
9VI4Vf+0+nuvleddc6VeAwDIlqqD+h7r49XOIuYWIeoyqmz7Q287NSm5QYtRH7F6NnzmbhXSDL52
Lpkz9nttNibamUhF3I6BrlSH8o+4LiU/fZQL005glcl5MSQ9BnPYu9ueiRK2YH3O5qqz34IX9Felldqs+v
EUNy94PTLd9njnodqSeM2Jc88lOPY0r3/C9xEgiqqPquqkAgCydeI4Xz7/hkcj66NW/u8idVABIE/H3k
od1AVBiLqk7KG3YUUMVeDS7wcDHlX3cLBmYrWVcx3NpCqy3W/5gZHNOTbvsAPDrOPxhlcr5bpsp
ZzL2dcl5LxWW9Z6d+j1VLI5L6Zkx2C1Z/A+3D80ejUmYNfnNIaSu+y6nznWQ/UUZXNvuFaoPYw+Q
aAb656LfezpXv9E7yNABDVTf/tX28zYDwA5UPVR7/uZx/VS0Cte9xL5hV8/oZcAAJk7Zv2GfPwdegHz
rtBXRewAxKNmNtcT86gwbG/anqHAOEfpntPHcqpFwIpwn+l9Rip/xM0BAHl441/fIKWHXqiXRK596
VXyW3/8z+yPAIAcFK4ROf5+66P/Xoz5Rk9UILaeNNbdmc3LcmaNABVZO1r3XPu8dSwZ9ODG0fGO
4juYsR8AcrL3um/J9655Ri+JVH/3tQSoAJCn4+8mQF0w9EQFxmrLxmANWFXOgK50yMzRu+d6jRU
p7ZyRbiplKXDUFf6fgn4GAMjStU9eKe9s/aS88723MYwfAPJ07O0M419AhKjAWAOBFgEqMsc9h+
WlJiiblx9NaEs42hKOtoSjLeHmqS0nrr1GDr/3pF6aLa5RONoSjraEoy3haAvSQIgKAADmBj/ghqMt4
WhLONoSjraEoy3haEs42hKOtoSjLeHmqS1IhpqoAAAAAAAABCBEBUAAAAAAAIhCiAgAAAAA
AAEAEQlTkSE2WU7DrfxRWGtLTa8fqNWRlo60XHO0NvZ3CijRibwjp6Uljxb0GG9aVTbJuGmH3UNr
7mJbRRv1Y4SYFAAAAAGChEaJirvUaK1Io1aSjl4H5pgJdY1Z9W1nOrBX1cwAAAAAAsIgIUTG/eg1Zrx
GfzqeibO717RkF+/1tqei1szFPbenKgXvLluvStdu0J5tkqAAAAAAALDRCVABIS+9Q9vVTOXVCyE4BA
AAAADgaCFGXmao1OlC7MazG6GD9UXuIvfE1A+VKNbNOpXokq1Vp79Mcxt9cdbYTvjOjjeoxqk7qY
JvCa1VOdrymMbVf2xvetob2H3JN4rTRY359ZEONcxFo46j1xj7t9ea5zKoOadx7KKotg9uYsD5pjOuS
9J4FAAAAAACLgxB1WakgL7TWaEdqpVEhpPpcQUoDQ+ybq4OvVyFiSYIva8pqYbBWZBqcNq0GNh
xyDHYINtgm65W1UnjQaYt7vIMqcrqqn3Z25OLAa9u7bmODtTKHQjgtuo2TKsrambLztHMgXeeZxR
iOHmh7W9xml8+s5dDDMoV7aKJrPizf6wIAAAAAAOYRIepS6knjnJuIuXUb+9Kt61BNOrIzmPx5qtL
Sr2+5QaEKG8/7ve16jXN+0FVt6VqVXfE2H0Nl2/qabl28L3G3sx1W8dJvU/gxWMe77oZg/mv77gF0
arI+MhUdf7xhKltu2wfPpR9GSvmMeBlqe8MPgsOuSWQbJ1NcO6PbuC+H7qbbu0ZI2ZEDN101hq
mfOpF9hDr9PTTNNTckuC7J7lkAAAAAALBICFGXkjERz96m16vQD9VEOl56FlRt+RP3+EGhqScXd9w
+e1VpeQGStc8LYa+fntmm0GPoXRS3SeX6lj/xUGXLC+U6OxdDexSOP94RimvidfQ0t22ElGaPTrN3av
2CcU02L/htrJ0fGKo+Ja+NftDbO3Si0mrVCRv3dbrau7jjBZKnvROYlRTuoSmuuWkm1wUAAAAAAM
wdQlRYdA3P0OH9SZmzk5+Ukn5qK56QU/ppespyMrCTEN0D77jsIdheXUtjqHdgSHsazOHy/rB4P5
SryllvyvYRvVNtxnbMHqOpKMoJfUHcwLlrXzzrnJ4+aYeVwfWW6mk/kMxMCvdQKtd8VtcFAAAAAA
DMG0LUZRWYKCdunckYgeWRMt3x+r1i3Z6eRig3URhpDK9PScUt3trctVrXE6cj6ik5UdFhZWC9anb
2EeriSf+6AAAAAACA+UKIupTasmH2OnVrPZr1HI+ocr3rlDEYevjD9lNT3JSzbglONXTcqCs6WRiZQYh

dOS164L4ctvUQeLv3pzs5lrF+QUP07K/5sv1xAQAAAACA5UOIuozMupwqYHLrohpDoCdXkpNuEjs
4XNoIEXNVcoamK6NqvWbF6+nZ2ZHz50fVFTXP2eBs/mZ90FMydk6nxNfQ3XdHDnadr3VrtZbsT/jr
h4e0ZyWFeyiVa57idQEAAAAAAAuNEHXJ+QGTMWP/VMxakU1Z3XCn3DFnS8+ZMcmTNFfFa1J7w
6+V6a1MmdfTsyPNpj76oaH8Rdl0u6xar6utN6yz5eg11r0anuYESU7AqVivP+8dkGx4U8nH5V+vZtP
5Wnf2/aIumOquNyfCylYK91Aq1zz5dQEAAAAAAEcTIeoy8oI9S3NVh0rGhDtTMmcun2r75iRC7nY
mCjuDs7o3V3WI5gWO5gzwaXOHxftCh/JXtqXl5XU1KdnnrCAlP6mTC95EVCojdOutWrxzHLe2bVBg
W9Yzb2i6eZ9Y68/k0w3VNv09lNI1T3hdAAAAAADA0USIupQqst1vGQGZUpVWv+8HRvaEQpMqyu
Ze1w/BbGr7g/scx2qn1yBt/9DrDZhIcVP2+oNtstj1YDOoh2rwhvQr1v62Ruysst0PrUtbbVnr3ZILLnU
8g6+ttiara2v22gwMTTeGs+c+ZD2Feyila57ougAAAAAAgCOp0FczrADITq8hK3oiL1WDdo+eiwAwk
urtPS8/mtCWcLQlHG0JR1vC0ZZwtCUcbQlHW8LRlnC0BWmgJyqQKbOOZ75D4gEAAAAAAJAOQlQ
gE23ZKAzU8ayeFTqhAgAAAAAALB5CVCAPql5pZpNXAQAAAAAAIEvURAUAAHODelXhaEs42hKOto
SjLeFoSzjaEo62hKMt4WhLuHlqC5KhJyoAAAAAAAAARCBEBQAAAAAAAIAIDOcHAAAAAAAAAgAj0R
EV2eg1Z2WjrhVnrSWNFzZavHhsyL60CAAAAAADA/CNERSZ6jRUplGrS0csAAAAAAADAoiJERfp6D
VmvEZ8CAAAAAADgaCBEBQAAAAAAAIAIhKhLqr3h1gddkUZPD7+3l51HaClTVePUeI3zcL7eZW/XH
MbfXHVepzc4uF+PuW1j58HXt2XDfY318F4Wo12TGjwv6jHpuVGSHr8S1obCSkPCD8+s/eo8VkaciG
TbBQAAAAAAWF6EqEuvI7VSQUoDw+
+bqwMhX3tjRI1T9fXpBJbR1H5WpamXpFyXrYr1MbN2OWHk4HlRmquFYNCZ4blRoWtYG6RTk9Lg
BFl2EFuSwZd3aqWhcDTRdgEAAAAAAJYcISosVWn1+9K3Hq2qXqUCwPNulNaTxjkdX5br0tWv7dbL
zjrrtTsXnYiusm19rlsX9zNSbdmv7W+rxHNafjv7e5tSTNCupHqNdS+MLNe7zj77LasFWvOcDkeza4NI
W3bd1Dh020055yW0VjvW3SDXOE/uBe3UZN17bZLtAgAAAAAAgBAVUm1tixtxVraMANRTlM09H
crZ4aVeu3bGe23noKufZadc3/La6ciqXT25uOP20qzK2U13yxXZsoNGFVLuibM6w3PTO5R9/VQ6O+
JmscXNPWd/1mPPbVvvorhNDpynypa42Whn56J1ZJYk2wUAAAAAAAAAhKiaha5OGDmHPzqkT44K
9tNrVlQN3A+WTUtJPFSdo9EPnYSmem+KanHGTWGtrquyCW7t0qM5p98Dbnz18X7+uYA7v7xxYR
2ZJsl0AAAAAAAAQoqIsJ82UcJTAxElGbdLcjGjnzNtlyawNRdm8ENYz2A9KQye5Giur7QIAAAAAABx
NhKiIoS0bZs9Kt46mWft0JuahXRm3obgpe3qIvV+v1tc8Nzybvl/DdfBh9KCdYLsAgLzp0Q3Ggz9yAQ
AAALNBiIrx2rte70o7oHNrfxpDyKc2ybYya1dJTroJqDsE3qVm4i8YM+6n1YaI16uZ9NUvzfakXXbw2f
XqnHrtK530Qtu4NVhjbRcAlpH9Xl+QwkwTy540VlZFWu57tPNHr+bqLILU+Qxze40Vpz0r+f/hT/0ba
p4P9zEP5wXDhq7XDO6ZmRvzvuZ9P7mPvG7mOO+3ebwnR+5jSf+gFfO8z/K9eGZinJuh951luGnG
nJfc34sTtofSdoiDEBWJ+AGdMSt9AiU/nTRm/7d+MFmdbhD8tO0KKsqaVzS0Kavem667baeO6OB7
cZw2JDp+/aavPhXsGRpSs9Wsc9pc9dvm/sOhHu7KJNtVjG3wDwuALMzsl/chKri09j/lv0npcCYu3Da
KcLuTP+4f5vte3N4IC3ONPyjOQq8h6zWRatX9x28G3NEnxsO8XrMy01/cAyWOhh+z+NZW52O1qS
YFda9TS6qdmpRmFPjk/0v7+Pc19R5csr6f6l3jHFk/T2Z778R5v83jPXncPtTnc/yDVmTok1eYm+C85/l
eHHFuht739CP98xP/vl3dH/g3Iot/IEadk9zfi8efl3zfiydoj/WNrUrbpf+eHO/7dn5+FsZY1g2DJWS9R/
TV5bd+A+hbPzD5uvW+9U+g87mq9ZZia/WtnxX060c8vNcqIa93P29uf9TD2NbIdtqStKvbr5fd9VXr
K8cxXx/yKNetVygJz83Exx/
+MDcdve3gMSfabqvqrS8PXwQAmEq3XrbeX8z3eP2+GngjyoffFv1vwAzaEEm/z8/8vXjm7XCuj9q/f
c28f5PzY/87OoP9RtP37dy1y/3eivPzV9rCv5dn1R7n5y9jv/pnrCy/l8a/rznvuYNtyPocjW9XvNdMa6J
9ZPIeqPdvbdd+hLRD3T/maud+Um3XK1IS/5w4n8/+vTjeucnjvS/
+fZv1+8v4cxImq7aNPy/h62fWnhHfw+nfR87+zd0737fBdX579Qr9vhz3uiJf9ERFDBXZVn8p0ksO56
821puAo7kr/t9KrNd7n9D2D52/MKlanIP1QqutCWuIJm1XEk4PIOsNTS/7rDdbf9h+0jYkPH57qP3g
ubQ5+wj8QdOuc2oMyXfZPWWMeqiWRNsFgMy05XytY71NXZBN503VUpEt9UY28fv35Iqbe9Lv7x
ltmTO69MupE/PRwFm1o9dYl1qnKmfn9kLNhnteWt7PKPPC+T6X6unAzyL5KMqJU9YH9+dQW08u
7sygPb2GqIFK5fqWv9/Ktv3zYmfnYgY9sRxj39d6h7JvfRj8fi7aJ64puxm9Ecd5v83jPXle3ved79+y1Ls
hP8tr6uf34dEJHdm5mO7dE/ec5PVeHOfc5GX8uXHeXwLf5xmY7Jxk9148/rzk+148tj0jfp6yR42mW

tYuzqii+fpZGOMRoi4pvw7mwJuLMeFQcMiBCgvdr1EPJ5TztxMM6dQPhf5rrYf5A725D/VQ+xmx35
Ht9MRtl/MGFlw3nvMG7H6d89gbakjCc5Pg+G2D59J+jDoG8zj1Y9QvU3G3a7xu+NgBYAoz+uV9M
enSL+W6bIX/A5CTnjTWa9KZVTvsoaMdqbbi/1ueGTUM0Rh6N9uSN/n84j6JXuOc9d1s/bI/oxu3sq2
HjNo17dWwypITNg/
+vJW13H5pT8huV1lOenWcNKPePgZk8AetuMFlmDTbEVuO78XTnJv86fJoB+cHhtOnWwJnknPCe7
FhxPub8/PnvuRaNYmfhRcOISoAAJgNfnmPTdUltX/5uTCLXoa6tpj9i6D+pWdGvR3b52vSqbZmPmL
C/0Op81AjV7KppRZXPr+4J+f2NDo7wwDEHTWkatqr7yOrObMI4efpl3ZMgT9oKfPyXhwwR3/Y6uy
flAvevxGqt6h6/9mYYa9C3osD9HwiwflBrO+pqedWiWHwjzD8LLxwCFEBAADmmDP5gRq2N6ueO
MFRDt36vqzO4hfU9oYzCcRc/dbuUL2Csh6WHcfc/eLePi/O6NHZXTNnsg5/YiAVeKtJgXKftGOWv7Q
jNfxByzKH78Xz9oet8pk147pY/4aeVWXUZtirkPfiAernGrdnrP89dXBKXadTkl3n7nn5IwymQYgKAA
Awp5wAVfXWmJ+hjMXNC3YNtk7tfK7hXHtXhU1NO8B1fuEpSEn9Vqh/CZrtcHptlsOyLfP1i7sOCGf5
y6Iecqzq2bt5jz0MViXezdWMZjYfZVa/tI9h93bqyMHgjTuqd9QS4w9ajkV4L56XP2x5ZtqrkPficIMl+f
qydXLfOk8nJau3vdn+EQZpIUQFAACzwS/vkVSvDSdAndMJ/zL8RSPMYE8j9bAngLR+Mexaz2dbt7sn
h6qoWc7nZKxZ/uLu9nw6O8NfFgeHTbpmdl7y/6V9rOIJceZ7CUZNPfuGnmG4O2f4g5Zvvt+LB+T+h
62S2GWOB8PbWf5cw3txTLq2eOCPkekZ+UcYfhZeOISoAABgNvjlPYKeRddiD3czevzkW+dSTf4wM
Bxc/0KW1S8ac6/XkJUVc0i2WqVmSp7lL6nz94u701utKjMcPSpSOW21wPoeCgyhV7dwzTovM26bL
dtf2uOpyGnVY6+27r+veL3G5m+islngD1qLaFZ/2NIjADo1Oe/9w6mHcM+oHinvxXGoUhklqUldLm
RwkSL/CMPPwounDwAAMCOtqvSt33L69a5e0a33y9aPJ2VvRX669bLVFtWewUe139KvWUZh56U
6JyfEblu53s/3bun26+Xg+ZiLe6RVHbg2rb71S2tfJT+5m+H38TB9HgKPefie1vdRxvdv3Pc1573Yf2R9
7eK0K4/35PH7CLt/3Ifxb1dq9H0x9H2r2jFw3Pp7Pu1rNcl5t78m8/fiEedGvd8M7Ns9hrTf/uKem6H
XZfY+POp+0XJ6L453XvJ7Lx7fnpB/xzO6RnHuxXn6WRjjFdT/rIsKAAAwE+5f6F3WD43zNRwQWBCq
x5xdm9BVbUl/Bl3nnO/pqrT6M5p5eS7pnk7G5ZnV9cH8Gfre9fjfR2Gvqc5r79gUjT83Id9bxnk7iuLc
LwrvxbOmRvOoOqhhgkP7+Vl4cRCiAgAAAAAAAEAEaqICAAAAAAAAQARCVAAAAAAAAACIQIgKA
AAAAAAAABEIUQEAAAAAAAAgAiEqAAAAAAAAAEQgRAUAAAAAAAACACISoAAAAAAAAABCBEBU
AAAAAAAAAIhCiAgAAAAAAAEAEQlQAAAAAAAAAiECICgAAAAAAAAARCFEBAAAAAAAAIAIhKgAA
AAAAAABEIEQFAAAAAAAAgAiEqAAAAAAAAAAQgRAVAAAAAAAAACIQogIAAAAAAAABABEJUAAA
AAAAAAIhAiAoAAAAAAAAAEQhRAQAAAAAAACACISoAAAAAAAAARCBEBQAAAAAAAIAIhKgAAA
AAAAAAEIEQFQAAAAAAAAiEKICAAAAAAAAQARCVAAAAAAAAACIQIgKAAAAAAAAABEIUQEAA
AAAAAAgAiEqAAAAAAAAAEQgRAUAAAAAAACACISoAAAAAAAAABCBEBUAAADAQmtvFKRQ0I+N
tl5raG94n19p9PTK2fDbuiEhLV0wbdlwz3vk+e1JY2Xca6xXNVb8ba00rK8CAGB+EKICAAAAODqa52
TGOemSUMHoqjT1kqMsZ9aK+rmpKJsX6tZnHZ3aesg1asv5Wkc/F6me3bS+CgCA+UGICgAAAOAI6
Ujt/OL38Zx/XTlwM89yXbr9vvT7e7I5KvksbsrZqn4eco16jXN+IFttyXZFPwcAYE4QogIAAAA4WuiNm
r3eoezrp3LqRKxeo5Xtlng5auAamb1Qq9IiQQUAzCFCVAAAAAABHTLzeqH590pVg6NpryIpbm9Orse
rX/7Rrehp1Vr119suC68NKtPqCtUJH1wEdeJ31CKsrGjyeYL3S6HYYzGPXj8F92fsp1ayzrDVXndeO3Ul
FtltujOpfo/aGXxagXN+yXjUo3vHbQto/dH0tcc5VoEar+6BWKwAsLUJUAAAAAEdEWep1HdJl2Bu1U
ytJYTVYDdRep0K2gfXN1VEBZlNWCyUxyoCqjUhpcMIpOxQceJ3F3t/IQK8jtZJRr7Rcl60YnTuHwlEtel
8JVbak7hZHba7KRqMh5/wEVS4M1gNIcvwqwA5pv3M+hoNUR/i5UueiNLhTJewaAQCWAiEqAAA
AgKNjzQ3p4vVGnVS53pW+qgPq9ax0hK1v7o5oh1dLtC/
+y5tyzkv7etJYd0PBqrT0a71td2qyPjIpNl6/F2OSpvaGePmv0a6um3ga+6psW5/r+hNFqRqm9n5iDc
MPTjLVrLnHV5b6hcF2Jjl+67VuGhvWfmsrOxfjnqu27Eadi8A1AgAsC0JUAAAAAEdIUTbdGYwy641al
bNuj8nKaWvJNWL9/mFoL05zBnqzXmhn56Lz+t5F2dGdIQPD3I3enN5rB4QPix+t7aeGgTCzuHnB31
ftfDo9MAOTTGnVs8OTUiU6fuu675lBqKO4dsYLbDsHXf0saOhcmfVeOzviZq/FzT1n+9Zjb+QMWgC
Ao4oQFQAAAMDR4oVsGfVGLZ+Ukn4aMGp9qKqcDiR3JTnpp31ix33dA90L01rllguwH8bwdve1A0
6dSBLymT0vz8ha4EuLsnbGbdi+HIYlthMITDJlPQudTGqK41fHZNc5DR3eHzR0ropr4h2yuodK7n4ja

rECAI48QlQAAAAAR8xAb9RD5+l8K8qJU/rp1MpyMn6am0BHRnTmnE6i8DlCYFIpo85ppLBzFSw5Y
HLD3NgTdQEAjgxCVAAAAABHj9kbtRYvTsvXYK/Onhx6Y8iHebVWhx7biYbtTyercHa88cfflg2z16lby9
Ss3ZpEcVP29D4Gyt7amueYpR8AIg0hKgAAAIAjyOiNmpQxjDw7g706u3Lg7tTtmVk66QWAo+p5ps
MsJeDXAHX05KJbmFROSaIqAdNKcvztXa/nqR24unVRp7iWaoZ+1ePUnkjLDlS7Xi3W0WUEAABHF
SEqAAAAgKPJmIAoTMlPDo3aqW3Z8Kapz1ZzdcObqKm94Q8/L59ZcwJAszZnc9UfQt7e8Gp0FlIZV2
4Gzta5WPd7WfYa61790aSTVU1twuP3A1djxv4k9PbVbRDscRoSdAMAlgYhKgAAAIAjKro3qjlzuwrp
nGAubi3NaZWlXG7Kqg4D/dzWmOFftd+ozdlc1cGh9+IREzJNorLtD1vv1KSk21XyE1S5kPuM9AmOv
3Lan6jKu5bGBFRJjDgX5r1RPat7ugIAlgYhKgAAAICjK6o3qqp7OVgzs9qavI5mIqfk7J4xPFyx63gO1Di
1a3MOvE4Je+2U7GHrIcdebVnr3eHxeYt9/BXZ7psz/itVaZk1TZu7Xs/fcexzEVYMVW8zrewaALA4Cn
1V3AUAAAAAAAAAEIqeqAAAAAAAAAAQgRAVAAAAAAAAAACIQogIAAAAAAABABEJUAAAAAAAA
AIhAiAoAAAAAAAAAEQhRAQAAAAAAACACISoAAAAAAAAARCBEBQAAAAAAAIAIhKgAAAAAAAA
AEIEQFQAAAAAAAABGEvn/AZM6nr53azj8AAAAAElFTkSuQmCC]]></redcap:Attachment>
</ItemDef>
<ItemDef OID="q21" Name="q21" DataType="text" Length="1" redcap:Variable="q21" redcap:FieldType="radio" redcap:RequiredField="y" redcap:BranchingLogic="[q20] = '2'">
<Question><TranslatedText>Do you prefer Life A or Life B, or do you think Life A and Life B are the same as one another?
</TranslatedText><redcap:FormattedTranslatedText>Do you prefer Life A or Life B, or do you think Life A and Life B are the same as one another?
 </redcap:FormattedTranslatedText></Question>
<CodeListRef CodeListOID="q21.choices"/>
</ItemDef>
<ItemDef OID="summary22" Name="summary22" DataType="text" Length="999" redcap:Variable="summary22" redcap:FieldType="descriptive" redcap:BranchingLogic="[q20] = '1'">
<Question><TranslatedText>
Alzheimer's disease - Description #[no]

If you have Alzheimer's disease…

1) You might forget recent events of importance to you. For example, if you are interested in hockey, then you might forget whether your favorite team won their last game, although you may remember watching the game itself.

2) You might forget the names of people you met recently, or the names of local streets.

3) You may no longer be able to handle your personal finances or plan leisure activities such as dinner parties or vacations. Simple decisions such as what to order in restaurants might become difficult to make. Your ability to concentrate may decrease and you might have difficulty driving.

4) You may start having trouble finding words to express your thoughts. You might need to be reminded to shower or brush your teeth, but you could do these activities on your own. You may need to post notes around the home to remind you of simple things like turning off the stove.

5) You may remain aware of the day and your surroundings. You might be able to travel to familiar locations like the supermarket, although probably with the help of a loved one or friend. If you are in a social gathering, then you might have difficulty following the conversation.

6) You may continue to enjoy hobbies such as puzzles or reading.

Now consider two possible lives. In Life A, you will live in perfect health for 10.5 years and then you will die of natural causes. In Life B, you will live in perfect health for the next 10 years, then you will live with Alzheimer's disease as described in the summary for another 10 years, and then you will die of natural causes.

</TranslatedText><redcap:FormattedTranslatedText>
Alzheimer's disease - Description #[no]

<p></p>

If you have Alzheimer's disease...
<p></p>
1) You might forget recent events of importance to you. For example, if you are interested in hockey, then you might forget whether your favorite team won their last game, although you may remember watching the game itself.
<p></p>
2) You might forget the names of people you met recently, or the names of local streets.
<p></p>
3) You may no longer be able to handle your personal finances or plan leisure activities such as dinner parties or vacations. Simple decisions such as what to order in restaurants might become difficult to make. Your ability to concentrate may decrease and you might have difficulty driving.
<p></p>
4) You may start having trouble finding words to express your thoughts. You might need to be reminded to shower or brush your teeth, but you could do these activities on your own. You may need to post notes around the home to remind you of simple things like turning off the stove.
<p></p>

5) You may remain aware of the day and your surroundings. You might be able to travel to familiar locations like the supermarket, although probably with the help of a loved one or friend. If you are in a social gathering, then you might have difficulty following the conversation.
<p></p>
6) You may continue to enjoy hobbies such as puzzles or reading.
<p></p>
<p></p>
<p></p>
<p></p>
Now consider two possible lives. In Life A, you will live in perfect health for 10.5 years and then you will die of natural causes. In Life B, you will live in perfect health for the next 10 years, then you will live with Alzheimer's disease as described in the summary for another 10 years, and then you will die of natural causes.
<p></p>
</redcap:FormattedTranslatedText></Question>
</ItemDef>
<ItemDef OID="a22" Name="a22" DataType="text" Length="999" redcap:Variable="a22" redcap:FieldType="descriptive" redcap:BranchingLogic="[q20] = '1'" redcap:InlineImage="1">
<Question><TranslatedText></TranslatedText></Question>
<redcap:Attachment DocName="105.png" MimeType="image/png"><![CDATA[iVBORw0KGgoAAAANSUhEUgAABVEAAADYCAIAAABtBTQzAAAAAXNSR0IArs4c6QAAAAR
nQU1BAACxjwv8YQUAAAAJcEhZcwAAEnQAABJ0Ad5mH3gAACpmSURBVHhe7d1fqB1HnthxJROC
hgRWLEvwbh5W2RAwGrHI6xflsmAFgm2WmcFgTISZwIYNl2tYs8IQyyxemAcJm1mD7gwCe2GQ/T
CJ/CBjhnVkIw/oYQzSMuD7YFgbZHEhMhZID9diMFdID5P+U1Vd9as/3ae6+/zp8/3wY/ee7qr6VdU5
atdP9rlz4HcAAAAAAGCKqPkBAHNydffqgR8fIAiC6BnfPf2d5//08F8+
+idf3/xSPV8AABHU/ACAedjd2z30+iFxxcCIgsIvv/jzP/qr//Qfi3j1qScf3L+vnjIAgBBqfgDA6Pb29x49/6
g4tRMEQWTENz37+3XBX8f5Z7F7bUgwYAEELNDwAY3dO/eFqc2gmCIDLiD1/4rl3w13Hl7QvqWQMA
8FDzAwDGdeqjU+LUThEi5gcAjOjiZxfFqZ0gCCIvzNf4/Xhp4/g
3d+6o5w4AwELNDwAYy7Vb1w6eOShO7QRBEPkhvsbvx09+9Lx69AAALNT8AIBR7O7tPvLG3/H78Gn+7tBEEQGRH7Gn8wPv34inoMAQAq1PwAgOGdv
HRSnNoJgiDyIvE1fj9efPyxu1/dUk8iAAA1PwBgcK/9+jVxxZCcIgsiL1q/x+/HjH37/wf376nkEAGuPmh
8AMKT3P39fHNkJgiDyouPX+P24cPPl9UgCgLVHHzQ8AGMzO7R1+UT9BEIPETF/j9+Oy6pBxMArD
dqfgDAMPb29w5vHxandoIgiLy6Wv8fmwdPXJzZ0c9ngBgjVHzAwAGsP9w/8Q7J8SRnSAIISSbbv
x+kTT3x77556SAHAuqLmBwAMYOuDLXFkJwiCyIvsr/H7cf6FLfWQAoB1B1Rc0PAOjr3LVz4shOEATRkRWz
2/xu/HL3/2U/WoAoC1RM2/EDe2Nw7UNGNv5XXh1gfGBhBQOBSbOmY9BTBEkK5ym9lumIlXUbOmX
MXm9eppPlp7MJGuDFb1q3OTSF1Xz+3Oj5l9Ru3u7h14/JI7sBEEQeTHI1/j9eGnj+N2vbqnnFACsH2p+
Wr+hQkW903xt8wlqizhAhVdSMdma2icarfbfi+m5g/NjzHHoIgiLwAoIlrqPX276eFf0cOWV8Rw6ZcxqV7hek83CS2s5LYOknOzE9lipad
u9Up3Sk3PGkT/GNSM2r5Yp4XVNZAgskJ7Q6xefra2yYipOE3MvXISsbl1ngmWxamPTokjO0EQRHa
M8TV+P/7pg/IvBVAmeWg7/tFohlEXalei+HSL2OSvXR+ew1XJ3TkdNVNqNH3S+dZpsW/eJuq+nfmldLCsAOkRWg7/tF1ohlEXalei+HSL2OSvXR+ew
jdQk/J9LBZiw6RcwtvZik1UKdebdMLDLKxve1fFNtjc+YYbCG202LvrGWQpUVGMS2cdyE2t04zwdK

4+NlFcV4nCILIjpG+xu/H1tEjX9/8Uj3IAGA9UPMvmqymvPLGVEL6juIhmuomVl+nyApRDXQXq95SP
fQI1UsxXPplTLqXnkEzhm6g5uhlCSzbJ3v5C22uNKml+PbMMD1/EMXr2mU000JfMcPrC4FhAxJLiw
4kJ6MbeB2iI3gXCh1mgmVx7da1g2cOiiM7QRBEXoz6NX4/Xn3qSb7YD2CtUPMvnlXqOCVQTZZXJV
kwBdqEaiqXalH3qV9sbpYD2Vd0fzFc+mVMSy/9Wg+iX6tViZelwCWPzGJ2u5mrbBIQ6NV0q9J3mJ4
ZRM7Xn0D7aH6f+lJogvF1Fdo3RGQuBS45ZGpvKt6FQt5bg/nb3dt95I1HxJGdIAgiL/7t//pX//17/0G
U5WPHP5z6G/VEA4A1QM2/DEIVpaJvucWVKLkCjToUS6pX1aD6eWP7ctWruWK6i+HSL2PaeukL6r
WagV6UXmRIIq/MYoZp+nSYvu7lvA1Ovw7TC6Su+RNoHy04I1eHdRUCA4mOHZZmcVur+95UQnN
rnwmWwP7D/WNvHRNHdoIgiLz4zt/9ix/8538vCvL5xJW3L6jnGgBMHTX/MkjUb+FbkaJsxmJJdSta
1K2LH+pL5orpLYZLv4xp7aWvVBea2dX0IkMSeWWWvL3SvZwWTr8O0wukrvkTaB8tOlijw7oKgYFE
xw5La7oI6r43ldDc2meCJXDy0klxZCcIgsiO4z/4A1GKzy22jh65ubOjHm0AMGnU/MsgUb+Fb+lSSF3
NLZbqfvpf71e9q0v6SjOgGC79Mqa9l75UXFFrau6Fd6KVzJK3V8HkTr8O04s28SfQPlqHfB3WVQgMJ
Dp2n0yhbiRTe1MJza19JIi01379mjivEwRBZMcf/89/I+rwOcfpE098c+eOesABwHRR8y+DRFFlih77n
qyEAv1NFZYqltQ4/tf46yvWeCJj+mVMl171vM2vs7dudUwiyW55hWVoO91uMwxiv5UVv2/7aKE
WZYLZ1lVo35DWcfwh5H55Q8SmX5p5CZiP9z9/X5zXCYIgsmMhX+P34yc/eI494wBguqj5l0Gg2Gm
Y8knfNXWQaW6u6NLI9GkplpqOoa7x2iv9MqZTLyu/uGPa68umaSqtzKI7zVZYBnZFZm+fXiB1LTCB
9tG8FvELzbABgVnJjm2TkXNr2ntX1Ovg3DrMBAuzc3uHX9RPEMRQscCv8fvxy5/9VD3pAGCiqPmX
QaDYsZmSymE3toosKV0sNR3NcCZbMEE9XPplTLdezWq97TA9HDMlzSwsw29BKTSOoxk0kLoWnE
D7YsMtrOHdWUdW12lDWiYT3x7VxB8xMLdOM8Ei7O3vHd4+LI7sBEEQ2bHAr/EH49OPr6jnHQB
METX/MggUO5JbdAUqILtBcbtrseS3C/YUF9MvYzr2Su+G6VVJ7Jgis+QVlnYv/XPB75CaXnRh0Qm0L
9Zt4TWx5hrsXui8IenJ2JnKbvp1PURoRG9unWeCudp/uH/inRPivE4QBJEdC/8avx8vPv7Y3a9uqace
AEwONT/QLlCOAuth64MtcV4nCILIjiX5Gr8fZ5979sH9+
+rBBwDTQs0PtKPmx3o6d+2cOK8TBEFkx1J9jd+PC6dfVs8+AJgWan6gHTU/1tDV3avivE4QBNEnlu1
r/H588t4l9QQEgAmh5gfaUfNj3ezu7R56/ZA4rxMEQWTHEn6N34+to0f+3z//s3oOAsBUUPMDABx
7+3uPnn9UnNcJgiCyY2m/xu/H6RNPfHvvnnoaAsAkUPMDABxP/
+JpcV4nCILIjiX/Gr8f51/YUk9DAJgEan4AQOPUR6fEeZ0gCKJPLP/X+P34v2+9qZ6JALD6qPkBAMrFzy
6KwzpBEESfWImv8Qfji+vX1ZMRAFYcNf9i6d8NVxnlF8Td2N7YvKx+nsbvonNXlFA0jK3V3vdOe+G8
UVq3aawxfvXhirl269rBMwfFeZ0gCCI7Vuhr/H68tHH8mzt31PMRAFYZNf8CqZpUG74wUgkmVPP7
K4oyuyvXKra90jZgsOSn5m9Dzb9Kdvd2H3njEXFeJwiCyI6V+xq/H2efe/bB/fvqKQkAK4uaf4FGrohM
bTuZmj+0ojC7rhdr1VtQj9FtSN2K2nU21PwrY//h/rG3jonzOkEQRJ9Yxa/x+/Hu2TPqQQkAK4uaf3G
6V7B5QuOvQ81vWinOWv3q3f07gLAubeCj5l8ZJy+dFId1giCIPrG6X+P349OPr6hnJQCsJmr+xdDFk
MWuJ92yVVRMViHVDCOq0dj4dhFmtfFrMmcGrSWbPazd06uR48M2d0wnM0ExW80bveDuXMVJ
o4dp+uoeiUXqJqGEPnszLIHL8d2oycUEV1JcbPamnmGyX0TXXE5Db0PEzchWYMm89uvXxGGdIAii
T6z01/j92Dp65OubX6onJgCsoNlqfnOijxc/wSZNTVLoe/5vyor4LJaesyM1vZrArYK1a4EG3p7Gxg8P
XrKGsAs3I/m2DTKsuV9fEy9jKxJ0r+Ke7mHP3Aza9A1cEkwLS7RxIZTYuxga1OkR3tOmReB+dTPcLzn
hnFxK+4xLdissmfc/f18c1gmCIPrEBL7G78erTz3JF/sBrK451PyiuOl7/LeHS9YxSy+4m6Zu0vtkWpmN
s0qr5AaExvf7euN73XSfxDvnD9tckXkSw9ptQllDKxLKJmIKbSPodtFBm7U44vuhs1gtxGS8ecjJyiFMB
zOmNSt75rGBmo6enFzNFZ3c9NJXmguJ3Fisnds7/KJ+giCGjWl8jd+Pfzj1N+rRCQCrZvCa3yeLkH6sQ
qKw0rVEaDdDmyXbmXKrZfWp8a1ropl+aQ0euCQMNqwZSHFTijFbdNnLUqidzetjJhnr4S9N5AgsPX
DJ0XEWpll0oA7iuZptk228PgVzrcdcMJ69/b3D24fFYZ0gCKJPTOlr/H5cefuCeoACwEoZv+bP6ROnR9
vYUP9/hYuJwM6Ey0+z6PpqoAALCu18IIFoZkYPiCZsrwk7D2s3lO9uaEVxgbUm96TToLXQ0A6dR7xl
OoW9SMmfhtvaG8PtYFbYiM4yoHMuuZO6iZMseBFLYf/h/ol3TojDOkEQRJ+Y2Nf4/dg6euTmzo56j
ALA6hj3v+13C4hKoGgodSwKdJ/Ny/qnFa4mArsZrpFEw66FVGD8UN/I6CHWQK4hh21aegsMrSgu
MKnQCLMNWmvvo1tUyfVMTOsuu2FyCPp+aHmVSMfU8jJyyS0ITic6Ryzc1gdb4rBOEATRM/7iz/9I
FMnTi1efelI9RgFgdSyo5g/WGO2Fge5WDqJ/Xt1yIrCb4RpJLLVrIRUYP9RXNMsq04Yb1nSriNahFcU

FU+qLzQh60FkW3KWPylQ00a2bnO27oVsU6kbe2lvHsIaodGnYPZdsE5xO6xyxGOeunRMndYIgiP7
xhy98V1TI04tP3ruknqQAsDrG/x1+gT5dSooQ3a/upl+tbD2R2hl7UbJd10IqMH6H4i3Uq9VQw+pW
G9vboUXONrfgPvlD6GaxMUNJ2/pUVKONzc16AKtx60L8uesrpovfxChuzZAsL5ccNZTFXAvMEYvE/x
o/QRAjxZ89+/uiSJ5SXDj9snqMAsBKWUTNr68ECohkbSAbdeq0xPydKZhyS66yWWagAAsKjT9L8aY
veBWgb5hhnS7mRWLMtPA+iTECQ8p+polu02FDKqZdxWncNqZM0bQ348hp1kzH5nK4YSMvl7dx
3pKscWKpsSD7D/eP//y4OKkTBEEMElP9L/x//MPv8z/XB2BFLaLmNzVGQHxgM1BTP/hXVkpkN8Pb
Y62xrYYz3JGqLF2Kt+aKI/HWDDKsua0HMdNvRg2sKC4wqUpoHvZQfj83rdaSvmD3k61bdiOcsqSbxJ
YX6xmfblYu//2NLKkk54jFu/3b2/zSfoIgxojvnv7O8396WBTMqx4vPv7Y3a9uqQcoAKya1an54wXFq
pYUid10Fyvux4q9AHurq+bdireCO4O2VP2HbW5Y7c30m4beiuICkzJSw4T72T1Cb1mQ6RSeanqT7
YxlPv1a5Q5Ps+ZOtsN0Z8/V/v4WN1JzxMLxP85PEMRIMb0v9n/68RX16ASAFbTImn+mQsAuJnzUF
FhG+lPL5xPL6eJnF8VJnSAIYpCY0hf733vj79VDEwBW0yK/z58YxWP6RFBUYemYDy2fTiyvV371ijipE
wRBDBLT+GL/T370vHpcAsDKyqz5g6oS3ivoAxV+M4y6Zv7L4tjfAgQGqZkblFVYHuYDXYl9qoHl8My
7z4iTOkEQRP+YwBf7X9o4/s2dO+pZCQArayE1f2ygaG2UqOwTt4AFsWp+Cn4svf2H+8feOiYO6wRB
EP1jpb/Yv3X0yM2dHfWgBIBVtqCav+QOlirZk3V98iYAoNXu3u6h1w+JwzpBEET/WN0v9l95+4J6RA
LAiput5gcATNLV3av8Gn+CIMaIVfxi//kXttTDEQBWHzU/AKD05m/eFCd1giCI/rFyX+x/9aknH9y/r56
MALD6qPkBAMqpj06JwzpBEET/WKEv9m8dPfL1zS/VMxEAJoGaf9FubG9Yv+lA//I3fjlBjPj9EOmXM
xF732eoWTm/53/09979yI1pnnuIwZx454Q4rBMEQfSPVfli/z998I/qaQgAU0HNv0iqJLIKImr+NqKM
TL+cyaJqfpOoNu5br5KNvSZlbnuIIe3t7x3ePiwO6wRBEP1j+b/Y/
+7ZM+pRCAATQs2/OKGCiJp/RmIT+xSZi9r7Oeaddw1Ozb+qPr/7Ob/GnyCIwWPJv9h/9rln+Ro/gEm
i5l8cav4BrH7NP8+6eN41+LzzYUAf3vhQHNYJgiD6x9J+sf+ljeN3v7qlnoAAMC3U/Iuh60tLVRbZdafV
xi9DTTVVErftQex28cIrUu4GLzuZO/Roqfv07aaHf0UPW14Rw6VfppnGhaK9mHtwKLuLPcEkp5PTS6
e0RCZuT07M2+HmsrPFcolll7ylW22aYdTNeMbAQFgp566dE4d1giCI/rGcX+z/4vp19ewDgMmh5l+
MtgLMFyrJbNb9ToO4rKJOXSn4F8MjB1Jbl9rqPn3f3DZJzCDqSvVaDJd+mRBeSkml9YYyF2zWQoPS
Oxa4G5l463RLyWSxXPq6NY639EDfunky4yxvB5bUyUsnxWGdIAiifyzbF/t/
+bOfqqceAEwRNf/ihAoiq4ZSV00rXUhFCzJTafmDNFdixZce1ar84uN6U/GaWMOElulQDXQXf/Z6h
OqlGC79Msq0kzkKaiJtIweW6umwY/7AQf6uNFfEEmbMFViH18zPXmnNGMqHlbL/cP/4z4+LwzpB
EETPWKov9p9/YUs98gBgoqj5FydVgFnXRDNZZxXkpfZBArxxvVowUBx64wbadE5d96lfbG6WA9lXd
H8xXPplTKiZuabm7rbRrwILsy4JXXbMvxCU8576DUJdurxlJnt8rRXZsXWKWAG3f3ubX+NPEMTgsSR
f7D994olv791TzzsAmChq/sXJK8BM+RWgBsqpD5smKrUexHQITK0Q6RWff4jqVTWoft7Yvlz1aq6Y7
mK49MuY4FrERXcofTMkliuYxYyrr3abssnftEp1dKdrGoS6BGbpNQtkl4IZu60NS2/n9s7BMwfFeZ0g
CKJnLPyL/VtHj9zc2VFPOgCYLmr+xckrwNzayqUG6lLFBeg2VTc9RtM+MGohMrsZU6tuRYu6dfFDfcl
cMb3FcOmXMcG1iIvuUPpmSCxXMIs3xW5TDozldzRXBL+BlavLyOGVFFoyhvJhNV387KI4rBMEQfS
PxX6x/5P3LqlnHABMGjX/4uQVYNHyq9GpPgxRHYt+un16agXdUF3NTV330/96v+pdXdJXmgHFcO
mXMcG1iIvuUMEeLcJ99Lj6arcpB8aSHXWTQt3KHzmUq8PIsdW3ZgzIw8p65VeviMM6QRBEz1jgF/
svnH5ZPd0AYOqo+RcnrwDrUEZ1qeLCVM+Nzc26ud3aDGEXfnLcQGpTGKZSq3H8r/HXV6zxRMb0y
5hQM3NN5XLbdBzYIUeseAN1G7n9PfVb+DsfytXlLQu0KbRn7LY2rI5n3n1GnNcJgiB6xkK+2P/jH37/
wf376tEGAFNHzb84HQsw2cy81he6VGhdiy8zVkU0Njf1wGZUkyk+uZbUTcdQ1/hK0i+jvHla+VWy2
Mi6h5leIlWHHZN5IvRI8Z2Q82km3IwcytW0UxetfdfNAtkL7RlD+bDK9h/uH3vrmDivEwRB9Izvnfw9
UZOPGi8+/tjdr26p5xoArAFq/gWyiqtCVRYFiiu/brJqK0tTVnUaJMKek9/WnbFmV4LhuVXSqZuOZjiT
LZigHi79MiE+UZXNGyrcoyXRDDuWHKnDexpOVWpGdtvU1+M7YToGspdaM3ZbG1bK7t7uodcPif
M6QRBEz/iv/
+URUZmPF59+fEU90QBgPVDzL5RdNFX1VKC4CtdNbqHm1mKdBwkxUxL1neFmDoxnNyhud03ttw
v2FBfTL9NM40LRXmxbOnsltkWC20nOrNuUu72n5s0rlFf1a2tou4kZzJ5h0dYbOZBdSWfstjasmqu7
V/k1/gRBDBv/

+m//5X879seiOB8j3j17Rj3LAGBtUPPDpcu0jvUsgDX05m/eFOd1giCInvHv/vrgXz76J6JEHzZ+8qPn1
VMMANYJNT9s5l/MUvIDSDn10SlxXicIgugZo36x/6WN49/cuaMeYQCwTqj5UbP/I23+W2wA7U68c
0Kc1wmCIHrGSF/s3zp65Ivr19XDCwDWDDU/aIbNT8EPoIO9/b3D24fFeZ0gCKJPjPTF/itvX1BPLgBY
P9T8AIBMn9/9nF/jTxDEsDH4F/vPv7ClnlkAsJao+QEA+T688aE4rxMEQfSMAb/Y/
+pTT3577556YAHAWqLmBwD0cu7aOXFeJwiC6BmDfLF/6+iRr29+qR5VALCuqPkBAH2dvHRSnNcJ
giD6xCBf7P/kvUvqIQUAa4yaHwDQ1/7D/eM/Py6O7ARBEH2i5xf73z17Rj2hAGC9UfMDAAZw+7e3
+TX+BEEMG9lf7D/73LMP7t9XjycAWG/U/ACAYezc3jl45sfhMEQfSJjC/2v/j4Y3e/uqUeTACw9qj5
AQCDufjZRXFeJwiC6BMZX+z/4vp19UgCAFDzL9yN7Y0Dls3L6roibh/Y2L6h7hQub3oXTXtroOQYNa
eJf7/DCACgvPKrV8SRnSAIok/M9MX+X/7sp+phBACoUPMvkCylFVOu65re1RTcXWr+8BipvxKoWE
O2jwAArmfefUYc2QmCIPpExy/2n/ur/6EeQwAAjZp/YUytretrU1zXF/R9fdtr36Xml028QWQr0frC
AAg7T/cP/bWMXFkJwiC6BOtX+w/feKJb+/dU48hAIBGzb8oXrGtLsX//XlreV4QbczLWIWuGwSGqC
+1jgAAIbt7u4dePySO7ARBENmR/mL/1tEjN3d21AMIEepxvXutf8VsluOAndgV3yrw0
Mqn8AXVzdvcqv8ScIYsBIfLH/6v/53+rRAwBwUfMvSoeaP1BuV2ao+aODqPvtNX/bCAAQ9+Zv3hR
HdoIgiD4R/GL/hdMvq4cOAMBDzb8orTW/VY7Xbbx6vlPNr8nqvu7UOgtLeAQASDr10SlxZCcIgugT
4ov9rz715IP799vrz715IP799UTBwDgoeZflFB1XlbVupL2q3FTcydqfq9NobhmvXLzxv+OwJYaAQDanHjnhDiyEw
RBZIf9xf4XH3/s7le31LMGABBCzb8wpnTWtbN7QVbvzV1Ta3sjmD76irng/82BuhIfo+sIgQsAYNvb3
zu8fVic2gmCILLDfLH/04+vqAcNACCCmn+BrDLepkpU217dM0fG6Ck28RGMWNEBmnut49AzQ+
gzed3P+fX+BMEMWB87+TvHf/BH6hHDAAgjpp/wUTF7ZbNdrldltj6dVNuO/2Ly+aI1cSr2u17NXcW
fu2eHoGaH0AHH974sDimqxcAAACYC45fAIA5OXftnPoJAAAAAc0HNDwAAAADANFHzAwDm5MCB
Of1Dh0TZSJSNRNlllI1E2UiUbXqJ1gFbCQCYE04k2UiUjUTZSJSNRNlllI1ESGArAQBzwokkG4mykSgbi
bKRKBuJspEICWwlAGBOOJFkI1E2EmUjUTYSZSNRNhIhga0EAMwJJ5JsJMpGomwkykaibCTKRiIksJ
UAgDnhRJKNRNlllI1E2UiUjUTZSIQEthIAMCecSLKRKBuJspEoG4mykSgbiZDAVgIA5oQTSTYSZSNRN
hJlI1E2EmUjERLYSgDAnHAiyUaibCTKRqJsJMpGomwkQgJbCQCYE04k2UiUjUTZSJSNRNlllI1ESGAr
AQBzwokkG4mykSgbibKRKBuJspEICWwlAGBOOJFkI1E2EmUjUTYSZSNRNhIhga0EAMwJJ5JsJMpG
omwkykaibCTKRiIksJUAgDnhRJKNRNlllI1E2UiUjUTZSIQEthIAMCecSLKRKBuJspEoG4mykSgbiZDAV
gIA5oQTSTYSZSNRNhJlI1E2EmUjERLYSgDAnHAiyUaibCTKRqJsJMpGomwkQgJbCQCYE04k2UiUjU
TZSJSNRNlllI1ESGArAQBzwokkG4mykSgbibKRKBuJspEICWwlAGBOOJFkI1E2EmUjUTYSZSNRNhIh
ga0EAMwJJ5JsJMpGomwkykaibCTKRiIksJUAgDnhRJKNRNlllI1E2UiUjUTZSIQEthIAMCecSLKRKBuJ
spEoG4mykSgbiZDAVgIA5oQTSTYSZSNRNhJlI1E2EmUjERLYSgDAnHAiyUaibCTKRqJsJMpGomwkQ
gJbCQCYE04k2UiUjUTZSJSNRNlllI1ESGArAQBzwokkG4mykSgbibKRKBuJspEICWwlAAAAAADTRM
0PAAAAAMA0UfMDAAAAADBN1PwAAAAAAEwTNT8AAAAAANNEzQ8AAAAAwDRR8wMAAAAA
ME3U/AAAAAAATBM1PwAAAAAA00TNDwAAAADANFHzAwAAAAAwTdT8AADM6vLmgcbmZXUV
AABg2VDzAwAmpC7Gx63Cb2xvNBnGTDjvv1koF1bY2L6hLgzJWUyFvyuZlbOH47xNo4v8gVGfvdq
wn4zEH9HErVmFh3LesWGXNa7kzoz6oBhRfFGr+j5FVuQsZ6i3qUOuFX0mrQlqfgDAuEY8zTusPPM
8tFVpxzjrFGcps47qXDXygapcyMbmZvF/RslTLmHuR0L7PDr8p8L5ZDdG+vRVa5nTJ8Let+GSRP+Eun
+G3IX2Es2YvDWr2FDldXOh3tK+qcKj2G9X7xStO1PeH/RBEVqUs6RKv3UlFlXdGvCPkr8cK7ltpBVV+c
2l6lXf1XXLNUCqeu8UsT/OLop76ICaHwAwIvc85Z5GBqUTVf9/ngcCd4VjGT1LmUBt3zhpyvd+9G2y
zeeNcVQpR/rwybHHyzX0OV7R74c/cfnRGGpp8YypW7PqOpRup17OrOpfc7MU22cu9H+72pZTXle
3e+WppRY1xPhKYlGBS/miyxH6J42vSF4ZMZe+oV72fdfK4czw1Se5eemmqm72WtM6ouYHAIxHng
G8U8Pgxs8gzOf4MfKyzPDlD/mHtpR+x8GZzf1zUBj3o+Bu4Gjrqwa236ih3zhv5v5SBt7IxF4NuI1tQ1
X3szdS9x43iyWcyFwtf+idRk82kGucx4WfaJB1KInluAb8fLduXetkuvOG8tYxYDKzm9UL+XEYMtHao
OYHAIzG/yfzgKedsDkfBqr1DHVmjHKOP8Oz9qz8cZxE1U41Rt6z8dYRNfKbVKi3sEhR/zDOp9z7E2p9
OgbhjVdmdPdt4JSJ4QbM1DaUt7FZ2rIMtqDQQNa18sfBPuyBXP6HYgh+oipP+UUFo3/W0NZZqttD
rS2Yq/qolSnqH+JTmY2XK7KdwyzN3qZwosEWtiao+QEAo/FPAMEjypBGT2CrDh5Dnd581VqUEZdU

rkIPX6Ycb0FavbAx81SfvIGP8mnz+eBVn7jKWKn8dfh/invxEvjjD7yXieEGzJQeqnrjBtjFdJbq7jDvVSB
RuQZ9pbw92IcikssyUCo/kXhfqgbx/e0msBxL+u6sYqM1+zdYqkCu6orcvYHeq2oFKlv5szvssLu4Hqj5
AQCjWcA/qud3FhCnxVENephylcto9qtMNJc1+R+NQYk3p96/MT8WVcJRP3f2Guqfx8lXjS23bsC3q
hrQnrn/UfCa9JMYbsBMqaHEx7GHYJbqojLUtnmJyjU0r8vbg30oUltXqJc3RDY/kffhG+APcnI5A4xvC
+Sqt6u+Vv88UL7guqr1aJubzoekh2pY88Z471J6jxFEzQ8AGM0C/lE9p7NAfdKZ45mjPrwNn9A5slm
GOGGnVHnH2z/vkzduvurdGXXL/Awjrsj5UAx3jq95f0L9P7Leu9ePn8BI3JpVdKhqN4daTsuEq9uDJJ
OJnM+EZYxcvoE+EX4ib+D2ubRKDFHdGuqzUPJy+Rmqd67fimrtW1O2GGJ18o+M//a3zwUSNT8AY
DT+P5gHOrvFzeMsUOWY84GjyjnqzlXKNONnGX85Yxzl4wY7Vcf5KUZdkWXoj4Q/b/lmDb2yxHgDp
goPVb1vAy6mdcJVgwEStiQqbw/2qWhd1FB/wvxE1RV7IfLDmCG+nIGWYfFy+Snad7ej1oHKBv0/F
NUK3GH8zAO8TWuHmh8AMB75T+bBjh9R42eozyTS4OePIou1jMA5aBTDnNp8xbjWsNWbNOq7
VGWwFzLmGbF6c8b9zPmfgLkkLQz+iajeGnfiztq8t663QEYtcWtWgaGqS8O+Sa0TrhoMsH0ticrbg7
1JC12U8wdpkD9V0eWM8GfWz+X8YSoMlzT9Ng3zHlWz9ZOUl+2x01NBEDU/AGBEzgFkmFNBWD
W2sNpnArGi+SymTDrKOzT31ThH3eHOvb4xP9Wu+jxszOETMeTaxCeg0izBXttQm5nImJ7MTOJDif
er1nNxVTZnpkUW63WVs0+OjjtTNhvsQ+8tqrhgDV5PKTCJztKLcu72SaN4y6lVl+fw2R78QRHP5d7p
vXX1cMFhnM/1oDu5Pqj5AQDjsg8g/HMac+McSHufR2Oqj/dooy/AwOd4DMJ9V2rqvRG3Vugdiy9
qJdeUeI8Kk3tQDE78VUWtOTJwkOiJmh8AAAAAgGmi5gcAAAAAYJqo+QEAAAAAmCZqfgAAAAAA
pomaHwAAAACAaaLmBwAAAABgmqj5AQAAAACYJmp+AAAAAACmiZofAAAAAIBpouYHAAAAAAG
CaqPkBAAAAAJgman4AAAAAAKaJmh8AAAAAgGmi5gcAAAAAYJqo+QEAAAAAmCZqfgAAAAAApo
maHwAAAACAaaLmBwAAAABgmqj5AQAAAACYJmp+AAAAAACmiZofAAAAAIBpouYHAAAAAGCa
qPkBAAAAAJgman4AAAAAAKaJmh8AAAAAgGmi5gcAAAAAYJqo+QEAAAAAmCZqfgAAAAAApom
aHwAAAACAaaLmBwAAAABgmqj5AQAAAACYJmp+AAAAAACmiZofAABguVzePKBsXlaXavrGxvYN
dWUcOo9IvwyavSl4+3BjeyN2K3kPAKaLmh8AAGC5WHWtW56ue83flO2V0DZEK/tmU5fwbzIAYDz
U/AAAAMvF+XfZdoW67jV/p/UHi/vmrwKo+AGsGWp+AACA5eLU/HaBu+Y1vyncW+Zl9s/sk7lCxQ9
g7VDzAwAALBe35rcKVa/m9y74hbFqUrawxlUdrCt2NawvF9es/5zeSqJZd7371tyaNC01tzOeM6A1
Uy0xlmldtTGvvBWk5l9y5yOaJBaY7AcAc0XNDwAAsFyaYnK7/kmXjFaVWb32L1jlpiqJm3K0lSmi432
sOlvWtRVrIoFBkqVvOKlcusWai6eZ3Oa2/lFkb5t/y4TiCwz3S04XAMZDzQ8AALBcdNFYVJC6MK0LR
utG1dC/kKr5VSOrJJVXdFnq9Wmu6AsyjWlhpmLl6VDuxhOYAf2cKaax0gxTaZ2/bhCfT2SBsYHkDAB
gPqj5AQAAlotTNaox9s9+2WmVk7KY1U2aujjR+RQ/jt5BtZEVckJfMIFabKDmDklyJfyHNTKAkunSYv
+RlDy/QNOu0bgAYHTU/AADAcgmX10Wp6VXGgVJZlqZ+k/Yr+rVdKLvj6hYhIrEzSoQ/pZJOqa/Kpb
WKT6HD/C1ua30/MrqZZcNdFgDMFTU/AADAcpEVsHptvt7f1JCyZWGsmt+9qF+ERBPHhhdvKpWTX
/P4UOsw/VLxX9P3o6JGOXWcNAIOi5gcAAFguXjEpi0hzI1B2ysLYb9J+Tn1Vbm
0VvEptE9OtyjUjbzsrWNYQ1TiyQBgPNT8AAAAyyVQVTLrlo7mRajlAzW8X126bDuW3nybODGc39nI
MWPO3DuV39TYlscDiljXwzPMGgAFR8wMAACyXUDFp6saSudFcVeWkKUy9K37xGr8SH8U0iWfW
ha2fJiWeoOk/c+2cmELb/OVymvYtC/QWkpwGAIyOmh8AAGC5tBST9g2rFJVELZsqQeUV83pDjq7r
3UI4ddPAT5Om27vs3kPW/K3zD0+npJvERo/17DprABgUNT8AAMByiRSTTTZHq3LBr16KslIWxP1j7F
f3aHs6bTsmtm90GkVUkuePJIlkurVXrFFLzb7qXypTWtti3Q6PbXUtdZwwAg6PmBwAAAABgmqj5A
QAAAACYJmp+AAAAAACmiZofAAAAAIBpouYHAAAAAAGCaqPkBAAAAAJgman4AAAAAAKaJmh8A
AAAAgGmi5gcAAAAAYJqo+QEAAAAAmCZqfgAAAAAApomaHwAAAACAaaLmBwAAAABgmqj5AQ
AAAACYot/97v8DXDSvEZ9i9ycAAAAASUVORK5CYII=]]></redcap:Attachment>
</ItemDef>
<ItemDef OID="b22" Name="b22" DataType="text" Length="999" redcap:Variable="b22" redcap:FieldType="descriptive" redcap:BranchingLogic="[q20] = '1'" redcap:InlineImage="1">
<Question><TranslatedText></TranslatedText></Question>

<redcap:Attachment DocName="B.png" MimeType="image/png"><!
[CDATA[iVBORw0KGgoAAAANSUhEUgAABVEAAAEJCAYAAACdcmdaAAAAAXNSR0IArs4c6QAAAA
RnQU1BAACxjwv8YQUAAAAJcEhZcwAAEnQAABJ0Ad5mH3gAAE9sSURBVHhe7d0PjCRnfef/b+/aY
PPHNmBskx+RHat7sZYlGJIg08PxL4egZ+6SJbnbYOVg5Oh3PVyi0zQSkwOxF+mX2wguG4lunS5iWrq
zliQyTEJugrLdIJRgckzHR4IxYTI/Z7rlc3T8wDYGjPljB3u3f/VUPVX1VHd1dVV3VXX39PtltaerpqfqqT/T
O/OZ5/k/k+hb5FAAAAAAAAAAChjumPAAAAAAAAAIAQhKgAAAAAAAAAAEIEQFQAAAAAAAAAAiEKIC
AAAAAAAAQARCVAAAAAAAAACIQIgKAAAAAAAAABEIUQEAAAAAAAAAgAiEqAAAAAAAAAAEQgRA
UAAAAAAACACISoAAAAAAAAABCBEBUAAAAAAAAAIhCiAgAAAAAAAEAEQlQAAAAAAAAAiECICg
AAAAAAAAARCFEBAAAAAAAAAIAIhKgAAAAAAAAABEIEQFAAAAAAAAAgAiEqAAAAAAAAAAAAQgRAVA
AAAAAAAACIQogIAAAAAAAABABEJUAAAAAAAAAAIhAiAoAAAAAAAAAAEQhRAQAAAAAAAACACISoA
AAAAAAAARCBEBQAAAAAAAAIAIhKgAAAAAAAAAAEIEQFQAAAAAAAAAAiEKICAAAAAAAAAQARCVAA
AAAAAAACIQIgKAAAAAAAAAABEIUQEAAAAAAAAAgAiEqAAAAAAAAAAAEQgRAUAAAAAAAACACISoAA
AAAAAAABCBEBUAAAAAAAAAIhCiAgAAAAAAAAEAEQlQAAAAAAAAAiECICgAAAAAAAAARCFEBAA
AAAAAAIAIhKgAAAAAAAABEIEQFAAAAAAAAgAiEqAAAAAAAAAAAAQgRAVAAAAAAAAACIQogIAAA
AAAABABEJUAAAAAAAAAIhAiLp0etJYKUih4DxWGj29PqjXWFWF+1Mxj3ZB2onXWFdL
MxLO7LSlg372PzHqPs4Vb2GrGzEO5vtDbdtK5JH08KNuQ9Cjmc+2g0AAAAAwPwhRF06Rdm8UJey
XurU1kPCkr3r3X0c5H1HH08KNuO9KNc5ee7bbHHkj5xr/wuNIh9E3Nw0wVzpfpP5UJjsdtvUg
D7TVk3fiEjwsI7ascDAAAAAEAOCFGXVGW7JV6O2/jcK4aYo+4KKp7Y6bqbVDjDSHbNIXSyOxnbQhTFXzK+Xpx4J6BHM7ZvIQHwCqOaGmpkwIxpPJMPNT66H0+te1
+kREu3HCSwHz4HSXB0dKibnnOtV70AUtW6Kc9TeGFHPNGS7E4vZbgAAAAAAlgQh6lVrLrnLnBTF
nqF0Imk2pv+KGKV4/zfdyx/OQ0lOJjt9gEfJ8GQzoaPvhxE4BtOlRl1XCBG4aZj8vErrjDFwu7SEq8oW2DBwAakxe
ISmZ0EodnzgrxDtyQe1+k/VpKD8tUQK4uJNZVmbgLDq6GRJvv7Pmk3iaa5kFGdbx/uZOBUmR5Gxh2Scy4h/dhDeU
UhTsiQR8IH6p/bn/Z8iGFGZ6aojm8FPpEyaNmWnTGJYlAYF8eVs6Ja1N/e0vlYBM6zA9eJBTKw/tDdszrT8kJRt
DmjdMNmzGmz4Ez3CA7o9I6e8Ju6FaNrkFXEhiW3wyfqiepsMGuzb2Gow6GSYxpOGHkCWyiR8j
bKd1b4/PfNf1x6WZDGOzXKJ6ekdzqEZ7hWXJexW0nGpiVmQ9krgO8rPXJ0oXZEFEdJzYagsGA2ApNS3Ip8A4u
BWqa5lx1kJb8ZDe3+f4UXjfiD4G8Ktub1GQqRBPPPpEk8uxdOpyAATWwlW6K5AcZF7KTF4BSyeobpWeZk
9ZG1bSf6Ps9EZ33G27Gn5UUXHBt4+M53Eq2dpySuVVbOE7d51wYr29iV2gTRkS2V4tbTzVEkJC3Ktny
8qZx1r20mgAeJJkRMk37RGB5aD5ph1H5gb5KvRKc6z/GKQQMFObgrOJZmk2qWmnjBLgcLXmWhtfFfY
7cYBdmGSIThAkPI8CU1YW1TTiA9m8b8H1LsmQWSuhdwMDDQiSBBkZuDHvIiHBK5Ka3b8L0GINP
DyCcZQhHjiB7/7d2dOqqpQjTj8u8bF6mMwCCPmiWC2BZdXzTcmOyrEIZCh8Z9+ljO7w8Tk7KnHQGNB
rSgyX2w2ABb+ht+mM1+N2aWfqLv73E6Ls5Z7JiEq/GVwFtKdb3Y7E3VfO1pVsaPV4ZO74yjU2jkHCtUmrq
Oi6u0A5wrE+DshYNkCpJ4v+38lvJ2PIK6Q7wGx9wNYx54xAqAfljPUzlm57Tc0OlCk9RDfd+9KfdOaC
VvBG/SMoALEcMvB0H6uqjTmbSuqRcbbtJ1oiCPxi0m17Yh7ZG9R9j1bv06zq5EsbH+5VQB/Rkg
bOzm23DZnPGzx63ZMw5R7qA8jv8mOZrRMoRrHkWgFyR9cATwlbZCtq18M5y3u9p8Ng4/3AGqmhL1
ejk8SFYrlp9LJFYmR2RU7YWwy5/7bDLsHBCu3OytU4kHhqWHaJ+2eiD0/IXX5MYhfA7F+oG/mSY
LnfNhxNyFKi61YJ6oLEa8yaA5QuPuqAkNBcLzjhSKaBjOB20pEkqK30A+jBVBJOWh9xvYLe9Tq2xSGR
IS4BsJfTgBXiyWEzw49TDxhhFIkXDc7hszc+HrbqmpzFHeNwq9iQ69eKVSojIr8x2vJ9X+8tjNkG4Qbn
jcy3dEhmGmLBdgZJKeJ/OiJuvVpFzqG+iFeT5a+V7lTMbpL+aagv43gTv9tKXQfQpCiDT0iNMkxJ7w6
Sgg0eaoaPJJLwz3C/iRS0yJ7+mbNtdbM1NXVmjJvnRuFkD9fM8mmKzWV0gxVL4FLGpzy46OJoLZQn
NYx2Ij2l8OEwDf8ryERDW+Gh5tBrDzvEwDpYnpr0nVrTr80X/t+NQjtKoobRoMDBBKfgpQK8Rr1e
4KX3WS/O6gNe4Pdc8+s8v8vUVrGyDfyfxk5nR79Bu5hWwfgBUJCcqXhF0PMkCyf6F0+Pso7a0PF8D
SJqs7l+TCVIDoTvs2gFdRLZcK4vl0wmp9tCDTcaW7NC0pLlx7h6aNU2/hDudb/QGxtsMt9Go8lHJFn+Bn
EIIp0M4kUnvjjaaRRLXCixnHgFCd4bW0/vVuTeKYgu2AbhxbW/33Xhdb6GvgrZwmyGGIRQBJvf6R
Dg1uC0ARHwqi40E0Z3ll4cCR/VTP/tR9GDH9wPkajzwI27ZYxtWS2HHGB6A6bLC98PfKrLc/tfbGXI
/3CFSmAJ12d+Y8lXwBWQEBcALGAlc1EvNT9iotQJbqrtR8pRzmtdOL4c/8X6AO/1nUNYIL5o7NdQJt
UHXsSXCxCdQT4lCsxlzHsvO9Emk+M72R7/fyeuDo6NCJfqTsjDfFNn/pOPWz2k3f/ASrfjOlVs5eHj
FR+nsJUnwx6NkERBj7SDT+i6Sc++p9YiFAzbzkhM7V0YQ/cvhEmxQUhQXsecqewJI+NVRYeHS
NlgFJ8VpbEtL2v6MXY/gKkIxd/XSEN2L4lx93c+r0YWqL19Mr+MFDFbtmftfAv7ftMPRWvSGqjAs
AAAA7mL0FgLrtGJRbwPMx3N0xf3n+SwJG+cHHwbydekBsHDsJC2b7tOy8wZ+MKLcfCG0h+7X2HsuRJ4
2U12QU0Gy7hQXVn7ZWh9EJE5PYpRmk+Sk/ZzB4Kl6lt+KAvOVQuTbE5cu9Lhg6Ko+7GmtD6zz6/S3Vo
Og6cQpX/oSs8jstUHh80Fl4szYA/AZKQ5fAJbIWfpqL5ah/c0Mj8Yk9cf3m/BEMqkA/47UmOIdvmq
gz+33bPqQxLEI1HTsvgN1ECjtCg6vsQngaMhFxLRvqqhlfYSZJHgpXtJmL7I1mbsLYOtD31Nx/gkGPXf
Jn7Yz1rxPPSOKivR68LsbVqmFtbg5xcF/Oa2iG4Nc/9vB8HyJuT5rLaC2hmOxi1Z5AbmHGb7WVxUL
KH8d7Nrlv5Mg80LuX/kn3U41yGAAAAAAAAAAANhvjJ5iVJjRM8ysXMjP+yQoh9tFTRPoAzYDlZwY4Qq
2VE2uwL28rv9cnwTB6lvAi6VsLKb6ULM74Zer7MwM1yFxLn2bKi1vrjrNmXLR+vx4NhvjVk56G7fIMp
C4XGXb7cHSVwNLyMbFnzxNNVwq8ZbE/MmaWy/+eBMqzJazYmdHJKCG8Nqyx4XZ0OKczA4w8TR
AMA/EXHuBO+/jZnbhDthWq3IXaVNC6Xo7/9VjJRhtxnxRFX3RntB/BqGz89BRlHO6bKcd6qhw7vB
eFJ0jeetqbldlkRmGy9O3d7m+6nLk9UbpB9vzjJaOb52Ppfv/dBrjGt2dCj68pfmT7P4Qk2yP8+rqZeY
+Pd4OZePNrt7cd/Iu8tYwB6jUJpH5N/pc5eKsVcpfbK5Vg98uRZwcOjAnYMlRkqEd4EAW9zJsovjuuSZ
WA2uZ+XdNF7iuuCiBtQ7Mq7OdeKsVdUCXE3w7t+/8qfpdC/d2rp8H4ltx5u18f88hOFRNmHBw2DK3QN
l5rmEfYN/CWFX/ykJNX8vt6gtqQI/N0JhbZ2w4zxK5mw/3bAQYwfhBXsOQfFnr4tKUBBpDzOH7X/Yv
ZW3HdV/ECQiPhzewYh55UW3Yad8p+4fZb4e9IyvEHF+AGvvjLJ0H5t+zbEKQuzmpO9ZFz6zdgR6Dja
GAfiqZbfbjldNpl1pwbqOf54qbPuPnxi9iTvEW5eW8xd6RmjxjHYDqRPpiYBP/yqgI8oWZd8EUrRe+1X
88JTqr/j7m9Kj1IqtYEntYWGbndiotw7cB4pgTQJUk+8KiMQHbYgCOZCIupW5g/+YfiOIGT2PNbOAxK
BqdQ4/PVkb4YWcW0ZLtM27gHtvcqpi1xCq9XBCxsmbVpSiKuSdM1NqtyW1hw6+N2B+uEZH0wsotTYr5s
eBY21z7OvCdOzbnw5E5zxHeNi3dGb+rmzz2/Pf4mUDxI+oZNvSsLNi4w4b22o7DqBzGnqXyr6ZbdnD
z7n3ayEJl2jrAYjhc66pTMUs3pAHXcpzm3iDeD4b5cxuemv8hAAAaIQOdkN31Gy3uPUVZnbdwfQKsP
5Ps/3ftKAnhVJfcvrTYljaMLrmuzMyQgnuf0ILd36h6a4SsxNQd4blT3Q/pIydLzQ3ZuiVTnecO1RsVQsv2
RxYxQWsnllcq6v+v2qL4WSv0nwaE9aF1fPGP16xuhLWBTNsn3s6e9b3G4d7w8+AdV+ZSyKOaFppEsF
E/6sHSy5ctlodJ83ngxu8yxrXQgqZiz7EGwzHsPn9LCJxrgvHn4KLzm0I01Dq9VZlvrG3PvUGuPgR
pXk5t5EHNEsuUxuVEvRbX7pVqI5b3kJ0YO+fmeeTI6fZVrlm2OJOxU96Y3uz8b9jiGrjSbumWW8Kk59
/sdsr37ttEsWrzK/r5OvYXn6rZRBqJOuQPZw8lYGu2Ln3Ff8rGy02gmjAeljz2axJSGZR0mRmXKj
KdNz/6J0wpnr09/2f6dYuMAtL2QnKWbCDtk4kKQRbQo69B0bI7Bqw0/ir8i+0ltfA3/nqkHJOh1yx
1ttyDSFOfGPiuiTdGbg8I9qkVKuDxVMzjx5x7S8/1tG/RKWE4I9Vw5Sh3+GLcdgkeMzu/aERoSGT
6zqFb9YBTSyPqiBlBe/7hgAsbWwBjoEbRfo8/vI6YEaYP6ZZmKl1Hre2EXlS5v8yo7DZhKswjBOcRL
OkfllD0vR09y8Sf8Cal2+WnWV/GaHx6T+EgvLw6zy8pK3BqrdZ9Z+WmrlY1fLqh9oIn8UW1uRzC0L8Ni
PN5NP+wQaB9ZbC8XLu0FrIwqoLKGNQV7nJ3f8MMp3ykEYnbNcRqMgHEjUPkgBc8+7Nb7bGgUHI5
d/b1VIL99PJf60VwRdMB55AxmW0lprOzUbfY+4jhcbq3kHU4Kj+Z86OnbjcXC7tnkEKgYYwZ8+2mr
m/vEvZjl7/uJtj1UTSoHGFeTsJhXDs7lb8hPv8g8xiqRfMwCRtMAhrYbfIZQ+SRu7fm4dVcDWHYhpP
TRasaTGGbr9UsTKSuf+v7Qz8TbVcJFBY5wCKmv/JjqJ5a4JhakvK0xAJ/+dodWxtqYeXR1Mu31PNZ9
gMQKhqNYW+b7bwBG86kGjn2M8ijMd75hWvxzAIc/5WiWaXf5R1BB1+tTIbpPWiH0gU7/DjYWg9zZ
ZGGmxk17SiWX0r3y3VRsJqYDlRy3Rg9HVJJeQBCT5ub2cdx0iW8Ir/jT/Fb+p9Oprm6dJIG/4sb3xqGx
eJ0jbsiJg2qcfALBq7mk+OuKMR6HnB4XiBLnl4LhJoc81zDNYqyJIEGTxX1F47u7OyOgzUZf4NzOhq5Q
dDmtoZGt1t+J+p2T5cfOHXQm0crj68bgsPpdabQZM6MadQYi3q+aDN7uXbwbXafk6TO5y+Tr1kkK9+0
+QlmC+a5xrD1F7SylZeaIt2JFNa0b4uyLlHBVGi0bPXBO5bZnnF6ZNvXvXNL1PSTa+c0PbZGsGkcT0M
Ir7NVbmAvfrsr18LvSyvyQ1zPJbT6OvfRh0UCyuX9Wt8vy6WdmoxpEJ6wJIT5Qhx8FpbAqIZ2IJjm/Lc
sX46eN++YKmxQFfn/+/cfjZsIWdP74GlMA7zwH57dzlbXs6CqKXvcCpxp73H1HuyXlxz6lYa7s8vt8Ik
VmMAAtvS/1SaSoZCsvMa7vdtcPFn4xF2C/D8qZqJqFsl4I1pHAkoZA8HPm9i2z+Cxul0U6vZqXRaS5b
7EijQ9vxCrTTvZWgbX9a6lFwSnbf7IHDR7d1tZVXxbR/y8dp4//9CKkxXOdzNqmpN2RR7TD6UBdUl7gl4
gOnT8vuvpmOc/ko9mjLvHQTVOB2+MRk1zpG7Y8z0iC9ZZaEJHjKN/d9NMi6TfJqMbOWvn/7FOmJ3
UUmCpe0t6tp/FWdMOJFvFqNfZ/W6UDw5VYrPvWd/sX7T0X+AX4Wi8dOOALyT+BBhfCQVOlW2RmZv/
WNNqjQXSEgGdzYsvCTbWORhdddN/w+x9GKjp9RdezMF9/FQz5LQ4H6ovvP3yNAm98xOZeDzFk5e3Tf
ovT16gNiRk9vTD9e+mGlWWKnd8OyLe1b1/AXqjBucRh1Qj8/GaUz3/wzWjTTSOp6DAnfw/mu5jbvk
JmUd5PODJnSbPUNKfdk0frUKb/d/dSIL04/m3vC1xmlNHK2O6pu1jnkbDbNa4zf8z+uO2Fj3aYG8W+2w9
tw6MRmNjuplSSZ3VsQwDfP8W/dNCTw/e9QyMMj6yOHWA1y55ZNlaDO6TtfkNhdG8/VPZlGc/8jmwA
eO0xxhGUY8A/gCHLCVSd1KuJLjJsFvuH3w0d6P3b/pj6jFvOdtnVzMjXmv48jFg5AfHV86Nqd9n/
FwKx2r8DaeVNj/8T3jHXEsRQv3DK7V1SKkeNTo6EGiWkmzMGQ2PH1hLMj/Lsn1HtjnsDmt47vkrb5vfP
bW/DP3l0jcf/OqrZ6ZXKyLf9vT2eFBN43j+5Lb+TNV8jhwSCOtSHMTd4jsKMh6dl4y5FaN3cHTRCiv
DMx4j/wAUXyD+PbL1w16ePFvjuSVp7s6NdTOhOpzr9vd98pLf+fnzKGk24n2qsHuPR5mnOo3PNK/
D6kUX2C9pH3N4BBiRpYMvhc0kd03YaxKAMdDs5GdA1t1gNbqDTHXT1QyuU6WzBX9U3a5Z/w/fn+w/
FFPjTd3xl3jmqmK8/s7TLeFTjSznyQF8gMCYXNjLkRsqgK+8I+bPkL7e+Z8xv0sCuxe7Eh6EKoySzR
+ojYhfmBXV4Sqv/k7Zz8fZzDPzm/zjP2c0dx3Pa2/PvfA3IFqsQ0F1/tfh9fzzbYvX9oZX+k73MtdOc
CV9AU8l2Q30Q/X86Dfv/P/SAPQrl0fe9iC8hq3u/6n1k1/HLxOejfVvC6D0r7cD//P+bn5u+ff8ZPu1V
D9XffuvNe7uMH/y7UDN5pjfU7lOzEzKPffP/vvt+g34/99rPT2z+/e0+Vl4ZxHVXfe2IqVW0tZsAW3
f/P+/zbnWWtgbbLoVOxvQXaKc+jdZ99H1dr4fwfX7TPG2Lw1t8tLWYfVAtn8Tdaqb1PxW24RDpwQG
/zW/+fr+v5kq0cEzBtM9JuLbv7RiWj/fnNzZ+yP4n1u6cVZ+79f1kb+2p+p+3ec8zKhlJF5lJNY4z0ynO
E9n0JAxdWoCTkT3VG4Gm/70Ef1/O+Fi99g+OH87rde8NxMbWtGc9JxS1PONQiYn1jB8PMs/iN/rKzjv9
1NP1WjdDzUSa5oDePWK90u/VsNmC1DnH+Xn0h73HO9Xe33Ul+Pn/uH4xFa6Ew2vwdpdeu9FfJyQ5WXq2
lbT4Vdxn6P8S1H+8fvrMzcV6zvprOz+ZL1xTs9Of1YoTpszmb7d7m1OZrk9j/cFV+V/wL+zr8Pa+Lle23
wYAK+mh8SVx3nG8T+jf3czDbqBtHE2SGLZSKf+vtr3sHP/NwJ0HlfcJ7L+Dzu1s/R/zs6/zcfyN/S5l5
iM5/ObPBn4cNsen2X4Op/mlg/nfVNTpT1qyvVGr1hYdPWD6/xv9rH1+tTrr19LJPiqcrKUz3s3TsH/Yo
c3/S/lxrS/ua9/5+6ko3u/88DT+fq6M/q3N3t6yu+Nx+uv2JHdaEGdj7b3ABKVUaznmd77FuOc4iCcU
vHlnblNfI/1UR8d+aqlBmbXY9t/h37n8rMdrx8/m9yrBR5Oj5fMt/OdV0NI1yhUfB66/fYKfYBY/dw97
/j/fq+tfn6LPn9X/ypjvVvX4z5MwtwsJ5qT9C+D1zuvDrpnP8nXv/a7eyzk/HfrPxzfs/AKMoAs3
Fm/5ozp7BxOdRmxWXqf/dc3brzOdcHsHUYEUWv0tjbwe2V3lnI/3L1yvjPGWb2DDGw0vGuMeF8vc/
ddaO9P1/v//6B+9XrjTz7+p5XOVnI33g4t7+lW/f7TiPaR7OZlBVFdBKqvhQQg/s1w77H/Y5gDT9qXe
ke+nuPfhxJ9oq5jbdyE3Sma3yNkb/Nuq++n0zeydXz18D+R7/TZL1EcaXH8/b37H7SF2/vUOv/uf9/pb
Xvfbr13vtf9Pf/9r//Vs+D/bdv/5Nt/9n9n7/j+W99Xv6+3R/wJRrr6TvsJX+6n/f2jz+1+cnuP8PDn3+5
X/ADF2v3/2zFmOT/D5XHvtv/u9q/uuu9fPJoAAQr6Pv+/uVJaIV1v/86v4G/u9F+3d5n+t6s/Xrvz8zVze
P/f95c/L+7+1+/iTC3s/WvD9aT+j7k/f3+ZuvP/nbx39+tv/X+Xq99/WP9u0/X/vzhr+n99d/17z9f/6P/
8DX//7vU

me+ta1Qq+NvI2oTnyA9c3XYDi+kT+5+Q+n11vQQAyIOqg/pzD7xELwX94W9/Tb73raf1EgAgc5f/RK
T/fb2AeUaIivgq29LyctSalHTA48w8bynX5YJR4NOfUMnSXNWB0GQ1HAPbsp55Q/a9of6KMUnTHP
Jqu9rKUt8KRKiWomxe8IdlN1d1gOaFzqMm/ZolJ3AMttM8tryOKdl+ipsXpO6/2HmtOZGTdS/7h6Br
7yrua1NNWBO03bzfw9odR+bHA8R339fvk7v+7C69BADIw/FLBXsYv/oYRgWozd+8Xy8BADLXf1Lk
8sf1AuYZISoSqWyH11+stqz17tBiV3FT9gZfW21NVr/R7K0XKBlgDM3OdKKjFJgB2KiyA+qc9bt+wOe
ya1/OW03PstS7fT9Yt1Wl1d8LTpaV1zEl2k9RNvf60h16sXq5MUzeVpHt4EGK7B96PbFTEbvtVlv6Lf
8+sqlzblwHu+5ulByOB4jBnYn/6Wfp7QQAeVr50kvl2iev1Evh/uFL35ZP//6hXgIAZO6y9RvZJcpbzbt
CX81aAgAxtTcK4nSSVCHqQGAKADGo4PT1/+318sAjD+g1AIA8lB56od0LNa6tu8vyiteFD/sHAGTgit
8QKfBL9ryiJyoAAMiVGsJPgAoA+VK9T1Uv1CTUsH7qowJAji79AfVR5xghKgAAyM1HvvgRezIpAEB+
VP3Tt33hZSProI6iAtS7/+NX9RIAIHN2fdR79ALmDSEqAADIxe6Du/LBv/igXgIA5OWOL18/tg7qKPv/
8zH53Mcf0ksAgMxd/n9FLn1BL2CeUBMVAABkTg3fV3VQmUgKAPJ189efL//8Czfppclc+dzjsnX36+
XWV79IrwEAZOtKXR/1Zr2MeUBPVAAAAkKknnn6CmfgBYAZe8MMrEk0kNcoz/3RJmu+/X3705DN6
DQAgW9b77aWPWx+fchYxFwhRAQBAZlRwqgLUh594WK8BAORB1T99yxdvlOf8OJ1f+R7/xo/k7g8
xKSAA5Kb/HZFL1EedJ4SoAAAgM+/77Pvk3ofv1UsAgLz83AMvkRsev0ovpeMrf/mI3PtJ/igGALm5/
DXr0dELmDVC1KXWlo1CQQrGY6XR05/LSa8hKxttveBob7jtWZG8mzM3Qs5LHL3GinPuVhoy6tT55
9d55H7NjwLuWyCW+n11+djffkwvAQDy8vJvPE9e+eC1eildn/zPB/LN//19vQQAyNylXZH+Y3oBs0S
IurR60lhZlaZecpTlzFpRP8+eHfiVasLfVIImPi+9hqzXor5KXfOCrAYvunRqJSlMENguK+5bIB7V+1T1Qg
UA5Ovqp47LG++bvg7qKKo+6u//
+y/bHwEAeVD1Uf+79fFZZxEzQ4i6tLpy4KZA5bp0+33p9/dkM68MdWzgt6QmPS+qZ+S4YK99XtxN
V1vqevelWy87K5qrQo4aA/ctEIuqf6rqoAIA8veWvRvtIDVLqifqH/721/QSACBz/UdFLv2xXsCsEKIuq
96h7OuncuqE5Nf/FGmL1zOyJ41zugtquS5bFedpcfOsVJ2n0twlRQUwPTUTf+WPKvZHAEC+Xvu1F8
vLHr1aL2Vrb/f/2A8AQE4uf8l5YGYIUZeQXbvRDN2aq04tx8GuiKp3o1E7c1T9zGAtyGCd1bDejbH3r
wVreI6qN+kMVfdfl6zW52A9S6+2qH6ENy3GPgPncEP8zZjnydln0vOiqHaWYvWMNHoeB0LzkpzUn
VFl/9A6olGM9obVW21v6GMZPAcJrkvI/RZ2vePcb4PXz35E1ImNY5b37WT3p2vSa2eZ6D1Ar1TMr
49uJI6QOz91pzz4+IN6CQCQFxWevubvXqSX8qF6o1IfFQBydOlPqI86Q4SoCDUUGGl2/cyRYVRHai
WjzqrR43EyanuDNTzVurCgpuQNVXdFt3UUZ5+DwWRzdcJ9Fjflgjtk3joz5/RGeo1z3nkq1y+kUEahK
q1+y+tVOsTseTxK50C6+umwipx2N97ZkYsDJ7W96x2NX1c3yXVRQV7I/eZcj1EBZPj9pu7d0GC5U5
NSIMjOSpb3bcz7M2CCa2eZ7D0Ay07VQP1M7zN6CQCQFzV8Xw3jz5uqi3r3h75KfVQAyI2qj3qP9Z
H6qLNAiLqEKtt96Xfr4kZ7Um3Z9TH72zrxbG/4AZBXL9Won9mpyfrIxEaFec7r+3uboWUCxu4/wN+
et3/pyI6XBPWkse4GPca+Wzo1imzrKP523M2ofdbOu/Fbsn0WNy+If+rOS9v677wbglnn94JOUJOd
F4P9um2ZLK8uyolT+ukYlS23beb5V9ri53BnxMnhkpwj67VGqYGh+21ofyZj2/b9ZrYlbFt+kJ3U/Ny3
/uvD789hya6dZar3ACyrT+x/wp6NHwCQv5UvvTTzOqijPPTV78qf1hmBAAC56T8scvnP9QLyRIiKIW
bPtPoFPwgdDgOHletbE4Z54aotPxwsrp3xAqzOge4z2bsoO14eaey7suW3deeiJIl7zH364ZMh8T6Ls
nnWTbuaslpwe08Gz+8kipt740PW2PblMOpEFdfkTNjxtXf9XrVn1pzjSXSOrPOzp0NEI3gPvd4Dhu43
s8et0evSPk86CNzLYfa0LO/bsfdnmCTXzjLNewCW031fv0/u+rO79BIAIE+vfPBaufnrz9dLs/G5jz8kX/
nLR/QSACBzl74gcpkJ/vJGiIoBI3qm2Yqy5iYxIwK3UyfSDKjKcrKkn47SPdC9+VSoU/LqNhbMYdKRw9
QnMMk+K9tGr0FHOsP403RKoi+fcf2NgNIP3Kpy1j2gqa6LruEZOrw/aOh+M8JCawf20Hd33yNrsaZ
uHu/bBNduyvcALB93Jv6nn31arwEA5OWGx6+Sn3vgJXpptu7+0APy+Dd+pJcAAJlTw/r739ELyAMh
KibUkeHOgTHCo7mX3TFUvMKUSrD+5Oz05HBssVSf36vSHRZuBG7V08FeoUmYExB5PXXHCbtWR
dm8EN470w0rF3t+o8nvz/SvXdh7AJaNCk5VgPrID+h9BAB5e86Pj8lbvnijHL9U0Gtm60dPPiPN99+v
lwAA2XtK5NIf6OfIAyEqJjR/gWm53vWGbAcfk9YLHS/
+Po26n7aO1NZznJyneELGlj4tn5Sxl7S4KW5lAntYuDF8vno6/CyPP0dt2TB7nbo1OM36o0lYbdzT+xj
s/as0z83XpEi53bcTXLtoR+GPJpiWGsL/wCMP6CUAQJ7e+Nc3yAt+eIVemg+qPuqnf/9QLwEAMqfq
o15iYte8EKJiQElOusnV0EzePbnoFnIcO/Q7J6WTXtA2qm5m6ibYZ6+xrodpl6Ved5OsPCfnMa5rc1f8

zphdOfAu6QmJc0m9HrXW/XH+/I4OP6sSyOGSnCOzLqcKFN26qMaQ96TUzPKqx6k9GZQdSHa9W
p7pDpOf0CzuW0usa5fme8AU1xDz7yNf/Ig9mRQAIH+lh1448zqoo3z6v/6D/MOXvq2XAACZu/xZkf
48dRU6ughRMcCcBCnYW9IPAlXYNTChz6yYNTCbq/5Q7faGHhpuPdIev510n72GrLsnrnpWNjeNyY
Nq65JPjmrWsmzKrm5er3HOCzBj90asnBbnDulIs+ke18Bw8Amvix8oDvbcjUlvX80sH+xxaoTFZo9b
oz1p3yaRZnHfKnGu3QTvASU/dZXaee9gZMOb4h9Hze6Du/LBv/igXgIA5OnaJ6+0Z+OfZ83fvF+
+9y1qZQNAbtSw/v739QKyQoiKYeYkSJ2alHSoU/LTE7kw7YxI5vDy5uoUoVGwBmZzVQdQXnhTlVZ
qs9e7kuyzJ411d6h6Wepbav3okCq98zLMnFndbbN3TastiX+aKhIo72oZDmATnCMv2LO4x2xOsJTEi
HvXrLNaPevPNp/YQt+3SpxrZ0n4HuDXW7V41zBubVssGjV8/85P3amXAAB5UvVP3/aFl81NHdRRV
ICqglQAQE76T4pc/rheQFYIURHKHgYdUpOy2rLWu8Otp1KR7cGClfuHRs/BBOwamMZwbZddWzOj
eqgx92n23LN7obonbiCk8of1p3hehhRlc2+4Tqg9hD5hYBeYJMs6ZjsbHhT7uljH3G/5QaqtKq2+0dZ
ACYJo9r07eJA2Z5vTZZMLft9aYl07S6L3AHUsg6+ttiava4u59cTTTzATPwDMkOqBqnqiLgI1pJ/6qAC
Qo8vWb6bUR81Uoa8KBgJAEmo2fT0ZlAph96btmTxL+lhOtaYNWBfEUbp2yJUKTit/VJF7H75XrwE
A5EnVQVWTSS2SK597XLbufr3c+uoX6TUAgGxdKXLFb4gUbtbLSBM9UQEkFCxRcGZtsUO49nnrWC
J6ZB4tR+vaIV/v+
+z7CFABYEbULPx3fPl6vbQ4nvmnS9J8//3yoyef0WsAANmy3m8vqWH9TzmLSBUhKoCY2rJh17o06
pWaJQoWUK+xIqv7demmUqJinh29a4d81e+ry8f+9mN6CQCQJ1X/9C1fvFGe8+PF/NXt8W/8SO7+
0AN6CQCQuf53RC7doxeQJkJUAJNRNS8XfPx7cXMvpRq/C+YIXDvkR/U+Vb1QAQCz8XMPvERueP
wqvbSYvvKXj8jnPv6QXgIAZO7y10QufUEvIC3URAUAAKEefuJhec32a+wJpQAA+bv568+Xf/6Fm/TS
YqM+KgDkjfqoaaMnKgAAGKKCUzWRFAEqAMyGqoO6aBNJRVH1Ue/
+0FftjwCAPKj6qGpY/7POIqZGiAoAAIbc+ak75cHHH9RLAIC8qQB1UeugjvLN//19+cPf/ppeAgBkrv+
oyKU/1guYFiEqAAAIUDVQP9P7jF4CAOTttV97sbzs0av10tGyt/t/t/7AcAICeXv+Q8MDVCVCTUk8aKm
uVbPTaknWjdOGltZ96MOYZeQ1Y2gmvbG+7rV6TR0yunpfZjb3NEO2zTne9M2m1bkPugvaHbWJC
BSwosjE/sf8KejR8AMBsqPH3N3x3tuqGqN6rqlQoAyMmlPxHpP6YXMClCVGCGeo0VKZRq0tHLWe
pd3DH205RdQj4AA+77+n1y15/dpZcAAHm7+qnj8pa9G/XS0aXqov7+v/8y9VEBIDeqPup/tz5SH3U
ahKhIqCibe33p99VjWyp6bXby3l8WRhxDryHrtTziU6UnF3eC+2qea1hrF8VRuA+A+aZm4n/nJ98pT
z/7tF4DAMibClBVkLoMVE/UT/7nA70EAMicXR91Vy9gEoSowDLoXRQ3Qy2Xy86Tzo5cXJwUdTFUt
qXfrYs+w8DCUMGpClAf+cEjeg0AIG8//ffX2UP5l8m9n3xYvvKX/NsDALm5vGc9mOBvUoSoS8eoLbl
i9kQctd6oc2mvz7s25fD+IutuGnU/VwKfNLcT9vko6Z8z+/PmMP7mqvP5EYU0/WNWj+T1Rv2h/FU
5e+GMDvk6shMnRQ3UUg17jG5PvHbHuTbD59DaumyYrzfqkQa2MbA+/BTHaYN5POpY/P2rh7vd
9nnrupbrsjXQXdYu3WC83n4M3DfArKgh/A888oBeAgDk7YbHr7Ink1pGd3/oAXn8Gz/SSwCAzF26R
6T/Hb2AJAhRl05R1s64PREPpOs8s3TlwE30Aj0U27LbdJ6Vz6xZXz17ldNV/awjB/4B2PywsCxn1nRr7
RCwJIMj5zu1UswQa5bnrCO1UkFW9fYcal2SINUYyl89LZXimniHs3Nx+hCvfEbcU+2L2e6pr43Dfn1w
Z846FVQOrG+uDgSpE7VBHcuqeFvWoakKSlf369Ld2wxcdxW+lsJKN3RqUprnibKwFD7yxY/Yk0kBA
GbjOT8+Jm/54o1y/FJBr1kuP3ryGWm+/37qowJAbp4SufQH1kfqoyZFiLqEimtuT8R9OXQTovauHwi
JEU72Dq1XOU6dmIcI1VI5LW6M2gzMjmSEhV6w15PGutvjsyotu6am9WjpLXRqsh4jjUz7nFW2rTa
Yw76rLadd22HVPv12d+vuV8TsRaoYQ/mrp9X2zVA4xpD+4qbsuefNfnTFa4Z1BPULwcDQN67d6V
wbV7neDX69Frbev2+maYPxeh2aFjf3vOc+P1RXYWtXf41/TppyLsFxAmnafXBXPvgXH9RLAIBZeONf
3yAv+OEVemk5PfTV78qf1h/USwCAzPUfFrn853oBcRGiLiOvJ6IfaPUOndivWnXCo32dFJrDwO38bS
5UZMsNoJq7fi8+s+6n2wPUXFff8ickqmx5QWCs3pgzPGfVlj+Rkh/mWi0Z7IY7gtk792TJfmJsJ0EYq/
Ua636vzepZ2QxPUMe3O61rY6vKWbchRsg+cv3+obPdKdoQeH0UI1Q3Q2s7cNWB6t6okwhkSA3f
v/NTd+olAMAsvPLBa+Xmrz9fLy23z338Idn/n4/pJQBA5i59QeQyf8BKghB1KRXlxCnnmRtode1x6W
U5efqkHXYF11vUMHDn2VzwQ7mmeJ0KuwdeWOgN5ffWWcfkDu+2H8bw7cAQ/VFmdc784HMy
Yb1zLRMP6W/LeT9BlVZoz1klRrtTuzaW8kkJ3d2o9a4p2hC7Z7Zxrq2N2WUO3P3Er8sLpOuJp59gJn
4AmLGXfPe58nMPvEQvQbn7P35Vvvct/m0CgNxcVvVRv68XMA4h6pLy6oraPTl74nSqPCUnKies/w

+uV3ngPEWoFiOYcodmt90x0xG9I6exkOfM6GmphqaXQkPC+LP0tzf8OqBVo6fp8kkSbhdl80L4jP1u
cDtiPjEgE+5M/A8/8bBeAwDIm6p/usx1UEdRAWrzN+/XSwCAzPWfFLn8cb2AcQhRl5U3tHlfDts6aL
N77VXEyQqN9VP3hsyCUdfTDi/9upOjwkuvNubQI2YYuIDnzB/KHyXmkP72hj9RVLUlIzuhTmDqa5O
CTNtg1JUdKNlqa55jln7k532ffZ/c+/C9egkAMAsrX3qpXPvklXoJpn/40rfl079/qJcAAJm7bP02eukze
gFRCFGXVklO2hlkRw52nSHNbh3Rkv0Jf3347OuzV9w864eaDXeSp4E6pCVnqL3i1eKc2KKdM2Mo/
xjjh/S3ZcNLUMtSV1PRTyvVazOhHNugZuhXPU7tScXsQNWYoCtu2QJgSvX76vKxv/2YXgIAzELpoRfa
D4z26f/6D3aYCgDIyeXPivTp2jMOIerS8ntyNptOOObWeCzq4p/uem+Sprnj9gDtSK2mA77BOqRmP
crmqj9sur3h1aUsxB5LvWDnzBzK787+H3iYIV70kP5e45w3jL9cv5BOuYRUr82E8miD3pbKoIM9Trvi
ls8dW7sVSMn/+v/
+l34GAJgF1ftU9ULFeHd/6AH9DACQi0v36CcYhRB1iZkzpqvehd7w88Ds5sYkTXPIq1NqC+shGaxH2
VzVwZg/Ll1GT440LNVzVtS1VJXmauqhoTmUvxxaW8AoiWC9cuSQ/l5D1r0CqtYrAxMwOY/JJkhK99
pMJoc2VLb9IfyBurRGfdmzm7MP3bEU7v7Fu+WOl9+hlwAAeXvjX99AHdSYfv7dP6WfAQByUXijfoJ
RCFGXWWDW8FPiTzbuDltXzPVzyAwvRw2ht+tRGr0uXeW6dJPWu0z1nFVke7BA5v5hSrUxzaH8o0
NdMxQeNaQ/Xl3VCaV5bSaVQxvsIfxhxVBVSNvvp1pfFohy1RVXyf/4lf8ht1x3i14DAMjT373yCf0M
UV7z1pvkbe+5VS8BADJ37FUix9+kFzBKoa/G9QIAgKXxwCMPyOv/2+vtmfoBAPm648vXyysfvFYvYd
D1P/E8+a1PvVGedw0TbwFALgovFrni/daTq51ljERPVAAAlsztN91uD+0HAOTvb27/tjx2PX/ECnPlc4
9L9fdeS4AKALmx3m+Pv8f6SIAaByEqAABL6F2n3iUfeMMH9BIAlC+Xjvfl8294VH78nMt6DVz/8t+V
5NZXv0gvAQAyd+ztIoWb9QLGmSpE7TVW9AQpBX9W65F60lhxJ1TZkODL27LhTbbiPCabqGZYeyO
43dBH1jOAAwAwhz788x+W07ed1ksAgLz84PnPyt7rvqWXoLzidS+R1X8bNhkqACATx4oix39eLyCO
OeiJqsJVf5ZqR84zwquZ0VcaKU3oAwDA4rjnl+
+xh/cDAPL10M0/kO6t39dLy+3al14l1d99rV4CAGSucI3IMTWMH0nkGKIWZXOvf5ukcIL9QLi
mn1P1N6h7OuncuqEZJedlqXedUNc/9Gtl/XnRZq7pKgAgOVzy3W32EGqClQBAPlR9VE/96Zv2h+X1
S/8xivsofwAgJzYdVDz7rl4NOQYog7XRLXrlZZq4nZEtYfVD9UoNb/OeaNb/RP0RP0YFAGA5vfm
WN8tH3/5RvQQAyIvqgbnoy5Uq/QQAyIvqibqs9VFVePoLv35CLwEAMmfXQuXoGB5WVM8uW3d46hJwTA7YddqEYTfT/4kcHneY0nPYU7fC+q9b2DCO//8rEiKf0cCAt7
7s+
+V2h01vQQAyIuqjbps9VGfd82VctfvUJMbAPJztzuy/9bt3vCVPtoGFghnqNlWAPfSBnqjeq6pUKAMjXfT/zuD1r/
7JQAer1P/E8vQQAyNzxO0UKL9YLFmMT89kTtXZQdnSKU61+v+RFSVLXHLmHZ2LqbU+64s9S0S1NFG
TQoGTGKR76eM295ryPpg13tgBlR9VFUnFQCQnx8/57J8/g2PLkV91Le951Z5zVtv0ksAgMwdf5PIsV
fpBUxqfkPU7oHXE8sevu/1HjWG93cOpKufTsfppZpmrVUAABaVmqm//attZuwHgJw9dv3T8je3f1sv
HU23vvpF8ku12/QSAyBzhVtEjEv0Lv/WYBpzHdN1FSFz85vlhPo1M7nWItxHgxO2jWqxuPoOpD20GPv6
/VjZG3Jwf1FBNd2Pdzga0fVgsysDVEya98oca+VK/5xJm1f2OsHyncO1O806o7arx19P/kG2p/K9Ux6
DsOEt32wXungORo8P2HiTbTn8/epHqPqpCY5P0DQbdffJvf88j16CQCQl7+/7Xvyjy//oV46WlQd1O
rvvVaufO5xvQYAkK2rRY6/2/p4hbOIqSxEiFqud4fDT/uRwnDa4qac1WVWRfblcGnyBRVsDU7a1ZTV
wqr1/3hUiFMKG3rcqUlpMKBKMklYe2NETUjVYzgYFmXWhihZtW+khNcqwXEma58TyIW9vrlaGBH
2qXNitLNcl/GVM9SxDbR/6us5/f0ej9OrffAcNVdHhZyTcPbh1XO2Dd97qd3vWGrvKL6DGfsBYAb+6v
WPHcn6qL/yH15JHVQAyNPx09RBTdH8hqilk34P0YN0Bu2Ha8tuuinKQug1zvnhkTuhV7/r1Zsdzzhv
5bp0dbDd9TbQlHNeotNLMEmY9dpzesOh2+3IzkX3tVm1IUpW7Rst2bVKcpzJ2tdrrHuBnP+HjZa1F
615bkRQaLRjb1OKem0koz1u06e5ntPf70n47fHb3pHa+ejIPHqivUH+PsLvvbTud0Ds2frfdepdegkAk
AdVH1UFqUfJyumftB8AgJwce53zQGpSC1HtnmjGkFHzEWco65DimpzxspxVfxuqF6C77UQbdnpv
me1yHmYvuTOyFivhWXQ9uejO2qUCFi+kKcrmBSPEidI7lH39VDo74mY3xc09J6yxHnub+mQmmiT
MmLjHCNyKa2eGQ/XM2hAlo/aNlPBaJTnORO0LtuOst74iW/aGVVC3J2GHE2hHTNWz/rmtbPtBrd

/2JNczhfs9gWrL7yFf2Up/+4q5j/B7L8+J+bAM7v7Fu+WOl9+hlwAAefjmjU/JV376u3ppsb3sp14o/
+a3mNAEAHJTuFHk+L/WC0jLHA/nDwYcXkjrjWE1w5B0mMHN0daVAzdTKp+Ukn5qK56QU/ppJDP
kHgioh2ouTjVJmK6nGTZ8Prc2REmpfSMlvFZJjjNR+0a3wwldR5fWOHUi6XdVVU4HNlaSk2473bYn
up4p3O9zpSwnAwcRIteJ+bAMrrriKmbsB4AZuP9V37HD1EWm6p/+
+n/5GeqgAkBurhQ5/mvWR+qgpm2+a6IWN2UvbMitPdQ3hXqoHtWLri8pZ7JH3OhefG5oM1EPZ
MWu5eiGPlE1KzNsQ5R5b19sebQvRuA3VlFOLEzSmcbxAvPpphfcZAepKlAFAOTn8yuPylNXX9JLi0f
1QFU9UQEAOTn+r0QKN+gFpGmqENUc9hv1cMLJoj8M2gxA7aBUrw9NMc2v04+4dRUtdp1B82tD
H2kGskvEuHZ+7Udf89zw5DXjJwlry4bZq9OtjWnWijRl0oYo2bcvLbGOc4btG21wgreeHHp1B4ZlOv
HcEcD5QZpuv+l2e2g/ACA/KkBVQeoiel3l/6IOKgDk6dhrrQd1ULMy3z1RkZGQ4dEus05mDGp2d9V
b0Q+rjZ7D7raTTBLW3vV6dtrhjxuYG8OTB6XehihZtW+khNdqguOM176Idth1itOdfT7Y9JDh+ImO
M737fWHkNjEflpGaZOoDb/iAXgIA5EEN6f+7Vz6hlxaDmoWfOqgAkCM1C7/qhYrMEKIupaKs+bN2
yao3Ztuc0XsMPcGXKlEb7K0YEnhNOEmYH/4YM+KbcmhDlFTbN1LCa5XkOBO1L6Id9rE7NVUTnsK
Rmqsb4jfdL5lQPrPmhNaJrmcK9/uiyeB+B0wf/vkPy+nbTuslAEAeVH3Ux65/Wi/NN1X/tPp7r5XnXX
OlXgMAyJaqg/oe6+PVziIyQYi6pIqbF/zehs1VHawYk86MU9n2h393alJygxmjRqg/UVew9mbkJGGV
09aSNq5dWbUhSmbtGy3ZtUpyrpO1b2w7ynXZSmV8eFnK5aas6vZ4Tbfaftab/j/Z9Zz6fs+LOdGV28
6Jws6U7ncgwj2/fI89vB8AkI9Lx/vy+Tc8Kj9+zmW9Zn79Uu02ufXVL9JLAIDMHVsTKdysF5AVQtSlVZ
TNvcFJu6rS6res/8djD/8OK6Rpb2dgoi679ubg/ixDk4RVZHuoDc72vF01d71eitm0IUqG7Rsp4bVKcJ
zJ2qfa0Zfu0IbVpo3SBlM7JWcHjzfsGiW6ntPf7/mw7q/B67F/aPQSTiCV+x0YzZ2x/7qrrtNrAABZ+8H
zn5W/ev1jemk+veatN8nb3nOrXgIAZO7Yq0SOv0kvIEuFviqCCAAAMIF7H75XKn9UkaefXYwhpgBw
FNzx5evllQ9eq5fmx7UvvUr+06ffzDB+AMhL4RqRK9R8BQzjzwM9UQEAwMTefMub5aNv/6heAgD
k4W9u/7Z875pn9NL8qP4udVABIFfH3239jwA1L4SoAABgKu/92fdK7Y6aXgIAZE3VR/3cm75pf5wX
v/Abr5BXvO4legkAkLljbxcppFNUD/EQogIAgKmp3qiqVyoAIB+qJ+re676ll2ZLhae/8Osn9BIAIHPHiiL
H36EXkBdCVAAAkAo10dQt192ilwAAWeve+n37MUuqDqoaxg8AyImqg3rsPXoBeSJEBQAAqVAz9b
d/tc2M/QCQI9UbdZb1Ue/6T6+2g1QAQE6O3SlSeKFeQJ4IUQEAQGpuu/42ueeX79FLAICsqbqon3
/DozOpj/q299wqp/7ZDXoJAJC5428SOXabXkDeCFEBAECq3lF8BzP2A0COvv2if7Jn7M/Tra9+kfxSjV
/kASA3hVtEjv0LvYBZIEQFcteWjUJBCsZjpdHTn1tSvYasbLT1gqO94Z6fFZnX09NrrDhtXGnIqCb6x+E8
lv5aY2mo2frfdepdegkAkLW/v+178o8v/6FeytbzrrlSqr/3Wrnyucf1GgBAtq4WOf5u6+MVziJmghAV
yFVPGiur0tRLjrKcWSvq58vHDiJLNeno5YXRa8h6LarV6loXZDV4saVTK0lhIDAGjqq7f/FuuePld+glAE
DW/ur1j8kPnv+sXsrOXb9zu1z/E8/TSwCAzB1XdVBfrBcwK4SoQK66cuDmbuW6dPt96ff3ZHNZM9
SxQeScUj1nxwW/7fPiHlq1pa5zX7r1srOiuSrkqFgGV11xFTP2A0COfvycy5nXR33zr9wir3nrTXoJAJC
5YyvW41V6AbNEiArkqXco+/qpnDohy9v/dHHF6znbk8Y53QW1XJetivO0uHlWqs5Tae6SomI53PSC
m+wgVQWqAIDsPXb903L/q76jl9L1sp96ofzKziplwAAmSvcKHL8tF7ArBGiLjGvnqP5CKntOLI2peqN
536d163Or/dp135sb+ivNdbZLwuuD/bKS2MbmtlG7zFcYzN4jMM1S+1HWN1Low3jal3a+zDDt+aq
87WDDQ9pc9i2o9o8tpfjROdl+J4ZuZ8YxxD7fGh+W9RjuK0OZwi9/7p0z506/lKsnrNGj+NAWF6Sk7
ozquwfDt9PNqM9ie65eMduS+H7wj1Pcd9HsNxuv+l2e2g/ACAff/fKJ+SbNz6ll9Kh6p/+
+n/5GeqgAkBurhQ5/mvWR+qgzgtC1CWlwpHQMKhTk1JhQ8ZlcHHYtR8HCkLa61TIMrC+uRoeXk2
1DRU2hfYY7EitNCqEU58zapaW61J3uw52duTiYMi0674ynbqmQ8GiZh/zyGBquM1uz8dQE5+X4X
umuToieEt8DFGcfQcvd0hb7WCw5A2hd6V67jxVafVbXq/SIWaP41E6B9LVT4MqcjrpPZfk2FP6vlDn
KY/3ERwdapKpD7zhA3oJAJC1z688Kk9dfUkvTe/f/Nar7J6oAICcHP9XIoUb9ALmASHqUmqLn8O4d
TmNeo3SlHPhSUpi5XrX3na/FYybwtaPGt482TaCw6mHj7EjO4PplEcFZM7r+3ubsrlVF+erBr/GPI9nZ
FyGWtm2ttd1t2Wptpx9bOvUrr3hB4Vhbe7UZH3kdQm2eXRT0jkv/invSO28cd0SHMPY8xHg7zu8r
dZxrbvBoHEu3Iamcu40u53bEitrHVKUE6f00wiVRPdckmNP7/uimOP7CI6OD//8h+X0bQxHAoA8q
ABVBalpWDn9k/YDAJCTY69zHpgrhKjLyOwZ/R0K27uOQGJ9dhLZaajqpx1t1M5bS25RqwPHd486T
aKsrlnBj6O4toZL7TrHIT3AyzXt4IBWXFNzugv6uxc9NvY3vV65pXPrI0P38YwexjWLxht3rwgfgZ5PrR3

31CbR5r8vFRbfnDoh3xB0xxDFHPfoW3tXZQd3SEycC4qW/5+zWtniH/u1HFY3yOhIe8k9uUwrEFKk
nsu0bGn+H2R2/sIjpp7fvkee3g/ACB7akj/V376u3ppMqr3qeqFCgDIiV0H9V/rBcwTQtRlZAQ0ImqY
bsGrZTiyhuIkyielpJ8GjFofJo1t2HQ9x9BhzEGnTgwGP0VZ8xMtLyzyA0Mj0J1YVK9WY/8jgrfhNscV/7
yMN90xjFaWk+MudvfAa79X7sF+GEPcRwyfn/zcTeuUjN51gntuimNPcv2HzlNe7yM4ctwZ+6+76jq
9BgCQJTXJ1KT1UVX907t+59XUQQWA3Kg6qHdaH6mDOo8IUZdSUTYvhPckdEOYqMl1FkZg8hyjn
mOk8MDO76nnDnc2AsPq6dg9GafXkeGOgjFCRlOK52UyYccwK2keVxw9ORxbLNWR2T2X2vVfkvc
RZOKW625hxn4AyNFfvf4x+fFzLuul+H6pdpvc+uoX6SUAQOaOrYkUbtYLmDeEqMuquCl7esjtQKlR
W/Pcos+u3ZYNs3edW7PRrMGZhHW+zurzZA+NNoYyV0/nF6FOH/qlfF4mkm1w6dXKHXpMWsd0
AsUTMrb06bje1BPcc+OPPf3vi6P9PoIsvfmWN8tH3/5RvQQAyNIPnv+sHaQm8Zq33iRve8+tegkAkL
ljrxI5/ia9gHlEiLrE1MzaqqeYPcGPHYR0vRqKo4f/GoxhxHPHrB2pgiW3/uMUba64U6Z3duT8+R29n
aqkk6GW5KR37gdnZO/JRbfoZeQQ8BgyOC+
+nI4hTOmkFwKOqumZL+NcNHfF75DZlQPvNJxwzn2EWPdckmPP4PpP/T6Cpfben32v1O6o6SUAQ
Jb+8eU/lL+/7Xt6Kdr1P/E8uet3qF8NALkpvFgP48c8I0RdRu0Ne6itmkU92FPMCHiMXnIlPxkzZmJvy
4Y3Dft884MlY2bySXgTWHWk2dQnKrWh/EXZdLsdqvO87l+XXmPdq22ZZBKkcVI7L578j8Fj1udsrvr
DyPW9bj9yHVtu1oBtyq7eda9xzgsxY/VgjnPPTXjsU1//hO8jZntyvRSYe6o3quqVCgDI3t/c/m157Pqn
9VI4Vf+0+nuvleddc6VeAwDIlqqD+h7r49XOIuYWIeoyqmz7Q287NSm5QYtRH7F6NnzmbhXSDL52
Lpkz9nttNibamUhF3I6BrlSH8o+4LiU/fZQL005glcl5MSQ9BnPYu9ueiRK2YH3O5qqz34IX9Felldqs+v
EUNy94PTLd9njnodqSeM2Jc88lOPY0r3/C9xEgiqqPquqkAgCydeI4Xz7/hkcj66NW/u8idVABIE/H3k
od1AVBiLqk7KG3YUUMVeDS7wcDHlX3cLBmYrWVcx3NpCqy3W/5gZHNOTbvsAPDrOPxhlcr5bpsp
ZzL2dcl5LxWW9Z6d+j1VLI5L6Zkx2C1Z/A+3D80ejUmYNfnNIaSu+y6nznWQ/UUZXNvuFaoPYw+Q
aAb656LfezpXv9E7yNABDVTf/tX28zYDwA5UPVR7/uZx/VS0Cte9xL5hV8/oZcAAJk7Zv2GfPwdegHz
rtBXRewAxKNmNtcT86gwbG/anqHAOEfpntPHcqpFwIpwn+l9Rip/xM0BAHl441/fIKWHXqiXRK596
VXyW3/8z+yPAIAcFK4ROf5+66P/Xoz5Rk9UILaeNNbdmc3LcmaNABVZO1r3XPu8dSwZ9ODG0fGO
4juYsR8AcrL3um/J9655Ri+JVH/3tQSoAJCn4+8mQF0w9EQFxmrLxmANWFXOgK50yMzRu+d6jRU
p7ZyRbiplKXDUFf6fgn4GAMjStU9eKe9s/aS88723MYwfAPJ07O0M419AhKjAWAOBFgEqMsc9h+
WlJiiblx9NaEs42hKOtoSjLeHmqS0nrr1GDr/3pF6aLa5RONoSjraEoy3haAvSQIgKAADmBj/ghqMt4
WhLONoSjraEoy3haEs42hKOtoSjLeHmqS1IhpqoAAAAAAAAABCBEBUAAAAAAAAIhCiAgAAAAA
AAEAEQlTkSE2WU7DrfxRWGtLTa8fqNWRlo60XHO0NvZ3CijRibwjp6Uljxb0GG9aVTbJuGmH3UNr
7mJbRRv1Y4SYFAAAAAGChEaJirvUaK1Io1aSjl4H5pgJdY1Z9W1nOrBX1cwAAAAAAsIgIUTG/eg1Zrx
GfzqeibO717RkF+/1tqei1szFPbenKgXvLluvStdu0J5tkqAAAAAAALDRCVABIS+9Q9vVTOXVCyE4BA
AAAADgaCFGXmao1OlC7MazG6GD9UXuIvfE1A+VKNbNOpXokq1Vp79Mcxt9cdbYTvjOjjeoxqk7qY
JvCa1VOdrymMbVf2xvetob2H3JN4rTRY359ZEONcxFo46j1xj7t9ea5zKoOadx7KKotg9uYsD5pjOuS
9J4FAAAAAACLgxB1WakgL7TWaEdqpVEhpPpcQUoDQ+ybq4OvVyFiSYIva8pqYbBWZBqcNq0GNh
xyDHYINtgm65W1UnjQaYt7vIMqcrqqn3Z25OLAa9u7bmODtTKHQjgtuo2TKsrambLztHMgXeeZxR
iOHmh7W9xml8+s5dDDMoV7aKJrPizf6wIAAAAAAOYRIepS6knjnJuIuXUb+9Kt61BNOrIzmPx5qtL
Sr2+5QaEKG8/7ve16jXN+0FVt6VqVXfE2H0Nl2/qabl28L3G3sx1W8dJvU/gxWMe77oZg/mv77gF0
arI+MhUdf7xhKltu2wfPpR9GSvmMeBlqe8MPgsOuSWQbJ1NcO6PbuC+H7qbbu0ZI2ZEDN101hq
mfOpF9hDr9PTTNNTckuC7J7lkAAAAAALBICFGXkjERz96m16vQD9VEOl56FlRt+RP3+EGhqScXd9w
+e1VpeQGStc8LYa+fntmm0GPoXRS3SeX6lj/xUGXLC+U6OxdDexSOP94RimvidfQ0t22ElGaPTrN3av
2CcU02L/htrJ0fGKo+Ja+NftDbO3Si0mrVCRv3dbrau7jjBZKnvROYlRTuoSmuuWkm1wUAAAAAAM
wdQlRYdA3P0OH9SZmzk5+Ukn5qK56QU/ppespyMrCTEN0D77jsIdheXUtjqHdgSHsazOHy/rB4P5
SryllvyvYRvVNtxnbMHqOpKMoJfUHcwLlrXzzrnJ4+aYeVwfWW6mk/kMxMCvdQKtd8VtcFAAAAAA
DMG0LUZRWYKCdunckYgeWRMt3x+r1i3Z6eRig3URhpDK9PScUt3trctVrXE6cj6ik5UdFhZWC9anb
2EeriSf+6AAAAAACA+UKIupTasmH2OnVrPZr1HI+ocr3rlDEYevjD9lNT3JSzbglONXTcqCs6WRiZQYh

dOS164L4ctvUQeLv3pzs5lrF+QUP07K/5sv1xAQAAAACA5UOIuozMupwqYHLrohpDoCdXkpNuEjs
4XNoIEXNVcoamK6NqvWbF6+nZ2ZHz50fVFTXP2eBs/mZ90FMydk6nxNfQ3XdHDnadr3VrtZbsT/jr
h4e0ZyWFeyiVa57idQEAAAAAAAuNEHXJ+QGTMWP/VMxakU1Z3XCn3DFnS8+ZMcmTNFfFa1J7w
6+V6a1MmdfTsyPNpj76oaH8Rdl0u6xar6utN6yz5eg11r0anuYESU7AqVivP+8dkGx4U8nH5V+vZtP
5Wnf2/aIumOquNyfCylYK91Aq1zz5dQEAAAAAAEcTIeoy8oI9S3NVh0rGhDtTMmcun2r75iRC7nY
mCjuDs7o3V3WI5gWO5gzwaXOHxftCh/JXtqXl5XU1KdnnrCAlP6mTC95EVCojdOutWrxzHLe2bVBg
W9Yzb2i6eZ9Y68/k0w3VNv09lNI1T3hdAAAAAADA0USIupQqst1vGQGZUpVWv+8HRvaEQpMqyu
Ze1w/BbGr7g/scx2qn1yBt/9DrDZhIcVP2+oNtstj1YDOoh2rwhvQr1v62Ruysst0PrUtbbVnr3ZILLnU
8g6+ttiara2v22gwMTTeGs+c+ZD2Feyila57ougAAAAAAgCOp0FczrADITq8hK3oiL1WDdo+eiwAwk
urtPS8/mtCWcLQlHG0JR1vC0ZZwtCUcbQlHW8LRlnC0BWmgJyqQKbOOZ75D4gEAAAAAAAJAOQlQ
gE23ZKAzU8ayeFTqhAgAAAAAALB5CVCAPql5pZpNXAQAAAAAAIEvURAUAAHODelXhaEs42hKOto
SjLeFoSzjaEo62hKMt4WhLuHlqC5KhJyoAAAAAAAAAARCBEBQAAAAAAAIAIDOcHAAAAAAAAAgAj0R
EV2eg1Z2WjrhVnrSWNFzZavHhsyL60CAAAAAADA/CNERSZ6jRUplGrS0csAAAAAAADAoiJERfp6D
VmvEZ8CAAAAAADgaCBEBQAAAAAAAIAIhKhLqr3h1gddkUZPD7+3l51HaClTVePUeI3zcL7eZW/XH
MbfXHVepzc4uF+PuW1j58HXt2XDfY318F4Wo12TGjwv6jHpuVGSHr8S1obCSkPCD8+s/eo8VkaciG
TbBQAAAAAAWF6EqEuvI7VSQUoDw+
+bqwMhX3tjRI1T9fXpBJbR1H5WpamXpFyXrYr1MbN2OWHk4HlRmquFYNCZ4blRoWtYG6RTk9Lg
BFl2EFuSwZd3aqWhcDTRdgEAAAAAAJYcISosVWn1+9K3Hq2qXqUCwPNulNaTxjkdX5br0tWv7dbL
zjrrtTsXnYiusm19rlsX9zNSbdmv7W+rxHNafjv7e5tSTNCupHqNdS+MLNe7zj77LasFWvOcDkeza4NI
W3bd1Dh020055yW0VjvW3SDXOE/uBe3UZN17bZLtAgAAAAAAgBAVUm1tixtxVraMANRTlM09H
crZ4aVeu3bGe23noKufZadc3/La6ciqXT25uOP20qzK2U13yxXZsoNGFVLuibM6w3PTO5R9/VQ6O+
JmscXNPWd/1mPPbVvvorhNDpynypa42Whn56J1ZJYk2wUAAAAAAAAAhKiaha5OGDmHPzqkT44K
9tNrVlQN3A+WTUtJPFSdo9EPnYSmem+KanHGTWGtrquyCW7t0qM5p98Dbnz18X7+uYA7v7xxYR
2ZJsl0AAAAAAAAQoqIsJ82UcJTAxElGbdLcjGjnzNtlyawNRdm8ENYz2A9KQye5Giur7QIAAAAAABx
NhKiIoS0bZs9Kt46mWft0JuahXRm3obgpe3qIvV+v1tc8Nzybvl/DdfBh9KCdYLsAgLzp0Q3Ggz9yAQ
AAALNBiIrx2rte70o7oHNrfxpDyKc2ybYya1dJTroJqDsE3qVm4i8YM+6n1YaI16uZ9NUvzfakXXbw2f
XqnHrtK530Qtu4NVhjbRcAlpH9Xl+QwkwTy540VlZFWu57tPNHr+bqLILU+Qxze40Vpz0r+f/hT/0ba
p4P9zEP5wXDhq7XDO6ZmRvzvuZ9P7mPvG7mOO+3ebwnR+5jSf+gFfO8z/K9eGZinJuh951luGnG
nJfc34sTtofSdoiDEBWJ+AGdMSt9AiU/nTRm/7d+MFmdbhD8tO0KKsqaVzS0Kavem667baeO6OB7
cZw2JDp+/aavPhXsGRpSs9Wsc9pc9dvm/sOhHu7KJNtVjG3wDwuALMzsl/chKri09j/lv0npcCYu3Da
KcLuTP+4f5vte3N4IC3ONPyjOQq8h6zWRatX9x28G3NEnxsO8XrMy01/cAyWOhh+z+NZW52O1qS
YFda9TS6qdmpRmFPjk/0v7+Pc19R5csr6f6l3jHFk/T2Z778R5v83jPXncPtTnc/yDVmTok1eYm+C85/l
eHHFuht739CP98xP/vl3dH/g3Iot/IEadk9zfi8efl3zfiydoj/WNrUrbpf+eHO/7dn5+FsZY1g2DJWS9R/
TV5bd+A+hbPzD5uvW+9U+g87mq9ZZia/WtnxX060c8vNcqIa93P29uf9TD2NbIdtqStKvbr5fd9VXr
K8cxXx/yKNetVygJz83Exx/
+MDcdve3gMSfabqvqrS8PXwQAmEq3XrbeX8z3eP2+GngjyoffFv1vwAzaEEm/z8/8vXjm7XCuj9q/f
c28f5PzY/87OoP9RtP37dy1y/3eivPzV9rCv5dn1R7n5y9jv/pnrCy/l8a/rznvuYNtyPocjW9XvNdMa6J
9ZPIeqPdvbdd+hLRD3T/maud+Um3XK1IS/5w4n8/+vTjeucnjvS/
+fZv1+8v4cxImq7aNPy/h62fWnhHfw+nfR87+zd0737fBdX579Qr9vhz3uiJf9ERFDBXZVn8p0ksO56
821puAo7kr/t9KrNd7n9D2D52/MKlanIP1QqutCWuIJm1XEk4PIOsNTS/7rDdbf9h+0jYkPH57qP3g
ubQ5+wj8QdOuc2oMyXfZPWWMeqiWRNsFgMy05XytY71NXZBN503VUpEt9UY28fv35Iqbe9Lv7x
ltmTO69MupE/PRwFm1o9dYl1qnKmfn9kLNhnteWt7PKPPC+T6X6unAzyL5KMqJU9YH9+dQW08u
7sygPb2GqIFK5fqWv9/Ktv3zYmfnYgY9sRxj39d6h7JvfRj8fi7aJ64puxm9Ecd5v83jPXle3ved79+y1Ls
hP8tr6uf34dEJHdm5mO7dE/ec5PVeHOfc5GX8uXHeXwLf5xmY7Jxk9148/rzk+148tj0jfp6yR42mW

tYuzqii+fpZGOMRoi4pvw7mwJuLMeFQcMiBCgvdr1EPJ5TztxMM6dQPhf5rrYf5A725D/VQ+xmx35
Ht9MRtl/MGFlw3nvMG7H6d89gbakjCc5Pg+G2D59J+jDoG8zj1Y9QvU3G3a7xu+NgBYAoz+uV9M
enSL+W6bIX/A5CTnjTWa9KZVTvsoaMdqbbi/1ueGTUM0Rh6N9uSN/n84j6JXuOc9d1s/bI/oxu3sq2
HjNo17dWwypITNg/
+vJW13H5pT8huV1lOenWcNKPePgZk8AetuMFlmDTbEVuO78XTnJv86fJoB+cHhtOnWwJnknPCe7
FhxPub8/PnvuRaNYmfhRcOISoAAJgNfnmPTdUltX/5uTCLXoa6tpj9i6D+pWdGvR3b52vSqbZmPmL
C/0Op81AjV7KppRZXPr+4J+f2NDo7wwDEHTWkatqr7yOrObMI4efpl3ZMgT9oKfPyXhwwR3/Y6uy
flAvevxGqt6h6/9mYYa9C3osD9HwiwflBrO+pqedWiWHwjzD8LLxwCFEBAADmmDP5gRq2N6ueO
MFRDt36vqzO4hfU9oYzCcRc/dbuUL2Csh6WHcfc/eLePi/O6NHZXTNnsg5/YiAVeKtJgXKftGOWv7Q
jNfxByzKH78Xz9oet8pk147pY/4aeVWXUZtirkPfiAernGrdnrP89dXBKXadTkl3n7nn5IwymQYgKAA
Awp5wAVfXWmJ+hjMXNC3YNtk7tfK7hXHtXhU1NO8B1fuEpSEn9Vqh/CZrtcHptlsOyLfP1i7sOCGf5
y6Iecqzq2bt5jz0MViXezdWMZjYfZVa/tI9h93bqyMHgjTuqd9QS4w9ajkV4L56XP2x5ZtqrkPficIMl+f
qydXLfOk8nJau3vdn+EQZpIUQFAACzwS/vkVSvDSdAndMJ/zL8RSPMYE8j9bAngLR+Mexaz2dbt7sn
h6qoWc7nZKxZ/uLu9nw6O8NfFgeHTbpmdl7y/6V9rOIJceZ7CUZNPfuGnmG4O2f4g5Zvvt+LB+T+h
62S2GWOB8PbWf5cw3txTLq2eOCPkekZ+UcYfhZeOISoAABgNvjlPYKeRddiD3czevzkW+dSTf4wM
Bxc/0KW1S8ac6/XkJUVc0i2WqVmSp7lL6nz94u701utKjMcPSpSOW21wPoeCgyhV7dwzTovM26bL
dtf2uOpyGnVY6+27r+veL3G5m+islngD1qLaFZ/2NIjADo1Oe/9w6mHcM+oHinvxXGoUhklqUldLm
RwkSL/CMPPwounDwAAMCOtqvSt33L69a5e0a33y9aPJ2VvRX669bLVFtWewUe139KvWUZh56U
6JyfEblu53s/3bun26+Xg+ZiLe6RVHbg2rb71S2tfJT+5m+H38TB9HgKPefie1vdRxvdv3Pc1573Yf2R9
7eK0K4/35PH7CLt/3Ifxb1dq9H0x9H2r2jFw3Pp7Pu1rNcl5t78m8/fiEedGvd8M7Ns9hrTf/uKem6H
XZfY+POp+0XJ6L453XvJ7Lx7fnpB/xzO6RnHuxXn6WRjjFdT/rIsKAAAwE+5f6F3WD43zNRwQWBCq
x5xdm9BVbUl/Bl3nnO/pqrT6M5p5eS7pnk7G5ZnV9cH8Gfre9fjfR2Gvqc5r79gUjT83Id9bxnk7iuLc
LwrvxbOmRvOoOqhhgkP7+Vl4cRCiAgAAAAAAAEAEaqICAAAAAAAAQARCVAAAAAAAAACIQIgKA
AAAAAAAABEIUQEAAAAAAAAgAiEqAAAAAAAAAEQgRAUAAAAAAAACACISoAAAAAAAAABCBEBU
AAAAAAAAAIhCiAgAAAAAAAEAEQlQAAAAAAAAAiECICgAAAAAAAARCFEBAAAAAAAAIAIhKgAA
AAAAAABEIEQFAAAAAAAAgAiEqAAAAAAAAAAQgRAVAAAAAAAAACIQogIAAAAAAAABABEJUAAA
AAAAAAIhAiAoAAAAAAAAAEQhRAQAAAAAAACACISoAAAAAAAAARCBEBQAAAAAAAIAIhKgAAA
AAAAAAEIEQFQAAAAAAAAAiEKICAAAAAAAAQARCVAAAAAAAAACIQIgKAAAAAAAAABEIUQEAA
AAAAAAgAiEqAAAAAAAAAEQgRAUAAAAAAAACACISoAAAAAAAAABCBEBUAAADAQmtvFKRQ0I+N
tl5raG94n19p9PTK2fDbuiEhLV0wbdlwz3vk+e1JY2Xca6xXNVb8ba00rK8CAGB+EKICAAAAODqa52
TGOemSUMHoqjT1kqMsZ9aK+rmpKJsX6tZnHZ3aesg1asv5Wkc/F6me3bS+CgCA+UGICgAAAOAI6
Ujt/OL38Zx/XTlwM89yXbr9vvT7e7I5KvksbsrZqn4eco16jXN+IFttyXZFPwcAYE4QogIAAAA4WuiNm
r3eoezrp3LqRKxeo5Xtlng5auAamb1Qq9IiQQUAzCFCVAAAAAABHTLzeqH590pVg6NpryIpbm9Orse
rX/7Rrehp1Vr119suC68NKtPqCtUJH1wEdeJ31CKsrGjyeYL3S6HYYzGPXj8F92fsp1ayzrDVXndeO3Ul
FtltujOpfo/aGXxagXN+yXjUo3vHbQto/dH0tcc5VoEar+6BWKwAsLUJUAAAAAEdEWep1HdJl2Bu1U
ytJYTVYDdRep0K2gfXN1VEBZlNWCyUxyoCqjUhpcMIpOxQceJ3F3t/IQK8jtZJRr7Rcl60YnTuHwlEtel
8JVbak7hZHba7KRqMh5/wEVS4M1gNIcvwqwA5pv3M+hoNUR/i5UueiNLhTJewaAQCWAiEqAAA
AgKNjzQ3p4vVGnVS53pW+qgPq9ax0hK1v7o5oh1dLtC/
+y5tyzkv7etJYd0PBqrT0a71td2qyPjIpNl6/F2OSpvaGePmv0a6um3ga+6psW5/r+hNFqRqm9n5iDc
MPTjLVrLnHV5b6hcF2Jjl+67VuGhvWfmsrOxfjnqu27Eadi8A1AgAsC0JUAAAAAEdIUTbdGYwy641al
bNuj8nKaWvJNWL9/mFoL05zBnqzXmhn56Lz+t5F2dGdIQPD3I3enN5rB4QPix+t7aeGgTCzuHnB31
ftfDo9MAOTTGnVs8OTUiU6fuu675lBqKO4dsYLbDsHXf0saOhcmfVeOzviZq/FzT1n+9Zjb+QMWgC
Ao4oQFQAAAMDR4oVsGfVGLZ+Ukn4aMGp9qKqcDiR3JTnpp31ix33dA90L01rllguwH8bwdve1A0
6dSBLymT0vz8ha4EuLsnbGbdi+HIYlthMITDJlPQudTGqK41fHZNc5DR3eHzR0ropr4h2yuodK7n4ja

rECAI48QlQAAAAAR8xAb9RD5+l8K8qJU/rp1MpyMn6am0BHRnTmnE6i8DlCYFIpo85ppLBzFSw5Y
HLD3NgTdQEAjgxCVAAAAABHj9kbtRYvTsvXYK/Onhx6Y8iHebVWhx7biYbtTyercHa88cfflg2z16lby9
Ss3ZpEcVP29D4Gyt7amueYpR8Alg0hKgAAAIAjyOiNmpQxjDw7g706u3Lg7tTtmVk66QWAo+p5ps
MsJeDXAHX05KJbmFROSaIqAdNKcvztXa/nqR24unVRp7iWaoZ+1ePUnkjLDlS7Xi3W0WUEAABHF
SEqAAAAgKPJmIAoTMlPDo3aqW3Z8Kapz1ZzdcObqKm94Q8/L59ZcwJAszZnc9UfQt7e8Gp0FlIZV2
4Gzta5WPd7WfYa61790aSTVU1twuP3A1djxv4k9PbVbRDscRoSdAMAlgYhKgAAAIAjKro3qjlzuwrp
nGAubi3NaZWlXG7Kqg4D/dzWmOFftd+ozdlc1cGh9+IREzJNorLtD1vv1KSk21XyE1S5kPuM9AmOv
3Lan6jKu5bGBFRJjDgX5r1RPat7ugIAlgYhKgAAAICjK6o3qqp7OVgzs9qavI5mIqfk7J4xPFyx63gO1Di
1a3MOvE4Je+2U7GHrIcdebVnr3eHxeYt9/BXZ7psz/itVaZk1TZu7Xs/fcexzEVYMVW8zrewaALA4Cn
1V3AUAAAAAAAAAAEIqeqAAAAAAAAAAAQgRAVAAAAAAAAAACIQogIAAAAAAAABABEJUAAAAAAAAA
AIhAiAoAAAAAAAAAAEQhRAQAAAAAAAACACISoAAAAAAAAAARCBEBQAAAAAAAAIAIhKgAAAAAAAAA
AEIEQFQAAAAAAAAABGEvn/AZM6nr53azj8AAAAAElFTkSuQmCC]]></redcap:Attachment>
</ItemDef>
<ItemDef OID="q22" Name="q22" DataType="text" Length="1" redcap:Variable="q22" redcap:FieldType="radio" redcap:RequiredField="y" redcap:BranchingLogic="[q20] = '1'">
<Question><TranslatedText>Do you prefer Life A or Life B, or do you think Life A and Life B are the same as one another?
</TranslatedText><redcap:FormattedTranslatedText>Do you prefer Life A or Life B, or do you think Life A and Life B are the same as one another?
 </redcap:FormattedTranslatedText></Question>
<CodeListRef CodeListOID="q22.choices"/>
</ItemDef>
<ItemDef OID="summary23" Name="summary23" DataType="text" Length="999" redcap:Variable="summary23" redcap:FieldType="descriptive" redcap:SectionHeader=" " redcap:BranchingLogic="[q2] = '1'">
<Question><TranslatedText>
Alzheimer's disease - Description #[no]

If you have Alzheimer's disease...

1) You might forget recent events of importance to you. For example, if you are interested in hockey, then you might forget whether your favorite team won their last game, although you may remember watching the game itself.

2) You might forget the names of people you met recently, or the names of local streets.

3) You may no longer be able to handle your personal finances or plan leisure activities such as dinner parties or vacations. Simple decisions such as what to order in restaurants might become difficult to make. Your ability to concentrate may decrease and you might have difficulty driving.

4) You may start having trouble finding words to express your thoughts. You might need to be reminded to shower or brush your teeth, but you could do these activities on your own. You may need to post notes around the home to remind you of simple things like turning off the stove.

5) You may remain aware of the day and your surroundings. You might be able to travel to familiar locations like the supermarket, although probably with the help of a loved one or friend. If you are in a social gathering, then you might have difficulty following the conversation.

6) You may continue to enjoy hobbies such as puzzles or reading.

Now consider two possible lives. In Life A, you will live in perfect health for 5 years and then you will die of natural causes. In Life B, you will live in perfect health for the next 10 years, then you will live with Alzheimer's disease as described in the summary for another 10 years, and then you will die of natural causes.

</TranslatedText><redcap:FormattedTranslatedText>
Alzheimer's disease - Description #[no]

<p></p>

If you have Alzheimer's disease...
<p></p>
1) You might forget recent events of importance to you. For example, if you are interested in hockey, then you might forget whether your favorite team won their last game, although you may remember watching the game itself.
<p></p>
2) You might forget the names of people you met recently, or the names of local streets.
<p></p>
3) You may no longer be able to handle your personal finances or plan leisure activities such as dinner parties or vacations. Simple decisions such as what to order in restaurants might become difficult to make. Your ability to concentrate may decrease and you might have difficulty driving.
<p></p>
4) You may start having trouble finding words to express your thoughts. You might need to be reminded to shower or brush your teeth, but you could do these activities on your own. You may need to post notes around the home to remind you of simple things like turning off the stove.
<p></p>

5) You may remain aware of the day and your surroundings. You might be able to travel to familiar locations like the supermarket, although probably with the help of a loved one or friend. If you are in a social gathering, then you might have difficulty following the conversation.
<p></p>
6) You may continue to enjoy hobbies such as puzzles or reading.
<p></p>
<p></p>
<p></p>
<p></p>
Now consider two possible lives. In Life A, you will live in perfect health for 5 years and then you will die of natural causes. In Life B, you will live in perfect health for the next 10 years, then you will live with Alzheimer's disease as described in the summary for another 10 years, and then you will die of natural causes.
<p></p>
</redcap:FormattedTranslatedText></Question>
</ItemDef>
<ItemDef OID="a23" Name="a23" DataType="text" Length="999" redcap:Variable="a23" redcap:FieldType="descriptive" redcap:BranchingLogic="[q2] = '1'" redcap:InlineImage="1">
<Question><TranslatedText></TranslatedText></Question>
<redcap:Attachment DocName="5.png" MimeType="image/png"><![CDATA[iVBORw0KGgoAAAANSUhEUgAABVEAAADYCAIAAABtBTQzAAAAAXNSR0IArs4c6QAAAARnQU1BAACxjwv8YQUAAAAJcEhZcwAAEnQAABJ0Ad5mH3gAACIfSURBVHhe7d1fqF7Vue9xY9Qdj0pyWk9I9WCiGw/BiKQNB+I6xUYQI0JAUGmQrXjYsohg2SFgI2IO50JRNoWsvRH0oljh9Jx4YZENJYot5ENJYot5KKFFArNhRfZkMq6sDRQL9aWUpUpY0Fz3z3xhzzGf8mfMd75jzfdec3w+D7vXOd4zxjDHeGZ0/9V37ur8BAAAAAAIAxIvMDAAZyYf3Cdf/7Ohrt4FP/Ofvf6r6tYAAAAB94u+4AIAhrG+s73pr1xxn4Ga8jY8x8V43ur8BAAAAAIAxIvMDAAZyYf3Cdf/7Ohrt4FP/Ofvf6rYAAAB94u+4AIAhrG+s73prIxn8aNNsN716/T/+t7/feerG6s4AAAB9IvMDAHq3sbmx/+39IvvMPPtkeeXjPP977908d1uuuvX39duTp7gOrpOgONqAs59+DT+ZsQN31lAmHWDAzEQNqgVCpTbNtP7PtqUN3mYE/ayfuv++Pn/+uI+AAAEPyPwAgL5c/OLijt3td76Gf+5Lw79OhxHm2Z76OhuEfjL9trRR/liPwAA/SHzAwAmGQRqpDYrSiztN8TrbFNXr67eGp9v+TVNsl+3+pScOk4BvkOQ9bl9Ti8r9sYhhyMA9tuRHsUNxiNSnNP8rsGHe/lMJqgXX9LRnf7zaIuxVu83+XbQA/a1QcCvDMEFuw4dW/MZ5I6hh/BUgZKjmYtTFJmHvnEkH4HuP14jfGEMKXRiAsRDeTRCCwLdvKI+PrCwkTHpvTpbrh4/lNo/jt/6T6+kvSRsyBp1DIG6VRB8X+AiCaBfCmDSAU9L3kWGtCb2SCWwkzgIPeVAjOyT+VJmgzlmhYFEuR/xAtiINWPbrLdlqQ7U7q6sAlnjk9i9WDbUtFs0E+5ZWzkzOAEJoMvJmPG+HJc8zHZ+lwhSfGJkxb5CU2FowFqOa19XNe3aP9+8MWRJW84DXPAhRtY9L0QGtzNjSthQFo9dtPjN3l4tbqhzfYD6Ae0onHSG11HZkOXYNO7Bno0SGBkSUjgA49dbiZS+Tp8TkrdOTpx6UWaS5Yhv9hDDaTM9BHTZ/ozpomN7DL8rRQe8HR1ewTbpHnuIgWl+dDnWZlB2P62D4Dhoeg5Y3GE7Xv8tgNHy7aurSKUZwaMKMBfNAovZMYRYaQgKgPBmW5xwzsTOJdsgqdZm7Kwgy4I5GZ+pN4ytmsTcnmCi6mgxI56dOOoSBwjPVM+RKTLeeR2P/NQYDU0B/XFYQ7z9Oqr9T0L2kW2bNyEZkqhNI4u8Dt3zvpKeyXsDXdO7YAlgYOIPIcZ5YQ5LU8EJH5nt7yeabHm6jGSHZe3Kke8Px2PMAWlcc3tQsYhFWOlywyBewi0HGqWQWtQtbN8aFrEGAgjM8oCHJ+sHFRwsWXTPH0b1AujkAsk0GL4lRN46BHKeX4PDg2c4CLyFn1XcZQHi6S5HmIhbHjQFmkEsrhT1Ye0J1mPdhSVh9jc+rvCWYE9FdHl3UVVB5rWUR9GjsAAfGvdpj60OJ4/Ml6ckLS1LYPE9Gu5TSxyZhVdELmEHFzB96Wl5zBbm/9U4FCQOgO84BU0pS2Ce0ZmTjGNMzx+mHV92mWYdpm+MOgTO7vzwkaDo0NpUDx9rmhtOfk7tDMWINIQvMnHc5zuvfjQbGQvU5Oh9RdVVVxFJ57kBCcBGdKMP9ac8tm/xPEXP8aCnQsKCfJSF2hl+R3KoKhWcqwCMp10L/8t3iahmrSk4zDPxIBSaO3RPFjyzDTrG0SdaaNEPPJmnXaWqyjTXVH9TB1oZ2Qyt6NZX+ZYNYDadGfs/sGgJ53nhzn5obFbMSYt8+lrFKjOvkO7P/QSkJOw3aX+H+/X8dGvJIIDAjrf2HvktVrZK3pV7ks3Y3hZijE2EQ87oTZ1OX/8G4B9KyUVaYBSUZSXPwTRAxEVHtaK0XGJKRTRnDUgtXXQ2TIzoDwR0/M3UKb7J+qdzx/AKaIjtPtfwQNGkOV8Tcm7rnPbjHrhqmTPmhTOOJgeaiK4o7s3HSZBRp/21hfTmmw3rOoVbWpsLM1jZWlPp6u6HZNXtVx+ZnL7+MK9GtKFxg+ZPOx9xJLu7Nnd8A9yIPNhzWaFZTIvOe1JzwCf0vnm7gz5P+OW0pJndKoclGF6IVgUubgwbaM5Mf3Qg+wUhrl/Ftvpdl/+ybv7F5gCxHl+/sIfJKEeBlgbwgwq7IXNI1mPADnC49WE/NSBydW1w2MXU/DvkT+0SZG16WtNyLrrxk9r0VeenoGahLxOhpqTPzEnHpx+0nxL0TktPCFDbIJ8BnUSy5Ih/+ubl5mBBOB/PrQcCsy8b7dHCKm+4sS7Y36MxZQTHYqNMs6e8SvyM5PJ2aB0vKWIE6F1GP4fNF2vlnzi5SPWbhWGU+MbS9mFM3GOaEfnMkZ/WpOh3Nu5Cdmv9ZnKPxpPYkLl2ofGgvlXHRq6fs6rRiSZRCavuGGIxW8Q9tWBm7wnfLT61+9Jzxgpfj3twO+XLwHiZxYxgaMyc/MywCgbyvXglS1BQ9grPu1U2lWtaAHNGnsS/S8sXA/3w4iuMtYvqmgcVm8k5uS6FgH+Z8Go96fdd7L1nsY3SSeQITx/vuaXWDgkJZhZbEX0tLj2l7NP6tgH7zHKeZmZSkeRXHePIRQnuXLvl/uGA7V7TOIzGfDQ3WDCgpD4VJYnY/Pm1Ox4qXRPiJEMBjNNZqgxVSTg08Zl5skhsbiVdrJMe+7eMxBZuZAAoqM29nl+HSpEFMuWoAnCT2G1+vG1BmVSzMS/v83X4c3c28chc+QqoMq+lOBSzTOyCcXiZmZRwDqL6xcUF6bhjTJe3FITqlGtGmmhskuj3WE2aBlwIdRnk3Sgu2eGt5A/ebD3bp3G9LxBv3AP+pnNAsYuI+sBaKdhTPN2CF3GwEvn1jkqE6zhZF07u6cKAOZm0q9Xh+5kw9tmlhpmnFyLUQrY7xDZm0PVyIfSmfNLjoaDgEnY8CrTPg7fqdBLpfo7FN7Z/JBskF5rMrxtiLi6cmaxTHp1BCUPsc8EIIH7rfkGXHuO6pHBD6ekQPTx5IyHHAlecZU3Il9K7ULnYtG54TKlWIXCqJmPNVkE/GpeqeeKRIZuaUOUBMD9QWdXu6ihc2Sdqwov40onh+SanrUxn0rYmTt3S2rrwWHzHDxXHX7LHyuYrWLVSgrrI4tZ3LnmqUkwIQ2AQ0QaWVZKX/QTKK1cnKpmUKRNKmw3bcvTVgPl4HIsOh9/ReVLw+RB6/xbEPi29GkU3WUXGD2r7gXNJL+ZtW+0OdFGcL8jv6iHwnIdB5FHehsvwYrm3LPCadoZg+Um6V8Gbq5HM7E4pX2PShZrC39jVlKgmnXC6DjYRm7ZSPzA1KWcxAUDZqg7HhfKn9xb/3xWw7xIFkLMwcP8o6lYVzSP7DPCacHJqqGDMSWKWhi7nqKmdOt79g6EyH30BYVo22rm6HeH2V0PvkDQmzlb0MDTTnmZbRRGTg6cI03jlLBJg8T+X+lmpJlGyDtj21/pE4zKkdWuB/5C1rmF/NJ56yFD28PjgD5ajiYBHLV4BXuOA+2IAf8J3aR+vkT6d22dnvp8fPVEuGSzgBqkP5v2BiN/2BxZeJ2HLSxkzrTM8MASMrHhKVdMu4SWKHhaprGEeA7rB6sRzHhJqJXNVtZqTmKhzNF+HRkZV8z6SlonoCc0MjG/PVWASGjpe6ItYeGjtKIPRVA/Xxo3YUgwSImfexCnU4Asp4VfkxYg/c/MKJ1nV7DgKQiVLS3x9YaCy3Ad8hlQ/6RxJxzbHBG/hvi5f9r6Rlxsk2Sj6YCOBdnmDhq+L+2ECEk8r+Y9K3U+1KsUHFyRevzS+LvGIyWZjT/vTlmCWz0JP7zgkHHgwnSXCi/lxPHOCcurMzJxW5ZhQL7ILNdGDc5Um0PTT3CAWjAHnx4IcNCZE6WCmSbf4rG5WTcUnMVk9/9L5lH/oa

0CY5S9c0pVA+1JD3CZGzaRa7NfZi50ESdRrUtzzHJytqafVEcj6mxRmcPcZwG82QNSbbmmUX3aHw
KvrV1WgmWxrnPzonIR5tyO3B8p0jpaRtf7AcAIBqZf9FkmrLijU5C6h09QndVXYyxjZDlUnVQQ4y8V
Y1QMxQvxXThlz7hUWoF9RyqQ7VGq4pj2zY5yt5ofaUuLfmPZ4bl2ZNUrKFdZtM91BU9vbrgmNYhs
DXvRHIxqoM1wDuDdSHTYSVYFhe/uLjj9R0i9dEm23a/tOP5/feIlJ688cV+AADikPkXz4g6jQhUkvEqJ
wOTo48rUzVVPcox5YvV1Xwi84oaL6YLv/RpGaVeq0nU62pX4mXOcckiq+jTrtcquzg4RtXDivIdlqcnke
u1F9A+mz2mvORaoH9fmfYDEZVzjksNsrS1FOtCJu6jwfDWN9b3/GiPSH20ybabXr3+
+wf3inzeU+OL/QAARCDzLwNXoqyot5rhSkQuR6cOYakaVXQofl5ZO1+Mqq/o4WK68EuftlHqQvW6
WoHalNqkS6CurKKnqcd0WL4a1fgYGuM6LM9RumQvoH0254qaOuwr45hIDOywNUOzd/W+tRTX
2tpXgiWweW3z4LsHReqjTbn1+jV+u/HFfgAAZkXmXwaB/OZ+yxPKZgxL1bCsR9k7+6G8pK/o0WK6
8Euf1lHqSnGhXl1JbdIlUFdWiTsrNarRozGuw/IcpUv2Atpn805W67CvjGMiMbDD1uohQvW+tRTX2
tpXgiVw/MPjIvLRptzufe42kcn7bj849O0v//BFdTsCAIAOyPzLIJDf3G+pKFRdjQ1L5Tj1r/eL0cUldaWe
UEwXfunTPkpdyq5Ue6rfc59EK1kl7qycxRvjOizP28VeQPtsHep12FfGMZEY2H0xmbKTLG0txbW29p
Vg0d781Zsi8tGm3HaeunGAr/Hb7Y2nn/zr119XNyUAAGhD5l8GgVClQ4/5nkxCjvE6hYXCUjWP/TX
+8ooxn6gYfunTZVS5bv3r7I23OhaR5LC4YOk6zuawGSYxP8qCPbZ9NlePvMBs+8q0H0jrPPYU8rysK
XzLz828BQzjo8sfichHm3LbfmbbU4fuEml8sPbBG69X9yUAAGhD5l8GjrBT0/FJvatzkO6ur6hopMe0
hKV6oGuoP3uFX/p0GmXUF+/o/uqy7hoqK6uoQbMFS8epyOrty3OULjkW0D6b1cN/oZ7WwbEqO
bBtMXJtdX/rSvXaubYOK8HCXLp6iV/UTzPbQ0d3ixw+cPvdLz6t7k4AABBE5l8GjrBj0pGqwexshCwp
HJbqgXo6Xc1ZoJwu/NKn26h6t9Zx6BENMxWNDJbujyDnmqehntRRuuRcQPtm3T2M6Zur9uyu04
G0LMZ/PFUXe0bH2jqtBIuwsbmxb22fiHy0Kbfhv8ZvN77YDwBAR2T+ZeAIO1IzdDkSkNkhe7trWLL7
OUeKi+GXPh1HhU9DjyoETqwiq8QFS3OU+jljDwgtz7sx7wLaN9vsYXUx1uocnul8IOHFmJXyYep1OY
VrRmttnVeCQW1e2zzy/hER+WhTbre+fMOzB+4WCXwhjS/2AwDQBZkfaOeIo8A0nPj5CRH5aFNu2
89sO/bgnSJ7L7DxxX4AAFqR+YF2ZH5M09mLZ0Xko028HT52u0jdC298sR8AgDAyP9COzI8JurB+Qe
Q92sTb3hduEXl7GdoPDn37j5//vrprAQCAhcwPtCPzY2rWN9Z3vbVLRD7alNvyfI3fbq8dfZQv9gMA
4EPmBwA0bGxu7H97v4h8tIm3x797h0jaS9XeO/3D6vYFAABNZH4AQMNjP31M5D3axNt3nvyGyN
hL2H79sw+rOxgAABjI/ACA2slPToq8R5t4+9aLN4t0vZztxP338cV+AABsZH4AQOXcZ+dE3qNNvN18
evszD+wT6XppG1/sBwDARuZfLPW74Qq9/IK4K2srq+ern8fxu+iaO3JoHKoSHoLut8b47igoF7+4uO
P1HSLy0Sbelvxr/Hbji/0AAAhk/gXKwlOZl0rpU1NVYEQJzd6RzRn5yfxtOt0a47ujoK1vrO/50R6R92g
Tbw88vUsk6i3R+GI/AAAmMv8C9RyX9D9SGE1Cc+3IojoRQmfT4dYY3x0FZfPa5sF3D4q8R5t42/3Sj
uf33yPi9JZofLEfAAATmX9xOiXYOUw086st9nSqo0Xmn7TjHx4XeY828XbTq9d//
+BekaW3UDt95Ht/
+eqr6v4GAGDayPyLoZKSwcyoOlwVRJwyUlY9jUi4vvnNhGb0sQNbYwWtec6c1hxp5W7/tPU7epBe
oFitYs2eU/M437SYCzc4LrcdSOP9TKOLMV+9j3KFwXEeXWs1OgY/i+xNz1Eo9bq1YkZzmNHHnqWx
aPF25zWjF2/+6k2R92i0Rx7eI1L0lmtvv3iiusUBAJi22TK/fhz3P4s7uzTygidTdFZngi2cCHwJyv1Wxjg1
RwfrTH3zuyfPGVM00pkS/NiSTKvfL6+Jl74dCa4ioftETdvYnrzYeiDu/dc9HO8Xb7rHhW/siFqV9hXnzF
4Gx4B0d9TMq0E6H13+SIQ9Gu3A8Z0iP2/R9ulP3qtudAAAJmyAzC+e9+d9iDenC2ajpec8TZ1+1Dn
pXvrgjIAUPADX/PZYa35rmBoT+OTsaesrsk5gWrOPq6prR4KxEpN/7WpOo4co3bpyOYUeoOc0VmU
u3TdRPdASU6u+oorrUepKfSFQu+5lbMIuZy3JGib33WnN6MOlq5f4Rf000b75T3+3Rb/Gb7cT99/3
+aVL1e0OAMBUJc/8NusBfy5GOMmkmXNBQgnK3Jjsp+NQy+5D8xvXRDf10pjccUlINq2eqNIsKeZ0
sHq0H5a1DDWkutBx5aaOq9DdvBN14K9VH5LsY43J6GuBtbjGtZdTLwMH2GHNSG9jc2Pf2j6R92gT
b9vPbHvq0F0iOW/pxhf7AQDoP/OnfXbXYWGlGRq2IsfJiLhZ0ZsurzoCkpPr5B0FRDc9u4O3YHtm6z
yt2VF+uq4dtXJsuclzvKpG55UXmr2tOZoD9H5q3lU6dK4lz011aRRzXmxynb9jmOjWXGaTWFBgzUh
s89rmkfePiLxHoz10dLfIzCNofLEfADBx/f63/Y6nfcdDfa5j1FFjVs+rn2bJSEvGcZru3CU6ujvZHPO7xnp
mdzEmako5bd3T2qBrR63aB6keRT1VXvfusnJdQ1DvO86n5BkY2l9ELXkEzuV411iT8+Tay6keLv4Fu
WohoRM/PyHCHo1273O3ibQ8msYX+wEAU7agzK97mQJho6KG5ZOon9tHLSvHabpzl9iqu5PNMX

+HcNV19oZ00+phBdHbtaNWalCocrW6rIvq3faZmFSPTNnJWmfrHMYUhS4du9eSfZzLaV2j+/zby3
WYuMOakdLZi2dF2KPRdp66cTRf47cbX+wHAExZ/7/DzzHGutQhFeTUuHKYetUyaHmFTsbclOzX8b
Rc83cIV65RrVJNq3qtrK25Ntk6iauDWluwcNVpZXW1nMDo3FrU3ry6ooc4zkfJ3pqhWFwtOaurir7
mWKPiGtdezjVK6LBmpMT/N36a3Y49eKfIySNrrx19tPoDAADAxCwi86srjgf8QN6wO3UatMTsk8no
CCd3WW/TEZCcXPPPEgjVBb0kc51NaaZtDNEvAnPaOlRx0/0Kjc5tc8oSdX89j+rS/Mj0wPqyu2Mtrp
Z1btaWjHl8pTPWPJkO5axqched1oyENq9tHv7xYRH5aBNve1+4RYTkkbVf/
+zD6g8AAAATs4jMr5/4HQIP+GqiOhfYV7YUz2m6j8fYoyMguTVnKqp0Clf6SkPgo0kyrX5bTaKXX8/q
2JHU7KKEFl8yx8neLSt3l8ypLqqH/Mh8I/3LjaplfxaeLeXkGk3N6sVsncq1HGDHNSOlq3+
+yi/tp4l2+NjtIiePpr13+ofVrQ8AwPRsnczvjg2lUExZXoHTbG5WvO8ISD7mURfdO4er5graSs0/bf2G
0V8vv+5o7cjF7OQ6XidHMVP4QMyKeT31uqqtXrqmbi62w3Jnr9X+WWRvhNZYM4sXPaPKySqdJ0F
K/D/np4m2/cy2Uf4X/q8dffSvX39d3fcAAEzPIjN/OFwIzcQgzTQV4KDuMO4lTMe5z86J1EebeLv15R
uePXC3yMxbup24/74/fv776o4HAGCSFvl9/sAsFj3Gg6CGuegbjDsJ0/LKL18RqY828XbP/7xVxOYt3f
gaPwAAkZnfqYjwVqB3JPx6muqa/g+Gff8UwDFJSb9BVEMc879W99+BwHg98cETIvXRJt4eOrpbJOc
t2vgaPwAAmYVkft9E3rwVSPaBt4AOjMxP4MckbV7bPPjuQZH6aFNu289se+rQXSI/b7nG1/gBACgt
KPPnmpOFInsw1wffBAC0Wt9Y3/XWLhH8aFNuO0/d+Pz+e0SK3kKNr/EDAKDNlvkBAKN0Yf0Cv8af
ZrZ7n7tNBOkt1H73i0+rOxsAgMkj8wMAcu/89h2R+mgTb1v0i/0fvPF6dU8DAAAyPwBAO/nJSZH6a
FNuN716/fcP7hWJesnbG08/ydf4AQAwkfkRJn4fQ/jlTNQvz6t+EcM8U82q8bv6e/9FENnOBthTbsg
zxGgdef+ICH60KbfdL+3YQl/s/8Ghb3/5hy+qWxkAABTI/AgLh/x5QuaiMr8uVOo38lfFBsrgg50hxmxj
c2Pf2j4R/GhTbgeO7xTRemkbX+MHAMBG5sdMRKqcJ2SKzD+YAesOncHJ/Ejj8peX+TX+NLM98vAe
ka6XsPE1fgAAnMj8mMnWz/xD5mIyP7asj698LFIfbcpt+b/Yz9f4AQDwIfMvhCfuOi/rHFdoH9GS+9
Tb9Qj7ipo2vyKmC78M050zWX+xdudU5hBzgUGNQY1RqqTBs3BzcWLdDc1aZjVfLbHtnLV1o089Tf
Wmv6JjIg8xR/dNZbqsPxOco9ToYr/fYQb06+zFsyL40abcvvXizSJmL0/ja/wAAASQ+RfDEZxcF+vAZzI
GdQxgJvW+flsX0ZNUV4rXYrrwywD3VnJVWWsqfcFkbNQpfGKOdz0Lb11uLljMV0tdN+axtu4YW3Y
PVuz2cTiP1RjRUiJ2/TljVW0fbvsMGMTxD4+L4EebcvvOk98QYXtJGl/jBwAggMy/ICrzOGKOvqRzj7qi
g5LVxZjGCmBS1UENMdJVNULNULwU04Vfeul+skamWkjbzI6tWjqcmD2xk30q9RWxhRlrOfZhdbO
rF1oruuo1WUPEkbWW6LJ+2cWaRA6wRrTOgIFsXts8/OPDIvjRptwe/
+4dIm8vvP3bv/5Ldb8CAAAXMv+iqBSjQ4yVpRzhqjVdZaw8Zal6lGPKF6ur+UTmFTVeTBd+6ePqpq9
Va2/2Ua8cGzMuCV1OzL7gpKYyerUOtDu4hnT5yHR1/14LcmDEEstLvv6BtfnXr1/6lq86OKYoL7XOg
AFd/fNVfo0/TbebT29/5oF9InUvsP3zPzxT3akAAMCDzL8wIvioKKXTlyNbZTyjjE46L/lzXDWq6FD8vL
J2vhhVX9HDxXThlz7OvYiLzanUmy6+Ws4qel51tduSdf26V2hgc7m6g2uIY5VWN0d1yVmxdW+O4n
7uTXVYv35daxRsTtxUTtE2A4Z16eqlHa/vENmPNtm2PF/sP7Vy+D/
+9KfqNgUAAB5k/sVRuaYIMyoG1WHNHc9EvOoQwFyqYVmPsnf2Q3lJX9GjxXThlz7OvYiLzanUmy6
+Ws4q1hK7Ldkxlz1QXxHsDkatLjO7d5Jpqeiq1+CduNa2qXlOpnpfTeGipgjPgMGd+
+ycCH60Kbf//sQ3RfxeSPv33/ymukEBAIAfmX+RquyTpSeVcIxI445nqmN1tVMAcyjHqX+9X4wuLqk
r9YRiuvBLH+dexMXmVM4RLdxj1LzqarclO+aSA1WXTNnLntlVq8PMvt23VnTVa/BMXGvfVJf1a8Z0h
XJQ6yoM7hmwCK/88hUR/GiTbdvPbDv24J0igQ/c+Bo/AAAdkfkXqgo0K6urZWgyM5MOUmbKken
KkZ50SPLlvlw1j/01/vKKMZ+oGH7p4+qmr1W1mn06TtwgZyxYE3Wb2XGwYqDdwz55V60uH5mjT
6a9YuvefAtSU7aXcHSx+2Sya8arZt3WZRZCM2BBnvjgCZH9aJNtt758w7MH7hY5fLDG1/gBAOiOzL9
YOi0VRJzRb6p4pXOPzlv6ihprTBgMR/VA11Ajz4msFX7pZa3TqF8V882sRujlBUp1ODFZx0PN5D8JuZ
56wfXMrIp1v+qice6qm6N6pr2iq16TVb15ocOmrBms9esL9fLFhvxzdJ3BcQED2Ly2efDdgyL70Sbb9
r5wi4jiwzS+xg8AwEzI/Aum001Gpaqa+W7NTDlGKJPs6Uz1QCtFuQuU04VfBvgXWlWzpnKPaCk0w
4kFZ3IkSjnQXSpXz9zsU173n4Qe6Kiea63YZW+e8lWpDpuaYf1SvSz3JPX77TN4zgh9W99Y3/XWLp
H9aJNth4/dLgL5AI2v8QMAMBMy/6LpcOPLLs10VGcezeyQva1fOrqa7H7OkeJi+GWY7pzJ+ovQFq5
e6BjvmoPkyrot2ZEoHQPNZJpfVa+Nqc0uejJzhVlfa2ZH9Uq4Yre9yRMSZcIlCm3rz5nT5OwlNVcR3m

uuOYN61x6Hvl1Yv8Cv8aeVbfuZbU8duktk8l7bpz95r7oRAQBAN2T+RVPBh+gCYKt457fviOxHm2zb
eerG5/ffI5J5T+3tF09UtyAAAOiMzL9Y+l91EvkBbCUnPzkpsh9tsu3e524T4byPdvrI9/7y1VfV/QcAA
Doj8y9K8z9ctv/DZwBYbkfePyKyH22y7aGju0VET9tO3H/f55cuVXceAACYBZl/UYzMT+AHsAVtbG7s
W9snsh9tmq3vL/bzNX4AAKKR+QEAkS5/eZlf408r2zf/6e96+mI/X+MHAGAeZH4AQLyPr3wssh9tsu
3A8Z0irs/f+Bo/AABzIvMDAOZy9uJZkf1ok22PPLxHhPZ5Gl/jBwBgfmR+AMC8jn94XGQ/2jTbTa9e//
2De0V0j258jR8AgPmR+QEA89q8tnn4x4dF/KNNs+1+aUeSL/a/d/qH1e0FAADmQOYHACRw9c9X+
TX+tLI98PQuEeBnba8dffSvX39d3VsAAGAOZH4AQBqXrl7a8foOEf9o02yPf/cOEeO7txP33/fHz39f3
VUAAGA+ZH4AQDLnPjsnsh9tmu3m09ufeWCfCPMd269/9mF1PwEAgLmR+QEAKb3yy1dE/KNNs3
3rxZtFmO/S+Bo/AABpkfkBAIk98cETIv7Rptm+8+Q3RKQPN77GDwBAcmR+AEBim9c2D757UMQ/
2jRb9y/28zV+AAD6QOYHAKS3vrG+661dIv7RJthuffmGZw/cLeK9s/E1fgAA+kDmBwD04sL6BX6NP
y1re1+4RcR7u/2f/3Wmum8AAEBSZH4AQF/e+e07Iv7RptkOH7tdhHyzvfH0k3yNHwCAnpD5AQA9
OvnJSRH/aBNs289sO/bgnSLql+0Hh7795R+
+qG4XAACQGpkfANCvI+8fEQmNsHm+2L/737xaXWjAACAHpD5AQD92tjc2Le2TyRA2gTbvc/dJgL
/B2+8Xt0lAACgH2R+AEDvLn95mV/jT8vaQ0d368DP1/gBABgAmR8AMISPr3ws4h9tgm37mW1PH
borC/zPHribr/EDADAAMj8AYCBnL54VCZA2wbbz1I1Z5t/7wi3VbQEAAPpE5gcADEfEP9o022MP3Z
H9b3VPAACAPvF3XADAQK67bqC/6VAoGoWiUSgahaJRKBqFoo2v0BRwlACAgfBEEo1C0SgUjULRK
BSNQtEohACOEgAwEJ5IolEoGoWiUSgahaJRKBqFEMBRAgAGwhNJNApFo1A0CkWjUDQKRaMQAj
hKAMBAeCKJRqFoFIpGoWgUikahaBRCAEcJABgITyTRKBSNQtEoFI1C0SgUjUII4CgBAAPhiSQahaJR
KBqFolEoGoWiUQgBHCUAYCA8kUSjUDQKRaNQNApFo1A0CiGAowQADIQnkmgUikahaBSKRqFoFI
pGIQRwlACAgfBEEo1C0SgUjULRKBSNQtEohACOEgAwEJ5IolEoGoWiUSgahaJRKBqFEMBRAgAGw
hNJNApFo1A0CkWjUDQKRaMQAjhKAMBAeCKJRqFoFIpGoWgUikahaBRCAEcJABgITyTRKBSNQtE
oFI1C0SgUjUII4CgBAAPhiSQahaJRKBqFolEoGoWiUQgBHCUAYCA8kUSjUDQKRaNQNApFo1A0CiG
AowQADIQnkmgUikahaBSKRqFoFIpGIQRwlACAgfBEEo1C0SgUjULRKBSNQtEohACOEgAwEJ5IolEo
GoWiUSgahaJRKBqFEMBRAgAGwhNJNApFo1A0CkWjUDQKRaMQAjhKAMBAeCKJRqFoFIpGoWg
UikahaBRCAEcJABgITyTRKBSNQtEoFI1C0SgUjUII4CgBAAPhiSQahaJRKBqFolEoGoWiUQgBHCUAYC
A8kUSjUDQKRaNQNApFo1A0CiGAowQADIQnkmgUikahaBSKRqFoFIpGIQRwlACAgfBEEo1C0SgUj
ULRKBSNQtEohACOEgAwEJ5IolEoGoWiUSgahaJRKBqFEMBRAgAAAAAwTmR+AAAAAADGicwPAA
AAAMA4kfkBAAAAABgnMj8AAAAAAONE5gcAAAAAYJzI/AAAAAAAjBOZHwAAAACAcSLzAwAAAA
AwTmR+AAAAAADGicwPAAAAAMA4kfkBAJjV+dXraqvnq6sAAADLhswPABiRMoz3m8KvrK3UFfos
OPQ/Wcg3lllZu1JdSKmxmQL/rGRWjTPs52PqnecPTHXvldLeGYE/ooG3ZuWeqvGJpd1Wv4In0+tfK
Hrk39RW/Zw8O2psJ9XH1KHWFv1r0kSQ+QEA/erxab7BqDPkQ1tRto9nnexZSu+jeK7q+YEq38jK6
mr2P73Uybcw+COh+Tya/q5o3Nm1nu6+Yi8D3RHmuaUr4v0T2vwz1NzoXLwVg2/NyjdVfl1fKI903l
LuWcyPa+4SrSeTv5/0LxSuTTW2VJhvX4FNFW8l/KNkb8cobuppR0V9faI4Ne/uutVKUKo8u4o4n8Yp
ivfQAZkfANCj5vNU82kkVWo+L9DPhA0d9iX3qvkBarj66dM/tn3fkymYT6YhqJkTzefnLu/Wqmf4yv
q87AXLm+NVFvzVwy9NauuU6l+1cuZFeNLzSrZ8ekL839cbdvJr1dvz1WnFNpUivkrgU05LsXzbkeYv6
h/R/JKj7XUG9XLeT+1fDo9fXEn1y+bpYo359rTFJH5AQD9kc8A1lNDcv1XEIZ5/Oh5W3r6/If4h7aQ+
R4HZzb4fZDp91ZoHmBv+ysmNj+o1B+ctXJ7K4kPMnBWCY+xbari/eiDVKP7rWJwF9JX8x/mLqMW
66jVz18u7EJJ9lEJbKcp4f3denSti+nOmsraR8Ji+jSLF/J2SFloMsj8AIDe2H9nTvi04zbww0Cxn1TPjF6N
x5/0jDPLf+ynUHFStZ7PrL99ePX8IWXKI8xKlD/0c5dbf0KNuyMJa768YvPcEpcMTJewUttU1sFGaauS
bEOuiYxr+Y/JbnZHLfumSMEuVNTJv6igzV/VdXSG4u1Ue3PWKm61vET5g38ps7FqeY4zzdbMY3IXSr
axiSDzAwB6Yz8BOB9RUuq9gKl48Ej19GYr9lLpcUv5LtT0ecn+NqSUG+uzTnHnJX6UDxvmxivuuEJfp
ex92H+K52IVsOdPfJaB6RJWCk9VfHAJTjFcpXg3zWflKJTvQV3J3052U3hqGRKVsguJz6Xo4D/fbhzb
MYTfnZVvtvr8kpVy1CquyNNL9FkVO6iq5T83p017itNA5gcA9GYBf6se7llAPC32KunDVFO+jfq88kK
D7Mm+NZISH055fn3eFkXBXu87cw/lz/3UK+aWR5fwoyomNFdu3wpWl/kEpktYKTSVuB3n4KxSXK

ykOjarUL6H+nX+drKbInR0mXJ7KarZhaybL8Ef5OB2EsxvctQqj6u8Vv6cqJ5zX8V+lNXVxk0yh2Ja/cF
Yn1L4jOFE5gcA9GYBf6se6FmgfNIZ8JmjfHhLX7DxyGZI8YQdUtTt7/ysO6/fesWn0+uR2RV63FHjpkj
3HF+y/oTaf2StT28+dgEt8NasvFMVp5lqOy0LLt5OUkwWatwThj5q2RLdEXYha+L2tbQKTFG8lepeyF
m17ArFJzffjkrtR5P3SLE7+UfG/vjb1wKJzA8A6I39N+ZEz25+QzwLFDUGfuAoavZ6coW8TP9V+t9OH
4/yfsmeqv3sEr3uyJD6lrDXLT+s1DsLzJewlHuq4nNLuJnWBRcdEhRsKZS/neyuaN1Uqj9hdqHiirkReT
NG8G8n0TYMVi27RPvpdtQ6Ud5h/pui2EFzGrtygo9pcsj8AID+yL8zJ3v88Oq/QvlMIiV//siqGNtwPA
f1Is1Tmy2b15i2+JB6/ZSKCuZG+nxGLD6cfu85+w4YpGgm+R1RfDTNhTf2Zn10c3NUVAJvzcoxVXEp
7YfUuuCiQ4LjaymUv53sQ1rophp/kJL8qfJup4c/s3atxh+mTLqi4Y8pzWdUrNYukl825w4vBU5kfgBA
jxoPIGmeCtyKuYWt/UwgdjTMZvKivXxCg+
+m8aib7rnX1udd3VQ+D2sD3BEp9ybugEK9BXNvqQ4zUDG8mJn4pxKfV2nOzRXVGivNqhivi5rz1O
h4Mnm3ZDe9tansgjF5uSTHIjoLb6rx7jxlKtZ2SsXlAe7t5H+h8NdqvjP30ZXTOadp3NdJT3I6yPwAgH
6ZDyD8fRqDaTyQzv086lPc3r3NvgCJn+ORRPNTKVWfjXhrC31i/k1tyT0FPqPM6P5CkZz4RxWl+pGB
B4k5kfkBAAAAABgnMj8AAAAAAONE5gcAAAAAYJzI/AAAAAAAjBOZHwAAAACAcSLzAwAAAAAwT
mR+AAAAAADGicwPAAAAAMA4kfkBAAAAABgnMj8AAAAAAONE5gcAAAAAYJzI/AAAAAAAjBOZH
wAAAACAcSLzAwAAAAAwTmR+AAAAAADGicwPAAAAAMA4kfkBAAAAABgnMj8AAAAAAONE5gc
AAAAAYJzI/AAAAAAAjBOZHwAAAACAcSLzAwAAAAAwTmR+AAAAAADGicwPAAAAAMA4kfkBAAA
AABgnMj8AAAAAAONE5gcAAAAAYJzI/AAAAAAAjBOZHwAAAACAcSLzAwAAAAAwTmR+AAAAAA
DGicwPAAAAAMA4kfkBAACWy/nV6yqr56tLJfXGytqV6ko/VB1RfhnUZ5OxzuHK2orvreB7ADBeZH4
AAIDlYuTaZjydeuavY3vBdQzeZF8f6hL+kwwA6A+ZHwAAYLk0/l22mVCnnvk77d8Z7ut/FEDiBzAxZH
4AAIDl0sj8ZsCdeObXwb1lXfr89DnpKyR+AJND5gcAAFguzcxvBFUr81sX7GBcdcl7GPNWA4wrZhp
Wl7Nrxn9ObxRRjHet94211WVaMndjvsaExkqVwFy6d9FHv7J2EFp/rrke0SWwweA4ABgUmR8AA
GC51GFyrfxJRUYjZRav7QtG3KwicR1HW+kQ7R9j5GyZawvGQhyTBKOvu6jcusFYi6Ve3Oqa+lFUb1t
/y4L8G3SPCy4XAPpD5gcAAFguKjRmCVIF0zIwGm8UHe0LocxfdTIiqbyiYqk1pr6iLsgyuodeilGnQ9z1
F9AT2jVDdOdKPU2hdf2qg389ng36JpIrAIBhkPkBAACWSyM1Vi/Mn+3YacRJGWZVlzqX+q+oaewes
o9MxBl5SU9i9PGSK8jJndgXwvQCcmJlh/VLVnX3BnW3TvsGgN6R+QEAAJaLO15nUdNKxo6oLKOp3
aX9inptBuXmvKqHiyjcmMXDXlJOlVRX5dZa+ZfQYf2GZm/1vmd2vcpac1sAMCgyPwAAwHKRCbh6r
b/eX2dI2TPTV+ZvXlQvXLyF/dx95VaiM7+9hA7rd4X3gnrfO7tnYNdVA0BSZH4AAIDlYoVJGSL1G47YK
YOx3aX9imNaMa+rh9Chi+buq0qqq3JrrfxLaF+c6pEpO1nVW+cwpij4iwFAf8j8AAAAy8URJpvxUb8
R6pkg85vhutmnQ/y2y/jp6czOVo2Emb91KnuodSiBDWZvGRPPvG4ASIjMDwAAsFxcYVLnxpx+o75
axUkdTK0rdnj1X/HPorv4K6tga5cJ8Reox8+cnQNLaFu/3E7dv2WD1kaCywCA3pH5AQAAlktLmDTf
MKKoJLJsKILKK/r1ipxd5d2Mu3TdwS4Tpvo3maNTZv7W9buXk1NdfLP7RnZdNQAkReYHAABYLp4w
WYfUxhtmds1ipQzG9mTtV9RrczprOblmbm528OwiqDmfDMlya61alxBafz08l5c0jsV82zW7OTTXd
cUAkByZHwAAAACAcSLzAwAAAAAwTmR+AAAAAADGicwPAAAAAMA4kfkBAAAAABgnMj8AAAA
AAONE5gcAAAAAYJzI/AAAAAAAjBOZHwAAAACAcSLzAwAAAAAwTmR+AAAAAADGicwPAAAAAM
A4kfkBAAAAABgnMj8AAAAAAGP0t7/9fyF3Z4FBI8m/AAAAAElFTkSuQmCC]]></redcap:Attachme
nt>
</ItemDef>
<ItemDef OID="b23" Name="b23" DataType="text" Length="999" redcap:Variable="b23"
redcap:FieldType="descriptive" redcap:BranchingLogic="[q2] = '1'"
redcap:InlineImage="1">
<Question><TranslatedText></TranslatedText></Question>
<redcap:Attachment DocName="B.png" MimeType="image/png"><!
[CDATA[iVBORw0KGgoAAAANSUhEUgAABVEAAAEJCAYAAACdcmdaAAAAAXNSR0IArs4c6QAAAA
RnQU1BAACxjwv8YQUAAAAJcEhZcwAAEnQAABJ0Ad5mH3gAAE9sSURBVHhe7d0PjCRnfef/b+/aY

PPHNmBskx+RHat7sZYlGJIg08PxL4egZ+6SJbnbYOVg5Oh3PVyi0zQSkwOxF+mX2wguG4lunS5iWrq
zliQyTEJugrLdIJRgckzHR4IxYTI/Z7rlc3T8wDYGjPljB3u3f/VUPVX1VHd1dVV3VXX39PtltaerpqfqqT/T
O/OZ5/k+hb5FAAAAAAAAAAChjumPAAAAAAAAAIAQhKgAAAAAAAAAEIEQFQAAAAAAAAAiEKIC
AAAAAAAAAQARCVAAAAAAAAAACIQIgKAAAAAAAAABEIUQEAAAAAAAAAgAiEqAAAAAAAAAAAEQgRA
UAAAAAAAACACISoAAAAAAAAABCBEBUAAAAAAAAAIhCiAgAAAAAAAAEAEQlQAAAAAAAAAiECICg
AAAAAAAAARCFEBAAAAAAAAAIAIhKgAAAAAAAAABEIEQFAAAAAAAAAgAiEqAAAAAAAAAAAQgRAVA
AAAAAAAACIQogIAAAAAAAABABEJUAAAAAAAAAAIhAiAoAAAAAAAAAAEQhRAQAAAAAAAACACISoA
AAAAAAAARCBEBQAAAAAAAAIAIhKgAAAAAAAAAAEIEQFQAAAAAAAAAiEKICAAAAAAAAAQARCVAA
AAAAAAACIQIgKAAAAAAAAABEIUQEAAAAAAAAAgAiEqAAAAAAAAAAEQgRAUAAAAAAAACACISoAA
AAAAAAABCBEBUAAAAAAAAAIhCiAgAAAAAAAAEAEQlQAAAAAAAAAiECICgAAAAAAAAARCFEBAA
AAAAAAIAIhKgAAAAAAAAABEIEQFAAAAAAAAAgAiEqAAAAAAAAAAAQgRAVAAAAAAAAAACIQogIAAA
AAAABABEJUAAAAAAAAAIhAiLp0etJYKUih4DxWGj29PqjXWPFeU1hpWF+1iMxj3ZB2onXjpLWdL
MxLO7LSlg372PzHqPs4Vb2GrGzEO5vtDbdtK5JH08KNuQ9Cjmc+2g0AAAAAwPwhRF06Rdm8UJey
XurU1kPCkracr3X0c5Hq2U3rq4B5oILBVWnqJUdZzqxle4faf1Qo1cT/rlhsR+14AAAAAADIGiHqMip
uytmqfi4dqZ0P9kbrNc75IVW1JdsV/XzhFGVzry/9vnpsS/aHkff+RpmXdmShKwdu8leuS9c+xj3ZzDJ
D7TVk3fijwsI7ascDAAAAAEAOCFGXVGW7JV6O2jxn9EY1e6FWpbW4CSqOot6h7OuncuoEPaQBA
AAAAEAuCFGXVkW2W26M6vdGbW/4Q6XL9a3wXoyqlqKuRek+wmpSjqyvaH59ZI1Jo6ZjoC7rqPX
GPu31edcGHd5fZI1J4zwEz5+5nbDPjxN+3INtCdS9tR4xy30ObGewraPOc7xjCm57uPZpYAh6c9VZF
2h4snM3eA7Uw9yc3Z6x+xzPPy71CLkXbAnaHvI9OHq7vqTHE6/dAAAAAAAcfYSoy6yyJXW3OGpz
VTYaDTnnJ6hyIWSM9FAIo3VqpQwmoCrK2hndwM6BdJ1nFmNId2dHLno7bcuubn/5zNpc9FKsnPa
D6gP/AGy9izv6PBo1Pe1wrCSDo63TPb8dqZUKUhrYSXM1aUimtjPY1qasDoZtEx2T2rZR+9S6H+te1
+kREu3HCSwHz4HSXB0dKibnnOtV70AUtW6Kc9TeGFHPNGS7E4vZbgAAAAAAlgQh6lILTjLVrLnBTF
nqF0Imk2pv+KGKV4+yL103ie3UZD3lhKW4dka3b18O3U23d42JhYxw0hjqferEnAz0rpz2yiY0d4M9
Ji/u6BisfEacDLUnjXX3GlSlpc9v3+0xnOr59bfvdUhWIdlAfdzx/O1494Hazrob/E1zTMbr9zZlc9v62PX
vV1Wv1/6cXXIi2X56jXUvsCzXu85r+8MlLiqR+4wr/BzteOl/krZbr3X/0hH2PRjY7rBkxzOu3QAAAAA
ALI+C9QtyXz/HklK9SwM9zlSwEhKq+K8rS71rTubTk8aK24tOBS/OZEYjX6963bk96Ubsy+dvW4Vde
9ZG1BBs1YOwWq1Ks9kcWu+3IaxdcdeNE387w+2yV3rnwG1/6DqbsV0VnO2FBNwB4e0wr3O11fc
nDEt0PSK2E9ivvuaS7JjMbQdfr41qa6JzF35+FOdanQqsS3p+lETnOlHbRwht44jjjDietO4RYBr3Pnyvv
OXCW/QSACAP99/xLnnNW2/SSwCAzB3/NZFjr9ILWBT0REVwkikVtoQGJP5Qeb/npMsYdm/2GE1F
UU6ccp51dJfTrj2WvywnT5+0e9QF11uqp/0AbA74vWmb4nVG7R44gZT1GW8ov7dOdT4sGbUo3S
BMfcIsazBr1jUo6ac28z7QPYSnOKZEvYkT7cec4f+kmIdQ3NyTvhmgTm3wHIWY6rrrurFuwJmaGO0
GMvDwEw/LOz/5Tr0EAMjDDY9fJXd/6AF5/Bs/0msAAJm7dI9I/zt6AYuCEBVBA6FScsO1P6fl1RVt7k
pbenJoj9k/JScqJ6z/D65XGeqc9ZIrrombLbpD+ttuIl09a/TozUtaAZl1DTJrOyHeSKpXqBe0GnVjgQX3x
NNPSOWPKvZHAEA+nvPjY/KWL94oP3ryGWm+/3555p8u6c8AALL1lMilP7A+PussYiEQoiJlGYRfXI3
RfTlsXxS7lKgd9lbEyVeN9XMZvhk9NO3A1+/VOyrwVcO67VqVQ480e0lOK1mv47yOaTHOXbjxbW/
Lhtnr1K2LatY5BRbUnZ+6Ux58/EG9BADIwxv/
+gZ5wQ+vsJ8/9NXvyp/WeR8GgNz0Hxa5/Od6AYuAEBUxleSkN1LbnBFfMSZJitM70Ri+HI+7744c7D
pf686+X7I/4a8fLjUwH4qbZ/0guOFOjFWVQIZacsoTKG6Jgvk22OvYvA/0seV1TIn2Y97LA8Pk1cz3hZ
xnoE/SdmNSNTtwdeukJv6eAubL+z77PvlM7zN6CQCQh1c+eK3c/PXn6yXH5z7+kHzlLx/RSwCAzF3
6gsjlr+kFzDtCVMRUlM2zbuVUc/Z1NbrYnOl8y+vt5wScijnre1s23JlrYvN7cqqJpBS3XmZRF0x117vh
6vxxe81a56Kmj3+wdqsx7F+aq7LhnTIV7Onh297K+dBcNQLH9nm/hqd7bHkdU6L9mLVbm7Lqre/p
me+ta1Qq+NvI2oTnyA9c3XYDi+kT+5+Q+n11vQQAyIOqg/pzD7xELwX94W9/Tb73raf1EgAgc5f/RK
T/fb2AeUaIivgq29LyctSalHTA48w8bynX5YJR4NOfUMnSXNWB0GQ1HAPbsp55Q/a9of6KMUnTHP
Jqu9rKUt8KRKiWomxe8IdlN1d1gOaFzqMm/ZolJ3AMttM8tryOKdl+ipsXpO6/2HmtOZGTdS/7h6Br

7yrua1NNWBO03bzfw9odR+bHA8R339fvk7v+7C69BADIw/FLBXsYv/oYRgWozd+8Xy8BADLXf1Lk
8sf1AuYZISoSqWyH11+stqz17tBiV3FT9gZfW21NVr/R7K0XKBlgDM3OdKKjFJgB2KiyA+qc9bt+wOe
ya1/OW03PstS7fT9Yt1Wl1d8LTpaV1zEl2k9RNvf60h16sXq5MUzeVpHt4EGK7B96PbFTEbvtVlv6Lf
8+sqlzblwHu+5ulByOB4jBnYn/6Wfp7QQAeVr50kvl2iev1Evh/uFL35ZP//6hXgIAZO6y9RvZJcpbzbt
CX81aAgAxtTcK4nSSVCHqQGAKADGo4PT1/+318sAjD+g1AIA8lB56od0LNa6tu8vyiteFD/sHAGTgit
8QKfBL9ryiJyoAAMiVGsJPgAoA+VK9T1Uv1CTUsH7qowJAji79AfVR5xghKgAAyM1HvvgRezIpAEB+
VP3Tt33hZSProI6iAtS7/+NX9RIAIHN2fdR79ALmDSEqAADIxe6Du/LBv/igXgIA5OWOL18/tg7qKPv/
8zH53Mcf0ksAgMxd/n9FLn1BL2CeUBMVAABkTg3fV3VQmUgKAPJ189efL//8Czfppclc+dzjsnX36+
XWV79IrwEAZOtKXR/1Zr2MeUBPVAAAkKknnn6CmfgBYAZe8MMrEk0kNcoz/3RJmu+/X3705DN6
DQAgW9b77aWPWx+fchYxFwhRAQBAZlRwqgLUh594WK8BAORB1T99WK8BAORB1T99yxdvlOf8OJ1f+R7/xo/k7g8
xKSAA5Kb/HZFL1EedJ4SoAAAgM+/77Pvk3ofv1UsAgLz83AMvkRsev0ovpeMrf/mI3PtJ/igGAALm5/
DXr0dELmDVC1KXWlo1CQQrGY6XR05/LSa8hKxttveBob7jtWZG8mzM3Qs5LHL3GinPuVhoy6tT55
9d55H7NjwLuWyCW+n11+djffkwvAQDy8vJvPE9e+eC1eildn/zPB/LN//19vQQAyNylXZH+Y3oBs0S
IurR60lhZlaZecpTlzFpRP8+eHfiVasLfVIImPi+9hqzXor5KXfOCrAYvunRqJSlMENguK+5bIB7V+1T1Qg
UA5Ovqp47LG++bvg7qKKo+6u//
+y/bHwEAeVD1Uf+79fFZZxEzQ4i6tLpy4KZA5bp0+33p9/dkM68MdWzgt6QmPS+qZ+S4YK99XtxN
V1vqevelWy87K5qrQo4aA/ctEIuqf6rqoAIA8veWvRvtIDVLqifqH/721/QSACBz/UdFLv2xXsCsEKIuq
96h7OuncuqE5Nf/FGmL1zOyJ41zugtquS5bFedpcfOsVJ2n0twlRQUwPTUTf+WPKvZHAEC+Xvu1F8
vLHr1aL2Vrb/f/2A8AQE4uf8l5YGYIUZeQXbvRDN2aq04tx8GuiKp3o1E7c1T9zGAtyGCd1bDejbH3r
wVreI6qN+kMVfdfl6zW52A9S6+2qH6ENy3GPgPncEP8zZjnydln0vOiqHaWYvWMNHoeB0LzkpzUn
VFl/9A6olGM9obVW21v6GMZPAcJrkvI/RZ2vePcb4PXz35E1ImNY5b37WT3p2vSa2eZ6D1Ar1TMr
49uJI6QOz91pzz4+IN6CQCQFxWevubvXqSX8qF6o1IfFQBydOlPqI86Q4SoCDUUGGl2/cyRYVRHai
WjzqrR43EyanuDNTzVurCgpuQNVXdFt3UUZ5+DwWRzdcJ9Fjflgjtk3joz5/RGeo1z3nkq1y+kUEahK
q1+y+tVOsTseTxK50C6+umwipx2N97ZkYsDJ7W96x2NX1c3yXVRQV7I/eZcj1EBZPj9pu7d0GC5U5
NSIMjOSpb3bcz7M2CCa2eZ7D0Ay07VQP1M7zN6CQCQFzV8Xw3jz5uqi3r3h75KfVQAyI2qj3qP9Z
H6qLNAiLqEKtt96Xfr4kZ7Um3Z9TH72zrxbG/4AZBXL9Won9mpyfrIxEaFec7r+3uboWUCxu4/wN+
et3/pyI6XBPWkse4GPca+Wzo1imzrKP523M2ofdbOu/Fbsn0WNy+If+rOS9v677wbglnn94JOUJOd
F4P9um2ZLK8uyolT+ukYlS23beb5V9ri53BnxMnhkpwj67VGqYGh+21ofyZj2/b9ZrYlbFt+kJ3U/Ny3
/uvD789hya6dZar3ACyrT+x/wp6NHwCQv5UvvTTzOqijPPTV78qf1hmBAAC56T8scvnP9QLyRIiKIW
bPtPoFPwgdDgOHletbE4Z54aotPxwsrp3xAqzOge4z2bsoO14eaey7suW3deeiJll7zH364ZMh8T6Ls
nnWTbuaslpwe08Gz+8kipt740PW2PblMOpEFdfkTNjxtXf9XrVn1pzjSXSOrPOzp0NEI3gPvd4Dhu43
s8et0evSPk86CNzLYfa0LO/bsfdnmCTXzjLNewCW031fv0/u+rO79BIAIE+vfPBaufnrz9dLs/G5jz8kX/
nLR/QSACBzl74gcpkJ/vJGiIoBI3qm2Yqy5iYxIwK3UyfSDKjKcrKkn47SPdC9+VSoU/LqNhbMYdKRw9
QnMMk+K9tGr0FHOsP403RKoi+fcf2NgNIP3Kpy1j2gqa6LruEZOrw/aOh+M8JCawf20Hd33yNrsaZ
uHu/bBNduyvcALB93Jv6nn31arwEA5OWGx6+Sn3vgJXpptu7+0APy+Dd+pJcAAJlTw/r739ELyAMh
KibUkeHOgTHCo7mX3TFUvMKUSrD+5Oz05HBssVSf36vSHRZuBG7V08FeoUmYExB5PXXHCbtWR
dm8EN470w0rF3t+o8nvz/SvXdh7AJaNCk5VgPrID+h9BAB5e86Pj8lbvnijHL9U0Gtm60dPPiPN99+v
lwAA2XtK5NIf6OfIAyEqJjR/gWm53vWGbAcfk9YLHS/
+Po26n7aO1NZznJyneELGlj4tn5Sxl7S4KW5lAntYuDF8vno6/CyPP0dt2TB7nbo1OM36o0lYbdzT+xj
s/as0z83XpEi53bcTXLtoR+GPJpiWGsL/wCMP6CUAQJ7e+Nc3yAt+eIVemg+qPuqnf/9QLwEAMqfq
o15iYte8EKJiQElOusnV0EzePbnoFnIcO/Q7J6WTXtA2qm5m6ibYZ6+xrodpl6Ved5OsPCfnMa5rc1f8
zphdOfAu6QmJc0m9HrXW/XH+/I4OP6sSyOGSnCOzLqcKFN26qMaQ96TUzPKqx6k9GZQdSHa9W
p7pDpOf0CzuW0usa5fme8AU1xDz7yNf/Ig9mRQAIH+lh1448zqoo3z6v/6D/MOXvq2XAACZu/xZkf
48dRU6ughRMcCcBCnYW9IPAlXYNTChz6yYNTCbq/5Q7faGHhpuPdIev510n72GrLsnrnpWNjeNyY

Nq65JPjmrWsmzKrm5er3HOCzBj90asnBbnDulIs+ke18Bw8Amvix8oDvbcjUlvX80sH+xxaoTFZo9b
oz1p3yaRZnHfKnGu3QTvASU/dZXaee9gZMOb4h9Hze6Du/LBv/igXgIA5OnaJ6+0Z+OfZ83fvF+
+9y1qZQNAbtSw/v739QKyQoiKYeYkSJ2alHSoU/LTE7kw7YxI5vDy5uoUoVGwBmZzVQdQXnhTlVZ
qs9e7kuyzJ411d6h6Wepbav3okCq98zLMnFndbbN3TastiX+aKhIo72oZDmATnCMv2LO4x2xOsJTEi
HvXrLNaPevPNp/YQt+3SpxrZ0n4HuDXW7V41zBubVssGjV8/85P3amXAAB5UvVP3/aFl81NHdRRV
ICqglQAQE76T4pc/rheQFYIURHKHgYdUpOy2rLWu8Otp1KR7cGClfuHRs/BBOwamMZwbZddWzOj
eqgx92n23LN7obonbiCk8of1p3hehhRlc2+4Tqg9hD5hYBeYJMs6ZjsbHhT7uljH3G/5QaqtKq2+0dZ
ACYJo9r07eJA2Z5vTZZMLft9aYl07S6L3AHUsg6+ttiava4u59cTTTzATPwDMkOqBqnqiLgI1pJ/6qAC
Qo8vWb6bUR81Uoa8KBgJAEmo2fT0ZlAph96btmTxL+lhOtaYNWBfEUbp2yJUKTit/VJF7H75XrwE
A5EnVQVWTSS2SK597XLbufr3c+uoX6TUAgGxdKXLFb4gUbtbLSBM9UQEkFCxRcGZtsUO49nnrWC
J6ZB4tR+vaIV/v+
+z7CFABYEbULPx3fPl6vbQ4nvmnS9J8//3yoyef0WsAANmy3m8vqWH9TzmLSBUhKoCY2rJh17o06
pWaJQoWUK+xIqv7demmUqJinh29a4d81e+ry8f+9mN6CQCQJ1X/9C1fvFGe8+PF/NXt8W/8SO7+
0AN6CQCQuf53RC7doxeQJkJUAJNRNS8XfPx7cXMvpRq/C+YIXDvkR/U+Vb1QAQCz8XMPvERueP
wqvbSYvvKXj8jnPv6QXgIAZO7y10QufUEvIC3URAUAAKEefuJhec32a+wJpQAA+bv568+Xf/6Fm/TS
YqM+KgDkjfqoaaMnKgAAGKKCUzWRFAEqAMyGqoO6aBNJRVH1Ue/
+0FftjwCAPKj6qGpY/7POIqZGiAoAAIbc+ak75cHHH9RLAIC8qQB1UeugjvLN//19+cPf/ppeAgBkrv+
oyKU/1guYFiEqAAAIUDVQP9P7jF4CAOTttV97sbzs0av10tGyt/t/7AcAICeXv+Q8MDVCVCTUk8aKm
uVbPTaknWjdOGltZ96MOYZeQ1Y2gmvbG+7rV6TR0yunpfZjb3NEO2zTne9M2m1bkPugvaHbWJC
BSwosjE/sf8KejR8AMBsqPH3N3x3tuqGqN6rqlQoAyMmlPxHpP6YXMClCVGCGeo0VKZRq0tHLWe
pd3DH205RdQj4AA+77+n1y15/dpZcAAHm7+qnj8pa9G/XS0aXqov7+v/8y9VEBIDeqPup/tz5SH3U
ahKhIqCibe33p99VjWyp6bXby3l8WRhxDryHrtTziU6UnF3eC+2qea1hrF8VRuA+A+aZm4n/nJ98pT
z/7tF4DAMibClBVkLoMVE/UT/7nA70EAMicXR91Vy9gEoSowDLoXRQ3Qy2Xy86Tzo5cXJwUdTFUt
qXfrYs+w8DCUMGpClAf+cEjeg0AIG8//ffX2UP5l8m9n3xYvvKX/NsDALm5vGc9mOBvUoSoS8eoLbl
i9kQctd6oc2mvz7s25fD+IutuGnU/VwKfNLcT9vko6Z8z+/PmMP7mqvP5EYU0/WNWj+T1Rv2h/FU
5e+GMDvk6shMnRQ3UUg17jG5PvHbHuTbD59DaumyYrzfqkQa2MbA+/BTHaYN5POpY/P2rh7vd
9nnrupbrsjXQXdYu3WC83n4M3DfArKgh/A888oBeAgDk7YbHr7Ink1pGd3/oAXn8Gz/SSwCAzF26R
6T/Hb2AJAhRl05R1s64PREPpOs8s3TlwE30Aj0U27LbdJ6Vz6xZXz17ldNV/awjB/4B2PywsCxn1nRr7
RCwJIMj5zu1UswQa5bnrCO1UkFW9fYcal2SINUYyl89LZXimniHs3Nx+hCvfEbcU+2L2e6pr43Dfn1w
Z846FVQOrG+uDgSpE7VBHcuqeFvWoakKSlf369Ld2wxcdxW+lsJKN3RqUprnibKwFD7yxY/Yk0kBA
GbjOT8+Jm/54o1y/FJBr1kuP3ryGWm+/37qowJAbp4SufQH1kfqoyZFiLqEimtuT8R9OXQTovauHwi
JEU72Dq1XOU6dmIcI1VI5LW6M2gzMjmSEhV6w15PGutvjsyotu6am9WjpLXRqsh4jjUz7nFW2rTa
Yw76rLadd22HVPv12d+vuV8TsRaoYQ/mrp9X2zVA4xpD+4qbsuefNfnTFa4Z1BPULwcDQN67d6V
wbV7neDX69Frbev2+maYPxeh2aFjf3vOc+P1RXYWtXf41/TppyLsFxAmnafXBXPvgXH9RLAIBZeONf
3yAv+OEVemk5PfTV78qf1h/USwCAzPUfFrn853oBcRGiLiOvJ6IfaPUOndivWnXCo32dFJrDwO38bS
5UZMsNoJq7fi8+s+6n2wPUXFff8ickqmx5QWCs3pgzPGfVlj+Rkh/mWi0Z7IY7gtk792TJfmJsJ0EYq/
Ua636vzepZ2QxPUMe3O61rY6vKWbchRsg+cv3+obPdKdoQeH0UI1Q3Q2s7cNWB6t6okwhkSA3f
v/NTd+olAMAsvPLBa+Xmrz9fLy23z338Idn/n4/pJQBA5i59QeQyf8BKghB1KRXlxCnnmRtode1x6W
U5efqkHXYF11vUMHDn2VzwQ7mmeJ0KuwdeWOgN5ffWWcfkDu+2H8bw7cAQ/VFmdc784HMy
Yb1zLRMP6W/LeT9BlVZoz1klRrtTuzaW8kkJ3d2o9a4p2hC7Z7Zxrq2N2WUO3P3Er8sLpOuJp59gJn
4AmLGXfPe58nMPvEQvQbn7P35Vvvct/m0CgNxcVvVRv68XMA4h6pLy6oraPTl74nSqPCUnKies/w
+uV3ngPEWoFiOYcodmt90x0xG9I6exkOfM6GmphqaXQkPC+LP0tzf8OqBVo6fp8kkSbhdl80L4jP1u
cDtiPjEgE+5M/A8/8bBeAwDIm6p/usx1UEdRAWrzN+/XSwCAzPWfFLn8cb2AcQhRl5U3tHlfDts6aL
N77VXEyQqN9VP3hsyCUdfTDi/9upOjwkuvNubQI2YYuIDnzB/KHyXmkP72hj9RVLUlIzuhTmDqa5O

CTNtg1JUdKNlqa55jln7k532ffZ/c+/C9egkAMAsrX3qpXPvklXoJpn/40rfl079/qJcAAJm7bP02eukze
gFRCFGXVklO2hlkRw52nSHNbh3Rkv0Jf3347OuzV9w864eaDXeSp4E6pCVnqL3i1eKc2KKdM2Mo/
xjjh/S3ZcNLUMtSV1PRTyvVazOhHNugZuhXPU7tScXsQNWYoCtu2QJgSvX76vKxv/2YXgIAzELpoRfa
D4z26f/6D3aYCgDIyeXPivTp2jMOIerS8ntyNptOOObWeCzq4p/uem+Sprnj9gDtSK2mA77BOqRmP
crmqj9sur3h1aUsxB5LvWDnzBzK787+H3iYIV70kP5e45w3jL9cv5BOuYRUr82E8miD3pbKoIM9Trvi
ls8dW7sVSMn/+v/
+l34GAJgF1ftU9ULFeHd/6AH9DACQi0v36CcYhRB1iZkzpqvehd7w88Ds5sYkTXPIq1NqC+shGaxH2
VzVwZg/Ll1GT440LNVzVtS1VJXmauqhoTmUvxxaW8AoiWC9cuSQ/l5D1r0CqtYrAxMwOY/JJkhK99
pMJoc2VLb9IfyBurRGfdmzm7MP3bEU7v7Fu+WOl9+hlwAAeXvjX99AHdSYfv7dP6WfAQByUXijfoJ
RCFGXWWDW8FPiTzbuDltXzPVzyAwvRw2ht+tRGr0uXeW6dJPWu0z1nFVke7BA5v5hSrUxzaH8o0
NdMxQeNaQ/Xl3VCaV5bSaVQxvsIfxhxVBVSNvvp1pfFohy1RVXyf/4lf8ht1x3i14DAMjT373yCf0M
UV7z1pvkbe+5VS8BADJ37FUix9+kFzBKoa/G9QIAgKXxwCMPyOv/2+vtmfoBAPm648vXyysfvFYvYd
D1P/E8+a1PvVGedw0TbwFALgovFrni/daTq51ljERPVAAAlsztN91uD+0HAOTvb27/tjx2PX/ECnPlc4
9L9fdeS4AKALmx3m+Pv8f6SIAaByEqAABL6F2n3iUfeMMH9BIAIC+Xjvfl8294VH78nMt6DVz/8t+V
5NZXv0gvAQAyd+ztIoWb9QLGmSpE7TVW9AQpBX9W65F60lhxJ1TZkODL27LhTbbiPCabqGZYeyO
43dBH1jOAAwAwhz788x+W07ed1ksAgLz84PnPyt7rvqWXoLzidS+R1X8bNhkqACATx4oix39eLyCO
OeiJqsJVf5ZqR84zwquZ0VcaKU3oAwDA4rjnl+
+xh/cDAPL10M0/kO6t39dLy+3al14ld99rV4CAGSucI3lMTWMH0nkGKIWZXOvL2oeq35gxuuuHH
iTiKvZsNXn92QzxwzV1qnJeTqkAgCWjDtj/3VXXafXAADyonqjfu+aZ/TS8lIBqgpSAQA5Of5ukcIL9QLi
mn1P1N6h7OuncqEZJedlqXedUNc/9Gtl/XnRZq7pKgAgOVzy3W32ENmfh4TC6UsfkuAwx0Zb1xFu3o
S/8xivsofwAgJzYdVDz7rl4NOQYog7XRLXrlZZq4nZEtYfVD9UoNb/OeaRVL1Uprp0RP0YFAGA5vfm
WN8jBxOwm8
+V2h01vQQAyIuqjbps9VGfd82VctfvUJMbAPJztcixO/VzTGKmlWIwpluy/9bt3vCVptOcPst1WY2ZPGu
htwVqXlDsFvVe01qobpeqIkoSO1khtC+I9Vd0Yra9/2bgFghnqNlWAPfSBnqjeq6pUKAMjXfT/zuD1r/
7JQAer1P/E8vQQAyNzxO0UKL9YLmMT89kTtSKU61v+RFSVLXHLmHZ2LqbU+64s9S0S1NFG
TQoGTGKR76eM295ryPpg13tgBlR9VFUnFQCQnx8/57J8/g2PLkV91Le951Z5zVtv0ksAgMwdf5PIsV
fpBUxqfkPU7oHXE8sevu/1HjWG93cOpKufTsfppZpmrVUAABaVmqm//attZuwHgJw9/3T3vpF8ku12/QSACBzhVtEjv0LvYBpzHdN1FSFz85vlhPo1M7nWltxHgxO2jWqxuPoOpD20GPv6
/VjZG3Jwf1FBNd2Pdzga0fVgsysDVEya98oca+VK/5xJm1f2OsHyncO1O806o7arx19P/kG2p/K9Ux6
DsOEt32wXungORo8P2HiTbTn8/epHqPqpCY5P0DQbdffJvf88j16CQCQl7+/7Xvyjy//oV46WlQd1O
rvvVaufO5xvQYAkK2rRY6/2/p4hbOIqSxEiFqud4fDT/uRwnDa4qac1WVWRfblcGnyBRVsDU7a1ZTV
wqr1/3hUiFMKG3rcqUlpMKBKMklYe2NETUjVYzgYFmXWhihZtW+khNcqwXEma58TyIW9vrlaGBH
2qXNitLNcl/GVM9SxDbR/6us5/f0ej9OrffAcNVdHhZyTcPbh1XO2Dd97qd3vWGrvKL6DGfsBYAb+6v
WPHcn6qL/yH15JHVQAyNPx09RBTdH8hqilk34P0YN0Bu2Ha8tuuinKQug1zvnhkTuhV7/r1Zsdzzhv
5bp0dbDd9TbQlHNeotNLMEmY9dpzesOh2+3IzkX3tVm1IUpW7Rst2bVKcpzJ2tdrrHuBnP+HjZa1F
615bkRQaLRjb1OKem0koz1u06e5ntPf70n47fHb3pHa+ejIPHqivUH+PsLvvbTud0Ds2frfdepdegkAk
AdVH1UFqUfJyumftB8AgJwce53zQGpSC1HtnmjGkFHzEWco65DimpzxspxVfxuqF6C77UQbdnpv
me1yHmYvuTOyFivhWXQ9uejO2qUCFi+kKcrmBSPEidI7lH39VDo74mY3xc09J6yxHnub+mQmmiT
MmLjHCNyKa2eGQ/XM2hAlo/aNlPBaJTnORO0LtuOst74iW/aGVVC3J2GHE2hHTNWz/rmtbPtBrd
/2JNczhfs9gWrL7yFf2Up/+4q5j/B7L8+J+bAM7v7Fu+WOl9+hlwAAefjmjU/JV376u3ppsb3sp14o/
+a3mNAEAHJTuFHk+L/WC0jLHA/nDwYcXkjrjWE1w5B0mMHN0daVAzdTKp+Ukn5qK56QU/ppJDP
kHgioh2ouTjVJmK6nGTZ8Prc2REmpfSMlvFZJjjNR+0a3wwldR5fWOHUi6XdVVU4HNlaSk2473bYn

up4p3O9zpSwnAwcRIteJ+bAMrrriKmbsB4AZuP9V37HD1EWm6p/+
+n/5GeqgAkBurhQ5/mvWR+qgpm2+a6IWN2UvbMitPdQ3hXqoHtWLri8pZ7JH3OhefG5oM1EPZ
MWu5eiGPlE1KzNsQ5R5b19sebQvRuA3VlFOLEzSmcbxAvPpphfcZAepKlAFAOTn8yuPylNXX9JLi0f
1QFU9UQEAOTn+r0QKN+gFpGmqENUc9hv1cMLJoj8M2gxA7aBUrw9NMc2v04+4dRUtdp1B82tD
H2kGskvEuHZ+7Udf89zw5DXjJwlry4bZq9OtjWnWijRl0oYo2bcvLbGOc4btG21wgreeHHp1B4ZlOv
HcEcD5QZpuv+l2e2g/ACA/KkBVQeoiel3l/6IOKgDk6dhrrQd1ULMy3z1RkZGQ4dEus05mDGp2d9V
b0Q+rjZ7D7raTTBLW3vV6dtrhjxuYG8OTB6XehihZtW+khNdqguOM176Idth1itOdfT7Y9JDh+ImO
M737fWHkNjEflpGaZOoDb/iAXgIA5EEN6f+7Vz6hlxaDmoWfOqgAkCM1C7/qhYrMEKIupaKs+bN2
yao3Ztuc0XsMPcGXKlEb7K0YEnhNOEmYH/4YM+KbcmhDlFTbN1LCa5XkOBO1L6Id9rE7NVUTnsK
Rmqsb4jfdL5lQPrPmhNaJrmcK9/uiyeB+B0wf/vkPy+nbTuslAEAeVH3Ux65/Wi/NN1X/tPp7r5XnXX
OlXgMAyJaqg/oe6+PVziIyQYi6pIqbF/zehs1VHawYk86MU9n2h393alJygxmjRqg/UVew9mbkJGGV
09aSNq5dWbUhSmbtGy3ZtUpyrpO1b2w7ynXZSmV8eFnK5aas6vZ4Tbfaftab/j/Z9Zz6fs+LOdGV28
6Jws6U7ncgwj2/fI89vB8AkI9Lx/vy+Tc8Kj9+zmW9Zn79Uu02ufXVL9JLAIDMHVsTKdysF5AVQtSlVZ
TNvcFJu6rS6res/8djD/8OK6Rpb2dgoi679ubg/ixDk4RVZHuoDc72vF01d71eitm0IUqG7Rsp4bVKcJ
zJ2qfa0Zfu0IbVpo3SBlM7JWcHjzfsGiW6ntPf7/mw7q/B67F/aPQSTiCV+x0YzZ2x/7qrrtNrAABZ+8H
zn5W/ev1jemk+veatN8nb3nOrXgIAZO7Yq0SOv0kvIEuFviqCCAAAMIF7H75XKn9UkaefXYwhpgBw
FNzx5evllQ9eq5fmx7UvvUr+06ffzDB+AMhL4RqRK9R8BQzjzwM9UQEAwMTefMub5aNv/6heAgD
k4W9u/7Z875pn9NL8qP4udVABIFfH3239jwA1L4SoAABgKu/92fdK7Y6aXgIAZE3VR/3cm75pf5wX
v/Abr5BXvO4legkAkLljbxcppFNUD/EQogIAgKmp3qiqVyoAIB+qJ+re676ll2ZLhae/8Osn9BIAIHPHiiL
H36EXkBdCVAAAkAo10dQt192ilwAAWeve+n37MUuqDqoaxg8AyImqg3rsPXoBeSJEBQAAqVAz9b
d/tc2M/QCQI9UbdZb1Ue/6T6+2g1QAQE6O3SlSeKFeQJ4IUQEAQGpuu/42ueeX79FLAICsqbqon3
/DozOpj/q299wqp/7ZDXoJAJC5428SOXabXkDeCFEBAECq3lF8BzP2A0COvv2if7Jn7M/Tra9+kfxSjV
/kASA3hVtEjv0LvYBZIEQFcteWjUJBCsZjpdHTn1tSvYasbLT1gqO94Z6fFZnX09NrrDhtXGnIqCb6x+E8
lv5aY2mo2frfdepdegkAkLW/v+178o8v/6FeytbzrrlSqr/3Wrnyucf1GgBAtq4WOf5u6+MVziJmghAV
yFVPGiur0tRLjrKcWSvq58vHDiJLNeno5YXRa8h6LarV6loXZDV4saVTK0lhIDAGjqq7f/FuuePld+glAE
DW/ur1j8kPnv+sXsrOXb9zu1z/E8/TSwCAzB1XdVBfrBcwK4SoQK66cuDmbuW6dPt96ff3ZHNZM9
SxQeScUj1nxwW/7fPiHlq1pa5zX7r1srOiuSrkqFgGV11xFTP2A0COfvycy5nXR33zr9wir3nrTXoJAJC
5YyvW41V6AbNEiArkqXco+/qpnDohy9v5Wqs5Tae6SomI53PSC
m+wgVQWqAIDsPXb903L/q76jl9L1sp96ofzKfziplwAAmSvcKHL8tF7ArBGiLjGvnqP5CKntOLI2peqN
536d163Or/dp135sb+ivNdbZLwuuD/bKS2MbmtlG7zFcYzN4jMM1S+1HWN1Low3jal3a+zDDt+aq
87WDDQ9pc9i2o9o8tpfjROdl+J4ZuZ8YxxD7fGh+W9RjuK0OZwi9/7p0z506/lKsnrNGj+NAWF6Sk7
ozquwfDt9PNqM9ie65eMduS+H7wj1Pcd9HsNxuv+l2e2g/ACAff/fKJ+SbNz6ll9Kh6p/+
+n/5GeqgAkBurhQ5/mvWR+qgzgtC1CWlwpHQMKhTk1JhQ8ZlcHHYtR8HCkLa61TIMrC+uRoeXk2
1DRU2hfYY7EitNCqEU58zapaW61J3uw52duTiYMi0674ynbqmQ8GiZh/zyGBquM1uz8dQE5+X4X
umuToieEt8DFGcfQcvd0hb7WCw5A2hd6V67jxVafVbXq/SIWaP41E6B9LVT4MqcjrpPZfk2FP6vlDn
KY/3ERwdapKpD7zhA3oJAJC1z688Kk9dfUkvTe/f/Nar7J6oAICcHP9XIoUb9ALmASHqEqUmqLn8O4d
TmNeo3SlHPhSUpi5XrX3na/FYybwtaPGt482TaCw6mHj7EjO4PplEcFZM7r+3ubsrlVF+erBr/GPI9nZ
FyGWtm2ttd1t2Wptpx9bOvUrr3hB4Vhbe7UZH3kdQm2eXRT0jkv/invSO28cd0SHMPY8xHg7zu8r
dZxrbvBoHEu3Iamcu40u53bEitrHVKUE6f00wiVRPdckmNP7/uimOP7CI6OD//8h+X0bQxHAoA8q
ABVBalpWDn9k/YDAJCTY69zHpgrhKjLyOwlZ/R0K27uOQGJ9dhLZaajqpx1t1M5bS25RqwPHd486T
aKsrlnBj6O4toZL7TrHIT3AyzXt4IBWXFNzugv6uxc9NvY3vV65pXPrI0P38YwexjWLxht3rwgfgZ5PrR3
31CbR5r8vFRbfnDoh3xB0xxDFHPfoW3tXZQd3SEycC4qW/5+zWtniH/u1HFY3yOhIe8k9uUwrEFKk
nsu0bGn+H2R2/sIjpp7fvkee3g/ACB7akj/V376u3ppMqr3qeqFCgDIiV0H9V/rBcwTQtRlZAQ0ImqY
bsGrZTiyhuIkyielpJ8GjFofJo1t2HQ9x9BhzEGnTgwGP0VZ8xMtLyzyA0Mj0J1YVK9WY/8jgrfhNscV/7

yMN90xjFaWk+MudvfAa79X7sF+GEPcRwyfn/zcTeuUjN51gntuimNPcv2HzlNe7yM4ctwZ+6+76jq
9BgCQJTXJ1KT1UVX907t+59XUQQWA3Kg6qHdaH6mDOo8IUZdSUTYvhPckdEOYqMl1FkZg8hyjn
mOk8MDO76nnDnc2AsPq6dg9GafXkeGOgjFCRlOK52UyYccwK2keVxw9ORxbLNWR2T2X2vVfkvc
RZOKW625hxn4AyNFfvf4x+fFzLuul+H6pdpvc+uoX6SUAQOaOrYkUbtYLmDeEqMuquCl7esjtQKlR
W/Pcos+u3ZYNs3edW7PRrMGZhHW+zurzZA+NNoYyV0/nF6FOH/qlfF4mkm1w6dXKHXpMWsd0
AsUTMrb06bje1BPcc+OPPf3vi6P9PoIsvfmWN8tH3/5RvQQAyNIPnv+sHaQm8Zq33iRve8+tegkAkL
ljrxI5/ia9gHlEiLrE1MzaqqeYPcGPHYR0vRqKo4f/GoxhxHPHrB2pgiW3/uMUba64U6Z3duT8+R29n
aqkk6GW5KR37gdnZO/JRbfoZeQQ8BgyOC+
+nI4hTOmkFwKOqumZL+NcNHfF75DZlQPvNJxwzn2EWPdckmPP4PpP/T6Cpfben32v1O6o6SUAQ
Jb+8eU/lL+/7Xt6Kdr1P/E8uet3qF8NALkpvFgP48c8I0RdRu0Ne6itmkU92FPMCHiMXnIlPxkzZmJvy
4Y3Dft884MlY2bySXgTWHWk2dQnKrWh/EXZdLsdqvO87l+XXmPdq22ZZBKkcVI7L578j8Fj1udsrvr
DyPW9bj9yHVtu1oBtyq7eda9xzgsxY/VgjnPPTXjsU1//hO8jZntyvRSYe6o3quqVCgDI3t/c/m157Pqn
9VI4Vf+0+nuvleddc6VeAwDIlqqD+h7r49XOIuYWIeoyqmz7Q287NSm5QYtRH7F6NnzmbhXSDL52
Lpkz9nttNibamUhF3I6BrlSH8o+4LiU/fZQL005glcl5MSQ9BnPYu9ueiRK2YH3O5qqz34IX9Felldqs+v
EUNy94PTLd9njnodqSeM2Jc88lOPY0r3/C9xEgiqqPquqkAgCydeI4Xz7/hkcj66NW/u8idVABIE/H3k
od1AVBiLqk7KG3YUUMVeDS7wcDHlX3cLBmYrWVcx3NpCqy3W/5gZHNOTbvsAPDrOPxhlcr5bpsp
ZzL2dcl5LxWW9Z6d+j1VLI5L6Zkx2C1Z/A+3D80ejUmYNfnNIaSu+y6nznWQ/UUZXNvuFaoPYw+Q
aAb656LfezpXv9E7yNABDVTf/tX28zYDwA5UPVR7/uZx/VS0Cte9xL5hV8/oZcAAJk7Zv2GfPwdegHz
rtBXRewAxKNmNtcT86gwbG/anqHAOEfpntPHcqpFwIpwn+l9Rip/xM0BAHl441/fIKWHXqiXRK596
VXyW3/8z+yPAIAcFK4ROf5+66P/Xoz5Rk9UILaeNNbdmc3LcmaNABVZO1r3XPu8dSwZ9ODG0fGO
4juYsR8AcrL3um/J9655Ri+JVH/3tQSoAJCn4+8mQF0w9EQFxmrLxmANWFXOgK50yMzRu+d6jRU
p7ZyRbiplKXDUFf6fgn4GAMjStU9eKe9s/aS88723MYwfAPJ07O0M419AhKjAWAOBFgEqMsc9h+
WlJiiblx9NaEs42hKOtoSjLeHmqS0nrr1GDr/3pF6aLa5RONoSjraEoy3haAvSQIgKAADmBj/ghqMt4
WhLONoSjraEoy3haEs42hKOtoSjLeHmqS1IhpqoAAAAAAAAABCBEBUAAAAAAAAIhCiAgAAAAA
AAEAEQlTkSE2WU7DrfxRWGtLTa8fqNWRlo60XHO0NvZ3CijRibwjp6Uljxb0GG9aVTbJuGmH3UNr
7mJbRRv1Y4SYFAAAAAGChEaJirvUaK1Io1aSjl4H5pgJdY1Z9W1nOrBX1cwAAAAAAsIgIUTG/eg1Zrx
GfzqeibO717RkF+/1tqei1szFPbenKgXvLluvStdu0J5tkqAAAAAAALDRCVABIS+9Q9vVTOXVCyE4BA
AAAADgaCFGXmao1OlC7MazG6GD9UXuIvfE1A+VKNbNOpXokq1Vp79Mcxt9cdbYTvjOjjeoxqk7qY
JvCa1VOdrymMbVf2xvetob2H3JN4rTRY359ZEONcxFo46j1xj7t9ea5zKoOadx7KKotg9uYsD5pjOuS
9J4FAAAAAACLgxB1WakgL7TWaEdqpVEhpPpcQUoDQ+ybq4OvVyFiSYIva8pqYbBWZBqcNq0GNh
xyDHYlNtgm65W1UnjQaYt7vIMqcrqqn3Z25OLAa9u7bmODtTKHQjgtuo2TKsrambLztHMgXeeZxR
iOHmh7W9xml8+s5dDDMoV7aKJrPizf6wIAAAAAAOYRIepS6knjnJuIuXUb+9Kt61BNOrIzmPx5qtL
Sr2+5QaEKG8/7ve16jXN+0FVt6VqVXfE2H0Nl2/qabl28L3G3sx1W8dJvU/gxWMe77oZg/mv77gF0
arI+MhUdf7xhKltu2wfPpR9GSvmMeBlqe8MPgsOuSWQbJ1NcO6PbuC+H7qbbu0ZI2ZEDN101hq
mfOpF9hDr9PTTNNTckuC7J7lkAAAAAALBICFGXkjERz96m16vQD9VEOl56FlRt+RP3+EGhqScXd9w
+e1VpeQGStc8LYa+fntmm0GPoXRS3SeX6lj/xUGXLC+U6OxdDexSOP94RimvidfQ0t22ElGaPTrN3av
2CcU02L/htrJ0fGKo+Ja+NftDbO3Si0mrVCRv3dbrau7jjBZKnvROYlRTuoSmuuWkm1wUAAAAAAM
wdQlRYdA3P0OH9SZmzk5+Ukn5qK56QU/ppespyMrCTEN0D77jsIdheXUtjqHdgSHsazOHy/rB4P5
SryllvyvYRvVNtxnbMHqOpKMoJfUHcwLlrXzzrnJ4+aYeVwfWW6mk/kMxMCvdQKtd8VtcFAAAAAA
DMG0LUZRWYKCdunckYgeWRMt3x+r1i3Z6eRig3URhpDK9PScUt3trctVrXE6cj6ik5UdFhZWC9anb
2EeriSf+6AAAAAACA+UKIupTasmH2OnVrPZr1HI+ocr3rlDEYevjD9lNT3JSzbglONXTcqCs6WRiZQYh
dOS164L4ctvUQeLv3pzs5lrF+QUP07K/5sv1xAQAAAACA5UOIuozMupwqYHLrohpDoCdXkpNuEjs
4XNoIEXNVcoamK6NqvWbF6+nZ2ZHz50fVFTXP2eBs/mZ90FMydk6nxNfQ3XdHDnadr3VrtZbsT/jr
h4e0ZyWFeyiVa57idQEAAAAAAAuNEHXJ+QGTMWP/VMxakU1Z3XCn3DFnS8+ZMcmTNFfFa1J7w

6+V6a1MmdfTsyPNpj76oaH8Rdl0u6xar6utN6yz5eg11r0anuYESU7AqVivP+8dkGx4U8nH5V+vZtP
5Wnf2/aIumOquNyfCylYK91Aq1zz5dQEAAAAAAEcTIeoy8oI9S3NVh0rGhDtTMmcun2r75iRC7nY
mCjuDs7o3V3WI5gWO5gzwaXOHxftCh/JXtqXl5XU1KdnnrCAlP6mTC95EVCojdOutWrxzHLe2bVBg
W9Yzb2i6eZ9Y68/k0w3VNv09lNI1T3hdAAAAAADA0USIupQqst1vGQGZUpVWv+8HRvaEQpMqyu
Ze1w/BbGr7g/scx2qn1yBt/9DrDZhIcVP2+oNtstj1YDOoh2rwhvQr1v62Ruysst0PrUtbbVnr3ZILLnU
8g6+ttiara2v22gwMTTeGs+c+ZD2Feyila57ougAAAAAAgCOp0FczrADITq8hK3oiL1WDdo+eiwAwk
urtPS8/mtCWcLQlHG0JR1vC0ZZwtCUcbQlHW8LRlnC0BWmgJyqQKbOOZ75D4gEAAAAAAJAOQlQ
gE23ZKAzU8ayeFTqhAgAAAAAALB5CVCAPql5pZpNXAQAAAAAAIEvURAUAAHODelXhaEs42hKOto
SjLeFoSzjaEo62hKMt4WhLuHlqC5KhJyoAAAAAAAAARCBEBQAAAAAAAIAIDOcHAAAAAAAAAgAj0R
EV2eg1Z2WjrhVnrSWNFzZavHhsyL60CAAAAAAADA/CNERSZ6jRUplGrS0csAAAAAAAADAoiJERfp6D
VmvEZ8CAAAAAADgaCBEBQAAAAAAAIAIhKhLqr3h1gddkUZPD7+3l51HaClTVePUeI3zcL7eZW/XH
MbfXHVepzc4uF+PuW1j58HXt2XDfY318F4Wo12TGjwv6jHpuVGSHr8S1obCSkPCD8+s/eo8VkaciG
TbBQAAAAAAWF6EqEuvI7VSQUoDw+
+bqwMhX3tjRI1T9fXpBJbR1H5WpamXpFyXrYr1MbN2OWHk4HlRmquFYNCZ4blRoWtYG6RTk9Lg
BFl2EFuSwZd3aqWhcDTRdgEAAAAAAJYcISosVWn1+9K3Hq2qXqUCwPNulNaTxjkdX5br0tWv7dbL
zjrrtTsXnYiusm19rlsX9zNSbdmv7W+rxHNafjv7e5tSTNCupHqNdS+MLNe7zj77LasFWvOcDkeza4NI
W3bd1Dh020055yW0VjvW3SDXOE/uBe3UZN17bZLtAgAAAAAAgBAVUm1tixtxVraMANRTlM09H
crZ4aVeu3bGe23noKufZadc3/La6ciqXT25uOP20qzK2U13yxXZsoNGFVLuibM6w3PTO5R9/VQ6O+
JmscXNPWd/1mPPbVvvorhNDpynypa42Whn56J1ZJYk2wUAAAAAAAAhKiaha5OGDmHPzqkT44K
9tNrVlQN3A+WTUtJPFSdo9EPnYSmem+KanHGTWGtrquyCW7t0qM5p98Dbnz18X7+uYA7v7xxYR
2ZJsl0AAAAAAAAQoqIsJ82UcJTAxElGbdLcjGjnzNtlyawNRdm8ENYz2A9KQye5Giur7QIAAAAAABx
NhKiIoS0bZs9Kt46mWft0JuahXRm3obgpe3qIvV+v1tc8Nzybvl/DdfBh9KCdYLsAgLzp0Q3Ggz9yAQ
AAALNBiIrx2rte70o7oHNrfxpDyKc2ybYya1dJTroJqDsE3qVm4i8YM+6n1YaI16uZ9NUvzfakXXbw2f
XqnHrtK530Qtu4NVhjbRcAlpH9Xl+QwkwTy540VlZFWu57tPNHr+bqLILU+Qxze40Vpz0r+f/hT/0ba
p4P9zEP5wXDhq7XDO6ZmRvzvuZ9P7mPvG7mOO+3ebwnR+5jSf+gFfO8z/K9eGZinJuh951luGnG
nJfc34sTtofSdoiDEBWJ+AGdMSt9AiU/nTRm/7d+MFmdbhD8tO0KKsqaVzS0Kavem667baeO6OB7
cZw2JDp+/aavPhXsGRpSs9Wsc9pc9dvm/sOhHu7KJNtVjG3wDwuALMzsl/chKri09j/lv0npcCYu3Da
KcLuTP+4f5vte3N4IC3ONPyjOQq8h6zWRatX9x28G3NEnxsO8XrMy01/cAyWOhh+z+NZW52O1qS
YFda9TS6qdmpRmFPjk/0v7+Pc19R5csr6f6l3jHFk/T2Z778R5v83jPXncPtTnc/yDVmTok1eYm+C85/l
eHHFuht739CP98xP/vl3dH/g3Iot/IEadk9zfi8efl3zfiydoj/WNrUrbpf+eHO/7dn5+FsZY1g2DJWS9R/
TV5bd+A+hbPzD5uvW+9U+g87mq9ZZia/WtnxX060c8vNcqIa93P29uf9TD2NbIdtqStKvbr5fd9VXr
K8cxXx/yKNetVygJz83Exx/
+MDcdve3gMSfabqvqrS8PXwQAmEq3XrbeX8z3eP2+GngjyoffFv1vwAzaEEm/z8/8vXjm7XCuj9q/f
c28f5PzY/87OoP9RtP37dy1y/3eivPzV9rCv5dn1R7n5y9jv/pnrCy/l8a/rznvuYNtyPocjW9XvNdMa6J
9ZPIeqPdvbdd+hLRD3T/maud+Um3XK1IS/5w4n8/+vTjeucnjvS/
+fZv1+8v4cxImq7aNPy/h62fWnhHfw+nfR87+zd0737fBdX579Qr9vhz3uiJf9ERFDBXZVn8p0ksO56
821puAo7kr/t9KrNd7n9D2D52/MKlanIP1QqutCWuIJm1XEk4PIOsNTS/7rDdbf9h+0jYkPH57qP3g
ubQ5+wj8QdOuc2oMyXfZPWWMeqiWRNsFgMy05XytY71NXZBN503VUpEt9UY28fv35Iqbe9Lv7x
ltmTO69MupE/PRwFm1o9dYl1qnKmfn9kLNhnteWt7PKPPC+T6X6unAzyL5KMqJU9YH9+dQW08u
7sygPb2GqIFK5fqWv9/Ktv3zYmfnYgY9sRxj39d6h7JvfRj8fi7aJ64puxm9Ecd5v83jPXle3ved79+y1Ls
hP8tr6uf34dEJHdm5mO7dE/ec5PVeHOfc5GX8uXHeXwLf5xmY7Jxk9148/rzk+148tj0jfp6yR42mW
tYuzqii+fpZGOMRoi4pvw7mwJuLMeFQcMiBCgvdr1EPJ5TztxMM6dQPhf5rrYf5A725D/VQ+xmx35
Ht9MRtl/MGFlw3nvMG7H6d89gbakjCc5Pg+G2D59J+jDoG8zj1Y9QvU3G3a7xu+NgBYAoz+uV9M
enSL+W6bIX/A5CTnjTWa9KZVTvsoaMdqbbi/1ueGTUM0Rh6N9uSN/n84j6JXuOc9d1s/bI/oxu3sq2

HjNo17dWwypITNg/
+vJW13H5pT8huV1lOenWcNKPePgZk8AetuMFlmDTbEVuO78XTnJv86fJoB+cHhtOnWwJnknPCe7
FhxPub8/PnvuRaNYmfhRcOISoAAJgNfnmPTdUltX/5uTCLXoa6tpj9i6D+pWdGvR3b52vSqbZmPmL
C/0Op81AjV7KppRZXPr+4J+f2NDo7wwDEHTWkatqr7yOrObMI4efpl3ZMgT9oKfPyXhwwR3/Y6uy
flAvevxGqt6h6/9mYYa9C3osD9HwiwflBrO+pqedWiWHwjzD8LLxwCFEBAADmmDP5gRq2N6ueO
MFRDt36vqzO4hfU9oYzCcRc/dbuUL2Csh6WHcfc/eLePi/O6NHZXTNnsg5/YiAVeKtJgXKftGOWv7Q
jNfxByzKH78Xz9oet8pk147pY/4aeVWXUZtirkPfiAernGrdnrP89dXBKXadTkl3n7nn5IwymQYgKAA
Awp5wAVfXWmJ+hjMXNC3YNtk7tfK7hXHtXhU1NO8B1fuEpSEn9Vqh/CZrtcHptlsOyLfP1i7sOCGf5
y6Iecqzq2bt5jz0MViXezdWMZjYfZVa/tI9h93bqyMHgjTuqd9QS4w9ajkV4L56XP2x5ZtqrkPficIMl+f
qydXLfOk8nJau3vdn+EQZpIUQFAACzwS/vkVSvDSdAndMJ/zL8RSPMYE8j9bAngLR+Mexaz2dbt7sn
h6qoWc7nZKxZ/uLu9nw6O8NfFgeHTbpmdl7y/6V9rOIJceZ7CUZNPfuGnmG4O2f4g5Zvvt+LB+T+h
62S2GWOB8PbWf5cw3txTLq2eOCPkekZ+UcYfhZeOISoAABgNvjlPYKeRddiD3czevzkW+dSTf4wM
Bxc/0KW1S8ac6/XkJUVc0i2WqVmSp7lL6nz94u701utKjMcPSpSOW21wPoeCgyhV7dwzTovM26bL
dtf2uOpyGnVY6+27r+veL3G5m+islngD1qLaFZ/2NIjADo1Oe/9w6mHcM+oHinvxXGoUhklqUldLm
RwkSL/CMPPwounDwAAMCOtqvSt33L69a5e0a33y9aPJ2VvRX669bLVFtWewUe139KvWUZh56U
6JyfEblu53s/3bun26+Xg+ZiLe6RVHbg2rb71S2tfJT+5m+H38TB9HgKPefie1vdRxvdv3Pc1573Yf2R9
7eK0K4/35PH7CLt/3Ifxb1dq9H0x9H2r2jFw3Pp7Pu1rNcl5t78m8/fiEedGvd8M7Ns9hrTf/uKem6H
XZfY+POp+0XJ6L453XvJ7Lx7fnpB/xzO6RnHuxXn6WRjjFdT/rIsKAAAwE+5f6F3WD43zNRwQWBCq
x5xdm9BVbUl/Bl3nnO/pqrT6M5p5eS7pnk7G5ZnV9cH8Gfre9fjfR2Gvqc5r79gUjT83Id9bxnk7iuLc
LwrvxbOmRvOoOqhhgkP7+Vl4cRCiAgAAAAAAAEAEaqICAAAAAAAAQARCVAAAAAAAAACIQIgKA
AAAAAAAABEIUQEAAAAAAAAgAiEqAAAAAAAAAAEQgRAUAAAAAAAACACISoAAAAAAAAABCBEBU
AAAAAAAAAIhCiAgAAAAAAAEAEQlQAAAAAAAAAiECICgAAAAAAAARCFEBAAAAAAAAIAIhKgAA
AAAAAABEIEQFAAAAAAAAgAiEqAAAAAAAAAAQgRAVAAAAAAAAACIQogIAAAAAAABABEJUAAA
AAAAAAIhAiAoAAAAAAAAAAEQhRAQAAAAAAACACISoAAAAAAAAARCBEBQAAAAAAAIAIhKgAAA
AAAAAAEIEQFQAAAAAAAAAiEKICAAAAAAAAQARCVAAAAAAAAACIQIgKAAAAAAAAABEIUQEAA
AAAAAAgAiEqAAAAAAAAAEQgRAUAAAAAAACACISoAAAAAAAAABCBEBUAAADAQmtvFKRQ0I+N
tl5raG94n19p9PTK2fDbuiEhLV0wbdlwz3vk+e1JY2Xca6xXNVb8ba00rK8CAGB+EKICAAAAODqa52
TGOemSUMHoqjT1kqMsZ9aK+rmpKJsX6tZnHZ3aesg1asv5Wkc/F6me3bS+CgCA+UGICgAAAOAI6
Ujt/OL38Zx/XTlwM89yXbr9vvT7e7I5KvksbsrZqn4eco16jXN+IFttyXZFPwcAYE4QogIAAAA4WuiNm
r3eoezrp3LqRKxeo5Xtlng5auAamb1Qq9IiQQUAzCFCVAAAAABHTLzeqH590pVg6NpryIpbm9Orse
rX/7Rrehp1Vr119suC68NKtPqCtUJH1wEdeJ31CKsrGjyeYL3S6HYYzGPXj8F92fsp1ayzrDVXndeO3Ul
FtItujOpfo/aGXxagXN+yXjUo3vHbQto/dH0tcc5VoEar+6BWKwAsLUJUAAAAAEdEWep1HdJl2Bu1U
ytJYTVYDdRep0K2gfXN1VEBZlNWCyUxyoCqjUhpcMIpOxQceJ3F3t/IQK8jtZJRr7Rcl60YnTuHwlEtel
8JVbak7hZHba7KRqMh5/wEVS4M1gNIcvwqwA5pv3M+hoNUR/i5UueiNLhTJewaAQCWAiEqAAA
AgKNjzQ3p4vVGnVS53pW+qgPq9ax0hK1v7o5oh1dLtC/
+y5tyzkv7etJYd0PBqrT0a71td2qyPjIpNl6/F2OSpvaGePmv0a6um3ga+6psW5/r+hNFqRqm9n5iDc
MPTjLVrLnHV5b6hcF2Jjl+67VuGhvWfmsrOxfjnqu27Eadi8A1AgAsC0JUAAAAAEdIUTbdGYwy641al
bNuj8nKaWvJNWL9/mFoL05zBnqzXmhn56Lz+t5F2dGdIQPD3I3enN5rB4QPix+t7aeGgTCzuHnB31
ftfDo9MAOTTGnVs8OTUiU6fuu675lBqKO4dsYLbDsHXf0saOhcmfVeOzviZq/FzT1n+9Zjb+QMWgC
Ao4oQFQAAAMDR4oVsGfVGLZ+Ukn4aMGp9qKqcDiR3JTnpp31ix33dA90L01rllguwH8bwdve1A0
6dSBLymT0vz8ha4EuLsnbGbdi+HIYlthMITDJlPQudTGqK41fHZNc5DR3eHzR0ropr4h2yuodK7n4ja
rECAI48QlQAAAAAR8xAb9RD5+l8K8qJU/rp1MpyMn6am0BHRnTmnE6i8DlCYFIpo85ppLBzFSw5Y
HLD3NgTdQEAjgxCVAAAAABHj9kbtRYvTsvXYK/Onhx6Y8iHebVWhx7biYbtTyercHa88cfflg2z16lby9
Ss3ZpEcVP29D4Gyt7amueYpR8Alg0hKgAAAIAjyOiNmpQxjDw7g706u3Lg7tTtmVk66QWAo+p5ps

MsJeDXAHX05KJbmFROSaIqAdNKcvztXa/nqR24unVRp7iWaoZ+1ePUnkjLDlS7Xi3W0WUEAABHF
SEqAAAAgKPJmIAoTMlPDo3aqW3Z8Kapz1ZzdcObqKm94Q8/L59ZcwJAszZnc9UfQt7e8Gp0FlIZV2
4Gzta5WPd7WfYa61790aSTVU1twuP3A1djxv4k9PbVbRDscRoSdAMAlgYhKgAAAIAjKro3qjlzuwrp
nGAubi3NaZWlXG7Kqg4D/dzWmOFftd+ozdlc1cGh9+IREzJNorLtD1vv1KSk21XyE1S5kPuM9AmOv
3Lan6jKu5bGBFRJjDgX5r1RPat7ugIAlgYhKgAAAICjK6o3qqp7OVgzs9qavI5mIqfk7J4xPFyx63gO1Di
1a3MOvE4Je+2U7GHrIcdebVnr3eHxeYt9/BXZ7psz/itVaZk1TZu7Xs/fcexzEVYMVW8zrewaALA4Cn
1V3AUAAAAAAAAAEIqeqAAAAAAAAAAQgRAVAAAAAAAAACIQogIAAAAAAABABEJUAAAAAAAA
AIhAiAoAAAAAAAAAEQhRAQAAAAAAACACISoAAAAAAAAARCBEBQAAAAAAAIAIhKgAAAAAAAAA
AEIEQFQAAAAAAAABGEvn/AZM6nr53azj8AAAAAElFTkSuQmCC]]></redcap:Attachment>
</ItemDef>
<ItemDef OID="q23" Name="q23" DataType="text" Length="1" redcap:Variable="q23" redcap:FieldType="radio" redcap:RequiredField="y" redcap:BranchingLogic="[q2] = '1'">
<Question><TranslatedText>Do you prefer Life A or Life B, or do you think Life A and Life B are the same as one another?
</TranslatedText><redcap:FormattedTranslatedText>Do you prefer Life A or Life B, or do you think Life A and Life B are the same as one another?
 </redcap:FormattedTranslatedText></Question>
<CodeListRef CodeListOID="q23.choices"/>
</ItemDef>
<ItemDef OID="summary24" Name="summary24" DataType="text" Length="999" redcap:Variable="summary24" redcap:FieldType="descriptive" redcap:SectionHeader=" " redcap:BranchingLogic="[q23] = '2'">
<Question><TranslatedText>
Alzheimer's disease - Description #[no]

If you have Alzheimer's disease...

1) You might forget recent events of importance to you. For example, if you are interested in hockey, then you might forget whether your favorite team won their last game, although you may remember watching the game itself.

2) You might forget the names of people you met recently, or the names of local streets.

3) You may no longer be able to handle your personal finances or plan leisure activities such as dinner parties or vacations. Simple decisions such as what to order in restaurants might become difficult to make. Your ability to concentrate may decrease and you might have difficulty driving.

4) You may start having trouble finding words to express your thoughts. You might need to be reminded to shower or brush your teeth, but you could do these activities on your own. You

may need to post notes around the home to remind you of simple things like turning off the stove.

5) You may remain aware of the day and your surroundings. You might be able to travel to familiar locations like the supermarket, although probably with the help of a loved one or friend. If you are in a social gathering, then you might have difficulty following the conversation.

6) You may continue to enjoy hobbies such as puzzles or reading.

Now consider two possible lives. In Life A, you will live in perfect health for 6 years and then you will die of natural causes. In Life B, you will live in perfect health for the next 10 years, then you will live with Alzheimer's disease as described in the summary for another 10 years, and then you will die of natural causes.

</TranslatedText><redcap:FormattedTranslatedText>
Alzheimer's disease - Description #[no]

<p></p>

If you have Alzheimer's disease...
<p></p>
1) You might forget recent events of importance to you. For example, if you are interested in hockey, then you might forget whether your favorite team won their last game, although you may remember watching the game itself.
<p></p>
2) You might forget the names of people you met recently, or the names of local streets.
<p></p>
3) You may no longer be able to handle your personal finances or plan leisure activities such as dinner parties or vacations. Simple decisions such as what to order in restaurants might become difficult to make. Your ability to concentrate may decrease and you might have difficulty driving.
<p></p>
4) You may start having trouble finding words to express your thoughts. You might need to be reminded to shower or brush your teeth, but you could do these activities on your own. You may need to post notes around the home to remind you of simple things like turning off the stove.

5) You may remain aware of the day and your surroundings. You might be able to travel to familiar locations like the supermarket, although probably with the help of a loved one or friend. If you are in a social gathering, then you might have difficulty following the conversation.
<p></p>

6) You may continue to enjoy hobbies such as puzzles or reading.
<p></p>
<p></p>
<p></p>
<p></p>
Now consider two possible lives. In Life A, you will live in perfect health for 6 years and then you will die of natural causes. In Life B, you will live in perfect health for the next 10 years, then you will live with Alzheimer's disease as described in the summary for another 10 years, and then you will die of natural causes.
<p></p>
</redcap:FormattedTranslatedText></Question>
</ItemDef>
<ItemDef OID="a24" Name="a24" DataType="text" Length="999" redcap:Variable="a24" redcap:FieldType="descriptive" redcap:BranchingLogic="[q23] = '2'" redcap:InlineImage="1">
<Question><TranslatedText></TranslatedText></Question>
<redcap:Attachment DocName="6.png" MimeType="image/png"><![CDATA[iVBORw0KGgoAAAANSUhEUgAABVEAAADYCAIAAABtBTQzAAAAAXNSR0IArs4c6QAAAARnQU1BAACxjwv8YQUAAAAJcEhZcwAAEnQAABJ0Ad5mH3gAACmoSURBVHhe7d1v6F3Vvedx02hvHJWE1glpHTQ6CEFF0spAzBSbQjEiCIKWBrmKw8iPCJYbBBspZpgHinIp5Hcvgj4oVpjOxAcWuVCi2EIetJBCoXngAy+k8ntgaaA+
+F0p5SfNg87+t9Ze67v+7H3W2Wufc/Z5v/gy8zv7rL2+a+2zf2Z/rCf3mr8DAAAAAIApIvMDAEZyYevCNf/7W6Gooaoe4+sfd/3Vlfdb11+ofNXQgAwJoh8wMAxrC1vbXvtX0imFFUjtr/3J6nD91hZv6ifvPzd5t7EQCAdULmBwBkt72zfej1QyKYUVUSO2n1m1P33SoCf1En77rT5/8obkAQBYG2R+AEB2D/3sIRHMKCpTPXBXQgMVKzC1SKsVeRY2/0SkmL9zR7RiZ5VgWOTtmBEGaVJDQxUVdkWYLIz2zKn1hZ3Kj9x3Mp2OJJi5DFbfZyrpZV3Mf2PXrThUbp4I8+FNY84IK7KMiB0Zdbq8QF35Vk8xCSCqGF
+LbMhV7BawWvf9pSqUw0cvN5+rfbTP/8+R+Mjmpk+fFfgDVl2HaXXbNwhyn5o8w8O8LUBr/yNRIRS
FJtZIBIeqwX3/vV5WJPYXBlf2S+LTW/OfPmN8NdWtp9kKHe0L77rNPRhcnjfw+dztSkD6pIuh9hOlmZI6ZEjfwDPYa7x4R8vU/uL6k9KUk2X8r71J7lTnbqmNGW4L7lIL8Y89vLCkrpNd/Ce9S3/GHepnkRcPtjI3M3LbDXLS9jFQGEqFNCwZQpW04JKI4Rl7H/zRtO/XJSFKt36LpjqkdbgqzLXyOwkTRGWTYaUIf66mTa0RbTJgHt+Nf63/lhzaJsnUjtXBJUzPmFaxYDRBGsM8hQn6MkrCWPvbBdKoEOhW0qx9JfBsdNeORCA0pSNqqH0UV2oLomGQU1YwVy/Z5O5tRaWNlG2z4uzEX/B9RQcjHZcijlOawK3mj4p/ezvQG5XLVQ3RMnTtlhFzckvavz/c3YDQG/nl0Bf/ixRIETDLPOOUKmfD2IIjdcGM0vxu+D0BgCo/Xuij0w9Ju5hf9/+uVmrjDgmmgNcgqf5DQqMS+99zJH1o/5Rs+JzhSmd8VjfW6x9AnqYyWlhcTc3wO1OTvcEE0vd7NlBmeMxT/LHUZavyFvmv7QCG6mLffVNfF1/vVgPcwQNQCr9R3+3nYDvyOiezhY2QhbIkz73Q+5PzBubfQpyEtkA/KgzW3aHmmFAtRFPcrHC+hmcZs3lgp94LKpYS2sqU/l8/+gVDiI/uWLK/qUqzQxJ7Chz4JgN7thRPTxdkVeWLyt4/iJmHTy+fbEJ5kBzT5KXRUwsAVZ

mQeUeeL6eIvQzrOUq/VJOp1syvxsuQ55JBd9NVu1yqHeHjOak+r2vdYnp5ErtddQPds7jn1Id8Cw/s
qdF8Q0bnkOWSRrZ2lOAcKaR8Nxre1vXXgxwdEJKOofLX3+evG+Rq/W69877G/ffFFc+sDALCayPzL
wJcoG+otO1yJyOUZ1CMsNWdVA6qfj26er85qj+jTxXTxIyFdZ6kDzetmBWpTapM+kb6yi56mPafH8t
VZ1sdgnddjeZ7WNXcB3bN5V2Trsa+CZyJxYo+tGezRzfvOUnxr614JlsDO1Z3Dbx4WkYyi8tXuM7sev
+9WEcXHrHdeebm5+wEAWE1k/mUQyW/
+twKhbMaw1JxWjKhHFz/Uh/QRfbaYLv4ypPMsdaQ60K6upjbpE+kru6RdK3WWNcI6r8fyPK1r7gK
6ZwtO1uqxr4JnInFij621pwjN+85SfGvrXgmWwIl3T4hIRlFZ64Hj+0UIH79+/8sPm18AAABWEJl/GU
Tym/8tFYWao6lhqT5P/c/71dnVIXWknVBMF38Z0n2WOlQcafbUvue/Ep1kl7Rr5W1unddjecEh7gK
6Z+vRr8e+Cp6JxIn9F1OoB8nWzlJ8a+teCRbt1V+/KvIYRWWthXyN3y2+2A8AWGlk/mUQCVU69Jjv
ySTkOV+nsFhYauZxv8ZfHzHmEx3jL0P6nFWvW/919sZbPZtI8rS0YOm7nPZpM0xifpQV99zu2Xwjyg
az7avQfUE653GnkNfLmSK0/NLMW8A43vv4PZHHKCpr3fjCtU/efbuI34sqvtgPAFhdZP5l4Ak7LR2f1
Ls6B+nh+oiKRvqcjrDUnug7NZy94i9Dep1l9Bfv6PHqsB4aayu7qJNmC5aeqyK7dy/P07rmWUD3bM
6l8IF2Wg/PquSJXYuRa2vHO0ea19619VgJFubSlUv8Rf3UmLX7zK5H7r9FBO/FFl/sBwCsKDL/MvCE
HZOOVBZzsBGypHhYak/U0+lu3gb1dPGXIf3OanfrXA59hmWmponB0v8RlHzzWNpJPa1r3gV0b9Y/
wpjeXnVgd70uSMdiwpenGeLO6Flbr5VgEbZ3tg9uHhSRjKKy1pFHbhaRexmKL/YDAFYRmX8ZeMKO
ZIcuTwIyBxRv9w1L7jjvmeJg/GVIz7PiV0OfVYlcsYbskhYszbPUzwX3hNjyghsLLqB7s/YIZ4ixVu/phd4
XJL4Ys1N5mnpdT+Gb0Vlb75VgVDtXd469fUzkMYrKWrc9c4MI20tSP7jvG3/65A/N7wYAACuCzA90
88RRYD2c/MVJkccoKmst1df43Xrp+IN8sR8AsFrI/EA3Mj/W09mLZ0Ueo6jc9fC3vi5i9rLVW6d/2PyG
AACwCsj8QDcyP9bQha0LIoxRVO765mNfEQF7Oes3P3+3+T0BAGDpkfmBbmR+rJut7a19r+0TeYyis
tbXnr1eROulrZP33MUX+wEAq4LMDwCwbO9sH3r9kMhjFJW1rj+9+4l7D4povczFF/sBAKuCzA8As
Dz0s4dEHqOo3LX8X+N3iy/2AwBWApkfANA69cEpEcYoKnfd+719Ik6vSvHFfgDA8iPzAwAa5z46J8lIY
ReWu/c/tefrQHSJLr0rxxX4AwPIj8y+W+rvhKln+grjLm0c3zjc/T+PvorN35Gdd187RKPS+NaZ3R0G5
+OnFPS/vEXmMorLWl3/0pe8fvk0E6dWq08e+/dfPP29+iwAAWD5k/gUqwlOdl2rDp6amwYQSmrs
jl7isFRJpp163xvTuKGhb21sHfnxA5DGKyl3f/c4BEaFXsV5/9mTziwQAwPIh8y9Q5riks+9kEppvR5K9
wzb/R/81AXrdGtO7o6DsXN05/OZhEcYoKnfdfWKvCM+rWx/
+9K3m1wkAgCVD5l+cPgl2HmuZ+dUG1Yj6lExXeFLI/GvtxLsnRBijqNz11X/6h9X9Gr9bJ+
+565NLl5rfKAAAlgmZfCPU0maCFU4EoQTlf6tgXDXPAOeahub3T14yprDSmRL92AaZVr9f
HxMvQzuy6HM2zrfje63cGiQPdl4Q//7bEZ73qzf958Vv7IReje4Vl8xRBs8Jw91RM68Gw3nv4/dEGK
OoEeqB4/tFYJ5G8cV+AMASGiHzi+f9eR/izemi2Wjpea+mTj/qOulR+sIZASl6AXzzu+c68zunqXMin5
w7bXtE9olMa47xdfXtyKIHSKET2lOMPqJ158rlFPoEPWd7NayVhCZqT3Sk9GqPqOb6LHWkPRDp3Y
4yNuG2c5bknCb33WvNyOHSlUv8Rf3U+HXnUzeJqDyl4ov9AIBlM3jmdzkP+HMxwklhmDkXJJagzI3
JcToOdew+Nr9xTAxTL43JPYeEwabVEzXslmJOlx6gz/SsTHCWoU5pDvRcuclZp16FdUa72tBEPYR7tT
uWY5xzCvpYZC2+87rbqZeRC9hjzRje9s72wc2DIoxRVO7a+/x1U/oav1t8sR8AsGzyZ/5hn911WDhq
h4ZV5LkyIm429Kbro56A5OW78p4GYpie3SPYsDuz9Z7WHCg/Xd+OLHpAO6LzHD1CXF41frYLYo92
5rBPaJeryS3H9O4lr4EaYjXzHrT5rqXnNDHMXqZNLCiyZgxs5+rOsbePiTBGUSPUI/ffIkLy9Oql4w82v
2kAACyBvP9tv+dp3/NQX+oZddQ5G+fVT7NkpCXjuZr+3CUG+ge5PPP7zg3M7mNMZBty2naks0Hfj
ix6gHGqmi+4eH1WdZIzvM/KdV9Bva/mCO/IFl5rUi99SjPGu5zgGltynlJ3OzXCJ7wgXy8M6OQvTook
RlHj1G3P3CAS8vTqNz9/t/lNAwBgCSwo8+tRpkjYaKjTyknUz91nLSvP1fTnLrFV/yCXZ/4e4arv7Jbhpt
WnVcRo345snjbqUPCcQjOmOEu1aEd3r1yNKNSDnHV2zmFMUekzsH8vOca7nM41+q9/d7seE/dY
M4Z09uJZEcMoasw68sjNIiRPqd46/cPmNw0AgOWQ/
+/w85zjHOqRCkrqvPo09arjpOUVuzLmpuS4nlfLN3+PcOU7q9NQ06pRRzc3fZvsMYlaiR7Rr3Fz2tGN

jXq0MbhzAnfz6og+xXN9lOKtGZql9ZKz+rroY541Kr7zutv5zhJ6rBlD4v8aP7XY2n1m11T/C/+Xjj/4ty+
+aH7TAABYDovI/OqI5wE/kjfcQb1OWmLulSnoCCd32W7TE5C8fPPPEgjVAb0kc522Yaa1TtEvInP6iG
k9K/PSp1Ws+btWLjfSjtfz+FehT2wP+we20no5183ZkjFP5Eo58xR6tHO6yV30WjMGtHN158hPjogY
RlFj1o0vXPvk3beLwLzqdfKeu/70yR+aXzMAAJbGIjK/fuL3iDzgq4naXOAeWSmBq+m/PMYePQHJz5
6p6tIrXOkjlshHM8i0+m01iV5+O6tnRy57UC22+Jp5lhzdsXJfw5oaokbIjyx0Zni5Sb3czyKwpZJco8nu
Xs3Wq13HBey5Zgzpyl+u8Jf2U4utO/7HjSIzr3rxNX4AwHJanczvjw21WExZXpGraW9WvO8JSCHmp
a6G9w5X9gq6Ws0/bfuGMV4vvx3o7MjLauNeXi9PM1P8gpjLKvup101v9dI3tXlqqXO5s/fq/iyKN2Jr
bJnNq5FJ7WSX3pNgSPwf56cWXg8c3y9i8+oWX+MHACytRWb+eLgQ7MQgzTQV4KHuMO4lrI9zH
50TGYyixqzdZ3Y9ft+tIjyvYvE1fgDAMlvk9/kjszj0OQEENcxF32DcSVgvL/7qRRHDKGrM2vv8dU8fuk
NE6NUqvsYPAFhyiZnfq4rwTqD3JPx2muaY/g+GQ/8WwDNJTb9BVEMa879WD9+BwHQ9+s6jIoZR
1Jh151M3iRS9WvX7X37Y/C4BALCUFpL5QxMF81Yk2UfeAnowMj+BH2tp5+rO4TcPixhGUWPW6n
6x/51XXm5+kQAAWFYLyvwle7JYZI/m+uibAIBOW9tb+17bJ2IYRY1WX/7Rl75/+DYRp5e/XvneY3yN
HwCw/GbL/ACASbqwdYG/xp9aYO1/bs9qfbH/B/d947M/ftr8/gAAsMTI/ACA0hu/e0PEMIoas+4+s
Vfk6mUuvsYPAFgVZH4AQOPUB6dEDKOoMeu73zkgovVyFl/jBwCsEDI/4sTfxxB/ORP1l+c1fxHDPFP
Nyvq7+rP/RRDFzkbYU2nMa4jJOvb2MRHDKGq0Wokv9vM1fgDAaiHzIy4e8ucJmYvK/LpRLW/kb5q
NlMFHu4aYsu2d7YObB0USo6jR6mvPXi8y9lIVX+MHAKwcMj9mIlLlPCFTZP7RjNh37AxO5scwPv7sY
/4af2qB9c3HviKS9vIUX+MHAKwcMj9msvqZf8xcTObHynr/8vsihlHUmPXwt74uwvvY1L/96780
AAKwOMv9CBOKu97DOcZXuMzpyn3q7PcM9oqYtj4jp4i/j9OBCMV6s3TuVeYq5wCjrJOss1dIQWLi
5OLFui93L7BbqJbZdcrZujGmnad4Md/RMFCDm6L+pQp/1F6Jz1Kwh7vs9ZkBeZy+eFTGMokar60/v
fuLegyJyL7b+
+R+faH43AABYKWT+xfAEJ9/BNvCZjJN6BjCTel+/rZvoSZoj1WsxXfxlhH8rpaatM5U+YDI26hW/Yp53
AwvvXG4p2izUSx035nG27jm3Hh7t2O/j8F5W44yOFqnrLxmr6vpwu2fAKE68e0IkMYoarZbqi/3PH
z3yH3/+c/OLAQDASiHzL4jKPJ6Yow/p3KOO6KDkDDGmcQKY1AxQpxjpqjlDzVC9FNPFXwbpcbJHoV
lI18yerTp6XDF3Yi/3qrRHxBZm7OXZhzPM7V7p7OjrZ3NOEZess0Wf9cshziTyBOeMzhkwkp2rO0d+
ckQkMYoarf7bo18V2XtR9e+//W3zWwEAwKoh8y+KSjE6xDhZyhOuOtNVwclTjmZEfU79YmOjnMg
8os4X08VfhviG6WPN2u0x6pVnY8Yhoc8Vcw94qamMUZ0nugN8p/T5yHT38F4r8sSEJdaHQuMjaw
uvX78MLV8N8ExRH+qcASO68pcr/DX+1KJq95ldj9x/i4jf4xdf4wcArDQy/8KI4KOilE5fnmxVCJxlDNJ5
KZzjmrOqAdXPRzfPV2e1R/TpYrr4yxDvXsRBeyr1pk+ol7eLnlcd7bdk3b8dFTvRXq4e4DvFs0pnmKe7
5O3YuTdP8zD/pnqsX79uWQ3tiW31FF0zYFyXrlza8/IeEcYoapy68YVrn7z7dhHCxyy+xg8AWHVk/sV
RuaYKMyoGtWHNH89EvOoRwHya04oR9ejih/qQPqLPFtPFX4Z49yIO2lOpN31CvbxdnCX2W7JnLvd
EfURwBxi9+szs30mho6OvnyU4catrU/NcmeZ9NYWPmiI+A0Z37qNzIolR1Gh12zM3iBw+WvE1fgD
ABJD5F6nJPkV6UgnHiDT+eKYGNkd7BTCP+jz1P+9XZ1eH1JF2QjFd/GWIdy/ioD2V94wO/nPUvOpo
vyV75pInqiGFepQ7s69Xj5lDu+/s6OtnCUzc6t5Un/VrxnSV+qTOVRj8M2ARXvzViyKJUdRodeSRm0U
aH6f4Gj8AYALI/AvVBJqjGxt1aDIzkw5SZsqR6cqTnnRICuW+UjOP+zX+
+ogxn+gYfxniG6aPNb3sMT0ntsgZK85E/Wb2XFhxojvCvfK+Xn0+Ms+YQnfHzr2FFqSm7G7hGeKOK
RTHjFd2385lVmIzYEEefedRkcQoapzafWbX4/fdKgJ57vrwp281tz4AAKuMzL9YOi1VRJzRb6p4pXOP
zlv6iDrXmDAajtoTfacaeU5krfjLIGedRv+mWWhmdYZeXqRVjysm+wSomcJXQq6nXXA7s69XO645a
Fx3NczTvdDd0dfP5nS3D/TYlDODs359oF2+2FB4jr4zeA5gBDtXdw6/eViEMYoap/Y+f93Th+4QsTxfv
f7syea+BwBgxZH5F0ynm4JKVS3z3ZaZcoxQJrnTmdoTnRTlb1BPF38ZEV5o082Zyn9GR6MZrlh0Jk+il
Cf6W5Xame0x9fHwldAnerqXOjv22VugfdOqx6ZmWL/ULss/Sft+9wyBa4Tctra39r22T4Qxihqn7nzq
JpHMM9XpY9/+6+efNzc9AAArjsy/aDrchLKLnY7azKOZA4q39UvPUJM7znumOBh/GacHF4rxIrTFu1
d6xjv7JLmyfkv2JErPiWYyLY+q18bU5hA9mbnCYqwzs6d7I96x397kFRJt4i0qXesvmdOU3CXZq4jvt
WTPoN51z0NuF7Yu8Nf4U4uqB47vF/l88Dp5z12fXLrU3O4AAKw+Mv+iqeBDdAGwKt743RsiiVHUO
DXCF/v5Gj8AYGLI/IuI/6dOIj+AVXLqg1MijFHUOPXVf/qHfF/s52v8AIDpIfMviv0fLrv/4TMALLdjbx8T

YYyixqm7T+wVWX2Q4mv8AIBJIvMvipH5CfwAVtD2zvbBzYMijFHUOPXd7xwQiX3O4mv8AICpIvM
DABJ9/NnH/DX+1ELqyz/60vcP3yZy+zzF1/gBAFNF5gcApHv/8vsijFHUOLX/uT1DfbH/rdM/bG5oAA
Amh8wPAJjL2YtnRRijqHHq3u/tE+k9oV46/uDfvviiuZsBAJgcMj8AYF4n3j0hwhhFjVMPf+vrIsPPVCfv
uetPn/yhuY8BAJgiMj8AYF47V3eO/OSICGMUNUJdf3r3E/ceFEm+f/3m5+82NzEAABNF5gcADODK
X67w1/hTC6mvPXu9SPI9i6/xAwDWAZkfADCMS1cu7Xl5j8hjFDVCffOxr4g831l8jR8AsCbI/ACAwZz
76JwIYxQ1Ts30xX6+xg8AWB9kfgDAkF781YsijFHUCHXjC9c+efftItuHiq/xAwDWB5kfADCwR995VO
QxihqhbnvmBpHtvfV//teZ5k4FAGANkPkBAAPbubpz+M3DIo9R1Ah15JGbRcIX9cr3HuNr/ACAtUL
mBwAMb2t7a99r+0Qeo6jctfvMrkfuv0XkfF0/uO8bn/3x0+YeBQBgPZD5AQBZXNi6wF/jT41/2//
6XHzZ3JwAAa4PMDwDI5Y3fvSHyGEWNUHc+dZNI+0W988rLz6+0W988rLzX0JAMA6IfMDADI69cEpkccoaoR64
Ph+M/DzNX4AwNoi8wMA8jr29jGRxygqd+0+s+s+26tAz9f4wcArDMyPwAgr+2d7YObB0Uko6jctf
f5654+dEeR+fkaPwBgnZH5AQDZffzZx/w1/tT4dedTN/E1fgDAmiPzAwDG8P7l90Ueo6gRqrn/AABY
V/xZCAAYydmLZ0Ueo6jc1dx8AACsK/4sBACMR+QxispdpdzZ0HAMC64s9CAMBIrrlmpD90aJSMRslolI
xGyWiUjEbJptdoHXApAQAj4YkkGY2S0SgZjZLRKBmNktEIEVxKAMBIeCJJRqNkNEpGo2Q0SkajZDRC
BJcSADASnkiS0SgZjZLRKBmNktEoGY0QwaUEAIyEJ5JkNEpGo2Q0SkajZDRKRiNEcCkBACPhiSQZjZL
RKBmNktEoGY2S0QgRXEoAwEh4IklGo2Q0SkajZDRKRqNkNEIElxIAMBKeSJLRKBmNktEoGY2S0SgZ
jRDBpQQAjIQnkmQ0SkajZDRKRqNkNEpGI0RwKQEAI+GJJBmNktEoGY2S0SgZjZLRCBFcSgDASHgiS
UajZDRKRqNkNEpGo2Q0QgSXEgAwEp5IktEoGY2S0SgZjZLRKBmNEMGlBACMhCeSZDRKRqNkNEp
Go2Q0SkYjRHApAQAj4YkkGY2S0SgZjZLRKBmNktEIEVxKAMBIeCJJRqNkNEpGo2Q0SkajZDRCBJcS
ADASnkiS0SgZjZLRKBmNktEoGY0QwaUEAIyEJ5JkNEpGo2Q0SkajZDRKRiNEcCkBACPhiSQZjZLRKB
mNktEoGY2S0QgRXEoAwEh4IklGo2Q0SkajZDRKRqNkNEIElxIAMBKeSJLRKBmNktEoGY2S0SgZjRD
BpQQAjIQnkmQ0SkajZDRKRqNkNEpGI0RwKQEAI+GJJBmNktEoGY2S0SgZjZLRCBFcSgDASHgiSUaj
ZDRKRqNkNEpGo2Q0QgSXEgAwEp5IktEoGY2S0SgZjZLRKBmNEMGlBACMhCeSZDRKRqNkNEpGo
2Q0SkYjRHApAQAj4YkkGY2S0SgZjZLRKBmNktEIEVxKAMBIeCJJRqNkNEpGo2Q0SkajZDRCBJcSAA
AAAIBpIvMDAAAAADBNZH4AAAAAAKaJzA8AAAAAwDSR+QEAAAAAmCYyPwAAAAAA00TmBwA
AAABgmsj8AAAAAABME5kfAAAAAIBpIvMDAAAAADBNZH4AAAAAAKaJzA8AwKzOb1zT2jjfHAUA
AFg2ZH4AwITUYTxvCr+8ebTtkLPh2P9modxY4ejm5ebAkKzNVPh3JbOyrmGejym7wC9Mc+/Vhr0z
Ir+ikbdm5Z/K+sSG3VZe0SuT9R8UGYU3taqfU2BH1naG+ph69FrRfyatCTI/ACCvjE/zFqPPmA9tVdsc
zzrFs5TeR/VclfmBqtzI0Y2N4v/J0qfcwuiPhObz6PB3hXVntzLdfdVeRrojzOs2XJPgb6j9O2RvdC7BjtG
3ZhWaqjyuD9SXdN5W/lnMj2vuFp1Xpnx/0H9Q+DZlbaky374im6reGvBXyd2O0dyUaUdVf32oejXv
7vr1GqBVfe0a4vpYV1G8hx7I/ACAjOznKftpZFCqUfX/j/lAYO8wl+xdygbN5cvTpvzss18m0zgfjKVqme
nmk3Pn6zX0c3xDfR7uwuWtMdTWwh1jb82q71RqXPNyZtX5NbtLcfn0gfk/rq7tlMebt+fqU4ttaoj5
G5FNeQ6lC25HmL9peEfySMZe6o3m5byfWjmdnr66k9uXdqvqzbn2tI7I/ACAfOQzgPPUMLj8HYRx
Hj8yb0tPX/6Q/tAWM9/j4MxGvw8KeW8F+wJm2181sflBDf3BOSt3tzLwhYxcqwEvY9dU1fvJF1Kdn
beLwd9IHy1/mLuNWqynV55/XLiNBtlHI7Id24D3d+eI61xMf85Uzj4GbKavZvVC3g5DNlobZH4AQDb
un8wDPu34jfwwUO1nqGfGIOvxZ3jGNSt/zNOoulKtzNcs3z6CMn9IhfoSFi3qH/Lc5c5vqHF3DMKZr
+xoX7eBW0amG7BT11TOhU3S1WWwDfkmMo6VPw52s3t6uTfFENxGVZ/yiwra/F19l85QvT3U3ry
9qlutbFH/EF7KbJxegcs5zNbMy+RvNNjG1gSZHwCQjfsE4H1EGVL2BqbqwWOopzdXtZdGxi2Vu1DT
ly3zbUipN5azT3XnDfwoHzfOjVfdcZVcrdx9uL/Fc3EauPMPfC0j0w3YKT5V9cENcBXjXap3h/msPI3K
Pagj5duD3RSBXoaBWrmNxOdSDQhf33482zHE351VaLb2+g3WytOrOiKv3kCfVbWDplv5sz3tsFdxP
ZD5AQDZLOCP6vGeBcTTYlaDPkzZym2016tsNMqe3FtjUOLDqa9fztuiapj1vjP3UP+cp181t7x0A35U
1YTmyt1bwRkyn8h0A3aKTSVuxzl4u1QHG0NdNqdRuYf2dfn2YDdF7NIV6u0N0c1t5Nx8A/wiR7cz
wPwmT6/6ctXH6p8H6ufdV7UfZWPDuknmUE2rPxjnU4pfY3iR+QEA2Szgj+qRngXqJ50Rnznqh7fhG
1qPbIYhnrBjqr75rp9z5+XtV306WS+Z2yHjjqybYrjn+JrzG+r+yjqf3nzcBlrkrVkFp6qu5lDb6Vhw9fYgz
WQj654w5OjlGuiOcBs5E3evpVNkiuqtoe6FktPL7VB9cvPtqNZ9acoRQ+xO/sq4H3/3WiCR+QEA2b

h/MA/07BY2xrNA1WPkB46qZ9YrVynb5O+Sfzs5HuXDBnuqDnNbZN2RYehbwl23/LCG3llkvgFb+ae
qPrcBN9O54GrAAA07GpVvD3ZXdG5qqN8wt1F1xNyIvBkThLcz0DYMTi+3RffV7alzonLA/DdFtQN7
GrfzAB/T2iHzAwDykX8yD/b4EZS/Q/1MIg3+/FF0MbbheQ7KYpinNlcxrzFt9SFl/ZSqDuZGcj4jVh9O
3nvOvQNGaVoY/I6oPhp74dbenI9ubp6OSuStWXmmqg4N+yF1LrgaMMDl62hUvj3Yh7TQTVm/SIP
8VgW3k+F31u1l/TIVhmsa/5iG+Yyq1bpNysPm3PGlwIvMDwDIyHoAGeapwK+aW1jtZwKxo3E2Uz
bN8gmNvhvrUXe4515XzrvaVj8PayPcEUPuTdwBlXYL5t6GupiRjvHFzCQ8lfi8anNurupmrbToYryues
7To+eVKYcNdtM7myoOGJPXS/Isorf4pqx352nTcLZTqw6PcG8P/g+KcC/7nbkvXT2ddxrrvh70Sq4PM
j8AIC/zAYQ/pzEa64F07ufRkOr2zjb7Agz8HI9B2J9KrflsxFsr9ImFN7WSe4p8RoXJ/YNicOJfVdTaRwYe
JOZE5gcAAAAAYJrI/AAAAAAATBOZHwAAAACAaSLzAwAAAAAwTWR+AAAAAACmicwPAAAAAMA
0kfkBAAAAAJgmMj8AAAAAANNE5gcAAAAAYJrI/AAAAAAATBOZHwAAAACAaSLzAwAAAAAwTWR
+AAAAAACmicwPAAAAAMA0kfkBAAAAAJgmMj8AAAAAANNE5gcAAAAAYJrI/AAAAAAATBOZHwA
AAACAaSLzAwAAAAAwTWR+AAAAAACmicwPAAAAAMA0kfkBAAAAAJgmMj8AAAAAANNE5gcAA
AAAYJrI/AAAAAAATBOZHwAAAACAaSLzAwAAAAAwTWR+AAAAAACmicwPAAAAAMA0kfkBAAAA
AJgmMj8AAAAAANNE5gcAAFgu5zeuaWycbw7V1BtHNy83R/JQfUT7ZdBem4JzHS5vHg29FX0PAK
aLzA8AALBcjFxrx9N1z/xtbK/4LkMw2bcXdQn/TQYA5EPmBwAAWC7W/5ZtJtR1z/y99u8N9+2/CiD
xA1gzZH4AAIDlYmV+M+CueebXwb1jXfr66eukj5D4AawdMj8AAMBysTO/EVSdzO8ccINxM6QcYcz
bnGAcMdOwOlwcM/5zeqOJYrzrvG+srW3Tkbmt+awJjZUqkbn06GqMfuXsILb+kr0eMSSyweh5AD
AqMj8AAMByacPkZv2TioxGyqxeuweMuNlE4jaOdtIhOnyOkbNlrq0YC/FMEo2+/qZy6wZjLY52cRub
6kfRvWv9HQsKb9B/XnS5AJAPmR8AAGC5qNBYJEgVTOvAaLxRDXQPxDJ/M8iIpPKIiqXOOe0RdUC2
0SP0Uow+PeJuuIGe0O0Zowc32mkqnetXA8LrCWwwNJFcAQCMg8wPAACwXKzU2Lwwf3ZjpxEnZ
ZhVQ9pcGj6ipnFHyDEyERfkIT2JMSZIrqAkd+IeiNMLKIlTeqxfcrr7N6iH9do3AGRH5gcAAFgu/nhdRE
0nGXuisoym7pDuI+q1GZTtedUIH9HYmiXAXVJJtVRH5dY6hZfQY/0Ge7R6PzC7XmXL3hYAjIrMDwA
AsFxkAm5e66/3txlSjizkyvz2QfXCJ9g4zD9WbiU587tL6LF+X3ivqPeDswdO7LtqABgUmR8AAGC5O
GFShkj9hid2ymDsDuk+4plWzOsbIfQYovnHqpbqqNxap/ASuhenRhTqQU73zjmMKSrhZgCQD5kfA
ABguXjCpB0f9RuxkQNkfjNc22N6xG+3TZiezhzs9Bgw83dO5Z7qXJTIBou3jIlnXjcADIjMDwAAsFx8Y
VLnxpJ+oz3axEkdTJ0jbngNHwnPooeEO6tg67aJCTdoz585O0eW0LV+uZ12fMcGnY1ElwEA2ZH5AQ
AAlktHmDTfMKKoJLJsLILKI/r1UTm7yrsFf+t2gNsmTo23mWcPmfk71+9fTkkNCc0eOrPvqgFgUGR+
AACA5RIIk21Itd4ws2sRK2UwdifrPqJem9M5yynZudkeENhFlD2fDMlya506lxBbf3t6qWxpXBbzbd/
s5qmlvisGgMGR+QEAAAAAmCYyPwAAAAAA00TmBwAAAABgmsj8AAAAAABME5kfAAAAAIBpIvMDAAAAADBNZH4AAAAAAKaJzA8AAAAAwDSR+QEAAAAAmCYyPwAAAAAA00TmBwAAAABgmsj
8AAAAAABME5kfAAAAAIBpIvMDAAAAADBFf//7/we7/2HzqQPjugAAAABJRU5ErkJggg==]]></redcap:Attachment>
</ItemDef>
<ItemDef OID="b24" Name="b24" DataType="text" Length="999" redcap:Variable="b24" redcap:FieldType="descriptive" redcap:BranchingLogic="[q23] = '2'" redcap:InlineImage="1">
<Question><TranslatedText></TranslatedText></Question>
<redcap:Attachment DocName="B.png" MimeType="image/png"><![CDATA[iVBORw0KGgoAAAANSUhEUgAABVEAAAEJCAYAAACdcmdaAAAAAXNSR0IArs4c6QAAAA
RnQU1BAACxjwv8YQUAAAAJcEhZcwAAEnQAABJ0Ad5mH3gAAE9sSURBVHhe7d0PjCRnfef/b+/aY
PPHNmBskx+RHat7sZYlGJIg08PxL4egZ+6SJbnbYOVg5Oh3PVyi0zQSkwOxF+mX2wguG4lunS5iWrq
zliQyTEJugrLdIJRgckzHR4IxYTI/Z7rlc3T8wDYGjPljB3u3/VUPVPVX1VHd1dV3VXX39PtltaerpqfqqT/T
O/OZ5/k+k+hb5FAAAAAAAAAAAChjumPAAAAAAAAAAAAIAQhKgAAAAAAAAAAAAAEIEQFQAAAAAAAAAAAAAAAiEKIC
AAAAAAAAQARCVAAAAAAAAACIQIgKAAAAAAAAAAABEIUQEAAAAAAAAgAiEqAAAAAAAAAEQgRA

UAAAAAAACACISoAAAAAAAAABCBEBUAAAAAAAAAIhCiAgAAAAAAAEAEQlQAAAAAAAAAiECICg
AAAAAAAAARCFEBAAAAAAAAIAIhKgAAAAAAAABEIEQFAAAAAAAAgAiEqAAAAAAAAAAQgRAVA
AAAAAAAACIQogIAAAAAAABABEJUAAAAAAAAAIhAiAoAAAAAAAAAEQhRAQAAAAAAACACISoA
AAAAAAAARCBEBQAAAAAAAIAIhKgAAAAAAAAAEIEQFQAAAAAAAAiEKICAAAAAAAAAQARCVAA
AAAAAAACIQIgKAAAAAAAAABEIUQEAAAAAAAAgAiEqAAAAAAAAAEQgRAUAAAAAAAACACISoAA
AAAAAAABCBEBUAAAAAAAAAIhCiAgAAAAAAAEAEQlQAAAAAAAAAiECICgAAAAAAAAARCFEBAA
AAAAAAIAIhKgAAAAAAAABEIEQFAAAAAAAAgAiEqAAAAAAAAAAAQgRAVAAAAAAAAAACIQogIAAA
AAAABABEJUAAAAAAAAAIhAiLp0etJYKUih4DxWGj29PqjXWPFeU1hpWF+1iMxj3ZB2onXjpLWdL
MxLO7LSlg372PzHqPs4Vb2GrGzEO5vtDbdtK5JH08KNuQ9Cjmc+2g0AAAAAwPwhRF06Rdm8UJey
XurU1kPCkracr3X0c5Hq2U3rq4B5oILBVWnqJUdZzqxle4faf1Qo1cT/rlhsR+14AAAAAADIGiHqMip
uytmqfi4dqZ0P9kbrNc75IVW1JdsV/XzhFGVzry/9vnpsS/aHkff+RpmXdmShKwdu8leuS9c+xj3ZzDJ
D7TVk3fijwsI7ascDAAAAAEAOCFGXVGW7JV6O2jxn9EY1e6FWpbW4CSqOot6h7OuncuoEPaQBA
AAAAEAuCFGXVkW2W26M6vdGbW/4Q6XL9a3wXoyqlqKuRek+wmpSjqyvaH59ZI1Jo6ZjoC7rqPX
GPu31edcGHd5fZI1J4zwEz5+5nbDPjxN+3INtCdS9tR4xy30ObGewraPOc7xjCm57uPZpYAh6c9VZF
2h4snM3eA7Uw9yc3Z6x+xzPPy71CLkXbAnaHvI9OHq7vqTHE6/dAAAAAAAcfYSoy6yyJXW3OGpz
VTYaDTnnJ6hyIWSM9FAIo3VqpQwmoCrK2hndwM6BdJ1nFmNId2dHLno7bcuubn/5zNpc9FKsnPa
D6gP/AGy9izv6PBo1Pe1wrCSDo63TPb8dqZUKUhrYSXM1aUimtjPY1qasDoZtEx2T2rZR+9S6H+te1
+kREu3HCSwHz4HSXB0dKibnnOtV70AUtW6Kc9TeGFHPNGS7E4vZbgAAAAAAlgQh6lILTjLVrLnBTF
nqF0Imk2pv+KGKV4+yL103ie3UZD3lhKW4dka3b18O3U23d42JhYxw0hjqferEnAz0rpz2yiY0d4M9
Ji/u6BisfEacDLUnjXX3GlSlpc9v3+0xnOr59bfvdUhWIdlAfdzx/O1494Hazrob/E1zTMbr9zZlc9v62PX
vV1Wv1/6cXXIi2X56jXUvsCzXu85r+8MlLiqR+4wr/BzteOl/krZbr3X/0hH2PRjY7rBkxzOu3QAAAAA
ALI+C9QtyXz/HklK9SwM9zlSwEhKq+K8rS71rTubTk8aK24tOBS/OZEYjX6963bk96Ubsy+dvW4Vde
9ZG1BBs1YOwWq1Ks9kcWu+3IaxdcdeNE387w+2yV3rnwG1/6DqbsV0VnO2FBNwB4e0wr3O11fc
nDEt0PSK2E9ivvuaS7JjMbQdfr41qa6JzF35+FOdanQqsS3p+lETnOlHbRwht44jjjDietO4RYBr3Pnyvv
OXCW/QSACAP99/xLnnNW2/SSwCAzB3/NZFjr9ILWBT0REVwkikVtoQGJP5Qeb/npMsYdm/2GE1F
UU6ccp51dJfTrj2WvywnT5+0e9QF11uqp/0AbA74vWmb4nVG7R44gZT1GW8ov7dOdT4sGbUo3S
BMfcIsazBr1jUo6ac28z7QPYSnOKZEvYkT7cec4f+kmIdQ3NyTvhmgTm3wHIWY6rrrurFuwJmaGO0
GMvDwEw/LOz/5Tr0EAMjDDY9fJXd/6AF5/Bs/0msAAJm7dI9I/zt6AYuCEBVBA6FScsO1P6fl1RVt7k
pbenJoj9k/JScqJ6z/D65XGeqc9ZIrrombLbpD+ttuIl09a/TozUtaAZl1DTJrOyHeSKpXqBe0GnVjgQX3x
NNPSOWPKvZHAEA+nvPjY/KWL94oP3ryGWm+/3555p8u6c8AALL1lMilP7A+PussYiEQoiJlGYRfXl3
RfTlsXxS7lKgd9lbEyVeN9XMZvhk9NO3A1+/VOyrwVcO67VqVQ480e0lOK1mv47yOaTHOXbjxbW/
Lhtnr1K2LatY5BRbUnZ+6Ux58/EG9BADIwxv/
+gZ5wQ+vsJ8/9NXvyp/WeR8GgNz0Hxa5/Od6AYuAEBUxleSkN1LbnBFfMSZJitM70Ri+HI+7744c7D
pf686+X7I/4a8fLjUwH4qbZ/0guOFOjFWVQIZacsoTKG6Jgvk22OvYvA/0seV1TIn2Y97LA8Pk1cz3hZ
xnoE/SdmNSNTtwdeukJv6eAubL+z77PvlM7zN6CQCQh1c+eK3c/PXn6yXH5z7+kHzlLx/RSwCAzF3
6gsjlr+kFzDtCVMRUlM2zbuVUc/Z1NbrYnOl8y+vt5wScijnre1s23JlrYvN7cqqJpBS3XmZRF0x117vh
6vxxe81a56Kmj3+wdqsx7F+aq7LhnTIV7Onh297K+dBcNQLH9nm/hqd7bHkdU6L9mLVbm7Lqre/p
me+ta1Qq+NvI2oTnyA9c3XYDi+kT+5+Q+n11vQQAyIOqg/pzD7xELwX94W9/Tb73raf1EgAgc5f/RK
T/fb2AeUaIivgq29LyctSalHTA48w8bynX5YJR4NOfUMnSXNWB0GQ1HAPbsp55Q/a9of6KMUnTHP
Jqu9rKUt8KRKiWomxe8IdlN1d1gOaFzqMm/ZolJ3AMttM8tryOKdl+ipsXpO6/2HmtOZGTdS/7h6Br
7yrua1NNWBO03bzfw9odR+bHA8R339fvk7v+7C69BADIw/FLBXsYv/oYRgWozd+8Xy8BADLXf1Lk
8sf1AuYZISoSqWyH11+stqz17tBiV3FT9gZfW21NVr/R7K0XKBlgDM3OdKKjFJgB2KiyA+qc9bt+wOe
ya1/OW03PstS7fT9Yt1Wl1d8LTpaV1zEl2k9RNvf60h16sXq5MUzeVpHt4EGK7B96PbFTEbvtVlv6Lf
8+sqlzblwHu+5ulByOB4jBnYn/6Wfp7QQAeVr50kvl2iev1Evh/uFL35ZP//6hXgIAZO6y9RvZJcpbzbt

CX81aAgAxtTcK4nSSVCHqQGAKADGo4PT1/+318sAjD+g1AIA8lB56od0LNa6tu8vyiteFD/sHAGTgit
8QKfBL9ryiJyoAAMiVGsJPgAoA+VK9T1Uv1CTUsH7qowJAji79AfVR5xghKgAAyM1HvvgRezIpAEB+
VP3Tt33hZSProI6iAtS7/+NX9RIAIHN2fdR79ALmDSEqAADIxe6Du/LBv/igXgIA5OWOL18/tg7qKPv/
8zH53Mcf0ksAgMxd/n9FLn1BL2CeUBMVAABkTg3fV3VQmUgKAPJ189efL//8Czfppclc+dzjsnX36+
XWV79IrwEAZOtKXR/1Zr2MeUBPVAAAkKknnn6CmfgBYAZe8MMrEk0kNcoz/3RJmu+/X3705DN6
DQAgW9b77aWPWx+fchYxFwhRAQBAZlRwqgLUh594WK8BAORB1T99yxdvlOf8OJ1f+R7/xo/k7g8
xKSAA5Kb/HZFL1EedJ4SoAAAgM+/77Pvk3ofv1UsAgLz83AMvkRsev0ovpeMrf/mI3PtJ/igGALm5/
DXr0dELmDVC1KXWlo1CQQrGY6XR05/LSa8hKxttveBob7jtWZG8mzM3Qs5LHL3GinPuVhoy6tT55
9d55H7NjwLuWyCW+n11+djffkwvAQDy8vJvPE9e+eC1eildn/zPB/LN//19vQQAyNylXZH+Y3oBs0S
IurR60lhZlaZecpTlzFpRP8+eHfiVasLfVIImPi+9hqzXor5KXfOCrAYvunRqJSlMENguK+5bIB7V+1T1Qg
UA5Ovqp47LG++bvg7qKKo+6u//
+y/bHwEAeVD1Uf+79fFZZxEzQ4i6tLpy4KZA5bp0+33p9/dkM68MdWzgt6QmPS+qZ+S4YK99XtxN
V1vqevelWy87K5qrQo4aA/ctEIuqf6rqoAIA8veWvRvtIDVLqifqH/721/QSACBz/UdFLv2xXsCsEKIuq
96h7OuncuqE5Nf/FGmL1zOyJ41zugtquS5bFedpcfOsVJ2n0twlRQUwPTUTf+WPKvZHAEC+Xvu1F8
vLHr1aL2Vrb/f/2A8AQE4uf8l5YGYIUZeQXbvRDN2aq04tx8GuiKp3o1E7c1T9zGAtyGCd1bDejbH3r
wVreI6qN+kMVfdfl6zW52A9S6+2qH6ENy3GPgPncEP8zZjnydln0vOiqHaWYvWMNHoeB0LzkpzUn
VFl/9A6olGM9obVW21v6GMZPAcJrkvI/RZ2vePcb4PXz35E1ImNY5b37WT3p2vSa2eZ6D1Ar1TMr
49uJI6QOz91pzz4+IN6CQCQFxWevubvXqSX8qF6o1IfFQBydOlPqI86Q4SoCDUUGGl2/cyRYVRHai
WjzqrR43EyanuDNTzVurCgpuQNVXdFt3UUZ5+DwWRzdcJ9Fjflgjtk3joz5/RGeo1z3nkq1y+kUEahK
q1+y+tVOsTseTxK50C6+umwipx2N97ZkYsDJ7W96x2NX1c3yXVRQV7I/eZcj1EBZPj9pu7d0GC5U5
NSIMjOSpb3bcz7M2CCa2eZ7D0Ay07VQP1M7zN6CQCQFzV8Xw3jz5uqi3r3h75KfVQAyI2qj3qP9Z
H6qLNAiLqEKtt96Xfr4kZ7Um3Z9TH72zrxbG/4AZBXL9Won9mpyfrIxEaFec7r+3uboWUCxu4/wN+
et3/pyI6XBPWkse4GPca+Wzo1imzrKP523M2ofdbOu/Fbsn0WNy+If+rOS9v677wbglnn94JOUJOd
F4P9um2ZLK8uyolT+ukYlS23beb5V9ri53BnxMnhkpwj67VGqYGh+21ofyZj2/b9ZrYlbFt+kJ3U/Ny3
/uvD789hya6dZar3ACyrT+x/wp6NHwCQv5UvvTTzOqijPPTV78qf1hmBAAC56T8scvnP9QLyRIiKIW
bPtPoFPwgdDgOHletbE4Z54aotPxwsrp3xAqzOge4z2bsoO14eaey7suW3deeiJIl7zH364ZMh8T6Ls
nnWTbuaslpwe08Gz+8kipt740PW2PblMOpEFdfkTNjxtXf9XrVn1pzjSXSOrPOzp0NEI3gPvd4Dhu43
s8et0evSPk86CNzLYfa0LO/bsfdnmCTXzjLNewCW031fv0/u+rO79BIAIE+vfPBaufnrz9dLs/G5jz8kX/
nLR/QSACBzl74gcpkJ/vJGiIoBI3qm2Yqy5iYxIwK3UyfSDKjKcrKkn47SPdC9+VSoU/LqNhbMYdKRw9
QnMMk+K9tGr0FHOsP403RKoi+fcf2NgNIP3Kpy1j2gqa6LruEZOrw/aOh+M8JCawf20Hd33yNrsaZ
uHu/bBNduyvcALB93Jv6nn31arwEA5OWGx6+Sn3vgJXpptu7+0APy+Dd+pJcAAJlTw/r739ELyAMh
KibUkeHOgTHCo7mX3TFUvMKUSrD+5Oz05HBssVSf36vSHRZuBG7V08FeoUmYExB5PXXHCbtWR
dm8EN470w0rF3t+o8nvz/SvXdh7AJaNCk5VgPrID+h9BAB5e86Pj8lbvnijHL9U0Gtm60dPPiPN99+v
lwAA2XtK5NIf6OfIAyEqJjR/gWm53vWGbAcfk9YLHS/
+Po26n7aO1NZznJyneELGlj4tn5Sxl7S4KW5lAntYuDF8vno6/CyPP0dt2TB7nbo1OM36o0lYbdzT+xj
s/as0z83XpEi53bcTXLtoR+GPJpiWGsL/wCMP6CUAQJ7e+Nc3yAt+eIVemg+qPuqnf/9QLwEAMqfq
o15iYte8EKJiQElOusnV0EzePbnoFnIcO/Q7J6WTXtA2qm5m6ibYZ6+xrodpl6Ved5OsPCfnMa5rc1f8
zphdOfAu6QmJc0m9HrXW/XH+/I4OP6sSyOGSnCOzLqcKFN26qMaQ96TUzPKqx6k9GZQdSHa9W
p7pDpOf0CzuW0usa5fme8AU1xDz7yNf/Ig9mRQAIH+lh1448zqoo3z6v/6D/MOXvq2XAACZu/xZkf
48dRU6ughRMcCcBCnYW9IPAlXYNTChz6yYNTCbq/5Q7faGHhpuPdIev510n72GrLsnrnpWNjeNyY
Nq65JPjmrWsmzKrm5er3HOCzBj90asnBbnDuIIs+ke18Bw8Amvix8oDvbcjUlvX80sH+xxaoTFZo9b
oz1p3yaRZnHfKnGu3QTvASU/dZXaee9gZMOb4h9Hze6Du/LBv/igXgIA5OnaJ6+0Z+OfZ83fvF+
+9y1qZQNAbtSw/v739QKyQoiKYeYkSJ2alHSoU/LTE7kw7YxI5vDy5uoUoVGwBmZzVQdQXnhTlVZ
qs9e7kuyzJ411d6h6Wepbav3okCq98zLMnFndbbN3TastiX+aKhIo72oZDmATnCMv2LO4x2xOsJTEi

HvXrLNaPevPNp/YQt+3SpxrZ0n4HuDXW7V41zBubVssGjV8/85P3amXAAB5UvVP3/aFl81NHdRRV
ICqglQAQE76T4pc/rheQFYIURHKHgYdUpOy2rLWu8Otp1KR7cGClfuHRs/BBOwamMZwbZddWzOj
eqgx92n23LN7obonbiCk8of1p3hehhRlc2+4Tqg9hD5hYBeYJMs6ZjsbHhT7uljH3G/5QaqtKq2+0dZ
ACYJo9r07eJA2Z5vTZZMLft9aYl07S6L3AHUsg6+ttiava4u59cTTTzATPwDMkOqBqnqiLgI1pJ/6qAC
Qo8vWb6bUR81Uoa8KBgJAEmo2fT0ZlAph96btmTxL+lhOtaYNWBfEUbp2yJUKTit/VJF7H75XrwE
A5EnVQVWTSS2SK597XLbufr3c+uoX6TUAgGxdKXLFb4gUbtbLSBM9UQEkFCxRcGZtsUO49nnrWC
J6ZB4tR+vaIV/v+
+z7CFABYEbULPx3fPl6vbQ4nvmnS9J8//3yoyef0WsAANmy3m8vqWH9TzmLSBUhKoCY2rJh17o06
pWaJQoWUK+xIqv7demmUqJinh29a4d81e+ry8f+9mN6CQCQJ1X/9C1fvFGe8+PF/NXt8W/8SO7+
0AN6CQCQuf53RC7doxeQJkJUAJNRNS8XfPx7cXMvpRq/C+YIXDvkR/U+Vb1QAQCz8XMPvERueP
wqvbSYvvKXj8jnPv6QXgIAZO7y10QufUEvIC3URAUAAKEefuJhec32a+wJpQAA+bv568+Xf/6Fm/TS
YqM+KgDkjfqoaaMnKgAAGKKCUzWRFAEqAMyGqoO6aBNJRVH1Ue/
+0FftjwCAPKj6qGpY/7POIqZGiAoAAIbc+ak75cHHH9RLAIC8qQB1UeugjvLN//19+cPf/ppeAgBkrv+
oyKU/1guYFiEqAAAIUDVQP9P7jF4CAOTttV97sbzs0av10tGyt/t/7AcAICeXv+Q8MDVCVCTUk8aKm
uVbPTaknWjdOGltZ96MOYZeQ1Y2gmvbG+7rV6TR0yunpfZjb3NEO2zTne9M2m1bkPugvaHbWJC
BSwosjE/sf8KejR8AMBsqPH3N3x3tuqGqN6rqlQoAyMmlPxHpP6YXMClCVGCGeo0VKZRq0tHLWe
pd3DH205RdQj4AA+77+n1y15/dpZcAAHm7+qnj8pa9G/XS0aXqov7+v/8y9VEBIDeqPup/tz5SH3U
ahKhIqCibe33p99VjWyp6bXby3l8WRhxDryHrtTziU6UnF3eC+2qea1hrF8VRuA+A+aZm4n/nJ98pT
z/7tF4DAMibClBVkLoMVE/UT/7nA70EAMicXR91Vy9gEoSowDLoXRQ3Qy2Xy86Tzo5cXJwUdTFUt
qXfrYs+w8DCUMGpClAf+cEjeg0AIG8//ffX2UP5l8m9n3xYvvKX/NsDALm5vGc9mOBvUoSoS8eoLbl
i9kQctd6oc2mvz7s25fD+IutuGnU/VwKfNLcT9vko6Z8z+/PmMP7mqvP5EYU0/WNWj+T1Rv2h/FU
5e+GMDvk6shMnRQ3UUg17jG5PvHbHuTbD59DaumyYrzfqkQa2MbA+/BTHaYN5POpY/P2rh7vd
9nnrupbrsjXQXdYu3WC83n4M3DfArKgh/A888oBeAgDk7YbHr7Ink1pGd3/oAXn8Gz/SSwCAzF26R
6T/Hb2AJAhRl05R1s64PREPpOs8s3TlwE30Aj0U27LbdJ6Vz6xZXz17ldNV/awjB/4B2PywsCxn1nRr7
RCwJIMj5zu1UswQa5bnrCO1UkFW9fYcal2SINUYyl89LZXimniHs3Nx+hCvfEbcU+2L2e6pr43Dfn1w
Z846FVQOrG+uDgSpE7VBHcuqeFvWoakKSlf369Ld2wxcdxW+lsJKN3RqUprnibKwFD7yxY/Yk0kBA
GbjOT8+Jm/54o1y/FJBr1kuP3ryGWm+/37qowJAbp4SufQH1kfqoyZFiLqEimtuT8R9OXQTovauHwi
JEU72Dq1XOU6dmIcI1VI5LW6M2gzMjmSEhV6w15PGutvjsyotu6am9WjpLXRqsh4jjUz7nFW2rTa
Yw76rLadd22HVPv12d+vuV8TsRaoYQ/mrp9X2zVA4xpD+4qbsuefNfnTFa4Z1BPULwcDQN67d6V
wbV7neDX69Frbev2+maYPxeh2aFjf3vOc+P1RXYWtXf41/TppyLsFxAmnafXBXPvgXH9RLAIBZeONf
3yAv+OEVemk5PfTV78qf1h/USwCAzPUfFrn853oBcRGiLiOvJ6IfaPUOndivWnXCo32dFJrDwO38bS
5UZMsNoJq7fi8+s+6n2wPUXFff8ickqmx5QWCs3pgzPGfVlj+Rkh/mWi0Z7IY7gtk792TJfmJsJ0EYq/
Ua636vzepZ2QxPUMe3O61rY6vKWbchRsg+cv3+obPdKdoQeH0UI1Q3Q2s7cNWB6t6okwhkSA3f
v/NTd+oIAMAsvPLBa+Xmrz9fLy23z338Idn/n4/pJQBA5i59QeQyf8BKghB1KRXlxCnnmRtode1x6W
U5efqkHXYF11vUMHDn2VzwQ7mmeJ0KuwdeWOgN5ffWWcfkDu+2H8bw7cAQ/VFmdc784HMy
Yb1zLRMP6W/LeT9BlVZoz1klRrtTuzaW8kkJ3d2o9a4p2hC7Z7Zxrq2N2WUO3P3Er8sLpOuJp59gJn
4AmLGXfPe58nMPvEQvQbn7P35Vvvct/m0CgNxcVvVRv68XMA4h6pLy6oraPTl74nSqPCUnKies/w
+uV3ngPEWoFiOYcodmt90x0xG9I6exkOfM6GmphqaXQkPC+LP0tzf8OqBVo6fp8kkSbhdl80L4jP1u
cDtiPjEgE+5M/A8/8bBeAwDIm6p/usx1UEdRAWrzN+/XSwCAzPWfFLn8cb2AcQhRl5U3tHlfDts6aL
N77VXEyQqN9VP3hsyCUdfTDi/9upOjwkuvNubQI2YYuIDnzB/KHyXmkP72hj9RVLUlIzuhTmDqa5O
CTNtg1JUdKNlqa55jln7k532ffZ/c+/C9egkAMAsrX3qpXPvklXoJpn/40rfl079/qJcAAJm7bP02eukze
gFRCFGXVklO2hlkRw52nSHNbh3Rkv0Jf3347OuzV9w864eaDXeSp4E6pCVnqL3i1eKc2KKdM2Mo/
xjjh/S3ZcNLUMtSV1PRTyvVazOhHNugZuhXPU7tScXsQNWYoCtu2QJgSvX76vKxv/2YXgIAzELpoRfa
D4z26f/6D3aYCgDIyeXPivTp2jMOIerS8ntyNptOOObWeCzq4p/uem+Sprnj9gDtSK2mA77BOqRmP

crmqj9sur3h1aUsxB5LvWDnzBzK787+H3iYIV70kP5e45w3jL9cv5BOuYRUr82E8miD3pbKoIM9Trvi
ls8dW7sVSMn/+v/
+l34GAJgF1ftU9ULFeHd/6AH9DACQi0v36CcYhRB1iZkzpqvehd7w88Ds5sYkTXPIq1NqC+shGaxH2
VzVwZg/Ll1GT440LNVzVtS1VJXmauqhoTmUvxxaW8AoiWC9cuSQ/l5D1r0CqtYrAxMwOY/JJkhK99
pMJoc2VLb9IfyBurRGfdmzm7MP3bEU7v7Fu+WOl9+hlwAAeXvjX99AHdSYfv7dP6WfAQByUXijfoJ
RCFGXWWDW8FPiTzbuDltXzPVzyAwvRw2ht+tRGr0uXeW6dJPWu0z1nFVke7BA5v5hSrUxzaH8o0
NdMxQeNaQ/Xl3VCaV5bSaVQxvsIfxhxVBVSNvvp1pfFohy1RVXyf/4lf8ht1x3i14DAMjT373yCf0M
UV7z1pvkbe+5VS8BADJ37FUix9+kFzBKoa/G9QIAgKXxwCMPyOv/2+vtmfoBAPm648vXyysfvFYvYd
D1P/E8+a1PvVGedw0TbwFALgovFrni/daTq51ljERPVAAAlsztN91uD+0HAOTvb27/tjx2PX/ECnPlc4
9L9fdeS4AKALmx3m+Pv8f6SIAaByEqAABL6F2n3iUfeMMH9BIAIC+Xjvfl8294VH78nMt6DVz/8t+V
5NZXv0gvAQAyd+ztIoWb9QLGmSpE7TVW9AQpBX9W65F60lhxJ1TZkODL27LhTbbiPCabqGZYeyO
43dBH1jOAAwAwhz788x+W07ed1ksAgLz84PnPyt7rvqWXoLzidS+R1X8bNhkqACATx4oix39eLyCO
OeiJqsJVf5ZqR84zwquuZ0VcaKU3oAwDA4rjnl+
+xh/cDAPL10M0/kO6t39dLy+3al14l1d99rV4CAGSucI3IMTWMH0nkGKIWZXOvL2oeq35gxuuuHH
iTiKvZsNXn92QzxwzV1qnJeTqkAgCWjDtj/3VXXafXAyDyonqjfu+aZ/TS8lIBqgpSAQA5Of5ukcIL9QLi
mn1P1N6h7OuncuqEZJedlqXedUNc/9Gtl/XnRZq7pKgAgOVzy3W3X1
S/8xivsofwAgJzYdVDz7rl4NOQYog7XRLXrlZZq4nZEtYfVD9UoNb/OeaRVL1Uprp0RP0YFAGA5vfm
WN8tH3/5Rv5RvQQAyIvqibHyEvqibqbs9VFVePoLv35CLwEAMmwd/f2Yra98/
SBzhVtEjv0LvYBpzF8ku12/QSACBzhVtEjv0LvYBpzHdN1FSFz85vlhPo1M7nWItxHgxO2jWqxuPoOpD20GPv6
/VjZG3Jwf1FBNd2Pdzga0fVgsysDVEya98oca+VK/5xJm1f2OsHyncO1O806o7arx19P/kG2p/K9Ux6
DsOEt32wXungORo8P2HiTbTn8/epHqPqpCY5P0DQbdffJvf88j16CQCQl7+/7Xvyjy//oV46WlQd1O
rvvVaufO5xvQYAkK2rRY6/2/p4hbOIqSxEiFqud4fDT/uRwnDa4qac1WVWRfblcGnyBRVsDU7a1ZTV
wqr1/3hUiFMKG3rcqUlpMKBKMklYe2NETUjVYzgYFmXWhihZtW+khNcqwXEma58TyIW9vrlaGBH
2qXNitLNcl/GVM9SxDbR/6us5/f0ej9OrffAcNVdHhZyTcPbh1XO2Dd97qd3vWGrvKL6DGfsBYAb+6v
WPHcn6qL/yH15JHVQAyNPx09RBTdH8hqilk34P0YN0Bu2Ha8tuuinKQug1zvnhkTuhV7/r1Zsdzzhv
5bp0dbDd9TbQlHNeotNLMEmY9dpzesOh2+3IzkX3tVm1IUpW7Rst2bVKcpzJ2tdrrHuBnP+HjZa1F
615bkRQaLRjb1OKem0koz1u06e5ntPf70n47fHb3pHa+ejIPHqivUH+PsLvvbTud0Ds2frfdepdegkAk
AdVH1UFqUfJyumftB8AgJwce53zQGpSC1HtnmjGkFHzEWco65DimpzxspxVfxuqF6C77UQbdnpv
me1yHmYvuTOyFivhWXQ9uejO2qUCFi+kKcrmBSPEidI7lH39VDo74mY3xc09J6yxHnub+mQmmiT
MmLjHCNyKa2eGQ/XM2hAlo/aNlPBaJTnORO0LtuOst74iW/aGVVC3J2GHE2hHTNWz/rmtbPtBrd
/2JNczhfs9gWrL7yFf2Up/+4q5j/B7L8+J+bAM7v7Fu+WOl9+hlwAAefjmjU/JV376u3ppsb3sp14o/
+a3mNAEAHJTuFHk+L/WC0jLHA/nDwYcXkjrjWE1w5B0mMHN0daVAzdTKp+Ukn5qK56QU/ppJDP
kHgioh2ouTjVJmK6nGTZ8Prc2REmpfSMlvFZJjjNR+0a3wwldR5fWOHUi6XdVVU4HNlaSk2473bYn
up4p3O9zpSwnAwcRIteJ+bAMrrriKmbsB4AZuP9V37HD1EWm6p/+
+n/5GeqgAkBurhQ5/mvWR+qgpm2+a6IWN2UvbMitPdQ3hXqoHtWLri8pZ7JH3OhefG5oM1EPZ
MWu5eiGPlE1KzNsQ5R5b19sebQvRuA3VlFOLEzSmcbxAvPpphfcZAepKlAFAOTn8yuPylNXX9JLi0f
1QFU9UQEAOTn+r0QKN+gFpGmqENUc9hv1cMLJoj8M2gxA7aBUrw9NMc2v04+4dRUtdp1B82tD

H2kGskvEuHZ+7Udf89zw5DXjJwlry4bZq9OtjWnWijRl0oYo2bcvLbGOc4btG21wgreeHHp1B4ZlOv
HcEcD5QZpuv+l2e2g/ACA/KkBVQeoiel3l/6IOKgDk6dhrrQd1ULMy3z1RkZGQ4dEus05mDGp2d9V
b0Q+rjZ7D7raTTBLW3vV6dtrhjxuYG8OTB6XehihZtW+khNdqguOM176Idth1itOdfT7Y9JDh+ImO
M737fWHkNjEflpGaZOoDb/iAXgIA5EEN6f+7Vz6hlxaDmoWfOqgAkCM1C7/qhYrMEKIupaKs+bN2
yao3Ztuc0XsMPcGXKlEb7K0YEnhNOEmYH/4YM+KbcmhDlFTbN1LCa5XkOBO1L6Id9rE7NVUTnsK
Rmqsb4jfdL5lQPrPmhNaJrmcK9/uiyeB+B0wf/vkPy+nbTuslAEAeVH3Ux65/Wi/NN1X/tPp7r5XnXX
OlXgMAyJaqg/oe6+PVziIyQYi6pIqbF/zehs1VHawYk86MU9n2h393alJygxmjRqg/UVew9mbkJGGV
09aSNq5dWbUhSmbtGy3ZtUpyrpO1b2w7ynXZSmV8eFnK5aas6vZ4Tbfaftab/j/Z9Zz6fs+LOdGV28
6Jws6U7ncgwj2/fI89vB8AkI9Lx/vy+Tc8Kj9+zmW9Zn79Uu02ufXVL9JLAlDMHVsTKdysF5AVQtSlVZ
TNvcFJu6rS6res/8djD/8OK6Rpb2dgoi679ubg/ixDk4RVZHuoDc72vF01d71eitm0IUqG7Rsp4bVKcJ
zJ2qfa0Zfu0IbVpo3SBlM7JWcHjzfsGiW6ntPf7/mw7q/B67F/aPQSTiCV+x0YzZ2x/7qrrtNrAABZ+8H
zn5W/ev1jemk+veatN8nb3nOrXgIAZO7Yq0SOv0kvIEuFviqCCAAAMIF7H75XKn9UkaefXYwhpgBw
FNzx5evllQ9eq5fmx7UvvUr+06ffzDB+AMhL4RqRK9R8BQzjzwM9UQEAwMTefMub5aNv/6heAgD
k4W9u/7Z875pn9NL8qP4udVABlFfH3239jwA1L4SoAABgKu/92fdK7Y6aXgIAZE3VR/3cm75pf5wX
v/Abr5BXvO4legkAkLljbxcppFNUD/EQogIAgKmp3qiqVyoAIB+qJ+re676ll2ZLhae/8Osn9BIAIHPHiiL
H36EXkBdCVAAAkAo10dQt192ilwAAWeve+n37MUuqDqoaxg8AyImqg3rsPXoBeSJEBQAAqVAz9b
d/tc2M/QCQI9UbdZb1Ue/6T6+2g1QAQE6O3SlSeKFeQJ4IUQEAQGpuu/42ueeX79FLAICsqbqon3
/DozOpj/q299wqp/7ZDXoJAJC5428SOXabXkDeCFEBAECq3lF8BzP2A0COvv2if7Jn7M/Tra9+kfxSjV
/kASA3hVtEjv0LvYBZIEQFcteWjUJBCsZjpdHTn1tSvYasbLT1gqO94Z6fFZnX09NrrDhtXGnIqCb6x+E8
lv5aY2mo2frfdepdegkAkLW/v+178o8v/6FeytbzrrlSqr/3Wrnyucf1GgBAtq4WOf5u6+MVziJmghAV
yFVPGiur0tRLjrKcWSvq58vHDiJLNeno5YXRa8h6LarV6loXZDV4saVTK0lhIDAGjqq7f/FuuePld+gIAE
DW/ur1j8kPnv+sXsrOXb9zu1z/E8/TSwCAzB1XdVBfrBcwK4SoQK66cuDmbuW6dPt96ff3ZHNZM9
SxQeScUj1nxwW/7fPiHlq1pa5sz7r1srOiuSrkqFgGV11xFTP2A0COfvycy5nXR33zr9wir3nrTXoJAJC
5YyvW41V6AbNEiArkqXco+/qpnDohy9vEKJetivO0uHlWqs5Tae6SomI53PSC
m+wgVQWqAIDsPXb903L/q76jl9L1sp96ofzKfziplwAAmSvcKHL8tF7ArBGiLjGvnqP5CKntOLI2peqN
536d163Or/dp135sb+ivNdbZLwuuD/bKS2MbmtlG7zFcYzN4jMM1S+1HWN1Low3jal3a+zDDt+aq
87WDDQ9pc9i2o9o8tpfjROdl+J4ZuZ8YxxD7fGh+W9RjuK0OZwi9/7p0z506/lKsnrNGj+NAWF6Sk7
ozquwfDt9PNqM9ie65eMduS+H7wj1Pcd9HsNxuv+l2e2g/ACAff/fKJ+SbNz6ll9Kh6p/+
+n/5GeqgAkBurhQ5/mvWR+qgzgtC1CWlwpHQMKhTk1JhQ8ZlcHHYtR8HCkLa61TIMrC+uRoeXk2
1DRU2hfYY7EitNCqEU58zapaW61J3uw52duTiYMi0674ynbqmQ8GiZh/zyGBquM1uz8dQE5+X4X
umuToieEt8DFGcfQcvd0hb7WCw5A2hd6V67jxVafVbXq/SIWaP41E6B9LVT4MqcjrpPZfk2FP6vlDn
KY/3ERwdapKpD7zhA3oJAJC1z688Kk9dfUkvTe/f/Nar7J6oAICcHP9XIoUb9ALmASHqUmqLn8O4d
TmNeo3SlHPhSUpi5XrX3na/FYybwtaPGt482TaCw6mHj7EjO4PplEcFZM7r+3ubsrlVF+erBr/GPI9nZ
FyGWtm2ttd1t2Wptpx9bOvUrr3hB4Vhbe7UZH3kdQm2eXRT0jkv/invSO28cd0SHMPY8xHg7zu8r
dZxrbvBoHEu3Iamcu40u53bEitrHVKUE6f00wiVRPdckmNP7/uimOP7CI6OD//8h+X0bQxHAoA8q
ABVBalpWDn9k/YDAJCTY69zHpgrhKjLyOwlZ/R0K27uOQGJ9dhLZaajqpx1t1M5bS25RqwPHd486T
aKsrlnBj6O4toZL7TrHIT3AyzXt4IBWXFNzugv6uxc9NvY3vV65pXPrI0P38YwexjWLxht3rwgfgZ5PrR3
31CbR5r8vFRbfnDoh3xB0xxDFHPfoW3tXZQd3SEycC4qW/5+zWtniH/u1HFY3yOhIe8k9uUwrEFKk
nsu0bGn+H2R2/sIjpp7fvkee3g/ACB7akj/V376u3ppMqr3qeqFCgDIiV0H9V/rBcwTQtRlZAQ0ImqY
bsGrZTiyhuIkyielpJ8GjFofJo1t2HQ9x9BhzEGnTgwGP0VZ8xMtLyzyA0Mj0J1YVK9WY/8jgrfhNscV/7
yMN90xjFaWk+MudvfAa79X7sF+GEPcRwyfn/zcTeuUjN51gntuimNPcv2HzlNe7yM4ctwZ+6+76jq
9BgCQJTXJ1KT1UVX907t+59XUQQWA3Kg6qHdaH6mDOo8IUZdSUTYvhPckdEOYqMl1FkZg8hyjn
mOk8MDO76nnDnc2AsPq6dg9GafXkeGOgjFCRlOK52UyYccwK2keVxw9ORxbLNWR2T2X2vVfkvc
RZOKW625hxn4AyNFfvf4x+fFzLuul+H6pdpvc+uoX6SUAQOaOrYkUbtYLmDeEqMuquCl5CI7esjtQKlR

W/Pcos+u3ZYNs3edW7PRrMGZhHW+zurzZA+NNoYyV0/nF6FOH/qlfF4mkm1w6dXKHXpMWsd0
AsUTMrb06bje1BPcc+OPPf3vi6P9PoIsvfmWN8tH3/5RvQQAyNIPnv+sHaQm8Zq33iRve8+tegkAkL
ljrxI5/ia9gHlEiLrE1MzaqqeYPcGPHYR0vRqKo4f/GoxhxHPHrB2pgiW3/uMUba64U6Z3duT8+R29n
aqkk6GW5KR37gdnZO/JRbfoZeQQ8BgyOC+
+nI4hTOmkFwKOqumZL+NcNHfF75DZlQPvNJxwzn2EWPdckmPP4PpP/T6Cpfben32v1O6o6SUAQ
Jb+8eU/lL+/7Xt6Kdr1P/E8uet3qF8NALkpvFgP48c8I0RdRu0Ne6itmkU92FPMCHiMXnIlPxkzZmJvy
4Y3Dft884MlY2bySXgTWHWk2dQnKrWh/EXZdLsdqvO87l+XXmPdq22ZZBKkcVI7L578j8Fj1udsrvr
DyPW9bj9yHVtu1oBtyq7eda9xzgsxY/VgjnPPTXjsU1//hO8jZntyvRSYe6o3quqVCgDI3t/c/m157Pqn
9VI4Vf+0+nuvleddc6VeAwDIlqqD+h7r49XOIuYWIeoyqmz7Q287NSm5QYtRH7F6NnzmbhXSDL52
Lpkz9nttNibamUhF3I6BrlSH8o+4LiU/fZQL005glcl5MSQ9BnPYu9ueiRK2YH3O5qqz34IX9Felldqs+v
EUNy94PTLd9njnodqSeM2Jc88lOPY0r3/C9xEgiqqPquqkAgCydel4Xz7/hkcj66NW/u8idVABIE/H3k
od1AVBiLqk7KG3YUUMVeDS7wcDHlX3cLBmYrWVcx3NpCqy3W/5gZHNOTbvsAPDrOPxhlcr5bpsp
ZzL2dcl5LxWW9Z6d+j1VLI5L6Zkx2C1Z/A+3D80ejUmYNfnNIaSu+y6nznWQ/UUZXNvuFaoPYw+Q
aAb656LfezpXv9E7yNABDVTf/tX28zYDwA5UPVR7/uZx/VS0Cte9xL5hV8/oZcAAJk7Zv2GfPwdegHz
rtBXRewAxKNmNtcT86gwbG/anqHAOEfpntPHcqpFwIpwn+l9Rip/xM0BAHl441/fIKWHXqiXRK596
VXyW3/8z+yPAIAcFK4ROf5+66P/Xoz5Rk9UILaeNNbdmc3LcmaNABVZO1r3XPu8dSwZ9ODG0fGO
4juYsR8AcrL3um/J9655Ri+JVH/3tQSoAJCn4+8mQF0w9EQFxmrLxmANWFXOgK50yMzRu+d6jRU
p7ZyRbiplKXDUFf6fgn4GAMjStU9eKe9s/aS88723MYwfAPJ07O0M419AhKjAWAOBFgEqMsc9h+
WlJiiblx9NaEs42hKOtoSjLeHmqS0nrr1GDr/3pF6aLa5RONoSjraEoy3haAvSQIgKAADmBj/ghqMt4
WhLONoSjraEoy3haEs42hKOtoSjLeHmqS1IhpqoAAAAAAAAABCBEBUAAAAAAAIhCiAgAAAAA
AAEAEQlTkSE2WU7DrfxRWGtLTa8fqNWRlo60XHO0NvZ3CijRibwjp6Uljxb0GG9aVTbJuGmH3UNr
7mJbRRv1Y4SYFAAAAAGChEaJirvUaK1Io1aSjl4H5pgJdY1Z9W1nOrBX1cwAAAAAAsIgIUTG/eg1Zrx
GfzqeibO717RkF+/1tqei1szFPbenKgXvLluvStdu0J5tkqAAAAAAALDRCVABIS+9Q9vVTOXVCyE4BA
AAAADgaCFGXmao1OlC7MazG6GD9UXuIvfE1A+VKNbNOpXokq1Vp79Mcxt9cdbYTvjOjjeoxqk7qY
JvCa1VOdrymMbVf2xvetob2H3JN4rTRY359ZEONcxFo46j1xj7t9ea5zKoOadx7KKotg9uYsD5pjOuS
9J4FAAAAAACLgxB1WakgL7TWaEdqpVEhpPpcQUoDQ+ybq4OvVyFiSYIva8pqYbBWZBqcNq0GNh
xyDHYlNtgm65W1UnjQaYt7vIMqcrqqn3Z25OLAa9u7bmODtTKHQjgtuo2TKsrambLztHMgXeeZxR
iOHmh7W9xml8+s5dDDMoV7aKJrPizf6wIAAAAAAOYRIepS6knjnJuIuXUb+9Kt61BNOrIzmPx5qtL
Sr2+5QaEKG8/7ve16jXN+0FVt6VqVXfE2H0Nl2/qabl28L3G3sx1W8dJvU/gxWMe77oZg/mv77gF0
arI+MhUdf7xhKltu2wfPpR9GSvmMeBlqe8MPgsOuSWQbJ1NcO6PbuC+H7qbbu0ZI2ZEDN101hq
mfOpF9hDr9PTTNNTckuC7J7lkAAAAAALBICFGXkjERz96m16vQD9VEOl56FlRt+RP3+EGhqScXd9w
+e1VpeQGStc8LYa+fntmm0GPoXRS3SeX6lj/xUGXLC+U6OxdDexSOP94RimvidfQ0t22ElGaPTrN3av
2CcU02L/htrJ0fGKo+Ja+NftDbO3Si0mrVCRv3dbrau7jjBZKnvROYlRTuoSmuuWkm1wUAAAAAAM
wdQlRYdA3P0OH9SZmzk5+Ukn5qK56QU/ppespyMrCTEN0D77jsIdheXUtjqHdgSHsazOHy/rB4P5
SryllvyvYRvVNtxnbMHqOpKMoJfUHcwLlrXzzrnJ4+aYeVwfWW6mk/kMxMCvdQKtd8VtcFAAAAAA
DMG0LUZRWYKCdunckYgeWRMt3x+r1i3Z6eRig3URhpDK9PScUt3trctVrXE6cj6ik5UdFhZWC9anb
2EeriSf+6AAAAAACA+UKIupTasmH2OnVrPZr1HI+ocr3rlDEYevjD9lNT3JSzbglONXTcqCs6WRiZQYh
dOS164L4ctvUQeLv3pzs5lrF+QUP07K/5sv1xAQAAAACA5UOIuozMupwqYHLrohpDoCdXkpNuEjs
4XNoIEXNVcoamK6NqvWbF6+nZ2ZHz50fVFTXP2eBs/mZ90FMydk6nxNfQ3XdHDnadr3VrtZbsT/jr
h4e0ZyWFeyiVa57idQEAAAAAAAuNEHXJ+QGTMWP/VMxakU1Z3XCn3DFnS8+ZMcmTNFfFa1J7w
6+V6a1MmdfTsyPNpj76oaH8Rdl0u6xar6utN6yz5eg11r0anuYESU7AqVivP+8dkGx4U8nH5V+vZtP
5Wnf2/aIumOquNyfCylYK91Aq1zz5dQEAAAAAAEcTIeoy8oI9S3NVh0rGhDtTMmcun2r75iRC7nY
mCjuDs7o3V3WI5gWO5gzwaXOHxftCh/JXtqXl5XU1KdnnrCAlP6mTC95EVCojdOutWrxzHLe2bVBg
W9Yzb2i6eZ9Y68/k0w3VNv09lNI1T3hdAAAAAADA0USIupQqst1vGQGZUpVWv+8HRvaEQpMqyu

Ze1w/BbGr7g/scx2qn1yBt/9DrDZhIcVP2+oNtstj1YDOoh2rwhvQr1v62Ruysst0PrUtbbVnr3ZILLnU
8g6+ttiara2v22gwMTTeGs+c+ZD2Feyila57ougAAAAAAgCOp0FczrADITq8hK3oiL1WDdo+eiwAwk
urtPS8/mtCWcLQlHG0JR1vC0ZZwtCUcbQlHW8LRlnC0BWmgJyqQKbOOZ75D4gEAAAAAAAJAOQlQ
gE23ZKAzU8ayeFTqhAgAAAAAALB5CVCAPql5pZpNXAQAAAAAAIEvURAUAAHODelXhaEs42hKOto
SjLeFoSzjaEo62hKMt4WhLuHlqC5KhJyoAAAAAAAAARCBEBQAAAAAAAIAIDOcHAAAAAAAAAgAj0R
EV2eg1Z2WjrhVnrSWNFzZavHhsyL60CAAAAAADA/CNERSZ6jRUplGrS0csAAAAAAADAoiJERfp6D
VmvEZ8CAAAAAADgaCBEBQAAAAAAAIAIhKhLqr3h1gddkUZPD7+3l51HaClTVePUeI3zcL7eZW/XH
MbfXHVepzc4uF+PuW1j58HXt2XDfY318F4Wo12TGjwv6jHpuVGSHr8S1obCSkPCD8+s/eo8VkaciG
TbBQAAAAAAWF6EqEuvI7VSQUoDw+
+bqwMhX3tjRI1T9fXpBJbR1H5WpamXpFyXrYr1MbN2OWHk4HlRmquFYNCZ4blRoWtYG6RTk9Lg
BFl2EFuSwZd3aqWhcDTRdgEAAAAAAJYcISosVWn1+9K3Hq2qXqUCwPNulNaTxjkdX5br0tWv7dbL
zjrrtTsXnYiusm19rlsX9zNSbdmv7W+rxHNafjv7e5tSTNCupHqNdS+MLNe7zj77LasFWvOcDkeza4NI
W3bd1Dh020055yW0VjvW3SDXOE/uBe3UZN17bZLtAgAAAAAAgBAVUm1tixtxVraMANRTlM09H
crZ4aVeu3bGe23noKufZadc3/La6ciqXT25uOP20qzK2U13yxXZsoNGFVLuibM6w3PTO5R9/VQ6O+
JmscXNPWd/1mPPbVvvorhNDpynypa42Whn56J1ZJYk2wUAAAAAAAAhKiaha5OGDmHPzqkT44K
9tNrVlQN3A+WTUtJPFSdo9EPnYSmem+KanHGTWGtrquyCW7t0qM5p98Dbnz18X7+uYA7v7xxYR
2ZJsl0AAAAAAAAQoqIsJ82UcJTAxElGbdLcjGjnzNtlyawNRdm8ENYz2A9KQye5Giur7QIAAAAAABx
NhKiIoS0bZs9Kt46mWft0JuahXRm3obgpe3qIvV+v1tc8Nzybvl/DdfBh9KCdYLsAgLzp0Q3Ggz9yAQ
AAALNBiIrx2rte70o7oHNrfxpDyKc2ybYya1dJTroJqDsE3qVm4i8YM+6n1YaI16uZ9NUvzfakXXbw2f
XqnHrtK530Qtu4NVhjbRcAlpH9Xl+QwkwTy540VlZFWu57tPNHr+bqLILU+Qxze40Vpz0r+f/hT/0ba
p4P9zEP5wXDhq7XDO6ZmRvzvuZ9P7mPvG7mOO+3ebwnR+5jSf+gFfO8z/K9eGZinJuh951luGnG
nJfc34sTtofSdoiDEBWJ+AGdMSt9AiU/nTRm/7d+MFmdbhD8tO0KKsqaVzS0Kavem667baeO6OB7
cZw2JDp+/aavPhXsGRpSs9Wsc9pc9dvm/sOhHu7KJNtVjG3wDwuALMzsl/chKri09j/lv0npcCYu3Da
KcLuTP+4f5vte3N4IC3ONPyjOQq8h6zWRatX9x28G3NEnxsO8XrMy01/cAyWOhh+z+NZW52O1qS
YFda9TS6qdmpRmFPjk/0v7+Pc19R5csr6f6l3jHFk/T2Z778R5v83jPXncPtTnc/yDVmTok1eYm+C85/l
eHHFuht739CP98xP/vl3dH/g3Iot/IEadk9zfi8efl3zfiydoj/WNrUrbpf+eHO/7dn5+FsZY1g2DJWS9R/
TV5bd+A+hbPzD5uvW+9U+g87mq9ZZia/WtnxX060c8vNcqIa93P29uf9TD2NbIdtqStKvbr5fd9VXr
K8cxXx/yKNetVygJz83Exx/
+MDcdve3gMSfabqvqrS8PXwQAmEq3XrbeX8z3eP2+GngjyoffFv1vwAzaEEm/z8/8vXjm7XCuj9q/f
c28f5PzY/87OoP9RtP37dy1y/3eivPzV9rCv5dn1R7n5y9jv/pnrCy/l8a/rznvuYNtyPocjW9XvNdMa6J
9ZPIeqPdvbdd+hLRD3T/maud+Um3XK1IS/5w4n8/+vTjeucnjvS/
+fZv1+8v4cxImq7aNPy/h62fWnhHfw+nfR87+zd0737fBdX579Qr9vhz3uiJf9ERFDBXZVn8p0ksO56
821puAo7kr/t9KrNd7n9D2D52/MKlanIP1QqutCWuIJm1XEk4PIOsNTS/7rDdbf9h+0jYkPH57qP3g
ubQ5+wj8QdOuc2oMyXfZPWWMeqiWRNsFgMy05XytY71NXZBN503VUpEt9UY28fv35Iqbe9Lv7x
ltmTO69MupE/PRwFm1o9dYl1qnKmfn9kLNhnteWt7PKPPC+T6X6unAzyL5KMqJU9YH9+dQW08u
7sygPb2GqIFK5fqWv9/Ktv3zYmfnYgY9sRxj39d6h7JvfRj8fi7aJ64puxm9Ecd5v83jPXle3ved79+y1Ls
hP8tr6uf34dEJHdm5mO7dE/ec5PVeHOfc5GX8uXHeXwLf5xmY7Jxk9148/rzk+148tj0jfp6yR42mW
tYuzqii+fpZGOMRoi4pvw7mwJuLMeFQcMiBCgvdr1EPJ5TztxMM6dQPhf5rrYf5A725D/VQ+xmx35
Ht9MRtl/MGFlw3nvMG7H6d89gbakjCc5Pg+G2D59J+jDoG8zj1Y9QvU3G3a7xu+NgBYAoz+uV9M
enSL+W6bIX/A5CTnjTWa9KZVTvsoaMdqbbi/1ueGTUM0Rh6N9uSN/n84j6JXuOc9d1s/bI/oxu3sq2
HjNo17dWwypITNg/
+vJW13H5pT8huV1lOenWcNKPePgZk8AetuMFlmDTbEVuO78XTnJv86fJoB+cHhtOnWwJnknPCe7
FhxPub8/PnvuRaNYmfhRcOISoAAJgNfnmPTdUltX/5uTCLXoa6tpj9i6D+pWdGvR3b52vSqbZmPmL
C/0Op81AjV7KppRZXPr+4J+f2NDo7wwDEHTWkatqr7yOrObMI4efpl3ZMgT9oKfPyXhwwR3/Y6uy

flAvevxGqt6h6/9mYYa9C3osD9HwiwflBrO+pqedWiWHwjzD8LLxwCFEBAADmmDP5gRq2N6ueO
MFRDt36vqzO4hfU9oYzCcRc/dbuUL2Csh6WHcfc/eLePi/O6NHZXTNnsg5/YiAVeKtJgXKftGOWv7Q
jNfxByzKH78Xz9oet8pk147pY/4aeVWXUZtirkPfiAernGrdnrP89dXBKXadTkl3n7nn5IwymQYgKAA
Awp5wAVfXWmJ+hjMXNC3YNtk7tfK7hXHtXhU1NO8B1fuEpSEn9Vqh/CZrtcHptlsOyLfP1i7sOCGf5
y6Iecqzq2bt5jz0MViXezdWMZjYfZVa/tI9h93bqyMHgjTuqd9QS4w9ajkV4L56XP2x5ZtqrkPficIMl+f
qydXLfOk8nJau3vdn+EQZpIUQFAACzwS/vkVSvDSdAndMJ/zL8RSPMYE8j9bAngLR+Mexaz2dbt7sn
h6qoWc7nZKxZ/uLu9nw6O8NfFgeHTbpmdl7y/6V9rOIJceZ7CUZNPfuGnmG4O2f4g5Zvvt+LB+T+h
62S2GWOB8PbWf5cw3txTLq2eOCPkekZ+UcYfhZeOISoAABgNvjlPYKeRddiD3czevzkW+dSTf4wM
Bxc/0KW1S8ac6/XkJUVc0i2WqVmSp7lL6nz94u701utKjMcPSpSOW21wPoeCgyhV7dwzTovM26bL
dtf2uOpyGnVY6+27r+veL3G5m+islngD1qLaFZ/2NIjADo1Oe/9w6mHcM+oHinvxXGoUhklqUldLm
RwkSL/CMPPwounDwAAMCOtqvSt33L69a5e0a33y9aPJ2VvRX669bLVFtWewUe139KvWUZh56U
6JyfEblu53s/3bun26+Xg+ZiLe6RVHbg2rb71S2tfJT+5m+H38TB9HgKPefie1vdRxvdv3Pc1573Yf2R9
7eK0K4/35PH7CLt/3Ifxb1dq9H0x9H2r2jFw3Pp7Pu1rNcl5t78m8/fiEedGvd8M7Ns9hrTf/uKem6H
XZfY+POp+0XJ6L453XvJ7Lx7fnpB/xzO6RnHuxXn6WRjjFdT/rIsKAAAwE+5f6F3WD43zNRwQWBCq
x5xdm9BVbUl/Bl3nnO/pqrT6M5p5eS7pnk7G5ZnV9cH8Gfre9fjfR2Gvqc5r79gUjT83Id9bxnk7iuLc
LwrvxbOmRvOoOqhhgkP7+Vl4cRCiAgAAAAAAAEAEaqICAAAAAAAAQARCVAAAAAAAAACIQIgKA
AAAAAAAABEIUQEAAAAAAAAgAiEqAAAAAAAAAAEQgRAUAAAAAAAACACISoAAAAAAAAABCBEBU
AAAAAAAAAIhCiAgAAAAAAAEAEQlQAAAAAAAAAiECICgAAAAAAAAARCFEBAAAAAAAAAIAIhKgAA
AAAAAABEIEQFAAAAAAAAgAiEqAAAAAAAAAAQgRAVAAAAAAAAAACIQogIAAAAAAABABEJUAAA
AAAAAAIhAiAoAAAAAAAAAEQhRAQAAAAAAACACISoAAAAAAAAARCBEBQAAAAAAAIAIhKgAAA
AAAAAAEIEQFQAAAAAAAAAiEKICAAAAAAAAQARCVAAAAAAAAAACIQIgKAAAAAAAAAABEIUQEAA
AAAAAAgAiEqAAAAAAAAAAEQgRAUAAAAAAAACACISoAAAAAAAAABCBEBUAAADAQmtvFKRQ0I+N
tl5raG94n19p9PTK2fDbuiEhLV0wbdlwz3vk+e1JY2Xca6xXNVb8ba00rK8CAGB+EKICAAAAODqa52
TGOemSUMHoqjT1kqMsZ9aK+rmpKJsX6tZnHZ3aesg1asv5Wkc/F6me3bS+CgCA+UGICgAAAOAI6
Ujt/OL38Zx/XTlwM89yXbr9vvT7e7I5KvksbsrZqn4eco16jXN+IFttyXZFPwcAYE4QogIAAAA4WuiNm
r3eoezrp3LqRKxeo5Xtlng5auAamb1Qq9IiQQUAzCFCVAAAAAABHTLzeqH590pVg6NpryIpbm9Orse
rX/7Rrehp1Vr119suC68NKtPqCtUJH1wEdeJ31CKsrGjyeYL3S6HYYzGPXj8F92fsp1ayzrDVXndeO3Ul
FtltujOpfo/aGXxagXN+yXjUo3vHbQto/dH0tcc5VoEar+6BWKwAsLUJUAAAAAEdEWep1HdJl2Bu1U
ytJYTVYDdRep0K2gfXN1VEBZlNWCyUxyoCqjUhpcMIpOxQceJ3F3t/IQK8jtZJRr7Rcl60YnTuHwlEtel
8JVbak7hZHba7KRqMh5/wEVS4M1gNIcvwqwA5pv3M+hoNUR/i5UueiNLhTJewaAQCWAiEqAAA
AgKNjzQ3p4vVGnVS53pW+qgPq9ax0hK1v7o5oh1dLtC/
+y5tyzkv7etJYd0PBqrT0a71td2qyPjIpNl6/F2OSpvaGePmv0a6um3ga+6psW5/r+hNFqRqm9n5iDc
MPTjLVrLnHV5b6hcF2Jjl+67VuGhvWfmsrOxfjnqu27Eadi8A1AgAsC0JUAAAAAEdIUTbdGYwy641al
bNuj8nKaWvJNWL9/mFoL05zBnqzXmhn56Lz+t5F2dGdIQPD3I3enN5rB4QPix+t7aeGgTCzuHnB31
ftfDo9MAOTTGnVs8OTUiU6fuu675lBqKO4dsYLbDsHXf0saOhcmfVeOzviZq/FzT1n+9Zjb+QMWgC
Ao4oQFQAAAMDR4oVsGfVGLZ+Ukn4aMGp9qKqcDiR3JTnpp31ix33dA90L01rllguwH8bwdve1A0
6dSBLymT0vz8ha4EuLsnbGbdi+HIYlthMITDJlPQudTGqK41fHZNc5DR3eHzR0ropr4h2yuodK7n4ja
rECAI48QlQAAAAAR8xAb9RD5+l8K8qJU/rp1MpyMn6am0BHRnTmnE6i8DlCYFIpo85ppLBzFSw5Y
HLD3NgTdQEAjgxCVAAAAAABHj9kbtRYvTsvXYK/Onhx6Y8iHebVWhx7biYbtTyercHa88cfflg2z16lby9
Ss3ZpEcVP29D4Gyt7amueYpR8Alg0hKgAAAIAjyOiNmpQxjDw7g706u3Lg7tTtmVk66QWAo+p5ps
MsJeDXAHX05KJbmFROSaIqAdNKcvztXa/nqR24unVRp7iWaoZ+1ePUnkjLDlS7Xi3W0WUEAABHF
SEqAAAAgKPJmIAoTMlPDo3aqW3Z8Kapz1ZzdcObqKm94Q8/L59ZcwJAszZnc9UfQt7e8Gp0FlIZV2
4Gzta5WPd7WfYa61790aSTVU1twuP3A1djxv4k9PbVbRDscRoSdAMAlgYhKgAAAIAjKro3qjIzuwrp
nGAubi3NaZWlXG7Kqg4D/dzWmOFftd+ozdlc1cGh9+IREzJNorLtD1vv1KSk21XyE1S5kPuM9AmOv

3Lan6jKu5bGBFRJjDgX5r1RPat7ugIAlgYhKgAAAICjK6o3qqp7OVgzs9qavI5mIqfk7J4xPFyx63gO1Di
1a3MOvE4Je+2U7GHrIcdebVnr3eHxeYt9/BXZ7psz/itVaZk1TZu7Xs/fcexzEVYMVW8zrewaALA4Cn
1V3AUAAAAAAAAAEIqeqAAAAAAAAAAQgRAVAAAAAAAAACIQogIAAAAAAABABEJUAAAAAAAA
AIhAiAoAAAAAAAAAAEQhRAQAAAAAAACACISoAAAAAAAAARCBEBQAAAAAAAIAIhKgAAAAAAAA
AEIEQFQAAAAAAAABGEvn/AZM6nr53azj8AAAAAElFTkSuQmCC]]></redcap:Attachment>
</ItemDef>
<ItemDef OID="q24" Name="q24" DataType="text" Length="1" redcap:Variable="q24" redcap:FieldType="radio" redcap:RequiredField="y" redcap:BranchingLogic="[q23] = '2'">
<Question><TranslatedText>Do you prefer Life A or Life B, or do you think Life A and Life B are the same as one another?
</TranslatedText><redcap:FormattedTranslatedText>Do you prefer Life A or Life B, or do you think Life A and Life B are the same as one another?
 </redcap:FormattedTranslatedText></Question>
<CodeListRef CodeListOID="q24.choices"/>
</ItemDef>
<ItemDef OID="summary25" Name="summary25" DataType="text" Length="999" redcap:Variable="summary25" redcap:FieldType="descriptive" redcap:SectionHeader=" " redcap:BranchingLogic="[q24] = '2'">
<Question><TranslatedText>
Alzheimer's disease - Description #[no]

If you have Alzheimer's disease…

1) You might forget recent events of importance to you. For example, if you are interested in hockey, then you might forget whether your favorite team won their last game, although you may remember watching the game itself.

2) You might forget the names of people you met recently, or the names of local streets.

3) You may no longer be able to handle your personal finances or plan leisure activities such as dinner parties or vacations. Simple decisions such as what to order in restaurants might become difficult to make. Your ability to concentrate may decrease and you might have difficulty driving.

4) You may start having trouble finding words to express your thoughts. You might need to be reminded to shower or brush your teeth, but you could do these activities on your own. You may need to post notes around the home to remind you of simple things like turning off the stove.

5) You may remain aware of the day and your surroundings. You might be able to travel to familiar locations like the supermarket, although probably with the help of a loved one or friend. If you are in a social gathering, then you might have difficulty following the conversation.

6) You may continue to enjoy hobbies such as puzzles or reading.

Now consider two possible lives. In Life A, you will live in perfect health for 7 years and then you will die of natural causes. In Life B, you will live in perfect health for the next 10 years, then you will live with Alzheimer's disease as described in the summary for another 10 years, and then you will die of natural causes.

</TranslatedText><redcap:FormattedTranslatedText>
Alzheimer's disease - Description #[no]

<p></p>

If you have Alzheimer's disease...
<p></p>
1) You might forget recent events of importance to you. For example, if you are interested in hockey, then you might forget whether your favorite team won their last game, although you may remember watching the game itself.
<p></p>
2) You might forget the names of people you met recently, or the names of local streets.
<p></p>
3) You may no longer be able to handle your personal finances or plan leisure activities such as dinner parties or vacations. Simple decisions such as what to order in restaurants might become difficult to make. Your ability to concentrate may decrease and you might have difficulty driving.
<p></p>
4) You may start having trouble finding words to express your thoughts. You might need to be reminded to shower or brush your teeth, but you could do these activities on your own. You may need to post notes around the home to remind you of simple things like turning off the stove.
<p></p>
5) You may remain aware of the day and your surroundings. You might be able to travel to familiar locations like the supermarket, although probably with the help of a loved one or friend. If you are in a social gathering, then you might have difficulty following the conversation.
<p></p>
6) You may continue to enjoy hobbies such as puzzles or reading.
<p></p>
<p></p>

<p></p>
<p></p>
Now consider two possible lives. In Life A, you will live in perfect health for 7 years and then you will die of natural causes. In Life B, you will live in perfect health for the next 10 years, then you will live with Alzheimer's disease as described in the summary for another 10 years, and then you will die of natural causes.
<p></p>
</redcap:FormattedTranslatedText></Question>
</ItemDef>
<ItemDef OID="a25" Name="a25" DataType="text" Length="999" redcap:Variable="a25" redcap:FieldType="descriptive" redcap:BranchingLogic="[q24] = '2'" redcap:InlineImage="1">
<Question><TranslatedText></TranslatedText></Question>
<redcap:Attachment DocName="7.png" MimeType="image/png"><![CDATA[iVBORw0KGgoAAAANSUhEUgAABVEAAADYCAIAAABtBTQzAAAAAXNSR0IArs4c6QAAAARnQU1BAACxjwv8YQUAAAAJcEhZcwAAEnQAABJ0Ad5mH3gAACmlSURBVHhe7d1fyF3Vue9xNeqORyWhdYe0HjS6EYKKpJUNMafYCGJEEASVBtmKmyMvESw7CDYi5nAuFGVTyLs3gl4UK5yeEy8U2VBUVIoNBdeuCHKe2ExUC/eLaW8Yi565r8x5pjP+DPnGmvMudYa7/fDwzlZc445njHmWqvOn3jhm+v3zfgZVQNHmTUVWEsdWZZS7QjCaUOpAJaL3kNZ9mvfkqKaZpD2l+zeR5N3lyqsYBp/jXfA7GNwrlL+cJV0WJkkYzkQtVYgkvG3BEVmF5Cii7RHQMlYe1x/j8O+BBFEJuMOvqvZGH6XhU9Y8BeRZ7qh9w9qH4XL/9CmHh/NHD1ZjKyRmZ/+6ssN9TnK1iZ8WY9n3Fnf2ebI/sdtrqW6TeC1BKbx9DAqUCAtKFf6O/hBcB5qTGE28NdXYM9Yp27/CrpVCkTojF5YwH4Apg0x7nZFrs4iHA/x1hxt/jEYyTBpyZUSf3FRq+GCl1QRPTBnyKcU6h+KkbaX4yTVD2sjxxBcZW7C/mdZc/hQsXfLLU2+mN2ABZdfxV+WIT7PK6bHnS/RZD5P9h0AMCZ4D3TZO8WKGK5XcB9eqDz4yQKK5pZgdbmRcBBYDYzDZLl9kmedwPBv9L1FOTOmoMgrNfF2Nbn0iAUaddRc/3Jo23NNEBqkzKA5hJJPTyR1cRVRWDzUzIwMlJtwDO9UTmSUQRcBCrSwLeBnRR93Ws6bfHqcGwi3sI2f8tgV8HbSz9XYd8d4gUEbyX6c6Nqa7aBJ+kKZQ7hmIdvT8mMNeqvWw99qwW1jc2FK91W+rXYCWpZCXHoVnYV0j4TPqJDFSfaQ9GlPjHLtVN5u2vfpiGIuKJ1EK9OXTqTZT3b9IOLoK4tcpfv+sxQWb8Po2WUM/fjy1G3O3ZbyNBJ8kXhYxYJOqLnNpDxl9FIXMI+kngZYDBL27TlKsYh6hr1C/hmxR2m2HNWvdsx0GNCUX2Eyy1WFpLkPcKWuf4BtH71qYvSLB7+RZ1ZyXRQQ1gSRqjtNkDb8UNZ8lMRMgY0RF5DJZgqaMj/DhdRqbdPN/p0z20T9Jx1A8nCKgTSkyhKYyfFy7TjK32SHnxTyVt4m+VzJe8CPz/5jDiM39Xhr3hKPwDM/VR0ttJMIb0YYMg/GDDqUpOkXZyfyQNn8+Em+yUZAZkQLqIUQ2c1i9OYV9dq6KbKGRL4vYNlkPv7i0TGqIwPqEc8aaBcNDy4BGfXJgGXfKiWG3S38zd+NZimf5Dx9oZsQuSGfiAZjsUrmrhjR7ojMVNQzLFtSlCSvvU5ecrTbpa3K5VrQaBv5vLLVFSpTGz9D48oJEYvxp8Z5u1fdBvZ0qB8Ct/xYtt89tjUbNKVv2KpzAyGDYnmNFqiUndRNMXmTGb4iPdMYsCsAA06IjKbCy7CUB0YDUndTbQH5WJ8EKJmTD9mfrlMqZOExqa4u8w9kGcpN7JS6SlNsh4XCnzwqmiG+DnYYpgTlTvapjpOyl0c6bx/B3QCvE8Eq5RFSsPp1gFC3tuIwjYrS0ZWWACFChJuFEm9gwVS60UDSUZAjvyNxMvFc0Au8EmftjWvPsHlmVhSOl+/cx9mmmDUW8xaQNvzAOoxPCZmn0PncJy94YW0ao/uc7wJtyaI+q8TIxrfzYo24mdGUhrb24fyJIo4PAq2ZMDxzGCujfa0r7xPjwdJ36ceY3iy8AhHHgahwy2yGQ4XxUgzMr/SS4NP3+qrHkg5XEVWahQZR4Rr4ZbhaowTKrIg/mgMzKqaZQWnV7rWLnIHl3WEn+7j/x9xD2zWjo/0v8Ml6h9gyZXnUIgndcRBkzLVVbH5VWiBYXgJ/CSCjGYfgWCibIUt0ZORu/6YY0CeqL/ZsLygDtA6BiJnaKtgt9jhjfvY6c5Ap1LXNbaolxFpH5iLZyMXI7kMDV19Hvm1OWHLd4whWC0Vq/qUnudrkGnhc6KKDj4WRQPrWjrbC0xZMpc9KP/FpmjCUHf3tekibL/OzOcjhJ1OzINx7lm+eK1C1m2S2CPVvvqEz72Q9dPw4NZSkaK2KQj6EnVjzSn/hIC5u2pxoKEuyF5W4Z3qIQJ9VS8DygHqPi5Zon5SbkTmhGh/b3Tk8HtLAgMO4/Pzb2GSx+4TS7VtqEWsDUWi5amzm4BsdaW36CS+3Rf5UPLMl0MmnEomw3i/c/bNhLYAxOuGjCq2hJ7aCJKMbl1idU4oQ4CMbg0Xz8rjhYCR8b9xtBS5ZnuCFbPhZkrKFBiqOPsQ8D+2gpg/P7gU5khWu4oeSYAiPSTUXL4kU0Sm6O6i+o2eIqD7XmYpQOXpszdo/SLg6BPbPd8Mxn1AhoEy3kNuu5R5RWyGGkktENXawXBiIMBkqaYcV5O18vhkuUPewuM4QX7KMXdeeTPiUE1itqQnamQQ8y+UDpSuGEOzF3EuPiRAtIPQkTmlTxJ1jCKIE4ZMGL7GDmuWU0YFN6JWM8XX8EKZ+aChpBhrOEbsVhzVvSHrfxNuLxsfFhfhqfKNWuW9tOSP84nMGhqu/w1F0x1w/pc7S1uFQJymhJVCsbdv7Sv7mgCGRa7E4Nn16SDFQu10Pr9T7oh0Vq9S8Lry/XlzYfnaRBKTGvJPI7A9LBpeIExamnDVtTD2zUC7i1yqxRy8wGUTKiSIDzH4hxp3I51bYsIP3VJIsGgH/RKH7qLOuVaXNGDIWTvWxCyJFlK6y2wHDL38+LHbcXAkuX6Xnt2sMHGLgsu2I+xxS5MCEjwRjfH2cRbeIYr9QC9yQAycgHtZdpJBWkGz0i6xUTBUKhS0BkQqdOgJIb9lrKQmtYlmuV9IWVD62Xu3Y6t7RRM26SxFf9d0NEalhWmXnm8fsKWTKyEZYmxeT7GJ5fqMTOdt+eSYsVsbh2c/1ZFdwbgSGkmhCGE6jNFO2R2trFHnl3Xm/6GH+c7P62iWKVXOpXjIpYd3Y9LLN2jKF6+FbDTcjz4dXVBXMbwsIiGjJxl98ITDj87cPvuBtcRjddWDZ5WfhOh98z+IqjuUHqLQeBNrPpRiwL/7wuUS4BxQz/XKsMwQ2z8YnpZPRIT4WNRAXWpXZM38bfL9S4fkGtfjbrKQHdhpIdnNRlYI4/0lqCZY4BdgQ9JWo2hOh3DtqvvkTAJDxlEmwyXrg2ikxXPpjVJ+DXnvL9krLLPQgpk9qAVVCVxlfaoRRF/ZH37sqahiuunSDqS09CC1XO0hNEKWBS59xQhrBc+pd6Bs3PuStQsxUeQDkRw/unSjH2VmFCqUfTa3cBrCOKf6YTFvd6Tw5YmlgGnrMO6IUNvhCNQ5UkuHAzzRYsyWH8gFl5ZeaPOqFGsO6P3v1nB0gWLd8BqfNJnlM7L7C3sI4dUj/CTYYUlO9RDrbcWiuVN9nITT7mqBqR3ne8Nbdqr2gP4qqwt4ZQnHsTGuN9gxeLvP2l67v2HQt9ft30m7yYJEdS3V0ubR/2nU3+J/9HF37DcQcthmeo8lDJ/uiaNVCfZn2fOwFbetMMK7ciO6nAqKP9W0Vj8eVX9ZD6GJuJ5wmlQdOdP4tmTfgGIn5w8vfUuS7u0e1PjmwoNfyjq1vzBXxWfaDwKj9eNX36A0Y3Q63O+XTeWm9umSPWGgDhYg0xJKm2aTTd1gg3NQ1ZkwzlYGNtxPugoJWlD8p8/6K0ya6glS9Kb9JiGjL+yaH8QAAt1JgSxM84T6m2xePbzOzB16IWZxlNmp42sCfP/9DYHyIsIhIbsfiKKq6d76Dzjb3lw+D/WsUh3G3LYE6Xvt6sPLkfaVOzyq5bsR2YqvgmIkcmrp3YgSU4A9fh3bdqhejbjQnhzEyDmjkzXtfhDcl/H6sFW2vs68RpMzYHs+6NGL1HdYWTvEOPDfzbgPJGk7bLAOEjzqSdTwg9lVsqK5cxxbJ8kELdi+Vx2zz+w21YOS85zbcz0Ljdc88zGwbdhpvrJjXS/hvWc3dZ5Bhax/ql2lA3G5J/sGC6tNAQoq1PGWJHTv9qsfN0NdCeTKkCRcxuHPJ/gNPYcu7BdH8EFfgB7AC9GmtBPg1afzv5yQ+5/HxCgiqGJNCHy9rsiF7kI3Ct6Kq10Xf8c13S7Lof6tmpyTQxuWR5ADzKSY7BNmkR1DTBbPfXCC4oKJ+JqyZPSBfE/2PcrPWjwNR2SOiU9s2PwQh/ZpM8OsQRmmHuCJ2zzr5mI/MbMrv7r6lGSp/3JHkd6jWFS29OGFOAcqcxbDQ6ZAhTT11YSb1Rl5j2hR2rKfA0s7PUNWAsRhYl5A8+LAFA8CKWXlKUJ2y+F4UXqaaCfSKi2Y8Bk9JvV3NG1wM7VsSDY3MHKyX3z1ZXkhOcoWzrpnnPZ3+P1P0O2dnD1fdJ3T0OvJ9hSprCzVHhOmT5rrX2NqbJVzr/jcdwfyo1fYKNUTlubDi2lDXDdFWdvZF4P8TYK3v/mPBQpWqlHytuWZdLr7nu/o8K2X0RbONHJ6PUPvsR0OTWKlq9MIdjn9g6ScO1WXOeOSF9nRw9hh+zNU+CjHE/NcdDdyLTOT3kKwTc1Dj6BABD2RGLlBc2EKX4A5e2kpsk1Qi+tHxhT7Ugeo1Rp0xk/uHWLpPMC8BwyMddlCeIWZfF7J+oNfRkVtr/gXGsOX+fAJRRxQvrMiyN/p3ecmLcHwHJ2Zrtf30HbfxDhkhWPcSP76FFxwB2gzNpHYjBwkDr/tvI+h/KAnlgAwUrHGcR6q0A3NfhQLbVTwhUgVT0i1lbaDyFRqbfMTWmZW8c/1ZfP69IoGSvCFp6yFhrnIZRyc5xdlVbzEHvR3WFuRlIzQbMr+8CgENTyoIeaWpNc5trqvcpc5P5DPs2tVSoGvsRKi/BCq20plBc7tjmP/pSQ0zaSxIWVy+BZP8YP/5ye4l4aD79WavBHxW3qCKI/OYbRJ8BWPjZz3uj/wO3qGLL+1yC8y+a1XgHeRUPEEhS9Et77qOCQQzoZQ8+9/+2gOAUvZpVFfVyXFvlgv4OYTJ/TRjdfU79fDt+KdVtrw32fdFR/4GAJvOrC5oR8t6BeoKhDK7G5PqYwE/0P/LapvKPu9UhWfnaRPK70F1yaEB5B81iZSq5AlS96GwwBpR7ddBbYGYzLm1ixyuq8B73Yfd1WiRxQeQrj0DMM0U2IYDU4zP8wcqN5g3MG1nbkXEE/wFHzrKqc0zoO8tzaEnLIOk8v6yWw9A1+3Zq2jA8d0kOr/kLLdgBOYDTOyDgSRY2cmAyBJo/vAhCtJBZcDYS1VjjWXvE7rrP05pDlmafxtxZ4+xbWVs/Vti9NpNwt3jgC/kJDrW0APTzVH8ND4pNhx2XkYsPWgvnmaXRBlO5Ze1E1nXF61QSzJwaPtcxf2TV4P4thFLcfEqA6ebUw7oHb8GJ6Xj1N2rOBmLcyrrV/TMPYtIXs20Nl9H2zEY/zQ4AWcR2nvCCRyQxB7YJGBb5Ur99xkFsu2O3s57r0u4NxJwAzn6yaEvXVaz0yLz7jeVq6GYPfAAY/RG6XdEhAgY3+v0VnTDrzqNkDhLhNsr+n6/4iqpdCYCK+Z2+WPW2XFB4/mf8t3+WLIgsSMHlVgKcCDgcRfhwK3fsB/MyzU/gc8Af2RoNSJ7r5kfyAXlpmLqRnVf3XHwDW4vdJUgTXZIFy5B9M0dWfXd5X5uBhKmN2zj2LIJI+d23lvM5Q1GvnpwFo0g4Mfi+hx4Etq8A6p4EJGGfN4c8n7vxF1qrD4/8hMbOZc8ATpdyBvh+AgYy9RfImPAE6jZPNNABZvo8GPAR7q6Wn8SujnmyjS7hVuPoAxwuE12pXWVfzG/c3u0qn4mkGkCiaBzGAPG/t39W5mbpKXGvKB9BQR2SU8Vb3B9Lk92u0e7Q/28lYt8jZOEoyR5h7kmvF4ofZXxlaPiHeshdN+y4RHQgKOAl+jTg3LUe0nMzCixm6UkhLhEJ8mYCzA+33XC9pnq2vdLmYa8ltLtuyOpmx6hefTcnGjBSYRy+yMKYpHWYEmr2rC9BYMv2Rj85NSE+A3mLb+oFo6Sp4kVQ+kxkxNLQl9VCMMm4KxMNbl8VQ9EFbhlrsPT3QUaT2r9ySHVhsn9OY0wjxlBRa6ssxz8Y8tTmMMR2xxAOpLWy4Blh9gMbdDTxh8B/1+yVNEb3mbR4y7aj04gk8mU9hWnUbQ8jdnkaMRBgWDxMO8kH5/1hk9DHbZN8WhGe/0wmO0SROQNHm8gMbE3yyO1ZM4b1Jb2CvwWlFD27lJSdwXhdvNXEwaXj9OmQRQFKBH/qiWFyz2xkmYmRb5uzuW3eaGfDnbPQA4tvDPbNhcrkPTmVmUsoKLUZqRT9mfaLfwXOnkIyPExM5fhu8Ku4/dh7XN3OHMIoNSAb96MggtutlehjsS40yyxQuyXUe+dJhFsDy26NMxuzcWxjvJMrdMSpVJ0VeY0PPHLW4v8Jq9fbnxdZYqVEPd05FaHq0CV/8KTXsC0b8dfc/XVH+8n0pxgPYs3yhC7SGXe/7h96q4HqdIEq6PQkTiQxuKpyY48H8mlbmHm5IxFwuzs0uBx28sjJ5HOkf4oQkKVWv4+2ou1+s7LT5W+t0Gu2K1eoD0aUrOJ49Szzo49SSWp5UiHqjsXU7OkBWpVh3HvIBGe4EMQExSEd8Y3QDgN9DHHYIkSd8KVL3ER+Z93rvHoQHxxfYWsiwmfhnkfUfzD+Xc2+pwQaZdxEwnoz+ruu7hwp1QPzZgtQSjz+W5f9ugCTvtlUYJYKxRxEXbTjuUtCo/AZRGFUOCXn0aCLqwFXPkrmbrLrTQc5UyHcfoZd8pmeeZB3dkV9D1CKfD4JkUTcsmF0YhGrGhS5Z8W+HUh/86JqsgPKBIqaeTB6hJFOiP+mWB/RfYxNIqh1EJW+ydi+QT9KpqBMx0zmx/9vtkuq5dEuNZiFbzmUtjCpZ7rHh2Mm6AM+4cx8RP+xojmOBbj4Itw0Q8M6lY5ER1JBXcpI2UZqvIS2BFpzcV2bLTNm4s+c7smUm0G+gGzvECQGbKYn2hMCg5wDMi9rtbS53p0EBZLK8XeCNWwjHHt39cyvkMQlRsqDBmaOtTD0FMrE2pvl32d3YR8eGIxWW2EbrhZlCwM1lfM6WoSF2l8/IJ4pmvgsF9XjZDtS6nWRr4T8TOu6j4M2/k3tYZJY3Gf2fHyA/TH6tWXZBFjhelR53kVRHkiV2OIT7DCDAumdz4Y8V8OuIPfufQm1xaBY+5Y56d2yHRjmSwtixa3+2CFW3cQAz6zWYzj3fqewsAjBLgTKHw7hj85BsK+h3rPCmEc7nn5t84z9vQTsMdrx8pYmhRM5JPQjlDNHybVwHmgWazm8fAiJcs7OUHvQWeKqUXSqQlHW4UVJ8HpBf5ee8l6v1MPsqw3ctKo7EjgP6Qj0+3HIJPvt5jDEPp3QIk1yFVwwH4lu/FR76RdCjNKgj4PXRYfAX0cPzyn7vBwnGH/OeQlbdAAz2QmLr8h2Li77eIr0Urt8WKDCT8Of97hubqP3yk3lUCPNE3Rtf/1+0TT9XQDuUHmAxtGvPKz2V1nG8mzJzFRYLi+qRUAUxO9vfSXOvCqwzEVWt5kh+HRB6eKufLnLd2VUbYbTzOGnQQa9bW9ZDzWxkKqKZnz8I7PK1R4oMtdE6CL8Qjz+8vMzB+xYVA3yRQjEO8OS7CbjXKQ0fp1yzNXPJ3GQvjZ7tCJTxZQQF6jB+XyOCZPJX+9LCtp9o2N9LUKjqzJ3gEeeEkYb97Db6rFNz7ZgZOI/Yp35yRAZ28Ji+sJ1D5uZ8BJOa9EmkUJjrXr2xr8wbL4RSgiM37hJ86IjuchC0nFmn9F8A7hEM7Gv3O+eCu5MbiitWOqnsNbbDwyN2Ae3eCuUSeK2ym0GZrrqOeA0CjmkhmtqKj3YbidcKnhtU9V8hNmm1sRTPnX2Dl/Ifs4x6wM8Udu7Vs+V0GyqPPxl05M57QwC2tXqPxTsQMnm6hyZdHpfW5e4mfl8l6RXGhYl0h0rG1qyw1ob3U6J9y+5mOQu3u5AlX8sxrDdBi6TqpkgY4BbfJ0Dq07XYO2VJ3F8nkRo7ZqPWgxVi5x5Bs1PCRqhEzZ1TUvqm25QR1qCNt9qzrEJuObrDzgTbkdpl0jmmEqcNExd1qfqgZWuj2n5O+KGS9nFRn0GBywkfbzzfxPmAkCEBMmXnUpJIYe9Qx4+pOoJXrkLQq3HHrW6xhZg9cVBQ/Wh8x1eVbf1OpqkApKDQTbVFTvtN/P4/4wb0W8Dp6sr1b1w4OECqIt1VyOCPWjQqnrvMD0g6ZcaUm2mdKXMoH3+WcOPgV6S/EinkXgD4Q07z5n3IUm4CiZW6QtCdlLPxt4cAYMbvkmJz1krqIGnEmXp9yJMyyc0kEVIhFkwbe2htSUQFc0KxrUwV2BUnGmKMzt2qhPDHwZz9Vnmg/8lZ2Mz0xZ2Jl4TrAhCIXkX1njTHOQoQYClGsEN2Jo+8TXEWtjhnB/QaEMYQeyWeDTHJT7ar9rWXsxkLXB5KhNkVVp5QAO8FVXsHvn6DJK2dx3DYc8y4Pb9wdBRKH1OEyt8uiYTnqEGDBbPVmb2Kpb4BqwAb5m7n+eVv3umHSG9HcqIa6uvVuRX9p+QKBrBHTUMoI0fXXIK9yoWJS5zDYkbdlw9zvXQ0RxlT4y1i3fQTuvwtOqPrE4tyGdWfx8q4FyEX7sSM6kPF0PwHNcJRxsDXLB6xQfzpbxsFtEpbvV5Ta6ODfFFNiXnl2qg+uFbdrGXOU/wR5zlTPg7KTbT2eIqFM/Ec7+fvnZGGS9oPnDy97ZlVOf8AJbBnd9NSPx18/lRBLHW48dZBmBZqKHymrW5rMtqrvcW/9ksGfkuKT5uTd0GJd1Lzd+9N0HdnTkPcDm1e3xCBnxnb3LvXNcFqrkl6oFvO5UtyIdxi3VChOs1tQ5Q2MeHJmSvHKLHtdLD0iHlsz0CEU0yeVAlK8kxbmMLpbNgwmTdFEcSBkE3pV86Ok6f8Lr1X2jhDbTdc4Ai07QLbc0a3G7d4eyjsU1iWmRh8ELD0HLtO4yS2XjeeN5nc8bJvRn8HMEq2K3gSNAbBvM9mJfaI3QmY5pvI0NZfAXRgR3bSVRY8tJWm3zdXTvLc1aEbgTrBnQtJFFMnYQwJtNGGXUjWLRn1WThnSeEw0VlyT0jrA8pKI9OfRzyLbYrT8RnMvpNz91uYOUcq0LIQ6uo9N6Lw4ed6I6iQgYArPFJwa3PNFW8PlPNNzh80jmo2RABLhlwgTWf08fKiRklpeXB1gCDCwXfzAl8p6r4dDYGNUWwH73aryjrn4iEBNNwC40afrkOhTK3Rv3Ga1aqQMUKU1eT6Y0WvXdtcJOxrKBLCdyIE4XIBZr6VmsaHOhpGBPUVBQrDX6I2WBnoZ2K68dS3O4Uq0gCg8I6hK0dWOhZbNtFJ1WgjGGKV7pGK0HxHaEJ27E0xSBb90C5cY8l6mFhT+pRO4AqHPBrEaJuBPTiOeLpBzbCx8jD6hlgK70rV6BN8O9WNH5RKqoJpwJtBB3ryHJ3pRUCdqFzObw4aVxEN/2IuFq6D1OgPr3rKYBuJ1GqS7BZ3EOvBPmGNLr4Hg8k8mRjRIEJ7Er3Plfx9FF0qp4oXV5mqYqdp2mbOu4NXRE+hb4UStfsE3KqhJ+kEi9kfHtNMVhsVeGyqjfwPVaPAyMZzjG8NjmHP1dD0wRFh8sohnFmeU8hC5mPQq4ntp1I2UzsFh6dIHNIakrOVr7sb8+PlDO6UQDxlnjsfbrx+eNkdKzKt7rZ8gY1EMEGV4LSVVdGImsaYPwzy9uKXFfIs7ezhhJvNPHqG7a+pwmxPl3bgWFcH2WH1RY6q0Yv+tQDrNqcfSpq8ka+HLXYq4l2FQ+SgPoQ8QGl4RBnUwkpSFBaZE1Snv7j3+oTUj6JW3OKahqSQaYK5jg6/SuGj7gbkJtYvyz35xwLF0BBTPBMH9JoXp7G1tuCQ27MGPK0PoDwIw9MPb5KvMkxnvBlZ7kkf6xG07OVOp+Db5KVNv+sTJwjgVUf4L/3xMMDWAqm0wuE4BiL5QIr54tc2tzmz0Zj1iqpBhjPk0iHnLP3dDZnF+IaJE+S1tlvz+xQl2UH+MEnF6S6pR4IMSbl+I+SBy0yaC7jffWqnG9LF8mj/vSY5H3JMTKTMMSQG2vh3IlCpPi4bqsntp8h0S6oj5skoZ4S9TBzSA3ZAqlQP+OP6W24IEbpQbSlVfxGP2FiNZ+SOSaebhhvbJD8mcZmOf4wIXZB1Wn54FLtvi1Mgmw3YjXnUsP+D1cszUMH4RQLAtrRtpukNfsZ4w59V1IdmJs16DzR5w+k88lxyfYW2OPSaVkfaKbQ/OHnDMYw9pYVNYGYmuRBNSUgkvrZHYWT84ZbpZPfNvzXnEi11oMjnnsUl0GbXlN3wRbMxhxg+xsGLvlAbtywlJpKXNwz0vrONG/UaQ3vS/S2P4dfJXp/BQWbbpvNaA8ftFRaozbY1dU6qDnCF2FkrhwQzbcPprQJXiWVMkiyt8R8EBPN+0PyeOXWArhxmaUk/WCGWb5DpW6egR5KRnQ/GDzfFVgtyR/NQnuEA5wxC53wShq9sNRIUfFExFNCMRohKHA4zq6pyyYKRMXtEZzDSKumZQTNw/Wxou7/aPL0wxijbd0zVqyxDpOWXzkVzxxsNaz8jgGkdxhZkSttX3B9YYBgTAbP5g4PzBw6/l2HJVbK6ThHSQW2aSS/Z0QKjkyLAUoOKR5lBIyq8WzeOrJyTldDL7sjm3rdpaIgZ2kJe5+CMxaU1PDX1Jy8p1IUbZ3P80HEOFSPHNGvt+N8Lv1bQbBMvaS4CXOcOT2Frb7EdJ9bqKEo1HYr9FUwmRR7Ph8YTTQ8j1UESMM4T7k1dTMvYMHIOYBvs+WfK5FmL7cV8x2jqKPyDGJAQMlCZl/DRFcqHtaybpU8Fby7W5C+oIUevtD0tmGOIQfdnPrRFzh0Rb2TC7mrqlP3rCGvDUh3umbmzqnsgvBGNkHpNWw2LkOgm5apymh6AiRbc8hd2E8Gkh2nvtbvK0gV1t7R5E5SrV9nVYFdvYWPEGjKK6KQT6+wcCmYD5ZcI6jJB0Dt5XgbNNvgDbqIMbrpZZmIpjCOGuXCSZVfLJWK+l7Bn0RsAhpBPzlswRYFEmVHDyWwEOZiGJaXq3FMP5K3vD3pv43sM57pYX88BZlf+nrRyqoZw47Hp3TCGzUoRd0NrtGxPwwrXfPp0aDk8zDkhHybBp/bPTUzBEQw8GpKU48ydGyXzUwqbRr2Dq3c6sdf6kdZvXgyKG1v/qIY8AeIiBJNRDlz8GDb89Pr1N0JfvnJvXV6Fc3P7tPFxgVl8GAqq+CVpCP4z1QqgPSdp5bspFhGCYuBFD6aBVbjdOLWWjqwDcD8LKvWgjvVNSOqdr9vT3AcPfldjLEKc6HO48G5JGWuPmtpKCjb7t5DBHzYujBFvhbnsXt0eT6VvJBLHgWEHBt4iYpTKnxuL9pW2BSEbnE8nZP7Nn3z5wE28tCJ5e6QGpPnLFsF6/8IzKZrM73tVsNR1EJ94LTi8sbSMP5PPrJDzIWN9ZFV+O7HoUvJmpF9EnIgpqE8oNg6PqPvdJWVJQ0z4kr9lPKZRM5O3wdGfCO2EYvIvY6BZ7YO/iZLTO4rHCm8aA7GjzFcoEPIl8UXwC8udQU+HyXrl5WZAb8eH7lo7T7C2H8xB09aolXy5cRDdtPqiV6ZJcLYzqhoKhhXGCEgwR4oH8cSvkv4K0fj+EzT9M7s8YRgNPs2vQMzAP9ELqF4GGXVUKa3XgwEWNRBhJy4SAT80Gfs81pHGFRXuFPYKWuH6V+6N6y6eeMDPmyWdnmCc2RxXSivGqHYD17rwwQCD8cSx4jcw4lvXnaNF+Wgv5SJfhD6Q25AxgUbLOYTMu+iHjBNKoXdSl2pgYc+tK9TnSoMPBLpnmKZ7Akza6AEYmJ7/I9QxfRBiKv7/s/9PdFzb13umHcL3ZTiXHHhk+xHHM8QXGvrmt7o8WbTOxgNJZuVNg56r3r14Jwxo4Sl5HiSuwNv5e/Nc8eYfw9lCrVgdcSHjO9tojUd7dtZ4BjupBT3WEtqNGdWGyz5MNaEAsgukO9SMU48Tdgv9eHNp/oEuL8IDwZq5ZOlscgHAZj/sV2MLVyPhymJuH5Y9X5CsSzKkGHg2CrZHbHUK2Er36kM8TfDVUp9fH+OaycS2GuS1u2VNwCjcbzFPsdOgrJG9m7bKm/ZM3krOQPcOtIcvUo6S2NGUnIVwF5w4Hc8eVuW0IG4HLJx5eCuSAJVw0rjlq8WCaUAUbYHmhH2wmEAsrrGx5jJ2RTIE4V+eo2PaJoX3BeYvvTwAeqfl5+HrG5i3F9cP1G6Tv0YfCYnOelO+nSQ+XODXFDFEHt3BM+P66r48PkzZ4OLoYMYKMxw7SNTbqGlGPS/KBYlo0Y+xlGzFK1YHyYRW8v3C7nVmmjoQf0VeeUO0ePSFqJJh94tXYTpzQcm1dUa24ciEsxwWqwEb8WsqHdyN9KEeXA+jZCkMWvv0x6mFFpIrSvI4qYqHqrVDRjjHPy3QGfLgjDHqvI47gqxZR0vYA6LFaS1mwEqwFbZH92mR+xgY5jqfBqwJaV5SLKnL19aJr3lZeLS/31vNtA4ywuHuyB3bK46Ke8sFP8lbHbFEEv8WQoOW1sG0hC1mvdbOGT3UVaUeJsMyfwcZhQs9vv6zmaIGnC4ahzGE+Br7dmM7/KVhwhF1k6CMlC9vNiN+Q2VIeOrOTHUzvzpOb4LfDPlNJnJQhmIcQYlcUnoEk79IjmNN7wXTuBy+3iBovE0VNRrvJ4ZiUFs4X5jmz5FVYD8e7mSfqfRWAkgRb4JqVfJW23I46nwaI80YD3rKdkB3SZeU+nbjXRLCOFIafJgrVZDlAgpfEJmHYpbAl5EIdPKmDJW6Q5pvE6ZAkTm3F+dH0hI5TTyjHzR3RDqHqHQ4G3dUM5mLnr+CjZSrDGyNPxVVGhGK9VS9yqX8QmcYVhhPFlvKuq3AkcW9xgJMZiwc6G8rwG0vpbgDQUWUOfeWsUWSEnSDDmUZMgGk9VxYlIHBWxYCZNI9Vh02AKy8VDuL6r1jdo4mRPqRhphxp+E5zfXo1EnbOw01C1Dwh5lw8j7ljHkmp+kqZVUJZ2Xu4d5uB8Xi1rXWnhTA8AQIm8JyFq5QlRXhgelZj8uQUuLzVpZ1D85I8ayxZz8BZdZhuwR7pAy4WpUNnl+FHG2qBYNNhBJq0EDJGY/ujM3lsMUAcC1xw0FgN7o8cNl+L+0L4RiYG+5/dzK2FNfMWnx8bCoHxhIb1N2h4RoJCNfnoChUkU6eR6TyhRTDIxxyFaS1vp2CrzG/T4f6J/6jNnx2gYN0m0JPZZj9nRzRbQPxWQ1+8ZAYYxmzEPrk3NQ5pzLdVnXR+rZT9D6F3L9xYwVntbG5x6qlSiF6IuMGW8iqtZAVx4mIsAMrHvvsqx9NwfYGLxqJKsgxVT13NyDPX1d9EZdtOofaBJYhbXOgmG+v0Yp4u0a60Rgps6DD3oVArHxEDFafC8oSjrXBq0aJq/TTwvDJ4UBhgeNZ8LyRnULyZg3JpvRZnb05RHVmA8DQ1Q7FtZkGXqLIL6HjYx7Gp2CW8GLJmfN88KvZwq2GqeQV2JkuHe88j8P0ohJW4dBJuHYqLvz0gRTdQLfZZ8V2sjCbj6eyf6U2pyZJaKaWvN2eX4EPgsqfLE9fPlT7Y9bVbfEokvnWtdHl/FcmRDPQ1MgE0jhlBu8DFYuOgqlCPC0l/X3TQMBm1pKlo9g8OtRrvFxJn14T2UzHHPOxDqojrVMjyVjrCaYKgNeYFRpavPs2tkHGjJj+SxEvW+ZjIVO1XN1/k3YHBTLwZfu0QSmxWYI97Zqa1GgZzP1wfr1lLaWyp/+KVUH5b62vRsXWyjvG/Fq7IL4NUtKXU7XWo/c2aulNmw7L8gTnUAYFCnUD/c1uYj2FjbLqwMb7N5RCbt+sdXhW4pW+rcFaCOKcDzAYyFVcOOJb0MFr0u1Ki+eQGoOwjvJnRb+5rRNALkb6Vh0VsINd3S8uW9lrwIYmHxsSqNsLNbtkjUCTwsv9uaBZhU7yfs08EG5tFa9PKIzRtuS7zejyUsp4Zoh6wFiZ6qJzVuTT2WfmZUqo2q1+FYUO7MPaRq/JjVQlTCOhA7wr3sRYxLXJCfuhU60jkafUDAAzvV2pz6odZF6ZE0xO/N9ZSnb8mjT9/VLwIffUwpEPRoZDMFAdzZ9V3GLMEqXP2jVFbqn0BR5RXMcNiYD2LPl9urF2xSxtXNUmV8dfCuDwbUkfxa1ehHGV1nqnqHcVTUPPqWaTPnq7LyZc3K9dhoc94mdvb1zTwOOaSiz1ckBK0QYyE0xwX5Sm4jBe8t7SpHnLqtoUbrqYv/mK5HXq1z+Ca0mGnEdYgOH8qezkf3wmBkFnx/SsDq3tPDoKh7mfx8+E8Iw9Z2CAmAaTOxRzbizU0v6tAELRgMhakp3Pu4AD9dq7HPP7KrbnzOmspXXyRwFuKYJ38Eqi6tEP3ke6GkHUa8dv/i3i/GIXR7NK76BVj6Tp6f1swi4Ut5L1CK5N9xqcnLS8qoJxwZ0nKuwr/Vj6wiRqnHXUUIBDvxUqVN/WYKHzYfLlsvvCjdKZcYj7iHZ+jUe+ODXAK2n2GfO9V69RfvkS5ZdqJ3yWcPHrCIfjhzJrZ+d3cY/LZTNTDAb6xTjlAyyIiLpCcd96VpJ+OZ7MvQKSC6yG+tpywjm+B5i/bQfnPVsLmRDNtKRm60ZLGk2Y5SVNuGlGQlwhYCTsJXAFhNy3HuvNzE6Hd8DuYnyvR6u4EdV3jp2q7aw09m8Uy+M2lMNaDr8yODJdKgvWrHN2dBDzKTp4P3IVqbTJcX8P/4UdfL3dB3XKsC7EL9eIv3rOjcCuaVNb1VANW+l4ijTTHZ7FEd63JiTYbsMHI46xzW3MfH9/3cGumhlG4ZkWI8ur7jkO1tThm8/nJF6OMPbP3yxmNB3+PSNHd8aSK/u1kEDuVyH+bXGjcULTxCHUgdi2Crm1nEf3cnqDjd52gN3dHG0vQ4lLPjG8gMbfJe8d0l6zYG0MgvDd9q4n6ajyU+aeZQ3Gu2b8H4u18hDcgVuCvI+eXKmnQlfK0iQKGmkrw5BqkDP73VVYtyMpmYU0vyFiucAdLb9jMcMg1Ci7p0xxAWZGkIu6kZjPQE5jN3KsfKR6Zw0oU1+yQ3Nqd6KQWm09yLhk+Syy9Pr0eTm0X3fWLb3aFQ6AltQzb/0kgAx3oHsAeqIa+9UxTIGSNiIcGrvrvKE9CXmTTqmntgTvQRmz8pIc8Qr5zk9lcpr7Ubt7Kh8A1XTPOdpNvvt6mC0nbX6WfnF1b+hFrEMzYoxDtAz/A3/h1MNpzmyTcmKk4UolAmBXNQsSxCf7oOHsLakyV5bXBAymfk3uvnRyv9T9rF60vxeEwAfXQXMaR+PfCNONBsgNfkCSMbDcmNvQkAb9FMlTpKLIySxm2mDjGAZWpvNNRHh7csPYexzqP8ORPWEQdLjRQ3jX3BLf60j/8Xn7rFsIWvO0WF3gMbXgAjAhKkcGW3TJFGMdMwbj4QlXwJQgIXH7PsG7TZhWrmnkE0zzWiD+i4PlVQbfZTjP3K9T3cDm3Y2j1CyMwSVpBZ3q6PTRCbVVfjH1ALdAWjj7aYCjk0fNvJCQDDt5MnnRxPrBd2AkabR5fj1cfKaYdlsnGZhFbk7gPe5A9ADkdPlQNKLJNG85uGQ9bNC2a2ktTxg0Twh7A3bk9gSw7KzvWL/cS2gzFOk5Tyx8xFFn6nJnAkVpuhqsCuMKjYY/2i4Ga1D8y6Nv9Gd9+ivIvXd0OBT4TmtMcK6WhX9HNsbDZMHZ3WQ6UeWDSK3tDD8p1OZKvTAzQjcbKcvMjQWTuD2xHVfpWEW2dCoS97h1GSwVvdBKoQVXodN0fzWybUwHSnNmY2oRBs6uhqLqSZQgqtkGTyzMvnmFSZ5Cy+vlfQKWYCNP5mXzPt/gEZuRxZQWOdeLhZWXtK9WS1fKgb8A0Fz2Yk+mfj5a3eOSI4fVUaH4cUjrqtJGhVA9S6VJ0F8r4Q+YgxZY/uyZ8iJL7xm68Ub1bqtgAoqqYeaE8TI63Qm8Gy1yc1J3Dmz8sEJvMy5EoJ0yQLgK9m3ZTQMV9tV2bgRw2KwNWgoMHzYi2fx9jqjvSpa4uHAWBuhiqhSc1NoUwYcEA1hCdt7o+bR5BGZfX1dKhqyb3dq+SOIUYg3FXJbUqcJpcvPBQhTwKw5GG2TFpF6IeeWzpJsaYxCZ+P+4O2dbpa0DN3tqDUg3h3tE3HI2b7YJpoXEt7fcp7hw0ogowB5rU5n19hHWFS5V8Rr1eW5oFR4mq6PvT7fJNfYRf79gq7+5F8+CxdLv7Ep7eM2KKPMGgDZ6L0Jx93Qb0lqSdRHTGMk3g0RkcEiDcfF9RxCcxK7pjUxovdjmGzzHLlZxmGTmmPlnKT8qtP0Ig9Wl9DdB6gZVUX/QUb4YZbBfSK9nqIX3ZGJW0ED9ZtrPiHjN4iUl8XqNZ+NJXwWvn3+OHyDm2eGMoh9bwmGamm0rNbGF5zhd6jUQnWuGm6l1LqSoAjD1SK31X1eeCj8zjnyIJVqW2e0pbexOS2mQUwEVDhG4xo9uM0IMT1PJVHGGJGMZYjBRQqRyfYRT8hBQCk9jqzjGzIQHLC0+KDoOGIsOIeW/0Ij6G31O5DKHPBShj4L7mzYTqQzXgXuJJ0LKczAvKsTpgqT3ZsQTkQGxjHhvR+OB7v3Ro8AYd2gJ2P1mN6cDhg8gS4V1ZfhXdL46MnGSOrIt4B0OJ3uqd1yR0kV66OE4iPqhzKBDsRjuuhaTpwF7s1xE2kD1GBhtQIHzVXImpwUOJROrBKX3bRfjz96dQ8S2GZYQaZi2lq6Rk5pMXoiSrh1+7cMqyyk9MbtmaePOeD3Fh6v0kmMzS79dOnRXcXSVS5UXJYBj9gR7rDnEiOsLd8DNaGLL9OAZQKkJdE0R0bdEhQ5v+i6iXsoC5PDhKJ+6Y+pSNTHN0XMMzPFgpmdo06mF2OTX7hd4V7jCu6wXYXXG+xrtOWX4oGtCGMbH45RvxfZ9PDrftKJQ2CGrJkWeyDBHBJ6GoUBc4BHwG8rb0g+K1TtcDmXHQ8hM5wq6wXcW41Sd4W4tBcTLGcMK5u5KmT7J1PmoN7cMZ/DW3LgCz0aMB0fUGMqHr9LnEJ9e0FA8ZmJxvdcM+XOjSLXuDEFjGPOBeqF6ebSKZbSGVEmydDBVDXwTVVXWgnxsJ0B3Wq48pMV5zRVJaCRFSO4TPNoDqw2Ly7lw5e2nc8nN18Vd3AI9GLwZ5pEfcuLZQubpHAEb0Hcnp6t4m1M2rZGPWMm3hQTTRAnbj4X0Shc2BKp27UXl99PdBwEYKvPTZjCHCDqpGRwNVErlm5VgJHp3iUnzEr0lXEL2WPrHW4rL4AzEtRmvvj0G1MVVGyHbC1o/MNQRdHJNmG9fRD9OXvP6tRx+1ZDSj1bMJSR8f2oRvN2G8CfK6Q7S9dH9ReGhE4HHOfrP+PX3GB8nBGIzjNODiNp4H4nBNyqb1srSgLdOSU4mJhYwMcShJtObvVuXDLMhHsl69zvg9QKr71JmHpXlMrbbkTYoJUU1DFm1CbcPvRzS2xd12Vc3vJnlmgm8BMfsuEvYb8WsoH6OfNw0tD8lXhnNWUAL8GQc7wK1eZdS3LQZG8HdCTdqPXSJyTxDhU8HGSVwZUZBLpUfBsA0jnz41lsuwE5R43jGXpMQIQjnxxMDkDOkZFNQVwR8CSSUlV6y2a3fHIWt3xgK8YfKkmc81u4mRcd6QOzHo5YvIFmiOK1+rhvE9dhr4K4JhlNvqzq0I4y4lD0gYhROi7Jw3H8vF0FyMDJx8SrNp3eFvExnXBXZiTWOc0qmDcEgXOpT1UE7krvGNXzy4A8O1S7mmm6Uh76NiTBoAqcEuryV68H8SNMTGLTaOHbQALuxzjZbjlQkhW17p3OHRSiw9RhPJ4TfNjgmUEKFdTGJ1zZBmI7KJY7k8eI7wlvOWbhgKPvJVz1AbVoLPWtpsCcqT3m4SBkXBECgPcyPrzTLcLIEQ9zq7Q61UFhH6ZmUmX2AVb2ZhJdpa8o9bTgLwAFPFuHyW2upo7eGKKK5KbOoYZxwRqH6FaZzOH6rX0YZlYa3d3K4wFs2ZB4c4w1lSMNwqvyK2P05dPv5ErYnKAKGthdQ2tyIyOsjawNDuozBcJo4eyNVDa5NdQGGGoutRBA4ObrBCB3AIbdsBK60lOrp5lkX25HHvMrUn8zkOSRm54hIUcZGWEmPBwXtGnTlCE9vyFZqwwxuHwd2Szqyb4aKCr5BuXNzOXHn0e1xtGDFvP87Xg8RCAQCT2Ip1LDmbTGsQG9ZxN6U2pCNB2aFlzXlHPOWoC3pVlhUzDzfspK3q1KIPw9PU7dFPCGnS4bjxbSDzd1JQbNj1XtVj8gnmz7KT6mjs0u9vS2kp2KWgbQ0PqBKyCLrGvX6AfUklVG6DxQzBxyJ4Ys4c8CJ2KcrrZIKQ8iqMEgQ5fVmtywdaJMdrx4SOlPIPNBNTT3FZlR3v5JxKJRCQVpOjJ0E/fuXR3FRqW29MmkA8vFfbhkNs7F8BrdIYJeL1EFKDiiotlXQcqSYlRaZtKAT1XCupITHPglhmT4LFgawhF6ByLTDtUtRtpxsVVG2gK0Jdk0JCnMnxGFmIwBgLXmKMndUSbawP7zzhTC5OZgUWpdPlvmOs9xfU1SC6M7+KYyXIVwYCw1FxQRDGHx7q+8bmBjIP3ZWrCbm0mC3ETNiHDZTpkJsgWJYYWoBKoFJMy4IKhSPnhMdv1Y4t4F9QQaZGjEo9x7vTNJvWpffBQVrMKc6EFeA5FvaXuHcLTEIZUe4sdBJnXfWHkm+rGXr5XAzCb3Ej5bkOAnM8BhFkS9hEAIcZsQGYs1KfVgHzTApLSHHhx+IDzB20tL/SiCxfn5JkZtBrPSPP8fqmoJxQm+Qtkih4yW7u9E1xVNoL4DTLwjUbEn9XrM+Jy3pQoWzYZQGe7KUl3c8w6yZr2Bq+w6evDQdpOMGGXtjTkPqx9Y4H5EVRX3rYqWu+/Z8QbBHzmGMDjw1r3IwEJ0R3bBDxVZT+zAM2GyLkJb9+lZyNqOeJUSNFsyTGvkdUWrTK3BiFXVu3nT3k4Sx4B/KpBPOnBo8ktrzlOX7K+afpU1RwKPQdA6FLXD+7TWAFJhV9JZ9D+aYr4m3wuYR5dO0LPJMdvAHXbHmNLEVPKWYMF1zxRXHBs7HN1YfGfFh0W6U6XgRplArDDgLdBqwtnHLBvDOVwYSM6dE8fO6eFPOUu9R3fZBHe37Pu0ZYNLR5MIrH7Rz5XjKf6KtYhLgUAq8sT1UwwlZn0GuiEeJ0cTErAmfSWqGVEKsq5ipA4NaJdSM0kq7U9ry0R5D7hJ3zAprSDc1IzomNKvSJQ8kXTjyfT6OZklXClT9s4RNGC04GhjBKOKoCtLO7RBEPSWt4HPZ0gsqnALf5CNSqnbUNuEBMKJJxK7xYIwu7HHpYKh4DL7tIlC7i0zPa8oYkjo0W4DnCWbRGUJJ8EMTuXpiC6COrmSfwP3mNLrXEmI7HS4rMMXBkkdiZIK9sNvE09e9bqKEtgcVAHEQtmjrWkmgq0zsJAKWmtpn1yyZGDPpY3a8ucUb8IMZaXumJl3AzxP0nMFjyR2qcQc3QSeEfE4k1KuLCsZ7D6JrXiWHQ8KE1pVDSGr2YuYiJKmeD8O6bPV35HEaWzSH6bN5KYxC7mUJpkNXxSlWtYrBOZyZ8lfG6hWhvb4UZlJaOb2dsaGgHzTVN8fM8XFq8e0DK0VBMZB4Ik5WrvqvaRmwUGzjZXnN5RdA9lqVG5LBPhMr2eNFv0XUSZ5U2zVdtVqwkMtWvsnsRTiNfN6SpR37D6J4gC2IagFmwf5HLmafjqGBaYKdKrT1ep6kOHJLJnFMwjy6KmHNkMP8gwhYshAQfJM3KNe16aJh9uX1L0Bmx9q6VY2tR0SjyyDtGMr4jKsVeYvcMQ9V3Qh1YS1a9ZphsBJEi2zCfjN5hc7ELsBGUhJJGaXSJuKOq2s5nbjWyE2DgmIZwiSdwKIlXpWTvrQ+aNAF3Fy5FIVvh6IS2wmEwsfnBJ2rH3x5mtqU3rE9ooLAPTsM8ZO2yY2TQbp3fGnmMmqWdXkOI1XxTC9gCOYm7HPo12Wc00kDfN1Id9jE0TOOQZTR+SLe2Q0AyXcVVlY0C5hnDFhOWvuUb7CcOYDdEBXO6p6e3A2DKBl0TU1hLW0F0Y1Q4F5NM0VfYUtlmMVUIOGWUJtkpB1uK9oDnCQ7bDoBKQJ3ODhAdLnwp3WhfzQWy5R0Vfy0KBlhoCoQEs2CpSOGQdPF3fHTD8H7KlHX1NqKUWUp3YyP6O8Lmxf1Wz1mHtSk0jLmDnPgQBXVjM6Sx6uNDhgOgXkHH6NWL40QDaaV0tX5lYvqzB9sWsb0tQUIK2fbeFPC8cJqXh9Sl0BvrWKpX0v2UNXmCiHzVJ9qE9kyD3R1MkSVpR0NnNpyY2i5qIHYIGK3YUSNNdZrzbbsnwuyuvJnlBrzHqnPQXV9HCw6P8GNnLqyMgbeTqCNsFhfPyZyt4OkCLU4V2iD7UNTmePONmJ0PMJS8MvXGUYd9vpFyn1NsCmBCIgJs5EV5NjZmPhSRuBDOA8sqX8t7UEZO5HAxR2GIIWCWumu2DdPpv9DOx19pZOg9SnO79i4xXvQrdkBPp6Mbz8aPxTCfpIs7j3UZLvbgzq3bnTqGCQ4jMC4HBKCnBRZMtKE5Kz61lN58vbF2b21NIWQgCCDCD3bZMXDSw2Iv4eBuXp1U9gbNtTHQwJ7U8c5KCLnO5m8lcKD3ZnFBGGe4pkCaSHt9XgVJKzVNpUOeYKktTGu9r5lkHkuiPHSDtsXXM2AIEgM6tLJjkAgIWqbXXzrT5djADGtrBf3e7Ddf6AMqxcb0hbxSmq5YnAPU0uCGWEwqsOrPc8wNOdpx5DbxNlZ2eIVCIYWBRUtmOHHa0kVhyxvVCmXDm0IlZhGd5D2q2fJHDxHIjCt0UFbMnUPfJ6yLJTiKPkOcAXmiFtOvGMqe9XqTfiYzTHOnUE8SCaIWPGwgpPGaFdUlVrTmYdVGLwBtPXNgQ2HsMnOGP3xrdFnNwbxGZPTqjTZPaqH0ZpCr4lOJhMnB3bErjY0WjzMvH9nOyfB6ZuKGCX1gSJiKAYHdKNX7HtKL44cPyGRc7x2ONEyVCbRiZzIBEO2tBjc0UmfUT2ZhsiHRPfPpRSAY1DaO0Ch7Gi7S5EX5YV7bb8Xv4mtvKTdWbJ2hxBCsPAHlxbGt0mTRN1CIUfP5tUjSW8CbQzDE0Os5PbQH7uA37dnmKwwLwlfH0MkULtA0N6Xy5QWYd2OkB6VG3EuLHWJx5xJbDGAr6KnvQAKCJtAuw0qyzYvfiqVxDIHwfQ7PjIOuaROJfpIp8y2aMvfhYUL2pAVW3BNwFzqL3mYOOHSrhVxVrDVaJVuhxQbtL3xvmV8t3r4EKa9ouHqSDIC9CvPqB6FEOBwLO5Yo2LtxTQ7qfmeDtCg6PjD6kYSBgr8ISDbVRV0MnNZMg3WfuZgcIKCTk23BNK4WPxkCwLTbPEqNGKsN8YWHyxn1mBQNcOLPeIMqFtONQH9Q2e6AE2lIwUm2sGJlRiZNVA7dfR2UOJaFR2W0A1JPqPPlLtm2Rn0BVJ54P16K2V6P1S7PCuvBjydrIkVIZv1uUK4QqU6bAkDSP4gyjAGfaSt4JZBBW7NjIyYH3SAW3AcJqZ1AYxrsmHp0w0K9S1UUXkXkWP0tmKQcMAjMLWp7wtQHJ71tBqc03Bo0K1nHBjj6JwjJQBXTCGIgkKr2D4rJIXdpWy0ALStT2mgwv6bXbA0WHmW9xlkvF6FF2fl5JhQK8Xqq0VhE3Cy7Z7ZuEmDxbhF2pJOjA3SPsmtNUAuh9DKW7qKQQ2XDwEyd2tHN5eWUOhOvHD3fVl12PZwJIUWZOQX95nfAnAEZTBYaLb9gJ5xlDBfmj7L0eN8OhwjRDKjjVQU2ZVKxVFRq2gAVYBjTW7jAQvkNSOq4ZyRT9JF6Iayk5KB8YAUH1i5XkDqK6HwN3yUYDZWZMPmZuLSmVhoP9i1vuMZ1zDcZk0Zd4fSXOWQw0lWErTjfsFPR4oZY6D3SWBQl9UTVBrEDnAgj0EYVSrnC7bPsnqhpxUuGAx3CQ5WjJbNtW6xKc0BMO4dAJqbUm9SGw6ZTuNTpeiv1FsPZCYvEuDWQFlQW2L85sNpVGJJQlDTu6cPVTL3AnnIKuwOJmVdWHjhYVtH4ZnSuEdA8cbs4cxoCvTO5lsHXIbbfSdYI6JDWkMI7QrRSEJOtZUBa6vDk7q6BRaOLZvEFBdn4GBvkQZK2mORvTWDvnSJ3kJTjXPctq8VOBTyMgT3FUl7w4ZBK7UEv8GXXLRoISXIL9XHIPt4nKBiAZHU2ihqKZHbR3uH1YJPv3JV1eTOK8ocl0H6E7kgEJTQ8oVtcbCwZrwi5QqiCGH1nmkGWxHNLmAKY0L8HF9Q4yNVwVw2Ov6NADKB0WjqXnEfVqHPIlD8GsXgnh3Mef5gI8PeDFmcnc4lTyNsG6qBWHgfYfQ8T3k0QS3Ok6eEYc0TuFEUzqT3MPSC1F9IpTvaxq3Jo3KcKdIMb3TJXwYW3E5M9WI8C57nAOnL02GiTTTTYb0efqg3wH38yWNWMDuBNvx2FvpQAqlwWcEgz7jLuFZFpbyGjZz3PjD1VSEe9r3yHH6kRISNr2cI4T1tZyoTICS5fX6NBV3RkLTZmq5SvkAgpa4IitZ9Jsu0Fx8UCJLxrgi1bLmdIL2xWxcdLIw5x2Ix5MD9qPPmUYoHR2n8pmCCnc7PjIZsZMjLAlJjMuSPaxMqObhZUHnmCV3PqFeSTeONA36nKWNe9Sz5bHcoTZCmfFvBRJUnybNUUM16cKhlMWMh4iyRUvgkakfyu9h0s1j7hbH6QEYRPjEgQAQ0WJZc6UhgnTZ8OTRLSNF0HsVyjXEOiYhy+

vLcW1tv6VYAlsXdw68MYBEY0oKr+67egukc9HKn7YDwAYG5l/GQTym/uUJ5TNGJaay4oR9ejiD/Uh
fURfLaYLv/TpvUodqQ60q6upTboE+soucfdKXdUZ0bluwPIcrWv2Avpn807WGrCvgmMiceGArbWX
CM15aymutfWvBEvg6DtHRTSiqPxqzzM7n9x/swjnI9VP7/zBV3/6ovmCAQAwAjL/MgjkN/cpFYWao
7Fhqb5O/df71dXVIXWknVBMF37p03+VOlQcafbUnnPfiV6yS9y9cjbvXDdged4h9gL6ZxvQb8C+Co
6JxIXDF1OoB8nW1lJca+tfCRbtld+9IqIRReVXV75w2U8O3CiS+aj18qMPf/vNN83XDACA1Mj8yyAQ
qnToMc/JJOS4XqewUFhq5rF/xl8fMeYTHcMvfYZcVa9b/3X2xqmBTSR5WVywdN3O7mUzTGK+lRX
72v7ZXCPKBrPtq9B/Q3rnsaeQ98uawrf80sxbwDTe+/Q9EY0oKsua4Gf8dr398kvNNw0AgNTI/MvA
EXZaOj6pszoH6eH6iIpG+pqesNRe6LrUn73CL30GXWX0F2f0eHVYDw21lV3URbMFS8ddkd37l+do
XXMsoH82a4T/QDutg2NV8sK+xci1teOtI81r59oGrAQLc+7COf6ifmo71C1PXCvS+GT1x9981HzfAA
BIisy/DBxhx6QjVYc52AhZUjgstRfq6XQ3Z4N6uvBLn2FXtbu1boe+omOmppHB0v0WlFzzdLSTOlrXn
Avo36x7hDF9d9We3Q26IT2L8d+eZog9o2Ntg1aCRdjc2ty3vk9EI4rKr3Y9e8VkP+O3ix/2AwBGQuZf
Bo6wI3VDlyMBmQOK00PDkj3OeaU4GH7pM/Cq8N3QV1UCd6whu8QFS/Mq9eeCfUFoed6NeRf
Qv9nuCGuIsVbn5YXBNyS8GLNTeZl6XU/hmtFa2+CVYFJbF7cOv3VYRCOKyq92nLz0kTtvEDl84uKH/
QCAMZD5gX6OOApsD8d+fUxEI4rKsu4+skck8IUUP+wHACRH5gf6kfmxPZ06e0rkIorKshb4M367+
GE/ACAtMj/Qj8yPbejMxhmRiygqy7rmucsfv+0mEbwXWD+98wdffv5Z8z0EAGBuZH6gH5kf283G5
sbuV3eLaERR+dWOk5c+eNf1InUvvF48ch8/7AcApELmBwB0bG5t7n9tv4hGFJVlHXzwOpG3l6TePP
Gz5gsJAMB8yPwAgI77f3W/yEUUlWXd+NTVImkvVX387jvNdxIAgDmQ+QEAreMfHhe5e5KKKyrUe+NH3RcZewuKH/QCA+ZH5F0v93XCVUf6CuPPrh9be
b/6cx99F191RR3Gq3p+D7xrUBn808vtEQTn7xdmdL+0UuYiisqwfPvwdka6XtvhhPwBgTmT+BRIJN
X1qahpklNDsHXWQ+eMN+mjk94mCtrG5sffne0UuoqgsFtPvwdka6XtvhhPwBgTmT+BRIJN
X1qahpklNDsHXWQ+eMN+mjk94mCtrG5sffne0UuoqgsVy7gXsV/9fLXP+wHAMyDzL9AI8clHYCzS
WiuHfVQGyby9xnw0cjvEwVl6+LWgTcOiFxEUVnWVSd2PHbHPhGQl7zY/J8k/sWJLAzIfMTSG
dA5t/Wjr5zVOQiisq1VuJn/HadOPzjvv379veCpuBBeNV4APjbDqcQwPbuDt2F/Zhs8rTlQvruuHbk59unIub2qx+CVV7qjrTm6F+j9tIY
IzxtiBrbOC3jxnTmteaWVz/7TtGX2RXqBYrWLNLuhL/APNhRsch/tuSOd8oTPEmK/dR72q4HUeQ3t1
Bgbfi+Kk51Yo7bq1akbzMmOMPUtn0eL04DVjFK/87hURiigq17rj0d0iS69Qvfb0seZLCwDALGbL/Pp
x3P8s7hzSyQueTDFYmwlWOBH4EpT7VMG4a44B1j31ze+evGRM0UlnSvBtSzKtPl8fEy99O/IS13uo
aTuD5MHeG+LefzvCcb466b4uvLGIXo3+FZfMUQbHBek+UTOvBum89+l7IhRRVK6155mdT+6/WQ
Tp1aqPfvlm89UFAGCwCTK/eN6f9yHenC4c+pad827q9KPukx6lb5wRkII3wDW/fa01v3WZuibwztn
Ttkdkn8C05hhXV9eOfIaOVeOMPqJ178rlFPoCPWd7NzrL8U3UXmiJ6dUeUc31VepIeyDQux1lbMJu
Zy3Jukzue9CaMYZzF87xF/VT26SufOGynxy4UUTolatjt9/6+blzzRcYAIBhkmd+m/WAPxcjnBTSzLkgo
QRlbkyO03GoZ/eh+Y1jYph6aUzuOCQkm1ZP1Oi2FHOGuG6km7UMcenAlZusdeptda7Qw7wTDeD
v5X0v7GsK+lhgLa7r+tupl4EbOGDNSG9za3Pf+j6Riygq17r3nr0iP69o8cN+AMCsxs/8aZ/ddVg41A0
Nq8hxZ0TcbOhN10cdAcnJdecdDcQwPbuDt2F/Zhs8rTlQvruuHbk59unlub2qx+CVV7qjrTm6F+j9tIY
sWBncS943NaTTzHmwy3X/HZeJYd1ldokFBdaMxLYubh1+67AIRRSVa912dJdIzitd/LAfADCTcf+3/Y6
nfcdDfWlg1FHXrL2v/jRLRloyjrvpzl1ioHuQzTG/61rP7C7GRF0pp21HWht07cjJsZ4ANW01Wl2qWw
xZuV6YoM571+O5MLS/iF7yvjmX411jS85T6m+nRrj4F+TqhYSO/fqYCEUUlWt991/+btV/xm8XP+w
HAAy3oMyvR5kCYaOhLisnUX/uv2pZOe6mO3eJrboH2RzzDwhXQ2fvSDetvqwiRrt25KLGDd5Ds7pi
vLq07z0xqRGFepC1zt45jCkqQwYO7yXHOJfTu0b3/e9vN2DiAWtGSqfOnhKhiKJyrR0nL33kzhtEYM6
g+GE/AGC48f8OP8c11qEBqaCkrqsvU696LlpeoTtjbkqOG3i3XPMPCFeuq3qlmlaNOrS+7trkwLVFbK
HZwKG1tfpS48re2ezNqyP6Esf9UYpTMzSL6yVndXXRxxxrVFzX9bdzXSUMWDNS4v8aP7V96u4je0R
azqZePHJf85UGACBoEZlfHXE84Afyhj1o0EVLzL4zBR3h5C7bbToCkpNr/lkCoTqgl2SusyvNtJ1L9IvAn
B5Db5BJr6bSmb9v5XIj7Xg9j3tF+sL2sHtgK66Xdd+sLRnzBO6aNU9hQDurm9zFoDUjoa2LWwd/cVB
EI4rKr2554lqRk3Oqj999p/lKAwAQtIjMr5/4HQIP+GqiNhfYR1aK5266b4+xR0dAcuvOVHUZFK70kY
7AW5NkWn1aTaKX387q2JGDGhVascWcWl7Ys/LuqkxqiBoh3zLflf6VR/Wy3wvPlkpyjaZu92q2Qe16
buDANSOlC3+5wF/aT+Vdu569Ir+f8et688TPmi8zAAB9Vifzu2NDLRRTllfgbnY3K847ApKPeaur4YPD

VXcFfa3mn7Y9YYzXy28HWjuyuTv3cjQzhW+IuayyrXrdLEG9dE1tXlrqXfXsvfrfi+JEaI0ts3k1Mqqd7DJ
4EqTE/3F+Ku968K7rRU7Opl48ct+333zTfJMBAOizyMwfDhdCNzFIM00FOKhPGJ8lbB+nPzktYhJFZ
VM3PnW1iMp51LHbb/3y88+a7zAAAAMs8vf8gVks+hoPghrmoj9gfJKwvTz/2+dFUqKobOrgg9eJwJ
xB8TN+AMCsIjO/UxXhrUDvSPjtNM0x/T8Y9v1bAMckNX2CqIY45v9a3f8JBPL10NsPiaREUXnUjpOX
Zva/8Odn/ACACAvJ/L6JvHkrkOwDp4ABjMxP4Me2tHVx68AbB0RYoqg86prnLn/8tptEcl7R4mf8AI
A4C8r8pe5kocgezPXBkwCAXhubG7tf3S3CEkXlUTf/8zUiPK9i8TN+AEC02TI/ACBLZzbO8Nf4U7nW3
Uf2iAi9cvXH33zUfFcBAJgRmR8AUHr9D6+LpERRedSOk5c+cucNIkWvUL398kvNtxQAgNmR+QEAje
MfHhdhiaLyqF3PXvHk/ptFll6JevnRh/kZPwBgHmR+hIm/jyH8cibqL89r/iKGeaaaVefv6h/9L4IodjbB
nkpT3kNk6/Bbh0VYoqg86pYnrhVxevnrp3f+4Ks/fdF8OQEAiELmR1g45M8TMheV+XWj2riRv2k2U
Qaf7B4iZ5tbm/vW94mwRFF51Mr9sJ+f8QMA5kfmx0xEqpwnZIrMP5kJ+06dwcn8SOPTrz7lr/Gnsq
wrX7jsJwduFLl6aYuf8QMAkiDzYyarn/mnzMVkfqysD85/IMISReVRe57ZuRI/7Odn/ACAVMj8C+GJu
87DOsdV+q/oyX3qdHuFfURNWx4R04VfhunBhWK8WLtzKvMSc4FBnYs6V6mWBs/CzcWJdXd0e5n
dfL3EtkvW1o0x7TTNSX9Hx0QeYo7hmyoMWX8hOEetM8Q+P2AGjOvU2VMiLFFUHnXb0V0iYC9b
8TN+AEBCZP7FcAQn18E28JmMiwYGMJM6r0/rJnqS5kj1WkwXfhng3kqpaWtNpQ+YjI06he+Y46xn
4b3LLQWb+Xqp48Y81tYd19bDgx2HvR3O22pc0dMidv0lY1V9b27/DJjE0XeOirBEUXnUvffsFTF7qYq
f8QMAEiLzL4jKPI6Yow/p3KOO6KBkDTGmsQKY1AxQlxjpqrlCzVC9FNOFX3rpcbJHoVlI38yOrVoG3D
F7Yif7rrRHxBZm7OXYhzXM7l7p7ejq12VdIm5Zb4sh65dDrEnkBdYVvTNgIlsXtw7+4qAISxSVQS3zD/
v/49//rfkGAgCQApl/UVSK0SHGylKOcNWbrgpWnrI0I+pr6hdra+VE5hF1vZgu/NLHNUwfa9beHaNe
OTZmHBKG3DH7gJOayhjVe6E9wHXJkLdMd/fvtSIvjFhifcg3PrA2//r1S9/y1QDHFPWh3hkwoQt/uc
Bf409lWd97+ioRtpeh/vWfHmu+ewAAJELmXxgRfFSU0unLka0KnquMQTov+XNcc1U1oPrzofX3q6v
aI/pyMV34pY9zL+Jgdyp10sXXy9lFz6uODluy7t+OCl3YXa4e4LrEsUprmKO75OzYuzdHcz/3pgasX79
udRp2J+6qp+ibAdM6d+Hczpd2irxEURnUDx/
+jojci61nDx38rz//ufniAQCQCJl/cVSuqcKMikFtWHPHMxGvBgQwl+ayYkQ9uvhDfUgf0VeL6cIvfZx7
EQe7U6mTLr5ezi7WEoct2TGXfaE+ItgDjF5DZnbvpNDT0dWvwztxq29T89yZ5ryawkVNEZ4Bkzv9y
WkRligqj3rgR98XwXuB9Z+//33zlQMAIB0y/yI12adITyrhGJHGHc/UwObooADmUF+n/uv96urqkDr
STiimC7/0ce5FHOxO5byih/saNa86OmzJjrnkhWpIoR5lz+zqNWBm3+57O7r6dXgmbvVvasj6NWO
6Sn1R7yoM7hmwCM//9nkRligqg7rqxI7H7tgnsvdCip/xAwBGQuZfqCbQHFpbq0OTmZl0kDJTjkxXj
vSkQ5Iv95Waeeyf8ddHjPlEx/BLH9cwfazp1R0zcOIOOWPFmmjYzI4bKy60R9h33tVryFvmGFPo79i7
N9+C1JT9LRxD7DGF4pjxqtu3d5mV0AxYkIfefkjkJYrKoJbhh/38jB8AMB4y/2LptFQRcUafVPFK5x6dt
/QRda0xYTActRe6LjXynMha4Zde1jqN/k0z38zqCr28QKsBd0z28VAz+e+EXE+74HZmV692XHPQuO
9qmKN7ob+jq1+X1b17YMCmrBms9esD7fLFhvxzDJ3BcQAT2Lq4deCNAyIvUVQG9Y8PfVeE8CmLn
/EDAEZF5l8wnW4KKlW1zLMtM+UYoUyypzO1F1opyt2gni78MsC/0KabNZX7ip5GM9yx4EyORCkv
dLcqtTN3x9TH/XdCX+joXurtOGRvnvZNqwGbmmH9Urss9yTt+f4ZPPcIY9vY3Nj96m6Rlyhq1WvHy
UsfvOt6EcUnK37GDwAYFZl/0XS48WWXbjpqM49mDihO65eOoSZ7nPNKcTD8MkwPLhTjRWgLd6
8MjHfdi+TKhi3ZkSgdF5rJtDyqXhtTm0P0ZOYKi7HWzI7ujXDHYXuTd0i0Cbeo9K2/ZE5TspfUXUV4r6
XuDOqsfR3GdmbjDH+NP5VfXfPc5Y/fdpNI4xPUR798s/lqAQAwDjL/oqngQ3QBsCpe/8PrIi9RVAZ14
1NXi0A+dr329LHmSwUAwGjI/Iul/6tOIj+AVXL8w+MiL1FUBnXwwetELB+vThz+8V+//rr5RgEAMBo
y/6J0/4fL9v/wGQCW2+G3Dou8RFGrXjtOXvrInTeIcD5GHbv91s/PnWu+SwAAjInMvyhG5ifwA1hB
m1ub+9b3ichEUateu5694sn9N4uInrz4GT8AYDJkfgBApE+/
+pS/xp/Kr2554loR0dMWP+MHAEyJzA8AiPfB+Q9EXqKoDOruI3tEUE9V/IwfADAxMj8AYC6nzp4Se
YmiVr1G+mE/P+MHAEyPzA8AmNfRd46KyERRq17f/Ze/S/7DwCYHpkfADCvrYtbB39xUEQi
lr1uu3oLhHa56k3T/ys+cIAADAhMj8AIIELf7nAX+NP5Vf33rNXRPe4evHIfd9+803zbQEAYEJkfgBAGu
cunNv50k4RmShqpevKFy77yYEbRYCftY7dfuuXn3/w/fE8AAJgWmR8AkMzpT06ntk55w
/7P373neYbAgDA5Mj8AICUnv/t8yIyUdSq1x2P7hYhgXHUqtcDP/q

+CPNDip/xAwAWjswPAEhs6+LWgTcOiMhEUStdV53Y8dgd+0SkDxc/4wcALAMyPwAgvY3Njd2v7h
apiaJWur739FUi1YeLn/EDAJYBmR8AMIozG2f4a/ypzOqHD39HBHtf/Z//dbL5JgAAsFBkfgDAWF7/
w+siMlHUqteQH/a//OjD/IwfALAkyPwAgBEd//C4iEwUtdJ1zXOXP37bTSLkm/XTO3/w1Z+
+aL4AAAAsGpkfADCuw28dFqmJola6bnzqapHzzfrjbz5qPvoAACwBMj8AYFybW5v71veJ1ERRK10H
H7xORP263n75peZzDwDAciDzAwBG9+lXn/LX+FM51Y6Tlz541/Ui8PMzfgDAEiLzAwCm8MH5D0R
qoqiVLvHD/uLP/IwfALCEyPwAgImcOntKpCaKWum65Ylrdea/8amrmw86AADLhMwPAJiOiEwUtep
195E9deYv/tx8ygEAWCb88wkAMJFLLpnoHzo0ikajWX37zTcvHrmvzPzculg0ikajaDSKll+j7YBbCQCY
CE8k0WgUbYJGX37+2bHbb+XWRaNRNBpFo1G0/BptB9xKAMBEeCKJRqNo0zT6+N13uHXRaBSNR
tFoFC2/RtsBtxIAMBGeSKLRKBqNotEoGo2i0SgajRDArQQATIQnkmg0ikajaDSKRqNoNIpGIwRwKwE
AE+GJJBqNotEoGo2i0SgajaLRCAHcSgDARHgiiUajaDSKRqNoNIpGo2g0QgC3EgAwEZ5IotEoGo2i0S
gajaLRKBqNEMCtBABMhCeSaDSKRqNoNIpGo2g0ikYjBHArAQAT4YkkGo2i0SgajaLRKBqNotEIAdxK
AMBEeCKJRqNoNIpGo2g0ikajaDRCALcSADARnkii0SgajaLRKBqNotEoGo0QwK0EAEyEJ5JoNIpGo2
g0ikajaDSKRiMEcCsBABPhiSQajaLRKBqNotEoGo2i0QgB3EoAwER4IolGo2g0ikajaDSKRqNoNEIAtxI
AMBGeSKLRKBqNotEoGo2i0SgajRDArQQATIQnkmg0ikajaDSKRqNoNIpGIwRwKwEAE+GJJBqNotE
oGo2i0SgajaLRCAHcSgDARHgiiUajaDSKRqNoNIpGo2g0QgC3EgAwEZ5IotEoGo2i0SgajaLRKBqNE
MCtBABMhCeSaDSKRqNoNIpGo2g0ikYjBHArAQAT4YkkGo2i0SgajaLRKBqNotEIAdxKAMBEeCKJRq
NoNIpGo2g0ikajaDRCALcSADARnkii0SgajaLRKBqNotEoGo0QwK0EAEyEJ5JoNIpGo2g0ikajaDSKRi
MEcCsBABPhiSQajaLRKBqNotEoGo2i0QgB3EoAwER4IolGo2g0ikajaDSKRqNoNEIAtxIAAAAAgDyR
+QEAAAAAyBOZHwAAAACAPJH5AQAAAADIE5kfAAAAAIA8kfkBAAAAAAMgTmR8AAAAAAgDyR+QE
AAAAAyBOZHwAAAACAPJH5AQAAAADIE5kfAAAAAIA8kfkBAJjV+2uXtNbeb44CAAAsGzI/ACAjdRg
fN4WfXz/Udhiz4dT/ZqHcWOHQ+vnmQEqdzVT4dyWz6tzDcd6m0Xm+MM1nr5b2kxH4igZOzco9V
ecdS7utcQXvzKj/QTEi/6ZW9X3y7KiznVRv04BeK/qfSdsEmR8AMK4Rn+Y7jD5TPrRVbcd41imepfQ+
queqkR+oyo0cWlsr/p9R+pRbmPyR0HweTf+p6HyyWyN9+qq9TPSJMO9buibeb2j3O9Td6Fy8HYO
nZuWbqjyuD9S3dN5W7lnMt2vuFr13pjyf9D8oXJvqbKky374Cm6pOJfwq2dsxmptG2lHVXx+qXs27
u2G9ErSq711D3J/OXRTnMACZHwAwou7zVPdpJCnVqPr/p3wg6O5wLKN3KRs0t2+cNuV7P/ptMk3
zxnRULUf68Mm5x+uV+jm+od4Pe+Hyo5Fqa/6OoVOzGjqVGte8nFl1fa3bpbh9+sD8b1ffdsrjzem5+
tRCm0oxfyOwKceheN7tCPM39e9IHhmxlzrRvJz3XSun09NXn+T2ZbdVdXKuPW1HZH4AwHjkM4D1
1JDc+B2EaR4/Rt6Wnr78Q/xDW8h8j4Mzm/xzUBj3o9C9gaPtr5rYfKNSv3HWyu2tJL6RgXuV8Db2T
VWdj76R6upxuxjcjfTR8g9zt1GLdfQa5z8u7EZJ9tEIbKcr4ee799b1LmY4ayprHwmb6btZvZAfh5SNtg
0yPwBgNPY/mRM+7bhN/DBQ7SfVM6NX5/EnPeOelX8cp1F1p1oj37Px9uE18ptUqG9h0aL+wzifcu
sbanw6krDmKzt271viIoHpEnbqm8q6sVH6uiTbkGsi41j5x2Qfdkcv+0ORgt2o6lP+UEGbv6vr1hmq0
6n25uxVfdTKFvUf/EuZjdXLczvTbM28Te5GyTa2TZD5AQCjsZ8AnI8oKY3ewFQ9eKR6erNVe2mMuK
VyF2r6suV4G1LqjY3Zp/rkJX6UD5vmg1d94ipjtbL3YX+L52I1sOdPfC8D0yXsFJ6qeuMS3MVwl+psm
vfK0ajcgzpSnk72ofD0MiRqZTcS70s1wH9/h3FsxxA+OyvfbO39S9bK0as6Iu9eoveq2kHTrfxzd9q0d3
F7IPMDAEazgH9UT/csIJ4WR5X0Yaqr3EZ7v8pGk+zJ/mgkJd6c+v6N+bGoGo76uTP3UP95nH7V3PL
WJXyrqgnNldsfBWvIfALTJewUmkp8HOfg7FIdbKS6bVajcg/t6/J0sg9F6NYV6u2l6GY3sj58Cb7Iwe0k
mN/k6FXfrvpY/edE/Zz7qvajrK11PiRzqKbVb4z1LoXvMZzI/ACA0SzgH9UTPQvUTzoTPnPUD2/pG3Y
e2QwpnrBDqr7j3T/rkzduv+rdGfWW2R1G3FHnQ5HuOb5mfUPtr6z17s3HbqAFTs3KO1V1N1Ntp2f
B1ekkzWSjzmfCMEYvW6JPhN3Imrh/Lb0CU1SnUn0WSlYvu0P1zs23o1r/rSlHpNid/MrYb3//WiCR+
QEAo7H/wZzo2c1vimeBqsfEDxxVz1HvXKVsM36X8bczxqO8X7Knaj+7xag7MqT+SNjrlm9W6p0F5k
vYyj1V9b4l3EzvgqsBCRr2NCpPJ/tU9G4q1TfMblQdMTciP4wR/NtJtA2D1ctu0X93B+qdqBww/4ei2
kF3Grtzgrdp2yHzAwDGI//JnOzxw2v8DvUziZT8+aPoYmzD8Rw0ijRPbbZiXmPa6k0a9V2qOpgbGfM
ZsXpzxv3M2Z+ASZoWkn8iqremu/DO3qy3bm6Ojkrg1KwcU1WH0r5JvQuuBiS4fT2NytPJ3qSFbqrz
RUryrfJuZ4TvrN2r82UqpGsafpvSvEfVau0m5WFz7vBS4ETmBwCMqPMAkuapwK2aW1jtZwKxo2k2

UzYd5R2afDedR910z722MT/VXfXzsDbBJyLl3sQnoNJuwdxbqpsZ6BhezEz8U4n3qzbn5qpunZUWX
YzXVc95egy8M+WwZB96a1PFAWPyekmORQwW3lTn7DxtGtZ2atXhCT7byf+Dwt+re2buW1dP55y
m87lOeie3DzI/AGBc5gMI/5zGZDoPpHM/j/pUH+/RZl+AxM/xSKL7rtSa90acWqF3zL+pldxT4D0qZP
cfFMmJf1VRax8ZeJCYE5kfAAAAAIA8kfkBAAAAAMgTmR8AAAAAgDyR+QEAAAAAyBOZHwAAAAC
APJH5AQAAAADIE5kfAAAAAIA8kfkBAAAAAMgTmR8AAAAAgDyR+QEAAAAAyBOZHwAAAACAPJH
5AQAAAADIE5kfAAAAAIA8kfkBAAAAAMgTmR8AAAAAgDyR+QEAAAAAyBOZHwAAAACAPJH5AQ
AAAADIE5kfAAAAAIA8kfkBAAAAAMgTmR8AAAAAgDyR+QEAAAAAyBOZHwAAAACAPJH5AQAAA
ADIE5kfAAAAAIA8kfkBAAAAAMgTmR8AAAAAgDyR+QEAAAAAyBOZHwAAAACAPJH5AQAAAADIE
5kfAAAAAIA8kfkBAAAAAMgTmR8AAGC5vL92SWPt/eZQTZ04tH6+OTIO1Ue0XwbtvSlY9+H8+iHfq
eA5AMgXmR8AAGC5GLm2G0+3e+ZvY3vFdRu8yb69qUv4bzIAYDxkfgAAgOXS+e+yzYS63TP/oP07
w337rwJI/AC2GTI/AADAculkfjPgbvPMr4N7z7r0/dP3SR8h8QPYdsj8AAAAy6Wb+Y2gamV+64AdjJs
h5Qhj3uYC44iZhtXh4pjxP6c3mijGWeu8sba2TU/m7szXmdBYqRKYS4+uxuhX1g5C6y911yOGBDYY
vA4AJkXmBwAAWC5tmFyv/6Qio5Eyq9f2ASNuNpG4jaO9dIj2X2PkbJlrK8ZCHJMEo6+7qdy6wViLp
V3c2rr6o+jet/6eBfk36L4uuFwAGA+ZHwAAYLmo0FgkSBVM68BonKgG2gdCmb8ZZERSeUTFUuua
9og6INvoEXopRp8BcdffQE9o9wzRgxvtNJXe9asB/vV4NuibSK4AAKZB5gcAAFgundTYvDD/bMdOI0
7KMKuGtLnUf0RNY4+QY2QiLshDehJjjJdcQUnuxD4QphdQEpcMWL9kdXdvUA8btG8AGB2ZHwAA
YLm443URNa1k7IjKMpraQ/qPqNdmUO7Oq0a4iMadWTzsJZVUS3VUbq2XfwkD1m/ojlbnPbPrVb
a62wKASZH5AQAAlotMwM1r/fP+NkPKkYWxMn/3oHrh4m3s5x4rtxKd+e0lDFi/K7xX1Hnv7J4Lh6
4aAJIi8wMAACwXK0zKEKlPOGKnDMb2kP4jjmnFvK4RwoAhmnusaqmOyq318i+hf3FqRKEeZHXvn
cOYouJvBgDjIfMDAAAsF0eY7MZHfSI0MkHmN8N1d8yA+G238dPTmYOtHgkzf+9U9qXWTQlssDhl
TDzzugEgITI/AADAcnGFSZ0bS/pEe7SJkzqYWkfs8Oo/4p9FD/F3VsHWbhPib9BeP3N2Diyhb/1yO+3
4ng1aGwkuAwBGR+YHAABYLj1h0jxhRFFJZNlQBJVH9OtDcnaVdwvu1u0Au02YGt9lXp0y8/eu372c
khrim9135dBVA0BSZH4AAIDl4gmTbUjtnDCzaxErZTC2J+s/oI6b01nLKXVzc3eAZxdB3flkSJZb69W7
hND628tLZUvjtpinXbObl5aGrhgAkiPzAwAAAACQJzI/AAAAAAB5IvMDAAAAAJAnMj8AAAAAAHki
8wMAAAAAkCcyPwAAAAAAeSLzAwAAAACQJzI/AAAAAAB5IvMDAAAAAJAnMj8AAAAAAHki8wM
AAAAAkCcyPwAAAAAAeSLzAwAAAACQJzI/AAAAAAA5+tvf/j8+23+IN6wWUQAAAABJRU5ErkJggg
==]]></redcap:Attachment>
</ItemDef>
<ItemDef OID="b25" Name="b25" DataType="text" Length="999" redcap:Variable="b25"
redcap:FieldType="descriptive" redcap:BranchingLogic="[q24] = '2'"
redcap:InlineImage="1">
<Question><TranslatedText></TranslatedText></Question>
<redcap:Attachment DocName="B.png" MimeType="image/png"><!
[CDATA[iVBORw0KGgoAAAANSUhEUgAABVEAAAEJCAYAAACdcmdaAAAAAXNSR0IArs4c6QAAAA
RnQU1BAACxjwv8YQUAAAAJcEhZcwAAEnQAABJ0Ad5mH3gAAE9sSURBVHhe7d0PjCRnfef/b+/aY
PPHNmBskx+RHat7sZYlGJIg08PxL4egZ+6SJbnbYOVg5Oh3PVyi0zQSkwOxF+mX2wguG4lunS5iWrq
zliQyTEJugrLdIJRgckzHR4IxYTI/Z7rlc3T8wDYGjPljB3u3f/VUPVX1VHd1dVV3VXX39PtltaerpqfqT/T
O/OZ5/k+hb5FAAAAAAAAAAAChjumPAAAAAAAAIAQhKgAAAAAAAAAAEIEQFAAAAAAAAAAAiEKIC
AAAAAAAAQARCVAAAAAAAAACIQIgKAAAAAAAABEIUQEAAAAAAAAgAiEqAAAAAAAAAEQgRA
UAAAAAAACACISoAAAAAAAABCBEBUAAAAAAAAIhCiAgAAAAAAAEAEQlQAAAAAAAAAiECICg
AAAAAAAARCFEBAAAAAAAAIAIhKgAAAAAAAABEIEQFAAAAAAAAgAiEqAAAAAAAAAQgRAVA
AAAAAAAACIQogIAAAAAAABABEJUAAAAAAAAAIhAiAoAAAAAAAAAEQhRAQAAAAAAACACISoA
AAAAAAAARCBEBQAAAAAAAIAIhKgAAAAAAAAAEIEQFQAAAAAAAAAiEKICAAAAAAAAQARCVAA
AAAAAACIQIgKAAAAAAAABEIUQEAAAAAAAAgAiEqAAAAAAAAAEQgRAUAAAAAAACACISoAA

AAAAAAABCBEBUAAAAAAAAAAIhCiAgAAAAAAAEAEQlQAAAAAAAAAAiECICgAAAAAAAAARCFEBAA
AAAAAAIAIhKgAAAAAAAABEIEQFAAAAAAAAgAiEqAAAAAAAAAAQgRAVAAAAAAAAACIQogIAAA
AAAABABEJUAAAAAAAAAIhAiLp0etJYKUih4DxWGj29PqjXWPFeU1hpWF+1iMxj3ZB2onXjpLWdL
MxLO7LSlg372PzHqPs4Vb2GrGzEO5vtDbdtK5JH08KNuQ9Cjmc+2g0AAAAAwPwhRF06Rdm8UJey
XurU1kPCkracr3X0c5Hq2U3rq4B5oILBVWnqJUdZzqxle4faf1Qo1cT/rlhsR+14AAAAAADIGiHqMip
uytmqfi4dqZ0P9kbrNc75IVW1JdsV/XzhFGVzry/9vnpsS/aHkff+RpmXdmShKwdu8leuS9c+xj3ZzDJ
D7TVk3fijwsI7ascDAAAAAEAOCFGXVGW7JV6O2jxn9EY1e6FWpbW4CSqOot6h7OuncuoEPaQBA
AAAAEAuCFGXVkW2W26M6vdGbW/4Q6XL9a3wXoyqlqKuRek+wmpSjqyvaH59ZI1Jo6ZjoC7rqPX
GPu31edcGHd5fZI1J4zwEz5+5nbDPjxN+3INtCdS9tR4xy30ObGewraPOc7xjCm57uPZpYAh6c9VZF
2h4snM3eA7Uw9yc3Z6x+xzPPy71CLkXbAnaHvI9OHq7vqTHE6/dAAAAAAAcfYSoy6yyJXW3OGpz
VTYaDTnnJ6hyIWSM9FAIo3VqpQwmoCrK2hndwM6BdJ1nFmNId2dHLno7bcuubn/5zNpc9FKsnPa
D6gP/AGy9izv6PBo1Pe1wrCSDo63TPb8dqZUKUhrYSXM1aUimtjPY1qasDoZtEx2T2rZR+9S6H+te1
+kREu3HCSwHz4HSXB0dKibnnOtV70AUtW6Kc9TeGFHPNGS7E4vZbgAAAAAAlgQh6lILTjLVrLnBTF
nqF0Imk2pv+KGKV4+yL103ie3UZD3lhKW4dka3b18O3U23d42JhYxw0hjqferEnAz0rpz2yiY0d4M9
Ji/u6BisfEacDLUnjXX3GlSlpc9v3+0xnOr59bfvdUhWIdlAfdzx/O1494Hazrob/E1zTMbr9zZlc9v62PX
vV1Wv1/6cXXIi2X56jXUvsCzXu85r+8MlLiqR+4wr/BzteOl/krZbr3X/0hH2PRjY7rBkxzOu3QAAAAA
ALI+C9QtyXz/HklK9SwM9zlSwEhKq+K8rS71rTubTk8aK24tOBS/OZEYjX6963bk96Ubsy+dvW4Vde
9ZG1BBs1YOwWq1Ks9kcWu+3IaxdcdeNE387w+2yV3rnwG1/6DqbsV0VnO2FBNwB4e0wr3O11fc
nDEt0PSK2E9ivvuaS7JjMbQdfr41qa6JzF35+FOdanQqsS3p+lETnOlHbRwht44jjjDietO4RYBr3Pnyvv
OXCW/QSACAP99/xLnnNW2/SSwCAzB3/NZFjr9ILWBT0REVwkikVtoQGJP5Qeb/npMsYdm/2GE1F
UU6ccp51dJfTrj2WvywnT5+0e9QF11uqp/0AbA74vWmb4nVG7R44gZT1GW8ov7dOdT4sGbUo3S
BMfcIsazBr1jUo6ac28z7QPYSnOKZEvYkT7cec4f+kmIdQ3NyTvhmgTm3wHIWY6rrrurFuwJmaGO0
GMvDwEw/LOz/5Tr0EAMjDDY9fJXd/6AF5/Bs/0msAAJm7dI9I/zt6AYuCEBVBA6FScsO1P6fl1RVt7k
pbenJoj9k/JScqJ6z/D65XGeqc9ZIrrombLbpD+ttuIl09a/TozUtaAZl1DTJrOyHeSKpXqBe0GnVjgQX3x
NNPSOWPKvZHAEA+nvPjY/KWL94oP3ryGWm+/3555p8u6c8AALL1lMilP7A+PussYiEQoiJlGYRfXl3
RfTlsXxS7lKgd9lbEyVeN9XMZvhk9NO3A1+/VOyrwVcO67VqVQ480e0lOK1mv47yOaTHOXbjxbW/
Lhtnr1K2LatY5BRbUnZ+6Ux58/EG9BADIwxv/
+gZ5wQ+vsJ8/9NXvyp/WeR8GgNz0Hxa5/Od6AYuAEBUxleSkN1LbnBFfMSZJitM70Ri+HI+7744c7D
pf686+X7I/4a8fLjUwH4qbZ/0guOFOjFWVQIZacsoTKG6Jgvk22OvYvA/0seV1TIn2Y97LA8Pk1cz3hZ
xnoE/SdmNSNTtwdeukJv6eAubL+z77PvlM7zN6CQCQh1c+eK3c/PXn6yXH5z7+kHzlLx/RSwCAzF3
6gsjlr+kFzDtCVMRUlM2zbuVUc/Z1NbrYnOl8y+vt5wScijnre1s23JlrYvN7cqqJpBS3XmZRF0x117vh
6vxxe81a56Kmj3+wdqsx7F+aq7LhnTIV7Onh297K+dBcNQLH9nm/hqd7bHkdU6L9mLVbm7Lqre/p
me+ta1Qq+NvI2oTnyA9c3XYDi+kT+5+Q+n11vQQAyIOqg/pzD7xELwX94W9/Tb73raf1EgAgc5f/RK
T/fb2AeUaIivgq29LyctSalHTA48w8bynX5YJR4NOfUMnSXNWB0GQ1HAPbsp55Q/a9of6KMUnTHP
Jqu9rKUt8KRKiWomxe8IdlN1d1gOaFzqMm/ZolJ3AMttM8tryOKdl+ipsXpO6/2HmtOZGTdS/7h6Br
7yrua1NNWBO03bzfw9odR+bHA8R339fvk7v+7C69BADIw/FLBXsYv/oYRgWozd+8Xy8BADLXf1Lk
8sf1AuYZISoSqWyH11+stqz17tBiV3FT9gZfW21NVr/R7K0XKBlgDM3OdKKjFJgB2KiyA+qc9bt+wOe
ya1/OW03PstS7fT9Yt1Wl1d8LTpaV1zEl2k9RNvf60h16sXq5MUzeVpHt4EGK7B96PbFTEbvtVlv6Lf
8+sqlzblwHu+5ulByOB4jBnYn/6Wfp7QQAeVr50kvl2iev1Evh/uFL35ZP//6hXgIAZO6y9RvZJcpbzbt
CX81aAgAxtTcK4nSSVCHqQGAKADGo4PT1/+318sAjD+g1AIA8lB56od0LNa6tu8vyiteFD/sHAGTgit
8QKfBL9ryiJyoAAMiVGsJPgAoA+VK9T1Uv1CTUsH7qowJAji79AfVR5xghKgAAyM1HvvgRezIpAEB+
VP3Tt33hZSProI6iAtS7/+NX9RIAIHN2fdR79ALmDSEqAADIxe6Du/LBv/igXgIA5OWOL18/tg7qKPv/
8zH53Mcf0ksAgMxd/n9FLn1BL2CeUBMVAABkTg3fV3VQmUgKAPJ189efL//8Czfppclc+dzjsnX36+
XWV79IrwEAZOtKXR/1Zr2MeUBPVAAAkKknnn6Cmfg
BYAZe8MMrEk0kNcoz/3RJmu+/X3705DN6

DQAgW9b77aWPWx+fchYxFwhRAQBAZlRwqgLUh594WK8BAORB1T99yxdvlOf8OJ1f+R7/xo/k7g8
xKSAA5Kb/HZFL1EedJ4SoAAAgM+/77Pvk3ofv1UsAgLz83AMvkRsev0ovpeMrf/mI3PtJ/igGALm5/
DXr0dELmDVC1KXWlo1CQQrGY6XR05/LSa8hKxttveBob7jtWZG8mzM3Qs5LHL3GinPuVhoy6tT55
9d55H7NjwLuWyCW+n11+djffkwvAQDy8vJvPE9e+eC1eildn/zPB/LN//19vQQAyNylXZH+Y3oBs0S
IurR60lhZlaZecpTlzFpRP8+eHfiVasLfVIImPi+9hqzXor5KXfOCrAYvunRqJSlMENguK+5bIB7V+1T1Qg
UA5Ovqp47LG++bvg7qKKo+6u//
+y/bHwEAeVD1Uf+79fFZZxEzQ4i6tLpy4KZA5bp0+33p9/dkM68MdWzgt6QmPS+qZ+S4YK99XtxN
V1vqevelWy87K5qrQo4aA/ctEIuqf6rqoAIA8veWvRvtIDVLqifqH/721/QSACBz/UdFLv2xXsCsEKIuq
96h7OuncuqE5Nf/FGmL1zOyJ41zugtquS5bFedpcfOsVJ2n0twlRQUwPTUTf+WPKvZHAEC+Xvu1F8
vLHr1aL2Vrb/f/2A8AQE4uf8l5YGYIUZeQXbvRDN2aq04tx8GuiKp3o1E7c1T9zGAtyGCd1bDejbH3r
wVreI6qN+kMVfdfl6zW52A9S6+2qH6ENy3GPgPncEP8zZjnydln0vOiqHaWYvWMNHoeB0LzkpzUn
VFl/9A6olGM9obVW21v6GMZPAcJrkvI/RZ2vePcb4PXz35E1ImNY5b37WT3p2vSa2eZ6D1Ar1TMr
49uJI6QOz91pzz4+IN6CQCQFxWevubvXqSX8qF6o1IfFQBydOlPqI86Q4SoCDUUGGl2/cyRYVRHai
WjzqrR43EyanuDNTzVurCgpuQNVXdFt3UUZ5+DwWRzdcJ9Fjflgjtk3joz5/RGeo1z3nkq1y+kUEahK
q1+y+tVOsTseTxK50C6+umwipx2N97ZkYsDJ7W96x2NX1c3yXVRQV7I/eZcj1EBZPj9pu7d0GC5U5
NSIMjOSpb3bcz7M2CCa2eZ7D0Ay07VQP1M7zN6CQCQFzV8Xw3jz5uqi3r3h75KfVQAyI2qj3qP9Z
H6qLNAiLqEKtt96Xfr4kZ7Um3Z9TH72zrxbG/4AZBXL9Won9mpyfrIxEaFec7r+3uboWUCxu4/wN+
et3/pyI6XBPWkse4GPca+Wzo1imzrKP523M2ofdbOu/Fbsn0WNy+If+rOS9v677wbglnn94JOUJOd
F4P9um2ZLK8uyolT+ukYlS23beb5V9ri53BnxMnhkpwj67VGqYGh+21ofyZj2/b9ZrYlbFt+kJ3U/Ny3
/uvD789hya6dZar3ACyrT+x/wp6NHwCQv5UvvTTzOqijPPTV78qf1hmBAAC56T8scvnP9QLyRIiKIW
bPtPoFPwgdDgOHletbE4Z54aotPxwsrp3xAqzOge4z2bsoO14eaey7suW3deeiJIl7zH364ZMh8T6Ls
nnWTbuaslpwe08Gz+8kipt740PW2PblMOpEFdfkTNjxtXf9XrVn1pzjSXSOrPOzp0NEI3gPvd4Dhu43
s8et0evSPk86CNzLYfa0LO/bsfdnmCTXzjLNewCW031fv0/u+rO79BIAIE+vfPBaufnrz9dLs/G5jz8kX/
nLR/QSACBzl74gcpkJ/vJGiIoBI3qm2Yqy5iYxIwK3UyfSDKjKcrKkn47SPdC9+VSoU/LqNhbMYdKRw9
QnMMk+K9tGr0FHOsP403RKoi+fcf2NgNIP3Kpy1j2gqa6LruEZOrw/aOh+M8JCawf20Hd33yNrsaZ
uHu/bBNduyvcALB93Jv6nn31arwEA5OWGx6+Sn3vgJXpptu7+0APy+Dd+pJcAAJlTw/r739ELyAMh
KibUkeHOgTHCo7mX3TFUvMKUSrD+5Oz05HBssVSf36vSHRZuBG7V08FeoUmYExB5PXXHCbtWR
dm8EN470w0rF3t+o8nvz/SvXdh7AJaNCk5VgPrID+h9BAB5e86Pj8lbvnijHL9U0Gtm60dPPiPN99+v
lwAA2XtK5NIf6OfIAyEqJjR/gWm53vWGbAcfk9YLHS/
+Po26n7aO1NZznJyneELGlj4tn5Sxl7S4KW5lAntYuDF8vno6/CyPP0dt2TB7nbo1OM36o0lYbdzT+xj
s/as0z83XpEi53bcTXLtoR+GPJpiWGsL/wCMP6CUAQJ7e+Nc3yAt+eIVemg+qPuqnf/9QLwEAMqfq
o15iYte8EKJiQElOusnV0EzePbnoFnIcO/Q7J6WTXtA2qm5m6ibYZ6+xrodpl6Ved5OsPCfnMa5rc1f8
zphdOfAu6QmJc0m9HrXW/XH+/I4OP6sSyOGSnCOzLqcKFN26qMaQ96TUzPKqx6k9GZQdSHa9W
p7pDpOf0CzuW0usa5fme8AU1xDz7yNf/Ig9mRQAIH+lh1448zqoo3z6v/6D/MOXvq2XAACZu/xZkf
48dRU6ughRMcCcBCnYW9IPAlXYNTChz6yYNTCbq/5Q7faGHhpuPdIev510n72GrLsnrnpWNjeNyY
Nq65JPjmrWsmzKrm5er3HOCzBj90asnBbnDulls+ke18Bw8Amvix8oDvbcjUlvX80sH+xxaoTFZo9b
oz1p3yaRZnHfKnGu3QTvASU/dZXaee9gZMOb4h9Hze6Du/LBv/igXgIA5OnaJ6+0Z+OfZ83fvF+
+9y1qZQNAbtSw/v739QKyQoiKYeYkSJ2alHSoU/LTE7kw7YxI5vDy5uoUoVGwBmZzVQdQXnhTlVZ
qs9e7kuyzJ411d6h6Wepbav3okCq98zLMnFndbbN3TastiX+aKhIo72oZDmATnCMv2LO4x2xOsJTEi
HvXrLNaPevPNp/YQt+3SpxrZ0n4HuDXW7V41zBubVssGjV8/85P3amXAAB5UvVP3/aFl81NHdRRV
ICqglQAQE76T4pc/rheQFYIURHKHgYdUpOy2rLWu8Otp1KR7cGClfuHRs/BBOwamMZwbZddWzOj
eqgx92n23LN7obonbiCk8of1p3hehhRlc2+4Tqg9hD5hYBeYJMs6ZjsbHhT7uljH3G/5QaqtKq2+0dZ
ACYJo9r07eJA2Z5vTZZMLft9aYl07S6L3AHUsg6+ttiava4u59cTTTzATPwDMkOqBqnqiLgI1pJ/6qAC
Qo8vWb6bUR81Uoa8KBgJAEmo2fT0ZlAph96btmTxL+lhOtaYNWBfEUbp2yJUKTit/VJF7H75XrwE

A5EnVQVWTSS2SK597XLbufr3c+uoX6TUAgGxdKXLFb4gUbtbLSBM9UQEkFCxRcGZtsUO49nnrWC
J6ZB4tR+vaIV/v+
+z7CFABYEbULPx3fPl6vbQ4nvmnS9J8//3yoyef0WsAANmy3m8vqWH9TzmLSBUhKoCY2rJh17o06
pWaJQoWUK+xIqv7demmUqJinh29a4d81e+ry8f+9mN6CQCQJ1X/9C1fvFGe8+PF/NXt8W/8SO7+
0AN6CQCQuf53RC7doxeQJkJUAJNRNS8XfPx7cXMvpRq/C+YIXDvkR/U+Vb1QAQCz8XMPvERueP
wqvbSYvvKXj8jnPv6QXgIAZO7y10QufUEvIC3URAUAAKEefuJhec32a+wJpQAA+bv568+Xf/6Fm/TS
YqM+KgDkjfqoaaMnKgAAGKKCUzWRFAEqAMyGqoO6aBNJRVH1Ue/
+0FftjwCAPKj6qGpY/7POIqZGiAoAAIbc+ak75cHHH9RLAIC8qQB1UeugjvLN//19+cPf/ppeAgBkrv+
oyKU/1guYFiEqAAAIUDVQP9P7jF4CAOTttV97sbzs0av10tGyt/t/7AcAICeXv+Q8MDVCVCTUk8aKm
uVbPTaknWjdOGltZ96MOYZeQ1Y2gmvbG+7rV6TR0yunpfZjb3NEO2zTne9M2m1bkPugvaHbWJC
BSwosjE/sf8KejR8AMBsqPH3N3x3tuqGqN6rqlQoAyMmlPxHpP6YXMClCVGCGeo0VKZRq0tHLWe
pd3DH205RdQj4AA+77+n1y15/dpZcAAHm7+qnj8pa9G/XS0aXqov7+v/8y9VEBIDeqPup/tz5SH3U
ahKhIqCibe33p99VjWyp6bXby3l8WRhxDryHrtTziU6UnF3eC+2qea1hrF8VRuA+A+aZm4n/nJ98pT
z/7tF4DAMibClBVkLoMVE/UT/7nA70EAMicXR91Vy9gEoSowDLoXRQ3Qy2Xy86Tzo5cXJwUdtTFUt
qXfrYs+w8DCUMGpClAf+cEjeg0AIG8//ffX2UP5l8m9n3xYvvKX/NsDALm5vGc9mOBvUoSoS8eoLbl
i9kQctd6oc2mvz7s25fD+IutuGnU/VwKfNLcT9vko6Z8z+/PmMP7mqvP5EYU0/WNWj+T1Rv2h/FU
5e+GMDvk6shMnRQ3UUg17jG5PvHbHuTbD59DaumyYrzfqkQa2MbA+/BTHaYN5POpY/P2rh7vd
9nnrupbrsjXQXdYu3WC83n4M3DfArKgh/A888oBeAgDk7YbHr7Ink1pGd3/oAXn8Gz/SSwCAzF26R
6T/Hb2AJAhRl05R1s64PREPpOs8s3TlwE30Aj0U27LbdJ6Vz6xZZ17ldNV/awjB/4B2PywsCxn1nRr7
RCwJIMj5zu1UswQa5bnrCO1UkFW9fYcaI2SINUYyl89LZXimniHs3Nx+hCvfEbcU+2L2e6pr43Dfn1w
Z846FVQOrG+uDgSpE7VBHcuqeFvWoakKSlf369Ld2wxcdxW+lsJKN3RqUprnibKwFD7yxY/Yk0kBA
GbjOT8+Jm/54o1y/FJBr1kuP3ryGWm+/37qowJAbp4SufQH1kfqoyZFiLqEimtuT8R9OXQTovauHwi
JEU72Dq1XOU6dmIcI1VI5LW6M2gzMjmSEhV6w15PGutvjsyotu6am9WjpLXRqsh4jjUz7nFW2rTa
Yw76rLadd22HVPv12d+vuV8TsRaoYQ/mrp9X2zVA4xpD+4qbsuefNfnTFa4Z1BPULwcDQN67d6V
wbV7neDX69Frbev2+maYPxeh2aFjf3vOc+P1RXYWtXf41/TppyLsFxAmnafXBXPvgXH9RLAIBZeONf
3yAv+OEVemk5PfTV78qf1h/USwCAzPUfFrn853oBcRGiLiOiOvJ6IfaPUOndivWnXCo32dFrJrDwO38bS
5UZMsNoJq7fi8+s+6n2wPUXFff8ickqmx5QWCs3pgzPGfVlj+Rkh/mWi0Z7IY7gtk792TJfmJsJ0EYq/
Ua636vzepZ2QxPUMe3O61rY6vKWbchRsg+cv3+obPdKdoQeH0UI1Q3Q2s7cNWB6t6okwhkSA3f
v/NTd+olAMAsvPLBa+Xmrz9fLy23z338Idn/n4/pJQBA5i59QeQyf8BKghB1KRXlxCnnmRtode1x6W
U5efqkHXYF11vUMHDn2VzwQ7mmeJ0KuwdeWOgN5ffWWcfkDu+2H8bw7cAQ/VFmdc784HMy
Yb1zLRMP6W/LeT9BlVZoz1klRrtTuzaW8kkJ3d2o9a4p2hC7Z7Zxrq2N2WUO3P3Er8sLpOuJp59gJn
4AmLGXfPe58nMPvEQvQbn7P35Vvvct/m0CgNxcVvVRv68XMA4h6pLy6oraPTl74nSqPCUnKies/w
+uV3ngPEWoFiOYcodmt90x0xG9I6exkOfM6GmphqaXQkPC+LP0tzf8OqBVo6fp8kkSbhdl80L4jP1u
cDtiPjEgE+5M/A8/8bBeAwDIm6p/usx1UEdRAWrzN+/XSwCAzPWfFLn8cb2AcQhRl5U3tFHl1fDts6aL
N77VXEyQqN9VP3hsyCUdfTDi/9upOjwkuvNubQI2YYuIDnzB/KHyXmkP72hj9RVLUlIzuhTmDqa5O
CTNtg1JUdKNlqa55jln7k532ffZ/c+/C9egkAMAsrX3qpXPvklXOJpn/40rfl079/qJcAAJm7bP02eukze
gFRCFGXVklO2Hw52nSHNbh3Rkv0Jf3347OuzV9w864eaDXeSp4E6pCVnqL3i1eKc2KKdM2Mo/
xjjh/S3ZcNLUMtSV1PRTyvVazOhHNugZuhXPU7tScXsQNWYoCtu2QJgSvX76vKxv/2YXgIAzELpoRfa
D4z26f/6D3aYCGDIyeXPivTp8Fp4jTp2jMOIerSS8ntyNpqRjnBObWeCzq4p/uem+Sprnj9gDtSK2mA77BOqRmP
crmqj9sur3h1aUsxB5LvWDnzBzK787+H3iYIV70kP5e45w3jL9cv5BOuYRUr82E8miD3pbKoIM9Trvi
ls8dW7sVSMn/+v/
+l34GAJgF1ftU9ULFeHd/6AF6ANZVDACCQi0v36CcYhRB1iZkzpqvehd7w88Ds5sYkTXPIq1NqC+shGaxH2
VzVwZg/Ll1GT440LNVzVtS1VJXmauqhoTmUvxxaW8AoiWC9cuSQ/l5D1r0CqtYrAxMwOY/JJkhK99
pMJoc2VLb9IfyBurRGfdmzm7MP3bEU7v7Fu+WOl9+hlwAAeXvjX99AHdSYfv7dAfP6WfAQByUXijfoJ

RCFGXWWDW8FPiTzbuDltXzPVzyAwvRw2ht+tRGr0uXeW6dJPWu0z1nFVke7BA5v5hSrUxzaH8o0
NdMxQeNaQ/Xl3VCaV5bSaVQxvsIfxhxVBVSNvvp1pfFohy1RVXyf/4lf8ht1x3i14DAMjT373yCf0M
UV7z1pvkbe+5VS8BADJ37FUix9+kFzBKoa/G9QIAgKXxwCMPyOv/2+vtmfoBAPm648vXyysfvFYvYd
D1P/E8+a1PvVGedw0TbwFALgovFrni/daTq51ljERPVAAAlsztN91uD+0HAOTvb27/tjx2PX/ECnPlc4
9L9fdeS4AKALmx3m+Pv8f6SIAaByEqAABL6F2n3iUfeMMH9BIAIC+Xjvfl8294VH78nMt6DVz/8t+V
5NZXv0gvAQAyd+ztIoWb9QLGmSpE7TVW9AQpBX9W65F60lhxJ1TZkODL27LhTbbiPCabqGZYeyO
43dBH1jOAAwAwhz788x+W07ed1ksAgLz84PnPyt7rvqWXoLzidS+R1X8bNhkqACATx4oix39eLyCO
OeiJqsJVf5ZqR84zwquZ0VcaKU3oAwDA4rjnl+
+xh/cDAPL10M0/kO6t39dLy+3al14l1d99rV4CAGSucI3IMTWMH0nkGKIWZXOvL2oeq35gxuuuHH
iTiKvZsNXn92QzxwzV1qnJeTqkAgCWjDtj/3VXXafXAADyonqjfu+aZ/TS8lIBqgpSAQA5Of5ukcIL9QLi
mn1P1N6h7OuncuqEZJedlqXedUNc/9Gtl/XnRZq7pKgAgOVzy3W32EGqClQBAPlR9VE/96Zv2h+X1
S/8xivsofwAgJzYdVDz7rl4NOQYog7XRLXrlZZq4nZEtYfVD9UoNb/OeaRVL1Uprp0RP0YFAGA5vfm
WN8tH3/5RvQQAyIvqibqbps9VFVePoLv35CLwEAMmfXQX2HXkBSc1ATNUKvISuFktS8lNXRqZVSq2H
aPu+HuNXTfpGB5WVM8uWd46hJwTA7YddqEYTfT/4kcHneY0nPYU7fC+q9b2DCO//8rEiKf0cCAt7
7s+
+V2h01vQQAyIuqjbps9VGfd82VctfvUJMbAPJztcixO/VzTGKmIWpluy/9bt3vCVptOcPst1WY2ZPGu
htwVqXlDsFvVe01qobpeqIkoSO1khtC+I9Vd0Yra9/2bgFghnqNlWAPfSBnqjeq6pUKAMjXfT/zuD1r/
7JQAer1P/E8vQQAyNzxO0UKL9YLmMT89kTtXZQdnSKU61v+RFSVLXHLmHZ2LqbU+64s9S0S1NFG
TQoGTGKR76eM295ryPpg13tgBlR9VFUnFQCQnx8/57J8/g2PLkV91Le951Z5zVtv0ksAgMwdf5PIsV
fpBUxqfkPU7oHXE8sevu/1HjWG93cOpKufTsfppZpmrVUAABaVmqm//attZuwHgJw9dv3T8je3f1sv
HU23vvpF8ku12/QSACBzhVtEjv0LvYBpzHdN1FSFz85vlhPo1M7nWItxHgxO2jWqxuPoOpD20GPv6
/VjZG3Jwf1FBNd2Pdzga0fVgsysDVEya98oca+VK/5xJm1f2OsHyncO1O806o7arx19P/kG2p/K9Ux6
DsOEt32wXungORo8P2HiTbTn8/epHqPqpCY5P0DQbdffJvf88j16CQCQl7+/7Xvyjy//oV46WlQd1O
rvvVaufO5xvQYAkK2rRY6/2/p4hbOIqSxEiFqud4fDT/uRwnDa4qac1WVWRfblcGnyBRVsDU7a1ZTV
wqr1/3hUiFMKG3rcqUlpMKBKMklYe2NETUjVYzgYFmXWhihZtW+khNcqwXEma58TyIW9vrlaGBH
2qXNitLNcl/GVM9SxDbR/6us5/f0ej9OrffAcNVdHhZyTcPbh1XO2Dd97qd3vWGrvKL6DGfsBYAb+6v
WPHcn6qL/yH15JHVQAyNPx09RBTdH8hqilk34P0YN0Bu2Ha8tuuinKQug1zvnhkTuhV7/r1Zsdzzhv
5bp0dbDd9TbQlHNeotNLMEmY9dpzesOh2+3IzkX3tVm1IUpW7Rst2bVKcpzJ2tdrrHuBnP+HjZa1F
615bkRQaLRjb1OKem0koz1u06e5ntPf70n47fHb3pHa+ejIPHqivUH+PsLvvbTud0Ds2frfdepdegkAk
AdVH1UFqUfJyumftB8AgJwce53zQGpSC1HtnmjGkFHzEWco65DimpzxspxVfxuqF6C77UQbdnpv
me1yHmYvuTOyFivhWXQ9uejO2qUCFi+kKcrmBSPEidI7lH39VDo74mY3xc09J6yxHnub+mQmmiT
MmLjHCNyKa2eGQ/XM2hAlo/aNlPBaJTnORO0LtuOst74iW/aGVVC3J2GHE2hHTNWz/rmtbPtBrd
/2JNczhfs9gWrL7yFf2Up/+4q5j/B7L8+J+bAM7v7Fu+WOl9+hlwAAefjmjU/JV376u3ppsb3sp14o/
+a3mNAEAHJTuFHk+L/WC0jLHA/nDwYcXkjrjWE1w5B0mMHN0daVAzdTKp+Ukn5qK56QU/ppJDP
kHgioh2ouTjVJmK6nGTZ8Prc2REmpfSMlvFZJjjNR+0a3wwldR5fWOHUi6XdVVU4HNlaSk2473bYn
up4p3O9zpSwnAwcRIteJ+bAMrrriKmbsB4AZuP9V37HD1EWm6p/+
+n/5GeqgAkBurhQ5/mvWR+qgpm2+a6IWN2UvbMitPdQ3hXqoHtWLri8pZ7JH3OhefG5oM1EPZ
MWu5eiGPlE1KzNsQ5R5b19sebQvRuA3VlFOLEzSmcbxAvPpphfcZAepKlAFAOTn8yuPylNXX9JLi0f
1QFU9UQEAOTn+r0QKN+gFpGmqENUc9hv1cMLJoj8M2gxA7aBUrw9NMc2v04+4dRUtdp1B82tD
H2kGskvEuHZ+7Udf89zw5DXjJwIry4bZq9OtjWnWijRl0oYo2bcvLbGOc4btG21wgreeHHp1B4ZlOv
HcEcD5QZpuv+l2e2g/ACA/KkBVQeoiel3l/6lOKgDk6dhrrQd1ULMy3z1RkZGQ4dEus05mDGp2d9V
b0Q+rjZ7D7raTTBLW3vV6dtrhjxuYG8OTB6XehihZtW+khNdqguOM176ldth1itOdfT7Y9JDh+ImO
M737fWHkNjEflpGaZOoDb/iAXgIA5EEN6f+7Vz6hlxaDmoWfOqgAkCM1C7/qhYrMEKIupaKs+bN2
yao3Ztuc0XsMPcGXKlEb7K0YEnhNOEmYH/4YM+KbcmhDlFTbN1LCa5XkOBO1L6Id9rE7NVUTnsK

Rmqsb4jfdL5lQPrPmhNaJrmcK9/uiyeB+B0wf/vkPy+nbTuslAEAeVH3Ux65/Wi/NN1X/tPp7r5XnXX
OlXgMAyJaqg/oe6+PVziIyQYi6pIqbF/zehs1VHawYk86MU9n2h393alJygxmjRqg/UVew9mbkJGGV
09aSNq5dWbUhSmbtGy3ZtUpyrpO1b2w7ynXZSmV8eFnK5aas6vZ4Tbfaftab/j/Z9Zz6fs+LOdGV28
6Jws6U7ncgwj2/fI89vB8AkI9Lx/vy+Tc8Kj9+zmW9Zn79Uu02ufXVL9JLAIDMHVsTKdysF5AVQtSlVZ
TNvcFJu6rS6res/8djD/8OK6Rpb2dgoi679ubg/ixDk4RVZHuoDc72vF01d71eitm0IUqG7Rsp4bVKcJ
zJ2qfa0Zfu0IbVpo3SBlM7JWcHjzfsGiW6ntPf7/mw7q/B67F/aPQSTiCV+x0YzZ2x/7qrrtNrAABZ+8H
zn5W/ev1jemk+veatN8nb3nOrXgIAZO7Yq0SOv0kvIEuFviqCCAAAMIF7H75XKn9UkaefXYwhpgBw
FNzx5evllQ9eq5fmx7UvvUr+06ffzDB+AMhL4RqRK9R8BQzjzwM9UQEAwMTefMub5aNv/6heAgD
k4W9u/7Z875pn9NL8qP4udVABIFfH3239jwA1L4SoAABgKu/92fdK7Y6aXgIAZE3VR/3cm75pf5wX
v/Abr5BXvO4legkAkLljbxcppFNUD/EQogIAgKmp3qiqVyoAIB+qJ+re676ll2ZLhae/8Osn9BIAIHPHiiL
H36EXkBdCVAAAkAo10dQt192ilwAAWeve+n37MUuqDqoaxg8AyImqg3rsPXoBeSJEBQAAqVAz9b
d/tc2M/QCQI9UbdZb1Ue/6T6+2g1QAQE6O3SlSeKFeQJ4IUQEAQGpuu/42ueeX79FLAICsqbqon3
/DozOpj/q299wqp/7ZDXoJAJC5428SOXabXkDeCFEBAECq3lF8BzP2A0COVFnD7fGh+W9RjuK0Ozwi9/7p0z506/lKsnrNGj+NAWF6Sk7
lv5aY2mo2frfdepdegkAkLW/v+178o8v/6FeytbzrrlSqr/3Wrnyucf1GgBAtq4WOf5u6+MVziJmghAV
yFVPGiur0tRLjrKcWSvq58vHDiJLNeno5YXRa8h6LarV6loXZDV4saVTK0lhIDAGjqq7f/FuuePld+gl
DW/ur1j8kPnv+sXsrOXb9zu1z/E8/TSwCAzB1XdVBfrBcwK4SoAK66cuDmnbuW6Wqs5Tae6SomH
5YyvW41V6AbNEi4ArkqXco+/qpnDohy9v/dHHF6znbk8Y53QW1XJetivO0uHlWqs5Tae6SomI53PSC
m+wgVQWqAIDsPXb903L/q76jl9L1sp96ofzKfziplwAAmSvcKHL8tF7ArBGivcKHL8tF7ArBGiLjGvnqP5CKntOLI2peqN
536d163Or/dp135sb+ivNdbZLwuuD/bKS2MbmtlG7zFcYzN4jMM1S+1HWN1Low3jal3a+zDDt+aq
87WDDQ9pc9i2o9o8tpfjROdl+J4ZuZ8YxxD7fGh+W9RjuK0OZwi9/7p0z506/lKsnrNGj+NAWF6Sk7
ozquwfDt9PNqM9ie65eMduS+H7wj1Pcd9HsNxuv+l2e2g/ACAff/fKJ+SbNz6ll9Kh6p/+
+n/5GeqgAkBurhQ5/mvWR+qgzgtC1CWlwpHQMKhTk1JhQ8ZlcHHYtR8HCkLa61TIMrC+uRoeXk2
1DRU2hfYY7EitNCqEU58zapaW61J3uw52duTiYMi0674ynbqmQ8GiZh/zyGBquM1uz8dQE5+X4X
umuToieEt8DFGcfQcvd0hb7WCw5A2hd6V67jxVafVbXq/SIWaP41E6B9LVT4MqcjrpPZfk2FP6vlDn
KY/3ERwdapKpD7zhA3oJAJC1z688Kk9dfUkvTe/f/Nar7J6oAICcHP9XIoUb9ALmASHqUmqLn8O4d
TmNeo3SlHPhSUpi5XrX3na/FYybwtaPGt482TaCw6mHj7EjO4PplEcFZM7r+3ubsrlVF+erBr/GPI9nZ
FyGWtm2ttd1t2Wptpx9bOvUrr3hB4Vhbe7UZH3kdQm2eXRT0jkv/invSO28cd0SHMPY8xHg7zu8r
dZxrbvBoHEu3Iamcu40u53bEitrHVKUE6f00wiVRPdckmNP7/uimOP7CI6OD//8h+X0bQxHAoA8q
ABVBalpWDn9k/YDAJCTY69zHpgrhKjLyOwlZ/R0K27uOQGJ9dhLZaajqpx1t1M5bS25RqwPHd486T
aKsrlnBj6O4toZL7TrHIT3AyzXt4IBWXFNzugv6uxc9NvY3vV65pXPrI0P38YwexjWLxht3rwgfgZ5PrR3
31CbR5r8vFRbfnDoh3xB0xxDFHPfoW3tXZQd3SEycC4qW/5+zWtniH/u1HFY3yOhIe8k9uUwrEFKk
nsu0bGn+H2R2/sIjpp7fvkee3g/ACB7akj/V376u3ppMqr3qeqFCgDIiV0H9V/rBcwTQtRlZAQ0ImqY
bsGrZTiyhuIkyielpJ8GjFofJo1t2HQ9x9BhzEGnTgwGP0VZ8xMtLyzyA0Mj0J1YVK9WY/8jgrfhNscV/7
yMN90xjFaWk+MudvfAa79X7sF+GEPcRwyfn/zcTeuUjN51gntuimNPcv2HzlNe7yM4ctwZ+6+76jq
9BgCQJTXJ1KT1UVX907t+59XUQQWA3Kg6qHdaH6mDOo8IUZdSUTYvhPckdEOYqMl1FkZg8hyjn
mOk8MDO76nnDnc2AsPq6dg9GafXkeGOgjFCRlOK52UyYccwK2keVxw9ORxbLNWR2T2X2vVfkvc
RZOKW625hxn4AyNFfvf4x+fFzLuul+H6pdpvc+uoX6SUAQOaOrYkUbtYLmDeEqMuquCl7esjtQKlR
W/Pcos+u3ZYNs3edW7PRrMGZhHW+zurzZA+NNoYyV0/nF6FOH/qlfF4mkm1w6dXKHXpMWsd0
AsUTMrb06bje1BPcc+OPPf3vi6P9PoIsvfmWN8tH3/5RvQQAyNIPPnv+sHaQm8Zq33iRve8+tegkAkL
ljrxI5/ia9gHlEiLrE1MzaqqeYPcGPHYR0vRqKo4f/GoxhxHPHrB2pgiW3/uMUba64U6Z3duT8+R29n
aqkk6GW5KR37gdnZO/JRbfoZeQQ8BgyOC+
+nI4hTOmkFwKOqumZL+NcNHfF75DZlQPvNJxwzn2EWPdckmPP4PpP/T6Cpfben32v1O6o6SUAQ

Jb+8eU/lL+/7Xt6Kdr1P/E8uet3qF8NALkpvFgP48c8I0RdRu0Ne6itmkU92FPMCHiMXnIlPxkzZmJvy
4Y3Dft884MlY2bySXgTWHWk2dQnKrWh/EXZdLsdqvO87l+XXmPdq22ZZBKkcVI7L578j8Fj1udsrvr
DyPW9bj9yHVtu1oBtyq7eda9xzgsxY/VgjnPPTXjsU1//hO8jZntyvRSYe6o3quqVCgDI3t/c/m157Pqn
9VI4Vf+0+nuvleddc6VeAwDIlqqD+h7r49XOIuYWIeoyqmz7Q287NSm5QYtRH7F6NnzmbhXSDL52
Lpkz9nttNibamUhF3I6BrlSH8o+4LiU/fZQL005glcl5MSQ9BnPYu9ueiRK2YH3O5qqz34IX9Felldqs+v
EUNy94PTLd9njnodqSeM2Jc88lOPY0r3/C9xEgiqqPquqkAgCydel4Xz7/hkcj66NW/u8idVABIE/H3k
od1AVBiLqk7KG3YUUMVeDS7wcDHlX3cLBmYrWVcx3NpCqy3W/5gZHNOTbvsAPDrOPxhlcr5bpsp
ZzL2dcl5LxWW9Z6d+j1VLI5L6Zkx2C1Z/A+3D80ejUmYNfnNIaSu+y6nznWQ/UUZXNvuFaoPYw+Q
aAb656LfezpXv9E7yNABDVTf/tX28zYDwA5UPVR7/uZx/VS0Cte9xL5hV8/oZcAAJk7Zv2GfPwdegHz
rtBXRewAxKNmNtcT86gwbG/anqHAOEfpntPHcqpFwIpwn+l9Rip/xM0BAHl441/fIKWHXqiXRK596
VXyW3/8z+yPAIAcFK4ROf5+66P/Xoz5Rk9UILaeNNbdmc3LcmaNABVZO1r3XPu8dSwZ9ODG0fGO
4juYsR8AcrL3um/J9655Ri+JVH/3tQSoAJCn4+8mQF0w9EQFxmrLxmANWFXOgK50yMzRu+d6jRU
p7ZyRbiplKXDUFf6fgn4GAMjStU9eKe9s/aS88723MYwfAPJ07O0M419AhKjAWAOBFgEqMsc9h+
WlJiiblx9NaEs42hKOtoSjLeHmqS0nrr1GDr/3pF6aLa5RONoSjraEoy3haAvSQIgKAADmBj/ghqMt4
WhLONoSjraEoy3haEs42hKOtoSjLeHmqS1IhpqoAAAAAAAAABCBEBUAAAAAAAAAAIhCiAgAAAAA
AAEAEQlTkSE2WU7DrfxRWGtLTa8fqNWRlo60XHO0NvZ3CijRibwjp6Uljxb0GG9aVTbJuGmH3UNr
7mJbRRv1Y4SYFAAAAAGChEaJirvUaK1Io1aSjl4H5pgJdY1Z9W1nOrBX1cwAAAAAAsIgIUTG/eg1Zrx
GfzqeibO717RkF+/1tqei1szFPbenKgXvLluvStdu0J5tkqAAAAAAALDRCVABIS+9Q9vVTOXVCyE4BA
AAAADgaCFGXmao1OlC7MazG6GD9UXuIvfE1A+VKNbNOpXokq1Vp79Mcxt9cdbYTvjOjjeoxqk7qY
JvCa1VOdrymMbVf2xvetob2H3JN4rTRY359ZEONcxFo46j1xj7t9ea5zKoOadx7KKotg9uYsD5pjOuS
9J4FAAAAAACLgxB1WakgL7TWaEdqpVEhpPpcQUoDQ+ybq4OvVyFiSYIva8pqYbBWZBqcNq0GNh
xyDHYlNtgm65W1UnjQaYt7vIMqcrqqn3Z25OLAa9u7bmODtTKHQjgtuo2TKsrambLztHMgXeeZxR
iOHmh7W9xml8+s5dDDMoV7aKJrPizf6wIAAAAAAOYRIepS6knjnJuIuXUb+9Kt61BNOrIzmPx5qtL
Sr2+5QaEKG8/7ve16jXN+0FVt6VqVXfE2H0Nl2/qabl28L3G3sx1W8dJvU/gxWMe77oZg/mv77gF0
arI+MhUdf7xhKltu2wfPpR9GSvmMeBlqe8MPgsOuSWQbJ1NcO6PbuC+H7qbbu0ZI2ZEDN101hq
mfOpF9hDr9PTTNNTckuC7J7lkAAAAAALBICFGXkjERz96m16vQD9VEOl56FlRt+RP3+EGhqScXd9w
+e1VpeQGStc8LYa+fntmm0GPoXRS3SeX6lj/xUGXLC+U6OxdDexSOP94RimvidfQ0t22ElGaPTrN3av
2CcU02L/htrJ0fGKo+Ja+NftDbO3Si0mrVCRv3dbrau7jjBZKnvROYlRTuoSmuuWkm1wUAAAAAAM
wdQlRYdA3P0OH9SZmzk5+Ukn5qK56QU/ppespyMrCTEN0D77jsIdheXUtjqHdgSHsazOHy/rB4P5
SryllvyvYRvVNtxnbMHqOpKMoJfUHcwLlrXzzrnJ4+aYeVwfWW6mk/kMxMCvdQKtd8VtcFAAAAAA
DMG0LUZRWYKCdunckYgeWRMt3x+r1i3Z6eRig3URhpDK9PScUt3trctVrXE6cj6ik5UdFhZWC9anb
2EeriSf+6AAAAAACA+UKIupTasmH2OnVrPZr1HI+ocr3rlDEYevjD9lNT3JSzbglONXTcqCs6WRiZQYh
dOS164L4ctvUQeLv3pzs5lrF+QUP07K/5sv1xAQAAAACA5UOIuozMupwqYHLrohpDoCdXkpNuEjs
4XNoIEXNVcoamK6NqvWbF6+nZ2ZHz50fVFTXP2eBs/mZ90FMydk6nxNfQ3XdHDnadr3VrtZbsT/jr
h4e0ZyWFeyiVa57idQEAAAAAAAuNEHXJ+QGTMWP/VMxakU1Z3XCn3DFnS8+ZMcmTNFfFa1J7w
6+V6a1MmdfTsyPNpj76oaH8Rdl0u6xar6utN6yz5eg11r0anuYESU7AqVivP+8dkGx4U8nH5V+vZtP
5Wnf2/aIumOquNyfCylYK91Aq1zz5dQEAAAAAAEcTIeoy8oI9S3NVh0rGhDtTMmcun2r75iRC7nY
mCjuDs7o3V3WI5gWO5gzwaXOHxftCh/JXtqXl5XU1KdnnrCAlP6mTC95EVCojdOutWrxzHLe2bVBg
W9Yzb2i6eZ9Y68/k0w3VNv09lNI1T3hdAAAAAADA0USIupQqst1vGQGZUpVWv+8HRvaEQpMqyu
Ze1w/BbGr7g/scx2qn1yBt/9DrDZhIcVP2+oNtstj1YDOoh2rwhvQr1v62Ruysst0PrUtbbVnr3ZILLnU
8g6+ttiara2v22gwMTTeGs+c+ZD2Feyila57ougAAAAAAgCOp0FczrADITq8hK3oiL1WDdo+eiwAwk
urtPS8/mtCWcLQlHG0JR1vC0ZZwtCUcbQlHW8LRlnC0BWmgJyqQKbOOZ75D4gEAAAAAAJAOQlQ
gE23ZKAzU8ayeFTqhAgAAAAAALB5CVCAPql5pZpNXAQAAAAAAIEvURAUAAHODelXhaEs42hKOto
SjLeFoSzjaEo62hKMt4WhLuHlqC5KhJyoAAAAAAAAAARCBEBQAAAAAAAIAIDOcHAAAAAAAAAgAj0R

EV2eg1Z2WjrhVnrSWNFzZavHhsyL60CAAAAAADA/CNERSZ6jRUplGrS0csAAAAAAADAoiJERfp6D
VmvEZ8CAAAAAADgaCBEBQAAAAAAAIAIhKhLqr3h1gddkUZPD7+3l51HaClTVePUeI3zcL7eZW/XH
MbfXHVepzc4uF+PuW1j58HXt2XDfY318F4Wo12TGjwv6jHpuVGSHr8S1obCSkPCD8+s/eo8VkaciG
TbBQAAAAAAWF6EqEuvI7VSQUoDw+
+bqwMhX3tjRI1T9fXpBJbR1H5WpamXpFyXrYr1MbN2OWHk4HlRmquFYNCZ4blRoWtYG6RTk9Lg
BFl2EFuSwZd3aqWhcDTRdgEAAAAAAJYcISosVWn1+9K3Hq2qXqUCwPNulNaTxjkdX5br0tWv7dbL
zjrrtTsXnYiusm19rlsX9zNSbdmv7W+rxHNafjv7e5tSTNCupHqNdS+MLNe7zj77LasFWvOcDkeza4NI
W3bd1Dh020055yW0VjvW3SDXOE/uBe3UZN17bZLtAgAAAAAAgBAVUm1tixtxVraMANRTlM09H
crZ4aVeu3bGe23noKufZadc3/La6ciqXT25uOP20qzK2U13yxXZsoNGFVLuibM6w3PTO5R9/VQ6O+
JmscXNPWd/1mPPbVvvorhNDpynypa42Whn56J1ZJYk2wUAAAAAAAAhKiaha5OGDmHPzqkT44K
9tNrVlQN3A+WTUtJPFSdo9EPnYSmem+KanHGTWGtrquyCW7t0qM5p98Dbnz18X7+uYA7v7xxYR
2ZJsl0AAAAAAAAQoqIsJ82UcJTAxElGbdLcjGjnzNtlyawNRdm8ENYz2A9KQye5Giur7QIAAAAAABx
NhKiIoS0bZs9Kt46mWft0JuahXRm3obgpe3qIvV+v1tc8Nzybvl/DdfBh9KCdYLsAgLzp0Q3Ggz9yAQ
AAALNBiIrx2rte70o7oHNrfxpDyKc2ybYya1dJTroJqDsE3qVm4i8YM+6n1YaI16uZ9NUvzfakXXbw2f
XqnHrtK530Qtu4NVhjbRcAlpH9Xl+QwkwTy540VlZFWu57tPNHr+bqLILU+Qxze40Vpz0r+f/hT/0ba
p4P9zEP5wXDhq7XDO6ZmRvzvuZ9P7mPvG7mOO+3ebwnR+5jSf+gFfO8z/K9eGZinJuh951luGnG
nJfc34sTtofSdoiDEBWJ+AGdMSt9AiU/nTRm/7d+MFmdbhD8tO0KKsqaVzS0Kavem667baeO6OB7
cZw2JDp+/aavPhXsGRpSs9Wsc9pc9dvm/sOhHu7KJNtVjG3wDwuALMzsl/chKri09j/lv0npcCYu3Da
KcLuTP+4f5vte3N4IC3ONPyjOQq8h6zWRatX9x28G3NEnxsO8XrMy01/cAyWOhh+z+NZW52O1qS
YFda9TS6qdmpRmFPjk/0v7+Pc19R5csr6f6l3jHFk/T2Z778R5v83jPXncPtTnc/yDVmTok1eYm+C85/l
eHHFuht739CP98xP/vl3dH/g3Iot/IEadk9zfi8efl3zfiydoj/WNrUrbpf+eHO/7dn5+FsZY1g2DJWS9R/
TV5bd+A+hbPzD5uvW+9U+g87mq9ZZia/WtnxX060c8vNcqIa93P29uf9TD2NbIdtqStKvbr5fd9VXr
K8cxXx/yKNetVygJz83Exx/
+MDcdve3gMSfabqvqrS8PXwQAmEq3XrbeX8z3eP2+GngjyoffFv1vwAzaEEm/z8/8vXjm7XCuj9q/f
c28f5PzY/87OoP9RtP37dy1y/3eivPzV9rCv5dn1R7n5y9jv/pnrCy/l8a/rznvuYNtyPocjW9XvNdMa6J
9ZPIeqPdvbdd+hLRD3T/maud+Um3XK1IS/5w4n8/+vTjeucnjvS/
+fZv1+8v4cxImq7aNPy/h62fWnhHfw+nfR87+zd0737fBdX579Qr9vhz3uiJf9ERFDBXZVn8p0ksO56
821puAo7kr/t9KrNd7n9D2D52/MKlanIP1QqutCWuIJm1XEk4PIOsNTS/7rDdbf9h+0jYkPH57qP3g
ubQ5+wj8QdOuc2oMyXfZPWWMeqiWRNsFgMy05XytY71NXZBN503VUpEt9UY28fv35Iqbe9Lv7x
ltmTO69MupE/PRwFm1o9dYl1qnKmfn9kLNhnteWt7PKPPC+T6X6unAzyL5KMqJU9YH9+dQW08u
7sygPb2GqIFK5fqWv9/Ktv3zYmfnYgY9sRxj39d6h7JvfRj8fi7aJ64puxm9Ecd5v83jPXle3ved79+y1Ls
hP8tr6uf34dEJHdm5mO7dE/ec5PVeHOfc5GX8uXHeXwLf5xmY7Jxk9148/rzk+148tj0jfp6yR42mW
tYuzqii+fpZGOMRoi4pvw7mwJuLMeFQcMiBCgvdr1EPJ5TztxMM6dQPhf5rrYf5A725D/VQ+xmx35
Ht9MRtl/MGFlw3nvMG7H6d89gbakjCc5Pg+G2D59J+jDoG8zj1Y9QvU3G3a7xu+NgBYAoz+uV9M
enSL+W6bIX/A5CTnjTWa9KZVTvsoaMdqbbi/1ueGTUM0Rh6N9uSN/n84j6JXuOc9d1s/bI/oxu3sq2
HjNo17dWwypITNg/
+vJW13H5pT8huV1lOenWcNKPePgZk8AetuMFlmDTbEVuO78XTnJv86fJoB+cHhtOnWwJnknPCe7
FhxPub8/PnvuRaNYmfhRcOISoAAJgNfnmPTdUltX/5uTCLXoa6tpj9i6D+pWdGvR3b52vSqbZmPmL
C/0Op81AjV7KppRZXPr+4J+f2NDo7wwDEHTWkatqr7yOrObMI4efpl3ZMgT9oKfPyXhwwR3/Y6uy
flAvevxGqt6h6/9mYYa9C3osD9HwiwflBrO+pqedWiWHwjzD8LLxwCFEBAADmmDP5gRq2N6ueO
MFRDt36vqzO4hfU9oYzCcRc/dbuUL2Csh6WHcfc/eLePi/O6NHZXTNnsg5/YiAVeKtJgXKftGOWv7Q
jNfxByzKH78Xz9oet8pk147pY/4aeVWXUZtirkPfiAernGrdnrP89dXBKXadTkl3n7nn5IwymQYgKAA
Awp5wAVfXWmJ+hjMXNC3YNtk7tfK7hXHtXhU1NO8B1fuEpSEn9Vqh/CZrtcHptlsOyLfP1i7sOCGf5
y6Iecqzq2bt5jz0MViXezdWMZjYfZVa/tI9h93bqyMHgjTuqd9QS4w9ajkV4L56XP2x5ZtqrkPficIMl+f

qydXLfOk8nJau3vdn+EQZpIUQFAACzwS/vkVSvDSdAndMJ/zL8RSPMYE8j9bAngLR+Mexaz2dbt7sn
h6qoWc7nZKxZ/uLu9nw6O8NfFgeHTbpmdl7y/6V9rOIJceZ7CUZNPfuGnmG4O2f4g5Zvvt+LB+T+h
62S2GWOB8PbWf5cw3txTLq2eOCPkekZ+UcYfhZeOISoAABgNvjlPYKeRddiD3czevzkW+dSTf4wM
Bxc/0KW1S8ac6/XkJUVc0i2WqVmSp7lL6nz94u701utKjMcPSpSOW21wPoeCgyhV7dwzTovM26bL
dtf2uOpyGnVY6+27r+veL3G5m+islngD1qLaFZ/2NIjADo1Oe/9w6mHcM+oHinvxXGoUhklqUldLm
RwkSL/CMPPwounDwAAMCOtqvSt33L69a5e0a33y9aPJ2VvRX669bLVFtWewUe139KvWUZh56U
6JyfEblu53s/3bun26+Xg+ZiLe6RVHbg2rb71S2tfJT+5m+H38TB9HgKPefie1vdRxvdv3Pc1573Yf2R9
7eK0K4/35PH7CLt/3Ifxb1dq9H0x9H2r2jFw3Pp7Pu1rNcl5t78m8/fiEedGvd8M7Ns9hrTf/uKem6H
XZfY+POp+0XJ6L453XvJ7Lx7fnpB/xzO6RnHuxXn6WRjjFdT/rIsKAAAwE+5f6F3WD43zNRwQWBCq
x5xdm9BVbUl/Bl3nnO/pqrT6M5p5eS7pnk7G5ZnV9cH8Gfre9fjfR2Gvqc5r79gUjT83Id9bxnk7iuLc
LwrvxbOmRvOoOqhhgkP7+Vl4cRCiAgAAAAAAAEAEaqICAAAAAAAAQARCVAAAAAAAAACIQIgKA
AAAAAAAABEIUQEAAAAAAAAgAiEqAAAAAAAAAAEQgRAUAAAAAAAACACISoAAAAAAAAABCBEBU
AAAAAAAAIhCiAgAAAAAAAEAEQlQAAAAAAAAAiECICgAAAAAAAAARCFEBAAAAAAAAIAIhKgAA
AAAAAABEIEQFAAAAAAAAgAiEqAAAAAAAAAAAQgRAVAAAAAAAAACIQogIAAAAAAABABEJUAAA
AAAAAAIhAiAoAAAAAAAAAEQhRAQAAAAAAACACISoAAAAAAAAARCBEBQAAAAAAAIAIhKgAAA
AAAAAAEIEQFQAAAAAAAAAiEKICAAAAAAAAQARCVAAAAAAAAACIQIgKAAAAAAAAABEIUQEAA
AAAAAAgAiEqAAAAAAAAAEQgRAUAAAAAAACACISoAAAAAAAAABCBEBUAAADAQmtvFKRQ0I+N
tl5raG94n19p9PTK2fDbuiEhLV0wbdlwz3vk+e1JY2Xca6xXNVb8ba00rK8CAGB+EKICAAAAODqa52
TGOemSUMHoqjT1kqMsZ9aK+rmpKJsX6tZnHZ3aesg1asv5Wkc/F6me3bS+CgCA+UGICgAAAOAI6
Ujt/OL38Zx/XTlwM89yXbr9vvT7e7I5KvksbsrZqn4eco16jXN+IFttyXZFPwcAYE4QogIAAAA4WuiNm
r3eoezrp3LqRKxeo5Xtlng5auAamb1Qq9IiQQUAzCFCVAAAAAABHTLzeqH590pVg6NpryIpbm9Orse
rX/7Rrehp1Vr119suC68NKtPqCtUJH1wEdeJ31CKsrGjyeYL3S6HYYzGPXj8F92fsp1ayzrDVXndeO3Ul
FtltujOpfo/aGXxagXN+yXjUo3vHbQto/dH0tcc5VoEar+6BWKwAsLUJUAAAAAEdEWep1HdJl2Bu1U
ytJYTVYDdRep0K2gfXN1VEBZlNWCyUxyoCqjUhpcMIpOxQceJ3F3t/IQK8jtZJRr7Rcl60YnTuHwlEtel
8JVbak7hZHba7KRqMh5/wEVS4M1gNIcvwqwA5pv3M+hoNUR/i5UueiNLhTJewaAQCWAiEqAAA
AgKNjzQ3p4vVGnVS53pW+qgPq9ax0hK1v7o5oh1dLtC/
+y5tyzkv7etJYd0PBqrT0a71td2qyPjIpNl6/F2OSpvaGePmv0a6um3ga+6psW5/r+hNFqRqm9n5iDc
MPTjLVrLnHV5b6hcF2Jjl+67VuGhvWfmsrOxfjnqu27Eadi8A1AgAsC0JUAAAAAEdIUTbdGYwy641al
bNuj8nKaWvJNWL9/mFoL05zBnqzXmhn56Lz+t5F2dGdIQPD3I3enN5rB4QPix+t7aeGgTCzuHnB31
ftfDo9MAOTTGnVs8OTUiU6fuu675lBqKO4dsYLbDsHXf0saOhcmfVeOzviZq/FzT1n+9Zjb+QMWgC
Ao4oQFQAAAMDR4oVsGfVGLZ+Ukn4aMGp9qKqcDiR3JTnpp31ix33dA90L01rllguwH8bwdve1A0
6dSBLymT0vz8ha4EuLsnbGbdi+HIYlthMITDJlPQudTGqK41fHZNc5DR3eHzR0ropr4h2yuodK7n4ja
rECAI48QlQAAAAAR8xAb9RD5+l8K8qJU/rp1MpyMn6am0BHRnTmnE6i8DlCYFIpo85ppLBzFSw5Y
HLD3NgTdQEAjgxCVAAAAABHj9kbtRYvTsvXYK/Onhx6Y8iHebVWhx7biYbtTyercHa88cfflg2z16lby9
Ss3ZpEcVP29D4Gyt7amueYpR8AIg0hKgAAAIAjyOiNmpQxjDw7g706u3Lg7tTtmVk66QWAo+p5ps
MsJeDXAHX05KJbmFROSaIqAdNKcvztXa/nqR24unVRp7iWaoZ+1ePUnkjLDlS7Xi3W0WUEAABHF
SEqAAAAgKPJmIAoTMlPDo3aqW3Z8Kapz1ZzdcObqKm94Q8/L59ZcwJAszZnc9UfQt7e8Gp0FlIZV2
4Gzta5WPd7WfYa61790aSTVU1twuP3A1djxv4k9PbVbRDscRoSdAMAlgYhKgAAAIAjKro3qjlzuwrp
nGAubi3NaZWlXG7Kqg4D/dzWmOFftd+ozdlc1cGh9+IREzJNorLtD1vv1KSk21XyE1S5kPuM9AmOv
3Lan6jKu5bGBFRJjDgX5r1RPat7ugIAlgYhKgAAAICjK6o3qqp7OVgzs9qavI5mIqfk7J4xPFyx63gO1Di
1a3MOvE4Je+2U7GHrIcdebVnr3eHxeYt9/BXZ7psz/itVaZk1TZu7Xs/fcexzEVYMVW8zrewaALA4Cn
1V3AUAAAAAAAAAEIqeqAAAAAAAAAAQgRAVAAAAAAAAACIQogIAAAAAAABABEJUAAAAAAAA
AIhAiAoAAAAAAAAAEQhRAQAAAAAAACACISoAAAAAAAAARCBEBQAAAAAAAIAIhKgAAAAAAAA
AEIEQFQAAAAAAAABGEvn/AZM6nr53azj8AAAAAElFTkSuQmCC]]></redcap:Attachment>

</ItemDef>
<ItemDef OID="q25" Name="q25" DataType="text" Length="1" redcap:Variable="q25" redcap:FieldType="radio" redcap:RequiredField="y" redcap:BranchingLogic="[q24] = '2'">
<Question><TranslatedText>Do you prefer Life A or Life B, or do you think Life A and Life B are the same as one another?
</TranslatedText><redcap:FormattedTranslatedText>Do you prefer Life A or Life B, or do you think Life A and Life B are the same as one another?
 </redcap:FormattedTranslatedText></Question>
<CodeListRef CodeListOID="q25.choices"/>
</ItemDef>
<ItemDef OID="summary26" Name="summary26" DataType="text" Length="999" redcap:Variable="summary26" redcap:FieldType="descriptive" redcap:SectionHeader=" " redcap:BranchingLogic="[q24] = '1'">
<Question><TranslatedText>
Alzheimer's disease - Description #[no]

If you have Alzheimer's disease...

1) You might forget recent events of importance to you. For example, if you are interested in hockey, then you might forget whether your favorite team won their last game, although you may remember watching the game itself.

2) You might forget the names of people you met recently, or the names of local streets.

3) You may no longer be able to handle your personal finances or plan leisure activities such as dinner parties or vacations. Simple decisions such as what to order in restaurants might become difficult to make. Your ability to concentrate may decrease and you might have difficulty driving.

4) You may start having trouble finding words to express your thoughts. You might need to be reminded to shower or brush your teeth, but you could do these activities on your own. You may need to post notes around the home to remind you of simple things like turning off the stove.

5) You may remain aware of the day and your surroundings. You might be able to travel to familiar locations like the supermarket, although probably with the help of a loved one or friend. If you are in a social gathering, then you might have difficulty following the conversation.

6) You may continue to enjoy hobbies such as puzzles or reading.

Now consider two possible lives. In Life A, you will live in perfect health for 5.5 years and then you will die of natural causes. In Life B, you will live in perfect health for the next 10 years, then you will live with Alzheimer's disease as described in the summary for another 10 years, and then you will die of natural causes.

</TranslatedText><redcap:FormattedTranslatedText>
Alzheimer's disease - Description #[no]

<p></p>

If you have Alzheimer's disease...
<p></p>
1) You might forget recent events of importance to you. For example, if you are interested in hockey, then you might forget whether your favorite team won their last game, although you may remember watching the game itself.
<p></p>
2) You might forget the names of people you met recently, or the names of local streets.
<p></p>
3) You may no longer be able to handle your personal finances or plan leisure activities such as dinner parties or vacations. Simple decisions such as what to order in restaurants might become difficult to make. Your ability to concentrate may decrease and you might have difficulty driving.
<p></p>
4) You may start having trouble finding words to express your thoughts. You might need to be reminded to shower or brush your teeth, but you could do these activities on your own. You may need to post notes around the home to remind you of simple things like turning off the stove.
<p></p>
5) You may remain aware of the day and your surroundings. You might be able to travel to familiar locations like the supermarket, although probably with the help of a loved one or friend. If you are in a social gathering, then you might have difficulty following the conversation.
<p></p>
6) You may continue to enjoy hobbies such as puzzles or reading.
<p></p>
<p></p>
<p></p>
<p></p>
Now consider two possible lives. In Life A, you will live in perfect health for 5.5 years and then you will die of natural causes. In Life B, you will live in perfect health for the next 10 years, then you will live with Alzheimer's disease as described in the summary for another 10 years, and then you will die of natural causes.
<p></p>

</redcap:FormattedTranslatedText></Question>
</ItemDef>
<ItemDef OID="a26" Name="a26" DataType="text" Length="999" redcap:Variable="a26" redcap:FieldType="descriptive" redcap:BranchingLogic="[q24] = '1'" redcap:InlineImage="1">
<Question><TranslatedText></TranslatedText></Question>
<redcap:Attachment DocName="55.png" MimeType="image/png"><![CDATA[iVBORw0KGgoAAAANSUhEUgAABVEAAADYCAIAAABtBTQzAAAAAXNSR0IArs4c6QAAAARnQU1BAACxjwv8YQUAAAAJcEhZcwAAEnQAABJ0Ad5mH3gAACm+SURBVHhe7d1fyF3Vue9xY9Qdj0pyWk9I9WCiGw/BiKQNB2JOsRHEiBAQVBpkKx62vEgEzGWeWkKUwxOCBDuVkyBfmSNdcSHvD99Sb7ccMKJrlyHFnVFUqVUGgLrUT9DQIUFhMe5WUDgSl9ROFNVMFCEeZvmUeaKkOeOeRGRb2DwIfmOXbLi5qHasJnEzUwT4DJDOSXOAE7rUVWDxTYuHUD4Pre8hgVy+5Uba3sgh6Tww8X1LDo6+pUuFcRXe8z2NhL1PNQz3PNyHaCw6SYPvQ8VZDU3V8C2YqdPMyMvdRpBe8dzUYSaPwPrNQuBgCqyUSSbEnzHxMa1D5kX8F6YmNb/uv1+JKKgRZR7Sxqe1hUjYHLS/h5JuY8PyX/7oGfEmZ1fQjo+hbqn8HV7J0sh3eTjKzO53xKzm48ZO8uUXGRNZBMSHgUfGYwmzOsPncwC9D6/oXOwl/6Mp51d85Oa0/f61ZPu2ivkL7/Ua5N4pRnCxh9Y5xMuFxL4FoL0nTwBjJ7+5CjTEpkjySzMzV4UVTz4YrqWkHzi+bZ+d8tedsRzhrHJEXQ7VTVmxPUUZqRSO1ZbRxdAp+SyqsvYrT91gM3E+O4b8ZhXEPX+Aa2o6XuMa1P2mwX2D4+9r1f7pAKJAYt79K0Iyr1sFLtt6+k6jrm8cFvwOvAxrqqAXyakiwX/OAAEVxpLBOjDnmqrGiNW4gxbyXcrz/vvX8PZUqVTL/KZnZR5HT8bMKMRBOtVkTKH9vDB8RRIh3EDd4UMJRyBk0IsLAPUsjnrz4krRWQqJtqOHX2ujpLu3u0ExRJ2PmB1fyHu7ST3ReK5n9kXJ/dDVoBUF8Svbl17ecHf/GJvvmeX/0TwfbsPIH8IbAdjpHODa9yrT0f1AfLgfnOzNEXZzrfOzZwebzEZM+/nGjSdNoVA5dFtXG9dW9njhWV4vHyf+nCYrCUfbh+tj/Ukp7rbmTqtmHB0gwvuWIlXYaKYlWXqwu5XEQT4BtxQ+SFXQ0obANYwHo/jX9XFc+GFMlctIRFtzrGIRqJtkH2hk+Nw4mCAK6LR2lyj3H3YVHyX81XfrtmRI8r5wqLvPv6YR+V3+HhRQK3v9LW5vT/UDN7kr+2sr+4XuKhibQVCdpaaNGzbGs/3HzMEepEgzpabg6x21HiD5mU8jlsM5sVj0zQ5x9N5jgfJT+bqL7o2dYzMl9FKvmQsbrO91TRgD1Jp1vvl5Uy1oPAQ8NZl/D8QzuxH/lzGh++aLqG+dJ2yCHWQw06vGrqP88hW8NCdhSdoY3MG3vi2dT1AGsPuz2RWyo5ik8zZJRHjrdzULBvnPSaSH5TTuLmN9dDXEK8DuXTC7gGRXDq4jKqFm8Zbz/mgkUnPoW6tQVxg4i9P8DQvY/ZdJvs9htzp8OJjKv8FUQhbSNEmqRxm+qO02tyx5a3aNR/gcC5zmVk/x9yG9/Pd+sy1jw0Sx7x0TB6+ryRvPmxn8OVaR9VT11hQI/Bfxwnga0lapa+dr1hgIM/0u/PZuUlhYoYOt/3mfZ5+MNRYLQvcIWymjDkRdMGcO8GdUqD86lqxB4QJtQaoDb5jFyx5Q9QU0W0IWIuYq0z/hMtEbHT8/9Xm4sbpWMTPbRiDHxSuNyazt9f4WI2dUgEsQ3zmdNLumqwFM8R5WzZjJOqa8j4T0Nh9pZtyiNKdq5gl4rpZYFt8eaOLSIzKaaHmvg8v0JGgovt8Lv0fVjBr06mFpq5xBRAUN/o9eU4fsqsVcwP/0YAtdnpRNWI2SDEJ5m6UzhuCs3TX3/f3P1SmXHIjHFI0AL1YUbuvUBT4jqgtC/1IRWFFXgNyzPDYBq51Dr19eNOf3akPbd9fLIq/IMLlS9N73q2MhyOMHpbjzcEFoHZJXVtKSeLZZ/PqRoQQDxBQb6xfeVPRA+i0/zR3zHO9oMQ7ttWS5FmEBmsXXpyB4eYEHpBuItdDJ3dAo/ajmd+TQWpZqM8WO3f0e2BpEf9pjq+2L1NVEsz+GfNz1iAzEfk/1/Z2RrpJjc5p2ivXqZwQ2r4GtmNrfEirLiK+4cAY8tmdD9+98rGstxDcaOQKxFjtqP2XWa+gm8GZIaOSi/uRr9xa/PRQzpVa0NnyiJEYmwBTpdOMMyKYpjdW67YOj0gEtoFx/P4KT0XG9W+qOqW6WmJgD8f60PaIjv7Y9LFszeN3aDwgB9lUjLAgCfpPR5uR2fOxiL9zz8pUe7CgW5tyEJIJ1rVn7JkJxywO9MvR4Y6pMLqXdgIBjF1BaX4p/PyJEHFIAdyf/T7b9KU0LhyvpX4tIsmUJZMmpgVTZoCLsNIDuaYDcFYBE2mZ7zZpavdU9iuvsb/P6I9mo3ztWbHXyNwWYuLbUqXkyR+Sy2AqFRmf/6DBAg4hONRCMp+QBBS5YLDAQMHtWj1hiPOZ1pxsPI7OkXOSxzAlrZPTjYSpYyJeeNvXwTP5Xls2bbBOedsAFCGHCkMPCrwzd2ie2rg33Lw0WHlrdcM9SDcRfBxYXzh2fuqDl7d8fywGk0vSMbtT9yh5HsdWL6oWYxrtiKWW9rvxVm9cL1Fl2R4rQrz8w6vDXg4CHzv33lWQAPYYgKMlzM0glA7+j9jT9g3+8jfnfdM3rP9ktXfO6BuTcqQQv3GDgBgBu4rDIBXRNVpb2wzyPB8oiSyx9LhQ1PCz+WNM/c3hA56D4KTetOovanv8pEmMoHCb+XmDqkDJ2Sr+oPaPJiUFXOkJyRAuxjBVPJvdMlz9D7aNv8wO6bBAXJBVZ0FNjnbrpRqXA4lG6Aev7+/CXa91SugO6t/2Mxw19xOXJNjMUfaoglHKMtvRAsYa1OYMAZ5SSPi8PiVo9tF6bX7w3eCDT/yuqnv1PNgr/YMzwTMCQoGnsOrg+mQYCZCoZT55Hb16zRe5KItP7Z1H3RozByqbxBwH+/9yu5ZGtj09+LC6vwXYuGNVBkoFbVNmGm/Uh0yBjr4m+yGUJtEv8lFmYZ0jVBmx7/4+5P0/vdA7oVKKMuvsDDGpO2pKa8DDpAQN8Y1zrjIbzSAvWqadHcBFDDPtnxFeGbOnD2sQbR/O9iN6/JwE4bcntgv1Qb+GVsWDYR5PN/ePVMIrSXFlhpgFzAxOF+I9G1S5ldXQLY8RR9EnnwzKvp9/bjvz+EHixiBc7ZXbNt/fUTpi7p2JT1WqVYVQ+8iVxwuUYTs4RG9JPqJZj9dygTczqtaVBRHjjYpl1TyKrQb2+ENMXFjLHFsuvD1lfBZf6oLN2A+SjJ7NtTc10qcfJzVY6S80VkCNsdTyFDvR69fH/4pZe9SSyFIOG4PJvFEgrsP4ngGHXjWGTcxMezJ9yeA2ID6jONMOTTUEvENJ4pXT3hBP28AD3+H4JxmD9lAjJEYp8s6e9BZlhXfOO+f7rU5P/7AQ7qoSzxEqkrAoQd+wtOCvR4+rMaHoEaAUZIqF5Xnm3zdJXjM5fXiWWvsV/HdkgPefGDKqAFOH3q1MhE8/LP89bWjRSsXOzxzPqZUxOPHIGKCIZXRrpBMKH4x5AWfp1wzlyY8MjS7oz0Fg5JAdtdPwOhrMX09xdKH4mFujHKhZ3aE1Nt6KBkwwj9Wj4Hv9Z4uvcgzDcbqBZFO7YlPeMX06fp0ROOz6tjnh8h29UdeyGQu+ZvSrhzzvHMEsdEYPmMzSfJTaOHvYG1eDCMv0jNy4lSsdHOi+Gbj/mrYtqSBt6S4WqIQ5W/f42Td8mm0m7/ziJNlUW8XazzlWy0QZUuMXEUsCalUhvWhIjmC1NH3oJgk2ou3owGrUaQaYHEqF9VjaYr0k+XumlTbqbgtfQb/3cixpeSDQHUiPkSzvR8P2VD1MtRq8UUMQohi58+b8wm0N56Qpm14jSH3UbmsXzOyObsdKFo9RkREAZ6MoFxF2yD9GgzW1KHVlO8pNj+yQrG2gBRmu7RbE8Y1cv6BIeAWGIU8KtQ9VNbM+8Jkj3N10pimE8tq86y+AbxfoDBs3odAc+kg06Rg59BH3HUPe71oh57Anmx0Zt3Nxm21efn2cGYL1z94Qv3xQzvABQbbZ6w+n/aLJJFP2eUMGs2pvjoDLNpJ4qBqQUx9vX+vtD9B9f0yjFz8p0GCWUN4xuTNRY1w5v+B2R/YehaQQWugkTKAO2vN1+T9X5XNGsYRA7Q8kqrZvU5fGGwFd3bRgaLuVQQ1YCVbsMhD1oA/9Ih5b3xWhHO8ILtpiB/t57eZBYMiKiGHhuDgu4lv0fQ7QI2E/cVGpf7ZwIlPHaHvPj2zGRF0DmNcQZqNd1dHeRdP+Z4upDXvP2Rz8oY69AS/yxQW9JHe3M9dbz+xh/tQOk0SzlAiBSpzF/gdj2xxV8FfOUfWtPqVzG+ls23wDmL9P7Dl7O+jUvhS9VmOQ1u2vjk5l5wCfUewrdS8k9lKGn9kefVbr0/62Owt/Zuj4eX6xFQ/PqgDtAgyTD0R41kCRsKvQKgv+QQpCf0g+VLaKVz/7nzm5V6bXJ4S29TWBMvUf9M5rC7t8+rrrwnmv9bjPpuvxQuabXsU/iJT+y5cfQCBFPedPuZpDs6ZsbBFhGnqMcXJfZhrFl0MXsWWjzGVS8DLVvvxTv6XLcRiOY+T1lXsHg6xw6wkY8zJSo88A1O6kk0t7F9QV5e+NbaPiwkH5f+S0qHBBrQA98cjWXsQRvwrnlsnQXN/MyZRiPtzDzNYtMnQOmWgnQvnSxtqhRTIp0DQqSPlrZz+ZJ2PVbGz9h2zR8aoKYgUVMwL5Gm+kQTgo+Ca2ZdyAwnMb3TXxHgnM5DSm4/VlJ9JEg5SlrRI6r8SoxfRpNvTWn+bn2lnZ+I8HRJlxeZe0DvxwjyLNp8jdETZ7UvjK0Vs3Mvc/SjLCM6nutE4cP/W4ysbAoM9Rmp5Nwfg6hqUzbfjb4SnLSNE5mlaM6+ELKa7CKySF3K/gj5LEQmvY8Uc2Zh1UrE5iQKUGQKimpETV9YhB1NXrzlVWRmQyu2bFlsRqoVT6s9CmYS2AQb3SNoj8M/FyQGLEVGp8oXmxYrc3xo+F7qO8z8ZCIdSXPc3ZTNvHq+GvELiIekPNmQ1P8nOQBfXOORTfBNsOdMkQwW3QfedjUJeNPyBH3j26zWh3fU1TeyiJt3KEXrx2FXyiyWPzn5c6lwdzTwqsAcrKFT2ODxg4enx3xTNq2lkDuOhk+CAw9fK2f2L9ecMtYeN6Z7OZ7YuLtgBy5ahKtw3MmAV5LCMvIpTmOtLQS+cqPK9B0vNdxhLk9XeM0MTnLHhi6GW8ArWnJhkAz2wHMYemF7/AvoMXKpPmm2bUBmuxuQLkjw4Pq2QZZRDnUm5KVJA4blfWrdK8sKZUeXHyO97urvjXJWvMbpdkhD/eiuB+WGgqUvr0MjCKeWxKrWc/ZHpRC2dO7yewKaj3+XaGypvBZMaI+rJHSF8dGKQ9nEgaSSOrgZTJBy1BOXF5+GyjLc/fEfGSXvUdvFfLJXuw9WBn1wSjbUhHdGUSYpJmUFlTbzrXfXL+ug5CyqKSQUJaN2DwDvGXxYqLFNS9f8rF7BiXxCFIzq1jXKZuhjXgO/aOczR6wtfAP2qsbP59y4BCy7uSMP0DiCsUUlLXfSoHSD+YLQdaUn7vTNMNySgeQqvG77ZnJkpZS7i61uw7ZnHz0+YSPx7RGufWW2RrdH6/1uvGFIcdX3FRoAyCQtHxHYUHYq46Rfi8XwM56rl3s7iwvVgDAn3F/Bh6Or7+GPKF6IjTZ7QuLWNWE5svCPpOBq6iUJKkeJ4nUVc3pPo3UMcp3ExZHH3SxsfvWp/YAi5S4Fd4v56o+1KObMcLG0jG+HwHsY0Wr7vU1HYCXSK/t9aqu4X0HbAb5KRUFuyGbD+OXTTXO5HRhI6ItHz3Yo5eGPfFJUhw8v1nSb4bT5CKcE4nLFNwDqD1hiTxv3uBZ4vpH1b1P2YFfvmrq2PsAQj3W1l/WrlbajVj/KtE5bz9yWmoqnGG8KbxJHK3YqURNcOGqjDK7NxqQdnPTxnOwOXz0Z5u6YgKXSFq5i4wRGF0zFKvVdhxs5Mbbp5vPx5+k3AhMe3LjLmCI0y+iOP+OWnXvk7J5nDAsUgO9pmqn5s9J1xlojqWqsTlaljEqZz2aNkxEcNyTlOcdYnNs12PGZoGVoDuxQnz8hAN3CbE2ugT4KkxdA1RIUvn/nTPNeJ7i2FaOapRP7FJSKLpvEJ7bmzXUSkDEtG1TnFmXOFcJAtSkqlzNvzyOOdu7Ip1rHCeG/2+D8pt8Xx4d2I1Fw3RPgEbM/F+X3+vfRULK/dspHLcfhyhiXXjlzjDR2NbqZqvTuMjk2HVmvPNg8Z2U+6M/k0Bq5qcy2Go1TzXoIjUbf4HM1T/OOuMJxZ9dATAP7Wq9vWoM28xuejV9/21a4nTsudHi1ly3jnzhkzZVoG+9C1cRYYSLixhV1HBiDaZh1+SuzeIm/iAkvNnEjlrbPtYf2PMxBxIAJg8FxcGMhSjBAx1Iq70z+FLTTn3aZvdSIPtw8GwsBBSt2n6ttvPVMbcbwO/jeZb9TOP+cMh+HdOwlxNQJaBQ5jnv7iKT66j9xu+Z8Ou4LOmTo5UYiEsmMaLNvHVqTrNB7FzlsZ7Bj0cy0jmjhLEV9bfD8f8L6gqPKy4uqwFxpxV0K8mEt4OzgwyijWPrI05HADdz4nmYhMp6wJgQpjQCAp8vAiPm7ZK91vbs/MM6b3jYdvEhZ25+HZfM7KoEROPxQwYg+y3bIOiSNrJxkXD+q7v/d3CvBWdFxeF6jJ5hdKrhQqDtyNMfWJNdqzwOQ57RfrYM0CJMDd1t9+zyHpQz4NhS5LuSuAkAZSufWUiIkKQEz0BHPhDRdrBIExd2zkXkwePcoPOBIPzl0tOjX0Q1DtAedeOrDzZOIgMqUzpzh55JnwxWlLXv2TgZ0HIPq3bPycGr3yCcAoDcTNpOu0Vr0IrzDlBLbzg4HJOkQy9gR+sLCUHXnj+qKAfmpjMHNxqimSfTxW/pSLK8XrF6OO1jj2pidIBNuiXX51IpQ6jhwPu8X7OYZjwkX2kt1H9jYAg3RiE4AQ3jt8GSMMBrdwbdeA3xtIk+36RnXSqbC0UWrlFa0fc3Yk+Trlqc79LlJlA1KPMTj0rPWYnXnXSgQuZtP2ARM/E/fl52nHRxf/CuWEZXsQIo/hHjuNmibdGRdhlkuJobYV6HzOZ2Je4aA6Khiy5y6kB2cNaKYLZjN2TqsvTG03vbtSZCk6zFbNGL+tVEytSnRQW4Cl/VCn9JDQnq9hPJ2mvvVY1mKsDuMsq43jRc9Isu7PhhMaK7bkHAzzfAMzrkj5R9X+pE+AKPuvWEVM3U/2Sxw7DyHIA+xt+t60lHXSVo+vfy+6smMq+ajRnrazEaxUl1r0mpW47ft3Pj3UwsvSdBGmTEuZJaYFYYp+3n9+5yvEjpqxsy2RKaxJ8iiAkkXrYx7vqF3WXK9ssIwmtiu2NPDphL0QOpCvCMcpUH7ihyAXb6d6ehn4SNufCKNQXBDIb0f/I+F3H9HTL+JKtLuOiF8uVetWtKKgrBh0CWBcvx7xO/+PM8iaXSZwxqXmsBJAl2MbEZqIGuyrNsfBhcJwRc+yzwfAd2gZcF6+S4gbv5g2kDKjSiZT1TmlFw+Lc8H3JuBmdZGfQ5Dt8gDEAp8o8Vn0+/KjWfb/Oz/aTDULMZ1e1UdNtkAFnQJ3dkzF96vymTOF1O8uC/0SDvH/BebSBkadqUu+EMOHm3m7nYXSBkJFTeqmHK3VnLfpZ11+7WzZnY1XIaeeaHHD6k9VHSP38+OO/OYe2S2IT0N0Ht4a9OmqSVSA51Y39KaxQ0H9a3RnUDQ7JhvGG2ndTFQxpYEAvwpmf2EGLlbcebZGBLMSO6Nxmm77h/bmDZdhMlZYm3mc2dQTe+4EHaqmAuqBCEFMQ9sEVtzFa2GBkZBhaH5T3ymgtiQQnRJfFXfy7Ds5VP9eWndHi2bXyvF95kLQGn9/wkPhd/NdV5wsU6XfT4Ic4NPaRoFxNwqiUjVtuT1GRCk96ddQ1KEQB6dbTBQX4c06NWJn7NC69hBfuRM/VoJ9P8TW/7aGX/rnrQOv7aTrWtZx5KoTXAE4ct/RN2sUOa4cB4GsmftEOJaN01uwfIRi46BGW1RdP7Vd7Sc3rGCP+BtLTqNm9d6y5rXeNuLCoxvhn43Tah5smsDyoyYt1b7BcSO/EuD0Ffr3J81jn+6GT0wMdnUH3PnI0RCjFsF9VjwU7M4FDkLwrwzWIH6ASpdxNB05JHpxVXFbEBW8BCn0Bt5hlyOFxQe6Ct1TU+SfP1fszKtGkmEL+ekUu81J1Rhek1K0IUcwsoJt6/5sH1HDe8BgjTIM5D6Uk/J8znbIZeP5JvdRQgL2MLmBXb4YPRNhG0x6Y3qgASLmd4AmvUI1+VPmHQ1gfuXpdbOkIBGNr1UBLDVuiMtN/16SmmIaO8s13Smzsbo/JEyiJsT7kf+n3l/k/aGmz3c33h6V5jf4y9v3TSZ2znLhHxCBxRnuG73KbT/5brr3Od3iwQy2CjQ8RjHOUJu2OqV0bTCzK/J8vbE8Vxn1oh7UXnN7LRnqBJ3HGEYq9qoTxRKJ/PlmJKuFCDHJuk1gTXjW4ydi8pnCdu4KeaoK9Sbo7kvo4bH8gPHo1a0Vh60Kcg3Rjyr8WmcOqR4XIJXZ3rq2ScRf7A5rJ15ZPSC+54kv1v0/PV/OeLiiTk2E+Vw2vGvKP5gS0pANh/1QIc9OYrprYP16AMPHLWZ8ToyBNBn2+pe1bVcC/YLXT/yhRFWTGRnNVO/MRdBkOtIcVAT7FOdm6RaIDUapr6pcY/3lBw9Ssl1ON/10OThY8sZU0HaRlCW1NNWgbm47DDmibM2dWvTUvKVu3+k79t46wHUgjW7vpdCw1kVDyY12dCIY/+9jytNHp6Xh07dC2WaLRHmU7zSIJkx+oJdWw3+yY5Ffb/V90K0pRBiVFd2/2oIm3hwbyGm5aO6GzM+ck9JJ5aSJJaw/8lVGyS1sHDS9/A7FJaLs6Jc8Zo4CiSAvWsfDy6LOW7F9/Ys1lNcN5ahnXVwkzTj5mhKxJzjc7Bpf+NgIlPGe03HX1F1f/MuWmBpQwEI0QXHvQLVeWtAzVnuBp7ZfJgvZx6sNJ5+Y02LuYQJTgPzUWGINjwyL1TG6+P6fvQPROsTx3QSqBrKcSt/jzlPBUHoBW4E4/hGGf7m1zqKxt9ihWaHEnqixEqFm4M5m6nDqhLpgVcJ8DqFkg8KqKO3wUuV6aM5SV8wuxF9Gb5/m8VmP1Vt9+EJ+eQNV1yMV3dCmyP9SUdg3dcA2q7rJ1pUV+XxTx5vNZbuC1WkX1qeh52rOJgOmpB+Jp3W+dGdnqAdNqk8l2jg6Y6SlX2qSsPpFDLrHJFqIkfJ7GvoEGqAfQjEi0xFUm79Fn4wY7PUnQpSX6vNC9ClX55PxGl1xLDGdZcAtrBLX8bv2pbAPO1+fXp/xBZ1HAEK8GEpcrvp+6ST6MSWjaYyqFn0r8XnO8Z2vFkbZfHW2bCvt48LYu7/n5RnjEd3Fx5L3/Kb3sozAXCX7jCcxYOx2o4RNcAeHwjzR+4PtB6bp4b8Gn33b+cQ+m8nu1Ncfaa7e2pFxfVNbakOwGqm9JzjIvH7HRyCKQYMFQSE7vmH/kvBcRRkV14eW6/qkL2FBGEZ+xJlfiRBJ3+9nMJ+2r9t+xLzbqeqnD4u0Qv3GOGSCO/r5fkCq7UcuqATncVdnnGBmYx1tmMVmq5pRQJC8HVy4Ri0+AoHuGhbb1jMp0Hs0wf0WgN1R9jQ4HNQp2tYbxc6hxrK4CdQvJNQvtq7x4sRDPyaNIgMd/XAxCD0CFbLy1nUuTHwEVPmpYGFV2ASLwJdIX1h6bSV6hZmQJ6fQPK6zu8J2sKnjS4/V2Y8fbB5LYhEJMlLZI2vXyU1yIO1XeLdvh/H5gjzo6Yw8NnoGRRHbgJLVZmPd6mrwtaN5ujFtTdgrPtLvTIEBZR/fTZaGUyCyrlGgHQzcoRwEkjXo21Amu5KYLSqyGPAPs+9xEtfkHfWhTA5gsi6NmMiXl52VzT3BJW3sbc2lBS/RmOfgr+DIXGGaI7bBkq4Zl4zY0Sj+8rHKIiF9VCsKbSvgcdTy9JhHySMe6RNT/xh6bRuygbkbvj8uGDGkYPejgjmdxKdq9hafr0kIcXl95lOJPOKBEkS2/Lfi7zqO1MbU4DoLSe9w0OR4SwxeQa71W1rzWlz+nYKZ4KZ73kTmO0T0lm5UoWGo5w4YXf6X5HvArkoMi/YaOMRc0mKSsaxN6KBTkZbDqyAzfGi1cZL2ZtJoiFbA01zqX3FIjMEfBp7hIYz7jMR5DN4OIuIVCKpiV6Ec0TfSjbjL0N3x1/OAJINcOt7KlIkflKp6eCAwYm6QmdoeGrwPtQlD7CCvaINVXaIj/Mfo1eFgpOYgaFAatP6vr+Wkxy2ZGFaXszzFXD0Ge4ISwbvf46qTiMIXBRb4HWGgBMdeQ4Dc6MCo3BDThF8P5XYKWrFDSLr2vJFmsP6sl8MVE2Xbdq3CxJj0jvFCR87qQy8vIp2Ahc2K2axOwyX8Rz1OXZSgFmXSN7NyJmx9qtQUuL/HnMsEuOV3ZUcDrKMOAd3fj8kVFeM6WHKWHV7aF2GAW8lPaUSQx8P8S37VTRHxeI7eNy7vq77T1BCNXmNKfgVYpm6cKDzhw9JyvIC0a0yUUVBiZyRXzDOvvMBQ+VBoj3oV6dnB/N0ou6gbrRJyNGNybq1d73Ur7V8lAGZM2Wl3xPKH3BmPrkcvCYnOE6W7dF4FnXGzfUuY6ZX2+EbJn8V0A5Vn6jOBK3ib7Ic99I96VThoGaiWVWeRdTdzQoeV+lV2ENPqwgiA3CAqemaXXJHZeJpKQbC/OoA45RbwWEJmCbMqZFQ3rEYDlqK8tRQWoEo0EH9R9ALpzOz7zvUg7FKv0RzIaqSEPjWSrk4JswrPvt2HnrZGo5BRC/NoKb8kBdavCu8ZSNGPOgFLTfvRbuHlf1vPHaZdHyIKsh60pEAQIDJcV+EMyZa2UmgiI0nsFvuydFCmsEqHPFDAQAgIKsMpFqgrBDBqT8P4WUM13sjkiIKUPxPGeOZzJk7tqD8M0q0hJsNT14WWALZoUHRvfbgZcJ6IXeV3FlhC0uxCaX8l1QCAsmsqNrtu6fhr2ysLqdsqEA3tIMcKoh9awVTUxjNEjA8DNF6VArNABYUHjT48I9fZ4Nk2jGIQB35uxR3tOf/KXNE3JqSaSUcnjmwr6VsIdhaPWEXnXqbdxqwPIYj95GGh3ThwlxtBCpYZKQm4ip53oXWgG6FLBy6eWJiXfhRGZXEbEmFn7TdrLTpAnVPB4iuyjrnpKF7m3f6HCKNmo4ziQRNUD61MsSqnCgP3N0gs4HgG6aq1kBuXaqtW3ALydwESl5bxI7uQX0LRpLQIvA1BH17mBAHo5Jzpay6i/ulD2kBvlR4AMlKxO6KhvJw14jIbDk2zbUsvOiFV3+JAEhbDIzLlGHKy+wHdNf3zaHuaCLtBiRbTmSYzQfMNbRS0tsPFJTYAcfgfbcZJSyhCJlYmQBrPTLSKIHMT3Z7tH+7KxDi/12awHRmUJZqNwJVWv9aL7DUXqvtOg16U30+c2GBkhuGaj7HyJAM8a/c00qpzGsjdzhVLL6G3K2+KQe3lhhwHXsfgq5NYzCZ4wHPAMgGG2v7iskmc8JSCpDrIvI+D2HcNnW9uzWRW0JLkAJpwDTFUxY6Y3vKbmcgH6KF9fwGSKU2BZUkmGgqhVAInJyMp6wu2CGk2YGq7hbe4A5dd+dJDiUXxCfQaQKVvYJM3FYwxCSjGtHQaZJ2cQGiAgG5/vzJRygkMAxPNa+kb6tYdSu0xu02xlhbrqoGunJp6M4gxn8BOjm0ySy2MmjcWPxvxvhXJDtN1bKK7oXQEQTqPAwKPHnDYTUMq1b0nXJLExwhXcMnd4nAF8JKqvHwH4sdaMn/0+uN5Nbes3nRM4cg/bWrL1nBixo2aB0nwwsnu5Omz/MUOqzTz4yCOw11vJ0f8rPBSOIHAEkhHKzILtqFxeC5yQ82nJyNxMXdnNUmjxKOy3c7bj3Nnb9UhmBWqMHTN7C3RBKmtZTm2okrnw9i/1J45oMCpr/Y7aIF30N3Ch9Od4NDNkJIDT0CnNPQUbwVTxjhIH4V58WOxbQIpGlapUJIqYnHrmVrCgnLIPfQi2uHNjw5n3I1P9r8oSAIJVvE8IdgXSR6ieP82jkhTWtnqzQpdbh1q6Q2ivfXDGc2Qlbmu/jOGoUc/7E9PqTQmXTqFu/DrsGH6qcLGn05b2V7kvzDINTd98m8qc3tSzk0WU7WDKdlTLuO1Fr46DWvk/DZmKx8AdrAPfY0/FVZwP8MG3P1I3sPG3dzlTL/05pkDgsSMUOAq+sMJ0A4f/6fPgWMadZIEmBTMGjLW3ndbDrnrvbnF+CDlOLz7NePKjl7hTT+tkhTCFuTe37fkF4QvvqphvCcrOYDHOdXmnqR6QWQiOCrngCyqqDp3lQ2jiDrXWeKlmGSK9tAyVDJ47RJU4+6sBkedTeFqdnzVZSNkJ3XuH+fjUAjhF9/5q0dJBXLx8UNyLtkDEcwK0G/0SrttI3ElUtgSaX+YgMxwZ8elxBRbmoB2zaRHzTmvUSBLKLdyhvgzI78sUGXUQh8bM/7nTgkP3CJYB4mIhm04fMcsCeqkDmy4CcFbG4kRxX4dqgE1ZxDhQ3SIuZZFz8fJuz9g6AR6kHsoHjcSNu6JmQ6Z1VPf4ogZBsaHV51iuHLfjgzp1AvZDi/uUfbXpuPTr3H6rQBv0WhMflHxxLrMsCJz00hZhmLlpQoVrSe1E53ZcqPMdPYs2eGlrB9Ptw7lTvxTgrwhX4kt9FS10fGYd5e2Ddyl6ryqwCjQSsh1CmZv69vTWHMpk5JE5y43WSQRv9AE2NcbovZrGC7YKmQFLcPyc5cHhaXZ6idNAx5XqxoXjaw4pjq7HPxRPJo5I2nrhxmGYomvmVWgQ25hGjHHE4WZg56OC7sDafrDMl4LJSB5bgH4dJu1X8dUytwQ8MBMuPo9Ob8ezmGQ+IbWiZs4dlYyXIdpbaW8tNrJbDeTewYUMLVAnMGiYtPjdBgYiwt4ZZqmPjZ8m1bXsEfDtNZ6ARrBhcDpkgAu7lzxTN8L5vZk24VpLLbSXW9E5cZUJNBTRDXF7WJeMVhSL3hxx5Gq1YcdCiW7YA8Ug6v0rEs4mJfrJEv+1m0BBvR3hYhDffQ4+jLEhcPlTIIQLpyU7cHJ0ky8aztBcfBkTHzfyLLuDwxYC5wXbQcxHaVUPcUWjjsnRqiCeH5OOpfGEi1iZPsjDHj+VsqxLUHeLvXWnMOdAGVUW+LzmJDs5x8HXqdBuE+NF9tbZUHnE2GWRNjYHtAODWKY4DPqbgIDw1y4nwYkqI4DIRTLsPDE7FZp5uO7vwNV2cdz7E6nRc2x+3z9Ne9WoUFj5XfJVgY31gG4NNJXfZ3EfK2q0fJaQWnZ0CX1/xEEfHSb7x0TrkFyuDkNDqyLB2m9r1H3Y3eS3sZ7jmvL9ZyfqOGyOe1cfK8GyTavRgHcRTV2AEN0ZvCkzGF/HNyYzvrkDmcpuqpKrgNoFomV4E7gbbE5gMRqm3AxjBUn1gJYXXrXTakp1cA6HPrtjZIo0Mt1LGZpV8uuuaFj7G4X5DpxmUQ2EVvyKfJlOt4bpcdlVXyDLM3Dn3Aq0+NBSCpKJJr6GdfG2t5FLWvHg6v6ogzuzdrCqtsNZ9HIf0pKtqSpgtyszVPbEqkwHLbzgTmE04BIyMzq6xEjsG3uBF7cIlzQAO9yB3iGbrgJ0a9I82VAcmuR1kbNvZlzsg1r9fYWtI5G5cNDI3Jq7p8J0RxZ4Shs6qp57PB19lI5VBqfm7kTLIZ5G+O9xHmoZw3NsIEKb7SBb3vBvU4oiy3XBaMkFKbNK8HpVgxKmD0pJ69c8nuVi2iP6ZqZxnu9Q/P1NuCl4MJMcUVuVPdzwVDsE8zD5HPqRi1AUrKKnHqRDmZJAQkjG+zaDkuYe5fTvKu2IbrHPbsxNfoT5EjKRlXmLSdvB/b4IFLh8L7+I6XJC0GOJkE1ZgwCD6M7aZrNgHDFLhlg04pqoXO/kOZm4NGr/pK3cBgyvVf4OP4gYZ6yMmz99F1kPNm/nUK0xD46M4hUxPh8YqT7DFBEKa2LvHmCkv2SBt0Ygq/Hf9ZTZ1aq4OdRzxt2vDzhbRkSoaDTKeBlkoWBdDoS9rdngXJ9iRxYABDK1hrpf7W4FttKMVgKbiyTGBErE15hzZqtSxE0Dx0VDbpNU1jHgCEsM72KUyCbbP4NehAmAHHCZrThMvjbmuGe1HsmPFZqiSpDLf4HxOHE9yjJ45ALd2hiG6i1Kl1vNtzjDzRKAqCpgzkoTQa5FDxMBJdYzrRvPijzPm2h8BbnsE9r8rPDhBAk+kWmyd9hlZPbkWaL6FdJF3XO9wlF2JSm4zq7Gw2w2f5hHiGxVYl3xYgAThdk80ATz0VaPYe6R0n2XeU6NE3OxbmMDjGzbRfnYNBZJPQrpDNBQxwxeeySdSeF75sGqMQrapcW5DPg7I9JXgMYh5HMnNBgDNs3ugfWjRRc7vEeGg7zWbA9tUmHD8UjbZOzSINkbUdUHk8/VNJVgEY1VNVhTM6I3sf4BdXpV8fw8DGs2kcMImbkCyFZtr1GEJhP3O79cRbpIC8dEQkuYZRIFzVUjdBoGtNJ8DNHzKNTAPcqVh9cXpwn1C2pB1DWyEMqHkyBMdXB5kDB1IgdEPxs7AhgzQ/7y+jqJNTwPD1DcmMWZmtLqTgJx4l06aPIvj9dXnF7dqFWcBsxvJuDT2AhLBvYeGX6JuKRHXLmBnBYiTl4eWJBvbmsF2V5ex2qAfzjXAwLSIUkh2xGJFgkBVVrTBHmnz6Vf2AgB0dRj/EWXpWTYMLb6ox7a3ylXcdOkg9Vs4FOTZRxl9UMm4fGfWm4CJ3DhcxmLbzAmNBEIMhUWz1eBmVnt0CuZMBHT2KZlGsxXNTVPrAjUp/bJN5t0uvkEiPEdfNxVSZxjVcFHH2SZ6XzzZCCAqEDHDZj4sLtNQZhWgPzWwY0hpZWVQlcZhUcGbEQnMqh6KjqrWBF6rktRp5OfTNsUU5P/l6x9ZGKD7xFW6S8KR3CLLXZJoNMK6Qe87nLVDYaJVTOjAU7MCVKdTdkhF57sJ5dsB9NjRoV3JrQ1RxmdWU02G1UgyTAnDlS6tmF5jHvBAukfDaPvtDmIN6fEKq8rhPBgx1y3F8J9MSUmZAK9z6mOpgjNKtLsgI6YdPVjQx2JsIJrjaAcuzb7J/vKvGeQMHi4nh5mW98PbgQvaQPqTiaT3EWvnNWW0oRqpMwIoNq6fzWqjMHjVXeSs3NoN0E6IgRx+kh4eEO2aJd2uNbmSN1jGcDX1XClE9zG6oHU1OkcX96Qw+W9E9ZcnSAKvAOzZ+SYM0Jjc7TjsP3GDs+A9ouBTu2o9xdzAUSqHeHxdfmqQXhCXz4r8npYqO3jaiMJN58Un0wZAlXWTS0ZplTKFVzDHlj0iEJAFTVA5pngwEr4I4CLr9JCKNpLI8xMbH/7cuGmaOOZqPDj8y1kvZVjcWb8PKaZWXx7L0sPYByx+CSO4YsDFOPLdSsRkzvzRlNbrmuUhfdoNzemItqQ1Mfwkn5D6vSxVwr4gMsOWM1BcZTqUs3JGLyAHrlrScF0w0gYOfzwkGa6Hkm4ZW7SOw8n4ujthF1n9HebvvHEG5kcb2i86EySZ9HQz11qIR7UjJjuwq5KdvsJtx26Amx8CmMEKgXzTb4kA4WOftgiDeovMSDvqFUk0GmY4nnBwTm96VUYcGMNg+I5N2HHTEBhS8wtbkm2ay5w7jPDP4x0jbE3hFJYP6yAPeuCtb4u4bdVEXdKPDpCu5SzqGv9r7+IM63dAQcQq5pxyC9eZhGMhl2p9ZpXPVLg2EzUTXrM7LQYE7LQDSXP5jKK5bbLCKxdcYdH7qByQr0GqYOdjpMTsk4Q9JMZVkNxjNl3oJwExVDL6nR7Kx5NDMGvE0vXj6hBk0ngmGfGdBa7bvDIUZaR4hPSlmvUIVDKhhx4vJKYMxSOyNE1/tqsqU1q5AAXa0NrXOJWQ0ck0lXP9TQ0p9ZZU0PDcrNs8XFvF3rCIAvjO3aT1JBPSt3kq0aBj7JvwFYoO3lHKJbp3kLWPKOzOdgJ6SeA5NrcSN3NvhwHWfXlU3vMzsnAyd7GXdUk7m27ANWgPtTjk4wy09T1bFcERSfuOKVKbrYbvCldxm2HC7B3lTFVYNDddJUf71xbGoS9yLmMB4C3gCSL1cgvYQhCWGmQjHS8hwYEDLzLNQjSAG1zp2IsE2XbJgPU8xHOMvR8RG3Y7DGrIuQ5u0Dvdz9YxZCT7xhjjfMRbgVXuPdRMn5WC5dWVpf0dq7E2hgMvSNTDtgsDkIg28DDPnABNB0w1qzN6CkHqRBGeuGm2ufHhu04CyPB1XnS00RRTyL5OHFgMczXzJl1mEF92gV7H6KJZVGnxK0XQQo5Cvxuge7JI89pANGVLWj8OxqIFz+aKIhp80Ej9QCOcQHlHagPYrWLkvKSO8wmkYGqDuhPKpzQ7GXWJRqXDTvbKa3Nc4uUl3OB1Q6EJz5+T9AJhFi8Y2xVtTzdDl6M1L8oZGD01ZqZw8q6YxsaXS3khhlVdvuL3lGHfMJTwUbkgnBdlPGjdxdwZcr0IaKNBGW3ffyZZQ1yBSj2gJ8YhaBy2G1xJkOaR1yfVzKZK9dH9bCG0RC2aqJjaXZbE8fCwpSWTO2YfT7KeKhRyt5Nhms6HiWx5rM9Y3Xi2P8NqOM9t0ndhxbfmJGldnWKOhsaNL5HC2rh8Pl5dgt2p7wqOOfOGBY1xtgVBM8DXwhYlXcVqEPCYUiY4Pkit8fZ42Vwbe2S8qkwVlUWwtZxFzr9N2wCgQvU30aMYU4RAJ6pwM6bOeG4pHSKv8CYR2Kvwd0pQXrD8Ezjn3AF6iCYDuNt9I9A2lPTYUSgzOgyMTs3fM3xNTKNcUqTvABGNa2zJc8pi8ZIc5kEOnpl0f/8MULv5nr0cEkNL7zwgIAJrEAJ1Kqz7IiEx0ShC1PIeUILI9bHdKrWXb3iMsqVc6n2tJGfP14fRl93aLkRKtNmQ02tyyGG3LwVwgdEp9jCHdpnT6SwPcbqT6atnxLIHGqlNqmLmTm2HhGnh+rt6I6fZBNtgjpoTjGc9CUdWn3BUt1V6ugKEEjvSKQfyw56cIYFn6uJ8Sg7ID5LX6M+aiI0Q9VbhQHLIZkCDQ0Zry6RfILw48BjnhVzzC7VGCjyB4ywlu2A7a42PyFfCmmMLkLu7qNkbyx6Bc6n64ap99WzvgR8rVOYWf6uuFzGEjm8ZWo9TcjHpP2T3r2FZNbgOfoNDUVX4RD3MKCoC1KjCDKgqwZTVNDHkuRxNhxCQNpAqQXfBbY8Eb9h8IXgY2qJ31SvIRKSU6oQzgvHeMM8Q7XS0GCMyHYl6ghh0WkUp4EwzPNbJbo9ZxBdgBb7v4yyNx0oEqaBuqPJi5vENMaIvhW9Ck5F6j0BUCUHiCHfOa1N3wk9KxnhPg8bqGlNIRqE73I6jGkMFpE91F8xFYe4vz1t9gHdBYsgeDB9TVLXYkSONuIlM8ysGnWI6rUP7w1YoqjlM9JzGN6sz7TvzqK3RjAWH2GSTvWWo2qNsdAk6Bm7UbQuLXNa1vU9w1iYuHrG3LYWmX4h4WJ6GN6UcCUiUrJIDtAoMfwQ6vAtDZT2rkE6CWN2fh0BIEcIYlGbKqOCUjTdKVOkvBkxVpWPyQkJIgK8nGnfg0dM0ImRxA6fvVWQuDzYjSDCeIAW3S9qaUG6bCn0GNNexljqEkmnQ0mrzL7MeTSwYeMCxkUNhDf0LaBfKVNNnR7fLddfvwjU7rKrvB16oxUYNZBRlmxOPbB4BFmlEzC6j0dL08E8hVmnRjOsPdFNUT8tyb0KIWFcF1OqpQlr3UtuQGgJzcYzdTWq62WVyoEUdUWyD3uC6yvzjJvRMnjH8WYOBEeG3OxTqKg7VyRaeyejnqYwbAINo2BpFhf0JuBKNRVrapx3GZD8vEIE9dLrcwzFNi5cAoNXJO8EVZcCF3IPPuvxFgYGJcXOqS3TyZOTtu3eXB3fhU7sC45pVexbMHMtzTDAS6YOTkhDfcqWnkzBwEMZjFOPrlO8nTmlr2mdnQxuqfEb9cXqZyEp7jYxj4kOVeZKo9fy5m97HcXm6LNQWgRrq7hRFrHBrCymcYvjymeJBqbtADvvZqFgzXKFOkk8tY9J8gtO9FuZnByTk9FHV5FcnQqGZKXhKArWrf0Ez+wn7Ug4pRA9Jrx8vbZ7krbrDPkn4S23ZUczkdDf7B5MBFmw6ttpuEQWZhj5X32SPQaUA9EQxTCbyDoTAU8Km1r69eVVRNPgF9Hj8G/q6VUH9kZsc0vMn4N2cNgrwbBbqUjkbn4O6wrv+LcZu4qjh9pt7jBMwfLOhL9bfr4lHXl2+o3ePgs2eRRPHAGm5o9w7iCOWfnH2JMR2jPWZAqAo9dkLtR16wu8gpSCG6C1qXU3Ss7XBF8jvIaYUkQw24KrV3AKa90l9FTvM9zRl2+k5jPCcqScs5mi5yEQ8F9cuVO4TPSYrzhbp0MFp36Xm9jD3PkzSqlIbVhdZZvCU4WeT2iDzvuD4B5GNGVU86xQDH3gXlTg8XDvbhc9pqvHMV3Vwz9lQfc9Q19ow6+gNqqEkr4pNfxQt8bzp1uAPzELv5LZeNafDwvFvhk10+NsR9FaagIWiKbTiDrR8CfM1rHIWEyO8Ynpo4vNIwqjLxnoG3Ybdtp6JIKP6BRq6O5C3WrCOSMYsy9ojuVGsJhdpiBb6uRI9YJiw7Q+NgvbGK2wQmv2xzz8KPlXKyT8dRtoHQMXkkMqdF8JzfhzSjBRaRYLbKfjIQjPEbTjlP0sVbFj9dnCqlRqxsXAQeXpPOZCbE7MDczPTC4zmwu7MNwQG6fKYe1RBi3e8s2hGQG5U69CVuHzG8HIbuMpVvGUDrHUmdHN1zkAAy4cXTXlyXwVYyb5HvrLr0mnqeIIs4YcQIXmOYzlIHJNjWU2HuOF40g5tG21nlkbnAKJGOr6umc6qaOCmLH5FFT7oDhqcm1GSkUu7yGfLF3dEamxp3aIgfNb3BklpByOMuUm2IXFKXXd1z7R1oYQD5FvLdH5KxmP4VRI4rZWyjHNbAUv0aQT7J3kq9ZhiO8iFZUbCk3gWeJmO7jY9p6xAeOQYSgtxg89UYtRNHyVCAvO4SCHDvpXFKFzqNnBhfATD6HRNWg7yprpd5a1H9sR3y5qH1O6aNqfP6aKnRxkMDyf6bMb5E7H1XOqL0b4vdLZYRmsO7oOUbYDe7RvyQ1g0CphqWzbQZUexXhZETu78bYQJNtkNCGTkJ6YFlVYqJ9rZfvQRmFdBqR7kqWdRNqxRTNPMiUmBHO4kUxR6WUvvjc7Jzv6iJQRrWP4MHsH7PjpeEq9pZKN9AgglDjjD0EDgEmO2rUL6dQrbO3NKOxSCXSM9M8JGZPqBu40uJPd19Ctm4Ps4xv1UGIr2aMFMG0ZUDEb9sPqxBmqm4Eh5XzkqKc9aL2sNL1SBgZ0IyRKtwfGP0VlQz6B1gdMNp3SzJ5qOHG9eNtTnGKPn0AtUv5WBmHHsSlcN97ZY3ytFgFmglwTpROIh2Q4nE1ezj3gyXfLKB2h9gk2a3Y8qtFHhXIm5aUwiP6Ta5DnQtdUHVyHIMOGUDADGn9c3sHkdrNMtvWjJq7HRazzgYZUIYRFJ8jpavbmpuVPpJ7KEOV7xamHvmIHEhsFMXV0nVVmcnEvjO7Gf8tyJNzeVJWSjE2TIqbYr0oSa5IKsH3AUCvxS48okHYHIyCy3t6RafNjBkvf6l8MbcEv85ldWWNsfO67rBcnzMq1tMIXB9PGRpYdAhh8vWMZS2tz6jxEWejLB0bdwBV2MV9DOH8WgWeVsJbIqBVaoPeNVJgxm1RqGQ3g4QVk1S2UE7W7IQNMUs0CQdbICnLL0sagB4dzdk3dKNzrDqW5U6w8EfSZgN1LlAvTh2Tmx8lHPRuYE0NIjlDGpuhMgQ6mgYCNxf3ATLGxsyzw9S9h3Xd7WHuavfbgOQxMJvE8H9sP5MQ7TqpqdIk9fT0CiLYhhvZPqrHFuUXVemzKiyV2rxFPbVTvmjFQIqJYdPqY1VUS1hOkv6x8OFtrjvmSGfFFB9g8fNBPF3B56HXGhFqfQUHoAD3CDD0JGyb4i5uNJjYyrHiBt2Xmk2qoCzFI3K6shDJPR1E3MiaTLDeVUozGcy6QFo6jiYDeYLy9JbTkwz6fsa8C3J8JKqxsH8Ls2WTTqTYKS3RoY9FyKuRe1TcZ2MMLNXXJXbGvaP24pvgZBzW0eHMLffdTT1wYnIRERuORzIIPkcrFcj1zhFGM6hX0PLnrn6pSy9iSj2ckmSqb8pbYjDTzvR8lZqwMYPuMMXAvhZdGBjwO5Y2B9rLYkvWk0JXtDqXTYr36LPqUFwYqJJlW1DWrsJeHSnCmVSlOR86qO0PADKz7qvvJg

nB0nc57dM6TGJ+lAX33ObZfCPyBt32lWm+Il3zuFPI6+VMEVp+rvMWMI4PP/tQxDaKSqvtZ7Y9ceg
OkcZHq/dff626pwEAWA1k/kXgCTs1HZ/UuzoH6eH6iIpG+pyGsFSf6Ds1nL3iL0NanWX0F+/o8eqw
HhprK7uok7oFS89Vkd2bl+dpXfIsoHk2Z0T4QD2th2dV8sSmxci11eOdI9Vr79parARzc+nKJf6gfqqve
uDYbpHDR67f/eKT6s4GAGAFkPkXgSfsmHSkspiDjZAlxcNSfaKeTnfzNiini78MaXdWvVvncugzLJ2aJg
ZL/0eQ881jqSf1tC55F9C8Wf8IY3p71YHdtbogDYsJX55qiDujZ22tVoJ52Nza3Le+T8Q2ikqr8b/G7xZf
7AcArBQy/yLwhB3JDl2eBGQOyN5uG5bccd4zxcH4y5CWZ8Wvhj6rELliFdklLViaZ6mfM+4JseUFNx
ZcQPNm7RHOEGOt3tMzrS9IfDFmp/w09bqcwjejs7bWK8Gotq5uHX3vqIhtFJVWN7903dMH7hQJf
C7FF/sBAKuDzA8088RRYDWc/PlJEdsoKq22n9l2/P7bRfaeY/HFfgDAiiDzA83I/FhNZy+eFbGNopLr8P
FbReqee/HFfgDAKiDzA83I/FhBFzYuiMxGUcm197mbRN5ehPrBoW//8fPfV3c8AAATReYHmpH5s
Wo2Njd2vblLxDaKSqvF+Rq/W68ee5gv9gMApo3MDwCwbG5t7n9rv4htFJVcj373NpG0F6rePf3D6
tYHAGCKyPwAAMsjP31EZDaKSq7vPP4NkbEXsH79sw+qux8AgMkh8wMAaqc+PiUyG0Ul17eev1G
k68Wsk/fewxf7AQBTReYHAFTOfXpOZDaKSq4bT29/6r59Il0vbPHFfgDAVJH550v92XCFQf6AuMvrR
9bOVz9P48+is3fkYV1UpfdT0P5umt5NOFEXv7i447UdIrZRVHIt+Nf43eKL/QCASSLzz1GWhMrwU+
o/AlUNJhS33B25vPm991PQ8m6a3k04VRubG3t+tEdkNopKrvue3CUS9VIUX+wHAEwPmX+OBs4+
+h8pTCZu+XbkUIM6bDHhFGRa3E3Tuwknauvq1sF3DorMRlHJtfuFHc/uv0vE6aUovtgPAJgeMv/8tE
qwM5he3Gp1xdQWO1zVhFOQIfNPx4kPTojMRlHJdcMr137/4F6RpZeoTh/93l++
+qr63QAAYPmR+edDxR6DTh1uiqi5UiqIbGREpnoaEVdD85txyjjpi9rBY3hzJzWPNMJ0eFp63f0SXqBYr
WKM3tOzeN906/bKeZeDZ7D4c2WrPcz1hBjvnrr5Qqj5wW07WUNdC6IeDNwKZR63Voxo3maMca
dxVq0eLv1mtHsjV+9ITIbRc1SDz24R6Topau3nj9Z/XoAALD8umV+/WwdfrD2DrEe/gMBobX6AX+J
H+9Dccj/Vsa4ap4BzjUNze+fPGdMYUUtJfqx9TKtfr88Jl6GdiT4mjTcJ11PUSuxrog82LTZwCWrR3jeL9
70nxffY0KvSvOKc+Yog+eE/m7CzqtBwIeffSgCG0XNUgdO7BT5eUnrk5+8W/2SAACw5EbI/OLhfdYn
cnO6aNBZeN6rqaOMuk56lL5wRtqJXgDf/O65zvzOaeqcyCfnTlsfkX0i05pjfF19OxKMlZgia+9+ilqGM
UKstnGzcgp9gp7TWJW529BE9YmOlF71EdVcn6WO1AcivetRxibcds6SnNPkvlutGY0uXbnEH9RP9Vj
f/Ke/W9Kv8bt18t57Pr90qfpVAQBgmfWe+V3O0/pMjKSR6WfOOYnFIXNjcpzONg27j81vHBPD1Etj
cs8hobdp9UQVu6WY08MZ0XyxZjhFD1CnVAc8O/McsrRchR4WnKiFcK/6usoxzjkZfSyyFt95ze3Uy8
gFbLFmNNjc2ty3vk9kNopKru1ntj1x6A6RnJe6+GI/AGAahs/8/T6I6yf/I3YCWEaeKyOyY0VvujzqSTte
vivvaSCG6dk9gg2bA1jrac2B8tP17aiRZ8tNGk8JfCJqWa03W7BHO3PYJ+hLUOuwsfa95KVWQ6xm3
oM230fmOU0Ms5dpEwuKrBkxW1e3jr53VGQ2ipqlHji2W2TmCRRf7AcATMCw/22/59Hd84Sea5lb
1Dlr59VPXQLPgvFcTX+IEgP9g1ye+X3nBmb3MSay9TltPdLZoG9HjRJOaj5FjSiWqFasR7fZrO4hqPc9l
7QUODG2u4Re8hJ4lxNcY03Ok2tup0b4hBfk64WQkz8/KQIbRc1Sdz9zi0jLkym+2A8AWHZzyvx6lC
mSHCrqtHwS9XPzWYvKczX9IUps1T/I5Zm/RVJqO7ulv2n1aQUx2rejRuqkDhtqc0q1oWyIGt30MZr
UiEw5yNla4xzGFIU2A9v3kmO8y2lco6dXprldi4lbrBlBZy+eFYGNomapnS9eP5mv8bvFF/sBAMtu+D
/Dz3OOc6jFI35OnVeepl41nLS4YlfG3JQc1/Jq+eZvkZR8ZzXqa1o16sj6um+TjZP4Bqi1Bc5JOKVUDTq
ytlZOYAxuXKd7vdQRfYo7RMve6tAsrZec1ddFH/OsUfGd19zOd5bQYs0I4v8bP9VvHb//dpGTJ1avHn
u4+uUBAGAJzSPzqyOep/VIeHAHtTppgblXJqPzmNxlvU1P2vHyzd8l3akDeknmOm39TGudol9E5nS
16CLX2uIUPz2uYA1umlO2qMfreeQyS/rE+rB/YC2tlx6lxjhbMuYJtc4482RatHO6yV20WjNCtq5uHf7
xYRHbKCq59j53kwjJE6tf/
+yD6pcHAIAlNI/Mrx/fPSJP62qi+iHfPbJUAlfTf3mMPXrSjp89U9GlVVLSRyyRj6aXafXbahK9/HpWz4
4ke4hiDHXX2nhKgHmeHN2wWX/LnBriLrMUOjO83KRe7scX2FJOrtFkdy9ma9Wu4QK2XDOCrvz5C
n9oP9VjHT5+q8jJk6l3T/
+w+rUBAGA5LU/m92eAUixzLK7I1bQ3K973pJ0Q81IXw1snJXsFTa1mn7Z+wxivl18PdHbkYw5qefmi
p4R41meKX0OzY95Pva56+5dZshfbYrndezV/fNkbsTXWzObFyKR2skvrSRDE/3N+qsfafmbbJP8L/1e
PPfzXr7+ufmcAAFhO88z88aQg2I//UqepgH6om5LbD0vq3KfnRHKjqOS6+aXrnj5wp8jMS10n773nj
5//vvptAQBgac3z+/yRWRz6nABBSF8am70luPiyxl3/5skhuFJVcd/3Pm0VsXuria/wAgGlIzPxeRYR3Ar

0n4dfTVMf0f/0b+qcAnklK+g1yF0Zj/tfq4ZsWWBKPvf+YSG4UlVwPHNstkvOSFl/jBwBMxlwyf2iiYHi
KJPvIW8AwjMxP4Mfy27q6dfCdgyK5UVRabT+z7YlDd4j8vHTF1/gBAFMyp8yfsyeLRfZoro+
+CQBotLG5sevNXSK8UVRa7Xzx+mf33yVS9BIVX+MHAExMt8wPAJikCxsX+GP8qb7q7mduEUF6iep
3v/ik+q0AAGASyPwAgNzbv31bJDeKSq4l/WL/
+6+/Vv0+AAAwFWR+AEDl1MenRHKjqLS64ZVrv39wr0jUC16vP/k4X+MHAEwPmR9x4s9jiL/sRP1Je
NUfxDDLVF1Zf/D+4H8QRLazEfaUG/MaYrKOvndUhDeKSqvdL+xYoi/2/+DQt7/8wxfVrwEAABNC5k
dcPOTPEjLnlfl1o9Kwkb9qNlIGH+0aYso2tzb3re8T4Y2i0urAiZ0iWi9s8TV+AMBUkfnRiUiVs4RMkflH
M2LfsTM4mR/9+OzLz/hj/Km+6qEH94h0vYDF1/gBABNG5kcny5/5x8zFZH4srY8ufySSG0Wl1eJ/sZ
+v8QMApo3MPxeBuOs9rHNcofmMhtyn3q7PcI+oafMjYrr4yzg9OJONF2v3TmWeYi4wyjrJOku1NA
QWbi5OrNti9zK7hXqJbeecrRtj6mmqN8MdPRMFiDnabyrTZv2Z6Bwla4j7fosZMKyzF8+K8EZRafWt
528UMXtxiq/xAwAmj8w/H57g5DtYBz6TcVLLAGZS7+u3dRM9SXWkeC2mi7+M8G8lV7V1ptIHTMZ
GveJXzPNuYOGNy81Fm4V6qePGPM7WPeeWw6Md230c3stqnNHQInX9OWNVTR9u8wwYxYkPT
ojwRlFp9Z3HvyHC9oIUX+MHAEwemX9OVObxxBx9SOcedUQHJWeIMY0TwKRqgDrFSFfVGWqG4q
WYLv4ySI+TPTLVQppm9mzV0eKKuRN7uVelPiK20LGXZx/OMLd7obGjr5/NOUVcssYWbdYvhziTyB
OcMxpnwEi2rm4d/vFhEd4oKq0e/e5tIm/Pvf7tX/
+lutcBAJguMv+8qBSjQ4yTpTzhqjFdZZw85ahGlOeUL9bW8onMI+p8MV38ZYhvmD5Wrd0eo155N
mYcEtpcMfeAl5rKGNV4ojvAd0qbj0x3D++1IE9MWGJ5KDQ+srbw+vXL0PLVAM8U5aHGGTCiK3+
+wh/jT/VSN57e/tR9+0TqnmP98z88Vd3lAABMGpl/bkTwUVFKpy9PtsoEzjIG6bwUznHVWcWA4ucj
6+eLs+oj+nQxXfxliHcv4qA9lXrTJ9TL20XPq462W7LuX4+KnWgvVw/wneJZpTPM013ydmzcm6d5m
H9TLdavX9eshvbEtnKKphkwrktXLu14bYfIbxSVUIvzxf4Xjxz+jz/9qbrFAQCYNDL//KhcU4QZFYPqsOa
PZyJetQhgPtVp2YhydPZDeUgf0WeL6eIvQ7x7EQftqdSbPqFe3i7OEtst2TOXe6I+IrgDjF5tZvbvJNPQ0
dfPEpy41rSpWa5M9b6awkdNEZ8Bozv36TkR3igqrf77Y98U8Xsu9e+/
+U11cwMAMHVk/nmqsk+WnlTCMSKNP56pgdXRVgHMozxP/ev94uzikDpSTyimi78M8e5FHLSn8p
7RwH+Omlcdbbdkz1zyRDUkU45yZ/b1ajFzaPeNHX39LIGJa82barN+zZiuUJ7UuAqDfwbMw8u/fF
mEN4pKqO1nth2//3aRwEcuvsYPAFgpZP65qgLNkbW1MjSZmUkHKTPlyHTlSU86JIVyX66ax/0af3n
EmE90jL8M8Q3Tx6pe9piWE1vkjAVnonYzey6sONEd4V55X682H5lnTKa5Y+PeQgtSUza38Axxx2Sy
Y8Yru2/jMguxGTAnj73/mMhvFJVQN7903dMH7hQ5fLTia/wAgFVD5p8vnZYKIs7oN1W80rlH5y19
RJ1rTBgNR/WJvlONPCeyVvxlkLNOo3/VLDSzOkMvL9KqxRWTfQLUTOErIddTL7ie2derHlcdNK67Gu
bpnmnu6Otnc7rbB1psypnBWb8+UC9fbCg8R9sZPAcwgq2rWwffOSjyG0Ul1N7nbhJRfJzia/wAgBV
E5p8znW4yKlXVzHdrZsoxQpnkTmeqT3RSlL9BOV38ZUR4oVU3Zyr/GQ2NOlyx6EyeRClP9LfK1TPbY
8rj4SuhT/R0zzV2bLO3QPuqVYtNdVi/VC/LP0n9fvMMgWuEoW1sbux6c5fIbxSVUIeP3yoC+QjF1/g
BACuIzD9vOtyEsoudjurMo5kDsrf1S89QkzvOe6Y4GH8ZpwdnsvEitMW7F1rGO/skubJ2S/YkSs+JZjL
Nj6rXxtTmED2ZucJsrDOzp3sl3rHd3uQVEm3iLQpN68+Z0+TcJdmriO81Z8+g3nXPw9AubFzgj/GnZq
/tZ7Y9cegOkckHrU9+8m51EwMAsErI/POmgg/RBcCyePu3b4v8RlEJtfPF65/df5dI5gPVW8+frG5fA
ABWDJl/vvS/6iTyA1gmpz4+JfIbRSXU3c/cIsL5EHX66Pf+8tVX1b0LAMCKIfPPi/0fLrv/4TMALLaj7x0V
+Y2iEuqBY7tFRO+3Tt57z+eXLlV3LQAAq4fMPy9G5ifwA1hCm1ub+9b3ifxGUV1r6C/28zV+AMCKI/
MDABJ99uVn/DH+1Oz1zX/6u4G+2M/X+AEAIPMDANJ9dPkjkd8oKqEOnNgp4vrsxdf4AQDIkPkBA
DM5e/GsyG8UlVAPPbhHhPZZiq/xAwBQIvMDAGZ14oMTIr9RVNe64ZVrv39wr4juycXX+AEAKJH5A
QCz2rq6dfjHh0WEo6iutfuFHb18sf/d0z+sbk0AAFYemR8A0IMrf77CH+NPzV73PblLBPiu9eqxh//69
dfVfQkAwMoj8wMA+nHpyqUdr+0QEY6iutaj371NxPj2dfLee/74+e+rOxIAAJD5AQA9OvfpOZHfKK
pr3Xh6+1P37RNhvmX9+mcfVPciAAAokPkBAH16+ZcviwhHUV3rW8/fKMJ8m+Jr/AAAuMj8AICeP
fb+YyLCUVTX+s7j3xCRPl58jR8AAC8yPwCgZ1tXtw6+c1BEOIrqWu2/2M/X+AEACCHzAwD6t7G5se
vNXSLCUVSnuvml654+cKeI997ia/wAAISQ+QEAg7iwcYE/xp+asfY+d5OI9279n/91prrnAACAg8wP
BjK2799W0Q4iupah4/fKkK+Wa8/+Thf4wcAIILMDwAY0KmPT4kIR1Gdavu11E/bJ+cOjbX/7

hi+pWAwAAPmR+AMCwjr53VKQ4iupUoS/2/+4Xn1Q3GQAACCDzAwCGtbm1uW99n0hxFNWp7n
7mFhH433/9teoOAwAAYWR+AMDgPvvyM/4Yf2rGeuDYbh34+Ro/AAAtkfkBAGP46PJHIsJRVKfafm
bbE4fuyAL/0wfu5Gv8AAC0ROYHAIzk7MWzIsVRVKfa+eL1Webf+9xN1S0FAACakPkBAOMREY6iut
YjD9yW/W91PwEAgCb8XRMAMJJrrhnpbzo0SkajZDRKRqNkNEpGo2TTa7QKuJQAgJHwRJKMRslolI
xGyWiUjEbJaIQILiUAYCQ8kSSjUTIaJaNRMholo1EyGiGCSwkAGAlPJMlolIxGyWiUjEbJaJSMRojgUgI
ARsITSTIaJaNRMholo1EyGiWjESK4lACAkfBEkoxGyWiUjEbJaJSMRslohAguJQBgJDyRJKNRMholo1E
yGiWjUTIaIYJLCQAYCU8kyWiUjEbJaJSMRslolIxGiOBSAgBGwhNJMholo1EyGiWjUTIaJaMRIriUAICR
8ESSjEbJaJSMRslolIxGyWiECC4lAGAkPJEko1EyGiWjUTIaJaNRMhohgksJABgJTyTJaJSMRslolIxGyWi
UjEaI4FICAEbCE0kyGiWjUTIaJaNRMholoxEiuJQAgJHwRJKMRslolIxGyWiUjEbJaIQILiUAYCQ8kSSjU
TIaJaNRMholo1EyGiGCSwkAGAlPJMlolIxGyWiUjEbJaJSMRojgUgIARsITSTIaJaNRMholo1EyGiWjES
K4lACAkfBEkoxGyWiUjEbJaJSMRslohAguJQBgJDyRJKNRMholo1EyGiWjUTIaIYJLCQAYCU8kyWiUjE
bJaJSMRslolIxGiOBSAgBGwhNJMholo1EyGiWjUTIaJaMRIriUAICR8ESSjEbJaJSMRslolIxGyWiECC4l
AGAkPJEko1EyGiWjUTIaJaNRMhohgksJABgJTyTJaJSMRslolIxGyWiUjEaI4FICAEbCE0kyGiWjUTIaJa
NRMholoxEiuJQAgJHwRJKMRslolIxGyWiUjEbJaIQILiUAYCQ8kSSjUTIaJaNRMholo1EyGiGCSwkAAA
AAwDSR+QEAAAAAmCYyPwAAAAAA00TmBwAAAABgmsj8AAAAAABME5kfAAAAAIBpIvMDAAA
AADBNZH4AAAAAAKaJzA8AAAAAwDSR+QEAAAAAmCYyPwAAAAAA00TmBwCgq/Nr19TWzldHA
QAAFg2ZHwAwIWUYHzaFX14/UncYsuHY/2Qh31jmyPrl6kCfrM0U+GclXVnXcJiPaXCBX5jq3iv1e2d
EfkUjb3Xln8r6xPrd1rCiV2bQv1AMKLypZf2cAjuyttPXx9Si15L+NWlFkPkBAMMa8GneYvQZ86GtaD
vEs072LKX3UTxXDfxAlW/kyNpa9j+D9Mm3MPojofk82v9dYd3ZtYHuvmIvI90R5nXrr0nwN9T+HbI3
OpNgx+hbXYWmyo/rA+UlnbWVfxbz45q5ReOVyd/v9S8Uvk1ZWyrMtq/Ipoq3evxVcrdjNDcNtKOiv
z5UvJp1d+169dCqvHYVcX2sqyjeQwtkfgDAgOznKftppFeqUfF/x3wgsHc4lMG75A2qyzdMm/yzH/
wymcb5YCxFy4FuPjn3cL36fo6vqM/DXbi8NfraWrhj7K2u2k6lxlUvOyvOL9ldssunD8z+cTVtJz9evT1
Tn1JsU33MX4lsynMoXXA7wuxNwzuSRwbspd6oXs76qeXT6emLO7l+abcq3pxpT6uIzA8AGI58BnC
eGno3fAdhnMePgbelp89/SH9oi5ntcbCz0e+DzLC3gn0BB9tfMbH5QfX9wTkrd7fS84WMXKseL2PT
VMX7yRdSnT1sF4O/kT6a/zBzG7VYT69h/nLhNuplH5XIdmw93t+Nl65xMe05Uzn76LGZvprFC3k79
NloZZD5AQCDcf/O3OPTjt/IDwPFfvp6ZgyyHn/6Z1yz/MdhGhVXqjbwNRtuH0EDf0iZ8hJmLcofhrnL
nd9Q4+7ohTNf3tG+bj23jEzXY6emqZwLm6SpS28b8k1kHMt/7O1m9/Ryb4o+uI2KPvkXFbTZu/oun
aF4u6+9eXsVt1reovwhvJRunF6By9nP1szL5G/U28ZWBJkfADAY9wnA+4jSp8EbmIoHj76e3lzFXioD
binfhZo+bznchpRyY0P2Ke68nh/l48a58Yo7rjBUK3cf7m/xTJwG7vw9X8vIdD12ik9VfHA9XMV4l+Ld
fj4rT6N8D+pI/nZvN0Wgl6GnVm4j8bkUA8LXtx3Pdgzxd7sKzVZfv95aeXoVR+TV6+mzKnZQdct/tqft
9yquBjI/AGAwc/hb9XjPAuJpcVC9PkzZ8m3U1ytvNMqe3FujV+LDKa/fkLdF0XDQ+87cQ/nzMP2Ku
eWl6/GjKiY0V+7eCs6Q2USm67FTbCpxO87A26U4WOnrsjmN8j3Ur/O3e7spYpcuU26vj25uI+fm6+
EXObqdHuY3eXqVl6s8Vv7cUz/vvor9KGtr1k0yg2Ja/cE4n1L8GsOLzA8AGMwc/lY90rNA+aQz4jNH+
fDWf0Prkc3QxxN2TNF3uOvn3HnD9is+nUEvmdthwB1ZN0V/z/El5zfU/ZV1Pr3ZuA20yFtdBacqrmZ
f22lYcPF2L81kI+ueMAzRy9XTHeE2ciZuXkujyBTFW33dCzmnl9uh+ORm21Gp+dLkI/rYnfyVcT/
+5rVAIvMDAAbj/o25p2e3sDGeBYoeIz9wFD0HvXKFvM3wXYbfzhCP8mG9PVWHuS0G3ZGh71vCX
bf8sPreWWS+Hlv5pyo+tx4307jgYkAPDRsa5W/3dlc0bqqv3zC3UXHE3Ii8GROEt9PTNgxOL7dF89V
tqXGifMDsN0WxA3sat3MPH9PKIfMDAIYj/87c2+NH0PAdymcSqffnj6yLsQ3Pc9Ag+nlqc2XzGtMW
H9Kgn1LRwdzIkM+IxYcz7D3n3gGjNM30fkcUH429cGtvzkc3M09HJfJWV56pikP9fkiNCy4G9HD5G
hrlb/f2Ic11U9YvUi+/VcHtDPA76/ayfpky/TWNf0z9fEbFat0m+WFz7vhS4EXmBwAMyHoA6eepwK+
YW1juZwKxo3E2kzcd5BMafTfWo25/z72uIe9qW/k8rI1wR/S5N3EHFOotmHvr62JGOsYX00l4KvF5l
WbcXNHNWmnWxXhd9JylR8srkw/r7aZ3NpUdMCYvl+RZRGvxTVnvztKm4mynVBwe4d7u/S8U4V
72OzNfunI67zTWfd3rlVwdZH4AwLDMBxD+Po3RWA+kMz+PhhS392Czz0HPz/Hohf2plKrPRry1RJ9
YeFNLuafIZ5SZ3F8oeif+UUWpfmTgQWJGZH4AAAAAAKaJzA8AAAAAwDSR+QEAAAAAmCYyPwAA

AAAA00TmBwAAAABgmsj8AAAAAABME5kfAAAAAIBpIvMDAAAAADBNZH4AAAAAAKaJzA8AAAA
AwDSR+QEAAAAAmCYyPwAAAAAA00TmBwAAAABgmsj8AAAAAABME5kfAAAAAIBpIvMDAAAA
ADBNZH4AAAAAAKaJzA8AAAAAwDSR+QEAAAAAmCYyPwAAAAAA00TmBwAAAABgmsj8AAAAA
ABME5kfAAAAAIBpIvMDAAAAADBNZH4AAAAAAKaJzA8AAAAAwDSR+QEAAAAAmCYyPwAAAAA
A00TmBwAAAABgmsj8AAAAAABME5kfAAAAAIBpIvMDAAAAADBNZH4AAIDFcn7tmsra+epQSb1
xZP1ydWQYqo9ovwjqa5NxrsPl9SOht6LvAcB0kfkBAAAWi5Fr7Xi66pm/ju0F32UIJvv6oi7gP8kAgO
GQ+QEAABaL9e+yzYS66pm/1f694b7+RwEkfgArhswPAACwWKzMbwbcFc/8Org3rEtfP32d9BESP4
CVQ+YHAABYLHbmN4Kqk/mdA24wrobkI4x5qxOMI2YaVoezY8Z/Tm80UYx3nfeNtdVtGjK3NZ81o
bFSJTKXHl2M0a+cHcTWn7PXI4ZENhg9DwBGReYHAABYLHWYXC9/UpHRSJnFa/eAETerSFzH0UY6
RIfPMXK2zLUFYyGeSaLR199Ubt1grMVRL25tXf0oujetv2FB4Q36z4suFwCGQ+YHAABYLCo0ZglSBd
MyMBpvFAPdA7HMXw0yIqk8omKpc059RB2QbfQIvRSjT4u4G26gJ3R7xujBlXqaQuP61YDwegIbD
E0kVwAA4yDzAwAALBYrNVYvzJ/d2GnESRlm1ZA6l4aPqGncEXKMTMQZeUhPYowJkivIyZ24B+L0A
nLilBbrl5zu/g3qYa32DQCDI/MDAAAsFn+8zqKmk4w9UVlGU3dI8xH12gzK9rxqhI9obM0S4C4pp1q
qo3JrjcJLaLF+gz1avR+YXa+yZm8LAEZF5gcAAFgsMgFXr/XX+
+sMKUdmhsr89kH1wifYOMw/Vm4lOfO7S2ixfl94L6j3g7MHTmy7agDoFZkfAABgsThhUoZI/YYnds
pg7A5pPuKZVszrGyG0GKL5x6qW6qjcWqPwEpoXp0ZkykFO98Y5jCkK4WYAMBwyPwAAwGLxhEk
7Puo3YiN7yPxmuLbHtIjfbpswPZ052OnRY+ZvnMo91bkokQ1mbxkTd143APSIzA8AALBYfGFS58acf
qM+WsVJHUydI254DR8Jz6KHhDurYOu2iQk3qM/vnJ0jS2hav9xOPb5hg85GossAgMGR+QEAABZL
Q5g03zCiqCSybCyCyiP69RE5u8q7GX/reoDbJk6Nt5ln95n5G9fvX05ODQnNHjqz7aoBoFdkfgAAgM
USCJN1SLXeMLNrFitlMHYnaz6iXpvTOcvJ2bnZHhDYRZQ9nwzJcmuNGpcQW399ei5vaVwW823f7
OapubYrBoDekfkBAAAAAJgmMj8AAAAAANNE5gcAAAAAYJrI/AAAAAAATBOZHwAAAACAaSLzAw
AAAAAwTWR+AAAAAACmicwPAAAAAMA0kfkBAAAAAJgmMj8AAAAAANNE5gcAAAAAYJrI/AAAA
AAATBOZHwAAAACAaSLzAwAAAAAwRX/72/8H2Y1tvWJkJYMAAAAASUVORK5CYII=]]></redcap:
Attachment>
</ItemDef>
<ItemDef OID="b26" Name="b26" DataType="text" Length="999" redcap:Variable="b26"
redcap:FieldType="descriptive" redcap:BranchingLogic="[q24] = '1'"
redcap:InlineImage="1">
<Question><TranslatedText></TranslatedText></Question>
<redcap:Attachment DocName="B.png" MimeType="image/png"><!
[CDATA[iVBORw0KGgoAAAANSUhEUgAABVEAAAEJCAYAAACdcmdaAAAAAXNSR0IArs4c6QAAAA
RnQU1BAACxjwv8YQUAAAAJcEhZcwAAEnQAABJ0Ad5mH3gAAE9sSURBVHhe7d0PjCRnfef/b+/aY
PPHNmBskx+RHat7sZYlGJIg08PxL4egZ+6SJbnbYOVg5Oh3PVyi0zQSkwOxF+mX2wguG4lunS5iWrq
zliQyTEJugrLdIJRgckzHR4IxYTI/Z7rlc3T8wDYGjPljB3u3f/VUPVX1VHd1dVV3VXX39PtltaerpfqT/T
O/OZ5/k+hb5FAAAAAAAAAAChjumPAAAAAAAAIAQhKgAAAAAAAAAAEIEQFQAAAAAAAAAAiEKIC
AAAAAAAAARCFEBAAAAAAAAIAIhKgAAAAAAAAABEIEQFAAAAAAAAAgAiEqAAAAAAAAAAAAAQgRAVA
AAAAAAAACIQogIAAAAAABABEJUAAAAAAAAIhAiAoAAAAAAAAAAAAEQhRAQAAAAAACACISoA
AAAAAAAARCBEBQAAAAAAAIAIhKgAAAAAAAAAEIEQFQAAAAAAAAAAiEKICAAAAAAAAQARCVAA
AAAAAAACIQIgKAAAAAAAAABEIUQEAAAAAAAAgAiEqAAAAAAAAAEQgRAUAAAAAAACACISoAA
AAAAAAABCBEBUAAAAAAAAAIhCiAgAAAAAAAEAEQlQAAAAAAAAAiECICgAAAAAAAARCFEBAA
AAAAAAIAIhKgAAAAAAAABEIEQFAAAAAAAAgAiEqAAAAAAAAAAAQgRAVAAAAAAAAACIQogIAAA
AAAABABEJUAAAAAAAAAIhAiLp0etJYKUih4DxWGj29Przv[illegible]
AAAABABEJUAAAAAAAAAAIhAiLp0etJYKUih4DxWGj29PqjXWPFeU1hpWF+1iMxj3ZB2onXjpLWdL

MxLO7LSlg372PzHqPs4Vb2GrGzEO5vtDbdtK5JH08KNuQ9Cjmc+2g0AAAAAwPwhRF06Rdm8UJey
XurU1kPCkracr3X0c5Hq2U3rq4B5oILBVWnqJUdZzqxle4faf1Qo1cT/rlhsR+14AAAAAADIGiHqMip
uytmqfi4dqZ0P9kbrNc75IVW1JdsV/XzhFGVzry/9vnpsS/aHkff+RpmXdmShKwdu8leuS9c+xj3ZzDJ
D7TVk3fijwsI7ascDAAAAAEAOCFGXVGW7JV6O2jxn9EY1e6FWpbW4CSqOot6h7OuncuoEPaQBA
AAAAEAuCFGXVkW2W26M6vdGbW/4Q6XL9a3wXoyqlqKuRek+wmpSjqyvaH59ZI1Jo6ZjoC7rqPX
GPu31edcGHd5fZI1J4zwEz5+5nbDPjxN+3INtCdS9tR4xy30ObGewraPOc7xjCm57uPZpYAh6c9VZF
2h4snM3eA7Uw9yc3Z6x+xzPPy71CLkXbAnaHvI9OHq7vqTHE6/dAAAAAAAcfYSoy6yyJXW3OGpz
VTYaDTnnJ6hyIWSM9FAIo3VqpQwmoCrK2hndwM6BdJ1nFmNId2dHLno7bcuubn/5zNpc9FKsnPa
D6gP/AGy9izv6PBo1Pe1wrCSDo63TPb8dqZUKUhrYSXM1aUimtjPY1qasDoZtEx2T2rZR+9S6H+te1
+kREu3HCSwHz4HSXB0dKibnnOtV70AUtW6Kc9TeGFHPNGS7E4vZbgAAAAAAlgQh6lILTjLVrLnBTF
nqF0Imk2pv+KGKV4+yL103ie3UZD3lhKW4dka3b18O3U23d42JhYxw0hjqferEnAz0rpz2yiY0d4M9
Ji/u6BisfEacDLUnjXX3GlSlpc9v3+0xnOr59bfvdUhWIdlAfdzx/O1494Hazrob/E1zTMbr9zZlc9v62PX
vV1Wv1/6cXXIi2X56jXUvsCzXu85r+8MlLiqR+4wr/BzteOl/krZbr3X/0hH2PRjY7rBkxzOu3QAAAAA
ALI+C9QtyXz/HklK9SwM9zlSwEhKq+K8rS71rTubTk8aK24tOBS/OZEYjX6963bk96Ubsy+dvW4Vde
9ZG1BBs1YOwWq1Ks9kcWu+3IaxdcdeNE387w+2yV3rnwG1/6DqbsV0VnO2FBNwB4e0wr3O11fc
nDEt0PSK2E9ivvuaS7JjMbQdfr41qa6JzF35+FOdanQqsS3p+lETnOlHbRwht44jjjDietO4RYBr3Pnyvv
OXCW/QSACAP99/xLnnNW2/SSwCAzB3/NZFjr9ILWBT0REVwkikVtoQGJP5Qeb/npMsYdm/2GE1F
UU6ccp51dJfTrj2WvywnT5+0e9QF11uqp/0AbA74vWmb4nVG7R44gZT1GW8ov7dOdT4sGbUo3S
BMfcIsazBr1jUo6ac28z7QPYSnOKZEvYkT7cec4f+kmIdQ3NyTvhmgTm3wHIWY6rrrurFuwJmaGO0
GMvDwEw/LOz/5Tr0EAMjDDY9fJXd/6AF5/Bs/0msAAJm7dI9I/zt6AYuCEBVBA6FScsO1P6fl1RVt7k
pbenJoj9k/JScqJ6z/D65XGeqc9ZIrrombLbpD+ttuIl09a/TozUtaAZl1DTJrOyHeSKpXqBe0GnVjgQX3x
NNPSOWPKvZHAEA+nvPjY/KWL94oP3ryGWm+/3555p8u6c8AALL1lMilP7A+PussYiEQoiJlGYRfXl3
RfTlsXxS7lKgd9lbEyVeN9XMZvhk9NO3A1+/VOyrwVcO67VqVQ480e0lOK1mv47yOaTHOXbjxbW/
Lhtnr1K2LatY5BRbUnZ+6Ux58/EG9BADIwxv/
+gZ5wQ+vsJ8/9NXvyp/WeR8GgNz0Hxa5/Od6AYuAEBUxleSkN1LbnBFfMSZJitM70Ri+HI+7744c7D
pf686+X7I/4a8fLjUwH4qbZ/0guOFOjFWVQIZacsoTKG6Jgvk22OvYvA/0seV1TIn2Y97LA8Pk1cz3hZ
xnoE/SdmNSNTtwdeukJv6eAubL+z77PvlM7zN6CQCQh1c+eK3c/PXn6yXH5z7+kHzlLx/RSwCAzF3
6gsjlr+kFzDtCVMRUlM2zbuVUc/Z1NbrYnOl8y+vt5wScijnre1s23JlrYvN7cqqJpBS3XmZRF0x117vh
6vxxe81a56Kmj3+wdqsx7F+aq7LhnTIV7Onh297K+dBcNQLH9nm/hqd7bHkdU6L9mLVbm7Lqre/p
me+ta1Qq+NvI2oTnyA9c3XYDi+kT+5+Q+n11vQQAyIOqg/pzD7xELwX94W9/Tb73raf1EgAgc5f/RK
T/fb2AeUaIivgq29LyctSalHTA48w8bynX5YJR4NOfUMnSXNWB0GQ1HAPbsp55Q/a9of6KMUnTHP
Jqu9rKUt8KRKiWomxe8IdlN1d1gOaFzqMm/ZolJ3AMttM8tryOKdl+ipsXpO6/2HmtOZGTdS/7h6Br
7yrua1NNWBO03bzfw9odR+bHA8R339fvk7v+7C69BADIw/FLBXsYv/oYRgWozd+8Xy8BADLXf1Lk
8sf1AuYZISoSqWyH11+stqz17tBiV3FT9gZfW21NVr/R7K0XKBlgDM3OdKKjFJgB2KiyA+qc9bt+wOe
ya1/OW03PstS7fT9Yt1Wl1d8LTpaV1zEl2k9RNvf60h16sXq5MUzeVpHt4EGK7B96PbFTEbvtVlv6Lf
8+sqlzblwHu+5ulByOB4jBnYn/6Wfp7QQAeVr50kvl2iev1Evh/uFL35ZP//6hXgIAZO6y9RvZJcpbzbt
CX81aAgAxtTcK4nSSVCHqQGAKADGo4PT1/+318sAjD+g1AIA8lB56od0LNa6tu8vyiteFD/sHAGTgit
8QKfBL9ryiJyoAAMiVGsJPgAoA+VK9T1Uv1CTUsH7qowJAji79AfVR5xghKgAAyM1HvvgRezIpAEB+
VP3Tt33hZSProI6iAtS7/+NX9RIAIHN2fdR79ALmDSEqAADIxe6Du/LBv/igXgIA5OWOL18/tg7qKPv/
8zH53Mcf0ksAgMxd/n9FLn1BL2CeUBMVAABkTg3fV3VQmUgKAPAJ189efL//8Czfppclc+dzjsnX36+
XWV79IrwEAZOtKXR/1Zr2MeUBPVAAAkKknnn6CmfgBYAZe8MMrEk0kNcoz/3RJmu+/X3705DN6
DQAgW9b77aWPWx+fchYxFwhRAQBAZlRwqgLUh594WK8BAORBBOW94WK8BAORB1T99yxdvlOf8OJ1f+R7/xo/k7g8
xKSAA5Kb/HZFL1EedJ4SoAAAgM+/77Pvk3ofv1UsAgMxd/mI3PtJ/igGALm5/
DXr0dELmDVC1KXWlo1CQQrGY6XR05/LSa8hKxttveBob7jtWZG8mzM3Qs5LHL3GinPuVhoy6tT55

9d55H7NjwLuWyCW+n11+djffkwvAQDy8vJvPE9e+eC1eildn/zPB/LN//19vQQAyNylXZH+Y3oBs0S
IurR60lhZlaZecpTlzFpRP8+eHfiVasLfVIImPi+9hqzXor5KXfOCrAYvunRqJSlMENguK+5bIB7V+1T1Qg
UA5Ovqp47LG++bvg7qKKo+6u//
+y/bHwEAeVD1Uf+79fFZZxEzQ4i6tLpy4KZA5bp0+33p9/dkM68MdWzgt6QmPS+qZ+S4YK99XtxN
V1vqevelWy87K5qrQo4aA/ctEIuqf6rqoAIA8veWvRvtIDVLqifqH/721/QSACBz/UdFLv2xXsCsEKIuq
96h7OuncuqE5Nf/FGmL1zOyJ41zugtquS5bFedpcfOsVJ2n0twlRQUwPTUTf+WPKvZHAEC+Xvu1F8
vLHr1aL2Vrb/f/2A8AQE4uf8l5YGYIUZeQXbvRDN2aq04tx8GuiKp3o1E7c1T9zGAtyGCd1bDejbH3r
wVreI6qN+kMVfdfl6zW52A9S6+2qH6ENy3GPgPncEP8zZjnydln0vOiqHaWYvWMNHoeB0LzkpzUn
VFl/9A6olGM9obVW21v6GMZPAcJrkvI/RZ2vePcb4PXz35E1ImNY5b37WT3p2vSa2eZ6D1Ar1TMr
49uJI6QOz91pzz4+IN6CQCQFxWevubvXqSX8qF6o1IfFQBydOlPqI86Q4SoCDUUGGl2/cyRYVRHai
WjzqrR43EyanuDNTzVurCgpuQNVXdFt3UUZ5+DwWRzdcJ9Fjflgjtk3joz5/RGeo1z3nkq1y+kUEahK
q1+y+tVOsTseTxK50C6+umwipx2N97ZkYsDJ7W96x2NX1c3yXVRQV7I/eZcj1EBZPj9pu7d0GC5U5
NSIMjOSpb3bcz7M2CCa2eZ7D0Ay07VQP1M7zN6CQCQFzV8Xw3jz5uqi3r3h75KfVQAyI2qj3qP9Z
H6qLNAiLqEKtt96Xfr4kZ7Um3Z9TH72zrxbG/4AZBXL9Won9mpyfrIxEaFec7r+3uboWUCxu4/wN+
et3/pyI6XBPWkse4GPca+Wzo1imzrKP523M2ofdbOu/Fbsn0WNy+If+rOS9v677wbglnn94JOUJOd
F4P9um2ZLK8uyolT+ukYlS23beb5V9ri53BnxMnhkpwj67VGqYGh+21ofyZj2/b9ZrYlbFt+kJ3U/Ny3
/uvD789hya6dZar3ACyrT+x/wp6NHwCQv5UvvTTzOqijPPTV78qf1hmBAAC56T8scvnP9QLyRIiKIW
bPtPoFPwgdDgOHletbE4Z54aotPxwsrp3xAqzOge4z2bsoO14eaey7suW3deeiJIl7zH364ZMh8T6Ls
nnWTbuaslpwe08Gz+8kipt740PW2PblMOpEFdfkTNjxtXf9XrVn1pzjSXSOrPOzp0NEI3gPvd4Dhu43
s8et0evSPk86CNzLYfa0LO/bsfdnmCTXzjLNewCW031fv0/u+rO79BIAIE+vfPBaufnrz9dLs/G5jz8kX/
nLR/QSACBzl74gcpkJ/vJGiIoBI3qm2Yqy5iYxIwK3UyfSDKjKcrKkn47SPdC9+VSoU/LqNhbMYdKRw9
QnMMk+K9tGr0FHOsP403RKoi+fcf2NgNIP3Kpy1j2gqa6LruEZOrw/aOh+M8JCawf20Hd33yNrsaZ
uHu/bBNduyvcALB93Jv6nn31arwEA5OWGx6+Sn3vgJXpptu7+0APy+Dd+pJcAAJlTw/r739ELyAMh
KibUkeHOgTHCo7mX3TFUvMKUSrD+5Oz05HBssVSf36vSHRZuBG7V08FeoUmYExB5PXXHCbtWR
dm8EN470w0rF3t+o8nvz/SvXdh7AJaNCk5VgPrID+h9BAB5e86Pj8lbvnijHL9U0Gtm60dPPiPN99+v
lwAA2XtK5NIf6OfIAyEqJjR/gWm53vWGbAcfk9YLHS/
+Po26n7aO1NZznJyneELGlj4tn5Sxl7S4KW5lAntYuDF8vno6/CyPP0dt2TB7nbo1OM36o0lYbdzT+xj
s/as0z83XpEi53bcTXLtoR+GPJpiWGsL/wCMP6CUAQJ7e+Nc3yAt+eIVemg+qPuqnf/9QLwEAMqfq
o15iYte8EKJiQElOusnV0EzePbnoFnIcO/Q7J6WTXtA2qm5m6ibYZ6+xrodpl6Ved5OsPCfnMa5rc1f8
zphdOfAu6QmJc0m9HrXW/XH+/I4OP6sSyOGSnCOzLqcKFN26qMaQ96TUzPKqx6k9GZQdSHa9W
p7pDpOf0CzuW0usa5fme8AU1xDz7yNf/Ig9mRQAIH+lh1448zqoo3z6v/6D/MOXvq2XAACZu/xZkf
48dRU6ughRMcCcBCnYW9IPAlXYNTChz6yYNTCbq/5Q7faGHhpuPdIev510n72GrLsnrnpWNjeNyY
Nq65JPjmrWsmzKrm5er3HOCzBj90asnBbnDulIs+ke18Bw8Amvix8oDvbcjUlvX80sH+xxaoTFZo9b
oz1p3yaRZnHfKnGu3QTvASU/dZXaee9gZMOb4h9Hze6Du/LBv/igXgIA5OnaJ6+0Z+OfZ83fvF+
+9y1qZQNAbtSw/v739QKyQoiKYeYkSJ2alHSoU/LTE7kw7YxI5vDy5uoUoVGwBmZzVQdQXnhTlVZ
qs9e7kuyzJ411d6h6Wepbav3okCq98zLMnFndbbN3TastiX+aKhIo72oZDmATnCMv2LO4x2xOsJTEi
HvXrLNaPevPNp/YQt+3SpxrZ0n4HuDXW7V41zBubVssGjV8/85P3amXAAB5UvVP3/aFl81NHdRRV
ICqglQAQE76T4pc/rheQFYIURHKHgYdUpOy2rLWu8Otp1KR7cGClfuHRs/BBOwamMZwbZddWzOj
eqgx92n23LN7obonbiCk8of1p3hehhRlc2+4Tqg9hD5hYBeYJMs6ZjsbHhT7uljH3G/5QaqtKq2+0dZ
ACYJo9r07eJA2Z5vTZZMLft9aYl07S6L3AHUsg6+ttiava4u59cTTTzATPwDMkOqBqnqiLgI1pJ/6qAC
Qo8vWb6bUR81Uoa8KBgJAEmo2fT0ZlAph96btmTxL+lhOtaYNWBfEUbp2yJUKTit/VJF7H75XrwE
A5EnVQVWTSS2SK597XLbufr3c+uoX6TUAgGxdKXLFb4gUbtbLSBM9UQEkFCxRcGZtsUO49nnrWC
J6ZB4tR+vaIV/v+
+z7CFABYEbULPx3fPl6vbQ4nvmnS9J8//3yoyef0WsAANmy3m8vqWH9TzmLSBUhKoCY2rJh17o06

pWaJQoWUK+xIqv7demmUqJinh29a4d81e+ry8f+9mN6CQCQJ1X/9C1fvFGe8+PF/NXt8W/8SO7+
0AN6CQCQuf53RC7doxeQJkJUAJNRNS8XfPx7cXMvpRq/C+YIXDvkR/U+Vb1QAQCz8XMPvERueP
wqvbSYvvKXj8jnPv6QXgIAZO7y10QufUEvIC3URAUAAKEefuJhec32a+wJpQAA+bv568+Xf/6Fm/TS
YqM+KgDkjfqoaaMnKgAAGKKCUzWRFAEqAMyGqoO6aBNJRVH1Ue/
+0FftjwCAPKj6qGpY/7POIqZGiAoAAIbc+ak75cHHH9RLAIC8qQB1UeugjvLN//19+cPf/ppeAgBkrv+
oyKU/1guYFiEqAAAIUDVQP9P7jF4CAOTttV97sbzs0av10tGyt/t/7AcAICeXv+Q8MDVCVCTUk8aKm
uVbPTaknWjdOGltZ96MOYZeQ1Y2gmvbG+7rV6TR0yunpfZjb3NEO2zTne9M2m1bkPugvaHbWJC
BSwosjE/sf8KejR8AMBsqPH3N3x3tuqGqN6rqlQoAyMmlPxHpP6YXMClCVGCGeo0VKZRq0tHLWe
pd3DH205RdQj4AA+77+n1y15/dpZcAAHm7+qnj8pa9G/XS0aXqov7+v/8y9VEBIDeqPup/tz5SH3U
ahKhIqCibe33p99VjWyp6bXby3l8WRhxDryHrtTziU6UnF3eC+2qea1hrF8VRuA+A+aZm4n/nJ98pT
z/7tF4DAMibClBVkLoMVE/UT/7nA70EAMicXR91Vy9gEoSowDLoXRQ3Qy2Xy86Tzo5cXJwUdTFUt
qXfrYs+w8DCUMGpClAf+cEjeg0AIG8//ffX2UP5l8m9n3xYvvKX/NsDALm5vGc9mOBvUoSoS8eoLbl
i9kQctd6oc2mvz7s25fD+IutuGnU/VwKfNLcT9vko6Z8z+/PmMP7mqvP5EYU0/WNWj+T1Rv2h/FU
5e+GMDvk6shMnRQ3UUg17jG5PvHbHuTbD59DaumyYrzfqkQa2MbA+/BTHaYN5POpY/P2rh7vd
9nnrupbrsjXQXdYu3WC83n4M3DfArKgh/A888oBeAgDk7YbHr7Ink1pGd3/oAXn8Gz/SSwCAzF26R
6T/Hb2AJAhRl05R1s64PREPpOs8s3TlwE30Aj0U27LbdJ6Vz6xZXz17ldNV/awjB/4B2PywsCxn1nRr7
RCwJIMj5zu1UswQa5bnrCO1UkFW9fYcal2SINUYyl89LZXimniHs3Nx+hCvfEbcU+2L2e6pr43Dfn1w
Z846FVQOrG+uDgSpE7VBHcuqeFvWoakKSlf369Ld2wxcdxW+lsJKN3RqUprnibKwFD7yxY/Yk0kBA
GbjOT8+Jm/54o1y/FJBr1kuP3ryGWm+/37qowJAbp4SufQH1kfqoyZFiLqEimtuT8R9OXQTovauHwi
JEU72Dq1XOU6dmIcI1VI5LW6M2gzMjmSEhV6w15PGutvjsyotu6am9WjpLXRqsh4jjUz7nFW2rTa
Yw76rLadd22HVPv12d+vuV8TsRaoYQ/mrp9X2zVA4xpD+4qbsuefNfnTFa4Z1BPULwcDQN67d6V
wbV7neDX69Frbev2+maYPxeh2aFjf3vOc+P1RXYWtXf41/TppyLsFxAmnafXBXPvgXH9RLAIBZeONf
3yAv+OEVemk5PfTV78qf1h/USwCAzPUfFrn853oBcRGiLiOvJ6IfaPUOndivWnXCo32dFJrDwO38bS
5UZMsNoJq7fi8+s+6n2wPUXFff8ickqmx5QWCs3pgzPGfVlj+Rkh/mWi0Z7IY7gtk792TJfmJsJ0EYq/
Ua636vzepZ2QxPUMe3O61rY6vKWbchRsg+cv3+obPdKdoQeH0UI1Q3Q2s7cNWB6t6okwhkSA3f
v/NTd+olAMAsvPLBa+Xmrz9fLy23z338Idn/n4/pJQBA5i59QeQyf8BKghB1KRXlxCnnmRtode1x6W
U5efqkHXYF11vUMHDn2VzwQ7mmeJ0KuwdeWOgN5ffWWcfkDu+2H8bw7cAQ/VFmdc784HMy
Yb1zLRMP6W/LeT9BlVZoz1klRrtTuzaW8kkJ3d2o9a4p2hC7Z7Zxrq2N2WUO3P3Er8sLpOuJp59gJn
4AmLGXfPe58nMPvEQvQbn7P35Vvvct/m0CgNxcVvVRv68XMA4h6pLy6oraPTl74nSqPCUnKies/w
+uV3ngPEWoFiOYcodmt90x0xG9I6exkOfM6GmphqaXQkPC+LP0tzf8OqBVo6fp8kkSbhdl80L4jP1u
cDtiPjEgE+5M/A8/8bBeAwDIm6p/usx1UEdRAWrzN+/XSwCAzPWfFLn8cb2AcQhRl5U3tHlfDts6aL
N77VXEyQqN9VP3hsyCUdfTDi/9upOjwkuvNubQI2YYuIDnzB/KHyXmkP72hj9RVLUlIzuhTmDqa5O
CTNtg1JUdKNlqa55jln7k532ffZ/c+/C9egkAMAsrX3qpXPvklXoJpn/40rfl079/qJcAAJm7bP02eukze
gFRCFGXVklO2hlkRw52nSHNbh3Rkv0Jf3347OuzV9w864eaDXeSp4E6pCVnqL3i1eKc2KKdM2Mo/
xjjh/S3ZcNLUMtSV1PRTyvVazOhHNugZuhXPU7tScXsQNWYoCtu2QJgSvX76vKxv/2YXgIAzELpoRfa
D4z26f/6D3aYCgDIyeXPivTp2jMOIerS8ntyNptOOObWeCzq4p/uem+Sprnj9gDtSK2mA77BOqRmP
crmqj9sur3h1aUsxB5LvWDnzBzK787+H3iYIV70kP5e45w3jL9cv5BOuYRUr82E8miD3pbKoIM9Trvi
ls8dW7sVSMn/+v/
+l34GAJgF1ftU9ULFeHd/6AH9DACQi0v36CcYhRB1iZkzpqvehd7w88Ds5sYkTXPIq1NqC+shGaxH2
VzVwZg/LI1GT440LNVzVtS1VJXmauqhoTmUvxxaW8AoiWC9cuSQ/l5D1r0CqtYrAxMwOY/JJkhK99
pMJoc2VLb9IfyBurRGfdmzm7MP3bEU7v7Fu+WOl9+hlwAAeXvjX99AHdSYfv7dP6WfAQByUXijfoJ
RCFGXWWDW8FPiTzbuDltXzPVzyAwvRw2ht+tRGr0uXeW6dJPWu0z1nFVke7BA5v5hSrUxzaH8o0
NdMxQeNaQ/Xl3VCaV5bSaVQxvsIfxhxVBVSNvvp1pfFohy1RVXyf/4lf8ht1x3i14DAMjT373yCf0M
UV7z1pvkbe+5VS8BADJ37FUix9+kFzBKoa/G9QIAgKXxwCMPyOv/2+vtmfoBAPm648vXvXyysfvFYvYd

D1P/E8+a1PvVGedw0TbwFALgovFrni/daTq51ljERPVAAAlsztN91uD+0HAOTvb27/tjx2PX/ECnPlc4
9L9fdeS4AKALmx3m+Pv8f6SIAaByEqAABL6F2n3iUfeMMH9BIAIC+Xjvfl8294VH78nMt6DVz/8t+V
5NZXv0gvAQAyd+ztIoWb9QLGmSpE7TVW9AQpBX9W65F60lhxJ1TZkODL27LhTbbiPCabqGZYeyO
43dBH1jOAAwAwhz788x+W07ed1ksAgLz84PnPyt7rvqWXoLzidS+R1X8bNhkqACATx4oix39eLyCO
OeiJqsJVf5ZqR84zwquZ0VcaKU3oAwDA4rjnl+
+xh/cDAPL10M0/kO6t39dLy+3al14l1d99rV4CAGSucI3IMTWMH0nkGKIWZXOvL2oeq35gxuuuHH
iTiKvZsNXn92QzxwzV1qnJeTqkAgCWjDtj/3VXXafXAADyonqjfu+aZ/TS8lIBqgpSAQA5Of5ukcIL9QLi
mn1P1N6h7OuncuqEZJedlqXedUNc/9Gtl/XnRZq7pKgAgOVzy3W32EGqClQBAPlR9VE/96Zv2h+X1
S/8xivsofwAgJzYdVDz7rl4NOQYog7XRLXrlZZq4nZEtYfVD9UoNb/OeaRVL1Uprp0RP0YFAGA5vfm
WN8tH3/5RvQQAyIvqibqs9VFVePoLv35CLwEAMmfXQX2HXkBSc1ATNUKvISuFktS8lNXRqZVSq2H
aPu+HuNXTfpGB5WVM8uWd46hJwTA7YddqEYTfT/4kcHneY0nPYU7fC+q9b2DCO//8rEiKf0cCAt7
7s+
+V2h01vQQAyIuqjbps9VGfd82VctfvUJMbAPJztcixO/VzTGKmIWpluy/9bt3vCVptOcPst1WY2ZPGu
htwVqXlDsFvVe01qobpeqIkoSO1khtC+I9Vd0Yra9/2bgFghnqNlWAPfSBnqjeq6pUKAMjXfT/zuD1r/
7JQAer1P/E8vQQAyNzxO0UKL9YLmMT89kTtXZQdnSKU61v+RFSVLXHLmHZ2LqbU+64s9S0S1NFG
TQoGTGKR76eM295ryPpg13tgBlR9VFUnFQCQnx8/57J8/g2PLkV91Le951Z5zVtv0ksAgMwdf5PIsV
fpBUxqfkPU7oHXE8sevu/1HjWG93cOpKufTsfppZpmrVUAABaVmqm//attZuwHgJw9dv3T8je3f1sv
HU23vvpF8ku12/QSACBzhVtEjv0LvYBpzHdN1FSFz85vlhPo1M7nWItxHgxO2jWqxuPoOpD20GPv6
/VjZG3Jwf1FBNd2Pdzga0fVgsysDVEya98oca+VK/5xJm1f2OsHyncO1O806o7arx19P/kG2p/K9Ux6
DsOEt32wXungORo8P2HiTbTn8/epHqPqpCY5P0DQbdffJvf88j16CQCQl7+/7Xvyjy//oV46WlQd1O
rvvVaufO5xvQYAkK2rRY6/2/p4hbOIqSxEiFqud4fDT/uRwnDa4qac1WVWRfblcGnyBRVsDU7a1ZTV
wqr1/3hUiFMKG3rcqUlpMKBKMklYe2NETUjVYzgYFmXWhihZtW+khNcqwXEma58TyIW9vrlaGBH
2qXNitLNcl/GVM9SxDbR/6us5/f0ej9OrffAcNVdHhZyTcPbh1XO2Dd97qd3vWGrvKL6DGfsBYAb+6v
WPHcn6qL/yH15JHVQAyNPx09RBTdH8hqilk34P0YN0Bu2Ha8tuuinKQug1zvnhkTuhV7/r1Zsdzzhv
5bp0dbDd9TbQlHNeotNLMEmY9dpzesOh2+3IzkX3tVm1IUpW7Rst2bVKcpzJ2tdrrHuBnP+HjZa1F
615bkRQaLRjb1OKem0koz1u06e5ntPf70n47fHb3pHa+ejIPHqivUH+PsLvvbTud0Ds2frfdepdegkAk
AdVH1UFqUfJyumftB8AgJwce53zQGpSC1HtnmjGkFHzEWco65DimpzxspxVfxuqF6C77UQbdnpv
me1yHmYvuTOyFivhWXQ9uejO2qUCFi+kKcrmBSPEidI7lH39VDo74mY3xc09J6yxHnub+mQmmiT
MmLjHCNyKa2eGQ/XM2hAlo/aNlPBaJTnORO0LtuOst74iW/aGVVC3J2GHE2hHTNWz/rmtbPtBrd
/2JNczhfs9gWrL7yFf2Up/+4q5j/B7L8+J+bAM7v7Fu+WOl9+hlwAAefjmjU/JV376u3ppsb3sp14o/
+a3mNAEAHJTuFHk+L/WC0jLHA/nDwYcXkjrjWE1w5B0mMHN0daVAzdTKp+Ukn5qK56QU/ppJDP
kHgioh2ouTjVJmK6nGTZ8Prc2REmpfSMlvFZJjjNR+0a3wwldR5fWOHUi6XdVVU4HNlaSk2473bYn
up4p3O9zpSwnAwcRIteJ+bAMrrriKmbsB4AZuP9V37HD1EWm6p/+
+n/5GeqgAkBurhQ5/mvWR+qgpm2+a6IWN2UvbMitPdQ3hXqoHtWLri8pZ7JH3OhefG5oM1EPZ
MWu5eiGPlE1KzNsQ5R5b19sebQvRuA3VlFOLEzSmcbxAvPpphfcZAepKlAFAOTn8yuPylNXX9JLi0f
1QFU9UQEAOTn+r0QKN+gFpGmqENUc9hv1cMLJoj8M2gxA7aBUrw9NMc2v04+4dRUtdp1B82tD
H2kGskvEuHZ+7Udf89zw5DXjJwlry4bZq9OtjWnWijRl0oYo2bcvLbGOc4btG21wgreeHHp1B4ZlOv
HcEcD5QZpuv+l2e2g/ACA/KkBVQeoiel3l/6IOKgDk6dhrrQd1ULMy3z1RkZGQ4dEus05mDGp2d9V
b0Q+rjZ7D7raTTBLW3vV6dtrhjxuYG8OTB6XehihZtW+khNdqguOM176Idth1itOdfT7Y9JDh+ImO
M737fWHkNjEflpGaZOoDb/iAXgIA5EEN6f+7Vz6hlxaDmoWfOqgAkCM1C7/qhYrMEKIupaKs+bN2
yao3Ztuc0XsMPcGXKlEb7K0YEnhNOEmYH/4YM+KbcmhDlFTbN1LCa5XkOBO1L6Id9rE7NVUTnsK
Rmqsb4jfdL5lQPrPmhNaJrmcK9/uiyeB+B0wf/vkPy+nbTuslAEAeVH3Ux65/Wi/NN1X/tPp7r5XnXX
OlXgMAyJaqg/oe6+PVziIyQYi6pIqbF/zehs1VHawYk86MU9n2h393alJygxmjRqg/UVew9mbkJGGV
09aSNq5dWbUhSmbtGy3ZtUpyrpO1b2w7ynXZSmV8eFnK5aas6vZ4Tbfaftab/j/Z9Zz6fs+LOdGV28

6Jws6U7ncgwj2/fI89vB8AkI9Lx/vy+Tc8Kj9+zmW9Zn79Uu02ufXVL9JLAIDMHVsTKdysF5AVQtSlVZ
TNvcFJu6rS6res/8djD/8OK6Rpb2dgoi679ubg/ixDk4RVZHuoDc72vF01d71eitm0IUqG7Rsp4bVKcJ
zJ2qfa0Zfu0IbVpo3SBlM7JWcHjzfsGiW6ntPf7/mw7q/B67F/aPQSTiCV+x0YzZ2x/7qrrtNrAABZ+8H
zn5W/ev1jemk+veatN8nb3nOrXgIAZO7Yq0SOv0kvIEuFviqCCAAAMIF7H75XKn9UkaefXYwhpgBw
FNzx5evllQ9eq5fmx7UvvUr+06ffzDB+AMhL4RqRK9R8BQzjzwM9UQEAwMTefMub5aNv/6heAgD
k4W9u/7Z875pn9NL8qP4udVABIFfH3239jwA1L4SoAABgKu/92fdK7Y6aXgIAZE3VR/3cm75pf5wX
v/Abr5BXvO4legkAkLljbxcppFNUD/EQogIAgKmp3qiqVyoAIB+qJ+re676ll2ZLhae/8Osn9BIAIHPHiiL
H36EXkBdCVAAAkAo10dQt192ilwAAWeve+n37MUuqDqoaxg8AyImqg3rsPXoBeSJEBQAAqVAz9b
d/tc2M/QCQI9UbdZb1Ue/6T6+2g1QAQE6O3SlSeKFeQJ4IUQEAQGpuu/42ueeX79FLAICsqbqon3
/DozOpj/q299wqp/7ZDXoJAJC5428SOXabXkDeCFEBAECq3lF8BzP2A0COvv2if7Jn7M/Tra9+kfxSjV
/kASA3hVtEjv0LvYBZIEQFcteWjUJBCsZjpdHTn1tSvYasbLT1gqO94Z6fFZnX09NrrDhtXGnIqCb6x+E8
lv5aY2mo2frfdepdegkAkLW/v+178o8v/6FeytbzrrlSqr/3Wrnyucf1GgBAtq4WOf5u6+MVziJmghAV
yFVPGiur0tRLjrKcWSvq58vHDiJLNeno5YXRa8h6LarV6loXZDV4saVTK0lhIDAGjqq7f/FuuePld+glAE
DW/ur1j8kPnv+sXsrOXb9zu1z/E8/TSwCAzB1XdVBfrBcwK4SoQK66cuDmbuW6dPt96ff3ZHNZM9
SxQeScUj1nxwW/7fPiHlq1pa5zX7r1srOiuSrkqFgGV11xFTP2A0COfvycy5nXR33zr9wir3nrTXoJAJC
5YyvW41V6AbNEiArkqXco+/qpnDohy9v/dHHF6znbk8Y53QW1XJetivO0uHlWqs5Tae6SomI53PSC
m+wgVQWqAIDsPXb903L/q76jl9L1sp96ofzKfziplwAAmSvcKHL8tF7ArBGiLjGvnqP5CKntOLI2peqN
536d163Or/dp135sb+ivNdbZLwuuD/bKS2MbmtlG7zFcYzN4jMM1S+1HWN1Low3jal3a+zDDt+aq
87WDDQ9pc9i2o9o8tpfjROdl+J4ZuZ8YxxD7fGh+W9RjuK0OZwi9/7p0z506/lKsnrNGj+NAWF6Sk7
ozquwfDt9PNqM9ie65eMduS+H7wj1Pcd9HsNxuv+l2e2g/ACAff/fKJ+SbNz6ll9Kh6p/+
+n/5GeqgAkBurhQ5/mvWR+qgzgtC1CWlwpHQMKhTk1JhQ8ZlcHHYtR8HCkLa61TIMrC+uRoeXk2
1DRU2hfYY7EitNCqEU58zapaW61J3uw52duTiYMi0674ynbqmQ8GiZh/zyGBquM1uz8dQE5+X4X
umuToieEt8DFGcfQcvd0hb7WCw5A2hd6V67jxVafVbXq/SIWaP41E6B9LVT4MqcjrpPZfk2FP6vlDn
KY/3ERwdapKpD7zhA3oJAJC1z688Kk9dfUkvTe/f/Nar7J6oAICcHP9XIoUb9ALmASHqUmqLn8O4d
TmNeo3SlHPhSUpi5XrX3na/FYybwtaPGt482TaCw6mHj7EjO4PplEcFZM7r+3ubsrlVF+erBr/GPI9nZ
FyGWtm2ttd1t2Wptpx9bOvUrr3hB4Vhbe7UZH3kdQm2eXRT0jkv/invSO28cd0SHMPY8xHg7zu8r
dZxrbvBoHEu3Iamcu40u53bEitrHVKUE6f00wiVRPdckmNP7/uimOP7CI6OD//8h+X0bQxHAoA8q
ABVBalpWDn9k/YDAJCTY69zHpgrhKjLyOwlZ/R0K27uOQGJ9dhLZaajqpx1t1M5bS25RqwPHd486T
aKsrlnBj6O4toZL7TrHIT3AyzXt4IBWXFNzugv6uxc9NvY3vV65pXPrI0P38YwexjWLxht3rwgfgZ5PrR3
31CbR5r8vFRbfnDoh3xB0xxDFHPfoW3tXZQd3SEycC4qW/5+zWtniH/u1HFY3yOhIe8k9uUwrEFKk
nsu0bGn+H2R2/sIjpp7fvkee3g/ACB7akj/V376u3ppMqr3qeqFCgDIiV0H9V/rBcwTQtRlZAQ0ImqY
bsGrZTiyhuIkyielpJ8GjFofJo1t2HQ9x9BhzEGnTgwGP0VZ8xMtLyzyA0Mj0J1YVK9WY/8jgrfhNscV/7
yMN90xjFaWk+MudvfAa79X7sF+GEPcRwyfn/zcTeuUjN51gntuimNPcv2HzlNe7yM4ctwZ+6+76jq
9BgCQJTXJ1KT1UVX907t+59XUQQWA3Kg6qHdaH6mDOo8IUZdSUTYvhPckdEOYqMl1FkZg8hyjn
mOk8MDO76nnDnc2AsPq6dg9GafXkeGOgjFCRlOK52UyYccwK2keVxw9ORxbLNWR2T2X2vVfkvc
RZOKW625hxn4AyNFfvf4x+fFzLuul+H6pdpvc+uoX6SUAQOaOrYkUbtYLmDeEqMuquCl7esjtQKlR
W/Pcos+u3ZYNs3edW7PRrMGZhHW+zurzZA+NNoYyV0/nF6FOH/qlfF4mkm1w6dXKHXpMWsd0
AsUTMrb06bje1BPcc+OPPf3vi6P9PoIsvfmWN8tH3/5RvQQAyNIPnv+sHaQm8Zq33iRve8+tegkAkL
ljrxI5/ia9gHlEiLrE1MzaqqeYPcGPHYR0vRqKo4f/GoxhxHPHrB2pgiW3/uMUba64U6Z3duT8+R29n
aqkk6GW5KR37gdnZO/JRbfoZeQQ8BgyOC+
+nI4hTOmkFwKOqumZL+NcNHfF75DZlQPvNJxwzn2EWPdckmPP4PpP/T6Cpfben32v1O6o6SUAQ
Jb+8eU/lL+/7Xt6Kdr1P/E8uet3qF8NALkpvFgP48c8I0RdRu0Ne6itmkU92FPMCHiMXnIlPxkzZmJvy
4Y3Dft884MlY2bySXgTWHWk2dQnKrWh/EXZdLsdqvO87l+XXmPdq22ZZBKkcVI7L578j8Fj1udsrvr
DyPW9bj9yHVtu1oBtyq7eda9xzgsxY/VgjnPPTXjsU1//hO8jZntyvRSYe6o3quqVCgDI3t/c/m157Pqn

9VI4Vf+0+nuvleddc6VeAwDIlqqD+h7r49XOIuYWIeoyqmz7Q287NSm5QYtRH7F6NnzmbhXSDL52
Lpkz9nttNibamUhF3I6BrlSH8o+4LiU/fZQL005glcl5MSQ9BnPYu9ueiRK2YH3O5qqz34IX9FeIldqs+v
EUNy94PTLd9njnodqSeM2Jc88lOPY0r3/C9xEgiqqPquqkAgCydel4Xz7/hkcj66NW/u8idVABIE/H3k
od1AVBiLqk7KG3YUUMVeDS7wcDHlX3cLBmYrWVcx3NpCqy3W/5gZHNOTbvsAPDrOPxhlcr5bpsp
ZzL2dcl5LxWW9Z6d+j1VLI5L6Zkx2C1Z/A+3D80ejUmYNfnNIaSu+y6nznWQ/UUZXNvuFaoPYw+Q
aAb656LfezpXv9E7yNABDVTf/tX28zYDwA5UPVR7/uZx/VS0Cte9xL5hV8/oZcAAJk7Zv2GfPwdegHz
rtBXRewAxKNmNtcT86gwbG/anqHAOEfpntPHcqpFwIpwn+l9Rip/xM0BAHl441/fIKWHXqiXRK596
VXyW3/8z+yPAIAcFK4ROf5+66P/Xoz5Rk9UILaeNNbdmc3LcmaNABVZO1r3XPu8dSwZ9ODG0fGO
4juYsR8AcrL3um/J9655Ri+JVH/3tQSoAJCn4+8mQF0w9EQFxmrLxmANWFXOgK50yMzRu+d6jRU
p7ZyRbiplKXDUFf6fgn4GAMjStU9eKe9s/aS88723MYwfAPJ07O0M419AhKjAWAOBFgEqMsc9h+
WlJiiblx9NaEs42hKOtoSjLeHmqS0nrr1GDr/3pF6aLa5RONoSjraEoy3haAvSQIgKAADmBj/ghqMt4
WhLONoSjraEoy3haEs42hKOtoSjLeHmqS1IhpqoAAAAAAAAABCBEBUAAAAAAAAAIhCiAgAAAAA
AAEAEQlTkSE2WU7DrfxRWGtLTa8fqNWRlo60XHO0NvZ3CijRibwjp6Uljxb0GG9aVTbJuGmH3UNr
7mJbRRv1Y4SYFAAAAAGChEaJirvUaK1Io1aSjl4H5pgJdY1Z9W1nOrBX1cwAAAAAAsIgIUTG/eg1Zrx
GfzqeibO717RkF+/1tqei1szFPbenKgXvLluvStdu0J5tkqAAAAAAALDRCVABIS+9Q9vVTOXVCyE4BA
AAAADgaCFGXmao1OlC7MazG6GD9UXuIvfE1A+VKNbNOpXokq1Vp79Mcxt9cdbYTvjOjjeoxqk7qY
JvCa1VOdrymMbVf2xvetob2H3JN4rTRY359ZEONcxFo46j1xj7t9ea5zKoOadx7KKotg9uYsD5pjOuS
9J4FAAAAAAACLgxB1WakgL7TWaEdqpVEhpPpcQUoDQ+ybq4OvVyFiSYIva8pqYbBWZBqcNq0GNh
xyDHYlNtgm65W1UnjQaYt7vIMqcrqqn3Z25OLAa9u7bmODtTKHQjgtuo2TKsrambLztHMgXeeZxR
iOHmh7W9xml8+s5dDDMoV7aKJrPizf6wIAAAAAAOYRIepS6knjnJuIuXUb+9Kt61BNOrIzmPx5qtL
Sr2+5QaEKG8/7ve16jXN+0FVt6VqVXfE2H0Nl2/qabl28L3G3sx1W8dJvU/gxWMe77oZg/mv77gF0
arI+MhUdf7xhKltu2wfPpR9GSvmMeBlqe8MPgsOuSWQbJ1NcO6PbuC+H7qbbu0ZI2ZEDN101hq
mfOpF9hDr9PTTNNTckuC7J7lkAAAAAALBICFGXkjERz96m16vQD9VEOl56FlRt+RP3+EGhqScXd9w
+e1VpeQGStc8LYa+fntmm0GPoXRS3SeX6lj/xUGXLC+U6OxdDexSOP94RimvidfQ0t22ElGaPTrN3av
2CcU02L/htrJ0fGKo+Ja+NftDbO3Si0mrVCRv3dbrau7jjBZKnvROYlRTuoSmuuWkm1wUAAAAAAM
wdQlRYdA3P0OH9SZmzk5+Ukn5qK56QU/ppespyMrCTEN0D77jsIdheXUtjqHdgSHsazOHy/rB4P5
SryllvyvYRvVNtxnbMHqOpKMoJfUHcwLlrXzzrnJ4+aYeVwfWW6mk/kMxMCvdQKtd8VtcFAAAAAA
DMG0LUZRWYKCdunckYgeWRMt3x+r1i3Z6eRig3URhpDK9PScUt3trctVrXE6cj6ik5UdFhZWC9anb
2EeriSf+6AAAAAACA+UKIupTasmH2OnVrPZr1HI+ocr3rlDEYevjD9lNT3JSzbglONXTcqCs6WRiZQYh
dOS164L4ctvUQeLv3pzs5lrF+QUP07K/5sv1xAQAAAACA5UOIuozMupwqYHLrohpDoCdXkpNuEjs
4XNoIEXNVcoamK6NqvWbF6+nZ2ZHz50fVFTXP2eBs/mZ90FMydk6nxNfQ3XdHDnadr3VrtZbsT/jr
h4e0ZyWFeyiVa57idQEAAAAAAAuNEHXJ+QGTMWP/VMxakU1Z3XCn3DFnS8+ZMcmTNFfFa1J7w
6+V6a1MmdfTsyPNpj76oaH8Rdl0u6xar6utN6yz5eg11r0anuYESU7AqVivP+8dkGx4U8nH5V+vZtP
5Wnf2/aIumOquNyfCylYK91Aq1zz5dQEAAAAAAEcTIeoy8oI9S3NVh0rGhDtTMmcun2r75iRC7nY
mCjuDs7o3V3WI5gWO5gzwaXOHxftCh/JXtqXl5XU1KdnnrCAlP6mTC95EVCojdOutWrxzHLe2bVBg
W9Yzb2i6eZ9Y68/k0w3VNv09lNI1T3hdAAAAAADA0USIupQqst1vGQGZUpVWv+8HRvaEQpMqyu
Ze1w/BbGr7g/scx2qn1yBt/9DrDZhIcVP2+oNtstj1YDOoh2rwhvQr1v62Ruysst0PrUtbbVnr3ZILLnU
8g6+ttiara2v22gwMTTeGs+c+ZD2Feyila57ougAAAAAAgCOp0FczrADITq8hK3oiL1WDdo+eiwAwk
urtPS8/mtCWcLQlHG0JR1vC0ZZwtCUcbQlHW8LRlnC0BWmgJyqQKbOOZ75D4gEAAAAAAJAOQlQ
gE23ZKAzU8ayeFTqhAgAAAAAALB5CVCAPql5pZpNXAQAAAAAAIEvURAUAAHODelXhaEs42hKOto
SjLeFoSzjaEo62hKMt4WhLuHlqC5KhJyoAAAAAAAARCBEBQAAAAAAAIAIAIDOcHAAAAAAAAgAj0R
EV2eg1Z2WjrhVnrSWNFzZavHhsyL60CAAAAAADA/CNERSZ6jRUplGrS0csAAAAAAADAoiJERfp6D
VmvEZ8CAAAAAADgaCBEBQAAAAAAIAIhKhLqr3h1gddkUZPD7+3l51HaClTVePUeI3zcL7eZW/XH
MbfXHVepzc4uF+PuW1j58HXt2XDfY318F4Wo12TGjwv6jHpuVGSHr8S1obCSkPCD8+s/eo8VkaciG

TbBQAAAAAAWF6EqEuvI7VSQUoDw+
+bqwMhX3tjRI1T9fXpBJbR1H5WpamXpFyXrYr1MbN2OWHk4HlRmquFYNCZ4blRoWtYG6RTk9Lg
BFl2EFuSwZd3aqWhcDTRdgEAAAAAAJYcISosVWn1+9K3Hq2qXqUCwPNulNaTxjkdX5br0tWv7dbL
zjrrtTsXnYiusm19rlsX9zNSbdmv7W+rxHNafjv7e5tSTNCupHqNdS+MLNe7zj77LasFWvOcDkeza4NI
W3bd1Dh020055yW0VjvW3SDXOE/uBe3UZN17bZLtAgAAAAAAgBAVUm1tixtxVraMANRTlM09H
crZ4aVeu3bGe23noKufZadc3/La6ciqXT25uOP20qzK2U13yxXZsoNGFVLuibM6w3PTO5R9/VQ6O+
JmscXNPWd/1mPPbVvvorhNDpynypa42Whn56J1ZJYk2wUAAAAAAAAhKiaha5OGDmHPzqkT44K
9tNrVlQN3A+WTUtJPFSdo9EPnYSmem+KanHGTWGtrquyCW7t0qM5p98Dbnz18X7+uYA7v7xxYR
2ZJsl0AAAAAAAAQoqIsJ82UcJTAxElGbdLcjGjnzNtlyawNRdm8ENYz2A9KQye5Giur7QIAAAAAABx
NhKiIoS0bZs9Kt46mWft0JuahXRm3obgpe3qIvV+v1tc8Nzybvl/DdfBh9KCdYLsAgLzp0Q3Ggz9yAQ
AAALNBiIrx2rte70o7oHNrfxpDyKc2ybYya1dJTroJqDsE3qVm4i8YM+6n1YaI16uZ9NUvzfakXXbw2f
XqnHrtK530Qtu4NVhjbRcAlpH9Xl+QwkwTy540VlZFWu57tPNHr+bqLILU+Qxze40Vpz0r+f/hT/0ba
p4P9zEP5wXDhq7XDO6ZmRvzvuZ9P7mPvG7mOO+3ebwnR+5jSf+gFfO8z/K9eGZinJuh951luGnG
nJfc34sTtofSdoiDEBWJ+AGdMSt9AiU/nTRm/7d+MFmdbhD8tO0KKsqaVzS0Kavem667baeO6OB7
cZw2JDp+/aavPhXsGRpSs9Wsc9pc9dvm/sOhHu7KJNtVjG3wDwuALMzsl/chKri09j/lv0npcCYu3Da
KcLuTP+4f5vte3N4IC3ONPyjOQq8h6zWRatX9x28G3NEnxsO8XrMy01/cAyWOhh+z+NZW52O1qS
YFda9TS6qdmpRmFPjk/0v7+Pc19R5csr6f6l3jHFk/T2Z778R5v83jPXncPtTnc/yDVmTok1eYm+C85/l
eHHFuht739CP98xP/vl3dH/g3Iot/IEadk9zfi8efl3zfiydoj/WNrUrbpf+eHO/7dn5+FsZY1g2DJWS9R/
TV5bd+A+hbPzD5uvW+9U+g87mq9ZZia/WtnxX060c8vNcqIa93P29uf9TD2NbIdtqStKvbr5fd9VXr
K8cxXx/yKNetVygJz83Exx/
+MDcdve3gMSfabqvqrS8PXwQAmEq3XrbeX8z3eP2+GngjyoffFv1vwAzaEEm/z8/8vXjm7XCuj9q/f
c28f5PzY/87OoP9RtP37dy1y/3eivPzV9rCv5dn1R7n5y9jv/pnrCy/l8a/rznvuYNtyPocjW9XvNdMa6J
9ZPIeqPdvbdd+hLRD3T/maud+Um3XK1IS/5w4n8/+vTjeucnjvS/
+fZv1+8v4cxImq7aNPy/h62fWnhHfw+nfR87+zd0737fBdX579Qr9vhz3uiJf9ERFDBXZVn8p0ksO56
821puAo7kr/t9KrNd7n9D2D52/MKlanIP1QqutCWuIJm1XEk4PIOsNTS/7rDdbf9h+0jYkPH57qP3g
ubQ5+wj8QdOuc2oMyXfZPWWMeqiWRNsFgMy05XytY71NXZBN503VUpEt9UY28fv35Iqbe9Lv7x
ltmTO69MupE/PRwFm1o9dYl1qnKmfn9kLNhnteWt7PKPPC+T6X6unAzyL5KMqJU9YH9+dQW08u
7sygPb2GqIFK5fqWv9/Ktv3zYmfnYgY9sRxj39d6h7JvfRj8fi7aJ64puxm9Ecd5v83jPXle3ved79+y1Ls
hP8tr6uf34dEJHdm5mO7dE/ec5PVeHOfc5GX8uXHeXwLf5xmY7Jxk9148/rzk+148tj0jfp6yR42mW
tYuzqii+fpZGOMRoi4pvw7mwJuLMeFQcMiBCgvdr1EPJ5TztxMM6dQPhf5rrYf5A725D/VQ+xmx35
Ht9MRtl/MGFlw3nvMG7H6d89gbakjCc5Pg+G2D59J+jDoG8zj1Y9QvU3G3a7xu+NgBYAoz+uV9M
enSL+W6bIX/A5CTnjTWa9KZVTvsoaMdqbbi/1ueGTUM0Rh6N9uSN/n84j6JXuOc9d1s/bI/oxu3sq2
HjNo17dWwypITNg/
+vJW13H5pT8huV1lOenWcNKPePgZk8AetuMFlmDTbEVuO78XTnJv86fJoB+cHhtOnWwJnknPCe7
FhxPub8/PnvuRaNYmfhRcOISoAAJgNfnmPTdUltX/5uTCLXoa6tpj9i6D+pWdGvR3b52vSqbZmPmL
C/0Op81AjV7KppRZXPr+4J+f2NDo7wwDEHTWkatqr7yOrObMI4efpl3ZMgT9oKfPyXhwwR3/Y6uy
flAvevxGqt6h6/9mYYa9C3osD9HwiwflBrO+pqedWiWHwjzD8LLxwCFEBAADmmDP5gRq2N6ueO
MFRDt36vqzO4hfU9oYzCcRc/dbuUL2Csh6WHcfc/eLePi/O6NHZXTNnsg5/YiAVeKtJgXKftGOWv7Q
jNfxByzKH78Xz9oet8pk147pY/4aeVWXUZtirkPfiAernGrdnrP89dXBKXadTkl3n7nn5IwymQYgKAA
Awp5wAVfXWmJ+hjMXNC3YNtk7tfK7hXHtXhU1NO8B1fuEpSEn9Vqh/CZrtcHptlsOyLfP1i7sOCGf5
y6Iecqzq2bt5jz0MViXezdWMZjYfZVa/tI9h93bqyMHgjTuqd9QS4w9ajkV4L56XP2x5ZtqrkPficIMl+f
qydXLfOk8nJau3vdn+EQZpIUQFAACzwS/vkVSvDSdAndMJ/zL8RSPMYE8j9bAngLR+Mexaz2dbt7sn
h6qoWc7nZKxZ/uLu9nw6O8NfFgeHTbpmdl7y/6V9rOIJceZ7CUZNPfuGnmG4O2f4g5Zvvt+LB+T+h
62S2GWOB8PbWf5cw3txTLq2eOCPkekZ+UcYfhZeOISoAABgNvjlPYKeRddiD3czevzkW+dSTf4wM

Bxc/0KW1S8ac6/XkJUVc0i2WqVmSp7lL6nz94u701utKjMcPSpSOW21wPoeCgyhV7dwzTovM26bL
dtf2uOpyGnVY6+27r+veL3G5m+islngD1qLaFZ/2NIjADo1Oe/9w6mHcM+oHinvxXGoUhklqUldLm
RwkSL/CMPPwounDwAAMCOtqvSt33L69a5e0a33y9aPJ2VvRX669bLVFtWewUe139KvWUZh56U
6JyfEblu53s/3bun26+Xg+ZiLe6RVHbg2rb71S2tfJT+5m+H38TB9HgKPefie1vdRxvdv3Pc1573Yf2R9
7eK0K4/35PH7CLt/3Ifxb1dq9H0x9H2r2jFw3Pp7Pu1rNcl5t78m8/fiEedGvd8M7Ns9hrTf/uKem6H
XZfY+POp+0XJ6L453XvJ7Lx7fnpB/xzO6RnHuxXn6WRjjFdT/rIsKAAAwE+5f6F3WD43zNRwQWBCq
x5xdm9BVbUl/Bl3nnO/pqrT6M5p5eS7pnk7G5ZnV9cH8Gfre9fjfR2Gvqc5r79gUjT83Id9bxnk7iuLc
LwrvxbOmRvOoOqhhgkP7+Vl4cRCiAgAAAAAAAEAEaqICAAAAAAAAQARCVAAAAAAAAACIQIgKA
AAAAAAAABEIUQEAAAAAAAAgAiEqAAAAAAAAAEQgRAUAAAAAAACACISoAAAAAAAAABCBEBU
AAAAAAAAAIhCiAgAAAAAAAEAEQlQAAAAAAAAAiECICgAAAAAAAAARCFEBAAAAAAAAIAIhKgAA
AAAAAABEIEQFAAAAAAAAgAiEqAAAAAAAAAAQgRAVAAAAAAAAACIQogIAAAAAAABABEJUAAA
AAAAAAIhAiAoAAAAAAAAAEQhRAQAAAAAAACACISoAAAAAAAAARCBEBQAAAAAAAIAIhKgAAA
AAAAAAEIEQFQAAAAAAAAiEKICAAAAAAAAQARCVAAAAAAAAACIQIgKAAAAAAAAABEIUQEAA
AAAAAAgAiEqAAAAAAAAAEQgRAUAAAAAAACACISoAAAAAAAAABCBEBUAAADAQmtvFKRQ0I+N
tl5raG94n19p9PTK2fDbuiEhLV0wbdlwz3vk+e1JY2Xca6xXNVb8ba00rK8CAGB+EKICAAAAODqa52
TGOemSUMHoqjT1kqMsZ9aK+rmpKJsX6tZnHZ3aesg1asv5Wkc/F6me3bS+CgCA+UGICgAAAOAI6
Ujt/OL38Zx/XTlwM89yXbr9vvT7e7I5KvksbsrZqn4eco16jXN+IFttyXZFPwcAYE4QogIAAAA4WuiNm
r3eoezrp3LqRKxeo5Xtlng5auAamb1Qq9IiQQUAzCFCVAAAAABHTLzeqH590pVg6NpryIpbm9Orse
rX/7Rrehp1Vr119suC68NKtPqCtUJH1wEdeJ31CKsrGjyeYL3S6HYYzGPXj8F92fsp1ayzrDVXndeO3Ul
FtltujOpfo/aGXxagXN+yXjUo3vHbQto/dH0tcc5VoEar+6BWKwAsLUJUAAAAAEdEWep1HdJl2Bu1U
ytJYTVYDdRep0K2gfXN1VEBZlNWCyUxyoCqjUhpcMIpOxQceJ3F3t/IQK8jtZJRr7Rcl60YnTuHwlEtel
8JVbak7hZHba7KRqMh5/wEVS4M1gNIcvwqwA5pv3M+hoNUR/i5UueiNLhTJewaAQCWAiEqAAA
AgKNjzQ3p4vVGnVS53pW+qgPq9ax0hK1v7o5oh1dLtC/
+y5tyzkv7etJYd0PBqrT0a71td2qyPjIpNl6/F2OSpvaGePmv0a6um3ga+6psW5/r+hNFqRqm9n5iDc
MPTjLVrLnHV5b6hcF2Jjl+67VuGhvWfmsrOxfjnqu27Eadi8A1AgAsC0JUAAAAAEdIUTbdGYwy641al
bNuj8nKaWvJNWL9/mFoL05zBnqzXmhn56Lz+t5F2dGdIQPD3I3enN5rB4QPix+t7aeGgTCzuHnB31
ftfDo9MAOTTGnVs8OTUiU6fuu675lBqKO4dsYLbDsHXf0saOhcmfVeOzviZq/FzT1n+9Zjb+QMWgC
Ao4oQFQAAAMDR4oVsGfVGLZ+Ukn4aMGp9qKqcDiR3JTnpp31ix33dA90L01rllguwH8bwdve1A0
6dSBLymT0vz8ha4EuLsnbGbdi+HIYlthMITDJlPQudTGqK41fHZNc5DR3eHzR0ropr4h2yuodK7n4ja
rECAI48QlQAAAAAR8xAb9RD5+l8K8qJU/rp1MpyMn6am0BHRnTmnE6i8DlCYFIpo85ppLBzFSw5Y
HLD3NgTdQEAjgxCVAAAAAABHj9kbtRYvTsvXYK/Onhx6Y8iHebVWhx7biYbtTyercHa88cfflg2z16lby9
Ss3ZpEcVP29D4Gyt7amueYpR8AIg0hKgAAAIAjyOiNmpQxjDw7g706u3Lg7tTtmVk66QWAo+p5ps
MsJeDXAHX05KJbmFROSaIqAdNKcvztXa/nqR24unVRp7iWaoZ+1ePUnkjLDlS7Xi3W0WUEAABHF
SEqAAAAgKPJmIAoTMlPDo3aqW3Z8Kapz1ZzdcObqKm94Q8/L59ZcwJAszZnc9UfQt7e8Gp0FlIZV2
4Gzta5WPd7WfYa61790aSTVU1twuP3A1djxv4k9PbVbRDscRoSdAMAlgYhKgAAAIAjKro3qjlzuwrp
nGAubi3NaZWlXG7Kqg4D/dzWmOFftd+ozdlc1cGh9+IREzJNorLtD1vv1KSk21XyE1S5kPuM9AmOv
3Lan6jKu5bGBFRJjDgX5r1RPat7ugIAlgYhKgAAAICjK6o3qqp7OVgzs9qavI5mIqfk7J4xPFyx63gO1Di
1a3MOvE4Je+2U7GHrIcdebVnr3eHxeYt9/BXZ7psz/itVaZk1TZu7Xs/fcexzEVYMVW8zrewaALA4Cn
1V3AUAAAAAAAAAEIqeqAAAAAAAAAAQgRAVAAAAAAAAACIQogIAAAAAAABABEJUAAAAAAAA
AIhAiAoAAAAAAAAAEQhRAQAAAAAAACACISoAAAAAAAAARCBEBQAAAAAAAIAIhKgAAAAAAAA
AEIEQFQAAAAAAABGEvn/AZM6nr53azj8AAAAAElFTkSuQmCC]]></redcap:Attachment>
</ItemDef>

<ItemDef OID="q26" Name="q26" DataType="text" Length="1" redcap:Variable="q26" redcap:FieldType="radio" redcap:RequiredField="y" redcap:BranchingLogic="[q24] = '1'">
<Question><TranslatedText>Do you prefer Life A or Life B, or do you think Life A and Life B are the same as one another?
</TranslatedText><redcap:FormattedTranslatedText>Do you prefer Life A or Life B, or do you think Life A and Life B are the same as one another?
 </redcap:FormattedTranslatedText></Question>
<CodeListRef CodeListOID="q26.choices"/>
</ItemDef>
<ItemDef OID="summary27" Name="summary27" DataType="text" Length="999" redcap:Variable="summary27" redcap:FieldType="descriptive" redcap:SectionHeader=" " redcap:BranchingLogic="[q25] = '2'">
<Question><TranslatedText>
Alzheimer's disease - Description #[no]

If you have Alzheimer's disease...

1) You might forget recent events of importance to you. For example, if you are interested in hockey, then you might forget whether your favorite team won their last game, although you may remember watching the game itself.

2) You might forget the names of people you met recently, or the names of local streets.

3) You may no longer be able to handle your personal finances or plan leisure activities such as dinner parties or vacations. Simple decisions such as what to order in restaurants might become difficult to make. Your ability to concentrate may decrease and you might have difficulty driving.

4) You may start having trouble finding words to express your thoughts. You might need to be reminded to shower or brush your teeth, but you could do these activities on your own. You may need to post notes around the home to remind you of simple things like turning off the stove.

5) You may remain aware of the day and your surroundings. You might be able to travel to familiar locations like the supermarket, although probably with the help of a loved one or friend. If you are in a social gathering, then you might have difficulty following the conversation.

6) You may continue to enjoy hobbies such as puzzles or reading.

Now consider two possible lives. In Life A, you will live in perfect health for 8 years and then you will die of natural causes. In Life B, you will live in perfect health for the next 10 years, then you will live with Alzheimer's disease as described in the summary for another 10 years, and then you will die of natural causes.

</TranslatedText><redcap:FormattedTranslatedText>
Alzheimer's disease - Description #[no]

<p></p>

If you have Alzheimer's disease...
<p></p>
1) You might forget recent events of importance to you. For example, if you are interested in hockey, then you might forget whether your favorite team won their last game, although you may remember watching the game itself.
<p></p>
2) You might forget the names of people you met recently, or the names of local streets.
<p></p>
3) You may no longer be able to handle your personal finances or plan leisure activities such as dinner parties or vacations. Simple decisions such as what to order in restaurants might become difficult to make. Your ability to concentrate may decrease and you might have difficulty driving.
<p></p>
4) You may start having trouble finding words to express your thoughts. You might need to be reminded to shower or brush your teeth, but you could do these activities on your own. You may need to post notes around the home to remind you of simple things like turning off the stove.
<p></p>
5) You may remain aware of the day and your surroundings. You might be able to travel to familiar locations like the supermarket, although probably with the help of a loved one or friend. If you are in a social gathering, then you might have difficulty following the conversation.
<p></p>
6) You may continue to enjoy hobbies such as puzzles or reading.
<p></p>
<p></p>
<p></p>
<p></p>
Now consider two possible lives. In Life A, you will live in perfect health for 8 years and then you will die of natural causes. In Life B, you will live in perfect health for the next 10 years, then you will live with Alzheimer's disease as described in the summary for another 10 years, and then you will die of natural causes.
<p></p>
</redcap:FormattedTranslatedText></Question>

</ItemDef>
<ItemDef OID="a27" Name="a27" DataType="text" Length="999" redcap:Variable="a27" redcap:FieldType="descriptive" redcap:BranchingLogic="[q25] = '2'" redcap:InlineImage="1">
<Question><TranslatedText></TranslatedText></Question>
<redcap:Attachment DocName="8.png" MimeType="image/png"><![CDATA[iVBORw0KGgoAAAANSUhEUgAABVEAAADYCAIAAABtBTQzAAAAAXNSR0IArs4c6QAAAAR
nQU1BAACxjwv8YQUAAAAJcEhZcwAAEnQAABJ0Ad5mH3gAACnDSURBVHhe7d1fyF3Vue9xY9Qd
j0pC6w5pPZjoQQhGJG04EHOKjSBGhICgYpCteDjyEsGyg2AjYg7nQlE2hbx7I+hFscLpOfHCIptothCL
lpID4Xmwgs3pPJeWBqoF++WUl5pLrrnvzHmGM/4M+cca8651prr+
+HhnHfNNeZ4xphrrdf5s653X/M3AAAAAAAwRWR+AMBILmxcuOZ/XUNRVHLtfmHH///5/6s+UQA
AtEDmBwCMYWNNzY9ebu0SAoSiqfd3wyrVPHtx78p67//j576vPFQAATcj8AIDBbW5t7n9rvwgwFEV
1qgcf2PM/7vovWb167KG/fv119ekCACCKzA8AGNzDP8GgNzDP31YpBeKojrVgRM7y8Bf1runf1h9ugAAiCLz
AwCGderjUyK9UBTXqXa/sOPZ/XeamT+rX//sg+ozBgKgerjUyK9UBTVqXa/sOPZ/XeamT+rX//sg+ozBgBAGJkfADCgc5+eE+mFoqhOtf3MtscP3S4Cf1
Z8sR8A0AaZHwAwlItfXNxz2g4RYCiK6lT3H9st0r4uvtgPAGhE5gcADGJjc2PPjz/aI9EJRVKe665lbRM4
XxRf7AQBxZH4AQP+2rm4dfOegwmcVWqnS9eOegSC8URXWqnS9e736N360L//f/VB88AAAcZH4AQP9OfHBCpBeKoj
pV6Gv8bp285+7PL12qPpsAANjI/ACCCzAAnr3xqzdzdEeqEoqmv9t0f+7PL12qPpsAANjI/ACCCzAAnr3xqzdzdEeqEoqmv9t0f+XmT7SJ0+
+v2/fPVV9QkEAMBA5gcA9OnDzz4UAEuqA9NDz4U0YWiqK6197mbRKpvrLeeP1l9CAEAMJD5AQC9uXTCM2Fot0f+
...

uzevDxP65JnAc2zOSPCB+ppPTyrkic2LUaurR7vHKkee9fWYiWYm0tXLvGH+imql7r/2G4Rwsev3/3i
k+qzDQBYAWT+ReAJOyYdqSzmYCNkSfGwVJ+op9PdvA3K6eIPQ9qdVe/WuRz6DEunponB0v8S5Hz
zWOpJPa1L3gU0b9Y/wpjeXnVgd60uSMNiwpenGuLO6Flbq5VgHja3Nvet7xO5haKohJrL1/jd4ov9A
LBSyPyLwBN2JDt0eRKQOSB7um1Ycsd5zxQH4w9DWp4Vvxr6rELkilVkl7RgaZ6lfs64J8SWF9xYcAH
Nm7VHOEOMtXpPz7S+lPHFmJ3y09TjcgrfjM7aWq8Eo9q6unX0vaMit1AUlVA3v3Td0wfuEPF7XsU
X+wFgdZD5gWaeOAqshpM/PylyC0VRCbX9zLbj990mgvd8iy/2A8CKIPMDzcj8WE1nL54VuYWiqLQ
6fPxWEbkXofhiPwCsAjI/0IzMjxV0YeOCCC0URaXV3uduEmF7QeoHh77zx89/X33mAQATReYHmp
H5sWo2Njd2vblL5BaKohJqob7G79arxx7ii/0AMG1kfgCAZXNrc/9b+0VuoSgqrR753rdFzF60evf0D6
sPPwBgisj8AADLwz99WIQWiqLS6ruPfUME7MWsX//sg+rzDwCYHDI/AKB26uNTIrRQFJVW33r+Rh
GtF7ZO3nM3X+wHgKki8wMAKuc+PSdCC0VRaXXj6e1P3btPROtFLr7YDwBTReafL/W34QqD/IG4y
+tH1s5XP0/jb9HZO/Kxrip/d6+d1m+N6b2joFz84uKO13aI3EJRVFot/tf43eKL/QAwSWT+OcrCU5mX
Sv2npqrBhBKauyNJXNRKw78kQMu3xvTeUdA2Njf2/GiPCC0URaXVvU/sEnF6WYov9gPA9JD552jgu
KTT72QSmm9HgtpgNUSfQSRt0uKtMb13FJStq1sH3zkoQgtFUWm1+4Udz+6/U2TpZSm+2A8A00P
mn58WCXYmq5j53RFk0pbI/CvtxAcnRGihKCqtbnjl2icP7hVBernq9NHv/
+Wrr6rfDgCA5Ufmnw+VlAxmjtXhqiDilJGy6mlECg7NbyY0Y4wb2KwVNOY5c1rzTLGq2LT1M/okvU
CxWsWZ3ZdK1XmewRlz4QbP4aYLYj2fsYYY89X7KFcUPS+gbS9roHMBxJOBS6HU69aKGc3TjDHuLN
aixdOt14xBvPGrN0RooSgquR58YI+I0MtYbz1/svoFAQBYft0yv74dD9+Le4dYeSGQKVqrM8ESJ4JQg
vI/lTGummeAc01D8/snzxlTWOlMib5svUyrny+PiYehHQn6rPJJ8dClprW2Jw82rTyw/3qE5/niSf95w
cUWEnpVmlecM0cZPCf0947qvBr058PPPhSJhaKo5DpwYqcIz8tbn/zk3erXBABgyY2Q+cX9/qw38e
Z00Wy08LxXU6cfdZ30KH3hjIAUvQC+
+d1znfmd09Q5kVfOnbY+IvtEpjXH+Lr6duTSo7TYcDXa6CNaN65cTqFP0HPWV8NaS2ii+kRHSq/6iG
quz1JH6gOR3vUoYxNuO2dJzmly363WjCFcunKJP9RPUX3VN//x75b3a/xunbzn7s8vXap+WQAAlln
vmd/l3ODPxAgnmX7mnJNYgjI3JsfpONSw+9j8xjExTD00JvccEnqbVk9UsVuKOQPkHJnYeGcZ6vzqQ
MuVm5x16iVZZ+hhwYlaCPeqNy3HOOdk9LHIWnznNbdTDyMXsMWa0b/Nrc196/tEaKEoKq22n9n
2+KHbRWxe9uKL/QAwDcNn/n7v3XVYOGKHhmXkuTIiblb0psujnoDk5bvyngZimJ7dI9iwObO1ntYc
KF9d344EuUH52CNweVWP1isv2KOdOewT9H5qkXU6WveS100NsZp5D9p8199zmhhmL9MmFhR
ZM3q2dXXr6HtHRWihKCq57j+2WwTmaRRf7AeACRj2v+333O17bupzLaOOOmftvPqpS0ZaMJ6r6c
9dYqB/kMszv+/cwOw+xkS2PqetRzob9O3Ipk6uBzSfo4cU/ZwZ2qxcNxHU82qO8I5s4bUm9ZLXwLu
c4Bprcp5cczs1wie8IF8v9Ojkz0+KxEJRVHLd9cwtIipPqfhiPwAsuzllfj3KFAkbFXVaPon6ufmsReW5m
v7cJbbqH+TyzN8iXLWd3dLftPq0ghjt25Gl5ZYd1ZhsiJqhnqD5fDUiUw5yltE4hzFFoc3A9r3kGO9yGtf
Y+vKKYS0mbrFm9OnsxbMisVAUlVw7X7x+Sl/jd4sv9gPAshv+b/h5znEOtUgFOXVeeZp61HDS4opd
GXNTclzLq+Wbv0W48p3VqK9p1agj6+u+TTZO4hnQrnG1gSNra+VoY3DjBO7m1RF9iuf6KNlTHZql9
ZKz+rroY541Kr7zmtv5zhJarBl94v8aP0X1WMfvu02E5OnVq8ceqn59AACW0DwyvzriucGP5A13UK
uTFph7ZTI6wsld1tv0BCQv3/xdAqE6oJdkrtPWz7TWKfpBZE5Xiy5+elzBGtw0p2xRj9fzqCH2S6ZPrA/
7B9bSejnXzdmSMU+odcaZJ9OindNN7qLVmtGjratbh398WOQWiqLSau9zN4mEPL369c8+qH59AA
CW0Dwyv77j94jc4KuJ6lzgHlkqgavpvzzGHj0Byc+eqejSKlzpI5bIS9PLtPppNYlefj2rZ0dS98WXzKnl6I
Y57VWZ1BDP9SmEzgwvN6mX+1oEtpSTazTZ3YvZWrVrelHarRl9uvLnK/zRforqqw4fv1WE5CnVu6d
/WP3iAAAsp+XJ/P7YUIrFlMUVuZr2ZsXznoAUYl7qYnjrcGWvoKnV7NPWTxjj9fLrgc6OfLotvuJpZorP
aS4r34B6XG1GPfRNbZ6as66XT/deza9F9kRsjTWzeTEyqZ3s0noS9In/4/wU1VdtP7Ntqv+F/6vHHvrr
119XvzUAAMtpnpk/Hi4EOzFInaYCPNQ7jPcSVse5T8+J6EJRVFrd/NJ1Tx+4QwTmZa+T99z9x89/X/2
+AAAsrXl+nz8yi0OfE0BQw0z0G4x3ElbLy798WUQXiqLS6s7/frPIzMtefI0fAKYhMfN7FRHeCfSehF9
PUx3T/8Fw6N8CeCYp6SeIakhj/tfq4XcgMF2Pvv+oiC4URaXV/cd2i9i8vMXX+AFgMuaS+UMTBfNWJ
NlHngJaMDI/gR8raevq1sF3DoroQlFUQm0/s+3xQ7eL8LyMxdf4AWBK5pT5c/ZkscgezfXRJwEAjTY2
N3a9uUukF4qiEmrni9c/u/9OEaGXq/gaPwBMTLfMDwCYpAsbF/gz/hTVS931zC0iRS9X/e4Xn1S/Fw

AAk0DmBwDk3v7t2yK6UBSVVsv7xf73X3+t+o0AAJgKMj8AoHLq41MiulAUlVA3vHLtkwf3iji9+PX6
E4/xNX4AmB4yP+LE32OIP+xE/fG86g8xzDJVV9bf6h/8D0FkOxthT7kxryEm6+h7R0V6oSgqoXa/sG
O5vtj/g0Pf+fIPX1S/CAAAE0LmR1w85M8SMueV+XWj0rCRv2o2UgYf7Rpiyja3Nvet7xPphaKohDp
wYqfI1YtcfI0fAKaKzI9ORKqcJWSKzD+aEfuOncHJ/OjHZ19+xp/xp6he6sEH9ohovZjF1/gBYMLI/Ohk
+TP/mLmYzI+l9dHlj0R0oSgqoZbii/18jR8Apo3MPxeBuOs9rHNcofmMhtynnq7PcI+oafMjYrr4wzg9
OJONF2v3TmWeYi4wyjrJOku1NAQWbi5OrNti9zK7hXqJbeecrRtj6mmqJ8MdPRMFiDnabyrTZv2Z6
Bwla4j7fIsZMKyzF8+K9EJRVEJ96/kbRcZeqOJr/AAweWT+
+fAEJ9/BOvCZjJNaBjCTel4/rZvoSaojxWMxXfxhhH8ruaqtM5U+YDI26hW/Yp5nAwtvXG4u2izUSx0
35nG27jm3HB7t2O7l8F5W44yGFqnrzxmranpxm2fAKE58cEKkF4qiEuq7j31DJO3FKb7GDwCTR+af
E5V5PDFHH9K5Rx3RQckZYkzjBDCpGqBOMdJVdYaaoXgopos/DNLjZI9MtZCmmT1bdbS4Yu7EXu5
VqY+ILXTs5dmHM8ztXmjs6Otnc04Rl6yxRZv1yyHOJPIE54zGGTCSratbh398WKQXiqIS6pHvfVuE7U
Wof/2Xf64+7QCA6SLzz4tKMTrEOFnKE64a01XGyVOOakR5TvlgbS2fyDyizhfTxR+G+IbpY9Xa7THqk
WdjxiGhzRVzD3ipqYxRjSe6A3yntHnJdPfwXgvyxIQllodC4yNrC69fPwwtXw3wTFEeapwBI7ry5yv8G
X+Kmr1uPL39qXv3icg93/qnf3iq+pwDACaNzD83IvioKKXTlydbZQJnGYN0XgrnuOqsYkDx85H188VZ
9RF9upgu/jDEuxdx0J5KPekT6uXtoudVR9stWfevR8VOtJerB/hO8azSGebpLnk7Nu7N0zzMv6kW69
ePa1ZDe2JbOUXTDBjXpSuXdry2QwQYiqK61kJ9sf/FI4f//U9/qj7kAIBJI/PPj8o1RZhRMagOa/54JuJV
iwDmU52WjShHZz+Uh/QRfbaYLv4wxLsXcdCeSj3pE+rl7eIssd2SPXO5J+ojgjvA6NVmZv9OMg0dff0
swYlrTZua5cpUz6spfNQU8RkwunOfnhPphaKohPqvj35TZO951b/95jfVxxsAMHVk/nmqsk+WnlTC
MSKNP56pgdXRVgHMozxP/c/7xdnFIXWknlBMF38Y4t2LOGhP5T2jgf8cNa862m7JnrnkiWpIphzlzu
zr1WLm0O4bO/r6WQIT15o31Wb9mjFdoTypcRUG/wyYh5d/
+bJILxRFda3tZ7Ydv+82Eb/HL77GDwArhcw/V1WgObK2VoYmMzPpIGWmHJmuPOlJh6RQ7stV87h
f4y+PGPOJjvGHIb5h+ljVyx7TcmKLnLHgTNRuZs+FFSe6I9wr7+vV5iXzjMk0d2zcW2hBasrmFp4h7p
hMdsx4ZPdtXGYhNgPm5NH3HxUBhqKornXzS9c9feAOEcLHLL7GDwCrhsw/XzotFUSc0U+qeKVzj8
5b+og615gwGo7qE32nGnlOZK34wyBnnUb/qlloZnWGXl6kVYsrJvsEqJnCV0Kup15wPbOvVz2uOm
hcdzXM0z3T3NHXz+Z0tw+02JQzg7N+faBevthQeI62M3gOYARbV7cOvnNQBBiKorrW3uduEjl8tOJ
r/ACwgsj8c6bTTUalqpr5bM1MOUYok9zpTPWJToryNyiniz+MCC+06uZM5T+joVGHKxadyZMo5Yn
+Vrl6ZntMeTx8JfSJnu65xo5t9hZoX7VqsakO65fqZfknqZ9vniFwjTC0jc2NXW/uEgGGoqiudfj4rSKNj
1N8jR8AVhCZf950uAllFzsd1ZlHMwdkT+uHnqEmd5z3THEw/jBOD85k40Voi3cvtIx39klyZe2W7Em
UnhPNZJofVY+Nqc0hejJzhdlYZ2ZP90q8Y7u9ySsk2sRbFJrWnzOnyblLslcR32vOnkE9656HoV3YuM
Cf8aeoGWv7mW2PH7pdBPKh65OfvFt9jAEAq4TMP28q+BBdACyLt3/7tggwFEV1rZ0vXv/s/jtFLB+
u3nr+ZPUBBgCsGDL/fOn/qZPID2CZnPr4lAgwFEV1rbueuUUk84Hq9NHv/
+Wrr6pPLwBgxZD558X+D5fd//AZABbb0feOigBDUVTXuv/YbpHPe6+T99z9+aVL1ecWALB6yPzzYm
R+Aj+AJbS5tblvfZ8IMBRFdaoRvtjP1/gBYMWR+QEAiT778jP+jD9FzVjf/Me/G+6L/XyNHwBA5gcAp
Pvo8kciwFAU1bUOnNgpsnovxdf4AQAZMj8AYCZnL54VAYaiqK714AN7RGKfsfgaPwCgROYHAMzqx
AcnRlChKKpT3fDKtU8e3Cty+yzF1/gBACUyPwBgVltXtw7/+LDIMBRFdardL+zo64v9757+YfXhBACs
PDI/AKAHV/58hT/jT1Ez1r1P7BLpPaFePfbQX7/+uvpkAgBWHpkfANCPS1cu7uZM5T+joVGHKxadyZMo5Yn
2R4TvVyXvu/uPnv68+kwAAkPkBAD069+k5EWAoiupUN57e/tS9+0SSb1+//tkH1acRAIACmR8AIAC
Xf/myyDAURXWqbz1/o0jyLYuv8QMAXGR+AEDPHn3/UZFhKIqrVN997BsizzcWX+MHAHHiR+QEAPd
u6unXwnYiw1AU1ak6fbGfr/EDAEL/ACA/m1sbux6fGwfKr7GDwAIfM
DAAZxYeMCf8afomapvc/dJLK9t/73/zxTfeoAAHCQ+QEAQ3n7t2/4+z7z5R+sHh4R+
wAQQeYHAAzo1MenRIahKKpKp9bT+z7fh9t4mcr+sHh77z5R+
+qD5sAAD4kPkBAMM6t+t5REWMoimpfkS/2/+4Xn1QfMwAAAsj8AIBBPPr+o1JhbW5t7vvJ2IMBRFdH
FpP6v3X3+t+owBABBBG5gcAADzO6zLzNQfMwAAAsj8AIBBHPr+o1JhbW5t7vvJ2IMBRFdH
NDtZeDna/wAgPbI/ACAkZy9eeFbEGIqi2tfOFF69/dv+dWebna/wAgPbI/ACA8YgMQ1FUp7rrmVv4Gj

8AoBMyPwBgJNdcM9I/dGiUjEbJaJSMRslolIxGyabXaBVwKQEAI+GOJBmNktEoGY2S0SgZjZLRCBFc
SgDASLgjSUajZDRKRqNkNEpGo2Q0QgSXEgAwEu5IktEoGY2S0SgZjZLRKBmNEMGlBACMhDuSZDR
KRqNkNEpGo2Q0SkYjRHApAQAj4Y4kGY2S0SgZjZLRKBmNktEIEVxKAMBIuCNJRqNkNEpGo2Q0Sk
ajZDRCBJcSADAS7kiS0SgZjZLRKBmNktEoGY0QwaUEAIyEO5JkNEpGo2Q0SkajZDRKRiNEcCkBACPh
jiQZjZLRKBmNktEoGY2S0QgRXEoAwEi4I0lGo2Q0SkajZDRKRqNkNEIElxIAMBLuSJLRKBmNktEoGY2
S0SgZjRDBpQQAjIQ7kmQ0SkajZDRKRqNkNEpGI0RwKQEAI+GOJBmNktEoGY2S0SgZjZLRCBFcSgD
ASLgjSUajZDRKRqNkNEpGo2Q0QgSXEgAwEu5IktEoGY2S0SgZjZLRKBmNEMGlBACMhDuSZDRKRq
NkNEpGo2Q0SkYjRHApAQAj4Y4kGY2S0SgZjZLRKBmNktEIEVxKAMBIuCNJRqNkNEpGo2Q0SkajZD
RCBJcSADAS7kiS0SgZjZLRKBmNktEoGY0QwaUEAIyEO5JkNEpGo2Q0SkajZDRKRiNEcCkBACPhjiQZj
ZLRKBmNktEoGY2S0QgRXEoAwEi4I0lGo2Q0SkajZDRKRqNkNEIElxIAMBLuSJLRKBmNktEoGY2S0S
gZjRDBpQQAjIQ7kmQ0SkajZDRKRqNkNEpGI0RwKQEAI+GOJBmNktEoGY2S0SgZjZLRCBFcSgDASL
gjSUajZDRKRqNkNEpGo2Q0QgSXEgAAAACAaSLzAwAAAAAwTWR+AAAAAACmicwPAAAAAMA0k
fkBAAAAAJgmMj8AAAAAANNE5gcAAAAAYJrI/AAAAAAATBOZHwAAAACAaSLzAwAAAAAwTWR+
AAAAAACmicwPAEBX59euqa2dr44CAAAsGjI/AGBCyjA+bAq/vH6k7jBkw7H/zUK+scyR9cvVgT5Zm
ynw70q6sq7hMC/T4AIfmOq9V+r3nRH5iEae6so/lfWK9butYUWvzKC/KAYU3tSyvk6BHVnb6etlatF
rSX8nrQgyPwBgWAPezVuMPmPetBVth7jXye6l9D6K+6qBb6jyjRxZW8v+n0H65FsY/ZbQvB/t/11hv
bNrA737ir2M9I4wr1t/TYKfUPszZG90JsGO0ae6Ck2VH9cHyks6ayv/LObLNXOLxiuTP9/rLwrfpqwtF
WbbV2RTxVM9fpTc7RjNTQPtqOivDxWPZt1du149tCqvXUVcH+sqiufQApkfADAg+37KvhvplWpU/P
9j3hDYOxzK4F3yBtXlG6ZN/toPfplM47wwlqLlQG8+Ofdwvfq+j6+o18NduHxr9LW1cMfYU121nUq
Nqx52Vpxfsrtkl08fmP3latpOfrx6eqY+pdim+pi/EtmU51C64HaE2ZuGdySPDNhLPVE9nPVVy6fT0xf
v5Pqh3ap4cqY9rSIyPwBgOPIewLlr6N3wHYRxbj8G3paePv8h/aYtZrbbwc5Gfx9khn0r2BdwsP0VE5
svVN8vnLNydys9X8jIterxMjZNVTyffCHV2cN2Mfgb6aP5DzO3UYv19Brm14XbqJd9VCLbsfX4/m68
dI2Lac+ZytlHj8301SweyLdDn41WBpkfADAY95/MPd7t+I18M1Dsp697xiDr9qd/xjXLfxymUXGlagNf
s+H2ETTwi5QpL2HWovxhmHe58wk13h29cObLO9rXreeWkel67NQ0lXNhkzR16W1DvomMY/mPv
b3ZPb3cN0Uf3EZFn/yLCtrsXX2XzlA83dfevL2Kt1reovwhvJRunF6By9nP1szL5G/U28ZWBJkfADAY9
w7Ae4vSp8EbmIobj77u3lzFXioDbinfhZo+bznchpRyY0P2Kd55Pd/Kx43zxivecYWhWrn7cD/FM3Ea
uPP3fC0j0/XYKT5V8cL1cBXjXYpn+3mtPI3yPagj+dO9vSkCvQw9tXIbidelGBC+vu14tmOIP9tVaLb6+
vXWytOrOCKvXk+vVbGDqlv+sz1tv1dxNZD5AQCDmcM/qse7FxB3i4Pq9WbKlm+jvl55o1H25L41ei
VenPL6Dfm2KBoO+r4z91D+PEy/Ym556Xp8qYoJzZW7bwVnyGwi0/XYKTaVeDvOwNulOFjp67I5jfI9
1I/zp3t7U8QuXabcXh/d3EbOm6+HD3J0Oz3Mb/L0Ki9Xeaz8uad+3n0V+1HW1qw3yQyKafUL47xK
8WsMLzI/AGAwc/hH9Uj3AuWdzoj3HOXNW/8NrVs2Qx932DFF3+Gun/POG7Zf8eoMesncDgPuyH
pT9HcfX3I+oe5H1nn1ZuM20CJPdRWcqriafW2nYcHF0700k42s94RhiF6unt4RbiNn4ua1NIpMUTzV
13sh5/RyOxSv3Gw7KjVfmnxEH7uTHxn35W9eCyQyPwBgMO4/mHu6dwsb416g6DHyDUfRc9ArV
8jbDN9l+O0McSsf1ttddZjbYtAdGfp+S7jrli9W3zuLzNdjK/9UxevW42YaF1wM6KFhQ6P86d7eFY2b
6usT5jYqjpgbkW/GBOHt9LQNg9PLbdF8dVtqnCgfMPubotiBPY3buYeXaeWQ+QEAw5H/ZO7t9iNo
+A7lPYnU+/1H1sXYhuc+aBD93LW5snmNaYsXadBXqehgbmTIe8TixRn2Pee+A0Zpmun9HVG8NPb
Crb05L93MPB2VyFNdeaYqDvX7IjUuuBjQw+VraJQ/3duLNNdNWR+kXj5Vwe0M8Jl1e1kfpkx/TeM
vUz+vUbFat0l+2Jw7vhR4kfkBAAOybkD6uSvwK+YWlvueQOxonM3kTQd5hUbfjXWr2999r2vId7W
tvB/WRnhH9Lk38Q4o1Fsw99bXxYx0jC+mk/BU4vUqzbi5opu10qyL8bjoOUuPllcmH9bbm97ZVHb
AmLxckmcRrcU3ZT07S5uKs51ScXiE93bvvyjCvexnZr505XTeaaz3da9XcnWQ+QEAwzJvQPjnNEZj3Z
DOfD8aUry9B5t9Dnq+j0cv7FelVL024qklesXCm1rKPUVeo8zkflH0TvyrilJ9y8CNxIzI/AAAAAAATBOZ
HwAAAACAaSLzAwAAAAAwTWR+AAAAAACmicwPAAAAAMA0kfkBAAAAAJgmMj8AAAAAANNE5
gcAAAAAYJrI/AAAAAAATBOZHwAAAACAaSLzAwAAAAAwTWR+AAAAAACmicwPAAAAAMA0kfkB
AAAAAJgmMj8AAAAAANNE5gcAAAAAYJrI/AAAAAAATBOZHwAAAACAaSLzAwAAAAAwTWR+AAA

AAACmicwPAAAAAMA0kfkBAAAAAJgmMj8AAAAAANNE5gcAAAAAYJrI/AAAAAAATBOZHwAAAAC
AaSLzAwAAAAAwTWR+AAAAAACmicwPAAAAAMA0kfkBAAAAAJgmMj8AAAAAANNE5gcAAAAAY
JrI/AAAAIvl/No1lbXz1aGSeuLI+uXqyDBUH9F+EdTXJuNch8vrR0JPRZ8DgOki8wMAACwWI9fa8XTV
M38d2wu+yxBM9vVFXcB/kwEAwyHzAwAALBbrf8s2E+qqZ/5W+/eG+/pfBZD4AawYMj8AAMBisT
K/GXBXPPPr4N6wLn399HXSR0j8AFYOmR8AAGCx2JnfCKpO5ncOuMG4GpKPMOatTjCOmGlYHc6
OGf85vdFEMZ51njfWVrdpyNzWfNaExkqVyFx6dDFGP3J2EFt/zl6PGBLZYPQ8ABgVmR8AAGCx1GF
yvfxJRUYjZRaP3QNG3KwicR1HG+kQHT7HyNky1xaMhXgmiUZff1O5dYOxFke9uLV19aPo3rT+hgW
FN+g/L7pcABgOmR8AAGCxqNCYJUgVTMvAaDxRDHQPxDJ/NciIpPKIiqXOOfURdUC20SP0Uow+Le
JuuIGe0O0ZowdX6mkKjetXA8LrCWwwNJFcAQCMg8wPAACwWKzUWD0wf3ZjpxEnZZhVQ+pcGj6
ipnFHyDEyEWfkIT2JMSZIriAnd+IeiNMLyIlTWqxfcrr7N6iHtdo3AAyOzA8AALBY/PE6i5pOMvZEZRlN
3SHNR9RjMyjb86oRPqKxNUuAu6ScaqmOyq01Ci+hxfoN9mj1fGB2vcqavS0AGBWZHwAAYLHIBFw
91l/vrzOkHJkZKvPbB9UDn2DjMP9YuZXkzO8uocX6feG9oJ4Pzh44se2qAaBXZH4AAIDF4oRJGSL1E
57YKYOxO6T5iGdaMa9vhNBiiOYfq1qqo3JrjcJLaF6cGpEpBzndG+cwpiiEmwHAcMj8AAAAi8UTJu3
4qJ+Ijewh85vh2h7TIn67bcL0dOZgp0ePmb9xKvdU56JENpg9ZUzced0A0CMyPwAAwGLxhUmdG3
P6ifpoFSd1MHWOuOE1fCQ8ix4S7qyCrdsmJtygPr9zdo4soWn9cjv1+IYNOhuJLgMABkfmBwAAWC
wNYdJ8woiiksiysQgqj+jHR+TsKu9m/K3rAW6bODXeZp7dZ+ZvXL9/OTk1JDR76My2qwaAXpH5AQ
AAFksgTNYh1XrCzK5ZrJTB2J2s+Yh6bE7nLCdn52Z7QGAXUfZ8MiTLrTVqXEJs/fXpubylcVnMp32zm
6fm2q4YAHpH5gcAAAAAYJrI/AAAAAAATBOZHwAAAACAaSLzAwAAAAAwTWR+AAAAAACmicwPA
AAAAMA0kfkBAAAAAJgmMj8AAAAAANNE5gcAAAAAYJrI/AAAAAAATBOZHwAAAACAaSLzAwAAA
AAwTWR+AAAAAACmicwPAAAAAMAU/e1v/wG7OzopdhE5FQAAAABJRU5ErkJggg==]]></redcap
:Attachment>
</ItemDef>
<ItemDef OID="b27" Name="b27" DataType="text" Length="999" redcap:Variable="b27"
redcap:FieldType="descriptive" redcap:BranchingLogic="[q25] = '2'"
redcap:InlineImage="1">
<Question><TranslatedText></TranslatedText></Question>
<redcap:Attachment DocName="B.png" MimeType="image/png"><!
[CDATA[iVBORw0KGgoAAAANSUhEUgAABVEAAAEJCAYAAACdcmdaAAAAAXNSR0IArs4c6QAAAA
RnQU1BAACxjwv8YQUAAAAJcEhZcwAAEnQAABJ0Ad5mH3gAAE9sSURBVHhe7d0PjCRnfef/b+/aY
PPHNmBskx+RHat7sZYlGJIg08PxL4egZ+6SJbnbYOVg5Oh3PVyi0zQSkwOxF+mX2wguG4lunS5iWrq
zliQyTEJugrLdIJRgckzHR4IxYTI/Z7rlc3T8wDYGjPljB3u3f/VUPVX1VHd1dVV3VXX39PtltaerpqfqT/T
O/OZ5/k+hb5FAAAAAAAAAAChjumPAAAAAAAAIAQhKgAAAAAAAAAAEIEQFCAAAAAAAAAAAAiEKIC
AAAAAAAAQARCVAAAAAAAACIQIgKAAAAAAAABEIUQEAAAAAAAAgAiEqAAAAAAAAEQgRA
UAAAAAAACACISoAAAAAAAABCBEBUAAAAAAAAIhCiAgAAAAAAAEAEQlQAAAAAAAAiECICg
AAAAAAAAARCFEBAAAAAAAAIAIhKgAAAAAAAABEIEQFAAAAAAAAAgAiEqAAAAAAAAAAAQgRAVA
AAAAAAAACIQogIAAAAAAAABABEJUAAAAAAAAAIhAiAoAAAAAAAAAAEQhRAQAAAAAAAACACISoA
AAAAAAAARCBEBQAAAAAAAAIAIhKgAAAAAAAAAAEIEQFQAAAAAAAAAiEKICAAAAAAAAAQARCVAA
AAAAAAACIQIgKAAAAAAAAABEIUQEAAAAAAAAgAiEqAAAAAAAAAAEQgRAUAAAAAAAACACISoAA
AAAAAAABCBEBUAAAAAAAAAIhCiAgAAAAAAAEAEQlQAAAAAAAAAiECICgAAAAAAAAARCFEBAA
AAAAAAIAIhKgAAAAAAAABEIEQFAAAAAAAAgAiEqAAAAAAAAAAAQgRAVAAAAAAAAAACIQogIAAA
AAAABABEJUAAAAAAAAAIhAiLp0etJYKUih4DxWGj29PqjXWXFUFQU+1iMxj3ZB2onXjZzZCdB2onXjpLWdL
MxLO7LSlg372PzHqPs4Vb2GrGzEO5vtDbdtK5JH08KNuQ9Cjmc+2g0AAAAAwPwhRF06Rdm8UJey
XurU1kPCkracr3X0fBPrqNLQs9VCpbXf5fa5Nl63OnQaSnCVqVeq/6J4yDa1xdGCuqAnBr/dyIDA8AAAAAADIGiHqMip
uytmqfi4dVJdS/VR/XzhFGVaKzry/9vvnpsS/aHkff+RmRdmXdmwu4JaTw6jk6wIHm
fvfrjo/VGHs/dm62x2Az4fv2HBjdn7Ayrsm9wRzQ5zWsMhzfbvkWqTNlrSXKXxUkijQt
2FgKQpv4ui62xmPd/wOxC3m4LuS/d0wHIsWA/IVsWOsA/rKvXkDqEsdv5KlrbLvyD

D7TVk3fijwsI7ascDAAAAAEAOCFGXVGW7JV6O2jxn9EY1e6FWpbW4CSqOot6h7OuncuoEPaQBA
AAAAEAuCFGXVkW2W26M6vdGbW/4Q6XL9a3wXoyqlqKuRek+wmpSjqyvaH59ZI1Jo6ZjoC7rqPX
GPu31edcGHd5fZI1J4zwEz5+5nbDPjxN+3INtCdS9tR4xy30ObGewraPOc7xjCm57uPZpYAh6c9VZF
2h4snM3eA7Uw9yc3Z6x+xzPPy71CLkXbAnaHvI9OHq7vqTHE6/dAAAAAAAcfYSoy6yyJXW3OGpz
VTYaDTnnJ6hyIWSM9FAIo3VqpQwmoCrK2hndwM6BdJ1nFmNId2dHLno7bcuubn/5zNpc9FKsnPa
D6gP/AGy9izv6PBo1Pe1wrCSDo63TPb8dqZUKUhrYSXM1aUimtjPY1qasDoZtEx2T2rZR+9S6H+te1
+kREu3HCSwHz4HSXB0dKibnnOtV70AUtW6Kc9TeGFHPNGS7E4vZbgAAAAAlgQh6lILTjLVrLnBTF
nqF0Imk2pv+KGKV4+yL103ie3UZD3lhKW4dka3b18O3U23d42JhYxw0hjqferEnAz0rpz2yiY0d4M9
Ji/u6BisfEacDLUnjXX3GlSlpc9v3+0xnOr59bfvdUhWIdlAfdzx/O1494Hazrob/E1zTMbr9zZlc9v62PX
vV1Wv1/6cXXIi2X56jXUvsCzXu85r+8MlLiqR+4wr/BzteOl/krZbr3X/0hH2PRjY7rBkxzOu3QAAAAA
ALI+C9QtyXz/HklK9SwM9zlSwEhKq+K8rS71rTubTk8aK24tOBS/OZEYjX6963bk96Ubsy+dvW4Vde
9ZG1BBs1YOwWq1Ks9kcWu+3IaxdcdeNE387w+2yV3rnwG1/6DqbsV0VnO2FBNwB4e0wr3O11fc
nDEt0PSK2E9ivvuaS7JjMbQdfr41qa6JzF35+FOdanQqsS3p+lETnOlHbRwht44jjjDietO4RYBr3Pnyvv
OXCW/QSACAP99/xLnnNW2/SSwCAzB3/NZFjr9ILWBT0REVwkikVtoQGJP5Qeb/npMsYdm/2GE1F
UU6ccp51dJfTrj2WvywnT5+0e9QF11uqp/0AbA74vWmb4nVG7R44gZT1GW8ov7dOdT4sGbUo3S
BMfcIsazBr1jUo6ac28z7QPYSnOKZEvYkT7cec4f+kmIdQ3NyTvhmgTm3wHIWY6rrrurFuwJmaGO0
GMvDwEw/LOz/5Tr0EAMjDDY9fJXd/6AF5/Bs/0msAAJm7dI9I/zt6AYuCEBVBA6FScsO1P6fl1RVt7k
pbenJoj9k/JScqJ6z/D65XGeqc9ZIrrombLbpD+ttuIl09a/TozUtaAZl1DTJrOyHeSKpXqBe0GnVjgQX3x
NNPSOWPKvZHAEA+nvPjY/KWL94oP3ryGWm+/3555p8u6c8AALL1lMilP7A+PussYiEQoiJlGYRfXl3
RfTlsXxS7lKgd9lbEyVeN9XMZvhk9NO3A1+/VOyrwVcO67VqVQ480e0lOK1mv47yOaTHOXbjxbW/
Lhtnr1K2LatY5BRbUnZ+6Ux58/EG9BADIwxv/
+gZ5wQ+vsJ8/9NXvyp/WeR8GgNz0Hxa5/Od6AYuAEBUxleSkN1LbnBFfMSZJitM70Ri+HI+7744c7D
pf686+X7I/4a8fLjUwH4qbZ/0guOFOjFWVQIZacsoTKG6Jgvk22OvYvA/0seV1TIn2Y97LA8Pk1cz3hZ
xnoE/SdmNSNTtwdeukJv6eAubL+z77PvlM7zN6CQCQh1c+eK3c/PXn6yXH5z7+kHzlLx/RSwCAzF3
6gsjlr+kFzDtCVMRUlM2zbuVUc/Z1NbrYnOl8y+vt5wScijnre1s23JlrYvN7cqqJpBS3XmZRF0x117vh
6vxxe81a56Kmj3+wdqsx7F+aq7LhnTIV7Onh297K+dBcNQLH9nm/hqd7bHkdU6L9mLVbm7Lqre/p
me+ta1Qq+NvI2oTnyA9c3XYDi+kT+5+Q+n11vQQAyIOqg/pzD7xELwX94W9/Tb73raf1EgAgc5f/RK
T/fb2AeUaIivgq29LyctSalHTA48w8bynX5YJR4NOfUMnSXNWB0GQ1HAPbsp55Q/a9of6KMUnTHP
Jqu9rKUt8KRKiWomxe8IdlN1d1gOaFzqMm/ZolJ3AMttM8tryOKdl+ipsXpO6/2HmtOZGTdS/7h6Br
7yrua1NNWBO03bzfw9odR+bHA8R339fvk7v+7C69BADIw/FLBXsYv/oYRgWozd+8Xy8BADLXf1Lk
8sf1AuYZISoSqWyH11+stqz17tBiV3FT9gZfW21NVr/R7K0XKBlgDM3OdKKjFJgB2KiyA+qc9bt+wOe
ya1/OW03PstS7fT9Yt1Wl1d8LTpaV1zEl2k9RNvf60h16sXq5MUzeVpHt4EGK7B96PbFTEbvtVlv6Lf
8+sqlzblwHu+5ulByOB4jBnYn/6Wfp7QQAeVr50kvl2iev1Evh/uFL35ZP//6hXgIAZO6y9RvZJcpbzbt
CX81aAgAxtTcK4nSSVCHqQGAKADGo4PT1/+318sAjD+g1AIA8lB56od0LNa6tu8vyiteFD/sHAGTgit
8QKfBL9ryiJyoAAMiVGsJPgAoA+VK9T1Uv1CTUsH7qowJAji79AfVR5xghKgAAyM1HvvgRezIpAEB+
VP3Tt33hZSProI6iAtS7/+NX9RIAIHN2fdR79ALmDSEqAADIxe6Du/LBv/igXgIA5OWOL18/tg7qKPv/
8zH53Mcf0ksAgMxd/n9FLn1BL2CeUBMVAABkTg3fV3VQmUgKAPJ189efL//8Czfppclc+dzjsnX36+
XWV79IrwEAZOtKXR/1Zr2MeUBPVAAAkKknnn6CmfgBYAZe8MMrEk0kNcoz/3RJmu+/X3705DN6
DQAgW9b77aWPWx+fchYxFwhRAQBAZlRwqgLUh594WK8BAORB1T99yxdvlOf8OJ1f+R7/xo/k7g8
xKSAA5Kb/HZFL1EedJ4SoAAAgM+/77Pvk3ofv1UsAgLz83AMvkRsev0ovpeMrf/mI3PtJ/igGALm5/
DXr0dELmDVC1KXWlo1CQQrGY6XR05/LSa8hKxttveBob7jtWZG8mzM3Qs5LHL3GinPuVhoy6tT55
9d55H7NjwLuWyCW+n11+djffkwvAQDy8vJvPE9e+eC1eildn/zPB/LN//19vQQAyNylXZH+Y3oBs0S
IurR60lhZlaZecpTlzFpRP8+eHfiVasLfVIImPi+9hqzXor5KXfOCrAYvunRqJSlMENguK+5bIB7V+1T1Qg
UA5Ovqp47LG++bvg7qKKo+6u//

+y/bHwEAeVD1Uf+79fFZZxEzQ4i6tLpy4KZA5bp0+33p9/dkM68MdWzgt6QmPS+qZ+S4YK99XtxN
V1vqevelWy87K5qrQo4aA/ctEIuqf6rqoAIA8veWvRvtIDVLqifqH/721/QSACBz/UdFLv2xXsCsEKIuq
96h7OuncuqE5Nf/FGmL1zOyJ41zugtquS5bFedpcfOsVJ2n0twlRQUwPTUTf+WPKvZHAEC+Xvu1F8
vLHr1aL2Vrb/f/2A8AQE4uf8l5YGYIUZeQXbvRDN2aq04tx8GuiKp3o1E7c1T9zGAtyGCd1bDejbH3r
wVreI6qN+kMVfdfl6zW52A9S6+2qH6ENy3GPgPncEP8zZjnydln0vOiqHaWYvWMNHoeB0LzkpzUn
VFl/9A6olGM9obVW21v6GMZPAcJrkvI/RZ2vePcb4PXz35E1ImNY5b37WT3p2vSa2eZ6D1Ar1TMr
49uJI6QOz91pzz4+IN6CQCQFxWevubvXqSX8qF6o1IfFQBydOlPqI86Q4SoCDUUGGl2/cyRYVRHai
WjzqrR43EyanuDNTzVurCgpuQNVXdFt3UUZ5+DwWRzdcJ9Fjflgjtk3joz5/RGeo1z3nkq1y+kUEahK
q1+y+tVOsTseTxK50C6+umwipx2N97ZkYsDJ7W96x2NX1c3yXVRQV7I/eZcj1EBZPj9pu7d0GC5U5
NSIMjOSpb3bcz7M2CCa2eZ7D0Ay07VQP1M7zN6CQCQFzV8Xw3jz5uqi3r3h75KfVQAyI2qj3qP9Z
H6qLNAiLqEKtt96Xfr4kZ7Um3Z9TH72zrxbG/4AZBXL9Won9mpyfrIxEaFec7r+3uboWUCxu4/wN+
et3/pyI6XBPWkse4GPca+Wzo1imzrKP523M2ofdbOu/Fbsn0WNy+If+rOS9v677wbglnn94JOUJOd
F4P9um2ZLK8uyolT+ukYlS23beb5V9ri53BnxMnhkpwj67VGqYGh+21ofyZj2/b9ZrYlbFt+kJ3U/Ny3
/uvD789hya6dZar3ACyrT+x/wp6NHwCQv5UvvTTzOqijPPTV78qf1hmBAAC56T8scvnP9QLyRIiKIW
bPtPoFPwgdDgOHletbE4Z54aotPxwsrp3xAqzOge4z2bsoO14eaey7suW3deeiJIl7zH364ZMh8T6Ls
nnWTbuaslpwe08Gz+8kipt740PW2PblMOpEFdfkTNjxtXf9XrVn1pzjSXSOrPOzp0NEI3gPvd4Dhu43
s8et0evSPk86CNzLYfa0LO/bsfdnmCTXzjLNewCW031fv0/u+rO79BIAIE+vfPBaufnrz9dLs/G5jz8kX/
nLR/QSACBzl74gcpkJ/vJGiIoBI3qm2Yqy5iYxIwK3UyfSDKjKcrKkn47SPdC9+VSoU/LqNhbMYdKRw9
QnMMk+K9tGr0FHOsP403RKoi+fcf2NgNIP3Kpy1j2gqa6LruEZOrw/aOh+M8JCawf20Hd33yNrsaZ
uHu/bBNduyvcALB93Jv6nn31arwEA5OWGx6+Sn3vgJXpptu7+0APy+Dd+pJcAAJlTw/r739ELyAMh
KibUkeHOgTHCo7mX3TFUvMKUSrD+5Oz05HBssVSf36vSHRZuBG7V08FeoUmYExB5PXXHCbtWR
dm8EN470w0rF3t+o8nvz/SvXdh7AJaNCk5VgPrID+h9BAB5e86Pj8lbvnijHL9U0Gtm60dPPiPN99+v
lwAA2XtK5NIf6OfIAyEqJjR/gWm53vWGbAcfk9YLHS/
+Po26n7aO1NZznJyneELGlj4tn5Sxl7S4KW5lAntYuDF8vno6/CyPP0dt2TB7nbo1OM36o0lYbdzT+xj
s/as0z83XpEi53bcTXLtoR+GPJpiWGsL/wCMP6CUAQJ7e+Nc3yAt+eIVemg+qPuqnf/9QLwEAMqfq
o15iYte8EKJiQElOusnV0EzePbnoFnIcO/Q7J6WTXtA2qm5m6ibYZ6+xrodpl6Ved5OsPCfnMa5rc1f8
zphdOfAu6QmJc0m9HrXW/XH+/I4OP6sSyOGSnCOzLqcKFN26qMaQ96TUzPKqx6k9GZQdSHa9W
p7pDpOf0CzuW0usa5fme8AU1xDz7yNf/Ig9mRQAIH+lh1448zqoo3z6v/6D/MOXvq2XAACZu/xZkf
48dRU6ughRMcCcBCnYW9IPAlXYNTChz6yYNTCbq/5Q7faGHhpuPdIev510n72GrLsnrnpWNjeNyY
Nq65JPjmrWsmzKrm5er3HOCzBj90asnBbnDuIls+ke18Bw8Amvix8oDvbcjUlvX80sH+xxaoTFZo9b
oz1p3yaRZnHfKnGu3QTvASU/dZXaee9gZMOb4h9Hze6Du/LBv/igXgIA5OnaJ6+0Z+OfZ83fvF+
+9y1qZQNAbtSw/v739QKyQoiKYeYkSJ2alHSoU/LTE7kw7YxI5vDy5uoUoVGwBmZzVQdQXnhTlVZ
qs9e7kuyzJ411d6h6Wepbav3okCq98zLMnFndbbN3TastiX+aKhIo72oZDmATnCMv2LO4x2xOsJTEi
HvXrLNaPevPNp/YQt+3SpxrZ0n4HuDXW7V41zBubVssGjV8/85P3amXAAB5UvVP3/aFl81NHdRRV
ICqglQAQE76T4pc/rheQFYIURHKHgYdUpOy2rLWu8Otp1KR7cGClfuHRs/BBOwamMZwbZddWzOj
eqgx92n23LN7obonbiCk8of1p3hehhRlc2+4Tqg9hD5hYBeYJMs6ZjsbHhT7uljH3G/5QaqtKq2+0dZ
ACYJo9r07eJA2Z5vTZZMLft9aYl07S6L3AHUsg6+ttiava4u59cTTTzATPwDMkOqBqnqiLgI1pJ/6qAC
Qo8vWb6bUR81Uoa8KBgJAEmo2fT0ZlAph96btmTxL+lhOtaYNWBfEUbp2yJUKTit/VJF7H75XrwE
A5EnVQVWTSS2SK597XLbufr3c+uoX6TUAgGxdKXLFb4gUbtbLSBM9UQEkFCxRcGZtsUO49nnrWC
J6ZB4tR+vaIV/v+
+z7CFABYEbULPx3fPl6vbQ4nvmnS9J8//3yoyef0WsAANmy3m8vqWH9TzmLSBUhKoCY2rJh17o06
pWaJQoWUK+xIqv7demmUqJinh29a4d81e+ry8f+9mN6CQCQJ1X/9C1fvFGe8+PF/NXt8W/8SO7+
0AN6CQCQuf53RC7doxeQJkJUAJNRNS8XfPx7cXMvpRq/C+YIXDvkR/U+Vb1QAQCz8XMPvERueP
wqvbSYvvKXj8jnPv6QXgIAZO7y10QufUEvIC3URAUAAKEefuJhec32a+wJpQAA+bv568+Xf/6Fm/TS

YqM+KgDkjfqoaaMnKgAAGKKCUzWRFAEqAMyGqoO6aBNJRVH1Ue/
+0FftjwCAPKj6qGpY/7POIqZGiAoAAIbc+ak75cHHH9RLAIC8qQB1UeugjvLN//19+cPf/ppeAgBkrv+
oyKU/1guYFiEqAAAIUDVQP9P7jF4CAOTttV97sbzs0av10tGyt/t/7AcAICeXv+Q8MDVCVCTUk8aKm
uVbPTaknWjdOGltZ96MOYZeQ1Y2gmvbG+7rV6TR0yunpfZjb3NEO2zTne9M2m1bkPugvaHbWJC
BSwosjE/sf8KejR8AMBsqPH3N3x3tuqGqN6rqlQoAyMmlPxHpP6YXMClCVGCGeo0VKZRq0tHLWe
pd3DH205RdQj4AA+77+n1y15/dpZcAAHm7+qnj8pa9G/XS0aXqov7+v/8y9VEBIDeqPup/tz5SH3U
ahKhIqCibe33p99VjWyp6bXby3l8WRhxDryHrtTziU6UnF3eC+2qea1hrF8VRuA+A+aZm4n/nJ98pT
z/7tF4DAMibClBVkLoMVE/UT/7nA70EAMicXR91Vy9gEoSowDLoXRQ3Qy2Xy86Tzo5cXJwUdTFUt
qXfrYs+w8DCUMGpClAf+cEjeg0AIG8//ffX2UP5l8m9n3xYvvKX/NsDALm5vGc9mOBvUoSoS8eoLbl
i9kQctd6oc2mvz7s25fD+IutuGnU/VwKfNLcT9vko6Z8z+/PmMP7mqvP5EYU0/WNWj+T1Rv2h/FU
5e+GMDvk6shMnRQ3UUg17jG5PvHbHuTbD59DaumyYrzfqkQa2MbA+/BTHaYN5POpY/P2rh7vd
9nnrupbrsjXQXdYu3WC83n4M3DfArKgh/A888oBeAgDk7YbHr7Ink1pGd3/oAXn8Gz/SSwCAzF26R
6T/Hb2AJAhRl05R1s64PREPpOs8s3TlwE30Aj0U27LbdJ6Vz6xZXz17ldNV/awjB/4B2PywsCxn1nRr7
RCwJIMj5zu1UswQa5bnrCO1UkFW9fYcal2SINUYyl89LZXimniHs3Nx+hCvfEbcU+2L2e6pr43Dfn1w
Z846FVQOrG+uDgSpE7VBHcuqeFvWoakKSlf369Ld2wxcdxW+lsJKN3RqUprnibKwFD7yxY/Yk0kBA
GbjOT8+Jm/54o1y/FJBr1kuP3ryGWm+/37qowJAbp4SufQH1kfqoyZFiLqEimtuT8R9OXQTovauHwi
JEU72Dq1XOU6dmIcI1VI5LW6M2gzMjmSEhV6w15PGutvjsyotu6am9WjpLXRqsh4jjUz7nFW2rTa
Yw76rLadd22HVPv12d+vuV8TsRaoYQ/mrp9X2zVA4xpD+4qbsuefNfnTFa4Z1BPULwcDQN67d6V
wbV7neDX69Frbev2+maYPxeh2aFjf3vOc+P1RXYWtXf41/TppyLsFxAmnafXBXPvgXH9RLAIBZeONf
3yAv+OEVemk5PfTV78qf1h/USwCAzPUfFrn853oBcRGiLiOvJ6IfaPUOndivWnXCo32dFJrDwO38bS
5UZMsNoJq7fi8+s+6n2wPUXFff8ickqmx5QWCs3pgzPGfVlj+Rkh/mWi0Z7IY7gtk792TJfmJsJ0EYq/
Ua636vzepZ2QxPUMe3O61rY6vKWbchRsg+cv3+obPdKdoQeH0UI1Q3Q2s7cNWB6t6okwhkSA3f
v/NTd+olAMAsvPLBa+Xmrz9fLy23z338Idn/n4/pJQBA5i59QeQyf8BKghB1KRXlxCnnmRtode1x6W
U5efqkHXYF11vUMHDn2VzwQ7mmeJ0KuwdeWOgN5ffWWcfkDu+2H8bw7cAQ/VFmdc784HMy
Yb1zLRMP6W/LeT9BlVZoz1klRrtTuzaW8kkJ3d2o9a4p2hC7Z7Zxrq2N2WUO3P3Er8sLpOuJp59gJn
4AmLGXfPe58nMPvEQvQbn7P35Vvvct/m0CgNxcVvVRv68XMA4h6pLy6oraPTl74nSqPCUnKies/w
+uV3ngPEWoFiOYcodmt90x0xG9I6exkOfM6GmphqaXQkPC+LP0tzf8OqBVo6fp8kkSbhdl80L4jP1u
cDtiPjEgE+5M/A8/8bBeAwDIm6p/usx1UEdRAWrzN+/XSwCAzPWfFLn8cb2AcQhRl5U3tHlfDts6aL
N77VXEyQqN9VP3hsyCUdfTDi/9upOjwkuvNubQI2YYuIDnzB/KHyXmkP72hj9RVLUlIzuhTmDqa5O
CTNtg1JUdKNlqa55jln7k532ffZ/c+/C9egkAMAsrX3qpXPvklXoJpn/40rfl079/qJcAAJm7bP02eukze
gFRCFGXVklO2hlkRw52nSHNbh3Rkv0Jf3347OuzV9w864eaDXeSp4E6pCVnqL3i1eKc2KKdM2Mo/
xjjh/S3ZcNLUMtSV1PRTyvVazOhHNugZuhXPU7tScXsQNWYoCtu2QJgSvX76vKxv/2YXgIAzELpoRfa
D4z26f/6D3aYCgDIyeXPivTp2jMOIerS8ntyNptOOObWeCzq4p/uem+Sprnj9gDtSK2mA77BOqRmP
crmqj9sur3h1aUsxB5LvWDnzBzK787+H3iYIV70kP5e45w3jL9cv5BOuYRUr82E8miD3pbKoIM9Trvi
ls8dW7sVSMn/+v/
+l34GAJgF1ftU9ULFeHd/6AH9DACQi0v36CcYhRB1iZkzpqvehd7w88Ds5sYkTXPIq1NqC+shGaxH2
VzVwZg/Ll1GT440LNVzVtS1VJXmauqhoTmUvxxaW8AoiWC9cuSQ/l5D1r0CqtYrAxMwOY/JJkhK99
pMJoc2VLb9IfyBurRGfdmzm7MP3bEU7v7Fu+WOl9+hlwAAeXvjX99AHdSYfv7dP6WfAQByUXijfoJ
RCFGXWWDW8FPiTzbuDltXzPVzyAwvRw2ht+tRGr0uXeW6dJPWu0z1nFVke7BA5v5hSrUxzaH8o0
NdMxQeNaQ/Xl3VCaV5bSaVQxvsIfxhxVBVSNvvp1pfFohy1RVXyf/4lf8ht1x3i14DAMjT373yCf0M
UV7z1pvkbe+5VS8BADJ37FUix9+kFzBKoa/G9QIAgKXXxwCMPyOv/2+vtmfoBAPm648vXyysfvFYvYd
D1P/E8+a1PvVGedw0TbwFALgovFrni/daTq51ljERPVAAAlsztN91uD+0HAOTvb27/tjx2PX/ECnPlc4
9L9fdeS4AKALmx3m+Pv8f6SIAaByEqAABL6F2n3iUfeMMH9BIAIC+Xjvfl8294VH78nMt6DVz/8t+V
5NZXv0gvAQAyd+ztIoWb9QLGmSpE7TVW9AQpBX9W65F60lhxJ1TZkODL27LhTbbiPCabqGZYeyO

43dBH1jOAAwAwhz788x+W07ed1ksAgLz84PnPyt7rvqWXoLzidS+R1X8bNhkqACATx4oix39eLyCO
OeiJqsJVf5ZqR84zwquZ0VcaKU3oAwDA4rjnl+
+xh/cDAPL10M0/kO6t39dLy+3al14l1d99rV4CAGSucI3IMTWMH0nkGKIWZXOvL2oeq35gxuuuHH
iTiKvZsNXn92QzxwzV1qnJeTqkAgCWjDtj/3VXXafXAADyonqjfu+aZ/TS8lIBqgpSAQA5Of5ukcIL9QLi
mn1P1N6h7OuncuqEZJedlqXedUNc/9Gtl/XnRZq7pKgAgOVzy3W32EGqClQBAPlR9VE/96Zv2h+X1
S/8xivsofwAgJzYdVDz7rl4NOQYog7XRLXrlZZq4nZEtYfVD9UoNb/OeaRVL1Uprp0RP0YFAGA5vfm
WN8tH3/5RvQQAyIvqibqs9VFVePoLv35CLwEAMmfXQX2HXkBSc1ATNUKvISuFktS8lNXRqZVSq2H
aPu+HuNXTfpGB5WVM8uWd46hJwTA7YddqEYTfT/4kcHneY0nPYU7fC+q9b2DCO//8rEiKf0cCAt7
7s+
+V2h01vQQAyIuqjbps9VGfd82VctfvUJMbAPJztcixO/VzTGKmIWpluy/9bt3vCVptOcPst1WY2ZPGu
htwVqXlDsFvVe01qobpeqIkoSO1khtC+I9Vd0Yra9/2bgFghnqNlWAPfSBnqjeq6pUKAMjXfT/zuD1r/
7JQAer1P/E8vQQAyNzxO0UKL9YLmMT89kTtXZQdnSKU61v+RFSVLXHLmHZ2LqbU+64s9S0S1NFG
TQoGTGKR76eM295ryPpg13tgBlR9VFUnFQCQnx8/57J8/g2PLkV91Le951Z5zVtv0ksAgMwdf5PIsV
fpBUxqfkPU7oHXE8sevu/1HjWG93cOpKufTsfppZpmrVUAABaVmqm//attZuwHgJw9dv3T8je3f1sv
HU23vvpF8ku12/QSACBzhVtEjv0LvYBpzHdN1FSFz85vlhPo1M7nWItxHgxO2jWqxuPoOpD20GPv6
/VjZG3Jwf1FBNd2Pdzga0fVgsysDVEya98oca+VK/5xJm1f2OsHyncO1O806o7arx19P/kG2p/K9Ux6
DsOEt32wXungORo8P2HiTbTn8/epHqPqpCY5P0DQbdffJvf88j16CQCQl7+/7Xvyjy//oV46WlQd1O
rvvVaufO5xvQYAkK2rRY6/2/p4hbOIqSxEiFqud4fDT/uRwnDa4qac1WVWRfblcGnyBRVsDU7a1ZTV
wqr1/3hUiFMKG3rcqUlpMKBKMklYe2NETUjVYzgYFmXWhihZtW+khNcqwXEma58TyIW9vrlaGBH
2qXNitLNcl/GVM9SxDbR/6us5/f0ej9OrffAcNVdHhZyTcPbh1XO2Dd97qd3vWGrvKL6DGfsBYAb+6v
WPHcn6qL/yH15JHVQAyNPx09RBTdH8hqilk34P0YN0Bu2Ha8tuuinKQug1zvnhkTuhV7/r1Zsdzzhv
5bp0dbDd9TbQlHNeotNLMEmY9dpzesOh2+3IzkX3tVm1IUpW7Rst2bVKcpzJ2tdrrHuBnP+HjZa1F
615bkRQaLRjb1OKem0koz1u06e5ntPf70n47fHb3pHa+ejIPHqivUH+PsLvvbTud0Ds2frfdepdegkAk
AdVH1UFqUfJyumftB8AgJwce53zQGpSC1HtnmjGkFHzEWco65DimpzxspxVfxuqF6C77UQbdnpv
me1yHmYvuTOyFivhWXQ9uejO2qUCFi+kKcrmBSPEidI7lH39VDo74mY3xc09J6yxHnub+mQmmiT
MmLjHCNyKa2eGQ/XM2hAlo/aNlPBaJTnORO0LtuOst74iW/aGVVC3J2GHE2hHTNWz/rmtbPtBrd
/2JNczhfs9gWrL7yFf2Up/+4q5j/B7L8+J+bAM7v7Fu+WOl9+hlwAAefjmjU/JV376u3ppsb3sp14o/
+a3mNAEAHJTuFHk+L/WC0jLHA/nDwYcXkjrjWE1w5B0mMHN0daVAzdTKp+Ukn5qK56QU/ppJDP
kHgioh2ouTjVJmK6nGTZ8Prc2REmpfSMlvFZJjjNR+0a3wwldR5fWOHUi6XdVVU4HNlaSk2473bYn
up4p3O9zpSwnAwcRIteJ+bAMrrriKmbsB4AZuP9V37HD1EWm6p/+
+n/5GeqgAkBurhQ5/mvWR+qgpm2+a6IWN2UvbMitPdQ3hXqoHtWLri8pZ7JH3OhefG5oM1EPZ
MWu5eiGPlE1KzNsQ5R5b19sebQvRuA3VlFOLEzSmcbxAvPpphfcZAepKlAFAOTn8yuPylNXX9JLi0f
1QFU9UQEAOTn+r0QKN+gFpGmqENUc9hv1cMLJoj8M2gxA7aBUrw9NMc2v04+4dRUtdp1B82tD
H2kGskvEuHZ+7Udf89zw5DXjJwlry4bZq9OtjWnWijRl0oYo2bcvLbGOc4btG21wgreeHHp1B4ZlOv
HcEcD5QZpuv+l2e2g/ACA/KkBVQeoiel3l/6IOKgDk6dhrrQd1ULMy3z1RkZGQ4dEus05mDGp2d9V
b0Q+rjZ7D7raTTBLW3vV6dtrhjxuYG8OTB6XehihZtW+khNdqguOM176Idth1itOdfT7Y9JDh+ImO
M737fWHkNjEflpGaZOoDb/iAXgIA5EEN6f+7Vz6hlxaDmoWfOqgAkCM1C7/qhYrMEKIupaKs+bN2
yao3Ztuc0XsMPcGXKlEb7K0YEnhNOEmYH/4YM+KbcmhDlFTbN1LCa5XkOBO1L6Id9rE7NVUTnsK
Rmqsb4jfdL5lQPrPmhNaJrmcK9/uiyeB+B0wf/vkPy+nbTuslAEAeVH3Ux65/Wi/NN1X/tPp7r5XnXX
OlXgMAyJaqg/oe6+PVziIyQYi6pIqbF/zehs1VHawYk86MU9n2h393alJygxmjRqg/UVew9mbkJGGV
09aSNq5dWbUhSmbtGy3ZtUpyrpO1b2w7ynXZSmV8eFnK5aas6vZ4Tbfaftab/j/Z9Zz6fs+LOdGV28
6Jws6U7ncgwj2/fI89vB8AkI9Lx/vy+Tc8Kj9+zmW9Zn79Uu02ufXVL9JLAIDMHVsTKdysF5AVQtSlVZ
TNvcFJu6rS6res/8djD/8OK6Rpb2dgoi679ubg/ixDk4RVZHuoDc72vF01d71eitm0IUqG7Rsp4bVKcJ
zJ2qfa0Zfu0IbVpo3SBlM7JWcHjzfsGiW6ntPf7/mw7q/B67F/aPQSTiCV+x0YzZ2x/7qrrtNrAABZ+8H

zn5W/ev1jemk+veatN8nb3nOrXgIAZO7Yq0SOv0kvIEuFviqCCAAAMIF7H75XKn9UkaefXYwhpgBw
FNzx5evllQ9eq5fmx7UvvUr+06ffzDB+AMhL4RqRK9R8BQzjzwM9UQEAwMTefMub5aNv/6heAgD
k4W9u/7Z875pn9NL8qP4udVABIFfH3239jwA1L4SoAABgKu/92fdK7Y6aXgIAZE3VR/3cm75pf5wX
v/Abr5BXvO4legkAkLljbxcppFNUD/EQogIAgKmp3qiqVyoAIB+qJ+re676ll2ZLhae/8Osn9BIAIHPHiiL
H36EXkBdCVAAAkAo10dQt192ilwAAWeve+n37MUuqDqoaxg8AyImqg3rsPXoBeSJEBQAAqVAz9b
d/tc2M/QCQI9UbdZb1Ue/6T6+2g1QAQE6O3SlSeKFeQJ4IUQEAQGpuu/42ueeX79FLAICsqbqon3
/DozOpj/q299wqp/7ZDXoJAJC5428SOXabXkDeCFEBAECq3lF8BzP2A0COvv2if7Jn7M/Tra9+kfxSjV
/kASA3hVtEjv0LvYBZIEQFcteWjUJBCsZjpdHTn1tSvYasbLT1gqO94Z6fFZnX09NrrDhtXGnIqCb6x+E8
lv5aY2mo2frfdepdegkAkLW/v+178o8v/6FeytbzrrlSqr/3Wrnyucf1GgBAtq4WOf5u6+MVziJmghAV
yFVPGiur0tRLjrKcWSvq58vHDiJLNeno5YXRa8h6LarV6loXZDV4saVTK0lhIDAGjqq7f/FuuePld+glAE
DW/ur1j8kPnv+sXsrOXb9zu1z/E8/TSwCAzB1XdVBfrBcwK4SoQK66cuDmbuW6dPt96ff3ZHNZM9
SxQeScUj1nxwW/7fPiHlq1pa5zX7r1srOiuSrkqFgGV11xFTP2A0COfvycy5nXR33zr9wir3nrTXoJAJC
5YyvW41V6AbNEiArkqXco+/qpnDohy9v/dHHF6znbk8Y53QW1XJetivO0uHlWqs5Tae6SomI53PSC
m+wgVQWqAIDsPXb903L/q76jl9L1sp96ofzKfzipIwAAmSvcKHL8tF7ArBGiLjGvnqP5CKntOLI2peqN
536d163Or/dp135sb+ivNdbZLwuuD/bKS2MbmtlG7zFcYzN4jMM1S+1HWN1Low3jal3a+zDDt+aq
87WDDQ9pc9i2o9o8tpfjROdl+J4ZuZ8YxxD7fGh+W9RjuK0OZwi9/7p0z506/lKsnrNGj+NAWF6Sk7
ozquwfDt9PNqM9ie65eMduS+H7wj1Pcd9HsNxuv+l2e2g/ACAff/fKJ+SbNz6ll9Kh6p/+
+n/5GeqgAkBurhQ5/mvWR+qgzgtC1CWlwpHQMKhTk1JhQ8ZlcHHYtR8HCkLa61TIMrC+uRoeXk2
1DRU2hfYY7EitNCqEU58zapaW61J3uw52duTiYMi0674ynbqmQ8GiZh/zyGBquM1uz8dQE5+X4X
umuToieEt8DFGcfQcvd0hb7WCw5A2hd6V67jxVafVbXq/SIWaP41E6B9LVT4MqcjrpPZfk2FP6vlDn
KY/3ERwdapKpD7zhA3oJAJC1z688Kk9dfUkvTe/f/Nar7J6oAICcHP9XIoUb9ALmASHqUmqLn8O4d
TmNeo3SlHPhSUpi5XrX3na/FYybwtaPGt482TaCw6mHj7EjO4PplEcFZM7r+3ubsrlVF+erBr/GPI9nZ
FyGWtm2ttd1t2Wptpx9bOvUrr3hB4Vhbe7UZH3kdQm2eXRT0jkv/invSO28cd0SHMPY8xHg7zu8r
dZxrbvBoHEu3Iamcu40u53bEitrHVKUE6f00wiVRPdckmNP7/uimOP7CI6OD//8h+X0bQxHAoA8q
ABVBalpWDn9k/YDAJCTY69zHpgrhKjLyOwlZ/R0K27uOQGJ9dhLZaajqpx1t1M5bS25RqwPHd486T
aKsrlnBj6O4toZL7TrHIT3AyzXt4IBWXFNzugv6uxc9NvY3vV65pXPrI0P38YwexjWLxht3rwgfgZ5PrR3
31CbR5r8vFRbfnDoh3xB0xxDFHPfoW3tXZQd3SEycC4qW/5+zWtniH/u1HFY3yOhIe8k9uUwrEFKk
nsu0bGn+H2R2/sIjpp7fvkee3g/ACB7akj/V376u3ppMqr3qeqFCgDIiV0H9V/rBcwTQtRlZAQ0ImqY
bsGrZTiyhuIkyielpJ8GjFofJo1t2HQ9x9BhzEGnTgwGP0VZ8xMtLyzyA0Mj0J1YVK9WY/8jgrfhNscV/7
yMN90xjFaWk+MudvfAa79X7sF+GEPcRwyfn/zcTeuUjN51gntuimNPcv2HzlNe7yM4ctwZ+6+76jq
9BgCQJTXJ1KT1UVX907t+59XUQQWA3Kg6qHdaH6mDOo8IUZdSUTYvhPckdEOYqMl1FkZg8hyjn
mOk8MDO76nnDnc2AsPq6dg9GafXkeGOgjFCRlOK52UyYccwK2keVxw9ORxbLNWR2T2X2vVfkvc
RZOKW625hxn4AyNFfvf4x+fFzLuul+H6pdpvc+uoX6SUAQOaOrYkUbtYLmDeEqMuquCl7esjtQKlR
W/Pcos+u3ZYNs3edW7PRrMGZhHW+zurzZA+NNoYyV0/nF6FOH/qlfF4mkm1w6dXKHXpMWsd0
AsUTMrb06bje1BPcc+OPPf3vi6P9PoIsvfmWN8tH3/5RvQQAyNIPnv+sHaQm8Zq33iRve8+tegkAkL
ljrxI5/ia9gHlEiLrE1MzaqqeYPcGPHYR0vRqKo4f/GoxhxHPHrB2pgiW3/uMUba64U6Z3duT8+R29n
aqkk6GW5KR37gdnZO/JRbfoZeQQ8BgyOC+
+nI4hTOmkFwKOqumZL+NcNHfF75DZlQPvNJxwzn2EWPdckmPP4PpP/T6Cpfben32v1O6o6SUAQ
Jb+8eU/lL+/7Xt6Kdr1P/E8uet3qF8NALkpvFgP48c8I0RdRu0Ne6itmkU92FPMCHiMXnIlPxkzZmJvy
4Y3Dft884MlY2bySXgTWHWk2dQnKrWh/EXZdLsdqvO87l+XXmPdq22ZZBKkcVI7L578j8Fj1udsrvr
DyPW9bj9yHVtu1oBtyq7eda9xzgsxY/VgjnPPTXjsU1//hO8jZntyvRSYe6o3quqVCgDI3t/c/m157Pqn
9VI4Vf+0+nuvleddc6VeAwDIlqqD+h7r49XOIuYWIeoyqmz7Q287NSm5QYtRH7F6NnzmbhXSDL52
Lpkz9nttNibamUhF3I6BrlSH8o+4LiU/fZQL005glcl5MSQ9BnPYu9ueiRK2YH3O5qqz34IX9Fellldqs+v
EUNy94PTLd9njnodqSeM2Jc88lOPY0r3/C9xEgiqqPquqkAgCydel4Xz7/hkcj66NW/u8idVABIE/H3k

od1AVBiLqk7KG3YUUMVeDS7wcDHlX3cLBmYrWVcx3NpCqy3W/5gZHNOTbvsAPDrOPxhlcr5bpsp
ZzL2dcl5LxWW9Z6d+j1VLI5L6Zkx2C1Z/A+3D80ejUmYNfnNIaSu+y6nznWQ/UUZXNvuFaoPYw+Q
aAb656LfezpXv9E7yNABDVTf/tX28zYDwA5UPVR7/uZx/VS0Cte9xL5hV8/oZcAAJk7Zv2GfPwdegHz
rtBXRewAxKNmNtcT86gwbG/anqHAOEfpntPHcqpFwIpwn+l9Rip/xM0BAHl441/fIKWHXqiXRK596
VXyW3/8z+yPAIAcFK4ROf5+66P/Xoz5Rk9UILaeNNbdmc3LcmaNABVZO1r3XPu8dSwZ9ODG0fGO
4juYsR8AcrL3um/J9655Ri+JVH/3tQSoAJCn4+8mQF0w9EQFxmrLxmANWFXOgK50yMzRu+d6jRU
p7ZyRbiplKXDUFf6fgn4GAMjStU9eKe9s/aS88723MYwfAPJ07O0M419AhKjAWAOBFgEqMsc9h+
WlJiiblx9NaEs42hKOtoSjLeHmqS0nrr1GDr/3pF6aLa5RONoSjraEoy3haAvSQIgKAADmBj/ghqMt4
WhLONoSjraEoy3haEs42hKOtoSjLeHmqS1IhpqoAAAAAAAAABCBEBUAAAAAAAAAIhCiAgAAAAA
AAEAEQlTkSE2WU7DrfxRWGtLTa8fqNWRlo60XHO0NvZ3CijRibwjp6Uljxb0GG9aVTbJuGmH3UNr
7mJbRRv1Y4SYFAAAAAGChEaJirvUaK1Io1aSjl4H5pgJdY1Z9W1nOrBX1cwAAAAAAsIgIUTG/eg1Zrx
GfzqeibO717RkF+/1tqei1szFPbenKgXvLluvStdu0J5tkqAAAAAAALDRCVABIS+9Q9vVTOXVCyE4BA
AAAADgaCFGXmao1OlC7MazG6GD9UXuIvfE1A+VKNbNOpXokq1Vp79Mcxt9cdbYTvjOjjeoxqk7qY
JvCa1VOdrymMbVf2xvetob2H3JN4rTRY359ZEONcxFo46j1xj7t9ea5zKoOadx7KKotg9uYsD5pjOuS
9J4FAAAAAACLgxB1WakgL7TWaEdqpVEhpPpcQUoDQ+ybq4OvVyFiSYIva8pqYbBWZBqcNq0GNh
xyDHYINtgm65W1UnjQaYt7vIMqcrqqn3Z25OLAa9u7bmODtTKHQjgtuo2TKsrambLztHMgXeeZxR
iOHmh7W9xml8+s5dDDMoV7aKJrPizf6wIAAAAAAOYRIepS6knjnJuIuXUb+9Kt61BNOrIzmPx5qtL
Sr2+5QaEKG8/7ve16jXN+0FVt6VqVXfE2H0Nl2/qabl28L3G3sx1W8dJvU/gxWMe77oZg/mv77gF0
arI+MhUdf7xhKltu2wfPpR9GSvmMeBlqe8MPgsOuSWQbJ1NcO6PbuC+H7qbbu0ZI2ZEDN101hq
mfOpF9hDr9PTTNNTckuC7J7lkAAAAAALBICFGXkjERz96m16vQD9VEOl56FlRt+RP3+EGhqScXd9w
+e1VpeQGStc8LYa+fntmm0GPoXRS3SeX6lj/xUGXLC+U6OxdDexSOP94RimvidfQ0t22ElGaPTrN3av
2CcU02L/htrJ0fGKo+Ja+NftDbO3Si0mrVCRv3dbrau7jjBZKnvROYlRTuoSmuuWkm1wUAAAAAAM
wdQlRYdA3P0OH9SZmzk5+Ukn5qK56QU/ppespyMrCTEN0D77jsIdheXUtjqHdgSHsazOHy/rB4P5
SryllvyvYRvVNtxnbMHqOpKMoJfUHcwLlrXzzrnJ4+aYeVwfWW6mk/kMxMCvdQKtd8VtcFAAAAAA
DMG0LUZRWYKCdunckYgeWRMt3x+r1i3Z6eRig3URhpDK9PScUt3trctVrXE6cj6ik5UdFhZWC9anb
2EeriSf+6AAAAAACA+UKIupTasmH2OnVrPZr1HI+ocr3rlDEYevjD9lNT3JSzbglONXTcqCs6WRiZQYh
dOS164L4ctvUQeLv3pzs5lrF+QUP07K/5sv1xAQAAAACA5UOIuozMupwqYHLrohpDoCdXkpNuEjs
4XNoIEXNVcoamK6NqvWbF6+nZ2ZHz50fVFTXP2eBs/mZ90FMydk6nxNfQ3XdHDnadr3VrtZbsT/jr
h4e0ZyWFeyiVa57idQEAAAAAAAuNEHXJ+QGTMWP/VMxakU1Z3XCn3DFnS8+ZMcmTNFfFa1J7w
6+V6a1MmdfTsyPNpj76oaH8Rdl0u6xar6utN6yz5eg11r0anuYESU7AqVivP+8dkGx4U8nH5V+vZtP
5Wnf2/aIumOquNyfCylYK91Aq1zz5dQEAAAAAAEcTIeoy8oI9S3NVh0rGhDtTMmcun2r75iRC7nY
mCjuDs7o3V3WI5gWO5gzwaXOHxftCh/JXtqXl5XU1KdnnrCAlP6mTC95EVCojdOutWrxzHLe2bVBg
W9Yzb2i6eZ9Y68/k0w3VNv09lNI1T3hdAAAAAADA0USIupQqst1vGQGZUpVWv+8HRvaEQpMqyu
Ze1w/BbGr7g/scx2qn1yBt/9DrDZhIcVP2+oNtstj1YDOoh2rwhvQr1v62Ruysst0PrUtbbVnr3ZILLnU
8g6+ttiara2v22gwMTTeGs+c+ZD2Feyila57ougAAAAAAgCOp0FczrADITq8hK3oiL1WDdo+eiwAwk
urtPS8/mtCWcLQlHG0JR1vC0ZZwtCUcbQlHW8LRlnC0BWmgJyqQKbOOZ75D4gEAAAAAAJAOQlQ
gE23ZKAzU8ayeFTqhAgAAAAAALB5CVCAPql5pZpNXAQAAAAAAIEvURAUAAHODelXhaEs42hKOto
SjLeFoSzjaEo62hKMt4WhLuHlqC5KhJyoAAAAAAAAARCBEBQAAAAAAIAIDOcHAAAAAAAAAgAj0R
EV2eg1Z2WjrhVnrSWNFzZavHhsyL60CAAAAAADA/CNERSZ6jRUplGrS0csAAAAAAADAoiJERfp6D
VmvEZ8CAAAAAADgaCBEBQAAAAAAAIAIhKhLqr3h1gddkUZPD7+3l51HaClTVePUeI3zcL7eZW/XH
MbfXHVepzc4uF+PuW1j58HXt2XDfY318F4Wo12TGjwv6jHpuVGSHr8S1obCSkPCD8+s/eo8VkaciG
TbBQAAAAAAWF6EqEuvI7VSQUoDw+
+bqwMhX3tjRI1T9fXpBJbR1H5WpamXpFyXrYr1MbN2OWHk4HlRmquFYNCZ4blRoWtYG6RTk9Lg
BFl2EFuSwZd3aqWhcDTRdgEAAAAAAJYcISosVWn1+9K3Hq2qXqUCwPNulNaTxjkdX5br0tWv7dbL

zjrrtTsXnYiusm19rlsX9zNSbdmv7W+rxHNafjv7e5tSTNCupHqNdS+MLNe7zj77LasFWvOcDkeza4NI
W3bd1Dh020055yW0VjvW3SDXOE/uBe3UZN17bZLtAgAAAAAAgBAVUm1tixtxVraMANRTlM09H
crZ4aVeu3bGe23noKufZadc3/La6ciqXT25uOP20qzK2U13yxXZsoNGFVLuibM6w3PTO5R9/VQ6O+
JmscXNPWd/1mPPbVvvorhNDpynypa42Whn56J1ZJYk2wUAAAAAAAAhKiaha5OGDmHPzqkT44K
9tNrVlQN3A+WTUtJPFSdo9EPnYSmem+KanHGTWGtrquyCW7t0qM5p98Dbnz18X7+uYA7v7xxYR
2ZJsl0AAAAAAAAQoqIsJ82UcJTAxElGbdLcjGjnzNtlyawNRdm8ENYz2A9KQye5Giur7QIAAAAAABx
NhKiIoS0bZs9Kt46mWft0JuahXRm3obgpe3qIvV+v1tc8Nzybvl/DdfBh9KCdYLsAgLzp0Q3Ggz9yAQ
AAALNBiIrx2rte70o7oHNrfxpDyKc2ybYya1dJTroJqDsE3qVm4i8YM+6n1YaI16uZ9NUvzfakXXbw2f
XqnHrtK530Qtu4NVhjbRcAlpH9Xl+QwkwTy540VlZFWu57tPNHr+bqLILU+Qxze40Vpz0r+f/hT/0ba
p4P9zEP5wXDhq7XDO6ZmRvzvuZ9P7mPvG7mOO+3ebwnR+5jSf+gFfO8z/K9eGZinJuh951luGnG
nJfc34sTtofSdoiDEBWJ+AGdMSt9AiU/nTRm/7d+MFmdbhD8tO0KKsqaVzS0Kavem667baeO6OB7
cZw2JDp+/aavPhXsGRpSs9Wsc9pc9dvm/sOhHu7KJNtVjG3wDwuALMzsl/chKri09j/lv0npcCYu3Da
KcLuTP+4f5vte3N4IC3ONPyjOQq8h6zWRatX9x28G3NEnxsO8XrMy01/cAyWOhh+z+NZW52O1qS
YFda9TS6qdmpRmFPjk/0v7+Pc19R5csr6f6l3jHFk/T2Z778R5v83jPXncPtTnc/yDVmTok1eYm+C85/l
eHHFuht739CP98xP/vl3dH/g3Iot/IEadk9zfi8efl3zfiydoj/WNrUrbpf+eHO/7dn5+FsZY1g2DJWS9R/
TV5bd+A+hbPzD5uvW+9U+g87mq9ZZia/WtnxX060c8vNcqIa93P29uf9TD2NbIdtqStKvbr5fd9VXr
K8cxXx/yKNetVygJz83Exx/
+MDcdve3gMSfabqvqrS8PXwQAmEq3XrbeX8z3eP2+GngjyoffFv1vwAzaEEm/z8/8vXjm7XCuj9q/f
c28f5PzY/87OoP9RtP37dy1y/3eivPzV9rCv5dn1R7n5y9jv/pnrCy/l8a/rznvuYNtyPocjW9XvNdMa6J
9ZPIeqPdvbdd+hLRD3T/maud+Um3XK1IS/5w4n8/+vTjeucnjvS/
+fZv1+8v4cxImq7aNPy/h62fWnhHfw+nfR87+zd0737fBdX579Qr9vhz3uiJf9ERFDBXZVn8p0ksO56
821puAo7kr/t9KrNd7n9D2D52/MKlanIP1QqutCWuIJm1XEk4PIOsNTS/7rDdbf9h+0jYkPH57qP3g
ubQ5+wj8QdOuc2oMyXfZPWWMeqiWRNsFgMy05XytY71NXZBN503VUpEt9UY28fv35Iqbe9Lv7x
ltmTO69MupE/PRwFm1o9dYl1qnKmfn9kLNhnteWt7PKPPC+T6X6unAzyL5KMqJU9YH9+dQW08u
7sygPb2GqIFK5fqWv9/Ktv3zYmfnYgY9sRxj39d6h7JvfRj8fi7aJ64puxm9Ecd5v83jPXle3ved79+y1Ls
hP8tr6uf34dEJHdm5mO7dE/ec5PVeHOfc5GX8uXHeXwLf5xmY7Jxk9148/rzk+148tj0jfp6yR42mW
tYuzqii+fpZGOMRoi4pvw7mwJuLMeFQcMiBCgvdr1EPJ5TztxMM6dQPhf5rrYf5A725D/VQ+xmx35
Ht9MRtl/MGFlw3nvMG7H6d89gbakjCc5Pg+G2D59J+jDoG8zj1Y9QvU3G3a7xu+NgBYAoz+uV9M
enSL+W6bIX/A5CTnjTWa9KZVTvsoaMdqbbi/1ueGTUM0Rh6N9uSN/n84j6JXuOc9d1s/bI/oxu3sq2
HjNo17dWwypITNg/
+vJW13H5pT8huV1lOenWcNKPePgZk8AetuMFlmDTbEVuO78XTnJv86fJoB+cHhtOnWwJnknPCe7
FhxPub8/PnvuRaNYmfhRcOISoAAJgNfnmPTdUltX/5uTCLXoa6tpj9i6D+pWdGvR3b52vSqbZmPmL
C/0Op81AjV7KppRZXPr+4J+f2NDo7wwDEHTWkatqr7yOrObMI4efpl3ZMgT9oKfPyXhwwR3/Y6uy
flAvevxGqt6h6/9mYYa9C3osD9HwiwflBrO+pqedWiWHwjzD8LLxwCFEBAADmmDP5gRq2N6ueO
MFRDt36vqzO4hfU9oYzCcRc/dbuUL2Csh6WHcfc/eLePi/O6NHZXTNnsg5/YiAVeKtJgXKftGOWv7Q
jNfxByzKH78Xz9oet8pk147pY/4aeVWXUZtirkPfiAernGrdnrP89dXBKXadTkl3n7nn5IwymQYgKAA
Awp5wAVfXWmJ+hjMXNC3YNtk7tfK7hXHtXhU1NO8B1fuEpSEn9Vqh/CZrtcHptlsOyLfP1i7sOCGf5
y6Iecqzq2bt5jz0MViXezdWMZjYfZVa/tI9h93bqyMHgjTuqd9QS4w9ajkV4L56XP2x5ZtqrkPficIMl+f
qydXLfOk8nJau3vdn+EQZpIUQFAACzwS/vkVSvDSdAndMJ/zL8RSPMYE8j9bAngLR+Mexaz2dbt7sn
h6qoWc7nZKxZ/uLu9nw6O8NfFgeHTbpmdl7y/6V9rOIJceZ7CUZNPfuGnmG4O2f4g5Zvvt+LB+T+h
62S2GWOB8PbWf5cw3txTLq2eOCPkekZ+UcYfhZeOISoAABgNvjlPYKeRddiD3czevzkW+dSTf4wM
Bxc/0KW1S8ac6/XkJUVc0i2WqVmSp7lL6nz94u701utKjMcPSpSOW21wPoeCgyhV7dwzTovM26bL
dtf2uOpyGnVY6+27r+veL3G5m+islngD1qLaFZ/2NIjADo1Oe/9w6mHcM+oHinvxXGoUhklqUldLm
RwkSL/CMPPwounDwAAMCOtqvSt33L69a5e0a33y9aPJ2VvRX669bLVFtWewUe139KvWUZh56U

6JyfEblu53s/3bun26+Xg+ZiLe6RVHbg2rb71S2tfJT+5m+H38TB9HgKPefie1vdRxvdv3Pc1573Yf2R9
7eK0K4/35PH7CLt/3Ifxb1dq9H0x9H2r2jFw3Pp7Pu1rNcl5t78m8/fiEedGvd8M7Ns9hrTf/uKem6H
XZfY+POp+0XJ6L453XvJ7Lx7fnpB/xzO6RnHuxXn6WRjjFdT/rIsKAAAwE+5f6F3WD43zNRwQWBCq
x5xdm9BVbUl/Bl3nnO/pqrT6M5p5eS7pnk7G5ZnV9cH8Gfre9fjfR2Gvqc5r79gUjT83Id9bxnk7iuLc
LwrvxbOmRvOoOqhhgkP7+Vl4cRCiAgAAAAAAAEAEaqICAAAAAAAAQARCVAAAAAAAAACIQIgKA
AAAAAAAABEIUQEAAAAAAAAgAiEqAAAAAAAAAEQgRAUAAAAAAACACISoAAAAAAAAABCBEBU
AAAAAAAAAIhCiAgAAAAAAAEAEQlQAAAAAAAAAiECICgAAAAAAAARCFEBAAAAAAAAIAIhKgAA
AAAAAABEIEQFAAAAAAAAgAiEqAAAAAAAAAAAQgRAVAAAAAAAAAACIQogIAAAAAAABABEJUAAA
AAAAAAIhAiAoAAAAAAAAAEQhRAQAAAAAAACACISoAAAAAAAAARCBEBQAAAAAAAAIAIhKgAAA
AAAAAAEIEQFQAAAAAAAAAiEKICAAAAAAAAQARCVAAAAAAAAACIQIgKAAAAAAAAABEIUQEAA
AAAAAAgAiEqAAAAAAAAAEQgRAUAAAAAAACACISoAAAAAAAAABCBEBUAAADAQmtvFKRQ0I+N
tl5raG94n19p9PTK2fDbuiEhLV0wbdlwz3vk+e1JY2Xca6xXNVb8ba00rK8CAGB+EKICAAAAODqa52
TGOemSUMHoqjT1kqMsZ9aK+rmpKJsX6tZnHZ3aesg1asv5Wkc/F6me3bS+CgCA+UGICgAAAOAI6
Ujt/OL38Zx/XTlwM89yXbr9vvT7e7I5KvksbsrZqn4eco16jXN+IFttyXZFPwcAYE4QogIAAAA4WuiNm
r3eoezrp3LqRKxeo5Xtlng5auAamb1Qq9IiQQUAzCFCVAAAAABHTLzeqH590pVg6NpryIpbm9Orse
rX/7Rrehp1Vr119suC68NKtPqCtUJH1wEdeJ31CKsrGjyeYL3S6HYYzGPXj8F92fsp1ayzrDVXndeO3Ul
FtltujOpfo/aGXxagXN+yXjUo3vHbQto/dH0tcc5VoEar+6BWKwAsLUJUAAAAAEdEWep1HdJl2Bu1U
ytJYTVYDdRep0K2gfXN1VEBZlNWCyUxyoCqjUhpcMIpOxQceJ3F3t/IQK8jtZJRr7Rcl60YnTuHwlEtel
8JVbak7hZHba7KRqMh5/wEVS4M1gNIcvwqwA5pv3M+hoNUR/i5UueiNLhTJewaAQCWAiEqAAA
AgKNjzQ3p4vVGnVS53pW+qgPq9ax0hK1v7o5oh1dLtC/
+y5tyzkv7etJYd0PBqrT0a71td2qyPjIpNl6/F2OSpvaGePmv0a6um3ga+6psW5/r+hNFqRqm9n5iDc
MPTjLVrLnHV5b6hcF2Jjl+67VuGhvWfmsrOxfjnqu27Eadi8A1AgAsC0JUAAAAAEdIUTbdGYwy641al
bNuj8nKaWvJNWL9/mFoL05zBnqzXmhn56Lz+t5F2dGdIQPD3I3enN5rB4QPix+t7aeGgTCzuHnB31
ftfDo9MAOTTGnVs8OTUiU6fuu675lBqKO4dsYLbDsHXf0saOhcmfVeOzviZq/FzT1n+9Zjb+QMWgC
Ao4oQFQAAAMDR4oVsGfVGLZ+Ukn4aMGp9qKqcDiR3JTnpp31ix33dA90L01rllguwH8bwdve1A0
6dSBLymT0vz8ha4EuLsnbGbdi+HIYlthMITDJlPQudTGqK41fHZNc5DR3eHzR0ropr4h2yuodK7n4ja
rECAI48QlQAAAAAR8xAb9RD5+l8K8qJU/rp1MpyMn6am0BHRnTmnE6i8DlCYFIpo85ppLBzFSw5Y
HLD3NgTdQEAjgxCVAAAAABHj9kbtRYvTsvXYK/Onhx6Y8iHebVWhx7biYbtTyercHa88cfflg2z16lby9
Ss3ZpEcVP29D4Gyt7amueYpR8Alg0hKgAAAIAjyOiNmpQxjDw7g706u3Lg7tTtmVk66QWAo+p5ps
MsJeDXAHX05KJbmFROSaIqAdNKcvztXa/nqR24unVRp7iWaoZ+1ePUnkjLDlS7Xi3W0WUEAABHF
SEqAAAAgKPJmIAoTMlPDo3aqW3Z8Kapz1ZzdcObqKm94Q8/L59ZcwJAszZnc9UfQt7e8Gp0FlIZV2
4Gzta5WPd7WfYa61790aSTVU1twuP3A1djxv4k9PbVbRDscRoSdAMAlgYhKgAAAIAjKro3qjlzuwrp
nGAubi3NaZWlXG7Kqg4D/dzWmOFftd+ozdlc1cGh9+IREzJNorLtD1vv1KSk21XyE1S5kPuM9AmOv
3Lan6jKu5bGBFRJjDgX5r1RPat7ugIAlgYhKgAAAICjK6o3qqp7OVgzs9qavI5mIqfk7J4xPFyx63gO1Di
1a3MOvE4Je+2U7GHrIcdebVnr3eHxeYt9/BXZ7psz/itVaZk1TZu7Xs/fcexzEVYMVW8zrewaALA4Cn
1V3AUAAAAAAAAAEIqeqAAAAAAAAAAAQgRAVAAAAAAAAACIQogIAAAAAAABABEJUAAAAAAAA
AIhAiAoAAAAAAAAAEQhRAQAAAAAAACACISoAAAAAAAAARCBEBQAAAAAAAIAIhKgAAAAAAAA
AEIEQFQAAAAAAAABGEvn/AZM6nr53azj8AAAAAElFTkSuQmCC]]></redcap:Attachment>
</ItemDef>
<ItemDef OID="q27" Name="q27" DataType="text" Length="1" redcap:Variable="q27" redcap:FieldType="radio" redcap:RequiredField="y" redcap:BranchingLogic="[q25] = '2'">
<Question><TranslatedText>Do you prefer Life A or Life B, or do you think Life A and Life B are the same as one another?

</TranslatedText><redcap:FormattedTranslatedText>Do you prefer Life A or Life B, or do you think Life A and Life B are the same as one another?
 </redcap:FormattedTranslatedText></Question>
<CodeListRef CodeListOID="q27.choices"/>
</ItemDef>
<ItemDef OID="summary28" Name="summary28" DataType="text" Length="999" redcap:Variable="summary28" redcap:FieldType="descriptive" redcap:SectionHeader=" " redcap:BranchingLogic="[q25] = '1'">
<Question><TranslatedText>
Alzheimer's disease - Description #[no]

If you have Alzheimer's disease...

1) You might forget recent events of importance to you. For example, if you are interested in hockey, then you might forget whether your favorite team won their last game, although you may remember watching the game itself.

2) You might forget the names of people you met recently, or the names of local streets.

3) You may no longer be able to handle your personal finances or plan leisure activities such as dinner parties or vacations. Simple decisions such as what to order in restaurants might become difficult to make. Your ability to concentrate may decrease and you might have difficulty driving.

4) You may start having trouble finding words to express your thoughts. You might need to be reminded to shower or brush your teeth, but you could do these activities on your own. You may need to post notes around the home to remind you of simple things like turning off the stove.

5) You may remain aware of the day and your surroundings. You might be able to travel to familiar locations like the supermarket, although probably with the help of a loved one or friend. If you are in a social gathering, then you might have difficulty following the conversation.

6) You may continue to enjoy hobbies such as puzzles or reading.

Now consider two possible lives. In Life A, you will live in perfect health for 6.5 years and then you will die of natural causes. In Life B, you will live in perfect health for the next 10 years, then you will live with Alzheimer's disease as described in the summary for another 10 years, and then you will die of natural causes.

</TranslatedText><redcap:FormattedTranslatedText>
Alzheimer's disease - Description #[no]

<p></p>

If you have Alzheimer's disease...
<p></p>
1) You might forget recent events of importance to you. For example, if you are interested in hockey, then you might forget whether your favorite team won their last game, although you may remember watching the game itself.
<p></p>
2) You might forget the names of people you met recently, or the names of local streets.
<p></p>
3) You may no longer be able to handle your personal finances or plan leisure activities such as dinner parties or vacations. Simple decisions such as what to order in restaurants might become difficult to make. Your ability to concentrate may decrease and you might have difficulty driving.
<p></p>
4) You may start having trouble finding words to express your thoughts. You might need to be reminded to shower or brush your teeth, but you could do these activities on your own. You may need to post notes around the home to remind you of simple things like turning off the stove.
<p></p>
5) You may remain aware of the day and your surroundings. You might be able to travel to familiar locations like the supermarket, although probably with the help of a loved one or friend. If you are in a social gathering, then you might have difficulty following the conversation.
<p></p>
6) You may continue to enjoy hobbies such as puzzles or reading.
<p></p>
<p></p>
<p></p>
<p></p>
Now consider two possible lives. In Life A, you will live in perfect health for 6.5 years and then you will die of natural causes. In Life B, you will live in perfect health for the next 10 years, then you will live with Alzheimer's disease as described in the summary for another 10 years, and then you will die of natural causes.
<p></p>
</redcap:FormattedTranslatedText></Question>
</ItemDef>
<ItemDef OID="a28" Name="a28" DataType="text" Length="999" redcap:Variable="a28" redcap:FieldType="descriptive" redcap:BranchingLogic="[q25] = '1'" redcap:InlineImage="1">
<Question><TranslatedText></TranslatedText></Question>

<redcap:Attachment DocName="65.png" MimeType="image/png"><!
[CDATA[iVBORw0KGgoAAAANSUhEUgAABVEAAADYCAIAAABtBTQzAAAAAXNSR0IArs4c6QAAAAR
nQU1BAACxjwv8YQUAAAAJcEhZcwAAEnQAABJ0Ad5mH3gAACmBSURBVHhe7d1fyF1VmudxY1S
S0WqD40i0GhMdhGBEMsoMqYxURZBShICgg8EeC4eRlwgOBMFWpAJzYaMMBXkvCvSiqPaiIHVh4
8UUUWoKclENYSiYXAjtRbp4YWroQNfFO00xvEXnomb/W2uv9w5Z7vh4eZ9+yz9nrW
2ue8Zv8sd/q2PwEAAAAAgCUi8wMAJnJl58pt//U2isq5/u8//uM7p06ee+Lxf/jt3zdfXAAA5ozvMDw
CYws7uzqGPD4l8RVG51X/7j6/958f+dVE/fP77//zHPzZfXwAAZovMDwAY3e7e7rEfHxPhiqJyq5Nn7
q8Df10/fe8vm28wAACzReYHAIzuhZ+9IMIVR9682bWlm7m7fcfv3f1R9r0vrNzh+CBX7AMDtUDAcWSwrNqt8sbNp+hgt
bu6d+PSECFcUlVs98PaBN449KuK9t3iwHwAwU2R+AMDwn5+VoQrisqqt7vrg9ldPHBHZPl8xxkFA
mCMyPwBgYB/9+iMRrigqw3r+ew+KVN9ZPNgPAJgdMj8AYEhffPOFSFYUlUlWE99fJ9s/3LB7sBwDMApkfADCiS19fEuGKonKrKr/Rf2vfL0wyLt6+LBfgDArJH5AQBjufq7qw
c+PCDyFUXlVt99/gGR80XxYD8AYL7I/ACAUezhIE6A2+rvcdmuTHwAwDDBTZH4AwPD2
bu6d+PSECFcUlVs98PaBN449KuK9t3iwHwAwU2R+AMDwzn5+VoQrisqqt7vrg9ldPHBHZPlI82A8A
mCMyPwBgYB/9+iMRrigqw3r+ew+KVN9ZPNgPAJgdMj8AYEhffPOFSFYUlUlWE99fJ9s/3LB7sBwDM
C5kfADCYazeu8Rf1U/nXg28dFEm+f/FgPwBgXsj8AIBh7O7tHt0+KsIVR9ZsqVfqKQF+oqYr+S2VRpUbh3BFTKKBvdwsE
eq12K6+MuQ6FmqvzmFGqGWpM8wGZv2kWvzX8xSbKJeZ3UtzzPJqe1t96C4PCZrjd4R4nIazCtrGG
RrgVn0COtTCK2t10qQjUtfXxLJiqIyrIPv7X/tyaMin49UPNgPAMgTmX/TZJpy4o1OuodfYYeqoYY51
ohy6cZoE4x8lZzhpqheimmi78MiZ+lVtDOoQY0a3S6eLbtkme5G22PtK2l8OVZYXnuJA3n1D6z6RHq
iJ5eHfBM6xHZWnAiuRg1wDkhOINzoNBjJcjF1d9dPfDhARGUkCrDevGZh0QY7H8DevZ3H4QK6RHq
+YZUceKQDUZr0oyMHnG+DKVrRlRn1O/2NoqJzKPqPPFdPGXIR1nqddqEvW62ZV4Wflccsgu+mq3
a5VDPDxntadV7XssT08i1+suoHs295z6kG+B4X0Vui+I6FzyHLLI1s5SnAOFtI8G09vZ3Tn8o8MiWVF
UhnXyzpP0ik49d5554/H/8k49/H//3d81vyoAAOSBzJ8DX6JsqLfscCUil2dQj2Jq8qUFtTfEpE+aV4kExnwZvR8Jh8cKxNbZqrHwHCAavV5HPxV
VfhnSdpQ40r5sVqE2pTfpE+souepr2nB7LV2dZH4N1Xo/leVrX3AV0z+Zdka3HvgqeicSJPbZmsEc37z
tL8a2teyXIwN7NvROfnhDJiqIyrCNv3i0C+TT13unv/b9/+qfmwV/b9/+qfmFwYAgAyQ/XMQyW/
+twKhbMWw1JxWjKhHFz/Uh/QRfbaYLv4ypPMsdaQ60K6upjbpE+kru6RdK3WWNcI6r8fyPK1r7g
K6ZwtO1uqxr4JnInFij621pwjN+85SfGvrXgkycPbzsyJZUVSGdc+7d7x+/BGRxierH791n791rvmFAQAgA2
T+HETYm/8tFYWao6lhqT5P/C/71dnVIBMF38Z0nWOlQcqfbUUue/Ep1ekjl7Rr5W1unddjecE
h7gK6Z+vRr8e+Cp6JxIn9F1OoB8nWzlJ8a+teCTbto19/JJIVRWVY+y/se/nf/bnI4RPX6cSw
m0bmz0EkVOnQY74nk5DnfJ3CYmGpmcd9+jL8+9VPB5t4XrmYUkZ3yL8Ghhd+/bogGHYsJX55miDujZ229VoJN2N3bPbp9VCQrisqwHnj7wBvHHRhEqBvwHhnxpbLFGrkkGCo5XdCfWyVCEq3Kaw
o0Y5fN/FoOAgByQ+XPgCTuSHbo8CcgcULzdNyy547xnioPxIyE9z4pfDX1WJXLFGrJLWrA0z2I1/F9wTYssL
biy4gO7N2iOcIcZavacXeI+Q+GLMTuVp6nU9hW9GZ229V4JJ7d3cO/3ZaZGsKCrDuuuDWcPi7ydQ/FgPwBg48j8Q
o0XD/YDADaOzA9088RR4NZw9RR4NZw7hfnRLKiqiqDrzruWcPi7ydQ/FgPwBg48j8QDcyP25NF69eeFLGKovKsp

16+T4TtfIoH+wEAm0XmB7qR+XELurJzRcQqisqzHnzroIjZuRUP9gMANojMD3Qj8+NWs7O7c+jjQyJ
ZUVSGdc+7d7z25FGRsTMsHuwHAGwKmR8AYNnd2z3242MiWVFUhrX/wr4z3/m2SNd5Fg/2AwA
2hcwPALC88LMXRLKiqDzr5Jn7RbTOuXiwHwCwEWR+AEDr/FfnRayiqDzr0f90jwjV+RcP9gMApkfm
BwA0Ln19ScQqisqz7n3nzjeOPSoS9SyKB/sBABMj82+W+rvhKqP8BXHXt09tXW5+XsbfRWfvyM+6r
p2jxXilx3m3tt7fpuV9CRfq6u+uHvjwgEhWFJVh7b+w75WnHxZZei7Fg/0AgImR+TeoSEJ1+KkNH4G
aBguKW+6OXOKyVjr26438ZP4uvb5Ny/sSLtXO7s7hHx0WyYqi8qznnj0sgvS8igf7AQBTIvNv0MjZR2
ffxcQt344ke4dt/o8FeDWKELqaHt+m5X0JF2rv5t6JT0+IWEVRedbxs/eKCD3H4sF+AMBkyPyb0yfBr
mN5cavHFVMbVCPqUzqusDwJ/ZD5l+Ps52dFrKKoPOuBtw/M9DF+t3iwHwAwDTL/ZqjYYzADp05K
FZGNjMjUTiPiamh+M24ZY9z0Za2gM5yZ05pnOiE6PG37jj5JL1CsVnFmtxfSm2rtmc8j0MJzuOsaWu
8XrCHGfO3W6xVGzwvo28saGP34ijcDl0Jp161VM5qnGWPcWaxFi7d7rxndPvr1RyJWUVSedfC9/a
89eVQk5/kWD/YDAKaxWubX99bhG2vvEOvmPxAQemtv8Gd8ex+KQ/63CsZV8wxwrmlofv/kJWM
KK2op0Y9tkGn1+/Ux8TK0I4s+Z+tyOz668oJvXbGvlprZmlce7Nps4JK1IzzvV2/6z4suOKVXo3vFJXOU
wXPCcF/ClVeDgC++
+ULEKorKtl585iERm+dePNgPAJjABJlf3Lyve0duThcNOtnzXk0dZdR10qP0hTPSTvQC+OZ3z3Xmd05
T50Q+OXfa9ojsE5nWHOPr6tuRRQ+QQieUjMWbwttVXYwRYrWdm5VT6BP0nMaqzMWHJmpPdK
T0ao+o5vosdaQ9EOndjjI24bZzluScJvfda83odO3GNf6ifmou9W9f+pciMC+jeLAfADC2wTO/y7lbX4
uRNArDzLkhsThkbkyO09mmY/ex+Y1jYph6aUzuOSQMNq2eqGG3FHO69AB9pmdlgjNp9/V1Vq5O
aQ54duY5ZOm5Cj0sOFEP4V7tRZJjnHMK+lhkLb7zutupl5EL2GPN6LC7t3t0+6iIVRSVZx15824RlZdU
PNgPABjV+Jl/2Btxfed/yk4Ac+S5MiI7NvSm66OetOPlu/KeBmKYnt0j2LA7gPWe1hwoP13fjix6QDui
8xwPz1WyqUnFJ6Ja9N5sxR7tzGGf0O5QC67So3cvednUEKuZ96DNd/k9p4lh9jJtYkGRNSNm7+be6
c9Oi1hFUXnWPe/e8frxR0ROXlLxYD8AYFTj/rf9nlt3zx16qWduUedsXVY/rRJ4MuO5mv4QJQb6B7k
88/vODczuY0xkG3LadqSzQd+OLHqAcaqaL7h4V2cfPaLq43Tos1ndQ1DvqznCF8EW215CL3kJvMsJr
rEl5yl1t1MjfMIL8vVCyLlfnBOxiqKyreU9xu/WD5//fvPLCQDA0DaU+fUoUyQ5NNRp5STq5+6zcuW5
mv4QJbbqH+TyzN8jKfWd3TLctPq0ihjt25HN00YdCp7jUn1ii22mLYao0W2D7s2qEYV6kLO1zjmMKS
p9BvbvJcd4l9O5Rv9H1t2ux8Q91oygi1cvikxFUTnXg28dFAl5efW3f/N58/sJAMDQxv87/DznOId63O
KX1Hn1aepVx0n5il0Zc1NyXM+r5Zu/R1LyndVpqGnVqFPb275N9phErUSP6DrH974ziU8z6NTWVj2
BMbhzne71Ukf0Ke4QrXhrhWZpveSsvi76mGeNiu+87na+s4Qea0YQ/9f4qdnVUy/fJ0Lykuqn7/1l8
8sJAMAINpH51RHP3XokPLiDep2UMffKFHQek7tst+lJO16+
+VdJd+qAXpK5Ttsw01qn6BeROX3EtO7K5JEeC/PT4yrW4K45ZYt2vJ5HLrOmTwzvR0rr5VxqZ0vGPK
HWBWeeQo92Tje5i15rRsjezb2TPzkpMhVFZV7Pf+9BEZWXUT98/vv//Mc/Nr+cAACMYBOZX9+
+e0Tu1tVE7U2+e2RWAlfTf3mMPXrSjp89U9WlV1LSRyyRj2aQafXbahK9/HZWz45c9qCaMdRdq+
+E+H5r5nlydMdm/S1Laoi7zFrozPByk3q5H19gSyW5RpPdvZqtV7uOC9hzzQi68Ycb/KX91Lzqrg9uf/
XEERGY517nnnj8H377982vJQAA45hP5vdngFosc+QrcjXtzYr3PWknxLzU1fDeScleQVer9adt3zDG6
+W3A50deVltel0+c17nlBDP+kzxa2h2LPup101v/zJr9mJ7LHf1Xt0fX/FGbI0ts3k1Mqmd7NJ7EgTxf5
yfml098PaBN449KmLzrIvH+AEAE9hk5o8nBcG+/ZdWmgoYhvpS8vXDTF36+pLIVBSVeR37iz8TsXm
+xWP8AIBpbPJ5/sgsDn1OAKkLU9PfSb58mLH3f/W+yFQUlXl99/kHRHieY/EYPwBgMomZ36uK8E6
g9yT8dprmmP6vf0P/FsAzSU2/Qe7CZMz/Wj38pQVm4qWfvyQyFUXlXPsv7Hvl6YdFhJ5X8Rg/AGB
KG8n8oYmC4SmS7CNvAeMwMj+BH/O3d3PvxKcnRKyiqJzrnnfveP34IyJIz6h4jB8AMKUNZf6SPVks
skdzffRNAECnnd2dQx8fErGKonKuI2/eLYL0XIrH+AEAE1st8wMAFunKzhX+Gn9qXnXyzP0iTudfPMY
PAJgemR8AUPrkN5+ITEVRmdeLzzwkQnXOxWP8AICNIPMDABrnvzovMhVF5VwH39/2pNHRbTO
tniMHwCwEWR+xIm/jyH+ciXqb8Jr/iKGdaZalfUX74/+F0EUO5tgT6UpryEW6/Rnp0Wsoqic68G3Dr
5x7FGRrjMsHuMHAGwKmR9x8ZC/TsjcVObXjWrjRv6m2UQZfLJriCXb3ds9un1UxCqKyrmOn71XBO
zcisf4AQAbRObHSkSqXCdkisw/mQn7Tp3ByfwYxje//4a/xp+aVz337GERs/MpHuMHAGwWmR8rm
X/mnzIXk/kxW19e/1JkKorKue764PZXnn5YhO1Misf4AQCbRebfiEDc9R7WOa7SfUZH7lNvt2e4R9S

05RExXfxlnB5cKMaLtXunMk8xFxhlnWSdpVoaAgs3FyfWbbF7md1CvcS2S87WjTHtNM2b4Y6eiQL
EHP03Veiz/kJ0jpo1xH2/xwwY18WrF0Wsoqic69537szwwX4e4wcAbByZfzM8wcl3sA18JuOkngHM
pN7Xb+smepLmSPVaTBd/GeHfSqlp60ylD5iMjXrFr5jn3cDCO5dbijYL9VLHjXmcrXvOrYdHO/b7OLy
X1Tijo0Xq+kvGqro+3O4ZMImzn58VsYqicq7HfvAtEbk3WzzGDwDIAZl/Q1Tm8cQcfUjnHnVEByVni
DGNE8CkZoA6xUhXzRlqhuqlmC7+MkiPkz0KzUK6ZvZs1dHjirkTe7lXpT0itrBiL88+nGFu90pnR18/m
3OKuGSdLfqsXw5xJpEnOGd0zoCJ7N3cO/mTkyJWUVTOdfLM/SJ4b6p4jB8AkAky/6aoFKNDjJOlPO
GqM10VnDzlaEbU59QvtrbKicwj6nwxXfxliG+YPtas3R6jXnk2ZhwS+lwx94CXmsoY1XmiO8B3Sp+P
THcP77UiT0xYYn0oND6ytvD69cvQ8tUAzxT1oc4ZMKEbf7jBX+NPzaj2X9h35jvfFvF7I8Vj/ACATJD5
N0YEHxWldPryZKtC4CxjkM5L4RzXnFUNqH4+tX25Oqs9ok8X08Vfhnj3Ig7aU6k3fUK9vF30vOpovy
Xr/u2o2In2cvUA3ymeVTrDPN0lb8fOvXmah/k31WP9+nXLamhPbKun6JoB07p249qBDw+IZEVR2d
Y9797x+vFHRAKfuHiMHwCQDzL/5qhcU4UZFYPasOaPZyJe9QhgPs1pxYh6dPFDfUgf0WeL6eIvQ7x
7EQftqdSbPqFe3i7OEvst2TOXe6I+IrgDjF59ZvbvpNDR0dfPEpy41bWpda5M876awkdNEZ8Bk7v09
SURqygq53rwrYMihE9ZPMYPAMgKmX+TmuxTpCeVcIxI449namBztFcA86jPU//zfnV2dUgdaScU08
Vfhnj3Ig7aU3nP6OA/R82rjvZbsmcueaIaUqhHuTP7evWYObT7zo6+fpbAxK3uTfVZv2ZMV6lP6lyF
wT8DNuH9X70vYhVF5VxPvXyfiOLTFI/xAwByQ+bfqCbQnNraqkOTmZl0kDJTjkxXnvSkQ1Io95Waed
zH+OsjxnyiY/xliG+YPtb0ssf0nNgiZ6w4E/Wb2XNhxYnuCPfK+3r1+cg8YwrdHTv3FlqQmrK7hWeIO6
ZQHDNe2X07l1mJzYANeennL4lYRVE514vPPCQC+QT1P3/x35tfGAAA8kDm3yydlioizug3VbzSuUfn
LX1EnWtMGA1H7Ym+U408J7JW/GWQs06jf9MsNLM6Qy8v0qrHFZN9AtRM4Ssh19MuuJ3Z16sd1
xw0rrsa5ule6O7o62dzutsHemzKmcFZvz7QLl9sKDxH3xk8BzCBvZt7Jz49IWIVRWVbd31w+6snjoh
MPmr9/K8+bH5bAADIBpl/w3S6KahU1TLfbZkpxwhlkjudqT3RSVH+BvV08ZcR4YU23Zyp/Gd0NFrhi
kVn8iRKeaK/Vamd2R5THw9fCX2ip3ups2OfvQXaN616bGqF9UvtsvyTtO93zxC4Rhjbzu7OoY8PiW
RFUdnWA28feOPYoyKZj1R/9R9e5jF+AECGyPybpsNNKLvY6ajNPJo5oHhbv/QMNbnjvGeKg/GXcXp
woRgvQlu8e6VnvLNPkivrt2RPovScaCbT8qh6bUxtDtGTmSssxjoze7o34h377U1eIdEm3qLStf6SOU
3JXZK9ivheS/YM6l33PIztys4V/hp/akZ1/Oy9IpyPUf/l6X/z+//zu+aXBACAnJD5N00FH6ILgLn45DefiF
hFUTnXd59/QET0wet//Y9fNr8eAABkhsy/Wfp/6iTyA5iT81+dF7GKorKt/Rf2vfL0wyKlD1g8xg8AyB
mZf1Ps/3DZ/Q+fASBvpz87LZIVRWVb975z5+vHHxFZfZDiMX4AQObI/JtiZH4CP4AZ2t3bPbp9VCQri
sq2jrx5t4jr6xeP8QMA8kfmBwAk+ub33/DX+FMzqpNn7hehfc3iMX4AQP7I/ACAdF9e/1LEKorKtvZf
2PfiMw+J3J5cPMYPAJgFMj8AYC0Xr14UyYqisq2D7+1/7cmjIr0nFI/xAwDmgswPAFjX2c/PimRFUdn
Wg28dFAF+1eIxfgDAjJD5AQDr2ru5d/In0Wyoqhs6/jZe0WMX6l4jB8AMCNkfgDAAG784QZ/jT81o
3ru2cMiyfcsHuMHAMwLmR8AMwLmR8AMIxrN64d+PCASFYUlWfd9cHtr544IvJ8Z/EYPwBgdsj8kk
hWFJVt3fvOnW8ce1Ske1Sk+kjxGD8AIYlI7I/ACAI7Iz/8MI7I/ACAIb3/q/q99/POX
RLKiqGzr37/4r0S29xaP8QMAZoTMDwAYxsXrF08+cdIkq4oKtvc8+6eLx5h3/P4jB8AEAmZ/dHHwhwWnqqKa2fwHHzrIAuO5u2uc2tG2LGD8AoBJ7QAwgqWbIcv8OXjjgIjvnE+IgSNDKxHmt8rojdAbZYq7Xj3fEyHRxPjvEAOB3Mv/LtHWjcbtYwlTkWaXQCV8fPSZLHQ9ZbO3e4H3x49XjiTqHn66P7X5adEWrbWYCYbAAW3d3m7qelU6V3yKVr/6E/T7nR0HkRoQyLXhnX+Y7OA9J8f8V6JKSD3IU2U5b0A9zY/u9
pGo2Q0SkajZDRCBJcSADAR7kiS0SgZjZLRKBmNktEoGY0QwaUEAEyEO5JkNEpGo2Q0SkajZDRKRiN
EcCkBABPhjiQZjZLRKBmNktEoGY2S0QgRXEoAwES4I0lGo2Q0SkajZDRKRqNkNEIElxIAMBHuSJLRK
BmNktEoGY2S0SgZjRDBpQQATIQ7kmQ0SkajZDRKRqNkNEpGI0RwKQEAE+GOJBmNktEoGY2S0Sg

ZjZLRCBFcSgDARLgjSUajZDRKRqNkNEpGo2Q0QgSXEgAwEe5IktEoGY2S0SgZjZLRKBmNEMGlBAB
MhDuSZDRKRqNkNEpGo2Q0SkYjRHApAQAT4Y4kGY2S0SgZjZLRKBmNktEIEVxKAMBEuCNJRqNkN
EpGo2Q0SkajZDRCBJcSADAR7kiS0SgZjZLRKBmNktEoGY0QwaUEAEyEO5JkNEpGo2Q0SkajZDRKRi
NEcCkBABPhjiQZjZLRKBmNktEoGY2S0QgRXEoAwES4I0lGo2Q0SkajZDRKRqNkNEIElxIAAAAAgGUi
8wMAAAAAsExkfgAAAAAIonMDwAAAADAMpH5AQAAAABYJjI/AAAAAADLROYHAAAAAGCZyP
wAAAAAACwTmR8AAAAAgGUi8wMAAAAAsExkfgAAAAAIonMDwDAqi5v3dbautwcBQAAyA2ZH
wCwIHUYHzeFX98+1XYYs+HU/2ah3Fjh1Pb15sCQrM1U+Hclq7Ku4Tgf0+gCvzDNd6827Dcj8isaeWt
V/qmsT2zYbY0remVG/QfFiMKbmuvnFNiRtZ2hPqYevWb6z6RbBJkfADCuEe/mLUafKW/aqrZj3OsU
91J6H9V91cg3VOVGTm1tFf/PKH3KLUx+S2jejw7/rbC+2a2Rvn3VXib6RpjXbbgmwd9Q+3fI3uhagh
2jb60qNFV5XB+oL+m6rfyzmB/X2i06r0z5/qD/oPBtytpSZb19RTZVvTXgr5K7HaO5aaQdVf31oerVu
rvr12uAVvW1a4jrY11F8R56IPMDAEZk30/ZdyODUo2q/3/KGwJ7h2MZvUvZoLl847QpP/vRL5Npm
g/GUrUc6csn5x6v19D38Q31ebgLl1+NobYW7hh7a1V9p1Ljmpcrq86v2V2Ky6cPrP9xdW2nPN68v
VafWmxTQ8zfiGzKcyhdcDvC+k3DO5JHRuyl3mhervupldPp6atvcvvSblW9udaebkVkfgDAeOQ9gHP
XMLjxOwjT3H6MvC09fflD+k1bzHq3gyub/HtQGPerYF/A0fZXTWx+UEN/cM7K3a0MfCEj12rAy9g1
VfV+8oVUZ4/bxeBvpI+WP6zdRi3W02ucf1y4jQbZRyOyHduA3+/OS9e5mP6cqZx9DNhMX83qhfw
6DNnolkHmBwCMxv2TecC7Hb+Jbwaq/Qx1zxhk3f4Mz7hm5Y/jNKquVGvkazbePoJG/pAK9SUsWt
Q/jPMtd35DjW/HIJz5yo72dRu4ZWS6ATt1TeVc2CRdXQbbkG8i41j542Bfdk8v90sxBLdR1ad8UEFbv
6vv0hmqt4fam7dX9VUrW9Q/hJeyGqdX4HIOszXzMvkbDbaxWwSZHwAwGvcOwHuLMqTRG5iqG
4+h7t5c1V4aI26p3IWavmw53oaUemNj9qm+eQPfysdN88WrvnGVsVq5+3B/i9fiNHDnH/haRqYbs
FN8quqDG+AqxrtU7w7zWXkalXtQR8q3B/tSBHoZBmrlNhKfSzUgfH378WzHEH93VaHZ2us3WCtPr
+qIvHoDfVbVDppu5c/2tMNexVsDmR8AMJoN/FE93b2AuFsc1aA3U7ZyG+31KhtNsif3qzEo8eHU1
2/Mr0XVcNTvnbmH+udx+lVzy0s34EdVTWiu3P0qOEPWE5luwE6xqcTXcQ3eLtXBxlCXzWlU7qF9Xb
492JcidukK9faG6OY2cr58A/wiR7czwPwmT6/6ctXH6p8H6ufdV7UfZWvL+pKsoZpWfzDOpxS/xvAi
8wMARrOBP6onuheo73QmvOeob96Gb2jdshmGuMOOqfqOd/2cb964/apPZ9RL5nYYcUfWl2K4+
/ia8xvq/so6n9563AZa5K1VBaeqruZQ2+lYcPX2IM1kI+s7YRijl2ugb4TbyJm4ey2dIlNUbw31XSg5vd
wO1Se33o5q3ZemHDHE7uSvjPvxd68FEpkfADAa9w/mge7dwqa4F6h6THzDUfUc9cpVyjbjdxl/O2
PcyocNdlcd5rYYdUeGob8S7rrlhzX0ziLzDdjKP1X1uQ24mc4FVwMGaNjRqHx7sG9F56aG+g1zG1VH
zI3IL2OC8HYG2obB6eW26L66PXVOVA5Y/0tR7cCexu08wMd0yyHzAwDGI/9kHuz2I2j8DvU9iTT4/
UfRxdiG5z5oFMPctbmKeY1pqw9p1E+p6mBuZMx7xOrDGfc7534DJmlaGPwbUX009sKtvTkf3do8
HZXIW6vyTFUdGvZD6lxwNWCAy9fRqHx7sA9po5uyfpEG+a0KbmeE31m3l/XLVBiuafxjGuYzqlbrNi
kPm3PHlwIvMj8AYETWDcgwdwV+1dzCvO8JxI6m2UzZdJRPaPLdWLe6w933usb8Vtvq+2Ftgm/Ek
HsT34BKuwVzb0NdzEjH+GJWEp5KfF61NTdXdbNWWnQxXIc91+nR88qUwwb70jubKg4Yk9dL8iyit
/imrHfXadNwtlOrDk/w3R78HxThXvY7a1+6ejrvNNb3etAreesg8wMAxmXegPDnNCZj3ZCufT8aUn
29R5t9Awa+j8cg7E+l1nw24q0ZfWLhTc1yT5HPqLC4f1AMTvyrilp7y8CNxJrI/AAAAAAALBOZHwAA
AACAZSLzAwAAAACwTGR+AAAAAACWicwPAAAAAMAykfkBAAAAAFgmMj8AAAAAAMtE5gcAAA
AAYJnI/AAAAAAALBOZHwAAAACAZSLzAwAAAACwTGR+AAAAAACWicwPAAAAAMAykfkBAAAAA
FgmMj8AAAAAAMtE5gcAAAAAYJnI/AAAAAAALBOZHwAAAACAZSLzAwAAAACwTGR+AAAAAAC
WicwPAAAAAMAykfkBAAAAAFgmMj8AAAAAAMtE5gcAAAAAYJnI/AAAAAAALBOZHwAAAACAZSL
zAwAAAACwTGR+AAAAAACWicwPAAAAAMAykfkBAAAAAFgmMj8AAAAAAMtE5gcAAAAAYJnI/A
AAAHm5vHVbY+tyc6im3ji1fb05Mg7VR7TPQXttCs51uL59KvRW9D0AWC4yPwAAQF6MXGvH01s9
87exveK7DMFk317UDP9NBgCMh8wPAACQF+t/yzYT6q2e+Xvt3xvu238VQOIHcIsh8wMAAOTFyvx
mwL3FM78O7h3r0tdPXyd9hMQP4JZD5gcAAMiLnfmNoOpkfueAG4ybIeUIY97mBOOImYbV4eKY
8Z/TG00U413nfWNtbZuOzG3NZ01orFSJzKVHV2P0K2cHsfWX7PWIIZENRs8DgEmR+QEAAPLShsnt
+icVGY2UWb12Dxhxs4nEbRztpEN0+BwjZ8tcWzEW4pkkGn39TeXWDcZaHO3itrbVj6J71/o7FhTeo

P+86HIBYDxkfgAAgLyo0FgkSBVM68BovFENdA/EMn8zyIik8oiKpc457RF1QLbRI/RSjD494m64gZ7
Q7RmjBzfaaSqd61cDwusJbDA0kVwBAEyDzA8AAJAXKzU2L8yf3dhpxEkZZtWQNpeGj6hp3BFyjEzE
BXlIT2KMCZIrKMmduAfi9AJK4pQe65ec7v4N6mG99g0AoyPzAwAA5MUfr4uo6SRjT1SW0dQd0n1
EvTaDsj2vGuEjGluzBLhLKqmW6qjcWqfwEnqs32CPVu8HZterbNnbAoBJkfkBAADyIhNw81o/3t9m
SDmyMFbmtw+qFz7BxmH+sXIryZnfXUKP9fvCe0W9H5w9cGLfVQPAoMj8AAAAeXHCpAyR+g1P7J
TB2B3SfcQzrZjXN0LoMUTzj1Ut1VG5tU7hJXQvTo0o1IOc7p1zGFNUws0AYDxkfgAAgLx4wqQdH/U
bsZEDZH4zXNtjesRvt02Yns4c7PQYMPN3TuWe6lyUyAaLt4yJV143AAyIzA8AAJAXX5jUubGk32iPN
nFSB1PniBtew0fCs+gh4c4q2LptYsIN2vNXzs6RJXStX26nHd+xQWcj0WUAwOjI/AAAAHnpCJPmG0Y
UlUSWjUVQeUS/PiVnV3m34G/dDnDbxKnxNvPsITN/5/r9yympIaHZQ2f2XTUADIrMDwAAkJdAmG
xDqvWGmV2LWCmDsTtZ9xH12pzOWU7Jzs32gMAuouz5ZEiWW+vUuYTY+tvTS2VL47KYb/tmN08
t9V0xAAyOzA8AAAAAwDKR+QEAAAAAWCYyPwAAAAAAy0TmBwAAAABgmcj8AAAAAAAsE5kfAA
AAAIBlIvMDAAAAALBMZH4AAAAAAJaJzA8AAAAAwDKR+QEAAAAAWCYyPwAAAAAAy0TmBwAA
AABgmcj8AAAAAAAsE5kfAAAAAIAl+tOf/j+jkEzIJcraqgAAAABJRU5ErkJggg==]]></redcap:Attachm
ent>
</ItemDef>
<ItemDef OID="b28" Name="b28" DataType="text" Length="999" redcap:Variable="b28"
redcap:FieldType="descriptive" redcap:BranchingLogic="[q25] = '1'"
redcap:InlineImage="1">
<Question><TranslatedText></TranslatedText></Question>
<redcap:Attachment DocName="B.png" MimeType="image/png"><!
[CDATA[iVBORw0KGgoAAAANSUhEUgAABVEAAAEJCAYAAACdcmdaAAAAAXNSR0IArs4c6QAAAA
RnQU1BAACxjwv8YQUAAAAJcEhZcwAAEnQAABJ0Ad5mH3gAAE9sSURBVHhe7d0PjCRnfef/b+/aY
PPHNmBskx+RHat7sZYlGJIg08PxL4egZ+6SJbnbYOVg5Oh3PVyi0zQSkwOxF+mX2wguG4lunS5iWrq
zliQyTEJugrLdIJRgckzHR4IxYTI/Z7rlc3T8wDYGjPljB3u3f/VUPVX1VHd1dV3VX39PtltaerpqfqqqT/T
O/OZ5/k+hb5FAAAAAAAAAAAAChjumPAAAAAAAAAAIAQhKgAAAAAAAAAAEIEQFQAAAAAAAAAAiEKIC
AAAAAAAAQARCVAAAAAAAAACIQIgKAAAAAAAAAABEIUQEAAAAAAAAgAiEqAAAAAAAAAAAEQgRA
UAAAAAAAACACISoAAAAAAAAABCBEBUAAAAAAAAAIhCiAgAAAAAAAEAEQlQAAAAAAAAAiECICg
AAAAAAAAARCFEBAAAAAAAAAIAIhKgAAAAAAAABEIEQFAAAAAAAAgAiEqAAAAAAAAAAQgRAVA
AAAAAAAACIQogIAAAAAAABABEJUAAAAAAAAAIhAiAoAAAAAAAAAEQhRAQAAAAAACACISoA
AAAAAAARCBEBQAAAAAAIAIhKgAAAAAAAAAEIEQFQAAAAAAAAiEKICAAAAAAAAQARCVAA
AAAAAAACIQIgKAAAAAAAAABEIUQEAAAAAAAAgAiEqAAAAAAAAAEQgRAUAAAAAAACACISoAA
AAAAAAABCBEBUAAAAAAAAIhCiAgAAAAAAAEAEQlQAAAAAAAAiECICgAAAAAAAARCFEBAA
AAAAAAIAIhKgAAAAAAABEIEQFAAAAAAAAgAiEqAAAAAAAAAAAQgRAVAAAAAAAAAACIQogIAAA
AAAABABEJUAAAAAAAAAIhAiLp0etJYKUih4DxWGj29PPFeU1hpMx3QkVLWdLxMpLdhUd5zq
MxLO7LSlg372PzHqPs4Vb2GrGzEO5vtDbdtK5JH08KNuQ9Cjmc+2g0AAAAAwPwhRF06Rdm8UJ
XurU1kPCkracr3x0c5Hq2U3Drq4B5oILLBVWnqJUdzZqxle4ef14faaf1Qo1cT/rlhsR+14AAAAAAADIGiHqMip
uytmqi4tmqfi4dqZ0P9kbrNc75IVW1JdsV/9vnpsS/aHkff+/vnpsS/aHkff+RpmXdmShKwdu8leuS9+c1c
D7TVk3fijwsI7ascDAAAAAAAEAOCFGXVPHM+WCwuXtFeiFx5N5B4KLYHXnbduAR/f6R6rcIpXb9zwgG
EPu31edcGHd5fZI4J+Z4a4+FhD7nzwjDBZdkd8Y+jVBDR3Wt1VVkvvLzqc4hOnZIhfH3Pu/ScrsGsu0XwpPPcBx
1zxN1ZW0k1QcHEBAAAAAAEOqm1RafiNB+L5Y8tPfdOEHnh1xkkpYSFp08qmNHvAz8tEr
y8DVQ0mtvDFAEZAmsJxZbr7xqcb5TY10SoKbfvgS8AfD9Av8ejWPdaJ0CnFcMx4n6ScdBBawtcVQsYx+q1
j1Q9PaGXKuTzotDSZsl/iTFHwM8YEEsFy9xHWkcrsw8PvQw4GumuqKuMEBBUZkb/kRTiWiV3yNq9Q9Bug+
2XDZUTY07hE9Wq8cgn51XGTB0J+3AC+hJiwnmcDgrOspVf9IaW5fg33yL+Ed0nQNWwVHX4jFfAF+nGrNOHiIH
ITL6Aaxa0Sn5GEhV/nFzpn7eVd5oHBhfSvmwTmjdQ+12V8d2jfo2s4p+h1+0GHvtJ+1b3GAyf0EiDVw+wmIWbC2k
D6gP/AGy9izv6PBo1Pe1wrCSDo63TPb8dqZUKUhrYSXM1aUimtjPY1qasDoZtEx2T2rZZ+9S6H+te1
+kREu3HCSwHz4HSXB0dKibnnOtV70AUtW6Kc9TeGFHPNGS7E4vZbgAAAAAAlgzQh6lLlLrLTrBF

nqF0Imk2pv+KGKV4+yL103ie3UZD3lhKW4dka3b18O3U23d42JhYxw0hjqferEnAz0rpz2yiY0d4M9
Ji/u6BisfEacDLUnjXX3GlSlpc9v3+0xnOr59bfvdUhWIdlAfdzx/O1494Hazrob/E1zTMbr9zZlc9v62PX
vV1Wv1/6cXXIi2X56jXUvsCzXu85r+8MlLiqR+4wr/BzteOl/krZbr3X/0hH2PRjY7rBkxzOu3QAAAAA
ALI+C9QtyXz/HklK9SwM9zlSwEhKq+K8rS71rTubTk8aK24tOBS/OZEYjX6963bk96Ubsy+dvW4Vde
9ZG1BBs1YOwWq1Ks9kcWu+3IaxdcdeNE387w+2yV3rnwG1/6DqbsV0VnO2FBNwB4e0wr3O11fc
nDEt0PSK2E9ivvuaS7JjMbQdfr41qa6JzF35+FOdanQqsS3p+lETnOlHbRwht44jjjDietO4RYBr3Pnyvv
OXCW/QSACAP99/xLnnNW2/SSwCAzB3/NZFjr9ILWBT0REVwkikVtoQGJP5Qeb/npMsYdm/2GE1F
UU6ccp51dJfTrj2WvywnT5+0e9QF11uqp/0AbA74vWmb4nVG7R44gZT1GW8ov7dOdT4sGbUo3S
BMfcIsazBr1jUo6ac28z7QPYSnOKZEvYkT7cec4f+kmIdQ3NyTvhmgTm3wHIWY6rrrurFuwJmaGO0
GMvDwEw/LOz/5Tr0EAMjDDY9fJXd/6AF5/Bs/0msAAJm7dI9I/zt6AYuCEBVBA6FScsO1P6fl1RVt7k
pbenJoj9k/JScqJ6z/D65XGeqc9ZIrrombLbpD+ttuIl09a/TozUtaAZl1DTJrOyHeSKpXqBe0GnVjgQX3x
NNPSOWPKvZHAEA+nvPjY/KWL94oP3ryGWm+/3555p8u6c8AALL1lMilP7A+PussYiEQoiJlGYRfXl3
RfTlsXxS7lKgd9lbEyVeN9XMZvhk9NO3A1+/VOyrwVcO67VqVQ480e0lOK1mv47yOaTHOXbjxbW/
Lhtnr1K2LatY5BRbUnZ+6Ux58/EG9BADIwxv/
+gZ5wQ+vsJ8/9NXvyp/WeR8GgNz0Hxa5/Od6AYuAEBUxleSkN1LbnBFfMSZJitM70Ri+HI+7744c7D
pf686+X7I/4a8fLjUwH4qbZ/0guOFOjFWVQIZacsoTKG6Jgvk22OvYvA/0seV1TIn2Y97LA8Pk1cz3hZ
xnoE/SdmNSNTtwdeukJv6eAubL+z77PvlM7zN6CQCQh1c+eK3c/PXn6yXH5z7+kHzlLx/RSwCAzF3
6gsjlr+kFzDtCVMRUlM2zbuVUc/Z1NbrYnOl8y+vt5wScijnre1s23JlrYvN7cqqJpBS3XmZRF0x117vh
6vxxe81a56Kmj3+wdqsx7F+aq7LhnTIV7Onh297K+dBcNQLH9nm/hqd7bHkdU6L9mLVbm7Lqre/p
me+ta1Qq+NvI2oTnyA9c3XYDi+kT+5+Q+n11vQQAyIOqg/pzD7xELwX94W9/Tb73raf1EgAgc5f/RK
T/fb2AeUaIivgq29LyctSalHTA48w8bynX5YJR4NOfUMnSXNWB0GQ1HAPbsp55Q/a9of6KMUnTHP
Jqu9rKUt8KRKiWomxe8IdlN1d1gOaFzqMm/ZolJ3AMttM8tryOKdl+ipsXpO6/2HmtOZGTdS/7h6Br
7yrua1NNWBO03bzfw9odR+bHA8R339fvk7v+7C69BADIw/FLBXsYv/oYRgWozd+8Xy8BADLXf1Lk
8sf1AuYZISoSqWyH11+stqz17tBiV3FT9gZfW21NVr/R7K0XKBlgDM3OdKKjFJgB2KiyA+qc9bt+wOe
ya1/OW03PstS7fT9Yt1Wl1d8LTpaV1zEl2k9RNvf60h16sXq5MUzeVpHt4EGK7B96PbFTEbvtVlv6Lf
8+sqlzblwHu+5ulByOB4jBnYn/6Wfp7QQAeVr50kvl2iev1Evh/uFL35ZP//6hXgIAZO6y9RvZJcpbzbt
CX81aAgAxtTcK4nSSVCHqQGAKADGo4PT1/+318sAjD+g1AIA8lB56od0LNa6tu8vyiteFD/sHAGTgit
8QKfBL9ryiJyoAAMiVGsJPgAoA+VK9T1Uv1CTUsH7qowJAji79AfVR5xghKgAAyM1HvvgRezIpAEB+
VP3Tt33hZSProI6iAtS7/+NX9RIAIHN2fdR79ALmDSEqAADIxe6Du/LBv/igXgIA5OWOL18/tg7qKPv/
8zH53Mcf0ksAgMxd/n9FLn1BL2CeUBMVAABkTg3fV3VQmUgKAPJ189efL//8Czfppclc+dzjsnX36+
XWV79IrwEAZOtKXR/1Zr2MeUBPVAAAkKknnn6cmfgBYAZe8MMrEk0kNcoz/3RJmu+/X3705DN6
DQAgW9b77aWPWx+fchYwBwhRAQBAZlRwV4/P8bZwFwhRAQBAZlRwqLLUh594WK8BAGS8BAORB1T99y
xKSAA5Kb/HZFL1EedJ4Sv6wAAAgM+/77Pvk3ofv1UsAgLz83ofv1UsAgLz83ofv1UsAgLz83ofv1UsA
DXr0dELmDVC1KXWlo1CQQrGY6XR05/LSa8hKxttveBob7jtWZG8mzM3Qs5LHL3GinPuVhoy6tT55
9d55H7NjwLuWyCW+n11+djffkwvAQDy8vJvPE9e+eC1eildn/zPB/LN//19vQQAyNylXZH+Y3oBs0S
IurR60lhZlaZecpTlzFpRP8+eHfiVasLfVIImPi+9hqzXor5KXfOCrAYvunRqJSlMENguK+5bIB7V+1T1Qg
UA5Ovqp47LG++bvg7qKKo+6u//
+y/bHwEAeVD1Uf+79fFZZxEzQ4i6tLpy4KZA5bp0+33p9/dkM68MdWzgt6QmPS+qZ+S4YK99XtxN
V1vqevelWy87K5qrQo4aA/ctEIuqf6rqoAIA8veWvRvtIDVLqifqH/721/QSACBz/UdFLv2xXsCsEKIuq
96h7OuncuqE5Nf/FGmL1zOyJ41zugtquS5bFedpcfOsVJ2n0twlRQUwPTUTf+WPKvZHAEC+Xvu1F8
vLHr1aL2Vrb/f/2A8AQE4uf8l5YGYIUZeQXbvRDN2tx8GuiKp3o1E7c1T9zGAtyGCd1bDejbH3r
wVreI6qN+kMVfdfl6zW52A9S6+2qH6ENy3GPgPncEP8zZjnydln0vOiqHaWYvWMNHoeB0LzkpzUn
VFl/9A6olGM9obVW21v6GMZPAcJrkvI/RZ2vePcb4PXz35E1ImNY5b37WT3p2vSa2eZ6D1Ar1TMr
49uJI6QOz91pzz4+IN6CQCQFxWevubvXqSX8vGLXiKZ8KP9/4o7zFLCgUQIPULM4SoCDUUGGl2/cyRYVRHai

WjzqrR43EyanuDNTzVurCgpuQNVXdFt3UUZ5+DwWRzdcJ9Fjflgjtk3joz5/RGeo1z3nkq1y+kUEahK
q1+y+tVOsTseTxK50C6+umwipx2N97ZkYsDJ7W96x2NX1c3yXVRQV7I/eZcj1EBZPj9pu7d0GC5U5
NSIMjOSpb3bcz7M2CCa2eZ7D0Ay07VQP1M7zN6CQCQFzV8Xw3jz5uqi3r3h75KfVQAyI2qj3qP9Z
H6qLNAiLqEKtt96Xfr4kZ7Um3Z9TH72zrxbG/4AZBXL9Won9mpyfrIxEaFec7r+3uboWUCxu4/wN+
et3/pyI6XBPWkse4GPca+Wzo1imzrKP523M2ofdbOu/Fbsn0WNy+If+rOS9v677wbglnn94JOUJOd
F4P9um2ZLK8uyolT+ukYlS23beb5V9ri53BnxMnhkpwj67VGqYGh+21ofyZj2/b9ZrYlbFt+kJ3U/Ny3
/uvD789hya6dZar3ACyrT+x/wp6NHwCQv5UvvTTzOqijPPTV78qf1hmBAAC56T8scvnP9QLyRIiKIW
bPtPoFPwgdDgOHletbE4Z54aotPxwsrp3xAqzOge4z2bsoO14eaey7suW3deeiJIl7zH364ZMh8T6Ls
nnWTbuaslpwe08Gz+8kipt740PW2PblMOpEFdfkTNjxtXf9XrVn1pzjSXSOrPOzp0NEI3gPvd4Dhu43
s8et0evSPk86CNzLYfa0LO/bsfdnmCTXzjLNewCW031fv0/u+rO79BIAIE+vfPBaufnrz9dLs/G5jz8kX/
nLR/QSACBzl74gcpkJ/vJGiIoBI3qm2Yqy5iYxIwK3UyfSDKjKcrKkn47SPdC9+VSoU/LqNhbMYdKRw9
QnMMk+K9tGr0FHOsP403RKoi+fcf2NgNIP3Kpy1j2gqa6LruEZOrw/aOh+M8JCawf20Hd33yNrsaZ
uHu/bBNduyvcALB93Jv6nn31arwEA5OWGx6+Sn3vgJXpptu7+0APy+Dd+pJcAAJlTw/r739ELyAMh
KibUkeHOgTHCo7mX3TFUvMKUSrD+5Oz05HBssVSf36vSHRZuBG7V08FeoUmYExB5PXXHCbtWR
dm8EN470w0rF3t+o8nvz/SvXdh7AJaNCk5VgPrID+h9BAB5e86Pj8lbvnijHL9U0Gtm60dPPiPN99+v
lwAA2XtK5NIf6OfIAyEqJjR/gWm53vWGbAcfk9YLHS/
+Po26n7aO1NZznJyneELGlj4tn5Sxl7S4KW5lAntYuDF8vno6/CyPP0dt2TB7nbo1OM36o0lYbdzT+xj
s/as0z83XpEi53bcTXLtoR+GPJpiWGsL/wCMP6CUAQJ7e+Nc3yAt+eIVemg+qPuqnf/9QLwEAMqfq
o15iYte8EKJiQElOusnV0EzePbnoFnIcO/Q7J6WTXtA2qm5m6ibYZ6+xrodpl6Ved5OsPCfnMa5rc1f8
zphdOfAu6QmJc0m9HrXW/XH+/I4OP6sSyOGSnCOzLqcKFN26qMaQ96TUzPKqx6k9GZQdSHa9W
p7pDpOf0CzuW0usa5fme8AU1xDz7yNf/Ig9mRQAIH+lh1448zqoo3z6v/6D/MOXvq2XAACZu/xZkf
48dRU6ughRMcCcBCnYW9IPAlXYNTChz6yYNTCbq/5Q7faGHhpuPdIev510n72GrLsnrnpWNjeNyY
Nq65JPjmrWsmzKrm5er3HOCzBj90asnBbnDulIs+ke18Bw8Amvix8oDvbcjUlvX80sH+xxaoTFZo9b
oz1p3yaRZnHfKnGu3QTvASU/dZXaee9gZMOb4h9Hze6Du/LBv/igXgIA5OnaJ6+0Z+OfZ83fvF+
+9y1qZQNAbtSw/v739QKyQoiKYeYkSJ2alHSoU/LTE7kw7YxI5vDy5uoUoVGwBmZzVQdQXnhTlVZ
qs9e7kuyzJ411d6h6Wepbav3okCq98zLMnFndbbN3TastiX+aKhIo72oZDmATnCMv2LO4x2xOsJTEi
HvXrLNaPevPNp/YQt+3SpxrZ0n4HuDXW7V41zBubVssGjV8/85P3amXAAB5UvVP3/aFl81NHdRRV
lCqglQAQE76T4pc/rheQFYIURHKHgYdUpOy2rLWu8Otp1KR7cGClfuHRs/BBOwamMZwbZddWzOj
eqgx92n23LN7obonbiCk8of1p3hehhRlc2+4Tqg9hD5hYBeYJMs6ZjsbHhT7uljH3G/5QaqtKq2+0dZ
ACYJo9r07eJA2Z5vTZZMLft9aYl07S6L3AHUsg6+ttiava4u59cTTTzATPwDMkOqBqnqiLgI1pJ/6qAC
Qo8vWb6bUR81Uoa8KBgJAEmo2fT0ZlAph96btmTxL+lhOtaYNWBfEUbp2yJUKTit/VJF7H75XrwE
A5EnVQVWTSS2SK597XLbufr3c+uoX6TUAgGxdKXLFb4gUbtbLSBM9UQEkFCxRcGZtsUO49nnrWC
J6ZB4tR+vaIV/v+
+z7CFABYEbULPx3fPl6vbQ4nvmnS9J8//3yoyef0WsAANmy3m8vqWH9TzmLSBUhKoCY2rJh17o06
pWaJQoWUK+xIqv7demmUqJinh29a4d81e+ry8f+9mN6CQCQJ1X/9C1fvFGe8+PF/NXt8W/8SO7+
0AN6CQCQuf53RC7doxeQJkJUAJNRNS8XfPx7cXMvpRq/C+YIXDvkR/U+Vb1QAQCz8XMPvERueP
wqvbSYvvKXj8jnPv6QXgIAZO7y10QufUEvIC3URAUAAKEefuJhec32a+wJpQAA+bv568+Xf/6Fm/TS
YqM+KgDkjfqoaaMnKgAAGKKCUzWRFAEqAMyGqoO6aBNJRVH1Ue/
+0FftjwCAPKj6qGpY/7POIqZGiAoAAIbc+ak75cHHH9RLAIC8qQB1UeugjvLN//19+cPf/ppeAgBkrv+
oyKU/1guYFiEqAAAAIUDVQP9P7jF4CAOTttV97sbzs0av10tGyt/t/7AcAICeXv+Q8MDVCVCTUk8aKm
uVbPTaknWjdOGltZ96MOYZeQ1Y2gmvbG+7rV6TR0yunpfZjb3NEO2zTne9M2m1bkPugvaHbWJC
BSwosjE/sf8KejR8AMBsqPH3N3x3tuqGqN6rqlQoAyMmlPxHpP6YXMClCVGCGeo0VKZRq0tHLWe
pd3DH205RdQj4AA+77+n1y15/dpZcAAHm7+qnj8pa9G/XS0aXqov7+v/8y9VEBIDeqPup/tz5U
ahKhIqCibe33p99VjWyp6bXby3l8WRhxDryHrtTziU6UnF3eC+2qea1hrF8VRuA+A+aZm4n/nJ98pT

z/7tF4DAMibClBVkLoMVE/UT/7nA70EAMicXR91Vy9gEoSowDLoXRQ3Qy2Xy86Tzo5cXJwUdTFUt
qXfrYs+w8DCUMGpClAf+cEjeg0AIG8//ffX2UP5l8m9n3xYvvKX/NsDALm5vGc9mOBvUoSoS8eoLbl
i9kQctd6oc2mvz7s25fD+IutuGnU/VwKfNLcT9vko6Z8z+/PmMP7mqvP5EYU0/WNWj+T1Rv2h/FU
5e+GMDvk6shMnRQ3UUg17jG5PvHbHuTbD59DaumyYrzfqkQa2MbA+/BTHaYN5POpY/P2rh7vd
9nnrupbrsjXQXdYu3WC83n4M3DfArKgh/A888oBeAgDk7YbHr7Ink1pGd3/oAXn8Gz/SSwCAzF26R
6T/Hb2AJAhRl05R1s64PREPpOs8s3TlwE30Aj0U27LbdJ6Vz6xZXz17ldNV/awjB/4B2PywsCxn1nRr7
RCwJIMj5zu1UswQa5bnrCO1UkFW9fYcal2SINUYyl89LZXimniHs3Nx+hCvfEbcU+2L2e6pr43Dfn1w
Z846FVQOrG+uDgSpE7VBHcuqeFvWoakKSlf369Ld2wxcdxW+lsJKN3RqUprnibKwFD7yxY/Yk0kBA
GbjOT8+Jm/54o1y/FJBr1kuP3ryGWm+/37qowJAbp4SufQH1kfqoyZFiLqEimtuT8R9OXQTovauHwi
JEU72Dq1XOU6dmIcI1VI5LW6M2gzMjmSEhV6w15PGutvjsyotu6am9WjpLXRqsh4jjUz7nFW2rTa
Yw76rLadd22HVPv12d+vuV8TsRaoYQ/mrp9X2zVA4xpD+4qbsuefNfnTFa4Z1BPULwcDQN67d6V
wbV7neDX69Frbev2+maYPxeh2aFjf3vOc+P1RXYWtXf41/TppyLsFxAmnafXBXPvgXH9RLAIBZeONf
3yAv+OEVemk5PfTV78qf1h/USwCAzPUfFrn853oBcRGiLiOvJ6IfaPUOndivWnXCo32dFJrDwO38bS
5UZMsNoJq7fi8+s+6n2wPUXFff8ickqmx5QWCs3pgzPGfVlj+Rkh/mWi0Z7IY7gtk792TJfmJsJ0EYq/
Ua636vzepZ2QxPUMe3O61rY6vKWbchRsg+cv3+obPdKdoQeH0UI1Q3Q2s7cNWB6t6okwhkSA3f
v/NTd+olAMAsvPLBa+Xmrz9fLy23z338Idn/n4/pJQBA5i59QeQyf8BKghB1KRXlxCnnmRtode1x6W
U5efqkHXYF11vUMHDn2VzwQ7mmeJ0KuwdeWOgN5ffWWcfkDu+2H8bw7cAQ/VFmdc784HMy
Yb1zLRMP6W/LeT9BlVZoz1klRrtTuzaW8kkJ3d2o9a4p2hC7Z7Zxrq2N2WUO3P3Er8sLpOuJp59gJn
4AmLGXfPe58nMPvEQvQbn7P35Vvvct/m0CgNxcVvVRv68XMA4h6pLy6oraPTl74nSqPCUnKies/w
+uV3ngPEWoFiOYcodmt90x0xG9I6exkOfM6GmphqaXQkPC+LP0tzf8OqBVo6fp8kkSbhdl80L4jP1u
cDtiPjEgE+5M/A8/8bBeAwDIm6p/usx1UEdRAWrzN+/XSwCAzPWfFLn8cb2AcQhRl5U3tHlfDts6aL
N77VXEyQqN9VP3hsyCUdfTDi/9upOjwkuvNubQI2YYuIDnzB/KHyXmkP72hj9RVLUlIzuhTmDqa5O
CTNtg1JUdKNlqa55jln7k532ffZ/c+/C9egkAMAsrX3qpXPvklXoJpn/40rfl079/qJcAAJm7bP02eukze
gFRCFGXVklO2hlkRw52nSHNbh3Rkv0Jf3347OuzV9w864eaDXeSp4E6pCVnqL3i1eKc2KKdM2Mo/
xjjh/S3ZcNLUMtSV1PRTyvVazOhHNugZuhXPU7tScXsQNWYoCtu2QJgSvX76vKxv/2YXgIAzELpoRfa
D4z26f/6D3aYCgDIyeXPivTp2jMOIerS8ntyNptOOObWeCzq4p/uem+Sprnj9gDtSK2mA77BOqRmP
crmqj9sur3h1aUsxB5LvWDnzBzK787+H3iYIV70kP5e45w3jL9cv5BOuYRUr82E8miD3pbKoIM9Trvi
ls8dW7sVSMn/+v/
+l34GAJgF1ftU9ULFeHd/6AH9DACQi0v36CcYhRB1iZkzpqvehd7w88Ds5sYkTXPIq1NqC+shGaxH2
VzVwZg/Ll1GT440LNVzVtS1VJXmauqhoTmUvxxaW8AoiWC9cuSQ/l5D1r0CqtYrAxMwOY/JJkhK99
pMJoc2VLb9IfyBurRGfdmzm7MP3bEU7v7Fu+WOl9+hlwAAeXvjX99AHdSYfv7dP6WfAQByUXijfoJ
RCFGXWWDW8FPiTzbuDltXzPVzyAwvRw2ht+tRGr0uXeW6dJPWu0z1nFVke7BA5v5hSrUxzaH8o0
NdMxQeNaQ/Xl3VCaV5bSaVQxvsIfxhxVBVSNvvp1pfFohy1RVXyf/4lf8ht1x3i14DAMjT373yCf0M
UV7z1pvkbe+5VS8BADJ37FUix9+kFzBKoa/G9QIAgKXxwCMPyOv/2+vtmfoBAPm648vXyysfvFYvYd
D1P/E8+a1PvVGedw0TbwFALgovFrni/daTq51ljERPVAAAlsztN91uD+0HAOTvb27/tjx2PX/ECnPlc4
9L9fdeS4AKALmx3m+Pv8f6SIAaByEqAABL6F2n3iUfeMMH9BIAIC+Xjvfl8294VH78nMt6DVz/8t+V
5NZXv0gvAQAyd+ztIoWb9QLGmSpE7TVW9AQpBX9W65F60lhxJ1TZkODL27LhTbbiPCabqGZYeyO
43dBH1jOAAwAwhz788x+W07ed1ksAgLz84PnPyt7rvqWXoLzidS+R1X8bNhkqACATx4oix39eLyCO
OeiJqsJVf5ZqR84zwquZZ0VcaKU3oAwDA4rjnl+
+xh/cDAPL10M0/kO6t39dLy+3aI14l1d99rV4CAGSucI3IMTWMH0nkGKIxbXOnbGKIWZXOvL2oeq35gxuuuHH
iTiKvZsNXn92QzxwzV1qnJeTqkAgCWjDtj/3VXXafXAADyonqjfu+aZ/TS8lIBqgpSAQA5OfUkcIL9QLi
mn1P1N6h7OuncuqEZJedlqXedUNc/9Gtl/XnRZq7pKgAgOVzy3W32EGqClQBAPlRQaoKVJfVtQ+/RFZ/eqNeAgBkTgWnKkBFMsFJmU98VEaxvRRjO4Jw/qy5ZUcw0SIAQjGtnw1uGtJJl5u0hlZpqsdAp0XcORKstRkAQRpFPgL/GTKzvtLZ9gHgAghgSIA5Of5ukcIL9QLi
mn1P1N6h7OuncuqEZJedlqXedUNc/9Gtl/XnRZq7pKgAgOVzy3W32EGqClQBAPlR9VE/96Zv2h+X1
S/8xivsofwAgJzYdVDz7rl4NOQYog7XRLXrlZZq4nZEtYfVD9UoNb/OeaRVL1Uprp0RP0YFAGA5vfm
WN8tH3/5RvQQAyIvqibqs9VFVePoLv35CLwEAMmfXQX2HXkBSc1ATNUKvISuFktS8lNXRqZVSq2H

aPu+HuNXTfpGB5WVM8uWd46hJwTA7YddqEYTfT/4kcHneY0nPYU7fC+q9b2DCO//8rEiKf0cCAt7
7s+
+V2h01vQQAyIuqjbps9VGfd82VctfvUJMbAPJztcixO/VzTGKmIWpluy/9bt3vCVptOcPst1WY2ZPGu
htwVqXlDsFvVe01qobpeqIkoSO1khtC+I9Vd0Yra9/2bgFghnqNlWAPfSBnqjeq6pUKAMjXfT/zuD1r/
7JQAer1P/E8vQQAyNzxO0UKL9YLmMT89kTtXZQdnSKU61v+RFSVLXHLmHZ2LqbU+64s9S0S1NFG
TQoGTGKR76eM295ryPpg13tgBlR9VFUnFQCQnx8/57J8/g2PLkV91Le951Z5zVtv0ksAgMwdf5PIsV
fpBUxqfkPU7oHXE8sevu/1HjWG93cOpKufTsfppZpmrVUAABaVmqm//attZuwHgJw9dv3T8je3f1sv
HU23vvpF8ku12/QSACBzhVtEjv0LvYBpzHdN1FSFz85vlhPo1M7nWItxHgxO2jWqxuPoOpD20GPv6
/VjZG3Jwf1FBNd2Pdzga0fVgsysDVEya98oca+VK/5xJm1f2OsHyncO1O806o7arx19P/kG2p/K9Ux6
DsOEt32wXungORo8P2HiTbTn8/epHqPqpCY5P0DQbdffJvf88j16CQCQl7+/7Xvyjy//oV46WlQd1O
rvvVaufO5xvQYAkK2rRY6/2/p4hbOIqSxEiFqud4fDT/uRwnDa4qac1WVWRfblcGnyBRVsDU7a1ZTV
wqr1/3hUiFMKG3rcqUlpMKBKMklYe2NETUjVYzgYFmXWhihZtW+khNcqwXEma58TyIW9vrlaGBH
2qXNitLNcl/GVM9SxDbR/6us5/f0ej9OrffAcNVdHhZyTcPbh1XO2Dd97qd3vWGrvKL6DGfsBYAb+6v
WPHcn6qL/yH15JHVQAyNPx09RBTdH8hqilk34P0YN0Bu2Ha8tuuinKQug1zvnhkTuhV7/r1Zsdzzhv
5bp0dbDd9TbQlHNeotNLMEmY9dpzesOh2+3IzkX3tVm1IUpW7Rst2bVKcpzJ2tdrrHuBnP+HjZa1F
615bkRQaLRjb1OKem0koz1u06e5ntPf70n47fHb3pHa+ejIPHqivUH+PsLvvbTud0Ds2frfdepdegkAk
AdVH1UFqUfJyumftB8AgJwce53zQGpSC1HtnmjGkFHzEWco65DimpzxspxVfxuqF6C77UQbdnpv
me1yHmYvuTOyFivhWXQ9uejO2qUCFi+kKcrmBSPEidI7lH39VDo74mY3xc09J6yxHnub+mQmmiT
MmLjHCNyKa2eGQ/XM2hAlo/aNlPBaJTnORO0LtuOst74iW/aGVVC3J2GHE2hHTNWz/rmtbPtBrd
/2JNczhfs9gWrL7yFf2Up/+4q5j/B7L8+J+bAM7v7Fu+WOl9+hlwAAefjmjU/JV376u3ppsb3sp14o/
+a3mNAEAHJTuFHk+L/WC0jLHA/nDwYcXkjrjWE1w5B0mMHN0daVAzdTKp+Ukn5qK56QU/ppJDP
kHgioh2ouTjVJmK6nGTZ8Prc2REmpfSMlvFZJjjNR+0a3wwldR5fWOHUi6XdVVU4HNlaSk2473bYn
up4p3O9zpSwnAwcRIteJ+bAMrrriKmbsB4AZuP9V37HD1EWm6p/+
+n/5GeqgAkBurhQ5/mvWR+qgpm2+a6IWN2UvbMitPdQ3hXqoHtWLri8pZ7JH3OhefG5oM1EPZ
MWu5eiGPlE1KzNsQ5R5b19sebQvRuA3VlFOLEzSmcbxAvPpphfcZAepKlAFAOTn8yuPylNXX9JLi0f
1QFU9UQEAOTn+r0QKN+gFpGmqENUc9hv1cMLJoj8M2gxA7aBUrw9NMc2v04+4dRUtdp1B82tD
H2kGskvEuHZ+7Udf89zw5DXjJwlry4bZq9OtjWnWijRl0oYo2bcvLbGOc4btG21wgreeHHp1B4ZlOv
HcEcD5QZpuv+l2e2g/ACA/KkBVQeoieI3l/6IOKgDk6dhrrQd1ULMy3z1RkZGQ4dEus05mDGp2d9V
b0Q+rjZ7D7raTTBLW3vV6dtrhjxuYG8OTB6XehihZtW+khNdqguOM176Idth1itOdfT7Y9JDh+ImO
M737fWHkNjEflpGaZOoDb/iAXgIA5EEN6f+7Vz6hlxaDmoWfOqgAkCM1C7/qhYrMEKIupaKs+bN2
yao3Ztuc0XsMPcGXKlEb7K0YEnhNOEmYH/4YM+KbcmhDlFTbN1LCa5XkOBO1L6Id9rE7NVUTnsK
Rmqsb4jfdL5lQPrPmhNaJrmcK9/uiyeB+B0wf/vkPy+nbTuslAEAeVH3Ux65/Wi/NN1X/tPp7r5XnXX
OlXgMAyJaqg/oe6+PVziIyQYi6pIqbF/zehs1VHawYk86MU9n2h393alJygxmjRqg/UVew9mbkJGGV
09aSNq5dWbUhSmbtGy3ZtUpyrpO1b2w7ynXZSmV8eFnK5aas6vZ4Tbfaftab/j/Z9Zz6fs+LOdGV28
6Jws6U7ncgwj2/fI89vB8AkI9Lx/vy+Tc8Kj9+zmW9Zn79Uu02ufXVL9JLAIDMHVsTKdysF5AVQtSlVZ
TNvcFJu6rS6res/8djD/8OK6Rpb2dgoi679ubg/ixDk4RVZHuoDc72vF01d71eitm0IUqG7Rsp4bVKcJ
zJ2qfa0Zfu0IbVpo3SBlM7JWcHjzfsGiW6ntPf7/mw7q/B67F/aPQSTiCV+x0YzZ2x/7qrrtNrAABZ+8H
zn5W/ev1jemk+veatN8nb3nOrXgIAZO7Yq0SOv0kvIEuFviqCCAAAMIF7H75XKn9UkaefXYwhpgBw
FNzx5evlIQ9eq5fmx7UvvUr+06ffzDB+AMhL4RqRK9R8BQzjzwM9UQEAwMTefMub5aNv/6heAgD
k4W9u/7Z875pn9NL8qP4udVABIFfH3239jwA1L4SoAABgKu/92fdK7Y6aXgIAZE3VR/3cm75pf5wX
v/Abr5BXvO4legkAkLljbxcppFNUD/EQogIAgKmp3qiqVyoAIB+qJ+re676ll2ZLhae/8Osn9BIAIHPHiiL
H36EXkBdCVAAAkAo10dQt192ilwAAWeve+n37MUuqDqoaxg8AyImqg3rsPXoBeSJEBQAAqVAz9b
d/tc2M/QCQI9UbdZb1Ue/6T6+2g1QAQE6O3SlSeKFeQJ4IUQEAQGpuu/42ueeX79FLAICsqbqon3
/DozOpj/q299wqp/7ZDXoJAJC5428SOXabXkDeCFEBAECq3lF8BzP2A0COvv2if7Jn7M/Tra9+kfxSjV

/kASA3hVtEjv0LvYBZIEQFcteWjUJBCsZjpdHTn1tSvYasbLT1gqO94Z6fFZnX09NrrDhtXGnIqCb6x+E8
lv5aY2mo2frfdepdegkAkLW/v+178o8v/6FeytbzrrlSqr/3Wrnyucf1GgBAtq4WOf5u6+MVziJmghAV
yFVPGiur0tRLjrKcWSvq58vHDiJLNeno5YXRa8h6LarV6loXZDV4saVTK0lhIDAGjqq7f/FuuePld+glAE
DW/ur1j8kPnv+sXsrOXb9zu1z/E8/TSwCAzB1XdVBfrBcwK4SoQK66cuDmbuW6dPt96ff3ZHNZM9
SxQeScUj1nxwW/7fPiHlq1pa5zX7r1srOiuSrkqFgGV11xFTP2A0COfvycy5nXR33zr9wir3nrTXoJAJC
5YyvW41V6AbNEiArkqXco+/qpnDohy9v/dHHF6znbk8Y53QW1XJetivO0uHlWqs5Tae6SomI53PSC
m+wgVQWqAIDsPXb903L/q76jl9L1sp96ofzKfziplwAAmSvcKHL8tF7ArBGiLjGvnqP5CKntOLI2peqN
536d163Or/dp135sb+ivNdbZLwuuD/bKS2MbmtlG7zFcYzN4jMM1S+1HWN1Low3jal3a+zDDt+aq
87WDDQ9pc9i2o9o8tpfjROdl+J4ZuZ8YxxD7fGh+W9RjuK0OZwi9/7p0z506/lKsnrNGj+NAWF6Sk7
ozquwfDt9PNqM9ie65eMduS+H7wj1Pcd9HsNxuv+l2e2g/ACAff/fKJ+SbNz6ll9Kh6p/+
+n/5GeqgAkBurhQ5/mvWR+qgzgtC1CWlwpHQMKhTk1JhQ8ZlcHHYtR8HCkLa61TIMrC+uRoeXk2
1DRU2hfYY7EitNCqEU58zapaW61J3uw52duTiYMi0674ynbqmQ8GiZh/zyGBquM1uz8dQE5+X4X
umuToieEt8DFGcfQcvd0hb7WCw5A2hd6V67jxVafVbXq/SIWaP41E6B9LVT4MqcjrpPZfk2FP6vlDn
KY/3ERwdapKpD7zhA3oJAJC1z688Kk9dfUkvTe/f/Nar7J6oAICcHP9XIoUb9ALmASHqUmqLn8O4d
TmNeo3SlHPhSUpi5XrX3na/FYybwtaPGt482TaCw6mHj7EjO4PplEcFZM7r+3ubsrlVF+erBr/GPI9nZ
FyGWtm2ttd1t2Wptpx9bOvUrr3hB4Vhbe7UZH3kdQm2eXRT0jkv/invSO28cd0SHMPY8xHg7zu8r
dZxrbvBoHEu3Iamcu40u53bEitrHVKUE6f00wiVRPdckmNP7/uimOP7CI6OD//8h+X0bQxHAoA8q
ABVBalpWDn9k/YDAJCTY69zHpgrhKjLyOwlZ/R0K27uOQGJ9dhLZaajqpx1t1M5bS25RqwPHd486T
aKsrlnBj6O4toZL7TrHIT3AyzXt4IBWXFNzugv6uxc9NvY3vV65pXPrI0P38YwexjWLxht3rwgfgZ5PrR3
31CbR5r8vFRbfnDoh3xB0xxDFHPfoW3tXZQd3SEycC4qW/5+zWtniH/u1HFY3yOhIe8k9uUwrEFKk
nsu0bGn+H2R2/sIjpp7fvkee3g/ACB7akj/V376u3ppMqr3qeqFCgDIiV0H9V/rBcwTQtRlZAQ0ImqY
bsGrZTiyhuIkyielpJ8GjFofJo1t2HQ9x9BhzEGnTgwGP0VZ8xMtLyzyA0Mj0J1YVK9WY/8jgrfhNscV/7
yMN90xjFaWk+MudvfAa79X7sF+GEPcRwyfn/zcTeuUjN51gntuimNPcv2HzlNe7yM4ctwZ+6+76jq
9BgCQJTXJ1KT1UVX907t+59XUQQWA3Kg6qHdaH6mDOo8IUZdSUTYvhPckdEOYqMl1FkZg8hyjn
mOk8MDO76nnDnc2AsPq6dg9GafXkeGOgjFCRlOK52UyYccwK2keVxw9ORxbLNWR2T2X2vVfkvc
RZOKW625hxn4AyNFfvf4x+fFzLuul+H6pdpvc+uoX6SUAQOaOrYkUbtYLmDeEqMuquCl7esjtQKlR
W/Pcos+u3ZYNs3edW7PRrMGZhHW+zurzZA+NNoYyV0/nF6FOH/qlfF4mkm1w6dXKHXpMWsd0
AsUTMrb06bje1BPcc+OPPf3vi6P9PoIsvfmWN8tH3/5RvQQAyNIPnv+sHaQm8Zq33iRve8+tegkAkL
ljrxI5/ia9gHlEiLrE1MzaqqeYPcGPHYR0vRqKo4f/GoxhxHPHrB2pgiW3/uMUba64U6Z3duT8+R29n
aqkk6GW5KR37gdnZO/JRbfoZeQQ8BgyOC+
+nI4hTOmkFwKOqumZL+NcNHfF75DZlQPvNJxwzn2EWPdckmPP4PpP/T6Cpfben32v1O6o6SUAQ
Jb+8eU/lL+/7Xt6Kdr1P/E8uet3qF8NALkpvFgP48c8I0RdRu0Ne6itmkU92FPMCHiMXnIlPxkzZmJvy
4Y3Dft884MlY2bySXgTWHWk2dQnKrWh/EXZdLsdqvO87l+XXmPdq22ZZBKkcVI7L578j8Fj1udsrvr
DyPW9bj9yHVtu1oBtyq7eda9xzgsxY/VgjnPPTXjsU1//hO8jZntyvRSYe6o3quqVCgDI3t/c/m157Pqn
9VI4Vf+0+nuvleddc6VeAwDIlqqD+h7r49XOIuYWIeoyqmz7Q287NSm5QYtRH7F6NnzmbhXSDL52
Lpkz9nttNibamUhF3I6BrlSH8o+4LiU/fZQL005glcl5MSQ9BnPYu9ueiRK2YH3O5qqz34IX9Felldqs+v
EUNy94PTLd9njnodqSeM2Jc88lOPY0r3/C9xEgiqqPquqkAgCydel4Xz7/hkcj66NW/u8idVABIE/H3k
od1AVBiLqk7KG3YUUMVeDS7wcDHlX3cLBmYrWVcx3NpCqy3W/5gZHNOTbvsAPDrOPxhlcr5bpsp
ZzL2dcl5LxWW9Z6d+j1VLI5L6Zkx2C1Z/A+3D80ejUmYNfnNIaSu+y6nznWQ/UUZXNvuFaoPYw+Q
aAb656LfezpXv9E7yNABDVTf/tX28zYDwA5UPVR7/uZx/VS0Cte9xL5hV8/oZcAAJk7Zv2GfPwdegHz
rtBXRewAxKNmNtcT86gwbG/anqHAOEfpntPHcqpFwIpwn+l9Rip/xM0BAHl441/fIKWHXqiXRK596
VXyW3/8z+yPAIAcFK4ROf5+66P/Xoz5Rk9UILaeNNbdmc3LcmaNABVZO1r3XPu8dSwZ9ODG0fGO
4juYsR8AcrL3um/J9655Ri+JVH/3tQSoAJCn4+8mQF0w9EQFxmrLxmANWFXOgK50yMzRu+d6jRU
p7ZyRbiplKXDUFf6fgn4GAMjStU9eKe9s/aS88723MYwfAPJ07O0M419AhKjAeEqMsc9h+

WlJiiblx9NaEs42hKOtoSjLeHmqS0nrr1GDr/3pF6aLa5RONoSjraEoy3haAvSQIgKAADmBj/ghqMt4
WhLONoSjraEoy3haEs42hKOtoSjLeHmqS1IhpqoAAAAAAAAABCBEBUAAAAAAAAAIhCiAgAAAAA
AAEAEQlTkSE2WU7DrfxRWGtLTa8fqNWRlo60XHO0NvZ3CijRibwjp6Uljxb0GG9aVTbJuGmH3UNr
7mJbRRv1Y4SYFAAAAAGChEaJirvUaK1Io1aSjl4H5pgJdY1Z9W1nOrBX1cwAAAAAAsIgIUTG/eg1Zrx
GfzqeibO717RkF+/1tqei1szFPbenKgXvLluvStdu0J5tkqAAAAAAALDRCVABIS+9Q9vVTOXVCyE4BA
AAAADgaCFGXmao1OlC7MazG6GD9UXuIvfE1A+VKNbNOpXokq1Vp79Mcxt9cdbYTvjOjjeoxqk7qY
JvCa1VOdrymMbVf2xvetob2H3JN4rTRY359ZEONcxFo46j1xj7t9ea5zKoOadx7KKotg9uYsD5pjOuS
9J4FAAAAAACLgxB1WakgL7TWaEdqpVEhpPpcQUoDQ+ybq4OvVyFiSYIva8pqYbBWZBqcNq0GNh
xyDHYINtgm65W1UnjQaYt7vIMqcrqqn3Z25OLAa9u7bmODtTKHQjgtuo2TKsrambLztHMgXeeZxR
iOHmh7W9xml8+s5dDDMoV7aKJrPizf6wIAAAAAAOYRIepS6knjnJuIuXUb+9Kt61BNOrIzmPx5qtL
Sr2+5QaEKG8/7ve16jXN+0FVt6VqVXfE2H0Nl2/qabl28L3G3sx1W8dJvU/gxWMe77oZg/mv77gF0
arI+MhUdf7xhKltu2wfPpR9GSvmMeBlqe8MPgsOuSWQbJ1NcO6PbuC+H7qbbu0ZI2ZEDN101hq
mfOpF9hDr9PTTNNTckuC7J7lkAAAAAALBICFGXkjERz96m16vQD9VEOl56FlRt+RP3+EGhqScXd9w
+e1VpeQGStc8LYa+fntmm0GPoXRS3SeX6lj/xUGXLC+U6OxdDexSOP94RimvidfQ0t22ElGaPTrN3av
2CcU02L/htrJ0fGKo+Ja+NftDbO3Si0mrVCRv3dbrau7jjBZKnvROYlRTuoSmuuWkm1wUAAAAAAM
wdQlRYdA3P0OH9SZmzk5+Ukn5qK56QU/ppespyMrCTEN0D77jsIdheXUtjqHdgSHsazOHy/rB4P5
SryllvyvYRvVNtxnbMHqOpKMoJfUHcwLlrXzzrnJ4+aYeVwfWW6mk/kMxMCvdQKtd8VtcFAAAAAAA
DMG0LUZRWYKCdunckYgeWRMt3x+r1i3Z6eRig3URhpDK9PScUt3trctVrXE6cj6ik5UdFhZWC9anb
2EeriSf+6AAAAAACA+UKIupTasmH2OnVrPZr1HI+ocr3rlDEYevjD9lNT3JSzbglONXTcqCs6WRiZQYh
dOS164L4ctvUQeLv3pzs5lrF+QUP07K/5sv1xAQAAAACA5UOIuozMupwqYHLrohpDoCdXkpNuEjs
4XNoIEXNVcoamK6NqvWbF6+nZ2ZHz50fVFTXP2eBs/mZ90FMydk6nxNfQ3XdHDnadr3VrtZbsT/jr
h4e0ZyWFeyiVa57idQEAAAAAAAuNEHXJ+QGTMWP/VMxakU1Z3XCn3DFnS8+ZMcmTNFfFa1J7w
6+V6a1MmdfTsyPNpj76oaH8Rdl0u6xar6utN6yz5eg11r0anuYESU7AqVivP+8dkGx4U8nH5V+vZtP
5Wnf2/aIumOquNyfCylYK91Aq1zz5dQEAAAAAAAEcTIeoy8oI9S3NVh0rGhDtTMmcun2r75iRC7nY
mCjuDs7o3V3WI5gWO5gzwaXOHxftCh/JXtqXl5XU1KdnnrCAlP6mTC95EVCojdOutWrxzHLe2bVBg
W9Yzb2i6eZ9Y68/k0w3VNv09lNI1T3hdAAAAAAADA0USIupQqst1vGQGZUpVWv+8HRvaEQpMqyu
Ze1w/BbGr7g/scx2qn1yBt/9DrDZhIcVP2+oNtstj1YDOoh2rwhvQr1v62Ruysst0PrUtbbVnr3ZILLnU
8g6+ttiara2v22gwMTTeGs+c+ZD2Feyila57ougAAAAAAgCOp0FczrADITq8hK3oiL1WDdo+eiwAwk
urtPS8/mtCWcLQlHG0JR1vC0ZZwtCUcbQlHW8LRlnC0BWmgJyqQKbOOZ75D4gEAAAAAAAJAOQlQ
gE23ZKAzU8ayeFTqhAgAAAAAAALB5CVCAPql5pZpNXAQAAAAAAAIEvURAUAAHODelXhaEs42hKOto
SjLeFoSzjaEo62hKMt4WhLuHlqC5KhJyoAAAAAAAAAARCBEBQAAAAAAAIAIDOcHAAAAAAAAAgAj0R
EV2eg1Z2WjrhVnrSWNFzZavHhsyL60CAAAAAAADA/CNERSZ6jRUplGrS0csAAAAAAAADAoiJERfp6D
VmvEZ8CAAAAAADgaCBEBQAAAAAAAIAIhKhLqr3h1gddkUZPD7+3l51HaClTVePUeI3zcL7eZW/XH
MbfXHVepzc4uF+PuW1j58HXt2XDfY318F4Wo12TGjwv6jHpuVGSHr8S1obCSkPCD8+s/eo8VkaciG
TbBQAAAAAAAWF6EqEuvI7VSQUoDw+
+bqwMhX3tjRI1T9fXpBJbR1H5WpamXpFyXrYr1MbN2OWHk4HlRmquFYNCZ4blRoWtYG6RTk9Lg
BFl2EFuSwZd3aqWhcDTRdgEAAAAAAAJYcISosVWn1+9K3Hq2qXqUCwPNulNaTxjkdX5br0tWv7dbL
zjrrtTsXnYiusm19rlsX9zNSbdmv7W+rxHNafjv7e5tSTNCupHqNdS+MLNe7zj77LasFWvOcDkeza4NI
W3bd1Dh020055yW0VjvW3SDXOE/uBe3UZN17bZLtAgAAAAAAgBAVUm1tixtxVraMANRTlM09H
crZ4aVeu3bGe23noKufZadc3/La6ciqXT25uOP20qzK2U13yxXZsoNGFVLuibM6w3PTO5R9/VQ6O+
JmscXNPWd/1mPPbVvvorhNDpynypa42Whn56J1ZJYk2wUAAAAAAAAAAhKiaha5OGDmHPzqkT44K
9tNrVlQN3A+WTUtJPFSdo9EPnYSmem+KanHGTWGtrquyCW7t0qM5p98Dbnz18X7+uYA7v7xxYR
2ZJsl0AAAAAAAAAAQoqIsJ82UcJTAxElGbdLcjGjnzNtlyawNRdm8ENYz2A9KQye5Giur7QIAAAAAAABx
NhKiIoS0bZs9Kt46mWft0JuahXRm3obgpe3qIvV+v1tc8Nzybvl/DdfBh9KCdYLsAgLzp0Q3Ggz9yAQ

AAALNBiIrx2rte70o7oHNrfxpDyKc2ybYya1dJTroJqDsE3qVm4i8YM+6n1YaI16uZ9NUvzfakXXbw2f
XqnHrtK530Qtu4NVhjbRcAlpH9Xl+QwkwTy540VlZFWu57tPNHr+bqLILU+Qxze40Vpz0r+f/hT/0ba
p4P9zEP5wXDhq7XDO6ZmRvzvuZ9P7mPvG7mOO+3ebwnR+5jSf+gFfO8z/K9eGZinJuh951luGnG
nJfc34sTtofSdoiDEBWJ+AGdMSt9AiU/nTRm/7d+MFmdbhD8tO0KKsqaVzS0Kavem667baeO6OB7
cZw2JDp+/aavPhXsGRpSs9Wsc9pc9dvm/sOhHu7KJNtVjG3wDwuALMzsl/chKri09j/lv0npcCYu3Da
KcLuTP+4f5vte3N4IC3ONPyjOQq8h6zWRatX9x28G3NEnxsO8XrMy01/cAyWOhh+z+NZW52O1qS
YFda9TS6qdmpRmFPjk/0v7+Pc19R5csr6f6l3jHFk/T2Z778R5v83jPXncPtTnc/yDVmTok1eYm+C85/l
eHHFuht739CP98xP/vl3dH/g3Iot/IEadk9zfi8efl3zfiydoj/WNrUrbpf+eHO/7dn5+FsZY1g2DJWS9R/
TV5bd+A+hbPzD5uvW+9U+g87mq9ZZia/WtnxX060c8vNcqIa93P29uf9TD2NbIdtqStKvbr5fd9VXr
K8cxXx/yKNetVygJz83Exx/
+MDcdve3gMSfabqvqrS8PXwQAmEq3XrbeX8z3eP2+GngjyoffFv1vwAzaEEm/z8/8vXjm7XCuj9q/f
c28f5PzY/87OoP9RtP37dy1y/3eivPzV9rCv5dn1R7n5y9jv/pnrCy/l8a/rznvuYNtyPocjW9XvNdMa6J
9ZPIeqPdvbdd+hLRD3T/maud+Um3XK1IS/5w4n8/+vTjeucnjvS/
+fZv1+8v4cxImq7aNPy/h62fWnhHfw+nfR87+zd0737fBdX579Qr9vhz3uiJf9ERFDBXZVn8p0ksO56
821puAo7kr/t9KrNd7n9D2D52/MKlanIP1QqutCWuIJm1XEk4PIOsNTS/7rDdbf9h+0jYkPH57qP3g
ubQ5+wj8QdOuc2oMyXfZPWWMeqiWRNsFgMy05XytY71NXZBN503VUpEt9UY28fv35Iqbe9Lv7x
ltmTO69MupE/PRwFm1o9dYl1qnKmfn9kLNhnteWt7PKPPC+T6X6unAzyL5KMqJU9YH9+dQW08u
7sygPb2GqIFK5fqWv9/Ktv3zYmfnYgY9sRxj39d6h7JvfRj8fi7aJ64puxm9Ecd5v83jPXle3ved79+y1Ls
hP8tr6uf34dEJHdm5mO7dE/ec5PVeHOfc5GX8uXHeXwLf5xmY7Jxk9148/rzk+148tj0jfp6yR42mW
tYuzqii+fpZGOMRoi4pvw7mwJuLMeFQcMiBCgvdr1EPJ5TztxMM6dQPhf5rrYf5A725D/VQ+xmx35
Ht9MRtl/MGFlw3nvMG7H6d89gbakjCc5Pg+G2D59J+jDoG8zj1Y9QvU3G3a7xu+NgBYAoz+uV9M
enSL+W6bIX/A5CTnjTWa9KZVTvsoaMdqbbi/1ueGTUM0Rh6N9uSN/n84j6JXuOc9d1s/bI/oxu3sq2
HjNo17dWwypITNg/
+vJW13H5pT8huV1lOenWcNKPePgZk8AetuMFlmDTbEVuO78XTnJv86fJoB+cHhtOnWwJnknPCe7
FhxPub8/PnvuRaNYmfhRcOISoAAJgNfnmPTdUltX/5uTCLXoa6tpj9i6D+pWdGvR3b52vSqbZmPmL
C/0Op81AjV7KppRZXPr+4J+f2NDo7wwDEHTWkatqr7yOrObMI4efpl3ZMgT9oKfPyXhwwR3/Y6uy
flAvevxGqt6h6/9mYYa9C3osD9HwiwflBrO+pqedWiWHwjzD8LLxwCFEBAADmmDP5gRq2N6ueO
MFRDt36vqzO4hfU9oYzCcRc/dbuUL2Csh6WHcfc/eLePi/O6NHZXTNnsg5/YiAVeKtJgXKftGOWv7Q
jNfxByzKH78Xz9oet8pk147pY/4aeVWXUZtirkPfiAernGrdnrP89dXBKXadTkl3n7nn5IwymQYgKAA
Awp5wAVfXWmJ+hjMXNC3YNtk7tfK7hXHtXhU1NO8B1fuEpSEn9Vqh/CZrtcHptlsOyLfP1i7sOCGf5
y6Iecqzq2bt5jz0MViXezdWMZjYfZVa/tI9h93bqyMHgjTuqd9QS4w9ajkV4L56XP2x5ZtqrkPficIMl+f
qydXLfOk8nJau3vdn+EQZpIUQFAACzwS/vkVSvDSdAndMJ/zL8RSPMYE8j9bAngLR+Mexaz2dbt7sn
h6qoWc7nZKxZ/uLu9nw6O8NfFgeHTbpmdl7y/6V9rOIJceZ7CUZNPfuGnmG4O2f4g5Zvvt+LB+T+h
62S2GWOB8PbWf5cw3txTLq2eOCPkekZ+UcYfhZeOISoAABgNvjlPYKeRddiD3czevzkW+dSTf4wM
Bxc/0KW1S8ac6/XkJUVc0i2WqVmSp7lL6nz94u701utKjMcPSpSOW21wPoeCgyhV7dwzTovM26bL
dtf2uOpyGnVY6+27r+veL3G5m+islngD1qLaFZ/2NIjADo1Oe/9w6mHcM+oHinvxXGoUhklqUldLm
RwkSL/CMPPwounDwAAMCOtqvSt33L69a5e0a33y9aPJ2VvRX669bLVFtWewUe139KvWUZh56U
6JyfEblu53s/3bun26+Xg+ZiLe6RVHbg2rb71S2tfJT+5m+H38TB9HgKPefie1vdRxvdv3Pc1573Yf2R9
7eK0K4/35PH7CLt/3Ifxb1dq9H0x9H2r2jFw3Pp7Pu1rNcl5t78m8/fiEedGvd8M7Ns9hrTf/uKem6H
XZfY+POp+0XJ6L453XvJ7Lx7fnpB/xzO6RnHuxXn6WRjjFdT/rIsKAAAwE+5f6F3WD43zNRwQWBCq
x5xdm9BVbUl/Bl3nnO/pqrT6M5p5eS7pnk7G5ZnV9cH8Gfre9fjfR2Gvqc5r79gUjT83Id9bxnk7iuLc
LwrvxbOmRvOoOqhhgkP7+Vl4cRCiAgAAAAAAAEAEaqICAAAAAAAAQARCVAAAAAAAAACIQIgKA
AAAAAAAABEIUQEAAAAAAAAgAiEqAAAAAAAAAEQgRAUAAAAAAAACACISoAAAAAAAAABCBEBU
AAAAAAAAIhCiAgAAAAAAAEAEQlQAAAAAAAAAiECICgAAAAAAAARCFEBAAAAAAAAIAIhKgAA

AAAAAABEIEQFAAAAAAAAgAiEqAAAAAAAAAAQgRAVAAAAAAAAAACIQogIAAAAAAABABEJUAAA
AAAAAAIhAiAoAAAAAAAAAAEQhRAQAAAAAAACACISoAAAAAAAAARCBEBQAAAAAAAIAIhKgAAA
AAAAAAEIEQFQAAAAAAAAAiEKICAAAAAAAAQARCVAAAAAAAAACIQIgKAAAAAAAAABEIUQEAA
AAAAAAgAiEqAAAAAAAAAEQgRAUAAAAAAACACISoAAAAAAAAABCBEBUAAADAQmtvFKRQ0I+N
tl5raG94n19p9PTK2fDbuiEhLV0wbdlwz3vk+e1JY2Xca6xXNVb8ba00rK8CAGB+EKICAAAAODqa52
TGOemSUMHoqjT1kqMsZ9aK+rmpKJsX6tZnHZ3aesg1asv5Wkc/F6me3bS+CgCA+UGICgAAAOAI6
Ujt/OL38Zx/XTlwM89yXbr9vvT7e7I5KvksbsrZqn4eco16jXN+IFttyXZFPwcAYE4QogIAAAA4WuiNm
r3eoezrp3LqRKxeo5Xtlng5auAamb1Qq9IiQQUAzCFCVAAAAAABHTLzeqH590pVg6NpryIpbm9Orse
rX/7Rrehp1Vr119suC68NKtPqCtUJH1wEdeJ31CKsrGjyeYL3S6HYYzGPXj8F92fsp1ayzrDVXndeO3Ul
FtltujOpfo/aGXxagXN+yXjUo3vHbQto/dH0tcc5VoEar+6BWKwAsLUJUAAAAAEdEWep1HdJl2Bu1U
ytJYTVYDdRep0K2gfXN1VEBZlNWCyUxyoCqjUhpcMIpOxQceJ3F3t/IQK8jtZJRr7Rcl60YnTuHwlEtel
8JVbak7hZHba7KRqMh5/wEVS4M1gNIcvwqwA5pv3M+hoNUR/i5UueiNLhTJewaAQCWAiEqAAA
AgKNjzQ3p4vVGnVS53pW+qgPq9ax0hK1v7o5oh1dLtC/
+y5tyzkv7etJYd0PBqrT0a71td2qyPjIpNl6/F2OSpvaGePmv0a6um3ga+6psW5/r+hNFqRqm9n5iDc
MPTjLVrLnHV5b6hcF2Jjl+67VuGhvWfmsrOxfjnqu27Eadi8A1AgAsC0JUAAAAAEdIUTbdGYwy641al
bNuj8nKaWvJNWL9/mFoL05zBnqzXmhn56Lz+t5F2dGdIQPD3I3enN5rB4QPix+t7aeGgTCzuHnB31
ftfDo9MAOTTGnVs8OTUiU6fuu675lBqKO4dsYLbDsHXf0saOhcmfVeOzviZq/FzT1n+9Zjb+QMWgC
Ao4oQFQAAAMDR4oVsGfVGLZ+Ukn4aMGp9qKqcDiR3JTnpp31ix33dA90L01rllguwH8bwdve1A0
6dSBLymT0vz8ha4EuLsnbGbdi+HIYlthMITDJlPQudTGqK41fHZNc5DR3eHzR0ropr4h2yuodK7n4ja
rECAI48QlQAAAAAR8xAb9RD5+l8K8qJU/rp1MpyMn6am0BHRnTmnE6i8DlCYFIpo85ppLBzFSw5Y
HLD3NgTdQEAjgxCVAAAAAABHj9kbtRYvTsvXYK/Onhx6Y8iHebVWhx7biYbtTyercHa88cfflg2z16lby9
Ss3ZpEcVP29D4Gyt7amueYpR8Alg0hKgAAAIAjyOiNmpQxjDw7g706u3Lg7tTtmVk66QWAo+p5ps
MsJeDXAHX05KJbmFROSaIqAdNKcvztXa/nqR24unVRp7iWaoZ+1ePUnkjLDlS7Xi3W0WUEAABHF
SEqAAAAgKPJmIAoTMlPDo3aqW3Z8Kapz1ZzdcObqKm94Q8/L59ZcwJAszZnc9UfQt7e8Gp0FlIZV2
4Gzta5WPd7WfYa61790aSTVU1twuP3A1djxv4k9PbVbRDscRoSdAMAlgYhKgAAAIAjKro3qjlzuwrp
nGAubi3NaZWlXG7Kqg4D/dzWmOFftd+ozdlc1cGh9+IREzJNorLtD1vv1KSk21XyE1S5kPuM9AmOv
3Lan6jKu5bGBFRJjDgX5r1RPat7ugIAlgYhKgAAAlCjK6o3qqp7OVgzs9qavI5mIqfk7J4xPFyx63gO1Di
1a3MOvE4Je+2U7GHrIcdebVnr3eHxeYt9/BXZ7psz/itVaZk1TZu7Xs/fcexzEVYMVW8zrewaALA4Cn
1V3AUAAAAAAAAAEIqeqAAAAAAAAAAQgRAVAAAAAAAAAACIQogIAAAAAAABABEJUAAAAAAAA
AIhAiAoAAAAAAAAAEQhRAQAAAAAAACACISoAAAAAAAAARCBEBQAAAAAAAIAIhKgAAAAAAAA
AEIEQFQAAAAAAAABGEvn/AZM6nr53azj8AAAAAElFTkSuQmCC]]></redcap:Attachment>
</ItemDef>
<ItemDef OID="q28" Name="q28" DataType="text" Length="1" redcap:Variable="q28" redcap:FieldType="radio" redcap:RequiredField="y" redcap:BranchingLogic="[q25] = '1'">
<Question><TranslatedText>Do you prefer Life A or Life B, or do you think Life A and Life B are the same as one another?
</TranslatedText><redcap:FormattedTranslatedText>Do you prefer Life A or Life B, or do you think Life A and Life B are the same as one another?
 </redcap:FormattedTranslatedText></Question>
<CodeListRef CodeListOID="q28.choices"/>
</ItemDef>

<ItemDef OID="summary29" Name="summary29" DataType="text" Length="999" redcap:Variable="summary29" redcap:FieldType="descriptive" redcap:SectionHeader=" " redcap:BranchingLogic="[q27] = '2'">
<Question><TranslatedText>
Alzheimer's disease - Description #[no]

If you have Alzheimer's disease...

1) You might forget recent events of importance to you. For example, if you are interested in hockey, then you might forget whether your favorite team won their last game, although you may remember watching the game itself.

2) You might forget the names of people you met recently, or the names of local streets.

3) You may no longer be able to handle your personal finances or plan leisure activities such as dinner parties or vacations. Simple decisions such as what to order in restaurants might become difficult to make. Your ability to concentrate may decrease and you might have difficulty driving.

4) You may start having trouble finding words to express your thoughts. You might need to be reminded to shower or brush your teeth, but you could do these activities on your own. You may need to post notes around the home to remind you of simple things like turning off the stove.

5) You may remain aware of the day and your surroundings. You might be able to travel to familiar locations like the supermarket, although probably with the help of a loved one or friend. If you are in a social gathering, then you might have difficulty following the conversation.

6) You may continue to enjoy hobbies such as puzzles or reading.

Now consider two possible lives. In Life A, you will live in perfect health for 9 years and then you will die of natural causes. In Life B, you will live in perfect health for the next 10 years, then you will live with Alzheimer's disease as described in the summary for another 10 years, and then you will die of natural causes.

</TranslatedText><redcap:FormattedTranslatedText>
Alzheimer's disease - Description #[no]

<p></p>

If you have Alzheimer's disease...
<p></p>
1) You might forget recent events of importance to you. For example, if you are interested in hockey, then you might forget whether your favorite team won their last game, although you may remember watching the game itself.
<p></p>
2) You might forget the names of people you met recently, or the names of local streets.
<p></p>
3) You may no longer be able to handle your personal finances or plan leisure activities such as dinner parties or vacations. Simple decisions such as what to order in restaurants might become difficult to make. Your ability to concentrate may decrease and you might have difficulty driving.
<p></p>
4) You may start having trouble finding words to express your thoughts. You might need to be reminded to shower or brush your teeth, but you could do these activities on your own. You may need to post notes around the home to remind you of simple things like turning off the stove.
<p></p>
5) You may remain aware of the day and your surroundings. You might be able to travel to familiar locations like the supermarket, although probably with the help of a loved one or friend. If you are in a social gathering, then you might have difficulty following the conversation.
<p></p>
6) You may continue to enjoy hobbies such as puzzles or reading.
<p></p>
<p></p>
<p></p>
<p></p>
Now consider two possible lives. In Life A, you will live in perfect health for 9 years and then you will die of natural causes. In Life B, you will live in perfect health for the next 10 years, then you will live with Alzheimer's disease as described in the summary for another 10 years, and then you will die of natural causes.
<p></p>
</redcap:FormattedTranslatedText></Question>
</ItemDef>
<ItemDef OID="a29" Name="a29" DataType="text" Length="999" redcap:Variable="a29" redcap:FieldType="descriptive" redcap:BranchingLogic="[q27] = '2'" redcap:InlineImage="1">
<Question><TranslatedText></TranslatedText></Question>
<redcap:Attachment DocName="9.png" MimeType="image/png"><![CDATA[iVBORw0KGgoAAAANSUhEUgAABVEAAADYCAIAAABtBTQzAAAAAXNSR0IArs4c6QAAAARnQU1BAACxjwv8YQUAAAAJcEhZcwAAEnQAABJ0Ad5mH3gAACqRSURBVHhe7d1fqF3V2e9x02jfeFQSWk9I60GjL0IwImnDgbhP0fQgjQgBQaUhqPjyyiaCpSFgI6V5ORcJihSyFUEPFBXenhMvLCL0RLG
FUFpIoOC+8EIhlX1haSBebEORLc1Fz/wzxphjPmPMecea865xr7+
+HhnKw5xxzPGHOtvZw/6857zT8BAAAAAMA8IvMDAEZybuXcNf/rGoqiUq7dh7b+

+53/Wtbbp06qn14AwMwi8wMAxrCyurLtxW0iXVAUlVRtf3bLU7vuMJk/q49+96H6GQYAzCYyPwB
gcKtrq7te3SXSBUVRSdXmE5se3XurHfizOraw78vLl9VPMgBgBpH5AQCDe/DXD4p0QVFUanXfge0i
8Jf10uOH1U8yAGAGkfkBAMM6+sFRES0oikqt7nzyJhH17XrvlZfVzzMAYNaQ+QEAAzrz8RkRLSiKSq
22HrtO/Bq/W59euKB+qgEAM4XMDwAYyvnPz285uUWkC4qikirvr/G7xS/2A8CMIvMDAAaxsrqy4
5c7RLqgKCq1+h8P/VcR75vq9L//m/rxBgDMDjI/AKB/a1fX9ry+R0QLiqJSq9uevkEE+3B9+OYb6occcA
DAjyPwAgP4deueQiBYURaVWNz537RO7bxepPlxH7r7rs+Vl9XMOAJgFZH4AQM9e+OMLIlpQFJVa
bT6x6eC9t4hI36WO77//qytX1E87ACB5ZH4AQJ/e/eRdES0oikqw9h28WYT57vXqM0fUDzwAIHlkf
gBAb5YvLfMX9VNU+vXfFv+LiPHrrXP/9/+oH3sAQNrI/ACAfqyure5c2imiBUVRqdX1xzcfvmenyPDrr
SN33/W3z/6ifvgBAAkj8wMAerB2dW3/W/tFtKAoKsF66AffFQE+rn5x4Ef/
+Ppr9RUAAEgVmR8A0IMjvz0icgVFUQnW9x/5lojuk9Qbx3+mvgIAAKki8wMAJnX6/GmRKyiKSrC+8
8z1IrRPXn/6zTvqiwAAkCQy/1RcXFq4prSwdFEd00LnpsYsavFs+8smcVdNaJwukzu7WC6z0Oc7n92
A4NZ140E/bQ3vQn1to6wEgzi3ck7kCoqiEqxefo3fLX6xHwASR+afEhOCZMKpsl9KIVVktvDLJnFXTW
icLhMyiyz1FnrVvMGtTyvzu2sj88+oldWVbS9uE9GCoqgE64Ef7hBxva869dgj/GI/ACSLzD813nBfhb+
UI6qMcJ5E59Nx2AY0TNrtdr+nk/l9ayPzz6LVtdVdr+4SuYKiqARr96GtIqj3W2+fOqm+FwAAiSHzT5FJ
/SbkmCOJp2KR2DqG+Y7DNp6Bbky3acn8mMSDv35Q5AqKohKs7c9ueWrXHSKl914f/e5D9dUAAE
gJmX+q6hnf/XcAFZORCmKAJyy15D19urrCPaKnzY+I6cIvm4Su8uc9PcS3sVx9tJdcm93Iniu8dOuq8
EWh5VmT6D96VbO2b7Y2wrrU06Bhh/YNsa5yu7UtRiylNsScyxfRtLbOK0Eqjn5wVOQKiqISrG/
+/Bs/3nObyOdD1E/2fu+Lv36uviAAAMkg809XlZMWl/QfnYzjyUgZa5gdlpRayPLQ592QaCZRR4rXYr
rwyybBq3R/ewo9Qi/JXGGzNu0j1+a/mbnQRJ2ualueZ5KFpSX3oLg9ttoavSPE7bTYd9bSy9YaZjEjau
9C09o6rQTJOPPxGZErKIpKs4b7NX63Tj32iPqOAAAkg8w/bTJNOfHGJCF9xlxhhuoh1rW1kOWjBuhL
rLylrtAzFC/FdOGXTcJX6RVUc+gBao1OF8+2XfIqd6PVkaq11Hx71rE8dxLFubTLbGaEPmKm1wc803
oEttY4kVyMHuBc0DiDcyDTYSVIxfnPz285uUXkCoqiEqw7n7xJxPKh671XXlbfFACANJD5p8+KOrUIVJ
LxKicDk2eML1PVqRHlNeWLxcV8IvuIvl5MF37ZpOUq/VpPol+rXYmXOc8hh+xi7na1VjnEw3NVdVnR
vsPyzCRyve4C2mdzrykP+RbYvK9M+w0RnXOeQzWytbMU50Am7q3B+FZWV3b8cofIFRRFJVhbj10
3wq/xu/XphQvq+wIAkAAyfwp8iVLRp+rhSkQuz6AOYUldVQwo/rywdLa4qjpiLhfThV82abtKH1Cv1
Qr0pvQmfQJ9ZRczTXVNh+Xrq2pvQ+26DsvztC65C2ifzbuiug77yngmEhd22JqlPlqdd5biW1v7SpCA
tatre17fI3IFRVEJ1uYTmx7de6tI4+PUsYV9X16+rL41AADTRuZPQSC/
+U81hLJ1hiV1WTaiHJ39oTxkjpirxXThl01ar9JHigPV6kp6kz6BvrJL3L3SV9VG1K7rsDxP65K7gPbZGie
rdNhXxjORuLDD1qpLBHXeWYpvbe0rQQIOvXNI5AqKotKs+w5sF1F8zHrp8cPqWwMAMG1k/hQE8
pv/lI5C6mhsWCqv0//zfnF1cUgfqSYU04VfNmm/Sh/Kjqg9Vef8d6KV7BJ3r7zNa9d1WF7jEHcB7bN
16NdhXxnPROLC7ovJlINka2cpvrW1rwTT9sIfXxChgqKoNGv8X+N3i1/sB4BEkPlTEAhVJvTY52QS8lx
vUlgoLKl53F/jL49Y84mO4ZdNulxVrtv8dfbWqY5NJHlZXLD03c76ZeuYxH4rC+617bP5RuQN1revTPs
NaZ3HnULeL2eKpuXn1r0FjOPdT94VoYKiqDTrxueufWL37SKBj19H7r7rs+Vl9Q0CAJgeMn8KPGGnY
uKTPmtykBlujuhoZK5pCUvVhb5Lm7NX+GWTTldZ/cUZM14fNkNDbWUXfdH6gqXnrsju7cvztC55Ft
A+mzOi+UA1rYdnVfLCtsXItVXjnSPqtXdtHVaCqVm+tMxf1E9RM1GbT2w6eO8tIn5Pq47vv/
+rK1fU9wgAYErI/CnwhB2biVQ19mArZEnhsFRdaKYz3bwNyunCL5t0u6rarXM7zBU162oaGSz9b0H
ON09NNamndcm7gPbN+kdY09dX3bC7TjekZTHNt0cNcWf0rK3TSjANq2urO5d2ilxBUVSate/gzSJ4
T7defeaI+ioBAEwJmT8FnrAj1UOXJwHZA7LTXcOSO857pTgYftmk41Xhu2GuKgTumCK7xAVL+yr954
x7QWh5jRtrXED7ZusjnCHWWr2XZzrfkPBi7E75Zfp1OYVvRmdtnVeCUa1dXdv/1n4RKiiKSrNue/oG
EblTqA/ffEN9oQAApoHMD7TzxFFgYzjy2yMiVFAUlWYl8mv8bvGL/QAwXWR+oB2ZHxvT6fOnRaigK
CrZeugH3xVhO536xYEf/ePrr9U3CwBgXGR+oB2ZHxvQuZVzIlRQFJVsff+Rb4mYnVq9cfxn6ssFADAu
Mj/QjsyPjWZldWXPxVv3pN+orxgAwAoKs36zjPXi4CdZv3pN+
+orxgAwIjI/ACAmtW11V2v7hKhgqKoNOv645sP37NTBPfU6+SP/1r9jdd/3ts7+oLxoAwFjI/ACAmgd//aAIF
RRFJVsp/xq/W/xiPwCMj8wPAKgc/eCoSBQBQURSVb9zy2TYTq9q9Os//

+OE+roBAIyCzA8AUM58fEYkCoqikq3tz255atcdIlHPRH30uw/Vlw4AYHhk/unSfzdcYZC/IO7i0sLiWf
Xn+fi76Oo78slGlNvMtYyF0vmjMX+fKGjnPz+/5eQWESooikqzvvnzb/x4z20iS89K/WTv97746+fqqw
cAMDAy/xTVoukQqUk1mKOE5u7IUfvXKCXyaAedPhrz94mCsbK6suOXO0SooCgq2XrghztEkJ6tOvX
YI/xiPwCMg8w/RQPHJfOvFOYmofl2JNQ3aC4gkbbq8NGYv08UtLWra3te3yMSBUVRydbuQ1tFhJ7F
evvUSfUdBAAYEpl/ejok2IlsxMyv92cGDH2T5weZf0M79M4hkSgoikq2vv3Tf5nRX+N36+M//EF9DQ
EABkPmnw6dlCx2KDXhqiDilJWyqmlEpG2a305o1hg3sNVW0Jrn7GntK52g3TxtdcZcZBYoVqs5s1eT
VKfspbkaznoOt92Q2vlMbYg1X7WPco3B6xp07VUbGHwvspMNt0Kr1m0UM9qXWWPcWWqLFqc
7rxmDeOGPL4hEQVFUsrX5xKZH994qkvPs1rGFfV9evqy+jAAAw1hf5jeP483P4t4htbzQkCk6qzLBD
CeCpgTlP5Wx7ppngHNPm+b3T56zpqilMy34tvUyrTlfHhMvm3ZUY66pTpnr/J8Wfbq2PXmw9Yb49
1+N8JwvTvqva1iqEtFLaV9xzh5l8VzQ3ydq3atBf9795F2RKCiKSrnuO7BdxOZZr5ceP6y+jwAAwxgh8
4vn/Ukf4u3pgtkoed67adKPvk9mlLlxVkAK3gDf/O61zvzOZfqawDvnTlsdkX0C09pjfF19O6pzrjKXNF
2jB1h9xCStK5dTmAvMnPqKjL2MpomqCx0xvaojurm5Sh+pDgR6V6OsTbjtnCU5l8l9d1ozhrB8aZm
/qJ+iZqjufPImEZjno9575WX1rQQAGEDvmd/lPOBPxAonmX7mnJJQgrI3JseZONSy+9D81jExTL+0Jv
ccEnqb1kyk1FuKOX3ESqz5mlbvLENfow50XLnNWadZRu0KM6xxog6ae1U3SY5xrsmYY4G1+K5rb6
dfBm5ghzWjf6trqzuXdopEQVFUsrX12HVz82v8bn164YL6bgIA9G34zN/vs7sJCwv10DCLPHdGxE3F
bLo86glIXr4772kghpnZPRobtme2ztPaA+W769uRZMZIjdfoK8Tt1ePXd0Pqo5056hd41iq3HNK5l7x
vekitmfdgne/
+ey4Tw+rLrBMLCqwZPVu7urb/rf0iUVAUlXIdvPcWkZPnqY7vv199PQEA+jbsf9vvedr3PNTnOkYdfc3
iWf2n9WSkxHjupj93iYH+QS7P/L5rG2b3sSaq63PaaqSzQd+OfOwpzrZfo6ct+uIrzfAuKzcLE/R5PUfzj
upC+4voJe+bdzmNa6zIeXLt7fQIn+YF+XqhR0d+e0TECYqiEq/bnr5B5OR5qg/ffEN9PQEA+jalzG9G2
QJhQ9GX5ZPoP7dflSrP3fTnLrFV/yCXZ/4O4arr7DX9TWsuK4jRvh210dcEG6vVZWP08Lb3xKZHZMp
Bzjpb57CmKHQZ2L2XHONdTusa/fe/vV2HiTusGX06ff60yBIURc1E7Tt4s4jK81GvPnNEfT0BAAYw/N
/h57nGOdQhFeT0deVl+lXLRekK3Rl7U3Jcx7vlm79DuPJd1aqvafWohaUl3ybbJ3FGVDMGb5bawML
iYjncatDa1N28PmIu8dwfLTu1jmZxveSsvi7mWOBO+a5rb+e7SuiwZvSJ/2v8FDWjtfnEpvn7L/yP77/
/qytX1NcTAGAA08j8+ojnAT+QN9xBnS5KmHtnMibCyV1W2/QEJC/f/OsJhPqAWZK9zrp+pq1dYl4E
5vQQs3oW5meuK9Tmb1u53Eg13szjX4a5sDrsH1iJ6+XcN2dL1jyBW+XMk+nQzukmd9FpzejR2tW1
fb/aJ7IERVEzUTc+d+0Tu28XsXl268jdd322vKy+mwAAw5hG5jdP/B6BB3w9UZUL3CMzpeFu+m+Pt
UdPQPKrz1R06RSuzJGawFvTy7TmtJ7ELL+a1bMjydel9U5l7KnlxC0rr6/Kpod47k+h6UrvzgpRvdz3o
mFLudDNqncvZuvUruUGdlwz+nTp75f4S/spakbrjn+7USTn2a3/9/pr6lsJADCY2cn8/thQCsWUdAXu
Zn2z4rwnIDWxb3UxvHO4qq+grdXk01YnrPFm+dVAZ0c+nQZJnma28A2xO+Yb0K/VZvRL39T2pbna
/fJZf6/29yI7EVpjxW5ejIxqJ7t0ngR94v84P0XNbt13YLsIz7NYLz1+WH0fAQCGNM3MHw4XQj0xSO
uaCvDQnzA+S9g4znx8RgQJiqJmojaf2PTo3ltFhJ6tOraw78vLl9WXEQBgSNP8ff7ALA5zTQOCGiZiPm
B8krCxPP/750WWoChqJmrrseue2nWHCNIzVJ9euKC+hgAAA4vM/F5FhHcCvSfhV9OoY+Y/GG76t
wCeSUrmBFENcez/Wr35EwjMr4ffflhkCYqiZqLufPImEaRnpd575WX1BQQAGN5UMn/TRI15K5DsA
6eADqzMT+DHhrR2dW3P63tElqAoaiZqFn+xn1/jB4CRTSnz5+qThSJ7MNcHTwIAWq2srmx7cZvIEh
RFpV/f/Pk3frznNhGqU66f7P3eF3/9XH31AABGsb7MDwCYS+dWzvHX+FPULNb2Z7fM0C/2f/S7D9
WXDgBgLGR+AEDutT+/JrIERVEzUbsPbRXROs16+9RJ9XUDABgRmR8AoBz94KjIEhRFzUQ98MMdI
mCnVqcee+QfX3+tvmsAACMi8yNM/H0M4Zfrov/yPPUXMUwy1XrV/q7+wf8iiGxnI+wpN+Y9xNza
/9Z+kSUoikq/Ev/Ffn6NHwCmiMyPsHDInyRkTivzm0alYSO/ajZSBh/tHmKera6t7lzaKeIERVHp13eeu
V4k7XTqT795R33FAABGR+bHuohUOUnIFJl/NCP2HTuDk/nRj0++
+IS/xp+iZrG+/8i3RNhOod44/jP15QIAmAYyP9Zl9jP/mLmYzI+Z9f7F90WWoChqJuqhH3xXRO7p1i8
O/Ihf4weA6SLzT0VD3PUeNjmu0H5FS+7Tp6sr3CN62vyImC78MswMzmTjxdq9U9mX2AsMql1Uu
0q3tDQs3F6cWHdNvZfdramX2HbO2bo1pppGnWzu6JmogZij+6YyXdafCc5Rqg1xz3eYAcM6ff60yB
IURaVf1x/ffPienSJ4T6uO3H3X3z77i/pOAQBMCZl/OjzByXewCnw266KOAcymz5vTpomZRB0pXovp

wi8D/FvJqbbOVOaAzdqoV/iOec42LLx1ublgs6Ze+rg1j7N1z7Xl8GDHbm+H97ZaV7S0iF1/zlpV25vb
PgNGceidQyJOUBSVfqXzi/38Gj8ApIDMPyU683hijjlkco8+YoKSM8SaxglgkhqgL7HSlbpCz1C8FNOF
XzYy42SPjFpI28yerTo63DF3Yi/3rlRHxBbW2cuzD2eY273Q2tHXr865RNyy1hZd1i+HOJPIC5wrWmf
ASNauru371T4RJyiKSr/+
+8PfFvF7/PrfR3+qvkoAAFNF5p8WnWJMiHGylCdctaarjJOnHGpEeU35YnExn8g+oq8X04VfNvENM
8fU2utj9CvPxqxDQpc75h7w0lNZo1ovdAf4LunylpnuzXstyAsjllgeahofWFvz+s3LpuXrAZ4pykOtM2
BEl/5+ib/Gn6Jmrjaf2HTw3ltECB+zju+//6srV9T3CABgqsj8UyOCj45SJn15slWm4SprkMlLzTlOXVU
MKP68sHS2uKo6Yi4X04VfNvHuRRysT6VP+jT18nYx8+qj3ZZs+lejQhfWl2sG+C7xrNIZ5ukueTu27s3
TvJl/Ux3Wb15Xag3rE9eVU7TNgHEtX1recnKLSBQURSVeNz537RO7bxdRfJw6cvddny0vq28QAMC
0kfmnR+eaIszoGFSFNX88E/GqQwDzUZdlI8rR2R/KQ+aIuVpMF37ZxLsXcbA+lT7p09TL28VZYrcle+Z
yLzRHBHeA1avLzP6dZFo6+vrVNE5cadvUJHdGnddT+OgpwjNgdGc+PiPiBEVR6ddtT98g0vg49eGbb
6jvDgBAAsj806SyT5aedMKxIo0/numB6minAOZRXqf/5/3i6uKQPlJNKKYLv2zi3Ys4WJ/Ke0UL/zV6X
n2025I9c8kL9ZBMOcqd2derw8xNu2/t6OtX0zBxpX1TXdZvWNMVyotaV2Hxz4BpeP73z4s4QVFU+
rXv4M0ikA9drz5zRH1rAADSQOafKhVoFhYXy9BkZyYTpOyUI9OVJz2ZkNSU+3JqHvfX+Msj1nyiY/hlE
98wc0z1qo/pOHGNnLHgTNRtZs+NFRe6I9w77+vV5S3zjMm0d2zdW9OC9JTtLTxD3DGZ7Jj1qt63d
ZmF0AyYkoffljECYqiEq/NJzY9uvdWEcuHq2ML+/g1fgBIDZl/ukxaKog4Y07qeGVyj8lb5oi+1powGI6
qC32XWnlOZK3wy0bOOq3+qlnTzPoKs7xAqw53TPZpoGdqvhNyPdWCq5l9vapx6qB13/UwT/dMe
0dfvzqne/1Ah005MzjrNweq5YsNNc/RdQbPAYxg7erantf3iERBUVTitfXYdU/tukOE84Hq0wsX1PcFA
CAZZP4pM+kmo1NVxT5bsVOOFcokdzpbdaGTovwNyunCLwOaF6q6OVP5r2hptI47FpzJkyjlhf5Wu
Wrm+pjyePOdMBd6uudaO3bZW0N71arDptaxfqlaln+S6nz7DA33CENbWV3Z9uI2kSgoikq87nzyJh
HOh6j3XnlZfVMAAFJC5p82E26asks9HVWZx7AHZKfNS89QmzvOe6U4GH4ZZgZnsvEitIW7FzrGu/p
FcmXdluxJlJ4L7WSaH9WvrantIWYye4XZWGdmT3cl3LHb3uQdEm3CLQpt68/Z0+TcJdVXEd5rrj6DP
uteh6GdWznHX+NPUTNX9x3YLiJ6v/XS44fVdwQAIDFk/mnTwYfoAmBWvPbn10ScoCgq8Rr0F/uPL
ez78vJl9QUBAEgMmX+6zP/USeQHMEuOfnBUJAqKohKvb//0Xwb6xf6P//AH9dUAAEgPmX9a6v/h
svsfPgNA2va/tV8kCoqiEq/dh7aKuD55vX3qpPpSAAAkicw/LVbmJ/ADmEGra6s7l3aKREFRVOL1wA
93iNA+SZ167JF/fP21+lIAACSJzA8AiPTJF5/w1/hT1GzVN3/+jR/vuU1E97j6yd7vffHXz9XXAQAgVWR
+AEC89y+
+LxIFRVGJ1/Znt/Tyi/0f/e5D9UUAAEgYmR8AMJHT50+LREFRVOJ1z2PbRIBfb/3nf5xQXwEAgLSR+Q
EAkzr0ziGRKCiKSrwe+sF3RYzvXr848CN+jR8AZgWZHwAwqbWra/t+tU8kCoqiUq7rj28+fM9OEea7
1JG77/rbZ39RP/wAgOSR+QEAPbj090v8Nf4UNVv1nWeuF3m+S/3pN+
+oH3sAwCwg8wMA+rF8aXnLyS0iVFAUlXJ9/5FviUgfrjeO/0z9wAMAZgSZHwDQmzMfnxGJgqKoxK
v7L/bza/wAMIvI/ACAPj3/+
+dFoqAoKuW68blrn9h9u4j3bh25+67PlpfVzzkAYHaQ+QEAPXv47YdFqKAoKuW67ekbRMJ368M33
1A/4QCAmULmBwD0bO3q2p7X94hQQVFUyrXv4M0i5Nv16jNH1I83AGDWkPkBAP1bWV3Z9uI2E
Sooikq2Np/YdPDeW0TUL+v4/vu/unJF/WwDAGYNmR8AMIhzK+f4a/wpaobK+4v9/Bo/AMw6Mj8
AYCiv/fk1ESooikq57nzyJpH533vlZfXzDACYTWR+AMCAjn5wVIQKiqJSrvsObDeB/6XHD6ufZADAzC
LzAwCGtf+t/SJUUBSVbG0+senRvbdmgf/Ywr4vbnqGbYuy/
yfXrigfoYBALOMzA8AGNwnX3zCX+NPUTNUD/zPHeqnFwAw48j8AIAxvH/xfREqKg9CgKpKudSPLgBgxvG
FDgAYYenzp0WooCgq2VI/twCAGccXOgBgPCJULHQAwkmuuGekfOjSKRqNo
NIpGo2g0ikajaPPXaCPgVgIARsITSTQaRaNRNBpFo1E0GkWjEQK4lQCAkfBEEo1G0WgUjUbRaBSNR
tFohABuJQBgJDyRRKNRNBpFo1E0GkWjUTQaIYbBcQAYCU8k0GgUjUbRaBSNRtFoFI1GCOBWAgB
GwhNJNBpFo1E0GkWjUTQaRaMRAriVAICR8EAG4lAGAkPJFEo1E0GkWjUTQaRaNRNBpFo1E0Gk
WjUTQaRaNRNBohgFsJABgJTyTRaBSNRtFoFI1G0WgUjUYI4FYCAEbCE0k0GgUjUbRaBSNRtFoFI1G0WgUjUYI4FYCAEbCE0k0GkWjUTQaRaNRNBpFo
xECuJUAgJHwRBKNRtFoFI1G0WgUjUbRaIQAbiUAYCQ8kUSjUTQaRaNRNBpFo1E0GiGAWwkAGAlP

JNFoFI1G0WgUjUbRaBSNRgjgVgIARsITSTQaRaNRNBpFo1E0GkWjEQK4lQCAkfBEEo1G0WgUjUbR
aBSNRtFohABuJQBgJDyRRKNRNBpFo1E0GkWjUTQaIYBbCQAYCU8k0WgUjUbRaBSNRtFoFI1GCO
BWAgBGwhNJNBpFo1E0GkWjUTQaRaMRAriVAICR8EQSjUbRaBSNRtFoFI1G0WiEAG4lAGAkPJFEo
1E0GkWjUTQaRaNRNBohgFsJABgJTyTRaBSNRtFoFI1G0WgUjUYI4FYCAEbCE0k0GkWjUTQaRaNR
NBpFoxECuJUAgJHwRBKNRtFoFI1G0WgUjUbRaIQAbiUAYCQ8kUSjUTQaRaNRNBpFo1E0GiGAWw
kAGAlPJNFoFI1G0WgUjUbRaBSNRgjgVgIARsITSTQaRaNRNBpFo1E0GkWjEQK4lQCAkfBEEo1G0W
gUjUbRaBSNRtFohABuJQAAAAAA84nMDwAAAADAfCLzAwAAAAAwn8j8AAAAAADMJzI/AAAAAA
DzicwPAAAAAMB8IvMDAAAAADCfyPwAAAAAAMwnMj8AAAAAAPOJzA8AAAAAwHwi8wMAAAA
AMJ/I/AAArNfZxWsqi2fVUQAAgNSQ+QEAc6QM48Om8ItLC1WHIRuO/W8W8o1lFpYuqgN9qm2
mwL8rWa/aPRzmbRpcww+M+uyV+v1kBH5EA6fWyz9V7R3rd1vDCt6ZQb8oBtS8qVl9nxp2VNtOX
29Th14z+p20QZD5AQDDGvBpvsbqM+ZDW9F2iGed7FnK7KN4rhr4gSrfyMLiYvb/DNIn38Loj4T282
j/n4raJ7sy0Kev2MtInwj7vvXXpPEntP4zVN/oRBo7Bk+tV9NU+XFzoLylk7byz2K/XRO3aL0z+flevyh8
m6ptqTDZvgKbKk71+KPkbsdqbhtoR0V/c6h4NenuuvXqoVV57xRxf2p3UZxDB2R+AMCA6s9T9aeR
XulGxf8/5gNBfYdDGbxL3kDdvmHa5O/94LfJNs4bU1O0HOjDJ+cerlffz/GKfj/chcuPRl9ba+4YOrVeXa
fS49TLdSuuL9W7ZLfPHJj87WrbTn5cnZ6oTym0qT7mVwKb8hyK17gdYfKmzTuSRwbspU+ol5O+a/l
0Zvrik1y9rLcqTk60p42IzA8AGI58BnCeGno3fAdhnMePgbdlps//EP/QFjLZ4+C6jf45yAz7UajfwMH2
V0xsv1F9v3HOyt2t9HwjA/eqx9vYNlVxPvpG6quH7WLxNzJH8z9M3EYv1tNrmK8Lt1Ev+1AC26nr8f
PdeutaF9OdM5Wzjx6bmbtZvJAfhz4bbRhkfgDAYNx/Mvf4tOM38sNAsZ+
+nhkb1R5/+mfds/yPwzQq7lRl4Hs23D4aDfwmZcpbmLUo/zDMp9z5CbU+Hb1w5ss71u9bzy0D0/X
YqW0q58ZGaevS24Z8E1nH8j/29mH39HI/FH1wGxV98l9UMCbv6rt1luJ0X3vz9io+anmL8g/NS1kfp
1fD7exna/Zt8jfqbWMbBJkfADAY9wnA+4jSp8Eb2IoHj76e3lzFXpQBt5TvQk+ftxxuQ1q5sSH7FJ+8n
h/lw8b54BWfuMJQrdx9uD/FE3EauPP3fC8D0/XYKTxV8cb1cBfDXYqz/bxXnkb5HvSR/HRvH4qGXp
aeWrmNxPtSDGi+v914tmMJn12vptmq+9dbK0+v4oi8ez29V8UOVLf8z/Vp+72LGwOZHwAwmCn8
o3q8ZwHxtDioXh+m6vJtVPcrbzTKntyPRq/Em1PevyE/FkXDQT939h7KPw/Tr5hb3roe36piQnvl7kf
BGTKZwHQ9dgpNJT6OE/B2KQ4qfd02p1G+h+p1frq3D0Xo1mXK7fXRzW3kfPh6+EEObqeH+W2eX
uXtKo+Vf+6pn3dfxX60xcXah2QCxbTmjXHepfA9hheZHwAwmCn8o3qkZ4HySWfEZ47y4a3/hrVHN
ksfT9ghRd/h7p/zyRu2X/HuDHrL3A4D7qj2oejvOb7k/IS6P7LOuzcZt4EROLVejVMVd7Ov7bQsuDjd
SzPZqPaZsAzRy9XTJ8Jt5EzcvpZWgSmKU319FnJOL7dD8c5NtqNS+63JR/SxO/kj47797WuBROYHAA
zG/QdzT89uzcZ4Fih6jPzAUfQc9M4V8jbDdxl+O0M8yjfr7am6mdti0B1Z+v5IuOuWb1bfOwvM12
Mr/1TF+9bjZloXXAzooWFLo/x0b5+K1k319RPmNiqO2BuRH8YIzdvpaRsWp5fbov3udtQ6UT5g8g9
FsYP6NG7nHt6mDYfMDwAYjvwnc2+PH42G71A+k0i9P39kXaxteJ6DBtHPU5srm9eatniTBn2Xig72
RoZ8RizenGE/c+4nYJSmmd4/EcVbU194bW/OWzcxT0ctcGq9PFMVh/p9k1oXXAzo4fa1NMpP9/Y
mTXVTtR+kXn6qGrczwM+s26v2w5Tpr2n4bernPSpW6zbJD9tzh5cCLzI/AGBAtQeQfp4K/Iq5hdl+Jh
A7GmczedNB3qHRd1N71O3vudc15Ke6rnweNkb4RPS5N/EJKFRbsPfW180MdAwvZl2apxLvV2nCz
RXdaivNulivi56T9Oh4Z/JhvX3onU1lB6zJyyV5FtFZeFO1s5O0UZztlIrDI3y2e/
+iaO5VPzPxrSun805T+1z3eic3DjI/AGBY9gMI/5zGaGoPpBM/jzYpPt6DzT4FPT/Hoxf1d6Wk3htxao
beseZNzeSeAu9RZu6+KHon/lVFqXpk4EFiQmR+AAAAAADmE5kfAAAAAID5ROYHAAAAAAGA+kfkB
AAAAAJhPZH4AAAAAAAOYTmR8AAAAAgPlE5gcAAAAAYD6R+QEAAAAAmE9kfgAAAAAAA5hOZHwA
AAACA+UTmBwAAAABgPpH5AQAAAACYT2R+AAAAAADmE5kfAAAAAID5ROYHAAAAAAGA+kfkBA
AAAAJhPZH4AAAAAAAOYTmR8AAAAAgPlE5gcAAAAAYD6R+QEAAAAAmE9kfgAAAAAA5hOZHwAA
AACA+UTmBwAAAABgPpH5AQAAAACYT2R+AAAAAADmE5kfAAAAAID5ROYHAAAAAAGA+kfkBAA
AAAJhPZH4AAAAAAAOYTmR8AAAAAgPlE5gcAAAAAYD6R+QEAAAAAmE9kfgAAgLScXbxGWTyrDpX
0iYWli+rIMHQf0T4F1b3JOPfh4tJC06ngOQCYX2R+AACAtFi5th5PN3rmr2J7wXcbGpN9dVMT/DcZ
ADAcMj8AAEBaav9btp1QN3rm77R/b7iv/lUAiR/ABkPmBwAASEst89sBd4NnfhPcW9Zl7p+5T+YIiR

/AhkPmBwAASEs981tB1cn8zgE3GKsh+QhrXnWBdcROw/pwdsz6z+mtJpp11jlvra1q05K5a/PVJrR
WqgXmMqOLMeaVs4PQ+nP19YghgQ0GrwOAUZH5AQAA0lKFyaXyTzoyWimzeO0esOKmisRVHG1
lQnTzNVbOlrm2YC3EM0kw+vqbyq1brLU4qsUtLuk/iu5t629ZUPMG/dcFlwsAwyHzAwAApEWHxix
B6mBaBkbrRDHQPRDK/GqQFUnlER1LnWuqI/qAbGNGmKVYfTrE3eYGZkK3Z4gZrFTTFFrXrwc0r6d
hg00TyRUAwDjI/AAAAGmppUb1wv6zGzutOCnDrB5S5dLmI3oad4QcIxNxRh4yk1hjGskV5ORO3A
NhZgE5cUmH9UtOd/8GzbBO+waAwZH5AQAA0uKP11nUdJKxJyrLaOoOaT+iX9tBuT6vHuEjGtdma
eAuKadb6qNya62al9Bh/Zb6aH2+YXazykp9WwAwKjI/AABAWmQCVq/Nr/dXGVKOzAyV+esH9Quf
xsbN/GPlVqIzv7uEDuv3hfeCPt84e8OFXVcNAL0i8wMAAKTFCZMyRJoTntgpg7E7pP2IZ1oxr2+E0G
GI4R+rW+qjcmutmpfQvjg9IlMOcrq3zmFNUWhuBgDDIfMDAACkxRMm6/HRnAiN7CHz2+G6PqZ
D/HbbNDPT2YOdHj1m/tap3EudmxLYYHbKmnjd6waAHpH5AQAA0uILkyY35syJ6qiKkyaYOkfc8Np
8pHkWM6S5sw62bpuQ5gbV9evOzoEltK1fbqca37JBZyPBZQDA4Mj8AAAAaWkJk/YJK4pKIsuGIqg
8Yl4vyNl13s34W1cD3DZhenydfXWfmb91/f7l5PSQptmbruy6agDoFZkfAAAgLQ1hsgqptRN2ds1ip
QzG7mTtR/RrezpnObl6bq4PaNhFUH0+GZLl1lq1LiG0/uryXN7Sui32ad/s9qW5risGgN6R+QEAAAA
AmE9kfgAAAAAA5hOZHwAAAACA+UTmBwAAAABgPpH5AQAAAACYT2R+AAAAAADmE5kfAAAA
AID5ROYHAAAAAAGA+kfkBAAAAAJhPZH4AAAAAAAOYTmR8AAAAAgPlE5gcAAAAAYD6R+QEAAAAA
mE9kfgAAAAAA5tE///n/AZ3ImNvxWh7NAAAAAElFTkSuQmCC]]></redcap:Attachment>
</ItemDef>
<ItemDef OID="b29" Name="b29" DataType="text" Length="999" redcap:Variable="b29"
redcap:FieldType="descriptive" redcap:BranchingLogic="[q27] = '2'"
redcap:InlineImage="1">
<Question><TranslatedText></TranslatedText></Question>
<redcap:Attachment DocName="B.png" MimeType="image/png"><!
[CDATA[iVBORw0KGgoAAAANSUhEUgAABVEAAAEJCAYAAACdcmdaAAAAAXNSR0IArs4c6QAAAA
RnQU1BAACxjwv8YQUAAAAJcEhZcwAAEnQAABJ0Ad5mH3gAAE9sSURBVHhe7d0PjCRnfef/b+/aY
PPHNmBskx+RHat7sZYlGJIg08PxL4egZ+6SJbnbYOVg5Oh3PVyi0zQSkwOxF+mX2wguG4lunS5iWrq
zliQyTEJugrLdlJRgckzHR4IxYTI/Z7rlc3T8wDYGjPljB3u3f/VUPVX1VHd1dVV3VXX39PtltaerpqfqqT/T
O/OZ5/k+k+hb5FAAAAAAAAAAChjumPAAAAAAAAIAQhKgAAAAAAAAAEIEQFQAAAAAAAAiEKIC
AAAAAAAAQARCVAAAAAAAACIQIgKAAAAAAAABEIUQEAAAAAAAgAiEqAAAAAAAAEQgRA
UAAAAAAACACISoAAAAAAAABCBEBUAAAAAAAAIhCiAgAAAAAAAEAEQlQAAAAAAAAiECICg
AAAAAAAARCFEBAAAAAAAIAIhKgAAAAAAAABEIEQFAAAAAAAAgAiEqAAAAAAAAAAQgRAVA
AAAAAAACIQogIAAAAAAABABEJUAAAAAAAAIhAiAoAAAAAAAAAEQhRAQAAAAAAACACISoA
AAAAAAAARCBEBQAAAAAAIAIhKgAAAAAAAAAEIEQFQAAAAAAAAiEKICAAAAAAAAQARCVAA
AAAAAACIQIgKAAAAAAAABEIUQEAAAAAAAgAiEqAAAAAAAAEQgRAUAAAAAAACACISoAA
AAAAAABCBEBUAAAAAAAAIhCiAgAAAAAAAEAEQlQAAAAAAAAAiECICgAAAAAAAARCFEBAA
AAAAAAIAIhKgAAAAAAAABEIEQFAAAAAAAAgAiEqAAAAAAAAAAQgRAVAAAAAAAAACIQogIAAA
AAAABABEJUAAAAAAAAAIhAiLp0etJYKUih4DxWGj29j+FFeU+1iXMXBaXtDXtDtdK
MxLO7LSlg372PzHqPs4Vb2GrGzEO5vtDbdtK5JH08KNuQ9CKNuQ9Cjmc+2g0AAAAAwPwhRF06Rdm8JJey
XurU1kPCkracr4+AHOGWdDDLLJDFVWnqAqdAx6bkdZANAAAAAwPwhRF06Rdm8JJey
XurU1kPCkracr3X0c5Hq2U3rq4B5oILBVWnqAJUdZzqxle4faf1Qo1cT/rlhsR+14AAAAAADlGiHqMip
uytmmX0yGb6X6rbm3rIYsCjqKTA8VXXKKQ4PqEJvB0k6M+Ey12kLR66Z3MgUmqjzbW6n3vYs7Fp
uytmqfi4dqZ0P9kbrNc75lVW1JdsV/9vnpsS/aHkff+RpmXdmShKwdu0OsaR3PJW3HSGLVK1yWQDCPDWgrqNV
GPu31edcGHd5J4J4zwWQN4sXZ0jHl78dUZ5o/7Z2d8QDL2s00zTZ8JK97XLk0d1b4CU09NxW3ZNv+Er8d02
GPu31edcGHd5fZI1J4zwEz5+5nbDPjxN+3INtCdS9tR4xy30ObGewraPOc7xjCm57uPZpYAh6c9VZF
2h4snM3eA7Uw9yc3Z6x+xzPPy71CLkXbAnaHvI9OHq7vqTHE6/dAAAAAAcfYSoy6yy6jZzv6AF7
VTYaDnJnUWRwM6oJwmoxlenMR+bx2+pUKnJ3ZwbS+mVeMDNbIHLmrsA2pKy9vLpHaIkjKFKjuoqdHpUpCLi0
VTYaDTnnJ6hyIWSM9FAIo3VqpQwmCrK2nnIjd2dHLno7bcuNn/FKsnPa

D6gP/AGy9izv6PBo1Pe1wrCSDo63TPb8dqZUKUhrYSXM1aUimtjPY1qasDoZtEx2T2rZR+9S6H+te1
+kREu3HCSwHz4HSXB0dKibnnOtV70AUtW6Kc9TeGFHPNGS7E4vZbgAAAAAAlgQh6lILTjLVrLnBTF
nqF0Imk2pv+KGKV4+yL103ie3UZD3lhKW4dka3b18O3U23d42JhYxw0hjqferEnAz0rpz2yiY0d4M9
Ji/u6BisfEacDLUnjXX3GlSlpc9v3+0xnOr59bfvdUhWIdlAfdzx/O1494Hazrob/E1zTMbr9zZlc9v62PX
vV1Wv1/6cXXIi2X56jXUvsCzXu85r+8MlLiqR+4wr/BzteOl/krZbr3X/0hH2PRjY7rBkxzOu3QAAAAA
ALI+C9QtyXz/HklK9SwM9zlSwEhKq+K8rS71rTubTk8aK24tOBS/OZEYjX6963bk96Ubsy+dvW4Vde
9ZG1BBs1YOwWq1Ks9kcWu+3IaxdcdeNE387w+2yV3rnwG1/6DqbsV0VnO2FBNwB4e0wr3O11fc
nDEt0PSK2E9ivvuaS7JjMbQdfr41qa6JzF35+FOdanQqsS3p+lETnOlHbRwht44jjjDietO4RYBr3Pnyvv
OXCW/QSACAP99/xLnnNW2/SSwCAzB3/NZFjr9ILWBT0REVwkikVtoQGJP5Qeb/npMsYdm/2GE1F
UU6ccp51dJfTrj2WvywnT5+0e9QF11uqp/0AbA74vWmb4nVG7R44gZT1GW8ov7dOdT4sGbUo3S
BMfcIsazBr1jUo6ac28z7QPYSnOKZEvYkT7cec4f+kmIdQ3NyTvhmgTm3wHIWY6rrrurFuwJmaGO0
GMvDwEw/LOz/5Tr0EAMjDDY9fJXd/6AF5/Bs/0msAAJm7dI9I/zt6AYuCEBVBA6FScsO1P6fl1RVt7k
pbenJoj9k/JScqJ6z/D65XGeqc9ZIrrombLbpD+ttuIl09a/TozUtaAZl1DTJrOyHeSKpXqBe0GnVjgQX3x
NNPSOWPKvZHAEA+nvPjY/KWL94oP3ryGWm+/3555p8u6c8AALL1lMilP7A+PussYiEQoiJlGYRfXl3
RfTlsXxS7lKgd9lbEyVeN9XMZvhk9NO3A1+/VOyrwVcO67VqVQ480e0lOK1mv47yOaTHOXbjxbW/
Lhtnr1K2LatY5BRbUnZ+6Ux58/EG9BADIwxv/
+gZ5wQ+vsJ8/9NXvyp/WeR8GgNz0Hxa5/Od6AYuAEBUxleSkN1LbnBFfMSZJitM70Ri+HI+7744c7D
pf686+X7I/4a8fLjUwH4qbZ/0guOFOjFWVQIZacsoTKG6Jgvk22OvYvA/0seV1TIn2Y97LA8Pk1cz3hZ
xnoE/SdmNSNTtwdeukJv6eAubL+z77PvlM7zN6CQCQh1c+eK3c/PXn6yXH5z7+kHzlLx/RSwCAzF3
6gsjlr+kFzDtCVMRUlM2zbuVUc/Z1NbrYnOl8y+vt5wScijnre1s23JlrYvN7cqqJpBS3XmZRF0x117vh
6vxxe81a56Kmj3+wdqsx7F+aq7LhnTIV7Onh297K+dBcNQLH9nm/hqd7bHkdU6L9mLVbm7Lqre/p
me+ta1Qq+NvI2oTnyA9c3XYDi+kT+5+Q+n11vQQAyIOqg/pzD7xELwX94W9/Tb73raf1EgAgc5f/RK
T/fb2AeUaIivgq29LyctSalHTA48w8bynX5YJR4NOfUMnSXNWB0GQ1HAPbsp55Q/a9of6KMUnTHP
Jqu9rKUt8KRKiWomxe8IdlN1d1gOaFzqMm/ZolJ3AMttM8tryOKdl+ipsXpO6/2HmtOZGTdS/7h6Br
7yrua1NNWBO03bzfw9odR+bHA8R339fvk7v+7C69BADIw/FLBXsYv/oYRgWozd+8Xy8BADLXf1Lk
8sf1AuYZISoSqWyH11+stqz17tBiV3FT9gZfW21NVr/R7K0XKBlgDM3OdKKjFJgB2KiyA+qc9bt+wOe
ya1/OW03PstS7fT9Yt1Wl1d8LTpaV1zEl2k9RNvf60h16sXq5MUzeVpHt4EGK7B96PbFTEbvtVlv6Lf
8+sqlzblwHu+5ulByOB4jBnYn/6Wfp7QQAeVr50kvl2iev1Evh/uFL35ZP//6hXgIAZO6y9RvZJcpbzbt
CX81aAgAxtTcK4nSSVCHqQGAKADGo4PT1/+318sAjD+g1AIA8lB56od0LNa6tu8vyiteFD/sHAGTgit
8QKfBL9ryiJyoAAMiVGsJPgAoA+VK9T1Uv1CTUsH7qowJAji79AfVR5xghKgAAyM1HvvgRezIpAEB+
VP3Tt33hZSProI6iAtS7/+NX9RIAIHN2fdR79ALmDSEqAADIxe6Du/LBv/igXgIA5OWOL18/tg7qKPv/
8zH53Mcf0ksAgMxd/n9FLn1BL2CeUBMVAABkTg3fV3VQmUgKAPJ189efL//8Czfppclc+dzjsnX36+
XWV79IrwEAZOtKXR/1Zr2MeUBPVAAAkKknnn6cfgMMrEk0kNcoz/3RJmu+/X3705DN6
DQAgW9b77aWPWx+fchYxFwhRAQBAZlRwqgLUh594WK8BAORB1T99yxdvlOf8OJ1f+R7/xo/k7g8
xKSAA5Kb/HZFL1EedJ4SoAAAgM+/77Pvk3ofv0ovpeMrf/mI3PtJ/igGALm5/
DXr0dELmDVC1KXWlo1CQQrGY6XR05/LSa8hKxttveBob7jtWZG8mzM3Qs5LHL3GinPuVhoy6tT55
9d55H7NjwLuWyCW+n11+djffkwvAQDy8vJvPE9e+eC1eildn/zPB/LN//19vQQQAyNylXZH+Y3oBs0S
IurR60lhZlaZecpTlzFpRP8+eHfiVasLfVIImPi+9hqzXor5KXfOCrAYvunRqJSlMENguK+5bIB7V+1T1Qg
UA5Ovqp47LG++bvg7qKKo+6u//
+y/bHwEAeVD1Uf+79fFZZxEzQ4i6tLpy4KZA5bp0+33p9/dkM68MdWzgt6QmPS+qZ+S4YK99XtxN
V1vqevelWy87K5qrQo4aA/ctEIuqf6rqoAIA8veWvRvtIDVLqifqH/721/QSACBz/UdFLv2xXsCsEKIuq
96h7OuncuqE5Nf/FGmL1zOyJ41zugtquS5bFedpcfOsVJ2n0twlRQUwPTUTf+WPKvZHAEC+Xvu1F8
vLHr1aL2Vrb/f/2A8AQE4uf8l5YGYIUZeQXbvRDN2aq04tx8GuiKp3o1E7c1T9zGAtyGCd1bDejbH3r
wVreI6qN+kMVfdfl6zW52A9S6+2qH6ENy3GPgPncEP8zZjnydln0vOiqHaWYvWMNHoeB0LzkpzUn

VFl/9A6olGM9obVW21v6GMZPAcJrkvI/RZ2vePcb4PXz35E1ImNY5b37WT3p2vSa2eZ6D1Ar1TMr
49uJI6QOz91pzz4+IN6CQCQFxWevubvXqSX8qF6o1IfFQBydOlPqI86Q4SoCDUUGGl2/cyRYVRHai
WjzqrR43EyanuDNTzVurCgpuQNVXdFt3UUZ5+DwWRzdcJ9Fjflgjtk3joz5/RGeo1z3nkq1y+kUEahK
q1+y+tVOsTseTxK50C6+umwipx2N97ZkYsDJ7W96x2NX1c3yXVRQV7I/eZcj1EBZPj9pu7d0GC5U5
NSIMjOSpb3bcz7M2CCa2eZ7D0Ay07VQP1M7zN6CQCQFzV8Xw3jz5uqi3r3h75KfVQAyI2qj3qP9Z
H6qLNAiLqEKtt96Xfr4kZ7Um3Z9TH72zrxbG/4AZBXL9Won9mpyfrIxEaFec7r+3uboWUCxu4/wN+
et3/pyI6XBPWkse4GPca+Wzo1imzrKP523M2ofdbOu/Fbsn0WNy+If+rOS9v677wbglnn94JOUJOd
F4P9um2ZLK8uyolT+ukYlS23beb5V9ri53BnxMnhkpwj67VGqYGh+21ofyZj2/b9ZrYlbFt+kJ3U/Ny3
/uvD789hya6dZar3ACyrT+x/wp6NHwCQv5UvvTTzOqijPPTV78qf1hmBAAC56T8scvnP9QLyRIiKIW
bPtPoFPwgdDgOHletbE4Z54aotPxwsrp3xAqzOge4z2bsoO14eaey7suW3deeiJIl7zH364ZMh8T6Ls
nnWTbuaslpwe08Gz+8kipt740PW2PblMOpEFdfkTNjxtXf9XrVn1pzjSXSOrPOzp0NEI3gPvd4Dhu43
s8et0evSPk86CNzLYfa0LO/bsfdnmCTXzjLNewCW031fv0/u+rO79BIAIE+vfPBaufnrz9dLs/G5jz8kX/
nLR/QSACBzl74gcpkJ/vJGiIoBI3qm2Yqy5iYxIwK3UyfSDKjKcrKkn47SPdC9+VSoU/LqNhbMYdKRw9
QnMMk+K9tGr0FHOsP403RKoi+fcf2NgNIP3Kpy1j2gqa6LruEZOrw/aOh+M8JCawf20Hd33yNrsaZ
uHu/bBNduyvcALB93Jv6nn31arwEA5OWGx6+Sn3vgJXpptu7+0APy+Dd+pJcAAJlTw/r739ELyAMh
KibUkeHOgTHCo7mX3TFUvMKUSrD+5Oz05HBssVSf36vSHRZuBG7V08FeoUmYExB5PXXHCbtWR
dm8EN470w0rF3t+o8nvz/SvXdh7AJaNCk5VgPrID+h9BAB5e86Pj8lbvnijHL9U0Gtm60dPPiPN99+v
lwAA2XtK5NIf6OfIAyEqJjR/gWm53vWGbAcfk9YLHS/
+Po26n7aO1NZznJyneELGlj4tn5Sxl7S4KW5lAntYuDF8vno6/CyPP0dt2TB7nbo1OM36o0lYbdzT+xj
s/as0z83XpEi53bcTXLtoR+GPJpiWGsL/wCMP6CUAQJ7e+Nc3yAt+eIVemg+qPuqnf/9QLwEAMqfq
o15iYte8EKJiQElOusnV0EzePbnoFnIcO/Q7J6WTXtA2qm5m6ibYZ6+xrodpl6Ved5OsPCfnMa5rc1f8
zphdOfAu6QmJc0m9HrXW/XH+/I4OP6sSyOGSnCOzLqcKFN26qMaQ96TUzPKqx6k9GZQdSHa9W
p7pDpOf0CzuW0usa5fme8AU1xDz7yNf/Ig9mRQAIH+lh1448zqoo3z6v/6D/MOXvq2XAACZu/xZkf
48dRU6ughRMcCcBCnYW9IPAlXYNTChz6yYNTCbq/5Q7faGHhpuPdIev510n72GrLsnrnpWNjeNyY
Nq65JPjmrWsmzKrm5er3HOCzBj90asnBbnDulls+ke18Bw8Amvix8oDvbcjUlvX80sH+xxaoTFZo9b
oz1p3yaRZnHfKnGu3QTvASU/dZXaee9gZMOb4h9Hze6Du/LBv/igXgIA5OnaJ6+0Z+OfZ83fvF+
+9y1qZQNAbtSw/v739QKyQoiKYeYkSJ2alHSoU/LTE7kw7YxI5vDy5uoUoVGwBmZzVQdQXnhTlVZ
qs9e7kuyzJ411d6h6Wepbav3okCq98zLMnFndbbN3TastiX+aKhIo72oZDmATnCMv2LO4x2xOsJTEi
HvXrLNaPevPNp/YQt+3SpxrZ0n4HuDXW7V41zBubVssGjV8/85P3amXAAB5UvVP3/aFl81NHdRRV
lCqglQAQE76T4pc/rheQFYIURHKHgYdUpOy2rLWu8Otp1KR7cGClfuHRs/BBOwamMZwbZddWzOj
eqgx92n23LN7obonbiCk8of1p3hehhRlc2+4Tqg9hD5hYBeYJMs6ZjsbHhT7uljH3G/5QaqtKq2+0dZ
ACYJo9r07eJA2Z5vTZZMLft9aYl07S6L3AHUsg6+ttiava4u59cTTTzATPwDMkOqBqnqiLgI1pJ/6qAC
Qo8vWb6bUR81Uoa8KBgJAEmo2fT0ZlAph96btmTxL+lhOtaYNWBfEUbp2yJUKTit/VJF7H75XrwE
A5EnVQVWTSS2SK597XLbufr3c+uoX6TUAgGxdKXLFb4gUbtbLSBM9UQEkFCxRcGZtsUO49nnrWC
J6ZB4tR+vaIV/v+
+z7CFABYEbULPx3fPl6vbQ4nvmnS9J8//3yoyef0WsAANmy3m8vqWH9TzmLSBUhKoCY2rJh17o06
pWaJQoWUK+xIqv7demmUqJinh29a4d81e+ry8f+9mN6CQCQJ1X/9C1fvFGe8+PF/NXt8W/8SO7+
0AN6CQCQuf53RC7doxeQJkJUAJNRNS8XfPx7cXMvpRq/C+YIXDvkR/U+Vb1QAQCz8XMPvERueP
wqvbSYvvKXj8jnPv6QXgIAZO7y10QufUEvIC3URAUAAKEefuJhec32a+wJpQAA+bv568+Xf/6Fm/TS
YqM+KgDkjfqoaaMnKgAAGKKCUzWRFAEqAMyGqoO6aBNJRVH1Ue/
+0FftjwCAPKj6qGpY/7POIqZGiAoAIbc+ak75cHHH9RLAIC8qQB1UeugjvLN//19+cPf/ppeAgBkrv+
oyKU/1guYFiEqAAAIUDVQP9P7jF4CAOTttV97sbzs7zx4CAOTttV97sbzs0av10tGyt/t/7AcAICeXv+Q8MDVCVCTUk8aKm
uVbPTaknWjdOGltZ96MOYZeQ1Y2gmvbG+7rV6TR0yunpfZjb3NEO2zTne9M2m1bkPugvaHbWJC
BSwosjE/sf8KejR8AMBsqPH3N3x3tuqGqN6rqlQoAyMmlPxHpP6YXMClCVGCGeo0VKZRq0tHLWe

pd3DH205RdQj4AA+77+n1y15/dpZcAAHm7+qnj8pa9G/XS0aXqov7+v/8y9VEBIDeqPup/tz5SH3U
ahKhIqCibe33p99VjWyp6bXby3l8WRhxDryHrtTziU6UnF3eC+2qea1hrF8VRuA+A+aZm4n/nJ98pT
z/7tF4DAMibClBVkLoMVE/UT/7nA70EAMicXR91Vy9gEoSowDLoXRQ3Qy2Xy86Tzo5cXJwUdTFUt
qXfrYs+w8DCUMGpClAf+cEjeg0AIG8//ffX2UP5l8m9n3xYvvKX/NsDALm5vGc9mOBvUoSoS8eoLbl
i9kQctd6oc2mvz7s25fD+IutuGnU/VwKfNLcT9vko6Z8z+/PmMP7mqvP5EYU0/WNWj+T1Rv2h/FU
5e+GMDvk6shMnRQ3UUg17jG5PvHbHuTbD59DaumyYrzfqkQa2MbA+/BTHaYN5POpY/P2rh7vd
9nnrupbrsjXQXdYu3WC83n4M3DfArKgh/A888oBeAgDk7YbHr7Ink1pGd3/oAXn8Gz/SSwCAzF26R
6T/Hb2AJAhRl05R1s64PREPpOs8s3TlwE30Aj0U27LbdJ6Vz6xZXz17ldNV/awjB/4B2PywsCxn1nRr7
RCwJIMj5zu1UswQa5bnrCO1UkFW9fYcal2SINUYyl89LZXimniHs3Nx+hCvfEbcU+2L2e6pr43Dfn1w
Z846FVQOrG+uDgSpE7VBHcuqeFvWoakKSlf369Ld2wxcdxW+lsJKN3RqUprnibKwFD7yxY/Yk0kBA
GbjOT8+Jm/54o1y/FJBr1kuP3ryGWm+/37qowJAbp4SufQH1kfqoyZFiLqEimtuT8R9OXQTovauHwi
JEU72Dq1XOU6dmIcI1VI5LW6M2gzMjmSEhV6w15PGutvjsyotu6am9WjpLXRqsh4jjUz7nFW2rTa
Yw76rLadd22HVPv12d+vuV8TsRaoYQ/mrp9X2zVA4xpD+4qbsuefNfnTFa4Z1BPULwcDQN67d6V
wbV7neDX69Frbev2+maYPxeh2aFjf3vOc+P1RXYWtXf41/TppyLsFxAmnafXBXPvgXH9RLAIBZeONf
3yAv+OEVemk5PfTV78qf1h/USwCAzPUfFrn853oBcRGiLiOvJ6IfaPUOndivWnXCo32dFJrDwO38bS
5UZMsNoJq7fi8+s+6n2wPUXFff8ickqmx5QWCs3pgzPGfVlj+Rkh/mWi0Z7IY7gtk792TJfmJsJ0EYq/
Ua636vzepZ2QxPUMe3O61rY6vKWbchRsg+cv3+obPdKdoQeH0UI1Q3Q2s7cNWB6t6okwhkSA3f
v/NTd+oIAMAsvPLBa+Xmrz9fLy23z338Idn/n4/pJQBA5i59QeQyf8BKghB1KRXlxCnnmRtode1x6W
U5efqkHXYF11vUMHDn2VzwQ7mmeJ0KuwdeWOgN5ffWWcfkDu+2H8bw7cAQ/VFmdc784HMy
Yb1zLRMP6W/LeT9BlVZoz1klRrtTuzaW8kkJ3d2o9a4p2hC7Z7Zxrq2N2WUO3P3Er8sLpOuJp59gJn
4AmLGXfPe58nMPvEQvQbn7P35Vvvct/m0CgNxcVvVRv68XMA4h6pLy6oraPTl74nSqPCUnKies/w
+uV3ngPEWoFiOYcodmt90x0xG9I6exkOfM6GmphqaXQkPC+LP0tzf8OqBVo6fp8kkSbhdl80L4jP1u
cDtiPjEgE+5M/A8/8bBeAwDIm6p/usx1UEdRAWrzN+/XSwCAzPWfFLn8cb2AcQhRl5U3tHlfDts6aL
N77VXEyQqN9VP3hsyCUdfTDi/9upOjwkuvNubQI2YYuIDnzB/KHyXmkP72hj9RVLUlIzuhTmDqa5O
CTNtg1JUdKNlqa55jln7k532ffZ/c+/C9egkAMAsrX3qpXPvklXoJpn/40rfl079/qJcAAJm7bP02eukze
gFRCFGXVklO2hlkRw52nSHNbh3Rkv0Jf3347OuzV9w864eaDXeSp4E6pCVnqL3i1eKc2KKdM2Mo/
xjjh/S3ZcNLUMtSV1PRTyvVazOhHNugZuhXPU7tScXsQNWYoCtu2QJgSvX76vKxv/2YXgIAzELpoRfa
D4z26f/6D3aYCgDIyeXPivTp2jMOIerS8ntyNptOOObWeCzq4p/uem+Sprnj9gDtSK2mA77BOqRmP
crmqj9sur3h1aUsxB5LvWDnzBzK787+H3iYIV70kP5e45w3jL9cv5BOuYRUr82E8miD3pbKoIM9Trvi
ls8dW7sVSMn/+v/
+l34GAJgF1ftU9ULFeHd/6AH9DACQi0v36CcYhRB1iZkzpqvehd7w88Ds5sYkTXPIq1NqC+shGaxH2
VzVwZg/Ll1GT440LNVzVtS1VJXmauqhoTmUvxxaW8AoiWC9cuSQ/l5D1r0CqtYrAxMwOY/JJkhK99
pMJoc2VLb9IfyBurRGfdmzm7MP3bEU7v7Fu+WOl9+hlwAAeXvjX99AHdSYfv7dP6WfAQByUXijfoJ
RCFGXWWDW8FPiTzbuDltXzPVzyAwvRw2ht+tRGr0uXeW6dJPWu0z1nFVke7BA5v5hSrUxzaH8o0
NdMxQeNaQ/Xl3VCaV5bSaVQxvsIfxhxVBVSNvvp1pfFohy1RVXyf/4lf8ht1x3i14DAMjT373yCf0M
UV7z1pvkbe+5VS8BADJ37FUix9+kFzBKoa/G9QIAgKXxwCMPyOv/2+vtmfoBAPm648vXyysfvFYvYd
D1P/E8+a1PvVGedw0TbwFALgovFrni/daTq51ljERPVAAAlsztN91uD+0HAOTvb27/tjx2PX/ECnPlc4
9L9fdeS4AKALmx3m+Pv8f6SIAaByEqAABL6F2n3iUfeMMH9BIAIC+Xjvfl8294VH78nMt6DVz/8t+V
5NZXv0gvAQyd+ztIoWb9QLGmSpE7TVW9AQpBX9W65F60lhxJ1TZkODL27LhTbbiPCabqGZYeyO
43dBH1jOAAwAwhz788x+W07ed1ksAgLz84PnPyt7rvqWXoLzidS+R1X8bNhkqACATx4oix39eLyCO
OeiJqsJVf5ZqR84zwquZ0VcaKU3oAwDA4rjnl+
+xh/cDAPL10M0/kO6t39dLy14d99rV4CAGSucI3IMTWMH0nkGKIWZXOvL2oeq35gxuuuHH
iTiKvZsNXn92QzxwzV1qnJeTqkAgCWjDtj/3VXXafXAyonqjfu+aZ/TS8lIBqgpSAQA5Of5ukcIL9QLi
mn1P1N6h7OuncuqEZJedlqXedUNc/9Gtl/XnRZq7pKgAgOVzy3W+EGqClQBAPlR9E/96Zv2h+X1

S/8xivsofwAgJzYdVDz7rl4NOQYog7XRLXrlZZq4nZEtYfVD9UoNb/OeaRVL1Uprp0RP0YFAGA5vfm
WN8tH3/5RvQQAyIvqibqs9VFVePoLv35CLwEAMmfXQX2HXkBSc1ATNUKvISuFktS8lNXRqZVSq2H
aPu+HuNXTfpGB5WVM8uWd46hJwTA7YddqEYTfT/4kcHneY0nPYU7fC+q9b2DCO//8rEiKf0cCAt7
7s+
+V2h01vQQAyIuqjbps9VGfd82VctfvUJMbAPJztcixO/VzTGKmIWpluy/9bt3vCVptOcPst1WY2ZPGu
htwVqXlDsFvVe01qobpeqIkoSO1khtC+I9Vd0Yra9/2bgFghnqNlWAPfSBnqjeq6pUKAMjXfT/zuD1r/
7JQAer1P/E8vQQAyNzxO0UKL9YLmMT89kTtXZQdnSKU61v+RFSVLXHLmHZ2LqbU+64s9S0S1NFG
TQoGTGKR76eM295ryPpg13tgBlR9VFUnFQCQnx8/57J8/g2PLkV91Le951Z5zVtv0ksAgMwdf5PIsV
fpBUxqfkPU7oHXE8sevu/1HjWG93cOpKufTsfppZpmrVUAABaVmqm//attZuwHgJw9dv3T8je3f1sv
HU23vvpF8ku12/QSACBzhVtEjv0LvYBpzHdN1FSFz85vlhPo1M7nWItxHgxO2jWqxuPoOpD20GPv6
/VjZG3Jwf1FBNd2Pdzga0fVgsysDVEya98oca+VK/5xJm1f2OsHyncO1O806o7arx19P/kG2p/K9Ux6
DsOEt32wXungORo8P2HiTbTn8/epHqPqpCY5P0DQbdffJvf88j16CQCQl7+/7Xvyjy//oV46WlQd1O
rvvVaufO5xvQYAkK2rRY6/2/p4hbOIqSxEiFqud4fDT/uRwnDa4qac1WVWRfblcGnyBRVsDU7a1ZTV
wqr1/3hUiFMKG3rcqUlpMKBKMklYe2NETUjVYzgYFmXWhihZtW+khNcqwXEma58TyIW9vrlaGBH
2qXNitLNcl/GVM9SxDbR/6us5/f0ej9OrffAcNVdHhZyTcPbh1XO2Dd97qd3vWGrvKL6DGfsBYAb+6v
WPHcn6qL/yH15JHVQAyNPx09RBTdH8hqilk34P0YN0Bu2Ha8tuuinKQug1zvnhkTuhV7/r1Zsdzzhv
5bp0dbDd9TbQlHNeotNLMEmY9dpzesOh2+3IzkX3tVm1IUpW7Rst2bVKcpzJ2tdrrHuBnP+HjZa1F
615bkRQaLRjb1OKem0koz1u06e5ntPf70n47fHb3pHa+ejIPHqivUH+PsLvvbTud0Ds2frfdepdegkAk
AdVH1UFqUfJyumftB8AgJwce53zQGpSC1HtnmjGkFHzEWco65DimpzxspxVfxuqF6C77UQbdnpv
me1yHmYvuTOyFivhWXQ9uejO2qUCFi+kKcrmBSPEidI7lH39VDo74mY3xc09J6yxHnub+mQmmiT
MmLjHCNyKa2eGQ/XM2hAlo/aNlPBaJTnORO0LtuOst74iW/aGVVC3J2GHE2hHTNWz/rmtbPtBrd
/2JNczhfs9gWrL7yFf2Up/+4q5j/B7L8+J+bAM7v7Fu+WOl9+hlwAAefjmjU/JV376u3ppsb3sp14o/
+a3mNAEAHJTuFHk+L/WC0jLHA/nDwYcXkjrjWE1w5B0mMHN0daVAzdTKp+Ukn5qK56QU/ppJDP
kHgioh2ouTjVJmK6nGTZ8Prc2REmpfSMlvFZJjjNR+0a3wwldR5fWOHUi6XdVVU4HNlaSk2473bYn
up4p3O9zpSwnAwcRIteJ+bAMrrriKmbsB4AZuP9V37HD1EWm6p/+
+n/5GeqgAkBurhQ5/mvWR+qgpm2+a6IWN2UvbMitPdQ3hXqoHtWLri8pZ7JH3OhefG5oM1EPZ
MWu5eiGPlE1KzNsQ5R5b19sebQvRuA3VlFOLEzSmcbxAvPpphfcZAepKlAFAOTn8yuPylNXX9JLi0f
1QFU9UQEAOTn+r0QKN+gFpGmqENUc9hv1cMLJoj8M2gxA7aBUrw9NMc2v04+4dRUtdp1B82tD
H2kGskvEuHZ+7Udf89zw5DXjJwlry4bZq9OtjWnWijRl0oYo2bcvLbGOc4btG21wgreeHHp1B4ZlOv
HcEcD5QZpuv+l2e2g/ACA/KkBVQeoiel3l/6IOKgDk6dhrrQd1ULMy3z1RkZGQ4dEus05mDGp2d9V
b0Q+rjZ7D7raTTBLW3vV6dtrhjxuYG8OTB6XehihZtW+khNdqguOM176Idth1itOdfT7Y9JDh+ImO
M737fWHkNjEflpGaZOoDb/iAXgIA5EEN6f+7Vz6hlxaDmoWfOqgAkCM1C7/qhYrMEKIupaKs+bN2
yao3Ztuc0XsMPcGXKlEb7K0YEnhNOEmYH/4YM+KbcmhDlFTbN1LCa5XkOBO1L6Id9rE7NVUTnsK
Rmqsb4jfdL5lQPrPmhNaJrmcK9/uiyeB+B0wf/vkPy+nbTuslAEAeVH3Ux65/Wi/NN1X/tPp7r5XnXX
OlXgMAyJaqg/oe6+PVziIyQYi6pIqbF/zehs1VHawYk86MU9n2h393alJygxmjRqg/UVew9mbkJGGV
09aSNq5dWbUhSmbtGy3ZtUpyrpO1b2w7ynXZSmV8eFnK5aas6vZ4Tbfaftab/j/Z9Zz6fs+LOdGV28
6Jws6U7ncgwj2/fI89vB8AkI9Lx/vy+Tc8Kj9+zmW9Zn79Uu02ufXVL9JLAIDMHVsTKdysF5AVQtSlVZ
TNvcFJu6rS6res/8djD/8OK6Rpb2dgoi679ubg/ixDk4RVZHuoDc72vF01d71eitm0IUqG7Rsp4bVKcJ
zJ2qfa0Zfu0IbVpo3SBlM7JWcHjzfsGiW6ntPf7/mw7q/B67F/aPQSTiCV+x0YzZ2x/7qrrtNrAABZ+8H
zn5W/ev1jemk+veatN8nb3nOrXgIAZO7Yq0SOv0kvIEuFviqCCAAAMIF7H75XKn9UkaefXYwhpgBw
FNzx5evllQ9eq5fmx7UvvUr+06ffzDB+AMhL4RqRK9R8BQzjzwM9UQEAwMTefMub5aNv/6heAgD
k4W9u/7Z875pn9NL8qP4udVABIFfH3239jwA1L4SoAABgKu/92fdK7Y6aXgIAZE3VR/3cm75pf5wX
v/Abr5BXvO4legkAkLljbxcppFNUD/EQogIAgKmp3qiqVyoAIB+qB+qJ+re676ll2ZLhae/8Osn9BIAIHPHiiL
H36EXkBdCVAAAkAo10dQt192ilwAAWeve+n37MUuqDqoaxg8AyImqg3rsPXoBeSJEBQAAqVAz9b

d/tc2M/QCQI9UbdZb1Ue/6T6+2g1QAQE6O3SlSeKFeQJ4IUQEAQGpuu/42ueeX79FLAICsqbqon3
/DozOpj/q299wqp/7ZDXoJAJC5428SOXabXkDeCFEBAECq3lF8BzP2A0COvv2if7Jn7M/Tra9+kfxSjV
/kASA3hVtEjv0LvYBZIEQFcteWjUJBCsZjpdHTn1tSvYasbLT1gqO94Z6fFZnX09NrrDhtXGnIqCb6x+E8
lv5aY2mo2frfdepdegkAkLW/v+178o8v/6FeytbzrrlSqr/3Wrnyucf1GgBAtq4WOf5u6+MVziJmghAV
yFVPGiur0tRLjrKcWSvq58vHDiJLNeno5YXRa8h6LarV6loXZDV4saVTK0lhIDAGjqq7f/FuuePld+glAE
DW/ur1j8kPnv+sXsrOXb9zu1z/E8/TSwCAzB1XdVBfrBcwK4SoQK66cuDmbuW6dPt96ff3ZHNZM9
SxQeScUj1nxwW/7fPiHlq1pa5zX7r1srOiuSrkqFgGV11xFTP2A0COfvycy5nXR33zr9wir3nrTXoJAJC
5YyvW41V6AbNEiArkqXco+/qpnDohy9v/dHHF6znbk8Y53QW1XJetivO0uHlWqs5Tae6SomI53PSC
m+wgVQWqAIDsPXb903L/q76jl9L1sp96ofzKfziplwAAmSvcKHL8tF7ArBGiLjGvnqP5CKntOLI2peqN
536d163Or/dp135sb+ivNdbZLwuuD/bKS2MbmtlG7zFcYzN4jMM1S+1HWN1Low3jaI3a+zDDt+aq
87WDDQ9pc9i2o9o8tpfjROdl+J4ZuZ8YxxD7fGh+W9RjuK0OZwi9/7p0z506/lKsnrNGj+NAWF6Sk7
ozquwfDt9PNqM9ie65eMduS+H7wj1Pcd9HsNxuv+l2e2g/ACAff/fKJ+SbNz6ll9Kh6p/+
+n/5GeqgAkBurhQ5/mvWR+qgzgtC1CWlwpHQMKhTk1JhQ8ZlcHHYtR8HCkLa61TIMrC+uRoeXk2
1DRU2hfYY7EitNCqEU58zapaW61J3uw52duTiYMi0674ynbqmQ8GiZh/zyGBquM1uz8dQE5+X4X
umuToieEt8DFGcfQcvd0hb7WCw5A2hd6V67jxVafVbXq/SIWaP41E6B9LVT4MqcjrpPZfk2FP6vlDn
KY/3ERwdapKpD7zhA3oJAJC1z688Kk9dfUkvTe/f/Nar7J6oAICcHP9XIoUb9ALmASHqUmqLn8O4d
TmNeo3SlHPhSUpi5XrX3na/FYybwtaPGt482TaCw6mHj7EjO4PplEcFZM7r+3ubsrlVF+erBr/GPI9nZ
FyGWtm2ttd1t2Wptpx9bOvUrr3hB4Vhbe7UZH3kdQm2eXRT0jkv/invSO28cd0SHMPY8xHg7zu8r
dZxrbvBoHEu3Iamcu40u53bEitrHVKUE6f00wiVRPdckmNP7/uimOP7CI6OD//8h+X0bQxHAoA8q
ABVBalpWDn9k/YDAJCTY69zHpgrhKjLyOwlZ/R0K27uOQGJ9dhLZaajqpx1t1M5bS25RqwPHd486T
aKsrlnBj6O4toZL7TrHIT3AyzXt4IBWXFNzugv6uxc9NvY3vV65pXPrI0P38YwexjWLxht3rwgfgZ5PrR3
31CbR5r8vFRbfnDoh3xB0xxDFHPfoW3tXZQd3SEycC4qW/5+zWtniH/u1HFY3yOhIe8k9uUwrEFKk
nsu0bGn+H2R2/sIjpp7fvkee3g/ACB7akj/V376u3ppMqr3qeqFCgDIiV0H9V/rBcwTQtRlZAQ0ImqY
bsGrZTiyhuIkyielpJ8GjFofJo1t2HQ9x9BhzEGnTgwGP0VZ8xMtLyzyA0Mj0J1YVK9WY/8jgrfhNscV/7
yMN90xjFaWk+MudvfAa79X7sF+GEPcRwyfn/zcTeuUjN51gntuimNPcv2HzlNe7yM4ctwZ+6+76jq
9BgCQJTXJ1KT1UVX907t+59XUQQWA3Kg6qHdaH6mDOo8IUZdSUTYvhPckdEOYqMl1FkZg8hyjn
mOk8MDO76nnDnc2AsPq6dg9GafXkeGOgjFCRlOK52UyYccwK2keVxw9ORxbLNWR2T2X2vVfkvc
RZOKW625hxn4AyNFfvf4x+fFzLuul+H6pdpvc+uoX6SUAQOaOrYkUbtYLmDeEqMuquCl7esjtQKlR
W/Pcos+u3ZYNs3edW7PRrMGZhHW+zurzZA+NNoYyV0/nF6FOH/qlfF4mkm1w6dXKHXpMWsd0
AsUTMrb06bje1BPcc+OPPf3vi6P9PoIsvfmWN8tH3/5RvQQAyNIPnv+sHaQm8Zq33iRve8+tegkAkL
ljrxI5/ia9gHlEiLrE1MzaqqeYPcGPHYR0vRqKo4f/GoxhxHPHrB2pgiW3/uMUba64U6Z3duT8+R29n
aqkk6GW5KR37gdnZO/JRbfoZeQQ8BgyOC+
+nI4hTOmkFwKOqumZL+NcNHfF75DZlQPvNJxwzn2EWPdckmPP4PpP/T6Cpfben32v1O6o6SUAQ
Jb+8eU/lL+/7Xt6Kdr1P/E8uet3qF8NALkpvFgP48c8I0RdRu0Ne6itmkU92FPMCHiMXnIlPxkzZmJvy
4Y3Dft884MlY2bySXgTWHWk2dQnKrWh/EXZdLsdqvO87l+XXmPdq22ZZBKkcVI7L578j8Fj1udsrvr
DyPW9bj9yHVtu1oBtyq7eda9xzgsxY/VgjnPPTXjsU1//hO8jZntyvRSYe6o3quqVCgDI3t/c/m157Pqn
9VI4Vf+0+nuvleddc6VeAwDIlqqD+h7r49XOIuYWIeoyqmz7Q287NSm5QYtRH7F6NnzmbhXSDL52
Lpkz9nttNibamUhF3I6BrlSH8o+4LiU/fZQL005glcl5MSQ9BnPYu9ueiRK2YH3O5qqz34IX9Felldqs+v
EUNy94PTLd9njnodqSeM2Jc88lOPY0r3/C9xEgiqqPquqkAgCydel4Xz7/hkcj66NW/u8idVABIE/H3k
od1AVBiLqk7KG3YUUMVeDS7wcDHlX3cLBmYrWVcx3NpCqy3W/5gZHNOTbvsAPDrOPxhlcr5bpsp
ZzL2dcl5LxWW9Z6d+j1VLI5L6Zkx2C1Z/A+3D80ejUmYNfnNIaSu+y6nznWQ/UUZXNvuFaoPYw+Q
aAb656LfezpXv9E7yNABDVTf/tX28zYDwA5UPVR7/uZx/VS0Cte9xL5hV8/oZcAAJk7Zv2GfPwdegHz
rtBXRewAxKNmNtcT86gwbG/anqHAOEfpntPHcqpFwIpwn+l9Rip/xM0BAHl441/fIKWHXqiXRK596
VXyW3/8z+yPAIAcFK4ROf5+66P/Xoz5Rk9UILLaeNNbdmc3LcmaNABVZO1r3XPu8dSwZ9ODG0fGO

4juYsR8AcrL3um/J9655Ri+JVH/3tQSoAJCn4+8mQF0w9EQFxmrLxmANWFXOgK50yMzRu+d6jRU
p7ZyRbiplKXDUFf6fgn4GAMjStU9eKe9s/aS88723MYwfAPJ07O0M419AhKjAWAOBFgEqMsc9h+
WlJiiblx9NaEs42hKOtoSjLeHmqS0nrr1GDr/3pF6aLa5RONoSjraEoy3haAvSQIgKAADmBj/ghqMt4
WhLONoSjraEoy3haEs42hKOtoSjLeHmqS1IhpqoAAAAAAAAABCBEBUAAAAAAAAAIhCiAgAAAAA
AAEAEQlTkSE2WU7DrfxRWGtLTa8fqNWRlo60XHO0NvZ3CijRibwjp6Uljxb0GG9aVTbJuGmH3UNr
7mJbRRv1Y4SYFAAAAAGChEaJirvUaK1Io1aSjl4H5pgJdY1Z9W1nOrBX1cwAAAAAAsIgIUTG/eg1Zrx
GfzqeibO717RkF+/1tqei1szFPbenKgXvLluvStdu0J5tkqAAAAAAALDRCVABIS+9Q9vVTOXVCyE4BA
AAAADgaCFGXmao1OlC7MazG6GD9UXuIvfE1A+VKNbNOpXokq1Vp79Mcxt9cdbYTvjOjjeoxqk7qY
JvCa1VOdrymMbVf2xvetob2H3JN4rTRY359ZEONcxFo46j1xj7t9ea5zKoOadx7KKotg9uYsD5pjOuS
9J4FAAAAAACLgxB1WakgL7TWaEdqpVEhpPpcQUoDQ+ybq4OvVyFiSYIva8pqYbBWZBqcNq0GNh
xyDHYlNtgm65W1UnjQaYt7vIMqcrqqn3Z25OLAa9u7bmODtTKHQjgtuo2TKsrambLztHMgXeeZxR
iOHmh7W9xml8+s5dDDMoV7aKJrPizf6wIAAAAAAOYRIepS6knjnJuIuXUb+9Kt61BNOrIzmPx5qtL
Sr2+5QaEKG8/7ve16jXN+0FVt6VqVXfE2H0Nl2/qabl28L3G3sx1W8dJvU/gxWMe77oZg/mv77gF0
arI+MhUdf7xhKltu2wfPpR9GSvmMeBlqe8MPgsOuSWQbJ1NcO6PbuC+H7qbbu0ZI2ZEDN101hq
mfOpF9hDr9PTTNNTckuC7J7lkAAAAAALBICFGXkjERz96m16vQD9VEOl56FlRt+RP3+EGhqScXd9w
+e1VpeQGStc8LYa+fntmm0GPoXRS3SeX6lj/xUGXLC+U6OxdDexSOP94RimvidfQ0t22ElGaPTrN3av
2CcU02L/htrJ0fGKo+Ja+NftDbO3Si0mrVCRv3dbrau7jjBZKnvROYlRTuoSmuuWkm1wUAAAAAAM
wdQlRYdA3P0OH9SZmzk5+Ukn5qK56QU/ppespyMrCTEN0D77jsIdheXUtjqHdgSHsazOHy/rB4P5
SryllvyvYRvVNtxnbMHqOpKMoJfUHcwLlrXzzrnJ4+aYeVwfWW6mk/kMxMCvdQKtd8VtcFAAAAAA
DMG0LUZRWYKCdunckYgeWRMt3x+r1i3Z6eRig3URhpDK9PScUt3trctVrXE6cj6ik5UdFhZWC9anb
2EeriSf+6AAAAAACA+UKIupTasmH2OnVrPZr1HI+ocr3rlDEYevjD9lNT3JSzbglONXTcqCs6WRiZQYh
dOS164L4ctvUQeLv3pzs5lrF+QUP07K/5sv1xAQAAAACA5UOIuozMupwqYHLrohpDoCdXkpNuEjs
4XNoIEXNVcoamK6NqvWbF6+nZ2Hz50fVFTXP2eBs/mZ90FMydk6nxNfQ3XdHDnadr3VrtZbsT/jr
h4e0ZyWFeyiVa57idQEAAAAAAAuNEHXJ+QGTMWP/VMxakU1Z3XCn3DFnS8+ZMcmTNFfFa1J7w
6+V6a1MmdfTsyPNpj76oaH8Rdl0u6xar6utN6yz5eg11r0anuYESU7AqVivP+8dkGx4U8nH5V+vZtP
5Wnf2/aIumOquNyfCylYK91Aq1zz5dQEAAAAAAEcTIeoy8oI9S3NVh0rGhDtTMmcun2r75iRC7nY
mCjuDs7o3V3WI5gWO5gzwaXOHxftCh/JXtqXl5XU1KdnnrCAlP6mTC95EVCojdOutWrxzHLe2bVBg
W9Yzb2i6eZ9Y68/k0w3VNv09lNI1T3hdAAAAAADA0USIupQqst1vGQGZUpVWv+8HRvaEQpMqyu
Ze1w/BbGr7g/scx2qn1yBt/9DrDZhIcVP2+oNtstj1YDOoh2rwhvQr1v62Ruysst0PrUtbbVnr3ZILLnU
8g6+ttiara2v22gwMTTeGs+c+ZD2Feyila57ougAAAAAAgCOp0FczrADITq8hK3oiL1WDdo+eiwAwk
urtPS8/mtCWcLQlHG0JR1vC0ZZwtCUcbQlHW8LRlnC0BWmgJyqQKbOOZ75D4gEAAAAAAAJAOQlQ
gE23ZKAzU8ayeFTqhAgAAAAAAALB5CVCAPql5pZpNXAQAAAAAAAIEvURAUAAHODelXhaEs42hKOto
SjLeFoSzjaEo62hKMt4WhLuHlqC5KhJyoAAAAAAAAARCBEBQAAAAAAAIAIDOcHAAAAAAAAAgAj0R
EV2eg1Z2WjrhVnrSWNFzZavHhsyL60CAAAAAAADA/CNERSZ6jRUplGrS0csAAAAAAAADAoiJERfp6D
VmvEZ8CAAAAAAADgaCBEBQAAAAAAAAIAIhKhLqr3h1gddkUZPD7+3l51HaClTVePUeI3zcL7eZW/XH
MbfXHVepzc4uF+PuW1j58HXt2XDfY318F4Wo12TGjwv6jHpuVGSHr8S1obCSkPCD8+s/eo8VkaciG
TbBQAAAAAAWF6EqEuvI7VSQUoDw+
+bqwMhX3tjRI1T9fXpBJbR1H5WpamXpFyXrYr1MbN2OWHk4HlRmquFYNCZ4blRoWtYG6RTk9Lg
BFl2EFuSwZd3aqWhcDTRdgEAAAAAAJYcISosVWn1+9K3Hq2qXqUCwPNulNaTxjkdX5br0tWv7dbL
zjrrtTsXnYiusm19rlsX9zNSbdmv7W+rxHNafjv7e5tSTNCupHqNdS+MLNe7zj77LasFWvOcDkeza4NI
W3bd1Dh020055yW0VjvW3SDXOE/uBe3UZN17bZLtAgAAAAAAgBAVUm1tixtxVraMANRTlM09H
crZ4aVeu3bGe23noKufZadc3/La6ciqXT25uOP20qzK2U13yxXZsoNGFVLuibM6w3PTO5R9/VQ6O+
JmscXNPWd/1mPPbVvvorhNDpynypa42Whn56J1ZJYk2wUAAAAAAAAhKiaha5OGDmHPzqkT44K
9tNrVlQN3A+WTUtJPFSdo9EPnYSmem+KanHGTWGtrquyCW7t0qM5p98Dbnz18X7+uYA7v7xxYR

2ZJsl0AAAAAAAAQoqIsJ82UcJTAxElGbdLcjGjnzNtlyawNRdm8ENYz2A9KQye5Giur7QIAAAAAABx
NhKiIoS0bZs9Kt46mWft0JuahXRm3obgpe3qIvV+v1tc8Nzybvl/DdfBh9KCdYLsAgLzp0Q3Ggz9yAQ
AAALNBiIrx2rte70o7oHNrfxpDyKc2ybYya1dJTroJqDsE3qVm4i8YM+6n1YaI16uZ9NUvzfakXXbw2f
XqnHrtK530Qtu4NVhjbRcAlpH9Xl+QwkwTy540VlZFWu57tPNHr+bqLILU+Qxze40Vpz0r+f/hT/0ba
p4P9zEP5wXDhq7XDO6ZmRvzvuZ9P7mPvG7mOO+3ebwnR+5jSf+gFfO8z/K9eGZinJuh951luGnG
nJfc34sTtofSdoiDEBWJ+AGdMSt9AiU/nTRm/7d+MFmdbhD8tO0KKsqaVzS0Kavem667baeO6OB7
cZw2JDp+/aavPhXsGRpSs9Wsc9pc9dvm/sOhHu7KJNtVjG3wDwuALMzsl/chKri09j/lv0npcCYu3Da
KcLuTP+4f5vte3N4IC3ONPyjOQq8h6zWRatX9x28G3NEnxsO8XrMy01/cAyWOhh+z+NZW52O1qS
YFda9TS6qdmpRmFPjk/0v7+Pc19R5csr6f6l3jHFk/T2Z778R5v83jPXncPtTnc/yDVmTok1eYm+C85/l
eHHFuht739CP98xP/vl3dH/g3Iot/IEadk9zfi8efl3zfiydoj/WNrUrbpf+eHO/7dn5+FsZY1g2DJWS9R/
TV5bd+A+hbPzD5uvW+9U+g87mq9ZZia/WtnxX060c8vNcqIa93P29uf9TD2NbIdtqStKvbr5fd9VXr
K8cxXx/yKNetVygJz83Exx/
+MDcdve3gMSfabqvqrS8PXwQAmEq3XrbeX8z3eP2+GngjyoffFv1vwAzaEEm/z8/8vXjm7XCuj9q/f
c28f5PzY/87OoP9RtP37dy1y/3eivPzV9rCv5dn1R7n5y9jv/pnrCy/l8a/rznvuYNtyPocjW9XvNdMa6J
9ZPIeqPdvbdd+hLRD3T/maud+Um3XK1IS/5w4n8/+vTjeucnjvS/
+fZv1+8v4cxImq7aNPy/h62fWnhHfw+nfR87+zd0737fBdX579Qr9vhz3uiJf9ERFDBXZVn8p0ksO56
821puAo7kr/t9KrNd7n9D2D52/MKlanIP1QqutCWuIJm1XEk4PIOsNTS/7rDdbf9h+0jYkPH57qP3g
ubQ5+wj8QdOuc2oMyXfZPWWMeqiWRNsFgMy05XytY71NXZBN503VUpEt9UY28fv35Iqbe9Lv7x
ltmTO69MupE/PRwFm1o9dYl1qnKmfn9kLNhnteWt7PKPPC+T6X6unAzyL5KMqJU9YH9+dQW08u
7sygPb2GqIFK5fqWv9/Ktv3zYmfnYgY9sRxj39d6h7JvfRj8fi7aJ64puxm9Ecd5v83jPXle3ved79+y1Ls
hP8tr6uf34dEJHdm5mO7dE/ec5PVeHOfc5GX8uXHeXwLf5xmY7Jxk9148/rzk+148tj0jfp6yR42mW
tYuzqii+fpZGOMRoi4pvw7mwJuLMeFQcMiBCgvdr1EPJ5TztxMM6dQPhf5rrYf5A725D/VQ+xmx35
Ht9MRtl/MGFlw3nvMG7H6d89gbakjCc5Pg+G2D59J+jDoG8zj1Y9QvU3G3a7xu+NgBYAoz+uV9M
enSL+W6bIX/A5CTnjTWa9KZVTvsoaMdqbbi/1ueGTUM0Rh6N9uSN/n84j6JXuOc9d1s/bI/oxu3sq2
HjNo17dWwypITNg/
+vJW13H5pT8huV1lOenWcNKPePgZk8AetuMFlmDTbEVuO78XTnJv86fJoB+cHhtOnWwJnknPCe7
FhxPub8/PnvuRaNYmfhRcOISoAAJgNfnmPTdUltX/5uTCLXoa6tpj9i6D+pWdGvR3b52vSqbZmPmL
C/0Op81AjV7KppRZXPr+4J+f2NDo7wwDEHTWkatqr7yOrObMI4efpl3ZMgT9oKfPyXhwwR3/Y6uy
flAvevxGqt6h6/9mYYa9C3osD9HwiwflBrO+pqedWiWHwjzD8LLxwCFEBAADmmDP5gRq2N6ueO
MFRDt36vqzO4hfU9oYzCcRc/dbuUL2Csh6WHcfc/eLePi/O6NHZXTNnsg5/YiAVeKtJgXKftGOWv7Q
jNfxByzKH78Xz9oet8pk147pY/4aeVWXUZtirkPfiAernGrdnrP89dXBKXadTkl3n7nn5IwymQYgKAA
Awp5wAVfXWmJ+hjMXNC3YNtk7tfK7hXHtXhU1NO8B1fuEpSEn9Vqh/CZrtcHptlsOyLfP1i7sOCGf5
y6Iecqzq2bt5jz0MViXezdWMZjYfZVa/tI9h93bqyMHgjTuqd9QS4w9ajkV4L56XP2x5ZtqrkPficIMl+f
qydXLfOk8nJau3vdn+EQZpIUQFAACzwS/vkVSvDSdAndMJ/zL8RSPMYE8j9bAngLR+Mexaz2dbt7sn
h6qoWc7nZKxZ/uLu9nw6O8NfFgeHTbpmdl7y/6V9rOIJceZ7CUZNPfuGnmG4O2f4g5Zvvt+LB+T+h
62S2GWOB8PbWf5cw3txTLq2eOCPkekZ+UcYfhZeOISoAABgNvjlPYKeRddiD3czevzkW+dSTf4wM
Bxc/0KW1S8ac6/XkJUVc0i2WqVmSp7lL6nz94u701utKjMcPSpSOW21wPoeCgyhV7dwzTovM26bL
dtf2uOpyGnVY6+27r+veL3G5m+islngD1qLaFZ/2NIjADo1Oe/9w6mHcM+oHinvxXGoUhklqUldLm
RwkSL/CMPPwounDwAAMCOtqvSt33L69a5e0a33y9aPJ2VvRX669bLVFtWewUe139KvWUZh56U
6JyfEblu53s/3bun26+Xg+ZiLe6RVHbg2rb71S2tfJT+5m+H38TB9HgKPefie1vdRxvdv3Pc1573Yf2R9
7eK0K4/35PH7CLt/3Ifxb1dq9H0x9H2r2jFw3Pp7Pu1rNcl5t78m8/fiEedGvd8M7Ns9hrTf/uKem6H
XZfY+POp+0XJ6L453XvJ7Lx7fnpB/xzO6RnHuxXn6WRjjFdT/rIsKAAAwE+5f6F3WD43zNRwQWBCq
x5xdm9BVbUl/Bl3nnO/pqrT6M5p5eS7pnk7G5ZnV9cH8Gfre9fjfR2Gvqc5r79gUjT83Id9bxnk7iuLc
LwrvxbOmRvOoOqhhgkP7+Vl4cRCiAgAAAAAAAAEAEaqICAAAAAAAAQARCVAAAAAAAAACIQIgKA

AAAAAAAABEIUQEAAAAAAAAgAiEqAAAAAAAAAEQgRAUAAAAAAAACACISoAAAAAAAAABCBEBU
AAAAAAAAAIhCiAgAAAAAAAEAEQlQAAAAAAAAAiECICgAAAAAAAARCFEBAAAAAAAAIAIhKgAA
AAAAAABEIEQFAAAAAAAAgAiEqAAAAAAAAAAQgRAVAAAAAAAAACIQogIAAAAAAABABEJUAAA
AAAAAAIhAiAoAAAAAAAAAEQhRAQAAAAAAACACISoAAAAAAAAARCBEBQAAAAAAAIAIhKgAAA
AAAAAAEIEQFQAAAAAAAAAiEKICAAAAAAAAQARCVAAAAAAAAACIQIgKAAAAAAAAABEIUQEAA
AAAAAAgAiEqAAAAAAAAAEQgRAUAAAAAAAACACISoAAAAAAAAABCBEBUAAADAQmtvFKRQ0I+N
tl5raG94n19p9PTK2fDbuiEhLV0wbdlwz3vk+e1JY2Xca6xXNVb8ba00rK8CAGB+EKICAAAAODqa52
TGOemSUMHoqjT1kqMsZ9aK+rmpKJsX6tZnHZ3aesg1asv5Wkc/F6me3bS+CgCA+UGICgAAAOAI6
Ujt/OL38Zx/XTlwM89yXbr9vvT7e7I5KvksbsrZqn4eco16jXN+IFttyXZFPwcAYE4QogIAAAA4WuiNm
r3eoezrp3LqRKxeo5Xtlng5auAamb1Qq9IiQQUAzCFCVAAAAAABHTLzeqH590pVg6NpryIpbm9Orse
rX/7Rrehp1Vr119suC68NKtPqCtUJH1wEdeJ31CKsrGjyeYL3S6HYYzGPXj8F92fsp1ayzrDVXndeO3Ul
FtltujOpfo/aGXxagXN+yXjUo3vHbQto/dH0tcc5VoEar+6BWKwAsLUJUAAAAAEdEWep1HdJl2Bu1U
ytJYTVYDdRep0K2gfXN1VEBZlNWCyUxyoCqjUhpcMIpOxQceJ3F3t/IQK8jtZJRr7Rcl60YnTuHwlEtel
8JVbak7hZHba7KRqMh5/wEVS4M1gNIcvwqwA5pv3M+hoNUR/i5UueiNLhTJewaAQCWAiEqAAA
AgKNjzQ3p4vVGnVS53pW+qgPq9ax0hK1v7o5oh1dLtC/
+y5tyzkv7etJYd0PBqrT0a71td2qyPjIpNl6/F2OSpvaGePmv0a6um3ga+6psW5/r+hNFqRqm9n5iDc
MPTjLVrLnHV5b6hcF2Jjl+67VuGhvWfmsrOxfjnqu27Eadi8A1AgAsC0JUAAAAAEdIUTbdGYwy641al
bNuj8nKaWvJNWL9/mFoL05zBnqzXmhn56Lz+t5F2dGdIQPD3I3enN5rB4QPix+t7aeGgTCzuHnB31
ftfDo9MAOTTGnVs8OTUiU6fuu675lBqKO4dsYLbDsHXf0saOhcmfVeOzviZq/FzT1n+9Zjb+QMWgC
Ao4oQFQAAAMDR4oVsGfVGLZ+Ukn4aMGp9qKqcDiR3JTnpp31ix33dA90L01rllguwH8bwdve1A0
6dSBLymT0vz8ha4EuLsnbGbdi+HIYlthMITDJlPQudTGqK41fHZNc5DR3eHzR0ropr4h2yuodK7n4ja
rECAI48QlQAAAAAR8xAb9RD5+l8K8qJU/rp1MpyMn6am0BHRnTmnE6i8DlCYFIpo85ppLBzFSw5Y
HLD3NgTdQEAjgxCVAAAAAABHj9kbtRYvTsvXYK/Onhx6Y8iHebVWhx7biYbtTyercHa88cfflg2z16lby9
Ss3ZpEcVP29D4Gyt7amueYpR8Alg0hKgAAAIAjyOiNmpQxjDw7g706u3Lg7tTtmVk66QWAo+p5ps
MsJeDXAHX05KJbmFROSaIqAdNKcvztXa/nqR24unVRp7iWaoZ+1ePUnkjLDlS7Xi3W0WUEAABHF
SEqAAAAgKPJmIAoTMlPDo3aqW3Z8Kapz1ZzdcObqKm94Q8/L59ZcwJAszZnc9UfQt7e8Gp0FlIZV2
4Gzta5WPd7WfYa61790aSTVU1twuP3A1djxv4k9PbVbRDscRoSdAMAlgYhKgAAAIAjKro3qjlzuwrp
nGAubi3NaZWlXG7Kqg4D/dzWmOFftd+ozdlc1cGh9+IREzJNorLtD1vv1KSk21XyE1S5kPuM9AmOv
3Lan6jKu5bGBFRJjDgX5r1RPat7ugIAlgYhKgAAAICjK6o3qqp7OVgzs9qavI5mIqfk7J4xPFyx63gO1Di
1a3MOvE4Je+2U7GHrIcdebVnr3eHxeYt9/BXZ7psz/itVaZk1TZu7Xs/fcexzEVYMVW8zrewaALA4Cn
1V3AUAAAAAAAAAEIqeqAAAAAAAAAAQgRAVAAAAAAAAACIQogIAAAAAAABABEJUAAAAAAAAA
AIhAiAoAAAAAAAAAEQhRAQAAAAAAACACISoAAAAAAAAARCBEBQAAAAAAAIAIhKgAAAAAAAAA
AEIEQFQAAAAAAAAABGEvn/AZM6nr53azj8AAAAAElFTkSuQmCC]]></redcap:Attachment>
</ItemDef>
<ItemDef OID="q29" Name="q29" DataType="text" Length="1" redcap:Variable="q29" redcap:FieldType="radio" redcap:RequiredField="y" redcap:BranchingLogic="[q27] = '2'">
<Question><TranslatedText>Do you prefer Life A or Life B, or do you think Life A and Life B are the same as one another?
</TranslatedText><redcap:FormattedTranslatedText>Do you prefer Life A or Life B, or do you think Life A and Life B are the same as one another?
 </redcap:FormattedTranslatedText></Question>
<CodeListRef CodeListOID="q29.choices"/>

</ItemDef>
<ItemDef OID="summary30" Name="summary30" DataType="text" Length="999" redcap:Variable="summary30" redcap:FieldType="descriptive" redcap:SectionHeader=" " redcap:BranchingLogic="[q27] = '1'">
<Question><TranslatedText>
Alzheimer's disease - Description #[no]

If you have Alzheimer's disease...

1) You might forget recent events of importance to you. For example, if you are interested in hockey, then you might forget whether your favorite team won their last game, although you may remember watching the game itself.

2) You might forget the names of people you met recently, or the names of local streets.

3) You may no longer be able to handle your personal finances or plan leisure activities such as dinner parties or vacations. Simple decisions such as what to order in restaurants might become difficult to make. Your ability to concentrate may decrease and you might have difficulty driving.

4) You may start having trouble finding words to express your thoughts. You might need to be reminded to shower or brush your teeth, but you could do these activities on your own. You may need to post notes around the home to remind you of simple things like turning off the stove.

5) You may remain aware of the day and your surroundings. You might be able to travel to familiar locations like the supermarket, although probably with the help of a loved one or friend. If you are in a social gathering, then you might have difficulty following the conversation.

6) You may continue to enjoy hobbies such as puzzles or reading.

Now consider two possible lives. In Life A, you will live in perfect health for 7.5 years and then you will die of natural causes. In Life B, you will live in perfect health for the next 10 years, then you will live with Alzheimer's disease as described in the summary for another 10 years, and then you will die of natural causes.

</TranslatedText><redcap:FormattedTranslatedText>
Alzheimer's disease - Description #[no]

<p></p>

If you have Alzheimer's disease...
<p></p>
1) You might forget recent events of importance to you. For example, if you are interested in hockey, then you might forget whether your favorite team won their last game, although you may remember watching the game itself.
<p></p>
2) You might forget the names of people you met recently, or the names of local streets.
<p></p>
3) You may no longer be able to handle your personal finances or plan leisure activities such as dinner parties or vacations. Simple decisions such as what to order in restaurants might become difficult to make. Your ability to concentrate may decrease and you might have difficulty driving.
<p></p>
4) You may start having trouble finding words to express your thoughts. You might need to be reminded to shower or brush your teeth, but you could do these activities on your own. You may need to post notes around the home to remind you of simple things like turning off the stove.
<p></p>
5) You may remain aware of the day and your surroundings. You might be able to travel to familiar locations like the supermarket, although probably with the help of a loved one or friend. If you are in a social gathering, then you might have difficulty following the conversation.
<p></p>
6) You may continue to enjoy hobbies such as puzzles or reading.
<p></p>
<p></p>
<p></p>
<p></p>
Now consider two possible lives. In Life A, you will live in perfect health for 7.5 years and then you will die of natural causes. In Life B, you will live in perfect health for the next 10 years, then you will live with Alzheimer's disease as described in the summary for another 10 years, and then you will die of natural causes.
<p></p>
</redcap:FormattedTranslatedText></Question>
</ItemDef>
<ItemDef OID="a30" Name="a30" DataType="text" Length="999" redcap:Variable="a30" redcap:FieldType="descriptive" redcap:BranchingLogic="[q27] = '1'" redcap:InlineImage="1">
<Question><TranslatedText></TranslatedText></Question>
<redcap:Attachment DocName="75.png" MimeType="image/png"><![CDATA[iVBORw0KGgoAAAANSUhEUgAABVEAAADYCAIAAABtBTQzAAAAAXNSR0IArs4c6QAAAARnQU1BAACxjwv8YQUAAAAJcEhZcwAAEnQAABJ0Ad5mH3gAACbhSURBVHhe7d1ByCxJnhDwRkF6TzYepPXiQxEaFXnqpXl4aMHD4GlgEZvFgyA8WlBovDh48jDLDLLQb2Vg5jDoHAZ6DgODsPQM60If9tALC/ZhDi008g4jNOwcPmEP32If1qrMyszIiMjIrKjMqzqfj/+6FeZEZEfGPiKz3Ov89Hd+

+8ecAAADALVLzA3Amn77+9I3/9IYQIox//bf/1m//89/8f3/2Z4c/JwCwKjU/AOfw+uH1W999K6p2h
Ljz+Gv/5jd2Nf8ufvLb3z78UQGAVan5Adjcw+PDO997J6p2hLjz+I3/8Bd/6+8/a2v+XfzP//H7hz8wAL
AeNT8Am/vGj78RVTtCiH/2j/96X/Dv4t/9o3/w6//zq8OfGQBYiZofgG19+IsPo1JHCPEPf/OvhAV/Gw
72A7A6NT8AG/r4lx9HpY4Qoj/Gn4aD/QCsS80PwFY+
+9Vnb377zajaEeLOIzrGn4aD/QCsSM0PwCZeP7x+
+3fejqodIUR0jD8NB/sBWJGaH4D1PX79+PwHz6NSRwiRPcafhoP9AKxFzQ/A+t7/6ftRqSOEKBzjT8P
BfgBWoeYHYGXf+cPvRKWOEGL2GH8aDvYDcDo1PwBr+tkXP4tKHSHELmaP8afhYD8Ap1PzA7Caz7
/63C/qFyKNhcf403CwH4ATqfkBWMfD48OzV8+iUkcIcdQx/jQc7AfgFGp+AFbw+PXjez96Lyp1hBAV
x/jTcLAfgGpqfgBW8MHvfRCVOkKIXVQc40/DwX4Aqqn5ATjVR599FNU5QohdVB/jT8PBfgDqqPkv4
stXL95ovXj15eFap3TvYvpJvfxk/uOUuI4nOk+W033ysp1mY80nv9uA4tK7xJt+2yaewnhuZ5kJm/j09
adRnSOE2MWJx/jTcLAfgApq/gvpi6C4whlqv2sqUqOarfxxSl2vE50ny4n6SbZWK3oP4xaXfqmaP52b
mv+Jev3w+q3vvvhWVOkKIVY7xp+FgPwDHUvNfTLa4H4q/ay5R4xIuU9HlLGx2h7apdpft92Vq/tzc1P
xP0cPjwzvfeycqdYQQu1jlGH8aDvYDcCw1/wX1VX9f5PRXrrwqjiq2hcX8wmb3Z6ONWTasmp9TfO
PH34jqHCHELlY8xp+Gg/0AHEXNf1HjGj/9dwCDvkZqRA0yxdJMvdHnqkV7ph91ei4cofp5R65eu9
rkIuYXvj1lnx3MJE4VjlqQe9yp1K0wsG6X7MGkadX+yoRdA1k2BiheGGBL3SbHOTiaYyatLf209iam6L
Z8K1+PAXH0Z1jhBiF6sf40/DwX4AllPzX9ZQJ7181f2Y1DiZGmknaBYWSwejIiuju58Wif0ghyvN52i48
scpxV5d/nCIrkU3pb5HKFh0Tjy3/GbuIQZa1GtueplBXrx6lV6Mtic0mmO2RbSdgXBnA6ssbWKUvsX
oKUzNbdFMuBof//LjqM4RQuxio2P8aTjYD8BCav5Li6uppLzpK6HuTt+jb9o1CfqOiqycQ4OuS1BvH
Xp0IzQfo+HKH6eUe3UzGMboGhzmmGTJLDsV90oXOlwZUsemt+eI6aWDHCRdl4zWt+iu9MN3FzL
DZhSWNjlQPJmuQdJhcoTkws6CmXAtPvvVZ29+
+82o1BFC7GKjY/xpONgPwEJq/ssLSp1RCdSKy6u9uGDKtMnVVGOHFm2f9sPLl/uBwitd/2i48scpM7
26z90g3efDqqKPe5lLiThLv9vDXOMmGZleQ7cm/YLp9YPE800nMD9a2qe9lJvg9Lp25jckyryXuTQSp
06mklzYqXs0nN/rh9dv/87bUZ0jhNjFpsf403CwH4Al1PzXIFdRHnS3xsVVVHJlGi0olg69mgbNzy9efd
L0Gq703aPhyh+nzPXqLhw+H2bQLapbZE4hb5ylH2bos2D6Xa/RYxj1WzC9TOpWOoH50bIzGluwrp
3MQFHHBUsLjFsf7idTyc1tfiZcgcevH5//4HlU5wghdnGGY/xpONgPwCw1/zUo1G/5WxNF2ZHF0qH
brkXbevdDe6m/0veOhit/nDLbq7vSXBhm1+oWmVPIG2ep26uu16jFqN+C6WVSt9IJzI82Odhgwbp
2MgNFHRcsbegSOdxPppKb2/xMuALv//T9qM4RQuzibMf403CwH4AyNf81KNRv+VtdKXS4Wlsstf2
6/3m/6d1c6q4MA0bDlT9Ome/VXdpdOaxpuJffiVlxlrq9yiYf9Vswvckm6QTmR1uQb8G6djIDRR2XT
2anbRSnTqaSm9v8TLi07/zhd6I6RwjRxtmO8afhYD8AZWr+a1AoqvqiJ7wXV0KZ/n0VViqWDuOkx/j
bK8F4UcbyxylLerXz7n+dfXBrYZJY3K2usMxt57jbEYOEj7KR9p0fLddin+C4de3Mb8jsOOkQ8X4lQ0x
Nf+/oJXAeP/viZ1GRI4Ro48zH+NNwsB+AAjX/NcgUO4O+fOru9nVQ37y/0pVGfZ+ZYmnomOs6XXu
VP05Z1CvIH93p23eX+6altHGWrtNxhWVmV+Ls89PLpG5lJjA/WtJi+sIwbEZmVnHHucnEcxvaJ1cOn
7NzWzATLubzrz73i/qFyMZFjvGn4WA/AFPU/NcgU+yE+pJqJGwcFFmxcrE0dOyH67NlE7TDlT9OWd
ZrWG2yHX2PkaOSVhaW+UewlxtnZBg0k7qVncD8YvMtguHHs55Y3aINmZnM9PYcmqQjZua2aCZc
wsPjw7NXz6I6Rwixiwse40/DwX4AstT81yBT7MTGRVemAgob7G4vLZbSdtme0cXyxykLe5V3o+/VK
OzYQZylrrAMe3U/76QdStObXNjkBOYXO26RNAnmmu2+s3hDypMJM+27dZ/bIXIjJnNbPBPO6vHr
x/d+9F5U5wgh2rjgMf40HOwHIEvND/My5Sjchw9+74OoyBFCtHHxY/xpONgPQErND/PU/Nynjz77
KCpyhBBtXMkx/jQc7AcgouaHeWp+7tCnrz+NihwhRBtXdYw/DQf7AQip+WGemp978/rh9VvffSuqc
4QQbVzVMf40HOwHIKTmB2Dk4fHhne+9ExU5Qog2rvAYfxoO9gPQU/MDMPKNH38jKnKEEG1c7T
H+NBzsB6Cl5gdg8OEvPoyKHCFEG1d+jD8NB/sB2FHzA3Dw8S8/Jto4efb+F104xWN7G6168uY6tMYPYdOsQdHfJtu70t4oz771WdvfvvNqMgRQrT
xJI7xp+FgPwBq/guKKtT1S6BDghsqt9lVjdTW/NmSX80/Z9G36fa+hLfq9cPrt3/n7aEUK08YSO8afh
YD/AnVPzX9DGtU9fAN9MuZVb0Yy+mi916cZVhB5nwbfp9r6EN+rx68fnP3geFTlCiDae3DH+NBzsB
7hnav7Lqahgj6LmX7crtlGD+Jmqflvx/s/fT8qcoQQfTy5Y/xpONgPcM/U/JfRlT2T2BsVBsFJd

MwTFSuTo0flltBm7T6Gs1gtjgLhw17JkX09LDDnb5TP8Fotp1k9EjfZaZhl3puvFa41kDm8tweju7vjJo
E4w1Lb2dY7Ddhaa5Rw2RDopsTW9EZ5t1rRgy7BW3SUUaTjm4vnjPzvvOH34kqHCFEH0/0GH8aD
vYD3K3jav7+3Xr6xTrbZPTyP1EgLDa84D/h1/upcih/ayfYtUyDZE+nxs8PvhcMMSq1OsXHtsqw/f32
WvRxakWTov7TcvMqDd3NZDRufHFusRNbNrTI3G9u5vuV96Ii18H8jPfCVoFMh/W+hEfPhgk/+
+JnUYUjhOjjSR/jT8PBfoD7dIaaP3p5P/WNPByuWOhcvexu9qVMt099q37jgmqnuAG58dO+yfhJt65
P4cmlww5X4jyFYcM2uay5FU1Z3jaYfGh6ud3QQYtotrOLjYfoO/RjBrMKVzA10NAxUZNruNIl73t1V4
YLhdxDq2ARabpkSkm3eN2L5sysz7/63C/qF2IqbuAYfxoO9gPcodVr/lTytn6SoNLYWWfMCymVQ+H
C4nZ9bTOz+tL4wbWoWfcxGDxzKbLasP1AB+OU0ZgluY3MSwad399k5lG2hYsNLZxF32xyoAWmcw
37GrdJ+uz01wpzyfWbT9d9LGzggjkz4+Hx4dmrZ1GRI4To4waO8afhYD/AHdq+5l/3Rbx/838xrgCeo
szORLXjQb/o9mqm2snK7XwmQdSsHz1jMuF8AbZ42LBh/HRzK8rLrPMIs70nnkg3rcWLbYxbJ2OM
O/RbMDhmjYtzxVvdNRkly14cyz2yTLeo2XiaY9GECnOm5PHrx/d+9F5U4Qgh+riZY/xpONgPcG+2/
W/7M6/umTf0vYV1S9fn5SfdT8cUPFcms5v5IipqmG+Uyoyf6zsxek4w0Niaww4tkwXmVpSVmc8x5
vN0LZoEXba+9ZLF9jki3f3JJUx0LG1JRa54C7LTmZzjIB5nbz5d1yJnekK5XEz54Pc+iCocIUQfN3aMPw
0H+wHuyoVq/r5VqFA5HHTd9oN0P8/3uIaZ3cwXUdFS841SmfEXVEpLRx9Zb9i+WyNqnVtRTtfuqD
UElvQ/LGjXpGs99xhDXYudtIGytNkxgiEaSxouzxW3yU5ndo6ZXDvz6RYMvGDOTPros4+iCkcI0cdNH
uOP4j//y986/HUAwB3Y/nf4ZfokIxa84u91/dpu3aeZTtertDPhouJ2C3crN/6CSinXa9Zaw3atXrx6lVv
kwrkdtYRc4245xf6HRi9evmwHCBrPTiDdr+5K3yWzpZ3drSOS1eWKR81l6a9l5tjJ9ZtPl+sVWTBnJv
m/xi9EIW7yGH8Y//7Fu//3T/7k8NcBAHfgEjV/dyXztl4oHtJGizpdsXRndvp6LF7lsMxMtZOVG/
+Y6q670E8pnOfYOsOOuvQfCmNOKG9QfHfBxPL6do1R47kx4xRD+36ceJqtvuNwOd9wUJcr2epkSc
E4U6l3knF2FqRLssWrWDRnpjx+/fjuD9+N6hwhxC5u+Bh/H//rj/7o8HcBAPfhEjV///qeUXhb7wYaX
vLTK0/KxG7mtydYY6bayRuP1GRZVCn1V0YKj2aVYfvb3SD99IdRMyvK6Frl76dzHY/aKa23FfaLW88s
Np9yr2uSTrM11XN6ulW50sc3saS9eI6hcfZmtEXpZjZw4ZyZ9NWffuWX9guRxj/9J29HFfKNxX//L79
7+FsAgLvxdGr+fA3QKtUc16uwm+PFRvcz1c6UcKub5osrpfEM5lKdPuxwI2jfT39omKwolc/cy8x1Jxx
3qmciM79QeQ/DjPt83edD7vw0W+PJLpju8bnmH9/uRmmOgzB507IqXZxl8SBM8n+cX4g0/tJ//Av
/4vnfiOrkmwnH+AHu0yVr/nKlEBm//seOGgrW0X0pff14oj7+5cdRwSOE+Kv/9s1/9c7fjKrlGwjH+AH
u1iXP8xdGSfR9Jqi6OLf+O+nLxxP2rT/4VlTwCCH+7vt/OSqYbyAc4we4W5U1f1ZTwicFfabCH4Y5XO
v/69+pfwuQGaTV31B3cTbhf60+/aWFJ+KbP/lmVPAIIW7sYL9j/AD37CI1/9RAk8VTobIv3IJtBDW/gp
+n7/Hrx+c/eB4VPELcedzSwX7H+AHu3IVq/r3xYKWSvVjXF28CMOv1w+u3vvtWVPMIcedxGwf7He
MH4LiaH4Cb9OnrT/0afyGiuIGD/Y7xA6DmB2Dv+3/8/ajgEUI86YP9jvEDsKPmB+Dgw198GBU8Qtx
5PN2D/Y7xA9BS81MW/T6G8sejdL8J7/CLGE4Z6lijX7y/
+S+C2K3sDGvaO+cecrPe+9F7Uc0jxJ3HUzzY7xg/AD01P2XlIv+UIvNSNX+fqLVtyX9IdqYa/Gx7yC17e
Hx49upZVPMIcefx5A72O8YPQE/Nz1GiqvKUIjOq+c/mjHnPXYOr+VnHF7/+wq/xFyKKJ3Sw3zF+AEJ
qfo7y9Gv+c9bFan6erJ9/+fOo4BHizuOpHOx3jB+AiJr/IibK3ezIvo5rzPeYqfu620OP9Eo37P5KNFz5Y
1nfeGfXPpp7dqiwSzjBolGnUa8uZWBi4uHkonmPjHOF2aZyRcveS5YetBmGOdyczpgZaEI0xvJF7SyZ
/05xjNaoSXp/wQhs66PPPopqHiHuPK7/YL9j/ACk1PyXkSmccheHgi8UdFpYgIW6+/3tPkk/yOFK8zk
arvyxIL+UvUPaZKj+QihYaFZ5xzJ3JyY+O929YrKpXN31YJxk6Zm+bfNixmWPI7utQY+ZFLXz3wtmNfd
w50fgLN7/6ftRzSPEnceVH+x3jB+AlJr/QrqaJ1Pm9Jf6uqe70hdKSZNgmKQAix0adF2C6urQoxuh+Rg
NV/44qW8X59g5TGRu5MxSEwt2LB04K92V4Uq0hCNzZdaRNEuzN2Yz5vKNJV2iLZtNsWT+cZNkkL
hD0mN2BM7k8evHd3/4blTzCHHncbUH+x3jByBLzX8pXRXTFzFJLZUprmarq52knkocWrR92g8vX+4
HCq90/aPhyh+n5Jr11w5zH7fpPmUWFlyKLNmx9EJWN1TQarZj2iDXZckj67NPr7URd6yYYntpqn1h
btPz7z9OTb9rkBmivTQ7Amf01Z9+5df4CxHGdR7sd4wfgClq/ouJCp+ulOqrr0xttTPRK2jU10vTddyh
V9Og+fnFq0+aXsOVvns0XPnjlOxaoovjobqbOVO5sln6cbury6bc5x9alTqOp9s3yHXJzDJplskey2acX
Vsm+bT8ohbMv/88GCUcDzzWDjE3Auf1+Vefv/ntN6OyR4h7jms72O8YPwAFav7L6eqappjpyqCh
WMuXZ1F5taAAyzl027VoW+9+aC/1V/re0XDlj1Oya4kujofqbuZM5cpmSaa4bMqZsdKO/ZVI2iDIt

WTk/Ep2ZjLm8o1MDjyYW9QpO3O43w2R0w1RHoGz+/iXH0c1jxB3Hld1sN8xfgAK1PyXdKh9dtVT
V+EEJU2+POsaHq4uKsAy2n7d/7zf9G4udVeGAaPhyh+nZNcSXRwPle0xI9+nG7e7umzKmbHijl2Tnb
ZVOnIu14KRp1Y/mzGXb2Ri4MH8opbMvxcM12g7zc4ikB+BS/jWH3wrqnmEuPO4koP9jvEDUKbm
v6hDQfPi5cu2aAprpr6QCqucuLrKVE99kTRV9+0dxkmP8bdXgvGijOWPU3LN+muHXOM2CwceiUd
sJAMtGzmzsVHHtEW687lcSx5Zps3OfMbZtU1NqBtyPkWmSdpmZ3ct+DTOOzvNRmkELuSbP/lmV
PMIcc9xDQf7HeMHYJaa/7L6aqkRlTP9za686uuevt7qr3R9gwGLxdHQMdc1qOeiWqv8cVIyzyD/Idn
UyF2PfnqFVAt2LM4zoRtpeifi+QwTHkbO5RraHS4G+941y2Tfmc+YyzeWZB9fWLCoZIRk/v2FYfrRgq
bHWDpC5gJn8Pj14/MfPI/KHiHuOS57sN8xfgCWUPNfWF/d7HRV1SC8OwirnKAoi6XDhYaOSRWVT
9AOV/5YMD3RQ7ZkqHyPmURH7FhxpExFGXfMp9obRh63aa9P70TfMZN9bzbjkrVNpD+kWrCoI+Y
fG6aVH2S4Pz/CxB6xtdcPr9/67ltR2SPEPccFD/Y7xg/AEmr+S+uLm6naZVwdDTVPL2ywu91/zDQNp
e2yPaOL5Y9lfeOdXfuoaCtnbyws78ad4pktm3Kmosx0DCvT/dXuczB02KQfLJzhrm0ycib7QTnjsrXFO
xSlKadozM1/LxxmL53SeBblte6NR+jupv3Y2qevP/Vr/IUI4yIH+x3jB2AhNf+ldYWP0gV4Kr7/x9+Pah
4h7jnOf7DfMX4AllPzX1b/P3Uq+YGn5MNffBiVPULcc5zzYL9j/AAcRc1/KeP/cDn9D58Brtt7P3ovKnu
EuOc428F+x/gBOIqa/1KCml/BDzxBD48Pz149i8oeIe45znCw3zF+AI6l5geg0he//sKv8Reij60P9jvGD
0AFNT8A9X7+5c+jskeIe47tDvY7xg9AHTU/ACf56LOPorJHiHuOjQ72O8YPQB01PwCnev+n70dljxD
3HKsf7HeMH4Bqan4ATvX49eO7P3w3KnuEuNtY92C/Y/wAnELND8AKvvvrTr/wafyH6WOtgv2P8AJx
IzQ/AOj7/6vM3v/1mVPkIcbexysF+x/gBOJGaH4DVfPzLj6OyR4h7jhMP9jvGD8Dp1PwArOlbf/CtqO
wR4m6jlIP9jvEDsAo1PwAr+
+ZPvhlVPkLcbdQd7HeMH4C1qPkBWNnj14/Pf/A8qnyEuNuoONjvGD8Aa1HzA7C+1w+v3/ruW1Hl
I8TdxlEH+x3jB2BFan4ANvHp60/9Gn8h2lh+sN8xfgDWpeYHYCvf/
+PvR5WPEHcbSw72O8YPwOrU/ABs6MNffBhVPkLcbcwe7HeMH4DVqfkB2NZ7P3ovqnyEuNsoHO
x3jB+ALaj5AdjWw+PDs1fPospHiPuMqYP9jvEDsBE1PwCb+
+LXX/g1/kK0kR7sd4wfgO2o+QE4h59/+fOo8hHibiM62O8YPwDbUfMDcCYfffZRVPkIcbfRH+x3jB+
ATan5ATifqOwR4m6jP9h/
+LMBANtQ8wNwJm+8caZ/6EhUTaJqFYn+9+eff/D3/s7hw2K2rppE1SSqJlG1syW6B7YSgDPxRlJNo
mpXnuj3/9t/Pfy0mK2rJlE1iapJVO1sie6BrQTgTLyRVJOomkTVJKomUTWJqklEga0E4Ey8kVSTqJpE1S
SqJlE1iapJRlGtBOBMvJFUk6iaRNUkqiZRNYmqSUSBrQTgTLyRVJOomkTVJKomUTWJqklEga0E4Ey8
kVSTqJpE1SSqJlE1iapJRlGtBOBMvJFUk6iaRNUkqiZRNYmqSUSBrQTgTLyRVJOomkTVJKomUTWJqkl
Ega0E4Ey8kVSTqJpE1SSqJlE1iapJRlGtBOBMvJFUk6iaRNUkqiZRNYmqSUSBrQTgTLyRVJOomkTVJKo
mUTWJqklEga0E4Ey8kVSTqJpE1SSqJlE1iapJRlGtBOBMvJFUk6iaRNUkqiZRNYmqSUSBrQTgTLyRVJ
OomkTVJKomUTWJqklEga0E4Ey8kVSTqJpE1SSqJlE1iapJRlGtBOBMvJFUk6iaRNUkqiZRNYmqSUSB
rQTgTLyRVJOomkTVJKomUTWJqklEga0E4Ey8kVSTqJpE1SSqJlE1iapJRlGtBOBMvJFUk6iaRNUkqiZR
NYmqSUSBrQTgTLyRVJOomkTVJKomUTWJqklEga0E4Ey8kVSTqJpE1SSqJlE1iapJRlGtBOBMvJFUk6i
aRNUkqiZRNYmqSUSBrQTgTLyRVJOomkTVJKomUTWJqklEga0E4Ey8kVSTqJpE1SSqJlE1iapJRlGtBO
BMvJFUk6iaRNUkqiZRNYmqSUSBrQTgTLyRVJOomkTVJKomUTWJqklEga0EAACA26TmBwAAgNuk
5gcAAIDbpOYHAACA26TmBwAAgNuk5gcAAIDbpOYHAACA26TmBwAAgNuk5gcAAIDbpOYHAACA
26TmBwAAgNuk5geAY33y8o3By08OVwEAro2aH4Ab0hbj21bhX756MWTYMuG5/83CfmE7L159e
biwptFiGv5dybFGe7jNY9rcxB+Yw3evte43o/BHtHDrWPmhRk9s3WVtq7gzm/5FsaHpRT3V5zSxotF
y1npMC3I90b+T7oSaH4Btbfg2PxLkOedLW5N2i3ed3btUv47mvWrjF6r9Ql68fLn7fzbJs1/C2V8Jw/f
R9b8Vo2/2YKNvX7OWM30jwn1bL8nkn9Dxn6HxQk8ymbF461hTQ+2v9xfaLT01VX6U8HGdnGJ2Z
/b3V/2LIreo0ZIap62rsKjm1op/lNLlBMlDG62oyd9faj6durpluVZI1e7dQbQ/o12M7rGAmh+ADY3fp
8ZvI6vqEjX//zlfCMYr3MrmWfYJDtu3TZr9s998m0LneTAjTcqNvnzx2NvlWvs9/qB7HunE46/GWkub
zli6daylQ3XtDh+P1vRvjbPstq+/cPrjmlvO/vrh9kl5WqVFrTH+QWFRmUv1JpcTOT3p9IriKxvm6m4c
Pp761PbD9cM33+Th4zhVc/OkNd0jNT8A24nfAZK3htVtnyFyntePjZfVD7//of6lreS018Gjnf17sLPtV

2G8gZutrxk4fFBrP7hk5ulSVt7Iwl6tuI1zQzX3qzey671tlkA+UX91/8PJabrJZnJt89dFmmiVdRwUljO2
4vd7dutmJ7NcMlSyjhWT9bvZfIi/DmsmuhtqfgA2k/6TecW3nbwzvww061nrnXHS6PVnfcGe7X/cJl
GzU4ON92y7dUza+CHttFu4S9H+sM23PPkTGnw7VpGMt8843reVUxaGWzHT3FDJxlaZy7LagnIDB
df2P672Zc/kSr8Ua0gTNXn2BxV6p2fNbV2gub3W2rK5mq/aPkX7w/RUjpPkmtjOdZYWblM+0WoL
uxNqfgA2k74BZF9R1rR5glDz4rHW21uqWcvBhkvar6Ibfp9yuwV12oVtmaf55q38Kl92ni9e841rbJU
qXUf6p/gkSYJ0/JX3sjDcipnKQzUPboVdLGdp7q7zrDKJ9mvoruxvr/almMgVWClVmih6Lk2D6f1dJrO
cQPnusaZGG/ZvtVSZXM2VePdWelbNCg7Z9j+Ph113F+
+Dmh+AzVzgH9XnexeI3hY3terL1Nh+GcN+7ROdZU3pV2NV0cNp92/Lr0WTcNPvXbiG9udt8jVjx1u
34qNqBgxnnn4VkianKQy3YqbSUNHX8QTZLM3Fg7W2LUm0X8PweX97tS9Faet22uWtkS1NlHz5Vv
iDXFzOCuOHMrna7WqvtT+vlC+7rmY9nZcvR1+SEzTD9g8meUrlPSZLzQ/AZi7wj+ozvQu0bzpnfOdo
X97WTzh6ZQus8YZd0uTdbv+Sb962+Zqns+mWpRk2XNHoS7Hee3wr+ROa/pFNnt5p0gS9wq1jTQ7
V7OZay5mZcHN7lWRxotF3IrBFrtRK34g0UTLw/FxmFYZobq31XdhLcqUZmid32opa81uzb7HG6uI/
Munjn58LMTU/AJtJ/8G80rvbtHO8CzQ5zvzC0eTcdOca+zTbZ9l+OVu8yk9b7a16Wppi0xUF1v5KpP
OOH9baKyuMt2Kq/FDNc1txMbMTbhqskHAm0f72at+K2UWt9ScsTdRcCRcSfxkrTC9npWUEklxpiv
ndXWh2oH2D078UzQrGw6SZV3hMd0fND8B24n8yr/b6MWn7DO07SWz1949dlmAZmfegTazz1p
bajRsM2zykTZ9SkyFcyJbviM3D2fY7l34DzpJ0Z/VvRPNoxhMfrS15dCfLZOwUbh0rM1Rzad2HNDvh
psEK2zeTaH97tYd00UWN/iCt8qdqcjkb/JlNc43+MO2sl7T8mNZ5Rs1s0yT7y+HY5amQpeYHYEOjF5
B13grymrEjT/udIFrReRazT7rJEzr7akavuuu996a2/FaPte/DvTN8I9ZcW/QNaAxLCNe21mYWMpYnc
5TpoaLn1TpxcU220Ux3WYLPTc5TcizcmX2z1b70yaJ2F4LB2yllJrFYeVGju6ekOUiW02oun+G7vfpfF
NO5xndO3rp2uOwwo+/1qjt5P9T8AGwrfAHxz2nOZvRCevL76JTm673Z6Bew8ns8qxg/ldbh2US3nt
ATm17Uk1xT4Rnt3NxfFKuL/lVFa3hl8CJxIjU/AAAA3CY1PwAAANwmNT8AAADcJjU/AAAA3CY1Pw
AAANwmNT8AAADcJjU/AAAA3CY1PwAAANwmNT8AAADcJjU/AAAA3CY1PwAAANwmNT8AAAD
cJjU/AAAA3CY1PwAAANwmNT8AAADcJjU/AAAA3CY1PwAAANwmNT8AAADcJjU/AAAA3CY1Pw
AAANwmNT8AAADcJjU/AAAA3CY1PwAAANwmNT8AAADcJjU/AAAA3CY1PwAAANwmNT8AAAD
cJjU/AAAA3CY1PwAAANwmNT8AAADcJjU/AAAA3CY1PwAAANwmNT8AwHX55OUbBy8/OVxqdT
devPrycGUbXZ4o/TUY9mYn2YcvX72YulW8B3C71PwAANclqGvH5em91/xD2d7IbcNkZT9s6hX+m
wyA7aj5AQCuy+h/yw4r1Huv+RetP1vcD/8qQMUP3Bk1PwDAdRnV/GGBe+c1f1+4z8yr379+n/orK
n7g7qj5AQCuy7jmDwrVpOZPLqSF8aHJvkUw7qFDcCWshrvLu2vBf04fJOkEd5P7wdyGNDM192i80
YDBTDuFsfrWTZv+U7KC0vz3xvOJmhQWWOwHcFZqfgCA6zIUk6/an7qSMagym8/phaDcPJTEQzk6
qy+ip/sEdXZc1zaCiWQGKZa+
+aTx0gPBXBLD5F6+6n6Mss/Nf2ZC0wvM9ytOF2A7an4AgOvSFY27CrIrTNuCMbjRNEwvlGr+Q6Og
JI2vdGVp0me40l2I0/Qt+qkEeRaUu9MJ+gHTnCV944NhmMbs/LsG0/OZWODUQPEMAM5DzQ8Ac
F1GVePhQ/hzWnYG5WRczHZNhrp0+ko3TNoibhNXxDvxpX6QoM2keAZ78UrSC2X9BPaiLgvmH0u
y5xfYN1u0boDNqfkBAK5LvrzelZpJZZwplePSNG0yf6X7HBbK43G7FjlR4tEoE9Ip7XUpu6vx0mZNT2
HB/APj1t39idH7WQ7GywI4KzU/AMB1iSvgw+f+eP9QQ8Ytd7aq+ccXuw85k4mn5dvGS6mu+dMp
LJh/rnhvdPcnR5/ouHTWAKtS8wMAXJekmIyLyP5GpuyMC+O0yfyVzLDRuLkWkQVNevm2Xcruary0
WdNTmJ9c12KnbZRknx0jGKIxnQxgO2p+AIDrkikmx+Vjf6PUcoWaPyyux20WlN9pmmn9cGHjJMe
KNf/sUGnXZFMKC9zdCgY+et4AK1LzAwBcl1wx2deNe/2N4eqhnOwL0+RKWrxOX5kepW8ynbkrbN
M0JdMJhv5H186FKczNP17O0H5mgclCitMA2JyaHwDguswUk+GNoBSNRbVsqQSNr/SfX8Sjd/XuTj
710CBNU9a1Hwt7r1nzz84/P529rsnU6FM9l84aYFVqfgCA6zJRTA5F6uhGWLvuysq4ME4Hm7/SfQ
6HS6azN66bxw0mVlE0Hi8ukuOlzZqdQmn+Q/e9fcpgW8LbudHDrntLZwywOjU/AAAA3CY1PwAA
ANwmNT8AAADcJjU/AAAA3CY1PwAAANwmNT8AAADcJjU/AAAA3CY1PwAAANwmNT8AAADcJj
U/AAAA3CY1PwAAANwmNT8AAADcJjU/AAAA3CY1PwAAANyiP//z/w/LdJCPLswYoQAAAABJRU5E
rkJggg==]]></redcap:Attachment>

</ItemDef>
<ItemDef OID="b30" Name="b30" DataType="text" Length="999" redcap:Variable="b30" redcap:FieldType="descriptive" redcap:BranchingLogic="[q27] = '1'" redcap:InlineImage="1">
<Question><TranslatedText></TranslatedText></Question>
<redcap:Attachment DocName="B.png" MimeType="image/png"><![CDATA[iVBORw0KGgoAAAANSUhEUgAABVEAAAEJCAYAAACdcmdaAAAAAXNSR0IArs4c6QAAAA
RnQU1BAACxjwv8YQUAAAAJcEhZcwAAEnQAABJ0Ad5mH3gAAE9sSURBVHhe7d0PjCRnfef/b+/aY
PPHNmBskx+RHat7sZYlGJIg08PxL4egZ+6SJbnbYOVg5Oh3PVyi0zQSkwOxF+mX2wguG4lunS5iWrq
zliQyTEJugrLdIJRgckzHR4IxYTI/Z7rlc3T8wDYGjPljB3u3f/VUPVX1VHd1dV3VV3VXX39PtltaerpqfqqT/T
O/OZ5/k+hb5FAAAAAAAAAAChjumPAAAAAAAAAAAIAQhKgAAAAAAAAAEIEQFQAAAAAAAAAAiEKIC
AAAAAAAAQARCVAAAAAAAAAACIQIgKAAAAAAAAABEIUQEAAAAAAAAAgAiEqAAAAAAAAAAEQgRA
UAAAAAAACACISoAAAAAAAAABCBEBUAAAAAAAAAIhCiAgAAAAAAAAEAEQlQAAAAAAAAAAiECICg
AAAAAAAAARCFEBAAAAAAAAAIAIhKgAAAAAAAAABEIEQFAAAAAAAAAgAiEqAAAAAAAAAAAAQgRAVA
AAAAAAAACIQogIAAAAAAAABABEJUAAAAAAAAAAIhAiAoAAAAAAAAAAEQhRAQAAAAAAACACISoA
AAAAAAAARCBEBQAAAAAAAAIAIhKgAAAAAAAAAEIEQFQAAAAAAAAAiEKICAAAAAAAAAQARCVAA
AAAAAAACIQIgKAAAAAAAAABEIUQEAAAAAAAAAgAiEqAAAAAAAAAAEQgRAUAAAAAAAACACISoAA
AAAAAAABCBEBUAAAAAAAAAIhCiAgAAAAAAAAEAEQlQAAAAAAAAAAiECICgAAAAAAAAAARCFEBAA
AAAAAAIAIhKgAAAAAAAAABEIEQFAAAAAAAAgAiEqAAAAAAAAAAAAQgRAVAAAAAAAAACIQogIAAA
AAAABABEJUAAAAAAAAAIhAiLp0etJYKUih4DxWGj29PqjXXWF+eU1hpWFn+1iMxj3xV6sdDdLnjonJB2onXXEfoo
MxLO7LSlg372PzHqHqPs4Vb2GrGzEO5vtdbUO5JH08O5vtDbdtK5JH08KNuQ9Cjmc+2g0AAAAAAwPwhRF06Rdm8UJey
XurU1kPqJUdKPcr3XAc5U3Zqxqxle4aB5B5oIlBVWnqJUdZzqxle4faf1Qo1cT/rlhsR+14AAAAAADIGiHqMip
uytmqfi8ilS2qz6j9Qr3bEV2ojPB5Cf8qkSjzrkShhnxu59S8XkrUFhPrFsh3ZnsTudVVVBfY4DvEZ6wC+k3IoaTiAgEkzwY
AAAAEAuCFGXVkW0W26w8SDhYSnHbcq91h8ns7P2+/aKGWQuDpqf/Z6WrIR8CJ2KhMzX+kLQi+RudR4JXIx7eE3GjgdF
GPu31edcGHd5fZI1J4zwEz5+5nbDPQ4+O3Bz66vPV/0ngUVcE/2bO6F/vSFZY
AAAEAuCFGXVkW26W26M6vdGbW/4Q6XL9a3wXoyqlqKuRek+wmpSjqvaH59Ybcpy+dvwY1GoA3+0T1f/mBDS
KhhU2xhoJhnyBNZWt9zcDA/vxB/pTDs6+rSYi4o6bYZbTdXbJsnvvqb0W6GSHGEpBeRRfAXFWvsr1zcKZyR4KGHZ4z
2fkE3jHwuh/VYHLKWyxLMOpVOBYfDo5+h9m0DtThRy72hd9EL4HmZEQUxPQM4rRE5hf9F1dSt79hGlEzi0trY5dvO7Mln
npwoBdTB3rydPYhgNNA8m8ipnFdAvf/Qw9ZmS/E5lOO8EXuXeTlJMg/HQNtgsSJYddfsA9rFxvPBU8B0MqSzZ
uVzfs0eeGxaryYfCPMk4pg9WdqSq2Ukh9WSf2kWo2f5P8BW0P3lQ7A4E80QHdXXZ+Fe+zUSvW+1L5AXwUFBR/F5HZ3bH
sn4vdTVAsHOr2TcuPLhoeSBCzOptKTzKRjwSZm5p8iL+VxkXPjKzAvF8IOAxp2S+5Xt2sQB63+2rvQ+a2vf6RMj3Yyo73gO
+w2wpndvN+90Dt7rH8ae9Gcu3ODd+G6kJoXQrQQYAs2x4ea6kF5XCa86vU6DQOm5yWc+gKjJ7A0fW3p7K/E7I5l
czuPaw9dIEJSaHW7j33JcrSfXyqwVGOt3Y61DmtA8xPl7DVXubj0dOnuCcQuv6qD/gaQXvH/ZLC/A7aKdNdZmEPbn
AEZJpBvB3f+TEnMBEC4iCKAPSfKGwKs7hz1ojZMeHd6ByTb19jZsbfE/XQ5l2TzDJQ8VlkxmsktS1eYP6WqVLj4t
WpD0NkOdRGVwXcY5V5PcMYL2Hmi8nxtBtbRnazGNTbYV07i0vqL1XoM5i8OBm6Vnw0Tw9X3LQcqGqqEOZxV/7PmN
xv69Gg9Xs8p8Qk2B2Q6x/dqe6gUazXjH0ETuWoT5/d3vtoAo/JjAfTLsKgDcqsV17ojd0ohn/Ijq2yvaANn0KL1BlpWW
LpFl3eZsZSvxCVJuhJK5qt2jyOKc2aO/DbnxTfQ3uj0ujFOqArsv0fzQUE7Fj0o/f+bFcwHIOvx8DpuyGq2JBNHCzTY
3hmMkTNB0Ej6u1wHmVIx9L6cJ3rK3eL0wNTsUyOIKOmNwx3XJH3a/zD8uHjNhT7Uf62C2KNjQ/OlHeP+xrWCzk6jS3ceOkzD
JjUwNQH8w+HZ7vo6+bOHgFSK0yKrq7oZuN8SSx5qKzlb25mEYrYnRxHdL1r9T+tp0jEjtsm2CZWj4K0+T9YSbGtvHl
OPbmaCOj0VZKTK7ESwQz5ziL+NtmuzdNrN5T4+Ogt7MFCCL0k3cw9x/R/D1WeLUgOIcsTLAXXx5sIX2ExZm5+/yjtpD
3+GHhvV4bjzTv5ZxgXK0Xbc1B35r0HeEOrfNNQOE+EOq3oFm+MrvEJFzRzKOEbVmO/zhVJ6H5GkVzhYjZeL4DFg+/gAzqJ
BQ19JHmlDyHaJtgDvNC6TWSyWuk/fOdz8PnuCo+lz9EbJdBZv7a3GBT3jTM6IX3bydKQ7EaVwaXxDW4YWbIHLsVHpDj
N0G3QdvvUXsbrMi8o6aIzVEnDT3eXm+hVoeG/XbQlODYrbdbb/dczrydrV2a8Gk+/6WVQfnPKfxJo7dlPOBBkzt3vO4gWGl+
A0nljPjaT37CybWTRsYjUoJkH3cK/yH9MM/3gUnF92P0G2uLvt0grxaIO7E8jJCzCY2lxIPXWpvPSE/EnEvLR+UKm+4pkzHu
T5F2nadH+jfgvPpWQCwiAeZtfPWb09+LuWT+7A/ALexXMrbimPWXp7F2SBBAbEqKEtW/qCg2sHDtq8fwNDtn+qvHBOsKZFtEcL

+gZ5wQ+vsJ8/9NXvyp/WeR8GgNz0Hxa5/Od6AYuAEBUxleSkN1LbnBFfMSZJitM70Ri+HI+7744c7D
pf686+X7I/4a8fLjUwH4qbZ/0guOFOjFWVQIZacsoTKG6Jgvk22OvYvA/0seV1TIn2Y97LA8Pk1cz3hZ
xnoE/SdmNSNTtwdeukJv6eAubL+z77PvlM7zN6CQCQh1c+eK3c/PXn6yXH5z7+kHzlLx/RSwCAzF3
6gsjlr+kFzDtCVMRUlM2zbuVUc/Z1NbrYnOl8y+vt5wScijnre1s23JlrYvN7cqqJpBS3XmZRF0x117vh
6vxxe81a56Kmj3+wdqsx7F+aq7LhnTIV7Onh297K+dBcNQLH9nm/hqd7bHkdU6L9mLVbm7Lqre/p
me+ta1Qq+NvI2oTnyA9c3XYDi+kT+5+Q+n11vQQAyIOqg/pzD7xELwX94W9/Tb73raf1EgAgc5f/RK
T/fb2AeUaIivgq29LyctSalHTA48w8bynX5YJR4NOfUMnSXNWB0GQ1HAPbsp55Q/a9of6KMUnTHP
Jqu9rKUt8KRKiWomxe8IdlN1d1gOaFzqMm/ZolJ3AMttM8tryOKdl+ipsXpO6/2HmtOZGTdS/7h6Br
7yrua1NNWBO03bzfw9odR+bHA8R339fvk7v+7C69BADIw/FLBXsYv/oYRgWozd+8Xy8BADLXf1Lk
8sf1AuYZISoSqWyH11+stqz17tBiV3FT9gZfW21NVr/R7K0XKBlgDM3OdKKjFJgB2KiyA+qc9bt+wOe
ya1/OW03PstS7fT9Yt1Wl1d8LTpaV1zEl2k9RNvf60h16sXq5MUzeVpHt4EGK7B96PbFTEbvtVlv6Lf
8+sqlzblwHu+5ulByOB4jBnYn/6Wfp7QQAeVr50kvl2iev1Evh/uFL35ZP//6hXgIAZO6y9RvZJcpbzbt
CX81aAgAxtTcK4nSSVCHqQGAKADGo4PT1/+318sAjD+g1AIA8lB56od0LNa6tu8vyiteFD/sHAGTgit
8QKfBL9ryiJyoAAMiVGsJPgAoA+VK9T1Uv1CTUsH7qowJAji79AfVR5xghKgAAyM1HvvgRezIpAEB+
VP3Tt33hZSProI6iAtS7/+NX9RIAIHN2fdR79ALmDSEqAADIxe6Du/LBv/igXgIA5OWOL18/tg7qKPv/
8zH53Mcf0ksAgMxd/n9FLn1BL2CeUBMVAABkTg3fV3VQmUgKAPJ189efL//8Czfpclc+dzjsnX36+
XWV79IrwEAZOtKXR/1Zr2MeUBPVAAAkKknnn6CmfgBYAZe8MMrEk0kNcoz/3RJmu+/X3705DN6
DQAgW9b77aWPWx+fchYxFwhRAQBAZlRwqgLUh594WK8BAORB1T99yxvdvlOf8OJ1f+R7/xo/k7g8
xKSAA5Kb/HZFL1EedJ4SoAAAgM+/77Pvk3ofv1UsAgLz83AMvkRsev0ovpeMrf/mI3PtJ/igGALm5/
DXr0dELmDVC1KXWlo1CQQrGY6XR05/LSa8hKxtteBob7jtWZG8mzM3Qs5LHL3GinPuVhoy6tT55
9d55H7NjwLuWyCW+n11+djffkwvAQDy8vJvPE9e+eC1eildn/zPB/LN//19vQQAyNylXZH+Y3oBs0S
IurR60lhZlaZecpTlzFpRP8+eHfiVasLfVIImPi+9hqzXor5KXfOCrAYvunRqJSlMENguK+5bIB7V+1T1Qg
UA5Ovqp47LG++bvg7qKKo+6u//
+y/bHwEAeVD1Uf+79fFZZxEzQ4i6tLpy4KZA5bp0+33p9/dkM68MdWzgt6QmPS+qZ+S4YK99XtxN
V1vqevelWy87K5qrQo4aA/ctEIuqf6rqoAIA8veWvRvtIDVLqifqH/721/QSACBz/UdFLv2xXsCsEKIuq
96h7OuncuqE5Nf/FGmL1zOyJ41zugtquS5bFedpcfOsVJ2n0twlRQUwPTUTf+WPKvZHAEC+Xvu1F8
vLHr1aL2Vrb/f/2A8AQE4uf8l5YGYIUZeQXbvRDN2aq04tx8GuiKp3o1E7c1T9zGAtyGCd1bDejbH3r
wVreI6qN+kMVfdfl6zW52A9S6+2qH6ENy3GPgPncEP8zZjnydln0vOiqHaWYvWMNHoeB0LzkpzUn
VFl/9A6olGM9obVW21v6GMZPAcJrkvI/RZ2vePcb4PXz35E1ImNY5b37WT3p2vSa2eZ6D1Ar1TMr
49uJI6QOz91pzz4+IN6CQCQFxWevubvXqSX8qF6o1IfFQBydOlPqI86Q4SoCDUUGGl2/cyRYVRHai
WjzqrR43EyanuDNTzVurCgpuQNVXdFt3UUZ5+DwWRzdcJ9Fjflgjtk3joz5/RGeo1z3nkq1y+kUEahK
q1+y+tVOsTseTxK50C6+umwipx2N97ZkYsDJ7W96x2NX1c3yXVRQV7I/eZcj1EBZPj9pu7d0GC5U5
NSIMjOSpb3bcz7M2CCa2eZ7D0Ay07VQP1M7zN6CQCQFzV8Xw3jz5uqi3r3h75KfVQAyI2qj3qP9Z
H6qLNAiLqEKtt96Xfr4kZ7Um3Z9TH72zrxbG/4AZBXL9Won9mpyfrIxEaFec7r+3uboWUCxu4/wN+
et3/pyI6XBPWkse4GPca+Wzo1imzrKP523M2ofdbOu/Fbsn0WNy+If+rOS9v677wbglnn94JOUJOd
F4P9um2ZLK8uyolT+ukYlS23beb5V9ri53BnxMnhkpwj67VGqYGh+21ofyZj2/b9ZrYlbFt+kJ3U/Ny3
/uvD789hya6dZar3ACyrT+x/wp6NHwCQv5UvvTTzOqijPPTV78qf1hmBAAC56T8scvnP9QLyRIiKIW
bPtPoFPwgdDgOHletbE4Z54aotPxwsrp3xAqzOge4z2bsoO14eaey7suW3deeiJIl7zH364ZMh8T6Ls
nnWTbuaslpwe08Gz+8kipt740PW2PblMOpEFdfkTNjxtXf9XrVn1pzjSXSOrPOzp0NEI3gPvd4Dhu43
s8et0evSPk86CNzLYfa0LO/bsfdnmCTXzjLNewCW031fv0/u+rO79BIAIE+vfPBaufnrz9dLs/G5jz8kX/
nLR/QSACBzl74gcpkJ/vJGiIoBI3qm2Yqy5iYxIwK3UyfSDKjKcrKkn47SPdC9+VSoU/LqNhbMYdKRw9
QnMMk+K9tGr0FHOsP403RKoi+fcf2NgNIP3Kpy1j2gqa6LruEZOrw/aOh+M8JCawf20Hd33yNrsaZ
uHu/bBNduyvcALB93Jv6nn31arwEA5OWGx6+Sn3vgJXpptu7+0APy+Dd+pJcAAJlTw/r739ELyAMh
KibUkeHOgTHCo7mX3TFUvMKUSrD+5Oz05HBssVSf36vSHRZuBG7V08FeoUmYExB5PXXHCbtWR

dm8EN470w0rF3t+o8nvz/SvXdh7AJaNCk5VgPrID+h9BAB5e86Pj8lbvnijHL9U0Gtm60dPPiPN99+v
lwAA2XtK5NIf6OfIAyEqJjR/gWm53vWGbAcfk9YLHS/
+Po26n7aO1NZznJyneELGlj4tn5Sxl7S4KW5lAntYuDF8vno6/CyPP0dt2TB7nbo1OM36o0lYbdzT+xj
s/as0z83XpEi53bcTXLtoR+GPJpiWGsL/wCMP6CUAQJ7e+Nc3yAt+eIVemg+qPuqnf/9QLwEAMqfq
o15iYte8EKJiQElOusnV0EzePbnoFnIcO/Q7J6WTXtA2qm5m6ibYZ6+xrodpl6Ved5OsPCfnMa5rc1f8
zphdOfAu6QmJc0m9HrXW/XH+/I4OP6sSyOGSnCOzLqcKFN26qMaQ96TUzPKqx6k9GZQdSHa9W
p7pDpOf0CzuW0usa5fme8AU1xDz7yNf/Ig9mRQAIH+lh1448zqoo3z6v/6D/MOXvq2XAACZu/xZkf
48dRU6ughRMcCcBCnYW9IPAlXYNTChz6yYNTCbq/5Q7faGHhpuPdIev510n72GrLsnrnpWNjeNyY
Nq65JPjmrWsmzKrm5er3HOCzBj90asnBbnDulIs+ke18Bw8Amvix8oDvbcjUlvX80sH+xxaoTFZo9b
oz1p3yaRZnHfKnGu3QTvASU/dZXaee9gZMOb4h9Hze6Du/LBv/igXgIA5OnaJ6+0Z+OfZ83fvF+
+9y1qZQNAbtSw/v739QKyQoiKYeYkSJ2alHSoU/LTE7kw7YxI5vDy5uoUoVGwBmZzVQdQXnhTlVZ
qs9e7kuyzJ411d6h6Wepbav3okCq98zLMnFndbbN3TastiX+aKhIo72oZDmATnCMv2LO4x2xOsJTEi
HvXrLNaPevPNp/YQt+3SpxrZ0n4HuDXW7V41zBubVssGjV8/85P3amXAAB5UvVP3/aFl81NHdRRV
ICqglQAQE76T4pc/rheQFYIURHKHgYdUpOy2rLWu8Otp1KR7cGClfuHRs/BBOwamMZwbZddWzOj
eqgx92n23LN7obonbiCk8of1p3hehhRlc2+4Tqg9hD5hYBeYJMs6ZjsbHhT7uljH3G/5QaqtKq2+0dZ
ACYJo9r07eJA2Z5vTZZMLft9aYl07S6L3AHUsg6+ttiava4u59cTTTzATPwDMkOqBqnqiLgI1pJ/6qAC
Qo8vWb6bUR81Uoa8KBgJAEmo2fT0ZlAph96btmTxL+lhOtaYNWBfEUbp2yJUKTit/VJF7H75XrwE
A5EnVQVWTSS2SK597XLbufr3c+uoX6TUAgGxdKXLFb4gUbtbLSBM9UQEkFCxRcGZtsUO49nnrWC
J6ZB4tR+vaIV/v+
+z7CFABYEbULPx3fPl6vbQ4nvmnS9J8//3yoyef0WsAANmy3m8vqWH9TzmLSBUhKoCY2rJh17o06
pWaJQoWUK+xIqv7demmUqJinh29a4d81e+ry8f+9mN6CQCQJ1X/9C1fvFGe8+PF/NXt8W/8SO7+
0AN6CQCQuf53RC7doxeQJkJUAJNRNS8XfPx7cXMvpRq/C+YIXDvkR/U+Vb1QAQCz8XMPvERueP
wqvbSYvvKXj8jnPv6QXgIAZO7y10QufUEvIC3URAUAAKEefuJhec32a+wJpQAA+bv568+Xf/6Fm/TS
YqM+KgDkjfqoaaMnKgAAGKKCUzWRFAEqAMyGqoO6aBNJRVH1Ue/
+0FftjwCAPKj6qGpY/7POIqZGiAoAAIbc+ak75cHHH9RLAIC8qQB1UeugjvLN//19+cPf/ppeAgBkrv+
oyKU/1guYFiEqAAAIUDVQP9P7jF4CAOTttV97sbzs0av10tGyt/t/7AcAICeXv+Q8MDVCVCTUk8aKm
uVbPTaknWjdOGltZ96MOYZeQ1Y2gmvbG+7rV6TR0yunpfZjb3NEO2zTne9M2m1bkPugvaHbWJC
BSwosjE/sf8KejR8AMBsqPH3N3x3tuqGqN6rqlQoAyMmlPxHpP6YXMClCVGCGeo0VKZRq0tHLWe
pd3DH205RdQj4AA+77+n1y15/dpZcAAHm7+qnj8pa9G/XS0aXqov7+v/8y9VEBIDeqPup/tz5SH3U
ahKhIqCibe33p99VjWyp6bXby3l8WRhxDryHrtTziU6UnF3eC+2qea1hrF8VRuA+A+aZm4n/nJ98pT
z/7tF4DAMibClBVkLoMVE/UT/7nA70EAMicXR91Vy9gEoSowDLoXRQ3Qy2Xy86Tzo5cXJwUdTFUt
qXfrYs+w8DCUMGpClAf+cEjeg0AIG8//ffX2UP5l8m9n3xYvvKX/NsDALm5vGc9mOBvUoSoS8eoLbl
i9kQctd6oc2mvz7s25fD+IutuGnU/VwKfNLcT9vko6Z8z+/PmMP7mqvP5EYU0/WNWj+T1Rv2h/FU
5e+GMDvk6shMnRQ3UUg17jG5PvHbHuTbD59DaumyYrzfqkQa2MbA+/BTHaYN5POpY/P2rh7vd
9nnrupbrsjXQXdYu3WC83n4M3DfArKgh/A888oBeAgDk7YbHr7Ink1pGd3/oAXn8Gz/SSwCAzF26R
6T/Hb2AJAhRl05R1s64PREPpOs8s3TlwE30Aj0U27LbdJ6Vz6xZXz17ldNV/awjB/4B2PywsCxn1nRr7
RCwJIMj5zu1UswQa5bnrCO1UkFW9fYcal2SINUYyl89LZXimniHs3Nx+hCvfEbcU+2L2e6pr43Dfn1w
Z846FVQOrG+uDgSpE7VBHcuqeFvWoakKSlf369Ld2wxcdxW+lsJKN3RqUprnibKwFD7yxY/Yk0kBA
GbjOT8+Jm/54o1y/FJBr1kuP3ryGWm+/37qowJAbp4SufQH1kfqoyZFiLqEimtuT8R9OXQTovauHwi
JEU72Dq1XOU6dmIcI1VI5LW6M2gzMjmSEhV6w15PGutvjsyotu6am9WjpLXRqsh4jjUz7nFW2rTa
Yw76rLadd22HVPv12d+vuV8TsRaoYQ/mrp9X2zVA4xpD+4qbsuefNfnTFa4Z1BPULwcDQN67d6V
wbV7neDX69Frbev2+maYPxeh2aFjf3vOc+P1RXYWtXf41/TppyLsFxAmnafXBXPvgXH9RLAIBZeONf
3yAv+OEVemk5PfTV78qf1h/USwCAzPUfFrn853oBcRGiLiOvJ6IfaPUOndivWnXCo32dFJrDwO38bS
5UZMsNoJq7fi8+s+6n2wPUXFff8ickqmx5QWCs3pgzPGfVlj+Rkh/mWi0Z7IY7gtk792TJfmJsJ0EYq/

Ua636vzepZ2QxPUMe3O61rY6vKWbchRsg+cv3+obPdKdoQeH0UI1Q3Q2s7cNWB6t6okwhkSA3f
v/NTd+olAMAsvPLBa+Xmrz9fLy23z338Idn/n4/pJQBA5i59QeQyf8BKghB1KRXlxCnnmRtode1x6W
U5efqkHXYF11vUMHDn2VzwQ7mmeJ0KuwdeWOgN5ffWWcfkDu+2H8bw7cAQ/VFmdc784HMy
Yb1zLRMP6W/LeT9BlVZoz1klRrtTuzaW8kkJ3d2o9a4p2hC7Z7Zxrq2N2WUO3P3Er8sLpOuJp59gJn
4AmLGXfPe58nMPvEQvQbn7P35Vvvct/m0CgNxcVvVRv68XMA4h6pLy6oraPTl74nSqPCUnKies/w
+uV3ngPEWoFiOYcodmt90x0xG9I6exkOfM6GmphqaXQkPC+LP0tzf8OqBVo6fp8kkSbhdl80L4jP1u
cDtiPjEgE+5M/A8/8bBeAwDIm6p/usx1UEdRAWrzN+/XSwCAzPWfFLn8cb2AcQhRl5U3tHlfDts6aL
N77VXEyQqN9VP3hsyCUdfTDi/9upOjwkuvNubQI2YYuIDnzB/KHyXmkP72hj9RVLUlIzuhTmDqa5O
CTNtg1JUdKNlqa55jln7k532ffZ/c+/C9egkAMAsrX3qpXPvklXoJpn/40rfl079/qJcAAJm7bP02eukze
gFRCFGXVklO2hlkRw52nSHNbh3Rkv0Jf3347OuzV9w864eaDXeSp4E6pCVnqL3i1eKc2KKdM2Mo/
xjjh/S3ZcNLUMtSV1PRTyvVazOhHNugZuhXPU7tScXsQNWYoCtu2QJgSvX76vKxv/2YXgIAzELpoRfa
D4z26f/6D3aYCgDIyeXPivTp2jMOIerS8ntyNptOOObWeCzq4p/uem+Sprnj9gDtSK2mA77BOqRmP
crmqj9sur3h1aUsxB5LvWDnzBzK787+H3iYIV70kP5e45w3jL9cv5BOuYRUr82E8miD3pbKoIM9Trvi
ls8dW7sVSMn/+v/
+l34GAJgF1ftU9ULFeHd/6AH9DACQi0v36CcYhRB1iZkzpqvehd7w88Ds5sYkTXPIq1NqC+shGaxH2
VzVwZg/Ll1GT440LNVzVtS1VJXmauqhoTmUvxxaW8AoiWC9cuSQ/l5D1r0CqtYrAxMwOY/JJkhK99
pMJoc2VLb9IfyBurRGfdmzm7MP3bEU7v7Fu+WOl9+hlwAAeXvjX99AHdSYfv7dP6WfAQByUXijfoJ
RCFGXWWDW8FPiTzbuDltXzPVzyAwvRw2ht+tRGr0uXeW6dJPWu0z1nFVke7BA5v5hSrUxzaH8o0
NdMxQeNaQ/Xl3VCaV5bSaVQxvsIfxhxVBVSNvvp1pfFohy1RVXyf/4lf8ht1x3i14DAMjT373yCf0M
UV7z1pvkbe+5VS8BADJ37FUix9+kFzBKoa/G9QIAgKXxwCMPyOv/2+vtmfoBAPm648vXyysfvFYvYd
D1P/E8+a1PvVGedw0TbwFALgovFrni/daTq51ljERPVAAAlsztN91uD+0HAOTvb27/tjx2PX/ECnPlc4
9L9fdeS4AKALmx3m+Pv8f6SIAaByEqAABL6F2n3iUfeMMH9BlhxJ1TZkODL27LhTbbiPCabqGZYeyO
43dBH1jOAAwAwhz788x+W07ed1ksAgLz84PnPyt7rvqWXoLzidS+R1X8bNhkqACATx4oix39eLyCO
OeiJqsJVf5ZqR84zwquZ0VcaKU3oAwDA4rjnl+
+xh/cDAPL10M0/kO6t39dLy+3al14l1d99rV4CAGSucI3IMTWMH0nkGKIWZXOvL2oeq35gxuuuHH
iTiKvZsNXn92QzxwzV1qnJeTqkAgCWjDtj/3VXXafXfAXAAADyonqjfu+aZ/Z/TS8lIBqgpSAQA5Of5ukcIL9QLi
mn1P1N6h7OuncuqEZJedlqXedUNc/9Gtl/XnRZq7pKgAgOVzy3W32EGqClQBAPlR9VE/96Zv2h+X1
S/8xivsofwAgJzYdVDz7rl4NOQYog7XRLXrlZZq4nZEtYfVD9UoNb/OeaRVL1Uprp0RP0YFAGA5vfm
WN8tH3/5RvQQAyIvqibqibqs9VFVePoLv35CLwEAMmfXQX2HXkBSc1ATNUKvISuFktS8lNXRqZVSq2H
aPu+HuNXTfpGB5WVM8uWd46hJwTA7YddqEYTfT/4kcHneY0nPYU7fC+q9b2DCO//8rEiKf0cCAt7
7s+
+V2h01vQQAyIuqjbps9VGfd82VctfvUJMbAPJztcixO/VzTGKmluy/9bt3vCVptOcPst1WY2ZPGu
htwVqXlDsFvVe01qobpeqIkoSO1khtC+I9Vd0Yra9/2bgFghnqNlWAPfSBnqjeq6pUKAMjXfT/zuD1r/
7JQAer1P/E8vQQAyNzxO0UKL9YLmMT89kTtXZQdnSKU61v+RFSVLXHLmHZ2LqbU+64s9S0S1NFG
TQoGTGKR76eM295ryPpg13tgBlR9VFUnFQCQnx8/57J8/g2PLkV91Le951Z5zVtv0ksAgMwdf5PIsV
fpBUxqfkPU7oHXE8sevu/1HjWG93cOpKufTsfppZpmrVUAABaVmqm//attZuwHgxO2jWqxuPoOpD20GPv6
/VjZG3Jwf1FBNd2Pdzga0fVgsysDVEya98oca+VK/5xJm1f2OsHyncO1O806o7arx19P/kG2p/K9Ux6
DsOEt32wXungORo8P2HiTbTn8/epHqPqpCY5P0DQbdffJvf88j16CQCQl7+/7Xvyjy//oV46WlQd1O
rvvVaufO5xvQYAkK2rRY6/2/p4hbOIqSxEiFqud4fDT/uRwnDa4qac1WVWRfblcGnyBRVsDU7a1ZTV
wqr1/3hUiFMKG3rcqUlpMKBKMklYe2NETUjVYzgYFmXWhihZtW+khNcqwXEma58TyIW9vrlaGBH
2qXNitLNcl/GVM9SxDbR/6us5/f0ej9OrffAcNVdHhZyTcPbh1XO2Dd97qd3vWGrvKL6DGfsBYAb+6v
WPHcn6qL/yH15JHVQAyNPx09RBTdH8hqiIk34P0YN0Bu2Ha8tuuinKQug1zvnhkTuhV7/r1Zsdzzhv

5bp0dbDd9TbQlHNeotNLMEmY9dpzesOh2+3IzkX3tVm1IUpW7Rst2bVKcpzJ2tdrrHuBnP+HjZa1F
615bkRQaLRjb1OKem0koz1u06e5ntPf70n47fHb3pHa+ejIPHqivUH+PsLvvbTud0Ds2frfdepdegkAk
AdVH1UFqUfJyumftB8AgJwce53zQGpSC1HtnmjGkFHzEWco65DimpzxspxVfxuqF6C77UQbdnpv
me1yHmYvuTOyFivhWXQ9uejO2qUCFi+kKcrmBSPEidI7lH39VDo74mY3xc09J6yxHnub+mQmmiT
MmLjHCNyKa2eGQ/XM2hAlo/aNlPBaJTnORO0LtuOst74iW/aGVVC3J2GHE2hHTNWz/rmtbPtBrd
/2JNczhfs9gWrL7yFf2Up/+4q5j/B7L8+J+bAM7v7Fu+WOl9+hlwAAefjmjU/JV376u3ppsb3sp14o/
+a3mNAEAHJTuFHk+L/WC0jLHA/nDwYcXkjrjWE1w5B0mMHN0daVAzdTKp+Ukn5qK56QU/ppJDP
kHgioh2ouTjVJmK6nGTZ8Prc2REmpfSMlvFZJjjNR+0a3wwldR5fWOHUi6XdVVU4HNlaSk2473bYn
up4p3O9zpSwnAwcRIteJ+bAMrrriKmbsB4AZuP9V37HD1EWm6p/+
+n/5GeqgAkBurhQ5/mvWR+qgpm2+a6IWN2UvbMitPdQ3hXqoHtWLri8pZ7JH3OhefG5oM1EPZ
MWu5eiGPlE1KzNsQ5R5b19sebQvRuA3VlFOLEzSmcbxAvPpphfcZAepKlAFAOTn8yuPylNXX9JLi0f
1QFU9UQEAOTn+r0QKN+gFpGmqENUc9hv1cMLJoj8M2gxA7aBUrw9NMc2v04+4dRUtdp1B82tD
H2kGskvEuHZ+7Udf89zw5DXjJwlry4bZq9OtjWnWijRl0oYo2bcvLbGOc4btG21wgreeHHp1B4ZlOv
HcEcD5QZpuv+l2e2g/ACA/KkBVQeoiel3l/6lOKgDk6dhrrQd1ULMy3z1RkZGQ4dEus05mDGp2d9V
b0Q+rjZ7D7raTTBLW3vV6dtrhjxuYG8OTB6XehihZtW+khNdqguOM176Idth1itOdfT7Y9JDh+ImO
M737fWHkNjEflpGaZOoDb/iAXgIA5EEN6f+7Vz6hlxaDmoWfOqgAkCM1C7/qhYrMEKIupaKs+bN2
yao3Ztuc0XsMPcGXKlEb7K0YEnhNOEmYH/4YM+KbcmhDlFTbN1LCa5XkOBO1L6Id9rE7NVUTnsK
Rmqsb4jfdL5lQPrPmhNaJrmcK9/uiyeB+B0wf/vkPy+nbTuslAEAeVH3Ux65/Wi/NN1X/tPp7r5XnXX
OlXgMAyJaqg/oe6+PVziIyQYi6pIqbF/zehs1VHawYk86MU9n2h393alJygxmjRqg/UVew9mbkJGGV
09aSNq5dWbUhSmbtGy3ZtUpyrpO1b2w7ynXZSmV8eFnK5aas6vZ4Tbfaftab/j/Z9Zz6fs+LOdGV28
6Jws6U7ncgwj2/fI89vB8AkI9Lx/vy+Tc8Kj9+zmW9Zn79Uu02ufXVL9JLAIDMHVsTKdysF5AVQtSlVZ
TNvcFJu6rS6res/8djD/8OK6Rpb2dgoi679ubg/ixDk4RVZHuoDc72vF01d71eitm0IUqG7Rsp4bVKcJ
zJ2qfa0Zfu0IbVpo3SBlM7JWcHjzfsGiW6ntPf7/mw7q/B67F/aPQSTiCV+x0YzZ2x/7qrrtNrAABZ+8H
zn5W/ev1jemk+veatN8nb3nOrXgIAZO7Yq0SOv0kvIEuFviqCCAAAMIF7H75XKn9UkaefXYwhpgBw
FNzx5evllQ9eq5fmx7UvvUr+06ffzDB+AMhL4RqRK9R8BQzjzwM9UQEAwMTefMub5aNv/6heAgD
k4W9u/7Z875pn9NL8qP4udVABIFfH3239jwA1L4SoAABgKu/92fdK7Y6aXgIAZE3VR/3cm75pf5wX
v/Abr5BXvO4legkAkLljbxcppFNUD/EQogIAgKmp3qiqVyoAIB+qJ+re676ll2ZLhae/8Osn9BIAIHPHiiL
H36EXkBdCVAAAkAo10dQt192ilwAAWeve+n37MUuqDqoaxg8AyImqg3rsPXoBeSJEBQAAqVAz9b
d/tc2M/QCQI9UbdZb1Ue/6T6+2g1QAQE6O3SlSeKFeQJ4IUQEAQGpuu/42ueeX79FLAICsqbqon3
/DozOpj/q299wqp7ZDC5428SOXabXkDeCFEBAECq3lF8BzP2A0COvv2if7Jn7M/Tra9+kfxSjV
/kASA3hVtEjv0LvYBZIEQFcteWjUJBCsZjpdHTn1tSvYasbLT1gqO94Z6fFZnX09NrrDhtXGnIqCb6x+E8
lv5aY2mo2frfdepdegkAkLW/v+178o8v/6FeytbzrrlSqr/3Wrnyucf1GgBAtq4WOf5u6+MVziJmghAV
yFVPGiur0tRLjrKcWSvq58vHDiJLNeno5YXRa8h6LarV6loXZDV4saVTK0lhIDAGjqq7f/FuuePld+glAE
DW/ur1j8kPnv+sXsrOXb9zu1z/E8/TSwCAzB1XdVBfrBcwK4SoQK66cuDmbuW6dPt96ff3ZHNZM9
SxQeScUj1nxwW/7fPiHlq1pa5zX7r1srOiuSrkqFgGV11xFTP2A0COfvycy5nXR33zr9wir3nrTXoJAJC
5YyvW41V6AbNEiArkqXco+/qpnDohy9v/dHHF6znbk8Y53QW1XJetivO0uHlWqs5Tae6SomI53PSC
m+wgVQWqAIDsPXb903L/q76jl9L1sp96ofzphlsX96ofzKfzsplwAAmSvcKHL8tF7ArBGicnqP5CKntOLI2peqN
536d163Or/dp135sb+ivNdbZLwuuD/bKS2MbmtlG7zFcYzN4jMM1S+1HWN1Low3jal3a+zDDt+aq
87WDDQ9pc9i2o9o8tpfjROdl+J4ZuZ8YxxD7fGh+W9RjuK0OZwi9/7p0z506/lKsnrNGj+NAWF6Sk7
ozquwfDt9PNqM9ie65eMduS+H7wj1Pcd9HsNxuv+l2e2g/ACAff/fKJ+SbNz6ll9Kh6p/+
+n/5GeqgAkBurhQ5/mvWR+qgzgtC1CWlwpHQMKhTk1JhQ8ZlcHHYtR8HCkLa61TIMrC+uRoeXk2
1DRU2hfYY7EitNCqEU58zapaW61J3uw52duTiYMi0674ynbqmQ8GiZh/zyGBquM1uz8dQE5+X4X
umuToieEt8DFGcfQcvd0hb7WCw5A2hd6V67jxVafVbXq/SIWaP41E6B9LVT4MqcjrpPZfk2FP6vlDn
KY/3ERwdapKpD7zhA3oJAJC1z688Kk9dfUkvTe/f/Nar7J6oAICcHP9XIoUb9ALmmASHqUmqLn8O4d

TmNeo3SlHPhSUpi5XrX3na/FYybwtaPGt482TaCw6mHj7EjO4PplEcFZM7r+3ubsrlVF+erBr/GPI9nZ
FyGWtm2ttd1t2Wptpx9bOvUrr3hB4Vhbe7UZH3kdQm2eXRT0jkv/invSO28cd0SHMPY8xHg7zu8r
dZxrbvBoHEu3Iamcu40u53bEitrHVKUE6f00wiVRPdckmNP7/uimOP7CI6OD//8h+X0bQxHAoA8q
ABVBalpWDn9k/YDAJCTY69zHpgrhKjLyOwlZ/R0K27uOQGJ9dhLZaajqpx1t1M5bS25RqwPHd486T
aKsrlnBj6O4toZL7TrHIT3AyzXt4IBWXFNzugv6uxc9NvY3vV65pXPrI0P38YwexjWLxht3rwgfgZ5PrR3
31CbR5r8vFRbfnDoh3xB0xxDFHPfoW3tXZQd3SEycC4qW/5+zWtniH/u1HFY3yOhIe8k9uUwrEFKk
nsu0bGn+H2R2/sIjpp7fvkee3g/ACB7akj/V376u3ppMqr3qeqFCgDIiV0H9V/rBcwTQtRlZAQ0ImqY
bsGrZTiyhuIkyielpJ8GjFofJo1t2HQ9x9BhzEGnTgwGP0VZ8xMtLyzyA0Mj0J1YVK9WY/8jgrfhNscV/7
yMN90xjFaWk+MudvfAa79X7sF+GEPcRwyfn/zcTeuUjN51gntuimNPcv2HzlNe7yM4ctwZ+6+76jq
9BgCQJTXJ1KT1UVX907t+59XUQQWA3Kg6qHdaH6mDOo8IUZdSUTYvhPckdEOYqMl1FkZg8hyjn
mOk8MDO76nnDnc2AsPq6dg9GafXkeGOgjFCRlOK52UyYccwK2keVxw9ORxbLNWR2T2X2vVfkvc
RZOKW625hxn4AyNFfvf4x+fFzLuul+H6pdpvc+uoX6SUAQOaOrYkUbtYLmDeEqMuquCl7esjtQKlR
W/Pcos+u3ZYNs3edW7PRrMGZhHW+zurzZA+NNoYyV0/nF6FOH/qlfF4mkm1w6dXKHXpMWsd0
AsUTMrb06bje1BPcc+OPPf3vi6P9PoIsvfmWN8tH3/5RvQQAyNIPnv+sHaQm8Zq33iRve8+tegkAkL
ljrxI5/ia9gHlEiLrE1MzaqqeYPcGPHYR0vRqKo4f/GoxhxHPHrB2pgiW3/uMUba64U6Z3duT8+R29n
aqkk6GW5KR37gdnZO/JRbfoZeQQ8BgyOC+
+nI4hTOmkFwKOqumZL+NcNHfF75DZlQPvNJxwzn2EWPdckmPP4PpP/T6Cpfben32v1O6o6SUAQ
Jb+8eU/lL+/7Xt6Kdr1P/E8uet3qF8NALkpvFgP48c8I0RdRu0Ne6itmkU92FPMCHiMXnIlPxkzZmJvy
4Y3Dft884MlY2bySXgTWHWk2dQnKrWh/EXZdLsdqvO87l+XXmPdq22ZZBKkcVI7L578j8Fj1udsrvr
DyPW9bj9yHVtu1oBtyq7eda9xzgsxY/VgjnPPTXjsU1//hO8jZntyvRSYe6o3quqVCgDI3t/c/m157Pqn
9VI4Vf+0+nuvleddc6VeAwDIlqqD+h7r49XOIuYWIeoyqmz7Q287NSm5QYtRH7F6NnzmbhXSDL52
Lpkz9nttNibamUhF3I6BrlSH8o+4LiU/fZQL005glcl5MSQ9BnPYu9ueiRK2YH3O5qqz34IX9Felldqs+v
EUNy94PTLd9njnodqSeM2Jc88lOPY0r3/C9xEgiqqPquqkAgCydel4Xz7/hkcj66NW/u8idVABIE/H3k
od1AVBiLqk7KG3YUUMVeDS7wcDHlX3cLBmYrWVcx3NpCqy3W/5gZHNOTbvsAPDrOPxhlcr5bpsp
ZzL2dcl5LxWW9Z6d+j1VLI5L6Zkx2C1Z/A+3D80ejUmYNfnNIaSu+y6nznWQ/UUZXNvuFaoPYw+Q
aAb656LfezpXv9E7yNABDVTf/tX28zYDwA5UPVR7/uZx/VS0Cte9xL5hV8/oZcAAJk7Zv2GfPwdegHz
rtBXRewAxKNmNtcT86gwbG/anqHAOEfpntPHcqpFwIpwn+l9Rip/xM0BAHl441/fIKWHXqiXRK596
VXyW3/8z+yPAIAcFK4ROf5+66P/Xoz5Rk9UILaeNNbdmc3LcmaNABVZO1r3XPu8dSwZ9ODG0fGO
4juYsR8AcrL3um/J9655Ri+JVH/3tQSoAJCn4+8mQF0w9EQFxmrLxmANWFXOgK50yMzRu+d6jRU
p7ZyRbiplKXDUFf6fgn4GAMjStU9eKe9s/aS88723MYwfAPJ07O0M419AhKjAWAOBFgEqMsc9h+
WlJiiblx9NaEs42hKOtoSjLeHmqS0nrr1GDr/3pF6aLa5RONoSjraEoy3haAvSQIgKAADmBj/ghqMt4
WhLONoSjraEoy3haEs42hKOtoSjLeHmqS1IhpqoAAAAAAAAABCBEBUAAAAAAAAAIhCiAgAAAAA
AAEAEQlTkSE2WU7DrfxRWGtLTa8fqNWRlo60XHO0NvZ3CijRibwjp6Uljxb0GG9aVTbJuGmH3UNr
7mJbRRv1Y4SYFAAAAAGChEaJirvUaK1Io1aSjl4H5pgJdY1Z9W1nOrBX1cwAAAAAAsIgIUTG/eg1Zrx
GfzqeibO717RkF+/1tqei1szFPbenKgXvLluvStdu0J5tkqAAAAAAALDRCVABIS+9Q9vVTOXVCyE4BA
AAAADgaCFGXmao1OlC7MazG6GD9UXuIvfE1A+VKNbNOpXokq1Vp79Mcxt9cdbYTvjOjjeoxqk7qY
JvCa1VOdrymMbVf2xvetob2H3JN4rTRY359ZEONcxFo46j1xj7t9ea5zKoOadx7KKotg9uYsD5pjOuS
9J4FAAAAAAACLgxB1WakgL7TWaEdqpVEhpPpcQUoDQ+ybq4OvVyFiSYIva8pqYbBWZBqcNq0GNh
xyDHYINtgm65W1UnjQaYt7vIMqcrqqn3Z25OLAa9u7bmODtTKHQjgtuo2TKsrambLztHMgXeeZxR
iOHmh7W9xml8+s5dDDMoV7aKJrPizf6wIAAAAAAOYRIepS6knjnJuIuXUb+9Kt61BNOrIzmPx5qtL
Sr2+5QaEKG8/7ve16jXN+0FVt6VqVXfE2H0NI2/qabI28L3G3sx1W8dJvU/gxWMe77oZg/mv77gF0
arI+MhUdf7xhKltu2wfPpR9GSvmMeBlqe8MPgsOuSWQbJ1NcO6PbuC+H7qbbu0ZI2ZEDN101hq
mfOpF9hDr9PTTNNTckuC7J7lkAAAAAALBICFGXkjERz96m16vQD9VEOl56FlRt+RP3+EGhqScXd9w
+e1VpeQGStc8LYa+fntmm0GPoXRS3SeX6lj/xUGXLC+U6OxdDexSOP94RimvidfQ0t22ElGaPTrN3av

2CcU02L/htrJ0fGKo+Ja+NftDbO3Si0mrVCRv3dbrau7jjBZKnvROYlRTuoSmuuWkm1wUAAAAAAM
wdQlRYdA3P0OH9SZmzk5+Ukn5qK56QU/ppespyMrCTEN0D77jsIdheXUtjqHdgSHsazOHy/rB4P5
SryllvyvYRvVNtxnbMHqOpKMoJfUHcwLlrXzzrnJ4+aYeVwfWW6mk/kMxMCvdQKtd8VtcFAAAAAA
DMG0LUZRWYKCdunckYgeWRMt3x+r1i3Z6eRig3URhpDK9PScUt3trctVrXE6cj6ik5UdFhZWC9anb
2EeriSf+6AAAAAACA+UKIupTasmH2OnVrPZr1HI+ocr3rlDEYevjD9lNT3JSzbglONXTcqCs6WRiZQYh
dOS164L4ctvUQeLv3pzs5lrF+QUP07K/5sv1xAQAAAACA5UOIuozMupwqYHLrohpDoCdXkpNuEjs
4XNoIEXNVcoamK6NqvWbF6+nZ2ZHz50fVFTXP2eBs/mZ90FMydk6nxNfQ3XdHDnadr3VrtZbsT/jr
h4e0ZyWFeyiVa57idQEAAAAAAAAuNEHXJ+QGTMWP/VMxakU1Z3XCn3DFnS8+ZMcmTNFfFa1J7w
6+V6a1MmdfTsyPNpj76oaH8Rdl0u6xar6utN6yz5eg11r0anuYESU7AqVivP+8dkGx4U8nH5V+vZtP
5Wnf2/aIumOquNyfCylYK91Aq1zz5dQEAAAAAAEcTIeoy8oI9S3NVh0rGhDtTMmcun2r75iRC7nY
mCjuDs7o3V3WI5gWO5gzwaXOHxftCh/JXtqXl5XU1KdnnrCAlP6mTC95EVCojdOutWrxzHLe2bVBg
W9Yzb2i6eZ9Y68/k0w3VNv09lNI1T3hdAAAAAADA0USIupQqst1vGQGZUpVWv+8HRvaEQpMqyu
Ze1w/BbGr7g/scx2qn1yBt/9DrDZhIcVP2+oNtstj1YDOoh2rwhvQr1v62Ruysst0PrUtbbVnr3ZILLnU
8g6+ttiara2v22gwMTTeGs+c+ZD2FeyiIa57ougAAAAAAgCOp0FczrADITq8hK3oiL1WDdo+eiwAwk
urtPS8/mtCWcLQlHG0JR1vC0ZZwtCUcbQlHW8LRlnC0BWmgJyqQKbOOZ75D4gEAAAAAAJAOQlQ
gE23ZKAzU8ayeFTqhAgAAAAAALB5CVCAPql5pZpNXAQAAAAAAIEvURAUAAHODelXhaEs42hKOto
SjLeFoSzjaEo62hKMt4WhLuHlqC5KhJyoAAAAAAAAARCBEBQAAAAAAAIAIDOcHAAAAAAAAAgAj0R
EV2eg1Z2WjrhVnrSWNFzZavHhsyL60CAAAAAAADA/CNERSZ6jRUplGrS0csAAAAAAAADAoiJERfp6D
VmvEZ8CAAAAAADgaCBEBQAAAAAAAIAIhKhLqr3h1gddkUZPD7+3l51HaClTVePUeI3zcL7eZW/XH
MbfXHVepzc4uF+PuW1j58HXt2XDfY318F4Wo12TGjwv6jHpuVGSHr8S1obCSkPCD8+s/eo8VkaciG
TbBQAAAAAAWF6EqEuvI7VSQUoDw+
+bqwMhX3tjRI1T9fXpBJbR1H5WpamXpFyXrYr1MbN2OWHk4HlRmquFYNCZ4blRoWtYG6RTk9Lg
BFl2EFuSwZd3aqWhcDTRdgEAAAAAAJYcISosVWn1+9K3Hq2qXqUCwPNulNaTxjkdX5br0tWv7dbL
zjrrtTsXnYiusm19rlsX9zNSbdmv7W+rxHNafjv7e5tSTNCupHqNdS+MLNe7zj77LasFWvOcDkeza4NI
W3bd1Dh020055yW0VjvW3SDXOE/uBe3UZN17bZLtAgAAAAAAgBAVUm1tixtxVraMANRTlM09H
crZ4aVeu3bGe23noKufZadc3/La6ciqXT25uOP20qzK2U13yxXZsoNGFVLuibM6w3PTO5R9/VQ6O+
JmscXNPWd/1mPPbVvvorhNDpynypa42Whn56J1ZJYk2wUAAAAAAAAhKiaha5OGDmHPzqkT44K
9tNrVlQN3A+WTUtJPFSdo9EPnYSmem+KanHGTWGtrquyCW7t0qM5p98Dbnz18X7+uYA7v7xxYR
2ZJsl0AAAAAAAAQoqIsJ82UcJTAxElGbdLcjGjnzNtlyawNRdm8ENYz2A9KQye5Giur7QIAAAAAABx
NhKiIoS0bZs9Kt46mWft0JuahXRm3obgpe3qIvV+v1tc8Nzybvl/DdfBh9KCdYLsAgLzp0Q3Ggz9yAQ
AAALNBiIrx2rte70o7oHNrfxpDyKc2ybYya1dJTroJqDsE3qVm4i8YM+6n1YaI16uZ9NUvzfakXXbw2f
XqnHrtK530Qtu4NVhjbRcAlpH9Xl+QwkwTy540VlZFWu57tPNHr+bqLILU+Qxze40Vpz0r+f/hT/0ba
p4P9zEP5wXDhq7XDO6ZmRvzvuZ9P7mPvG7mOO+3ebwnR+5jSf+gFfO8z/K9eGZinJuh951luGnG
nJfc34sTtofSdoiDEBWJ+AGdMSt9AiU/nTRm/7d+MFmdbhD8tO0KKsqaVzS0Kavem667baeO6OB7
cZw2JDp+/aavPhXsGRpSs9Wsc9pc9dvm/sOhHu7KJNtVjG3wDwuALMzsl/chKri09j/lv0npcCYu3Da
KcLuTP+4f5vte3N4IC3ONPyjOQq8h6zWRatX9x28G3NEnxsO8XrMy01/cAyWOhh+z+NZW52O1qS
YFda9TS6qdmpRmFPjk/0v7+Pc19R5csr6f6l3jHFk/T2Z778R5v83jPXncPtTnc/yDVmTok1eYm+C85/l
eHHFuht739CP98xP/vl3dH/g3Iot/IEadk9zfi8efl3zfiydoj/WNrUrbpf+eHO/7dn5+FsZY1g2DJWS9R/
TV5bd+A+hbPzD5uvW+9U+g87mq9ZZia/WtnxX060c8vNcqIa93P29uf9TD2NbIdtqStKvbr5fd9VXr
K8cxXx/yKNetVygJz83Exx/
+MDcdve3gMSfabqvqrS8PXwQAmEq3XrbeX8z3eP2+GngjyoffFv1vwAzaEEm/z8/8vXjm7XCuj9q/f
c28f5PzY/87OoP9RtP37dy1y/3eivPzV9rCv5dn1R7n5y9jv/pnrCy/l8a/rznvuYNtyPocjW9XvNdMa6J
9ZPIeqPdvbdd+hLRD3T/maud+Um3XK1IS/5w4n8/+vTjeucnjvS/
+fZv1+8v4cxImq7aNPy/h62fWnhHfw+nfR87+zd0737fBdX579Qr9vhz3uiJf9ERFDBXZVn8p0ksO56

821puAo7kr/t9KrNd7n9D2D52/MKlanIP1QqutCWuIJm1XEk4PIOsNTS/7rDdbf9h+0jYkPH57qP3g
ubQ5+wj8QdOuc2oMyXfZPWWMeqiWRNsFgMy05XytY71NXZBN503VUpEt9UY28fv35Iqbe9Lv7x
ltmTO69MupE/PRwFm1o9dYl1qnKmfn9kLNhnteWt7PKPPC+T6X6unAzyL5KMqJU9YH9+dQW08u
7sygPb2GqIFK5fqWv9/Ktv3zYmfnYgY9sRxj39d6h7JvfRj8fi7aJ64puxm9Ecd5v83jPXle3ved79+y1Ls
hP8tr6uf34dEJHdm5mO7dE/ec5PVeHOfc5GX8uXHeXwLf5xmY7Jxk9148/rzk+148tj0jfp6yR42mW
tYuzqii+fpZGOMRoi4pvw7mwJuLMeFQcMiBCgvdr1EPJ5TztxMM6dQPhf5rrYf5A725D/VQ+xmx35
Ht9MRtl/MGFlw3nvMG7H6d89gbakjCc5Pg+G2D59J+jDoG8zj1Y9QvU3G3a7xu+NgBYAoz+uV9M
enSL+W6bIX/A5CTnjTWa9KZVTvsoaMdqbbi/1ueGTUM0Rh6N9uSN/n84j6JXuOc9d1s/bI/oxu3sq2
HjNo17dWwypITNg/
+vJW13H5pT8huV1lOenWcNKPePgZk8AetuMFlmDTbEVuO78XTnJv86fJoB+cHhtOnWwJnknPCe7
FhxPub8/PnvuRaNYmfhRcOISoAAJgNfnmPTdUltX/5uTCLXoa6tpj9i6D+pWdGvR3b52vSqbZmPmL
C/0Op81AjV7KppRZXPr+4J+f2NDo7wwDEHTWkatqr7yOrObMI4efpl3ZMgT9oKfPyXhwwR3/Y6uy
flAvevxGqt6h6/9mYYa9C3osD9HwiwflBrO+pqedWiWHwjzD8LLxwCFEBAADmmDP5gRq2N6ueO
MFRDt36vqzO4hfU9oYzCcRc/dbuUL2Csh6WHcfc/eLePi/O6NHZXTNnsg5/YiAVeKtJgXKftGOWv7Q
jNfxByzKH78Xz9oet8pk147pY/4aeVWXUZtirkPfiAernGrdnrP89dXBKXadTkl3n7nn5IwymQYgKAA
Awp5wAVfXWmJ+hjMXNC3YNtk7tfK7hXHtXhU1NO8B1fuEpSEn9Vqh/CZrtcHptlsOyLfP1i7sOCGf5
y6Iecqzq2bt5jz0MViXezdWMZjYfZVa/tI9h93bqyMHgjTuqd9QS4w9ajkV4L56XP2x5ZtqrkPficIMl+f
qydXLfOk8nJau3vdn+EQZpIUQFAACzwS/vkVSvDSdAndMJ/zL8RSPMYE8j9bAngLR+Mexaz2dbt7sn
h6qoWc7nZKxZ/uLu9nw6O8NfFgeHTbpmdl7y/6V9rOIJceZ7CUZNPfuGnmG4O2f4g5Zvvt+LB+T+h
62S2GWOB8PbWf5cw3txTLq2eOCPkekZ+UcYfhZeOISoAABgNvjlPYKeRddiD3czevzkW+dSTf4wM
Bxc/0KW1S8ac6/XkJUVc0i2WqVmSp7lL6nz94u701utKjMcPSpSOW21wPoeCgyhV7dwzTovM26bL
dtf2uOpyGnVY6+27r+veL3G5m+islngD1qLaFZ/2NIjADo1Oe/9w6mHcM+oHinvxXGoUhklqUldLm
RwkSL/CMPPwounDwAAMCOtqvSt33L69a5e0a33y9aPJ2VvRX669bLVFtWewUe139KvWUZh56U
6JyfEblu53s/3bun26+Xg+ZiLe6RVHbg2rb71S2tfJT+5m+H38TB9HgKPefie1vdRxvdv3Pc1573Yf2R9
7eK0K4/35PH7CLt/3Ifxb1dq9H0x9H2r2jFw3Pp7Pu1rNcl5t78m8/fiEedGvd8M7Ns9hrTf/uKem6H
XZfY+POp+0XJ6L453XvJ7Lx7fnpB/xzO6RnHuxXn6WRjjFdT/rIsKAAAwE+5f6F3WD43zNRwQWBCq
x5xdm9BVbUl/Bl3nnO/pqrT6M5p5eS7pnk7G5ZnV9cH8Gfre9fjfR2Gvqc5r79gUjT83Id9bxnk7iuLc
LwrvxbOmRvOoOqhhgkP7+Vl4cRCiAgAAAAAAAEAEaqICAAAAAAAAQARCVAAAAAAAAAACIQIgKA
AAAAAAAABEIUQEAAAAAAAAgAiEqAAAAAAAAAAEQgRAUAAAAAAACACISoAAAAAAAAABCBEBU
AAAAAAAAAIhCiAgAAAAAAAEAEQlQAAAAAAAAAiECICgAAAAAAAARCFEBAAAAAAAAIAIhKgAA
AAAAAABEIEQFAAAAAAAAgAiEqAAAAAAAAAAAQgRAVAAAAAAAAACIQogIAAAAAAABABEJUAAA
AAAAAAIhAiAoAAAAAAAAAEQhRAQAAAAAAACACISoAAAAAAAAARCBEBQAAAAAAAIAIhKgAAA
AAAAAAEIEQFQAAAAAAAAAiEKICAAAAAAAAQARCVAAAAAAAAAACIQIgKAAAAAAAAABEIUQEAA
AAAAAAgAiEqAAAAAAAAAEQgRAUAAAAAAACACISoAAAAAAAAABCBEBUAAADAQmtvFKRQ0I+N
tl5raG94n19p9PTK2fDbuiEhLV0wbdlwz3vk+e1JY2Xca6xXNVb8ba00rK8CAGB+EKICAAAAODqa52
TGOemSUMHoqjT1kqMsZ9aK+rmpKJsX6tZnHZ3aesg1asv5Wkc/F6me3bS+CgCA+UGICgAAAOAI6
Ujt/OL38Zx/XTlwM89yXbr9vvT7e7I5KvksbsrZqn4eco16jXN+IFttyXZFPwcAYE4QogIAAAA4WuiNm
r3eoezrp3LqRKxeo5Xtlng5auAamb1Qq9IiQQUAzCFCVAAAAABHTLzeqH590pVg6NpryIpbm9Orse
rX/7Rrehp1Vr119suC68NKtPqCtUJH1wEdeJ31CKsrGjyeYL3S6HYYzGPXj8F92fsp1ayzrDVXndeO3Ul
FtltujOpfo/aGXxagXN+yXjUo3vHbQto/dH0tcc5VoEar+6BWKwAsLUJUAAAAAEdEWep1HdJl2Bu1U
ytJYTVYDdRep0K2gfXN1VEBZlNWCyUxyoCqjUhpcMIpOxQceJ3F3t/IQK8jtZJRr7Rcl60YnTuHwlEtel
8JVbak7hZHba7KRqMh5/wEVS4M1gNIcvwqwA5pv3M+hoNUR/i5UueiNLhTJewaAQCWAiEqAAA
AgKNjzQ3p4vVGnVS53pW+qgPq9ax0hK1v7o5oh1dLtC/
+y5tyzkv7etJYd0PBqrT0a71td2qyPjIpNl6/F2OSpvaGePmv0a6um3ga+6psW5/r+hNFqRqm9n5iDc

MPTjLVrLnHV5b6hcF2Jjl+67VuGhvWfmsrOxfjnqu27Eadi8A1AgAsC0JUAAAAAEdIUTbdGYwy641al
bNuj8nKaWvJNWL9/mFoL05zBnqzXmhn56Lz+t5F2dGdIQPD3I3enN5rB4QPix+t7aeGgTCzuHnB31
ftfDo9MAOTTGnVs8OTUiU6fuu675lBqKO4dsYLbDsHXf0saOhcmfVeOzviZq/FzT1n+9Zjb+QMWgC
Ao4oQFQAAAMDR4oVsGfVGLZ+Ukn4aMGp9qKqcDiR3JTnpp31ix33dA90L01rllguwH8bwdve1A0
6dSBLymT0vz8ha4EuLsnbGbdi+HIYlthMITDJlPQudTGqK41fHZNc5DR3eHzR0ropr4h2yuodK7n4ja
rECAI48QlQAAAAAR8xAb9RD5+l8K8qJU/rp1MpyMn6am0BHRnTmnE6i8DlCYFIpo85ppLBzFSw5Y
HLD3NgTdQEAjgxCVAAAAABHj9kbtRYvTsvXYK/Onhx6Y8iHebVWhx7biYbtTyercHa88cfflg2z16lby9
Ss3ZpEcVP29D4Gyt7amueYpR8AIg0hKgAAAIAjyOiNmpQxjDw7g706u3Lg7tTtmVk66QWAo+p5ps
MsJeDXAHX05KJbmFROSaIqAdNKcvztXa/nqR24unVRp7iWaoZ+1ePUnkjLDlS7Xi3W0WUEAABHF
SEqAAAAgKPJmIAoTMlPDo3aqW3Z8Kapz1ZzdcObqKm94Q8/L59ZcwJAszZnc9UfQt7e8Gp0FlIZV2
4Gzta5WPd7WfYa61790aSTVU1twuP3A1djxv4k9PbVbRDscRoSdAMAIgYhKgAAAIAjKro3qjlzuwrp
nGAubi3NaZWlXG7Kqg4D/dzWmOFftd+ozdlc1cGh9+IREzJNorLtD1vv1KSk21XyE1S5kPuM9AmOv
3Lan6jKu5bGBFRJjDgX5r1RPat7ugIAIgYhKgAAAICjK6o3qqp7OVgzs9qavI5mIqfk7J4xPFyx63gO1Di
1a3MOvE4Je+2U7GHrIcdebVnr3eHxeYt9/BXZ7psz/itVaZk1TZu7Xs/fcexzEVYMVW8zrewaALA4Cn
1V3AUAAAAAAAAAEIqeqAAAAAAAAAAAQgRAVAAAAAAAAAACIQogIAAAAAAAABABEJUAAAAAAAAA
AIhAiAoAAAAAAAAAAEQhRAQAAAAAAAACACISoAAAAAAAAAARCBEBQAAAAAAAIAIhKgAAAAAAAA
AEIEQFQAAAAAAAAABGEvn/AZM6nr53azj8AAAAAElFTkSuQmCC]]></redcap:Attachment>
</ItemDef>
<ItemDef OID="q30" Name="q30" DataType="text" Length="1" redcap:Variable="q30" redcap:FieldType="radio" redcap:RequiredField="y" redcap:BranchingLogic="[q27] = '1'">
<Question><TranslatedText>Do you prefer Life A or Life B, or do you think Life A and Life B are the same as one another?
</TranslatedText><redcap:FormattedTranslatedText>Do you prefer Life A or Life B, or do you think Life A and Life B are the same as one another?
 </redcap:FormattedTranslatedText></Question>
<CodeListRef CodeListOID="q30.choices"/>
</ItemDef>
<ItemDef OID="summary31" Name="summary31" DataType="text" Length="999" redcap:Variable="summary31" redcap:FieldType="descriptive" redcap:SectionHeader=" " redcap:BranchingLogic="[q29] = '2'">
<Question><TranslatedText>
Alzheimer's disease - Description #[no]

If you have Alzheimer's disease...

1) You might forget recent events of importance to you. For example, if you are interested in hockey, then you might forget whether your favorite team won their last game, although you may remember watching the game itself.

2) You might forget the names of people you met recently, or the names of local streets.

3) You may no longer be able to handle your personal finances or plan leisure activities such as dinner parties or vacations. Simple decisions such as what to order in restaurants might become difficult to make. Your ability to concentrate may decrease and you might have difficulty driving.

4) You may start having trouble finding words to express your thoughts. You might need to be reminded to shower or brush your teeth, but you could do these activities on your own. You may need to post notes around the home to remind you of simple things like turning off the stove.

5) You may remain aware of the day and your surroundings. You might be able to travel to familiar locations like the supermarket, although probably with the help of a loved one or friend. If you are in a social gathering, then you might have difficulty following the conversation.

6) You may continue to enjoy hobbies such as puzzles or reading.

Now consider two possible lives. In Life A, you will live in perfect health for 9.5 years and then you will die of natural causes. In Life B, you will live in perfect health for the next 10 years, then you will live with Alzheimer's disease as described in the summary for another 10 years, and then you will die of natural causes.

</TranslatedText><redcap:FormattedTranslatedText>
Alzheimer's disease - Description #[no]

<p></p>

If you have Alzheimer's disease...
<p></p>
1) You might forget recent events of importance to you. For example, if you are interested in hockey, then you might forget whether your favorite team won their last game, although you may remember watching the game itself.
<p></p>
2) You might forget the names of people you met recently, or the names of local streets.
<p></p>
3) You may no longer be able to handle your personal finances or plan leisure activities such as dinner parties or vacations. Simple decisions such as what to order in restaurants might become difficult to make. Your ability to concentrate may decrease and you might have difficulty driving.
<p></p>
4) You may start having trouble finding words to express your thoughts. You might need to be reminded to shower or brush your teeth, but you could do these activities on your own. You

may need to post notes around the home to remind you of simple things like turning off the stove.
<p></p>
5) You may remain aware of the day and your surroundings. You might be able to travel to familiar locations like the supermarket, although probably with the help of a loved one or friend. If you are in a social gathering, then you might have difficulty following the conversation.
<p></p>
6) You may continue to enjoy hobbies such as puzzles or reading.
<p></p>
<p></p>
<p></p>
<p></p>
Now consider two possible lives. In Life A, you will live in perfect health for 9.5 years and then you will die of natural causes. In Life B, you will live in perfect health for the next 10 years, then you will live with Alzheimer's disease as described in the summary for another 10 years, and then you will die of natural causes.
<p></p>
</redcap:FormattedTranslatedText></Question>
</ItemDef>
<ItemDef OID="a31" Name="a31" DataType="text" Length="999" redcap:Variable="a31" redcap:FieldType="descriptive" redcap:BranchingLogic="[q29] = '2'" redcap:InlineImage="1">
<Question><TranslatedText></TranslatedText></Question>
<redcap:Attachment DocName="95.png" MimeType="image/png"><!
[CDATA[iVBORw0KGgoAAAANSUhEUgAABVEAAADYCAIAAABtBTQzAAAAAXNSR0IArs4c6QAAAAR
nQU1BAACxjwv8YQUAAAAJcEhZcwAAEnQAABJ0Ad5mH3gAACfDSURBVHhe7d1NqC3JfRjwSRTCi
AQymGDGzsIvnwyyMBNnM3kY8gLBFgaDwIRMHAKBmMcYHJhNLBEcyEJGIhjmLQTSwjhaGEaLM
VpEjIRjmIUNQzBkFgLPYmweRMGz0OISTLjCs3DO6dMf1VXVH7dOd59z+/5+/LHv6a6qf1Wdc+/r/5
tXVy/8JQAAALBHan4ANvLe8/de+C8vCCF2HJ/+wqd+
+R/9gz/70z+pv+0BuDQ1PwBbeH7z/KWvvBSVB0KIncXP/8yP/+td//ed+9i9+
+MP6mx+Ai1LzA7C6m9ubV7Sj+PhP8dtf+LX6+x+Ai1LzA7C6z/3O56LaQaAixs/ixX
/l0W/Cf4g9/9536RwAAl6PmB2Bdb373zag2EELsLD79hU/90k89imr+Nz77GQf7f7AS5OzQ/Ait7+3tt
RbSCE2F+cjvGn4WA/wMWp+QFYFYy/vff//FL70Y1QZCiJ1Feiw/DQf7AW5PzQ/AKj7Afn4+jaApJ11Fe/
xM4iPcafhoP9ABek5gdgdgdgebef3L769Vej2kAIsbP4+zcvpvLXlvZqLWuvvvtI/d/Oc/f1dzcOtp3Fbt/xrB0
fIXQRD6XP9N4x/Trr/9EvpjkrquPaBL0AWEKMfajxdStnJgGyVtPsIqy3Dxf9eskGL7F0dZXGbHLb6Jy6D7+
gPcClqfgAW9uU/
+HJUGAgh9hfjx/jTcLAf4CLU/AAs6VsffisqisqDIQ+4s5x/jTcLAfYHtqfgAW88HHH/hF/ULsPmYe40/Dw
X6A7an5AVjGze3No2ePotpApACLG/mH+MPw0H+wE2puYHYAG3n9w+
+caTqDAQQuwv7nqMPw0H+wG2pOYHYAFvfPuNqDAQQuwvyo7xp+FgP8Bm1PwAnOtD
MhxP6i+Bh/Gg72A2xGzX8RHz17/MLJ42cf1dcaY/cupp3U03enXw4p63WmbbKc792np2lWlnznDx
swuvQm8aqftoF3oT+3TWbCKt57/l5UGAghdhnnHONPw8F+gG2o+S+kLQAn9Oi+S9S8EMKbxjgcRzByPafUNZmo6o9KLF4VbUw3kPmTaEdtu6qoB85ePHTRIgfV1hRlmqy+1cbsmW3SB1GqnqJHhr9lkCBmU9kbg8U5ra9/8HsGzR9s7xePVE/PJ6Yf5JDPw4o5q1/4M9lbYIgh9pZ7nUa7n2mWfWPEpr5CMKADx8hYwX6G8s2JLTLmmApVqZTrhSNgTqHSy9uklkuUyJ45hFpK8+5s9XIKnU5zuBl9+Vo9ZoGZlvB2kfyEfzpQMjDmyUfNirAUB9L/CyyT9y+zw+7UPxuUUGF8Bn4B2FDvjSS03oKcJrR/OvgeG29d6V86m50T23TyS/gRv/oWmXFMhgtcm1N9MWzd46vI2g1+QF35tjfJjn+/f8OHDfw8SN+gMnc/2D6xa+HhBvgG+7P/0Av7vKm5n/qr4/6tDZmK2uhBrf3Dw8bH3v3z8/oP/fO7f2xtMbCJv3pRBrFvmR//0XDwXgbf/5Re/MprXn/76+IRsG56u47jdQ4Onf/v4f4df7vn/5v3ll3+8/fzt1/9Wf/n//t/nf9Me/6T//1/9+8/+Nfvdf/wfv/+37/v/7//3//v5D43/+R/X95/+c/+T/+V/+t//B/yf/mf/f/B/wz/yf/u//3f//8X//n//j/4P//X/9/Hv/9//t/+L//Dv/yf/7f/8X//j/6L//Pf/3/+H/8P/+P/4f/y//n/+v//f/3/+H/9//9//P//1/+L/8Xf/3//v/1/+7//b/8H/+f/8f/6v/+f/6//x//3//L//V//1/+r//J//l/+L/83/93//X//r/+b//7//P//X/+3//p//n//7/83//n//7/+3/+r//v//3/+H/+f/+3//n/v///vr//r/+b//L/+r/+T//3//7/8X//z/8P/8P/8X//X/8v//X//r//N//z/8H/8f/8//w//o/+o/+w/+s/9v/f//5/6v/9/+7/+b/+//9v//n//+f//v/ef//7/8X/+///f/+P//r/+v/zf/+//J/+b/8//h//9//7/83/+r/+D/+j/4/+v/4/+A//7/8X/+P/9f/9P/zf/8//1/+7/+j/+b/8v/1f/6//t//8//1//p//p//5//w//y//Z/+Z//b/+D/+n/+f/2/+p/+P/+f/1f/rf/r//V//P//T/9H/4f//f/7//f/+X/+f/+3/x//f/8/+r/+X/8f//v/8H/5//j//D/+j/9P//r//f/7//J/+f/6//U/+n/6//3//D//X//v/8f/9f//n/+n/+b//D//P//v/5P/5//1/+//8f/0//7//p/+f/8v//f/4//h/+L/8X/5//x/+T/+T/+X/+n/9//l/+n/9v/6//j/9v/4//5//7/+3//n/+v//d/+T/+n//f/3f/+3/8X//X/+f/+3/+P/+f/+f/+H/9//7/+n//v//v/6//p/+//2/+n//f/6//t/8X/+//1//7/9f/7f/9P//P/8//L//z//f//X//f/7f/p/8//x//H/6//D//f/9H/9//5/+///b/+z/+X//X/+f/2/+f/+f/9//T/9H/9//x/+f/1f/6/+X/8f/+v/8v/6//p/+//1//v/+H/8v/4//0/+P/8//l//T//f/2//P/+f//n/+T/+b/4/+z/+j//v/8P/0/+//p//X//H/9P/7f/+f/6//T//v/6//T/+//9//p/+P/6//d//n//z/+T/+f//v/+f/+v/+f/+P/8//0/+f/6/+P/8P/5f/x/+v/+f/8/+//5//p//r/+P//v/7//3/9f/8P/+f//f/z/+j/+v/7f/8//T/+f/1/+//+//+/+D/+L/8v/1f//f/+//x/9P/1f/9P/9P/5f//P/9//7/+b/+P/+/+r/8f/7//z/+H/8v/5f/+f/2/+X/8//5//H//P/9f/9//3/+v/9f/+3/+v/9//n/9//v//P/+X/+v/7f/+X/5f/6//J//n/+P/8//f//P/2/+X/9//9//r/+X//H/+P/+/+3/+P/y/+X/8P//P/8//X/8f//X//n/+P/+/+f//v/+f/9P/9//3/8f/+n/+f//H/+P/9f/+v/+P/+3/+j/+n/6//T/+j/+X/+X/9f/+f/+P/+v/+H/9//5//r/+P/+f//P/9P/+P//f/6//X/+v//z/+j/+f/8//z/+P/+H/+v/7f/9f/+v/9P/+v/+P/+P/7//+f//v/+f//H/8//+P/8f/9f/9//z/+b/9//v/9//3/+r/8P/+n/+f/+n/9P/+v/7f/6//3//P/+T/8v/7//z/+f//P/9P/+/+3/+f/+/+f/+v/+n/+//z/+f/9//9//9f/+v/6//f/9//+f//f/7//+/+r//P/7f/+P//v/9f//r/+P/6/+v/+v/+P/9P/+X/+f/+P/+f/9f/+P/+//+v/+P/9P/+f/9f/9f/7//6//3/+f/+v/+//3//f/+v/+P//f//v/+P/+//7//9//z/+f/7//3/+v/9//+f//P//P/7/+//+//7/+f/+P/+v//3/8v/+P/+f/+v/+v/+P/+f/+f/+//+v/+n/9//9f/9f//f/+//+f/+f/+f/+v/+f/8//+v/+n/+f/7f/+P//v/+f/+f/9//7//7/+v/+f/+f/+//9/+v/+f/+v/+/+f//f/+f//P/+f/+P/+f//v/+f/+f/+f/+f/+v/+P/+/+f/+f/+P/+f/+//+//+P/+f/+f/+//+f/+f/+f/+f/+//+f/+P/+f/+//+f/+f/+f/+//+f/+f/+//+f/+f/+//+f/+//+f/+//+f/+/+f/+f/+//+/+f/+/+f/+/+/+f/+/+f/+/+f/+/+f/+/+/+/+/+/+/+f/+/+f/+f/+/+/+f/+/

wSPNlVjfq9GJ7LYKumQQDKww3JOiVZpuaTDSVXpP23nESQ3ObPROuxZvffTMqDIQQu4xlj/Gn4W
A/wKrU/JfV1UlPnzVfJjVOpkY6CJqFxVKtV2RlNPfTIrEdpL5SvY6GG385ZLRXkz8comnRTKntEQoWnR
PPLb+ZR2MDzeo1Nb3MII+fPUsvRtsT6s0x2yLazkC4s4FFljYwStui9y4MzW3WTLgab3/v7agqEELsM
tY4xp+Gg/0A61HzX1pcTSXlTVsJNXfaHm3TpknQt1dk5dQNmi5BvVX3aEaoXkbDjb8cMt6rmUE3Rt
OgnmOSJbPsVNwrXWh3pUsdG96eO0wvHaSWdJ0zWtuiudIO31zIDJsxsrTBgeLJNA2SDoMjJBcOZs
yEa/H+999/8UsvRoWBEGJ/sd4x/jQc7AdYiZr/8oJSp1cCncTl1VFcMGXa5GqqvrrFqc/pxdOnx4HCK0
3/aLjxl0MmejWvm0Ga1/WqopdHmUuJOEu7291c4yYZmV5dtyr9jOm1g8TzTScwPVra53QpN8Hh
dR1Mb0iU+ShzqSdOnUwluXBQ9tawvec3z1/+zZejwkAIsb/4m//xr/3bn/y7UWW+Xrzx2c/87z/
+4/oHDQDLUfNfg1xFWWtu9YurqOTKNJpRLNW9qgbV14+fvVv16q603aPhxl8OmerVXKhf1zNoFt
UsMmckb5ylHabrM2P6Ta/e29DrN2N6mdQn6QSmR8vOqG/Gug4yA0UdZywt0G9d30+mkpvb9Ey
4Aref3L769VejwkAIsb/41H/+K7/wT/9OVJavHV948s/
+3//9v/WPGwAWoua/BiP1W/7WQFF2x2Kp7nZocWp9+OJ0qb3S9o6GG385ZLJXc6W60M3upFlkz
kjeOEvZXjW9ei16/WZML5P6JJ3A9GiDg3VmrOsgM1DUccbSui6R+n4yldzcpmfCFXj9ndejwkAIsct4
7Rf+dlSQbxNf/ZU36h83ACxEzX8NRuq3/K2mFKqvlhZLp37Nf96veleXmivdgNFw4y+HTPdqLh2u1G
vq7uV3YlKcpWyvssl7/WZMb7BJOoHp0Wbkm7Gug8xAUcf5kzk4NYpTJ1PJzW16Jlzal//gy1FVIITYZf
zEL/
+NqBTfMn7vv/12/UMHgCWo+a/BSFHVFj3hvbgSyvRvq7CxYqkeJz3Gf7oSjBdlHH85ZE6v07zbX2cf3
JqZJBZ3Kyssc9vZ73aHQcK3spL2nR4t1+KY4G7rOpjekMlx0iHi/UqGGJr+0Z2XwDa+9eG3oqpACLHL
2PgYfxpvfPYzf/rBB/WPHgDOpua/Bplip9OWT83dtg5qm7dXmtKo7TNRLHUdc12Ha6/xl0Nm9QryR
3fa9s3ltulY2jhL0+luhWVmV+Ls09PLpD7JTGB6tKTF8IVu2IzMrOKOU5OJ59a1T67Ur7NzmzETLuaD
jz/wi/qFeAhxkWP8aTjYD7AgNf81yBQ7obak6gkbB0VWbLxY6jq2w7XZsglOw42/HDKvV7faZDvaHj
13SlpYWObfgqPcOD3doJnUJ9kJTC823yIYvj/rgdXN2pCJyQxvT90kHTEzt1kz4RJubm8ePXsUFQZCiF
3GpY7xp+FgP8BS1PzXIFPsxPpFV6YCChscbs8tltJ22Z7RxfGXQ2b2Gt+NtldlZMdqcZaywjLs1Xx9kHY
Ym97gwgYnML3YfoukSTDXbPeD2RsyPpkw07Fb8/o0RG7EZG6zZ8Kmbj+5ffKNJ1FVIITYZVz2GH8a
DvYDLELND9My5Sg8DG98+42oKhBC7DIufow/DQf7ARah5odpan4eprfefyuqCoQQu4wrOcafhoP9
AOdT88M0NT8P0HvP34uqAiHEXuN6jvGn4WA/wJnU/DBNzc9D8/zm+UtfeSmqCoQQu4xrO8afho
P9AOdQ8wPQc3N788pXX4mqAiHELuMKj/Gn4WA/wDnU/AD0fO53PhdVBUKIXcbVHuNPw8F+gG
JqfgA6b373zagqEELsNa75GH8aDvYDlFHzA1B7+3tvRyWBEGKvcf3H+NNwsB+ggJr/sprfDVdZ5RfEff
Ts8dN366/38bvo+ivKObQ4LfNoou1J731ozOr5kM3+NO3vQ7hT73///Re/9GJUFQghdhn34hh/Gg7
2AxRQ819QrzRdowSqE+yo3EpXlMiU75OrzZb8av4psz5N+/sQ7tXzm+cv/
+bLUVUghNhl3KNj/Gk42A9wV2r+C1q59mn/SmE35VZuRZH+AtsO4+ttmilC72bGp2l/H8Kduv3k9t
WvvxpVBUKIvcb9OsafhoP9AHei5r+cGRXsWfZXbk3vWLO+tsGsTU56MYuafz9ef+f1qCQQQuw17u
Mx/jQc7AeYT81/GU3ZEwgLzrZSqkS1UVAydcNE5erQ+GG5FbRJq6/eDCaLs3DYsGdSRA8P291pO7
UTjGbbSEbvBuluhVMb0PRKx8sZGDBzeXixJ737B70mwXjd0k8zHO03YG6uXsNkQ6KbA1vR6Obdqk
YMuwVt0lF6k45uz54z0778B1+OSgIhxF7jnh7jT8PBfoD57lbzt8/Www/W2Sa9h/
+BAmG27gH/Hj/eD5VD+VsHwa5lGiR7OjR+fvCjYIheqdUYfdsWGba9f7oWvRxaUU/bp7vV9hv8tOT
mNdj4oBmxtyPxxanFDmxZ1yJzv7qZ7zc64ZJctekZH4WtApkO1TRnDTS1gXeeDQO+9eG3opJACLH
XuNfH+NNwsB9gpg1q/ujh/dwn8nC40ULn6mV3sy1lmn1qW7UbF1Q7oxuQGz/tm4yfdGv6jLxz6b
DdlTjPyLBhm1zW3Ir6kl5tl+k+keHlNmMGLaK8k4uNh2g7tGMGswpnPjRQ1zFRkqu70iRvezVXugsju
btWwSLSdMmUkm7xumfNmUkffPyBX9QvxMOJ+36MPw0H+wHmWLzmTyVP62cJKo2DZca8kLFy
KFxY3K6tbSZWPzZ+cC1q1rwMBs9ciiw2bDtQrZ8yGjMnmkkw3tDsk0HbPoPrTWbedKkvzFxsaOYs2
maDA80wnKvb17hN0uegvTYyl1y/6XTNy5ENnDFnJtzc3jx69igqCYQQe419HONPw8F+gEnr1/zLPoi
3T/6P+xXAfZTZmah2rLWLPl3NVDtZuZ3PJIiataNnDCacLsBmDxs2jN/d3IpibZvYSJ9YZpf6miTRO9Kk
mL3YSr91Mka/Q2Z5g7PMmJ0r3uqmSS9Z9mJf7i3LdIua9afZF01oZM6Muf3k9sk3nkQlgRBir7GbY/

xpONgPMGndf9ufeXTPPKEfzaxbmj5P322+ukvBc2Uyu5kvoqKG+UapzPi5vgOj5wQD9S05bNcyW
WBuRTnhEO/O7BOaztO0qKbYpGtbz1lsmyPS3G/GGN6EvrHlFeSKtyA7ncE5duJxjqbTNS1yhieUy8
WQN779RlQSCCF2HD//Mz8elcp7il//uZ+tf7QBkHOhmr9tFRqpHGpNt+MgzdfTva5VZjfzRVS01Hyj
VGb8GZXS3NF7lhu27VaJWudWNKXpc5f1zOlTL+jQpGk99TaGmhYHp0bJ0ibHCIaozGk4P1fcJjudyTl
mch1Mp5sx8Iw5M+it99+K6gEhxL7jx37l01GdvKf4w999p/7pBkDO+r/DL9MnuTTjEf+o6Xfq1rya6H
S9xnYmXFTcbuZu5cafUSnlek1aatim1eNnz3KLnB4kadGNOLBZuTGb5YzOtW70+OnT0wBB48l5pvv
VXGm7ZLa0cbh1h2RlueJRc1naa4Obm+83nS7XKzJjzgzyv8YvxAOMn/7FH4lK5X3Eb3/h1+ofbQAM
uETN31zJPK2PFA9po1mdrli6MwdtPRavsltmptrJyo1/l+quudBOKZxn3zLD9rq0L0bGzIhGzUwsvjRjY
nltu0qv8dSYcYqufTtOPM2TtmN3Od+wU5Yr2epkScE4Q6kPknEOZqRLssWrmDVnhtx+cvvab70W1
QNCiN3H/v6F/6//3M/
+xQ9/WP9oA2DAJWr+9vE9Y+RpvRmoe8hPr9wrA7uZ355gjZlqJ68/UpVlVqXUXukZeWsWGba93Q
zSTr8bNbOiWC5Lb6fSufZHbYyt9yTsF7eeWGw+5VHTJJ3myVDP4ekW5UrfvoElHcVzDPWzV6PNSje
xgTPnzKCP//xjv7RfiIcWn/7Cp37ppx5FZfP9jTc++5k/
+9M/qX+oATDs/tT8+RrgZKzmuF4ju9lfbHQ/U+0MCbe6aj67UurPYCrV+cN2N4L27fS7hsmKckYbZe
Z6EHbJvSNZmfmFxvcwzHjM17yuc+enedKf7Izp3j3X9Nt3uDE2x06YvGpZlC7OMnsQBvkf5xfiAcaeD
vY7xg8w0yVr/vFKIdJ//I/daShYRvOh9PHjnnr7e29H9YAQYvexj4P9jvEDzHfJ8/wjoyTaPgNUXWyt/U
z68HGPffH3vxjVA0KI3cd9P9jvGD/AnRTW/FlVCZ8U9JkKvxumvtb+69+hvwXIDHLS3lB3sZnwX6sPf2
jhnvj8Nz8f1QNCiH3HvT7Y7xg/wF1dpOYfGmiweBqp7EduwTqCml/Bz/13+8ntq19/NSoJhBD7jvt7s
N8xfoC7ulDNf9QfbKxkH63rR28CMOn5zfOXvvJSVBIIIfYd9/Fgv2P8AAXuVvMDsEvvPX/Pr/EX4qHF/
TrY7xg/QBk1PwBHX/ujr0X1gBBi33GPDvY7xg9QTM0PQO3N774ZlQRCiH3HfTnY7xg/QDE1P+Oi38
cw/vJOmt+EV/8ihnOGuqveL95f/RdBHFa2wZqOttxDduvJN55EJYEQYt9x/Qf7HeMHOIean3HjRf45
Realav420cm6JX+dbKMafLM9ZM9ubm8ePXsUlQRCiH3HNR/sd4wf4Exqfu4kqirPKTKjmn8zG+bdu
gZX87OMD3/woV/jL8SDiqs92O8YP8D51Pzcyf2v+besi9X83Fvf+eg7UUkghNh3XOfBfsf4Ac6n5r+Ig
XI3e7mt4yrTPSbqvuZ21yO90gx7vBINN/5yXNv44NA+mnt2qLBLOMFRvU69Xk3KwMDEw8lF8+7p
5wqzDeWKln2ULD1o0w1T3xzOmBloQDTG/EUdzJn/wegYJ70m6f0ZI7Cut95/KyoJhBD7jms72O8Y
P8Ai1PyXkSmcche7gi8UdJpZgIWa+
+3tNkk7SH2leh0NN/5yRH4pR3XaZKj2QihYaNb4jmXuDkx8crpHo8mGcjXXg3GSpWf6npqPZpz3d
mS3NegxkaJ0/kfBrKbe3OkR2MTr77welQRCiH3H9Rzsd4wfYClq/gtpap5MmdNeauue5kpbKCVNg
mGSAixWN2i6BNVV3aMZoXoZDTf+clDbLs5xUE9kauTMUhMzdiwdOCvdle5KtIQ75sqsI2mWZq9
MZszl60u6RFs2mWLO/OMmySBxh6TH5Ahs5PaT29d+67WoJBBC7Diu5GC/Y/wAC1LzX0pTxbRFTF
JLZYqryerqIKmnEnWLU5/Ti6dPjwOFV5r+0XDjL4fkmrXX6rn32zSvMgsLLkXm7Fh6IasZKmg12TFtk
Osy5y1rsw+vtRJ3LJji6dJQ+5G5Dc+/fTk0/aZBZojTpckR2NDHf/6xX+MvxIOKazjY7xg/wILU/BcTFT5
NKdVWX5na6mCgV9CorZeG67i6V9Wg+vrxs3erXt2Vtns03PjLIdm1RBf7QzU3c4ZyZbO04zZX5025z
d+1GuvYn27bINclM8ukWSZ7LJtxcm2Z5MPyi5ox//Z1p5ewP3DfaYipEdjWBx9/8OKXXoyqAiHEjuO
yB/sd4wdYlpr/cpq6pipmmmjKoK9by5VlUXs0owHLqbocWp9aHL06X2itt72i48ZdDsmuJLvaHam7m
DOXKZkmmOG/KmbHSju2VSNogyDVn5PxKDiYy5vL1DA7cmVrUOTtT32+GyGmGGB+Bzb39vbejkk
AIse+41MF+x/gBFqfmv6S69jlUT02FE5Q0+fKsaVhfnVWAZZz6Nf95v+pdXWqudANGw42/HJJdS3S
xP1S2x4R8n2bc5uq8KWfGijs2TQ5OrdKRc7lmjDy0+smMuXw9AwN3phc1Z/6tYLjKqdPkLAL5EbiEL
/7+F6OSQAix47jIwX7H+AHWoOa/qLqgefz06aloCmumtpAKq5y4uspUT22RNFT3HdXjpMf4T1eC8
aKM4y+H5Jq11+pc/TYzB+6JR6wkA80bObOxUce0RbrzuVxz3rJMm4PpjJNrG5pQM+R0ikyTtM3B4
Vrwqp93cpqVsRG4kM9/8/NRVSCE2HFsf7D/f377v9c/bgBYjpr/stpqqRKVM+3Nprxq65623mqvNH
2DAUeLo65jrmtQz0W11vjLQck8g/x1sqGRmx7t9EZSzdixOM+AZqThnYjn0024GzmXq2tXXwz2vW
mWyX4wnTGXry/J3r8wY1HJCMn82wvd9KMFDY8xd4TMBTZw+8ntq19/NaoKhBA7jp98/W9FZfl6
8c3f+FL9swaARan5L6ytbg6aqqoT3u2EVU5QlMXS4UJdx6SKyic4DTf+csTwROtsyVD5HhOJ7rBjoyN

lKsq4Yz7VUTdyv83p+vBOtB0z2Y8mM85Z20D6OtWMRd1h/rFuWvlBuvvTIwzsEWt7fvP8pa+8FFU
FQogdx7/45y9Hxfka8Rv/8hcd4wdYiZr/0triZqh26VdHXc3TChscbrcvM01Dabtsz+ji+MtxbeODQ/u
oaBvPXplZ3vU7xTObN+VMRZnpGFamx6vN62DosEk7WDjDQ9tk5Ez22njGeWuLdyhKM56iMjX/o3
CYo3RK/VmMr/WoP0JzN+3H2t57/p5f4y/Ew4m//p/
+6r969SeiEn3Z+A//5B//4P98v/4RA8DS1PyX1hQ+ShfgvvjaH30tqgqEEDuOH/3VF//dK38vKtQXjP/
1P36v/uECwArU/JfV/qdOJT9wn7z53TejqkAIseNY72C/Y/wAa1PzX0r/Hy6n//AZ4Lo9+caTqCoQQu
w41jjY7xg/wAbU/JcS1PwKfuAeurm9efTsUVQVCCH2Gosf7HeMH2Aban4ACn34gw/9Gn8hHk4se
7DfMX6Abaj5ASj3nY++E1UFQogdx1IH+x3jB9iMmh+As7z1/ltRVSCE2HGcf7DfMX6ALan5ATjX6+
+8HlUFQoi9xpkH+x3jB9iYmh+Ac91+cvvab70WFQZCiL3GOQf7HeMH2JiaH4AFfPznH/s1/kI8nCg72
O8YP8D21PwALOODjz948UsvRoWBEGKvcdeD/Y7xA1yEmh+Axbz9vbejqkAIsde408F+x/gBLkXND
8CSvvj7X4wKAyHEXmP+wX7H+AEuRc0PwMI+/83PR4WBEGKvMedgv2P8ABek5gdgYbef3L769Vej
wkAIsdcYP9jvGD/AZan5AVje85vnL33lpagwEELsMkYO9jvGD3Bxan4AVvHe8/f8Gn8hHkgMHex3jB
/g4tT8AKzla3/0tagwEELsNdKD/Y7xA1wDNT8AK3rzu29GhYEQYq8RHux3jB/gSqj5AVjXk288iQoDIc
Quoz3Y7xg/wPVQ8wOwrpvbm0fPHkW1gRBil/Gjv/rioeZ3jB/geqj5AVjdhz/40K/xF+KBxH/9N/
+6/s4H4Aqo+QHYwnc+
+k5UGAghdhn19zwA18HPZQA28tb7b0W1gRBif1F/wwNwHfxcBmA7UW0ghNhf1N/tAFwHP5cB2
MgLL2z0h45ExSQqJlExiYpJVEyiYvtL9BDYSgA24omkETFJComUTGJiklUTCJG2EoANuKJpJhExSQqJl
ExiYpJVEwiRthKADbiiaSYRMUkKiZRMYmKSVRMIkbYSgA24omkmETFJComUTGJiklUTCJG2EoANuK
JpJhExSQqJlExiYpJVEwiRthKADbiiaSYRMUkKiZRMYmKSVRMIkbYSgA24omkmETFJComUTGJiklUT
CJG2EoANuKJpJhExSQqJlExiYpJVEwiRthKADbiiaSYRMUkKiZRMYmKSVRMIkbYSgA24omkmETFJCo
mUTGJiklUTCJG2EoANuKJpJhExSQqJlExiYpJVEwiRthKADbiiaSYRMUkKiZRMYmKSVRMIkbYSgA24o
mkmETFJComUTGJiklUTCJG2EoANuKJpJhExSQqJlExiYpJVEwiRthKADbiiaSYRMUkKiZRMYmKSVRM
IkbYSgA24omkmETFJComUTGJiklUTCJG2EoANuKJpJhExSQqJlExiYpJVEwiRthKADbiiaSYRMUkKiZR
MYmKSVRMIkbYSgA24omkmETFJComUTGJiklUTCJG2EoANuKJpJhExSQqJlExiYpJVEwiRthKADbiiaS
YRMUkKiZRMYmKSVRMIkbYSgA24omkmETFJComUTGJiklUTCJG2EoANuKJpJhExSQqJlExiYpJVEwi
RthKADbiiaSYRMUkKiZRMYmKSVRMIkbYSgA24omkmETFJComUTGJiklUTCJG2EoANuKJpJhExSQq
JlExiYpJVEwiRthKAAAA2Cc1PwAAAOyTmh8AAAD2Sc0PAAAA+6TmBwAAgH1S8wMAAMA+qfkBA
ABgn9T8AAAAsE9qfgAAANgnNT8AAADsk5ofAAAA9knNDwB39e7TFzpP362vAgBcGzU/ADtyKsbX
rcI/eva4y7Bmwq3/ZuG4sIPHzz6qLyypt5iKvyu5q94ervM2rW7gG6b+7J0s+8kY+RYduXVX+aF679iy
y1rX6M6s+oNiRcOLuq/v08CKestZ6m2akeue/kx6INT8AKxrxaf5niDPlg9tVdo1nnUOz1LtOqrnqpUf
qI4Lefz06eH/rJLnuITNHwnD59HlPxW9T3ZnpU9ftZaNPhHhvi2XZPA7tP891F/oWQYzjt66q6Ghjtfb
C6ctPTdVfpTw7To7xeTOHO8v+oMit6jekirnrWtkUdWtBb+V0uUEyUMrrajK316qXp27unm5Fkh12
rtatD+9XYzuMYOaH4AV9Z+n+k8ji2oSVf9/yweC/grXsnqWY4J6+9ZJc3zvV9+m0DZvTE+VcqUPXzz2
ermWfo6vNe9HOvH4o7HU0oYzjt26q7lDNe3ql3dW9T/pZzIsX3vh/LdrajnH6/Xts/KcjC1qifFrI4vKXC
o3uJzI+UmHVxRfWTFXc6N+ee67dhyuHb76JHcv+6mqm2et6SFS8wOwnvgZIHlqWNz6GSLbPH6sv
Kx2+OMX5Q9tY857HLyzzT8HB+t+FPobuNr6qoHDN2rpNy6ZebqUhTdyZK8W3Mapoar7xRvZ9F43
SyCfqL16/OLsNM1kM7nW+XGRJlpkHbWR5fQt+Pme3LrJycyXDJWsY8Fk7W5WL+KPw5KJHgw1P
wCrSf9kXvBpJ2/jh4FqPUs9Mw7qPf4sL9iz45frJKp2qrPynq23jkErv0kHpy08pDh9sc6nPPkODT4di0j
GO2bs79vCKUeGWzDT1FDJxhaZyrLYgnIDBdeOXy72Yc/kSj8US0gTVXmOBxVa52fNbV2gur3U2rK5
qo/aMcXpi+Gp3E2Sa2A7l1laUE35RIst7IFQ8wOwmvQJIPuIsqTVE4SqB4+lnt5S1VpqKy7puIpm+GP
K9RbUOC1szTzVJ2/hR/lx23zwqk9cZa1U6TrS7+KzJAnS8Rfey5HhFsw0PlT1xi2wi+NZqrvLvFeZRMc1
NFeOtxf7UAzkCiyUKk0UvS9Vg+H9nSeznMD43bsaGq3bv8VSZXJVV+LdW+i9qlZQZzt+3R922V18G
NT8AKzmAn9Ub/csED0trmrRh6m+4zK6/Tom2mRN6UdjUdGbc9q/NT8WVcJVP3fhGk5fr5OvGjve
ugXfqmrAcObpRyFpcp6R4RbMNDZU9HE8QzZLdbG21LYliY5r6F4fby/2oRjbuoPT8pbIliZKPnwLfCO

PLmeB8UOZXKftOl07fb1Qvuy6qvU0nj7tfUjOUA3bvjHJuzS+x2Sp+QFYzQX+qN7oWeD0pLPhM8fp
4W35hL1HtsAST9hjqrzr7V/yyVs3X/XurLplaYYVV9T7UCz3HH+SfIem37LJu3eeNEFr5NZdDQ5V7eZ
Sy5mYcHV7kWRxot5nIrBGrtRCn4g0UTLw9FwmjQxR3Vrqs3CU5EozVO/ceSs6md6aY4slVhd/y6Rv
//RciKn5AVhN+gfzQs9uw7Z4FqhybPzAUeVcdecqxzTrZ1l/OWs8yg9b7Kl6WJpi1RUFlv5IpPOO36yl
VzYy3oKp8kNV79uCi5mccNVggYQTiY63F/tUTC5qqe+wNFF1JVxI/GEsMLychZYRSHKlKaZ3d6bJgY4
Nzv9QVCvoD5NmXuBtenDU/ACsJ/6TebHHj0HrZzg9k8QWf/44ZAmWkXkOWsUyT22pw7jBsNWbt
Oq7VGUIF7LmM2L15qz7mUs/AZskPVj8E1G9Nf2J99aWvHVny2RsjNy6q8xQ1aVl36TJCVcNFti+iUT
H24u9SRddVO8baZHvqsHlrPA9m+bqfTMdLJd0/G1a5j2qZpsmOV4Oxx6fCllqfgBW1HsAWeapIK8a
O3K/nwmiFW2zmGPSVd6hzVfTe9Rd7rk3teanuu/0PNza4BOx5NqiT0ClW0K4tqU2cyTj+GTuZHio6
P06OXNxVbbeTA9ZgtdVznNyzNyZY7PFPvTJog4XgsFPU8pMYrbxRfXunpOmliznpLq8wWd78R8Uw
7n6d87eutNw2WF6n+tFd/LhUPMDsK7wAcSf02ym90B69vPokOrjvdroF7DwczyL6L8rJ/V7E926R+
/Y8KLu5ZpG3qOD3f2gWFz0VxUn3SODB4kzqfkBAABgn9T8AAAAsE9qfgAAANgnNT8AAADsk5ofA
AAA9knNDwAAAPuk5gcAAIB9UvMDAADAPqn5AQAAYJ/U/AAAALBPan4AAADYJzU/AAAA7JOaH
wAAAPZJzQ8AAAD7pOYHAACAfVLzAwAAwD6p+QEAAGCf1PwAAACwT2p+AAAA2Cc1PwAAAOyT
mh8AAAD2Sc0PAAAA+6TmBwAAgH1S8wMAAMA+qfkBAABgn9T8AAAAsE9qfgAAANgnNT8AAA
Dsk5ofAAAA9knNDwAAAPuk5gcAAIB9UvMDAADAPqn5AQCuy7tPX6g9fbe+dNLcePzso/rKOpo8U
fpr0O3NQbIPHz17PHRr9B7Afqn5AQCuS1DX9svTh17zd2V7JbcNg5V9t6lX+DcZAOtR8wMAXJfef8s
OK9SHXvPPWn+2uO/
+KkDFDzwwan4AgOvSq/nDAveB1/xt4T4xr3b/2n1qr6j4gQdHzQ8AcF36NX9QqCY1f3IhLYzrJscWw
bh1h+BKWA03lw/Xgn9OHyRpBHeT+8HcujQTNXdvvN6AwUwbI2O1ras27atkBWPzP+rPJ2oyssDRf
gCbUvMDAFyXrph8dvqqKRmDKrN6nV4Iys26JO7K0UltET3cJ6iz47q2EkwkM8ho6ZtPGi89EMwl0U
3u6bPmyyj71PwnJjS8wHy/0ekCrEfNDwBwXZqi8VBBNoXpqWAMblQN0wtjNX/dKChJ4ytNWZr06
a40F+I0bYt2KkGeGeXucIJ2wDTnmLZxrRumMjn/psHwfAYWODRQPAOAbaj5AQCuS69qrF+EX6dlZ
1BOxsVs06SrS4evNMOkLeI2cUV8EF9qBwnaDIpncBSvJL0wrp3AUdRlxvxjSfb8Attms9YNsDo1PwD
AdcmX14dSM6mMM6VyXJqmTaavNK/DQrk/btMiJ0rcG2VAOqWjJmVzNV7apOEpzJh/oN+6uT8w
ejvLTn9ZAJtS8wMAXJe4Aq5ft8f7uxoybnmwVs3fv9i8yBlMPCzfNl5Kcc2fTmHG/HPFe6W5Pzj6QMe
5swZYlJofAOC6JMVkXES2NzJlZ1wYp02mr2SGjcbNtYjMaNLKt21SNlfjpU0ansL05JoWB6dGSfbJMYI
hKsPJANaj5gcAuC6ZYrJfPrY3xlouUPOHxXW/zYzyO00zrB0ubJzkWLDmnxwq7ZpsysgCD7eCge88b4
AFqfkBAK5Lrphs68aj9kZ3tS4n28I0uZIWr8NXhkdpmwxnbgrbNM2Y4QRd/zvXziNTmJp/vJyu/cQCk
4WMTgNgdWp+AIDrMlFMhjeCUjQW1bJjJWh8pX39OB69qXcP8qm7BmmacU37vrD3kjX/5Pzz0zl
qmgyNPtRz7qwBFqXmBwC4LgPFZFek9m6EteuhrIwL43Sw6SvN63C4ZDpH/bq532BgFaP648VFcry
0SZNTGJt/1/3omDLYlvB2bvSw69HcGQMsTs0PAAAA+6TmBwAAgH1S8wMAAMA+qfkBAABgn9T8
AAAAsE9qfgAAANgnNT8AAADsk5ofAAAA9knNDwAAAPuk5gcAAIB9UvMDAADAPqn5AQAAYJ/U/
AAAALBPan4AAADYo7/8y/8PnM7zPX+h0m8AAAAASUVORK5CYII=]]></redcap:Attachment>
</ItemDef>
<ItemDef OID="b31" Name="b31" DataType="text" Length="999" redcap:Variable="b31"
redcap:FieldType="descriptive" redcap:BranchingLogic="[q29] = '2'"
redcap:InlineImage="1">
<Question><TranslatedText></TranslatedText></Question>
<redcap:Attachment DocName="B.png" MimeType="image/png"><!
[CDATA[iVBORw0KGgoAAAANSUhEUgAABVEAAAEJCAYAAACdcmdaAAAAAXNSR0IArs4c6QAAAA
RnQU1BAACxjwv8YQUAAAAJcEhZcwAAEnQAABJ0Ad5mH3gAAE9sSURBVHhe7d0PjCRnfef/b+/aY
PPHNmBskx+RHat7sZYlGJIg08PxL4egZ+6SJbnbYOVg5Oh3PVyi0zQSkwOxF+mX2wguG4lunS5iWrq
zliQyTEJugrLdlJRgckzHR4IxYTI/Z7rlc3T8wDYGjPljB3u3f/VUPX1VHd1dVV3VXX39PtltaerpqfqqT/T

O/OZ5/k+hb5FAAAAAAAAAAChjumPAAAAAAAAAIAQhKgAAAAAAAAAEIEQFQAAAAAAAAAiEKIC
AAAAAAAAQARCVAAAAAAAAAACIQIgKAAAAAAAAABEIUQEAAAAAAAAgAiEqAAAAAAAAAAEQgRA
UAAAAAAACACISoAAAAAAAAABCBEBUAAAAAAAAAIhCiAgAAAAAAAEAEQlQAAAAAAAAAiECICg
AAAAAAAAARCFEBAAAAAAAAAIAIhKgAAAAAAAABEIEQFAAAAAAAAgAiEqAAAAAAAAAAQgRAVA
AAAAAAAACIQogIAAAAAAABABEJUAAAAAAAAAIhAiAoAAAAAAAAAEQhRAQAAAAAAACACISoA
AAAAAAAARCBEBQAAAAAAAIAIhKgAAAAAAAAAEIEQFQAAAAAAAAiEKICAAAAAAAAAQARCVAA
AAAAAAACIQIgKAAAAAAAAABEIUQEAAAAAAAAgAiEqAAAAAAAAAAEQgRAUAAAAAAACACISoAA
AAAAAAABCBEBUAAAAAAAAAIhCiAgAAAAAAAEAEQlQAAAAAAAAAiECICgAAAAAAAAARCFEBAA
AAAAAAIAIhKgAAAAAAAABEIEQFAAAAAAAAgAiEqAAAAAAAAAAQgRAVAAAAAAAAAACIQogIAAA
AAAABABEJUAAAAAAAAAIhAiLp0etJYKUih4DxWGj29PqjXWPFeU1hpWF+1iMxj3ZB2onXjpLWdL
MxLO7LSlg372PzHqPs4Vb2GrGzEO5vtDbdtK5JH08KNuQ9Cjmc+2g0AAAAAwPwhRF06Rdm8UJey
XurU1kPCkracr3X0c5Hq2U3rq4B5oILBVWnqJUdZzqxIe4faf1Qo1cT/rlhsR+14AAAAAADIGiHqMip
uytmqfi4dqZ0P9kbrNc75IVW1JdsV/XzhFGVzry/9vnpsS/aHkff+RpmXdmShKwdu8leuS9c+xj3ZzDJ
D7TVk3fijwsI7ascDAAAAAEAOCFGXVGW7JV6O2jxn9EY1e6FWpbW4CSqOot6h7OuncuoEPaQBA
AAAAEAuCFGXVkW2W26M6vdGbW/4Q6XL9a3wXoyqlqKuRek+wmpSjqyvaH59ZI1Jo6ZjoC7rqPX
GPu31edcGHd5fZI1J4zwEz5+5nbDPjxN+3INtCdS9tR4xy30ObGewraPOc7xjCm57uPZpYAh6c9VZF
2h4snM3eA7Uw9yc3Z6x+xzPPy71CLkXbAnaHvI9OHq7vqTHE6/dAAAAAAAcfYSoy6yyJXW3OGpz
VTYaDTnnJ6hyIWSM9FAIo3VqpQwmoCrK2hndwM6BdJ1nFmNId2dHLno7bcuubn/5zNpc9FKsnPa
D6gP/AGy9izv6PBo1Pe1wrCSDo63TPb8dqZUKUhrYSXM1aUimtjPY1qasDoZtEx2T2rZR+9S6H+te1
+kREu3HCSwHz4HSXB0dKibnnOtV70AUtW6Kc9TeGFHPNGS7E4vZbgAAAAAAlgQh6lILTjLVrLnBTF
nqF0Imk2pv+KGKV4+yL103ie3UZD3lhKW4dka3b18O3U23d42JhYxw0hjqferEnAz0rpz2yiY0d4M9
Ji/u6BisfEacDLUnjXX3GlSlpc9v3+0xnOr59bfvdUhWIdlAfdzx/O1494Hazrob/E1zTMbr9zZlc9v62PX
vV1Wv1/6cXXIi2X56jXUvsCzXu85r+8MlLiqR+4wr/BzteOl/krZbr3X/0hH2PRjY7rBkxzOu3QAAAAA
ALI+C9QtyXz/HklK9SwM9zlSwEhKq+K8rS71rTubTk8aK24tOBS/OZEYjX6963bk96Ubsy+dvW4Vde
9ZG1BBs1YOwWq1Ks9kcWu+3IaxdcdeNE387w+2yV3rnwG1/6DqbsV0VnO2FBNwB4e0wr3O11fc
nDEt0PSK2E9ivvuaS7JjMbQdfr41qa6JzF35+FOdanQqsS3p+lETnOlHbRwht44jjjDietO4RYBr3Pnyvv
OXCW/QSACAP99/xLnnNW2/SSwCAzB3/NZFjr9ILWBT0REVwkikVtoQGJP5Qeb/npMsYdm/2GE1F
UU6ccp51dJfTrj2WvywnT5+0e9QF11uqp/0AbA74vWmb4nVG7R44gZT1GW8ov7dOdT4sGbUo3S
BMfcIsazBr1jUo6ac28z7QPYSnOKZEvYkT7cec4f+kmIdQ3NyTvhmgTm3wHIWY6rrrurFuwJmaGO0
GMvDwEw/LOz/5Tr0EAMjDDY9fJXd/6AF5/Bs/0msAAJm7dI9I/zt6AYuCEBVBA6FScsO1P6fl1RVt7k
pbenJoj9k/JScqJ6z/D65XGeqc9ZIrrombLbpD+ttuIl09a/TozUtaAZl1DTJrOyHeSKpXqBe0GnVjgQX3x
NNPSOWPKvZHAEA+nvPjY/KWL94oP3ryGWm+/3555p8u6c8AALL1lMilP7A+PussYiEQoiJlGYRfXl3
RfTlsXxS7lKgd9lbEyVeN9XMZvhk9NO3A1+/VOyrwVcO67VqVQ480e0lOK1mv47yOaTHOXbjxbW/
Lhtnr1K2LatY5BRbUnZ+6Ux58/EG9BADIwxv/
+gZ5wQ+vsJ8/9NXvyp/WeR8GgNz0Hxa5/Od6AYuAEBUxleSkN1LbnBFfMSZJitM70Ri+HI+7744c7D
pf686+X7I/4a8fLjUwH4qbZ/0guOFOjFWVQIZacsoTKG6Jgvk22OvYvA/0seV1TIn2Y97LA8Pk1cz3hZ
xnoE/SdmNSNTtwdeukJv6eAubL+z77PvlM7zN6CQCQh1c+eK3c/PXn6yXH5z7+kHzlLx/RSwCAzF3
6gsjlr+kFzDtCVMRUlM2zbuVUc/Z1NbrYnOl8y+vt5wScijnre1s23JlrYvN7cqqJpBS3XmZRF0x117vh
6vxxe81a56Kmj3+wdqsx7F+aq7LhnTIV7Onh297K+dBcNQLH9nm/hqd7bHkdU6L9mLVbm7Lqre/p
me+ta1Qq+NvI2oTnyA9c3XYDi+kT+5+Q+n11vQQAyIOqg/pzD7xELwX94W9/Tb73raf1EgAgc5f/RK
T/fb2AeUaIivgq29LyctSalHTA48w8bynX5YJR4NOfUMnSXNWB0GQ1HAPbsp55Q/a9of6KMUnTHP
Jqu9rKUt8KRKiWomxe8IdlN1d1gOaFzqMm/ZoIJ3AMttM8tryOKdl+ipsXpO6/2HmtOZGTdS/7h6Br
7yrua1NNWBO03bzfw9odR+bHA8R339fvk7v+7C69BADIw/FLBXsYv/oYRgWozd+8Xy8BADLXf1Lk
8sf1AuYZISoSqWyH11+stqz17tBiV3FT9gZfW21NVr/R7K0XKBlgDM3OdKKjFJgB2KiyA+qc9bt+wOe

ya1/OW03PstS7fT9Yt1Wl1d8LTpaV1zEl2k9RNvf60h16sXq5MUzeVpHt4EGK7B96PbFTEbvtVlv6Lf
8+sqlzblwHu+5ulByOB4jBnYn/6Wfp7QQAeVr50kvl2iev1Evh/uFL35ZP//6hXgIAZO6y9RvZJcpbzbt
CX81aAgAxtTcK4nSSVCHqQGAKADGo4PT1/+318sAjD+g1AIA8lB56od0LNa6tu8vyiteFD/sHAGTgit
8QKfBL9ryiJyoAAMiVGsJPgAoA+VK9T1Uv1CTUsH7qowJAji79AfVR5xghKgAAyM1HvvgRezIpAEB+
VP3Tt33hZSProI6iAtS7/+NX9RIAIHN2fdR79ALmDSEqAADIxe6Du/LBv/igXgIA5OWOL18/tg7qKPv/
8zH53Mcf0ksAgMxd/n9FLn1BL2CeUBMVAABkTg3fV3VQmUgKAPJ189efL//8Czfppclc+dzjsnX36+
XWV79IrwEAZOtKXR/1Zr2MeUBPVAAAAkKknnn6CmfgBYAZe8MMrEk0kNcoz/3RJmu+/X3705DN6
DQAgW9b77aWPWx+fchYxFwhRAQBAZlRwqgLUh594WK8BAORB1T99yxdvlOf8OJ1f+R7/xo/k7g8
xKSAA5Kb/HZFL1EedJ4SoAAAgM+/77Pvk3ofv1UsAgLz83AMvkRsev0ovpeMrf/mI3PtJ/igGALm5/
DXr0dELmDVC1KXWlo1CQQrGY6XR05/LSa8hKxttveBob7jtWZG8mzM3Qs5LHL3GinPuVhoy6tT55
9d55H7NjwLuWyCW+n11+djffkwvAQDy8vJvPE9e+eC1eildn/zPB/LN//19vQQAyNylXZH+Y3oBs0S
IurR60lhZlaZecptTlzFpRP8+eHfiVasLfVIImPi+9hqzXor5KXfOCrAYvunRqJSlMENguK+5bIB7V+1T1Qg
UA5Ovqp47LG++bvg7qKKo+6u//
+y/bHwEAeVD1Uf+79fFZZxEzQ4i6tLpy4KZA5bp0+33p9/dkM68MdWzgt6QmPS+qZ+S4YK99XtxN
V1vqevelWy87K5qrQo4aA/ctEIuqf6rqoAIA8veWvRvtIDVLqifqH/721/QSACBz/UdFLv2xXsCsEKIuq
96h7OuncuqE5Nf/FGmL1zOyJ41zugtquS5bFedpcfOsVJ2n0twlRQUwPTUTf+WPKvZHAEC+Xvu1F8
vLHr1aL2Vrb/f/2A8AQE4uf8l5YGYIUZeQXbvRDN2aq04tx8GuiKp3o1E7c1T9zGAtyGCd1bDejbH3r
wVreI6qN+kMVfdfl6zW52A9S6+2qH6ENy3GPgPncEP8zZjnydln0vOiqHaWYvWMNHoeB0LzkpzUn
VFl/9A6olGM9obVW21v6GMZPAcJrkvI/RZ2vePcb4PXz35E1ImNY5b37WT3p2vSa2eZ6D1Ar1TMr
49uJI6QOz91pzz4+IN6CQCQFxWevubvXqSX8qF6o1IfFQBydOlPqI86Q4SoCDUUGGl2/cyRYVRHai
WjzqrR43EyanuDNTzVurCgpuQNVXdFt3UUZ5+DwWRzdcJ9Fjflgjtk3joz5/RGeo1z3nkq1y+kUEahK
q1+y+tVOsTseTxK50C6+umwipx2N97ZkYsDJ7W96x2NX1c3yXVRQV7I/eZcj1EBZPj9pu7d0GC5U5
NSIMjOSpb3bcz7M2CCa2eZ7D0Ay07VQP1M7zN6CQCQFzV8Xw3jz5uqi3r3h75KfVQAyI2qj3qP9Z
H6qLNAiLqEKtt96Xfr4kZ7Um3Z9TH72zrxbG/4AZBXL9Won9mpyfrIxEaFec7r+3uboWUCxu4/wN+
et3/pyI6XBPWkse4GPca+Wzo1imzrKP523M2ofdbOu/Fbsn0WNy+If+rOS9v677wbglnn94JOUJOd
F4P9um2ZLK8uyolT+ukYlS23beb5V9ri53BnxMnhkpwj67VGqYGh+21ofyZj2/b9ZrYlbFt+kJ3U/Ny3
/uvD789hya6dZar3ACyrT+x/wp6NHwCQv5UvvTTzOqijPPTV78qf1hmBAAC56T8scvnP9QLyRIiKIW
bPtPoFPwgdDgOHletbE4Z54aotPxwsrp3xAqzOge4z2bsoO14eaey7suW3deeiJIl7zH364ZMh8T6Ls
nnWTbuaslpwe08Gz+8kipt740PW2PblMOpEFdfkTNjxtXf9XrVn1pzjSXSOrPOzp0NEI3gPvd4Dhu43
s8et0evSPk86CNzLYfa0LO/bsfdnmCTXzjLNewCW031fv0/u+rO79BIAIE+vfPBaufnrz9dLs/G5jz8kX/
nLR/QSACBzl74gcpkJ/vJGiIoBI3qm2Yqy5iYxIwK3UyfSDKjKcrKkn47SPdC9+VSoU/LqNhbMYdKRw9
QnMMk+K9tGr0FHOsP403RKoi+fcf2NgNIP3Kpy1j2gqa6LruEZOrw/aOh+M8JCawf20Hd33yNrsaZ
uHu/bBNduyvcALB93Jv6nn31arwEA5OWGx6+Sn3vgJXpptu7+0APy+Dd+pJcAAJlTw/r739ELyAMh
KibUkeHOgTHCo7mX3TFUvMKUSrD+5Oz05HBssVSf36vSHRZuBG7V08FeoUmYExB5PXXHCbtWR
dm8EN470w0rF3t+o8nvz/SvXdh7AJaNCk5VgPrID+h9BAB5e86Pj8lbvnijHL9U0Gtm60dPPiPN99+v
lwAA2XtK5NIf6OfIAyEqJjR/gWm53vWGbAcfk9YLHS/
+Po26n7aO1NZznJyneELGlj4tn5Sxl7S4KW5lAntYuDF8vno6/CyPP0dt2TB7nbo1OM36o0lYbdzT+xj
s/as0z83XpEi53bcTXLtoR+GPJpiWGsL/wCMP6CUAQJ7e+Nc3yAt+eIVemg+qPuqnf/9QLwEAMqfq
o15iYte8EKJiQElOusnV0EzePbnoFnIcO/Q7J6WTXtA2qm5m6ibYZ6+xrodpl6Ved5OsPCfnMa5rc1f8
zphdOfAu6QmJc0m9HrXW/XH+/I4OP6sSyOGSnCOzLqcKFN26qMaQ96TUzPKqx6k9GZQdSHa9W
p7pDpOf0CzuW0usa5fme8AU1xDz7yNf/Ig9mRQAIH+lh1448zqoo3z6v/6D/MOXvq2XAACZu/xZkf
48dRU6ughRMcCcBCnYW9IPAlXYNTChz6yYNTCbq/5Q7faGHhpuPdIev510n72GrLsnrnpWNjeNyY
Nq65JPjmrWsmzKrm5er3HOCzBj90asnBbnDulIs+ke18Bw8Amvix8oDvbcjUlvX80sH+xxaoTFZo9b
oz1p3yaRZnHfKnGu3QTvASU/dZXaee9gZMOb4h9Hze6Du/LBv/igXgIA5OnaJ6+0Z+OfZ83fvF+

+9y1qZQNAbtSw/v739QKyQoiKYeYkSJ2alHSoU/LTE7kw7YxI5vDy5uoUoVGwBmZzVQdQXnhTlVZ
qs9e7kuyzJ411d6h6Wepbav3okCq98zLMnFndbbN3TastiX+aKhIo72oZDmATnCMv2LO4x2xOsJTEi
HvXrLNaPevPNp/YQt+3SpxrZ0n4HuDXW7V41zBubVssGjV8/85P3amXAAB5UvVP3/aFl81NHdRRV
ICqglQAQE76T4pc/rheQFYIURHKHgYdUpOy2rLWu8Otp1KR7cGClfuHRs/BBOwamMZwbZddWzOj
eqgx92n23LN7obonbiCk8of1p3hehhRlc2+4Tqg9hD5hYBeYJMs6ZjsbHhT7uljH3G/5QaqtKq2+0dZ
ACYJo9r07eJA2Z5vTZZMLft9aYl07S6L3AHUsg6+ttiava4u59cTTTzATPwDMkOqBqnqiLgI1pJ/6qAC
Qo8vWb6bUR81Uoa8KBgJAEmo2fT0ZlAph96btmTxL+lhOtaYNWBfEUbp2yJUKTit/VJF7H75XrwE
A5EnVQVWTSS2SK597XLbufr3c+uoX6TUAgGxdKXLFb4gUbtbLSBM9UQEkFCxRcGZtsUO49nnrWC
J6ZB4tR+vaIV/v+
+z7CFABYEbULPx3fPl6vbQ4nvmnS9J8//3yoyef0WsAANmy3m8vqWH9TzmLSBUhKoCY2rJh17o06
pWaJQoWUK+xIqv7demmUqJinh29a4d81e+ry8f+9mN6CQCQJ1X/9C1fvFGe8+PF/NXt8W/8SO7+
0AN6CQCQuf53RC7doxeQJkJUAJNRNS8XfPx7cXMvpRq/C+YIXDvkR/U+Vb1QAQCz8XMPvERueP
wqvbSYvvKXj8jnPv6QXgIAZO7y10QufUEvIC3URAUAAKEefuJhec32a+wJpQAA+bv568+Xf/6Fm/TS
YqM+KgDkjfqoaaMnKgAAGKKCUzWRFAEqAMyGqoO6aBNJRVH1Ue/
+0FftjwCAPKj6qGpY/7POIqZGiAoAAIbc+ak75cHHH9RLAIC8qQB1UeugjvLN//19+cPf/ppeAgBkrv+
oyKU/1guYFiEqAAAIUDVQP9P7jF4CAOTttV97sbzs0av10tGyt/t/7AcAICeXv+Q8MDVCVCTUk8aKm
uVbPTaknWjdOGltZ96MOYZeQ1Y2gmvbG+7rV6TR0yunpfZjb3NEO2zTne9M2m1bkPugvaHbWJC
BSwosjE/sf8KejR8AMBsqPH3N3x3tuqGqN6rqlQoAyMmlPxHpP6YXMClCVGCGeo0VKZRq0tHLWe
pd3DH205RdQj4AA+77+n1y15/dpZcAAHm7+qnj8pa9G/XS0aXqov7+v/8y9VEBIDeqPup/tz5SH3U
ahKhIqCibe33p99VjWyp6bXby3l8WRhxDryHrtTziU6UnF3eC+2qea1hrF8VRuA+A+aZm4n/nJ98pT
z/7tF4DAMibClBVkLoMVE/UT/7nA70EAMicXR91Vy9gEoSowDLoXRQ3Qy2Xy86Tzo5cXJwUdTFUt
qXfrYs+w8DCUMGpClAf+cEjeg0AIG8//ffX2UP5l8m9n3xYvvKX/NsDALm5vGc9mOBvUoSoS8eoLbl
i9kQctd6oc2mvz7s25fD+IutuGnU/VwKfNLcT9vko6Z8z+/PmMP7mqvP5EYU0/WNWj+T1Rv2h/FU
5e+GMDvk6shMnRQ3UUg17jG5PvHbHuTbD59DaumyYrzfqkQa2MbA+/BTHaYN5POpY/P2rh7vd
9nnrupbrsjXQXdYu3WC83n4M3DfArKgh/A888oBeAgDk7YbHr7Ink1pGd3/oAXn8Gz/SSwCAzF26R
6T/Hb2AJAhRl05R1s64PREPpOs8s3TlwE30Aj0U27LbdJ6Vz6xZXz17ldNV/awjB/4B2PywsCxn1nRr7
RCwJIMj5zu1UswQa5bnrCO1UkFW9fYcal2SINUYyl89LZXimniHs3Nx+hCvfEbcU+2L2e6pr43Dfn1w
Z846FVQOrG+uDgSpE7VBHcuqeFvWoakKSlf369Ld2wxcdxW+lsJKN3RqUprnibKwFD7yxY/Yk0kBA
GbjOT8+Jm/54o1y/FJBr1kuP3ryGWm+/37qowJAbp4SufQH1kfqoyZFiLqEimtuT8R9OXQTovauHwi
JEU72Dq1XOU6dmIcI1VI5LW6M2gzMjmSEhV6w15PGutvjsyotu6am9WjpLXRqsh4jjUz7nFW2rTa
Yw76rLadd22HVPv12d+vuV8TsRaoYQ/mrp9X2zVA4xpD+4qbsuefNfnTFa4Z1BPULwcDQN67d6V
wbV7neDX69Frbev2+maYPxeh2aFjf3vOc+P1RXYWtXf41/TppyLsFxAmnafXBXPvgXH9RLAIBZeONf
3yAv+OEVemk5PfTV78qf1h/USwCAzPUfFrn853oBcRGiLiOvJ6IfaPUOndivWnXCo32dFJrDwO38bS
5UZMsNoJq7fi8+s+6n2wPUXFff8ickqmx5QWCs3pgzPGfVlj+Rkh/mWi0Z7IY7gtk792TJfmJsJ0EYq/
Ua636vzepZ2QxPUMe3O61rY6vKWbchRsg+cv3+obPdKdoQeH0UI1Q3Q2s7cNWB6t6okwhkSA3f
v/NTd+olAMAsvPLBa+Xmrz9fLy23z338Idn/n4/pJQBA5i59QeQyf8BKghB1KRXlxCnnmRtode1x6W
U5efqkHXYF11vUMHDn2VzwQ7mmeJ0KuwdeWOgN5ffWWcfkDu+2H8bw7cAQ/VFmdc784HMy
Yb1zLRMP6W/LeT9BlVZoz1klRrtTuzaW8kkJ3d2o9a4p2hC7Z7Zxrq2N2WUO3P3Er8sLpOuJp59gJn
4AmLGXfPe58nMPvEQvQbn7P35Vvvct/m0CgNxcVvVRv68XMA4h6pLy6oraPTl74nSqPCUnKies/w
+uV3ngPEWoFiOYcodmt90x0xG9I6exkOfM6GmphqaXQkPC+LP0tzf8OqBVo6fp8kkSbhdl80L4jP1u
cDtiPjEgE+5M/A8/8bBeAwDIm6p/usx1UEdRAWrzN+/XSwCAzPWfFLn8cb2AcQhRl5U3tHlfDts6aL
N77VXEyQqN9VP3hsyCUdfTDi/9upOjwkuvNubQI2YYuIDnzB/KHyXmkP72hj9RVLUlIzuhTmDqa5O
CTNtg1JUdKNlqa55jln7k532ffZ/c+/C9egkAMAsrX3qpXPvklXoJpn/40rfl079/qJcAAJm7bP02eukze
gFRCFGXVklO2hlkRw52nSHNbh3Rkv0Jf3347OuzV9w864eaDXeSp4E6pCVnqL3i1eKc2KKdM2Mo/

xjjh/S3ZcNLUMtSV1PRTyvVazOhHNugZuhXPU7tScXsQNWYoCtu2QJgSvX76vKxv/2YXgIAzELpoRfa
D4z26f/6D3aYCgDIyeXPivTp2jMOIerS8ntyNptOOObWeCzq4p/uem+Sprnj9gDtSK2mA77BOqRmP
crmqj9sur3h1aUsxB5LvWDnzBzK787+H3iYIV70kP5e45w3jL9cv5BOuYRUr82E8miD3pbKoIM9Trvi
ls8dW7sVSMn/+v/
+l34GAJgF1ftU9ULFeHd/6AH9DACQi0v36CcYhRB1iZkzpqvehd7w88Ds5sYkTXPIq1NqC+shGaxH2
VzVwZg/Ll1GT440LNVzVtS1VJXmauqhoTmUvxxaW8AoiWC9cuSQ/l5D1r0CqtYrAxMwOY/JJkhK99
pMJoc2VLb9IfyBurRGfdmzm7MP3bEU7v7Fu+WOl9+hlwAAeXvjX99AHdSYfv7dP6WfAQByUXijfoJ
RCFGXWWDW8FPiTzbuDltXzPVzyAwvRw2ht+tRGr0uXeW6dJPWu0z1nFVke7BA5v5hSrUxzaH8o0
NdMxQeNaQ/Xl3VCaV5bSaVQxvsIfxhxVBVSNvvp1pfFohy1RVXyf/4lf8ht1x3i14DAMjT373yCf0M
UV7z1pvkbe+5VS8BADJ37FUix9+kFzBKoa/G9QIAgKXxwCMPyOv/2+vtmfoBAPm648vXyysfvFYvYd
D1P/E8+a1PvVGedw0TbwFALgovFrni/daTq51ljERPVAAAlsztN91uD+0HAOTvb27/tjx2PX/ECnPlc4
9L9fdeS4AKALmx3m+Pv8f6SIAaByEqAABL6F2n3iUfeMMH9BIAIC+Xjvfl8294VH78nMt6DVz/8t+V
5NZXv0gvAQAyd+ztIoWb9QLGmSpE7TVW9AQpBX9W65F60lhxJ1TZkODL27LhTbbiPCabqGZYeyO
43dBH1jOAAwAwhz788x+W07ed1ksAgLz84PnPyt7rvqWXoLzidS+R1X8bNhkqACATx4oix39eLyCO
OeiJqsJVf5ZqR84zwquZ0VcaKU3oAwDA4rjnl+
+xh/cDAPL10M0/kO6t39dLy+3al14l1d99rV4CAGSucI3IMTWMH0nkGKIWZXOvL2oeq35gxuuuHH
iTiKvZsNXn92QzxwzV1qnJeTqkAgCWjDtj/3VXXafXAADyonqjfu+aZ/TS8lIBqgpSAQ5Of5ukcIL9QLi
mn1P1N6h7OuncuqEZJedlqXedUNc/9Gtl/XnRZq7pKgAgOVzy3W32EGqClQBAPlR9VE/96Zv2h+X1
S/8xivsofwAgJzYdVDz7rl4NOQYog7XRLXrlZZq4nZEtYfVD9UoNb/OeaRVL1Uprp0RP0YFAGA5vfm
WN8tH3/5RvQQAyIvqjfu+ZtOYAkK2rRY6/2/p4hbOIqSxEiFqud4fDT/uRwnDa4qac1WVWRfbIcGnyBRVsDU7a1ZTV
wqr1/3hUiFMKG3rcqUlpMKBKMklYe2NETUjVYzgYFmXWhihZtW+khNcqwXEma58TyIW9vrIaGBH
2qXNitLNcl/GVM9SxDbR/6us5/f0ej9OrffAcNVdHhZyTcPbh1XO2Dd97qd3vWGrvKL6DGfsBYAb+6v
WPHcn6qL/yH15JHVQAyNPx09RBTdH8hqiIk34P0YN0Bu2Ha8tuuinKQug1zvnhkTuhV7/r1Zsdzzhv
5bp0dbDd9TbQlHNeotNLMEmY9dpzesOh2+3IzkX3tVm1IUpW7Rst2bVKcpzJ2tdrrHuBnP+HjZa1F
615bkRQaLRjb1OKem0koz1u06e5ntPf70n47fHb3pHa+ejIPHqivUH+PsLvvbTud0Ds2frfdepdegkAk
AdVH1UFqUfJyumftB8AgJwce53zQGpSC1HtnmjGkFHzEWco65DimpzxspxVfxuqF6C77UQbdnpv
me1yHmYvuTOyFivhWXQ9uejO2qUCFi+kKcrmBSPEidI7lH39VDo74mY3xc09J6yxHnub+mQmmiT
MmLjHCNyKa2eGQ/XM2hAlo/aNlPBaJTnORO0LtuOst74iW/aGVVC3J2GHE2hHTNWz/rmtbPtBrd
/2JNczhfs9gWrL7yFf2Up/+4q5j/B7L8+J+bAM7v7Fu+WOl9+hlwAAefjmjU/JV376u3ppsb3sp14o/
+a3mNAEAHJTuFHk+L/WC0jLHA/nDwYcXkjrjWE1w5B0mMHN0daVAzdTKp+Ukn5qK56QU/ppJDP
kHgioh2ouTjVJmK6nGTZ8Prc2REmpfSMlvFZJjjNR+0a3wwldR5fWOHUi6XdVVU4HNlaSk2473bYn
up4p3O9zpSwnAwcRIteJ+bAMrrriKmbsB4AZuP9V37HD1EWm6p/+
+n/5GeqgAkBurhQ5/mvWR+qgpm2+a6IWN2UvbMitPdQ3hXqoHtWLri8pZ7JH3OhefG5oM1EPZ

MWu5eiGPlE1KzNsQ5R5b19sebQvRuA3VlFOLEzSmcbxAvPpphfcZAepKlAFAOTn8yuPylNXX9JLi0f
1QFU9UQEAOTn+r0QKN+gFpGmqENUc9hv1cMLJoj8M2gxA7aBUrw9NMc2v04+4dRUtdp1B82tD
H2kGskvEuHZ+7Udf89zw5DXjJwlry4bZq9OtjWnWijRl0oYo2bcvLbGOc4btG21wgreeHHp1B4ZlOv
HcEcD5QZpuv+l2e2g/ACA/KkBVQeoiel3l/6IOKgDk6dhrrQd1ULMy3z1RkZGQ4dEus05mDGp2d9V
b0Q+rjZ7D7raTTBLW3vV6dtrhjxuYG8OTB6XehihZtW+khNdqguOM176Idth1itOdfT7Y9JDh+ImO
M737fWHkNjEflpGaZOoDb/iAXgIA5EEN6f+7Vz6hlxaDmoWfOqgAkCM1C7/qhYrMEKIupaKs+bN2
yao3Ztuc0XsMPcGXKlEb7K0YEnhNOEmYH/4YM+KbcmhDlFTbN1LCa5XkOBO1L6Id9rE7NVUTnsK
Rmqsb4jfdL5lQPrPmhNaJrmcK9/uiyeB+B0wf/vkPy+nbTuslAEAeVH3Ux65/Wi/NN1X/tPp7r5XnXX
OlXgMAyJaqg/oe6+PVziIyQYi6pIqbF/zehs1VHawYk86MU9n2h393alJygxmjRqg/UVew9mbkJGGV
09aSNq5dWbUhSmbtGy3ZtUpyrpO1b2w7ynXZSmV8eFnK5aas6vZ4Tbfaftab/j/Z9Zz6fs+LOdGV28
6Jws6U7ncgwj2/fI89vB8AkI9Lx/vy+Tc8Kj9+zmW9Zn79Uu02ufXVL9JLAIDMHVsTKdysF5AVQtSlVZ
TNvcFJu6rS6res/8djD/8OK6Rpb2dgoi679ubg/ixDk4RVZHuoDc72vF01d71eitm0IUqG7Rsp4bVKcJ
zJ2qfa0Zfu0IbVpo3SBlM7JWcHjzfsGiW6ntPf7/mw7q/B67F/aPQSTiCV+x0YzZ2x/7qrrtNrAABZ+8H
zn5W/ev1jemk+veatN8nb3nOrXgIAZO7Yq0SOv0kvIEuFviqCCAAAMIF7H75XKn9UkaefXYwhpgBw
FNzx5evllQ9eq5fmx7UvvUr+06ffzDB+AMhL4RqRK9R8BQzjzwM9UQEAwMTefMub5aNv/6heAgD
k4W9u/7Z875pn9NL8qP4udVABIFfH3239jwA1L4SoAABgKu/92fdK7Y6aXgIAZE3VR/3cm75pf5wX
v/Abr5BXvO4legkAkLljbxcppFNUD/EQogIAgKmp3qiqVyoAIB+qJ+re676ll2ZLhae/8Osn9BIAIHPHiiL
H36EXkBdCVAAAkAo10dQt192ilwAAWeve+n37MUuqDqoaxg8AyImqg3rsPXoBeSJEBQAAqVAz9b
d/tc2M/QCQI9UbdZb1Ue/6T6+2g1QAQE6O3SlSeKFeQJ4IUQEAQGpuu/42ueeX79FLAICsqbqon3
/DozOpj/q299wqp/7ZDXoJAJC5428SOXabXkDeCFEBAECq3lF8BzP2A0COvv2if7Jn7M/Tra9+kfxSjV
/kASA3hVtEjv0LvYBZIEQFcteWjUJBCsZjpdHTn1tSvYasbLT1gqO94Z6fFZnX09NrrDhtXGnIqCb6x+E8
lv5aY2mo2frfdepdegkAkLW/v+178o8v/6FeytbzrrlSqr/3Wrnyucf1GgBAtq4WOf5u6+MVziJmghAV
yFVPGiur0tRLjrKcWSvq58vHDiJLNeno5YXRa8h6LarV6loXZDV4saVTK0lhIDAGjqq7f/FuuePld+gIAE
DW/ur1j8kPnv+sXsrOXb9zu1z/E8/TSwCAzB1XdVBfrBcwK4SoQK66cuDmbuW6dPt96ff3ZHNZM9
SxQeScUj1nxwW/7fPiHlq1pa5zX7r1srOiuSrkqFgGV11xFTP2A0COfvycy5nXR33zr9r9wir3nrTXoJAJC
5YyvW41V6AbNEi+/qpnDohy9v/dHHF6znbk8Y53QW1XJetivO0uHlWqs5Tae6SomI53PSC
m+wgVQWqAIDsPXb903L/q76jl9L1sp96ofzKziplwAAmSvcKHL8tF7ArBGiLjGvnqP5CKnteOLI2peqN
536d163Or/dp135sb+ivNdbZLwuuD/bKS2MbmtlG7zFcYzN4jMM1S+1HWN1Low3jal3a+zDDt+aq
87WDDQ9pc9i2o9o8tpfjROdl+J4ZuZ8YxxD7fGh+W9RjuK0OZwi9/7p0z506/lKsnrNGj+NAWF6Sk7
ozquwfDt9PNqM9ie65eMduS+H7wj1Pcd9HsNxuv+l2e2g/ACAff/fKJ+SbNz6ll9Kh6p/+
+n/5GeqgAkBurhQ5/mvWR+qgzgtC1CWlwpHQMKhTk1JhQ8ZlcHHYtR8HCkLa61TIMrC+uRoeXk2
1DRU2hfYY7EitNCqEU58zapaW61J3uw52duTiYMi0674ynbqmQ8GiZh/zyGBquM1uz8dQE5+X4X
umuToieEt8DFGcfQcvd0hb7WCw5A2hd6V67jxVafVbXq/SIWaP41E6B9LVT4MqcjrpPZfk2FP6vlDn
KY/3ERwdapKpD7zhA3oJAJC1z688Kk9dfUkvTe/f/Nar7J6oAICcHP9XIoUb9ALmASHqUmAn8O4d
TmNeo3SlHPhSUpi5XrX3na/FYybwtaPGt482TaCw6mHj7EjO4PplEcFZM7r+3ubsrlVF+erBr/GPI9nZ
FyGWtm2ttd1t2Wptpx9bOvUrr3hB4Vhbe7UZH3kdQm2eXRT0jkv/invSO28cd0SHMPY8xHg7zu8r
dZxrbvBoHEu3Iamcu40u53bEitrHVKUE6f00wiVRPdckmNP7/uimOP7CI6OD//8h+X0bQxHAoA8q
ABVBalpWDn9k/YDAJCTY69zHpgrhKjLyOwlZ/R0K27uOQGJ9dhLZaajqpx1t1M5bS25RqwPHd486T
aKsrlnBj6O4toZL7TrHIT3AyzXt4IBWXFNzugv6uxc9NvY3vV65pXPrI0P38YwexjWLxht3rwgfgZ5PrR3
31CbR5r8vFRbfnDoh3xB0xxDFHPfoW3tXZQd3SEycC4qW/5+zWtniH/u1HFY3yOhIe8k9uUwrEFKk
nsu0bGn+H2R2/sIjpp7fvkee3g/ACB7akj/V376u3ppMqr3qeqFCgDIiV0H9V/rBcwTQtQloV4YhBjUq0ImqY
bsGrZTiyhuIkyielpJ8GjFofJo1t2HQ9x9BhzEGnTgwGP0VZ8xMtLyzyA0Mj0J1YVK9WY/8jgrfhNscV/7
yMN90xjFaWk+MudvfAa79X7sF+GEPcRwyfn/zcTeuUjN51gntuimNPcv2HzlNe7yM4ctwZ+6+76jq
9BgCQJTXJ1KT1UVX907t+59XUQQWA3Kg6qHdaH6mDOncIUZeeWpZg+bhyjn

mOk8MDO76nnDnc2AsPq6dg9GafXkeGOgjFCRlOK52UyYccwK2keVxw9ORxbLNWR2T2X2vVfkvc
RZOKW625hxn4AyNFfvf4x+fFzLuul+H6pdpvc+uoX6SUAQOaOrYkUbtYLmDeEqMuquCl7esjtQKlR
W/Pcos+u3ZYNs3edW7PRrMGZhHW+zurzZA+NNoYyV0/nF6FOH/qlfF4mkm1w6dXKHXpMWsd0
AsUTMrb06bje1BPcc+OPPf3vi6P9PoIsvfmWN8tH3/5RvQQAyNIPnv+sHaQm8Zq33iRve8+tegkAkL
ljrxI5/ia9gHlEiLrE1MzaqqeYPcGPHYR0vRqKo4f/GoxhxHPHrB2pgiW3/uMUba64U6Z3duT8+R29n
aqkk6GW5KR37gdnZO/JRbfoZeQQ8BgyOC+
+nI4hTOmkFwKOqumZL+NcNHfF75DZlQPvNJxwzn2EWPdckmPP4PpP/T6Cpfben32v1O6o6SUAQ
Jb+8eU/lL+/7Xt6Kdr1P/E8uet3qF8NALkpvFgP48c8I0RdRu0Ne6itmkU92FPMCHiMXnIlPxkzZmJvy
4Y3Dft884MlY2bySXgTWHWk2dQnKrWh/EXZdLsdqvO87l+XXmPdq22ZZBKkcVI7L578j8Fj1udsrvr
DyPW9bj9yHVtu1oBtyq7eda9xzgsxY/VgjnPPTXjsU1//hO8jZntyvRSYe6o3quqVCgDI3t/c/m157Pqn
9VI4Vf+0+nuvleddc6VeAwDIlqqD+h7r49XOIuYWIeoyqmz7Q287NSm5QYtRH7F6NnzmbhXSDL52
Lpkz9nttNibamUhF3I6BrlSH8o+4LiU/fZQL005glcl5MSQ9BnPYu9ueiRK2YH3O5qqz34IX9Felldqs+v
EUNy94PTLd9njnodqSeM2Jc88lOPY0r3/C9xEgiqqPquqkAgCydel4Xz7/hkcj66NW/u8idVABIE/H3k
od1AVBiLqk7KG3YUUMVeDS7wcDHlX3cLBmYrWVcx3NpCqy3W/5gZHNOTbvsAPDrOPxhlcr5bpsp
ZzL2dcl5LxWW9Z6d+j1VLI5L6Zkx2C1Z/A+3D80ejUmYNfnNIaSu+y6nznWQ/UUZXNvuFaoPYw+Q
aAb656LfezpXv9E7yNABDVTf/tX28zYDwA5UPVR7/uZx/VS0Cte9xL5hV8/oZcAAJk7Zv2GfPwdegHz
rtBXRewAxKNmNtcT86gwbG/anqHAOEfpntPHcqpFwIpwn+l9Rip/xM0BAHl441/fIKWHXqiXRK596
VXyW3/8z+yPAIAcFK4ROf5+66P/Xoz5Rk9UILaeNNbdmc3LcmaNABVZO1r3XPu8dSwZ9ODG0fGO
4juYsR8AcrL3um/J9655Ri+JVH/3tQSoAJCn4+8mQF0w9EQFxmrLxmANWFXOgK50yMzRu+d6jRU
p7ZyRbiplKXDUFf6fgn4GAMjStU9eKe9s/aS88723MYwfAPJ07O0M419AhKjAWAOBFgEqMsc9h+
WlJiiblx9NaEs42hKOtoSjLeHmqS0nrr1GDr/3pF6aLa5RONoSjraEoy3haAvSQIgKAADmBj/ghqMt4
WhLONoSjraEoy3haEs42hKOtoSjLeHmqS1IhpqoAAAAAAAABCBEBUAAAAAAAAIhCiAgAAAAA
AAEAEQlTkSE2WU7DrfxRWGtLTa8fqNWRlo60XHO0NvZ3CijRibwjp6Uljxb0GG9aVTbJuGmH3UNr
7mJbRRv1Y4SYFAAAAAGChEaJirvUaK1Io1aSjl4H5pgJdY1Z9W1nOrBX1cwAAAAAAsIgIUTG/eg1Zrx
GfzqeibO717RkF+/1tqei1szFPbenKgXvLluvStdu0J5tkqAAAAAAALDRCVABIS+9Q9vVTOXVCyE4BA
AAAADgaCFGXmao1OlC7MazG6GD9UXuIvfE1A+VKNbNOpXokq1Vp79Mcxt9cdbYTvjOjjeoxqk7qY
JvCa1VOdrymMbVf2xvetob2H3JN4rTRY359ZEONcxFo46j1xj7t9ea5zKoOadx7KKotg9uYsD5pjOuS
9J4FAAAAAACLgxB1WakgL7TWaEdqpVEhpPpcQUoDQ+ybq4OvVyFiSYIva8pqYbBWZBqcNq0GNh
xyDHYlNtgm65W1UnjQaYt7vIMqcrqqn3Z25OLAa9u7bmODtTKHQjgtuo2TKsrambLztHMgXeeZxR
iOHmh7W9xml8+s5dDDMoV7aKJrPizf6wIAAAAAAOYRIepS6knjnJuIuXUb+9Kt61BNOrIzmPx5qtL
Sr2+5QaEKG8/7ve16jXN+0FVt6VqVXfE2H0Nl2/qabl28L3G3sx1W8dJvU/gxWMe77oZg/mv77gF0
arI+MhUdf7xhKltu2wfPpR9GSvmMeBlqe8MPgsOuSWQbJ1NcO6PbuC+H7qbbu0ZI2ZEDN101hq
mfOpF9hDr9PTTNNTckuC7J7lkAAAAAALBICFGXkjERz96m16vQD9VEOl56FlRt+RP3+EGhqScXd9w
+e1VpeQGStc8LYa+fntmm0GPoXRS3SeX6lj/xUGXLC+U6OxdDexSOP94RimvidfQ0t22ElGaPTrN3av
2CcU02L/htrJ0fGKo+Ja+NftDbO3Si0mrVCRv3dbrau7jjBZKnvROYlRTuoSmuuWkm1wUAAAAAAM
wdQlRYdA3P0OH9SZmzk5+Ukn5qK56QU/ppespyMrCTEN0D77jsIdheXUtjqHdgSHsazOHy/rB4P5
SryllvyvYRvVNtxnbMHqOpKMoJfUHcwLlrXzzrnJ4+aYeVwfWW6mk/kMxMCvdQKtd8VtcFAAAAAA
DMG0LUZRWYKCdunckYgeWRMt3x+r1i3Z6eRig3URhpDK9PScUt3trctVrXE6cj6ik5UdFhZWC9anb
2EeriSf+6AAAAAACA+UKIupTasmH2OnVrPZr1HI+ocr3rlDEYevjD9lNT3JSzbglONXTcqCs6WRiZQYh
dOS164L4ctvUQeLv3pzs5lrF+QUP07K/5sv1xAQAAAACA5UOIuozMupwqYHLrohpDoCdXkpNuEjs
4XNoIEXNVcoamK6NqvWbF6+nZ2ZHz50fVFTXP2eBs/mZ90FMydk6nxNfQ3XdHDnadr3VrtZbsT/jr
h4e0ZyWFeyiVa57idQEAAAAAAAuNEHXJ+QGTMWP/VMxakU1Z3XCn3DFnS8+ZMcmTNFfFa1J7w
6+V6a1MmdfTsyPNpj76oaH8Rdl0u6xar6utN6yz5eg11r0anuYESU7AqVivP+8dkGx4U8nH5V+vZtP
5Wnf2/aIumOquNyfCylYK91Aq1zz5dQEAAAAAAEcTIeoy8oI9S3NVh0rGhDtTMmcun2r75iRC7nY

mCjuDs7o3V3WI5gWO5gzwaXOHxftCh/JXtqXl5XU1KdnnrCAlP6mTC95EVCojdOutWrxzHLe2bVBg
W9Yzb2i6eZ9Y68/k0w3VNv09lNI1T3hdAAAAAADA0USIupQqst1vGQGZUpVWv+8HRvaEQpMqyu
Ze1w/BbGr7g/scx2qn1yBt/9DrDZhIcVP2+oNtstj1YDOoh2rwhvQr1v62Ruysst0PrUtbbVnr3ZILLnU
8g6+ttiara2v22gwMTTeGs+c+ZD2Feyila57ougAAAAAgCOp0FczrADITq8hK3oiL1WDdo+eiwAwk
urtPS8/mtCWcLQlHG0JR1vC0ZZwtCUcbQlHW8LRlnC0BWmgJyqQKbOOZ75D4gEAAAAAAJAOQlQ
gE23ZKAzU8ayeFTqhAgAAAAAALB5CVCAPql5pZpNXAQAAAAAAIEvURAUAAHODelXhaEs42hKOto
SjLeFoSzjaEo62hKMt4WhLuHlqC5KhJyoAAAAAAAAARCBEBQAAAAAAAIAIDOcHAAAAAAAAAgAj0R
EV2eg1Z2WjrhVnrSWNFzZavHhsyL60CAAAAAAADA/CNERSZ6jRUplGrS0csAAAAAAAADAoiJERfp6D
VmvEZ8CAAAAAADgaCBEBQAAAAAAAIAIhKhLqr3h1gddkUZPD7+3l51HaClTVePUeI3zcL7eZW/XH
MbfXHVepzc4uF+PuW1j58HXt2XDfY318F4Wo12TGjwv6jHpuVGSHr8S1obCSkPCD8+s/eo8VkaciG
TbBQAAAAAAWF6EqEuvI7VSQUoDw+
+bqwMhX3tjRI1T9fXpBJbR1H5WpamXpFyXrYr1MbN2OWHk4HlRmquFYNCZ4blRoWtYG6RTk9Lg
BFl2EFuSwZd3aqWhcDTRdgEAAAAAAJYcISosVWn1+9K3Hq2qXqUCwPNulNaTxjkdX5br0tWv7dbL
zjrrtTsXnYiusm19rlsX9zNSbdmv7W+rxHNafjv7e5tSTNCupHqNdS+MLNe7zj77LasFWvOcDkeza4NI
W3bd1Dh020055yW0VjvW3SDXOE/uBe3UZN17bZLtAgAAAAAAgBAVUm1tixtxVraMANRTlM09H
crZ4aVeu3bGe23noKufZadc3/La6ciqXT25uOP20qzK2U13yxXZsoNGFVLuibM6w3PTO5R9/VQ6O+
JmscXNPWd/1mPPbVvvorhNDpynypa42Whn56J1ZJYk2wUAAAAAAAAhKiaha5OGDmHPzqkT44K
9tNrVlQN3A+WTUtJPFSdo9EPnYSmem+KanHGTWGtrquyCW7t0qM5p98Dbnz18X7+uYA7v7xxYR
2ZJsl0AAAAAAAAQoqIsJ82UcJTAxElGbdLcjGjnzNtlyawNRdm8ENYz2A9KQye5Giur7QIAAAAAABx
NhKiIoS0bZs9Kt46mWft0JuahXRm3obgpe3qIvV+v1tc8Nzybvl/DdfBh9KCdYLsAgLzp0Q3Ggz9yAQ
AAALNBiIrx2rte70o7oHNrfxpDyKc2ybYya1dJTroJqDsE3qVm4i8YM+6n1YaI16uZ9NUvzfakXXbw2f
XqnHrtK530Qtu4NVhjbRcAlpH9Xl+QwkwTy540VlZFWu57tPNHr+bqLILU+Qxze40Vpz0r+f/hT/0ba
p4P9zEP5wXDhq7XDO6ZmRvzvuZ9P7mPvG7mOO+3ebwnR+5jSf+gFfO8z/K9eGZinJuh951luGnG
nJfc34sTtofSdoiDEBWJ+AGdMSt9AiU/nTRm/7d+MFmdbhD8tO0KKsqaVzS0Kavem667baeO6OB7
cZw2JDp+/aavPhXsGRpSs9Wsc9pc9dvm/sOhHu7KJNtVjG3wDwuALMzsl/chKri09j/lv0npcCYu3Da
KcLuTP+4f5vte3N4IC3ONPyjOQq8h6zWRatX9x28G3NEnxsO8XrMy01/cAyWOhh+z+NZW52O1qS
YFda9TS6qdmpRmFPjk/0v7+Pc19R5csr6f6l3jHFk/T2Z778R5v83jPXncPtTnc/yDVmTok1eYm+C85/l
eHHFuht739CP98xP/vl3dH/g3Iot/IEadk9zfi8efl3zfiydoj/WNrUrbpf+eHO/7dn5+FsZY1g2DJWS9R/
TV5bd+A+hbPzD5uvW+9U+g87mq9ZZia/WtnxX060c8vNcqIa93P29uf9TD2NbIdtqStKvbr5fd9VXr
K8cxXx/yKNetVygJz83Exx/
+MDcdve3gMSfabqvqrS8PXwQAmEq3XrbeX8z3eP2+GngjyoffFv1vwAzaEEm/z8/8vXjm7XCuj9q/f
c28f5PzY/87OoP9RtP37dy1y/3eivPzV9rCv5dn1R7n5y9jv/pnrCy/l8a/rznvuYNtyPocjW9XvNdMa6J
9ZPIeqPdvbdd+hLRD3T/maud+Um3XK1IS/5w4n8/+vTjeucnjvS/
+fZv1+8v4cxImq7aNPy/h62fWnhHfw+nfR87+zd0737fBdX579Qr9vhz3uiJf9ERFDBXZVn8p0ksO56
821puAo7kr/t9KrNd7n9D2D52/MKlanIP1QqutCWuIJm1XEk4PIOsNTS/7rDdbf9h+0jYkPH57qP3g
ubQ5+wj8QdOuc2oMyXfZPWWMeqiWRNsFgMy05XytY71NXZBN503VUpEt9UY28fv35Iqbe9Lv7x
ltmTO69MupE/PRwFm1o9dYl1qnKmfn9kLNhnteWt7PKPPC+T6X6unAzyL5KMqJU9YH9+dQW08u
7sygPb2GqIFK5fqWv9/Ktv3zYmfnYgY9sRxj39d6h7JvfRj8fi7aJ64puxm9Ecd5v83jPXle3ved79+y1Ls
hP8tr6uf34dEJHdm5mO7dE/ec5PVeHOfc5GX8uXHeXwLf5xmY7Jxk9148/rzk+148tj0jfp6yR42mW
tYuzqii+fpZGOMRoi4pvw7mwJuLMeFQcMiBCgvdr1EPJ5TztxMM6dQPhf5rrYf5A725D/VQ+xmx35
Ht9MRtl/MGFlw3nvMG7H6d89gbakjCc5Pg+G2D59J+jDoG8zj1Y9QvU3G3a7xu+NgBYAoz+uV9M
enSL+W6bIX/A5CTnjTWa9KZVTvsoaMdqbbi/1ueGTUM0Rh6N9uSN/n84j6JXuOc9d1s/bI/oxu3sq2
HjNo17dWwypITNg/
+vJW13H5pT8huV1lOenWcNKPePgZk8AetuMFlmDTbEVuO78XTnJv86fJoB+cHhtOnWwJnknPCe7

FhxPub8/PnvuRaNYmfhRcOISoAAJgNfnmPTdUltX/5uTCLXoa6tpj9i6D+pWdGvR3b52vSqbZmPmL
C/0Op81AjV7KppRZXPr+4J+f2NDo7wwDEHTWkatqr7yOrObMI4efpl3ZMgT9oKfPyXhwwR3/Y6uy
flAvevxGqt6h6/9mYYa9C3osD9HwiwflBrO+pqedWiWHwjzD8LLxwCFEBAADmmDP5gRq2N6ueO
MFRDt36vqzO4hfU9oYzCcRc/dbuUL2Csh6WHcfc/eLePi/O6NHZXTNnsg5/YiAVeKtJgXKftGOWv7Q
jNfxByzKH78Xz9oet8pk147pY/4aeVWXUZtirkPfiAernGrdnrP89dXBKXadTkl3n7nn5IwymQYgKAA
Awp5wAVfXWmJ+hjMXNC3YNtk7tfK7hXHtXhU1NO8B1fuEpSEn9Vqh/CZrtcHptlsOyLfP1i7sOCGf5
y6Iecqzq2bt5jz0MViXezdWMZjYfZVa/tI9h93bqyMHgjTuqd9QS4w9ajkV4L56XP2x5ZtqrkPficIMl+f
qydXLfOk8nJau3vdn+EQZpIUQFAACzwS/vkVSvDSdAndMJ/zL8RSPMYE8j9bAngLR+Mexaz2dbt7sn
h6qoWc7nZKxZ/uLu9nw6O8NfFgeHTbpmdl7y/6V9rOIJceZ7CUZNPfuGnmG4O2f4g5Zvvt+LB+T+h
62S2GWOB8PbWf5cw3txTLq2eOCPkekZ+UcYfhZeOISoAABgNvjlPYKeRddiD3czevzkW+dSTf4wM
Bxc/0KW1S8ac6/XkJUVc0i2WqVmSp7lL6nz94u701utKjMcPSpSOW21wPoeCgyhV7dwzTovM26bL
dtf2uOpyGnVY6+27r+veL3G5m+islngD1qLaFZ/2NIjADo1Oe/9w6mHcM+oHinvxXGoUhklqUldLm
RwkSL/CMPPwounDwAAMCOtqvSt33L69a5e0a33y9aPJ2VvRX669bLVFtWewUe139KvWUZh56U
6JyfEblu53s/3bun26+Xg+ZiLe6RVHbg2rb71S2tfJT+5m+H38TB9HgKPefie1vdRxvdv3Pc1573Yf2R9
7eK0K4/35PH7CLt/3Ifxb1dq9H0x9H2r2jFw3Pp7Pu1rNcl5t78m8/fiEedGvd8M7Ns9hrTf/uKem6H
XZfY+POp+0XJ6L453XvJ7Lx7fnpB/xzO6RnHuxXn6WRjjFdT/rIsKAAAwE+5f6F3WD43zNRwQWBCq
x5xdm9BVbUl/Bl3nnO/pqrT6M5p5eS7pnk7G5ZnV9cH8Gfre9fjfR2Gvqc5r79gUjT83Id9bxnk7iuLc
LwrvxbOmRvOoOqhhgkP7+Vl4cRCiAgAAAAAAAEAEaqICAAAAAAAAQARCVAAAAAAAAAACIQIgKA
AAAAAAAABEIUQEAAAAAAAAgAiEqAAAAAAAAAAEQgRAUAAAAAAAACACISoAAAAAAAAABCBEBU
AAAAAAAAAIhCiAgAAAAAAAEAEQlQAAAAAAAAAiECICgAAAAAAAAARCFEBAAAAAAAAAIAIhKgAA
AAAAAABEIEQFAAAAAAAAgAiEqAAAAAAAAAAQgRAVAAAAAAAAACIQogIAAAAAAAABABEJUAAA
AAAAAAIhAiAoAAAAAAAAAEQhRAQAAAAAAACACISoAAAAAAAAARCBEBQAAAAAAAIAIhKgAAA
AAAAAAEIEQFQAAAAAAAAAiEKICAAAAAAAAQARCVAAAAAAAAACIQIgKAAAAAAAAABEIUQEAA
AAAAAAgAiEqAAAAAAAAAEQgRAUAAAAAAAACACISoAAAAAAAAABCBEBUAAADAQmtvFKRQ0I+N
tl5raG94n19p9PTK2fDbuiEhLV0wbdlwz3vk+e1JY2Xca6xXNVb8ba00rK8CAGB+EKICAAAAODqa52
TGOemSUMHoqjT1kqMsZ9aK+rmpKJsX6tZnHZ3aesg1asv5Wkc/F6me3bS+CgCA+UGICgAAAOAI6
Ujt/OL38Zx/XTlwM89yXbr9vvT7e7I5KvksbsrZqn4eco16jXN+IFttyXZFPwcAYE4QogIAAAA4WuiNm
r3eoezrp3LqRKxeo5Xtlng5auAamb1Qq9IiQQUAzCFCVAAAAAABHTLzeqH590pVg6NpryIpbm9Orse
rX/7Rrehp1Vr119suC68NKtPqCtUJH1wEdeJ31CKsrGjyeYL3S6HYYzGPXj8F92fsp1ayzrDVXndeO3Ul
FtltujOpfo/aGXxagXN+yXjUo3vHbQto/dH0tcc5VoEar+6BWKwAsLUJUAAAAAEdEWep1HdJl2Bu1U
ytJYTVYDdRep0K2gfXN1VEBZlNWCyUxyoCqjUhpcMIpOxQceJ3F3t/IQK8jtZJRr7RcI60YnTuHwlEtel
8JVbak7hZHba7KRqMh5/wEVS4M1gNIcvwqwA5pv3M+hoNUR/i5UueiNLhTJewaAQCWAiEqAAA
AgKNjzQ3p4vVGnVS53pW+qgPq9ax0hK1v7o5oh1dLtC/
+y5tyzkv7etJYd0PBqrT0a71td2qyPjIpNl6/F2OSpvaGePmv0a6um3ga+6psW5/r+hNFqRqm9n5iDc
MPTjLVrLnHV5b6hcF2Jjl+67VuGhvWfmsrOxfjnqu27Eadi8A1AgAsC0JUAAAAAEdIUTbdGYwy641al
bNuj8nKaWvJNWL9/mFoL05zBnqzXmhn56Lz+t5F2dGdIQPD3I3enN5rB4QPix+t7aeGgTCzuHnB31
ftfDo9MAOTTGnVs8OTUiU6fuu675lBqKO4dsYLbDsHXf0saOhcmfVeOzviZq/FzT1n+9Zjb+QMWgC
Ao4oQFQAAAMDR4oVsGfVGLZ+Ukn4aMGp9qKqcDiR3JTnpp31ix33dA90L01rllguwH8bwdve1A0
6dSBLymT0vz8ha4EuLsnbGbdi+HIYlthMITDJlPQudTGqK41fHZNc5DR3eHzR0ropr4h2yuodK7n4ja
rECAI48QlQAAAAAR8xAb9RD5+l8K8qJU/rp1MpyMn6am0BHRnTmnE6i8DlCYFIpo85ppLBzFSw5Y
HLD3NgTdQEAjgxCVAAAAABHj9kbtRYvTsvXYK/Onhx6Y8iHebVWhx7biYbtTyercHa88cfflg2z16lby9
Ss3ZpEcVP29D4Gyt7amueYpR8Alg0hKgAAAIAjyOiNmpQxjDw7g706u3Lg7tTtmVk66QWAo+p5ps
MsJeDXAHX05KJbmFROSaIqAdNKcvztXa/nqR24unVRp7iWaoZ+1ePUnkjLDlS7Xi3W0WUEAABHF
SEqAAAAgKPJmIAoTMlPDo3aqW3Z8Kapz1ZzdcObqKm94Q8/L59ZcwJAszZnc9UfQt7e8Gp0FlIZV2

4Gzta5WPd7WfYa61790aSTVU1twuP3A1djxv4k9PbVbRDscRoSdAMAlgYhKgAAAIAjKro3qjlzuwrp
nGAubi3NaZWlXG7Kqg4D/dzWmOFftd+ozdlc1cGh9+IREzJNorLtD1vv1KSk21XyE1S5kPuM9AmOv
3Lan6jKu5bGBFRJjDgX5r1RPat7ugIAlgYhKgAAAICjK6o3qqp7OVgzs9qavI5mIqfk7J4xPFyx63gO1Di
1a3MOvE4Je+2U7GHrIcdebVnr3eHxeYt9/BXZ7psz/itVaZk1TZu7Xs/fcexzEVYMVW8zrewaALA4Cn
1V3AUAAAAAAAAAEIqeqAAAAAAAAAAQgRAVAAAAAAAAAACIQogIAAAAAAABABEJUAAAAAAAA
AIhAiAoAAAAAAAAAEQhRAQAAAAAAACACISoAAAAAAAAARCBEBQAAAAAAAIAIhKgAAAAAAAA
AEIEQFQAAAAAAAABGEvn/AZM6nr53azj8AAAAAElFTkSuQmCC]]></redcap:Attachment>
</ItemDef>
<ItemDef OID="q31" Name="q31" DataType="text" Length="1" redcap:Variable="q31" redcap:FieldType="radio" redcap:RequiredField="y" redcap:BranchingLogic="[q29] = '2'">
<Question><TranslatedText>Do you prefer Life A or Life B, or do you think Life A and Life B are the same as one another?
</TranslatedText><redcap:FormattedTranslatedText>Do you prefer Life A or Life B, or do you think Life A and Life B are the same as one another?
 </redcap:FormattedTranslatedText></Question>
<CodeListRef CodeListOID="q31.choices"/>
</ItemDef>
<ItemDef OID="summary32" Name="summary32" DataType="text" Length="999" redcap:Variable="summary32" redcap:FieldType="descriptive" redcap:SectionHeader=" " redcap:BranchingLogic="[q29] = '1'">
<Question><TranslatedText>
Alzheimer's disease - Description #[no]

If you have Alzheimer's disease...

1) You might forget recent events of importance to you. For example, if you are interested in hockey, then you might forget whether your favorite team won their last game, although you may remember watching the game itself.

2) You might forget the names of people you met recently, or the names of local streets.

3) You may no longer be able to handle your personal finances or plan leisure activities such as dinner parties or vacations. Simple decisions such as what to order in restaurants might become difficult to make. Your ability to concentrate may decrease and you might have difficulty driving.

4) You may start having trouble finding words to express your thoughts. You might need to be reminded to shower or brush your teeth, but you could do these activities on your own. You may need to post notes around the home to remind you of simple things like turning off the stove.

5) You may remain aware of the day and your surroundings. You might be able to travel to familiar locations like the supermarket, although probably with the help of a loved one or friend. If you are in a social gathering, then you might have difficulty following the conversation.

6) You may continue to enjoy hobbies such as puzzles or reading.

Now consider two possible lives. In Life A, you will live in perfect health for 8.5 years and then you will die of natural causes. In Life B, you will live in perfect health for the next 10 years, then you will live with Alzheimer's disease as described in the summary for another 10 years, and then you will die of natural causes.

</TranslatedText><redcap:FormattedTranslatedText>
Alzheimer's disease - Description #[no]

<p></p>

If you have Alzheimer's disease...
<p></p>
1) You might forget recent events of importance to you. For example, if you are interested in hockey, then you might forget whether your favorite team won their last game, although you may remember watching the game itself.
<p></p>
2) You might forget the names of people you met recently, or the names of local streets.
<p></p>
3) You may no longer be able to handle your personal finances or plan leisure activities such as dinner parties or vacations. Simple decisions such as what to order in restaurants might become difficult to make. Your ability to concentrate may decrease and you might have difficulty driving.
<p></p>
4) You may start having trouble finding words to express your thoughts. You might need to be reminded to shower or brush your teeth, but you could do these activities on your own. You may need to post notes around the home to remind you of simple things like turning off the stove.
<p></p>
5) You may remain aware of the day and your surroundings. You might be able to travel to familiar locations like the supermarket, although probably with the help of a loved one or friend. If you are in a social gathering, then you might have difficulty following the conversation.
<p></p>
6) You may continue to enjoy hobbies such as puzzles or reading.
<p></p>
<p></p>

<p></p>
<p></p>
Now consider two possible lives. In Life A, you will live in perfect health for 8.5 years and then you will die of natural causes. In Life B, you will live in perfect health for the next 10 years, then you will live with Alzheimer's disease as described in the summary for another 10 years, and then you will die of natural causes.
<p></p>
</redcap:FormattedTranslatedText></Question>
</ItemDef>
<ItemDef OID="a32" Name="a32" DataType="text" Length="999" redcap:Variable="a32" redcap:FieldType="descriptive" redcap:BranchingLogic="[q29] = '1'" redcap:InlineImage="1">
<Question><TranslatedText></TranslatedText></Question>
<redcap:Attachment DocName="85.png" MimeType="image/png"><![CDATA[iVBORw0KGgoAAAANSUhEUgAABVEAAADYCAIAAABtBTQzAAAAAXNSR0IArs4c6QAAAAR
nQU1BAACxjwv8YQUAAAAJcEhZcwAAEnQAABJ0Ad5mH3gAAColSURBVHhe7d1fqF7Vue9xY9Qdj
0pC6w5pPZjoQQhGJG04ENcpNoIYEQKCikG24uHIIoJlB8FGxBzOhaJsCll7I+hFscLpOfEiRTanRLGFXL
SQHgrNhRfZkMq6sDRQL9aWUpY0F93z3xhzzGf8mfMd75zjne9c3w8P5P5+Sdc8zxjDHfdy3nz/pmX/c
3AAAAAAAwRWR+AEAiF9YvXPe/rqMoKn3tfmnH///5/6t+FAEAWwmZHwCQwvrG+q63d4kcQlFU
grrpteufPrj3xH33/vHz31c/kACALYPMDwAY3MbmxXN9T9T0/9oscQlFUFUmqGT13e7nt9t9/t5
n5s/r1z85VP5wAgC2AzA8AGNDZz86KEEJRVRaf4W1rl75fkg/QP7e/ADCUi19c3PHDpF
KIpKUw8e3S3Svi6+2A8AWweZHwAwiPWN9T0/2iNCCEAy8zF8cfWFdfgCYHjL
/QlxZW7mutLJ2pTqmhM4tjF7U6vn2lz5xV80pTZf5nV8tl1no853PbkBw66rxoJ82z7vQXFuSlWAQF
9YviARCUVTKeux73xbRPbpO3Hfvv/3mN9gBgEsj8C6JDkEw4dfYbU0gVmS380ifuqjmlI6Invch
Sb6G3mje49UVlfnttZP4ltb6xvuvtXSKBUBSVrL77xdDdEbp+zXl45/O9/+lP1Ew4AWH5k/oVxhvs/I05
osoI50h0Lh2HbUHDpN1u93sxmd+1NjL/MtrY3/N2qjmVI/FIrF1DPMdh209A92YbtOS+TGPR3/6qEggFEUlqx6/xm/Xv/7LP1c/5w4fkt
rf/HUBNZ6SCGOAISy15T52ur7CPqGnzI2K68Euf0FXuvKeGuDaWa452kmszG5lzhZduXBW+
L1R6d61vbNNkYYlzoaeHZo3hDjKrtb22LEUhpD9Ll8Eb61dV4JxuLkJydFAqEoKmU9/NAeEdT7Lb7Y
DwDTQOZfrDonra6pP1ZGRMsYxQ6KepDpSvBbThV/6BK9S1Ai1JH2Fydi0i
1yb+2bmQhN1uqpteY5JVtbW7IPi9pgaa3SOELfTYN5ZQy9b88yiRzTeBd/aOq0Eo3H2s7MiflAUlbIO
HN8pInrvxRf7AWAayPyLJtOUFW90ElJn9BV6qBpiXNsWqxAAWAWAMAyPyLLtOUFW90ElJn9BV6qBpiXNsIWS7VAHWJkbeqK9QMxUsxXfilT/gqtYJ6Dj
WgWqPVxbFtm7zK3mh9pG4t+W/PDMuzJ6lYl3aZTY9QR/T06oBjWofA1rwTycWoAdYF3hmsA5kO
K8FYXPzi4o43dogEQlFUstr90o7n7/n7i4o43dogEQlFUstr90o7n998tIvoQxRf7AWACyPyLZ0SdRgQQqyXiVk4HJMcaVqZqqEeU15Yv
V1Xwi84i6XkwXfunTcpV6rSZRr6tdiZc5xyGL7KLvdr1WOcTBcVV9WdG+w/L0JHK99gLaZ7OvKQ+5F
ujfV6b9hojOOcehBtnaWop1IBP31iC99Y31PT/aIxIIRVHJ6qbXrn/64F4Rzocrvtgrtg/PAMuOzD8GrkRZU
aea4UpELsegDmGpuqoYUPx5Ze18cVV9RF8upgu/9Gm7Sh2oXlcrUJtSm3QJ9JVd9DT1NR2Wr65q
vA2N6zosz9G6ZC+gfTbnipo67CvjmEhc2GFrhubo6ry1FNfa2leCEdi8tnnwvwYMigVAUlbKG/hq/XXyx

HwCWGpl/DAL5zX3KE8pmDEvVZdmIcnT2h/KQPqKvFtOFX/q0XqWOFAfq1ZXUJl0CfWWXuHuIrm
qMaFzXYXmO1iV7Ae2zeSerddhXxjGRuLDD1upLhOq8tRTX2tpXghE4fu64iB8URaWse567TQTyBP
WDQ9/58g9fVL8FAADLhsw/BoH85j6lolB1NDYsldep/3m/uLo4pI7UE4rpwi992q9Sh7Ij1Z7qc+470
Up2ibtXzuaN6zoszzvEXkD7bB36ddhXxjGRuLD7YjLlINnaWoprbe0rwaK99au3RPygKCpl7Xz5xjRf47
frzaee+OvXX1e/CwAAS4XMPwaBUKVDj3lOJiHH9TqFhcJSNY/9Nf7yiDGf6Bh+6dPlqnLd+q+zN051
bCLJy+KCpet2Ni+bYRLzrSzY17bP5hqRN5htX5n2G9I6jz2FvF/WFL7l52beAtL46PJHIn5QFJWytp/e9
uShO0UUT1kfvvlG9esAALBUyPxj4Ag7NR2f1Fmdg/RwfURFI31NS1iqL3Rd6s9e4Zc+na4y+oszerw6
rIeG2sou6qLZgqXjrsju7ctztC45FtA+mzXCf6Ce1sGxKnlh22Lk2urx1pHqtXNtHVaChbl09RJ/UT9FLbY
ePLpbhPD09btffFr9UgAALA8y/xg4wo5JR6oGc7ARsqRwWKov1NPpbs4G5XThlz7drqp3a90OfUXD
TE0jg6X7Lci55mmoJ3W0LjkX0L5Z9whj+uaqPbvrdENaFuO/PdUQe0bH2jqtBIuwsbmxb22fiB8URa
WshXyN3y6+2A8Ay4jMPwaOsCM1Q5cjAZkDstNdw5I9znmlOBh+6dPxqvDd0FcVAnesIrvEBUvzKv
XnjH1BaHnejXkX0L7Z5ghriLFW5+WZzjckvBizU36Zel1O4ZrRWlvnlSCpzWubRz44IuIHRVEp69ZXbn
j2wF0ifi+q+GI/ACwdMj/QzhFHga3hxM9PiPhBUVTK2n5627EH7hDBe7HFF/sBYLmQ+YF2ZH5sTWc
unhHxg6KoxHX42O0ico+h+GI/ACwRMj/QjsyPLejC+gWRPSiKSlx7X7hFhO2R1A8OfeePn/++
+mUBABg3Mj/QjsyPrWZ9Y33X27tE/KAoKmWN6mv8dr1+9BG+2A8AS4HMDwBo2Njc2P/OfhE/KI
pKXI9979siZo+t3j/1w+q3BgBgxMj8AICGR3/6qMgeFEUlru8+8Q0RsMdZv/7ZueoXBwBgrMj8AIDa
yU9OiuxBUVTi+taLN4toPdo6cd+9fLEfAEaOzA8AqJz97KzIHhRFJa6bT21/5v59IlqPufhiPwCMHJl/sd
TfDVcY5C+Iu7K2snq+
+vM0/i665o5cGne1016bV1RauqDzp2l6H8KJuvjFxR1v7BDxg6KoxDX+r/HbxRf7AWDMyPwLlCWh
MvyU+o9AVYMJxS17R5K4qZWZ/iWBQuZv0enTNL0P4VStb6zv+dEekT0oikpc9z+1S8TpZSm+2A8A
o0XmX6CBs49Ov5OJW64dCTq9l0P0FV1iKSF0Rh0+TdP7EE7U5rXNg+8dFNmDoqjEtfulHc/vv1tk6
WUpvtgPAKNF5l+cDgl2LtOLW+13zB7RZcdqzEBvxGSR+afj+LnjIntQFJW4bnrt+qcP7hVBernq1JHv/
+Wrr6pfKwCA0SDzL4aKPQYzcOqkVBDZyIhM9TQirvrmN+OWMcZOX40VtIYzc1rzSitE+6etz+iL9ALF
ahVrdlfEVNc5BivqosAQg7lXg+Owf7OlxvlMY4gxX731coXB6zy69moMtG6IOOm5FUq9bq2Y0bzM
GGPP0li0ON15zWj31q/eEtmDoqj09fBDe0SEXsZ658UT1W8WAMBozJb59bO1/8HaOaTx8O8JCJ3
VD/hL/Hjvi0PuUxnjrjkGWPfUN7978pwxRSNqKcG3rZdp9fnymHjp25GgrypPipdOrnUFhuuVNO6IP
OiatHGF+5bVIxzni5Pu64ILjulVaV9xzhxlcFxQLLPTRG03cObVwOOjyx+J4EFRVPo6cHynCM/LW5/+5
P3q9wsAYBwSZH7x8D7vE7k5XTDojJ7zbuooo+6THqVvnJF2gjfANb99rTW/dZm6JvDO2dPWR2Sfw
LTmGFdX145sepQWHG4u3uTfrmpgjBCrbd2snEJfoOc0VmUu3zdRfaElpld9RDXXV6kj9YFA73qUsQ
m7nbUk6zK5705rRqtLVy/xF/VT1MLrm//4d8v7NX67Ttx37+eXLlW/ZQAAI9B75rdZT+tzMZJGpp85
FyQUh8yNyXE627TsPjS/cUwMUy+NyR2HhN6m1RNVmi3FnB5yjkxovDVp+/21Vq4uqQ503Kyp4yr
0MO9EHfh71fdJjrGuyehjgbW4rmtvp14GbmCHNaPFxubGvrV9IntQFJW4tp/e9uShO0VsXvbii/0A
MCrDZ/5+H8T1k/9KMwEsI8edEdmxojddHnWkHSfXnXc0EMP07A7ehu0BrPO05kD57rp2JMgNyte
dtF7keUfUssw9SPbSm6OtOZoX6FtQm2VrnXvJW62GNJo5Dza53jLHZWJYc5lNYkGBNSNk89rmkQ+
OiOxBUVT6evDobhGYp1F8sR8AxmPY/7bf8ejueELPdcwt6prV8+pPswSekXHcTXeIEgPdg2yO+V3Xe
mZ3MSZq6nPaeqS1QdeOmtTF9YD2a2zt16gRxRKtpl02q3sI6ryaw38TmkLbi+glb4FzOd411uQ8ufZ
2aoSLf0GuXvA58fMTInhQFJW+7nnuNhGVp1R8sR8ARmJBmV+PMgWSQ0Vdlk+i/tx+1Vg57qY7RI
mtugfZHPN3SEpdZ2/ob1p9WUGMdu2ooeOW26hpQpdU02ZD1Oi6Z3tLNSJTDrJW3jqHMUWhy8
DuveQY53Ja19j5HRHDOkzcYc3wOnPxjAgeFEWlr50v3zilr/HbxRf7AWAkhv87/BzXWIc6POLn1HXlZ
epVy0XjFboz5qbkuI53yzV/h6TkuqpVX9OqUStra65Ntk7iGNB2jeu82k5wrdWgldXVcgJjcOs67fulju
hLHLdUyU7N0CyuI5zV1UUfc6xRcV3X3s51ldBhzfDi/xo/RY2hjj1whwjJ06vXjz5S/d4BACzOIjK/OuJ4
Wg+EB3tQp4tGzL4zGZ3H5C7rbTrSjpNr/lnSnTqgl2Sus6mfaRuX6BeBOW0dusi1drjETY8rNAa3zSlb
1OP1PHKZJX1hfdg9sBbXS49SY6wtGfP4WmeseTId2lnd5C46rRk+m9c2D//4sIgfFEUlrr0v3CIS8vTq
1z87V/3eAQAsziIyv358dwg8rauJ6od8+8hS8dxN9+0x9uhIO27NmYounZKSPtIQeGt6mVafVpPo5d

ezOnYktS7eXmtzViW035J5nRzdsgx3y5waYi+z5LvSv9yoXvbb59lSTq7R1OxezNapXdv72G3N8Lr656
v8pf0UtfA6fOx2EZKnVO+f+mH1GwcAsFDLk/ndGaAUyhzjFbibzc2K846042Pe6mJ456TUXEFbq/m
nrU8Y4/Xy64HWjlyCi3esNWPO63pHnBzrM4Xvodkx76deV73dyyw1F9thubP3an/7shOhNdbM5s
XIqHayS+dJ4MX/cX6KWnhtP71tqv+F/
+tHH/nr119Xv24AAAu1yMwfTgpC8/FfmmkqoB/qQ8nHD0vq7GdnRQKhKCpx3frKDc8euEsE5mW
vE/fd+8fPf1/9ogEALNoiv88fmMWir/EgdSE1/Znkw4cl9uovXxUJhKKoxHX3f79VZOZlL77GDwCjEpn
5nYoIbwV6R8Kvp6mO6f/61/dvARyTlPQJcheSMf9rdf+HFlgSj3/4uEggFEUlrgeP7haxeXmLr/EDwNg
sJPP7JvKGp0CyD5wChmFkfgI/lt/mtc2D7x0UCYSiqJS1/fS2Jw/dKcLzMhZf4weAEVpQ5s81JwtF9mC
uD54EALRa31jf9fYuEUIoikpZO1+
+8fn9d4sIvVzF1/gBYJxmy/wAgEm6sH6Bv8afohZb9zx3m0jRy1W/
+8Wn1S8UAMCYkPkBALl3f/uuSCAURSWu5f1i/4dvvlH9KgEAjAyZHwBQOfnJSZFAKIpKWTe9dv3TB
/eKOD3+evOpJ/gaPwCMFpkfYeLvYwi/nIn6m/Cqv4hhnqlm1fiL9wf/iyCynSXYUy7lPcRkHfngiAghFE
WlrN0v7ViuL/b/4NB3vvzDF9VvEADA+JD5ERYO+fOEzEVlft2oNGzkr5olyuDJ7iGmbGNzY9/aPhFCKI
pKWQeO7xS5eszF1/gBYOTl/JiJSJXzhEyR+ZNJ2Dd1Bifzox+Xv7zMX+NPUYuthx/aI6L1OIuv8QPA+JH
5MZPlz/wpczGZH0vr4ysfiwRCUVTKWoov9vM1fgBYCmT+hfDEXedhneMK7Ve05D51ur7CPqKmzY
+I6cIvw/TgTDZerN05lXmJucCgxkWNq1RLg2fh5uLEuhuavcxuvl5i2zlr68aYeprqpL+jYyIPMUf3TWW
6rD8TnKPUGGKf7zADhnXm4hkRQiiKSlnfevFmkbFHVXyNHwCWBZl/MRzByXWwDnwm46KOAcyk
zuvTuomepDpSvBbThV8GuLeSq9paU+kDJmOjTuE75jjrWXjrcnPBZr5e6rgxj7V1x7Xl8GDHbm+H87
YaV7S0iF1/zlhV25vbPgOSOH7uuAghFEWlrO8+8Q2RtMdTfI0fAJYFmX9BVOZxxBx9SOcedUQHJW
uIMY0VwKRqgLrESFfVFWqG4qWYLvzSS4+TPTLVQtpmdmzV0uGO2RM72XelPiK2MGMvxz6sYXb3
QmtHV78m6xJxy1pbdFm/HGJNIi+wrmidAYlsXts8/OPDIoRQFJWyHvvet0XYHkP967/8c/VrAgAwe
mT+RVEpRocYK0s5wlVruspYecpSjSivKV+sruYTmUfU9WK68Esf1zB9rFp7c4x65diYcUjocsfsA05qK
mNU64X2ANclXd4y3d2/14K8MGKJ5SHf+MDa/OvXL33LVwMcU5SHWmdAQlf/fJW/xp+iFlg3n9r+
zP37RORebP3TPzxT/YIAACwDMv/CiOCjopROX45slfFcZQzSecmf46qrigHFn1fWzhdX1Uf05WK68E
sf517EweZU6qSLr5ezi55XHe22ZN2/HhW6sLlcPcB1iWOV1jBHd8nZsXVvjuZ+7k11WL9+XWs0bE7c
VE7RNgPSunT10o43dogcQlFUshrVF/tfXjn873/6U/XbAQCwDMj8i6NyTRFmVAyqw5o7nol41SGAu
VSXZSPK0dkfykP6iL5aTBd+6ePcizjYnEqddPH1cnaxlthtyY657Av1EcEeYPTqMrN7J5mWjq5+Dd6Ja
22bmufOVOfVFC5qivAMSO7sZ2dFCKEoKmX918e/KbL3ourffvOb6vcCAGBJkPkXqco+WXpSCceIN
O54pgZWRzsFMIfyOvU/7xdXF4fUkXpCMV34pY9zL+JgcyrnFS3c16h51dFuS3bMJS9UQzLlKHtmV6
8OM/t239rR1a/BM3GtfVNd1q8Z0xXKi1pXYXDPgEV49ZevihBCUVSy2n5627EH7hDxO33xNX4AW
EZk/oWqAs3K6moZmszMpIOUmXJkunKkJx2SfLkvV81jf42/PGLMJzqGX/q4huljVa/mmI4TN8gZC9
ZE3WZ23FhxoT3CvvOuXl3eMseYTHvH1r35FqSmbG/hGGKPyWTHjFfNvq3LLIRmwII8/uHjIodQFJ
Wsbn3lhmcP3CVCeMria/wAsKTI/Iul01JBxBl9UsUrnXt03tJH1LXGhMFwVF/outTIcyJrhV96Wes0+lf
NfDOrK/TyAq063DHZx0PN5L8Tcj31guuZXb3qcdVB476rYY7umfaOrn5NVvfmgQ6bsmaw1q8P1M
sXG/LP0XUGxwEksHlt8+B7B0UOoSgqWe194RaRw5MVX+MHgOVF5l8wnW4yKlXVzLM1M+UYoU
yypzPVF1opyt2gnC78MsC/0KqbNZX7ipZGM9yx4EyORCkvdLfK1TM3x5TH/XdCX+jonmvt2GVvnvZ
Vqw6bmmH9Ur0s9yT1+fYZPPcIQ1vfWN/19i6RQyiKSlaHj90u0nia4mv8ALC8yPyLpsONL7s001Gde
TRzQHZav3QMNdnjnFeKg+GXYXpwJhsvQlu4e6FjvGteJFfWbcmOROm40Eym+VH12pjaHKInM1eY
jbVmdnSvhDt225u8Q6JNuEWhbf05c5qcvaTmKsJ7zTVnUGft6zC0C+sX+Gv8KWpRtf30ticP3SkC+d
D16U/er37+AQBLiMy/aCr4EF0ALIt3f/uuyCEURSWrnS/f+Pz+u0UsH67eefFE9ZMPAFhOZP7F0v9TJ
5EfwDI5+clJkUMoikpW9zx3m0jmA9WpI9//y1dfVT/2AIDlROZflOZ/uGz/h88AMG5HPjgicghFUcnq
waO7RT7vvU7cd+/nly5VP/AAgKVF5l8UI/MT+AEsoY3NjX1r+0QQoSgqTSX4Yj9f4weAaSDzAwAiXf
7yMn+NP0Utqr75j3833Bf7+Ro/AEwGmR8Ao/jKx+LHEJRVLI6cHynyOq9FF/jB4ApIfMDADOzy5uIZ
kUMoikpWDz+0RyT2OYuv8QPAxJD5AQDzOn7uuMghFEWlqZteu/7pg3tFbp+n+Bo/AEwMmR8A

MK/Na5uHf3xYRBGKotLU7pd29PXF/vdP/bD6qQYATAWZHwDQg6t/vspf409Ri6r7n9ol0ntEvX70kb
9+/XX1Iw0AmAoyPwCgH5euXtrxxg4RRSiKSlOPfe/bIsPPVCfuu/ePn/++
+mEGAEwImR8A0Juzn50VOYSiqDR186ntz9y/TyT57vXrn52rfowBANNC5gcA9OnVX74qoghFUWn
qWy/eLJJ8x+Jr/AAwYWR+AEDPHv/wcRFFKIpKU9994hsiz7cWX+MHgGkj8wMAerZ5bfPgewdFFKE
oKk3N9MV+vsYPAJNH5gcA9G99Y33X27tEFKEoKkHd+soNzx64S2R7X/E1fgCYPDI/AGAQF9Yv8Nf4
U9RCau8Lt4hs76z//T9PVz+uAIDpIvMDAIby7m/fFVGEoqg0dfjY7SLhi3rzqSf4Gj8AbAVkfgDAgE5+cl
JEEYqiEtT209uOPXCHyPm6fnDoO1/+4YvqpxQAMGlkfgDAsI58cEskEYqiElTgi/2/+8Wn1c8nAGDqy
PwAgGFtbG7sW9sn0ghFUQnqnuduE2k/qw8AbAFkfgDA4C5/eZm/xp+iFlPHt1tBn6+xg8
AWw2ZHwCQwsdXPhZRhKKoBLX99LZBn6+xg8AWxCZHwCQyJmLZ0QaoSgqQe18+cbn99+d
ZX6+xg8AWxCZHwCQjogiFEWlqXueu42v8QPA1kTmBwAkct11if6hQ6NoNlPGo2g0ikajaDSKNr1G
WwG3EgCQCE8k0WgUjUbRaBSNRtFoFI1GCOBWAgAS4YkkGo2i0SgajaLRKBqNotEIAdxKAEAiPJFE
o1E0GkWjUTQaRaNRNBohgFsJAEiEJ5JoNIpGo2g0ikajaDSKRiMEcCsBAInwRBKNRtFoFI1G0WgUjU
bRaIQAbiUAIBGeSKLRKBqNotEoGo2i0SgajRDArQQAJMITSTQaRaNRNBpFo1E0GkWjEQK4lQCARHgiiUajaDSKRqNoNIpGo2g0QgC3EgCQCE8k0WgUjUbRaBSNRtFoFI1GCO
BWAgAS4YkkGo2i0SgajaLRKBqNotEIAdxKAEAiPJFEo1E0GkWjUTQaRaNRNBohgFsJAEiEJ5JoNIpGo
2g0ikajaDSKRiMEcCsBAInwRBKNRtFoFI1G0WgUjUbRaIQAbiUAIBGeSKLRKBqNotEoGo2i0SgajRD
ArQQAJMITSTQaRaNRNBpFo1E0GkWjEQK4lQCARHgiiUajaDSKRqNoNIpGo2g0QgC3EgCQCE8k0
WgUjUbRaBSNRtFoFI1GCOBWAgAS4YkkGo2i0SgajaLRKBqNotEIAdxKAAAAAACmicwPAAAAAMA
0kfkBAAAAAJgmMj8AAAAAANNE5gcAAAAAYJrI/AAAAAAATBOZHwAAAACAaSLzAwAAAAwTWR
+AAAAAACmicwPAAAAAMA0kfkBAAAAAJgmMj8AALM6v3pdbfV8dRQAAGBsyPwAgAkpw/iwKfz
K2krdYciGqf/NQr6xzMralepAnxqbKfDvSmbVuIfDvE2D8/zAVJ+9Ur+fjMCPaODUrNxTNd6xfrc1rOC
dGfQXxYD8m1rW98mzo8Z2+nqbOvRa0t9JWwSZHwAwrAGf5huMPikf2oq2QzzrZM9Seh/Fc9XAD
1T5RlZWV7P/Z5A++RaSPxKaz6P9fyoan+zaQJ+
+Yi+JPhHmfeuvifcntPkz1NzoXLwdg6dm5ZsqP64PlLd03lbuWcy3a+4WrXcmP9/rLwrXphpbKsy3r8C
milM9/ijZ2zGamwbaUdFfHypezbu7br16aFXeu4q4P427KM6hAzI/AGBAzeep5tNIr1Sj4v9P+UDQ3
OFQBu+SN6hu3zBt8vd+8NtkSvPGNBQtB/rwybmH69X3c3xFvR/2wuVHo6+t+TuGTs2q61RqXPVyZ
sX1pWaX7PbpA/O/XW3byY9Xp+fqUwptqo/5K4FNOQ7F825HmL+pf0fyyIC91Inq5bzvWj6dnr74J
Ncvm62Kk3PtaSsi8wMAhiOfAaynht4N30FI8/gx8Lb09Pkf4h/aQuZ7HJxZ8s9BZtiPQvMGDra/YmLzj
er7jbNWbm+l5xsZuFc93sa2qYrz0TdSXT1sF4O7kT6a/2HuNmqxjl7D/LqwG/Wyj0pgO009fr5bb13r
YrqzprL20WMzfTeLF/Lj0GejLYPMDwAYjP1P5h6fdtwSPwwU+
+nrmdGr8fjTP+Oe5X8cplFxp2oD37Ph9uE18JuUKW9h1qL8wzCfcusn1Ph09MKaL+/YvG89twxM12
OntqmsGxulrUtvG3JNZBzL/9jbh93Ry/5Q9MFuVPTJv6igzd/VdesMxem+9ubsVXzU8hblH/xLmY3V
y3M7+9maeZvcjXrb2BZB5gcADMZ+AnA+ovRp8Aam4sGjr6c3W7GXyoBbynehps9bDrchpdzYkH2
KT17Pj/JhaT54xSeuMFQrex/2T/FcrAb2/D3fy8B0PXYKT1W8cT3cxXCX4mw/75WjUb4HdSQ/3duH
wtPL0FMru5F4X4oB/vvbjWM7hvDZWflmq+9fb60cvYoj8u719F4VO6i65X9uTtvvXdwayPwAgMEs
4B/V6Z4FxNPioHp9mGrKt1Hfr7xRkj3ZH41eiTenvH9DfiyKhoN+7sw9lH8epl8xt7x1Pb5VxYTmyu2P
gjVkPoHpeuwUmkp8HOfg7FIcrPR126xG+R7q1/np3j4UoVuXKbfXRze7kfXh6+EHObidHuY3OXqVt
6s8Vv65p37OfRX7UVZXGx+SORTT6jfGepfC9xhOZH4AwGAW8I/qRM8C5ZNOwmeO8uGt/4aNRzZ
DH0/YIUXf4e6f9ckbtl/x7gx6y+wOA+6o8aHo7zm+ZP2E2j+y1rs3H7uBFjg1K+9Uxd3sazstCy5O99J
MNmp8JgxD9LL19ImwG1kTt6+lVWCK4lRfn4Wc1cvuULxz8+2o1H5r8hF97E7+yNhvf/taIJH5AQCD
sf/B3NOzm1+KZ4GiR+IHjqLnoHeukLcZvsvw2xniUd6vt6dqP7vFoDsy9P2RsNct36y+dxaYr8dW7qm

K963HzbQuuBjQQ8OWRvnp3j4VrZvq6yfMblQcMTciP4wR/NvpaRsGq5fdov3udtQ6UT5g/g9FsYP
mNHbnHt6mLYfMDwAYjvwnc2+PH17DdyifSaTenz+yLsY2HM9Bg+jnqc2WzWtMW7xJg75LRQdzI0
M+IxZvzrCfOfsTkKRppvdPRPHWNBfe2Jv11s3N0VEJnJqVY6riUL9vUuuCiwE93L6WRvnp3t6khW6q
8YPUy0+VdzsD/MzavRo/TJn+mobfpn7eo2K1dpP8sDl3eClwIvMDAAbUeADp56nArZhbWO5nArGj
NJvJmw7yDiXfTeNRt7/nXtuQn+qm8nlYS/CJ6HNv4hNQqLdg7q2vmxnoGF7MTPxTiferNOfmim6Nl
WZdjNdFz3l6dLwz+bDePvTWprIDxuTlkhyL6Cy8qcbZedpUrO2UisMJPtu9/6Lw92qemfvWldM5p2l
8rnu9k1sHmR8AMCzzAYR/TiOZxgPp3M+jPsXHe7DZF6Dn53j0ovmulKr3RpxaonfMv6ml3FPgPcp
M7hdF78S/qijVjww8SMyJzA8AAAAAwDSR+QEAAAAAmCYyPwAAAAAA00TmBwAAAABgmsj8AA
AAAABME5kfAAAAAIBpIvMDAAAAADBNZH4AAAAAAAKaJzA8AAAAAwDSR+QEAAAAAmCYyPwAA
AAAA00TmBwAAAABgmsj8AAAAAABME5kfAAAAAIBpIvMDAAAAADBNZH4AAAAAAKaJzA8AAAA
AwDSR+QEAAAAAmCYyPwAAAAAA00TmBwAAAABgmsj8AAAAAABME5kfAAAAAIBpIvMDAAAA
ADBNZH4AAAAAAKaJzA8AAAAAwDSR+QEAAAAAmCYyPwAAAAAA00TmBwAAAABgmsj8AAAAA
ABME5kfAAAAAIBpIvMDAAAAADBNZH4AAAAAAKaJzA8AADAu51evq6yerw6V1ImVtSvVkWGoP
qL9GNT3JmPdhytrK75TwXMAMF1kfgAAgHExcm0znm71zF/H9oLrNniTfX1TR/hvMgBgOGR+AACA
cWn8b9lmQt3qmb/T/p3hvv5XASR+AFsMmR8AAGBcGpnfDLhbPPPr4N6yLn3/9H3SR0j8ALYcMj8
AAMC4NDO/EVStzG8dsINxNSQfYcxbXWAcMdOwOpwdM/5zeqOJYpy1zhtrq9u0ZO7GfI0JjZUqgb
n06GKMfmXtILT+XHM9Ykhgg8HrACApMj8AAMC41GFyrfyTioxGyixe2weMuFlF4jqOttIh2n+NkbNl
ri0YC3FMEoy+7qZy6wZjLZZ6catr6o+ie9v6Wxbk36D7uuByAWA4ZH4AAIBxUaExS5AqmJaB0ThRD
LQPhDJ/NcIIpPKIiqXWNfURdUC20SP0Uow+HeKuv4Ge0O4ZogdX6mkKretXA/zr8WzQN5FcAQCk
QeYHAAAYl0ZqrF6Yf7ZjpxEnZZhVQ+pc6j+iprFHyDEyEWfkIT2JMcZLriAnd2IfCNMLyIlLOqxfsrq7N6i
Hddo3AAyOzA8AADAu7nidRU0rGTuisoym9pD2I+q1GZSb86oRLqJxYxYPe0k51VIdlVtr5V9Ch/Ub
mqPVec/sepW15rYAICkyPwAAwLjIBFy91l/vrzOkHJkZKvM3D6oXLt7Gfu6xcivRmd9eQof1u8J7QZ3
3zu65sOuqAaBXZH4AAIBxscKkDJH6hCN2ymBsD2k/4phWzOsaIXQYornHqpbqqNxaK/8S2henRmT
KQVb31jmMKQr+ZgAwHDI/AADAuDjCZDM+6hOhkT1kfjNcN8d0iN92Gz89nTnY6tFj5m+dyr7Uui
mBDWanjIlnXjcA9IjMDwAAMC6uMKlzY06fqI9WcVIHU+uIHV79R/yz6CH+zirY2m1C/A3q62fOzoEl
tK1fbqce37JBayPBZQDA4Mj8AAAA49ISJs0TRhSVRJYNRVB5RL9ekbOrvJtxt64H2G3C1Pgm8+o+M
3/r+t3Lyakhvtl9V3ZdNQD0iswPAAAwLp4wWYfUxgkzu2axUgZje7L2I+q1OZ21nFwzNzcHeHYR1JxP
hmS5tVatSwitv748l7c0bot52jW7eWmu64oBoHdkfgAAAAAAponMDwAAAADANJH5AQAAAACYJj
I/AAAAAADTROYHAAAAAGCayPwAAAAAAEwTmR8AAAAAgGki8wMAAAAAME1kfgAAAAAApon
MDwAAAADANJH5AQAAAACYJjI/AAAAAADTROYHAAAAAGCayPwAAAAAAEzR3/72H+jnQL8iM1J
nAAAAAElFTkSuQmCC]]></redcap:Attachment>
</ItemDef>
<ItemDef OID="b32" Name="b32" DataType="text" Length="999" redcap:Variable="b32" redcap:FieldType="descriptive" redcap:BranchingLogic="[q29] = '1'" redcap:InlineImage="1">
<Question><TranslatedText></TranslatedText></Question>
<redcap:Attachment DocName="B.png" MimeType="image/png"><![CDATA[iVBORw0KGgoAAAANSUhEUgAABVEAAAEJCAYAAACdcmdaAAAAAXNSR0IArs4c6QAAAA
RnQU1BAACxjwv8YQUAAAAJcEhZcwAAEnQAABJ0Ad5mH3gAAE9sSURBVHhe7d0PjCRnfef/b+/aY
PPHNmBskx+RHat7sZYlGJIg08PxL4egZ+6SJbnbYOVg5Oh3PVyi0zQSkwOxF+mX2wguG4lunS5iWrq
zliQyTEJugrLdIJRgckzHR4IxYTI/Z7rlc3T8wDYGjPljB3u3f/VUPVX1VHd1dVV3VXX39PtItaerpfqqT/T
O/OZ5/k+hb5FAAAAAAAAAAAChjumPAAAAAAAAAAIAQhKgAAAAAAAAAAEIEQFQAAAAAAAAAAAiEKIC
AAAAAAAAAQARCVAAAAAAAAACIQIgKAAAAAAAAABEIUQEAAAAAAAAAgAiEqAAAAAAAAAAAEQgRA
UAAAAAAACACISoAAAAAAAAABCBEBUAAAAAAAAAIhCiAgAAAAAAAEAEQlQAAAAAAAAAAiECICg

AAAAAAAAARCFEBAAAAAAAAAIAIhKgAAAAAAAABEIEQFAAAAAAAAgAiEqAAAAAAAAAAQgRAVA
AAAAAAAACIQogIAAAAAAABABEJUAAAAAAAAAIhAiAoAAAAAAAAAEQhRAQAAAAAAACACISoA
AAAAAAAARCBEBQAAAAAAAIAIhKgAAAAAAAAAEIEQFQAAAAAAAAiEKICAAAAAAAAQARCVAA
AAAAAAACIQIgKAAAAAAAAABEIUQEAAAAAAAAgAiEqAAAAAAAAAEQgRAUAAAAAAACACISoAA
AAAAAAABCBEBUAAAAAAAAAIhCiAgAAAAAAAEAEQlQAAAAAAAAAiECICgAAAAAAAARCFEBAA
AAAAAAIAIhKgAAAAAAAABEIEQFAAAAAAAAgAiEqAAAAAAAAAAQgRAVAAAAAAAAACIQogIAAA
AAAABABEJUAAAAAAAAAIhAiLp0etJYKUih4DxWGj29PqjXWPFeU1hpWF+1iMxj3ZB2onXjpLWdL
MxLO7LSlg372PzHqPs4Vb2GrGzEO5vtDbdtK5JH08KNuQ9Cjmc+2g0AAAAAwPwhRF06Rdm8UJey
XurU1kPCkracr3X0c5Hq2U3rq4B5oILBVWnqJUdZzqxle4faf1Qo1cT/rlhsR+14AAAAAADIGiHqMip
uytmqfi4dqZ0P9kbrNc75IVW1JdsV/XzhFGVzry/9vnpsS/aHkff+RpmXdmShKwdu8leuS9c+xj3ZzDJ
D7TVk3fijwsI7ascDAAAAAEAOCFGXVGW7JV6O2jxn9EY1e6FWpbW4CSqOot6h7OuncuoEPaQBA
AAAAEAuCFGXVkW2W26M6vdGbW/4Q6XL9a3wXoyqlqKuRek+wmpSjqyvaH59ZI1Jo6ZjoC7rqPX
GPu31edcGHd5fZI1J4zwEz5+5nbDPjxN+3INtCdS9tR4xy30ObGewraPOc7xjCm57uPZpYAh6c9VZF
2h4snM3eA7Uw9yc3Z6x+xzPPy71CLkXbAnaHvI9OHq7vqTHE6/dAAAAAAAcfYSoy6yyJXW3OGpz
VTYaDTnnJ6hyIWSM9FAIo3VqpQwmoCrK2hndwM6BdJ1nFmNId2dHLno7bcuubn/5zNpc9FKsnPa
D6gP/AGy9izv6PBo1Pe1wrCSDo63TPb8dqZUKUhrYSXM1aUimtjPY1qasDoZtEx2T2rZR+9S6H+te1
+kREu3HCSwHz4HSXB0dKibnnOtV70AUtW6Kc9TeGFHPNGS7E4vZbgAAAAAAlgQh6lILTjLVrLnBTF
nqF0Imk2pv+KGKV4+yL103ie3UZD3lhKW4dka3b18O3U23d42JhYxw0hjqferEnAz0rpz2yiY0d4M9
Ji/u6BisfEacDLUnjXX3GlSlpc9v3+0xnOr59bfvdUhWIdlAfdzx/O1494Hazrob/E1zTMbr9zZlc9v62PX
vV1Wv1/6cXXIi2X56jXUvsCzXu85r+8MlLiqR+4wr/BzteOl/krZbr3X/0hH2PRjY7rBkxzOu3QAAAAA
ALI+C9QtyXz/HklK9SwM9zlSwEhKq+K8rS71rTubTk8aK24tOBS/OZEYjX6963bk96Ubsy+dvW4Vde
9ZG1BBs1YOwWq1Ks9kcWu+3IaxdcdeNE387w+2yV3rnwG1/6DqbsV0VnO2FBNwB4e0wr3O11fc
nDEt0PSK2E9ivvuaS7JjMbQdfr41qa6JzF35+FOdanQqsS3p+lETnOlHbRwht44jjjDietO4RYBr3Pnyvv
OXCW/QSACAP99/xLnnNW2/SSwCAzB3/NZFjr9ILWBT0REVwkikVtoQGJP5Qeb/npMsYdm/2GE1F
UU6ccp51dJfTrj2WvywnT5+0e9QF11uqp/0AbA74vWmb4nVG7R44gZT1GW8ov7dOdT4sGbUo3S
BMfcIsazBr1jUo6ac28z7QPYSnOKZEvYkT7cec4f+kmIdQ3NyTvhmgTm3wHIWY6rrrurFuwJmaGO0
GMvDwEw/LOz/5Tr0EAMjDDY9fJXd/6AF5/Bs/0msAAJm7dI9I/zt6AYuCEBVBA6FScsO1P6fl1RVt7k
pbenJoj9k/JScqJ6z/D65XGeqc9ZIrrombLbpD+ttuIl09a/TozUtaAZl1DTJrOyHeSKpXqBe0GnVjgQX3x
NNPSOWPKvZHAEA+nvPjY/KWL94oP3ryGWm+/3555p8u6c8AALL1lMilP7A+PussYiEQoiJlGYRfXl3
RfTlsXxS7lKgd9lbEyVeN9XMZvhk9NO3A1+/VOyrwVcO67VqVQ480e0lOK1mv47yOaTHOXbjxbW/
Lhtnr1K2LatY5BRbUnZ+6Ux58/EG9BADIwxv/
+gZ5wQ+vsJ8/9NXvyp/WeR8GgNz0Hxa5/Od6AYuAEBUxleSkN1LbnBFfMSZJitM70Ri+HI+7744c7D
pf686+X7I/4a8fLjUwH4qbZ/0guOFOjFWVQIZacsoTKG6Jgvk22OvYvA/0seV1TIn2Y97LA8Pk1cz3hZ
xnoE/SdmNSNTtwdeukJv6eAubL+z77PvlM7zN6CQCQh1c+eK3c/PXn6yXH5z7+kHzlLx/RSwCAzF3
6gsjlr+kFzDtCVMRUlM2zbuVUc/Z1NbrYnOl8y+vt5wScijnre1s23JlrYvN7cqqJpBS3XmZRF0x117vh
6vxxe81a56Kmj3+wdqsx7F+aq7LhnTIV7Onh297K+dBcNQLH9nm/hqd7bHkdU6L9mLVbm7Lqre/p
me+ta1Qq+NvI2oTnyA9c3XYDi+kT+5+Q+n11vQQAyIOqg/pzD7xELwX94W9/Tb73raf1EgAgc5f/RK
T/fb2AeUaIivgq29LyctSalHTA48w8bynX5YJR4NOfUMnSXNWB0GQ1HAPbsp55Q/a9of6KMUnTHP
Jqu9rKUt8KRKiWomxe8IdlN1d1gOaFzqMm/ZolJ3AMttM8tryOKdl+ipsXpO6/2HmtOZGTdS/7h6Br
7yrua1NNWBO03bzfw9odR+bHA8R339fvk7v+7C69BADIw/FLBXsYv/oYRgWozd+8Xy8BADLXf1Lk
8sf1AuYZISoSqWyH11+stqz17tBiV3FT9gZfW21NVr/R7K0XKBlgDM3OdKKjFJgB2KiyA+qc9bt+wOe
ya1/OW03PstS7fT9Yt1Wl1d8LTpaV1zEl2k9RNvf60h16sXq5MUzeVpHt4EGK7B96PbFTEbvtVlv6Lf
8+sqlzblwHu+5ulByOB4jBnYn/6Wfp7QQAeVr50kvl2iev1Evh/uFL35ZP//6hXgIAZO6y9RvZJcpbzbt
CX81aAgAxtTcK4nSSVCHqQGAKADGo4PT1/+318sAjD+g1AIA8lB56od0LNa6tu8vyiteFD/sHAGTgit

8QKfBL9ryiJyoAAMiVGsJPgAoA+VK9T1Uv1CTUsH7qowJAji79AfVR5xghKgAAyM1HvvgRezIpAEB+
VP3Tt33hZSProI6iAtS7/+NX9RIAIHN2fdR79ALmDSEqAADIxe6Du/LBv/igXgIA5OWOL18/tg7qKPv/
8zH53Mcf0ksAgMxd/n9FLn1BL2CeUBMVAABkTg3fV3VQmUgKAPJ189efL//8Czfppclc+dzjsnX36+
XWV79IrwEAZOtKXR/1Zr2MeUBPVAAAkKknnn6CmfgBYAZe8MMrEk0kNcoz/3RJmu+/X3705DN6
DQAgW9b77aWPWx+fchYxFwhRAQBAZlRwqgLUh594WK8BAORB1T99yxdvlOf8OJ1f+R7/xo/k7g8
xKSAA5Kb/HZFL1EedJ4SoAAAgM+/77Pvk3ofv1UsAgLz83AMvkRsev0ovpeMrf/mI3PtJ/igGALm5/
DXr0dELmDVC1KXWlo1CQQrGY6XR05/LSa8hKxttveBob7jtWZG8mzM3Qs5LHL3GinPuVhoy6tT55
9d55H7NjwLuWyCW+n11+djffkwvAQDy8vJvPE9e+eC1eildn/zPB/LN//19vQQAyNylXZH+Y3oBs0S
IurR60lhZlaZecpTlzFpRP8+eHfiVasLfVIImPi+9hqzXor5KXfOCrAYvunRqJSlMENguK+5bIB7V+1T1Qg
UA5Ovqp47LG++bvg7qKKo+6u//
+y/bHwEAeVD1Uf+79fFZZxEzQ4i6tLpy4KZA5bp0+33p9/dkM68MdWzgt6QmPS+qZ+S4YK99XtxN
V1vqevelWy87K5qrQo4aA/ctEIuqf6rqoAIA8veWvRvtIDVLqifqH/721/QSACBz/UdFLv2xXsCsEKIuq
96h7OuncuqE5Nf/FGmL1zOyJ41zugtquS5bFedpcfOsVJ2n0twlRQUwPTUTf+WPKvZHAEC+Xvu1F8
vLHr1aL2Vrb/f/2A8AQE4uf8l5YGYIUZeQXbvRDN2aq04tx8GuiKp3o1E7c1T9zGAtyGCd1bDejbH3r
wVreI6qN+kMVfdfl6zW52A9S6+2qH6ENy3GPgPncEP8zZjnydln0vOiqHaWYvWMNHoeB0LzkpzUn
VFl/9A6olGM9obVW21v6GMZPAcJrkvI/RZ2vePcb4PXz35E1ImNY5b37WT3p2vSa2eZ6D1Ar1TMr
49uJI6QOz91pzz4+IN6CQCQFxWevubvXqSX8qF6o1IfFQBydOlPqI86Q4SoCDUUGGl2/cyRYVRHai
WjzqrR43EyanuDNTzVurCgpuQNVXdFt3UUZ5+DwWRzdcJ9Fjflgjtk3joz5/RGeo1z3nkq1y+kUEahK
q1+y+tVOsTseTxK50C6+umwipx2N97ZkYsDJ7W96x2NX1c3yXVRQV7I/eZcj1EBZPj9pu7d0GC5U5
NSIMjOSpb3bcz7M2CCa2eZ7D0Ay07VQP1M7zN6CQCQFzV8Xw3jz5uqi3r3h75KfVQAyI2qj3qP9Z
H6qLNAiLqEKtt96Xfr4kZ7Um3Z9TH72zrxbG/4AZBXL9Won9mpyfrIxEaFec7r+3uboWUCxu4/wN+
et3/pyI6XBPWkse4GPca+Wzo1imzrKP523M2ofdbOu/Fbsn0WNy+If+rOS9v677wbglnn94JOUJOd
F4P9um2ZLK8uyolT+ukYlS23beb5V9ri53BnxMnhkpwj67VGqYGh+21ofyZj2/b9ZrYlbFt+kJ3U/Ny3
/uvD789hya6dZar3ACyrT+x/wp6NHwCQv5UvvTTzOqijPPTV78qf1hmBAAC56T8scvnP9QLyRIiKIW
bPtPoFPwgdDgOHletbE4Z54aotPxwsrp3xAqzOge4z2bsoO14eaey7suW3deeiJIl7zH364ZMh8T6Ls
nnWTbuaslpwe08Gz+8kipt740PW2PblMOpEFdfkTNjxtXf9XrVn1pzjSXSOrPOzp0NEI3gPvd4Dhu43
s8et0evSPk86CNzLYfa0LO/bsfdnmCTXzjLNewCW031fv0/u+rO79BIAIE+vfPBaufnrz9dLs/G5jz8kX/
nLR/QSACBzl74gcpkJ/vJGiIoBI3qm2Yqy5iYxIwK3UyfSDKjKcrKkn47SPdC9+VSoU/LqNhbMYdKRw9
QnMMk+K9tGr0FHOsP403RKoi+fcf2NgNIP3Kpy1j2gqa6LruEZOrw/aOh+M8JCawf20Hd33yNrsaZ
uHu/bBNduyvcALB93Jv6nn31arwEA5OWGx6+Sn3vgJXpptu7+0APy+Dd+pJcAAJlTw/r739ELyAMh
KibUkeHOgTHCo7mX3TFUvMKUSrD+5Oz05HBssVSf36vSHRZuBG7V08FeoUmYExB5PXXHCbtWR
dm8EN470w0rF3t+o8nvz/SvXdh7AJaNCk5VgPrID+h9BAB5e86Pj8lbvnijHL9U0Gtm60dPPiPN99+v
lwAA2XtK5NIf6OfIAyEqJjR/gWm53vWGbAcfk9YLHS/
+Po26n7aO1NZznJyneELGlj4tn5Sxl7S4KW5lAntYuDF8vno6/CyPP0dt2TB7nbo1OM36o0lYbdzT+xj
s/as0z83XpEi53bcTXLtoR+GPJpiWGsL/wCMP6CUAQJ7e+Nc3yAt+eIVemg+qPuqnf/9QLwEAMqfq
o15iYte8EKJiQElOusnV0EzePbnoFnIcO/Q7J6WTXtA2qm5m6ibYZ6+xrodpl6Ved5OsPCfnMa5rc1f8
zphdOfAu6QmJc0m9HrXW/XH+/I4OP6sSyOGSnCOzLqcKFN26qMaQ96TUzPKqx6k9GZQdSHa9W
p7pDpOf0CzuW0usa5fme8AU1xDz7yNf/Ig9mRQAIH+lh1448zqoo3z6v/6D/MOXvq2XAACZu/xZkf
48dRU6ughRMcCcBCnYW9IPAlXYNTChz6yYNTCbq/5Q7faGHhpuPdIev510n72GrLsnrnpWNjeNyY
Nq65JPjmrWsmzKrm5er3HOCzBj90asnBbnDulls+ke18Bw8Amvix8oDvbcjUlvX80sH+xxaoTFZo9b
oz1p3yaRZnHfKnGu3QTvASU/dZXaee9gZMOb4h9Hze6Du/LBv/igXgIA5OnaJ6+0Z+OfZ83fvF+
+9y1qZQNAbtSw/v739QKyQoiKYeYkSJ2alHSoU/LTE7kw7YxI5vDy5uoUoVGwBmZzVQdQXnhTlVZ
qs9e7kuyzJ411d6h6Wepbav3okCq98zLMnFndbbN3TastiX+aKhIo72oZDmATnCMv2LO4x2xOsJTEi
HvXrLNaPevPNp/YQt+3SpxrZ0n4HuDXW7V41zBubVssGjV8/85P3amXAAB5UvVP3/aFl81NHdRRV

lCqglQAQE76T4pc/rheQFYIURHKHgYdUpOy2rLWu8Otp1KR7cGClfuHRs/BBOwamMZwbZddWzOj
eqgx92n23LN7obonbiCk8of1p3hehhRlc2+4Tqg9hD5hYBeYJMs6ZjsbHhT7uljH3G/5QaqtKq2+0dZ
ACYJo9r07eJA2Z5vTZZMLft9aYl07S6L3AHUsg6+ttiava4u59cTTTzATPwDMkOqBqnqiLgI1pJ/6qAC
Qo8vWb6bUR81Uoa8KBgJAEmo2fT0ZlAph96btmTxL+lhOtaYNWBfEUbp2yJUKTit/VJF7H75XrwE
A5EnVQVWTSS2SK597XLbufr3c+uoX6TUAgGxdKXLFb4gUbtbLSBM9UQEkFCxRcGZtsUO49nnrWC
J6ZB4tR+vaIV/v+
+z7CFABYEbULPx3fPl6vbQ4nvmnS9J8//3yoyef0WsAANmy3m8vqWH9TzmLSBUhKoCY2rJh17o06
pWaJQoWUK+xIqv7demmUqJinh29a4d81e+ry8f+9mN6CQCQJ1X/9C1fvFGe8+PF/NXt8W/8SO7+
0AN6CQCQuf53RC7doxeQJkJUAJNRNS8XfPx7cXMvpRq/C+YIXDvkR/U+Vb1QAQCz8XMPvERueP
wqvbSYvvKXj8jnPv6QXgIAZO7y10QufUEvIC3URAUAAKEefuJhec32a+wJpQAA+bv568+Xf/6Fm/TS
YqM+KgDkjfqoaaMnKgAAGKKCUzWRFAEqAMyGqoO6aBNJRVH1Ue/
+0FftjwCAPKj6qGpY/7POIqZGiAoAAIbc+ak75cHHH9RLAIC8qQB1UeugjvLN//19+cPf/ppeAgBkrv+
oyKU/1guYFiEqAAAIUDVQP9P7jF4CAOTttV97sbzs0av10tGyt/t/7AcAICeXv+Q8MDVCVCTUk8aKm
uVbPTaknWjdOGltZ96MOYZeQ1Y2gmvbG+7rV6TR0yunpfZjb3NEO2zTne9M2m1bkPugvaHbWJC
BSwosjE/sf8KejR8AMBsqPH3N3x3tuqGqN6rqlQoAyMmlPxHpP6YXMClCVGCGeo0VKZRq0tHLWe
pd3DH205RdQj4AA+77+n1y15/dpZcAAHm7+qnj8pa9G/XS0aXqov7+v/8y9VEBIDeqPup/tz5SH3U
ahKhIqCibe33p99VjWyp6bXby3l8WRhxDryHrtTziU6UnF3eC+2qea1hrF8VRuA+A+aZm4n/nJ98pT
z/7tF4DAMibClBVkLoMVE/UT/7nA70EAMicXR91Vy9gEoSowDLoXRQ3Qy2Xy86Tzo5cXJwUdTFUt
qXfrYs+w8DCUMGpClAf+cEjeg0AIG8//ffX2UP5l8m9n3xYvvKX/NsDALm5vGc9mOBvUoSoS8eoLbl
i9kQctd6oc2mvz7s25fD+IutuGnU/VwKfNLcT9vko6Z8z+/PmMP7mqvP5EYU0/WNWj+T1Rv2h/FU
5e+GMDvk6shMnRQ3UUg17jG5PvHbHuTbD59DaumyYrzfqkQa2MbA+/BTHaYN5POpY/P2rh7vd
9nnrupbrsjXQXdYu3WC83n4M3DfArKgh/A888oBeAgDk7YbHr7Ink1pGd3/oAXn8Gz/SSwCAzF26R
6T/Hb2AJAhRl05R1s64PREPpOs8s3TlwE30Aj0U27LbdJ6Vz6xZXz17ldNV/awjB/4B2PywsCxn1nRr7
RCwJIMj5zu1UswQa5bnrCO1UkFW9fYcal2SINUYyl89LZXimniHs3Nx+hCvfEbcU+2L2e6pr43Dfn1w
Z846FVQOrG+uDgSpE7VBHcuqeFvWoakKSlf369Ld2wxcdxW+lsJKN3RqUprnibKwFD7yxY/Yk0kBA
GbjOT8+Jm/54o1y/FJBr1kuP3ryGWm+/37qowJAbp4SufQH1kfqoyZFiLqEimtuT8R9OXQTovauHwi
JEU72Dq1XOU6dmIcI1VI5LW6M2gzMjmSEhV6w15PGutvjsyotu6am9WjpLXRqsh4jjUz7nFW2rTa
Yw76rLadd22HVPv12d+vuV8TsRaoYQ/mrp9X2zVA4xpD+4qbsuefNfnTFa4Z1BPULwcDQN67d6V
wbV7neDX69Frbev2+maYPxeh2aFjf3vOc+P1RXYWtXf41/TppyLsFxAmnafXBXPvgXH9RLAIBZeONf
3yAv+OEVemk5PfTV78qf1h/USwCAzPUfFrn853oBcRGiLiOvJ6lfaPUOndivWnXCo32dFJrDwO38bS
5UZMsNoJq7fi8+s+6n2wPUXFff8ickqmx5QWCs3pgzPGfVlj+Rkh/mWi0Z7IY7gtk792TJfmJsJ0EYq/
Ua636vzepZ2QxPUMe3O61rY6vKWbchRsg+cv3+obPdKdoQeH0UI1Q3Q2s7cNWB6t6okwhkSA3f
v/NTd+olAMAsvPLBa+Xmrz9fLy23z338Idn/n4/pJQBA5i59QeQyf8BKghB1KRXlxCnnmRtode1x6W
U5efqkHXYF11vUMHDn2VzwQ7mmeJ0KuwdeWOgN5ffWWcfkDu+2H8bw7cAQ/VFmdc784HMy
Yb1zLRMP6W/LeT9BlVZoz1klRrtTuzaW8kkJ3d2o9a4p2hC7Z7Zxrq2N2WUO3P3Er8sLpOuJp59gJn
4AmLGXfPe58nMPvEQvQbn7P35Vvvct/m0CgNxcVvVRv68XMA4h6pLy6oraPTl74nSqPCUnKies/w
+uV3ngPEWoFiOYcodmt90x0xG9I6exkOfM6GmphqaXQkPC+LP0tzf8OqBVo6fp8kkSbhdl80L4jP1u
cDtiPjEgE+5M/A8/8bBeAwDIm6p/usx1UEdRAWrzN+/XSwCAzPWfFLn8cb2AcQhRl5U3tHlfDts6aL
N77VXEyQqN9VP3hsyCUdfTDi/9upOjwkuvNubQI2YYuIDnzB/KHyXmkP72hj9RVLUlIzuhTmDqa5O
CTNtg1JUdKNlqa55jln7k532ffZ/c+/C9egkAMAsrX3qpXPvklXoJpn/40rfl079/qJcAAJm7bP02eukze
gFRCFGXVklO2hlkRw52nSHNbh3Rkv0Jf3347OuzV9w864eaDXeSp4E6pCVnqL3i1eKc2KKdM2Mo/
xjjh/S3ZcNLUMtSV1PRTyvVazOhHNugZuhXPU7tScXsQNWYoCtu2QJgSvX76vKxv/2YXgIAzELpoRfa
D4z26f/6D3aYCgDIyeXPivTp2jMOIerS8ntyNptOObWeCzq4p/uem+Sprnj9gDtSK2mA77BOqRmP
crmqj9sur3h1aUsxB5LvWDnzBzK787+H3iYIV70kP5e45w3jL9cv5BOuYRUr82E8miD3pbKoIM9Trvi

ls8dW7sVSMn/+v/
+l34GAJgF1ftU9ULFeHd/6AH9DACQi0v36CcYhRB1iZkzpqvehd7w88Ds5sYkTXPIq1NqC+shGaxH2
VzVwZg/Ll1GT440LNVzVtS1VJXmauqhoTmUvxxaW8AoiWC9cuSQ/l5D1r0CqtYrAxMwOY/JJkhK99
pMJoc2VLb9IfyBurRGfdmzm7MP3bEU7v7Fu+WOl9+hlwAAeXvjX99AHdSYfv7dP6WfAQByUXijfoJ
RCFGXWWDW8FPiTzbuDltXzPVzyAwvRw2ht+tRGr0uXeW6dJPWu0z1nFVke7BA5v5hSrUxzaH8o0
NdMxQeNaQ/Xl3VCaV5bSaVQxvsIfxhxVBVSNvvp1pfFohy1RVXyf/4lf8ht1x3i14DAMjT373yCf0M
UV7z1pvkbe+5VS8BADJ37FUix9+kFzBKoa/G9QIAgKXxwCMPyOv/2+vtmfoBAPm648vXyysfvFYvYd
D1P/E8+a1PvVGedw0TbwFALgovFrni/daTq51ljERPVAAAlsztN91uD+0HAOTvb27/tjx2PX/ECnPlc4
9L9fdeS4AKALmx3m+Pv8f6SIAaByEqAABL6F2n3iUfeMMH9BIAlC+Xjvfl8294VH78nMt6DVz/8t+V
5NZXv0gvAQAyd+ztIoWb9QLGmSpE7TVW9AQpBX9W65F60lhxJ1TZkODL27LhTbbiPCabqGZYeyO
43dBH1jOAAwAwhz788x+W07ed1ksAgLz84PnPyt7rvqWXoLzidS+R1X8bNhkqACATx4oix39eLyCO
OeiJqsJVf5ZqR84zwquZ0VcaKU3oAwDA4rjnl+
+xh/cDAPL10M0/kO6t39dLy+3al14l1d99rV4CAGSucI3IMTWMH0nkGKIWZXOvL2oeq35gxuuuHH
iTiKvZsNXn92QzxwzV1qnJeTqkAgCWjDtj/3VXXafXAADyonqjfu+aZ/TS8lIBqgpSAQA5Of5ukcIL9QLi
mn1P1N6h7OuncuqEZJedlqXedUNc/9Gtl/XnRZq7pKgAgOVzy3W32EGqClQBAPlR9VE/96Zv2h+X1
S/8xivsofwAgJzYdVDz7rl4NOQYog7XRLXrlZZq4nZEtYfVD9UoNb/OeaRVL1Uprp0RP0YFAGA5vfm
WN8tH3/5RvQQAyIvqibqs9VFVePoLv35CLwEAMmfXQX2HXkBSc1ATNUKvISuFktS8lNXRqZVSq2H
aPu+HuNXTfpGB5WVM8uWd46hJwTA7YddqEYTfT/4kcHneY0nPYU7fC+q9b2DCO//8rEiKf0cCAt7
7s+
+V2h01vQQAyIuqjbps9VGfd82VctfvUJMbAPJztcixO/VzTGKmIWpluy/9bt3vCVptOcPst1WY2ZPGu
htwVqXlDsFvVe01qobpeqIkoSO1khtC+I9Vd0Yra9/2bgFghnqNlWAPfSBnqjeq6pUKAMjXfT/zuD1r/
7JQAer1P/E8vQQAyNzxO0UKL9YLmMT89kTtXZQdnSKU61v+RFSVLXHLmHZ2LqbU+64s9S0S1NFG
TQoGTGKR76eM295ryPpg13tgBlR9VFUnFQCQnx8/57J8/g2PLkV91Le951Z5zVtv0ksAgMwdf5PIsV
fpBUxqfkPU7oHXE8sevu/1HjWG93cOpKufTsfppZpmrVUAABaVmqm//attZuwHgJw9dv3T8je3f1sv
HU23vvpF8ku12/QSACBzhVtEjv0LvYBpzHdN1FSFz85vlhPo1M7nWItxHgxO2jWqxuPoOpD20GPv6
/VjZG3Jwf1FBNd2Pdzga0fVgsysDVEya98oca+VK/5xJm1f2OsHyncO1O806o7arx19P/kG2p/K9Ux6
DsOEt32wXungORo8P2HiTbTn8/epHqPqpCY5P0DQbdffJvf88j16CQCQl7+/7Xvyjy//oV46WlQd1O
rvvVaufO5xvQYAkK2rRY6/2/p4hbOIqSxEiFqud4fDT/uRwnDa4qac1WVWRfblcGnyBRVsDU7a1ZTV
wqr1/3hUiFMKG3rcqUlpMKBKMklYe2NETUjVYzgYFmXWhihZtW+khNcqwXEma58TyIW9vrlaGBH
2qXNitLNcl/GVM9SxDbR/6us5/f0ej9OrffAcNVdHhZyTcPbh1XO2Dd97qd3vWGrvKL6DGfsBYAb+6v
WPHcn6qL/yH15JHVQAyNPx09RBTdH8hqiIk34P0YN0Bu2Ha8tuuinKQug1zvnhkTuhV7/r1Zsdzzhv
5bp0dbDd9TbQlHNeotNLMEmY9dpzesOh2+3IzkX3tVm1IUpW7Rst2bVKcpzJ2tdrrHuBnP+HjZa1F
615bkRQaLRjb1OKem0koz1u06e5ntPf70n47fHb3pHa+ejIPHqivUH+PsLvvbTud0Ds2frfdepdegkAk
AdVH1UFqUfJyumftB8AgJwce53zQGpSC1HtnmjGkFHzEWco65DimpzxspxVfxuqF6C77UQbdnpv
me1yHmYvuTOyFivhWXQ9uejO2qUCFi+kKcrmBSPEidI7lH39VDo74mY3xc09J6yxHnub+mQmmiT
MmLjHCNyKa2eGQ/XM2hAlo/aNlPBaJTnORO0LtuOst74iW/aGVVC3J2GHE2hHTNWz/rmtbPtBrd
/2JNczhfs9gWrL7yFf2Up/+4q5j/B7L8+J+bAM7v7Fu+WOl9+hlwAAefjmjU/JV376u3ppsb3sp14o/
+a3mNAEAHJTuFHk+L/WC0jLHA/nDwYcXkjrjWE1w5B0mMHN0daVAzdTKp+Ukn5qK56QU/ppJDP
kHgioh2ouTjVJmK6nGTZ8Prc2REmpfSMlvFZJjjNR+0a3wwldR5fWOHUi6XdVVU4HNlaSk2473bYn
up4p3O9zpSwnAwcRIteJ+bAMrrriKmbsB4AZuP9V37HD1EWm6p/+
+n/5GeqgAkBurhQ5/mvWR+qgpm2+a6IWN2UvbMitPdQ3hXqoHtWLri8pZ7JH3OhefG5oM1EPZ
MWu5eiGPlE1KzNsQ5R5b19sebQvRuA3VlFOLEzSmcbxAvPpphfcZAepKlAFAOTn8yuPylNXX9JLi0f
1QFU9UQEAOTn+r0QKN+gFpGmqENUc9hv1cMLJoj8M2gxA7aBUrw9NMc2v04+4dRUtdp1B82tD
H2kGskvEuHZ+7Udf89zw5DXjJwlry4bZq9OtjWnWijRl0oYo2bcvLbGOc4btG21wgreeHHp1B4ZlOv

HcEcD5QZpuv+l2e2g/ACA/KkBVQeoiel3l/6IOKgDk6dhrrQd1ULMy3z1RkZGQ4dEus05mDGp2d9V
b0Q+rjZ7D7raTTBLW3vV6dtrhjxuYG8OTB6XehihZtW+khNdqguOM176Idth1itOdfT7Y9JDh+ImO
M737fWHkNjEflpGaZOoDb/iAXgIA5EEN6f+7Vz6hlxaDmoWfOqgAkCM1C7/qhYrMEKIupaKs+bN2
yao3Ztuc0XsMPcGXKlEb7K0YEnhNOEmYH/4YM+KbcmhDlFTbN1LCa5XkOBO1L6Id9rE7NVUTnsK
Rmqsb4jfdL5lQPrPmhNaJrmcK9/uiyeB+B0wf/vkPy+nbTuslAEAeVH3Ux65/Wi/NN1X/tPp7r5XnXX
OlXgMAyJaqg/oe6+PVziIyQYi6pIqbF/zehs1VHawYk86MU9n2h393alJygxmjRqg/UVew9mbkJGGV
09aSNq5dWbUhSmbtGy3ZtUpyrpO1b2w7ynXZSmV8eFnK5aas6vZ4Tbfaftab/j/Z9Zz6fs+LOdGV28
6Jws6U7ncgwj2/fI89vB8AkI9Lx/vy+Tc8Kj9+zmW9Zn79Uu02ufXVL9JLAIDMHVsTKdysF5AVQtSlVZ
TNvcFJu6rS6res/8djD/8OK6Rpb2dgoi679ubg/ixDk4RVZHuoDc72vF01d71eitm0IUqG7Rsp4bVKcJ
zJ2qfa0Zfu0IbVpo3SBlM7JWcHjzfsGiW6ntPf7/mw7q/B67F/aPQSTiCV+x0YzZ2x/7qrrtNrAABZ+8H
zn5W/ev1jemk+veatN8nb3nOrXgIAZO7Yq0SOv0kvIEuFviqCCAAAMIF7H75XKn9UkaefXYwhpgBw
FNzx5evllQ9eq5fmx7UvvUr+06ffzDB+AMhL4RqRK9R8BQzjzwM9UQEAwMTefMub5aNv/6heAgD
k4W9u/7Z875pn9NL8qP4udVABIFfH3239jwA1L4SoAABgKu/92fdK7Y6aXgIAZE3VR/3cm75pf5wX
v/Abr5BXvO4legkAkLljbxcppFNUD/EQogIAgKmp3qiqVyoAIB+qJ+re676ll2ZLhae/8Osn9BIAIHPHiiL
H36EXkBdCVAAAkAo10dQt192ilwAAWeve+n37MUuqDqoaxg8AyImqg3rsPXoBeSJEBQAAqVAz9b
d/tc2M/QCQI9UbdZb1Ue/6T6+2g1QAQE6O3SlSeKFeQJ4IUQEAQGpuu/42ueeX79FLAICsqbqon3
/DozOpj/q299wqp/7ZDXoJAJC5428SOXabXkDeCFEBAECq3lF8BzP2A0COvv2if7Jn7M/Tra9+kfxSjV
/kASA3hVtEjv0LvYBZIEQFcteWjUJBCsZjpdHTn1tSvYasbLT1gqO94Z6fFZnX09NrrDhtXGnIqCb6x+E8
lv5aY2mo2frfdepdegkAkLW/v+178o8v/6FeytbzrrlSqr/3Wrnyucf1GgBAtq4WOf5u6+MVziJmghAV
yFVPGiur0tRLjrKcWSvq58vHDiJLNeno5YXRa8h6LarV6loXZDV4saVTK0lhIDAGjqq7f/FuuePld+glAE
DW/ur1j8kPnv+sXsrOXb9zu1z/E8/TSwCAzB1XdVBfrBcwK4SoQK66cuDmbuW6dPt96ff3ZHNZM9
SxQeScUj1nxwW/7fPiHlq1pa5zX7r1srOiuSrkqFgGV11xFTP2A0COfvycy5nXR33zr9wir3nrTXoJAJC
5YyvW41V6AbNEiArkqXco+/qpnDohy9/dHHF6znbk8Y53QW1XJetivO0uHlWqs5Tae6SomI53PSC
m+wgVQWqAIDsPXb903L/q76jl9L1sp96ofzKfziplwAAmSvcKHL8tF7ArBGiLjGvnqP5CKntOLI2peqN
536d163Or/dp135sb+ivNdbZLwuuD/bKS2MbmtlG7zFcYzN4jMM1S+1HWN1Low3jal3a+zDDt+aq
87WDDQ9pc9i2o9o8tpfjROdl+J4ZuZ8YxxD7fGh+W9RjuK0OZwi9/7p0z506/lKsnrNGj+NAWF6Sk7
ozquwfDt9PNqM9ie65eMduS+H7wj1Pcd9HsNxuv+l2e2g/ACAff/fKJ+SbNz6ll9Kh6p/+
+n/5GeqgAkBurhQ5/mvWR+qgzgtC1CWlwpHQMKhTk1JhQ8ZlcHHYtR8HCkLa61TIMrC+uRoeXk2
1DRU2hfYY7EitNCqEU58zapaW61J3uw52duTiYMi0674ynbqmQ8GiZh/zyGBquM1uz8dQE5+X4X
umuToieEt8DFGcfQcvd0hb7WCw5A2hd6V67jxVafVbXq/SIWaP41E6B9LVT4MqcjrpPZfk2FP6vlDn
KY/3ERwdapKpD7zhA3oJAJC1z688Kk9dfUkvTe/f/Nar7J6oAICcHP9XIoUb9ALmASHqUmqLn8O4d
TmNeo3SlHPhSUpi5XrX3na/FYybwtaPGt482TaCw6mHj7EjO4PplEcFZM7r+3ubsrlVF+erBr/GPI9nZ
FyGWtm2ttd1t2Wptpx9bOvUrr3hB4Vhbe7UZH3kdQm2eXRT0jkv/invSO28cd0SHMPY8xHg7zu8r
dZxrbvBoHEu3Iamcu40u53bEitrHVKUE6f00wiVRPdckmNP7/uimOP7CI6OD//8h+X0bQxHAoA8q
ABVBalpWDn9k/YDAJCTY69zHpgrhKjLyOwlZ/R0K27uOQGJ9dhLZaajqpx1t1M5bS25RqwPHd486T
aKsrlnBj6O4toZL7TrHIT3AyzXt4IBWXFNzugv6uxc9NvY3vV65pXPrI0P38YwexjWLxht3rwgfgZ5PrR3
31CbR5r8vFRbfnDoh3xB0xxDFHPfoW3tXZQd3SEycC4qW/5+zWtniH/u1HFY3yOhIe8k9uUwrEFKk
nsu0bGn+H2R2/sIjpp7fvkee3g/ACB7akj/V376u3ppMqr3qeqFCgDIiV0H9V/rBcwTQtRlZAQ0ImqY
bsGrZTiyhuIkyielpJ8GjFofJo1t2HQ9x9BhzEGnTgwGP0VZ8xMtLyzyA0Mj0J1YVK9WY/8jgrfhNscV/7
yMN90xjFaWk+MudvfAa79X7sF+GEPcRwyfn/zcTeuUjN51gntuimNPcv2HzlNe7yM4ctwZ+6+76jq
9BgCQJTXJ1KT1UVX907t+59XUQQWA3Kg6qHdaH6mDOo8IUZdSUTYvhPckdEOYqMl1FkZg8hyjn
mOk8MDO76nnDnc2AsPq6dg9GafXkeGOgjFCRlOK52UyYccwK2keVxw9ORxbLNWR2T2X2vVfkvc
RZOKW625hxn4AyNFfvf4x+fFzLuul+H6pdpvc+uoX6SUAQOaOrYkUbtYLmDeEqMuquCl7esjtQKlR
W/Pcos+u3ZYNs3edW7PRrMGZhHW+zurzZA+NNoYyV0/nF6FOH/qlfF4mkm1w6dXKHXpMWsd0

AsUTMrb06bje1BPcc+OPPf3vi6P9PoIsvfmWN8tH3/5RvQQAyNIPnv+sHaQm8Zq33iRve8+tegkAkL
ljrxI5/ia9gHlEiLrE1MzaqqeYPcGPHYR0vRqKo4f/GoxhxHPHrB2pgiW3/uMUba64U6Z3duT8+R29n
aqkk6GW5KR37gdnZO/JRbfoZeQQ8BgyOC+
+nI4hTOmkFwKOqumZL+NcNHfF75DZlQPvNJxwzn2EWPdckmPP4PpP/T6Cpfben32v1O6o6SUAQ
Jb+8eU/lL+/7Xt6Kdr1P/E8uet3qF8NALkpvFgP48c8I0RdRu0Ne6itmkU92FPMCHiMXnIlPxkzZmJvy
4Y3Dft884MlY2bySXgTWHWk2dQnKrWh/EXZdLsdqvO87l+XXmPdq22ZZBKkcVI7L578j8Fj1udsrvr
DyPW9bj9yHVtu1oBtyq7eda9xzgsxY/VgjnPPTXjsU1//hO8jZntyvRSYe6o3quqVCgDI3t/c/m157Pqn
9VI4Vf+0+nuvleddc6VeAwDIlqqD+h7r49XOIuYWIeoyqmz7Q287NSm5QYtRH7F6NnzmbhXSDL52
Lpkz9nttNibamUhF3I6BrlSH8o+4LiU/fZQL005glcl5MSQ9BnPYu9ueiRK2YH3O5qqz34IX9Felldqs+v
EUNy94PTLd9njnodqSeM2Jc88lOPY0r3/C9xEgiqqPquqkAgCydeI4Xz7/hkcj66NW/u8idVABIE/H3k
od1AVBiLqk7KG3YUUMVeDS7wcDHlX3cLBmYrWVcx3NpCqy3W/5gZHNOTbvsAPDrOPxhlcr5bpsp
ZzL2dcl5LxWW9Z6d+j1VLI5L6Zkx2C1Z/A+3D80ejUmYNfnNIaSu+y6nznWQ/UUZXNvuFaoPYw+Q
aAb656LfezpXv9E7yNABDVTf/tX28zYDwA5UPVR7/uZx/VS0Cte9xL5hV8/oZcAAJk7Zv2GfPwdegHz
rtBXRewAxKNmNtcT86gwbG/anqHAOEfpntPHcqpFwIpwn+l9Rip/xM0BAHl441/fIKWHXqiXRK596
VXyW3/8z+yPAIAcFK4ROf5+66P/Xoz5Rk9UILaeNNbdmc3LcmaNABVZO1r3XPu8dSwZ9ODG0fGO
4juYsR8AcrL3um/J9655Ri+JVH/3tQSoAJCn4+8mQF0w9EQFxmrLxmANWFXOgK50yMzRu+d6jRU
p7ZyRbiplKXDUFf6fgn4GAMjStU9eKe9s/aS88723MYwfAPJ07O0M419AhKjAWAOBFgEqMsc9h+
WlJiiblx9NaEs42hKOtoSjLeHmqS0nrr1GDr/3pF6aLa5ROsSjraEoy3haAvSQIgKAADmBj/ghqMt4
WhLONoSjraEoy3haEs42hKOtoSjLeHmqS1IhpqoAAAAAAAAABCBEBUAAAAAAAAAIhCiAgAAAAA
AAEAEQlTkSE2WU7DrfxRWGtLTa8fqNWRlo60XHO0NvZ3CijRibwjp6Uljxb0GG9aVTbJuGmH3UNr
7mJbRRv1Y4SYFAAAAAGChEaJirvUaK1Io1aSjl4H5pgJdY1Z9W1nOrBX1cwAAAAAAsIgIUTG/eg1Zrx
GfzqeibO717RkF+/1tqei1szFPbenKgXvLluvStdu0J5tkqAAAAAAALDRCVABIS+9Q9vVTOXVCyE4BA
AAAADgaCFGXmao1OlC7MazG6GD9UXuIvfE1A+VKNbNOpXokq1Vp79Mcxt9cdbYTvjOjjeoxqk7qY
JvCa1VOdrymMbVf2xvetob2H3JN4rTRY359ZEONcxFo46j1xj7t9ea5zKoOadx7KKotg9uYsD5pjOuS
9J4FAAAAAACLgxB1WakgL7TWaEdqpVEhpPpcQUoDQ+ybq4OvVyFiSYIva8pqYbBWZBqcNq0GNh
xyDHYINtgm65W1UnjQaYt7vIMqcrqqn3Z25OLAa9u7bmODtTKHQjgtuo2TKsrambLztHMgXeeZxR
iOHmh7W9xml8+s5dDDMoV7aKJrPizf6wIAAAAAAOYRIepS6knjnJuIuXUb+9Kt61BNOrIzmPx5qtL
Sr2+5QaEKG8/7ve16jXN+0FVt6VqVXfE2H0Nl2/qabl28L3G3sx1W8dJvU/gxWMe77oZg/mv77gF0
arI+MhUdf7xhKltu2wfPpR9GSvmMeBlqe8MPgsOuSWQbJ1NcO6PbuC+H7qbbu0ZI2ZEDN101hq
mfOpF9hDr9PTTNNTckuC7J7lkAAAAAALBICFGXkjERz96m16vQD9VEOl56FlRt+RP3+EGhqScXd9w
+e1VpeQGStc8LYa+fntmm0GPoXRS3SeX6lj/xUGXLC+U6OxdDexSOP94RimvidfQ0t22ElGaPTrN3av
2CcU02L/htrJ0fGKo+Ja+NftDbO3Si0mrVCRv3dbrau7jjBZKnvROYlRTuoSmuuWkm1wUAAAAAAM
wdQlRYdA3P0OH9SZmzk5+Ukn5qK56QU/ppespyMrCTEN0D77jsIdheXUtjqHdgSHsazOHy/rB4P5
SryllvyvYRvVNtxnbMHqOpKMoJfUHcwLlrXzzrnJ4+aYeVwfWW6mk/kMxMCvdQKtd8VtcFAAAAAA
DMG0LUZRWYKCdunckYgeWRMt3x+r1i3Z6eRig3URhpDK9PScUt3trctVrXE6cj6ik5UdFhZWC9anb
2EeriSf+6AAAAAACA+UKIupTasmH2OnVrPZr1HI+ocr3rlDEYevjD9lNT3JSzbglONXTcqCs6WRiZQYh
dOS164L4ctvUQeLv3pzs5lrF+QUP07K/5sv1xAQAAAACA5UOIuozMupwqYHLrohpDoCdXkpNuEjs
4XNoIEXNVcoamK6NqvWbF6+nZ2ZHz50fVFTXP2eBs/mZ90FMydk6nxNfQ3XdHDnadr3VrtZbsT/jr
h4e0ZyWFeyiVa57idQEAAAAAAAuNEHXJ+QGTMWP/VMxakU1Z3XCn3DFnS8+ZMcmTNFfFa1J7w
6+V6a1MmdfTsyPNpj76oaH8Rdl0u6xar6utN6yz5eg11r0anuYESU7AqVivP+8dkGx4U8nH5V+vZtP
5Wnf2/aIumOquNyfCylYK91Aq1zz5dQEAAAAAAEcTIeoy8oI9S3NVh0rGhDtTMmcun2r75iRC7nY
mCjuDs7o3V3WI5gWO5gzwaXOHxftCh/JXtqXl5XU1KdnnrCAlP6mTC95EVCojdOutWrxzHLe2bVBg
W9Yzb2i6eZ9Y68/k0w3VNv09lNI1T3hdAAAAAADA0USIupQqst1vGQGZUpVWv+8HRvaEQpMqyu
Ze1w/BbGr7g/scx2qn1yBt/9DrDZhIcVP2+oNtstj1YDOoh2rwhvQr1v62Ruysst0PrUtbbVnr3ZILLnU

8g6+ttiara2v22gwMTTeGs+c+ZD2Feyila57ougAAAAAAgCOp0FczrADITq8hK3oiL1WDdo+eiwAwk
urtPS8/mtCWcLQlHG0JR1vC0ZZwtCUcbQlHW8LRlnC0BWmgJyqQKbOOZ75D4gEAAAAAAJAOQlQ
gE23ZKAzU8ayeFTqhAgAAAAAALB5CVCAPql5pZpNXAQAAAAAAIEvURAUAAHODelXhaEs42hKOto
SjLeFoSzjaEo62hKMt4WhLuHlqC5KhJyoAAAAAAAAARCBEBQAAAAAAAIAIDOcHAAAAAAAAAgAj0R
EV2eg1Z2WjrhVnrSWNFzZavHhsyL60CAAAAAAADA/CNERSZ6jRUplGrS0csAAAAAAAADAoiJERfp6D
VmvEZ8CAAAAAADgaCBEBQAAAAAAAIAIhKhLqr3h1gddkUZPD7+3l51HaClTVePUeI3zcL7eZW/XH
MbfXHVepzc4uF+PuW1j58HXt2XDfY318F4Wo12TGjwv6jHpuVGSHr8S1obCSkPCD8+s/eo8VkaciG
TbBQAAAAAAWF6EqEuvI7VSQUoDw+
+bqwMhX3tjRI1T9fXpBJbR1H5WpamXpFyXrYr1MbN2OWHk4HlRmquFYNCZ4blRoWtYG6RTk9Lg
BFl2EFuSwZd3aqWhcDTRdgEAAAAAAJYcISosVWn1+9K3Hq2qXqUCwPNulNaTxjkdX5br0tWv7dbL
zjrrtTsXnYiusm19rlsX9zNSbdmv7W+rxHNafjv7e5tSTNCupHqNdS+MLNe7zj77LasFWvOcDkeza4NI
W3bd1Dh020055yW0VjvW3SDXOE/uBe3UZN17bZLtAgAAAAAAgBAVUm1tixtxVraMANRTlM09H
crZ4aVeu3bGe23noKufZadc3/La6ciqXT25uOP20qzK2U13yxXZsoNGFVLuibM6w3PTO5R9/VQ6O+
JmscXNPWd/1mPPbVvvorhNDpynypa42Whn56J1ZJYk2wUAAAAAAAAhKiaha5OGDmHPzqkT44K
9tNrVlQN3A+WTUtJPFSdo9EPnYSmem+KanHGTWGtrquyCW7t0qM5p98Dbnz18X7+uYA7v7xxYR
2ZJsl0AAAAAAAAQoqIsJ82UcJTAxElGbdLcjGjnzNtlyawNRdm8ENYz2A9KQye5Giur7QIAAAAAABx
NhKiIoS0bZs9Kt46mWft0JuahXRm3obgpe3qIvV+v1tc8Nzybvl/DdfBh9KCdYLsAgLzp0Q3Ggz9yAQ
AAALNBiIrx2rte70o7oHNrfxpDyKc2ybYya1dJTroJqDsE3qVm4i8YM+6n1YaI16uZ9NUvzfakXXbw2f
XqnHrtK530Qtu4NVhjbRcAlpH9Xl+QwkwTy540VlZFWu57tPNHr+bqLILU+Qxze40Vpz0r+f/hT/0ba
p4P9zEP5wXDhq7XDO6ZmRvzvuZ9P7mPvG7mOO+3ebwnR+5jSf+gFfO8z/K9eGZinJuh951luGnG
nJfc34sTtofSdoiDEBWJ+AGdMSt9AiU/nTRm/7d+MFmdbhD8tO0KKsqaVzS0Kavem667baeO6OB7
cZw2JDp+/aavPhXsGRpSs9Wsc9pc9dvm/sOhHu7KJNtVjG3wDwuALMzsl/chKri09j/lv0npcCYu3Da
KcLuTP+4f5vte3N4IC3ONPyjOQq8h6zWRatX9x28G3NEnxsO8XrMy01/cAyWOhh+z+NZW52O1qS
YFda9TS6qdmpRmFPjk/0v7+Pc19R5csr6f6l3jHFk/T2Z778R5v83jPXncPtTnc/yDVmTok1eYm+C85/l
eHHFuht739CP98xP/vl3dH/g3Iot/IEadk9zfi8efl3zfiydoj/WNrUrbpf+eHO/7dn5+FsZY1g2DJWS9R/
TV5bd+A+hbPzD5uvW+9U+g87mq9ZZia/WtnxX060c8vNcqIa93P29uf9TD2NbIdtqStKvbr5fd9VXr
K8cxXx/yKNetVygJz83Exx/
+MDcdve3gMSfabqvqrS8PXwQAmEq3XrbeX8z3eP2+GngjyoffFv1vwAzaEEm/z8/8vXjm7XCuj9q/f
c28f5PzY/87OoP9RtP37dy1y/3eivPzV9rCv5dn1R7n5y9jv/pnrCy/l8a/rznvuYNtyPocjW9XvNdMa6J
9ZPIeqPdvbdd+hLRD3T/maud+Um3XK1IS/5w4n8/+vTjeucnjvS/
+fZv1+8v4cxImq7aNPy/h62fWnhHfw+nfR87+zd0737fBdX579Qr9vhz3uiJf9ERFDBXZVn8p0ksO56
821puAo7kr/t9KrNd7n9D2D52/MKlanIP1QqutCWuIJm1XEk4PIOsNTS/7rDdbf9h+0jYkPH57qP3g
ubQ5+wj8QdOuc2oMyXfZPWWMeqiWRNsFgMy05XytY71NXZBN503VUpEt9UY28fv35Iqbe9Lv7x
ltmTO69MupE/PRwFm1o9dYl1qnKmfn9kLNhnteWt7PKPPC+T6X6unAzyL5KMqJU9YH9+dQW08u
7sygPb2GqIFK5fqWv9/Ktv3zYmfnYgY9sRxj39d6h7JvfRj8fi7aJ64puxm9Ecd5v83jPXle3ved79+y1Ls
hP8tr6uf34dEJHdm5mO7dE/ec5PVeHOfc5GX8uXHeXwLf5xmY7Jxk9148/rzk+148tj0jfp6yR42mW
tYuzqii+fpZGOMRoi4pvw7mwJuLMeFQcMiBCgvdr1EPJ5TztxMM6dQPhf5rrYf5A725D/VQ+xmx35
Ht9MRtl/MGFlw3nvMG7H6d89gbakjCc5Pg+G2D59J+jDoG8zj1Y9QvU3G3a7xu+NgBYAoz+uV9M
enSL+W6bIX/A5CTnjTWa9KZVTvsoaMdqbbi/1ueGTUM0Rh6N9uSN/n84j6JXuOc9d1s/bI/oxu3sq2
HjNo17dWwypITNg/
+vJW13H5pT8huV1lOenWcNKPePgZk8AetuMFlmDTbEVuO78XTnJv86fJoB+cHhtOnWwJnknPCe7
FhxPub8/PnvuRaNYmfhRcOISoAAJgNfnmPTdUltX/5uTCLXoa6tpj9i6D+pWdGvR3b52vSqbZmPmL
C/0Op81AjV7KppRZXPr+4J+f2NDo7wwDEHTWkatqr7yOrObMI4efpl3ZMgT9oKfPyXhwwR3/Y6uy
flAvevxGqt6h6/9mYYa9C3osD9HwiwflBrO+pqedWiWHwjzD8LLxwCFEBAADmmDP5gRq2N6ueO

MFRDt36vqzO4hfU9oYzCcRc/dbuUL2Csh6WHcfc/eLePi/O6NHZXTNnsg5/YiAVeKtJgXKftGOWv7Q
jNfxByzKH78Xz9oet8pk147pY/4aeVWXUZtirkPfiAernGrdnrP89dXBKXadTkl3n7nn5IwymQYgKAA
Awp5wAVfXWmJ+hjMXNC3YNtk7tfK7hXHtXhU1NO8B1fuEpSEn9Vqh/CZrtcHptlsOyLfP1i7sOCGf5
y6Iecqzq2bt5jz0MViXezdWMZjYfZVa/tI9h93bqyMHgjTuqd9QS4w9ajkV4L56XP2x5ZtqrkPficIMl+f
qydXLfOk8nJau3vdn+EQZpIUQFAACzwS/vkVSvDSdAndMJ/zL8RSPMYE8j9bAngLR+Mexaz2dbt7sn
h6qoWc7nZKxZ/uLu9nw6O8NfFgeHTbpmdl7y/6V9rOIJceZ7CUZNPfuGnmG4O2f4g5Zvvt+LB+T+h
62S2GWOB8PbWf5cw3txTLq2eOCPkekZ+UcYfhZeOISoAABgNvjlPYKeRddiD3czevzkW+dSTf4wM
Bxc/0KW1S8ac6/XkJUVc0i2WqVmSp7lL6nz94u701utKjMcPSpSOW21wPoeCgyhV7dwzTovM26bL
dtf2uOpyGnVY6+27r+veL3G5m+islngD1qLaFZ/2NIjADo1Oe/9w6mHcM+oHinvxXGoUhklqUldLm
RwkSL/CMPPwounDwAAMCOtqvSt33L69a5e0a33y9aPJ2VvRX669bLVFtWewUe139KvWUZh56U
6JyfEblu53s/3bun26+Xg+ZiLe6RVHbg2rb71S2tfJT+5m+H38TB9HgKPefie1vdRxvdv3Pc1573Yf2R9
7eK0K4/35PH7CLt/3Ifxb1dq9H0x9H2r2jFw3Pp7Pu1rNcl5t78m8/fiEedGvd8M7Ns9hrTf/uKem6H
XZfY+POp+0XJ6L453XvJ7Lx7fnpB/xzO6RnHuxXn6WRjjFdT/rIsKAAAwE+5f6F3WD43zNRwQWBCq
x5xdm9BVbUl/Bl3nnO/pqrT6M5p5eS7pnk7G5ZnV9cH8Gfre9fjfR2Gvqc5r79gUjT83Id9bxnk7iuLc
LwrvxbOmRvOoOqhhgkP7+Vl4cRCiAgAAAAAAAEAEaqICAAAAAAAAQARCVAAAAAAAAACIQIgKA
AAAAAAAABEIUQEAAAAAAAAgAiEqAAAAAAAAAEQgRAUAAAAAAAACACISoAAAAAAAAABCBEBU
AAAAAAAAAIhCiAgAAAAAAAEAEQlQAAAAAAAAAiECICgAAAAAAAAARCFEBAAAAAAAAAIAIhKgAA
AAAAAABEIEQFAAAAAAAAgAiEqAAAAAAAAAAQgRAVAAAAAAAAACIQogIAAAAAAAABABEJUAAA
AAAAAAIhAiAoAAAAAAAAAEQhRAQAAAAAAACACISoAAAAAAAAARCBEBQAAAAAAAIAIhKgAAA
AAAAAAEIEQFQAAAAAAAAiEKICAAAAAAAAQARCVAAAAAAAAAACIQIgKAAAAAAAAABEIUQEAA
AAAAAAgAiEqAAAAAAAAAEQgRAUAAAAAAAACACISoAAAAAAAAABCBEBUAAADAQmtvFKRQ0I+N
tl5raG94n19p9PTK2fDbuiEhLV0wbdlwz3vk+e1JY2Xca6xXNVb8ba00rK8CAGB+EKICAAAAODqa52
TGOemSUMHoqjT1kqMsZ9aK+rmpKJsX6tZnHZ3aesg1asv5Wkc/F6me3bS+CgCA+UGICgAAAOAI6
Ujt/OL38Zx/XTlwM89yXbr9vvT7e7I5KvksbsrZqn4eco16jXN+IFttyXZFPwcAYE4QogIAAAA4WuiNm
r3eoezrp3LqRKxeo5XtIng5auAamb1Qq9IiQQUAzCFCVAAAAAABHTLzeqH590pVg6NpryIpbm9Orse
rX/7Rrehp1Vr119suC68NKtPqCtUJH1wEdeJ31CKsrGjyeYL3S6HYYzGPXj8F92fsp1ayzrDVXndeO3Ul
FtltujOpfo/aGXxagXN+yXjUo3vHbQto/dH0tcc5VoEar+6BWKwAsLUJUAAAAAEdEWep1HdJl2Bu1U
ytJYTVYDdRep0K2gfXN1VEBZlNWCyUxyoCqjUhpcMIpOxQceJ3F3t/IQK8jtZJRr7Rcl60YnTuHwlEtel
8JVbak7hZHba7KRqMh5/wEVS4M1gNIcvwqwA5pv3M+hoNUR/i5UueiNLhTJewaAQCWAiEqAAA
AgKNjzQ3p4vVGnVS53pW+qgPq9ax0hK1v7o5oh1dLtC/
+y5tyzkv7etJYd0PBqrT0a71td2qyPjIpNl6/F2OSpvaGePmv0a6um3ga+6psW5/r+hNFqRqm9n5iDc
MPTjLVrLnHV5b6hcF2Jjl+67VuGhvWfmsrOxfjnqu27Eadi8A1AgAsC0JUAAAAAEdIUTbdGYwy641al
bNuj8nKaWvJNWL9/mFoL05zBnqzXmhn56Lz+t5F2dGdIQPD3I3enN5rB4QPix+t7aeGgTCzuHnB31
ftfDo9MAOTTGnVs8OTUiU6fuu675lBqKO4dsYLbDsHXf0saOhcmfVeOzviZq/FzT1n+9Zjb+QMWgC
Ao4oQFQAAAMDR4oVsGfVGLZ+Ukn4aMGp9qKqcDiR3JTnpp31ix33dA90L01rllguwH8bwdve1A0
6dSBLymT0vz8ha4EuLsnbGbdi+HIYlthMITDJlPQudTGqK41fHZNc5DR3eHzR0ropr4h2yuodK7n4ja
rECAI48QlQAAAAAR8xAb9RD5+l8K8qJU/rp1MpyMn6am0BHRnTmnE6i8DlCYFIpo85ppLBzFSw5Y
HLD3NgTdQEAjgxCVAAAAABHj9kbtRYvTsvXYK/Onhx6Y8iHebVWhx7biYbtTyercHa88cfflg2z16lby9
Ss3ZpEcVP29D4Gyt7amueYpR8Alg0hKgAAAIAjyOiNmpQxjDw7g706u3Lg7tTtmVk66QWAo+p5ps
MsJeDXAHX05KJbmFROSaIqAdNKcvztXa/nqR24unVRp7iWaoZ+1ePUnkjLDlS7Xi3W0WUEAABHF
SEqAAAAgKPJmIAoTMlPDo3aqW3Z8Kapz1ZzdcObqKm94Q8/L59ZcwJAszZnc9UfQt7e8Gp0FlIZV2
4Gzta5WPd7WfYa61790aSTVU1twuP3A1djxv4k9PbVbRDscRoSdAMAlgYhKgAAAIAjKro3qjlzuwrp
nGAubi3NaZWlXG7Kqg4D/dzWmOFftd+ozdlc1cGh9+IREzJNorLtD1vv1KSk21XyE1S5kPuM9AmOv
3Lan6jKu5bGBFRJjDgX5r1RPat7ugIAlgYhKgAAAICjK6o3qqp7OVgzs9qavI5mIqfk7J4xPFyx63gO1Di

1a3MOvE4Je+2U7GHrIcdebVnr3eHxeYt9/BXZ7psz/itVaZk1TZu7Xs/fcexzEVYMVW8zrewaALA4Cn1V3AUAAAAAAAAAEIqeqAAAAAAAAAAQgRAVAAAAAAAAACIQogIAAAAAAABABEJUAAAAAAAAAIhAiAoAAAAAAAAAEQhRAQAAAAAAACACISoAAAAAAAAARCBEBQAAAAAAAIAIhKgAAAAAAAAEIEQFQAAAAAAABGEvn/AZM6nr53azj8AAAAAElFTkSuQmCC]]></redcap:Attachment>
</ItemDef>
<ItemDef OID="q32" Name="q32" DataType="text" Length="1" redcap:Variable="q32" redcap:FieldType="radio" redcap:RequiredField="y" redcap:BranchingLogic="[q29] = '1'">
<Question><TranslatedText>Do you prefer Life A or Life B, or do you think Life A and Life B are the same as one another?
</TranslatedText><redcap:FormattedTranslatedText>Do you prefer Life A or Life B, or do you think Life A and Life B are the same as one another?
 </redcap:FormattedTranslatedText></Question>
<CodeListRef CodeListOID="q32.choices"/>
</ItemDef>
<ItemDef OID="summary33" Name="summary33" DataType="text" Length="999" redcap:Variable="summary33" redcap:FieldType="descriptive" redcap:BranchingLogic="[q23] = '1'">
<Question><TranslatedText>
Alzheimer's disease - Description #[no]

If you have Alzheimer's disease…

1) You might forget recent events of importance to you. For example, if you are interested in hockey, then you might forget whether your favorite team won their last game, although you may remember watching the game itself.

2) You might forget the names of people you met recently, or the names of local streets.

3) You may no longer be able to handle your personal finances or plan leisure activities such as dinner parties or vacations. Simple decisions such as what to order in restaurants might become difficult to make. Your ability to concentrate may decrease and you might have difficulty driving.

4) You may start having trouble finding words to express your thoughts. You might need to be reminded to shower or brush your teeth, but you could do these activities on your own. You may need to post notes around the home to remind you of simple things like turning off the stove.

5) You may remain aware of the day and your surroundings. You might be able to travel to familiar locations like the supermarket, although probably with the help of a loved one or friend. If you are in a social gathering, then you might have difficulty following the conversation.

6) You may continue to enjoy hobbies such as puzzles or reading.

Now consider two possible lives. In Life A, you will live in perfect health for 4 years and then you will die of natural causes. In Life B, you will live in perfect health for the next 10 years, then you will live with Alzheimer's disease as described in the summary for another 10 years, and then you will die of natural causes.

</TranslatedText><redcap:FormattedTranslatedText>
Alzheimer's disease - Description #[no]

<p></p>

If you have Alzheimer's disease...
<p></p>
1) You might forget recent events of importance to you. For example, if you are interested in hockey, then you might forget whether your favorite team won their last game, although you may remember watching the game itself.
<p></p>
2) You might forget the names of people you met recently, or the names of local streets.
<p></p>
3) You may no longer be able to handle your personal finances or plan leisure activities such as dinner parties or vacations. Simple decisions such as what to order in restaurants might become difficult to make. Your ability to concentrate may decrease and you might have difficulty driving.
<p></p>
4) You may start having trouble finding words to express your thoughts. You might need to be reminded to shower or brush your teeth, but you could do these activities on your own. You may need to post notes around the home to remind you of simple things like turning off the stove.
<p></p>
5) You may remain aware of the day and your surroundings. You might be able to travel to familiar locations like the supermarket, although probably with the help of a loved one or friend. If you are in a social gathering, then you might have difficulty following the conversation.
<p></p>
6) You may continue to enjoy hobbies such as puzzles or reading.
<p></p>
<p></p>
<p></p>
<p></p>

Now consider two possible lives. In Life A, you will live in perfect health for 4 years and then you will die of natural causes. In Life B, you will live in perfect health for the next 10 years, then you will live with Alzheimer's disease as described in the summary for another 10 years, and then you will die of natural causes.

<p></p>
</redcap:FormattedTranslatedText></Question>
</ItemDef>
<ItemDef OID="a33" Name="a33" DataType="text" Length="999" redcap:Variable="a33" redcap:FieldType="descriptive" redcap:BranchingLogic="[q23] = '1'" redcap:InlineImage="1">
<Question><TranslatedText></TranslatedText></Question>
<redcap:Attachment DocName="4.png" MimeType="image/png"><![CDATA[iVBORw0KGgoAAAANSUhEUgAABVEAAADrCAIAAADv60EwAAAAAXNSR0IArs4c6QAAAARnQU1BAACxjwv8YQUAAAAJcEhZcwAAEnQAABJ0Ad5mH3gAACoMSURBVHhe7d1fqGVXnSfwxFK7MhoSbCdEHUyZIRCSIKVhINaIXYIYEQKBKBbSBoeRogJKh4BGxAzzEFGahlQ3gj40aWF6Jj5EpBmJYjfkoYU4NHQ9+JCGGOrBxoA+VEvT3GAees7ZZ/9da+0/d9+9z9nn1OfDovuefdZav7XW2ZXsb5Jzve7fdfuKvSm8nF2mDFDCh+5HFtpjIJAYNx7eYwI99JVx6tmZH14HoQrDbQ1wKTpgqfw4V56ArXzASqmr2S+SHTULvwyJa3s8pFcYTRSazpV7NJXx8vsuaXd5dhP6IPDGVbADF+Wi7b97Z3JEzYGRnxQ5gRXQKiJzAWDc3lXEWr7Ez4JMj0gsxVfGmhwVE9v0ee+XRasnm4c4KXtFdQu0DqvsFDvS5fTNgf8IDCBUhRfQgaR95sPYPNOUZD0CCN9L8bo+B5a6O6dRdwyOmpH64c1SHUN4kvQAh9AAKMtkxXlV9NezVSu6Jz1G33Rz+HDKJj+63/M33nN6+3CuAAIDKMHNP8yPDE/K/4zUsXm0lbQwDQvjdYxvTQBxodiB3tA9xtQ+LCTZRxTiN/3YS5oMxPsF+V3BgCMs3Cmgd2DCPLPH69aG+O8QgsiTCeOvv//5anyPH1nYoeiE+0n0Vx1HEQngO3h1+8UcyIBXLB1mmqQhS6dAL3OyQzlSZvACgbfe5UuxOHb6awgAGxOH

wdQTifr/liPwAslsy/BKlEmSveaoarIHIlOg0IS/morEP287nLL2Sjqivl8GC67pdt+kYVF/LX+QqKTRWbT
OmoG1Ypp6nGDFh+MarxMTTGDVheovRGvID+2ZIrahqwr5XERMHAAVurafbO34+Wklpb/0pYgK
M3js5+92wQL7UFtrm/xh83X+wHgGWS+ZegI7+l32oJZccMS/mwVY9N79UPm0vllXJ0MF33yza9o4
or2YVqdRvFJlM66oZVxp1VMarRozFuwPISpTfiBfTP1jpZZcC+VhITBQMHbK0aEsjfj5aSWlv/SliAC89f
CLKltsB216M3B4F8C+1L93/gt//8q/xGAQAWQ+Zfgo78ln6riEL51bFhaTOu+Nf72ejsUnGlmjCYrvtl
m/5RxaXVlXxP1Xvpk+gVVhl3VsnijXEDltfaJV5A/2wD6g3Y10piomDg8MWsbDqFpaOlpNbWvxJ27Z
t//80gW2oLbLc88ZbtfI0/bt/49CO/f/31/HYBAJZB5l+CjlBVhp76e2ESSowvU1hXWMrnib/Gv7lSmy+
o2P2yzZBRm3WXv86+9tbAIqFw2LhgmTrO5rBjTFL/KDPx2P7ZUj3WBY63r5X+A+mdJ54iPK9oirblrx
17C2zHD1/+YZAttQW2U0/d+Kn73xtE8W2273/j6fyOAQCWQeZfgkTYqZTxqXi3zEFl9/JKEY3KMT1h
qRqYGtqevbpfthk0qlY/eKfsX1wuu3aVDasUg44XLBOnElbvX16i9EZiAf2zRT3aL1TTJiRWFQ7sW0y4t
qp/dCV/nVzbgJWwM1deu+lX9e9F+8iDtwUhfPvtH//2p/l9AwAsgMy/BImwU1dGqoZ651rICnWHp
WpgOV1ZLVlgM133yzbDRlW7jY6jHNFwrKIjg2X6I1hLzdNQTZoovZFcQP9m0z1q0zdX3bK7QQfSs5j
248m7xDMm1jZoJezCtaNrZy6fCbKltsC2k6/xx80X+wFgUWT+JUiEnVAzdCUSUL3D6u2hYSnulxwZX
Ox+2WbgqO7TKEdlOk4sF1YZFyzro4qfV+IBXctr3VjrAvo32+wRdamtNTl8ZfCBdC+mXmk9rHi9mSI1
Y7S2wSthq47eODr/vfNBttQW2N7+5Td/7t73BfF7V80X+wFgOWR+6JeIo3B9uPSjS0G21BbYTj1140
Mfek8QvHfbfLEfABZC5od+Mj/Xp2deeibIltoy2wMPvTOI3EtovtgPAEsg80M/mZ/r0ItXXwyCpbbMd
scX3haE7YW0L93/gV+/
+sv8fgIAdkTmh34yP9ebq9eu3vqtW4NsqS2wLepr/HH7+oMf98V+ANgtmR+AhmtH1+7+9t1BttSW
2T754XcHMXtp7dknv5LfWADALsj8ADR84q8/EQRLbZntg4+8IwjYy2w/
+8Hz+b0FAGydzA9A5fGfPB4ES22Z7V2P3RRE68W2S/fd44v9ALArMj8Aued+8VwQLLVltpuePPXZ9
58JovWSmy/2A8CuyPy7VfxuuMwsvyDuIcvnLr6Q/3wYv4uuuaNOfvXecIPP6vDuKAov/eql00+fDrKl
tsy2/K/xx80X+wFgJ2T+HVqFp01e2pg+NeUFDiihxTvqUJ2vQNpv0K1xeHcUpavXrt7+Z7cHwVJbZnv
/p28N4vS+NF/sB4Dtk/l3aOa4VEbeg0loqR21qhK/QDrEgFvj8O4oCkdvHJ397tkgWGrLbLd98fTn77
4zyNL70nyxHwC2T+bfnWMl2BGu78xf7DQjkPaT+a9rF56/EARLbZntrV9702fO3hEE6f1qT57/o3/7
3e/yOw8AmJ/MvxuNRLpRz7FluMoEcaqWsqppghTcNn89odX6xIGtsYLePFeftj4yyubt01bvlIPKBQa
rLUSzVza9z53L52zdQH3hNYnLfQfSeH+l0aU2X7WPzfI7x7UYWqvRsfOzWL3ZchSFat2lbMb6sFqfeJ
bGooO3B6+ZWXzz778ZBEttse1jH709iND72L792KX85gMA5ne8zF8+jrc/iye7NPJCS6YYrMoEe5wI2
hJU+q2V2qklOkRn2jZ/evK12hSNdFbo/NgmmbZ8f3MteNm2o5RN39W4YlCtSiDZI7zYeyCJxa1UPR
LvZ2+mx3Xf2CNq5fpXvFbvVZMYMN0ddezVMJ0fvvzDIFVqi233XrglCM/72376V8/mtyAAMLMtZP
7gef+kD/H16Tqz0eIlT7NMP8U5lb3Kg6sFpM4DSM0fj43mj4YVYzo+uXja6kpYp2Paep9U1dSOYnmv
dZ8BSy/mrHUJRvWuPJyiHFDOWYxYqS+9baJqYGRMrepKUbwcVVypLnTUrnrVNhGXi5YUDQv3PWj
NzOHKa1f8ov59aX/4J3+wv1/jj9ul+
+559cqV/EYEAOY0eeaPRQ/4J1ILJyvTzLkjXQmqvrGwXxmHenbfNX/tWtCteFmbPHEpMNm05US5Z
slgzqS8z6ZH6jhD0TKCQQNXXhets9xWY0TZrXWiAdprVYcU9onGrJTXOtaSGtdfrnjZcYAD1sz0rh1dO
3P5TBAstWW2U0/d+Kn73xvE5n1vvtgPANsxf+af9tm9DAv5N7VPEJZ2LnEyQdzMlZveXE0EpKTUyS
cKBN3K2RNaC/ZntsHT1juGn25qR4HN8GJgMVk4UUPL8RY1Bq880+wdzdEcUO6n0rnSwOBa4bkV
XRrFkhebUuefGBZ0ay6zKVhQx5qZ2NEbR+e/dz4Iltpi20cevC0IzIfRfLEfALZg3v+2P/G0n3ioXxsYdYo
xF18ofjpORlqYxGmmc1fQMd0plpg/NbZl9pTaRE1TTlv1jDaY2lHDZmw1LrGuhGLarFcxpCwxZOXlw
gLF+63raBnYtr+1EbXCc0sup3WNlXCetf5yRY+U9gWlajGhSz+6FKRKbbHtrkdvDqLyITVf7AeAue0o8
5e96jrCRq4Ytp6k+Ll/1FIlTjOdu4KtpjvFEvMPCFdDZ2+YbtpyWCbondpRXVEkraN0PnDVoyjR95nU1
cpuOkXr7J0jXPmQjsNrhX2Sy+ldY/r8+8sNmHjAmpnSMy89E6RKbbHtlifeckhf44+bL/YDwNzm/x1+
iTHRpQGpYK0YtxlWvOoZtFxdJ1PfVNhv4Gml5h8QrlKjek01bdHr3OXLqU32TVIsI63rvPKR5y5e3FSo
Fehdebz5ch3FkMT5FFZvHaPYuFrhrKkq5bWOY0qN6y+XGhUYsGam5H+Nf4/aQx96TxCSD699/cGP
57cmADCDXWT+4kriAb8jb8SdBg1asPhkVsoIF+6y2mYiICWl5j9OICwulEuqr7NpmmkbQ8oXHXP2

GnpUtdVkGvP3rTzcSNW/nCe9jnJgdTndsTKuVnRu0ZZq83ScVTTPyoByUbVwF4PWzISO3jh64C8fCL
Kltsx2xxfeFiTkw2s/
+8Hz+a0JAMxgF5m/fOJP6HjALyaqckF8Za+0nGb6eGp7TASktOZMWZVB4aq80tDx0Uwybfl2MUm
5/GrWxI46JdbVpj51OHHPypurqiu6tK2jbWT7zkbVij+LIi2tdZ1Vs3o226ByPQc4cM1M6bV/fc0v7d+
X9sBD7wxC8iG1Z5/8Sn5TAgDz2J/Mn44NG10xZbk6TrO52eD9REBqUz/qrPvgcNVcQV+pk09bvVHr
Xy6/6hjtqFNiXa0Sxeq6D6S+rPUGitf5ZrrWUR+61jivlOPX6v8sVm90rbFSL571HFUurDJ4Eqbkf5x/X9
qpp2481P/C/
+sPfvz3r7+e35EAwDx2mfm7w0WgmRhCx5oKEoo7zL3E9eO5XzwXxEttme3tX37z5+59XxCY971du
u+eX7/6y/xeBABms8vv83fMEinHtBDUOJHyBnMncX356t99NYiX2jLbnf/t7UFm3vfma/wAsB0jM3
9SFuGjQJ9I+NU0+bXyPxhu+6cAiUk2yjdENcap/9fq7XcgHK6Hv/9wEC+1ZbaPPHhbEJv3t/kaPwBsz
U4yf9tErXmrI9l3vAUD1DK/wM916eiNo7PfPRvES22B7dRTN37q/vcG4Xkfm6/xA8A27SjzrzUn64rs
nbm+800Ael29dvXWb90aJExtge2WJ97y+bvvDCL0fjVf4weALTte5gfgIL149UW/xn8v2l2P3hyk6P1
q//i3P83vOQBgK2R+ANa+8w/fCeKltsy2v1/s//43ns7vNgBgW2R+AHKP/
+TxIF5qC2xv/dqbPnP2jiBOL79949OP+Bo/AGyfzE+34PcxdL88luKX5+W/iOEkUx1X43f1z/6LIFY728
Ke1rZ5hhys8987HyRMbYHtti+e3q8v9n/p/g/89p9/ld9kAMAWyfx06w75JwmZu8r8ZaGNeSN/Xmx
LGXxrZ8ghu3Z07czlM0HC1BbY7r1wS5Crl9x8jR8AdkXm51iCVHmSkBlk/q3ZYt1tZ3CZn2m8/NuX/R
r/vWgf+
+jtQbReZvM1fgDYIZmfY9n/zL/NXCzzs7d+/MqPg3ipLbDtxRf7fY0fAHZL5t+JlribvFzmuEz/iJ7cV7xdj
YivFNOurwTTdb/sVnZeWfUP1p6cqj6kvsBOjUGNUUXJmpaF1xcXrLuhWatera1WsO21aOu1PtU0+
ZvtFRMTtQjmGL6plSHrX+mcY6PRJX5/wAzM65mXngkSprbA9q7Hbgoy9qKar/EDwM7J/LuRCE6pi
1Xgq6sNGhjA6or3y7fLIuUk+ZXsdTBd98sO6a2s5WWjqcoLdbWNJnWfWOLdloX3Lnets1hbreJ6bZ
5o64mxm+6dFYd9HMljrY3oKTF2/Wu1VfV9uP0zsBUXnr8QJExtge2Dj7wjSNrLab7GDwA7J/PvSJF5
EjGnvFTmnuJKGZSiLrVpogAWyjsUQ2rpKh9RzJC9DKbrftmq7BfWWMkX0jdzYquRAScWT5wUn0p1
JdjCMWsl9hF1i6tneium6jVFQ4Ij6y0xZP1hl2iScEA0oncGtuTojaMH/vKBIGFqC2yf/PC7g7C9hPY3f/
Hn+Z0EAOyOzL8rRYopQ0yUpRLhqjddrUR5KpL32IzZvLh4cT1R/UoxPpiu+2WbVLfyWr72Zp/iVWJjt
UuBIScWX0gqpqr16h0Yd0gNGfKRldXb95oJB45Y4uZSW/
+OtbWvv3zZtvyiQ2KKzaXeGdii1/71Nb/Gf/ntpidPffb9Z4LIvdv2p3/82fweAgB2SubfmSD4FFGqTF+
JbLXSMqrWqcxL7TkuH5V1yH4+d/mFbFR1pRweTNf9sk1yL8HF5lTFmylttZJVynmLq8OWXNavenU
NbC637JAaklhl1C1RPZSs2Lu3RPF26U0NWH/5utIo2Jy4aTNF3wxs15XXrpx+
+nQQMrWltUV9sf+Jcw/8y29+k99AAMBOyfy7U+SaLMwUMagKa+l4FsSrAQEsJR+26rHpvfphc6m
8Uo4Oput+2Sa5l+Bic6rizZS2Wskq0RKHLTkxVzywvBKIO9RqDZk5vZOVnoqpeg2tE1f6NnWSk8nfL6
ZIKabonoGte+4XzwUJU1tg+y8P/2GQvXfV/unnP89vHQBg12T+Xcqzzyo9FQmnFmnS8azomF8dFM
ASNuOKf72fjc4uFVeqCYPpul+2Se4luNicKjmiR3pMMW9xddiSE3OFA4suK5te8cypWgNmbtt9b8V
UvYaWiSv9mxqy/lJtusxmUO8qatIzsAtf/buvBglTW1o79dSND33oPUH83n7zNX4AWBSZf6fyQHPu4
sVNaKpnpjJI1VNOmK4S6akMSW25by2fJ/4a/
+ZKbb6gYvfLNqlu5bW8VrPPwIkbwhkz0UTDZk4cbDAw7hGffKrWkI8s0Welv2Lv3toWVEzZXyLRJe6
zsrpWe9Ws27vMTNcM7MjD3384CJna0trbv/zmz937viCEb7P5Gj8ALI3Mv1tlWsoEcaZ8s4hXZe4p
81Z5pRhbm7AzHFUDU0NreS7IWt0vW0XrrNXPi7XNXIwol9dRasCJhXVaFDO1n0S4nmrB1cypWlW
//GLt3Ituieor/RVT9Zqi6s0LAzYVzRCtv7xQLT/YUPscQ2dIXGALjt44Ovvds0HI1JbW7vjC24IcvrXma/
wAsEAy/46V6WalSFWV+ruVesqphbJQPF1dNTBKUekCm+m6X3ZoX2heLZoqPaKn0DFOrHOmRKI
MB6ZLrVUzN/tsrrefRDkwUX2tt+KQvbWUz0sN2NQx1h+qlpWepHq/f4aWM2JuV69dvfVbtwYhU1
tae+ChdwZpfDvN1/gBYIFk/l0rw01bdmmmoyrzlOodVm+XLxNd6+J+yZHBxe6X3crOK6v+QWjrrp4
ZGO+ag8KVDVtyIlEmBtaT6fpq8bo2db1LOVl9hau+0cyJ6rnuisP2Fp5QUKa7RKZv/Wv1adbiJTVX0b
3XteYMxbvxOOb24tUX/Rr/hbdTT934qfvfGwTyudtP/+rZ/BYBAJZE5t+1IviILsC+

+M4/fCcImdrS2i1PvOXzd98ZxPL52rcfu5TfHADAwsj8u1X+q06RH9gnj//k8SBkaktrdz16c5DMZ2pP
nv+jf/vd7/I7AwBYGJl/V5r/4XL8Hz4DLNv5750PQqa2tPaRB28L8vnk7dJ997x65Up+TwAAyyPz70ot
8wv8wB66dnTtzOUzQcjUFtW28MV+X+MHgIWT+QEY6eXfvuzX+C+8/eGf/MF8X+z3NX4AWD6ZH
4DxfvzKj4OQqS2t3XvhliCrT9J8jR8A9oLMD8CJPPPSM0HI1JbWPvbR24PEfsLma/wAsC9kfgBO6sLz
F4KQqS2qvfVrb/rM2TuC3H6S5mv8ALAvZH4ATurojaMH/vKBIGdqi2q3ffH0VF/sf/bJr+QfPACweDI/
ABN47V9f82v8F97e/
+lbg/Q+on39wY///vXX808dAFg8mR+AaVx57crpp08HOVNbVPvkh98dZPhjtUv33fPrV3+Zf94AwD
6Q+QGYzHO/eC4Imdqi2k1Pnvrs+88ESX54+9kPns8/aQBgT8j8AEzpq3/31SBnaotq73rspiDJD2y+xg
8A+0jmB2BiD3//4SBnaotqH3zkHUGe722+xg8Ae0rmB2BiR28cnf/Gf8ntji+8Lcj2yfa//sdT+ScKA
OwhmR+AuXznH74T5ExtUe2Bh94ZJPpygePTj/gaPwDsNZkfgBk9/pPHg/gaPwDsNZkfgBk9/pPHg5ypLaedeurGhz70niDnl+1L
93/gt//8q/yDBAD2k8wPwPLzOf+98EDW15bSOL/b/49/+NP8/XD4TRE1tOe2u
R28O0v6qff8bT+efHwCwz2R+AGb38m9f9mv8l9w+8uBt9cDva/wAcDBkfgCW3HDDlv6mo9BoCo2m0GgKjabQaAqNdniFrgeOEoAt8UQymkKjKjKTSaQqMpNJpCoylEB0cJwJZ4IhlNodEUGk2h0RQaTaHRFKKDowRgSzyRjKbQaIqNJpCoyk0mkKjKTSaQqMpNJpCoylEB0cJwJZ4IhlNodEUGk2h0RQaTaHRFKKDowRgSzyRjKbQaIqNJpCoyk0mkKjKTSaQqMpNJpCoylEB0cJwJZ4IhlNodEUGk2h0RQaTaHRFKKDowRgSzyRjKbQaIqNJpCoyk0mkKjKTSaQqMpNJpCoylEB0cJwJZ4IhlNodEUGk2h0RQaTaHRFKKDowRgSzyRjKbQaIqNJpCoyk0mkKjKTSaQqMpNJpCoylEB0cJwJZ4IhlNodEUGk2h0RQa
TaHRFKKDowQAAIDDJPMDAADAYZL5AQAA4DDJ/AAAAHCYZH4AAAA4TDI/AAAAHCaZHwAAAA6T
zA8AAACHSeYHAACAwyTzAwAAwGGS+QEAAOAwyfwAcFwvXLyhcvGF/CoAwNLI/AAckE0YnzeFv3
L5XFVhzoLb/icL642tnLv8Sn5hSo3NZPyzkuNqnOE8H9PsWv7A5PfexrR3Rscf0Y63jis9VeMTm3Zb8
+o8mVn/QjGj9k3t6+fUsqPGdqb6mAbU2tO/Jl0nZH4A5jXj03xDrc42H9qysnM866yepcp9ZM9VMz
9QrTdy7uLF1f+Zpc56C1t/JKw/j05/VzTu7MpMd1+2ly3dEfVzm65I65/Q5p+h5kZPpLVi51vH1TbV+n
p5YXOkJy2VnqX+cZ24RO/JrN+f9C8UqU01tpQ52b46NpW9NeEfpXg7teJ1M+0oq19eyl6ddHfDak1
QanN2ueB8GqcYvMcAMj8AM2o+TzWfRiZVFMr+/zYfCJo7nMvsVdYF8uObp8z6s5/9mOq288E0ZC
VnuvnCueerNfVzfK74POKFh7fGVFtrr9j11nENnarol788tmz8RrPK6vjKCyf/uPq2s76ev32iOhtdm5pi
/lzHphKXxmvdTuDkRdt3FF6ZsVbxRv7ypJ/aerpy+uxOrl42S2VvnmhP1yOZH4D5hM8A0VPD5OavE
NjO48fM2yqnX/8w/qGty8keB49t6/fByry3QvMAZ9tfNnH9g5r6g4tWHm9l4oPsOKsJj7Fvquz90Qd
ZjJ63Sk26UHl1/cOJyxSLTdSa5y8XcaFJ9pHr2E7ThPd379H1Lma4aKpoHxMWK08zexHeDlMWum7I
/ADMJv4784RPO2lbfhjI9jPVM2OrxuPP9Gpntv5xnkLZSVVmPrP59tFq5g9pZXOEqxKbH+a5y6M/ob
W7YxLRfOuKzXObuGTHdBNW6psqOthR+qpMtqHURLVr6x8nu9kTteKbYgpxoazO+osKpZNXTR1dT
fb2VHtL1sputXWJzQ/tSzmeqFbLcU6ztfoxpQtNtrHrhMwPwGziJ4DkI8qUZi9Qlz14TPX0Fsv2kptxS+
tdFNOvS863ocJmY3PWye68iR/lu23nxsvuuMxcpeJ9xH+KTyQqEM8/8Vl2TDdhpe6psg9uglPsrpK9
O81nlSi03kNxZf32ZDdFS62aiUrFhYLPJevQfr7DJLZT0/3ucbXNVp3fZKUStbIr4elN9FllO8irrX9uTjvtK
V4fZH4AZrODv1Vv71kgeFqc1aQPU03rbVTntS60lT3Ft8akgg9nc35z3hZZwVnvu/oeNj/PUy+bOzy6
CT+qbML6yuNbIepyMh3TTVipa6rgdjyBZJXsYm6qY4sKrfdQvV6/PdlN0XV0K5vtTVEtLhTdfBP8Qe7
czgTz1yVqbY5rc23z80T1kvvK9lO4eLFxk5xANm35wUSfUvcZkyTzAzCbHfytekvPApsnnS0+c2we3qY

v2Hhkq5niCbtLVne+84vuvHnrZZ/OrEcWV5hxR42bYrrn+I3oT2j8Rzb69E4mLlDqeOu4WqfKTnOq7f
QsOHt7kmJhocY9UTNHrdhEd0RcKJq4fy29OqbI3prqXliLasUVsk/uZDva6D+adY8pdhf+kYk//v61EJ
L5AZhN/DfmiZ7d2m3jWSCrseUHjqzmrCeXWZeZv8r825njUb7dZE/V7eISs+6oZupbIl53+GFNvbOO
+SYslZ4q+9wm3EzvgrMOExTsKbR+e7K7ondTU/0JiwtlV+obCW/GEdq3M9E2aqJacYn+0x2od6J1h5
PfFNkOmtPElSf4mK47Mj8A8wn/zjzZ40er+StsnklCkz9/rKrUtpF4DprFNE9tsdW8tWmzD2nWTymr
UN/InM+I2Ycz7z0X3wFbKboy+R2RfTTNhTf2Fn10J5aoWOh467gSU2WXpv2QehecdZjg+HoKrd+e
7EPa6aYaf5Am+VPVup0Z/szGtRp/mFamK9r9MU3zGWWrjYusL9fn7l4KSTI/ADNqPIBM81SQls0d2
O9ngmBH29nMuugsn9DWd9N41J3uuTc2513dtHkeLm3hjphyb8EdkKm2UN/bVIfZUbF7McfSPlXw
eW2ccHNZtcZKV1Vqr7OaJ6kx8GTW3Sa76aNNrS7UJt8sKbGIwbo31Xj3JGVy0XY2sstbuLcn/wtFe6
3mOyc+us10yWka9/WkJ3n9kPkBmFf9AcTfp9maxgPpiZ9H22S392yz78DEz/FMovmpbOSfTfDWH
n1i7Zvayz11fEYrB/cXiskF/6hio3pk8CBxQjI/AAAAHCaZHwAAAA6TzA8AAACHSeYHAACAwyTzAwA
AwGGS+QEAAOAwyfwAAABwmGR+AAAAOEwyPwAAABwmmR8AAAAOk8wPAAAAh0nmBwAAg
MMk8wMAAMBhkvkBAADgMMn8AAAAcJhkfgAAADhMMj8AAAAcJpkfAAAADpPMDwAAAIdJ5g
cAAIDDJPMDAADAYZL5AQAA4DDJ/AAAAHCYZH4AAAA4TDI/AAAAHCaZHwAAAA6TzA8AAACHSe
YHAACAwyTzAwAAwGGS+QEAAOAwyfwAAABwmGR+AAAAOEwyPwDAsrxw8YbcxRfySxvFG+cuv
5JfmUdRJyi/BNXZrETn8Mrlc21vdb4HcLhkfgCAZanl2mY8vd4zfxXbM6ljaE321aEu8J9kAMxH5gcA
WJbGv8uuJ9TrPfMP2n8y3Ff/KEDiB64zMj8AwLI0Mn894F7nmb8M7j3rKs+vPKfyisQPXHdkfgCAZ
Wlm/lpQjTJ/dCEOxnmXdY/avPmA2pV6Gi4ur67V/nP6WpFC7d3o/draqjI9mbsxX2PC2koLHXOVvb
M+5atoB13rX2uuJ+jSscHOcQBbJfMDACxLFSYvb34qImMtZWav4wu1uJlH4iqO9ipDdPuYWs4Oc22
mtpDEJJ3RN1003HpNbS2RanEXLxc/BtX71t+zoPYNpsd1LhdgPjI/AMCyFKFxlSCLYLoJjLU3so7xha7
Mn3eqRdLwShFLozHVleJCWKbsUS6lVmdA3G0vUE4Y1+xSds5V02R61190aF9PywbbJgpXALAdMj
8AwLI0UmP+ov5zHDtrcTIMs0WXKpe2XymmiXuEfcJEvBJeKiep9WkVrmAt3El8oVu5gLVgyID1h6Lq
6Q2W3QbtG2B2Mj8AwLKk4/UqakbJOBGVw2gad+m/UryuB+XmvEWPlKBwY5YW8ZLWipLF1XBrv
dqXMGD9Nc3exfsts5errDS3BbBVMj8AwLKECTh/XX69v8qQYc+VuTJ/82LxIqW1cLt033ArozN/vIQ
B60+F90zxfuvsLQOHrhpgUjI/AMCyRGEyDJHlG4nYGQbjuEv/lcS0wbypHoEBXUrpvkXJ4mq4tV7tS+
hfXNFjZdMpqt47R22KTHsxgPnI/AAAy5IIk834WL7R1XOCzF8P180+A+J3XKZdOV29c1RjwszfO1U8
NDqUjg2u3qpNfOx1A0xI5gcAWJZUmCxz41r5RnU1j5NlMI2uxOG1/Ur7LGWX9spFsI3LdGkvUI0/d
nbuWELf+sPtVP17NhhtpHMZALOT+QEAlqUnTNbfqEXRUJBluyJoeKV8fS6cvci7K+nSVYe4TLeif1N9
9JSZv3f96eWsFV3aZm8bOXTVAJOS+QEAlqUlTFYhtfFGPbuuYmUYjOPJ+q8Ur+vTRctZa+bmZoeWX
XRqzheG5HBrvXqX0LX+avjaumTtWOpvp2avD10bumKAycn8AAAAcJhkfgAAADhMMj8AAAAcJpkf
AAAADpPMDwAAAIdJ5gcAAIDDJPMDAADAIfr3f///VL6AMJyu9K8AAAAASUVORK5CYII=]]></redc
ap:Attachment>
</ItemDef>
<ItemDef OID="b33" Name="b33" DataType="text" Length="999" redcap:Variable="b33"
redcap:FieldType="descriptive" redcap:BranchingLogic="[q23] = '1'"
redcap:InlineImage="1">
<Question><TranslatedText></TranslatedText></Question>
<redcap:Attachment DocName="B.png" MimeType="image/png"><!
[CDATA[iVBORw0KGgoAAAANSUhEUgAABVEAAAEJCAYAAACdcmdaAAAAAXNSR0IArs4c6QAAAA
RnQU1BAACxjwv8YQUAAAAJcEhZcwAAEnQAABJ0Ad5mH3gAAE9sSURBVHhe7d0PjCRnfef/b+/aY
PPHNmBskx+RHat7sZYlGJIg08PxL4egZ+6SJbnbYOVg5Oh3PVyi0zQSkwOxF+mX2wguG4lunS5iWrq
zliQyTEJugrLdIJRgckzHR4IxYTI/Z7rlc3T8wDYGjPljB3u3f/VUPVX1VHd1dVV3VXX39PtltaerpqfqqT/T
O/OZ5/k+hb5FAAAAAAAAAAChjumPAAAAAAAAAAAIAQhKgAAAAAAAAAAEIEQFQAAAAAAAAAAiEKIC
AAAAAAAAAAQARCVAAAAAAAAAAAAgAiEqgRAAAAAAAAAAgAiEqgRAAAAAAAAAAAAgAiEqgRAAAAAAAAAAAAgAiEqgRA

UAAAAAAACACISoAAAAAAAAABCBEBUAAAAAAAAAIhCiAgAAAAAAAEAEQlQAAAAAAAAAiECICg
AAAAAAAAARCFEBAAAAAAAAIAIhKgAAAAAAAABEIEQFAAAAAAAAgAiEqAAAAAAAAAAQgRAVA
AAAAAAAACIQogIAAAAAAABABEJUAAAAAAAAAIhAiAoAAAAAAAAAEQhRAQAAAAAAACACISoA
AAAAAAAARCBEBQAAAAAAAIAIhKgAAAAAAAAAEIEQFQAAAAAAAAiEKICAAAAAAAAQARCVAA
AAAAAAACIQIgKAAAAAAAAABEIUQEAAAAAAAAgAiEqAAAAAAAAAEQgRAUAAAAAAACACISoAA
AAAAAAABCBEBUAAAAAAAAAIhCiAgAAAAAAAEAEQlQAAAAAAAAAiECICgAAAAAAAARCFEBAA
AAAAAAIAIhKgAAAAAAAABEIEQFAAAAAAAAgAiEqAAAAAAAAAAQgRAVAAAAAAAAACIQogIAAA
AAAABABEJUAAAAAAAAAIhAiLp0etJYKUih4DxWGj29PqjXWPFeU1hpWF+1iMxj3ZB2onXjpLWdL
MxLO7LSlg372PzHqPs4Vb2GrGzEO5vtDbdtK5JH08KNuQ9Cjmc+2g0AAAAAwPwhRF06Rdm8UJey
XurU1kPCkracr3X0c5Hq2U3rq4B5oILBVWnqJUdZzqxle4faf1Qo1cT/rlhsR+14AAAAAADIGiHqMip
uytmqfi4dqZ0P9kbrNc75IVW1JdsV/XzhFGVzry/9vnpsS/aHkff+RpmXdmShKwdu8leuS9c+xj3ZzDJ
D7TVk3fijwsI7ascDAAAAAEAOCFGXVGW7JV6O2jxn9EY1e6FWpbW4CSqOot6h7OuncuoEPaQBA
AAAAEAuCFGXVkW2W26M6vdGbW/4Q6XL9a3wXoyqlqKuRek+wmpSjqyvaH59ZI1Jo6ZjoC7rqPX
GPu31edcGHd5fZI1J4zwEz5+5nbDPjxN+3INtCdS9tR4xy30ObGewraPOc7xjCm57uPZpYAh6c9VZF
2h4snM3eA7Uw9yc3Z6x+xzPPy71CLkXbAnaHvI9OHq7vqTHE6/dAAAAAAAcfYSoy6yyJXW3OGpz
VTYaDTnnJ6hyIWSM9FAIo3VqpQwmoCrK2hndwM6BdJ1nFmNId2dHLno7bcuubn/5zNpc9FKsnPa
D6gP/AGy9izv6PBo1Pe1wrCSDo63TPb8dqZUKUhrYSXM1aUimtjPY1qasDoZtEx2T2rZR+9S6H+te1
+kREu3HCSwHz4HSXB0dKibnnOtV70AUtW6Kc9TeGFHPNGS7E4vZbgAAAAAAlgQh6lILTjLVrLnBTF
nqF0Imk2pv+KGKV4+yL103ie3UZD3lhKW4dka3b18O3U23d42JhYxw0hjqferEnAz0rpz2yiY0d4M9
Ji/u6BisfEacDLUnjXX3GlSlpc9v3+0xnOr59bfvdUhWIdlAfdzx/O1494Hazrob/E1zTMbr9zZlc9v62PX
vV1Wv1/6cXXIi2X56jXUvsCzXu85r+8MlLiqR+4wr/BzteOl/krZbr3X/0hH2PRjY7rBkxzOu3QAAAAA
ALI+C9QtyXz/HklK9SwM9zlSwEhKq+K8rS71rTubTk8aK24tOBS/OZEYjX6963bk96Ubsy+dvW4Vde
9ZG1BBs1YOwWq1Ks9kcWu+3IaxdcdeNE387w+2yV3rnwG1/6DqbsV0VnO2FBNwB4e0wr3O11fc
nDEt0PSK2E9ivvuaS7JjMbQdfr41qa6JzF35+FOdanQqsS3p+lETnOlHbRwht44jjjDietO4RYBr3Pnyvv
OXCW/QSACAP99/xLnnNW2/SSwCAzB3/NZFjr9ILWBT0REVwkikVtoQGJP5Qeb/npMsYdm/2GE1F
UU6ccp51dJfTrj2WvywnT5+0e9QF11uqp/0AbA74vWmb4nVG7R44gZT1GW8ov7dOdT4sGbUo3S
BMfcIsazBr1jUo6ac28z7QPYSnOKZEvYkT7cec4f+kmIdQ3NyTvhmgTm3wHIWY6rrrurFuwJmaGO0
GMvDwEw/LOz/5Tr0EAMjDDY9fJXd/6AF5/Bs/0msAAJm7dI9I/zt6AYuCEBVBA6FScsO1P6fl1RVt7k
pbenJoj9k/JScqJ6z/D65XGeqc9ZIrrombLbpD+ttuIl09a/TozUtaAZl1DTJrOyHeSKpXqBe0GnVjgQX3x
NNPSOWPKvZHAEA+nvPjY/KWL94oP3ryGWm+/3555p8u6c8AALL1lMilP7A+PussYiEQoiJlGYRfXl3
RfTlsXxS7lKgd9lbEyVeN9XMZvhk9NO3A1+/VOyrwVcO67VqVQ480e0lOK1mv47yOaTHOXbjxbW/
Lhtnr1K2LatY5BRbUnZ+6Ux58/EG9BADIwxv/
+gZ5wQ+vsJ8/9NXvyp/WeR8GgNz0Hxa5/Od6AYuAEBUxleSkN1LbnBFfMSZJitM70Ri+HI+7744c7D
pf686+X7I/4a8fLjUwH4qbZ/0guOFOjFWVQIZacsoTKG6Jgvk22OvYvA/0seV1TIn2Y97LA8Pk1cz3hZ
xnoE/SdmNSNTtwdeukJv6eAubL+z77PvlM7zN6CQCQh1c+eK3c/PXn6yXH5z7+kHzlLx/RSwCAzF3
6gsjlr+kFzDtCVMRUlM2zbuVUc/Z1NbrYnOl8y+vt5wScijnre1s23JlrYvN7cqqJpBS3XmZRF0x117vh
6vxxe81a56Kmj3+wdqsx7F+aq7LhnTIV7Onh297K+dBcNQLH9nm/hqd7bHkdU6L9mLVbm7Lqre/p
me+ta1Qq+NvI2oTnyA9c3XYDi+kT+5+Q+n11vQQAyIOqg/pzD7xELwX94W9/Tb73raf1EgAgc5f/RK
T/fb2AeUaIivgq29LyctSalHTA48w8bynX5YJR4NOfUMnSXNWB0GQ1HAPbsp55Q/a9of6KMUnTHP
Jqu9rKUt8KRKiWomxe8IdlN1d1gOaFzqMm/ZolJ3AMttM8tryOKdl+ipsXpO6/2HmtOZGTdS/7h6Br
7yrua1NNWBO03bzfw9odR+bHA8R339fvk7v+7C69BADIw/FLBXsYv/oYRgWozd+8Xy8BADLXf1Lk
8sf1AuYZISoSqWyH11+stqz17tBiV3FT9gZfW21NVr/R7K0XKBlgDM3OdKKjFJgB2KiyA+qc9bt+wOe
ya1/OW03PstS7fT9Yt1Wl1d8LTpaV1zEl2k9RNvf60h16sXq5MUzeVpHt4EGK7B96PbFTEbvtVlv6Lf
8+sqlzblwHu+5ulByOB4jBnYn/6Wfp7QQAeVr50kvl2iev1Evh/uFL35ZP//6hXgIAZO6y9RvZJcpbzbt

CX81aAgAxtTcK4nSSVCHqQGAKADGo4PT1/+318sAjD+g1AIA8lB56od0LNa6tu8vyiteFD/sHAGTgit
8QKfBL9ryiJyoAAMiVGsJPgAoA+VK9T1Uv1CTUsH7qowJAji79AfVR5xghKgAAyM1HvvgRezIpAEB+
VP3Tt33hZSProI6iAtS7/+NX9RIAIHN2fdR79ALmDSEqAADIxe6Du/LBv/igXgIA5OWOL18/tg7qKPv/
8zH53Mcf0ksAgMxd/n9FLn1BL2CeUBMVAABkTg3fV3VQmUgKAPJ189efL//8Czfppclc+dzjsnX36+
XWV79IrwEAZOtKXR/1Zr2MeUBPVAAAkKknnn6CmfgBYAZe8MMrEk0kNcoz/3RJmu+/X3705DN6
DQAgW9b77aWPWx+fchYxFwhRAQBAZlRwqgLUh594WK8BAORB1T99yxdvlOf8OJ1f+R7/xo/k7g8
xKSAA5Kb/HZFL1EedJ4SoAAAgM+/77Pvk3ofv1UsAgLz83AMvkRsev0ovpeMrf/mI3PtJ/igGALm5/
DXr0dELmDVC1KXWlo1CQQrGY6XR05/LSa8hKxttveBob7jtWZG8mzM3Qs5LHL3GinPuVhoy6tT55
9d55H7NjwLuWyCW+n11+djffkwvAQDy8vJvPE9e+eC1eildn/zPB/LN//19vQQAyNylXZH+Y3oBs0S
IurR60lhZlaZecpTlzFpRP8+eHfiVasLfVIImPi+9hqzXor5KXfOCrAYvunRqJSlMENguK+5bIB7V+1T1Qg
UA5Ovqp47LG++bvg7qKKo+6u//
+y/bHwEAeVD1Uf+79fFZZxEzQ4i6tLpy4KZA5bp0+33p9/dkM68MdWzgt6QmPS+qZ+S4YK99XtxN
V1vqevelWy87K5qrQo4aA/ctEIuqf6rqoAIA8veWvRvtIDVLqifqH/721/QSACBz/UdFLv2xXsCsEKIuq
96h7OuncuqE5Nf/FGmL1zOyJ41zugtquS5bFedpcfOsVJ2n0twlRQUwPTUTf+WPKvZHAEC+Xvu1F8
vLHr1aL2Vrb/f/2A8AQE4uf8l5YGYIUZeQXbvRDN2aq04tx8GuiKp3o1E7c1T9zGAtyGCd1bDejbH3r
wVreI6qN+kMVfdfl6zW52A9S6+2qH6ENy3GPgPncEP8zZjnydln0vOiqHaWYvWMNHoeB0LzkpzUn
VFl/9A6olGM9obVW21v6GMZPAcJrkvI/RZ2vePcb4PXz35E1ImNY5b37WT3p2vSa2eZ6D1Ar1TMr
49uJI6QOz91pzz4+IN6CQCQFxWevubvXqSX8qF6o1IfFQBydOlPqI86Q4SoCDUUGGl2/cyRYVRHai
WjzqrR43EyanuDNTzVurCgpuQNVXdFt3UUZ5+DwWRzdcJ9Fjflgjtk3joz5/RGeo1z3nkq1y+kUEahK
q1+y+tVOsTseTxK50C6+umwipx2N97ZkYsDJ7W96x2NX1c3yXVRQV7I/eZcj1EBZPj9pu7d0GC5U5
NSIMjOSpb3bcz7M2CCa2eZ7D0Ay07VQP1M7zN6CQCQFzV8Xw3jz5uqi3r3h75KfVQAyI2qj3qP9Z
H6qLNAiLqEKtt96Xfr4kZ7Um3Z9TH72zrxbG/4AZBXL9Won9mpyfrIxEaFec7r+3uboWUCxu4/wN+
et3/pyI6XBPWkse4GPca+Wzo1imzrKP523M2ofdbOu/Fbsn0WNy+If+rOS9v677wbglnn94JOUJOd
F4P9um2ZLK8uyolT+ukYlS23beb5V9ri53BnxMnhkpwj67VGqYGh+21ofyZj2/b9ZrYlbFt+kJ3U/Ny3
/uvD789hya6dZar3ACyrT+x/wp6NHwCQv5UvvTTzOqijPPTV78qf1hmBAAC56T8scvnP9QLyRIiKIW
bPtPoFPwgdDgOHletbE4Z54aotPxwsrp3xAqzOge4z2bsoO14eaey7suW3deeiJIl7zH364ZMh8T6Ls
nnWTbuaslpwe08Gz+8kipt740PW2PblMOpEFdfkTNjxtXf9XrVn1pzjSXSOrPOzp0NEI3gPvd4Dhu43
s8et0evSPk86CNzLYfa0LO/bsfdnmCTXzjLNewCW031fv0/u+rO79BIAIE+vfPBaufnrz9dLs/G5jz8kX/
nLR/QSACBzl74gcpkJ/vJGiIoBI3qm2Yqy5iYxIwK3UyfSDKjKcrKkn47SPdC9+VSoU/LqNhbMYdKRw9
QnMMk+K9tGr0FHOsP403RKoi+fcf2NgNIP3Kpy1j2gqa6LruEZOrw/aOh+M8JCawf20Hd33yNrsaZ
uHu/bBNduyvcALB93Jv6nn31arwEA5OWGx6+Sn3vgJXpptu7+0APy+Dd+pJcAAJlTw/r739ELyAMh
KibUkeHOgTHCo7mX3TFUvMKUSrD+5Oz05HBssVSf36vSHRZuBG7V08FeoUmYExB5PXXHCbtWR
dm8EN470w0rF3t+o8nvz/SvXdh7AJaNCk5VgPrID+h9BAB5e86Pj8lbvnijHL9U0Gtm60dPPiPN99+v
lwAA2XtK5NIf6OfIAyEqJjR/gWm53vWGbAcfk9YLHS/
+Po26n7aO1NZznJyneELGlj4tn5Sxl7S4KW5lAntYuDF8vno6/CyPP0dt2TB7nbo1OM36o0lYbdzT+xj
s/as0z83XpEi53bcTXLtoR+GPJpiWGsL/wCMP6CUAQJ7e+Nc3yAt+eIVemg+qPuqnf/9QLwEAMqfq
o15iYte8EKJiQElOusnV0EzePbnoFnIcO/Q7J6WTXtA2qm5m6ibYZ6+xrodpl6Ved5OsPCfnMa5rc1f8
zphdOfAu6QmJc0m9HrXW/XH+/I4OP6sSyOGSnCOzLqcKFN26qMaQ96TUzPKqx6k9GZQdSHa9W
p7pDpOf0CzuW0usa5fme8AU1xDz7yNf/Ig9mRQAIH+lh1448zqoo3z6v/6D/MOXvq2XAACZu/xZkf
48dRU6ughRMcCcBCnYW9IPAlXYNTChz6yYNTCbq/5Q7faGHhpuPdIev510n72GrLsnrnpWNjeNyY
Nq65JPjmrWsmzKrm5er3HOCzBj90asnBbnDulIs+ke18Bw8Amvix8oDvbcjUlvX80sH+xxaoTFZo9b
oz1p3yaRZnHfKnGu3QTvASU/dZXaee9gZMOb4h9Hze6Du/LBv/igXgIA5OnaJ6+0Z+OfZ83fvF+
+9y1qZQNAbtSw/v739QKyQoiKYeYkSJ2alHSoU/LTE7kw7YxI5vDy5uoUoVGwBmZzVQdQXnhTlVZ
qs9e7kuyzJ411d6h6Wepbav3okCq98zLMnFndbbN3TastiX+aKhIo72oZDmATnCMv2LO4x2xOsJTEi

HvXrLNaPevPNp/YQt+3SpxrZ0n4HuDXW7V41zBubVssGjV8/85P3amXAAB5UvVP3/aFl81NHdRRV
ICqglQAQE76T4pc/rheQFYIURHKHgYdUpOy2rLWu8Otp1KR7cGClfuHRs/BBOwamMZwbZddWzOj
eqgx92n23LN7obonbiCk8of1p3hehhRlc2+4Tqg9hD5hYBeYJMs6ZjsbHhT7uljH3G/5QaqtKq2+0dZ
ACYJo9r07eJA2Z5vTZZMLft9aYl07S6L3AHUsg6+ttiava4u59cTTTzATPwDMkOqBqnqiLgI1pJ/6qAC
Qo8vWb6bUR81Uoa8KBgJAEmo2fT0ZlAph96btmTxL+lhOtaYNWBfEUbp2yJUKTit/VJF7H75XrwE
A5EnVQVWTSS2SK597XLbufr3c+uoX6TUAgGxdKXLFb4gUbtbLSBM9UQEkFCxRcGZtsUO49nnrWC
J6ZB4tR+vaIV/v+
+z7CFABYEbULPx3fPl6vbQ4nvmnS9J8//3yoyef0WsAANmy3m8vqWH9TzmLSBUhKoCY2rJh17o06
pWaJQoWUK+xIqv7demmUqJinh29a4d81e+ry8f+9mN6CQCQJ1X/9C1fvFGe8+PF/NXt8W/8SO7+
0AN6CQCQuf53RC7doxeQJkJUAJNRNS8XfPx7cXMvpRq/C+YIXDvkR/U+Vb1QAQCz8XMPvERueP
wqvbSYvvKXj8jnPv6QXgIAZO7y10QufUEvIC3URAUAAKEefuJhec32a+wJpQAA+bv568+Xf/6Fm/TS
YqM+KgDkjfqoaaMnKgAAGKKCUzWRFAEqAMyGqoO6aBNJRVH1Ue/
+0FftjwCAPKj6qGpY/7POIqZGiAoAAIbc+ak75cHHH9RLAIC8qQB1UeugjvLN//19+cPf/ppeAgBkrv+
oyKU/1guYFiEqAAAIUDVQP9P7jF4CAOTttV97sbzs0av10tGyt/t/7AcAICeXv+Q8MDVCVCTUk8aKm
uVbPTaknWjdOGltZ96MOYZeQ1Y2gmvbG+7rV6TR0yunpfZjb3NEO2zTne9M2m1bkPugvaHbWJC
BSwosjE/sf8KejR8AMBsqPH3N3x3tuqGqN6rqlQoAyMmlPxHpP6YXMClCVGCGeo0VKZRq0tHLWe
pd3DH205RdQj4AA+77+n1y15/dpZcAAHm7+qnj8pa9G/XS0aXqov7+v/8y9VEBIDeqPup/tz5SH3U
ahKhIqCibe33p99VjWyp6bXby3l8WRhxDryHrtTziU6UnF3eC+2qea1hrF8VRuA+A+aZm4n/nJ98pT
z/7tF4DAMibClBVkLoMVE/UT/7nA70EAMicXR91Vy9gEoSowDLoXRQ3Qy2Xy86Tzo5cXJwUdTFUt
qXfrYs+w8DCUMGpClAf+cEjeg0AIG8//ffX2UP5l8m9n3xYvvKX/NsDALm5vGc9mOBvUoSoS8eoLbl
i9kQctd6oc2mvz7s25fD+IutuGnU/VwKfNLcT9vko6Z8z+/PmMP7mqvP5EYU0/WNWj+T1Rv2h/FU
5e+GMDvk6shMnRQ3UUg17jG5PvHbHuTbD59DaumyYrzfqkQa2MbA+/BTHaYN5POpY/P2rh7vd
9nnrupbrsjXQXdYu3WC83n4M3DfArKgh/A888oBeAgDk7YbHr7Ink1pGd3/oAXn8Gz/SSwCAzF26R
6T/Hb2AJAhRl05R1s64PREPpOs8s3TlwE30Aj0U27LbdJ6Vz6xZXz17ldNV/awjB/4B2PywsCxn1nRr7
RCwJIMj5zu1UswQa5bnrCO1UkFW9fYcal2SINUYyl89LZXimniHs3Nx+hCvfEbcU+2L2e6pr43Dfn1w
Z846FVQOrG+uDgSpE7VBHcuqeFvWoakKSlf369Ld2wxcdxW+lsJKN3RqUprnibKwFD7yxY/Yk0kBA
GbjOT8+Jm/54o1y/FJBr1kuP3ryGWm+/37qowJAbp4SufQH1kfqoyZFiLqEimtuT8R9OXQTovauHwi
JEU72Dq1XOU6dmIcI1VI5LW6M2gzMjmSEhV6w15PGutvjsyotu6am9WjpLXRqsh4jjUz7nFW2rTa
Yw76rLadd22HVPv12d+vuV8TsRaoYQ/mrp9X2zVA4xpD+4qbsuefNfnTFa4Z1BPULwcDQN67d6V
wbV7neDX69Frbev2+maYPxeh2aFjf3vOc+P1RXYWtXf41/TppyLsFxAmnafXBXPvgXH9RLAIBZeONf
3yAv+OEVemk5PfTV78qf1h/USwCAzPUfFrn853oBcRGiLiOvJ6IfaPUOndivWnXCo32dFJrDwO38bS
5UZMsNoJq7fi8+s+6n2wPUXFff8ickqmx5QWCs3pgzPGfVlj+Rkh/mWi0Z7IY7gtk792TJfmJsJ0EYq/
Ua636vzepZ2QxPUMe3O61rY6vKWbchRsg+cv3+obPdKdoQeH0UI1Q3Q2s7cNWB6t6okwhkSA3f
v/NTd+olAMAsvPLBa+Xmrz9fLy23z338Idn/n4/pJQBA5i59QeQyf8BKghB1KRXlxCnnmRtode1x6W
U5efqkHXYF11vUMHDn2VzwQ7mmeJ0KuwdeWOgN5ffWWcfkDu+2H8bw7cAQ/VFmdc784HMy
Yb1zLRMP6W/LeT9BlVZoz1klRrtTuzaW8kkJ3d2o9a4p2hC7Z7Zxrq2N2WUO3P3Er8sLpOuJp59gJn
4AmLGXfPe58nMPvEQvQbn7P35Vvvct/m0CgNxcVvVRv68XMA4h6pLy6oraPTl74nSqPCUnKies/w
+uV3ngPEWoFiOYcodmt90x0xG9I6exkOfM6GmphqaXQkPC+LP0tzf8OqBVo6fp8kkSbhdl80L4jP1u
cDtiPjEgE+5M/A8/8bBeAwDIm6p/usx1UEdRAWrzN+/XSwCAzPWfFLn8cb2AcQhRl5U3tHlfDts6aL
N77VXEyQqN9VP3hsyCUdfTDi/9upOjwkuvNubQI2YYuIDnzB/KHyXmkP72hj9RVLUlIzuhTmDqa5O
CTNtg1JUdKNlqa55jln7k532ffZ/c+/C9egkAMAsrX3qpXPvklXoJpn/40rfl079/qJcAAJm7bP02eukze
gFRCFGXVklO2hlkRw52nSHNbh3Rkv0Jf3347OuzV9w864eaDXeSp4E6pCVnqL3i1eKc2KKdM2Mo/
xjjh/S3ZcNLUMtSV1PRTyvVazOhHNugZuhXPU7tScXsQNWYoCtu2QJgSvX76vKxv/2YXgIAzELpoRfa
D4z26f/6D3aYCgDIyeXPivTp2jMOIerS8ntyNptOObWeCzq4p/uem+Sprnj9gDtSK2mA77BOqRmP

crmqj9sur3h1aUsxB5LvWDnzBzK787+H3iYIV70kP5e45w3jL9cv5BOuYRUr82E8miD3pbKoIM9Trvi
ls8dW7sVSMn/+v/
+l34GAJgF1ftU9ULFeHd/6AH9DACQi0v36CcYhRB1iZkzpqvehd7w88Ds5sYkTXPIq1NqC+shGaxH2
VzVwZg/Ll1GT440LNVzVtS1VJXmauqhoTmUvxxaW8AoiWC9cuSQ/l5D1r0CqtYrAxMwOY/JJkhK99
pMJoc2VLb9IfyBurRGfdmzm7MP3bEU7v7Fu+WOl9+hlwAAeXvjX99AHdSYfv7dP6WfAQByUXijfoJ
RCFGXWWDW8FPiTzbuDltXzPVzyAwvRw2ht+tRGr0uXeW6dJPWu0z1nFVke7BA5v5hSrUxzaH8o0
NdMxQeNaQ/Xl3VCaV5bSaVQxvsIfxhxVBVSNvvp1pfFohy1RVXyf/4lf8ht1x3i14DAMjT373yCf0M
UV7z1pvkbe+5VS8BADJ37FUix9+kFzBKoa/G9QIAgKXxwCMPyOv/2+vtmfoBAPm648vXyysfvFYvYd
D1P/E8+a1PvVGedw0TbwFALgovFrni/daTq51ljERPVAAAlsztN91uD+0HAOTvb27/tjx2PX/ECnPlc4
9L9fdeS4AKALmx3m+Pv8f6SIAaByEqAABL6F2n3iUfeMMH9BIAIC+Xjvfl8294VH78nMt6DVz/8t+V
5NZXv0gvAQAyd+ztIoWb9QLGmSpE7TVW9AQpBX9W65F60lhxJ1TZkODL27LhTbbiPCabqGZYeyO
43dBH1jOAAwAwhz788x+W07ed1ksAgLz84PnPyt7rvqWXoLzidS+R1X8bNhkqACATx4oix39eLyCO
OeiJqsJVf5ZqR84zwquZ0VcaKU3oAwDA4rjnl+
+xh/cDAPL10M0/kO6t39dLy+3al14l1d99rV4CAGSucI3IMTWMH0nkGKIWZXOvL2oeq35gxuuuHH
iTiKvZsNXn92QzxwzV1qnJeTqkAgCWjDtj/3VXXafXAADyonqjfu+aZ/TS8lIBqgpSAQA5Of5ukcIL9Qi
mn1P1N6h7OuncuqEZJedlqXedUNc/9Gtl/XnRZq7pKgAgAOVzy3W32EGqClQBAPlR4VE/96aZ2TS
S/8xivsofwAgJzYdVDz7rl4NOQYog7XRLXrlZZq4nZEtYfVD9UoNb/OeaRVL1Uprp0RP0YFAGA5vfm
WN8tH3/5RvQQAyIvqibqs9VFVePoLv35CLwEAMmfXQX2HXkBSc1ATNUKvISuFktS8lNXRqZVSq2H
aPu+HuNXTfpGB5WVM8uWd46hJwTA7YddqEYTfT/4kcHneY0nPYU7fC+q9b2DCO//8rEiKf0cCAt7
7s+
+V2h01vQQAyIuqjbps9VGfd82VctfvUJMbAPJztcixO/VzTGKmIWpluy/9bt3vCVptOcPst1WY2ZPGu
htwVqXlDsFvVe01qobpeqIkoSO1khtC+I9Vd0Yra9/2bgFghnqNlWAPfSBnqjeq6pUKAMjXfT/zuD1r/
7JQAer1P/E8vQQAyNzxO0UKL9YLmMT89kTtXZQdnSKU61v+RFSVLXHLmHZ2LqbU+64s9S0S1NFG
TQoGTGKR76eM295ryPpg13tgBlR9VFUnFQCQnx8/57J8/g2PLkV91Le951Z5zVtv0ksAgMwdf5PIsV
fpBUxqfkPU7oHXE8sevu/1HjWG93cOpKufTsfppZpmrVUAABaVmqm//attZuwHgJw9dv3T8je3f1sv
HU23vvpF8ku12/QSACBzhVtEjv0LvYBpzHdN1FSFz85vlhPo1M7nWItxHgxO2jWqxuPoOpD20GPv6
/VjZG3Jwf1FBNd2Pdzga0fVgsysDVEya98oca+VK/5xJm1f2OsHyncO1O806o7arx19P/kG2p/K9Ux6
DsOEt32wXungORo8P2HiTbTn8/epHqPqpCY5P0DQbdffJvf88j16CQCQl7+/7Xvyjy//oV46WlQd1O
rvvVaufO5xvQYAkK2rRY6/2/p4hbOIqSxEiFqud4fDT/uRwnDa4qac1WVWRfblcGnyBRVsDU7a1ZTV
wqr1/3hUiFMKG3rcqUlpMKBKMklYe2NETUjVYzgYFmXWhihZtW+khNcqwXEma58TyIW9vrlaGBH
2qXNitLNcl/GVM9SxDbR/6us5/f0ej9OrffAcNVdHhZyTcPbh1XO2Dd97qd3vWGrvKL6DGfsBYAb+6v
WPHcn6qL/yH15JHVQAyNPx09RBTdH8hqilk34P0YN0Bu2Ha8tuuinKQug1zvnhkTuhV7/r1Zsdzzhv
5bp0dbDd9TbQlHNeotNLMEmY9dpzesOh2+3IzkX3tVm1IUpW7Rst2bVKcpzJ2tdrrHuBnP+HjZa1F
615bkRQaLRjb1OKem0koz1u06e5ntPf70n47fHb3pHa+ejIPHqivUH+PsLvvbTud0Ds2frfdepdegkAk
AdVH1UFqUfJyumftB8AgJwce53zQGpSC1HtnmjGkFHzEWco65DimpzxspxVfxuqF6C77UQbdnpv
me1yHmYvuTOyFivhWXQ9uejO2qUCFi+kKcrmBSPEidI7lH39VDo74mY3xc09J6yxHnub+mQmmiT
MmLjHCNyKa2eGQ/XM2hAlo/aNlPBaJTnORO0LtuOst74iW/aGVVC3J2GHE2hHTNWz/rmtbPtBrd
/2JNczhfs9gWrL7yFf2Up/+4q5j/B7L8+J+bAM7v7Fu+WOl9+hlwAAefjmjU/JV376u3ppsb3sp14o/
+a3mNAEAHJTuFHk+L/WC0jLHA/nDwYcXkjrjWE1w5B0mMHN0daVAzdTKp+Ukn5qK56QU/ppJDP
kHgioh2ouTjVJmK6nGTZ8Prc2REmpfSMlvFZJjjNR+0a3wwldR5fWOHUi6XdVVU4HNlaSk2473bYn
up4p3O9zpSwnAwcRIteJ+bAMrrriKmbsB4AZuP9V37HD1EWm6p/+
+n/5GeqgAkBurhQ5/mvWR+qgpm2+a6IWN2UvbMitPdQ3hXqoHtWLri8pZ7JH3OhefG5oM1EPZ
MWu5eiGPlE1KzNsQ5R5b19sebQvRuA3VlFOLEzSmcbxAvPpphfcZAepKlAFAOTn8yuPylNXX9JLi0f
1QFU9UQEAOTn+r0QKN+gFpGmqENUc9hv1cMLJoj8M2gxA7aBUrw9NMc2v04+4dRUtdp1B82tD

H2kGskvEuHZ+7Udf89zw5DXjJwlry4bZq9OtjWnWijRl0oYo2bcvLbGOc4btG21wgreeHHp1B4ZlOv
HcEcD5QZpuv+l2e2g/ACA/KkBVQeoiel3l/6IOKgDk6dhrrQd1ULMy3z1RkZGQ4dEus05mDGp2d9V
b0Q+rjZ7D7raTTBLW3vV6dtrhjxuYG8OTB6XehihZtW+khNdqguOM176Idth1itOdfT7Y9JDh+ImO
M737fWHkNjEflpGaZOoDb/iAXgIA5EEN6f+7Vz6hlxaDmoWfOqgAkCM1C7/qhYrMEKIupaKs+bN2
yao3Ztuc0XsMPcGXKlEb7K0YEnhNOEmYH/4YM+KbcmhDlFTbN1LCa5XkOBO1L6Id9rE7NVUTnsK
Rmqsb4jfdL5lQPrPmhNaJrmcK9/uiyeB+B0wf/vkPy+nbTuslAEAeVH3Ux65/Wi/NN1X/tPp7r5XnXX
OlXgMAyJaqg/oe6+PVziIyQYi6pIqbF/zehs1VHawYk86MU9n2h393alJygxmjRqg/UVew9mbkJGGV
09aSNq5dWbUhSmbtGy3ZtUpyrpO1b2w7ynXZSmV8eFnK5aas6vZ4Tbfaftab/j/Z9Zz6fs+LOdGV28
6Jws6U7ncgwj2/fI89vB8AkI9Lx/vy+Tc8Kj9+zmW9Zn79Uu02ufXVL9JLAIDMHVsTKdysF5AVQtSlVZ
TNvcFJu6rS6res/8djD/8OK6Rpb2dgoi679ubg/ixDk4RVZHuoDc72vF01d71eitm0IUqG7Rsp4bVKcJ
zJ2qfa0Zfu0IbVpo3SBlM7JWcHjzfsGiW6ntPf7/mw7q/B67F/aPQSTiCV+x0YzZ2x/7qrrtNrAABZ+8H
zn5W/ev1jemk+veatN8nb3nOrXgIAZO7Yq0SOv0kvIEuFviqCCAAAMIF7H75XKn9UkaefXYwhpgBw
FNzx5evllQ9eq5fmx7UvvUr+06ffzDB+AMhL4RqRK9R8BQzjzwM9UQEAwMTefMub5aNv/6heAgD
k4W9u/7Z875pn9NL8qP4udVABIFfH3239jwA1L4SoAABgKu/92fdK7Y6aXgIAZE3VR/3cm75pf5wX
v/Abr5BXvO4legkAkLljbxcppFNUD/EQogIAgKmp3qiqVyoAIB+qJ+re676ll2ZLhae/8Osn9BIAIHPHiiL
H36EXkBdCVAAAkAo10dQt192ilwAAWeve+n37MUuqDqoaxg8AyImqg3rsPXoBeSJEBQAAqVAz9b
d/tc2M/QCQI9UbdZb1Ue/6T6+2g1QAQE6O3SlSeKFeQJ4IUQEAQGpuu/42ueeX79FLAICsqbqon3
/DozOpj/q299wqp/7ZDXoJAJC5428SOXabXkDeCFEBAECq3lF8BzP2A0COvv2if7Jn7M/Tra9+kfxSjV
/kASA3hVtEjv0LvYBZIEQFcteWjUJBCsZjpdHTn1tSvYasbLT1gqO94Z6fFZnX09NrrDhtXGnIqCb6x+E8
lv5aY2mo2frfdepdegkAkLW/v+178o8v/6FeytbzrrlSqr/3Wrnyucf1GgBAtq4WOf5u6+MVziJmghAV
yFVPGiur0tRLjrKcWSvq58vHDiJLNeno5YXRa8h6LarV6loXZDV4saVTK0lhIDAGjqq7f/FuuePld+glAE
DW/ur1j8kPnv+sXsrOXb9zu1z/E8/TSwCAzB1XdVBfrBcwK4SoQK66cuDmbuW6dPt96ff3ZHNZM9
SxQeScUj1nxwW/7fPiHlq1pa5zX7r1srOiuSrkqFgGV11xFTP2A0COfvycy5nXR33zr9wir3nrTXoJAJC
5YyvW41V6AbNEiArkqXco+/qpnDohy9IiWqs5Tae6SomI53PSC
m+wgVQWqAIDsPXb903L/q76jl9L1sp96ofzKfziplwAAmSvcKHL8tF7ArBGiLjGvnqP5CKntOLI2peqN
536d163Or/dp135sb+ivNdbZLwuuD/bKS2MbmtlG7zFcYzN4jMM1S+1HWN1Low3jal3a+zDDt+aq
87WDDQ9pc9i2o9o8tpfjROdl+J4ZuZ8YxxD7fGh+W9RjuK0OZwi9/7p0z506/lKsnrNGj+NAWF6Sk7
ozquwfDt9PNqM9ie65eMduS+H7wj1Pcd9HsNxuv+l2e2g/ACAff/fKJ+SbNz6ll9Kh6p/+
+n/5GeqgAkBurhQ5/mvWR+qgzgtC1CWlwpHQMKhTk1JhQ8ZlcHHYtR8HCkLa61TIMrC+uRoeXk2
1DRU2hfYY7EitNCqEU58zapaW61J3uw52duTiYMi0674ynbqmQ8GiZh/zyGBquM1uz8dQE5+X4X
umuToieEt8DFGcfQcvd0hb7WCw5A2hd6V67jxVafVbXq/SIWaP41E6B9LVT4MqcjrpPZfk2FP6vlDn
KY/3ERwdapKpD7zhA3oJAJC1z688Kk9dfUkvTe/f/Nar7J6oAICcHP9XIoUb9ALmASHqUmqLn8Ln8O4d
TmNeo3SlHPhSUpi5XrX3na/FYybwtaPGt482TaCw6mHj7EjO4PplEcFZM7r+3ubsrlVF+erBr/GPI9nZ
FyGWtm2ttd1t2Wptpx9bOvUrr3hB4Vhbe7UZH3kdQm2eXRT0jkv/invSO28cd0SHMPY8xHg7zu8r
dZxrbvBoHEu3Iamcu40u53bEitrHVKUE6f00wiVRPdckmNP7/uimOP7CI6OD//8h+X0bQxHAoA8q
ABVBalpWDn9k/YDAJCTY69zHpgrhKjLyOwOwBVW1jnmtyCWpjXtcAzlx2qpy1i6jVQeqlNqY6FcWxEcRJPMEq0Uw74Ey0w0GDH3d8yWm9UapB4Xkj0fy4eb
aKsrlnBj6O4toZL7TrHIT3AyzXt4IBWXFNzugv6uxc9NvY3vV65pXPrI0P38YwexjWLxht3rwgfgZ5PrR3
31CbR5r8vFRbfnDoh3xB0xxDFHPfoW3tXZQd3SEycC4qW/5+zWtniH/u1HFY3yOhIe8k9uUwrEFKk
nsu0bGn+H2R2/sIjpp7fvkee3g/ACB7akj/V376u3ppMqr3qeqFCgDIiV0H9V/rBcwTQtRtRlZAQ0ImqY
bsGrZTiyhuIkyieIpJ8GjFofJo1t2HQ9x9BhzEGnTgwGP0VZ8xMtLyzyA0Mj0J1YVK9WY/8jgrfhNscV/7
yMN90xjFaWk+MudvfAa79X7sF+GEPcRwyfn/zcTeuUjN51gntuimNPcv2HzlNe7yM4ctwZ+6+76jq
9BgCQJTXJ1KT1UVX907t+59XUQQWA3Kg6qHdaH6mDOo8IUGRIUZdSUTYvhPckdEOYqMl1FkZg8hyjn
mOk8MDO76nnDnc2AsPq6dg9GafXkeGOgjFCRlOK52UyYccwK2keVxw9ORxbLNWR2T2X2vVfkvc
RZOKW625hxn4AyNFfvf4x+fFzLuul+H6pdpvc+uoX6SUAQOaOrYkUbtYLmDeEqHuqClCl7esjtQKlQR

W/Pcos+u3ZYNs3edW7PRrMGZhHW+zurzZA+NNoYyV0/nF6FOH/qlfF4mkm1w6dXKHXpMWsd0
AsUTMrb06bje1BPcc+OPPf3vi6P9PoIsvfmWN8tH3/5RvQQAyNIPnv+sHaQm8Zq33iRve8+tegkAkL
ljrxI5/ia9gHlEiLrE1MzaqqeYPcGPHYR0vRqKo4f/GoxhxHPHrB2pgiW3/uMUba64U6Z3duT8+R29n
aqkk6GW5KR37gdnZO/JRbfoZeQQ8BgyOC+
+nI4hTOmkFwKOqumZL+NcNHfF75DZlQPvNJxwzn2EWPdckmPP4PpP/T6Cpfben32v1O6o6SUAQ
Jb+8eU/lL+/7Xt6Kdr1P/E8uet3qF8NALkpvFgP48c8I0RdRu0Ne6itmkU92FPMCHiMXnIlPxkzZmJvy
4Y3Dft884MlY2bySXgTWHWk2dQnKrWh/EXZdLsdqvO87l+XXmPdq22ZZBKkcVI7L578j8Fj1udsrvr
DyPW9bj9yHVtu1oBtyq7eda9xzgsxY/VgjnPPTXjsU1//hO8jZntyvRSYe6o3quqVCgDI3t/c/m157Pqn
9VI4Vf+0+nuvleddc6VeAwDIlqqD+h7r49XOIuYWIeoyqmz7Q287NSm5QYtRH7F6NnzmbhXSDL52
Lpkz9nttNibamUhF3I6BrlSH8o+4LiU/fZQL005glcl5MSQ9BnPYu9ueiRK2YH3O5qqz34IX9Felldqs+v
EUNy94PTLd9njnodqSeM2Jc88lOPY0r3/C9xEgiqqPquqkAgCydel4Xz7/hkcj66NW/u8idVABIE/H3k
od1AVBiLqk7KG3YUUMVeDS7wcDHlX3cLBmYrWVcx3NpCqy3W/5gZHNOTbvsAPDrOPxhlcr5bpsp
ZzL2dcl5LxWW9Z6d+j1VLI5L6Zkx2C1Z/A+3D80ejUmYNfnNIaSu+y6nznWQ/UUZXNvuFaoPYw+Q
aAb656LfezpXv9E7yNABDVTf/tX28zYDwA5UPVR7/uZx/VS0Cte9xL5hV8/oZcAAJk7Zv2GfPwdegHz
rtBXRewAxKNmNtcT86gwbG/anqHAOEfpntPHcqpFwIpwn+l9Rip/xM0BAHl441/fIKWHXqiXRK596
VXyW3/8z+yPAIAcFK4ROf5+66P/Xoz5Rk9UILaeNNbdmc3LcmaNABVZO1r3XPu8dSwZ9ODG0fGO
4juYsR8AcrL3um/J9655Ri+JVH/3tQSoAJCn4+8mQF0w9EQFxmrLxmANWFXOgK50yMzRu+d6jRU
p7ZyRbiplKXDUFf6fgn4GAMjStU9eKe9s/aS88723MYwfAPJ07O0M419AhKjAWAOBFgEqMsc9h+
WlJiiblx9NaEs42hKOtoSjLeHmqS0nrr1GDr/3pF6aLa5RONoSjraEoy3haAvSQIgKAADmBj/ghqMt4
WhLONoSjraEoy3haEs42hKOtoSjLeHmqS1IhpqoAAAAAAAABCBEBUAAAAAAAAIhCiAgAAAAA
AAEAEQlTkSE2WU7DrfxRWGtLTa8fqNWRlo60XHO0NvZ3CijRibwjp6Uljxb0GG9aVTbJuGmH3UNr
7mJbRRv1Y4SYFAAAAAGChEaJirvUaK1Io1aSjl4H5pgJdY1Z9W1nOrBX1cwAAAAAAsIgIUTG/eg1Zrx
GfzqeibO717RkF+/1tqei1szFPbenKgXvLluvStdu0J5tkqAAAAAAALDRCVABIS+9Q9vVTOXVCyE4BA
AAAADgaCFGXmao1OlC7MazG6GD9UXuIvfE1A+VKNbNOpXokq1Vp79Mcxt9cdbYTvjOjjeoxqk7qY
JvCa1VOdrymMbVf2xvetob2H3JN4rTRY359ZEONcxFo46j1xj7t9ea5zKoOadx7KKotg9uYsD5pjOuS
9J4FAAAAAACLgxB1WakgL7TWaEdqpVEhpPpcQUoDQ+ybq4OvVyFiSYIva8pqYbBWZBqcNq0GNh
xyDHYINtgm65W1UnjQaYt7vIMqcrqqn3Z25OLAa9u7bmODtTKHQjgtuo2TKsrambLztHMgXeeZxR
iOHmh7W9xml8+s5dDDMoV7aKJrPizf6wIAAAAAAOYRIepS6knjnJuIuXUb+9Kt61BNOrIzmPx5qtL
Sr2+5QaEKG8/7ve16jXN+0FVt6VqVXfE2H0Nl2/qabl28L3G3sx1W8dJvU/gxWMe77oZg/mv77gF0
arI+MhUdf7xhKltu2wfPpR9GSvmMeBlqe8MPgsOuSWQbJ1NcO6PbuC+H7qbbu0ZI2ZEDN101hq
mfOpF9hDr9PTTNNTckuC7J7lkAAAAAALBICFGXkjERz96m16vQD9VEOl56FlRt+RP3+EGhqScXd9w
+e1VpeQGStc8LYa+fntmm0GPoXRS3SeX6lj/xUGXLC+U6OxdDexSOP94RimvidfQ0t22ElGaPTrN3av
2CcU02L/htrJ0fGKo+Ja+NftDbO3Si0mrVCRv3dbrau7jjBZKnvROYlRTuoSmuuWkm1wUAAAAAAM
wdQlRYdA3P0OH9SZmzk5+Ukn5qK56QU/ppespyMrCTEN0D77jsIdheXUtjqHdgSHsazOHy/rB4P5
SryllvyvYRvVNtxnbMHqOpKMoJfUHcwLlrXzzrnJ4+aYeVwfWW6mk/kMxMCvdQKtd8VtcFAAAAAA
DMG0LUZRWYKCdunckYgeWRMt3x+r1i3Z6eRig3URhpDK9PScUt3trctVrXE6cj6ik5UdFhZWC9anb
2EeriSf+6AAAAAACA+UKIupTasmH2OnVrPZr1HI+ocr3rlDEYevjD9lNT3JSzbglONXTcqCs6WRiZQYh
dOS164L4ctvUQeLv3pzs5lrF+QUP07K/5sv1xAQAAAACA5UOIuozMupwqYHLrohpDoCdXkpNuEjs
4XNoIEXNVcoamK6NqvWbF6+nZ2ZHz50fVFTXP2eBs/mZ90FMydk6nxNfQ3XdHDnadr3VrtZbsT/jr
h4e0ZyWFeyiVa57idQEAAAAAAAuNEHXJ+QGTMWP/VMxakU1Z3XCn3DFnS8+ZMcmTNFfFa1J7w
6+V6a1MmdfTsyPNpj76oaH8Rdl0u6xar6utN6yz5eg11r0anuYESU7AqVivP+8dkGx4U8nH5V+vZtP
5Wnf2/aIumOquNyfCylYK91Aq1zz5dQEAAAAAAEcTIeoy8oI9S3NVh0rGhDtTMmcun2r75iRC7nY
mCjuDs7o3V3WI5gWO5gzwaXOHxftCh/JXtqXl5XU1KdnnrCAlP6mTC95EVCojdOutWrxzHLe2bVBg
W9Yzb2i6eZ9Y68/k0w3VNv09lNI1T3hdAAAAAADA0USIupQqst1vGQGZUpVWv+8HRvaEQpMqyu

Ze1w/BbGr7g/scx2qn1yBt/9DrDZhIcVP2+oNtstj1YDOoh2rwhvQr1v62Ruysst0PrUtbbVnr3ZILLnU
8g6+ttiara2v22gwMTTeGs+c+ZD2Feyila57ougAAAAAAgCOp0FczrADITq8hK3oiL1WDdo+eiwAwk
urtPS8/mtCWcLQlHG0JR1vC0ZZwtCUcbQlHW8LRlnC0BWmgJyqQKbOOZ75D4gEAAAAAAJAOQlQ
gE23ZKAzU8ayeFTqhAgAAAAAALB5CVCAPql5pZpNXAQAAAAAAIEvURAUAAHODelXhaEs42hKOto
SjLeFoSzjaEo62hKMt4WhLuHlqC5KhJyoAAAAAAAAARCBEBQAAAAAAAIAIDOcHAAAAAAAAAgAj0R
EV2eg1Z2WjrhVnrSWNFzZavHhsyL60CAAAAAAADA/CNERSZ6jRUplGrS0csAAAAAAADAoiJERfp6D
VmvEZ8CAAAAAADgaCBEBQAAAAAAAIAIhKhLqr3h1gddkUZPD7+3l51HaClTVePUeI3zcL7eZW/XH
MbfXHVepzc4uF+PuW1j58HXt2XDfY318F4Wo12TGjwv6jHpuVGSHr8S1obCSkPCD8+s/eo8VkaciG
TbBQAAAAAAWF6EqEuvI7VSQUoDw+
+bqwMhX3tjRI1T9fXpBJbR1H5WpamXpFyXrYr1MbN2OWHk4HlRmquFYNCZ4blRoWtYG6RTk9Lg
BFl2EFuSwZd3aqWhcDTRdgEAAAAAAJYcISosVWn1+9K3Hq2qXqUCwPNulNaTxjkdX5br0tWv7dbL
zjrrtTsXnYiusm19rlsX9zNSbdmv7W+rxHNafjv7e5tSTNCupHqNdS+MLNe7zj77LasFWvOcDkeza4NI
W3bd1Dh020055yW0VjvW3SDXOE/uBe3UZN17bZLtAgAAAAAAgBAVUm1tixtxVraMANRTlM09H
crZ4aVeu3bGe23noKufZadc3/La6ciqXT25uOP20qzK2U13yxXZsoNGFVLuibM6w3PTO5R9/VQ6O+
JmscXNPWd/1mPPbVvvorhNDpynypa42Whn56J1ZJYk2wUAAAAAAAAhKiaha5OGDmHPzqkT44K
9tNrVlQN3A+WTUtJPFSdo9EPnYSmem+KanHGTWGtrquyCW7t0qM5p98Dbnz18X7+uYA7v7xxYR
2ZJsl0AAAAAAAAQoqIsJ82UcJTAxElGbdLcjGjnzNtlyawNRdm8ENYz2A9KQye5Giur7QIAAAAAABx
NhKiIoS0bZs9Kt46mWft0JuahXRm3obgpe3qIvV+v1tc8Nzybvl/DdfBh9KCdYLsAgLzp0Q3Ggz9yAQ
AAALNBiIrx2rte70o7oHNrfxpDyKc2ybYya1dJTroJqDsE3qVm4i8YM+6n1YaI16uZ9NUvzfakXXbw2f
XqnHrtK530Qtu4NVhjbRcAlpH9Xl+QwkwTy540VlZFWu57tPNHr+bqLILU+Qxze40Vpz0r+f/hT/0ba
p4P9zEP5wXDhq7XDO6ZmRvzvuZ9P7mPvG7mOO+3ebwnR+5jSf+gFfO8z/K9eGZinJuh951luGnG
nJfc34sTtofSdoiDEBWJ+AGdMSt9AiU/nTRm/7d+MFmdbhD8tO0KKsqaVzS0Kavem667baeO6OB7
cZw2JDp+/aavPhXsGRpSs9Wsc9pc9dvm/sOhHu7KJNtVjG3wDwuALMzsl/chKri09j/lv0npcCYu3Da
KcLuTP+4f5vte3N4IC3ONPyjOQq8h6zWRatX9x28G3NEnxsO8XrMy01/cAyWOhh+z+NZW52O1qS
YFda9TS6qdmpRmFPjk/0v7+Pc19R5csr6f6l3jHFk/T2Z778R5v83jPXncPtTnc/yDVmTok1eYm+C85/l
eHHFuht739CP98xP/vl3dH/g3Iot/IEadk9zfi8efl3zfiydoj/WNrUrbpf+eHO/7dn5+FsZY1g2DJWS9R/
TV5bd+A+hbPzD5uvW+9U+g87mq9ZZia/WtnxX060c8vNcqIa93P29uf9TD2NbIdtqStKvbr5fd9VXr
K8cxXx/yKNetVygJz83Exx/
+MDcdve3gMSfabqvqrS8PXwQAmEq3XrbeX8z3eP2+GngjyoffFv1vwAzaEEm/z8/8vXjm7XCuj9q/f
c28f5PzY/87OoP9RtP37dy1y/3eivPzV9rCv5dn1R7n5y9jv/pnrCy/l8a/rznvuYNtyPocjW9XvNdMa6J
9ZPIeqPdvbdd+hLRD3T/maud+Um3XK1IS/5w4n8/+vTjeucnjvS/
+fZv1+8v4cxImq7aNPy/h62fWnhHfw+nfR87+zd0737fBdX579Qr9vhz3uiJf9ERFDBXZVn8p0ksO56
821puAo7kr/t9KrNd7n9D2D52/MKlanIP1QqutCWuIJm1XEk4PIOsNTS/7rDdbf9h+0jYkPH57qP3g
ubQ5+wj8QdOuc2oMyXfZPWWMeqiWRNsFgMy05XytY71NXZBN503VUpEt9UY28fv35Iqbe9Lv7x
ltmTO69MupE/PRwFm1o9dYl1qnKmfn9kLNhnteWt7PKPPC+T6X6unAzyL5KMqJU9YH9+dQW08u
7sygPb2GqIFK5fqWv9/Ktv3zYmfnYgY9sRxj39d6h7JvfRj8fi7aJ64puxm9Ecd5v83jPXle3ved79+y1Ls
hP8tr6uf34dEJHdm5mO7dE/ec5PVeHOfc5GX8uXHeXwLf5xmY7Jxk9148/rzk+148tj0jfp6yR42mW
tYuzqii+fpZGOMRoi4pvw7mwJuLMeFQcMiBCgvdr1EPJ5TztxMM6dQPhf5rrYf5A725D/VQ+xmx35
Ht9MRtl/MGFlw3nvMG7H6d89gbakjCc5Pg+G2D59J+jDoG8zj1Y9QvU3G3a7xu+NgBYAoz+uV9M
enSL+W6bIX/A5CTnjTWa9KZVTvsoaMdqbbi/1ueGTUM0Rh6N9uSN/n84j6JXuOc9d1s/bI/oxu3sq2
HjNo17dWwypITNg/
+vJW13H5pT8huV1lOenWcNKPePgZk8AetuMFlmDTbEVuO78XTnJv86fJoB+cHhtOnWwJnknPCe7
FhxPub8/PnvuRaNYmfhRcOISoAAJgNfnmPTdUltX/5uTCLXoa6tpj9i6D+pWdGvR3b52vSqbZmPmL
C/0Op81AjV7KppRZXPr+4J+f2NDo7wwDEHTWkatqr7yOrObMI4efpl3ZMgT9oKfPyXhwwR3/Y6uy

flAvevxGqt6h6/9mYYa9C3osD9HwiwflBrO+pqedWiWHwjzD8LLxwCFEBAADmmDP5gRq2N6ueO
MFRDt36vqzO4hfU9oYzCcRc/dbuUL2Csh6WHcfc/eLePi/O6NHZXTNnsg5/YiAVeKtJgXKftGOWv7Q
jNfxByzKH78Xz9oet8pk147pY/4aeVWXUZtirkPfiAernGrdnrP89dXBKXadTkl3n7nn5IwymQYgKAA
Awp5wAVfXWmJ+hjMXNC3YNtk7tfK7hXHtXhU1NO8B1fuEpSEn9Vqh/CZrtcHptlsOyLfP1i7sOCGf5
y6Iecqzq2bt5jz0MViXezdWMZjYfZVa/tI9h93bqyMHgjTuqd9QS4w9ajkV4L56XP2x5ZtqrkPficIMl+f
qydXLfOk8nJau3vdn+EQZpIUQFAACzwS/vkVSvDSdAndMJ/zL8RSPMYE8j9bAngLR+Mexaz2dbt7sn
h6qoWc7nZKxZ/uLu9nw6O8NfFgeHTbpmdl7y/6V9rOIJceZ7CUZNPfuGnmG4O2f4g5Zvvt+LB+T+h
62S2GWOB8PbWf5cw3txTLq2eOCPkekZ+UcYfhZeOISoAABgNvjlPYKeRddiD3czevzkW+dSTf4wM
Bxc/0KW1S8ac6/XkJUVc0i2WqVmSp7lL6nz94u701utKjMcPSpSOW21wPoeCgyhV7dwzTovM26bL
dtf2uOpyGnVY6+27r+veL3G5m+islngD1qLaFZ/2NIjADo1Oe/9w6mHcM+oHinvxXGoUhklqUldLm
RwkSL/CMPPwounDwAAMCOtqvSt33L69a5e0a33y9aPJ2VvRX669bLVFtWewUe139KvWUZh56U
6JyfEblu53s/3bun26+Xg+ZiLe6RVHbg2rb71S2tfJT+5m+H38TB9HgKPefie1vdRxvdv3Pc1573Yf2R9
7eK0K4/35PH7CLt/3Ifxb1dq9H0x9H2r2jFw3Pp7Pu1rNcl5t78m8/fiEedGvd8M7Ns9hrTf/uKem6H
XZfY+POp+0XJ6L453XvJ7Lx7fnpB/xzO6RnHuxXn6WRjjFdT/rIsKAAAwE+5f6F3WD43zNRwQWBCq
x5xdm9BVbUl/Bl3nnO/pqrT6M5p5eS7pnk7G5ZnV9cH8Gfre9fjfR2Gvqc5r79gUjT83Id9bxnk7iuLc
LwrvxbOmRvOoOqhhgkP7+Vl4cRCiAgAAAAAAAEAEaqICAAAAAAAAQARCVAAAAAAAAACIQIgKA
AAAAAAAABEIUQEAAAAAAAAgAiEqAAAAAAAAAEQgRAUAAAAAAAACACISoAAAAAAAAABCBEBU
AAAAAAAAAIhCiAgAAAAAAAEAEQlQAAAAAAAAAiECICgAAAAAAAAARCFEBAAAAAAAAIAIhKgAA
AAAAAABEIEQFAAAAAAAAgAiEqAAAAAAAAAAQgRAVAAAAAAAAACIQogIAAAAAAAABABEJUAAA
AAAAAAIhAiAoAAAAAAAAAEQhRAQAAAAAAACACISoAAAAAAAAARCBEBQAAAAAAAIAIhKgAAA
AAAAAAEIEQFQAAAAAAAAiEKICAAAAAAAAQARCVAAAAAAAAACIQIgKAAAAAAAABEIUQEAA
AAAAAAgAiEqAAAAAAAAAEQgRAUAAAAAAACACISoAAAAAAAAABCBEBUAAADAQmtvFKRQ0I+N
tl5raG94n19p9PTK2fDbuiEhLV0wbdlwz3vk+e1JY2Xca6xXNVb8ba00rK8CAGB+EKICAAAAODqa52
TGOemSUMHoqjT1kqMsZ9aK+rmpKJsX6tZnHZ3aesg1asv5Wkc/F6me3bS+CgCA+UGICgAAAOAI6
Ujt/OL38Zx/XTlwM89yXbr9vvT7e7I5KvksbsrZqn4eco16jXN+IFttyXZFPwcAYE4QogIAAAA4WuiNm
r3eoezrp3LqRKxeo5XtIng5auAamb1Qq9IiQQUAzCFCVAAAAAABHTLzeqH590pVg6NpryIpbm9Orse
rX/7Rrehp1Vr119suC68NKtPqCtUJH1wEdeJ31CKsrGjyeYL3S6HYYzGPXj8F92fsp1ayzrDVXndeO3Ul
FtltujOpfo/aGXxagXN+yXjUo3vHbQto/dH0tcc5VoEar+6BWKwAsLUJUAAAAAEdEWep1HdJl2Bu1U
ytJYTVYDdRep0K2gfXN1VEBZlNWCyUxyoCqjUhpcMIpOxQceJ3F3t/IQK8jtZJRr7Rcl60YnTuHwlEtel
8JVbak7hZHba7KRqMh5/wEVS4M1gNIcvwqwA5pv3M+hoNUR/i5UueiNLhTJewaAQCWAiEqAAA
AgKNjzQ3p4vVGnVS53pW+qgPq9ax0hK1v7o5oh1dLtC/
+y5tyzkv7etJYd0PBqrT0a71td2qyPjIpNl6/F2OSpvaGePmv0a6um3ga+6psW5/r+hNFqRqm9n5iDc
MPTjLVrLnHV5b6hcF2Jjl+67VuGhvWfmsrOxfjnqu27Eadi8A1AgAsC0JUAAAAAEdIUTbdGYwy641al
bNuj8nKaWvJNWL9/mFoL05zBnqzXmhn56Lz+t5F2dGdIQPD3I3enN5rB4QPix+t7aeGgTCzuHnB31
ftfDo9MAOTTGnVs8OTUiU6fuu675lBqKO4dsYLbDsHXf0saOhcmfVeOzviZq/FzT1n+9Zjb+QMWgC
Ao4oQFQAAAMDR4oVsGfVGLZ+Ukn4aMGp9qKqcDiR3JTnpp31ix33dA90L01rllguwH8bwdve1A0
6dSBLymT0vz8ha4EuLsnbGbdi+HIYlthMITDJlPQudTGqK41fHZNc5DR3eHzR0ropr4h2yuodK7n4ja
rECAI48QlQAAAAAR8xAb9RD5+l8K8qJU/rp1MpyMn6am0BHRnTmnE6i8DlCYFIpo85ppLBzFSw5Y
HLD3NgTdQEAjgxCVAAAAABHj9kbtRYvTsvXYK/Onhx6Y8iHebVWhx7biYbtTyercHa88cfflg2z16lby9
Ss3ZpEcVP29D4Gyt7amueYpR8Alg0hKgAAAIAjyOiNmpQxjDw7g706u3Lg7tTtmVk66QWAo+p5ps
MsJeDXAHX05KJbmFROSaIqAdNKcvztXa/nqR24unVRp7iWaoZ+1ePUnkjLDlS7Xi3W0WUEAABHF
SEqAAAAgKPJmIAoTMlPDo3aqW3Z8Kapz1ZzdcObqKm94Q8/L59ZcwJAszZnc9UfQt7e8Gp0FlIZV2
4Gzta5WPd7WfYa61790aSTVU1twuP3A1djxv4k9PbVbRDscRoSdAMAlgYhKgAAAIAjKro3qjIzuwrp
nGAubi3NaZWlXG7Kqg4D/dzWmOFftd+ozdlc1cGh9+IREzJNorLtD1vv1KSk21XyE1S5kPuM9AmOv

3Lan6jKu5bGBFRJjDgX5r1RPat7ugIAlgYhKgAAAICjK6o3qqp7OVgzs9qavI5mIqfk7J4xPFyx63gO1Di
1a3MOvE4Je+2U7GHrIcdebVnr3eHxeYt9/BXZ7psz/itVaZk1TZu7Xs/fcexzEVYMVW8zrewaALA4Cn
1V3AUAAAAAAAAAEIqeqAAAAAAAAAAQgRAVAAAAAAAAACIQogIAAAAAAABABEJUAAAAAAAA
AIhAiAoAAAAAAAAAEQhRAQAAAAAAACACISoAAAAAAAAARCBEBQAAAAAAAIAIhKgAAAAAAAA
AEIEQFQAAAAAAAABGEvn/AZM6nr53azj8AAAAAElFTkSuQmCC]]></redcap:Attachment>
</ItemDef>
<ItemDef OID="q33" Name="q33" DataType="text" Length="1" redcap:Variable="q33" redcap:FieldType="radio" redcap:RequiredField="y" redcap:BranchingLogic="[q23] = '1'">
<Question><TranslatedText>Do you prefer Life A or Life B, or do you think Life A and Life B are the same as one another?
</TranslatedText><redcap:FormattedTranslatedText>Do you prefer Life A or Life B, or do you think Life A and Life B are the same as one another?
 </redcap:FormattedTranslatedText></Question>
<CodeListRef CodeListOID="q33.choices"/>
</ItemDef>
<ItemDef OID="summary34" Name="summary34" DataType="text" Length="999" redcap:Variable="summary34" redcap:FieldType="descriptive" redcap:SectionHeader=" " redcap:BranchingLogic="[q33] = '2'">
<Question><TranslatedText>
Alzheimer's disease - Description #[no]

If you have Alzheimer's disease...

1) You might forget recent events of importance to you. For example, if you are interested in hockey, then you might forget whether your favorite team won their last game, although you may remember watching the game itself.

2) You might forget the names of people you met recently, or the names of local streets.

3) You may no longer be able to handle your personal finances or plan leisure activities such as dinner parties or vacations. Simple decisions such as what to order in restaurants might become difficult to make. Your ability to concentrate may decrease and you might have difficulty driving.

4) You may start having trouble finding words to express your thoughts. You might need to be reminded to shower or brush your teeth, but you could do these activities on your own. You may need to post notes around the home to remind you of simple things like turning off the stove.

5) You may remain aware of the day and your surroundings. You might be able to travel to familiar locations like the supermarket, although probably with the help of a loved one or friend. If you are in a social gathering, then you might have difficulty following the conversation.

6) You may continue to enjoy hobbies such as puzzles or reading.

Now consider two possible lives. In Life A, you will live in perfect health for 4.5 years and then you will die of natural causes. In Life B, you will live in perfect health for the next 10 years, then you will live with Alzheimer's disease as described in the summary for another 10 years, and then you will die of natural causes.

</TranslatedText><redcap:FormattedTranslatedText>
Alzheimer's disease - Description #[no]

<p></p>

If you have Alzheimer's disease...
<p></p>
1) You might forget recent events of importance to you. For example, if you are interested in hockey, then you might forget whether your favorite team won their last game, although you may remember watching the game itself.
<p></p>
2) You might forget the names of people you met recently, or the names of local streets.
<p></p>
3) You may no longer be able to handle your personal finances or plan leisure activities such as dinner parties or vacations. Simple decisions such as what to order in restaurants might become difficult to make. Your ability to concentrate may decrease and you might have difficulty driving.
<p></p>
4) You may start having trouble finding words to express your thoughts. You might need to be reminded to shower or brush your teeth, but you could do these activities on your own. You may need to post notes around the home to remind you of simple things like turning off the stove.
<p></p>
5) You may remain aware of the day and your surroundings. You might be able to travel to familiar locations like the supermarket, although probably with the help of a loved one or friend. If you are in a social gathering, then you might have difficulty following the conversation.
<p></p>
6) You may continue to enjoy hobbies such as puzzles or reading.
<p></p>
<p></p>

<p></p>
<p></p>
Now consider two possible lives. In Life A, you will live in perfect health for 4.5 years and then you will die of natural causes. In Life B, you will live in perfect health for the next 10 years, then you will live with Alzheimer's disease as described in the summary for another 10 years, and then you will die of natural causes.
<p></p>
</redcap:FormattedTranslatedText></Question>
</ItemDef>
<ItemDef OID="a34" Name="a34" DataType="text" Length="999" redcap:Variable="a34" redcap:FieldType="descriptive" redcap:BranchingLogic="[q33] = '2'" redcap:InlineImage="1">
<Question><TranslatedText></TranslatedText></Question>
<redcap:Attachment DocName="45.png" MimeType="image/png"><![CDATA[iVBORw0KGgoAAAANSUhEUgAABVEAAADYCAIAAABtBTQzAAAAAXNSR0IArs4c6QAAAAR
nQU1BAACxjwv8YQUAAAAJcEhZcwAAEnQAABJ0Ad5mH3gAACnFSURBVHhe7d1fyF/Vne9xY9SJR
yU5rRNiPZjoIAQjEhsOxJxiI4gRISBoaZBRPBx5iGCZINiImOFcKMpQyDODoBfFCqfnxAuLDJQotpCLFlI
oNBdeOJDKc2FpoF48I6U80lz07H9r7bW/68bW/68/ev/Vbe/9+z3reLxad57f3Wuu71t47uj/q75lr/gYAAAA
AAHJE5gcATOTC2oVr/vc1tPza/U/
+1+J/m9sMAACWCX+HBgBMYW19bdebu8ygSMuj3fDKtf/r7n/Y+eL1zZ0GAADLhMwPABjd+sb6/r
f2i6xIy6M9/NCeIvM/eeiOv379AQBA0Oc+PSeCIi2PtV8xAACw
NMj8AYFynPj4lgiItj7b7hR3P7r9LZ/6i/fpnHzR3HQAALAcyPwBgROc+PSeCIi2Ptv3MticP3WEG/qKd
vPeeP37++
+beAwCAJUDmBwCM5eIXF3e8tkNkRVoe7cFju0Xgr9urxx7hi/0AACwPMj8AYBRr62t7frRHBEVaHu
3uZ24RUd9sfLEfAIDlQeYHAKS3cXXj4DsHRVCk5dF2vni9+Bq/3S78v//5SxzY2vUGMQzmWqeBgAAsDhkfgBAYm/86g0RFGnZtP/x2N+LeO9rp49+9y9y9ffdU8EwAAY
EHI/ACAlD787EOREmnZtL3P3SSCfbi99fzJ5rEAAAALQuYHACRz6colfF/ru3ml657+sCdItX3tk9+8m
7zcAAAgEUg8wMA0ljjWN+3uk8ERVoebfuZbccfuF3k+SGNL/YDAYZH4AQAIbVzeOvndUBEVaNu
3w8VtFmB/e+GI/AAALROYHACRw8ucnRUqkZdP+28p/ETF+1sYX+wEAWBQyPwBgXmcvnr/Nu/H/feaJYXUJGzZZPVyc0wJnVsYvaiV8/0k/fe8x+/
+U3z0AAAgKmQ+RdEhyCZcNrst0whVVS28EefuFFzmqbKnPQia8lCbzNvcOuLyvz22sj8m9Ta+tquN
3eJlEjLpn37iW+I6D5Pe/HI4f/805+aRwcAAAEyCzL8wznDfhr9ljqgywjkSncvAblvQOGl32PVeTOZ3rY3
Mvxmtb6zvf2u/V/2u/SIm0bNptz98oV/
+8anm6QEAJMg8y+QTv065OgjjS56KRWIbGOYHdtt6RrowwYl82/70UZESadm0VF/jt9u
Ng8QAAAYH5l/oboZ3/5nAC2dkSqigyMs9eQ9dbodYR9R05ZHxHThjz6hUe68p7q4Nlbq9naSazML
mXOFl26MCg8KLc+YRP3o1M7av9lOD2Ooo4Bnh+YFMUbZ1foWI5bS6aLPlYvwrW3wSrAsTn18SqR
EWk7t4Yf2iKyesPHFgAAJkPmX6w2J62sqh+tjOPISAWjmxZU4ZtCUF+o/ptxbYaOVZnVqV6pD4Hp5x1T
RTThT/6hEepFbRzqA7NGq0qjm3b5Ch7o+2RtrTkvzwLM+2RtrTkvzwLM+epGENHTKb7qGO6OnVAce0DoGteSeS
i1EdrAHeGawDhQErwbK4+MXFHa/tECmRlk3b3/cKOZ/ffJVJ68sYX+wEAmAaZ/GMqNOJQDUZr0oy
MDn6uDJVV9OjHlN/WFkpJzKPqPFiuvBHn55R6rOaRH1udiU+lhyHLLKKvtrtWmUXB8eodlhVfsDy9
CRyvfYC+mezx9SHXAv076vQf0FE5ZLjUIcsbS3FOlCIuzWY3tr62p4f7RFRkZZN++vX36w8E/+7v+/PHD+/54e7DJzP/rz71fe/TB8/+ffe/+HK/I5yM1v

tgPAMAEyPzLwJUoG+pUN1yJyOXoNCAsNaOqDtXPR1bPV6PaI3q4mC780advlDrQfG5WoDalNukS
qCur6GnaMQOWr0Z1bkNn3lDlOUrX7AX0z+ZcUdeAfRUcE4mBA7Zm6PZuzltLca2tfyVYAhtXNw6+
c1CkRFpObdSv8duNL/YDADA2Mv8yCOQ39ylPKJsxLDXDih517+KH+pA+okeL6cIffXpHqSPVgXZ1N
bVJl0BdWSXuWqlRnR6dcQOW5yhdsxfQP5t3staAfRUcE4mBA7bWDhGa89ZSXGvrXwmWwIkPToiI
SMup3f3MLSKTj91+cOj+L//wRfN4AQCAEZD5l0Egv7lPqSjUHI0NS/U49a/3q9HVIXWknVBMF/7o0
z9KHSqONHtqz7mvRC9ZJe5aOYt3xg1YnreLvYD+2QbUG7CvgmMiMXD4Ygp1J1naWoprbf0rwaK9
8as3RESk5dR2vnj9BF/jt9vr33vir19/3TxkAAAgNTL/MgiEKh16zHMyCTnG6xQWCkvNPPbX+Osjxnyi
Yvijz5BR9br1r7M3Tg0sIslhccHSdTm7w2aYxLyVFXts/2yuHmWB2fZV6L8gvfPYU8jrZU3hW35p5i1g
Gh9+9qGIiLSc2vYz2548dIdI45O1919/rXnOAABAamT+ZeAIOy0dn9RZnYN0d31ERSM9picstQNdQ
/3ZK/zRZ9Aoo744o/urw7prqKysogbNFiwdV0VW71+eo3TNsYD+2awe/gPttA6OVcmBfYuRa2v7W
0eaz861DVgJFubSlUv8ov6824PHdoscPnH73S8+aZ42AACQFJl/GTjCjklHqg6zsxGypHBYagfq6XQ1Z
4F6uvBHn2Gj2t1al0OP6JipaGSwdN+CkmuejnZSR+macwH9m3X3MKbvrtqzu0EXpGcx/svTdLFndK
xt0EqwCOsb6/tW94mISMupTf81frvxxX4AAEZC5l8GjrAjdUOXIwGZHYrTQ8OS3c85UhwMf/QZOC
p8NfSoSuCKNWSVuGBpjlI/F+wBoeV5N+ZdQP9muz2sLsZancMLgy9IeDFmpXKY+lxP4ZrRWtvglW
BSG1c3jr53VEREWk7t5peue/rAnSKBL6TxxX4AAMZA5gf6OeIosDWc/PlJERFpObXtZ7Ydf+B2kb0X2
PhiPwAAyZH5gX5kfmxNZy+eFRGRllk7fPxWkboX3vhiPwAAaZH5gX5kfmxBF9YuiHxIy6ztfe4mkbeX
of3g0P1//Pz3zVMIAADmRuYH+pH5sdWsra/tenOXiIi0nNryfI3fbq8ee4Qv9gMAkAqZHwDQsb6xvv
+t/SIi0jJrj33nWyJpL1V79/QPm8cRAADMh8wPAOh49KePinxIy6x9+4lviIy9hO3XP/ugeSIBAMAcyP
wAgNapj0+JfEjLrN32/I0iXS9nO3nvPXyxHwCA+ZH5AQCNc5+eE/mQllm78fT2p+7bJ9L10ja+2A8A
wPzI/IulfjdcZZRfEHd59cjK+ebnPH4XXXdHQYO327kPytAqW9bgy5vfQ5ipi19c3PHaDhERaZm1Jf8a
v934Yj8AAHMi8y9QkYTq8FNLH4GaAhnFLXtHAe317duuM/KT+fsMepryewhztba+tudHe0Q+pGX
W7vveLpGoN0Xji/0AAMyDzL9AI2cfHXmziVuuHXm1ib93u6orIXQ2A56m/B7CTG1c3Tj4zkGRD2m
Ztd0v7Hh2/10iTm+Kxhf7AQCYB5l/cWZKsBHyi1uzXDG100rPdlXfkW5Etsj8+TjxwQmRD2mZtRteu
fb7B/eKLL2J2umj3/3LV181zysAAJgFmX8xOom0ZgZOnZQqIhsZkamdRsRV3/xm3DL62Omrs4LecG
ZOa460QrR/2vaMHqQXKFarWLO36t5HjjRzhjegSgfmM5h7NTgO913DzvlCp4sxX7v1eoXBcR5Da3
U6Bm9fcdJzKZR23Vo1oznM6GPP0lm0OD14zej3xq/eEPmQlI97+KE9IkVvuvbW8yebRxYAAMxitsyv
3639L9bOLp2Xf09AGKx9wd/Er/e+OOQ+VTCumqODdU1987snLxlTdKKWErxtSabV5+tj4qNvRy513
2KcGmRUsbnWFXq0nJPKg73X0LGfQtvDcb466R4X/rMQUavRv+KS2cvgGJDuIZx5NfD48LMPRTik5
dcOnNgp8vMmbZ/85N3mwQUAAINNkPnFy/u8b+TmdMGgs/ScV1NHGXWddC994Yy0E7wArvnts
db81jA1JnDn7GnbI7JOYFqzj6uqa0e2plfZZ8DSO4s3+ceoZRg9RKHezcop9AA9p7Eqc7e+idqBlpha7
RFVXI9SR9oDgdptL2MTdjIrSdYwue9Ba0avS1cu8Yv6s2/f/Ke/26Rf47fbyXvv+fzSpebxBQAAwyTP/D
brbX0uRtIopJlzQUJxyNyY7KezTc/uQ/Mbx0Q39dGY3HFISDatnqjRLSnmdGr61D1cl1OyJu2/vtbKRR
3HzhyHOgauQnfzTjSAv1Z7XWUfa0xBHwusxTWuv5z6GLiAA9aMHusb6/tW94l8SMusbT+z7clDd4j
kvKkbX+wHAGBW42f+tC/i+s2/+ab2HMln4RxXRmTHht50fdSRdpxcV95RQHTTszt4C/YHsMHTmh3
l3XXtSKiHq4FqMjlRj95hnjuilmXuQbKX3u1tzdEdoC9Ba5bNDa4lL7Xq0inmPNjlumWOYaJbd5ldYkG
BNSNk4+rG0feOinxIy689eGy3yMwZNL7YDwDATMb9b/sdr+6ON/TSwNyixqycVz/NEniWjONqukO
U6OjuZHPM7xrrmd3FmKgr5bRtT2uDrh111GPbcY51DdFbR/eoJlZVdO8hm9U1BHXeu3TPQO9SCx
G15CVwLse7xpacp9RfTvVw8S/IVQs+J39+UoRDWn7t7mduEWk5m8YX+wEAGG5BmV/3MgWSQ0
MNKydRP/ePWlaOq+kOUWKr7k42x/wDktLQ2TvSTauHVURv145Mqojb8B2pOqERTa2ii+rddxtNxk
rrTtbWeueQmx3ScXgt2ce5nN41um9Zf7kBEw9YM7zOXjwrwiEtv7bzxeuz+Rq/3fhiPwAAw43/O/w
cY6xDA17xS2pcPUx96hm0vEJXxtyU7DfwarnmH5CUXKN6pZpW9TqyuuraZN8kahlu7uvlmlPNE1xr
0+nIyko9gdG5d7P29dJLV0PsLlpxaoZicbXkrK4q+pj7ylZc4/rLuUYJA9YML/6/8W+FdvyB20VOzqy9e
uyR5oEGAABBi8j86ojjbT0QHuxOgwYtMfvKFHQek7tst+lIO06u+WdJd+qAXpK5zq4003aG6A+BOX
u5LpU8NmBhbrpfpdO5b05Zou2v55HLrOmB7WF3x1ZcLetSW1sy5vGVLljzFAaUs6rJXQxaM3w2rm

4c/vFhERFpmbW9z90kQnJm7dc/
+6B5oAEAQNAiMr9+fXcIvK2ridqXfPvIpuK5mu7LY+zRkXbcujNVVQYlJX2kI3BrkkyrT6tJ9PLbWR07C
nKsy3GsO6syfPaS7N2zWXfJkuriWnrJN9K/3Kha9u3zbKkk12jqVq9mG1Su5wIOXDO8rvz5Cr+0P/t2
+PitIidn0949/cPmUQYAAH02T+Z3Z4BaKHMsr8DV7G5WnHekHR/zUlfdByel7gr6Ss0/bXvC6K+X33
a0dhTkWJfzWHde1x1xcqzPFL6GZsWynvrc1HYvs9Zd7IDlzl6r//YVJ0JrbJnFq55R5WSVwZPAi//n/N
m37We2Zflf+L967JG/fv118xwDAIA+i8z84aQgdF//pZmmAtJQDyWPHzapc5+eEymRllm7+aXrnj5w
p8jMm7qdvPeeP37++
+YJBgAAAyzy+/yBWSx6jAepC1PTzyQPHzaxl3/5skiJtMzaXf/zZhGbN3Xja/wAAMwqMvM7VRHeCv
SOhN9O0xzT//Wv758COCap6RPkLkzG/K/V/Q8tsEk8/v7jIiXSMmsPHtstkvMmbXyNHwCACAvJ/L6J
vOEpkOwDp4BxGJmfwI/Nb+PqxsF3DoqUSMupbT+z7clDd4j8vOkaX+MHACDOgjJ/qTtZKLIHc33wJ
ACg19r62q43d4mgSMup7Xzx+mf33yVS9CZqfI0fAIBos2V+AECWLqxd4Nf4593ufuYWEaQ3UfvdLz
5pnlQAADAjMj8AoPT2b98WKZGWWdukX+x///XXmmcUAADMjswPAGic+viUSIm0nNoNr1z7/YN
7RaJe8vb6957ga/wAAMyDzI8w8fsYwh9non4TXvOLGOaZaladX7w/
+i+CKHY2wZ5KU15DZOvoe0dFUKTl1Ha/sGMTfbH/B4fu//IPXzSPJgAAiELmR1g45M8TMheV+XWh
2riRvyk2UQaf7BoiZ+sb6/tW94mgSMupHTixU0TrpW18jR8AgPmR+TETkSrnCZki809mwrpTZ3AyP
9L47MvP+DX+ebeHH9oj0vUSNr7GDwBAEmR+zGTzZ/4pczGZH5vWR5c/EimRllNb/i/28zV+AABSIf
MvhCfuOg/rHFfpH9GT+9TpdoR9RE1bHhHThT+G6c6For9Yu3Mqc4i5wKDOoM4oVdLgWbi5OLHu
jm4ts5qvlth2ydq60aedpjnpr+iYyEPMMXxThSHrLwTnqHW62OcHzIBxnb14VgRFWk7ttudvFDF7eR
pf4wcAICEy/2I4gpPrYBv4TMaggQHMpM7r07qInqQ5Un0W04U/Bri3UmrKWlPpAyZjo07hK+Y461
l473JLwWK+Wuq4MY+1dcfYunuw4rDb4bysxoieErHrLxmr6ru5/TNgEic+OCGCIi2n9u0nviHC9pI0v
sYPAEBCZP4FUZnHEXP0IZ171BEdlKwuxjRWAJOaDmqIka6aEWqG6qOYLvzRS/eTNQrNQvpmdmzV
MuCK2RM72VelPSK2MGMtxz6sbnb1Sm9FV70ua4i4ZL0lhqxfdrEmkQOsEb0zYCIbVzcO//iwCIq0n
Npj3/mWyNsLb//
+b//aPH8AACAFMv+iqBSjQ4yVpRzhqjddFaw8ZWl61GPqDysr5UTmETVeTBf+6OPqpo81a+/2UZ8c
GzMOCUOumH3ASU1l9OodaHdwDRlyy3R1/14rcmDEEutDvv6BtfnXrz/6lq86OKaoD/XOgAld+fM
Vfo1/xu3G09ufum+fSN0LbP/yj081Tx4AAEiEzL8wIvioKKXTlyNbFTyjjE46L/lzXDOq6lD9fGT1fDWqP
aKHi+nCH32cexEHu1Opky6+Ws4qel51dNiSdf22V2hgd7m6g2uIY5VWN0d1yVmxd2+O4n7uTQ1Y
v/7c6hTsTtxVT9E3A6Z16cqlHa/tEFmRlk1bni/2v3jk8H/
+6U/NYwcAABIh8y+OyjVVmFExqA1r7ngm4tWAAObSDCt61L2LH+pD+ogeLaYLf/Rx7kUc7E6lTrr4a
jmrWEsctmTHXPZAfUSwOxi1hszs3kmhp6KrXod34lbfpua5Ms15NYWLmiI8AyZ37tNzIijScmr//fFvi
vi9kPYfv/lN88ABAIB0yPyL1GSfIj2phGNEGnc8Ux2bo4MCmEM9Tv3r/Wp0dUgdaScU04U/
+jj3Ig52p3KO6OEeo+ZVR4ct2TGXHKi6FOpe9syuWgNm9u2+t6KrXodn4lb/poasXzOmq9SDeldhc
M+ARXj5ly+LoEjLpm0/s+34A7eLBD5x42v8AACMhMy/UE2gObKyUocmMzPpIGWmHJmuHOlJhyR
f7is189hf46+PGPOJiuGPPq5u+lhTq9tn4MQdcsaKNdGwmR0XVgy0e9hX3lVryC1z9Cn0V+zdm29B
asr+Eo4udp9Cccz41K3bu8xKaAYsyOPvPy6yIi2bdvNL1z194E6RwydrfI0fAIDxkPkXS6eliogz+qSKVzr
36Lylj6ixxoTBcNQOdA018pzIWuGPXtY6jfpNMd/MaoReXqDUgCsm63iomfxXQq6nXXA7s6tW268
5aFx31c1RvdBf0VWvy6rePTBgU9YM1vr1gXb5YkP+OYbO4DiACWxc3Tj4zkGRFWnZtL3P3SSi+DSN
r/EDADAqMv+C6XRTUKmqZZ5tmSnHCGWSPZ2pHWilKHeBerrwxwD/Qptq1lTuET2FZrhiwZkciVIO
dJcqtTN3+9TH/VdCD3RUL/VWHLI3T/mm1IBNzbB+qV2We5L2fP8MnmuEsa2tr+16c5fIirRs2uHjt4
pAPkHja/wAAIyKzL9oOtz4sks3HbWZRzM7FKf1R0dXk93POVIcDH8M050LRX8R2sLVKwPjXXeQXN
mwJTsSpWOgmUzLo+qzMbXZRU9mrrDoa83sqN4IVxy2N3mFRJlwiUrf+kvmNCV7Sd1VhPda6s6gz
trjMLYLaxf4Nf65tu1ntj156A6RyUdtn/zk3ebBAgAA4yDzL5oKPkQXAJvF2799W2RFWjZt54vXP7v/L
pHMR2pvPX+yeaQAAMBoyPyLpf9VJ5EfwGZy6uNTIivSsml3P3OLCOdjtNNHv/uXr75qnicAADAaM
v+idP/DZfs/fAaA5Xb0vaMiK9KyaQ8e2y0ietp28t57Pr90qXmSAADAmMj8i2JkfgI/gE1ofWN93+o+k

RVpebSxv9jP1/gBAJgMmR8AEOmzLz/j1/jn2r75T3830hf7+Ro/AABTIvMDAOJ9dPkjkRVp2bQDJ3a
KuD5/42v8AABMjMwPAJjL2YtnRVakZdMefmiPCO3zNL7GDwDA9Mj8AIB5nfjghMiKtDzaDa9c+/2
De0V0j258jR8AgOmR+QEA89q4unH4x4dFXKTl0Xa/sCPJF/vfPf3D5nEBAAATIvMDABK48ucr/Br/X
Nt939slAvys7dVjj/z166+bZwUAAEyIzA8ASOPSlUs7Xtsh4iItj/bYd74lYvzwdvLee/74+e+bpwQAAEy
LzA8ASObcp+dEVqTl0W48vf2p+/aJMD+w/fpnHzTPBwAAmByZHwCQ0su/fFnERVoe7bbnbxRhfkjj
a/wAACwWmR8AkNjj7z8u4iItj/btJ74hIn248TV+AAAWjswPAEhs4+rGwXcOirhIy6MN/2I/X+MHAG
AZkPkBAOmtra/tenOXiIu0DNrNL1339IE7Rbx3Nr7GDwDAMiDzAwBGcWHtAr/GP8u297mbRLy32
//55zPNcwAAABaKzA8AGMvbv31bxEVaHu3w8VtFyDfb6997gq/xAwCwJMj8AIARnfr4lIiLtAza9jPb
jj9wu4j6dfvBofu//MMXze0HAACLRuYHAIzr6HtHRWKkZdB8X+z/3S8+aW48AABYAmR+AMC41jf
W963uE4mRlkG7+5lbROB///XXmrsOAACWA5kfADC6z778jFIN5vSVaJnL54ViZGWQdv54vVF5t/73E3NbQYAAM
uEzA8AmI6Ii7Q82qMP3lb8b3OPAQDAMuHv0ACAiVxzzzUR/06FQNApFo1A0CkWjUDQKRcuv0FbA
pQQATIQ3kmgUikahaBSKRqFoFIpGIQRwKQEAE+GNJBqFolEoGoWiUSgahaJRCAFcSgDARHgjiUaha
BSKRqFoFIpGoWgUQgCXEgAwEd5IolEoGoWiUSgahaJRKBqFEMClBABMhDeSaBSKRqFoFIpGoWg
UikYhBHApAQAT4Y0kGoWiUSgahaJRKBqFolEIAVxKAMBEeCOJRqFoFIpGoWgUikahaBRCAJcSADA
R3kiiUSgahaJRKBqFolEoGoUQwKUEAEyEN5JoFIpGoWgUikahaBSKRiEEcCkBABPhjSQahaJRKBqFol
EoGoWiUQgBXEoAwER4I4lGoWgUikahaBSKRqFoFEIAlxIAMBHeSKJRKBqFolEoGoWiUSgahRDApQ
QATIQ3kmgUikahaBSKRqFoFIpGIQRwKQEAE+GNJBqFolEoGoWiUSgahaJRCAFcSgDARHgjiUaha
BSKRqFoFIpGoWgUQgCXEgAwEd5IolEoGoWiUSgahaJRKBqFEMClBABMhDeSaBSKRqFoFIpGoWgUik
YhBHApAQAT4Y0kGoWiUSgahaJRKBqFolEIAVxKAMBEeCOJRqFoFIpGoWgUikahaBRCAJcSADAR3k
iiUSgahaJRKBqFolEoGoUQwKUEAEyEN5JoFIpGoWgUikahaBSKRiEEcCkBABPhjSQahaJRKBqFolEoG
oWiUQgBXEoAwER4I4lGoWgUikahaBSKRqFoFEIAlxIAMBHeSKJRKBqFolEoGoWiUSgahRDApQQAT
IQ3kmgUikahaBSKRqFoFIpGIQRwKQEAE+GNJBqFolEoGoWiUSgahaJRCAFcSgAAAAAA8kTmBwAA
AAAgT2R+AAAAAADyROYHAAAAACBPZH4AAAAAAPJE5gcAAAAAIE9kfgAAAAAA8kTmBwAAAAAg
T2R+AAAAAADyROYHAAAAACBPZH4AAAAAAPJE5gcAYFbnV65prZxvjgIAACwbMj8AICN1GB83hV
9ePdJWGLPg1P9kodxY4cjq5eZASp3NVPhnJbPqXMNxbtPoPH9gmmevIvbJCPwRDZyalXuqzh1Lu6
1xBa/MqH+hGJF/U5v1PnI21NlOqts0oNYm/WvSFkHmBwCMa8S3+Q6jzpQvbVXZMd51incpvY/qv
WrkF6pyI0dWVor/GaVOuYXJXwnN99H0T0XnyW6N9PRVe5noiTCvW7oi3j+h3T9D3Y3OxVsxeGp
WvqnK4/pAfUnnLeWexbxdc5fovTLl+aR/oXBtqrOIynz7CmyqOpXwj5K9HaO4aaQdVfX1oerTvLsbVi
tBqfraNcT16VxFcQ4DkPkBACPqvk9130aSUoWq/zvlC0F3h2MZvUpZoLl845Qp7/3ol8k0zY3pqEqO
9PDJucerlfo9vqHuh71w+Wik2pq/YujUrIZOpfo1H2dWja91qxSXTx+Y/3b1bac83pyeq04ttKkU8zcC
m3IciufdjjB/Uf+O5JERa6kTzcd571o5nZ6+epLbj91S1cm59rQVkfkBAOOR7wDWW0Ny41cQpnn9G
Hlbevryh/iXtpD5XgdnNvlzUBj3UehewNH2V01s3qjUN85aub2VxBcycK0SXsa+qarz0RdSjR63isFdS
B8tf5i7jFqso9Y4f7mwCyXZRyOwna6Ez3fvpetdzHDWVNY+EhbTV7P6IB+HlIW2DDI/AGA09t+ZE77
tuE38MlDtJ9U7o1fn9Sc945qVP45TqLpSrZGv2Xj78Br5JhXqS1iUqH8Y5ym3/oQaT0cS1nxlxe51S1w
yMF3CSn1TWRc2Sl+VZBtyTWQcK39M9rA7atkPRQp2oapO+UUFbf6qrktnqE6n2puzVvWolSXqH/x
LmY1Vy3M502zNvEzuQsk2tkWQ+QEAo7HfAJyvKCmNXsBUvXikenuzVXtpjLilchdq+rLkeBtS6o2N
Wad68hK/yodN8+BVT1xlrFL2Puw/xXOxCtjzJ76WgekSVgpPVd24BFcxXKU6m+ZeOQqVe1BHytPJH
gpPLUOiUnYhcV+qDv7rO4xjO4bw2Vn5ZmuvX7JSjlrVEXn1Et2ragdNtfLn7rRpr+LWQOYHAIxmAX+
rnu5dQLwtjirpy1RXuY32epWFJtmT/WgkJW5Off3GfCyqgqM+d+Ye6p/HqVfNLS9dwltVTWiu3H4U
rC7zCUyXsFJoKvE4zsFZpTrYSHXZrELlHtrP5elkD0Xo0hXq7aWoZheyHr4Ef5CD20kwv8lRq75c9bH65
0T1nPuq9qOsrHQekjlU0+obY92l8DWGE5kfADCaBfyteqJ3gfpNZ8J3jvrlLX3BziubIcUbdkhVd7zrZz1
549ar7s6ol8yuMOKOOg9Fuvf4mvUn1P4ja929+dgFtMCpWXmnqq5mqu30LLg6naSYLNR5Jgxj1LIl
eiLsQtbE/WvpFZiiOpXqWShZtewK1Z2bb0e1/ktT9kixO/lHxr79/WuBROYHAIzG/htzonc3vyneBaoa

E79wVDVHvXKVssz4Vcbfzhiv8n7J3qr97BKj7siQ+pGw1y1vVuqdBeZLWMo9VXXfEm6md8FVhwQF
ewqVp5M9Fb2bSvUnzC5UHTE3Ih/GCP7tJNqGwapll+i/ugP1TlR2mP+hqHbQncaunOA2bTlkfgDAe
OTfmZO9fniNX6F+J5GSv38UVYxtON6DRpHmrc1WzGtMW92kUe9SVcHcyJjviNXNGfeZs5+ASYoW
kj8R1a3pLryzN+vWzc1RUQmcmpVjqupQ2pvUu+CqQ4LL11OoPJ3sJi10U50/SEn+VHm3M8KfWbt
W5w9TIV3R8G1Kc4+q1dpFysPm3OGlwInMDwAYUecFJM1bgVs1t7C53wnEjqbZTFl0lDs0+W46r7r
p3nttYz7VXfX7sDbBE5Fyb+IJqLRbMPeW6mIGKoYXMxP/VOJ+1ebcXFWts9KiivG5qjlPjYFXpuyW7K
G3NlUcMCavl+RYxGDhTXXOzlOmYW2nVh2e4NlO/hcKf63umbkvXT2dc5rOc530Sm4dZH4AwLjM
FxD+Po3JdF5I534f9ake79FmX4DE7/FIontXas29Eac20R3zb2pT7ilwjwrZ/YUiOfGPKmrtKwMvEnMi
8wMAAAAAkCcyPwAAAAAAeSLzAwAAAACQJzI/AAAAAAB5IvMDAAAAAJAnMj8AAAAAAHki8wM
AAAAAkCcyPwAAAAAAeSLzAwAAAACQJzI/AAAAAAB5IvMDAAAAAJAnMj8AAAAAAHki8wMAAA
AAkCcyPwAAAAAAeSLzAwAAAACQJzI/AAAAAAB5IvMDAAAAAJAnMj8AAAAAAHki8wMAAAAAkC
cyPwAAAAAAeSLzAwAAAACQJzI/AAAAAAB5IvMDAAAAAJAnMj8AAAAAAHki8wMAAAAAkCcyPw
AAAAAAeSLzAwAAAACQJzI/AAAAAAB5IvMDAAAAAJAnMj8AAAAAAHki8wMAAAAAkCcyPwAAA
AAAeSLzAwAALJfzK9c0Vs43h2rqxJHVy82Rcag6ovwyaK9NwboOl1eP+E4FzwFAvsj8AAAAy8XItd14
utUzfxvbK67L4E327UVdwn+SAQDjIfMDAAAsl86/yzYT6lbP/IP27wz37T8KIPED2GLI/AAAAMulk/n
NgLvFM78O7j3r0tdPXyd9hMQPYMsh8wMAACyXbuY3gqqV+a0DdjBuupQ9jHmbAcYRMw2rw8U
x4z+nN4ooxlnrvLG2tkxP5u7M15nQWKkSmEv3rvroT9YOQusvddcjugQ2GBwHAJMi8wMAACyXNk
yu1j+pyGikzOqzfcCIm00kbuNoLx2i/WOMnC1zbcVYiGOSYPR1F5VbNxhrsbSLW1lVP4rqfevvWZB/
g+5xweUCwHjI/AAAAMtFhcYiQapgWgdG40TV0T4QyvxNJyOSyiMqllpj2iPqgCyje+ilGHUGxF1/AT2
hXTNEd26001R61686+Nfj2aBvIrkCAJgGmR8AAGC5dFJj88H82Y6dRpyUYVZ1aXOp/4iaxu4h+8hEX
JCH9CRGHy+5gpLciX0gTC+gJIYMWL9kVXdvUHcbtG8AGB2ZHwAAYLm443URNa1k7IjKMpraXfqPq
M9mUO7Oq3q4iMKdWTzsJZVUSXVUbq2XfwkD1m/o9lbnPbPrVba62wKASZH5AQAAlotMwM1n/
fX+NkPKnoWxMn/3oPrg4i3s5+4rtxKd+e0lDFi/K7xX1Hnv7J6BQ1cNAEmR+QEAAJaLFSZliNQnHLFT
BmO7S/8Rx7RiXlcPYUAXzd1XlVRH5dZ6+ZfQvzjVo1B3sqr3zmFMUfEXA4DxkPkBAACWiyNMduOj
PhHqmSDzm+G622dA/LbL+OnpzM5WjYSZv3cqe6h1UQIbLE4ZE8+8bgBIiMwPAACwXFxhUufGkj7
RHm3ipA6m1hE7vPqP+GfRXfyVVbC1y4T4C7TjZ87OgSX0rV9up+3fs0FrI8FlAMDoyPwAAADLpSd
MmieMKCqJLBuKoPKI/nxEzq7ybsFduu1glwlT/bvM0Skzf+/63cspqS6+2X0jh64aAJIi8wMAACwXT5
hsQ2rnhJldi1gpg7E9Wf8R9dmczlpOqZubux08uwjqzidDstxar94lhNbfDi+VJY3LYp52zW4OLQ1dM
QAkR+YHAAAAAACBPZH4AAAAAAAPJE5gcAAAAAIE9kfgAAAAAA8kTmBwAAAAAgT2R+AAAAAADyR
OYHAAAAAACBPZH4AAAAAAAPJE5gcAAAAAIE9kfgAAAAAA8kTmBwAAAAAgT2R+AAAAAADyROYHA
AAAACBPZH4AAAAAAHL0t7/9f/aWfln3syR4AAAAAElFTkSuQmCC]]></redcap:Attachment>
</ItemDef>
<ItemDef OID="b34" Name="b34" DataType="text" Length="999" redcap:Variable="b34" redcap:FieldType="descriptive" redcap:BranchingLogic="[q33] = '2'" redcap:InlineImage="1">
<Question><TranslatedText></TranslatedText></Question>
<redcap:Attachment DocName="B.png" MimeType="image/png"><![CDATA[iVBORw0KGgoAAAANSUhEUgAABVEAAAEJCAYAAACdcmdaAAAAAXNSR0IArs4c6QAAAA
RnQU1BAACxjwv8YQUAAAAJcEhZcwAAEnQAABJ0Ad5mH3gAAE9sSURBVHhe7d0PjCRnfef/b+/aY
PPHNmBskx+RHat7sZYlGJIg08PxL4egZ+6SJbnbYOVg5Oh3PVyi0zQSkwOxF+mX2wguG4lunS5iWrq
zliQyTEJugrLdlJRgckzHR4IxYTI/Z7rlc3T8wDYGjPljB3u3f/VUPVX1VHd1dV3VVV3VXX39PtltaerpqfqqT/T
O/OZ5/k+hb5FAAAAAAAAAAChjumPAAAAAAAAAIAQhKgAAAAAAAAAEIEQFQAAAAAAAAAiEKICAEKIC
AAAAAAAAQARCVAAAAAAAAACIQIgKAAAAAAAABEIUQEAAAAAAAgAiEqAAAAAAAAEQgRA
UAAAAAAACACISoAAAAAAAABCBEBUAAAAAAAAIhCiAgAAAAAAEAEQlQAAAAAAAAiECICg

AAAAAAAAARCFEBAAAAAAAAAIAIhKgAAAAAAAABEIEQFAAAAAAAAgAiEqAAAAAAAAAAQgRAVA
AAAAAAAACIQogIAAAAAAABABEJUAAAAAAAAAIhAiAoAAAAAAAAAEQhRAQAAAAAAACACISoA
AAAAAAAARCBEBQAAAAAAAIAIhKgAAAAAAAAAEIEQFQAAAAAAAAiEKICAAAAAAAAQARCVAA
AAAAAAACIQIgKAAAAAAAAABEIUQEAAAAAAAAgAiEqAAAAAAAAAEQgRAUAAAAAAACACISoAA
AAAAAAABCBEBUAAAAAAAAAIhCiAgAAAAAAAEAEQlQAAAAAAAAAiECICgAAAAAAAARCFEBAA
AAAAAAIAIhKgAAAAAAAABEIEQFAAAAAAAAgAiEqAAAAAAAAAAAQgRAVAAAAAAAAACIQogIAAA
AAAABABEJUAAAAAAAAAIhAiLp0etJYKUih4DxWGj29PqjXWPFeU1hpWF+1iMxj3ZB2onXjpLWdL
MxLO7LSlg372PzHqPs4Vb2GrGzEO5vtDbdtK5JH08KNuQ9Cjmc+2g0AAAAAwPwhRF06Rdm8UJey
XurU1kPCkracr3X0c5Hq2U3rq4B5oILBVWnqJUdZzqxle4faf1Qo1cT/rlhsR+14AAAAAADIGiHqMip
uytmqfi4dqZ0P9kbrNc75IVW1JdsV/XzhFGVzry/9vnpsS/aHkff+RpmXdmShKwdu8leuS9c+xj3ZzDJ
D7TVk3fijwsI7ascDAAAAAEAOCFGXVGW7JV6O2jxn9EY1e6FWpbW4CSqOot6h7OuncuoEPaQBA
AAAAEAuCFGXVkW2W26M6vdGbW/4Q6XL9a3wXoyqlqKuRek+wmpSjqyvaH59ZI1Jo6ZjoC7rqPX
GPu31edcGHd5fZI1J4zwEz5+5nbDPjxN+3INtCdS9tR4xy30ObGewraPOc7xjCm57uPZpYAh6c9VZF
2h4snM3eA7Uw9yc3Z6x+xzPPy71CLkXbAnaHvI9OHq7vqTHE6/dAAAAAAAcfYSoy6yyJXW3OGpz
VTYaDTnnJ6hyIWSM9FAIo3VqpQwmoCrK2hndwM6BdJ1nFmNId2dHLno7bcuubn/5zNpc9FKsnPa
D6gP/AGy9izv6PBo1Pe1wrCSDo63TPb8dqZUKUhrYSXM1aUimtjPY1qasDoZtEx2T2rZR+9S6H+te1
+kREu3HCSwHz4HSXB0dKibnnOtV70AUtW6Kc9TeGFHPNGS7E4vZbgAAAAAAlgQh6lILTjLVrLnBTF
nqF0Imk2pv+KGKV4+yL103ie3UZD3lhKW4dka3b18O3U23d42JhYxw0hjqferEnAz0rpz2yiY0d4M9
Ji/u6BisfEacDLUnjXX3GlSlpc9v3+0xnOr59bfvdUhWIdlAfdzx/O1494Hazrob/E1zTMbr9zZlc9v62PX
vV1Wv1/6cXXIi2X56jXUvsCzXu85r+8MlLiqR+4wr/BzteOl/krZbr3X/0hH2PRjY7rBkxzOu3QAAAAA
ALI+C9QtyXz/HklK9SwM9zlSwEhKq+K8rS71rTubTk8aK24tOBS/OZEYjX6963bk96Ubsy+dvW4Vde
9ZG1BBs1YOwWq1Ks9kcWu+3IaxdcdeNE387w+2yV3rnwG1/6DqbsV0VnO2FBNwB4e0wr3O11fc
nDEt0PSK2E9ivvuaS7JjMbQdfr41qa6JzF35+FOdanQqsS3p+lETnOlHbRwht44jjjDietO4RYBr3Pnyvv
OXCW/QSACAP99/xLnnNW2/SSwCAzB3/NZFjr9ILWBT0REVwkikVtoQGJP5Qeb/npMsYdm/2GE1F
UU6ccp51dJfTrj2WvywnT5+0e9QF11uqp/0AbA74vWmb4nVG7R44gZT1GW8ov7dOdT4sGbUo3S
BMfcIsazBr1jUo6ac28z7QPYSnOKZEvYkT7cec4f+kmIdQ3NyTvhmgTm3wHIWY6rrrurFuwJmaGO0
GMvDwEw/LOz/5Tr0EAMjDDY9fJXd/6AF5/Bs/0msAAJm7dI9I/zt6AYuCEBVBA6FScsO1P6fl1RVt7k
pbenJoj9k/JScqJ6z/D65XGeqc9ZIrrombLbpD+ttuIl09a/TozUtaAZl1DTJrOyHeSKpXqBe0GnVjgQX3x
NNPSOWPKvZHAEA+nvPjY/KWL94oP3ryGWm+/3555p8u6c8AALL1lMilP7A+PussYiEQoiJlGYRfXl3
RfTlsXxS7lKgd9lbEyVeN9XMZvhk9NO3A1+/VOyrwVcO67VqVQ480e0lOK1mv47yOaTHOXbjxbW/
Lhtnr1K2LatY5BRbUnZ+6Ux58/EG9BADIwxv/
+gZ5wQ+vsJ8/9NXvyp/WeR8GgNz0Hxa5/Od6AYuAEBUxleSkN1LbnBFfMSZJitM70Ri+HI+7744c7D
pf686+X7I/4a8fLjUwH4qbZ/0guOFOjFWVQIZacsoTKG6Jgvk22OvYvA/0seV1TIn2Y97LA8Pk1cz3hZ
xnoE/SdmNSNTtwdeukJv6eAubL+z77PvlM7zN6CQCQh1c+eK3c/PXn6yXH5z7+kHzlLx/RSwCAzF3
6gsjlr+kFzDtCVMRUlM2zbuVUc/Z1NbrYnOl8y+vt5wScijnre1s23JlrYvN7cqqJpBS3XmZRF0x117vh
6vxxe81a56Kmj3+wdqsx7F+aq7LhnTIV7Onh297K+dBcNQLH9nm/hqd7bHkdU6L9mLVbm7Lqre/p
me+ta1Qq+NvI2oTnyA9c3XYDi+kT+5+Q+n11vQQAyIOqg/pzD7xELwX94W9/Tb73raf1EgAgc5f/RK
T/fb2AeUaIivgq29LyctSalHTA48w8bynX5YJR4NOfUMnSXNWB0GQ1HAPbsp55Q/a9of6KMUnTHP
Jqu9rKUt8KRKiWomxe8IdlN1d1gOaFzqMm/ZolJ3AMttM8tryOKdl+ipsXpO6/2HmtOZGTdS/7h6Br
7yrua1NNWBO03bzfw9odR+bHA8R339fvk7v+7C69BADIw/FLBXsYv/oYRgWozd+8Xy8BADLXf1Lk
8sf1AuYZISoSqWyH11+stqz17tBiV3FT9gZfW21NVr/R7K0XKBlgDM3OdKKjFJgB2KiyA+qc9bt+wOe
ya1/OW03PstS7fT9Yt1Wl1d8LTpaV1zEl2k9RNvf60h16sXq5MUzeVpHt4EGK7B96PbFTEbvtVlv6Lf
8+sqlzblwHu+5ulByOB4jBnYn/6Wfp7QQAeVr50kvl2iev1Evh/uFL35ZP//6hXgIAZO6y9RvZJcpbzbt
CX81aAgAxtTcK4nSSVCHqQGAKADGo4PT1/+318sAjD+g1AIA8lB56od0LNa6tu8vyiteFD/sHAGTgit

8QKfBL9ryiJyoAAMiVGsJPgAoA+VK9T1Uv1CTUsH7qowJAji79AfVR5xghKgAAyM1HvvgRezIpAEB+
VP3Tt33hZSProI6iAtS7/+NX9RIAIHN2fdR79ALmDSEqAADIxe6Du/LBv/igXgIA5OWOL18/tg7qKPv/
8zH53Mcf0ksAgMxd/n9FLn1BL2CeUBMVAABkTg3fV3VQmUgKAPJ189efL//8Czfppclc+dzjsnX36+
XWV79IrwEAZOtKXR/1Zr2MeUBPVAAAkKknnn6CmfgBYAZe8MMrEk0kNcoz/3RJmu+/X3705DN6
DQAgW9b77aWPWx+fchYxFwhRAQBAZlRwqgLUh594WK8BAORB1T99yxdvlOf8OJ1f+R7/xo/k7g8
xKSAA5Kb/HZFL1EedJ4SoAAAgM+/77Pvk3ofv1UsAgLz83AMvkRsev0ovpeMrf/mI3PtJ/igGALm5/
DXr0dELmDVC1KXWlo1CQQrGY6XR05/LSa8hKxttveBob7jtWZG8mzM3Qs5LHL3GinPuVhoy6tT55
9d55H7NjwLuWyCW+n11+djffkwvAQDy8vJvPE9e+eC1eildn/zPB/LN//19vQQAyNylXZH+Y3oBs0S
IurR60lhZlaZecpTlzFpRP8+eHfiVasLfVIImPi+9hqzXor5KXfOCrAYvunRqJSlMENguK+5bIB7V+1T1Qg
UA5Ovqp47LG++bvg7qKKo+6u//
+y/bHwEAeVD1Uf+79fFZZxEzQ4i6tLpy4KZA5bp0+33p9/dkM68MdWzgt6QmPS+qZ+S4YK99XtxN
V1vqevelWy87K5qrQo4aA/ctEIuqf6rqoAIA8veWvRvtIDVLqifqH/721/QSACBz/UdFLv2xXsCsEKIuq
96h7OuncuqE5Nf/FGmL1zOyJ41zugtquS5bFedpcfOsVJ2n0twlRQUwPTUTf+WPKvZHAEC+Xvu1F8
vLHr1aL2Vrb/f/2A8AQE4uf8l5YGYIUZeQXbvRDN2aq04tx8GuiKp3o1E7c1T9zGAtyGCd1bDejbH3r
wVreI6qN+kMVfdfl6zW52A9S6+2qH6ENy3GPgPncEP8zZjnydln0vOiqHaWYvWMNHoeB0LzkpzUn
VFl/9A6olGM9obVW21v6GMZPAcJrkvI/RZ2vePcb4PXz35E1ImNY5b37WT3p2vSa2eZ6D1Ar1TMr
49uJI6QOz91pzz4+IN6CQCQFxWevubvXqSX8qF6o1IfFQBydOlPqI86Q4SoCDUUGGl2/cyRYVRHai
WjzqrR43EyanuDNTzVurCgpuQNVXdFt3UUZ5+DwWRzdcJ9Fjflgjtk3joz5/RGeo1z3nkq1y+kUEahK
q1+y+tVOsTseTxK50C6+umwipx2N97ZkYsDJ7W96x2NX1c3yXVRQV7I/eZcj1EBZPj9pu7d0GC5U5
NSIMjOSpb3bcz7M2CCa2eZ7D0Ay07VQP1M7zN6CQCQFzV8Xw3jz5uqi3r3h75KfVQAyI2qj3qP9Z
H6qLNAiLqEKtt96Xfr4kZ7Um3Z9TH72zrxbG/4AZBXL9Won9mpyfrIxEaFec7r+3uboWUCxu4/wN+
et3/pyI6XBPWkse4GPca+Wzo1imzrKP523M2ofdbOu/Fbsn0WNy+If+rOS9v677wbglnn94JOUJOd
F4P9um2ZLK8uyolT+ukYlS23beb5V9ri53BnxMnhkpwj67VGqYGh+21ofyZj2/b9ZrYlbFt+kJ3U/Ny3
/uvD789hya6dZar3ACyrT+x/wp6NHwCQv5UvvTTzOqijPPTV78qf1hmBAAC56T8scvnP9QLyRIiKIW
bPtPoFPwgdDgOHletbE4Z54aotPxwsrp3xAqzOge4z2bsoO14eaey7suW3deeiJIl7zH364ZMh8T6Ls
nnWTbuaslpwe08Gz+8kipt740PW2PblMOpEFdfkTNjxtXf9XrVn1pzjSXSOrPOzp0NEI3gPvd4Dhu43
s8et0evSPk86CNzLYfa0LO/bsfdnmCTXzjLNewCW031fv0/u+rO79BIAIE+vfPBaufnrz9dLs/G5jz8kX/
nLR/QSACBzl74gcpkJ/vJGiIoBI3qm2Yqy5iYxIwK3UyfSDKjKcrKkn47SPdC9+VSoU/LqNhbMYdKRw9
QnMMk+K9tGr0FHOsP403RKoi+fcf2NgNIP3Kpy1j2gqa6LruEZOrw/aOh+M8JCawf20Hd33yNrsaZ
uHu/bBNduyvcALB93Jv6nn31arwEA5OWGx6+Sn3vgJXpptu7+0APy+Dd+pJcAAJlTw/r739ELyAMh
KibUkeHOgTHCo7mX3TFUvMKUSrD+5Oz05HBssVSf36vSHRZuBG7V08FeoUmYExB5PXXHCbtWR
dm8EN470w0rF3t+o8nvz/SvXdh7AJaNCk5VgPrID+h9BAB5e86Pj8lbvnijHL9U0Gtm60dPPiPN99+v
lwAA2XtK5NIf6OfIAyEqJjR/gWm53vWGbAcfk9YLHS/
+Po26n7aO1NZznJyneELGlj4tn5Sxl7S4KW5lAntYuDF8vno6/CyPP0dt2TB7nbo1OM36o0lYbdzT+xj
s/as0z83XpEi53bcTXLtoR+GPJpiWGsL/wCMP6CUAQJ7e+Nc3yAt+eIVemg+qPuqnf/9QLwEAMqfq
o15iYte8EKJiQElOusnV0EzePbnoFnIcO/Q7J6WTXtA2qm5m6ibYZ6+xrodpl6Ved5OsPCfnMa5rc1f8
zphdOfAu6QmJc0m9HrXW/XH+/I4OP6sSyOGSnCOzLqcKFN26qMaQ96TUzPKqx6k9GZQdSHa9W
p7pDpOf0CzuW0usa5fme8AU1xDz7yNf/Ig9mRQAIH+lh1448zqoo3z6v/6D/MOXvq2XAACZu/xZkf
48dRU6ughRMcCcBCnYW9IPAlXYNTChz6yYNTCbq/5Q7faGHhpuPdIev510n72GrLsnrnpWNjeNyY
Nq65JPjmrWsmzKrm5er3HOCzBj90asnBbnDuIIs+ke18Bw8Amvix8oDvbcjUlvX80sH+xxaoTFZo9b
oz1p3yaRZnHfKnGu3QTvASU/dZXaee9gZMOb4h9Hze6Du/LBv/igXgIA5OnaJ6+0Z+OfZ83fvF+
+9y1qZQNAbtSw/v739QKyQoiKYeYkSJ2alHSoU/LTE7kw7YxI5vDy5uoUoVGwBmZzVQdQXnhTlVZ
qs9e7kuyzJ411d6h6Wepbav3okCq98zLMnFndbbN3TastiX+aKhIo72oZDmATnCMv2LO4x2xOsJTEi
HvXrLNaPevPNp/YQt+3SpxrZ0n4HuDXW7V41zBubVssGjV8/85P3amXAAB5UvVP3/aFl81NHdRRV

ICqglQAQE76T4pc/rheQFYIURHKHgYdUpOy2rLWu8Otp1KR7cGClfuHRs/BBOwamMZwbZddWzOj
eqgx92n23LN7obonbiCk8of1p3hehhRlc2+4Tqg9hD5hYBeYJMs6ZjsbHhT7uljH3G/5QaqtKq2+0dZ
ACYJo9r07eJA2Z5vTZZMLft9aYl07S6L3AHUsg6+ttiava4u59cTTTzATPwDMkOqBqnqiLgI1pJ/6qAC
Qo8vWb6bUR81Uoa8KBgJAEmo2fT0ZlAph96btmTxL+lhOtaYNWBfEUbp2yJUKTit/VJF7H75XrwE
A5EnVQVWTSS2SK597XLbufr3c+uoX6TUAgGxdKXLFb4gUbtbLSBM9UQEkFCxRcGZtsUO49nnrWC
J6ZB4tR+vaIV/v+
+z7CFABYEbULPx3fPl6vbQ4nvmnS9J8//3yoyef0WsAANmy3m8vqWH9TzmLSBUhKoCY2rJh17o06
pWaJQoWUK+xIqv7demmUqJinh29a4d81e+ry8f+9mN6CQCQJ1X/9C1fvFGe8+PF/NXt8W/8SO7+
0AN6CQCQuf53RC7doxeQJkJUAJNRNS8XfPx7cXMvpRq/C+YIXDvkR/U+Vb1QAQCz8XMPvERueP
wqvbSYvvKXj8jnPv6QXgIAZO7y10QufUEvIC3URAUAAKEefuJhec32a+wJpQAA+bv568+Xf/6Fm/TS
YqM+KgDkjfqoaaMnKgAAGKKCUzWRFAEqAMyGqoO6aBNJRVH1Ue/
+0FftjwCAPKj6qGpY/7POIqZGiAoAAIbc+ak75cHHH9RLAIC8qQB1UeugjvLN//19+cPf/ppeAgBkrv+
oyKU/1guYFiEqAAAIUDVQP9P7jF4CAOTttV97sbzs0av10tGyt/t/7AcAICeXv+Q8MDVCVCTUk8aKm
uVbPTaknWjdOGltZ96MOYZeQ1Y2gmvbG+7rV6TR0yunpfZjb3NEO2zTne9M2m1bkPugvaHbWJC
BSwosjE/sf8KejR8AMBsqPH3N3x3tuqGqN6rqlQoAyMmlPxHpP6YXMClCVGCGeo0VKZRq0tHLWe
pd3DH205RdQj4AA+77+n1y15/dpZcAAHm7+qnj8pa9G/XS0aXqov7+v/8y9VEBIDeqPup/tz5SH3U
ahKhIqCibe33p99VjWyp6bXby3l8WRhxDryHrtTziU6UnF3eC+2qea1hrF8VRuA+A+aZm4n/nJ98pT
z/7tF4DAMibClBVkLoMVE/UT/7nA70EAMicXR91Vy9gEoSowDLoXRQ3Qy2Xy86Tzo5cXJwUdTFUt
qXfrYs+w8DCUMGpClAf+cEjeg0AIG8//ffX2UP5l8m9n3xYvvKX/NsDALm5vGc9mOBvUoSoS8eoLbl
i9kQctd6oc2mvz7s25fD+IutuGnU/VwKfNLcT9vko6Z8z+/PmMP7mqvP5EYU0/WNWj+T1Rv2h/FU
5e+GMDvk6shMnRQ3UUg17jG5PvHbHuTbD59DaumyYrzfqkQa2MbA+/BTHaYN5POpY/P2rh7vd
9nnrupbrsjXQXdYu3WC83n4M3DfArKgh/A888oBeAgDk7YbHr7Ink1pGd3/oAXn8Gz/SSwCAzF26R
6T/Hb2AJAhRl05R1s64PREPpOs8s3TlwE30Aj0U27LbdJ6Vz6xZXz17ldNV/awjB/4B2PywsCxn1nRr7
RCwJIMj5zu1UswQa5bnrCO1UkFW9fYcal2SINUYyl89LZXimniHs3Nx+hCvfEbcU+2L2e6pr43Dfn1w
Z846FVQOrG+uDgSpE7VBHcuqeFvWoakKSlf369Ld2wxcdxW+lsJKN3RqUprnibKwFD7yxY/Yk0kBA
GbjOT8+Jm/54o1y/FJBr1kuP3ryGWm+/37qowJAbp4SufQH1kfqoyZFiLqEimtuT8R9OXQTovauHwi
JEU72Dq1XOU6dmIcI1VI5LW6M2gzMjmSEhV6w15PGutvjsyotu6am9WjpLXRqsh4jjUz7nFW2rTa
Yw76rLadd22HVPv12d+vuV8TsRaoYQ/mrp9X2zVA4xpD+4qbsuefNfnTFa4Z1BPULwcDQN67d6V
wbV7neDX69Frbev2+maYPxeh2aFjf3vOc+P1RXYWtXf41/TppyLsFxAmnafXBXPvgXH9RLAIBZeONf
3yAv+OEVemk5PfTV78qf1h/USwCAzPUfFrn853oBcRGiLiOvJ6lfaPUOndivWnXCo32dFJrDwO38bS
5UZMsNoJq7fi8+s+6n2wPUXFff8ickqmx5QWCs3pgzPGfVlj+Rkh/mWi0Z7IY7gtk792TJfmJsJ0EYq/
Ua636vzepZ2QxPUMe3O61rY6vKWbchRsg+cv3+obPdKdoQeH0UI1Q3Q2s7cNWB6t6okwhkSA3f
v/NTd+olAMAsvPLBa+Xmrz9fLy23z338Idn/n4/pJQBA5i59QeQyf8BKghB1KRXlxCnnmRtode1x6W
U5efqkHXYF11vUMHDn2VzwQ7mmeJ0KuwdeWOgN5ffWWcfkDu+2H8bw7cAQ/VFmdc784HMy
Yb1zLRMP6W/LeT9BlVZoz1klRrtTuzaW8kkJ3d2o9a4p2hC7Z7Zxrq2N2WUO3P3Er8sLpOuJp59gJn
4AmLGXfPe58nMPvEQvQbn7P35Vvvct/m0CgNxcVvVRv68XMA4h6pLy6oraPTl74nSqPCUnKies/w
+uV3ngPEWoFiOYcodmt90x0xG9I6exkOfM6GmphqaXQkPC+LP0tzf8OqBVo6fp8kkSbhdl80L4jP1u
cDtiPjEgE+5M/A8/8bBeAwDIm6p/usx1UEdRAWrzN+/XSwCAzPWfFLn8cb2AcQhRl5U3tHlfDts6aL
N77VXEyQqN9VP3hsyCUdfTDi/9upOjwkuvNubQI2YYuIDnzB/KHyXmkP72hj9RVLUlIzuhTmDqa5O
CTNtg1JUdKNlqa55jln7k532ffZ/c+/C9egkAMAsrX3qpXPvklXoJpn/40rfl079/qJcAAJm7bP02eukze
gFRCFGXVklO2hlkRw52nSHNbh3Rkv0Jf3347OuzV9w864eaDXeSp4E6pCVnqL3i1eKc2KKdM2Mo/
xjjh/S3ZcNLUMtSV1PRTyvVazOhHNugZuhXPU7tScXsQNWYoCtu2QJgSvX76vKxv/2YXgIAzELpoRfa
D4z26f/6D3aYCgDIyeXPivTp2jMOIerS8ntyNptOOObWeCzq4p/uem+Sprnj9gDtSK2mA77BOqRmP
crmqj9sur3h1aUsxB5LvWDnzBzK787+H3iYIV70kP5e45w3jL9cv5BOuYRUr82E8miD3pbKoIM9Trvi

ls8dW7sVSMn/+v/
+l34GAJgF1ftU9ULFeHd/6AH9DACQi0v36CcYhRB1iZkzpqvehd7w88Ds5sYkTXPIq1NqC+shGaxH2
VzVwZg/Ll1GT440LNVzVtS1VJXmauqhoTmUvxxaW8AoiWC9cuSQ/l5D1r0CqtYrAxMwOY/JJkhK99
pMJoc2VLb9IfyBurRGfdmzm7MP3bEU7v7Fu+WOl9+hlwAAeXvjX99AHdSYfv7dP6WfAQByUXijfoJ
RCFGXWWDW8FPiTzbuDltXzPVzyAwvRw2ht+tRGr0uXeW6dJPWu0z1nFVke7BA5v5hSrUxzaH8o0
NdMxQeNaQ/Xl3VCaV5bSaVQxvsIfxhxVBVSNvvp1pfFohy1RVXyf/4lf8ht1x3i14DAMjT373yCf0M
UV7z1pvkbe+5VS8BADJ37FUix9+kFzBKoa/G9QIAgKXxwCMPyOv/2+vtmfoBAPm648vXyysfvFYvYd
D1P/E8+a1PvVGedw0TbwFALgovFrni/daTq51ljERPVAAAlsztN91uD+0HAOTvb27/tjx2PX/ECnPlc4
9L9fdeS4AKALmx3m+Pv8f6SIAaByEqAABL6F2n3iUfeMMH9BIAIC+Xjvfl8294VH78nMt6DVz/8t+V
5NZXv0gvAQAyd+ztIoWb9QLGmSpE7TVW9AQpBX9W65F60lhxJ1TZkODL27LhTbbiPCabqGZYeyO
43dBH1jOAAwAwhz788x+W07ed1ksAgLz84PnPyt7rvqWXoLzidS+R1X8bNhkqACATx4oix39eLyCO
OeiJqsJVf5ZqR84zwquZ0VcaKU3oAwDA4rjnl+
+xh/cDAPL10M0/kO6t39dLy+3al14l1d99rV4CAGSucI3IMTWMH0nkGKIWZXOvL2oeq35gxuuuHH
iTiKvZsNXn92QzxwzV1qnJeTqkAgCWjDtj/3VXXafXAADyonqjfu+aZ/TS8lIBqgpSAQA5Of5ukcIL9QLi
mn1P1N6h7OuncuqEZJedlqXedUNc/9Gtl/XnRZq7pKgAgOVzy3W32EGqClQBAPlR9VE/96Zv2h+X1
S/8xivsofwAgJzYdVDz7rl4NOQYog7XRLXrlZZq4nZEtYfVD9UoNb/OeaRVL1Uprp0RP0YFAGA5vfm
WN8tH3/5RvQQAyIvqibqs9VFVePoLv35CLwEAMmfXQX2HXkBSc1ATNUKvISuFktS8lNXRqZVSq2H
aPu+HuNXTfpGB5WVM8uWd46hJwTA7YddqEYTfT/4kcHneY0nPYU7fC+q9b2DCO//8rEiKf0cCAt7
7s+
+V2h01vQQAyIuqjbps9VGfd82VctfvUJMbAPJztcixO/VzTGKmIWpluy/9bt3vCVptOcPst1WY2ZPGu
htwVqXlDsFvVe01qobpeqIkoSO1khtC+I9Vd0Yra9/2bgFghnqNlWAPfSBnqjeq6pUKAMjXfT/zuD1r/
7JQAer1P/E8vQQAyNzxO0UKL9YLmMT89kTtXZQdnSKU61v+RFSVLXHLmHZ2LqbU+64s9S0S1NFG
TQoGTGKR76eM295ryPpg13tgBlR9VFUnFQCQnx8/57J8/g2PLkV91Le951Z5zVtv0ksAgMwdf5PIsV
fpBUxqfkPU7oHXE8sevu/1HjWG93cOpKufTsfppZpmrVUAABaVmqm//attZuwHgJw9dv3T8je3f1sv
HU23vvpF8ku12/QSACBzhVtEjv0LvYBpzHdN1FSFz85vlhPo1M7nWItxHgxO2jWqxuPoOpD20GPv6
/VjZG3Jwf1FBNd2Pdzga0fVgsysDVEya98oca+VK/5xJm1f2OsHyncO1O806o7arx19P/kG2p/K9Ux6
DsOEt32wXungORo8P2HiTbTn8/epHqPqpCY5P0DQbdffJvf88j16CQCQl7+/7Xvyjy//oV46WlQd1O
rvvVaufO5xvQYAkK2rRY6/2/p4hbOIqSxEiFqud4fDT/uRwnDa4qac1WVWRfblcGnyBRVsDU7a1ZTV
wqr1/3hUiFMKG3rcqUlpMKBKMklYe2NETUjVYzgYFmXWhihZtW+khNcqwXEma58TyIW9vrlaGBH
2qXNitLNcl/GVM9SxDbR/6us5/f0ej9OrffAcNVdHhZyTcPbh1XO2Dd97qd3vWGrvKL6DGfsBYAb+6v
WPHcn6qL/yH15JHVQAyNPx09RBTdH8hqilk34P0YN0Bu2Ha8tuuinKQug1zvnhkTuhV7/r1Zsdzzhv
5bp0dbDd9TbQlHNeotNLMEmY9dpzesOh2+3IzkX3tVm1IUpW7Rst2bVKcpzJ2tdrrHuBnP+HjZa1F
615bkRQaLRjb1OKem0koz1u06e5ntPf70n47fHb3pHa+ejIPHqivUH+PsLvvbTud0Ds2frfdepdegkAk
AdVH1UFqUfJyumftB8AgJwce53zQGpSC1HtnmjGkFHzEWco65DimpzxspxVfxuqF6C77UQbdnpv
me1yHmYvuTOyFivhWXQ9uejO2qUCFi+kKcrmBSPEidI7lH39VDo74mY3xc09J6yxHnub+mQmmiT
MmLjHCNyKa2eGQ/XM2hAlo/aNlPBaJTnORO0LtuOst74iW/aGVVC3J2GHE2hHTNWz/rmtbPtBrd
/2JNczhfs9gWrL7yFf2Up/+4q5j/B7L8+J+bAM7v7Fu+WOl9+hlwAAefjmjU/JV376u3ppsb3sp14o/
+a3mNAEAHJTuFHk+L/WC0jLHA/nDwYcXkjrjWE1w5B0mMHN0daVAzdTKp+Ukn5qK56QU/ppJDP
kHgioh2ouTjVJmK6nGTZ8Prc2REmpfSMlvFZJjjNR+0a3wwldR5fWOHUi6XdVVU4HNlaSk2473bYn
up4p3O9zpSwnAwcRIteJ+bAMrrriKmbsB4AZuP9V37HD1EWm6p/+
+n/5GeqgAkBurhQ5/mvWR+qgpm2+a6IWN2UvbMitPdQ3hXqoHtWLri8pZ7JH3OhefG5oM1EPZ
MWu5eiGPlE1KzNsQ5R5b19sebQvRuA3VlFOLEzSmcbxAvPpphfcZAepKlAFAOTn8yuPylNXX9JLi0f
1QFU9UQEAOTn+r0QKN+gFpGmqENUc9hv1cMLJoj8M2gxA7aBUrw9NMc2v04+4dRUtdp1B82tD
H2kGskvEuHZ+7Udf89zw5DXjJwlry4bZq9OtjWnWijRl0oYo2bcvLbGOc4btG21wgreeHHp1B4ZlOv

HcEcD5QZpuv+l2e2g/ACA/KkBVQeoiel3l/6IOKgDk6dhrrQd1ULMy3z1RkZGQ4dEus05mDGp2d9V
b0Q+rjZ7D7raTTBLW3vV6dtrhjxuYG8OTB6XehihZtW+khNdqguOM176Idth1itOdfT7Y9JDh+ImO
M737fWHkNjEflpGaZOoDb/iAXgIA5EEN6f+7Vz6hlxaDmoWfOqgAkCM1C7/qhYrMEKIupaKs+bN2
yao3Ztuc0XsMPcGXKlEb7K0YEnhNOEmYH/4YM+KbcmhDlFTbN1LCa5XkOBO1L6Id9rE7NVUTnsK
Rmqsb4jfdL5lQPrPmhNaJrmcK9/uiyeB+B0wf/vkPy+nbTuslAEAeVH3Ux65/Wi/NN1X/tPp7r5XnXX
OlXgMAyJaqg/oe6+PVziIyQYi6pIqbF/zehs1VHawYk86MU9n2h393alJygxmjRqg/UVew9mbkJGGV
09aSNq5dWbUhSmbtGy3ZtUpyrpO1b2w7ynXZSmV8eFnK5aas6vZ4Tbfaftab/j/Z9Zz6fs+LOdGV28
6Jws6U7ncgwj2/fI89vB8AkI9Lx/vy+Tc8Kj9+zmW9Zn79Uu02ufXVL9JLAIDMHVsTKdysF5AVQtSlVZ
TNvcFJu6rS6res/8djD/8OK6Rpb2dgoi679ubg/ixDk4RVZHuoDc72vF01d71eitm0IUqG7Rsp4bVKcJ
zJ2qfa0Zfu0IbVpo3SBlM7JWcHjzfsGiW6ntPf7/mw7q/B67F/aPQSTiCV+x0YzZ2x/7qrrtNrAABZ+8H
zn5W/ev1jemk+veatN8nb3nOrXgIAZO7Yq0SOv0kvIEuFviqCCAAAMIF7H75XKn9UkaefXYwhpgBw
FNzx5evllQ9eq5fmx7UvvUr+06ffzDB+AMhL4RqRK9R8BQzjzwM9UQEAwMTefMub5aNv/6heAgD
k4W9u/7Z875pn9NL8qP4udVABIFfH3239jwA1L4SoAABgKu/92fdK7Y6aXgIAZE3VR/3cm75pf5wX
v/Abr5BXvO4legkAkLljbxcppFNUD/EQogIAgKmp3qiqVyoAIB+qJ+re676ll2ZLhae/8Osn9BIAIHPHiiL
H36EXkBdCVAAAkAo10dQt192ilwAAWeve+n37MUuqDqoaxg8AyImqg3rsPXoBeSJEBQAAqVAz9b
d/tc2M/QCQI9UbdZb1Ue/6T6+2g1QAQE6O3SlSeKFeQJ4IUQEAQGpuu/42ueeX79FLAICsqbqon3
/DozOpj/q299wqp/7ZDXoJAJC5428SOXabXkDeCFEBAECq3lF8BzP2A0COvv2if7Jn7M/Tra9+kfxSjV
/kASA3hVtEjv0LvYBZIEQFcteWjUJBCsZjpdHTn1tSvYasbLT1gqO94Z6fFZnX09NrrDhtXGnIqCb6x+E8
lv5aY2mo2frfdepdegkAkLW/v+178o8v/6FeytbzrrlSqr/3Wrnyucf1GgBAtq4WOf5u6+MVziJmghAV
yFVPGiur0tRLjrKcWSvq58vHDiJLNeno5YXRa8h6LarV6loXZDV4saVTK0lhIDAGjqq7f/FuuePld+glAE
DW/ur1j8kPnv+sXsrOXb9zu1z/E8/TSwCAzB1XdVBfrBcwK4SoQK66cuDmbuW6dPt96ff3ZHNZM9
SxQeScUj1nxwW/7fPiHlq1pa5zX7r1srOiuSrkqFgGV11xFTP2A0COfvycy5nXR33zr9wir3nrTXoJAJC
5YyvW41V6AbNEiArkqXco+/qpnDohy9v/dHHF6znbk8Y53QW1XJetivO0uHlWqs5Tae6SomI53PSC
m+wgVQWqAIDsPXb903L/q76jl9L1sp96ofzKfziplwAAmSvcKHL8tF7ArBGiLjGvnqP5CKntOLI2peqN
536d163Or/dp135sb+ivNdbZLwuuD/bKS2MbmtlG7zFcYzN4jMM1S+1HWN1Low3jal3a+zDDt+aq
87WDDQ9pc9i2o9o8tpfjROdl+J4ZuZ8YxxD7fGh+W9RjuK0OZwi9/7p0z506/lKsnrNGj+NAWF6Sk7
ozquwfDt9PNqM9ie65eMduS+H7wj1Pcd9HsNxuv+l2e2g/ACAff/fKJ+SbNz6ll9Kh6p/+
+n/5GeqgAkBurhQ5/mvWR+qgzgtC1CWlwpHQMKhTk1JhQ8ZlcHHYtR8HCkLa61TIMrC+uRoeXk2
1DRU2hfYY7EitNCqEU58zapaW61J3uw52duTiYMi0674ynbqmQ8GiZh/zyGBquM1uz8dQE5+X4X
umuToieEt8DFGcfQcvd0hb7WCw5A2hd6V67jxVafVbXq/SIWaP41E6B9LVT4MqcjrpPZfk2FP6vlDn
KY/3ERwdapKpD7zhA3oJAJC1z688Kk9dfUkvTe/f/Nar7J6oAICcHP9XIoUb9ALmASHqUmqLn8O4d
TmNeo3SlHPhSUpi5XrX3na/FYybwtaPGt482TaCw6mHj7EjO4PplEcFZM7r+3ubsrlVF+erBr/GPI9nZ
FyGWtm2ttd1t2Wptpx9bOvUrr3hB4Vhbe7UZH3kdQm2eXRT0jkv/invSO28cd0SHMPY8xHg7zu8r
dZxrbvBoHEu3Iamcu40u53bEitrHVKUE6f00wiVRPdckmNP7/uimOP7CI6OD//8h+X0bQxHAoA8q
ABVBalpWDn9k/YDAJCTY69zHpgrhKjLyOwlZ/R0K27uOQGJ9dhLZaajqpx1t1M5bS25RqwPHd486T
aKsrlnBj6O4toZL7TrHIT3AyzXt4IBWXFNzugv6uxc9NvY3vV65pXPrI0P38YwexjWLxht3rwgfgZ5PrR3
31CbR5r8vFRbfnDoh3xB0xxDFHPfoW3tXZQd3SEycC4qW/5+zWtniH/u1HFY3yOhIe8k9uUwrEFKk
nsu0bGn+H2R2/sIjpp7fvkee3g/ACB7akj/V376u3ppMqr3qeqFCgDIiV0H9V/rBcwTQtRlZAQ0ImqY
bsGrZTiyhuIkyielpJ8GjFofJo1t2HQ9x9BhzEGnTgwGP0VZ8xMtLyzyA0Mj0J1YVK9WY/8jgrfhNscV/7
yMN90xjFaWk+MudvfAa79X7sF+GEPcRwyfn/zcTeuUjN51gntuimNPcv2HzlNe7yM4ctwZ+6+76jq
9BgCQJTXJ1KT1UVX907t+59XUQQWA3Kg6qHdaH6mDOo8IUZdSUTYvhPckdEOYqMl1FkZg8hyjn
mOk8MDO76nnDnc2AsPq6dg9GafXkeGOgjFCRlOK52UyYccwK2keVxw9ORxbLNWR2T2X2vVfkvc
RZOKW625hxn4AyNFfvf4x+fFzLuul+H6pdpvc+uoX6SUAQOaOrYkUbtYLmDeEqMuquCl7esjtQKlR
W/Pcos+u3ZYNs3edW7PRrMGZhHW+zurzZA+NNoYyV0/nF6FOH/qlfF4mkm1w6dXKHXpMWsd0

AsUTMrb06bje1BPcc+OPPf3vi6P9PoIsvfmWN8tH3/5RvQQAyNIPnv+sHaQm8Zq33iRve8+tegkAkL
ljrxI5/ia9gHlEiLrE1MzaqqeYPcGPHYR0vRqKo4f/GoxhxHPHrB2pgiW3/uMUba64U6Z3duT8+R29n
aqkk6GW5KR37gdnZO/JRbfoZeQQ8BgyOC+
+nI4hTOmkFwKOqumZL+NcNHfF75DZlQPvNJxwzn2EWPdckmPP4PpP/T6Cpfben32v1O6o6SUAQ
Jb+8eU/lL+/7Xt6Kdr1P/E8uet3qF8NALkpvFgP48c8I0RdRu0Ne6itmkU92FPMCHiMXnIlPxkzZmJvy
4Y3Dft884MlY2bySXgTWHWk2dQnKrWh/EXZdLsdqvO87l+XXmPdq22ZZBKkcVI7L578j8Fj1udsrvr
DyPW9bj9yHVtu1oBtyq7eda9xzgsxY/VgjnPPTXjsU1//hO8jZntyvRSYe6o3quqVCgDI3t/c/m157Pqn
9VI4Vf+0+nuvleddc6VeAwDIlqqD+h7r49XOIuYWIeoyqmz7Q287NSm5QYtRH7F6NnzmbhXSDL52
Lpkz9nttNibamUhF3I6BrlSH8o+4LiU/fZQL005glcl5MSQ9BnPYu9ueiRK2YH3O5qqz34IX9Felldqs+v
EUNy94PTLd9njnodqSeM2Jc88lOPY0r3/C9xEgiqqPquqkAgCydel4Xz7/hkcj66NW/u8idVABIE/H3k
od1AVBiLqk7KG3YUUMVeDS7wcDHlX3cLBmYrWVcx3NpCqy3W/5gZHNOTbvsAPDrOPxhlcr5bpsp
ZzL2dcl5LxWW9Z6d+j1VLI5L6Zkx2C1Z/A+3D80ejUmYNfnNIaSu+y6nznWQ/UUZXNvuFaoPYw+Q
aAb656LfezpXv9E7yNABDVTf/tX28zYDwA5UPVR7/uZx/VS0Cte9xL5hV8/oZcAAJk7Zv2GfPwdegHz
rtBXRewAxKNmNtcT86gwbG/anqHAOEfpntPHcqpFwIpwn+l9Rip/xM0BAHl441/fIKWHXqiXRK596
VXyW3/8z+yPAIAcFK4ROf5+66P/Xoz5Rk9UILaeNNbdmc3LcmaNABVZO1r3XPu8dSwZ9ODG0fGO
4juYsR8AcrL3um/J9655Ri+JVH/3tQSoAJCn4+8mQF0w9EQFxmrLxmANWFXOgK50yMzRu+d6jRU
p7ZyRbiplKXDUFf6fgn4GAMjStU9eKe9s/aS88723MYwfAPJ07O0M419AhKjAWAOBFgEqMsc9h+
WlJiiblx9NaEs42hKOtoSjLeHmqS0nrr1GDr/3pF6aLa5RONoSjraEoy3haAvSQIgKAADmBj/ghqMt4
WhLONoSjraEoy3haEs42hKOtoSjLeHmqS1IhpqoAAAAAAAABCBEBUAAAAAAAAIhCiAgAAAAA
AAEAEQlTkSE2WU7DrfxRWGtLTa8fqNWRlo60XHO0NvZ3CijRibwjp6Uljxb0GG9aVTbJuGmH3UNr
7mJbRRv1Y4SYFAAAAAGChEaJirvUaK1Io1aSjl4H5pgJdY1Z9W1nOrBX1cwAAAAAAsIgIUTG/eg1Zrx
GfzqeibO717RkF+/1tqei1szFPbenKgXvLluvStdu0J5tkqAAAAAAALDRCVABIS+9Q9vVTOXVCyE4BA
AAAADgaCFGXmao1OlC7MazG6GD9UXuIvfE1A+VKNbNOpXokq1Vp79Mcxt9cdbYTvjOjjeoxqk7qY
JvCa1VOdrymMbVf2xvetob2H3JN4rTRY359ZEONcxFo46j1xj7t9ea5zKoOadx7KKotg9uYsD5pjOuS
9J4FAAAAAACLgxB1WakgL7TWaEdqpVEhpPpcQUoDQ+ybq4OvVyFiSYIva8pqYbBWZBqcNq0GNh
xyDHYINtgm65W1UnjQaYt7vIMqcrqqn3Z25OLAa9u7bmODtTKHQjgtuo2TKsrambLztHMgXeeZxR
iOHmh7W9xml8+s5dDDMoV7aKJrPizf6wIAAAAAAOYRIepS6knjnJuIuXUb+9Kt61BNOrIzmPx5qtL
Sr2+5QaEKG8/7ve16jXN+0FVt6VqVXfE2H0Nl2/qabl28L3G3sx1W8dJvU/gxWMe77oZg/mv77gF0
arI+MhUdf7xhKltu2wfPpR9GSvmMeBlqe8MPgsOuSWQbJ1NcO6PbuC+H7qbbu0ZI2ZEDN101hq
mfOpF9hDr9PTTNNTckuC7J7lkAAAAAALBICFGXkjERz96m16vQD9VEOl56FlRt+RP3+EGhqScXd9w
+e1VpeQGStc8LYa+fntmm0GPoXRS3SeX6lj/xUGXLC+U6OxdDexSOP94RimvidfQ0t22ElGaPTrN3av
2CcU02L/htrJ0fGKo+Ja+NftDbO3Si0mrVCRv3dbrau7jjBZKnvROYlRTuoSmuuWkm1wUAAAAAAM
wdQlRYdA3P0OH9SZmzk5+Ukn5qK56QU/ppespyMrCTEN0D77jsIdheXUtjqHdgSHsazOHy/rB4P5
SryllvyvYRvVNtxnbMHqOpKMoJfUHcwLlrXzzrnJ4+aYeVwfWW6mk/kMxMCvdQKtd8VtcFAAAAAA
DMG0LUZRWYKCdunckYgeWRMt3x+r1i3Z6eRig3URhpDK9PScUt3trctVrXE6cj6ik5UdFhZWC9anb
2EeriSf+6AAAAAACA+UKIupTasmH2OnVrPZr1HI+ocr3rlDEYevjD9lNT3JSzbglONXTcqCs6WRiZQYh
dOS164L4ctvUQeLv3pzs5lrF+QUP07K/5sv1xAQAAAACA5UOIuozMupwqYHLrohpDoCdXkpNuEjs
4XNoIEXNVcoamK6NqvWbF6+nZ2ZHz50fVFTXP2eBs/mZ90FMydk6nxNfQ3XdHDnadr3VrtZbsT/jr
h4e0ZyWFeyiVa57idQEAAAAAAAuNEHXJ+QGTMWP/VMxakU1Z3XCn3DFnS8+ZMcmTNFfFa1J7w
6+V6a1MmdfTsyPNpj76oaH8Rdl0u6xar6utN6yz5eg11r0anuYESU7AqVivP+8dkGx4U8nH5V+vZtP
5Wnf2/aIumOquNyfCylYK91Aq1zz5dQEAAAAAAEcTIeoy8oI9S3NVh0rGhDtTMmcun2r75iRC7nY
mCjuDs7o3V3WI5gWO5gzwaXOHxftCh/JXtqXl5XU1KdnnrCAlP6mTC95EVCojdOutWrxzHLe2bVBg
W9Yzb2i6eZ9Y68/k0w3VNv09lNI1T3hdAAAAAADA0USIupQqst1vGQGZUpVWv+8HRvaEQpMqyu
Ze1w/BbGr7g/scx2qn1yBt/9DrDZhIcVP2+oNtstj1YDOoh2rwhvQr1v62Ruysst0PrUtbbVnr3ZILLnU

8g6+ttiara2v22gwMTTeGs+c+ZD2Feyila57ougAAAAAAgCOp0FczrADITq8hK3oiL1WDdo+eiwAwk
urtPS8/mtCWcLQlHG0JR1vC0ZZwtCUcbQlHW8LRlnC0BWmgJyqQKbOOZ75D4gEAAAAAAJAOQlQ
gE23ZKAzU8ayeFTqhAgAAAAAALB5CVCAPql5pZpNXAQAAAAAAIEvURAUAAHODelXhaEs42hKOto
SjLeFoSzjaEo62hKMt4WhLuHlqC5KhJyoAAAAAAAAARCBEBQAAAAAAAIAIDOcHAAAAAAAAAgAj0R
EV2eg1Z2WjrhVnrSWNFzZavHhsyL60CAAAAAAADA/CNERSZ6jRUplGrS0csAAAAAAAADAoiJERfp6D
VmvEZ8CAAAAAADgaCBEBQAAAAAAAIAIhKhLqr3h1gddkUZPD7+3l51HaClTVePUeI3zcL7eZW/XH
MbfXHVepzc4uF+PuW1j58HXt2XDfY318F4Wo12TGjwv6jHpuVGSHr8S1obCSkPCD8+s/eo8VkaciG
TbBQAAAAAAWF6EqEuvI7VSQUoDw+
+bqwMhX3tjRI1T9fXpBJbR1H5WpamXpFyXrYr1MbN2OWHk4HlRmquFYNCZ4blRoWtYG6RTk9Lg
BFl2EFuSwZd3aqWhcDTRdgEAAAAAAJYcISosVWn1+9K3Hq2qXqUCwPNulNaTxjkdX5br0tWv7dbL
zjrrtTsXnYiusm19rlsX9zNSbdmv7W+rxHNafjv7e5tSTNCupHqNdS+MLNe7zj77LasFWvOcDkeza4NI
W3bd1Dh020055yW0VjvW3SDXOE/uBe3UZN17bZLtAgAAAAAAgBAVUm1tixtxVraMANRTlM09H
crZ4aVeu3bGe23noKufZadc3/La6ciqXT25uOP20qzK2U13yxXZsoNGFVLuibM6w3PTO5R9/VQ6O+
JmscXNPWd/1mPPbVvvorhNDpynypa42Whn56J1ZJYk2wUAAAAAAAAhKiaha5OGDmHPzqkT44K
9tNrVlQN3A+WTUtJPFSdo9EPnYSmem+KanHGTWGtrquyCW7t0qM5p98Dbnz18X7+uYA7v7xxYR
2ZJsl0AAAAAAAAQoqIsJ82UcJTAxElGbdLcjGjnzNtlyawNRdm8ENYz2A9KQye5Giur7QIAAAAAABx
NhKiIoS0bZs9Kt46mWft0JuahXRm3obgpe3qIvV+v1tc8Nzybvl/DdfBh9KCdYLsAgLzp0Q3Ggz9yAQ
AAALNBiIrx2rte70o7oHNrfxpDyKc2ybYya1dJTroJqDsE3qVm4i8YM+6n1YaI16uZ9NUvzfakXXbw2f
XqnHrtK530Qtu4NVhjbRcAlpH9Xl+QwkwTy540VlZFWu57tPNHr+bqLILU+Qxze40Vpz0r+f/hT/0ba
p4P9zEP5wXDhq7XDO6ZmRvzvuZ9P7mPvG7mOO+3ebwnR+5jSf+gFfO8z/K9eGZinJuh951luGnG
nJfc34sTtofSdoiDEBWJ+AGdMSt9AiU/nTRm/7d+MFmdbhD8tO0KKsqaVzS0Kavem667baeO6OB7
cZw2JDp+/aavPhXsGRpSs9Wsc9pc9dvm/sOhHu7KJNtVjG3wDwuALMzsl/chKri09j/lv0npcCYu3Da
KcLuTP+4f5vte3N4IC3ONPyjOQq8h6zWRatX9x28G3NEnxsO8XrMy01/cAyWOhh+z+NZW52O1qS
YFda9TS6qdmpRmFPjk/0v7+Pc19R5csr6f6l3jHFk/T2Z778R5v83jPXncPtTnc/yDVmTok1eYm+C85/l
eHHFuht739CP98xP/vl3dH/g3Iot/IEadk9zfi8efl3zfiydoj/WNrUrbpf+eHO/7dn5+FsZY1g2DJWS9R/
TV5bd+A+hbPzD5uvW+9U+g87mq9ZZia/WtnxX060c8vNcqIa93P29uf9TD2NbIdtqStKvbr5fd9VXr
K8cxXx/yKNetVygJz83Exx/
+MDcdve3gMSfabqvqrS8PXwQAmEq3XrbeX8z3eP2+GngjyoffFv1vwAzaEEm/z8/8vXjm7XCuj9q/f
c28f5PzY/87OoP9RtP37dy1y/3eivPzV9rCv5dn1R7n5y9jv/pnrCy/l8a/rznvuYNtyPocjW9XvNdMa6J
9ZPIeqPdvbdd+hLRD3T/maud+Um3XK1IS/5w4n8/+vTjeucnjvS/
+fZv1+8v4cxImq7aNPy/h62fWnhHfw+nfR87+zd0737fBdX579Qr9vhz3uiJf9ERFDBXZVn8p0ksO56
821puAo7kr/t9KrNd7n9D2D52/MKlanIP1QqutCWuIJm1XEk4PIOsNTS/7rDdbf9h+0jYkPH57qP3g
ubQ5+wj8QdOuc2oMyXfZPWWMeqiWRNsFgMy05XytY71NXZBN503VUpEt9UY28fv35Iqbe9Lv7x
ltmTO69MupE/PRwFm1o9dYl1qnKmfn9kLNhnteWt7PKPPC+T6X6unAzyL5KMqJU9YH9+dQW08u
7sygPb2GqIFK5fqWv9/Ktv3zYmfnYgY9sRxj39d6h7JvfRj8fi7aJ64puxm9Ecd5v83jPXle3ved79+y1Ls
hP8tr6uf34dEJHdm5mO7dE/ec5PVeHOfc5GX8uXHeXwLf5xmY7Jxk9148/rzk+148tj0jfp6yR42mW
tYuzqii+fpZGOMRoi4pvw7mwJuLMeFQcMiBCgvdr1EPJ5TztxMM6dQPhf5rrYf5A725D/VQ+xmx35
Ht9MRtl/MGFlw3nvMG7H6d89gbakjCc5Pg+G2D59J+jDoG8zj1Y9QvU3G3a7xu+NgBYAoz+uV9M
enSL+W6bIX/A5CTnjTWa9KZVTvsoaMdqbbi/1ueGTUM0Rh6N9uSN/n84j6JXuOc9d1s/bI/oxu3sq2
HjNo17dWwypITNg/
+vJW13H5pT8huV1lOenWcNKPePgZk8AetuMFlmDTbEVuO78XTnJv86fJoB+cHhtOnWwJnknPCe7
FhxPub8/PnvuRaNYmfhRcOISoAAJgNfnmPTdUltX/5uTCLXoa6tpj9i6D+pWdGvR3b52vSqbZmPmL
C/0Op81AjV7KppRZXPr+4J+f2NDo7wwDEHTWkatqr7yOrObMI4efpl3ZMgT9oKfPyXhwwR3/Y6uy
flAvevxGqt6h6/9mYYa9C3osD9HwiwflBrO+pqedWiWHwjzD8LLxwCFEBAADmmDP5gRq2N6ueO

MFRDt36vqzO4hfU9oYzCcRc/dbuUL2Csh6WHcfc/eLePi/O6NHZXTNnsg5/YiAVeKtJgXKftGOWv7Q
jNfxByzKH78Xz9oet8pk147pY/4aeVWXUZtirkPfiAernGrdnrP89dXBKXadTkl3n7nn5IwymQYgKAA
Awp5wAVfXWmJ+hjMXNC3YNtk7tfK7hXHtXhU1NO8B1fuEpSEn9Vqh/CZrtcHptlsOyLfP1i7sOCGf5
y6Iecqzq2bt5jz0MViXezdWMZjYfZVa/tI9h93bqyMHgjTuqd9QS4w9ajkV4L56XP2x5ZtqrkPficIMl+f
qydXLfOk8nJau3vdn+EQZpIUQFAACzwS/vkVSvDSdAndMJ/zL8RSPMYE8j9bAngLR+Mexaz2dbt7sn
h6qoWc7nZKxZ/uLu9nw6O8NfFgeHTbpmdl7y/6V9rOIJceZ7CUZNPfuGnmG4O2f4g5Zvvt+LB+T+h
62S2GWOB8PbWf5cw3txTLq2eOCPkekZ+UcYfhZeOISoAABgNvjlPYKeRddiD3czevzkW+dSTf4wM
Bxc/0KW1S8ac6/XkJUVc0i2WqVmSp7lL6nz94u701utKjMcPSpSOW21wPoeCgyhV7dwzTovM26bL
dtf2uOpyGnVY6+27r+veL3G5m+islngD1qLaFZ/2NIjADo1Oe/9w6mHcM+oHinvxXGoUhklqUldLm
RwkSL/CMPPwounDwAAMCOtqvSt33L69a5e0a33y9aPJ2VvRX669bLVFtWewUe139KvWUZh56U
6JyfEblu53s/3bun26+Xg+ZiLe6RVHbg2rb71S2tfJT+5m+H38TB9HgKPefie1vdRxvdv3Pc1573Yf2R9
7eK0K4/35PH7CLt/3Ifxb1dq9H0x9H2r2jFw3Pp7Pu1rNcl5t78m8/fiEedGvd8M7Ns9hrTf/uKem6H
XZfY+POp+0XJ6L453XvJ7Lx7fnpB/xzO6RnHuxXn6WRjjFdT/rIsKAAAwE+5f6F3WD43zNRwQWBCq
x5xdm9BVbUl/Bl3nnO/pqrT6M5p5eS7pnk7G5ZnV9cH8Gfre9fjfR2Gvqc5r79gUjT83Id9bxnk7iuLc
LwrvxbOmRvOoOqhhgkP7+Vl4cRCiAgAAAAAAAEAEaqICAAAAAAAAQARCVAAAAAAAAACIQIgKA
AAAAAAAABEIUQEAAAAAAAAgAiEqAAAAAAAAAEQgRAUAAAAAAACACISoAAAAAAAAABCBEBU
AAAAAAAAAIhCiAgAAAAAAAEAEQlQAAAAAAAAAiECICgAAAAAAAAARCFEBAAAAAAAAIAIhKgAA
AAAAAABEIEQFAAAAAAAAgAiEqAAAAAAAAAAQgRAVAAAAAAAAACIQogIAAAAAAAABABEJUAAA
AAAAAAIhAiAoAAAAAAAAAEQhRAQAAAAAAACACISoAAAAAAAAARCBEBQAAAAAAAIAIhKgAAA
AAAAAAEIEQFQAAAAAAAAAiEKICAAAAAAAAQARCVAAAAAAAAACIQIgKAAAAAAAAABEIUQEAA
AAAAAAgAiEqAAAAAAAAAEQgRAUAAAAAAACACISoAAAAAAAAABCBEBUAAADAQmtvFKRQ0I+N
tl5raG94n19p9PTK2fDbuiEhLV0wbdlwz3vk+e1JY2Xca6xXNVb8ba00rK8CAGB+EKICAAAAODqa52
TGOemSUMHoqjT1kqMsZ9aK+rmpKJsX6tZnHZ3aesg1asv5Wkc/F6me3bS+CgCA+UGICgAAAOAI6
Ujt/OL38Zx/XTlwM89yXbr9vvT7e7I5KvksbsrZqn4eco16jXN+IFttyXZFPwcAYE4QogIAAAA4WuiNm
r3eoezrp3LqRKxeo5Xtlng5auAamb1Qq9IiQQUAzCFCVAAAAAABHTLzeqH590pVg6NpryIpbm9Orse
rX/7Rrehp1Vr119suC68NKtPqCtUJH1wEdeJ31CKsrGjyeYL3S6HYYzGPXj8F92fsp1ayzrDVXndeO3Ul
FtltujOpfo/aGXxagXN+yXjUo3vHbQto/dH0tcc5VoEar+6BWKwAsLUJUAAAAAEdEWep1HdJl2Bu1U
ytJYTVYDdRep0K2gfXN1VEBZlNWCyUxyoCqjUhpcMIpOxQceJ3F3t/IQK8jtZJRr7Rcl60YnTuHwlEtel
8JVbak7hZHba7KRqMh5/wEVS4M1gNIcvwqwA5pv3M+hoNUR/i5UueiNLhTJewaAQCWAiEqAAA
AgKNjzQ3p4vVGnVS53pW+qgPq9ax0hK1v7o5oh1dLtC/
+y5tyzkv7etJYd0PBqrT0a71td2qyPjIpNl6/F2OSpvaGePmv0a6um3ga+6psW5/r+hNFqRqm9n5iDc
MPTjLVrLnHV5b6hcF2Jjl+67VuGhvWfmsrOxfjnqu27Eadi8A1AgAsC0JUAAAAAEdIUTbdGYwy641al
bNuj8nKaWvJNWL9/mFoL05zBnqzXmhn56Lz+t5F2dGdIQPD3I3enN5rB4QPix+t7aeGgTCzuHnB31
ftfDo9MAOTTGnVs8OTUiU6fuu675lBqKO4dsYLbDsHXf0saOhcmfVeOzviZq/FzT1n+9Zjb+QMWgC
Ao4oQFQAAAMDR4oVsGfVGLZ+Ukn4aMGp9qKqcDiR3JTnpp31ix33dA90L01rllguwH8bwdve1A0
6dSBLymT0vz8ha4EuLsnbGbdi+HIYlthMITDJlPQudTGqK41fHZNc5DR3eHzR0ropr4h2yuodK7n4ja
rECAI48QlQAAAAAR8xAb9RD5+l8K8qJU/rp1MpyMn6am0BHRnTmnE6i8DlCYFIpo85ppLBzFSw5Y
HLD3NgTdQEAjgxCVAAAAABHj9kbtRYvTsvXYK/Onhx6Y8iHebVWhx7biYbtTyercHa88cfflg2z16lby9
Ss3ZpEcVP29D4Gyt7amueYpR8Alg0hKgAAAIAjyOiNmpQxjDw7g706u3Lg7tTtmVk66QWAo+p5ps
MsJeDXAHX05KJbmFROSaIqAdNKcvztXa/nqR24unVRp7iWaoZ+1ePUnkjLDlS7Xi3W0WUEAABHF
SEqAAAAgKPJmIAoTMlPDo3aqW3Z8Kapz1ZzdcObqKm94Q8/L59ZcwJAszZnc9UfQt7e8Gp0FlIZV2
4Gzta5WPd7WfYa61790aSTVU1twuP3A1djxv4k9PbVbRDscRoSdAMAlgYhKgAAAIAjKro3qjlzuwrp
nGAubi3NaZWlXG7Kqg4D/dzWmOFftd+ozdlc1cGh9+IREzJNorLtD1vv1KSk21XyE1S5kPuM9AmOv
3Lan6jKu5bGBFRJjDgX5r1RPat7ugIAlgYhKgAAAlCjK6o3qqp7OVgzs9qavI5mIqfk7J4xPFyx63gO1Di

1a3MOvE4Je+2U7GHrIcdebVnr3eHxeYt9/BXZ7psz/itVaZk1TZu7Xs/fcexzEVYMVW8zrewaALA4Cn1V3AUAAAAAAAAAAEIqeqAAAAAAAAAAQgRAVAAAAAAAAAACIQogIAAAAAAABABEJUAAAAAAAAAIhAiAoAAAAAAAAAEQhRAQAAAAAAACACISoAAAAAAAAARCBEBQAAAAAAAIAIhKgAAAAAAAAAEIEQFQAAAAAAABGEvn/AZM6nr53azj8AAAAAEIFTkSuQmCC]]></redcap:Attachment>
</ItemDef>
<ItemDef OID="q34" Name="q34" DataType="text" Length="1" redcap:Variable="q34" redcap:FieldType="radio" redcap:RequiredField="y" redcap:BranchingLogic="[q33] = '2'">
<Question><TranslatedText>Do you prefer Life A or Life B, or do you think Life A and Life B are the same as one another?
</TranslatedText><redcap:FormattedTranslatedText>Do you prefer Life A or Life B, or do you think Life A and Life B are the same as one another?
 </redcap:FormattedTranslatedText></Question>
<CodeListRef CodeListOID="q34.choices"/>
</ItemDef>
<ItemDef OID="summary35" Name="summary35" DataType="text" Length="999" redcap:Variable="summary35" redcap:FieldType="descriptive" redcap:SectionHeader=" " redcap:BranchingLogic="[q33] = '1'">
<Question><TranslatedText>
Alzheimer's disease - Description #[no]

If you have Alzheimer's disease...

1) You might forget recent events of importance to you. For example, if you are interested in hockey, then you might forget whether your favorite team won their last game, although you may remember watching the game itself.

2) You might forget the names of people you met recently, or the names of local streets.

3) You may no longer be able to handle your personal finances or plan leisure activities such as dinner parties or vacations. Simple decisions such as what to order in restaurants might become difficult to make. Your ability to concentrate may decrease and you might have difficulty driving.

4) You may start having trouble finding words to express your thoughts. You might need to be reminded to shower or brush your teeth, but you could do these activities on your own. You may need to post notes around the home to remind you of simple things like turning off the stove.

5) You may remain aware of the day and your surroundings. You might be able to travel to familiar locations like the supermarket, although probably with the help of a loved one or friend. If you are in a social gathering, then you might have difficulty following the conversation.

6) You may continue to enjoy hobbies such as puzzles or reading.

Now consider two possible lives. In Life A, you will live in perfect health for 3 years and then you will die of natural causes. In Life B, you will live in perfect health for the next 10 years, then you will live with Alzheimer's disease as described in the summary for another 10 years, and then you will die of natural causes.

</TranslatedText><redcap:FormattedTranslatedText>
Alzheimer's disease - Description #[no]

<p></p>

If you have Alzheimer's disease…
<p></p>
1) You might forget recent events of importance to you. For example, if you are interested in hockey, then you might forget whether your favorite team won their last game, although you may remember watching the game itself.
<p></p>
2) You might forget the names of people you met recently, or the names of local streets.
<p></p>
3) You may no longer be able to handle your personal finances or plan leisure activities such as dinner parties or vacations. Simple decisions such as what to order in restaurants might become difficult to make. Your ability to concentrate may decrease and you might have difficulty driving.
<p></p>
4) You may start having trouble finding words to express your thoughts. You might need to be reminded to shower or brush your teeth, but you could do these activities on your own. You may need to post notes around the home to remind you of simple things like turning off the stove.
<p></p>
5) You may remain aware of the day and your surroundings. You might be able to travel to familiar locations like the supermarket, although probably with the help of a loved one or friend. If you are in a social gathering, then you might have difficulty following the conversation.
<p></p>
6) You may continue to enjoy hobbies such as puzzles or reading.
<p></p>
<p></p>
<p></p>
<p></p>

Now consider two possible lives. In Life A, you will live in perfect health for 3 years and then you will die of natural causes. In Life B, you will live in perfect health for the next 10 years, then you will live with Alzheimer's disease as described in the summary for another 10 years, and then you will die of natural causes.
<p></p>
</redcap:FormattedTranslatedText></Question>
</ItemDef>
<ItemDef OID="a35" Name="a35" DataType="text" Length="999" redcap:Variable="a35" redcap:FieldType="descriptive" redcap:BranchingLogic="[q33] = '1'" redcap:InlineImage="1">
<Question><TranslatedText></TranslatedText></Question>
<redcap:Attachment DocName="3.png" MimeType="image/png"><![CDATA[iVBORw0KGgoAAAANSUhEUgAABVEAAADYCAIAAABtBTQzAAAAAXNSR0IArs4c6QAAAARnQU1BAACxjwv8YQUAAAAJcEhZcwAAEnQAABJ0Ad5mH3gAACk8SURBVHhe7d1fyF3Vue9x02h33CoJrSekdaPRgxCMSFo5ELOLTaEYEQKCSoNsxcORlwiWHQQbKeZwLhRlU8i7N4JeFCvsnhMvLLKhRLGFXLSQQqG58MINUd4Li4F68W4p5ZXmomf+G2OO8Yw/c6yx5pxrvXN9Pwz2ftdcY4xnjDFXdP7U/P9faavvEAAAAAgCki8wMARnJh48I1/+ca2kzttWdO/q87/3tzzggAAADMi8wMAxrCxubHn1T0i0NLi7eCJ3UXgL9pnn3zcnCMAAMAsyPwAgMFtbm0eeO2ACLS0eCNv77w77d2bVK0x/pe81q7DIjdHsNAPKuJdWNR7XqrnmRcAOg7U8YzY/jnt/Eb3DkvRn3uF5WafpPBpePTNi1E4KuOdubbD3+hZAdAEZuzxAGgo4qJ2F/O3YHyp3nbo/51J1Z+rLRvJNbJq2hAplf4mC6Pf5hqUfAZYE5EVsbyS4uXzpXSFmHS8yQGEcJ+u6A8XgWHPTJV1NdCyz/ytu7FRCHWU7tEEhMVDpXGf/jXdRe3zpy2MbdVGmr5x6SXwLgFRo2Kxx/3UfIwnvQBI/rVu5e6ZfR0p92dwhMp6bvAIrc2pe3hz5Dzt0XCbDSQ7nPqsFhgKSo0YADW84O/6frqrR4qLYF58RLoItHzMR+tRFzajYaRA2bJ3UzUAvuBlzkSGiSyR7SGeAJQrUySPhrD+cXn5p8XKd5PE0DfpTZAejtq1mKlL2v8CUC2EXvWJ/rQzdVRs90PnQ5fOeZRLU2vwPcd2gs2ku6/5Xm3/rBxlYxL2cfx//xt6Pt7RNwR+RmMHJhBO7sJSbcUPEN7MZdBuefvpqhrxOcsk7LuuYr5Fx3kGZWgcvyZYNv4IvBKUysCxvtDGj9dKKiQoYNvOR/8cFMZPoNsMuv6fwzb+bUzzY24FPsZC2zBVCGcOZ1IZAz3lQYRvhZLjbUbbd0pc6gQ8j8o8tBVcqedXuXq4bdQJP6JPlpXYaPkI2vGYuJvsdAXMD8ObTxApQbWFiVh8OTIUGuYR1Zjs3kMZGYzBLN0Ag/P2BGGaDe73/EiwwHkdaIkzYiHg9Az1kOp0zI2r1s60EYnL5vjI2Zeo/D9Bk2J92dHYlQoivX9UdfLmgSCmuRGuxY8N3HT2MKFYaHyTnDHV5Xw/NTDxtR+Hfye2hhW7ofjmlELO8uNtdcWoXOi1rcAA==

0z+ZdkS1hXwXPRGJgwtYMdu/mfWcpvrV1rwRLYOvq1qE3DokoS0tpu5+7buiv8buNL/YDAAAy/zKI
5Df/W4FQNmNYaoYVPerexQ/1JX1FjxbTxV+GdI5SV6oL7epqapM+kbqySt5ZqVFWD2tcwvI8pWvu
ArpnC07WSthXwTORGJiwtXaI0LzvLMW3tu6VYAmceOeEiLK0lLbzzI5H771VBPIR2g/v/dbnf/y0uXk
AAGAlkfmXQSS/
+d9SUai5mhuW6nHqX+9Xo6tL6ko7oZgu/jKke5S6VFxp9tS+5z+JTrJK3ll5i1vjEpYX7OIuoHu2hHoJ+
yp4JhID0xdTqDvJ0s5SfGvrXgkW7ZXfvCKiLC2x3X9sr0jjo7WXH3vkr19+2dxCAACwesj8yyASqnToM
d+TScgzXqewWFhq5nG/xl9fMeYTFeMvQ1JG1evWv87eeCuxiCSH5QVL33Haw2aYxLyVFXds92y+H
mWB2fZV6D6QznncKeR5OVOEll+aeQsYx7sfvStyLC2xjfw1fre9/fJLzV0EAACrh8y/DDxhp6Xjk3pX5y
DdXV9R0UiP6QhL7UDf0HD2ir8MSRpl1Bfv6P7qsu4aKyurqEGzBUvPqcjq3cvzlK55FtA9m9MjfKGd1
sOzKjmwazFybW1/50rz2ru2hJVgYS5ducQv6s9rNz5/7RMHbxchfPz2h1990NxLAACwYsj8y8ATdkw
6UlnMzkbIkuJhqR2op9PVvAXq6eIvQ9JGtbt1jkOPsMxUNDNY+m9ByTePpZ3UU7rmXUD3Zv09jOn
tVQd2l3QgHYsJH0/TxZ3Rs7aklWARNrc296/vF1GWltJ2ntlx/L5bRPxeSOOL/QAArCwy/zLwhB3JDl2
eBGR2KN5ODUtuP+9IcTH+MiRxVPw09KhK5MQaskpesDRHqZ8L7oDY8oIbCy6ge7N2D6eLsVbv8E
LygcQXY1Yqh6nX9RS+GZ21Ja8Eo9q6unX0raMiytIS2+HjN4vsvcDGF/sBAFhNZH6gmyeOAqvh5C9P
ihxLS2y3PX2DSN0Lb3yxHwCAFUTmB7qR+bGazl48K3IsLbEtydf43cYX+wEAWDVkfqAbmR8r6MLG
BZFjaentoe98U4TtJWk/vPdbn33ycXOPAQDACiDzA93I/Fg1G5sbe17dI3IsLbF9+5GviaS9VO3FYw/
wxX4AAFYHmR8AYNnc2jzw2gGRY2mJ7RvPXC8y9hK2N0//qLnZAABg6sj8AADLgz9/UORYWmK7/v
TOx+/ZLwL2crbf/uKd5n4DAIBJI/MDAFqn3j8lciwtvS3t1/jddvLuu/hiPwAAq4DMDwBonPvwnAixtP
R2z2N7RK5e8sYX+wEAWAVk/sVSvxuuMsgviLu8fmTtfPPzNH4Xnb0jn6JHvc0Sv3YvTfJHY3qfKCgXP
72466VdIsfSEtveZ3c9deAOEaqXv/HFfgAAJo/Mv0BWNB0iNTUFJpTQ3B1J4lBrHf+MAIWkj8b0PlH
QNjY39v1kn8ixtMT21R9/5QeHbhNxers0vtgPAMC0kfkXaOC4pNPvZBKab0eC2CCJNFnCUU3vEwVl
6+rWoTcOiRxLS2/f/94+EaS3UeOL/QAATBuZf3ESEuxcVjHzqx66A5E0FZl/pZ1454QIsbT0dvDEbpGit
107ffS7f/nii+bTAAAApoXMvxgqKRnMHKvDVUXEKSNltdOIFBya30xoRh83sFkr6Mxz5rTmSLGq2LTt
O3qQXqBYreLM7qGGBfqaCzd4LncdiPV+wepizNfuo15SdFxAai2rY/ReFG8GjkJp161VM5rDjD7uLN
aixdvJa8YgXvnNKyLE0tLb1//577bj1/jd9tozJ5sPBAAAmJbZMr9+HA8/i3u7WHkhkCmStZlgGyeCUIL
yv1UwTs3TwTnT0Pz+yUvGFFY6U6K3rZdp9fv1NfEytKMoPUewq5rW2p682Hkg/v23PTzvV2/6x8U
3llGr0b3iktnL4BnQ3ydq5tWgP+9+9K4IsbT0tvPMjkfvvVWE5+3bPvjZm83HAgAATMgImV8878/7E
G9OF81GS897mjr9qHPSvfTBGQEpegC+
+d2xzvzOMDUmcufcadsrsk5kWrOPr6pvR0HtCiILb+c0OonSnSuXU+gBes52LdbSQxO1Ax05tdorqr
gepa60FyK1217GJtxyzpKcYXLfSWvGEC5ducQv6p+n3X9sr4jN27qdvPuuTy5daj4cAABgKnrP/C7nA
X8uRjgp9DPngsQSlLkx2U/HoY7dx+Y3rolu6qUxueeS0Nu0eqKGXVLMGaX7lmL9nWWIe5C4cpOzT
v8ta5cYmihBuFa7adnHGVPQ1yJr8Y3r5dYnrBn929za3L+
+X4RYWnq788mbRGaeQOOL/QAATM/wmb/fZ3cdFo7YoWE78pyMiJsNven6qicgeflO3lNAdNOze
wQLdme25GnNjvLu+nbUwbNhKXC8qkbyyit2b2cOe4DeTyuyTkdyLXluqotVzHvR5jt/zzDRzV6mTS
wosmb0bOvq1tG3jooQS0tvu5+7bhpf43cbX+wHAGBihv1v+z1P+56H+lJi1FFj1s6rn2bJSEvGc5r+3C
U6+ju5PPP7xgZm9zEmsvU5bdvT2aBvR10SxqguVT1VXndPWbkuIqj3PedTCwyM7S+jljwD73KCa2z
JeUrd5VQPn/CCfLXQo5O/PClCLG2mdvy+W0RUnlLji/0AAEzJgjK/7mWKhI2GGlZOon7uHrWsPKfpz
11iq/5OLs/8CeEqdXZLf9PqYRXR27ejTimVmz5FF1Wi656YVI9C3clZZ+ccxhSVlI7ptWQf73I61+g//
+5yCRMnrBl9OnvxrEiwtFnbbU/fIHLylBpf7AcAYEqG/x1+njHOpYRUUFLj6mHqVceg5RU7GXNTsl/ia
fnmTwhXvlGd+ppW9Tqyvu7bZOckng5phZsNHFlbq3sbnTsncDevrughnvNRirdmKJZXS87qq6Kveda
o+MZ1l/ONEhLWjD7xv8bfSzt8/GYRlafUXjz2QPNxAQAA29wiMr+64nnAj+QNt1PSoCXmnkxBRzi5y
3abnoDk5Zt/lkCoLuglmeu09TOtNUS/iMzpkqOcqiF6NRWrszOHWLncSNtfz+M5n4Ie2F72d2zl1dK
9VB9nS8Y8odIFZ55CQjmnmtxF0prRo62rW4d/elgkWNqsbeeZHRP+L/x/
+4t3mo8LAADY5haR+fUTv0fkAV9N1OYC98q2EjhN//EYe/QEJD97pqpKUrjSVyyRW9PLtPptNYlefju

rZ0eS3aXReVT2ODlxx8q9JSuqi+oh1xEa6d1ZJauWey8CWyrFzsquXs2WVK7jABPXjD5d+fMVfmn//
O3G56994uDtIi1PoL15+kfNBwUAAGx/2yfz+2NDLRZTIlfkNO3Nivc9ASnEPOqqe3K4slfQVWr+ads3
jP56+W1HZ0c+Ziff8Xp5ipniB2JWLOup101t9dI3tb3YhOXOXqv7XhRvxNbYMotXPbPKySrJk6BP/I/z
99Lu+J83isC83duLxx7465dfNp8SAACw/S0y88fDhWAnBmmmqQAP9Qnjs4TVce7DcyLB0jLa/cf2iti
8fdvJu+/67JOPm88HAACYhEV+nz8yi0OPCSCoYS76A8YnCavlhV+/IBIsbda288yOR+
+9VYTnbdr4Gj8AANOTmfm9qgjvBHpPwm+naa7p/2A49E8BPJPU9BtENeQx/2v18CcQmK6H335Y
hFjarG33c9c9deAOkZ+3XeNr/AAATNJCMn9oomDeiiT7yFtAAiPzE/ixkraubh1645AIsbRZ251P3iQi9
PZqfI0fAICpWlDmL9mTxSJ7NNdH3wQAdNrY3Njz6h4RYmmztu37xX6+xg8AwITNlvkBAJN0YeMCv
8Z/zvbVH3/lB4duE3F6W7Q//OqD5nMAAAAmh8wPACi9/vvXRYilzdr2Prtr232x/
+2XX2o+AQAAYIrI/ACAxqn3T4kQS5u1HTyxW4TqZW4vP/YIX+MHAGDayPyIE7+PIf5yJuqX5zW/iG
GeqWZl/a7+wX8RRLGzEfZUGvMMMVlH3zoqQixt1vb97+0T0Xo52w/v/dbnf/y0ufEAAGCiyPyIi4f8
eULmojK/LlQbNvI3xUbK4KOdIaZsc2tz//p+EWJpM7Xt8sV+vsYPAMAqIPNjJiJVzhMyReYfzYh1x87g
ZH7046PPP+LX+M/ZvvHM9SJgL1vja/wAAKwIMj9msv0z/5i5mMyPbeu9y+
+JEEubtX37ka+JmL08ja/xAwCwOsj8CxGIu97LOsdVukd05D71djvCvaKmLa+I6eIv43TnQtFfrN07lTn
EXGCUNcgapUoaAgs3FyfWbbFrmdVCtcS2S87WjT7tNM2b4YqeiQLEHOmbKqSsvxCdo2Z1cd9PmA
HDOnvxrAixtFnbQ9/5pgjby9D4Gj8AACuFzL8YnuDku9gGPpMxKDGAmdT7+m1dRE/SXKlei+niLyP8
Wyk1ZZ2p9AWTsVGv+Il53g0svHO5pWixUC113ZjH2bpnbN09WjHtdniP1RjRUSJ3/SVjVV03t3sGjO
LEOydEiKXN1K4/vfPxe/aLyL3wxtf4AQBYKWT+BVGZxxNz9CWde9QVHZScLsY0TgCTmg5qiJGumhF
qhuqlmC7+Mkj3kzUKzUK6ZvZs1ZFwYu7EXu6ptFfEFmas5dmH082tXums6Ktnc4aII+sskbJ+2cWZR
A5wRnTOgJFsXd06/NPDIsfSZmrL9sX+//i3f23uLgAAWA1k/kVRKUaHGCdLecJVZ7oqOHnK0fSox9Q
v1tbKicwraryYLv4yxNdNX2vWbvdRrzwbMy4JKSfmXvBSUxm9Oge6HXxDUm6Zrh7ea0UOzFhifSn
UP7K28Pr1y9DyVQfPFPWlzhkwoit/vsKv8Z+z/Y/+Hvy6C96Lav/zT4819BQAAK4PMvzAi+KgopdOXJ1
sVAqOMTjovhXNcM6rqUP18ZP18Naq9ooeL6eIvQ7x7ERftqdSbPqFa3ip6XnU1bcm6ftsrNtBeru7g
G+JZpdPNU13yVuzcm6d4mH9TCevXr1tWQXtiWz1F1wwY16Url3a9tEvkWFp623lmx/H7bhHxe/z2
3JHD//WnPzU3FQAArAwy/
+KoXFOFGRWD2rDmj2ciXiUEMJ9mWNGj7l38UF/SV/RoMV38ZYh3L+KiPZV60ydUy1vFWWLakj1z
uQP1FcHtYNRKmdm/k0JHRV89S3DiVtem5jmZ5n01hY+aIj4DRnfuw3Mix9Jmajc+f+0TB28XIXzk9p
+/
+11zOwEAwCoh8y9Sk32K9KQSjhFp/PFMdWyuJgUwj3qc+tf71ejqkrrSTiimi78M8e5FXLSn8o7o4B
+j5lVX05bsmUsOVF0KdS93Zl+thJlDu+
+s6KtnCUzc6t5Uyvo1Y7pKPahzFQb/DFiEF379gsixtJnabU/fIEL4mI2v8QMAsLLI/AvVBJoja2t1aDIzk
w5SZsqR6cqTnnRICuW+UjOP+zX+
+ooxn6gYfxni66avNbXsPokTW+SMFWeitJk9BysGuj3ck/fVSrllnj6F7oqdewstSE3ZXcLTxe1TKK4Zr+
y6ncusxGbAgjz89sMix9JmaoeP3yyi+DiNr/EDALDKyPyLpdNSRcQZ/aaKVzr36Lylr6ixxoTRcNQO9A0
18pzIWvGXQc46jfpNsdDMaoReXqRUwonJOgFqpvBJyPW0C25n9tVq+zUXjXNX3TzVC90VffVsTnX7
QsKmnBmc9esL7fLFhsJzpM7guYARbF3dOvTGIZFjaelt55kdj957qwjkQze+xg8AwIoj8y+YTjcFlapa5r
stM+UYoUxypzO1A50U5S9QTxd/GRFeaFPNmco/oqPQDCcWncmTKOVAf6lSO7Pdp74ePgk90FO9
1FkxZW+B8k2phE3NsH6pXZZ/kvb97hkCZ4ShbWxu7Hl1j4iytPS2+7nrnjpwh4jlgza+xg8AwIoj8y+a
Djeh7GKnozbzaGaH4m390tPV5PbzjhQX4y/jdOdC0V+Etnj1SmK8swfJlaUt2ZMoPQPNZFpeVa+Nq
c0uejJzhUVfZ2ZP9Ua8Ytre5AmJMvESla71l8xpSu6S7FXE91qyZ1DvuuMwtAsbF/g1/vO0O5+8ScTy
4doHP3uzuW0AAGBVkfkXTQUfoguA7eL1378ucixtpnb/sb0inA/RXnvmZHPDAADACiPzL5b+V51Ef
gDbyan3T4kcS0tvI3yx//TR7/7liy+auwUAAFYYmX9R7P9w2f0PnwFguR1966iIsrT09vV//rvhvth/8u
67PrI0qblPAABgtZH5F8XI/AR+ANvQ5tbm/vX9IsrS0tvBE7tFVu+r8TV+AACgkfkBAJk+
+vwjfo3/PO3739sn4vr8ja/xAwAAE5kfAJDvvcvviRxLS29f/fFXfnDoNhHa52l8jR8AAAhkfgDAXM5eP

CuiLC297X12V19f7Odr/AAAwEXmBwDM68Q7J0SUpaW3ex7bI9J7XuNr/AAAwEXmBwDMa+vq1u
GfHhZRlpbeHvrON0WAn7W9efpHzc0AAAAwkPkBAD248ucr/Br/7Hb96Z2P37NfxPj09uKxB/765Zf
NnQAAADCQ+QEA/bh05dKuI3aJNEtLbN945nqR5BPbybvv+uyTj5t7AAAAYCPzAwB6c+7DcyLK0tLb
tx/5msjzKe23v3inOX0AAAAHmR8A0KcXfv2CiLK09DbrF/v5Gj8AAIgj8wMAevbw2w+LKEtLbDc+f+
0TB28XwT7U+Bo/AADoROYHAPRs6+rWoTcOiTRLS2y3PX2DyPbextf4AQBACjI/AKB/G5sbe17dI9Is
LbEdPn6zSPhu42v8AAAgBZkfADCICxsX+DX+eW3nmR3H77tFhHyz/fv/PtOcMgAAQBSZHwAwlNd
//7pIs7TEFvli/8uPPcLX+AEAQCIyPwBgQKfePyXSLC2x3fnkTSLtF+2H937r8z9+2hwuAABAFzI/AGBY
R986KtIsLbHdf2yvyPx/
+NUHzbECAAAkIPMDAIa1ubW5f32/SLO0lLbzzI4Xjz2gA//bL7/UnCkAAEAaMj8AYHAfff4Rv8Y/r332
ycd14Odr/AAAIAOZHwAwhvcuvyfSLC2lFUdXBH6+xg8AAPKQ+QEAIzl78awItLTOVpxbkfn5Gj8AAM
hD5gcAjEcEWlpnKw6Nr/EDAIBsZH4AwEiuuWakv+lQKBuFslEoG4WyUSgbhbJNr9Aq4CgBACPhiSQ
bhbJRKBuFslEoG4WyUQgRHCUAYCQ8kWSjUDYKZaNQNgplo1A2CiGCowQAjIQnkmwUykahbBTK
RqFsFMpGIURwlACAkfBEko1C2SiUjULZKJSNQtkohAiOEgAwEp5IslEoG4WyUSgbhbJRKBuFEMFRA
gBGwhNJNgplo1A2CmWjUDYKZaMQIjhKAMBIeCLJRqFsFMpGoWwUykahbBRCBEcJABgJTyTZKJS
NQtkolI1C2SiUjUKI4CgBACPhiSQbhbJRKBuFslEoG4WyUQgRHCUAYCQ8kWSjUDYKZaNQNgplo1A
2CiGCowQAjIQnkmwUykahbBTKRqFsFMpGIURwlACAkfBEko1C2SiUjULZKJSNQtkohAiOEgAwEp5I
slEoG4WyUSgbhbJRKBuFEMFRAgBGwhNJNgplo1A2CmWjUDYKZaMQIjhKAMBIeCLJRqFsFMpGo
WwUykahbBRCBEcJABgJTyTZKJSNQtkolI1C2SiUjUKI4CgBACPhiSQbhbJRKBuFslEoG4WyUQgRHCU
AYCQ8kWSjUDYKZaNQNgplo1A2CiGCowQAjIQnkmwUykahbBTKRqFsFMpGIURwlACAkfBEko1C2
SiUjULZKJSNQtkohAiOEgAwEp5IslEoG4WyUSgbhbJRKBuFEMFRAgBGwhNJNgplo1A2CmWjUDYK
ZaMQIjhKAMBIeCLJRqFsFMpGoWwUykahbBRCBEcJABgJTyTZKJSNQtkolI1C2SiUjUKI4CgBACPhiS
QbhbJRKBuFslEoG4WyUQgRHCUAYCQ8kWSjUDYKZaNQNgplo1A2CiGCowQAAAAAYJrI/AAAAAAA
TBOZHwAAAACAaSLzAwAAAAAwTWR+AAAAAACmicwPAAAAAMA0kfkBAAAAAJgmMj8AAAAAA
NNE5gcAAAAAYJrI/AAAAAAATBOZHwAAAACAaSLzAwAwq/Nr17TWzjdXAQAAlg2ZHwAwIXUYHza
FX14/0lYYsuDY/2Sh3FjhyPrl5kKfrM1U+GcIs7LOcJjbNLjAH5jms1fr95MR+SMaeWtW/qmsO9bvtoY
VPZlB/0IxoPCmtut9CuzI2k5ftymh1jb9a9KKIPMDAIY14NO8xagz5kNbVXaIZ53iWUrvo3quGviBqtzI
kbW14v8MUqfcwuiPhObzaP+fCuuT3Rro01ftZaRPhHlu/RUJ/gm1/wzZG51LsGL0rVmFpiqv6wv1kc
5byj+LebvmLtF5MuX7vf6Fwrcpa0uV+fYV2VT1Vo9/lNztGMVNA+2oqq8vVa/m3V1arR5K1WfXEO
djnaJ4DwnI/ACAAdnPU/bTSK9Uoer/j/lAYO9wKINXKQs0xzdMmfLeD35MpnFujKUqOdCHT849XK
2+n+Mb6n64C5cfjb62Fq4Ye2tWqVOpfs3LmVXja3aV4vj0hflvV9d2yuvN23PVqcU21cf8jcimPJfyBb
cjzF80vCN5ZcBa6o3m5bx3rZxOT199ktuXdqnqzbn2tIrI/ACA4chnAOepoXfDVxDGefwYeFt6+vKH/I
e2mPkeB2c2+uegMOxHwT7AwfZXTWzeqL5vnLNydys9H2TkrHo8xq6pqvezD1KNHraKwV9IXy1/m
LuMWqyn1jB/uXAL9bKPRmQ7th4/351H17mYdM5Uzj56LKZPs3ohPw59FloZZH4AwGDcvzP3+LTj
N/LDQLWfvp4Zg6zHn/4ZZ1b+OEyh6qRaA5/ZcPsIGvgmFeojLErUPwzzKXf+hBqfjl4485UV7XPruW
Rkuh4rdU3lHGyWriq9bcg3kXGt/LG3D7unlvuh6INbqKpTflFBm7+q7+gM1dt97c1bq/qolSXqH8JLm
Y1TK3Cc/WzNPCZ/od42tiLI/ACAwbhPAN5HlD4NXsBUPXj09fTmqvbSGHBL5S7U9GXJ4Tak1Bsbsk7
1yev5UT5unA9e9YmrDFXK3Yf7p3guTgF3/p7PMjJdj5XiU1U3rodTjFep3u3nXnkKlXtQV8q3e/tQBG
oZeirlFhL3peoQPt80nu0Y4u/OKjRbe369lfLUqq7I0+vpXlU7aKqVP9vT9nuKq4HMDwAYzAL+Vj3es4
B4WhxUrw9TtnIb7XmVhUbZk/vR6JW4OfX5DfmxqAoO+rkz91D/PEy9am55dD3eqmpCc+XuR8Hp
Mp/IdD1Wik0lPo5z8FapLjb6OjanULmH9nX5dm8fitjRFert9VHNLeR8+Hr4gxzdTg/zmzy16uOqr9U
/91TPu69qP8ramvUhmUM1rb4xzI2KnzG8yPwAgMEs4G/VIz0L1E86Iz5z1A9v/Re0HtkMfTxhx1R1
hzs/55M3bL3q7gx6ZG6FAXdkfSj6e46vOX9C3T+yzt2bj1tAi7w1q+BU1Wn2tZ2OBVdv91JMFrI+E4Y
harI6+kS4hZyJu9fSKTJF9VZfn4WSU8utUN25+XZU6z6askcfu5N/ZNzb370WSGR+AMBg3L8x9/TsFj
bGs0BVY+QHjqrmoCdXKcsMX2X47QzxKB/W21N1mFti0B0Z+v5IuOuWN6vvnUXm67GUf6rqvvW

4mc4FVx16KNhRqHy7t09F56b6+hPmFqqumBuRH8YM4e30tA2DU8st0X26iTonKjvM/6GodmBP4
1bu4TatHDI/AGA48u/MvT1+BA1foX4mkXp//iiqGNvwPAcNop+nNlcxrzFtdZMGvUtVBXMjQz4jVjd
n2M+c+wkYpWih909EdWvshVt7c27d3DwVlchbs/JMVV3q9yZ1Lrjq0MPxdRQq3+7tJi10U9YfpF7+
VAW3M8CfWbeW9Yep0F/R+G3q5x5Vq3WLlJfNueNLgReZHwAwIOsBpJ+nAr9qbmF7PxOIHY2zm
bLoIHdo9N1Yj7r9Pfe6hvxU2+rnYW2ET0SfexOfgEq7BXNvfR1mpGJ8MTMJTyXuV23OzVXVrJUWVY
zXVc15aiSeTNmttw+9s6nigjF5vSTPIpLFN2W9O0+ZhrOdWnV5hM9273+hCNey35n76OrpvNNYn+
teT3J1kPkBAMMyH0D4+zRGYz2Qzv08GlJ9vAebfQF6fo5HL+y7UmvujXhrG92x8Ka25Z4i96gwub9
Q9E78o4pa+8jAg8ScyPwAAAAAAEwTmR8AAAAAgGki8wMAAAAAME1kfgAAAAAAponMDwAAA
ADANJH5AQAAAACYJjI/AAAAAADTROYHAAAAAGCayPwAAAAAAEwTmR8AAAAAgGki8wMAAAA
AME1kfgAAAAAAponMDwAAAADANJH5AQAAAACYJjI/AAAAAADTROYHAAAAAGCayPwAAAAAA
EwTmR8AAAAAgGki8wMAAAAAME1kfgAAAAAAponMDwAAAADANJH5AQAAAACYJjI/AAAAAAD
TROYHAAAAAGCayPwAAAAAAEwTmR8AAAAAgGki8wMAAAAAME1kfgAAAAAAponMDwAAAAD
ANJH5AQAAAACYJjI/AAAAAADTROYHAAAAAGCayPwAAADL5fzaNY21882lmnrjyPrl5sowVB1Rfh
m0Z1NwzuHy+pHQW9H3AGC6yPwAAADLxci1djxd9czfxvaK7xiCyb491CX8JxkAMBwyPwAAwHKx
/l22mVBXPfMn7d8b7tt/FEDiB7BiyPwAAADLxcr8ZsBd8cyvg3vHuvT56XPSV0j8AFYOmR8AAGC52J
nfCKpO5ncuuMG46VL2MOZtBhhXzDSsLhfXjP+c3iiiGO867xtra8t0ZG5rPmtCY6VKZC7du+qjXzk7iK
2/ZK9HdIlsMDoOAEZF5gcAAFgubZhcr39SkdFImdVr94IRN5tI3MbRTjpEh8cYOVvm2oqxEM8k0ejr
Lyq3bjDW4mgXt7aufhTVu9bfsaDwBv3jossFgOGQ+QEAAJaLCo1FglTBtA6MxhtVR/dCLPM3nYxIKq
+oWOqMaa+oC7KM7qGXYtRJiLvhAnpCt2aM7txop6l0rl91CK8nsMHQRHIFADAOMj8AAMBysVJj8
8L82Y2dRpyUYVZ1aXNp+Iqaxu0h+8hEXJCX9CRGnyC5gpLciXshTi+gJIYkrF9yqvs3qLsl7RsABkfmBw
AAWC7+eF1ETScZe6KyjKZuI+4r6rUZlO15VQ8fUdiaJcBdUkmVVFfl1jqFl5CwfoPdW70fmF2vsmVvC
wBGReYHAABYLjIBN6/11/vbDCl7FobK/PZF9cInWDjM31duJTvzu0tIWL8vvFfU+8HZAwNTVw0AvS
LzAwAALBcnTMoQqd/wxE4ZjN0u3Vc804p5fT2EhC6av68qqa7KrXUKL6F7capHoe7kVO+cw5iiEi4
GAMMh8wMAACwXT5i046N+I9azh8xvhmu7T0L8dsuE6enMzk6NHjN/51TuUOdQIhss3jImnnndA
NAjMj8AAMBy8YVJnRtL+o32ahMndTB1rrjhNXwlPIvuEq6sgq1bJiZcoB0/c3aOLKFr/XI7bf+ODTobiS
4DAAZH5gcAAFguHWHSfMOIopLIsrEIKq/o10fk7CrvFvyl2w5umTjV32aO7jPzd67fv5yS6hKaPTQyd
dUA0CsyPwAAwHIJhMk2pFpvmNm1iJUyGLuTdV9Rr83pnOWU7NxsdwjsIsqeT4ZkubVOnUuIrb8d
XipLGsdivu2b3RxaSl0xAPSOzA8AAAAAwDSR+QEAAAAAmCYyPwAAAAAA00TmBwAAAABgmsj8A
AAAAABME5kfAAAAAIBpIvMDAAAAADBNZH4AAAAAAKaJzA8AAAAAwDSR+QEAAAAAmCYyPwA
AAAAA00TmBwAAAABgmsj8AAAAAABME5kfAAAAAIAp+tvf/j+m/KqvuTE3LAAAAABJRU5ErkJggg
==]]></redcap:Attachment>
</ItemDef>
<ItemDef OID="b35" Name="b35" DataType="text" Length="999" redcap:Variable="b35"
redcap:FieldType="descriptive" redcap:BranchingLogic="[q33] = '1'"
redcap:InlineImage="1">
<Question><TranslatedText></TranslatedText></Question>
<redcap:Attachment DocName="B.png" MimeType="image/png"><!
[CDATA[iVBORw0KGgoAAAANSUhEUgAABVEAAAEJCAYAAACdcmdaAAAAAXNSR0IArs4c6QAAAA
RnQU1BAACxjwv8YQUAAAAJcEhZcwAAEnQAABJ0Ad5mH3gAAE9sSURBVHhe7d0PjCRnfef/b+/aY
PPHNmBskx+RHat7sZYlGJIg08PxL4egZ+6SJbnbYOVg5Oh3PVyi0zQSkwOxF+mX2wguG4lunS5iWrq
zliQyTEJugrLdIJRgckzHR4IxYTI/Z7rlc3T8wDYGjPljB3u3f/VUPVX1VHd1dVV3VXX39PtltaerpqT/T
O/OZ5/k+hb5FAAAAAAAAAChjumPAAAAAAAAAIAQhKgAAAAAAAAAAEIEQFQAAAAAAAAAAiEKlC
AAAAAAAAAQARCVAAAAAAAAACIQIgKAAAAAAAAAABEIUQEAAAAAAAAAgAiEqAAAAAAAAAEQgRA
UAAAAAAACACISoAAAAAAAABCBEBUAAAAAAAAAIhCiAgAAAAAAAEAEQlQAAAAAAAAiECICg

AAAAAAAAARCFEBAAAAAAAAAIAIhKgAAAAAAAAABEIEQFAAAAAAAAgAiEqAAAAAAAAAAQgRAVA
AAAAAAAACIQogIAAAAAAABABEJUAAAAAAAAAIhAiAoAAAAAAAAAEQhRAQAAAAAAACACISoA
AAAAAAAARCBEBQAAAAAAAIAIhKgAAAAAAAAAAEIEQFQAAAAAAAAAiEKICAAAAAAAAQARCVAA
AAAAAAACIQIgKAAAAAAAAABEIUQEAAAAAAAAgAiEqAAAAAAAAAEQgRAUAAAAAAAACACISoAA
AAAAAAABCBEBUAAAAAAAAAIhCiAgAAAAAAAEAEQlQAAAAAAAAAiECICgAAAAAAAAARCFEBAA
AAAAAAIAIhKgAAAAAAAABEIEQFAAAAAAAAgAiEqAAAAAAAAAAAQgRAVAAAAAAAAAACIQogIAAA
AAAABABEJUAAAAAAAAAIhAiLp0etJYKUih4DxWGj29PqjXWPFeU1hpWF+1iMxj3ZB2onXjpLWdL
MxLO7LSlg372PzHqPs4Vb2GrGzEO5vtDbdtK5JH08KNuQ9Cjmc+2g0AAAAAwPwhRF06Rdm8UJey
XurU1kPCkracr3X0c5Hq2U3rq4B5oILBVWnqJUdZzqxle4faf1Qo1cT/rlhsR+14AAAAAADIGiHqMip
uytmqfi4dqZ0P9kbrNc75IVW1JdsV/XzhFGVzry/9vnpsS/aHkff+RpmXdmShKwdu8leuS9c+xj3ZzDJ
D7TVk3fijwsI7ascDAAAAAEAOCFGXVGW7JV6O2jxn9EY1e6FWpbW4CSqOot6h7OuncuoEPaQBA
AAAAEAuCFGXVkW2W26M6vdGbW/4Q6XL9a3wXoyqlqKuRek+wmpSjqyvaH59ZI1Jo6ZjoC7rqPX
GPu31edcGHd5fZI1J4zwEz5+5nbDPjxN+3lNtCdS9tR4xy30ObGewraPOc7xjCm57uPZpYAh6c9VZF
2h4snM3eA7Uw9yc3Z6x+xzPPy71CLkXbAnaHvI9OHq7vqTHE6/dAAAAAAAcfYSoy6yyJXW3OGpz
VTYaDTnnJ6hyIWSM9FAIo3VqpQwmoCrK2hndwM6BdJ1nFmNId2dHLno7bcuubn/5zNpc9FKsnPa
D6gP/AGy9izv6PBo1Pe1wrCSDo63TPb8dqZUKUhrYSXM1aUimtjPY1qasDoZtEx2T2rZR+9S6H+te1
+kREu3HCSwHz4HSXB0dKibnnOtV70AUtW6Kc9TeGFHPNGS7E4vZbgAAAAAlgQh6lILTjLVrLnBTF
nqF0Imk2pv+KGKV4+yL103ie3UZD3lhKW4dka3b18O3U23d42JhYxw0hjqferEnAz0rpz2yiY0d4M9
Ji/u6BisfEacDLUnjXX3GlSlpc9v3+0xnOr59bfvdUhWIdlAfdzx/O1494Hazrob/E1zTMbr9zZlc9v62PX
vV1Wv1/6cXXIi2X56jXUvsCzXu85r+8MlLiqR+4wr/BzteOl/krZbr3X/0hH2PRjY7rBkxzOu3QAAAAA
ALI+C9QtyXz/HklK9SwM9zlSwEhKq+K8rS71rTubTk8aK24tOBS/OZEYjX6963bk96Ubsy+dvW4Vde
9ZG1BBs1YOwWq1Ks9kcWu+3IaxdcdeNE387w+2yV3rnwG1/6DqbsV0VnO2FBNwB4e0wr3O11fc
nDEt0PSK2E9ivvuaS7JjMbQdfr41qa6JzF35+FOdanQqsS3p+lETnOlHbRwht44jjjDietO4RYBr3Pnyvv
OXCW/QSACAP99/xLnnNW2/SSwCAzB3/NZFjr9ILWBT0REVwkikVtoQGJP5Qeb/npMsYdm/2GE1F
UU6ccp51dJfTrj2WvywnT5+0e9QF11uqp/0AbA74vWmb4nVG7R44gZT1GW8ov7dOdT4sGbUo3S
BMfcIsazBr1jUo6ac28z7QPYSnOKZEvYkT7cec4f+kmIdQ3NyTvhmgTm3wHIWY6rrrurFuwJmaGO0
GMvDwEw/LOz/5Tr0EAMjDDY9fJXd/6AF5/Bs/0msAAJm7dI9I/zt6AYuCEBVBA6FScsO1P6fl1RVt7k
pbenJoj9k/JScqJ6z/D65XGeqc9ZIrrombLbpD+ttuIl09a/TozUtaAZl1DTJrOyHeSKpXqBe0GnVjgQX3x
NNPSOWPKvZHAEA+nvPjY/KWL94oP3ryGWm+/3555p8u6c8AALL1lMilP7A+PussYiEQoiJlGYRfXl3
RfTlsXxS7lKgd9lbEyVeN9XMZvhk9NO3A1+/VOyrwVcO67VqVQ480e0lOK1mv47yOaTHOXbjxbW/
Lhtnr1K2LatY5BRbUnZ+6Ux58/EG9BADIwxv/
+gZ5wQ+vsJ8/9NXvyp/WeR8GgNz0Hxa5/Od6AYuAEBUxleSkN1LbnBFfMSZJitM70Ri+HI+7744c7D
pf686+X7I/4a8fLjUwH4qbZ/0guOFOjFWVQIZacsoTKG6Jgvk22OvYvA/0seV1TIn2Y97LA8Pk1cz3hZ
xnoE/SdmNSNTtwdeukJv6eAubL+z77PvIM7zN6CQCQh1c+eK3c/PXn6yXH5z7+kHzlLx/RSwCAzF3
6gsjlr+kFzDtCVMRUlM2zbuVUc/Z1NbrYnOl8y+vt5wScijnre1s23JlrYvN7cqqJpBS3XmZRF0x117vh
6vxxe81a56Kmj3+wdqsx7F+aq7LhnTIV7Onh297K+dBcNQLH9nm/hqd7bHkdU6L9mLVbm7Lqre/p
me+ta1Qq+NvI2oTnyA9c3XYDi+kT+5+Q+n11vQQAyIOqg/pzD7xELwX94W9/Tb73raf1EgAgc5f/RK
T/fb2AeUaIivgq29LyctSalHTA48w8bynX5YJR4NOfUMnSXNWB0GQ1HAPbsp55Q/a9of6KMUnTHP
Jqu9rKUt8KRKiWomxe8IdlN1d1gOaFzqMm/ZolJ3AMttM8tryOKdl+ipsXpO6/2HmtOZGTdS/7h6Br
7yrua1NNWBO03bzfw9odR+bHA8R339fvk7v+7C69BADIw/FLBXsYv/oYRgWozd+8Xy8BADLXf1Lk
8sf1AuYZISoSqWyH11+stqz17tBiV3FT9gZfW21NVr/R7K0XKBlgDM3OdKKjFJgB2KiyA+qc9bt+wOe
ya1/OW03PstS7fT9Yt1Wl1d8LTpaV1zEl2k9RNvf60h16sXq5MUzeVpHt4EGK7B96PbFTEbvtVlv6Lf
8+sqlzblwHu+5ulByOB4jBnYn/6Wfp7QQAeVr50kvl2iev1Evh/uFL35ZP//6hXgIAZO6y9RvZJcpbzbt
CX81aAgAxtTcK4nSSVCHqQGAKADGo4PT1/+318sAjD+g1AIA8lB56od0LNa6tu8vyiteFD/sHAGTgit

8QKfBL9ryiJyoAAMiVGsJPgAoA+VK9T1Uv1CTUsH7qowJAji79AfVR5xghKgAAyM1HvvgRezIpAEB+
VP3Tt33hZSProI6iAtS7/+NX9RIAIHN2fdR79ALmDSEqAADIxe6Du/LBv/igXgIA5OWOL18/tg7qKPv/
8zH53Mcf0ksAgMxd/n9FLn1BL2CeUBMVAABkTg3fV3VQmUgKAPJ189efL//8Czfppclc+dzjsnX36+
XWV79IrwEAZOtKXR/1Zr2MeUBPVAAAkKknnn6CmfgBYAZe8MMrEk0kNcoz/3RJmu+/X3705DN6
DQAgW9b77aWPWx+fchYxFwhRAQBAZlRwqgLUh594WK8BAORB1T99yxdvlOf8OJ1f+R7/xo/k7g8
xKSAA5Kb/HZFL1EedJ4SoAAAgM+/77Pvk3ofv1UsAgLz83AMvkRsev0ovpeMrf/mI3PtJ/igGALm5/
DXr0dELmDVC1KXWlo1CQQrGY6XR05/LSa8hKxttveBob7jtWZG8mzM3Qs5LHL3GinPuVhoy6tT55
9d55H7NjwLuWyCW+n11+djffkwvAQDy8vJvPE9e+eC1eildn/zPB/LN//19vQQAyNyIXZH+Y3oBs0S
IurR60lhZlaZecpTlzFpRP8+eHfiVasLfVIImPi+9hqzXor5KXfOCrAYvunRqJSlMENguK+5bIB7V+1T1Qg
UA5Ovqp47LG++bvg7qKKo+6u//
+y/bHwEAeVD1Uf+79fFZZxEzQ4i6tLpy4KZA5bp0+33p9/dkM68MdWzgt6QmPS+qZ+S4YK99XtxN
V1vqevelWy87K5qrQo4aA/ctEIuqf6rqoAIA8veWvRvtIDVLqifqH/721/QSACBz/UdFLv2xXsCsEKIuq
96h7OuncuqE5Nf/FGmL1zOyJ41zugtquS5bFedpcfOsVJ2n0twlRQUwPTUTf+WPKvZHAEC+Xvu1F8
vLHr1aL2Vrb/f/2A8AQE4uf8l5YGYIUZeQXbvRDN2aq04tx8GuiKp3o1E7c1T9zGAtyGCd1bDejbH3r
wVreI6qN+kMVfdfl6zW52A9S6+2qH6ENy3GPgPncEP8zZjnydln0vOiqHaWYvWMNHoeB0LzkpzUn
VFl/9A6olGM9obVW21v6GMZPAcJrkvI/RZ2vePcb4PXz35E1ImNY5b37WT3p2vSa2eZ6D1Ar1TMr
49uJI6QOz91pzz4+IN6CQCQFxWevubvXqSX8qF6o1IfFQBydOlPqI86Q4SoCDUUGGl2/cyRYVRHai
WjzqrR43EyanuDNTzVurCgpuQNVXdFt3UUZ5+DwWRzdcJ9Fjflgjtk3joz5/RGeo1z3nkq1y+kUEahK
q1+y+tVOsTseTxK50C6+umwipx2N97ZkYsDJ7W96x2NX1c3yXVRQV7I/eZcj1EBZPj9pu7d0GC5U5
NSIMjOSpb3bcz7M2CCa2eZ7D0Ay07VQP1M7zN6CQCQFzV8Xw3jz5uqi3r3h75KfVQAyI2qj3qP9Z
H6qLNAiLqEKtt96Xfr4kZ7Um3Z9TH72zrxbG/4AZBXL9Won9mpyfrIxEaFec7r+3uboWUCxu4/wN+
et3/pyI6XBPWkse4GPca+Wzo1imzrKP523M2ofdbOu/Fbsn0WNy+If+rOS9v677wbglnn94JOUJOd
F4P9um2ZLK8uyolT+ukYlS23beb5V9ri53BnxMnhkpwj67VGqYGh+21ofyZj2/b9ZrYlbFt+kJ3U/Ny3
/uvD789hya6dZar3ACyrT+x/wp6NHwCQv5UvvTTzOqijPPTV78qf1hmBAAC56T8scvnP9QLyRIiKIW
bPtPoFPwgdDgOHletbE4Z54aotPxwsrp3xAqzOge4z2bsoO14eaey7suW3deeiJIl7zH364ZMh8T6Ls
nnWTbuaslpwe08Gz+8kipt740PW2PblMOpEFdfkTNjxtXf9XrVn1pzjSXSOrPOzp0NEI3gPvd4Dhu43
s8et0evSPk86CNzLYfa0LO/bsfdnmCTXzjLNewCW031fv0/u+rO79BIAIE+vfPBaufnrz9dLs/G5jz8kX/
nLR/QSACBzl74gcpkJ/vJGiIoBI3qm2Yqy5iYxIwK3UyfSDKjKcrKkn47SPdC9+VSoU/LqNhbMYdKRw9
QnMMk+K9tGr0FHOsP403RKoi+fcf2NgNIP3Kpy1j2gqa6LruEZOrw/aOh+M8JCawf20Hd33yNrsaZ
uHu/bBNduyvcALB93Jv6nn31arwEA5OWGx6+Sn3vgJXpptu7+0APy+Dd+pJcAAJlTw/r739ELyAMh
KibUkeHOgTHCo7mX3TFUvMKUSrD+5Oz05HBssVSf36vSHRZuBG7V08FeoUmYExB5PXXHCbtWR
dm8EN470w0rF3t+o8nvz/SvXdh7AJaNCk5VgPrID+h9BAB5e86Pj8lbvnijHL9U0Gtm60dPPiPN99+v
lwAA2XtK5NIf6OfIAyEqJjR/gWm53vWGbAcfk9YLHS/
+Po26n7aO1NZznJyneELGlj4tn5Sxl7S4KW5lAntYuDF8vno6/CyPP0dt2TB7nbo1OM36o0lYbdzT+xj
s/as0z83XpEi53bcTXLtoR+GPJpiWGsL/wCMP6CUAQJ7e+Nc3yAt+eIVemg+qPuqnf/9QLwEAMqfq
o15iYte8EKJiQElOusnV0EzePbnoFnIcO/Q7J6WTXtA2qm5m6ibYZ6+xrodpl6Ved5OsPCfnMa5rc1f8
zphdOfAu6QmJc0m9HrXW/XH+/I4OP6sSyOGSnCOzLqcKFN26qMaQ96TUzPKqx6k9GZQdSHa9W
p7pDpOf0CzuW0usa5fme8AU1xDz7yNf/Ig9mRQAIH+lh1448zqoo3z6v/6D/MOXvq2XAACZu/xZkf
48dRU6ughRMcCcBCnYW9IPAlXYNTChz6yYNTCbq/5Q7faGHhpuPdIev510n72GrLsnrnpWNjeNyY
Nq65JPjmrWsmzKrm5er3HOCzBj90asnBbnDulIs+ke18Bw8Amvix8oDvbcjUlvX80sH+xxaoTFZo9b
oz1p3yaRZnHfKnGu3QTvASU/dZXaee9gZMOb4h9Hze6Du/LBv/igXgIA5OnaJ6+0Z+OfZ83fvF+
+9y1qZQNAbtSw/v739QKyQoiKYeYkSJ2alHSoU/LTE7kw7YxI5vDy5uoUoVGwBmZzVQdQXnhTlVZ
qs9e7kuyzJ411d6h6Wepbav3okCq98zLMnFndbbN3TastiX+aKhIo72oZDmATnCMv2LO4x2xOsJTEi
HvXrLNaPevPNp/YQt+3SpxrZ0n4HuDXW7V41zBubVssGjV8/85P3amXAAB5UvVP3/aFl81NHdRRV

lCqglQAQE76T4pc/rheQFYIURHKHgYdUpOy2rLWu8Otp1KR7cGClfuHRs/BBOwamMZwbZddWzOj
eqgx92n23LN7obonbiCk8of1p3hehhRlc2+4Tqg9hD5hYBeYJMs6ZjsbHhT7uljH3G/5QaqtKq2+0dZ
ACYJo9r07eJA2Z5vTZZMLft9aYl07S6L3AHUsg6+ttiava4u59cTTTzATPwDMkOqBqnqiLgI1pJ/6qAC
Qo8vWb6bUR81Uoa8KBgJAEmo2fT0ZlAph96btmTxL+lhOtaYNWBfEUbp2yJUKTit/VJF7H75XrwE
A5EnVQVWTSS2SK597XLbufr3c+uoX6TUAgGxdKXLFb4gUbtbLSBM9UQEkFCxRcGZtsUO49nnrWC
J6ZB4tR+vaIV/v+
+z7CFABYEbULPx3fPl6vbQ4nvmnS9J8//3yoyef0WsAANmy3m8vqWH9TzmLSBUhKoCY2rJh17o06
pWaJQoWUK+xIqv7demmUqJinh29a4d81e+ry8f+9mN6CQCQJ1X/9C1fvFGe8+PF/NXt8W/8SO7+
0AN6CQCQuf53RC7doxeQJkJUAJNRNS8XfPx7cXMvpRq/C+YIXDvkR/U+Vb1QAQCz8XMPvERueP
wqvbSYvvKXj8jnPv6QXgIAZO7y10QufUEvIC3URAUAAKEefuJhec32a+wJpQAA+bv568+Xf/6Fm/TS
YqM+KgDkjfqoaaMnKgAAGKKCUzWRFAEqAMyGqoO6aBNJRVH1Ue/
+0FftjwCAPKj6qGpY/7POIqZGiAoAAIbc+ak75cHHH9RLAIC8qQB1UeugjvLN//19+cPf/ppeAgBkrv+
oyKU/1guYFiEqAAAIUDVQP9P7jF4CAOTttV97sbzs0av10tGyt/t/7AcAICeXv+Q8MDVCVCTUk8aKm
uVbPTaknWjdOGltZ96MOYZeQ1Y2gmvbG+7rV6TR0yunpfZjb3NEO2zTne9M2m1bkPugvaHbWJC
BSwosjE/sf8KejR8AMBsqPH3N3x3tuqGqN6rqlQoAyMmlPxHpP6YXMClCVGCGeo0VKZRq0tHLWe
pd3DH205RdQj4AA+77+n1y15/dpZcAAHm7+qnj8pa9G/XS0aXqov7+v/8y9VEBIDeqPup/tz5SH3U
ahKhIqCibe33p99VjWyp6bXby3l8WRhxDryHrtTziU6UnF3eC+2qea1hrF8VRuA+A+aZm4n/nJ98pT
z/7tF4DAMibClBVkLoMVE/UT/7nA70EAMicXR91Vy9gEoSowDLoXRQ3Qy2Xy86Tzo5cXJwUdTFUt
qXfrYs+w8DCUMGpClAf+cEjeg0AIG8//ffX2UP5l8m9n3xYvvKX/NsDALm5vGc9mOBvUoSoS8eoLbl
i9kQctd6oc2mvz7s25fD+IutuGnU/VwKfNLcT9vko6Z8z+/PmMP7mqvP5EYU0/WNWj+T1Rv2h/FU
5e+GMDvk6shMnRQ3UUg17jG5PvHbHuTbD59DaumyYrzfqkQa2MbA+/BTHaYN5POpY/P2rh7vd
9nnrupbrsjXQXdYu3WC83n4M3DfArKgh/A888oBeAgDk7YbHr7Ink1pGd3/oAXn8Gz/SSwCAzF26R
6T/Hb2AJAhRl05R1s64PREPpOs8s3TlwE30Aj0U27LbdJ6Vz6xZXz17ldNV/awjB/4B2PywsCxn1nRr7
RCwJIMj5zu1UswQa5bnrCO1UkFW9fYcal2SINUYyl89LZXimniHs3Nx+hCvfEbcU+2L2e6pr43Dfn1w
Z846FVQOrG+uDgSpE7VBHcuqeFvWoakKSlf369Ld2wxcdxW+lsJKN3RqUprnibKwFD7yxY/Yk0kBA
GbjOT8+Jm/54o1y/FJBr1kuP3ryGWm+/37qowJAbp4SufQH1kfqoyZFiLqEimtuT8R9OXQTovauHwi
JEU72Dq1XOU6dmIcI1VI5LW6M2gzMjmSEhV6w15PGutvjsyotu6am9WjpLXRqsh4jjUz7nFW2rTa
Yw76rLadd22HVPv12d+vuV8TsRaoYQ/mrp9X2zVA4xpD+4qbsuefNfnTFa4Z1BPULwcDQN67d6V
wbV7neDX69Frbev2+maYPxeh2aFjf3vOc+P1RXYWtXf41/TppyLsFxAmnafXBXPvgXH9RLAIBZeONf
3yAv+OEVemk5PfTV78qf1h/USwCAzPUfFrn853oBcRGiLiOvJ6IfaPUOndivWnXCo32dFJrDwO38bS
5UZMsNoJq7fi8+s+6n2wPUXFff8ickqmx5QWCs3pgzPGfVlj+Rkh/mWi0Z7IY7gtk792TJfmJsJ0EYq/
Ua636vzepZ2QxPUMe3O61rY6vKWbchRsg+cv3+obPdKdoQeH0UI1Q3Q2s7cNWB6t6okwhkSA3f
v/NTd+olAMAsvPLBa+Xmrz9fLy23z338Idn/n4/pJQBA5i59QeQyf8BKghB1KRXlxCnnmRtode1x6W
U5efqkHXYF11vUMHDn2VzwQ7mmeJ0KuwdeWOgN5ffWWcfkDu+2H8bw7cAQ/VFmdc784HMy
Yb1zLRMP6W/LeT9BlVZoz1klRrtTuzaW8kkJ3d2o9a4p2hC7Z7Zxrq2N2WUO3P3Er8sLpOuJp59gJn
4AmLGXfPe58nMPvEQvQbn7P35Vvvct/m0CgNxcVvVRv68XMA4h6pLy6oraPTl74nSqPCUnKies/w
+uV3ngPEWoFiOYcodmt90x0xG9I6exkOfM6GmphqaXQkPC+LP0tzf8OqBVo6fp8kkSbhdl80L4jP1u
cDtiPjEgE+5M/A8/8bBeAwDIm6p/usx1UEdRAWrzN+/XSwCAzPWfFLn8cb2AcQhRl5U3tHlfDts6aL
N77VXEyQqN9VP3hsyCUdfTDi/9upOjwkuvNubQI2YYuIDnzB/KHyXmkP72hj9RVLUlIzuhTmDqa5O
CTNtg1JUdKNlqa55jln7k532ffZ/c+/C9egkAMAsrX3qpXPvklXoJpn/40rfl079/qJcAAJm7bP02eukze
gFRCFGXVklO2hlkRw52nSHNbh3Rkv0Jf3347OuzV9w864eaDXeSp4E6pCVnqL3i1eKc2KKdM2Mo/
xjjh/S3ZcNLUMtSV1PRTyvVazOhHNugZuhXPU7tScXsQNWYoCtu2QJgSvX76vKxv/2YXgIAzELpoRfa
D4z26f/6D3aYCgDIyeXPivTp2jMOIerS8ntyNptOOObWeCzq4p/uem+Sprnj9gDtSK2mA77BOqRmP
crmqj9sur3h1aUsxB5LvWDnzBzK787+H3iYIV70kP5e45w3jL9cv5BOuYRUr82E8miD3pbKoIM9Trvi

ls8dW7sVSMn/+v/
+l34GAJgF1ftU9ULFeHd/6AH9DACQi0v36CcYhRB1iZkzpqvehd7w88Ds5sYkTXPIq1NqC+shGaxH2
VzVwZg/Ll1GT440LNVzVtS1VJXmauqhoTmUvxxaW8AoiWC9cuSQ/l5D1r0CqtYrAxMwOY/JJkhK99
pMJoc2VLb9IfyBurRGfdmzm7MP3bEU7v7Fu+WOl9+hlwAAeXvjX99AHdSYfv7dP6WfAQByUXijfoJ
RCFGXWWDW8FPiTzbuDltXzPVzyAwvRw2ht+tRGr0uXeW6dJPWu0z1nFVke7BA5v5hSrUxzaH8o0
NdMxQeNaQ/Xl3VCaV5bSaVQxvsIfxhxVBVSNvvp1pfFohy1RVXyf/4lf8ht1x3i14DAMjT373yCf0M
UV7z1pvkbe+5VS8BADJ37FUix9+kFzBKoa/G9QIAgKXxwCMPyOv/2+vtmfoBAPm648vXyysfvFYvYd
D1P/E8+a1PvVGedw0TbwFALgovFrni/daTq51ljERPVAAAlsztN91uD+0HAOTvb27/tjx2PX/ECnPlc4
9L9fdeS4AKALmx3m+Pv8f6SIAaByEqAABL6F2n3iUfeMMH9BIAIC+Xjvfl8294VH78nMt6DVz/8t+V
5NZXv0gvAQAyd+ztIoWb9QLGmSpE7TVW9AQpBX9W65F60lhxJ1TZkODL27LhTbbiPCabqGZYeyO
43dBH1jOAAwAwhz788x+W07ed1ksAgLz84PnPyt7rvqWXoLzidS+R1X8bNhkqACATx4oix39eLyCO
OeiJqsJVf5ZqR84zwquZ0VcaKU3oAwDA4rjnl+
+xh/cDAPL10M0/kO6t39dLy+3al14l1d99rV4CAGSucI3IMTWMH0nkGKIWZXOvL2oeq35gxuuuHH
iTiKvZsNXn92QzxwzV1qnJeTqkAgCWjDtj/3VXXafXAADyonqjfu+aZ/TS8lIBqgpSAQA5Of5ukcIL9QLi
mn1P1N6h7OuncuqEZJedlqXedUNc/9Gtl/XnRZq7pKgAgOVzy3W32EGqClQBAPlR9VE/96Zv2h+X1
S/8xivsofwAgJzYdVDz7rl4NOQYog7XRLXrlZZq4nZEtYfVD9UoNb/OeaRVL1Uprp0RP0YFAGA5vfm
WN8tH3/5RvQQAyIvqibqs9VFVePoLv35CLwEAMmfXQX2HXkBSc1ATNUKvISuFktS8lNXRqZVSq2H
aPu+HuNXTfpGB5WVM8uWd46hJwTA7YddqEYTfT/4kcHneY0nPYU7fC+q9b2DCO//8rEiKf0cCAt7
7s+
+V2h01vQQAyIuqjbps9VGfd82VctfvUJMbAPJztcixO/VzTGKmIWpluy/9bt3vCVptOcPst1WY2ZPGu
htwVqXlDsFvVe01qobpeqIkoSO1khtC+I9Vd0Yra9/2bgFghnqNlWAPfSBnqjeq6pUKAMjXfT/zuD1r/
7JQAer1P/E8vQQAyNzxO0UKL9YLmMT89kTtXZQdnSKU61v+RFSVLXHLmHZ2LqbU+64s9S0S1NFG
TQoGTGKR76eM295ryPpg13tgBlR9VFUnFQCQnx8/57J8/g2PLkV91Le951Z5zVtv0ksAgMwdf5PIsV
fpBUxqfkPU7oHXE8sevu/1HjWG93cOpKufTsfppZpmrVUAABaVmqm//attZuwHgJw9dv3T8je3f1sv
HU23vvpF8ku12/QSACBzhVtEjv0LvYBpzHdN1FSFz85vlhPo1M7nWItxHgxO2jWqxuPoOpD20GPv6
/VjZG3Jwf1FBNd2Pdzga0fVgsysDVEya98oca+VK/5xJm1f2OsHyncO1O806o7arx19P/kG2p/K9Ux6
DsOEt32wXungORo8P2HiTbTn8/epHqPqpCY5P0DQbdffJvf88j16CQCQl7+/7Xvyjy//oV46WlQd1O
rvvVaufO5xvQYAkK2rRY6/2/p4hbOIqSxEiFqud4fDT/uRwnDa4qac1WVWRfblcGnyBRVsDU7a1ZTV
wqr1/3hUiFMKG3rcqUlpMKBKMklYe2NETUjVYzgYFmXWhihZtW+khNcqwXEma58TyIW9vrlaGBH
2qXNitLNcl/GVM9SxDbR/6us5/f0ej9OrffAcNVdHhZyTcPbh1XO2Dd97qd3vWGrvKL6DGfsBYAb+6v
WPHcn6qL/yH15JHVQAyNPx09RBTdH8hqilk34P0YN0Bu2Ha8tuuinKQug1zvnhkTuhV7/r1Zsdzzhv
5bp0dbDd9TbQlHNeotNLMEmY9dpzesOh2+3IzkX3tVm1IUpW7Rst2bVKcpzJ2tdrrHuBnP+HjZa1F
615bkRQaLRjb1OKem0koz1u06e5ntPf70n47fHb3pHa+ejIPHqivUH+PsLvvbTud0Ds2frfdepdegkAk
AdVH1UFqUfJyumftB8AgJwce53zQGpSC1HtnmjGkFHzEWco65DimpzxspxVfxuqF6C77UQbdnpv
me1yHmYvuTOyFivhWXQ9uejO2qUCFi+kKcrmBSPEidI7lH39VDo74mY3xc09J6yxHnub+mQmmiT
MmLjHCNyKa2eGQ/XM2hAlo/aNlPBaJTnORO0LtuOst74iW/aGVVC3J2GHE2hHTNWz/rmtbPtBrd
/2JNczhfs9gWrL7yFf2Up/+4q5j/B7L8+J+bAM7v7Fu+WOl9+hlwAAefjmjU/JV376u3ppsb3sp14o/
+a3mNAEAHJTuFHk+L/WC0jLHA/nDwYcXkjrjWE1w5B0mMHN0daVAzdTKp+Ukn5qK56QU/ppJDP
kHgioh2ouTjVJmK6nGTZ8Prc2REmpfSMlvFZJjjNR+0a3wwldR5fWOHUi6XdVVU4HNlaSk2473bYn
up4p3O9zpSwnAwcRIteJ+bAMrrriKmbsB4AZuP9V37HD1EWm6p/+
+n/5GeqgAkBurhQ5/mvWR+qgpm2+a6IWN2UvbMitPdQ3hXqoHtWLri8pZ7JH3OhefG5oM1EPZ
MWu5eiGPlE1KzNsQ5R5b19sebQvRuA3VlFOLEzSmcbxAvPpphfcZAepKlAFAOTn8yuPylNXX9JLi0f
1QFU9UQEAOTn+r0QKN+gFpGmqENUc9hv1cMLJoj8M2gxA7aBUrw9NMc2v04+4dRUtdp1B82tD
H2kGskvEuHZ+7Udf89zw5DXjJwlry4bZq9OtjWnWijRl0oYo2bcvLbGOc4btG21wgreeHHp1B4ZlOv

HcEcD5QZpuv+l2e2g/ACA/KkBVQeoiel3l/6IOKgDk6dhrrQd1ULMy3z1RkZGQ4dEus05mDGp2d9V
b0Q+rjZ7D7raTTBLW3vV6dtrhjxuYG8OTB6XehihZtW+khNdqguOM176Idth1itOdfT7Y9JDh+ImO
M737fWHkNjEflpGaZOoDb/iAXgIA5EEN6f+7Vz6hlxaDmoWfOqgAkCM1C7/qhYrMEKIupaKs+bN2
yao3Ztuc0XsMPcGXKlEb7K0YEnhNOEmYH/4YM+KbcmhDlFTbN1LCa5XkOBO1L6Id9rE7NVUTnsK
Rmqsb4jfdL5lQPrPmhNaJrmcK9/uiyeB+B0wf/vkPy+nbTuslAEAeVH3Ux65/Wi/NN1X/tPp7r5XnXX
OlXgMAyJaqg/oe6+PVziIyQYi6pIqbF/zehs1VHawYk86MU9n2h393alJygxmjRqg/UVew9mbkJGGV
09aSNq5dWbUhSmbtGy3ZtUpyrpO1b2w7ynXZSmV8eFnK5aas6vZ4Tbfaftab/j/Z9Zz6fs+LOdGV28
6Jws6U7ncgwj2/fI89vB8AkI9Lx/vy+Tc8Kj9+zmW9Zn79Uu02ufXVL9JLAIDMHVsTKdysF5AVQtSlVZ
TNvcFJu6rS6res/8djD/8OK6Rpb2dgoi679ubg/ixDk4RVZHuoDc72vF01d71eitm0IUqG7Rsp4bVKcJ
zJ2qfa0Zfu0IbVpo3SBlM7JWcHjzfsGiW6ntPf7/mw7q/B67F/aPQSTiCV+x0YzZ2x/7qrrtNrAABZ+8H
zn5W/ev1jemk+veatN8nb3nOrXgIAZO7Yq0SOv0kvIEuFviqCCAAAMIF7H75XKn9UkaefXYwhpgBw
FNzx5evllQ9eq5fmx7UvvUr+06ffzDB+AMhL4RqRK9R8BQzjzwM9UQEAwMTefMub5aNv/6heAgD
k4W9u/7Z875pn9NL8qP4udVABIFfH3239jwA1L4SoAABgKu/92fdK7Y6aXgIAZE3VR/3cm75pf5wX
v/Abr5BXvO4legkAkLljbxcppFNUD/EQogIAgKmp3qiqVyoAIB+qJ+re676ll2ZLhae/8Osn9BIAIHPHiiL
H36EXkBdCVAAAkAo10dQt192ilwAAWeve+n37MUuqDqoaxg8AyImqg3rsPXoBeSJEBQAAqVAz9b
d/tc2M/QCQI9UbdZb1Ue/6T6+2g1QAQE6O3SlSeKFeQJ4IUQEAQGpuu/42ueeX79FLAICsqbqon3
/DozOpj/q299wqp/7ZDXoJAJC5428SOXabXkDeCFEBAECq3lF8BzP2A0COvv2if7Jn7M/Tra9+kfxSjV
/kASA3hVtEjv0LvYBZIEQFcteWjUJBCsZjpdHTn1tSvYasbLT1gqO94Z6fFZnX09NrrDhtXGnIqCb6x+E8
lv5aY2mo2frfdepdegkAkLW/v+178o8v/6FeytbzrrlSqr/3Wrnyucf1GgBAtq4WOf5u6+MVziJmghAV
yFVPGiur0tRLjrKcWSvq58vHDiJLNeno5YXRa8h6LarV6loXZDV4saVTK0lhIDAGjqq7f/FuuePld+glAE
DW/ur1j8kPnv+sXsrOXb9zu1z/E8/TSwCAzB1XdVBfrBcwK4SoQK66cuDmbuW6dPt96ff3ZHNZM9
SxQeScUj1nxwW/7fPiHlq1pa5zX7r1srOiuSrkqFgGV11xFTP2A0COfvycy5nXR33zr9wir3nrTXoJAJC
5YyvW41V6AbNEiArkqXco+/qpnDohy9v/dHHF6znbk8Y53QW1XJetivO0uHlWqs5Tae6SomI53PSC
m+wgVQWqAIDsPXb903L/q76jl9L1sp96ofzKfziplwAAmSvcKHL8tF7ArBGiLjGvnqP5CKntOLI2peqN
536d163Or/dp135sb+ivNdbZLwuuD/bKS2MbmtlG7zFcYzN4jMM1S+1HWN1Low3jal3a+zDDt+aq
87WDDQ9pc9i2o9o8tpfjROdl+J4ZuZ8YxxD7fGh+W9RjuK0OZwi9/7p0z506/lKsnrNGj+NAWF6Sk7
ozquwfDt9PNqM9ie65eMduS+H7wj1Pcd9HsNxuv+l2e2g/ACAff/fKJ+SbNz6ll9Kh6p/+
+n/5GeqgAkBurhQ5/mvWR+qgzgtC1CWlwpHQMKhTk1JhQ8ZlcHHYtR8HCkLa61TIMrC+uRoeXk2
1DRU2hfYY7EitNCqEU58zapaW61J3uw52duTiYMi0674ynbqmQ8GiZh/zyGBquM1uz8dQE5+X4X
umuToieEt8DFGcfQcvd0hb7WCw5A2hd6V67jxVafVbXq/SIWaP41E6B9LVT4MqcjrpPZfk2FP6vlDn
KY/3ERwdapKpD7zhA3oJAJC1z688Kk9dfUkvTe/f/Nar7J6oAICcHP9XIoUb9ALmASHqUmqLn8O4d
TmNeo3SlHPhSUpi5XrX3na/FYybwtaPGt482TaCw6mHj7EjO4PplEcFZM7r+3ubsrlVF+erBr/GPI9nZ
FyGWtm2ttd1t2Wptpx9bOvUrr3hB4Vhbe7UZH3kdQm2eXRT0jkv/invSO28cd0SHMPY8xHg7zu8r
dZxrbvBoHEu3Iamcu40u53bEitrHVKUE6f00wiVRPdckmNP7/uimOP7CI6OD//8h+X0bQxHAoA8q
ABVBalpWDn9k/YDAJCTY69zHpgrhKjLyOwlZ/R0K27uOQGJ9dhLZaajqpx1t1M5bS25RqwPHd486T
aKsrlnBj6O4toZL7TrHIT3AyzXt4IBWXFNzugv6uxc9NvY3vV65pXPrI0P38YwexjWLxht3rwgfgZ5PrR3
31CbR5r8vFRbfnDoh3xB0xxDFHPfoW3tXZQd3SEycC4qW/5+zWtniH/u1HFY3yOhIe8k9uUwrEFKk
nsu0bGn+H2R2/sIjpp7fvkee3g/ACB7akj/V376u3ppMqr3qeqFCgDIiV0H9V/rBcwTQtRlZAQ0ImqY
bsGrZTiyhuIkyielpJ8GjFofJo1t2HQ9x9BhzEGnTgwGP0VZ8xMtLyzyA0Mj0J1YVK9WY/8jgrfhNscV/7
yMN90xjFaWk+MudvfAa79X7sF+GEPcRwyfn/zcTeuUjN51gntuimNPcv2HzlNe7yM4ctwZ+6+76jq
9BgCQJTXJ1KT1UVX907t+59XUQQWA3Kg6qHdaH6mDOo8IUZdSUTYvhPckdEOYqMl1FkZg8hyjn
mOk8MDO76nnDnc2AsPq6dg9GafXkeGOgjFCRlOK52UyYccwK2keVxw9ORxbLNWR2T2X2vVfkvc
RZOKW625hxn4AyNFfvf4x+fFzLuul+H6pdpvc+uoX6SUAQOaOrYkUbtYLmDeEqMuquCl7esjtQKlR
W/Pcos+u3ZYNs3edW7PRrMGZhHW+zurzZA+NNoYyV0/nF6FOH/qlfF4mkm1w6dXKHXpMWsd0

AsUTMrb06bje1BPcc+OPPf3vi6P9PoIsvfmWN8tH3/5RvQQAyNIPnv+sHaQm8Zq33iRve8+tegkAkL
ljrxI5/ia9gHlEiLrE1MzaqqeYPcGPHYR0vRqKo4f/GoxhxHPHrB2pgiW3/uMUba64U6Z3duT8+R29n
aqkk6GW5KR37gdnZO/JRbfoZeQQ8BgyOC+
+nI4hTOmkFwKOqumZL+NcNHfF75DZlQPvNJxwzn2EWPdckmPP4PpP/T6Cpfben32v1O6o6SUAQ
Jb+8eU/lL+/7Xt6Kdr1P/E8uet3qF8NALkpvFgP48c8I0RdRu0Ne6itmkU92FPMCHiMXnIlPxkzZmJvy
4Y3Dft884MlY2bySXgTWHWk2dQnKrWh/EXZdLsdqvO87l+XXmPdq22ZZBKkcVI7L578j8Fj1udsrvr
DyPW9bj9yHVtu1oBtyq7eda9xzgsxY/VgjnPPTXjsU1//hO8jZntyvRSYe6o3ququVCgDI3t/c/m157Pqn
9VI4Vf+0+nuvleddc6VeAwDIlqqD+h7r49XOIuYWIeoyqmz7Q287NSm5QYtRH7F6NnzmbhXSDL52
Lpkz9nttNibamUhF3I6BrlSH8o+4LiU/fZQL005glcl5MSQ9BnPYu9ueiRK2YH3O5qqz34IX9FeIldqs+v
EUNy94PTLd9njnodqSeM2Jc88lOPY0r3/C9xEgiqqPquqkAgCydeI4Xz7/hkcj66NW/u8idVABIE/H3k
od1AVBiLqk7KG3YUUMVeDS7wcDHlX3cLBmYrWVcx3NpCqy3W/5gZHNOTbvsAPDrOPxhlcr5bpsp
ZzL2dcl5LxWW9Z6d+j1VLI5L6Zkx2C1Z/A+3D80ejUmYNfnNIaSu+y6nznWQ/UUZXNvuFaoPYw+Q
aAb656LfezpXv9E7yNABDVTf/tX28zYDwA5UPVR7/uZx/VS0Cte9xL5hV8/oZcAAJk7Zv2GfPwdegHz
rtBXRewAxKNmNtcT86gwbG/anqHAOEfpntPHcqpFwIpwn+l9Rip/xM0BAHl441/fIKWHXqiXRK596
VXyW3/8z+yPAIAcFK4ROf5+66P/Xoz5Rk9UILaeNNbdmc3LcmaNABVZO1r3XPu8dSwZ9ODG0fGO
4juYsR8AcrL3um/J9655Ri+JVH/3tQSoAJCn4+8mQF0w9EQFxmrLxmANWFXOgK50yMzRu+d6jRU
p7ZyRbiplKXDUFf6fgn4GAMjStU9eKe9s/aS88723MYwfAPJ07O0M419AhKjAWAOBFgEqMsc9h+
WlJiiblx9NaEs42hKOtoSjLeHmqS0nrr1GDr/3pF6aLa5RONoSjraEoy3haAvSQIgKAADmBj/ghqMt4
WhLONoSjraEoy3haEs42hKOtoSjLeHmqS1IhpqoAAAAAAAAABCBEBUAAAAAAAAAIhCiAgAAAAA
AAEAEQlTkSE2WU7DrfxRWGtLTa8fqNWRlo60XHO0NvZ3CijRibwjp6Uljxb0GG9aVTbJuGmH3UNr
7mJbRRv1Y4SYFAAAAAAGChEaJirvUaK1Io1aSjl4H5pgJdY1Z9W1nOrBX1cwAAAAAAsIgIUTG/eg1Zrx
GfzqeibO717RkF+/1tqei1szFPbenKgXvLluvStdu0J5tkqAAAAAAALDRCVABIS+9Q9vVTOXVCyE4BA
AAAADgaCFGXmao1OlC7MazG6GD9UXuIvfE1A+VKNbNOpXokq1Vp79Mcxt9cdbYTvjOjjeoxqk7qY
JvCa1VOdrymMbVf2xvetob2H3JN4rTRY359ZEONcxFo46j1xj7t9ea5zKoOadx7KKotg9uYsD5pjOuS
9J4FAAAAAACLgxB1WakgL7TWaEdqpVEhpPpcQUoDQ+ybq4OvVyFiSYIva8pqYbBWZBqcNq0GNh
xyDHYINtgm65W1UnjQaYt7vIMqcrqqn3Z25OLAa9u7bmODtTKHQjgtuo2TKsrambLztHMgXeeZxR
iOHmh7W9xml8+s5dDDMoV7aKJrPizf6wIAAAAAAOYRIepS6knjnJuIuXUb+9Kt61BNOrIzmPx5qtL
Sr2+5QaEKG8/7ve16jXN+0FVt6VqVXfE2H0Nl2/qabl28L3G3sx1W8dJvU/gxWMe77oZg/mv77gF0
arI+MhUdf7xhKltu2wfPpR9GSvmMeBlqe8MPgsOuSWQbJ1NcO6PbuC+H7qbbu0ZI2ZEDN101hq
mfOpF9hDr9PTTNNTckuC7J7lkAAAAAALBICFGXkjERz96m16vQD9VEOl56FlRt+RP3+EGhqScXd9w
+e1VpeQGStc8LYa+fntmm0GPoXRS3SeX6lj/xUGXLC+U6OxdDexSOP94RimvidfQ0t22ElGaPTrN3av
2CcU02L/htrJ0fGKo+Ja+NftDbO3Si0mrVCRv3dbrau7jjBZKnvROYlRTuoSmuuWkm1wUAAAAAAM
wdQlRYdA3P0OH9SZmzk5+Ukn5qK56QU/ppespyMrCTEN0D77jsIdheXUtjqHdgSHsazOHy/rB4P5
SryllvyvYRvVNtxnbMHqOpKMoJfUHcwLlrXzzrnJ4+aYeVwfWW6mk/kMxMCvdQKtd8VtcFAAAAAA
DMG0LUZRWYKCdunckYgeWRMt3x+r1i3Z6eRig3URhpDK9PScUt3trctVrXE6cj6ik5UdFhZWC9anb
2EeriSf+6AAAAAACA+UKIupTasmH2OnVrPZr1HI+ocr3rlDEYevjD9lNT3JSzbglONXTcqCs6WRiZQYh
dOS164L4ctvUQeLv3pzs5lrF+QUP07K/5sv1xAQAAAACA5UOIuozMupwqYHLrohpDoCdXkpNuEjs
4XNoIEXNVcoamK6NqvWbF6+nZ2ZHz50fVFTXP2eBs/mZ90FMydk6nxNfQ3XdHDnadr3VrtZbsT/jr
h4e0ZyWFeyiVa57idQEAAAAAAAuNEHXJ+QGTMWP/VMxakU1Z3XCn3DFnS8+ZMcmTNFfFa1J7w
6+V6a1MmdfTsyPNpj76oaH8Rdl0u6xar6utN6yz5eg11r0anuYESU7AqVivP+8dkGx4U8nH5V+vZtP
5Wnf2/aIumOquNyfCylYK91Aq1zz5dQEAAAAAAEcTIeoy8oI9S3NVh0rGhDtTMmcun2r75iRC7nY
mCjuDs7o3V3WI5gWO5gzwaXOHxftCh/JXtqXl5XU1KdnnrCAlP6mTC95EVCojdOutWrxzHLe2bVBg
W9Yzb2i6eZ9Y68/k0w3VNv09lNI1T3hdAAAAAADA0USIupQqst1vGQGZUpVWv+8HRvaEQpMqyu
Ze1w/BbGr7g/scx2qn1yBt/9DrDZhIcVP2+oNtstj1YDOoh2rwhvQr1v62Ruysst0PrUtbbVnr3ZILLnU

8g6+ttiara2v22gwMTTeGs+c+ZD2Feyila57ougAAAAAAgCOp0FczrADITq8hK3oiL1WDdo+eiwAwk
urtPS8/mtCWcLQlHG0JR1vC0ZZwtCUcbQlHW8LRlnC0BWmgJyqQKbOOZ75D4gEAAAAAAJAOQlQ
gE23ZKAzU8ayeFTqhAgAAAAAALB5CVCAPql5pZpNXAQAAAAAAIEvURAUAAHODelXhaEs42hKOto
SjLeFoSzjaEo62hKMt4WhLuHlqC5KhJyoAAAAAAAAARCBEBQAAAAAAAIAIDOcHAAAAAAAAAgAj0R
EV2eg1Z2WjrhVnrSWNFzZavHhsyL60CAAAAAADA/CNERSZ6jRUplGrS0csAAAAAAADAoiJERfp6D
VmvEZ8CAAAAAADgaCBEBQAAAAAAAIAIhKhLqr3h1gddkUZPD7+3l51HaClTVePUeI3zcL7eZW/XH
MbfXHVepzc4uF+PuW1j58HXt2XDfY318F4Wo12TGjwv6jHpuVGSHr8S1obCSkPCD8+s/eo8VkaciG
TbBQAAAAAAWF6EqEuvI7VSQUoDw+
+bqwMhX3tjRI1T9fXpBJbR1H5WpamXpFyXrYr1MbN2OWHk4HlRmquFYNCZ4blRoWtYG6RTk9Lg
BFl2EFuSwZd3aqWhcDTRdgEAAAAAAJYcISosVWn1+9K3Hq2qXqUCwPNulNaTxjkdX5br0tWv7dbL
zjrrtTsXnYiusm19rlsX9zNSbdmv7W+rxHNafjv7e5tSTNCupHqNdS+MLNe7zj77LasFWvOcDkeza4NI
W3bd1Dh020055yW0VjvW3SDXOE/uBe3UZN17bZLtAgAAAAAAgBAVUm1tixtxVraMANRTlM09H
crZ4aVeu3bGe23noKufZadc3/La6ciqXT25uOP20qzK2U13yxXZsoNGFVLuibM6w3PTO5R9/VQ6O+
JmscXNPWd/1mPPbVvvorhNDpynypa42Whn56J1ZJYk2wUAAAAAAAAhKiaha5OGDmHPzqkT44K
9tNrVlQN3A+WTUtJPFSdo9EPnYSmem+KanHGTWGtrquyCW7t0qM5p98Dbnz18X7+uYA7v7xxYR
2ZJsl0AAAAAAAAQoqIsJ82UcJTAxElGbdLcjGjnzNtlyawNRdm8ENYz2A9KQye5Giur7QIAAAAAABx
NhKiIoS0bZs9Kt46mWft0JuahXRm3obgpe3qIvV+v1tc8Nzybvl/DdfBh9KCdYLsAgLzp0Q3Ggz9yAQ
AAALNBiIrx2rte70o7oHNrfxpDyKc2ybYya1dJTroJqDsE3qVm4i8YM+6n1YaI16uZ9NUvzfakXXbw2f
XqnHrtK530Qtu4NVhjbRcAlpH9Xl+QwkwTy540VlZFWu57tPNHr+bqLILU+Qxze40Vpz0r+f/hT/0ba
p4P9zEP5wXDhq7XDO6ZmRvzvuZ9P7mPvG7mOO+3ebwnR+5jSf+gFfO8z/K9eGZinJuh951luGnG
nJfc34sTtofSdoiDEBWJ+AGdMSt9AiU/nTRm/7d+MFmdbhD8tO0KKsqaVzS0Kavem667baeO6OB7
cZw2JDp+/aavPhXsGRpSs9Wsc9pc9dvm/sOhHu7KJNtVjG3wDwuALMzsl/chKri09j/lv0npcCYu3Da
KcLuTP+4f5vte3N4IC3ONPyjOQq8h6zWRatX9x28G3NEnxsO8XrMy01/cAyWOhh+z+NZW52O1qS
YFda9TS6qdmpRmFPjk/0v7+Pc19R5csr6f6l3jHFk/T2Z778R5v83jPXncPtTnc/yDVmTok1eYm+C85/l
eHHFuht739CP98xP/vl3dH/g3Iot/IEadk9zfi8efl3zfiydoj/WNrUrbpf+eHO/7dn5+FsZY1g2DJWS9R/
TV5bd+A+hbPzD5uvW+9U+g87mq9ZZia/WtnxX060c8vNcqIa93P29uf9TD2NbIdtqStKvbr5fd9VXr
K8cxXx/yKNetVygJz83Exx/
+MDcdve3gMSfabqvqrS8PXwQAmEq3XrbeX8z3eP2+GngjyoffFv1vwAzaEEm/z8/8vXjm7XCuj9q/f
c28f5PzY/87OoP9RtP37dy1y/3eivPzV9rCv5dn1R7n5y9jv/pnrCy/l8a/rznvuYNtyPocjW9XvNdMa6J
9ZPIeqPdvbdd+hLRD3T/maud+Um3XK1IS/5w4n8/+vTjeucnjvS/
+fZv1+8v4cxImq7aNPy/h62fWnhHfw+nfR87+zd0737fBdX579Qr9vhz3uiJf9ERFDBXZVn8p0ksO56
821puAo7kr/t9KrNd7n9D2D52/MKlanIP1QqutCWuIJm1XEk4PIOsNTS/7rDdbf9h+0jYkPH57qP3g
ubQ5+wj8QdOuc2oMyXfZPWWMeqiWRNsFgMy05XytY71NXZBN503VUpEt9UY28fv35Iqbe9Lv7x
ltmTO69MupE/PRwFm1o9dYl1qnKmfn9kLNhnteWt7PKPPC+T6X6unAzyL5KMqJU9YH9+dQW08u
7sygPb2GqIFK5fqWv9/Ktv3zYmfnYgY9sRxj39d6h7JvfRj8fi7aJ64puxm9Ecd5v83jPXle3ved79+y1Ls
hP8tr6uf34dEJHdm5mO7dE/ec5PVeHOfc5GX8uXHeXwLf5xmY7Jxk9148/rzk+148tj0jfp6yR42mW
tYuzqii+fpZGOMRoi4pvw7mwJuLMeFQcMiBCgvdr1EPJ5TztxMM6dQPhf5rrYf5A725D/VQ+xmx35
Ht9MRtl/MGFlw3nvMG7H6d89gbakjCc5Pg+G2D59J+jDoG8zj1Y9QvU3G3a7xu+NgBYAoz+uV9M
enSL+W6bIX/A5CTnjTWa9KZVTvsoaMdqbbi/1ueGTUM0Rh6N9uSN/n84j6JXuOc9d1s/bI/oxu3sq2
HjNo17dWwypITNg/
+vJW13H5pT8huV1lOenWcNKPePgZk8AetuMFlmDTbEVuO78XTnJv86fJoB+cHhtOnWwJnknPCe7
FhxPub8/PnvuRaNYmfhRcOISoAAJgNfnmPTdUltX/5uTCLXoa6tpj9i6D+pWdGvR3b52vSqbZmPmL
C/0Op81AjV7KppRZXPr+4J+f2NDo7wwDEHTWkatqr7yOrObMI4efpl3ZMgT9oKfPyXhwwR3/Y6uy
flAvevxGqt6h6/9mYYa9C3osD9HwiwflBrO+pqedWiWHwjzD8LLxwCFEBAADmmDP5gRq2N6ueO

MFRDt36vqzO4hfU9oYzCcRc/dbuUL2Csh6WHcfc/eLePi/O6NHZXTNnsg5/YiAVeKtJgXKftGOWv7Q
jNfxByzKH78Xz9oet8pk147pY/4aeVWXUZtirkPfiAernGrdnrP89dXBKXadTkl3n7nn5IwymQYgKAA
Awp5wAVfXWmJ+hjMXNC3YNtk7tfK7hXHtXhU1NO8B1fuEpSEn9Vqh/CZrtcHptlsOyLfP1i7sOCGf5
y6Iecqzq2bt5jz0MViXezdWMZjYfZVa/tI9h93bqyMHgjTuqd9QS4w9ajkV4L56XP2x5ZtqrkPficIMl+f
qydXLfOk8nJau3vdn+EQZpIUQFAACzwS/vkVSvDSdAndMJ/zL8RSPMYE8j9bAngLR+Mexaz2dbt7sn
h6qoWc7nZKxZ/uLu9nw6O8NfFgeHTbpmdl7y/6V9rOIJceZ7CUZNPfuGnmG4O2f4g5Zvvt+LB+T+h
62S2GWOB8PbWf5cw3txTLq2eOCPkekZ+UcYfhZeOISoAABgNvjlPYKeRddiD3czevzkW+dSTf4wM
Bxc/0KW1S8ac6/XkJUVc0i2WqVmSp7lL6nz94u701utKjMcPSpSOW21wPoeCgyhV7dwzTovM26bL
dtf2uOpyGnVY6+27r+veL3G5m+islngD1qLaFZ/2NIjADo1Oe/9w6mHcM+oHinvxXGoUhklqUldLm
RwkSL/CMPPwounDwAAMCOtqvSt33L69a5e0a33y9aPJ2VvRX669bLVFtWewUe139KvWUZh56U
6JyfEblu53s/3bun26+Xg+ZiLe6RVHbg2rb71S2tfJT+5m+H38TB9HgKPefie1vdRxvdv3Pc1573Yf2R9
7eK0K4/35PH7CLt/3Ifxb1dq9H0x9H2r2jFw3Pp7Pu1rNcl5t78m8/fiEedGvd8M7Ns9hrTf/uKem6H
XZfY+POp+0XJ6L453XvJ7Lx7fnpB/xzO6RnHuxXn6WRjjFdT/rIsKAAAwE+5f6F3WD43zNRwQWBCq
x5xdm9BVbUl/Bl3nnO/pqrT6M5p5eS7pnk7G5ZnV9cH8Gfre9fjfR2Gvqc5r79gUjT83Id9bxnk7iuLc
LwrvxbOmRvOoOqhhgkP7+Vl4cRCiAgAAAAAAAEAEaqICAAAAAAAAQARCVAAAAAAAAACIQIgKA
AAAAAAAABEIUQEAAAAAAAAgAiEqAAAAAAAAAEQgRAUAAAAAAACACISoAAAAAAAAABCBEBU
AAAAAAAAAIhCiAgAAAAAAAEAEQlQAAAAAAAAAiECICgAAAAAAAAARCFEBAAAAAAAAIAIhKgAA
AAAAAABEIEQFAAAAAAAAgAiEqAAAAAAAAAAAQgRAVAAAAAAAAAACIQogIAAAAAAAABABEJUAAA
AAAAAAIhAiAoAAAAAAAAAAEQhRAQAAAAAAACACISoAAAAAAAAARCBEBQAAAAAAAAIAIhKgAAA
AAAAAAEIEQFQAAAAAAAAAiEKICAAAAAAAAQARCVAAAAAAAAAACIQIgKAAAAAAAAAABEIUQEAA
AAAAAAgAiEqAAAAAAAAAEQgRAUAAAAAAACACISoAAAAAAAAABCBEBUAAADAQmtvFKRQ0I+N
tl5raG94n19p9PTK2fDbuiEhLV0wbdlwz3vk+e1JY2Xca6xXNVb8ba00rK8CAGB+EKICAAAAODqa52
TGOemSUMHoqjT1kqMsZ9aK+rmpKJsX6tZnHZ3aesg1asv5Wkc/F6me3bS+CgCA+UGICgAAAOAI6
Ujt/OL38Zx/XTlwM89yXbr9vvT7e7I5KvksbsrZqn4eco16jXN+IFttyXZFPwcAYE4QogIAAAA4WuiNm
r3eoezrp3LqRKxeo5Xtlng5auAamb1Qq9IiQQUAzCFCVAAAAAABHTLzeqH590pVg6NpryIpbm9Orse
rX/7Rrehp1Vr119suC68NKtPqCtUJH1wEdeJ31CKsrGjyeYL3S6HYYzGPXj8F92fsp1ayzrDVXndeO3Ul
FtltujOpfo/aGXxagXN+yXjUo3vHbQto/dH0tcc5VoEar+6BWKwAsLUJUAAAAAEdEWep1HdJl2Bu1U
ytJYTVYDdRep0K2gfXN1VEBZlNWCyUxyoCqjUhpcMIpOxQceJ3F3t/IQK8jtZJRr7Rcl60YnTuHwlEtel
8JVbak7hZHba7KRqMh5/wEVS4M1gNIcvwqwA5pv3M+hoNUR/i5UueiNLhTJewaAQCWAiEqAAA
AgKNjzQ3p4vVGnVS53pW+qgPq9ax0hK1v7o5oh1dLtC/
+y5tyzkv7etJYd0PBqrT0a71td2qyPjIpNl6/F2OSpvaGePmv0a6um3ga+6psW5/r+hNFqRqm9n5iDc
MPTjLVrLnHV5b6hcF2Jjl+67VuGhvWfmsrOxfjnqu27Eadi8A1AgAsC0JUAAAAAEdIUTbdGYwy641al
bNuj8nKaWvJNWL9/mFoL05zBnqzXmhn56Lz+t5F2dGdIQPD3I3enN5rB4QPix+t7aeGgTCzuHnB31
ftfDo9MAOTTGnVs8OTUiU6fuu675lBqKO4dsYLbDsHXf0saOhcmfVeOzviZq/FzT1n+9Zjb+QMWgC
Ao4oQFQAAAMDR4oVsGfVGLZ+Ukn4aMGp9qKqcDiR3JTnpp31ix33dA90L01rllguwH8bwdve1A0
6dSBLymT0vz8ha4EuLsnbGbdi+HIYlthMITDJlPQudTGqK41fHZNc5DR3eHzR0ropr4h2yuodK7n4ja
rECAI48QlQAAAAAR8xAb9RD5+l8K8qJU/rp1MpyMn6am0BHRnTmnE6i8DlCYFIpo85ppLBzFSw5Y
HLD3NgTdQEAjgxCVAAAAABHj9kbtRYvTsvXYK/Onhx6Y8iHebVWhx7biYbtTyercHa88cfflg2z16lby9
Ss3ZpEcVP29D4Gyt7amueYpR8AIg0hKgAAAIAjyOiNmpQxjDw7g706u3Lg7tTtmVk66QWAo+p5ps
MsJeDXAHX05KJbmFROSaIqAdNKcvztXa/nqR24unVRp7iWaoZ+1ePUnkjLDlS7Xi3W0WUEAABHF
SEqAAAAgKPJmIAoTMlPDo3aqW3Z8Kapz1ZzdcObqKm94Q8/L59ZcwJAszZnc9UfQt7e8Gp0FlIZV2
4Gzta5WPd7WfYa61790aSTVU1twuP3A1djxv4k9PbVbRDscRoSdAMAIgYhKgAAAIAjKro3qjlzuwrp
nGAubi3NaZWlXG7Kqg4D/dzWmOFftd+ozdlc1cGh9+IREzJNorLtD1vv1KSk21XyE1S5kPuM9AmOv
3Lan6jKu5bGBFRJjDgX5r1RPat7ugIAIgYhKgAAAICjK6o3qqp7OVgzs9qavI5mIqfk7J4xPFyx63gO1Di

1a3MOvE4Je+2U7GHrIcdebVnr3eHxeYt9/BXZ7psz/itVaZk1TZu7Xs/fcexzEVYMVW8zrewaALA4Cn
1V3AUAAAAAAAAAEIqeqAAAAAAAAAAQgRAVAAAAAAAAACIQogIAAAAAAABABEJUAAAAAAAA
AIhAiAoAAAAAAAAAEQhRAQAAAAAAACACISoAAAAAAAAARCBEBQAAAAAAAIAIhKgAAAAAAAA
AEIEQFQAAAAAAAABGEvn/AZM6nr53azj8AAAAAElFTkSuQmCC]]></redcap:Attachment>
</ItemDef>
<ItemDef OID="q35" Name="q35" DataType="text" Length="1" redcap:Variable="q35" redcap:FieldType="radio" redcap:RequiredField="y" redcap:BranchingLogic="[q33] = '1'">
<Question><TranslatedText>Do you prefer Life A or Life B, or do you think Life A and Life B are the same as one another?
</TranslatedText><redcap:FormattedTranslatedText>Do you prefer Life A or Life B, or do you think Life A and Life B are the same as one another?
 </redcap:FormattedTranslatedText></Question>
<CodeListRef CodeListOID="q35.choices"/>
</ItemDef>
<ItemDef OID="summary36" Name="summary36" DataType="text" Length="999" redcap:Variable="summary36" redcap:FieldType="descriptive" redcap:SectionHeader=" " redcap:BranchingLogic="[q35] = '2'">
<Question><TranslatedText>
Alzheimer's disease - Description #[no]

If you have Alzheimer's disease...

1) You might forget recent events of importance to you. For example, if you are interested in hockey, then you might forget whether your favorite team won their last game, although you may remember watching the game itself.

2) You might forget the names of people you met recently, or the names of local streets.

3) You may no longer be able to handle your personal finances or plan leisure activities such as dinner parties or vacations. Simple decisions such as what to order in restaurants might become difficult to make. Your ability to concentrate may decrease and you might have difficulty driving.

4) You may start having trouble finding words to express your thoughts. You might need to be reminded to shower or brush your teeth, but you could do these activities on your own. You may need to post notes around the home to remind you of simple things like turning off the stove.

5) You may remain aware of the day and your surroundings. You might be able to travel to familiar locations like the supermarket, although probably with the help of a loved one or friend. If you are in a social gathering, then you might have difficulty following the conversation.

6) You may continue to enjoy hobbies such as puzzles or reading.

Now consider two possible lives. In Life A, you will live in perfect health for 3.5 years and then you will die of natural causes. In Life B, you will live in perfect health for the next 10 years, then you will live with Alzheimer's disease as described in the summary for another 10 years, and then you will die of natural causes.

</TranslatedText><redcap:FormattedTranslatedText>
Alzheimer's disease - Description #[no]

<p></p>

If you have Alzheimer's disease...
<p></p>
1) You might forget recent events of importance to you. For example, if you are interested in hockey, then you might forget whether your favorite team won their last game, although you may remember watching the game itself.
<p></p>
2) You might forget the names of people you met recently, or the names of local streets.
<p></p>
3) You may no longer be able to handle your personal finances or plan leisure activities such as dinner parties or vacations. Simple decisions such as what to order in restaurants might become difficult to make. Your ability to concentrate may decrease and you might have difficulty driving.
<p></p>
4) You may start having trouble finding words to express your thoughts. You might need to be reminded to shower or brush your teeth, but you could do these activities on your own. You may need to post notes around the home to remind you of simple things like turning off the stove.
<p></p>
5) You may remain aware of the day and your surroundings. You might be able to travel to familiar locations like the supermarket, although probably with the help of a loved one or friend. If you are in a social gathering, then you might have difficulty following the conversation.
<p></p>
6) You may continue to enjoy hobbies such as puzzles or reading.
<p></p>
<p></p>
<p></p>
<p></p>

Now consider two possible lives. In Life A, you will live in perfect health for 3.5 years and then you will die of natural causes. In Life B, you will live in perfect health for the next 10 years, then you will live with Alzheimer's disease as described in the summary for another 10 years, and then you will die of natural causes.
<p></p>
</redcap:FormattedTranslatedText></Question>
</ItemDef>
<ItemDef OID="a36" Name="a36" DataType="text" Length="999" redcap:Variable="a36" redcap:FieldType="descriptive" redcap:BranchingLogic="[q35] = '2'" redcap:InlineImage="1">
<Question><TranslatedText></TranslatedText></Question>
<redcap:Attachment DocName="35.png" MimeType="image/png"><![CDATA[iVBORw0KGgoAAAANSUhEUgAABVEAAADYCAIAAABtBTQzAAAAAXNSR0IArs4c6QAAAARnQU1BAACxjwv8YQUAAAAJcEhZcwAAEnQAABJ0Ad5mH3gAACm8SURBVHhe7d1/qB7Vve9xbdre5LQHQ28R216OaS/liIqk9Z90U44plCoFQciRBrGH9p6y2ULLCYE2Uurh/JGgSCFb8aIXigqn98Y/LCK3N0oshNJCAgX3H/6hkMr+Q2kg/rENRXYwf/Q+M7PWmjXf9WPmWc+seZ5nnnveLxTn7mVlrfdeame h81Gf3hr8BAAAAAIAxIvMDAAZyfvv8Df9xA22A9n//5zPqogMAgNVG5gcADGF7Z3v/E/tFNKXlaPtO7PnRP371g/ffU5ceRrg/ffwdBm...

onvHbxVxPLc7dWnn1I3GwAAjAKZfxFE8pv/lI5C6mhqWKrG6X+9X44uD+kj9YRiuvjHkPZR+tDkiNpT
fc5/JVrJKmnXylu8Ma7D8oJd3AW0z9ahXod9TXgmEgO7L2ai6iRLO0vxra19JZi3x//wuAiltKzt29+6R
QTyYdo7Fy+qWw4AAJYfmX8RREKVCT32OZmEPONNCouFJTWP+zX+6og1n6gY/xjSZVS1bvPr7K1T
HYtIclhasPRdzuawKSaxb2XJHds+m69HUWC6fU20X5DWedwp5PVypggtvzD1FjCMV95+RSRSWtZ2
x9GbRBQfrB1fO/ThlSvqxgMAgCVH5l8EnrBTM/FJnzU5yHQ3R3Q0MmNawlI90Dc0nL3iH0M6jbLqi
zOmvz5susbKyip60HTB0nNVZPX25XlKVzwLaJ/N6RE+UE/r4VmVHNi2GLm2ur9zRH32rq3DSjA3W
5e3+EX9Q7abf7x3yK/xu+3Jhx9S9x4AACw5Mv8i8IQdm4lUDXZnK2RJ8bBUDzTTmWreAtV08Y8h3
UbVu3UuhxnRMFXRxGDpvwUF3zwN9aSe0hXvAto36+9hTd9cdWB3nS5Iy2LCl0d1cWf0rK3TSjAP
O7s7BzYPiFBKy9fm8jV+t/HFfgAAxoHMvwg8YUdqhi5PArI7TE53DUtuP+9IcTD+MaTjqPjVMKNKkS
umyCppwdIepX+ecAfElhfcWHAB7Ztt9nC6WGv1Dp/ofEHii7ErFcP052oK34zO2jqvBIPavb57+MXDI
pTSsrZ5fY1ftI07b393a0s9BwAAYGmR+YF2njgKrIaN326IRErL2ub4NX63nTh8z0dXr6pHAQAALCcy
P9COzI/VdPrCaZFIaVnb3L/G77ZnHtlQTwMAAFhOZH6gHZkfK+j89nmRSGlZ274Texbha/xuO/fC8+q
ZAAAAS4jMD7Qj82PVbO9s739ivwiltKztu9/8ogjbC9L4Yj8AAEuNzA8AaNjZ3bntmdtEIqVlbV8/8jm
RtBeq/eLe73x87Zp6PgAAwFIh8wMAGu779X0ikdKyti88sk9k7AVsz5/4mXo+AADAUiHzAwBqx14/J
hIpLWvbd2LPQ3cdEAF7Mdsff/OyekoAAMDyIPMDAJQzb50RiZSWuy3s1/jdtnHn7X9598/qWQEAA
EuCzD9f+nfDlbLVvfwnC9P+sfFr0VCLaV9xQW7l8UzoL+HcOrVIOCVt18RcZSWu936o8+I/Lyk7aVTJ9Vj
BAAAFtgAmV+8vM/6Rm5PFw06C897NU2U0dfJ9DIXzko70Qvgm98d68zvDNNjInfOnbY+IutEprX7
+Kr6dhRUryCy8IK1eFt4lF6G1UOstnWzcgozwMxprcrebWiieqAjpVZ9RBc3o/SR+kCkdt3L2oRbzlmS
M0zuu9Oa0Wrr8ha/qH/g9tmffvL7d3xZhOflbW/9/vfqYQIAAIuq98zvct7WZ2IljYl+5pyTWByyNyb7
mWzTsvvY/NYx0U1/tCb3HBJ6m9ZMpDRLijmjTN9CrL8zafv1dVauh6gDnp15DjV0XIXpFpyog3Ct+jr
JPs6YCXMsshbfuPZy+mPkAnZYM1rs7O4c2DwgEikta1v2r/G77fjaoQ+vXFGPFAAAWEj5M3+/L+Lm
zX+tmQCWkefKiOyomE1XRz1px8t35T0FRDczu0ewYHsA6zyt3VHeXd+OWng23K51UOCO6GV13m
yp2duZoznAXILaNFvrXEteat2lUcx7sMl3yzzDRLfmMpvEgiJrRszu9d3DLx4WiZSWu43ga/xue/Lhh9R
TBQAAFlLe/7bf8+rueUMvdMwtesz6Wf3TNIFnwXiupj9EiY7+Ti7P/L6xgdl9rIma+py27uIs0LejNnnG
6B7lEvWKTe8umzU1BH3ec0krgYGx7SXUkpfAu5zgGmtynkJ7Od3DJ7wgXy2EbPx2Q8RRWu42mq/
xu+3Vp59SDxYAAFg8c8r8ppctkhwUPayYRP/cPmpRea6mP0SJrfo7uTzzd0hKXWdv6G9aM6wkevt2
1CphQ7pObIiadtJF9267jTbdY6Lq5GytdQ5rilKXjt1ryT7e5bSu0X/L2st1mLjDmhF0+sJpEUdpudvIvs
bvtncuXlSPFwAAWDD5f4efZ4xzqMMrfkGPq4bpTy2DFlfsytibkv06Xi3f/B2Skm9Uq76m1b3WNjd9
m2ydxNOhbYzvvN5OdK2q09r6ejWB1bl1ne710kfMEM8l1SanpiiWVkvO6qtijnnWqPnGtZfzjRI6rBl
B/K/xD9++
+80vipA8snbi8D3q8QIAAAtmHplfH/G8rUfCg9up06AF5l6ZCZPH5C7rbXrSjpdv/mnSnT5glmSvs6m
faRtDzIfInC45yqnqrtXp0mG/JdOv1OjcNqcsUfc388hlVszA+rC/Yy2tluml+zhbsuYJlZ5w5pnoUM6pJ
nfRac0I2b2+e+hXh0QopWVtX3hknwjJI2vnXnhePV4AAGDBzCPzm9d3j8jbup6ofsl3jyyVwNX0Xx5r
j56049ecqazSKSmZIw2RW9PLtOa0nsQsv57VsyOp2UWxL5W7Vu+Q6H4r9jjZu2Wz/pIF3cVdZiU0
MrzcpFru7QtsqSDXaGtWL2frVK7lAnZcM4Iu//Uyv7R/4Pb1I58TOXk07ZlHNtSDBQAAFs/yZH5/Bqj
EMsfiilzN5mbFeU/aCbEvddm9c1JqrqCt1OzT1ies/mb5dUdnRz52p46XLzokxLM+W/wa2hWLevqz

qu1fZqW52A7Lnb5W+
+2bnIitsWYXL3smlZNVOk+CIP7H+Ydvo/wv/E8cvuejq1fVUwUAABbPPDN/PCkIzdd/aaqpgH7oh5L
HD0vqzFtnRCilZW37Tux56K4DIjMvddu48/Z3t7bU8wQAABbSPL/PH5nFYcYEkLowNPNM8vBhiT36
u0dFLqVlbSP7Yv//e+5Z9SQBAIBFlZj5vcoI7wR6T8Kvp1HHzH/9G/qnAJ5JKuYEuQuDsf9r9fBDCyyJB
156QORSWtY2mi/2P/nwQ+oZAgAAC2wumT80UTA8RZJ95BSQh5X5CfxYfrvXdw8+d1DkUlrWNoIv
9h9fO/ThlSvqGQIAAAtsTpm/0JwsFtmjuT56EgDQantne/8T+0UupeVrI/hi/zsXL6qnBwAALLbpMj8A
YJTOb5/n1/gP2W7+8d4f3PYVEaSXpb369FPquQEAAAuPzA8AKDz7p2dFLqVlbXccvUlk6aVofI0fAID
lQuYHACjHXj8mcikta/v2t24RiXrB20/u/toH77+nHhcAALAMyPyIE7+PIf5xKvo34alfxDDLVNNq/OL9
7L8IYrKzAfZUGPIaYrQOv3hY5FJavvbpn3/iewdvFbl6kdubb5xTwoAAFgSZH7ExUP+LCFzXpnfFKrkj
fyq2EAZfLBriDHb2d05sHlARFNavrZEX+x/6dRJ9ZQAAIDlQebHVESqnCVkisw/mAHrDp3Byfzox9sfv
M2v8R+yLcUX+089eOTja9fUIwAAJYHmR9TWf7MP2QuJvNjab126TWRS2lZ24J/sZ+AMAsLzI/H
MRiLvewybHldpHtOQ+fboe4R7R0xZHxHTxj3Gm88Skv1i7dyp7iL3AqMagxihd0hJYuL04se6GZi27
WqiW2HbB2brVp55GnQxX9EwUIObovqmJLuufiM5RaXRxz3eYAXmdvnBa5FJavrbgX+z/429eVo8F
AABYNmT+
+fAEJ9/BOvDZrEEdA5hNnzenTREziTpSfhbTxT9G+LdSUGWdqcwBm7VRr/gV85wNLLx1uYVosVAtfd
yax9m6Z2zVPVqx2+3wXlZrREuJ1PUXrFW13dz2GTCIoy8fFdGUlq8t7Bf7nz/xM/VAAACAJUTmnxO
deTwxxxwyuUcfMUHJ6WJN4wQwSXXQQ6x0pUboGcqPYrr4xyDTT9aYUAtpm9mzVUeHK+ZO7OV
elfqI2MKUtTz7cLq51UutFX31mpwh4pK1luiyftnFmUQOcEa0zoCB7F7fPfSrQyKa0vK1r/7L34u8Pff2
i3u/w9f4AQBYamT+edEpxoQYJ0t5wlVruppw8pRD9ajGVB/W14uJ7CN6vJgu/jHE180cU2tv9tGfPB
uzDgldrph7wEtPZfVqHeh28A3pcstM9fBeS3JgwhKrQ6H+kbWF128+hpavO3imqA61zoABXf7rZX6
N/5Dtn+69WaTuObaNO2//y7t/Vo8CAABYTmT+uRHBR0cpk7482WoiMMrqZPJSOMepUWWH8ue
1zbPlqPqIGS6mi38M8e5FHGxOpU/6hGp5q5h59dFuSzb1616xgc3lmg6+IZ5VOt081SVvxda9eYqH
+TfVYf3mc61RsDlxUzVF2wwY1tblrb0n94poSsvU9jx24z/f/Q8ie8+r8TV+AABGgMw/PzrXlGFGx6A
6rPnjmYhXHQKYjxo26VH1nvxQHTJHzGgxXfxjiHcv4mBzKn3SJ1TLW8VZYrcle+ZyB5ojgtvBqtVlZv9O
Jloq+uo1BCeutW1qliujzuspfPQU8RkwuDNvnRHRlJav3XT8U4vwxf7/dezf1O0HAADLjMw/Tyr7TN
KTTjhWpPHHM91RHe0UwDyqcfpf75ejy0P6SD2hmC7+McS7F3GwOZV3RAv/GD2vPtptyZ655EDd
ZaLq5c7sq9Vh5tDuWyv66jUEJq61b6rL+g1rulI1qHUVFv8MmIdHf/eoiKa0fG3uX+w/cfiej65eVfceA
AAsMzL/XKlAs7a+XoUmOzOZlGWnHJmuPOnJhKRQ7iuoedyv8VdHrPlExfjHEF83c0zVavbpOHGDn
LHkTNRtZs+FFQPdHu6V99Xqcss8fSbaK7buLbQgPWV7CU8Xt8/E5Jj1qVm3dZml2AyYkwdeekBEU
1q+Nscv9m/cefu7W1vqrgMAgCVH5p8vk5ZKIs6Ykzpemdxj8pY5osdaE0bDUT3QN9TKcyJrxT8GOe
u06qtioZn1CLO8SKkOV0zWCdAzha+EXE+94HpmX626nzpoXXfdzVN9or2ir16TU715oMOmnBmc9
ZsD9fLFhsJzdJ3BcwAD2L2+e/C5gyKa0jK1OX6x/9wLz6tbDgAAlh+Zf85MupnQqapmn63ZKccKZZI7
na0e6KQof4FquvjHiPBCVTVnKv+IlkJTXLHoTJ5EKQf6SxXqmZt9quPhK2EGeqoXWit22VugvCrVYVN
TrF+ql+WfpD7fPkPgGiG37Z3t/U/sF+mUlqnN5Yv9zzyyoW42AAAYBTL/vJlwE8ouzXRUZx7D7jA5bT
56utrcft6R4mD8Y5zpPDHpL0JbvHqpY7xrDpIr67ZkT6L0DLSTaXFUf7amtruYyewVTvo6M3uqK/GK
3fYmr5AoEy9Ralt/wZ6m4C6puYr4XgvNGfRZdxxyO799nl/jP1j7yg8/KzJ51nZ87RBf4wcAYGTI/POm
gw/RBcCyePZPz4poSsvXDt3/eZHM87V3Ll5U9xgAAIwFmX+
+zL/qJPIDWCbHXj8moiktU9vz2I33f+NLIpznaK8+/ZS6uwAAYETI/PPS/A+X3f/wGQAW2+EXD4t0SsvAyhnd2dA5sHRDqlZWq3/ugzIqX32I6vHfrwyh
V1XwEAwLiQ+QEAid7+4G1+jf9gLd8X+9/6/e/VHQUAAKND5gcApHvt0msimtKxUxf7H/p1El1LwEA
wBiR+QEAMzl94bRI+QEAMzl94bRIp7RMrfcv9p968MjH167plgMAgDEg8wMAZnLtk2tHzxwVAZWW2MjH166pGwkAAMaAzA8AMI13r7x7/E+Ig2G7Dogy37394t7v8DV++
+/p24hAAAYCTI/ACBm/ZtqaidLDDEJ4BGagZGfhtYAAnK5LW3Dn6wQR+bbBg7Mj8AoE+P/u5ZfwAr
CbM2+dEemUlql94ZF9Isl3bBt33v6Xd/+sbhgAABg7Mj8AoE+P/u5RkU5pmVraF/v/

+JuX1a0CAAArgMwPAOjZAy89lNIpLVOb9ov9z5/4mbpJAABgNZD5AQA9272+e/C5gyKd0nK0qb7Y
z9f4AQBYQWR+AED/tne29z+xXwRUWo7W8Yv9G3fe/u7Wlro9AABgZZD5AQBZnN8+z6/xH6bd9e
B+kfDddu6F59WNAQAAq4TMDwDI5dk/PSvSKS1T+/a3bhEh327PPLKhbgkAAFgxZH4AQEbHXj8m
0iktR/v0zz/xvYO3iqhftROH7/no6lV1PwAAwIoh8wMA8jr84mERUGk52s0/3vuD274iAj9f4wcAYM
WR+QEAee3s7hzYPCACKi1Hu+PoTSLzv/r0U+o2AACAlUTmBwBk9/YHb/Nr/Idp9hf7n3z4IXUDAAD
AqiLzAwCG8Nql10Q6peVo5ov9x9cOfXjlirr6AABgVZH5AQADOX3htAiotBzt5h/vnWT+dy5eVNcdA
ACsMDI/AGA4Ip3SMrXT/
+OH6ooDAIDVRuYHAAzkhhsG+psOhZJRKBmFklEoGYWSUSjZ+AqtAi4lAGAgvJEko1AyCiWjUDIKJa
NQMgohgksJABgIbyTJKJSMQskolIxCySiUjEKI4FICAAbCG0kyCiWjUDIKJaNQMgoloxAiuJQAgIHwRp
KMQskolIxCySiUjELJKIQILiUAYCC8kSSjUDIKJaNQMgolo1AyCiGCSwkAGAhvJMkolIxCySiUjELJKJSM
QojgUgIABsIbSTIKJaNQMgolo1AyCiWjECK4lACAgfBGkoxCySiUjELJKJSMQskohAguJQBgILyRJKNQ
Mgolo1AyCiWjUDIKIYJLCQAYCG8kySiUjELJKJSMQskolIxCiOBSAgAGwhtJMgolo1AyCiWjUDIKJaMQ
IriUAICB8EaSjELJKJSMQskolIxCySiECC4lAGAgvJEko1AyCiWjUDIKJaNQMgohgksJABgIbyTJKJSMQs
kolIxCySiUjEKI4FICAAbCG0kyCiWjUDIKJaNQMgoloxAiuJQAgIHwRpKMQskolIxCySiUjELJKIQILiUAY
CC8kSSjUDIKJaNQMgolo1AyCiGCSwkAGAhvJMkolIxCySiUjELJKJSMQojgUgIABsIbSTIKJaNQMgolo
1AyCiWjECK4lACAgfBGkoxCySiUjELJKJSMQskohAguJQBgILyRJKNQMgolo1AyCiWjUDIKIYJLCQAYC
G8kySiUjELJKJSMQskolIxCiOBSAgAGwhtJMgolo1AyCiWjUDIKJaMQIriUAICB8EaSjELJKJSMQskolIx
CySiECC4lAGAgvJEko1AyCiWjUDIKJaNQMgohgksJABgIbyTJKJSMQskolIxCySiUjEKI4FICAAAAADBO
ZH4AAAAAAMaJzA8AAAAAwDiR+QEAAAAAGCcyPwAAAAAA40TmBwAAAABgnMj8AAAAAACME
5kfAAAAAIBxIvMDAAAAADBOZH4AAAAAAMaJzA8AAAAAwDiR+QEAmNbZ9Rtq62fVUQAAgEVD5
gcAjEgVxvOm8Euba3WFnAWH/icLxcYm1jYvqQN9amymxD8rmVbjGua5TdkF/sCoZ6/S75MR+SMa
OTUt/1SNO9bvtvKKXpmsf6HIKLypZb1PgR01ttPXbepQa0n/mrQiyPwAgLwyvs03WHWGfGkry+Z4
15m8S5l9lO9VmV+oio2sra9P/k+WOsUWBn8ltN9H+38qGk92LdPTV+5loCfCvm79FQn+CW3+GW
pudCbBitFT0wpNVRw3B6pLOmsp/yz27Zq5ROuVKc73+hcK36YaWyrNtq/IpspTPf5RcrdjFbdl2lFZ3
xwqP826u261eihVXTtFXJ/GVRTn0AGZHwCQUfN9qvk20itdqPz/Q74QNHeYS/YqRQF1+fKUKe599s
tkG+bGNJQlMz18cu58tfp+j1f0/XAXLh+NvrYWrhg7Na2uU+l+6uPUyvGVZpXJ5TMHZr9dbdspjqvT
M9WpxDbVx/xKZFOeQ+mC2xFmLxrekTySsZY+oT7OeteK6cz05ZNcf2yWKk/OtKdVROYHAOQj3w
Gct4be5a8gDPP6kXlbZvrih/SXtpjZXgenNvhzMJH3UWhewGz7Kye2b1TfN85ZubuVni9k5Fr1eBnbp
irPJ19IPTpvFYu/kDla/DBzGb1YT608f7lwC/WyDyWynaYen+/WS9e6mO6cqZx99FjMXM3yg3wc+iy
0Msj8AIBs3L8z9/i24zfwy0C5n77eGYMarz/9s65Z8WOeQuWVqmW+Zvn2EZT5Jk1Ul3BSovohz1Pu
/Am1no5eOPMVFZvXreeSkel6rNQ2lXNhk7RV6W1DvomsY8WPvT3snlruQ9EHt1BZp/iigjF7Vd+ls5
Sn+9qbt1b5qBUlqh/CS5mOUytwOfvZmn2Z/IV629iKIPMDALJx3wC8ryh9yl7AVr549PX25ir3omTc
UrELPX1RMt+GtGpjOeuUT17Pr/Jxwzx45RNXylXK3Yf7p3gmTgF3/p6vZWS6HivFpypvXA9XMV6lP
NvPvfIUKvagjxSne3soArUsPZVyC4n7UnYIX99uPNuxxM9OKzRbff16K+WpVR6RV6+ne1XuQFUrfm
5O2+9VXA1kfgBANnP4W/Vw7wLibTGrXl+mmopt1NerKDTIntxHo1fi5lTXL+djURbM+tzZe6h+zlOv
nFteuh5vVTmhvXL3UXC6zCYyXY+VYlOJx3EG3irlQaWvy+YUKvZQfy5O9/ZQxC7dRLW9Pqq5hZyHr4
c/yNHt9DC/zVOrulzVsernnup591XuR1tfbzwkMyinNTfGuUvxawwvMj8AIJs5/K16oHeB6k1nwHeO
6uWt/4KNVzZLH2/YMWXdfNfPefLy1ivvTtZL5lbIuKPGQ9Hfe3zF+RPq/pF17t5s3AJG5NS0glOVV7O
v7bQsuDzdSzFZqPFMWHLUcvX0RLiFnInb19IqMkV5qq9noeDUciuUd262HVXaL03Ro4/dyT8y7u1
vXwskMj8AIBv3b8w9vbuFDfEuUNYY+IWjrJn1ypWKMvmr5N9Ojlf5sN7eqsPcEll3ZOn7kXDXLW9
W3zuLzNdjKf9U5X3rcTOtCy479FCwpVBxurenonVTff0JcwuVR+yNyIcxQXg7PW3D4tRyS7Rf3Y5aJy
o6zP5QlDtoTuNW7uE2rRwyPwAgH/l35t5eP4LyV6jeSaTe3z8mVaxteN6Dsujnrc01mdeatrxJWe9S
WcHeSM53xPLm5H3m3CdgkKITvT8R5a1pLryxN+fWzcxTUYucmpZnqvJQvzepdcFlhx4uX0uh4nRv
N2mum2r8QerlT1VwOxn+zLq1Gn+YJvorGr9N/dyjcrVukeKwPXd8KfAi8wMAMmq8gPTzVuBXzi0s9

zuB2NEwmymKZrlDg+
+m8arb33uvK+dT3VS9DxsDPBF97k08AaV6C/be+rqYkYrxxUwlPJW4X5UZN1dWa6x0UsX6XNacpU
bHK1N06+2hdzY1OWBNXi3Js4jO4ptqnJ2ljOJsp1IeHuDZ7v0vFOFazTMzX7pqOu80jee61yu5Osj8A
IC87BcQ/j6NwTReSGd+Hw0pH+9ss89Bz+/x6EXzrlTUvRGnluiOhTe1lHuK3KOJ0f2FonfiH1VU6lcGX
iRmROYHAAAAAGCcyPwAAAAAAIwTmR8AAAAAgHEi8wMAAAAAME5kfgAAAAAAxonMDwAAAA
DAOJH5AQAAAAAYJzI/AAAAAADjROYHAAAAAGCcyPwAAAAAAIwTmR8AAAAAgHEi8wMAAAAAA
ME5kfgAAAAAAxonMDwAAAADAOJH5AQAAAAAYJzI/AAAAAADjROYHAAAAAGCcyPwAAAAAAIw
TmR8AAAAAgHEi8wMAAAAAME5kfgAAAAAAxonMDwAAAADAOJH5AQAAAAAYJzI/AAAAAADjRO
YHAAAAAGCcyPwAAAAAAIwTmR8AAAAAgHEi8wMAAAAAME5kfgAAAAAAxonMDwAAAADAOJH
5AQAAAAAYJzI/AAAAAADjROYHAABYLGfXb1DWz6pDFX1ibfOSOpKHriPKL4L62kw41+HS5lroVPQc
AIwXmR8AAGCxWLm2GU9XPfPXsb3kuwzBZF9f1AX8JxkAkA+ZHwAAYLE0/l22nVBXPfN32r833Nf/
KIDED2DFkPkBAAAWSyPz2wF3xTO/Ce4t6zLXz1wnc4TED2DlkPkBAAAWSzPzW0HVyfzOATcYqy5F
D2teNcA6YqdhfXhyzPrP6a0imnXWOW+trS7Tkrkb8zUmtFaqReYyvcs+5pOzg9j6C831iC6RDUbHAc
CgyPwAAACLpQ6Tm9VPOjJaKbP87B6w4qaKxHUcbWVCdHiMlbNlri1ZC/FMEo2+/qJy6xZrLY56ceu
b+kdRvW39LQsKb9A/LrpcAMiHzA8AALBYdGicJEgdTKvAaJ0oO7oHYplfdbIiqTyiY6kzpj6iD8gypodZi
lWnQ9wNFzATujVjTGelnqbUun7dIbyewAZDE8kVAMAwyPwAAACLpZEa1Qf7Zzd2WnFShlndpc6l4
SN6GreH7CMT8YQ8ZCax+gTJFRTkTtwDcWYBBTGkw/olp7p/g6Zbp30DQHZkfgAAgMXij9eTqOkkY
09UltHU7dJ+RH+2g3JzXt3DRxRuzBLgLqmgS+qjcmutwkvosH5Ls7c+H5jdrLLW3BYADIrMDwAAsFh
kAIafzdf76wwpe07kyvzNg/qDT7BwmL+v3Epy5neX0GH9vvBe0ueDswcGdl01APSKzA8AALBYnDA
pQ6Q54YmdMhi7XdqPeKYV8/p6CB26GP6+uqQ+KrfWKryE9sXpHhNVJ6d66xzWFKVwMQDIh8wP
AACwWDxhshkfzYlYzx4yvx2um306xG+3TJiZzu7s1Ogx87dO5Q51Lkpkg5NT1sRTrxsAekTmBwAAW
Cy+MGIyY8GcqI+qOGmCqXPEDa/hI+FZTJdwZR1s3TIx4QL1+Kmzc2QJbeuX26n7t2zQ2Uh0GQCQH
ZkfAABgsbSESfuEFUUlkWVjEVQeMZ/X5Ow67074S9cd3DJxun+TPbrPzN+6fv9yCrpLaPbQyK6rBoB
ekfkBAAAWSyBM1iG1ccLOrpNYKYOxO1n7Ef3Zns5ZTqGZm5sdAruIas4nQ7LcWqvWJcTWXw8vFC
Wty2Kf9s1uDy10XTEA9I7MDwAAAADAOJH5AQAAAAAYJzI/AAAAAADjROYHAAAAAGCcyPwAAAA
AAIwTmR8AAAAAgHEi8wMAAAAAME5kfgAAAAAAxonMDwAAAADAOJH5AQAAAAAYJzI/AAAAA
ADjROYHAAAAAGCcyPwAAAAAAIwTmR8AAAAAgDH629/+P6A6KAOf5JOPAAAAAElFTkSuQmCC]]
></redcap:Attachment>
</ItemDef>
<ItemDef OID="b36" Name="b36" DataType="text" Length="999" redcap:Variable="b36"
redcap:FieldType="descriptive" redcap:BranchingLogic="[q35] = '2'"
redcap:InlineImage="1">
<Question><TranslatedText></TranslatedText></Question>
<redcap:Attachment DocName="B.png" MimeType="image/png"><!
[CDATA[iVBORw0KGgoAAAANSUhEUgAABVEAAAEJCAYAAACdcmdaAAAAAXNSR0IArs4c6QAAAA
RnQU1BAACxjwv8YQUAAAAJcEhZcwAAEnQAABJ0Ad5mH3gAAE9sSURBVHhe7d0PjCRnfef/b+/aY
PPHNmBskx+RHat7sZYlGJIg08PxL4egZ+6SJbnbYOVg5Oh3PVyi0zQSkwOxF+mX2wguG4lunS5iWrq
zliQyTEJugrLdIJRgckzHR4IxYTI/Z7rlc3T8wDYGjPljB3u3f/VUPVX1VHd1dV3VX39PtltaerpqfqqT/T
O/OZ5/k+hb5FAAAAAAAAAAChjumPAAAAAAAAIAQhKgAAAAAAAAAAEIEQFQAAAAAAAAAAiEKICAAAAAAAAAAiEKIC
AAAAAAAAAQARCVAAAAAAAAAACIQIgKAAAAAAAAABEIUQEAAAAAAAAgAiEqAAAAAAAAAAAQgRA
UAAAAAAACACISoAAAAAAAABCBEBUAAAAAAAAIhCiAgAAAAAAAEAEQlQAAAAAAAAAiECICg
AAAAAAAARCFEBAAAAAAAAIAIhKgAAAAAAABEIEQFAAAAAAAAgAiEqAAAAAAAAAAQgRAVA
AAAAAAAACIQogIAAAAAAABABEJUAAAAAAAAAIhAiAoAAAAAAAAAEQhRAQAAAAAAACACISoA
AAAAAAARCBEBQAAAAAAAIAIhKgAAAAAAAAAEIEQFQAAAAAAAAAiEKICAAAAAAAAQARCVAA

AAAAAAACIQIgKAAAAAAAAABEIUQEAAAAAAAAgAiEqAAAAAAAAAEQgRAUAAAAAAAACACISoAA
AAAAAAABCBEBUAAAAAAAAAIhCiAgAAAAAAAEAEQlQAAAAAAAAAiECICgAAAAAAAARCFEBAA
AAAAAAIAIhKgAAAAAAAABEIEQFAAAAAAAAgAiEqAAAAAAAAAAQgRAVAAAAAAAAACIQogIAAA
AAAABABEJUAAAAAAAAAIhAiLp0etJYKUih4DxWGj29PqjXWPFeU1hpWF+1iMxj3ZB2onXjpLWdL
MxLO7LSlg372PzHqPs4Vb2GrGzEO5vtDbdtK5JH08KNuQ9Cjmc+2g0AAAAAwPwhRF06Rdm8UJey
XurU1kPCkracr3X0c5Hq2U3rq4B5oILBVWnqJUdZzqxle4faf1Qo1cT/rlhsR+14AAAAAADIGiHqMip
uytmqfi4dqZ0P9kbrNc75IVW1JdsV/XzhFGVzry/9vnpsS/aHkff+RpmXdmShKwdu8leuS9c+xj3ZzDJ
D7TVk3fijwsI7ascDAAAAAEAOCFGXVGW7JV6O2jxn9EY1e6FWpbW4CSqOot6h7OuncuoEPaQBA
AAAAEAuCFGXVkW2W26M6vdGbW/4Q6XL9a3wXoyqlqKuRek+wmpSjqyvaH59ZI1Jo6ZjoC7rqPX
GPu31edcGHd5fZI1J4zwEz5+5nbDPjxN+3INtCdS9tR4xy30ObGewraPOc7xjCm57uPZpYAh6c9VZF
2h4snM3eA7Uw9yc3Z6x+xzPPy71CLkXbAnaHvI9OHq7vqTHE6/dAAAAAAAcfYSoy6yyJXW3OGpz
VTYaDTnnJ6hyIWSM9FAIo3VqpQwmoCrK2hndwM6BdJ1nFmNId2dHLno7bcuubn/5zNpc9FKsnPa
D6gP/AGy9izv6PBo1Pe1wrCSDo63TPb8dqZUKUhrYSXM1aUimtjPY1qasDoZtEx2T2rZR+9S6H+te1
+kREu3HCSwHz4HSXB0dKibnnOtV70AUtW6Kc9TeGFHPNGS7E4vZbgAAAAAAlgQh6lILTjLVrLnBTF
nqF0Imk2pv+KGKV4+yL103ie3UZD3lhKW4dka3b18O3U23d42JhYxw0hjqferEnAz0rpz2yiY0d4M9
Ji/u6BisfEacDLUnjXX3GlSlpc9v3+0xnOr59bfvdUhWIdlAfdzx/O1494Hazrob/E1zTMbr9zZlc9v62PX
vV1Wv1/6cXXIi2X56jXUvsCzXu85r+8MlLiqR+4wr/BzteOl/krZbr3X/0hH2PRjY7rBkxzOu3QAAAAA
ALI+C9QtyXz/HklK9SwM9zlSwEhKq+K8rS71rTubTk8aK24tOBS/OZEYjX6963bk96Ubsy+dvW4Vde
9ZG1BBs1YOwWq1Ks9kcWu+3IaxdcdeNE387w+2yV3rnwG1/6DqbsV0VnO2FBNwB4e0wr3O11fc
nDEt0PSK2E9ivvuaS7JjMbQdfr41qa6JzF35+FOdanQqsS3p+lETnOlHbRwht44jjjDietO4RYBr3Pnyvv
OXCW/QSACAP99/xLnnNW2/SSwCAzB3/NZFjr9ILWBT0REVwkikVtoQGJP5Qeb/npMsYdm/2GE1F
UU6ccp51dJfTrj2WvywnT5+0e9QF11uqp/0AbA74vWmb4nVG7R44gZT1GW8ov7dOdT4sGbUo3S
BMfcIsazBr1jUo6ac28z7QPYSnOKZEvYkT7cec4f+kmIdQ3NyTvhmgTm3wHIWY6rrrurFuwJmaGO0
GMvDwEw/LOz/5Tr0EAMjDDY9fJXd/6AF5/Bs/0msAAJm7dI9I/zt6AYuCEBVBA6FScsO1P6fl1RVt7k
pbenJoj9k/JScqJ6z/D65XGeqc9ZIrrombLbpD+ttuIl09a/TozUtaAZl1DTJrOyHeSKpXqBe0GnVjgQX3x
NNPSOWPKvZHAEA+nvPjY/KWL94oP3ryGWm+/3555p8u6c8AALL1lMilP7A+PussYiEQoiJlGYRfXl3
RfTlsXxS7lKgd9lbEyVeN9XMZvhk9NO3A1+/VOyrwVcO67VqVQ480e0lOK1mv47yOaTHOXbjxbW/
Lhtnr1K2LatY5BRbUnZ+6Ux58/EG9BADIwxv/
+gZ5wQ+vsJ8/9NXvyp/WeR8GgNz0Hxa5/Od6AYuAEBUxleSkN1LbnBFfMSZJitM70Ri+HI+7744c7D
pf686+X7I/4a8fLjUwH4qbZ/0guOFOjFWVQIZacsoTKG6Jgvk22OvYvA/0seV1TIn2Y97LA8Pk1cz3hZ
xnoE/SdmNSNTtwdeukJv6eAubL+z77PvlM7zN6CQCQh1c+eK3c/PXn6yXH5z7+kHzlLx/RSwCAzF3
6gsjlr+kFzDtCVMRUlM2zbuVUc/Z1NbrYnOl8y+vt5wScijnre1s23JlrYvN7cqqJpBS3XmZRF0x117vh
6vxxe81a56Kmj3+wdqsx7F+aq7LhnTIV7Onh297K+dBcNQLH9nm/hqd7bHkdU6L9mLVbm7Lqre/p
me+ta1Qq+NvI2oTnyA9c3XYDi+kT+5+Q+n11vQQAyIOqg/pzD7xELwX94W9/Tb73raf1EgAgc5f/RK
T/fb2AeUaIivgq29LyctSalHTA48w8bynX5YJR4NOfUMnSXNWB0GQ1HAPbsp55Q/a9of6KMUnTHP
Jqu9rKUt8KRKiWomxe8IdlN1d1gOaFzqMm/ZolJ3AMttM8tryOKdl+ipsXpO6/2HmtOZGTdS/7h6Br
7yrua1NNWBO03bzfw9odR+bHA8R339fvk7v+7C69BADIw/FLBXsYv/oYRgWozd+8Xy8BADLXf1Lk
8sf1AuYZISoSqWyH11+stqz17tBiV3FT9gZfW21NVr/R7K0XKBlgDM3OdKKjFJgB2KiyA+qc9bt+wOe
ya1/OW03PstS7fT9Yt1Wl1d8LTpaV1zEl2k9RNvf60h16sXq5MUzeVpHt4EGK7B96PbFTEbvtVlv6Lf
8+sqlzblwHu+5ulByOB4jBnYn/6Wfp7QQAeVr50kvl2iev1Evh/uFL35ZP//6hXgIAZO6y9RvZJcpbzbt
CX81aAgAxtTcK4nSSVCHqQGAKADGo4PT1/+318sAjD+g1AIA8lB56od0LNa6tu8vyiteFD/sHAGTgit
8QKfBL9ryiJyoAAMiVGsJPgAoA+VK9T1Uv1CTUsH7qowJAji79AfVR5xghKgAAyM1HvvgRezIpAEB+
VP3Tt33hZSProI6iAtS7/+NX9RIAIHN2fdR79ALmDSEqAADIxe6Du/LBv/igXgIA5OWOL18/tg7qKPv/
8zH53Mcf0ksAgMxd/n9FLn1BL2CeUBMVAABkTg3fV3VQmUgKAPJ189efL//8CzfppclC+dzjsnX36+

XWV79IrwEAZOtKXR/1Zr2MeUBPVAAAkKknnn6CmfgBYAZe8MMrEk0kNcoz/3RJmu+/X3705DN6
DQAgW9b77aWPWx+fchYxFwhRAQBAZlRwqgLUh594WK8BAORB1T99yxdvlOf8OJ1f+R7/xo/k7g8
xKSAA5Kb/HZFL1EedJ4SoAAAgM+/77Pvk3ofv1UsAgLz83AMvkRsev0ovpeMrf/mI3PtJ/igGALm5/
DXr0dELmDVC1KXWlo1CQQrGY6XR05/LSa8hKxttveBob7jtWZG8mzM3Qs5LHL3GinPuVhoy6tT55
9d55H7NjwLuWyCW+n11+djffkwvAQDy8vJvPE9e+eC1eildn/zPB/LN//19vQQAyNylXZH+Y3oBs0S
IurR60lhZlaZecpTlzFpRP8+eHfiVasLfVIImPi+9hqzXor5KXfOCrAYvunRqJSlMENguK+5bIB7V+1T1Qg
UA5Ovqp47LG++bvg7qKKo+6u//
+y/bHwEAeVD1Uf+79fFZZxEzQ4i6tLpy4KZA5bp0+33p9/dkM68MdWzgt6QmPS+qZ+S4YK99XtxN
V1vqevelWy87K5qrQo4aA/ctEIuqf6rqoAIA8veWvRvtIDVLqifqH/721/QSACBz/UdFLv2xXsCsEKIuq
96h7OuncuqE5Nf/FGmL1zOyJ41zugtquS5bFedpcfOsVJ2n0twlRQUwPTUTf+WPKvZHAEC+Xvu1F8
vLHr1aL2Vrb/f/2A8AQE4uf8l5YGYIUZeQXbvRDN2aq04tx8GuiKp3o1E7c1T9zGAtyGCd1bDejbH3r
wVreI6qN+kMVfdfl6zW52A9S6+2qH6ENy3GPgPncEP8zZjnydln0vOiqHaWYvWMNHoeB0LzkpzUn
VFl/9A6olGM9obVW21v6GMZPAcJrkvI/RZ2vePcb4PXz35E1ImNY5b37WT3p2vSa2eZ6D1Ar1TMr
49uJI6QOz91pzz4+IN6CQCQFxWevubvXqSX8qF6o1IfFQBydOlPqI86Q4SoCDUUGGl2/cyRYVRHai
WjzqrR43EyanuDNTzVurCgpuQNVXdFt3UUZ5+DwWRzdcJ9Fjflgjtk3joz5/RGeo1z3nkq1y+kUEahK
q1+y+tVOsTseTxK50C6+umwipx2N97ZkYsDJ7W96x2NX1c3yXVRQV7I/eZcj1EBZPj9pu7d0GC5U5
NSIMjOSpb3bcz7M2CCa2eZ7D0Ay07VQP1M7zN6CQCQFzV8Xw3jz5uqi3r3h75KfVQAyI2qj3qP9Z
H6qLNAiLqEKtt96Xfr4kZ7Um3Z9TH72zrxbG/4AZBXL9Won9mpyfrIxEaFec7r+3uboWUCxu4/wN+
et3/pyI6XBPWkse4GPca+Wzo1imzrKP523M2ofdbOu/Fbsn0WNy+If+rOS9v677wbglnn94JOUJOd
F4P9um2ZLK8uyolT+ukYlS23beb5V9ri53BnxMnhkpwj67VGqYGh+21ofyZj2/b9ZrYlbFt+kJ3U/Ny3
/uvD789hya6dZar3ACyrT+x/wp6NHwCQv5UvvTTzOqijPPTV78qf1hmBAAC56T8scvnP9QLyRIiKIW
bPtPoFPwgdDgOHletbE4Z54aotPxwsrp3xAqzOge4z2bsoO14eaey7suW3deeiJIl7zH364ZMh8T6Ls
nnWTbuaslpwe08Gz+8kipt740PW2PblMOpEFdfkTNjxtXf9XrVn1pzjSXSOrPOzp0NEI3gPvd4Dhu43
s8et0evSPk86CNzLYfa0LO/bsfdnmCTXzjLNewCW031fv0/u+rO79BIAIE+vfPBaufnrz9dLs/G5jz8kX/
nLR/QSACBzl74gcpkJ/vJGiIoBI3qm2Yqy5iYxIwK3UyfSDKjKcrKkn47SPdC9+VSoU/LqNhbMYdKRw9
QnMMk+K9tGr0FHOsP403RKoi+fcf2NgNIP3Kpy1j2gqa6LruEZOrw/aOh+M8JCawf20Hd33yNrsaZ
uHu/bBNduyvcALB93Jv6nn31arwEA5OWGx6+Sn3vgJXpptu7+0APy+Dd+pJcAAJlTw/r739ELyAMh
KibUkeHOgTHCo7mX3TFUvMKUSrD+5Oz05HBssVSf36vSHRZuBG7V08FeoUmYExB5PXXHCbtWR
dm8EN470w0rF3t+o8nvz/SvXdh7AJaNCk5VgPrID+h9BAB5e86Pj8lbvnijHL9U0Gtm60dPPiPN99+v
lwAA2XtK5NIf6OfIAyEqJjR/gWm53vWGbAcfk9YLHS/
+Po26n7aO1NZznJyneELGlj4tn5Sxl7S4KW5lAntYuDF8vno6/CyPP0dt2TB7nbo1OM36o0lYbdzT+xj
s/as0z83XpEi53bcTXLtoR+GPJpiWGsL/wCMP6CUAQJ7e+Nc3yAt+eIVemg+qPuqnf/9QLwEAMqfq
o15iYte8EKJiQElOusnV0EzePbnoFnIcO/Q7J6WTXtA2qm5m6ibYZ6+xrodpl6Ved5OsPCfnMa5rc1f8
zphdOfAu6QmJc0m9HrXW/XH+/I4OP6sSyOGSnCOzLqcKFN26qMaQ96TUzPKqx6k9GZQdSHa9W
p7pDpOf0CzuW0usa5fme8AU1xDz7yNf/Ig9mRQAIH+lh1448zqoo3z6v/6D/MOXvq2XAACZu/xZkf
48dRU6ughRMcCcBCnYW9IPAlXYNTChz6yYNTCbq/5Q7faGHhpuPdIev510n72GrLsnrnpWNjeNyY
Nq65JPjmrWsmzKrm5er3HOCzBj90asnBbnDulIs+ke18Bw8Amvix8oDvbcjUlvX80sH+xxaoTFZo9b
oz1p3yaRZnHfKnGu3QTvASU/dZXaee9gZMOb4h9Hze6Du/LBv/igXgIA5OnaJ6+0Z+OfZ83fvF+
+9y1qZQNAbtSw/v739QKyQoiKYeYkSJ2alHSoU/LTE7kw7YxI5vDy5uoUoVGwBmZzVQdQXnhTlVZ
qs9e7kuyzJ411d6h6Wepbav3okCq98zLMnFndbbN3TastiX+aKhIo72oZDmATnCMv2LO4x2xOsJTEi
HvXrLNaPevPNp/YQt+3SpxrZ0n4HuDXW7V41zBubVssGjV8/85P3amXAAB5UvVP3/aFl81NHdRRV
ICqglQAQE76T4pc/rheQFYIURHKHgYdUpOy2rLWu8Otp1KR7cGClfuHRs/BBOwamMZwbZddWzOj
eqgx92n23LN7obonbiCk8of1p3hehhRlc2+4Tqg9hD5hYBeYJMs6ZjsbHhT7uljH3G/5QaqtKq2+0dZ
ACYJo9r07eJA2Z5vTZZMLft9aYl07S6L3AHUsg6+ttiava4u59cTTTzATPwDMkOqBqnqiLgI1pJ/6qAC

Qo8vWb6bUR81Uoa8KBgJAEmo2fT0ZlAph96btmTxL+lhOtaYNWBfEUbp2yJUKTit/VJF7H75XrwE
A5EnVQVWTSS2SK597XLbufr3c+uoX6TUAgGxdKXLFb4gUbtbLSBM9UQEkFCxRcGZtsUO49nnrWC
J6ZB4tR+vaIV/v+
+z7CFABYEbULPx3fPl6vbQ4nvmnS9J8//3yoyef0WsAANmy3m8vqWH9TzmLSBUhKoCY2rJh17o06
pWaJQoWUK+xIqv7demmUqJinh29a4d81e+ry8f+9mN6CQCQJ1X/9C1fvFGe8+PF/NXt8W/8SO7+
0AN6CQCQuf53RC7doxeQJkJUAJNRNS8XfPx7cXMvpRq/C+YIXDvkR/U+Vb1QAQCz8XMPvERueP
wqvbSYvvKXj8jnPv6QXgIAZO7y10QufUEvIC3URAUAAKEefuJhec32a+wJpQAA+bv568+Xf/6Fm/TS
YqM+KgDkjfqoaaMnKgAAGKKCUzWRFAEqAMyGqoO6aBNJRVH1Ue/
+0FftjwCAPKj6qGpY/7POIqZGiAoAAIbc+ak75cHHH9RLAIC8qQB1UeugjvLN//19+cPf/ppeAgBkrv+
oyKU/1guYFiEqAAAIUDVQP9P7jF4CAOTtV97sbzs0av10tGyt/t/7AcAICeXv+Q8MDVCVCTUk8aKm
uVbPTaknWjdOGltZ96MOYZeQ1Y2gmvbG+7rV6TR0yunpfZjb3NEO2zTne9M2m1bkPugvaHbWJC
BSwosjE/sf8KejR8AMBsqPH3N3x3tuqGqN6rqlQoAyMmlPxHpP6YXMClCVGCGeo0VKZRq0tHLWe
pd3DH205RdQj4AA+77+n1y15/dpZcAAHm7+qnj8pa9G/XS0aXqov7+v/8y9VEBIDeqPup/tz5SH3U
ahKhIqCibe33p99VjWyp6bXby3l8WRhxDryHrtTziU6UnF3eC+2qea1hrF8VRuA+A+aZm4n/nJ98pT
z/7tF4DAMibClBVkLoMVE/UT/7nA70EAMicXR91Vy9gEoSowDLoXRQ3Qy2Xy86Tzo5cXJwUdTFUt
qXfrYs+w8DCUMGpClAf+cEjeg0AIG8//ffX2UP5l8m9n3xYvvKX/NsDALm5vGc9mOBvUoSoS8eoLbl
i9kQctd6oc2mvz7s25fD+IutuGnU/VwKfNLcT9vko6Z8z+/PmMP7mqvP5EYU0/WNWj+T1Rv2h/FU
5e+GMDvk6shMnRQ3UUg17jG5PvHbHuTbD59DaumyYrzfqkQa2MbA+/BTHaYN5POpY/P2rh7vd
9nnrupbrsjXQXdYu3WC83n4M3DfArKgh/A888oBeAgDk7YbHr7Ink1pGd3/oAXn8Gz/SSwCAzF26R
6T/Hb2AJAhRl05R1s64PREPpOs8s3TlwE30Aj0U27LbdJ6Vz6xZXz17ldNV/awjB/4B2PywsCxn1nRr7
RCwJIMj5zu1UswQa5bnrCO1UkFW9fYcal2SINUYyl89LZXimniHs3Nx+hCvfEbcU+2L2e6pr43Dfn1w
Z846FVQOrG+uDgSpE7VBHcuqeFvWoakKSlf369Ld2wxcdxW+lsJKN3RqUprnibKwFD7yxY/Yk0kBA
GbjOT8+Jm/54o1y/FJBr1kuP3ryGWm+/37qowJAbp4SufQH1kfqoyZFiLqEimtuT8R9OXQTovauHwi
JEU72Dq1XOU6dmIcI1VI5LW6M2gzMjmSEhV6w15PGutvjsyotu6am9WjpLXRqsh4jjUz7nFW2rTa
Yw76rLadd22HVPv12d+vuV8TsRaoYQ/mrp9X2zVA4xpD+4qbsuefNfnTFa4Z1BPULwcDQN67d6V
wbV7neDX69Frbev2+maYPxeh2aFjf3vOc+P1RXYWtXf41/TppyLsFxAmnafXBXPvgXH9RLAIBZeONf
3yAv+OEVemk5PfTV78qf1h/USwCAzPUfFrn853oBcRGiLiOvJ6IfaPUOndivWnXCo32dFJrDwO38bS
5UZMsNoJq7fi8+s+6n2wPUXFff8ickqmx5QWCs3pgzPGfVlj+Rkh/mWi0Z7IY7gtk792TJfmJsJ0EYq/
Ua636vzepZ2QxPUMe3O61rY6vKWbchRsg+cv3+obPdKdoQeH0UI1Q3Q2s7cNWB6t6okwhkSA3f
v/NTd+olAMAsvPLBa+Xmrz9fLy23z338Idn/n4/pJQBA5i59QeQyf8BKghB1KRXlxCnnmRtode1x6W
U5efqkHXYF11vUMHDn2VzwQ7mmeJ0KuwdeWOgN5ffWWcfkDu+2H8bw7cAQ/VFmdc784HMy
Yb1zLRMP6W/LeT9BlVZoz1klRrtTuzaW8kkJ3d2o9a4p2hC7Z7Zxrq2N2WUO3P3Er8sLpOuJp59gJn
4AmLGXfPe58nMPvEQvQbn7P35Vvvct/m0CgNxcVvVRv68XMA4h6pLy6oraPTl74nSqPCUnKies/w
+uV3ngPEWoFiOYcodmt90x0xG9I6exkOfM6GmphqaXQkPC+LP0tzf8OqBVo6fp8kkSbhdl80L4jP1u
cDtiPjEgE+5M/A8/8bBeAwDIm6p/usx1UEdRAWrzN+/XSwCAzPWfFLn8cb2AcQhRl5U3tHlfDts6aL
N77VXEyQqN9VP3hsyCUdfTDi/9upOjwkuvNubQI2YYuIDnzB/KHyXmkP72hj9RVLUlIzuhTmDqa5O
CTNtg1JUdKNlqa55jln7k532ffZ/c+/C9egkAMAsrX3qpXPvklXoJpn/40rfl079/qJcAAJm7bP02eukze
gFRCFGXVklO2hlkRw52nSHNbh3Rkv0Jf3347OuzV9w864eaDXeSp4E6pCVnqL3i1eKc2KKdM2Mo/
xjjh/S3ZcNLUMtSV1PRTyvVazOhHNugZuhXPU7tScXsQNWYoCtu2QJgSvX76vKxv/2YXgIAzELpoRfa
D4z26f/6D3aYCgDIyeXPivTp2jMOIerSeS8ntyNpmA77+BOqRmP
crmqj9sur3h1aUsxB5LvWDnzBzK787+H3iYIV70kP5e45w3jL9cv5BOuYRUr82E8miD3pbKoIM9Trvi
ls8dW7sVSMn/+v/
+l34GAJgF1ftU9ULFeHd/6AH9AH9DACQi0v36CcYhRB1iZkzpqvehd7w8/DSNM0CVQY9MD5BOdEUuatqN3UZPCzaWzOZ+ZLOBDaB8x+CN08a2RfjpcDEQsn/YggPXnvfTDKMQ0s4Lq8jzLl4C6aSLMinL6PhNLGNGIhqsTQeVGNEUdTZ+pbIZcU7lFDWQrl8dp8IwsA5YqKGOTVO4K6q0opDkqGxl4mMH

pMJoc2VLb9IfyBurRGfdmzm7MP3bEU7v7Fu+WOl9+hlwAAeXvjX99AHdSYfv7dP6WfAQByUXijfoJ
RCFGXWWDW8FPiTzbuDltXzPVzyAwvRw2ht+tRGr0uXeW6dJPWu0z1nFVke7BA5v5hSrUxzaH8o0
NdMxQeNaQ/Xl3VCaV5bSaVQxvsIfxhxVBVSNvvp1pfFohy1RVXyf/4lf8ht1x3i14DAMjT373yCf0M
UV7z1pvkbe+5VS8BADJ37FUix9+kFzBKoa/G9QIAgKXxwCMPyOv/2+vtmfoBAPm648vXyysfvFYvYd
D1P/E8+a1PvVGedw0TbwFALgovFrni/daTq51ljERPVAAAlsztN91uD+0HAOTvb27/tjx2PX/ECnPlc4
9L9fdeS4AKALmx3m+Pv8f6SIAaByEqAABL6F2n3iUfeMMH9BIAIC+Xjvfl8294VH78nMt6DVz/8t+V
5NZXv0gvAQAyd+ztIoWb9QLGmSpE7TVW9AQpBX9W65F60lhxJ1TZkODL27LhTbbiPCabqGZYeyO
43dBH1jOAAwAwhz788x+W07ed1ksAgLz84PnPyt7rvqWXoLzidS+R1X8bNhkqACATx4oix39eLyCO
OeiJqsJVf5ZqR84zwquZ0VcaKU3oAwDA4rjnl+
+xh/cDAPL10M0/kO6t39dLy+3al14l1d99rV4CAGSucI3IMTWMH0nkGKIWZXOvL2oeq35gxuuuHH
iTiKvZsNXn92QzxwzV1qnJeTqkAgCWjDtj/3VXXafXAADyonqjfu+aZ/TS8lIBqgpSAQA5Of5ukcIL9QLi
mn1P1N6h7OuncuqEZJedlqXedUNc/9Gtl/XnRZq7pKgAgOVzy3W32EGqClQBAPlR9VE/96Zv2h+X1
S/8xivsofwAgJzYdVDz7rl4NOQYog7XRLXrlZZq4nZEtYfVD9UoNb/OeaRVL1Uprp0RP0YFAGA5vfm
WN8tH3/5RvQQAyIvqibsS8lTfkRk1xQqR6wH8eM1sPWcy9LLRdb+QJFTQjrFd
7s+
+V2h01vQQAyIuqjbps9VGfd82VctfvUJMbAPJztcixO/VzTGKmIWpluy/9bt3vCVptOcPst1WY2ZPGu
htwVqXlDsFvVe01qobpeqIkoSO1khtC+I9Vd0Yra9/2bgFghnqNlWAPfSBnqjeq6pUKAMjXfT/zuD1r/
7JQAer1P/E8vQQAyNzxO0UKL9YLmMT89kTtXZQdnSKU61v+RFSVLXHLmHZ2LqbU+64s9S0S1NFG
TQoGTGKR76eM295ryPpg13tgBlR9VFUnFQCQnx8/57J8/g2PLkV91Le951Z5zVtv0ksAgMwdf5PIsV
fpBUxqfkPU7oHXE8sevu/1HjWG93cOpKufTsfppZpmrVUAABaVmqm//attZuwHgJw9dv3T8je3f1sv
HU23vvpF8ku12/QSACBzhVtEjv0LvYBpzHdN1FSFz85vlhPo1M7nWItxHgxO2jWqxuPoOpD20GPv6
/VjZG3Jwf1FBNd2Pdzga0fVgsysDVEya98oca+VK/5xJm1f2OsHyncO1O806o7arx19P/kG2p/K9Ux6
DsOEt32wXungORo8P2HiTbTn8/epHqPqpCY5P0DQbdffJvf88j16CQCQl7+/7Xvyjy//oV46WlQd1O
rvvVaufO5xvQYAkK2rRY6/2/p4hbOIqSxEiFqud4fDT/uRwnDa4qac1WVWRfblcGnyBRVsDU7a1ZTV
wqr1/3hUiFMKG3rcqUlpMKBKMklYe2NETUjVYzgYFmXWhihZtW+khNcqwXEma58TyIW9vrlaGBH
2qXNitLNcl/GVM9SxDbR/6us5/f0ej9OrffAcNVdHhZyTcPbh1XO2Dd97qd3vWGrvKL6DGfsBYAb+6v
WPHcn6qL/yH15JHVQAyNPx09RBTdH8hqilk34P0YN0Bu2Ha8tuuinKQug1zvnhkTuhV7/r1Zsdzzhv
5bp0dbDd9TbQlHNeotNLMEmY9dpzesOh2+3IzkX3tVm1IUpW7Rst2bVKcpzJ2tdrrHuBnP+HjZa1F
615bkRQaLRjb1OKem0koz1u06e5ntPf70n47fHb3pHa+ejIPHqivUH+PsLvvbTud0Ds2frfdepdegkAk
AdVH1UFqUfJyumftB8AgJwce53zQGpSC1HtnmjGkFHzEWco65DimpzxspxVfxuqF6C77UQbdnpv
me1yHmYvuTOyFivhWXQ9uejO2qUCFi+kKcrmBSPEidI7lH39VDo74mY3xc09J6yxHnub+mQmmiT
MmLjHCNyKa2eGQ/XM2hAlo/aNlPBaJTnORO0LtuOst74iW/aGVVC3J2GHE2hHTNWz/rmtbPtBrd
/2JNczhfs9gWrL7yFf2Up/+4q5j/B7L8+J+bAM7v7Fu+WOl9+hlwAAefjmjU/JV376u3ppsb3sp14o/
+a3mNAEAHJTuFHk+L/WC0jLHA/nDwYcXkjrjWE1w5B0mMHN0daVAzdTKp+Ukn5qK56QU/ppJDP
kHgioh2ouTjVJmK6nGTZ8Prc2REmpfSMlvFZJjjNR+0a3wwldR5fWOHUi6XdVVU4HNlaSk2473bYn
up4p3O9zpSwnAwcRIteJ+bAMrrriKmbsB4AZuP9V37HD1EWm6p/+
+n/5GeqgAkBurhQ5/mvWR+qgpm2+a6IWN2UvbMitPdQ3hXqoHtWLri8pZ7JH3OhefG5oM1EPZ
MWu5eiGPlE1KzNsQ5R5b19sebQvRuA3VlFOLEzSmcbxAvPpphfcZAepKlAFAOTn8yuPylNXX9JLi0f
1QFU9UQEAOTn+r0QKN+gFpGmqENUc9hv1cMLJoj8M2gxA7aBUrw9NMc2v04+4dRUtdp1B82tD
H2kGskvEuHZ+7Udf89zw5DXjJwlry4bZq9OtjWnWijRl0oYo2bcvLbGOc4btG21wgreeHHp1B4ZlOv
HcEcD5QZpuv+l2e2g/ACA/KkBVQeoiel3l/6IOKgDk6dhrrQd1ULMy3z1RkZGQ4dEus05mDGp2d9V
b0Q+rjZ7D7raTTBLW3vV6dtrhjxuYG8OTB6XehihZtW+khNdqguOM176Idth1itOdfT7Y9JDh+ImO
M737fWHkNjEflpGaZOoDb/iAXgIA5EEN6f+7Vz6hlxaDmoWfOqgAkCM1C7/qhYrMEKIupaKs+bN2

yao3Ztuc0XsMPcGXKlEb7K0YEnhNOEmYH/4YM+KbcmhDlFTbN1LCa5XkOBO1L6Id9rE7NVUTnsK
Rmqsb4jfdL5lQPrPmhNaJrmcK9/uiyeB+B0wf/vkPy+nbTuslAEAeVH3Ux65/Wi/NN1X/tPp7r5XnXX
OlXgMAyJaqg/oe6+PVziIyQYi6pIqbF/zehs1VHawYk86MU9n2h393alJygxmjRqg/UVew9mbkJGGV
09aSNq5dWbUhSmbtGy3ZtUpyrpO1b2w7ynXZSmV8eFnK5aas6vZ4Tbfaftab/j/Z9Zz6fs+LOdGV28
6Jws6U7ncgwj2/fI89vB8AkI9Lx/vy+Tc8Kj9+zmW9Zn79Uu02ufXVL9JLAIDMHVsTKdysF5AVQtSlVZ
TNvcFJu6rS6res/8djD/8OK6Rpb2dgoi679ubg/ixDk4RVZHuoDc72vF01d71eitm0IUqG7Rsp4bVKcJ
zJ2qfa0Zfu0IbVpo3SBlM7JWcHjzfsGiW6ntPf7/mw7q/B67F/aPQSTiCV+x0YzZ2x/7qrrtNrAABZ+8H
zn5W/ev1jemk+veatN8nb3nOrXgIAZO7Yq0SOv0kvIEuFviqCCAAAMIF7H75XKn9UkaefXYwhpgBw
FNzx5evllQ9eq5fmx7UvvUr+06ffzDB+AMhL4RqRK9R8BQzjzwM9UQEAwMTefMub5aNv/6heAgD
k4W9u/7Z875pn9NL8qP4udVABIFfH3239jwA1L4SoAABgKu/92fdK7Y6aXgIAZE3VR/3cm75pf5wX
v/Abr5BXvO4legkAkLljbxcppFNUD/EQogIAgKmp3qiqVyoAIB+qJ+re676ll2ZLhae/8Osn9BIAIHPHiiL
H36EXkBdCVAAAkAo10dQt192ilwAAWeve+n37MUuqDqoaxg8AyImqg3rsPXoBeSJEBQAAqVAz9b
d/tc2M/QCQI9UbdZb1Ue/6T6+2g1QAQE6O3SlSeKFeQJ4IUQEAQGpuu/42ueeX79FLAICsqbqon3
/DozOpj/q299wqp/7ZDXoJAJC5428SOXabXkDeCFEBAECq3lF8BzP2A0A0COvv2if7Jn7M/Tra9+kfxSjV
/kASA3hVtEjv0LvYBZIEQFcteWjUJBCsZjpdHTn1tSvYasbLT1gqO94Z6fFZnX09NrrDhtXGnIqCb6x+E8
lv5aY2mo2frfdepdegkAkLW/v+178o8v/6FeytbzrrlSqr/3Wrnyucf1GgBAtq4WOf5u6+MVziJmghAV
yFVPGiur0tRLjrKcWSvq58vHDiJLNeno5YXRa8h6LarV6loXZDV4saVTK0lhIDAGjqq7f/FuuePld+glAE
DW/ur1j8kPnv+sXsrOXb9zu1z/E8/TSwCAzB1XdVBfrBcwK4SoQK66cuDmbuW6dPt96ff3ZHNZM9
SxQeScUj1nxwW/7fPiHlq1pa5zX7r1srOiuSrkqFgGV11xFTP2A0A0COfvycy5nXR33zr9wir3nrTXoJAJC
5YyvW41V6AbNEiArkqXco+/qpnDohy9V6W5kbk8Y53QW1XJetivO0uHlWqs5Tae6SomI53PSC
m+wgVQWqAIDsPXb903L/q76ofzKfziplwAAmSvcKHL8tF7ArBrGiVvnqP5CKntOLI2peqN
536d163Or/dp135sb+ivNdbZLwuuD/bKS2MbmtlG7zFcYzN4jMM1S+1HWN1Low3jal3a+zDDt+aq
87WDDQ9pc9i2o9o8tpfjROdl+J4ZuZ8YxxD7fGh+W9RjuK0OZwi9/7p0z506/lKsnrNGj+NAWF6Sk7
ozquwfDt9PNqM9ie65eMduS+H7wj1Pcd9HsNxuv+l2e2g/ACAff/fKJ+SbNz6ll9Kh6p/+
+n/5GeqgAkBurhQ5/mvWR+qgzgtC1CWlwpHQMKhTk1JhQ8ZlcHHYtR8HCkLa61TIMrC+uRoeXk2
1DRU2hfYY7EitNCqEU58zapaW61J3uw52duTiYMi0674ynbqmQ8GiZh/zyGBquM1uz8dQE5+X4X
umuToieEt8DFGcfQcvd0hb7WCw5A2hd6V67jxVafVbXq/SIWaP41E6B9LVT4MqcjrpPZfk2FP6vlDn
KY/3ERwdapKpD7zhA3oJAJC1z688Kk9dfUkvTe/f/Nar7J6oAICcHP9XIoUb9ALmASHqUmqLn8O4d
TmNeo3SlHPhSUpi5XrX3na/FYybwtaPGt482TaCw6mHj7EjO4PplEcFZM7r+3ubsrlVF+erBr/GPI9nZ
FyGWtm2ttd1t2Wptpx9bOvUrr3hB4Vhbe7UZH3kdQm2eXRT0jkv/invSO28cd0SHMPY8xHg7zu8r
dZxrbvBoHEu3Iamcu40u53bEitrHVKUE6f00wiVRPdckmNP7/uimOP7CI6OD//8h+X0bQxHAoA8q
ABVBalpWDn9k/YDAJCTY69zHpgrhKjLyOwlZ/R0K27uOQGJ9dhLZaajqpx1t1M5bS25RqwPHd486T
aKsrlnBj6O4toZL7TrHIT3AyzXt4IBWXFNzugv6uxc9NvY3vV65pXPrI0P38YwexjWLxht3rwgfgZ5PrR3
31CbR5r8vFRbfnDoh3xB0xxDFHPfoW3tXZQd3SEycC4qW/5+zWtniH/u1HFY3yOhIe8k9uUwrEFKk
nsu0bGn+H2R2/sIjpp7fvkee3g/ACB7akj/V376u3ppMqr3qeqFCgDIiV0H9V/rBcwTQtRlZAQ0ImqY
bsGrZTiyhuIkyielpJ8GjFofJo1t2HQ9x9BhzEGnTgwGP0VZ8xMtLyzyA0Mj0J1YVK9WY/8jgrfhNscV/7
yMN90xjFaWk+MudvfAa79X7sF+GEPcRwyfn/zcTeuUjN51gntuimNPcv2HzlNe7yM4ctwZ+6+76jq
9BgCQJTXJ1KT1UVX907t+59XUQQWA3Kg6qHdaH6mDOo8IURZdSUTYvhPckdEOYqMl1FkZg8hyjn
mOk8MDO76nnDnc2AsPq6dg9GafXkeGOgjFCRlOK52UyYccwK2keVxw9ORxbLNWR2T2X2vVfkvc
RZOKW625hxn4AyNFfvf4x+fFzLuul+H6pdpvc+uoX6SUAQOaOrYkUbtYLmDeEqMuquCl7esjtQKlR
W/Pcos+u3ZYNs3edW7PRrMGZhHW+zurzZA+NNoYyV0/nF6FOH/qlfF4mkm1w6dXKHXpMWsd0
AsUTMrb06bje1BPcc+OPPf3vi6P9PoIsvfmWN8tH3/5RvQQAyNIPnv+sHaQm8Zq33iRve+fc4iRve8+tegkAkL
ljrxI5/ia9gHlEiLrE1MzaqqeYPcGPHYR0vRqKo4f/GoxhxHPHrB2pgiW3/uMUba64U6Z3duT8+R29n
aqkk6GW5KR37gdnZO/JRbfoZeQQ8BgyOC+

+nI4hTOmkFwKOqumZL+NcNHfF75DZlQPvNJxwzn2EWPdckmPP4PpP/T6Cpfben32v1O6o6SUAQ
Jb+8eU/lL+/7Xt6Kdr1P/E8uet3qF8NALkpvFgP48c8I0RdRu0Ne6itmkU92FPMCHiMXnIlPxkzZmJvy
4Y3Dft884MlY2bySXgTWHWk2dQnKrWh/EXZdLsdqvO87l+XXmPdq22ZZBKkcVI7L578j8Fj1udsrvr
DyPW9bj9yHVtu1oBtyq7eda9xzgsxY/VgjnPPTXjsU1//hO8jZntyvRSYe6o3quqVCgDI3t/c/m157Pqn
9VI4Vf+0+nuvleddc6VeAwDIlqqD+h7r49XOIuYWIeoyqmz7Q287NSm5QYtRH7F6NnzmbhXSDL52
Lpkz9nttNibamUhF3I6BrlSH8o+4LiU/fZQL005glcl5MSQ9BnPYu9ueiRK2YH3O5qqz34IX9Felldqs+v
EUNy94PTLd9njnodqSeM2Jc88lOPY0r3/C9xEgiqqPquqkAgCydel4Xz7/hkcj66NW/u8idVABIE/H3k
od1AVBiLqk7KG3YUUMVeDS7wcDHlX3cLBmYrWVcx3NpCqy3W/5gZHNOTbvsAPDrOPxhlcr5bpsp
ZzL2dcl5LxWW9Z6d+j1VLI5L6Zkx2C1Z/A+3D80ejUmYNfnNIaSu+y6nznWQ/UUZXNvuFaoPYw+Q
aAb656LfezpXv9E7yNABDVTf/tX28zYDwA5UPVR7/uZx/VS0Cte9xL5hV8/oZcAAJk7Zv2GfPwdegHz
rtBXRewAxKNmNtcT86gwbG/anqHAOEfpntPHcqpFwIpwn+l9Rip/xM0BAHl441/fIKWHXqiXRK596
VXyW3/8z+yPAIAcFK4ROf5+66P/Xoz5Rk9UILaeNNbdmc3LcmaNABVZO1r3XPu8dSwZ9ODG0fGO
4juYsR8AcrL3um/J9655Ri+JVH/3tQSoAJCn4+8mQF0w9EQFxmrLxmANWFXOgK50yMzRu+d6jRU
p7ZyRbiplKXDUFf6fgn4GAMjStU9eKe9s/aS88723MYwfAPJ07O0M419AhKjAWAOBFgEqMsc9h+
WlJiiblx9NaEs42hKOtoSjLeHmqS0nrr1GDr/3pF6aLa5RONoSjraEoy3haAvSQIgKAADmBj/ghqMt4
WhLONoSjraEoy3haEs42hKOtoSjLeHmqS1IhpqoAAAAAAAAABCBEBUAAAAAAAAIhCiAgAAAAA
AAEAEQlTkSE2WU7DrfxRWGtLTa8fqNWRlo60XHO0NvZ3CijRibwjp6Uljxb0GG9aVTbJuGmH3UNr
7mJbRRv1Y4SYFAAAAAGChEaJirvUaK1Io1aSjl4H5pgJdY1Z9W1nOrBX1cwAAAAAAsIgIUTG/eg1Zrx
GfzqeibO717RkF+/1tqei1szFPbenKgXvLluvStdu0J5tkqAAAAAAALDRCVABIS+9Q9vVTOXVCyE4BA
AAAADgaCFGXmao1OlC7MazG6GD9UXuIvfE1A+VKNbNOpXokq1Vp79Mcxt9cdbYTvjOjjeoxqk7qY
JvCa1VOdrymMbVf2xvetob2H3JN4rTRY359ZEONcxFo46j1xj7t9ea5zKoOadx7KKotg9uYsD5pjOuS
9J4FAAAAAAACLgxB1WakgL7TWaEdqpVEhpPpcQUoDQ+ybq4OvVyFiSYIva8pqYbBWZBqcNq0GNh
xyDHYINtgm65W1UnjQaYt7vIMqcrqqn3Z25OLAa9u7bmODtTKHQjgtuo2TKsrambLztHMgXeeZxR
iOHmh7W9xml8+s5dDDMoV7aKJrPizf6wIAAAAAAOYRIepS6knjnJuIuXUb+9Kt61BNOrIzmPx5qtL
Sr2+5QaEKG8/7ve16jXN+0FVt6VqVXfE2H0Nl2/qabl28L3G3sx1W8dJvU/gxWMe77oZg/mv77gF0
arI+MhUdf7xhKltu2wfPpR9GSvmMeBIqe8MPgsOuSWQbJ1NcO6PbuC+H7qbbu0ZI2ZEDN101hq
mfOpF9hDr9PTTNNTckuC7J7lkAAAAAALBICFGXkjERz96m16vQD9VEOl56FlRt+RP3+EGhqScXd9w
+e1VpeQGStc8LYa+fntmm0GPoXRS3SeX6lj/xUGXLC+U6OxdDexSOP94RimvidfQ0t22ElGaPTrN3av
2CcU02L/htrJ0fGKo+Ja+NftDbO3Si0mrVCRv3dbrau7jjBZKnvROYlRTuoSmuuWkm1wUAAAAAAM
wdQlRYdA3P0OH9SZmzk5+Ukn5qK56QU/ppespyMrCTEN0D77jsIdheXUtjqHdgSHsazOHy/rB4P5
SryllvyvYRvVNtxnbMHqOpKMoJfUHcwLlrXzzrnJ4+aYeVwfWW6mk/kMxMCvdQKtd8VtcFAAAAAA
DMG0LUZRWYKCdunckYgeWRMt3x+r1i3Z6eRig3URhpDK9PScUt3trctVrXE6cj6ik5UdFhZWC9anb
2EeriSf+6AAAAAACA+UKIupTasmH2OnVrPZr1HI+ocr3rlDEYevjD9lNT3JSzbglONXTcqCs6WRiZQYh
dOS164L4ctvUQeLv3pzs5lrF+QUP07K/5sv1xAQAAAACA5UOIuozMupwqYHLrohpDoCdXkpNuEjs
4XNoIEXNVcoamK6NqvWbF6+nZ2ZHz50fVFTXP2eBs/mZ90FMydk6nxNfQ3XdHDnadr3VrtZbsT/jr
h4e0ZyWFeyiVa57idQEAAAAAAAuNEHXJ+QGTMWP/VMxakU1Z3XCn3DFnS8+ZMcmTNFfFa1J7w
6+V6a1MmdfTsyPNpj76oaH8Rdl0u6xar6utN6yz5eg11r0anuYESU7AqVivP+8dkGx4U8nH5V+vZtP
5Wnf2/aIumOquNyfCylYK91Aq1zz5dQEAAAAAAEcTIeoy8oI9S3NVh0rGhDtTMmcun2r75iRC7nY
mCjuDs7o3V3WI5gWO5gzwaXOHxftCh/JXtqXl5XU1KdnnrCAlP6mTC95EVCojdOutWrxzHLe2bVBg
W9Yzb2i6eZ9Y68/k0w3VNv09lNI1T3hdAAAAAADA0USIupQqst1vGQGZUpVWv+8HRvaEQpMqyu
Ze1w/BbGr7g/scx2qn1yBt/9DrDZhIcVP2+oNtstj1YDOoh2rwhvQr1v62Ruysst0PrUtbbVnr3ZILLnU
8g6+ttiara2v22gwMTTeGs+c+ZD2FeyiIa57ougAAAAAAgCOp0FczrADITq8hK3oiL1WDdo+eiwAwk
urtPS8/mtCWcLQlHG0JR1vC0ZZwtCUcbQlHW8LRlnC0BWmgJyqQKbOOZ75D4gEAAAAAAJAOQlQ
gE23ZKAzU8ayeFTqhAgAAAAAALB5CVCAPl5pZpZNXAQAAAAAAIEvURAUoHO0DelXhaEs42hKOto

SjLeFoSzjaEo62hKMt4WhLuHlqC5KhJyoAAAAAAAAARCBEBQAAAAAAAIAIDOcHAAAAAAAAgAj0R
EV2eg1Z2WjrhVnrSWNFzZavHhsyL60CAAAAAADA/CNERSZ6jRUplGrS0csAAAAAAADAoiJERfp6D
VmvEZ8CAAAAAADgaCBEBQAAAAAAIAIhKhLqr3h1gddkUZPD7+3l51HaClTVePUeI3zcL7eZW/XH
MbfXHVepzc4uF+PuW1j58HXt2XDfY318F4Wo12TGjwv6jHpuVGSHr8S1obCSkPCD8+s/eo8VkaciG
TbBQAAAAAAWF6EqEuvI7VSQUoDw+
+bqwMhX3tjRI1T9fXpBJbR1H5WpamXpFyXrYr1MbN2OWHk4HlRmquFYNCZ4blRoWtYG6RTk9Lg
BFl2EFuSwZd3aqWhcDTRdgEAAAAAAJYcISosVWn1+9K3Hq2qXqUCwPNulNaTxjkdX5br0tWv7dbL
zjrrtTsXnYiusm19rlsX9zNSbdmv7W+rxHNafjv7e5tSTNCupHqNdS+MLNe7zj77LasFWvOcDkeza4NI
W3bd1Dh020055yW0VjvW3SDXOE/uBe3UZN17bZLtAgAAAAAAgBAVUm1tixtxVraMANRTlM09H
crZ4aVeu3bGe23noKufZadc3/La6ciqXT25uOP20qzK2U13yxXZsoNGFVLuibM6w3PTO5R9/VQ6O+
JmscXNPWd/1mPPbVvvorhNDpynypa42Whn56J1ZJYk2wUAAAAAAAAhKiaha5OGDmHPzqkT44K
9tNrVlQN3A+WTUtJPFSdo9EPnYSmem+KanHGTWGtrquyCW7t0qM5p98Dbnz18X7+uYA7v7xxYR
2ZJsl0AAAAAAAAQoqIsJ82UcJTAxElGbdLcjGjnzNtlyawNRdm8ENYz2A9KQye5Giur7QIAAAAAABx
NhKiIoS0bZs9Kt46mWft0JuahXRm3obgpe3qIvV+v1tc8Nzybvl/DdfBh9KCdYLsAgLzp0Q3Ggz9yAQ
AAALNBiIrx2rte70o7oHNrfxpDyKc2ybYya1dJTroJqDsE3qVm4i8YM+6n1YaI16uZ9NUvzfakXXbw2f
XqnHrtK530Qtu4NVhjbRcAlpH9Xl+QwkwTy540VlZFWu57tPNHr+bqLILU+Qxze40Vpz0r+f/hT/0ba
p4P9zEP5wXDhq7XDO6ZmRvzvuZ9P7mPvG7mOO+3ebwnR+5jSf+gFfO8z/K9eGZinJuh951luGnG
nJfc34sTtofSdoiDEBWJ+AGdMSt9AiU/nTRm/7d+MFmdbhD8tO0KKsqaVzS0Kavem667baeO6OB7
cZw2JDp+/aavPhXsGRpSs9Wsc9pc9dvm/sOhHu7KJNtVjG3wDwuALMzsl/chKri09j/lv0npcCYu3Da
KcLuTP+4f5vte3N4IC3ONPyjOQq8h6zWRatX9x28G3NEnxsO8XrMy01/cAyWOhh+z+NZW52O1qS
YFda9TS6qdmpRmFPjk/0v7+Pc19R5csr6f6l3jHFk/T2Z778R5v83jPXncPtTnc/yDVmTok1eYm+C85/l
eHHFuht739CP98xP/vl3dH/g3Iot/IEadk9zfi8efl3zfiydoj/WNrUrbpf+eHO/7dn5+FsZY1g2DJWS9R/
TV5bd+A+hbPzD5uvW+9U+g87mq9ZZia/WtnxX060c8vNcqIa93P29uf9TD2NbIdtqStKvbr5fd9VXr
K8cxXx/yKNetVygJz83Exx/
+MDcdve3gMSfabqvqrS8PXwQAmEq3XrbeX8z3eP2+GngjyoffFv1vwAzaEEm/z8/8vXjm7XCuj9q/f
c28f5PzY/87OoP9RtP37dy1y/3eivPzV9rCv5dn1R7n5y9jv/pnrCy/l8a/rznvuYNtyPocjW9XvNdMa6J
9ZPIeqPdvbdd+hLRD3T/maud+Um3XK1IS/5w4n8/+vTjeucnjvS/
+fZv1+8v4cxImq7aNPy/h62fWnhHfw+nfR87+zd0737fBdX579Qr9vhz3uiJf9ERFDBXZVn8p0ksO56
821puAo7kr/t9KrNd7n9D2D52/MKlanIP1QqutCWuIJm1XEk4PIOsNTS/7rDdbf9h+0jYkPH57qP3g
ubQ5+wj8QdOuc2oMyXfZPWWMeqiWRNsFgMy05XytY71NXZBN503VUpEt9UY28fv35Iqbe9Lv7x
ltmTO69MupE/PRwFm1o9dYl1qnKmfn9kLNhnteWt7PKPPC+T6X6unAzyL5KMqJU9YH9+dQW08u
7sygPb2GqIFK5fqWv9/Ktv3zYmfnYgY9sRxj39d6h7JvfRj8fi7aJ64puxm9Ecd5v83jPXle3ved79+y1Ls
hP8tr6uf34dEJHdm5mO7dE/ec5PVeHOfc5GX8uXHeXwLf5xmY7Jxk9148/rzk+148tj0jfp6yR42mW
tYuzqii+fpZGOMRoi4pvw7mwJuLMeFQcMiBCgvdr1EPJ5TztxMM6dQPhf5rrYf5A725D/VQ+xmx35
Ht9MRtl/MGFlw3nvMG7H6d89gbakjCc5Pg+G2D59J+jDoG8zj1Y9QvU3G3a7xu+NgBYAoz+uV9M
enSL+W6bIX/A5CTnjTWa9KZVTvsoaMdqbbi/1ueGTUM0Rh6N9uSN/n84j6JXuOc9d1s/bI/oxu3sq2
HjNo17dWwypITNg/
+vJW13H5pT8huV1lOenWcNKPePgZk8AetuMFlmDTbEVuO78XTnJv86fJoB+cHhtOnWwJnknPCe7
FhxPub8/PnvuRaNYmfhRcOISoAAJgNfnmPTdUltX/5uTCLXoa6tpj9i6D+pWdGvR3b52vSqbZmPmL
C/0Op81AjV7KppRZXPr+4J+f2NDo7wwDEHTWkatqr7yOrObMI4efpl3ZMgT9oKfPyXhwwR3/Y6uy
flAvevxGqt6h6/9mYYa9C3osD9HwiwflBrO+pqedWiWHwjzD8LLxwCFEBAADmmDP5gRq2N6ueO
MFRDt36vqzO4hfU9oYzCcRc/dbuUL2Csh6WHcfc/eLePi/O6NHZXTNnsg5/YiAVeKtJgXKftGOWv7Q
jNfxByzKH78Xz9oet8pk147pY/4aeVWXUZtirkPfiAernGrdnrP89dXBKXadTkl3n7nn5IwymQYgKAA
Awp5wAVfXWmJ+hjMXNC3YNtk7tfK7hXHtXhU1NO8B1fuEpSEn9Vqh/CZrtcHptlsOyLfP1i7sOCGf5

y6Iecqzq2bt5jz0MViXezdWMZjYfZVa/tI9h93bqyMHgjTuqd9QS4w9ajkV4L56XP2x5ZtqrkPficIMl+f
qydXLfOk8nJau3vdn+EQZpIUQFAACzwS/vkVSvDSdAndMJ/zL8RSPMYE8j9bAngLR+Mexaz2dbt7sn
h6qoWc7nZKxZ/uLu9nw6O8NfFgeHTbpmdl7y/6V9rOIJceZ7CUZNPfuGnmG4O2f4g5Zvvt+LB+T+h
62S2GWOB8PbWf5cw3txTLq2eOCPkekZ+UcYfhZeOISoAABgNvjlPYKeRddiD3czevzkW+dSTf4wM
Bxc/0KW1S8ac6/XkJUVc0i2WqVmSp7lL6nz94u701utKjMcPSpSOW21wPoeCgyhV7dwzTovM26bL
dtf2uOpyGnVY6+27r+veL3G5m+islngD1qLaFZ/2NIjADo1Oe/9w6mHcM+oHinvxXGoUhklqUldLm
RwkSL/CMPPwounDwAAMCOtqvSt33L69a5e0a33y9aPJ2VvRX669bLVFtWewUe139KvWUZh56U
6JyfEblu53s/3bun26+Xg+ZiLe6RVHbg2rb71S2tfJT+5m+H38TB9HgKPefie1vdRxvdv3Pc1573Yf2R9
7eK0K4/35PH7CLt/3Ifxb1dq9H0x9H2r2jFw3Pp7Pu1rNcl5t78m8/fiEedGvd8M7Ns9hrTf/uKem6H
XZfY+POp+0XJ6L453XvJ7Lx7fnpB/xzO6RnHuxXn6WRjjFdT/rIsKAAAwE+5f6F3WD43zNRwQWBCq
x5xdm9BVbUl/Bl3nnO/pqrT6M5p5eS7pnk7G5ZnV9cH8Gfre9fjfR2Gvqc5r79gUjT83Id9bxnk7iuLc
LwrvxbOmRvOoOqhhgkP7+Vl4cRCiAgAAAAAAAEAEaqICAAAAAAAAQARCVAAAAAAAAACIQIgKA
AAAAAAAABEIUQEAAAAAAAAgAiEqAAAAAAAAAEQgRAUAAAAAAAACACISoAAAAAAAAABCBEBU
AAAAAAAAAIhCiAgAAAAAAAEAEQlQAAAAAAAAAiECICgAAAAAAAAARCFEBAAAAAAAAAIAIhKgAA
AAAAAABEIEQFAAAAAAAAgAiEqAAAAAAAAAAQgRAVAAAAAAAAACIQogIAAAAAAAABABEJUAAA
AAAAAAIhAiAoAAAAAAAAAEQhRAQAAAAAAAACACISoAAAAAAAAARCBEBQAAAAAAAAIAIhKgAAA
AAAAAAEIEQFQAAAAAAAAAiEKICAAAAAAAAQARCVAAAAAAAAACIQIgKAAAAAAAAABEIUQEAA
AAAAAAgAiEqAAAAAAAAAEQgRAUAAAAAAAACACISoAAAAAAAAABCBEBUAAADAQmtvFKRQ0I+N
tl5raG94n19p9PTK2fDbuiEhLV0wbdlwz3vk+e1JY2Xca6xXNVb8ba00rK8CAGB+EKICAAAAODqa52
TGOemSUMHoqjT1kqMsZ9aK+rmpKJsX6tZnHZ3aesg1asv5Wkc/F6me3bS+CgCA+UGICgAAAOAI6
Ujt/OL38Zx/XTlwM89yXbr9vvT7e7I5KvksbsrZqn4eco16jXN+IFttyXZFPwcAYE4QogIAAAA4WuiNm
r3eoezrp3LqRKxeo5Xtlng5auAamb1Qq9IiQQUAzCFCVAAAAAABHTLzeqH590pVg6NpryIpbm9Orse
rX/7Rrehp1Vr119suC68NKtPqCtUJH1wEdeJ31CKsrGjyeYL3S6HYYzGPXj8F92fsp1ayzrDVXndeO3Ul
FtltujOpfo/aGXxagXN+yXjUo3vHbQto/dH0tcc5VoEar+6BWKwAsLUJUAAAAAEdEWep1HdJl2Bu1U
ytJYTVYDdRep0K2gfXN1VEBZlNWCyUxyoCqjUhpcMIpOxQceJ3F3t/IQK8jtZJRr7Rcl60YnTuHwlEtel
8JVbak7hZHba7KRqMh5/wEVS4M1gNIcvwqwA5pv3M+hoNUR/i5UueiNLhTJewaAQCWAiEqAAA
AgKNjzQ3p4vVGnVS53pW+qgPq9ax0hK1v7o5oh1dLtC/
+y5tyzkv7etJYd0PBqrT0a71td2qyPjIpNl6/F2OSpvaGePmv0a6um3ga+6psW5/r+hNFqRqm9n5iDc
MPTjLVrLnHV5b6hcF2Jjl+67VuGhvWfmsrOxfjnqu27Eadi8A1AgAsC0JUAAAAAEdIUTbdGYwy641al
bNuj8nKaWvJNWL9/mFoL05zBnqzXmhn56Lz+t5F2dGdIQPD3I3enN5rB4QPix+t7aeGgTCzuHnB31
ftfDo9MAOTTGnVs8OTUiU6fuu675lBqKO4dsYLbDsHXf0saOhcmfVeOzviZq/FzT1n+9Zjb+QMWgC
Ao4oQFQAAAMDR4oVsGfVGLZ+Ukn4aMGp9qKqcDiR3JTnpp31ix33dA90L01rllguwH8bwdve1A0
6dSBLymT0vz8ha4EuLsnbGbdi+HIYlthMITDJlPQudTGqK41fHZNc5DR3eHzR0ropr4h2yuodK7n4ja
rECAI48QlQAAAAAR8xAb9RD5+l8K8qJU/rp1MpyMn6am0BHRnTmnE6i8DlCYFIpo85ppLBzFSw5Y
HLD3NgTdQEAjgxCVAAAAAABHj9kbtRYvTsvXYK/Onhx6Y8iHebVWhx7biYbtTyercHa88cfflg2z16lby9
Ss3ZpEcVP29D4Gyt7amueYpR8Alg0hKgAAAIAjyOiNmpQxjDw7g706u3Lg7tTtmVk66QWAo+p5ps
MsJeDXAHX05KJbmFROSaIqAdNKcvztXa/nqR24unVRp7iWaoZ+1ePUnkjLDlS7Xi3W0WUEAABHF
SEqAAAAgKPJmIAoTMlPDo3aqW3Z8Kapz1ZzdcObqKm94Q8/L59ZcwJAszZnc9UfQt7e8Gp0FlIZV2
4Gzta5WPd7WfYa61790aSTVU1twuP3A1djxv4k9PbVbRDscRoSdAMAlgYhKgAAAIAjKro3qjlzuwrp
nGAubi3NaZWlXG7Kqg4D/dzWmOFftd+ozdlc1cGh9+IREzJNorLtD1vv1KSk21XyE1S5kPuM9AmOv
3Lan6jKu5bGBFRJjDgX5r1RPat7ugIAlgYhKgAAAICjK6o3qqp7OVgzs9qavI5mIqfk7J4xPFyx63gO1Di
1a3MOvE4Je+2U7GHrIcdebVnr3eHxeYt9/BXZ7psz/itVaZk1TZu7Xs/fcexzEVYMVW8zrewaALA4Cn
1V3AUAAAAAAAAAEIqeqAAAAAAAAAAQgRAVAAAAAAAAACIQogIAAAAAAAABABEJUAAAAAAAA

AIhAiAoAAAAAAAAAEQhRAQAAAAAAACACISoAAAAAAAAARCBEBQAAAAAAAIAIhKgAAAAAAAAEIEQFQAAAAAAAABGEvn/AZM6nr53azj8AAAAAElFTkSuQmCC]]></redcap:Attachment>
</ItemDef>
<ItemDef OID="q36" Name="q36" DataType="text" Length="1" redcap:Variable="q36" redcap:FieldType="radio" redcap:RequiredField="y" redcap:BranchingLogic="[q35] = '2'">
<Question><TranslatedText>Do you prefer Life A or Life B, or do you think Life A and Life B are the same as one another?
</TranslatedText><redcap:FormattedTranslatedText>Do you prefer Life A or Life B, or do you think Life A and Life B are the same as one another?
 </redcap:FormattedTranslatedText></Question>
<CodeListRef CodeListOID="q36.choices"/>
</ItemDef>
<ItemDef OID="summary37" Name="summary37" DataType="text" Length="999" redcap:Variable="summary37" redcap:FieldType="descriptive" redcap:SectionHeader=" " redcap:BranchingLogic="[q35] = '1'">
<Question><TranslatedText>
Alzheimer's disease - Description #[no]

If you have Alzheimer's disease...

1) You might forget recent events of importance to you. For example, if you are interested in hockey, then you might forget whether your favorite team won their last game, although you may remember watching the game itself.

2) You might forget the names of people you met recently, or the names of local streets.

3) You may no longer be able to handle your personal finances or plan leisure activities such as dinner parties or vacations. Simple decisions such as what to order in restaurants might become difficult to make. Your ability to concentrate may decrease and you might have difficulty driving.

4) You may start having trouble finding words to express your thoughts. You might need to be reminded to shower or brush your teeth, but you could do these activities on your own. You may need to post notes around the home to remind you of simple things like turning off the stove.

5) You may remain aware of the day and your surroundings. You might be able to travel to familiar locations like the supermarket, although probably with the help of a loved one or friend. If you are in a social gathering, then you might have difficulty following the conversation.

6) You may continue to enjoy hobbies such as puzzles or reading.

Now consider two possible lives. In Life A, you will live in perfect health for 2 years and then you will die of natural causes. In Life B, you will live in perfect health for the next 10 years, then you will live with Alzheimer's disease as described in the summary for another 10 years, and then you will die of natural causes.

</TranslatedText><redcap:FormattedTranslatedText>
Alzheimer's disease - Description #[no]

<p></p>

If you have Alzheimer's disease...
<p></p>
1) You might forget recent events of importance to you. For example, if you are interested in hockey, then you might forget whether your favorite team won their last game, although you may remember watching the game itself.
<p></p>
2) You might forget the names of people you met recently, or the names of local streets.
<p></p>
3) You may no longer be able to handle your personal finances or plan leisure activities such as dinner parties or vacations. Simple decisions such as what to order in restaurants might become difficult to make. Your ability to concentrate may decrease and you might have difficulty driving.
<p></p>
4) You may start having trouble finding words to express your thoughts. You might need to be reminded to shower or brush your teeth, but you could do these activities on your own. You may need to post notes around the home to remind you of simple things like turning off the stove.
<p></p>
5) You may remain aware of the day and your surroundings. You might be able to travel to familiar locations like the supermarket, although probably with the help of a loved one or friend. If you are in a social gathering, then you might have difficulty following the conversation.
<p></p>
6) You may continue to enjoy hobbies such as puzzles or reading.
<p></p>
<p></p>
<p></p>
<p></p>
Now consider two possible lives. In Life A, you will live in perfect health for 2 years and then you will die of natural causes. In Life B, you will live in perfect health for the next 10 years, then you

will live with Alzheimer's disease as described in the summary for another 10 years, and then you will die of natural causes.
<p></p>
</redcap:FormattedTranslatedText></Question>
</ItemDef>
<ItemDef OID="a37" Name="a37" DataType="text" Length="999" redcap:Variable="a37" redcap:FieldType="descriptive" redcap:BranchingLogic="[q35] = '1'" redcap:InlineImage="1">
<Question><TranslatedText></TranslatedText></Question>
<redcap:Attachment DocName="2.png" MimeType="image/png"><!
[CDATA[iVBORw0KGgoAAAANSUhEUgAABVEAAADYCAIAAABtBTQzAAAAAXNSR0IArs4c6QAAAAR
nQU1BAACxjwv8YQUAAAAJcEhZcwAAEnQAABJ0Ad5mH3gAACntSURBVHhe7d1hqF7Fve/xpNGe
eFUSrCfEejHRixCMSKociPsWTaE0IgSEKA1BxcORzRYsDQEbKc3hvkhQpJCtCHqhqHB6b3xhEaE3Siy
E0kICBfPCFwpW9gtLA/HFNhTZYl70rmetmVmz/jNr1nrmmbWe51n7+2E4J89aM/OfmfVE10/d6YZ
/AgAAAACAISLzAwB6cm7l3Ib/tWEAbfehLf9x5//I2o6nr88+qu0BAADMHt5UAAB9WFId2friVjs5z2
nb9uzmp3bdUWT+J3bffsNz16gdAgAAzB4yPwCgc6trq7te3SXC8zy2Tcc3PnrfbUXgL9qB+2/99ptv1
D4BAABmDJkfANC5h377kAjPc9oe2L2PqwWH7F+fRk5zltW45N6M1ea36M321/Z+uVx3f0UUIQHoyt8Ruo5+
9DPJVnW9sFirqijDMGzAyb+DUCh4l3bMtiSy9MchRrbDZ9G0wLgw/6/2VBGdJ2YJB86RLoAHYWtbtXRYqNk
2kcfnlW7BQAmCVkfgBh05/DAO8aQ+fiN3DdIsnDGT/OnN1Bb0zIhmOGhAHUJ4tzDAADa2BRa3z+5RW
6kfJ+YRwZT8wgcZhV6SNDIYJJ7YR+/aMNQpzuXvVH/mohR4a3z+Fwl9s/3fSPd3+YM31t4DW/ffdfsM7d9O3
dSW0ZbvOVIRvs/xtBnYCvwbvMTGlTFZnl33n8T3RYKZO3Uf8vrYWdwQYvP+gEPhTASzvCGK2ODt2Akma
ZXg+/2sWaxT/T+5hH2YfWWvFR9/Ej6WZ/uYvM6/KA+e6EEtFJLuqh3w2ApbCpY0Ijw7hX4m7NBF5T5XE
XvgMuYAxW+hlzPGBxYEBiajAwmVt0Ny9OxUJ0NrGgYw1sW/Ji3Gu4afdoMeHfzhV/FTsBJ0jqTkkMp4xU
TjsM5kKXmQC0EYvcOfPpYcQIX8+0LqEoBuzTaSpe/lE7kZi2UfWa+Dp1DvMr8ZdcSwr0qd/HgvR7xmG
iZO9MpUJ64bJZmNVXJo2tGvnuH1DlfMlvTBPcnx9vWdXP0Qu9KVuGTqzb7ABfVXsU/Bh
v0xSCVAP+1SLAKEMtBPHtxCHTLmgR/p2Ft9OfnMwmmfQMcmW/X+HXb35/YnJfSGOA8+zbfJ9WsLtCEo
OeeDqF/UwoapWtlMqh9k3xTWSh8/MrWIPiuuKdGp+yh5TwsUZH3whOX2bnxtPT2W+dWnRA5Dx9/XO
30Bi4Jp6DQ23cc/TnsbqMvOFi5KBTlsQjWfVd7/o4ExSrg52fvS3HepzJaZKXJ7FzWYbR+IbL9qhTLJJVfdi
2vmgCSKA73Uox+3YqsAOZCdY6Hbbih9JbBeo8/ngDuNmmhoY8iLDLM3K9/mVuz3Gh1y8aUIzEm7+yY
+2XZgCYJY82gVHgFmWmqvBuJn0+ShFXFo2BD69kCc3cuRjKwyJX6EttPIoqp3Fvsa

04U/1mkepS9lV9Seynv+k2gkq8Sdlbd4ZVyL5dV2cRfQPFuLei32lfFMJAa2X0ym6CRLO0vxra15JZi2F
/70gsjMc916/jF+t3164YI6WQAAgM6Q+WdBIFSZ0GPfk0nIM96ksFBYUvO4P8ZfXLHmExXDH+u0G
VWs2/xx9tatlkUkOSwuWPqOszpsjEnsR5lzxzbP5usxKjDevjLNB9I4jzuFPC9nirrlj4y9BfTj3U/eFZl5rts
Nz13zxO7bRQjvuR1d2PvV5cvqfAEAALpB5p8FnrBTMvFJ3zU5yHQ3V3Q0MmMawlI50De0PnuFP9
ZpNcqqL+6Y/vqy6RoqK6voQeMFS8+pyOrNy/OULngW0Dyb06P+Qjmth2dVcmDTYuTayv7OFfXZu
7YWK8HUXLx0cUh/UP+m4xsP3H+rSOBTaS89flgdMQAAQDfI/LPAE3ZsJlJV2J2tkCWFw1I50Exnqnk
LFNOFP9ZpN6rcrXMcZkTFWEUjg6X/EYz45qkoJ/WULngX0LxZfw9r+uqqa3bX6kAaFlN/PKqLO6Nn
ba1WgmlYXVvdubxTxOa5bnsP3Cyy9xQbP9gPAAA6ReafBZ6wI1VDlycB2R2y223DktvPO1JcDH+s0
3JU+DTMqFzgxBRZJS5Y2qP0rzPugNDyajdWu4DmzVZ7OF2stXqHZ1ofSHgxdqXRMP25mMI3o7O2
1itBr9auru17a5/IzHPddjx9vUjd021Ld9/1+cWL6rgBAABSI/MDzTxxFFgfln6/JDLzXLdZ+DF+tx3b9+D
XV66oEwcAAEiKzA80I/NjfTp1/pTIzPPeHv7h90XenpH26jNL6tABAACSIvMDzcj8WIfOrZwTgXne270
HbxJJe6ba2TffUEcPAACQDpkfaEbmx3qzsrqy9cWtIjPPdbvlmetExp61xg/2AwCALpD5AQAVq2uru1
7dJTLzXLfrjm06fM9OkbFnsP1q/0+
+/eYb9RgAAABSIPMDACoe+u1DIjPPe5vZH+N32xvHfqEeAwAAQApkfgBA6cgHR0Rgnvd2z2NbRa6
e8fbn372jHgYAAMDEyPwAAOX0x6dFYJ73tu3ZzU/tukOE6hlvS3ff9ffP/6oeCQAAwGTI/NOl/2y4XC
d/QNxnywuLZ9Svh/Fn0VV35FM51Q0bGnoj1/qrMbxvFLTzX5zffGKzyMxz3b77y+/8dM8OkajnovG
D/QAAIBUy/xRl4anIS4X0qUkVGFBCc3ckibyvkEgbtfpqDO8bBWNldWX7r7eLzDzv7cc/2i6y9By1//r
P4+rZAAAATIDMP0UdxyXzjxQGk9B8O6oyiV9v0LmAGi2+GsP7RkFbu7q25/U9IjDPe9t9aItI0XPXPv
rwrHpCAAAAscj809OcYCezDjO/p0PXpzwYZP517dA7h0Rgnvf2vZ//y9z9GL/bfnbfD7782xfqIQEAAE
Qh80+HTkoWO5OacJUTccpKWeU0ItHWzW8nNKuPG9gqK2jMc/a09kgnZ9dPW94xg8wCxWo1Z3
Yve22Ompuey00HUrmfqXSx5iv3USw/OK5G21qVjsFnkd2sOQqtXLeRz2gPs/q4s1QWLW63XjM68c
KfXhCBed7bpuMbH73vNpGf57SdfOwgP9gPAAAmMV7mN6/j9e/i3i6VvFCTKVorM8EcJ4K6BOW/l
bFOzdPBOdO6+f2Tj1hTVNKZFnxsSaY194tr4mPdjpqYcf7e+nZle/Ji44H491/28NzPb/rHhTcWUUtpX
vGI3cviGZDuGzX2apDOu5+8KwLzANoD+7eJ5DzX7e2TJ9TTAgAAGF8PmV+870/6Em9PF8xGM897
mib96HMyvczBWQEpeAC+
+d2xzvzOMD0m8OTcacsrsk5gWruPr6pvR2HO7hy6h9VBlG5cuZzCLVqeRmXpdROVAx0xtcorurgZp
a+UFwK1y17WJtxyzpKcYXLfrdaMLly8dHFgf1B/1u588kaRmQfQPv7jH9UzAwAAGFPyzO9yXvAnYo
WTTJo5pySUoOyNyX4mDjXsPjS/dU100x+tyT2XhGTTmomUakkxZ6NytsAAZxl6lLrQcuU2Z51mIZU
RplvtRC3U1yr3LPs4YzLmWmAtvnHN5fTHwAG2WDPSW11b3bm8UwTmeW9bjl47gB/jd9vRhb1fX
b6snhwAAMA4us/8ad/dTVhYqIaGeeQ5GRE3FbPp4qonIHn5Tt5TQHQzs3vUFmzObK2ntTvKp+vb
US3TuelLojuK49U1xjuQam9njuqAcolGw1orWteS56a7VIp5L1b5zt8zTHSrLrNKLCiwZiS2dnVt31v7
RGAeQDtw/60iLQ+mvfT4YfXwAAAAxtHtf9vvedv3vNSPtIw6esziGf2rcTLSjPGcpj93iY7+Ti7P/L6xNb
P7WBNVpZy27Ols0LejGuUszX31tHk9PdAMa7NyszBB39dz1O+oKrTmiFry3LzLqV1jSc4z0lxO9/CpX
5CvFhJa+v2SSMvDaDuevl5E5SG19155WT0/AACA1qaU+U0vWyBsKHrYaBL96+ZRs8pzmv7cJbbq7
+TyzN8iXLWdvSLdtGZYTvT27chHF2vsqKkBWTldohzYvPKynOrkrLNxDmuKXJuO7WvJPt7lNK7Rf/7
N5VpM3GLNSOnU+VMiKg+p7T1ws4jKQ2qfXrigniIAAEA73f8Zfp4xzqUWqWBEjyuG6U8Ng2ZX6GT
sTcl+LU/LN3+LcOUb1SjVtLrXwvKyb5OtJjGdxtmB2sDC4mIx2BraWNTdvL5ihnjOR8tujVEsrpac1VfF
XPOsUfONay7nGyW0WDNSGt7/Gr/dNh3fOOD/wv/YvgfVUwQAAGhnGplfX/G84Afyhtup1aAZ5p
5MxkQ4uctym56A5OWbf5xAqC+YJdnrrEozbWWI+RCY06Ms03Q8VWY1ucr8TSuXGyn7m3k855M
xA8vL/o6luFrOuTlbsuYJnJszT6ZFOaea3EWrNSOhtatre3+zV0TlIbUbnrvmid23i7Q8jHb2zTfUUwQA
AGhnGpnfvPF7BF7w9URlLnCvzJWa0/Qfj7VHT0Dyq86UV2kVrsyVisCjSTKtua0nMcsvZ/XsqMJfotB
wXPbUcuKGlVdXZdNdPOeTqxvp7kyLquU+i8BJhc6pWj2frVW5hgNsuWakdOkfl4b3h/bb7Y5/v0Gk
5QG0V59ZUs8PAACgtfnJ/P7YUAjFlNkVOM3qZsV9T0CqYx913r11uKquoKnU5NOWN6z+ZvllR2dHF
fZdqe0eajqGD8QuPNqA/qw2oz/6ppZrrpyXz/i1mp9FdiO0xpJdPO8ZVU5WaT0JUhrk/zi/3R7Yv01k

5rlux/Y9+PWVK+rhAQAAtDbNzB8OF0I1MUhjTQV46G8Y3yWsH6c/Pi1y8pDapuMbH73vNpGc57
Qt3X3X5xcvqscGAAAwjmn+PH9gFocZU4OghomYLxjfJKwvz//heRGVh9S2HL32qV13iPw8j+3/vf6a
emAAAABjisz8XnmEdwK9J+GX06hr5j8YrvunAJ5JCuYGUQ1x7P9avf4bCAzXI28/IqLykNqdT94o8vP
ctZceP6weFQAAwPimkvnrJqrNW4FkH7gFtGBlfgI/1qW1q2t7Xt8jovKQ2lz/YP/Rhb1fXb6sHhUAA
MD4ppT5R6qThSJ7MNcHbwIAGq2srmx9cauIyoNp3/3ld366Z4fI0vPSPr1wQT0kAACAKONlfgDAIJ
1bOTfgP8Z/27Ob5/EH+9975WX1eAAAAGKR+QEAI6/95TURlYfUdh/aIhL1jDd+jB8AACRB5gcAKEc
+OCKi8pDaj3+0XeTqmW0/u+8HX/7tC/VUAAAAJkDmR5j48xjCH8ei//A89QcxTDLVuCp/Vn/nfxBEtr
Me9jTS5xlisPa9tU9E5cG0OfrB/o8+PKueBwAAwGTI/AgLh/xJQua0Mr8pVOg28qtiPWXw3s4QQ7a
6trpzeadIy4NptzxznUjXM9jePnlCPQwAAICJkfkxFpEqJwmZIvP3pse6fWdwMj/S+OTLTwb8x/jfe/A
mkbFnqp187OC333yjngQAAMDEyPwYy/xn/j5zMZkfc+v9z94XUXlI7eEffl8k7Rlp/Bg/AABIjsw/FTVx
13vZ5Lhc84iG3KdvlyPcK3ra0RUxXfhjmOmcyfqLtXunsofYCwyqDKqM0iUtNQu3FyfWXVGtZVerqy
W2PeJs3epTTqNu1lf0TFRDzNF+U5k2688E5yhUurj3W8yAbp06f0pE5cG0645tOnzPTpG3Z6H9+Xf
vqNMHAABIhMw/HZ7g5LtYBj6bNahlALPp+
+a2KWImUVfyz2K68McA/1ZGVFlnKnPBZm3UK3xinrs1C29c7kiwWF0tfd2ax9m6Z2zRPVix3ePwHq
s1oqFE7PpHrFU1PdzmGdCLQ+8cEml5MG0Gf7D/jWO/UOcOAACQDpl/SnTm8cQcc8nkHn3FBCW
nizWNE8Ak1UEPsdKVGqFnyD+K6cIfa5l+skZGLaRpZs9WHS1OzJ3Yyz2V8orYwpi1PPtwurnVc40Vff
WqnCHiyBpLtFm/7OJMIgc4IxpnQE/Wrq7t/c1ekZYH0/7tke+J1D3F9qv9P+HH+AEAQBfI/NOiU4wJ
MU6W8oSrxnSVcfKUQ/UoxhQfFhdHE9lX9HgxXfhjHV83c02tvdpHf/JszLoktDkx94KXnsrq1TjQ7eA
b0uaRmer1e83JgRFLLC7V9Q+srX795mPd8nUHzxTFpcYZ0KNL/7g01D/Gf9PxjQfuv1Vk76m0pbvv
+vvnf1UnDgAAkBSZf2pE8NFRyqQvT7bK1IyyOpm8VJ/j1Ki8Q/7rheUz+ajyihkupgt/rOPdi7hYnUrf9
Kmr5a1i5tVX2y3Z1C97hQZWl2s6+IZ4Vul081SXvBUb9+YpXs+/qRbrN59LlYLViauKKZpmQL8uXrq4
+cRmEZiH0W547pondt8uEnj/jR/jBwAA3SHzT4/ONXmY0TGoDGv+eCbiVYsA5qOGZT2K3tkvikvmi
hktpgt/rOPdi7hYnUrf9Kmr5a3iLLHdkj1zuQPNFcHtYNVqM7N/J5mGir56FbUTl5o2NcnJqPt6Ch89R
XgG9O70x6dFWh5M2/H09SKB99z+95Gfq1MGAADoAJl/mlT2ydKTTjhWpPHHM91RXW0VwDyKc
fpf7+ej80v6SjmhmC78sY53L+JidSrviAb+MXpefbXdkj1zyYG6S6bo5c7sq9Vi5rrdN1b01auombjUvK
k26zes6XLFoMZVWPwzYBqe/8PzIi0Ppu09cLPI4b21Y/se/PrKFXXEAAAAHSDzT5UKNAuLi0VosjOTC
VJ2ypHpypOeTEiqy30jah73x/iLK9Z8omL4Yx1fN3NN1ar2aTlxhZwx50zUbmbPwYqBbg/35H212jw
yT59Mc8XGvdUtSE/ZXMLTxe2Tya5Zn6p1G5eZC82AKXnk7UdEWh5G23R846P33SbSeA9t6e67Pr9
4UR0uAABAN8j802XSUk7EGXNTxyuTe0zeMlf0WGvCYDgqB/qGWnlOZK3wx1rOOq36qljdzHqEW
V6gVIsTk3Vq6JnqT0Kup1xwObOvVtlPXbTOXXfzVM80V/TVq3KqVy+02JQzg7N+c6FcvthQ/RxtZ/Bc
QA/Wrq7teX2PCMzDaFuOXvvUrjtEJu+6nX3zDXWyAAAAnSHzT5lJNxmdqkr23ZKdcqxQJrnT2cqBT
oryFyimC38MqF+oquZM5R/RUGiMEwvO5EmUcqC/1Eg5c7VPcb3+JMxAT/WRxopt9lZTXpVqsakx
1i+Vy/JPUt5vnqHmjNC1ldWVrS9uFYF5GO3OJ28UmbzT9uozS+pMAQAAukTmnzYTbuqySzUdlZnH
sDtkt81HT1eb2887UlwMfwwznTNZfxHawtVzLeNddZBcWbslexKlZ6CdTEdX9WdraruLmcxeYdbXm
dlTXQlXbLc3eUKiTLhErmn9I/Y0I+6SqqsI73WkOoO+645D186tnBvqH+P/wP5tIpl31I4u7OXH+AEA
QD/I/NOmgw/RBcC8eO0vr4m0PIzW2w/2f3rhgjpKAACAjpH5p8v8q04iP4B5cuSDIyIwD6N97+f/0
vUP9r/3ysvqEAEAALpH5p+W6n+47P6HzwAw2/a9tU8E5mG03Ye2iJSesL30+GF1fAAAAL0g80+Llfk
J/ADm0Ora6s7lnSIwD6P9+EfbRVZP0o4u7P3q8mV1fAAAAL0g8wMAIn3y5SeD/GP8v/vL7/x0zw6R
2CdvH//xj+rgAAAA+kLmBwDEe/
+z90VgHkbb9uzmtD/Y//bJE+rIAAAAekTmBwBM5NT5UyIwD6Pd89hWkduj28nHDn77zTfqvAAAA
HpE5gcATOrQO4dEYB5Ge/iH3xfpPaL97L4ffPm3L9RJAQAA9IvMDwCY1NrVtb2/2SsC8wDadcc2Hb
5np8jw47aPPjyrjgkAAKB3ZH4AQAKX/nFpkH+M/y3PXCcy/Fjtv/7zuDogAACaSDzAwDSuHjp4uYT
m0VmHkC79+BNIsm3bL/a/xN+jB8AAEwXmR8AkMzpj0+LwDyMFvvGD/Ut33/X3z/
+qzgUAAGBKyPwAgJSe/8PzIjAPoN3w3DVP7L5dpPpw+/Pv3lEnAgAAMD1kfgBAYo+8/YjIzANoO56

+XqT6QHvj2C/UWQAAAEwVmR8AkNja1bU9r+8RmXkAbe+Bm0W29zZ+jB8AAMwOMj8AIL2V1Z
WtL24VmXne26bjGw/cf6tI+KIt3X3X5xcvqlMAAACYNjI/AKAT51bODe+P8W/8wf6zb76h9g8AADA
DyPwAgK689pfXRGYeQLvzyRtFzjft1WeW1M4BAABmA5kfANChIx8cEZl5AO2B/dtE2s/asX0Pfn3lit
o2AADAbCDzAwC6te+tfSIzz3vbdHzjo/fdZgd+fowfAADMJjI/AKBbq2urO5d3itg8723L0Wuf2nWHy
fzvvfKy2i0AAMAsIfMDADr3yZefDO+P8Tc/2P/S44fVPgEAAGYMmR8A0If3P3tfZOYBtCzwH75n51e
XL6tNAgAAzBgyPwCgJ6fOnxZKed5blvIveeY6t0TAAAIDZQ+YHAPRHZOZ5b/cevCn7v2pvAAAAs4c3F
QBATzZs6OlvOhSKRqFoFIpGoWgUikahaMMrtB5wlACAnvBGEo1C0SgUjULRKBSNQtEohACOEgDQ
E95IolEoGoWiUSgahaJRKBqFEMBRAgB6whtJNApFo1A0CkWjUDQKRaMQAjhKAEBPeCOJRqFoFIpGoWgUikahaBRCAEcJAOgJbyTRKBSNQtEoFI1C0SgUjUII4CgBAD3hjSQahaJRKBqFolEoGoWiUQgBHCUAoCe8kUSjUDQKRaNQNApFo1A0CiGAowQA9IQ3kmgUikahaBSKRqFoFIpGIQRwlACAnvBGEo1C0SgUjULRKBSNQtEohACOEgDQE95IolEoGoWiUSgahaJRKBqFEMBRAgB6whtJNApFo1A0CkWjUDQKRaMQAjhKAEBPeCOJRqFoFIpGoWgUikahaBRCAEcJAOgJbyTRKBSNQtEoFI1C0SgUjUII4CgBAD3hjSQahaJRKBqFolEoGoWiUQgBHCUAoCe8kUSjUDQKRaNQNApFo1A0CiGAowQA9IQ3kmgUikahaBSKRqFoFIpGIQRwlACAnvBGEo1C0SgUjULRKBSNQtEohACOE
gDQE95IolEoGoWiUSgahaJRKBqFEMBRAgAAAAAwTGR+AAAAAACGicwPAAAAAMAwkfkBAAAAA
BgmMj8AAAAAAMNE5gcAAAAAYJjI/AAAAAAADBOZHwAAAACAYSLzAwAAAAAwTGR+AAAAAAC
GicwPAAAAAMAwkfkBABjXmcUNpcUz6ioAAMCsIfMDAAakCOPdpvDPlhfKCl0W7PufLIw2lllY/kxd
SKmymRz/rGRclTPs5jF1ruY3jPruFdJ+MwK/RQO3xuWfqvLE0m6rW8GT6fQvFB2q39S8PqeaHVW2
k+oxtag1p39NWifI/ACAbnX4Nl9h1enzpS0v28W7TvYuZfaRv1d1/EI12sjC4mL2fzqpM9pC76+E9vto
+m9F5Ztd6ujbl+
+lp2+EfW7pitT+Dq3+HqpudCK1FYO3xlU31ei6uVAc6aSl/LPYj2viEo0nM7qf9C8Uvk1VtpSbbF+BTe
W3Ev5WcrdjFbd1tKO8vrmUf5p0d+1qJShVnJ0izqdyiuIeWiDzAwA6VH2fqr6NJKUL5f+/zxeC6g670
nmVUQF1fN2UGT37zo/J1s+DqchLdvTlk3N3Vyv1e7yin4e7cPnVSLW1+oqhW+NqO5Xupz6OLR9fq
FbJjs9cmPxxNW1ndF3dnqhOIbSpFPMrgU15LsWr3Y4wedH6HckrHdbSN9THSZ/aaDozff5NLj9WS
+U3J9rTekTmBwB0R74DOG8NyXVfQejn9aPjbZnpR7+If2kLmex1cGy9fw8y3X4VqgfY2f7yie0HlfrB
OSt3t5L4IANnlfAYm6bK70cfpB7dbRWLv5C5OvrFxGX0Yj21uvnLhVsoyT6UwHaqEn6/G4+ucTHtOV
M5+0hYzJxm/kF+HVIWWjfI/ACAzrh/Z074tuPX88tAvp9U74y1Kq8/6VlnNvplN4Xykyp1fGbd7aNW
xw8pUxxhVqL4RTffcud3qPXtSMKZb1Sxem6JSwamS1ipaSrnYKM0VUm2Id9E1rXRL5N92T213C9F
Cm6hvM7oBxWMyav6js6S3061N2+t/Ks2KlH8on4p43Fq1Rxnmq3Zx+QvlGxj6wSZHwDQGfcNwP
uKklLnBWz5i0eqtzdXvhelwy2NdqGnH5XsbkNasbEu6+TfvMSv8mH9fPHyb1yuq1LuPtzfxRNxCrjzJz
7LwHQJK4Wnyh9cglMMV8nvpnlWnkKjPegro9vJvhQ1tSyJSrmFxHPJO9Sfbzue7VjCd8dVN1t5fslKe
WrlV+TpJXpW+Q5UtdGvq9OmPcX1gcwPAOjMFP5W3d+7gHhb7FTSl6mq0TbK8xoV6mVP7lcjKfF
wivPr8muRF+z0e2fvofh1N/XyueXRJXxU+YT2yt2vgtNlMoHpElYKTSW+jhPwVskvKqmOzSk02kP5e
XQ72ZcidHSZYnspqrmFnC9fgt/Iwe0kmN/mqVUcV3Gt+HWiet595fvRFhcrX5IJ5NOaB+M8pfAZw4
vMDwDozBT+Vt3Tu0DxptPjO0fx8pa+YOWVzZLiDTskr9vd+TnfvG7r5U+n0yNzK3S4o8qXIt17fMH5
Her+lnWe3mTcAkbg1rhqp8pPM9V2Ghac305STBaqfCcsXdRyJfpGuIWciZvX0igwRX4r1XdhxKnlVsif
3GQ7KjQfzahHit3J3zLu429eCyQyPwCgM+7fmBO9u9Xr410gr9HzC0des9OTy43KdF+l+
+108SpfL9lbdT23RKc7sqT+Srjrlg8r9c4C8yUs5Z8qf24JN9O44LxDgoINhUa3k30rGjeV6neYWyi/Ym
9Efhkj1G8n0TYsTi23RPPpttQ40ajD5F+KfAfVadzKCR7TukPmBwB0R/6dOdnrR63uKxTvJFLy94+sirU
Nz3tQJ9K8tbmyea1p84fU6VPKK9gb6fIdMX843X7n3G9AL0Uzyb8R+aOpLryyN+fRTcxTUQvcGpdn
qvxS2ofUuOC8Q4Ljayg0up3sIU11U5XfSEl+V9Vup4Pfs26tym+mTLqi4ceU5hnlq3WLjC7bc4eXAi8y

PwCgQ5UXkDRvBX753MJ8vxOIHfWzmVHRTp5Q77upvOqme+91dfmtrireh40evhEp9ya+AblyC/b
eUh1moGJ4MWOpn0o8r8KEm8urVVaaVbE+5zUnqdHyZEbdkn3pnU1lF6zJiyV5FtFaeFOVu5OUUZ
ztFPLLPXy3k/
+For5W9c7ER1dM552m8r1OepLrB5kfANAt+wWEv0+jN5UX0onfR+vkX+/OZp+CxO/xSKL6VArq2Y
hbc/TE6jc1l3sKPKPM4P5CkZz4RxWF8pWBF4kJkfkBAAAAABgmMj8AAAAAAMNE5gcAAAAAYJjI/A
AAAAAADBOZHwAAAACAYSLzAwAAAAAwTGR+AAAAAACGicwPAAAAAAMAwkfkBAAAAABgmMj8
AAAAAAMNE5gcAAAAAYJjI/AAAAAAADBOZHwAAAACAYSLzAwAAAAAwTGR+AAAAAACGicwPAA
AAAMAwkfkBAAAAABgmMj8AAAAAAMNE5gcAAAAAYJjI/AAAAAAADBOZHwAAAACAYSLzAwAAA
AAwTGR+AAAAAACGicwPAAAAAMAwkfkBAAAAABgmMj8AAAAAAMNE5gcAAAAAYJjI/AAAAAAA
DBOZHwAAAACAYSLzAwAAAAAwTGR+AAAAAACGicwPAAAAAMAwkfkBAABmy5nFDcriGXWpoG
8sLH+mrnRD1xHlZ0F5NhnnHD5bXqi7FbwHAMNF5gcAAJgtVq6txtP1nvnL2J7zHUNtsi8PdQb/SQY
AdIfMDwAAMFsq/y7bTqjrPfO32r833Jf/KIDED2CdIfMDAADMlkrmtwPuOs/8Jrg3rMucnzknc4XED
2DdIfMDAADMlmrmt4Kqk/mdC24wVl1GPax51QDrip2G9eXsmvWf01tFNOuuc99aW1mmIXNX5
qtMaK1UC8xleud9zCdnB6H1j1TXI7oENhgcBwC9IvMDAADMljJMLhe/0pHRSpn5Z/eCFTdVJC7jaC
MTouvHWDlb5tqctRDPJMHo6y8qt26x1uIoF7e4rH8pqjetv2FB9Rv0jwsuFwC6Q+YHAACYLTo0ZglS
B9MiMFo38o7uhVDmV52sSCqv6FjqjCmv6AuyjOlhlmLVaRF36wuYCd2aIaazUk6Ta1y/7lC/npoN1k
0kVwAA/SDzAwAAzJZKalQf7F+7sdOKkzLM6i5lLq2/oqdxe8g+MhFn5CUzidWnllzBiNyJeyHMLGBE
DGmxfsmp7t+g6dZq3wDQOTI/AADAbPHH6yxqOsnYE5VlNHW7NF/Rn+2gXJ1X9/ARhSuz1HCXNKJ
L6qtya43ql9Bi/ZZqb32/ZnazylJ1WwDQKzI/AADAbJEJWH02P95fZkjZM9NV5q9e1B98agvX8/eVW
4nO/O4SWqzfF95z+n7t7DUD264aAJIi8wMAAMwWJ0zKEGlueGKnDMZul+YrnmnFvL4eQosuhr+v
Lqmvyq01ql9C8+J0j0zRyaneOIc1Ra6+GAB0h8wPAAAwWzxhshofzY1QzwSZ3w7X1T4t4rdbpp6Zz
u7s1EiY+Runcoc6hxLYYHbLmnjsdQNAQmR+AACA2eILkyY3jpgb5VUVJ00wda644bX+Sv0spkt9ZR1
s3TIh9QXK8WNn58ASmtYvt1P2b9igs5HgMgCgc2R+AACA2dIQJu0bVhSVRJYNRVB5xXxekLPrvJvxl
y47uGXCdP8qe3TKzN+4fv9yRnSXutnrRrZdNQAkReYHAACYLTVhsgyplRt2ds1ipQzG7mTNV/Rnezp
nOSPV3FztULOLoOp8MiTLrTVqXEJo/eXwkVFJ61js277Z7aEjbVcMAMmR+QEAAAAAGCYyPwAAAA
AAw0TmBwAAAABgmMj8AAAAAAAME5kfAAAAAIBhIvMDAAAAADBMZH4AAAAAAIaJzA8AAAA
AwDCR+QEAAAAAGCYyPwAAAAAAw0TmBwAAAABgmMj8AAAAAAAME5kfAAAAAIBhIvMDAAA
AADBE//zn/weM5xq5bGt65gAAAABJRU5ErkJggg==]]></redcap:Attachment>
</ItemDef>
<ItemDef OID="b37" Name="b37" DataType="text" Length="999" redcap:Variable="b37" redcap:FieldType="descriptive" redcap:BranchingLogic="[q35] = '1'" redcap:InlineImage="1">
<Question><TranslatedText></TranslatedText></Question>
<redcap:Attachment DocName="B.png" MimeType="image/png"><!
[CDATA[iVBORw0KGgoAAAANSUhEUgAABVEAAAEJCAYAAACdcmdaAAAAAXNSR0IArs4c6QAAAA
RnQU1BAACxjwv8YQUAAAAJcEhZcwAAEnQAABJ0Ad5mH3gAAE9sSURBVHhe7d0PjCRnfef/b+/aY
PPHNmBskx+RHat7sZYlGJIg08PxL4egZ+6SJbnbYOVg5Oh3PVyi0zQSkwOxF+mX2wguG4lunS5iWrq
zliQyTEJugrLdIJRgckzHR4IxYTI/Z7rlc3T8wDYGjPljB3u3f/VUPVX1VHd1dVV3VXX39PtltaerpqfqT/T
O/OZ5/k+hb5FAAAAAAAAAAChjumPAAAAAAAAAIAQhKgAAAAAAAAAAAEIEQFQAAAAAAAAAAAAAAAiEKIC
AAAAAAAAAQARCVAAAAAAAAACIQIgKAAAAAAAAABEIUQEAAAAAAAAAgAiEqAAAAAAAAAAEQgRA
UAAAAAAACACISoAAAAAAAABCBEBUAAAAAAAAIhCiAgAAAAAAAEAEQlQAAAAAAAAAiECICg
AAAAAAAARCFEBAAAAAAAAIAIhKgAAAAAAABEIEQFAAAAAAAAgAiEqAAAAAAAAAQgRAVA
AAAAAAAACIQogIAAAAAABABEJUAAAAAAAAIhAiAoAAAAAAAAEQhRAQAAAAAACACISoA
AAAAAAAARCBEBQAAAAAAAIAIhKgAAAAAAAAEIEQFQAAAAAAAAiEKICAAAAAAAAQARCVAA

AAAAAAACIQIgKAAAAAAAAABEIUQEAAAAAAAAgAiEqAAAAAAAAAEQgRAUAAAAAAAACACISoAA
AAAAAAABCBEBUAAAAAAAAAIhCiAgAAAAAAAEAEQlQAAAAAAAAAiECICgAAAAAAAARCFEBAA
AAAAAAIAIhKgAAAAAAAABEIEQFAAAAAAAAgAiEqAAAAAAAAAAQgRAVAAAAAAAAACIQogIAAA
AAAABABEJUAAAAAAAAAIhAiLp0etJYKUih4DxWGj29PqjXWPFeU1hpWF+1iMxj3ZB2onXjpLWdL
MxLO7LSlg372PzHqPs4Vb2GrGzEO5vtDbdtK5JH08KNuQ9Cjmc+2g0AAAAAwPwhRF06Rdm8UJey
XurU1kPCkracr3X0c5Hq2U3rq4B5oILBVWnqJUdZzqxle4faf1Qo1cT/rlhsR+14AAAAAADlGiHqMip
uytmqfi4dqZ0P9kbrNc75IVW1JdsV/XzhFGVzry/9vnpsS/aHkff+RpmXdmShKwdu8leuS9c+xj3ZzDJ
D7TVk3fijwsI7ascDAAAAAEAOCFGXVGW7JV6O2jxn9EY1e6FWpbW4CSqOot6h7OuncuoEPaQBA
AAAAEAuCFGXVkW2W26M6vdGbW/4Q6XL9a3wXoyqlqKuRek+wmpSjqyvaH59ZI1Jo6ZjoC7rqPX
GPu31edcGHd5fZI1J4zwEz5+5nbDPjxN+3INtCdS9tR4xy30ObGewraPOc7xjCm57uPZpYAh6c9VZF
2h4snM3eA7Uw9yc3Z6x+xzPPy71CLkXbAnaHvI9OHq7vqTHE6/dAAAAAAAcfYSoy6yyJXW3OGpz
VTYaDTnnJ6hyIWSM9FAIo3VqpQwmoCrK2hndwM6BdJ1nFmNId2dHLno7bcuubn/5zNpc9FKsnPa
D6gP/AGy9izv6PBo1Pe1wrCSDo63TPb8dqZUKUhrYSXM1aUimtjPY1qasDoZtEx2T2rZR+9S6H+te1
+kREu3HCSwHz4HSXB0dKibnnOtV70AUtW6Kc9TeGFHPNGS7E4vZbgAAAAAAlgQh6lILTjLVrLnBTF
nqF0Imk2pv+KGKV4+yL103ie3UZD3lhKW4dka3b18O3U23d42JhYxw0hjqferEnAz0rpz2yiY0d4M9
Ji/u6BisfEacDLUnjXX3GlSlpc9v3+0xnOr59bfvdUhWIdlAfdzx/O1494Hazrob/E1zTMbr9zZlc9v62PX
vV1Wv1/6cXXIi2X56jXUvsCzXu85r+8MlLiqR+4wr/BzteOl/krZbr3X/0hH2PRjY7rBkxzOu3QAAAAA
ALI+C9QtyXz/HklK9SwM9zlSwEhKq+K8rS71rTubTk8aK24tOBS/OZEYjX6963bk96Ubsy+dvW4Vde
9ZG1BBs1YOwWq1Ks9kcWu+3IaxdcdeNE387w+2yV3rnwG1/6DqbsV0VnO2FBNwB4e0wr3O11fc
nDEt0PSK2E9ivvuaS7JjMbQdfr41qa6JzF35+FOdanQqsS3p+lETnOlHbRwht44jjjDietO4RYBr3Pnyvv
OXCW/QSACAP99/xLnnNW2/SSwCAzB3/NZFjr9ILWBT0REVwkikVtoQGJP5Qeb/npMsYdm/2GE1F
UU6ccp51dJfTrj2WvywnT5+0e9QF11uqp/0AbA74vWmb4nVG7R44gZT1GW8ov7dOdT4sGbUo3S
BMfcIsazBr1jUo6ac28z7QPYSnOKZEvYkT7cec4f+kmIdQ3NyTvhmgTm3wHIWY6rrrurFuwJmaGO0
GMvDwEw/LOz/5Tr0EAMjDDY9fJXd/6AF5/Bs/0msAAJm7dI9I/zt6AYuCEBVBA6FScsO1P6fl1RVt7k
pbenJoj9k/JScqJ6z/D65XGeqc9ZIrrombLbpD+ttuIl09a/TozUtaAZl1DTJrOyHeSKpXqBe0GnVjgQX3x
NNPSOWPKvZHAEA+nvPjY/KWL94oP3ryGWm+/3555p8u6c8AALL1lMilP7A+PussYiEQoiJlGYRfXl3
RfTlsXxS7lKgd9lbEyVeN9XMZvhk9NO3A1+/VOyrwVcO67VqVQ480e0lOK1mv47yOaTHOXbjxbW/
Lhtnr1K2LatY5BRbUnZ+6Ux58/EG9BADIwxv/
+gZ5wQ+vsJ8/9NXvyp/WeR8GgNz0Hxa5/Od6AYuAEBUxleSkN1LbnBFfMSZJitM70Ri+HI+7744c7D
pf686+X7I/4a8fLjUwH4qbZ/0guOFOjFWVQIZacsoTKG6Jgvk22OvYvA/0seV1TIn2Y97LA8Pk1cz3hZ
xnoE/SdmNSNTtwdeukJv6eAubL+z77PvlM7zN6CQCQh1c+eK3c/PXn6yXH5z7+kHzlLx/RSwCAzF3
6gsjlr+kFzDtCVMRUlM2zbuVUc/Z1NbrYnOl8y+vt5wScijnre1s23JlrYvN7cqqJpBS3XmZRF0x117vh
6vxxe81a56Kmj3+wdqsx7F+aq7LhnTIV7Onh297K+dBcNQLH9nm/hqd7bHkdU6L9mLVbm7Lqre/p
me+ta1Qq+NvI2oTnyA9c3XYDi+kT+5+Q+n11vQQAyIOqg/pzD7xELwX94W9/Tb73raf1EgAgc5f/RK
T/fb2AeUaIivgq29LyctSalHTA48w8bynX5YJR4NOfUMnSXNWB0GQ1HAPbsp55Q/a9of6KMUnTHP
Jqu9rKUt8KRKiWomxe8IdlN1d1gOaFzqMm/ZolJ3AMttM8tryOKdl+ipsXpO6/2HmtOZGTdS/7h6Br
7yrua1NNWBO03bzfw9odR+bHA8R339fvk7v+7C69BADIw/FLBXsYv/oYRgWozd+8Xy8BADLXf1Lk
8sf1AuYZISoSqWyH11+stqz17tBiV3FT9gZfW21NVr/R7K0XKBlgDM3OdKKjFJgB2KiyA+qc9bt+wOe
ya1/OW03PstS7fT9Yt1Wl1d8LTpaV1zEl2k9RNvf60h16sXq5MUzeVpHt4EGK7B96PbFTEbvtVlv6Lf
8+sqlzblwHu+5ulByOB4jBnYn/6Wfp7QQAeVr50kvl2iev1Evh/uFL35ZP//6hXgIAZO6y9RvZJcpbzbt
CX81aAgAxtTcK4nSSVCHqQGAKADGo4PT1/+318sAjD+g1AIA8lB56od0LNa6tu8vyiteFD/sHAGTgit
8QKfBL9ryiJyoAAMiVGsJPgAoA+VK9T1Uv1CTUsH7qowJAji79AfVR5xghKgAAyM1HvvgRezIpAEB+
VP3Tt33hZSProI6iAtS7/+NX9RIAIHN2fdR79ALmDSEqADIxe6Du/LBv/igXgIA5OWOL18/tg7qKPv/
8zH53Mcf0ksAgMxd/n9FLn1BL2CeUBMVAABkTg3fV3VQmUgKAPJ189efL//8Czfppclc+dzjsnX36+

XWV79IrwEAZOtKXR/1Zr2MeUBPVAAAkKknnn6CmfgBYAZe8MMrEk0kNcoz/3RJmu+/X3705DN6
DQAgW9b77aWPWx+fchYxFwhRAQBAZlRwqgLUh594WK8BAORB1T99yxdvlOf8OJ1f+R7/xo/k7g8
xKSAA5Kb/HZFL1EedJ4SoAAAgM+/77Pvk3ofv1UsAgLz83AMvkRsev0ovpeMrf/mI3PtJ/igGALm5/
DXr0dELmDVC1KXWlo1CQQrGY6XR05/LSa8hKxttveBob7jtWZG8mzM3Qs5LHL3GinPuVhoy6tT55
9d55H7NjwLuWyCW+n11+djffkwvAQDy8vJvPE9e+eC1eildn/zPB/LN//19vQQAyNylXZH+Y3oBs0S
IurR60lhZlaZecpTlzFpRP8+eHfiVasLfVIImPi+9hqzXor5KXfOCrAYvunRqJSlMENguK+5bIB7V+1T1Qg
UA5Ovqp47LG++bvg7qKKo+6u//
+y/bHwEAeVD1Uf+79fFZZxEzQ4i6tLpy4KZA5bp0+33p9/dkM68MdWzgt6QmPS+qZ+S4YK99XtxN
V1vqevelWy87K5qrQo4aA/ctEIuqf6rqoAIA8veWvRvtIDVLqifqH/721/QSACBz/UdFLv2xXsCsEKIuq
96h7OuncuqE5Nf/FGmL1zOyJ41zugtquS5bFedpcfOsVJ2n0twlRQUwPTUTf+WPKvZHAEC+Xvu1F8
vLHr1aL2Vrb/f/2A8AQE4uf8l5YGYIUZeQXbvRDN2aq04tx8GuiKp3o1E7c1T9zGAtyGCd1bDejbH3r
wVreI6qN+kMVfdfl6zW52A9S6+2qH6ENy3GPgPncEP8zZjnydln0vOiqHaWYvWMNHoeB0LzkpzUn
VFl/9A6olGM9obVW21v6GMZPAcJrkvI/RZ2vePcb4PXz35E1ImNY5b37WT3p2vSa2eZ6D1Ar1TMr
49uJl6QOz91pzz4+IN6CQCQFxWevubvXqSX8qF6o1IfFQBydOlPqI86Q4SoCDUUGGl2/cyRYVRHai
WjzqrR43EyanuDNTzVurCgpuQNVXdFt3UUZ5+DwWRzdcJ9Fjflgjtk3joz5/RGeo1z3nkq1y+kUEahK
q1+y+tVOsTseTxK50C6+umwipx2N97ZkYsDJ7W96x2NX1c3yXVRQV7I/eZcj1EBZPj9pu7d0GC5U5
NSIMjOSpb3bcz7M2CCa2eZ7D0Ay07VQP1M7zN6CQCQFzV8Xw3jz5uqi3r3h75KfVQAyI2qj3qP9Z
H6qLNAiLqEKtt96Xfr4kZ7Um3Z9TH72zrxbG/4AZBXL9Won9mpyfrIxEaFec7r+3uboWUCxu4/wN+
et3/pyI6XBPWkse4GPca+Wzo1imzrKP523M2ofdbOu/Fbsn0WNy+If+rOS9v677wbglnn94JOUJOd
F4P9um2ZLK8uyolT+ukYlS23beb5V9ri53BnxMnhkpwj67VGqYGh+21ofyZj2/b9ZrYlbFt+kJ3U/Ny3
/uvD789hya6dZar3ACyrT+x/wp6NHwCQv5UvvTTzOqijPPTV78qf1hmBAAC56T8scvnP9QLyRIiKIW
bPtPoFPwgdDgOHletbE4Z54aotPxwsrp3xAqzOge4z2bsoO14eaey7suW3deeiJll7zH364ZMh8T6Ls
nnWTbuaslpwe08Gz+8kipt740PW2PblMOpEFdfkTNjxtXf9XrVn1pzjSXSOrPOzp0NEI3gPvd4Dhu43
s8et0evSPk86CNzLYfa0LO/bsfdnmCTXzjLNewCW031fv0/u+rO79BIAIE+vfPBaufnrz9dLs/G5jz8kX/
nLR/QSACBzl74gcpkJ/vJGiIoBI3qm2Yqy5iYxIwK3UyfSDKjKcrKkn47SPdC9+VSoU/LqNhbMYdKRw9
QnMMk+K9tGr0FHOsP403RKoi+fcf2NgNIP3Kpy1j2gqa6LruEZOrw/aOh+M8JCawf20Hd33yNrsaZ
uHu/bBNduyvcALB93Jv6nn31arwEA5OWGx6+Sn3vgJXpptu7+0APy+Dd+pJcAAJlTw/r739ELyAMh
KibUkeHOgTHCo7mX3TFUvMKUSrD+5Oz05HBssVSf36vSHRZuBG7V08FeoUmYExB5PXXHCbtWR
dm8EN470w0rF3t+o8nvz/SvXdh7AJaNCk5VgPrID+h9BAB5e86Pj8lbvnijHL9U0Gtm60dPPiPN99+v
lwAA2XtK5NIf6OfIAyEqJjR/gWm53vWGbAcfk9YLHS/
+Po26n7aO1NZznJyneELGlj4tn5Sxl7S4KW5lAntYuDF8vno6/CyPP0dt2TB7nbo1OM36o0lYbdzT+xj
s/as0z83XpEi53bcTXLtoR+GPJpiWGsL/wCMP6CUAQJ7e+Nc3yAt+eIVemg+qPuqnf/9QLwEAMqfq
o15iYte8EKJiQElOusnV0EzePbnoFnIcO/Q7J6WTXtA2qm5m6ibYZ6+xrodpl6Ved5OsPCfnMa5rc1f8
zphdOfAu6QmJc0m9HrXW/XH+/I4OP6sSyOGSnCOzLqcKFN26qMaQ96TUzPKqx6k9GZQdSHa9W
p7pDpOf0CzuW0usa5fme8AU1xDz7yNf/Ig9mRQAIH+lh1448zqoo3z6v/6D/MOXvq2XAACZu/xZkf
48dRU6ughRMcCcBCnYW9IPAlXYNTChz6yYNTCbq/5Q7faGHhpuPdIev510n72GrLsnrnpWNjeNyY
Nq65JPjmrWsmzKrm5er3HOCzBj90asnBbnDulIs+ke18Bw8Amvix8oDvbcjUlvX80sH+xxaoTFZo9b
oz1p3yaRZnHfKnGu3QTvASU/dZXaee9gZMOb4h9Hze6Du/LBv/igXgIA5OnaJ6+0Z+OfZ83fvF+
+9y1qZQNAbtSw/v739QKyQoiKYeYkSJ2alHSoU/LTE7kw7YxI5vDy5uoUoVGwBmZzVQdQXnhTlVZ
qs9e7kuyzJ411d6h6Wepbav3okCq98zLMnFndbbN3TastiX+aKhIo72oZDmATnCMv2LO4x2xOsJTEi
HvXrLNaPevPNp/YQt+3SpxrZ0n4HuDXW7V41zBubVssGjV8/85P3amXAAB5UvVP3/aFl81NHdRRV
lCqglQAQE76T4pc/rheQFYIURHKHgYdUpOy2rLWu8Otp1KR7cGClfuHRs/BBOwamMZwbZddWzOj
eqgx92n23LN7obonbiCk8of1p3hehhRlc2+4Tqg9hD5hYBeYJMs6ZjsbHhT7uljH3G/5QaqtKq2+0dZ
ACYJo9r07eJA2Z5vTZZMLft9aYl07S6L3AHUsg6+ttiava4u59cTTTzATPwDMkOqBqnqiLgI1pJ/6qAC

Qo8vWb6bUR81Uoa8KBgJAEmo2fT0ZlAph96btmTxL+lhOtaYNWBfEUbp2yJUKTit/VJF7H75XrwE
A5EnVQVWTSS2SK597XLbufr3c+uoX6TUAgGxdKXLFb4gUbtbLSBM9UQEkFCxRcGZtsUO49nnrWC
J6ZB4tR+vaIV/v+
+z7CFABYEbULPx3fPl6vbQ4nvmnS9J8//3yoyef0WsAANmy3m8vqWH9TzmLSBUhKoCY2rJh17o06
pWaJQoWUK+xIqv7demmUqJinh29a4d81e+ry8f+9mN6CQCQJ1X/9C1fvFGe8+PF/NXt8W/8SO7+
0AN6CQCQuf53RC7doxeQJkJUAJNRNS8XfPx7cXMvpRq/C+YIXDvkR/U+Vb1QAQCz8XMPvERueP
wqvbSYvvKXj8jnPv6QXgIAZO7y10QufUEvIC3URAUAAKEefuJhec32a+wJpQAA+bv568+Xf/6Fm/TS
YqM+KgDkjfqoaaMnKgAAGKKCUzWRFAEqAMyGqoO6aBNJRVH1Ue/
+0FftjwCAPKj6qGpY/7POIqZGiAoAAIbc+ak75cHHH9RLAIC8qQB1UeugjvLN//19+cPf/ppeAgBkrv+
oyKU/1guYFiEqAAAIUDVQP9P7jF4CAOTttV97sbzs0av10tGyt/t/7AcAICeXv+Q8MDVCVCTUk8aKm
uVbPTaknWjdOGltZ96MOYZeQ1Y2gmvbG+7rV6TR0yunpfZjb3NEO2zTne9M2m1bkPugvaHbWJC
BSwosjE/sf8KejR8AMBsqPH3N3x3tuqGqN6rqlQoAyMmlPxHpP6YXMClCVGCGeo0VKZRq0tHLWe
pd3DH205RdQj4AA+77+n1y15/dpZcAAHm7+qnj8pa9G/XS0aXqov7+v/8y9VEBIDeqPup/tz5SH3U
ahKhIqCibe33p99VjWyp6bXby3l8WRhxDryHrtTziU6UnF3eC+2qea1hrF8VRuA+A+aZm4n/nJ98pT
z/7tF4DAMibClBVkLoMVE/UT/7nA70EAMicXR91Vy9gEoSowDLoXRQ3Qy2Xy86Tzo5cXJwUdTFUt
qXfrYs+w8DCUMGpClAf+cEjeg0AIG8//ffX2UP5l8m9n3xYvvKX/NsDALm5vGc9mOBvUoSoS8eoLbl
i9kQctd6oc2mvz7s25fD+IutuGnU/VwKfNLcT9vko6Z8z+/PmMP7mqvP5EYU0/WNWj+T1Rv2h/FU
5e+GMDvk6shMnRQ3UUg17jG5PvHbHuTbD59DaumyYrzfqkQa2MbA+/BTHaYN5POpY/P2rh7vd
9nnrupbrsjXQXdYu3WC83n4M3DfArKgh/A888oBeAgDk7YbHr7Ink1pGd3/oAXn8Gz/SSwCAzF26R
6T/Hb2AJAhRl05R1s64PREPpOs8s3TlwE30Aj0U27LbdJ6Vz6xZXz17ldNV/awjB/4B2PywsCxn1nRr7
RCwJIMj5zu1UswQa5bnrCO1UkFW9fYcal2SINUYyl89LZXimniHs3Nx+hCvfEbcU+2L2e6pr43Dfn1w
Z846FVQOrG+uDgSpE7VBHcuqeFvWoakKSlf369Ld2wxcdxW+lsJKN3RqUprnibKwFD7yxY/Yk0kBA
GbjOT8+Jm/54o1y/FJBr1kuP3ryGWm+/37qowJAbp4SufQH1kfqoyZFiLqEimtuT8R9OXQTovauHwi
JEU72Dq1XOU6dmIcI1VI5LW6M2gzMjmSEhV6w15PGutvjsyotu6am9WjpLXRqsh4jjUz7nFW2rTa
Yw76rLadd22HVPv12d+vuV8TsRaoYQ/mrp9X2zVA4xpD+4qbsuefNfnTFa4Z1BPULwcDQN67d6V
wbV7neDX69Frbev2+maYPxeh2aFjf3vOc+P1RXYWtXf41/TppyLsFxAmnafXBXPvgXH9RLAIBZeONf
3yAv+OEVemk5PfTV78qf1h/USwCAzPUfFrn853oBcRGiLiOvJ6IfaPUOndivWnXCo32dFJrDwO38bS
5UZMsNoJq7fi8+s+6n2wPUXFff8ickqmx5QWCs3pgzPGfVlj+Rkh/mWi0Z7IY7gtk792TJfmJsJ0EYq/
Ua636vzepZ2QxPUMe3O61rY6vKWbchRsg+cv3+obPdKdoQeH0UI1Q3Q2s7cNWB6t6okwhkSA3f
v/NTd+olAMAsvPLBa+Xmrz9fLy23z338Idn/n4/pJQBA5i59QeQyf8BKghB1KRXlxCnnmRtode1x6W
U5efqkHXYF11vUMHDn2VzwQ7mmeJ0KuwdeWOgN5ffWWcfkDu+2H8bw7cAQ/VFmdc784HMy
Yb1zLRMP6W/LeT9BlVZoz1klRrtTuzaW8kkJ3d2o9a4p2hC7Z7Zxrq2N2WUO3P3Er8sLpOuJp59gJn
4AmLGXfPe58nMPvEQvQbn7P35Vvvct/m0CgNxcVvVRv68XMA4h6pLy6oraPTl74nSqPCUnKies/w
+uV3ngPEWoFiOYcodmt90x0xG9I6exkOfM6GmphqaXQkPC+LP0tzf8OqBVo6fp8kkSbhdl80L4jP1u
cDtiPjEgE+5M/A8/8bBeAwDIm6p/usx1UEdRAWrzN+/XSwCAzPWfFLn8cb2AcQhRl5U3tHlfDts6aL
N77VXEyQqN9VP3hsyCUdfTDi/9upOjwkuvNubQI2YYuIDnzB/KHyXmkP72hj9RVLUlIzuhTmDqa5O
CTNtg1JUdKNlqa55jln7k532ffZ/c+/C9egkAMAsrX3qpXPvklXoJpn/40rfl079/qJcAAJm7bP02eukze
gFRCFGXVklO2hlkRw52nSHNbh3Rkv0Jf3347OuzV9w864eaDXeSp4E6pCVnqL3i1eKc2KKdM2Mo/
xjjh/S3ZcNLUMtSV1PRTyvVazOhHNugZuhXPU7tScXsQNWYoCtu2QJgSvX76vKxv/2YXgIAzELpoRfa
D4z26f/6D3aYCgDIyeXPivTp2jMOIerS8ntyNptOOObWeCzq4p/uem+Sprnj9gDtSK2mA77BOqRmP
crmqj9sur3h1aUsxB5LvWDnzBzK787+H3iYIV70kP5e45w3jL9cv5BOuYRUr82E8miD3pbKoIM9Trvi
ls8dW7sVSMn/+v/
+l34GAJgF1ftU9ULFeHd/6AH9DACQi0v36CcYhRB1iZkzpqvqWpWG1YT1CqtYrAxMwOY/JJkhK99

pMJoc2VLb9IfyBurRGfdmzm7MP3bEU7v7Fu+WOl9+hlwAAeXvjX99AHdSYfv7dP6WfAQByUXijfoJ
RCFGXWWDW8FPiTzbuDltXzPVzyAwvRw2ht+tRGr0uXeW6dJPWu0z1nFVke7BA5v5hSrUxzaH8o0
NdMxQeNaQ/Xl3VCaV5bSaVQxvsIfxhxVBVSNvvp1pfFohy1RVXyf/4lf8ht1x3i14DAMjT373yCf0M
UV7z1pvkbe+5VS8BADJ37FUix9+kFzBKoa/G9QIAgKXxwCMPyOv/2+vtmfoBAPm648vXyysfvFYvYd
D1P/E8+a1PvVGedw0TbwFALgovFrni/daTq51ljERPVAAAlsztN91uD+0HAOTvb27/tjx2PX/ECnPlc4
9L9fdeS4AKALmx3m+Pv8f6SIAaByEqAABL6F2n3iUfeMMH9BIAIC+Xjvfl8294VH78nMt6DVz/8t+V
5NZXv0gvAQAyd+ztIoWb9QLGmSpE7TVW9AQpBX9W65F60lhxJ1TZkODL27LhTbbiPCabqGZYeyO
43dBH1jOAAwAwhz788x+W07ed1ksAgLz84PnPyt7rvqWXoLzidS+R1X8bNhkqACATx4oix39eLyCO
OeiJqsJVf5ZqR84zwquZ0VcaKU3oAwDA4rjnl+
+xh/cDAPL10M0/kO6t39dLy+3al14l1d99rV4CAGSucI3IMTWMH0nkGKIWZXOvL2oeq35gxuuuHH
iTiKvZsNXn92QzxwzV1qnJeTqkAgCWjDtj/3VXXafXAADyonqjfu+aZ/TS8lIBqgpSAQA5Of5ukcIL9QLi
mn1P1N6h7OuncuqEZJedlqXedUNc/9Gtl/XnRZq7pKgAgOVzy3W32EGqClQBAPlR9VE/96Zv2h+X1
S/8xivsofwAgJzYdVDz7rl4NOQYog7XRLXrlZZq4nZEtYfVD9UoNb/OeaRVL1Uprp0RP0YFAGA5vfm
WN8tH3/5RvQQAyIvqjbqs9VFePoLv35CLwEAMmfXQX2HXkBSc1ATNUKvISuFktS8lNXRqZVSq2H
aPu+HuNXTfpGB5WVM8uWd46hJwTA7YddqEYTfT/4kcHneY0nPYU7fC+q9b2DCO//8rEiKf0cCAt7
7s+
+V2h01vQQAyIuqjbps9VGfd82VctfvUJMbAPJztcixO/VzTGKmIWpluy/9bt3vCVptOcPst1WY2ZPGu
htwVqXlDsFvVe01qobpeqIkoSO1khtC+I9Vd0Yra9/2bgFghnqNlWAPfSBnqjeq6pUKAMjXfT/zuD1r/
7JQAer1P/E8vQQAyNzxO0UKL9YLmMT89kTtXZQdnSKU61v+RFSVLXHLmHZ2LqbU+64s9S0S1NFG
TQoGTGKR76eM295ryPpg13tgBlR9VFUnFQCQnx8/57J8/g2PLkV91Le951Z5zVtv0ksAgMwdf5PIsV
fpBUxqfkPU7oHXE8sevu/1HjWG93cOpKufTsfppZpmrVUAABaVmqm//attZuwHgJw9dv3T8je3f1sv
HU23vvpF8ku12/QSACBzhVtEjv0LvYBpzHdN1FSFz85vlhPo1M7nWItxHgxO2jWqxuPoOpD20GPv6
/VjZG3Jwf1FBNd2Pdzga0fVgsysDVEya98oca+VK/5xJm1f2OsHyncO1O806o7arx19P/kG2p/K9Ux6
DsOEt32wXungORo8P2HiTbTn8/epHqPqpCY5P0DQbdffJvf88j16CQCQl7+/7Xvyjy//oV46WlQd1O
rvvVaufO5xvQYAkK2rRY6/2/p4hbOIqSxEiFqud4fDT/uRwnDa4qac1WVWRfblcGnyBRVsDU7a1ZTV
wqr1/3hUiFMKG3rcqUlpMKBKMklYe2NETUjVYzgYFmXWhihZtW+khNcqwXEma58TyIW9vrlaGBH
2qXNitLNcl/GVM9SxDbR/6us5/f0ej9OrffAcNVdHhZyTcPbh1XO2Dd97qd3vWGrvKL6DGfsBYAb+6v
WPHcn6qL/yH15JHVQAyNPx09RBTdH8hqilk34P0YN0Bu2Ha8tuuinKQug1zvnhkTuhV7/r1Zsdzzhv
5bp0dbDd9TbQlHNeotNLMEmY9dpzesOh2+3IzkX3tVm1IUpW7Rst2bVKcpzJ2tdrrHuBnP+HjZa1F
615bkRQaLRjb1OKem0koz1u06e5ntPf70n47fHb3pHa+ejIPHqivUH+PsLvvbTud0Ds2frfdepdegkAk
AdVH1UFqUfJyumftB8AgJwce53zQGpSC1HtnmjGkFHzEWco65DimpzxspxVfxuqF6C77UQbdnpv
me1yHmYvuTOyFivhWXQ9uejO2qUCFi+kKcrmBSPEidI7lH39VDo74mY3xc09J6yxHnub+mQmmiT
MmLjHCNyKa2eGQ/XM2hAlo/aNlPBaJTnORO0LtuOst74iW/aGVVC3J2GHE2hHTNWz/rmtbPtBrd
/2JNczhfs9gWrL7yFf2Up/+4q5j/B7L8+J+bAM7v7Fu+WOl9+hlwAAefjmjU/JV376u3ppsb3sp14o/
+a3mNAEAHJTuFHk+L/WC0jLHA/nDwYcXkjrjWE1w5B0mMHN0daVAzdTKp+Ukn5qK56QU/ppJDP
kHgioh2ouTjVJmK6nGTZ8Prc2REmpfSMlvFZJjjNR+0a3wwldR5fWOHUi6XdVVU4HNlaSk2473bYn
up4p3O9zpSwnAwcRIteJ+bAMrrriKmbsB4AZuP9V37HD1EWm6p/+
+n/5GeqgAkBurhQ5/mvWR+qgpm2+a6IWN2UvbMitPdQ3hXqoHtWLri8pZ7JH3OhefG5oM1EPZ
MWu5eiGPlE1KzNsQ5R5b19sebQvRuA3VlFOLEzSmcbxAvPpphfcZAepKlAFAOTn8yuPylNXX9JLi0f
1QFU9UQEAOTn+r0QKN+gFpGmqENUc9hv1cMLJoj8M2gxA7aBUrw9NMc2v04+4dRUtdp1B82tD
H2kGskvEuHZ+7Udf89zw5DXjJwlry4bZq9OtjWnWijRl0oYo2bcvLbGOc4btG21wgreeHHp1B4ZlOv
HcEcD5QZpuv+l2e2g/ACA/KkBVQeoiel3l/6IOKgDk6dhrrQd1ULMy3z1RkZGQ4dEus05mDGp2d9V
b0Q+rjZ7D7raTTBLW3vV6dtrhjxuYG8OTB6XehihZtW+khNdqguOM176Idth1itOdfT7Y9JDh+ImO
M737fWHkNjEflpGaZOoDb/iAXgIA5EEN6f+7Vz6hlxaDmoWfOqgAkCM1C7/qhYrMEKIupaKs+bN2

yao3Ztuc0XsMPcGXKlEb7K0YEnhNOEmYH/4YM+KbcmhDlFTbN1LCa5XkOBO1L6Id9rE7NVUTnsK
Rmqsb4jfdL5lQPrPmhNaJrmcK9/uiyeB+B0wf/vkPy+nbTuslAEAeVH3Ux65/Wi/NN1X/tPp7r5XnXX
OlXgMAyJaqg/oe6+PVziIyQYi6pIqbF/zehs1VHawYk86MU9n2h393alJygxmjRqg/UVew9mbkJGGV
09aSNq5dWbUhSmbtGy3ZtUpyrpO1b2w7ynXZSmV8eFnK5aas6vZ4Tbfaftab/j/Z9Zz6fs+LOdGV28
6Jws6U7ncgwj2/fI89vB8AkI9Lx/vy+Tc8Kj9+zmW9Zn79Uu02ufXVL9JLAIDMHVsTKdysF5AVQtSlVZ
TNvcFJu6rS6res/8djD/8OK6Rpb2dgoi679ubg/ixDk4RVZHuoDc72vF01d71eitm0IUqG7Rsp4bVKcJ
zJ2qfa0Zfu0IbVpo3SBlM7JWcHjzfsGiW6ntPf7/mw7q/B67F/aPQSTiCV+x0YzZ2x/7qrrtNrAABZ+8H
zn5W/ev1jemk+veatN8nb3nOrXgIAZO7Yq0SOv0kvIEuFviqCCAAAMIF7H75XKn9UkaefXYwhpgBw
FNzx5evllQ9eq5fmx7UvvUr+06ffzDB+AMhL4RqRK9R8BQzjzwM9UQEAwMTefMub5aNv/6heAgD
k4W9u/7Z875pn9NL8qP4udVABIFfH3239jwA1L4SoAABgKu/92fdK7Y6aXgIAZE3VR/3cm75pf5wX
v/Abr5BXvO4legkAkLljbxcppFNUD/EQogIAgKmp3qiqVyoAIB+qJ+re676ll2ZLhae/8Osn9BIAIHPHiiL
H36EXkBdCVAAAkAo10dQt192iIwAAWeve+n37MUuqDqoaxg8AyImqg3rsPXoBeSJEBQAAqVAz9b
d/tc2M/QCQI9UbdZb1Ue/6T6+2g1QAQE6O3SlSeKFeQJ4IUQEAQGpuu/42ueeX79FLAICsqbqon3
/DozOpj/q299wqp/7ZDXoJAJC5428SOXabXkDeCFEBAECq3lF8BzP2A0COBZP2A0COvv2if7Jn7M/Tra9+kfxSjV
/kASA3hVtEjv0LvYBZIEQFcteWjUJBCsZjpdHTn1tSvYasbLT1gqO94Z6fFZnX09NrrDhtXGnIqCb6x+E8
lv5aY2mo2frfdepdegkAkLW/v+178o8v/6FeytbzrrlSqr/3Wrnyucf1GgBAtq4WOf5u6+MVziJmghAV
yFVPGiur0tRLjrKcWSvq58vHDiJLNeno5YXRa8h6LarV6loXZDV4saVTK0lhIDAGjqq7f/FuuePld+glAE
DW/ur1j8kPnv+sXsrOXb9zu1z/E8/TSwCAzB1XdVBfrBcwK4SoQK66cuDmbuW6dPt96ff3ZHNZM9
SxQeScUj1nxwW/7fPiHlq1pa5zX7r1srOiuSrkqFgGV11xFTP2A0COfvycy5nXR33Tir3nrTXoJA
5YyvW41V6AbNEiArkqXco+/qpnDohy9v/dHHF6znbk8Y53QW1XJetivO0uHlWqs5Tae6SomI53PSC
m+wgVQWqAIDsPXb903L/q76jl9L1sp96ofzKziplwAAmSvcKHL8tF7ArBGiAgAAAMA7Ah+dpGN+qpEDZPBCJB3qpeaN
536d163Or/dp135sb+ivNdbZLwuuD/bKS2MbmtlG7zFcYzN4jMM1S+1HWN1Low3jal3a+zDDt+aq
87WDDQ9pc9i2o9o8tpfjROdl+J4ZuZ8YxxD7fGh+W9RjuK0OZwi9/7p0z506/lKsnrNGj+NAWF6Sk7
ozquwfDt9PNqM9ie65eMduS+H7wj1Pcd9HsNxuv+l2e2g/ACAff/fKJ+SbNz6ll9Kh6p/+
+n/5GeqgAkBurhQ5/mvWR+qgzgtC1CWlwpHQMKhTk1JhQ8ZlcHHYtR8HCkLa61TIMrC+uRoeXk2
1DRU2hfYY7EitNCqEU58zapaW61J3uw52duTiYMi0674ynbqmQ8GiZh/zyGBquM1uz8dQE5+X4X
umuToieEt8DFGcfQcvd0hb7WCw5A2hd6V67jxVafVbXq/SIWaP41E6B9LVT4MqcjrpPZfk2FP6vlDn
KY/3ERwdapKpD7zhA3oJAJC1z688Kk9dfUkvTe/f/Nar7J6oAICcHP9XIoUb9ALmASHqUmASHqUmqLn8O4d
TmNeo3SlHPhSUpi5XrX3na/FYybwtaPGt482TaCw6mHj7EjO4PplEcFZM7r+3ubsrlVF+erBr/GPI9nZ
FyGWtm2ttd1t2Wptpx9bOvUrr3hB4Vhbe7UZH3kdQm2eXRT0jkv/invSO28cd0SHMPY8xHg7zu8r
dZxrbvBoHEu3Iamcu40u53bEitrHVKUE6f00wiVRPdckmNP7/uimOP7CI6OD//8h+X0bQxHAoA8q
ABVBalpWDn9k/YDAJCTY69zHpgrhKjLyOwlZ/R0K27uOQGJ9dhLZaajqpx1t1M5bS25RqwPHd486T
aKsrlnBj6O4toZL7TrHIT3AyzXt4IBWXFNzugv6uxc9NvY3vV65pXPrI0P38YwexjWLxht3rwgfgZ5PrR3
31CbR5r8vFRbfnDoh3xB0xxDFHPfoW3tXZQd3SEycC4qW/5+zWtniH/u1HFY3yOhIe8k9uUwrEFKk
nsu0bGn+H2R2/sIjpp7fvkee3g/ACB7akj/V376u3ppMqr3qeqFCgDIiV0H9V/rBcwTQRlZAQ0ImqY
bsGrZTiyhuIkyielpJ8GjFofJo1t2HQ9x9BhzEGnTgwGP0VZ8xMtLyzyA0Mj0J1YVK9WY/8jgrfhNscV/7
yMN90xjFaWk+MudvfAa79X7sF+GEPcRwyfn/zcTeuUjN51gntuimNPcv2HzlNe7yM4ctwZ+6+76jq
9BgCQJTXJ1KT1UVX907t+59XUQQWA3Kg6qHdaH6mDOo8IUZdSUTYvhPckdEOYqMl1FkZg8hyjn
mOk8MDO76nnDnc2AsPq6dg9GafXkeGOgjFCRlOK52UyYccwK2keVxw9ORxbLNWR2T2X2vVfkvc
RZOKW625hxn4AyNFfvf4x+fFzLuul+H6pdpvc+uoX6SUAQOaOrYkUbtYLmDeEqMuquCl7esjtQKlR
W/Pcos+u3ZYNs3edW7PRrMGZhHW+zurzZA+NNoYyV0/nF6FOH/qlfF4mkm1w6dXKHXpMWsd0
AsUTMrb06bje1BPcc+OPPf3vi6P9PoIsvfmWN8tH3/5RvQQAyNIPnv+sHaQm8ze8+tegkAkL
ljrxI5/ia9gHlEiLrE1MzaqqeYPcGPHYR0vRqKo4f/GoxhxHPHrB2pgiW3/uMUba64U6Z3duT8+R29n
aqkk6GW5KR37gdnZO/JRbfoZeQQ8BgyOC+

+nI4hTOmkFwKOqumZL+NcNHfF75DZlQPvNJxwzn2EWPdckmPP4PpP/T6Cpfben32v1O6o6SUAQ
Jb+8eU/lL+/7Xt6Kdr1P/E8uet3qF8NALkpvFgP48c8I0RdRu0Ne6itmkU92FPMCHiMXnIlPxkzZmJvy
4Y3Dft884MlY2bySXgTWHWk2dQnKrWh/EXZdLsdqvO87l+XXmPdq22ZZBKkcVI7L578j8Fj1udsrvr
DyPW9bj9yHVtu1oBtyq7eda9xzgsxY/VgjnPPTXjsU1//hO8jZntyvRSYe6o3quqVCgDI3t/c/m157Pqn
9VI4Vf+0+nuvleddc6VeAwDIlqqD+h7r49XOIuYWIeoyqmz7Q287NSm5QYtRH7F6NnzmbhXSDL52
Lpkz9nttNibamUhF3I6BrlSH8o+4LiU/fZQL005glcl5MSQ9BnPYu9ueiRK2YH3O5qqz34IX9Felldqs+v
EUNy94PTLd9njnodqSeM2Jc88lOPY0r3/C9xEgiqqPquqkAgCydel4Xz7/hkcj66NW/u8idVABIE/H3k
od1AVBiLqk7KG3YUUMVeDS7wcDHlX3cLBmYrWVcx3NpCqy3W/5gZHNOTbvsAPDrOPxhlcr5bpsp
ZzL2dcl5LxWW9Z6d+j1VLI5L6Zkx2C1Z/A+3D80ejUmYNfnNIaSu+y6nznWQ/UUZXNvuFaoPYw+Q
aAb656LfezpXv9E7yNABDVTf/tX28zYDwA5UPVR7/uZx/VS0Cte9xL5hV8/oZcAAJk7Zv2GfPwdegHz
rtBXRewAxKNmNtcT86gwbG/anqHAOEfpntPHcqpFwIpwn+l9Rip/xM0BAHl441/fIKWHXqiXRK596
VXyW3/8z+yPAIAcFK4ROf5+66P/Xoz5Rk9UILaeNNbdmc3LcmaNABVZO1r3XPu8dSwZ9ODG0fGO
4juYsR8AcrL3um/J9655Ri+JVH/3tQSoAJCn4+8mQF0w9EQFxmrLxmANWFXOgK50yMzRu+d6jRU
p7ZyRbiplKXDUFf6fgn4GAMjStU9eKe9s/aS88723MYwfAPJ07O0M419AhKjAWAOBFgEqMsc9h+
WlJiiblx9NaEs42hKOtoSjLeHmqS0nrr1GDr/3pF6aLa5RONoSjraEoy3haAvSQIgKAADmBj/ghqMt4
WhLONoSjraEoy3haEs42hKOtoSjLeHmqS1IhpqoAAAAAAAAABCBEBUAAAAAAAAIhCiAgAAAAA
AAEAEQlTkSE2WU7DrfxRWGtLTa8fqNWRlo60XHO0NvZ3CijRibwjp6Uljxb0GG9aVTbJuGmH3UNr
7mJbRRv1Y4SYFAAAAAGChEaJirvUaK1Io1aSjl4H5pgJdY1Z9W1nOrBX1cwAAAAAAsIgIUTG/eg1Zrx
GfzqeibO717RkF+/1tqei1szFPbenKgXvLluvStdu0J5tkqAAAAAAALDRCVABIS+9Q9vVTOXVCyE4BA
AAAADgaCFGXmao1OlC7MazG6GD9UXuIvfE1A+VKNbNOpXokq1Vp79Mcxt9cdbYTvjOjjeoxqk7qY
JvCa1VOdrymMbVf2xvetob2H3JN4rTRY359ZEONcxFo46j1xj7t9ea5zKoOadx7KKotg9uYsD5pjOuS
9J4FAAAAAAACLgxB1WakgL7TWaEdqpVEhpPpcQUoDQ+ybq4OvVyFiSYIva8pqYbBWZBqcNq0GNh
xyDHYINtgm65W1UnjQaYt7vIMqcrqqn3Z25OLAa9u7bmODtTKHQjgtuo2TKsrambLztHMgXeeZxR
iOHmh7W9xml8+s5dDDMoV7aKJrPizf6wIAAAAAAOYRIepS6knjnJuIuXUb+9Kt61BNOrIzmPx5qtL
Sr2+5QaEKG8/7ve16jXN+0FVt6VqVXfE2H0Nl2/qabl28L3G3sx1W8dJvU/gxWMe77oZg/mv77gF0
arI+MhUdf7xhKltu2wfPpR9GSvmMeBlqe8MPgsOuSWQbJ1NcO6PbuC+H7qbbu0ZI2ZEDN101hq
mfOpF9hDr9PTTNNTckuC7J7lkAAAAAALBICFGXkjERz96m16vQD9VEOl56FlRt+RP3+EGhqScXd9w
+e1VpeQGStc8LYa+fntmm0GPoXRS3SeX6lj/xUGXLC+U6OxdDexSOP94RimvidfQ0t22ElGaPTrN3av
2CcU02L/htrJ0fGKo+Ja+NftDbO3Si0mrVCRv3dbrau7jjBZKnvROYlRTuoSmuuWkm1wUAAAAAAM
wdQlRYdA3P0OH9SZmzk5+Ukn5qK56QU/ppespyMrCTEN0D77jsIdheXUtjqHdgSHsazOHy/rB4P5
SryllvyvYRvVNtxnbMHqOpKMoJfUHcwLlrXzzrnJ4+aYeVwfWW6mk/kMxMCvdQKtd8VtcFAAAAAA
DMG0LUZRWYKCdunckYgeWRMt3x+r1i3Z6eRig3URhpDK9PScUt3trctVrXE6cj6ik5UdFhZWC9anb
2EeriSf+6AAAAAACA+UKIupTasmH2OnVrPZr1HI+ocr3rlDEYevjD9lNT3JSzbglONXTcqCs6WRiZQYh
dOS164L4ctvUQeLv3pzs5lrF+QUP07K/5sv1xAQAAAACA5UOIuozMupwqYHLrohpDoCdXkpNuEjs
4XNoIEXNVcoamK6NqvWbF6+nZ2ZHz50fVFTXP2eBs/mZ90FMydk6nxNfQ3XdHDnadr3VrtZbsT/jr
h4e0ZyWFeyiVa57idQEAAAAAAAuNEHXJ+QGTMWP/VMxakU1Z3XCn3DFnS8+ZMcmTNFfFa1J7w
6+V6a1MmdfTsyPNpj76oaH8Rdl0u6xar6utN6yz5eg11r0anuYESU7AqVivP+8dkGx4U8nH5V+vZtP
5Wnf2/aIumOquNyfCylYK91Aq1zz5dQEAAAAAAEcTIeoy8oI9S3NVh0rGhDtTMmcun2r75iRC7nY
mCjuDs7o3V3WI5gWO5gzwaXOHxftCh/JXtqXl5XU1KdnnrCAlP6mTC95EVCojdOutWrxzHLe2bVBg
W9Yzb2i6eZ9Y68/k0w3VNv09lNI1T3hdAAAAAADA0USIupQqst1vGQGZUpVWv+8HRvaEQpMqyu
Ze1w/BbGr7g/scx2qn1yBt/9DrDZhIcVP2+oNtstj1YDOoh2rwhvQr1v62Ruysst0PrUtbbVnr3ZILLnU
8g6+ttiara2v22gwMTTeGs+c+ZD2Feyila57ougAAAAAAgCOp0FczrADITq8hK3oiL1WDdo+eiwAwk
urtPS8/mtCWcLQlHG0JR1vC0ZZwtCUcbQlHW8LRlnC0BWmgJyqQKbOOZ75D4gEAAAAAAJAOQlQ
gE23ZKAzU8ayeFTqhAgAAAAAALB5CVCAPql5pZpNXAQAAAAAAIEvURAUAAHODelXhaEs42hKOto

SjLeFoSzjaEo62hKMt4WhLuHlqC5KhJyoAAAAAAAAARCBEBQAAAAAAAIAIDOcHAAAAAAAAgAj0R
EV2eg1Z2WjrhVnrSWNFzZavHhsyL60CAAAAAADA/CNERSZ6jRUplGrS0csAAAAAAADAoiJERfp6D
VmvEZ8CAAAAAADgaCBEBQAAAAAAAIAIhKhLqr3h1gddkUZPD7+3l51HaClTVePUeI3zcL7eZW/XH
MbfXHVepzc4uF+PuW1j58HXt2XDfY318F4Wo12TGjwv6jHpuVGSHr8S1obCSkPCD8+s/eo8VkaciG
TbBQAAAAAAWF6EqEuvI7VSQUoDw+
+bqwMhX3tjRI1T9fXpBJbR1H5WpamXpFyXrYr1MbN2OWHk4HlRmquFYNCZ4blRoWtYG6RTk9Lg
BFl2EFuSwZd3aqWhcDTRdgEAAAAAAJYcISosVWn1+9K3Hq2qXqUCwPNulNaTxjkdX5br0tWv7dbL
zjrrtTsXnYiusm19rlsX9zNSbdmv7W+rxHNafjv7e5tSTNCupHqNdS+MLNe7zj77LasFWvOcDkeza4NI
W3bd1Dh020055yW0VjvW3SDXOE/uBe3UZN17bZLtAgAAAAAAgBAVUm1tixtxVraMANRTlM09H
crZ4aVeu3bGe23noKufZadc3/La6ciqXT25uOP20qzK2U13yxXZsoNGFVLuibM6w3PTO5R9/VQ6O+
JmscXNPWd/1mPPbVvvorhNDpynypa42Whn56J1ZJYk2wUAAAAAAAAhKiaha5OGDmHPzqkT44K
9tNrVlQN3A+WTUtJPFSdo9EPnYSmem+KanHGTWGtrquyCW7t0qM5p98Dbnz18X7+uYA7v7xxYR
2ZJsl0AAAAAAAAQoqIsJ82UcJTAxElGbdLcjGjnzNtlyawNRdm8ENYz2A9KQye5Giur7QIAAAAAABx
NhKiIoS0bZs9Kt46mWft0JuahXRm3obgpe3qIvV+v1tc8Nzybvl/DdfBh9KCdYLsAgLzp0Q3Ggz9yAQ
AAALNBiIrx2rte70o7oHNrfxpDyKc2ybYya1dJTroJqDsE3qVm4i8YM+6n1YaI16uZ9NUvzfakXXbw2f
XqnHrtK530Qtu4NVhjbRcAlpH9Xl+QwkwTy540VlZFWu57tPNHr+bqLILU+Qxze40Vpz0r+f/hT/0ba
p4P9zEP5wXDhq7XDO6ZmRvzvuZ9P7mPvG7mOO+3ebwnR+5jSf+gFfO8z/K9eGZinJuh951luGnG
nJfc34sTtofSdoiDEBWJ+AGdMSt9AiU/nTRm/7d+MFmdbhD8tO0KKsqaVzS0Kavem667baeO6OB7
cZw2JDp+/aavPhXsGRpSs9Wsc9pc9dvm/sOhHu7KJNtVjG3wDwuALMzsl/chKri09j/lv0npcCYu3Da
KcLuTP+4f5vte3N4IC3ONPyjOQq8h6zWRatX9x28G3NEnxsO8XrMy01/cAyWOhh+z+NZW52O1qS
YFda9TS6qdmpRmFPjk/0v7+Pc19R5csr6f6l3jHFk/T2Z778R5v83jPXncPtTnc/yDVmTok1eYm+C85/l
eHHFuht739CP98xP/vl3dH/g3Iot/IEadk9zfi8efl3zfiydoj/WNrUrbpf+eHO/7dn5+FsZY1g2DJWS9R/
TV5bd+A+hbPzD5uvW+9U+g87mq9ZZia/WtnxX060c8vNcqIa93P29uf9TD2NbIdtqStKvbr5fd9VXr
K8cxXx/yKNetVygJz83Exx/
+MDcdve3gMSfabqvqrS8PXwQAmEq3XrbeX8z3eP2+GngjyoffFv1vwAzaEEm/z8/8vXjm7XCuj9q/f
c28f5PzY/87OoP9RtP37dy1y/3eivPzV9rCv5dn1R7n5y9jv/pnrCy/l8a/rznvuYNtyPocjW9XvNdMa6J
9ZPIeqPdvbdd+hLRD3T/maud+Um3XK1IS/5w4n8/+vTjeucnjvS/
+fZv1+8v4cxImq7aNPy/h62fWnhHfw+nfR87+zd0737fBdX579Qr9vhz3uiJf9ERFDBXZVn8p0ksO56
821puAo7kr/t9KrNd7n9D2D52/MKlanIP1QqutCWuIJm1XEk4PIOsNTS/7rDdbf9h+0jYkPH57qP3g
ubQ5+wj8QdOuc2oMyXfZPWWMeqiWRNsFgMy05XytY71NXZBN503VUpEt9UY28fv35Iqbe9Lv7x
ltmTO69MupE/PRwFm1o9dYl1qnKmfn9kLNhnteWt7PKPPC+T6X6unAzyL5KMqJU9YH9+dQW08u
7sygPb2GqIFK5fqWv9/Ktv3zYmfnYgY9sRxj39d6h7JvfRj8fi7aJ64puxm9Ecd5v83jPXle3ved79+y1Ls
hP8tr6uf34dEJHdm5mO7dE/ec5PVeHOfc5GX8uXHeXwLf5xmY7Jxk9148/rzk+148tj0jfp6yR42mW
tYuzqii+fpZGOMRoi4pvw7mwJuLMeFQcMiBCgvdr1EPJ5TztxMM6dQPhf5rrYf5A725D/VQ+xmx35
Ht9MRtl/MGFlw3nvMG7H6d89gbakjCc5Pg+G2D59J+jDoG8zj1Y9QvU3G3a7xu+NgBYAoz+uV9M
enSL+W6bIX/A5CTnjTWa9KZVTvsoaMdqbbi/1ueGTUM0Rh6N9uSN/n84j6JXuOc9d1s/bI/oxu3sq2
HjNo17dWwypITNg/
+vJW13H5pT8huV1lOenWcNKPePgZk8AetuMFlmDTbEVuO78XTnJv86fJoB+cHhtOnWwJnknPCe7
FhxPub8/PnvuRaNYmfhRcOISoAAJgNfnmPTdUltX/5uTCLXoa6tpj9i6D+pWdGvR3b52vSqbZmPmL
C/0Op81AjV7KppRZXPr+4J+f2NDo7wwDEHTWkatqr7yOrObMI4efpl3ZMgT9oKfPyXhwwR3/Y6uy
flAvevxGqt6h6/9mYYa9C3osD9HwiwflBrO+pqedWiWHwjzD8LLxwCFEBAADmmDP5gRq2N6ueO
MFRDt36vqzO4hfU9oYzCcRc/dbuUL2Csh6WHcfc/eLePi/O6NHZXTNnsg5/YiAVeKtJgXKftGOWv7Q
jNfxByzKH78Xz9oet8pk147pY/4aeVWXUZtirkPfiAernGrdnrP89dXBKXadTkl3n7nn5IwymQYgKAA
Awp5wAVfXWmJ+hjMXNC3YNtk7tfK7hXHtXhU1NO8B1fuEpSEn9Vqh/CZrtcHptlsOyLfP1i7sOCGf5

y6Iecqzq2bt5jz0MViXezdWMZjYfZVa/tI9h93bqyMHgjTuqd9QS4w9ajkV4L56XP2x5ZtqrkPficIMl+f
qydXLfOk8nJau3vdn+EQZpIUQFAACzwS/vkVSvDSdAndMJ/zL8RSPMYE8j9bAngLR+Mexaz2dbt7sn
h6qoWc7nZKxZ/uLu9nw6O8NfFgeHTbpmdl7y/6V9rOIJceZ7CUZNPfuGnmG4O2f4g5Zvvt+LB+T+h
62S2GWOB8PbWf5cw3txTLq2eOCPkekZ+UcYfhZeOISoAABgNvjlPYKeRddiD3czevzkW+dSTf4wM
Bxc/0KW1S8ac6/XkJUVc0i2WqVmSp7lL6nz94u701utKjMcPSpSOW21wPoeCgyhV7dwzTovM26bL
dtf2uOpyGnVY6+27r+veL3G5m+islngD1qLaFZ/2NIjADo1Oe/9w6mHcM+oHinvxXGoUhklqUldLm
RwkSL/CMPPwounDwAAMCOtqvSt33L69a5e0a33y9aPJ2VvRX669bLVFtWewUe139KvWUZh56U
6JyfEblu53s/3bun26+Xg+ZiLe6RVHbg2rb71S2tfJT+5m+H38TB9HgKPefie1vdRxvdv3Pc1573Yf2R9
7eK0K4/35PH7CLt/3Ifxb1dq9H0x9H2r2jFw3Pp7Pu1rNcl5t78m8/fiEedGvd8M7Ns9hrTf/uKem6H
XZfY+POp+0XJ6L453XvJ7Lx7fnpB/xzO6RnHuxXn6WRjjFdT/rIsKAAAwE+5f6F3WD43zNRwQWBCq
x5xdm9BVbUl/Bl3nnO/pqrT6M5p5eS7pnk7G5ZnV9cH8Gfre9fjfR2Gvqc5r79gUjT83Id9bxnk7iuLc
LwrvxbOmRvOoOqhhgkP7+Vl4cRCiAgAAAAAAAEAEaqICAAAAAAAAQARCVAAAAAAAAACIQIgKA
AAAAAAAABEIUQEAAAAAAAAgAiEqAAAAAAAAAEQgRAUAAAAAAAACACISoAAAAAAAAABCBEBU
AAAAAAAAIhCiAgAAAAAAAEAEQlQAAAAAAAAAiECICgAAAAAAAAARCFEBAAAAAAAAIAIhKgAA
AAAAAABEIEQFAAAAAAAAgAiEqAAAAAAAAAAAQgRAVAAAAAAAAAACIQogIAAAAAAABABEJUAAA
AAAAAAIhAiAoAAAAAAAAAEQhRAQAAAAAAACACISoAAAAAAAAARCBEBQAAAAAAAIAIhKgAAA
AAAAAAEIEQFQAAAAAAAAAiEKICAAAAAAAAQARCVAAAAAAAAAACIQIgKAAAAAAAAAABEIUQEAA
AAAAAAgAiEqAAAAAAAAAEQgRAUAAAAAAACACISoAAAAAAAAABCBEBUAAADAQmtvFKRQ0I+N
tl5raG94n19p9PTK2fDbuiEhLV0wbdlwz3vk+e1JY2Xca6xXNVb8ba00rK8CAGB+EKICAAAAODqa52
TGOemSUMHoqjT1kqMsZ9aK+rmpKJsX6tZnHZ3aesg1asv5Wkc/F6me3bS+CgCA+UGICgAAAOAI6
Ujt/OL38Zx/XTlwM89yXbr9vvT7e7I5KvksbsrZqn4eco16jXN+IFttyXZFPwcAYE4QogIAAAA4WuiNm
r3eoezrp3LqRKxeo5Xtlng5auAamb1Qq9IiQQUAzCFCVAAAAAABHTLzeqH590pVg6NpryIpbm9Orse
rX/7Rrehp1Vr119suC68NKtPqCtUJH1wEdeJ31CKsrGjyeYL3S6HYYzGPXj8F92fsp1ayzrDVXndeO3Ul
FtltujOpfo/aGXxagXN+yXjUo3vHbQto/dH0tcc5VoEar+6BWKwAsLUJUAAAAAEdEWep1HdJl2Bu1U
ytJYTVYDdRep0K2gfXN1VEBZlNWCyUxyoCqjUhpcMIpOxQceJ3F3t/IQK8jtZJRr7Rcl60YnTuHwlEtel
8JVbak7hZHba7KRqMh5/wEVS4M1gNIcvwqwA5pv3M+hoNUR/i5UueiNLhTJewaAQCWAiEqAAA
AgKNjzQ3p4vVGnVS53pW+qgPq9ax0hK1v7o5oh1dLtC/
+y5tyzkv7etJYd0PBqrT0a71td2qyPjIpNl6/F2OSpvaGePmv0a6um3ga+6psW5/r+hNFqRqm9n5iDc
MPTjLVrLnHV5b6hcF2Jjl+67VuGhvWfmsrOxfjnqu27Eadi8A1AgAsC0JUAAAAAEdIUTbdGYwy641al
bNuj8nKaWvJNWL9/mFoL05zBnqzXmhn56Lz+t5F2dGdIQPD3I3enN5rB4QPix+t7aeGgTCzuHnB31
ftfDo9MAOTTGnVs8OTUiU6fuu675lBqKO4dsYLbDsHXf0saOhcmfVeOzviZq/FzT1n+9Zjb+QMWgC
Ao4oQFQAAAMDR4oVsGfVGLZ+Ukn4aMGp9qKqcDiR3JTnpp31ix33dA90L01rllguwH8bwdve1A0
6dSBLymT0vz8ha4EuLsnbGbdi+HIYlthMITDJlPQudTGqK41fHZNc5DR3eHzR0ropr4h2yuodK7n4ja
rECAI48QlQAAAAAR8xAb9RD5+l8K8qJU/rp1MpyMn6am0BHRnTmnE6i8DlCYFIpo85ppLBzFSw5Y
HLD3NgTdQEAjgxCVAAAAAABHj9kbtRYvTsvXYK/Onhx6Y8iHebVWhx7biYbtTyercHa88cfflg2z16lby9
Ss3ZpEcVP29D4Gyt7amueYpR8Alg0hKgAAAIAjyOiNmpQxjDw7g706u3Lg7tTtmVk66QWAo+p5ps
MsJeDXAHX05KJbmFROSaIqAdNKcvztXa/nqR24unVRp7iWaoZ+1ePUnkjLDlS7Xi3W0WUEAABHF
SEqAAAAgKPJmIAoTMlPDo3aqW3Z8Kapz1ZzdcObqKm94Q8/L59ZcwJAszZnc9UfQt7e8Gp0FlIZV2
4Gzta5WPd7WfYa61790aSTVU1twuP3A1djxv4k9PbVbRDscRoSdAMAlgYhKgAAAIAjKro3qjlzuwrp
nGAubi3NaZWlXG7Kqg4D/dzWmOFftd+ozdlc1cGh9+IREzJNorLtD1vv1KSk21XyE1S5kPuM9AmOv
3Lan6jKu5bGBFRJjDgX5r1RPat7ugIAlgYhKgAAAICjK6o3qqp7OVgzs9qavI5mIqfk7J4xPFyx63gO1Di
1a3MOvE4Je+2U7GHrIcdebVnr3eHxeYt9/BXZ7psz/itVaZk1TZu7Xs/fcexzEVYMVW8zrewaALA4Cn
1V3AUAAAAAAAAAEIqeqAAAAAAAAAAQgRAVAAAAAAAAACIQogIAAAAAAABABEJUAAAAAAAA

AIhAiAoAAAAAAAAAEQhRAQAAAAAAACACISoAAAAAAAAARCBEBQAAAAAAAIAIhKgAAAAAAAAEIEQFQAAAAAAAABGEvn/AZM6nr53azj8AAAAAElFTkSuQmCC]]></redcap:Attachment>
</ItemDef>
<ItemDef OID="q37" Name="q37" DataType="text" Length="1" redcap:Variable="q37" redcap:FieldType="radio" redcap:RequiredField="y" redcap:BranchingLogic="[q35] = '1'">
<Question><TranslatedText>Do you prefer Life A or Life B, or do you think Life A and Life B are the same as one another?
</TranslatedText><redcap:FormattedTranslatedText>Do you prefer Life A or Life B, or do you think Life A and Life B are the same as one another?
 </redcap:FormattedTranslatedText></Question>
<CodeListRef CodeListOID="q37.choices"/>
</ItemDef>
<ItemDef OID="summary38" Name="summary38" DataType="text" Length="999" redcap:Variable="summary38" redcap:FieldType="descriptive" redcap:SectionHeader=" " redcap:BranchingLogic="[q37] = '2'">
<Question><TranslatedText>
Alzheimer's disease - Description #[no]

If you have Alzheimer's disease...

1) You might forget recent events of importance to you. For example, if you are interested in hockey, then you might forget whether your favorite team won their last game, although you may remember watching the game itself.

2) You might forget the names of people you met recently, or the names of local streets.

3) You may no longer be able to handle your personal finances or plan leisure activities such as dinner parties or vacations. Simple decisions such as what to order in restaurants might become difficult to make. Your ability to concentrate may decrease and you might have difficulty driving.

4) You may start having trouble finding words to express your thoughts. You might need to be reminded to shower or brush your teeth, but you could do these activities on your own. You may need to post notes around the home to remind you of simple things like turning off the stove.

5) You may remain aware of the day and your surroundings. You might be able to travel to familiar locations like the supermarket, although probably with the help of a loved one or friend. If you are in a social gathering, then you might have difficulty following the conversation.

6) You may continue to enjoy hobbies such as puzzles or reading.

Now consider two possible lives. In Life A, you will live in perfect health for 2.5 years and then you will die of natural causes. In Life B, you will live in perfect health for the next 10 years, then you will live with Alzheimer's disease as described in the summary for another 10 years, and then you will die of natural causes.

</TranslatedText><redcap:FormattedTranslatedText>
Alzheimer's disease - Description #[no]

<p></p>

If you have Alzheimer's disease...
<p></p>
1) You might forget recent events of importance to you. For example, if you are interested in hockey, then you might forget whether your favorite team won their last game, although you may remember watching the game itself.
<p></p>
2) You might forget the names of people you met recently, or the names of local streets.
<p></p>
3) You may no longer be able to handle your personal finances or plan leisure activities such as dinner parties or vacations. Simple decisions such as what to order in restaurants might become difficult to make. Your ability to concentrate may decrease and you might have difficulty driving.
<p></p>
4) You may start having trouble finding words to express your thoughts. You might need to be reminded to shower or brush your teeth, but you could do these activities on your own. You may need to post notes around the home to remind you of simple things like turning off the stove.
<p></p>
5) You may remain aware of the day and your surroundings. You might be able to travel to familiar locations like the supermarket, although probably with the help of a loved one or friend. If you are in a social gathering, then you might have difficulty following the conversation.
<p></p>
6) You may continue to enjoy hobbies such as puzzles or reading.
<p></p>
<p></p>
<p></p>
<p></p>
Now consider two possible lives. In Life A, you will live in perfect health for 2.5 years and then you will die of natural causes. In Life B, you will live in perfect health for the next 10 years, then

you will live with Alzheimer's disease as described in the summary for another 10 years, and then you will die of natural causes.
<p></p>
</redcap:FormattedTranslatedText></Question>
</ItemDef>
<ItemDef OID="a38" Name="a38" DataType="text" Length="999" redcap:Variable="a38" redcap:FieldType="descriptive" redcap:BranchingLogic="[q37] = '2'" redcap:InlineImage="1">
<Question><TranslatedText></TranslatedText></Question>
<redcap:Attachment DocName="25.png" MimeType="image/png"><![CDATA[iVBORw0KGgoAAAANSUhEUgAABVEAAADYCAIAAABtBTQzAAAAAXNSR0IArs4c6QAAAARnQU1BAACxjwv8YQUAAAAJcEhZcwAAEnQAABJ0Ad5mH3gAACjkSURBVHhe7d1f6GbVfe9xzdicsTXN0LQebUIzySEwqIRJpQczLXUCQSUgCFY6pE3I4ZQfIyR0CJiRoIdzoSglML8WQS+CEU7OGS8MUk5QMYG5SGBSApkLLywY+V0YMhAvfpUQfpUPK5SPe/tfZa3/VvP+tzTez/Zee33XWvsZ3Z3+Jz+Jz++57ncAAAAAAGCOyPwAgIlc2rt03f9f+bu+c/d0W+Oo2v2aa2LNDLm3B2f5T5sh0Ak9Nt8mwe6cUlrXVHW3QrAR2nydkMPxG8Lrt3xJ/Rm4x8R5V3+zzcP71+tv+YNdXGVswN/a5u72d2Zjv4tm9vEZs+6iRhEgxN+2Q8gPdG/QjxEHvfSjHmu5X4Uw+6hsDgEufjhHzm+S16u4FpjsLhvP0e3VRj31Fgy2++eBNXO2KXCQbPZ78gGlGPPAOGc3Xg7H8rvXGV+Qf2g/r7N9aN0BCXDJ1sY2ikiGeVnTjrqaeyy93q4b6L2pXVVbMfsaPIUKVwmbowLwRoMdNzJF5GfSqnPt9IeW8oyj2F0VBXh2WHzxhOwUlqc1/cfq4z/ue4ZHmVQYpNXr0wIfLZTmFAG8cCVrKldiONlSkt8pGI9f2v4hVPjVUXfc+mxXJRLUTD76BXNupAbkHPUFWOX6OxBbxXwBAJedDGaMhXTGRmGGCZhxK+L/Ewhq/zEXa5y6lTO6UMuk0/7NsBWu7TM4y6pPzMgaqm+jT15WRB5ydA2QkFSfV4+4TJnkFSSP4BDvtqJxZ7x1NEuZiNjGqbjNK7Tl03nCt3mBvYQJqCZEYYBhN5WEq2ToVTwO+p+XFrjG34u8Zq7+vsV0DfA/tyO0Ys4rqHbQZ8+6+FgzfOGqPX1l/HaY27VAhwPS0v/ZI8BaBTZ6MJCwrCQ4QYgGJQm7OuoUoGdfUoebeTMr6G6GUOCjGumI7yWvBGePQAIKn4cuFVthm+z3FO0X+AR5HlFDbv41YWh6MBHqWWxUDXHzhBeDmgcM7Imnm4+SJ9mE6ZSuBsz3fEp8TWqsQw8CAlHvmTvelWTnKPYTT3jSZfawrc94M+WjN64ElIhbowk3GxQVTmGDHqLjMCwjLg0gfb/2vQy/IKlpI+HXK+f0vXOKGf5anSF/D71y9lKUpgTJA2h6FTupOrVFw7rjMsx1GCVAF2vpmZO5m3tMtpjO80FKbrirmkBWHt7Jp7PJ12d1SyaaekJ7WmrhgEdBJNEkOK9pYpy+grx0dV+Ap6j4nc/RaPqrnXXBa9Ip/MAKJSKvuqGwQ0MZHHsW/SwRVNKlAoS8Zfj4/PDpsbDMr+SV/AGy57hijV7enWSAM2M1bR4IeGK5FrpHC7GGDkRdD/vgnLGMYwQ04Q3AgeE+q+b+hhnYvyRLS53eg01q9JEjhIG5vj7+4Ygd0N/PFRqcgMM5Ce8NNgA2DqhcUtm7jNlAAAAYipAK6lTPtIxUwL6rUVtDyCKPlOp6hhO0BRd4q7L/SUsEMAUdbehrZqI/tIY6zgRZdWpnpEMCuZpo9RpVXEllx8ZvGLlMr6rAjP2zHp3NwxQnv+6x6NNnvgpjpoeVqmZMT7ZiEnTZODd9OMOXyBcNMn2k5sQBUzkvqoiDFTvrHUMdGKXMj1e25ulXZc4DavsBpHwR4vUJG6x+mXg5mVdP1HZVc4k+B7LeAnLd0hBcKl2SGGHMprdp9POlAT5TUqdvK7qPdjrjUzyPGo8yBmAsdmeqFdZmjdwjWq+6xLsyQoRjvkIUJh1rafHvCFBDCp7zZQzkC1mNSsfTTIYBTOBfebhi4QobNdOjdZT6oPH2eTbiqtkDlbNTY0rWsR0DFmRr4BMpL8EIERKggodiOWZBuhaVMbL29d93aBVMxOjZ/bXB3bWkpyjMrLSDBHBGvo+3pfI7EvptqrB0/0u0MQzU5dsZgrSAlaU3+R9YNTpWH9lpSgvRhxmWFSr/GMQ3QZTUkSvUVEDiVeq+/OYCIG5KiwnabD6zvZjFQdH4M3hVgZ/y8K2MSPsHT3Ra0cExeJaB9w8iMLDg+6u5G0kn1t4gjRFYC1qL7USPsU0GZFdPxoD5VOpUE7MwUAEIeiaaKfOYgSL/N7aJR58N8ooRXF+OBCh4akKNVYZrWvFCF7m3sEWJGR5XR7R2ZSFAtH5MY9BQWdE3xwNGYtjEAD7kwaoIC4GRo2JzeaOuMUcy20m5lC+KxXw9zl/QklmRwUDoWa1TX0UTomppOdnSfoEe4RVXqJ0F/eEgFz4U58hIQJV8PBSfI0ukqJEv66pAnnQdH24QOuUsF9EZgFUjRU9mz/6bodWcDmpgvVODL6E23WJOGO2GTRN/R3Dih8+QEzMU99lkW/LkINi1gsm0i5gyh6OaZxAcdHRkJNbAqaLoCRNkTyeh/pl4MsNGLTasSwYcPBbJBu3ZMxy8+tcnzUlFY0/zWWrHRVSmQnxzHOzXqB7L8rYqxv7GSpRz27dUgHkd3QqX6jLsHAG8FsI2nLJRjHuIVM/fbfSpdSNCwX8LBBqrGyahR0p4BZyNxgVjK+EFrRFuDotC6yh9qbO2b1gJI1FYbnhxr/Hl8Cc1u0W8gb5bDqGjltw3zDjvcXCS0yI2bh7UxhqpwE0FalCaSrFeaBRNglSaoxHkOnFmWmkuxqmM6RnBBvcOxM0oXV7W5BJ3cUdehLdaJBsq74NCztSiqeVqZH/ZiR0Y9xH+bsFaVWLlVK4NZLnZTAK4zLQd7OEzwGbcU9h9EXoKWtdQ0gf2EC2N+yv+ePHHO5dsOzi5Te+Y0VXPZ0cFfgc+eXrYcttb5Lk8R8p3htLs3Zi2rxM6r3W8tcMWz+nC4VW/eT6zOl+pL4xMiMWCkpjpcZi1iYhGKr85Ed6gRE6ycl7HMj2d1rNBfl1tuE/HTv/pjUsmOdbiwR/1bpCHeODs41GzBspqDVUJ+l6+5kQd7E0/tzcQUBhWGdkFGRPpMFFzBMtS8iIgnX4sU1eoDnOxBs6dVQsw6i9iuJrkFy1ir12sn0dMtDf84RlzSVx3F8AP6Wb6T5D5lq2NIe5BMQyXnwiuFnHjZXuVvzdmVtWxnrfn1Or+Q4XMN9CqhaJMF7FJ0Kw0tK7Fpoh13pu2Ak1cPa1qbkwm5Ugts34/7uQ6FR99KlE/20KsvnV0w1snghw0TQIjsvjNW0wl3h4Ks1Tc2k6HH7/HXY+/4qvOaxGF6NHN4HgHXqKb6xV98gaptGp1EqAhsHRALBf8nyVn4Ugsd0mQDvyA+4vM4RMN7P5hF00i8asJDp2AW8VBgNNzFr8UtAGIuyNHt0qfkQ3LNdNI5RXQXgEKFFOV5mRXJ5cWy9/vKFmWVKwa8cnOwJrwRnW9ap1+fv/tVrbZzQS4kb3MUwuwmNGrTo2WtXaySi8Y8mbp+/O3SlUTF1MCl9jGefrr3MfUWtdlCDLs4HnrfFW3RvWIj5xTIRX7E7bB2gRqhWX1RWZb9QaYzgWGv+i/h4n2JpUJKb4OYoKtMk5qvMBAlrmlMttJy6W0orImlMr+CKh1I7YHTjtQYrgRU7a6aUz0GHW7Nx30w/Yc2OWExkExiPCiVCw6c/ZjIRgjX9hZ+pfUwA1vQW82nHZD8dEG+JoXhjRTI1OrMtJJIQZQPmB9uXq1wcbc89BjB4Spq2GvG4pQt9s5tSH2gjUYwXw8Qx0aLz+9xZoHs4DAxfpiV13sGdYiVqfLYPm7ZStHHXJebVvSXx3RmNHAd3UUhaEH42Fy1bu8JRzoH4jQ8PtCCvijeOFGGu7l1ZEYBX9TftJAO/8WWCaY/wGSjJbNX++6KDXctaVNbe9qmPgQxPzDLj3qMK6n0OTxbvcpOOavSgNNyHiP0qUDpWZgvqBmwvhsbuh3wdzHcAUP1gGwf/P4yEBhG7Lwks05UTyfKy4i2RTl7n/RDBWt55DdXMzlBQ3WK+xvrQHk3/NXnP3+xOe5C/zG+bi4OfS8HOSnSfNBQNCo3ibx5Y9UkiV1jOd1Akj8NPGrf/CvJ9nOwaE0r2ZkUlVSlMVDzoHvvvB1e0sm/fMLb0k0S39sQgbqM7vw6GnIMdrE+SyFIiICeKPFc9X2KnMNt0DsrEAWzYJRL6cNlQV5Eu1zJ0fgjiOn/CdCRQ/3fYhU3OFDxNzj9OvsYeL98bY4gTNs6GEPafo4I3SIdd9YI8lunmbTDEBpdEEMYQPZmjzPVVQ37qLUpoBVWXm6NIt1R3tPy0SIRkC+kevVBAhb+lWpjSCV9C7/U3vtvsZIgoG3uYm/ExfTvTVmAjdp8bhlPdrjYIQn4NtB1rMnSNmEr85l4mdEsCU7ZFVcNPkHThqtBWPlrZv/NK1NOh/H9vFKt/sNOaTIgzmafxPBhDQ5ZKlVDhHGfQAPJ0nQHfYbXLt9YJiVZD+61Ff09fDZ/1j5sUSz+oTLZ/9rAxUQ7oJPf2EwoBqQsq5bWb+tJ6nEmyW3ZTWEHyL5oDfqkvqzTVl8xi67P9dNlibVY2WncKP11/u35z8G/hHdynR3GlF6H1Dj7XVtd5Xn9DfGN+dSgTnn5l+CxGYpxJWA4ywvhW4t0/dJMq/GwNvGW9wu3vIzWSgkl3KMQvA7lfHtp9U0JjPjyFhqmO7ct+fwrW8/bH/0iNN/ysjAtdftr2aUtZTUDtJXl0aIeOLfXT/lNxmGW5+iZNG/rD8wk3dgTGb3Tt4OkJnoNOzaG2W0d/n4ZsrmdFcodYOzbqsjIFkefPA6dqhEwXXzlQiBNXbDtzG7L1wPbodSj7ucMa+tkcaJl0Ol0Ph3JL1Bzw96zxmWs5qbxp41Ly4lcJKNgM4bgCCXQ8+Za5NewIjB4RB4dwWSYWbiXyl2/VKwhGqj2r0lvU4o0C3WWSvhbOuyWqvkjrVfj6CkpcN7KvTYrxnRbcRUk8R8PH/GaSnbU61OuUV6pvHTWV/F7IdpFY0Jg/QmWl+PDmVtjjRIhxWgmgjrwfMwQnL6u3bYUAAYz9Yg2+vrUrr3/Rl5/FJ2ddelXEOPsB5Tk6qrqCmFEgSwVLe53AfG6C7yYmeL5Xn3fZ2PfmGgvjgLQyWzBxnBwXvgaJyehPDeBzkQ8DpRf8LL+gLbUa4zfntHQGL+xaA6W3Aq+J9BDZ1ppv7b5JV1Fpl9PvxLqYXvr5Zo4JyVNsW8QXnbBoyP/QVJfc6CP1o/g4C8dLmUWGDDwydkFU6ptnULIH5zLWBfSmI4vsrkkIunvZfe0dy3BYPEmBuTr0YaGzYs0Wk2/RkLnxcHq+lgeTddQkMjrzf2oiKRvbxaBdcpfH8Uz8H1BOJ4VB2d2KhtHk7tPRJ/qv8abCDozE4hEuy3tahATR6cG/ZRbxXHBp9FG9WVBGMj74WCW9N7E1HMlB1TDE/D6wfXzG6N7HP/t8ds7v7WZ1tjVu7ymMNeqmCbXbZuCnn+U/AWc3lpdJmwXq7Ap4GsxLXQ1tXm8nyU2L/rFg9mxiELxPxGXjUqZ8Ksb2UXpc/ErXhaQDVbkhaiK4PqnAGvz3ggg3PntlaPOVnVN3tgnlWbw6Mm4G+u3+fr1/vvf+G/zmvOV0fFS+QhsVvVLQAAVRF4Lp+nXgS5ZpfUT63YNyN7yXh+Ltbrq70d2qP1DNSBFnbgYwXezY8XWyeI3gGZlIeHe1/5cOZgHu9C99R8q2/mePDmlqm+ku6WZ6nbd5EqtpoHt7DXy7Vse7TzMBNtmVv2MpJPOVfHpx6GwA3wA3EqoInUhNpJVX3vKSMbwjZuXfyrIoZEpmXo+Xgv93t8i+FXx15pxZ9l2X61YJvVGGKHkwyZyIWFNkyuqqyHHs9kfS1yf/75/XJoZmCAJLoy0UYlpsSDBR33AAAD0MSA2D2s+NSUZD4cBDq/PITp6ibmLmWhnKZMRBAAAYSh5+CRWPeTKJkQO7FkvR0f6v2c9v44/u5O0g7AAfV+BO/pHz5p7HX/22euOLPx7ffbLqafdM+f0Rqrtu+/nX9qu3/z4ebI8VDRc3EZebrW2PDvT9r1lxGqNJt7T8YwRyR6DGPKfNwgI0fl7d3YVGF/6B/4ujeZ5E/5QMlTJ+UYXNt1d3+OzcX6sNqtT0ub47pfuIsG4T8k5S1RDAxiZyXhiGfYR4nUTZ5b5lmPJjU8BCgkwnWpLLlwczAb9gaT8skdjQ8Dh4Jqw9SCw8Kt44Lfp4bywfDtzEBcNMUg6Hkfzdzlgd6i1DwwzSKoFqPnp8eOyzVnhUcPyAc5Zh3rvo1WVfHMoKTcbPIHKu3mQqnUwdQs+p3ejscw+bc4d5dAAhxjaSF3zY/+wN3ubaeAeVpFZoEuCF7j/UgyT3pedelfoYEBG7W1stdEKrHbmm4j2+9WTU+gPOrcpV+KcP/Xw8k6ADObe2uCTM/jA/Bgc8AaAzvq3k45LUw/Dn4jmntGUNPCAPdHT3fy5jNQ9AABrwgAOpeBbAIWXZqFHrLvsDKY2nd/cTIdxt1nfwtPLkNz8bv3R9HAFJ2TD7wLH/xaMAUsnCMxLAKK03v+Mo9zptwFmcnDDzLIVxoYt/uveWtMZbYbSb3fmC51X3QHY0+KFsLaVAbXVRdlKYEEx4uLlCjwqtHmEytVpf3IHZL1b1npwB3pf55PGeHsZhkZFd9uQCvL5N+PvsfCS9Oz5wqJxrBjR/VyebMMGlSsySgRwcq8sB3v5sRx/ANyjUJMWyXbhpJsCuMaj4dyf8+e/Ws/Y8MyOKvXS4Vn94e0rFfvEQaFoWlVWk1eNJNJLlNpdUZx1dSoDUdqBYV+Lt3ppNsyz8xYmaQQ5hsGIjmX3AxD1vmKB7K/ruhBsYnZ2lU48T/yYcRcb4uI9C5pcBAuTdNEEIMbF//33NhTNzzxUXeLcSWOEyVgnYZuYSxP+FBoOQ8lRFqJTtmBiGpUFIqdkU/Nlr7sMTjT6uzxMGhUKwLKTUsvX2LFJxfXwvb8yL9RLflR2H8BtaFKQZZYdgTSuc8QVvJqqn4XEPtgV+F4XnvNgeV0R04aZ4c9dn9uXPn/3TTNnnL62D0/NW2t9m2WiDNTAr6PKRwU+AD7bu7PtJhD2REEu4eBjV8TUFdSyKs5zYUA+B0MjSHY4Z+6nqmv4Cp4mwBsi+i7eJwCnU8nXKuMs3xNnRrEJkPXjn4kFzIxcAKQ6u9AxZYwEqMiQHnsKpJFYkbEbn1kmBn+IKYNLVdTPh3/ZwZXOeyhKfhgPCtKlWxrrFrjSHv4btCh0JvEAcDVv96bPoJ56k+YZ7aA8A8ncfGR0wlCpIdNqj4JqYEmxqfWhYEAAC7lfl2w/A5YXbm8TjGh80TJl58sfjxnbwlzBTMiaCt/Nj53zZHYqp2XT/AU9bVqTkCOdQDB17LRJTyoHfZkExfMlWo9VA7RubhJiFFzepNd+EJxbLwSN8hHS1cvfxDiYJaRiARUgfWk1z/Ymo3zcsxyU8A34sXUvHk9CDlFUpT87lh9nSRb8yc25MK8iSRD8JzCyWWHn0eQt71CVh+A7idyEhepWgQbSpJWlf0srtbOOS2e51OZ3tfYIbgL4gFeLFNkiwzxZ1kTXtSgm8WTxhiEptmkb8CiDt9+7PiGk/T6fh70uhGZhi3u30rJUCd8xO2vRhgSJ/jf/XN4rQaCd4cnrsmmfUwmOeFKAWZEAfD5N6mHaQ+66Cgh9gDnydvTLfcdT+DM7NyOZ8OxhlVSrNWB+4X8WXuAtNJyi9L0bEBf9VUwmwzSLFJaNjfpxyjMWLWhKQx2kRmM9l7Y+RoDxe10F8bu8ANqTr9iRkZx7GeKDyjuhyEMtDeyC70kmN2EeqlPTNHeiDi7+N5P4QoE+KIbqSmrTjt+U+lyibEnqFs1n1Ht+7krvNLMVBOBQ4M5KxVKLR1vgOVxwfj8xdTTN4vOb1yYOg+kHRyVEdI5s2tuswLt0M4Q0gcNBhl43XkQrQRMRNljKrMcXJnc18aqYv+lRGA0j0HwH1A6KvyajOyjm8rXkPPDL8FpD13N23tq9OY9Pwvfek9fTtWdX7YTzeWGcBNx3a+D0V4H9V5r6Ld29bqTKn2SK0mZGmRtsEaZcmLbjMpzJqFW7r+v/3xL9aL3r8YBZXLzNpibE8uyPh1zrjNuTq+nQD69TvG2DQsNxbDbDfmK2YErjf9LMKn/S1rcdg2L2ySzatHP7tk2gWr7rcf8QbVdEqwmfk+Q8LsS/mUEtzhJPR1Oa5LsxlALvuAqm6yJFiyEh3HaS/8NFtjQcJfE2/lLPL7OhFm+PHrtMxWvoY4nCE2lK0b5bKdDeY2v6vmQXcn8gk5vFXkP5PTnf5iuG8rnuuTW3H8nz1qn6gQQ/X2Zt7BbyHW3yrz4VUmuzG6a5Fh5NkqtOqZXj4Gp1DrcQjvt8BiC7lJvwz/BMHdlkoxz4o39XnjGx+Rz5R+6IeJtuSL9w5UCvqknOHSmfwR0WsnJ3zD4hMm7ylfk8aWAmJcf8dqrJEbXlSUmEtwd6i+W3G2A4hcZCdd3ypHlNqEMgAKzDRhB4SkP5nB3Eev2uxanx2Nfk+AJUnFvJn6F8trCi+mDGB5uvtgIgc3bwdRrq7R5ojPUunTfhjn1Gxi9e5d2d0NpkJEkUyvbVhNZ1gAqH4fyKWZbjYV2d8x7DMb4wYTGPd3RALjDiOuIyJqvunEBCbuuS3EjgpBTYu99P+F1LnQkXzY93l7ZGwjDgCZOvnQaOaCNaW/sqMV5qH2XpJuTkHHVZ7Ol2o0NWGN2hhjOYhV6uQymhIVVvdfEN8GMbkfzQrrSa0qmZbhmz5U5EDw3vVuvSjFhAizZzCNdNc/KO60rVdQCYFNkmj2XtwgqEajPmFmWv5CKdQ8y/i6fmoN4r/CLnf+gMV9UJBhZH2FNhRTRzpSFbGbNGqLt0vYRZHlxULuBN0nQz4xl3b5FZFzs8rzOndAcmXQzKFRzmBGy5HvEBsZO5yWhn3/YOp3m+KuI6kA9/s8vR6E9LaTJZG39+XyFoLlwXG4PXYnHc8FlhvhzsEzOEp7m8TxrjdDxL2/w6N8ToELmp30L+nGuq+NphxBAG9/+vbCaeY7e3H3eVbBRQ0yK5ccBx1ubhjZpSTzjA9+CQgEeWTf8FZSJZXVvDY+Hc+QIQ3NWrfSgR3ekFCAymzTXJSuajndCm+dA9Kr8/NfYM82IH6tXMTOhYCYRi6sUB9B5dJwRZRkP1YQkpprUyD4mlUuKUHL0TwhalA3s8Fy5YzF+pf6NuCt2GZ+MltpJAh9YrGKyDC8ltvTWfXsvlLeamNa0S7ZC3tWbw9wq7FVhhd+6zEVzhaD9Jn2uSD+3vwWuwjv3Mvr/L8IfmA/xYdyYuK4MzJa6UV4aR9eEkHHiPdh4fhYBFGQ/pO0Ct7cOf+hB9HEhYLSBNVNmiF/wSgd8PlnlK28ZDAUA7O94zDxeKRSsd28WL5E0WU4XZT0VXpHFtfXpbz81q93e7AyB9ln4VwDiOTK+5Yiuu6Mcd67/P1eQvXzL2TJfK2pBmrX5GzxjEgPOGOj2Yb1cC+LdFOrZt7aR71pMu26OH9IzYxLK8bTZGkWJHfdYMJBb5YTiv6EFJkYBTrLGfabkmLHj6rzCnSybEhWbL0Wj0Wnui9jIg+c+qDNo1HjeP1UfAJi7kKXrqcvKw+ZyDvKI7dZ6WhWfLTBSWX6HhQZ0+eENrkkNQxgOd2fCJ+z3lZwb3Uk0yPeq6cGLdGkbhbdV2K3VErSjI+xdgFXjPhCtSnNdWFI94+/vwxtsmAIuwyLo8fVDldtw7o8vv8pkNrS3X8XtkG2UsUBxLnJGGVfEGuQbtKVmOUUx1Od4i1e39rUSGAUEw6LrUm5tKkhvPi9T9QSZfg70pUBk3XD+XdJRtF71A8bAGwMjWf1ZvdYWmPuG2KNCGvXa7ymHqW9hBivLqA7DhPHQO3AB8ESDQdZJq2rf3ZS81xKAKgJFhS5b0VR7rmk9g4+jgVZGqm3CA4ms2EhG1FUjnjPeGkQbDLgQAtaKHEiMUZHC0HDBk3ZE8eVLvSmXALeg5XnAtDjAU3Ax+VFdJavyl0HhlhrcYsY5mPFuZVMVHRm6E47TJyzdL6MxQzyUl6nAjEAV6BmMoXvfdhKBoBRbyhmQQSbKKm0gNyCS7u1tRvqHHQzbp9sfuMQZJYE66P4rHBGC9xYHtEVUBkqlSpHhKLuA+1T1dp4dTdXsiDiDvGUB/MBkEzYVPhP7gT5XoJAnm2t/fQ6W0u1ZkrZy9vQm2mTw0TMEGZqnpmaqNpQtt72KdsF7Ff3UqnAHd6RtVcnDVEKcI1gKVWuq3h1tjUWxBYpf5ULuXIMsS2V9UrP9bE5xtDL83E7K4D7+XF9vNd2SpIVbDTDbE16uLrxQrhmnb9p9yIQ4KtQgoEX8YkrzjAhnRbUG9Q1ycRpOgFXCCMYMHDKzRLEb2Jl7LcJRFYzPxs1qAyFYj5bqvMp8HtoWyIzWuWXY5ug9A0JRKxU3BhodoLRqz9k8k8pRSHhnbT4H5DqsA4nL7JPsI2LhD1hXtcUF9prBUG1hWl3V1LK33GJcfm6ZHqwU7rXIPW+kyEVgdJqchuGm4NOmTb7o93kN5VYoJtM6aWXsAWPCFO5mZjHpumYSXOBnGsD8W5rUf7JSDzbjGeOaqYqrjlCvrafwwOxYYrGaMuGmzV08CT1PVV1gcgeewrgKv/1LY8uuZfHDH/0ZIyEN4iOHEYLJ54BW3nFBkz7l0w5D09HPVi5yJmxh5GnzbYmfBW4jq6HeFh9ktl6+dU2zKi+qp2ey3f8Q3lHLdt8rcYh0RSI6pzZ4GbzxC+cFVRM2xkgsBH3TNk0RuSSrUAL9ic5dcgMcvKc4oZCPQaBVDnwVUEgEQ1YATi4CGe7vYv6iVSQmBJ9Jwx2m6ryPk3QEh0NZfmsHePd9anDnLMeFKiQyWutnapbpwd7d2gWlI8Ypr/R0sVjtCgKKPn8c6F9XkEIp6ZYVPMrxS7UxGYaGHgVgQ3OHmN4QoSoK5Lrxy4m1eVb2ZXq0nyf4ZMyPv3Jh8cEq5lLlO2GTtxr0Hax8OCu8G3gQFBN2F3Svd0ELqHlHlQPVIx4Ly7BZ46YDzyuFRNoYRIihpPmAPIbfVAuItj9gb8vsq6vdvw+Cv4iPTZH3J+NZRCo7XbKBgW8nvVmUk06Q37yyz8D07q0tVAr5vEQW5qZ2t7aGPWoX5SqSgfWGyoKZZ5O2b2KcnJ3GFaHmzl2q/98BLsSMKbk3jhtGs6bFcQvu3KdGyEUbdLWE8xo3ZMLJhb2KnnNyZKtXz31OQ2KV4FNeZs2vq2Oyk8f0wmGwH/QoeKCSnLjqgKqXaIQzxoKV1OO1Dq+OMAJvz1WqnEvyl1ZTn+PoVgB8iOTWHfPqqS2rE1+XRnTbfe+cRHvVw8Qz9oewujRdBm5MaUhOxXHrAvL9pN7gR5JqRhUiTqcTH3HOB9w2d5a1VhkK4mtPxSnPNrHVpuYHnlrEP3WlFa5vjxHWLi62vdJQ2qA+sLHFEI4gS1KRy3q6l9uChmXtmTzjb2wgXJjSUp66rB1L3OmbL3ZhHWVnnYZ5eY0LPVCx11fIPbuHTZ8Qml3wCZjpjl5aNLU1yi3DyAPrdHTBFc7Z3b4Hr13JAgAbSnt2dMIhLdgCzZ6twTHtPPhOh8ZnFPB8AvFZ5K5ljJU8OJ6jfyl1wpe3hjL4Ed0Zk4rQMBQ8RlZhnaXl0j4lF7UOV6a0URmo47XPoeSDc6D0OUYcvhUUA3PR6AYB02NjwGyU8f0E6n9xtcQfn4YQb5PZc1WRVjLwmg7ZD8Oq9dBkMdhDbRENd5dDKq0vn9AqJpEjRPEV5V0uBzN8n6Se1PX4dZUh5YSzjnxO4VdjoVYa7WntwZnXXIvfKT5KDrCMwHGL3kvtlEbNT7pVuU6sFQdbZlGD6xpGA10HT4vnRpC6HPbp3bQpP+Mti94xf81vO9BnBDwUJiI5gM+0YqBlEDTJyyOK2Y1b2aqJaMCRsOmBvb81tYBVcWW8Wdl0PknUUeePMFbdXGfr9jrAOYz38GsEOhH0JWHRzK2LmOgxNXC+Fa4W5pPgh7VTnZeQjdf6yESM8VqWbuR6Cz9hhhO8VFUPXBgafVm8sD7+q2WSwJA03Crdh1pWYSQwBsSL4LXm2oU5VJSTndi6nvDnjDgMRvDRsRRPUf6xXXq4WDkEkjjv6gXu8vn0UXdMwJbaVqjJqNaowPhmqtfSiZFtcfwiYbVH4fxVTeDuKD9SHV9C4GBWPsrILpB7Q20jLQCLqQXSV83rrZD1mEAfP3MH5w2bvlKyJTGyz17PBGA3KLe8PLLyngsqfBkVuvnKZpBZ3pAn2fEdLFLeGt12yrnZ2dxqQOSQoeyM4CN96WTFFXG9g1UrHW0DBnI2uMxTmUU8W4X7TpaqbXtzgtGx13c60D89OkdwaRc7VQu8QpbgQPXB7x3Hr9V34XWfb/ObHhcpB5k4SmdsT40A94HaLTzPgvnMT5bHKMs6lAhg2wTtqdiAN8xDpHZgujdzb9PAqrc6s7LSVrjeSJ2aDoCbXnSMFHBqSyYQGv1pI9hgNqdXbVmlqPqRHjSh64S1Ai1G68dJX+u6vjB8dBMm7lkmwxvTl9ht0Rc6NE7WqEPBk80cwgzs18VXiMKfTAUAnM9RIEqXLqvwEv0NrQWkAbzz4lVAQRABTQ6c58TBq1QQUUYcLOZNPaW5XyMWz45uUFHb8WPhGVLXMOl8anVtCyMVFMVWbDQX2QaG1R69j9yrFbzcBl6EErsEJhMV5/8Xp5T+4fQVXZpPqh8vqAMc8kSJTZtaYV0gZ0QZwH3HTd7umXp7VUmfLVRjSxMfxHiWRHfZUjeL8rJv3JlmD4PwIkPIu4ZMyEgB3rzWpd0gSJZLCg8LF1GmZyxcq4ZKXPWhvBpa4k6U+Pw1C1vo5bPXpKgPsfaNFv+D1yYq0oswHXe2+RhXCKR1xnQRyDuNdPYPVcFMz2pmnN0a5SFfP3VCtyV68w0Gxd0ay39G+SnDqqZaRbm2v5oynmqUD0hP2apHrW8tMsiSQl2GkuUxYqWUbv7aeTJxcxKT1Ac8hSpKDoqXv+jxWbQ4yEvbT/jN+AV9Lz9C7t3RG9lSXvr2f4ziPNZpOrp6LCb9g7GUxIENpXKxFvzbvN0P5L1hK4KROpVhHoMfLUMjQsBHV5W4Xj+hgtWVkW8SSCuG6b4a6RwnKd1Vp7sfKfu+KfrHLyN1hQOd/bAfiCxdamfAz9ZENUUKOFXYBw1NqDVdjQX8Ea5S8EKtukfEwBoYyLWOqZYeHJLVDtZfEjv3PnFsUOpHrGxLTxmQmKvGkf07mhQbm6XzVsBSGOMUJG36TtmqxvTpXq6MvChNk2EfXjvkwtPGtiJeMIfEYUd6T8jqXjB8+9YZPtnYDCQtbmoNUWlNBUhIMIbRiO5fY4ZhTc2r5cUYmOyRIlFPS9+iBXJWJRAaxV7nBOPTMpkSP7Co8oyObgaxLQgzmSXbdDrbZtsbqCp9VFEiXeH2AyyPsRNqN8UVXb/z6UsvNF8TXepXh8vcbTFDjBR04SfjF5x2L7uqsQwA7b4afC0XQXl0AUvRtvYEBqzrUVjD3mwCAEphQPJ7jXLCbVPCL78vL6vxkWMPO6nsQBQUI0tWIh6YfxGJBdI5f3Ke/UEagPsM2sgEFkBpPB6IS0/2AVexsNNOPZ5LFD/HmPJOgkAYvnr1lLwnKcwq9DRbH4aC6ELcFWIXw/LpRp8ofTDaNI6HeWAOD3XQXdy54i05cf17KBkOLqZ0lG45vWTgMJMT3ZAKn7yBGBCAhkvxz0GMhIPrpvCEBz2hJfZTlzyHgXmkYjwfR6z9dqmQHdcaM1D2mnhzqbLKZcwV7TZxV4D9ztv9pnq5ZULnvPUyKAmGABvSyR71pTG3TOXNqOmQRpRCR6GTzKqPFjJMvvCZK1oH4Gm66PnYnSUc0NQAIeLvrzVE0DMs2hOzYyXuXPF07GaCYp44RyuiPhJDg7xEcvWTpyIpC6i4oJAVq5s8ae3uP4XiyWGZP1lMHcXq6xCpDkMxc87fcjC4h8iOYuMkjr5rrv99uJPh3WqgfI01ywvvefrP5u1o0v6GJ14M1vFNIkYL8VoGvsUo3Lp2HDjmeiTSFSQWdkQdnBT8A0HzCRsWrsMUyLk0Xq1ZChSDXtoHqg0ZqqF2tAGdUI12BD8lP9uf7q2jTtVVsDqfWUUfH5aqJ0J8ng9MLBSqnJqadB2iSxdxSlq1xMVA4mmHk4BVNEG8GTvB8iTaV2RsaFNwLxLgfEdj9rWDRAA0BL9J7EH0LvUETmm86vmzxnk6SQCIGMkqQIDuxPZlJE8gbdNXB5C6Y1nrzNeKtiu9XdE3NWhtaNUw+PNr6u7Jjg4hwAGkHNkMVwkXh6IOFs4jZZHUG4Qm7m5mP7vE5VDyj4WB5TEu1uU7CgRngTvtbqGNgrcYy4a0aE8SkjUTydqqBS0N2TIJp39ywFlUiU5FCzZcW9uBXO5r3zD3YOiPTRbUx02TB8hhhKwnPWDTuHzGF1BEzxU5fX9tfd0d5LZYmKj3iq03nLgYo7nIhjj5Iwn7CBZLHk7wUqUtx3NWImK4xJ5Fw6lNZaHu4E0s3oPYb5fLWLOkEf7iivFiE1BKqr4HRhjLd7tMRC2wPGfBqMOS9H3DfpttlGUXW8d9a0nUg23cZGq1h2Pn98jNmlsgrM8DmJc1xaFzxhH3EqKpfHtLXJP0nOZ93Mk2rRg9vBwKO5Y0LzHhmPKBqBcXKYDrlIT80J1WAj3R0Hb7AVCUxzsBaRHQ9gSP1qHDazLDDmrYIMcDqkT3jjQMq38M8Y5ZgPWXqO3mXy7OBsW9MbpqbJy1hSTjtvu9Yh6rVhYVKGrWm1KEQK3I4MyRoRNQYIxm5j0

QnfemYpvbumZYAuceemMiL7LbKN+OV+8fe3Oz7z7i3e6+wEAAJBC5t8GkfzmP6WiUHc0Nyy116n
/eb+5ujmkjvQDiuHiL0PSV6lD1ZFuTf05/04kySp5e+Utbl03YHrBLu4E0qMNqDdgXRXPQOLC4ZOptJ
1kaWcqvrmlZ4JNe+pHT4nou9h27923iig+ZXvyoQd/
+/773V0BAACIIvNvg0io0qHHPCeTkOd6ncJiYakbx/0Yf3vEGE9UjL8MGXJVO2/96+yNUwOLSPKyvG
Dp2077shUGMW9lw702PZqvR11gtXVV0huSHMcdQu6XM0Ro+rWVl4BpvPzmyyL3LrbdfubDIoRP
31588onuxgAAAESR+beBJ+z0dHxSZ3UO0t31ERWN9DWJsNRf6Ls0nL3iL0MGXWXUF2d0f3VYd42
VlVXURasFS8+uyOrp6XlKtzwTSI/m9Agf6If18MxKXpiajJxb39850r32zm3ATLAxV65e4Rf1t22yL+dLt
p/94PXu9gAAAISR+beBJ+yYdKSymJ2NkCXFw1J/oR5OV/MWaIeLvwwZdlW/Wmc79BWWlYpmBkv
/Laj5xrH0g3pKt7wTSC/W38MY3p51YHWDNiQxmfD2dF3cET1zGzQTbML+wf7x3eMi+i6zTfzlfPHG
B/sBAMAQZP5t4Ak7kh26PAnI7FCdHhqW3H7eK8XB+MuQgVfFd0Nf1YjsWEdWyQuW5lXq54p7Q
Wx6wYUFJ5BerN3D6WLM1Xt5ZfCGxCdjVqovU6/bIXwjOnMbPBNM6uDawekXTovou9j2l1/4ExG8
N9v4YD8AAEgi8wNpnjgKLMPZ758VuXex7eP/8Acicm9D44P9AAAgjswPpJH5sUwXLl8QuXexbYNfz
pdsfLAfAABEkPmBNDI/FujS3iWRe5fcvvBXfyqS9va0r935mV+
+/fPutgEAANjI/EAamR9Ls7e/d+zpYyL3LrZtw5fzxdtj997DB/sBAIAXmR8AYNk/2D/xzAmRexfbbv7q
0S35cr54e/78N7r7BwAAYCDzAwAs9333PpF7F9u26sv5ku3H33uppu4UAAAAKmR8A0Dv32jmRe5f
c/vrem0Wu3uZ29o7b+GA/AAAQyPwAgM7FNy6K0Lvktp1fzhdvfLAfAAAIZP7NUr8brjHKL4h7a/fUz
ivdz/P4XXT2inysXb3uukTvmriiNeC6ZRv8bprfm3CmLr9z+egTR0XuXWy78fyRL93+CZGoD0Xjg/0A
AMBE5t+gKgm14adVPgJ1BWYUt9wVSd70nlqv/yIyf8Kgd9P83oRztbe/d8u3bhG5d8ltm7+cL9n4Y
D8AANDI/Bs0cvbRf6Uwm7jlW5FNh3e1QOeAjxqXELqaAe+m+b0JZ+rg2sHJ506K0Lvk9umHjokUfb
gaH+wHAAAamX9z0gl2PQvM/J4OQ3ZZ7cpIN2K2yPzzcealMyL0Lrkdli/ni7fzp+/
+zXvvdTcYAAAsGJl/M1TsMZiBUyelhshGRmTqhxFxNTS+GbeMPm76smaQDGfmsOaVTogOD9uf0R
fpCYrZKs7oXubc/FTptcbzHE7toXW+YnUxxuuX3s4wel3A0FpWx+jtq04GtkLp5601I5qXGX3cUaxJi9
OD54y0p370lAi9S25HHr/
+b09+XOTnQ9qeefhsd48BAMCCrZb59bN1+MHa28V6+A8EhMH6B/xD/HgfikP+UxVj1zwdnD0Nje
8fvGYMYUUtJXrbigyrz7fHxMvQilL0dcHevnnFhlYjWjsiDyb30L9lfQ/P+eak/7r4XmTU6qRnXDN7GT
wXlHsTrjwbBLz85ssi9C68Ha4v50u217/zfHenAQDAUk2Q+cXD+7pP5OZw0aCz9by7qaOM2ifdS2+c
kXaiG+Ab373WGd+5TF0TuXPusP0RWScyrNnHV9W3ojhndR7G5E3hK9SgRg8x2+Ri5RDuPI1Zmas
NDdRf6Mip1R9RxfVV6kh/IFK772Uswi3nTMm5TK570JyRdOXqFX5Rv9k+
+T9uEpn5sLezd9z29pUr3f0GAACLVDzzu5yn9bUYSaNSZswNicUhc2Gyn842idXHxjeOiW7qpTG455
BQbFg9UMcuKcZM6keLXOAMmt5fZ+bqku7AwMWaBs5CdwsONEC4Vr9Nso9zTUUfi8zFd126nHo
Z2cABc0bC/sH+8d3jIvQuud30yA2H9Mv54o0P9gMAsHDjZ/6yD+L6yf+UnQAOI8/OiOzY0Ytuj3rSjp
dv5z0FRDc9ukewYDqADR7W7Cjvrm9FQbrz6m8Szy7Z1NjijqhpDV5sw+7tjGFf0K9KW2V5g2vJrVZ
drGLegzbfLfNcJrrZ07SJCUXmJJiDawenXzgtQu/C26H+cr5444P9AAAs2bj/bb/n0d3zhF4bmFvUNTu
vqJ9WCTxbxrOb/hAlOvo7uTzj+64NjO5jDGQrOWzf01mgb0UB/Sjpvo50HdWjmaKqpXsPWayuIajz
aozwJthiy8yoJbfAO53gHHtynFq6nOrhE56QrxZCzn7/rEi8C29//uAfiZw8s8YH+wEAWKwNZX7dyxR
JDh11WT2I+jl91bby7KY/RIml+ju5POMPSEpDR7eUG1Zf1hC9fSvyUcWSHQNUndhkuxpVF9W7r5V
ebD/DrpOztOQYxhCNIR2H15J9vNNJztF/y9LlBgw8YM4IunD5gki8tM9/7hYRkmfW+GA/AACLNf7v
8PNc4xwa8IhfU9e1l6lXiYu2V2xnzEXJfgN3yzf+gKTkuyqp1LCq16ndXd8iBw2iOw1bgW9MtZzoAF2
nUzs77QBG5+Q83f1SR/Qlni1VqlMrFMurJUf1VdHHPHNUfNely/muEgbMGUF8G7/bPvjND8zmK/
pC7bF77+neAQAAYEk2kfnVEc/TeiQ8uJ0GXbTF3J2p6DwmV9kv05N2vHzjr5Lu1AE9JXOetjLDWpfo
F5ExPfoyoe2Rcx0wMT/dr2F1To0pS/T99Thymi19YX/Y37GXV0v3Un2cJRnjhEpXnHEqA8o51eQqBs
0ZIQfXDu769l0i9NJuffhGEZJn1n78vZe6dwAAAFiSTWR+/fjuEXlaVwP1D/nukUMlsJv+7THW6Ek7fv
ZITZVBSUkfsURuTZFh9Wk1iJ5+P6pnRRZ/iZYcNjSqEltvy7xO9k4s1l+yprq402yFrgxPN6uWe/simyvn
aLKrN6MNKpfYwIFzRtDVX1/ll/a7bcaf6n/

+/De6ew8AABbm8GR+fwZoxTLH9orspr1Ycd6TdkLMrW66D05K9gxSpdYftj9h9NfT7zs6K7KYZyXV2
zPXin2le0e8PPMzxffQrFjXU6+72v5ptuzJDpju6rXSt686EZtjzyze9MwqJ6sMHgRBfDm/2448fv39n/
2oSMszaI/de89v33+/u/EAAGBhNpn540lBsB//pZWGAspQb0refjikLr5xUYRe2k2P3PCVE58UmflQt
7N33PbLt3/e3XIAALA8m/w8f2QUh74mgNSFqen3JG8+HGKP/vBREXppn/ryh0RsPtSNj/EDALBwm
Znfq4nwTqD3JPx+mO6Y/q9/Q38L4BmkpU+QuzAZ879WD79pgUPigRcfEKGX9tf33iyS8yFtfIwfAAB
sJPOHBgqGp0iyj5wCxmFkfgI/Dr+DawcnnzspQu/C2zy+uo+P8QMAgMqGMn/NHiwW2aO5PnoSA
JC0t7937OlJIvcuvN381aMiQh+uxsf4AQBAa7XMDwCYpUt7l/g1/qId6q/u+9kPXu9uLQAAWDYyPw
Cg9uxPnxWhl3ZIv7rvxSef6G4qAABYPDI/AKBz7rVzIvQuvN30yA1fuv0tIlFveXvyoQf5GD8AANDI/Ig
Tv48h/nIl6jfhdb+IYZ2hVmX94v3RfxFEtbIJ1lSbcg8xW6dfOC1y78Lb4frqvq/d+Zl3f/FOdy8BAADI/Ei
Jh/x1QuamMr8u1Bo38nfFJsrgk+0h5mz/YP/47nGRexfeDtFX9/ExfgAAIJD5sRKRKtcJmSLzT2bCulNn
cDI/ynjz3Tf5Nf5mO/L49Yfiq/v4GD8AAHCR+bGSw5/5p8zFZH4cWq+
+9arIvQtvN3/16FdOfFJk7K1qfIwfAAB4kfk3IhB3vYd1jmukr0jkPnW6v8I9ooatj4jh4i/jdOdK1V/M3T
uUeYk5wSjrIusqVdIQmLg5OTFvi13LrBaqJZZdc5Zu9OmH6U6GK3oGChBjDF9UZcj8K9ExWlYX9/yA
ETCuC5cviNy78LbNX93Hx/gBAEAImX8zPMHJd7APfCbjooEBzKTO69O6iB6kO9K8FsPFX0b4l1Lryjp
D6QMmY6Fe8R3znA1MPDndWrRYqJY6bozjLN1zbds9WnHY7fBuq3FFokTu/GvGrFI3Nz0CJnHmpT
Mi9y68feGv/lSE7S1pfIwfAACEkPk3RGUeT8zRh3TuUUd0UHK6GMM4AUzqOqhLjHTVXaFGaF6K4
eIvg3Q/WaPSTSQ1smepjgE75g7s5e5Kf0QsYcVannU43dzqjWRFXz2bc4nYsmSJIfOXXZxB5AXOFck
RMJGDawd3ffsukXuX3Lbzq/v+9V/
+ubthAAAADjL/pqgUo0OMk6U84SqZripOnnJ0Pdpr2hc7O/VA5hF1vRgu/jLE100f6+Zu91GvPAszD
glDdsw94KWGMnolL3Q7+C4Zcst09fBaG/LCjCm2h0L9I3MLz1+/DE1fdfAM0R5KjoAJXf31VX6Nv9k
+/g9/ICL3Zts//f0Xu1sFAADgQ+bfGBF8VJTS6cuTrSqBq4xOOi+Fc1x3VdOh+fnU7ivNVf0RfbkYLv4yx
LsWcdAeSp30CdXyVtHjqqPDpqzr971iF9rT1R18l3hm6XTzVJe8FZNr8xQP8y9qwPz1655V0B7Y1g6
RGgHTunL1ytEnjorou+S2PV/d9/VTd/3Hr37V3ScAAAAfMv/mqFzThBkVg/qw5o9nIl4NCGA+3WVV
j7Z39UN7SB/RV4vh4i9DvGsRB+2h1EmfUC1vFWeKw6bsGcu9UB8R3A5GrSEj+1dSSVT01bMEB+6l
FrXOznTn1RA+aoj4CJjcxTcuity75Hbk8ev/5s4/E/F7I+3ff/KT7g4BAAAEkPk3qcs+VXpSCceINP54pjp
2RwcFMI/2OvU/7zdXN4fUkX5AMVz8ZYh3LeKgPZT3igT/NWpcdXTYlD1jyQtVl0rbyx3ZV2vAyKHVJ
yv66lkCA/fSixoyf80YrtFelJyFwT8CNuHRHz4qou+S2zZ8dR8f4wcAAEOQ+TeqCzSndnba0GRmJh2kz
JQj05UnPemQFMp9tW4c92P87RFjPFEx/jLE100f62rZfQYObJEjNpyBho3s2VhxodvD3XlfrSG3zNOn
kq6YXFtoQmrIdAlPF7dPpTpmvLLrJqfZiI2ADXngxQdE9F1y+/RDx0QIn7LxMX4AADAQmX+zdFpqiDi
jT6p4pXOPzlv6iLrWGDAajvoLfZcaeU5krfjLIGeeRv2uWGhkdYWeXqTUgB2TdQLUSOGdkPPpJ9yP7K
vV9+sOGvuuunmqV9IVffVsTnX7wIBFOSM489cH+umLBYXHGDqC5wAmcHDt4ORzJ0X0XXLb1Ff38
TF+AAAwHJl/w3S6qahU1TPP9syUY4QyyR3O1F/opCh/gXa4+MuI8ES7as5Q/isShVbYsehInkQpL/S
XqvUj233a4+Gd0Bd6qteSFYesLVC+KzVgUSvMX+qn5R+kP58eIbBHGNve/t6xp4+J6LvYduP5Ixv56j4
+xg8AAIYj82+aDjeh7GKnoz7zaGaH6rR+6eIqcvt5rxQH4y/jdOdK1V+Etnj1xsB4Z18kZzZsyp5E6bnQ
TKb1UfXaGNrsogczZ1j1dUb2VO/EKw5bm9whUSZeopGaf80cpuZOyZ5FfK01ewR11r0OY7u0d4lf4
6/b9F/d9/p3nu/uBAAAwABk/k1TwYfoAuCwePanz4rou+T2l1/4ExHLx2vPPHy2uwcAAADDkPk3S/
9PnUR+AIfJudfOiei72DbZV/edP333b957r7sBAAAAw5D5N8X+D5fd//AZALbb6RdOi/S72PaRf/wvY
39139k7bnv7ypVu6wEAAAYj82+KkfkJ/AAOof2D/eO7x0X6XWy7/cyHRUov2/gYPwAAyEPmBwBke
vPdN/k1/rqN99V9fIwfAABkI/MDAPK9+tarIvoutt14/sgXP31cxPX1Gx/jBwAA6yDzAwDWcuHyBZF+
F9s+tvP7IrGv2fgYPwAAWBOZHwCwrjMvnRHpd7Htrvv/WOT2dRof4wcAAGsi8wMA1nVw7eCub9
8l0u8yW8Gv7nv+/De6/QUAAMhF5gcAFHD111f5Nf5t+/DXf2/9r+577N57fvv+
+93mAgAA5CLzAwDKuHL1ytEnjooAvMy25lf3nb3jtl+
+/fNuWwEAANZA5gcAFHPxjYsi/S623Xv3rSLJD28/fxMX
4AAFAQmR8AUNgDLz4gAvAy260P3yjyfLLxMX4AAFAWmR8AUNjBtYOTz50UAXiZbaWv7uNj/AAA

oDgyPwCgvL39vWNPHxMBeIHtyOPXP/DfPyayfajxMX4AAFAcmR8AMIpLe5f4Nf5VG/jVff/nfz3ebR
wAAEA5ZH4AwFie/emzIgAvs534uz8UCV+0Jx96kI/xAwCAMZD5AQAjOvfaORGAl9k+/7lbRM7X7
Wt3fubdX7zT7RcAAEBRZH4AwLhOv3BaBOAFtg9+8wOhr+772Q9e73YKAACgNDI/AGBc+wf7x3eP
iwy8wOb96r4Xn3yi2yYAAIARkPkBAKN78903+TX+VfuLBz5iBn4+xg8AAMZG5gcATOHVt14VAXiB7
cjj19//2Y+2gZ+P8QMAgAmQ+QEAE7lw+YLIwAts+qv7+Bg/AACYAJkfADAdEYCX2T715Q/xMX4AA
DANMj8AYCLXXTfRv3QolI1C2SiUjULZKJSNQtnmV2gJ2EoAwER4IslGoWwUykahbBTKRqFsFEIEWw
kAmAhPJNkolI1C2SiUjULZKJSNQohgKwEAE+GJJBuFslEoG4WyUSgbhbJRCBFsJQBgIjyRZKNQNgplo
1A2CmWjUDYKIYKtBABMhCeSbBTKRqFsFMpGoWwUykYhRLCVAICJ8ESSjULZKJSNQtkolI1C2SiECL
YSADARnkiyUSgbhbJRKBuFslEoG4UQwVYCACbCE0k2CmWjUDYKZaNQNgploxAi2EoAwER4IslGo
WwUykahbBTKRqFsFEIEWwkAmAhPJNkolI1C2SiUjULZKJSNQohgKwEAE+GJJBuFslEoG4WyUSgbh
bJRCBFsJQBgIjyRZKNQNgplo1A2CmWjUDYKIYKtBABMhCeSbBTKRqFsFMpGoWwUykYhRLCVAICJ
8ESSjULZKJSNQtkolI1C2SiECLYSADARnkiyUSgbhbJRKBuFslEoG4UQwVYCACbCE0k2CmWjUDYKZa
NQNgploxAi2EoAwER4IslGoWwUykahbBTKRqFsFEIEWwkAmAhPJNkolI1C2SiUjULZKJSNQohgKwE
AE+GJJBuFslEoG4WyUSgbhbJRCBFsJQBgIjyRZKNQNgplo1A2CmWjUDYKIYKtBABMhCeSbBTKRqFs
FMpGoWwUykYhRLCVAICJ8ESSjULZKJSNQtkolI1C2SiECLYSADARnkiyUSgbhbJRKBuFslEoG4UQwV
YCACbCE0k2CmWjUDYKZaNQNgploxAi2EoAwER4IslGoWwUykahbBTKRqFsFEIEWwkAmAhPJNkol
I1C2SiUjULZKJSNQohgKwEAAAAAmCcyPwAAAAAA80TmBwAAAABgnsj8AAAAAAADME5kfAAAAAI
B5IvMDAAAAADBPZH4AAAAAAOaJzA8AAAAAwDyR+QEAAAAAmCcyPwAAAAAA80TmBwAAAAB
gnsj8AACs6pWd63o7r3RHAQAAtg2ZHwAwI20YHzeFv7V7qq8wZsGp/2ahXljl1O5b3YGSrMU0+Lu
SVVl7OM5tGl3gD0z33muVfWdE/ohGTq3KP5R1x8oua1zRnRn1HxQjCi/qsN6nwIqs5ZS6TQNqHdJ/
Ji0EmR8AMK4Rn+YtRp0pH9qasmM861TPUnodzXPVyA9U9UJO7exU/2eUOvUSJn8kNJ9Hy78rrH
d2b6R3X7OWid4R5r6VKxL8E2r/GbIXupZgxeipVYWGqo/rA+2WrlvKP4p5u9YukdyZ+nzRf1D4FmUt
qbHeuiKLak4V/KPkLscobhppRU19fah5te7qhtUqUKrdu47YH2sXxTkMQOYHAIzIfp6yn0aKUoWa/z
/lA4G9wrGMXqUu0G3fOGXqez/6NpmmuTGWpuRIbz459ni1Sj/Hd9T9cCcu3xqllhauGDu1qqFDqX
7dy5U117fsKtX26QPr367Ucurj3em16rRiiyoxfieyKM+hfMHlCOsXDa9IHhmxljrRvVz3rtXD6eGbd3L
/0i7VnFxrTUtE5gcAjEc+AzhPDcWNX0GY5vFj5GXp4esf8h/aYtZ7HFzZ5O+DyrhvBXsDR1tfM7B5o0r
fOGfm7lIKb2RkrwpuY2qo5nz2Rqqrx61i8BfSR+sf1i6jJuupNc4/LtxCRdbRiSzHVvD9ndy65GSGc4Zy
1lGwmN7N5oV8O5QstBhkfgDAaNx/Mxd82vGb+GGgWU+pZ8Yg6/GnPGPP6h/HKdTsVG/kPRtvH
UEj36RKu4VVifaHcd7lzp9Q491RhDNeXdHet8IlI8MVrJQaytnYLKkqxRbkG8g4Vv9Y7M3uqeW+KU
pwCzV16g8qaOtX9W2doTldam3eWs1brS7R/hCeymqcWoHtLLM0c5v8hYotbCHI/ACA0bhPAN5Hl
JJGL2BqHjxKPb25mrV0RlxSvQo1fF1yvAUp7cLGrNO88wo/ysdN88Zr3nGNsUq563D/FK/FKeCOX3
gvI8MVrBQfqrlxBXYxXqU5W+ZeeQrVa1BH6tPF3hSBWoZCpdxC4r40HcL7O4xnOYb42VWFRuv3r1
gpT63miNy9QveqWUFXrf7ZHrbsLi4DmR8AMJoN/Kt6umcB8bQ4qqIPU7Z6Gf1+1YUmWZP71ihK
3Jx2/8Z8WzQFR33fmWtofx6nXjO23LqCt6oZ0Jy5+1ZwuqwnMlzBSrGhxNtxDd4qzcFOqW1zCtVr6
F/Xp4u9KWJbV2mXV6KaW8h58xX4gxxdToHxTZ5a7Xa1x9qfC9XzrqtZj7KzY71J1tAMq2+Mc5fiew
wvMj8AYDQb+Ff1RM8C7ZPOhM8c7cNb+YLWI5uhxBN2TFN3vP1z3nnj1mvuzqhb5lYYcUXWm6Lc
c3zL+RPq/pF17t563AJa5NSqgkM1u1lqOYkJN6eLFJOFrPeEYYxarkLvCLeQM3B6LkmRIZpTpd4LNae
WW6G5c+utqJXemrpHidXJPzLu7U/PBRKZHwAwGvdfzIWe3cKmeBZoakz8wNHUHHXnGnWZ8au
Mv5wxHuXDij1Vh7klRl2RofRbwp23vFmlVxYZr2Ap/1DNfSu4mOSEmw4FCiYK1aeLvSuSiyr1J8wt1
BwxFyLfjBnCyym0DINTyy2R3t2BkgPVHdZ/UzQrsIdxKxe4TYtD5gcAjEf+m7nY40fQ+BXaZxKp+PNH
VcVYhuc5aBRlntpc1bjGsM1NGvUuNRXMhYz5jNjcnHHfc+47YJKileLviObW2BO31ubcurV5KiqRU6
vyDNUcKnuTkhNuOhTYvkSh+nSxm7TRRVl/kIr8qQouZ4Q/s24t6w9TpVzR+G0qc4+a2bpF6sPm2P
GpwIvMDwAYkfUAUuapwK8ZWzjczwRiRdMspi46yh2afDXWo265517XmO9qW/s8rE3wjii5NvEOa
PRLMNdWajMjFeOTWUl4KHG/WmsurqlmzbSqYrxuaq5TY+DO1N2KvemdRVUHjMHbKXkmMVh8

UdbZdcp0nOW0msMTvLeL/4MiXMs+s/bWtcN5h7He10V3cjnI/ACAcZkPIPx7GpOxHkjXfh4Nad7e
o42+AYWf41GEfVda3b0Rpw7RHQsv6lCuKXKPKrP7B0Vx4q8qWv0jAw8SayLzAwAAAAAwT2R+AA
AAAADmicwPAAAAAMA8kfkBAAAAAJgnMj8AAAAAAPNE5gcAAAAAYJ7I/AAAAAAAzBOZHwAAAA
CAeSLzAwAAAAAwT2R+AAAAAADmicwPAAAAAMA8kfkBAAAAAJgnMj8AAAAAAPNE5gcAAAAAYJ
7I/AAAAAAAzBOZHwAAAACAeSLzAwAAAAAwT2R+AAAAAADmicwPAAAAAMA8kfkBAAAAAJgn
Mj8AAAAAAPNE5gcAAAAAYJ7I/AAAAAAAzBOZHwAAAACAeSLzAwAAAAAwT2R+AAAAAADmicw
PAAAAAMA8kfkBAAAAAJgnMj8AAAAAAPNE5gcAAAAAYJ7I/AAAAAAAzBOZHwAAAACAeSLzAwA
AbJdXdq7r7LzSHWqpE6d23+qOjEPVEeW3Qb83FWcf3to9FToVPQcA80XmBwAA2C5GrrXj6dIzfx/
bG75tCCb7flO38G8yAGA8ZH4AAIDtYv1v2WZCXXrmH7R+b7jv/yqAxA9gYcj8AAAA28XK/GbAXXj
m18E9MS+9f3qf9BESP4DFIfMDAABsFzvzG0HVyfzOATcYd13qHsa43QXGETMNq8PVMeM/pzeKK
MZZ57wxt75MInNb41kDGjNVImPp3k0f/cpZQWz+NXs+oktkgdHrAGBSZH4AAIDt0ofJ3fYnFRmNl
Nm8dg8YcbOLxH0cTdIhOnyNkbNlrm0YE/EMEo2+/qJy6QZjLo5+cju76kdRPTX/xITCC/RfF50uAIyHz
A8AALBdVGisEqQKpm1gNE40Hd0DsczfdTIiqTyiYqlzTX9EHZBldA89FaPOgLgbLqAHdGvG6M6dfph
Gcv6qQ3g+gQWGBpIzAIBpkPkBAAC2i5Uauxfmz27sNOKkDLOqS59Lw0fUMG4P2Ucm4oo8pAcx+
gTJGdTkStwDcXoCNXHJgPlLTnX/AnW3QesGgNGR+QEAALaLP15XUdNJxp6oLKOp2yV9RL02g7I9ru
rhIwpbowS4U6qpkuqoXFpSeAoD5m+we6vzgdH1LHv2sgBgUmR+AACA7SITcPdaf7y/z5CyZ2WszG
8fVC98goXD/H3lUrIzvzuFAfP3hfeGOh8cPXDh0FkDQFFkfgAAgO3ihEkZIvUJT+yUwdjtkj7iGVaM6+s
hDOii+fuqkuqoXFpSeArpyakeIbaTUz05hjFEI1wMAMZD5gcAANgunjBpx0d9ItazQOY3w7XdZ0D8d
suE6eHMzk6Ngpk/OZR7qbMpkQVWp4yBV543ABRE5gcAANguvjCpc2NNn+iPdnFSB1PniBtew0fC
o+gu4coq2LplYsIF+utXzs6RKaTmL5fT908s0FlIdBoAMDoyPwAAwHZJhEnzhBFFJZFlYxFUHtGvT8nR
Vd6t+Ev3Hdwycaq/zby6ZOZPzt8/nZrqEho9dOXQWQNAUWR+AACA7RIIk31ItU6Y2bWKlTIYu4OlJ
6jX5nDOdGp2brY7BFYRZY8nQ7JcWlJyCrH595fX6pLGtpinfaObl9aGzhgAiiPzAwAAAAAwT2R+AAA
AAADmicwPAAAAAMA8kfkBAAAAAJgnMj8AAAAAAPNE5gcAAAAAYJ7I/AAAAAAAzBOZHwAAAAC
AeSLzAwAAAAAwT2R+AAAAAADmicwPAAAAAMA8kfkBAAAAAJgnMj8AAAAAAPNE5gcAAAAAYI5
+97v/BGT2wDQGr4t1AAAAAElFTkSuQmCC]]></redcap:Attachment>
</ItemDef>
<ItemDef OID="b38" Name="b38" DataType="text" Length="999" redcap:Variable="b38" redcap:FieldType="descriptive" redcap:BranchingLogic="[q37] = '2'" redcap:InlineImage="1">
<Question><TranslatedText></TranslatedText></Question>
<redcap:Attachment DocName="B.png" MimeType="image/png"><![CDATA[iVBORw0KGgoAAAANSUhEUgAABVEAAAEJCAYAAACdcmdaAAAAAXNSR0IArs4c6QAAAA
RnQU1BAACxjwv8YQUAAAAJcEhZcwAAEnQAABJ0Ad5mH3gAAE9sSURBVHhe7d0PjCRnfef/b+/aY
PPHNmBskx+RHat7sZYlGJIg08PxL4egZ+6SJbnbYOVg5Oh3PVyi0zQSkwOxF+mX2wguG4lunS5iWrq
zliQyTEJugrLdlJRgckzHR4IxYTI/Z7rlc3T8wDYGjPljB3u3f/VUPVX1VHd1dVV3VXX39PtltaerpqfqfqT/T
O/OZ5/k+hb5FAAAAAAAAAAAChjumPAAAAAAAAIAQhKgAAAAAAAAAEIEQFAAAAAAAAAiEKIC
AAAAAAAAARCVAAAAAAAAACIQIgKAAAAAAAABEIUQEAAAAAAAgAiEqAAAAAAAAAEQgRA
UAAAAAAACACISoAAAAAAAAABCBEBUAAAAAAAAAIhCiAgAAAAAAAEAEQlQAAAAAAAAAiECICg
AAAAAAAAARCFEBAAAAAAAAIAIhKgAAAAAAABEIEQFAAAAAAAAAgAiEqAAAAAAAAAAAQgRAVA
AAAAAAAACIQogIAAAAAAABABEJUAAAAAAAAAIhAiAoAAAAAAAAAAEQhRAQAAAAAAACACISoA
AAAAAAAARCBEBQAAAAAAAIAIhKgAAAAAAAAAEIEQFAAAAAAAAiEKICAAAAAAAAQARCVAA
AAAAAAACIQIgKAAAAAAAABEIUQEAAAAAAAgAiEqAAAAAAAAAEQgRAUAAAAAAACACISoAA
AAAAAABCBEBUAAAAAAAAAIhCiAgAAAAAAAEAEQlQAAAAAAAAiECICgAAAAAAAARCFEBAA
AAAAAIAIhKgAAAAAAABEIEQFAAAAAAAgAiEqAAAAAAAAAAQgRAVAAAAAAAACIQogIAAA

AAAABABEJUAAAAAAAAAIhAiLp0etJYKUih4DxWGj29PqjXWPFeU1hpWF+1iMxj3ZB2onXjpLWdL
MxLO7LSlg372PzHqPs4Vb2GrGzEO5vtDbdtK5JH08KNuQ9Cjmc+2g0AAAAAwPwhRF06Rdm8UJey
XurU1kPCkracr3X0c5Hq2U3rq4B5oILBVWnqJUdZzqxle4faf1Qo1cT/rlhsR+14AAAAAADIGiHqMip
uytmqfi4dqZ0P9kbrNc75IVW1JdsV/XzhFGVzry/9vnpsS/aHkff+RpmXdmShKwdu8leuS9c+xj3ZzDJ
D7TVk3fijwsI7ascDAAAAAEAOCFGXVGW7JV6O2jxn9EY1e6FWpbW4CSqOot6h7OuncuoEPaQBA
AAAAEAuCFGXVkW2W26M6vdGbW/4Q6XL9a3wXoyqlqKuRek+wmpSjqyvaH59ZI1Jo6ZjoC7rqPX
GPu31edcGHd5fZI1J4zwEz5+5nbDPjxN+3INtCdS9tR4xy30ObGewraPOc7xjCm57uPZpYAh6c9VZF
2h4snM3eA7Uw9yc3Z6x+xzPPy71CLkXbAnaHvI9OHq7vqTHE6/dAAAAAAAcfYSoy6yyJXW3OGpz
VTYaDTnnJ6hyIWSM9FAIo3VqpQwmoCrK2hndwM6BdJ1nFmNId2dHLno7bcuubn/5zNpc9FKsnPa
D6gP/AGy9izv6PBo1Pe1wrCSDo63TPb8dqZUKUhrYSXM1aUimtjPY1qasDoZtEx2T2rZR+9S6H+te1
+kREu3HCSwHz4HSXB0dKibnnOtV70AUtW6Kc9TeGFHPNGS7E4vZbgAAAAAAlgQh6lILTjLVrLnBTF
nqF0Imk2pv+KGKV4+yL103ie3UZD3lhKW4dka3b18O3U23d42JhYxw0hjqferEnAz0rpz2yiY0d4M9
Ji/u6BisfEacDLUnjXX3GlSlpc9v3+0xnOr59bfvdUhWIdlAfdzx/O1494Hazrob/E1zTMbr9zZlc9v62PX
vV1Wv1/6cXXIi2X56jXUvsCzXu85r+8MlLiqR+4wr/BzteOl/krZbr3X/0hH2PRjY7rBkxzOu3QAAAAA
ALI+C9QtyXz/HklK9SwM9zlSwEhKq+K8rS71rTubTk8aK24tOBS/OZEYjX6963bk96Ubsy+dvW4Vde
9ZG1BBs1YOwWq1Ks9kcWu+3IaxdcdeNE387w+2yV3rnwG1/6DqbsV0VnO2FBNwB4e0wr3O11fc
nDEt0PSK2E9ivvuaS7JjMbQdfr41qa6JzF35+FOdanQqsS3p+lETnOlHbRwht44jjjDietO4RYBr3Pnyvv
OXCW/QSACAP99/xLnnNW2/SSwCAzB3/NZFjr9ILWBT0REVwkikVtoQGJP5Qeb/npMsYdm/2GE1F
UU6ccp51dJfTrj2WvywnT5+0e9QF11uqp/0AbA74vWmb4nVG7R44gZT1GW8ov7dOdT4sGbUo3S
BMfcIsazBr1jUo6ac28z7QPYSnOKZEvYkT7cec4f+kmIdQ3NyTvhmgTm3wHIWY6rrrurFuwJmaGO0
GMvDwEw/LOz/5Tr0EAMjDDY9fJXd/6AF5/Bs/0msAAJm7dI9I/zt6AYuCEBVBA6FScsO1P6fl1RVt7k
pbenJoj9k/JScqJ6z/D65XGeqc9ZIrrombLbpD+ttuIl09a/TozUtaAZl1DTJrOyHeSKpXqBe0GnVjgQX3x
NNPSOWPKvZHAEA+nvPjY/KWL94oP3ryGWm+/3555p8u6c8AALL1lMilP7A+PussYiEQoiJlGYRfXl3
RfTlsXxS7lKgd9lbEyVeN9XMZvhk9NO3A1+/VOyrwVcO67VqVQ480e0lOK1mv47yOaTHOXbjxbW/
Lhtnr1K2LatY5BRbUnZ+6Ux58/EG9BADIwxv/
+gZ5wQ+vsJ8/9NXvyp/WeR8GgNz0Hxa5/Od6AYuAEBUxleSkN1LbnBFfMSZJitM70Ri+HI+7744c7D
pf686+X7I/4a8fLjUwH4qbZ/0guOFOjFWVQIZacsoTKG6Jgvk22OvYvA/0seV1TIn2Y97LA8Pk1cz3hZ
xnoE/SdmNSNTtwdeukJv6eAubL+z77PvlM7zN6CQCQh1c+eK3c/PXn6yXH5z7+kHzlLx/RSwCAzF3
6gsjlr+kFzDtCVMRUlM2zbuVUc/Z1NbrYnOl8y+vt5wScijnre1s23JlrYvN7cqqJpBS3XmZRF0x117vh
6vxxe81a56Kmj3+wdqsx7F+aq7LhnTIV7Onh297K+dBcNQLH9nm/hqd7bHkdU6L9mLVbm7Lqre/p
me+ta1Qq+NvI2oTnyA9c3XYDi+kT+5+Q+n11vQQAyIOqg/pzD7xELwX94W9/Tb73raf1EgAgc5f/RK
T/fb2AeUaIivgq29LyctSalHTA48w8bynX5YJR4NOfUMnSXNWB0GQ1HAPbsp55Q/a9of6KMUnTHP
Jqu9rKUt8KRKiWomxe8IdlN1d1gOaFzqMm/ZolJ3AMttM8tryOKdl+ipsXpO6/2HmtOZGTdS/7h6Br
7yrua1NNWBO03bzfw9odR+bHA8R339fvk7v+7C69BADIw/FLBXsYv/oYRgWozd+8Xy8BADLXf1Lk
8sf1AuYZISoSqWyH11+stqz17tBiV3FT9gZfW21NVr/R7K0XKBlgDM3OdKKjFJgB2KiyA+qc9bt+wOe
ya1/OW03PstS7fT9Yt1Wl1d8LTpaV1zEl2k9RNvf60h16sXq5MUzeVpHt4EGK7B96PbFTEbvtVlv6Lf
8+sqlzblwHu+5ulByOB4jBnYn/6Wfp7QQAeVr50kvl2iev1Evh/uFL35ZP//6hXgIAZO6y9RvZJcpbzbt
CX81aAgAxtTcK4nSSVCHqQGAKADGo4PT1/+318sAjD+g1AIA8lB56od0LNa6tu8vyiteFD/sHAGTgit
8QKfBL9ryiJyoAAMiVGsJPgAoA+VK9T1Uv1CTUsH7qowJAji79AfVR5xghKgAAyM1HvvgRezIpAEB+
VP3Tt33hZSProI6iAtS7/+NX9RIAIHN2fdR79ALmDSEqAADIxe6Du/LBv/igXgIA5OWOL18/tg7qKPv/
8zH53Mcf0ksAgMxd/n9FLn1BL2CeUBMVAABkTg3fV3VQmUgKAPJ189efL//8Czfppclc+dzjsnX36+
XWV79IrwEAZOtKXR/1Zr2MeUBPVAAAkKknnn6CmfgBYAZe8MMrEk0kNcoz/3RJmu+/X3705DN6
DQAgW9b77aWPWx+fchYxFwhRAQBAZlRwqgLUh594WK8BAORB1T99yxdvlOf8OJ1f+R7/xo/k7g8
xKSAA5Kb/HZFL1EedJ4SoAAAgM+/77Pvk3ofv1UsAgLz83AMvkRsev0ovpeMrf/mI3PtJ/igGALm5/

DXr0dELmDVC1KXWlo1CQQrGY6XR05/LSa8hKxttveBob7jtWZG8mzM3Qs5LHL3GinPuVhoy6tT55
9d55H7NjwLuWyCW+n11+djffkwvAQDy8vJvPE9e+eC1eildn/zPB/LN//19vQQAyNylXZH+Y3oBs0S
IurR60lhZlaZecpTlzFpRP8+eHfiVasLfVIImPi+9hqzXor5KXfOCrAYvunRqJSlMENguK+5bIB7V+1T1Qg
UA5Ovqp47LG++bvg7qKKo+6u//
+y/bHwEAeVD1Uf+79fFZZxEzQ4i6tLpy4KZA5bp0+33p9/dkM68MdWzgt6QmPS+qZ+S4YK99XtxN
V1vqevelWy87K5qrQo4aA/ctEIuqf6rqoAIA8veWvRvtIDVLqifqH/721/QSACBz/UdFLv2xXsCsEKIuq
96h7OuncuqE5Nf/FGmL1zOyJ41zugtquS5bFedpcfOsVJ2n0twlRQUwPTUTf+WPKvZHAEC+Xvu1F8
vLHr1aL2Vrb/f/2A8AQE4uf8l5YGYIUZeQXbvRDN2aq04tx8GuiKp3o1E7c1T9zGAtyGCd1bDejbH3r
wVreI6qN+kMVfdfl6zW52A9S6+2qH6ENy3GPgPncEP8zZjnydln0vOiqHaWYvWMNHoeB0LzkpzUn
VFl/9A6olGM9obVW21v6GMZPAcJrkvI/RZ2vePcb4PXz35E1ImNY5b37WT3p2vSa2eZ6D1Ar1TMr
49uJl6QOz91pzz4+IN6CQCQFxWevubvXqSX8qF6o1IfFQBydOlPqI86Q4SoCDUUGGl2/cyRYVRHai
WjzqrR43EyanuDNTzVurCgpuQNVXdFt3UUZ5+DwWRzdcJ9Fjflgjtk3joz5/RGeo1z3nkq1y+kUEahK
q1+y+tVOsTseTxK50C6+umwipx2N97ZkYsDJ7W96x2NX1c3yXVRQV7I/eZcj1EBZPj9pu7d0GC5U5
NSIMjOSpb3bcz7M2CCa2eZ7D0Ay07VQP1M7zN6CQCQFzV8Xw3jz5uqi3r3h75KfVQAyI2qj3qP9Z
H6qLNAiLqEKtt96Xfr4kZ7Um3Z9TH72zrxbG/4AZBXL9Won9mpyfrIxEaFec7r+3uboWUCxu4/wN+
et3/pyI6XBPWkse4GPca+Wzo1imzrKP523M2ofdbOu/Fbsn0WNy+If+rOS9v677wbglnn94JOUJOd
F4P9um2ZLK8uyolT+ukYlS23beb5V9ri53BnxMnhkpwj67VGqYGh+21ofyZj2/b9ZrYlbFt+kJ3U/Ny3
/uvD789hya6dZar3ACyrT+x/wp6NHwCQv5UvvTTzOqijPPTV78qf1hmBAAC56T8scvnP9QLyRIiKIW
bPtPoFPwgdDgOHletbE4Z54aotPxwsrp3xAqzOge4z2bsoO14eaey7suW3deeiJIl7zH364ZMh8T6Ls
nnWTbuaslpwe08Gz+8kipt740PW2PblMOpEFdfkTNjxtXf9XrVn1pzjSXSOrPOzp0NEI3gPvd4Dhu43
s8et0evSPk86CNzLYfa0LO/bsfdnmCTXzjLNewCW031fv0/u+rO79BIAIE+vfPBaufnrz9dLs/G5jz8kX/
nLR/QSACBzl74gcpkJ/vJGiIoBI3qm2Yqy5iYxIwK3UyfSDKjKcrKkn47SPdC9+VSoU/LqNhbMYdKRw9
QnMMk+K9tGr0FHOsP403RKoi+fcf2NgNIP3Kpy1j2gqa6LruEZOrw/aOh+M8JCawf20Hd33yNrsaZ
uHu/bBNduyvcALB93Jv6nn31arwEA5OWGx6+Sn3vgJXpptu7+0APy+Dd+pJcAAJlTw/r739ELyAMh
KibUkeHOgTHCo7mX3TFUvMKUSrD+5Oz05HBssVSf36vSHRZuBG7V08FeoUmYExB5PXXHCbtWR
dm8EN470w0rF3t+o8nvz/SvXdh7AJaNCk5VgPrID+h9BAB5e86Pj8lbvnijHL9U0Gtm60dPPiPN99+v
lwAA2XtK5NIf6OfIAyEqJjR/gWm53vWGbAcfk9YLHS/
+Po26n7aO1NZznJyneELGlj4tn5Sxl7S4KW5lAntYuDF8vno6/CyPP0dt2TB7nbo1OM36o0lYbdzT+xj
s/as0z83XpEi53bcTXLtoR+GPJpiWGsL/wCMP6CUAQJ7e+Nc3yAt+eIVemg+qPuqnf/9QLwEAMqfq
o15iYte8EKJiQElOusnV0EzePbnoFnIcO/Q7J6WTXtA2qm5m6ibYZ6+xrodpl6Ved5OsPCfnMa5rc1f8
zphdOfAu6QmJc0m9HrXW/XH+/I4OP6sSyOGSnCOzLqcKFN26qMaQ96TUzPKqx6k9GZQdSHa9W
p7pDpOf0CzuW0usa5fme8AU1xDz7yNf/Ig9mRQAIH+lh1448zqoo3z6v/6D/MOXvq2XAACZu/xZkf
48dRU6ughRMcCcBCnYW9IPAlXYNTChz6yYNTCbq/5Q7faGHhpuPdIev510n72GrLsnrnpWNjeNyY
Nq65JPjmrWsmzKrm5er3HOCzBj90asnBbnDulIs+ke18Bw8Amvix8oDvbcjUlvX80sH+xxaoTFZo9b
oz1p3yaRZnHfKnGu3QTvASU/dZXaee9gZMOb4h9Hze6Du/LBv/igXgIA5OnaJ6+0Z+OfZ83fvF+
+9y1qZQNAbtSw/v739QKyQoiKYeYkSJ2alHSoU/LTE7kw7YxI5vDy5uoUoVGwBmZzVQdQXnhTlVZ
qs9e7kuyzJ411d6h6Wepbav3okCq98zLMnFndbbN3TastiX+aKhIo72oZDmATnCMv2LO4x2xOsJTEi
HvXrLNaPevPNp/YQt+3SpxrZ0n4HuDXW7V41zBubVssGjV8/85P3amXAAB5UvVP3/aFl81NHdRRV
ICqglQAQE76T4pc/rheQFYIURHKHgYdUpOy2rLWu8Otp1KR7cGClfuHRs/BBOwamMZwbZddWzOj
eqgx92n23LN7obonbiCk8of1p3hehhRlc2+4Tqg9hD5hYBeYJMs6ZjsbHhT7uljH3G/5QaqtKq2+0dZ
ACYJo9r07eJA2Z5vTZZMLft9aYl07S6L3AHUsg6+ttiava4u59cTTTzATPwDMkOqBqnqiLgI1pJ/6qAC
Qo8vWb6bUR81Uoa8KBgJAEmo2fT0ZlAph96btmTxL+lhOtaYNWBfEUbp2yJUKTit/VJF7H75XrwE
A5EnVQVWTSS2SK597XLbufr3c+uoX6TUAgGxdKXLFb4gUbtbLSBM9UQEkFCxRcGZtsUO49nnrWC
J6ZB4tR+vaIV/v+

+z7CFABYEbULPx3fPl6vbQ4nvmnS9J8//3yoyef0WsAANmy3m8vqWH9TzmLSBUhKoCY2rJh17o06
pWaJQoWUK+xIqv7demmUqJinh29a4d81e+ry8f+9mN6CQCQJ1X/9C1fvFGe8+PF/NXt8W/8SO7+
0AN6CQCQuf53RC7doxeQJkJUAJNRNS8XfPx7cXMvpRq/C+YIXDvkR/U+Vb1QAQCz8XMPvERueP
wqvbSYvvKXj8jnPv6QXgIAZO7y10QufUEvIC3URAUAAKEefuJhec32a+wJpQAA+bv568+Xf/6Fm/TS
YqM+KgDkjfqoaaMnKgAAGKKCUzWRFAEqAMyGqoO6aBNJRVH1Ue/
+0FftjwCAPKj6qGpY/7POIqZGiAoAAIbc+ak75cHHH9RLAIC8qQB1UeugjvLN//19+cPf/ppeAgBkrv+
oyKU/1guYFiEqAAAIUDVQP9P7jF4CAOTttV97sbzs0av10tGyt/t/7AcAICeXv+Q8MDVCVCTUk8aKm
uVbPTaknWjdOGltZ96MOYZeQ1Y2gmvbG+7rV6TR0yunpfZjb3NEO2zTne9M2m1bkPugvaHbWJC
BSwosjE/sf8KejR8AMBsqPH3N3x3tuqGqN6rqlQoAyMmlPxHpP6YXMClCVGCGeo0VKZRq0tHLWe
pd3DH205RdQj4AA+77+n1y15/dpZcAAHm7+qnj8pa9G/XS0aXqov7+v/8y9VEBIDeqPup/tz5SH3U
ahKhIqCibe33p99VjWyp6bXby3l8WRhxDryHrtTziU6UnF3eC+2qea1hrF8VRuA+A+aZm4n/nJ98pT
z/7tF4DAMibClBVkLoMVE/UT/7nA70EAMicXR91Vy9gEoSowDLoXRQ3Qy2Xy86Tzo5cXJwUdTFUt
qXfrYs+w8DCUMGpClAf+cEjeg0AIG8//ffX2UP5l8m9n3xYvvKX/NsDALm5vGc9mOBvUoSoS8eoLbl
i9kQCtd6oc2mvz7s25fD+IutuGnU/VwKfNLcT9vko6Z8z+/PmMP7mqvP5EYU0/WNWj+T1Rv2h/FU
5e+GMDvk6shMnRQ3UUg17jG5PvHbHuTbD59DaumyYrzfqkQa2MbA+/BTHaYN5POpY/P2rh7vd
9nnrupbrsjXQXdYu3WC83n4M3DfArKgh/A888oBeAgDk7YbHr7Ink1pGd3/oAXn8Gz/SSwCAzF26R
6T/Hb2AJAhRl05R1s64PREPpOs8s3TlwE30Aj0U27LbdJ6Vz6xZXz17ldNV/awjB/4B2PywsCxn1nRr7
RCwJIMj5zu1UswQa5bnrCO1UkFW9fYcal2SINUYyl89LZXimniHs3Nx+hCvfEbcU+2L2e6pr43Dfn1w
Z846FVQOrG+uDgSpE7VBHcuqeFvWoakKSlf369Ld2wxcdxW+lsJKN3RqUprnibKwFD7yxY/Yk0kBA
GbjOT8+Jm/54o1y/FJBr1kuP3ryGWm+/37qowJAbp4SufQH1kfqoyZFiLqEimtuT8R9OXQTovauHwi
JEU72Dq1XOU6dmIcI1VI5LW6M2gzMjmSEhV6w15PGutvjsyotu6am9WjpLXRqsh4jjUz7nFW2rTa
Yw76rLadd22HVPv12d+vuV8TsRaoYQ/mrp9X2zVA4xpD+4qbsuefNfnTFa4Z1BPULwcDQN67d6V
wbV7neDX69Frbev2+maYPxeh2aFjf3vOc+P1RXYWtXf41/TppyLsFxAmnafXBXPvgXH9RLAIBZeONf
3yAv+OEVemk5PfTV78qf1h/USwCAzPUfFrn853oBcRGiLiOvJ6IfaPUOndivWnXCo32dFJrDwO38bS
5UZMsNoJq7fi8+s+6n2wPUXFff8ickqmx5QWCs3pgzPGfVlj+Rkh/mWi0Z7IY7gtk792TJfmJsJ0EYq/
Ua636vzepZ2QxPUMe3O61rY6vKWbchRsg+cv3+obPdKdoQeH0UI1Q3Q2s7cNWB6t6okwhkSA3f
v/NTd+oIAMAsvPLBa+Xmrz9fLy23z338Idn/n4/pJQBA5i59QeQyf8BKghB1KRXlxCnnmRtode1x6W
U5efqkHXYF11vUMHDn2VzwQ7mmeJ0KuwdeWOgN5ffWWcfkDu+2H8bw7cAQ/VFmdc784HMy
Yb1zLRMP6W/LeT9BlVZoz1klRrtTuzaW8kkJ3d2o9a4p2hC7Z7Zxrq2N2WUO3P3Er8sLpOuJp59gJn
4AmLGXfPe58nMPvEQvQbn7P35Vvvct/m0CgNxcVvVRv68XMA4h6pLy6oraPTl74nSqPCUnKies/w
+uV3ngPEWoFiOYcodmt90x0xG9I6exkOfM6GmphqaXQkPC+LP0tzf8OqBVo6fp8kkSbhdl80L4jP1u
cDtiPjEgE+5M/A8/8bBeAwDIm6p/usx1UEdRAWrzN+/XSwCAzPWfFLn8cb2AcQhRl5U3tHlfDts6aL
N77VXEyQqN9VP3hsyCUdfTDi/9upOjwJkwDqa5O
CTNtg1JUdKNlqa55jln7k532ffZ/c+/C9egkAMAsrX3qpXPvklXoJpn/40rfl079/qJcAAJm7bP02eukze
gFRCFGXVklO2hlw52nSHNbh3Rkv0Jf3347OuzV9w864eaDXeSp4E6pCVnqL3i1eKc2KKdM2Mo/
xjjh/S3ZcNLUMtSV1PRTyvVazOhHNugZuhXPU7tScXsQNWYoCtu2QJgSvX76vKxv/2YXgIAzELpoRfa
D4z26f/6D3aYCGDIyeXPivTp2jMOIerSvrnzFnj3VyX5Sp4E6pCVnqL3i1eKc2KKdM2Mo/
crmqj9sur3h1aUsxB5LvWDnzBzK787+H3iYIV70kP5e45w3jL9cv5BOuYRUr82E8miD3pbKoIM9Trvi
ls8dW7sVSMn/+v/
+l34GAJgF1ftU9ULFeHd/6AH9DACQi0v36CCYhRB1Zkzpqvehd7wzpCqC+shGaxH2
VzVwZg/Ll1GT440LNVzVtS1VJXmauqhoTmUvxxaW8AoiWC9cuSQ/l5D1r0CqtYrAxMwOY/JJkhK99
pMJoc2VLb9IfyBurRGfdmzm7MP3bEU7v7Fu+WOl9+hlwAAeXvjX99AHdSYfv7dP6WfAQByue6dJBXf
RCFGXWWDW8FPiTzbuDltXzPVzyAwvRw2ht+tRGr0uXeW6dJPWu0z1nFVke7BA5v5hSrUxzaH8o0
NdMxQeNaQ/Xl3VCaV5bSaVQxvsIfxhxVBVSNvvp1pfFohy1RVXyf/4lf8ht1x3i14DAMjT373yCf0M

UV7z1pvkbe+5VS8BADJ37FUix9+kFzBKoa/G9QIAgKXxwCMPyOv/2+vtmfoBAPm648vXyysfvFYvYd
D1P/E8+a1PvVGedw0TbwFALgovFrni/daTq51ljERPVAAAlsztN91uD+0HAOTvb27/tjx2PX/ECnPlc4
9L9fdeS4AKALmx3m+Pv8f6SIAaByEqAABL6F2n3iUfeMMH9BIAIC+Xjvfl8294VH78nMt6DVz/8t+V
5NZXv0gvAQAyd+ztIoWb9QLGmSpE7TVW9AQpBX9W65F60lhxJ1TZkODL27LhTbbiPCabqGZYeyO
43dBH1jOAAwAwhz788x+W07ed1ksAgLz84PnPyt7rvqWXoLzidS+R1X8bNhkqACATx4oix39eLyCO
OeiJqsJVf5ZqR84zwquZ0VcaKU3oAwDA4rjnl+
+xh/cDAPL10M0/kO6t39dLy+3al14l1d99rV4CAGSucI3IMTWMH0nkGKIWZXOvL2oeq35gxuuuHH
iTiKvZsNXn92QzxwzV1qnJeTqkAgCWjDtj/3VXXafXAADyonqjfu+aZ/TS8lIBqgpSAQA5Of5ukcIL9QLi
mn1P1N6h7OuncuqEZJedlqXedUNc/9Gtl/XnRZq7pKgAgOVzy3W32EGqClQBAPlR9VE/96Zv2h+X1
S/8xivsofwAgJzYdVDz7rl4NOQYog7XRLXrlZZq4nZEtYfVD9UoNb/OeaRVL1Uprp0RP0YFAGA5vfm
WN8tH3/5RvQQAyIvqibqs9VFVePoLv35CLwEAMmfXQX2HXkBSc1ATNUKvISuFktS8lNXRqZVSq2H
aPu+HuNXTfpGB5WVM8uWd46hJwTA7YddqEYTfT/4kcHneY0nPYU7fC+q9b2DCO//8rEiKf0cCAt7
7s+
+V2h01vQQAyIuqjbps9VGfd82VctfvUJMbAPJztcixO/VzTGKmIWpluy/9bt3vCVptOcPst1WY2ZPGu
htwVqXlDsFvVe01qobpeqIkoSO1khtC+I9Vd0Yra9/2bgFghnqNlWAPfSBnqjeq6pUKAMjXfT/zuD1r/
7JQAer1P/E8vQQAyNzxO0UKL9YLmMT89kTtXZQdnSKU61v+RFSVLXHLmHZ2LqbU+64s9S0S1NFG
TQoGTGKR76eM295ryPpg13tgBlR9VFUnFQCQnx8/57J8/g2PLkV91Le951Z5zVtv0ksAgMwdf5PIsV
fpBUxqfkPU7oHXE8sevu/1HjWG93cOpKufTsfppZpmrVUAABaVmqm//attZuwHgJw9dv3T8je3f1sv
HU23vvpF8ku12/QSACBzhVtEjv0LvYBpzHdN1FSFz85vlhPo1M7nWItxHgxO2jWqxuPoOpD20GPv6
/VjZG3Jwf1FBNd2Pdzga0fVgsysDVEya98oca+VK/5xJm1f2OsHyncO1O806o7arx19P/kG2p/K9Ux6
DsOEt32wXungORo8P2HiTbTn8/epHqPqpCY5P0DQbdffJvf88j16CQCQl7+/7Xvyjy//oV46WlQd1O
rvvVaufO5xvQYAkK2rRY6/2/p4hbOIqSxEiFqud4fDT/uRwnDa4qac1WVWRfblcGnyBRVsDU7a1ZTV
wqr1/3hUiFMKG3rcqUlpMKBKMklYe2NETUjVYzgYFmXWhihZtW+khNcqwXEma58TyIW9vrlaGBH
2qXNitLNcl/GVM9SxDbR/6us5/f0ej9OrffAcNVdHhZyTcPbh1XO2Dd97qd3vWGrvKL6DGfsBYAb+6v
WPHcn6qL/yH15JHVQAyNPx09RBTdH8hqilk34P0YN0Bu2Ha8tuuinKQug1zvnhkTuhV7/r1Zsdzzhv
5bp0dbDd9TbQlHNeotNLMEmY9dpzesOh2+3IzkX3tVm1IUpW7Rst2bVKcpzJ2tdrrHuBnP+HjZa1F
615bkRQaLRjb1OKem0koz1u06e5ntPf70n47fHb3pHa+ejIPHqivUH+PsLvvbTud0Ds2frfdepdegkAk
AdVH1UFqUfJyumftB8AgJwce53zQGpSC1HtnmjGkFHzEWco65DimpzxspxVfxuqF6C77UQbdnpv
me1yHmYvuTOyFivhWXQ9uejO2qUCFi+kKcrmBSPEidI7lH39VDo74mY3xc09J6yxHnub+mQmmiT
MmLjHCNyKa2eGQ/XM2hAlo/aNlPBaJTnORO0LtuOst74iW/aGVVC3J2GHE2hHTNWz/rmtbPtBrd
/2JNczhfs9gWrL7yFf2Up/+4q5j/B7L8+J+bAM7v7Fu+WOl9+hlwAAefjmjU/JV376u3ppsb3sp14o/
+a3mNAEAHJTuFHk+L/WC0jLHA/nDwYcXkjrjWE1w5B0mMHN0daVAzdTKp+Ukn5qK56QU/ppJDP
kHgioh2ouTjVJmK6nGTZ8Prc2REmpfSMlvFZJjjNR+0a3wwldR5fWOHUi6XdVVU4HNlaSk2473bYn
up4p3O9zpSwnAwcRIteJ+bAMrrriKmbsB4AZuP9V37HD1EWm6p/+
+n/5GeqgAkBurhQ5/mvWR+qgpm2+a6IWN2UvbMitPdQ3hXqoHtWLri8pZ7JH3OhefG5oM1EPZ
MWu5eiGPlE1KzNsQ5R5b19sebQvRuA3VlFOLEzSmcbxAvPpphfcZAepKlAFAOTn8yuPylNXX9JLi0f
1QFU9UQEAOTn+r0QKN+gFpGmqENUc9hv1cMLJoj8M2gxA7aBUrw9NMc2v04+4dRUtdp1B82tD
H2kGskvEuHZ+7Udf89zw5DXjJwlry4bZq9OtjWnWijRl0oYo2bcvLbGOc4btG21wgreeHHp1B4ZlOv
HcEcD5QZpuv+l2e2g/ACA/KkBVQeoiel3l/6IOKgDk6dhrrQd1ULMy3z1RkZGQ4dEus05mDGp2d9V
b0Q+rjZ7D7raTTBLW3vV6dtrhjxuYG8OTB6XehihZtW+khNdqguOM176Idth1itOdfT7Y9JDh+ImO
M737fWHkNjEflpGaZOoDb/iAXgIA5EEN6f+7Vz6hlxaDmoWfOqgAkCM1C7/qhYrMEKIupaKs+bN2
yao3Ztuc0XsMPcGXKlEb7K0YEnhNOEmYH/4YM+KbcmhDlFTbN1LCa5XkOBO1L6Id9rE7NVUTnsK
Rmqsb4jfdL5lQPrPmhNaJrmcK9/uiyeB+B0wf/vkPy+nbTuslAEAeVH3Ux65/Wi/NN1X/tPp7r5XnXX
OlXgMAyJaqg/oe6+PVziIyQYi6pIqbF/zehs1VHawYk86MU9n2h393alJygxmjRqg/UVew9mbkJGGV

09aSNq5dWbUhSmbtGy3ZtUpyrpO1b2w7ynXZSmV8eFnK5aas6vZ4Tbfaftab/j/Z9Zz6fs+LOdGV28
6Jws6U7ncgwj2/fI89vB8AkI9Lx/vy+Tc8Kj9+zmW9Zn79Uu02ufXVL9JLAIDMHVsTKdysF5AVQtSlVZ
TNvcFJu6rS6res/8djD/8OK6Rpb2dgoi679ubg/ixDk4RVZHuoDc72vF01d71eitm0IUqG7Rsp4bVKcJ
zJ2qfa0Zfu0IbVpo3SBlM7JWcHjzfsGiW6ntPf7/mw7q/B67F/aPQSTiCV+x0YzZ2x/7qrrtNrAABZ+8H
zn5W/ev1jemk+veatN8nb3nOrXgIAZO7Yq0SOv0kvIEuFviqCCAAAMIF7H75XKn9UkaefXYwhpgBw
FNzx5evllQ9eq5fmx7UvvUr+06ffzDB+AMhL4RqRK9R8BQzjzwM9UQEAwMTefMub5aNv/6heAgD
k4W9u/7Z875pn9NL8qP4udVABIFfH3239jwA1L4SoAABgKu/92fdK7Y6aXgIAZE3VR/3cm75pf5wX
v/Abr5BXvO4legkAkLljbxcppFNUD/EQogIAgKmp3qiqVyoAIB+qJ+re676ll2ZLhae/8Osn9BIAIHPHiiL
H36EXkBdCVAAAkAo10dQt192ilwAAWeve+n37MUuqDqoaxg8AyImqg3rsPXoBeSJEBQAAqVAz9b
d/tc2M/QCQI9UbdZb1Ue/6T6+2g1QAQE6O3SlSeKFeQJ4IUQEAQGpuu/42ueeX79FLAICsqbbqon3
/DozOpj/q299wqp/7ZDXoJAJC5428SOXabXkDeCFEBAECq3lF8BzP2A0COvv2if7Jn7M/Tra9+kfxSjV
/kASA3hVtEjv0LvYBZIEQFcteWjUJBCsZjpdHTn1tSvYasblLT1gqO94Z6fFZnX09NrrDhtXGnIqCb6x+E8
lv5aY2mo2frfdepdegkAkLW/v+178o8v/6FeytbzrrlSqr/3Wrnyucf1GgBAtq4WOf5u6+MVziJmghAV
yFVPGiur0tRLjrKcWSvq58vHDiJLNeno5YXRa8h6LarV6loXZDV4saVTK0lhIDAGjqq7f/FuuePld+glAE
DW/ur1j8kPnv+sXsrOXb9zu1z/E8/TSwCAzB1XdVBfrBcwK4SoQK66cuDmbuW6dPt96ff3ZHNZM9
SxQeScUj1nxwW/7fPiHlq1pa5zX7r1srOiuSrkqFgGV11xFTP2A0COfvycy5nXR33zr9wirr3nrTXoJAJC
5YyvW41V6AbNEiArkqXco+/qpnDohy9v/dHHF6znbk8Y53QW1XJetivO0uHlWqs5Tae6SomI53PSC
m+wgVQWqAIDsPXb903L/q76jl9L1sp96ofzKfziplwAAmSvcKHL8tF7ArBGiGjGvnqP5CKntOLI2peqN
536d163Or/dp135sb+ivNdbZLwuuD/bKS2MbmtlG7zFcYzN4jMM1S+1HWN1Low3jal3a+zDDt+aq
87WDDQ9pc9i2o9o8tpfjROdl+J4ZuZ8YxxD7fGh+W9RjuK0OZwi9/7p0z506/lKsnrNGj+NAWF6Sk7
ozquwfDt9PNqM9ie65eMduS+H7wj1Pcd9HsNxuv+l2e2g/ACAff/fKJ+SbNz6ll9Kh6p/+
+n/5GeqgAkBurhQ5/mvWR+qgzgtC1CWlwpHQMKhTk1JhQ8ZlcHHYtR8HCkLa61TIMrC+uRoeXk2
1DRU2hfYY7EitNCqEU58zapaW61J3uw52duTiYMi0674ynbqmQ8GiZh/zyGBquM1uz8dQE5+X4X
umuToieEt8DFGcfQcvd0hb7WCw5A2hd6V67jxVafVbXq/SIWaP41E6B9LVT4MqcjrpPZfk2FP6vlDn
KY/3ERwdapKpD7zhA3oJAJC1z688Kk9dfUkvTe/f/Nar7J6oAICcHP9XIoUb9ALmASHqUmqLn8O4d
TmNeo3SlHPhSUpi5XrX3na/FYybwtaPGt482TaCw6mHj7EjO4PplEcFZM7r+3ubsrlVF+erBr/GPI9nZ
FyGWtm2ttd1t2Wptpx9bOvUrr3hB4Vhbe7UZH3kdQm2eXRT0jkv/invSO28cd0SHMPY8xHg7zu8r
dZxrbvBoHEu3Iamcu40u53bEitrHVKUE6f00wiVRPdckmNP7/uimOP7CI6OD//8h+X0bQxHAoA8q
ABVBalpWDn9k/YDAJCTY69zHpgrhKjLyOwlZ/R0K27uOQGJ9dhLZaajqpx1t1M5bS25RqwPHd486T
aKsrlnBj6O4toZL7TrHIT3AyzXt4IBWXFNzugv6uxc9NvY3vV65pXPrI0P38YwexjWLxht3rwgfgZ5PrR3
31CbR5r8vFRbfnDoh3xB0xxDFHPfoW3tXZQd3SEycC4qW/5+zWtniH/u1HFY3yOhIe8k9uUwrEFKk
nsu0bGn+H2R2/sIjpp7fvkee3g/ACB7akj/V376u3ppMqr3qeqFCgDIiV0H9V/rBcwTQtRlZAQ0ImqY
bsGrZTiyhuIkyielpJ8GjFofJo1t2HQ9x9BhzEGnTgwGP0VZ8xMtLyzyA0Mj0J1YVK9WY/8jgrfhNscV/7
yMN90xjFaWk+MudvfAa79X7sF+GEPcRwyfn/zcTeuUjN51gntuimNPcv2HzlNe7yM4ctwZ+6+76jq
9BgCQJTXJ1KT1UVX907t+59XUQQWA3Kg6qHdaH6mDOo8IUZdSUTYvhPckdEOYqMl1FkZg8hyjn
mOk8MDO76nnDnc2AsPq6dg9GafXkeGOgjFCRlOK52UyYccwK2keVxw9ORxbLNWR2T2X2vVfkvc
RZOKW625hxn4AyNFfvf4x+fFzLuul+H6pdpvc+uoX6SUAQOaOrYkUbtYLmDeEqMuquCl7esjtQKlR
W/Pcos+u3ZYNs3edW7PRrMGZhHW+zurzZA+NNoYyV0/nF6FOH/qlfF4mkm1w6dXKHXpMWsd0
AsUTMrb06bje1BPcc+OPPf3vi6P9PoIsvfmWN8tH3/5RvQQAyNIPnv+sHaQm8Zq33iRve8+tegkAkL
ljrxI5/ia9gHlEiLrE1MzaqqeYPcGPHYR0vRqKo4f/GoxhxHPHrB2pgiW3/uMUba64U6Z3duT8+R29n
aqkk6GW5KR37gdnZO/JRbfoZeQQ8BgyOC+
+nI4hTOmkFwKOqumZL+NcNHfF75DZlQPvNJxwzn2EWPdckmPP4PpP/T6Cpfben32v1O6o6SUAQ
Jb+8eU/lL+/7Xt6Kdr1P/E8uet3qF8NALkpvFgP48c8I0RdRu0Ne6itmkU92FPMCHiMXnIlPxkzZmJvy
4Y3Dft884MlY2bySXgTWHWk2dQnKrWh/EXZdLsdqvO87l+XXmPdq22ZZBKkcVI7L578j8Fj1udsrvr

DyPW9bj9yHVtu1oBtyq7eda9xzgsxY/VgjnPPTXjsU1//hO8jZntyvRSYe6o3quqVCgDI3t/c/m157Pqn
9VI4Vf+0+nuvleddc6VeAwDIlqqD+h7r49XOIuYWIeoyqmz7Q287NSm5QYtRH7F6NnzmbhXSDL52
Lpkz9nttNibamUhF3I6BrlSH8o+4LiU/fZQL005glcl5MSQ9BnPYu9ueiRK2YH3O5qqz34IX9Felldqs+v
EUNy94PTLd9njnodqSeM2Jc88lOPY0r3/C9xEgiqqPquqkAgCydeI4Xz7/hkcj66NW/u8idVABIE/H3k
od1AVBiLqk7KG3YUUMVeDS7wcDHlX3cLBmYrWVcx3NpCqy3W/5gZHNOTbvsAPDrOPxhlcr5bpsp
ZzL2dcl5LxWW9Z6d+j1VLI5L6Zkx2C1Z/A+3D80ejUmYNfnNIaSu+y6nznWQ/UUZXNvuFaoPYw+Q
aAb656LfezpXv9E7yNABDVTf/tX28zYDwA5UPVR7/uZx/VS0Cte9xL5hV8/oZcAAJk7Zv2GfPwdegHz
rtBXRewAxKNmNtcT86gwbG/anqHAOEfpntPHcqpFwIpwn+l9Rip/xM0BAHl441/fIKWHXqiXRK596
VXyW3/8z+yPAIAcFK4ROf5+66P/Xoz5Rk9UILaeNNbdmc3LcmaNABVZO1r3XPu8dSwZ9ODG0fGO
4juYsR8AcrL3um/J9655Ri+JVH/3tQSoAJCn4+8mQF0w9EQFxmrLxmANWFXOgK50yMzRu+d6jRU
p7ZyRbiplKXDUFf6fgn4GAMjStU9eKe9s/aS88723MYwfAPJ07O0M419AhKjAWAOBFgEqMsc9h+
WlJiiblx9NaEs42hKOtoSjLeHmqS0nrr1GDr/3pF6aLa5RONoSjraEoy3haAvSQIgKAADmBj/ghqMt4
WhLONoSjraEoy3haEs42hKOtoSjLeHmqS1IhpqoAAAAAAAAABCBEBUAAAAAAAAIhCiAgAAAAA
AAEAEQlTkSE2WU7DrfxRWGtLTa8fqNWRlo60XHO0NvZ3CijRibwjp6Uljxb0GG9aVTbJuGmH3UNr
7mJbRRv1Y4SYFAAAAAGChEaJirvUaK1Io1aSjl4H5pgJdY1Z9W1nOrBX1cwAAAAAAsIgIUTG/eg1Zrx
GfzqeibO717RkF+/1tqei1szFPbenKgXvLluvStdu0J5tkqAAAAAAALDRCVABIS+9Q9vVTOXVCyE4BA
AAAADgaCFGXmao1OlC7MazG6GD9UXuIvfE1A+VKNbNOpXokq1Vp79Mcxt9cdbYTvjOjjeoxqk7qY
JvCa1VOdrymMbVf2xvetob2H3JN4rTRY359ZEONcxFo46j1xj7t9ea5zKoOadx7KKotg9uYsD5pjOuS
9J4FAAAAAACLgxB1WakgL7TWaEdqpVEhpPpcQUoDQ+ybq4OvVyFiSYIva8pqYbBWZBqcNq0GNh
xyDHYlNtgm65W1UnjQaYt7vIMqcrqqn3Z25OLAa9u7bmODtTKHQjgtuo2TKsrambLztHMgXeeZxR
iOHmh7W9xml8+s5dDDMoV7aKJrPizf6wIAAAAAAOYRIepS6knjnJuIuXUb+9Kt61BNOrIzmPx5qtL
Sr2+5QaEKG8/7ve16jXN+0FVt6VqVXfE2H0Nl2/qabl28L3G3sx1W8dJvU/gxWMe77oZg/mv77gF0
arI+MhUdf7xhKltu2wfPpR9GSvmMeBlqe8MPgsOuSWQbJ1NcO6PbuC+H7qbbu0ZI2ZEDN101hq
mfOpF9hDr9PTTNNTckuC7J7lkAAAAAALBICFGXkjERz96m16vQD9VEOl56FlRt+RP3+EGhqScXd9w
+e1VpeQGStc8LYa+fntmm0GPoXRS3SeX6lj/xUGXLC+U6OxdDexSOP94RimvidfQ0t22ElGaPTrN3av
2CcU02L/htrJ0fGKo+Ja+NftDbO3Si0mrVCRv3dbrau7jjBZKnvROYlRTuoSmuuWkm1wUAAAAAAM
wdQlRYdA3P0OH9SZmzk5+Ukn5qK56QU/ppespyMrCTEN0D77jsIdheXUtjqHdgSHsazOHy/rB4P5
SryllvyvYRvVNtxnbMHqOpKMoJfUHcwLlrXzzrnJ4+aYeVwfWW6mk/kMxMCvdQKtd8VtcFAAAAAA
DMG0LUZRWYKCdunckYgeWRMt3x+r1i3Z6eRig3URhpDK9PScUt3trctVrXE6cj6ik5UdFhZWC9anb
2EeriSf+6AAAAAACA+UKIupTasmH2OnVrPZr1HI+ocr3rlDEYevjD9lNT3JSzbglONXTcqCs6WRiZQYh
dOS164L4ctvUQeLv3pzs5lrF+QUP07K/5sv1xAQAAAACA5UOIuozMupwqYHLrohpDoCdXkpNuEjs
4XNoIEXNVcoamK6NqvWbF6+nZ2ZHz50fVFTXP2eBs/mZ90FMydk6nxNfQ3XdHDnadr3VrtZbsT/jr
h4e0ZyWFeyiVa57idQEAAAAAAAuNEHXJ+QGTMWP/VMxakU1Z3XCn3DFnS8+ZMcmTNFfFa1J7w
6+V6a1MmdfTsyPNpj76oaH8Rdl0u6xar6utN6yz5eg11r0anuYESU7AqVivP+8dkGx4U8nH5V+vZtP
5Wnf2/aIumOquNyfCylYK91Aq1zz5dQEAAAAAAEcTIeoy8oI9S3NVh0rGhDtTMmcun2r75iRC7nY
mCjuDs7o3V3WI5gWO5gzwaXOHxftCh/JXtqXl5XU1KdnnrCAlP6mTC95EVCojdOutWrxzHLe2bVBg
W9Yzb2i6eZ9Y68/k0w3VNv09lNI1T3hdAAAAAADA0USIupQqst1vGQGZUpVWv+8HRvaEQpMqyu
Ze1w/BbGr7g/scx2qn1yBt/9DrDZhIcVP2+oNtstj1YDOoh2rwhvQr1v62Ruysst0PrUtbbVnr3ZILLnU
8g6+ttiara2v22gwMTTeGs+c+ZD2FeyiIa57ougAAAAAAgCOp0FczrADITq8hK3oiL1WDdo+eiwAwk
urtPS8/mtCWcLQlHG0JR1vC0ZZwtCUcbQlHW8LRlnC0BWmgJyqQKbOOZ75D4gEAAAAAAJAOQlQ
gE23ZKAzU8ayeFTqhAgAAAAAALB5CVCAPql5pZpNXAQAAAAAAIEvURAUAUAAHODelXhaEs42hKOto
SjLeFoSzjaEo62hKMt4WhLuHlqC5KhJyoAAAAAAAARCBEBQAAAAAAAIAIDOcHAAAAAAAAgAj0R
EV2eg1Z2WjrhVnrSWNFzZavHhsyL60CAAAAAAADA/CNERSZ6jRUplGrS0csAAAAAAADAoiJERfp6D
VmvEZ8CAAAAAADgaCBEBQAAAAAAAIAIhKhLqr3h1gddkUZPD7+3l51HaClTVePUeI3zcL7eZW/XH

MbfXHVepzc4uF+PuW1j58HXt2XDfY318F4Wo12TGjwv6jHpuVGSHr8S1obCSkPCD8+s/eo8VkaciG
TbBQAAAAAAWF6EqEuvI7VSQUoDw+
+bqwMhX3tjRI1T9fXpBJbR1H5WpamXpFyXrYr1MbN2OWHk4HlRmquFYNCZ4blRoWtYG6RTk9Lg
BFl2EFuSwZd3aqWhcDTRdgEAAAAAAJYcISosVWn1+9K3Hq2qXqUCwPNulNaTxjkdX5br0tWv7dbL
zjrrtTsXnYiusm19rlsX9zNSbdmv7W+rxHNafjv7e5tSTNCupHqNdS+MLNe7zj77LasFWvOcDkeza4NI
W3bd1Dh020055yW0VjvW3SDXOE/uBe3UZN17bZLtAgAAAAAAgBAVUm1tixtxVraMANRTlM09H
crZ4aVeu3bGe23noKufZadc3/La6ciqXT25uOP20qzK2U13yxXZsoNGFVLuibM6w3PTO5R9/VQ6O+
JmscXNPWd/1mPPbVvvorhNDpynypa42Whn56J1ZJYk2wUAAAAAAAAhKiaha5OGDmHPzqkT44K
9tNrVlQN3A+WTUtJPFSdo9EPnYSmem+KanHGTWGtrquyCW7t0qM5p98Dbnz18X7+uYA7v7xxYR
2ZJsl0AAAAAAAAQoqIsJ82UcJTAxElGbdLcjGjnzNtlyawNRdm8ENYz2A9KQye5Giur7QIAAAAAABx
NhKiIoS0bZs9Kt46mWft0JuahXRm3obgpe3qIvV+v1tc8Nzybvl/DdfBh9KCdYLsAgLzp0Q3Ggz9yAQ
AAALNBiIrx2rte70o7oHNrfxpDyKc2ybYya1dJTroJqDsE3qVm4i8YM+6n1YaI16uZ9NUvzfakXXbw2f
XqnHrtK530Qtu4NVhjbRcAlpH9Xl+QwkwTy540VlZFWu57tPNHr+bqLILU+Qxze40Vpz0r+f/hT/0ba
p4P9zEP5wXDhq7XDO6ZmRvzvuZ9P7mPvG7mOO+3ebwnR+5jSf+gFfO8z/K9eGZinJuh951luGnG
nJfc34sTtofSdoiDEBWJ+AGdMSt9AiU/nTRm/7d+MFmdbhD8tO0KKsqaVzS0Kavem667baeO6OB7
cZw2JDp+/aavPhXsGRpSs9Wsc9pc9dvm/sOhHu7KJNtVjG3wDwuALMzsl/chKri09j/lv0npcCYu3Da
KcLuTP+4f5vte3N4IC3ONPyjOQq8h6zWRatX9x28G3NEnxsO8XrMy01/cAyWOhh+z+NZW52O1qS
YFda9TS6qdmpRmFPjk/0v7+Pc19R5csr6f6l3jHFk/T2Z778R5v83jPXncPtTnc/yDVmTok1eYm+C85/l
eHHFuht739CP98xP/vl3dH/g3Iot/IEadk9zfi8efl3zfiydoj/WNrUrbpf+eHO/7dn5+FsZY1g2DJWS9R/
TV5bd+A+hbPzD5uvW+9U+g87mq9ZZia/WtnxX060c8vNcqIa93P29uf9TD2NbIdtqStKvbr5fd9VXr
K8cxXx/yKNetVygJz83Exx/
+MDcdve3gMSfabqvqrS8PXwQAmEq3XrbeX8z3eP2+GngjyoffFv1vwAzaEEm/z8/8vXjm7XCuj9q/f
c28f5PzY/87OoP9RtP37dy1y/3eivPzV9rCv5dn1R7n5y9jv/pnrCy/l8a/rznvuYNtyPocjW9XvNdMa6J
9ZPIeqPdvbdd+hLRD3T/maud+Um3XK1IS/5w4n8/+vTjeucnjvS/
+fZv1+8v4cxImq7aNPy/h62fWnhHfw+nfR87+zd0737fBdX579Qr9vhz3uiJf9ERFDBXZVn8p0ksO56
821puAo7kr/t9KrNd7n9D2D52/MKlanIP1QqutCWuIJm1XEk4PIOsNTS/7rDdbf9h+0jYkPH57qP3g
ubQ5+wj8QdOuc2oMyXfZPWWMeqiWRNsFgMy05XytY71NXZBN503VUpEt9UY28fv35Iqbe9Lv7x
ltmTO69MupE/PRwFm1o9dYl1qnKmfn9kLNhnteWt7PKPPC+T6X6unAzyL5KMqJU9YH9+dQW08u
7sygPb2GqIFK5fqWv9/Ktv3zYmfnYgY9sRxj39d6h7JvfRj8fi7aJ64puxm9Ecd5v83jPXle3ved79+y1Ls
hP8tr6uf34dEJHdm5mO7dE/ec5PVeHOfc5GX8uXHeXwLf5xmY7Jxk9148/rzk+148tj0jfp6yR42mW
tYuzqii+fpZGOMRoi4pvw7mwJuLMeFQcMiBCgvdr1EPJ5TztxMM6dQPhf5rrYf5A725D/VQ+xmx35
Ht9MRtl/MGFlw3nvMG7H6d89gbakjCc5Pg+G2D59J+jDoG8zj1Y9QvU3G3a7xu+NgBYAoz+uV9M
enSL+W6bIX/A5CTnjTWa9KZVTvsoaMdqbbi/1ueGTUM0Rh6N9uSN/n84j6JXuOc9d1s/bI/oxu3sq2
HjNo17dWwypITNg/
+vJW13H5pT8huV1lOenWcNKPePgZk8AetuMFlmDTbEVuO78XTnJv86fJoB+cHhtOnWwJnknPCe7
FhxPub8/PnvuRaNYmfhRcOISoAAJgNfnmPTdUltX/5uTCLXoa6tpj9i6D+pWdGvR3b52vSqbZmPmL
C/0Op81AjV7KppRZXPr+4J+f2NDo7wwDEHTWkatqr7yOrObMI4efpl3ZMgT9oKfPyXhwwR3/Y6uy
flAvevxGqt6h6/9mYYa9C3osD9HwiwflBrO+pqedWiWHwjzD8LLxwCFEBAADmmDP5gRq2N6ueO
MFRDt36vqzO4hfU9oYzCcRc/dbuUL2Csh6WHcfc/eLePi/O6NHZXTNnsg5/YiAVeKtJgXKftGOWv7Q
jNfxByzKH78Xz9oet8pk147pY/4aeVWXUZtirkPfiAernGrdnrP89dXBKXadTkl3n7nn5IwymQYgKAA
Awp5wAVfXWmJ+hjMXNC3YNtk7tfK7hXHtXhU1NO8B1fuEpSEn9Vqh/CZrtcHptlsOyLfP1i7sOCGf5
y6Iecqzq2bt5jz0MViXezdWMZjYfZVa/tI9h93bqyMHgjTuqd9QS4w9ajkV4L56XP2x5ZtqrkPficIMl+f
qydXLfOk8nJau3vdn+EQZpIUQFAACzwS/vkVSvDSdAndMJ/zL8RSPMYE8j9bAngLR+Mexaz2dbt7sn
h6qoWc7nZKxZ/uLu9nw6O8NfFgeHTbpmdl7y/6V9rOIJceZ7CUZNPfuGnmG4O2f4g5Zvvt+LB+T+h

62S2GWOB8PbWf5cw3txTLq2eOCPkekZ+UcYfhZeOISoAABgNvjlPYKeRddiD3czevzkW+dSTf4wM
Bxc/0KW1S8ac6/XkJUVc0i2WqVmSp7lL6nz94u701utKjMcPSpSOW21wPoeCgyhV7dwzTovM26bL
dtf2uOpyGnVY6+27r+veL3G5m+islngD1qLaFZ/2NIjADo1Oe/9w6mHcM+oHinvxXGoUhklqUldLm
RwkSL/CMPPwounDwAAMCOtqvSt33L69a5e0a33y9aPJ2VvRX669bLVFtWewUe139KvWUZh56U
6JyfEblu53s/3bun26+Xg+ZiLe6RVHbg2rb71S2tfJT+5m+H38TB9HgKPefie1vdRxvdv3Pc1573Yf2R9
7eK0K4/35PH7CLt/3Ifxb1dq9H0x9H2r2jFw3Pp7Pu1rNcl5t78m8/fiEedGvd8M7Ns9hrTf/uKem6H
XZfY+POp+0XJ6L453XvJ7Lx7fnpB/xzO6RnHuxXn6WRjjFdT/rIsKAAAwE+5f6F3WD43zNRwQWBCq
x5xdm9BVbUl/Bl3nnO/pqrT6M5p5eS7pnk7G5ZnV9cH8Gfre9fjfR2Gvqc5r79gUjT83Id9bxnk7iuLc
LwrvxbOmRvOoOqhhgkP7+Vl4cRCiAgAAAAAAAEAEaqICAAAAAAAAQARCVAAAAAAAAACIQIgKA
AAAAAAAABEIUQEAAAAAAAAgAiEqAAAAAAAAAEQgRAUAAAAAAACACISoAAAAAAAAABCBEBU
AAAAAAAAAIhCiAgAAAAAAAEAEQlQAAAAAAAAAiECICgAAAAAAAARCFEBAAAAAAAAIAIhKgAA
AAAAAABEIEQFAAAAAAAAgAiEqAAAAAAAAAAQgRAVAAAAAAAAACIQogIAAAAAAABABEJUAAA
AAAAAAIhAiAoAAAAAAAAAEQhRAQAAAAAAACACISoAAAAAAAAARCBEBQAAAAAAAIAIhKgAAA
AAAAAAEIEQFQAAAAAAAAAiEKICAAAAAAAAQARCVAAAAAAAAACIQIgKAAAAAAAAABEIUQEAA
AAAAAAgAiEqAAAAAAAAAEQgRAUAAAAAAACACISoAAAAAAAAABCBEBUAAADAQmtvFKRQ0I+N
tl5raG94n19p9PTK2fDbuiEhLV0wbdlwz3vk+e1JY2Xca6xXNVb8ba00rK8CAGB+EKICAAAAODqa52
TGOemSUMHoqjT1kqMsZ9aK+rmpKJsX6tZnHZ3aesg1asv5Wkc/F6me3bS+CgCA+UGICgAAAOAI6
Ujt/OL38Zx/XTlwM89yXbr9vvT7e7I5KvksbsrZqn4eco16jXN+IFttyXZFPwcAYE4QogIAAAA4WuiNm
r3eoezrp3LqRKxeo5Xtlng5auAamb1Qq9IiQQUAzCFCVAAAAAABHTLzeqH590pVg6NpryIpbm9Orse
rX/7Rrehp1Vr119suC68NKtPqCtUJH1wEdeJ31CKsrGjyeYL3S6HYYzGPXj8F92fsp1ayzrDVXndeO3Ul
FtltujOpfo/aGXxagXN+yXjUo3vHbQto/dH0tcc5VoEar+6BWKwAsLUJUAAAAAEdEWep1HdJl2Bu1U
ytJYTVYDdRep0K2gfXN1VEBZlNWCyUxyoCqjUhpcMIpOxQceJ3F3t/IQK8jtZJRr7Rcl60YnTuHwlEtel
8JVbak7hZHba7KRqMh5/wEVS4M1gNIcvwqwA5pv3M+hoNUR/i5UueiNLhTJewaAQCWAiEqAAA
AgKNjzQ3p4vVGnVS53pW+qgPq9ax0hK1v7o5oh1dLtC/
+y5tyzkv7etJYd0PBqrT0a71td2qyPjIpNl6/F2OSpvaGePmv0a6um3ga+6psW5/r+hNFqRqm9n5iDc
MPTjLVrLnHV5b6hcF2Jjl+67VuGhvWfmsrOxfjnqu27Eadi8A1AgAsC0JUAAAAAEdIUTbdGYwy641al
bNuj8nKaWvJNWL9/mFoL05zBnqzXmhn56Lz+t5F2dGdIQPD3I3enN5rB4QPix+t7aeGgTCzuHnB31
ftfDo9MAOTTGnVs8OTUiU6fuu675lBqKO4dsYLbDsHXf0saOhcmfVeOzviZq/FzT1n+9Zjb+QMWgC
Ao4oQFQAAAMDR4oVsGfVGLZ+Ukn4aMGp9qKqcDiR3JTnpp31ix33dA90L01rllguwH8bwdve1A0
6dSBLymT0vz8ha4EuLsnbGbdi+HIYlthMITDJlPQudTGqK41fHZNc5DR3eHzR0ropr4h2yuodK7n4ja
rECAI48QlQAAAAAR8xAb9RD5+l8K8qJU/rp1MpyMn6am0BHRnTmnE6i8DlCYFIpo85ppLBzFSw5Y
HLD3NgTdQEAjgxCVAAAAABHj9kbtRYvTsvXYK/Onhx6Y8iHebVWhx7biYbtTyercHa88cfflg2z16lby9
Ss3ZpEcVP29D4Gyt7amueYpR8Alg0hKgAAAIAjyOiNmpQxjDw7g706u3Lg7tTtmVk66QWAo+p5ps
MsJeDXAHX05KJbmFROSaIqAdNKcvztXa/nqR24unVRp7iWaoZ+1ePUnkjLDlS7Xi3W0WUEAABHF
SEqAAAAgKPJmIAoTMlPDo3aqW3Z8Kapz1ZzdcObqKm94Q8/L59ZcwJAszZnc9UfQt7e8Gp0FlIZV2
4Gzta5WPd7WfYa61790aSTVU1twuP3A1djxv4k9PbVbRDscRoSdAMAlgYhKgAAAIAjKro3qjlzuwrp
nGAubi3NaZWlXG7Kqg4D/dzWmOFftd+ozdlc1cGh9+IREzJNorLtD1vv1KSk21XyE1S5kPuM9AmOv
3Lan6jKu5bGBFRJjDgX5r1RPat7ugIAlgYhKgAAAICjK6o3qqp7OVgzs9qavI5mIqfk7J4xPFyx63gO1Di
1a3MOvE4Je+2U7GHrIcdebVnr3eHxeYt9/BXZ7psz/itVaZk1TZu7Xs/fcexzEVYMVW8zrewaALA4Cn
1V3AUAAAAAAAAEIqeqAAAAAAAAAAQgRAVAAAAAAAAACIQogIAAAAAAABABEJUAAAAAAAA
AIhAiAoAAAAAAAAAEQhRAQAAAAAAACACISoAAAAAAAAARCBEBQAAAAAAAIAIhKgAAAAAAAA
AEIEQFQAAAAAAAABGEvn/AZM6nr53azj8AAAAAElFTkSuQmCC]]></redcap:Attachment>
</ItemDef>

<ItemDef OID="q38" Name="q38" DataType="text" Length="1" redcap:Variable="q38" redcap:FieldType="radio" redcap:RequiredField="y" redcap:BranchingLogic="[q37] = '2'">
<Question><TranslatedText>Do you prefer Life A or Life B, or do you think Life A and Life B are the same as one another?
</TranslatedText><redcap:FormattedTranslatedText>Do you prefer Life A or Life B, or do you think Life A and Life B are the same as one another?
 </redcap:FormattedTranslatedText></Question>
<CodeListRef CodeListOID="q38.choices"/>
</ItemDef>
<ItemDef OID="summary39" Name="summary39" DataType="text" Length="999" redcap:Variable="summary39" redcap:FieldType="descriptive" redcap:SectionHeader=" " redcap:BranchingLogic="[q37] = '1'">
<Question><TranslatedText>
Alzheimer's disease - Description #[no]

If you have Alzheimer's disease...

1) You might forget recent events of importance to you. For example, if you are interested in hockey, then you might forget whether your favorite team won their last game, although you may remember watching the game itself.

2) You might forget the names of people you met recently, or the names of local streets.

3) You may no longer be able to handle your personal finances or plan leisure activities such as dinner parties or vacations. Simple decisions such as what to order in restaurants might become difficult to make. Your ability to concentrate may decrease and you might have difficulty driving.

4) You may start having trouble finding words to express your thoughts. You might need to be reminded to shower or brush your teeth, but you could do these activities on your own. You may need to post notes around the home to remind you of simple things like turning off the stove.

5) You may remain aware of the day and your surroundings. You might be able to travel to familiar locations like the supermarket, although probably with the help of a loved one or friend. If you are in a social gathering, then you might have difficulty following the conversation.

6) You may continue to enjoy hobbies such as puzzles or reading.

Now consider two possible lives. In Life A, you will live in perfect health for 1 year and then you will die of natural causes. In Life B, you will live in perfect health for the next 10 years, then you will live with Alzheimer's disease as described in the summary for another 10 years, and then you will die of natural causes.

</TranslatedText><redcap:FormattedTranslatedText>
Alzheimer's disease - Description #[no]

<p></p>

If you have Alzheimer's disease...
<p></p>
1) You might forget recent events of importance to you. For example, if you are interested in hockey, then you might forget whether your favorite team won their last game, although you may remember watching the game itself.
<p></p>
2) You might forget the names of people you met recently, or the names of local streets.
<p></p>
3) You may no longer be able to handle your personal finances or plan leisure activities such as dinner parties or vacations. Simple decisions such as what to order in restaurants might become difficult to make. Your ability to concentrate may decrease and you might have difficulty driving.
<p></p>
4) You may start having trouble finding words to express your thoughts. You might need to be reminded to shower or brush your teeth, but you could do these activities on your own. You may need to post notes around the home to remind you of simple things like turning off the stove.
<p></p>
5) You may remain aware of the day and your surroundings. You might be able to travel to familiar locations like the supermarket, although probably with the help of a loved one or friend. If you are in a social gathering, then you might have difficulty following the conversation.
<p></p>
6) You may continue to enjoy hobbies such as puzzles or reading.
<p></p>
<p></p>
<p></p>
<p></p>
Now consider two possible lives. In Life A, you will live in perfect health for 1 year and then you will die of natural causes. In Life B, you will live in perfect health for the next 10 years, then you will live with Alzheimer's disease as described in the summary for another 10 years, and then you will die of natural causes.
<p></p>
</redcap:FormattedTranslatedText></Question>

</ItemDef>
<ItemDef OID="a39" Name="a39" DataType="text" Length="999" redcap:Variable="a39" redcap:FieldType="descriptive" redcap:BranchingLogic="[q37] = '1'" redcap:InlineImage="1">
<Question><TranslatedText></TranslatedText></Question>
<redcap:Attachment DocName="1.png" MimeType="image/png"><![CDATA[iVBORw0KGgoAAAANSUhEUgAABVEAAADYCAIAAABtBTQzAAAAAXNSR0IArs4c6QAAAAR
nQU1BAACxjwv8YQUAAAAJcEhZcwAAEnQAABJ0Ad5mH3gAACjUSURBVHhe7d1/qB7Vve/xxG17
k6s9Btubo55iflwKwUjZVU7J2Vc0gjQSEAJRDB5Tcjmy2YLlhICNSHO4fyQoUshWBL1QVDi9N/5hEaE
3SiyE0kICBfcf/qGQyv5DaSD+sQ1FdjB/eOfHmpk137VmzTzrmTXPPPO8XyzO2c/MWuu71nqebeYT
3bsbvgUAAAAAAENE5gcAdOT86vkN/2tDN+32pzerqgAAADOMzA8A6MLq2uqWF7eIZB6obWT/SNi4OTT4+98
SPt6vCAAAAAM4zMDwAIbm19bderu0QyD9TmTmzcf98d//aj/65qAwAAzDAyPwAguId/
+7BI5uHankd+EAX+qH35xeeqPAAAwKwi8wMAwjr6wVERy8O1O1A3166pRQAA
MwkMj8AIKAzH58RsTxcu+XYd47s2pln/qi9feqkWgcAAMBMIvMDAEK58PmFmFTSc3iWQeqM2d2d2Pjo
vXfqgT9tH314Tq0GAABg9pD5AQBBrK6t3vbr20QyD9ceevA2kfbTdmxhz1dXrqg1AQAAzBgyPwCgf
evX1+dfxeWEfX19tKTT6hlAQAAzBgyPwCgfYfeOSRiebh2xfOWFzvqqvUBAADMDDI/PVqxtr62fXm4a
g/2AwCAGUTmBwC0ZuXySEo2GJ27Pzz3x8m+0i4XVbpxfOfjkxBcg794h8xfVvlWfnxtnVSkUqzxVF8yZNNqN
/2AwCAGUTmBwC0ZuXyjKZbVoEw/QAAAOEAT//5c3ZdEfqm3G1pqFuSH5+7kxhVFBrQ+gBC7ABgDMBRNK...

baiv3FFvbaurcFKMDErl1e6/EX9Udt/3x0iinfZPr14Ue0cAABgypH5+8ASdnR5pCrRO2shS3KHpWJg
Pl1ezVognc79skqzUcVujePIR5SMVNQzWNrfgphtnpJiUkvplHUB9Zu199CmL6+6YneNDqRmMdXH
o7qYM1rW1mglmIS19bXty9tFJg/a/vnA90UI77gdW9jz1ZUrav8AAADTjMzfB5awI5VDlyUB6R2i20
3DktnPOlJcdL+s0nCU+zTyUQnHiSmyil+w1EdlX0fMAa7lVW6scgH1my33MLpoa7UOjzQ+EPdi9Erx
sOx1OoVtRmNtjVeCTq1fX9/71l6RyYO2bU/dJBL4RNpLTz6hjgAAAGCakfmBepY4CsyGpd8viUwetN
387I2Hd+8Q8XtSjR/sBwAAA0DmB+qR+TGbTl84LTJ50DZ3YuOj994pgvcE29Ldd322sqLOAgAAYDq
R+YF6ZH7MoPOr50UmD93u37dVpO6Jt+N7H/j66lV1IgAAAFOIzA/UI/Nj1qyurW55cYvI5EHb7kO3i
Lzdk/bq00vqUAAAAKYQmR8AULK2vrbr1V0ikwdtW5/ZdGTXThG2+9POvfmGOhoAAIBpQ+YHAJQ
8/NuHRSYP2jYfn3t8fpuI2b1q/GA/AACYXmR+AEDh6AdHRSYP3fbfd4fI2D1sv9r3s2+uXVNnBAAAM
D3I/AAA5czHZ0QgD93uOXirSNe9bW8c/6U6JgAAgOlB5p+s7HfDJcL9grhLywthC0yTxr+PLzq1xbPq
a36NXz31KduwIT0198vmxMF7z4N6Fz6/sOnkJpHJg7ZtT90kcnXP259/9446LAAAgClB5p+gPL2kgo
XJvA5xNdYovKszI/OPQKRx98vmyPwdWV1bve3Xt4lMHrTd/OyNh3fvEKG6523p7rv+9tlf1ZEBAAB
MAzL/BHWSIvOIFCGuxhocuy1XkvlHFCjzI4j16+vzr8+LTB60zZ3YeOCnPxSJeioaP9gPAACmC5l/csL/C
0s978dITTEyfzfI/NPk0DuHRCYP3e7ft1Vk6Slq//kfJ9TBAQA9B6ZfzKyHKPRo1A5rIuwo2WgYhozS
MnAH3GlpopkZblcmtgypSxc6tJw8UrTqUodjdnEzYqdZopl5ZIZ9WFaH3MW1/k0XnOuYrXWy+Xjqh
+R97eXz24XI8wr2bTxFTGd+6VD3jMSdRYLt86jD9FXh0Ze+NMLIpCHbrv+9R9Eip669tGH59TxAQA
A9Ntomb/BY7u1S/bUnhj3ibx4vK8ND/1VOpFUthvLrYiZsnS2M83OKZpXpCY7ayd5sZStMvqIutU3X
HzMYyqlfkGxirqWAclb02iiuvMZeTXFkFIH86J9Zktp7ZL1m1WjfYRSeZF8EnUleS2mc7+sYt9HTNU05
skv6LRdwu3dT94VgTx02/rMpiO7dooIPXXtF/f+5MsvPleHCAAA0GMdZH7xSD7uw7g+nTs99J31NI
1UlffKD05LRa4DiAeq+9kI99lnlbReYqCxYjmxnMJ78X5TFVeyufNR2ZXiQjGRKe+lrdEsZyzJGCbPp9G
apWxWbb3V8xpLMbpo09i2WaI6ZEPM1WczJC/FdO6XdnknWSCiVlE3rWWfqLRyeaXjX9T/3edveH
x+m8jPU9pOPXaQH+wHAAD913rmN7X7DK5lgMhUP9fbTtN2WLJfnrwa777hW5AVyruJccZ966US
Y4+jLz5TPVVxfrKPMSaSX3OswDauvlz20nE+DdZsMuYV74vlQkTOa+nTuHQ6Jn2xuBhPpF/Jxovp3C
+tbH3ya2rh5T7ZK8uutEuwWltf2768XWTy0G3/fXeI5DzV7e1TJ9VpAgAA9FX4zO8zplr+PL8w/c/1lp
OxZTcjxFhyYw37tKaKQlmdvLCFuZZyb2OOxouPNJ5KHmnWpbRx68Uyy1tjGya6lZdZJhbkWLNFzftS
saOKUdXrt1Gjkg7J1wvLZ5NRxZV8uJjO/dLKuhFxsTxPdtPGUQjxL+rf+9ZeEchDt3sO3ioy8wDax3/8o
zpTAACAXgr73/ZbHsiLkXnPWDmtVMrGLJ7Nvmo4sI8sp2nNPLKjvZNL4xGlU81G5cuzvJ25rFO+ViG
7P8LiPaaSR2qt1mAJcp5Yfbmsh031gmy1pKxPMiybo+hv31HF6kYsrYZFPdLe0RfppfxKPlpM535pZd
2IuFieJ7tp4yiEb5d+vyQCeei27ambRFoeRju2sOerK1fUsQIAAPTPhDJ/3ktXTiw22bB4kuzr+lF9ZTlN
a+aRW7V3chlhhOoa9dQPO1U/TdYjknYy9th4KV5TyT7Wag2WYNSK1JdrMHGDNdupgdG4rL97aZ
Gso7rqWzodl/3r/WR0cim7UkwopnO/tLJuRFwsz2MdgTqnL5wWgTx0u/nZGw/v3iHS8mDaS08+o
U4WAACgf8L/Dj/LGONSwwf3bFw6LHs1tU/7rpPRNyX7jR5zRhmh+i4sLuqHnbAsuMysk13JhzRdit9
UcoW2FVvPWLCNqy9nGyU0WHMFNdL2vti3JOe1lM4uOUurecwf40+vaPOJiu6XVrY+
+TVVqNynyawwdP+/xj+wH+M323uvvKwOFwAAoGcmkfmzK1r4sFwyyU6NBvWYeTKRPIbJXRbbtI
S3GiONyFeQKL3T+VqyyyI0ipda/3yepkvxm8o4UmPF2jyOJRjzRBqUM6rJXTRac4V8roTonN/MJs5n
zStVL66mdDHQNrR6J+6XdsYiteKqUtW02Yh8ba46s279+vqe3+wRsTxou/3pzSIkD699evGiOl8AAIA
+mUTmL4eXMsdzejZRETLMK1Ol4jTtx6PtMevQfNujjdAXIN+PfM0lDd7arEvjpXhNZTlS+4pjriWUqy
ezNSpXcz4N12ynr8nsW15xRt9j9UnUlC4G5tPl1awF0uncL6tUr1KVMuaxj3BXwbeX/36541/aP8hf4
Ke343sfUIcLAADQJ9OT+e1P9ik9eEwPx2mWNyvuW3JjjRFHWANdobw42Ud/d+OFZ6/VJkZZyuhT2
Y9UX3B0o9kS9OJJT69yskrjSWzyJVWtvFzZMp84iaalzX7WkeKi+6VD3jMSdRZn5i6dqDoflHX/P87/0
IO3iZw8pHbuzTfUyQIAAPTJJDP/SE/m5Yd6iYf8NmVnzan2C+8L2nbm4zMilgdt333+hsfnt4moPIz26
tNL6kwBAAB6ZpI/z+
+YxZCPqUAMak1+1Jxpr/C+IIjn/vCcSOZB2/f//b8c2bVTBOZpb8f3PvD11avqQAEAAHrGM/NbJRHe
CPTGBX0adS3/j5ar/hbAMkkqv0EQGl/

+NiSq3gt0jfcFYR14+4BI5kHbj37+PZGZp7ot3X3XZysr6igBAAD6ZyKZv2qiyjTjSPaOWxiRli0Jlj3C+4K
w1q+vz78+L5J50PY/9v83kZynt/2/119T5wgAANBLE8r8sfJkrsjuzPXOmwCAWqtrq1te3CKSebg2d2L
jI//yTyI8T2N76ckn1AkCAAD01WiZHwAwSOdXz3f5a/xvfvbGw7t3iAg9Xe3Ywp6vrlxRxwcAANBXZ
H4AQOy1v7wmknnQdvvTm0WKnq726cWL6uAAAAB6jMwPAFCOfnBUJPOg7Z6Dt4ogPS3tvVdeV
kcGAADQb2T+/iv92vawv7Xg0vJCR78lrvLXPACYrL1v7RXJPGh76MHbRJzuf+PH+AEAwBQh8/dc+Tc
dhoz8qhKZH5hta+tr25e3i2Qern33+Rsen98mQnWf2y/u/cmXX3yuDgsAAKD3yPw9l/1L/tD/qwRd
Z3AyP9Bfn3z5SZe/xn/rM5uO7NoponVv20cfnlPHBAAAMA3I/P3WWTQm8wPQvH/pfZHMg7Yf/fx
7Ilr3s7196qQ6IAAAgClB5p+0PPwm9H+dX/o5/pQtIOv/JYA+mexbLqSXqipk+W8MjKiu9SmmUTerK
46U+cU0zfcVabKFiHOOVKmLeb/BDMCUOX3htEjmQdv9+7aKgN23duqxg99cu6ZOBwAAYEqQ+SfJ
ErYjWVy03LUFZPskMS13OitVFcqua/MYgdkyNu3urGhP3hYySiv5oJoqvluIaQuzrkKbsn4GYDodeue
QSObh2tyJjY/8yz+JmN2fxo/xAwCAKUXmn5w8KWbpMY+WRZxsEI21wKk6FVfUhWyWEQtl82jp1u
hmVk/UVrTVMxijxJHVVmmyBdnFmEQOMEbUzgBMq/Xr63t+s0eE83Dt5mdvPLx7hwjbPWl//t076
lAAAACmCpl/Yixx1IyXxgVTNo/WpXaU2cE2xLJCo1tevSbfyoG1S4xY+iSXqodULq96C/nLqh1kHSxTp
JdqZwCm2eW/X+7y1/j/cPG/irDdh/bG8V+q4wAAAJg2ZP5JsUV+M2DmebI6546Y+fPuibyDbUh9YL
ZWl6wVG2zMVr+afV8NtpC/LpQKlicuS6eomwGYciuXVzad3CTCebh2z8FbReSebPvVvp/xY/wAAGB
6kfknxR5oKwNpdTRuEGutsTRhdtAKNZnZvo1ITUVbPaly7kLdvsY5HHU/m8Imm8I9AzD9znx8RiTzo
G3fA7eL4D2ptnT3XX/77K/qFAAAAKYQmX9S7IE2C4/ZVTOgGhrEWi22pr3MaW2FmgRm+zbqK9rq
SRVzF+r31WQLOW26RDqodhUa+wzAIDz3h+dEMg/Xvvv8DY/PbxeyeKNH+MHAADTjsw/KXn01H
OhkUcbROP6WGv2yLNpPq2tkGVmY6ClT6S+YoONVa4pm7W+iqWL2ScSXdNeles2Wal7BmAoDrx
9QITzcG3rM5uO7NopEnjH7X/7zFOgSW3+bWy2Y+iSXqodULq96C/nLqh1kHSxTp
JdqZwCm2eW/X+7y1/j/cPG/irDdh/bG8V+q4wAAAJg2ZP5JsUV+M2DmebI6546Y+fPuibyDbUh9YL
ZWl6wVG2zMVr+afV8NtpC/LpQKlicuS6eomwGYciuXVzad3CTCebh2z8FbReSebPvVvp/xY/wAAGB
6kfknxR5oKwNpdTRuEGutsTRhdtAKNZnZvo1ITUVbPaly7kLdvsY5HHU/m8Imm8I9AzD9znx8RiTzo
G3fA7eL4D2ptnT3XX/77K/qFAAAAKYQmX9S7IE2C4/ZVTOgGhrEWi22pr3MaW2FmgRm+zbqK9rq
SRVzF+r31WQLOW26RDqodhUa+wzAIDz3h+dEMg/Xvvv8DY/PbxPxeyKNH+MHAADTjsw/KXn01H
OhkUcbROP6WGv2yLNpPq2tkGVmY6ClT6S+YoONVa4pm7W+iqWL2ScSXdNeles2Wal7BmAoDrx
9QITzcG3rM5uO7NopEnjH7X8f/Xe1cwAAgKlF5p+cPHxmgTRPikVCbRAeLbFWjJIpN7+tTWsrVPRTF
/OJim6W6pH6irZ6BmMB5QsN9mXMYGwhv1DsQOypeo6mM1guANNo/fr6/OvzIpyHaz/6+fdECO
+yHd/7wNdXr6qdAwAATC0y/yTlabFEz4V53sxCrMkSJ+Uoe51YMW25T3pdC9FSNrAizNZWbLCxW
MUKVLUG+xphC1KxMvskxf36GSqOCZg6q2urW17cIsJ5uHb/vq0iinfTlu6+67Ovq9Ven6+67OVFbVnAACAaUbmn
7RynixSYqpBNLbEScsoPZbGV7PX2rx6l3wyfXlRX2Pm6jDrrthgYxl9CZFyJXeVRN0WYvo0MXNV5VW
4txsrz5DdNccBU+f86vnOfo3/3ImNB376QxHG296WNAsD1+D0mV8GI2XndhoKAcvVtNU3AtZI1C0qjjS2TU1HXXS+ev4eHGRfT6BY+H64ZbhtsdDPOKBEKVCxV7tWcljGZHtlX0tIGHyyLI6mvnbdKmK2U5Pnv+pS4N8xV6jA12tUDJWeBJq5NHg+GJnTDZ1W+j
fgCAp0+
+/KSzX+P/3edveHx+m0jpLbaXnnxC7QoAAGBAyPwAAH/vX3pfhPNwbeszm47s2imyeivt2MKer65c
UVsCAAAYEDI/AGAspy+cFuE8XNv1r/8g4nor7eM//jLtBgAAYFjI/ACAcR1655AI5/+Ha/fu2isQ
Em1DQAAgMEh8wMAAxrV+fX3Pb/aIcB6ozZ3YeOCnPxRZvYX26cWL6uAAAABjfdduQVW0AAAAAMDpkfANCCy3
+/3Nmv8b/52RsP794h0nqr7eM//jLtBgAAYFjI/ACAcR1655AI5+Ha/fu2isQ
SwcAABgoMj8AoDVnPj4wnm4dw/BW0GH6n953+cUIsGAAAYLjI/AKBNz/3hORlhORHOw7X9990hkn
zD9qt9P+PH+AEAwCwg8wMAAWnbg7QMinAdqm4/PPT6/TeT52rZ0911/+/Ry0UAAB6Mj8j8D2ra6tbntbdr
uQ7eIVO9obAbwbxz/pVoiAADADCDzN1AAAAAQDzAwCCOL96vrNf43/+vq0i21sbP9/bRrqP+0YsybvPkFM1
xMZH771TJHzRlu6+67OVFbUyACA2UDmBwAAAAAA2UDmBwAEdPSCowAAA4UjJKf/B2o3P3vj4d07RHpvpH3/4w/q
/lkfgBAWvhJivyeaC27ambRM7P26tPL6nVAAAAAzIbyvUBrAgAAAzBIyPwAgkOwBAWHvf2ivyeaC27ambRM7P26tPL6nVAAAAzBIyPwAgrLX1te3L20
B
+cD3RdqP2vG9D3x99apaDQAAAwCwh8wMAgvvky
+7Qw/8/Bg/AACYZWR+AEAX3r/0vgjngdrm43OPz2/LM/97r7ysVgAAAADB7yPwAgI6cvnBa5PNAbe
szm9LA/9KTT6jaAAAAM4nMDwojgjn4VoU+I8t7PnqyhVVGAAAYCaR+QEAHdmwoaM/dKJCUeb
/9OJF9TqYLnekvgqMQt4o5I1C3ijkjkjULehldoFnCUAICOdPmg0M2P8fOM5Y1C3ijkjkjULeKOSNQt46Kz
QLOEoAQEd4IvFGIW8U8kYhbxTyRiFvFIIDRwkA6AhPJN4o5I1C3ijkjkjULeKOSNQnDgKAEAHeGJxBuJ1BuFl
vFHIG4W8UcgbhbxRCA4cJQCgIzyReKOQNwp5o5A3CnmjkjDcKwYGjBAB0hCcSbxTyRiFvFvFPJGIW8U

8kYhOHCUAICO8ETijULeKOSNQt4o5I1C3igEB44SANARnki8UcgbhbxRyBuFvFHIG4XgwFECADrCE4
k3CnmjkDcKeaOQNwp5oxAcOEoAQEd4IvFGIW8U8kYhbxTyRiFvFIIDRwkA6AhPJN4o5I1C3ijkjULe
KOSNQnDgKAEAHeGJxBuFvFHIG4W8UcgbhbxRCA4cJQCgIzyReKOQNwp5o5A3CnmjkDcKwYGjBA
B0hCcSbxTyRiFvFPJGIW8U8kYhOHCUAICO8ETijULeKOSNQt4o5I1C3igEB44SANARnki8UcgbhbxRy
BuFvFHIG4XgwFECADrCE4k3CnmjkDcKeaOQNwp5oxAcOEoAQEd4IvFGIW8U8kYhbxTyRiFvFIIDR
wkA6AhPJN4o5I1C3ijkjULeKOSNQnDgKAEAHeGJxBuFvFHIG4W8UcgbhbxRCA4cJQCgIzyReKOQN
wp5o5A3CnmjkDcKwYGjBAB0hCcSbxTyRiFvFPJGIW8U8kYhOHCUAICO8ETijULeKOSNQt4o5I1C3ig
EB44SANARnki8UcgbhbxRyBuFvFHIG4XgwFECADrCE4k3CnmjkDcKeaOQNwp5oxAcOEoAQEd4Iv
FGIW8U8kYhbxTyRiFvFIIDRwkAAAAAwDCR+QEAAAAAGCYyPwAAAAAAw0TmBwAAAABgmMj8A
AAAAAAME5kfAAAAAIBhIvMDAAAAADBMZH4AAAAAAIaJzA8AAAAAwDCR+QEAAAAAGCYyPwA
AAAAAw0TmBwBgVGcXNxQWz6qrAAAAfUPmBwAMSBrGw6bwS8sLRYWQBbv+m4V4Y5GF5Uvq
QptKm0nwdyWjKp1hmLcpuIpvGPXZS7X7yXB8izpujco+Vekda3dbYTlPJug/KAKq3tS0vk8VOyptp62
3qUGtKf1n0owg8wMAwgr4NF+i1enyoS0pG+JZJ3qWyveRPFcFfqCKN7KwuBj9nyB14i10/kioP4+2/
6kofbILgT59yV46+kTo59Zekcrv0PL3UHmjY6ms6Lw1qqqp4uv5hfRIxy1ln0V/u8YuUXsy8f1W/0Fh2
1RpS4nx9uXYVHKrxW8lcztacV2gHSX180vJq3F316xWC6XSs1PE+ZROUdxDA2R+AEBA5eep8tNIq7
JCyf/v8oGgvMNQgleJC6jjC1Mmfu+DH5OumzemJCkZ6MMn5w5Xq+3neCV7P8yFy49GW1urrui6
NaqmU2X91MuRJeNT5SrR8eUXxn+76rYTX1e3x6qTcm2qjfkVx6Ysl/xVbkcYv2j1juSVgLWyG+rluO9
aPF0+ffJJLl6WSyU3x9rTLCLzAwDCkc8AxlND68JXELp5/Ai8rXz6+Av/hzaX8R4HR9b55yAS9qNQPsB
g+0sm1t+ott84Y+XmVlo+SMdZtXiMdVMl970PMhsdtorGXii/Gn8xdplssZZaYf5xYRZqZR+KYztlLX6+
a4+udjHNGVMZ+2ixWH6ayQv5cWiz0Mwg8wMAgjH/ZG7xaceu44eBZD9tPTNWKj3+tE87s/jLMI
WSkyoEPrNw+6gU+E2KpEcYlUi/CPMpN75DtU9HK4z54orlc2u5pGO6FivVTWUcrJe6Kq1tyDaRdi3
+srUPu6WW+aFog1koqRP/oEJu/Kq2o9Mkt9vam7VW8lGLS6RfVC9lNEatiuNsZ2v6MdkLtbaxGUH
mBwAEYz4BWB9R2hS8gC558Gjr6c2U7EUJuKV4F9n0cclwG8qkGwtZJ/nktfwo79bNBy/5xCVClTL3
YX4Xj8UoYM7f8lk6pmuxknuq5I1r4RTdVZK77bxXlkLxHrIr8e3WPhQVtTQtlTILifcl6VB9vs1YtqNx3x
1V1WzF+bVWylIruSJPr6X3KtmBqhZ/XZ623VOcDWR+AEAwE/ijurtnAfG0GFSrD1Nl8TaK84oLdbIn
86PRKvHmpOcX8mORFAz6udP3kH4dpl4ytzy6Ft+qZEJ95eZHwegyHsd0LVZyTSU+jmOwVkkuKm0
dm1Eo3kPxOr7d2ofCdXSRdHttVDMLGR+
+Fr6RndtpYX6dpVZ6XOm19OuW6ln3lewns7hY+pCMIZk2f2OMd8l9xrAi8wMAgpnAH9UdPQukTz
odPnOkD2/tFyw9smnaeMJ2SeqGOz/jkxe2XvLuBD0ys0LAHZU+FO09x6eM71DzW9Z498ZjFsg5bo
2qcqrkNNvaTs2Ck9utFJOFSp8JTYhappY+EWYhY+L6tdRyTJHcauuzEDNqmRWSd268HaXqjybu0cb
u5LeM+fbXrwUSmR8AEIz5B3NLz27VungWSGp0/MCR1Ax6com4TPgq4bcT4lG+WmtP1dXMEkF3
pGn7I2GuW75Zbe/MMV+LpexTJe9bi5upXXDSoYWCNYXi2619Kmo31dZ3mFkouaJvRH4YPVRvp6
VtaIxaZon6022odqK4w/gfimQH5WnMyi28TTOHzA8ACEf+ydza40el8BXSZxKp9eePqIq2DctzUBDt
PLWZonm1aZM3Kei7lFTQNxLyGTF5c8J+5sxPQCdFI61/IpK3przw0t6Mt25slooZx61RWaZKLrX7JtU
uOOnQwvHVFIpvt/YmTXRTpW+kVr6rKrcT4HvWrFX6Zoq0V9T9NrXzHiWrNYvEl/W53UuBFZkfABB
Q6QGknacCu2RuYbqfCcSOutlMXDTIO9T5bkqPuu0995pCfqrL0ufhXAefiDb3Jj4BiWIL+t7aOkxHRfd
iRlI9lXi/UmNuLqlWWmlURXud1BynRsOTibu19qE3NhVd0CZPl2RZRGPuTZXujlNGMbaTSi538Nlu/
R8U1bXKd8Y+unQ66zSlz3WrJzk7yPwAgLD0BxD+nEZnSg+kYz+PVkk+3sFmn4CWn+PRivK7klLvjbg
1Re9Y9aamck+O9ygyuH9QtE78VUWqeGTgQWJMZH4AAAAAAIaJzA8AAAAAwDCR+QEAAAAAGC
YyPwAAAAAAw0TmBwAAAABgmMj8AAAAAAAME5kfAAAAAIBhIvMDAAAAADBMZH4AAAAAAIa
JzA8AAAAAwDCR+QEAAAAAGCYyPwAAAAAAw0TmBwAAAABgmMj8AAAAAAAME5kfAAAAAIBhI
vMDAAAAADBMZH4AAAAAAIaJzA8AAAAAwDCR+QEAAAAAGCYyPwAAAAAAw0TmBwAAAABgm
Mj8AAAAAAAME5kfAAAAAIBhIvMDAAAAADBMZH4AAAAAAIaJzA8AAAAAwDCR+QEAAAAAGCY
yPwAAAAAAw0TmBwAAAABgmMj8AAAAAAAME5kfAAAAAIBhIvMDAAAAADBMZH4AAIB+Obu4

QVk8qy6lshsLy5fUlTCyOqJ8HxRnEzHO4dLyQtUt5z0AGC4yPwAAQL9oubYcT2c98xexPWE7hspkXx
xqD/8mAwDCIfMDAAD0S+nfZesJddYzf6P9W8N98VcBJH4AM4bMDwAA0C+lzK8H3BnP/Hlwr1lXf
n75OeVXSPwAZg6ZHwAAoF/KmV8LqkbmNy6YwVh1iXto86oB2hU9DWeXo2vaf06vFclod4372tqK
MjWZuzRfaUJtpRnHXHnvpE/
+ytiBa/2x8npEF8cGneMAoFNkfgAAgH4pwuRy+lUWGbWUmbw2L2hxU0XiIo7WykN09RgtZ8tcm
9AWYpnEGX3tReXWNdpaDMXiFpezL0X1uvXXLKh6g/ZxzuUCQDhkfgAAgH7JQmOUILNgmgZG7U
bS0bzgyvyqkxZJ5ZUslhpjiivZBVkm75EvRavTIO5WF8gnNGu65J2VYppE7fqzDtXrqdhg1URyBQDQD
TI/AABAv5RSo3qhf23GTi1OyjCbdSlyafWVbBqzh+wjE3FEXson0fpUkiuIyZ2YF9zyBcTEkAbrl4zq9g3
m3RrtGwCCI/MDAAD0iz1eR1HTSMaWqCyjqdml/kr2Wg/K5XmzHjaicGmWCuaSYlnJ7KrcWq3qJT
RYv6bcO7tfMXu+ykJ5WwDQKTI/AABAv8gErF7nP95fZEjZMxIq85cvZi9sKgtXs/eVW/HO/OYSGqzfF
t4T2f3K2SsGNl01ALSKzA8AANAvRpiUITK/YYmdMhibXeqvWKYV89p6CA265Ox9s5LZVbm1WtVLq
F9c1iOSdjKq186hTZGoLgYA4ZD5AQAA+sUSJsvxMb/h6tlC5tfDdblPg/htlqmWT6d3Nmq0mPlrpzK
HGofi2GB0S5t45HUDQlvI/AAAAP1iC5N5bozlN4qrKk7mwdS4YobX6ivVs+RdqitnwdYs41JdoBg/cn
Z2LKFu/XI7Rf+aDRobcS4DAIIj8wMAAPRLTZjUb2hRVBJZ1hVB5ZX89YKcPcu7EXvpooNZxi3rX6aPbj
Pz167fvpxY1qVq9qqRTVcNAK0i8wMAAPRLRZgsQmrphp5do1gpg7E5Wf2V7LU+nbGcWDk3lztU7
MKpPJ8MyXJrtWqX4Fp/MTwWl9SORb9tm10fGmu6YgBoHZkfAAAAAIBhIvMDAAAAADBMZH4AA
AAAAIaJzA8AAAAAwDCR+QEAAAAAGCYyPwAAAAAAw0TmBwAAAABgmMj8AAAAAAAME5kfAA
AAAIBhIvMDAAAAADBMZH4AAAAAAIaJzA8AAAAAwDCR+QEAAAAAGCYyPwAAAAAAQ/Ttt/8fGf
gb9+u7XWAAAAAASUVORK5CYII=]]></redcap:Attachment>
</ItemDef>
<ItemDef OID="b39" Name="b39" DataType="text" Length="999" redcap:Variable="b39"
redcap:FieldType="descriptive" redcap:BranchingLogic="[q37] = '1'"
redcap:InlineImage="1">
<Question><TranslatedText></TranslatedText></Question>
<redcap:Attachment DocName="B.png" MimeType="image/png"><!
[CDATA[iVBORw0KGgoAAAANSUhEUgAABVEAAAEJCAYAAACdcmdaAAAAAXNSR0IArs4c6QAAAA
RnQU1BAACxjwv8YQUAAAAJcEhZcwAAEnQAABJ0Ad5mH3gAAE9sSURBVHhe7d0PjCRnfef/b+/aY
PPHNmBskx+RHat7sZYlGJIg08PxL4egZ+6SJbnbYOVg5Oh3PVyi0zQSkwOxF+mX2wguG4lunS5iWrq
zliQyTEJugrLdIJRgckzHR4IxYTI/Z7rlc3T8wDYGjPljB3u3f/VUPVX1VHd1dVV3VXX39PtltaerpqfqqT/T
O/OZ5/k+hb5FAAAAAAAAAAAChjumPAAAAAAAAAIAQhKgAAAAAAAAAEIEQFQAAAAAAAAAAiEKIC
AAAAAAAAQARCVAAAAAAAAACIQIgKAAAAAAAAABEIUQEAAAAAAAAgAiEqAAAAAAAAAAAEQgRA
UAAAAAAAACACISoAAAAAAAAABCBEBUAAAAAAAAAIhCiAgAAAAAAAEAEQlQAAAAAAAAAiECICg
AAAAAAAAARCFEBAAAAAAAAIAIhKgAAAAAAAABEIEQFAAAAAAAAgAiEqAAAAAAAAAAAQgRAVA
AAAAAAAACIQogIAAAAAAABABEJUAAAAAAAAAIhAiAoAAAAAAAAAEQhRAQAAAAAAACACISoA
AAAAAAAARCBEBQAAAAAAAIAIhKgAAAAAAAAAEIEQFQAAAAAAAAAiEKICAAAAAAAAQARCVAA
AAAAAAACIQIgKAAAAAAAAABEIUQEAAAAAAAAgAiEqAAAAAAAAAAEQgRAUAAAAAAAACACISoAA
AAAAAAABCBEBUAAAAAAAAAIhCiAgAAAAAAAEAEQlQAAAAAAAAAiECICgAAAAAAAAARCFEBAA
AAAAAAIAIhKgAAAAAAAABEIEQFAAAAAAAAgAiEqAAAAAAAAAAAQgRAVAAAAAAAAACIQogIAAA
AAAABABEJUAAAAAAAAAIhAiLp0etJYKUih4DxWxWPB29Gj29PqXWPF29Fu+PqqjXjWB1hpWF+1iMxj3ZB2onL
MxLO7LSlg372PzHqPs4Vb2GrGzEO5vtDbdtK5JH08KNuQ9CCjmc+2g0AAAAwPwhRF06RdmdK8Jjey
XurU1kPCkracr3ZhM8LqsTrM/sZE6cTGJc5TRoFrUG7+NIefp1GZpOOuOT4fe4ZJYU35i+ChBaMoo4
uytmqfi4dqZ0P9kbrNc75IVW1JdsV/XzhZfF+mO+rVh/mWf7jW4QLU+1+xg+rBRf1mmiO/l2Obmk+nl/Xm1GcZ
D7TVk3fijwsI7asDAAAAAEAuCFaQBA
AAAEAuCFGXVkW2W2W26W26M6vdGbW/4Q6XL9a3oXoPgrr1mlGsIEpXsaHa1kmXp7
AAAAAEAuCFGXVkW2W26M6vdGbW/4Q6XL9a3wXoyqlqKuRek+wmpSjqvaH59ZI1Jo6jZivaH59ZI1Jo6jZjoC7rqPX

GPu31edcGHd5fZI1J4zwEz5+5nbDPjxN+3INtCdS9tR4xy30ObGewraPOc7xjCm57uPZpYAh6c9VZF
2h4snM3eA7Uw9yc3Z6x+xzPPy71CLkXbAnaHvI9OHq7vqTHE6/dAAAAAAAcfYSoy6yyJXW3OGpz
VTYaDTnnJ6hyIWSM9FAIo3VqpQwmoCrK2hndwM6BdJ1nFmNId2dHLno7bcuubn/5zNpc9FKsnPa
D6gP/AGy9izv6PBo1Pe1wrCSDo63TPb8dqZUKUhrYSXM1aUimtjPY1qasDoZtEx2T2rZR+9S6H+te1
+kREu3HCSwHz4HSXB0dKibnnOtV70AUtW6Kc9TeGFHPNGS7E4vZbgAAAAAAlgQh6lILTjLVrLnBTF
nqF0Imk2pv+KGKV4+yL103ie3UZD3lhKW4dka3b18O3U23d42JhYxw0hjqferEnAz0rpz2yiY0d4M9
Ji/u6BisfEacDLUnjXX3GlSlpc9v3+0xnOr59bfvdUhWIdlAfdzx/O1494Hazrob/E1zTMbr9zZlc9v62PX
vV1Wv1/6cXXIi2X56jXUvsCzXu85r+8MlLiqR+4wr/BzteOl/krZbr3X/0hH2PRjY7rBkxzOu3QAAAAA
ALI+C9QtyXz/HklK9SwM9zlSwEhKq+K8rS71rTubTk8aK24tOBS/OZEYjX6963bk96Ubsy+dvW4Vde
9ZG1BBs1YOwWq1Ks9kcWu+3IaxdcdeNE387w+2yV3rnwG1/6DqbsV0VnO2FBNwB4e0wr3O11fc
nDEt0PSK2E9ivvuaS7JjMbQdfr41qa6JzF35+FOdanQqsS3p+lETnOlHbRwht44jjjDietO4RYBr3Pnyvv
OXCW/QSACAP99/xLnnNW2/SSwCAzB3/NZFjr9ILWBT0REVwkikVtoQGJP5Qeb/npMsYdm/2GE1F
UU6ccp51dJfTrj2WvywnT5+0e9QF11uqp/0AbA74vWmb4nVG7R44gZT1GW8ov7dOdT4sGbUo3S
BMfcIsazBr1jUo6ac28z7QPYSnOKZEvYkT7cec4f+kmIdQ3NyTvhmgTm3wHIWY6rrrurFuwJmaGO0
GMvDwEw/LOz/5Tr0EAMjDDY9fJXd/6AF5/Bs/0msAAJm7dI9I/zt6AYuCEBVBA6FScsO1P6fl1RVt7k
pbenJoj9k/JScqJ6z/D65XGeqc9ZIrrombLbpD+ttuIl09a/TozUtaAZl1DTJrOyHeSKpXqBe0GnVjgQX3x
NNPSOWPKvZHAEA+nvPjY/KWL94oP3ryGWm+/3555p8u6c8AALL1lMilP7A+PussYiEQoiJlGYRfXl3
RfTlsXxS7lKgd9lbEyVeN9XMZvhk9NO3A1+/VOyrwVcO67VqVQ480e0lOK1mv47yOaTHOXbjxbW/
Lhtnr1K2LatY5BRbUnZ+6Ux58/EG9BADIwxv/
+gZ5wQ+vsJ8/9NXvyp/WeR8GgNz0Hxa5/Od6AYuAEBUxleSkN1LbnBFfMSZJitM70Ri+HI+7744c7D
pf686+X7I/4a8fLjUwH4qbZ/0guOFOjFWVQIZacsoTKG6Jgvk22OvYvA/0seV1TIn2Y97LA8Pk1cz3hZ
xnoE/SdmNSNTtwdeukJv6eAubL+z77PvlM7zN6CQCQh1c+eK3c/PXn6yXH5z7+kHzlLx/RSwCAzF3
6gsjlr+kFzDtCVMRUlM2zbuVUc/Z1NbrYnOl8y+vt5wScijnre1s23JlrYvN7cqqJpBS3XmZRF0x117vh
6vxxe81a56Kmj3+wdqsx7F+aq7LhnTIV7Onh297K+dBcNQLH9nm/hqd7bHkdU6L9mLVbm7Lqre/p
me+ta1Qq+NvI2oTnyA9c3XYDi+kT+5+Q+n11vQQAyIOqg/pzD7xELwX94W9/Tb73raf1EgAgc5f/RK
T/fb2AeUaIivgq29LyctSalHTA48w8bynX5YJR4NOfUMnSXNWB0GQ1HAPbsp55Q/a9of6KMUnTHP
Jqu9rKUt8KRKiWomxe8IdlN1d1gOaFzqMm/ZolJ3AMttM8tryOKdl+ipsXpO6/2HmtOZGTdS/7h6Br
7yrua1NNWBO03bzfw9odR+bHA8R339fvk7v+7C69BADIw/FLBXsYv/oYRgWozd+8Xy8BADLXf1Lk
8sf1AuYZISoSqWyH11+stqz17tBiV3FT9gZfW21NVr/R7K0XKBlgDM3OdKKjFJgB2KiyA+qc9bt+wOe
ya1/OW03PstS7fT9Yt1Wl1d8LTpaV1zEl2k9RNvf60h16sXq5MUzeVpHt4EGK7B96PbFTEbvtVlv6Lf
8+sqlzblwHu+5ulByOB4jBnYn/6Wfp7QQAeVr50kvI2iev1Evh/uFL35ZP//6hXgIAZO6y9RvZJcpbzbt
CX81aAgAxtTcK4nSSVCHqQGAKADGo4PT1/+318sAjD+g1AIA8lB56od0LNa6tu8vyiteFD/sHAGTgit
8QKfBL9ryiJyoAAMiVGsJPgAoA+VK9T1Uv1CTUsH7qowJAji79AfVR5xghKgAAyM1HvvgRezIpAEB+
VP3Tt33hZSProI6iAtS7/+NX9RIAIHN2fdR79ALmDSEqADIxe6Du/LBv/igXgIA5OWOL18/tg7qKPv/
8zH53Mcf0ksAgMxd/n9FLn1BL2CeUBMVAABkTg3fV3VQmUgKAPJ189efL//8Czfppclc+dzjsnX36+
XWV79IrwEAZOtKXR/1Zr2MeUBPVAAAAkKknnn6CmfgBYAZe8MMrEk0kNcoz/3RJmu+/X3705DN6
DQAgW9b77aWPWx+fchYxFwhRAQBAZlRwqgLUh594WK8BAORB1T99yxdvlOf8OJ1f+R7/xo/k7g8
xKSAA5Kb/HZFL1EedJ4SoAAAgM+/77Pvk3ofv1UsAgLz83AMvkRsev0ovpeMrf/mI3PtJ/igGALm5/
DXr0dELmDVC1KXWlo1CQQrGY6XR05/LSa8hKxttveBob7jtWZG8mzM3Qs5LHL3GinPuVhoy6tT55
9d55H7NjwLuWyCW+n11+djffkwvAQDy8vJvPE9e+eC1eildn/zPB/LN//19vQQAyNylXZH+Y3oBs0S
IurR60lhZLaZecptTlzFpRP8+eHfiVasLfVIImPi+9hqzXor5KXfOCrAYvunRqJSlMENguK+5bIB7V+1T1Qg
UA5Ovqp47LG++bvg7qKKo+6u//
+y/bHwEAeVD1Uf+79fFZZxEzQ4i6tLpy4KZA5bp0+33p9/dkM68MdWzgt6QmPS+qZ+S4YK99XtxN
V1vqevelWy87K5qrQo4aA/ctEIuqf6rqoAIA8veWvRvtIDVLqifqH/721/QSACBz/UdFLv2xXsCsEKIuq

96h7OuncuqE5Nf/FGmL1zOyJ41zugtquS5bFedpcfOsVJ2n0twlRQUwPTUTf+WPKvZHAEC+Xvu1F8
vLHr1aL2Vrb/f/2A8AQE4uf8l5YGYIUZeQXbvRDN2aq04tx8GuiKp3o1E7c1T9zGAtyGCd1bDejbH3r
wVreI6qN+kMVfdfl6zW52A9S6+2qH6ENy3GPgPncEP8zZjnydln0vOiqHaWYvWMNHoeB0LzkpzUn
VFl/9A6olGM9obVW21v6GMZPAcJrkvI/RZ2vePcb4PXz35E1ImNY5b37WT3p2vSa2eZ6D1Ar1TMr
49uJI6QOz91pzz4+IN6CQCQFxWevubvXqSX8qF6o1IfFQBydOlPqI86Q4SoCDUUGGl2/cyRYVRHai
WjzqrR43EyanuDNTzVurCgpuQNVXdFt3UUZ5+DwWRzdcJ9Fjflgjtk3joz5/RGeo1z3nkq1y+kUEahK
q1+y+tVOsTseTxK50C6+umwipx2N97ZkYsDJ7W96x2NX1c3yXVRQV7I/eZcj1EBZPj9pu7d0GC5U5
NSIMjOSpb3bcz7M2CCa2eZ7D0Ay07VQP1M7zN6CQCQFzV8Xw3jz5uqi3r3h75KfVQAyI2qj3qP9Z
H6qLNAiLqEKtt96Xfr4kZ7Um3Z9TH72zrxbG/4AZBXL9Won9mpyfrIxEaFec7r+3uboWUCxu4/wN+
et3/pyI6XBPWkse4GPca+Wzo1imzrKP523M2ofdbOu/Fbsn0WNy+If+rOS9v677wbglnn94JOUJOd
F4P9um2ZLK8uyolT+ukYlS23beb5V9ri53BnxMnhkpwj67VGqYGh+21ofyZj2/b9ZrYlbFt+kJ3U/Ny3
/uvD789hya6dZar3ACyrT+x/wp6NHwCQv5UvvTTzOqijPPTV78qf1hmBAAC56T8scvnP9QLyRIiKIW
bPtPoFPwgdDgOHletbE4Z54aotPxwsrp3xAqzOge4z2bsoO14eaey7suW3deeiJll7zH364ZMh8T6Ls
nnWTbuaslpwe08Gz+8kipt740PW2PblMOpEFdfkTNjxtXf9XrVn1pzjSXSOrPOzp0NEI3gPvd4Dhu43
s8et0evSPk86CNzLYfa0LO/bsfdnmCTXzjLNewCW031fv0/u+rO79BIAIE+vfPBaufnrz9dLs/G5jz8kX/
nLR/QSACBzl74gcpkJ/vJGiIoBI3qm2Yqy5iYxIwK3UyfSDKjKcrKkn47SPdC9+VSoU/LqNhbMYdKRw9
QnMMk+K9tGr0FHOsP403RKoi+fcf2NgNIP3Kpy1j2gqa6LruEZOrw/aOh+M8JCawf20Hd33yNrsaZ
uHu/bBNduyvcALB93Jv6nn31arwEA5OWGx6+Sn3vgJXpptu7+0APy+Dd+pJcAAJlTw/r739ELyAMh
KibUkeHOgTHCo7mX3TFUvMKUSrD+5Oz05HBssVSf36vSHRZuBG7V08FeoUmYExB5PXXHCbtWR
dm8EN470w0rF3t+o8nvz/SvXdh7AJaNCk5VgPrID+h9BAB5e86Pj8lbvnijHL9U0Gtm60dPPiPN99+v
lwAA2XtK5NIf6OfIAyEqJjR/gWm53vWGbAcfk9YLHS/
+Po26n7aO1NZznJyneELGlj4tn5Sxl7S4KW5lAntYuDF8vno6/CyPP0dt2TB7nbo1OM36o0lYbdzT+xj
s/as0z83XpEi53bcTXLtoR+GPJpiWGsL/wCMP6CUAQJ7e+Nc3yAt+eIVemg+qPuqnf/9QLwEAMqfq
o15iYte8EKJiQElOusnV0EzePbnoFnIcO/Q7J6WTXtA2qm5m6ibYZ6+xrodpl6Ved5OsPCfnMa5rc1f8
zphdOfAu6QmJc0m9HrXW/XH+/I4OP6sSyOGSnCOzLqcKFN26qMaQ96TUzPKqx6k9GZQdSHa9W
p7pDpOf0CzuW0usa5fme8AU1xDz7yNf/Ig9mRQAIH+lh1448zqoo3z6v/6D/MOXvq2XAACZu/xZkf
48dRU6ughRMcCcBCnYW9IPAlXYNTChz6yYNTCbq/5Q7faGHhpuPdIev510n72GrLsnrnpWNjeNyY
Nq65JPjmrWsmzKrm5er3HOCzBj90asnBbnDulIs+ke18Bw8Amvix8oDvbcjUlvX80sH+xxaoTFZo9b
oz1p3yaRZnHfKnGu3QTvASU/dZXaee9gZMOb4h9Hze6Du/LBv/igXgIA5OnaJ6+0Z+OfZ83fvF+
+9y1qZQNAbtSw/v739QKyQoiKYeYkSJ2alHSoU/LTE7kw7YxI5vDy5uoUoVGwBmZzVQdQXnhTlVZ
qs9e7kuyzJ411d6h6Wepbav3okCq98zLMnFndbbN3TastiX+aKhIo72oZDmATnCMv2LO4x2xOsJTEi
HvXrLNaPevPNp/YQt+3SpxrZ0n4HuDXW7V41zBubVssGjV8/85P3amXAAB5UvVP3/aFl81NHdRRV
ICqglQAQE76T4pc/rheQFYIURHKHgYdUpOy2rLWu8Otp1KR7cGClfuHRs/BBOwamMZwbZddWzOj
eqgx92n23LN7obonbiCk8of1p3hehhRlc2+4Tqg9hD5hYBeYJMs6ZjsbHhT7uljH3G/5QaqtKq2+0dZ
ACYJo9r07eJA2Z5vTZZMLft9aYl07S6L3AHUsg6+ttiava4u59cTTTzATPwDMkOqBqnqiLgI1pJ/6qAC
Qo8vWb6bUR81Uoa8KBgJAEmo2fT0ZlAph96btmTxL+lhOtaYNWBfEUbp2yJUKTit/VJF7H75XrwE
A5EnVQVWTSS2SK597XLbufr3c+uoX6TUAgGxdKXLFb4gUbtbLSBM9UQEkFCxRcGZtsUO49nnrWC
J6ZB4tR+vaIV/v+
+z7CFABYEbULPx3fPl6vbQ4nvmnS9J8//3yoyef0WsAANmy3m8vqWH9TzmLSBUhKoCY2rJh17o06
pWaJQoWUK+xIqv7demmUqJinh29a4d81e+ry8f+9mN6CQCQJ1X/9C1fvFGe8+PF/NXt8W/8SO7+
0AN6CQCQuf53RC7doxeQJkJUAJNRNS8XfPx7cXMvpRq/C+YIXDvkR/U+Vb1QAQCz8XMPvERueP
wqvbSYvvKXj8jnPv6QXgIAZO7y10QufUEvIC3URAUAAKEefuJhec32a+wJpQAA+bv568+Xf/6Fm/TS
YqM+KgDkjfqoaaMnKgAAGKKCUzWRFAEqAMyGqoO6aBNJRVH1Ue/
+0FftjwCAPKj6qGpY/7POIqZGiAoAAIbc+ak75cHLVC8qQB1UeugjvLN//19+cPf/ppeAgBkrv+

oyKU/1guYFiEqAAAIUDVQP9P7jF4CAOTttV97sbzs0av10tGyt/t/7AcAICeXv+Q8MDVCVCTUk8aKm
uVbPTaknWjdOGltZ96MOYZeQ1Y2gmvbG+7rV6TR0yunpfZjb3NEO2zTne9M2m1bkPugvaHbWJC
BSwosjE/sf8KejR8AMBsqPH3N3x3tuqGqN6rqlQoAyMmlPxHpP6YXMClCVGCGeo0VKZRq0tHLWe
pd3DH205RdQj4AA+77+n1y15/dpZcAAHm7+qnj8pa9G/XS0aXqov7+v/8y9VEBIDeqPup/tz5SH3U
ahKhIqCibe33p99VjWyp6bXby3l8WRhxDryHrtTziU6UnF3eC+2qea1hrF8VRuA+A+aZm4n/nJ98pT
z/7tF4DAMibClBVkLoMVE/UT/7nA70EAMicXR91Vy9gEoSowDLoXRQ3Qy2Xy86Tzo5cXJwUdTFUt
qXfrYs+w8DCUMGpClAf+cEjeg0AIG8//ffX2UP5l8m9n3xYvvKX/NsDALm5vGc9mOBvUoSoS8eoLbl
i9kQctd6oc2mvz7s25fD+IutuGnU/VwKfNLcT9vko6Z8z+/PmMP7mqvP5EYU0/WNWj+T1Rv2h/FU
5e+GMDvk6shMnRQ3UUg17jG5PvHbHuTbD59DaumyYrzfqkQa2MbA+/BTHaYN5POpY/P2rh7vd
9nnrupbrsjXQXdYu3WC83n4M3DfArKgh/A888oBeAgDk7YbHr7Ink1pGd3/oAXn8Gz/SSwCAzF26R
6T/Hb2AJAhRl05R1s64PREPpOs8s3TlwE30Aj0U27LbdJ6Vz6xZXz17ldNV/awjB/4B2PywsCxn1nRr7
RCwJIMj5zu1UswQa5bnrCO1UkFW9fYcal2SINUYyl89LZXimniHs3Nx+hCvfEbcU+2L2e6pr43Dfn1w
Z846FVQOrG+uDgSpE7VBHcuqeFvWoakKSlf369Ld2wxcdxW+lsJKN3RqUprnibKwFD7yxY/Yk0kBA
GbjOT8+Jm/54o1y/FJBr1kuP3ryGWm+/37qowJAbp4SufQH1kfqoyZFiLqEimtuT8R9OXQTovauHwi
JEU72Dq1XOU6dmIcI1VI5LW6M2gzMjmSEhV6w15PGutvjsyotu6am9WjpLXRqsh4jjUz7nFW2rTa
Yw76rLadd22HVPv12d+vuV8TsRaoYQ/mrp9X2zVA4xpD+4qbsuefNfnTFa4Z1BPULwcDQN67d6V
wbV7neDX69Frbev2+maYPxeh2aFjf3vOc+P1RXYWtXf41/TppyLsFxAmnafXBXPvgXH9RLAIBZeONf
3yAv+OEVemk5PfTV78qf1h/USwCAzPUfFrn853oBcRGiLiOvJ6IfaPUOndivWnXCo32dFJrDwO38bS
5UZMsNoJq7fi8+s+6n2wPUXFff8ickqmx5QWCs3pgzPGfVlj+Rkh/mWi0Z7IY7gtk792TJfmJsJ0EYq/
Ua636vzepZ2QxPUMe3O61rY6vKWbchRsg+cv3+obPdKdoQeH0UI1Q3Q2s7cNWB6t6okwhkSA3f
v/NTd+olAMAsvPLBa+Xmrz9fLy23z338Idn/n4/pJQBA5i59QeQyf8BKghB1KRXlxCnnmRtode1x6W
U5efqkHXYF11vUMHDn2VzwQ7mmeJ0KuwdeWOgN5ffWWcfkDu+2H8bw7cAQ/VFmdc784HMy
Yb1zLRMP6W/LeT9BlVZoz1klRrtTuzaW8kkJ3d2o9a4p2hC7Z7Zxrq2N2WUO3P3Er8sLpOuJp59gJn
4AmLGXfPe58nMPvEQvQbn7P35Vvvct/m0CgNxcVvVRv68XMA4h6pLy6oraPTl74nSqPCUnKies/w
+uV3ngPEWoFiOYcodmt90x0xG9I6exkOfM6GmphqaXQkPC+LP0tzf8OqBVo6fp8kkSbhdl80L4jP1u
cDtiPjEgE+5M/A8/8bBeAwDIm6p/usx1UEdRAWrzN+/XSwCAzPWfFLn8cb2AcQhRl5U3tHlfDts6aL
N77VXEyQqN9VP3hsyCUdfTDi/9upOjwkuvNubQI2YYuIDnzB/KHyXmkP72hj9RVLUlIzuhTmDqa5O
CTNtg1JUdKNlqa55jln7k532ffZ/c+/C9egkAMAsrX3qpXPvklXoJpn/40rfl079/qJcAAJm7bP02eukze
gFRCFGXVklO2hlkRw52nSHNbh3Rkv0Jf3347OuzV9w864eaDXeSp4E6pCVnqL3i1eKc2KKdM2Mo/
xjjh/S3ZcNLUMtSV1PRTyvVazOhHNugZuhXPU7tScXsQNWYoCtu2QJgSvX76vKxv/2YXgIAzELpoRfa
D4z26f/6D3aYCgDIyeXPivTp2jMOIerS8ntyNptOOObWeCzq4p/uem+Sprnj9gDtSK2mA77BOqRmP
crmqj9sur3h1aUsxB5LvWDnzBzK787+H3iYIV70kP5e45w3jL9cv5BOuYRUr82E8miD3pbKoIM9Trvi
ls8dW7sVSMn/+v/
+l34GAJgF1ftU9ULFeHd/6AH9DACQi0v36CcYhRB1iZkzpqvehd7w88Ds5sYkTXPIq1NqC+shGaxH2
VzVwZg/Ll1GT440LNVzVtS1VJXmauqhoTmUvxxaW8AoiWC9cuSQ/l5D1r0CqtYrAxMwOY/JJkhK99
pMJoc2VLb9IfyBurRGfdmzm7MP3bEU7v7Fu+WOl9+hlwAAeXvjX99AHdSYfv7dP6WfAQByUXijfoJ
RCFGXWWDW8FPiTzbuDltXzPVzyAwvRw2ht+tRGr0uXeW6dJPWu0z1nFVke7BA5v5hSrUxzaH8o0
NdMxQeNaQ/Xl3VCaV5bSaVQxvsIfxhxVBVSNvvp1pfFohy1RVXyf/4lf8ht1x3i14DAMjT373yCf0M
UV7z1pvkbe+5VS8BADJ37FUix9+kFzBKoa/G9QIAgKXxwCMPyOv/2+vtmfoBAPm648vXyysfvFYvYd
D1P/E8+a1PvVGedw0TbwFALgovFrni/daTq51ljERPVAAAlsztN91uD+0HAOTvb27/tjx2PX/ECnPlc4
9L9fdeS4AKALmx3m+Pv8f6SIAaByEqAABL6F2n3iUfeMMt6DVz/8t+V
5NZXv0gvAQAyd+ztIoWb9QLGmSpE7TVW9AQpBX9W65F60lhxJ1TZkODL27LhTbbiPCabqGZYeyO
43dBH1jOAAwAwhz788x+W07ed1ksAgLz84PnPyt7rvqWXoLzidS+R1X8bNhkqACATx4oix39eLyCO
OeiJqsJVf5ZqR84zwquZ0VcaKU3oAwDA4rjnl+

+xh/cDAPL10M0/kO6t39dLy+3al14l1d99rV4CAGSucI3IMTWMH0nkGKIWZXOvL2oeq35gxuuuHH
iTiKvZsNXn92QzxwzV1qnJeTqkAgCWjDtj/3VXXafXAADyonqjfu+aZ/TS8lIBqgpSAQA5Of5ukcIL9QLi
mn1P1N6h7OuncuqEZJedlqXedUNc/9Gtl/XnRZq7pKgAgOVzy3W32EGqClQBAPlR9VE/96Zv2h+X1
S/8xivsofwAgJzYdVDz7rl4NOQYog7XRLXrlZZq4nZEtYfVD9UoNb/OeaRVL1Uprp0RP0YFAGA5vfm
WN8tH3/5RvQQAyIvqibqs9VFVePoLv35CLwEAMmfXQX2HXkBSc1ATNUKvISuFktS8lNXRqZVSq2H
aPu+HuNXTfpGB5WVM8uWd46hJwTA7YddqEYTfT/4kcHneY0nPYU7fC+q9b2DCO//8rEiKf0cCAt7
7s+
+V2h01vQQAyIuqjbps9VGfd82VctfvUJMbAPJztcixO/VzTGKmIWpluy/9bt3vCVptOcPst1WY2ZPGu
htwVqXlDsFvVe01qobpeqIkoSO1khtC+I9Vd0Yra9/2bgFghnqNlWAPfSBnqjeq6pUKAMjXfT/zuD1r/
7JQAer1P/E8vQQAyNzxO0UKL9YLmMT89kTtXZQdnSKU61v+RFSVLXHLmHZ2LqbU+64s9S0S1NFG
TQoGTGKR76eM295ryPpg13tgBlR9VFUnFQCQnx8/57J8/g2PLkV91Le951Z5zVtv0ksAgMwdf5PIsV
fpBUxqfkPU7oHXE8sevu/1HjWG93cOpKufTsfppZpmrVUAABaVmqm//attZuwHgJw9dv3T8je3f1sv
HU23vvpF8ku12/QSACBzhVtEjv0LvYBpzHdN1FSFz85vlhPo1M7nWItxHgxO2jWqxuPoOpD20GPv6
/VjZG3Jwf1FBNd2Pdzga0fVgsysDVEya98oca+VK/5xJm1f2OsHyncO1O806o7arx19P/kG2p/K9Ux6
DsOEt32wXungORo8P2HiTbTn8/epHqPqpCY5P0DQbdffJvf88j16CQCQl7+/7Xvyjy//oV46WlQd1O
rvvVaufO5xvQYAkK2rRY6/2/p4hbOIqSxEiFqud4fDT/uRwnDa4qac1WVWRfblcGnyBRVsDU7a1ZTV
wqr1/3hUiFMKG3rcqUlpMKBKMklYe2NETUjVYzgYFmXWhihZtW+khNcqwXEma58TyIW9vrlaGBH
2qXNitLNcl/GVM9SxDbR/6us5/f0ej9OrffAcNVdHhZyTcPbh1XO2Dd97qd3vWGrvKL6DGfsBYAb+6v
WPHcn6qL/yH15JHVQAyNPx09RBTdH8hqilk34P0YN0Bu2Ha8tuuinKQug1zvnhkTuhV7/r1Zsdzzhv
5bp0dbDd9TbQlHNeotNLMEmY9dpzesOh2+3IzkX3tVm1IUpW7Rst2bVKcpzJ2tdrrHuBnP+HjZa1F
615bkRQaLRjb1OKem0koz1u06e5ntPf70n47fHb3pHa+ejIPHqivUH+PsLvvbTud0Ds2frfdepdegkAk
AdVH1UFqUfJyumftB8AgJwce53zQGpSC1HtnmjGkFHzEWco65DimpzxspxVfxuqF6C77UQbdnpv
me1yHmYvuTOyFivhWXQ9uejO2qUCFi+kKcrmBSPEidI7lH39VDo74mY3xc09J6yxHnub+mQmmiT
MmLjHCNyKa2eGQ/XM2hAlo/aNlPBaJTnORO0LtuOst74iW/aGVVC3J2GHE2hHTNWz/rmtbPtBrd
/2JNczhfs9gWrL7yFf2Up/+4q5j/B7L8+J+bAM7v7Fu+WOl9+hlwAAefjmjU/JV376u3ppsb3sp14o/
+a3mNAEAHJTuFHk+L/WC0jLHA/nDwYcXkjrjWE1w5B0mMHN0daVAzdTKp+Ukn5qK56QU/ppJDP
kHgioh2ouTjVJmK6nGTZ8Prc2REmpfSMlvFZJjjNR+0a3wwldR5fWOHUi6XdVVU4HNlaSk2473bYn
up4p3O9zpSwnAwcRIteJ+bAMrrriKmbsB4AZuP9V37HD1EWm6p/+
+n/5GeqgAkBurhQ5/mvWR+qgpm2+a6IWN2UvbMitPdQ3hXqoHtWLri8pZ7JH3OhefG5oM1EPZ
MWu5eiGPlE1KzNsQ5R5b19sebQvRuA3VlFOLEzSmcbxAvPpphfcZAepKlAFAOTn8yuPylNXX9JLi0f
1QFU9UQEAOTn+r0QKN+gFpGmqENUc9hv1cMLJoj8M2gxA7aBUrw9NMc2v04+4dRUtdp1B82tD
H2kGskvEuHZ+7Udf89zw5DXjJwlry4bZq9OtjWnWijRl0oYo2bcvLbGOc4btG21wgreeHHp1B4ZlOv
HcEcD5QZpuv+l2e2g/ACA/KkBVQeoiel3l/6IOKgDk6dhrrQd1ULMy3z1RkZGQ4dEus05mDGp2d9V
b0Q+rjZ7D7raTTBLW3vV6dtrhjxuYG8OTB6XehihZtW+khNdqguOM176Idth1itOdfT7Y9JDh+ImO
M737fWHkNjEflpGaZOoDb/iAXgIA5EEN6f+7Vz6hlxaDmoWfOqgAkCM1C7/qhYrMEKIupaKs+bN2
yao3Ztuc0XsMPcGXKlEb7K0YEnhNOEmYH/4YM+KbcmhDlFTbN1LCa5XkOBO1L6Id9rE7NVUTnsK
Rmqsb4jfdL5lQPrPmhNaJrmcK9/uiyeB+B0wf/vkPy+nbTuslAEAeVH3Ux65/Wi/NN1X/tPp7r5XnXX
OlXgMAyJaqg/oe6+PVziIyQYi6pIqbF/zehs1VHawYk86MU9n2h393alJygxmjRqg/UVew9mbkJGGV
09aSNq5dWbUhSmbtGy3ZtUpyrpO1b2w7ynXZSmV8eFnK5aas6vZ4Tbfaftab/j/Z9Zz6fs+LOdGV28
6Jws6U7ncgwj2/fI89vB8AkI9Lx/vy+Tc8Kj9+zmW9Zn79Uu02ufXVL9JLAIDMHVsTKdysF5AVQtSlVZ
TNvcFJu6rS6res/8djD/8OK6Rpb2dgoi679ubg/ixDk4RVZHuoDc72vF01d71eitm0IUqG7Rsp4bVKcJ
zJ2qfa0Zfu0IbVpo3SBlM7JWcHjzfsGiW6ntPf7/mw7q/B67F/aPQSTiCV+x0YzZ2x/7qrrtNrAABZ+8H
zn5W/ev1jemk+veatN8nb3nOrXgIAZO7Yq0SOv0kvIEuFviqCCAAAMIF7H75XKn9UkaefXYwhpgBw
FNzx5evllQ9eq5fmx7UvvUr+06ffzDB+AMhL4RqRK9R8BQzjzwM9UQEAwMTefMub5aNv/6heAgD

k4W9u/7Z875pn9NL8qP4udVABIFfH3239jwA1L4SoAABgKu/92fdK7Y6aXgIAZE3VR/3cm75pf5wX
v/Abr5BXvO4legkAkLljbxcppFNUD/EQogIAgKmp3qiqVyoAIB+qJ+re676ll2ZLhae/8Osn9BIAIHPHiiL
H36EXkBdCVAAAkAo10dQt192ilwAAWeve+n37MUuqDqoaxg8AyImqg3rsPXoBeSJEBQAAqVAz9b
d/tc2M/QCQI9UbdZb1Ue/6T6+2g1QAQE6O3SlSeKFeQJ4IUQEAQGpuu/42ueeX79FLAICsqbqon3
/DozOpj/q299wqp/7ZDXoJAJC5428SOXabXkDeCFEBAECq3lF8BzP2A0COvv2if7Jn7M/Tra9+kfxSjV
/kASA3hVtEjv0LvYBZIEQFcteWjUJBCsZjpdHTn1tSvYasbLT1gqO94Z6fFZnX09NrrDhtXGnIqCb6x+E8
lv5aY2mo2frfdepdegkAkLW/v+178o8v/6FeytbzrrlSqr/3Wrnyucf1GgBAtq4WOf5u6+MVziJmghAV
yFVPGiur0tRLjrKcWSvq58vHDiJLNeno5YXRa8h6LarV6loXZDV4saVTK0lhIDAGjqq7f/FuuePld+glAE
DW/ur1j8kPnv+sXsrOXb9zu1z/E8/TSwCAzB1XdVBfrBcwK4SoQK66cuDmbuW6dPt96ff3ZHNZM9
SxQeScUj1nxwW/7fPiHlq1pa5zX7r1srOiuSrkqFgGV11xFTP2A0COfvycy5nXR33zr9wir3nrTXoJAJC
5YyvW41V6AbNEiArkqXco+/qpnDohy9v/ofzKziplwAAmSvcKHL8tF7ArBGiLjGvnqP5CKntOLI2peqN
536d163Or/dp135sb+ivNdbZLwuuuD/bKS2MbmtlG7zFcYzN4jMM1S+1HWN1Low3jal3a+zDDt+aq
87WDDQ9pc9i2o9o8tpfjROdl+J4ZuZ8YxxD7fGh+W9RjuK0OZwi9/7p0z506/lKsnrNGj+NAWF6Sk7
ozquwfDt9PNqM9ie65eMduS+H7wj1Pcd9HsNxuv+l2e2g/ACAff/fKJ+SbNz6ll9Kh6p/+
+n/5GeqgAkBurhQ5/mvWR+qgzgtC1CWlwpHQMKhTk1JhQ8ZlcHHYtR8HCkLa61TIMrC+uRoeXk2
1DRU2hfYY7EitNCqEU58zapaW61J3uw52duTiYMi0674ynbqmQ8GiZh/zyGBquM1uz8dQE5+X4X
umuToieEt8DFGcfQcvd0hb7WCw5A2hd6V67jxVafVbXq/SIWaP41E6B9LVT4MqcjrpPZfk2FP6vlDn
KY/3ERwdapKpD7zhA3oJAJC1z688Kk9dfUkvTe/f/Nar7J6oAICcHP9XIoUb9ALmASHqUmqLn8O4d
TmNeo3SlHPhSUpi5XrX3na/FYybwtaPGt482TaCw6mHj7EjO4PplEcFZM7r+3ubsrlVF+erBr/GPI9nZ
FyGWtm2ttd1t2Wptpx9bOvUrr3hB4Vhbe7UZH3kdQm2eXRT0jkv/invSO28cd0SHMPY8xHg7zu8r
dZxrbvBoHEu3Iamcu40u53bEitrHVKUE6f00wiVRPdckmNP7/uimOP7CI6OD//8h+X0bQxHAoA8q
ABVBalpWDn9k/YDAJCTY69zHpgrhKjLyOwlZ/R0K27uOQGJ9dhLZaajqpx1t1M5bS25RqwPHd486T
aKsrlnBj6O4toZL7TrHIT3AyzXt4IBWXFNzugv6uxc9NvY3vV65pXPrI0P38YwexjWLxht3rwgfgZ5PrR3
31CbR5r8vFRbfnDoh3xB0xxDFHPfoW3tXZQd3SEycC4qW/5+zWtniH/u1HFY3yOhIe8k9uUwrEFKk
nsu0bGn+H2R2/sIjpp7fvkee3g/ACB7akj/V376u3ppMqr3qeqFCgDIiV0H9V/rBcwTQtRlZAQ0ImqY
bsGrZTiyhuIkyielpJ8GjFofJo1t2HQ9x9BhzEGnTgwGP0VZ8xMtLyzyA0Mj0J1YVK9WY/8jgrfhNscV/7
yMN90xjFaWk+MudvfAa79X7sF+GEPcRwyfn/zcTeuUjN51gntuimNPcv2HzlNe7yM4ctwZ+6+76jq
9BgCQJTXJ1KT1UVX907t+59XUQQWA3Kg6qHdaH6mDOo8IUZdSUTYvhPckdEOYqMl1FkZg8hyjn
mOk8MDO76nnDnc2AsPq6dg9GafXkeGOgjFCRlOK52UyYccwK2keVxw9ORxbLNWR2T2X2vVfkvc
RZOKW625hxn4AyNFfvf4x+fFzLuul+H6pdpvc+uoX6SUAQOaOrYkUbtYLmDeEqMuquCl7esjtQKlR
W/Pcos+u3ZYNs3edW7PRrMGZhHW+zurzZA+NNoYyV0/nF6FOH/qlfF4mkm1w6dXKHXpMWsd0
AsUTMrb06bje1BPcc+OPPf3vi6P9PoIsvfmWN8tH3/5RvQQAyNIPnv+sHaQm8Zq33iRve8+tegkAkL
ljrxI5/ia9gHlEiLrE1MzaqqeYPcGPHYR0vRqKo4f/GoxhxHPHrB2pgiW3/uMUba64U6Z3duT8+R29n
aqkk6GW5KR37gdnZO/JRbfoZeQQ8BgyOC+
+nI4hTOmkFwKOqumZL+NcNHfF75DZlQPvNJxwzn2EWPdckmPP4PpP/T6Cpfben32v1O6o6SUAQ
Jb+8eU/lL+/7Xt6Kdr1P/E8uet3qF8NALkpvFgP48c8I0RdRu0Ne6itmkU92FPMCHiMXnIlPxkzZmJvy
4Y3Dft884MlY2bySXgTWHWk2dQnKrWh/EXZdLsdqvO87l+XXmPdq22ZZBKkcVI7L578j8Fj1udsrvr
DyPW9bj9yHVtu1oBtyq7eda9xzgsxY/VgjnPPTXjsU1//hO8jZntyvRSYe6o3quqVCgDI3t/c/m157Pqn
9VI4Vf+0+nuvleddc6VeAwDIlqqD+h7r49XOIuYWIeoymz7Q287NSm5QYtRH7F6NnzmbhXSDL52
Lpkz9nttNibamUhF3I6BrlSH8o+4LiU/fZQL005glcI5MSQ9BnPYu9ueiRK2YH3O5qqz34IX9Felldqs+v
EUNy94PTLd9njnodqSeM2Jc88lOPY0r3/C9xEgiqqPquqkAgCydeI4Xz7/hkcj66NW/u8idVABIE/H3k
od1AVBiLqk7KG3YUUMVeDS7wcDHlX3cLBmYrWVcx3NpCqy3W/5gZHNOTbvsAPDrOPxhlcr5bpsp
ZzL2dcI5LxWW9Z6d+j1VLI5L6Zkx2C1Z/A+3D80ejUmYNfnNIaSu+y6nznWQ/UUZXNvuFaoPYw+Q

aAb656LfezpXv9E7yNABDVTf/tX28zYDwA5UPVR7/uZx/VS0Cte9xL5hV8/oZcAAJk7Zv2GfPwdegHz
rtBXRewAxKNmNtcT86gwbG/anqHAOEfpntPHcqpFwIpwn+l9Rip/xM0BAHl441/fIKWHXqiXRK596
VXyW3/8z+yPAIAcFK4ROf5+66P/Xoz5Rk9UILaeNNbdmc3LcmaNABVZO1r3XPu8dSwZ9ODG0fGO
4juYsR8AcrL3um/J9655Ri+JVH/3tQSoAJCn4+8mQF0w9EQFxmrLxmANWFXOgK50yMzRu+d6jRU
p7ZyRbiplKXDUFf6fgn4GAMjStU9eKe9s/aS88723MYwfAPJ07O0M419AhKjAWAOBFgEqMsc9h+
WlJiiblx9NaEs42hKOtoSjLeHmqS0nrr1GDr/3pF6aLa5RONoSjraEoy3haAvSQIgKAADmBj/ghqMt4
WhLONoSjraEoy3haEs42hKOtoSjLeHmqS1IhpqoAAAAAAAAABCBEBUAAAAAAAAAIhCiAgAAAAA
AAEAEQlTkSE2WU7DrfxRWGtLTa8fqNWRlo60XHO0NvZ3CijRibwjp6Uljxb0GG9aVTbJuGmH3UNr
7mJbRRv1Y4SYFAAAAAGChEaJirvUaK1Io1aSjl4H5pgJdY1Z9W1nOrBX1cwAAAAAAsIgIUTG/eg1Zrx
GfzqeibO717RkF+/1tqei1szFPbenKgXvLluvStdu0J5tkqAAAAAAALDRCVABIS+9Q9vVTOXVCyE4BA
AAAADgaCFGXmao1OlC7MazG6GD9UXuIvfE1A+VKNbNOpXokq1Vp79Mcxt9cdbYTvjOjjeoxqk7qY
JvCa1VOdrymMbVf2xvetob2H3JN4rTRY359ZEONcxFo46j1xj7t9ea5zKoOadx7KKotg9uYsD5pjOuS
9J4FAAAAAACLgxB1WakgL7TWaEdqpVEhpPpcQUoDQ+ybq4OvVyFiSYIva8pqYbBWZBqcNq0GNh
xyDHYlNtgm65W1UnjQaYt7vIMqcrqqn3Z25OLAa9u7bmODtTKHQjgtuo2TKsrambLztHMgXeeZxR
iOHmh7W9xml8+s5dDDMoV7aKJrPizf6wIAAAAAAOYRIepS6knjnJuIuXUb+9Kt61BNOrIzmPx5qtL
Sr2+5QaEKG8/7ve16jXN+0FVt6VqVXfE2H0Nl2/qabl28L3G3sx1W8dJvU/gxWMe77oZg/mv77gF0
arI+MhUdf7xhKltu2wfPpR9GSvmMeBlqe8MPgsOuSWQbJ1NcO6PbuC+H7qbbu0ZI2ZEDN101hq
mfOpF9hDr9PTTNNTckuC7J7lkAAAAAALBICFGXkjERz96m16vQD9VEOl56FlRt+RP3+EGhqScXd9w
+e1VpeQGStc8LYa+fntmm0GPoXRS3SeX6lj/xUGXLC+U6OxdDexSOP94RimvidfQ0t22ElGaPTrN3av
2CcU02L/htrJ0fGKo+Ja+NftDbO3Si0mrVCRv3dbrau7jjBZKnvROYlRTuoSmuuWkm1wUAAAAAAM
wdQlRYdA3P0OH9SZmzk5+Ukn5qK56QU/ppespyMrCTEN0D77jsIdheXUtjqHdgSHsazOHy/rB4P5
SryllvyvYRvVNtxnbMHqOpKMoJfUHcwLlrXzzrnJ4+aYeVwfWW6mk/kMxMCvdQKtd8VtcFAAAAAA
DMG0LUZRWYKCdunckYgeWRMt3x+r1i3Z6eRig3URhpDK9PScUt3trctVrXE6cj6ik5UdFhZWC9anb
2EeriSf+6AAAAAACA+UKIupTasmH2OnVrPZr1HI+ocr3rlDEYevjD9lNT3JSzbglONXTcqCs6WRiZQYh
dOS164L4ctvUQeLv3pzs5lrF+QUP07K/5sv1xAQAAAACA5UOIuozMupwqYHLrohpDoCdXkpNuEjs
4XNoIEXNVcoamK6NqvWbF6+nZ2ZHz50fVFTXP2eBs/mZ90FMydk6nxNfQ3XdHDnadr3VrtZbsT/jr
h4e0ZyWFeyiVa57idQEAAAAAAAuNEHXJ+QGTMWP/VMxakU1Z3XCn3DFnS8+ZMcmTNFfFa1J7w
6+V6a1MmdfTsyPNpj76oaH8Rdl0u6xar6utN6yz5eg11r0anuYESU7AqVivP+8dkGx4U8nH5V+vZtP
5Wnf2/aIumOquNyfCylYK91Aq1zz5dQEAAAAAAEcTIeoy8oI9S3NVh0rGhDtTMmcun2r75iRC7nY
mCjuDs7o3V3WI5gWO5gzwaXOHxftCh/JXtqXl5XU1KdnnrCAlP6mTC95EVCojdOutWrxzHLe2bVBg
W9Yzb2i6eZ9Y68/k0w3VNv09lNI1T3hdAAAAAADA0USIupQqst1vGQGZUpVWv+8HRvaEQpMqyu
Ze1w/BbGr7g/scx2qn1yBt/9DrDZhIcVP2+oNtstj1YDOoh2rwhvQr1v62Ruysst0PrUtbbVnr3ZILLnU
8g6+ttiara2v22gwMTTeGs+c+ZD2Feyila57ougAAAAAAgCOp0FczrADITq8hK3oiL1WDdo+eiwAwk
urtPS8/mtCWcLQlHG0JR1vC0ZZwtCUcbQlHW8LRlnC0BWmgJyqQKbOOZ75D4gEAAAAAAJAOQlQ
gE23ZKAzU8ayeFTqhAgAAAAAALB5CVCAPql5pZpNXAQAAAAAAIEvURAUAAHODelXhaEs42hKOto
SjLeFoSzjaEo62hKMt4WhLuHlqC5KhJyoAAAAAAAAARCBEBQAAAAAAAAAIDOcHAAAAAAAAAAgAj0R
EV2eg1Z2WjrhVnrSWNFzZavHhsyL60CAAAAAADA/CNERSZ6jRUplGrS0csAAAAAAADAoiJERfp6D
VmvEZ8CAAAAAADgaCBEBQAAAAAAAIAIhKhLqr3h1gddkUZPD7+3l51HaClTVePUeI3zcL7eZW/XH
MbfXHVepzc4uF+PuW1j58HXt2XDfY318F4Wo12TGjwv6jHpuVGSHr8S1obCSkPCD8+s/eo8VkaciG
TbBQAAAAAAWF6EqEuvI7VSQUoDw+
+bqwMhX3tjRI1T9fXpBJbR1H5WpamXpFyXrYr1MbN2OWHk4HlRmquFYNCZ4blRoWtYG6RTk9Lg
BFl2EFuSwZd3aqWhcDTRdgEAAAAAAJYcISosVWn1+9K3Hq2qXqUCwPNulNaTxjkdX5br0tWv7dbL
zjrrtTsXnYiusm19rlsX9zNSbdmv7W+rxHNafjv7e5tSTNCupHqNdS+MLNe7zj77LasFWvOcDkeza4NI
W3bd1Dh020055yW0VjvW3SDXOE/uBe3UZN17bZLtAgAAAAAAgBAVUm1tixtxVraMANRTlM09H

crZ4aVeu3bGe23noKufZadc3/La6ciqXT25uOP20qzK2U13yxXZsoNGFVLuibM6w3PTO5R9/VQ6O+
JmscXNPWd/1mPPbVvvorhNDpynypa42Whn56J1ZJYk2wUAAAAAAAAhKiaha5OGDmHPzqkT44K
9tNrVlQN3A+WTUtJPFSdo9EPnYSmem+KanHGTWGtrquyCW7t0qM5p98Dbnz18X7+uYA7v7xxYR
2ZJsl0AAAAAAAAQoqIsJ82UcJTAxElGbdLcjGjnzNtlyawNRdm8ENYz2A9KQye5Giur7QIAAAAAABx
NhKiIoS0bZs9Kt46mWft0JuahXRm3obgpe3qIvV+v1tc8Nzybvl/DdfBh9KCdYLsAgLzp0Q3Ggz9yAQ
AAALNBiIrx2rte70o7oHNrfxpDyKc2ybYya1dJTroJqDsE3qVm4i8YM+6n1YaI16uZ9NUvzfakXXbw2f
XqnHrtK530Qtu4NVhjbRcAlpH9Xl+QwkwTy540VlZFWu57tPNHr+bqLILU+Qxze40Vpz0r+f/hT/0ba
p4P9zEP5wXDhq7XDO6ZmRvzvuZ9P7mPvG7mOO+3ebwnR+5jSf+gFfO8z/K9eGZinJuh951luGnG
nJfc34sTtofSdoiDEBWJ+AGdMSt9AiU/nTRm/7d+MFmdbhD8tO0KKsqaVzS0Kavem667baeO6OB7
cZw2JDp+/aavPhXsGRpSs9Wsc9pc9dvm/sOhHu7KJNtVjG3wDwuALMzsl/chKri09j/lv0npcCYu3Da
KcLuTP+4f5vte3N4IC3ONPyjOQq8h6zWRatX9x28G3NEnxsO8XrMy01/cAyWOhh+z+NZW52O1qS
YFda9TS6qdmpRmFPjk/0v7+Pc19R5csr6f6l3jHFk/T2Z778R5v83jPXncPtTnc/yDVmTok1eYm+C85/l
eHHFuht739CP98xP/vl3dH/g3Iot/IEadk9zfi8efl3zfiydoj/WNrUrbpf+eHO/7dn5+FsZY1g2DJWS9R/
TV5bd+A+hbPzD5uvW+9U+g87mq9ZZia/WtnxX060c8vNcqIa93P29uf9TD2NbIdtqStKvbr5fd9VXr
K8cxXx/yKNetVygJz83Exx/
+MDcdve3gMSfabqvqrS8PXwQAmEq3XrbeX8z3eP2+GngjyoffFv1vwAzaEEm/z8/8vXjm7XCuj9q/f
c28f5PzY/87OoP9RtP37dy1y/3eivPzV9rCv5dn1R7n5y9jv/pnrCy/l8a/rznvuYNtyPocjW9XvNdMa6J
9ZPIeqPdvbdd+hLRD3T/maud+Um3XK1IS/5w4n8/+vTjeucnjvS/
+fZv1+8v4cxImq7aNPy/h62fWnhHfw+nfR87+zd0737fBdX579Qr9vhz3uiJf9ERFDBXZVn8p0ksO56
821puAo7kr/t9KrNd7n9D2D52/MKlanIP1QqutCWuIJm1XEk4PIOsNTS/7rDdbf9h+0jYkPH57qP3g
ubQ5+wj8QdOuc2oMyXfZPWWMeqiWRNsFgMy05XytY71NXZBN503VUpEt9UY28fv35Iqbe9Lv7x
ltmTO69MupE/PRwFm1o9dYl1qnKmfn9kLNhnteWt7PKPPC+T6X6unAzyL5KMqJU9YH9+dQW08u
7sygPb2GqIFK5fqWv9/Ktv3zYmfnYgY9sRxj39d6h7JvfRj8fi7aJ64puxm9Ecd5v83jPXle3ved79+y1Ls
hP8tr6uf34dEJHdm5mO7dE/ec5PVeHOfc5GX8uXHeXwLf5xmY7Jxk9148/rzk+148tj0jfp6yR42mW
tYuzqii+fpZGOMRoi4pvw7mwJuLMeFQcMiBCgvdr1EPJ5TztxMM6dQPhf5rrYf5A725D/VQ+xmx35
Ht9MRtl/MGFlw3nvMG7H6d89gbakjCc5Pg+G2D59J+jDoG8zj1Y9QvU3G3a7xu+NgBYAoz+uV9M
enSL+W6bIX/A5CTnjTWa9KZVTvsoaMdqbbi/1ueGTUM0Rh6N9uSN/n84j6JXuOc9d1s/bI/oxu3sq2
HjNo17dWwypITNg/
+vJW13H5pT8huV1lOenWcNKPePgZk8AetuMFlmDTbEVuO78XTnJv86fJoB+cHhtOnWwJnknPCe7
FhxPub8/PnvuRaNYmfhRcOISoAAJgNfnmPTdUltX/5uTCLXoa6tpj9i6D+pWdGvR3b52vSqbZmPmL
C/0Op81AjV7KppRZXPr+4J+f2NDo7wwDEHTWkatqr7yOrObMI4efpl3ZMgT9oKfPyXhwwR3/Y6uy
flAvevxGqt6h6/9mYYa9C3osD9HwiwflBrO+pqedWiWHwjzD8LLxwCFEBAADmmDP5gRq2N6ueO
MFRDt36vqzO4hfU9oYzCcRc/dbuUL2Csh6WHcfc/eLePi/O6NHZXTNnsg5/YiAVeKtJgXKftGOWv7Q
jNfxByzKH78Xz9oet8pk147pY/4aeVWXUZtirkPfiAernGrdnrP89dXBKXadTkl3n7nn5IwymQYgKAA
Awp5wAVfXWmJ+hjMXNC3YNtk7tfK7hXHtXhU1NO8B1fuEpSEn9Vqh/CZrtcHptlsOyLfP1i7sOCGf5
y6Iecqzq2bt5jz0MViXezdWMZjYfZVa/tI9h93bqyMHgjTuqd9QS4w9ajkV4L56XP2x5ZtqrkPficIMl+f
qydXLfOk8nJau3vdn+EQZpIUQFAACzwS/vkVSvDSdAndMJ/zL8RSPMYE8j9bAngLR+Mexaz2dbt7sn
h6qoWc7nZKxZ/uLu9nw6O8NfFgeHTbpmdl7y/6V9rOIJceZ7CUZNPfuGnmG4O2f4g5Zvvt+LB+T+h
62S2GWOB8PbWf5cw3txTLq2eOCPkekZ+UcYfhZeOISoAABgNvjlPYKeRddiD3czevzkW+dSTf4wM
Bxc/0KW1S8ac6/XkJUVc0i2WqVmSp7lL6nz94u701utKjMcPSpSOW21wPoeCgyhV7dwzTovM26bL
dtf2uOpyGnVY6+27r+veL3G5m+islngD1qLaFZ/2NIjADo1Oe/9w6mHcM+oHinvxXGoUhklqUldLm
RwkSL/CMPPwounDwAAMCOtqvSt33L69a5e0a33y9aPJ2VvRX669bLVFtWewUe139KvWUZh56U
6JyfEblu53s/3bun26+Xg+ZiLe6RVHbg2rb71S2tfJT+5m+H38TB9HgKPefie1vdRxvdv3Pc1573Yf2R9
7eK0K4/35PH7CLt/3Ifxb1dq9H0x9H2r2jFw3Pp7Pu1rNcl5t78m8/fiEedGvd8M7Ns9hrTf/uKem6H

XZfY+POp+0XJ6L453XvJ7Lx7fnpB/xzO6RnHuxXn6WRjjFdT/rIsKAAAwE+5f6F3WD43zNRwQWBCq
x5xdm9BVbUl/Bl3nnO/pqrT6M5p5eS7pnk7G5ZnV9cH8Gfre9fjfR2Gvqc5r79gUjT83Id9bxnk7iuLc
LwrvxbOmRvOoOqhhgkP7+Vl4cRCiAgAAAAAAAEAEaqICAAAAAAAAQARCVAAAAAAAAACIQIgKA
AAAAAAAABEIUQEAAAAAAAAgAiEqAAAAAAAAAEQgRAUAAAAAAACACISoAAAAAAAAABCBEBU
AAAAAAAAAIhCiAgAAAAAAAEAEQlQAAAAAAAAAiECICgAAAAAAAAARCFEBAAAAAAAAIAIhKgAA
AAAAAABEIEQFAAAAAAAAgAiEqAAAAAAAAAAQgRAVAAAAAAAAACIQogIAAAAAAAABABEJUAAA
AAAAAAIhAiAoAAAAAAAAAEQhRAQAAAAAAACACISoAAAAAAAAARCBEBQAAAAAAAIAIhKgAAA
AAAAAAEIEQFQAAAAAAAAAiEKICAAAAAAAAQARCVAAAAAAAAACIQIgKAAAAAAAAABEIUQEAA
AAAAAAgAiEqAAAAAAAAAEQgRAUAAAAAAACACISoAAAAAAAAABCBEBUAAADAQmtvFKRQ0I+N
tl5raG94n19p9PTK2fDbuiEhLV0wbdlwz3vk+e1JY2Xca6xXNVb8ba00rK8CAGB+EKICAAAAODqa52
TGOemSUMHoqjT1kqMsZ9aK+rmpKJsX6tZnHZ3aesg1asv5Wkc/F6me3bS+CgCA+UGICgAAAOAI6
Ujt/OL38Zx/XTlwM89yXbr9vvT7e7I5KvksbsrZqn4eco16jXN+IFttyXZFPwcAYE4QogIAAAA4WuiNm
r3eoezrp3LqRKxeo5Xtlng5auAamb1Qq9IiQQUAzCFCVAAAAAABHTLzeqH590pVg6NpryIpbm9Orse
rX/7Rrehp1Vr119suC68NKtPqCtUJH1wEdeJ31CKsrGjyeYL3S6HYYzGPXj8F92fsp1ayzrDVXndeO3Ul
FtltujOpfo/aGXxagXN+yXjUo3vHbQto/dH0tcc5VoEar+6BWKwAsLUJUAAAAAEdEWep1HdJl2Bu1U
ytJYTVYDdRep0K2gfXN1VEBZlNWCyUxyoCqjUhpcMIpOxQceJ3F3t/IQK8jtZJRr7Rcl60YnTuHwlEtel
8JVbak7hZHba7KRqMh5/wEVS4M1gNIcvwqwA5pv3M+hoNUR/i5UueiNLhTJewaAQCWAiEqAAA
AgKNjzQ3p4vVGnVS53pW+qgPq9ax0hK1v7o5oh1dLtC/
+y5tyzkv7etJYd0PBqrT0a71td2qyPjIpNl6/F2OSpvaGePmv0a6um3ga+6psW5/r+hNFqRqm9n5iDc
MPTjLVrLnHV5b6hcF2Jjl+67VuGhvWfmsrOxfjnqu27Eadi8A1AgAsC0JUAAAAAEdIUTbdGYwy641al
bNuj8nKaWvJNWL9/mFoL05zBnqzXmhn56Lz+t5F2dGdIQPD3I3enN5rB4QPix+t7aeGgTCzuHnB31
ftfDo9MAOTTGnVs8OTUiU6fuu675lBqKO4dsYLbDsHXf0saOhcmfVeOzviZq/FzT1n+9Zjb+QMWgC
Ao4oQFQAAAMDR4oVsGfVGLZ+Ukn4aMGp9qKqcDiR3JTnpp31ix33dA90L01rllguwH8bwdve1A0
6dSBLymT0vz8ha4EuLsnbGbdi+HIYlthMITDJlPQudTGqK41fHZNc5DR3eHzR0ropr4h2yuodK7n4ja
rECAI48QlQAAAAAR8xAb9RD5+l8K8qJU/rp1MpyMn6am0BHRnTmnE6i8DlCYFIpo85ppLBzFSw5Y
HLD3NgTdQEAjgxCVAAAAAABHj9kbtRYvTsvXYK/Onhx6Y8iHebVWhx7biYbtTyercHa88cfflg2z16lby9
Ss3ZpEcVP29D4Gyt7amueYpR8AIg0hKgAAAIAjyOiNmpQxjDw7g706u3Lg7tTtmVk66QWAo+p5ps
MsJeDXAHX05KJbmFROSaIqAdNKcvztXa/nqR24unVRp7iWaoZ+1ePUnkjLDlS7Xi3W0WUEAABHF
SEqAAAAgKPJmIAoTMlPDo3aqW3Z8Kapz1ZzdcObqKm94Q8/L59ZcwJAszZnc9UfQt7e8Gp0FlIZV2
4Gzta5WPd7WfYa61790aSTVU1twuP3A1djxv4k9PbVbRDscRoSdAMAIgYhKgAAAIAjKro3qjlzuwrp
nGAubi3NaZWlXG7Kqg4D/dzWmOFftd+ozdlc1cGh9+IREzJNorLtD1vv1KSk21XyE1S5kPuM9AmOv
3Lan6jKu5bGBFRJjDgX5r1RPat7ugIAlgYhKgAAAICjK6o3qqp7OVgzs9qavI5mIqfk7J4xPFyx63gO1Di
1a3MOvE4Je+2U7GHrIcdebVnr3eHxeYt9/BXZ7psz/itVaZk1TZu7Xs/fcexzEVYMVW8zrewaALA4Cn
1V3AUAAAAAAAAAEIqeqAAAAAAAAAAQgRAVAAAAAAAAACIQogIAAAAAAAABABEJUAAAAAAAA
AIhAiAoAAAAAAAAAEQhRAQAAAAAAACACISoAAAAAAAAARCBEBQAAAAAAAIAIhKgAAAAAAAAA
AEIEQFQAAAAAAAAABGEvn/AZM6nr53azj8AAAAAElFTkSuQmCC]]></redcap:Attachment>
</ItemDef>
<ItemDef OID="q39" Name="q39" DataType="text" Length="1" redcap:Variable="q39" redcap:FieldType="radio" redcap:RequiredField="y" redcap:BranchingLogic="[q37] = '1'">
<Question><TranslatedText>Do you prefer Life A or Life B, or do you think Life A and Life B are the same as one another?

</TranslatedText><redcap:FormattedTranslatedText>Do you prefer Life A or Life B, or do you think Life A and Life B are the same as one another?
 </redcap:FormattedTranslatedText></Question>
<CodeListRef CodeListOID="q39.choices"/>
</ItemDef>
<ItemDef OID="summary40" Name="summary40" DataType="text" Length="999" redcap:Variable="summary40" redcap:FieldType="descriptive" redcap:SectionHeader=" " redcap:BranchingLogic="[q39] = '2'">
<Question><TranslatedText>
Alzheimer's disease - Description #[no]

If you have Alzheimer's disease...

1) You might forget recent events of importance to you. For example, if you are interested in hockey, then you might forget whether your favorite team won their last game, although you may remember watching the game itself.

2) You might forget the names of people you met recently, or the names of local streets.

3) You may no longer be able to handle your personal finances or plan leisure activities such as dinner parties or vacations. Simple decisions such as what to order in restaurants might become difficult to make. Your ability to concentrate may decrease and you might have difficulty driving.

4) You may start having trouble finding words to express your thoughts. You might need to be reminded to shower or brush your teeth, but you could do these activities on your own. You may need to post notes around the home to remind you of simple things like turning off the stove.

5) You may remain aware of the day and your surroundings. You might be able to travel to familiar locations like the supermarket, although probably with the help of a loved one or friend. If you are in a social gathering, then you might have difficulty following the conversation.

6) You may continue to enjoy hobbies such as puzzles or reading.

Now consider two possible lives. In Life A, you will live in perfect health for 1.5 years and then you will die of natural causes. In Life B, you will live in perfect health for the next 10 years, then you will live with Alzheimer's disease as described in the summary for another 10 years, and then you will die of natural causes.

</TranslatedText><redcap:FormattedTranslatedText>
Alzheimer's disease - Description #[no]

<p></p>

If you have Alzheimer's disease...
<p></p>
1) You might forget recent events of importance to you. For example, if you are interested in hockey, then you might forget whether your favorite team won their last game, although you may remember watching the game itself.
<p></p>
2) You might forget the names of people you met recently, or the names of local streets.
<p></p>
3) You may no longer be able to handle your personal finances or plan leisure activities such as dinner parties or vacations. Simple decisions such as what to order in restaurants might become difficult to make. Your ability to concentrate may decrease and you might have difficulty driving.
<p></p>
4) You may start having trouble finding words to express your thoughts. You might need to be reminded to shower or brush your teeth, but you could do these activities on your own. You may need to post notes around the home to remind you of simple things like turning off the stove.
<p></p>
5) You may remain aware of the day and your surroundings. You might be able to travel to familiar locations like the supermarket, although probably with the help of a loved one or friend. If you are in a social gathering, then you might have difficulty following the conversation.
<p></p>
6) You may continue to enjoy hobbies such as puzzles or reading.
<p></p>
<p></p>
<p></p>
<p></p>
Now consider two possible lives. In Life A, you will live in perfect health for 1.5 years and then you will die of natural causes. In Life B, you will live in perfect health for the next 10 years, then you will live with Alzheimer's disease as described in the summary for another 10 years, and then you will die of natural causes.
<p></p>
</redcap:FormattedTranslatedText></Question>
</ItemDef>
<ItemDef OID="a40" Name="a40" DataType="text" Length="999" redcap:Variable="a40" redcap:FieldType="descriptive" redcap:BranchingLogic="[q39] = '2'" redcap:InlineImage="1">
<Question><TranslatedText></TranslatedText></Question>

<redcap:Attachment DocName="15.png" MimeType="image/png"><![CDATA[iVBORw0KGgoAAAANSUhEUgAABVEAAADYCAIAAABtBTQzAAAAAXNSR0IArs4c6QAAAARnQU1BAACxjwv8YQUAAAAJcEhZcwAAEnQAABJ0Ad5mH3gAACf9SURBVHhe7d1fqC3XfR9wtS5FpoWKUIKSPuS2pSAUEa7jF/US8C0U2wQCBhMqTAqFlss1xCAMsUxxSh9kbEJAN8FgPwTZ0LTyg4wJNbJRAvchBrkEch/8oIAs7oODBfbDiQnCOvB3Xv2/Fmz1po/Z87M3rPnfj4Mydkza63fWmv2leYraY4f+zkAAACwRTI/AEdy/7nY6s63/Y3Ve/c7/oNttG1lyn8DOnxqZ5XnZ8Bsxu4p/Xw8BZ1H23FFP0gjm4bXyMpafsPvvs8wyS+j7iExVDUOHd5NvwtTJeYgKcLtXiwt35BHAMjRXkOLNJAx9PcSSwGwmmsqO2fT8KWJ7Kf2XBi5d+f/gbTtjKCWsg57lu4qnf0tc9ylg1kiApV9dgk+2AWJX7YngWDPbbNIP/T9JfkdqEA6r/w+s2tn3EOMW4XeAQAGXERRx8n44KZXVXP3+gsGb/OhK2ewtPsUABuHMrvDqGVrUDnrb2n44cuqBPnhvwm5O+kh8HHSf+DqzW1i99NPl+b3BAjO/OxAXA7ovwzC/Dy9fs8L38rzx6zKcpmn7TDA/GBaSGIPJ/3e/Y2IHsZk/6BzbkEoLFQfQZXxTLNJmDhDg7ERtCEOXAdj0KVt3A3WIrCMT6K11htb8vYD+kWmXORL1+E16zK+WQVl7/MQtRlNNDf5Q9gUIwaKWbXVWG2GqKHjuBDLfUoWp36uZ+cqRJnjMrDwFZDUWqBwZaaSs7BQw2vGfdugm8vlRx42ZDOKyMXQYN9AiZ6V70Rl3fHO0Fi0a8yGtmnlcjSRGLumyjHJRcf3z4EEwmltuO9Pap0CNHGw7OA99mpVL0izpmDmzhzk7fWMfO5P6njP9ui8Ahv8TvY9G8Yn6fftm3ltudUq7lOvhJlw6Q54x+MBbDhuBi+XbyhPKhVyNCpDfS4LMt3vPTN0q/mUVdPd3U1/TsVBU/xA3bI3iXytjaRQ7UgDGRRhQ8lDtrGS7KrFxatTbYbrtH74zzbRO8uLkuvvzqjKpaSSz2FWDf1QhF7GwGKVmOfYNxmvhqUM7nzUzZwDAXeVqffXIwcP4gvjwGU2TuJTZBaXnwqc6I5AhaYBewCn5dbTF5TakWOmSuAdw5NxtNmB3nO8N7jPJ0hKDmo+SNzSeY6Gnc5Fr6X+HcnMxpzAZXPmSKzVNbV5o9AkJWNmBR4m9Ba4DvwuttsPgB/17jJazh2ToTpDPpIo6MSEgbJ1xutYoPu+DE8vSxdeKfS4i4BbxXc2Gng0DwHUqdp+O8Rk4jAnXJEfsd+3A9u3bn9O2GNzD1wRIZSFxfPIXm/D8zBp+XJ6eg5yOsY1sPA6aO0POL6T1iKcvRymd3LGZa7NXoUOsWRO1pNmwfZIjwWZLmcujDspzTOVZzEwW5OsKLhqPRfRkvOUsm+TGvhA6LOtNJ92PE58DvfjlvvAjj/fTTTb+2HlThRXSbbXAaH8YIrXOHNBYfozRWmCaJq2//VxN6ORqfKC8mfaDKkmyA3FZcFPBsvtE+CfRvh+dV0C+1fbc/W0Y2bXr0ypuUT5WPeo85EE30S9czaCL6Ya1p+G8ndWDy3xAbc/4cPfVNbFOK+ZKSWtyncfCsPE55ndn0z8H/Yn1Hy9egrOI1B/+r7JIc0fGEbF/I1AiHBvZoAkw04HrNPm5ztVecR0NT41A/MzFtoQxvQ4PE6FI5AHwEhuJxFdk/kk2UO4Ce6ofYh//GvR8Xg+MhQyQN/3sVfHqYx8bvRC+HGHmjkT3Iv/eT6N+83nMWPq8jGl2M4XnEIGDP0FSiZr3ONX+5QQQvR2yAWtvBtEb/N0ZuaoCEkaX6SbqfVZSlzcGvZ8BAsHaI84vVpVyHXAM+WxuXeY88EagHCZrXw7vzKqsPPBDw7+F2k1DJSLqlqtWEDs1Oqr75efVlhe5j4yj9i+NPtfcQLvD9pcIbvJRCqF9m7O1RdNjdzLjUYRxNCrwqi7+iZzXnkJMxXkuitLUh+xqM/aVYtSpqN6sQsnqNqaMAAN4tEbpw6OZ9sS3+Azp0Y6+g5bSizhwYYgDtA46k9h5FYRTfrkE9ciebHG/95kv+ROYpHKR5YA/xYSBi1kY6HAbg0ZWAkPFfuZRUh/RSNuP7shIPrlwcjJ0ia8X9PvT3e7PV5eYKfHFn5Mm8JIaPWGBIqWUaCKTBiCdmUAgTk6c6IvCnBgsjfvO2sHkHzUy7eoJsMp3gkeGpr7HqEH7S/C6TuJJjCuKsF+xYS6SOOS4wrAebgvbHEyxeR7oZGoEdD0+dJACwpYCoizAcGtuiUZaSiAW0GZuFz2VaiM1AIE/0qlDTJPgaJCBHRTZ+tHF5Ex04YqnjX8wOuFCYwf5Ruj28aImuoZl0vzi7cpgVK/fE6mYD6lqGDz0BaAIR0nwXq3vBWmCwJBjMbAjojE7H5TnojePGjKOSvnXa/rIuf2nEIKNaCg4Pvxvi1XhqNaLqzq7vyexuPxzrG66pNkyDPLO1IiIpOGeKFIdMKFdPzhrT4/wC5ssMvd3bxKWK7xCh0Yz+W5/NwaoYGp3SIiBUJnfmgKkdGe8ZmIzFM07pN+8kH/GJMftyN7thbp1SgbJf+H/tIAtiDJQ1OHSzJfNvfwtbq7ymNn92SCHsGVFZx/7gPSwHDMIZ2JlHpftuPuKxd0wDyaYfXfqnpi+sCafH8FGvxpTVt27EpGq/iPxoXXVrmntxlj/CfAv2nFL8J66ZjPDg6aDyBLj7FQjo5IhNrqSJwvtlZvJDGwX08W+sRDWpSHjmyiycgjTcKRRRcACvqD79XqTsUW5wQ0hxPAdPnCOrK3y+Y3jNWyN5OdC3bT1cnAyhYhh2JFHKjg5A3E3AMV/WNcCwNrpdbywAuIRMoA3BB9eq86xFHUvi3W/Wen1lStWzWwkHH/5u0lw5rPgnxeVsv9frYsh3RgYR7Sx/OmAm8ZZvvAOzIXDDjIKYk1F/BwOqKhMymUs4l4uZU0+2dEvEfUY4b5J1d8hiEy07Ql4Ip8tn5SohNRVeYt7Cc5EzjtI1EzNG5/7HlJSZnqA4T3jm9sybVaSZ8tycU9HMShtnEEGeCBMEF3ieiYp8iDc3uhvbkypMjC11eb6dqg9HO7hqAsBDOeOMCrXb3BPNTTiT+tvx5RVTCzUu5krDk+DJ5FyqhnbKhWsYRTQ6ZuJ8Ze5jkz/9hlZK/

+3ah7FXp2rBRXmRYsw17Vzztph77pdS6scwLDi223SJoEc8123xm9If2TCSvtu1WfD0PkRkzmNnom
HNXle5e3v3Y7StorOU77Gn96eLEfAFghmR+GZeIoPBrufutulLRXcqzhNf708GI/ALA2Mj8Mk/l5NL3
0xktR0l7JsZ7X+NPDi/0AwKrI/DBM5ucRdP/h/Shpr+dYz2v86fGpD37gR2//oNxEAIBTk/lhmMzPo+b
hxcMnvvhElLRXcqztNf70+NxHPuzFfgBgJWR+AFouLi+e+tJTUdJeybHO1/jT4+UXPlPuJgDAScn8ALR
89E8/GiXtlRxrfo0/Pb77jVfLDQUAOB2ZH4DG8995Pkra6znW/Bp/etx95mkv9gMAJyfzA1B65fuvRD
F7Pcf6X+NPDy/2AwAnJ/OfVvW74QqL/IK4t+7duvNa+fM2fhdde0U9dg3HrrV1HyrjqjzCRn+btvcl3
Kg3fvjG4y8+HiXtlRzn8hp/enixHwA4LZn/hMpMWpk/ApUFNhS30hV1qnd3xFqzkV/mHzLq27S9L+
FWPbx4+OQfPhkl7ZUc5/Uaf3p4sR8AOCGZ/4QWzj516N1M3MqtKK9uuTO81qq1EHo1I75N2/sSb
tTle5c3v3IzStrrOc7rNf708GI/AHBCMv/pjE+w02wvbo3bspbpVaXit1a4sdCM2S+bfjudefS6K2es5zvE
1/vR44faH/uGnPy23GwDgiGT+06hiTyAMnO3YGmWjIDI1w0RxtWv8MG4FbdL01ZrBYDgLhw17Ji
G6e9jmSt2pnmA020oy+k575wqDs6/65MZLhWsNZE53L/YgnmurSTBes/TDDHv7dRhbq9Ww9/bt
LnZsRaWZd60YMewWtElHaU06ujx6zgz7wl9+IYrZ6znO9zX+9PjSJ+
+WOw4AcERXy/z1s3X3g3W2SevhvyMgjNY84J/x431XHMpf2gl2LdMg2dOu8fOD7wVDtKJWpfe2z
TJsff1wLvrYtaJI1Wt3rerRO/Od3Lz6vlrZceOTQ4vt2LKmReZ6cTHfr3fCU2qVhme8F7YKZDrM9yW88
mzo8M03vxnF7PUc5/4af3q8/tWXy30HADiWI2T+6OH9uk/k4XC9QWf1srtZR5lqn+pW9cYFaad3A
3Ljp32T8ZNuVZ+eO5cO25yJ6/QMG7bJVc2tKLJvEk2hZ96FYPKh7m7VNIIWUa3BxcZD1B3qMYNZh
avtGqjpmJhSqzlTFa97VWeaEz21m1bBItJyyZSSbvG6R82ZQQ/eebDaX9S/O879Nf70uPvM028/eF
DuPgDAUcye+VPJ0/q1BEljZ54xT6QvDoULi9vV2WZg9X3jB+eiZtXHYPDMqchsw9YDldoIozEH5PYyI
xl0eH+TmUelRi42NHIWdbPOgUbortXsa9wm6bNTn+uZS67fcLnqY88GjpgzAy4uL27cuxHF7PUc23i
NPz282A8AHNnymX/eB/H6yf9WOwGco8zORNmxVC/6cDaTdrJyO58pEDWrR8/oLDgcwEYPGzaM
725uRd0yax1psGfHHammNXqxhXbrZIx2h3oLGldZ3+ha8VZXTVrFsifbcrcs0y1q1p5mWzShnjnT5/K
9y9tfux3F7PUcW3qNPz282A8AHNOy/21/5tE984S+NzK3VH3uvFb9dJXAszKZ3cyHqKhhvlEqM36u
b8foOcFAbXMO27RMFphbUbfMpEYarlO1KMauCtWtxyy2rhGprnfOvqNj35ZMqBVvQXY6nXNsxO
PsDZerWuR0TyhXiy53v3U3itmrOn7zN345yskbO7zYDwAczYkyf90q1JMcSlW3/SDVz8O91iqzm/kQ
FS013yiVGX9EUho7est8w9bdClHr3Iq6TVpJoarT17Mcfdekaj10G0NVi51Do2Rpg2MEQxTGNBxfK2
6Tnc7gHPO3bLjciIFHzJlOL73xUpSx13b80iffH4XkjR1e7AcAjmb53+GX6ZOcGvGIv1f1O3SrPg10Wq
+
+nQkXFbcbuVu58UckpVyvQXMNW7W6de9ebpFXm9u4fcqNWfXsLVM2unXnzmGAoPHgPNOpV
WfqLj2z3126QrFpteJRc1Xqcz07nOs3XC7XKzJiznRa8/8af338+sd/IcrJGzs+95EPl/cDAGBJp8j81ZnM
03pPeEgbjeq0YunO7NR5LF5ls8xM2snKjX+VdFedqKcUzrNtnmFbXeoPPWP269in+PSIieXV7Qqtxk
NjxiWa9vU48TQP6o7N6XzDxrRayVYnSwrG6Sq9k4yzM6JcUi1exag50+Xyvctn/
+TZKGOv8Nj2f+H/3W+8Wt4PAIAlnSLz14/vGT1P69VAzUN+euasdOxmfnuCNWbSTl57pKLKqKRUn
2npuTWzDFtfrgapp9+MmllRt8yk9tLT7VErA6PvhP3i1gOLzZfcq5p0zL6zZ/d0J9VKb1/HkvbiOYba1Y
vRRpUb2MCRc6bTO3//zpp/af/heP8L7/vEr92IovI2jpdf+Ex5JwAAFnY+mT+fAQ76Msd69exme7HR
9Uza6RJuddF8dFJqz2Co1PWHbS4E7evpNw2TFXXLTGovfzocN3dHsjLzC/XvYVhxX6/6XNbOT/OgPd
kR0716reHbt7vQN8dGWLxoOalcXGX0IHRa+f84/+HY5Iv9n/vIh3/27rvIbQAAWNgpM39/Uoi0H/9j
VxoK5lF9KX39OFOvfP+VKGOv8NjYi/13n3n6R2//oLwBAADLO+X7/D2jJOo+HaQujq3+TvryccY+
+xefjTL2Co8tvdjvNX4A4A4MgmZv6sIsIngT6T8JthynP1f/3b9U8BMoMc1BfkLo4m/K/Vu7+0cCY+9vW
PRRl7bcdmXuz3Gj8AcHwnyfxdA3WGp55k33MJlhFkfoGf83f53uXNr9yMYvbajg282O81fgDgJE6U+
ffag/VF9t5c33sRgEEPLx4+8cUnopi9tuOsX+z3Gj8AcCpXy/wABNL9h/fX/AbE9h/fX/2v8z/fF/r/
+89fLjQYAOC6ZH4C9L//Vl6OMvbbjTF/s//rnXyy3GADg6GR+AErPf9f5/KGav7/AHBC
Mj/9ot/H0P/xSqrfhFf+IobrDHVVrV+8v/gvgtit7Ahr2jvmHrJZt792O4rZazavO6MX+T33wAz/52x+W
OwsAcAoyP/36QuapMn9d6GP9oesmUXlxc37t2IYvbajnN5sd9r/ADAycn8XEm8TMfzRHrHvsDC7zM483f/Lmyn+N/1m82O81fgBgDWR+ruT8TM8/8xc7HMz9n69lvfjmL22

o6Vv9jvNX4AYCVk/pPoiLvZ03WOKwz3GMh91eWmR3qmGnZ/Jhqu/2O/uvHOrn009+xQYZdwgr1
anVq9qpKBjomHk4vm3dKuFVbrqhUtey9ZetCmGaa82F0xM1CHaIzxi9oZM/
+d3jEOWk3S6yNGYFkvvfFSFLPXdqz2xX6v8QMA6yHzn0YmOOVONoEvFHQaGcBC1fX6cl2kHqQ8U
3yOhuv/2CO/lL2ybDJUfSIULDSrf8cyVzsmPjjdvd5iXbWq88E4ydIzfQ/NeyuOux3ZbQ16DJSYOv+9Y
FZDN3d4BI7iuVefi2L22o51vtjvNX4AYD1k/hOpMk8m5tSn6txTnamDUtIkGCYJYLGyQdUlSFdlj2qE4
mM0XP/HTnW7uMZOOZGhkTNLTYzYsXTgrHRXmjPREq5YK7OOpFlavTBYMVevLekSbdlgiTHzj5skg
8Qdkh6DI3AkI+9dPvsnz0Yxe1XHCl/s/7M//qNy+wAAVkDmP5UqxdQhJslSmXA1mK52kjyVKFsc+h
[truncated: 4,352,025 more chars]
